# Identification and in silico bioinformatics analysis of PR10 proteins in cashew nut

**DOI:** 10.1002/pro.3856

**Published:** 2020-05-16

**Authors:** Shanna Bastiaan‐Net, Maria C. Pina‐Pérez, Bas J. W. Dekkers, Adrie H. Westphal, Antoine H. P. America, Renata M. C. Ariëns, Nicolette W. de Jong, Harry J. Wichers, Jurriaan J. Mes

**Affiliations:** ^1^ Wageningen Food and Biobased Research Wageningen University and Research Wageningen The Netherlands; ^2^ Institute of Life Technologies HES‐SO Valais‐Wallis Sion Switzerland; ^3^ Wageningen Seed Lab, Laboratory of Plant Physiology Wageningen University Wageningen The Netherlands; ^4^ Biochemistry Wageningen University and Research Wageningen The Netherlands; ^5^ Wageningen Plant Research Wageningen University and Research Wageningen The Netherlands; ^6^ Allergology, Department of Internal Medicine Erasmus MC Rotterdam The Netherlands

**Keywords:** *Anacardium occidentale*, Bet v 1‐like, cashew nut, in silico allergenicity analysis, oral allergy syndrome (OAS), PR10, RNA‐seq

## Abstract

Proteins from cashew nut can elicit mild to severe allergic reactions. Three allergenic proteins have already been identified, and it is expected that additional allergens are present in cashew nut. pathogenesis‐related protein 10 (PR10) allergens from pollen have been found to elicit similar allergic reactions as those from nuts and seeds. Therefore, we investigated the presence of *PR10* genes in cashew nut. Using RNA‐seq analysis, we were able to identify several PR10‐like transcripts in cashew nut and clone six putative *PR10* genes. In addition, PR10 protein expression in raw cashew nuts was confirmed by immunoblotting and liquid chromatography–mass spectrometry (LC–MS/MS) analyses. An in silico allergenicity assessment suggested that all identified cashew PR10 proteins are potentially allergenic and may represent three different isoallergens.

**Abbreviations**
aa: amino acids
APC: antigen‐presenting cells
BAT: basophil activation test
Borax: sodium borate decahydrate
bp: base pairs
ELISAs: enzyme‐linked immunosorbent assays
FAO/WHO: Agriculture Organization/World Health Organization
MHC: major histocompatibility complex
MT: melting temperatures
Mw: molecular weights
nsLTPs: nonspecific lipid transfer proteins
OAS: oral allergy syndrome
ORF: open reading frame
PDB code: protein data bank code
pI: isoelectric point
PR10: pathogenesis‐related protein 10
PSM: the total number of identified peptides to spectrum matches.
RACE: rapid amplification of cDNA ends
SPT: skin prick test
TLPs: thaumatin‐like proteins

## 1 INTRODUCTION

The cashew tree (*Anacardium occidentale* L.) is a tropical perennial tree native to South America.^1^ In the harvest season of 2017/2018, cashew nut production reached near 790,000 metric tons (on kernel basis), with Western Africa as lead producer representing 43% of the world share (International Nut and Dried Fruit Council; https://www.nutfruit.org/). Cashew nuts are appreciated for their taste and nutritional properties (such as high lipid and essential amino acids (aa) content, and rich in minerals like potassium, magnesium, and calcium).^2^, ^3^, ^4^ In addition, they are suggested to have positive health effects, as consumption of the cashew nut kernel has been linked to reduction of cholesterol levels and coronary heart disease risks.^1^, ^5^, ^6^ Some cashew nut proteins however, may induce adverse reactions in tree nut allergic individuals, with symptoms ranging from mild (like nausea, diarrhea, eczema, and asthma) to severe reactions^7^ which are associated with a high risk of anaphylaxis.^8^ Three allergens have been identified and characterized in cashew nut; Ana o 1 and Ana o 2 from the cupin family and Ana o 3 belonging to the albumin family.^9^, ^10^ Importantly, the pathophysiology of cashew nut allergic responses of some patients indicates mild oropharyngeal symptoms (i.e., symptoms in the middle throat area, including the oral cavity)^11^, ^12^, ^13^, ^14^ that match the oral allergy syndrome (OAS): oral tingling or itching (pruritus) with or without swelling of the lips, oral mucosa, and throat (angioedema).^13^, ^15^ According to studies of Li et al.^11^ and Hasegawa et al.,^13^ between 100 and 75% of respectively studied patients' cohorts showed OAS associated to cashew nut consumption. Also 64% of patients in a cohort of 176 children manifested typical OAS during a cashew nut food challenge test.^14^ Proteins typically responsible for OAS include proteases, α‐amylase inhibitors, peroxidases, profilins, seed‐storage proteins, pathogenesis related proteins (PRs), thiol proteases, and lectins in vegetables.^16^, ^17^, ^18^, ^19^, ^20^

Bet v 1 from birch pollen is a main elicitor of pollen allergy symptoms and the first identified allergenic member of the family 10 of pathogenesis‐related proteins (PR10).^21^ Bet v 1 cross‐reactive homolog that act as elicitors of a food‐mediated OAS allergic immune response have been found in various fruits, vegetables, nuts (hazelnut, walnut, almond, and peanut) and seeds.^20^, ^21^, ^22^, ^23^ For instance, Ara h 8, the Bet v 1‐homolog in peanut, is most likely responsible for the cross‐reactivity observed between birch and peanut and its associated OAS symptoms,^24^ while the PR10 protein Jug r 5 is evidently associated with the manifestation of a birch pollen‐associated walnut allergy.^25^

Despite the fact that cashew nut allergy is often accompanied by symptoms consistent with OAS associated with a PR10‐allergen hypersensitivity, no information is available on the presence of cross‐reactive *PR10* genes in cashew nuts. Therefore, we employed an RNA‐seq analysis to identify *PR10‐like* transcripts in cashew nut. Subsequent cloning and sequence analysis enabled us to identify multiple *PR10* genes in cashew nut and allowed us to perform an in silico prediction analysis for allergenic potency of the identified putative cashew PR10 proteins.

## 2 RESULTS

### 2.1 *Identification of putative cashew nut PR10‐like genes by transcriptome analysis*

Next‐generation sequencing of RNA extracted from cashew nut resulted in an RNA‐seq library of 65,599,531 trimmed reads with an average length of 112.3 base pairs (bp). A summary of statistics after sequencing is presented in Table 1. Genome alignment of reads for transcript assembly was not possible due to the lack of an existing reference genome database for cashew nut. Therefore, we used a de novo transcriptome assembly approach which generated a BLAST library consisting of 53,114 contigs with a minimum and maximum contig length of 126 and 12,132 bp, respectively. Fifty percent of the entire assembly is contained in contigs ≥804 bp.

**TABLE 1:** Summary of statistics of the RNA‐seq library and de novo transcriptome assembly

	Count (no.)	Average length (bp)	Total bases (bp)
Reads	65,599,550	112.33	7,368,725,189
Matched reads^a^	58,971,799	112.27	6,620,625,613
Non matched reads^b^	6,627,751	112.87	748,099,576
Reads in pairs^c^	55,271,842	124.93	
Broken paired reads	3,699,957	125.31	
Contigs	53,114	599	31,860,598

a Number of reads that showed an overlap with each other.

b Number of reads that contained unique transcript sequence.

c Reads that have been sequenced from both ends.

Next, we used a BLAST query in the cashew nut transcriptome to identify putative PR10 proteins. Since PR10 protein sequences (derived from nut/seed) are not available for members within the cashew family (family of Anacardiaceae), we used the nut‐derived PR10 allergen Pru du 1 from almond from the phylogenetically related Rosaceae family.^26^ This BLAST search identified nine contigs within the cashew RNA‐seq paired reads dataset, that shared 32–55% sequence identity with Pru du 1 isoforms (Table 2). Sequence alignment revealed that only 3 of the 9 contigs identified contained a complete open reading frame (ORF) sequence. These were contig #18220, #25355, and #25514, whose sequences were subsequently used for cloning. The ORF in contig #25355 showed the highest sequence identity to Pru du 1.

**TABLE 2:** Identified contigs using the PR10 allergen Pru du 1 from almond as BLAST query, ranked according to total score value

Contig no.	Contig mapping			BLAST results			
	Consensus length (bp)	No. of reads	Average coverage	Total score	Min. E‐value	Max identity (%)	Identity with
Contig #25355	529	34	7.26	368–423	5.15^−44^–2.33^−52^	49.69–54.72	Pru du 1.01 t/m Pru du 1.06
Contig #25514	626	76	13.88	322–353	6.47^−37^–4.01^−41^	40.52–43.23	Pru du 1.01 t/m Pru du 1.06
Contig #18220	711	277	43.52	344–376	8.04^−40^–1.74^−44^	44.87–50.00	Pru du 1.01 t/m Pru du 1.06
Contig #16127	421	539	142.77	195–229	5.21^−19^–6.24^−24^	39.39–41.84	Pru du 1.01 t/m Pru du 1.06
Contig #18221	236	28	11.80	139–171	2.79^−11^–4.57^−16^	37.66–55.36	Pru du 1.01 t/m Pru du 1.06
Contig #16128	456	315	74.49	106–164	2.39^−06^–1.55^−14^	36.36–48.98	Pru du 1.01 t/m Pru du 1.06
Contig #25317	718	234	35.74	104–113	2.39^−06^–8.83^−07^	36.36–39.39	Pru du 1.01 t/m Pru du 1.06
Contig #4938	732	3,835	578.90	104	4.84^−06^	31.88	Pru du 1.01
Contig #25513	429	54	13.93	93	7.37^−05^	38.89	Pru du 1.01

*Note* Identified contigs using the PR10 allergen Pru du 1 from almond as BLAST query, ranked according to total score value. Putative PR10 amino acid sequences corresponding to each contig were aligned to Pru du 1 using Clustal W (1.7) multiple sequence alignment for comparison reasons.
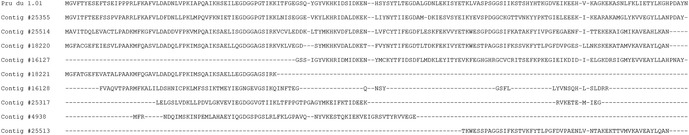

**FIGURE 1:**
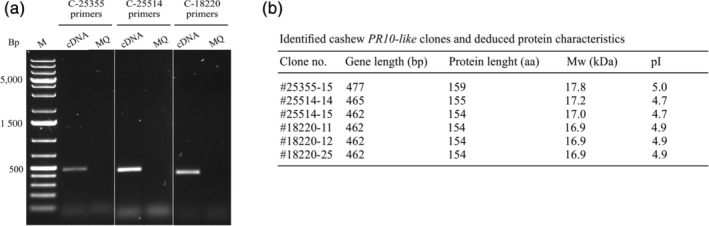
Cloning of cashew PR10‐like genes. (a) PCR amplification of PR10‐like genes identified in contigs #25355, #18220, and #25514; (b) characteristics of the identified cashew PR10‐like clones and their different variants. aa, amino acids; bp, base pairs; kDa, kilo Dalton; pI, isoelectric point

To confirm the presence of the identified putative *PR10* ORFs in cashew nuts we used PCR‐based cloning using contig‐specific primers (Table S1). Sequence analysis of amplified full‐length ORFs (Figure 1a) confirmed the *PR10‐like* gene sequences that were predicted by the de novo transcript assembly. In addition, one or more genetic variants for two of the *PR10‐like* ORFs were identified which differed slightly in length and sequence. These multiple allelic variants were found in *PR10* contig #25514 (clones #14 and 15) and *PR10* contig #18220 (clones #11, 12, and 25) (Figure 1b). The deduced proteins of the identified variants ranged in length between 154 and 159 aa and the molecular weights (Mw) were predicted to be in range of 16.9–17.8 kDa while isoelectric point (pI) values ranged from 4.7 to 5.0, as observed for other PR10 proteins.^27^

Sequence comparisons between the isolated clones and the assembled RNA‐seq contigs showed a high level of sequence similarity. For example, clone #25355‐15 showed 99% aa‐homology with contig #25355 while clones #25514‐14 and ‐15 are 100 and 98% homologous to contig #25514, respectively. Clones representing contig #18220 showed 99% (#18220‐11), 100% (#18220‐12), and 99% (#18220‐25) homology with the original contig ORF sequence. Thus, in this study the RNA‐seq approach proved to be an accurate and powerful approach to identify the presence and genetic variants of *PR10‐like* sequences.

### 2.2 *Bioinformatics analysis of the putative PR10‐like proteins of cashew*

To further verify that the putative PR10 proteins identified in cashew are indeed related to pathogenesis‐related proteins belonging to the PR10 family, a general NCBI‐BLAST was performed using their deduced aa sequence as query (FASTA search). As shown in Table S2, the top 5 BLAST results corresponded to other PR10 proteins and all putative cashew PR10 proteins display a high identity to the PR10 proteins Pru av 1 and Pru ar 1 from cherry and apricot, respectively. Moreover, all identified clones contain the Prosite PS00451 “pathogenesis‐related proteins Bet v I family signature” G‐x(2)‐[LIVMF]‐x(4)‐E‐x(2,3)‐[CSTAENV]‐x(8,9)‐[GNDS]‐[GS](2)‐[CS]‐x(2)‐[KT]‐x(4)‐[FY] (for cellular localization, membrane‐protein and protein–protein interactions) as well as the PFAM Bet v 1 domain (PF00407).

**FIGURE 2:**
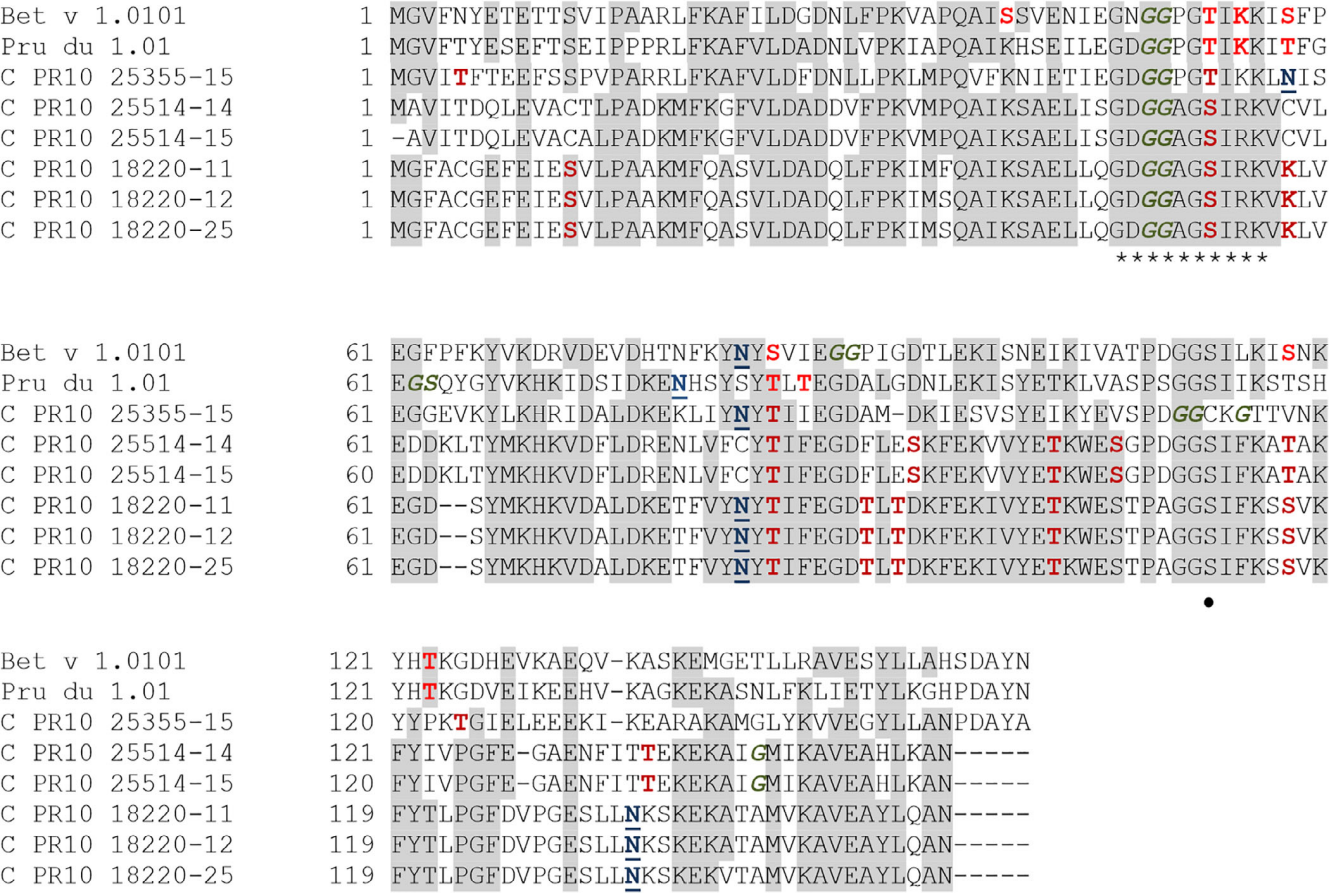
Clustal alignment of the cashew PR10‐like proteins, Bet v 1.0101 (P15494) from birch pollen and Pru du 1.0101 (ACE80939.1) from almond. Cashew nut AA‐regions that are identical to the PR10 proteins of birch and/or almond are shaded in grey. Putative phosphorylation sites are indicated in bolt red, putative N‐myristoylation sites are indicated in bold italic green and predicted N‐glycosylations sites are blue underlined. Stars underneath the alignment mark the p‐loop region in Bet v 1.0101. The • indicates Ser112 essential for IgE cross‐reactivity between Bet v 1 and Mal d 1

Next, the putative cashew PR10 protein sequences and Pru du 1 were aligned to the PR10 reference protein Bet v 1 from birch pollen and their predicted co‐ and post‐translational modification sites were analyzed (Figure 2 and Table S3). All identified sequences contain the Bet v 1 characteristic common feature of a glycine‐rich P‐loop motif (GxGGxGxxK),^28^, ^29^ although variants of clones #25514 and #18220 contain an additional arginine before the lysine in the P‐loop region (GxGGxGxxxK). The structural P‐loop element facilitates nucleotide‐binding interactions in some proteins.^28^ Clone #25355‐15 shows a similar deduced aa‐sequence length as Pru du 1 and Bet v 1, while the other cashew PR10‐like proteins are five aa shorter at the C‐terminal end.

All clones contain putative co‐translational myristoylation sites, allowing for membrane targeting and protein–protein and protein–lipid interactions,^30^ and post‐translational phosphorylation sites which may greatly define the structural conformation of a protein, its signalling pathways and metabolism.^31^, ^32^ Compared to a single predicted N‐glycosylation site in Bet v 1 and Pru du 1, two N‐glycosylation sites were predicted for clones #18220 and #25355, while these sites are lacking in clones #25514‐14 and ‐15.

**FIGURE 3:**
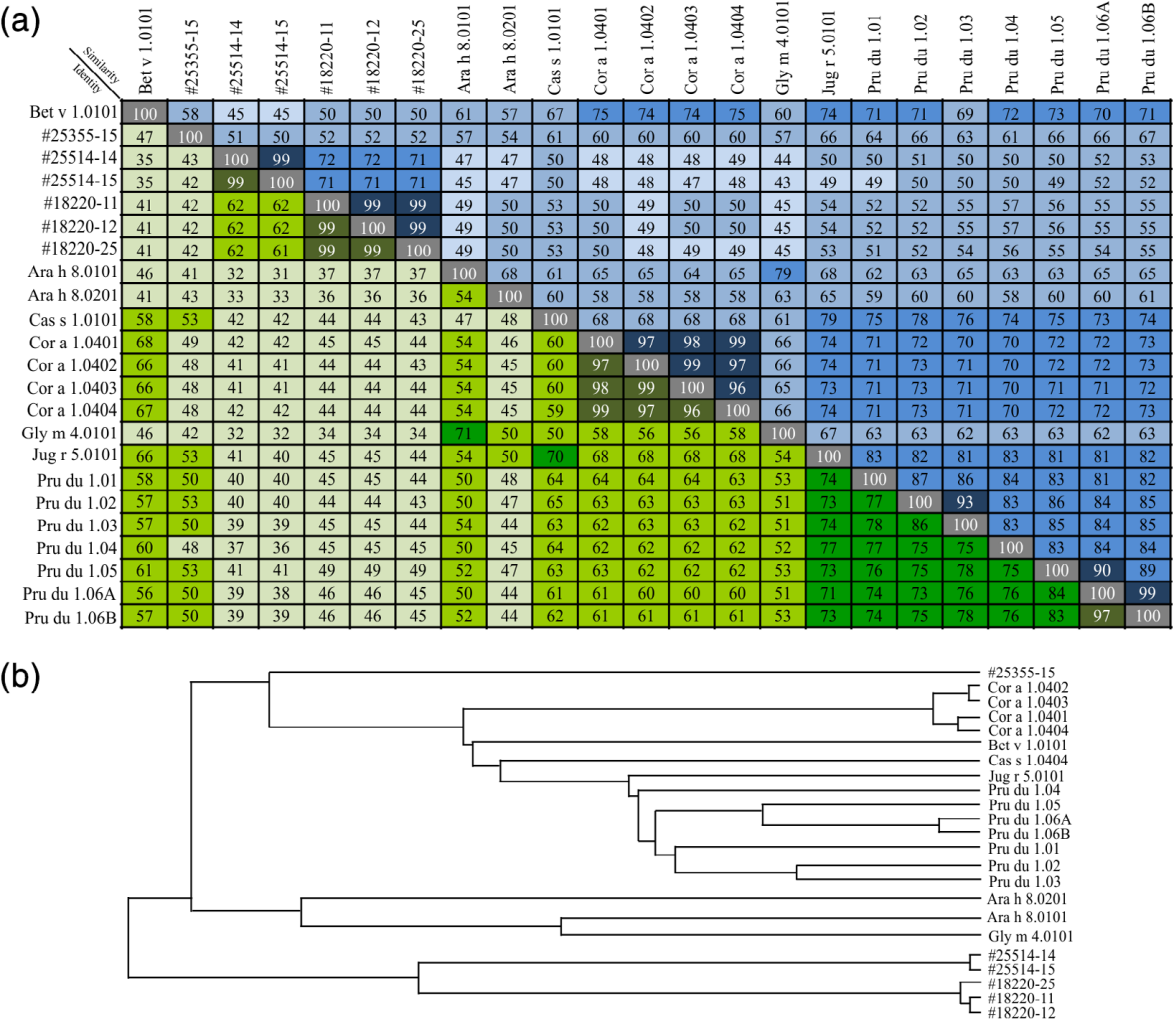
Similarity and identity analysis (a) and phylogenetic clustering (b) of cashew PR10 proteins, Bet v 1 from birch pollen (Bet v 1A; 4bkd‐1bv1) and the well‐studied PR10 allergens from almond, chestnut, hazelnut, peanut, soybean, and walnut. Pru du 1.01 (ACE80939.1), Pru du 1.02 (ACE80941.1), Pru du 1.03 (ACE80943.1), Pru du 1.04 (ACE80945.1), Pru du 1.05 (ACE80947.1), Pru.du.1.06A (ACE80951.1), and Pru du 1.06B (ACE80949.1) from almond; Ara h 8.0101 (AAQ91847.1), and Ara h 8.0201 (ABP97433.1) from peanut; Cas s 1.0101 (ACJ23861.1) from sweet chestnut; Cor a 1.0401 (AAD48405.1), Cor a 1.0402 (AAG40329.1), Cor a 1.0403 (AAG40330.1), and Cor a 1.0404 (AAG40331.1) from European hazelnut; Gly m 4.0101 (CAA42646.1) from soybean; Jug r 5.0101 (APD76154.1) from English walnut; Bet v 1 (Bet v 1A; 4bkd‐1bv1) from birch pollen

A similarity and identity analysis of the deduced aa between the PR10‐like proteins from cashew and various tree nuts and legumes is shown in Figure 3a. The cashew PR10‐like proteins show the highest sequence identity with PR10 allergens from almond, chestnut, and hazelnut (36–53%) as compared to leguminous PR10 allergens Ara h 8 and Gly m 4 (31–43%). Cluster analysis visualized a similar trend in phylogenetic relationships as the similarity and identity analysis (Figure 3b). The sequence identities to Bet v 1 are in the expected range of 35–47%^27^ where a low aa‐identity does not exclude the ability to cross‐react with Bet v 1‐specific IgE antibodies, as in vitro demonstrated for Dau c 1 (PR10 from carrot) which displays only 38% sequence identity with Bet v 1.^33^

**FIGURE 4:**
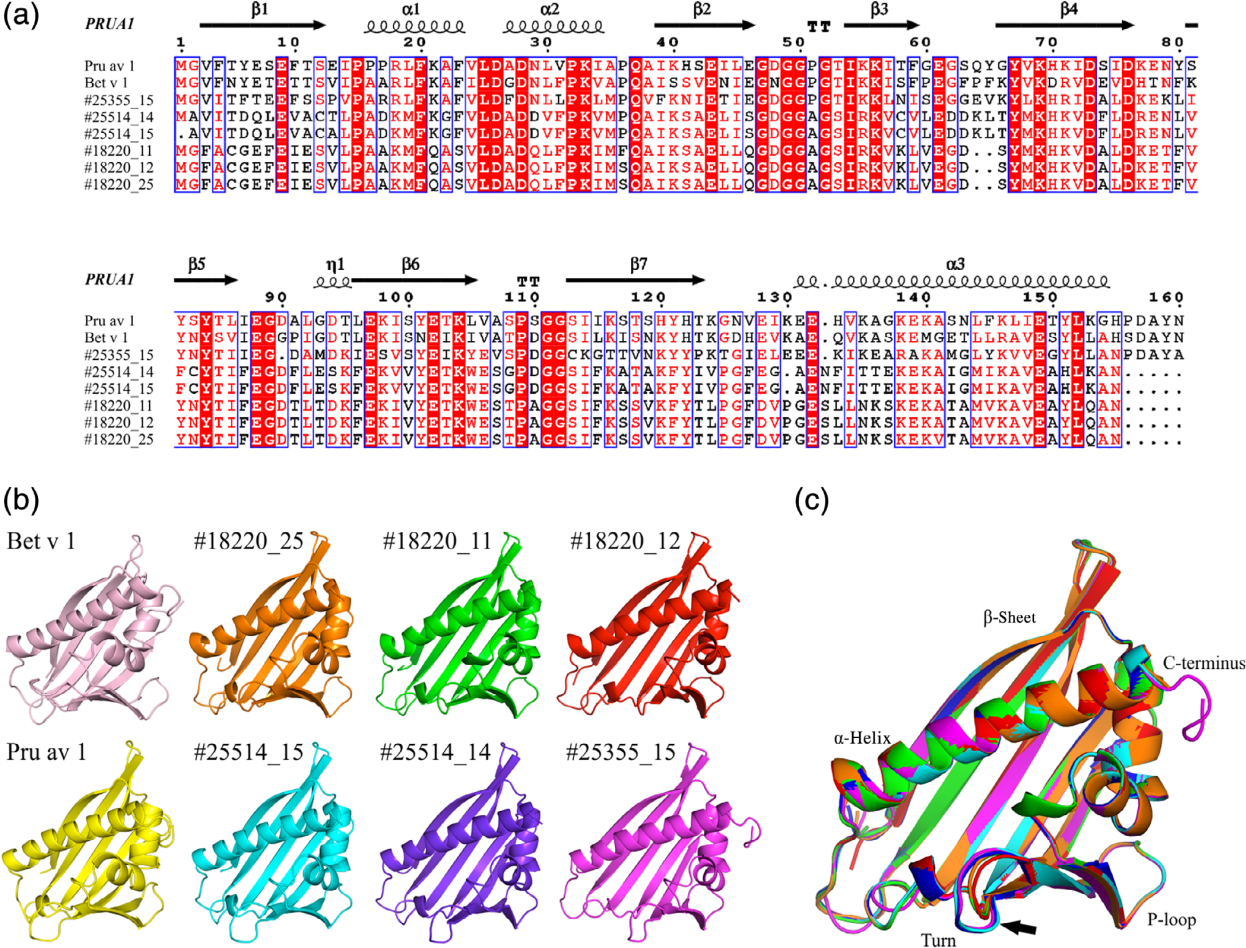
Structural modelling of the putative cashew PR10‐like proteins using the PRUA1 NMR structure as template. (a) ClustalW alignment of the cashew PR10‐like proteins and PR10 allergens from Bet v 1 and Pru av 1using the software Esprit. The α‐helices, β‐sheets, and turns (TT) of Pru av 1 (PRUA1) are indicated above the alignment. (b) Structural modelling of tertiary structure using the program Modeller and Pymol. (c) Superimposed view of models generated for #25355‐15, #25514‐14, #25514‐15, #18220‐11, #18220‐12, and #18220‐25. The arrow indicates a difference in the predicted turn area

Based on the deduced protein sequence of the identified *PR10‐like* clones, a prediction was made of the structural features of the cashew PR10‐like proteins. Since the protein crystal structure for Pru du 1 is lacking, we used the NMR structure of the major cherry allergen from *Prunus avium*, Pru av 1 (PruAV1; PDB code 1E09), as template as all cashew PR10 clones displayed a high sequence identity to Pru av 1 (42–52%; see Table S2). Structural modelling (Figure 4) shows that the predicted cashew PR10‐like protein structures are highly similar to the Bet v 1A^29^ and Pru av 1^34^ crystal structures. All displayed the characteristic basket‐like hydrophobic cavity formed by two V‐shaped short α‐helices wrapped around a long C‐terminal α‐helix and a folded seven‐stranded antiparallel β‐sheet.^33^ Some small differences in α‐helix bending could be observed as well as the length of the turnaround residue 65, which is shorter in the structures of the #18220 proteins (indicated by an arrow).

The NCBI BLAST results as well as the other bioinformatics analyses, including the high similarity between the predicted cashew PR10‐like protein conformational structures and the crystal structure of Bet v 1, strongly suggest that the identified *PR10* genes in cashew nut indeed belong to the family of *PR10* genes.

### 2.3 *Presence of PR10 proteins in cashew nut extract*

**FIGURE 5:**
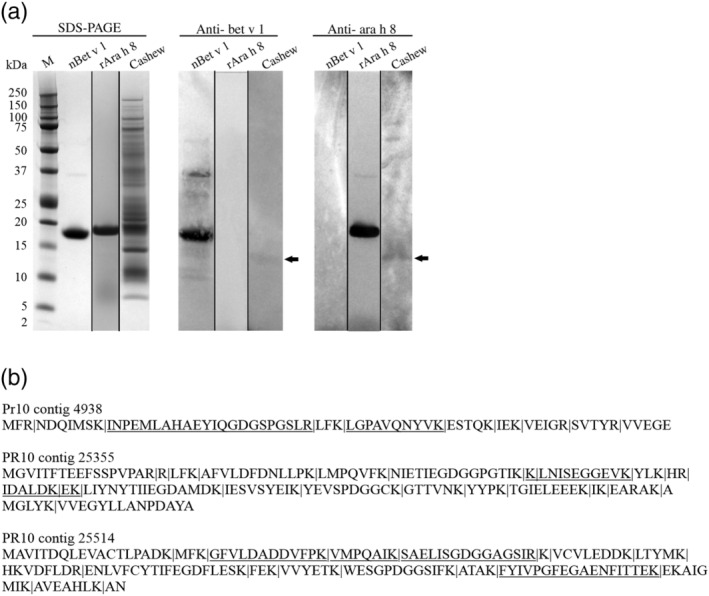
Identification of PR10 proteins in cashew nut total protein extract. (a) SDS PAGE gel electrophoresis and western blot of the positive controls nBet v 1 and rAra h 8 and raw cashew nut extract using anti‐bet v 1– and anti‐ara h 8–specific antibodies. The arrow points towards a positive band in cashew nut extract; (b) LC–MS/MS peptide identification in raw cashew nut extract after trypsin digestion. Identified peptides in contigs 4,938, 25,355, and 25,514 are underlined. Trypsin cleavage sites are indicated by the symbol |. Sequence coverage for contig 4,938 was 47%, 12% for contig 25,355 while for contig 25,514, sequence coverage was 34%

The presence of *PR10* RNA in cashew nuts does not mean that the corresponding proteins are also present. Two approaches have been applied to demonstrate the presence of PR10 proteins in cashew nut: immunoblotting using commercial IgG antibodies against Bet v 1 and Ara h 8 (PR10 protein from peanut^35^), and LC–MS/MS peptide identification using the identified cashew *PR10* RNA‐seq contig sequences as well as the cloned *PR10* gene variances as database‐query (Figure 5). Both anti‐Bet v 1 and anti‐Ara h 8 antibodies showed some binding affinity to a cashew nut protein, resulting in a very faint band of around 13–14 kDa in size (Figure 5a). The polyclonal antibodies used seem to be highly selective based on the positive control results, which could explain their weak binding to cashew nut protein. Based on the deduced aa‐sequence, the expected size of cashew PR10 proteins would lay between 16.9 and 17.8 kDa, as also visible for native Bet v 1. Detection of a slightly smaller protein in the cashew nut protein extract could indicate potential proteolytic hydrolysis during the extraction procedure. The fact that PR10‐like protein peptides, corresponding to RNA‐seq contigs #4938, #25355, and #25514, were identified in the cashew nut protein extract by LC–MS/MS, confirms that *PR10* genes are indeed expressed in cashew nut although likely much less than Ana o 3 (Figure 5b, Tables S4a and S4b).

### 2.4 *In silico analysis of potential allergenicity*

As PR10 proteins from fruits, vegetables and nuts are commonly associated with a birch pollen–related allergy,^18^ we performed several in silico prediction analyses using online available software tools to examine the potential allergenicity of identified cashew PR10 proteins (see Tables S5–S7), for which the results are summarized in Table 3. First, the Food and Agriculture Organization/World Health Organization (FAO/WHO) CODEX Alimentarius guidelines (2001) were assessed. These state that a sequence is potentially allergenic if it either has an identity of at least six contiguous aa OR ≥ 35% sequence identity over an alignment length window of ≥80 aa when compared to known allergens.^36^ The allergenicity prediction criteria were assessed using the software tools AllergenOnline and SDAP, as listed in Tables S5a and S5b, respectively. In particular, clone #25355 was predicted to contain multiple 6‐mers and even 8‐mers peptide sequences identical to peptides in existing allergens. In addition, each of the cashew PR10 proteins showed 179 hits in the 80‐mers sliding window alignment analyses. According to the FOA/WHO guidelines, all identified cashew PR10‐like proteins would be labelled as potential allergens (Table 3).

**TABLE 3:** Summary of performed in silico allergenicity prediction analyses using several online prediction servers

Software	Link	Prediction
SDAP	http://fermi.utmb.edu/	For each cashew PR10‐like protein, multiple 6‐mers and 80‐mers sliding windows have been identified suggesting cross‐reacting characteristics.
AllergenOnline	http://www.allergenonline.org/	Multiple 8‐mer hits for #1‐15 and one to two hits for #2‐14/15 and #3‐11/12/25. All showed 179 hits of 80‐mers sliding windows suggesting cross‐reacting characteristics.
AllerTOPv.2	http://www.ddg‐pharmfac.net/AllerTOP/	All cashew PR10‐like proteins, except clones #25514‐14 and ‐15, are predicted to be probably allergenic with nearest allergen matches being bet v 1‐like allergens.
AllergenFPv.1.0	http://www.ddg‐pharmfac.net/AllergenFP/	All cashew PR10‐like proteins are predicted to be probably allergenic with nearest allergen matches being Bet v 1‐like allergens.
BepiPred 1.0	http://www.cbs.dtu.dk/services/BepiPred‐1.0/	Each cashew PR10‐like protein contains several predicted linear B‐cell epitopes
BPAP	http://imed.med.ucm.es/Tools/antigenic.pl	Each cashew PR10‐like protein contains several predicted linear B‐cell epitopes
ElliPro	http://tools.iedb.org/ellipro/	Multiple continuous as well as discontinues B‐cell epitopes have been predicted for each cashew PR10‐like protein
NetCTL‐1.2	http://www.cbs.dtu.dk/services/NetCTL/	Three to six MHC‐class ligands and 146–151 T‐cell epitope peptides have been predicted using the cashew PR10‐like proteins as query.

Furthermore, we used the web‐based computational system AllergenFP and AllerTOPv.2. The AllerTOPv.2 program predicted that all cashew PR10 proteins are possible allergens and to be cross‐reactive with IgE antibodies recognizing homologous allergens (Table S6). The AllergenFP prediction indicted that four out of the six PR10 proteins of cashew nut are potentially allergenic. In this case PR10 #25514 clones 14 and 15 were not ranked as potential allergens and these small differences are likely due to the use of different computational methods.

When a protein is predicted to be allergenic or to be cross‐reactive, it should contain antigenic epitope regions that allow for binding to secreted antibodies or antigen‐specific cell membrane receptors.^37^ Antigenic B‐cell epitopes, the aa‐region that is recognized by an IgE‐antibody, can be linear (continuous, ~10%) or conformational (partial continuous or discontinuous, ~90%). T‐cell epitopes on the other hand (the aa‐region presented on antigen‐presenting cells [APC] by the major histocompatibility complex [MHC] molecules) are commonly continuous. Using epitope prediction software tools, several continuous and discontinuous B‐cell epitopes were predicted for each of the cashew PR10‐like protein clones identified (Table S7). In addition, MHC‐class peptides and T‐cell epitopes have been predicted.

**FIGURE 6:**
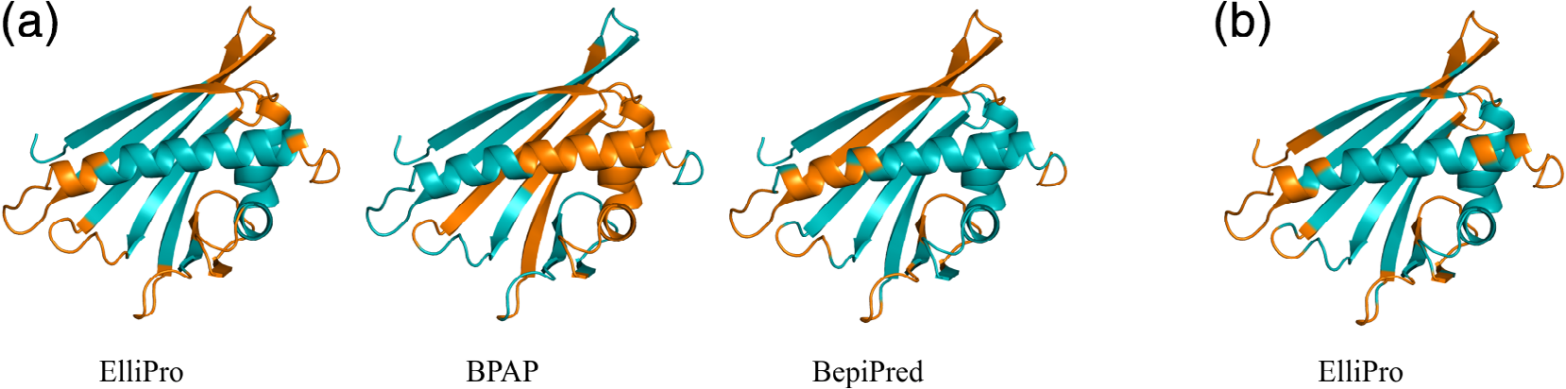
Predicted epitopes for #25355‐15 as indicated on the modelled tertiary structure. (a) Continuous epitopes predicted by the software tools ElliPro, BPAP and BepiPred 1.0; (b) Discontinuous epitopes predicted by ElliPro 1.0

Predicted B‐cell epitopes where annotated on the structural model of PR10 #25355‐15 to evaluate the prediction value of the three software tools used (Figure 6). ElliPro 1.0 predicts almost all epitopes in the flexible regions (i.e., links between the structural elements) which are generally the most antigenic.^38^

The epitope region ENIEGNGGPG recognized by Bet v 1–specific IgE antibodies within the p‐loop region (E43‐G52) is predicted in each cashew *PR10‐like* clone (underlined in Table S7) with 80, 60, and 50% identical aa in #25355, #25514, and #18220, respectively. Whether two or more amino substitutions in this epitope region might affect the level of Bet v 1–specific IgE cross‐reactivity will have to be determined. Also, aa S112 shown to be crucial for IgE binding of Mal d 1 and Pru av 1 and cross‐reactivity with Bet v 1^39^, ^40^ is present in the sequence of both #25514 and #18220 (Figure 2).

Thus, we employed a range of analyses (AllergenOnline, SDAP, NetCTL‐1.2, BPAP, BepiPred, AllergenFP, and AllerTOPv.2) and the results combined show that the identified PR10 proteins from cashew nut are possibly allergenic and may indeed cross‐react with Bet v 1–specific IgE antibodies.

## 3 DISCUSSION

Cashew nut is solely consumed after proper shelling and roasting, which significantly improves the sensory characteristics (smell, flavour, texture, and taste) and eliminates the risks associated with traces of irritating substances derived from the shell (anacardic acid, cardanol, and cardol).^1^, ^41^ In general, PR10 family proteins are considered heat‐labile and their allergenicity is destroyed or strongly reduced upon heating, at least in fruits and vegetables (reviewed by Fernandes et al.^42^). However, Ara h 8 and Gly m 4, the Bet v 1‐allergenic homologs from peanut and soy respectively, have shown to be thermally resistant to some extent and able to provoke clinical responses even after heat treatment.^43^, ^44^ Similarly, roasted hazelnuts can still provoke allergic reactions in Cor a 1‐monosensitized individuals.^45^ Thus, since medically relevant OAS complaints, consistent with a PR10 sensitization, are often reported in a patient's anamnesis after consumption of cashew nut, although consumed in processed form, suggests that clinically reactive PR10 proteins may still be present in the kernel. This was the underlying reason for demonstrating the presence of PR10 proteins in cashew nut in this study.

Using RNA‐seq transcriptome profiling and sequence‐specific cloning, we were able to identify three different isotypes of PR10 proteins in cashew nut with several allelic variances. Sequence identity analyses and structural modelling confirmed their identity as Bet v 1 homologous proteins belonging to the PR10 protein family. Six partial ORFs identified in the RNA‐seq contig BLAST point out the presence of various other isotypes or isoforms of *PR10‐like* sequences in cashew nut, which might be elongated and extracted using Rapid amplification of cDNA ends (RACE) techniques in the future. In addition to the presence of PR10 mRNA, two independent LC–MS/MS analysis experiments and immunoblotting assays indicated the presence of PR10 protein in cashew nut as well. Using LC–MS/MS, we were able to detect three PR10‐coding contigs out of nine contigs identified. Possibly, trypsin inhibitors limiting the efficiency of the LC–MS/MS sample preparations might have been present in our protein extract,^46^ which could be one of the reasons why peptides of only three contigs were traced back. Another reason might be a possible low concentration of some of the PR10 contigs in our extract. When comparing the protein iBAQ scores of the detected PR10 contigs with the score for Ana o 3.0101, which has more or less the same protein mass, the PR10 proteins are presumably at 99 times (for #25355) to 2,970 times (for #25514) a lower concentration (Table S4b). However, proper protein quantification using spiked standards in multiple biological replicates should confirm this.

The existence of multigene *PR10* copies in cashew nut is in line with findings for the *PR10* gene Gly m 4 for which multiple copies exist in the soybean genome.^47^ Chromosome studies in cashew nut populations^48^, ^49^ suggest an overall diploidic genotype but does not rule out the existence of polyploid species. However, it is also likely that seeds pooled for the RNA extraction procedure originated from different trees and thus represent different genotypes.

To assess the possible allergenicity of the cashew PR10 proteins, a preliminary in silico‐prediction analysis was performed. The presence of multiple 6‐mers, 8‐mers, and 80‐mers sliding window peptides with cross‐reacting characteristics, the potential allergenicity predictions by the online software tools AllerTOPv.2 and Allergenv1.0 as well as the presence of various predicted B‐cell epitopes has led us to conclude that the identified cashew PR10 proteins should be considered as potential allergens that are predicted to exhibit IgE cross‐reactivity with Bet v 1. Thus, cashew PR10 proteins might have been the causative agents for observed OAS symptoms in cashew allergic patients in earlier studies^11^, ^12^, ^13^, ^14^ or even be responsible for more severe symptoms. Severe cases of OAS aggravating to systemic reactions, have been observed in allergic reactions to peanut and pistachio^17^, ^44^, ^50^ estimated that around 5% of OAS patients have symptoms progressing to systemic responses including nausea, vomiting, abdominal pain, upper respiratory obstruction, or anaphylaxis.

Most importantly, clinical relevance of identified PR10 proteins in processed cashew nuts still needs to be demonstrated through IgE‐immunoassays [e.g., basophil activation test (BAT), skin prick test (SPT) and/or ELISAs] to actually identify these proteins as real allergens. It might be however, that not all of the *PR10‐like* genes present in cashew nut are clinically relevant and thus their individual and possibly their combined allergenicity should be quantified. Expression levels of the different PR10 isoforms and isoallergens might even fluctuate per genus, origin or per season, depending on climate and environmental or geographical factors/influences.^1^ Thus, influence of variation in exposure levels should be taken into account in future risk assessments as well as tolerance thresholds per isoallergen.

However, cashew nut‐provoked OAS symptoms should be carefully interpreted especially when symptoms emerge at low doses of cashew nut exposure. Oral allergy symptoms are frequently reported by peanut allergic individuals, especially when exposed to very low doses between 100 μg and 5 mg of peanut protein.^51^ This implies that seed storage proteins, which are commonly seen as major allergens causing severe allergic reactions, can also provoke subjective reactions (oral itching) and mild objective reactions (lip swelling) that correspond to OAS symptoms associated with a PR10 sensitization. Besides, OAS symptoms might also be caused by other PR‐family members, such as nonspecific lipid transfer proteins (nsLTPs; PR‐14) or thaumatin‐like proteins (TLPs; PR‐5), or by proteins belonging to the profilin family.^18^ Current investigations are ongoing to investigate whether such allergen family members are also expressed in cashew nut.

Lastly, the mechanism behind how some seed/nut PR10 proteins retain their allergenicity after heating is still an intriguing question. Seeds are plant organs that usually have a low water content and that have several protective adaptations to cope with dehydration which protects cellular integrity and stabilizes proteins, RNA and DNA. Further, seeds contain high levels of storage compounds, like sugar, fat and proteins. In this sense, seeds are different from fruit and vegetable tissues and the seed matrix can play a role in the protection of PR10 allergenic proteins from thermal destruction. Interestingly, this protection from thermal destruction has been observed in fat/oil‐rich leguminous seeds (peanut and soy) and nuts (hazelnut).^35^, ^43^, ^45^ The total fat content in cashew nut is high as well and accounts for 48.3% of the total weight,^52^ which is comparable to the lipid content reported for peanut (40–50%).^53^ In addition, PR10 stability has also been linked to binding to their ligands. The characteristic structure of Bet v 1 and its homolog, comprising of seven‐stranded β‐sheets flanked by three α‐helices forming a central basket‐like hydrophobic cavity,^34^ allows binding of a variety of lipophilic ligands.^54^ Like Bet v 1,^55^ Ara h 8 is hypothesized to bind flavonoids (quercitin, apigenin, and daidzein), and lipid sterols.^24^, ^43^, ^53^ This ligand binding provided increased thermal proteolytic stability to the Bet v 1^56^ and Ara h 8^43^ structure. Thus, it seems possible that cashew nut PR10‐like proteins may function as flavonoid or sterol carriers. Whether thermal degradation of cashew PR10 proteins is influenced by the seed matrix and its ligands, and thereby their allergenic cross‐reactivity, remains an important issue to be investigated.

## 4 MATERIALS AND METHODS

### 4.1 *Sample preparation and RNA isolation*

Technical details about sample preparation before RNA isolation, the RNA‐seq transcriptome profiling and the RNA‐seq data analysis and BLAST analyses specifications can be found in Data S1.

### 4.2 *Cloning of PR10‐like sequences*

*PR10‐like* sequences were amplified from cashew nut RNA using contig‐specific primers (Table S1). First, extracted RNA was converted by Oligo(dT)20 primers included in the iScript Select cDNA Synthesis Kit after which *PR10‐like* sequences were amplified by contig‐specific primers (see Table S1) using the MT platinum SuperFi DNA proofreading polymerase kit according to manufacturer's instructions. Amplified PCR products were A‐tailed and sub‐cloned into the plasmid pGEM‐T easy for sequencing (BaseClear B.V.; Leiden, The Netherlands). A minimum of four clones per construct were subjected to sequence verification. Cloned *PR10‐like* sequences have been deposited into the NCBI GenBank database with the following accession numbers: MN258363 (#25355‐15), MN258364 (#25514‐14), MN258365 (#25514‐15), MN258366 (#18220‐11), MN258367 (#18220‐12), and MN258368 (#18220‐25).

### 4.3 *Property analysis*

#### 4.3.1 *Sequence alignments*

A phylogenetic tree based on the deduced protein sequences of the cashew nut *PR10‐like* genes and PR10 allergens from nuts and legumes was created in the Clustal Omega program of UniProt (https://www.uniprot.org/align/). Protein sequence alignments were conducted in ClustalW 1.7 (http://www.ch.embnet.org/software/ClustalW.html). Pairwise sequences identity and similarity were calculated via SIAS (http://imed.med.ucm.es/Tools/sias.html).

#### 4.3.2 *Co‐ and post‐transcriptional modifications*

The intra‐domain feature scan in PROSITE database (https://prosite.expasy.org/) was used to predict putative phosphorylation sites, N‐myristoylation sites and N‐glycosylation sites in the deduced protein sequences of PR10‐like cashew proteins. The Simple Modular Architecture ResearchTool (SMART, http://smart.embl-heidelberg.de/) was used for the PFAM domain search.^59^

#### 4.3.3 *Structural modelling*

For structure predictions, alignments of the deduced protein sequences of each of the cloned cashew PR10 proteins, the major birch pollen allergen Bet v 1.0101 (PDB‐id: 4bkd and 1bv1) and the major cherry allergen Pru av 1.0101 (PruAV1; PDB‐id:1E09) were created. The structure 1E09 was used as modelling template. For prediction of tertiary structure, structural modelling was performed using the Modeller program (version 9.16).^60^ Two‐hundred comparative models were generated for each sequence, after which the models with lowest corresponding DOPE scores were selected for image generation using Pymol (version 1.4). Secondary structure prediction was performed as described by Offermann et al.^61^ using ClustalW and ESPrit3.0 (http://espript.ibcp.fr/ESPript/ESPript/) to extract and visualize sequence alignments.

### 4.4 *Detection of PR10 protein in cashew nut by Western blot*

Protein extract was prepared from fresh milled raw cashew nuts as described by Wangorsch et al.^25^ and its concentration was determined by Bradford according to manufacturer's instructions. SDS‐PAGE protein separation was carried out on NuPAGE 1 mm 10% Bis‐Tris gels (Novex by Life Technologies) under non‐reducing conditions by loading 10–100 μg of denatured cashew protein in NuPAGE LDS sample buffer alongside a Precision Plus Protein Dual Xtra molecular weight marker (Bio‐Rad Laboratories Inc., CA). Gels were either stained with Bio‐Safe™ Coomassie Stain (Bio‐Rad Laboratories Inc.) or subjected to western blotting as previously described.^62^ Blotting was carried out using specific Bet v 1 (BETVIA, rabbit polyclonal antibody, orb51330; dilution 1:1,000; Biorbyt, Cambridge, United Kingdom) and Ara h 8 (rabbit polyclonal antibody, PA‐AH8, dilution 1:1,000; Indoor Biotechnologies, Cardiff, United Kingdom) antibodies alongside 10 μg of a native Bet v 1 and recombinant Ara h 8 positive control (NA‐BV1‐1 and RP‐AH8, respectively; Indoor Biotechnologies). Imaging and analysis were performed using a Universal Hood III and Image Lab 4.1. software (Bio‐Rad Laboratories Inc.).

### 4.5 *LC–MS/MS protein identification*

#### 4.5.1 *Sample preparation*

Of each protein sample, 100 μg was suspended in 100 μL 2% (wt/vol) SDS in 20 mM dithiothreitol. Suspensions were sonicated for 10 min followed by incubation at 60°C for 30 min. After cooling to room temperature Iodoacetamide was added from a 0.5 M stock to a final concentration of 50 mM, and suspensions were incubated in the dark for 30 min. From each suspension 50 μg of protein, according to the Bradford analysis carried out on the original protein extract, was used for trypsin (1:10) digestion according to the S‐Trap™ Micro Spin Colum Digestion Protocol from ProtiFi (Huntington, NY). After digestion, peptides were eluted with 50% acetonitrile in 0.1% formic acid. Eluates were dried by Speedvac and subsequently dissolved in 40 μL 2% acetonitrile in 0.1% formic acid.

Two different processing methods were carried out in a repeat experiment. One aliquot was incubated with addition of 1% RapiGest (Waters Corporation, Milford, MA) in Tris/HCl pH 7.4 and 1 μg of Trypsin (1:50; Promega Gold Sequencing grade). After overnight digestion at 37°C, peptides were acidified with 1% TFA (trifluoric acid) and the digest was centrifuged at 16,000 rpm. The supernatant was loaded onto an OASIS HLB SPE microcolumn (Waters Corporation), washed twice with 100 μL 2% acetonitrile in 0.1% formic acid and eluted with 50 μL 50% acetonitrile in 0.1% formic acid. Another 50 μg aliquot was again processed according to the S‐Trap™ Micro Spin Colum Digestion Protocol from ProtiFi. Eluates were dried and dissolved as described above.

#### 4.5.2 *LC–MS/MS*

The first set of peptide eluates were injected onto a nanoAcquity UPLC (Waters Corporation), trapped onto a Symmetry C18 2 cm × 180 μm trap column. Using a 60‐min gradient from 4 to 16 to 30% and final to 85% acetonitrile in 0.1% formic acid, peptides were separated on an analytical charged surface hybrid CSH column, 15 cm × 75 μm, 1.8 μm particle size at 50°C at a flow rate of 400 nL/min. Column effluent was on‐line connected to a QexactivePlus using a nanoFlex electrospray.

For the independent replicate experiment (RapiGest and S‐trap digests) peptide eluates were loaded onto an Easy‐nLCII (ThermoFisher Scientific, Waltham, MA) equipped with a PepSep trap column 2 cm × 100 μm and separation column 8 cm × 75 μm, 3 μm particle size at 24°C at a flow rate of 200 nL/min. Elution was a 24‐min gradient from 10 to 30 to 45% and final to 85% acetonitrile in 0.1% formic acid. Column effluent was on‐line connected to a QexactivePlus using a nanoFlex electrospray (ThermoFisher Scientific).

In both experiments, MS acquisition was performed using a DDA method with alternating MS1 scan at resolution 70,000 profile mode, AGC target 3e6, maxIT 50 ms, scan range 500–1,400 m/z, and subsequently 10 MS2 scans centroid mode, resolution 17.500 AGC target 5e4, maxIT100 ms, with isolation window 1.6 m/z at NCE = 28 on with preferred peptide match ions of charges 2, 3 or 4 and a dynamic exclusion window of 30 s.

#### 4.5.3 *Data processing*

LC–MS/MS spectra were processed using MetaMorpheus version 0.0.295^63^ for the first sample set. Peptide identification was performed using a protein sequence database composed of all PR10 RNA‐seq contig sequences including additional identified allelic variants, plus 111 proteins from Anacardium taxon A171928 as present in UniProt database (on December 2017), plus a set of frequent contaminant proteins (e.g., trypsin, keratins, BSA, etc.). The combined search database contained 12 non‐decoy protein entries including 490 contaminant sequences. The following search settings were used: protease = trypsin; maximum missed cleavages = 2; minimum peptide length = 4; maximum peptide length = unspecified; initiator methionine behavior = variable; fixed modifications = carbamidomethyl on C, carbamidomethyl on U; variable modifications = oxidation on M; max mods per peptide = 2; max modification isoforms = 24; precursor mass tolerance = ±5 PPM; product mass tolerance = ±20 PPM; report the total number of identified peptides to spectrum matches (PSM) ambiguity = True. A minimum of two peptides were required for protein identification.

The two samples belonging to the replicate experiment were processed using MaxQuant (version 1.6.5.)^64^ using the same protein sequence database and a set of contaminant proteins as default in MaxQuant. Search parameters included a minimum peptide length of 6, fixed modifications = carbamidomethyl on C, variable modifications = oxidation on M. A minimum of one peptide per protein was accepted at PSM FDR 1% and protein FDR 1%. For visualization and evaluation purposes an example msms.txt result file from MaxQuant for each of the detected cashew nut PR10 contigs was loaded into the software Skyline,^65^ together with the .raw files. Identified peptides peaks were integrated in MS profiles, and the peptide spectra matches were exported as presented in Figure S1.

Ion intensity and PEP scores for peptides identified in each of the two LC–MS/MS experiments are visualized in Table S4a. iBAQ scores for Ana o 3.0101 and each of the PR10 contigs in cashew nut as detected by MaxQuant protein identification analysis are listed in Table S4b for semi label‐free quantification. Ana o 3.0101 was chosen for this comparison as the protein mass of this 2S albumin is close to the protein mass of the PR10 proteins.

### 4.6 *Assessment for potential allergenicity*

#### 4.6.1 *80‐aa sliding window and 6‐mer and 8‐mer component analysis*

The 6‐mer and 8‐mer component analysis was performed by assessing the deduced aa sequence of cashew PR10‐like proteins using the online available software tools SDAP and AllergenOnline v12, respectively.^66^, ^67^ Both software tools also assessed the 80‐aa sliding window alignment.

#### 4.6.2 *Analysis of allergenicity*

The computational predictive tools AllerTOPv.2 and AllergenFPv.1.0 were applied to predict protein allergenicity and cross‐reactivity. The AllerTOPv.2 and AllergenFP are alignment‐free allergen prediction models based on various aa descriptors, taking into account residue hydrophobicity, size, abundance, and α‐helix and β‐strand forming propensities.^68^, ^69^

#### 4.6.3 *Prediction of B‐ and T‐cell epitopes*

MHC subtype A1 T‐cell epitopes were predicted using the NetCTL‐1.2 online prediction tool (http://www.cbs.dtu.dk/services/NetCTL/) applying a threshold of 0.75.^70^ The structure based tools Ellipro (http://tools.iedb.org/ellipro/),^71^ BPAP (http://imed.med.ucm.es/Tools/antigenic.pl) and BepiPred 1.0 with threshold 0.35 (http://www.cbs.dtu.dk/services/BepiPred-1.0/)^72^ were used for the prediction of B‐cell epitopes.

## CONFLICT OF INTEREST

The authors declare no conflicts of interest.

## AUTHORS CONTRIBUTIONS

Shanna Bastiaan‐Net designed and conducted most of the experiments and took the lead in acquiring funding and writing of the manuscript. Maria C. Pina‐Pérez acquired the cashew total protein isolates and performed the immunoblotting experiments. Bas J. W. Dekkers assisted with the RNA isolation procedures and Renata M. C. Ariëns with the RNA‐seq data analysis. Structural modelling was performed by Adrie H Westphal while the LC–MS/MS analyses were conducted by Antoine H. P. America. Nicolette W. de Jong, Harry J. Wichers, and Jurriaan J. Mes critically revised the manuscript.

## Supporting information


**Data S1.** Supplementary materials.
