# Supplementary material for: Identification and in silico bioinformatics analysis of PR10 proteins in cashew nut
Source: Protein Sci. 2020 May 16;29(7):1581–95. doi: 10.1002/pro.3856 (PMC7314402; doi:10.1002/pro.3856)
Supplement: Supplementary file 1 — Data S1. Supplementary materials. [file PRO-29-1581-s001.zip › PRO_3856_Supplementary figure and tables_JPS.pdf]

Supplementary tables belonging to the manuscript

Identification and *in silico* bioinformatics analysis of PR10 proteins in cashew nut.

Shanna Bastiaan-Net, Maria C Pina-Pérez, Bas JW Dekkers, Adrie H Westphal, Antoine HP America, Renata MC Ariëns, Nicolette W de Jong, Harry J Wichers and Jurriaan J Mes

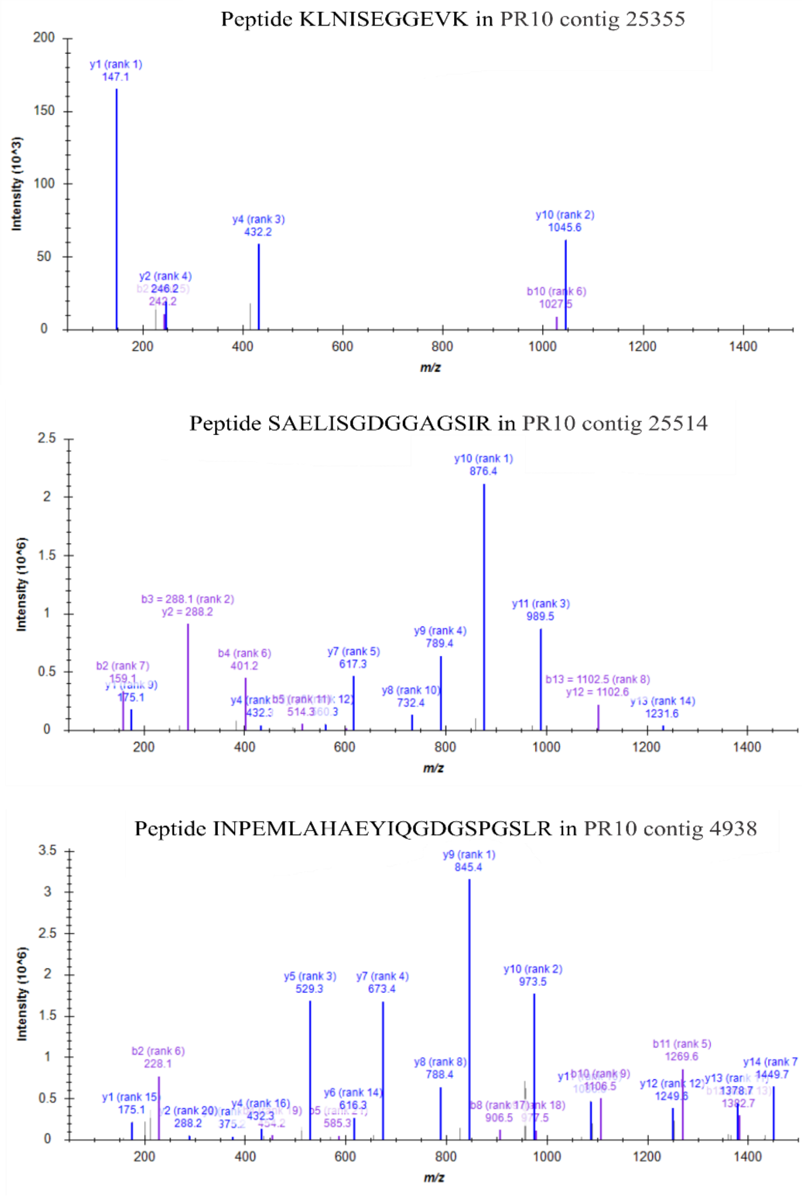

Supplementary Figure 1. Example peptide MS-MS spectra for each of the detected cashew nut PR10 contigs in MaxQuant as visualized in the program Skyline.

Supplementary Table 1. PCR primer sequences and their corresponding polymerase specific melting temperatures (MT) used for PCR amplification of *PR10-like* genes identified in contigs #25355, #18220 and #25514.

**Primer**	**Sequence '5-3' (bp)**	**MT platinum SuperFi DNA polymerase (°C)**
Contig-25355-F	ATG GGT GTC ATC ACT TTC ACT G	64.2
Contig-25514-F	ATG GCA GTT ATC ACT GAT CAA C	60.9
Contig-18220-F	ATG GGA TTT GCC TGC GGT GAA T	72.2
Contig-25355-R	TTA AGC ATA GGC ATC AGG ATT T	61.8
Contig-25514-R	TCA GTT GGC CTT GAG GTG TGC T	70.5
Contig-18220-R	TTA ATT GGC CTG GAG GTA TGC T	65.7

Supplementary Table 2. Output of an NCBI-BLAST search in the UniProtKB/Swiss-Prot (swissprot) database by blastp algorithm. The top 5 results are shown. The identified cashew PR10-like proteins are indeed related to pathogenesis-related proteins belonging to the PR10 family. All display the highest homology to the PR10 allergens Pru av 1 and Pru ar 1 from cherry and apricot, respectively.

Clone 25355-15

No.	Allergen	Source	Accession	Score	E-score	Identity
1	Pru ar 1	Apricot	O50001.1	180	1e-57	53%
2	Pru av 1	Cherry	O24248.1	172	1e-54	52%
3	Mal d 1	Apple	P43211.2	166	4e-52	52%
4	Mal d 1	Apple	Q40280.3	163	3e-51	51%
5	Pyr c 1	Pear	O65200.1	163	4e-51	50%

Clone 25514-14

No.	Allergen	Source	Accession	Score	E-score	Identity
1	Pru av 1	Cherry	O24248.1	141	1e-42	43%
2	Pru ar 1	Apricot	O50001.1	141	1e-42	44%
3	Mal d 1	Apple	Q40280.3	128	2e-37	42%
4	Aln g 1	Alder pollen	P38948.2	124	7e-36	41%
5	Mal d 1	Apple	P43211.2	121	1e-34	39%

Clone 25514-15

No.	Allergen	Source	Accession	Score	E-score	Identity
1	Pru av 1	Cherry	O24248.1	139	1e-41	42%
2	Pru ar 1	Apricot	O50001.1	139	1e-41	44%
3	Mal d 1	Apple	Q40280.3	125	2e-36	42%
4	Aln g 1	Alder pollen	P38948.2	121	9e-35	40%
5	Mal d 1	Apple	P43211.2	119	1e-33	39%

Clone 18220-11

No.	Allergen	Source	Accession	Score	E-score	Identity
1	Pru av 1	Cherry	O24248.1	139	1e-41	42%
2	Pru ar 1	Apricot	O50001.1	139	1e-41	44%
3	Mal d 1	Apple	Q40280.3	125	2e-36	42%
4	Aln g 1	Alder pollen	P38948.2	121	9e-35	40%
5	Mal d 1	Apple	P43211.2	119	1e-33	39%

Clone 18220-12

No.	Allergen	Source	Accession	Score	E-score	Identity
1	Pru av 1	Cherry	O24248.1	142	5e-43	46%
2	Pru ar 1	Apricot	O50001.1	136	1e-40	45%
3	Cor a 1	Hazel pollen	Q08407.3	129	6e-38	42%
4	Car b 1	Hornbeam pollen	P38950.2	128	2e-37	43%
5	Mal d 1	Apple	Q40280.3	127	3e-37	44%

Clone 18220-25

No.	Allergen	Source	Accession	Score	E-score	Identity
1	Pru av 1	Cherry	O24248.1	140	2e-42	46%
2	Pru ar 1	Apricot	O50001.1	134	8e-40	44%
3	Cor a 1	Hazel pollen	Q08407.3	127	3e-37	42%
4	Car b 1	Hornbeam pollen	P38950.2	126	1e-36	42%
5	Mal d 1	Apple	Q40280.3	126	1e-36	44%

Supplementary Table 3. Prediction of putative co- and post-translational modifications of Bet v 1.0101 (P15494), Pru du 1.01 (ACE80939.1) and the putative PR10-like proteins of cashew nut.

**Type of modification**	**Bet v 1.0101**<br>160 aa	**Pru du 1.01**<br>160 aa	**#25355 -15**<br>159 aa	**#25514-14**<br>155 aa	**#25514-15**<br>154 aa	**#18220 -11**<br>154 aa	**#18220 -12**<br>154 aa	**#18220 -25**<br>154 aa
Casein kinase II phosphorylation site	SsvE(40-43)<br>SfpE(58-61)<br>SviE(85-88)<br>TkgD(123-126)	TfgE(58-61)<br>TltE(85-88)<br>TegD(87-90)<br>TkgD(123-126)	TftE(5-8)<br>TiiE(85-88)<br>TgiE(124-127)	TifE(85-88)<br>SkfE(94-97)<br>TkwE(103-106)<br>SgpD(107-110)<br>TekE(136-139)	TifE(84-87)<br>SkfE(93-96)<br>TkwE(102-105)<br>SgpD(106-109)<br>TekE(135-138)	SvlD(23-26)<br>TifE(83-86)<br>TltD(89-92)<br>TkwE(101-104)	SvlD(23-26)<br>TifE(83-86)<br>TltD(89-92)<br>TkwE(101-104)	SvlD(23-26)<br>TifE(83-86)<br>TltD(89-92)<br>TkwE(101-104)
N-myristoylation site	GGpgTI(49-54)<br>GGpiGD(89-94)	GGpgTI(49-54)<br>GSqyGY(62-67)	GGpgTI(49-54)<br>GGckGT(110-115)<br>GCkgTT(111-116)<br>GTtvNK(114-119)	GGagSI(49-54)<br>GMikAV(143-148)	GGagSI(48-53)<br>GMikAV(142-147)	GGagSI(49-54)	GGagSI(49-54)	GGagSI(49-54)
Protein kinase C phosphorylation site	TiK(53-55)<br>SnK(118-120)	TiK(53-55)	TiK(53-55)	SiR(53-55)<br>TaK(118-120)<br>TeK(136-138)	SiR(52-54)<br>TaK(117-119)<br>TeK(135-137)	SiR(53-55)<br>TdK(91-93)<br>SvK(116-118)	SiR(53-55)<br>TdK(91-93)<br>SvK(116-118)	SiR(53-55)<br>TdK(91-93)<br>SvK(116-118)
N-glycosylation site	Nysv(83-86)	Nhsy(79-82)	Nise(58 – 61)<br>Nyti(83 – 86)			Nyti(81-84)<br>Nksk(134-137)	Nyti(81-84)<br>Nksk(134-137)	Nyti(81-84)<br>Nksk(134-137)
cAMP- and cGMP-dependent protein kinase phosphorylation site	KKiS(55-58)	KKiT(55-58)						
Tyrosine kinase phosphorylation site						Klv.EgdsY(58-65)	Klv.EgdsY(58-65)	Klv.EgdsY(58-65)

Supplementary Table 4A. Cashew nut PR10 LC-MS/MS peptide identifications using MetaMorpheus and the MaxQuant analysis for the RapiGest and S-trap purifications.

			MetaMorpheus		MaxQuant			
			Intensity		Intensity			
Protein	Peptide sequence (AA)	Lenght	Old	Score[a]	RapiGest	S-trap	Score[b]	PEP Score[c]
PR10 contig 25355	KLNISEGGEVK	11	ND	---	4.05^{+08}	3.00^{+08}	52.59	1.73^{-2}
	IDALDKEK	8	1.84^{+07}	7.073	ND	ND	---	---
PR10 contig 25514	SAELISGDGGAGSIR	15	8.16^{+06}	16.264	2.05^{+07}	7.09^{+08}	99.50	5.64^{-08}
	GFVLDADDVFPK	12	ND	---	1.35^{+07}	ND	111.65	1.82^{-06}
	VMPQAIK	7	7.39^{+06}	9.101	ND	ND	---	---
	FYIVPGFEGAENFITTEK	18	2.17^{+07}	9.056	ND	ND	---	---
PR10_Contig_4938	INPEMLAHAEYIQGDGSPGSLR	22	2.87^{+07}	32.200	3.00^{+08}	6.20^{+08}	30.246	3.06^{-45}
	LGPAVQNYVK	10	1.18^{+07}	14.249	4.11^{+08}	3.49^{+08}	143.94	7.06^{-4}

a. Score in MetaMorpheus = combination of ion score (reflects how well the observed mass spectra matched to amino acid sequences within the peptide) and expect value (frequency that this match would occur by chance)
b. Andromeda score for the best associated MS/MS spectrum in MaxQuant
c. Posterior Error Probability score for the best associated MS/MS spectrum = probability of a peptide being wrongly identified ($P < 0.05$)

Supplementary Table 4B. iBAQ scores for the cashew nut PR10 contig protein sequences and Ana o 3.0101 (Q8H2B8) in the RapiGest and S-trap LC-MS/MS identifications.

Protein	iBAQ[a] RapiGest	iBAQ[a] S-trap	(Mean Ana o 3)/(mean PR10)[b]
PR10 contig 25355	$3.12^{+07} \pm 0.20^{+07}$	$2.31^{+08} \pm 0.17^{+08}$	99.2
PR10 contig 25514	$2.83^{+06} \pm 0.12^{+06}$	$5.91^{+06} \pm 0.19^{+06}$	2970
PR10 Contig 4938	$2.37^{+08} \pm 0.11^{+08}$	$3.23^{+08} \pm 0.13^{+08}$	46.4
2S albumin Ana o 3.0101	$1.40^{+10} \pm 0.20^{+10}$	$1.20^{+10} \pm 0.03^{+10}$	1

a. Mean iBAQ-values (intensity-based absolute quantification of proteins) = the ratio of the sum of all peptides
b. Mean between the RapiGest and S-trap values.

Supplementary Table 5A. AllergenOnline prediction results of putative PR10-like cashew proteins for the identification of homologous 8-mers and 80-mers sliding window alignments. The software only depicts the number of hits identified.

**Cashew PR10-like clone**	**No. of 8-mers hits**	**No. of 80-mers sliding windows hits**
#25355-15	127	179
#25514-14	2	179
#25514-15	2	179
#18220-11	1	179
#18220-12	1	179
#18220-25	1	179

Supplemental Table 5B. Allergenicity prediction results of putative PR10-like cashew proteins using the SDAP prediction software for the identification of homologous 6-mers and sliding 80-mer windows

PR10-like	Identification of 6-mer					Identification of 80-mers sliding window alignments (top 5 types are indicated)				
**Clone**	**6-mer (aa)**	**Homology to**	**Accession**	**Source**	**Identity**	**Homology to**	**Accession**	**Source**	**Identity**	**Homology**
**#25355-15**	VLDFDN	Lyc e 4.0101	CAA75803	Tomato	Bet v 1-like	Pru p 1.0101	Q216V8	Peach	Bet v 1-like	59% over 47 aa
	GEVKYL	Jug n 2	AAM54366	Black walnut	Vicilin	Pru av 1	O24248	Cherry	Bet v 1-like	60% over 48 aa
	GEVKYL	Jug r 2	AAF18269	English walnut	Vicilin	Que a 1.0301	ABZ81046	White oak	Bet v 1-like	59% over 47 aa
	KHRIDA	Que a 1.0201	ABZ81045	White oak	Bet v 1-like	Que a1.0201	ABZ81045	White oak	Bet v 1-like	58% over 46 aa
	HRIDAL	Que a 1.0201	ABZ81045	White oak	Bet v 1-like	Rub i 1.0101	Q0Z8U9	Red raspberry	Bet v 1-like	61% over 49 aa
	RIDALD	Que a 1.0201	ABZ81045	White oak	Bet v 1-like					
	EGDAMD	Asp n 25	P34754	Aspergillus	3 phytase B					
	LANPDA	Pru ar 1	O50001	Apricot	Bet v 1-like					
**#25514-14**	AIKSAE	Cas s 1	CAD10374	Chestnut	Bet v 1-like	Pru ar 1	O50001	Apricot	Bet v 1-like	46% over 37 aa
	YMKHKV	Cor a 1.0401	AAD48405	Hazelnut	Bet v 1-like	Mal d 1	CAA96534	Apple	Bet v 1-like	44% over 35 aa
	YMKHKV	Cor a 1.0402	AAG40329	Hazelnut	Bet v 1-like	Cas s 1	CAD10374	Chestnut	Bet v 1-like	44% over 35 aa
	YMKHKV	Cor a 1.0403	AAG40330	Hazelnut	Bet v 1-like	Rub i 1.0101	Q0Z8U9	Red raspberry	Bet v 1-like	49% over 39 aa
	YMKHKV	Cor a 1.0404	AAG40331	Hazelnut	Bet v 1-like					
	TIFEGD	Tar o RAP	AAB92255	Cacao	Bet v 1-like					
**#25514-15**	VACALP	Blo t 21.0101	ABH06350	Mite	Allergen group 5/21	Mal d 1	CAA96534	Apple	Bet v 1-like	44% over 35 aa
	VACALP	Blo t 21.0101	ABH06348	Mite	Allergen group 5/21	Cas s 1	CAD10374	Chestnut	Bet v 1-like	45% voer 36 aa
	VACALP	Blo t 21.0101	ABH06347	Mite	Allergen group 5/21	Rub i 1.0101	Q0Z8U9	Red raspberry	Bet v 1-like	51% over 41 aa
	VACALP	Blo t 21.0101	ABH06344	Mite	Allergen group 5/21					
	VACALP	Blo t 21.0101	ABH06346	Mite	Allergen group 5/21					
	AIKSAE	Cas s 1	CAD10374	Chestnut	Bet v 1-like					
	YMKHKV	Cor a 1.0401	AAD48405	Hazelnut	Bet v 1-like					
	YMKHKV	Cor a 1.0402	AAG40329	Hazelnut	Bet v 1-like					
	YMKHKV	Cor a 1.0403	AAG40330	Hazelnut	Bet v 1-like					
	YMKHKV	Cor a 1.0404	AAG40331	Hazelnut	Bet v 1-like					
	TIFEGD	Tar o RAP	AAB92255	Cacao	Bet v 1-like					
**#18220-11**	AIKSAE	Cas s 1	CAD10374	Chestnut	Bet v 1-like	Mal d 1	CAA96534	Apple	Bet v 1-like	58% over 46 a
	YMKHKV	Cor a 1.0401	AAD48405	Hazelnut	Bet v 1-like	Rub i 1.0101	Q0Z8U9	Red raspberry	Bet v 1-like	59% over 47 aa
	YMKHKV	Cor a 1.0402	AAG40329	Hazelnut	Bet v 1-like	Pru av 1	O24248	Cherry	Bet v 1-like	55% over 44 aa
	YMKHKV	Cor a 1.0403	AAG40330	Hazelnut	Bet v 1-like	Que a 1.0301	ABZ81046	White oak	Bet v 1-like	45% over 36 aa
	YMKHKV	Cor a 1.0404	AAG40331	Hazelnut	Bet v 1-like					
	KHKVDA	Tar o RAP	AAB92255	Cacao	Bet v 1-like					

	TIFEGD	Tar o RAP	AAB92255	Cacao	Bet v 1-like					
	AYLQAN	Chi t 7	P84298	Midge	Globin CTT-VIIB-3					
	AYLQAN	Chi t 7	P84299	Midge	Globin CTT-VIIB-3					
**#18220-12**	AIKSAE	Cas s 1	CAD10374	Chestnut	Bet v 1-like	Mal d 1	CAA96534	Apple	Bet v 1-like	58% over 46 aa
	YMKHKV	Cor a 1.0401	AAD48405	Hazelnut	Bet v 1-like	Rub i 1.0101	Q0Z8U9	Red raspberry	Bet v 1-like	58% over 47 aa
	YMKHKV	Cor a 1.0402	AAG40329	Hazelnut	Bet v 1-like	Pru av 1	O24248	Cherry	Bet v 1-like	55% over 44 aa
	YMKHKV	Cor a 1.0403	AAG40330	Hazelnut	Bet v 1-like	Que a 1.0301	ABZ81046	White oak	Bet v 1-like	45% over 36 aa
	YMKHKV	Cor a 1.0404	AAG40331	Hazelnut	Bet v 1-like					
	TIFEGD	Tar o RAP	AAB92255	Cacao	Bet v 1-like					
	AYLQAN	Chi t 7	P84298	Midge	Globin CTT-VIIB-3					
	AYLQAN	Chi t 7	P84299	Midge	Globin CTT-VIIB-3					
**#18220-25**	AIKSAE	Cas s 1	CAD10374	Chestnut	Bet v 1-like	Mal d 1	CAA96534	Apple	Bet v 1-like	58% over 46 aa
	YMKHKV	Cor a 1.0401	AAD48405	Hazelnut	Bet v 1-like	Rub i 1.0101	Q0Z8U9	Red raspberry	Bet v 1-like	59% over 47 aa
	YMKHKV	Cor a 1.0402	AAG40329	Hazelnut	Bet v 1-like	Pru av 1	O24248	Cherry	Bet v 1-like	55% over 44 aa
	YMKHKV	Cor a 1.0403	AAG40330	Hazelnut	Bet v 1-like	Que a 1.0301	ABZ81046	White oak	Bet v 1-like	45% over 36 aa
	YMKHKV	Cor a 1.0404	AAG40331	Hazelnut	Bet v 1-like					
	KHKVDA	Tar o RAP	AAB92255	Cacao	Bet v 1-like					
	TIFEGD	Tar o RAP	AAB92255	Cacao	Bet v 1-like					
	AYLQAN	Chi t 7	P84298	Midge	Globin CTT-VIIB-3					
	AYLQAN	Chi t 7	P84299	Midge	Globin CTT-VIIB-3					

Supplementary Table 6. Results of allergenicity prediction of cashew PR10-like proteins using AllerTOPv.2 and AllergenFP allergen prediction servers and nearest allergen match.

		Nearest matching allergen		
**Clone**	**Prediction**	**Accession**	**Source**	**Definition**
**#25355-15**	Probable allergen[a]	CAA96549	*Corylus avellana* (Hazelnut)	Cor a 1 (Bet v 1-like)
	Probable allergen[b]	Q941P6	*Malus domestica* (Apple)	Mal d 1.0109 (Bet v 1-like)
**#25514-14**	Probable non-allergen[a]			
**& -15**	Probable allergen[b]	ABZ81046	*Quercus Alba* (White oak)	Que a 1 (Bet v 1-like)
**#18220-11,**	Probable allergen[a]	Q43549	*Malus domestica* (Apple)	Mal d 1 (Bet v 1-like)
**-12 & -25**	Probable allergen[b]	Q43551	*Malus domestica* (Apple)	Mal d 1 (bet v 1-like)

a. As predicted by AllerTOPv.2

b. As predicted by AllergenFP.

Supplementary Table 7. Prediction of MHC ligands and t-cell and B-cell epitopes using various software tools. The region containing the Bet v 1-specific IgE epitope ENIEGNGGPG is underlined.

PR10-like clone	Type of analysis	Software	Location of the prediction sites
**#25355-15**	B cell epitope prediction (Continuous)	ElliPro<br>BPAP<br>BepiPred 1.0	9-17, 35-54, 60-67, 76-80, 89-96, 105-113, 121-134,152-159<br>10-40, 64-73, 76-84, 94-106, 140-154<br>9-15, 44-54, 59-65, 91-94, 105-128, 131-136, 138, 156-159
	B-cell epitope prediction (Discontinuous)	ElliPro 1.0	K40:N41:S60:E61:G62:G63:E64:V65:Y67,<br>M1:G2:V3:I4:T5:D93:K94:E96:Y121:P122:K123:T124:G125:I126:E127:L128:E129:E130:E131:K132:K134,<br>S11:P13:V14:E105:V106:S107:P108:D109:G110:G111:K113,<br>G149:L152:A153:N154:P155:D156:A157:Y158:A159,<br>E43:T44:I45:E46:G47:D48:G49:G50:P51:G52:T53:I54,<br>D76:E78:K79:L80
	MHC and T-cell ligands	NetCTL-1.2	6 MHC ligands<br>151 T-cell epitope peptides
**#25514-14**	B cell epitope prediction (Continuous)	ElliPro<br>BPAP<br>BepiPred 1.0	9-18, 33-53, 60-66, 74-80, 92-97, 106-114, 122-132<br>4-14, 20-44, 53-61, 66-74, 77-85, 95-101, 114-126, 143-150<br>30-32, 37, 39, 44-53, 103-114, 127-130, 132-140,
	B-cell epitope prediction (Discontinuous)	ElliPro 1.0	E61:D62:D63:K64:L65:T66,<br>A11:E106:S107:G108:P109:D110:G111:G112:S113:I114,<br>E128:G129:A130:E131:N132:T135,<br>M1:A2:V3:I4:T5:L92:E93:S94:K95:Y122:I123:V124:P125:G126:F127,<br>E43:L44:I45:S46:G47:D48:G49:G50:A51:G52:S53,<br>T13:L14:P15:D17:K18:K33:A150:H151:L152:K153:A154:N155,<br>F74:D76:R77:E78:N79:L80,<br>M35:P36:Q37:A38,<br>I39:K40:S41:L60
	MHC and T-cell ligands	NetCTL-1.2	3 MHC ligands<br>147 T-cell epitope peptides
**#25514-15**	B cell epitope prediction (Continuous)	ElliPro<br>BPAP<br>BepiPred 1.0	8-16, 34-52, 59-65, 73-79, 91-96, 105-113, 121-134<br>4-15, 19-43, 52-60, 65-73, 76-84, 94-100, 113-125, 142-149<br>29-31, 36, 38, 43-52, 102-113, 126-129, 131-139,
	B-cell epitope prediction (Discontinuous)	ElliPro 1.0	E127:G128:A129:E130:N131:F132:T134,<br>A1:V2:I3:T4:L91:E92:S93:K94:E96:Y121:I122:V123:P124:G125:F126,<br>A10:A12:L13:P14:D16:F73:D75:E77:N78:L79:E105:S106:G107:P108:D109:G110:G111:S112:I113:A149:H150:L151:K152:A153:N154,<br>E42:L43:I44:S45:G46:D47:G48:G49:A50:G51:S52,<br>M34:P35:Q36:A37:I38:K39:S40:L59:E60:D61:D62:K63:L64:T65

	MHC and T-cell ligands	NetCTL-1.2	3 MHC ligands<br>146 T-cell epitope peptides
**#18220-11**	B cell epitope prediction<br>(Continuous)	ElliPro<br>BPAP<br>BepiPred 1.0	9-15, 33-53, 56-65, 74-78, 87-95, 104-112, 120-134<br>9-44, 53-61, 66-72, 75-82, 112-130, 140-149<br>44-53, 64-65, 72-73, 89-92, 102-112, 124-140
	B-cell epitope prediction<br>(Discontinuous)	ElliPro 1.0	E11:S12:V13:L14:P15:K18:E104:S105:T106:P107:A108:G109:G110:S111:I112:A149:Y150:L151:Q152:A153:N154,<br>M1:G2:F3:A4:C5:G87:D88:T89:L90:T91:D92:K93:T121:L122:P123:G124:F125:D126:V127:P128:G129:E130:S131:L132:N134,<br>D74:K75:E76:T77:F78,<br>M35:F36:Q37:A38:I39:K40:S41:E43:L44:L45:Q46:G47:D48:G49:G50:A51:G52:S53:K56:K58:V60:E61:G62:D63:S64:Y65:E86,<br>D26:Q29:K33
	MHC and T-cell ligands	NetCTL-1.2	4 MHC ligands<br>146 T-cell epitope peptides
**#18220-12**	B cell epitope prediction<br>(Continuous)	ElliPro<br>BPAP<br>BepiPred 1.0	9-15, 33-38, 40-53, 58-64, 74-78, 90-95, 104-112, 121-131<br>9-44, 53-61, 66-72, 75-82, 112-130, 140-149<br>44-53, 64-65, 72-73, 89-92, 102-112, 124-140
	B-cell epitope prediction<br>(Discontinuous)	ElliPro 1.0	E11:V13:L14:P15:E104:S105:T106:P107:A108:G109:G110:I112:A149:Y150:Q152:A153:N154,<br>M1:G2:F3:T89:L90:T91:D92:K93:T121:L122:P123:G124:F125:D126:V127:P128:G129:E130:S131:N134,<br>M35:S36:Q37:A38:K40:S41:K58:V60:E61:G62:D63:S64,<br>E43:L44:L45:Q46:G47:D48:G49:G50:A51:G52:S53,<br>D74:K75:E76:T77:F78,<br>D26:Q29:K33
	MHC and T-cell ligands	NetCTL-1.2	4 MHC ligands<br>146 T-cell epitope peptides
**#18220-25**	B cell epitope prediction<br>(Continuous)	ElliPro<br>BPAP<br>BepiPred 1.0	9-15, 33-53, 58-65, 74-78, 87-95, 104-112, 120-131<br>9-44, 53-61, 66-72, 75-82, 112-130, 140-149<br>44-53, 64-65, 72-73, 89-92, 102-112, 124-134, 137-139,
	B-cell epitope prediction<br>(Discontinuous)	ElliPro 1.0	E11:S12:V13:L14:P15:K18:E104:S105:T106:P107:A108:G109:G110:S111:I112:A149:Y150:L151:Q152:A153:N154,<br>M1:G2:F3:A4:C5:G87:D88:T89:L90:T91:D92:K93:Y120:T121:L122:P123:G124:F125:D126:V127:P128:G129:E130:S131:N134,<br>E43:L44:L45:Q46:G47:D48:G49:G50:A51:G52:S53,<br>M35:S36:Q37:A38:I39:K40:S41:K58:V60:E61:G62:D63:S64:Y65<br>D74:E76:T77:F78,<br>D26:Q29:K33
	MHC and T-cell ligands	NetCTL-1.2	4 MHC ligands<br>146 T-cell epitope peptides
