# Supplementary material for: Identification and in silico bioinformatics analysis of PR10 proteins in cashew nut
Source: Protein Sci. 2020 May 16;29(7):1581–95. doi: 10.1002/pro.3856 (PMC7314402; doi:10.1002/pro.3856)
Supplement: Supplementary file 1 — Data S1. Supplementary materials. [file PRO-29-1581-s001.zip › PRO_3856_Supplementary Materials and methods_JPS.pdf]

Supplementary Materials and methods belonging to the manuscript

Identification and *in silico* bioinformatics analysis of PR10 proteins in cashew nut.

Shanna Bastiaan-Net, Maria C Pina-Pérez, Bas JW Dekkers, Adrie H Westphal, Antoine HP America, Renata MC Ariëns, Nicolette W de Jong, Harry J Wichers and Jurriaan J Mes

Sample preparation and RNA isolation

Samples & chemicals

Raw in-shell cashews nuts were obtained from Intersnack B.V. (Doetinchem, The Netherlands). Chemicals were purchased from Sigma-Aldrich Inc. (St. Louis, MO, USA) unless stated otherwise. RNeasy Mini Kit and RNase-Free DNase Set for on-column DNA digestion were from Qiagen (Hilden, Germany). Primers were from Biolegio B.V. (Nijmegen, The Netherlands). MT platinum SuperFi DNA polymerase was from Invitrogen (Carlsbad, CA, USA), the pGEM®-T easy vector system was purchased form Promega Benelux B.V. (Leiden, The Netherlands) while the iScript Select cDNA Synthesis Kit was from Bio-Rad Laboratories B.V. (Veenendaal, The Netherlands). Coomassie Bradford Protein Assay kit was from Thermo Fisher Scientific (Breda, The Netherlands)

RNA isolation

Ten raw cashew nuts were mixed to acquire a representative sample. Total RNA extraction was performed using the Hot-Borate procedure described by Maia et al. [59] with some small adjustments. In short, to a spatula tip of cashew nut powder, 800 µL of Hot-borate buffer (0.2M sodium borate decahydrate (Borax); 30 mM EGTA; 1% (w/v) SDS and 1% (w/v) sodium deoxycholate, containing 1.6 mg DTT (dithiothreitol) and 48 mg PVP40) heated to 80˚C was added and samples were rigorously mixed. After the addition of 1 mg Proteinase K the suspension was incubated for 15 min at 42˚C while mixing every 5 minutes. After adding 64

μL of 2 M KCl, the samples were incubated on ice for 30 min and subsequently centrifuged for 20 min at 16,100xg. The supernatant was extracted with an equal volume of phenol/chloroform/IAA (24:25:1), followed by the addition of an equal volume of isopropanol. The samples were stored in the freezer at -20˚C for 1 hour. Hereafter, the samples were centrifuged for 20 min at 16,100xg at 4˚C. Pellets were re-suspended in 600 μL MQ after which 200 μL ice-cold 8 M LiCl was added and the tubes were incubated overnight on ice. Pellets were obtained by centrifugation for 20 min at 16,100xg at 4˚C, washed with 150 mL ice-cold 2 M LiCl and re-suspended in 100 μL MQ and further purified with RNeasy spin columns, including on-column DNase treatment following manufacturer's instructions. RNA quality and concentration were assessed by agarose gel electrophoresis and UV spectrophotometry using Nanodrop (Thermo Fisher Scientific; Waltham, MA, USA). The RIN-value of the cashew RNA sample used for RNA-seq transcriptome profiling as checked by BioAnalyzer (Agilent Technologies; Amstelveen, The Netherlands) was 8.6.

RNA-seq transcriptome profiling

RNA-seq library construction, including quality control and filtering (PhiX and adaptor sequences removal), quantification and sequencing using the Illumina Hiseq2500 (2x 125 cycles) were performed by BaseClear B.V. (Leiden, The Netherlands).

The raw RNA-seq sequence data has been deposited to the NCBI Sequence Read Archive (SRA) database in fastq format (SRA accession code PRJNA566328).

RNA-seq data analysis and BLAST analyses

Raw sequencing reads were uploaded into Gx CLC Genomics Workbench version 10.1.1 (Qiagen; Hilden, Germany), and subsequently paired and trimmed. Paired reads were *de novo* assembled and BLAST analyses were performed on paired assembly consensus databases for

contig extraction using BLOSUM62 (Expectation value 10; word size 3; threads 8; low complexity filter without mask lower case; existence 11; extension 1). BLAST tblastn (protein sequence and translated DNA database) analyses based on the Protein Data Bank (pdb)were performed by using the PR10 Bet v 1-like allergens of almond (*Prunus dulcis*) as query: Pru du 1.01 (ACE80939.1), Pru du 1.02 (ACE80941.1), Pru du 1.03 (ACE80943.1), Pru du 1.04 (ACE80945.1), Pru du 1.05 (ACE80947.1), Pru du 1.06A (ACE80951.1) and (Pru du 1.06B_ACE80949.1). Selection of reliable homologs was based on a minimal E-value of 10^{-8} as specified by Silvanovich et al [60].
