# Supplementary material for: Identification and in silico bioinformatics analysis of PR10 proteins in cashew nut
Source: Protein Sci. 2020 May 16;29(7):1581–95. doi: 10.1002/pro.3856 (PMC7314402; doi:10.1002/pro.3856)
Supplement: Supplementary file 1 — Data S1. Supplementary materials. [file PRO_3856_C6_paired assembly consensus.pdf › PRO_3856_C6_paired assembly consensus.pdf]

```
>contig_1_consensus
TTTTTTTTTTTTAAGTAACACAAATCTTTTATTTAGATAGGTAATGATTTAACAGCACTA
TGTCTGCAAATCTAAGAACATACATCCAGAGTATCTAAAACATAGAGAACCAGCAAGCAA
AGTTGCTAATACTAAAACCCTACAATCATTACATAACCCCACAAAACACCACTTGTTTTT
TATTGAAAGCAAGAAAGGAAAAATTAAAAACGACAGACAATATTAAATGATGGATTATTT
CCGTCAGACTTCCTCGTCCTTCTCGTCCTCGCTCCAGCAGCATTTCAGAATGGACCTGGG
AGATGAACTGTCAGCTTTGGTAAAGCTGTAGTATATCAGCGAGCCACCATTACCAAGCAC
TTGAAAACTCTGTTCTCCAAGAGCATATCCCTTCATATCCACAGAGAACTTAGTTGCAAG
TTCTTCAACAATGTCAGAATGCAAAGAAACAGAAAACTCAGCAGGCTCAAAGCAGGCCAG
GACCCTTTCAAGCATGGGGACCAACTCGACAACCTCAAAGTCATATCCAACAGACTCGAA
GCTTGCATAACTATAACCATCTTCAGGTGTGACATGTATGGTAGATACAGCATCCCTTTC
AATACTATTCATGGAGTAGCCACAAGGGTCAAACTCAAAATCAATTATGTCAGATTTCGG
AAGAATCTTCCTGATACCAGATTTATCAGTCATTAGAGCAGCTGAGCTTGCCTCAGTTTT
GTAGAAGACAGATGCCCTCTTCTTGTCTAAGCCAGTCATGCACATCTCAAGAGTGTAAAC
TGGGTCTAAGTGTTTCCCCATTTTGGCAGAGGCAGAGTAAACATGCCATTTCTTAGTGCA
GTCAGGACTACCCATCACATAAACAGTGCTCTCCAAACCAAACTTGCAGAAATGACCATC
CAGGACAGCTACTTCTTCAGAAAAGCTACGATGTGGAAAAGGCTGGGCTCCAGGAAATAT
GAAGCTTCCACGAGTGTACCTCACAGATTGAACCTTGAGGGATAGTGAATCAGCCAACTT
AAAAATGGCAGGGATTGAGAGAAGCAACTTTGTAGTACCACAAGTTTTAATGATAACTTT
GTAGGGGTACACAAAGAGGCTAGATTCAGAAAGCACATACGAATCAAGAAACTCGTTAGA
CAATGAGGAAACAATAGTGCATGCAGCTGGTTTCAGTATCTCATCCAATTGATCTTTGGA
GAACGAGCGGAAACCAGCACCTTCAGGATCAGCAAAGACACCAGGCTCAAAAAATGAAAC
CTCGAGCCTCTTTTCGAAACCTTCAAATCCAATTGCAGAGACTGGCAAGTCCATCTTGCC
TCACAGAGAGCAAAAACAGAAACGGTTGCAAAGAGTGCAAGAGCGAGTACAGGAGAGAAC
GAGGGAAGCAGAAAAGAAACAGGACAAGTAAATTGAAGAATTGAAAAGAAAAGCGAAAAT
CAAAATAGCGGAAAGAGAAAGCTTAATGGGGTTATGTCAGAACTTAGGGAACCTCAGGAT
GGCTTTCGGACGCAGTTAGAGTATGCAGCAGATCTGAATTTCTTGATTCCACCGTGCGGC
CTGACGTCTTCAATGCTGTAACCAAGAGGAGCTTCGTATAACAAGGATTTACTACTACTG
CTAGACTTCTTTTTACCACCTTTGGTTTCCATTAGCTCATTCAATCTCAATATCTAAAAG
ACGCCAGAGAAGAAACCTAAGAAGCGACGAACCGCCAAATAAACCCTAGAAAATATTAGA
AGATAGAGAGAGCCGAGAGTTGGTTTAAATATATATATATATATATGAAAGAGACTTATT
GAGACAAGCCGCTGCTG
>contig_2_consensus
GAATGTCTCAACCAGTAGAGAATAGGTCATCTTATTTTTAATGAAAACAACAAAGGAAAA
ATAAAAAACAACAACATCAAATCGGGGATTGTTTCCCTTCGACTTCCTCGTCCTTCTCGT
CCTCGCTCCAGCAGCATTTCAGAATGGACCTGGGAGATGATGAAATGTGAGCTCTAATAA
AGCTGTAGTAGACCAGCGAGCCACCGTTACCAAGCACTTCAA
>contig_3_consensus
GAATGTCTCAACCAGTAGAGAATCGGTCATCTTATTTTTAATGAAAACAACAAAGGAAAA
ATAAAAAACAACAATATCAAATCAGGGATTGTTTCCCTTCGACTTCCTCGTCCTTCTCGT
CCTCGCTCCAGCAGCATTTCAGAATGGACCTGGGAGATGATGAAATGTGAGCTCTAATAA
AGCTGTAGTAGACCAGCGAACCACCGTTACCAAGCACTTCAAA
>contig_4_consensus
GGATGGACTTTGTCATTTAAAGAAGCCTTGCGTTGGCATTAGTAGTTGGGCCTAATCTCT
GCTTGGGGACACGTCTTTCCACTTATGTGGTCCTAAACAAAATTAGCTAGATAATTTTCT
GGCTTGCGAAACCATACTTTCGGTTTAAATTAAATCGTCAGAACCAATCAATTGGCCATT
GACTCAATTATTTCACTAACAAAATACACCACCAACTTAACAATTATCCATTCAGAGCTT
AGCAAGCAGAGCATTCTGGATAGGCTTTTCAGCCATGCCATTGCTGTCTTTGATATCCGA
ATCGCTGGAACTGGGAGGAACATCCACTGCTGGTGAGCAATTGAAGAACCCATGTGGCAT
GAGCATAAAACCAATGCGATCCACTGGCATTACAGGCCAATCTTCGAGTCGAGGAATGTA
AGTCACTCCAAATACATACCTGTATAGCACCATAGAACTATATCAGTTTCTTCCAGAGAT
CGATTTTGCTTAACCCATGTTGCCAATCCTTCTCCATCTCGTGGATTCTGATTAGGAAAC
TCTCCCCCAGGGTACATTTCATCATGTGCATAAGGAGTGACCCAAAGATTATGCTTCAAG
AAAGCAGCTCTTCTCAAAAATTTGGCCTCAGAACCAGCTAATGGTAAACAGTTTGAACCA
GGCACTAGCTTATATCCAGTTAATTGCCCAGTCCGGTTAACATTTCTTGTGTTTCTTATA
ATCCAATGGCGAGCAGATAAAGGATCACAATCACGCATTGCTTGCAATTCTGATCTTAGT
AGTTCCTCTTGAGCGTAGAATGCATTATTATGAACATTATTCTTTCCAGGTTCTTCAACT
TTAACATTCACCTCAACAATCTGATTAAATGCTTCACCAGGCTTGCAGTCAACAGCCATG
```

TCCATGCGAGCCACAAAAAAATGTTGATGAACGGGTGCATATAACCCAGGTGCAATAGTT
GTACCATATTTTCGTACTTCCCCAGGTTGAATTGCACCTAAGCTGAGAATTCCTGTGAGC
TTAACCTCAGCTTCAATTTTCCCATCCTGATAAAAATGCCAGAAAAATCCATACTCATAA
TTTGCAACAGTACATATAAATGACACTGTTAGCCTTCTAGACCTTCGAACTTCTGCTAAA
CCTGTTCTCCAGTCTTGATGCTTCCATAAGATACCATGGTCCTCCTCATGCAAACAAACA
CAATTTTCAATTGTTTCAACACCTCCAGTGAAATTTGTAAAGTGTGCATCAAAGTACTTG
ACGTAACCCAAACAATCACACCCCTTCTTAAGAGAATGGGCATTTTTGCCTAGCCCATCT
TCCCCTGCATCAAATGCATTTTTTCTATAATGTGGATCATTTGGATCTCCATGAGGGACC
ACCATCTCAACAAAACTTAACCTGTGGGCGACAGGCCTCAGACCTCGACTACCATCAACA
TATGCGACAGAGTAGATTATTAAACCCTCCCTTGGAGTAAAGCCAATACGAAAATTCCAC
TTCTGCCATTCAACAAAATGTCCATTAACACGAAAGCTTGGACCTTCTGGCTGAAGAATC
TGAAGAGGTTTCACATCACTTCGATCAACACCTCCTCGTGTTTCACCAGCAGTGTAGTTT
CTTAATGGATCAGCTGGCGGTAGATGAACAAGTTTACGGTCTTCAAACTCAATTACCACC
ATATTTTGCATATCCACAAGAACATGGATTCCTTCAACCGGACGAGCATAACCATTTTCC
ATGGGACAGTCACTCTCAGTTCTGCAGAAAATGAGCGGTTTAGCAAGTCTGCGACTTGGA
GCATCAGCTTCACTGTGATATCCCACGCACCAGGGATCGACCATAACAAGATCCATGTCC
TCAATACCCCTCCTCTTCATTGCCTC
>contig_5_consensus
TTTTTCTGGCATTTTTATCAGGATGGGAAAATTGAAGCTGAGGTTAAACTTACAGGAATT
CTCAGCTTAGGGGCACTTCAACCTGGGGAAGTAAGGAAATATGGCACCACTATTGCACCT
GGGTTATATGCCCCTGTTCATCAACACTTTTTTGTGGCTCGCATGGATATGGCAGTTGAT
TGTAAGCCTGGGGAAGCGTTTAATCAGGTTGTTGAGGTGAATGTTAAAGTTGAAGAACCG
GGAAAGAATAATGTTCATAATAACGCATTCTATGCCGAAGAGGAATTACTAAGATCAGAA
TTACAAGCTATGCGTGATTGTAATCCTTTAACTGCTCGCCATTGGATTATAAGGAACACA
AGAAATGTTAATCGGACTGGGCAGTTAACTGGATATAAACTAGTGCCTGGTTCAAACTGT
TTACCATTAGCGGGTTCTGAGGCCAAATTTTTGAGAAGAGCAGCTTTCTTGAAGCATAAT
CTTTGGGTCACTCCTTATGCATATGATGAAATGTATCCTGGGGGAGAGTTTCCTAATCAA
AATCCACGAGTTGGGGAAGGATTGGCTACTTGGGTTAAGCAAAATCGATCACTGGAAGAA
GCTGATATAGTTCTTTGGTACGTATTTGGAGTGACTCACATTCCTCGACTGGAGGACTGG
CCTGTAATGCCAGTGGATCGCATTGGTTTTATGCTCATGCCGCATGGGTTCTTCAATTGC
TCACCAGCCGTGGATGTCCCTCCCTGTGCGAGCGATTTGGATATCAAAGACAGCAATGGA
ATAACTGAGAAGCCTATGCAGAATGCTCTGCTTGCTAAGCTCTGAGTGGATAATTTTTCA
GTGGGATTGCGTTCAGATGCTCCTTTGTTGAGTATGTTTGCGTGTGTGTGTGTGTGTGTG
TGTGTATTTTGTTAAATAATTGAGTCCGATTGCCAACTTCTGACACAAACATTTCGCCAT
TATTTCCAACGAGTTCAGTCGTTTGTTATGATAGCATAGTATTAGGTGGCAGTTCTTTGA
ATCAAGGTAAAATATGTGGACGGTAGGGCGGTAGGCCATATGTCATGAAATTGAGTGTTG
TTTGGTTGGCGTCGGAAATGTCCGATTGTAACGCTCCAAATTCTTGGAAAGACGCGCTCT
CAATGGGAGTTTATTTTAAGTACTTG
>contig_6_consensus
GTAGAGCTCATGATTTATGTAGCAAGCAATGATATAACATTCTGCTAAGCAAGAGAATAA
AACTTCATGTGGGAACTCAGGAACAGGAATTAATCCAATCAAAATAAAGATTTTGATTGG
CTGAAATTGATTCATACAACTGATAAGTCGTTCAGCACCAAAACAGAGGTCTTTAGAGGT
GTGGTGAGTAGCTCAGTTCAGAATCAAAACACAAGTTTAATGAGTACAACCTCAATCACA
AACAGATAAATTGATTCCCAAACTAAAATCGGGGTGTATGCACTGCAGTGAGCTCAAACA
CAACTGAAGTCAGCCAAGATTGTTTATAAAACGATTTTCTCCTCGAGCTCATAGTCGACT
AATGACTGCCTCCACCTCAGCTGGAGTATAGAGATGGTATGTTGGAGCTACCTTTGCAAT
ATCAACATTGTTCGGAGTAACCTTCTCTTCCATAACTTGCTTCAGAATGGACAGAGCAAT
TGTCTCCGCTTCTTGAAGAGTAAGGTCCTTGTTAAATTGTTCTTGAAGAGAGCTGTCGGC
ACCTTCTGAACCAGAACCAATAGCTTTTGCATTGCATTGCCAGAATGTGCCAGATGGATC
TGTATAATACAGGCTGGGCCCATTCTCATCATGACCAGCAATAAGAAGAGATACCCCAAA
CGGACGAGACATGGATTCCTCATCACCTTCACCAAAACGTAGTGCCAGATCACAAAGTGC
TTGAGTTGTAGATTCCACCGTCATTGGCTCACCATATGAGAATCTATGGTTCTGAGTTTC
CACCCGTGCATGCTCAACAAGTGTACGAGCATCAGCAATCAAACCACTCATAGCACATCC
TATATGATTATCTATTTCCATGATTTTTTCAACACTGCTAGGTTCCAGCAGAGGTGAAGT
GATCCGTTTCTCCACAGCGAGCACAACCCCTTCTTTCGTCTTCAACCCAATTGCAGTTGA
ACCCAGCTTGATTGCCTCAATAGCATATTCAACTTGAAACAACCTGCCCTCTGGAGAGAA
AGTGTTCACTCCTCTATCATACTCAGTCCTTGTAAGGAACATTTTCTACCCTTTGATTG
>contig_7_consensus

CAGCAAAACCTGCGGGAATTTGCTCTATCTCAGGTGGATTTTCATTAAAGATCAAGAGAA
TGTATTATACAATCCACAAACGCTCATAGTTCAAGAACCTCTCCTATGTCTCTCTTAGCA
ATCTATCCACATAAAAACCCAATAACCGCGTTCAAAAGCAGCGAAAAAAGATGGGCTAGC
TCATGTACACACCCCATCTAATGAGTTCTCATACAAGCATTAGCCTCAAAAGCAACCATG
CCCATCAAAATGAAGCAGGACAATGAGAGATCAACTACAATCTTCACACTAAAAGCACAA
AGCCAAGCCCCAAAAAGAGGATTTTACTGTTGAGTCACTTTCATGGAAAATCTTGAAATA
GTGGTTCTTAGCCACGCCTAATCCCCATGAAGTAATCGAAGTCGTGGTGCTGGACCCTCA
GCTGCTTTAGCCACGAATCTGCTACACTGAAAAGTGACATTAGTCTTTCTTGGTCCCGCC
CACAATTGCCCGAGACCCACACATTCCCTTGCATCTTGTAAGTGGCCAAACCAAATGGAG
GGAGAGAGATCCCTTCCCCTTCCTTACGCTTCCTTTCAGCACCCTCAATGTCATCATCAG
GGTCCATATCTTGAAAAGAAGATGAAAGTGTGTGGTACGTGAGGAAGCATGTAGACAAGT
CCTTTATGGTCCTTCCCATGGGAATATGATAAATTGGATACCAAGCTACTGCCATCCAAC
TAGCTGGCGAAAGATCAACACTTCTCAATGACATCAAACCAGGGTATCTGCGAGCTAGTC
CATTAATCTTATCCATGAGAGGAACTCTTCCATAGGGAGTTGATCTCTCGAAGTACTGAA
AATAAAGGTAACCCAATCTATCATTCACATGCAAAAGATTGTCTTGCTCAAATCCTCCTT
CCTCAGAAGAGCATCCGTCCCATCTCCATAACTTGTCACTCTCACCCTCATCGCTACAAG
AATCACTAAATGAATCCCTTGTCTCGCCGTCATCTGTCTCTTCCCTTAACCCATTCACAG
ATGAATTGCTGGTAAAGATTTGAATTGCAGATAGATAAGGCACATAGTACTGAACCAAGG
TGTCACCGTTATTCAAAGAAACAGGAACCCCAGCCCCATACGCACTCCATTCATCATAAC
AATTCCATAGATCACCTAATGTGAAATACTCAATCTTTTCCCTCTCCCATGGATGCCATA
ACGTATTAAGGTTCCTAATCTCTGTCTTGGGGAGAAATTGAGATGAAACTAAGGGTGTGG
TGCAATGGATGAAGCACTCCAGATTAGATTGAGTTGACCCTTTGTCAAGAAACATGGTTA
CAACCGAACGAGAGAGGGAGGGAGAGATTCAAAGGGCTCCCAGCACCAACTTTGAAAAGA
AATGAAAGCGAAAAACAGAGAACAATGACAAAGAAGTGAGAGGGAGAGAGAGATTATAGG
GATTTTGATTTCTA
>contig_8_consensus
CATAAATTGACATTTGCAAAATTAAAGAAAGCAAGACACATAAGTTATTTGCTGAGTCAA
ACAAAATATAGAACACAACAAATAGAAATAGAGAATGCAGTAAACTGAAATCCAAGATTA
ACCCAAAATAGCAGACCCAATACTTCCAATCTAAATATTAGAGAGATAATACTTAAAAAA
GCACAGGCAATAACTGAAGTAGAATTAGAGCAAGAACTTCATCTTTGAAGCCAGCTGCAC
AACTTTTTAGCACTTCACCTCCTTCAAGCCATGGGCAAAATACAAAAATGTTGGGTCAGT
GGCACCGTCCTTGTAGTAGGCAAACACCAGGGAACCATCATCATGCATGCTCTCACCAAC
AAAGAATTGAAGGTCACTGAGCTTTGAGAGCAAGAACTTAGTTGCTCCCTCAATGTTTTT
CTTGAACTCCTCTTGCTTCTCTCCTTCCAACTTGGGTGTCAACAACTTGATGTACCTCTT
CATGAATGTGACAAATTGCTTCTTATCAAAAGCAGGTTGTTCCTGGAGCCTGAATGTGTC
AACAATGTCAACCACCTTAACAGCTTGGTCATCAACACCCTCATCCTCATCAGCACCTTC
AGCAGAAGGATTGGCGCCAATGTCTACATCAACTGATCCTTGAACAACCCACTTTCCCTC
AACTTCCCAAAGCACGCCATTTTGGATTTCCTTGTATGGAAAGGAGTCAGAGAGGAGCTC
GTCACCGGTGAGAAGATCCTGGTAAACTAACATGATTGCAGTCTCTCCCTCTCTGTGAGA
TCGAACAAGAAAATAAATCAGCAGAGCGGGAGCCGAGAGAGACGCAGCGTCGAGAGAGCG
AGAGGGCTTGGCCGTGTGCGGACAGCAGGATTATATAGGCTAGGGTTCGGCTGAGGATCG
GGAAAATTACGCAACTTCTAAA
>contig_9_consensus
ATTAAGTGCATGACTTAGAATCATCAGAACACAGAAACTGCCAAATCACTGTCATCAAAA
TTAGATTTGTTTACTCCATTTATGCCTTACTGTCAATGCTCATGGGCTCTGCCTCCTTTC
CTTCTGCTTTCTGCAAATTCCCAAAAAAAAAAAAGATAAAAGGATCATTCATAAGAATAT
AATATAATAAGAACAGGATCGGAGTCTCAAGTACAATATCATCGTGAAACATTGATACTG
CAATCATTAAATTTGTTCTTCCAAGAAGGCCTAAATAGCATTCTAGGCTATAAGTTTGAT
GCACAAATAGTGGAATAATGATCAACTCCCAGCGCCTCCAAGCCCAATTATTGCTTGTCT
TTCTATTCTTTTATTCTTTTTTAAAAAAGAAAAAAAAGTAAAAAATAGATTGCAGCCATT
TCTTTTTTTATGAGCCAAAAGATTCCGATCATTGCTTATTTATTCGTTTTGAACGACGTT
GTGACTTACATTAGCTTCAGAGCCCAATTTACTCATCTTGGCAAACATGTTGCCGTAAAA
CTTCGCCTCCTTTTTGTTGAACTCCTTCATCTTCTCCTTCAAAGTTTTGTATTCCAGCTT
GACATCCCTGTTNNNNNNNNNNNNNNNNNNNNNNNNNNNNNNNNNNNNNNNNNNNNNNNN
NNNNNNGCCTGGGCCCTTCTATACAGGGCTTTAACATTTCTGCTCTCTGAGTCAAGCACC
TTTGTGCACAACTTTTCTGCCTGCTTGTATTCTCTTAATTTCAGCTTGCAAGCGGCATCA
TTCAGATTGCAAGCAACCTTCAGTGCCTTAGACTGCTTTTTCTCCTCTTCACTGAAAGAA
GAGTCATACTCTATGTACTTCACAGCTTTTTCATATCTCTTTGAGGCTCTTGCATATTTG

CCAGCCTTGAACAATATATTGCCTTCTTCCTTCTTCTTACCAGCGGCTTCAATCTTTTCC
TCGGTGTTCATGTCCCATGATTCCTTCTCCTTCTCAAATGATACCAGCTCCACCTCATAG
TACACTGTTGAGTTAGCAGGTACCACAGCCAGGTCCTGCTTTGACACAGATGAGCCAAAA
GCATACTCCGGAGCTATAGTCAGCAGTGCTAACTCACTTTTCTTCATGGTCATCACAGCT
TTATCAAGCCCTTCAATAACTTGTTCTTCGTCTGTCTTAAACTCAAACAGTTCCTCTTCT
TCACCATGACCCTTTTTAAAAAACACAGTACCATCTTGCAGTTTCCCAATCAATTTCACT
TTAACAACCGATCCATCATTGGGACGCTCATATCCCTCCCCTTCTTTCAGGATCTTTTTG
ATAACCTTCTTGTCATCAGTTACCTCAGACACAATCTTCCATGAAATTAACTCCAAAGTA
ATTTGAAGATTGCTATTTGGTGGAACTGCACCTTCATTGCCCGATGCAGGTTTGCCCTGC
TCTCCAAATCCATATTGTGGCTTCACTGTAAGAAGTACTTTCTCTCCCTTCTTCATTGTC
TTAACAGCCCTTGATAATGCAGGACAGAGATGACCATCCTTGACTGTGAACTCTACAGCA
TCTGCTTTTGCAATAACTGTACCATCCTCCAGTCTTGCTTCATAATTCACTAACACTTCG
TCCAAATCCTTTGGATTCTCCCATTTCTCTCCTTCAACGAGAACTTTTTTGAAAATCCCA
CCATCCTTGCAAATGTCCTTGACACTAGTCCAAGACAGCAATTCAACGTCGAACTGAAGT
GTTGAATTAGGTGGAATGGTTGGCGGGGAACCCGACTCACCATAAGCAAGCTCAGGAGGA
ATGGTGAAAAGTGCATTTTCACCTTTCTTCATCGTCTTAATACCAAGGTCCCATCCTTTA
ATAACAGTTCCTTGACCAAGCGTGAACTTGAAAGGTGTGCCCCTGTCACGACTGGAATCA
AACTTGCTACCATCCAGCAGAGTTCCGGTGTAATGAACTTCAACTTCATCGCCGTTATCA
GGAGTGTCCCAGCCTTCACCCTCTTTGAGGAGCTTCTTTTTGAGTCCTTGGTTCCCGATC
TCCTTCTCTTCGCCCACCTTCAAAATCGGAGATTCGTCAGGAAGATCCATGTCCTCATCC
ATCATCTCATTGGCAGCAGGAATATCGAAGTCTTCATCCATGGCTCTCTTGATTTGCTAA
CTTACAAGGAAGGATCTAGATAGACAGAAGGAGGCCAAAAAGTTAACAAGAATGCGAATG
AAATGTTGTGAATGGGGTCTTGAAAGAAGATGGGGCTTTATGAAGGTGATAGAGTGAGAG
ATCTTGGGTTCTAGAGACTTCTAAGATAATATAAGGGCTTCTAGTGAAGGCAGAAATTAA
CTGATACGTTAACGGAAGGCGAGTTGCCTTCCAGACTCTACTATTGTTAATTTCTTATTC
TATTAACTTTTCTGTGC
>ontig_10_consensus
GACCACCGAGATCTACACTCTTTCCCTACACGACGCTCTTCCGATCTGGGAGGGACGAAT
CGGAGCGACGTAGGGCTGAATCTCAGTGGATCGTGGCAGCAAGGCCACTCTGCCACTTAC
AATACCCCGTCGCGTATTTAAGTCGTCTGCAAAGGATTCTACCCGCCGCTCGGTGGGAAT
CACGATTCAAGGCGGCCCTCGCGGCGCTTCCGCCGCGGGGGCCTGACCGACGACACGTGC
CTCTGGGGACCGGGAGGTCCCTACTGCAGGTCGGCAATCGGGCGGAGGGCGCACGCGTCG
CTCTAGCCCGGATTCTGACTTAGAGGCGTTCAGTCATAATCCAGCGCACGGTAGCTTCGC
GCCACTGGCTTTTCAACCAAGCGCGATGACCAATTGTGCGAATCAACGGTTCCTCTCGTA
CTAGGTTGAATTACTATCGCGACGCCGGCATCAGTAGGGTAAAACTAACCTGTCTCACGA
CGGTCTAAACCCAGCTCACGTTCCCTATTGGTGGGTGAACAATCCAACACTTGGTGAATT
CTGCTTCACAATGATAGGAAGAGCCGACATCGAAGGATCAAAAAGCAACGTCGCTATGAA
CGCTTGGCTGCCACAAGCCAGTTATCCCTGTGGTAACTTTTCTGACACCTCTAGCTTCAA
ATCCCGAAGGTCTAAAGGATCGATAGGCCACGCTTTCACGGTTCGTATTCGTACTGAAAA
TCAGAATCAAACGAGCTTTTACCCTTTTGTTCCACACGAGATTTCTGTTCTCGTTGAGCT
CATCTTAGGACACCTGCGTTATCTTTTAACAGATGTGCCGCCCCAGCCAAACTCCCCACC
TGACAATGTCTCCCGCCTCCGATTCACGGAATAAGTAAAATAACGTTAAAAGTAGTGGTA
TTTCACTTTCGCCTCTCGGCTCCCACTTATACTACACCTCTCAAGTCATTTCACAAAGTC
GGACTAGAGTCAAGCTCAACAGGGTCTTCTTTCCCCGCTGATTCTGCCAAGCCCGTTCCC
TTGGCTGTGGTTTCGCTGGATAGTAGACAGGGACAGTGGGAATCTCGTTAATCCATTCAT
GCGCGTCACTAATTAGATGACGAGGCATTTGGCTACCTTAAGAGAGTCATAGTTACTCCC
GCCGTTTACCCGCGCTTGGTTGAATTTCTTCACTTTGACATTCAGAGCACTGGGCAGAAA
TCACATTGCGTGAGCATCCGTGGGGACCATCGCAATGCTTTGTTTTAATTAAACAGTCGG
ATTCCCCTTGTCCGTACCAGTTCTGAGTCGGCTGTTCGACGCCCGGGGAAGGCCCCCGAG
GGGGCCGTTCCCAGTCCGTCCCCCGGCCGGCACGCGGCGACCCGCTCTCGCCGCGGGAGC
AGCTCGAGCAGTCCGCCGACAGCCGACGGATTCGGGACTGGGACCCCCGTGCCCAGCCCT
CAGAGCCAATCCTTTTCCCGAAGTTACGGATCCGTTTTGCCGACTTCCCTTGCCTACATT
GTTCCATCGACCAGAGGCTGTTCACCTCGGAGACCTGATGCGGTTATGAGTACGACCGGG
CGCGGGCGGCACTCGGTCCTCCGGATTTTCAAGGGCCGCCGGGGGCGCACCGGACGCCGC
GCGACGTGCGGCGCTCTTCCGGCCGCTGGACCCTACCTCCGGCTGAGCCGTTTCCAGGGT
GGGCGGGCCGTTAAACAGAAAAGATAACTCTTCCCGAGGCCCCCGCCGACGTCTCCGGAC
TCCCTAACGTCGCCGTCAGCCGCCGCGTCCCGGTTCGGGAATTTTAACCCGATTCCCTTT
CGAAGCTCGCGCGTCTCGGCGCTGTCGGACGGGCTTCCCCCGTCTCTTAGGATCGACTAA

CCCATGTGCAAGTGCCGTTCACATGGAACCTTTCCCCTCTTCGGCCTTCAAAGTTCTCAT
TTGAATATTTGCTACTACCACCAAGATCTGCACCGACGGCCGCTCCGCCCGGGCTCGNNN
NNNNNNNNNCTTGCCCCGACGGCCGGGTGTAGGTCGCGCGCTTCAGCGCCATCCATTTTC
GGGGCTAGTTGATTCGGCAGGTGAGTTGTTACACACTCCTTAGCGGATTTCGACTTCCAT
GACCACCGTCCTGCTGTCTTAATCGACCAACACCCTTTGTGGGTTCTGGGTTAGCGCGCA
GTTGGGCACCGTAACCCGGCTTTCGGTTCATCCCGCATCGCCAGTTCTGCTTACCAAAAA
TGGCCCACTTGGAGCTCTCGATTCCGTGGCGCGGCTCAACGGAGCAGCCGCGCCGTCCTA
CCTATTTAAAGTTTGAGAATAGGTCGAGGGCGTTGCGCCCCCGATGCCTCTAATCATTGG
CTTTACCCGATAGAACTCGCCCGCGGGCTCCAGCTATCCTGAGGGAAACTTCGGAGGGAA
CCAGCTACTAGACGGTTCGATTAGTCTTTCGCCCCTATACCCAAGTCAGACGAACGATTT
GCACGTCAGTATCGCTGCGGGCCTCCACCAGAGTTTCCTCTGGCTTCGCCCCGCTCAGGC
ATAGTTCACCATCTTTCGGGTCCCGACGGGCATGCTCGCACTCGAACCCTTCACAGAAGA
TCGAGGTCGGTGGGCGGTGCACCCGCGAGGGATCCCGCCTGTCAGCTTCCTTGCGCCTTA
CGGGTTTGCTGGCCCGTCGACTCGCACGCACGTCAGACTCCTTGGTCCGTGTTTCAAGAC
GGGCCGAATGGGGAGCTCGCGGGCCGACGCCGGGAGCACGCAGGTGCCGAGGCACGCCTT
GCGGCGCGTGCTGCCGACCACGATCGNNNNNNNNNNNNNNNNNNNNNNNNNNNNNNNNNN
NNNNNNNCGCGTCGGTCCGCGCCCCGAGCCGATCGGCGGACCGGCTCGACGCCGTTCCGC
ATCCGACCGGGGCGCATCGCCAGCCCCCATCCGCTTCCCTCCCGACAATTTCAAGCACTC
TTTGACTCTCTTTTCAAAGTCCTTTTCATCTTTCCCTCGCGGTACTTGTTCGCTATCGGT
CTCTCGCCCGTATTTAGCCTTGGACGGAATTTACCGCCCGATTTGGGCTGCATTCCCAAA
CAACCCGACTCGCGGACAGCGCCTCGTGGTGCGACAGGGTCCGGGCGCGACGGGGCTCTC
ACCCTCTCCGGCGCCCCTTTCCAGGGGACTTGGGCCCGGTCCGCCGCTGAGGACGCTTCT
CCAGACTACAATTCGGACGTCGGGGACGCCCGATTCTCAAGCTGGGCTCTTCCCGGTTCG
CTCGCCGTTACTAGGGGAATCCTGGTAAGTTTCTTTTCCTCCGCTTATTGATATGCTTAA
ACTCAGCGGGTGATCCCGCCTGACCTGGGGTCGCGACGCGATGCGGGCGGGCATAGGCCC
GCCATCGAGGGTCAAGGAGCCCGCGGCACAGAGAGAGCGCGTCCGCGCGACGGGATCATC
GGGTTCGTTCCGAACGCCACCGACCGTCGCGGGAAGCGCCGCCGAGGACTCGCGTTTGGG
CCAACCGCACGGGCGAGCGCACGGGAGGCCACCTTCCGCCCAGCGCCGCGGAACCGGAGG
ATCCGGGGAGGGCGACGATGCGTGACACCCAGGCAGGCGTGCCCTCGGCCTGACGGCTTC
GGGCGCAACTTGCGTTCAAAGACTCGATGGTTCACGGGATTCTGCAATTCACACCAAGTA
TCGCATTTCGCTACGTTCTTCATCGATGCGAGAGCCGAGATATCCGTTGCCGAGAGTCGT
CACTGACAATGAAAGGAGGCAACGCTTCCCGCACGCGCACCGAGCACGGGGCGACGGGAG
CGAGCCCTCTCAAGTAAGATTCCTTGGCGCG
>ontig_11_consensus
AAGAAGGAAAGGCTTCAATTTCAAAGCCAAAATTAATTCTTTATTTGCTTGGATGTCAAT
AACCCATTTGCAGCCGCAGTGCCGTATATATGCTTACTATTGACAAATGTCATGAAAAAA
ATTGACTGATTTAGCACCACAAACAAATTGACATCAGTTATAAAGTAATGTAACACCATT
AACAAGCTGACTGAAATAAAAAAAATATACACCAAGACCAATTACTTCTTTCCAGCCTGA
AAAGAAGTATGAATGTCATCATTTCTATCTGTAAGAAAAATGAGTACATAAAAGTATGGG
CC
>ontig_12_consensus
TGACGGAACATATCGTATAAAAACTTGGTAAAATCAAATACTAAAACAAATACAATTAAA
AGCTATAAACGGTTAGATCTACCTAATAATAGAAGCCCAAATCTATTTTCTTCTGCCTCT
CTTGGTCACAGAGACCAACTCCATATTCCAGTAAAATAATCTTTAATGTAAATAAGCTTT
TTCTTACAAGCACTAGAGATCTTACAACCTAGAAATCACTGTCTCGCAACCTTTTGACAT
TAGAAGCACACACAAACATCCACATCCACTTTCCACATCTATACACATACAGCGAAAGAA
TTTTACAACAGATGTAGAGATCAAAACCACTCGCTAATTCTAGATGGAAATGGAAGACTG
GCATTTCATGAAGGACGTAGAGATCAATTCAGTTGATAATTTTAGATAGAAATGGGAGGT
CGACCTCGTCCCATGGTGAACCCTCTGGTTCCATCGGGCATTCTTGGGCCAGGAGGAGGC
TTTGCAGCTTCATAACTGGAGGAAAGTGTTCCATGGCCCTGCCCATTGCTTCCCCGGTAT
CTTTGTCTCCTAGGTCGTCCCCGGTTTCTACCTTTCTGCCCACTTTTCTCCTTGGATAAG
AGGTCTCCATCCTCTTCATCAGGGGTATCATCATGATTCTCATTTAAGTTGTTCTCTTCA
TCACCCAAAGGATCAGATACTCGTCCAGTGTTACTCCTTTCAGAATCAACCCTCCAGCCT
TGCTTTCTCTGGCCATACTTGGCCAGTCGTTTAGGAAGCTTAACCCGCATGCCATTTCTC
CAGTCTTGTTCATTATTTAACATTACCACTGCTTTCTCAGCATCTTCCACAGTCTCATAC
TCCACCAATGCATGTAACTTATTACCAATCATAAACTCAGTCTTGTTGCCTTTCTTTGAC
TCTTCCATGGCACTTGGATCATGGACGCAAACCTTTCTTACATTTCCAGCTTGAGCAAAT
ATTCTCCGGATATTTTCCACCGAGTGATCCTCTGGCAGATTTTCTACCAAAACAGTTAAC

AACTTTGAATCCTTGACCTCAGTGACTGGAAGAGGATTAAGGCGCTTCACTTTCTTCCCA
TTTGAACTCACAACAAGCAAGGATGATTCCCTGAGTGCAGCCACTATCGATTTGTTGTCT
CGAGTAAGTTTCTTCATTTTCCTAAAAGATGCAATAACCGATATAGCAACAAACCCTTCT
TTGTTCTTCTTAATCCAACTCATCATATACTTGTCAGTGGACAAATTTTCATCACTAAAG
TAATATTCCACCTGCTTAATAATCTTGAGTTTCAGATCATCAGTGAGAACGGGTGCAGCA
GATGAGAGTTCGGGATCTTTCACTTCAACTTCAGCGGGAACGTGATCAGGATCATCATCA
GACGGTGGAACGGGAACACCATCGGACTCCGGTGATCCAACTGGAGAGAAATCAGGATCA
TGCGGAGGAGACGGTGGTAAGACATCAACGGTTTCACCGACAACAGCGGTCATGGCCATG
TCCGCAGCCGTGGAGGAACCGTTAACGCCGTCCATTAGGAGAGAAGAAGGAGACGAAGAA
GAAGAAAGAAGAAGACTACTAACGCAAGAAACGAGTTGAGATCTTTTCTTACTTAGAAAA
CCCTCCCGCCCATTGCTCCTCTCTCTCTCTCTCCCTCTCTCTCTGCACATTGCAATAAGA
ATACAGAGATGGGTACAGTGCGTGAAGTTGCAATATCCCTTGATGGGGTTAGGGACAAGA
ACATCATGCAGCTCAAGAAGCTTAACACCGCGCTCTTTCCTGTTCGCTACAACGATAAGT
ACTATGCTGATGCCCTTGCCTCTGGCGAATTTACCAAACTAGCATATTACAGTGACATCT
GTGTCGGTTCAATTGCATGTCGGCTGGAGAAGAAGGAGGGTGGGAACATCTGTGTTTACA
TCATGACATTGGGTGTTTTGGCACCATATCGTGGGCTAGGCATTGGTACAAAGCTGTTGA
ACCATGTTCTCGATCTCTGTTCCAAGCAAAATATATCAGAGGTTTACTTGCATGTGCAGA
CAAACAATGAAGATGCTATCAACTTCTACAAGAAATTTGGGTTTGAAATCACAGACACCA
TCCAGAACTATTATACAAACATCACCCCACCAGACTGCTTTGTTCTTACCAAGCTCATTA
CTCAACATCAAGTGAAGAAATAACAGTGAAAAACCGTTATCAACAACCGTTGTCAATATG
CAAGTCCTGGTTTATTGTTGTTGCATTTAAACTTTACCCTGCGCCAATTATACTCAGAGT
TTTAGTACCACAATGTTGAGGTCATACAAATGCTAGAATGCATAGATGAGTTGAATTATT
TTACCGCTATCTACTGTTTTTGATGCACAAAATTTTCCTTGTTGATCAGGTCAGTTGTAA
ACAATTGAAAATTTTGCCAGAGACTGGATAAGAGTTTTTGTGTAATATCATGAAG
>ontig_13_consensus
AAGACCATCCCGCTTCCTTTCTCCTGCAAAAGCTAAACTAGTATAATCTGTATGGAAAGA
CCTATTGGATTTATTCCATTAATTTTTTTAATCACCAGTTTGACGGACAGCTGGTGAAC
TAGTTTACCTAAATTTACATAAGTGTGACAACAAAAAATGTGTTCAAGCATATGCTTACA
TCCTGATACAGGTGTTTTAGAGATTCTGGGAGCGTTCCTTGGACGATTTGTAGATGCGAA
ATGCAGCATAACACATAGTAGCATTACCTATCACTGTAAGAGCAGCTTGCAGAGCCAC
>ontig_14_consensus
TGATAAAATTCAAATCTTTATTTGTAGCAATCTCCCCACTTTGTTTTATTTCCAAACAGA
ACACTAAATTTCAACTTTTGCGGGAAAATATATACTACTAACTACATATTTTCACTGAGC
CTCAGCTAGCGACTTCAACGGTAAGAGCCACCACCGGTGGCTCCGGGATGATAACGGGGA
TCGGTGGCAGGGTTGGTGTAATCATCTACATAGTCTTCGGTGGCAGTGTTGGTTTTGGGA
GACTCAGTGCCCCGTGTCTTGTATCCCGGCTGAGGTTCACGGGACTCCGCCTCCAGCTCC
GCATCGTTTTTTCTCATCTCCTGCTCCACAGTAGCGGCTTCTTTCTTCTCCGGGTCTCTC
GTAGTCAACTTCTCTACCTTCTCTTTGGTGGTGGCCTTGGCCTTCTCTATGCCGGCCTTG
GTGGAGGCGGCAGCGTTGGCGGCCGATTCCTTCACGGACTCTACGGCTTTCTTACCAAGA
TCGGAAGAGCACAACGTCTGAACTCCAGTCACGCCA
>ontig_15_consensus
CTATCTAGCATCTCAAGCTTCCCACAAAGTCGAACAATTGGTGGCAAATAATGCTTAGGT
ATTAAAGTATCAACCTCTTCTGCATGAAGCTGGCTGAGATATGGATGATTGCATATATTT
CGAAGCTCCATAACAGAGTTATGAACCGACCGAGCCTTTGGATTTCCAATTGAACCAAGA
TTTTCTTCAACTCTCTTCATTAACAGCTTCTGATAGGCGGAAGCCTCACATCTTATAAGT
CTCTCAATCTTTTCAGGCAGTTCATTTTCAACCTTGTGTTTTAGCCTCCGAAGTACAAAT
GGCCGCAGAACCTGGTGCAAACGGTTTATGATTAACAGATTTTCCTCCTCAGACAACAAA
GCTTCATCAGGTGAACTGTCTCCATTACTTTGAAATGGTTTGTTGAACCATTGAGAAAAA
TCCTCTGATGAATTGAATATATTTGGCAACAAAAAGTTGAGTAAAGCCCAAAGTTCTTCA
AGATTATTCTGTAGTGGTGTTCCAGTTAACAACAATCTGTGAGAACTCTGATAGTGCTTC
AAGTCCGCATTCAACTTGCAAGATGCATTTTTGATGCGATGGCCTTCATCAATTATAATA
TAATGCCAATGTATTTTACTAAGTTTTGGTCTATCATGCTTGTTCATCAGATACTCATAT
GTTGTCAGGAGAACATTGAATTTCTGATGCACAATTTTTTCCTTGAATAATCTGCGTCTC
TCCTCTGGAGGCCCAGAATATACAATTTTGTGTATTCCAGGAGCCCAAAAGTTGATTTCC
GAGTCCCACCCGGGTAGAACTGATGATGGTACAACCACTAAAAAGGGTCCTCTATCATTT
TTGGCTTCCATAAGGTAACAAAGCAAAGCAATAACCTGAACAGTTTTTCCAAGGCCCATT
TCATCAGCTAAGATTCCATTCAAATGATTGTTGTAAAGTGAAACCAACCACCTTAGTCCA
CTCATCTGGTACTCCCTCAGTTTTCCACCATGAAGACAAGTTGGCTGCTCTGAAATACTT

TCTTTAATACTATGAGCCATCAAATAGTACTT
>ontig_16_consensus
CTGAGAAACATCAATTTGTTCACCTGCTTCACCTACAGAACATTTTGAATCCTCCACTAA
CACTAAGCTGCTAACTGGATCTTTTTCACGGGATCCCTCAAGTTTTTCCTCAATCCCATC
AATTTGATCACCTGCTACACCAACAGAAGCTTTTGAATCCTCCCCTGACACTAAGTTGTA
AACTATACTCTTCTCAGAGGAATCATCTATCTTTCCTTCCTCAACTACCAAGGAGGATAA
AGGCAGACCCAAATCCTCAGATAAGGTCTCTTGCATAAACCCAGCCCCTGAAGAAACATA
AATTTGTTCACCTGCATCATCTAAAGAAGCTTTTGAATCCTCTGCTGACACTAAGCTGCT
ACCTGGATCTTTTTTAGAGGATCCCTCAATTAATTCTTCCTCAATCACAATGGGGGATGA
AAGCAGATCTAAGTCCTTAGATAATGTCTCTGGCTGAGCAATGTCAATTTGTTCACCTGT
TTCACCTACGGAAGCTTTCAAATCCCCAGCTGGTACTAAGTTGCTAACTAGATTCTTCTC
AGAGGAACCATCTATCTTTTCTTCCTCAATCACCCTGGAGGGTAAAGGCAGACCCGAATT
ATCAGATAAGTTCTCTTGCTCAGCCCCAGCACCCAAAGAAACATTCATTTGATTATTTGC
TTCACCTACAGAACCTATTAAATCCTCTACTGACACCAAGCTGCTAAGTGGAACCTTTCC
AGAGGAACCCTCAATATTTTCTTCCTCAATCGCCATGGAGGATGAAGGCAGATCTAAGTC
CCTTGGTAAGTTCTCTGAAATTACATTACTACCCTGAGAAACATCATTTTGTAAACCTTC
TTTACCTACAGAACCTTTCGAATCCTCCACTGACACTAAGCTGCTAACTGGATCTTCTTC
AGAGGATCCCTCAATTTTTTCCTCCTCAATCACCATGGATGATGAAACCAGATCTAAGTC
CTTTGTTAAGTTCTCTGGCATAAAGTCACTTACAGAACCTTTTGAAACCTCCACTGACAC
CAAGCTGCTAACTGGATCTTTTCCAGACAATCCCTCAATTTTTTCCTCAGTCACCATGGA
GGATGGAGACAGATCTATGTCATTTGATAAGGTCTCCGGTATGACATTACCACCCTGAGA
AACATAAATTTGGACACCTGCATCACCTACAGAAAACTTTGAATCCTCTGCTGACACTAA
GTTGCCAACTGTATCCATCTCAGAGGAACCATATATCTGTCCTTTCTCAGTCACCAAGGA
TGAAGGCAGATCCAAAGCTCTGCCACCCTGAGAAACATTAATATGGTCATCTGCTTCAAC
TATTGACCCTTTTCCTTTTAACTCATCCAGGGCCACCACAAAGCTGCTATCTATATCCTG
CTGAGTTGAATCATTAATCTTTTCTTCCTCAATCTCAGCGGATGAAGGCAGACCCAAATC
CAAAGATAAGCTCTCTGGTAAAGCATCACCACTCTGAAAAACATTCTTTTTATTAACTGC
TTCAGACCCCTTCGAATTATCTAGTACCACCAAGTTGCAAACAGAATCTTTCTCTGGGCA
GCCCTCAATCTTATCTTCCTCAATTGCTAGTGCTGATGAAGGCGGACTCAACTCCTCACA
CAAGTTTCCTGGCACTACCCCGCTATCTTTAGAAACAGCAATTTGATCACATGTCAGAAT
TGAGGAAGCCTCAATGCTGCTAACAGCAAGAAGAGAAGTTTCTAGAGACTCTTTTGAGGA
AGGCTCACTTAAGCTAGGAGGTTCTGTTGCAATGACAACAAGTGGGCTTGTTCTGTCATG
ATCACCAGTATCACAATCATTCATGGATATTGCATGAACAAAAGATGATTTTGGAGAATC
ATTTGATGAAGGTAATTTTGAGTTTCCAAAATTATTCTCAGTACTATCAGAATGTGGCAC
GACACCTGTGCAATCACTGTCAGGCAAAAGTAACTCCTGAGCATGAACATCCTTCTTTTG
GCCTGCATAGAACTGATCTGTACAACTGATCTCTGAGGTACTTCCCATCATTGTTCTGTC
ATCAGGATGCTGTGTTGGCTCTCTTAAATTACCCATGGTCTCAGCAGACCCATCATCAAT
CTTACTTTCAGGTTTGGAAGCTTCAGTCTGTAAGGCCTGATGGTCACAAGAGAAAGGAGA
ATTGTCACCCTTAATTTCTCCATCCACACCTTCGTTACTAGGATAATCTCCAGCTATAGC
TTTTGTTGAGCAGGTTTCTGAGATAATCTCATTGTTACCTTCAATAATTGCATGACTTTC
AGTCTCAGACTGTTCTTTATGCTTGTTAATTTCAATGTCAAAACCCAGAGAGGCTGTTCC
CAATGTAGACATCTCTGGACAAGTTTTAACCACTGCACTCTGGTTTTGGACCACCTCCAT
GATGGTGCTCCTGGATAAAGCTGTAACAGGTGCATTCTTATTGTCACTCACAGAGTAAAC
GGCTTTGGCTTTTGATGAGAACGTTTCAGAGGAAATCTCCTTGTCAACTGTAACTTTCGT
ATCACTTTCAGTCCTATACTGGTCTTCAGGCTGGCTGATTGGAATGTCAAAACCAAGTGG
GTCAATTTCCAAAGTTGTCAAATCAGGACAGGCTTTATCATCTGAATTTTGTATTTTTGC
AATATGGGTAACAGCTTTAGCTTTTGATGAGAAAGTTTTAGAGAATATCTCCTTGTCACC
CATAACTTTTGCTTCACTTTCAGTCCAGGACTCCTTATGCTTTTTCATTAGAATGTCAGA
ACCAGGAGGGTCAGTTTCCAAAGTCGGAATATCTGGATAGGTTTTCTCATCTGCACTTTG
ATTATGGGACACCTCTTGAACAGGTTCACTGGCTACAGCCATAATAGGATCATCATTACC
TTTACTCTCAAAAGGTACACCAGCTTTAGATGAGCACGTTTCAGATGTAATCTCCTTGTC
ACTTTTATCTTTTGCGTCATTTCCATTTTCAAATTGGTCCTCATGTGTATTGATTACAAA
GTCAAAACTCAGTTGTGCTGTTGCCAAAGTTGGCATCTCTGGACATGTTTTGTCCTCTGA
ACCTGGGTTTTGGACAACCTCTGCATCATTGTTACTAGATACAGCTACAATAGAAGCATT
CATAGCTTCACTACCACAATGGTCAAAAGGTTTATCTTTGGATGGGACTGCTTCAGAGAA
AATCTCAGTGTCACCTTCAAAAGTTGCATCAATTCCTGTTGCAGGCTGCTTTTTATTCTC
ATTGATGGGAATGTCCAAACCCAGAGGGACTATTTCCAAAGCTGGCATCTCTGGACATGC
TTTATCTTTTGAAATTTGGCTTCGCACCTCCCCTGCAATGTTACTGGATATAGGCATAGT

ATTATCCCTAGCTTCAATCCCAGAACATTCACCAGCTTTAGCTTTTGCTGAGCAAGTTTC
AGAGAAAATCTCCTTCATGACACGAGCAACATCATTTACAGCAGTCTGAATTCGTCCAAA
AACTGGGTTTGTACATGTTTCTTTTCGTTTTGGATCCAGACTAGCCAAACCACCCACAGA
TGGGACCTCAGATGCAGATGTCTGGATAATCTCAGTCAATTCCTTAGCATCAGTGTCTTG
CTTACTACTTGGGTCAACGGTTTTGCTTCCTCTCATTTCCCTAGGTTGGCTATGAGATTG
CTGATTTAAATTTGGATCTAGGCCAACAGACCCATCAGGAATTGCAGATAATCCCAAAGC
CTGTCTCTTTCCCCTGCGCCTAGGTGCTTCTCCTCTGCTAAGAGTTTTACGACCTTGTCT
TTTCATGGGAACAGACTCTGGAGCCTGAGCAGGGGTCAATTGATAAGGTGCAGCAACAGA
TGGGTCAGTAAAAATTGACTGAGACCCAGGAGCTATAGCCCCAGGAACAGGCTGCATCAT
TTTATTGGTATCTTCCACTGCAGTCGAACCACGAAGAGAGTCAGCTACAGAGCCAGCAGC
TTGAGATGCAGTCTTCAAAGTTGATATATCGGTACAAGCTTTGTCCACCGAATTTTGGTT
CTTGATCACCTCAACAACAGTTTTACTTGATACAGTCAACGTTGGAAGTGCCCCACCTTT
TGAAGAGTTTTTTCCAGATAAATCCTCCCTCGACTCTGAAGCAACTTCAGATGAAGTAGC
ACTTGGAACTTGTAGAGAAGCAGGAACAGAAGATATGCCACTGATATCATGCAACAACAA
CGAGCTTGTTGGAAGTGGGTGCTGGACAGCATTATCTGATTGCTCAAGGGATTTTTTTTC
CAGACCAGAAACATCAGCAGGTCCACCAACACCATGCGGTTGCTCCTGAATCGCATTTGA
CAATTCCTGATGCACGATATCTTGTTTACTTCTCATTGAAACAGCTTTACTTCCTGACAA
ATTACCAGAGTTATCCTCTGATTGAAGATCTGATTTCATATTCAAACCAGTAGGAACAGG
AAGTGATATCACCGCCTGTCTCTTTCCTCTACGACGGGGGGTCACCACTCCACTTTGTGT
TTTCCTGCTCTGCCCTTTAGTTTGCATAGGTGCTGGAGGACAGGGGAGGGAGGGTTGCAT
TGCAGCAGGAACAGATGTTTCATACGTGACTGGTACCAAATTTAAAGAATCCCCTACAAC
AGAAGGATCACTAGTTCCACCAGCAGAACTAGGCAAAGAATCAGAAACAGGAGCAATTTG
GATGCTAGAACCAGTTGCATCAATGGTGATAGGCTGGCTCCCTATGGGATTCACAGATTT
ACTCTGGGATTGCACATTTATTTTTCGATCAGTACCAGGACTTGATAAACCATCTGCAAC
AGCAGTTAACTCTGAATTATGTTTCTTTCCCCTACGCCGGGTTGTTAGCTCACCACTCTG
AATCTTTCGGCCTCGTCCCCTTGATTGCACAGATATAGAAGGGCATCCAGATGCAGATTG
TGAGCCAGGAGGAGCAGGTTGGAACTCCGAGGTTGGCTGAGAACTAGAAGCAATCCCTAC
ATGTTGCAAAGATCCACTGACAGCAGTAGGTCCAGGCAATGAAACAGGACCCAAGGTTGT
TGAATGGCTGGTCACTGTGTCTTTCTGCAACCCTGTCTCCACTTTCCCTGTTCCAGAAGA
TGCTGGGATCACCATTGCAATTGGAGATTTATCTGCCCTCCTTGGCCTTCCACGGCCCCG
CTTAGATGGTGGTGTTGCCTCTTTGCTTTGCTGTAACTGTGGAGGATCCACAGACGGCGG
TGGAGGGTGTGGAGAGGGTACAGGGGGTGAAGAGGGTTGAGGGATACCAACAGGAGGTTC
TGTGCTATCAGGAGCTACTGTGGAGCTGCTGATGACTGTTGGCAAGTTCTTTTCTGTTCC
TTCCTCCTTCACCTTAGGGGATTCAGGAGACTCAGCCTGACACATCTTTTCAAATTCCTC
TTCTGTCCATTGCTCTTCATAGGACCTCACCTCTCTTGCTCTCTTGCCTCTTCCATATTG
TTTAGTATCAAGTGCCCCAAGATATTCATTCTTCCGCTTTACACCAACTTGAGGTGTTAA
CACAGTCTTTGCTACATCAAAGATCTTCATTGCTTCATAGAATGCTTGCAAATCATCATC
AGTAACAAGGCGAGAAGGCAAGGGAGGTAAAGGCTCAGAACCATCCATCCCGTGTCTGCC
AACCAGCTTTTTCCACATTGCCATTTCTTGTTCCCGCCTTTGCTTGTCAACTGATTCAAA
CACATCAATCTCCGATTCGCTGCGGGCTAAAACATCATTTAAAGCATCATCGTCTAACAC
AGGTGCAGCTTCCTCTTTCTTACACTCCCGCAGTAAAGACTCTAAGTATTCCCTACGATC
TTCAGCACTCGTATTATTATCAAAGAAACCAGCAGTAATGCTCTGATTAGCAACTCCGAG
TTTGTGCTCAGCTGAAGCTCTAACTTGTTCCTCAACAGTTTGAACTGTTTCAAATCTAAG
AACAAGCACGTCCCTCTTCTGGCCAATCCTATGAGCCCTTGCCTGTGCTTGCAGATCAAC
CTGTGGGTTCCAGTCAGTGTCAAATATAATCACAGTATCAGCAGCTTGAAGATTAACTCC
AACACCACCAGCACGAATGCTTAGAAGAAATATAAAAAAGGGTGAATCTTGTTGGTTGAA
TCTGTCAATGAGGGCACCTCGGTCACCCCCAGATGTATGCCCATCCAACCTAAGGTATCG
GTATTGTTTAAGGGTGAGATACTCTTCCATGACATCAAGCAGCCTAGTCATGGTGGAAAA
GAAAAGAACCCGATGATCAGTCGCTTTCAGTTTTGGCAGTAACCTATCTAGCATCTCAAG
CTTCCCACAAAGGCGAACAATTGGTGGCAGAAAATGCTTAGGTATTAGAGTATCAATCTC
CTCTGCATGAAGCTGGCTGAGATACGGATGATTGCATATATTTCGAAGCTCCATAACAGA
GTTATGAACCGACCGTGCCTTTGAATTTCCAATTGCACCAAGATTTTCTTCAACCCTCTT
CATTAAGAGCTTCTGATAGGCAGAAGCCTCACATCTTATAAGTCTCTCAATCTTTTCAGG
CAGTTCATTTTCAACCTTGTGTTTCAGCCTCCGAAGTACAAATGGTCGTAGAACTTGGTG
CAAACGGTTTATGATTAACAGATTCTCCTCCTCAGATAACAAAGCTTCATCAGGTGAACT
GTCTCCATTACTTTGAAATGGTTTGTTGAACCATTGAGAAAAATCTTCAGATGAATTGAA
TATATTCGGCAACAAAAAGTTGAGTAACGCCCAAAGTTCTTCAAGATTATTCTGCAGTGG
TGTTCCAGTTAACAACAATCTGTGAGAACTCTGATAGTGCTTCAAGTCTGCATTCAACTT

GCAAGACGCATTTTTTATGCGATGGCCTTCATCAATTATAATATAATGCCAATGTATCTT
GCAAAGTTTAGGTCTATCGTGTTTGTTCATCAAATACTCATATGTTGTCAGAAGAACATT
GAATTTCTGATGCACAATTTTTTCCTTGAATAATCTACGCCTCTCCTCTGGAGGCCCAGA
ATATACAATTTTATGTATTCCAGGGGCCCAACAGTTGATTTCTGAGTCCCACCCAGGTAG
AACTGAAGATGGTACAACCACTAAAAAAGGTCCTCTATCATTTTTGGTTTCCATAAGGTA
ACAAATTAGAGCAATAACCTGGACAGTTTTTCCAAGGCCCATTTCATCAGCTAAGATTCC
ATTCAAATGATTGTTGTAAAGTGATACCAACCACCTCAGTCCATTCATCTGGTACTCCCT
CAATTTTCCACCCTGGAGACAAGTTGGCTGCTCTGAAATACTCTCTTTAACACTATGAGC
CATCAGATAATACTTCTCATTGCTTTCCAAGTAATGCTTTGCTTGGTCACTTTCATCTTC
ATTTTCCACAACAGGCTCGTACTTCTCAATTATTGGAGCTCTTGTTTCATCCATATCATC
TTCAAAACGACTCGACGCAGCCTTAGCCTCTTGCAGCTTGGACCCAAGCTTTTGAAGATA
TTTCTCAGTCTCTTTGAGGAGTTGCTTTACCCGATCGGATTTAGCATCCTGTACCATTCG
CAAATATCCCTCCACATCATTTATCTTCAATAAATTAATCTTCTCACGCTGAATTCTGTC
AATCTTCTCACGATGAATACGTTCCTTTCTTTTATGGAACTCCTTAACAAATTTATTAAT
ACCCCTCCATCGTTCTCTCTTCACTTTAAATACATCATCGAGCCTCTCCTTGTGGATTTC
TATCTCACTGAAGAACTCCTTCTGCCTCTCACGAATTCTCTTTTGTCGCTCTTCCTTCAT
TTTCTGCTCATATTTTTCAAGTTGTTTTATCCTTCTACCATGTCTATGCTTCTTACATGA
TTTCAGCCGATCCATGTCAGTTGAAACTGGCTTAAAAAAGTCATTTAGAAAATCACTCCT
GAGACGACGTTGAAGTCCCAACAATTGCAGCTTCTTCAGTTCTATGACACTTTTAGTTTT
GGCAGATATATCTTCAGATGAGCTCACGTTTTCCTTTAACTTATCATAACAAGCTGAAAT
TTTTTGCTTTGTTTTTTGCTGCTTTACTATCCAACTTTGCTCAGCCAATAGTTTTCTTTT
TTGCATATCCATAATCCATTTCTCTGACATGGTATACTTTGGAGCTGGTGGCAAATTAGA
ACGCAGTGACTTATCTTCCTCATTATTCTCTGTTGTGAAAGCAATGCCATTTCTAAATGA
GTTATCAACTGGAAGTGCTTTGATCCCATCTGAAAACTTTGAATCATCCTGAGACACGTG
TTTTGACATCAAACTAGCATCCTTCATTGAAATCATTGCACGGTGATCATTGTTAGCTCC
TGAAACTGGTTTCCACTGTTCCCTCATTGACAAAGAACACAAGTGACCATCAGAAAGACC
AGTGTTAACAATACTTTGAAACTGACAAGGAACATTATCTTTTCTTTCTGGGCCCGATTC
CTGCTGAACAGCAGAGGCTGGTAGGGGTCTTCTTGATACATCATTCTGACTGCCAATACC
AGTCCAGCTATTCATCTCAGAGTTCATAAATTCAGGCTGCTGTGATGTAGTTGCTAAAAA
TGTCTGAGATTCCAGTGTGTCTTGATTCTTCATTTCAGCCTCTGATTTTCTTGTAGCCGG
TAGTTGTTTCGCCTCTGCAAGTGCAAAATGATCAGCAGCTAAGCCACTTTTTTCCTCCAC
CAACTTTGAGTTCTCAGCCTCCCTAGACAAAGAGTCAGCCTCTAGGATTCTTCCAGAAGA
TGAACCACCTGAAGGGATTCGATCAGTTTCCCTGATATTACTCAGCCTTCCAGATGGTGC
CACAACTCCAGGAATGCTACTTTGATCATTCAAGGATTGTTGTTTTGCTGTATGGTGGTC
CATGAGCTCCTTGCGTGATACATCTACATTGCCACCTTCTTTTGGAAAGACGTTTCCAAG
TGCAATTTCAAGATGCAGTTTCTTTGGCATCAAACCGTTTCTAAAAGCTAAAAACACAAG
GCACTGGGCTCGGAGTTGCTTCAGTTGTTGTTCCTTGAAAGGCATGCCAGAGAATGCAAG
GCCCTGGGCAACAGAAGATTTACCTGTATCTCTAGAAGGTGTGGTTCTAAGCATAGTTGT
ACCTGAAATGCTATTTTGGCGGCCAACCTGAACAATCCTATTTGAATCTGTTAAGGCATT
TGGACTTCCTTCATGCTCCATAACCTTTCCACTAGGATTCACAGTAGATACTTCATCCCC
TCGATTATTAGACAGTTGGGCTTCTGCTGGGTTAGGCATTGAGGAGACTGATTCTCCTGT
TGGGAATGAATTTGAACTAACCGGTAACCCTCCATGAACATTTCCAAAAGCACTTCCTGG
ATACTGCATTGCAGATTCTGACATCATGTTACTGGGAATAACATTTGAAGAAAACTTGGG
AACATGAGATCTTTCAAATGGAAATCCGCCTCTATTCCATACACCCCTGGAAGCAAAAAT
GTCATTTGTTGAAGGAAGGGCACTCCCTTGTTGCGGTCCTGAAGTGTTGTGAGAAGAGGA
AACTTCCACCTCTTCAGGGAATTTTGAAGCTGAAGCCCTTGTAAGTGAATTACTAATATT
AGCTGAATCCATTGGCTTTTCAGAAATATTTTGACCATCTTGCTTAACTCTGAGTACCGA
ACTCATGTTACCAGACAAAGATGAAAAATGTTCCATTTGACCACCACTTGGAATCATGTT
AAAGTTTGAAAGCTCACCGCCTTTAACTGAAAAAACCCCAGGAGATTCAACCTTATTCAT
CTTCCCCCTCCTAGCATTAACCACAGGGTTCCAGGAATCATGTTGCTGGGGGTTCTCATT
TTGTATTTCCACTGGAATTGACGAATCACCTCTCTTTCTCTTAGTTGTAGGCTTTTTACC
ATCTTTTTGCCTTTCTTGT
>ontig_17_consensus
TTTTTTTTTTTATTAAATGGATTTTATAATGTAAGTTGGGAAATACAACTTGTCCGGGT
TAACTAGTTTTTTACATAAATGTACGGAAATATTAACTAGTCTCACAAACTGTAATATCA
AATCCTTGAGGTCCAACGGTTTAATTTAACTACCCAGCAGTCTAAACTACTCTTACATAT
CCTTTTGGCTTCTGAAAGCCAACAAAGAAGGCCACAACAAAACAATCAAGCAGCAGCCAC
AACCTCAGCAACAGCAGCCTGGGAGGGGGCAGCTGAAGGAGCAGCCTGAGAAGTTTTAGC

ATCCTTGGCAGGGGAATGTGCTGGAGGTGGAGTCGGCTTCAAATGGGCTGGGGTATTGTG
CTTCAATCTTATGAGTATCTGCCGCATCCTTGATAGATCAGTGGGTTTCCCAGGTTTTGG
ACCTTGGATCACAACGACAGTAGGCTTCTTTTCCTGATCCTTCTTTGCTTTCTTCTTCTC
CATAGCTGGCACTTCATTAGGAATGAGCTCTGCAATTGCTTTCCAGAAATTCTTATCAGC
TTCAGCATGAAACTTCTGGTGGTAGGCTACAAATAACTTCTCTCTTTCCCTGTTAGTTTC
TTTTTTATTTTCACAAGTCACCGCCCTCTTCCTGTAGAATTCAACTTTGTATTTCTCTGC
TTCTTCAATTATTTGGTTTAACATCTCTTTCTCTTTCTTCTCCTTCTCCTCCAATCGAAT
TGCATTTTCCCTTCTCCATTCTCTGAGAGCAAAGCCCTCCTCCGGAGTCATCTCACAAGG
AGGCGGCAAAACTGGCTCGTCAGATTCACCGTTCGCATAGTCGCCGTAGATCGACGGCTG
CGATTGGAACACGTCATCTTCAGTGTCTAAAGACGAGTTATTGTTGTTGAAGAACGGCGA
ATCAACGGCCGTGTCAGCAGGATGGGAGCCATAGCCTTGATAACCGTGGTCGTGAAAGGG
ATCAGGTGACTCCGCGACCGGGACCGAGTCAAAGGATTCGTCGAAATTTGACATTGGTTT
TGGGTAAGGTTTGGTCTGATCTTCCACGCCGTTCTCAACAAC
>ontig_18_consensus
ACGAGATATTAAAAGGGATTTTATAATTAAGTTGGGAAGTACAACCTGTCGAGGTTAGCA
AGTTTTTTACATAAATGAACAACAAATATTAACTAGTCTCACAAACTGTAATATCAAATC
CCTGAGGTTCAACGGTTTAATTTAACAAGCCAGCAGTCTAATAAACTATTCTTACATATC
CTTCTGGCTTCTGAAAGCCAACAAAGAAGGCCACAACGAAACAATCAAGCAGCAGCCACA
ACCTCAGCAACTGCAGCCTAGGAGGGGGCAGCTGAAGGAGCAGCCTGAGAAGTCTAGCAT
CCTTGGCAGGGGAATGTGCTTGAGGTGGGGTCGGCTTCAAATGGGCTGGAGCACTGTGCT
TCAATCTTATGAGTATCTGCTGCATCCTTGATAGATCAGTGGGTTTCCCGGGTTTTGGAC
CTTGGATCACAACAATGGCAGGCCTATTTTCGTGATCCTTCTTTGCTTTCTTCTTCTCCA
TAGCTGGCACTTCATTAAGGATGAGCTCTGCAATTGCTTTCCAGTAATTCTTATGGGCTT
CAGCATGCAACTTGTGTTGGTTGGCCACAAACAACATCTCTCTTTCCCTGTCAGTTTCTT
TTTTAATTTTCACGAGTCACCACACTCTTCCTGTAGAATTCAACTTTGTATTTCTCTGCT
TCTTCAATTATTTGGTTCAACATCTCTTTCTCTTTCTTCTCCTTCTCCTCCAATCGAATT
GCATTTTCCCATTTCCGTTCTCTGAGAGCGAA
>ontig_19_consensus
GGTGTCTGAAATTTCAAAAGAAATAAGAACGCTATGATTTACTAATATTATTTCCCATAG
ATATCAACGTCTACTTCATATACAAAGTACCAACTATCTTACAGACTCCATAATCTGTAC
AAAAAGTAGCCTTATTCTTAAGAATATAGCATTCAACTTTCTTCCATCCACAAACAAGCA
GAGAGCACACATATATACTACTGTTCTGCAATATTTCAGTATGTAGCTATTTTGCCAGGT
TTACAGTTTCCCATTCCAGATGTATCAATATCAACCCAAACTTCATTAACTCGACCAATA
AACTGCAAAGGAGAACTATATTGTGCAATGGAGTAAGCATAATCACTGTCAGAAGAAGTA
CAGTTAGCCCAAGGAGACCTACTCAAGCTGATGGCAAGTTTCTCAGCCTCGCGCACAATG
TTTTCGTCCTTTGCGAATCCAGAGAACTTCCTTACAGCAACATTGTGACTACCCCATACA
TAGGGCTTCAAATGAAGTTCAGGGAGGGGAGTTGGTGGGTCAGCTTGGAACTTCACAGGC
AGGTAAAATCTCACAACATAGGCAGATGAGTGAAGGGGCCCAGCCCCAGGAACTATGCTA
GTCACAACAGGAGACGTCATTGGAATTCGAGAGTAATTAAGATTGGCACCTTCGATGAAC
TGGAACAACCGGTGAAAGCCAAACAAGGTGGCCTTCTCGAAGGAGAGTTCACGGACAGAG
GCGGACATCCACGTGGATTCTGTGTAACGTCTAACATCGAAATCCGATTCTGAGTGTACC
ACCTCATAATTCGGAGAATCGATTGCGTTGCTAAGTACCACCAAACACAGCAGGCTCACC
ACGTTCATCAACATCTTCCTTCTCTCCATTTCTTTCTCCCCCTTCCTCCAGAAACATCAG
ACAAGAAGCGTCAGCCCTCAAAAATAGCGGAGGTTATATAATTTTACAATTTTA
>ontig_20_consensus
GAGGGGAGTGGGTGGGTCAGCTTGGAACTTCACAGGCAGGTAAAATCTCACAACATAGGC
AGATGAGTGAAGGGGCCCAGACCCAGGAACTATGCTAGTCACAACAGGAGATGTCATTGG
AATTCGAGAGTAATTAAGATTGGCACCTTCGATGAACTGGAACAACCGGTGAAAGCCAAA
CAAGGTGGACTTCTCGAAGGAGAGTTCACGAACAGAGGCGGACATCCACGTGGATTCTGC
GTAAAGTCTAACTTCGAAATCCGATTCTGAGTGTACCACCTCATAATTCGGAGAATCGAT
CGCGTTGCTAAGTACCACCAAACACAGCAGACTCACCACCTTCATCAACATCTTCCTTCT
CTCGATTTCGTTCTCCCCCTTCCTATCATCAGACGAGAAGCGTCAGCCCTCAAAAACAAA
CGGAGGCTATCTAATTTTACAATTTTGTCCCTGTTCAAAGTC
>ontig_21_consensus
GCAAGATGATTGTATTGACCTTATAAATTACATAGCAAGGAGAGTTTAAACAGCTACAAC
AATGGCAACGATAGGGATCTTATAATTCCATGATAAGCTACATGCTAATGCTGATGTTGG
ACCATGCTGTTGACCAAAACCTGGGAAGGGGTGTCGACCACAATAATATACAAACCTTTG
TTTTTTCAAAACCAGTCTCAAGCAAAGGAGCTGATAACTCAAGCAACTAAAGCTTTAATT

CATGATTACAAGAATTCAGTTAGTGTCTTTGAGGCAGCTTCCATCGTCTAAGCACCATTC
ATTGCAGTATCAACATGGAGGACTCCATTTCCATCTTCCATAATCTTGCCATTGATCTGT
GTTCTCAGCCTCCAATCTGAAGGATCAGAGTTCCCATTCAAGAGCTTGTGATTTTCCTCT
TTAGTTCTACCATCCTTGTCTTTGGTGCTCATGGGCAGTAACAGAGGTGAAATTCCACTC
GTACGATCAGGCAGTTCAGGAAATTTCTTGTCTAAAAAGAACACCACTAAATTGACAGTG
TACC
>ontig_22_consensus
CTCTTTCCCTACACGACGCTCTTCCGATCTCAACATACTTAAGAAACAAGATTATCATTC
AATGACAGGGAGGCCAGTTGATCTGATGGGCTGTTAGCTTGCTGAGCAGCAACACTCCTC
AGTACCTCCATGGCTTCTGCTACTTTTGCCTTCAATGCCTCCGGAGACTCAAGAAGATGC
AAGACCTCAGTCTGGTCCATCTCCAGAAGCATGCCTGTAACCTTGGCTGCAGCATCCCTT
TCAAGCTGGTCCACTAGAGGGTATAAGCTTTCACCCAGCAACGTCCGCTGCTGTTCTGGT
GATGCATTTGCAAGGGCAGTTGACAATGTTGTAATTGGCATAGGTTGTCCAACAGCAGCA
TCTCGCATGGTCATGCCCCCACCCATGTCATATGGAACAGGAAGCATACCTCCAGCAACA
CCAGGCATTGGAGCTTCCGGCATGCTACGACCAGCTGGGTATCGATACATACGTCCTCTA
GGAAGCATCTGCTGCTGCATTACAGGTAGTGACTGCTGAGGTTGTTGCACAGAACCTGCC
ACCCGTCGCCCTCCAGGTCGCTGGCCATGCTGGCCCTGCTGAACAACTGGAACAAAGAAA
TTTGGCATTGGAGCCCCACCAGGTCTCATTCCTGGAACAAGTTGCTGCTGATAACCAAAT
CCTGCCTGTGGAGGAATCATAGCTGGTGGTCCTTGGCCATATAAAAATTGTTGGCCAAGG
CCAGGTGCACCAGGAGGATACATTGGCATTCGAGGTGCAACCGAAGGGGCCATAGCAACT
GGTCTCATTTGTGAAAACTGAGCCTGAAGCCTTGCTCTCCTGTCTTCTTTCCTCTGGGCA
AGTGCAACATAAAGTGGCTTGCTAACAACCATTTTACCATTCATCTCCATCAGGGCTCGA
GATGCTTCATCAGGAGTTGTAAATGCCACAAATCCAGAGCCTTTACTGATTCCACTTGGA
TCACACATAACCTTGCAGGAAGTAATTGTGCCAAACTCTGAGAACAATTCCTTTAACTTT
TCATCATCGATACTATCATCCAAGTTCTTTACATACAAATTCAAACCTTGAAATTTGTCT
ACAGTGTCCTTTATGCTTTGCTCAAATCGCTCTTTCAATTCAAGCTCTCTTTCAGATTTT
TTCTGGGCCTTTCCAACATACCACTCTTTATCATCAAACTTCTTACCATTAAGAGCATCA
ACAGCACTAGCAGCATCATCTGCATTTTCAAAATTGACAAATCCAAAACACTTGGATTTT
CCATCACCATCCCTCATCACTACGGCACTAGTAATTGTTCCGTATTCACCGAAAACATTC
TTTAAATCCTCATCTGTTATTGTTTCAGAAAGATTTTTCACATAGACATTATTGAATTTT
GCTTTACTCAGTGTACTATCCCTTTCATGCTTACGAAGAAAGTGTCCAACATAAACTTGT
TTATCATTAATAAGCATGCCATTTAGCTTATCAATAGCATTTTGAGCAGCTTCTTCAGTG
TCAAATTGAACAAACCCATAGCCTTTGGATTGCCCAGAAGCACCAGTAGCTATCTTACAA
GACAGAATGTTTCCAAATGAAGAAAATGTATCATGTAATGCTTTGTGGTCAATAGTCTTA
TCCAGATTCTTAATAAATATGTTTCCAGTACCACTCTTGCGAATACTAGGATCACGATGA
GAATACATTATTCTAATAGGCTTGTTATTCAGAGGAGTGAAGTTCAGAACATCCAATGCC
CTTGCCGCGTCAGTGGGATTGCTATAGTTAACATAACCATAGCCAAGTGATCTCCTGGTG
CTCAGGTCATGACAAACCCTGACCGATACGACTTGACCGACCTGATTAAACAGATCGTAG
AGTTGCGAATCGGTCACATTAATATGTAGATCGCCAACATAAAGCGAAGTTGTAAGAAAC
TGGTTCCCAGCCGCCACTCCGTTTGGCCCCAATATTGGCGCCTGATGCTGAATCTGAATC
TGCGCCATGGCCAAAATTCTAGATTCCTTGTTCTCTTTTTTTTTTTTTTTTCTTTTTCCG
CTTTTTCTCTGCTTTTTTCCCTCTCCTCTCTTAACTTTTCAAGCAAAAACTTGGAGAGAT
AACAAGATAATAATTCTGACTCTAACAATTATATTCAAGAGAACAAATCGCTAAACTGAG
GATTTTGGTAGTCCACTTTGATTTTTTTTTTTTTTTTTGGGAGATTTTAGAAGATAAGAG
AGGAGAGGGAAAGGGGGGGGGG
>ontig_23_consensus
ACCTGCTGAGGTACAACAGTCCTCAGAACCTCCATAGCCTCTCCAACTTTCGCCTTTAAT
GCCTCCGGTGACTCAAGAAGATGCAAGACCTCAGTCTGGTCCATCTCCAGAAGCATGCCG
GTAACCTTTGCTGCCGCATCTCTTTCCAGCTGTTCCACTAGAGGGTACAAGCTTTCACCC
AGCAACGTCCTTTGCTGTTCTAGTGATGCATTTGCAAGGGCAGTAGACAATGTTGTAATT
GGCATAGATTGTCCAACAGCTGCATCTCGCATAGGCATGCCCCCACCAATGTCATACGGA
ACAGGAAGAACAAAACCAGTAACACCAGGAATTGGACCTTCTGTCATGCTGCGGCCCGGT
GGATATCGATACATACGTCCCCTAGGAAGCATCTGCTGCTGCATCACAGGCATTGGCTGC
TGGGACTGCTGCACAGGAGCTGCCCCACGTCGCCCGCCAGGTCGCTGTCCTTGCTGACCC
TGCTGAACAACTGGACCAAAGAAATTTGGCATTGGTGCCACACCAGGTCTCATTCCAGGA
ACAAGTTGCTGCTGATAACCAAATCCAGCCTGTGGTGGTATCATAGCTGGGGGTCCTTGG
CCATATAAAAATTGTTGGCCAAGGCCTGGTGCACCAGGAGGGTACATTGGCAATCGAGGT
GCAACAGAGGGGGCAATTGCAACTGGTCTCATTTGTGAAAACTGAGCCTGAAGCCTTGCT

CTTCTATCTTCTTTCCTCTGGGCAAGAGAAACATAAAGTGGCTTGCTAACAACCAATTTA
CCATTCATCTCTGCCAGAGCTCGAGATGCTTCTTCAGGAGTTGAAAATGCCACAAATCCA
GAGCCTTTACTGATTCCACTTGGATCAAGCATAACCTTGCAGGAAGTAATTGTACCAAAC
TCTCTGAATAAATCCTTCAATTTTTCATCATCAATACTATCATCCAAGTTCTTAATATAC
AGATTCAAACCTTGAAATTTGTCTACATTTTCCTGCATGCTTTGCTCATATCGCCCTTTC
AATTCAAGTTCTCTTTCAGATTTTTTCTGAGCTTTTCCAACATACCACTCTTTATCATCA
AATTTCTTACCATTAAGCGCATCAACAGCTTTAGCAGCATCATCTGCATTTTCAAAATTG
ACAAATCCAAAACACTTGGATTTTCCATCTCCATCCCTCATCACTACAGCACTCGTAATT
GTTCCATATTCACCGAAAATTTTCTTCAAATCTTCATCTGTTGTTGTTTCAGAAAGATTT
TTCACATAGACATTGTTGAATTTTGTTTTACTCAATGTACTATCCCTTTCATGCTTCCGA
AGAAAGTGCCCAACATATACTTGTTTATCATTAATAAGCATGCCATTAAGCTTATCAATA
GCACTTTGAGCAGCTTCTTCAGTGTCAAATTGAACAAAACCATAGCCTTTGGATTGACCA
GAAGCATCAGTGGCTATCTTGCAAGAAAGAATGTTTCCAAATGAAGAGAACGTATCATGT
AATGCTTTTTGGTCAATAGTCTTATCCAGATTCTTAATAAATATGTTTCCAGTACCACTC
TTGCGAATACTAGGATCCCTATGAGAATACATGATCCTAATGGGCTTGTTATTCAGAGGA
GTGAAGTTCAGCAAATCCAATGCCCTTGCCGCATCTGTGGGATTGCTATAGTTAACATAT
CCATAGCCAAGTGATCCCCTGGTGCTGAGGTCTCGACAAACCCTGACCGATACGACTTGG
CCGACCTGGTTAAACAGATCGTAGAGTTGCGAATCGGTCACATTAAAATCGAGATCGCCA
ACATAAAGCGACGTCGTAAGGAACTGGTTCCCAGCCGCCACTCCGTTTGGCCCC
>ontig_24_consensus
TTTTTTTTTATAGAAAAGTGATATTTATATTACTTAGGATAAAAAAATATTTAAAACCAA
ACCTTAGATGGTAAAATATAATTCTCGTTCCCTTTCCTAAAGCCATTTTTTGTCGCAAAT
ATCACAACTCTTTATTTGATTTCAAGTAAAGACACAACAACCAAACAGAAAAAACAGTTC
CAAACAATAATCTCCCACAGGAAAAGACAAATATCTAGTGAACCCCTTCATATTCACCAA
CCATTTAACTATACGAGCCACCGGTACCGTGGTATGGACCCGGTGGATTCCCTCCGGCCC
GAGTGTTATGAGCCATGGTTCGCCCCGTCCCACTATTTACTCCGATCGGGTGAGACTCCA
CCTTCCCTTCAGCCACGTGCCCACCCATGGGCTGCCCAGTGCCATGTCCGGGCATTGCGG
ACATCTGGTGACCCCCAGTGGAGTAGCCCAGGTCCCCACTGGTTGCACCAGCAGTAGCAG
CAGGAGTGCTGTGACTTTTTTCTACTCCGGTAGGGCTGGTTGCTGTGAAACCTGGATTGC
TGTCACCACCTGAGGGTGAATGTCTTGCAACTGCATTCTGCTTCTTAGCCTCCAGCTTGT
TGATTTCTGCTTGTGTTAGCTTCTCTTCTTTCCTTTCAGTTGCAATTGCTTTCTCCATTG
GAGTATGAGCTTGCATCTTCTCCACCTTCTCTTGCACAGTGGCCTTCGTCTTATCCATTC
CAGCTTTTGCAGACGCTGCCATGTTGGCTGCTGTTTCCTTCATTGACTGCATTTGCGCTT
TCAGTTGTCTCGAGCTTCGATCACTCTGAATTTCGATCGCTTGGTTTGTATGAATAAATA
GCAGCTTGGGGTTATGGTTGGATAGACGAAGGAGGAATCATGGATTAGAGAAACGTGTCA
AGGAGACATAGCACGTG
>ontig_25_consensus
TTGTTTCTTCCTTCCTTAAGCACAAAGATGCCTTTATCTTATCAATGATACTGTGTATAC
ATAAAATATTATACCAGTTATTGTTTTACACAATACTATTTTTACTTTATAACTTACCAT
CGAACTAAATTCGAACCTTGAATTGCTCGAAACTGAACTTCAGAATAATAAGTCAAACCT
AATTTAACAAGAACCAAGCCACGAGTAAATCTATGTAACAAAAATGTATTGGTACTTGAA
ACATTCAATTGTTTTTTTACAATTTTCGGTTCTTTTTTTTTTTTTTTATGACAGATAAAA
TAAAATAAAATAAAATAAAATAAAAAAGTTAACCTAATACGACCATTGATTATCTTCTTC
TTCTTCTTCTTCTTCTTCTTCTTCTTCTTGCAGATCTGCAAGAGAGAAACAACTTTGTGA
CTTGAAACTAATCTTGAAACCGCTTCCATTTAACGTTTTTGTTGGTGGGTCTTTCATCAC
TCGTGCTTCTTCTTCGTCTTCATCCCTTGTTTTTTGTTTAAAAGAAGACAGATTTTGAAG
CTGATGAGGATGAGGATGATGATCTTCTTCGTCTTCTTCTTCAAGAGCAGACAGAATGGG
CATAGATATTGGGTTCTGCCATGAGTAAAAAGACGATCTTCTACTGTCTCTGGATAACTT
GTTCACCATCAGCAATCTTCTTCTTTTGTTAAACGGATTCTCTTCCTTCTCCAGATCCTT
CACTGAGCTCACAACTGATAGATTCGCAAACGATTTCGATCTCCCATTATAGTGATTCGA
TAATCCTCTCTTTATCGGAAGAGAGTCTTCAAATGAATCCAAAGAAAGCGTCGTCTTTTT
CAAGTTTGACGACGATGAAACGTCATCGTTTTTGTTTTTGTTTTCGTTTTCGTCTTCGCT
GTCACCTGGATCCCCGATCGACGACGAAGTGTCCGTATCCGATCCATATCCGGAACCGAA
TTTGTCTCCGCTGGTCTCACGACGATCTTCTTCTTTCCCACCGCCGTTACTCTTCAAGAA
AAAACAGGTCGACTTATCAACACCGCTTCGGTCTCGGACGCGCGACGACGGAGACGGTGA
CGGTGACAGTGACGGCACCGACACAGACACCTCCATTCTATAAGTGGGACCCACCAGGAC
TTCCATTACTTCCTCCTTTTTCTCACCTCCTCCTCCTCCTCCGCCTTCGTGCTCGTCGTC
TCTTTCTCTGGTTCTCCCTCAAAGGATCTCTTCTCATCAATTGGCCCAAAACGACTTCGT

GTTGCGCATAGACACACGGAGTGCTAAAGAAGAACGCATACAGAAGAGAAAAGAGAAGGG
TGTGTGTAGCATAAGGATGAGGTGGGTGTCGGTTAAGGAATGGAGGTTGGCTAGCTGAGA
GATAATGCCACGAGAAACTGTATATATGGAGACTCATCCC
>ontig_26_consensus
CTGATATCTCCTTTTCAATCTGTATTCTAATAAATGAAACCAATGGGACTCATCATAGCA
TTGAATTAGATACCCTTCTGAGCCCATCCGAGAAACACACAAGTTTTGGCCATCAAACAC
TTCTGCTTTCAATAATATTTGTTCAACAAAGTCTACTTAACATCACGGAGTTCTTGTATT
GGATGATAACAAGATTAACATATGAGCAAAACAATTTAACAAACATAAGCAATGCAATGG
AATGCAATGCTCTTGTGCACTTCCAGCCCTCTTCATTTGTAAAATTCAAAGTCAGTGTCC
GGTTTCTTCACATTGGTTCGATAACCCTTCTCAAAGTCCTTCGGGAGTATTACATATCTG
TTCTTGCGCACTGCATGCATTCCAGCTTCTTGACAGATAGCAGCAATCTCTGCAGCACTG
ATTTTGTCAGGCCGAGAGACATAATCCTCCAAGTCCACCTCATCACTTAGGTTCATTTTA
GCAGTGCAAACCTGAAAAACGAGCCTCTTTTGCCGTCTATCAGGCAATGGGAACTCAATT
TTTCTATCAAGCCTCCCAGGACGTAAGAGTGCAGGGTCCAAAGTGTCGGCACGATTGGTG
GCCATAATAACCTTAACATTAACTGTCTGGTCAAACCCATCCATCTGATTCAGTAACTCC
ATGAGAATACGCTGAACCTCTCTATCAGCACCAGTTTGAGCATCAAATCTAGCAGTGGCA
ATAGCATCTACCTCATCTATAAAGATAATGGCAGGGGCATTCTCTTTGGCAAGACGGAAA
ACATCACGAACCATCCGTGGACCCTCACCCAGATACTTCTGAACAAATTCAGAGCCAACA
ACTCTAATGAATGCTGCAGTCGTGTGATTAGCAACTGCCTTGGCAAGCATAGTTTTTCCA
GTTCCAGGTGGTCCATAAAGCAAAACTCCACGAGGAGGGTCTATCCCAATTTGCTTGTAT
AATTCATGATGAGTAAGAGGCAACTCAACAGCCTCACGAATTTCCTGCTTTTGAATATCA
CATCCTCCAATATCATTATAAGTAACATCAGGCTTCTCCGACTGGCTAAGAAGCGAAATA
CTGGAATCAGCTTCGGGTGGAAGAACATCAACCAGTGCATTAGAATGTCGGTGCAAAGCC
ACAGAGGCCGAGGGTTTCAAAAGCTCCCGATTAATCGTGCTGAGAATTCTAACATAATAA
TTGGAGCCGGTAGTGGAGCCCACGATGCCGTTGTTCTGGTCAACCATCTCCATGAACTGG
CCGATAACCAAAGGCACAGATTGGATCCGCTTGACCTCCTCTTGAGCACGGAGGAGTTCG
CGTTTCAAATTCTTCTGTTCATCTTTGACGTACTCTTCTTGGATATCGATGAACTCGAGC
TGACGTTGCAGGGATTTAAGGCGGCTGTAGAGGTCGTCTTCGTCGGAGCATTGGTGGTCG
CTAGAGCCGCCGTGGAGGTCGGATCTTGTGGAAGGGAGTGAAGGTGGCGGGTCGGAGGAG
AGTTTAGGGTCGAGAACCATCGCGGCTGCCATTGTTGGAGATTGAGAAAAGGGAGAGTCT
GGAGTAAGAAGCAAGAAATTGATGGAGGGCGTTTTTGTAATCCCCA
>ontig_27_consensus
GTTCCTTGCAGTTGCTGCTGCTGCATCTCTTGCTGTCGGAGCAACTTTGAACACTCTGGT
TCTCCCTCAATTGAACCAGCTTCCCCAAAGGTCAGTGGATGCAATTGCTATCAAACAGCA
GCTTTTATCACAATATGACATGCTTCAGTCTCGTATCAAGGACCTAAAAGAAGCTGCTGA
AAAAGAGGTTTGGATGTTGGCTCGGATGTGCCGGCTAGAGAACAAAATTTATGCTGTAGG
AGAACCTTCTTATCGTGCTCGGAAAAGTAAAGTTAAGAGGGTGCGTGAAGGCTTGGAAAA
TTCCCTCAAGGGACGGATCGAGCTGATTGACAGCTATGCAAGG
>ontig_28_consensus
ATAAAATCGATTTCATTAACATTTGAAGAGCCTTCCAGCATATAATTTATCTTATATTAT
CTTCCAAGTCCCGAACCATGAGACACATTTTATGACACCCAAGCCTGTAGCTTGTTTATT
TGCATCCACATACACAAACTATTGTACACACCATACAGCACAAAGCTTAGAGCATAAGAA
GGAAAGAAAGCACTTGGAATACTTCAGTATTATTTATCACGCATAGGGTTTATAGAGACA
AGCTGGATCTTCAAGAATTCACAATCACCCCCCCATTAGGGTGGAGGACTTGGCCAGTGA
TGTAGGAGGAGCAATGGTTACAAGCCAGAAACACATAGCAAGGCGCAACTTCAATGGGCT
GACCAGCTCTCTTCTTTGGAACTTCGGACCCGAACTTGGCAGTTTCGTCTTCGCTGAATG
ATGAAGGAATCAATGGAGTCCAGATGGGTTCAGGCGCCACACCGTTCACTCGTATTCCTC
TGTTGACAAGTTGCAGCGCAAGCCCTCTTGTGAACGCCGTGATCGCTCCCTTTGTTGCTG
TGTAGTCGAGCAGTTTGGGGTTTCCCCGGTATGAAAGCACCGATGTGGTGTTGATTATGG
CGCTTCCTTCCTTCATATGCTTCAACGCATGCCTAGTCACGAGGAAGTGGGAGAAAATAT
TAGTCCTAAAGACTTTGTCAAGCCTTTGCTCATCAATCTCCTCAATGGAGCCTGCTTTGT
ACTGCACAGCCGCATTGTTAACCAGAATGTCGATTTTTCCATACGCATTCACCACTTCAT
CAACAACCCTTTTGCAGTTCTCATCATATCCTAAATCTGCCGCTATTGCCACGGGATCCC
TCTGTAAGCCATCTAACAAGTATGTTATCCTATGATAACAAAACAAATTTTACAGATTTT
AGATTCGAAAGCGTA
>ontig_29_consensus
GCTTCTGCAGGTAAATCATCCTTTTCCCTGAAAATATAGACGCTCCGAAGATCGACACAT
CAAATATACGTTCTTATGAGGTGGAGTTCCTGGTCTCAACATGTTGTTGCCAAATCCAAT

TTTCCCTTTCTTCTCAGAAGCTTTAAGGATCTGAAAAGAACACGTCAATGTATCATCCAC
ACTCATCATGGCACCGGCATTATCAAATTCACCACAGTAGTTTGGTGCAGAGAATATGGT
GACCAGCTGCCGTTTTGCAAAGAACTCATAACCATCTTCCACAACCTGATGAGCTCGACA
GACAAGATCGAGGTCATGCTTCTCAAGAAATTCAGCAACTTTGTCAGCTCCGAATGTGAA
TGACACACCCCTGTCATTCTCTCCCCATCCCTGAATATCTTTATCAGGATCAGCCCACAA
GAGATCACAGAGAAGGCCCTGATCTGGCACATCAACAGGGCGCGCAATATTCCTGATCTG
ATCTAAATTTTTCAAATCAGGAGATAACCCACCATGCATGCAAAGGATCTTTTCATCTAT
AAGAGCAGCAACAGGAAGACAATTAAAGCACTCAGTAAACGTCTTCCACACACGAACATT
GAACCTCCTCTTGCACTCATCATAGAAACCGTATATTCGATTGATAGAGGCACATTCATG
GTTGCCTCTAAGAAGAAAAAAGTTCTCCTTATACTTGATCTTGTATGCAAGAAGTAGGCA
TATAGTCTCTATGCTCTGCT
>ontig_30_consensus
CTGATGAGCCCGACAGACAAGATCGAGGTCATGCTTTTCAAGAAATTCAGCAACTTTGTC
AGCACCGAATGTGTATGACACACCCCTGTCATTCTCACCCCAGCCCTCAATATCTTTATC
AGGATCAGCCCATAAGAGATCACAGAGAAGGCCCTGATCCGGCACATCAACAGGGCGAGC
AATATTCCTGATCTGATCTAAGTTTTTCAAATCAGGAGACAACCCACCATGCATGCAAAG
GATCTTTTCATCTATGAGAGCAGCAACAGGCAGACAATTAAAGCACTCGGTAAATGTCTT
CCACACACGAACATTAAACCTCCTCTTGCACTCATCATAGAAACCGTATATTCGGTTGAT
AGAAGCACATTCATGGTTGCCCCTAAGAAGAAAAAAGTTCTCCTTATACTTAATCTTGTA
TGCCAGAAGCAAGCATATAGTCTCTATGCTCTGCT
>ontig_31_consensus
GGGGGGTCATTCAGAGTAGAGACTAGAATACAAACGCAAAATTATTTTTAGTGATATTTC
ACAAAAACATAAGACAACATGTAACACGTTACCAGCAGCCTATCATCCTACAAACTCATG
ATAGATCTGTCAAGCACAAAAAAAAATGCGTAAGAAGATAGCCCATTTATACATGTTGTTC
AAGTCATCAACTCTCAAAAATCCAAAACCTGAGACTGGCAACCCAGACGTGCCAACTCAA
CTTGGTACATTAGATCATGAATTTGTTATTAACACAAACAAACCATATTGGTGAATCCCC
ACTCCCGACAGGATTTCTTGAACCGTTGCTCATGAGAAAAGTTTCTGGCCTCCACCCCTT
CGCCAAACACCACGTACAGGCAATCCTTCAGCGAGGTTCTGGTTGCCATCTAAGCTGTTA
TCCAATGGATTGGGATCAGGATTAGGGTCTGAATTTTTAAGATCCAAAAGAGCAGCCATT
GAGCCATCACCAAGCCGTACTCTGTGAAACTTAGAGGTTTTCTTTTTCTGCTTATTCCCA
CCACCCCCATCCGCCAAAATTTGATCTAATTCACCAGCAGCAGATGATTGGTCATTCTGA
TCCGTATTCTCAACAATTACCTTTGATTTGCCTTTAGCACCACGGTAGCCATCCTTGGTT
AGCACCTCCACCTCTTCTTCTGAAGGCTTGTAACTTGCTTCCAGTTCTTGAACAGTGCTT
ATGAAACTATTTGCTAAAGTATCCCTTGAATTACTCAGTTCAGCACCAACAGACTTGCCT
TTATAACCATCTGCTAACATATCTTTAGAATTACTCTGTTCAGTACCCAAAGACTTACCT
TTACCTTTTTTACGGCCATTAGTCTCTCTCCACTGGGAACCATTCTCTTGCCCATCACTG
CCTTGCAAACTAGCATTGTAGGTCTCAATTAGCTCTCTCTGCTTCTGACCATCAGGAAGA
AGACTAGCCAACTCAAGAACTAAATGTGACAGGCCATATTTCTGCACATTATCCAGGTAT
CTCTTCGCGTCAATCGAGCCTTGTCGGTATTGTCCAGATATGTCTTTAAAAGCAGAAAAT
TTTTCTTCATCATATTGAAGGGAAGCACGCATCTTTTCTACTAAAGATCTGTTTGCAGCT
TGAACATTCTCCACTCTTGGTGGAGGCTGATTACTTGAAAGCAATTTTCGTGTTTGCCGA
ACAGAAACTGGTGGGAAATCAGAAATAGAAGGTTCTAGTGATCCATTATCAGCAAGATTC
GGGGCAGAAGCAGAGTGGCTTATCCTACTGCTATTGCCAGAAGTTGGTTGTGAACTAGAT
GACACGAGTCCATGCACTGTGCTTGGTCGAGTCTGAGCCTTGATTTCAGTAGAAGCTGCA
AAACTTGATAGTGTAGGCCCACTAACAGAACTCCATGATGTTACAGGTGCAACATTACTT
GTAGCCCTAAGTTGGATTGAGTTATTTGATGGCTGCAATTGCCCACGACTAGCAGATGGC
CATGCTTGACCTGAGCGGAGAACAGTAACTTTTCTGTTAATTTTGCGTCGGAGATGTGCT
GCCATGGTGTTGTCAGGTAAACCTGTATTTCTTGGCTTTTGTTGACTGCTGCCGGAAGCC
ATGGGGAGGGGAGGAAAGGAAGATTGTTCTAGTGGTGCCATCCTTGAGTTTTGCCCCAAG
GCTTGAAGGTATCTTGAAGGTTCCGAATCTGTAGTAGCTAATGATTCAAAAGGCTGGACG
AGTGGGTCAATATTGTCTGCATCTCTGAGATCTGAGACTACTTGTTCACCGGATGAAGAT
GGATAATAAGATGAGCTATCAGCACCAACAGTCTCCAAACTTGCCTGAAGAACTGCATTA
CGTTTTGCACGAGACATACGCCCACCATGTTCAATAGCATTATGCCTCTTCATTTCTGCT
TCAGATTGAAATACAACAAATTTTTTGGCAAGGCAGGCTTCATCCTCACAAAGAAAATGA
TCCCTGCGGAAATGAATCTCCAGGTCATCATAATTCTTATAATATTCATACTGCCCAGGA
TGCTGCCTTTGACATATATGACAGGTATAGTGTTCTGTGGACATATGCGAATACAGTTCA
TTATCCCCATAGAACGGGGTTTTGCAAAATTCACATAT
>ontig_32_consensus

AGGCCCGCGGCTCCCCGACGTGAGGGCACATGTTCGCGGGTCGTCCTGGCGGGCAGGTTT
CGACAATGATCCTTCCGCAGGTTCACCTACGGAAACCTTGTTACGACTTCTCCTTCCTCT
AAATGATAAGGTTCAGTGGACTTCTCGCGACGTCGCAGGCGGCGAACCGCCCGCGTCGCC
GCAATCCGAACACTTCACCGGACCATTCAATCGGTAGGAGCGACGGGCGGTGTGTACAAA
GGGCAGGGACGTAGTCAACGCGAGCTGATGACTCGCGCTTACTAGGAATTCCTCGTTGAA
GACCAACAATTGCAATGATCTATCCCCATCACGATGAAATTTCAAAGATTACCCGGGCCT
GTCGGCCAAGGCTATAGACTCGTTGAATACATCAGTGTAGCGCGCGTGCGGCCCAGAACA
TCTAAGGGCATCACAGACCTGTTATTGCCTCAAACTTCCTTGGCCTGAACGGCCATAGTC
CCTCTAAGAAGCTGGCCACGAAGGGTCACCTCCGCATAGCTAGTTAGCAGGCTGAGGTCT
CGTTCGTTAACGGAATTAACCAGACAAATCGCTCCACCAACTAAGAACGGCCATGCACCA
CCACCCATAGAATCAAGAAAGAGCTCTCAGTCTGTCAATCCTTACTATGTCTGGACCTGG
TAAGTTTCCCCGTGTTGAGTCAAATTAAGCCGCAGGCTCCACTCCTGGTGGTGCCCTTCC
GTCAATTCCTTTAAGTTTCAGCCTTGCGACCATACTCCCCCCGGAACCCAAAGACTTTGA
TTTCTCATAAGGTGCCAGCGGAGTCCTAAAAGCAACATCCGCTGATCCCTGGTCGGCATC
GTTTATGGTTGAGACTAGGACGGTATCTGATCGTCTTCGAGCCCCCAACTTTCGTTCTTG
ATTAATGAAAACATCCTTGGCAAATGCTTTCGCAGTTGTTCGTCTTTCATAAATCCAAGA
ATTTCACCTCTGACTATGAAATACGAATGCCCCCGACTGTCCCTGTTAATCATTACTCCG
GTCCCGAAGGCCAACACAATAGGACCGAAATCCTATGATGTTATCCCATGCTAATGTATA
CAGAGCGTAGGCTTGCTTTGAGCACTCTAATTTCTTCAAAGTAACAGCGCCGGAGGCACG
ACCCGGCCAGTTAAGGCCAGGAGCGCATCGCCGGCAGAAGGGACGAGTCCACCGGTGCAC
ACCGCGAGGCGGACCGGTCGACCCAACCCAAGGTCCAACTACGAGCTTTTTAACTGCAAC
AACTTAAATATACGCTATTGGAGCTGGAATTACCGCGGCTGCTGGCACCAGACTTGCCCT
CCAATGGATCCTCGTTAAGGGATTTAGATTGTACTCATTCCAATTACCAAGCTCGAAGAG
CCCGGTATTGTTATTTATTGTCACTACCTCCCCGTGTCAGGATTGGGTAATTTGCGCGCC
TGCTGCCTTCCTTGGATGTGGTAGCCGTTTCTCAGGCTCCCTCTCCGGAATCGAACCCTA
ATTCTCCGTTACCCGTCACCACCATGGTAGGCCCCTATCCTACCATCGAAAGTTGATAGG
GCAGAAATTTGAATGATGCGTCGCCGGCACGATGGCCGTGCGATCCGTCGAGTTATCATG
AATCGTCAGAGCGACGGGCAAAGCCCGCGTCGACCTTTTATCTAATAAATGCGTCCCTTC
CGGAAGTCGGGGTTTGGTGCACGTATTAGCTCTAGAATTACTACGGTTATCCGAGTAGCA
GATACCATCAAACAAACTATAACTGATTTAATGAGCCATTCGCAGTTTCACAGTCTGAAT
TAGTTCATACTTACACATGCATGGCTTAATCTTTGAGACAAGCATATGACTACTGGCAGG
ATCAACCAGGTAGCATTCCTCGACGACGCCGGCGACCGCATGAGTCCCGTCCGCCACGCG
GGCCGCGACGGGATCAAGACGGCCGCGGCGGTCGTTCGCGTCAAGGGACAAACGCAAGGC
TCGGGCGAGCGGGGTCGAGACCCCGCACCCGCGCACGACGTTCCGCATCCGAGAGAGCGG
GCAACAGCTCCGCGGGCCTCAACAGCCCCGCAACGATGCGAGGCGTGCGAGGAACGCGCA
GCGCGCCTGTCGTCCACCCCGCACCCCCTCGAGGGCGTGGGCGGAAAATGGCAACGATAC
GATCGAATTCCGTTTCGTCGGTAGGCAACACAGGATATCCGGAGCACCCGCGAGGCAGCG
TCTGCGCTCGGGGAAAGACTGAGAAAGACGGGCGGTCGACAGTTCGATGCGCGAGCACGG
AGCCTGCCAACCCACGCCGCCAAAACACCACTCACACGCCCGTTGCGTTCACTTGGAAGC
AGCCCCGCCAAACGCACCGCACCCCCACGACAGTAGCCGTCAAGCGTACGGAAGCATCGA
GCAAGGCCGCCCCAGCACCGCGGGACGCGAAAAGAACACCGAGAGACGAGGGACAGCCTG
CCTCAAGCACGCGGGCATCGCACCCCATCCGGGATCGACCTTTTGCACGTCGCGCACTCT
GTGCACGACAGATGCTCAGGCTCACTCGAGCAGGGTGTTTCTCCGCACAAGCCTTCAACA
GGCACGGGGCAGCAACGTCGGGCCATCACGGGTGTTCGCGTTAGCCCCCTGTGCGCGGGG
ATCCATCGCGAGATCACTGACATGAGGCAAGGACGTTGAACCGTGTCCGGGATACCGCAG
CTTAGCCATGCGTCAGCACTGTGTAGGCACCAAACGCCACGTGCCTGGCATGGGTGATCG
CAACGGCAGCAGGTGCACGGGGCAAGGGCATCGAACCATGCCAAATTTTCTGCTGCTCAC
TCGCACAGTGACGCACAAGCTTGCATTGGAGAATTAAAACACAGCGCTCAACCCCCTTCC
CGTGGCTTCAACGAACTAGCATCGATTCCCACATTTTTGCAGATTGACCCCACAGAGGGG
CCCACCGTCGTCGACCGATCGTCAGAGCAATGTCGGTTACCAATGTGTCATCGGCAACCT
ACGATGGCTGATACGAGGGGCAAGCAACGTATCGTCCGCGCCATCGAGGGGGGATTAGCT
CTGAGGGCGCCCAGTTGAGACTACCACCTCCC
>ontig_33_consensus
CTCTAACAGGTGACAATTTGGCAAAGAGGCAAACAATGGGTGAGACCTTTGGCCTTTTTC
CTGGTATTTGTACGCAATTAATTTCGCCTTATTTTGCTCTTGTGCTTAATATTCTTGATA
CCCACAACATCAAAACGAGAGAATGGTTTCTTTTATTTCCATTAATTCCATCACATAAAT
AAATAATTGAAGAAGAAATTTTGGATGACATAATCAAATACTTAATTCAGAACAAAAAGG
ATATTACATTGTTATTTATTAGCCAATTTCAATACCAGCACGCAATATACATAAACAAGA

CCATCCATTGCATTGTTCTCGAACACAATACTATATGCAGTGGTTTCAGCCGGAGATTTC
AATGGCCTTAACCTCAGGCTTCTTAACCTCAACTTTAGGAGCAGTAACAGTCAGAACTCC
GTTCTCCATCGAAGCCTTAATCTCCTCCAGTTTCACATTCTCCGGCAGCTTGAACCTCCT
GGAGAATTTCCCACAACTACGTTCCACACGATGCCAAGTGTCGTTCTTGTCTTCCTTCTC
TATATTCCTCTCTCCACTAATTTGAAGCACACTGTTCTCTTCGATCTCCACCTTCACTTC
CTCTTTCTTGAGCCCTGGAAGATCGGCCTTGAACACGTGAGCCTCGGGGGTTTCCCTCCA
GTCTATCCGGGTGTTGACGAAAGTAGATGTTTCTTGAGGGAAACGAACGGAAGGTGAAGA
AAAATCCTTGAATGGATCCCAAACGTCTAGGGAGAAGGGATCGAAAATACTGCCGCGCTT
GTTGCCGAAGAAGCTCGGAATTAGCGACATATTGTTTCACAGGATGCGAGAGTACTGTAC
TGATTGCTAAATCTGATACCCAAACTTTGAAGAATTGTGTCCGATGCTCTGAAACGAGAG
TGTGTTGGGCGTTTATAAGGAGAGTGGAGGATTCTAGTAATTTCGTGCACCATCTTTTAT
GTTTAGTTAATGAAGGCTGTAGGACTTTCTAGTGAATCCTGGAAATTGTCAAGTTCCTGG
GCTTTGTAGAAAG
>ontig_34_consensus
GCGTGCACCTCCTCCTTTGGATCTTCCTCCATCTGATCCTCCTTCTCAACTTCTTCAGGC
TTTGCTTCCTCTGGACTGGCCACCCCATTATTCTCTTCTGCTGAGTCAAGCTGCAACGAG
CGGATATCCTCCTCAATATCCATAGTGACGAGGCCTGAGGTAGCAAAATTAAAATTAGGG
TTTTCTGAATGAAAAGATGAAAGCAATAGAAGAGAAGAGAAAAGAGGCGTGTGGCAGCGT
CGTTGCGGAAGCTCGCCTTAGCCTTAGCGCATAGATCAAGGAGTAAAATGTGAAAAGTCA
AAATAGGGG
>ontig_35_consensus
CCTCCAGTAACAAAATTGGTTATGCGCTAGCACAAAGATCAGTCACCTTCCCAACTGCCA
CAGTTTTCCCTTCTGTGCGAAGAGTGAACCTTCCAAGCTGCGGAAACTCTGAGAACTTCT
CAATGCATATCAAGTTATTCACCTGAATACGGCATACAACAACAGCACCATTCTTCACAA
AGAGAACTTTCTTTTTCATGGGTTTCTTTGTCTTTGGATCAATTTGCTGTCGTAGCTCAA
CAATCTCACATTCCTCAACAATGGAATGAATGTGCAAGACTGCCTTATAGCCAGCAGTAA
AAATTGCATTGTCCAATAGCTCAAGGATCTGCAACTGGGCAACAAATTCAGTGACTGCAA
CTATTGGTTTGGCAACACTTGACAAGACAAAACCCATTAATATATCCTCTTCTTCAATCC
CACCCAATCTCAGCCGTAGATTTTCGCCAGGTCCTGCACATCTAACCTTATCTTCATCAC
AGTATACAGCAATAACTTTTACTTGAGCCTTATTTGGCATGACCAACAAGGACTCACCCT
CTCGCACACTACCGGATTCTACTTTTCCCATCACAACAGTTCCCATATCTTTGAATTTGT
CAATTATAGGCATCCTAAATGGACCTTTAGGATCTCGTGAAGGAACCTCAATGGTATCAA
GGGTTTCAAAGAGACAAGGACCATTCCACCATGGGCAGATGCTTTTTTCTACTCTTGTTT
TCATGTTTGAGCCAATCAAACCAGAGATTGGAAGAAACTGGACATCTTTCTTCACGTTGT
ACCCTGAGGACTTTAAGAATGGTATCAAAGATGACTCAATTTCATCATACCTCTCTTTTG
ACCAGTTCACAGTAGGATCATCCATTTTGTTCACAACAATGAGCAGCTTAGAGACACCCA
AGGTTTTTGCCAGCATCACATGCTCACGGGTTTGTCCACCTTTCTCATATCCAGTTTCGA
ATTCCCCTTTTCTAGCTGATATTACCAGTACGCCAATATCAGCTTGAGATGCTCCACTAA
TCATATTTGGAACATAACTCTTGTGACCAGGTGCATCCAAAATTGTAAACCTAGTTGTCT
CCGTTTCAAAGTGTGCTCTTCCAACTTCTACTGTTTTACCCTTGACTCTCTCCTCTTCAT
TAGTGTCCATAATATATGCCATATACCAGCTTTCCCTACTTTTTTCCTTTGCTTCTTTCT
CATACTTTTGGATTGTACGGTCATCAACCTGACCACTAAGGAATAGTGTCTGACCTCCAG
TTGTGGACTTACCAGCATCAACATGACCAATAAATACAACATTCAAGTGTCGCTTCTTAA
TTCCTTCAGCCTCCTCCAGTTCAACATCTTGTGGTGCAGATATTTCCTCATCTTGCACTT
TTGGCTCAGGATTAACCAGATGAGAAACCGTGCACACCTCCTCCTTTGGATCTTCCTCCA
TCTTAACATCCTTCTCAACTTCTTCAGGCTTTGCTTCTTCAGGATTGGGCAACCCATCAT
TCTCTTCTGCTGCTGAGTCAAGCTGTAAGGAGCGGATATCCTCCTCAATATCCATAGTGA
TGAGGAAATGAGGACGCAAAATTAGGGTACTCTGAATGAAAGGATGTATGCAAAAGAAGA
GAAAAGAGGCGGGCGGTAATGTTTGCTCTCAAATCTTTACCTTAACGGCTTAACCTCAGC
TTAGTGTACTATTTTGAGACTAAACTGTGGAAAATAAAAA
>ontig_36_consensus
AGTATTTACCTCTGCTTTACATTTTTTTTTTTGTAATTAAATGATATATAAGCAAAAAAC
AACAGATAAACCCGAGACAACTTAAACGCAACGAAAAAACTCACCCACACCCAAAAGCGA
GGGATTACAAAAAGTACAAATCCAAGCTTTTGAAGTATCTATTTACAAGCAAAAAGAACA
CTAATCTAAGCCTAACCAAATACAAAATCAAACACAAAGCTATTATCTTGTGTAATTATT
ATCCACAAGTTAACAAAAAATGCCCTAATCCTACTTGTGTCATTGCAAGCTATGAGCCCA
ATGGGGTTGATCAAATAAGCTGGTTTACAAACCCAAGAACAAGAAGATTCAGCTGAACGA
AGATCAGCTTGATACCGCGGCATAACCACTCCTTGCTTGCCCAACTCCTCACAACCGCTA

AGAGTAAGCACCCTCCAATTTGTTTTCCAATCTTAGTTGAGAACCACCCTTGGTTGACTC
CATTCCACCCAGGGTGAGTGTCCTTAGCTCCAGAATTAGTGCATGTAAAATCAGAAGTAA
CTTGGCCCCACAAAACAATAAGACAGCCGGCTTTGTACATGGTGAACCTGTGATTTTTCT
GAATATTCCACAGGCCAAAAACATTCTTTTTTCCTTGCAGCGTAGTCACCAAAATTAGCC
CAAATATAGCATGCAACATAATATTTTCAATGGTCAACAGCCTGCCCCAAAAGGCCTTCC
TAAATACATGCAAGACAACCTGGAAAGAGAGGAGAAACTGGAGTAGAACCTGGAAAATTT
TCCAGATTTTACTGTTTACAGAGCAGTACCGTAAACAGTAAAATATGGTTTTAACCAAGC
AAAAACCCTGACCAAAAGCGCTCGGATCCCATCGAAACAGCTCTCTGCAAGTGTCATTTG
CTCTAATCTTTGCAACCGAAGGCGTTCAAAGCTGATCGGAATTAAAATGCCACAAATAGA
TAGTAAAAACCCAAACATGAGGCCAAATGAAAAAAGGAGCAGATCCTTTAGTAATGCAAG
GCTGGATAAACCTTATCCTCCGTATCATTCCACTACCCTCGACACCAACTGTAGCTTTAA
CCATTTTAAATTTTGGAAATGATGTAACTAAAAGATAAACCCAAAATCCTGCAAGTTAGT
TAACACCGAGTGAAGAATAGAGTAACTCCTGCAAAATAGAAAATTAGTTAGCACAAGTAT
CCTCATCATGATTCTCTCTTTACCAACCCTCTTATCAAGGGACGTGTTAAAGAAAAAACA
ATCCCAAATCCCCTTGGCCCTAATGGTCTACCAAAACCCAACGTTTCTTAGATAGCAAAA
TCTCAGTACACTCACTCTTGGTATTATTGACCAAACAGTAACAAAACAAAGTTTTTTGTA
CAACCTAAACGAGTGTTGCCTTATTAGTATTTACCTCGGCTTTACATTATTGAACAAAGA
GTATTTACAAAATCCTCAGGATTAGACACAGAACTACTTTGAGGATTGATCATAACTTAG
AGGACGACTCTTGAAGACTGAAATTTCTCACACAAAAACCCTAAAGAACTATTCAACGTC
AATATATCTAACATTTTTTGGAGTCTGCTCCACGTTCTTCTTCGGCACCGTCACTGTCAA
CACGCCGTGATCCAGTGCACACTTGATCTCATTCAAGTTGACATTCTCCGGCAACCGGAA
ACGCCTTACGAAGCTGCCTCGCTGCCTTTCGACGCGGTGCCACTTGTCGTTTTCGTCCTT
CTGATTTTTCGTCCTCTCACCACTGATCTGAAGCACGTTGTTGTCTTCGACATGCACTTT
TACCTCCTCTTTCCTCACCCCTGGGATATCGGCATGAAATATGTGGGAGTCCTCAGTTTC
ACGCCAGTCGACATGAGCATGAGCCAGAGCGCTGGCTTCATCAGAACGCTGAAGTTGGCC
TCTACATGCGCCAGCGGTAAAAGGGTCCCATATTTCAGGGGTAAACCATGGATCATAGTC
GCCTCTGCGTCCTCCGAACCAGTGTCCCAATGAAGTCATATTTTTTTTTCTTAGGCAGAA
GGGAGAAATGACTAATGAGGTTTGGTTTTGTTTATCTATGGCTTCATAACTTGAGTCGTT
ACACTGTTTTGGAGGACGCA
>ontig_37_consensus
AGATCAGCATCACGCTGGTTAACGGCTTTTGATGATGGCCTCAAGGAATTTTCATCAATA
AAAGTGTAATTCTCATTTGCAGGATTCGTACCTAAGTACATGAAGAGATTGTTCTTGCTA
AGTCCAATATCACCATATTGCATGACATGAGAGCCAAAGATAGAATTGTCATTTGCAGTC
CTCCTTTTAACCAGTTCATACTGCTGGTACAAAGTTTCTGTCCTCAGGTTGTGTATGTCA
CTATCCTCCATCCAAGCAACACTATACAAGTCACCCAAACAGGTTTCATATTCAGGAGGA
GGGCTAGGATTCTCCCCAGGGCAATAGGTCCCCCAACTGCTCTCTTCTGCATTAGCTGCT
GTAGTTGCATATATATTCAAACCCTCAGGAAGAAGACCCTCAAAGATACTTCCAGACTCG
CAGGCTTCAAGATAAAATACCAAGCTTTTGTAGGTCCCAGAAGCATGCTTCTTCTTTAAG
ACATCAATCAAATCGTTTGCATACATAAATGGGCTGGTTGGCATGCCGAGCACCCCAGGA
CCCCCATGATCGGTGTAGTAGATGAAAATATGATCGTTTGGACCACTATCCACAACCTTC
CCGCTGCCCCCTGTAAGAGCAGTTTTGTCTCCAAGGATAACAGCAAAGAAGTTTCCAACA
GTAACATCTTCCCCAACATAATCCTTTGGAACTCCGTCGTAAACATCATCGCCATGCGGA
CTGTTAATGATTACTCCAGGCCTAGGGTTTTCCTCATTCCAAGCTATATCATCGTACATG
AAAACAATAATATTTTCCTCTTTCAAACCGCCTTTCCTCAAGAGTTGATAAGCATGACAA
ACGTCAGCCTGGTGCCT
>ontig_38_consensus
AGATCAGCATCACGCTGGTTAACAGCTTTTGATGATGGCCTCAAGGAATTTTCATCAATA
AAAGTGTAATTCTCATTTGCAGGATTTGTACCTAAGTACATGAAGAGATTGTTCTTGCTA
AGTCCAATATCACCATATTGCATGACATGAGAGCCAAAGACAGGATTGTCATTTGCAGTC
CTCCTTTTAACCAGTTCATACTGCTGGTACAAAGTTTCTGTCCTCAGGTTGTGTATGTCA
CTGTCCTCCATCCAAGCAACACTATACAAGTCACCCAAACAGGTTTCATATTCAGGAGGA
GGGCTAGGATTCTCCCCAGGGCAATAGGTCCCCCAACTGCTCTCTTCTGCATTAGCTGCT
GTGGTTGCATATATATTCAAACCCTCAGGAAGAAGACCCTCAAAGATACTTCCAGATTCG
CAGGCTTCAAGATAAAATACCAAGCTTTTGTAGGTCCCCGAAGCATGCTTCTTCTTTAAG
ACATCAATCAAATCGTTTGCATACATATATGGGCTGGTTGGCATGCCGAGTACCCCAGGA
CCCCCATGATCGGTGTAGTAGATGAAAATATGATCGTTTGGACCACTATCCACAACCTTC
CCGCTGCCCCCTGTAAGAGCAGTTTTGTCTCCAAGGATAACAGCAAAGAAGTTTCCAACA
GTAACATCTTCCCCAACATAATCCTTTGGAACTCCGTCGTAAACATCATCACCATGCGGA

CTGTTAATGATTACTCCAGGCCTAGGGTTTTCCCCATTCCAAGCTATATCATCGTACATG
AAAACAATAATATTTTCCTCTTTCAAACCGCCTTTCCTCAAGAGTTGATAAGCGTGACAA
ACGTCAGCCTGGTGCCT
>ontig_39_consensus
TCAAGTCTTTCGGCCTATTTCTAAAGCCTTCATCTATTTCGGAGTGGCTCTAATTTGATC
TGTGCCCGGTTAGAAAATGCCTACCACTGCCACTGGTAAAACATTTGGGCCCCTATCGTT
GCCAGCATAGAGATTTGGATATATATATACTTTCAAATAACAACTACTGTTCGGCAGAAA
AGTGCACCAACTTCATATTTACACCAAAGCAATCAAAATGCATTAGGGTATTAGTCTGTC
TATGCTGTCACACTGACACTATCACAGCCACGGACNNNNNNNNNCCTTTGTGAAGAGAAC
TCCAAGGATTTAGAGGAACACTATCACAAGCTTGGGCAGATGCTTCAGCCATCTTGTCCT
TGTCAATTCCTGCATTGCAAAGGTTCGCGAGGGAACGCATATGTTTCATTCCATATTGGG
ACAGGGATCCACAGTGACTTTCAAAAGTCCTCACCAGCGATTTGAGGCAGTCCCAGTCGT
CCACTAGTGATTGACCTGCCGGTTGTATAGTGTTCAAGACCTCTGGACCTTTTTCAATTC
CAAATAAGAGCTTCCCTATGAGTTTGATGCTGTGGTCTATATGCACTCTATGGGACATTG
CTTCAACAAACTGCTTCTGAGCTTCAACCTTCCTCAAAGAACCCTCTGGGGCCTTGCGGT
ACTTATACCAGAAGTGTACAAGATCAGCATCACGCTGGTTAACAGCTTTTGATGATGGTC
TCAAGGAATTGTCAGCGATAAAAGTGTAATTATCATTTGCAGGATTTGTACCCAAATACA
GGAAGAGATTATTCTTGCTAAGCCCAATATCACCATATTGCATGACATGAGAGCCAAAGG
CAGAATTGTCATTAGCAGTCCTTCTTTTAACCAGTTCATACTGCTGGTGCAAAGTTTCCA
TCTTCAGGTTGTGTATGTCACTGTCCTCCATCCAAGCAACACTATACAAGTCACCCAAAC
AGGTTTCGTATTCAGGAGGAGGGCTAGGATTCTCCCCGGGGCAATAGGTTCCCCAACTGC
TCTCTACTGAGTTAGCAGCTGTGGTTGCATAGATATTCAAACCCTCAGGAAGAAGACCCT
CAAAAATACTTCCAGATTCACAGGCTTCAAGATAAAATACCAAGCTTTTGTAGGTCCCAG
AAGCATGCTTCTTCTTTAAGACATCAATCAAATCGTTTGCATAAAGATATGGGTTGGTTG
GCATCCCCAGGACCCCAGGACCCCCATGATCTGAGTAGTATATGAAAATATGATCGTTTG
GACCGCTATCCACAACCTTCCCGCTGCCCCCTGTTAGAGCCGTTTTGTTACCAAGGATAA
CAGCAAAGAAGTTTCCAACAGTAACATCTTCCCCAACATAATCCTTTGGAACTCCGTCGT
AAACATCATCCCCATGCGGACTGTTAATTATTACTCCAGGCCTAGGGTTTCTTCATTCG
AAGCTATATCATCATACATGAAAACAATAATATTTTCCTCTTTTAAACCGCCTTTTCTCA
AGAGTTGGTAAGCATGACAAACATCAGCCTGGTGCCTGTAATTCCAGTAGCCATTCGATC
CTGCAATCAGCACCGCCCATCTCGTCCCTACAGTATCATCGACGCCGTTGACCGCACCGG
GACGGAAGAACCTCGATGCTTCCGAGGGTAATCGGAGAACCTTATCGGATATGTCTCTGG
TTCCGACGACAATTACGGAAAGGGCGGCAAGAACCGTGAACAACACGCCAGCTGTAGGGC
GCATCATAGTCAGGCGGAGCTAGAGAC
>ontig_40_consensus
GTCTTAGATTAAAATTTAAAATAGTAAATGCGTATAATAAGCTAATAAAAGACGGATTTA
CTGCCAGCGTTGCCAGCGGCAGCGAGAAACTGGACAACCGCAGGTGGAAAAGAATAAGAA
GGCGTGCAGAAAGAGAGATGAGGCAGCAGGGAGTGTTGGGGAAAGGGGTGGTGAGAAGTG
ATAGATAGGGATTGTGTGTAAGGCAATATTAAGCCATTTTTCTTCAGTTTGCATAGCCAT
ATCACTCTCAGAAACTCAGGCAGGCAGGCAGGTAGCTTTTATCTTTGTACAAGTATAGAA
AGTGGTCACCTGAAAGTTGAAGGAAGAGTAGAAAACCAATATCTGTCTTTGTTCGAGATT
CCTTGCTGCTGTTTTAGTGATCATCAGGATTGGGTGGGTCTTATAAATGGAGAAGTTGAT
GAAATTCTTTGCATGAGTAGGAAAAGTAGTTTTTTATTGGACAGAACTCATCAAGAGTTC
ATTCACATCGTAGTAGTAGTAGTAGTAGTAATATTAACTTAACTACATACAAGCTCTCTGCTC
TCTGCTCCCTGCTTTCTTCTCCCCTCTCACAGCAGAGACTATCCAAGCTCTCCTTTATTA
TTCATTACCTATCCCCCTTACAAAGCAAAACAAAAAACATAGTACGTACACCACATGCAT
TCGGCTTTAAATCACCACTAGCTCAGCTCAAATTACTTAAAATTTAAGCATCATCCCTCA
TGTGGTGGCTTGACTCTCCACTTGTCAAAGTTGTCTGCTGATTGTTGAACTTGATCTTCC
TAGCATCTTCTCTTGAGATCTGGAACGCATTGGCTAACACTTCCTCTGGCATGCCACCAA
GCACCGAGGTGCGTCCAGCGAGAGGACTCGTCATGGCCCGATCATTGGTCTTGAAAGAAA
TCCATTCGAATCTTTCCTCTCTTGCACGTTTCACTACTGCAAAGTTTTGTGGCACCACCA
ACATCTGTCCCTCGCGGACTTCGCCGTCGAACACTCTGTTGCCGAAGTTGTCTACTACTT
GAACCTGGCCTTTACCCTTGCACCCGTATATTATGCTGTGCGAGTTGAGGTTCCAGTGTG
GCAGCACTAGAGCATTTTTGTATAGCACACCCTTTTCAACACTGAGTTGAAGCCATTTGA
GGATTGGGAGGTTGAGGCTGTTGAGTGTGGTAAGACGACCGACTTCTGGGGTGTAAATGT
CAGCGCGAGCAGGATCATTGATATTCTCTTTGAGTCTCATAGTGCAAATGGTCTCTTCAA
TCCCATTGTCACGCTGTCCCCATCGGCGTTTTTCATCCTCACTTTCCTCTTCACTCTCAC
TTCCACGCTCGCTCTGACTCCTTGATGGGCGGATCACCCGAAGTTCGTCATCCTTCACCT

TAACAATGCCACCCCTGTTGTCCTCGCTTTTGAGCTGCTTTATGAGACGTTCGTCCACTT
GGAAAGCCTCAGCCAATAACTCTGTATCGAAGCCAGAAAAAAGGTTACGCCCGCGAGATT
GGTGTTGTTGCTGCTGCTGGAACACATCTTTTGGGTTACCAGCCAGATGGAATTTTCGTG
GGGTCCTATCAAGCTGATTTTGACTGTTTGAGACGTCTAGAAGAGTAACAGTGACGACCG
GGGAATTGCCCTCGTTGTAGCACCAGTGTGCTACTCCGGCGGGGATTGCGATGATATCGC
CTCGACGGAAGCGTCGAATCTTTTGATGCCGGTCCTGGAACCTACCACTCTGTCCCTGTT
GTCGTCCCTGTTGGGGCGCTTGGTAAGTTTCTGGGCATCCTGGATATGATATTCCTGTCA
TACCCTCACCCTGGACAACGTAAATAAGTTGAGGAGCATTAGAATATTGAGGCAAGAGAA
GGCCATTAGGTTGGATGGTATGCCTAACCAAGGCAACACCAGCGCATCGGAATTGCTCAT
GGTTAGGATCCCAGGCTTCCACCGTACCGGCTTCATACTCAACTCGGTTATCGGGTTCAA
GGGCATCCAGCCTATCGATTTGGCACTCATCTTGTTGTTGCCATTCCTGGCGAGAAGCTA
GGCAACCATGAAAGAGAATTAAAAAGCAAACAGAAAGAGAGAGCAGAGAAGTGTTAGCCA
TGGAGAAGATGAATTGATTGTGTCTGAGATGA
>ontig_41_consensus
CCAGCAAGAACATCAAAGTTCTTAAGCAAAACCTTTAAGAAGCTTCCGGCCCCAGATCCC
ATATTCAGTCTCAAGCGGTTTATTTCCTTATTTTTCTTGTTCTGATCTCCAAATTTGAGC
AGAAGTTTGTTTGTTTGTCCTGGTGAGAGAACGTTGGGTTGTATCGATACTTAAGAGCAA
CGGGCAGTGCAGACAAGCGCTCAGAAACATTAAAGTAGTGGTAAAGTAAAGATAACACCG
ATGGTACTGAAAAAAGAGCCAGTCATCACGTAAAATAAGGGGAAACTAATCTGAGTCCCA
>ontig_42_consensus
CTATATTACAAAAACATCTTTATACATTATTACGGCACCGTAATTTATACCTCGAAATAT
TACCAAATATGATACCAAGTAACGTTACCGTGGCGACGATGACCTGAAGCTATGGAGATG
AATAAGGATTCCAAAGAGTGCCAGTGAAGTCATTTTCCAGGGATGAGAGCATGGAGAAAT
AGTCTGAAGATTTTGTGAGACACTGTAATGAAAAAGAAGCCAATGCTTGTTCTTCATGAG
TTCCATCATAGCTTGAGGACTCCAGCAGACAAGGAGCAAGAATTGAGCATGCCATTAACA
ACAAATCTTCATCACCAAGAGAGTGCAGAAATTTGCTCAGCCATGCAACTGTGAAGAGGC
TTGCTCTTGCTAAACTTGGAATCCCTTTGCCTATGCAATCTGAAAGAGCTGAAAATAAAC
TCTTGTTGCCACTCTTCACCAGCACAATTGCAGCCACTTTTTGCCAAATTTCTGTTGCCT
CTTCCTCTTCATTCTTGTCATTCAAGGTTTCACTGAAGGCTATTCTCTTGCTGCAGAATG
AATCCTCTTTCTCATGAAAGCTTGCTTGCTTTAACAGCCAATTTTCTGGTGTTGCCTCGG
AAAAATAACCTCCCAACATCATGAGAGCTCTTGCTGATTGTTCTTGAACCTTTTCACCAC
AGGTTTGGCAATCCATTGCTGCTATAATTGTCTCAATTGCTTCCTCTCTGTAAATGCTGC
ACTTCAATGGATCCTCCAGAAGATCAAGCTGCAATAAAATTGCTGCAACCAGTGGCCGTT
CTTCTGGTGGAGCTCTTTGCAGATAAACTAGCAAAATGTGCATAGTGTTGAAGCCACCCC
ATCCTCTACTTAATCCGTTTAAAAATTTCATCATCTGGGTTCTTCTGCAAATGTAATATT
CTTTTAAATTTGCTATTTTTTTGAGCCTGATTATGGACCTGAAATGAATTTTTAGAAGGA
AATATTCATACCTTTGTAATGAG
>ontig_43_consensus
CTTGAAGTTCTCTGGTGTTGATGTATAATCTGGCATCCCAATTGTCATAAGGCCACAAAA
CTCGAGGTTTGGGCAGTTTTGACTAACATGTTGTGCAAGTTCCCCACACCCTGAGGGCTC
AACACCAGATTTTGATTCTTCTCCACTGGTATTAACTTGGATAAAGACTTTTAATGGCTT
TCTTCCCATGCTTGCAACCATACGATCAAGACGATCAGCAATCTTCTTGTCATCCACAGT
TTCTACCATCGCAAGGTTTGGCACACCAGTCAGTAAAGTTTTCACTTTATTGCTCTGTAA
ATTCCCGATAAAATGCCACTCTATATCATCTGGAAGCTGAGGAGCTTTCTCGACGAGTTC
TTGAACATAATTTTCACCGAACGATCGATGGCCGGCATCATAAACTTGACGGAGGAGAGG
TACCGGCTTGGTTTTGCTAACGGCGACGATTCGGATTCGGTCCGGCGCGCGAGCACACCG
ATCGGCTGCCTGGTGAACTCTTTGAATAACGGAACCGAGCGCCGCCGCGGCCAGGCCTTC
TGTGGCGGCTGAGGACGTGGCCATTGCTTCTTCGTTGATGTTAGTGTTGTCTCTTTCGTT
GCCTTCTCCAAGTTCTAGATTCTTCATTCACTCGTTTCAGTAACAGATTGTACCAAAACT
C
>ontig_44_consensus
CTTCATGTTGAATCAATGGAATTATGTAAGTCATTACATCATAAATTAAAAGGAAAGCAA
ACGGGTAATTCTAATTTAAACATATTCTCACCTGCAAAGGGGATTTGAAATAGATAGATT
ATCTCACGCCAAACAACAAATGAGCAGAGATCCATGAGGATGCTCGATTCTTGACTGCAA
AAAGAGCTGGAAACATATTACACCAAATCTTTCTGTTGACCAAATTTACTTGAATATTGT
TTGTACTAATGCATCAATCAGAAACCGAGAAAATTTTTCAGCAACCAATACATGTCACAG
AGCAGTTTCAATCTCAGAAGGACTTCTTTTCATATGAAACCAGCCCATGTATGCCACCTT
CAACTTGTGCAGCTCCTGTTCGGACCTCCAGCCTTAGTGTTCTTGACCTTAAAAGCTCGT

GACCTGATTGCAGAGAATAAGCACCAAGATCCTGAAACCCTTGCTTGACTGCTTGCATTG
TGTATGGTATAAACTTCAAAATTGAACCTTTGTCTGCAACAGCGCCAACAACCCCCTGAG
CAATCTTTAGCTTAGCTGTATCACCCAAGTATCTCGCATCACTCCCCTTAGTCATTGCTT
CAAGGGAACCCATGCCACGATATTTTTTTACCCGTAGGCCTTTCTGATTATACTGATAAG
TTCCAGGGGCCTCTGTACTTCCGGCTAAGAAGCTTCCCATCATTACAGTAGATGCCCCCA
GCATCAAAGCCTTAACAATATGTCCAGAGTTTGAAATACCACCATCCGCGATGACAGGTA
CACCATCTTGTGCAGAAATAGATGAAACTTTGTAAACAGCGGTTGCCTGTCCTCGCCCAA
CCGCACAAACCTCTTGTGTGGTACATATGGACCCAGACCCCATCCCTACTCTTAGCCCAT
CCACACCAGCCTGAATCAAATTCTGTGCCTGATACATTGTCACAACATTTCCACCAATCA
CATCCAACTCTGGATATGTCTTCTTTACATACTTGATCATCTCAATCTGATAACTTGAAT
TACCTTGAGAACTATCCAACACTATCACATTCACCCCAGCCTTCACCAAATGCTCTAGCC
TCTGCTTATCAGATTCCCTAGTTCCAATTGCTGCTCCAACCATCCACTTTCCATCAGGTC
CCACTGTGGCCTTTGCCAATCTTGGATACCCCTCTATTCTCTCCACATCCTCCTTCAGAA
CCACATCTAGCACCTCACCATTATTCTCCAAAACAACAAAATCAAGCTCATTTTTCTCCA
GAACTCCACTAATTTGACCCAAATCATATTCTGAGGGGACATAAACCTTAGAACTGCAAT
CACGCATATAATCAAAAACTTTTACTTTCTTATCATTCAAATTCTCCCAATCAGATTTGG
CCACGTAACCCAATATTTTGGATCTTGCAGTACCTGACTCAGTGACAAAAAGATGATTGT
AATCCCCAAACTCCGTTACGTCATCAATAAAGGCGTCCCGCGTCCTAAAAATCGCCGAAT
TTGATGAAAAGATCGGAACACGGCGCGACTTGGCTGAGGCAACGAGACTAGCCTGGTCGG
AGGGCGTGCAGTTGGAGTGAATAATACCGATGCCTCCAAGAGCAGCCATGGCGGCGGCCA
TGTAGTCCTCGGTGACAGTATCCATTGGCGAAGCAACACAGGGGAGGGAGAGGTCAATGT
TGCGAGTGAGACGAGTAGAGAGGGAGACAGCATCAGTTGGAAAGTCGATGTAGTGAGGAA
GGAAGATAACGTCATCGTAAGTATAGGAGTAGCCTTGGTTGAAAAGCTTCTCGGCTGAGA
AACCGTCTTCAATCGGAGCTGGTGAGAAGTCCATGACGGTGGCGGTTGTAGTGACGGAGA
CTTGAATTTAGGGTTTGACGCGAAAGGATGGTAAATATGGAGGGAAATGGACAAACGTCG
TGTTTGACTTTTAATACTAATCCCACCACCACGTGGCGGACAACG
>ontig_45_consensus
GCATCCTTCAGAAACCTTGCTCCAAATTACAAGTGGCTTAAATTTTATAAAATACAGTTT
TCACCAACATGGCTACTTTCGAAGTTTAATATTAAATTAATACAGAGATGTAGTGTTGGC
TATATTGGATGTATACTTGAATTGGCTGGGTCTGATTTGGGACATCCATGAGTTCAAAAG
AAAAAATAACAATAAAAGAGACTTTGATGAAGAAAGTAGTGAGCAAGGGCGCTGCAAAGT
TCCCAGCAAACAGATTTTACTTGCATGTACTCTCTCTCGCTCTCTCTTCTGGAGCAATTA
CACCCACAAGGCCTCCAAAACCTCAAGGGAAAAAATGAAATTACATAAGAGGTATTGGGA
TTCAAAATTGAAAACAAAAACAAAGAAGTTTCCCAGGCTCAAGCAAATGCCCAGGGGCTT
CTGCACCATCATAAGTTCATGACCTTCTTTAGTCAGGACTATAACCCAAAATTTTAAGCT
CCAACAAGTGATGCTCTGTACTGTTAATTGGGCAGCGATACGAAAATTGTCAAAACAAAC
ATCAAAATTTGGCAAGTAGCCCACTTGGTTTTGGTTCCAAAACCCTCTGAAACTGCTCCT
CTATTAGCTGAAAAAAATCATCAGGCCACCTCTGATATACAGAATTGGGATGGATACTCA
GATAACAGGGTTTTATAGTTAGTGCCTTGGTTAAGCAGATTACTCAATCTAAGTCCTTTA
AAGAACCTGAAGATTCCGATCCATTCGGAGATTCCTCTCTGTTTGCAGAAGCAGAAGCTG
AAGCTGAAGGGACCCCTTGGTGATGGCTCTGGCTCTCCTCATTACCAATGCTTCGAGGTT
TGCCCAATCTTGACCGAATATTGGTACCCAGGGGAAATGGCTCCGGATCACCAGCATTAG
GACCATCAGTATGAAAAAGGATAGGTCGGCAACGTTTATCCTCATTCATCAAGCTTGAAT
TCTGAAAATGAGCAATTAGAGCAGCTTTTCCCTGAATACGAGCATAAGCAAGAGACGCCA
CCTTTTCACTGTTGAACTTTTCCCATTTTTTGCCATTGAATGCCTCTTGAAGTCAATTGG
CAGATAAATAAAATCGTATGTTCCTTGGCACTGCTCATCAATTGCAGCCAAAAGCATCTT
CGAAGTATACTTGTTGGGAATGTTTTTTATCATCAGTGTAGTTCGGTTGTCTTCACCCCG
CAATATCCGGTCAATATCAAGCTCATACTGTTTCTTGTCAGCATGATTAGAATTAGTTTC
ATTTCTACGATATGAGAAGTTTCTCACACGCTCATTAGGAGAATCAAAAGAGGCTTGCAT
TGAAACCATTGGATTCCTTCCAGGAAAAAGGTGGCACATCTGCTGAGGAGAACGCAATCC
AACATTTTTAGTCATGTCAATGCAATTTCCACCAACATGAGACAAGATGTTGTGTGAAGC
AATATCCATAGGATGTGATGGAGAACGAACAGGGAAGCCAACACTTCCAAGAGAACCCAA
GTGGAAATTAGAAATTTCAGGAGACTCCCCTGCATAAATCTGTTGCCTGTCCCAGGGAGA
GGGATTAACTGCGGGTGCTGATCCAATATGGTGGTGTACAGGCGATGCTGCATTCAGCAT
CAAAGTAGGTCCTCCAGGAAATCCAGGCAAATGAGGAACACGACTGGCATGCAGACCATT
GAGAAATGATGGTGAATTTGGCCAAACCATGGGACTTGAAGGATGTTGTTGATGTGAGTT
GGAGTTGTTCCACACAAAAGGATTTCCATGAAGGGAGTAATTTCCATTTCCAGAAGATCC
AAAAACCCCTCCACTAGGCTCCATCAAGTGGTCATTTGAACTCACTCCACCAATATGCCT

ATTGTCAAGTCCTTCAGTAATATTCTGACCAACATTACCAGTAGAGTTAGGGGGGTTGTA
TGGAATACCATTGGCCAAAGTACCATACTCAGGTAAAGAATGAGGATGGAAATTTGGACG
TTGTTTATTTAACTTTATTTCACTCAAAGAGAGGTTGGCCTCATGAAGGTCAAAATTGTT
GCCAATAGAGGCCAATCTTGCTGGAGGGGGCAAGTTGTTTGGCACACCCGAGGTCTGGTG
AGATTCAATAAATGCACTTGCAGGCGACTGAAAAGCAGAATGTATAGCCTGGAGTGATCC
AGTATCCATGGAGATAGATGCAATTACTCCAGGTGAAACCATGTGTCCTGATGGCATGTC
ATCAAAAGTACTTTGGCAAAGATTTGGTTCATCTTGCTCCTGCTCTGACTGCAGCATGAA
ACGTCTTGCACCCCCAGGACGGCTTGCTTCAAGCTTGATTTGCTTCCCAGCAATGTCACT
CCTATTCAATGCACAAAGTGCAGCCTCTGCAGCTCTAACGTCATAAAACTCTATAAATTT
GTGATTATGCTTATTTGGAGTCTCACGGATTTCTCTAATTTCTCCATAAGCACCGAAAAT
TTGACGAAGCTCATCAGTTGAAACAGAGGAATCAAGATTGAAAACCACCAAAGTGCCCTG
GTTCGTATCTTTTTCTGATGGATTATCCTTGGGAATTGAATAGTGTATGTCAAGTTTCCT
ACGCCTCAAGGGCTTATTTTGAAGTGCTCTCATTGCATTGCGGGCTGCCCTTATATCATA
ATAAGAAATCATAACAAATCCGCGATGCTTGCAGGCTGTATAGATGGTTCGGATCTCCCC
ATATTGCTCAAAAAGAGCCTTTAGTTCAAAATCTTCCACATTGCTATTGATGTTTCTGAC
AAAAAGTGTTCTAGAAGGGTGTTCACCATATGGATGCTCACCAACAACAGAACCACTTGA
ACCCCCTTGACCAGCAACATCATAATTTTTTAGTCCTGCATATATTCGATCATCCCCTTC
TAATTCAATGCCTCCACTGCAGCTAAAAAGGTCAAAATCTTCTAAATCATCTCCAATATT
GGCATGAAAATCATGTCCCAGATCATCAATCACTCCAGAAAATAGATCATCTTCATCAGG
AAGGAGATTTCCAATGGTCTGTGCCTCAACTTCTTCCAAGGATTCAAATGGCTCTCCTTC
CCCATGGTGAGTAACAACTGTTTTGAAGGGCTGATTAGATAGAGCATCATTTACTGACAA
CTTCAATTTTCGACTAAAAATATCAGACAATGAGCTTGAGAACAGACTGCTTTCAGACTG
TATCCCTTTAATACCGTTTCTGTTTCCACTTAAACTATAACATGCTGGTTGCATGGACAA
ATTTGACCATGATTTTGGATGGTGATTCACAGAATTCCATGAACTCTCAGACAAATTTGC
AATTCCTTCCTCTCCACCAATGCCCAGTTTTTTCATATGGTCTCTGGCTAAAATTGACTG
TCGCATCTCCATCCAATCCACAGATGGGATCCCAATCGGCGAAAATTTTTCCAGCGGTGA
TGAAGCAACCAATTTGTTGCCAGACATAGCTACCATTCCATCTGTTCCTTGGTGATCAGA
CATGGTATTCGACTTCCAAAATCCAATCTGTCTCTCCGTAGGAATACAAATTTCCTCAAA
GAAGTGGGACGATGCAGATCCACCCCTTGTGTCCATTATTCCAAATGGCATCTTGTAACT
TAGATACTCGATTAAAAAAAAAAAAAAACCAACACAGAGCCGAAAACTCAAGCTTTCTCGC
TCACTAGCTCTGCCAATGCTTCATGACCTTCTCCTTTAACAGGTCTTCAAATTCAGCTTT
CTCTCTTAAAAATTCAGAAACTAAACGGAGCCATATAAGAGAGAAAGCAAAAGACAGAAG
AATCAACAACAATGGAGAGATGAGAGAGAGAAATAGATTTATAAAGGACAGAATGGAAGG
GATCTCAGCATCTTTTCTTCTTCTTTTCGCTTCATTACAAACTATAACTCACCACACAAG
AGAGAGAATATATTTCTCTCCTTTTGTTTTCTCTTACACATCAAATCATCAGAATTCACA
AATCATTTCTTAAAAAAAAAAAAAAACACTCACTCTCTCTCTCTTGAATGGAATCATTTGA
ACCGGGGATTAAAAGTTTCAAACCGGAGAGCAAATTCCCGGCATATTACCGGCGAGATTT
CGCTCGCCGGTCTCAATTACAGAGAAAACCAAGATCGGCAAACGCAAGAAGTTTGAGAGA
AGCAGAGAGAGAGAGAGAGAGAGAGAAAAAAGGGTGGGAAGAGTTTATTTCCCCGGT
>ontig_46_consensus
ATAGTTATGAAATAATAATAGATGATGTTTTTTAATATAATGAAGTTGGTGTGCTTCAAA
GTTTTATAATATTTTTTAGTATATTATATATTTGTATCCGTATGTGTGTGATGCTACTAC
ATATTAAAGATGCATAGGTAACTGATACAGGTGGATTTAGGAGATGGGGACTCGAAACAA
ACTAAAATTAAATCAGGATAATCCCTGTATACGTGGAAGCCGGAGAAAGAATGCAGGGAG
AAGTCAACATATTCTTTGGCTAAAGCCTTTTCTTTTTATTTAGTTTTGCTTAATGCAGTT
GGGAAGATGATGTCCGCTGCTCACATTTACATACATATATACGTTATCCATATATAACGT
TCACTAGTCAGTAGCTCAGCCTTTCTCAGACCCCAAACAACCTTCCCATTTCTTCTCATT
CATCAGCACGCCCTTCTTTTTCCTTTCGCCATTCCGGCCCCTGGAAGAAAAACTCTTCAT
CTTGTTTTCCAAACACTTTGTCCACTTCTTCTCCTTCCATTTTGAATGCCAACTCTTTCG
CTTCCTTCTCCATGACCTTTATAATGTTCTTCTTCCCCGCAAGTGTGTACCTTATGTTGC
CTTCTGCGTTTACTTCAAAGCACACGATTTCCAAGTTTTCATTTCCCGAAGCAACAGTCG
CGAAAGGGTGACCCGCCGGGACAATGAACACTGTGTCCTTTCTTATCCGTGCCCTCAATT
TCTTGTAACTTGGGTGTGAGCTTTTCGAAGAGGATAGATGAGGACACGCTATTTCAACGC
ATCCTTCTCCTGAAACAACAATGGCTATTTTCGTTGCCCGTGAGTTGTAGAATGGAACAG
ACATTCCTCCCTTGGTGATGTTCGCGTAGGAGACAACCATGTCAAGCTTTTCAAGCGGCG
GATAATCTATACGTTCAGCTTCAAAGAGTTGGCCGTATTTATTGGATTGAGAGGGATCCT
TTTTGAAAAGTTTGAATGATCCCGTTGATTCCTCTGTAAATGGCCAAATTTTAGGGCCTT
CGCCTCTCCGGCTCATAGCCCGAATTTGTTCTTTGGAGGCTTTCATGATAGTTCCTTGGT

CCTGTTTCTCGAAAAGTTTCTCAAGTGTGTCCTTTGAGGTCTTCAGTGCGGCTTCTAGTA
TTTCCCAGCTGAAAGCTCTGTAGAAAGACTCTGGATTTTCACCGCCTGGTCCATGAAACA
CTTCGAAATGCCCTGGAAGATTGACTGGTCGGAGGAATTGGACGAGGTAAAGCTTCTCGT
TTTCGTCGTTATTGGCGATATAAAAAGGAGTACCAGAACTAATGCTGACGATGTCTCCCT
GTCTGACATTAATGCTCTCTCGTTTGTTCTCAAGGATCTTGGTGATCGTTCCTCGTCCCC
AAGAAACGAAGAAAATACTGTCAGCATCCATGTGGCTTGGAACTACAAAAGCCTGAGGAT
TCGCAACGAGAACGGCTAGACGGTATTTCTCCAGGGCATGAAGAAGCTTCGATTTTTGAG
TGAACTTGGGAAGAAGAACAACTTTTCCTTGCTCAGTCTTGACTTTGGTGGTGAAATCTT
CGTCTTCGAATACATAGGGATTCTCATCTTCTTCCTCGGCTTCGTCTTCGTCTTCGTCTT
CATCGTCTTCTCTCTTGTAATTATGTTGTCCTCTCTCTTTCTTATACCTCTCCTGACACC
TAAAGCGGCATAGTTGCTTTTGTTGTCCTCCTTCTTGTCTCTCGCACTGCCTCATGCACT
GACTGAGATGCTTTTCAGCTGGTTCATGAGTGCTGGGTTCATCAACGCCACCAGTTCCCC
ATTCTTCTTCTTCCTCCTCATGCTCTCGTTCCCGTCCTTTCTTCTCTTTGTAGTACTTTT
CACACTCTTTCACACACTGCTCCTTCTGTTGCTCGTCATACTGCCTCTGGACTTTGCACT
GGTGCTTGCACTGTTTCAGCTCCGGGTCTATTTTAGCCAAAGCAAAACCTAAACACAGGA
CCAAAACAGAAACGAGAAAAAGAGAAAAAGAAAACTTTGTAGGCGGACCCA
>ontig_47_consensus
CTCACAGTCATTCTATTTATACATTTGGTAATGATAAAAGTTATATATAGACAACATAAA
ATCTAAATCATGATTTTAAATTGTTTATATAAACATTTGTTAGAAGTTGTTTAATCAACG
GTATTAACTGTGAAAAGTCGTTCTGTGTTGGTCACATTTTTCCAAGTAGCTGTCCTAAAA
ATTAAGAGGAACATACGCAAGACTTGAAGAAAAAAGATAGATAATTATAAACAAAGACCA
CGACATAAGTAGATCTGAAGTCTCCCACGGTGGAAAGACATGCGTACTTTATTTCATTAA
GAAAAACGTACAACCGGAACCAAACATGGCGTCTTATTACTTGCATCAGCAATTCTTGGT
GCAAAAACAGCGTCTGTGTATTCCTTGAGTTTTGCATCTGCCACCAGAGAAACCTTCATT
CCTGCACACTGAAGCGCAGTTGTGGCTGCTAATGCATGTTCCATGAAACGAATGGCTTTG
TGACTCACACATCCTGCCTTCTGATGGCACCACCATCACTTCAGAAATCAATACAATAAG
CAGCAACAAGGAAAGCCGTATTGATTTCATCTCCATATCGTTCACTTTTAAACTAACTGA
ATATAAGATCTAAGCTTGCATTGATCTGATATATACACACACACACACACACACACACAC
AGACATGATATTAATCACGGGCATCGAGGGAGTGCATGCATGTCCAGGGTTCCCATATAT
TTGACACGCAAGCTG
>ontig_48_consensus
CGCACGATTTTTCATTTCTATGGGTTCCTTCTTCCAAACCAGAATCGGTATTAAAAGAAT
ATATCTGAACGCAATCTAACTGGTTTCACCAAAATGTTTATTCAAATATAAATAACCAGG
GACTACTGTCCAGATGCGATTAGGGCGACGGCCCTTCTTCTGAGACTACTTTTTGTCGGC
CTTTCCACCAATTCATCACCTATCTAACTCAGTACTCTAGATTTGAACCAACTCCACCCC
TATCTAGTTTGAATTTGTTCTTGCATCCAACAAATATGGAACAATCAAACAGCATCAACT
AAAGAAGAGAAAACCCCATTTCCAATCTCTTCTCCATGCCAGCATAATAAAAACAAGTTT
GAGTAATGCATGAAACGGTTCTAGCTTCTGTTAAACATGCAGGGCATTTTCCCACATTTT
ACCTGATCCTAAAAGAAATTCTAATACACTGATTTGTAAATAAGATAATGTTAATGCTAG
TCCTCATAGCAAAAACAACGATAATAAGAGATATAAGCATATGAAGCAGAGAGTCAGAAG
CAAAGTCACAACTTCTTGTTCATTCTCGTCCACTGTCTTCATCTGGCCCGCCAGCTTCTT
TAACATCGACAGTGAAACCATATTCTTGGTCACAACCCATATATGAATCACACATAAAAT
ACAGAGTATATGATTTCTTCCCAGCATCTGTAGGAGCAGCAAAATCAAGCTTGACCCTTG
ACTTCCTTTGGAGCGACACTCTCTTAATGGCAAGCAACTGGTTAGTCTTGGCATCGCCAA
CAACGAGCCACCAGCCTTCCTCTTTGGCTTTAGGGTATCTAGGAGCATCAACAGGTCCCA
CCTCTGTCCTCCCCTCCACATCCCGTTCAAGGGTGACTTGCAATGTAATGTCTTCTCCAG
CCCTTACATTATCACCATCCAGCACCTCGTAAGACATGTCTATATTGGGGAAACGATTGC
AAAACCTTGCAATATCAGTTAACTGTGAATCGGACATTTGCAGAAGCTCACGCCTCTCAT
CATCCTCCATCTCCACCAAATCAAACACTGTCTCTATGCTCTTTCCAGGATTCTCTAGAC
ATCTTTTCGCCAACTCTTTTGTAAAATGTGGGAGCTGGAGAAGCATGGAATCACGTTCCC
ACATGCCCTGAGTTACCATTTGGCTCACTTCCATTGCAAGGAGTGCAAGGCTCAACCAAC
CATTACTTGAAATAACATCAACCATAGCCTGAAGCAATCTACTAGCAGAAAGGAGAACCT
CCCGCTGGTCCAATGCAAGGTTCCCACCCACTGATTGCCTCGAGAAATGTGCTTGAAGAA
GAGCATTCGCCTTAACGTGTGGATCCGTGCATCTTGGATTCTCAAATGAAAATCTCTGGT
GATTAATCAGCTTCCGAACCGCCTCTTCCTCCCCAGGTCTTATAGGTAGTTGTGCATACT
CTGAAGCTGAAGCAAGAATCTCCAGAAGACCCTTCATCTTTGTCTTTGGAGCCAAAGAAG
AACTGAACCGTTCAATAGTAGTGTAACTAATGTAATAGTAAGAAGCAATCATACCATGAT
TTGAAGGAGAAAGGTCCATGTCATCTTCTATAATAATGCACTTGCTTGCTTCCAGATCAC

TTAATGTGTTCTCAACCAGCTCTGAGAGGTGATCAGAGAGATGTCTGTGACTGACACCCT
GGAGATTGTAGTAGTTTGGATTTTGAGTAAGCCTTCTGTACATGAATGTCCAAGTGAGAT
AATCCACAGCATCTTGCTTGTTCTCAATAACTCCTGCAACAACTTCTGCATTGAAATTAT
CGTGTAGGAAATGATGCAAATGACTTTCAACAGGAAAAGCTTCATACAAGAACTTCTTGT
AGTATTCTTTACGAGGGGCATGACAAAGGATGACACATTTCCCAGAATTATCCAGCAACG
GTCGACTGGCATGACCCATCATCTGCAATAGATCTGTTACAGGATAGTCCGTGTGAGCAT
TTTCCTGCCCATCATAATACTGTGTTCCCATCACAACCACCAAATGTGCTGTCAATGGCA
TTCCCCAACAGAGCAAACTACTCATAACACAAACCTGAATGAATCCACCTTTAAATAGCT
CTGAAACAACTTCTTGGTCATAACTGTTTAGACCCTCATGCAAGTACCCCACTCCTTGAC
GTAAAGTGTCTCTCAACATCTCTTCACTGATACCACTGAGATATTCCTCAAAGTTTTCAT
GGGATCGCAATAGAAAAGCTGAATTCTGATCAGTGTCCACACTTGAATATGTCATCAGAT
CAACTGCAGTGAGTCGGACATATTTTCTTGAAGGGACAAATACAAGTGCAGGCTTTCCAT
TCTTAGCATGTTGAACAATTGCAGTATATGTAGGTTTTGTCATAGCTTGCATCCTTGCAT
CAAAATTGGCTATATCCACTCCCTGAATATGTATTTCCAGAGGCACAGGACGAACGCCAG
GAGGAAAATTAAAAAGACCATGCGAGGTAGCCCCTATCCATTCCCCAAGATCCTTGGCAT
TTGCAAGAGAAGTAGACAGAGCAACAAGACGGATCTTGTTCTCGACCTGGCTAGCAATAT
ACCTCATCCGAGAAACAATCACCTCTAAGACAGGACCACCTTGACCCCCAATCAGGTGGA
GTTCATCAACAATGAAAAGACTAACCTGCTGCACATACTTCCTCTGTTTCCAGCGGCGAG
ATAAAGCATCCCATTTCTCTGGGGTACTGATAATTATTTGACCTTTCTCAAGCAGTTTCA
GATCCATAGCAGTTTCCCCAGTTAATTCAACCACCCGCATTCCAAGAAGGCGGCCAAACT
TCTCCTCCCAATCACGTAAGCGTTCTTTAGCAAGAGCTGCAATGGGCGCTATATATACAG
CACGCAAGACACTATCAGATGCTTTTTGATGATTCCTCAAAATGGCAAACTCTGCACAAA
TTGTCTTCCCGCTCCCTGTTGGAGCAGCAACTAAGACATTATCATCTGTATTATACAACA
CAGTAAACACCTGAGTCTGAACAGGATTAAAATGCTTAAAATCTTTATAAAGATTTTCAT
ATGATGGATTGCGCAATGCTGTCACCGGGAGAGGCTGTAAGTCTAAGAGTTCTGTAGGTG
GAGGATACTTTTCAGGCAAAATAAGGTGTCTGAACGATACAGGTAAAACAGTTTGTGAGC
CCAGCCACTTATCTGACACAACACGTATAAAATACTGTGGTGGCAATGGTTCATAGATTG
GCACAGTGAAATTCAATGCGTGGTCCTCATCTATGTACTGCTTCTTCAACATAAAATATT
CATGATGAAGAATGTATTCACCATCATTATCCTCCACAATTACCCAAAATGGCTCCACAT
ATCCATGGACCCTGTCATCCCACTGGAAGTCTGGTGTTATTGTAAGTTCAACCCTTAAAA
TCGTGCGAGTTATTGGTTGAACATGTGCTGCAAGGTTTAGTTTTGGGAATTGATGAACAA
ATCTGTGAAGTGTTCTACCCATCTTTGAGTAACGAATGAGCTCCCCAAGCTCTGCAGCTG
AGAGATCATAATACCTCTCCCATGAAATATCTTTCTTCTCCAACTTCATTAAAATTTCAT
TTGGGATTCCATTGAACTGGCGGAGAGGTGTTTGGACATTCCACATCCGTTTGGTAACCA
TTTTGCTCAAGTTCAAAGCTTTCTCTGCTATTTGTGCCCAACCCCGTTTCAGCACAATCT
CAAAAAGAGCTCTCATAAGACGTCCAGCACTCTGAGTTATGAACACCATGTCAGAAGTTA
ATGAAAGCCCTTCAAGCTTTAGCTGAGAAATATATGCCTGCAGCAAAACATTAATCTTGG
CACTAGGCTCTTCCAGGCTTTCCTTTATAGGAATGGGAACACGGTCCAACAGCTTGGCTA
GCTCCATCTTCTCATCTTGCCTAACTGTGACGTATTTGAATTCTTCACTAAGAGAAAATA
GCCGGCAAAGCTCAATATCGCCCATGGTTGGCTTCAAATGTTCATTATATGTGGCTATAG
TCCCATGAGTTATATAGTAATAGCTTGCAATGCGGCCCAAGTCTGTAACCTGGAAATATC
CACTTTTCCTATCATACTTGATCAAGTTATTTCTGTCCAAGATGGTGGCTGCAGAATGAA
TCAAATCAGCTCTTCTCTCCCCCAATGTTATATCTTCTTTCAAAACATCAGGCGACAAAC
CATAAAGCAAAGGATTTCGTAACATACGAATGTACAGGTAAGTGTATCCAATCCAATTGC
AGGCCTCTTTAGCATTTTGGACAGTGCCCAGGACAATTTCAGCATTTAGCTGATCAGCTA
GTTTGGACACAAACTGACTTTCGATAGGGAGTTGCTGATTCATTAAAGAGAGATAGTATT
GAAGTTCACTATGACCAGTAATGATAATCCCTTCCCCATATGAATCATATTGAGGCCTTC
CAGCACGACCAAGCATCTGCATAATATCCAGAGGACTAAGTTCAGTCCATGCCCCTTTTT
CTGGATTGTAAATCTGGGTTCCCTTAATGATCACTGTATGAGCAGGCAAATTCACACCCC
AAGCAAGAGTTGCAGTGGAAACCAACACCTGAACATGTCCATCACCAAAAAGATCCTCAA
CAAGTTGACGATCACCCCTTGTCATTCCAGCATGATGAATTGCAAAACCATACGGCAAAA
GGTCTTTAAGATCATTGCTCTTGACCATATCAGTGTGACTTTGGAGAATCTCACGGCTGA
CACTGTCCTCTTTCAAGAATCTACCAAGGGTATCGTTCTCAAGTGCTGCATCTCGTATTG
CACGAGCTGTTTTTGCTGTTTCCTTCCTGGAATGGACGAAAATAAGAACTTGATGCTTTC
CAGCAACTGCCATAACCTTTTCATAGCACAAATCATTCATCAACTGGAACCTTTGCAGAG
GCTTCTTGACCGTGATCCCAATATACTGCTGAGAAAGAGGGACAGGTCTATAACTATTGT
CAAAGTGGAACAAGCCTTTTCCCAAATCCACTCGTAAAAATAAAGCCACATCTTCATAAT
TTGGGAGTGTAGCTGACAACCCAACCAATCGAATATGCTCTTTTGTAGTTTCAATTTGCC

TAACAGTTCTAGCAACAATGCTTTCAAGCACAGGACCTCTATTGTCATGAAGCAAATGAA
TTTCATCAATAATAAGAAGCTTCACAAGCTGTGTATAGGTCCGATCACCTGATTTTCTGG
TAATGATATCCCACTTCTCAGGAGTGGTCACAATTATTTGTGTTTCTTCAATTTGTTGGC
GAGTGAGTGTCTGGTCTCCACTTAGCTCCCTCACCTTGACATCATATACCTGCAAACGAT
TAGAAAGATTACCGACGACTTCAGCAACAAGAGCTTTCATTGGTGCCACATAGACAATCT
TATAGTTGCCATGGTTGAAAGAACCATCTGGGTTCCGATTCAATGCTAACTGCTGAAGTA
TGGTAAGCACAGCAACATTAGTTTTCCCAGCCCCAGTAGGAGCACACAACAGTATATTAT
CTGCTGAGAAGAGGGCAGTCTCATACACTCTACTCTGCACCCTGTTCAACTGGGTCATTC
CTTTGAAAGCTGGGTGTGCCCACTCTGGCATTTCGGTTATCTTTATCAACTTCTCATTGG
GATCCAGAGGTTTTGGTTTCAATGCTGGCACATGAACTTCTTCATATCCCTTGCTAAGAT
GTCTATATGACCCAGTCGGAAGATCGCACTTTTTATTTGCCATCAAAAGACCACCCTGTT
CAAAAGCAAGGCTGTCTAGATCAAGTAACTGGCGCTGACCCTGCAACCATCCACCATCTG
CTTCTCTATCAACAAGACCCCTCCTATCCCTGTTGCCATCTCCACTGCTCTCATCCTTTA
GCCTGCGAGCCTCTTCTCTAATACTCTTCTCCAGATTCTTCTGCCTTTCCTTGGCAGTTG
CCCTTGTTGCATGCAACTGCTCCAAAATTGCAATCAAATCAGGACCCGCACCCATCATTT
CCACCTCAATCTTCTTCCTCTCTTCTTGGTCTTCAGACCTCGCCAAACGAGTACACCACA
CAATCTTCAGGCGGTTATGCAATAAAAACTTAATGAGACTAAATTTCTCAAACTGAAGAT
GCACAAGCAGATTAGTCTCAACTTCCCTGTCATCCCCCTCAGCAAGTATCTTCAGAACCT
CTTCCGCAAGTTTTTGACACTGTTGTGGGTCAATTTGTTGCTCATAAGCTTGAGAGATTT
TCCTTTGAAGCCAATATGCATCAATATCCTGGACATTAAGACTCATGCCCTCATTTGCAT
CATGTGAGTCATCATCGTCAATCCCACCACCCATTTGCATAGCTGCAGAACCATTGGGCT
CAGCCACATCATCATCATCCTCTTCATCCTCCTGAACCATATCCAGATCACTCTCTTCCT
CCTCATCATCATTCTCCTCGAATTCAACAGCTACACCCATATCATCATCAAGCGCCTCAT
CACCATTAGCAGTGGAAACACCAGCATCACCCCCATCTTGATAATCAGTAATAAGCCTTC
CAATGGAAACAAGCTGATCAAAGACATGATTTGGTATGGGGTTCAACAACTTCTCAATAT
CCTTCTTCTTATTAGGGTTTTTGACAGTGTCATTCTTAAGAACAGCCAAAATCTCATCAG
CAGCAGCACTAACAATATTAAGAGGTTGACCACCCAACTGTTGTTGAATGACACTGAGCA
TAGCCTCATAGGCAGCTCTGGTTTCCTTAGTCTTGGGCTGGTAAAGCCCTTCCTCAGTGG
ATGTCAGAACGCTTTCTTGACGAAGACGACGTCTCTTGCTCTGCCGAGGAACAGGCTCAG
AAAGTGGCTCACGCTCCTTCTTCTTCTTAGATTTCTTTAATTTCTCATCTAACTCGGGGG
GTCTGCCCCTGTAAGCACGGTCACCAAAGCTTCTGGGATCAATCTTACCCCAGAGAGACT
CAGGCTCGCCAGTGGGCTCGTGAGTATCACGTGGGCGAGAATCCGTCGTTAGTACAAGAC
TCGAGTTTGCTCTATACTCATATTGCTTGAATCGGGCGTGTGCTTCCGCACCACCACCGA
GATGTGCCATATCGGAAGTAGATTTCAAATCAATTCAAACCGGTTAGAGAAAGACTGACA
AACCGCCTGAGATTATAAGCCCAATACGTCGTAGCGGCAGCTTCACGTTCACTCACCGCC
CGAGTCCAGAGAATTTTGTTTTGCATCCAAGGACTCCTAAACCGCCTTAAAACTCCAAAG
CGGCGGTCAGATAATAAAAATGAAACGACG
>ontig_49_consensus
GGGCTTTACCAGCCCAAGACCAAGGAAACCAGAGCTGCCTATGAGGCTATGCTCAGTGTC
ATTCAACAACAGTTGGGTGGTCAGCCTCTTAATATTGTTAGTGCTGCTGCTGATGAGATT
TTGGCTGTTCTTAAAAATGATGGTGTTAAAAACCCTGATAAGAAGAAGGATATTGAGAAG
TTGTTGAACCCAATACCAAATCATGTCTTTGATCAGCTTGTTTCAACTGGAAGGCTTATT
ACTGATTATCAAGATGGGGGTGATGCTGGGGCTCCCGCTGCTAGTGGCGATGAGGCACTT
GATGATGATATGGGTGTGGCTGTTGAATTTGAAGAGAATGATGATGAAGAGGAAGAGAGT
GATCTGGATATGGTTCAGGAGGATGAAGAGGATGATGATGATGTGGCTGAGCCCAATGGT
TCTGCGGCTATGCAAATGGGTGGAGAGATTGATGATGATGACTCACAGGATGCCAATGAG
GGCATGGGTCTTAATGTCCAGGATATAGATGCTTATTGGCTTCAAAGGAAAATCTCTCAA
GCTTATGAACAACAAATTGACCCACAGCAGTGTCAGAAGCTTGCAGAAGAGGTTCTGAAG
ATACTTGCTGAGGGAGATGACCGGGAAGTTGAGACTAATCTACTGGTGCATCTTCAGTTT
GAGAAATTCAGCCTCATTAAGTTTTTATTGCATAACCGTCTAAAGATTGTGTGGTGTACT
CGGTTAGCGCGGTCTGAAGACCAAGAGGAGAGGAAGAAGATTGAGGAGGAAATGATCGGC
TTGGGTCCTGATCTGATTGCAATTTTGGAGCAGTTGCATGCAACAAGGGCAAGTGCCAAG
GAAAGGCAGAAGAATCTGGAGAAGAGTATTAGAGAAGAGGCTCGCAGACTGAAGGATGAG
AGTGGTGGAGATATTAACAGGGATAGGAGGGGTCATGTTGATAGAGATGCAGATGGTGGC
TGGTTGCAGGGTCAGCGCCAGTTACTTGATCTAGATAGCCTTGCCTTTGAACAGGGTGGT
CTTTTGATGGCAAATAAAAAGTGTGATCTTCCTACAGGGTCATATAGACATCTTAGCAAG
GGATATGAAGAAGTTCATGTGCCAGCATTGAAACCAAAACCACTGGATCCCAATGAGAAG
TTGATAAAGGTAACTGAAATGCCGGAGTGGGCACATCCAGCTTTCAAAGGAATGACACAG

TTGAACAGAGTGCAAAGTAGAGTGTATGAGACTGCCCTTTTTTCAGCAGATAATATACTG
TTGTGTGCTCCTACTGGGGCTGGGAAAACTAATGTTGCCGTGCTTACCATACTTCAGCAG
TTAGCATTGAATCGGAACCCAGATGGTTCGTTCAACCATAGCAACTATAAGATTGTCTAT
GTGGCACCGATGAAAGCTCTTGTTGCTGAAGTTGTTGGTAATCTTTCTAATCGTTTGCAG
GTATATGATGTCAAGGTGAGGGAGCTAAGTGGAGATCAGACTCTCACTCGGCAACAAATT
GAAGAAACACAAATAATTGTGACTACTCCTGAAAAGTGGGATATCATTACCAGAAAATCA
GGGGATCGAACCTACACGCAACTTGTGAAACTGCTGATCATTGATGAAATTCATTTGCTT
CATGATAATAGGGGGCCTGTGCTTGAAAGTATCGTTGCTAGAACTGTTAGGCAAATTGAA
ACTTCAAAAGAGCATATTCGGTTGGTTGGGTTGTCTGCTACACTCCCCAATTATGAAGAT
GTTGCTTTATTTTTACGAGTGGATTTGGGAAAAGGGTTGTTCCACTTTGACAATAGTTAT
AGACCTGTCCCTCTTTCTCAACAGTATATTGGGATCACAGTCAAGAAGCCACTGCAAAGG
TTCCAGTTGATGAATGATTTGTGTTACGAAAAGGTTATGGCAGTTGCTGGAAAGCATCAA
GTTCTTATTTTCGTCCACTCCAGGAAGGAAACAGCAAAAACAGCTCGTGCAATACGAGAT
GCTGCACTTGAGAATGATAGCCTTGGTAGATTCTTAAAGGAGGACAGTGTCAGCCGGGAG
ATTCTCCAAAGTCACACAGATATGGTCAAAAGCAACGATCTTAAAGACCTTTTGCCTTAT
GGTTTTGCAATTCATCATGCTGGGATGACTAGGGGTGACCGTCAACTTGTTGAGGATCTT
TTTGGTGATGGGCATGTGCAGGTGTTGGTTTCCACTGCAACTCTTGCTTGGGGAGTGAAT
TTGCCTGCTCATACAGTGATCATTAAGGGAACACAGATTTACAATCCAGAGAAAGGAGCA
TGGACTGAGCTTAGTCCTCTGGATATTATGCAGATGCTTGGTCGTGCTGGAAGGCCTCAG
TATGATTCGTATGGAGAAGGGATTATCATTACTGGTCATAGTGAACTTCAATATTATCTT
TCTTTAATGAATCAGCAGCTTCCTATCGAAAGTCAGTTTGTGTCGAAACTAGCTGATCAG
CTAAATGCTGAAATTGTTCTTGGGACTGTCCAAAATGCTAAAGAAGCCTGCAATTGGATT
GGATACACTTACCTCTACATTCGTATGTTAAGAAATCCTCTGCTTTATGGTTTATCCCCT
GGGGTTCTAAAAGAAGATATAACATTGGGGGAGAGGAGAGCTGATTTGATTCATTCTGCT
GCCACCATCTTGGACAGAAGTAACTTGATCAAGTATGATAGGAAAAGCGGATATTTCCAG
GTTACAGACTTGGGTCGCATTGCAAGTTATTATTATATAACTCATGGGACAATAGCTACA
TATAATGAGCATTTGAAGCCAACAATGGGTGATATTGAGCTTTGCCGGCTATTTTCTCTT
AGTGAAGAATTCAAATATGTTACAGTTAGGCAAGATGAGAAGATGGAGCTGGCTAAGCTG
TTGGACCGTGTTCCCATTCCCATAAAGGAAAGCCTGGAAGAGCCTAGCGCCAAGATTAAT
GTTTTGCTCCAGGCATACATTTCTCAGTTAAAGCTTGAAGGGCTTTCATTGACTTCTGAC
ATGGTGTTCATAACACAGAGTGCGGGACGTCTTATGAGAGCTCTTTTCGAGATTGTATTG
AAACGAGGTTGGGCACAACTAGCAGAGAAAGCTTTGAACTTGAGCAAAATGGTTACTAAA
CGGATGTGGAATGTCCAAACACCTCTTCGGCAGTTCAATGGAATCCCAAATGAAATATTA
ATGAAATTGGAGAAGAAAGATTTCGCATGGGAAAGGTATTATGATCTCTCACCTGCAGAG
CTTGGGGAGCTCATACGTTACTCAAAGATGGGTAGAACACTTCACAGATTTGTTCACCAA
TTCCCAAAACTAAACCTTGCAGCACAGGTTCAGCCAATAACTCGCTCGGTTTTAAGGGTT
GAACTAACAATAACGCCTGACTTCCAGTGGGATGAAAAGGTCCATGGATATGTGGAGCCG
TTTTGGGTAATTGTAGAGGATAACGATGGTGAATACATTCTTCATCATGAGTATTTCATG
CTGAAGAAGCAGTACATAGATGAGGACCACACGCTGAATTTCACTGTGCCTATCTATGAA
CCATTGCCGCCACAGTATTTTATACGTGTTGTGTCAGATAAGTGGCTGGGTTCACAAACA
GTTTTGCCTGTCTCTTTCAGGCACCTTATTTTGCCAGAGAAGTATCCTCCACCTACAGAG
CTCTTGGACTTGCAGCCTCTCCCCGTGACAGCATTGCGCAATCTAGCATATGAAAGCCTT
TATAAGGATTTTAAGCATTTTAATCCTGTCCAGACTCAGGTCTTTACCGTGTTGTATAAT
ACAGATGATAATGTCTTAGTTGCGGCACCAACAGGGAGCGGGAAGACCATATGTGCAGAA
TTTGCCATTTTAAGGAATCATCAAATAGCATCAGATAGTGTCATGCGTGCTGTATATATA
GCACCCATTGCAGCTCTTGCTAAAGAACGCTTCCGTGATTGGGAGGAGAAGTTTGGCCAT
CATCTTGGAATGCGAGTTGTTGAGTTAACTGGGGAAACAGCAATGGATCTGAAACTGCTT
GAGAAAGGGCAAATAATTATTAGTACTCCAGAGAAATGGGATGCTTTATCCCGCCGCTGG
AAGCAGAGGAAGTATGTGCAGCAGATTAGTCTTTTCATCGTTGATGAGCTCCACCTGATT
GGGGGGCAAGGTGGTCCTGTCTTGGAGGTGATTGTTTCTCGAATGAGATACATTGCCAGC
CAGGTTGAGAACAAGATCCGTCTTGTGGCTTTGTCTACTTCTCTTGCAAATGCCAAGGAT
CTTGGAGAATGGATAGGAGCTACCTCTCATGGCCTTTTCAATTTTCCTCCTGGGGTTCGT
CCTGTGCCTCTGGAAATACATATTCAGGGGGTGGATATAGCCAATTTTGATGCAAGGATG
CAGGCTATGACAAAACCGACGTACACTGCAATTGTTCAATATGCCAAGAATGGAAAGCCT
GCACTTGTATTTGTCCCTTCAAGAAAATATGTCCGACTCACTGCTGTTGATCTGATGACA
TATACAAGTATGGACAGTGATCAGAATTCAGCTTTTCTATTGCGGTCCCATGAAAACTTT
GAGGAATATCTTAGTGGTATCAGTGAAGAGATGTTGAAAGCCACTTTACCTCATGGAGTG
GGTTACTTGCATGAGGGTTTAAGCAGTTATGATCAAGAAGTTGTTTCAGAGCTATTCAAA

GGTGGATTCATTCAGGTTTGTGTTATGAGTAGTTCCCTCTGTTGGGGAGTGCCCTTGACA
GCACATTTGGTGGTTGTGATGGGAACTCAGTATTATGATGGGCAGGAAAATGCTCATACG
GACTATCCTGTTACAGATCTATTGCAGATGATGGGTCATGCCAGTCGACCTTTGCTGGAT
AATTCTGGGAAATGTGTCATCTTATGTCATGCTCCTCGTAAAGAATACTACAAGAAGTTC
TTGTATGAAGCTTTTCCAGTCGAAAGTCATTTGCATCATTTCCTACATGATAATTTCAAT
GCAGAAGTTGTTTCGGGAGTTATTGAGAACAAGCAAGATGCAGTGGATTATCTCACTTGG
ACGTTCATGTACAGAAGGCTCACTCAAAATCCAAATTACTACAATCTCCAGGGTGTAAGT
CACAGGCATCTCTCTGATCACCTCTCAGAGCTAGTTGAAAACACATTAAGTGATCTGGAA
GCAAGCAAGTGCATCATTATAGAAGATGACATGGACCTTTCTCCTTCAAATCATGGCATG
ATTGCTTCTTACTATTACATCAGTTATACTACTATTGAACGGTTCAGTTCTTCTTTGACT
CCGAAGACAAGGATGAAGGGTCTTCTGGAGATTCTTGCTTCAGCTTCAGAGTATGCACAA
CTACCTATACGTCCTGGGGAGGAAGAGGTTGTTCGCAGGTTAATTAATCATCAGAGATTT
TCATTTGAGAATCCAAGATGCACTGATCCACATGTAAAGGCAAATGCTCTTCTTCAAGCA
CACTTCTCTAGGCAATCTGTGGGTGGGAACCTTGCATTGGACCAGCGGGAGGTACTGCTT
TCTGCTAGTCGCTTGCTCCAGGCAATGGTTGATGTTATTTCAAGTAATGGTTGGTTGAGC
CTTGCACTCCTTGCAATGGAAGTGGGTCAAATGGTGACTCAAGGCATGTGGGAACGCGAT
TCCATGCTTCTCCAGCTCCCACATTTTACAAAAGATTTGGCGAAGAAATGCCAAGAGAAT
CCTGGAAAGAGCATAGAGACAGTGTTTGATTTGGTGGAGATGGAGGATGATGAGAGGCGT
GAGCTCCTGCAGATGTCTGATTCACAGTTGACTGATATTGCAAGGTTTTGCAATCGTTTC
CCCAACATAGACCTGTCTTATGTGGTGCAGGATGGTGATAATGTAAGGGCCGGGGAAGAC
ATTACATTGCAAGTCACCCTTGAGCGGGATGTGGAGGGGAGGACAGAGGTGGGACCTGTT
GATGCTCCCAGATATCCTAAAGCCAAGGAGGAAGGATGGTGGCTTGTTATTGGTGACACC
AAGACCAACCAGTTGCTTGCCATCAAGAGGGTGTCACTCCAAAGGAGGTCGAGAGTCAAG
CTTGATTTTGCTGCTCCCTCGGATGCTGGAAAGAAATCATATACTTTGTATTTTATGTGT
GATTCATATATGGGCTGTGACCAAGAATATAGTTTCATTGTTGATGTCAAAGAAGCTGGC
AGCCTTGATGAAGACAGTGGACGAGAATAAAGAAATAGTTGCAACTCTACGTCTGACTTT
CTGCTTTTAGTGCAGAGTAAAACACTATTTTATATTGTTTATGTCTCTTGTTGACTCTTA
CTGTTTTTGCAACGAGGACTAGTATAAGAAGAACGTCTGCTAAAAATTGGGCTGCCCTGC
ATATTTCACAGAAGCTACGACTCTGCTATGCATCACTCTAACTTGTATTAATTATATAGG
CATGGAAAAGAGACTGGGAATCGGGATCTTCTATCTGTTGGTGCCGTTTGATTGTTCCAT
GGTTGCTTGATGCAGAGATAGATTCAAAATCTGTACATTTTGCTGAAACTAGTCAATTCT
GCATAGAGAAGTCTGTCTGTATTAGAATTGGATTCAAATTGAATTATTCAAGTTTGAATT
AAACTAATTGAGTTTGAGTCAATGAGTTAGCTGAGTTTGAGTTTATCCAAACATTTGAGT
TTGAGTTTTTTTAGATCGAATTCAAAGTTAGATGTTTGTCCTAAGCTAGTTTTATGTTAT
TTTTTTGGATTTGAATCAATCTCCAATTGATATTTATTCAGGCTTGATCTCCAGTGAATC
CAGCCTAGTCTGGTTTTCTTCTATGATGCATACACTTCCCCCTTGCAGATATGTTTCTGT
AATGTGAGTCTAGTTTGAATTAGCAGCAGATTCAAT
>ontig_50_consensus
CATAGGCCATCTATCATGTCTGTTGCAGGTCGTGTAAATTTTGGAACAATTCTTGATGTG
GTAAGTGACTTGAAGCCATGAACTTTATCCTCGTCTTCGTCTTCTAATCTACCACTACCA
CTACCAGTATCAGTATCTATAGCAATTGCAACTTGATCATTGTTGACTTTACGCAAACTG
CGAACTTGAGATGTCGTAGAGGGTCCAGGTGCTGCCCACTTGTTTATCAATGGTTCAACT
TCTAAAATTTCAGATGTCAAACTAGTTTCAAGGCCTGAATCTTCCACTCCTTTAAGCATG
TTTATCTGTGACTCCTTCTCACTTAAAGACTTCTGAAGAATCTCCTCCCTGCGCGTTAAC
TCTTGTACTTTACTCTTTTCTACTTGCAACAATGCATCCCGGCTTTGAGCCACTACCTGC
AGTTCCTTGAATTCAGATATAATTGCTGCTATCTTCTTCTGAAGTATTTCCTTGTATTCA
TGCAGTTGATCAGTTTTCCTATCAATAAGCAAATCATGGGCCCCAAACCGAGAAATCATA
GAGTCAAACCAAGACAAAAACTCCTGGACCTCTTCTAAATCTCTCTTGTTGCTTATCTGT
GATGCAACAAGAACATCGTTGGTGCATCTAGTGACCTCCTGCCTCAAGAAAGAAATCTCA
GAATCCCGATCTTGCAATTGTGATTGTAGTTTTTCAACCTCAGCAAGAAGATTTTCAGAC
ATATGATGAAGCTCATCAAACTTATTCACAGTAACAAAAAGCCTTTTTGAAATCTTTCCA
CGTGAAGCTTCAAGATTCTCCAACTCTATGTTCTTTTGTTGCACCACTTTTTCCAATTCC
TCAAGTTTGTTCTTCAACTCTTCCATTTGAGTCTCCTCCTCATCAAGTGCTTGCATTAGT
GCTTCAATTTCTTGGTCCTTGGAAACTAGCACATCAGACAGTGATTTGACCCTCTCCCGC
AACTCCACTAAGGCGGCTTGATCATCTAGCAACTCATTCACTCTCTGCTTCAATAGGCTC
CGTTCTTCCTCCATCATTTTCATTTGCTGTTCCATATCATACAACCGAGTTTGCGAAGAT
TGAATATCTAGTGAGTAAGTGCAAACAGCAGCTTCAGCTTCCTTGATTTGACTAACAAGC
TCTGCACAAATCCTATCCTTTTGGATGTCTTTCTCCTGAAGCTCTCTCTGCAAACTTCCA

ATGGTGATTTTCATTTCCTCTAGATTACCTTCTACAATTTCAGTTTTCATAATAGCAAAA
TCCTTGACAGCTGATAATAGTTTCTCTGCAGTAGCCTTGACATTTTCTTCAGAGAAACAG
GTCTGTCCACCAAAAGAAAGTCTATCCACAAATAATGCAGGATTCAAGTTAGTTCCCAGG
TCTTGAACAACTAATTTATTTCCAATCAGCTCAGCTTTTCTATTTTCAATTTCCATAACA
GAATTAGCACATGCATCATAAAGCAAGGCAAAATTTCTGCGCAACGTGACAATTTCCATA
TCCTTCGCTTTTTCACTTGATTCCATGTGCAGAACATCTCTTTTCAAGGCTTCAGATAAA
GTTATCTGATTAGACATTTCTCTGCTAAGAATCTCCATTGCTTTAGACAGGTTGCTTGCT
TTATCATGTAATGCTATAGGGTGTCTATGCAACTTCTCCTTCAAAACACCAATATCAGTC
TTCAATTCTTGCAGGGAACACTGAAAAGCACTATAAACCTCATTTATTACACTATCATCA
ACATAGGCTGGTACTTTGGGTCCTAACCATGAACCCACAGATAGAAGTTTCTCCTTGTGT
TCTGAATTGGTGGAAGTACTGCCTCCATATGTACTTAAATTAGGCATGTCAATCACACTA
GTAGCATCCCCTTGCTTCAGACAAGACTCCATGAAATTTTCCACATGTTGCAGCAATTCC
AAGTCCTTGGAGAAAATATCAGCTAATATATTATTAATATCAGAAAAGTGTTTCTTCAGT
TCGTCAACAACAATTTTTAAGGCCATCAATTCAGAATATTGTTTCTGTTTTCCCTCAGAA
TTAAGTGTTGATAACTGTTCAAGATGTGCGAGTGCTTCAATTTTGGCAGCTTCAGCTACC
TCCTTTTCCTTGGAGATATCCACAAGTTCGTTGGCAGTATTTGCCAACTCTTCCTGGAGA
GAATCATTTCTCTCTTGAACTTCATTTAACTCCGCAAGTAGTAACTCTGCCGCTCTTCTA
GATTTCCTTGATTCTTGTTCTGCACAAGCCAGAGCCGCATGCAAATCACGAAGTTGTTTC
CCAATCTGTTCTACCTTCTGAACTGGGTCACTGATTGTAATTTCACCCCCAATGTCAATG
TCACCTATGGCATTAATAGTCACATTCAAAACATGGTCTCTCTCCTGCAATAGCTGCTCA
GTTTCTGTCAGGCGATTCCTCAAGAACAGTCTCTCAGATTCTAGGGCTTCCACTGTTTCA
GGATATGTAGATAAGTCGCTAATCTCAGATTTCAAGCGCTCAAGTTCATTATTCATCTCT
TCAACAGTTTGTTTCAGGCTATCCCTCTGTTGAACCAATGACTTTCCTTTTCTAACTGCA
ACATTTAACTTCTCTCTCAGCGAAGCTGATTTCTGCTCCTCCTGAATAAGCAACTCTTGC
AACTCTCCTCTTTTTCTATCAAGAGATTCAACTTCACAAAGCAAAGATTTTTGCCTCTCC
ATATATGCATCTCTCTCTTCCTTCACATGCATTAGATTCTGAAGAGCCTCCTCCAGGTCT
TTCTTCATACCTGATATGGAAAGATCCTCAGTAATTAGCATATCTCTACTTTGTGATTCA
CCACAATCTGCATGAGTTTCCTCAGAGTAGTGCTCATCTGCTGCACCCTCAGCGCTGGGT
TTTGCAGTAGCAAGGGCTGAGTAGCGCTCTATAAGTTTACTCAGCAATACCTCCAAGTAC
TCAGTTGTGCTCTCACCAGAAACAAGCTCTTTTGCACCAGGGTCAAGCAATGCATCACTA
ACCAAGTCCTCCAATCTATGTATCTGATCTTCAATGTTGCGAATGCAGTCCTCATTACTA
AACTTCTCAGCCAATTTCTCTTGTAAACCCGTCACTTCACTCTGCAGTTTTTCATTCTCA
ACTTCAAACTGGACTGCCTTCGCTGAAATTTTCTCATGATCAGAGATCAGAATCCTCAAT
CTTTCAGAAAGATTTTTCCTCTCATCCATAATGGCTTGGAGTTCACCCTCAAGGCCGGAT
ATCCTCTTTTGTGACTCTTCCAAATCAGCACTTACTGATCCACAATAGTTTTCCAGGTTA
TCAATCTTCTGCTGGAGAGTACTACTTTCCTGATTAGCTTCCAAAAATGCAGTTCCCAAC
CACTCAATCCTATCCTCAGGTTCCATAGATTGCATGTGTGAGGGCATGTCAATTCTGTCC
AGAACTTCTTCCCATCGTTGCACCAAAAGGTTTCTCTCCATTAACGACTGTTCCAGCATT
TCATTTTGTTCAGCTAACCCATAGAACTTACCTTGAAGCTCCTCATACTTTCTTCTCATA
TCTTCCCCTGAACTAGAGCTCAGTGGTGCATTTTCTTTCCAGGCATCCATAACCACAAAC
CCACTATCATTGTAGGACACTCCTACAGAATTCTTCTGATCCCAGTCAGTGACAGCCAAA
GAATTACCAGCAGCTGATCTTGCTAACCACTCAACCTTTTCAATTATGCCCCTTGAATGA
AAATGCTCAGGTAGATCTAGATCTTCCAGGATCTCTTCAATTCTCTGAAGTACAGAGTCC
TTGAGAAGAAATGATTCTCTTAAGGCAGTAGCTGAGTTTCGAATGTATGAAAGCTCAGAT
TCAAGAGCTTCCACACGCTCACCTGCCTCCATATAGGACTTGAGTTTTGTTTCTAGCTCA
TGAAGCCAAGAATCCTTCAACTGTAACTCCTGAGAGCATCGCTCCAGTTCCTTGGATGTC
TCAGCCAGAGACTGCTTGAAACTATCACGCTGAACGACCAAACCTTTCCCCTTGGCAACA
GCAATGCTGAGCTTCTCTCTGACAGAAGATACCCGCTGGTCTGACTGTTCAAGTTCATTT
ATCTTTTCCTTCAACTCAGAACGTGTGACAGCAAGGACTTCTTCTGCCCAACTTAAACTT
TCCTTCAGGGCAAGGATTTCAATTTCATAACTAATGTTCAAAGCAGATAACTGCTGCACC
ATCTCCTGAAGTTCTGTCAATTCCATCACCTTGGATCCCAACTCTTCTGTTGATGAAGTC
ACATGCTCACCAGCCTGTTTGTACTTCTGAACAAGTGAAGACACCAATGATTCCAGGTGT
GAAGATGGTGTTTTATCTAAGTTGGTTCTAGTGCAATCCAGCTCCACCACACCCTCCACA
TCTTCTATTAACTTCTGAATGGCGTCTAAATCAAGACACCTCTGGTTCAATTCCTCAATA
TCATTTGCCTTCCTGATCAACTCAGAATTGAGTTCACTATTTAAGGATTGAAGTTGCAGT
CTCTCACCTAGAAGAGTTCCCAGTTGATCCACAATGGTTTTACACTTGATGTAGTCGATC
GAATCAGGCAGAGTCCCACCTTCAAAGTTCATCTCGTGTTCATCATCCACAGCACCACAT
GAATCAATTAAAAGTTCCCTAAGATCATCATATATCTTCTGCAATATAACAGAAGTTGAT

TTATTCTTCCGAAGCAAATCATTGAACTTCTCATTCACTTCTTCATA
>ontig_51_consensus
TCCAAAACACACGAAATTTAAGTTAATTTCAAGCCTCTCAAAGTTTCTTCTTGTCCCAAC
CTTTTCTACTTTCTGCAACTCTTCATCAATCAAACACACGAACTCAGATACAGAGGCGAG
GTAATAGTATGGCGAGCTGCAACATGGCTTCTGCTGCCTCTGGCTTCTTGCTGACACCCA
GTGTTGCAACCAATGCAAACTCAAATTCTAGGAACAATATGGTGTTTTTCCCTTCAAAGA
ACACCGCCTCCACCAACAACAATTCAAGGCTTGTTGTAAGAGCAGCTGAAGAAGGAGTTG
CCCCCCCAGCCACCACTACTGCCCCGGCGCAAGGTGAAGCTCCGAAGCCCAAGCCACCTC
CAATTGGACCCAAGAGAGGCACTAAGGTGAAGATTCTGAGGAGGGAGTCTTACTGGTACA
ATGGAATTGGATCTGTTGTAGCTGTTGACCAGGATCCAAAGACTCGTTACCCAGTTGTGG
TTCGATTCAACAAGGTTAATTATGCAAATGTATCCACAAACAACTACGCATTGGATGAGA
TCGAAGAAGTTAAATGAGTTAACCCAATAGCTGTGTAATTTTGATTTTCACATTATTATT
CTCTTCCTATTCGTAGCTTAAGCTTTTGTGTGCCAGGAATGTGTATTATATATTCTTCCC
>ontig_52_consensus
AACTGGTAAAATAGCCTTTGTTATTCAACTACTATTGTAACTCACTAGGATTCAGAGAAG
AAAAATCCATTAAATCCTCAAAGCTTGACAAATGTTTGTAAGGCCAACAATCATATCAAT
ACTAAATCCAAATCTTAGTCTTAAGATACGAAAGGAAATAATCCAGTAACATGATAGGCC
ATGAAATTGAATAATCCTATAGATAAACATTCAGAAGTAGCATTAGAGTTTTTATCCCTG
TGTTCGAATTGGTACTTTCAATTGGACTCCGCAGAATTTGAAAGCAACTGAATTTTCTTG
TCATCTGGGTCGGTATGTGGAATTCTGTCTTCATCCAAGCGATGGTGATGGTTACCAGAA
TCACCATCAAAGTGATTTTGCTTATGTTTGTGATGATGGTGGTGATGATGCCGGCTGCAT
TTGTGCCTTGTTTGTTTATCTGCTTCTCCACAGTTGTCATCTGATGACCGTCCTAAAGAT
CTGCTTCTCTGATCATGCCTGTTGCCATCAGACTCAGATGATTTTGAGTCCACACTGTGC
GACCGTCGGTGACGTTTATGATGCTTTGCATGACTTCTCCTTCGGACTCTACCGTCCTCG
AAAGAAGAATCGGAAGCATCAGAATCTGAACTGGAATCGTTATGTCTGTGTCTTCTATGG
TTTCGCTTCTTCCTCTCTTCATAATCACTCTCAGAATCTGACCTTGAATTGCTTTGCCTG
TGTCTTCTATGGCTTCGCTTTCTCTTCCTCTCTTCTTCAGATCCGCTGTCATATTCATCA
CTGCTATCATCACTTGTGCTTGAGGATCGTCGCTTTGGCTTTCTTTTCGATTTCTTCTCC
CTCTGCTTCTCATTGTCACCTGAATCAGTGTGGCCATGCTTTCTGTGCTTCTTGTGGCTT
CTTCTTCTTCTCCTTCTACCCCCATCACTGCTATCACTATCACTATCACTATCAGAATCT
GATACGGACCTTCTTTTATGCTTTGCAGCCTTTTTCTTTTCCTTGGCTTCAGCATCGGCC
TTTGCCTTTGCAGCAGCTTCTAACTTCTGCTCCCATTTTAGAGGGGCTTCTTTCCTCTCC
AAAGCTCTCTTCTTTTTCTCCTCGACCAACTGGATGAACTTCTTGCAGTCCATTTATATA
TAAAAACCAGCTCAATTCCTGCATTTTGGTACGATGATGACCAAATGCACATCTTATATG
AACTGGCACAATCTTAAAATGGAAGGGCTAGCTCGAAGGCCCAAAATCAGAATTTCCCAC
ACCAACCAAACAGCTCTACCTTGGCTGGAGATCAACCGAAGAGCTATAGAGGTCTGCTGA
ATTGGGGCTTCTTCGCTTTAAAAACTAGGGCTTGACTTCGCTTCCTTCCAGAAATTTCTA
TGCTCTAATGATTAATTTATTAATTTCCTTATATGAAAATT
>ontig_53_consensus
AAAAGGACATCGATACTTCATAGTAGCTAGCTATGGCGGCTACCTAAGGCTTACACAAGC
TAACACACACCACACAATATTATTAAACTCTACATGCTTTAAGCCGGTAAAACATTACGG
GTGATGGTGATGGTGATGCGGTTTGTGTGGGTCGTGGGGGCCTGCAGGTGGAGGCGGTCC
ATGAGGTCCTGGTGGAGGAGGGCCTGGCTGGTGAGGAGGCGGTGGTCCAGGTGGTGGTGC
TTGTGGAGGTGGCGGTCCAGGGGGTCCTGGAGGAGGCGGATCGTGTGGTGGACCATGGGG
TGGAGGAGGATGATGCATCTTCTTGTTGCTTTGGTGACTCCTTTTTTTCCTTTTCGTTTT
TTAATAACTGGTTTGTGGCGAAATGGCGATGAGAAGTGAGAAAAGGTGAAAGGATAAGGT
TCGAGATTTATAGCATAATCTCTTCCGATATTCAGCTACCAGTCAGTTCTCACCTACAAT
TCACATGATGTGTGGATCCTTCTGTGGCCATGGCTGATTGGACCGGCTGATTGAAAAGAG
TGAAGTTTCTGGTAAAGAAGAAATAAATAACATTATCTTCAACACTACCTTGCCCTTGAA
AAATTAGGGCCATGAAAGGTTCTAGACATGGCAAGTGGCAAGGTGGGGCCTAATTTCTCA
TTTAATGTACAGGATCGATGAAATCCTCGTCCTATGAAACAGTATCAACCTCTTTCTCTA
TCGTTTCCTTGCCCTCGATCACATCATTATCCCCG
>ontig_54_consensus
GAAAAGCACTTCCACCTTCTCTACTTTTTTTTTTTTTTTTTCAAAAGAAAGAGCAGTTTCA
GAAATTTGATATTGCAGTTCGACTCCCAAGTTTTGATGACTGGGGTCTTTGAGAAGAAAC
TGTTTTGAAGCTCTTTTCAACTTGTGGGTTTGACAGAGAGTTGGATTCTGCATTGAAAGT
TGCTGCTATATCTGATTGTGGAGATGACAACACGACATCCATAGCGAGTATCGCCAGCTA
CCAACTCCAATATATAAGAAACAACGTATAATCAATTGAGAAAAGCTATCGGAAAGTTTC

AGTTCAAAAGCAGCTTTATTATTATGCTACCAACGAAGGTACTTATATACATCCAGAGGC
ATATCAAAAGAAAGGCTCGGCCACAACCTGAGAGTCGTATCAACGACCCAGAACCAGAAC
CTGAAAACTGATACCGATACATAAAGTACGGCTAAACTCACGAACAGAAGCTAGTACAAA
ACGGGAAACTGAAACTTAAATACAACTTCTTCAGCTATAACCTTTCATCAACTACTAAAA
CACAGCTTAACTCTTGGTCCTGAACTTGGATTCGTCAATCGAGACTCCCTCCTGAGCTGC
ACGTTCCCC
>ontig_55_consensus
GTGTAATTTATCCAGAAATAAAGGCATCCAATCCTGCAAAATACCTCTGAATTGATACAA
GAGAATAAAACACATAAAACTTGTACAAGTACTTGTAGAATGCAGCAGTTCAGCCAGAGC
GACTAAAGCAAACTCGATAATTTAATACCCTGAGTAGTAAAATAGTTGTAGTATATAAGT
TTCAAGTAGAGAAATGCATGCCACTGAAATATCAGATTATTTGAGACTACTTAGAGCATG
TTGTCTGGAAAGATTCCGAACTGTGTACCAGACAACCTAGTTTTTTCATTCTACATGAAG
ATTACCTGTGATCATGTACAAGGAAATTGCACATCGCTGTAAAATCTGGCAATCTGACAA
CAACTTATCCCTAGTTCGAGCAGCACCCTATTCTCTTTAGAATGGAAGCGTCCTCATTGA
CATTTATTGTCTCTCTTTTTGAAGGAAGTGTGGAAGAAGCTCCATCAATGCCTGCCTCAA
CTGCCCTCTTGCTAACAACTTTGTATATCTGATTAATAACTTCAGTGAAAGCCTTTTCCA
CATTTGTTGCATCTAATGCTGATGTTTCCATGAAATATACGGACTCGTCCTCAGCAAAAG
CTTTACCGTCGTCAGTGGAGACGGCTACAAGGTGCCGAAGATCTGACTTGTTGCCAATCA
GCATGACAACAATGTTGGGGTCAGTGTGCTCTCTCAATTCCTTCAACCACCTCCTAACGT
TCCCGAAGGTGGAAATTTGTGTTACATCATAAACAAGCAAAGCACCAACAGCACCTCTAT
AATAAGCACTTGTAATGGCACGGTACCTTTCTTGGCCAGCAGTGTCCCAAATCTGAGCCT
TGACGACCTTGCCATCAATATTCAAACTTTTGGTAGCAAACTCAACACCAATAGTGGACT
TGGACTCGAGATTAAACTCGTTTTTGGTAAAC
>ontig_56_consensus
CAAAAAAAAAAAAAAAAAATACAGTATCATCCAATATCACAAAATAATTTAAATTAAAATC
AAACAGAATTACCCAATCAAATCAAGTCAAACGCCTAGTCATTGCTCGGATCCAGTGCTA
CGCATACCAATTCAGCAGAAACAGTGGGAAAATTCTGACATGGACACTTGACGAAATAAA
GAAGATAATTATAAATAAATACAGAACAGAAACTAACATTGGAAATGAAATGAACTACGA
AGAAGAGTGCCTTTCAAAACAGTAATAACACGAAATTAAACGTTACAAATAACCCAGTAC
AACACTCTCTTTATTAACAACACACACATACAACCAATTGAATTCTCAATTACAACACTT
GATTGTATAATAGCTGCTTGATCTCCACCCAATTATCTATTAACAGCCTCCATCAAAAGG
TAGAGATCACATCAACATCACACGGCTTAAGGGCCCTGATGCTGCTGCGTCCCCGCTTGC
TGCTGTCCATATTGCTCAACCCTGTCCTTCATTTCTCTCGCTTTACCAGCCAGCTTATTG
CGTGCATAATCAAACTGATCGGCCCCAGGTGGCTGCTTTCCAGTAACAAACCTGTAGATC
CATGATAAAACAGTGATGGCTGCAACACCGAAACCACCCGAGGCAAGGAAGCCGGTCGTC
AAAAGAGCCATTGTAATGACTGCAGGAACAAGAACAGGACTGAATATGACGAAGAGTGGA
GTGGCAATGCTCAGTGCAATGACGGTACCAGCCATAGTCAAACACGAGAGAAGCAAAAGA
GAGCCACCAGCGGTGACAGCAGTTGCCCCCTTCACAACCTGGTAGGACAGAGGTTGTTGT
TGGATGTGCTGCTGGTAATGTTCAGCCATAATGTGACGGTATCAAAGAGGTTTAGATGAA
TTTGAGAAGGAAATCGATGATTTTGCTGAGTTGAGGTTGAGGGCCAGAGAGAGTGATGAA
TTAAATAAGAGGAAGAGTGGGAGAGTGGAAGGCACGTGTTTAAAAAAGGAGGCACTTGTT
GAAGAGGTTATCATGCAGGGGTTGCATG
>ontig_57_consensus
AACCAAAACTTCAGCACCATTCATTTCATATTTGTTGCTGTTGGTGACCCATTGTAGAC
AGACAACACCGGGAGGGTGTGATTGGGATCTATCCTATCTAAACCCTTACAACTCTAGTT
GTTCCACTTCTTTCATCCTGCCAATGTGAAATACTGACTGCCAAACAATCAAGACATTTG
TGCACAAAATGGTGATTACCTGACTCAGGGTATGCAAATCCATAAACCAACTCGACACAT
CCAAATGCATGCANNNNNNNNNNNNNNNNNNNNNNNNNAACTTGGCCCATTCAGCAGATTCCT
TAATCATATGATCTTCTGCTGATAAACATTCATTTAGAAATTCTCTACTTGACATGGATT
GCTGAATCAAGGGACCTGCACTACTACCAAGGGTATCTGCTAGATCTCCAAGAACCCCGA
TTGCTGTCTTAGTCACCACCTCATCCATGTCTTTCTCCCGGTACAAACTATCTAAGAACT
GAAGAATATGAGGTGCATAGGGCATTAATAATTGGGTTTTCGGAGAACCCTTGAAACCTT
GAAAGATACCCGAGTATGCCTCCAAGATTCCATTTCTCAAAGAATTGGTATACTCCATCA
TATCTTCATCAGCACCTGATGTACGTACAGATAGCTCAGCTGCACTCTGAAGCATGGGCA
TAGCATACATCAAGTACTTCTCAAAGCTTTCACCAATTGCCAAAGCAATGTCACCAAAGC
ATGAAAAAAATTGGAGGCTTCACAGATCTATGCAACTGATTGCTTGACAAATCCTTCAGGA
GTTGGGTCATAATCCCATCACAGTAAGGCAATATCTTTTCCTCCAGAGCCCTGCAGATAT
CCCCCACCACACCTACTGTGATGGCACAGACCTGGTAATCCTCAAAATTTTGGAGACCCA

TTTCCAAAAACTTGTAAAACTCTGGCATGTATTTTGCAAAATCTGGACCTGTCACATAGG
CAAGAGCTCCAATGACAAGCATAGCCTCCTCATGAGCTGTGGCACTTTGAACAGCAAATA
CTCTTAAGAAGAGGCCCATCATCTGGTCTGCATACTGCATGAAGACATACTTTGTTTGCT
CAGATGATCCTAGCTTCTGTATGATGACCTGCAAGCAACCACAAAGAAGG
>ontig_58_consensus
AGTGATAATGCCTGATTTGTGAAGTCTTCCAAAACACGTTTTAATTCAGCCTTAGCTCTC
TTGACAGACTGTTCAGTAGGGCCCTCAATAAACAAATAAAGTTTGCGTTCCCCAGGTCCT
GTGACCCGGCTTGGTGGAAAATACTGACCCCTGGTGGTAATGGCAGCACCAGTCCATTCT
GATATTGGACCCAACGTTTCCTTGTGAGTAACTTTCCATCGAGCATTCTGGGGAAAATCG
TTGATCTCCAGTTCTGCTTCATAGTGTTCAGGCATTGCATCAGCTTGAATTTTTGCCAAA
TTATGCTGCAAGTTGATGGCAGCTGCCAACGCCGCTGCCCGAGCTGCCCCATCATTGGGC
ACCACAGACAACCCGGTACCAGGAACAACTGGTCCTGCTCCGGGTATCGGTAGTCCGAGA
ACACCTGGCAGAGATATGGGTAATCCAGCATTGGGAAGCATTTGGGCGGCCGAGATAGGA
GTTGGCAACGACACAGAGGCTTTGGAGGAAGCAGCAATAGCTGCAATCTTAGCAAGTGCA
TCTTGTTGCGAGATGTCAGCACCTGCCTTCCGCACGCCTTCGTCTTCATCTTCTGAATCT
GACTTATCTTCCTCAAATCCATACTCTTTTGCCTGTGCTTTCTTCGCAGCTTTCCTCTTC
TCATCCTCCTCCTCATTAAATTTAAAACCACTTCCACCATAACCTGTTCCATGGGCTTGC
TCAAGACCCTGATTCACTTTTGCCATGAAACTATCAGCAAGAGCCTTAAGGTCATCAGGA
ACAACTTGCTCAGAGAGCTCCAATGCCTTCACAAGATCTGGTGCATATCTTGCGTCCTCC
TCAGAGATGAATGTGATTGCACAGCCTTTTCTACCAGCTCGGCCTGTCCTACCAACACGG
TGTACATAATCCTCATAGTGGTTTGGAGCATCAAAGTTGATCACTAATTCAAGCTCTTTC
ACATCTAAACCTCTAGCTGCAACACTAGTTGCAATCAACAAATTGCAGACATTGCTCTTG
AAATCAGAAATAGTGGACTCACGATCTGTCTGATCCTTGGCCCCATGAAGTGAAAGACAT
GGATAACCATGTCTAAGCAAATCCTTAAACAAAACATCACATTTTTCCTGTGAGTGTACA
AATATCAAAATTTTTCCCTTCTCATACCATTCACCTAGTAATTCTAACAGTCTTAAAAAT
CTCTCACTTTCTGGCCTCACTTCAACCAACTGTGTTATGTCTTTGTTTACAACGCTTCTG
CCACCAACCTGCATCTCCACAGGTTTGTTCAAAACCTTGCGTGCCAAAATTTCAACCTGA
CGCGGGAAAGTAGCAGAAAATAGTACAGTTTGACGATCTGGCCTAATATTTTGAACAATC
CGAGTGATCTGAGGTTCAAAACCCATGTCAAACATTCTATCAGCTTCGTCCATGACCAAA
TAAGTAACTCTTCTCAAATTGGTAATTTTCCCTCCGCTTGTGCAAAGTATATCAATCATC
CGACCTGGAGTACAAACAACAATTTCAGTACCTCGTTTTAAGTCACTAATTTGCTGAGCA
ACACCAGAACCACCATAGACAGGCACACATCTGATACCCATTACCTTGGCAAACTTCTTG
ATATCACTGTGGATCTGCTGGACAAGCTCCCTTGTAGGTGCCATTATAAGTCCAATTGGC
CCATCGCCAGCTATCACAGGTGGCTGGTCCTTGATATGCCTCAACATCGGCAGCACAAAA
GCAAGGGTCTTTCCAGACCCAGTTTTTGCAATGCCTATGCAATCTCGCCCACTCATAATT
ACTGGTAGTGCCTGGGCTTGGATTGGCATAGGCTTTTCAAAGTTAAGCTTCTTTATCGTC
TCCATAATTTTGCTTGTAAGTCCAGTTTGGTGCCAGGATTTGACTGGTTTGGGGACATCC
TTTCCGTGTATTTTCAATTCCAGCTGTTTCCTGTATGCAACAACCTCTTCAGGAGTCATT
CTTGAAATTTCTTTCACTTCAATATAGAAATTTTTCCTGAAAGGTTTGTAATCAATTTTT
GAGTGGTCAACAATAGATAATTTCTCAGCTTTTGTCTTTTTCACTCTCTTCATAAATTCA
TCATCATCTTCATCTTCAACTGTTTCCTCACCATTCTCAAGGTCCTTAGAATCAGAATCA
GAATCAGAATCTTCACCAGGAATTATTCTCCCCAGAGACTTATTTAAACCTCTCTTTGGC
TGCTCACGATTAATCCTATCACCCTTTTGATCTTTCTTTTTTGGGTCCACGCCATTAGCA
TCCACTATCGTCAATTCCACAACATTGTTTAGCTTCTCAACTTCAGGTAAAACCATTGAG
TTCATAAATGCATCAAGTGG
>ontig_59_consensus
AACTTGTGATGCCTTAGGCGGGAGCTGAACCTATATTATATATTTGATCCGTATAATTAA
TTACTTGTATCTAATTACAAAGCAGCCAGCCATTTCCATCGCTTCCCCTACAAAAAAGCA
TGTATATTCATTTACAATCTCATGTCCCCCTTTCCCTATCACTACCCAACCGTATTAACA
AGAGAGCTGGAAAATTTGGAATGAAATTAGAAAATCAAGGCAAATGTAATTTATTCAAAA
AATCTCTCCTTCCTCCCCATCCCCATTCAGATTCAGTATCTCCTCATCATCATCCCCCAA
ATAATCATTTTTCATAAACAAACCAATGCGCTCCAATGGGTTCTCATGATCATGCCCCTT
GAATGCTCTCTTCGACATCGCTGACCTTTTATCACCCTCTAGGAGATGAGGGGATAAGCA
AAACCCCGTTAGCATTTCAAGTTCCTTCAGAATCTCCAGGTTGTGCTCTATCTCCACAGT
TCTTTCCATCTTTTGCAAGGCAATTCGAGCAGCTTCTCTTTCTTTCTCTCGTTGCCTTTT
CAACTCAATTTCTGCCTTAATTCGTGAGGCAGCTTCAGCAGCCTGGATCTGTGCTTCAAT
CCTAGCCTTCTCTTCACGTTGCCTCCTTTCTAATCTTTCTTTCTCTTGCTGCATCTTCGC
AGGGTCAGGTTTGTCACCATGATCAAGGAGTGTCCTCTGCTGCGCTTTCAAAATTGTGTC

TGCAAAACGCTTCTTCAGCATAGCAGCACGGAGAGCCTTCTTTGGTGACATTTGTGCATC
TAGGAGTGATGCTGTCCAATCTTCAGCAGATGCAGAATCTGCTGCCAGAGGTGTAAGCTG
AGAACTAGGACATAAATTCCCATCATCCAAGGCACTTACAGCCCCATCAGAATCAGGATC
TGACTTGCTTGTCAGTGATGTTGACGTGCTTTTTGCTTGACAATCCCATTTAGAAGCATC
AGCACTACATGCACGATGACCTCCACCCAATGATCTCTCTGAAGTGATATCACTCGAGGA
CGCATGAGTAGAATCACTAGGAAGGTTGCATCGACAAGTTGTGTTGTCACATTTGAGACA
TTTTTGAGAGGCAACTGGGCTCAATGGTGGCTTGGCATTAGCAATGCCACAAGCATGACT
AGCACCACCATCGGTGCCTTTTTGTAAATGTTTCCCTGCTATCTTGTTTGAGCTCTGTGA
TGACCTAGGACGCTCTACCTTTGCATCCACCGTATATGTGGGATGCCTTACAGCCCTTTC
TTCTGCTGTCATTGACTTCTTTGATGACAAAGGGTTATGCAAGGGAGGTGTTTTGGGACA
ATTATCTCTTGTATCATTGACTTCCATCATTTTTCCACTTACAACTTTCCCCAGGCCAGC
TTTTGGTCTTTCATGGGTCCAATTATCCTCCAAGGATTCCCATTTCATAAGAAAGAGATC
ATTCAATTCCTCTGCCATTTTATGAACGTTATTCTCTGGAGGATTATAGAGCATGGCATT
AGAAAATGTCAATCTGACATCAGCCACAAACTCTTCAGTTCCATGGTACATATTTTTGTT
CAGCTTCGTTTTTATTGTTCCCAAATCCATTGGCTTAGATATAATTGAAAAGTAATCAGG
TATATTCAATTTCACTGGATCCACAGGTTGATTGAAAACCCAGCCAGCTGGATGGGCCAT
CAGTGATTTCAAAAGGACAGAACACTGGTGGGTTACACCACGGTCCATCTTTCGCCTTTT
ATCCTTCAGACCTGCTATCACATCTTGGGCTTCACGCTTGTTACCAGATAGCACAAGAGG
TCTCTCCTTTGAAGAAGTAGAAGCAAACATCCTTTTCAAATCAGATGAAGGATGTGATTT
ACTCATCTCCAATTGTTTACTTTCATTAGCAGAGGTTTTGTGATGCCAATTCTGGCCATA
TTCACAGGATTGTGTCCCAGGAACAACTTCAATTCTTTGGTTTCCAAACTTAATCTTTAA
CTTCTTCTTAGTTATTACAGCCTCAGCAGCAATCATGGTTGAAAC
>ontig_60_consensus
CTTTTCCTTCACTACCAACGTATTAACAAGAGACCTGGAAAACTGAAAAGAAAATAGTAC
AGGAAATAAAAAGGCAAAACCTATATTCATCAAAAAATCTCCCCTTCTTCCCCATCCCGA
TTCAGATTCAATATCTCATCATCATCTCCCAAATAATCGTTTTTCATAAACAAACCAATG
CGCTCCAGTGGGTTCTCATAATCGCCTCCCCTGAATGCTCTGTTTGACATTGCTGACCCC
TTTTCACCCTCCAAGAGATGAGGGGATAAGCAAAACCCAGTTAGCATTTCAAGTTCCTTA
AGAATCTCCAGGTTGTGTTCTATCTCAACAGTTCTTTCCATCTTCTGCAAGGTAATTCGA
GCAGCTTCTCTTTCTCTCTCTCGTTGCCTTTTCAACTCAATTTCAGCCTTCATTCGTGAG
GCAGCTTCAGCAGCCTGGATTTGGGCTTCAATCCTAGCCTTCTCTTCTCGTTGCCTCCTT
TCTAATCTTTCTTTCTCTTGCAGCATCTTCACAGGGTCAGCTTTGTCACCATGATCAAGG
AGTGTCTTCTGCTGCGCTTTCAAAATTGTGTCCGCAAAACGAGTCTTCAGCATAGCAGCA
CGGAGTGCCTTCTTAGGTGACATTTGTGCCCCTAGGAGTGATGCTGTCAAATCTTCTGCA
GATGCAGAATCTGTTGCAGGAGGTGTAAGCTGAGAACTAGGACATAGATTCCCATCATCC
AATGCACTTACAGCCCCATCAGAATCTGGATCTGACTTGCTTGTCTGTGATGTTGACATG
CTTTTTTCTTGACAAGCCAGTGTAGAAGCATCAGCACTACAAGAACGATGATCTCCATCC
AACATTTCTGAAGTGATATCACTTAAGGATGTATGAGTTGAATCACTAGGAAGGTTGCAT
TGACAAGTAGTGCTGTCACATTTGCCACATTTTTTGCAGGCAACTGGGCTCAATGATGGC
ATGGAATTAGCAGTTCCACAAGCACGTTTACCACCACCATCAGTACCTTTTTGTGAATGT
TTCCCCTCTGTCTTGATTGAACAGCTCTGTGACAGTCTAGGACGCTCAACCTCTGTATCT
CTGGTATGAGGGGGATTCCTTACAGCCCTCCCTTCAACTGTTTTTGACTTCTTTGATGAC
AAAGAATTATGCAAGGGAGGTGTTTTGGGACAATTATGTCTTGCATCATTTACTTTCATC
ATTCTTCCACTTACGGTGTTCCCCAGTCCAGCTTTCGGTCTTTCACGGGTCCATTTATCC
TCCAAGGATTTCCATTCCATAAGAAAGAAATTATTCAATTCCTCTGCCATTTTATGAACA
TTATTCTCTGGAGGATTATAAAGCATGGCATTAGAAAAAGTCAGTCTGACATCAGCCACA
AACTCTTCAGTTCCATAATACATATTTTTATTCAGCTTTGTCTTTATTGTTCCCAAATCC
ATGGGCCTGGATATGATTGAGAAGTAATCTGGTATATTCAATTTCACTGGATCCACTGGT
TTATTGAAAACCCAGCCAGCTGGATGGGCCATCAGTAATTTCAAAAGGACAGAACAATGA
TGAGTTACACCACGTTCCATCTTTCGTCTTTTATCATTTGGACCCTCTATCACATCCTGG
GCTTCACGCTTGTTACCAGCTATTATGAGAGGTCTCTCCTTTGAAGATGCAGATGCAAAC
ATCCTTTTTGAATCAGATGAAGGATGTGATTTGCTCATCTCTAATTGCTTACTTTCATTC
CCAGAGGTTTTGTGATGCCAATTTTCAGTAACTGTAAACTGCTGTCCATATTCACATGGT
CGTGTCCCAGGAACAGCTTCAATTCTCTGGGTTCCAAACTTAATCTTCAACTTCTTCTTC
GTTACTACAGCTTCAGTAGCAATCATGGTTGAAAC
>ontig_61_consensus
TGATTATAACAAAAGATTTAGAATAGCCATTTGAATAAAGACTAAATGAATTTACAGTAT
TTTTACAGGGTATAAATCAGTTTCATCTGCTCATTGATAGGCTTTTTCCAAATAAATCTT

AAGAGAGTAAAATCCAAGACTACAGAAACTCTGCCACTGGGACAAAAGGGTCAAGGAAGA
TGGGCAAGCCCCATTCGAAGCATTACTAATTTCTTTGCATACTTAGCTAAGAACCTAGCA
AGAAACAACAAAATTGTACAATAAAGCTAACATCATGAGGAAGAAAATCAACAGTAGCGT
TAACCTGCACGAAGGTAACTTCTTGCCAATGATGAACACTACTTCCGATGTCTCCTCTTC
GGTTCAGCTCTTTGCCGCTTCTCATCTTCCCACTCTCGGTCATAATTTGGATCTGAGTCA
CGACGAGTCATCTTGGCTGCTACTTCTTTTCCATGGACCCTTATATTTTGTTCCTCCAAG
TAACCACCACGCTGCATCATGGCTCCCTTTCGGTCTCCCCTGTCTCGTCCATCGTACGAC
TGTGAGACACCAATGGCAAGGGTTGGAGGTGGTGGAGCCGCAGGAGAATACTCAACAGCT
TCCCCTGATGGAAGATGTTCTCTTTTTAGTTTCCGTCTCTTAGAGGCAACATTAGCATCC
ATATCCTCCTTCGAAAGATTTGCTTTGTCCCTATCCCTCTCCCGATCTCTCTCTCTTTCA
TCTGCCTTCAGTGACAAAATCTCTCGTTCTTCTCTCTTTCTCTCTCGAAAATCATCTTCC
CTCCTACGTTTGGTATCATCTTGTGATACCAAGTTATTTTCTTCTGATCGTCTCCGCTCC
TTTTCATCATGTCTTGGAGAAAGCCTTTGTGAATGTCTGGCATTTCCAAACCTTCTATCA
GCATCTTCATCTCGTCTACCAGTATTAACAGATTGGGGGATCATATGAGGGGGCAGGGGA
GGTGGTGGAGGTAAATTTTGACCATGAAATCGATCATCATAGGACTTTTCTGAAGATGAG
TCGCTGTACCGTATTTTACTTCTGTCATCTTTGTTTCTATCATCCTTAACTTTGTCAGAA
ATTCTATCAAAATTCCTGTCATTACCTCTATCTTGCCCCCTCTCAACTGAACGCTCCGTT
CCATACCTTTCCATGGATCTATCTCTAGGTTTTTCAGCTATAATATCATCTCCTCGGGAC
TTATCTAGGCGGTCAACTCTTTCCCTATGTTCTCTATCATATCTATCATAGCCTTTATCT
TTTGACCTATCCAAAGGCTTGTCCATGGACCTATACATATTTGGTTCATCTGTGCCTGTT
TTCTCAAGATCCACAAATCGAGGATCCTGGGATCTCTCTCTGTCAGATAATCTAACTTCA
ATATCAACATCCCTTGGTTCAGAGTCCCCTTTATAGCGTTTACTTGGTCTGTCAGGATCT
TCAGCGGGGCTGCTTCTTTTTTGAAGTTTGTCACTAGATTTTGAAACAGCAGCAGAGTTA
TCATGCCTTGGAGAATGAACAATTCGGGAAGAAGAGGCCCTTGGAGCATCAGACACCTCA
TTGCCATCATTTTTACCACCAGAAAACCTCATGTCAGAAGACTTGACAACTCCACTATCC
GTCTTTGAGTCAACAGCATGCATCTCTGATCTTGATGAAGAAACATTAGAAGTCCCAACT
TGCTTGCTGTCTGAAGCATGAGAAATCGAATCTCTTTCATTAATGGAAGCGCCAGAATTT
CTGCTAACAGCTTTTCCAGACTTTTCATCTTTTGTAAGATCCTGTTTTGTAGCTTTATTG
AGTGATCCAGTTGGCAGGGATCGCTTTGCTGAAGCTTTGGACTCAGAGTCCGCAGAATTC
TTTGAAGCAACTTTGGCAATGTTTTCATCCAATGTTCTATTTACAGATTCATCCACCTGT
TTCTGATTTTCCACTGATCTTGATGTTCCTCCTTGTACAGAAGCTGACATGGATGAATGA
ACATCAGAACCATTGGCGATTGAACCACCTTTTAGTTTTAGATGTCCTGGATCAGATTTT
CCCAGTGTGACATTTTCTGTTCTTTCCAACCTCCCATCAGCAGGTTTTGTTCTTGAAATC
GGGTCTTTAAGTGAACTTCCAGAATCTAAATGTTGTGTTACAGCAGACACTGTTTTTCCC
CAGATAAAGATTTGGAAGCCAGTGAAGCAGCTGGTTTCAGTTCCAGGTATCCCATGCCAA
ATTCTTCATCTGTAACCCATGAAGGTTTTCTAGCAGCCAAAGCAGCAGCAACACCAGTTG
CCAACACTTTGAGATCCTCTCGTTCGTCACTTTTAATTTTAGCTACCCGCTTTTCAAGGT
TGATTCCACTTTTTCTAGTGACTGGGAAAACAGCAGAGATTTTTGTCAACATTATAAGAG
CATTACGAATTTCCATGTACTCTGTGGATTCCAAGCACTGAATCAATAAACGAGTAATTC
TTTGACTCCACTTCCAGTGTACCTTAATGAACTGGCCATATGTAACACGCTGACTGTTTG
GATATCTGTAATAAACAGCAAAGCCCGGCATGTTCCCACACTCACGTTCATATATTGATT
CATCACTCTTCCAGTAGTAAGCAATCTTTAGCGTCTCATACAAAAACCTCCCAAGTCTAC
CGGCTTCATACTCAGTGCAACAGCATATCATAGGTTGTAAGGTTTTGCAAATCAGAACAT
CGATGTGGTTGACAGTGTTAAAAAATGGTGTCCCAAGGGAATGAAGTGTATGTACAAACA
TGGCACAGTACACAGCGTCTGGCATACTGAAAGTACAGCGTGGAAAAATACAACGCTGGA
GGAATTCCATATTAATCTTCAGAGTATCAGGGCAGGAACTCAACCATTTGTCTTTTTCAT
GTGAAAGCCGCTGACGAACTGATGCCACATTTTCTTCATGCTTCTGGAGTTCACTGGTCA
GACGATCTAGAGACTCTTGAATTCTTTCTTTGTCTTTTTTCCTCTTGGTTATTGCTGAAC
TTGAATTATCTGAAAGCTCTTCCAGAGCTTTAAGTGCAGCATGCTGCTTGGCGATTTCAG
ATTCATAACGGTTTCTAGGAACATAAAGATCATAGAGTGTGAGCCCCCAAAAAGTAGTGT
AGAGATCAGGAGAAAGGCTATTCCAGGCTTTTGAGGGTAACATGGTTTTAACCGTATCCA
GAAGATCTGACCACATTATAGGCCTCTGAGAACCAAGATCCAGAACAACTTTTCCAGGAC
ATCCTATAGGTTCAGACTCCAAGTTTGCAGTTGTGACATTTGAATTGTCATTATCATCCA
AAGGCCAGAAAACATCAGAACCACCTTCACACTTAAAGAGCCTCATCACAGGACGATATA
TTAAGAAAGCGACCTCAGGATCAAGGTGGTATTGGTGCACAAGATCATTGAGTGAGGGTA
CCAGCTGAGCGTAAGCAGCAGACGGATTCATAGCGCTGCAAAGAAATTCCACATACTGAA
GAAGAGTTCCATGGCATCTATCAAACTGTTCACTAACCATTTTTATGTAAGGTGCATCTG
CAGTTATAACAACCACAGAACGGTGTTGAGCAATAAGCAACAGAAGGGGGATTGCTAACT

TTGGTTCATCCTTTGGAAGCAGTGAATCTTTTAGCCTATTGGTGGATTTGATCAATGCCT
TGTTATTTCTAGTTACTCCAAACGAAGTAGCCTGATATCGAAGGGTCTCACTCCCTGCCA
TGGCGTCCAACTGTTCCTCAGTCAGGTTCTCCGTGTATTGAACATTAGCCATTTGTTGAA
TAAGTTCCTGAAGGAGAACAAGCTCAATTCCTTGCCCTTTTTTCAACTGGTTTACAAGAT
ACTGGAAAAGGCCTCTCAGTTCCATTGATGGATACTTCTTACACAGGTGACCCCAAAATG
AAGCT
>ontig_62_consensus
AATTCGGTTGTTGTTTTATATAACTGATTTCAAATCTCAATCCATGTTTTGATTATAGGT
TATGTCAAATTCAAAATTATCCTGCTAATATTATGATTGGCCGCTGCATGCCACCTAAAG
TTTTTTCGATATTGATGATTGGCCTGCGTGTTAACAATCCTGCTTCACAACTTTCGGCAT
TCATCCATGGATGTTATTTTTGGTGTTCATTGACCATAATTATCTGCTTTTTACACAAAT
CCAACAATAACATTGAAAAGCAAACACATAATGTAAACATACATGCATGCATGACGGAAG
ATTAAGGATGATGATGATGACGAAGATCAACACGGAAACAGAACCAAAACTCAGTAACTC
TGAGCAGCCTCTTGAGCCTTGTGTTGCACATACAGACCGTACTCCTTAGCATTTTCCTTC
ATATCCCTAGCCGTCTCAGCTATCTTCATCCTAGCATAATCCAGCTGGTCAGCCCCCGGT
GGGTGCTTTCCCGTCACGTACCCGTATACCCACGATAACAACGTTATCGCAGTCAAACCA
CACCCACCGGAGAACAAGAGCCCAGCGGCAGCCAAGCAAATCACTATCCCAGCCGGGACT
AGAATTGGACTGAACAGGACTAGTAGTGGAGTAGCCAAAACGAGAGCTATCACCGTCCCG
GTTGTGGTTAAACCTGCTAAAAATAACAGTGACACACCTATTGCGGCCGCCGTGAAAAAC
TTCACCGTCACGTATGACGTCGGTGCTCTGTCGTAGAAGTCCTGGGTGGTAACGGACCTT
GGATGATCAGACATGGTGGTCGAATGTTGTAGTGCTTGATGATCACAAAAGGGGGGGAGA
ACGGTGAAAGATCGTGAATGGAAAGCAGAGAGCGGAGGCGATATAAAGAGGGGAGCAAGG
TGACGTGGCGGCAGAACATCATACAAGATTTTATGGTGATGTGTGTTGACGGTGATGAGA
TTGTCTAGACGGTGGCATGCGTTGGCAGCTTTGCGG
>ontig_63_consensus
CTTGAGAGACTCTCTTATCTCATCATTAAACTTCTTGCCCCATATGCTGGTCCCACTTTT
GCCTGTTCCTGTCGGGTCTCCACCCTGAATCATAAAACCTTTAATGTTCCGGTGAAATAT
CGTCCCATCATAGTAACCACTGGCACATAGTGCCAAAAAATTCTGTAACCAGTTACACTC
ATAAGATTACTAACCATATTATTCGCCAATTAAGAACCCAGACAGCACTTCTACGCTCAT
TGTGGCACACTAAAATGCTTCTTTAGTAGTAAACCCTAACTGACACTGAAATAATATTAT
CAGGCAAAGAAAATTTAAACCCAGAAATATTATTTAATATTGAATCGCTCAAGCATGTAA
TTTCCAAAAAATAAAAATAAAAATAAAAAGC
>ontig_64_consensus
CCAGGTCGCCGCAAAGGAGATCTAGCAAACAAACAGGCAACCTTCATAAACAAGGTATGG
CAACAGCTAGCCCACAAATCCTTCTCTCAATTTTCCCCATTGAACTTTAAAAATAAAGAC
TTTGTAGGGCATGCCAGGGTTGCAGCACAAAGGTATTATTACAAAAAAATAAAACAGAAT
GAATAATTATAGTAAGACTTCTCATAAAGAAACAGAAAGGTGACGTTGGAATACTTCTCA
GAAGTCCAGCACAAGATAATGTCAGGGTACGTTTAGGAAAAACAAGAAAATTTCAAATTG
AGATGCACAAATCATGTACTGGAATAAAAGCAATATTTGATAAGAGGGAATAAAAGCAGT
AAATTTAATAAAGGTATCATTGCCACAAGTTACTGAAATTGCAATGGAAAGATGCAGACT
TTTAAACACTTGAAAGAGAAAAAACCTCACTCATGAGTCAATCCTGAAGAATATGTAATA
ATAGGCAAGCATCGCAACTATATCACTGTCAGGAATCACACTGTAAAGATTTTTCAAGCA
ACCATCCAGTAAGCAACAATGTATCAATCAAATATAGAGGGGTGTAAATTACTGGGTAGC
TGAAGTGACCAAAAGCACAAGAACTCACACAATTTAACGTTACCCATTCTATAACAGAGT
ACACTCATCGATCACCACGTCATTTGAGGTACCAACTTGAATAGGAGCGGGCTGGTGATC
TTGAATAAGAACGCTGAGAGACAGACCTAGACCTAGCAGGAGATGCAGATGGAGACCTAC
GTGAATATTTGGCCCTGGGAGAAATGCT
>ontig_65_consensus
ATCTTGAATAGGAGCGGGCTGGTGATCTTGAATAAGAACGCTGAGAGACAGACCTAGACC
TAGCAGGAGATGCAGATGGAGACCTACGTGAATATTTGGCCCTGGGAGAAATGCTCCTGC
TCCTATCGCTGTAGCTGCGGCTGTGACTTCTGCTTCTTGAGTAATATGGGCTACGGCTTC
GGCTCCTTGAATAATATGGACTGCGACTAGGGCTTCTGGAGTAGCTTCTCCTTGAATCAT
ACTCCCTCACCCGTACATAAGACTGAGAAAATGCATTACGAAACTCAGATCCATCAAGCT
TCTTTATAGCATATTTCATATCATCATAGGTTGTGTAATCCACAATTCCAGTCATACCAC
CACGATCACGAAACACTTGAGAAAAACAGACATCACCAGCTCTACGCATGTGATCCTTAA
GATCTTGCCAAGAAGCAGAAGAAGGTAATCCAGTCACCAAAACACGATAGTCAGAGCGCC
TAGAAACTCCTCGACTACCTCCACTACCACTGTAACTGCTGTATCGATCTACTGATGATG
GACGCCTGCGCCCCCCATGTGCAAGCTCAACCCGTAATCTGTATCCATCAAAATTATAGC

CATCCCGACCACGGATAGCATCTTCAGCATCACGGTAATCCTCAAACTCAACAAAGGCAT
AACCTGGAGGCCTTGGAGGGATCTTCAAGTCAATATCAACAATGGTCCCATACTTGTAAA
ACAAATCCTCAACTTCTCTCATTCGAGTATCTCCTGGGAGATTCCCAACGTAGAGTGTAC
GGCTCGATCGGCTGCTCATTTTCTCT
>ontig_66_consensus
CTCGGTATTGAATGCTAGTATTACAAGGAATCAACTGCCCGCGCTCAGTTTCCAGTTTAA
GTTGGAGAAATTCTAATCGTTTCTTTACAGGTTTAACTTCAGCCCGGACGAAAACATGT
CTTCAGATCAACAACGCGCTGAACTAGACGCCAGGGCCAGGAAGGGAGAGACTGTTGTGC
CTGGTGGAACTGGTGGCAAAAGCCTGGAAGCCCAAGAACACCTTGCAGAAGGGAGGAGCC
GTGGAGGGCTGGCAAGGAAGGAGCAACCGGGGACAGAAGAGTACCAGGAAATGGGCCAGA
AGGGCGGGCTCAGCACGGGTGACCAGTCCAGCGGGGAACGTGCAGCTCAGGAGGGAGTCT
CGATTGACGAATCCAAGTTCAGGACCAAGAGTTAAGCTGTGTTTTAGTAGTTGATGAAAG
GTTATAGCTGAAGAAGTTGTATTTAAGTTTCAGTTTCCCGTTTTGTACTAGCTTCTGTTC
GTGAGATTAGCCGTAC
>ontig_67_consensus
CTGAATCTGACCCGACAACGATATAGTCCTTCTGAGAGCCAGTAAGACGGAACTGAGCGA
GGGAGCGAATGGCACCAAAAATTTCAGTGGAAAGAACACTTTCGATTTTCCCAGAACTTT
CGGGGCGTAGAAGCTCGAGGATTTTACCACGCGCGACGACGATTTCGGGAGTTTTGGTTC
CCGAGAAGTTTCCATTGATTGCACAGAGTATGCCAGTGGATTGTTGGAGAGTGAGACTGT
AGAGATACATAGTGGTTAGTATTAGGGTTAGGGTTTTGAGAGGAAAGTTGTGGGATGAAG
AGGGGTTAGGGTTTTGAAAA
>ontig_68_consensus
AAATGAAAAAAAAAAAAAAAAAAAAAAAAAAAAAGCAGACAAGGGAAAAAAAAAAAAAAAAA
AAACTATAATTCAGACAAAAAAAAGTGGAAAATAGATGTTCTCCCTCTTTGAAATGAGAA
ACTGTCTATAAGCAACACATTACTTCCCTGGGAAAGGTGTAAGGTACTGAAAGGCAGGAA
CTAAGATTAAACATTCACATAAAGCTCTTTGACGCCTTGAATTTCTGAAAATGGATATAA
TATAATACAAGTATGGTACACAACCTACACAAAGTTGAGGCAAAGAAATGAAAGAAAAAA
GGAATATATGTATATATATTCTTATATAAATTAAAAATTAAAAGGCTAATTAATACCAGA
GCCACAAGGATTCCATCTAACTGAGACCAGTGACCAAAAATTTGGTGAACAAGCTTTTCA
ACCCACAGAAACAAGTTAGGTGAACACACCTGAATTACAAACAAACTAATATGACTCTAC
TTACGCTGCTTCTTTTGCGAAGGCTCCGAATGGGGCTCAGCAGCATTAAAAAGCTGTTCC
CGGATGTAAGCCATTGATCTCAACATAGCACCTAAGGTATCCCTTGAAACTTGAAATTTA
ACCTCTGACTCTCCAGATGTTGTCTCAGTATCTTGAAG
>ontig_69_consensus
CCAGCATAAACCACTTACAGGAATCAGAGCACATTCTAGCCTTACAGATGGAGATGCTCC
ATCATGTTCAACCTCACCTTCTACTAATAATTGTCAGGTTTCTCCATCAAACTACCTGAA
TAGAAACCAGCAAAGTCCAGTCATACTGATGGGGGATTCAGTGGTTGAGCCTGCAAGCAA
TTTGGTGCAGGAGCTTTATAGCAAGTCTGATATCAGGGTCAAACATGAGTTACCCAACTC
AAAAGGATCAGAACATCTGAAGTACAAAGGCAATTTGACTGATCAATTAGAAGCTTCCTC
TTCTGGAACATCATATTGCCTGGATCCTGGCAACATCCAGCAGAATTTCTCTCTCCCAAC
CTATTGTTTGGATGGTGACGCCCAGTCACACACTCGGAACAATCTTCCTTTTACTGCTAA
TCTTGATGGATTGGCACCTGACACATTATTGTCAAGGGGATATGACTCTCAAAAGGATCT
CCAAAACTTGCTTTCTAACTATAGTAGTACTCCAAGAGATATTGAGACAGAGTTGTCTAC
TGCTGCATTGAGTTCTCAGTCATTTGGAGTGCCAAACATCCCTCTCAAGCCTGGTTGTTC
CAATGATGTTGCCATCAATGAAGCTGGGGTATTGAGTGGTGGACTATGGGCCAACCAGGC
TCAGCGTATGCGGACATATACAAAGGTCCAAAAGCGTGGCTCTGTTGGAAGATCTATAGA
TGTCACACATTACAAAGGTTACGACGAGCTGAGGCATGATTTGGCTCGCAGGTTTGGAAT
TGAAGGGCAGCTGGAAGATCCTCAAAGCTCTGATTGGAAGCTAGTATACGTGGATCATGA
AAATGACATGTTGCTTGTTGGTGATGATCCATGGGACGAATTTGTAAGCTGCGTTCAGAG
CATAAAAATACTTTCATCAGCTGAAGTACAGCAAATGAGCTTAGATGGAGATCTGGGTAA
TGTGCCAGTTTCAAATCAAGCTTGTAGTGGAACTGACAGTGGCAATGCATGGAGAGGTCA
CTATGATGATAACTCGGCTGGCTCGTTTAATCGATAGGGACTTATTTATAACTGAGAATA
TGAGCTGTGCATGACCGATAGCAGTGAAGAGCAGTATCATGACATGATTATAGTTTGGAA
CCCTCATCTCGCTCTTCGAAGGGGTGTAGGACAGTAGTTGCTGGTTGACAGAAAGACCAA
TTCTCCTAATACTCTCTTCCACCGAGTGGCCTTTGGTAACAGGTAGCCATACCTAGGCGC
ACAGAGCATATGATATGGTTGTATAATTAAATCTTACCCTCATTGAAAGCTTATCTGACA
TCCACAATTATTCTACTTGTAAATGCTGTGACTTAGAGCAACAATGAACAGGAACATGTA
GTGACAGCTACTTACATTCCTCAACGAAATATACCTGTAGAATCATTCTTCCTTGTATTG

CAATCATTCCCTAAATATTTGTACTATCTACTTCTGTAAACATCACTGCCGAAAAATTGG
AAAATTGATTCATTCCTGCCTCCTAAAACATAAATTAGAAAGCATCGCCTACTACTCTAG
TTGCTGACGATCAAGGTTGGACCCACCCATCTTCAAAGGCAATTCAGGTATTCCATTGAA
TTGTAACCCAAATGGCAACGTAAAATCCTAGCAACGAAGGAAAGAGCTACAATGCGATAT
ACTCAAAAGCAAGATTACATTCAAAATCCGTATGCCTTGAGGCAACTCATCCATAAAAAC
TCAGTTAATACATATAAAATTGCGAAGACAAACAGCTAAGAGAGGACAGCCATCACATCA
GATGCTCTTAAAGCAATGTAGTTGGTACCATCGTTGCCCTTGAAGTCGGTTCCTGCATAT
TTAGAGTACAACACTGTGTTCCCTGGGTTTAAGGGTAGTGCTTTTCTGTTACCCTCCTCA
TCTATAAGACCAGGCCCAACAGCAATTACCGTGCCAATTGAAGGTTTTTCCTTGGTTGCC
TCCGTCAGCAACAGACCTCCAGCAGTTTTCTCCTCAGCCTCAGCAATCTTTATGAATACT
CGATCATTCAGAGGCTTGAGATCCTTGATATCATCAGTCTCAAGAATGCCAACAATGTCA
TCCTCCTTCAGAATNNNNNNNNNNNNNNNNNNNNNNNNNNNNNNNTCCAATTTGGTTTTC
CCAATTGACTTTCCCTCGCCAACAGCCACCACCTCACCTGATTGAGGCTTTGACTGAGCT
GTTGTTGGTAGTAAAATACCACCATCGGTTTTCTCCTCTACCGTCTTTATCTTAACCAAC
ACTCTATCACCCAAAGGTTTAAGTGAAGTGTACTTGGGAGCAACCGCAGTGGCCGCTTTA
ACGACGAAAGAGGGAAAGGAGCGCTGACTAAAAGTAGTCCTAACAGAAGAGAGCCTGACG
GAGGGAGACCTTAACCCTTCAAATGATGCTAGACCCCTTGCTGGAACCCCAACTGATAAT
GCTGTTAGCTGAGAGCTCGACATTTTTTAAAAACAAAGAAGGGGAATGCGTCTGGCTTGT
TAAGGTCGCGCAAGAAGTAAGAATAGAGAAGATAAAACCCTAGATTCAGTAAGTAGAGTG
TTCTTTTTTT
>ontig_70_consensus
AATAATCCACATCAAAGCTATACAATAATAGCCAAAACAAATTAATAATAACAGCATAAA
AACCAGATTTACTTCTAATAATAAGAAGCCTAACTACCACTACTAGATGAAGACATGGAT
ATAGGATGAACCCAATCATCCTTCTACCACTTCATATAACCTCCCTCAAGGATACTTCCC
ATATGCGTTGTCCCTCCTGTTGTTCCTGAACGCGCAGCATCCGACGGAGTAGACGATGAT
GAGTGCGATGAGGAAGACAATGTTGATGATGGCCACCTTCTTCCAGTCAGTTTTGATTTG
GTCCAGCACCCCAGCTTTG
>ontig_71_consensus
CCATTAGTTGTTGATACATAGCCTCCGCTATTATCTCATTAGGTTTTAGATGCGTATATT
ACAAAGATTATAGTAACGTTGATATGCTCCAACTGAGTAGGAGACCTGTCCTAAACACAG
CCAAGTGCGCGCCCATATGCATGCATTAAGAGTTCTAGTGCTGTCAGAAAGACCGTGAGT
CATTTTTATGAAGCAAACTATCAAGGAATCCAGCAATTGCAGAAACAAGATTGAAGCAAA
AAGACACAACAAGATTTTGGTAAGGATGTGGTATGGGATGTCAAACTACAACTAGTTCTA
CTGCGGGATAGCAGACACAATCCCACCACAATTGGCTGAAAGCAACAGATTCAGCAGCAT
TTGGGGGAAACATCAATCCATCAATTGCATAAAAAGAAAACAACTGACAATAAAGAAAGC
AAAACTAATCCATTAAACTTCTATAGACTCAAACTCATTCGCAATATTCAAAAACCAAAT
GAATCCTCAAAATTAGTTTGTCACACTTATCCATATCTTAAATCAAAAAACAAAAATACT
AATACATCCAAATGTAAACTACACTTAAGATGACCAAAACATACTGAAAGAAGCTCCTAA
TCAAACAAACTGAATCCCATATCATCATCACTCTCTTCCTTGGGCTCCTCCTTCTTCTCC
TCTGGTGGAGGGGCAGCAGCAGCAGCCCCACCAGCACCACCAGCAGGGGCGGCTACAGCA
GCTGGAGCACCACCACCGCCAGCACCCACATTCATAACAAGGTCATCAATGCTTCGCTTC
TCGGCAAGTTTCGCAAACAAGCTAGGCCAGTAAGATTCAATGGTGATATTAGATGCTTTC
ACCAACTGTGCAATCTTATCTGCAGTGATGGCTATACCATCATCATGGAGGGCCAAACAA
GCGTAAGTGCAAGCAAGCTCTGCAACCGACATCGTTTTATTTGACTGAGAAAAATAAAAG
AACAAATTAATATAGCTAACAATCATAATATGATCAACCGACTAGCCAAAAAGGGACAAC
ATGAAAATGAACTTCAAAGGGCAAATGTGTTACCTGAGAAAATGTCTGCGACTG
>ontig_72_consensus
ACATGTTGATGAACTATCATCCAATAAACTCCTTTTTTCAGCCATACCATTTGGTACAGA
TGACAGACCACAAATTCCACTGCTAGTGGCTTCCATGGGAGGTGTAACTTCGGATGTATC
AGTATCCCTGTCTTCTGAATCATGCTGAAGCACTTCAGCAACACCATCAACTGAATCAGA
AACGTCTGAAATATCTTCCAGCACATCAGGCTTTTCAAGCACAGGCCGTGCTTCTTCTGG
AATAGATTCCTTTTTCTCATCACTAGGGTTTTCTTCCTCAAGCTTGTCCTGATTACCAGC
ACTGGGCCTTTCATCCCTCTTTTTTTCTTTCCCTTAGCGATTGTTCCGTTTCTGCTTGGT
CTGCTTTTTCTTTGTTTTCTTCTTTTCAGCAGCTCCAAGTTTAGCCTTCTGCAAACTTTC
AGCCCGCCATGCGGCTTCCTCTTCACGAATTAACTCTTCTTGCCTCTTCAATGCAACAAC
TTCCCCATAGGCAACATCAATTTTATTGCTGAATATATGGGCAAGGACAAAAATTTCCAC
AGTCCTACGCCCCAATTCTGTAAGACGCCTTTCATCCCGCTCAATGGAATCCTTGTTAAA
GTCCTCCCCAGAGCTACCATCCCTCATACGATTTTGAGGACCTTTCACATCTTTTGGAGG

CAATGGTTCCAATGCAGCCCTCTCTAGTAGTGGCGGCACATCCACCAAAACAAACATATC
ATTCTCTACATGAATGAAAGGAGCTGGAGCTGGAGTTTCCTCAGCATCCAACAACTTTGG
TCTCACTTTGTTGCACTTAGCCTGCCCTTCAAGAGCCTTCAATCCACTATACAAGGAATC
CATTACCAAAGCAGATGTGACATCCTTTTCTATAAAAAAGTGCTTTACAACTTCTTTCAG
AATTACATCTGTTTTCTCTCTAGACATCCGGTGCCTAGCATTTTGGTCAATTCCCAACCA
GAAAGCACAGAAGCTTGACCACCGAGCTTTATCTTCTATCAACCTCCCAAGCTTCCCTCG
CCTTTCTTCTAAAAAACGCTGACATATTTCCTCTACATTTGTCATATATACCCTAACAAG
TTCTCTCCTATACTGACGATCAAGGCATCGAAAAGGGCGATCTGATTTCTCCCTGATGAC
TTGAACTTGTGCCTTTATAATCAGAGTGTCACCATCTTTAAACCCATCAGACACTTTTGA
AAGCTCCATAAACTTTTTCCAACCCCAGTCATGCTCTTTCTTCCCAAACCGATGCAAAGT
ATCAGAAT
>ontig_73_consensus
TCCCCAGAGCTACCATCCTTCATACGATTTTGAGGACCTTTCTCATCTTTTGGGGGCAAT
GGTTCCAATGCAGCCCTCTCTAGTAGTAGCACCACATCATCCACCAAAACAAACATATCA
TTCTCTACATGAATGATAGGAGCTGGAGTTTCCTCAGCATCCAACAACTTTGGTCTCACC
TTGTTGCACTTAGCCTGCCCTTCAAGAGCCTTCAATCCACTATACAAGGAATCCATTACC
AAAGTAGATGTGACTTCCTTTTCTATAAAAAAGTGCTTTACAACTACTTTCAGAATTACA
TCTGTCTTCTCTCTAGACATCCGGTGCCTAGCATTTTGGTCAATTCCCAACCAGAAAGCA
CAGAAGCTTGACCACCGAGCTTTATCATCTATCAACCTCCCAAGCTTCCCTCGCCTTTCT
TCTACAAAACGCCGACATATTTGCTCTACATTTGTCAAATATACCCTAACAAGTTCTCTC
CTATACTGACAATCAAGGCATCGAAAAGGGCGATCTGATTTCTCCCTGATGACTTGAACT
TGAGCCTTAATAATTAGAGTGTCACCATCTTTAAACCCATCAGACACTTTTGAAAGCTCC
ATAAACTTTTTCCAACCCCAGTCATGCTCTTTCTTCCAAAACCGATGCAAAGTATCAGAA
T
>ontig_74_consensus
TTTTTTTTTTGGTAATCAAAGAAATTTAATATCCATACAAATTAAACCATAACTTGATTC
AGACGTTAAAACCTTAAATCGAAATTGTCCAGAAAACAAAACATTTGTATCGAACAAACT
ACACTAAGGCCGACCAAAATTCAAGAAACCTAAAGTAGCTCATGCATCAATTCCATGATC
CTCAGCAATGAGCAGCTTTCCAGTAGGCTTTGCCAAGAGATCGGTGTACTCTTGGAAGGG
AGATTTGATGAATCGGGTCTCCCTCCAAAAGTCAGGAGTGAGGAATCCATAGGTCTTCAA
CAAGCACTCAAAAGTTGCCTTCACAAAGTTTCCAAGAGTCTTGGTGGAGCCGCGTGAGGA
AGTGAAAACATCTTCAATCCCAGCGAACTGAAGAACTTTCTTTGGAACCCTAGCAGCCAC
GATCCCAGCTCCACGAGGTGCCGGCACCATTCTCACGGTAACGCTACCACACTTCCCGGT
AACCTTGCAGGGTACAGTGTGGGGCTTACCGATTTTGTTACCCCAGTATCCTCTCCTAAC
AGGAATGACGGAGAGTTTAGCCAATATAATGGCCCCTCGAATAGCTGTGGCCACTTCCTT
CGAGCATTTAACACCGAGCCCCACATGACCGTTACCATCTCCAACGACGACAAAAGCTTT
GAACCTGGTTCGCTGACCGGCACGAGTTTGCTTCTGAACCGGCATGATTTTCATGACCTC
ATCCTTCAAGGAGGGACCGACGAGTTGATCAACGATTTGGTACTCCTTGATCGGCAAAGA
GTGGAGATAGATCTGCTCAAGGCTTCTGATCTTATTCTCCTTAACCAAACGACCGAGCTT
AGTGACTGGAACCCATTTCTCTTCCTCCTCTCTACGGCCAGCGCGGCGGCGGCCACGAGG
GCCTCCACGGTCACCACGGCCTCGCCCGAATCCACGTCCGAAGCTTCCACGTTCGCCACC
ACGCTCTGCCATACTTGATTGGTTAGAATAGGTTTCGCAAGGGTTTTAGGTTAACCAGGG
CTTCCCGCAGAGAAAAAAATGCTGAGCGAGAGGCAGAGTAATGGGT
>ontig_75_consensus
CGTTATTTTAATCAATTCGAATTAAATTTTTTCGAATTTGTAAGCTTAGCAAGCCAAACA
CTTTCCAAACTTGAGTCAGTTTAGCTCAAATCGATTCCTATATATTCCTAGTTCATTTTT
CTCCTTCACCAAGCCACTAGGCAAAAACTTCTATTGGGAAAAATCTTTTACACCAACCAG
TCATCAGGATTGCATATTGGTTCAACAGAAATATGACTTCAATGCTTTAAATTTCATCCT
ACATAGCAACTTTATTCATGCATGGTTCAGTTAAGTGACCACTTGAGGAATTTTGGCAAG
AGATTCCTGTATCTTCTTTTCTTCAAAAGATAAATCCACCATGGCTCCTTGCAAATACTT
CTCATAGGATGGCAAGTCAAAATGACCATGGCCGCACATTGCAGTGAGAATAACCTTTGC
TTCTCCAGTCTCTTTACACTGGAGAGCTTCCCTGATGGTTGCTGCAATAGCATGCGTTGG
CTCTGGCGCTGGTATTATTCCTTCGCTCCGAGCAAATTGTATGGCACCTTGAAAGCACTC
TGTCTGAGGGATTGAAATCGCTTCCATGAGACCCAATTCATAGACATGTGAAAGTAATGG
TGCCATACCATGATATCGTAGTCCTCCAGCATGAATTGGATCAGGAATGAACTCATGTCC
AAGTGTATGCATCTTCATCAATGGAGTCATCCCAGCTGTATCACCAAAATCATATGCATA
AACACCTTTTGTAAGAGACGGACATGCTGCAGGCTCAACTGCTCTTATGACAGGGTTGAT
TTTTCCGCCAAGTTTCTCACGGATGAATGGGAAACTAAGCCCAGCAAAATTGGATCCACC

ACCAGTACACCCTATGATAATGTCAGGGGTTTCACCAGCAGCCTCCATTTGTTTTATACA
TTCTTCACCTATAACAGTCTGGTGTAGCAAGACATGATTTAGAACACTTCCAAGGCAGTA
CTTAGTATCAGCGTTTGTAGCTGCAACCTCTACAGCTTCTGAAATAGCTATTCCTAAACT
TCCTGGGGTGGACGGATCCATTTGAAGGATTCTACGACCTGCTTCAGTGATGTTAGATGG
AGATGGGTATACCTGTGCACCCCAAGTCTGCATCATTAATCTCCGGTAAGGTTTTTGATC
ATAAGAAGCACGGACTTGCCAGACCCCGCAGTCCACACCAAATAAGCTGCATGCAAAGGC
CAGTGAACTTCCCCACTGGCCAGCCCCAGTTTCAGTCACAACTTTTTTGATGCCTTGCTG
TGCATTATAGTAAACTTGTGGAACTGCAGAGTTAGGTTTATGTGATCCAGCCGGGCTGAC
ACCTTCATATTTGTAGTAAATTCTGGCTGGTGTACCAAGAAGCTTCTCCAATCTCCTAGC
TCTGATTAGAGGTGTTGGGCGCCAAAGCTTATAGATATCAAGAACTTCGTCAGGGATATC
TATGAACCTCTCACTGCTAACTTCTTGCTTTATCAATTCATCAGCAAACAAGGGAGCTAA
ATCATCAGGTTTGATTGGTTCAAAAGTTTGAGGATTCAAAAAAGGAGGAGGCTTTACTGA
TAAATCTGCAATCAGATTGTACCATTGATGTGGAATTTCGACTGGTTTGGCATTATCGTT
CAGAGCTGCTCTCACTTTACCACCATTAGAAAGTTTCACATGTGCTATCTTGGTCTTTGG
CACGAAACTCCCAAGAAATTGTTCTGCAGCCTGAAAGTGAATTCTTTTAGAACTCGAAAA
GGGAAGGGGGTTGGCATAGAAAAGTGACAGCGAGGCCATTCCTAGAAACTCTCAGTTCTC
CT
>ontig_76_consensus
GCCTCAGAATTTGCAGGACTTAATTACAAAGTAGATTGTTTAGTATTTATTTGCAATTAT
AATATTTAAAAGGCATGTACAGACTTTCAATGTCTTGGCTTGGCCAAGGGTTCCAAATTC
ATTTCCAATCCAGCATGTCTAATAGCCACAGGACCTCTTCCTTGGAGTTTGCAGCATCCC
AGAAATCCATTTCCAGCCCAAAAACATCTAGGAAACTTTCATTTCCATCTTCCATGAAAT
GCAAAGAAATCCATGCTCGGATTCCAGAAGTGAAAGGGTAAGCAGGGGCAAAGTCAAGAT
ACTCAAATGCATGGGCCCTGTGGGATGATCGATCTACATAATCAAACCTGGATTTATTAA
TTATACAAGCAACTCTATTGGAATCCTTCCGTCCCACAAGCACTGAAGCACTCACTGTCT
GGCTCCAAGGAATTTTGAACCTTGGCTCAACAGGTATGGTCATCATGTACTCACGGTAGT
CCTCAAGATGAAATTGTGCCATGTCTGAAATTCCAAATGGTGGGATCTTGATTCCATTCT
GGAAGACAGGGCCTGGGACCACTTCGGAGAAAATCAATTTATCCTCAGTCCAAATATCAA
TGTAAAATTCCAAATCCTCAAAGGAAAGCCTTGGTGGCAAAAGAGTTCGACACTGCTGCC
GCCGTTTATAAAAGGTATGATATAGTTTGTAGTAGGTTACAGTGGGAGGATTAGGTCGCT
TGCAAATTGAAGGCCACCTCTTTGCACATAAACATTTCCAGAAGTAATCATCCCGGGCTA
TGTCTCTCCATTGCTTACACACGACCATACAGGAAGCAAGGTCTACTCCATCCAATAATA
GGAAAACAGCCTTTAAAATTTCTTCATTTGTCATTGTCGGCTAGAGAACAACAATCAAGA
GCAGAATAATAAACATTCCAGCAACCATTTACAGCATTGCAAGATTGACAGGTGAATGCA
GTTGATTCTGAGGAACTTTCCT
>ontig_77_consensus
ACCCAGATATCATTTATCGTAGTATTAAATAAAAACTAAATAGAATAATAATAATAATAA
TAATAATAATATTCTTATGTAATTCAAGCCGTCCGATTCATCATCTACCATTAAATTCGC
CCTTACATAACCCAAACGGGCCAGGCCCATCCCAAAAAAAACAACAATTAAACCATGAAC
ACTCGTCATCGTGATCATCATCATCATGGTGATAATAAGAATAAACAGGACGACGACAAT
CACGACAGTCTCTGCAACTGCATCATCTTGGAGAAGTCCTCGAAGCACACAAAACCATTA
TCATTTTTACTCACCAACGCTATCATGTGCCGGCAATCGTCTAACGTGCACCACTTGTCC
CCCAGTACCGTGAACACACGCAACAACTCCTCAGCCGTTATTTTCCCATCATGATCCGTG
TCGAAAAAATCAAACGTCTCTCTCAGCTCCGTTTCAAGGGACGGCTCGTAAGCCGAGCCA
ACCACCCTGCTCATCAGCGAGTCCAAGCTTATACAACCAACACCATCAGAACCCACCTCA
CTGAGCATCAAAGACACCTCTTCTTGGGTGGGTGGCTCAGTTCCCAGCCGGATCAAGAGA
GACTCCAGCTCGGTCCTCGAAACCAACCCATCGTTATCCCTGTCGAGCAACTTGAACGCC
TCGACAAGCTCGCAGTGAATATCGGCCGAAATATCCGACCAATCGCCCGATCTTTCGGGT
AAAACGCTGGTGGGGGTGGAGGTAACGTCAGGGCCGGAACTGGGTTTACGACGATGGTTA
GAAGAGGAAGAATCCGAAGAAGAGGTGGAACTAAAAGAAGAAGGGTGGGAACGAGTGACT
CCGTCTTTTTTCTTCTTTGAGAAGAAACTTTTGGAGCCGAGTTTGTGGATGATTTGCTTC
ATTGGAGGAAGTGAAAATGAGAGGAGGAAGATGAAAGGAAGGTTGAGATTTGAGGAAAAG
AAGGAAGGAGAAGAGGTTAAAAGGAGAAAGAGAGGAGTCCATCAGGAAAGAGGGTGAGCT
TCCGGGAACTTTCGCAAAAGGGTATTAAATGGTGATATTTATAATTACGTTGACGGATGT
GGAAGGACTTATTACATGCGGTGGGC
>ontig_78_consensus
ATCTCATGATAATGTGTCAACTCTTTGACACACGAATAATTACTAAATATTTTATATTAA
ATTGAAGGTAAATAGATATAAAAGAAAGACTACATCTAGAGGTATAAGTGAGTCAAACCA

CTCATGAGCTACTGGCAGCTTGACTTGCGTTGAATAGGGCCTGATTCTAGAAATACCAAC
AAGATATTGAAAGAAAACCATTACAAGAAAACTAGTAAAGCAGAAATCCAAAATTCTCTT
CCGACAAAAAATTCCAGAAAAACAAACTATAGTTTTCACCTTAGGGTCCAAGCAAGAACC
AAAAACTCTCATTTAATTAAGGTAACATAAAATTTGATAGTTCATCATTCTGCATCAAGA
ATGGTGCCCTTTTCACTGACATAGATACCGTCAAGGAATTTCCTGATATCCTTGTTCTTG
ACATGGCATTTCTGATTAATTAAAGCACATGACCGAGACACAAGCTCAATGTCACTCCCA
TCCAATATGATCTCATCCTTAACCTTTTCGGATCGGACAATGCTTACACCCTCAAGCATA
TCCACTTTGCGAACCTTTTTCTCTCCAAGAAAATTACGGATCTCAATCGACTTGTTTCCG
TTAGTAATCGAAGCGTTGATCGGAAAGTGAGCGTACACGAATCTCATCTTGTACCGGTAA
CCTTTAGTAACACCAGTAATGAGATTCTCCACGTGACTGAGCGCGGTACGAATGGCGGCG
CTGGTCTTGCGGGAGCCGAACCAGGCATCGATCTTGAGCTTGCGCTTTCCGGTCTCCTCG
TCGGTCATGAGGTGGAAGTCGAGGTTGAGGTGCTTGAAGTTCCGGGTGAGCTTGCCTCGG
GGGCCCTCCACTTCGATAATTTTGGCATTGACTTTGATCTTCACCCCATCAGGGATATCC
ATCGTCTCCGAAGAAAGAATCGTCTTCATCTCCCTCTCCCTCTTCTTCTTCTTCTTCTTC
TTCTTCTTCTTCTTCTTCTAGCTGTGGCTGCGAATCGAAACCAGCGAGGGCACAGTGACG
AT
>ontig_79_consensus
CTGGCTAGTCTCATCATGCACCTGATACTTTGCTAAGGACATTCTTCTCTCCTCCATGGA
CATTGCCTCATCATCCCAAACAAGATAGACCTCATTAACAGCTGGCTGAGTGGCAGGGGC
TTTATTTGCTATTACAGGGGGTGGTCCAATTGAAGGACCACCTGTGTTGGGACCTGAGGC
ATAAGAATGTGAATTGGATAATGTCCCACCTTGCATGCCTACGACATAATAATTGCTGGT
CATAGTTGAGTTGGCACCTGAATGCACATCAACTGAGCCTTTAATTTCCGCTGAAGAGCT
AACTGGAATAGTGGTTGATGGAATGGGAACAGAAAATGGTGAACTTTGAGTAGGTGTACT
GTTATTAACAACAGGGAACAAGGGTTGAGATACAGCAACAGGTGGTGTGGACGAGGGGAT
TCCAGGAGGAATTACTTGTGATGGTTGAAGTGAAGATGTACTTGATGGTAAAGGAGGTCT
CACATTTTGCACGGGAAACAAAGGTTGTTGGACATAACCTGCTGAAGCAGGAGGAATTGA
AACAGCCGGATGCTGTGGATACCAAGGCTGTGGGCGGGGAGGCACTGGCCAACCAGTGGG
AGGTACTGGTACTGCAGAACTGTAGATTGGTTGCATCGCCCCTAAAGTTGGTTGAGGATA
GCCAGCACCTAATTGTCCAGGCACCACTCCAGCTACCTGAGTGTAAGGAGTCTCGACTTT
GGCCACTTTGGAGGGAGCCTCTTCTTCTTCTTCTCCATAGTGAGCAGCTAATATGTCAGG
TGGAATTCCTTGCATTCCATATATTTCAATATCAGTCGACTCTCTTCCAGGTTTTGCATT
GGGAACTTTGGTGACGGACTCCTTGTGAACCTGGAGGACGTGAATGGCCATGCCACCGGC
AGTGGAGAGCTTCTTATGGCAGACGTGGCACTTGAAGTGCTTAGCCTTCTGGTGCTGAAC
TAGAATCTTCTCGTCGTCGAATTCTCTGTCACAGTAATAGCACCATACCTTCGACGATAC
TCTCTTCTTCTTCTTCCCCATTCTCCTTCCCCTAGCAATCCAAATCGAAATCAATTCGAA
TCGAAACCGAGGGATTTAAGATTCTAGGGTTTCTCGGCTTTGCTGAGTCGCTGGCTGGGG
GCTTGTGAGATCTAATAGTAATATTAATGGACGGATGAGATTTTGTGTTAAGTGTTTTCT
TTCGAACGACGAGTAGAAACGGGAATTGAAGTCTGTCAATTGTGTCTCAGAT
>ontig_80_consensus
AACATTGGCCGCATAACTTGGGAAGTAGACAAGTTCCTTTTCCTTCTGCAAATCTTCACT
TATTTTCATAATAACCAGGAAAACTAAACGTCATAAGAGTAAAATGCAGCTGCTGATGTA
TCTCTGGCCTTCCTCAGAGCATGCAATCAGTTCGAGCTGGCAACCTCCTGAAGAAATTCA
GTGGCACAGAAGGAAGCCTATGACGGAACCTTGGATGTCTTAACACATAAAATCCTGTGA
GAAGAGCCACAACTTGGAATGGTGTAACATAAAGAACAATGGCAGCAATCAAACAAAAAA
TCACAAACAGAGCAGTGGCTCTCGGATCTCTCCAGCTCAACAAAGATTGCAGCCTCTCCC
CTTGCGTAGCCAAATCACCAACCACAGTCTGAATTCTCCCAGCAATGCTTCTCAATCGAT
CATATCTCATCCTTACAATATCAGCAGGCCTAGAGGTCGGAAATGTATCAAATTCTTCAT
CCAGTTCATCAGGATGAGCAATATCTGCATGAGAGAGACGAGTGTCCATGTGAGGAGGAT
GCCTTGGTCTCCACCTATAATACCAAACTCCAATCAAGAACAGGTATAGGAAAATTGTAG
GCAAAATAAGCTCTGGGTACAACACCAATATGATAAACAAGATGTGAATCAACACTGTTG
TAATGGGGTTTTTCCAATTGCAGATCTGATCAAACCATCTTCCCACAGCAATTAACCCAC
TCAAGACTCCCATGATCCTGAAAAAATTAGCTTTACTTCTTCTCATGCTCCACATGTGAG
AACCCACATCCAGCATAAATTCCACAACCTCCTTCCTCAATGGTGGTTCGGCTCGGCTAA
GCCTCATTGACACAATCTGAGTGGCCTGATGCCTCAAGTTATCCAGTTGGCTAACAGTTA
AAGGATGCAGATAATGCATCTTCGGCAACAATGGTTGTGAGTACATATGCATCATGTTAA
GCAATGATGAGCACGTGAATCTCACAGCCAAATGAATTTCACCCATCTTCTTGACGCCAT
TTGTATATAGAACCAGAAGTGGATAGGAATGAGTATAAACACGATCTGTTTCGAGGGTAG
ACAGACGAATCCTTACCTTTCCAATCTTTGTATCCTTTAACCCTCCAGATTTATCTCCTC

CATGCACGTGCCCATTATCAAACACACCAATTGTTATAACAGTACAAGGGTCAAAAACTT
CCCATGTGTATTGCTCATTCCACTTCGGAGTAAAGCTATCTATGATTGTTCTGGTTCGAA
CCCATTTCTGCCCATACTTTGCCACACAATAAGCATCTGTAGTGTTCCGCCCATCTTTAT
TCTTCATTGGCATCAAACCTTGAGCATTCAAAATTCCCAATTCTAGTACCCCTATGTTGG
ACTTCCATAACCGTTTGTCTGTTGGCCTAAGATCACTACTATAGTGTGTGGACTCATCCA
AGACATGATAACCACCTTCCAAACAGATCCTCACGTGAATCCTGCTAGCAAACTTTGTGT
CCTTTTTCTCCCCTTCCACAATCATATGCTTTTCAAGATTAAACCACCTAGTGTTTACAG
GTTTATGATCAAGCCTCCTTTCCACATACTGCAAAGGAATCACACACCTTCCCAAAACTT
CATCCTTGTTAGGTGCAACTCTATCTTCTACGCTCAAAATCAAGTGCTCCTCAAATGGTT
CTGCAGCAACAAACATCAGATCCTCATTCCACAAAGGATTTATAGTCCTACTAGCAGAAC
TTCTAGTTCTCAAAGCCTGATTTCCAAGTATGGCCTTCACAAAAACATCTGGATACCTGC
CCTTGTCAGTGGGTTGCAGGTCCTGAGCTTCAATCACATTAACCCTCAAATACCAAAGCT
TAGGAGAGAGATACACCTTGGACCTGATATTTGCTAGACCATCCATGCCACTAACAGTGG
CTGCATCCGAACGCCATGCCTCAGGAAATGCCTCATCTGCTTGAGTACCCATCCAAACAG
CCAACATTAATTCTCCTTTAACTTTATCCCCCTTCCTATCCTCCAACCTATACCACTGTG
GAGCCAAAGGACTATCTGGGGGAACCCGCTTAGGAATCTCATTCAAATCAAAAAAAACTC
TACCCATAAAATCATCCTTCACAAAGTCCTTATCCTTCACAATAACCTCAAGCACAGATG
CCTGAATCCGATCCTTTGAGAATGCAAACACCTGTTTCCACTCAGGATTTGATTTCTTCT
CAAAATGCCGAGTGGTACCCTTATAATTGCCAAGCTTAACTTCAACATATGGGTCACAAG
TACCAGTAACATCTTTTCCAGGTAAGTCCTTAGCCTTCTCAACCCGGACATAAAGATATT
GCATCTGCTCAACAAGGTCATAGGTACTGGTGAGCTTGTCACCGGTGACCTTTCCCCCAC
CCAAATGGGGATTGGTCTCTTTCAACGAAAAATCTTCTGGAGGTGGTCTCTGCATCATCT
TTTATGGAGAGAGAGAGAGATCGAAATTCCAAAACTTCAAATGGACTCGAACAGTTGCGT
TAGAAAACTGAAGAGTTGTAGTTTTCTAATTAATTCAAAAAGTTTCCGGAAACCTTACAA
GCGCTTTTTTAAAG
>ontig_81_consensus
CTATCCTCCAACCTATACCACTGTGGAGCCAAAGGACTATCAGGAGGAACCCGCTTTGGA
ATCTCATTCATATCAAAATAAACTCTACCCATAAAATCATCTTTCACAAAGTCCTTATCC
TTCACAATGACTTCAAGTACAGATGCCTGAATCCGATCCTTAGAGAAGGCAAACACCTGG
TTCCACTCAGGATTTGATTTCTTCTCAAAATGCCGAGTAGTACCCTTATAATTGCCAAGC
TTAACTTCAACATAAGGGTCACAACTACCAGAGACATCTTTTCCAGGTAAGTCCTTGGCC
TTGACAACCCGGACATAGAGGTACTGCATCTGCTCCACAAGGTCATAGGTGCTGGTGAGC
TTGTCACCGGTGATCTTGCCCCCACCCAAATGGGGATTGGTCTCTTTCAAAGAAAAATCT
TCTGGAGGTGGTCTGTGCATCATGTTTTACGGGGAGAAAACCGAAGAGTTGTATCGTTTT
GAATTCAAAATGCTTCCGGAAAACCTTAAAGGGGGTTTTTAAAGCTTCAGATCGGTGAG
CTTAACCATGAACGATCAAAGCAGCGAAACAAACTTCGTTTTTTTTACTTTTAGTGTTCG
TTGTTTCAGAGT
>ontig_82_consensus
GTTCTAACAAGTTCAAAGACAGATATCCAGATTCACCCAGCAACAGAGAAGACGAGAACA
AAACCATTTGGAAATTAAGGGGCTAATTTTCCTGCTGAAATCACAAATCCAGAAAAAAAT
TTTGGAGCATCATAGCTCAGCAAAATTCGTAATGCAGCAATCACATTCACCATCTGCTTC
CACCACATGTAAACAGCATTATTGACAATTTTTGATAAGTTGAAGATAACATTACGAATT
TTGACACCACTAACACAGTACTGTAATGCTTATATTCTGCAACTAATCTGGGTAGTGTTG
ACTAATATCTTAAACTCATGATCATCGTTGCAGCTGAGATTTGTGAGGTGTAAACAGCAG
AAAACAGCTAGTTCACAGCAGGATCATAAATTCAATACATTAAAATGCATGAAGATTGAA
GACTCTGTGCTATCCCAAAATTCAGTCTTCGCTAGGCTCCTGAATTTGGATCGATGTCTT
TGCTAAGCTTGCCTCCAGTTCATTTAGCTCATCAATGTCCAACTCATCATCGTCATCATC
AGGAAGTAATTCATCATCCTGAACTGCTGTTGCTGAGCTACTTGGTCCATCTCCAGCAGA
ATTGCCACTGACCTTCTGTTCCTTAGTATCAGATTGTTCCTCTCTCTGGTACTTCTCATA
TGCCTCAGCATCATCCACAAACACACTAGAGTCTGACAGGAATAGTTCACGCCCACTCAT
ACGGTCATTCTTAGCTCTCTCTGCCCTCTGTGCAGCCAAACCAGCATCTCTTTCAGCAAT
CTTCTTCTTCTTCCACTCCATGAACAACTCAGGAGTCATGGGCGTTGAAGAAGTTACTTT
AGCACGCTGATTTTCAATCTCCTCCTCAATAGTTATCTTTTCACTCTCTTCCTCTAACAG
AGCCTTCATCTGAGATTTCAAAACATATCCAGGAGGAAGAGCATGTCTGTAGTGGCAGTC
TTTACCACCATTAGGGCAGATCCAAAACCATCCATATTGTTTCTTCTCCACAGCTTCCAA
GAAGAATTTACAAACAATATCAGTAGGTTTGTTCTGTTGATACTCTTTATTCTTTGATTC
CACGACCTTCTCCAAAGTTTCTTGATCCCAATCCTCCAT
>ontig_83_consensus

GTGGGAAACAGATTAAGCCGATAAATTTAATTTAATTAGGTCACAAAATATTCAAATACG
TACTTTAGACAAACCACTGACAATATCAATTCACACTTCTGCCAAGTTGAATTATAAATT
TCATTGAATAGCCTTCAGGAACGAACACCAGGAAACAACGGCAAAAATAGTCTCCTGATT
TCTAACTATGCGGCATGGCAACGTTCATATGCCCATATTTTTGCATCACTTTCTTCATTG
CAAGGGCTGCCGCTTCACTTGCTCGTATTGTCCCAGTTGCCATGATTGCAGTAAATTCAC
GGCCAATCTCCTCTTGCACCTTACTTCGGGCATCTCCTAGAGGATCTTCTGTTACCCCAT
TAACCCCCGGCTTCTCTTGAGCTTCCACAGGTTTGACAGCAGGATGCTCCGCATTATCCA
GTGCCTTTGGCTCATCAGAACTTGCAGGTTGCTCTTCAGGTTTCACTTTACCATTGGCCT
CTCTCACAGTCTTTTGCTCAGCGGGAGTGCTACGACTAAAGCTAGAGCCTTTGTTGACAG
TAGCT
>ontig_84_consensus
GTGCTCAACTACCTGATGAATATAAAAGAAATAATAATCTTGAAGGCCTTGAAATCTACT
CAAAAGCCACAAAGAAAATACTAGCATCCACTAACTACTAACAGTTGATACATATCAGTT
AAAGGAAAGCATTTCTACAGCACAGCTTTTTAGTCTTGCAGAGATACTGAAATCCACTAC
AGTACAGAAAAGCAAAACTAACATATATAGTTGATCATGAAATCCTCCAAAATCAACTTC
AGACACTAACGAAACGCAAGTAATCCTTCTTGGAAGGAAGTTCTACAGTCCTGTAACCTT
TGGGGAACATTTCTTTGGCTTGCTCATTGGTCACACTGGGGGAGATCACCACAGACTCTC
CCGGTTTCCAGTTGGCAGGTGTCGCTATCTTGTGCTTTGCCGCCAACTGCAGAGAGTCTA
TCGCTCTCAACACTTCATCCATGTTCCGCCCCGTCGTTGCAGGGTATAGAAAGCTCAGCT
TAATCAGCTTGTCGGGGCCTACGATATGAAGGGCTCGAGATGGCATATGTTGTCCTTTTG
AATCTCTCTCATCTGGGTCCATCATGTTGAGTTGTTTGATGAGCTCTCGGTTGGGATCTG
CAATGATCGGGTACGTCACCTTGCATCCAGGAACATAGGCTTCAATGTCCTTGGTCCAAT
CATTATGGGACTGCACATCGTCACAAGACAAACCCAGAAGCTTCACCCCTCTCTTTTCGA
ACTCCGGTATGTATTGGGCCATTTTAGCAAGTTCAGTTGTACACACAGGTGTGAAATCAC
CCAGCCATCGCCGACGTAGTCATGGATCTTCATCTTCCCTTGTGTGGTTTCAACCTCAAG
GTTGGGACAGGTATCTCCAATTGTGAGGCCTGGCATTTTCAACAACACACTCTGGACTCT
GCAAATTCAACCTTCCAGATCGGAAGAGC
>ontig_85_consensus
AAAAAAAAGGCACCTTTCCTCTCCTCAGCCATTTCGGGTTAAGAAGATGTGAAAGCACCT
TTCTCTCTATAAGAATAAGAACACGGTGCGTTACGAGGTGCGAAGTGGGAGAGAAGGGAT
TTCATAATTGGGGTTTTGAATAAGACGACCTTTTCATTTTTCATTTTTTTTCATATTGCA
AAAGTAATAAGAATGAGAGGTGTTAAGCTTTTTATCATCCTGGCGTCGAGCTATTTTTCC
GCAGGACCTCCCCTACAGTATCGTCACCGCAGTAGAGTTTAACCACCAAGTTCGGGATGG
ATTGGTGTGGTTCCTCTACGCCTAGGACACCAGAATATCGAACCATGAACGAAGAAAGGC
ATGCGAGAAAAGCATATTGGCTAGTGATTGTGAGGCCCCAATTCTTAACTGGAGGGGACA
CCAAAGGCCTCTGCCCTTCCATCCCTTGGATAGATAGAGAGGGAGGGCAGAGCTTTTGGT
TTTTTCATGTTGTCAAAGAGTTGAACAATGGTTTTTCGTGTTGTCAAAAAGTTGAACAAT
GAAAATAGATGGCGAGTGCCTGATCGAATTGATCGAGTCATGTAGGAACAAGGTTCAAGT
CTACCGGTCTGTTAGGGTGCCTCAGCTGCATACATCACTGCACTTCCACTTGACACCTAT
CGTAATGATAAACGGCTCGTCTCGCCGTGACCTTCTCTTGAATTCTCAAAACTTCTGTCG
CTCCATCCCCGCAGGGGCAGAGAACCCGTCGCTTTCTCGGCTGTGCTAGCGGAGGCTCTG
GGGAAGTCGGAATAGGAGAGCACTCATCTTGGGGTGGGCTTACTACTTAGATGCTTTCAG
CAGTTATCCGCTCCGCACTTGGCTACCCAGCGTTTACCGTGGGCACGATAACTGGTACAC
CAGAGGTGCGCCCTTCCCGGTCCTCTCGTACTAGGGAAAGGTCCTCTCAATGCTCTAACG
CCCACACCGGATATGGACCGAACTGTCTCACGACGTTCTGAACCCAGCTCACGTACCGCT
TTAATGGGCGAACAGCCCAACCCTTGGAACATACTACAGCCCCAGGTGGCGAAGAGCCGA
CATCGAGGTGCCAAACCTTCCCGTCGATGTGAGCTCTTGGGGAAGATCAGCCTGTTATCC
CTAGAGTAACTTTTATCCGTTGAGCGACGGCCCTTCCACTCGGCACCGTCGGATCACTAA
GGCCGACTTTCGTCCCTGCTCGACGGGTGGGTCTTGCAGTCAAGCTCCCTTCTGCCTTTG
CACTCGAGGGCCAATCTCCGTCCGGCCCGAGGAAACCTTTGCACGCCTCCGTTACCTTTT
GGGAGGCCTACGCCCCATAGAAACTGTCTACCTGAGACTGTCCCTTGGCCCGTAGGTCCT
GACACAAGGTTAGAATTCTAGCTCTTCCAGAGTGGTATCTCACTGATGGCTCGGGCCCCC
CCGGAAGGAGGCCTTCTTCGCCTTCCACCTAAGCTGCGCAGGAAAAGCCCAAAGCCAATC
CCAGGGAACAGTGAAGCTTCATAGGGTCTTTCTGTCCAGGTGCAGGTAGTCCGCATCTTC
ACAGACATGTCTATTTCACCGAGCCTCTCTCCGAGACAGTGCCCAGATCGTTACGCCTTT
CGTGCGGGTCGGAACTTACCCGACAAGGAATTTCGCTACCTTAGGACCGTTATTGTTACG
GCCGCCGTTCACCGGGGCTTCGGTCGCCGGCTCCCCTGTCATCAGGTCACCAACTTCCTT
GACCTTCCGGCACTGGGCAGGCGTCAGCCCCCATACATGGTCTTACGACTTTGCGGAGAC

CTGTGTTTTTGGTAAACAGTCGCCCGGGCCTGGTCACTGCGACCCCCTTTGTGAGGAGGC
ACCCCTTCTCCCGAAGTTACGGGGCTATTTTGCCGAGTTCCTTAGAGAGAGTTGTCTCGC
GCCCCTAGGTATTCTCTACCTACCCACCTGTGTCGGTTTCGGGTACAGGTACCCTTTTGT
TGAAGGTCGTTCGAGCTTTTCCTGGGAGTATAGCATGGGTTACTTCAGCGCCGTAGCGCC
TGGTACTCGAACATTGGCTCGGGGCATTTTCTCTACCCCTTCTTACCCTGAAAGGGCAGG
GGCACCTTGCGTCCTTGAACCGATAACCATCTTTCGGCTAACCTAGCCTCCTCCGTCCCT
CGGGACCAACAAGGGGTAGTACAGGAATATTCACCTGTTGTCCATCGACTACGCCTTTCG
GCCTGATCTTAGGCCCTGACTCACCCTCCGTGGACGAACCTTGCGGAGGAACCCTTGGGT
TTTCGGGGCATTGGATTCTCACCAATGTTTGCGTTACTCAAGCCGACATTCTCGCTTCCG
CTTCGTCCACCGCCGCTCGCGCGGGTGCTTCCCCCTAAGGCGGAACGCTCCCCTACCGAT
GCATTTTTACATCCCACAGCTTCGGCAGATCGCTTAGCCCCGTTCATCTTCGGCGCAAGA
GCGCTCGATCAGTGAGCTATTACGCACTCTTTCAAGGGTGGCTGCTTCTAGGCAAACCTC
CTGGCTGTCTCTGCACCCCTACCTCCTTTATCACTGAGCGGTCATTTAGGGGCCTTAGCT
GGTGATCCGGGCTGTTTCCCTCTCGACGATGAAGCTTATCCCCCATCGTCTCACTGGCCG
ACCTTGACCCCTGTTATTTTGAGGTCATATCTAGTATTCAGAGTTTGCCTCGATTTGGTA
CCGCTCTCGCGGCCCGCACCGAAACAGTGCTTTACCCCTAGATGTCCAGTCAACTGCTGC
GCCTCAACGCATTTCGGGGAGAACCAGCTAGCTCTGGGTTCGAGTGGCATTTCACCCCTA
ACCACAACTCATCCGCTGATTCTTCAACATCAGTCGGTTCGGACCTCCACTTAGTTTCAC
CCAAGCTTCATCCTGGTCATGGATAGATCACCCAGGTTCGGGTCCATAAGCAGTGACAAT
TGCCCTATGAAGACTCGCTTTCGCTACGGCTCCGGTGGTTCCCTTAACCAAGCCACTGCT
TATGAGTCGCCGGCTCATTCTTCAACAGGCACGCGGTCAGAGCCCTGGTCTCGTCTCCTC
CCACTGCTTGGGAGCTTGCGGTTTCATGTTCTATTTCACTCCCCGATGGGGGTTCTTTTC
ACCCTTCCCTCACGGTACTACTTCGCTATCGGTCACCCAGGAGTATTTAGCCTTGCAAGG
TGGTCCTTGCTGATTCACACGGGATTCCACGTGCCCCATGCTACTCGGGTCAGAGCGTAA
GCTAGTGATGCTTTCGGCTACTGGACTCTCGCCATCTAGGGTGCAGCACTCCATCGCTTC
GCCTAGCAGCACGACGCTTGTATTGCTCTCCCACAACCCCGTTTTCACGGTTTAGGCTGC
TCCCATTTCGCTCGCCGCTACTACGGGAATCGCTTTTGCTTTCTTTTCCTCTGGCTACTA
AGATGTTTCAGTTCGCCAGGTTGTCTCTTGCCTGCCCATGGATTCAGCAGCAGTTCGAAA
GGTTGACCTATTCGGGAATCTCCGGATCTACGCTTATTTTCAACTCCCCGAAGCATTTCG
TCGCTTACTACGCCCTTCCTCGTCTCTGGGTGCCTAGGTATCCACCGTAAGCCTTTCCTT
GTTTGAACCTCGCCCTTCACTTTACTTTAAGGCTATGCCATCCTAAGGTGCTGCTAAATG
GAAGGATCTTATCAACGTCCATGAATGATAAACCATCGATCGAACTGCCGAATCGGAAAA
ATTGGGTGCTATCATATTCATATAGCTTTGTATCGGCTAAGTTCACGAGTTGGAGATAAG
CGGACTCGAACCGCTGACATCCGCCACAGGGTAAACCACCGCCTCTCAGGCCCCCACTGA
TTCTACCATAGAGGCCAACGATAGACAATAACTCCCCCCCGAACACAGCTTACAACTTTC
ATCGTACTGTGCTCTCCAAAGAGCAACTCTTCTCAAAATCTCAAAGGGTGCTGAGTTGGA
ATCCCATTCTAAGTAAGGATTCTTGTGGTTCCGGAGGATCCAGCTACAGAAGAACCGGGA
ACGGAGAGCTTTCCCCCCTTTTCCGCCCCGACTCTTTGGTCTTAAGAATGCTGGTTTTAA
GAATGAGTGATTGCTCCTTCTCCGACCGCCGGTTTTAAGAATGAGTAATTGCCCTTCTCC
GACCCTTAACTGCCCAACCTGAGAGTGGACAGCTAATGCAGTCCACTTATTGAGCAGGGT
TCTATGGTCGGTCCGCGACCCCTGGATACCGAAGGCGTCCTTGGGGTGATCTCGTAGTTC
CTACGGGGTGGAGACGATGGGGTCGGTCCATGGATTTTCCTTCCTTTTGTCGCATTTCGC
TCAAAGGGTTGAAGGGAGATAGTGCATCAAGCTGTTCGCAAGGGCCAACTTGATCCTCTT
CCCCAGGGATCCCAGATGAGGGAACCCTAAGAGAGCCGTCGACTCCAACTACCGTTCATG
TACGATCCATACTAGATCTGACCAACCGCCCATCCTACCTCTTCTACGTTCTTGACAGCC
CATCTTTGTCTCAGTAGAGTCTTTCAGTGGCATGTTTCGGTCCTCTTCCCCATTACTTAG
AAAAAGTGAGCCACCGGTTCAGGTACAAGATACTATCATTACCGCCTGGACAATTAGACA
TCCAACCCGTAATCGCAACGACCCAATTGCAAGAGCGGAGCTCTACCAACTGAGCTATAT
CCCCCCGAGCCAAGTGGAGCATGCATGAAGGAGTCAGATGCTTCTTCTATTCTTTTCCTT
GGCGCAGCTGGGCCATCCTGGATTTGAACCAGAGACCTCACCCGTGAAGTAAATCATCGC
ACCTACGGTCCAACCAATTGGGAGAGAATCAACAGATTCCTTTTCGGGAGCGATTCATCC
TTCCCGAACGCAGCATACAACTCTCCGTTGTACTGCGCTCTCCAAGTGTGCTTGTTCCCC
CCTTCTTCCTTACCATGGCAAGTATTTGTGAAATAACTCCGATGAGAAGAAAAAAGAAGG
CATTAAGAGACCCTCCCGGCCCAACCCTAGACACTCTAAGATCCTTTTTCAAACCTGCTC
CCATTTCGAGTCAAGAGATAGATAAATAGACACATCCCATTGCACCGATGGGGGGCGTTC
GTAGTGCCTGAGGGGGTCGAAGACCAAGAAGTGAGTTATTTATCAGCCAAGCATTCTTCT
TACGGCTAGATCCAATCTCCTGGTCCCTGCGGAAAGGAAAAGGAAAAAGAATTTCACGTT
CTTCCTTTCGGGAAGGGAGGATTAGGAAAATCCTATTGATTGCAGCTTTCTCCAGACCTC

CGGGAAAAGCATGAAAAAAAAGGCTCGAATGGTACGATCCCTCCGTCACCCCAGAATGAA
ATGAAAGGGGCGATCTCGTAGTTCTTGGTCTGTGAAGATACGTTGTTAGGTGCTCCATTT
TTTTTCCCATTGAGGCCGAACCTAAACCTGTGCTCGAGAGATAGCTGTCCATACACTGAT
AAGGGATGTATGGATTATCGAGAAGAGAGGAGCCGTGGTGGTCCCCCCCGGACCGCCCGG
ATCCCACGAGTGAATAGAAAGTTGGATCTACATTGGATCTCACCTGAATCGCCCCATCTA
TCCTCCTGAGGAGGAGTTTGGTTTCAAACCCCCGGTTCGAACAGGAGAAGTACGCCATGC
TAATGTGCCTTGGATGATCCACATCTCAGGGTCAGGCGCTGATGAGCACATTGAACTATC
CATGTGGCTGAGAGCCCTCACAGCCCAGGCACAACGACGCAATTATCAGGGGCGCGCTCT
ACCACTGAGCTAATAGCCCGTCGTGCGGGCCTCCCGCTGGGGGCCCACTATGCCAAAAGC
GAGAGAAACCCCATCCCTCTCTTTCCTTTTTACGCCCCCATGTCGCCATAAGGGAGGGAC
ATGGGGGCGTAAAAGGGGGATCCTATCAACTTGTTCAACTTGTTCCGACCTAGGATAATA
AGCTCATGGGCTTGGTCTTACTTCACCGTCGAGAAATGAAAGAAGACTTCCATCTCCAAG
TTTCACTCAGGCGTAGCTCGCTTCTTTTTGGGTGTGAAGCGGTGTCAAACCAAAATACTC
AACAAGCATTAGCTCTCCCTGAAAAGGAGGTGATCCAGCCGCACCTTCCAGTACGGCTAC
CTTGTTACGACTTCACTCCAGTCACTAGCCCTGCCTTCGGCACCCCCCTCCTTGCGGTTA
AGGTAACGACTTCGGGCATGGCCAGCTCCCATAGTGTGACGGGCGGTGTGTACAAGGCCC
GGGAACGAATTCACCGCCGTATGGCTGACCGGCGATTACTAGCGATTCCGGCTTCATGCA
GGCGAGTTGCAGCCTGCAATCCGAACTGAGGACGGATTTTTGGAGTTAGCTCCCCCTCGC
GGGATCGCGACCCTTTGTCCCGGCCATTGTAGCACGTGTGTCGCCCAGGGCATAAGGGGC
ATGATGACTTGACGTCATCCTCACCTTCCTCCGGCTTATCACCGGCAGTCTGCTCAGGGT
TCCAAACTCAACGGTGGCAACTAAGCACGAGGGTTGCGCTCGTTGCGGGACTTAACCCAA
CACCTTACGGCACGAGCTGACGACAGCCATGCACCACCTGTGTCCGCGTTCCCGAAGGCA
CCCCTCTCTTTCAAGAGGATTCGCGGCATGTCAAGCCCTGGTAAGGTTCTTCGCTTTGCA
TCGAATTAAACCACATGCTCCACCGCTTGTGCGGGCCCCCGTCAATTCCTTTGAGTTTCA
TTCTTGCGAACGTACTCCCCAGGCGGGATACTTAACGCGTTAGCTACAGCACTGCACGGG
TCGATACGCACAGCGCCTAGTATCCATCGTTTACGGCTAGGACTACTGGGGTATCTAATC
CCATTCGCTCCCCTAGCTTTCGTCTCTCAGTGTCAGTGTCGGCCCAGCAGAGTGCTTTCG
CCGTTGGTGTTCTTTCCGATCTCTACGCATTTCACCGCTCCACCGGAAATTCCCTCTGCC
CCTACCGTACTCCAGCTTGGTAGTTTCCACCGCCTGTCCAGGGTTGAGCCCTGGGATTTG
ACGGCGGACTTAAAAAGCCACCTACAGACGCTTTACGCCCAATCATTCCGGATAACGCTT
GCATCCTCTGTATTACCGCGGCTGCTGGCACAGAGTTAGCCGATGCTTATTCCCCAGATA
CCGTCATTGCTTCTTCTCCGGGAAAAGAAGTTCACGACCCGTGGGCCTTCTACCTCCACG
CGGCATTGCTCCGTCAAGCTTTCGCCCATTGCGGAAAATTCCCCACTGCTGCCTCCCGTA
GGAGTCTGGGCCGTGTCTCAGTCCCAGTGTGGCTGATCATCCTCTCGGACCAGCTACTGA
TCATCGCCTTGGTAAGCTATTGCCTCACCAACTAGCTAATCAGACGCGAGCCCCTCCTCG
GGCGGATTCCTCCTTTTGCTCCTCAGCCTACGGGGTATTAGCAGCCGTTTCCAGCTGTTG
TTCCCCTCCCAAGGGCAGGTTCTTACGCGTTACTCACCCGTCCGCCACTGGAAACACCAC
TTCCCGTCCGACTTGCATGTGTTAAGCATGCCGCCAGCGTTCATCCTGAGCCAGGATCGA
ACTCTCCATGAGATTCATAGTTGCATTACTTATAGCTTCCTTGTTCGTAGACAAAGCCGA
TTCGGAATTGTCTTTCATTCCAAATTCCAAGGCATAACTTGTATCCATGCGCTTCATATT
CGCCTGGAGTTC
>ontig_86_consensus
CTTTGTTGGCGAAGCTTTTCAGCTACAGGATCCAATGGCACTTCCTTTGCTACTTCAGCT
ACTTTTTTATTTTTCTCACTAGGTTTTTTGTCAGCCTTTTTGGAGACTTTTTCAGCAGGA
GCAGCTGGAGCAGGTGCAGGAGCGGGTTCATCTTCATCCTCCCACGAATCTTTTACATCG
CTCTCTTCTACATCTTCATCATCCCATGCATTTTTAACTGGCTCCTTTTCAGGAAGAGGT
GGAATTACATCATCTTCCCAATCTTCCATCGTCGACGCAGAATCTAAAGCTTAAATATCA
CCGAGGCGGAGAAATTATTAGAGTCAAAACCCTAAAAACCGAAACGAGACGGAAAGAAAA
GAAATCGAGAGAGAAAATATAGGGGACTCGAATCTTCTAAGAAGAATCGAAAAAGTCTGA
ATGGTCAGTAAGAGTTGTGAGCGCCAGAGGTCGAAAGCTGAAGACGTTTCAAGTTTGTGC
TGTGTTTCGAAGACTGAACCAGCCGACGAGTTTAGGGTTAAAATCATTTCACCCCTTAGG
CTTTCCCAAAATGTTGTTTGGGAAACTGCTGAACACGT
>ontig_87_consensus
AGCTCAAAAAAAAAAATGCTCAAATCTCTGGAAAACCATCTCAGACTCTCAGCCATTCACT
AAACAAGAAAATTACTGGTTGAGTAAAAAGATTAACAAAATTAAAATCATACAACTAATT
TTAAAGACATCTCCATCAAATATTAACTTTGCATCCAAAAAACAACCTTGTTTCTAATGT
AAAATGTGTTTAAATAAACATATCTGCCAGTGCAGTAGCTCTTCAAAGAAAACTAATACG
GATACAAATGCATCCAAATAAGCTGATTCAGTTCACAAAACTTTTAAACCAAATAACAAA

ACAGCGAGAACAAAACAAAGAAAAAGCCTAGTAACCTCCCTTGAGATCGTTAACAACATC
ATAAGGAATTGGCGTGACAGTTTCCAATGTTGCACCCTCCACCATGAAACCTGGTTTTGG
CCCCTCTGGTTGCCTCTTTGTACTGATGACCTCACCCTTTGCCTTCGCTTCAGCCTTGAG
TTCATCATTCTTCTTCTTCCTCAGCTTGAATTCCTCGGTACACCTAGAGGGCTGCACATG
CTCCACACGGACATGGATCCTCTTCCTAATTATCCTGTTACCCACCTGCTTGTTGACCTC
GACACCAATGGCGCGTTTGGTGACGTTCCAAACGCGGCCAGTGCGGCCGTGGTAGAATTT
GTGGGGCATACCCTTGTGAACGGCACCATTAACTTTGACGTCAACGTAATCACCGATCTT
GTAAGTTCTAAGGTAGGTTGATAACGGGATGTAACCCTTCTTTCTGAATCCCCGGGCAAA
GAGATCCCTTGTTCTGGCTCTCACACCATGACCAGCCGGCATTTTCACTCACCTACTGAG
AGCTTTGCCTCTCTGTGAA
>ontig_88_consensus
TGAGCTTTCAAACAATTATAATCTTTCCAGATAATACTTGGATAATATTAATGGGAAAGC
ATCATTCAGTCATGAATGTCAACAACCATTGATGAGCCACATTAAGCTTCAGTATACAAA
TACATTATTTTCAACTCTATGTTTTCCTTTCCCAGATTCCCCTTCCTGCATATTCAGGGT
TTGGTCAATTCTTCCGCCCTTCAACCAAAATTATGTGGTATCCATGCGTTGATTTGAAGG
GTGCACTGGTAGCTCCAACTGGTGTACTGAAGGCAACTTCCTGAAATGGGCCGGCCATTT
TTCCACGTGGAAACCATCCAAGGTCTCCACCCTTCTTACCAGATGGGCATTCTGAATACT
CCGCAGCTATCTTGGCAAACTCAGCTGGTGGAACCTTATCTCCATTGCTAAGCCAACCAT
CTTGCAACTTCTTGTATGCTTCATTGATCTTCCCTTGTTTCTCACATAAAAGGTGTCTTG
CTTTGACATATGTGCAGGTGCCAAGCCCATCAGCTGACTTCCCACCTTTTCCTTTACCCT
TTGAACCATTCTCATCAC
>ontig_89_consensus
TCCCAGATTCCCCTTCCTGCATATTCAGGGTTTGGTCAATTCTTCCGCCCTTCAACCAAA
ATTATGTGGTATCCATGCCTGCATTGCAAGGTGCAAAATAAGCTAGTATATGCGATGGAT
AATATTGCTTGTCCACAAAATATGCCTAGAACAAGTTGGTATAGAAGGTTAAATGAAAAA
AAAATTAAGAGCATACGTTGATTTGAAGGGTGCACTGGTAGCTCCAACTGGTGTACTGAA
GG
>ontig_90_consensus
CTGATTTAAAGAAAACTTTTAACAAATTAGACTCTTATAAGAATCAAAACCCCAAAAACA
TAGTTTAATTTCTATCAACACAAGACAAGTTAAAATCAATAGGATCAGTATCCAAATTCC
CCTTCCTACCTTGTCAACCCATGATAGTCTTAATTTTGCTAAATTTAATAATCTTAATTT
TATGATAACTTTAAGATTCCCTCAATCAAACACAGCCTTAAAAGACATCTCCATCAGATA
TTAACTTAGAATTCGGAAAACACCCTGGTTTCTAATGTAAAATGTGATTAAATAAACATC
TCTACTAGTGCAGTATGACTTCCAAAAAGAGACTGATTCAGATAAAAATGTATCCAAATA
AGCTGATTTAGTTCTCAAAAGGTTTTCTGCTGGATAGCAAAACACCAAGAACATAACAAT
GAAAAAAAAACTAGTAACCTCCCTTCAGATCATTAACAACATCATATGGAATGGGTGTGA
CAGTTTCCAATGTTGCAGCTTCCACCATGAAACCTGGTTTAGGTCCCTCTGGTTGTCTCT
TTGTACTGATGACCTCACCCTTGGCCTTGGCTTCAGCCTTGAGTTCATCATTCTTCTTCT
TCCTCAGTTTGAATTCCTCCGTACATCTGGATGGCTGCACATGCTCCACACGTACATGGA
TCCTCTTCCTAATTATCCTGTTACCCACCTGCTTGTTGACCTCGACACCGATGGCGCGCT
TGGTGATGTTCCAAACGCGGCCAGTGCGACCATGGTAGAACTTGTGAGGCATTCCCTTGT
GAACGGCGCCATTAACCTTGACGTCAACGTAATCACCGATCTTGTAAGTTCTAAGATAGG
TCGATAATGGGATGTAACCCTTCTTTCTGAATCCCCGGGCAAAAAGATCCCTTGTTCTAG
ATCTCACACCATGACCGGCTGGCATTTTTACTCACCTTCTGAGAGCTTTCTCTCTCTACG
GTTTGAAGAAGAAGAAGAGGTGGATGGGTGATAGCTAAAACCCTAGACTTTCGAAGTGCT
ATTTATATACGAAGTTTCTAGGGTTT
>ontig_91_consensus
CTTGCACCAAACATTAACGCCGGAGCAGAATAAGGTCTGTATCGATACCAGCAGTAATCG
AAGTTATCACATCTAAAGACGCTACTGTCAGTTAAATGTGAAAAATTGAAATACAGAAAG
TGTGTATTAACATTATATCTTGCCTCTGGGGGACTCAACTGTGCTGAATCTAAGACCTAT
TATTGGAAATGAAAATGTCAAAGTGCCAAGACACTAATTTGCCGATAGGACTGACACAAA
CATTGCATGAAAGGAGTTTTAAACAAGAATCTGGTTACATAGCAACACAAAAACATCTTT
GCAGAACTCCATAATATATATTTCCCTACCCTTCTACAACCACGGGAAAAATAAAGCATA
ACAAAGCAAACATACTCCATAAAATACGGTCAATATTGTAAAACCATCATCCCATGGCAT
ACTTCTGAGTCCAGCTGCGTGCAGTGGCTTCATATTTAGCCCTGTCTGACTTATACATGT
GGGCAATCTCGGGTACAAGAGGATCGTCAGGATTAGGGTCAGTAAGTAATGAGCAGATAG
AGAGCAGGACCTTCGAAATTGTAAGAGCTGGGCTCCATTGTTCCTTAAGAATATCAAGGC
AAATGCTTCCGTTGCTGTTGATGTTTGGATGGAACACCTTGGTCCTAAAAGCAACCTTGG

GGGGCTTAAAAGGATAATCTGGAGGAAAATGAATAGTAACCAAAAATACACCTCCTGCAT
ATGGGCTATCAGATGGCCCCATAATTGTCGCTTGCCAATGGAACATGTCTTCAGC
>ontig_92_consensus
AAGCAGGCCAGGACCCTCTCCAGCATGGGGACCAATTTAACAACCTCAAAATCATATCCA
ACAGACTCGAAACTTGCATAACTGAAACCATCTTCAGGTGTGACATGAATAGTAGATATA
GCATCCCCTTCAATACTATTCATGGAGTAGCCACAAGGGTCAAACTCAAAATCACATATC
TCAGATTTCGGAAGAATCTTTCTGATACCAGAATCATCAGTCATCAGAGCAGCTGAGCTT
GCCTCAGTTTTATAGAAGATAGATGCCCTCTGCTTGTCTAAGCCAGTCATGCACATCTCA
AGAGTGTAAACTGGGTCTAAGCGTTTTTCCAGTTCAGCAGAAGCAGAGTAAACATGCCAC
TTTTTGGTATGATTACCCGTCACATAAGCTGTCTTGTCCAATCCAAACTTGCCAAAGTGA
CCATCCAGGACAGCTACTTCTTCAGTGAAGCTACGATGTGGAAATGGCTGGGCTCCAGGA
AATATGAAGCTTCCACGAGTGTACCTCACAGATTGAACCTTGAGGGAGAGTGAATCAGCC
AACTTAAGTATGGCAGGGATTGAGAGAAGCAACTTTGTTGTACCACAAGTTTTGATGATA
ACTTTGTACGGGTACACAAAGAGGCTAGATTCCGACAGCACATATGAATCAAGATACTCA
TTAGACAATGAGGAAACAATAGTGCATGCGGCTGGTTTCAATATCTCATCCAATTGATCC
CTGGAGAATGAGCGGAAACCTGCTCCTTCAGGATCAGCAAAGACACCAGGCTCGAAAAAT
GAAATCTCAAGCCTCTTTTCAAAACCTTCAAATCCAATTGCAGAGACTGGCAATTCCATC
TTGCCTTACAGAGAGCAGCAAACAAACAACAATTGCAAGAGAGAGCGAGAACGAGCGAAC
GAGGGAAGCAGAAAAGAAACAGGCCCAGTAAATTGCAGAACAGAGGACAATAACTAAAAA
TTAAATTGCACAGAATGAAAGAACCTAATGGGTTATAACAAAACTTAGGGAATCTCAGGA
TGGCTTTCGGACGCAGTTAGAGTATGCAGCAGATCTAAATTTCTTAATTCCACCGTGAGG
CCTAACGTCTTCAATGCTGTAACCGAGGGGAGCTTCGTAAAACAAGGATTTACTACTACT
GCTAGACTTCTTCTTACCACCTTTGGTTTCCATTAGCTCATTCAAACTC
>ontig_93_consensus
GGGGAAGCCTAATGTTTGTCTCAAGGAGGCCAGTTTCTTCGGGGTCGCAATCCCATCATA
CCGTAAAGCTGAGTTCAAGTCTTCCCAGATTAGTATAAAGAATTTCAGAAAAATAGAGCT
ACACATTGTGACTGATAAGGCTCAAACAGCTACTACAGCTCCTTCAATCACACAGGCTGC
ACCAGATGGAAAAAAGACTTTGAGAAAGGGCAATGTAATAATCACTGGAGCCTCCTCTGG
ACTAGGTCTGGCCACAGCCAAGGCACTTGCTGATACAGGAAAATGGCACATTGTTATGGC
GTGCAGGAATTTTCTCAAGGCTGAGAGGGCAGCTAAATCAGTTGGCATGGATAAGGAGAA
TTATACTGTCATGCATCTTGACCTTGCTTCCTTTGACAGTGTCCGGCAATTTGTTGATGC
CTTCCAACATTCTGATAGGCTGCTTGATGCACTGGTTTGTAATGCTGCTGTGTACTTACC
CACCGCCAAGGAGCCTACTTACACGGCTGAAGGATTTGAGCTTAGTGTTGGGACAAACCA
TCTTGGCCACTTTCTCCTCTCAAGGCTGCTGCTTGATGACTTAAAAAAATCTGACTACCC
CTCAAAGCGTCTCATCATTGTTGGCTCCATCACAGGAAACACTAACACCTTAGCTGGTAA
TGTGCCTCCAAAGGCCAACCTTGGGGATCTGAGAGGTCTTGCAGGAGGCTTGAATGCGCT
GAACAGCTCACCTATGATAGATGGAGGAAAATTTGATGGTGCCAAGGCCTACAAAGATAG
CAAAGTCTGCAACATGCTAACCATGCAAGAGTTCCATAGACGCTACCACGAGGAGTCAGG
GATCACCTTTGCTTCTCTCTACCCTGGTTGCATAGCTACAACAGGCCTGTTCAGGGAGCA
CATTCTCTTGTTCAGGCTTCTTTTCCCTCCCTTCCAAAAGTACATAACTAAAGGCTATGT
GTCAGAAGAGGAAGCAGGAAAACGGCTAGCACGGGTTGTGAGTGATCCAAACCTAACAAA
ATCTGGAGTTTACTGGAGCTGGAACAAGAACTCTTCTTCATTTGAAAACCAGCTATCTCA
AGAAGCCAGTGATCCAGAGAAGGCACGTAAGGTGTGGGAGATTAGCGAGAAGCTCGTTGG
ATTGACCTAAAGTAGATCATGAACCACAAAAACCTCTCTCTTTCAAGAGAAGATGTACTT
TGAGCTTCATATCAAATAGGAAAACCAAGATACTAGTGTCTACTATTTTCTGCAGCATTC
TCCTCCTTTCCAAGTGCAACAACTACTTTCTTCAAGATTGCTTCAGTTGGCTGGTGAGAA
TTCTCCACAGCCTTGGAAATTGCTTGCCATTTCTCACCGTGCTTTGGTTCAGCAGCAATG
CATCTTTTCAACACATCCTTCTGAGTGCCTTCAGTTCCATGCTGCAATTCAAACTTATAG
TATAATGCCCAGAAATCCCCAATATCCGGAGCAAGAGTAACTGCCCTGTTCAGCCAGTTC
CTAGCTTTGTCAACCTTCCTATCATGCCAAAACAACTTTGCCACAGCAGCAATAACATGA
GGATCATGATCGCACTTCTTCAGGGCATCAGTACTCTTGGTTTTCCTCTGAGGCCGGGAA
ACCATCTCAATTGATGCAGCCCACAATATGCCACTATTGGGACACTCCTGCAATGCCTTT
GCCATCAATATATCAGCTTCCCTCTTGTTTCCATGCCTAGATTCGGCTCGAACTGCAGCA
AGCCAAAGTTCTGGATTGTGAGGATTCTTCTTCCTAGCCATCGTGAGCACAGCCCGTGCT
TTGCCCAACCCATTCATTTTCTCCTCCAGATTAGCAAGTGAAAGCCATAGAGGAATACAA
TTAGGACAGTGCTTAAGACCAGACTCATAAGCTTCTTTGGCTTGCTCTGGGTGATCAAGT
CGTTCCTCTAGCTGCCCCATCATCAACCACAACTTGAAGAAAGACGGAAATTGTTTCAAT
CCTTCATTAAGCAACCTTCTCTCCTCAGCGTTGTTCCCTAATTCTCTTTCAACAATAGCC

GATTTCATCCAAACTCTTTCTGTCCCACCCATATCCCTTGCCTTAGCAAGCAGCATTCTT
GCTCTTTCAGGCTCATGGTTCTCAAATTCAAGCTTAAATGCTGCAAGCCAAATCTCCTCC
GAATTAGGTATAGCTGCATAGGCTTCTTGAAGAATAGCTCGTGCTGCAGGCACATCCCCA
GCAAGCCATTTCTCTTTTGCACCCATAAGCCAAAGAACTTCAGCCTGTGGGCAATAAGTA
ACAGCTTTTCTAAGTAAAGCATCAAGACTTTCCCTAGTCCCATGGCTTTTCTCAAGCTGT
GCCGCCTTAAGCCAAATACTCTTCTTGGTCAAAAACACAGTGAGTGCATGTGCATAAATG
GCTCTGGCAGTCTCGATGGACCCTCTTTTCTTACACTCTTCAGCGTCTGCCACCCAAGTC
CTTTTCCTATCCTCTTCTTCCACACCAATCCCGATTGTATTCTTAATAATTGCTTGACAA
GTTACCACAGACCCCGCTCTCTCAGCAGCCTCAGCCTCTTTCATCCATGACTCTCGGTCA
ATAACAAACCCCTCTCTTTGCAATGCCCTAACTCCCCTCTCGATAAGCTTCCCCACCATG
GTAGTATTGCCATTGGCTTCCTCCAACTTAGCAGCAGTTATCCAAATTGCAGGTTCTTTA
GCCAACTTTTCCCTAGCCCTATTAAGGACTTTCTTTGCGTTAACATAAGTTTCCAACCTG
GCTAAGGCTAACCACAACTCAACATGTAAAGGACAACACTCAACCGCCCTATGCAATAAC
AACCTCGCATCCTCCTCATTTGCTAACTCCACAACGGCCTTCCACAACCTAACTGAGTCA
GGTATATTCTCCAATCCTTTCCTCAAAACCCTACTCTTATTTACATCATCATGCTCCAAT
TTCGCAGCCTGTAACCACAACTTAACAGAATTAGGAATCATCTTTACACCTTGAGCGATT
ACAGCCTTGGCCTCCTCAGGATTTGCAAGCCTACAAGCTTCTAGCCAAACATCTTCATTT
TTTGGACACTCCTCACAGCCCCTTTGAATCAGATGTCTGGCAGCAACAATCTTCCCAGCC
ACTTCTTCCAATCTGGCAGCTGCAATCCAACCAGGTGGATGCTTTGGGTTTGTTTGAATT
ACACTCTTCAACAACAACCTAGCCTTCTTAATATCTGAGATTTCTGCATCACTGGTTATC
TTCATGCTCTTTAAATCAGTCAAGTACCCTTTCGGGTCTACCACCGTCAAACCCGAAACA
GAATCAGACAAACGGTCCAATTTCAATGACAAAACTGTTCCTCTCCCTTCACCAACTGCC
GTCAAATCAGTAACAGGGGTCTGACCCCAAGGAGTTTCAGTTCCACCAGCTGCTCTGCTC
TTGGGATCTAAAGCAGTCACATGCTCTTGCTCCTGTCTAGCTTTCTCTAACAATGTATCA
GGTACGGGCACGAAACTTTCAAACCGTTTCTTCTTGTTTCTCAATGAATAATCTCCGATT
TCAGGGATACTATCCCACTCATCAGCTGACAATGTATACAATTTCCTCTTTAAATCAGCG
AACTGCTCCGTGATTTTAGGATTAGAAGCACGATACTTTTCAATCTCTTGCTTTAACCTA
GCTTCTCTCCTATCTTTTCGTCGCGAATCCATTCTTTTATCAATAGCTTCCCAAACAGCA
TCAGCTTCTTTATCATCTTCATCATACTCAGAATTCGCAAACAACCCGACATCATTTCCC
TCAAATTCATCGAACTTTTGATTCTCATCATACCCTTTATCATCTCCCTCATCATCTTCT
TCGTCCTCTCCTGGTTTCCCACGCCCTCTACCCGCTCCAGCCGAACTAGCTGCACCGCCG
ATTGTAGTAGCAGACCGGTCAGGGAGGTCAGGAGCAGCCCGGGCCGGCCCAATATCAGAT
CGGGTGGTGAACCCGGTAGCTCCACGACCCAACCCGGCAACATAATTGGGAGGCGGCTTG
GAATTGAGGAAATCAAGGCGAGGCTTGCTTGGTGGAGGCGGAGGAGCTGCAGGAGTGGTC
CCACCGACACCACCAAGGAGAGGAGCGTGGAGGGTCAGGGTTGAGTAACGGGTGATACCC
AGATGAGAGAGAAGGGTAGAATCGCCATAAGTGGAATTGTAGGAAGGACTTAAAAATTGT
TGAAGAGAAATCGGGATGCATGTCTGGTGTTGAAGGGATTGTTTGAGGGAGTAAAGAGTA
GTGGTATTAGGATTTAGGTTCAAAAAAATTGTTCTGGAATTGGGCGTTGTTACGAATACC
ATTATTGATTCAGCG
>ontig_94_consensus
AACATCTCCCCTACATCACCATCTACAACAAAAAAAAAAAAAAAGGCTGCATGTGAAAGGG
TTTCAAGCTACCAATACACTACTGTATCAATATCCTAAAATTAAACATTAACACTGCCAG
TTGGTGACAAAGGATAAATACGGAAGAGATAATTAGACATAAGATGGGTAAGGCAACAAT
CAGACGTTTCCATCACATTCAAGGGAGAACCCCCAAGTCTCAAGCTGCTGTCTGGATCGG
TCCATTTGATCTGTAGCCACGACCTCTTCCGCGGTATCCTCTCCCACGACCACGACCACT
GCCTTGAGCAGGGGCTTCATTATTGTAGTTTCCATCTTGCTTTCCATCAACGTAAGGTCC
ATTGTATCCTCCCCTCCCACGGCCACGGATACTACGTCCTCTCCCTCGACCCCTACCTCT
AGCATAACCTCGATTGTTGTAGTCCCAACCTCCATCTTCATACTCAACCGATATGAAACT
ATCCCCTCTTCCTCTTGACCTTCCTCTACCAGATCGACCCCTCCGCCGACCACTAGGTGA
ACCCTCTCCATCATAATCAAATTCCGTGGATGCCTTGACCTGTTCACTTGGCAATGGTGG
CTGATACCCAACAGAAGAGGTATTCAGTTCCTTCTTTGACAGGGTTATCGTAATCATTGA
AACATGCCTTGTGGTTTCCAAAGGAAGGAGGCCTTCTTCCAGAGGCTCCCACATATCAGT
TATGTCTGTGGAACCTATAGCTGTATTCTGATGAAGACCAACAATTCTTCTTTTAATTAA
CTCCACTATGGTCACAGTTTTATTGATAGCTCTTCCCATTGCCTTGAACACAACTTCAGT
TGAACCCCTTTCTTGAAGTAGACTCATAGCATAAGTGATATAGCTGCGCATCCTCCCTTG
ACTAGTAATTCGAATCTCATTTTCGTCAATCGGAGTCTCTGCTTTTGGCTTCTCCACTCT
CTGGTACCGATCCATCCTCCTTCGCTCAAGATAAAAGAGCCCTCTTCTACGGCTTTGTTT
GTTCTGTTATGCCCCAAGCCAATTAATGGGATTTCCCCGCATTATATAGCCACGCTCAGT

TTCTAAAACTACCCTAACCGATTCCTTTATCCGGTCCAACCGGTTTGTCACCCGGTTCGA
CGGTCTCCATGCCGGCCTATTAGAACATTC
>ontig_95_consensus
ATCGCCTTGAACAGGTGCTTCATTATTGAAGTTTCCATCTTGCTTTCCATCAACATAAGG
CCCATTGTATCCTCCCCTCCCACGGCCACGGAAACTACGTCCTCTCACTCGACCCCTACC
TCTAGCATACCCCCGATTGTTGCGGTCCCAACCTCCATCTTCATATTCAACAGGCAAAAA
ACCATTCCCTCTTCCTCTTGACCTTCCTCTACCTGATCGACCCCTACGCCGACTACTAGG
TGAACCCTCTCCATCATAATCAAATTCTGTGGATGTCTTGACTTGTTCAGCAGGTAATGG
AGGCTGATAACCAATAGAAGAGGTATTCAGTTCCTTCTTTGAAAGGGCTATTGTAATCAT
TGACACATGCCTTGTGGTCTCCAAAGGAAGGAGGCCTTCTTCCAGGGGCTCCCACATATC
AGTTATGTCTGTGGAACCAATAGCTGTATTCTGGTGAAGACCAACAATTCTTCTCTTAAT
TAACTCCACTATCGTCACAGTCTTATTGATAGCCCTTCCCATCGCCTTGAATACAACTTC
ATTTGAACCTTTTTCTTGAAGTAGACTCATAGCATAAGTGATATAGCTGCGCATCCTCCC
TTGACTGGTAATTCGAATCTCATTTTCGTCAATTGGAGTCTCTGCTTTTGGCTTCTCTAC
TCTCTGGTACCGATCCATCCTCTTTCGCTCAAAGATAAGGAGCCCTCTTCTACGGCTTTG
TTTCTTCTTTTATGCCCCAAGCCGGTTACTGGGAATTTTCTGCTTTATATAGCCCCTCTC
AGCCTCTGAAACTACCCTAATCGATTCTCTTAATCCGGTTCAACCGGTTTCTCCCCCGGT
TCATATAG
>ontig_96_consensus
TATACCACAAAGCAGAACCAAAACCAGGGTAGAACAGAACATCTATAGATGGCATCCCTT
TCTTCCTTCAGTCAACTTCCTCAATCTTAGGCCCGGCACCGGTGCCACCAGAACTAGCCT
GGCCATAGCCTTTCGGCATCTCTGCACCACCACCCATTGGAACATCCCCACCAGCTCCTT
GATACATTTTGGCAATTATTGGGTTGCACAGACCCTCCAGTTCTTTCAACTTGTCCTCAA
ATTCTTCAACTTCAGCCAATTGGTTCCTGTCAAGCCACTCAATGGCCTCGTCAATAGCCT
TCTCGATCTTCTTCTTATCTGCAGGATCAAGTTTTCCAGCGAACTTCTCATCTTTCACTG
TGTTCCTCATGTTGTAGGCATAATTCTCCAGAGCATTTTTCGCATCCACTTTCTTCTTGA
CTTCCTCATCCTCCGC
>ontig_97_consensus
TATACCACAAAGCAGAACCAAAAACAGGGTAGAACAGAACATCTATAGATGGCATCCCTT
TCTTCCTTCAGTCAACTTCCTCAATCTTAGGCCCGGCACCGGTCCCACCAGAACTAGCCT
GGCCATAGCCTTTCGGCATCTCTGCACCACCACCCATTGGAACATCCCCACCAGCTCCTT
GATACATTTTGGCAATTATTGGGTTGCACAGGCCCTCCAGTTCTTTCAACTTGTCCTCAA
ATTCTTCAACTTCAGCCAATTGGTTCCTGTCAAGCCACTCAATGGCCTCGTCAATAGCCT
TCTCAATCTTCTTCTTATCTGCAGGATCAAGTTTTCCAGCGAACTTCTCATCTTTCACTG
TGTTCCTCATGTTGTAGGCATAATTCTCCAGAGCATTTTTCGCATCCACTTTTTTCTTGA
CTTCCTCATCCTCCGC
>ontig_98_consensus
GCCTAGAGTAAAATTAATTTAATATTTGATAGTTAAATTTTTATGGATATTTGATTTAAA
AAAAAATAAAAGAACAAGACTTGTACGTTTAGAAAAACCTTACGTGGGATTAAATCTTTT
GACCTTAAATCTAGTGAAATCAAATCTTCTGCCCCATCAGAGACGCAAGAATCGGATTGC
TTGTAGAAATACGTGGCAGAATATAATTTGTCGCAGTGATTCACCACCCACTACTTTCCA
CAAAGATGGCTCGACATGACTTCGACTAGTCCTGGATTTCAAGTTCAATGGCGGTGAAAT
AGTCTTCTCTTGGCATTGGAATCGATCCAAATAGAGCCAAACTCCATATTCAAGTCAAAC
TAACATCAAGTTCAAAACATCTTGATTCAAATCCGATCAAATCAGACTAACAATTGAAAT
CAAAACGATTTGATTCAAATCCAACCCTAATAGTTTCACCACTACCCCAGCATCTTTCTC
AACCAATTTCTAACAAATTAAAAAGAAGGATAATTTTCAGTTCAGAATTACAGATGAAAG
GTCTATTTTAAAGCAAGCAGATTGAAAAACTTTGATTAACATGACTGCTTCTTATTCTGC
TTTTTCATGGTTCATAAGCATAAACAAAACCATATTTCTATCATTGGAACACTTATTCAT
AGCCAAAAACAGTCTCAGGAAAAAGATTTACATCAGAAAGGCCCTGTATAAACTTCAATT
CCTCCCAATTTTCTCTGACTCCTCTCGTCAATTTCTTCTTTCTTTCTGTATCGTTTCAAC
GTACTCAACTACTCACTATCTGGTGTATTTGTATTAGTAGAGGACTGCTTCATTCAGGTT
TACAGCTTCAGGGTCTCGTTCCTCGAATATGTCCAAGAGTGAAAGTGCCTCTGGGACACT
GAGCTCAAGGCGAAACTTGGGGCCATCATAGTGATGGAGAATTGGCCTGAAGGAATCCTC
TTCCAGTGGCGCACAATTCATCCTCTTGAAAACTCGGACCTGAGCCGGGAAGCGTGATTC
GCCAGGGCATTTCTTGTCCGCCCAGGCTGTTGGATCAATGTTTGTTCCACCAAAACTAGC
AGCCTCAAAAATTCCATGGAGTTGGTGGGTGGTGTAGTTGTAGAGGAAAAGGGGCAAGCC
TGGGGTTATAGCCCGCACTGAATCGCGGTAACGTGGAGGCAAGCCAAAGAGCTCTCTCTT
GAGATTCTCAGCCATGGTATCGTTGTTGCAGACAAATATATACCCACCAATAGTTTCGTT

CCTGGGCAAAGCTTCCGATGGCGGCAGTGTCTTAAACCTCTTATCAGCTGCACTTTTCTC
CTTGGTTTCATTATTATTGTTGCTCTCTTTGTTCTGATTCTTCTTGTGGCCGTTCTTCTT
GCCGCCCTTGCCTCCATATTCATGCTCTTCTTTGCTCTGTTTCCCCTTCAAATTAGTATT
CATATAACTGTTGTAAGAAATACCAAAATTTCCCGGGTTTGAATAAATTCCTTTGTTGAA
TCCTCCATTGATCCCAACGTTCTTGAGAGAGGCGTCGCCAACAAGTGGACCAATCGGACC
AAAACCAAAATCGTTGAACCCATGTAAATCGGATCCTTTGGTTGGCTTCAAACTACTGTT
GAGTGAACCGCCGACTCTTATATCGAAATTCCTCCTCTCATCCGGCCTCTTGGACAGATA
ATTATTGCTCCAAATCGGATCATTGAGCGACAAATTATCCAAGTTTGACGTTTGAAGCCT
GAGTTGGTCGCTGAACTGCCAAAACGATTGCTGATTGTTCTCCATCTGAAACACCCAAAT
TCACTTCCAACACAGCAAAATAAGAAATGGGTATCGCTTATTTATGCTTTATATGCGTAA
TTCATGTGAGAAAAAGCTTAGAACAGCGGAAGAAATAACAGAGGAAAGGTAAATTGAGGT
GAAAGTTGGTGAAGAGACGAGACTTAAATATGGAGAGTTCGGATGTTTAAGGCGAAATAA
AACAAAAATTTCACAGGGTGTGAAAACAATTAGAGTAAAGGCAGTTTAAGGTGGTGACGT
GGCGGAAGAAAGCGAG
>ontig_99_consensus
TGAAACGTATAATATCAGAGGATAACATGACATTAATAAAGTATTCTATAATCTGAACTT
ATTTATGTATGAGCCAAACAGAAGAACAAAAAGGGCAAACATGACAGCAGTCCAGGAAAA
AGTTATACATCATAAGGCTGTGTATACAACTCTTGAATCTTCCAAATTCCCTTCGGACTC
CTCCTCCCACAATATTGAATACATAACTCAATTTCTTCTTTCTCTGCATTGTTTCAACCG
ACTCTACTTCTTCGCTAACTGGTGAATCTTGTATTAGTAGAGGATTGCTTGGTTTTGGTC
ATTTGCTTCAGGGTCCTGTTCTTCGAATATGTCCAAGAGTGAAAGTGCCTCTGGCACGTT
AAGTTCAAGGCGAAACTTGGGGCCGTCGTAGTGGTGAAGAATTGGCCTGAATGAGTCCTC
TTCCAGTGGAGCACAATTTTTTCTCTTGAAAACTCGGACCTGAGCCGGGAAGCGTGATTC
GCCAGGGCATTTCTTGTCATCCCAGGCTGTAGGATCAATGTTTGTTCCTCCAAAACTAGC
AGCCTCAAAAACTCCATGGAGTTGGTGGGTGGAGTAGTTGTAGAGGAAAAGAGGCAAGCC
TGGGGTTATAGCCCGCACTGAATCGCGGTAACGCGGTGGCAAGCCAAAGAGCTCTCTTCT
GAGATTCTCAGCCATGGTATCGTTGTTGCAGACGAAGATATACCCACCAATAGTTTCATT
TTTGGGCAGAGCTTCCGATGGTGGCAGCGTCTTAAACCTCTTATCCGCTGCACTTTTCTC
CTTGGTTTCGTTATTATTGTCGGCATCTTTGTTCTGATTCTTTTTGTGGCTGTTCTTCTT
GCCGGTTTTGCCTCCAGATTCATACTCTTCCTTATTCTGTTTCCCCTTCAAATTAACATT
CATATAATTGTTGTTAGTAATACCCAAATTTCCAGGCTTTGAATACATTCCCTTGTTAAA
TCCTCCATTAATCCCAACGTTCTTGAGAGAGGCGCTGGCGCCGCCAACAAGCGTCCCGAT
CGGACCCAAGCCAAAATCGTTGACGTTAATGTTTCTGTTGTTTTTGAAAGCCATCTTGTA
CCCTTCACTGATGCCCATCTTGTACCCATCGTTGAACCCGTCATTGAACTCGTTTAAATC
GGATCCCTTCATCGGCTTGATCGAATTCACTTCGCCGCCGACTCTTATATCGAAATTCCT
CCTCTCATCCGGCCTCCTAGACACATAATTATTGCTCCAAATTGAATCATTAAGCGACAG
ATTAGCCAAGTTTGACGTTTGAAGCCTGAGCTGGTCACTGAACTGCCAAAACGATTGCTG
ATTGTTCTCCATCTGAAACACCGGGAAAAAAAAACCCACTTCTAACACAAGAAAAAACGAT
ATGGGTATTGCTTATTTAAGCGTTATATGTGGAAATAATTCCCCGGAAAAAAAAAAAAAA
AAAAA
>ntig_100_consensus
CTTGAATATTTTAGATTTTAATTTAAGTTTAGGTCAAATAGAAATTGAGGATGAATCGCA
AACCAATGTTGTCAAGTCTACCCTCAGATAAGGTATTATTTCAATCGACCTTCACTTATC
ATCCAACCTTTACTCCTTTGCTGATCAAACGAAGCCAGCGCTACTTCAACTTTTATTGCT
AAAGTGTTCATACACAAAAGGATGACGCAAATTAAAGTCTCTGGACCCATAAAAGATCTT
ATCATTAACACAGAAACCGAGCCATCCATTAACACAGAAACCGAGCTCACACATGGCTAT
GTTGCACAAACAAATACAACTTTTTTTACTTTATTCATATCAGAAACTCTGTATATACAC
TCCACAGACAGCCCTTAAATTATATTACTACTACTTCTTAGTTTGCTCCTTGGGCGGCTT
CACGATCGTGATAGGGCACACAGCGTGATGGGCACAATAGTCACTTACACTGCCCAAAAG
AGCTCTCTTAATCTTGCCGAGGCCGCGGCTTCCCAGAACAAGAAGATCCATGTGCATTTG
TTCTGCGGCTTGACAAATCATGTCTTTGGGATCTCCTTCGAGTATCAGGGTTTCTGCTTT
CACCAGCTTATCATTGCACATTTGTAACGCGCGGGATAGTAGAGCAGCAGAATTTTCTTC
CTGGGCTTTCCTCATTGATTNNGGACGATGAACTGCTGAAAAGGATGTTGAACATGAACA
AGAGTGAACTTGCAGCCATCGGAGCCATCCTCGCTTGCTTCCGGGGTTATGCCGGCGATA
CAATAAAGATTATCGAGACTCCATTTTAGTGCATAGAAGCTCTCTTCACTGTCATCAATG
CCCACCATCACCTTCAGCTTCTTCTTCTTCTCCGGTTGCAGTATCGCCGCTGACGTATCT
GCAGCTGCACTCGTCGTCTCCATGATCGGTATCCGTTCTCTGAATCAACCAGGAAGCTTG
ATTACATAAAGAGAGAGGGCACTTCGGTGGGTGTGATATGGAGCTAAGTGTGATGTCCAA

ATCAGCATCAAGGTCA
>ntig_101_consensus
GGCGTCATAAAAAAAAATTCTCGCGCAGATAATCAAGTTCTTTATTGCTCTTTCCAACAA
CTCAAAAAACCCTGTTCATTCCTTTTGTTTCTATTAGGCTGATACTAATTATTTTGTTTT
ATGGCGCCAAAGACTGAAGGCAAAGCCATTGGCATTGATCTCGGCACCACTTACAGCTGC
GTCGGAGTATGGCAAAACGACCGCGTGGAAATCATTGCCAACGATCAAGGCAACCGGACT
ACTCCTTCTTATGTTGCCTTCACTGATACTGAACGTTTGATCGGAGATGCGGCAAAAAAC
CAGGTTGCCATGAACCCTCAGAACACTGTCTTCGACGCTAAACGTCTCATCGGTAGGCGG
TATTCTGACCCGGCCGTTCAGAGCGACATGAAACTCTGGCCTTTTAAGGTCGTGGCGGGT
CCTGGAGATAAGCCTATGATTGTGGTTACGTATAAAGGGGAGGAGAAACAGTTCGCCGCT
GAAGAGATTTCTTCAATGGTGTTGACGAAGATGAGGGAGATAGCTGAGGCTTATCTGGGT
CAGAATGTGAAGAGCGCTGTGATTACTGTTCCTGCTTATTTTAATGACTCGCAAAGACAA
GCTACAAAGGATGCTGGAGCTATAGCCGGGCTGAATGTTATGAGAATTATTAATGAACCG
ACTGCTGCAGCTATTGCTTATGGTTTGGACAAGAAGGCGTCGAGATCTGGTGAGAAGAAT
GTGCTTATTTTTGATCTTGGTGGAGGGACTTTTGATGTTTCCTTGTTGACGATTGAAGAG
GGGATTTTCGAAGTGAAGGCGACTGCTGGAGATACTCATCTCGGAGGTGAAGATTTTGAT
AATAGGCTTGTCAATCATTTTGTAGCGGAGTTCAAGAGGAAGCATAAGAAAGATATCAGT
GGCAATGCAAGAGCTTTGAGGCGTTTGAGGACGGCGTGTGAGAGGGCGAAGAGGACTTTG
TCTTCCACCACGCAGACGACGATTGAGATTGATTCGCTCTATGAAGGTATTGATTTCTAT
TCTACCATTACTAGAGCAAGATTTGAAGAACTGAACATGGATTTGTTCAGAAAGTGTATG
GAGCCTGTGGAGAAATGTCTTCGGGACTCCAAGATTGACAAGAGTCAGGTTCACGATATT
GTTCTTGTTGGTGGGTCAACAAGAATTCCCAAAGTTCAGCAACTATTGCAAGATTTCTTC
AATGGCAAGGAACTCTGCAAGAGCATAAACCCCGATGAGGCGGTCGCTTATGGAGCGGCT
GTCCAGGCTGCTATTTTGAGCGGTGAAGGGAATGAGAAGGTTCAAGATTTGCTGCTTCTT
GACGTCACCCCTCTCAGCCTTGGAATTGAAACTGCTGGTGGTGTAATGACAGTTTTGATA
CCGAGAAACACGACGATTCCCACCAAAAAAGAGCAAGTCTTTTCAACGTATTCTGATAAT
CAGCCTGGAGTTTTGATTCAAGTTTATGAAGGTGAAAGGACTAGAACAAAAGACAACAAT
CTGCTGGGAAAATTTGAGCTCACGGGGATTCCACCGGCTCCCAGAGGTGTTCCACAGATC
AATGTGTGCTTTGACATTGATGCAAATGGGATATTAAATGTGTCAGCTGAAGATAAAACT
GCGGGAGTGAAGAACAAAATTACAATAACAAATGATAAGGGAAGACTGAGCAAGGAGGAA
ATTGAGAGACTGGTTCAAGAAGCGGAAAAGTACAAGGCGGAGGATGAGGAAGTCAAGAAG
AAAGTGGATGCGAAAAATGCTCTGGAGAATTATGCCTACAACATGAGGAACACAGTGAAA
GATGAGAC
>ntig_102_consensus
TCCTTTCTGGGCGAAGAAGGTGGGCCCATAGCGTAACAAAATATCGATTTACCCAAAACT
GATATTGCAGAAACAATCCCAGCTACATAAAACACCATAACCAATCCAAGAACCCAAGGC
ATCAACATGAACCCAATAAAGAACGTCACGGATCCACACAACATCAAAGCCAACGAAATC
CCCAGCATCAACGACGCGAATCCTACCGGAGTGATCTGCGACGACGACCTCCTCGTCGAC
GACACAACCGCTGTCGATGATTGATCGGAGTAACGCATCGGCGACGGCGGCGATCGCAGG
ATGTTCAACACCAGAGCTGATAACTCGTAAAAGACCCTAGACTGTTGATCTTGATGCAGG
AGATCGGAAGAGCCACACGTCTGAACTCCAGTCACGCCAATATCTCGTATGCCGTCTTCT
GCTTGA
>ntig_103_consensus
GCTAAAAATTAATTAATTAACAACTAAACTGGAACAAATGATTGGAAAGAAGAAAGACAC
AACATCACCACTACTCATCAACAATAATTCACAGAGGCAAACAACAATTCAAAGATTAAA
TTACAGATAAAAGAAAAATGAAAAAGAGACATAATAAGCCCCTCTCATACACATACCAAC
AAATAGAAAAGAAAAAGCTATTTTGGTTTAGTGGTGTTTTGATTTCCATTTTGTTTTCTT
AGTTGGAAGGGTTGCTGCGGAATGATCCCAAAGCCTTGATAGCAGCCGCTCTCAAAATGT
ACTGGTGGTATGCCGCTGCAGCCAGTGCTCCAACGAATGGTCCAACCCAGAAGATCCATT
GATCATCCCAGGCTTTGTCATTGTTGTAAATGATAGCAGCACCGAAACTCCTAGCGGGAT
TGATGCCAGTTCCAGTGATGGGGATTGTGGCCAAGTGAACCATGAACACAGCGAACCCAA
TTGGGAGAGGGGCCAAAACAGGAACGTGAGAGTCACGAGCGCTTCTCTTGGGGTCAGTGG
CGGAGAAGACAGTGTAGACAAGCACAAAGGTGCCAATAATCTCAGCGCCCAAAGCTGTGC
CCTTGCTGTAGCCGGGAGCCACGGTGTTTGTACCACCACCAAGAGAGTTGTACGCGTGCT
TCATGAAAGCCTTCACCAACCCGACACCACAAATAGCTCCCAAACACTGAGCGATCATGT
ATGCCAAAGCCCTGATCAGGGACACCTTTCTGGCCAAGAACAACCCAAATGTAACTGCAG
GGTTGATGTGACCACCAGAAATACCAGCAGTGCAATAAACAAGAACAAAGATCATGCCAC
CAAAGGCCCATGCAATACCCAAAAGACCGACTCCATCACATGGGCCATTTTGTTTCTTAT

GGCCGATAACGGTGGCAATGGTAACGTAAAGGAAGAGGAGGGTGGCGATAAACTCCGCGA
TAAGCGCTCTGTAGAAAGACCAGAGCTTGATCTCACCAAAGTCAAGGAGAGGAGCTGGTG
GTGGATCTACATAGTCCTTTCTGTGGTGAGTTTGCACTTCTTCACTCACTTCCTTAGACA
TTTTTCAATCCTTTCTTCCGGATTCAAAGTAATCTCTACAGAAAGAAGAACTCCTAGAAG
CCTTCTCTCTTGGGAAGTGTACCTGTATGTGTCTTGCTCTTAGGTCTCTCTTTTCAAAGT
AGGATGAAGCTTCATTTAAGGATAAAGCTAAGGG
>ntig_104_consensus
GCTGATAACCAAATCCTGCCTACAATGGAACATTGAGAATAAAAAATCAAAGCTCTTGTG
CCAGATTTAAAATAAAAAAAAATACGTATACAGCCAATGCAGACAAAAAGTTATGTAAAA
TAGCTGAGCTACAAAGGTTTAGCTGGGTCATTTTTCTTTCAAATAGTCTTCCACAAACAA
AAGTTCATATCTGGACCATGACAAATTATTTGTCCTCAAGCTTAAATGAAAAAGGAATAA
AGTTTCAGAAAGTCACAGAAATAGCATGTTGAGACGATATATTTTGAAAAGAATAATGGA
AGATAGTCTGTTGCAATGTACAACCAGTAAACAAGACATGAAAAGAAAAAGAAGAGCCTG
GATTGGAATCCTCTTCCAACACCATTTATAGTTGGCCATAGAGATCATACCAATAACAGA
ACCTAGGTGAACCATCAATGTTGGATCAGAAAAGAAAAATCAGTACAAATAGTCAAAAGT
AAAATATGAAAACTAAAGATTCCTATCATACATCCAATTTTCCTTGAAAGAGGATGAGTC
CTACAGTACACCTTAACTTGTTATAAATTCACCTGTGGAGGAATCATAGCTGG
>ntig_105_consensus
CTCCAAACCTTTCCTTAGTTTTTCAAGGATTCCGGATGGGAAATGTATCCAGCAAGTCAA
CATTTAGGACATTTTGGGACACCCGTAGAAAATTTCATACAGCAACAAAACAAAACAGTA
CAACCAGTTCTCAAAAGGTATCTCACAGCAAGAAATATGCTTGTTTCAGAAGAAATTCAA
TTAAAAATGCCCTAAACCTTTCATTTGTTTTCAAGGATTCCAGATGGGAAATGTATCCAG
CAAGTAAACATTTAGGACATTGTGGGACACCCGTAGCAAATGTCATGCAGCATCAAAGCA
AAACAGTAAAACCAGTTTTTAAAAGGCATCTCACAGCCTTGGAATAAAGCAAATGTCTAT
ATTTTTTTCTTTTTTAGGGCTTTCAATACCCTCTAACAGTAGAACATAACCCTCTTGACA
TTCTCCTTCAGTGCAGGAAGTGGTCTCACAGCAGCACCAGGACTGGGTCCTCGAGTGCTC
CTGGCACCTGCCCCTCCACCCATGCCTCCGCGTCCAGCAGCAGTGCCACTTGTCATTGAA
CCGCTATCTGATGTCTCTGGTTCCATGTAGAAACGAGCTCTGAATGCAGCAAGATGGGCA
TAGTATGCTGGTGGAACAATGGAAACAGAACGTGTGCACCTCGCATATGTGTAGCAGAGG
TTATTTGTGAGGGACTGTAGTCCATCAGCTGAAAATTTGTTCTCATCCCATAGTACATGG
TAATGAGCTGGCCGGCTTGTACCCTGTATTCCAGCATGGCTACAGAGGTAAAAGTCAAAT
TCAGTTGGATGACAAATTTTTGAGTCCACAACAGTACCAGGCAATATATTCCCACTCTTG
TCAACTGCACTGCGATCATGATGGTTGTTGGCAAACAACCTTGTGTGGTGCCGTTTCTGA
ACCACTACAAAAGTCACAGGAGGCTGATAGTTTGGTTCCAAGGAAGCGCATGCCTTTCGA
ATAGCATCTAGTTCATAGAGTAACACTTGATAGAACTGCCCTTCACTGACACCATCCCTG
TAAAAGATTATGCGCTGTGGCTTCTGCCCAGTTGCTCTACGGAAAGAAATAAGGAGTTCC
TTGATCATGCCTCCGGACACTGTCCCTCTAAAAGGATCCTGCCATGTCTTGAATAAATCT
TGGATGAGCTCCTGCCGATGAGCTTGAGCACAAACCAATCCAGCATACTTTGTGACCTCT
GGCCAATCTTGAGATGCCACAACAGCCGCAATTGATGGGCTGGAATCCTCTCCAGGGTGA
GGATGGGTAACATCAGCCCCAAAAATTATGGTAGGTCGGTCACTGACTAAAGGTATACGC
CTAGATATTGCATCAACAAGCACAGTATTCCTTCCTCCAACCTTTACATTTATCTTCAAT
GCAACATTAGCTAAATACTGCTTATTCATCTTGAAAACATGCTTAGTCAAACAGCACTGG
GAAACAAGACCGAGATCTGTTTCACAAATTCGCTTCAAATCACCATAAAGAGAGCCATTA
TTAT
>ntig_106_consensus
CCCTTTGCTAGGACGGGGCCATCTGTGCCACGAAGACAACCCAGTTGACCAATAAAAATT
CGACACCTAAACGACAACCAAATTGGATAAGCACGCCGCGTAAGATTTGGTGAGAGAGTT
GCTTAGAAAACGATCATTGCAGAGCAACAGGAATGGAAAAGCCCGCCTATGACATCGGCA
TCTCATGTCAGATATTCCATCGCTAATGGGCCTAAACCTAGGCCCACAAAAGACACCATC
ACAAACCAAAGCGTTGTCGTTTTCTACGATTACAATACCCACGCCATACGGCGACCAAGT
CAAGTCAAGATATTTTTTTATTCTCATTTTCATCTTCTTTCTAGATCTTTATGGAAACGA
GCAAACAATCAAGCCCCGGAAAACCGGCGATAAAAAGCGTTAGAATTTGCAATGATGATC
AATATCAGATTCAGAGTTAGAATTAAGCCCATTCCTTATTGATAACGCAGCTCTATTAAA
GACAGGAAACAGAGTCCGCTCCACGGAACTACCAAAGACTCAACCCACAAAAACAAGAGA
AATTAAATCCTACACACACGTACATCATAGATACCATGGTCGTCCACCATGATTCACTAT
ACTTATGATCATCATCGAAATCTAGGCTTTGTTTCTTTGGAATCTAAGATAGTTGACCGC
AGTCAGCAATCGCAACGGGCTTCGAAGTCCTGCCGGAGCTTGATCCCACTTTCTCAATAG
CCTTCACGACGTCCATTCCCTCAACAACCTGACCGAACACCACGTGCTTCCCGTCAAGCC

ACTCGGTCTTGGCGGTACAGATAAAGAACTGAGACCCGTTGGTTCCCGGTCCGGCATTGG
CCATGGAGAGGATACCGGGTCCCGTGTGCTTCTTGACGAAATTCTCGTCGGCGAACTTGG
CGCCGTAGATTGATTCTCCTCCGGTACCGTTTCCGGCAGTAAAGTCACCTCCCTGGCACA
TGAACCCAGGGATCACACGGTGGAAGGATGATCCTTTGTAGTGGAGAGGCTTTCCGGAGC
GACCGACTCCTTTCTCACCAGTGCAGAGAGCTCGGAAATTCTCCGCCGTCCGGGGAGTGG
TGTCCTTAAAGAGCTCCATCACGATCCTCCCGGCCGGCTGACCGCCGACGGTCATGTCGA
AGAAAACCTTAGGGTTAGCCATTACTGAAATCGACAAAGAGAGAGAAACAAGTAGACTTT
GTGTAGTGTTGTTTGTATGTGGAGAATTATTAGGGTTTGGATGGGCTTTATATAGGGGGG
AGCGGTGGGGGAAGATGAGAAACAGAGAATCTGGAGCGTCGAGGGAGGACACGTGGACGA
AGATTGAAGCGAATTGAAGTAACCGTGGTTAGGTTTAGGGTTAAGTGAAGGAACCGAGGC
TGGGAGCTGGAGTGTCGTTATCCATCCTGTAGCAATATTTCGTTCTGACCTTTAGTTGTG
CCCGTCCTCGTGAAGAAGCAAATGTGTTTCTCCTTCACTGCCTCTTTCTCATATGATATT
GGAACAAAA
>ntig_107_consensus
CTAGATCTTTATGGAAACAAGCAAACAATCAAGCCCCGGAAAACCGGCGATAAAAAGCAT
TAGAATTTGCAATGATGATCAATATCAGATTCAGAGTTAGAATTAAGCCCATTCCTTATT
GATAACGCAGCTCGATTAAAGACAGGAAACAGAGTCCGCTCCACGGAACTACCAAAGACT
CAACCCACAAAAACAAGAGAAATTAAATCCTACACACACGCACATCATAGATACCATGGT
CGTCCACCATGATTCACTATATTTATGATCATCATCGAAATCTAGGCTTTGTTTCTTTGG
AATCTAAGATAATTGACCGCAGTCAGCAATCGCAACGGGCTTCGAAGTCCTGCCGGAGCT
TGATCCCACTTTCTCTATAGCCTTCACCACGTCTAAACCCTCAACCACCTGACCGAACAC
CACGTGCTTCCCGTCGAGCCACTCGGTCTTGGCGGTGCAGATAAAGAACTGAGAACCGTT
GGTTCCAGGTCCAGCATTAGCCATGGAGAGGACTCCCGGTCCGGTGTGCTTCTTGACGAA
ATTCTCGTCGGCGAACTTGGCGCCGTAGATGGATTCTCCTCCGGTACCGTTTCCGGCAGT
AAAGTCACCTCCCTGGCACATGAATCCGGGGATCACGCGGTGGAAGGTTGATCCCTTGTA
GTGGAGAGG
>ntig_108_consensus
CTGATAGCTTATTTTCATTTTCTTCAACTGCTTCAATAAATTCTGACAGCTTATTTCTTC
AACTCTAGCAGCTTTCTCCAACCACTTCTAATAGCCGGCAACTTATTTTCAATTTTTCCA
ACTGCTTCTGACAATGACAAGGTGAGAGTGTTCAAGTAGATTTCAAACTAAAATACAAAT
GAATTTTGAAAAAAAAAAAAAAAATGGTTGAGGAGAGGGGTTGGGGCAGTTTAGAGTCCAA
AGCCAAACACGAAAGAGATAAGTCAAGGGGGGTTTAAATGTCCTTCCTCAAAAGACGAAT
TTGACATTTCTCTATCTCCAAAAGGACAATTACGGTTGATAAGGAATGGTAAAACGATTG
ATGCAACAGATGATGCGAGCTTCAACATTTCTCCAAACACCTGAGAC
>ntig_109_consensus
GACATTTCTCTATCTCCAAAAGGACAATTACGGTTGTTAAGGAATGCTAAAACGATTGAT
GCAACAGATGATGCGAGCTTCAACATTTCTCCAGGCTCCTGAGACGTTTCAGCGCGATTG
TTTTCAGCCTTAATTTTTTTGTCAATCAATGATGCATAAATCTGTATAAATCTCTCAAGT
GTCTTCCGGATGGTTGAAGNNNNNNNNNCAGCGCATTCTCTGTCAAACTCAAGCACGCAAG
ATTGACTTAAAGTGTCAACTGACTCAGCAAAATTTTTCCCCTCGTTCAGTGATTGTTCCA
AGCTGGTCTTAAAAGTATTTATGGCTTTCTCACAAAGACGATTCAGCATGTCACCATACA
TAGGGTGGAAAAGTTCTAATACTTTTGATTTCAAGTATTCTCTTCTAGCAGTTCTTATAC
CTTTATCCAAGTCTTTTGTCTCCTCGTCGTACTCTGACAAGCAAGTCTTCAGAATAGGGA
CAAGGCGTTCAGCAAAATTTTTTGGCCCCGCTTGAATAGCCTCCCTAATCTCCGACTGAA
GCTTAGCATCAGACAAACTATCCAACTTCCGTTCGATAATTTCGTTGCACCGATAAGAGG
AAAACATTACCTGTG
>ntig_110_consensus
TTTTTTTTTTTCTAAAAAGCAAGAATTCTTGAAAGGATTCATTTAATGTTTTGTTGGAGT
TTAATAGCTTTAATACAAAGTAGGGCATTGGCGTGAGCTTTGTCATACTTGGAAGGAAGA
GGAGTGATGTCTTCAAGGGTGCTGATGATAAAAGCAAGTGTCTACAGCTTTTATAGAGAT
GTTAGCGATGATCCTGCCTTACATCAAAAGTTATCGTCTCCTACTTTTATATATTTAGCA
TATTAAAAACCATACATGTCTGTCTTCTGAACAAAACAAAACGAAACAGCCTTGCTTTCA
TTCTGCAGTGTCCGTCACCATTCCTTTCTCTTCAAACTTCACCGCCACCAAGAACCTTAT
GCTTGTCTGTTTTGGATCATATACAACATACTCATTGTATTCAAGTGAGCTGTCTTTATG
CTCCGAGGGGATTAGCCGGCCGCAAGGGACTTTAATGTCATCTTTCCAAGTGAAATGTTC
TGATTCATCTGTTTTCTTTCTACCCAAACCTTTCACTCCAATCTTCTTGTCTTCTAACGG
CTTCGTATCCTCAGGCGGGTTCTTGAGCTCTGTTACTTCATTTCCTAAAGATAAAACAGC
CAAAACTAGAAATCCCTCTGGCCTGTCAACTGCTGTAAACCCATACCTTGCAGCTTCTGC

AGCCGCATCGGAACAAACTATTGCCTTGCCAAACATGTAGCCAGGTGCTGGGAGAGAGCA
AACCGCAGGCAAGAACCCTTTATGCAAATGCCTTAAAAGGTTTGAACTTCGGGTACCACA
CCAGAGGAGGACCTTGTTGGGCAGCTTCTTCATTTCATCCAAGGACGGGGCAACACTTGA
TTCGACAGCAAATATGCTGTCAACTGACACTCCATACTCTATATCTGCAAGTTTGACAGG
CTCATAAGTTTTCTCCAGGTAGCTCAGAATCATCTTGTATTCATCAGAATCTTTGTCCAG
AGCTGAGATGGAGCAGCCTAGTTTCTCATAGCGGTCGGACAGTGGATCATCAAGCGTCGA
ACCACTCATGTCTCCAATAAGATGCGAAGCCTCTGTTATGTCCCGAACAGTCTCGAATGA
AGCTGCAGCATAGTCTGCAAGTTCTTGATAATCCCTAAAGACGAAAGGTCTGGTGGAATG
CATGAGGGTAAACCATCTATTGCTGAAATCTGCCCAAACAGCTTCAGCACTCTGCCCTGT
CTCTTTCATCGATTTCAATGTTTCTGTAAATTTTAGGATTGTCTCTTTACATCTTTCAAG
GTGCACGTTAGAAAGCATTCCTATTGGGAGGTCCGGAGCATCTAATCCCATTTCCATCAG
AGCATACCTGTAAATCTCTTGACTACATAAGACTTTCATAAAATTTGCAACCACTGGCTC
GATTTTGCAATGTGTTACCGCAACCCCAAGTTGTCTCATGGCAAGCCCTCCATGTCTCGC
ATCAACTCCATCATCCATGTCCATTGGATAGAATTTTAACGGCTTCTTCTCAAATTTCTT
TTCACGTTCCCACGGCTCAAACTCGTTACCAGTTATATCCTCAAACAGTCTCACAAACTC
CATAATTGCATCATCCACATTAGGCCATTCTTCAAGCCTCTCTTCAGCATTAGCATCGTC
ACCCACTTTTCCTTTCTTGTAATACAGATTCAAGTTACTTTCTGGAACTGTTATCAGTTG
CATGACACAGTAGTGATTCAGTCCACGCCCTTGGTCGCAAATGGAAAAAGCACAGTTGTA
CAATATTCCATCTTTCTCAAAGATCTTGCCTCCTTTCTCATGCAGTTTGGAATCCTTGTA
GACTCCTCTCTTTCCAAATAACTTGAGTTCTGCTGTAATTGACTCGAGTGCCTCTTCATT
CGGATCCATTTTATCCCATGGAATTCCTTTGCCTTCGGGAGCAAGATCAGTGACGATATC
GTAGGCTTCTGGAGGTTGAGCTTCTTGTTTCTCTATACTGTCAGCCAACCAAGCTTCTCT
CACGACCTGTGTACCTCGCTCCATGGCCTCCACCAATTTGGATGTGCCACCGCGTTCTCT
TTCAGCAGGTGAAGCAACTAGGCAGGTCGCACCTATAACCGAGTTGGCGACCTTTCCTCC
ATGTTTTTCAATTGCCCTCTTCCAATATTGATGTGTACGAGAAAGACGACCCATTAGCGA
AATCGTCATCCCTGTGAAGGGTTTCTCAGCAGCAACTGGATACCTTCTGGGCCGGCGATT
GGGATCTTGATACTTCTTTATCAAGTCCGAATCTACGGTTCTGAGGACAGAATCTGGTAT
TTTGACTGCTTCTTCCCTCCTGGGTGGATCTTTGGTTCTGAATGTGCAAGAAGACCATTC
ACTGTACATTCCTTTGCAAGTATATCTTTTACCGTCAAATTCCAATTTGCTATTGCAAAT
TGTACATCTTCCTAACGGCCCATAAAACAGCATATCTTGGCATTGAGTGACCATAAAGGG
GTCAGGGCCAGATGAGTCTTGGTCATTAGCTTCAAGAATCTCTCTCATTTGTTCAATAGA
GAGATTAGACTCAATAGCTTTACAAAATTCTTCAAACTCCACATCTATAACATCAGATTC
ACCAGCTTTTCCATCAGTCTTGCCGTTGTCATTTTCTTTCTTTGCCTTCTTTGGAGATTG
CTCCGCATCATGTGTTCTGTTTTCAGCTTTCTGCTTCCTTGTCATCACTTTCTCTTCATC
AACAGATGCTTGCTCCGCATCATGTGTTCTGTTTTCAGCTTTCTGCTTCCTTGTCATCAC
TTTCTCTTCATCAACAGATGCATG
>ntig_111_consensus
TTTCAAATAATATAATAATGCTTGCATATAATTTCCATTGGTTTAAGCATCGAGTTAACG
TATAAAAATCATTTTGCTGATATACATATATCAACTTAATGTTTAGAACATTATTAGGAA
ATGGAGGAAGAACAAAAATCCTCTTCCCCCACAAGTAACTATCTTATAATGAAGGTCCTC
CTCCTCCCAAGAAAGGAAGACCACCAGTGCTTTTAACCAATCAAAAAAAGAAAAAAGGGG
GAAAAATATACAAAAGAAACTGAACCCACTAAAATAAAAACATGGATCTTCAGTCTTCAT
AGAGAATCTGCAACCATTAGGCGGCAGATGGGGCTGGGATATAGTGGACAACACCAGCCT
CCGACAAGGCTGCCATTAACTGCTCCATGTTCACCGGCTCTGCTTCAGGCACACTTGCTT
CAATGTTCTTGTAGTCAAGGGATTCATCATCGAATATATCCCTCCATTTCTTCACTAGCA
TCTTTATATCTTCTCTCTCCATGTTCTCTTCTTTTCCAGTATACCTCCATGGCTTAGAAC
CAGCAGCACAGTAGTGCACAACTTTGACTTGGTCAAGCTGGATGTTTTCAGGATGACGCC
ATAACAAAGCCAAAACAAGGTTGTAAATCGGAGGAATAGGCTTGTAGATATCCTTGAAGA
ACATGTTCAAGAAGTCCTGCTCAGCAAAAGGAGTGGGAGGGGTAATTTCAAGGGTCCTCA
AGAGATCATCGTAAGTGGGCAAGCTGGGCTCATACACAAACATGCCAGCATTGAAATAAA
GGGGAGGTTTAGGACCCAACTCGGCCGGCCAGTTCACCCTGTCAGGACACTGTTGGCAAT
AACCAATCTTGTATTGAGGAGTGTGAGTCCAGGTTTTCTCACAGAAACAGTCCATCACGG
CATAGAAATAACCATCAGCCATGTCGAAGAGATGATCTATGTTCTCAAATACTTGGATAT
CGCCGTCCAAATATATCATCTTACTGTACTCAACAAACTCCCAAATGCGAAGCTTGGAGT
AGTTGATGACATAATAGGCCAT
>ntig_112_consensus
TTTCAAATAATATAATAATGCTTCATATAATGTCCATTAGTTTAAGCATCGAGTTAATGT
ATACAAATCATTTTGCTGATATATACATATTAACTTAATTTACAGAACATTAGGAAATGG

AGGAAGAACAAAAATCCTCTTCCTCCACAAGTAACTATCTTATAATGAAGGTCCTCCTCC
TCCCAAGAAAGGAAGTCCACCAGAACTTTTAACCAATCAAAAATTTAAAAAAAAAGGGAAA
AAATATGCGAAAGAAACTGAACCCAATAAAATAAAATCATGGATCTTCAGTCTTCATAGA
GAATCTGCAACCATCAGGCGGCAGATGGGGCGGAATTATAGTGGAAAACACCAGCCTCCG
ACAAGGCTGCCATTAACTGCTCCAAGTTCACCGACACTGATTCAGGCACACTTGCTTCAG
TGTTCTTGTAGTCAAGTGATTCATCATCGAATATATCCCTCCATTTCTTCACTAGCATCT
TTATATCTTCTCTGTCCATGTTCTCTTCTTTCCCAGTATACCTCCATGGCTTAGAACCAG
CAGCACAGTAGTGAACAACTTTGACTTGGTCAAGCTGGATGTTTTCAGGATGACGCCATA
ACATAGCCAAAACAAGGTTATAAATTGGAGGAATAGGCTTGTAAATATCCCTGAAGAACA
TATTCAAGAAGTCCTGCTCAGCAAAAGGAGTGGGAGGGGTAATTTCGAGGGTCCTCAAGA
GATCATCGTAAGTGGACAAGCTGGGCTCATACACAAACATGCCAGCATTGAAATAAAGGG
GAGGTTTAGGACCCAACTCGGCCGACCAGTTCACCCTGTCAGGACACTGTTGGCAATAAC
CAATCTGGTATTGAGGAGTGTGAGTCCAGGTTTTCTCACAGAAACAGTCCATCACGGCAT
AGAAATAACCATCAGCCATGTCGAAGAGATGATCTATGTTCTCAAATACTTGGATATCAC
CGTCCAAATATATCATCTTACTGTACTCAACAAACTCCCAAATTCGAAGCTTGGAATAGT
TGATGACATAATAGGCCAT
>ntig_113_consensus
CGGGATACCCAAGTCACCAAGCAAATTATCAGCTTCTTCCGCCTCAATCCTAGCAACTTC
CTCAATTGGACCATCTAAAGTTATTTGACCGTCAAGGCCAAAAGCCTGAGGATGGTCGAA
TTTGAGATTCAAATCCCCTTTTGTGCTCTGTCCTAGATAATCAACATCGTACTTACTAGA
TGTCGTAACTGAAAACAATTCAACCCCGGCACTGTCACTGCTACTGTTACTGCAATCACA
CTTTAATGAAGTCGCCGGGCTTCTTGGTGTGATAGTAAGAGAATCCATGTTGAAAAACCC
AACTGCGTTTCTGAAACCGTTGGAGTTTGGGGCGGCCATTGAAAGATTAGACAACTTGAA
GTTGCCCATAATCTCAGCCGAACGATACATGTTTCTTTGTTATTGTGTGGATAATTATCG
TATGGAAT
>ntig_114_consensus
AAAAAAAAAAAAAAAAAAAACGGCTCAAGTATCTTTCATTAGCGACTAATAAAAAACTAAT
AACAATGAAAGCTTAAGCACTCCCAATACTAGATTTCACGTGAATAACAACTCAGTCTTA
CGCCACTGGGAGTTGGACAGGGACCTCAATATCAGTAGCAACCACTGATGACCTGATATA
CTCCTCTTCATCCTTAGGAGGATGGATGGTGACCAGATCGGGTAGAGGAGTTGTCGGGCC
CTGCTTGCCCTTGGGATCCCAGTCAAGCATGATCTTGACCTTGATACCAAGCACACCCTG
TCTAAGAAGAACATGCCTCACAGCTGAGTCGATGTATTCATTGACAGGTTGACCAGAGGA
GATCATATATCCATCCTTGAACTTCATTGATTTGGCACGCTGAGCCCTCAACTTCCCACT
GACAATTACCTCACAACCCTTGGCTCCACTCTCCATGATAAACCTCAGTACACCATAGCA
AGCCCTTCTAACTGCAAGACCTCCTAGAAGCTTATAACGCAGAGACTCGGCCTGAGCTAT
TGCACAGAGTCCCCTGTTGTTGACTTTCTCAGCATAGAGCTCCACACTGTTCTCTGGAAA
CTTGAACCGTTTCTGAACAACGGAAGTCAGCTCCCTAATCCTCCTTCCTTTCTCACCGAG
AACGTTCTGGGTGCGAGTGGCTCGGATGATGATCTCGGTGCGCATGGGAGTTACCCTGAC
TTCTACACCAGAGTATCCGTCCTCCGCCAACTCTCTGGTCAATACCTCATTCAACTCGGC
GAAAAACACTCCATCGGCCACAAACTTACGCTTCTTGCTGATTTGAGTAGCCATGTTGCT
TTCTTTTGTTGAGTTGCAGTTATTAGTTATCAGTCAGCCAGCAAGGTCGGTGGCTCCCGT
GCAACCCTCTAAACCCTAAGATGTTGAGAAGGGCTTGCGACTCACTCGGATGGGTTTTAT
GGATTGGATGAGATGGATCGGACGGCAGGATTTGTTTTTAGGGTTTGG
>ntig_115_consensus
CCCTAATCATATCTGTATAAACGATTTAAGAAATGATGGTCTTATTTAGTAACCTTTAAT
CTGCAAAATCAACACAATTGCTCATAATAAACAAATTTTAAACATTACTACTGACTACAG
TATTATCAAAATTTGAGAAAATTTTGAACATAACAAATAAAACCCAGCTCTCATGTCTCC
GCTAATATCCTTCATTACAAACAAAAAGAAACGTTTAAGCACTACCAATGCCAGAGAGTT
CACATGAATTACAACTCAGCCTTAAGCCACCGGCAGTTGGATCGGCACCTCGATATCAGT
AGCAACTATTGATGGCCTGACATATTCCTCTTCATCCTTGGGAGGATGGATAGTGACAAG
ATCTGGTAGAGGGGTTGTAGGGCCTTGCTTTCCCTTAGGATCCCAATCAAGCATGATTTT
AACCTTAATACCAAGTACACCCTGTCTCAAAAGAACATGTCTCACAGCAGAATCAATATA
CTCATTGACTGGCTGACCAGAAGAGATCATGTATCCATCCTTGAACTTCATTGATTTGGC
ACGCTGAGCCCTCAACTTCCCACTGACAATTACCTCACAACCCTTTGCTCCACTCTCCAT
GATGAACCTCAATACACCATAGCAAGCCCTGCGAACAGCAAGGCCACCGAGGAGCTTGTA
GCGGAGGGACTCGGCCTGAGCAATAGCACAGAGTCCCCTGTTATTAACTTTCTCAGCGTA
AAGCTCCACACTGTTCTCTGGGAACTTAAAACGCTTCTGCACCACCGAGGTCAGCTCTCT
AATTCTTTTTCCTTTCTCACCGAGAACGTTCTGGGTGCGAGTGGCTCGGATTATGATCTC

GGTACGCATTGGAGTTACCCTGACCTCCACACCAGAGTATCCATCCTCTGCCAGCTCTCT
AGTCAATACCTCATTCAACTCAGCGAAAAAAACTCCATCGGCCACAAACTTTCGCTTTTT
GCTGATTTGAGTAGCCATATTTTTTGTTTTGTTGGGTTGCAGAGACTAGATCACAGAAAG
TGAGAAAGGTGGCTGGTCCCAGTGCAATTCTGCAAACCCTAATTTTGAAAGGGGTTTGCG
ACTTATAAGGATGGGAGGAGATGCATCGGACGGCAGGATTTCCTTTTAGGGCTTGGTAAA
GGACGGATCAGATTGTGTGTGATTTGTTCGGTTACGG
>ntig_116_consensus
CACGCTAATTATTCCTTTTATTGGCCCAGAAACAAGGCTTATAATGCTAAACTTTGTCGT
ATCATATAAACAAAAAAAGAGTGACGAAATTGTAAAACTTTTGCATAGCCAGAATCATGT
CAAAGCTAAAAACCTTGATAAGGACAAGTAAATTTTTCCCAAAGAAAGTTCATAATCATC
TTCTGAGGTTACACATCTAAATAGAACTGACATAAGCATGACAAAAATGCAAAGAGCGGA
GCATTCATCCATAGCATATAGCCCCAAACAGACAATATGAGGAAGTGAGAAACACGAGAG
TTACATGAACAAGTTGGGCCTTTTTGCCTTGTATGTAGTCTTGAGCTTCCTTGTAGGTGG
CCTAACTTTCTTGAAAACCAGAGGGAACTTGATTTTGGAGTTGTGGAACTGCTTGGTGCT
TTCCCTCTTGCAAAGCTTGGCAGGTATGGTGGCTGTCTTGATGATCTGAATACATGGATA
CCTCACTCGGTGTCGAGAAGCCATCTCAGTGTACATTTGTTCTACTGCACCGTTAAGAGT
AGTGTCTCGGTATTCCTTGTACATATTGTGATACCCAGTTCTGCTTTGATATCGCAACCA
GATACCATAGTTCTTAACCTTGGTAGGATTCTTCTCAAAGATCTCATTGATTGCTAACAT
TTGCCCGTTGCTCTTCTTGACTTTTTTAAGCTTCCTCAAGAAATACCAAAACTTGGATTT
TGCACGAACTTCATTGGTGGCCCAGAGCTTCATTCTATAGATTTTGGGATGCTCATCGCT
TTCTGTAGGCAAAGCCCTTCCCACCACCTGGTACTGGTGAAACCTGAAAGTAACCATTTT
GATTCTGCAGATGAAATGAGAAGGGTCTGTGGCTCTTTGCAGGAAGAGGAAGAAGAAGAG
GGTTTATATGCGGTGCTGGTTCTAAAACCCTAAATCGTGTTTGCTTGCTTGTAACATTTG
GGCCTTGTT
>ntig_117_consensus
GCCCAACCAAATCCAAAAAATGGACATTCATTTATTCATCAAACAAAACCAAAGTGTACA
GACAATAACATGTCCATAATGGACTAAACAATGAACCCACATGTTTAACCTTAGATGCAT
CAAGAAGCATAGCAAAACATCAAGCTGCAAAATAAACCACCTGTGAACATCTCAACCGAT
GTGGACATAACAAATATAATAATATATTGTTAAGGAACCATTAAAGAATATAAATTATTA
GATAAGACATCTAAAATACTAAACATGCAACATGGCACTAATGTATGATTCAGCTCAAGT
GAACAACTATAGAACTGTCAACCATTCGGTCTATATTATGCAGTTTGAACCAAAATGTAC
CAAAAAAAAGGTTGAAAAATACTCTCCCGCAGTATAGACTTTCTTTCAACAACAACAATA
ACAAAAAAGATTGGACTCTTTTAACTCTTTACTACATAAGGTTTTAAGAATAACCAGACT
CTGAACATGATTCCTTTTCTCCACAAAAGAACAAGGACAGTAGCCAAGTGGGATGGGACG
ATCGCAAGTAGATTATATGTCAAAGGACAGCTTTCAATCATCTGAGGCTTTGGGGGAATT
TTCTGGTGTTTTCAAAACCGCTGTTTGCGTTTTTCCTTTATGTGCCATAACAGCTGCCCG
AAACTCTTCCACAATGGAACCATCCTTGAACTTCAACGCAAATGTAGAAAGACCATCCTT
CCCTTCACCAGCACTATTTATGCAGGCAAAAGTGATGCCTTTTTTATCCATATTTGTGAG
CTTCATATCAGGATAAAGGCTAGCATTCAAGATCAACCTGTAATTTCCCCTGGTTCGCAT
GAGAAGCCTAGCTCTTCCTGACACAGTTGTGGACATATTCACCTTAATTTCTCCCTTCCC
ACGCTCCTTCCATGCTCCATCAAAAAACTCAAAAAGCACTGAATCAGCAGAGAAAACCAC
AGTCTCATTCTCTTCTCCTGTCTCAACAACTACTTCCTGCATCAATGGGAAGCCAACCCC
CTCACTCTTTGAAATAATAGAAGCCCCAGATGCGTTAAAGAGAGAAGAATTTCCATTATT
AAGTTGTCCAAAAACAGAACTAGAGCCAGAACTTAATGCAGATCCATCATTTGGAATTGA
CCCAAAGGAGAATGTTGAAGTGGAAAACCCAGTTCCAGCAAGTCCTGTGAAGGCATTTTG
ACCACTTGAAAGTTGCCCAAATGAACTGAGTGAACCTGAACCTGCATTTTCATGATTGCT
ATCTTTCTCATCTTCATTCTCAGTCTTCTCTGCACCAGCTTCCTTTTCATTATGAACCTC
AGATGCCTCATTAACAATAGCCCCTTCCTGCTTAGCCTCAGTACTCTCCTTGTCAGCAGC
AACCTCAGATGATTGCTCATCAATCTTGCTTTCTGACAGCTCAGTACCCTGATCTTTATC
CTCTTCCACTTCATTAGCAGCAGAAGCTTCAGCCACTGGCTCATCAATCTTGCTCTCAGA
CTGCTTCCTACTCTCATCTTTACCCTTTTCAGCTCCACTGTTTGAAGATGCTTCAGCCAC
AGGTTCATCAATCTTGTTCTCAGATGGCTTACTACTCTCTTCTTCCCCCTTCTCACTTTC
ATCATCAGCATCATTTTTGCTGCCATCTGAAACCTTCTTCTCACTAGAAGTTGGCACTCC
AGTTGTAACAGCAGTAACTGTTGCACTAGGACCAGTTGGGGGAATTAACTTGATAGCAGC
AAAAGGATTGGATGAGGGAGTAGATGTGGTACTACGACGAACTTTGACTATTCTTCTGTT
TGCAAGCACCTCGTCACTGGCCCTCTTGAAAGTTCCATTTTCTTGTTCAGTATCTTCATC
ATCATCAAGACCAGGGTTATCTCGAGAGAGTTCCCTTCCTGCGGCCCTCTTCTTTGAAGG
AGGAAGGTTTTCTGCATCTCCCATTGAAGATTTGCACCAAGAACAAAAACTTGGAATTC

>ntig_118_consensus
TGCACAACTCGATCGCTCAAGCCTCGACTCTTATTCCGTATCCAGAAACCCAAAAATAAA
ATAAATTCAGAAAAGTAAACTATAAATTAATAAATAAATAAAAATAAGAACCCTCTTATT
ACCGGCTCGCCCTTTCTCTGTTCCTCCCTCTGTTTAGGGTTTTACTTCTTCTACGTCCTC
CTTTACGCATCACTGTTTATACCTTTCAATTGCCACAAATTCCAAGTTTTTGTTCTTTGG
TGAAAATCTTCAATGGGGGATGCAGAAATCCTTCCTTCTTCAAAGAAGAGGGCTGCCGGA
AGAGAACTCTCTCGAGATAACCCTGGCCTTGATGATGAGGAAGATGTTGAACAAGAAAGT
GGAACTTTCAAAAGGGCCAGTGACGAGGTGCTTGCAAATAGAAGAATAGTCAAAGTTCGT
CGTGGTACAACATCTAATCCCTCCTCCAATCCTTTTGCTGCTATCAAATTGATCCCCCCA
ACTGATCCTAGTGTAACGGTTACCACTGCTATTACAACTGGAGAGCCAACTTGTAGTGAG
AAGAAGGTTTCAGATGGTGACAAAAATGGTGCTGATAACGAAATTGAGAAGGGGAATGAA
GAGAGTAGTAAGCCTTCTGAGAGCAAGGTTGATGAGCCAGTGGCATCTGCTGCTAATGAT
GCAAAAGATGACAGCAGTAAGCATTTCGAGAGCAAGATTAATGAGCCATTAGTTGAAGCA
TCTGCTGTTAATGAAGCTGAAAAGGGTAAAGGTGAGAGTACTGAGCAGTTAGAAAGCAAG
ATTGATGAGCAATCATCTGAGGCTGCTGTAGACAAGGAGAGTATTGATGCTAAGAAGGGA
GGGACTATTGTCAGTGAGGCAGGCAAATCCGAGGTTGATAATGGAAAGGAAGCTAGTGCA
GAGAAGACTGAGAATGAAGATGAGAAGCATAGCAGTCATGAAAGCACAGATTCAGGTTCA
CTCAGTTCATTTGGGCAGCTTTCAAGTAGTCAAAATGCCTTCATGGGACTTGCTGGAACT
GGGTTTTCCACTTCAACATTCTCCTTTGGGTCAATTTCAAAGGGTGGATCTACATTTAGT
TCTGGCTCTAGTTCTGTTTTTGGACAACTTAACAATGGAAATTCTTCTCTCTTTGGCACA
TCTGGGGCTTCTATTGTTTCAAAGAATGAGGAAGCTGGCTTCCGATCTATGCAGGAAGTT
GTTGTTGAGACAGGTGAAGAGAATGAGACTGTGGTTTTCTCTGCTGATTCGGTGCTTTTT
GAGTTTTTTGATGGAGCATGGAAGGAGCGTGGGAAGGGAGAAATTAAGGTGAATATTTCC
ACAACCGTGGCAGGAAGGGCTAGGCTTCTCATGAGAACCAGGGGAAATTACAGGTTGATC
TTGAATGCAAGCTTGTATCCTGATATGAAGCTAACAAATATGGATAAAAAAGGCATCACT
TTTGCCTGCATAAATAGTGCCGGTGAAGGGAAGGATAGTCTTTCTACACTTGCCTTGAAG
TTCAAGGATGGTTCCATTGTGGAAGAATTTCGGGCAGCTGTAATGGCACATAAAGGAAAA
GCACAAACAGCGGTCTTGAAAACACCAGAAAATTCCCCCAAAGCATCAGATGATTGAAAG
CTGTCCTACTTTGACACAGAATTTTCTTGGGATGGTCCCTTCGCACTTCCTAACTGTCCT
CTTTCTTCTTTGGAAGAAAAGGAATCACGTTGGAGTCTGGTTATTCCCAAAACCTTATGT
AGCAAAGAGTTAACTCTAATTTTTTTAGTTTAAAGTCTAGTCTGCGGCAGATTATTTTTC
AACTTTCTTTTGGGTACATCTTTGGTTTAAACTGCATCATTTAAACCTGTAAGGACATGG
ATTGGTTGTCAGATATGTAGTTGTTCCCTTTACCTGAACTTCAGATTGAATGCAATGTTA
CATGTTTAGTATTTCACAGGTCTTTCCAATAATTTAAATGATCTTTCTTGCTTTCTTAAC
A
>ntig_119_consensus
TACAGTGCAGATATTACAGCAAACAAAACTTGAGATCTTTATTCTTGCACTTTCAGCAAA
TAATTTCTCTATACCCATTCTACAATGACAGTAGTATTTATCTAGTAGTAAAAGTTTTAA
AATCCCATCCTCCAATATTCCAAATATAATTATGTGCTACAGCGAAGCATTATTCCGATT
TCTACATTCAATAAATTTTACACCTAACATTTCCCATGCTCTCTACACTCATGTTGTTAT
AGTTCAAACAACCAAACAATGATTCGAAGGGCAAGAACCGCATAAAACCACTATGTTATC
AAAGCAATGGAAAAAACTCGTAATTTTCCGTTGTTCCTCAATTAAAAAAAATTCAGAATA
TCAAAATAAATAACAAGTAACATAAAATGGTAAAACCTAGATGGTAGTAGTATCGAGTTC
CATGACGCTCTTAACTTTGGGGGCGTAGAGGGAGTACACAACAGACTGTCGTTTTGCAAC
GGTCAACTGGTTCTTCCCCTCCCGATAAGCTGACAATATCGACTCTGAGTCAGTAAGGTT
CTTATTGTCGCGGAACCCGTCGATAGTACGGCGCTTTGCGTACTCTCTTATGTTGTAGTC
GCTAAACTCACGCGCCACGCGTAGTAGAGAGCGATACAACCCTAATATATCGGTTCGCGT
GGGTGCTGCTACGCCGGAACACACCATCTCTAATCGCTGCTCCGGAGCTAGGGTGGAGGT
CGGTTCTTTTAGCTTCTGGATGTCTGTGCAAGAGAAACCGATTTATGACTTTCGTAACAC
GCACCGTTTTTGG
>ntig_120_consensus
TCACAAGCACATGTCCAAAAACAACAGCAAGGGCTAGAACCATCATCAAATAGTTTTACC
GACTTTATTCTGAATGGCTAACGCATTTGGGTCAATCCAAGGCATAAAAAAACAACTTGA
ATGACAAAATACCTGATAAACAATAACATCCCATAGGAAAACAATGATGGCAAGCAAAAC
ATAAACTTCAAAATGCAGAAAGAAAAAAAGGTTCACTCAGTTTATAGCCTAATATTCTCA
ACAAAAAACAGGTATCCAGTGAAGAAAATAACTGACAAGACAACTACCAAAAAACCCTCT
GAATTTCCTTGACTCCTAAATTATCAAATGATTTGAGTTACATAAACAAGTTGGGCCTCT
TTGCCTTGTATGTAGTTTTCAGCTTCCTAGTTGGTGGCCTAACCTTCCTGAACACCAAGG

GGAACTTGATTTTGGAGTTGTGGAACTGCTTGGTGCTCTCTCTCTTGCATAGCTTGGCGG
GAATGGTGGCAGTCTTGATAATTTGAATACAAGGACTTCTGACCCTGTGACGAGAAGCCA
TCTCAGTGTACATTTGTTCCACAGCCCCATTCAAAGTGGTGTCACGGTATTCCTTGTACA
TGTTGTGGTAACCAGTTCGGCTTTGATAACGCAACCAAATACCATAGTTCTTGATCTTAG
TTGGATTCTTCTCAAAGATCTCGTTGATAGCAAGAACTTGACCAATGCTCTTCTTTACCT
TCTTCAGCTTCCTTAGAAAGTACCACCTCATTGGTGGCCCACAACTTCATCCTGTAAATC
TTAGGATGCTCATCGGACTCTGATGGGAGAGCTCTCCCAACAACCTGGTACTGGTGAAAC
TTGAAAGTGACCATTTTCGAGGGAAGAGAAGATGTAAAGATCGAAGCAGCCACGCTCTTC
CTCAGTGGAACGTAGAAGAAGAACTAGGGTTTTCTTACGCTTATATCGCAATGAAAAACC
TAAAATGAATAATATGTTAAAGTGACG
>ntig_121_consensus
ATTACCAGGCCAGTACAACAAAAAGTAAAGGAACACAGCCCAAATATTACAACTAATCAA
ACAGAATCTAGGGTTCTGTCACCAGTCACACATCATAACGCCGCATATAATTAAGCCCTC
TTCTTCGTTCTTCCTGTAGTGAGCCAGATACCCTTCTCATTTCATCTGCAGAATCAAAAT
GGTTACTTTCAAGTTTCATCAGTACCAGGTGGTGGGAAGAGCTTTGCCCACCGAAAATGA
TGAACATCCCAAGATCTACAGAATGAAGCTCTGGGCCACCAATGAAGTTCGCGCCAAGTC
TAAGTTTTGGTATTTCTTGAGGAAGCTGAAGAAAGTTAAGAAGAGCAATGGGCAGATGTT
GGCAATCAACGAGATCTTTGAGAAGAATCCTACCAAAGTTAAGAACTATGGTATCTGGTT
GCGATATCAAAGCAGAACCGGGTATCACAATATGTACAAGGAATATCGAGACACAACCCT
TAATGGTGCTGTAGAACAAATGTACACCGAGATGGCTTCTCGTCACCGAGTGAGGTGTCC
ATGCATTCAGATCATCAAGACAGCCACCATTCCTGCCAAGCTTTGCAAGAGGGAAAGCAC
CAAGCAGTTCCACAACTCCAAAATCAAGTTCCCTCTGGTTTTCAAGAAAGTTAGGCCACC
AACAAGGAAGCTCAAGACTACATACAAGGCAACAAGGCCTAACTTATTTATGTAACTCCC
CTATGTTTCTCACTTCCTCCTAAGTTGCCTGTTTGGATGCTTTGGATTATTGCCTTTCCA
TTTTTGTCATGCTTATATTGGTACTATTAGATGTGACTCAAGAAGATGATTCTGAACTTT
CTGTGGCAAAAAAAAATTAATTTCTCA
>ntig_122_consensus
ACACCAAATATATTAATATCCCATCAAAAATCTGAAATCCCTCTTGAACCAATAAATGGA
GCCATTAAGAAATCCAACAAAGATAAAATATCACCTTTGATAGTTATGAAACTTCCAAAA
CTTGACAGTAATCCTCAAATAGTCTAAAAAGATCAACATAAACTTTTAGAACCTCCTACA
CAATACTCAAGTTCTGGAAAAGCATTACAGCAAATGGACAAAAACTAAAAGACTATTCAT
GGGCGAAGGGGGTTTCCAACTCCAAGTCCAGAAACTGGGCGTGGTGCAGCACCAGGAGCA
CCCTGAGCAGGCTTTGGCATATCATCCCTGCGTCTGATGAAACGCTTTGGTGGCTCACGG
TTCCTACGGTCAGTGCGAGAGCGAACCCTCACCACTCGGGAGTGAATTGCACACGAAACG
CAGTATTGCATCTTCGCATACAACTTAGGCAGCACATACCCGTCATAGATACAGGCCTCC
TGCACATCCCTGACAGCGGCTTGCTCCACGATGTTCCTCACCAGAAACCTCTTGATGGCC
TTGTCCTTGGGGCAGCACTTCCCGCAGTTTGAGCATCTCACAAAGTTGACATGTCCACGC
CCGTGCTTGTTGCGCCCACCGTTTCTACGCTTGAAAGTCATTGTAGGTGAATTGATGCCG
CGGCGAGTTCTTGCTCTCAAGCTCTCAGTGGCGCTAGGGTTTTCCGAACCCTCGGATTTT
ACCCTTTTATATAGAAGAAGTAATTGCCGTGATTATGAGGCCCCTATAACTCTCTAATAT
TTTAACTTCCACCCCTGCTTGTAATATCAC
>ntig_123_consensus
CCTTCAATTTGTATTCAGAAACAGAAATGAATCTTCAACAACAATTATTTTCTAAATTGC
TCTTGTTCATATTGTTTCTCACAGAGAACAGAAAACAATTGTTTGCAAAAGTTAAAAACA
GCACAAATGACATATCGTTCATAACTAGGGGCTAAAAACTAGAAAAAAAACAGTTCACTT
ATAGGTGCAGATAAATTCAACCGTTCGTCTTTAACGCATATGATTTCATGCCCTTTTGGC
AAAGTTGTCTGTGTCATCTTCATCCCTTCCATACAAAGGTTCATCCCGGTCAAAACCTGC
TGGGCCAGCATTTTCACCACTAGCAAATTGATTACTGTCATCACCGGCAGAAAAGTTGAA
ACCATCACTGTTATTGCTAAAACTTCCAGTATTTCCACTTTCAAAACTGTTTCCGCCAGA
AAAGTTTCCAAACCCAGTACTTCCGTCTTGTACACTGTCAACACCACCACTACCAGCAGC
AACACCATAATTGCCACTGTTAAATCCACCAGCATTTCCACCACCATAGTTATCAGCTGT
CTGATAGCCAGTATTACCGCCACCAAAACTATTGGGGTAATTTCTTCCACTTCCATAGTT
GTTACCATCACCATAACCAGCATTATCACCTCCATAACCTCCACCAGGAGCCCCATAAGC
ATTGTTGTAATTACCCCCATAACCACCTCCGCCTCCATAACCACCTCCACCTCCAAAACC
ACCACCTCGAGCTCTATCAGCCGCATAATTCACTCTTACACGACGGCCATGAAGATCCTG
TCCATCCAAGGCCTGGATGGCACTAGAGGCCTCCTCTGCGGAGCTGTATGTAACAAAACC
AAATCCTCTAGATCTACCAGTCTCACGGTCCAAAATCACTCTAGCTTCAGCTACATAACC
ATATTTACTGAATGCTTCCTTTAGACTTTGGTCATCCGTGCTATATGAAGTCCTCCAAC

GAAAAGTTTTGAAGATGACATGAATCGTATTGCTTGGTAGATGGAAAGTTTTGATGCAGA
TGGTTCACCCACAATCTGCCTGCTAGCAGCCTGCCTAAGTATACTCCCAACC
>ntig_124_consensus
CTGCAGGAAATCTTCTAGCTGTAAGTGTGGACTTGGTAGTATCACAAATTATATCCCAAT
TAAGGTTCAAATCCCTACAGTATGACTTTACAATCAAGATCCTCTGCATATTTTGATCTC
TATATCAAGGTGGGACAAATCTTTAACATAAGCATGACACTCTGAACAACAGGGACATTA
TACAATGTATAAGCTTTGCTTTCAACTTCGTCATTGTTCTGCACGACTCTGCTTGAACAA
AAGCCAGACTGCTATAATACATGGAGCTGAAACCATAACGAGAGACTGAAAGATGTTGGG
TCTGGTTATGTCAGGGAATAGGATGCTTCCGTACTTAATCACAGCTGCACCCACAAATGA
GCCAGCTCCTACTTTCAACAAATAGTTGACATCGTCCTGAGTGAATGAATCTTGGCCTTC
TGACTGAGGATGGTTGGAGGCTTGGACTGTGAATGAACGTCGTAGACTTGACACAGAAGA
GTGATTTGTGATGTTAATGAGGAATTTAGAGGAACTTGTGCTCTTCTTTGTCAGATTTAA
ACGACGGCGGGTTACGGCGGTGGAGCTATGCCTGAGCTTCGGTGCTCCTCCGGATGCGGT
GGCTGAAGTAACCGTATCTGTCAGAGCTCGAGTGGCTATGATTGTCATTGTTGCCACCAA
CTTAAGCCCGCTTTAAATTTGGGAATTTTGGTAAATCAAATTTAGCAATTTTAGAG
>ntig_125_consensus
GGCATTGCAATCTCTGGTTGCTCCCTGACAACAAAAATGTCATGGAGCGCCTAATGGCAT
AAACTTTCCCCATGTGATAAGCAGAAGCAAGGACAAATGTCTTTGACAAAGAGAGAGATT
CTAAACAAGGATGATTATGGAGATTTTTCACTGCCATGGCATACTCTTATTCTTCTATGT
GCTTCAACTTTAAAGAGAAATTCTCAACTTGGAGAGAACTGAGAGATTAAGAGAATTTCT
AGGCCATATGAAGCAACTCCCCACCCAACTTAACCAGGACAGCCCAGATGTCAATGAAAT
CAAGGAGTGTAAAAGTAAACTGAAAATTATAAGGCATGCGAAATTATTTAAGCCCATAAT
AGGGCGGCAAATACAATTGAAAAGGAACATGGATTCAACAAAAAGGAAAAAAGAGTGATT
TACATAAAAGATGCACAGTAGAACACAATCTAACACTCAATTACTTTTAGCAAACACACC
AAACTGGTAAATTTCATGTCACAAACCAATCCTGCTTGTAATCAAAGATTTAGTATAGTT
GGAACCACTGAGTTATGATCTGAACAAGCAGCAAATTGCGTTTTTCAACATATGGGCTCT
AGTCAAAAGGCCATCCAGTGTAACCCATCTGGTGGTTATTCTCATGCATTGCTGGTGAGC
TACTTCCAGCCGTTCCGTTATGGTTTGAGCTAGAACTAGATGAACGAAATCGCCAGAAAT
TAGAGAATATATTCCGTCTTCCATGATTCTCCCTTCCACTGGCATTGCCACCAAAACTGC
TGCTTTGACTTGGACCACTTGAGCTTTGATTACGACTGCTACTTGACCCTTGTGCTGGTA
ATTCCTGGCGGCAAACTGGGCATGAATTGTGCTGCACTAGCCATGGAACAATACAATCAG
AATGGTATATATGGTTACAAGGCATTTGTCTCGCTTCTGAACCAAGCTCAAACTGGTCTT
TGCAAACAGGACAGTGGGAATCAGAACGAAGATGCCTCTGTGTGATCTTAATAGTAGGCA
TAGCATTGATTGAAGATTGGGCTGCAGGGGGAGGGCCACGTCGGTCATTAGCTGAAAGCT
GCTCAAAAAGTTCTTCTAGTCCAGGACCTATAAAATAATGACCAGCATTACCCCGTGCAA
TACCAATTCCTGGAGTCCCCCCAAAAAGTGCTTCAACACCATTTCCAGACAATCTAAAAG
GAATTTGGCCACCAAAAACCAATAAAGGCCCAAAGCCCTGACCATGTTGATGAAATGAAT
CAGATCTACCCCTGATATCATGATTATGTCTTTCCGCCATTCGCTGCCTCATAATGGCTG
AGAAAGCTTCCATAAGAGCGAACCTCTGGTCATGTTGTTCATCAGTGTCCAATCCAAAGT
AATCCAGTGGGCCAATCGGCGTCATATCATCAAGCTCTTGAACAAAGCCTCCACTACAGT
AAAGGCAGACTGCATCTCGTCCTTGCAGACGAACTGGCCTCCTGCATCTGTAACACCAGT
GGGTGTTTCTGCCGCTTGACATTGTGTCCTCTCTTTCTCCTTCTCAAGAAGATCGAGAGG
AATTTTCAAGAATTACGAAAGTTAAAAGGGGCGAGAAACCCTAAATGTAAAATTCGAGAA
TTTTGTAGCTTTTCATATTTTAGAT
>ntig_126_consensus
AAGCAGCAAATTGTGTTTCTCACAATAAGAGCTTAGTCAAACGGCCAACCTGTGTACCCC
ATCTGGTGGTTATTCTCATGCATTGCTGGTGAGCTGCTTCCAGCTGTTCCATTATGGTTA
GAGCTTGAACTTGATGAGCGAAAAGGCCATAAATTAGAGAATATATTCCGTCTTCCTTGA
TTCTCCCTTCCACTAGCATTGCCACCAAAATTGCTGCTTCGACTTGGACCACTTGAGCTC
TGATGACTACTTCGACTGCTACTTGATCCTCGTGGTGGTAATTCCTGGCGGCAAACCGGG
CANNNNNNNNNNNNNNNNCTTCTGAACCAAGTTCAAATTTGTCTTTGCAAACAGGACAGT
GTGAATCAGAACGAAGATGTCTCTGTGTGATCTTAATAGTAGGCATAGCATTGATTGAAG
ATTGAGCTGCAGGAGGAGGGCCCCGTTGATCATTAGCTGAAAGCTGCTCAAAAAGTTCTT
CTAGTCCAGGACCTATAAAATAATCACCAGCATTACCCCGTGCAAAACCAACTCCTGGAG
CCCCACCGAAAAGTGCTTCAAACCCACCATTGCCAGACAATCTAAAAGGTATTTGNNNNG
TCTTTCAGCCATTCGCTGCCTCATAAAGGCAGAGAAAGCTTCCATAAGACCAAACCTGTG
GTCACGTTCTTCATCACTGTCGAACCCAGAGAAATCCACTGGGAGGCTGTGAACCATATC
ATCAAGCTCTTGAACAAAGCCTCCACTACAGTACAGGCAGACTGCATCTCGCCCTTGCAG

ACGAACTGGCCTCCTGCATCTGTAACACCAGTGGGTGTTTCTGCTACTTGACATTTTCTC
GTCTCTTTCTCCCTTTGCAAACTCTCAGAAGTCTGAAACAAAAACTTGTCCCCAGAACTT
CCTCACTATTCAACTAGTGTCTCTGCATATTGGCTCTGAAGATCGAAAGGAATTTTGCAG
AAGAGTGAAAGCTAAAGGGGGAGAAAGAAACCCTAAATGCAAAATTTGAGATTGGTATTT
TTTTAATTATTATGACTTTGTT
>ntig_127_consensus
CGCATTTGCAAAGCCTCCGCTGGTTATTCTTGACGTGCCAAGTAGAGTTACGCTTTCAAG
TCTTGGTGTTCTTTCGGCAATGTAAAGGAGATGCTGGTCTGACATCTCGAGGTATTTTGG
AATGCAAATACTTTTTAGGGACAATCTCCAATGCTCCAGAGAAATTCCAAACGCATCCTT
ACCTTGCATTATAGTCTTTAATAGCTTCATCAATTCAATATCTATATGCTTAAAACCTTC
CAAAAACTGGACATCTTTAGGAATCAAATGGCGAAGTTTGAATGATCTACCAGTAAAAGA
ATCAGCACTACCGAGAGCAGCCATTACAGGACTCAAATCAATGTCATTGTGCTCCCAAAA
CAAAATATCCCAACAGCATAATCGCCACGAGTGGCACACTCTTACAACATTACCAGGCAA
GTCAGTGAAGGAAAATATTTTTTTGAGAACATGAGTATCTAAATCCTCCCATCTTCTCGA
GGACCCTTCCGTCATCTCACCGCTAGTCTTCTTTTGATCAAACTCAGTCTTCTCCATCTG
CGAAACACAGTCACATATTCTTGTCAAACACACCCACAAATTTCAGCCAGCATCACCATAT
CTAGTTAAAAACCCACAACATATTCACTAAGTTGGAGAAGTTACAGATTCCATAGTTTCA
CCCCACATATAACCCCAATTTAAAGAATACCTTAAGCCCTCTCTCGGTCCGTATCCCTTA
AAATCTTTCTTCTGTATTTCTATCAACCTAATGATGTTTGTCTACTTATGATTTTGAACC
ATACTACATTGTTCTTCGTTTTCAGAAACACCATTGAGATGCAAAACCTCTTGAAGTCTA
GAAAGAACAAACAATAAAAACCCAAAAACTTCAGTCTACTCATGGAAACTCTCTATATTA
TAACTAGTTTGATGAGGAGTGAAGGATTTAAACTAAAAAAAAATCAGTAATACAATAATC
AAATTACATCATGAAACTTCAACTTTTATTATAATTTTATAACAAAG
>ntig_128_consensus
ATCAGTGCGCACATGAGACTTCAAGAGACCCATATAAGGTGTTAGACAAAATCAAAAACA
AAGATAAAGCATTTAAGATATTTAAAATCACACAAGACTTTTTATTTCAAAAACTGCTAC
TTCTCACAATTCACACAAATAGTACAGACGTAACTCGAAAACAAATCCATGCTTTATTTG
GTGACAATCAACCGGAAACCAAAACAGATGTCATAAAGACAATCTCGCAACAGTCATTCT
GATATGCTTTAGCCAGAAATCTCAATGGCCTTAACTTCAGGCCTTTTCTCCTCCTCCTTA
GGAATGGTCACAGTGAGCACTCCATTCTCCATGGTAGCTTTCACTTTCTCCATCGTCGCA
TTCTCGGGCAGCCTGAACCTCCTCAGGAACTTCCCACTGCTCCTCTCCACCCGGTGCCAC
TTGTCATTCTTCTCTTCTTGCTCCTTGCTCCTTTCTCCACTTATCTGCAGTATTCTACCT
TCTTCAATTTCTACTTTCACCTCTTCCTTCTTCAGCCCTGGAAGATCGGCTTTGAAAATA
TGAGCTTCCGGGGTCTCCTTCCAGTCGATTCTTGCATTCGCAAATGCAGATGTTTCAGGA
GCTGAAGAGGGCACATTGGACACGGCTGAGGTGAAGAACCCATCAAAAGGATCCCATATG
TCAAGAGAAAATGGATCAAATACGTTGGTTCTTCGCCCACCGAAAATGCTTGGAATCAGT
GACATGATCGGAAAATTTGAAGCTAGTGAAATGTTGAAAAAGTGCTGAGGATATGTAGTT
TTGAATACTTTGATCAGTTTCAAGATAAGATGTGAAGTGGGATGACGAGCCTATATATAG
GATTAGGAGGATGATTGATCAACTTACTATTAAAGCGAAAGAAATATCTGGTTCTTTCGT
TGGCCTCCTCTTCTCTTTTTATCATTCCACTTC
>ntig_129_consensus
CTGGAATACTCACTATGACAACTCTTCTCTTTGCTAATCTTTCTTAATCAATTCAATAAG
AGAAAAAGGAACACCGTGTGGGGCTGGGAGCATTATTTCAGACGCAAATATCAAAAGCAT
TAACAGAAAGCATTCGATAATTCTGCTCCAATATCCTTTCTCACAACATATAAGACTCTT
TAACGTCGACGCTTCAATGTAAATACATATACAGAACTCACATATTAAGTATATATGAAC
CAAAAGCATAAACCAAAGAAACAGATACAAAATCATAACAAATCAATCAAAACACACAAC
ACAACGATCATCTTTCTTAATGGCAAGCACTTTCTTTCTCTTTCTCTTTCTCCTTGTCCT
CTTCAGTCTTGGTGTGATACCCAGGAAGCTTCTCCTTGATTTCTCCAAAAGGCCTTTCT
TTTCCTTTGTCTCTCCTTCATGGGATGGTGCCTCTGGGGTGGCAACCTCAACTGCAGGTG
CAGGAGGAGGAGTCTGAAATTCCTCAGATTTCTTATTATGTCCTGGTAGTTTCTCTTTAA
TTTTATCAAGAAAACCCTTTTTTTCTTCAGGATGAGATGTTTCCTGATCCACTACTTCAA
CAGGAATCACCGTGTCTGTGTCCTCGTGCTTTTTCTCTTCCTTTTCTCCTGATATCTTCT
CTTTCAACTTCTCCTTCAATCCCTTCTTTTCCTTCTTCTTCTTCTTCTTCTCTTCTCCTT
CACCTTCCTCTTCATCGCTTGAAGAGCTTGAACTGCTGCTTCCAGATCGGTTAAGCTTCT
CTAACAGACAAGGGTTTTTCTCCTCTTCAACCTTTGGCTCCGATTCAGATACCTGAACCT
TCTCAAACTCTGTAACGATCACCTCCTCTTCAGGCTTCTCCTCTTCTTTCTTCCCCAAGA
AATCAAACACCCCACGATCCTTGATCTCCACTGCACCCTCGTCAACAGCAGTGGGCTCAG
ACTCATGGCTCTTGCTGTGGTGCTCTTCAGCCATATTCTTAAAATCAGCAATTAATACAA

GACGCAAAGAGAGGAACTTGCTATGAGTGTTGTTACCAGATGCTTTCTTGAAGATATGGA
TTTCTTCACCGAGACTCGGTGGCTTTTATAGTGTCTCTCCACGCGCATACGACGCGAGTA
AGAGATTGCAAAGCCAAAAGGGATCAGCTTGACACTGTAACCGACGTAAGCT
>ntig_130_consensus
CTCAAGCAAAGCAAATAAAACAGCCATTTGGATCAAGCGCGATCACAAATATATATTTAT
ACACAATAAGCACCAGAATAGGTATTTATTTAATAAGTTTTTTAAACCGAATACATGACC
AAAAGAAGGCCAGTATCAGCGTCCAAGAAACACAGCAACAAACAAGCACAAGACCTAGAA
GAGTGCTTATACAACTTATTCAAGACATCGCACATTGACTGGCAGCATCAAACACACCAC
ATAAACCACAGATCCAACAAAACAGAAATAAACCATAACATGGCCAACTCAGAAACCCCC
GCGGAGACGAAGCACAAGATGCAAGGTCGACTCCTTCTGAATGTTGTAATCAGCAAGGGT
CCTTCCATCCTCCAGCTGCTTCCCAGCGAAAATCAACCTCTGCTGGTCTGGTGGGATCCC
CTCCTTATCCTGGATCTTAGCCTTAACATTGTCAATTGTATCTGAACTCTCAACTTCTAA
GGTGATGGTCTTTCCAGTGAGGGTCTTCACAAATATCTGCATCCCACCCCGGAGACGCAG
CACCAGATGAAGTGTGGATTCCTTCTGGATATTATAGTCTGCAAGTGTCCTCCCATCTTC
AAGCTGCTTACCAGCAAAAATGAGCCTCTGCTGATCTGGTGGGATACCCTCTTTGTCTTG
GATTTTGGCTTTCACATTGTCAATTGTGTCAGAACTCTCAACCTCCAAGGTAATAGTTTT
CCCAGTGAGGGTCTTTACAAAAATCTGCATACCACCACGGAGGCGAAGAACCAAGTGGAG
GGTTGACTCCTTTTGGATGTTGTAGTCTGCAAGAGTACGACCATCCTCGAGTTGCTTGCC
AGCAAAGATAAGCCTCTGTTGGTCTGGTGGGATTCCTTCCTTATCTTGAATTTTGGCTTT
GACATTATCAATGGTGTCAGAACTTTCCACTTCAAGGGTGATTGTCTTTCCAGTGAGAGT
TTTTACGAATATCTGCATACCACCTCTGAGTCGGAGAACCAAGTGAAGGGTGGACTCCTT
CTGAATGTTATAGTCAGCGAGTGTTCGTCCATCCTCAAGTTGTTTGCCTGCAAAGATAAG
CCTTTGCTGGTCTGGAGGAATCCCTTCCTTGTCCTGAATTTTGGCTTTGACATTGTCAAT
GGTGTCAGAGCTTTCCACCTCCAGGGTGATAGTCTTACCAGTGAGAGTCTTTACAAAGAT
CTGCATACCACCCCTTAGACGGAGGACCAAGTGAAGGGTTGACTCTTTCTGGATATTATA
ATCAGCAAGTGTACGCCCATCCTCTAGTTGTTTCCCGGCAAAAATAAGTCTTTGCTGGTC
TGGGGGGATCCCTTCCTTGTCTTGAATTTTTGTCTTGACGTTATCAATGGTGTCAGAGCT
TTCCACCTC
>ntig_131_consensus
TATCACAAAAGCAATGTTGTCAGATGGCATGAAAGAGGTGTTGTCAATATGCTATTTATT
TTATCTATCTTCTAGTTCCGCTAAACAACTTGAATTTCTGGCTGAGTGTAACTTTTCCAA
TATATCAGCTCTCTCATTCCTTCACTAAGCAGTTTGGTAAGTTCCGGGGTAAAAAAATAA
TGGCTCTTACCTGGCTCATCCTCCTGGGACAGGATGAATCACAAGTGGTTCTGTAATGCA
TATATTTAATGTAAATAGCCACGAAAGCAGTGTTGCTATTCTGAATTCTTCAGTCCAAGT
GATCACGGAAACGGATGATTTTGTTGATAAAAGTCCTCTCACCGCGATAGATTACAATGG
CTGAGATGCAGAAGCAAGTTAACCCGATGGAAAAATGAATTAAGAAGGTTGGCCAGAAAT
TTTGCCGGTCCAGAAATATTTTCTCAGAAAGGAGAGTAATTACACCTGAAAACAACAGAA
ACGGCCAACCTGAAGGCAAGCAGCTGCGGAAGGAAAAAAAGGTAGAATCCTGGAAATAAT
GCCTGATAAATAGTGCAGAATACATAATCCTGAGAGACATATTTATAGAATTTGCCAGAA
TTAATCCAAC
>ntig_132_consensus
GTTTTATATCAATTATAGAAAGTAACTACCCATACATAACTTCAAAACACAAGGTTAATA
AGGTATGTATGCATTGTAAGACATAAGCCATCAAAATACATCCTCTAACAAACACCAAAG
ATTCAGTAGTTGACATCCATGAAAATAAATCAACAATTCACTTCAGTAGAAATTGCATGT
CCTAACAAGAAATTTAATACTAAATGCAAATCCGAATAAAAAAGTCTCAAACGACAACAG
TAACAAAATTTAATGTCAGAACCACATCTCCAACTCAAATTGCTGTGAATGCCTTCTTCG
CACCACCAGAAGACCCAGCTGGTATCACTTTCAAGACATTAAATCTGACTGTCTTTGATA
AAGGCCTGCACTGGCCGATGATAACATGGTCTCCCTCTTTCACACGGAAGCATGGTGATA
TGTGTGCAGGAATGTTGGAATGTCTCTTCTCATACCTCTGATATTTCTTCACAAAATGAA
GGTAATTGCGTCTAACAATGATGGTCCTGTTCATTTTAGCACTGTGGCAAGTACCAGCCA
AAATACGACCCCTGATAGAAACAGTGCCAGTAAATGGGCACTTCTTATCAATATAAGTAC
CTTCAATTGCCTCTCTGGGTGTCTTGAATCCCAATCCAATACTCTTCCAGAATCGATTCC
CTCCCTTTCCGGGTCTCTTCCCCTTCGCTGGCTTCTTCGAACTCAAAAACACTTTGGGCT
GCTTCAGAAACGCCTTCTCAGTCTGCTCCGCCATGGCTAGATTTTTCGGATAACTCGCAG
CTGACGAATGAATAGAGGAGGCCAAGTAGTGAGAAACCCTAAAACGCGCCGAGAGTGAAA
AAGTTGCGAACCTCTAAATAGGGTTTGTAACTGAAGTGAGAG
>ntig_133_consensus
TGCATCTGGACAATACTACTATAATGTCATAAACTTGTCCAAATAAAACAATTTGTGAAG

CTGCCAAGTCAAGAGACACCAATTTCTGCGCCTAAAAGAGAAGCAATTCAAAGCCTCTCA
CAGCCCATATTATACTGGTCACAGAATTTATTCGCTCGAAACATAACATAGGGAGCCATC
TCATCAGCTGTACATGATAATTATTTCAAACTCACAATTCCTGATCCAAATATGGATCTC
TTGAAGCATTTCCTCCTCTCAGATATCCTCCAACACTAGAAACTCCGGAAACAAAGTTGC
TCATCAAGGAAGTTCCCCATGACAGCCATCCAGTCTCATCTTGTTGCTGCTTGTTTGCTT
CTTCCGTGCTTGTCCGCTGTGCCTCAGCTAAGAGATCAAGCCACCTCTTTGCCATAAAAT
CCTTGACAGCCTCAACCCCTCTATTTGCAATCTCTGTTGAGGGAATAATGAGTGGATTCT
GTTCCAGGTTGAGCTTGGTAAGATTTTCAAGCCTGAAAAATGTGTCAGGAAGAGCTTGTA
TTTGGTTATTGCTAATATCCAATTCCCTGAGGTTGATTAGATCACCAAGAGTTTCAGGAA
GCTCGGTCAAGTCACTAAAATTACTGCTCAGGTTCACAACTTCAAGATTTGTCAATCTCC
CAATTGCATATGGCAGGCCTCGAAGCTCATTGAAATGAACATCCAGGTATTTCAGCGACC
TCATTTCACAAATAGAAGCGGGAAGAGCGCGGATTTTATTCAGCTGAATTGAGAGCCTCT
GAAGATTCAGTAGTCCATATCCCATATTTGCGGGCAAGAATGCCAAATTGTTAAAGCTTG
CATCTAACTCCACCAGTGAACTGCAGCCAGCAATACTTTCAGGAAGGTTATTCAGCTTGT
TTCCTGACAAATTAAGGACCTTCAGACTAAGCAACAATCCAATTGAGTCGGGCAGGAATT
TTAGCAGGTTGGAAGAAACATCAAGCACTTCAAGTTTCTTTAATCCTGCTATAGAGTCAG
GAATGACCTCTAATTCATTGCGAGAAAGATTAAGCACAAGCAACCCACGAAGCTTTCCAA
AATCCTCAGGCAAGAACCTCAACTGACGTTCAACAAGCTCGATTCTCTCCACAACAACAC
CACTTTTCGCTTCCGTCAGTATCTTCACCAAGTCCTCATTCACATCTTCCAACTCGACAG
CAGCCATCTCGTAAACCCGACCTAGCATCTCCTCCGCTTCTCCCAATTTACTCCCATACT
CTTCATGCATCTCCTCCAATTTCACAACCGCCTTGTACGCTTCAAGGTCTGCCGAAAACG
ATGGCGTTTCATGGATCTGGGCAATCTTAAATCGAGCAGCAGAGACAGCATCGGGGTCGG
GTCGAGGTCCAAGGGAATTGAAGATGAGAAAGTAAGTCTGAACAATTTGGTGGGGAAGGG
CTTGCGTTAGTGAAGTGAGAATTTTAGGATGGTTGAAATGAGGATACTTTGTGGTCAAAT
AAATGCAAGTTTCAGGTGGAAGGGAATGAGC
>ntig_134_consensus
GTCCGATTTCTAATCATAAAAAGTGTAAACTGAAACAAAATTCCATGCTCTGATACAACC
AGTTCAACACTTCAGAAAGAAAATTGAAGTCCCTTTAGTTGAAAAAGGTATCTGAATATG
GAAATCAATAATAATCTACCAGTCATTTTAAGTGCAACAAGAACCAGTTCAAAAAATCAA
AAGCAACAAAATGTTCTTGTATTGCAATGTCTCTGGCAGCCACACCACCCAAAAGAGCCT
TCAACCTGGAAAACCTTCTAAGTTGCAACTGCCCTTGTAAATTCTTTCCACAAAATCCTC
ATAGATGCTTGTAAATTAAAACCAAATGACCACATTAGGGAAGAGGAATACCGCCTCCGG
AATATTCAACCTCCTGAGCAGGGGATAACGATGAAACCCAATCTGAAGCATGTGTCGGAA
GAAGCCCCAACGTGTTAAGCTTCCAAACACCCAGTGCTAAACCTCCCAAATTAAGGGCAA
TGAAAAGCAGCTTAGGCCCCAAAAGATCTACTTTACTGTCTTTGTAAGGCTCAAAAACCT
TTCCAACACCTTGAAGAGCACTTATAGGTTGCCAGAGAGCTGAAAAAGTAATACCAATGC
TGAAAAGATGTACAGTACTTCCAGCCATCCACATCATAAACCCCATCATTAGCAAATTCT
TGAAAGGAGCCTGTGCCACTTCCCAGGCTTTCTGAGCCTTCCAATTAGCTTCAGCATCTT
TCTTTTGGCGACTGGTGGTTGAATCTTCCTGATCTTGAGAAGCGCGAGTGAAGCCAGGAG
GATCAGGGATGTCACGTGAGGAAGGAGTGGTTGAATTGTCGGTGAAATCAATGGCCCATC
TTCGCCCCGAACCCACCACTGCTTTACCCTTCTCCATTAGACTCGATATAATTCCCTGCA
GTTTCCGCTGGGCTTCTTCTCTTAACTATCTTTCATTTCTTGTTTCACC
>ntig_135_consensus
TTCACCTAACCACATCCATTTATGTATAGATATGTTAATAAATGTATTATTATAACATTT
ATCAATAAATTTCAACACCCCCAAGATTTTGCAGAACAAAAGGTTAACCTGAAGATGGGC
ATCAATAATACTCTACCAACTCATAATGAAGTGCTATAGAAACCAGTTCCAGAGATGGAA
AACAGGATTTGTTGTTGTACTGTGTAAGGCAGCCAGCCACACCCACACAAAACAAGCATT
CAAACAGGAAGTTCCCCTTGATTGCTCTTGTAAGTTCTTTCCACAAAAAAACTCATAGAA
CAGTTTTAAATTAAAATCCAAGGAGCACATCAGTGCAGAGGAATACCTCCTCCTGAATAT
TCAACCTCCTGAGCAGGTGGTAATGATGAAACCCAGTCTGATGCATGTGTTGGAAGAAGC
CCCAACGTGTTAAGCTTCCAAACACCCAGTGCTAAACCTCCCAAATTAAGGGCAATGAAA
AGCAACTTAGGCCCCAAAAGATCTACTTTACTGTCTTTGTAAGGCTCAAAAACCTTTCCA
ACTCCTTGAAGAGCACTTATAGGTTGCCAGAGAGCTGAAAAAGTAATACCAATGCTGAAA
AGATGTACAGTGCTTCCGGCCATCCACATCATAAACCCCATCATCAGTAAATTCTTTAAA
GGAGCTTGTGCCACTTCCCAAGCTTTCTGAGCCTTCCAATTAGCTTCGGCATCTTTCTTC
TGGCGACTGGTGGTTGAATCATCCTGGTCTTGAGAAGCACGAGTGAAGCCAGGAGGATCA
GGGATCTCACGTGAGGAAGGAATGGTTGAATTGTCAGTGAAATCAACGGCCCATCTTCTC
CCCGATCCCATCACTGCTTTACCCTTCTCCATTAGATTCGATGGAATTCACTGTTCTTCG

TCGTCGGATTGGAACTCTTTTTTTTTTTTTTCC
>ntig_136_consensus
ATCTGCCGGCATTTGACATCGCAGAAAAAGACAAAACAGATAAGGAAAATTTTCAAACCA
AGTATTTAAATATAATAATTTCAATAAGAGAGATTACGGCAAGCAAAACACTACGACGAA
GTTTTCTCATACAATAGCTATTAACATTAAGGTTCCCAGGGAAAAAGAAAAAAGCCCTTC
AAATATGAAAATTTTAGAAAGGGAAAAGCATCACAACCACTCCTCATCTCCAGCTACAAG
CATTCCTACTAAAAAAGACAATATGGTTGAACCCCAAGATGATGCGATGCAAGGCAAGGG
TAAGTGTGAACCATTCAAATTAAAAGACTAGAGGAGAGGCTTATATGTTACAACAATATA
GCAATTATACTCTCCCTTGAATGGCAACCCCAATCCTGACCTCGACACTTCTCCACGTTA
ACCAGCATGAGCCCACATGACACAGACACAAAACAACCGACTAAATGCAAAGGGAACTCA
CCATCAAAGAACTTCGACAACTTAGAAGCACTTCCTGTGGACAATGGACGGACCTGACTC
ATCATACTCACCCTTAGAAATCCACATCTGCTGGAAGGTGCTAAGCGATGCAAGGATTGA
TCCTCCAATCCAGACACTGTATTTTCTCTCTGGTGGAGCTACAACCTTGATCTTCATGCT
GCTAGGAGCAAGGGCTGTGATTTCCTTGCTCATTCGATCAGCAATACCAGGGAACATGGT
TGATCCACCACTGAGAACAATGTTACCATAGAGATCCTTTCTGATATCAACATCACACTT
CATGATGGAGTTGTAGGTGGTCTCATGGATACCAGCAGCTTCCATACCGATGAGTGATGG
CTGGAAGAGGACTTCTGGGCAACGGAATCTCTCAGCTCCAATGGTGATGACTTGGCCATC
TGGCAGCTCATAGTTCTTCTCAACAGAAGAGCTGCTCTTAGCAGTCTCAAGCTCTTGCTC
ATAGTCCAAGGCAACGTAAGCAAGCTTCTCCTTCATGTCACGAACAATTTCCCGTTCTGC
AGTGGTGGTGAACATGTACCCTCTCTCAGTAAGAATCTTCATCAGTGCATCAGTGAGATC
ACGACCAGCAAGATCCAAACGAAGAATTGCATGTGGGAGTGCATAACCCTCATAAATTGG
CACAGTGTGAGACACACCATCACCAGAATCCAGCACAATACCAGTTGTTCGACCACTGGC
ATAGAGGGACAGAACGGCCTGAATAGCAACATACATGGCAGGGACGTTGAATGTTTCAAA
CATAATCTGGGTCATCTTTTCTCTGTTAGCCTTGGGGTTTAAGGGTGCCTCAGTGAGAAG
CACTGGGTGCTCTTCAGGGGCAACACGAAGCTCATTGTAGAAAGTGTGATGCCAGATCTT
TTCCATATCATCCCAATTGCTCACAATGCCATGCTCGATAGGGTACTTCAAGGTAAGAAT
ACCTCTTTTGGATTGGGCCTCATCACCTACATAGGCATCTTTCTGCCCCATCCCAACCAT
CACACCTGTGTGCCTTGGCCTACCCACAATACTAGGAAACACTGCTCTGGGAGCATCATC
ACCAGCAAACCCAGCCTTCACCATGCCAGTTCCATTGTCACAAACGAGAGGCTGAATATC
CTCTGCATCGGCCATTTTTTAGAAGAAAAGAGACGACGACAACAGCGGAGAGTGTGTGAG
GCGGCGAGGTTCTCCCTCTCTGATGGGGGATGAGAAAGAGAAAATATTTGAAAGTGTATA
GAGTGAGAGAAGATGAAGAGGAAGGTAGAGACTATAAATATTCAGGCCAGAAATTTTTAA
ATTCACATGAAATTTCCACTAAATTTTGGAGGAATCATT
>ntig_137_consensus
TTGCATGTATTTTCTTTTTTAAGAACTACGATCAAAAGACGAAATCAGGTATTGGATTCG
AAGATAAGGATTAAAGTTGTAAAATCCATTTCATATTTTGAGAGTGTACGTATCTGACTT
CAGACCTGATGAATCTTTTTTTTTCCTTGATTTTCCAGGCAAACAAACAAGAGTTACGCT
GTTCTTAATCTGAATACAAAATCTTTTACTGAAGACTTTAATAATAAGAAACAAGAAATA
TTCCCTCGTTTTGATTTGTAGAAACATCAAAACAGATAATTAATACGTACTGACAATCAT
CGTGAAAACAACACGAAGAAAAACCTGACAGATTTTCTTAGTTGCAAAAATACGAGGACT
GATCTGATCTTGACGCTCTTCGGTTACTATATACACACATATATCATCGTCTTCTTCATT
CGTACATGTCAGTTTGAAAGCTACTAAAATACCAAACAAAACTCATACAACTTCGGGCGG
TGGTGGGAGGTTAAGATCGAGATTTAGATTCATCAATGACCTC
>ntig_138_consensus
AAAGATCCATTCAATATGACGACTTTGGAATACTTTTTTATCCAAAGATCTACAATCTTA
AACGAATCCCTTTTATTGATGGATACAATCTAATAAAAACTTAAACAAAATTCCAACTAT
AATGACAATTTGCACAGGAATCACGATCTGACAGTGACAATAGACGACAGCAAGAAAAAT
GTATATTATAATAGAAAGTTGAAAGAGATTCTTGATAAAATATTAAGGCTTAATCTTTAG
CACCAGAAAATGGAACAGTCAAGTCCTAGCCAGATTGGATTCTATCCAAACCGAACCCAA
AACAAACCTAATTCAAGTGAGGTCAATAAACTGAGACTCGAATATATTCAAATTAACTTA
AACTTGGCTCATTCTATCTCAACTTGAATACAGGCTCGAAAAACTCATATAAATCCTGCA
CTAGGTCCAAGTGCAAGAGGTAGGGAGCCTTAGTAACAGGACACAAGGGAGACAATGTCG
AAAGATCCAAAGTAAGAAATGGTCCACATGTCCCTCCTATATCTAGCCCTTCAACGCCTA
ACACCAAAGCAAAATATAACAAAAATCATAAAAGCTGAAAAGATTTGGCGGGATATTAAT
ATATGGAATTCATAAACACACACACACACACACACACACAATACAGTAAAAGTCTGTAACCC
AGTGGTTGCCACCTAAGCATTTCACTTAAAGGCCTGAACTCTCCATGTGCGTCTGTGTCA
TGCACTACCAGATAAAAAAAACGTTCACCTCTTCCACCTCTTTCTTACCAGAAAACAAAA
ATTCACCTCTTCCTCACCTGCAAATACTTCCAAGAATCCCATCCAAATAGATCATTGATC

TACACCGTTAATTTTTTTGCTTGGCGGAAAATCCGAGAAGGATATTCTTTAGACAAAACC
AGCTTATATTTTCTTTTTTGATGGGTTCTTCATCCTGTTCACAACTTATGCTGTTCCTCC
AAGAGCCAAAGATCCTGCAACTTCACTCTAACAGTTCAAAAACAACACCCTAAACCCATA
ATCCAACTGTTTTGAAGAACAAATATTTTAAGTTAAAAAGTCATAAAGGGACCTCTCCTT
TGCTGTCAAATAATTTTAAGATGAAAATCCCACAACAGAATTTAAATCAAATTTAGAAAA
AGGATACATGGTGATGATAATAAGTAAATTAACAGAAAAACAAAACCCTAGAAAAAATAA
GAAAACGAAAAACTGGAGGCGGAACAGAGATGCTCCAAAACACACCTCAAAACATTTGGC
TTAGGATGGTTTGGCTAAGATGAAGATTTCGGAGTCTCAGGCTTTCTGAGGAGAATCATG
TGCATCGGAGTAAAAATTTCAGTTTCGCCGCCTCTGGTTAAATAGTCAGCTGTCTTGAAG
AGCATCTCATGGACATCGACAGTTCCTTTTGGTGCAATCCCCAAGGCGGCGAGTACTGTA
ACCAGAATGTGATTCCTCCAGTAAGCGATCCTGCCCATCTTCAGTCTGGTCCACCACGGC
TGAGCCGGCGGTTTAGCCAGATCCTTCTCCTTCACCACTTCAAACCCAACCTTTTTAGCT
GTCTCAGCAATGTCAGCGTAGCTTCTAAGCCCAGGCAAAGCGTCTCCTCTTTCAATCCCT
TGAATAATCTCAACGTGTTCGGGGTTGTCGCCTCTGTATTTGTCAGTTGTAACCCACTCG
TACGAGACGTAGAGAGATCCAGGCTTCAGAACCCTAAACACCTCGCCGTATACATCTTCA
AGCTTCGGCGCGTGACATGTTGCTTCGATGGAGTATGCGCCGTCGAAGCTGTTGTCAGGG
AACGGCATCTCAAGAAAATTTCCACAAACAACCTCACAAAGCGAATCTAAACCCGCTTTC
TTGTTATGTAAACGAGCGCGATTCACCTGGTAGTCATTGATTGTGATTCCCACCACTTTA
GCCCGCGAGTGGGCGGCGATGGCACGCATAGGCCCACCAACGCCGCATCCAACGTCCAGA
ATACGCTGTCCCGGCTTGACATCGATGAGATCGACAGCCATCTCTTCGTGGAGGCGCGTG
GCATCACGATGGGACTTACCAGGGATGGAAGGAGAGAAATGAAAGGACTGACCCCACCCC
CATTCGTAAATGTCGGTAACTAAGTTATAAAAAGTATCCACAAAATCCGGAACTTTCTCG
GCGGTCTCGATCTCTTTGGGGCGGCGGAAGAAAGACCAGTATTGCTTGTAATTGTCTTGT
ACTTTCTCAGCGGAGATTGAACCACCCGATAGATCGACGGCTCGTTTGCCCTTTTGTTCA
GCAGGGCCTAAAATGCAAACGAACCAGAAGAGACCACCGGCTAAGAGAGCCCCGGTGCAG
AACAGAGCAAGAGAGTCCATTAGAGGCA
>ntig_139_consensus
AATGCAACATATAAGACTAGGAACATTAACATTTCCATTATTCTACAAGTACACAAACAG
TAAGTTGCCAAACATAGAACCAGTAAATTACCAAACTTGAAAAATTAAAAGTGAAGTTTT
CAATCAAATCCCTTAAAGCAGGGATTTTATTCCAGGATCCAGTTCTGACATTCCAAACAA
GGCGTCTAGCACACACATATCAATACAAAACTAAACATCTTAAGAGTCAGAATGCCTGAA
GACCAAAATCAACTTCCATCAAAATCGATACCTCAAGTAATATGCTCTGCCAAAATAAGG
ATCTTGACATGCTTTGTTCCACAGATCTTGGCATGCTTTTACTTCCTCGGTACTCTGGCC
CCAAGTGGCCGGCCTTGCATTGTGACCCAAATTGTCTTATACACATGATAAGTCAGGGCT
AAGGCAGCAATCTCAGAGATGATGATAAGTTTATCTTTATCAGAGAAAAATTCGAAAATG
CGGTAAAAACTCTCATAGGTGAAGCAAATAGAAGGCATTTTCACAAAAAGAAGATAATAA
AACAAAACCACAATGGTTAGGATAACCCCGGAGATCCTATTCAAGATTGACATTGTTGAA
GTGAGCTG
>ntig_140_consensus
ATTGACATTGTTGAAGTGAGCTGTGGCTTATAAATAGAAAGATGAGGAGACAGTGGGCGC
TGGATTACTTTAAAACTTGGATTACTTGCATCAATGCTATCCCCCTTTTCTCCCTTCAAA
GCTTGTGTGCTCATCAACTTTTTGGCTCCACAGTTGTTCACAGCGTTGAAAACAGTCCCC
ATCTGTATGAAAGCCCTTTGCTGAAAAAAGAAATTGTGGGCTGTCATCTCATTTGATGTG
CTCAATAAACCAGACAAATTGTTTCCTAAGCCCCCCAACAATGAATGCCTCGCATTCAGC
ATAGCTTCCTTTACTGATGCATCAATGCTATCCTCCTTTTCTCCCTTCAGTGCTTGTGTG
CTTATCAGCTTTTTTGTGCCAGAATTGTCCACAACGTTGGGAACAGTCCCCATCTGTATG
AAAGTCCTTTGCTGAGATAAGAAATTGCAGGCTGTCATATCATTTGAAGTGCACAATAAA
CTAGACAAATTGTTTCCAAAGCCCCCTGACAATGAGCGTCTTGCTTCGCGGGACAATCTT
AAAGCCAAACTTGCGGCCATCTCTGAAACTAAGATCCCAGCCTGGAATACTACAACCGAA
GCAAGATTGATCTAATAGAGCAGCAGACAATACCTAAAAACCTACTGCAGCTTACAGCTG
CAGAGAGTGAAATAATGAGGTATTTGGATTTGGGCCACAGCCCAGAAGTTGCAGGTCCAG
TCCAACCACAAAGAAGGAACCCTAATAGAAAAAGTCAAGTCATTAACTAAAAAACAAATA
AACCCACTGCTAGGATTCCCAAACTCTCTCAGTTCTCTCCACGTG
>ntig_141_consensus
ATATAATGAAGTCTGTGTTCTTTTCTTGTGCACATCCATGTATGTATAATAACTAAGCTT
TTTTATTTTTTCTGTTGACAATATAAAAACTAAGAAAGTTATGGAGGTCATAAAATCTCT
TTAAAAATTGTATGGTAAATTAATTAATAATGGAGCTACCGATGGATACATAATATTTTT
TTATATATGCATATGTAAACTGCCAAATAAAGAAAAATATATGCATATATAGCTACAGAA

TCTGTTCCATTATTCATCGCCCTGAAATTCTGAAAACCTAGTCACCCNNNNNNNNNNNNN
NNNNNNNNNNNNNNNNNNNNNNNNNNNNNNNNNNNNNNNNNNNNNNNNNNNNNNNAGTCA
GTACATGAAACAAAGATCAATCTCTACTTAATAGTGAGAAGACAATACTTAAAACAAACA
AAAAACTAACAAAATGCTGAAAAAAAGAGTCAGTACATGAAACAAAGATCAAATCTCGCT
CCACAGTAGACTAGCAGAGGTTTAAACAACAAGAATACTCGTAAAAAAAGACTTTAACGA
AAATTTAAGTACTGAAAAAGCAATAATAACAATAATAATACACACCAATTCACATATATA
TAATGTGATATATACGGCTGGTGCAGCAGGCAGTGTATGTACACTCATCTTCTTCATCTA
CATCAAGAGCTCATTCACCCAACCCAGATCAGGATCGGGACAACCCAGCTGCCCGCCATT
AATATCATTGGTCTTCTGATCATCAACCGAACATTTGTCTGAACAATCGCCAGCAAAAGG
ATTTATAGACCCTGATGGGGTTGGAGTTGAAGGAGAAAGAACAAATTGTTGGTGATCGTC
AATATTAAAAGTATTTGCATCGATCCATGGAACATTAGCATTCATGCTACTGCCAACAGG
GACAGACAAAGGAGAAGAAACTTCATTAAAATTCATTGCATCCAAAGAACTCATCAACTC
ATTAAGCACATCTTTGTAACTCATGGCCGCATCAGAAGGACTGCTAAACTTCGTCAAACA
ATCACTGTAGCGCGAAATAGGTGAGAATCTATGATTTACTGGTGACAGTGGCGGTGACAT
AGACGGTGACCGTGACAAATGAGACATACACATCAAAGTACTAGTTGGAGAGGAATTCAC
AGAATGATTGCAAGGCATGCAACAGTGATGATTCTTATGATTGTTCACCGGAGAATTGCC
ACAGAGAGGGTGACTGTACTTGGGAGATGATGAAATCTCAGGCAAAATGCGAAGCTGACG
TGCAGAGTGAGCAAAAAAACACACCTTTCGTTTGCAATTCTTGCCGTCTTTGCAAGCTTC
AGTTCTATATCGTGACGGATGGAGCCAACACTCAAAGACTCCATGAGCAAACTCACAATC
ATCTCCACGGCTACACCCGCCTCCCCTACGAAACTCAGAACAAACAGTACCAGAATAATG
AAACCTGAGAGGATCACGACGGCGAGCCTTCTCCGCAGGATGAGCAAAAGGACAGTCAGT
CCAGTCATGGCTTCGGCTGCGAGTACAACGACGGATCTTAAACTCATACATGCGAAAGTG
ATCAGAGGCGTAAGGATCAGAATCAGAGTCGGGATCAGAATCAACATCAGTGTTGTTGAA
AGGCAAGAACTTATGAAGATTGTTGAGTTCTTCAAGAAACAACATGTTATTGATTTCTTG
ATGATGGTTATGGTGCAACAATTTCTTGCTATTGAGAAGAAGTTTCCTGGGAGGAACCTC
AATATTTTTGAAGGGCTTGGTGGGACCTTTAGGGGAAAGAAGATGGTGGAGTTGGTGTTG
ATGCTGGTCAGCACAAATAGTGCTCATAGCTGAATATATGAATCAAATGAAATTAATGTA
CAAGTGAAGAGAGATGGAATGAAGGTTGGCAATGGTATTGAGGGAGAAAGGAAACAAGAG
AAGAACTAGTTAAATAGGTTAGAGAGAAAGAGGGGTATGGGAGGAAGGGGGATAAGGTGT
GGAGTTAGAGATGCG
>ntig_142_consensus
ACCGAGCTCTACACTCTTTCCACACGACGCTCTTCCGATCTCCCCACCATTACAGCTGTA
TTTCGGATTCCATCAGTAACTTTTAAATTCTTTTTCTTTCTTTTTTACTTGGTGTTTAAT
TAATTTTCCCTCCAATAAAATCCAAATTTATGTGGTGAATCCATGTATATTAAATCCTCC
ATTATAACAATTTGAAACTTTTACATGGACGACAGATTATTATGTTTTTGTTGCATCTGT
AAACTTCCAGATAAAAAATTTAAATATGTAAATCCTGAAATTCTCAAAACCTAGTCACAA
TTCTTCCGCATGTCAGTCACGTATCCCCACAAGGTCATCCTCAATGCAAGCTAAGCCAAC
AACTGGCGGGTTTAAAAAAATATCGAAGTCCTGATTTTCAGACGTTGTTGTTAGGTTAAA
CATGACACTTGTATACACATACATTCATCCGAGAAAAAGGATTAAGAAAGAGAAAGATTC
TTCATTATCGCACCTTGCTTACAGTTGAAGTCAGTACATGAAACAAAGATCATATTTCTT
CCCTACTTAACAGAGAGATGACAGTAGTTCAAACAAAACAATCCAAAAAAAATGCTAAAG
GACAGTCAGTGCATGAAACAAAGGTATTAAATCTCGCTCCACTGCAGACCTAGCAGAACT
TTAAGCAAAAGGAATACTACTTGGAAAGATAGAAGACATTAACAAGGAAATGAAGTACTG
AAAAGCTAGTAGTAATAATCATGCTGCACGCCAAATCACATACATATATATACGGCTGGT
GCAGGCAGTGGATGACACATCTTTCTTCATCTACATCAAGAGCTCATTCACCCAACCCAG
ATCAGGATCCGGACAACCCAGCTGCCCATCATTAATGTCATTAATCCTCTGATCAAAACC
CAAACATTTGTTTGAACAATTCCTACCGAAAGGATTCTGTGACCCTGATGGGTTTGGAGT
TGAAGGAGAAAGAACAAATTGTTGCTGATCTTCAATGTCAACAGAATTTACATAAATCCA
AGGAACATTATTATTCATGTTGCTAACAGCGGGGACAGGCAAAGGAGAAGAAACTTCTTT
GAAATTCATTGCATCCAAAGAAGTCATCAGCTCATTAAGCACATCTTTGTAACTCATGGC
CGCATCAGCAGTAGTGCTCAACTTTGTCAAACGCTCAAGGTACCGAGTAATTGGTGAGAA
TCCTTGATTCACAGGCGACAATGGCGGTGAAACAGACGGTGATCGTGACAAATGAGACAT
ACCCAACAAAGTACTAGTTGGAGAGGAATTTACAGAATGAAGGCAAAACATGCAACAATG
ATTATTACTATGATTGTTCACCGGAGAATTGCTGGAAAGAAGGTGACTATACTTTGGAGG
TGATGAAATCTCAGGCAGAATGCGGAGCTGACGTGTAGAGTGAGCGAAAAAACACACCTT
TCGTTTACAAATCTTGCCGTCTTTGCATGCTTCGGTTCTATATCGTGAAGGATGGAGCCA
GCACTCAAAGACTCCGTGAGCAAACTCACAATCATCCCCACGGCTACACCCACCTCCCCT
ACGGAACTCAGAACAAACAGTTCCAGAGTAATGAAACCTTCGAGGATCACGGCGGCGAGC

CTTCTCTCCTGGATGAGCAAAAGGACAGTCAGTCCAGTCATGGCTACGGCTTCGAGTACA
GCGACGGATCTTAAATTCGTACATGCGAAAGTGATCGGAGGCATAAGGATCCGCATCACA
GTCAGGATCAGAATCAGCATCNNATCTTGATGATGGCTATGGTGCGAGAGTTTCTCGCTG
TTTAGAGGAATTTTCCTAGGAGGAACCTCAATATCTTTGAAGGGTTTGGTGGGAGTTTTA
GGGGAAAGAAAATGATGGAGTTTGTCTTGATGTTGATCAGCACAAATGGTGGTCATAGCT
GAATATATCAAAATGAGAAAACAAAAGGAGAAGATAGTTAGAGAGAATGAATAAAAATGA
ACATGGTGGTGAAGAAGGT
>ntig_143_consensus
ACTCTTCTTCTTCCTTGAGGCAAAGCTGCTTGGTCCAATTTCTATGGACATTCTCTTTGT
TCGAGCAGAAAGCTCTTCTGATTCCTTCATACCATCTTCAACTTCGCCATCTGTTTCATT
TTCTTCATCACTTTTCTCCCCTGACTTTGAATCTGAACCCCATCGATCTCCACCATCATC
ATGGTCCCACCGTTCTCCAACCGAAGGCATCCAAAAATCAGCTTCTTCCAAATCACTCTC
CTTATCTGAAGACCCCCTCAGTTCAGAGATAATTTCTTCAAACTCTTTTCTCTTCTTCAT
TTTCTTATTCTTCTTATTTAAACTATTGGCACTTGTCTTTCCTTCCTCTTCTTCCTTAGT
TCCATTTGATTCAAGTTTTTTCGTTTCCATTTGCTC
>ntig_144_consensus
TCACCATTCTAAGAGATACAAATCTCAAAATCTTGAGATGGCATATATATAGCAATTCAG
TTAGCATTGGATATCACCAATAAAAACAACAAACAAGTTTGTTCCATCCCAAAACAACAA
ACCTAATTTTCCTTTCCTCACAAATAACAAGTAGTTTTAACATCTAGTTATTTCTTGGAG
GTAATCTTCAATAGAAAGACAACGAGTTGATGAGAAAATGACACACCACTCAGTAGTACG
CTTTTCTGGGGTTGTTCATAGGATTGGGTCTATAACGGTAACCAAGCATCATTCGCTTCT
TGTACTGTTCATATATATCATCATCAGGAGTCACCTCACCGGGATTATGTGCACCAACCC
CCAAATTGTTTGTCTTTACATCACCTGCCGTGATTGGATCTGCAATTCCACTTCTGGAGC
TCCCCAGACCCTCACCTTCTTTCCAACCCATTTTGGACAGAAGCTTATGCCCAATGTTGT
CAGCCTGAATTCTAGCCTTATCTGCAGCCTCTTTTGCAGCTTTCTGAGCAGCTACATCAT
TACAGGTAGACATGAACTTCTCAAGCTCTTCTAGTGGAATGTAATCACCCATGTGATGAC
CTCTTTTAGTTGGTGGACCTTGAAGGGAAGGAGGTGGGGGCATCTCATCCTTCGATTGTT
TAGGCTGTCTTCTCTTCTCTTCCTGAGCAGCCTTCTTCATGTAAAACTCCATCATAGCTA
TAGGATCAGCAGTCGGTGGTCCACTAGATTCAACAGTGCCAGGTCTTCCAGTTGAAGCTG
CCTGAGTTGAAGACAAAGAAGCTCTTGGCTCTTCAGATGTTTCATATAAAGCTGAGGCAG
GGATTTGATAATTTTGCTGCTGAAGAGACCTTTGGGAAACATTAGCAGATTTAGAGGCTG
AGGTGCTCATGCCACCGCCTTGAGGAGTCTGAGTTTCTGTGGTCTGTGAAAGAGTTTTTT
CCTCTTCAGCAAGCCGATATTCATAATACTTGTAATCCGCACAACTCTCATCAAAGAGGA
ATTTAAAAGGTGTGTCTCCAGGATTCTTTTGACGAGTTACATGCTCAAATTGCCTTCCAT
GTTTGGCAACAAAACTTGCCAATTTGTCAGCTACTTTCTTCACAGTAGGATCACTTGGAG
GAAGTGGTGCAACTGCAGCTTGTCTTGATGATAGCTCAGATGCCTCTGATTGACCCAACT
TCTGCCGTTTCATTGGTCCATCACCTGAACCAATTTCAGCATCTGTCTCATCTTCATCCT
CATCTACACCAAACTTAACAGGAGGTGCCACAACCTTAGACTTTTGTTTCAAGCTGAATG
CAAATTTGCCACCTGATGTAGCAGGGCCAGATTTATGCGTATCCACAATGGGTTTTTTTA
AAGATATTTTAGGAGTTGAAGTCCCTGATACAATCTTGACTGGTTTAGACTCCTCTACTA
ATGTAGTGCTTTTCTCCCTCTCTTTTTCCTTGTCTTTGCCTTGTTGAAGCTGTTTAAACC
TCTCCATGAAGGAGCCATCATTAACAAAAAGACTAGGAGGTGTTCCTTTCTCCATTGACT
AGGAGCTAAAAATATTTAGGCTGTTCAACAAGCAAGCTTCCAAGATATTCAGATATTGAA
CCGGAACTTTTAATCCATCCCTTTATAAATACACAAAAATTACACTAATTGCATAACATG
GCAATCAAGCATCAACACCCATATCCTATAATGACAAAATATAAAATAAAATAAAATAAC
ATCTGCCACAGCATAATTCATTCCAAAATTGAATATACCTCCCAAGACAACCTAAAAGTA
AAAAC
>ntig_145_consensus
GAAAATAGAAAAAAAAAAAAAAAATACCAATACAAGATGAGCCGTGTGAAGCCTTGTGTCA
GAACTGTTGAAAAAATTAATCAAATCTTGAGATTGCATACATATACCAATTCTATAAGCA
TCCAATTGCACCAAAACAAACACCAGATTGGTCTGTTCTGTTGCGAAACAACAAGCCTAA
TTTTCCTTTTCTCACAGATAACAAGTAGCTTTCACATCTAGTTATTTCTTTAAGGCAAAA
GTTGATGAGTTGATGACAAAATAAAATGCCACTCAGTAGTATGCTTTTCTGGGATTGTTC
AAAGGATTGGGTCTGTAACGGTAGCCAAGCATCATTCGCTTCTTGTACTGTTCATATATA
TCATCGTCAGGAGCTACCTCCCCAGGATTATGTGCACCAACCCCTAGATGGTTTGTCTTT
ACATCACCTGCCATGATTGGATCTGCAATTCCACTTCTAGAACTCCCCAGACCCTCACCT
TCTTTCCAACCCATTTAGACAAAAGCTTATGCCCAATGTTGTCAGCCTGAATTCTTGCC
CTATCTGCAGCCTCTTTTGCAGCTTTCTGTGCAGCTGCATCATTACAGGCAGCCATGAAC

TTCTCAAGCTCTTCTAATGGAATGTAATCACCCATGTGATGACCTCTTTTAGAAGGTGGA
CCTGCACCTTGAAGGGAAGGAGGAGGGGGCATCTCATCCTTTGACTGTTTAGGCTGTCTC
CTCTTTTCTTCCCGAGCAGCCTTCTTCATGTAGAATTCCATCATAGCTATAGGATCAGCA
CTAGGTGGTCCATTAGATTCACCACTGCCAGGCCTTCCTGAGGAAAATGCTTGAGTTGAA
GCCAGAGAAGCTTTTGGCTCTTCAGCAGTTTCATATAAAGCTGAGGCAGGTGTTTGATAA
TTTTTCTGTTGAAGAGACTTTTGAGAAACATGATTAGATTTAGAGGCTGAAGTGCTCGTG
CCACCATTGTGAGGTGTCTGAGTCTCCGTGACCCGTGAAAGAGTTTTTTCCTCTTCAGCA
AGTCGATATTCATAATACTTGTAGTCTGCACAACTCTCATCAAAAAGGAATTTAAATGGT
GTGTCTCCAGGATTCTTTTGTCGGGTTACATGCTCAAATTGCCTTCCGTGATTGGCAACA
AAACTTGCCAATTTGTCTGCAACTATCTTCACGGTAGGATCACATGGGGAAGGTGGTGCA
ACTGCAACTTGTCTTGATGATTGCTCAGACGCCTCTGATTGGCCCAACTTCTGCCGTTTC
ACTGGTGCATCACCTGAACCATTTTCAGCATCTATCTCATCTTCATCCTCATCTGAACCA
AACTTAACAGGAGGTGCCGCAACTTTAGACTTTTGTTTCAAGCTGAACGCAAGTTTGCCA
CCTGATGCAGCAGAGTTGATCTTCTGTGTATCCACAATGGGTTTTCTTATAGATACTTTA
GGAGTTGAAGTCCCTGATACAATCTTGATGGGTTTAGACTCCTCTATTGATGTAGTGCCT
TTCTCCCTCTCCTTTTCCTTGTCTTTGCCTTGTTGAAGCTGTTTAAACCTCTCCATGAAG
GAGCCATCATTAACAAAAAGGCTAGGGGGTGCTCCTTTCTCCATTGGCGGCCAAACAACA
GTAAACTTTGATTTTACGAAAGAGATCAGATTCGCAAACGAGAGAGATATTTTCTCGACC
GGTTTCTGGTGGAATCGAGCGGAAACTGGATTAGTGAATTACGTGATTATAAATTATAAT
GAGAAAGAGGACACCGGTGGCCTTCAATAAGGGCTGCTCTGTTTACGTGGGAGATTTTGC
AATTGAGGACTTACTCTTTCGAAGTTTTGCAAACTCTTCCTTCCAACTTTCTACGAT
>ntig_146_consensus
GACCCAATTTCTTTGAAAACAAATCCATACAATAATTAGCAAACACAAACACAAACACGA
ACACGAATATTTTAAGATTGCCAGTACAAGCTTGAACACAAATATAACATAGAAATAATC
CATATATCAAGCAAACTATTTGTATGTTGAAATGAAATGGGGAAAAAAAAAAGGACGCCA
TTTTGGGTATGAATTATACCCAACATCGCCATTTTGGATATGATTTATTCCCAAAACATA
TGCAAACACAAACATATTTGCTGAATACAAATATGTTTGCATTTGTTTGCACAATAATGA
ATAAACCAAACCAAATCCAAACATAATTACCGTTTGCGAACGCAAACACAAATGCCCAAC
TAATCGTGTTCAATGAACACGAATACTATCCATTAACAAACAAACCAAACACGAATATAT
CTCTGCTCGTATTCAGTTCAACTCAAATTCAATTCTAGGCAAAACAGATTGAATATTCAA
ACTAGATTTCTTTATTCACAACCAAAGCCTGAATAATAACACAGTATTATTACATTCTCA
GTCCTTACAGCCCATTTATTTACATGCAAACTGTTGTACACAACGACAATGTGAACCCAT
AATCAAATATGAATTCAGCTTTCATGATGTCCGGTGTATATCCAGTAGTAGTAAAGACAA
ACACTCTTTCTTCACTAGTCACTAGGTAGTAAATACTAGAAACTCATATGGGTTCTACTA
GTGACAGAAGAGTATTACCTTTGCAAGCTCTAGTGCTTTGTCGCATCCAAAAAACTTCTC
CACAATTGCCAGTGCAAACTCCATGGAAGTTCCTGGCCCTCGGCTAGTAATGAGGTTGCC
ATCAACTACAACTCTGTTATTTATTTCACTCTTATCTGACAACTTGTCACACATTGCTGG
AAAAGCTGTAGCTTTTTTACCCTTGAGTAAGCCATTGGGCTCCAAGACTAGAGCTGGGGA
TGCACATATAGCACCATATGGTCTATTTGATTCCCTTTGTTTCTTAAGCATATTCACAAG
TTTCTCTGAATTAGCAAATGCTTGAGCACCACCGAGCCCGCCTGGCAATACAATTAGGTC
GAATGAAAGTTTAGCAGCCTCATCAAGAAGCATATCTGCCACTAATTTAACTTTTCGAGA
AGCCAGAATTTCTAGTTTATCCCCAACTGAGGCAACTACCACATTTGCTTTAGCCCGTCG
CAAAATGTCAATGATCATAACAGCTTCCATTTCTTCTGAACCATCTGCAATAGGTACAAG
AATCTGAGGAAAATTATCAAATGTCCACTGCATTGGATTGAACTCAGTTATAGTATACTG
ATCCCCATGGTTAGGACGCATCACCAGCGGCCCAGAAACTTCATCAGCTTTCTCTTTCCC
ATACAATTGCTCAACTAGTGCCACAGCAAACTCCATCGTAGTGCCAGGTCCACGACTTGT
CACAACTTTACCATCTAGCTGCACTCTTGATTCAACAGTTGTCGCACAAGCAGGTGCTAA
TTGCTCCATAAATGCTGGATAACATGTGGCCTTTAATCCTTTCAACAAGCCCCAAGATCC
TAGTGCCACAGCAGGTGAAGCACAGACAGCGGCATAAGGTCGCCCATCTGCAGCCTGCTT
CTTCACCATGCTTTCTAATATTCCACAATCTTTCAGATTAGTAGCACCAGGCATACCTCC
AGGAAGAGTAATAAGATCAAAAACAGCGTCGCTGCAGTCAGAAATGAGAGCATCGGCGAC
AATCTTGACGGCATGGCACGCGTCAACACGAAGCTGTTTCTCCACAGAAGCGACCGTCAC
GTCAGCTCCGGACCTCCTCAAAACATCGATGGTTATGACCGCCTCCATCGGTTCACTCCC
GTTCGCGATCGGCACCAAAACCTTCTTTGTGGAGGACGACATTGCAGCGGTTGTCGTATT
GGCGAAGAAGCGACGAGACTTGAGAGATGGAAGTTGCTTGGGAAGAAGAGGAGAGTGACG
GAGAAGAAAGTGCGGAGTGAGGTAGCGCAAGTTCATTAATACCATGTTTATTTATGTATT
TATTTTCAGTTATTCTGGGTTGTCTCTGGAGACATCCATTCGGTGGTGGCAGTACAGTAT
GGTATCGACACGATGGATATGAAAGTTGGTGGGCCGGTCCGCCCTGGCCTGGATCTGTGT

TAATCGGGTTGGGTTCAGGCTTTGTTCTAATTTGGTAACCATTCCCCAAGAGTTGCCCGT
AGAAATATTGCATAAGCTGGAAAGGGGGAGAGGCTATAGATGGCAATTTGATCTGTCAAA
GCGGGTCTTTGGCCCGCTCCTTCCTAAACGAGGAGAAAATTTTTCAATAGGAAGAGAGAT
AGAGATGAGGGCATACATTAGAC
>ntig_147_consensus
TGGAATGTAGGCTATGTCCGCCAAACTAAATTGACCAAGGAAGAATGGGCCATCATCAAA
TTTATTAAGAGCATTTTCTAAGTAATCGAAAGCTGCACCAGCTTGTTTTACAGGGTCTCC
TTTGAATGCAGTATACACATCCCTGTTAAATGTATCAGTGTATGATAATAATTCTTCACC
AAAGTTCCTTTTGTCAAGATCATTGGGGAAAAGAGAAGGTCCT
>ntig_148_consensus
CATTGGGGAAAAGAGAAGGTCCTCCAAAGCTGCTGTCAACATATTTAATCAAATCAATAC
TCTCTCCAATGATTTTGCCATTGTGTTCCAATGCTGGAACCTTGTTAACAGAGTAAACTT
TTTCTGCGTACCAAGCAGGCCTGTCTTGGAGGTTTAACGGAACTAACTTAATATCGTCTT
GTAATCCCTTATAGTTTCTGGTGATCCACACTCGTTGGGCAAAAGGACACGTGTAACAAG
TATACAGCCTGGTGGTTCCATCAAAAAGTGGCGGTTGTTCGGCTTTGGAGTCCAAAACCG
GAGGGAGAATCTCTTGCACG
>ntig_149_consensus
CGAGAGAAATAATCATGTATTGCCAAGAGGGCACGAGCTGACTGGCGCGTTGTCAATATT
CGATGCATTTGTTGTAATGTTTGCTGCCGCAAATTGTCAGCCTGACGAATGAAACCCTCC
AAAGTCCCAAGCTTTCCCATGGCCATAGCCATTTGCCCCATATAGTTTGCCACATTCCCA
GATGAACCTGAGGAGCCAAGAGATCCACTGGAAAGTGTCTCAGCGAGGGACTGTTGCAAT
GCCTCCATCCCTTGAGACAATGCATCCTCTGCCTGTTGGGAGGATTGCTGCAAGTTGCCA
ATGCCGACAAGCTGCTGCTCTGTTAAAGGCTCCAATTGATTCACAAGAAGCTTGAGAAGT
TCGGATGAACGGAAACCACCAAGCCACAAGAAACACCTCTCAGCAGGTGTTTTCCACATG
CCCGATAACAAATGGAAAACATCAGCTTTGGCTGCATTGGACTTCAGCCTAAAAATTTCA
TCATAGTGTGCCATTACACCATCAATGATAATTCGTAATTCTGTGTCACTTGCATGAGAA
TTAACTGCTGATCTAAGCTCGTTGATTTGTCGGTTATGCTCTTCAAGCCATCGTGCATAC
TCTACATCAAATGCCATGGCTCCATTTCCACTCATTGAATGAGCTTGATCTCCTGAGCTT
GATATAAAGATTCCCTGCTGCCGTGCTCGTTGAAGTTCCTGCTCAAGTTGGGTCAGCTTC
AAACGGCTACTTTCCAACTGCTGGACATATGCTTTCTTCCTTAATCGGCTTTTTCTAGCA
GCCTCGCGATTTTGAGCAAGCCTACGAAGAGTCTTTTGATCCATTTTGTCTTTTGATCTA
TCACTAGAATCATCAGCCACGAGAGCTGCAGATTGACCTCTATCAAACCTTTGACTTTTT
TCATCGGTGTCTGCATCGGTTGAGATATCAGTCCTGGGACTAGCATCAGCCATATTAGAT
TCTCCCCAATTCCCTGTATTACCACTAGAAATAGAAACTGGATTCAATTGTTGACTCTTT
TCCAACAAGAGCCTCTGGGTCTCCACTTGGATGGTAGATGGATTTATGTTAAATGAAGTT
GATATCTTATCAAAAGTGCCAATTTGGAAGGGATCAGAAACAACTGCTTGGTCGCCTGCT
TTGGTTGAATTAATCACCGTGTTTCCACTTAGGTTGACAGCATCCTCTAAGCGAAATCTG
GCAGAGTGCTCAAATACCCCAAAATCATTAACACGATAATTCTGGATTGTGCTACCCTCT
GCATAAAATATGTTGGAAATGGGCAATTGAGAATCAAAACTTGGCATTCTGCTAGATGCT
GCTTTCCCATCTTCAGCTCCAGACCTCACAGTCCTACTCCCCATGCATCACCATCATCAT
CATCACCACCTCCTTAGTCACCCTCAAACAAATTAGAATTCCCAACCCTTTCCCTAAGAA
AACCAAAAATGCAAACTAAAGATACCACGTTCTTAGAAAATTCTTTCGTTCTACTTGCTA
AAACAACGAGAGAAGCAAAGAGAAAAGTAGAAAATATCAATGATCTAATGTATAAGAACA
ATACTACTCTAAAACAACAATCTTAACTAAAAAAGAATTCCATTTACATGACTGCATAAA
CCAAAAACCATATGAAAGTCCATCAGAAGCAGCAATCTCCAAATAGTTCATCATCCAGAA
GAAGTACACGTTCAGAGCACCAAACAAACAGCATTCAGAGACAGAATGAGGTAGTACTGA
GAAAATCATCCTATTACAGGGAATACTACATACTGAATCACAAAAATAATACCCAGAAGA
AACCTAGCAAAAGATAAATAAAAATCCAGCTGCAGAAGCAACAGAATCAAATACCTAATT
TTGATTCATATTCTTTCTTCAACTAAAAG
>ntig_150_consensus
CCTTCTACCCCTGATTTTATCCATTTCCCCCCCCCCCCCCCTCTCCCTTTCCCCAAAAAA
AAAAATATTGTGGCATTTTTAAAAAATTTTTTTTTTTTTTTATTTTTTTTGACAACACTA
AAGAACCAAGAATCTTTGTCAGAACTGTCAATTCAGATTTTCATGCACGGCCCATATATT
ACATTGTGTTTTCACTCTCTTGGGCGGGCTAGCCAGAGCGAACTAAGAGCACGAAGTCGA
GAGAAATAATCATGTATTGCCANNTGCCGTAAATTGTCAGCCTGTCGAATGAACCCCTCC
AGAGTCCCTAGCTTTCCCATGGCCATGGCCATTTGCCCCATGTAGTTTGCCACATTCCCA
GATGAACCTGAGGAGCCAAGGGATCCACTGGAAAGAGTCTCCGCAAGGGACTGTTGCAAT
GCCTCCATCCCTTGAGACAATGCATCCTCTGCCTGTTGCGAAGATTGCTGCAAGTTGCCA

ATGCCAACAAGCTGCTGCTCTGTTAAGGGCTCCAATTGATTCACAAGAAGCTTGAGAAGC
TCAGATGAACGGAAACCACCAAGCCACAAGAAACACCTTTCAGCAGGTGTTTTCCACATG
CCCGATAACAAATGGAAAACATCAGCTTTGGCTGCATTGGACTTTAGCCTAAAAATTTCA
TCATAGTGAGCCATTATACCATCAACAATAATACGTAGTTCTGTGTCACTAGCATGAGAA
TTAACTGCTGATCTAAGCTCATTGATTTGTCTGTTATGATCTTCAAGCCATCGTGCATAT
TCTACATCAAATGCCATGGCCCCATTTCCACTCATTGAATGAGC
>ntig_151_consensus
CCAGGGGCAACTGGGGAGATTCCACCAGAGAAGTGGGATCCAAATCCATGGCCAAGCAGG
GCACCAACACCACCATTGCTGCAAACATTGGAAGAACTCAATCCAAGAGTGGATGTGAAG
CAGCTCCTAAGCAGCTGAACCACAGCATCACTACTATGGTGGAAGACTGTTCGAGAAGCA
GAAAACACAAGGAAGTCATTCCAACGCTTTGCTTTCTGCGCCCATAAGGAGGCTATGATG
GGCATACATGGCCAGGGGCAACTAATTGCAAGAGAACTTAAGGCAGGGGCAACTAGAACA
AGAAAACGCTCCGACGCCTTATCAAGTTTGTATGTTATAGTGAGACTAACCAGGGCCGCC
AAGGGCAAAGGAAGTGTAGCTGGAGAACTGCCCCCTGCAGCAAGACTTGGGACATCAACA
CCAGTGGCAGCTAGGATTTTCTTGATCTGTTGCTCAACGCTGATTAAATTTACAGCAGGA
CTCGGCCAGTCAGTTCCATTCATAAAAGCAGGCTTCCATATACCTCGTGTTACTTCTGCT
GAAAAGTAGCTAACTATGGTTGCCAGAGTAGCTGGAAGAAAATCAGCAAGTTCTTTGAGC
CCTGTAGCTAGTTCACGAGGTGAGAGTCTTCCATGGGCACAGGCAGTCAGAGCAGCATCA
AGAACAAAAGGAGTTGCTTCCAGGATATCCCATGCAGGTACTCTCAGCCTAACAAACACA
TCCTCCACTCCAGACCCAGATGAATTAGTACTACCAGAAGTTGTAGGAGTTAAAGGAGTC
CCACTTCTATTAATCTTCCTAAGCATCATATTGAGAAGCACATCAACAAGCTGATGAACA
GGTGTCCCATGTTCAAGACCAGAGAGGGTGGAAGCAATACATTCTTGATGTTGTTGGTAC
CAAAACTTTAATTTGGGAAAAGAATCCAAAAAAATGGGACCTAAAGACATAGTAAAAATT
TTTGAAAATCTATTGGTTTTGAATTGATCCTTGGGAGACTTTCCGAAAGATGCTAATGTA
GAGTTCCGAACTACTAATAGATATTCTGGACTTAGTTGGGATCTCACAGGCGCTGCCATG
TCTCCTATTACCTGTTCAACAGAAGGATGATCAAACCTCCATAACCGCAGTAAAAGGGTA
AATGCAGTGGAGAAAACTGCATGACAAGACAACTCCTCCCCTGTCGGAAGAGTCCATGAG
ACACTGGGAATGCTTGATCCAAAAACCTCACAAATGGGCATCAGTGCACCTGCAAGTAGT
GGAACCACCAAGCCATGTAGGGCCAAAATCTGCACACAATCTACAGATGATATTCCAACA
AGAAGAACATATAAAAATGGAGCACAACCAATTAAATGGTTTTCAATCCCAGAATGATCT
ACAGGAACTTCCGGTGACAACAATTTTATAATGAGAAGAATGGTGTTTTCCTGAATACTC
CAACCACGAACTAAAGATGCTCCACAAAGAATCATAGCAGCTGATATCTTCTCATCATCT
GAACCATTGACAGCAATCTCATATACTCTCTCAATCTCTGCTAAACTAGAAGCTGGAGCA
GCAATCAAAGCATTAATCAATGATGGAGTTAGTGGGGACCCCTTCATGAGCGAAGACCAC
CCTGGTGATTGGGTAGAAACATTTCGGGGTATTTGGTTGCTGTGTCCATTCACATTAGCT
GACCAGAGATACGCTGAAGTATCAAGCAGATTCCTAGCAATACAAGACTCAACAATTAGA
TGTCGCATGTTTCCAGAACAGTTAATTGGCAAGTCATTCATGCTCACACTTTCACAGTAA
CCATTACCAACTGTGAGACCTGAAGCAAACATTATTGCTTTGGCAGCAGCCTGATTGGCA
ATTGAGCGAACAGATTGCGGTGGAGTCAACAAGCTCTCATAATCAGCCAACAATTGTAGG
CTAGCAACTAATTCCTTCCGCCGTTGTCCTGGAGCATGCTTCTCTTGTCCATGATTGTTT
GGGCTTTGTTCAGTTTCCTCTATCAGTACACTTTCATCTTCCTCAATAATCTCAGCAACC
GCAAGCGTTGTAATAGACAACAACATGGACAAGCAACTATCAAGACGAGGTACAGGACCT
TCACTTGGATCCCTTTCCCTTTGAACAAGCCTTAAAGCCGCAATCCATAGACCTAAGAAT
GTATCAAGCCATGATGTACCATTAACCGCCTGCAGAGCCTTTACTAAACCAGTAAGAATT
TCAATGGAACTATATGCTGCAACTTGGCTTCCATTTATAGCATCTTCCAGAAACAAATCA
ATTGGAAGCCACAATGCCGACTGTGTAGATCCATTACATTGACCAGCGGAAGATATTAAC
GAACCAGAAGTCATTATTGCATGAACCTCTTTTGATGATGTTTTACATTCCCTAGACAAA
ACTTTATGAGTATCAGACAGCAGCTGCAGAAGGGCCTCTGGTGTAACATGTTTAGAATTT
CTCAATGCAGTTGACTTCATTATAAGCAGTTTTAGCCGCTGAACAAAAATTCCCCAATGT
GATGGCATGTTTTGACATGCCAAGCAAAGAATTTTTGAAGTGACCTTGTATCGCAGAAAT
TCTCCGATTAGCTCTATGGCCATGGCAGTATTTGCTTTGCACAGTCTTTCATGGTGCTCA
CTTCTCTTCTCATTGAAGCTATCAAGACCATCTATTTCCATGTCTTGTGGCTTGGTTGAC
CATCTAAAATTCTTTTCCGGAGAAAGTTCCAATAGGCTCTCGTCGTCTAATGAAGCATCT
AGTAATTGCAACACAATTAGCGTAATTTGGTCCATTGACAAGTGACAAAAATGAAAAGGC
ATGCTGCTTGACGAGTTCCATATAAAGCCTATATGCTGCAGGGTAGAGCTGCCGGTTTGG
AATGACCCTGGAGGAGAGAATCGCGAGTACGAGTAAGGGAGGGACGACCTTGACAGTCAA
GGCCTTGTCGAGAAACTTCCAAGCAATAGGGACGTGATTGTCCCAGCAGATGTGAGAGAC
GAGTAGATAAGCGAGTTGAGTTGAAGGGAGAGAAACTCCCGCTGAGTTAAGAGTTGAAGT

AAGTTGAACCGCCCAAACGAGGGGGTCACAATTCTTCTCCTGCGCCGCCTTCGTCGTCTC
CAACACCGTGTCCCATAAGCTACGTTCATTAGGATGTACGGATACCACCATCACTACGGT
ACAACCTCTTTGTCTCTGTCTTCTCTTCCTTTTTGTTTTAGAGACAACTGAGAGAGGAGG
AAGAGCAAGAACAATAACAATTAGGGAGAGAGAACAAGAGAAGAGACAATCACTGATTAG
ACTTCAGAAGCTAAAGTCAGCTTCGGAGGCAGGTCTCAATTTCTTCCCCTTTCCCCTTTC
CGCTTTCCCTGTGTGTAGGGTTTA
>ntig_152_consensus
CAGTATAAATATAACACGAAAGGCTAGGCCATACACCAATATTTCCAATCCAGAAAACCA
ACTAAGCCCATCTCAAGTCATACCGATTCTGAGCAGTATTATATGACCAACCCAGTATTC
AAAAATGGATCCTATTCATCATCCACACCAACATTTCCATATCAAACCGAATCATCTTTC
ACTTCAAAGATGTCTAAATCATTCATACAAAACACAAAAACTTAGCAAATTATCATAAAT
TTAAATTTCAGACCACCACAATTAAAAAAAGAGTCTTTAAGATTCAAATCTGAACAAAAT
TTTCTACTATAAAATATGCTAAACAATAGCCTTTGATAGAACTCCATAAGATTGATAACA
GAATTTGGATAAGAGTGCAGAAAATAAAACTAAGAAAACTACGCCTTATCAGCCTTGGCA
GCAGTTGCAGCAGCTTGTCCTCTAAGACGACCAGTTCTCCTTGCAGCAATGAGACCAACC
TTCTGGCCAGGAGGTGCATCACGCCTGACAGTGCTGGCATGTCCAATGTGCTGGTGGTTA
CCTCCTCCGTGAGGATGCTCCACTGGATTCATTGCCACACCACGTACCTTAGGCCAGCAG
TTTCTCTTCACTCTGAATTTGTGGTATGCATTTCCAGCTTTCAGGAGGGGTTTCTCAGTC
CTTCCACCACCAGCGACCTGCCCAATCATGGCTCTGCACCCACTTGGAACAATCTTCTTG
GCACCAGATGGAAGCTTGATTCTGGTGGTGTCGTTATCGGGGTTATGACTGATGATGACA
GCGTAATCACCAGAAGCCCTAGCAAAGACGCCACGATCACCGACATGATGCTCAACGTTA
CAAACAACAGCACCTTCAGGGATAGATCTAACAGGCAGCACGTTACCGACCATGAGAGTG
GCCTTCTTGCCACAGTACACAAACTGCCCAGTGTAAACACCCTCAGCAGCAATGAAAAGC
TCCTTCTGTTTCATGTATCGAAATGGGTGGCGGAAAGTGACTCGGGCGAGTGGAGCGCCA
CGACCAGGATCGTGGATAATTTCAGTGACAACTCCCTTGAGATACCCATTGCGTTCACCA
AAATCAAGGCTCCGGAATTGGGCCGGCCCCTTACGGTGGTGGGTGTGGGACTTGAAGACA
GAACCAGCACCCTTACGCTGAGCGCGAATAACTCTACCCATCGTTCCTTAGAGAATGAAG
CTCAGGCGGCAGGTTAGCAAGATTAGGGTTTGCAGAGGATGGACTGGGTTTTCTCGCTTA
TATTTATAGACAAGGTTGTTCTATA
>ntig_153_consensus
AAAAAAAAAGACAAGCATGTGATCTAAGACAGATTCTTCAGATTCAAGAAACTTGTGTTC
TTCCTATTCAAATTAAAGTGAATCTCCCTTCAAAATGGCTCTACTTTTTAGTATCTCCAA
GAAAATAAATATCTAAAATTTTCTAACACATGCTACAACAGCAAGGCAAGTCCTCTGTGG
CCTAAATTTCCAAAACTACTTCCACTTATGAACTCTCTTAAAATTTCAAGATGCCTAGTG
TGAGTTACAAGAGTCCACACTTCTCCGAAGCGCCTCATGCAGCTGAACTTGGCAACAACA
TTTGTTTACTTCTTCTTCTTTGCTTTGCTGGATAGCGACTGCGTATAAGCAGTTCCCTGA
AGCCATTCATCCATTGAGGCATCTGTAGATCTTGTTCCAACTTCCACCTTTGGAGCTTTC
TTGGTTTTCTTAGATAATTGTTTTACTTGACCCTGGATCCATAAACCAGCGAAGACAAGA
AGTGCAGCTGCAAGGCTGAAAAGAAACACGGATTCAACACTCAGGAAACCACCAGACTCC
ACAACTTCAAGGGTACCATTATAGAAGGTAGTAACATATGGATGCTGGTCTATCTCATAG
ATGACAGAGCCCACAAGATCAAAAGTTCCAGGCTGCAAGTACTTGCTGACAGCAAAAATG
TATGGGAATGTAGCTTGAGCCAAAGCTGGGACAGATGCATTGTTAAAGGCCTGTGAAGTC
AGATTCTGAACCAGCAGCTTATGATCAAAAGGCAGATGAATACTGGCCCGAATCGCAACC
ACATTGACAACTGACTCCCCATCGTTTTTCATCCCAACAAGAAGGTCAGTCTCTTCTCCA
GCTGTCACCGATCGTGCGCTATTCTTAGGGAAAATGCAAACGGTGTCAACTCCTGGAGCT
GGGCCAAAACTACTATCAACAAATTCTTGGGCATCTTCCCCAACAATCCCAAGATCACCG
CCTTCAACAGTCTCCACAGCTTCCTCAGAATCCGACTGACACCAAGCCACTTGGAGAAGA
GGAGAGGCGAGGAGGAGGAGAGCGAGGAAAAGAACCCTAAAATTCTTCATCGAGATGTTC
CGTACGACGCCGCTGCGGCTGAAGACGATCAGTGACGAAGACCAAGCTTTTGTAGCTGGG
GCTTTCTCAGGTCAACAGTACTGTCTCTGAACATGAGAAACGCCTATCTATCCTTTCACT
CTTGTGGCGGATCGACCATACGACACCGTTTCAATCGTTGAAATTTC
>ntig_154_consensus
CTAACATCATATAGTGGAGCGGTCGATTAAGCAGCATCACATCATACCGCCCCAATCAAC
ATAGTACTCCTTGACCTGACCCTAGAGCACCAACTTTATACATTAGATGGGATCATAAGA
AGCTCCAGCAGATGATCACCGAAAAAACGAAAACATCTCAAGGAATCCAACCATCCCATA
TCAGCTATCTATCCGCCTTTCAAATTTTCATTTAACAGTACATGAGGAGGTAATGGGCAA
CAACGTTCAATGACAGACACAAAAAGAGAGCATATAAACTTCTCTTCTCAAGTAGCTGCT
AGTTCCGTGATGCTGGAAGCATCTGCGGCCAATGAACCAAAGGATGACAGGAGCCACCCC

TTCTTCTGGGCATACTCAACATAGAAACTGAATTTCTCCTTGGCATTTGGAATGTCAAGG
GCCAGATCATCAAGACCATCCTTGATCCTGGTAAACCCTTTGGTCATCTGATTGATAGTG
ATTAACCCTTCACTGAAGCATTCCTGCAACAGGTCAAGCATTCTATCATTCTTTTTCTCC
ATAGCCATAACTAGTGCCTTCTTCACCACTTCATGGTTGAAAAAAGGCATTCCAATATCA
CGGATACACTGGCAAGCTTCACCTACAACACCCCCACTTTCATACTCTTCCAATAGCTTC
ATTATCTTGTCCTTTGCATCCTCCACAGCCCAGCCAGTCCCACCTCCCCAGCACCGAAGG
ATCCTCTCACCAGCATGGCGGGCCGCAATGAGTGATCGAGCCATTTGCACAGTCTCACTC
CCACTACAATTTGGGGGCAACTTGCTGCCAATCTCTTCTAAATTCAGAGGAGCCAGGACA
TCGTCAATCACGGCCCTAGCCAGAAAAAGTGCAAGCTCATTTGAAGCATCCAGAATGTCC
AATGCTGTATCTTCTGCAGATTCAAGGAGTAAGACAAAGCCATTTACAATATCTTCAGTT
GAAAAGATCTCAATGTGAAGAGCAGAGAGCAAAACTGATGCCATTTCTTTCTCTCTGTTC
TTTCTGTCTAAAGCAAGGGTGATCAGCTTCTTCAGAAATATTGGGTTCAATTCAGGAGCC
CCCAGATCTTCAAGACTCCTGATTAGTTCAGGAATGTCATCTGAAAGGAAATACTCATGA
ATTATGGTCACAACTTCCTCCTTGTACCGCCTCACCTTTTCACTTTTGTTTTGTACATTT
CCATCTTCACCTGATGATTTCATGAAAGAAGCATCAAGCCATCCTTCAGAGATAGCTATG
TGGATAAGAGATTGGAACAAGGTTTTCGCAGATGGAATGTCAAGAGCAAGATCATCAAGA
CTCTCATCCAACCTAGCAAACCCTTTTCCCATTTGACTAGAACTAATCAGCCCTTCCTCA
GCAGCTTCCTTTAACAACTTTAATATAAGTGGTTCAGCTGTTCGAATCTCCATGGCAAGA
ACCAAAGCCCTCTTCACTACTTCGTGATGGAAGAATGAAACGCCTAGCTCCCTTATGCAC
CTACATGCTTCAACAGTATCGCCACTCTCTACATATTCCCTCAGCAGATCAGCTATCTTT
TTCTTCACTTCTTCAACAGTAATGTGAGTGCTACCTCCCCACCTCCTTTCCACAAGTTCT
GCATGATGTGGAGCTGAGAGGTAGCTCTTTTCTGCAGTTTGGATAACCTGAAATCCTTTG
GAGGATTCTGGAAGTGTCTTCTTTGCTCGTGTGATGAAAGCCGGAGGAAGAATATCATCA
ACTACAGCACGAGCTACAAACAAAGCAAGGATGTCAACTGCATCTAGTATGTCAACAGCA
AGGTCATCAGCAGAGTCAAGAAGAATGACAAAGCCATCCCTAATCTGAGCTGGGCTGAAA
ACATCTGCATATAGTGCTGAAAGCAGAACTGAAGCCATTTCTTTCTCCTTATCATGTCTA
TCCATGGCCACGGAAACAAGCCGCTTTATGAAGTAAGGATGATATTCACTGAAGCCAAGT
TCTCTGAGATCAGATGCAGCCCCCTCCACATCACCAGCACTGAAATATTCCTCTATGATG
GTAACAACAGATTTTTTGTAATCATCCAAGGGGTCAGAGATCGTTGCCCCCACAAGTTGA
TAAGGTTCCTCTCCACTGTCATAGTTAGGATCATTCCTGTCAATATGAGAATCAACGTTA
GTGTCAAGAAGTTTGCCCCATGTGCCCTTGCCACCGGCACCATCCTTCTTCACCCGCCCG
AACTTTCCAGAATGTGACCTACGCACATGCCTAACCGCAATTCCAGCAGTAGGTGCTTTA
CCCGCAACAGGAACTTTCAACTGATGGTCAGACAGTAAAGATGATGGAGACTTTGGAGAT
GAAGACAAAATCTCCACATTTTGGCAAGCTATTTTCAACATTTCTCTCTGCTCATCAGTT
AGAAATCCCTCACTTGAAGCCATTTTCACTCACCAAATCAGTGTCTATGCTCGACGAAGA
CTGAATATAAAACCCGAATATGACTCTTCTGTGCTTGGCCCGGATCTCGAGCCAAGACAG
GAGAAGAAAATCGGATCTCCAAGTCGGACACAAACTCAAGCGTCAGAAGACTAAACGAGA
AAAGAAGAGATAAATAGATAAAATGAAACAAATGAATGGGAATCTTCCAATCAGAAACCC
TAGATCAAAAGAAAACAACGGTCCGATTAAGTGTAACTGCGGACCCAAATTCTAGTGTGA
GTCTCAATCCCTTTCAAGAGTA
>ntig_155_consensus
GCCTGTATCCGTGTTGTTGGGCCGTCCATCGGAAGATGTCGAGGGCGAGATCGGGGTCTG
ACTGGGCTTGCAAGGCGTAGACGACGTCGGGTGGGGTGAAGCCAGGCTTGAGGTGTTGGA
CCCAGGCTTCGAATTGTTTTTCAAGGGGAGTTCGGGTTCGGGTTGGATTAAGTGCGTCGA
TTGAGGAAGATAACAATCGATTGTGATAAACCGATGGGTGGGTGGAGGGAGCGTTGGAAA
TTAGGGGTACATGCAGAGAAAAGAG
>ntig_156_consensus
TAGCTGCTGTGTCCATGACCAGGATTTGGTGCTTCAGTGGGTATCACGGGTTTTGGGGTG
TTTCTTTTTGAGGCACCAGCAAGTGAATAAGACGTGGACAGCATCTGTGCATCACTCACA
GTATTCTTGTTGACAGTATAATCAGGACTCAAATGCTTGCTTCTTGGTGATCTGAAGTGC
ACACGTGTGCCAGGACCAGTTACGTTGCTTGATCTAAGGTGCTCTGTGACATCAGCTGAT
TGTGGAGCATTAACAACTTGGTGATTGATATCCACTGGCGTTGCATGACTGCTGCTCCCC
CCAGGAACTGGAGATACTCGAGGCTGACTTCTACTTTTCTGTGCCTGCTGGAACTTTATG
ATGGTCCAATCAAAAATATAATCAAACTCATATCCTTCACGAGCAAACAAGTCACGAAAA
AGACGCTTTAAGAATCCATAATCAGGCCGCTGATCAAATGTCAAAGAATGGCAGTAATGG
ATATATGATGCAAACTCGACAGGATGAGCTTTGCACAGAACCTCAATAGGAGTTGATAAT
TTCTTCTCGCATATTTTGTCATATTTCTGCTTTTTTGTGGCAGCTTTTAACCCCTGCCAA
GGAAGGCTTCCTCTTAGGAAATATAGAAGTACATAACCAAGGGACTCCAAATCATCCCGT

CGACTTTGCTCAATCCCAAGATGTGTATTGCAACTTGCATATCGTGCCGTCCCTGTTAAG
TTTTTATTTTCCCTGTAAGGTATATGCCGATTAGTTGTAGCATCACGATATCTTTTTGCA
AGTCCAAAATCAATTATGTAAACCTGACTTGCTTTACGACCAAGACCCATGAGGAAATTA
TCAGGCTTTATGTCTCTATGCAAAAATCCTTTAGAGTG
>ntig_157_consensus
CGTTGGATCACTTCGCTGAAGAAATACGCTGCAACGATGAAATCCAACTACTTGAAGGAC
CAACCCTGGCGCTGTTACCAGTACCAGGATTTGGTGGTTCAGTTGGCATCCCTGGTTTTG
GGGTGTGTCTTTTTGAGGCACCAGCAAGTGAGTAAGAAGTGGATGGCATGTGTCCATCAC
TCACAATATTCTTGTCAACAGTATAATCAGGACTTAAATGCTTTCCCATAGGTGATCTGA
AGTGTACACGTGTCCCAGGACCAGTAACATGGCTTGATCGAATGTGCTCTGTGACATCAG
CTGAGTGCGGAACATTAACACCTCCTTGGTGATTGATATCCATTGGCATCGCATGACTGC
TGCTCCCCCCAGGAATCGGAGATAATCGAGGCTGACTTCTACTTTTCTGTGCTTGCTGGA
ACTTTATGATGGTCCAATCAAAAATATAATCAAATTCATATCCTTCACGAGCAAACAAGT
CACGAAACAGACGTTTTAAGAATCCATAATCAGGCCGCTGATCAAAGGTCAAAGAATGGC
AGTAATGGATATATGATGCAAACTCAACAGGGTGAGATTTGCACAAAACCTCAATAGGAG
TTGATAACTTCTTCTCACATATTTGTCATATTTCTGCTTTTTTGTTGCAGCTTTTAACC
CCTGCCATGGAAGGCTTCCTCTTAGAAAATATAAAAGCACATAACCAAGAGACTCCAAAT
CATCTCGTCGACTTTGCTCAATCCCAAGATGTGTATTGCAGCTTGCATATCGTGCAGTCC
CTGTTAAGTTTTTATTTTCCCTGTAAGGTATATGCCGATTAGTTGTAGCATCACGATATC
TTTTTGCAAGTCCAAAGTCAATTATATAAACCTGATTCGCTTTGCGGCCAAGGCCCATTA
AGAAGTTGTCAGGCTTTATGTCTCTATGCAAAAATCCTTTAGAGTGCACAAATTCTATTC
TTGTAATCAT
>ntig_158_consensus
GAGAGGGGGATTTATCCCAATGAGATTTACAAATAGAGGCGTCGTATCCGAGTGCCAACA
ATCCATCAGTCACAATCTTCCTGCAATTATCGTCTTTCCTTTTACAGATCTTGTTCTTCT
CTATGATCTTCGCCGTGTCCGCTAGTAAATTCCGTTCCAAAACGCTCGCGGAAGACACCA
AACCCTTGAGAATTTCACACGCTTCGCCGGAGGAAGCGATGTTGGAGTCGCAGAAATATG
AGTCCAATTCGTCTTCGGAGCTGTCGTTACAGCTGCCATTAAAGCAATTGCAACGGCTGC
GGCCACACCTAATAGCACCGGATTGTTGCTTCTCGTTGTTCTCCTCGATGAAGCTCAGGA
CCATCTTCGCCAAGCAGACTGAGCTAGGCTCGAAGTCAGCCGAGCTGCCACCATTGTAGC
TGCTGTCCTTGCTGAAATGTACCGGCGGCTCCTCAATAACGACAACACCGGCCTTTTCTG
CGGCTGAATTTCTCAAAAACTGTCGCTCAAACAGCCGCTTTAGTCGCGATTTCGCTACCG
GCTTTACTGGTTCCGAAAGAACCGATTCTTCCGCTGTGTGAGAATCGATTGGTTGGATCT
TCATCGGAAAAAGACGTGATCACCGTCGATCAAGAAATACTATGATCGATCTTTAAAAAA
ATCATAAGAATTTCTCAATCTTTTCCAAACCTAATTTTGTTTCATGAGTTAACAAAATCC
GTATTTGGTTCATAAAACCAACATTACACAATCAAACAAAATCTCTCAACTCCTCGACAC
GAGGAGTCCTCCTTAATTTTTTACCTTTAATTCCAAAATACAAGAATGAAGAAAGGTTAT
AAAGCTTTAAAGTAAACTATAGGTCAGTTGAACATTTAGTGAACCCAAAAACAAAAGAAG
GGAGGCTTCTACTCTCTACACATCAATCGAAACAATCTATCTCTCTCTTTTATTCAGATC
AGCGAACCGAATCCACACAGAAACCCAAAATTTCTTGAAAATCCAAAGCGTTTAGCCAAA
ATCCAGAGTCTTAAGCTAAAATAAGTACCTTTAATCTGCGATTATTATATACTAATATGC
CTCTGATTTGCTGGACAAAACGTGGAATAACTTCGAAACTCAGCCAAGTATAGTCGCTTT
TGCTTAGGGTTTCTAAATAAGGTTAAAGTAACCCAAATACGTGACGACACGAGAACGCAA
CGTTTTGGCCTTCGCAATCGAAACAACGAAGAGTCGCTGAAGCCTCCATTTTTTCCGGAC
AAGAGTTTTAACGTCCGGTGAGTTTTCACCGGACCGGCGACGGGAAATTGAACTAGAAAA
GAGGGGAGAGAAAGAAGAAGAAAAAATGGTTTAAAAAAGTTATAAAATGGAATTGAGAGC
TGCGATTGTGCGTGTTTTTGGACTTGGAGAGGGAACGATCTAGAGATTCTCCCTCTTCCA
CGCACAAAATTCAATCTAGAGTTTCTAGATGGATGGTCTTGATGCCTCTCCC
>ntig_159_consensus
TGTTTTAATCTCTCCACCAGAAAATAGAAAATATATATGCGCTTAAAAACAAAAATTCAC
GTCTCCCAATAAGATAAGAAGATTAGCCAAGCGAGGACTTTCAGCCCCACCTAACTTTGA
ATGATCAACACAATATAACTCAAACAAAATTATTGTAGCTGTAAAGTGCAGCCATAATTG
AAAGGAGATTGTATTCCTTTATTGATTTCACAGATTTCAGCCACTCCTCTCGGAAATACA
AACACAATGAAGTCCCATATAAAAGCAGCGGTTAATCAAACCTTACAAAGACACTGACAA
GCCAAGGAGTTTCAACAAAACCCAACAGAGGATTTTTGAATTAGCCTTGACTTTCTTATT
TACAGAAAAGAGACCTAAGGTTTCTTCTTATTTAGGATAAGATAGCATAACCATGGCTGC
TTTATCTGGGTAGGTTCAAATTTCACTTCCCACATTTGCATGGATTGCAGTTGCAGTTAG
CACCGCAGCCGCAGTTCTCTGCTCCATAGTTCATCTCAGATCCTTCAGAGAACGTCTTCA

CAGGAGCCACCCCGACGATGAGGGTCTCGGTGGTTGTGGTCTCCGAGAAAGACAAGTCAG
GATACATGCCGCATCCGCCGCAGCCGCTGCCGCACTTGCAGCCGGAGCCACAGCCACAAT
TTCCACCACAGCAAGACATTTTCTTAGTGAAAAAGAGATCTGGAGAGACTAAACTGGGAA
TTAGGATTTACTCGAAGACAAGGCGCAGATTCAGGCTGGTCAACTGGGACAATGGCACTA
TTATCTAGGCCTATCAGATCACCTAGTAAATCAGGCACTGG
>ntig_160_consensus
CATCATATCAATTTGCTCTTATAAGTAAAAGGTAGATTTATATAGCATATTTGTATCTCA
ATCTGTGTCAATTAGAGCGAACTGGCTGGAAACAGTGACAGAAGCAAGAGTATGGAGCTT
TTATTTTTATTGTAATGACATAGGATAATAGTTGCACAAAGTGGACAAACTCAGAAGCCA
CCAACCCAATTAAAGACAAAGTGCAGAGAAAGAAGACACAAAGGGATGCCAGACTACAAA
CAAAGAGTAAAGCAAAAAACAAAGCATTCATGTGCAGAAGATACAATAGAAAAACTAGTA
ATCTTCAGGAGCCAAGGGCTGGTGGGTGTGATGTGGTGGGGGCTCTGTTGGAATCACCAT
GTACTCATAAATGAGTGCTGCTAGCCCTGATCCAATTAAAGGACCAACCCAGTAGATCCA
CTGGTTTTTCCATCTCCACCCAACAAGTGCTGGGCCAAATGCTCTTGCAGGGTTCATGGC
TGCTCCATCAAATGGCCCCCCTGTCAAGACATTTGCTCCATAAATGAACCCAATGGCCAG
AGGTGCNNNNNNNNNNNNNNNNNNNCCAGATGCTACCCAAAATCCCACCGGTCTCATGCCG
TTGGTGACAAGTCTCAACAAGACAGTTCCAACAACAGCAGCCAGAAGCTGAGCAATCCAG
TAATATATGGCACGGAGGACAGTGATTCTTCCTCCAAGAAGAGCACCAAAGGTAACAGCA
GGATTCACATGGCCACCTGAGATGTTGATACTCGCTGACACGGCTGCGAACAGCGCCAAC
GCGTGTGCTAGTGCTATCGCAACGAGATCTGAAGGTGTTGTCGTTGAAGCCTTATATACC
TTGGCTAGAGCTAGGAGAGAGCCTTCGCCGGCAAAGACAAAGATGAGAGTGGAGATGAAT
TCAGCTAAGGTGGCTCTGACAGTATCCGGGTGAGCCGCTTCATCAGCTCTCCCAAACGCA
TATCTGCGAGGTGGCATGGTGGGCTCTCTATACAATCTTGGCTTTGGTAGTCTCTCCACT
ACCAACAACACTACCGGAGATCTCTAGTCTTTATAAGTAATATATTATACTACTTCTGTG
CTAATTCTTAAGCTTTATGGTGCAATAATACTTTCATTTGGAGGTGCAAAATAAGAGAGA
TGGTCAGTTTGAGTTCGAATGTCTGCATGTATGTGAAGTGGAAGAATGATG
>ntig_161_consensus
CAGAAAGTGAAGAGTTATTGAACCTATTATGCACGTAGTTTGAAGTGCTGAGTAGTTATA
AACATCACATATACTCCTGATATGAAGCCTACTAGCAATAATGACATGATAATGCAACTC
TTCAAAACATGCAAACAAAAGCACACTAATAAAAAGACTACCTGTGCCAGAACCCTTACA
CCATACCACCCAGTGCACACAGGTACAGTAGCACACATCACTAGACCTCAGACCAGAGCT
TACATCAATAGCTATCCAACCACGATCCACATCAGAGCAAAAAAATGTGATGCGTCTTTC
AAGTTCAATATGTATCGATCAAAGACACTTCATCCACAGACTCAACTACAGTTAAGAGTC
TATTTATGTGAGTCGATAAATTAAAAACAAATCATTCTCCGAGATAAGAAAAACTCACAG
ATAGCATACTGAGTATCTCTATGGAAGAATTTGAAACATAATACCAGCACCTGAA
>ntig_162_consensus
TTTTAAAGGTATACATTGTTAAAACCTTCATTAAAGGTGTCTGTAATACAACTTAAGGTC
CCAGTTTTTTTCTAAACAAAATCAACAATTTTGCAGGGTTTTCTTTACAGCTGCCTATCT
GGTTGGCCTGCTTATGGCTTCCTTTTTCATCATCAATCAAACCCCCATGTTACAATATTT
GCCACATCTCATTTCCAAAGCAACACAAAAACAACCCAAATTGATAAATATGATGCTTCT
TCTCCTGGCCTTTTATCGGTCCAATTGCTGGCAAAACTTTCTTATGATCTTGTAGTCCTT
TGGCAAAAATTTTGATTTATTTACCTTTCTTTCTGATTTGATCAAAGTATATCATATTCC
TTGCTTCATTGACTTGTTCACGTATATAAATAAAGCATTGATGGCTCACTTAGTCAATCT
CGCCTTCCTCTACGTCGTTTAGAGGGTTTGTATTCGGAATGGTTGGTGGTTCGGCTTCTT
CCTCCTCGTCATCCTGTTTCATGTATAAACCAAGTTGTTCCAAGGGGTTGCTGCCTCCAA
ATTTGAAACTACCCAAGCCATCCTGAGAGTGGTCCGGGCTTGTCTCATCAACAGAGCTTG
GTAAATGCTCGGCAGGGACAGTCCTAAGCATTTCTAAGTCCTCGAGGAAACGAGAATTCT
CATTTATTTCAACGGTCTTCTCCATCTTCAGCAATGCCTGCCGTGCAGCTTCTCTCTCTA
ACTCTCTCTTTCGTCTAGCCTCAGCTGCAGCTTCTTCTTCAGCTTGCCTTCTAGCCTCTT
CAGCAGCCTTTGCTTCTGCTTGCAACCGAGCCTTTTCTTTCCGTCGCTGAAGTTCAAGTT
CCTCCCTCTCCTTACGCAATTTCTCAGGGTCTCCCTTGTCACCCTGTGAAAGAGTTTTTT
CCCGAGCTTTTAAAATGGTATCAGCAAATCGATTCTTCAATAAAGCAGCCCGATAAAGCT
TTCCGGGTGAGACCTGCCTCTCACTGGGAGCACTGTCACCATCTTGACAACAATCAGACT
CAATTGAGCTCGGTTTTTGCTGGGAAGTATGTTCAACCTGATCCAACTCACTGACAGATT
GATTTCCATCAAAGGGGTTCCCAGTACTTGTCTTCTCATCTAATTGAGCTCCAGAACTCA
AGGCTTCTGGTTCCTTTGAGACATTGATGGGACTTGACACTTTAACACCACTAGATTCAC
TACCAGAAGAACCGCTCGAGTCAGAATCACTGGAGCTGTCTGAACCAACACATTTACTGC
TTTTAATCCCTGTATCCTTTTCAATCTCCACGGGAGGATAGCTTGTGACAGGAGGTTCAT

TTCCACCT
>ntig_163_consensus
CTTCTCTCTGAACCTCTCTCTTTCGTCTAGCCTCAGCTACAGCCTCTTCTTCAGCTTGCC
TTCTAGCCTCTTCAGCAGCCTTTGCTTCTGCTTGCAATCTAGCCTTTTCTTTCTGTCACT
GTAGTTCGAGTTCCTCCTTCTCCTTGCGCAATTTTTCTGGGTCCCCTTTATCACAAAATG
CTCACAGAACAAAAACGTGTACGTGGGTAAGTCAAACAGTAGCCCCTAGTATATAACCAA
CCTGTGAAAAGAGTTTTTTCCTGAGCTTTTAAAATGGTATCTGCAAATCGATTCTTTAAT
AAAGCAGCCCGATAAAGTTTTCCAAGAGTAACCTGCTTCTCACCGCAACCACTGTCAC
>ntig_164_consensus
AGCTGCAGAAAAGGGTTAAAATAGCCCTTCAATTATTCAATTATGCTTTCAATGGAGGTT
CTCCAAGCCACAGCCACTTCATAACCGTGAAACTCAAAGATTAAATTCAATATTCATATA
GACAATTATTTCATCATTAAGATCAATGTCAAGAGAATATATCCAACAATTTGGAGAAGA
AAAATCCTCAAGCAGTAAACAAATGTCATCGTCATACAGAAACTAGAAAACATCAAGTAG
TCTTTGAGCAAGTTGAATATTATTCTGTGGTCTATGCCATGCAGCCTAGGCTTAGTTTTT
GGTAACACTTATAACAGTATTAGACTCCAGTGCCCAATGAAGCATGTAAGTCATGAACAT
GGCAATGGACACCAAGGAAAAATATGGAAATAAAACCAACGCAAGTCTCACTTATAACAA
CAACCTAGCTAGAAAGGAAAATGCAACAAGCTCAGTAGTTTTCGGACCAGTTTGCAGCAC
TACCAACTCTTGGCATGCCCGACTCTTTCTTAGCCTCGGCATCCACCTTGAATGTTGCAC
CACAAACATGGCCAGAACTGTCCACCCTAGGCATTGGCTTCAATTCATTCAGAGGGTGCT
CAAAGCTGCTCAATCCTCCAGAAGATGTAAAGAAGCACCCTTTAGGGAATGGTGCAAAAG
ACTTGCCACAGCCCTTCTTCACAATCTCTACATCATCTGAAATCACAAGATGGCCTTCAG
AATCGGTACCCCAGAAAAAGGGAACACTCCCATCAGCATCCCCGGCTAAGAACGTGACTT
TTGAAGAACTGTCGTAAAGAATAAATGCAAATTTCCCTTGGAAGTCTCTAACAACCTGAT
CGGCTGGATAAGGACCCCGATCTCTCAGAGTTCTGTATGCCTCAATAATAATAATTGCTT
CATTTGCCATTTTGTTAAGTCCATACTGTTGCTTAAGAAGAGCAACATTCTCAATGTGGC
CTTGAAACAAGCAGAAAATGTCGTCCACAACTGCAAATAATCTGGGTAGAAGTGGGTTCT
GCTTGTCAAGAGAATAGGCCATTAGACCAGCGGAGCCAAGATTGATAGTTATAGAGCCAG
AGCGAACAGAGGCAAAGTGTTTACCTAATGACCCATCTTTGAATGCGGTAACCGAGTCTG
GTTGTGGGCTACGCAGAGCCTCCGGGCTCTTGGCCACTGATTTTTCAAAAACTGCCAACA
TTTTTTTACTCTAATCAGCAAACAACACGAACAAAAAACAAGATTCGATTGCCAATGAAG
ACAAATGCTAAGTAGCTGATGTTAAAGTTCTACAAAATGATTATGCTGAAGGCGACGATA
TGACTTGTTAGTCAGGACGAAGGATGT
>ntig_165_consensus
CAACATTGTTTAGATTTAGCACAATAGAAATCAGCCCTTTCAAATAGTTATAAATATGAA
AGTTTACATTATTCTGACACCCAGGAGAGAAGGAAGCAATGAACCCACCTTGCCAAACTC
AAATACAAGGGCAGACACTGAAACACAACTAGGTATACTATTAATGAAACACATGGAATG
GGTGCTAGTAAAACATAGTTCTTTTCTGACTCATCACTCATGACATCCAATGAAGACAGC
AAACCAGTACCCCAGCCATGGTTTAAACAATCATTTACTTCAACAGAATGGTGGTTTCAG
CAACCATAGAAGTGACCACATATGGATCCATGTTGGAAGCAGGCCTCCTGTCCTCGAAGT
AACCTTTTCCGTCTTTCTCTGTGTCTCGTCCAACACGAATGGATGCACCACGGTTTGCCA
CGCCCCATTTGAATGTGTTGATGTTAGCAGTTTCATGTCGACCAGTGAGACGGCGCTCAT
TGCCTTCACCATAAGCAGCAATGTGCTCTTTGTGCCTATGCCCAAGCTTTTCAATTGCCT
TCTTGATAACCTCAAATCCTCCTTCACTCCTCATTGACTTGGTGCTATAATTTGTGTGAG
CGCCGGCACCGTTCCAGTCACCCTCAATTGGCTTCGGATCGAAGGAAAGGACAACTCCTG
CAATTTCTGTAATCCTCTCCAAAATATAGCGAGCAACCCACAATTGGTCACCAGCAGAGA
TACCGACAGAAGGACCCACTTGAAATTCCCATTGGCCAGGCATGACTTCTCCATTGATAC
CACTAATGTTGATTCCAGCGTATAAACAAGCTTTGTAATGGGCATCAACGATGTCCCGTC
CCCAGGCTTTGTCAGCACCAACGCCGCAGTAGTACGGTCCCTGAGGTCCAGGGTAACCAC
CAACCGGCCAGCCGATGGGCCATTTAACATCTTTCTGAAGCAGGGTGTATTCTTGTTCGA
TACCATACCAAGGCTCTTCTGCAAGAACATCTGGATGGCTGAAGATCTTGGCTGCTGCAT
GTCTCTTGTTGGAAGAAATTGGTTCTCCAGCAGGATTATAAGCATCACACATAACAAGAA
TATTGTTGCCTCTTCTGAACGGATCCTTAAAAATTGCTTGGGGGTAGAGGATCACTTCAC
TATCCTCACCAGAAGCTTGACCAGTGCTCGAACCATCATAGTTCCACTTCGGTAACTTAG
CAGGATCACTAACAGGTCCATTAAGAGTTCTTGCTTTGCTCCTCAAATCCATCCCAGATC
CACCGATCCATATGTACTCGGCGATGATCTTGTTCGTGGTTTCTGAAAGGTTAAGGTTGA
GGAGATCCGTAAGAAGAGACATGTTTCACCAACAAAAACTCAGACTCAATTCAATCAAAA
CCCCTTTTGCACTCGCTCCGCCTGTATTAATGATTTGTTTTTGTAGAGACGTGCACAAAA
GAGGATTTTGCGTGCCACTTTATAGTGCCG

>ntig_166_consensus
ATCAAAACAAAATTAAGTATTATCATATAAATCAATCATTTTATCAAAACTGTGTTAAAC
TATGTATACAAAGGAAGAACAAAGTCATTATACAAGAACACTGGAAAGAGGACAATATAA
ATGGAAGGGAAACCATTATATATATAAATATATAGTGCTTCATAATTTATTTCCATAGTG
GATCATCATCTCCATCTCTTAACATAGTAGAAAAGGACATCGATACTTCATAGTAGCTAG
CTATGGCGGCTACCTAAGGCTTACACAAGCTAACACACACCACACAATATTATTAAACTC
TACATGCTTTAAGCCGGTAAAACATTACGGGTGATGGTGATGGTGATGCGGTTTGTGTGG
GTCGTGGGGGCCTGCAGGTGGAGGCGGTCCATGAGGTCCTGGTGGAGGAGGGCCTGGCTG
GTGAGGAGGCG
>ntig_167_consensus
TAAAAACTTAAACATCCCATTATTGAAAAGAAATGGTGCCAATAAGAAATCCAATTAAAA
CAAATTATCATTTGTTATACCCATGAAACTTGCAAAACTCAACAGTAATGATCAAAATCG
TCTTAAGAGATCATTATATAATATTAAAAATCCCTACACAATACTCAAGTTCTAGGAAAG
AAGAAAAGCAAAGGGAAGAATGTAAGACTCTTCATGGGCGAAGGGGATTCCCAACACCAA
GTCCACCAGCTGGACGAGGTGCAGCTCCAGGAGCACCCTGAGCGGGCTTTGGCATATCAT
CCCTGCGTCTGATGAATCGCTTTGGTGGTTCACGGTTCCTGCGGTCAGTGCGAGAGCGAA
CCCTCACAACACGGGAGTGAATTGCACACGAAACGCAATACTGCATCTTCGCATACAACT
TAGGCAGCACATACCCATCATAGATACAGGCCTCTTGCACATCTCTAACAGCTGCTTGCT
CCACGATGTTTCTCACCAGAAACCTCTTGATGGCCTTATCCTTTGGACAGCACTTTCCGC
AGTTTGAGCATCTCACGAAGTTGACGTGTCCACGCCCATGCTTGTTACGCCCAGCATTTC
TACGCTTGAAAGTCATTGTTAATGCTGCGATGAGTTCTTGCTCTCAAGCTCACAGTTTCG
GCTGCGCAGCACTAGGGTTTTTCGAATTCTCCCATTTTACCATTTTATATAGAGGAGCG
>ntig_168_consensus
CCTCCATATTATAAATCTCTATAAAAAAGCCCTCTGTCTTAAAATTCAGGGACGATATGG
TCCTCCAGGTTTGAAAATAGAAAAACACATCCAAAGTTTTTGGATAATAACACACCCAAA
AAAATTGTTACTTCACCATTATACCCTTGAGAAGCTATCAATTTATGTACGAATAGAGCA
ACACATCAAGAAATAAAATTGCTAAAAATGGCAAAACAAATAATTAGAACAAAGCCGGTC
TCAATAACAGCAAAAGATGGCTGTAGATTGTTAACAACTCTCAAAATGTTTTGGCAAAGG
TCTGAGACCATTCTAAATTTTATTTTTACAAATCCAAAACTGCTTCGACAGAAGAAAATA
TCATCAAGACATCAGTGTTCAGCAAACAATTACTCAACAATTACCCTGAGCTACTAATAC
GAACCAGTCGGTTCTCCCTCAGGGTTTAAGAGCCAGGGCGAGACCAATCTTGGCACTCTT
CTCAATGGCCTTGCTGTCTACCTCACCAGAAATGGTGAAGAGTGACTTCGGACGCCACTC
ATGCTGGATGAGTGCGCTCACCTTGCCAGCATTGTTCAAACGTGCCTTCACTGTGGTCAA
TGGATCCAATGCATGCTGGGTTCCAACAGTGATGGTATTCTCGTTGGTTGAGAAGCTATG
GGTTACTTCTGCACCAACAGCAGTGCTTGTCAAAGGGTTAACAATGTGGTAGTATGACCC
ACTGAGAGAGTCACCCTTGTTGTTCAAAGTCAATGCAGCAATCAGGTCTGCGTTGCAGAA
GCTGACTCCAGCATTGCATTTTGTGAAGTCCCCAGATTTGGAGTCAAATGACACATCAGT
TCCAAGTGAGAGACCATTAGTGCCAACCACACCTGAGAAGTTAACAATGGGGTTTGCGGT
CAACCCAATGCTTGTGCTGATACCCGCATAGTCATGCAAATACTGGAGTTCAATCTTGCC
TGACCTTTGATCAGGAACTTTGAAGCTCAGTATGGTTTTCAGCCCAGGAGCAGGTTCATC
AACAGTGATAGTTGTAAACAGATTCGAGCTTGTATCCACTTTGACATCAGTGGTGATATT
CTTGTTCTTCAGCTGAGTATTAACATCAGCCAGAAACAGTTCTCCTTTTTTCGTTCCTGA
AGAAGTAATAGCAACTCCCGTTGGAGAGTAGGTAGTGATAGTGAACTTCTGGTCACTCTG
ATAATCCTTGTAAAGAAGATCTCTGGCTTTTTTGCCAATATCAGAGTAAAGACCAGGACC
CTTGCCCATGATTGAATTTTTTTCTTGTTTTTGAAACAAGTAGCCGGGACCCAGAAAAAT
CCAGAAGGGAAAAGCTACAAGGAAAGCCAAGATGATGGCAGATTTAATATATA
>ntig_169_consensus
AACAACAATTAAAATGTAAAACATCCCAAAATGTTAAACATGAACAGTCAGGCAAGAAAA
ATCTACCGATTGATGTGAAAATAATATAATGTAGTAAATACTGTCTCCTTAAACTTGTTT
TAATCTCAATACTAATAGAAAACATTCCAAAATAAAATCTTGCAGAACATTTATAACAAA
ATTGGAACGTACATATCAGACAACACTTATGAAGCATTCTCCTTTTTCAGCTTGGAAAGC
TCCTGCCTGACCAATGCTCCCTTCTTGATCTTAGCCAACATAATCTTGAACCTGTCAAAG
TCATTAAGCGATGCCCTTCTCTTCTGGACAATCAGCTTTCTGCCCCAGGAGCTGTTCTCC
CACTTGTTCTTAACATCAGCCTTCTCCATTGCCTCAATCAACGTCTTCTTCTTTGGGACA
CGGTTAATGTCAATCTTGATATCAGTTAGGGAAAGCCTCTTGAAATTCATCTGGCTTCTC
ACCATATCAGGGGCATCAACTAGGGCTCTGTTCTGATCGATAACATCGACGATGACGACA
AGCCTGCCGTAGTCTTTGCCGTAGTTGACGAGGGCGACCCTCCCGATCTCCACGTACCTC
TTGAAAGGCATTTTGGGTGTGTGTTTTCTGTGTGAGTGTGCAAGGAAGAGAGTGCAAGAG

GCGG
>ntig_170_consensus
TGGGACATTAAGAATTTTAAGATGGTCTAAAGTAGCCTAACAGTCTTTTACCAACCCAAC
GACACAAGTCCACCCTGGCAAACACATTCAACATTCAAGAATACAACGCCAAAATGAGAA
TCGTACTTTCAACATTTAGAAATACAACACCAAATCAGGCTAAATTTGTCCACAGTTCAT
TTTATTGAAATTGATGCCAACCATTGGTAGATAACAAATCTAATTTCGTCATTAACACAT
ATGATGTTAGGAGCAAGCACTAGACGAGACCGAGTGACCTAGCCACTGAAAACTTTACTC
GGATACAACACAAACACCAGAACAATCGCCTATTAATCTTCAGAAGTGCTTGTAGTTATC
TAAGACACTGGCCAAATGATTGGCTAGTTTTTTATATAAATTTCGCAAATATGCTCACAC
TGTGCCAACAAAGAACTCTTAAGTAAAATAGTCCAAAAAAGTAACATGAGCATAGCACAG
AAACGGGGAAGAATCTATCAATTGAACTGACTACAATATCATTAAGTAAAAAGTGCCTCC
TTAAACTTGTCTCAATCTCAAACTATCAGAAAACACTCCAAATAACACCTTTCATAACAC
TGATAACAAAATTGAGAGTTACATATCAGACAACACTTAGGAAGCATTCTGCTTTTTAAG
CTTTGCAAGCTCCTGCCTAACCAACCCTCCCTTCTTGATCTTGGCCAACATAATCTTGAA
CCTGTCAAAGTCATTAAGAGAAGCTCTTCTCTTCTGGACAATCAGCTTTCTGCCCCAGGA
GCTGTTTTCCCACTTGTTCTTAACATCAGCCTTCTCCATAGCCTCAATCAAAGTCTTCTT
CTTTGGAACACGGTTAATGTCAATTTTGATATCAGTCAATGAAAGCCTCTTGAAATTCAT
CTGGCTTCTGACCATATCAGGTGAATCAACTAGAGCTCTGTTCTGATCGATAACATCAAC
GATGACGACAAGCCTGCCATAATCCTTGCCGTAGTTGACGAGGGCAACCCTTCCGATCTC
CACGTACCTCTTGAATGGCATTTTGGTTTTGGGTGCGCGTGAGGAAGAGACTACAAGAGG
CGGAGATGCAC
>ntig_171_consensus
CCTACACATGTAATTTGTTTTTTGCTACTATGCATTTGTGCACAAATCCCAAACTCATAA
AGAAATCAATATAAAGAATAAAAAGTGCACATGAAATATCTGACAATGGATTTCTAAACT
GTCATGTACTTGAGAACAGCTGCACGGACCTTTTGCATGAAAGCTGCAGCTTGCTCCTGG
TTTCCCCGCTGTGCATTTGCTATGTTTTCGTCTAGAAGTGTCAATAAGGATCTATTGATC
TCATTTTGTTCAATCATTTCCAATAAAGTTCCTTTCACATCCTTAGACGTCAAGATTTTT
GTTAGACTTTTCCTTGCTGTTATAAGGTCAGACTGCATTTTGTCATAGGCTTCTGTTCCT
TCTTGCAGAGCCTTCTGTAATGCTTCCAGCTCAATCAATCTGTCTTCAATATCCTGAGTT
TTTGAAACAGCAAAA
>ntig_172_consensus
GGCCTTTCTGGAGCGACGGTGAGCGCGGCGGCAGCGGTGATGGGCGTTGGATGAGAAAGT
AGATGAAAAGAAGGCGAAGAAGATGAGAAAGAGGAGGGGTTTTAGAGTTTTTGCTGGGCT
CTTAATGGACTGAAAAGTTTTGCTTATTGGGCCTATTCGGCCCACCGTTTTCGTCTAATG
CTGATTTTGTGAACTTCATAGTTGAATGGATAATGCCAAGAACTTATTTTGTACATAAAA
TCTATACAACACAAATCCAAACAACCATGAATCTAGCATCTTCATGGAGCTTCAGCTGTT
TGCCTTTATTAAGGAACACATATATAAACATAATCACTAAATATAATCTTTGAGATTGTA
AAACTTTAATCCTCATCGCCCCTCCTGCGGTAATCAGCTCCTTCCTCATGCTCTTCGAAC
CTCTTCTTCAGAGTCTCAACTTTAGCCTCACCAATGGAGGCCTCTTCCGCTCCATCATAC
AACTTATCACTCGTCTGACTCGCCACCCCTTGCTCCGCTGCTTGAAGCTTCTGCTTCTCC
TCCGCAACCCTTTCGAACTCTTTAGCTGTCGATGGCGCTCTCTCCTCCATTTTGTGTCTT
TCTTGACCCAGTCGGCTATCTGGGCCATACCTCCTGAAGGGCTTGTAAAGGCAGGCGGCG
AAGGGCTTGATGGAAGATGGTCGAGCAGATTTGTGGAAGAGAAAGATGAAGCCTCTCACT
TGAAATAGCGGCGACATGGATGAAAGCTTTCTAATTTCAGTAAAGTGGGACTGAGAACAG
GAGGAGACCAGATCGCAAGAACTTCAAATTGTGGAAAATTGGGAACAAAATAGAGGTTTG
GTGTCACATCATGTCGGCAATTGAATGGACACGTGGTGCTCTGTGCATTTGCCACTTCGT
TTTAACTTTTAATCTTGCGACCTGTTTGGACATTGTAACTTTGGTTTCTCTGAACTGAAC
TGAACTTCGCCGTCGCTCTTTGCGATATGGAGATCAATGGAGATAGTTCTGAAGTGCCTC
GAATCCTCAGAGAACTCGAAACCTTAAAAGCTTCGAAGAGGGACATTGAGCATCGAATTT
CCTTTCTCGAAGCTCAGCTTCATGAGAAACAAAACGCCGTTAGTAACGGTTCTTGTGCTT
CAATCTCCACCGTTGATTCCAATGGCTTATCACCGGACATGATCTACCGTTACAGTCGCC
AACTCTTGCTTCCTTCATTTGGAGTTCAAGGGCAGTCGAATCTATTAAAATCTTCAATTC
TAGTCGTTGGAGCTGGTGGTTTGGGCTCGCCAGCTCTTCTCTATCTGGCAGCTTGTGGGG
TTGGGGGCGATTGGGTGTTGTGGATCATGATGTAGTTGAGCTTAACAACATGCACAGGCA
GGTTATCCACACTGAAGCATATATTGGTCAGCCCAAAGTGAGATCAGCCGCTGCTGCTTG
TCACTCGATCAATTCCACTATTCAAATTGTTGAACACCAGGAAGCATTACGTACATCAAA
TGCTTTGGAGATTTTCAGCAAATATGACATAGTAGTAGATGCAACTGATAATGCACCAAG
CCGATACATGATCAGTGATTGTTGTGTGGTGTTGGGGAAGCCTCTTGTATCCGGTGCTGC

AGTGGGATTGGAAGGGCAGCTTACAGTTTACAACTACAATGGAGGTCCATGCTATCGTTG
CCTATTTCCAACTCCACCACCTACAACAGCATGCCAAAGATGTTCTGATAGTGGAGTCTT
AGGAGTAGTTCCTGGAATTATCGGCTGTCTCCAAGCTTTAGAGGCCATTAAAATTGCTAG
TGCTATTGGTGAGCCACTCTCAGGAAGGATGCTTCTATTTGATGCTTTGTCAGGACGAAT
TCGTATTCTTCCCTTGAAGTTGAATTTGCTTCCAGCAGATTCCAGAATAAGCAGCACAGA
ATATAAAGAAAAAGTCGTTAAAGGGGAACCACATGTATTGGTGGATGTACGGCCAGCACA
TCACTTCAAGATTGTTTCTCTTCCAAACTCTATAAACATCCCACTCACAAGTTTGGAGGC
AAGATTACCCGAGGTTTCATCAGCATTGAAAGAAAAAGAAGAGCAGAATGGAACTAATCC
TGGTTTCGGTGTTAGCCTATATGTGGTATGTAGAAGGGGCAACGATTCACAGAGAGCTGT
GCAGCGTCTCCAGACCATGGGTTTCACTTCAGCTAAGGATATCATTGGGGGGATCGAGTT
CTGGGCACGTGATGTGGATCCAAACTTTCCTACCTATTAGCAGGCATTCTCAAGAGAGGC
AGGTTTGTACCATTATTAATTTGTCACTCTACTTATATGTTCCATTTTGTATTGTTTTTG
CATAAATTACATGAAACCAACCATTAAAGTCTCCCAGTTGCTACCTTGAAGTAGCCTATA
ATTTTGATTCTTTTCCCGTTGTATACATGTAGAAGAACATAATAAGTAATGTTCTGGGTG
TGGGC
>ntig_173_consensus
GTATGTACTCCTCTTCCAACATGTCATTATCATACAGCGCTTTGAGAACCAATGCTACCT
CCTTCGCCATCTCAGGGCTTGCCTTTCCAAAGAAAGACTCAATGGAATGCAGGAGAACCA
TCTGGGATCCCTCCTCTTGAGTTGCAACAGCCAGGTAGTTCTTCTTTTTCAACACCTCCT
TAGCAAATCCTTTCTCAACACCATCAAAGAGAGCTGCAAATATGGCATCCATCACATCTT
GTGGAGTACCATTGAGTGTACTCAGAAATGTTTTAAGTTGGCTACCAGAAGAGCCCTTGT
TCAGCCATTCCTTTATCTCATTCACAAGCCTTTCATGGGTATTAGTACCATTAGTTCCAT
TATCATGTCCCTTCTTCACCGACTTATTCTTCTCTTCACTGGTAGAAAGCATAACCATAT
CTGCAGTAACAGCACTCAACTGCTCCTGGATACGCTGCTTGGCTGCCTCCAGTGAAGTGT
CTGTTTGCCACTGAACATCGTCACCATCGTCATCAGTAACTGCTTGCTCATTTTCATCAC
CCTGGCTGTGAGAAGGGGAGTGATCCTCGTCAGAAGCATGGGATTTCTTCTTTGTTGATG
CACCTTTGGTAGAAACATCCTTTGAACCAGTAGAAGAACCCTTTTTCTTTCCTGTTTCTT
TTTTAAGCTTCCTCATCTCCTCATCTGCAGCCTCACCCTCCTTGAGCCGTTCCTTCTCAG
CTCTCCTCATTGCTTTCTTGTCCTTGGAAGATTTCTTTTGCTCGGGGGGGTTCTTGATAA
TAAATGTAGTAAGCTTGTCCCTCATATCTACATCTGATACAAACCCACAAGCAGCACATT
TCAGTTGGACCATCTGCATCTTAGTAATGACTATCTCAGTCTCTGGGTTTCCACATCCAT
AGCACTGCACGTATTTCTTAATAAAATTCTCAAGGAGCCCAGCAAGCTTAGCAGTATCAT
GGGCTCCATTAACAAGTGAAGTTCCAGTCTTCTCATCAAATTTGGACTGAGCCCCAAGCT
CATTCCCAAAATACTTAGTAGTGTACGATGCAGGTCTAGCCAAGGCCTTAGCAATCTCAA
CCATGTTAACCACATTGGTCTTGATTCCATTGCCTCTTCCTTCAATCTTGGTGATCATTT
TTGGCATCTTATACCTATAAAAGGCATCATCACTGTTTGAAGCACCAATATTTTGCAAAG
CCATTTTAATTCAACCGTTAAAGAGCAACGAGTCAAACTAATCAGAAAGAGCAAAAGACA
GCAACAGCTTCTTCCTTGGTGTGAAATCCTCCTTTACCAAACAAGTTCCCAGCAATAGGA
AAAGGTCGCATAAATTGGCAACCAAAACAGTGTGTATTATTTGCCTCAGCTAACTCAAGA
TGCATGCAGCCAACTCCCAGGGATGTATCTCTGTTCTTTTTTCTGGACGAAGGACAATAT
TCACCAAGCAAACCAATTGACGTCAAAAAGAAAGATGCCCGTTCAGACATAAAAGAAGTT
TCAAACTTGACACCCTCCACTTTGTCGGAGCTTCAACCCAGCAGACTACACTGCCAACTT
CCTTGAGATGATAAGAATAATTAAAAACGAGAGACAGACGTTGAAGAAGGAGCTCTTTTG
TGAGGTGAAGAGATATTGTGAGACAATATTGAGCGGAGACGGCTTTTATATACGAGGCTT
TACGAGTTTAGGGCAAACTCA
>ntig_174_consensus
CTTAACACGTTGTGCTTCAGCCAAGAGCGACATTCTCCTAGCAGTCTTAGCATACGGGTT
GAGCCTGAGCATAGTGTTAAGATTCTTCAGTGGGTTCTTCTTCATTGGTGCTCTCTTCAC
CTCCTTCTTAATCGGCCTCACTACAGATTGCACCTCATCAGAGTTAATGATCCTAGCCAA
ATCAGCATTCACCATCTTGGCACGGGGCAATACGTAGCCCTTCTTCTTCTCAGACCCCTT
ATCGAAGGTTCCATAGATTGAATCTAACTTCTCAACGGCTGACTTGGTCCAGATAACAAA
TCGTCCAAGGTGTCCACCTGGAGCGAGCTTCAAAAGATTCAGCCTCTCAACGTTAACGAT
CTCAACACCAGGAATGTTCCTGAAGGCCTTAACGAGCTTCGCGCCTTCAGTTGAGTACAC
AACAAGTGGGCCCTTCCTGGAAATGTAACGACGGTTTCTCATTTTACCTTTTCCAGGACG
AATTGACTCACTCTTCTTAGCTTTCTCAGCATCATCATAAGCACCAATCTGTTTCAAAAC
CTTCAAAGCAGCGGATGTTTTCTCAACACCCTCGACTGAATCGCTGACGACCAGAGGCAA
CTCGGGAACTTTGTCGATCTTGTGGCCACGTGCCATCACGAGGGACGGGATGGCGGAAGC
GGCGATGGCGGACACGATAGCGTACCTCTTCTGGTTCACGTTGATCTTCCGGTGCCAGCG

GCGCCAGATCTTGGTCGGAGCAAACATGCGTCCGCCACGACACATGTTACCAAAGGCTCC
CTGGCCAGCACGGTGAGTACCGCCACCGGGAACACGGGGGATACGGGAGACCGCACGACC
GGTTCCCCAAGATTCTGCAGATGTCTGGTGCCCAGCTCTCTTGGAGACAGCGTAGGGCTG
GCGGCTGTTCTTGGAGATATTTGAGTGGACGAAAGTGACGATGTCGGGACGGATTGAAGC
CTTCATGACGGCGGGTAATTGTAGGGTGGGGGCTGCATCCGTGGCCATGTCACCTTCCAA
TGACTGGACGGACACCAAAGGACGAGCAGCGGCGGCCATTGGTTTAGGGGAGTATGGGAA
GGCTTAAAGGAACACGGGGAGGCTAGGGTTTAGTCTGTGAGACTGTCCATACTCCCCA
>ntig_175_consensus
ATACAGTTTCTTCATAAAAACAAACAATCAATCCATTTAGTCAAATCATTGACATTCACA
TTCACATTCACTTTACCACAAAAAGGAGTAGCAGTAAAAAGGAGCCCATAGACACCAGTA
ACAGTAGCTTGACAGCAACGGAGATAAAGTTGGATAATAAATTACATCATCCACTTACGA
GAATGCTTCGGGAGAAAGCTGTACGAGTGTCAATGCACCCACCCAACCCATAGAGCACCA
ACGAACAGAAAATGCACAAGCTACTAAGGCCATTGTATTAATTTTTCATCCGCGTAGATG
CGTTGATGAATTTGGGCATAGTCCCAGATTACAAAACATTATAAGAACACCAAAGAATAT
AAAAACCAAAAAAGAAAAATCGATTGCCTCGAAGGGGCAATATTATGTCTAATATAACCC
TTTTTTTCATTGTTAACCAGCAATTCAAATAATAAGGTGGGAATTTCTTTTTCTTTGCTC
GGAGGTAAGAATCAATAGGTTGGGGAGGGATCAAACCCGTATCTCCTAAAGCACTCACTT
GGCACTGGCAAGCTGGCTGCGGATGAGCTTGGCAGCAGTAACCAAGTTTTTGAGGGCAGG
CTTCACCTCAGAGTACTTGCGAGTCTTGAGACCACAGTCAGGGTTCACCCACAAGATGTT
GGTTTCGAGCACAGCAAGCATCTTGTTGATTCTGTCAGCAATCTCTTCAGTTGATGGTAT
TCTAGGAGAGTGGATGTCATAGACACCAGGGCCAATTCCAGCACCATACTTAACTCCCTC
ACGGAAAACTGAGAGGAGCTTCTCATCAGATCGAGAGTTCTCAATGGTGACCACATCAGC
ATCCATGTCAATAATGGAGTGAATAATATCATTGAAGTTGGAGTAGCACATGTGAGTGTG
GATCTGGGTAGTGTCTTGAACACCAACGTTGGTGATCCTGAAGGAGTGGACAGCCCATTC
AAGGTAGAAAGCATGCTCGGATTTCCTAAGAGGCAATCCTTCTCTCAAAGCAGCCTCATC
AATTTGGATAACAGTGATGCCCGCCTTCTCAAGATCCTCAACTTCATCCTTCATAGCCAA
AGCAATCTGGTAGCAGGTCTCAAACCTGGGCTGGTCATTTCGGACAAAGGACCAGTTGAG
AATAGTGACAGGGCCAGTAAGCATTCCCTTCATAGGGCGAGCAGTCATACTCTGAGCTGC
TGAGGACCAGAATACAGTCATAGGCTTGGGTCGGCTCACATCACCATAGATGATTGGTGG
CTTCACACAACGAGATCCATAGGATTGCACCCATCCATTGGCAGTGAAGGCAAAACCAGA
CAGCTGCTCTCCAAAGTACTCAACCATATCGTTCCTCTCAGGCTCTCCATGAACCAGAAC
ATCAATATCAAGCTCTTCTTGGAGTTTGACAACTTTCCTGATTTCCTCTTTGATGGAATT
AACATATTCCTCCTCAGAGATCTTCTTAGCCTTGTACTCACGGCGAACTCTCCTAAGTTC
AATGGTTTGTGGGAAGGACCCAATGGTGGTGGTTGGGAGCACTGGAAGATTAAGCTTCTT
CTGTTGAGCATCTAATCTAGCACTAACATTGGTAGCACGTCGGTGGTCTGAACCCTTCAA
AGCAGCAGCCGCCTTTTGAACTTCCTCATTTGTCACCCTTGGGGAGGATTTCCTTGAAGC
CTGAGCAGCAGCATTGGCAGAGAAGAATGCCTCATCCTTCTGACCAGCCAAAGCCTTGGC
TAGGGCATTCACTTCAACAACTTTCTGGGCAGCAAATGCGAGCCATGACTTGATTTCCTT
GTCCAACTTAGGCTCATTAACAAGATCAACAGCTGTATGGAGAAGTGAGCAGGATGTGGA
GACAACAAGCTTGTCTTTACCAACAATGCCCTCAAGTTCCTGCAGGTTGCTAAGAGAAGC
AGCAAGATCATTGGCCCAAATGTTCCTTCCGTCAACCACTCCAGCAAAGAGGTATTTTCC
AGGAGGGAATTCACCCTTGATCAGGTCAAGGGTCTTGGTTCCACGAACTAAATCAAAACC
ATAGGCTGTGACACCCTTCAAACCAATCAGGGTCTTGAATGCCTCAGCTGGAATATCAGC
AAAGTAGGTCTCAATGAGAACATTCAAGCCAGAGAGAGTTGATTCCAGTTCAGCATAGGC
CTCAGTGAATGCTTGCAATTTGTGGGATTCAAGATCCATGACAAGCATGGGCTCATCAAA
CTGAATCCATGTGGCACCAGCAGCTTTAAGCTCAGAAATTACTTTCTTGTAGACAGGGAG
GACTTCGGGGAGGAGAGAGAGAAGAGAGAAGGACTTCTCAACACCCTTGCTGGGTTTAGA
GAGCAACAAGTAAGACACAGGGCCAACAATGACTGGAACAGTCTCTACTCCAAGCGCCTT
AGCCTCCTTGTATTCACTAACAGCCTTGTGAGAAGCATAAGAGAATTTGGTTTCCGGAGA
CAACTCAGGAACAATGTAGTGGTAGTTTGTGTCAAACCACTTGGTCATTTCCATAGCGGG
AACAGCGGCATTTCCTCTGGCCATAGAGAAGTAAACATCGAACCCAATCTCACCACCGTT
CCAGTTGTACCTATCTGGAATTGCGCCAAGCATTGCGGTGGTGTCGAGCACTTGATCATA
GTAAGAGAAAGTGTTGCTAGGAATGTACTTGATCCCAGCCTCAGACATCTGTTTCCAGAT
GGCGCATCTCAGCTCGGCCGCAACCTTCTGCAAATCCTCAGCGCTGCTCTTGGCATCCCA
GAATGACTCCAATGCAAACTTGAGCTCTCTCTTGGGGCCCATGCGTGGGTATCCAACAAT
GTGAGACGCCATTTTTCAAACAGAGATAAAACCGGAGAAATTTGAGAAAGAGTCTGGCCA
GAGAGTGGAGAGAAGAGAGCGGCACG
>ntig_176_consensus

CAATAATTAGATTTACATCCACAAATACACAGCCTGAAAATGAAGGTACATTAATCTGAT
AATCAAAAGGCTTGTAACCTCCATTAGAAAAAAACCATAAACACAAAGAGAAACTCATAA
GAAAGCATTCTATGAAATGTCACCAAACAAGGTAGATGCAAACAGAAAACCAATTTATGT
AGACAACAAATGAAATAATGCAAAAAATGACATTGGTCAGACTTTACGCACACCTGGACC
ACTTAAATGGTCCAAACTGAGTAAATTCTGGACAACATTTCATTGGTGTGACTTAACACT
TTGGTGCACAATAACAAAACTTAAAATAAATAAATATTGGTCACATATAACCAAAGAAAC
TTGGGTACAGAATTTCACAAATTGCTATCAAATATCACAAGGAAAACCCAAAACAAATCT
CACATGTTATCTGTTATAAAGAAG
>ntig_177_consensus
TCACAAAGAACTTGAGTAACATCATGCAATAATCCTCTGCGGTCAAGGCAAAAGAACTTT
AATAAGTAAACTGGAGAAGATGAAGGGCATGTAGACTGCTGAGTTAAGTAAAATGACACT
AAACATGAGGGGCATATAGACGATAGGCGGTTCTTTAAATTTGACCATCTTACAATGATC
GGACCAGGCCGAGGAAACACCCATAATACTATGTAGCACCATTTTCCATCAGTTGAGATA
TCTCCTTTTGCAATGTAAAGTCCGAAATCAAGAATTATTCTGCAAACATCGCATCCGAGG
CCTGTTTTATCAGGACAATTAATTGTAATGACATGAGGATCTCGCGGAACTTTTCCCTTC
TGGATTACAACGACGTCGTCGCCCGGCACTCCCATACTGAAAACAGGAAAGTTCCGCCAC
GATTTTCTGGAAGCGCTGGACCGTTCGTTCTATTACCTAAAAAGGAATGGTCAAGAAGAT
AGTCGAAAGCTGTTTCTTCTTTCGACTAAAATCGCTAGAGAGAGAGAGAGGAAGTCGTGG
AA
>ntig_178_consensus
TTGCCTTGACCGCAGAGGATTATTGCATGGTAATATAGATATCTACACTCTTAATTTTTT
TTCTCTGATTGTAAAATGTGATGGTACATTAGTGGTTCTGAAATGGTTACAATTTCTTAT
GTCAGATGTTACTCAAGTTCTTTGTGAGCTTGAGCTTACGATTCAAAGGGTGAAAGTGAT
GACAACACCAGATGGAAGAGTCTTGGACCTCTTCTTT
>ntig_179_consensus
AAGAAAACACAGAAGATTTTGTTTTAAGCACTTTAAAATGTACTTTTCACATTTACTGTT
AGTAGGTGTTAGAAAGTTTATAACAGTTCCAGTAAATAGTCTATAAAGGAAGAAAACTAT
GGAGGAATAACTCATAATTAGATTAGATGAACATACAACATGAATTTTCTGGGAAACGAA
AAAAAATTGAATTACTGTAATGGGAAAGTTGAGAAGGAGAGAAGCTGACTTTTTTTCTTT
TTTTTTTTCTGTTTTGACTCTTTGGATGGGTCAGAAGATGGTGAAGAGGCAAGCGTTGCT
TTGAAATTTCAGAATCTGAAATTGGGGTCGACGTCCATTGCCCAAGCAAACTTTGCGAGA
AGTGACTTGTTGAACATCTTCTCAATAGGAACTTCATGTCGCTTGCACCATGCGACTGTT
ATCCTAGGGTCAAGGTAATTGATCTTGGACGTGCCCAATGCCACAGTTTTTAAATCCTCT
TTGGTCTTCATATCTCGTTCCATTTTTTCAATCTTCACATTGGTTTGAGCTATCTTCCTC
TCTATTGCTTCAGGGGTCAGGTTCCTCTTTCGCTTTCCATCGGCATCCTTCAATGGGGGC
TTCCCTTTTCTAGCCCTTTCCAAATCGATTTTCAGGTCCTTCAGAGCAGCCTTGAGCTCT
TCTATTTTCTCGGTCAACCTTGTCATCTGCGCACCATGTGATTTTGAAACAGACCGCTGA
TGATTACAAATAATTGCAACCTCTTTGTTTGCAGTGTTATAAACAACAATCTTTTCCGCA
ACATCACCTTCTTTAGTTCCCTTACTCAACATCTCATCCAATGTGATCGAGGCATTATAA
GTACGGAAAACTTTTGCTGTCAGGCCAGGCATGAGTTCCTTCAGATGAGCATTCAGTTTA
GCTGTATCCAGCTTGTCAAAAAGATCATCACTTTGCTTCTTTCCAGTTTGAAACTGCCCA
ATTGCCTTGTACACAGGAAGCTCCACTTCAACTGTGTTCACATATTGAATTGAATCTTTA
CCCAGGAAGTCAAACTTTAACTGATTTGGTGGGATACACTCTACATTACCCACTTTCAAT
GTACAGCAACCAACAGTATCAGCTTCATCATCATCCTTCTCATTACCCGCCCTAAGAGCT
AGTTTATCAATAAGATAGGTTGCGACTGCTATCTGCCTCCTTGCACTATCTTTGCTAGTA
AAATCCTTGGTGTATGATGCTCTAATGCTCTCTATGTAGTCCTTCAGCTTTCTTGCTTTC
TCATATTTCTCTTTGTCACTTTGCCCCTTCAAGGAACTGCTAGCAGCCAGGAACACGTAC
TTGAATTCCCTAGGATTAATTGGATCATTCCAGAAAGCTAACCATGTGACAGTGTTATCA
TGCTTTACTTCCTTCCAGCTTTCACCAGGAATAGGACATTCGGGAATTGGGACATCCTCC
CCAATGTTTATGGTAATGTCACTTGGACGGATGCGTTTTTTCAGCTTCCCCATCTTTGGA
TGCTCTCCCCTGCCTCGAAACAGCCCAGGGGGTTCAACTCTGAAATTCCCAACCTTCTCT
TTAACACCATCAACTACAGCCCACATATACTTCTCCTCTTGTTTCAATTTCTCTTCTCTC
ATGGCCTTCTTCTCTTCAGAACTCATCTGTTTCTTAATCTCCTTCTGCTCCTGATGCCAA
TCATATATTGGCTTAAAATCACAATCTGCCAAATTCTGAATTACATGGTTTTTACCCAGC
ATTTTACGCCAATCAGTCCAAAAGTTCTCCTTGAATTTTGGTTTCTGCATGTAATCTGTT
TCTAGCATCACTGCATACATTGTTGCAACCTCCTCTTGCTCAGGAGTTAGGTCAACTGGT
TTCCCCTTGTAGAGCATCTTAACCCCATGAGGCGTGTATGGAGGAGGAAAAATGACACCA
TTATGAACCAGAGTAGTCCATTTCTTTTGCCCATCACCAGCACTAGGAAGCATTTTGGTT

GATTTCGAATATTTTGAATTTTTCATTATTTTCTTGGACTTCTTGTTAATTTTATTTGAC
GACGCAGAAACAACTTTTGTTGATTTTGTCGTCTTTGTAGGAACTACTTTTTTATCAGAT
GATATTGATTTCTTCATTCTCTGGGCTATTGGAATATGGTCATCATCATCTGCTTTCTGT
TCTTTTTTTACTGTTACTTGTTTAACTTTCAGTTCTGTAGAACTATTCCCGACCTTTGGC
TTTTTAGCTGATAATGTAGCCAATGAACCCACATTATCTAAAGGTCTCTTGATTGGCACG
GAAGAAGACTTTTGCTGCTTGTCAAACGTGGTCATACCATTTGGCAGAACTTTTGAAGTC
AGTGGTTTCTTTGTATTGGGATTGATAGGCTTTTCACCTGATGCTCCAGCAGAAGACTTT
GAGAACCTTGATGATAATGGAACTTCATCATCAGAATCATCGGGAGCACTTTTAACTTCA
ACCTTAGATATTGGCGATGCATTCACTTTAGAAGCATTGAGCTCTCTATTGGCATTGCTG
TTGTTTCCCTTAAGTCTAGCACTCAAAGGTTTGTCATCGTCAGAATCCTCATCATCATTT
TTCTCGCCATTACTAACCGAATCATCTTCCTTCTTCACAGAAATTGATGCATTTGTCTGC
GGAGATTGTTTTAACCTATCATCCAATGGACTTGGTGTTTTTGAATTAGGCACTGGTGAC
TTCACTGCCCCTGATGACCCCTTGGAAGATGAAGATGATGCTTTTAGATTTGGTGGAGGA
GATCTCAAATGTGATGCCTTGAAAGATGGACCTGTTGTCTTACCCTTTTGAGCATTGACA
TTTTGACCATTAGCTGTGGATGATGATGGTCTTCTCCCTTCAGGATTCACTTGGTTCTGG
TTAGCGGCTGTGTTACTCCTTTTGAAAACTACTGGACCATCGTAGTCATCATCAAAATCA
TTGACTATGTTCGGTTCAGTAGAAGATTCAATAGCCATTATCCCACAACAACACTTGTTG
GCATGTATTCACAATTCTTTCAAGGAACAGATCGAGCTGGTGATTGACTAGATCAAATCG
TCTCCTTTAATCGCTCTCTCTCTCTCTCTCGCTTTTGCTCTCGCTCCTCCACTGTCCCTT
GTGGTGC
>ntig_180_consensus
TTTTTTTTTTGGCACAAAACCCAGATCAAAGTCTATATTTTTACTATTGACACAAATGAT
AGATTTACATATAAACAATTCCTCCAGGTTTTACACAAACCACAATCATATCTTACCCTC
TAGATGGTGAGCAACATCTTGCTACTCGTATAAATAAAACACACAACCTTTTACAGAGAA
TATATAGAATACATACACATATATATGGCATTATTCTATCTCATCATAAGGGCCAAAATG
GCATAACAGGAGCATCATCTGCGGTTACTTAGCTACCTGGTTCTGTGGTTGTGATTTAGT
CTGGCATGCTGCAGACATCTCTCGTAAACGTCTGGCAATCTCTGTGAATGATGGGCGCGC
CACAGGATCTGGAGCCCAACATTGCTCCATGAGCAATCTCCATTCATAGTCACAGTAGCT
TGGAACTGGGGGCCTCAATGTGTTGTTGACGATACCTCCTATAATGGCTCCATAATGCAT
ATTAGCATAAGGCTCCTCACCAGTGAGAATCTCCCAAAGGACAATACCAAAAGAGAACAC
ATCAACCTTCTCGGAAACCTTGCTACTGCTACCATTCAAGAGCTCTGGAGCCATCCAAGG
AAGAGTTCCCCTCACACCACCAGTAACCAAGGTATTTCTTTTAATCTTTGACAAGCCAAA
ATCACCAACCTTGCAAATGGGCCGTAAAGGATCTTTTAAGTTGACGAGCAAATTGTCACA
TTTCAAATCAAAATGCACGATATTCTTTGAATGCAAATATTCCATTCCAAATGCTGCATC
CATTGCAATAATAAGCCTCTTACGGCGATCAAGATGCCTGTCCTTGCTAAGTAAAACATG
TCTAAGAGACCCATTCACCATGAATTCAGTCACAGTGGCTAGTGTTCCTCCCGGTCCATC
CTGCACCACACCATAAAAGGCCACAACATTTGGATGATGGAGCTTTGAAAGAATTTCAGC
TTCACGCCAGAACTCCAGAGTCAATCTTTCTTGCTCTGATGAACGACCAGTGAAACAGCT
CTTTTTTATTCGCTTAATGGCAACATCTGTTCCCCTCCATTTCCCATGGTAAACAGTCCC
AAATGTGCCAGAACCCAGTTCTCTAAGCTCTTCCAGATCTTCATTTTTTATTATCTGCAA
TGTACTGATATCAAAATCTCCCAAAGCAAAATCAACAAAAGGTGCACTAGTACTTGGGAG
ATCCAACCTTGTCTCCTCATAATCTGATTCTGGAATTCTAGAGTTCATTCCCAGTAAAGG
CATACTTTGATCACCCTTGAGATGAGAATGCATGTTATCTTGACGCATATTCATGGTATT
GGATCCGATGGTACCAGATGATTCTCGCTGAATGTCCTCATCAAAATTGATATGTGGGTC
ATTTTGGGTCATTGCAACTCCATCATGTTTTAAAGGGGTATAACTGTTATCAGCAGGAGT
GCCCTCCTCAATCTTTGTAAGTGGAGAAGAAAAGTCCAAGTGATCTTGGTCCATAAGGGA
AACATCCTTTCTAGAAAACTCATCTTGAGCCAAGTTCAGAAAGTATGACCACCGCTTAGC
ATCATGATTCTCCACATTCCAGCTTGCAACTGCTCCATCTCCATGCATTGGATTGATACC
ACCAAGGGTTTCAGAGATTCTTGCTTTTGAGAACATGTCAGTTAGAAAATCACGAGGAAA
TCGATCATTGATGTCAATAAGTATGTCTCCATGCTCACTATTGCCAATGCCAACACTGGA
ATCCCTTACTGCAGCAGCTCTAACTGGATTTTCCATCCGTAGAAAAGGCTGTGCATTCCC
CTTAGATTCATTCCCATTGTTAATTTTTTCTGCAGTATCACTCCATTCATATTTTGATGG
TTTCAATGTGGAATCTTCCAGATGATTACTATGAGCTCCATGCCTTGCTGTAGGATGCTG
CATGCCAACTGCTTCTATGTCACTAATAGACTGCTCTTTATAAGTGTCTTTGATTCCAGT
TATTTCCTGACCAACCACTGAATTTGGAACCATCTGCCTTGGTTCGGTTTTCTCTACATT
TGGCTCAAGCCCCTCTTGAGAAAAGTTTGAACCTGTTTTGTTCATTTTATCAGCGAATTC
TTTTGGCTTTCTCAATTGAGTAAGTTCACCTTCAACCGACTGAGGATTTATATACAGCTT
TTTCCCAGATGTGGTGGACTGCTCAGTCTGGGAAGCCATGTTACTGTCATGCGATTTATC

AACTGATTCTGTGACAGGTTGAGAACAATCACCATGCGCTTGGGTAATTAGATACTGAGA
GCCAAAGGAATCATTAGATTTTGACAAACGGTTCATTTCGGCCTGCTCTCTTGGAATTCG
CTCAGAATGATAAACTCTTTGAGAAATCACAGAAGGTTCAAGGTAGCTAAAGTCAGTCAA
CCCAGCCTCCACATCACTATATGCAGACGCAAAAACTCCATTAGTCGCATAGAAACGGTT
ATCATCATTGTCCTTCCTCCCCTCATTTACAGTTTCAGGGGGCCAGTTCTGTAGAGGTTC
CTGAGCCTTTTTATTACTTTTTGAGGGCAATAATGGTGAACCTATGTCTCGGGATAAATC
AGTTGATACCTCTTCCCTAGGAATATAGTTCGAAACAGTAGTACACGGATGTAAAAAAAC
AGCATGCTCTTTTTCAGAAATTCGAATTTTTTCAGTTTCATTAATCTTTGGCAGCGAGCT
ATCTCTTTTCATTTTGGGATCCTTTATTGATGTTTCCTTATCCAGGGGCCAAATCTTCTC
AGATTCATTTATTTTAGGAGCTGATCCAGCATTTTTGAGTTTAGCCTCCTTAAGAAATAC
TTCCTGACCTTCAGCATGGAAGCCACCATGCATCTGCTCTTCAGGCAAAGGTTGATTCAG
ATGTCCATAGAGCGGCATGGAGATTTCCTCACGTGTTGCTAAGTTTGAACCATAGTCATA
TTGCAGTGGAGCAGAAAAAAGAGTAGTGCTCTTCACATCTATCTGGGGGAAGCCCTCTGT
AGAGTGCAAAGAGGATGGCACATGCTGGC
>ntig_181_consensus
CACTCTTTCCCACACGACGCTCTTCCGATCTCACATTTACTATTGACAGAAATAAAAAAT
TTACATATTTGACAAGTCCATCAGGTTCTGCACAAACCACAACCATATCTCACCCTTTCA
ATGGTGAGCAACAACTTGCTACTTTGTATAAAAATACACACAACTTTATATGGAGAACAT
ATATATAAATATGTATATATGTGAGTGTGTATAAAACTGGCATTATTGTATCTCATCATA
ATGGCTAAATTGGCATAAAAAGAGAATTATTCGCGGTTACTTGGCTACCTGGTTTTGTGG
TCGTGATTTAGTCTGGCATGCTGCAGACATAACACGTAATCGTGTGGCTATCTCTGTAAA
TGATGGGCGTGCCACGGGATCTGGAGCCCAACATTGTTCCATTAGTATTCTCCATTCATA
GTCACAGTAGCTTGGAACTGGGGGCCTCAAAGTGTTATTGACGATACCTCCTATGATCGC
TCCATAATGCATATTAGCATAAGGCTCCTCACCAGTGAGAATCTCCCAAAGGACAATACC
AAAAGAGAACACATCAACCTTCTCGGAAACCTTGCTACTGCTACCATTCAAGAGTTCTGG
AGCCATCCAAGGAAGGGTTCCCCTCACACCACCAGTAACCAAGGTATTTCTTTTAATCTT
TGACAAGCCAAAATCACCTACCTTGCAAATAGGCCGGAAAGGATCTTTTAAATTGACAAG
CAAATTGTCACATTTCAAATCAAAATGCACGATATTTTTTGAATGTAAATATTCCATTCC
AAATGCTGCGTCCATTGCAATAATGAGCCTCTTGCGGCGATCAAGATGCCTGTCCTTGCT
AAGTAAAACATGTCTAAGAGACCCATTCACCATGAATTCTGTCACAGTGGCCAGTGTTCC
TCCCGGTCCATCCTGCACCACACCATAGAAGGCCACCACATTTGGATGATGGAGCTTTGA
AAGAATTTCAGCTTCACGCCAGAACTCCACGGTCAATCGTTCTTGCTCAGATGAACGACC
AGTAAAACAGCTCTTTTTTATTCGCTTAATGGCAACATCTGTTCCTCTCCATTTCCCATG
GTAAACAGTCCCAAATGTGCCAGAACCCAGTTCTCTAAGCTCTTCCAGATCTTCATTTTT
TATAATCTGCAACGTACTTATATCAAAATCTCCCAAACCAAGATCAACAAAAGGTGCACT
GGTACTTGGGAGATCCAACCTCCCCTCCTCATAATCTGATTCTGGTATTCTAGTGTTGAT
GGCCTGTGAAGGCACACTTTGATCGCCCTTGAGATGAGAATGCATGTTATCTTGATGCAT
ATCTATGGTATTGGATCTAACAGTAGCAGACGACTCTCGCCGGATGTCCTCATCAAAATT
GATATGCGATTCATTTTGAGTCATTGCACTTCCATCAGGTTTTAAAGGGGGATAGCTGTA
ATCAGTAAGAGTCCCCTCCTTGATGTTTGTAAGTGGAGGGGTGAAACCCAGGTGATCTTG
GTCCATAAGGGAAACATCTTTTCTAGAAAACTCATCTTGAGCCAGGTTTCGGAAATACGA
CCACCGCTTAGGATCATGATTCTCCACATTCCAGCTTGCAACTGGTCCATCTCCGTGCGT
TGGACTCATACCAGCCAGGTTTTCAGAAATACTTGCTTTTGAGAATATATCAGTTAGGAA
ATCGCGAGGAAATCGGTCATTGATGTCAATAAGTATGTCCCCCTGCTCACTATTGCCAAC
ATCCCTTGTAGGACCAGCTCTAACTGGATTTTCTATCCTTGCAAAAGGTTGTGCATGTCC
CTTAGGTTCATTCCCATTGTTAATTTTTTCCGCAATATTACTCCGCTCATATTCTGATGG
TTTCAAAATGGAATCTTCCGGATGCTTACTAGTTTCATGACTTGCTGTAGGATGATGCAT
ACCAATTGCTTCTTTGTCACTGACAGAGTGGTCTTTATATGTATCTTTGATCCCAGTTAC
TTCCTGATCAACCACAGAGTTTGGGCCCACCTGCCTTGGTTCAGATTTCTCTAAATTTGA
CTCAACCACATCTTCTGAAATGTTGGAATTTGTTTTGTTCATTTTATCAGCAACTACTTT
AGGCTTTCTTAATTGAGCAAGTTCAGCTTCAACTGACTGAGGATTTGTATACAACTCTTT
GGCAGATCTGACAGACTGCTCAGTCTGGGAAGCCATATTGCTTTCATGCAATTTATCAAC
TGAGTCTGCAATAGGTTGAGAACAATCACTATGTGCTTGAGGAATTAGATATTGAGAGCC
AAAGGAATTATCAGATTTCGACAAACGGTTCATTTCGGCCTGCTCTCTTGGAATTCGTTC
AGAATGATAAACCCTTTGGGGAATCATAGATTGTTCAAGGTAGCTAAATTCAGTTGGCCC
AGTCTCTACATCACTATAACCAGATGTAAAAACTCCATTAGTTGCATAAAAACGGTCATC
ATCATTGTTCTTCCTCCCCTCATTTACAGCCTCCAGGGGCATTGGGTTCTGTATAGGTTC
CTGACCCTTTTTATTACTTTTTGAGGGCAATGACGGTGAACCTATGTCTGCAGATGAATC

GATTGATATCTCTTCCCTAGGAATATGGCTTGAAACAGTAGTATAAGGGTGTGAAGAAAC
AACATGCTCCTTTTCAGAAATTCGAAGCTTTTCCGTTTCATTAATCTTTGGCAGCGAGCT
ATCTCTTTTCATTTTCGGATCCTTTATTGATGCTTCCTTATCCAAGGAGCGAATCTTCTC
AGGTTCATTTATTTTAGGAGAAGATCCAACTTTTTTGAGTTTAGCCTCCTTCAGAAATAC
TTCTTGACCTTCAGCGTGGAAGCCACCATGCATCAGCTCTTCAGCCAGAGGTTGATCCAG
TTGCCCATAGACAGCCACAGGGACTAAATTTTCTCCACGCGTTGCTAACTTTGAACCATA
GTCACATTGCAGTGGAGCAGAGAAAAGAGCGGTGCTCTTCACATCTATCTGGGAAAAGCT
CTCTGTAGAGTGCAAAGTGGATGATATATGCTGGCCAACTTCTCCATGGTTTATTTTCAG
TCCCTGGTAAGGCTGTGAATTTGACTGATAAGCACTTGAGGAACTTACCAGCCCTGGTTG
AGAAGATTGAATTTTAGATGCAGGTGCACTAAATGTCATAGTTGTGGTGCTAGCACCAGC
CAATTCAGCTGCAGCCCGACCAGTTTCACTTTCAACATTTAGACTGAGCAACTCATCCAA
ATTATTTTGTGAAGTGCTTGCTAAAGCAATTGAGTTTTTTCGTGATCCTAGGTCCATACC
ATTAACAGCAACAACGTATTGAATCTCAGAATCAACCTCCATGTTTTCCAGACCAAACTG
AGCATCATCTAGATCTCTACTAGAAAATAGAAACATACGAGGTTTCTGTTGTCCCCTGTT
ATCTAATGTATTAAATTCTTCCATCATGTTCTGCAGATCCTCATCAGAGGATACAGAAAC
CAACGCATCAAGATCCTCACCAGGAAGTTGATATTTTATTGTATGTGTTTCACTATATAT
TGCCAAAGCTTTCTGCATAAGCTCCTGCCATGAGATATCTTTAGGTAGCCGGATAATACG
TGTTTCTCCTCCCACATACCGTAGCTTTCCATCAATAGGACGAGGCAAAATTTTTCCACC
AAAGCTACAGAGGAACTTCACCTTAGTTGATGGGCAATCAGGAGCTACTGATGAAGCATA
ACCATGAACCCCTCGACTCACTTCATTTCTTGATGAGGTGTGTGGCACTGAATGCACAGA
ATCGTAGTAACTTCTGTCTTCATGTGCAGAAGATCCCTTTCTCTCAAATTCCTGAGCGCG
GCCTGTCTCTGCCGTGTTAAGCATTGTGATATCAGACCCACTCTCTGGACCAGTCTGGGG
AATTCCTAACATTCCCGACAGACCCATATAAACAGGTGGATTGTTAAAATCACCATAAGT
ATTTGGAACAATATGCTGCCTAGGAATCACCCTGTCCCGCATAAATTCTAGAGAAAACTC
CTCACCAGTCTGAATGGAGTAGTTCAGCACTGGTGGTCGAGCTCCCATAGACATTGTGTA
ATCTGGAGGCCGGATGTTAGTGTTGATATTACTAGAGGGGTCATGGAGGAACCTTTGATT
TGCTGAACCAGGTCCCTCATTTCCAGATTCGACATTAGTGTATCGGACCTGTTCATAATT
TTTTGGCTGATTCATTGTACCCTTTCCAAAATTTCTCTCCATTTTATTCAACTTCACCTG
GGCCAAACACACCCAGGCAAGCTGCCATGAAAATCACACCAATAACATCTCAAAACTAGA
ATTCTCCCAAATCCACTATGAAGGTGAGTAGCTTGAAAAAACAAGATCTTCTTACGCGGA
AACTAGAGTCTTTTAACCAGGAACCTGTGCCAAGCAGCAAAGACAGCGGCTTCTCTCTCT
CTCTCTCTCTAAAGCAGTGAACTTTAGTTCGAAATAAACACTATTATCTCTGTAATTTTG
TCCGCTAAATTAGATTGTTGCTTGTCAGCGTTCCCACTCTAGATCTACCACTTACAGCTA
CAAAGCTTACAATTTTTAGGACTTTTTTCACACGCTAATCATCATCTTTTTTGTTGGGAT
TCTGATGTGAGAGCCCTGGTGGGGCATTG
>ntig_182_consensus
TCCTATAATTTATTACGGGATATTGATATATATCTAACATGTCTCGTGAGTTGGGTGTTA
TGTGTTTGTTCATCAATAAATAGTTCTTGTAACCTTACCGCAGTGCCTTTCTTCCCTTAC
TCTCCCTCGCTCCCAAAATAAGAAGACCCATTTCTTTTTCTTCTTCCCTTAAAACCTCCT
TGCAAGCTTAGAGATGTCGAGTAGAGCCAGTAAGCGTGTGAGTTTCAGCCCTGATGTCAA
TATGAAACCGACGGTTTTTCTCAAACACGGAGGCAGCACAAGATCAGCGGGAAAACGTAG
GCGAGTCATCGTTGGAATTTTCAGTTTTAGAGCCACCAGAAGTTCAAGATTTTCACCAGG
AATCTTACTCAGGCGTCTGGGTTCTAAAGTGGCAAGAGCTCTCCGTTTTGTGTCTACGCC
AAACCCGAAGAGGTCTTCCCGGAGAGTTTCTTCATCTGGTTTGCAAAGATCGAGATCATT
AGCAGACTCAATAGA
>ntig_183_consensus
CCGGAGGAGGAATTTCTTTGCAAAATACACATCATAGTAAGAACATGTATTGTTATCTAA
GCTGCCCCCAAGTTGATAGTGGTGTAGCTTAGAGAAGACCAGATAAATCCAAAGAAGATA
AAAAAGGTTTGTATATATTACTTAGAACGGAATGTAAAAGTGTAACACTACAAAACAAGG
CTGGCAAAGACACAGATAACATTTTACCAGAGACTCGGAGACCTCACCCATTCAACGGAG
ACATGCAAGCAAAAACCATACATTAGCATTTAAATGCCAGTAACCATACAACATCTTATT
AGAATACTTATGTAGTAGATGTACTAGGACAGACACCGTTGATCATGCATTAACGTTCTC
ATTGAGCCTGGCAGCTGCAGCTACAGAAGACGCCACTCCACCGGGATGTGTAGCGACGTT
CGGGTTATTCCTTAGCTCTGCGCTCGCCACTCCTTCAGCATCCTGTCGAGTCACTGCTTT
GTCTGCTGGCAGTTTTGCAGTTGCACCCGAGATCACTTCTTTGAGCTTTATTTTTTCTTC
TTCACGGGTCGTTGTTTCATTATGAACCGCAGCAGACTGAGCTGAAGCTGCGAGGCCACC
TGGGGTTATGACATTGGTCCCAGTGGCTCGCACTTCAGCTGCTTGTATTGCAGCCGCATC
GCTTTGATCTATTGGCTTATCACCGGCTGTTTCTGCAGTAGCTTCAAGTGCTTCTCCAAT

TGTAATTGCACCTTGCTGGACAGCCAAAGCTGCAACTGTCTGTGGCACTGGCCTAGCCTG
TCCACCAACTGATTCAGTGACCACCCGAGCACCAGGCACATCAGTTTCAATAACAGTGAC
GCCTTCGCCTCCAGCGATATCAGTGACATCGCGGTGACCAACAAAACCTGCTTTCTCATT
TTGGTCAGCAGCAGACTGCATGACAGCAGCAGGTCCTCCCTTCTGGGTCTGCCCGAACAC
CGTGCTCTCCGCCGACTGCATCATTGCAGCATCTTCTGGTGCAACAGGTCTCCTAGCAAG
ATCCCCAGAGACGTTGAATATGTTACCGTATCTGACGGGATCTTGATCCACCTGAGGCCT
CCATGGCTGATCTTGACTCATCTTGAACTTTCAAGCTATAAAAGCTGAAACCAACAAAAC
TTGGGTTAGAAACAGTAATGCTTGCCTTTTGGAATTGCAAGTTGGCAACGGGAAGGTGAT
AAATAAAGCTGATTAAAGTCGGAAGTGTTTCGACACGTTGATACAAAGACACGCGTTGAA
TAAGAAATTAGCA
>ntig_184_consensus
AAAAACACTTGTAGATGGTGAGGTTTCTTCAGACAAATAACAGTAGCGTCCAACTTACCA
TGCAATTTAGAGAAAAAGATAAAAGAGCAGAAGAGGGTGCCAGTATCTTGGCTTGAGATG
TTCAAAATAAGAATTTGATCACAATAATTTGCATAATCAATAGCCACATATCAATTTCGA
TTAAACCATAACATTGTAATTGACATAATAAGGTTTTCAGACAAACCTGGATGAGTCATG
TTATCGCTTCAAATGGATTTATTTGATAGAGTGAAAATTACAGTAGAATATTAAATTTCA
CATTTGTTAAGAGATTTTAAAGAGGCATTCGACAATATTAAGATCGAGGATAAAATATTT
TAAAGCTGCTTAAAATCACACAAATTTGTTATTTCAGAAGCTAGTGACATATGCAGACTT
GCATTTATGCTCTTACAATCACTCACACAGAAACATCACTCCAAAACAAACTCACTCATG
GTTTGCCTCCACGGAGGCATTTTATTTGCTTAGCCAGAGATGTCAATGGCCTTAACTTGA
GGCTTCTTCACCTCCTCCTTGGGTACGGTAACAGTAAGCACTCCATCCTCCATGCTAGCT
TTCACTTGCTCCGTCTTTGCATTCTCAGGCAACCTAAACCTCCTCAGGAACTTCCCACTG
CTCCTCTCTACGCGGTGCCACTTATCATTTTTCTCTTCTTGCTCTTTGCTTCTCTCTCCA
CTTATCTGCAGAATTCTGTCCTCTTCAACTTCAACTTTCACCTCTTCCTTCTTCAGCCCT
GGAAGATCAGCTTTGAAGATGTGAGCTTCTGGGGTCTCCTTCCAGTCGATTCTTGCATTA
GCAAATGCAGATGTTTCACGAGCTGAAGAAGGCATATTGGCCACAGCCGAGGTGAAGGAC
CCATCGAAAGGATCCCATATGTCTAAAGAAAAGGGATCAAAAACGTTGGTTCTTCGCCCA
CCAAAAATGCTAGGAATGAGTGACATGTTCTTTGCTTTATATTGAAGAAATATAGAAGCT
TTTGATCGGTTTCCGGATGCAAACCAAGACGGATGAGCAGACTATATATAGGATCAGGAG
GAGAATTCCAGTAACTTGATCGAAATTTCTGGCTCTCTCGTGGGCCTTCTATTTTTCTTT
TCATATAATATTTTTTTAATCTGCACAGGTACAGTCCTATCTTCTAAAACCTTCTACCAT
CC
>ntig_185_consensus
TTTTTGTCTTTGACAGTTTTAACACTATTCCTTGTTACCCCACAAATTTTGCTTCAGATT
CTAAAAAATATCATGTTCATACATGCTGGATGTCACAATTAAATTTGTATGTACAATGAG
GATCAGTAAATATTTGTTTTAACTACTGAGAGAAAGGGACCAGAACTGGCAAAATTTGTC
TTTGGCCGTATACCGCGTCTAGTTATATCATTAACTAGCCCGCTATATATTATTCTGAGC
ATGCTGTGGGCTGAAGATAGGATTGAACTTGGGAAGAGGATCCTCACGAAACCAATCGTG
ATTGAGGGCATCATCAACTGTTATCCTCTGATCAGGATTGTAAGTTAGAAGTTTATTTAA
CAAGTCGAATCCTGAATCGGAAAGAACTGGAGATCCTATGAAAGATGTCGCAGGAAACCT
TCTCCGCAACAAATTAAACGGTTGGTTAACAAATTTTGCTTTGGCTCGTCCTGATAGTTC
AGATAATCCAGGCCAATCTTTCACAGTTGGCGTCCCAAGCGTTTTGAATATCTTGTCAAT
CTGGTCAGCTTCACTTGTCCCACTGAATAGTGGTTTCTTGGCTAGCATTTCAGCCATTAT
GCAACCCACTGACCACATGTCAACTGCTGTCGAATACTTCTTTGCTCCTAACAAAAGTTC
AGGAGCCCTGTACCATAGAGTAACCACCATTGATGTGTATGGCTTAAGTGGGCTTCCATA
CTGGCGTGACATCCCAAAGTCACAGATTTTTAACTCACCCGGATTGTTCAAAAGAAGATT
TGATGTTTTCAAATCCCTGTGAAGAATCCAATTGTCATGCAGATACTTGACACCCTCCAG
AAGCTGTAGCATCAAGCATTTGACATCACTAGTACTAAATCGGAACTTCATTGACTCCAT
TAGCCCTTTCAGGTCGTGTTCCATGTACTCCATAACCATAAAAACGCTATCAAAGTCATC
CATAACAACTTCCTTAACCCTAACAATTGAAGGGTGATTAAGAGACAAGAGAATGTTGAT
TTCCCTCAAAGATGAAGTAGGAAAACCATATTCTAAATAATCCTCTTTCCTACCAACATT
CATCTTCACCTTCTTCAATGCCACAATTTCTCCTGTCTTCTTGTCCCTAGCTCTATACAC
AACGCCATATGTGCCTTCACTTACTTTGCTGAGATACTCATACTCAAGCACATGTCTACA
ACTCTGAAGCATGTTATTGCGTCTTTGTCCAAAAACAGTAACCTCCTCAATAGCAATATC
CTCATCAGATGTATTATCAACGTCCAAGGCATCATCAAGCTCCAACTCGTCTTCAGAATC
TTCATTTTTGGAAGAATGCTCCACATCAATAACATCAGATTCCGACAATCTGACCCCATC
TGCAGAGTTTTCTCTAAAAAACTCTCCACTCTCGGGAGTAGACCTCTTTGAAGGCTGATC
CCCCTTATCAGGTGCATCTTCACCATCAGATAGTGGAATATTCTCCAAATCACTGTCAGA

TGCCCAGCGTGATGTTGACGTACTCTTTGCTTGAACAAACTCCTGCAACTCTGACTGAAC
CTGGTCATCAAACCCAATATCTTGAGGCAATGGAGACAATTCCACTGACAACTCAGACTC
AATGGCCCCCACTGAACCACCGTCAGTATTCTCTGAAACCAAGTCCAACCCTTTAGCTTT
ATTTTCCAACAAGGGGCTTTTCACAGAAACCCCATTAGAGGCAACATTAGGCACCTCACT
AACCAAGCGCACTGAAGATTTCAAAGTTGGTAAAGCAGAACATGTAGGTGCAATTACGTT
CTTAGACGAAATATCCACTTTCTTTTCATCTAGATCCCATATGACGGGTGAAAACTTCCT
CTTCTTCTCAGGAGGCAAGTGAATCCCATCATCACATTTTAGCACTTCACCACTTTTACA
AAGTGCATCTTCACACCTCGCTTCACCACGTTTTGGGCCAGAATTTTGCCCATTCGACAA
GTACTGGGACCCATGTTTATACTTGTGGTCAACCCAGTCCAAACTCACTTTGGGATCACG
AGTACCGTTTCTGTAATGAACAATTTCTCTTTTGGAGTAACCATAATAATACCTCTCTCT
TCTCTTGGAAACATTGGAACGCCCTGCCGCCATATTATTAACACCACAACTCTTCTCAAT
AAAAAATGAAACTCAAGAAACAAAAATACGATAAAAGAGCTAACCAATCCCAAACACTCA
AATATTTAATAATAAAACGTATCCCTAATTCTTAAAATTGAATATACTATTTTCCCAACA
ATTCTCCAACAAAAAACCTAATCAAATATTTCAACCAAGAGACGTACAGGAAGAACAAGA
ATCAGTAACAGATATCAAAGACAGATGATGATGATGAATCAATAGAAAAAATAAAATTGA
GTAATGAATCAGAGCAGAATCAAAACCCTAGAACTTTCAAATTGGTGCTTACTTCTGTCT
CTCCTTCGAAATTCACAGGTAATTAAAGGTCTTACCGGTGAGCGAAAGGAAAGACA
>ntig_186_consensus
AATCCCTCCAAGTAGACAATTCCAATGGCAAAACATATTACGCATTGTTCCTCTAAGAAT
AGCTCTAAAACCAAAAATAAATATAAGATTCTGTGACTACTATAATAATTAATAACAGAA
CACACGGATACTAAAAGTAATGATATAAAGTTAAAATCAAGGGCAGCAAAATTTATGCTT
TAAATGATTTGACAGCAACTTAATCCCTTCCCCCTAAAAAAAAAAAATAATAATAAACACA
GCTTACAGCTTGTCTTCGAATTCTGACAGTGGGGTCATCTGCTCCTTCAAGCCCTTCCTC
TTACGGATATCCATGACAAGCTGTGCAGCCTGTGATCCAGCCTCCATTGGGTCTGACGAC
ATCATGTCCCAGTGATCAAACACAGATTGTGGGAAAGCCTGTCCTGAGGTTGCAGCTCTC
AAAGTGCCAGAGAATCCAAATGACTCAATGACAGGAAGGTATGCCTTAATGTTGTAGAGC
GGGGTACCTGGCCTCTGCATCTCCTCAAAGACATGACCACGTTTCTGGTTAAGAACACTG
TAAATACCACCAAGGGCTTGCTCAGGAGCTTGGATTTCAACAAGGTACACAGGTTCAAGT
AGCCTAGGCTTAGCTGTCAGCTGGGCAGCATATATAACCCTACGAGCGGTCGGAATGACC
TGACCACCTCCTCTGTGAATGGCATCAGCATGAAGAACCACATCACAGACTTCGAAGCAG
ATACCTCTCATGTTCTCTTCTGCCAATGCACCTTCCTTTGATGCCCATTGGAACCCAGCA
ACAACAGAATCCTTGATTTCATTCAAGTACTGAACTCCCTTACACATGTCCACAACCATG
TTCGGGCCAGTGGTCTCAGGGCCAAAACACCAGATTTTCTTGGCCAGATCCTTGTCCCAA
CCAAACTCCTCAGAAAGGATCTTAGAACGAGCCTTAGGATCATCCCTAGGACCAATACGA
CCTTCATCAATGGCCTCAGCAAGGCCATCCTCCAATGGCCGGGCTTCCATATAGAGACGG
TTGTGTTTGTTGGGGGACTTACTCATGACAGTGCGGCATGACTTCTCAAGGACAGTCTCA
CGGAATGATACAACAGGATCAGACTTAATGATTTCAGCACCACCCATGAAATCTTCCTGC
AAATCCTTCAAACAGATCTCAAGGTGGAGTTCACCAGCACCTGCAATGATGTGCTCTCCA
GACTCCTCCATGGTACATACAACCATAGGATCTGACTTGGCCAGACGTTTAAGACCTTCA
ACAAGCTTGGGGAGATCAGATGCAACTTTGCACTGAACTGCCACACGAACAACAGGCGAG
ACGGAAAACTTCATTGCTCGAATAGGATGAGCGTCCACTTCCTTCTCATTCGTCAGGGTG
GCATTCTTTGTGATGAACTGATCCAAACCAACCATGGCCACTGTGTTACCACAAGGCACA
TCCTCAACAGTTTCCTGCTTCTTTCCCATCCAAATAACAGTCCTTTGTACACTCTTCACA
TACAAATCTTTCTTTTCACCAGGTACATAATTTGGACCCATAATCCTGACCTTCAAACCA
GTTGAGACCTTACCAGAGAACACACGACCAAAAGCAAAAAACCTACCCTTATCAGATGCA
GGAATCATCTTAGATACGTAAAGCATGAGAGGACCATCGGGATCACAGTTCCTGATAGCA
TTAGCATATTGATCATCAAGGGGACCCTCATACAAGTTCTCAACACGGTACTTCTGTGCC
TTGGCAGGAGAGGGAAGGTGAAAGATCATCATTTCAAGTAGTGCAGTACTTGCAGGAAGC
CATGTCTGCATAACTCGCTTCATCAACGCTTTCCCCATCAAGTCTTTTTCCTCTGCCTTC
ATGCTCACATTAAGTTTTTGCAGCATAGGCCAGAGCTTATCTTTCTGATCATTCATGCAA
GTGTTGATGATCTGCTTAATGGGTTCATAGCAAAACTGAACAAAACCACGCTTGCACGTC
GGAGAGCCAGTCTTCTTAGTAGTCCACTTCCTCGTAGCAGGGTCAAAGTAGTTCTCACCC
CAGAGCCTCTCCATCATCTTTGATTCATCAACACCAAATTTGGATGCATACATCTCTGCA
AAGTTTGTCAAAGTAAAAGCCCAACCGTGCAAACCAGCAGAGAAAGCGACTGTTCCTTTC
TCGGGGTAGACCTGAACATCACCAAGAAGTGGATCCTCATATGTAGCCATAATGACATTA
GCATTCTCAATCACTCTCTGGAATGTCTGGTAAGCCTCTTCTCCATCAACCTGAAGCTCG
AGGAAGCACCTGTCCATCTTGTTGACAGTCAAAACAGGCCTAATCCTTTCACCCAGAGCC
TGTCTTAGAACAGTTTCAGTCTGGACACAGACACCCTCAACACAGTCTACCACCACAAGT

GCACCATCAGTAATACGAAGAGCAGCTGTAACTTCAGATGAGAAATCAACATGCCCAGGG
GAATCAATAAGATTGATCAAGTACTCGTTTCCTTGGCGTTCTCCCTTGTAGTTCTTTAAA
GCATCATCACTCATCTCATAGTAGAGAGAGATACCAGTAGACTTGATCGTAATGCCACGC
TCAGCTTCATCAGCACGAGTATCAGTCATACGGACATCNNNNNNNNNNNNNNNNNNNNAC
ATATTACGGATGTTGTGTTTGTAGTCCATAATGCGGCGAAGCTCGTCAGCTGTGAACTTC
ACCATCTTGTCTGCTCTTCAAATATCACCAACAAATATTCTTTCCTTGAAGGCGGCGAGA
GAGAAAGGCGGGACTGGGAATGGTGCGGGCAAGTTGAGTAGATATAGCGAGAGAAGTGAA
AGGGTAAATGCGCC
>ntig_187_consensus
AAATCATGAGATTTTTTAAATGCAAGACCACTGAAACGATTTTTTATTATCGACAGAAAA
TATAACGAAATAGGGGCACTTAGCCTGTGCTGAAATTAACCACAAAGGCGGCATAACCAC
ACAAAGTCGAACAAAGAAGCTCAGACAACTAATCGGCAGACCCATCAATTAACTATAATA
ATGTCCCACAGTTTGACATGTCAGACTAAATTTTCAAAATGCATCAAAAGTCCTTCGACT
GGAACACCAAAACCGCCGCCCCTCTCGTCCAGAACATGTTGCTTTTATCCTTGCGAAGTA
CAACTTCTCTCAGCACTTAAGACACTAAAAAAAAAAAAACACAAACACCTCCTCAACATA
ATGGAACCTAGTTACAAAAACAAATATCCTTCACATTAGGGGCACGATTGTTAATATAGA
GTATCCATATATTAACTTAGGGAATCCATTTGATGTTAGGAACAATAGCTTTGATTTTTT
TTTCAGACAAAAGTTCAAGATATGAAGTATCAAGCAAACTTAAATGATTAGTTATTGGGC
AAGAAACTATCATTTAACTGGTTGCTGGAAGGAAGACTCTAATGAGTTCTTTTGCTGTGA
CTTTTTTCCTACATGTGGGGCATTTGGCCTGCGCTGAAATTGCAGCCCGAATGCATGCCT
TACAGAATATGTGACCACACTTTGTTGACATCTCCTCAACCAATGGGCCCATGCAAATTG
GGCAAGTGAATATAGGCTCCTTAGGTGGTGGCGGTGGCGGTTGAGGTGTTGGTGCATGCT
TCTTAACACTCTCTCTCTACAGATAGAGTATTAAATGAGTCAAAAACTTGGACATCATTT
TGAATTGAACAACTTATTACAGAGTTTCTTAATTTTCAGTGCAATAAATGCATTGATAGC
AATGAAGATAACAGTTCTTACCATAGAGTTGCTAGTGCCTTCC
>ntig_188_consensus
CTATTCATATGCCTTAGTTAAAGCATTGTTTACATTTGAGAATATAGTGATTTGGATTAG
TCTAATTTTGTTAGTTTAATTCATTTTAGTTAATCAAATTCTCAATGTTTAACTTAAAAT
AGCTAATTCCAGAATTCCCAGCCATTCTTGAAATTTTTGCACTTCATTCCCAGCCCAAAC
AAGCTACCATGAATTTCACCATCCTAACAGCAATTTTGTTTACCAAAACATAAAATTGAC
ATGCATAACAAACATCTTGAAATAGAGAAAACTCGTTGTGACTAAAGATGAATATTTGAC
CCAAAAAATTAGGACCATGCCCTACACATGAAGGGGATTCCTTAAATATGCAGGGATGGG
GATATCCATATCCCTGCCCTGCCACATACCGGCTGCCACTATCCAATTTACTACCAATCT
ACTAAAATATATTGTATTTTTAATTACAAAAATACTATTAATATATAAAACTCAACCTCA
GTTCATCTTCTTCCCAAACTATTCAGCATCGAAACCCCTTTATTTTCATCATCCAGACAT
GTTAATACTTGTTAATATATGAGGAAAATGTATGATATTTTAGCTTACTCGACACTCAAC
TTCGTCTATTGGACGGGGTAGGGACAAGGAGGGGGAATGGAATATTCAGCCCGGCCCCAC
CACCCCAGCCTGTTACCATCCCTATTCATGGCTGCATGAGATTGGGCTTCCTTGAAGATC
CTTTAGAACACTCCATATGCAAAACAAATTATTTTTTCTTTTTCAAGTAGTAGTACATCA
CAAACAGAATCTCATTACCTAAGCAGTTCAAACACACCCAGTACATTACAGTTTGCCATC
TAGGGAACAACTGAAGATCATGACACAAGTAAAAAGCATTCTGCAAGCACATAAAATAAC
TATATCCATATTTTAAAACAGAGAAACTCTACAAACCTACTAGATTTAACTGGCTCGTAC
TAGTTCTTTCCCAACCTAGCTCCTTAGGGCTTAATACAATTCTCAGACAATTTTACTCCT
TAATTCAGTTCATGGCTAGCACTGAGACAGCCAAAGAAGAAAAAAGACAAAGGTTGAGCA
CTTTATAAATGGCAGGTATCATATTTGATATGATTGATTTTAAAACAGACGATAGACTGT
GATCAAAGCCTTAAAACATCAGCTACCACACATAAGTTCCATTTGCATAGCAGTCTGTTT
ATCCAAGTTTCAATATTTGTGTATTTCAATTTCTTATAGAAGTCAAACTGTGAATTGTAA
AGATGGATCTCTGGTTCAATACCTCTTGTAAAGCTTCTATTGTTCCAAGAAATTATTACT
TCTGACAAAAGAAGAAAGACACTCCTTTTAACTCATAAAAAAAAACAATGAATCAATAATC
ATAGATACTGAGTCAACCACAATGAAAAAGTGAAGTCACCATGGGAAAAAAGGGTTCCTT
ATTTTCTATATTAGTTTGATAGTACATGTATATTGAGTCGACTGCACCAATAAAGTTCAT
ACATCCCCTTTCAGTTAAGTGTATTACTAAAAGAGTTGTGGATACTTCTGTAAATAAGGG
CAGGCAAATCACTCTCTCTTATTGCACTTTTCTTATTCTTGCACTTCTCCCTTGTTCTCA
TTCATATCAGTATTCACAATTATTGGGAAGAACAACAAATAATTAATTCTTAATTTCTCA
TATCCCTAACTTTTCAGTATATTCTACAATAATTGTGGTATCTGGCTGAAGAACCCTAAC
TCTCAAACCAATGGCAAATGTTATACGGGAAGATATTCATACTCGAGAAGCTAGATATTC
AACAATACAATTCACAGCCATAAATCAATCAAACTAGGATTTTAAGTTATTAATCCTAAC
CAACAGGCCAAATATTACAAGCACAAATTTCATCTTCCTGGCTCTGCAATTGCGGTATAA

AACCAGTATTTCTTCTATAATTTTTCATCACACAACAAGAAGACATGAATAAAGCCAAAA
CAACATAAATTTAGCAAACAGAAGAATATGTATCATGAACAAAACGGCAGAGCTGATGAA
CCAAGAGAATTGCAAATAAATAGCTAGCTTTTGAAATTGTATCAAATCAACAGAAAGAAA
AACTAATAATTGATAATCTATTCATAGCTGTTCACCGCATACGAAAATTGAACTTCTTAA
TCCAAAACAAAGTTATTAAGAAACCTGAAAATTCACCTCATATAACCCGGGATCTTAATA
CGAATAAAGAGCATACTCATAACATAAGCGATAATAAGGGATGAGATCCCAGCAGAGGCA
TAAGAAACCTTGACACTTTTAGCCCGGTTCTTATCAAGAGCCAGATCCAAGAGCACGATC
CCCATCCCACCAAGCACGAACATGAACCCAGACGAAAGACCCTCGATGATGTACTGACCG
TTGACACGTCCTGGAAGGAACACAACTGGCCGAACCGAACCCGTGGTAGGGTCCTGGGTG
GACCCGATGCCTGGAGGCTCCACAATCACATCGTAAACGATCCCAGAAACCACCATGAAG
TAGGTGAGGAGAACGAGGGCGAAGACGGTCATGGGTGAAGGGAGAGTGAAAGAGGGGAGC
TTCAGGCGGAGTCTGGGTGGGCGTAGAATGGTGTAGGGGAGGATCCTCAGGATGTGGAAG
AGAGGGTCCATGGATGTAGAAGAAGGATCTGCTTGTGTAGAGAGGCTCTGTGATTGTTCT
GATTTGGGGGCCATGGCCGTGTTGGGGTTCTAGAGGACTGTTTGCGAAGTAAATAATCCC
AACTCTAATATTTACTTCTAAATCATATTCATCCCTGGTAAG
>ntig_189_consensus
CTTCAATTTACCAAAGATGGTATCATTATTCGTTCAAAACTTTATTTTTCTACCGTAAGA
TTTAACATCCAAATAGTACTTAAGAATAGTTTGGATGACAAATTGTTATAAGACTTGAGC
CGAATTCTTCAACGTTAAAAAAAAAATAAAATAAAATTATGTGAACAAAAATATAATGAC
TTCATCTGAGCCTTCGCCCACTTCACGAACTCCATCCACTGCATATCCCAATTCCCTTGT
GCACAAAAGCTCCTCCTCTGAACAAGCCGCCGGAAAAGTCAATGCATTGTCCTACTAACC
TCAGCCAAACCAAAGTTCCATGTCACGGGAGGAACACCCAACGCTCACCCTGTGCGGCAC
TTAACTCAGTCCGTCGAGCTAAGTAAGCCTGACCCTGGTTTGCGATGCGAGAAATATCGT
TGCGCTCCGGTTGCGATAATCGAACTTAGAAAGGTAGAAATGAGAGAGCAATATCAAAGA
TGCTGGGAAAACTTTCTTTCTCTATACAATTGATGCTACAGGCTTAATCTATACACTTAA
TTCTTACTTTGCTGCAACATGACTTATACATAAGTCTATCTCCCTATTTATAGCCTTTCA
ATGGAGGTGCTTGACCTGCTGTGGATAAGATTTCAGTCAGCTGCCTGTTGCTGATTTGCT
GGGCAAGAGGTGGGCGGCAAGAGGTGGCGCACATGCATGCTAGCATCGCAAGGTAGGTTA
GCAGGGTTCTCAGTAGGAGGTGGCGTACATGCACGTTGACAGGCAAGGTTGGTTGGTAGG
GTTGTAAGCAGGAGGTGGCGTACATGCAGGGCCTCAGCCACCGCCCCTACTCAGCTGCTT
ACTCACTTGGTGCTCACTCAGCTGCTTGCTTGCGTCCCTGGGTGCTCACTCAGCTACCTG
CTTGCTGCGTGTGTGCGTCACCTAGCTGCTCACTCAGCTGCTTGCTTGCTGCCCTGGGTG
CTCATTCAGCTGCCTGCTTGCCGCGTGCATGCGTTACCAAGCTGCTCACTCAGCTGCTTG
CGTGCGTCACCTAGCTGCTCACTCAGCTGCCTGCTTGTTGCGTGAGCTGCTTCCGCACAT
GGCCTGCGTGCGCTGCTAGCTGCTCACGCACCTGTTTACGTGCGCACCCAGCAGCTTACG
CAACTGGTCTACATGGCCAAGCTGCTGCGTCATGCTTATGCGTGCATCCTGCTTGCTGGC
CGCACCTGCGTTCACACCAGCTTGCTGACACTCTTAGGCAGCTGCCTACGCACCCGCATT
CACACCTGGTCCGCCTGCGTTCACACCAGCTCGCTGTCGCACCTGGGCAGCTGCATACGC
ACTTGCGTTCACACTTGGTCCTCCCCTGATTGCGGAGCTACGTGCTTTAGCCTTTGCGGG
AATTTACGGGTTGTGGCACTCTCCCCCGCCTAATTCGTGATGCCCTCATCATGGGTTCAC
AGCTTTTTGCAGACCTCGTTGCTTGGCCGCTTGTGTTGCCATCAGTCTGTCGTGGCTTCC
AAGCTTGCTTTGGCATTCGTCTGTCCCTCTTCGGTTGCACTCCCGAATAGGTTGGCCCCT
GTTGCTTCAATCTTTTGCAGTCTTGTAAAGGCTTTGCGGTCTGTCGGTATTGTGGTAATG
CTGTTCCAGTCTCCCCTTGGTGTGATGGACTACTCCTTCTGAGCATGGTCACCTCCCGCG
TACCTCCGCTGACATCACATCCTAGAACTCCTTGTGCTTCTCCTAAACCTTCCTCCGCAG
TTCAGCTGTGTGCCAAGTCTGGTCCTCCCCCTTGGTGGCCAGAATTGTATCTTCTGCCTC
CTTCAGCTCCGTGCTGAGTCTCATGGCAGAAATCTTCTTGGCGGTGATGGTGCTTTCCCT
GGCTAATGGCACCAATCCTGGGTTCCCCTCCTCCAAGATCATCATGGTGTCAGTGAAAGG
AATTGGTAAAGCTCTGACTTGATCCATGAACTCCATCTCGAGTACAATTGGATAATCATC
CATCGAAACTATTGAGAAATTTAACTTACCTTTCCATTGCCCTATGTGGAT
>ntig_190_consensus
CAGGTATTCTACATCATGAACCACTTTGTACTGTCGGCTTCGTTGACTAAATTCTGTGGA
AATTCTGGTATAACGTCAGATATGATTGCCTGATAAATTTTACTTTTGCTTCTTGGACAG
CTCAGTGGACATCCATCTGCATTTTAGTCAAAAGTACACAGCAATATTTAAATACGTCTT
TGAGGATTCTAAACGTATCTGTCTTTGACTAAAGCCAATAAATGACAGAAGGCAGATGGG
AGCATTTTCATTTCTATATTTTACAAGCCTATATTTTACAAGCAAATATAGTAAGCCCCA
ACTAGAATAGAGCTTACTCAGAGGCATTATTTCAGGACGCAAGATTAAGGTGAGGCCTAC
AAAGAAAATAAAAGAACATAAGAAGGAATTGGATTCTATTAAGAGACGATAACCATAAGA

GCAACTTCAACCATTTGAGATATTGAGTATGGATGGTTAACGGTTGAACTGGTTGGTGTT
TGAGGGAGAATCCTGGAATTTCCCCTTCCAACTTTGACAGATTATTTCTGTATTTCCATC
AAATACAACAAATACAAAGCTGCTGAATATTACAAGCACCTACATATTTTACCCAAAAGA
AAGGAATAACAAGGGGAGGGAAAAAAAAAGGATCTGAGAAACTGTCAACTGCCCTATCAAA
TTCATTGAACAATACAAAAACTAATATACAGGGCGCAGCAAAGAAAGACATCTGCCGCGG
GAGGGCTTCTTTCCAGGCCCTCAAAGAACAACTACTACCTGCCTATGTCCCCATTACTTC
TCCAAGATTCTTCTTCCCGAATAATGTTCCCACTCTGCCCACCATACTGGTCAGCTTTAG
CATAGTGCATTTTAATGGCTGCCACTGGATCTGAAACACCCGCGCATGGTCCACCCACGA
GTATACCTCCCCGTCCTACAACATTCACTCCGACCATCCCTCTTCCTGGCTGCGGAAGAG
TTGAGAGTGCTTCAAACCCATTTCGTCTTTGCATCTCGGAGAATAACAAGTCCTGCCTCT
GCTTTACATCTACATTAAATGGCTGTACTTTACTAATATCACGACTCCTATACACCTGAT
AGTCCACCACCCCATTACTGCTACTGATTTCCCTTTGCGGAACAACTGCTACACCATTCA
AATTGCGCTCACTACTCTTGACTTGCAAAGCCTGCTGCTCCATTTTCAATGAGGCGGCAG
GATAATTGCCGAAGGCAGCAGGATACTTGGCAAGCAAAATGGTCGAATCAGGCAAAGATG
AAAAACCAGTTTGTATTCTGTTCCCATCCCAAAATCCATAACTCCTCACAGGAACATTCT
CCAGACCTAGGTAGTCATTTCGATCAAACTTCAAAATCTCAGCAGGGTGAGCGGCGGTGA
ATTTCAAATTAGATACCTTGCAGCTTTGTTTATGATGACGGGCCCCCTTTTCTTCGTTCT
CATGATTGACGGAATTTGGCTTCTGAGAAGATGAGGGATGGCTAAGTATCTTGCCAAACA
ATTTAACATCACCATTCTTGCATGGTCTTTCCGTGTCAGACAAACTTCGTGAATGGGATC
TTGGAGGATCATTCTTTTGGTCTAGGTTTGGAGAGAGGAGTGGAAGATCGCAAACTGATG
CGTGAGACATTGAACTACTACATTTCTGAATGTAGCAATCCTGAGCCATATAGTGCACCC
CATTACTCCTGTCTGACTTAGAAATGCCGTGAACTTCGGAAAGCTGTCTGCAACTAGTAT
CACCATTCATCTCTTTCTTTGCAGAAATCTGCAATGGATAACCCCTGAGAATCTGGGAGT
GTTCAACATGGTTCAATGCAGAATGCCCCAACAGATTATGGTGATAGTCTTCACTGCTAA
CAGATATACAAGCATTTTTATCTTTATTCCCCTGAAAGTCAAATGTAGTCGACAGCCTGT
TTCGTTTAACTGTTTTTTCGCATTGACTGGCCACAGAAACTTGTGGCACAGAAGTTACAG
AGGCAAGAGTTTTCTCCAGTGGCAACAACGTTACTGGAAGCATGCCCCCACCATCTGACT
TCAGAGGAACAGAACAAGAACTTGTATCTACAGTCAGATCAACAATATTCCCACATGCAT
TTGAACCTCGCACCATATCCCTGCCATCATTTTTATCAGCTCTATTATGGTCAATTCGAT
GCTCCAAACCATTTTCACATCCCTTAACAGGAGCCTCAGTAGTCTCAACTGCAATATTTA
AACCTACCGGGCATGATTTCATGGCTTCTCCACTAGTGACTGTCTCTGCCAATTTATCTT
TTCCTCCTTCCTTCTCAACAAATACAACTGGAATTTTATGGGCCTGCAGTGACTCGGACT
GGCAGTCAACATCATCCATTTTGTAACTTTCCAATTCAGAAACCACCTCAGGATTATGCA
GTACCTTACATTCATCAGAAACCAAAGGACTGACATCCTTCGAATCTTGATCATCAAGAA
GTCTCACCCCATCGTCATCCCCTGATTTATTCAGGTCAGTTTGCAAGTTCATGGTTCCCA
AATCACAATTGTCCTGATTTTTGTTGACCCCAGAAGATGGCAAGTCATCCACTTTTGAGT
CCACCTTATCATTGAAGACCGAGCTAGTCTCCAAGACACATGCATCTTCTGTGTCACTCC
CTCCACCATTGACATCATCACTTACAGAGGTTCCTGCAGTTGCACGTCCAGTATGTATTA
AATCCAGTCCGAGGCACTTCCGTGCCTTGCTAAAAAAAACCTTGCATTGATCTCTAGACC
TTGTTCTAACACATCGTGAGATCATGACAAAATCCTTACCATAAGATGACACTGCCTGCA
TAAAGATGGATTTCTCCTCATCTGTCCAGTCAGCAGGATCCATTTCCCCACAACTCTCAT
CAGAGCAAGTGTCATCATCAACATTCTGAGTAACATCAGAAGTTGAATGTCTCCTAAAAG
AATCCATTTTCTGATGCTTCCATTCCCGTTGGCCCTCTCCAGGATCAACAGAACTTGTGA
TGCAAGAACTCATAGCCTCAGACGACAAGGAACCACATATTCCCGCTAAAACATCTGCAG
CAGCTGTTTCTCTCTCATTCCCATGTACATCAACACTGCTTGACCTCTCTGTCATGCCAT
CATCACCAAAAGAAAATTTTGAATCACTGCACCCTCCCAAGGGGATTCTACCAGACATCT
GTCGTGCATTGTCAGCTTGGAATGCAGCTGCCATCTCAGTAGCTTCACCCAAAATATCAA
GGGACGCTGCGTTCATCTGCCGATCCCATTTTTTGCCTGGTCTTATCAAGTAGGTATTAG
AACATGACTTCACTGGTTCAGGCCTTTTCTTTATTTTCACGAAGCAATCGGACTTGTGGT
TTTTATAGTAGAATTCAACACAATCAGCAGTTGTCTTATGATCAAGAAATGAAGCAATTT
TCCTAAAATCTTTCCCAAAGGTGGCCAATTTATCCATGAAAATTTCTCTCTCTTCCACAG
TCCAGGGATTAATCATAGCTCTTTCCTTCTCCACAGCACATGGATCTTCAACCAATCCAT
TACTAGAGACAAACTTAGACGCCATCTTTTCCTTTTTGTCCAATATCAATGCTGGCATCT
TCAAAGAATCCCTATAAACCTTGATCTGGGAATCTGAAAGCAGTTTGCTAGTGAAATTAA
TTATCTCCGTGGTTGGTACAAGGCTTAAATTTCCTGTTGCAGGAGAAGAAAATCGAGAGC
GAATAGAAGATCGGTGCTTCTGATATCCACTATGTGTTATCCTTGAGATTAATTGACATT
TCTTTTGAGACATCACACGATGTTTCCTTATCGAAAGCAATCGCAGATCTTCCTTCCAAA
GATGTTGAAAGGCTTTAAACTTAAGGGTCAATACTCTCTCCTTAAATCTTAAAAACTGCT

TCCTCTTTACAAATTTTTCTTTGACAGAGGAGTTGTCCTGACAACGCAAGACACTGGTAA
CTCCCCAAAAATCAACATTACAGTGATCTCTTGGCAATAACTTATTAAATGCTTCAGAAG
CCCTATCAGCATGTTCTTTGTTGCAACCCAAAATAATATCACACAAATTTTTTCCATCAG
CAAACAGATTCAAATCATCAGAAACAGCTGCAGCACCTTCATTCTCTGGAAGGATATTAG
CATTCCTGCAAGCAGTGCTACCAGTCACCTCTTCCTTACAGGAGACAGGAGTTGTACATT
TAAGTTCAACATTTTCCGACTGAATGGCATCCAAATCTCCAGATTCACCATTTTTAACCA
TGTAAGACAGAATGTCAGCCTTTAACTCAGTCAACGGCTCAACAAATTTAGAAGTAGCAG
TTCCAGGACTATCTACATCTTCATCTTTAACATTAGCATGAGCTCCTTCCAAGACATGAC
TACAATCAGGCATCTTATCCAACAAACCTTCACCACAAGAATCAACTTGCAAAGGAGCAG
GTCGGGCAATCAAATCGGAGGCAGTCCCCTGCTCCTTTAAATTATCTGGATTTCCCTCCA
CTGACAAAGAACTGGATGTTACAGGACAAGGACATCTACTTCCCGATTCAGACTTCAGTG
ATTTAAGTTCAATTTCAAGTGAATCGATTGCAGACTCGGTCACCTCCAGGGCCTTCAAAA
TATTGCCTTTCAATATAAGCAACTTATTCATTGCAGTAGATGGAACAAAACTAGAATCTA
AAAAACTAGAATCATCAGGATGAAGCATTTCCACAAGTGAAGAGCCTAAATTAGTTATTG
AATTGCTATCCAACTTCTCTAGATTAAATAAAAATCCCTCAAGATGACTCTGAGACCCAA
TACTGGGGGAGCCACATAAATTACTGTTATCATTATCAACACTCATTGCCTTGCTGAACG
TTTTCTCCTCTACACCTGATGACGAACTGCAAGCAACAGAGGATGGAGTTGTAGGTGATG
CACAATCTGACAATCCTATGACTCTAGGGCTTTTCTCGGCCAAGTTTGAAGAGAGAGAAT
GGGCAGGTTCTATATTACTAGTCATAACAACAGCCCCATCTTTGTTCACACTCACATCAG
GACCTTCAACTTTCTTTTTCTCATATTTTGCCAATCCCTCACCCCATCCGAGCCGTGGCT
TCTTCCTAGAAGTAGTCTCCTCAGATTGTGTAGCAGAAGTCACATAGGTTGCTGCATCCC
CTGATGGAGACCGGACAGGAGTTGCATTTTTTTGCTGTAACTCCGCCTTCCCTTCATTAG
AATCTATGCCACCCAGGCTCTTTGAGCTGCTTGAATGACTGAACCCTGAACCCCGAGAAG
ACAAACTTCCAGAACGAGTCCACTTAAGTGGCTTCCAATCCAGTGAATTATCCCTCTCAC
ATCTTTGGCCTGTGCCAAACCCATTGACACCACCCATTTTATTATCATTTTGATCTTTCA
ACTGAAGCTGATCCCATGTGTTTACAAAGTCAGCTTGAGGATGAGATGGATATGAAATCA
TATCATCAACTGACTTATGATCATTACTCAGGTCTTGAAGCCTCCCAGGACCATTGGATG
ACACATTGCTTGTTTCCCATGAATGCCCTTTCCAGTCTTTCTGACTAAGAGAACCTTTAT
TCTCCCTACCATTCCTGCCATATTTCCCATCTCCACGCGAAACAGACATGCGGGTGCTCT
CATCTTCAAACATCTTGTCACCAGTTCGAGAAGGCACATAGCCATGACAAGATTCTTCAC
CAAACGAGTACCAGCCACACTGCTTACCATGACCTGGTGGTCTGCGATAGTCGACAGACC
CCAACCGGGGGAGATCGCGACAGTGTGAAGAGGACGAGGAGTAATCTCTCCATCTGGTCA
GGGACCCGCCGCCACCTCCTAGCAAGGAGTCTGACGCCGTCCTCTCGTGGTGGTTATTAT
TGTTATTGTGCTTCCTCTCCTTGAAGAAGTCTTTCCGATCCCAAGGCAGTGGTTCTGGCG
GCATGAAATTATAATTCCACTCAAACGCATCGGCAGGAAACACTAATAATCTAAGATTTT
CAAAAGCTGATTAATGGCCAGATCGTCGAAAAACTTGAGAGGGCAGCAGCACAGAACGAA
CTGATATTCTCAAGATCGAACTAGAATCGATAGAAACTGCTCACGAACTGTAAGCTCTAA
ACCACGGAAAACCTAAGGTTCTGAGAGGGTGAAAGCAGACCTAAATGATAGCAAGAACCT
GATTTCAAACTAAGCCGTGATAAAGCGGCTGATGTCTGCGGTTTGAGAATTGGATCCACC
ACGCCAGACGATGATAGTTTTCTAGAGCTTCAACACAACACCTCTGTGTCTTGTAGTAAT
AGAGGGGAGGATTTTATCGCCAATTTGCTCTGCCTTGTGGCGTTTGGAAGAAAGAGATGT
GAAAACGTTAGACGATAAGGGACAAAGA
>ntig_191_consensus
CTCTGTTTTACAGGCAAAAGCAATCATCAATATCCAATTATTGGCCGTGCCTGGACGTTT
TGATAGAAGCCCTCGTTGTGGTACATTAGGCTTACAGGAATCGTTAGTTGGCGAATCAGC
AGGTGTTGGGACTTGGAATTGCCCCTTCTAACTTTGACAGATTATTTCTGTATTCCCCAA
ATACAACACATACAAGGCTGCTGCGAGCGGAGGATATTACAAGCACCTATAATATTTTGC
CCGAAAAAGAAAAAAGATCTGAGAAACTGTCGGCTGCCCTACCATTTTCATTGAACAATA
CAAAAACTATTATACAGGGTGCAGCAAAGAAAATCTTTTGCTGTCTGCAGAGGGCTTCTT
TCCGGAGCCCTCAAAGAACCAACTACTACCTTCCTAAGTCCCCTCCATTACTTTCTCCAA
GATTCTTCTTCCTGAACAATGCTACCACTCTGCCCACCATACTGATGGTCAGCTTTAGCA
TAGTGCATTTTAATGGCTGCCACTGGATCTGAAACACCACCTAACAGTATCCCCCCTCCT
CTTCCTACAACATTCACTCCAACCATCCCCCTTCCAGGCTGTGGAAGACTCGAGAGGCCT
TCAAATCCATTTTGTCTTTGCATCTCAGAGAATAACAACTCCTGCCTCTGATTTACATCT
ACGGTAAATGGCTGTACTTTACTATTATCACGACTCCTATACACCTGATAGTCCACTACT
CCATTACTGCTGCTAATTTCCCTTGAAGGAACAACTGCTACACTGTTCAAATTGCACTCA
CTACTCTTGACTTGCAGAGCCTGCTGCTCCATTTTGGATGAGGAGGCAGGATAATTGCCC
AAGGCTGCAGGATACTTGGCCAGCAAAATAGCTGAATCAGGCAAAGATGAAAAACCAGTT

TGTATTCTTCTCCCGTCCCAAAATCCATAACTCTTCACAGGAACATTCTCCAGACCTGGA
TAGTTATTACGATCAAACTTCAACATCTCAGTAGGGCGAGGAGCAGTTAATTTCAAATTT
GATACCTTGCAGCTTGGTTTATGATGATGAGCCCCCTTTTCTTCAGTCTCATGACTACCA
AAATTTTGCTTTTGTGAAGATGAGGGATGACTAAGTATCTTACCAAATAATTTTACATCA
CCAATCTTGCACGGTCTATCCATTTCTGGTAACCCCTGTGAATAGGTTCTTGGAGGATCA
TTCTTCTGGTCTAAGTTTGGCAACAGCAGCGGAAGCTCAGGAACTGCTGCATGAGATGTT
GAACTAGTGCACTTCTGAACGTAGCAATCCTGAGCTATGTTCACACTGTTACTCCTGTCT
GGTTTAGAAGCACCTTTATCTTGGGACACTTGTCTGCAGACAATATTGCCGTTCATCTCT
TTCTTTGCTGAAATTTGCAACGGGTAACCCTTGAGAATCTGGGAGTGTTCAACATGGTGC
AGCATGGAATGCCCCATAAGGTTATGCTGATAGTCTTCACTGCCAACAGATATATGAGCA
TTCTTATCTTTATTTCCCTGAAAGTCAAGTGCAGTAGACAATCCATTTTGTTCAACTGTT
TTTTCAGGTAGACTGGCAGCAGGATCATGTGTAATACAAGTTACAGGAGCAAGAGAGTTC
TCTGGTGGCAATGGCATTCCAGGAAGTTTATTCCCATTTTCCAACTTTAGAGGAACAGAA
CAAGAACTTGCATCCACAGCCAGATCAAGAATATCCCCAACTACATTTGAACATCTCATC
ATATCTCTGTCACTACTATCGGCTGAGCATGTTTTATTATGCTCATTTCGTTGTTCCAAA
CCATTTTCAAATCCCTCAACAGGAGCCTCAGTAGTCTCAAATACAACATTTAAACCTGGT
GGGCATGATTTCAAGGCTTCTTTAATGGTTGCTGGTACTGGCACACAAACAGCTTGTTCT
GTCAATTTATCCTTTTCACCTTCCTTTTCAAGCAATAAAACAGAAATTTTATGGGCCTGC
AGTGCCTTGGACGGGCTATCAACATCATCCATTATCTTTCTTTCCAATTCAGAAACTACC
TGTGGATTTTGCAGTATCTTACATTCATCAGAAGCCAAAGGAGTTACATCCTTGGATCCT
GGGTCATCAAGATGTCTCACCCCATTGCCGTCCTCTGTTTTATTCAGGTCACTTTGCAAG
CTTATGGCTTCCGTACTGGTTTTTTCCTGATTTTTGTTAACCACAGAGGATGGCAAGTCA
TTCATTTGAGAGTCCACCTTATCACTGCAGATAACCGAGCTTGCCTCTAAGACACATGCA
TCTTCACTGTCACTTTCTCCACCATTGTCATCATCACTTACAGAGGTTCCCGCAATTCCA
CATCCAGTAGGTATCAAATCCAGTCCAAGGCACTTCCGTGCCTTGCTAAAGAAAACTTTG
CATTGATCCCTGGACCGCGTTCTAACACATCGCGAGATCATGACAAAATCCTTACCATAG
GATGACAAAGCCTGTATAAAGATGGATTTCTCTTCATCTGTCCAATCGGCAGGATCCATT
TCCCCACAACTGTCATCCGAGCAAGTGTCATCATCAATATTCTGAGTAACATCAGATGTT
GACTGCCGCTTCCTCACAGAATCCATTTTTAGGTGCATCCCCTCCCATTGGCCCTCCCCA
GGTTCAACAGAACTTGTGATGCAAGAACTTATTGCCTCAGAAGACAGGGAACCACATATT
CCCGCTAAAACATCCGCAGCAGCAGTTTCCCTTTCGTTCCCATGCACGTCAAAACTACTT
GACCTTTCTGTCATGCCATCATCACCCAAAGATAACTTTGAATCACTACGCCCTCCCAAA
GAGATTCTACCACAACACATCTGACGAACATTGTTGGCTTGGAATGCAGCTGCCATCTCA
GAAGCTTCACCCAATATATCAAGGGATGCTGCATTCATCTGCCGATCCCATTTTTTGCCT
GATGCAATTAAGTAGGTATTAGAGCACTGCTTCAGTTGTTGAGGCCTTTTCTTTATTTTC
TCAAAACAATCAGACTTGTGGTTTTTATAGTAGAACTCAACACAATCAGCAGTCGTCTTG
TGATCAAGAAATGAAGCAATTTTCCTGAAATCTTTCCCAAAAGTGGCTAATTTATCCATG
AAAATTTCTCTCTCTTCCACAGTCCAAGGATTGATCATAGCTCTTTCCTTCTCCACAGCA
CAAGGATCTTCAACCAATCCATTACTAGAGATAAACCTTGACATCATCTTCTCCTTCTTG
TCCAATATCAAGGCTGGCATCTTCAAAGAATCCCTATAAACCTTGATCTGAGAATCTGAA
AGCAGCTTGCTAGTGAAATTAATTATCTCTATGGTTGGAACAAGGCTTAGGTTTCCCGTT
GCAGGAGAAGAAAATCGAGAGCGTATAGAAGACCGGTGCTTCTGATAGCCACTATGGGTT
GTCCTCAAGCTTAATTCACATTTCTTTTGAGACTTAACACTGTATTTCCTTATTGAAAGC
AAGCGCATATCTTCCTTCCAAAGATGTTGAAATGCTTTAAACTTAAGAGTCAATACTCTC
TCCTTAAATCTTAAAAGCTGCTTCCTCTTTACAAACTTTTCTTTGACTAATGAGTTATCC
TGACAACACAAGACATTGACAACTTCCCAAACATCAACATTACAGTTATCTCTTGGCAAT
AGCTTATTAAACTCTTCAGAAGCCCTAGTAGCATATTTTCTATTGCAACCCAAAATTATA
TCGCACAAATTTTCTCCATCAGCACGTAGACTCAAATCACCAGAGAAAAGTGCACCAACT
TCATTCTCTGTAATTGTAATGCCATTTCTGCCAGTGGACCCACCCGTTACCTCTTCCTTA
GGCTGTGTCCATTTATGTTCAACATTTTCTGACTTAATAGAAACTAAATCTCCAGAACAT
TCACCATTTTTAACCATGTCAGATACAGACTCTAACTTCATCTTGACTGATGGTTCAACA
AATTTAGAAGTAGCAGTTCCAGGACTATCTACATCCTCATCTTTACCATTAGCATGAGCT
TCTTCCAAGACACGACCACAACCAGGGATCTCACCCAACAAACCTTCAACGCAAGAATCA
ATTTGTAAAAGTGCAGGCCGTGCAATTGAATTGGAGGCAGTCCCCCGTTCCTTTAAATTA
TCTGTGTTAGCCTCTACCGACAAAGAACTAGATGTTGCAGGATAAGGACATCTGATTCCC
AATTCAGATTTCAATGACTTAAGTTCAGTTTCCAGTGAATCAATTTTGGACTCAGTCATC
TCCAGCGTCTTCAAAATATCACCTTTCAATATAAGCAACTTATTCATTGCAGTAGATCTA
ACAAAACTAGAATCTAAAAAAGTAGGATCATCAGAATGGGGCAATTCCATAAGTGAAGCA

CCTAAATTAGAAATTGAACTGCCATCCAACTTCTCTAAATTAAATAAAAATCCCTCAAGA
TGATTCTGCAACCCAATACTGGGTGAGCCACATAAATTACTGTTGTCATTATCAACACTC
GCTGCCTTGCTATACACTTTCTCCTCTATACCTGACGAAGAACTGCAAGCAACAGAGGAT
GGAGTTGCAGGTGATGCACAGTCTGACAAGCCCATAACTCTAGGACTTTTTTCAGTATAG
TTTGAAGAGAGAGAATGAGCAGGTTCTGCGTTACTAGTCAAAACAACAGCTCCATCTTTG
TTCCCACTCACATCAGGACCTTCAACTTTCTTTTTCTCATACTTTGCCAATCCCTCACCC
CATCCGAGCCGAGGCTTCTTCCTAGAAGTAATCTCCTCAGATGCTGTGGCAGACATCAGA
CATGTTACTGCATCTCCAGAAGGAGACTGGACTGGAGTTGCATTTTTCGGCTGTAACTCA
GTCTTCCCTTCATTGGAATCTACACCACCCAGGCTCTTTGAACTGCTTGAATGGCTGAAC
CCCAAACCCCGGGAAGACAAACTTCCAGATCGAGTCCACTTAAGTGGCTTCCAATCCAGT
GAATTCTCTCTCACACATCTTTGGCCAGTGCCCAACCCATTGACACCACCCATTTTATTT
TCATGCTGATCTTTCAACTGAGGGTGATCCCATGTGTTTACAAAGTCAGCTTGAGGATGA
GATGGATATGTTAGCATATCATCAACTGACCTCTGATCATTGCTTAGGTCTTGTAGCCTT
TCAGGAATGCTGGATGACCCATTGCTCGCTTCCAATGAATGCCCTTTCCAATCTTTCTGA
TTAAAAGAACCTTTATTCTCCCTAGTATTCCGTCCATAATTCCCATCTCCACGCAAAAAA
GAAAAACGAGTACTCTCATCTTCCAGCATCTTGTCACCAGTTCGAGAAGGCACAAAACCA
TGACAAGATTCTTCGGCAATCGAGTACCAGCCTCCCTGCTTACCATAACCTGGAGGTCTG
CGATAGTCGACAGATCCCCACCGAGGGGGCTCTCGACTGTGAGAAGAGGACGAGGAGTAA
TCTCTCCATCTGGTCAGGGGCCCGCTGCCATCTCCTAGCAAGGAGTCAGACGGTGTCCTA
TCGTGGTGGTTATTATTGTTATGGTGCTTCCTCTCTTTGAAGAAGTCTTTCCGATCCCAA
GGCAGTGGTTCTGGCGGCATGAAATTACGACTCCACCCAATCGCATCGGCAGGAAACACC
AATTCCCCACTACTAGCGTAACCAAACACTGGTATTCTCAAGATCGAAGAAGAACCGACA
GAAACTGCTCAGAAATGTAATCTCTGAACCGGAGAAAAACCTACGGTTCTGGGAGAGTTA
AGACCTGCCAAAACTGATAGCAGTAGCCTCATTTCAAATGAAGCCACGATAAAGTGGCTG
ATGCCTACGGCTTGAGAATCAAATCCGCTCCTTCAGATGATAATAGTTTTCTAGGGTTTC
AAGGCACACCTCTGTGTATTGCAGTTATAGATGGAGGTGGATTTTCTCGCGAATTTTCCC
TGCCTTGTGGTGTTTGGAAGAAAAAGAAAGTGAAAATGCTAGCCGATAAGGAATGAAGAG
CGCCTTCAGAGAGA
>ntig_192_consensus
CTTCATATTAACTAATACACTTCCCCAACCTTGTTTTGAAGATTAAAACAACACCCCTAC
ACTTAATCAAGCGTGACAATTTGTGGCAGATGATATACAATTTCTCTGATTTATTCGTTA
AAATTTTATACCATTCAACTTTCTTTGACGGAATAAAGAAAACCCTTTCATTATTCTTT
TGGGATTTTCTATCTGAACAGAGTAAGAAAAAGTAAACTAGATTATGTAGGAACATGGCA
TGTGTTCATAGCAAACAAACCACACAGTTTGAACTAATACAACCACCCAAACCAAAATAC
AGAACAAAGTTTCAGCCTTTTTATTATAGACATAGACACTAACTTCATGCTGCTTGGAGC
CCAATGACACCGCAAGCTACTCTACCACCAGCATTTCCAGTGGATTTGCTCAGTTCATGC
CCACCCTTGCCAAGATCATCAGGATCTGCATGGACAACAATAGCCCTTCCGACAATTGAA
TTTGGTCCAGTAAGAGGAATCTGCTTGTCGACCACCGTGAAGCTAACTGTGCCATCGTCA
CCAGCATTGACGTTTCCTAGATCACCAGCATGACGATTATCATCCTCTGGGGCGCCATGT
TCTTTGCCAGCAGGGTTAAAATGAGGTCCAGTTGACATACAACCATTTGTGGTGTCACCA
AGGGCATGAACATGGAATCCATGAAGCCCAGGCTTAAGACCAGAAAGGGTTCCAGTTACA
GTAGTTGGACCATCTTCTTCCTGGGCAAAATAGACTGTTCCCTTGACACCCTCGCTGCTG
TTAAGTACGGCCACAGCTTTCACCATTGTTCTACGTGATCTCAGAGCACCCCCAACAGTG
AGTTGTTGGCCGATGGGGATTCTGTACCGTTTTATACAACAAGAGTGAGTGTGCCACGTG
TAGCGGTGGAGGTCTTTTATCTGTTCCAAATGGAGCCCTTCTAGCCTGCCT
>ntig_193_consensus
CTTAAATAACACCTTACCTCAATATCCTTGATTCTGAAATTGTTTTCACCTGTTCCAATG
GAAAGCTACAAAATTAAAATTTGATTAATAAAGCACTTGAACTGTATTGTAAAGAATCTG
AGGTATCAATGCCCGAAACCTTTACCCCTCAAAAGACTACTATGACTTGACCAAATTTTG
TCATTACTTGGTTTTACATGCTACCCTTGATCTTAGCGAAAAGATGGAGTGGTATAAATG
ACTTCTCTTTAAATGTCTTAAATTTAATTAAACCTTCCCTAGTTTCAGCATCTCTGCGCA
AATAATTCACAAGTAATTAAATCATTCATGGATGCAGCAGACTTCCCAGCACCTATGACC
GGAACAAGGTTAAACATGGTTTCCCCATCACTTTTAAGCCTCTGATCCATTTCGGCGAGT
TGATTGAATGCACTGGTGAAGATGGCCAACAGTGTCTCCATGGACATGCCAAGGCTTGTT
GGGAAGTAGATTCTCAAGTTCATTACATCTCCCCAAGAGAATTTGTTTGCCACGATTGTT
TTATTGAGAAGACAAACACAAAACTGTGATACTCTTTCCATTTGTCCTTCCGTCAGAGAA
GTTAGATGAGTCACTTTATGACTGGCACCTTGAGCCTCGGAACAGATATTGCCAGCAACT
TCACCAGTGATACTTAAAAACACAGCACATATCTTTTCACCAATGACACATTTCTGAAGG

CAAGAATCATGCCAATCTGCAAGCTGGAAACCCCAATAATTAGGTGTGTTCATACAATAT
GGGTCTTGCACTGTTTCACTCTCTGTTTCCAAACCCTCCGTGACATGAAGAATGGGTTTT
ATTTCTACAAGTGCTCTTTTTGGTAGGTCAGAGACAAGGACATATAGGAAAATGGGATCG
AGTACTTTAGACATCCTTCCTTCTAAATAAGAGACTCTCAATTGCTTTAGAAAACTGTCC
AGCTTGTCCTGTACTTTTTGTCTCTCAGATGATGCAACACGAGCTGAACAGTAAATAACA
AAGAAAATTGTTGATGCAGATATTGAACAATTAAAGCTTTTTGCCACAGCTTCACTGTTT
CGTAGTGCTTGCTCCAACTCTGCAGCAGGGCCTCCATCACAGAGATTCATGGTGGGAGGA
TCAAGGCCTAACTGTCCAGCCATGTGAAGTACTTCCTTATGCAAGGTCGCCTGACTATAT
GGTCCAATGCAACTCGGTGCCCAGCAGGATATACTTTGTACATGCAAAACCCTTTTGCAT
TGATCCTTTCCCACTAGAACTTCAATGTATGCCTTTCCAAAGCCAGATTCCAACAAAGGC
AATTCAATTGTACTGCGGGACGGAACACCAAATGGGCACCTCTCAACAAAGAGTGGACAA
TCGAGTGTCAATGTTTCATATTCCCCTCCCTCTCCACAGACATTAATCCCATACAAATCT
TTCAACTTATGCAAATAGGAATCCAGGAACGCTATTTCTTTGCCCAAGTGTTTTGCAGGG
TCCAAACCCATAGCTGCAACCTTTACTGTGATAGCCTTAATACCACTGGTTATCATTTCA
TGAAGGAGAACTAACTGATCTTGCTTCCACAAGAATGCCAGAGAAACTAATCCTAACCTA
GAACAAACACTCTCCACACATAGCCTTTGATAATCTGAAGCAATAGCACCTGAATATACT
ACTGTGACAGATGGTATTCGTCTTTTCACTTCATTCAACAAAATAAACATATCTTCAACT
TCATCTCCTGGCGTCAATTTGTAGGTAAGCTTTGATCCCAAAAAAATTCCCAAATAAAACT
GGCTTGGCTTGTGGAGCCTTTTATCCTTCTTCTGAACAATGGCAACCCCTATGCACTCAGC
ATAGCCCACAGTTATTTGGTGTCCAACAGTTTGATACATGTAGCTGTCCAACTCATCCAC
CGAATCATCAGACGGTAACAAATTCGCCAAAGCAACAACCTCGTGGCCATATTCGATGCA
TTTCATCATGGCGTAACAGCTGTCCTTGCCGCCGCTGACTAACGCCACGACCTTCATCTT
TCTTCCGACACTTAACTGCGCCTCGGCCTTTGGTTTAGGGTTACTGCTATCTCACTGGAG
ACTGA
>ntig_194_consensus
CATAAAATGAAAAGGGGAAAAATAAGCATCTTACAGCCCCCAGAACCAAAATCCTGACAT
TATATAATGGCCTTCATTAACTGAAAATTTGCAACAATAAATCGCCATGACACCAAATCT
GGCATGGCATCACTGGGTTTCAGATTGCGAGCCCATATATCCAAACATCTTATAAAATTA
TGACACTAGACCTATAAAACGGGACTCAAGCTAGAGACAAGGAAAAAGCACAGATAATCA
CACTTGCCAACGTCAAAATTCCTGATCCCATACTAATAATTAAGACAAAATCTAATCAGA
GGAGATCAGCCACGTTTGATAGCAGCTCCTCAATGATCACATTATAGAACTTCGGGATGT
CAAATAGCATCATCCTCTCATCGTCCCTAGTGATAAAATTGATTGCAACACCTTTTCTCC
CAAATCTGCCACTTCGGCCTATACGGTGAAGATAGTTCTCCGGCTGTGTAGGCAAGTCAT
AGTTTATTACCAGGGACACTTGCTGTACATCGATACCAAGAGCCAAAAGATCAGTGGTGA
TCAGAACACAAGATTACCCAGACCGGAATTCACGCATGATGATATCTCTTGTATTCTGGT
CCATGTCTCCATGGGTGGCAGAGAAAGTGTGATCACGGCTCCGCATTTTGTCGGTCAACC
AGTCCACCTTGCGTCGGGTATTCACAAAAATGACACTCTGGGTGATGGCTAAGGTTTCAT
AAAGATCACAGAGTGTCTCAAGCTTCCACTCATCTTTTTCAACATTAACATAAAATTGCT
TTATACCTTCCAAAGTAAGCTCATCACGCTTAACTAGAATTTGCACTGGTTTGTTCATAA
ACTTCCTGGTAATTTCGAGGGCCTCAGGTGGCATGGTAGCAGAGAAGACTCCAACCTGAA
CTTTCGATGGCAGAAGCTGAAAATATCATAGATCTATCTGATCCTTGAAACCTCTGGAAA
GCATTTCATCAGCCTCATCCAACACAAACATCTTGATGTAGTCAGGGCGAAGTGACTGCC
TCCACAGCATATCAAACACACGACCAGGAGTGCCCATGACAACATGCACACCATTTGATA
GAATGCGTTGATCTTCACAAACACTGGTTCCACCAACACAAGCATGTACCCTGACACCAA
GGTAGTCTCCCAATGCTCGCATAACCTTTTCAATCTGCTGTGCAAGCTCACGAGTGGGTG
CCAAAACCAAGGCCTCTTTGTTGTATTGCCGACGGTTTTTCAAAACCCACAAGAAGGAAC
ATTGCCATACGCGTAAATGTCTCTCAGAAGGTTCTCTTGCAGTCCCATACTATCAAATGT
ATCATGTACCTCATCATAAGAGGTAAAGAAATCTTGTCCATAAGCTGTAAGCAAGTCATT
CATCTTAGCATCAAACTGACGTGCATCAAATTGCGATCCTTCGGCTGCCAATCCTGCCAT
GACTGGAGATGATCGAAGACAAAAAGAATCGTGAAGAGTTTTGTTAAATTTAGACGTCAT
TAGGGTTTTCCCAAACTCCTCGGATCTATGCCGTGGAGTTAAGGAACGACTGTGCTTGAG
GGAGGGTGAACGAGAGCAAGTGCAAGTTTATATTTATTTATTTTAGTCTGCG
>ntig_195_consensus
GTTAATCTGGAAAAATAAATTTGTATATGAAAAAGCATCAATGAGTCACAACTGGAAACA
CCACAAAAGTATTCTATGTTCAACGAGTAACAAGCGCATTTCCAAGAAAGGGAAAATTTA
AATAGGATCTAATTACCTATAAAACACTGAATCTGTCCCAAATCTTATTCATATCCAAAA
CTTGAATCACCATCCTCTGATTGCATGTTAGATTTAACGGGTGGGTTTCTTTTTCCCAGG
AATTGCAACCCTCTTACCCTTCCGGGTCCTGGCATTTGTGCTGGTTCTCTGTCCTCTGCA

AGGCAAGCCTTGTTGATGTCTTATCCCTCGGTAGCATTGTATGTCTACCAATCTTCCTAT
ATCGTTTTTTATTAATCGCCTCAAATCTTCTCCAGTCAGGTATCTTGAAACTTCTTCACG
AAGATTGTTGAGCTCCACCCCAGTTAAATCCTTAGTAAGTTTGTTCCCTATGCTGAGGTC
ACAAAGGATTTGGCGAGCTCTTGAGAGACCTACTCCATAAATGTGCTGAAGTGAGATTTC
AAGTCTCTTGTCGTTTGGGATCTCTGTGTTCCCCACTCGAAGGCATTGAAAGCGTAGACC
ACGAGATGAAACAGTCTGAAGGAGTCGCTGACTGATATTAGAAAGTAGCCCGACTGAATT
CTGTAAACCAATCATTGTAAATATCCAGTAATGTGGCGGTGGTTCCCGGCGGCGACGGCT
AAGAGGGCTGAACAGAAGTGGCAGCAAGTGAATTGGAGTGAAGACTGAATAAGAGGGTAA
GTCGAGA
>ntig_196_consensus
GTGCAACATCCAATCCCCAAGCTCAATTTATTAATTCTAGTTTATTTTATTCAACTGTAT
TCACATAAAAGCAAGTGAACTTAAAGCGTATGAGGCTTACAGATTAAACATGACCTTAAT
GGCAGCACCACCACCAGCACTAGTTTCAAAGGCCTCTTCCACCTCCTTCTGAGAGAATCC
AAATCGATGTGTTATCAGTGGCTTAACGTTGATTTTACCAGTTCTTATGAATTCCAGACA
TAGCGGCCATGTGTTCTTGTATCGAAAGATCCCAATTACATCAACCTCCCTTGCAGCTGC
TGGAGTTAGAGGTACGGTCATCTCATGATGGCCCATTCCCACGAGGCACACTTTGCCGCC
TGAATGAGTAGCAGATAAAGCTGTTGACATGGTTTTGTTAAAGCCTGCACAATCAAAGCT
CACATCCACTCTCCCCATGACTTTATGTATCTGTGCTACTTCTTCTGCAACATCCTCCAA
ATTTGTTGAAACTTTGACAAGATCATCTGCACCAAGCCCCTTGGCAACAGATAAACGATA
GTCATCCACATCCACAATGACAATTCTTGGAGCTCCGAATGCCCGAGCTGCTAGCATTGT
GACAAGCCCTATGGGCCCGGCTCCCATGATCAATACATTTGTCTCTGGACCAATATTAGC
TCGGCGGCAAGCATGGACACCAACGCTTAAGGGCTCACACATAGCTCCTTCCTCCAAGCT
CACATTTTCAGGCAGTTTAAAACATAAGTCTGCAGGATGCACCACCTGGTTGGCAAGAGA
ACCATGAACCGGAGGAGTCGCAAAGAACTTCATTTCAGGGCATAGATTGTATCGACCTTC
TTTGCACTGATTGCATCGCCAGCAGCTGATCCCTGGCTCCAGGGCTACACGATCACCAGG
CACTAAAGTCTTCACTTCACTTCCCACCTCCTCTATGATCCCAGCACACTCATGACCAAT
CACCATAGGCTCTTTCACAACAAAATCTGCACATCTCAAGGTCTTGAGATAGTGAACATC
ACTTCCACATATACCAACAGCTTTCATTGTAACTCTAACATCATATGGTCCCAGAGGAGG
AAGGTTGAAAGGTTGAATCTTGATGGTATTAAGACCCACAAGCCAAGCTGCCATGTTCAC
TTCCTCTCCATCCTTAATTTCTCCATGTGACATTCCTCCTTTGCCCATCTTTGCTTTGCT
TCTTCAAAGACACACACACACACACAGTGTAGACCAAGGTCTTAATCTTAAGGCCACAGAAA
CTGATGATTGATTAATAATTGAGGATGTTATATGTTTAAAGGTGTATTTTGATTCTAGAA
AATGTGAAGAAGAAGAGGAGTATATGGG
>ntig_197_consensus
TTCTGATTTTCAATTGTACGTGTTTATTTTAAAATGCAAGTATATTCGTTCCTTCTGGGT
ATTTGGGGTTTGGACGCACTCTTGTACATTCAAGATTGTTTTGTTGAGATTATTTGAAAT
TTTGAGTTCATTTATCCAAACCATTCTTGTGAGTCACTTCCATGTTGAGAATTAGTAGGT
AGGATTCATTGTTACTCGGGTGGACATGGGTGTCTTCACCAGTCCCGACTACTTCAGGTT
TGGTATCCTCTCCTGTAGAATCTATCAACTCAATAGACCAGAAAAAAGTTCCAAGGGTGG
GCTCCAGTCAAACATACAAAAGCATACGCAAGCTAACTCCATTAAAAGTAAGACTTTAGA
GTAATATTCAACCGACAGCAATTTTCAAAAAAATAAGCCAATGCCCCCTACTAAGAAGAG
GTTTAAACAGTACCCATGACACAACTAAAAAGTTAAAAGAGTAAAAATGTAGAAGGTGCA
GCAGCTTAGTCCACTTCCTCCATCTTGCTGCCCTCCGCATCCTCAGCTGCCTCCTCCAGT
GGTGGCATGTCAGAGTCTTCCACAGCATCTTCATCAATGCTCAGCCCAAGTTTGAGCATT
CTGTGGATTCTGTTGCCGAAGGTGTTTGGATCATCAAGGCTGAATCCAGAGGTGAGAAGG
GCAGTCTCGAAAAGGAGAAGGACGAGGTCCTTAACAGATTTGTCGTTTTTGTCAGCATCT
GCCCTCTTCCTCAGCTCCTCCATGATGGGGTTCTCCGGGTTGATCTCCATGGTCTTCTTG
CTGGACATGTATCCTGCCATGCTGTTGTCCCTCAAGGCCTGAGCCTTCATGATCCTTTCC
ATGTTGGCAGTCCAGCCATACTCACCTGTCACCAAACAGCAAGGAGAATCCACAACACGG
TCGGATACGACAACCTTCTCAACACGGTCACCCAAAACATCCTTCATCACCTTGCACAGA
CCCTCAAACTTCTCCTTCAAGGTTTCCTTCTTTTTCTTCTCCTCTTCGCTCTCATCAAGC
TTCAGACCTTCTTTGGTTGCAGAGACAAGCTTCTTGCCCTCAAATTCCTTGAGTTGTCCA
ACAGCATACTCATCAATGGCGTCAACCATGAAAAGCACTTCATAACCCTTCTTCTTCAGT
TTCTCAAGGAATGGAGAGTTTTCCACCGCCTTCTTGCTCTCACCAGTGATGTAATAGATG
TCATTCTGCCCTTCCTTCATTCTGGTCACGTAATCTTTCAAGCTTGTCATCTCATCCCCG
CTCTTGGTAGAATGGTATCTAAGCAATTCAGCAAGCTTGGTCTTGTTCGTGGAATCCTCG
TGGATACCAAGCTTGAGGTTTTTAGAGAAGGCCTCATAGAATTTGTTGTAGTCTTCCTTG
TTCTCAGCAATCTCAAAGAAAAGCTCGATGCATTTCTTCACCAAATTCTTTCGGATGACC

TTGAGAATCTTGTTCTGTTGAAGCATTTCTCTTGAGATGTTGAGAGGAAGATCCTCAGAG
TCAACGATACCCTTCACAAAGCTGAGGTACTCTGGGATCAACTCCTCACAGTTGTCCATG
ATGAAGACACGGCGAACATAGAGCTTAATGTTATTGGGTTTCTTTCTGGTGTCAAATAGA
TCAAAGGGTGCCCTCTTGGGGACGAAGAGGATAGCCTTGAACTCAAGTTGACCCTCAACT
GAGAAGTGTTTCACAGCCAAATGCTCCTCCCAATCATTGGTCAGACTCTTGTAGAAAGCA
GCATACTCTTCCTTCGTGATTTCTTCAGGCTTCCTCATCCAGATTGGCTTCTGCTTGTTA
ACCAAGGACCACTCATGTGACACCTCCTTGATCTTCTTCTTCTTTTTCTCTTCTTTCTCC
TTCTCCTCATCCACTTCCTCAACCTTACCCTCCTCATCCTTCTTCTCTTCATCATCTTCA
TCATCAGAAATTTCCTTCTCAGTGGTCTTCTCAATCCAGAGAGAAATTGGATAACTAATG
AACTCAGAGTGCTTCTTAATGAGATCCTTCAGTCGGCGCTCCTCAAGGTACTCAAGCTGG
TCCTCCTTAAGATGGAGGGTGATCTTTGTACCTCGGCCAAGAGGCTCGCCAGAAGTGTCT
CTGGTAACAGTGAATGACCCCCCAGCTTGGGATTCCCAGATATATTGCTCATCATCGTTG
TGCTTGGTGGTGACAATAACCTTCTCTGCAACCAAGTAAGCTGAGTAGAAACCAACACCA
AATTGACCAATCATACTGACATCAGCACCAGCAGCCAATGCTTCCATAAACTCCTTGGTT
CCAGACCTCGCAATGGTACCAAGATTGTTGACCAAATCAGCCTTGGTCATGCCGACACCA
CTGTCAATAATGGACAGTGTGTTATTAGCCTTGTCTGGAACAATGTGGATGAACAGCTCT
GGTTGAGCATCAAGCTTACTCTTGTCAGTCAAGCTTTCAAATCTGATCTTGTCCAGAGCA
TCGGAGGCGTTACTGATGAGCTCACGAAGAAAGATCTCCTTGTTACTGTAAAAGGTGTTG
ATGATCAAACTCAACAGTTGGTTGATCTCCGCCTGAAAGGCAAACGTCTCTGTCTCGGCA
GCCATCTCAACCTGACCAAACGCCTTGTTGTTCACTTTTCAAAAGCACTTCCGATCAAAA
CACGGGAATCAGAGCGTCAGAGAGAAAGAGAGGGAAGCAAGAATGGAAACTGAGGACTTT
GTAGAAGATGAGTGAGGAAATGTAAATTATA
>ntig_198_consensus
CTGCTCAGGCCAAAATCAGTCGATATTTATTTGAGCACTGGCACCTTCCATCATTCCATT
GCGTATCTGCTCAGTGATATCCCTAACATGGCCAGGACCATGGGGAATGAATACAGTGGT
GTTCTTTGATGAGTTTCCAAGGTCTTTAATTGTGTCAAAATACTGAGTAACCATGATGAG
ATCCATCACTTCCTTAGCTGAGGTGCCTTCCACCTTGTGAGAGAAGTTCAAGATGTTTTC
TCTCAAACCATCTGTAATTGCCTGGCGTTGTCTCGCAACTCCTAATCCACCAAGGTACTT
GGCTTCAGCTTCTGCTTCTGCTTTTTTAACTTGGAGAATCTTCTCAGCTTCCCCTTTGTA
TACACTAGCAAGCTGAAGCCTTTGAGCTGCATTTATCTCATTCATTGCTTTGCGTACAGC
AGCATCAGGTATAATGTCAACCATCAATATATGTTCAATGCTATATCCATATGCTCCCAT
CACCTTTTCAAGCTCCTCCAAAACAGATTTAGCAACTTCACCCTTTTGTTCAAAGAGCTC
ATCCAATGTCATTCTCGGAACAAGAGCACGAACCACATCAAATACATAAGCCTGGATCTG
CTCTTTGGGGTTTTGCAACTCGTAAAATGCATCATCAGCATTTTGTCTAACGATCCGGTA
TTGGATTGAGCAAAGCAATTGCACAAAGACATTATCCTTGGTCTTGGTTTCGATACGGAC
ATCGAGGGAACTGATCCTTGTGGACATAATTCCAGCGAGCCATTCACCCGCGAAAGGGTT
GAAAAAATTGAGCCCCGGCTGAGCCAACTTGTCAAACCTTCCCCATTTCTCGATCACACC
GATACTGGCCTGGTCTATACACCCGACAAATATGCAAAACGTATTCCCCATCTCTCCTCT
CTTTCACTCCCTTCTCTGTTTCTTCCTTTAGAAGTCTTCTACTCTTTACAATAACTTTTA
TTTGATTGATTTCTCTCCTTTGTGAAGGCCGAAGACCAGTGCTAGCTAGAGGTGTCATGC
CGGGCCGAGTCG
>ntig_199_consensus
CAGTAAGTTGGCTGGTGCCATCACTCTCACACTTGAATATTTTCTTCAAAATAGCTGGAC
CTTTAAAGTCTGCACATCTCTTCTGCAAAGCTTGTTCCACTTTAATACACTACAAAGCCA
GTAACCTACTTTATATCCAATTCTGCATATCCTGTACAATCAAAGCTTCACTACAAACTG
TGCAACAAAAATTGTACTAAGAACAGCTTGTGTGATCGTGGACAATATTTAGGCTTTGGC
AGTAATGTCCATCTTCCGAGTCTGAGATTGAAACAATTCAGCTATATCTGCAGATGTACT
ACATTCCTCCGGATTTCTATCAGTTACAGTGCGTATGAATCTAGGAAATCTGATTGATAT
GCCACGGGATGGATGAACAATGCCTATAGCAGCCTGGTGAACTGGTGACACTGTAAAATC
CGCACCTCTTATTTCCCAAACAAGATCCGGAGAGAACCACATATCGGCAACCTCACCTGT
TCTGTAGTAAGATGGCTTTTTTGATAAGATCTTATCACCAGAAAAATATTCCTTCATCTC
AACATAAAAGGAATCAGAGAATCCAGACATGACACGACACACACTTTGGTATTCCTCAGT
CTCAGGGTTGTAACATGCCATGAGGAATGGACTGAACCATCCTGCTTTCCTCCCATTGCC
ATGCCAAGCACCAATAGGGACTAGATCAAGAGAGTCATTCAATCCTTCCACATAATCTCG
TTTCACCTTTAACCATGTGTCGCTACGCTTTGATGGAGAATATTGAGCATCAATGTCTAA
AGATTTAACCATAATCCCTTCACATGAAGAGTGGAGTGCATTTTCAAGAAAATCACTCAT
CCGAGCTAACGTGTCTTTGCTGGTCAAAATAGCATCCTCTGCTTCAATAGTCATTTCTTT
TGCATACTCAAAATAACCCAACTTTTCATCACAGAACAAATCTTTCAAGCATTTTCGTCT

CTGACGAAGACTGCAACCCAACAGCTGCTCTCCATTAGCAAACATTATATCGAAGACGAA
GACACAAATATCAACCTTTATGCTAGTCATTGTGATCAAGGAATCTTTGCTCCCCCTCTC
TCTTGAAGATAGTTCTTGGAAAGACATGAGCTTGAAGCCATTCTTCCTATCAATAGCAAC
TACCTCTGCATCCAGCACAAAAGACTCTGCAGCAGGCTTACTAGATTCCTTAACTATACT
AATTAAATCAGGAAATCTTGCTGTTGTTTCATCCCCATTTCGCGAAAAAATGCACACAGT
GCCATCAGATGATTTATGAATTTGGGCTCGCTGACCATCATATTTGTATTCACAAGTAAA
AGCTTTATTTTGGAAGAGATTCAGCACTTGTGGAAGTCCATTGGTAATT
>ntig_200_consensus
CTATATGTTAACTTCACAACTCTGCTTTCGATTCCAAGTCTTTTTCAGATGGCGGCATTT
CTTTGGAGGAAGCATCAGTTTTTGAAGTCTCTGATTGAATAGGGGACACTGTTGTTTCAT
GGCGCTCCGCTTCATCACCTAAGATTTTTGCAGTGAACTGTCGAGCCTCGGCATCAAGAG
ATGGCCACTCCGAAACTATTCTTCTAGCACCCTGAAGCTTTGGTTCTTTTGTCATAGGAT
TGTTGCAAAGATCAATGGTTTTTGCCTTGGACTTTGTAACATTACCACACCATGACTCAC
ACCATAGCCATTCCTGGGGCAGTGAGAAAATATGTACTGTATGCTGGGCATAGTTTGGAA
GATCCTGATCTAGATTGGCTAGACTGTTTGGATCCTTGCTAAGCGTTTCATAAATAACTC
TTAAGTTATCTCCCGCAGCAGTTTGGCGAAACTTTTTCAAGTCAACAACATATAAAGCAC
TAATATGGTATGGTTTACCACGTAAATGTTCTTTCCAGAAACCTTGTCTCCAAAATCGAT
ATCCATCCATATCCTTGTTGTTATCACAAAATGGAGTATATGCAAGAGGTCTTCCCTTTA
AATCCATGTCATAGAGTTCTCCCATGTCAGCCCTGACAACTTGATCCGCATCGACGAATA
TAACCTTGTCCAATGAGAGGGGAAAAATAACATCAAGGAATAAGATCTTATAAGCCCAGA
TGATTCGCTGTTTTTCAGTCTGCTTATGCAACCATGTCGGCCATTTATAGGTGACCAGTT
CATACTCGAAACCATATTCTTGTGCCATATGTGGAATCACATCCTTAAACTGGGGCGAGA
GATAGTTTTTTATAAACCAGAATTTCACTGGTCGACGGGTGTTCTTTAAGACACTTAGAA
TCATGATTTTGAGAAAGCGCTCGTATAAGTGTCCTGAAGCAATAGAAAATATATTAATTG
TTTCCCCGTGTCGTTCGCCTTTCCTATGGTCCACTGAAGTTCTATTCTTCTTTTTTGTTT
TCTCGCTACCAACGATAATACCAGAAGCCCATTTGAGAATGTTAGAGTTCCAGCTACCTT
TCTTTTCCTGAGAATGATCATCATCAGAAACCAGCAACTTTTCATGCTCCTTGCCCTTCT
TCTTTACAACTTCCATATGAACGACTTTACCCCGCAAGTCATTTATGGTGATACGTTTTG
ACATCAATTTGCCCTGGCTTGCATCACCATCTTCCTTAAGGTCATAAATCTCAGAACTTC
TTCCAGGAGCAAGTTGCAGGTACCAGACCCCAGGAGAGACTTTCATCTGCCAATAACCCA
AATTGGCCATCACAAGAGTATCAACCAAGTGAGGCCTACTCTTTGTTCCAAGAATTAGCT
GAAGGCCTCGAGGAGGATCACGATCTTTCTCCGAACAATGACCAGTAAGGACAAGAGCTT
CCAGCTCAAAAACTGCTGGTAATGTCCTTGTCTCACCCAGCTTTTCAAGCAAGATATTAT
CCAAATCATGTACAGCAACAACAGGCTCAACAAGCCAGGGCTCTGGTACATCAAGATTCA
TGGTGAGGGTTTTGGACAACGGCATATTTGCAAAAAAGGCTTTTGGGCCATTTATTGATA
AATCACTACTGCTGAAATCATCCATAGTTGGAAAAACATATCTGTAGTAATTCTTCAGAG
GAAGATCCACGAGGGAACTCATTGGGTTCAGTATAATTCTCATGCTTGATCGAGTGTATT
TCTGCAGAACTCGAAGAATGGAAGAAAGCTTCTGGCCAATGGAACTTAAAGGATCAATAA
CCGCATCAACATGAATAACAGAATTCTCACTGTTTAAAACAACAGCACTATGCTCCGCAC
TTAAAATCTCAAAACGTGCTCTTTCAGAACCTCGATCCCGCATAGCCAAGGAAGATGACA
CAAGCATAATGATATCACTTACAAATTTGCTTGTGAGCAAGTCTGGGTCTATGTCCGGAT
ACTCATCAAGCCAGTTAACTCCTTCTAATATCTCCCAGACGTACTTTACTCTGTGCTTGA
ATTCCACTGACTCCAAAAGATCCAGATCATGACTCATAAATGTGCTTACATCAATAGGAG
ACAAAACTCTCCCATTAGTAATGACAGCATTAACACCAGATTCAGCTCCAAGCTCCCTAT
ACAAAAATTGTGCCACCTTATTCAAATACTTTCTCATGCTCCCATCATTATATTCAGGAA
GGGAGGAAATAAAACCTTTTAATGACAAGCCATTTGCCTCAGCAAACTCACAGATCTTAT
CAATAAATGCTTGAGTGTTTTCAGCAGAAACTGGAGATGCAAGGGTACGCTCATAATATG
AGCACAGTTGATCCAAAAATTCAAGTACCTTTTTCTTATGGCTATATGTGGATGCAGTGA
CTTCAAAAGCCTTTACAAAAAGGAGACTAGATGAATCTAAGTGCTGACTAGCACTAAAGA
GAACACCTAAACGAGCACCTTTAGATCCTCTGATCAGAAAATGAATTCCTTCACGGAGTA
GCTTTATCCCATTCTTTGATGTGACATCAACAGCAAGCAGATGTGTAACAGGCTTCACAT
CGTCCATAGTTTCAGGAGAATGCAAATAGTGAAGATCTTTGAGCAAAGACTCCTCTCCAA
GAAATGAAGAAGCCAAAGAAATAAACCTATGTTTAAACTTGGCATCGTTGATAATCTGTG
GATTATAGCGAATAATACCATTTTCTGATAGGAATTTGTCCAGCACATCAGTATGAGAAT
TTATATGTCCATAATAAACTTGCTCTTGTATTCTTGGAAGCTCATCATTCATGGCATTGA
TAAGGGTCTCCTCATTTGAATCATATACAAGACCATTCATCAAGAGGCAAGACTTTAGCT
TGGCCAAACCCAGTTTGAAAACAAACATTGTAGTCTCTTGAGATTGCTCCTTAAAAGTTT
TCTCCTTTTCCAGCTTTAGTAATAAATCTTGAGGTGGAGATTTTGCCTTTGATAATATGG

TTTCTACAAAAGCTCCCTCAATATGATGCATTTCGAGAGCATCATCTGCAGAATCCTCTG
ACTCCATCCTCAATCTGTTCACGTTGCTCAAAAACTGGAAAGCTGTTGGGGTCCCATGGT
TTTCCTTAATGTAACTGAAAAGACGTATAATCAAGCTGGATATATCCTCCCTGACTTGAC
TGTCATCTTCCACAGCAGAAGACTCGAGCTCACCACCACTTACTTCAAGCTTATTGATAA
ACTTGGTTGAATAGAGAATCACACCAAATCTCAAAGGAAAATTATTCTCATACAAAGATG
TAATCATGTCAATAATCTTAAGACCTTGGACTGTGGCCGGATCAAGAACATAAACAGCAT
GAAAGAGGTTCTTCCGAATATAACGTAGTTGTCCAGGGTAGACAGGCATCAAAATCTCAT
TTATATTGCTCCGCCATCGCTTATACATAGCATCTTCTTCCAGGTTATTGAGATAATGAA
CATGAGTAGAACGAAAATCAACACGGAACATATTGGACTCAGAAGGAGGCACAACTGATA
GAAGTTTCTTTATAACGCTATGTGGAATCTTAAATTTTGAAAATTGATCAGCCAAGGATA
AGTCCTGATGAACCAAGTCAATCAACAGGTAAAGGTCAATATCTTCAACATTGATCAAAG
CACCATTAAGAGCCATTATGGACTTCCCCGGGGGCACCATACGCTGGTTTGAAGTTATTT
CTTCTTTAATTGAATCATTGAGCTTCATGCGAGATAATGAAGAAACTACACTTGGAAAAT
TTTGGCTAATTTCTTGCATTGACTGTAGAGGGTCAGAGGCATGAACTATTCTCTGAGCAG
TTTGATGCCCCAAATCCTTTAGTTCCCAAACATCAAGGGTGTCTGATATTGTGGATGACA
ACAGATTATCCCTGAAAGCCATTATCTCAGATGTAAGCTCTGGTTTGCGTTCCAGAATTT
TGGAAAATATGAACCCTCTAACTTCTTGGCTAAGATCTTCTGTTCGAGGATCTTCCAAGG
TGACACCTTCTTTTACTGCACTGTCATCCATAGCCTTATATTCCATGTTCTTCAAAGCAA
GCTCAACACCATAGCCACCCAAATTTAAGGAATCTCTAGCACCAACAGAACCACAAATGC
CAGTTTTTGCCTCACAGCCAGAAGGTAAAACTGGTCTAACTACATACTTCACTTTTCCCT
CTTTGGCAGCTTGAACTAAAGTGATATGAAACTCCTTGAAGCAATCAGTTCCAAGAGCAC
CATAAAGAATAGCAATAGGACTTGCAGTACTTGAATCAAAGTGAATGTGATCAAAATCAA
ACAATTCAAGCTGCTGAAATGAGTCTCCAGCAATTTCAGTGGGATCCGAAAGCCACAATA
ACAGTTCCTTAACATCAAAGAACAACGCCCCACCAGTATCCACCCAACAACATTTCCCAC
CAGGACTTTTTGGATTTATGCCGACAAGTAAGGAATCCGATATTCTCGTTTCCAGATTTT
CACTTGGTTCTGAATTACTCTCTTCCACCAGTGGAAAAGAAGAAAGAGACTCCTCTGCTA
GCTGGCGATAAAGCACCAATCTTGGAGATGCTGATCTTAGCATAAGGGAGAATTCATATA
GTGACGCCAATGATTCACCTAAAAAAGAGCTACCATGTTTCAGAATTTTTTTGAGGCAAC
CTTTCGCTGTATAAGTATCGGTATCATCTTCATCATGATACCAGATATCAATGAACTTCC
AAAAAAGGTCTTTCCTTTCATTAGATAGTAATTCTCCAGCTTCTAGAAGCAAAGGAGTGC
CCGACCACTTAGCCCGAACAGCGACCTGAACATTCTTAGGTTTCCGAATCTCCGCAGAAA
CAGAACCAAACCCAAGAAGACTTACACAAACAAGGAGGACAAGAACGCAAAACCCAGATC
TAAAACGGCGCTCCATTTCCGGAGAAACCCTGAAAAACGACATTTAATTTGAAAGCTTTT
ACAGTTTCAGGGGTTCGAACGAAGGAAACATTGATTAAAGGGAGAAGTGAAAGTGAAGTC
AAGTCGGAGTATGGACGTGCACAAAAGTCCGGGTGGAAGCGACCACTGGCGTAGACCAAG
ACTTTTGCCCAGGGAAGGGAGACTCCGAAGCGTGAGGGAGCTTTGATAGGTTCAGTGGTG
GTCTATTCAAATGAGACAATGGTTTTATTAGAGAT
>ntig_201_consensus
CTCCCACATTTATTATAAACATAACTTTGTCTTTTCTTTTCCTGGGTAATACACTGTTCA
TCTTACATGATCTTTGTATAAATCCATCCAATGTTCATAGCTAGTATTGACCTCGGTTTG
GTAAGGACAAGTTACACTCATAAAATTATAACAAATTAAAGAGTCTTATACATAACAATG
ACAACATGGATGACTTCATATTAATATGTTTCTGAATTCATCAGAGTCCACGTATAAGCT
GATGTCCCTCCTAAGTGCATGGTTTCCATTGATGGAAGGCCGGCCAACAGAAANNNNNNN
NNNNNNNNNNNNNNNNNNAGTCACTGTCGGCCGTATGCGGAAGACATGTTGTGGCCATA
TTGAGAAGACATATTATCGGTGGGAGGCATTGGTGGGGCTCTGGGTCTTGCATATTCTGC
AAATATAACCCACCCATCCAAAAACTTGCCATCCATCCCCTCTATACCTTTTGCAGCATC
CTCCAAGGTAGCATATCTTACAAAACCAAATCCCTTGGAATACCCAGACACTCGATCTGT
CACTACCCTAGCTTGAACCACTTCACCAAACTGAGAAAAGGCTTCCTTCAGTCTCTCAGT
AGTTGTGCGCTTACTAAGTCCTGAGACAAAAAGATTGGTGCTGGGTTCGACCTTCGGTGG
CTGAGATGCCTCCGCCGCGGACGGAGGAGGGTTGAAAGAAGGAAAAGGAGAAGTGGAGAA
GAGCCGTCTCAAGCCGCGCTGTGCAGCCACCGCCCTCATAGCCATTATGGGTTTAAGAAG
CCTTCGAGCTAAAACCCCTTGATACTAATGGAGACAAATCAATTGATTTTAAAAAAAAAA
AAAAAA
>ntig_202_consensus
GCCTTATGCAAACTTATCCATTATGCACCGCTACTTTTCACTTCACTGTAGTGAGAACAA
GCTACAATTTGATAAAATTACAAAAAAAATGATAACATAATCAAGACTGTCTTGTACATG
ATAATGCAAACATGGAATGGATCAGTTCAAATCAATATGTTTCTGGGTTCAGATACTGGT
TTCAGGTTGATGGAAGACTGATAAGTTTCTCCAAACAGAAAATAGAGGATAATTCATAAA

TAATACTCACTGATGAAGGACAGTATGAAGGATGACCAGCTTGTTTTGTGATCGTATGAG
TCATTGGCGGCCGTATGGGGAAGACGTGTTCTGGCCATATTGAGAAGACATATTCTCCGA
TGGAGGTGTTAATGGCGATCTGGGTCTTGCGTACTCTGCAAATATAACCCACCCATCCAG
AAACTTGCCATCCATGCCCTCTATTCCTTTCGCAGCTTCCTCCAAAGTAGCATATCTAAC
AAAACCAAATCCCTTGGAATATCCAGACACTCGATCAGTCACTACCCTAGCTTGGACAAC
TTCACCAAATTGAGAAAAGGCTTCCTTGAGTCTCTCAGTAGTTGTGCGCTTACTAAGTCC
TGAGACAAAAAGGTTGGTGCTGGGCTCGGCCTTGGGTTGCTGAGCAGCCGCCGTCGCGGA
TGGAGGAGGATTGAAAGACGGAAAAGGAGAAGTGGAGAAGAGCCGCCTCAAGCCGCGCTG
CGCCGCCACTGCCCTCACAGCCATTATGGGTTTAAGCTAAAACCCCTCGATTGTGTTAGA
GACAATTTTTTTTCCGTCCAG
>ntig_203_consensus
ATAACAGTGCAGTCTTTTTATTCTAAAACAACCACAAGGCACATCCCTATTCACCAACGG
AACCGTTTAACTCATCTTAGCTGTGGCGCAGTCTTTTTATTCTAAAACAACCACAACAAG
GCACATCCCTATTCACCAACGTAACCGTTTAACTCATCTTAGCTGTGGCGCCCGTGTTCA
GTTTTGCACAGTACTCGAATAGACTTCCATCGAAACAGCGTCTCACAGCTTCTTTTGATA
GGAAACCTTCCTCGTCCCTCGCCAGAATGTACAAAACTAACCATTCCCCTTTACTTGCAA
TCCAGCCAAAGAAGTCCATAACTACACGGTTTCCCTCCGTCATGTCCCAGAGCTCTCCCA
GTGTTAACTTATCGGGCACTGTATGAGAATATTTGCTAAACATGTTTTCCAGGTTTGCAG
GCAGGTACCTTCCTTCAGTGTCGTATGTTCCTGAGTCGCTGCCATGCTTGGCTTTGTGTA
TGTTGTGCATGTATATGGGTAAGAAAGGAGATGGGATCCACCCCGGAAGGGTAGGATAAC
TGAGTGCCAAGTTGATGATAACGGCCAATATCACAGAAGCTATCATGTTGAAACCGATTG
CCCTTAGTCCTGTGTAAGTCTCCCAAGGGTAAATGATCCCATTGTCATCTTGGTCAAAAA
AGGCTACATGCTGCTGAAGAACACTCAGGTGGTTATGATTGTGGCCGGGTGTTCCATTCG
GATGGTTCTTATCTAGAGCTTCCAGTGCTCTTGGCATATAAGGCTTGGGAATTTTGTCTT
CCAAGTTAGTGGCGACTCTTCTCTCGTAAGTGACTGGTGCATAAGGAGCCTCAGTTGCCA
TTGCATCTCGATCCATGATCTCCCAAAAAAGCTGCTTAAATTGATCACAAATTGAGCTTA
CTGCAAGCAAGATAGATGCTTATAAAAGAATAGTAATGGGCGTTGATGGAAGCAAGAGAA
GAGGAGAGCCAAACTGCCGAACCAAGCTTACGCGTGGCTTGCATGCAAAAGAATGATAGG
>ntig_204_consensus
CTGATGGCCAACAGGCTTAGAATTATAAACAATAAAGGAGAAGGGATAATAGATAGTCCT
CTTTGTTTTAAGAGTTCGTAAGCTCTTGGCATGCAAAATTCCTCCAAATATCCTTCTCTG
TGTCATTAGATGTTCACTGACTCTCAAATAATCTTGTGCACCTTCCTTGTGCAAACATGA
CTGCTGCTCATGGAGCAAAGGTCCATCTTGACCTTCTATAGATTGTACCAGACTGTCATT
CTCAGCGGCACATACCACAAGTCCAAACTGGCAATATCCAGAAGAAGTAGGTTGAAACCA
GGCAAGATTAACATGAATACCATATCTACTTAAAATAGGATTTGCATGTGTTTCAAGCCA
ACTGATAACATGACCTAAAGTAATCTTTAAGTGTCCACAGCGAACCAAGCGCAGTTGGGC
ATTTAGACCTGCCACTAGTCGATACCATATGGTTGGTGGGACAGACTGACTCATGAGGCT
AGTAAGAATGTTATCAGTCTGAAGAAAGAAGGGAGCCATGTAGCTTCCATCTCCTCCAAA
AATTAGTGACATTGGCAACCTTTGTTGAAGACGAGGAGGAAGATCAGATCTCTTCTCATC
ACCACCAAGGAAAAAGTCAACATATGCCAGCATTAGATCAGCAGTTGCAGCTACCTTAAG
CCCTTCGTAGAGAGCACGTGAACGGCAAGAACGTAAGCAAGCATGATCATATTCTGAACG
AACAAATTCACGAAGTTGCTGTAATTTCCTTTTTCGGCAACGTTGTAGCCAGGACCATGC
CAGTGGATAAGCAAGAATGGAAAGAATGCTGTAGATAGATCCTTCCCACCACTGATATGC
AGCTAAACCATTGATCTCATCCACAAATCTATTGAATGCATCCTCATATACAATACCCAT
TACTTGTTCTGGAGGGGAGTGAGGCAAATACCAAGGTTCACTGAAAGTATTGGGCCCCAT
AAAATACATTCTATGTATATGACTCTGGGACTCTTCAGTCCTACTTGTCTCCAAAACCTC
ATTCAAAGACTCTAAGAAAGGGAAGGAGTGATCTATTCTAGAGGCCCGAGGAGCAGGCAC
CAGAGTTGGCAGTTCATCCCCACCCACATATTTCATCCGTGCAACAC
>ntig_205_consensus
GATCTCGATAACCATATAATACCTGGCAATAAAGCACTGGTACGCATAAAATACTTCACT
GAAATACAAGAAGAAACAGAAGATGTAAACATAATATAATCTTACTCGCCCTTGAGATGC
GTTTCTCGAGCTCAAAAAACACCTGCATGCAGCTGGAAAAGACAGATTCTGAGCCCGAGG
AAGTGAAAATGCCAAAGGATCGAAGTTGCCCTACAAGAGAAGAACTCCCAAGAAATCCTT
GAAAAGGATTTATGGGAAAGTACCCATAACACCATCACTTAAAGATGACACTAATAAGAG
CATCAAGGCCACCCCTTGCCATTTTGGTCCGAGAAAGTGAGGCATGGGGAGGGGTGGCCT
TGCAGGGATGGTGCAGTTGGAAGAGGGTTTGATAATGAAGGATGAGGAAGGCTGTTATAG
GGGTTAACTTTTGGTTTGTCTTGTTGGTCGCTGGAGCAATTAAAGGCTGAGATGACTTAA
GTAGGCGTGTGTGGTCACTTTTGTTTAAAGGAAAGAGAAAGAAATTAAAGTAGAG

>ntig_206_consensus
ATCCATCTTCTTGCAATTTATACAAGTAAATCTTGTTAGCATATTGTTTTCTTGAAGCAA
GTGTTTATACAGCAAACAAATAGCAAGACTCCTTACCTTGAACAACCTGGTGCTCGTCGT
TGAAACATCCCAACTTCGCCTCTTCAGAGGTAATAAGACACCTTTTTCATTGGTAGTTGT
TAAAAAAAAAAATCACATCAATCGTATTCGTTAATCAACTTGCTCTACTTGCAACTACAC
ACCCCAATAAACCAAAAAGAGCTCAGATGATTGTTTTAAAAGGTGCTGAACTTGTGGTTT
TACATAGACAATGGAAGAAGCATCCTTCGTTTCTTTTTCCTAGCCAGTCTCACTTGCTTT
GTAATTTTCATGCATAAAAGCAATAGCAATGTGATGCATACTAAGATAAAAGCAAGTTGC
AAATTCCTAAAATATAACGAAACTGCTGAAAAGACCAGAAAGGCTGCAATGATAAGTTCC
CATATTACGAAGATCACAACATCAACCCTTGAAGAAGGTAGATTAGAAGTGGAAGAGAAA
AATGATACAGAAGCCCAATCGTGCTTGGATCGATTCTGGTATTCTCTCAACTGCTCCGCC
AGATTCGATCGCGTTATCATTGCTGCCTTGGCTTTTGTTTCTCTCCCCGGAGCCGCTTTA
CGACTCGTATATCAATGGAGATTTCGGAGAGGAAGGAATTGAACGATGAACTGGCAGATA
GGCATCGAAGAGAGCATCGACGTTCAAATTCAATTGGTTTCTGCTTCTGGCTTTCTTTCA
CCTGAACCTTCTCTCACGATAAATTTTCCCTTTTTG
>ntig_207_consensus
ATCCTTTGCACACAATGGTTATGAGTTGTGTTTATTTTTTCACGATGAATTTTGAATACC
GTGTGGAGAGTTAAAAGTTAATTCCGATACTCTTTGGCGATACCTGGTGTAACAAGTTAT
TACACTTTCTTCCACACAAATACTGGCAAGTCTTCCCTGTCTGCGCTCGGCATACATTAG
GGGGACACTGCTACAAACAACCTCAAAAGAACTAAAATCCCAAAGCCAGTCTCCAAACGT
GTCTTTGCCTTAATAATTTATATTATCAACCCTGTCTATGCGACAAGTCAGCAGATCTAG
ATCTAGAACCAGACGGTGACCTTGATGCTCCCCCACCCGGTGGAGACCTCCAATGAGCCC
GCGACCCTTCCTCGTACTCAGGTTTCCTGCCATAGTCATTGCCATGGTTCCGATCAGCAA
CAGCATCATCATAAGCAGGAGAATAAGACCGGTGAGTGTATTCAAAGTCTCGTGCTTGTG
GAAGACCTCTAGGTGATCTTGGGTGTTCAGTATGTCTTCTCCTCGGCGAAACAGGGTAAT
GTTCATGTTGTCTTGGGACAGGTGTGTAGGACCTTGAGCGATATCGACCTCTAGAACCTT
GAGGATGTCGAGGGGAACGTGAATGGGATACCTGAACCTAGCTCTCTGGCGCATCTCCTC
TGGTCTTTTCCTTGTATCTGCAGCAACAACAACAAATATTTCCCTTCCAGCTAAACGCTT
CCCATTCATGTGGTGCTGAGCTTCTGCAGCATCATCAGGATCCACAAATTGCACAAAAGC
AAACCCTCGAGGTTCACCAGTGTAATAATCCTTGGGAATGTACACATCCCTAACAAGCCC
AAATCTCTCAAATGGAACTCGAAGTTCTTCTGGCCTGCAATCAAGAGGAATATTTCGAAC
CAAAAGACTTCCATGGTTCTGCTCCTTGCGTCTTCCATAACCTCCACC
>ntig_208_consensus
GTCTCCATTATCATTTAATCCAATAATTTGCGTATCATCTAAACAATAATCAAACACTAG
CGATACTGGTGCAATAAGTTACAGTTTCTTCCACATATACAAGCAAACCTATGAACGTGG
CTTTCGCTGTCTGCGAGGGGAATACATTTGAGGAGACAATGCAGGAAACTGCTTCTTGAC
AACCTGAAAACAGCTGATGAATCTGACCTAACATCTAAAAGCCAGTCTCCAAAAATGTCT
TCACCTTAATGGCGTATTTTATCTGCTGCGTCTAGGAGACAAGTCAGCAGATCTAGATCT
AGAACCAGACGGTGACCTTGATGCTCCATCACCTGGTGGAGACCTCCAATGAGTCCGGGA
TCCCTCATAGGCAGATTTCTCGCCATAATCATTGCCATGGTTTGGATCAGCAGCGTCATC
ATAAGCAGCAGAATATGGCCTGCGAGTGTATTCAACACCTCGCCCTTGTGGAGGACCTCC
AGGTGATCTTGGGTATTCAGCATGTCTTCTACTTGGGGAAATAGAGTAATGTTCATGTTG
TCTTGGGACAGGAGAGTAGGACCTTGAGCGATAATGACCTCTAGACCCTTGACGATGTCG
AGGGGAGCGTGAACGGGATACTGAACGTGAC
>ntig_209_consensus
CTGTTATGATTGGTTGTTTTTAAAATAAACAACTACAACAAAGAATTTATGGATATGTAC
CTCAAACTTCAGGAATCTGAATTCATAATTCTGGCAGAAACTTGATTCACTGAGCTATTT
CATTGGCTAGAAGGCCAAGTTTCTTCATAAGGTCAAATCTAATCCAAGTTGTTTGCCTTA
CAAAACTTCAATACAGCAAGATAAAGACCAGGCTCGTCTACGTATTTAGCTATCACAAAA
GAATGATTGGCTGCAACTTTCTGTAAAGTCATTTCATCAGGCAAGTCAGAGATTACAACA
TCTGCAAACTGTTCCTGTTGGTGCTTCAAAATTTCCCTCCCTTGGGGGTGGCTTATAACC
ACTCGTGCACCT
>ntig_210_consensus
ACCTGGGGAACAATGACCAGCAAGTGCCCTGAAAACCTGATCAAGTGTGAAGGGCAAAGC
AGGCAGAAAATAGAGAAATACAACATCTAAAGGTGCCCACTCCTCTGGCACATATATCAG
TTCCCCTTGCCAACACTTAACCTCATCATATTTCGCTTTAATGCAAGCTAACACAAAAAG
TGAATCATGCACAACAAGAAAAAAGCTGCAGGGTGATGAATCAACCACCCGATCCACAAA
ACCTTCAGAACCAATTGAAACCAAAACCCTGGAAGAATGATCAATCTCTCCAGCGGAAAT

TATATGGTCAATCTTCTGGATATTCTCATCGGAGTTTAGCTCATCCGAGTCAAGAAATGA
CCAATCTTTTTCAATTAAGTCCATTACAGATACAACTCCCTCATCAGAAGGAACTGTAGA
ACCAATGGATGTCTGCTGAGTGCTTACTGAAAAAAGTGATTTGGGTTTTAGCAAGTGTGA
AAGGGGAGCATGGCGGTATCCATTAAAGCAAAGATTTGAAGAGAAAACATACCGAGGTTT
TGATATATGACAAAGTGAAGACTTGCAAGACAGGGATGAGATAAGTGGAGAAAAAGAAAA
GAAAAGAGAATTCATCGACGAGAGCCAGCTAATGAAATGAACCAAAAAATGGGTTGTAAA
ACTCTATTATATCTTCTAGGTTAAATAAAGGTTCAATATTTGCTAATTTCAATGGGTTGC
CAAAGCCAGATAATGAATTCAACCAAAATGAAGCCTACCTAGTCTGTTTAACCAATTAG
>ntig_211_consensus
TATACACTATACAACCTGAAACCGGAAATCACTGAGTATCCAAAGTTGAGTCGAACTCCA
TGTACAAGAAAACACAAACCCCTCAACCTTTATTTATGGTTTCCTGTCTCTCTTATCAGT
TTTCCTGTAGCTTATACACAGAGTCTACGAATTCTTCTGTGTCCCAAAGAAACGAACCAA
AGGCAATTGCCTCCAAAACCAGCCTCTTCAGGCTTGAAAATGATGTCGAGATCAACTCAT
CAGTTTCAAGCAAACCCTTTCCATCCCCAAACAGTGCAAGGCTGTGTTTCTCAACCAAAT
GCACTGCTTC
>ntig_212_consensus
GGGTACAAAAACAAATAAAGTACTTTGTGGCTGACGTAAAGTTTAGTAGCAGCTAACATA
AAACGCAACCGTTTTGGTCTATTGTAATCCTATCCGCAACATAATCTACTTGAAAACAAA
TATAAACATCTAATTAGATAGAATCTTGATTAAAGGGTAAATTTGCCTGCCTTACTTTAC
ATTACAAGTTGGCTCAAAAAGCAAACCCTACTAATCCTTGTTCCCATATTGTTTTGGGTA
TACACGACTCATCAAGCAACTCCATAAATTCACCATCATATTATGCACAAACACATTCAG
GTGATAAACAGAAATATTACATTAATCATCATCATATGAGACATGGAAGCTAGTGGTGCA
CCCAGATGCTGTCGGATCCTCAGCCCCGCAACAATCATAGAACTTGGCAGCATATGGTGG
ATCATCAGGTCCCAATTCATAGTAC
>ntig_213_consensus
CCACTCAATAAATCAAATTCCGTTTTACACCTTTATTGCAGCTATAATTCTTTATAGCCT
TTAGTGCAACAAACATTTATTAAAAAGGTTAACAAATTTGAAGGACTCTTCAGCAAAAAT
ATTAAATGTGAAGGAACATAAAATTAAAGAAAAATAAAGCGGTATGTACATGAGCTGAGC
CTGAGTTATTAAATATCTCCTGTGCATTTAACAAACATTGTTTTCTTTTTTTTTTTTTTT
TTGGGTCATTTTCTTTTCGTTGGAGTCTGAAGCTAAATTCAGCAAGCCTAATATGCTCTT
CGTAAATGGGTGATGAACTACAAAGAATTTCGGCCAAGTACTTGATACTTCTTAAACATA
ATTATGTAGTTTAGTAGAGCTTAATTCTGGTGTTGATCGGGCTTCGACATAAGGGGCATA
ATTCAAGGTCCTGTCCACATTCACAACATGTCTGATGACCGCAGCCAAAGGCCATATCCT
TCACATTGGTGAGGCAAATAACACATAGCTGATTATCATATGTAGAACTTGAAGCAGGAG
GAGCTGAGCTAACAGGCTGACTGTCCCTTTCATAAGGAGCATTTGGCTTGAAACTGGTAG
AGGTAGAATAGGAAGGTTTCAAGCTGCTGATAGACGTTGCACCGTATACAGGTGGAGGGA
GGGGAACCCTCTCTGGAATATTTCCTGTCTGGCGACCCAGTATATTAAGCTCTATAGTTG
CCTTATATTGAGAAGGAATCTCCATTAAAGCAGCAAGTGCAAATTCTGTTTCCTTTCGAG
ATTGTTCATGATTCTTTAACATTATCTCTGTAAAGTTCACAAACTGGAAATTATCAAAAG
CTCGGGCAGGGATATTATCATCAAATTCCTTCATCATGTCCCAGGGCCCATCCCCTACCC
CAACTAGAACAATTGACAGAGGAAGCTTGCTTGCTTCTACTATTGCCTCAACTGTCTTCT
GCTCCTGTGGACTTAATTGGCCACGCTGAGTATCAACACTTCTTGTTACCTGTCCATCAG
CAATTATCAATAATACATGATACTGGCCACCACTCTGCTCAACAATGGTCATGGCCATTT
CAATTATAGGAGCAAATGATGTCGGTCCTGCAAGTCGTAAATGAGGCACAATTTCCCTAT
ACCGACTTAACACCTCCTCAAATCCATTACAAAATCTCTCATCTGGAAAGAAACTGAAGA
CATCTCGATCGTGTGTTGATGCATCCCCAAATCCATAGCATGGAATCAAGTTATCCTCAT
CAAATGCAGCTAATGTTTTCCCAATAATGGATATTGCTTGTTCATAAGGATTTAAACCAT
CTCCAATATGATGCAAGCTTCGTCTGTTATATGATCGTGAACCTGTCCACTCATTGCTCT
TTGNNACCTGATCTAAGGATTTGTAATCATCAGCGATTCTTGAATACCTCCTATCAAGGT
TCCTTGTGTTACGACTGTATTGCTCAGAAGAAGAATAATACTGTTGTGATGTATATGTTT
GCTGTGGTGGATAAGGCTGACATTCTTCACCATACTCTGGCTGAGAATCCCATGACGTTG
ATCGAAATAATGAATTCTCCCTCCAACTGGACCTCGAGCTTCCGGATCCCATCAACTAAC
TTATCTA
>ntig_214_consensus
AAAAAAAAAAAAAAAAGTTTAAATTAACTTAAAAGTATCACCTTCACCATTTCACAAATAG
TACAACAGAAAATATTGAAGGCAAAGAGGAATAAAAAACAAAACTAAAACCCAGGACCAA
TGCGGGCAGCAAAAGCATCCTATATCAAGTACAATAGAAAGAGTTTTAGATTTTGTTGAA
TGCAACAATGTCACAACTCTGGACATATTCATTGCATGGCTCAGCTGTGAGATGTTCCTC

GATGAGTGAATCCACAGAAACAAGGTCATCAACAATTGTAAGCATGATTTGCAACTTCTT
AATCCCATAACCAACTGGAACCAGTTTCGATGCTCCCCACAAGAGACCTGGCATGTTAAC
ATTCCTAACGGCCTCTTCAAGCTTCTTCATGTCTGTCTCATCATCCCAAGGTTTCACATC
CAACAAAACAGATGATTTCCCACTTTCTTTCTTCTTAGCAGACCTGGCTTTGGCTGCCTC
CCTCTCCTCTGCTGCCTTTTTATCCTCCTCTGTCTCATCTCCAAAGAGATCCAAATCATC
ATCATCATCTCCAGCAGGAGCCTTGGCTGGAGTAGCTTCAACTGGAGTACCTTTACCACC
AATTCTCACACCAACTGCTTTGCCAGGAAAGGCTGTGGCGAGATGCGAAGAGACGCATTT
GTACCACTTACTGGCATTAGGGAAGGAACTACCTGGCTTCTCTAAAACGGCACCGTAGAC
CTTGATGTCATCCTTCGTAATCTTATCCCCAGAGATATAAGTTTTTCCAGAGAGATAGTC
GTCGAGAGACTTGAGGCCCGATTCATTGTGGAGATCTGAGAAAGTGATGGCCATTTGTGA
TTGAATTTATGAAAACGGAGAAGGACGGGGAGAAAAGCGGATTGGGTGTCCGAAAGGAAG
GACGATTTTATGAAGGAGTGGTATTTTTATCTGCTTAAGAAACCCTGGATAAGACTCATG
TACAAACCCCAAAAGACCTAACAGCACGAATGGATCCCAATGGAGTTAAT
>ntig_215_consensus
CAAGTATATTTTATTTATAAATACAATATATTTCTACAATATACAGTGACATGGGGTGAG
GGTCGCTATATTCATCTCCGATCTTTACGTGATCTTCAACTAGAGTTATTGGTGTTAGAG
CTTTGAAACTTTCCTCCACGTTCAGCTTGATTACGATCATGAGGTCGAGGATGCAACTGA
GATTGATAAGTTTCAGAAGAAGGATGATGACGTTCAGGATGAGATGAATCATTAATGTTA
GTATCATTATTTGAAGTGTTGGGATTCTTAATGTTGTGGGCGCCGGAGAAGAGATCGACT
GGTTCGGAATCAGCAATCTTTCCTCCTTCAAGTGGCGTGCCGTGCTTGTAACTGACCCGC
AGAGTTTCGTCTTCAGATAGCTTCGCCTGTGCAGTACCGTACTCTATATCGTCTTTGCTC
TTCGCCATTGATTCTTTCTATGTAAGCTCTTTACGTTTCTCAATTCCAATTCCTACTGGT
GGC
>ntig_216_consensus
CAAATTGAATCACACTTAATTTAAACTTAATTCAATTCAAATTCACCCTTACGTGTGAGA
GATTGCAATGTCTTCAAGTAAACTGCCAAAAATTTCTAGCACTTAAAAAACTTTCACTCT
AAAAAAATGTTTATTACAAAAAAAAAAAAAAATTCATACAAAAACTTAAACGTATTCAAGC
TGAACTCAGCATTCAAAAGGAGTAAAGAAAAGAGAACAAGGATTTAAAAAGTAGAATAAG
AAAAAAAAAAAGAAAAGGAATAACAAAAGCACATCTTCATCCACTTCTGTATTTAACAAG
CTCAGCATGTAAAATAACTCGTTTGTTTGATCTAAAGGTCCACGCAGAGGTAAGTGATAA
CAGACCCTGCCCTCCTTTGGGTCCTCTCACTAACCTTCCACTTTTCAACCATCACCTTTT
TTATGTTTACACAACTGGAAACCACAGTGGCAACTCCAATTGCAGTAATCCCCGGGCAAT
ACACCATGTGACACGACTCCAGCATCGTGCAGTTTTTAACAAGATGGGCTAAACCAATGT
CTGTGATTTGACGGCAGTGTGATAACACTATGTCCTTCAGTAATGGACAACCTTCTCCTA
ACTCTGCCATTGCCATATCTCCCAAGTTCTGGAGAACGCTTACATCTAGGTAATTCAGTT
CAGGGCACCCTCTTGCAATGGCAATAATTCCAGTATCACCAATTTGGTGGCAACCACTTA
CATTAAGATGTTTCAAGGAGCAGCCCTTACCTATGGCAATGAGAGCTTCATCCCCCACTC
TGTCACAAAATCGAAGACTAAGCTCAGTCAGAAACTTGCAATTCTCACCTACAGCTACAA
TTCCATTGTTCCCAATCTTATAGCATCGACGGATGTGAAGTTTCTTTAAATTCTGGCAGC
CTCTAGCTATACTGCCAATGGCCGCATCTCCAATGTTAGAGCAATCTACCAAGTGAAGAG
CTTGCAAGAACTTACAGCCCCTTCCAAGTTCAAGAAAAGCAGAATTACCAATTCTCTGGC
AGTATAATAAAGCTAATTCAGTGAGGCTCTGGCAAGATTGTCCAATGGACTCCAGTCCCC
CAGTCCCGATATTGTGGCAACCATTGACTTCAAGATGTGTAAGTTCTTTGCAGCCCGCAG
CTATTGCTTCCAAACCCTTGTCACTCAGAAAATAGCAATCACTTAGAGTGAGATTTTTTA
ACTTCTTACATCCATTTCCTATAGCATGCAAACCCATGTCTGTAAATTGCTGGAAACTGC
ATAAAGCCAATAACTCCAAAGCAACACAATGAGTTCCCACAGCTATCAAAGCCTCATCTG
TAACGTTAATACACTGAAGCTTCAAAACTTTTAGATCAGGACATCCTTGGGCCACAGCAA
GCACCCCTTTATTGTGGATGAATTCTGAATCTAATGACAATGTTTCAAGAGCTTTACAGT
GAGAACCCACAGCTTCTAATGATATATCAGTAATTTTTACACAAGCTGCAACACCAATAG
ATTTTAAAGATTTGCCACAACCAAGAGCCAAGTCAGCTAAACCCGTGTCAGTCAAACCTT
CACAAAAACGTAAATTCAGATCTTCAAGTTGCTTGCAACATTTCCCAACAGCAGATAGCC
CTTGATCTCCAATGTAACAACCCTGTAAATCCAAAGATTTCAAGTACATACACTTGCAAG
CAAGAGACATTAAACCCACGGAGGATATGTTAGAGCACCAGATTAAGCTCAATTTTTCAA
GCTTCGAAAAGCCTTCAGCAAGGGCGGTCAACCCAGCATCTGATAAACAATATGATTCAA
CTTGACCTTCCTCGGATCCACTTATTTCACCAACATATTGCAGCTTAGGCGATGAAAGAG
CCAATTGATCACTGGCACGCCTTCTTACACAGCTAATAGGGGCAGTATATCCATTCAATC
TTCCTTGGAGTGTTGCTAGAATTTGCCCGGCGTTTGGTCCTCTCACTTACCTTACCACTC
TCAACCATGATCTTTCTTATGTTGGGACAACTGGTGACAACAGAGGCAACTCCAGCTGAA

GTAATTCGAGGGCATCGAACCAGGTAACATGACTCCAGCCCTGTGCAATTTCTAACAAGT
TGGCCTAAACCAGCATCTGTTATTTGAGGGCACCGCCTCAGTACTAAATTCTTGAGTAAT
GGGCAACCTTCCCCAATCTCAGCCATTGACACATCTCCCATATTCGCAAGTCCAGTTATG
CTTAGCTGATGGAGTTGAGGAGATCCTCTTGCAATGGCTGTTATTCCAGCATCACCAACT
TCATGACAGCCACTGATACTGAGAAATCTTAAGGAGCAACCTTCACCAACTGCGATCAAG
CCCTCATCCCCCACTCTATCACAAAGTTCAACCCTAAGGTCAGTAAGAGATTTGCAGTAT
TCACCGACTGCTATAATTCCTCCATTACCAATCTCATCACATCGACATATCTGAAGTTTC
TTCATTTTCCGACAGCCTCTAGCAATGGCACAGATGGCATCATCATTGATAACAGAACAT
TCCACTAAGAGAAGAGATTGCAAGAACTCACAACCCCTTCCAACTTCAAGAAAAGCACGA
TTACCAATTAGTGGGCATCGGACCACAGCTAAGTTTGTGAGGTGCCGGCATGATCGTCCT
ATTGACTCTAATCCTACAGTTCCAATATCTTGGCAACAGTAAATTTCAAGATTTGTAAGT
TCCTTGCAACCTGCAGCAATTGCTTCCAAACTCGAGTCGGTCAGAAAACGGCAATCACTT
AGACTGAGATTTTTTAACCTCTTACACCCATTCCCTATAGCACGCAAACTCTTGTCAGTA
AGTCTTCGGAAAGTGTTTAATTCCAGCAACTCCAGAGACAAACAATGAGTTCCCACAGCT
ATCA
>ntig_217_consensus
TGCCTTCATTTTTTTGCTATAAGGAGAATCATATATAACATGGTAGTGAATTTAATAAAT
GAAATTGGTGGATGCTATTGAGTCAGAATGCTTAGTCAAGCAAGGTGTTAATTTACATTT
TGATGTGGAGCCAAATCTATGCTCTGTTCTTTTGTGTGGACTAGAATCTCCTCTTCTCCT
TGCTTTTCCTTGCCGAAAATCTCTTCATTTTGTTTGAGAATTATTTCCTCTTCCACTTCA
AGCTGTTCCATGCATTCATCAAAGGCAGCTATCAATTCTGGATCAAAGATAGTGCCCTCA
ATGATTTTATTTTCTGGCTCTGATACCGCTATAGCATAGCTACCGAATTTTGGAGTAGCA
TAAACTCCTGGGGAATAAACTTGTCCTCCAACATGTGTCCATTCCCTAAGGCTTGCAACT
CTTGGAAATTCAATGGTTGTTGGGATCTCTGTCAGCTCTAGCCAGTCTGCAATGGCCTTT
CTCTTCGACCCCTTCTTAAAGATGCTTGTCTCTCTAGTAGCATCCACAACATTTTGTTCC
TCTGTTATCTTAGGACTTGACATAACTACTGTGGTTTCTGTTCTTGAACTAAAAAGTATA
CCCTTTTGTTCAGTTACAGTTTTTGCTTCTACTTGGAATTCGTCTTCTGGTTCTGACAGC
TTAGAATGGTGATGACATTGCAAATGAGTCTCTTCCTTGCCATTAAACCATATTTGCTCT
GCATCTGCCATCGCCCTGGATGAGCGGAGCTTTTCAACCAGTGCATCATATTCCTCTACC
GTGTGGAAGCTCCTACTCCTAGCACAAATAATACCCGTATTGTTTCCATTGTATCTTTCT
GTAACAAGAGAGAACATTTCATAGTCATCTTCATCTTCTAACCAATGACAACTCTTTGAC
CATCGACCTGGATCAGCAATGAAGATGCCGGTTCTTTGGTCTTTTTTTGACAGTATTAAC
TGTCAGTGCCCTTGTTTTGTTTACTTCATTTCTCATCAGCTCAAAAGCTTCTTGTTTACG
GCGTATATTTGGTTCTGAAGAGACTCTGTGAGATAGTTGTTCATTGGCTACAGGATTGGT
GGGGCAAACTAGTACATGGAATAGGTCCTTGCTGCCACTAATAGAGATGCCATTTGCCCT
CTGAAGGCTTTGGTTCAGCTCCTCCCTCAAACTCACAGATTTCGTCACCAATTTTGAAGA
TATACATCCCATTTTTATGAGCTTCAAACCTGTTGAAATGCTAAACTTGATTGTATATGA
ATCAGTCACAACTCAATTATCCCAAGGCTAAAGCTGGACACTTCTTCTCATCGTTGCCCC
CACAACTTCAGTTGAGACAAACTAGGCCAGACTTGTTAAAAGTTGTCAATTTTAACTAGG
TTATTCAGCGTTGTTGAACAAGCCTACTTTGGAGGAGAAAATGGAAACCGAACCCAGAGA
GATGAGCTTCACCAACTGATGGGAAATTATCTTTATATAGTTAGTCAATGATATGTTGGA
AACCAAACCAAG
>ntig_218_consensus
GCATCGTGTCATATTACACTTCTTTGCAGTAGCTGAAACTCTCTTCCATTTGTATGAGAA
GTATTTCCTCCTCTTCAAAGTTGTTCCCTGCATTCAATAAAGGCAGCCACCAATTCTGGA
TCAAAGATAGTGCCCTCAATAAATTTGTTTTCCGACTCCATTATCGTAAAGCATAGGCTT
TGCAACTCCTGGAGAATAAACTTTTCCACCAACATGTAACCATTCACTAAAGGTTTCAAC
TCTCACAAATTCAATGATTGTTGAGATTTCTAATACACTATTTTCAATGGCTTTTCTCTT
CAGTTCCTTGTCAAATATACTTGTTTCTTTAGTGGCATCCTCAACATTTGATTCCTCTGT
TGTCCTAGAGCTAGTAGGTTGTGGTTTCTGATCTCAAACTAAAGACGTCACCCTTTTCTT
CAACTACAATGTTTTTTTCTACTGCAGCTTCATCCTCTGATTGTGATTTGTTAGAATGGT
GAGCACTAAACATAGTGGATCTGGATGAGCAGAGCTTGTCAACAGTTCATCATATTCCTC
TACTGTGTTGAAGCTCCTACTTCAAGCATGTGTTTGACATGTAATACCCTTATTGTTCTT
ATGGTATCTCTTTGTAACAAGAGAAAACATATCATACTCATTTTTTAACCAATGACAACT
CTTCAACCATCGAGCTAGATCAGCATCCCAATAGAGATTCCGTAGGTTTTGGAAGCAATG
ACTATGCTTGTTCTTCCTCTTCTTTCTCGGCATTAGGTGTCAATTCCCATGTTCTGTTGA
CTTCATTTGTTATGAGCTCAAAAACTTCTGCTTTGGGGCTTGTATTTGGTTCTGAAGAGA
AACTATCAGATAGGAGTTCACTGGCTACAGGATTAGCAGGGCAGACTTGTGAATGGAAAT

GATCCTTGCCACAACTAGAGATGCCATGTGCTTTCCTATGAAAACTCTGGTTCGGCTCTT
CCCCCAAATTCAATGATCTTGCCACCAAATTTGAACAATTACATCCCATTTTTAAGAGCA
CCTAAAAGCATTGCCTTCACT
>ntig_219_consensus
AGGAGGTGCTAAAGAATAACATAATTTATGGATGAATGAGATAAAATATAAGGCTGACCA
TCTCCATGCGCTATCCAATTGTCAAGTCGAAAGAGTAAGCATTTTGCAAGGCTAGGAATC
AATGATCCGGAAGTCACCAAAACATTTACATGTTGGAATTTTGCACTAGTGGAGTCAGAG
ATTCCACTGGTTGAATGGAAGGAAGAAGTTGAGTTGTCTTCCCCACTTGAACACTGCTCC
AGAAAGGCCCTTGCTGAAGATAGCCATCTATCTGTATATTCATCAGTCATACTATACAAT
TCATCCAAGAAACTCAACATTTCTTGATCAAAAATACTTTGCAATCCCTGTTTATACTTA
TATCCAACTGCTCGGAGCCTGTATGCTAGTTTCTTGAGAGAGGGATCATCAAATGAAGGA
CTAAACCCATCTCGGTTAAAATCATAACCAGAAAGATCTAACCCATCATCGTATTGCTTC
AATGCATCAAGAAAAGGCTTATTGTAGTTCTCAACTTGCACATAGAAGAAAT
>ntig_220_consensus
AAAGGTTAGTGATTACAAACCATTAAGAAAGCAAACGAGATCTTGGGTCATCATATCAGT
GGTTACCATCTTCAAAGAGTAGTCATGTCTATCGGTAGTAAGCCTGCTGATAAGCTGGTG
CCAAACCCGTGCCATATCCCCCGTATGCAGGATAGTTCATGGGAGCTACTGGATAAGATC
CACCAAGAGGGGCTGCTTCTGGTGAGTATGGATATGGATTGGTTGGTGGGCTCCTGTTGC
CATACACGGCAGGTGGGGATGTGTATGGTGGGACCCCAGCTGGGTATTGAGTGTGTGAAG
AAGACCTAACAAAGGTGGGTGGTGCAGGGAAAGATGACACATAGGCATTTGTCAAACGAC
CAGCCTTGGCAGGAGGCATTGGACCACCATTACTAGCCCGTGTACGTTTAGTAGCAGGGA
CAGCAGCTGGCTTTTTCTTCTCGATCTTGGCCTTCTCCAGCTGCCCAAGACGCTTCTTGA
GATTTTCTGGAGGAAACTCAACTTCAAGTTTGTACTCTTCAATGCACTTGATGACTGCCC
GGAGAGCTGACTGCTCTTTGCGTGCAGCAAGGTGACCAGCTCGGCCAGTGTTATTTGGAT
CCTCCATAATAGATGCAGAAGCCTTCTTTGCATCCTTTAGAAAAGCTTTAAGTAGAGGAA
CTGGGGGGAACTTGTCCACCAGGCCAACCTCATATGTGAAATGCACTGCATCAAGCAGTT
GTCCCCTACTGATTAATTCTTCAATCATCTCAGGCATTTTATCACCCAAACCAAGCGAAA
GGGCGAGTTTTGGCATCTGCTTGCGCCAGGCAGAGCCAACCACAAGCTTCCGATACAAGT
TGACATCGTCTTTCTTAACAATCCCAAACGTAACGAGATGCTGTAAAAAGGTATGCACAT
CCGGCGTCTTCACGTTCTCAATCCCACCTCTCTCTTCTAAGCTCACCTTCCAAGTCTCAG
CGATCTTGTTGGCCTTTGCCGTCACACTAGGCGTCACCAACAACCTCGACTTACCAATAA
CCGGGTCAACCATCACTGGTATCAAACTCTCCAACACCAACACGCATGCCCATCCCAAAT
CATTGCTACTCTTATCTTTCCTCTTGTCCACGGGAAAAACCTCCGATATAGCTTCCATAA
CAAACTTGGCGGGATCAACACATTCAGTTAGCGCCACCGGCAATACATTTCTCATCTCGT
CTATTTCTTTCTTTTTAGTAGTCATAAACTTCCAGAATTCTCTTGCCTCCATTTTAAGAC
AATAATCCTTCAGCATCATCAACAATGCGTCGCTGTCGTCGACTTCGCCGTCGGGGTTGT
TGAATTCCTTCTGTTGCGAGAGAGAATTCAGGTTACCCAAAGCAGCCTCAGTGCGTTCTT
CCAGCTTTTCTAAGGCGATCTCGACGCTGCCATCGATGGTGACCTCCCGCTTCTTCAGAA
CATCCAAGGAAGCTTTTGTTTCAGAGTCCAGGGTTTGTATCTTGTGTTTAAGCGCAGCAG
ATTTTTTCAGGATGTTCTGTTCAAGGGAAGTGAAGTGATCAGAAAGCTCCTTCCAAAGGA
GAGTGCAGCTGGTCATTAACGAGGCTTGACGCTGGAACTCGTCAAAATTCGGCGGAGTCA
ACTCGGTTAACTCGCCTGGATCGGGGATCGACCCCATTGATTCGATTTTCTTACTTTGAT
GACCCAGAAACCGAGTTAACAACCAGAACTAAAAAATAAAACAAAACAAAACCGGACCCT
AGAAATGTACAGAAAATCGAA
>ntig_221_consensus
CCGGGCGAAAAAAAACTTGTTAAAAAAAAACCCACTATTTAAAAAAAAAAAAAAAAAAAA
AAAAAAACGGCCCACCCTTCAAAGATGGGCCATACTACTTGGATTCTGGAAAGCCTAGCC
TGCCACACCAATTTTCGAAGACTTCTAAGTGAACTTCCAGCCCTCAATGATATATTGATC
TCAAAGCAGCAGGATAGATCCAATATGGCGGCCCTGTGTTAGACAAATGTTCAGAAGTTG
GATTCTGTACCATGCCTCAAAACTTCTGTTGAAACTCCTCCCAAGTAGGCTCCAGTTAAC
CAGACCGCCTTCCAGTACCAGCCAGGGCTAGCAACCATTTGCCGAAATCACCTTTTCTGT
GACAACTGGTTTACCACCCTTGTAGGCATCTCCAGTTTTTGCAACAGATCCATTCTCAGA
AATCTTCACATCTCCATTGGCCTGAGGAGTGCAATTAGGTTCTTTGCTCAAACGTGTGGC
AACCTCCAATGATTTTTGTGACTCCAGATCACCATTCAAAGCTTCTTTCTTTTCCTCTGA
GTGCTTTCCTTCCCATACCTGTTTCTGGAACTGCTGTTGTTTAGCCTTCTTTTCTTGAAT
GGTCTTTTGCCGAGCTTCATAAAACCCAAAGTCATCCAAAATACAAGTTCTGCTAGAATG
TTCCTTGAATATTTTGATCATTTTAAGTCCCTGTTCTAACTTGATCTCTTGGGTATCCCT
GCTGTTAGTGACAGGCTTGTTCTCATTGTTCTCAAGAGTAATGTGCTTCAACAAACTATT

GGGTACATCCTTCACAACATGCCATGTCACAGGGAAACAGCCAGTCCATTTATCTTGTTG
CCAGTACTCCACATTTTTGTTAAAATCAACAGGTCCTGTCATCTCCGCAAGGCCAACAAA
TTGCCCACTTGTATTGACCGAGAAGAAAAGAAATAAAGGGCAGCCACCAGACTTTTGCTG
AGCCTCCTGATATGCAGCATCAAGCTTCTTGTTTCCATTTGGGGTGCTAGCCCAGACATT
ATACTTGATGCTCTTGTGAACATCATCCTCACTGTATGACTTTATAACAAAAAATTTTGC
ATCAGAATACTCCTCTGGAAAATCAGCTTTATTGTATTGTTCTCTATCTGGAATCACAGT
AGTTGCCTTCTCTTCATCAGTTGTCCCATTTGTCTGCACATTCTGCTCTTTAACTGCTAA
TGCAACAGGTGCTGAGCCCTTTTGGTTCTTGGAACCCTTGGCCCTTGGTCCTCTGTTAAG
CTCATTCAAACCATCTACATTCTCATTACCATAACCAAAGTAACCACGTCCCCTGGTTTT
ATACTTGCCATCAACAGCCAACCATCCACCACGCCCATTTGTTCTAAAATCATACCCATT
AGAGCCAAAGCCCATTCCAGATCTAAATGGGTTACCATACTGACCATACAACTTGTTTGG
ATACAATCTATTCACATTCATAAAACCCTGTGCAGTACCCATTGGTCGAGGTTGGTGCAA
ACCCATGTAATGAGAATTTGGTCGATAATTATGATTTCTTGAAGAAGGAACATTGTTGCC
ATTAGAAATTGAAGTACTGTATGTGGTGCTATTAACTGGCCTAAGCTGCAAATCTGAGAT
CATTGAGCCATCCACCCATGGGATAGGAGACCGTACCCCATCAAAGCTAAATCTTGGATC
TTGATAGCCAGATGCAGGAACACGCCCAGGCAATGAACCTCTTCCATAGGCATTATTAGA
GTTAAAGGAAGAAGTTTGATATGATGGTTTAAAAGGAGTAGCACCATTACTTCCCTTAAC
TCCTCCATTTGTAACAGTATTGGATTTCCCATTAGCTGTTTCTACAGGTAAAGGCTTCTG
CTCAGGAGCTGCAGAGGTGGATGCATCCAAGGGAGGTGCAGCAATAGGATTGGGGTATGG
AGCACCGTTTGGAGTAAGTGGCTGGAAATAATGTGGATACTGGTAGTGCTGAGGTCCATA
TAGCTGACCATCAGTCCCCACGGTTGGAACAGGTGAAGTTGCTGGTGAATATGGTCCATA
CGGTGCATATCCATAACCATGGTGATACATAAGAGATCCATTATCCCCATACACTCCAGA
TGTCATCTCAATTCCATCTTGACCTGCAAATCTGTAGTCATCAAACTCATTCACATTCCC
ATCATACCCTCCATAGTAGAAAGTAGAAGGATAACCATTAGGCACAAAGCACACGTTGGG
ATCCACAAAATCACTAAGAAATGGGGTTCCCAGCCGCTCTGATGGGATCTGACCAGTTGC
AGCATTGCCTGAATCAACAGACCCGAATTGACTGGCAGGAGCCTTCTTGTTAGGTTCAGG
AATTTCAAGAGTCTTGGTCTGCGAATCTAATGACAACTTCTGCAGCAAATCTGAAGCTTG
ATCTACATGAGGAGCAACTGTAGGAGCCATGGATTACACAACAACAAAACTAAAACAACA
ACAAAGAACGGATCTTGATGCCTTGTAATCTTATAAAATCCTAAATATAAACAAATAGGG
GCTTTAGAACCCTAAAAGACGAGAAATTCAAACTGGGTTACTCTTCTCACCAAAGCTCGC
TAACACAGCCGCCAAGGGTTTGGGTTTAGCGAAGAAAGAGAGGGTTTTGCCAATCCAATG
GCGGTGATTCAAATAGCACCACGGACGCCTCCTTTCGGG
>ntig_222_consensus
CAAAGTCATCCAAAATACAAGTTTTACTAGAATGCTCCTTGAATATTTGGATCATTTGGA
GTCCCTGCTCTAACTTGACCTCTTGGGTGTCCCTGCTGTTAGTGACAGGCTTATTCTCAT
TGTTCTCAAGAGTAATGTGTTTCAACAAACTATTTGGTACATCCTTCACTATATGCCATG
TAACTGGGAAACAACCAGTCCATTTATCTTGTTGCCAATACTCCACATTCTTGTTAAAAT
CAACAGGGCCTGCCATCTCAGCAAGGCCAACAAATTGCCCACTTGTGTTAACCGAGAAGA
AAAGAAATACAGGGCAGCTACCAGACTTTTGCTGAGCTTCCAGGTATGCTGCATCAAGCT
TCTTGTTTCCATTTGGGGTACTAGCCCAGACATTATACTTGATGCTCTTATGGACATCAT
CCTCACTGTATGATTTAATAACAAAGAATTTAGCATCAGTATAGTCCTCCGGGAAATCAG
CTTTATTGTATTGTTCTCTGACTGAAATTGCAGTAGTTGCCTTCTCTTCATCAGTGGTCC
CATTTGTCTGCATTTTTTGCTCTTTAAGTGCTAAGGCAACAGGTGCTGGGCCCCTTTGGC
TCTTAGAAACCCTGGCCCTTGGTCCTCTGTTAAGTTCATTCATACCATCCATGCTCTCAT
TACCATAACCAAAATAGCCACGTCCCCGGGTTTTATACTTTTCATCAACAGCCAACCATC
CACCACGCCCACTTGTTCGTAAATCATAACCATTAGAACCAAAGCCCATCCCAGATCTAT
ATGCGTTACCATACTGACCATATAACTTGTTTGAATACATCCTATTCACATTCATAAAAC
CATGTGCAGTACCCATTGGCCGTGATTGGTGCAAGCCCATAAAATGAGAATTTGGCCGGT
AATTTTGATTTCTTGAAGAAGTAATATTGTTCCCATTTGAAATTGTAGTACTGTTTGTGC
TATTCACTGGCCTATGCTGTGAATCTGAAACCATTGAGCCATCCAACCATTGGTTCGGAG
ACCGCATCCCATCAAAGCTAAATCTTGGATCTTGATAGCCAGATGCTGGAATACAGCCAG
GCAATGAACCCCTTCCGTATGTAGTATTTGAATTAAATGAAGGGGTTTGATATGATGATT
TGAATGGAGTAGCACCATTACTCCCCTTCACTCCTCCATTTGCACCAGCAATGGTTTTCC
CATTAGCTGTTTCTACAGGCAAAGGCTTCTGGTCTGGAGCTGCAGGAGTGGAAACATCTG
AAGGAGGTGAAGCAACAGGATTAGGGGAGTATGGACCACTGGTTGGAGTCAGTGGTGGGA
AATAATGTGGATACTGGTAGTGCTGAGGTCCATATAGCTGACCATCAGTTCCTATGGTTG
GAACAGGTGAAGTTGCTGGTGAATATGGCCCATATGGTGCATATCCAAAACCATGGTGAT
ACATAATTGACCCGTTATCCCCATACACTCCAGACGTCATCTCCACTCCATCTTGACCTA

CAAATCTGCCGTCCTCCCATTCATTCCCCTCATACCCCCCATAGTAGAAAGCAGAAGGAT
AACCATTAGGGACAAAGCACACGTTGGGATCCATAAAATCATTCAGAAAAGGAGTTCCTG
ACCGCTCTGTTGGAATCTGACCTGATACAGCATTGGCCAAATCAACAGACCCAGGTTGAT
TGGCTGAAGGCTTTTTGGTGGGTTCAGGAATTTCAAGAGTTTTGGCCTCTGAATCCAATG
ACAACTTCTGCAGCACATCAGAAGCTTGATCTACAGGAGAAGCAACTGTAGGAGCCATGA
ATAACACAACAACACAACTACAACAAGAACAAATAACGGATCTTGATGCTTTGTAATCTT
ATAAAAGCCTAAAAATATAAAATTGGGAAAAATTAGGGGTTCTCGAACCCTAAACGCCAA
GCAACTGGAAATGGGTTAACACTGATAACACAGCCGCCAAGAGTTTGGGTTTAGCGAAAG
AGAGAGGGGTTTGCGAAGGACAAAGCGGTGATTCAAATAGCATCACAGACGCCACCTGCG
GGTTATTTTATGATCTCACTTGGGCAGATTCATAACGTGGCGAGCCTTGATTGGGTAAAT
TGTCCCACTATTTTGTCTTTATTGGGGACGGAGGCGGGTTT
>ntig_223_consensus
GGTCACTATATGAACAATCAGATTGATATAAAATTCAACTACTGAGTTGATGCTCTAGTT
TAATATCGACTTGCTTGCCCCAAGACATGGATTCAATCTACCCCTAATTCAGATGCCACC
TCAATTATGCTATGAAACAGCATAATCTAAATGTTATACAGTAAAATGGATTCGCATAGC
CGTCAATAATCTACTAACTCAATTCACATAGCGGCCAAGATCGCTAAAACACAAATGTTT
AACTAGAGTTCTGATTCAACGAGAGATCAGCAATGCCAGCAATCAAGTTATCAAGTGAAT
CATCTTTCATGTCCTTCCACGTCCCCGAATCATCGAAGGCTGATGGTGTTGATTTAAAAG
AAGTTCCTAACAGGGCGTCGTTCAATTGCATAAACAGCTCTTCTTCCCCACCTGAAACAC
CCAAATCCCAGACCCTGAATATATCCTCTAACCGAGGCTCCATTTCAACCGCTGAAGGAT
CTAAATCAAGCCCGGAACCAATGGGTTCGCTATCTTTCTTGCCATCCTCGTGAATTTCCT
TTCTCTCCTTGTAACTCTGCAGAAAAGGATCTCCCACAAAAGCTTCTGTAGCATCATCAC
CCGGAAAATTTTGTGCAAGTAAAATACCATTATTCTGTTTAATTGGATAATTATCACTGT
TTGTTTCTGATTCTGCTAGGATTGGAACAACACCATCATCATCATTTGGTACAGGGAACT
GCTCTGCCTCTTTACTTGGAGTATGTATCATAATGTCAGTTTCTCTGGGAGAATCACTGC
TTCCATGTGCAGAATAAGGAAGTGATCCAGGCTCAGGACTAACAATTGCTAGTAAAGCAT
CTTTCAGAGCATTCCAGCCACTATCCGAAACTTCAGCATCTTTAATCAAGCAAGAATCAA
CCCAGGCTAAATCTTCAGGGGAAAGCACATCTCCACTCTGATTGGACTGCAAAGATTCAA
TAATAAGCGAAGAAATAGACCCTTCCATCATGTCATCCTCTAGAGCCATGG
>ntig_224_consensus
ATGGCAATTTTAAGCATTCGATACAAATACTCACATCCAACTTGGGAGTTCCTTGTCTTA
ATGTTTCCAATCATCATTGTACGCTGATAACTAATTACATGAGTATACCTGATACACACC
CCTTTCTTGTTCTTTTAAACACAACAATAATTCACCCTGCTAGTAACTTTGGAAGAAAAC
AGACCAAGGGATTAAGAAACTTGTAGGGCTCTGACACGGGCAGTCAATTCTCGGACCTGG
CTCCTCAAACGTTCGGGATTGCAGAAGAAGTCAACAAAAACCCGCCTTTGAAGCCCAACC
TCCTGAGAATGATGCTTAATAATCTCATTTTTCTTACAGTGCTCCAGTGTAACTTCTTCT
ACAGCAGCAGATAGTTTTGCATTGATCTTTTCCATCTCATCAAGCTCTTTCATTAATGGC
CGAGGTGTCAATATTGGTTGTGCAGTTGTTGAAGAAGCAAATAATAGTTGCTGCAAACTC
TGAATAAGTGTGCACAGCTCATTGATGCATCTATTTCTGTCCCCTGGAAGAGAATTTTCA
AGATCAGATTGAAGGGTCATTAAGTCTGAACGAAGCGCAGATATCTGCTGAACAAGCCCA
GGAGCAGAAACATATGTTGACAGCCCAGCTTGAGCAT
>ntig_225_consensus
TTTGAGGTCATACTATGCTGCATATCAGCCAATATAAGCACTTGAACCTGCTTTGCTTCC
CTCATTCTCCGAGAAAACTTCACCGAACCAGTCGGTTTTCTCTGAAAGTTATCCAAATCA
ACCCATGTGGGATAATGCAACTTTTGCAACACTTTAGCCTCCATTATCTATCTCACAGGT
AATTTAATCACTTCTTAAATTACTCCAAAGCTCAAATTATTT
>ntig_226_consensus
TCATTTGTCTATGTGCGAAAGAGCGAGCGAGTGAGAAAGACAGAGATACCTCAATTGCAA
TGGTAGCTGGTGCAAGAGCTGATACATCTCTGTGACCAAGTTTGGCTGCAATATCTTCAT
AATAGCAAAAATACACACAGAAATTTCACAAGCAAAAGCATCAAAAAAGAAAAAATCTCT
TAACACAAAAGAGAAGAAAATATAACAGTAAAAATCGATTTATACATACGAAAATACTTA
CTTTCCAAGCAGACCCTAGCATTGGCATTCTTATATGCTGTAGACCTCTCTTCCCATAGA
GTTGATATGCGCTTTGTGTATGCATCGCCTGATTCATGATGAAAAAGGGGGCGAGAATTG
GTTCCTACAGGTGCAATTCTTCATGGCAATGCTTCTAAAGGTACATCCAAAAAGTCACTA
ATGCCCTTATGCCTATATTCCCAGTTGATGTTGTGCGTAACTGCACCTCCACTGGTAGAA
ACAAAAGTCGATGCATTATAGACAGCTTCCAGAGTACCTCAGTCTGGCAAAATTTGTTCT
TGTTGCTTGGACTTTGACCTCTTGGTAACCGGAAGGAAAGTGTGTCAAATGCCTGATTAA
CACTTAGATATTTTGATCATGCACTCAGAGCATTAACTCACCACGACAGTGATAAACCAG

ATCATAAGATACACGAAGAATCTATATGTGTAGTGCAAGAGAGAGAGTTTTATTCCTAGC
CTCTTTATCTCTGAAGAAACCCTCCCCATAAAGTTTAAATATGTCAGCTACAGTAGTTCC
ACCCACAGATTGCTCTATCAAGGAGTCACTGTCAGTAAATGAATAACCAAGCACTCCAGA
CAGAACCTGCCAACAGTAGTTTTTCCAGAACCCATCATTCCTTCAAAATCAAAGCTTCAT
CAAGAGCAGCATGAAAATCTCTGCGTTCCAATATTGGAGCTGGAAAGCCCTTGAAAGCAC
AGGAAACCTCCGAGTCAACAGATCTAATCCAGTTTGAGGTCATACTATGCTGCATATCAG
CCAATATAAGCACTTGAACCTGCTTTGCTTCCCTCATTCTCCGAGAAAACTTCACCGAAC
CAGTCGGTTTTCTCTGAAAGTTATCCAAATCAGCCCATGTGGGATACTGTAACTTTTGCA
ACACTTTAGCCTCCATTATCTATCTCACAGGTAATTTAATCACTTCTTAAATTACTCCAA
AGCTCAAATTATTTGCAACCCAATTTCACT
>ntig_227_consensus
CGCCACTCTCTCTTAACTTAATTTAACTGGGGAGGACAATAAGTAATGACTTCATATTAC
AGAGAGATTACAGACCCAGATGGTGGTGGTTCCACAGTTTCCAGTATTGAAAATCAAATT
AACATATATAAAGGTTGAGCATTTATAAATGAGAGCTACTTATATATGTTGATATATTGT
ATATTAAGAAATTGGAGAGGAAGAAAAACATGCTCCTCCCTTCCATTATTTTCTTCTATG
GCTTGCCAACTTTTAACATCTTTGCAAGCCTTATTTGAAGAAAGTTTCTAAAACACTATC
AAATAATTTAAAAAAAAAAAAAAGAGGGAGAGAGAAAGAGAGAGGGAGAGAGAAGGGTGAC
AGATTTTATTGACAAAAAACAAATCAATCTTTGTCAAGCTAGCCACTCGCTGTCCATTCC
TTCAAATTCCCCAATATCCATGCAGGGCAAGGCAGGATCTTGGAGGTAGTCATCTGTGTA
GTTATGGCTAAGATGTTGTTGCAAGCAGCTCTCATCTGCGTTCAACCAGGAGGGGAACTC
CATGACTTCATCGAACGGATGGAAAGCATCATCCTCTGGCTTCATTTCTTCAGGTCCAAG
TTCCATTGTCTTAGAGAACTTGAACCACCAAGCTGATGTCATCAAATTCATTGTATCATT
CCACTCTATCTGATGTTGTTCTCCTATTGATCTGATCTCAGCCATTTTCTCATCATCCAT
CGCTGGATGTAACCCAGTTCCGGTACCACAGGATTCCAAAACATCAGAAACGGCAGATGG
GCCCCCAACGTCAACTGATTTTGCAACTGAAGGATGTTCTTCATTGGCAAATGAAGGAGT
AGGAGAAGAAGTTGATGATGGAGATGAGGAAGAGTAGATTGATGGGTTGTTGCTGCTCTG
GTAAAGAGACGTGTCTAAGTCGTTGAAGTCATGAAGACTGAAGTTTAGGCCTAAGGTTTG
GTTTGGAAGAGTAAAATTAAGGGTTTCAGGTAAAGGAGGAACTTGTGAAACAGGCCAAGA
ATATGGGTTAGGAGGAGGAGGAGGAGGAGGAGGAAGAGGAAAATTGGAGAAAGATGAGTA
TGAAAGATTGTCTAAATAATTTGAAAAATTGGAAGTGTTTGATGGGTATATTCTAGGATT
TCTCCTAGATTTTATCTTGCCTTCCTGTTCAAAACATTGTTGGGGTGATAGTTGTAAAGG
TCGTGATTGTTGTTTTTGTTGTTGTTCTAGCTGTTGTTGCTCACTGGCTTGTTTCATAGC
AGCCCTATGGAACTTAAGTGCGGTGACAATTTCTCTCCTAGCCTCAGCCATGTTTAGAAG
CCTTTCTTGGTAAGGCCTACTGGTGTGAAGTCTCCTCCTAACTTGTTTTTTATGTTGTTG
AGGTTGTTGGGGTGTCTGATTTTTGGGGGCTTCACTAAATCCTTCACCAAATTTGGTTAA
ATTTTGGACGGAAACACTATGATCACCATCAACACACTCAGGGTCTTTAGGATTCATGGT
GTCTTTGGTTCTTGATGAGGTAAGTTGTTTCACAAGGCTACGGATATAAGCACCAGAGAG
TTGAGGCTCATCTTTGAGCTTACTATTACTAAGCTTGCTGTCAATTTCCATTTCTTCTCT
GCTCTCTGGCTTTGAAGATCTACCCCTCATCTCTCTCTCTCTCTCTCTCTCTCTCTCTCT
CTCTCTCTCAAACACTCAATTTAGAAATGCAGAGGGAGAAGGTTTGGGAAGGTCATGGTT
TTTTTTTTTTTTTCTATATTCTGAGAGACAAAGTACAGAATGCCGAGAGGCAGAGGCTGA
TACGGAGTACCCCCGAGGTCCTCCTGGGTGTAATAAATACTGACTTAGGGACAGTCAAGG
GTTTTTAAGTTCTTTTTTATATTTCCCTCATTTAGCAAACCCCTTATATTACTGTTTTAT
TAACCGCA
>ntig_228_consensus
CATAAACTATCCACTATTTGGTTATTTGGCCATAAGATGTTTCCAAACATGATTTGTAAT
AATTATTTAGAAACGTTCTTGCTTACAAGAACAAAAACTCCAATCCAAATCCAAAGTAGC
TTGTTTGACTAGGAAAGTTTCTTATGCCCCGGAGGACGAGGGTTGCCGCCGAGAGGATAC
GTAGGCACAGTGGTCTCAGGCACAGGAGCAGCCAATCCAACTGGATGGTGCTGGTGTCCC
TTGTCCGCCCCAACCACCGGCTCCTTTATCTGCCCATACTGAGTGGCAGCAGGATAGGGG
TCGTCATGATGATGCTGGGGCTGGGCGTAACCGAGTACATGCGAGTGCTTGGCTCGTAAT
TTCTCAGCAGCATGCTCGGCCTTGGCCTGGTGTAACTCCAGCTTCGCTTGAGCCTCCTTC
GCCTTCCTCCGCTGGTGCGCTATCTCCTTCTCTTCCTCTGTCCTTGCTGCCGCCTGCTCC
ACCTGCTCTTCAACTTTTGCTTTGTAGATATCGACGTGAGCCTTGCCTGAGCTGGCGGCA
TTACTCACTTTTTCCTTCACTGTATGCATGATTCAATCTCTCTGCTTGTCTATCTGTGTA
GTTATTTA
>ntig_229_consensus
CATAAACTATCCACTATTTGGTTCTTTGGCCATAAGATGTTTCCAATCATGATTTGTTAT

AATTATTTAGAAACGTTCTTGCTTACAAGAACAAAAATTCCAATCCAAATCCGAAGTAGC
TTGTTTGATTAGGAAAGTTTCTTATGCCCCGGAGGACGAGGGTTGCCGCCGAGAGGATAC
GTAGGCACAGTGGTCCCAGGCACAGGAGCAGCCAATCCAACTGGATGGTGCTGGTGTCCC
TTGTCCGTTCCAACCACCGGCTCCTTTATCTGCCCATACTGAGTGGCAGCAGGATAGGGG
TCGTCATGATGATGCTGGGGCTGGGCGTAACCCAGTACATGGGAGTGCTTGGCTCGTAAT
TTCTCAGCAGCATGCTCAGCCTTGGCCTGGTGTAACTCCAGCTTCGCTTGAGCCTCCTTC
GCCTTCCTCCGCTGGTGCGCTATCTCCTTCTCTTCCTCTGTCCTCGCTGCCGCCTGCTCC
ACCTGCTCTTCAACTTTTGCTTTGTAGATATCGACGTGAGCCTTGCCTGAGCTGGCGGCA
TTACTCACTTTTTCCTTCACTGTATGCATGATTCAATCTCTCTGTTTGTCTATCTGTGTA
GTTATTTA
>ntig_230_consensus
CCTTTTTCCCTCCCCGACGCTCTTCCGATCTACCCAAGCAATTTGCCCTCCTTCAAATCT
TTTTTTTTTTTTTTTTTTCCAAACTTTTTACAGAAGTTGCATATATTAGTCCTCAACC
ATCTCCTCCTCATCATCGATCTCCTCAATCTTCATCTTCTTAACTGCCTGAGCAGCATCA
CTTCTTGCTACACTCGGGATTTGAATCAACACATCGGATTTTTGTCCCGTGGCTGCATTG
ACAATTCCTCCGTTTCTGTCCACCCGGTACGCCAAATCATTCCCCTGTGGCCCTACGTCG
ATGTATACCGTTGAGTTCTCATCGATCTCCTCCCTCACCAGCATTCTCGATAGCTCCGTT
ACCACCTTCTTCTCGAGCCATCTCCTTATAGGCCTTGCACCATAAACTGGATCATAGCTC
TCTGCCAATACGATATCCAGAGCAGCATCGGTAACTGCCAATGCGACGCCCCTCTCAGCA
AGACGGACAGCCACGTCTTTCATTTGCAACCTTGCAACTTGCCTCAGTTGGTCATGCGAG
AGAGGATCGAACACTACTATCTCATCGAGCCGATTCAGGAGCTCTGGCTTGAAGTGCTTC
CTCACCTCTTGCATAACTCGATCGCGGGCAACATGCATTGGCACCTTGCCCATTAAGCCA
GATAAGAGGTGCTCAGCGCCAAGGTTTGAAGTCATGATGATCACTGTATTTCTGAAGTCA
ACGGTGCGGCCTTGGCCATCTGTTAATCTTCCATCATCCAGAACTTGAAGGAGAGTGTTA
AACACAGCAATGTGTGCTTTCTCCACTTCATCAAACAGTACAACACTATAGGGCCTCCGC
CTGACAGCTTCAGTGAGTTGTCCACCTTCTTCGTGTCCGACGTAACCAGGTGGAGCACCA
ATTAGCCGTGATACAGAGTGCTGTTCCATGTACTCCGACATGTCTATTCTCACCAGAAGA
TTTTCTTCATCGAAGAGTTGCTCAGCAAGTGCCTTAGCAAGCTCGGTTTTACCAACACCA
GTTGGACCCAAGAAGAGGAATGAGCCAGTAGGTTGTTGTGGCCTACCAAGTCCAGCCCTT
GACCTCAACACGGCCTCAGCAACAGCATTAACTGCATCATCTTGTCCAACCACTCTCTTA
TGCAATCTCTCAGCTAGCCCAATTAACCTTTCTTTCTCATTCTGTCCAAGCCTTGTAACA
GGAATGCCAGTCCAACGGCTCACCACCTCGGCTATGTGTTCTGGTCCAACAGTTTCCGTA
AGCATTACATTTTCACCAGCGTTTCCATTAGCGTTTCCTTCAAGCTGCGCTATGGCAGAT
TCCACTTCTTGAATTGCCCCATATCGCAGATCAGCAACTCTTGCTAAATCGTATCTTCTT
TCAGCCTCTTGTAATGCAAAGAGGAGCTCTTCTCTCTTTTGTTTAAGCCGTCTAATCTCA
TCAATTCTTTCCTTCTCTTTTCTGTACTTCATCATTAGTGGTTGAAGCTTGTCCCTCAGA
TCATCAAGTTCTTTCCGAACTTCAACAAGACGGGCTTTGCTAGCTTTATCTTTCTCCTTT
TCTAGTGCATGAAGTTCAACTTCCAATTGCATTCTTTTCCTCTCAAGGTTGTCTATTTCC
TCAGGCTGACTATCAAGCTGCACCCTCACGTTAGCACAGGCTTCATCAACCAAATCGATT
GCCTTATCAGGGAGATGTCGCCCAGTAATGTATCGAGATGAGAGCTGAGCAGCAACAACA
AGAGCACGATCCTGAATCCGGACACCATGGTGACCCTCATACCTCTCTTTCAATCCTCTA
AGGATGCTAATAGTATCAGGGACACTAGGCTCGGCCACAAAAACTTGTTGAAACCTTCTC
TCAAAGGCAGCATCTTTCTCCACGTACTTCCTATACTCCTCAAGTGTGGTAGCACCAATG
CACCGCAGCTGGCCTCTTGCAAGCATGGGCTTGAAGAGGTTAGCGGCATCCATTGATCCC
TCAGTTCGACCAGCTCCAAGAACCAGGTGAATTTCATCAATAAACAAAATCACTTTGCCC
TCGGCTTCCTCCACTTCTTTCAAAACAGCCTTTAACCTCTCCTCAAATTCTCCCCGGTAC
TTAGCACCCGCTACCAGGGCGCCCATATCCAACGCAACAAGCCTCACATCAGCAAGATTA
CTAGGAACATCCCCTCTTACAATTCTCTGAGCCAACCCTTCTACCACAGCAGTTTTACCA
ACTCCTGGCTCTCCAATGAGGACTGGATTGTTTTTTGTCCTCCTTGATAGGATTCTAATG
ACCCGCCTAATTTCTTCATCTCGTCCAATGACTGGATCAAGCTTTCCTGCCTGTTCAACA
AGGTCTCTTCCATAAGTCTTCAAAGCTTGAAATGTAGTATCACCAGAAGCACTCTCCACC
TTCTTGCCTTCCTTTCCTCGAAGTTTTTCTACCTCAGATTTCACTTTCGCTGTGGCAACC
CCAGCTTCCTTTAACAAGTCCCCAATCTGAGAATCCTCCAGGAGTCCTAGAATTAGCTGA
TCAACAGCAAGATGGGTGTCTCCACGTGACTTTTGTGCAGCTTGAGCCCGTCTAATTGCT
TTGATAAGAGTAGTGCTAGCTGGAATTTCATCAGGCGGCGGGGTTTGAGAAGGGAGTTTC
TTCATGGCTTGTTTTAACACTCTTTCTGCAGACTGAACAGCATTTTCACCCCCCAGCATTG
CTAATCGCTTGGGAGAAGATGCCGCTGCGTTCAGATAACAAAGCGATAGCAAGGTGTAGA
GGAGTGAACTGTGCATGACCAGCACTCATTGCCAGTTCATGCGCCCCAGCAATAGCTTCA

TTTGTCTTGTGGGTGAACTTATCAGGATTCATTGGCTCTTTTTCTAAAGTATCTACAAGA
ACTTGAGTATAAAACTTCCTTTGAAAGACAGTTAGACAGAAGAAGTGTTAGATTTCCTTC
TTGCTTTGTAGTGTTAAGCGAGTTAGCCTTCCTGTTTAGGACCTGCGAGCAAATTTTCTG
ATTTCTGTGCGTGAGAAGAGAGAGCAAGAGATGGCCTTTTGTTAAGCTAAGAGAGACGTC
TAGAC
>ntig_231_consensus
TTTTTTTTATAAGACCTGTCTCATTATATACGACGAGTAAAGTAATGTGATTACAAATTA
CAATCTAATAATCTTCCATCTCTCTCTTTTAAAGTATGAAATATGAATACCACCTTACAT
TGCTTACGTATCAGAACTCCAATTTCCAGAGCCAAAAATCAGAATTCATGACCCACGAAG
AACTTAAGACGGCCGGTTTGGTGACCGCATCTGAATCTGAACGGGGATGGGTCGACCCGT
ACCTGATCCTCCACAGCTGTTACACGCCATGCGACCCGATCCCGAACAAGTTCGGCAGCC
AACATCACCGTCGGACCACCGGGTACACATACACATGACTCGGCCCGTACCGTTGCAACG
GGGACACCTTGGAAATTGTCCGGCCATGGGTTTCTGGTCCATGACCGATTTGACGAGAAC
GGCTCCGGCCAAAACGCTTAGCCCAGTGGCCAACTGCGTCAACACAAGCGGACCCATAAT
AAGTACCTGGAAATAAATGGATTCAAGAACAGAGCAAAGAAGCCTGCACAGAGAGAGGGA
GAGAGAAAAATGAAGATCTTTCAAAGTGATATATCCAGG
>ntig_232_consensus
AATTACAGCATTTTATAACCAGTTTGTAAATAGGCATGACCAGTATTCACATTACTATTA
ATTTTAGAAGTCGAAGCTTGTAATCAACTGATAATTGATCATATAAGTTTTCTTCACATG
GTTTTGTAATTACCACCATCTTCCTCAGAGATTAATTTGTTTAGTCTCTGCAGCAGCATG
TCAAAATCATCTTGGGTTCTGATCAGGCTTAGCCTTACATGGCTATGATCTTCTGCACTA
AAGATGTTACCTTCTCGCCCATCAATCTTTCCAGCTCTCAGAACTTCATAGCAATCGTTG
TCCTTTTCTGTCTCACATCTCAACCATGCATAAGCTGGTGAAGCTGGCCTGACTTTGTGG
AAGAAGTTGCAGTACTGAGGAGCAATTTCCTGGAAAGAAAATCTCTTTGATAGAGATACA
ATCTTGCTCAACTTTTCCCACCGGCTCCTCATTGTTTTGTATCCGAATTCAAATATTTCC
TTTCCTCTGCTTTCTAGCACTACTTGCACAAGCTTCAAGGCTCTTAGCTGACTGTCCCGA
GAAACTCCTTGGGTGTTTAAACGCATGTAAGTTGTCATTCTTTCGTACACACTCTCTTCC
TTTATCAATGCCCATCCAAACCTGGAGCCAGCATGGCCAGTTATCTTTGAGAGTGTGAAA
ATCATAACATCTCCATCTGCAGCAGCTGGAATTGGTGTAAAATGAGGCCAATAATAAGCA
TGATCATAAATTTGTTTGACATTTGGGCCCTGAAGAACTGCCTTGTTCATCTTTCCATCA
GGATTGTTTGGTGCCGTTACAAACTCAATCAAATTCATCCTGGTATCTGAGCTATCTGAG
CTATTCTCCCACAGAGACGTATCTCCATAGAACTCGAAATCCACTGATCGGAAATAGTCT
GTTTGCCCTTGGTAAACCGGGTAAAAAGGAGCTGTAGCCACAACTTTTGCAGGTGAGGAA
GAGTTAACAGGAGAAAGGGCATGGACTGCAGCATTAAGGAGTTGGGTTGAACCAGCACCC
AAAATGATGAATCTGTTTTTTGTAACTGCATTTCCTACAACGGCATGGAGTTTTCGGATG
TGCCTGTCAAGCTCTTGAGAGATGTAAGAGAGGTCTGCGTATGTATAACTCATTCTGTGC
CACCCTGCTACCAGGATTGCACTGCTCGCTGCTTTTTGCATCCAGAAAGGCTCCAAGAAC
AACGGATCCCCGCTGTCGGCGTTAGCAGGACAATCAGGCAAAAGCTGAGAGCAGTCAGGT
CCAGAGTAGCAAGAATTACACTCACAAACTGGCGGTTTTCCGTCGACAACAACCCCATCC
AAGTAAGCTCTTCCATGGCCGGAACATGAAATTGCAGCCACAGTCTCAGCTTCTCTTGCT
GCTCTGCTGCTCCAACCAAGATTCCACTCATTGGAGCTTTTAACTTTGGCCATGCTTTTT
CAACCGCCTAGGTCACTGGCAATAGTTGCGACAGCCGCCTTGTTCGGATAAAGAGAAACA
GGTCAAAATAATATAACAATTCTATTGGCCTATAGAAATGATTCCTTGTATGGTGTAGAT
TAGCAGAGGTGGAGATGCTACTAGAGGAAAGAAAAAGAATGAGGCGGCTAAGGAAGATTT
TTCGTTCTTGGCCTCCTTGAAAGG
>ntig_233_consensus
TTTTTTTTTAAAACTAAAGGATGCTATATTACAAATTTCACCAAGAAGGACTGCAATGCA
GCCGAACAAGTTCATTTGTCATATTGTCTACAGAAAATGAGAACGCAATAATTCATTGGA
AATACATAAGTAGTGCAAATTACAGCATTTTATAACCAGTCTGTAAATAGGCATGACCAG
CCTTCACATTACTATTAATTTTAGAAGTCGAAGCTTGTAATCAACTGATAGTTGATCATA
TAAGTTTTCTTCACATGGTTTTGTAATTACCAGCATCTTCCTCAGAGATTAATTTG
>ntig_234_consensus
TGCAAAATTTGGAAACTACTAGAGTAATACAGGAGGGACCGGCAAAGGAACATGGATTAC
GCAATTAAACAAGCTACTAAAAACTAACTTTTCCTACATATTACATGGGGATCTAACTAC
TTAAGTTGTAGAAAACATAACCTTCTGCAGTGGACCTGATTTTGGGTAACTTAGGTCAGA
TTAAAAAAAAGTATGTACTAGTTTCCTCCCAAGGCAAAGATAATTACAAAATGACATCAT
AATGGATTACAAATCTGCCATCATCAAAGTCCCCACAATTACTGATCCCCTCTCTCCCAC
TTTCCCCAGTTTTGTGCACAAAATAACGGAGCAAATCAAATTACATTAGATAAAAAATCA

CTAAAGTACGACATGATGACAAATAGAATAGTCTCTAAAATATGTGGTATAAATCAGGAA
GTAGAATAGTAATACCCTGCTGGTGGTCCCATCCCATTTACTGGTGGCATTCCATAAGGC
ATCACAGTCATAGGACCTGCATTGTAGTATCCAGTACCGGAGATCTTGGCTACACTAGCT
TGGCCCTGCATGCCATCCCTTGCATACTGCTGCCATACCAGCTGCTCCTGCACAAGCAAG
TGCTGTTTCTTTTCCATATCGGACATTTGCACATATGAAGGAGGTGGAATGCTCAATGAT
GCAGCAAATGGGTCCTGATTAACTGTCTGAACTGTTCCATCTGGAGCCGGAAGAGCCAAA
ACTGGTGTTGAAGTCTTCCCTAAACCTGGCAATGCAACACTACTTGCACTCCCACCACTC
AATTGAGAAGTGCTCACATGCTGCCTCACCATTCCCTGATCATACATGCCATTCAACAAC
AATGGATCAAACCCACCACCCAATGCTGCTTTCTGTCTCGACAAATTGCTGGCAGACTCA
ACCAATGCCAATTCCCAATCTGCCTTGCCAGGTTCAGCAGCAGGGGTTTGCCAACCCGAG
GTAACCTGCTCCCCATTTGATGGAAATGCTTCCCATGATCCATTACCATTAGCTGCAGGC
CCATTAAACAAAGCCAAGGCCAACTTATTCCCCTGTTCATCGGCTGTAACTGTATCATCC
CTCAAATTCACCAAATCTTCTGTCACTCGTGGTTTTTGAGGCTCAGGTTCAGGTTCAGGC
TCTGGTTCAGGTGGTGGCAGAGGAGCGTAATTCTCTGGAGGAGGCAATGCCTTTATTTCA
TTCATATCCGGTACTGGTTCCTCATGTGGAGGAGTATCCAATTCTTTCCTCTCTGGACTC
TTAGGCCTATTGGCTCTATCCCTCAAAAACTCCTGCAAAGTCTCTAACAACTTGCTTGTA
ATCCTCTGAACATCCGGATACTCAGAGGACCTTGCCACACCATTATCCTTGCACCAATTA
TAAAAAGCAATCAGCTCATCAATCTGCTTTGCTGCACTAGCATATGCATCAAAGGCCTTA
ACACAGTCTGTATACTCCATATCAAAAAACTTATCAAGCAACACAGCCAAAACTTCACAA
ATATTTGAATAAATCTGAAAACTCTCTTTCACCATTGGATACAAAGTAATCAAAATCATC
CTATTATTTTTAGCCAGACCCATGGGCCTACATGACAGGAGCCTATCGAGCAGTCTCTGC
AAATGCCCCATTTTTTCAAATATCCTCTCCGGCTTCATTTCTCTCAATGGAGTCACTACG
CCTTTTTTCTCTTCCCCACCACTAGCCTCACTCATATCTCCATACGATCTTGATCTCCTG
GTCATTCCATAATTGCCATAACCACCATTGCCTCTAGAGTCTCCATAATCATACTCATTC
AGTCTGGGAGGAGGAGACCGAAAATCATCCCCATATCTAGATCCAAATCCATCATGATAA
CTACCAGAACCTGAAACAGACTTAGAACCGCGCTTTCTATCAAACAAAACAAGTTCTAGT
CTTTGATCTAAATACATGGCATAAGTTCTCACAAAAGCTGACTGGTCCCACGAGCTCGAA
TGAGCTTCATCTCTAAAATCCGACATGTTCAAGAGCCTTGTTCCTCTTCTTGTTGCATAC
AAGATCTCCTCTTGAAATATTGGATCCCCCTCGTTTAATAGCCGGTGTACAAGCGTCAGA
GTCTTAAGTGCCACAATCCAGTCACGTGTCTTGCCCAGACGCTTTGAAACGGCCGACACA
CAAGCGTGAACATAGACACGTGAGTAAGAGGTTAAGTTGAGGATCTCGCGAATGTATTTC
TCGTCGGCCGGGTCGTCGTCGTGACTCGTGGCTTTGACTATTGCAACTTCGAGGTCTGGA
GCCATGTTACTTGACACCTTAGCTATGCCTATGCTAGTCTGGTCTTTAACAGCCCCGATC
GCTTTCCGAATTGTGCTCGGCGCCATATCTACCGTTGATTTAACTCTCACACCTTTTCCG
TTTCACCGAGTTGTTTAAAGTCGAATAGTAAATGATCAAGGGACCGTCGTCAGATGTTGG
AGAAGATGC
>ntig_235_consensus
AATGAAACCACCAGTCCATCCCGGTAAGTGCTTTCTAGCACTTCCATTCCAGGCTTCCTC
AAAACCAGCAAGTTCATCTTCATTCAGATTGTGCAAAGTCTGAAGTGTATCCACCTTACC
ATCAGTATTAAGCTTTCTTGTAACAGAATGCCCCTTGTGGTGAAGTCCCCTTGAAATCAT
GTGAGTCGCTTGCCTGGTAGCACTATCAGCTTCTTTGCTTTCTTCAATTGATACACCATC
ACTTCCTGTCCTCCTTGTATTAGAGAAAGTATGGTAAGCTCCATTGGCACCACTGTAAGT
TACAGTAGAACTTGAAAAGGTGAAGTTATGAGCTTGGGGCTGGGGCTGGGGCTGGGGCTG
GGCACCATTAAACAGACTATAATCACTGCTGTGTTGCAAATACTTGTTCTTTCTTCCTTC
AACTACATCATCTGGATCCTCAACAAAAGGCTCTGTAATAGACCTTCCATGCTTCCTAGG
ATTTTCTTTCTTCTCTTCAACTGCTTTTTCATTTTCATCATCAGAGTTAAGCTCTTCTAT
AACTGGCCCCCTTGACCTTTTGGGCTCTGGAGGTTGATGCTCAATAAAAGCAGATGGTTG
CATATTAGGGAAAGGACTCCCGATGGAACCAAAGAAGCTTGACTCAAGCATACCACCAAA
AGGTCTTGTGAAGAAAGGATCATCAAATGGGTCCCTTCCGCCAAAGAAACTAGACATTAA
ACTCCTGGGATCCCCAAAGCTATCAAAACCTCCGAACCCAGCAAAAGGATCCCCAAAATT
ATAGAAAGGATCCCTGCCTCTTCTCCCTCTTTGCATTTTCCCACAGCTATAAATTTTCAA
AACT
>ntig_236_consensus
TAAAAAGAGAACATAGACAAACTTCACATCCAGCAAGACACCACAATCCAATGTACAAGT
TGATATATATATATATATATATATATATATATATATATATATATATATATATTATAAACATC
TTATGCATTAATGGGAATGATGACCACCAGGAAGTTTCTCCTTGATCTTCTCCATCACAC
TTTTCTTCTCATGATGCCCTTGTTGCTGTTCTGGAGCTTCGGTGGAGATGATTGTGGTCT
GTGAATGCTCCTCCCTCTGTTGCTTTCCATTTCCGCCAAGTTTCTCTTTTATTTTCTCCT

TCAGCCCCTTCTTCTTCCTCCTCCCACCTTGCCCATCATCCTCAGACGAGCTAGAGCTTG
AACTGCCAGAGCGCTGCATGGCCTCTCCAGCACCCACATGTTGATCACGCTGGCGGTCAT
GTTCCAGCTCTTGCTTCAACTCTCGATGCTCATGATGACGTTCCGCACAAGACAGCTGAG
CCTGAGACTGAGCTCCGGCATAATGACCACCAGGCACATGAGCAGCAGCGGGTGGAAAAC
CAATACCAACGCCGCGGACTTGGCTCGCAGCAGGTGCACCTTGGGTTAGAGCAACACCGG
TGAGGTGCATGGGGTTACCGTATTCGTCGGTTAACTGAACTGGGTTCCCAAGTTCATCAG
TCAGTTGAATTGGGTTCCCAAACTCATCACGTAAATCAGCCATTTTGGTTCAATATTTTG
ATTTGAAAAGTATTGCTTGCTGTATATTTGGATATTGATGAGTACATTCAGGCTTTATTA
AGAGTTTAAAGAGTGAAAGAGTGACGGTGGAGCAAGAGAAACAGTATTGGTTTTTGCACG
TCAATCTGGTGTTTACGTTGACACGTAGCCGCAGCGGCAAGGCAGGTGTCGCTTGAGAGA
TTAACAACGATATCCTTCGGATAATATTTATCGCGATTCCGTGTCAAACTGGCATGAAAG
GCCAAAAAATATGCGGCACGAGATGGATTATTTGAGCACCGCCTCAGTTTTCACTGCCCGA
TGAAGAGAATCAAGTTCGAGAACAAAGACAGCATCGACCCAAACATAATAAATGAACAAC
AAATACGCTTCATGGCTCCGCAAAATGGGAAAGCAAAAGTGGCTAAAACCATTAATCTAA
CCCAAAAAATATTTACAGATTAAAAAAGAAAGGCAATCAAATAGCGCATATAACCTGTATT
TGCTTCGATATGTTCTTCATTTACAAGAGAGATTATCCTGGAGGTCATCCAACAACAAAC
CCATCAAGTGAAGGAAGGAATCCAGCCAGCCATACACAGTTTCAATAGAATGAATTCATT
TTTGTCCAAGCAAGAAACAAAATTATCTGTACACTAGGACCAATAGTTCCACAATGCATT
TTCATTCTACTAACTTCTTTCTTTTGCATAATTACCTTCTTTACCTATTTCTTTTTCCTT
CTTTACAAAGTAAATCCAGAAACTAATTCTGCTTAAGTTTCCGAGTTTGTATGAAGATTT
GTTCTTTAAACATCTTGATGAAAAACATCCACCACTCTTCATCAAACTGAAGCTCCACAG
AAAATCAAACATTGGCATTCTGTAATTATGAACCTTCTTTTAGAAAAGTCACAAAGACAA
AAAGAGGGCTAGAAATACTAAATTATGCTTAGTCCGAAATTCGACCTAAATTTGTATGAA
TATGTATGCGTGTGTATGTGTCTATGTGCGGCCCTCGAATATCTGTTGGACGCACAATGG
GTCTCATGGTGCCAAGTCAAAGGTACACAAATTTAGCAATGATGCCCAAGCATGGAAATG
GATAATTTCAGTGTACACAGAAATAAAACAAGAGAAAATTTATCACCTCTTACAGTTGAG
AAGCAGTT
>ntig_237_consensus
GATGTGGAATCTATAGAGGTGAATGTATCAAAGAAGAAGGTGATTCTTACTTGTAAACCT
GTCAATGAAGGATCTTCATCAAGAAAATCTTCTACTGTATGCAACAATTTATCCAGCAAA
CCCACAACAACTGCATGGCTTCCTTCCTATATGTGATCTCGAATCAATGCTATCTACAAC
TATGAGCCTTGTAACAGCTTGTAGAATCACTATCCAAGCCATGAAATAAATGTAAGGTTT
TTGTTGGGTCATAGTTTCCCTTTGATGAACAAGCCTCTGGAACCAAGAAAGGGATGCTCG
CTTCAAGGGGAAATGCAATTTTATACTTGATATTGATCTATCTTTATTCTCAACTTCATT
TCACAAAAGCTAAAGCTTAAATCATACTACATTTGTATATATATATNNNNNNNNNNNNNN
NNNNNNNNTGCCATGTTTGAACTTGCCTCCATGAGCCCCAAAGGGCTTGTGACCACCATG
AGACATATGCCCACCATGTGCTAAGTGAGATGCGCCATAAGCAGCTGCACCGGCAGCAGC
ACCCCCAGCTAGCATAGCCCCCATGCCGCCATGTCCTGAATGATGTGAATCTGACGGGCC
ATGTGCAGCTGGAGGGTATCCAGCAGGAGGATAGCCTTGCTGGGGAGGGTAACCTTGCTG
TGGAGGATACTGTCCTGGAGGTGGAGGGTATCCATGGCCTCCTCCATGACCTGCCAGATG
ACCAGCTACATGACCAGCAACATTTGAAAGTAGACCCTTACCGGATTCTTCCTTTTTGCC
GCCTCCCATGCTTTCTCGATCCCACAATAACTGTAT
>ntig_238_consensus
CCCAATTCTGCCGTAGGCCCAGATGATTTATGTACAATAATTGATAAAAGCCAGAAGGTA
ATCTGTATAGATATATAATTTAGTAAAAGCATGTCAAATCCAGTTAAATAAACAGAGAGC
TTAAAGTCATCATAGAATGTATGAATTATTGAATAAAACCAACATCTGAGTTACTTGATA
TATGTAGAGTCAAGAGTCATATACTTCAGTAGATCAAGAAGAGCAAATCACTTCCACTTC
TTGAACTTGCCTCCGAACATTCCATGCTTTCCATGCTTGAATTTGCCATGCTTACCATGC
TTGAATTTGCCGTGCTTACCGTGTTTGAACTTGCCTCCATGAGCCATATGAGCTCCAAAA
GGCATGTGACTGTGAGACCCATGTGCCAAGTGAGATGCACCATAAGCAGCTGCAGCTGCT
GCGGCACCACCAGCAAGCAGTGCCCCCATACCGCTGTGCCCTGAATGGTGTGAGCCTGAT
GGGCCATGAGCAGCAGGAGGGTATCCAGCAGGGGGGTAACCAGATGGTGGATAGCCAGCA
GGAGGGTAACCTTGCTGTGGTGGATAGCCTTGTGGGGGGTAACCTTGCTGTGGTGGATAA
CCTTGTGGGGGGTAACCTTGCTGCGGTGGATAACCTTGAGGAGGATATGCACCGGGTGGT
GGAGGGTTCTGAGGAGGATATCCATGACCTCCAACATAACCAGCTAGATGTGAAAACAGC
CCCTTATCAGATTCTTCGTTCTTTCCACCTCCCATATTTTCCCGATCCAAGAATAACTAT
ATCTTTCTAAAAAGGTGATTCCGAATTCGAAAAGTAGAGGCAATAATAGAGGGAGAGATG
CAATTGGGAATAATATAACAGCTATCGTAAGCAACTTCCGAGCAAGTTCTGACTCTAAAA

ATGAGGCGAAAAGGGGATTCCTTCTACCCAGTTTTTAAGCGTTTTATTCGTGTGTATCT
TCAAATCAAACTTTATCCTAAAACGAAAACGTTCACATCAGGCCGGATGCCTATGTCCCA
CCCAAACTTTAAGGATGCCGTCGTCCGACCATTTAAGACTGGAAAATTTAGACATACGGT
TACCCTGGTTCCGTCTAAGCCCCCTTGTTTTCCTTTTAATTTTTATTAAAGAATCCGTTT
TCTAATTTAAAAAAAGGTGTCAAATATTCGAT
>ntig_239_consensus
AGTACTGGAGGAGTAGCCAACATATCCACCTCTTGAACCACGGCCTCGGCCTCTACCGCG
ACTGCCAGGGTTATAGTACCTCTCTCCTTCAGCAGGTTTCAAAAACTCATTTATGCTGAC
AGACTTTTTAGCTTTCTCTTCTTTATCAGCAGCATCTTTGCGTTTGTCCTTATCAGAACC
CAGTTTGATAAAGATGTCATTGTTGCTCTTCTTGCTTGAAAGTTGTTGCATGGACTTGAA
ATCTTTGTCCACGTCAACCTTCCTTTCCTCAATCTTCAAGGCAAGCAAAGATTTTCTCTT
CTCTTCAAGAACCTTTTCATACTCTTCCAACGTCATTTCCTTTTCCTCAGGCTCCTTCTC
TTCTTGCTCATTAGCAGGATTCTCCTTATTAGCATCTCCAGCATCTTCTTCTCCAGCAGG
CTTGTCATCACTAACATTCTTATCAATTTCAACAACAGGTTCTTCGGTCTCCGGTGCAAT
TTCATCCGCTGGAGTTCCCCAATTACCACGACCAGCCCCATCACGCTTTATCTCATAGCC
ACGCCCTGTCCCACTACGACGTTCAAACTGTCTACGTGGTTGTGCTCCATCACCTGATTC
TCCATCGCCAAAGCCACCACGACGACCCCCACGGAATGAACCACGAGGAGCCCCATAACT
ACGTCTTTCAGATGGCTTTCCTCCTTCTCCCTCTTCAGATGGTCTATATCCACCAGAAAA
TCCATTATTGCCACCAAATGTGTCACTGCTTCTTGAATCTCGGTCAAAGCCACGACCTCC
TCCACGCCCAAAACCACGTCCACCACCCCGGGCCCCACCACGACCACCTTCAGTCTTTGA
ATCCCTCACAGCTTGAGTGGGAGGGGGTGGCTTGGTAGGAAACTTAGCAGCCGGCTTAGC
AGCTGTCTGACCTTGAGGAGCCGACACAGGTTTCTTAGGCTTGTCAGCGAGCTTCGGCGG
TTGAAGAGAGACGAGCTGACTTGGGTCTTCGTTATCATTGTCACCCAGTAGATCAAAGGG
GTTCACCGTGGCCATTGGTATGGATAAGCGTATAAACTACCGAGACAAAAAAAATGAACT
TAGAACGAAATGGACCCTTATTAATTATTGAAAGCCATGACGGAATCGATCTAAAAAAAA
AAAAAA
>ntig_240_consensus
GAAAAACCTGAAGATGAATAATTTGTGAATAACGGCAAGTTTATTTTATTAATCTTGAAT
ATCAATATAATGAGAATCCTTTTCCATCCAAATCATATATAATTTACGAGTAAAGGTAGA
AAATAAAAACTCTAATTCTTTTATACCAATTTCCCCACTTTATCAATTACCAAAAAAAAA
GAAGTTTCCCCACTTTATTCAATTCAAACAGATTTACAGCCAAATTCAAGACTAAACAGA
TTTAAAAAAAAAAAAACCTTCAGCGGGGAAATATATACTACTAGCTAGAGACCTTAACTA
AGCCCCAGCTAGCTACTTCAACGGTAAGCGCCGCCGGAGGTGGGTCCGGGGTGATAATAG
GGATCAGAGGCAGGGGTGGTGTAATCATCGACACAGTAATCGGTGGCAGTGGTGGTTTTG
GTAGGTTCACTGCCCCATGTCATGTATCCAGGGTGAGGTCCGCCGGACTCAGCCAGCCGC
CGGGCCTCTTCGTTGTGCGCCTTGGCGGCTTCCTTGTTGAGTTCCGCACCAGCTTTTCTT
ATCTCTTTTTCTACTGTGGCCGCTTCTTTATCTGCCGGGTCTCTGCTTGTCAACTTCTCC
ACCTTCTCCTTGGTGGCGGCCTTGGCCTTCTCCATGCCGGCCCTGGCAGAGGCGGCAGTA
TTGGCGGCCGATTCCTTCACAGACTCTACGGTTTTTTTACCTGATTGCATTTTTCCAGAT
TTCTAAAGAATATGAACTTTTGCCCGAAGAGATCGGAAGAGAGTCGTGTAGGGAAAGAGA
ATAGATAACGGTGGAGGCAGTGTCAATAA
>ntig_241_consensus
GCAGAAGTTTCAGAAACAGCCTTTTCAAACTCTCAGATAAAACTCCTTTCAAAATTATAA
GGGAAAAGACCTTGGGGTCACGTAATGACTAACTGCCAACCCGGTAACACAAAAGTTTAA
CACCAATCGCTCGAACTCTAGTATGGTTCGACAGGCGTGCGGAAGTTTGCTCCTGCATAA
ACTGCTTCAGCACCAAGCTCTTCCTCAATTCTTAGAAGCTGGTTGTACTTTGCAAGTCGC
TCGGACCTGCAAGGTGCTCCGGTTTT
>ntig_242_consensus
GCAGAAGTTTCAGAAACAGCCTTCTCAAACTCAGATAAAACTCCTTTCAAAATTATAAGG
GAAAAGACCTTGGGGTCACGTAATAACTAACTGCCAACCCGGTAACACAAAAGTTTAACA
CCAATCGCTCGAACTCTAGTATGGTTCGACAGGCGTGCGGAAGTTTGCTCCTGCGTAAAC
TGCTTCAGCACCAAGCTCTTCCTCAATTCTTAGAAGCTGATTGTACTTTGCAAGTCGCTC
TGACCTGCAAGGTGCTCCGGTTTT
>ntig_243_consensus
AACACGCTCACGGTATCTGTCGAACCAACAAAGCACACCGATTTGATAAAGCAAAACCCA
ACTAATCATTAATCGGACGGCTCTTATTAACCTCCCCTCTCTATAAACAGTCCGCAGCCA
ACATTTCGTTCATTCTCTCTCAATCCCTCTCCTTCTAGATCCATCTATAAGCTTTAGATA
TGGCTATTACTATTGTCTCCGTCAAGGCTAGGCAGATCTTCGATAGCCGTGGCAATCCGA

CGGTCGAGGTTGATGTGAAGACATCAGATGGTCACTTGGCAAGAGCTGCTGTTCCAAGTG
GTGCTTCAACTGGAATTTATGAGGCTCTTGAGTTAAGAGATGGGGGCTCAGACTACCTTG
GAAAAGGTGTATTGAAGGCTGTCGAGAATGTCAATGCAATCATTGGCCCGGCTCTTGTTG
GCAAGGACCCAACCAAGCAGACTGAAATTGACAATTTCATGGTTCAACAACTTGATGGAA
CCGTAAATGAGTGGGGTTGGTGCAAACAGAAGCTTGGAGCTAACGCCATCTTGGCAGTGT
CTCTTGCTGTCTGCAAGGCTGGTGCTCATATCAAGAAGATTCCACTATACAAGCATATTG
CCGAACTCGCTGGTAACAAGAAGTTAGTGTTGCCAGTACCTGCTTTCAATGTCATCAATG
GAGGATCACATGCAGGCAACAAACTTGCCATGCAAGAGTTTATGATTCTTCCTGTTGGAG
CTTCTACTTTCAAGGAGGCTATGAAGATGGGTGTTGAAGTGTACCACCATTTGAAGGCTG
TCATCAAGAAGAAGTATGGTCAAGATGCAACAAACGTTGGTGATGAAGGTGGCTTTGCAC
CTAACATTCAGGAAAACAAAGAAGGACTTGAGTTGCTCAAGACAGCCATTGCCAAGGCTG
GTTACACCGGCAAAGTTGTCATTGGAATGGATGTTGCTGCATCTGAGTTCTATGGTTCAG
ATAAGACTTACGATTTGAACTTCAAAGAAGAGAACAATGATGGTTCTCAAAAGATCTCAG
GAGATGCACTAAAAGATCTTTACAAGTCATTTGTTTCTGAGTATCCTATTGTATCAATCG
AAGATCCATTTGACCAGGATGACTGGGAGCACTATGCGAAGATGACTAGTGAAATTGGAG
CAAATGTACAAATCGTGGGAGATGATCTATTGGTCACCAATCCCAAGAGGGTTGAGAAGG
CTATCAAAGAGAAGACTTGCAATGCCCTTCTCCTTAAGGTGAACCAAATTGGATCTGTAA
CTGAGAGCATTGAAGCTGTCAAGATGTCCAAGAGAGCTGGGTGGGGTGTGATGGCTAGCC
ACCGCAGTGGGGAGACTGAAGATACTTTCATTGCTGATCTCTCAGTTGGCTTGGCCACGG
GTCAAATTAAAACCGGAGCACCTTGCAGGTC
>ntig_244_consensus
CGCTAAGCACTAAAAGAGAAAACAAAACTTGGAAATGACTAGTATCCATTACAGTCCTTG
CTAATATCAAAATCCACAGAAAGAGATAAGGAATTATTAGAGAAACTGGAAGCAAAGGTC
TATAAAAGTCTCGTCTTTGGGAAATGAAACTACAAACAACAACTCCTAAAAGGTAAATTG
AAAACTGCATATGAATCTGTGATACATTCATTCCTGACCTAACTGTCAATGATTTTGTGT
GTAATCAAAGCTCAGAGGTTGCTGACAGAATGGAGCCTGAAAAACAGCATGTCTAAACTG
CTCATATGAATTTGCATACATCATTGCAGCACTTGGCCATTGCACATATGCAGCTTCCAT
AGATGTTTGTTGTCTGGTGGCACAATAGGGGCTCATAATAGGATTTGCTGGAGGCCTATA
ATTTAGGGAAGCTTGAGTCCCAGGGATCTGACTCAACTGGGGAAAACACGTGTCATACTG
TATGAATGGAAGTTGAACTTTCTGCATGTTGAAGGGTGCCAAGCCAAAGCCCTGATTAGC
TGGAAGGTTCCTAACATACCTATCATAACTTCGATCATAATCAGGATCTGTACGATCCTT
CTCCCTATCCCTGAAAATAGCAACTCGAGAAGAACTGCCATCCCTGATGCTCACAATCTT
TTCTGTATCAATAACAGAATTCCTGCTACCTTCATTCTCATCTCTGCTCAAGACTATAGC
TTTCCCATCATTAGGGACCTGAGAAAATGAATCCTCTGAGTCAGGACTGCCAGGACTACT
AAATATGCGGGCACGTGCCCTGTCATACTCCTCCTTCCTCTCCTCAACACTTCTGACAGG
ACTTCGCTTGACCCCAAATTCATTAGCTTCACTAATCGATCCCCTATTGGGCCTAGGCCT
AATGACAATTTTAATCTGATCAAGCTTCTCACTTTCTTCGGACTGTTTAGCAGGAATTTT
AGATAAACAAACAGCTGGATATTTGCTTTCTGCTGTTTTCCTCACCAGAATTCTATTACC
TGACCCATCTAAGCCATTCTCCTGGACCATGGTTACTAGCCCATAGTGTTGAGAAACACG
GTGTGCAGCCAGTCGAAGATAAGAAATAGGAAAATGCTGGAACTCGAAATACTGCTGATC
AGGATTTTGCAAAAATCTCTGGATATCAAGTTCCATCCGCAGAATGGTGAGACGATGACG
AGGGTTTTGAAGAGCCTCAACCAAAAATGGATCCACCATTGACTCTTTATCATTGAAAAC
TGTTGCCGTGGCTGTAGTTGTGGAATCCATGAATAGCTAGCTTCTTCTCTATCTCTCTCT
TGAATTCACGCAGTTTTCTTCCCGTATAATCTTCTTCTCAAAATCTCTTGTCCAATTCCC
CAATAAGAGAAAAAAGAATCAAGAAGAAGAAGAAGAAGAAGCAGGTGTTTTAGATTTGAG
CATTAGACTAGCAAACAAGACAAGAGAAAAGAGAAGGAAGCGTGCGTGGGAGACTGGAAG
GGATGGGTGGCTTTAGAATTGGATTTGGTTTTTGTTAAATTTATCCTTCTCAACCGAGAA
AGAA
>ntig_245_consensus
CGAACTTTGAGCCGTAGAAATGGAATAGCAAGCCGGAAGTTTGATCAATTGCTGATTCAA
AGAAGGGTTCTTAAGTCCAGCACATAATGAAGATCAGATAAGTTGGATGTTCAAACGTCT
CATCATGCAACACTCGATAAGGTATACTTATCCTCTACTTTTCCTATAATCAACCAAAGG
TTCTGGTAAACTGATTACATGATGACTTTAAGCAAAATACGAAGAAAAAAAGAGACAGAA
ATATCACAACAATATATGATTTGGAAACACTACTTATGTTATTGAAAATCTAATAAGCAA
TAAATACGCCGTACACGTGTTATATTATATTACACTAATGCTATACACAAGTTTCAAGAC
TCAACCCAGCTGAAGAGCATAGATAAATATTACCATCCAACTTTCTCTAGTTATAACGCC
TCTTGGAACTTTAGAGTGTGCTCAGACACCAGCTGAGACTTCATTTGCGACTGGATTATC
ATTTTTCTTTGGTGGTTCTTGTGTCTTCTGTAAAAGAGCCTTATTTGGAACCAAAACAAT

GAATATGTTCCTGTCTCTGAAGTTTTTGCTCTCCTCAATTGCAGCCTCTCCAACATCATT
CTGAAAACGTTTAATAAGCTCAATAGCGATATTTCTGAACTCATTTTCCCGGCCTTTGAG
GTTAACGATGACCTTAACCTTGTTGCCATCTTTCAAGAACCTCTGTGCAGCTTTCAAGCG
AACAGTATAGTCATGTTGGTCAATGTTGTAACCCATTTTTAACTCCTTCATATCCATCCG
ATTGGCAGCACTTTTCTTCTGCTGTCCTCTTTTCTTCTTTTGTTGTTCATATCTATATTT
GTTGTAATCCATTATTCTGACGACTGGCGGATCAGCTTCTGGCGAAATTATTACCAGGTC
AAGCTCAGCATCTTCAGCCGTTTGAATGGCTTCACTTTTAGAAACAACACCAACCATATT
CTGCTGAGCATCGATAAGTCTCACTGTAGCTGACCTACCTGATGCCATCGATGTCCAAGG
CTTGTTCAGCGTCAGAGTCGCTCCTTCGGGACCCCCTGGAATCGAAGCGGGGCCCTCCAC
CGCCGTAGCGGGCGATTATGGAGACGCTGTGGTGATGATCTGAGGGCGACGAGATAGAGA
CGAGAAAGAGATTATGAGGACTACATAAGTGAAAGTGGAGACCGAAGAATTTTGATTCGA
GCGAGGAGAAAGACGACGTCATTTTGGGGACAGCAAATGCCGTCGTGGTCATCAGACCAA
CCATTGTGCTGGCGTAGATCAAAGCGCCTGAACTTCTTATCCAGATTTTTTTTAAAGCT
GGAGAGAGCGATTATCGCATTATGGGCTTCGTTGGGCTGATTTTGGGCTTAATCTTGAGA
CCCAATTAAAAATTACTTATATTTCACAATTCATGCCATATGCCTAGCTTCAAATGCGCT
ATAGTCTTAATCTTGCGTCATTGCAAGCTGAATTAATTAAACAGAAAAACCTTTATTTTA
AAAAGAGTAATTATATTTACCAAGAAAAAAAAGAGTAATTATACTCTTACATAATAGAAA
GTAAACCTCCCAAAACTTCTCTATTTTACAAATAAAACCTTAATATCCATGCGCGAAGCC
CCGTGGATCAACCCACGAGCCGCCAAAACAGAGCAAACCAGCTTTGTTCTTCAAGGCCCT
CACGCATCAACTTTAATCAACCTCTTCAATCTTCGGCCCAGCACTAGCTCCGGACGCAGT
AGAGCCACCACCGGGCATATCATCATCCATTGGCATGTCACCACTGGCACCGCCCTGATA
CATCTTAGCAATTATGGGATTACAAAGCCCCTCCAGCTCCTTCAACTTGTCCTCAAGCTC
ATCAACTTCAGCCAACTGGTTCCTATCCAGCCACTCTATGGTCTCATCCACTGCTTTCTC
GATTTTCTGCTTCTCCTCCGGTTTCAATTTCCCGGCAATCTTCTCATCCTTAATGGTGTT
CCTCATGTTATAAGCATAGTTCTCTAACGCATTCTTCGCCTCCACCTTCTTCTTCACTTC
CTCATCTTCCTCCTTGTACCTTTCCGCCTCCTTCACCATCTTCTCAATCTCCTCCTTGCT
CAACCTTCCTTTATCATTCGTAATGGTGATCTTGTTCTTCACACCAGCCGTTTTATCTTC
CGCGCTGACATTGAGAATGCCATTAGCATCTATGTCAAAACACACATTGATTTGTGGAAC
TCCTCTCGGCGCAGGAGGGATTCCTGTAAGCTCAAACTTTCCGAGAAGATTATTATCCTT
CGTCCGCGCCCTCTCGCCTTCATAAACTTGGATCAAAACCCCAGGTTGATTATCCGAATA
AGTTGAAAAAATTTGCTCTTTCTTGGTGGGGATCGTTGTGTTCCTCGGAATCAAAGTAGT
CATCACTCCACCGGCCGTTTCCAGGCCAAGACTCAGAGGCGTAACATCAAGAAGCAATAA
ATCTTGAACTTTCTCGTTTCCTTCTCCGCTCAGGATTGCCGCCTGAACGGCCGCGCCATA
TGCCACTGCTTCATCAGGGTTAATACTCTTACAGAGCTCCTTCCCGTTGAAGAAATCCTG
CAAAAGTGACTGAACTTTCGGGATTCTGGTCGATCCACCGACAAGTACCACTTCATGAAC
CTGACTTTTATCGATCTTGGCGTCTCTCAAACACTTTTCCACCGGCTCCATACACTTCCT
GAACAAATCCATGTTCAGCTCCTCGAACCTGGCTCTGGTAATCGTCGCATAAAAATCAAT
CCCTTCGTATAAAGAATCAATCTCGATTGTCGTTTGGGTGGTGGAAGATAAAGTTCTCTT
GGCCCTTTCGCACGCAGTTCTCAATCTCCTCAAAGCGCGAGCGTTGCCGCTGATATCTTT
CTTATGCTTTCTTCTGAACTCCGCCACAAAATGGTTCACCATCCTATTATCAAAATCTTC
ACCGCCCAAATGGGTATCGCCCGCCGTGGCTTTAACTTCAAATATCCCTTCTTCGATCGT
CAACAACGAGACGTCAAATGTTCCACCGCCGAGGTCGAAAATAAGCACGTTTTGTTCGCC
TTTTCTCGAGGCTTTCTTGTCCAAACCATAAGCAATGGCGGCAGCAGTAGGCTCATTAAT
GATTCTCATTACGTTGAGCCCTGCAATGACTCCGGCGTCCTTCGTAGCCTGCCGCTGAGA
GTCGTTAAAGTATGCCGGAACTGTGACCACAGCGTTCTTTATACTCTGACCCAGATAAGC
TTCAGCTATCTCTCTCATTTTCGTCAGCACCATGGACGATATTTCTTCCGGAGAGAATTG
TTTTTCTTCTCCTTTGTACTGAACGACGATCATCGGCTTCTCTGCCAGCCCAGGAATAAC
CCTAAAAGGCCATAGCTTCATGTCGCTTTGCACCGACGGGTCAGAGAAACGACGACCGAT
GAGACGTTTAGCATCGAACACTGTATTTTGCGGGTTCATGGCCACTTGGTTCTTCGCAGC
ATCGCCGATTAAGCGCTCAGTGTCGGTGAATGCGACGTAAGACGGCGTGGTGCGGTTGCC
CTGGTCGTTGGCTATGATCTCTACGCGGTCGTTTTGCCACACTCCCACGCAGCTGTAAGT
CGTGCCAAGGTCTATGCCAATAGCTTTGCCTTCGCTTGACATATTTTTAATCACCAAAAA
CAAATAAAATCGATCGGAGAAAGAGTGAAAGAAGATATTTTATGAGTGTTTTGAAGGTAT
ATGTCAAGCGAAGGAGATA
>ntig_246_consensus
CTTCATAAATTGCAAAGTTGTTTCTGTAAAATCAAAAGCTTGATGAGAATGAATTAGTAA
GAACAAATATATTATAATCACGTGTTCAGACAGCAGGGCGGTTTGAGAAGAACTAGATTA
TTGGCAAATAATTAAGATACAGATTGATTCACTACCACATTTCAGTATTCCCTTCTACAT

TGTGAGATGGGATGGTTGCTCCTCTCTCTCNNAAAACCAAAAAAAAAAAAGAAAAAAAAATG
CTACTCGCACCAAAAATTACTCCCAGACTTTTAGTTAAAATTTCCGGTTCTTCTAGCCAA
ATAATTGAATCTCTACCTTTCAAAACTTACAGGATGTTACTTCAGCTCTTGTTTTTCTGC
TTTTGTTTTCTTTTCTTGGAATCTAAGGATGTTAAACCCTTGCCCAATCCAGCCGGTGGT
TGACCTTGCTCCGATGGTTTTGCATCCTTTTTGGCATCTGCTGCTGCTACTGGGGCATTT
GAAGGGTCACCGTTGGCTTCCTTTGGGACATCAGCGGCCCCGCTACCATTGGCATCAGGG
GTAATTCCTGAGTTCATAACGTTGAGAAGTTGGGTAAGCGGTGGTAAAGCTTGGGCTGGA
AGACCATGTGGGCGAATCAAGAGACCCTTTGCTGCAGCCTTTTTTCTGATTTCAGCTGCT
GCACGAGCTGCCCTTTCATCAAAGCCCCTTCCTCGAGGAATGGTCTCACCAAGTGCTGCT
GCATTAAGTGAATTGTTGTTGACAGAAGTGCCAGAAGCTCCAGCTGCAGCAACAGCTTGC
AAAGCATGTACCAGATCAGGATTGGCCTTCAAGATATCAATAGCCTTTTGCGTTGAAGCT
GCATTAAAAGCCTGTCCTTTCTGTTTTTGTACATTCATCTGATGTTCCCGCATCTTAAAT
GTCTTCATCCAGTTTTGAGAGTCCTTTGTCCTTGAATCCTCCTCACCAAGTTGTTTGACA
AGTATATCATAGGTTTTCTTTTCATGCTGGTGAGACAGTTTGAAGGCACCCATGCAGTTG
AATGCAATTGCAAGAGCATGATAGCAAACAGCGGTCTGTATATGCTCCTCACCTAGAAGC
CTCTCATTCTTTTTCAAGGCCTCTTGCAAATAGCGGAGAGCTGTGTCCATCTTCCCAATG
TCCTGGTACATCATCGCAACATTGATGAAAGTTGCAGCCACATCAGGATGATCTGGGCCA
GATGAAAGACTCAGCAAGAGCAATGCCCGGGACATGTGCCGTAGTGCAAGCTCTGTTTGG
TTAAGTCCATGATAGAAAAGAGCCATATTGCCATAGCTGTGAGCAGTATCTGGATGGTCA
AGACCCAAACAACGTTCATTTATAATTAGTTCCTTGTGCTGTTGCATAATTGCTCCAGCC
ATGTCTCCTGCATGATATAAGACCATGGCAAGGTACCGACAGCAGTTGGCAACCTCCCGA
TGCATTGGACCGGTCACCTGTTGAAGTATTGAAAACGCCTCAGAAAACAATGTGTAGGCT
TCACTAAGCAGACCCTCAGACAACTGAGCTTTCCCCATTTCAACAAGGTTCTTAGCTTCA
GAACATACAGGAACTGAATGCTTCACTACTGGTTGAAGATTAAGGATGTCTGAAGTTTGA
AAAGGTGCTGCAGCATTAAAATCATATTTGCGAGCCGCAATGCTTATGCCCACCTTTTGG
CAAAGATTACGCATAACTGAAACTTTCTTTACTCGCAATCTTGCATCCTCAGGCAACTCA
AACTGATATTTCAACTTTGCAAATTCTTTAAGGTCAGACCAAAGAGTTTCTGAGGTAATA
TTCACATAAGAAGAATGATTGTTTCTTGCAGACGCTTTAGATTTCCATCTGGCTTGATCC
TTGGAGGACTTACCCAAAGCTTGATGACTTGCATGATCTTTCTTTTGAGTTCTGGACTGC
ACAGCAGCAACTTTGCCCCCAATAGTTTGACAACTTCCAAAGAAACAGTTAAAAAAATGT
GCAATTGCTGGGCCAAGATCATGATCCTCTACATCTCTTAAAACATCCTTTATGATATGC
TTTGCGGACCTGACTACAATTTCATTAGAACAGAGATCCCATAGATGAGGTAAATGCTTG
GTCCCCTCAGCTACTTTTCCAATATATCGAATATTAATTCCATGCGCGTGAAGTGCTTCA
GTGAATGTCTGGCCATCCATTGGTGAAGCTTCAAGCGTACAAAGATCTTGTATAAATTTT
GGAAGCACAACATTTGCAAGGTAAGAACTAACTCTCCTCACATTTTCTTCATCTGCTGCT
ATCTCCTCTTGACTCCCAGCCAACTTAAATTCAGTTAAAACATTGGGATTAAATGCTATC
TCCTCACACGATTCAGAACTTTCTATAGGTGTAGAAGCACATTCTTGGACAGCCTCAACC
TTCTCTTTTGTTGTATCTGGCTCTTCAGGAACAGCCACTTTTATAGAATCAGAGGTCCGA
TCAGCCTTGCCCTCAGATTTAGACACAGATTTTGATTTTCCAGAAGCTTCAGCCTGGAAA
AAGGCAGTAATTAGTTCTGGCCTCAAAATACAAAATCGGGACCCAGGTCCAGAGTAGTTT
GCATCCCGAGGAGTTACTCTCATCAAGTCCAGAAGATAATGCCTGTCATCACTCCCAATG
ATGCCCTTGCATTCTACTGGAGCAGCTAATTTGAAAACATTTCCAGATCCATCAAGGACA
CTGTGTTCCTTCAAATGAAGGCATTTAGCAGCTTCAAGAACCTTGGAATGGAAATCTTCA
TTCCAACATATTTTCTTGCCATTGTCAACAGAACCATACAAAAGGGAATCAGATTTGTCA
CCTTGAAGAATTCCAGGTAAAACACTCTGAGCTACAACTCTATGACCCCTGTAATCAATT
ATAGCCATAGCAAGATTATAAAGTCCAGGGACATCAGCTTCCTGGTATGCCTTGGTGCCT
TTCAAATCGTTATTTGCAGATGCATATGTGGCCTGTTCACTCTCAGCCAACTGGGCTTCA
GCAGGCACTTGAGTCAATTCTACAACACCATTGGGTTCCCCCAGACTCACACCATTGGGT
TCCCCCAGACTCTCACCTTTGGAAATTCCACCATCCTCTTGTACAACATCATTAGAAACC
TTCTCAGAAGACTTGGTTGAAGAACCAATGCCCTCAACGTTTAAATTGGTAACAGATGGA
CGTTTCCTAGACAGATGTTCAAGGTCTGCATCCACCGCAAAACTGAAGAATATATTGTTG
TGAACGTAC
>ntig_247_consensus
GAAGCCATATATTCACAGTATTTTTTGTGATCAGTTCGAGAGAAATGAATACAAGCTACC
TCTATTTTATCGATCAATTATTCTGATTTTATTTATGGCTGCTGTACAAACTAGGACATT
CTGAGCACAAGCCTTAAACTACATAATACAAGTCCATACTCAGCTAGCTGGCTGTCTATA
TTTTACTCAACAAGAGACGCTACCAAATCCCCTCGTTGTTTCAGGCAGCACAGGCTGGGC
AAGTACAGCCAGTACCACACTTACAAAATGCCCTCCCTCTTCCACTAATCTCAGCCTTGG

AGCAGCTACAGGGATTGCAGCTGCAGTGCTCCCCGCACCCGCACGTCTTGTGCTCATACT
CGCTCCCACCCTTGCTTGCGCAACTGC
>ntig_248_consensus
ATTTGATATAGGACTGCAAATTGAGTTGTCTACACTTGAAACATGGTTATCTTTTGCCGA
AGAATAGACTATGAGAAAGAAGATGGCGGGGATTTAATCACATGTTTCCACTTGAACGGT
AGTTTTCGCTTGTGCCAAAACGGGTGATTTTTTGCACTTTTGCTTGTTAGGTGGGAACAG
TTACATGAAGAAATAGTATGGAGAACCAAGTGGATATTAACATGAAACCTTTTATGTATA
AGCCGAAAGCATAAAACTGACTGAGAAATCCATACACAGACTGTTACGAGAGGGCAAGTT
GGTAACACCAATCGAGACATCCACACCAAACCCACCCAGTTACTCCACTTACAGACAACA
ATGTGATGTGGGGCAAAAATTTAGACATCTTCCCACACATCTCCTGTAACATCAACTGGA
GAAAAAAGCTCTAAATACATTGATTAACATGTTCCATACACATGCTGCACTGGGGTTTCC
TGTTCCAATTACCTCTTTTAACTGTCTCCTTCAACCACATTATTGAATTGAAGCATTCAG
TTTCACATCAAAAACTACAAGAGTGTGACACAGGACAAGCAACAACAGTCAAAGTTGGAT
AAACGCATGAAGCTCTTATTCCGTGGGTGGGGGAAATTGAAACCAAGATGGCAAGTGACA
GTGTATTTGCAAAGTTTTCCTTGTCATGACCCTCAGAAATGGTAAACCGTTTCTTCCCTG
TTGCTATTACTACTCATTTGTACCATCAAAGCTCCCCATTGCGTTTGCCCGCTTTTTCTC
CCTCCAGTTCTCACCCCATACTTTTGCCTCTGCCACAGCGCGCTCTCTATCTAAATCACG
ATTCCTTGGAGTGTCCCTTGCTCTCCCAGTGCTTACAGGACTATCCCTCTGAGATTCCTG
TTGCTCTAAATCATCCACACCCCATTTTCTCCCCGATCCACCTCCAGTTTCATTTCTGCC
CAAGTCTACACCATTACTCAATTCCCTAACAAGGTCCCTCTTCCTGTAATCAGGAGAATT
TTTATGGGTGGAAGGTGACCCATGCCTCCCAACAATTCCAAGCTCATCTGGGCTATCTAG
CCCATCTCCCATATTCCTTCGGTCATCCCTATTTCTTGGGGTAGATGAACCAACTCTGTT
ACTCAGATTGCTGGCTGAATTTGGGTCATAGGCTTGGGAGGCTAAATATGTAAGTGCAGT
CACAACATCACCAATAAGAGGTCTTGTAGCAGCTTGCTCCTGCAAACACATGGCTGCAAC
TGCAAGAGCTTGGTAAAGTCCTCGCATTGGGTAGCAACCTTGCAATAGAGGGTCAGCCAT
CTTGGGAAACTTCCTACGATCCTTGAAGAGTGGTCGTGCCCAGGCAACAAGATTATGCTC
TCCATGATCCCGTTTATTATCGATAGCTTTGCGCCCTGTGATCAACTCAAGAAACACAAC
TCCAAAACTGTACACATCAGACTTTAAAGTGAGTTGGCCAGTCATAGCATATTCCGGCGC
ACAATAACCATAGGTACCCATCACTCGAGTTGATACATGGGTTTTGTCACCAACAGGGCC
CAACTTAGCAAGCCCAAAATCAGATAACTTAGGGTGATAGCCCTCATCAAGGAGAATGTT
TGATGATTTTAGGTCCCTATATATAACAGGAGGGCTCGCTTTATCATGTAGGTATTCTAA
CCCCTTAGCTGCACCAGCTGCAATCTTCATCCGTGTATTCCAGTCCAATGGCTCTTTGTC
TGGTGGGAGATCATGTAAATGATCTTCCAATGATCCCAGGGGCATGAATTCATAAACAAG
GAGCCGTTGGTCCCCATCCGCACAATAACCGATCAAATTGACAAGGTTAGAGTGGTGTAA
AAGGCTAAGCATGAGAACTTCCACTAGAAATTCTCTATTTCCTTGAAGACCATTTCGGTC
AAGCTGTTTGACAGCAACTACCTGTCCTGTGCTTTCCAAGCAGCCCTTGTAAACACGTCC
AAAACCGCCTTCACCCAATAGACATTCTGGTCTAAAGTTTCTAGTGGCAGCAGCAAGCTC
TCTAAATGTAAATGTTTGTGCAGCAATTTTTGCAGTTGTTTCCTTAGGAACTACCGGTTC
CTTCTTAAGATCAGAAACGCTCCCAGATTTTGACTTGTCTGAAATAACTCTATTAACATG
ATGAGACTGAGAAATTGAACCATCTTTAGTTGAGTCCTTGTTCAGTTCCTTCACTACAAC
GCTAGCAGCTTCCTCGTTATTGGATGATCCAAAACAAGGAAAACACCCACCCATATCAAA
TCCCAACAAATCAATCAAAACTTGAACAAAGTTTCAAACTTTGATTACAACCAAAAATGT
TATTTTAACCATGACTCACTCTCCAGCTTCTTCTCTTCATGAAAAAAAAAAAAAAATCTAA
AAAACCCATCTCAAGATTTACCCATCAGATCACTCTTATCTTTCTATGATCTTGAGTGTC
CATTTAAGACAAAGATTGAGGCCTAAAAGGCAGATCAACAAATCGAAGAAAATCTGTGTT
ATATGAGAT
>ntig_249_consensus
CAAAGAGGATGGCACATGCTGGCTAGCTTCTCCATGGTGCATTTTCAATCCCTGGTAAGG
CTGTGAATTCGACTGAGAAGCATTAGAGGAGCTTACCAGCCCCGGTCGAGAAGATTGGAT
TGTAGACGTGGGTGCACTAAGTGTCAAAGTTGTGGTGCTAGTACCAGCAAATTCAGCTGC
TGCTTGACCAGTGTCTCTTTCAACATTTAGACTGAATAACTCATCCAAATTATTTTGTGA
AGTGCTTGCCAAAGCAATTGAATTCTTTCGTGATCCTAGTTCCATGCCATTCACAAAAAC
AACATATTGAATCTCAGAATCAACCTCCATGTTCTCTAGACCAAACTGACCATCCTCTAG
ATCTGTACTAGAAAATAGGAAAATGCGAGGTTTTTGTTGTCCCCTATCTTCTAATACATT
ACATTCTTCCATCATGTTCTGCAGATCTTCATCACAAGATACAGAAACCAATGCATCAAG
ATCCTCACCAGGAAGTTGATATTTAATTGTATGTGTTTCACTATAAATAGCCAAAGCTTT
CTGCATAAGCTCTTGCCATGAGATATTTTTACTTAGTCGGAAAATGCGTGTTTCTCCTCC
CACATAACGCAGCTTCCCATCAATAGGACGGGGCAAAATTTTTCCGCCAAAGCTGCAGAG

GAACTTCACCTTGGTTGATGAGCTATCATGATCGCTTGAAGAGCCATAACCATGAACCCC
TTGACTCACTTCATTTCTTGAGGAGGTACATGGCACTGAATGCACTGAATCAGAATAACT
TTTGTCTTTATGAGCGGAAGATCCCTTTCTATCAAACTCCCGGGTACGGCCTGTCTCTGC
CGTGTTAAGCATTTTGATATCAGACCCACTCTCTGGACCTGTATGGGGAATTCCTAACAT
TTCTGGCTGACCCATATAAACAGGTGGATTGTTAAAAACACCATAAGCATTAGGAACAAT
GTGCTGCCTGGGAATCACCCTTTCCCGCATAAATTCTAGAGAAAACTCCTCACCAGTCTG
AATGGAGTAGTTAAGCACAGGTGGTTTAGCTCCCAAAGACATGGTGTAATCTGGAGGTCG
GACGTTAGTGTTGATATTACTAGAGGGGTCATGAAGGAATTTTTTATTTGCTGAACCAGG
TCCCTCATTTCTAGTTTCGGCATTAGTGGATCGGACATGATCATAAGTTTTTTGCTGATC
CATTGTACCTTTTCCAAAATTTCTCTCCATTTCATTCAACTTCACCTGGGCTAAACAGAC
CCAGACAAGATGCCATGGAAATCACTCCAATACCATCTCAACACCAGCATTCTTCATTTC
CATTAATGAAGTTCAGTAGCTTTAAAAAACAAGATCATCCCACGTGAAAATTTCAGCCTT
TAACCAGAAACCCGGGCCAAACACCAAAGACAGAGGCTTCTCTGTCTCTCTCCAAAAGCA
CTGAACTTTAGTTAAAAAAATCACCAGAGTCTGTAATTTTGTCTGCTAAATTAGATTGTT
GTTCTAGAGCATTCCTAGTCTAGATCTACCACTTAAAATCTATGAATTTTACGACTTTTT
TCACACGCTAATCATCATGACGTTGTTGAGATTCCGGTATGGTTGCCGTGGGGTTCTGGT
ACTGTTAATGATTCCAATGTCAT
>ntig_250_consensus
AATCCTTCCAAAACCTGAAACTGTTTTACTACCATGCTATTAGTAAGATAGGCATAAATA
ACCAAAAGAAAACGATTAACTGAAAATTTAAACATATTAGAAATTAAGAACTTTGGGGCC
GCCATGTCATTTCAAGTTAGCATCACTTCTCTCAATGAGCAGTAAAAAGTTTACTCAACC
ATCATGCAGTAGTAAGCATATTCATTACAATTTGTTTTCAGGTTTATCAAATATAACCAT
ACATATCTTTTTCATACAGTGATTCGAGTTAGACTAGAAATTGGCTGCAAACAAGCATGC
ATGGAAAAAAATAAGGCACACAGGCAGAAACACCGATACCACCAGTGCAGCTGGAATATTG
ATTTGCTTAGGGTATAAAAGGTGCACCAGAGCTAAGTAAATAATATATACAGGTAGCATG
CATGTAATGTAACATTTCAACATAACATTTATGCCTCATCCGTACTCAATCAGCCCTACT
TTTCAAAGAATTCAAAACATATTAGATCACATATATTTCAAATCATATAAGAGACTCACC
AGCTGACAAACTGACAACTTATTGAATTATTATGATTATG
>ntig_251_consensus
GATGTAAGAGGAGCTTTGTGATCTTTCAACAATCTCAACTGCATCCATATTCCCGGGAGT
AGGAAGATATTTGTTATCATTTGTGGAATGACTTTTACCATTCCTAAACAGCTGCACTGC
TGACAAAAAGGGAACAAAATAGGCACGGAATGAGAACCGATCAACACCCAATCTATTAGA
CTGCTCAAAATCCTCTGCCCTTATTTCTAACCCGTAGCTCCCATGCTTTTCATACCACTG
CCACAGGCTTCCCAACAAGATATTAGGTGCCTCGTGTCTGCACAAAGATGCCCCAACAAC
CTGGTCCTGTGAACATTTATTACAGCTCTTTATATTTGTTGAATGACAAATGACTGGAGA
GGAAAAGTGTAGAAGTCTTTCAAACTCAGCAATTGGAGAACCAGTGGCCATCTGAACAGC
TGCAGATGCCAATTGCATCCTACAGGCATGATTCACTGATAGTAAAATCTTGTCCAAATC
AGTTTCAACTACAGAAAAGTTTTCATCTTTAGATTCAATAGTCAAGCAATTGGC
>ntig_252_consensus
GCGCACTTCTTCTATCAAACCGAAACGATACTGAGAGTAAGATTGTTGTAGACACTCGGT
TTGGCGAAAACCCGTCACAATTGTAAATAAAGCAAATTCTTAATAATCATTTTTGAAACA
CAACCATATTATAGTAATACATAGACAAGTTACTTCATCAAGAGGCAGAGCAAGAACAGA
GTCACTAAGCAGCGCAATGAGGCCGAAGAATGCCCAGAAGGAGTAATGACGCTAATCACA
TTGAATTATTGAACACATTATTTCTTCACGATAAGACATTTCCTTCAAATAGCCCTCTCA
CATTCTTGCCTTAGCAACCACCACATTTCCTTCATCAATATTCATTGCCCCTGGTCTAGT
CCATGTTTCCTTGAATTGCTCCTCCGTCACTCCTTTGCCCATAGCAGCAGTTGAAGAAAT
GCCTCCCTGGACAGTCTTGAGGTACCTATCATAATAGTTTGCACCAGACCATTTTTGGTG
AGCAAGTGTATCAACTCCATGTTTTCGTTCTTCCCTCTGGATCCTCTCCACGTAAGCCAG
CATACCTCTCCTTGCATAATCTCCAGCAAATGTGTCTGTCACAAGCGCATCAGCATGGAA
ACCTGCCAGTGTTATGAACTGCCAGCTGTAGCCAAGCCTAGCTATTCTTGGAATGAAATC
TTTCATTTCTTCATCAGTCATTCCTGATGCATCCCAATTGAAAGATGGGGAAAGATTATA
GGCCAACATCATCTCTGGCTGCTTTGATTTCACTCCTTTTGCAAAGTTGGTGCACTCAAC
CAAGTCAGGGCTAGCAGTCTCCATCCAAATAAGATCAGCATGAGGAGCAAAAGCCCAGCC
GCGAACAACTGCAGCCATAACCGACCCTTTGAACCTGTAGAATCCTTCTCTAGTTCTGGG
CAAGTCCCAGTCCCAAAAGAGATTCTTCAGCCCCAATCTCTCAGCAATCTCTCTGCCTTG
TTCATTCGATAGGCACTTGTCATAGCTACAATGGTTCATCCATTCATTCATTCTCCTTCT
TTTCTCGTCCTCACCAACATTCATATTCTTGATTGCATCCGTTACACATTCAGAGAATGT
CTTGAGCTGTGCCGTGGCCAGCCAATTTTCCTCAATGGCTTGAAGTTCAGCACCTGTTTT

GCCGGCATCCATTGCTTCTTCCAAAATGTTAGCCAAAGCGTTCCTTCTAAGATTTGGATT
AGTAGCACCGAGAATGAACTGATGGTCTCTAGTGTCGACATTGGTTTGGATCAAATTAGC
CGCAACTGCATCAGTCCTAGCCACCAAAACAGTCTCCACTCCCATGACATCAAATTGCAA
CCTCGCAGCCACAAGCCTATTAATGTGTTCACTGACCGCAACAAGCACCTTTCCAGCCAT
ATGACCACATTTTTTGGTCACTGAAGACTGGTCTTCAATATGGACACCTGCAGCCCCACG
CTCAACGAAAAGTTTGCAAAGCTTAACTGTTGCTGTAGTACCACCAAACCCAGTATCGCC
ATCAGCTATAATTGGCTTCAGNNTTCTATCATGGTATTGCTGAGCAAAGAAAAGATGTTC
CACTTTGTTTGGAACTGTGTCATAGGGGTAATCAGCAAGGTCTGGGCCTGGCTCATTGGT
TGAAGTGTGGGTGGATGAACACTGCCAACCGGAGACATAAATGGAGTCCAAGTGCTTGGC
CATCATGGTTACCTGGACAGGATCAAGTGCACCAAAAGTCCTGGAGGCAGTGCCATTTGC
TTGGTGTGTTTTAAGAGTTCTCCAAAGCTTTTTGGCCATCTCGTTGGAGCCATAGCCTTG
TCTAAGTGTCCCTCGAAGAGCTACAACATCTCTTGCTGAATATGGTCTCCGGGTTAGCCT
GAACCTCTCTGTGTTCCACCAAGCTTGCACCTCTGCAACTTCTGCTTCAAATCTGCCTTC
TTCCTCCATTATCATTGAAGGCACTGAGTAAGATGCAGCCATGGTAGAACTTCGTATTTC
TGCAGAAAAGCTTCTGTAATGTAAATGCACTCTTAACAGCTAAGAG
>ntig_253_consensus
AAGAAATCGCCAGAAAAACTCCTGCTTCCACACCCTACAGATCTGGATCTTGAGACTTTA
CGCTCAAAAGAGGAAGCTGAAGCGGTTGCTTGACTGTTGGGACTATCATCTTCCTCAATG
ACAATATCACTTTTCTTTGAAAGGGAAGATCCCGAGCATCTCTCTTTTGACTTCACATGT
AAGCCATTTGTGGTATTTGATGTTAAAGTTGCGATCTTATCC
>ntig_254_consensus
TATAGGATGAACCCAATCATCCTTCTACCACTTCATATAACCTCCCTCAAGGATACTTCC
CATATGCGTTGTCCCTCCTGTTGTTCCTGAACGCGCAGCATCCGACGGAGTAGACGATGA
TGAGTGCGATGAGGAAGACAATGTTGATGATGGCCACCTTCTTCCAGTCAGTTTTGATTT
GGTCCAGCACCCCAGCTTTGCATGACTGGCAGTCATAGCACAGCTGATTTTGGTCATTGG
ACCATAAGGAACAGTCAGAATTGCTCGAAGTTGTGCCATTGTTGGTCCATTGTGTTGGGG
CGACATATGTGAATCCGCAATCATTTGATGGCTTACAACAACCAGACTGAACTGAAGATA
A
>ntig_255_consensus
TCTGCAGCCTCCTTGGAATGCATGGTAATTATACCTCGGTGCGTGGCTTGAGAATAGATA
TTATAATTTGAATTTCTGAATAATAATGTAGTTAATGGCTTGAATGTTCCGAAGATCGAG
GTGACCTTTCCAGAATGGGATATAAATAGTCAAAATTTCCAAAATAAAATATAACCTATA
ATTTGCTAAGATTATCTGTGCTAATTTTACATGCACTAACCATACTAACTGAAAGCGTAC
ACACAAAGTTAATCAGCAGCTTAAGCTATTATATGAATAATGAATTCAAATCCCACGCCC
CACAATGAGCTCAGCAGCTGCACCCATTGCACCAACACCACCAATTCCCAGAAGGGCTAA
AGCTAATTCTTCCTCCTTCCATTGCCTCAGGCCATTACTCAGCCTTTTCAGTACATCAAC
ATTCACCTCCTGCATCCATGTTGGAGTGCATGCGATCATGAAGTTAACAAAACCTGACAC
ATATGCCCGCCAAGTTGCACCATCACAACCAAGTGATATCTTCCCGTCCAGGGCACTAGC
AAGAAATTCTAGGTGAGCCCCAATAACCATGGATCGTCGCTTTGAATGCCGTGATTCTGC
TTCCACACCCCAAGCAAATGACCCACTAAGCACTGCAAAGTACGCTAGTGCATAACCCCC
CAGCATTGCAACCATGCCTCCACATTCTCCACCTTCCTGCTCCAGTCCATGAACAGATAT
AAACCATGAAGGTAAAGTTTCGATAATCAAAGATTGCACCAAACTTAATCCACCAGACAT
CCAAACTAAGTAAGCCCCTAGTGAAGCAGCAATCTTGACACGCATCATTGCTGCAGCAAT
AGAAACTTGTCCATATCTCAATCCATACTTGGTCTTCTTCAATTTCTCTGATTTTTCCCT
AGGTAACCCACCACTAGCAATATCTCTTACAGAAAGCATCAAAAGAGAGACAATTTCATC
TTTCATAAACATTACATCTCTGATCGATTGATGTACTCGTAAGTAGAGAATACCAGGGGC
AACTGGGGAGATTCC
>ntig_256_consensus
TGTTGGAAAGTCCCACAGGAAAAGAATAGTTCCTTGCAAAAAGTTTGGTGCTTAGTTAAA
TTGACTGATTAAAGAATGCTTAAAGCAAATTATAGAATCACTTCTAAAAGCCCTAAATAA
AAGGAGTCTAAGACGCTTTATTACAGCCAGTACCAATTATATTAGCAGCGAAGAATGGAC
AAGAGGCATAACCTACCAGAAAGCCAGTTAAGCACAATTTTTTAAGGAAAACCATACAAC
CAGGCATGTACAAGGTGAGGATAAAATCTATCACTGTGATTAATAGCAATCACAGTTTTT
ACAGCATTCATATTTACATCTGCAAAACGTTAATAAATGAAAAACATTCAGGTAATAAGA
GAAACAGAATCTCAGCAATCATCATCAACTGTCAGCAGCCCTGAAGATAGTTAGCAGGGA
AAGAAAAAGATTAATGATGTCCAGGTAGAGTGAAACCGCAGCCCATATGTACTCGTCATA
AGAGTAGCGCTTGATCAGGTTGTCTGTGTCGTATATAATGTACCCACAGAATATGATTGA
TGCCAAGCACCCATAGATCATTACTGAGATCCTACCCAAAGGAAATAAAATCTG

>ntig_257_consensus
AATACTCCTGAAGGGCATCTCAAACACTTCATTGCAGTTGGCGCCAATCAAACTACAGCA
AAGAACGGAGAAAAAGGCATATCCTACAGCTAATAAAGCAAATTTTATGAAGAAACTCCT
AAAAGAAGAATTAGCAGACACGATATGAGAAGATTTGCCAGTTTTTATTTCCAACAATTT
TCATCAAGATTGAGATGCATATAATTGAAGACAAAGGCATGTACAAAATATATTGCAGTT
AACATTCAAGCTTTTACAACATGCACTATACATCTGCAAAACGCTAATACATGTTAAAAA
GAATCTCTTATATTCAAAAACCAAGAGAAACAGAACCTCAACTATCATCTTCAACTGTCG
GCAGCTCTGAAGATAGTTAGCAGCGAAAGGAAAAGATTAATGATGTCAAGATAGAGTGAA
ACAGCAGCCCATATATACTCATCATAGGTATAGCGCTTGATCAGGTTGTCTGTGTCGTAT
ATAATGTACCCACAGAATATGATTGAGGCCAAGCACCCATAGATCATCACTGAGATCTTA
CCCAAGGGAAAAAGAATCTGAATGAAAGCAAACAGCATTAGAACAATAATAGCACCAAAC
AGGAAGGGCCCCAGAAAGTTGAAGTCATGGCCTCTTTTGGCAGCCCAGAATGTGTAAAGA
GTGAGAGACACAACCACCGCAGAAGTCAGAATAACTGATTCTAGAATAACTTTCC
>ntig_258_consensus
TATCTGCTTCTGTTGCACCTTCCAGTACAAACGAGGAGCATCATGCAGGAGTACATCTCC
ACCAGAGACTACCCAGTAAAATCCAACTATCCACCAGAGAAATGATGCCATTGTATTTAT
TGACTCACATCGTTTAGTGACACTGGATCGACTTGAACCCAGGGATTCTCCACCATCCTC
CTCATCCTCATTCTCACTATCATTGTTCACATCCCCCGTACTGCCACCACCCCTCTCAGC
ATCCCTGACTCTCCTAGTGTTCCTCCTCCGGTACTCCAACCACACCAGCACCACATGCAC
CAAACACTGCAATGCATACCCGCAGATCCATAACCTAATCGGTGTATTAGGTTTCTCATC
AATAGTAACCATCAACATCGCCACGGAAACCACCACGAATGCGGTGTTCCACAACATATC
CAGCGCAACAACCGGCTTCGAGTAGCCCCAATCAGCACGCCGCTCTTCCAGCTCACGTGC
CGCAGTTTCTCGCACGAGCATGG
>ntig_259_consensus
CATTCTCACTATCATTGTTCACATCCCCCGTACTGCCACCACCCCCCTCAGCATCCCTGA
CTCTCCTAGTGTTCCTCCTCCGGTACTCCAACCACACCAGCACCACATGCACCAAACACT
GCAATGCATACCCGCAGATCCATAACCTAATCGGTGTATTAGGTTTCTCATCAATAGTAA
CCATCAACATCGCCACGGAAACCACCACGAATGCGGTGTTCCACAACATATCCAGCGCAA
CAACCGGCTTCGAGTAGCCCCAATCAGCACGCCGCTCTTCCAGCTCACGTGCCGCAGTTT
CTCGCACGAGCATGGACGGGCCTCGGCCACGGCCGGCGACGCGGCCGAGCAAGAGCGCAA
GAGTCGCGGGGCGGGCGGCAACAGCAGTAGGTGATGGAGAGGATTCTCGCGGACGGAGGA
GAGGCGCGTGACTCTCAGAGCCATCGCCCGCCGCCGCGGCGGAGGTCTGTGAAGTCGACA
TGAAAGCGAACAAAACGAAGCAAACGAATTGAACTTGAAGCTTAAAGAAAGAAGACGAAG
>ntig_260_consensus
AACCCAATCATCCTTCTACCACTTCATATAACCTCCCTCAAGGATACTTCCCATATGCGT
TGTCCCTCCTGTTGTTCCTGAACGCGCAGCATCCGACGGAGTAGACGATGATGAGCGCGA
TGAGGAAGACAATGTTGATGATGGCCACCTTCTTCCAGTCAGTTTTGATTTGGTCCAGCA
CCCCAGCTTTGCATGACTGGCAGTCATAGCATAGCTGATTTTGGTCATTGGACCATAAGG
AACAGTCAGAATTGCTCGAAGTTGTGCCATTGATGGTCCATTGTGTTGGGGCGACATATG
TGAATCCGCAAACATCTGATGGCTTACAGCAACCAGACTGAACTGAAGATAA
>ntig_261_consensus
CTGGGGACATCCTTAATTTAAAAGAAAAAACTGAAATATAATTTAATTTTTTCTTTCTTA
ATTTAAACAAACCCACAATTTATCACCAGCCTCCGATGAGTTTTCAGTAGTTGAAGAAGT
AGTAATAGTAGTAGCAGTGGTGGTAGTACTAGTAGTAGTAACGATCAACGGCGGAAATTC
ATTTCTATTCGGCGTGAACGGCGGTGATGGATTAGATCATAAGATGTTCCGCTAGACCTC
GGAAGATAAACTATGGATTCTGAAACTGCACAAGTTCTTTTACAAGAAGTGTAAAGATTG
ACAAATCCGAACCTGTCACTTATACTTCTTGATACTTTCCCCAAATTCCAGAACAGCTTG
CTGCTTGTTGAATAGTCATCTTCGTCGCCGTCGTCATCGCCTTTTGAACATTCAACCAAG
GCAGCTACGAACGGATCCTTTCTAAAGCTGTGGTCGCCATTGCAGCTGAATTTCTTGTTC
ACTCCGATGTCGGTCCTGGAATGTAACTTTTTCAAGTTTTCGGGTGTGACAGGAGGTGGA
AGGGGGAGTTGTTTCAGGAAAGATTCCTTCTTTTTGAGCACGTTGTTGTTGTTGTTGTTG
CTGTTATTAATACCATTTTTCTTGGGAATTCCAGGCAATTGTTCCCAAGAAAATGGCACC
CCGGAAAATCGTAGTGGGGTGGCAGGACTAAGAGGAGAATCGTCGGGAAAGTATGACGAA
GAGGATGAGAATGAAGATGAAGACGAAAAGGAAAGCGTTCGTCGAGAAGGAGTGTTTTTC
TTCGTTGGAAATATGGTTTCACCATTTTTCCCAACAATTTGAGCTCCGACGTTATGTCTG
GTCTTGGAATTC
>ntig_262_consensus
GAGGGCTGCAACAAAAGTACACAACAAGGCATCTGCATTAATTGTCAATAACCATAACTC

TCCATCAAGAAGCATTAGTTACGGCCTAAGTTCAGATTTTTAGTTGCCATTACAACATGA
ATAGATCCAAGACAAGGGTTTATAAAAGCCAACGTTAATCTATTTTTTACCCTCAAAATG
TATAAAGTGCCTTAGGCCTAAAAACTTTCCAGACAACTATCACAACACAATTACATTAAC
TCGACACCCAAACATACAGATAACATTATAGATACGATATTGAGGGAACAAATGAGACTC
ACAAGAGCAGAAACCGGATGAAAGATCCATAAGATAGACAACCATAGTGTATATAAAATT
AGGATCGAATTCAATTAAATTTACACATAATAAGTTCTTCCAACTTTTAACATGATCCAA
TGACCATGCATGTAATAACACATCACATACTCCTTTAACCCCTAGCCTTAAGCTCAGCAA
GACGCCTTGACAGATCATCCTCATCAACTTTCTCCTGTTGCTTGGTTGGTACAGCATGAG
CTGCTGGTTGTGGGAGCCCAACAGAGACCTCAAGACCATAATCATCCGCCACTTGCTGCA
TCAAGCTGTTGACCTCCCCTTCCGGAGTAGACAGTGAGGTGGAGCCTGCCATTGAGCTCT
CCATAAACTCTGCCTGCACCTCCATGTTCACAAACTGCTTCTCAAATTGATCCATTGTTT
CTGACATCTTCTGCAGATTACCAGTAGCAAGAGTTGATTCGAGCGATTTAACGATAGAAG
CCATGGACTTATTAATATTGGTCATTTTGGCTTGGGTGTCGAGCCGAGCGACGACGGCGT
CGAGACGAGAAGCGAGTCGGAGATAGTTCATCTGCTCGGTGCGCTTACGGATGGCGTTCT
CAGCGTAGATTCTGGCGCCGTCCATGTTACCTTTCTCAATGGCCTTTTTGACCTTCAATT
TCTCTTCTTTCTCGGCCTTCTCGCACTTCTTCGCCTGCCGTTGCAGAGACTTCGACGTGA
ATTTCAAGTCCATGATCTGATTTAACAGCTTATCCGTGTTACCCATGATGGATCTGAGAA
TTAGAAAATTAGGGTTTCCCAGAGAGAGATTGCGATTTGGGGATTTGCCTTTGGATATTT
GTTTTGTATTCGTTTCGATGTGTAAATTACTGAACCCCCCTC
>ntig_263_consensus
CCCGGGTTTTACACTAATTTCTTTTCTAATTAATTATGTCATCTGAGTCGCCATCGGATT
CCGGGGAATGTTCTGCAAATCCGTCTCCGCCCGATGTATCCACCGCCGCCACTGTTGATT
GCTCTAATGGGAACGGAAACATTTCTCCCTCGTCCCAGCAAGCCCAGATCCCTCTCACCT
GGCCTCATGATGGGAAGCTGTCATTTGAATGGATCGAGCGTCTCATGTTCACCTTCGATT
GGTCTTCTAAAAATCTTCCTCCTTCTGATTTTCCCTCTGTTTTACCTGTTGATGTGTTTG
ACACTTTGATCCTCACTGCTTCCAAGATTCTTCATAAAGAGCCTAATTGTGTGAGAATCG
ACGCTCTTCAACCCGAATCAACTGTTGTTGTTGTGGGTGATGTTCATGGGCAATTGCATG
ATGTCCTTTTTCTTCTCAGTGATGCTGGCTTTCCTTGTGAAAATCGCGTCTTCGTCTTCA
ACGGTGATTACGTGGACAGAGGCGCTTGGGGTCTTGAAACCTTCTTGTTATTGTTAGCTT
GGAAGGTTCTTATGCCACATAGAGTGTATCTACTCCGAGGAAACCATGAATCTAAATACT
GCACATCTGTCTATGGCTTTGAGAAGGAAGTTTTAGCTAAGTATGGTGATAAAGGAAAAC
ACGTATACCGGAAATGTTTGGGATGCTTTGAAGGGCTTCCTTTGGCCTCTATAATAGCAG
GAAATGTTTATACAGCCCATGGGGGGCTTTTTCGCAGTGTTTCTATTAGTTCATCGAAGA
GATCAAAAGGAAAGAAGAACCGCAGGATAAGTCTTAGTACCGAAGACAATTCTTTAACTC
TTGGTTCATTGGAGGAATTATCTAAAGCTCGGAGATCAGTTCTTGATCCCCCATGGGAAG
GTTTGAACTTGATTCCTGGTGATGTACTATGGTCAGATCCTTCAATGAAATATGGTCTTT
CACCAAATAAGGAGAGAGGAATTGGCCTATTGTGGGGACCTGATTGCACTCAGGACTTCT
TGAATAAGTATGACTTAAAGTTAATTATCAGATCACATGAAGGTCCAGATGCGAGAGAAA
AGAGGCCTGGTTTAGCAGGAATGGATAATGGATACACTATAGATCATGAAGTTGGATCAG
GGAAGTTGATTACACTCTTTAGTGCTCCAGACTATCCACAATTTCAGGCAACAGAAGAAA
GGTACAGAAACAAAGGGGCATATATCGTTTTGAGACCTCCTAACTTTGATGAACCAGATT
TCCATAGCTTTGAAGCAGTCACTCCGAGACCAGAGGTGAAACCATACTATGATTTTGAAG
ATGTCATTGATTCTGATGAAGAGTTGGACTTGGCATCAATGGAGACCACTATATAAAGTG
TCTATATATATATATATATATATATATATGGTTTAATGATATAGCATAAAAGATCTACAAGA
TATCACATAAATGGTACCAGCATCGAAGATGCTACAACCCTTGCGAAAGTAAAATTTGGA
AAACCCAATTCCGTTAAATTTACACATATANNNNNNNNNNNNNTCATTTATAGCACTTCCT
TAACCTCTAGCCTTAAGCTCAGCAAGACGCCTTGACAGATCATCCTCATCAACTTTCTCC
TGTTGCTTGGTTGGTATAGCATGCGCTGCTGGTTGTGGGAGCCCAACGGAGACCTCAAGA
CCGTAATCATCTGCCACTTGCTGCATCAAGCTGTTGACCTCCCCTTCCGGAGTAGACAGT
GAGGTGGACCCTGCCATTGAGCTCTCCATGAACTCTGCCTGAACCTCCATGTTCACAAAC
TGCTTCTCAAATTGATCCATTGTCTCTGACATCTTCTGCAAATTACCTGTAGCAAGAGTT
GATTCGAGCGATTTAACGATAGAAGCCATGGACTTATTAATATTGGTCATTTTTGCTTGG
GTGTCAAGCCGAGCGACGACGGCGTCGAGGCGAGAAGCAAGTCGGAGATAATTCATCTGC
TCGGTACGCTTTCGAATGGCGTTCTCGGCGTAGATTCTGGCGCCATCCATGTTGCCTTTC
TCAATGGCCTTTTTGACCTTTAATTTCTCTTCTTTCTCGGCCTTCTCGCACTTCTTAGCC
TGCCGTTGCAACGACTTCGCCGTAAACTTCAAGTCCATGATCTGATTCAACAGCTTGTCC
GTGTTTCCCATGCTGGATCTGAGAATTTGAAAGTTGGGGTTTTCCAAGAGAGAGGTTGAG
ATTTGGGGATTTTGAATAAACAAAAAAATCTCCTCCGTCTTCTAACTTAATTGTTCTCTC

TTTCTTTCAAGGAAAAGTCTCCGC
>ntig_264_consensus
TATACTTTTAAGTCAAATAAGTAGAAGCTCAATCTTAAGCTGACCATTTTGAATTCAAAT
CGAACTCGAGCTTACCTTTGTTTAAGGCATCCAATAGGCATGGATAAATGATAAGGCAGG
AAACTGAATGGGAACCCCGACTGTGAAAGCATGTCCAATGCAAGCTCCTACCTATATGAG
AAATGCATGGAGTACACAACCGTACCATCAAACCAGGGAACGAACGGGGTTCTTAAAGCA
GAAAGATAATATTTCTTATTATTACACATGACAAGAAAAATAATCCTTATGTAAAGGCTA
TATGTGGATGCCATTTTTCTGTATAACATCAGGTAAAGAACATATTAAAAAGAATGCTCT
AACAAAACCAACACTATTCATCCCTACAAACTACTTTTCTCCGTGCATAAGGGATTAAAG
ACAGACCTCCCATCTCTGCAGTAGATCAACACCTGCCAACCTTTCAGCTAACCTCTGATT
GCAGCATGGGGAAGAAATTCCAGTTGTTGGAAGGTCTAGCTTCCTTCCCAACAACATTCT
CATTGGCCCACCACTCAGAGCTGATTGAGGGCTTATCGGAGACTTCTCCCTCAGCGTTGT
CAAAGTTGAACTCTTTGATTGACCCAAGAGTGATTGATCGATGCTTTCGATACAAATGCT
CCTCCTCTACTTCTCCCAAAGCCTTTTCAGGCATTTTATTGGAAGTATCTGCAGTATACA
GCTCAAAACAACTTTCACTCTCTGTTAATTTCCCTTCTCTTTGTATTTGGCCCTCTGCCA
CTATATCCTTAGGACACTCTGCTACAATGCGTGGTGATAAAACTGATTTATTAGCAAGAC
AGCGTGCAACCTCTTCACCACTAAGCTCAAATGATACTCTGTGATGAATTATATGTTCTT
CATTTTTTGATTTATTCTCTGACTCAGCACTTGATGATACCTCAGAAATCTGGTTCTCCC
AAAGAAATCCATCCCGTGAGGCAGGCGTCAAAGATCCCGAACCCAGCCTTGAACCANNNT
CAGAGATCCAGAACCTAACCTTGAACCCCATTTCAGAGTAGTAAAATGTTCAAAGCCCAA
GAGCTTGGGAGCCTCCCCCATACAGAACTCAAGAATAGGGAGTTTATCTGGGAAAGGAGA
AGAAGTGCCAGAATTAGATATTGCTGTGCCTGGGGATATGAGTTGACCACCTGGGCTTCC
TGGGTACAGCGGATATGACTGAAATCCATACTGAGATAATGAAAATTTCTGATTGATCCC
ACTGTTTCTTCGAGCACGTTCCAGTGAAGATGCCAGCATTTCAGCAAATGGCACTTCAGG
GGATGAAGGGGTTGTCAACTGAACAGATTCAGGTGGGGGAGTGCAAAAAGCAGTAGAAGG
TTCAGTAGTGAAAGTCGAAAATACAGGTGGTGTGACTAGCTGGGTTTCATGTGCATAAGG
GCCTATGGCAAAAATGGATCTAGGCCCACTAGGAGAGAAGGCATTGATGGAGAGGGAAGT
AAGGGACAGCAATCCAGCAGGTGATTGGGTAGCAGATGGAGGATCAGATTGAAGGAAAGA
TGCAGGCGAAGAGGGTGGAGCAATAAAGGGAAGAACAATGGCAGTTGAGTGGGTCAGATT
TTCTGGAACTGGGGCTGCAGCTCCTGTTACAACTGGTTCAGGAACTAGGACAGCATCGCT
AATCCGCTTGCTACTTTTATGCGACCCAAAACACCAGTAAAGGCTCCAGCAGCTGCCCCA
TCTTCTTTTCTGGACGGTTGTGGTCGGCTGGGGACGAGACTCAGCCGAAACTATGGCGGT
TGCGGCGGCGTTGACGGTGTCGACACTGTTGTTGTTGTTGTTCACACTACCCATCTTCGA
TTTCGAAACATGAAACGAAACCGAAGCTGCAATAACATCTACTACGTTTCGATTTCGATC
TCTTTTGCAAGAGAGAGAGAATTTAAGTTGCCAATTATAATAAAATAAACGATAAAATGAAT
CAAGCTGAAGAGAAATACAACGTAAATAAAAGATCAGATCTGGTTCCTGATCTCTTCGGG
TTTAGTCTCTCTCCAGTCGTTATCCTCCAGTTTTCATCCATTTTGCTCTGAAGTTTTCGA
CACGTCAGCTGATTGGACGGTACGGACCCGCCTATCACAACGGCTTTCTATTTGTTTGAG
CCTACTCTACCC
>ntig_265_consensus
ATTCTGAAAGACTAGATGATTATATTGCTGGAGAAGAATAGGCCCTGTGGTACATTTCTA
TTTTTCACTTCCTACAAGAAACAATAGACTTGTCAATCCCTAACCGGCTATAAAAGAACT
ATCTCCGCACAACATTTACCCAACAAATGGAACTGGTGTCTCTCAACAGTCAATACATAT
CAACAACAGCTGAAATGTTTGACAGCAAACTCAGTTCACACTTTTTCAGCACCGCAAATA
ACTAACAGTGCTCAACTTTACTGGAAATTTTAGCTCCTGGGTTTATTTTCCTGACTCAGT
ATCAGTATGGCACAGTGAATGGTAGAGCATATATATCAGCCTCAGGCAAGTAACAGACGT
GCGGCAAAGCAGTTGAGTTAGGTAAACCAAGGTTACTCGCTTCAGCAATTATGGCTCCAC
ATTTCTTGTCCTGCTTTCTCCCAAAAAGCCATTCCACATCGTCAGAAGTGCGGTCAGGTT
CCATTGGAGGTGCAACCCAGTTCTCAATAAAATCTCTGAATTTTAATGATTGAGGCGAGC
AGTGGCATGGCACAACTGTTTCAGTTGTCTCAGCATAACTTTCAGACACTCTTGTAGACT
CACAAGGCTTTTCTACATTGATCCTTAGTTCTGCACATGTAGCTGTGGAAATAGAACAAG
GCAGTTCTCCCTCTTCCAAGCCTGTTCTATGAGCCTGTTCCTGCATCACTTGAGTACATG
CATCATAAGTCCCCCCAGAGGCAGTGCAAGGCTGTTCCTTGCTGGGTAACACTTCTGGAT
CATTTTGCCTTTGGAGAGGCAACCGGATCCGAATAATGCTTCCAGAATTATTCTCACCAC
CAGG
>ntig_266_consensus
CATTATGATATGATTTTCTCTTCAAGATACCATTCTGGGAATCGACTTCGGTTCAGTTCA
GTTCAGCTACGGCTGTGCAGTCTGGAGCCCGAACGTCTTCTTAATAATAAGTGTCAAAGC

GCACTAAGCCAAATGTACACAATTCACCGCCTCACTCCTAAGATCCTGATCGAAACCCTA
AAATGCCGATACTAGTAAACACCGACTTCTCTAACTCAACTCAACCTCCTGCTCTAACCG
GAACAAGAAGAAGAAGAAGAGATGGCGAAGCACTCGGCTGGGTGGTGGCTGCCGCTGCAG
AAACGATGGCTTCTGACTCTGTTGATCATGCTCTCGGTCTCGACAGTGATTGCTTTCTTG
ATCAGAGCTGCCTTCGATCCCTGCGATCACCACCTCGAACTTGCCGAGAAACGAGTTAAC
TCAGTTCCGAAAATATCCGCAAAACCTAGTCCTCTCAGTTTCATGAAGTCCAAGCTTGTT
CTCTTGGTTTCTCACGAGCTATCACTTTCTGGTGGTCCGTTGCTGCTGATGGAACTAGCA
TTTCTTTTAAGGAGTGTTGGTACTGAGGTGTACTGGATGACAATCCAGAAGCCGTCCAAA
GAAGACGAGGTGATATACAGTTTGGAACATAAGATGTTGAATCGAGGGGTACAGGTCATC
TCTGCAAAAGGTCAAGCAGCTGTAGATATGGCTCTTAAATCAGATTTGGTTGTTTTGAAC
ACTGCTGTTGCTGGGAAATGGCTGGATGTTGTTGTCAAGGAAGCTTTTCCTCGTGTTCTT
CCAAAGGTATTATGGTGGATCCATGAAATGCGAGGGCATTACTTCAAACCTGAGTATGTC
AAGCACCTTCCTTTTGTTGCTGGTGCAATGATTGATTCACATGTAACAGCAGAATACTGG
AAGAATAGGACTCGGGAGCGATTGAGGATTAAAATGCCTGACACCTATGTTGTTCATCTT
GGAAATAGCAAGGAACTTATGGAAGTTGCTGAAGACAGTGTCGCGAAAAGAGTTTTGCGT
GAGCATGTCAGGGAATCTCTTGGAGTGCGAAATGAAGACTTACTTTTTGCTATAATAAAC
AGTGTTTCACGTGGAAAGGGACAGGATCTATTTCTCCGTTCCTTCTACGAGAGCTTGGAA
TTGATAAAAGGAAAGAAGCTACAGGTGCCATTGATGCATGCAGTGATTGTAGGAAGTGAC
ATGAATGCACAGACTAAGTTTGAGTCAGAATTAAGGAACTTTGTAGCACAGAAGAAAATT
CAAGATCAAGTTCATTTTGTGAATAAAACTTTGACAGTAGCTCCATATCTTGCTGCAATC
GATGTTCTCGTTCAGAATTCCCAGGCACGAGGTGAATGTTTTGGGAGGATAACTATTGAA
GCTATGGCATTTCAGTTACCAGTACTGGGAACAGCAGCCGGCGGCACCACAGAGATTGTC
GTGAATGAAACGACTGGTTTGTTGCATTCTGCTGGAAAAGAAGGGGTGACTCCTCTTGCT
AAAAACATTGTGAAGCTTGCAACTCACGTTGAGAGGAGACTTACAATGGGGAAGAGAGGG
TACGAGAGGGTCAAGGAAATGTTTTTAGAAGAGCACATGGCCGAGAGAATAGCTGCAGTA
CTGAGGGAAGTCTTACAGAGGTCTAAGAGTCACTCACATTAGATGCCTTTGCAGTTTAGG
TCTTTTATTTTAACCACCAGGCAGAGCCAATTTTAAGCAGGGGTCTAGTCGTCTAGTGTT
GAACATTGTGTCCAAAATTATGTGGACTAGAACGAGGGTGCCATTTTCTATTTCTGCTTA
CATAAAGCAATGTAAATCATGTACATAAAACTTGACATCACATGTTTATGATTATTTAAC
TATACGATGCACTAACATTACATAAATACGAAACCGAAAGACTAGCTAGCCTGGCCATAT
ACTAGTACTTTATAAGGCTCAGTAGTGGTGGACGAAGGCTGGCTGTATATGCCATGGACC
TGAACCTAGTGGTGATGATGGTGTCCGGGAAGTTTTTCCTTTATCTTCTCCATCATTCCT
TTTTTCTCATGGTGATGGTGCTGCCCTTCTCCATCCACATCGTAACCACCAGGAGTGGTG
GTTGCTGTGCCATGATGTCTTTCATCTTTGTGCCCACTGCCTGGCAACTTTTCCTTAATC
TTCTCCTTAAGCCCCTTCTTCTTCCTTCTCCCACCGTGTCCATCATCCTCCGAAGAGCCA
GAGCTACTTGAGCTCCCTGAACGTTGAAGGCCGTGATGCCTTTGGTCATGGTATCCCGTA
CCTAGGTGGGTGGCGCCACTGCCAGCCACTGCTGCAGTTCCATAATCGCCAGTAGTAGTG
CTAGACGTCTGATAAACTGGGTTCCCATATTCATCATTCTGACGAATTGGGTTGCCATAT
TCATCTACATGTGGAACTGCCCCATAGTGGTTCTGAAATTGTGCCATTTCCAACTGAAGC
TATGTATATGAAACAGAAATCTCTGGATTGAATTCTTGCTTCCACGTAACTAAGCCAGTT
TGTACTTCAAAACATGACATGGGTTTCTGTATTTATATACAGTAGGGCAAGTAAACAACG
ATTAATGGCCATGGAACCCTTTCATTATGCGTGGCGTGTATGTATGTTCATAAGCTGCCA
CGTTACCTCTGAAGGACT
>ntig_267_consensus
GTTCGTTAGTTGATTATGACGGGTAATCGTATCACATAATACAATAAAATGCCATTATTT
CAATTATGTTATACAAGAATAAAACTAGCAGCATAAGTACGCCGGTAAATTATGGTCTAA
TCAGGAACCATAGGAATTGGAAAAAGAACAACTTACACTGCACAAACTGAACATTCAATG
GATTTCAAGGTGTCCAGATACTCAGCTTGTTCAATACATCCATGACAGGTCCTGTCGAAA
AGAAACAGACCTTGCTGCTCCTTGAACACCCCTTTCGGAAATTCGAGGACAATCTTCCAC
AACCAGCCGGTCCAATTTGTATGAACCCACAAGTGGCTTTAATCCATCATTTGTTACCCC
CAAACATTTTTTCAACCGCAATATACACAACCGAGGGAACTGGCTCACTAGCTGCAGCCC
TTCGTCGCTAATTTCTTGACATCCGACAAGTTCCAAGATTTCAAGATACTCAGCTGAGCA
AAGTGTTTCCATCCCGGCATCATTGAAGGAACATACATGGTCGAGAGCAAGTTCTTTAAT
GGGGCACTTGTGAATCAAAGTTAGGATTCCATCCAGAGTAAATGATGAAAAAGAAGGAAA
CTCGCCATCAGAGAACGATAACCTGACAGATTCAAGCATCGTACAGTTGTGCGCCAAGGC
TATTAGACTGTTATCAGTCAAGCTCAATGGGTTGTTCAACAGAAGTGGTAAGGAGAAATC
TGAAGGCACCCGGAGGGAAATAGACCGAAGTTTACTTGAGCTTTGGGCCAGGTTTGTCAT
GTCAGAGTCCCTTAGACCAAAACACATGTCAAAATGAATCTTCTCCAAGTTTTTGCACTT

CCCCAATACACAAGCAAGCCCTTTCCCCGGGCTGATAATACAATTACTTAAGCTAAGCTC
CACCATGTTTTCACATGGAACCAACTGTTTTTGCCAATGATCTACAGCTAATCGATCATA
AACTTTCATATATCTGTAATTAGCATCCACTTCAAATTGCAAGTGCTTAAGCTTCCGCCA
GCTAGGGCCTAACTTTATTAAATCCCCCTCTCCAATTGATCTACAATTCTTAATCAAAAG
ATCTTCGAGCGTTCCAAGCTTGCCAAGATATTCCAGCCATTCAACACTGTAATTCTTGGA
GAGAAATTCAACTTCAAAGCCAATAGGTTGAAGCAAGCAGCTAAGTACCGGAGACCCACA
TCGGTGATGTAAGTGCAGTAGTTTAACGTGAGATCCGTTAGACACATACAGTTGCTAGAA
AGAATGGAAAGCCCTTGATCATCCAGTTGTTTTCCTAGTCTTGACATCCAACCAGAGTAT
GTAATTTCCACTTTTGTTAAGTTTGGAAATCTATTACAAAGGGAAGTCAGTGCTTCATTT
GCAGGGTCTAGTCCACAGCCAACACGAAGAAAATGTCTCTGTTCATTATCCAGCTCATAT
AAGCGTTTACATGCCAATGAAGCAGAGTTTCTGTCCACAATTTTCTCAATCCTGCACAAA
ATCTCCCAAACTAGATGGTCTGGTAATGCCTCCATCACAGCTTTCTTCTCACCCTAATAC
TGGCACATTAGTAGCCTCTGG
>ntig_268_consensus
CATGAAAATCCATATTCCATACTGGAAATAACGAAAGAAAGAAAATGATATCTAGCCTTT
ATTTAAAGCATCTCCGGTATATAACAGTAAACTACATTTCTAGGTCTCAGTCTGGCGATG
TCACATTTACATTCATAAAAATAGATATTAACCTGTTCATATGGCTTCATGTGACCCAGC
AGATGTTCCCCGACGGTAGAAAATACCAAAACATGTGCAATAAAAAAGGCAATCGAGTTT
TTAAAGAAGATATTTTCAAATCATAGAACATGACCCATAAGTTCTCTATGGTGTTAAATT
TATTGCCGCTCAGAATTTCAAATCCGTTGTCCATGTACCTTGAACACTAGGGCGTTTGGT
TACATGTCTTGTCTTCAGTTTTATCACCTTTCTTCCCAATGCATTCCTTAGCCGGGTTGC
ATGCAGAAGTATCTCTTCTGGAGTCAAATTCCTCACACATACCACTCGCTCATTATGGTT
CCGGTAAAAAGCCTTCAAATGTGGATGCTGACCACGAATAAGTTCCGTAACCACCTCCAG
CTGAGGGTTGCTCTCTTTAAATGCTGGCAAATCTGACTCTATAAATGCCCTGAGATCGGA
AGAGACACGTCTGAACTC
>ntig_269_consensus
CTTTCCGCCACTAGCCTCCTGATCTTTAATAGTAAAAGAATAGAATATTTATTAAGTATC
AAAGTATATTTATAATAATTCAAAGCTTTTAAAAGTAATTTACTATTTAAGGGTTTATGA
AATATGTAGGAGAGAAAAAAACCACAGAGGGAACAATTCAGGGATATATCTATTCCCATTC
CGACTATAGAGATTTTAACCATTCTTGTCCCCATCCATGTCCTCATCCTTGTCTTCATCC
CTGTTTCTATCGGAAAATCTCCTTCCTATTTGGGTTTTGCAAGTTGAATTGCCATCTCTA
TCAAACATTGTTATCCCTTGGTATTTATTTAAAGTTCATACATTGTGTAACCATAAACTA
CATATCTTAGGTCTGAATCAACAAAACCCCATTGCACTATTAAAGCAGAATATACCAGAA
CAATGTTCTGCTTGCCAAAGAGATAAAAAAGACTATTAAGCCTAAAAATAAACATCTCCA
GAAGACATTGAATCATAACACACAACCCAGAAGTTACGCAGATTCACTTACAAGAGTATC
TTCCATGGAATTGTCACAATGTGGATGTACACAAGAAAGAGATAAATGAAACAGAGCTTT
CTACAGGGTTCATTTTCTTGTCACTCTTTTTTTCATGTCAGCCTTCCATGTACTTTGAAC
AGTAGGGTGCTTACTTACATGTCTTGTCTTCAGTTTTAACACCTTTGTTCCCAATGCATT
CCTTAGCCTGGTTGCATGCAAAAGTATCTCTTCTGGAGTCATATTCTACACACATACCAC
CCACTTGTTATGGTTCCCTGGATATGTGGAAATGGAAACATTCATGAAACTAGGAACAGA
AGAATGTTAGAGCCAAGGATTATATAATCCACAGGAAGAAGGAATTGGGAGCACGGGTGG
AACACATAGGAGATGATGGACCACAAGTACAACAAACAACACACGCAGCGAAAAACATGG
GCAATTATGAAATGGGCAAGAATAAGAGTGGGATTGAAGAATGAGAGAAGTAAGAAAACG
TGGAAAGTGACAAAAACATGAATAGACAGTTATGAAATGAGCAAGAATAAGAGAGAGACT
GGGGAAGGGGTGAAAGAGGGAAAAATTACGAGAAAACACAAGGGAAGGGACAGTGCTGGC
CTGCAAAATGGGATTATGGTGGAGCATACGTGGAACACATAAGAGATAATGGAACACAAG
GACAACAAACAACACAAGCAATGGAAAACATGGGAAATTATGAAATGGGAAAACTAGAAG
TGGGATTAAGGAGAGAAGTAAGGAATTGTGGAATGTGGCGAAATCATGTTTGGACAGTTT
TATAATGGGCAAGAATAAGAGAGCGGTCGAGGAAGGGAGAAAGAAGGAAAAGTAAAGAGA
AAACACAAGAGAAGGGACAGTGTTGGCCTCTTGAAGGCTAACGAGATTATGGTGTTACTT
TGTTTCTGTAAATATTGCAATCAATCAATAAAAACATCTAATTTTCATCCATTTCTGTTT
ATGCTACTTGGGTTCCTAATAGAACACTTTAAGACATGCATGGAAGATTGGGGAAGAGGG
AGAGAGAAGACTTACTGTAAAAGGCCTTCAAATGTGGATGCTGTCCCCGAATAAGTTCAG
TAACCACTTCGAGCTGTGGGTTGCTCTCTTTAAACGCTGGCAACTTCGACCCCATAAGTG
CC
>ntig_270_consensus
GTCAAATAACAAAAAAATTACAAATCAGACATCACACGCACTGCAATTCACAGAAGTAAT
TGTAAAGTGTCACACGCTCCTCCGATGCTATAATTCACAGTAGTAATCACAAAAGAAGAG

ATGCTGAACAATCAAACAAAAGTAAAAGAAAAGGAAAACTCCAAACATCCCAGTTTTTCT
AGCAAACATTAAGACATATGAGTTGAGTAGCCAGCAGAATTTCTATTCAAAGCTCTCTCC
AGCCTCGTCTCAAATGGGGTAGCGTACCAATTTACTGCCTTGTCCTCTCTGTACTTGCTA
GTTGTGTTGGGTATGCCTTTGTAAGGAGACACTGAGCCAGAATCCTTGGCACTGCTTGTG
GGACTCCAATAGCTCCCAACACTCCCTATTATGGCCATCTCATCAGGGCTTCGACTTGGT
GACTTCCTTGGTGAAGAAGACGAAAACTGCTTGATTTCTTCCAGAGTCAGAGCACCTAAA
ATAGGTCTATCTTCTTGGCTCCTCAGTGAGTTTGATCCTTGTGACATGCTTTTCTCCGGC
GTAGTGTTCAAAGAAATTACGTTACTCTTGTTAATCGGTGTAGTTTCTGATGAAGACAAC
CAGTTTGAGAGGCTAGCATCAACTGGGACTTCTTGGTTTGGCTTCTTGGATTGATTAACA
GATTTTTTTTTGAAGCTGAAAGGTAATTCTTTGAAACTCCGCTCCAAGCTAAATGAAGCC
CGGGGTTCTTCATCCTCTGTGAAATTCTCTTTCTGGTGCTTCAGTTGCGATTTCCCCTTA
TTTTTCACAGTTTTCCATTGAGTGAGATTTTCAACCGGGTTCAAAACAGAATGGACATAA
GCACTTCTATCTCTTACTCTTTGATTATCCTGAATTGGCTTCACACCTCCCTCATCCAAA
TCACTTGTTACCGAAGAACTGTCAACAACATTCTTTGTATCATCCTTACCCACAGCTATT
CTTGATTTAGCAACCATATCATAATCATCATCCTCATAAATATCATCATATTCCACTGCC
CCGTCATCCTCCTCGTCCTCGTCACAATCAAGATCACTACCCAATTCCAGTTCATCTTCT
TCATCATCGCTTTCTCTGCAGTTTTGGTATCTATAATTAGAAGGATAAGAGCCCGAGCTC
GATGTAACGGAACTAGCTTCAGAAGAACACCGAGTTTGCTTTGATTTCACTAAATTCTCC
TCCTCTTCCTTCTTCTCC
>ntig_271_consensus
GTCCGAGCAAGTACTCATTCAACAAAACACATTAACTTAAGGTCCAGAATAGTCATGAAC
TCTTAAAATGATAGATCGGCAGATCCAAAAATAATAACAATCAAACCCAAAAACCACTAC
ATAAAGACTAATAAGNNCCTCTTGCCATAAGCTCTCTAATTGCTTTCCTTGCAAGTGATC
CATTGATCCTTAAACGATCAGAGAGAATGGAAGGAGTGATGAGCTTGTACTTGGGAGCCT
CAGAGAGAAGCTTATCATAAGTTGCTTGATCAAACAACACCATGTTGTTCACCTTCTCCT
TTTGCTTCCCCTTGCTCCACTTCTTCTTCTTCTGCTTTCCGCCACCGGATTTCGCAGGCT
TGGACGATGGTGGGGGAGCCTTGTCCTTCTTTGGAGCCATTGGATGTGATCACAGAGAGA
TGAACTTCAAGGTGGTGAAGAAGTAAGATGAAAAAGAGCAAAGCGAGGCGGACAGGCTGA
GAATGTATATCTAACCCTAGGGTATCCCATAGATCGGTTTTGTATGAGATTACACGGCGT
CGTTCTAAAAAGACTTTACTTGCTTTACTTTTGGGCTGCTTCTGAATCATCTTCCACTCC
CAGGTCAGGCCGGGGGGACAAAAGAAATAATTTTTGTTTCTTCCATGCCTTATCTCCTTC
TCAAAAGCAAGAGAGAAAGTGGCCATTTTTA
>ntig_272_consensus
GCTCACTTTGATCCCATGAATTGTCTTCCTCAACAATGTGGATTGATAACAAGATTGATC
CAAATAGCCAACTATTTTGTTCATCCTATATTTTTATTTAAAATGTTGACATTTTCAAAG
TCTATACGAGTACTCTTTCGACAAAAAACTTTTTAATGTCCGAAACTGTAGCTAATAAAC
TCTTAAAAAGATAGGCGTGAACAAAAATAATAACAAACAAGCTCCAGGAACCACTGCATA
ATAACTAATAAACAAAAATATTGAAAGACCTAGGTATTTGTTGCCCTGGTGTAGATCTGC
TGGCTTGCATGGGCAGACACCATTCTGATCGAACCTCTTGCCATTAGCTCTCTTATTGCC
CTCCTAGCAAGTGATCCATTAATCCTTAAACGATCAGAGAGAATGGAAGGAGTTATGAGC
TTGTACTTAGGAGCCTCAGAAAGAAGCTTATCATAAGTTGCTTGATCAAACAACACCATG
TTGTTCACCTTCTCCTTTTGCTTCCCCTTGCTCCACTTCTTCTTCTTCTGCTTTCCGCCG
CCGGATTTCGCTGGCCTGGAAGACGGTGGAGGAGCCTTGTCCTTCTTTGGAGCCATTGGA
TCTGATCACAGAGAGACGAGCCGCGGGGTGGTGCAGAAGCGAGAAGAAAAAGAGAGGAGC
GAGGCGTGGAGG
>ntig_273_consensus
ACACTTAATAATTATTAAGTGTGGGCTCTCATCTGCCCCTCATGCCCACGTCCGACGCCC
CACAACTGCCAACCCACCAGAATCAGCCTCAGACATACCCAACACTAGGCATTCAGCATC
CTCCTTGGATCATCAAGATTCACCAACAAACAAGGTAAAAATGACAAGGACATGAAAAGA
AAAGATTAAATTGAAAAAATTTATGAGGGCTAATCTCGTATGTGCTAGACATATACATAC
ATATACACCCTCACTGCATATACATGTCAACAACTAAACTCTATCTGGAAAATAAAAATC
CCAAGAAAAAAATTACATCCCCCAAAGAAGGAAAGGGGTTACTTTGCTTATGTCTTTTAC
CCACTTTCCCTTTCTTTTCCTTCTACAAAACGTTCTCTCATCTTCTCTCACCGTCAAACC
AATCAAAAATTATAATTAGGGTTCGACGTCAAGTTTGTCAGATTATAAACACATGTATAC
ATAATTCGATACACATTGCAAAGCAACTCAACTGTTGCAATTTCTGTTGCAATCGATCCG
AATGTTGAGTCTGCTTTGTTGGTTGATCCGAACTTTGTATCCATGACTCATCTAACCAGA
GGGCAGCAAGGGATTTTTCTACTCACCGGCAGGTTGTTTCTGTTCTGCTGGTTTCACTTG
AACTGCCGTGGGAACAGCATGAACATATGAAAAACCAGGAGGAAAAGGCTGATGTGGCTT

CCCGGAGGCCTGTGCATTAGCAAAATTGGCAGCATGTATAAGACCCTGTGACGTTAAGGG
ACCAGTTTTAATATTGGTAGCAGCAGCAACAGCCTTGGCAGGATCTGAAGTGCTGGTGTT
AGAATGAGTAACTGGAGGGCACAGTGATAGTATCCCAGCTGAGGAAGTCCCTGAAGTGCC
AGGCAGCTGCTGTTGGTCTCGGTGTCGTACCGTGAAAAACCCACTGCCAGCCGAGGTAGA
TGAAGTTGTATTCGCTACACTTTGAGGTTGGGCTTGCATACATGCATTAGAGAATAACAG
TTGGGCTTGCTGAATTGCATGCTTTGATATCTGCTGCTGCTGCTGCTGCTGATGCTGTTG
TGACATTTGAGGCTTAACTCCAGTTCCTGGAGAAGTGATAACAGGGCTTCCAAGTATTGA
TGGAACAGGAGATGACTTCCTGCCTGTGGACGCAGTTGCACAAGTCCTTGGGCTTCCACC
AGCACTAGTCTTGGAAATTGAAGTTGCTGGGGAACCAACTAGCACTGGTGGAGATGCAGA
CTGGTTATTACCAGATGGTGGCTGCCCCTGTGTTGCTGCTGATGATTTGGTGTTAGCAGC
AAATGATATTTGTGTGTGGCTTTGCTGGGTTCTGCCATGTTGTTGGGGAAGGTTTCTGTG
CGATGATGTGGTTGATGGAGCAAGAGATTGAGAACTAACTTGAGAGGCAGTTGTCCTTCC
AGAATTCTTCCACTGAGGAGCTTGCACTGAGCTCCAGTTATTTTGGACAAAATTTTGGGG
AAAAACAGATGAAGCACCAGGAAACTTTGCAGCCATAGATGTTGAAGGTTGTAGGTGGTC
AGAATAAACACTTCCATTGCTTGTAGCTGGTATTTTGCTTCGAGCAACGGCGGCAGCAGC
AGTTGCAAATTGCTGTTTTTGAAGTTGAATCATTTGCTGCTGCTGATGTTGACTCCGCTG
CAGTTGCTGCTGTGCAGGAGAAGCATTATCATTCCTAATAGAAGAAGGCATAACAGAAAC
CGTCGTTCGTGCAGGTTCAGAACCAAGGTTTAGACTTCGTGTTGAACCATCAATGACTGT
GTTGCCTGTCATAGCAGATACTGGTCGATTGGCTAAATCTGGCCTGGAAAAAGCAATGGA
CGGCCCAACAGTTGCCGGTGGTTTTCCTGCCATTGGCTTTCTTTCTTCCTCCACATTAGA
GGTGTCACTACCTCCAGCTTTCCCTTCTTCCGATACACCATAATTCTTTTTCTGCTGTGC
CGCCTGGGCAGCTGCCATATACTGATAGCTCTGGAAAATAGCAGGATTCTGTGCAATGGT
AGAAAGGTCAACGCCAGGAGCTGTAGTACCATTAATAGAAGCAAATGTCATTGCAAACGT
TTGAGGCATCATAGTTTCAACTCCAACCTTGGTACCCTGCTGTTGCAGCTGTTGCTGAGG
CTTCTTTTCATCGTAATTACCACTCAAACTAGAGGCACCTCCCATTGAAGTAGTAGTCAT
CAAAGCAAAGTTTGGTGGGTGTAAAGCCATTGGAAAATTCTGTCCATAGAAAGTCATATT
TGCACGAGAGGCACGGCTATCAGCTGTTGATGGGCTCTCTTCACCACCCATTTCATTTTC
AAGCTGGCGAGATTGATGAGGAACCTGTTGGTTTTGCTGTTGCTGTCGTGGCTGCAGTTG
CAGTTGCTGAGAAGCCTGGTTCTTTGGAGCAGGAAAACCTTGCAAGGTTCCACCTCCACC
GCCGCTACCGTTATGCAGCTTTGGGTGTTGGCTCTGTAAATGTTTCTGGGATGAAGAGGA
ACCATTGGAAATGCTGGCGTTTTGAAGACCTTGTTGGCTTTGGTGTGACTGAGTAGGTAC
TGGTTGTTGCTGCTGCTGAAGCTGTGAAGGATGTAGCATTTGAGAAGAATAGAAGGACCC
ACTAAAGAATGGCATACTCTGAGCATTGGTTCCCCTGTAAACAGGAGTTGCTCCCATATG
GCCAGACATAGGAAATGGGTAGGCACCGTTCTGCAAAATTGCGAAGTACTGAGTTTGTGT
CTCATTGCCTGGTATCTTTGGGTAGTTCAAGCCCATTGCCGTGGCTGCAGCTCCTCCTGT
TGCAGTGGCACTCACTGAGACAGAATTAGACCCACTTGATGAACCAGCATTAGCAGCAGC
TGGAGGTGCCGAGACAGAACCAGGTTGAACAGCTGCAGCAGAAGCTTGATGTTGGCTCAA
TGGGAAGGTAAAAGCAGGAGCCAGTATATTACTAGGAGCTCCCGGTGGTAGAGCTTGCTG
AATCAAAACCTTTCTTTGAGACGAATCGAGTATGTTGGCATGTTGAGAAACTTTGTTGTC
TTTTCCAGAAGGACCAGGAAATATTGCCAGACCCTGCTCCTTATCTTGTGCATCGTTCAC
ACCTTTTCCTGGAGCGGTTCCATGCAATTCTGAGGGAGGCACAACATTGAGATTGCAAGC
CATTGCCCCATATAGTGAAGCAGAACCGGCAGCAGCTGGCCAGAAAGGATTCATCCTCGT
AAATTGCTGAAGGTAGTGTATGTTCCTTGCAATGTAGCAATGAGTTGCACACCTCTTTGG
TCTGGGTTGAGAAAACAACAAATTTGGAGGTGGTATGGAAGCAGAAGAGACAGTGCTACC
ATCCATGGATACAACTCCTTGTAAAGGTGCGGCCATATATCCCATAGGAGGAAGTCCACC
AGGCCAACCAGCCACAGTCAGAGGCAAAGATAGTGAAGTAGATTGGGCATTTTTCTCAGG
GACCGAAGTCGGTTGTTGCTGCTGCTGTTGCTTTTGGAAATTCTGTTGCAGCTTGTTGCC
GTTTAAGTTAACAGTACCACTATCTCTATCATTCTTCTCCAAGTCAAGCTGGAGGTCAAT
GTTCCTCTCCTTTTCAACAACTGGCTTCTGGGATTCACTTTCTTCTCCTATTGTTTTTGC
CTTCTTTTGTTCACCTTCAACAGCTATATCTTCCTTGACAACTTTGACTACCTTCTCATC
GTCATCTACCACAGACTTCATTTCCCTTTGAACATGAACAGCCACACAGTCAATCTCGCC
GTCAATTTCAGGTGATGATCTCGATGGAGGAGGAGCCATCAAATCTATCTGGAATTTTTC
TTCTCGCTGGTTCTCAATTTCAGATGTCGTTGAGTTCGCTTTAGTAACTGCCAAATGATC
TCTACAATCATCATCTAATCTGAGACTAGCAGTACATTCCTTCTTAGGCGACTGAGGTTG
AGGTTGAGGCTGAGGCCCGTCTTTTGCCACAGCCACATCTCTTCCACACTCTGATTCTTC
TATCAAAACCTTCGAATCCGGTAATAAAAGGTTGTTCTCTGGCTTAACAGACTCAACCTT
CGGCTCAGAGGCTTGAGAGTTCACCACATCGTAGGAAGTTGAACCGTTCTCAGCTGCTGC
AGATCCCGAATTCTTGTCCAAATTCGCCGAACAGGTTTCTGTCTTAGAAGATTGATCCAT

CTCAACCTTTGTGGTAGACGAAATGGGAACATTACGCACGGTAAGCGACGAAGGATTCTC
ATCTTCGTACTTGACAGGTCGTGGTCTTTTCCTCTTCGGCGCAATAGCAGACATAGGAGC
AGTTGAAGTGCTAGAATTTGTGGGCAAAATTGAAGAAGACTGAGGAAGTGTAGATGGGGA
ATTCGAGATCGGAGAAGAAACTCGGGATTTAGAGTCACTCGTGGACTTATTATTATTATT
ATTATTATTATTAATCTCTTTCGAATTCAAATCCCCCATAATTTCTTGCTGAGATGGACC
CTGCGGTTGCCTCATCCCGTACAAAACTTCAGCAATCTCGATCTCAATCTCGTCCTGAGC
CGACGATGAAGACTTGTTGCTCGACGATTTTGGTGGCCTTAGTTTGGGTCCGCTAGGTTT
CATCTTCTTCCGAACTGAAAAAATGGAAGAAGGCTCAATCGGTGTGGAAGTTGCTAACGC
AGTTGCAACACTCGTTCTCACAGGTGAAGATGACGCTTGTCTCTGTATTTGCTCTCCACC
ACCACCAACACCACCACTTGAAGTCCAACATTCATGGGACCTTTTTGTAGAGGCTGACCG
AGCTTTTCTAGGCACCGACACACCAATCATCTCATCGGCGACTTTCCAAGTAGAGGTAGA
GGTGGTGGTCACGGCGGTCGTAGCCGTCGGCGCGGTTCTAAAAACTTTGGCCAGCGGTGG
AAAGCTTTTCCGACTGTTGTGATTATTATGATTATTATTATTACTGTGGCTATTACTATT
ATGATTGTGATGATGATGATGATGGTGATGATGAAGATTCAACATCGACGATGTTGATGT
TGATGGATTGGGCGGAAGCGTCCTTATAGAAGAATCTCCACCTCCACCTCCATCGTCATC
ATCGTCTTCTTCATCATCATTTACACTCTCCTCTGAACTGTCATCCCCTCCGTCATCTCT
ATTGGTTCCGAGCATAAATTTATCGCTTCTCCTTCTCTTGCTACTCCTACTTATTCGATC
TCTCTCTCTATCACGGTCTATGTCACGGTCACGGTCTCGGTCTCGCTCGCGGTCTCGGTC
ACGGTCTTTCTTTCGATCTCTTAGCCTAGCCGTTTCCTGTAACTCCAATGGACCGTCTTC
TTCTGGAGAGTCTCTGAGACTGC
>ntig_274_consensus
GCCAGAATATATCTAATTGGCACTTAACCTGTACTGAGTAGTAGTTTGATATTTATAATC
CGGGTCAATTTTGGACCAACAGAAAAATAGTACAAAGCAGAAATCCTCGGCATGCCCTCT
ACAGCAGCCAAAAATGCCTCNNAAAAAAAAAAAAAAAGATATTACAACACATATCAACACA
CCTAATTCAAAAAAAATGCCATGTTACTTCGACTAGACCTTCACCATAAGACAAAGGAAC
ACAAAAAACCAATGACAGGCTTCAACGCTTCCAGAAACACAACTCAATGGAAAACCTGAA
ACTCGTGCTGCTACATTTGGTCTCTCTCCAATCACTGTGGAATCACTCCTAATTTTCACA
CCAACTGCCAAGTTAGTAGCTACCCATCGGGGGCATGCCAAAAGGTGGCATCGGAGGCAT
TCCCATGGCACCCATAGGTGGTGGTGGCACAAAACCTCCTCCCATTGAAGGTGATCCTCC
CATTCCAGGCATTGGTGGTGCGGGCAAAGCAAGAGGCAGCAACTGGGCATACATATTCTG
>ntig_275_consensus
GTAAAGAATTTTAAGTTTAAATTAGTAAAAGATTTAGATGTTGCTGGGTGTTAATTGTAA
AGCCCTGCAAGAGTGCAAGTACCCAAATCTTGAATTTGATCAACTTTGTAGCCAAACATG
AAGCATTCAACCACAAAGTCGATTATTTCATATTCTTAATTCCAGTATTTACAGCTCACA
TACAGCACTGTTTGGATATTCAAGAAAAAAAAAAATTCTGAAGACATAGAAACTGATAGAA
GATCCTGCTCAATAGACCCCCACCATGCCACGATACAGCTAACTGCTGAACATCGACCAT
GAGGTGTTGTTTTCAAACCACCTTAACCTTTCATTGCCTATAAAACTGCTGGACCAAGTG
GTCCTCCAGGTGGCAGAATATCCGGTGGCACAGTCGGTGGCGGGTCAATATTAGGCGGAG
TAGAAGGCGGTGGGATGATGTCCGGAGGTGGGTCAGGTGGAAGTTTATCAGGCGGTGGTG
GACGCGGTGTGCCTGGTGGAGGCAAAGGATTATCAGGAGGTGTTTGTGGTGCAGGTGAGG
GCGGAATTGGATATTCGTGGCCTGGGTCAGTATTGATTTCCGGCGGAATAGGTTCTATCT
CTATATCTGGAGTAGTCTTGGGAATTTCTGATGGAAAAGGACTCGGTTGATAGTCCGGAA
CTTTAGAAGGAAACTCAGGCGGAGCTTTTCCCGGAAACTCAGGTGTAGTAGAGGCGGCAT
AATTGGCAACCGAAAGACGTGTTTTGTTTCTCCCGTTTCCAATAAATCCAGTGGCGATCC
TACACTTATGCGGCATTGCAGCAGAAGAAAGTGAGAGCATTGATGGCGTTTTAGGTATGG
ATGGACACAGTGAGATGATTTTTCTCATTACTATAGGAATGGATGGGATTGCAGAGACAG
TAGCATTGGGCATTTTTCACTTCGGCCAAGATGAGCAAATCAGGCCAGAAATAAAAGCAC
GTACTTGACTGAATGCATGAAGGTAGAGTGACACGTGGGAGCACGTGTCATTGGTACGAC
ACGTGTGCGTCTTGCATGCACAATATCAATACCCCTGCTAGACACGTCAAAGGGGGTCCA
TTAATCTGAAGCTTCAATGAAAGACTACCAGTGATTACTATTATAAGTACATTTAACAGA
CCAGAAAAAACATAAAATTCTTATCAGAACTGCACGATACAAGATTAATATCTAGAAAAG
ATGTGAACAATACATGGTAATTATACATCAGGAGGCACAGGAGGACGCGGTTGCGGTGGA
ACCTCTGGTGGCGGCACTTTATCAGCTGGACAGGGAGGCGGAACCTCGTCTGGCTGTTTA
GGCGGCTGATTTTGCTTCGGTTTCAGAGGAACGCCCCATGACGGTGGTGAAGGCAACGGT
GGACGTGGAAACTCTGGGTTTGGGTTTGGGTCTGGTCCAAGCGGCGGTGCATCGGACTTA
GTAATGATGATCGAACCCCATTCTAGTGGTGTAATATCTCTCCGGGCTTGACGTCCGGTT
GTCTTGGGAATCTCTAGCCACGCAGGTTTCGGCGCGGAATCTGTCTCTGGTGGGTTAGAT
TGGTGGTGAAAGTGGAACTGAAATGAGAAATTTCGAGTGCTTTCTGAAGCCATTGTTGAT

ATTTTTTTCGTGAGATCGCTGTGCATGGAGCTTTAACTGTGGCTGAATAAAATCGACTGG
GTTTCTTCATCAGGTTGAATGAGATCGCATACAGTTGCAGAGGCAAAATCCGAAATTTAT
ATGATGAAAAATGTATTGGTGGGACGACGCGTGTATCCACGTGGAAGTGGGGACGCCGTC
TATGTCGCATTACTTGATTCCACAGTTCGACACGTAAGCATCCATCGCCCATCAACTCGA
ATGGAATAAATTTCATACCATTTACCAGAAATTTAGTAGACTTTCTTGGAAAAAAATTAT
GGGATTTCTAAGATAACAGGGCTAGTACAGGATTATATGAAAGTGTCTACGTGTCTTTGC
AGAGACTTGCTTGGATTTGGAAAAA
>ntig_276_consensus
CAATTCAAAAGAGCAGCCAGAGTCTTCCTCAATTTTTTTGTTTTTGTTTTTGTTTGTCAC
TTCAGTTTTATGAACAAAGGAAGAGAGATAGCAATCTATGAGAATGCTGCAAAGTTCTAA
GATACATATAAATATTTTCACAGTTAGATATTTTGTCAAACATCTCTCCAGCTCATACTC
GAGCCTTTTTCACAGGTTGTAAATCATCATCATGATGCCCCCCTTCACTTGCGTCCTTCT
CCATCTTCTTTCTAGATAATATCTTATTAATCTTGTGCAGCTCATTATTAAGTTTCTGGT
CCTTGTTCTGTTTTCGTGTCTTCCGCCCATCTTGCATTTTCACACCAAACTGAAAGGCAG
CCTTTGGCATGGCTTCCTTCTGCTCATTGTATGTCGCCCATTCCTCTTCAGTCTCAAAGT
CCCACCTATGAAGACGACCCTTTGCCCTTCCACCCATATCCATTTTTGACAAGTCATCTT
CATCATCACTGTCCACAATCTCACGATTATATTCTTGATACCCAGGGTAACATTCAGAAT
AACTCTCGGAGATGAAGTTGGGATCCTTCTCTCGGGCATCCCTTTCTCTCAATTGTTGAA
GCCTTTGATCATCTCGCTTGAACACAGAACCTAAGCCCCGATCCTTTTCTTCTTGAGTCA
TGAAGCGCGGGTCTTGTGGCATGTTCAAACCTGCTTGAATATCATAACCCTGCATGTCTG
GTTGTAGGTATTGTTCAGAATAAGCCAATTGTTCATCATATTGGTAGTCCTGCCACTCTC
CCTGATAGCCACCAGCCAAAACTTGTGCTTGCATGGCATCATATCCATTCATATCTTGCC
AGACTTGAGGTGCAGCAGGTGGAGCAGGACCATAAGCAGGTTCATTGAAGTAGGATACTT
TTTCCTTATTTCGAGGAGACTCTTCCATATCCTCAGAAAGAGGGCTTTGGCTGATGTCCT
TACCAGGAACGTCGTAGTCAACACCATCCCCCACAAATATATCTTCTTCTTCTGCCCTGG
CAACAGATGGGCCTTGCTTCTCTCTTGAATCAGCATGACTTTTTCTAGGAGGTGGTGGAG
GAGGAGGCAAGATCTCTCTTTCAGTTCTATTATTTAGTACTCCACCATTAGGTTTTAATG
GTTTATCATCTTCATCATATTCATTACCAATAACAGTTGTAGTCTTTCCTTTTGCATCTC
TTTCCTTCTTTTTCTTCTTGAGAACCTTTCCAGATGATCCAAGGCGAAGATATGTCATGA
TTTTGGCAATTCGATCTAGCACAGAACCATCAACACTAACAGTAACCATTTCATCTGGAA
CAGGACAATCAGCTTTACTCCTTTGCAAGGTAGTTGGTATATCACTAGAGTATCCACCCT
CCATGTTAAATATAAACGACATTCGACCTGGAAGGAACATCTCATTTGTCTTGATAACAG
TTTGAGGCTTGACTATAGATTGATACACTGACTACTAGACTTTCCATCAGCATCATCTCC
AGCATCAGGCTTCTTGTCAATCTCACTTCTCACTTTGTTAAGTAATGCATAATCCAACCC
TTTCACCAAATGAGTGTGCTCCACATCACCTCCAAGATACTTGCTCTTCTCGATGGATAA
TTTGTGCGCATCAGCCGACCGAAGATCAACGGTGCCCGGAGGAGCGACAGCGTGAAAAGA
GCCCAATTCTGTAGGTTCATAATCAGGATTCTGATCCTCTCTTCTCTCCTTGGCTCGATC
CCGATATTTCGGTTGCTCCAGCTCCTCCGGTTTCTCCTCTTTGCGACGGGCGATTTTCTC
CTTGTAATATTTCTTTGCAGACGTCATTGGAGAGAACTTTCAGTATTATCCTAGCTGTTC
TGGTTAGGGTTTAGAGTGACTCGAAGGGAGGGAGATTTCATATGCCC
>ntig_277_consensus
TATTAAGAGATGATGAGTAAAAACATAAAACGTCTTTTATAAATTGAAAACTAAAAAAGG
CTGCCATAAACCTTGCTCTTCTCTTTTGTAAATGCATTTATTCTATGCCAAACCATAAAC
AGGAAGTTAATTCATATAAAAGTAAAATCATCATGCATGCTCCTTTTTCAATTGCAGAAC
AAGGGAGTTACGAACTCCCTTCAAAGAGAACACTTACCCTTCCACCCATATCCATTTTTG
ACAAGTCATCTTCATCATCACTGTCCACAATCTCACGATTAT
>ntig_278_consensus
GGGCCTGTAAAACTTTTACATTTCAAAGGAAAATATAGCTGCGATGAATCATTTCCAGCA
ACGACCAGCGAACAAATTCTCCTGTAGAAGAAACCTGCTACTAAAGAAACTAAGAAGGCG
ATGCAGCATTTTAATTCAGTAACTAACCATAGGATAAAGAAAGTTCACAACTCTACAAAC
TAGGATCCTGTACAATACAACATAAGGTGGCTGCACCATCTACTGCCTAGTCTAATGTCA
TAATACAGACTGGTTTCTTCCTACACAATTAAATCTAGGAACACCATCAACTAGTTGAAT
GTCACAGTCATCCAAGGCGTCTCAAAAAGTTACAGGAAAAATGACAAAATATAGGAGTAT
TATATGGACTAAAGTTGTTAACTAAACACTACAGCACATTTAACAGCTCACTGGAGCTGG
CTGTTGTGGGCCGTCAGTGCGCCCCATTTGGTCAACTGTCCCATTTTCAATAACCTCTGC
ATTGATTTCTTTTCCTTTCTCATTGAACTTCACAGTAGAATGCTCTTCATTCTTTGGCAG
GTCTTGCTTCTCCTTGATCTCGAGATGGGATGCAGGAGCTGATCTGTTGGCTTGCTCTTT
TTCAGCTCCCCCATTTGCATCCATGGTCACAGTATCAGGGTTGTCCACTTTGCTGTCATC

ACTGGCAGGGTCTCTCTCACTCCAAGGAACCTTTTGTGCAGAGTCTCCAACCTTTTCTTT
TTCGGTTGGAGTTACTTCAGCAGCTGTCTCTTCAGTTTCTGGCTTTTGCACTTCTGTTGC
ACTTTCATTTATTTTAGCATCAGGAACTTCTTTTTCTTTGTTCTCCTTCTTGGTTTCCTC
AGCAGGTTTTTCATTCTCTGCACCAGTTTTCTCCAGATCAACAGTCTTGCTCCCTCGTGT
TTTCTCATCTTTCTCAGTACCTTCTGCATCTTGCATTTCGACATCTTTCTCCCCCTCAGT
TTTCTCTGGAGTAGCTTCAGCTTCTTTGTTATCCTTCTTTGAGCCTGACAATGATTTTCG
GCCTCGCTTCTTTAGAGGCTCCTTTTCTGGAGTTGGAGTTGCCTTGGCCTTCTCACTTAT
CCTTGCTGATCTCCTTGGGGTCTCACCAGTGCCCCAATCGAACTCTGAGATTGCAGGATT
ATCAGGGTGAGATTTTAGATATTGCTCCAGTTGTTTTCTGTTATTAATCTCCTCCCCGGT
ATGTGCAATAAACATGATCTCACTTTTCTTAGGGGTACCCGCTTTCTTGGGGTAGTACAT
TTTCTTCCAAGCAGGAGGTGCTGGTAGCTCAACAGAAAAAACTTCCTCTTTACTCTCCAT
TTTTCCGCCTGTAAAACCCAAAAAAAAAAAA
>ntig_279_consensus
CCCTTATCAAGGCACATTATAAATTGCCTTCTACCAAATAATTTATTGTGAATTCAAGCA
AGAACAGAGGATAAAACAGAGGATAAAATTCATATCCATTTCTTTTATGATACATAGAAA
AAAATAAAAAAGTACAGAAAAGGATCCATGCCCAACCTGAAACCCCTATCAACTAAAATA
CAGCACAAATGTTCATAGAATTTGCTAGTAAAACTGTTACAAAATTGTGACAACTCATAC
CTGGCAGTGTAACCATAAATTTTTTTTTTTTCCCCAGAAGAAAGTTGTAGAGGAGAGAAA
TCATGACGTATTTGAAGACCTTGCAATCCTATATTTGCCACCACCAAACAATTCCAGGTG
ACTAACCCAAGGCAAAGCTCATGCTGTTAATCTCTTATCCTTAAATTATTTTTTTCCAGC
ATTTGAAATAACCATGGCCTCCTAAAGGAAACCAAATTACCTGTCAGAATTGTTGTATTT
ACTTCTTCCTGTACCTGCTCTTGGCTCTCACGATTTTTCTTTGAGACTTGTCAACAGTGC
TGCCCAATGAGAAGCTACCTCCTCCACCATTGAAGTTTAAACTACCAGAGGCCTGAAATG
GAGAAGGGTTCTGGGGGTTGGCCAGACTCTGTTGGCTTCCAAATTGGAAAGAATTTGCTC
CTGAGGGAGTTGTTGAACCAAAGGCAAAGCCACTCGAAGGTGTCATGGGTTGACCAAACA
CAGGAACTGGAGTGGATGCTTGAACTGTGTCCTCAGCCATGCTGTCGTCCATACCCACTT
GATCATTATTATTATTTGAGGAAGTGGAACCAAAAGCAAATACAGAACCCGTGTTACCAA
AGGCAGGTTCGGATAAGGTGGCTGCTGCAGCTGAAGAAAATGGAAAAATGGAACTTGATG
TGGTATTAGCAGATGATCCAAACAAAGAAGCACTGCTAGCAGCAGCAGACAAAGCTGATG
CTCCAAACTGAAACCCAGTAGAGGGAGATAACGAGTTGAACGTCGAAGCAAATGAAGAAG
TCTTAGGAGCTTGCCAACTGGAACCAAAAACATTGTTAGTAGAGCCACTGCTGGAGCTGA
TCGAGTTAGCCTCTGATGAAGAACTGGAATTCAATCCAAATGTAGGAGTTGAACCAAAAG
GTTTAGCCACAGAAGTTGCGGAGCCAAATAAAGAACTGCCAGAGGAAAAGGCAGGATTCC
CAACCAACCCAAATGACATTGATGTTGAAGAAGTTGATCCAAATTGATTAGGCATGCTCT
GAGATGAAGTTGATACACCAGTCACATTGACAGATGCCTGAGCACTACCAGCACTGAAGG
GGTTGGAAACTTGAGCGTTGGTAGAAGCCAAAGTAGCTCCAGAACTGAATCCAAAGATAC
CACTTCCAGCGCTGGAGACTGCAGAAGATGTACCACCAAAAATACCACTTCCAGTACTTG
CAACAGGTGGAGTTACAGAACTAAAAATGCTACTTCCAGTGCTTGTTGATGGCATACTGC
CAAGAATGCTAGTTCCTGTGCCAATTGCAGCTGGCGATGTCCCAATAGAAGTACTACTTC
CCATGCTTGCAAAAGTTGACGTCCCCCCAAAAAGCATGCTGGTAGAATTACCAAAGCTGG
GCTCCTTAATCTTCTGTTCTGTTGATTCAACACCAGACGGAGCCGTAGCCAATGTAGGTG
AAGCTGAGAAAGATGTGACTGCAGATGCTCCAAACTTGAAAACAGGTGAAGAAGCAACCA
TGAAAGGTGTTGCAGTTGATGTAGATGTCGTGTGAGAGGTACTGTGGCTGATAGAAACAA
CTGAATTAGCACTACTAAATAAGTTCTGATTGGTTCCAGTGCTGGAAACAAGAGGAGTGA
GGGAAGAAGTTGAAGTTGAGCTTGAAGCAAGAGAGCCATTGTCTAAACTTGCACTACTAG
CCGATTGGGAGAATATACTAGCTGATGACAATGACGTAGATGCAGCTGAAGGAAGTGCAG
TTTCAGGTGTTTTAGAAAAGATTTCAGCTTTTGGAATAGTTCCATTGTCAGCTATATCTG
ATTCAGGCACTTTTGGTACAGAACCAGTTGCAACACCAGGAACAGAACAAAAGCTTTTTA
AGCTTTCTGGCTTTTCTTGCAAATGGGTGCCAAATTTTGGAGTAGAAGATTCCTTAACCA
CCAATGTTTCAGATTTTATAGTAAATTGTGGTGCTTTATCAACATTTGTTTTGCTAGTTC
CAAACATGGGAGAAACAACATTTGAATCCTTTTGTGAAGTGAATTTCTCTCCAAAGCCAA
ATATAGGAGCACCACTTGGTTCATTTGGTAAAGTTGCTTTATCAGAAGTCGAGGTTGATG
AAGTAGCTACCACAGTTTTCTGTTCAGTAATGCTGGAGGAAGGTGCAATGGGAAAGGTAA
ATAGTGAGTTCTTTTCAACCACTACAGATCCATCATGAGTTCCAATATCAGATGTTTTGT
TCAAAACAGAATTTGGTGTCTTAACTTCAGAAAGAGCTGAAGCCTTCTCCACTGCAATTA
CTTCATCAGCAGCAGACTTATTCTCAACCACAGGAACATCCGGTTTATCCTTCCCTTTCA
TCAAAGGAGTAGAAGCAGTCCCATTAGAATAATCATCATCATCTAGCTCCACATAATCCT
CATGTGCACTCATCTGGAAACCTCGTTTCTTCTGTGGATAAGGATGTGCAATGCAGTTTG

TCAAAGAAGAATCTGTAGTTTTTGTGCTAAGCACATCATCCTTGACAACACCAGTAGCAT
CTAACCCATTCATAACAGAAACAGACTTATCCAAAGGAGAAACAAACTTGGTTGGGCCAT
TTTCTTCAACCTTATCTTGTTTTTGGGATGTAGAATCTCTAGCATCAGGTAAAAAGGTGT
CATGTAAGCCACATGACCCATTATTATCTTGAATGTTTTCCAGAAAATTTGATGAATCAA
CATTCTCCAGGCTTTTACGAGCTGCTCCATTTAGCATTGATGGCGACAACTTTGTAGGCA
ATTTCTCCCTTGTAGAAACCAATTTATCAAGCTGCTGCAGTATTTTAAAAGCTGTCTCAC
TAGACTTTGAGGGAACAGGGTTATAACTTGTACTAGGAATGTTGTTATCCCCATTTTCTG
TTAAACTTTTTGTAAATCCATGCTTCTCTGCATCAGATAGCTGTGGTTTCTGTATTGAAG
ACAAGGGCTGTTGAACACTAGAAACATCACTTCCACGCATGGAGAGAGAACTTCCACAAG
CTGGCAAGCTTAAGTTTCTTGAAGTCAGAAGGTTAGGCTTCCGTCGAATTGTACGTATAG
GACCAACAGAACCTATGTCATTGTCCAGGACTGAACTCCTGCGTTTAAGTGTCCCTTGTT
CAGGTTCAGTAAGTCTGTCCTTCTGCCAGATGCTCTGAGTTGATGATGATGGCCTACTAC
GAGAATCAACTGCAAGCCCAACACCCTTGAGGGTAGTGGTTGAGTGAATTTGAGAATATG
GTGTTCGTGCCATATTGTATATTGCAGATCTTCCACGGGATCTAGGAGTCAGATAACTAT
TTTCAGCAACACCAAGATGGCCAGATGATCGTGGAACAAGAGACATAATGGGTGCTTCAG
AAGGTAACACACTGTTGTTTAGCACGGTTGACTCTTCCTTCAATGCCTGACTGCACACAC
CTAGCATTGATGGGGATACTTTAAAAGACCTACTTCCCATGTAAGCTTTAGCAAGCTCTG
CAGGTGAAGCAACATCCTCATCAACAACACAACAGGAGTTGAACTGTGTTTGCTTATAAT
CCCATTCTCTTTTACAGGAGATTTTAATGCTGGCTCCTTCCCCTGATGAAATGAAAGTTG
ATTTGAAGGGATCACTTCAGACCTATCCCGCTCATTGTCAAGAGGCTTATCAATGATTCT
TGATCTCAGAAGGGCAGTCAATTGATCAATCTCGGATCTTGTGAAAGTCTTCTGCTTCAA
TAATTGCTCTAGTTCTTTAAGCCCTCTATCGTGATCGTTGGTAAGGCCATCAGGACAATT
AATGGCTCCTTGAGCACTAGGATCATCTGCAGATACTAGTTCCTGGTGCATTTCTTGTGC
TTCATGATTTCCTGTTGTAGCTGGTTCGCACGTTGTCGACGGTGCTGGTGGAGGTGGCGG
CGGCGGAAGTTGCTTACGAAAAACAGAGCCGAATAGTCGGTGAGCGCTTGAAACAATGAG
TTTATGGGCTGGATCAACAAGTTTCGATAGCCAACCATTATTATTACTAAGATTGACATT
GGCAGTGTTTCTAAGCACCGCCGATGTGGGAGGGCGATCGTACGGCGTCGATTGCGTAGT
TCTCCGAAATGGACGCTTTCTGAACTTTCCTCCGGTGCCGAATCCGCTCTCATCACGAGC
TGTCGCCATGAGAGAGAGGGCCAAAATTAGGGTTTGTGATTTCAGGGATTTTAGAAAAGG
AAAGAACGAGAGAGCAGGGATAATTAGGGCTTTTAAAATTGCCC
>ntig_280_consensus
TTTTTTTTTTTTTTTTTTCACACAACCCAAATTCATTTTATTAATCCTTTTATTCGTTA
TCACACACAATCCTTCCAACCCAACACCCACATAACTTTACTACAAAGTTAACATCTTTT
AAACAAACTTAGACATTCCAGGGCATAAAATCCCTATTACCACAAACAAACGAGAAATTA
TATGCGTATGCTCAACCATAGTAATCATAATATGACAAAGGGCCTAACTTTAAGAAGCAA
CACGACCAACAACTGGGTACTTAGCTTCGAACTTCTTCTCCCAATCATCAAGAACGCCGA
TCTCTTTCTCAGACAGACCATCGAGTGAGGGAGTGATATCCTCATCATTCTTGCTCATTT
TTGCCAAAGCTCTGCTCGCATCTTTGCCCGCGAACATCACGTACGCACCACCTGGGCCGT
AAAAGGATTTGCCAGTCGTCACATCAAACACGCGGCCTTTGATAGCAACATAGATGGGTT
TTGAAGGGTCGGTACCGTCGTACTGGAGAAGTTGCTGGGGCGTGAACTCCATTGTTGTTG
ATGTTGTTGTTTAGACTGGTTGGTCCTCCC
>ntig_281_consensus
GGGGGCGAAAAATTCGATGGCAAAGTCTGTGATACAACAATTAAGTAGAAAACAAGCAGC
TTAGCAAGTATAAAGGCGTCCCTTATGGAGTACACGGTACTATGTAATCCTACCCAGAAG
TGGAAGGCCCAAAAAAAAAAAAAGATCAAAATGCATAGATTACAAGGACTGTACATCTTT
TCTCTACGAAACCAAAAGAAAATTAAATTATGCCCCTTGGAACGGGACAAAAAGAACTAG
AAAAGAAGATACAAGAAAAATTTACAGCAGAAGCACCTTAACACTGAGAAAGTGAGTAGC
TCTTGCTCTTCAAGAAAAAGCTAAAAGCTCTCCCCACCTTTCTTCTTGAGACCGATTTAC
ATAGTGGTTTTGATTTTGCCTCGGGAGAAGGATCAGGTGCAGCAGAAATTGTTGGTGTGG
TCTTTTCGCCATTATCAGCCTGTTGTTGTTGTCTAATGTCAACAGGAGGATGATCCCGTT
GCCTTTGCTCCCGGAACAACATCAGAAGTTTCTGTGCCTTGTCTCGCCCTCTCGTGGTCC
CATTCACTGATATTGACACCAGTGCGGGTATGACACCCTCTTGTAAGACCATTTGACTGC
ATTTCTCACTCCCATTACACAAAATTAAAAGGCATGCAACAGCTTGCTCCTGTTCAGTGG
GTTCACCAGTGTCTAACACCGTTGCAAGTCCAGCAATTAGGTCAGGGGCTGAAAGCATTT
CATTTTTTCCCGCTTGACTTGTTGCCAAATTTAGCAAAACAGCCAAGGACTTTTCTGTCC
ACATGCGGTCACCAACAATCGCTAGAGATTGGAGGCCATTTATAATACCAGCAGAAAGGA
GGTTTGGAATATTGGAGGGGACAGTGGAAAGATTGAAAAGGGCATGAAGGGCATCCAGCT
TGCACTGTATTTCAGTTTTACCTTGACAAAGCTGAACTAAAAATTGGACAGCTTGACTTG

AGCCAATTATGGACTTCGCATCTTCAAAGCAGGAGAGATTCAGATAAACAGCTGTTGCCG
ATCCATGAGAGTTGGGGTTGGAGATCATTTTCTCCAGCAATTGAATTATTCCTGCCGCTA
ACATCAATTCCTTGTTCCTGTTATTGTTGACAGCAAGGTTGAAAAGAGCCATTGCTCCAA
CTTCTTGAGCATAAGAATCCCCCTGATGCACAGCTGATTCTAGAAACCTCAACAACACTT
CAACAAACCCATTTGCCCCCA
>ntig_282_consensus
GCTTCTAGGACCTTAATAAATTTACATAGATTTTTACTGAGTAGAAACATCCAGGTTAGG
AAATACAAGATGTTAATCAGAAGGCTTCCCCTATGAAATGCATTGTATGTAGATGAGGAA
GAGTGAGAAAAGAAACCTGGGAGGGCTGTACATCTCTTCTCTACCAAACCAAAAGAAAAT
CAAATTATGCCCCCGGAAGGGGAGGAAAAGAACTAGAAAAGAAGATTCAGGTATGACACT
GAGAAATCGAGTAGCTCTTTCTCCTCCAGAAAATGCTCAAAGCTCTGCCCATCTTTCTTC
CTGAGACTGATTTACATAGTGGTTTTGATCTAGAAGAAGGAGCAGCAGCAATTTTTGGTG
TGGTTTTTTCACTATTATCAACCTGTTGTCCAATGTCAACTGGAGGATGATCTCGTTGCC
GTTTCTCACGGAACAGCATCAAAAGTTTCTGTGCCTTATCTCGCCCTCTCATGGTCCCGT
TCACTGATATTGAAACCAGTGCTGGTATGACGCCCTCTTGTAAGACCATTTGACTGCATT
TCTCACTCCCTTTACATAATACTAAAAGGCATGCAACAGCTTGCTCCTGTTCCATGGGTT
CACCAGCGTCTAACACTGATGCCAGCCCAGCAATTAGGCCAGGGGTTGAAAGCATTTCAT
TTTTCCCTGCCTCACTTGAAGCCAAATTTATCAGAACAGCCAAGGATTTTTCTGTCCACA
TGCGGTTACCAGCAATTGCTAGAGATTGGAGGACACTAATAATACCAGCAGAAAGGAGGT
GTGGAATGTTGGAGGGGACAGTGGACAGGTTGAAAAGGGCATGAAGGGCATCAAGCTTGC
ATTGTACTTGAGTTTCACCTTTACAAAGCTGAACTAAAAATTGGACAGCCTGACTAGTGC
CAATTATGGACTTGGCATCTTCAAAGCAGGAAAGATTAAGATACACAGCTGTTGCTGGTC
CATAAGAGTTGGGGTTGGAGATCATTTTCTCCAGCAACTGAAGTATTCCTGCTGCTAACA
ACAATTCCTTGTTTCTGTCATTGTTGACAGCAAGGTTAAAAAGAGCCATTGCTCCAATTT
CTTGAGCATAAGAATACCTCTGATCCACAGCACACTCTAGAAACGTCAACAATGCTTCAA
CAAACCCATTTGCCCCCACAAAATTTCTGGCCTCTTCATCATCCTTCAGCAAGAACCGTA
TTTGTTCTACAACATTGCATTTGTTTCTCAAGTCTTCTTCTCCATTCAAGACATTCAAAA
AATCCTGACATCTTTCAAGCATACTAAGTTCAGACTCTTCCTCCTGTGCATATGCATTTT
CAAGTTCATAGTATTCAGAATCCTCTATAGTTCCACTGTCCTCCACAGGCACAACCTTCA
CCTCCTTCAACTTACAAGAACTGACACTACCAATTGATCTCAAATTAGTGGAGTCAGACT
CAGACAAAGCCAGTCTCCAATAATTCAGATCAAGAGATTCAGGGGGACAGTCAGGAATAG
AAACTCCATTCTGTTCACACCAGCTGGCAATCAGACCCTT
>ntig_283_consensus
TACTGTCTCTGCAATCCCATCCAGTCCTATAGTAATGAGAAAAATCATCTCAGTGTCCAT
CCATACCTAAAACGCCATCAATGCTCTCACTTTCTTCTGCTGCAATGCCGCATAAGTGTA
GGATCGCCACTGGATTTATCGGAAACGGGAGAAACAAAACACGTCTTTCGGTTGCCAATT
ATGCCGCCTCTACTACACCTGAGTTTCCGGGAAAAGCTCCGCCAGAGTTCCCTTCTAAAG
TTCCGGACTATCAACCGAGTCCTTTTCCATTAGAAATTCCCAAGACTACTCCAGATATAG
AGATAGAACCTATTCCGCCGGAAATCAATACCGACCCAGGCCACGAATATCCAATTCCGC
CCTCACCTGCACCACAAACACCTCCTGATCATCCTTTGCCTCCACCAGGCACACCGCGTC
CACCACCGCCTGATAAGCTTCCACCTGAACCACCTCCGGACATCATCCCACCGCCTTCTA
CTCCGCCTAATATTGACCCGCCACCGACTGTGCCACCGGATATTCTGCCACCTGGAGGAC
CACTTGGTCCAGCAGTTTTACAGGCAAAGAAAGGTTAAGGTGGTTTGAAAACAACACCTC
ATGGTCGATGTTCAGCAGTTAGCTGTATCGTGGCATGGTGGGGGTCTATTGAGCAGGATC
TTCTCTCAGTTTCTATGTCTTCAGAATTTTTTTTTCTTGAATATCCAAACAGTGCTGTAT
GTGAGCTGTAAATACTGGAATTAATAATATGAAATAATCGACTTTGTG
>ntig_284_consensus
ATTAAATTTCATTCACTCCAATTGATTTTTGTCCAATAATATGATTTTACAACTGATTTT
TTCGCAGGCAAATCAGTTCTTGTGACCAGACCTCAGAACAGCAACTGCAACTTGGCCCAT
TTGATTCATTCCAGTCAGTTAGACTTGAGTTGTCTCCTGAGAGGAAAGTCTCGCCGGCCA
TGAGCTCTTTACAAGGTTACTATGGCCTCAAACTGGCAGCTAAAAGACCAGCATCGGAAC
ATTTTGAGATGATGGTTTACAGGAATGGAGAAGCTCAAAATTTAGGGGATTGGCATTCAC
TCGAATCATTCCCAACTTCCAGTCTGCCTTTGGACAATAATAGAAGATCTAAAAGCTATG
CGAATGTAAATCTAGCTTACCCTGATGAACTGAATGATATCGTTAATGTTGCAGACGTTA
CAAAAAGCGAACCTGATGATAGGAATAAGAAACTAGTTAGACGGCGTCAAAAATCTGACG
CCAACTTTGCTTCTCAGCCCCCAGAAATGGTGTTGAAGGAGGAAAACAGTAGCCCAGACG
GGCTCGTGCTATCAGCAATTACTGGAAAGTGTTCAGCCCCGAGATCCAAAACCACCAGCC
GACCTGTATTGGCACCAGAGGATGCGAAAACTGGGCTTAGTCCAGTACATTCTGTCTTGG

GAGATTGTCTTCTGGGCGACCAGCTCTCTGGAGTGAGGAAGTCATCAAGTACATCTAATT
TACAGCATCAGCCATCCATCTGGCCTGCTTCAAAATGGGATTTGCAAGCTCTTTCAACTG
TGGCCATTGCCAAACCAATCTTGGATGGACTGCCAAGCACTATAAGTGGTCGAAGAAACA
AAACTGCACTTGATTAAATTTCAGAAACTCAACTGTTGATGTTGATCCTCACTTGTCGGG
CCTGCAAATGTTATGTGATCAAATTTATGTCCAAAATTTCAATATACTTGAAGTTGACAA
AAAGTAGTTATGTTGATGCTTCGAGCTTCAGCCGCAATTCTTTGTTTATCTCTTAACTAA
AGCTGCATTCTTCTGCAACTCGGTATTAATAAAATATGCACAAACCAGCCCCAGAATTCT
GACTTCTGCAATTATGGCTTTCCCCTCCAGGTTTCAATGTAGTTGGCCTTCCAGGAAATT
CAGACATTATTCCTCAATGGGAAACCAGATAAAAACTCAATGAATTAAGAAAGGTCAGCT
TTTGTGATATGCCCAGACCAAGTCAGAAACATGAGCCTACACTTGGAAGCATATTTTCCC
TGGATACATTTGTCAGGAAAAATTACATATACACAGTCCTGCCAATAGTCTCCACAAGCT
CTAACACTGCCTTCTGTAAATGGATTTGTTCACAAGATGAACCCCCTTTTCGAGAAGAAT
CTAATGTGAGATTTTAAGTTGTCTCACAATCCACACATTAAAGCAGTTAAAAAGGCTTTA
AGTTCATTCTTAAGCTGGTCTCCAACAATTAAGTGTTGACATGTCTGGTCTACATGGCAA
AATCTGGGAAACATTGTGACCTGGTATAGGAGGTGCAGCTGGTAAATTGTTTGTGGCGGG
TAGCTGAAAACCCATATTATTTGTGGGTGGTCTTTCAACAGGTGGCTGAAAGTAACCTTC
TTGGACAAATGTAGGTCCTGATTGAGGGGGGGTTCTCCTCCCACCCATCCACACACCATG
CTGACTCTCTGAATTAAATTCACCTGATGACATCCTCCACTGTTCATCACCTACATATCG
CCTTTGGTTATCAGGGTGTGATGGCATTACATATGGGTGGGGGTAATGCTGATGAGGATG
TTGCTGAATCCCTGGCTTTGTAAATGAGAAATGATTGGAAGGGGTTTGAGAGAGGGCAGG
ATGTAGAGGTCTTTGCACATATGGTGTATTACTTTGCTGAAATTGCTGGTTTGTTTGAGA
AGCTTGGGGATTTAAAAATATATCATTATGACTGTATTCCAACTGCCTTGAAGAACTATA
TCCAGAAGGTTCTCGTGAACTGCATATTCCTGTTGGTACAAAACAAGGTGATTGCTGTGT
AAATAATTCATTTTTCACAGTATTATCAATATGACCTCCATGTGGAGTATTTCCAGGCAT
TTGGACAATTTGATTACCACTGGCTGTGTTGCAATATTCACGAGGAACAGATGATTGATA
TGCCAACTGAGGTGAAGACTGAACTGATGACTGTGGTGGCATTAACGGTTGAGACACTAA
TGAAGATTGTGACTGTACCACTGATGATTGTGGTACAATAGATGGTGGCAGCCCCAAAGG
AGGAGGAGGTGGTGGTGGTGGTGGTGGTGGTGGCTGTGATGGAAAAGGAGGAGGTGGCGG
TGGAGGTGAAGGAAATGACATTGGAGGAGGTGGCGGCGGTGACGGAGGCAAGGGAGGCAG
TGGAGGAGGAGAATCTGGAGGCAATGGAGGAGAACCCTCAGGTAAAGGGGGTAACTCACT
GAAATTATTAACTGACTCCAAGATCCTATCTGAGCCATGCTGTTGTGATTCCATTTCAGA
GGAATCATTTTCCTCCTTTGGATGACCAGAAACATCTTCCATTTCAAGCTCGCCATCCAC
ATCCTCCAAGATGCAATGACGCCTATCGTTGACAGTAACGATGCATGTTTCTGACTCTCC
TGAAGCACGTCTTTGCTCTACTGGTGATGCATCACCCAGTTCTTTATATGTACTGCTTGG
AAGATCATCTTCTTCTTCATCCTCAAAGACATGGGAAGATAGAAAGCCAGGCAACTGAAA
TGTAGCATTACTGCCATACTCATCAACAAGCATGCCTTCCATTTCTCTAATGGGATCATC
TATAGCACGCTCAGCTCGTGATGGTCGCTTTAAGGAAAATCCAGAAGTCGACTCATCATT
TGAAACTCCAAT
>ntig_285_consensus
CCGGCTTAAAGCACAACATGTTAGTAATTACATAAATGATTGTATAAACAGTACACTACA
GTTCATATAAAGCCACTTGGAAGCTGCAGGACAGACCACATATAAGATACAGGAAACAAC
ATCTCTTCACTAGAGAGGTGAAAGGCGCATGTAAGAGGGAAAACATGCGCACAAGATGTG
TGGATTCCAGGCTGACTTGTGATGTAGAAATCTAAGCAAGAGACAGACCCAGGTAACTAC
TGGATCCCACAAGCTATATGGTCCATCAAAGTCCACCAACCCCCCTAGTTCACAGGGGTG
TCACATAGCATACACAATCTCTGCTGTTGATGTCGAGATCGAGAGCAATTTCAAAGTCCT
CCATATCATCATCGAGGTCGAGGCAACCTATCAAATAATGGTTGAGACCATGTGTTCCAC
CAGCTGGTATAGTGGCCTCAGCAATGATTTGCAGGATCTCAGCAAGGATTTGCATGATCT
CATCAATGATGTGCTTGATCTCATTATTGATTTGCATGGGCTGATCAGGCTCTTCAAACT
GGTTGCTCATTGTGTTTTCAACCATGAGATCAGCTGGAAGGTTCTTTAAGAACCACTCAT
GGTTTCTAATCTCAGGAATAGTTATCCTCTTTGCAGGATCAGCCACAAAAATCCTGGAGA
TCAGATGGCGGCACTCAGGAGATATATGAACATAATCTGGAATTGAGTACTGAACATTCA
GAATACGGTGTATTGTCTTGCGGAAGTTCTTTGGCTCCTCAGGATCCTCAAAAGGATATG
CTCCCACCAACATCACATATAATGTTACCCCACATGACCATACATCAGCAATCTTGCCAT
CATACTCTTGCCTCAGCAACACTTCTGGGGCAATGTATGCTGGAGTACCAAC
>ntig_286_consensus
TTAACTCAAAATTTGTTTTTCTTCTGAACTGGCAATACATCTGTAACCAAATCAAGTTCA
TGTGGATGATCATTTCCAGGCTTTCCTTCCTCGGAACTGCTTGCCAGATCGGTTATGTTT
TTTCTTCTTCTCCTCGCGAGATTTTGCCGCCTTGGCTCTTTTGGCAGCAAGGGGCATAGC

CTTCTTGTGTTCCTTCTGACGATTGCTCAAACCACCCGTCTTTTTCTGTTTTACAGCAGT
TCGAGCCATAAATTTTCCTCTGTCTTCCCTGCCTGCTCTAACCAAGGCAAGTCTTTCTTC
CTTGCTCAACTTTTGCCGTAGATGAGCTTCAAGCTTGGCTGGATCCACACGCTTAACACT
CAGTTGATCTGAACTTGGCATCTTAAACCCTTGCTGAGCCAGAGCAAGCTTTGCATCTTT
CTTTGCCTTCAGTTCCTTGATCCTTTGGAAGTCTTCGTTAGAAAGAATACCATCAGCTGA
GTCAGAGGAGGTACAGCCTATTTTTGCTTCTGCCAATTTCTTCAGAGCCCGAAGACTAGT
ATCAGCAGCAACAAGTTGTCCATTAAAATCACAATATTTTCTCTTCCTTGCTTTAGATTC
TTTAGAACACAGTCCAGTGTCTCCAGCATCATTGTCATCATTTGTAGGAAGAAGATTACC
TTCTTCAAGTTCCTCATCTTCCTCTGTCTCTACATCCTCATCCTCCTCTTCCCCATCCTC
TTCCTCCTCATTATCACTATCATCAACATCATCATCACTGACACTAATACCATCATCATC
TTCATCTACTGTATCATCACCTTCACCATCATCTTCCTCATTTCCAGTATGATCACTATG
TAATTCACTTTCTTCATCAATATCAACAATAGACTTCTCATAATTATCATGATTGTCATC
ATAGTCACCAAATGTAGATTCATCAGCGTCGTCCCTGCTGTCATCACTGCCTTCATCATG
TTGTAACAACTCAACACCGGGAACATCGCTAGCAACACTGACTTCTCCATATGCTTTTGG
CCTTGCCTTAGGGTCAATAGGGCGCCCACGATCCTTCTTCACCAGCAATGAAGGGCAAAC
CTCTCTAAATAAAGTGATAAGTGAACGAGCTGCTGCTGAAATTGCCTTCTCATGTGATTT
CTTATATAATACAAGATCTTGCAGCAAGTCTTCTGTCATCAACAAAGGCATCCGTAAGCA
TATTTCCCTAACCACATTCAGCCCAACAGCAATGGCCTCTGTACGTGAACGATCATGTAC
AAATTGATTCACAATCTGTTTGAATAAAGGTTCAACAGCATCAGGAGGCACCATATCATG
GCAAGCCTGAACAGCTGCTGCAAGTAAATTGGTGATATCACGTTGATGGGGCTGAACATA
CTTCTGGAGGAAAGGGTAAAAATTTAGCAAAATCAATCGGTGAAGTCCAATAGTTCGAGC
AATCACTTTCAACATCATCATCTTTACCTCAAAACGTTCATTGCAAGTTTGAAGACGAGA
AAATAGCTTCTCAGCAAAACCCTGTGCATCAATTAGATGATTAAGAGGCAAGTAATAACT
TGTAGAGTTCTTTTCTGATGACAGGCGTTGCTGCTTCTTCATGCTACGCATGGCACGCTG
TAATTTTGCTTTCTTTTTTTTCTTGCTAGATGTTGTACCTTTATGGTGCGCCTTATAGAC
AGCTTCCTTAGTAAGGATGCTTTGCGGTGTTTGAGTGGTCACATCATCTTCATCACTTGA
AGCATCAATATCATCATCATCATCTTCAATATTCTCACAGTCAAGAAGAAATGATAGTGC
AGCGATCATGATCCTTGATGATGAATGGAAACAAGCTTTACATATAGCATTTGCTGTTCT
ATCATCAAACCACACCTTTCTCCGATGAAGTTCACACAAAGTAGTAAGTGACCTCTTGGC
TCTTGTTTCATCCTCTGCCTGTAGAATTGAAAACAAAATATTCTGAAGTGCTCGATTCTT
GGGCTCATGCTTATGCTTCTGATTCATGCGCTTAATGCTATGAACAACATGAGAAAATGC
CAACTTCCTCAGATTCCTATCGCCTAGGGTTTGAAGCTCCATGAATAATGCAAGGATGTC
TTGGATATCAACTATCTTCCGATTAACAAGAAGAATAAGAGCTTGAGTGACATGGCACCT
AAGTCCCGACGGTAATGTTGCCGCAGACGATTTTAAGAACTCAGCAAGCTGACTCGGGAA
GTCGGCGAGTTGCTTGCGATAAAAGGGCGTGACATGCGCTAAAAACATAGCGCGGTCACC
GAGGTCTTTTGCTATGGTGGAATCACCACCAACGCCGCTGATAGTGCCGAAGTTGAGAGC
TGCTTGCTGCTGGAAGAGATCGAGAGCCGAATTGAATTGCTTGTAAATGAGTAAGAGCTC
CGTTTCGTAGCCATCCGGGTCGCATTTCATTTTCGATTGTAGTGTTGGTAAACTTAATTT
CTCAGAGCTTCGGCCGGAGGCTAAGAGCGAATCCGGCGAGAGGCCGGCGACGACGTGCTG
AGTTGACATTAGCCACTAAGCAGAAGTTGTAAGGTTTTGGGGAAATTTGAGAAACCCTAG
CAAAACCCTGCAAAGTTGTAAACTATGCTTTCTTTAAAGGCCAACCGCCATATGCC
>ntig_287_consensus
CACATTAGCCGTGGCAATTTCCTGAAAGCAAGCAATGCATATCAAACTTTAAAGGGTCAT
GACCATAGCATCTCTTTATACAACCGCATTAAAAAGACAACATGATGAGAGACTTACACA
ACATTATTACTATAATTCAGAGCAGATAGACCTACCGGTAACACTAAAAATACCACCTAT
TATCTGCAACTCAACCGTATATGCTACTCATTGTGTAATCTAATACAGCCCCTTGTAAAC
CCACCACCAATAATTCCAATTCCACTGCTATGTAGCCCTTCCCAGAAAATAGAGACTACG
ATTATATAGCCGGTTCAAAATGACCCAAAAACAACAGAAGTACAAACAACATATCAAATA
GATGGCTTAAACCACTGGAAGTGGGTCTGGCAATGAATCGGGGGCAGCACCGCTGCTGCC
ATTGAGCACTGTAACATTTCCATCCGAATCTACATCTACAATTACTGAATCACCCTCTTT
AATTTCTCTTGCAAGCATCTTCTCAGCCATGCTGTCCTCCAAAAGTCTCATAATTGCTCT
TCTCAAAGGCCTAGCACCATAACTTGGGTTGTACCCTTCCTCAACCACCCTCTCTCTAAA
TCTCTCTGTAACTTGAAGCTCTATATCTTTTGTCTTCAATCTATCAAGCACTTCCTTTAG
CATTATATCCGCAATTTCTTTTACCTCGATCTTAGTGAGCTGCCGGAAAACAATCATCTC
ATCCAACCTGTTCAAGAACTCAGGCCTGAAATATTGCTTCAATTCTTCTGTCACCAGACT
TTTAATCCTGTTATAACTGCTATCCTTCTCATCATAATCAAGATCAAACCCTATGCGGCG
TCCTCCCTTCTCAATAACACTGCTACCAACATTTGATGTCATAATTAGAAGGGTATTTTT
GAAATCTACAGTTCTTCCCTTGCTGTCTGTCAACCTTCCATCCTCGAGAATCTGAAGCAT

CATGTTGAAAACATCAGGGTGAGCCTTCTCAATCTCATCAAAGAGTACTACTGTGTATGG
ACGACGCCGAACAGCCTCAGTTAGCTGACCACCCTCAGTGTAACCAACATAACCAGGGGG
AGAACCAATGAGCTTGGAGACTGTGTGTCTTTCCATGAACTCACTCATATCAAGCCTAAT
CATGGCCTCTTCTGAGCCGAAATAATAAGCAGCAAGTGCTTTTGCCAGTTCTGACTTCCC
TACACCAGTGGGACCAGAAAATATGAAACTAGCAATTGGTCGGTTGGGGTTCTTTAGTCC
AACACGAGCTCGGCGGATGGCACGGCTAATGGCTTTAACGGCTTCATCCTGACCAATGAC
TCGCTTATGGAGGGTCTCCTCCATCTTGAGTAGGCGATCAGATTCATCAGTTGAAACTTT
CTCAACAGGAATGCCAGTCCACGCAGAGACAATGTGTTGAATGTCCACTTCGGTTACAAC
AGGACCCACATCACCCGCTTCCGTCTCTGCCTTACTCATCTCCTTACCTTTATCAACAAG
GGCTGTGATCTGAGCCTTAAGGTCCATTTCTCTATCACGTAACTCCCCCGCCTTTTCAAA
GTCTTGGCCACGAACAGCTTCATTTTTCTCCTTAGTGATCTGCCTAAGCTCCTTTTCAAG
CTCTCTAGCTTCCTCAGGAAGCTGAGCATGACGAAGACGAACTCGAGAACCAGCTTCATC
AATCAAATCAATTGCTTTATCAGGCAGGAAGCGATCACTGATGTATTGGTAAGATAGCTG
TGCTGCAGAAATTAGTGCTTCATCAGTGTAACGAAGTTTGTGGTGAATCTCATATCGCTC
TCGGAGTCCTTTCAAAATCTGAATGGTTTCATCAACTGATGGTTCAGGAACTTTGACAGG
CTGAAAACGTCTTTCCAATGCTGGGTCCTTCTCAATGTGCTTTCTATACTCGTCGAGTGT
TGTGGCTCCAATACACTGCAATTCTCCTCTTGCAAGAGCTGGCTTTAAAATGTTAGCAGC
ATCAATTGCCCCTTCTGCTGCTCCTGCTCCAATTAAGGTGTGCACCTCATCAATGAAAAG
AATTATTTCATCACTCTGTTTGATTTCCTCCATTAGTTTCTTCAATCTTTCTTCAAACTC
TCCACGATATTTTGTCCCAGCGACAAGAAGACCCATATCCAGAGTTATAACCTTCTTTCC
TTCAATTGTATCAGGAACATCACCACTTGCAATTCGTTGTGCCAGGCCTTCTGCAATAGC
CGTTTTACCCACACCAGGTTCTCCAATAAGACATGGGTTGTTCTTAGTCCTACGGCCCAA
AATTTGGATAACACGTTCTATTTGTGGCTGTCTTCCAACAACAGGATCCAATTTACCCTC
TTCTGCTAACTTGGTCAAATTAGTTCCATACTCTTCCAGGGTTGGCATCTTATTACCACT
GCTTCCTCCTCCAACACCAGCACCAACAGCTTCTGTGCTCTCACCAACCATTCGAATAAC
CTGTGTGCGTATGTTACTAGGGTCAGCACCTAAATTCTCGAGAACACGGGCTGCTACACC
TTCCCCTTCACGAAGAAGGCCAAGAAGCAAGTGCTCAGATCCAATATAGTTATGGCCCAA
TTGTCGGGCTTCCTCCAGGGAGAGTTCCAAAACACGCTTTGCACGAGGAGTAAATGGAAT
TTCCACAGCAACGAAACCGCTTCCCCTTCCAATTATCTTCTCCACCTCTACACGTGCATC
TTTAAGATTGATTCCCATTGATTTTAGAACCTTTGCAGCAATGCCAGTACCTTCACCAAT
AAGACCCAAGAGAATCTGCTCTGTGCCAACAAAATTGTGACCAAGCCTTCTTGCTTCCTC
TTGGGCAAGCATAATGACTTTAATTGCTTTTTCTGTGAAGCGCTCAAACATGGCTTTAAC
AACCATTCTGCCAGTCCTCCTACGTCCAGAATAGAGAGAAAGACCCACCCTGGAGTGGAA
ATTTATGCCAGGTTTTGCCAGGTTATCTAAAGCATTGGAGCCTCGCAACCCCGAAAAATT
TCTCATTCTTAACACAGGTGCTTGTAAACAGCTCATCATTCTAACATTCCTTCTTGACTT
TAAAGATTCTTGCGACCGCCCAAGCCTTCTGCCAGGGGGAACCAAGGAAGGGATATTAGC
TGTGTGCACCAGAATCGCCATGGTATCCACTAAGTGATCT
>ntig_288_consensus
CAGCAGCCATTTATATAAAATTTCTTGTTATTTATGGAGAAAAGTCTATTTTAAATATTA
CAATATCCATTTAAACAGCTCTGTAGGAATTTTATCTAGAGATATACAACACTCAATGCA
CATATCATACAGAATATCTTCAGCAGCCAGTAGGCTATGTTTTGTTTATATAATTATCTT
CCACAGCTACTAGGCAATGCAAGTTTCAAGGGAGGACTTGGCAAGCCACAATAAGCCTGG
ATCATTCCGTTTCAAAACTATGTAAGTTTCTGCTTTCCCGATTTCATCCAAAACAACATC
TTCATTGAAACCAATCAAATTCAGAATCTCCATGGCAGCTCTATAATTTGCAACATTCCT
TTGGATTATGGGGTTAGCCTTCCGGAGTCTTCTGTACTTCATCTCCTCAGGGTGCTCAAT
CACATTCCTAATTATCTTAAAAAGGGTTTGCCGGACTGTATTTGCCTCCAAGGGATTAAC
TTCAGCTTGCAGCATCTCAATCGCCTTCTGCAGACGATTACAAACAATGGTCACTGGGTC
TTGGATTCTCCTCAATTCTTGGGCATCTGGATCAGGTTCATCAATTTTCATTCGGGATAT
ACCATGTGGATGTACCAGAGTATCATCAGGATCAGGCTCACCCTGTACAATTCCATTGGT
CTTAGATTCATCTGGATCGGGTTCTCTATGAGCCTTTATATTGCCTTGATCTTCCCTGGT
TTGCTTCACTAAAGAATGTGCAATGTTTGTACATCCCGAAATTCTCCCCTTAGCCTCTGA
ATCATCTGGATCAGGCTCATCCACTCTCTTCCTTGGCTGAGGAATGCTTTCCACCTCCAT
TACCTGACAGTGATGAGAATAAGGTTCAGCCACAACTCCAGAATTCAAGAGAGCTTTAGA
GTCATCTGGATCAGGTTCTTCAAATGAATTTGTTGAAGCCAAAGTTG
>ntig_289_consensus
GCTGCTGGCGAGGAGCGGGTGGACCGAGGCTGGCGAGGAGGACCCCACACACACTTTCCA
GTTTGTATTTTATTTTCAGTTTGGACCTGTGAGGTCTTGTACTTGTACGTTTTGTAAATG
TATATGTACTCAGATGTAAAACAGTTGTACAGAGCAGTTATATATATATATATAGTAAAG

CCAGTTTGGGCTCAATGTATATTTCAGTTTCAGCTTCCGCTTGCAGTGTAAATTAGATCA
GCAGTAGTTATTTTATTTTCAGTAGGATTTCCGTTGCGGGTACCCAAGTTTTCAGATGAT
TAATAGAAAGCTCCCCAGATATTGTTATTATTCAGCAGCAGAGGGTACTCGTGGCGGCGG
GTCCTACAGAATTATATATGAAACAGGCATTTAATTCCCTCACAAGGCTGCTCTGCCACT
ACCATGGACTCCTATCCGTAAACTCCTGATGCTCCAATTTTACCTCCATTGCGTAAGATC
TGCTGTTCCAATCATAGCAATGACCTTGGCAATAAGCCAGATTCTTCATCCTCAATATCT
GCCAATTCTGCTCCACCGCCTCCACTGGCAGCAACTGCTTTGTTTTCAGAAACTGTAGCA
CCCTTGCTTTCACTGCCTTTACATGCCTGAAGATCCGAACTCACATCAGAAGTATTTGGC
ACACAATATTTCTGCAGCCATCTATCAGTTTCCCATAGAACATGCATGATGCTCTCTCGT
GCAGCATAGCCATGGCTCTCGTAGGGGAGAAGGACTAGGCGACAGAGAGCACCATGACCT
TTCAAAGCATTAAAGAAACGATCTGACTGCATAGTTAGTGTTCCTGGATTATTGTCTTCT
TCTCCATGGATAATTAAGATTGGCTTCTTAATTTTATTCGCAGACATGAAAGGGCTCATT
TCTACATAGGTAGAAGTGGCCTCCCAAAGAGTTCTATCCTCATTCTGAAAACCAAAAGGT
GTAAGTGTTCTGTTGTAAGCTCCAGAACGAGCAATTCCACAACAGAAAAGATGAGCAGCA
TGTGCTAGGAGGTTTGCAGTCATGAATGCACCATAGGAATGTCCTCCAACAGCAATTTTT
CTTGGATGAGCCACTCCACGTTGGACAACTTCCTCAACAGCAGCTTCTGCACTTGCAACC
AGTTGCTCCACATACCTATCATTTGCCTCCTCATCACCTTCACCAATAATAGGAATTGTT
GGCCCAGATAAAATAGCAAACCTTCTAGCCAGCCAAAGAAGGGGTGACGTTGGACCTATG
CCCGGAAATTCATTAGGAGAACCGCGAACTTGTCCAGCAGCATCTTTGCTTTTAAATTCT
CCTGGATAAGACCAGAACAAACATGGAAGAGGGCCATCTTTTGATGGATCATAACATGGT
GGCAAGTATAATGTTGCGGTAAGTTGAACCCCATCCTTTCTCTGATATTTGATCATCTCT
TTGTGCAATGATGCCAACTGCGGATATGGGTGAGGAAATTTTGTAATTTGACAAGATTTC
TTATCTGGCCAACTCTGGATATAATATTGGGTATTTTCAGTTTTTGACTCTTTGGATGTC
AGTATTTTCAACTGATTAATGTGTATATCCCCTTCTGCCTGGTCAGACATTAAAGCAACA
GTGGTTTCATAAAATTTTTCCTTATCGCTCTCCCATATTCGTTCTTTGTTGCCTGTATTT
ATGTCAAACAAATCAAGAAAGGGAATGTTCCCTTCTGGTGTAGCACCACTTCCATTAAGT
AAAAGATAAGTGCTTTCATCATTTTCTTTCTTTATTTTTGCAATCACATAAGTTCCAGCA
GGTGTTCTCCTCATCATGGGAGAGCCAGGATCAGAGTACACATCTTCTGATGACCGATCA
AATAATATACGGGGACTCACATCTCTAGATCCGGGAGATATTATCCAGGTTCTTGTTCGC
CTTGTTTTATACCAAGATTCATAAACTAGGGCAAGTGAATCATTACACCAAGAAATCCCT
CCGTAGCGAAGATCAAGTTGATGTAATATTGCTGGCTCTTCACCTTCTAGAGGCTCAGCA
GGCTGTGTATAAATTATATCACGTGGAGTAACTTCTACTTTTGCATCACCTCCATCTTGT
GTCTCCGCCCAGTAGAGGGTAGATGGCTTATCTGCTCTCCAATTGATTGATCGCATCCCT
TTCCGGACACTACTGAATGCAATGGGGATGTCCTCCGCAAGTGGCAAGTCGCAAAGTTCC
CTGACGAATTTCCCATCAGTTGTCCAAACTTCGACCTTCTTTGGAAATCTTCCGCATGGT
ACAACAAAAGAATAAGGTCGGTGGATTGAACTAATCAAAAGGTACTTCTGATCTGGGGAA
GGATCCATTGATGTGTATACAGCTGGAGGCCCAATTTCTTTCACAGTCCCATTCAAAGAA
ACCAAAATAAGTTGTGTGGTGGCATAGTAGTCAAACAAATCTTCATCATACTCATCCTTC
AGTAGATCCTGGAAGGTTCTAACCTGAATAATATTTTTCTGTTCATTAGATTGGATCTTT
GGGCCAGAGGGAACCAAAGGTTTCTTTGGTGGGTCGCCACGTGATAACGGGATGATACAA
ACTAACAAAGTAGAGTCATCTACCCAAACAAAGTTCTCAAACACTGCATTAAGATATATA
TCAGGTGATTGAAACAATGGTCTAGCTTTTGCAGTTTCCACATCAGCAACCCAAACCCTA
AGCTTACTACTTCCACCATCCTCCTCATCACCTCGGATGCTGAAGGCTAGATGCCGCCCA
TCATGTGACCAGATCACAAAATTGATCTTGGAACCATCAGGAAAACCATGTACCTCTCTT
TCTGGCCCCAGCATACCATCCGGCAATAATTGATGTATCCCAATACCAGTGTAAAATGAC
ATCCTACTCCTAGTATTGCATTTGCCATCAATCCGAATACCAGCTAGCTTTTCCTCTGGT
CTTGCTAGCTCTACCAATGGAGGCAAAGCTCTGCGCTTGAGAAATAGTATTTTATCCCTT
TGCGGAGAGAATGACAATGCAGGAAGTGGTGGAGCATCAACAATATCTCTAATCTCAGGT
GGAGGAAGGCGATATCCACCTCCCAGGTAGTCTTCATCTTCGGTGGCAGTAGCATTAGAA
GCAGAAGAGGATACAGAGCCATTAGAGCCACCGTCTTCGGGAGCAACTGCGTTGATAGGG
ACAAGATGGTGCAACCTACCGGCGGCTGTCATGGTGATGGTTTTGAGGGACTTAGATGAA
TGAGTTCTCAGGTGGCCAGGTGTACGGATGGATGAGAATCGACCGAGTTTTAGAGGGGGA
AAGAGCTGTGGACAAAGAGGAGACGGACAGACGAGAGAGAGGCGATGATAGACTTTGTTA
>ntig_290_consensus
AGATCTTCCAGAAATTTAATATTATGAATCATTTAATAAAAAATTTGATGTGCTTTTTCC
TATGATACATCAAAAGAATATGATACATTTAAGAATTTACATCACTTTTGGCAAACCATA
AGCAAACCGTGTGTCAGTGATCAAGGTTTAGTTTCTAATTTCCCCAGCGAGATGTAATAA
AGGAGCGAATCCGGTTCCAGTGTGCGAACCTGGAACCGTGGAATTGTACTCTTACTGATT

TTTTTGATCCTTCTCTGACAAATTCCAAGTCCTTGACAATGCTATCCTGAATGGTTTCTC
TATCATCAAGCAGAGGTAAAGCAACTAAACCCTTGGACTCCAAGTTTTTTATCCACCCAC
GCAGTAAATAGTTCACAACCTCAGGGACTTCATCGTGAGGGCAGTGACCAGCTGGACTGA
GTTCATAGTATGGAGCTTCAGGTAGCTGCCTTTTCACCTGAAGGCCCCAGATAGGCTTAA
CCCAGGGGTCTTCTTTTCCATACATGAGACAGATTGGAACATCATTCATCTGACACCCAG
ATAATGCCTCCCTGAATGATAGTTGTCCCTGTGGAGCAAACATAATTGACGCAAATGATG
CGGCAGCAGCAGGATGCTCGGTTGTCTCAAGAATACGAGAGAAAACTTTGTCTATGTTAG
TAGAATGATCTGCATAAACNNNNNNNNNNNNNNNNNNNATCAATTCCGTGAGCTTTCGCACA
CTTGCAGGTAGAGGAAATGTTCCAGACCATGGAAAAATCTTTGCTAGTCTTGGGGACTTC
ACAGGATTAGGTAAAAATCCCCAGAAGGGGGGTTGCGTTAAGCAATGTAACACCCCTTCACT
AGTTGAGGGTTGCAAGCAGCAAAGTAAACCGCAACAAATCCTCCAAGCGAGTTCCCCACA
ACATAGACTGGTTCACGAATAATCTCTTCTATGAAATTGCGAACCTGATCCTGCCATAAA
TCCAAAGAAAAAACGAGCTCACTCGCCCATGGTTCAGCATCATCTCCAAATCCCCACACT
TTATCCTTCCCTTCTGTTGTAACTCCTTCCTCAGACAGTGCAGTAGGATCTTCAAATGGC
AATGACATGCCTTGCCCAAGAAAATCAATTGCCCATACTCTGTAGTCCCTGCCCAAATCT
TTTAGTTGCTTCTCATAATGAAAAGAGCCAACACCAAAGCCAGGAAGAAACAGGACAGGT
GGGGAATTTACATTTTCGCACCCTNNNNNNNNNNNNNNNNNNNNNNNNNNNNNNNNNNNN
NCACCCCCTTCACCATCAATTACATATCCGTCACAGTTCCCACTTGATGCCTTTGAACAG
ACCTTCTTAAACCCTTCAACAACTTTTAATGAATGAGAACCTTTTCCATGATCAACATTC
TTTAAGAATAACTGGTTACTATCAGAATATCTTGGGAACACACTTCTGAAATAAATCCTA
GTACACAAAATATTATGTTTCCTGCATGAGCTCGAACCTCTCTTGACAAATTTCCACCTG
AAGTTTACAACATGACTGCTGGGCGGGCAATTGTATGATATAAT
>ntig_291_consensus
AGCCTCCTGTCCTCTAGATTTGGCTTCTGCAGCCTCCTGTCCTCTAGATTTGGCTTCTGC
AGCTTTCTTCTTTAATTCAGGTCCCACCTCAGGGCAATCATTCTTGGGTTTTATATCCTC
TGGTAAATTATATTCCTTCATGTTTCTTCGTTCTTCCTGTTTGCTTGTTTCAGACTGCAT
ATATTCAAGAATTCCATCAACCGTCCATTCTGGGACAGATTTAATTGCTGATGTTAAGGG
AAAAAGGATCTCAACAGCCCCACGGTTGCCTCTATCAGCTGCAGCTTGTATGGGCTTCTG
ATCATCCTCATCCGTGACATTAGCATCAGCTCCAGCTTTCAATAAGGATTCAATAATTTC
TGTGCTCCCAATATCAGCAGCAATATGCAATGGGGTTGCTCCACCAGCAATAATATTCAC
ATTAGCACCTGCCTGAATTAATAAGTCTAAGCATGCTAGAGAACCAGCTGCAACTGCTGA
CAACAGTGGAGTAATGTTATCTTCAGTTTCTGCATTAGGATTGGCATGGTGATCTAACAA
AACTTTCACAGCATCTTGTTGAGCATGACCAGCTGCCCAAACTAAAGGAGTACCAGAATC
ACTTTGCGAATCAACCTCGACACCTTTGGAGAGTAAATATTTCAGCAATTCAATATTTCC
TGTTCCTGCAGAATGATGCAAAGCTGTGGCCCCTAGGTTACTGGGTATGGCAGGATTGGC
CCCATGCTCAAAGAGATATTTAACAGTTTCAGTATGTCCCTGACGAGCAGCATGAAGAAT
AGGAGTCTCACCATCTTCATCTTGTGTATTAACATCGAGATTCAATTCCTCCAACAAATA
CTTGCACACATCAGTCTTGCCCTCTCTAGCTGCAAAATGTAGCGCCCCTCGTTGGTTGGC
ATCTTTTATATCAGCAACGGTTGTTGCCATTCCTTTCCCCTGATCATCTAGTTGCTGCGC
TACCCTTTTGAGAAGATCAAGATTCCCAGTTTGAGCAGCATTTAAAAACAGTTGAACACT
TTGCCTCACTTTAAGAGCATGAGAAGCATGAGAAGCATCAGGAGACATTGTTTCGAAGTG
GGGTTTTCGGTAAGCGGAGAGAAAATCTTAATTTGGTGCGGAAGATTCTGGGGTTTGTAT
TTGAGAAAGTTGGGTTTAATCAAATTGACTCGCCGCCGAAAGAAACTGATATTCACCTTT
ACCTTCCTGGCTGCCCTATAATTTTCCCCATATAATCATAGGTGAGG
>ntig_292_consensus
TTGTCGAATATTTATTTATTTATTCATTAATTTTGAAGCATATACAATACAATACAACAC
TCGAGAGAAAGAGATAAGAAAAAGAGTTCGAAAGCATCTGAGACCAGAGCCTGAAAGTGA
GAGCACAAGGACATCTTTGTCCTTAGCGGGGTCTGGGTTCGGGTTTTTACGAACTATGAT
TCGAACGGATGTTAAGAAGGTTTCAAGGGAAGAACACCGATTAATACATGGAAACTTGTA
TGAAATTAATATCCTAATATTTCCAGTTGTAGAAAACATATCTTCATATTACAGTTTCCT
TTCCTGTATGTGCTACTTTCTGATAATTTTCACACTTTACTGCACTACACACTAGAGACT
TCTTATGTTTCAAGCTTTTTTTTTTTTCTTCATATTTGTGTTGGGGACTTAGAATGGGGC
GCGATGAGGGACACCCTTTCTTCTCCTGGCATTGCGGCGGTTGTAAATGGGAGGAGTGGT
TGGCGAGAGCTTGTTGGTGGCTTCATCTCCAGAGCTCCAAGACCTTTCTCCGCCTTCGTT
TTGGTGGGTGTCTGAGTAGATTGAAAGTCCTTCCATCAGACGGTTGAAGACAAGTGATCT
TCGAAGCTTCTTTGGAGTTGAGGATTCATCTGATTCTGACCCACCATGGATCCCATCCTT
GTGCTTGGCTCTCAGTGTAAATGTGAGGTCCGGATCACCCCTCTTCATACCTCCAAGCTT
ACAGCCAAGAGAACAGAATCTGAAGGAATCAAGGAGGCCTCTGCAACAAATTTCGCAAGT

ATTGGTGACCCCTTTGCCAGGCCTAGGCTGGGGGCGTTCATTCAAGAACACAATCTTAGC
GCTGTTAATAATGTAGGTCTGCACACAAGAAATGTCTATATGCTTCTGTATCTCATTGAC
CCTGACCACGTTGTGGTAGGAGGATCTTCTTATCTGAACAACACGGTGATCTTTGTGGCT
AATCAAACAGTAAGAACAGAGAGCATTTCCCATACAATCTAAACAAAACATGTTACATTC
GCTTTTATTGGAGTC
>ntig_293_consensus
CGGGGTCTGAAATTCTTTTTTTTTTATGAGTTTAATATAATTTAGTAAATTATAATATTT
AAAAAAAACAAAAAATACATATGGATGTTCTTTTTATTTCCCGCCACGTTTAGAAACCGC
GACTTAGTGTTTATCATACGGCGTCGTTTAAATTTCCGCCGGCATTGCTTTCTTTCAACT
GTGCATCGTCTCCAGGTATTGCTGACGCTTTCCGAACTCTCTTTTTCCTTGTACGAATTT
ATTAATGGTGACGAGGGTTCTCACAGAAGTCGATGACAAAATCGTGCGCAGTATATATGG
CCGTAGGGCATTTTGAAGGGAGAGAATAGAAGATATAGGAGACACAGAACCAAATGGCTG
CATTGAAGAAGAAACTCACAGCTACAATACACCAACATACACATTCAAGTACACTAAATA
CGTACATTAAAAACACCCCAAACAAGAACCACACATTCATACGTTATTCCTTCTCATTTC
CTCAGTCCTGGGGACTAACTCAAGTCCTCACTGTTCCAGTCTTCCCAACTTCATGGGCCT
TGTTCTGGGTATCCTGGCCTACTTCTTTGGTCTTCTGGCCCACATATCCCGCCATGTCCG
CCATGCGCCGCTTTGCCTGGTCCGCTATCTCTGGCACCGATCCAGTGGACTGCCGGAAGC
AGTTCAAGATCCATGATAATGAAGTCAAGCCGGTCAACCCAAAAGCCCCAGAAGTGAAAA
AGCCCGTCACTGCAAGACCGATAGCAATAGCCGCCGGGACGATGACTGGGCTGAATATTA
TAAACAGCGGTGTGGTGACCAAAACCCCAATTAAAGAACCGACCAGAGTCAAGCCAGCCA
GTGCGAGCAGGGTGCCAGCCACGGGCAGGAGTGTGACCACTGCAAGAACTTGGCTGGCTG
AAGGTCCGCTTCTTGGCAAGAGTGGCTTGCCACCGGCTCCGAGATGATCATAACGGTGCT
GTGGGTGCACCTGAATCTGGGTTGGATGTGGACGATCACGATCTCGATCACGTTCGGCCA
TTGGTGAAGAAGAAGACGAATAAGAAGGAGATGATGATGATGATGATGATGATGATGATG
ATTACAAGGAGAGAT
>ntig_294_consensus
AGAGCTTAAGAAAAGCAGCAAAGGATGGGACTGCGAAGGTAGCTGATGAAAGACAAGAGG
TGATTACAGTTGCAAGTAGCTAGAAACAGGAATCGTGGAAAAAAGTCTCAACCTTTCAGA
CATGAAGATGGCAAAGCCGTCAGTTGCATTCATTCTACTTGTGTTCTGGTCAATTAATCT
GTTGTAGATTTGTATCTCTATCTTAGCCCAGACCTTTATCTCCTAATCTTCCGCCGTCTG
AATCTCCCCCATCATGATACGGTTGCTGACCACATTAAATCCCAAAACTGAAGGACTAAC
CTTTTTCCCTCTCTTCCCTTTTTTCTTGCCGCCGCCGCCTCCTCCCTTTGATGATCCATC
AGGACCAACTGAATTATCACGCTCAAAGTCTCCGATGCCAGCATTGTCAGAACTCATATC
TCCGGAACTGAACCCAGTGACCTTTCGATCATTCTGACTTTGAAAGGCAATTTCAAGTAC
GTCAGCAGGCAACAACTCCTTATAGTTGAGAAACTTGTCGATGAATTCATGGTTGGGATC
ACGCAATCCAAGGTTCTCTATCAGAAGCATCTCAGCCTCTGATGTGGACTGCTTCAAACA
AAATTCCAAGAAACTCGTGTCTTTTTCCCCAATAAGCCTAACACACTCACTCTCACACCA
ATCTCTGAAGTCCGTGGCCTCTGAATACTTGGCCATGCTATCTTTTCTCCCCTTAAGGGA
AGACTGAGAAGAAGCAGGCGAAGATGAAAGTGCACGCTCAGCAGTTCTGCCACCCATAGA
TTTTTGTCGGTTCAAGGACCCGCTTAGAGTTGATTTTACAGGAGTGTTTTTGGACCCCCA
ACTGCCCTGATTTGAAAGAAGAGGAAAATCTACCTGTTTAGTTTCTTTCTTTGATTGATC
CATTGGACCCCAGAATAGATCATCATCTCCTCTATATTTTGACTGCAAGGCAGAGTGGGA
ATTTATCAGGATTGGAGAAGCACTCTTGGATGGAGAAGCAGAATGCGACAATGAGATGTT
TGAAGGATCAGCAGCCTGAGTTGGCTGGGATTTTTGAGGAGTTGGTATTTGATTCTGGGA
CTGCAAAGAAGTCTTCTTCCCCTGCTCCTTTTGGATGTCCCGCAATGATGTGGGCTTTGA
GATCTTGGAGTCAGTAGACCAAGCAAAAGCAGCGGAAGGATTTGTAGATTCCCCCTTCCA
TAGGACAAAATCTCCCAAAGATGGCCCTGGTGGTTTTGCTGGCAATACTTCCTTCTCTGG
CTGTACATTACGAGAGTTCTTAGTTTTCTCAACGGGACTCGTATCAACAGGAACATCAGT
AGGTGTTGAGACCTTAGCTCCAGCACTTTTGGCTTTGACAGACTTTTTACGACTCTTTTT
AGTTTCTTTAGCCTGAATAAAACTACCATCATTTACAGGTTCACCATGCATTGCAGCATC
CTGCAAACTTGGCAAACTAGACTTACTGTCAAGAGCAGCATCCACAACTCCTTCATCGGA
CTTTGCCAAAACTTCTTCTGCCAATAGATCATGTAACTGGCTCTTCTTGCTCTTTGTATT
CAGAGAAATTTCAGGTTCCTCCACATTTGACAAAGAAGTGCCTTTATGAATTTCTGCAGA
ACCCTTTGGGTCCGAATTGGCAACAACCCCTGCCCAATGAGATGACAAATTGATAGAGCT
GACAGAAGTAGTGATCTCAGACACCACCAACTCTGCCTGTGCTTTCCTCTGTTCTTCCTG
TTGAATTTCTAATAATGATTTAGGCTTGAAACCAGGAGCAGGTCTCCAAGCTCTCTGCCC
AGAATGTAATTGGTTGTTTTGGACTGAGACAGACCCAACCAATCTGGATTCATCCTTGGT
CTCCACAATTTCAACATCATCCTTGGAAATCCTTTCAGGTAAGCCCTGAGCATTTTGAGA

ATCTGGATTTTCTGCAGTAGAAAACCCAGAATTGCTCTCTCTTGTCTTTTGTAGAGATGC
TCCCCCCTGTGTTTCTCTAGCACCGAAATTTGCCTCAAACTCTGCATCAGGAGTAACTGC
CCCCTCTGTTTCTGATTGATTAGATTGCTGCAAAGATGAAGTTTTTGATACTCCCTTTGA
TTGGTCAGACGAATGTGACTTGTTAGACTTTTGCTTTCTGGACTTCTTCTCAGAAGTTTT
CTTGACCTCAGGAACTTCTACATTCTTTGATTCTGTCACCATGGAGGGTCCATCATTGCT
TCTTTTGATTTCAACCTGCAGTCCTTCACAGGCAATATCTACTGGAGCTTTCACATCATT
GGCATGCTCAGGCACTGATAGTTTATTCTCAGAAGTATCAATAGATGATACAGTAACAGA
CATTTCATGGGAATCTAAAGGTGAAGAGTCCACAGCACCTTCCAACGTTGCAACTCTGAA
AGGTTCATCTGTCTTCCACATGTCTTCTGATGCCTTCACAAGGGTAACAGGAGCATGACA
TTCTGAAGCAAAAACAGGTTTCACTAAGAATGATTCCTGCTGAGACTTATTAATGGCCTC
CAGAGAGGAGAAGCCTTCAGTAACTGTTGAGGCTGCCAATTCTTCCTTCTGAGTGATATC
ATCTATTTGCTCTGGAAGAGCAGCATTCCATTTTAGACTATTAAACATCTGATTTGGCAA
TGGTAAGACAGAGTAATCAGACACACCAGCATGACTTACATCCTGCAAGACTTGTGGGGG
CAAATTCAGAAAGTCAGAGGTCCGTTCATCTTGGATCTTGGGAACATGAATCTGCAAACC
ACTCTGAAACAACTCTTGTACTGGTTGAAGCCGAGAAGAATCAACAGATGCATTCCCTGT
TGTTATACCAGCCTGTAACTGTGCATATGACTGCTCACCTGGAACCTTTTGAGAATGATG
CTCAGACAGAACCTGAGAGAGCAACTGCTGTTGCCAAAGTAACTGTTGCTGCTCTTCCTG
TTGCTGTTGCTGCTTAAGCAACAATAGTTTATCCAACAACAGCAGTTGCTGTGCTGGAAC
AGATGCCTGAGAATGTGCCTGCAACAAGTACTGCTGCTGCAACAAACTTAATAATTGAGG
ATCTTGTGATAAACCAGAGGACAGTACCTTTTCAGGTGCTGAGATACCAGCAGGATTTTC
CACAGTTTGACCCAATGAACATACTAATGGGGTTAGGTTCTGTGCTTGCAGCCTCTGTTT
TTGTATACCAAAGGGGGATTGAGGAGTCAAGCTTTGAGCATGATGCAAATCAATTTTATT
CTGAAGTGGATCCAAGCCACTTTGGACCGAAAAATTTGACCAGCCAGGAACACCATTATT
TATGCTAGAGCCAGACCTGTCTGACAAGCCTTGAAGGACGGACATCAACTCATGACTTTG
CGAATGAAGCTGGTGAGAACCATCACTCATTGAAGATAAGAGGTTTGCTGGAGTTGGTGA
TTCAGATAACATATTTGCCTTTGAAACAATAGATGTAGCATCTCTCCCTGGCCAGTAAGG
ATAAGGACAAGGTAAAGACCTCTGCTGCTCAAGTGTCATTCTGTTCACCATGAGGTATGG
GTCACTTGAACCATCTACACCTGAAGGTGGAACAACGCTGGAAGTATTATTTCCACCATA
ACCTTGAAAACCTCCCATCAGTGACTCCAGAAACCTATTTTCAGCTTCTGTAGTCGAACC
ATCTTTATGCCTTGTCTCATTTCTCAACACATCAAAGCTACTGTAGTTTGGTTTACTTGA
TACATCTGTGATTTCATTCTGTTTTGGCACATTAAATCCAGGTGGTGGTCTGGCTTTAGC
TCGTAAATGGGGCATAACATCACCGAGTAGTGAAAATGGTGAATCCTTTGATGCACTTGC
AACACGAACTGGCAAATCAATACCAAAATATCCAGCTTCAAACCATCCAATGATATCAAT
CCCACTAAAAGGTCCTTGAATCTCACCCTGGGGATCTTTATAATATAATATTAAATCTTC
TGGAGAGGGCTGTGCGAATTTCCTGGTTTCCTGCTCCCTATCCATAACCATTGATGCCTG
CCTTTTAATAACAGAATCTTCACTGGTCTTCCATTTAGACAAATCACCATCACAATGGCT
AGTGGTATCCTTCCGTGGCATTGACCAAGCAATGTCAGAGGTTACTGAATTAACATCACT
TGGAATGTCCATTCGAACATGTAAAGCCGTATGTGACTGATCTCCCAGTGATGAGGCCCT
CCACACAGCCGCAGAACAGACAGAATTATTCTCCTGCACACTCAATTCCCTATTGATGGC
ATCAACCTTCCTAAGAGGACCACCATCTTCTCTAAAAGCTTCAGCTCTAACCATGTAATC
AGGGTAAGCCTTCCGATCTTGGATAGTTTCCATTGTCGAGTTGGTACCACAAATGCGTGC
TTGCTTTTCCTGCGATGAACCATCTGAGTAATTCACATAACCACCCTTTGAATTCTCAGA
ACTTTCATCTTTATAATCATCAACAGCAAGTGGTAAATCTTCTTTACTATCATGTTTGGT
TCGCCTTGATGAAGTAAAATCAACTGAGTTCCGCCCAACAGATCCATCTTTTGATACCTG
TGGTGCACCACTACTTATGATGTCCCCCTTGTCAATCCCCTTCAATATAACCATTTCTTC
AGGATTTGGTGAATAAAATGCAAGAGGTTCTACAGGATCTTCTTGTGTGAGAGAAGGAAC
CTGAGCAAGCCCTTCTAAGAGTTTCCGATATGATGTCATATCAGTTGCCCTATACACATC
AAGCAATTTTGTTCTGCTATACCTTAACTGATGATTTTCTCCATGGCTACTTTCACTCTT
ATCAGAGAGAACTCCCAGAGGCTGAGCATGACTTGAGACACTGTTCAGAAAGTTCCCACC
GGAATTCAGCTTTCCACGACCAGCAGAAAAAGTAGGTGTGCTTTCCCCACGTCCCCGGCT
ATAAGAAAATATAGGAACTTGTTTGTTAGGTGTTACAGTATGGTGATGAGCTGAGTCTCC
CCTTCCACGATTTTGTAATGAGTTAGATCTCCATGGTCGATAGCTATCCCCATCCCTCTC
ATCCTTTCCATGACT
>ntig_295_consensus
TCCATCAGTTGCTAGTTTGTAAATAGAACATAAAACTTACGTGAAATGAATTAATAAATA
ATAATTTATAAAGAATTATGAACCTGAGAGGGTCAGACAAGTTGATACAACACACCACAG
GAGAAACAAAGACAAGGTAAAAACCAGGCAAACCCCTAGAGAAGAAACTAAAAAACCTTT
CCCCGTATAATGCCCCTAAAACATGTCGAAGCCCATATTACAGTAACATGATTTAAGTCC

TATTGGAATCTGATCGTCTTCAGATCTCATTATGGATATCCAAAATACAGCTTCCTGAGC
TACAATGAGCTATTTACTACCGACACAGACAAAACTGTAGCATATATTTGAATCAACCAT
TCAGATACATTTAAGATTTCCAGAAAAAAAGAATTTGAACATTTGCCACAAGCACACCTA
AGAGAAAATCCTCCTTACATTATGCATCTCAACTCTCCTAACCATTTAGCTGGATGCAAC
TGTTTTTGACATGGACTTCTTAATGGCTTCGGCATATGTCCTGTGCTGATAGGTAAGGGT
TGATTCCAGAAAAAAAGAATTTGAACATTTGCCACAAGCACACCTAAGAGAAAATCCTCC
TTACATTATGCATCTCAACTCTCCTAACCATTTAGCTGGATGCAACTG
>ntig_296_consensus
TTAAAGGGGAAAAACAGGGAGGACAAAAAAAAAAAAAAAAACACAAAGAGCAGAAGATTCT
TGTCAATGAGCATGGATGTTGTCCTGTACTTGCTAGTCCAACTACTAGTTATACACTGCA
TTAAATAAACACCCTATAGATTCTAGAGAGAGAAACAAGCAAGACCAACTAAAGGTTTCT
ACTTCTTACCATAATCTCTATTTTCTTCTATTCACATGCCAATTTGGTGTCGAAACTGCT
CAAGCAAATTGCGAAAGCCTGAAATGCAGATAAAGGATACCTGTAGTCCATTGTGAACGT
ATCCTTGGCAACCTTACCAAATTGAAGAATGATCTTGTCGTGATCTGACTGGGGTGGCTG
TGAAGGAGTGGGTGCACCAGCAGCAGGCTGGGTTGAAGCAATTAGCTGGAAATTTTTCAC
AGAAGCAACAGTTACTCTTCCTCGAAAGTTGAGGCACCAACACTGCAGCTGCTCGTGCCA
TCTCGGAGCTTTGTTCCGAAGAATTAGAGGACGCTCCTTTCCCCCCTCCTCATCATCACG
AATGCCAACTATGTCAGAAAATCGGGAGCTACTAAACTCAGTTGAACTATCCATTGATTT
TGAGAGGTTCCGGAAGGAATCTTCAAGGGAGCGAGGATTAAGCTCTGCCTGGCCTGGGAC
ACTACCACCAAGCTCAAGGGATGAGGCTGGAATTGAGTGCATGGTGCAATGCATCCTCCG
AGGGCCCCTGGTACCTAAAACGTTCAGTTCATATGTGACCTGAGCAATGTTGTAGCTACC
AGTGGGAACCTTCGGTGAAACCTTTTTTGAATAGAACCTGCGGCTTCGGCCAGGTGGAGA
AAGCTGGGCATTGTTGTAAGGAGGTTGTGTGTCATAAATGATGAATTTTGTGCCAAGAAA
ATTTGACCTCAATTTCCCAATGTAAGTACTGCTTGATCTTGATATGTTATCAGCATCCAT
GGAGATTACATACTCTGTGCAAGTGGTTCTCCTAGTCCTTTTGGCAGAAAGAAGAAACTT
GCCACTTTCAACAAGCGAAGAAGTGCTAAGGCACAGGAAAAGATGGTAAGTCAAATTAGA
TTTATCCCTCTTAATTAAGCACTGAATAGCCCCATCCCTGGGCCCTGGCTGNNTTCCCAC
AGAATTCAGGGCTTCTAACAATCTCTTTGCACATTTCCCTCCATGATCTACACACGGCAG
CACATGCAACAACATGTTTGCGAGCAGGCCACATACTCTCACTTGCCTCCAATCTCTTGA
TCACATCACGCAAAAGCTCAGGTGGGAG
>ntig_297_consensus
CACCAAAGTTACAATCCAATTTTTACAATACAATAAAAATATTGACTACAATTTTCTATC
CTAATAAAAATGGCAAAAAACAAGGAACACTAAGAAACGGTTGTTATTACTGTTTTCTCA
ATTTCTGCTGTTACAACACTTTTTTTGTATGTGTTTTTATTGCTCTATTCACAAGCAAGT
TTGGTGTCAAAGCTGCTCAAGCATATAGCAAAAGCCTGAAACGCTGAGAGGGGATATCGA
TAATCCATGGTGAATATGTCTTTTCTAACTTTGCCAAACTGCAGAATTATCTTATCATGG
TCCAGTGGAGCTGGTTGTGATGGATTGGCTGCACTGGCTGCTGGCTGTGACGCAGCAATC
AATTGAAAATTCTTAACAGAGGCAACGGTGACCCTTCCCCTGAAATTCAGACACCAACAC
TGTAGTTGTTCATGCCATCTTGGGGCCTTGTTTTTGAGAATCAGGGGCCTCATTTTTCTA
TCCCCATCATCACCGATCTCAGAAAATCGTGCACTGCTGAATTCAACAGAGTGGCCAAGA
GACTTTGAGAAGGAGATGCTCCGAAATGAGTCCTCGAAAGTCTGGGGAAGAAGCTCTTGC
TGTTCAGGCACAGTGCCACCAGCCTCAAGTGCTGAGGCTGGAACTGAGTGCATGATGCAA
TGCATTCTTCGTGGGCCACGGGTTCCAAGCACATTTAGCTCATATGCGATCTGGGCTACA
TTATAGCTTCCAGTTGGGACTTTTGGGGAAACCTTTTTGGAATAAGATCTACGACTTGTA
CGCCCATGAGGAGAGATATTAGCAAAGGAATATGGTGGCTGTGTGTCATATGTTATGAAT
TTTGTCCCAAGGAAATTCGATCTCAATTTCCCAATGTAAGTACTGCTTGACCTTGAAATG
TTGTCAGCATCCATTGAGATTACATACTCTGTGAAAGTAGTTCGACGGGTCCTTTTGGCA
GAAAGAAGAAATTTCCCATTTTCAACTAATGAGGCATCAATAAGAATACAATATCCTGAA
GTTGAACGTCTATCTGATGGGGACCTGGCCCAATCTGCATCAGAGTATCCAA
>ntig_298_consensus
AGAGACACATTTTAGCAAGCCTGACATTCTATTTACCACCCTAACTTGTAAGTAACCTAG
TGCAATATTTATGACAGCATACAGATGCCAATGGCAAGAAGGGAAAGCGAAAGACCAGGC
CGAAGCCTCGCAACACGTAGCAATAACCTACCTGCAGACAGAAAATGTATACAAAGAAAT
ATACCCAAATATTTTTGTTTTTTTAATCTTTTTTACAATCCATAGGTTCAAGACAGTGCC
ATTCCGCATGCAAGTAGTTTTTCATTTTCAAGTAATTTACTTTTACCTTTTAGGACAAGA
ACACTGGGACGCTGGCATTTTTCACAAACTGGCAGATAGGACAAACACCGACTAACACAT
CACAATCTTTACATAAACAAAGGTGCCTACAAGGCATCAACAAAATAGAGACTTCCCTTG
TCTTGCACGCCCTGCAGGTCATGTGATGATTCCTAGAACCTAATTTTGCAGATCCACGGG

GAATGCTTAGATAATTGTTTGGATCAATGTATGATGCGGCATCATCAGCTTCATTATCTC
CAAATCCTTCCTTCCCTTGATCAGCACCCTGTGAAATTGCCTGCTGCAGGTTGTTCTTCA
GGAAATTAACAACAGACTCATTGTATTTGGCTCTGTAGTGCCAATTTTGGGCTTCTGAAG
CCACCTGCTTTATTTTCTCAATCAGTTCCCTATTCTTGCGATTCATGTTTTCTATCTCTA
TGTCTTTCTCTTGGAGTTTCTTACCCACACCTTTCTCTATAGCTGAAAGGAATGAAGCCA
CGTGTCTCTGCTTCAAGTCCCTAACCCCCTTTGCCAAGTGTTCCTCCTGAATTTTGATAT
ACTGGTCAAATTCTTCATTTTGACGATCAAGCTCAGTCCTAATATTGTCACCAAGAGACA
AGATGATTGGTGGCGCAGCATTCAAACTACCACTTGCAGAAGTTACAGATGAGTTACGTT
CATCATCATCATATGATAGCTTTAACCCTGTTGATACAAGGTTTGGTTTTGGAATACTTG
CAGAATGATCAGCCTCATCTTGGCAGATGTTTGAATTCAAGGAGATCTGCAGCTTCTGCT
GCCTTGAAAAATTTTCCATTTCCCTGCCTCGCTTATTGGGCCGAAGCATAGGAGTAATAT
GCTCATTTCCAAAATGATTTACTGGATCGATGTTACACCCAA
>ntig_299_consensus
GAAAACTTGTAAGCATCTCTGTCCATCGGGCTGTGCAAAAACAGGTCCGCGTTCGGATAG
ACTTGTAGAACGTTCTCCACTATCGACGGTCCAGTGAGTTCGAACCTCCTGGCCGCACCG
ACCAAACATACAGCCATTCTCGACCGAAGCAACTCATCTTTCCACTGATCCACCGATGAT
TCCAGCCGATTCTGCCCCCCACTCGGCGGCGAATTCCCGAAATCGTATGAATTAAAACTA
GAGAGGGTATGGTTAAAAATAAAAGATCGAAGCGAAGAAAACGAGTATAAAGATGTAGAA
GATAAAGAAAGAAACACGATAATAGATAAAGGAGGGATTATCAACAAAAGAAAACGCCTA
TCAAGATCCGAAACCCATTTGGTAACCTGCATGGTGGCTTTTTGTGGACTCTTTAGCATC
TGTTTCGTTTCTTTGTCGATTTAT
>ntig_300_consensus
ACGAACGCTCTTCCGATCTAGCACACCCCAGGAGAGACAAAGATGGGAGAAAGGCCAGGC
AAACCCCTAAGGAAAAAACGACAACTTATTTCCTCGCATAATTGCCCCTAAAACATATGG
TAGGCATCTTACAGTAAACATGATTTAATTTCAGCTTAAATCTAATCGTCTTCTGATCTC
AACATGGATATCCAAAACAGACTCCTGAGCTACAATGAGCTATCTATTACCGACACTCAG
AGAAAAACCCAAAAAAAAAAAGTACTGTATATTTGTATCAACCATTCAGATATATTTAAT
ACTTGCTCAAAAAAGAACTTAAACATTTGCCACAAGCATATCTAAGTGAAAATTCCCCCT
GCATTATGTTTCACAACTCTGCTATCCACTTAGCTTGATGCAACTGGTTTTGATATGGCC
TGCTTAACGGCCTCCGCATATGTCCTGTGCTGGTAGGTGAGGGTTGATTCCAGCAAGTCC
CAGAGTGATGTCTTTGGGTTCCAACCAAGTTGTTTATTTATTATGGTCATGTCCGGAATC
CTCTTGTCACTATCATCATATCCTTCACCATAAAATTCTTTGGAGCTGACATCAATGGTT
GGCTCCTCCAGAGCAGGTTCTCCACTCACCTTTGAGTAGATCTCAGTCATCATTTCAGCA
AGCTGTCGAACTGTTACTTCATTGTGGGGATTGCCCACATTGAAGATCTGACCGTTAGCC
CTCGCTGGATTTTCAATCATCAACAGAACAGCTTCAATAGCATCCTTTATAAAAACAAAA
GTTCTCTGAGACTGGCCACCGTCCACCAGCTTGAGTGGCTGCCGACGTAGGAGATTATTA
CTAAAGCATGCCAGAACCCTAGGAACACCCTCACTAGGACCATCAATGCCAGGTATGAAA
TCCATCCTGGGTCCAATCCAGTTGAAAGGTCTCACAATGGTGAATTCAAGACCATTCTCA
GCACCCTCAGCATAAATGAGCCTCTCAATCAATTGCTTTGCACATGCATATGACCATCGC
TGCTTCTCAATGGAGCCAAAAATGCAGGGGGAGGCATCTTCCTTAAGGACATAGTAGGCA
GGATCCTGACGAAGTGGGCTATCCTTAGGAAGGAAGCTTCCTATAGTTTTCCCATATACT
TCACAAGTTGAGAAGTGAATCAGACGCTTGTTGTTCTCCGAACAGTATTTGACCACAGGA
AGCGCGTCAATAAAGTTACTGTAGATTGTGTCAAGAGGACGCGTGTTGTAATCAGCCGGC
GTACAGATTGCAGCTAGATTAATCGTCAGATCTGCCATCTTGATGAGGCCCTCGAGACGC
GAATCGTGCTTAATATTAAGTCGATGGAACTGGATCCGGTCGGAACAAGTAGCCGACGCA
CGCTCAAACAAGTGCTTGATCTTGTCATTATAAACATCGAGCGCCAGAATCTTGTGAGGA
GTCTCGAAGAGAATCTTCTCGCATAGGTGAGAGCCGATAAACCCTCCGGCACCAATCATG
CATATCGTCATCGGCTTTATAGGCCCCCCATCCAGATCCAATCTCACGGCTGCCGTCGCC
ATTTCCGTAAACGACGAGATAACTGACGTAGAACTATTCTATCAGTCGCAAAATGCGTGT
GAGTAGGTGCAAGCGACTAACGTGCACCGAGAGTGTAGAGTTACGAAAGAGGAGATACTG
TGTTCTCGTTTGGGAGA
>ntig_301_consensus
TCTACCTTTTTATACTAAATAACCGAATACAACCTCTAAAATGCAGGGGATGACTTAGCA
ACAAGGCTCCCTAACTGTAGAACTATTTTCATGATGGTATGTGGGCATTGCTGAATATAA
TCAAAGAGTCTCTACAATTTTACAGATTCATTTCATGACGTATTATCACAAAAGCAAAAA
GTGATCAGCCAAAATAGGTGGCCAACATAGACTACGGGAAAAACACAGATAAAAAGCAAT
AAGGGTCCATGAGAATTGGGGGGGTGGTGGGAAACAAAACTAGATGCATAATTTGCAACT
GTTACTTCAACTGTATCTTTCGACAGAGGCAGGAAAGTCAGAATCAACCCAGCAAGTTAA

TGGAGAAAGATTCTCAACCAAATCTTTCAACTCTTCGTCGCCGTAATTCTTCCAAAGAAA
TAACTCCAGCGTCAGATTGTAATTCATTGCCACCTCTATCTGTTCCACACATCTCACACG
AGTTTAACCAGCCAGAATTATCAAATGTACAAGCTCGGCATCTCCATGTGCTGGGTGAAG
CACCTCTGTTATGAGCCCGACCAACAGTTCCCCTACCAGAAATCCGTGGTCGTAGAAACA
GATACCGTACAAACCTCGAAGGCCATCTCAGTGCACCAGTAACACCTCTAATGAACATGG
TCAGCGGGTTTGGACCTGAATACAGAGTTTTCAACTTCAAATAGACAATCCCAGCAAGGA
TGCCACCAAGATGGCCCAGAAATGAGACACCAGGTACAACCATCTGAATAAGAATCAATT
CAGCCCAAGCAGCATAACGAGATGGGACTACGAATCCATAGACATTAGTAAAGTTTTCTG
AGTGAGAACAGAGGACGACTTTCATGGCAAAGAGAACACCAGAAAATCCAACAGCATACT
CAGAGTAATAAGGTCTTCCATAGTCAAGAAACAAAAGAAGAGATTTGGCTAGCAGCAATG
TGATGCCCTGAGACAAGCCAACTAGTGCAACAACCATGGAGGCAAATTCCGTGCTTCCCA
TTGAAGTTTCCAATCTGATTCCCTTCCATAAGAGTGACATCATGTTGTAGAACAAGTGAG
ATTCACCCACATGATAGAATGCGGACAAGAATAAGCGTTTCAGGTCCCTGTGCT
>ntig_302_consensus
CGAAAGCCGGGTTACGGTGCCCACCTGCGCGCTAACCCAGAACCCACAAAGGGTGTTGGT
CGATTAAGACAGCAGGACGGTGGTCATGGAAGTCGAAATCCGCTAAGGAGTGTGTAACAA
CTCACCTGCCGAATCAACTAGCCCCGAAAATGGATGGCGCTGAAGCGCGCGACCTACACC
CGGAGATCGGAAGAGCGTCGGTAGGGAAAGAGTGTAGATCT
>ntig_303_consensus
CGCACATATATGAAAACCTTCATTAGAGATAACAGAATAAAAGTCACACCACTGCAATTC
ATTACTTTCCAGTGCCCATATCCTGTAAAAATGCCAAGGATTGGGCTTCAAAGTCACCTT
CAGAAACCCCAATGCACGACCCTGGGAACTGCAAACTCAGCCTATCTAACTAATATCTAA
CAAACATTACTCATTTACTTGAGTCCATTTGCCTCACTTAAATTTACACCTAATTCCCTA
CAATCGAACTTTTTTGGCCACAACTTCTCTCCATTTGAAAGGCTATGGCGGTTACCTCAC
TAACATAACTCCTCCACTATATCTTTTATTCTTTCTTGCTTGCTTCACTTGCTTAATGCG
TAGAGTTAATGCACTAGATGCAACATAAAATTAACCCCTCCAACTTGTGGTTGAAATTGT
AGGAGAACAAACAGCTTCAACAGATAAACGCTTCAAGACATTCGGGCATGGGTCTTGGCC
AAAGTTACTGTTGGATATGGCCACTGTGCATTTCTGCTTTCCAATACATGTCCTCTGCAA
GATGTCATAAGATGTAGAAGCATGGCAATGTCCTTGCTGGTAAGTCCCACAAGTTCCCAA
AGGAGTTCCAAAGCTTGCAAATTTAATTGATGAAATGGTGTGGCCTAGACTACATCGCAG
GTGAGCTTTGGGGCTGTGGAAATCTTCTGGTTTTCCATAGCTTTCGATGTGCCAGTTCTT
GATAGTTGGATGATACTCAGAAACCTCAGCGCAAACACTGGTTACTGATCTCTTCACAAG
AGAAATTCTTGAAGGATCCCCTCCAAGCTCTTCAAAAAGGACCAACAGATTTTGGGTTGG
CTTCAACCAGGACCAAGGCACATGGTACCATCTTTGGGTTGGTTGGCCACAACCAAGCTG
ACACTTTGGAGGTTTGTATGCCCCAGCATAATTACAAGGACCACAGTTTCCATTAGCATT
TGCAGTCCAGTATCTGCCAATACTCTGCCCATTTATCCATATTTGACCCTTTCCCATACC
CTCCATATCCAAAGCCAAAGGCTCATCTCCTTCAGGTGCATTGAAATAAGCCTTATGCCA
TGTTAACGGCTGAGGCCTTTGTGCAGCTAATGATCCCTCCACCCACTCAACAGAGGAAAA
AGCATTTGGGGAAACGAGATTCATTGCTTCTCCTTTCAGACCAACCTGATAGGTCCACTT
CTGCCAGGACAAGTCCCGCATTCCCTGATCAAGTCCATGCAATGCAACTGGACCAAGGAT
TCCCGTGTTCCAAGTCTCAAAATGTTCACCCACATTAGCCAATCCAACAGCAACGCTCAG
CAGTGCAATTCTGTTTGTTCCAGCATGCAAATTCGCCTTTCCAGTGTATGTGAATCTCCT
ATTCTGTCTTGTTCCAAAGGCAGAGCCTGACAGCTGTCCATTAATAAAGACATGCATGAC
ATGACCTGTTGATTGAACAATGAGAGTGGGGAGTTCCCCTCCATGGAGGAAGGATTCTGA
AGAACTGATATCAACACTAGTTATATACCATAAATAATCACTCACATCCCTAGTGACATT
TATCTGATCCAATAGACCCTGAGTGGTGAAAGTCGAGGTATCATCTATTGAAGAAATATC
CTCATTGTAACTCTCCCAAGAAAACATCCCAGCATTAGTTGGCAACATTTCCATTTGTGA
CGTCTGAACTCCAACCTTGGCAGTATTAAAGACCACATTCCTGCAGTCAGGGAGGACACT
GATGGACCAAGGTGGCAAGTTGTAGTGCCTGTTATTGAACATCACCCTTGCACCAGACTT
CGTATCATAGTTTGAGAGAAAAGCAGCACAATCTCCTGATTGTGAAGAAAATACATGAGC
CTGTTGAAAGCTCCCTAATGAAGTAACTATTGGATCAGCAGAAACTAAAGCTTGCTCACA
CATCTTAATAGACCTGTGAAGCTCTTTCAGATGACCATACTTCGGTTGCCTGATCAGACC
ATATTCATCTATTGGAGCATCATAATCATAGCTAGTGGTGATGAAAGGGCCTCCAGCTGT
CCGTCCGAAATTTGTCCCTCCATGGTACATGTAGTAGTTTATAAATGATCCTCCCTTCTG
AATAAATCGAGCAACTGCATATGCCAAATCTTGAACTGGGCGCTGATGAATTGGGCCACC
AAACTCCGAGAACCAGCCACTCCAAGCCTCTGTCCAGATTGCAGGTTTATATGGTTTGTT
GGGAGTGAATGTGTCACAGTAAAGACCATTACATGTGTTTATCACCGGATCTGGGGCATC
ATCTTCCTTGCACATCACCCAGGGCACCCCAGTTCCCATTTCTACAGCCATCTTGGCCGC

CCAAGTCATGTAATTGTAGCCAAAAGAACCAAGCAACTTGCCTTGTGTTCCATACTCATT
CTCGATCTGAGAGAGAATAATGGGGCCTCCCTGGGACTCAAACAGTTTTTCGCTCTTCAT
CAGCCCCACAATTTTCTCAGTGAAGCCTTGCATTGCCCTCTTGAAAGGTTCATTGTCTGT
TCTGAAACTGATGCCGGGAACATACTTCAACCAAACGGGAAATCCTCCAAAATTCCATTC
TGCACAAACATAAGGTCCAATGCGAAGCTGAGCGTAGAGCCCTGCTTTTTGTATTGCTTT
TATGAACCTCACCAGGTCATATCTCCCTTCAAAATTGTAATTGCCAGGAGAAGGCTCATG
GACATTCCAGAAAACATAAGTGTCGATGACATCAAGACCTCCGTCTTTTGCCTTCTGTAT
TAGATCCTCCCACATATCAGGGGTGCTTCTTGGGTAATGAATGGAACCAGAGAAGAGAAT
TCTTCTTTGCCCATTAATCACAATTGCCTTCCTATCGTAGGTCACGCTACACTGGATCAG
CTGAGAACCCAGATACAAACACAAGCATAACACAATGAACAACCTGGAAACCACCAAAGT
TCCCATTTCTTCTCTTCAATCCCAGGTATCTTACACTGACACTTAACATACAAAAAAAGC
TGTCTCCTTGTAGCGGTGGAGGGAAACTTGAAGC
>ntig_304_consensus
GCTTAAATAGAGAGAACAATTCCTAATTTGAAAATAAGTTTATTTATACCAAAAATATAA
TTTTAGAAATAATTAATTATTTCAAAATTCTTTGTAGATAAATAAAAATTTAGAGTAGAA
TAAACCAACAACACATGCTTGAAGCAAACCACATAATAATTCCAGTAGTTCAAATATTTC
AACCCATTAAAACAAAAATGCATCCTTAATTCAAAATGAACTGCTCGCTACAAGCTTTAA
CTTATTGATAGCTAGTAATCCCCCCCTCTTCCGAAATTGCCTTCACCACGAAAATTGTTA
TTAAATGATCGAGGCCCACTTGGTCTATCATTGGCATAACTAACACGGATGTTTCTACCA
TTTAGCTCCTGGCCATCCATAGCTGAAAGTGCCGAACTGGCAGAGTCATCGCTGGCAAAA
TTAACAAACCCAAATCCTCTTGATCTCCCAGTGTCTCTGTCAGTTATAACTCTTGCTTCA
ACCACTTCACCAAAGCTGGAAAAAGCTTCCTTGAGTGACATGTCATCCGTCCCATAGGAA
AGTCCACCAATAAAAAGCTTAGATGATGAAATGCTGCGGATAGAGTTGAGCATAGATGGC
AATGAAGCTTGCCCATTTTGCCTAACAAGCATACCAAGTCTGCTACTGAAAGCCATCTCA
ACCCACCACTCTTATCGTGAAGCAGAACCTGAGAATAGGGTTTATGAGCCAAGCGTTCTG
AAAAACATAGAAGCGGCTAGGGTTTAAGGCCTTTTATAGTGCCGTCGGCCACTCAAAATA
TCTTTTTCTCCAGAGAAAAGATCTCGGGGTGAATATCAGAAACTAC
>ntig_305_consensus
TTTTTTTTCATGTGTGAAGCTTTTCTATTAAATTCAGAAGTTAAATAGAAACATTTCACA
TGAAGCTCTGAAAATACATTAAAATTTTGATTTCTTGTTATAGACAACGAAAGGCATTAA
ATGTTATAAATCAGAAACATGATACAAGGTTTATCCTTTCCCATTGGCAAGCAGTTCCCC
CTGATGGCAGGATTGTCTAACTACCAGAATAATTATCAGGCTTCACATTTGAAGCAAATC
TTGAGCTTTGGGCCAATGTCTCTAGATAAGACCTATAATGCATGAAGAAATTTGCATTCT
TTTACCAGCGCTCTCTTGGCCTACCATCAGGAAGCACAGGGACTAGCACCTTCTCTTCAT
TCTTCATTGAGCTATCTACCTGTTTAACTGATTTGCCAACTTCAATCTTCACAGTTCCAT
CATCTTCACTGAAGTCTGGGGGTTGATCATCATCTTCATCGCTCCAATCATCCCCAAGCT
CATCATCATCTTCTTCATCTTCGTCATCATCATCAAACTGCCCATCAGTCATATAATCAT
CAATATCGGACAATCCCTCTCCATCCTCATAGCTTCCATCTTCTGGCTCATCAGCACTCT
CATCAACTTTGCTGGGAGCATTTCTCCGTGGGAATCTAGGAACGGAGACAAGTGCACGTA
ATTTCTCTTTGATTACCAACAGTCGATCTTTATCTATCAATTGAGAATCACGATATGCTT
CCCTGAGGAACACTGAGTCCCTATCACCTTTCAAGGACACATAAAACAAATCAGGATGCC
TTATCAGCATCCCCCTCAGCTGCTGAGAGAATCTAAACTCCTCTCGAAAATGAGTGAGGT
GATCCACAAGAGTTCTCTTCTCAACAGTCAGGCTCAAAATCTCATGCACAACCCCGCAAG
CATGCTTTTCCTTCTCTGGTGTACCTGATTTCAATT
>ntig_306_consensus
GCAGGTTGCAAAATACCCACAGAATTTGATGGTAGTAGTTGTCCAGAGGCAACCCCAGAA
ACTGATTTAGGATTAACTGATTTCTTCTCAGAAAATTCTCCAACATCATCTTCAGACTCA
TAACTCGGATCCATGCATGGGTCAATATGAACTACTTCCACTCCAAGAAGCCTGTCCTTG
TGAATATCAGGAGAGTTCAAAAGCTCTAACTTCTCCACACATTCTCTGATCTCGTTTTCG
TGTCCCTTTTCACTAGCTTGATCATGGAGATGTACAAGCCGCAGTTTCTGTGCTTCAAGT
AAATCATCAATTCTCTGTGATTGTAACTTTAATGCTATCTCCAGAATCTCTACCACTGTA
AAACTTTTCATGAATCCACATTTGATGCTCTGGCACAACTGTCTGCATTCATCCTCAGAG
AACTCCTCATTTGAAATCGCATCAATTGTAAGCAAATCACTCTTTTTCAAATCTTGAACT
TCTATCATTAAATCAGTTTTTCTGTCTCCAATTACATATGGTTCAGCTGCCTTGCCTGTA
CTGATGCTTGATTTCGTAAAGCACTATTTTCCATCATCATGATAGTTTTCATGCATGCTG
CGCAAAGCCCCTTGTTCTCTCGTATGCAAAAATAATCAGCATCCTTGATGCATCCCTTGC
ATAAAGAATATGTACAAGTATAGCACACATAATGTGAAGCCTTCCGACAGGTACTACAGA
TATGCCACCC

>ntig_307_consensus
GCTTCAGTAAATGCGATAAACAAGGCGTTCAAAGCAGTGGTGTAGAGAGAAATAAAAGAA
GATAACGAGATTGTTTGTCTGATCCATATTTGACTGGATCCTTTCTCTGTTTTGAGTACT
TGTTCATGTATAACTCTTCCACGTCCACCGTAATCTGAGTAACCATTTTATATAATACAT
GATTGCCTATCGAGAAACATAGCAGCAAGAAGAAGAACCCAGATTTTCTGAAAACCCCAA
GAGATAAAAGGCACACCTAAATATGATTTCTGCTAGTAAAGCTGCCTTTACGGTTCTATA
CCCTTGGAAGCTGGCCAAGACAGATCATGAGAGCATAAAGGTCAAACACAACACACACAG
TGTACAAAAATAACTAAGTAGGTTTTAGTACATTATTTCCAACAAGTTTGGAGGAGAAAC
AATCAGAATGCCCATGATGCATTACATTCTCATATTCAGACCAACATTCAAAATATTAAA
CCAAGTTGGCTTCAGAGACAACCTTATTAACTGCATTATATAGTATAAGATTGCTGCATA
ATAATTGCTCATACAGCTGGTGCTGCTTCTTCAATTTGTCTGGCTTCAAGTGTCTTCTCG
TATTCCTCTATGGACTTGAAGAGCTCTGAAAAGTTGCCCTTCCCAAAACCTCCACAGCCC
CCCTTTTGGTAAGTCTTCCCTTCATCATCCTTAAGCATGCATCCTACTCTCTGAATTATC
TCTATAAAGATTGTTGGCCTATCACCAACAGGCTTAGTAAAGATCTGGAGAAGAGTGCCC
TGATCATCTCTATCAACTAAAATCCCCAATTCTTCACACTCTTTAATCTGCTCATCAGTC
AACACGTCACCAGCACGATTCTTCAAATTCTTGTAATAAGTGGGCGGTGGTGATGGCATA
AATTCAAACCCACCAACTCCACTTCTCTTCCTCATTTCTCTTAACGTTCTGAATATATCT
TCACTCACAAGCGCCAAATGTTGCAACCCAGCTCCCTCATTGTGCTCCAAATAAGTCTGT
ATCTGACTCTTACGCTTCGTCCCAAAAACTGGCTCATTCAACGGAAGTAACACCATTTCG
TCATTGCTAGCTAAAACCATTGAGTTCAGTCCACTCTCACTCGTCCCAACATCTTCCGCT
GTGAACTCAGCGAACTCATGAAACCCAGTAAACCCTTTCACATAGGAAACAGCGGGGCCC
AGTTCAGGCACGTTCCCCACAGCGTGATCAAGACGCCTGATCCCGTAATCCAACGGGAAC
GACGAAATCTCATCCGTCCGTTCAAAGCCAGGTAAGAATAAGCAAGAATCAAAATTATTC
CCACTTTTGTAACTCACATATCGCAAAACGACATCACCGTAGAGATGAACCTCAGAAATA
GCAGCACGGTTGTCGAGAATCTTAGGAGAAGAAACGGGCTTAGCACCATGGGCCACACTC
GTAGTAAAAGCAATCTCAGCATCTTCAACTTCAACACCAACTGACCTCACAGCAAGTCCA
TGAGATAGAGCAAAGGCCCGGCAAGAATCAATGCTAAAAGTTGGAATAGAAGCGGTGTTT
TTGTAATCATCACCAGAAATCGAAGGCGAATACGGCGCAGTAAAGACGAAATTAAGTTCA
CCAGAGCGTAAAAGATACGATGCATGAACCATGTTACCGGTGGATAAATCGGATTTGGCG
ATAATGGGCATGCCAAGTCCCCAGGAGAAACGGCGTGCCGCATTGATAGCATCGGTACAC
CAGAATTCGATGTGGTGGAACTTCTTTACCTTAAATCGGTCTGATTTTGGGTTGTGGCGG
ACGAAGTTTGAAAAACCGACAAGCTTGAACGAACTGTCGTCCTGTTTGCCCATTGTGGGA
GGTTTTGTGGCTTCGTTCTGAGAGCGGTGATCAAGACAAGGAAGATTTTGGTTTTGACTT
GACACGCCAATCCTTATATGTCCGTTTGAG
>ntig_308_consensus
GGTTCGCTGTCTAAGGTAAAAGACTGATGAAATATAGAAACTGAACAAGTTAATGAACAA
TCAATTAACCTATTTATAATTGAAATTTTAATAGAATTGCAGAAACGTCGTCAATGAAGT
AACTCTCACAAATCTTCCTCGGTGGAATCAATTCACAACACCATCCATGCATTTGGATGT
GCGGGAAGTGACAGGCAGATCGTCATTTATAGCATCTACCATGTACTTGCCCCTGATTCC
TATGAATTAAACTAGCTCCAATGATGATCCGTTGGTAAACTTTCTTTGATCTTTTCAAGC
TTTTTCAGAATTTCCAAGAATGAAGGTCTTGCATTCATGTCAGCATCCCAGCACCTCTCC
GTTAAGTCTCTCAGTTCAGGGATATATCCTTTAGCATGACAAGTAGGCCTATGTCCTTTT
GCCACATATTTGGCTGCTTCATGTGGACCCAAATGTGAAAGTGGTGGTTCTCCTTCAAGC
ATCTCATAAAGAATCATTCCAAAAGAGAAGACGTCAACCTTTTTATCATACTTTCGATGC
TTGAAGACCTCAGGAGCCATATACCGGTAACTTCCAGTCTCCCCAGTCATTTTGTACACA
TCATGAGAATTTTGAACCTTGATGAGCTTGCTTAGTCCAAAGTCTCCAACTTTCAAGTGG
TCTGCGCTTGTGTTGACCAAAAGAATATTCCTCGGCTTCAAGTCTCGATGAATTATGACA
TTAGGCTCATTGTGAAGATAAGCCATTCCTCTGGCGATGTCTAATGCAAAGTTAACTGCT
GTCGTGGGATTGAGTGCACCTTTTTCCTTAAGGTAGTTATGAAGATCACC
>ntig_309_consensus
AATGAACAATCAATTAACCTATTCTCATAATTGAAATTTGAATAGAAGTGCAGAGAGGTA
GTTAAACAAGTAGAAACTCTCACAAATCTTCCTCAATTGAATCTATTCACAACACCATCT
ATGCGATCGGATGTGCGGGGAAGCGCCCTCGAATCCTATGAAGTAAATAAGCTCCAATGA
TGATCTGTTGGTAAAGTTTCCTTGATCTTTTCAAGCCTTTTCAGAATTTCCAAGAATGAA
GGTCTTGCGTTCATGTCAGCATCCCAGCACCTCTCCGTTAAGTTTCTCAATTCAGGGGTA
TACCCTTTAGCACGACAAATAGGCCTTTGTCCCTCTGCCACATATTTAGCTGCTTCATAA
GGCTCAAAATGTGACAGTGGAGGTTCTCCTTCAAGCATCTCATAGAGAATCATTGCAAAA
GAGAAAACATCAACCTTCTTATCGTATTTTCGATGCTTGAAGACCTCAGGAGCCATATAC

CGGTAACTTCCAGTCTCCCCAGTCATTTTGTACACATCATGAGAATTTTGAACCTTGATG
AGCTTGCTTAGTCCAAAGTCCCCAACTTTCAAGTGGTCTGCATTTGAGTTGACCAAAAGT
ACATTCCTAGGCTTCAGATCTCTATGAATTATGACATTTGGCTCATTGTGAAGATAAGCC
ATTCCTCTGGCAATGTCCAATGCAAAGTTAACTGCTGTTAAGGGGCTGAGTGCACCCCTTT
TCCTTGAGGTAATTATGGAGGTCACCCCCTCGTAGATACTCAGTAATTAACATAAGGGGC
TTCTTCTCAGTGACAGCTCCCAAAAATTGGACTATATTAGGATGGCGAAGTTTCACTAGC
AAATTAACCTCATGCCTGAAGTCCTGAATCACCAATCTATCATCTGAAAGAGATGGGAGA
ATACGTTTAATAGCAACTGGTGTTCCTCGCCAACTGGCTTTTAAAATCTCACCAAAAGAA
CCCTTGCCAATAATTGTTGAGCTTGAGAAGTCCAGCTCTGTAGGATCAATTTCCCAATCA
CACTTATTTGGAAGTGGGGGAGGGACAGGCTTTGGTTCAAAGTGACTTCCAGTTTGACCA
AAAGATAAGCCACCATAAGACTGTAAAAGCTCAACCATTTTATATTTTTTAGCTCCTTCT
GCATCAGCAAGAGGAGTATTTTTCCAACGATCCTGGGCGTTGACGTCAGCACCAAACTCG
ATCAAGGTTTTGGCGACTTCTGTCCAGCCATGGAGGGAGGCAACGTGAAGCGGAGTCCGA
TTATCATAGTCCCTTGCCTGGACCAAAGATCGATCTTCTTCGAGAAGTTTCCGGACAGCA
GAGCAGTCGTTTTGATGAGCGTGCCAGAGTATAAGTGACGTCCTGCTCACTCTGGCCTTA
TCTTTCTGCTTGTTGTTGGATGACGTGGCAGAATTGCCCTTCTCGGATGATCCCGCGCAG
GGGGAATCACCGCCGCCGGATGAATTGGAGCTCATGACCAAGGATCAGATCGGATCAGAT
CTTGTTATTCTTATTAGACTTTTTTATGCAAAAAATAAAATATTGATTTATTAGTTTCAG
TTTAAATTGGGTACGATTCACAGGTTGCAGAAGGCTGGAAGGGAAGGGAAGGGAAGGGAA
GGGAGG
>ntig_310_consensus
TACGTGTTTTCCTCTTGCGCTGCATGTAATCATGAATTAAAGAATTCACAGCATTATGCC
AAACCACATCATAATTATGTTTAGAGAAGAAAACCTTAAAAACTAAGTTAGTAGCATCTA
CATTAAAAGCCAAAAAGAAAAATGACTAGAAGAATAATCTAAAGCTTGTGGATACTAGTA
TGAGAATATCTTGAAAACGTAAATAGTTATAGATCAAATAAGAAGAAACAAATTCATATC
ATGAAACATCAAGCAACTCTAATGTATTAAAGGCATGAAGATAAGCTTGAGAATATGCAT
AAAAGACAACAACAATATAATTGTTTGGACCCGCACAAGGAACTTTAAGACACAGAGACA
GCCTGGTTCTGACAAATCATGATATTACAACATCAACAAAAAATAATTTTACAAAGACAT
TTTGAAAGTACTATACAAATTGAATGCTTCAGCATTATTATGTAGCAATTGAGCACAAAA
ATGATTAATAAAGTCAAGAATTCAAAACTTCTTTAAACATAAAAAAAATCGGGGAAAAAG
GATATTTTAAATATATTTCCATGTTG
>ntig_311_consensus
CAGATGCATAAACAAAGATAAAAACTTTCATACACTGCTCTATTTGAGCTTTCTTCTAGC
TAAGAACCTAGCTACAGGAGGCGTAAGCGCGATCGTTATCGGAACCCTAATAGGAAAAAG
CACTTTATTACATAACAAGGCCAAAGCCAGAGCTCCACCAGACGACGCCACAAGCTCGGC
CGTCCGATTCCTCTGCTTCTTCTCCTCATTTAAGTCCGTATTTTCATGAGTGGAAATAAA
CCCATCAGAGGGTTGAGGTGCTTGAGTCTCTCCCCGGTTTTTCGAGAGTCCAGGGAGGTG
CCAAGTTTCGAAGATTGATTCCACGTCGACGTTGCTCTTGATGGCAATATAGAGGCCCGT
TATTGAGGCGGCGGAGACGGACAAATGAACCCCAAGAGCTACTTTTCCGTACTTTTTGAC
TAGCTCTTTGAATCGGCCACCTGCCATTAATCGAATTTCTTCCGACGTGCACTGGGGTTT
TGCGGTGGCTTTGTTAAGCTTACAGTTTAGGGTTACCAAGTAAAAGTAAGGCGGCTGTAT
GATTCCTACGAGTGGGAGTAACGGCGCTTCAGCATTTTGCTACTTGATATACCTTCCAGA
CATGTCAAAGGGGCGGCACCGGGCCGGCCCGTAGCACAGCTGTGGAGCACCAGTCACAAT
TTCACCAATTGTGTCAGACCTCTACACTGTATGGGTCATACTAGAAGCTTTAGAATCTGG
CCC
>ntig_312_consensus
CAGATGCATAAACAAAGATAAAAGCTTTCATACACTGCTCTATTTGAGCTTTCTTCTAGC
TAAGAACCTAGCTACAGGAGGCGTAAGCGCGATCGTTATCGGAACCCTAATAGGAAAAAG
CACTTTATTACATAACAAAGCCAAAGCCAGGGCTCCACCAGACGACGCCACAAGCTCGGC
CGTCCGATTCCTCGGCTTCTTCTCCTCATTTAAGTCCGTATTTTCATGAGTGGAAATAAA
CCCATCAGAGGGTTGAGGTGCATGAGTCTCTCCCCGGTTTTTCGA
>ntig_313_consensus
CAACGATTGAAACCTTTTTATATGATCATTGAGATAATAGGCTTTTTTAGTGGAAACAAA
AATGTCTCCAATAGGCCAGAAAGCAACCTTTATTTACCTCAAAAGTGCACCAGATACGCC
ATAAAGTACAGTAACGAAGACACTCATACAAGAAAATACAAACAGCAATACGAGGGGCAC
AGAAGCCTTAATAATTATACTATTACCAGGAAGCTCAGCATGCTCCAGTTCTTCTGCCAT
CCACAAACCTCTTCCACCGTATGGTGACTTCCTCCTGGATCTGTCTCTCACTCTTTTTCA
GAGGCTTCCCACCTTGAGTTTCATTGATTATAGCAGTGACATGTGCAGCTTTGGTCGCTA

TGCGACTTGGGAGCGCGTCTATTTCCTTTAGGACTTCCTCAATGTTGGCAACAAGCCTCT
CTGCCAAGCTCCGGCTGAAATCCTCCCTAATCACCACTCGGAGAACAGCAATGTGCTCAG
CATTAGGAGGCATTGTATAGGCTGGAATTATCCATCCAAACCTTCTCAAGCTGTCGGATA
CCTCAAACACAGTGTGTTTGCTGCTGTCCTTCAATGCAAAGGCTACCAGAGGCACTCCAA
CATCCTTGGAGACAATGTCAAACCGCCCCGTTTTTCTGAGTCCTTCTTTCAGCACCTTTG
CATTCTCCATGCAGTTTTCCATAATGTTCTTGTAGCCCTCAGAGCCAAGTCGTATAAACT
GATAATATTGAGCAATTATTTGACTAGAGCCTTTGGAGAAGTTCAGGGTGAAAGTTGGTT
GATCAGTTCCAAGATAGTTGATATGAAAGACAAGTTCTTCTGGCAGGTCATCTTTGGTCC
TCCACACAACCCAGCCAACACCAGCATAGACAAGGCCATACTTGTGGCCACTGACATTAA
TACTCTTCACCAGTGGCAAACGGAAATCCCATTCAAGTTCTGGGTATAGGAAAGGAGCAA
TAAATCCTCCGCTAGCAGCATCAACATGAATTGGAGTGTCCCAACCAGTCTCCTCATTCT
TTTTGGTAAGAAGTTCATTAAGAAGTTTCACATCCTCAAACTCTCCGGTCAGGGTTGATC
CCAGAATGGCTGCAACACAAATAGTGTTCTCATCTACCAGCTCGACAGCTTTCACAGGGT
CCATAACATAGTATCCCTCCCTCAGCTTCACCTCCTTTAGCTCAACCTCAAAGTATCTTG
CAAACTTCTCCCAGCAAACCTGCACGTTAGCTCCAGTGACTATGTTGGGCTTATCATATG
GTTTGCCTTGTGCTTTTCTCCTCTGCTGCCACTTCCGTTTGAAAGCTAAACCTGCCAGCA
TTATTGCCTCCGATGAACCCACAGTCCCAACACCAATTGCAGTCTCATCATCTCCAACTG
GAGCATTGAATAGGTGGGCAATCATATTCACACACCGATTCTGAAGCTCTGTTGTAACAG
GGTACTCATCCATGTCAACATAGTTCTTATTAATCGAAGCCATGATAAGGCGATCACACT
CAGGCTCCATCCATGTGGTCACAAAGGATGCTAAGTTCAGCCTTGGATTTCCATCTAGCA
TCAGCTCATCATTTATTACCTGGTATGCTGCTTCCTTGGGCATCGAATTCTCAGGAATCT
TGAATCTTGGAACAGGAGAGCGGACATATCTGGAAGCAAACGTGGAGTGCAGATGCTCCG
CAGAGTCGGTTCTCGTAGAAGAGATCACCATTTGTATGTCAGAGAAAATTCAATTCAGAC
GGTGAAAGAGTGAAACTTGAATGAGGTGGAAGAAGATAGAGTGCTTTATAGGTTCACAGA
AAAAAGGACGGAGACAGACACCGTGGTCCATTAAGGGAGTGTGACGTGATGGGTTACCTA
TCTAAGGCTTGTCTGCCATTTGGCTTGG
>ntig_314_consensus
ATCTCTCATTAATTACAGGAAAAATGGGAAAATAATATTTAATGGTATCTTGTACTATAG
CCCTGACTTGGATAATTAGGATTTTTGTCTCACTAAATTTGGCAACAACTCCTTCTAGAC
TTTGCCTTATCTTAAAACTGCTTTTAGTCTAATCACATCCAAAACCTCACCCAAAAAGCG
CCCCCAGCCATCCACAGATATGGCAGCTCAATCTGTTAGCCACTTGTTAATGATGGGAAC
ACCAAACTTATCCACCATGTGAGATTTTGGGAAGCATACAACAATATCAAATATCAATCT
TTTCAGAATTCCCTACCATCTCATAATTAAAGTTGATGCTGACAATTAAAAGCTATATGG
AGCTGATTAGCAATCTTGAAGGCAAACAAACAACTTAAGAAACTGAAATTGAAACCAAAT
CAAAAGACAGTAGGTATTATACATCACTCAGGTTATAAGTTTTGATGGACGTAGACTAAG
CCTAGCTAATGTCATATACATGATAGTATAACAGCAGAAAATTTCACAACCTAAAACAGC
AAACAAACAGAGGACTGTACACTAAAAGATGAGGTGCAAAAAAGGGGGATTTATTAACAT
TGTATAGAAAGAAAACAGACCATAAATCCCTAGTCTTGAAAATTGGGTAAAATATGTATG
CTCTTGCTTGCTACTCCTGCAATTTTCAACTGGTCTATGCAGAGCCATGCTTGCACGTTA
TCTCCATATCTTCACCATTTTATGAGCAACAATATCCCAGATGAGGTTAACAAACGCCTC
AAAGCATCAGTCCAACGCAACCAAGCTCCACAAGCTCAGTTACATATCCAGGCCAACCAC
ACGGCTTCTCTATCAAATCTTCCAGCTCATGAAATAGTTGAGTGTCGTTAACAGCCACTT
CTTCATCAACCATATTTTCAAGAACCGATCTGTCCCCAAGTAGGCTTCCTACCAACAAGC
GGTATGCAGATGATGTATCTTCATCACTTGTTTCACTCATCAAATTGCCCTTAAGATCCT
CAACATGACTGTACAATTCATTTTCACCATCCATAGCACCTGGGGATGGAGCCTTTTTAA
TTGTATCAGTAATTACAGTACTATCTGGTAACATATCAGCGAGCTCGGACTGCATTGAAG
CCAATTTTTCCTGAGCTTGGTTTCTACAATTGACACTTTCAGGCAAGTTTTCAGCCTCTT
GGGCTGCAAGCAGTCTCCTTCTCTTTCTAATTTCACCTCCATCAAGCTCTCTGTGTTGCT
TTCTCTTCTGAATTAAATGTTGAACGAATTTGGGGTATTTGGCTACTTTTGCAAAGAAAC
TCAACATCTGTTGCTGCTTACACTCTGCACATCGTATACGTTCTTCAACAGCAGTAATTT
GATTCAGTGAATCCTCTTGCTGTTGTCTGAGCTTCAAAATTTCCAATCTCAGTGTACTCT
GATCATCTCTTAAATTCTCCAGCTCAGCTTCCAATCCAGATTTGGTCAAATCCACACAAG
TTCTACTTCTTCTTTTGATGCTTCTAAGTAAATGCTTCTTCCCTCCCTGAAACTTTTCAT
TTGCGAATTCCCATCTATCTGAATGAATCTTCCTAAACCCATAAGTGTTTAACTGGCGAA
TGAAGCTCGAGAAATTCTTGTGCTTAAAATACTTAGGAAGAAGATTTTCAGAGAAGCCAT
GCGAGTCCCAAACAATAAAGCTACTACGATTAGCACCCCACGAAACAATCGAGTCGGTCT
CCGGATTCTCCACAATCTCATATATCTTCCTCAAAAAAGGCGGGGGACCTACCTCATTCA
GCCCTTGCATTGGCTTTGGCAAACTCATCGATGAAGTCGAAGAAAACGACGATCCATTTG

AATCAATG
>ntig_315_consensus
GATTTCTCTTTATAGCAACAGTGCTTTTAAAATTTTCTTCAAGTACAAAGGTCTATACTT
AAATCCAACCCATCAAACAAAACCACATTGTTGCCATCAACGTACATCATAATCAAGTAA
AACCGTTTTATTAGCAACAAAATAAAAATCATTAACTAATTCATTACTGCTAATTGTTGA
ACCCACGCTTTTGGTAAGCAGCCACCTCACCCTCGTTTGGGAAAAACCCCATGAGTCGAC
CGGCCGAGTTCTGGTACGCGTACATGAACCCACCCATCAGCCCGATCAAGCCACCGGTCA
CCATCGACGGCCCAGGAAGACCCGGCTTTAACCCGGAAAGGTAGCCGACGACAACGGAGA
CGCCGGTGATGGTGGAGAAACGGAAGTAATCGAGGGTGTTGAAATTGCCAACAACTTTGG
TGAATGGCGGGTTTCGATCTATTACTGGGTATTGTGGCTTCTCCGAGGCTGTGATGTCTG
TGTTCATCCTGTTGCTGTCACTCTATCACTCTTCTTCTCTCAGTGTGTTCTTTGAAGGTG
GTGGCTGGTTTTTGGCTACTGGTTGTGGTCACCGATTTCAACGCCTCTGCGTGGATGAGG
AAGGGGTTACTTTGGGGCTGCTGCTGATGGGCCAGTAATGGGCTCTTCCCAATATAGGAA
TCCCATGGGCTACTTTTCATTGGAATTAAAAGAACCAACAATTTGCATCATAAACTATGA
TAATATCTCTTTAGTATGAGCTTATTGTGGTTC
>ntig_316_consensus
CTGATCTGATCCCATGCTGTCTCTTTCCATGAAATGTCATTAGCTTGGTATAGAGGGCAA
AAATCCTTCAATACTTTATTTTCAGAAGATTTGCGTCCATGGTGCGTCTCCCATACGTTG
AACTCATCATCAAACATATATTAACAATAGCAACTTCGACTACAGACTACGTAATTAATA
AAAGAAAACAGGACACTGGAATAACAAATTGCCACCATGGTTGTGGTGGCGCTACGTACA
CCATTCTACCTAAATGGAACATGACATAAAGAAGCTAAGAAGCTGACAACTACGACAGAA
AAAAAAAGCTGAAACAACACAAGCCTTAAGAGCACCCACAACTGCATGTCAAGCACCTAC
TTCAAGNNNNNNNNNNNNNNNNNNNNNNNNNNNAAAATCCTCAGCCCCTTGTTACACGAGTGT
TGTAGGACTTGCAATTTGGGCACTTCTGAGCCACCACATGGAAATGTACATTCGAGGTCT
TCCCACAATCGTTGCAAAGAATCCAAACCATTTTGTTTAGGTATGATTCTGGCATTGGTC
TAGCTGCGATCTCCATGTCATATTTCTCCCAGACCTTGGACATGTCACAGACCGACTTGG
AACAGAGCGGGCAAGCAAATTGATAATGTTCTCTCATCTCCTTCAAGCAGTTTTTGTGAA
TGGTGTGCCCACATGGCATGANNTGATGCATTGCACCTTCAACACATGGGTGCCTGTTCT
TCAAAAGGATTGAGTAGCAGCAACCACACTTATAGCAATGGAAGAAATTCTCCCGTCCAC
CAATTCTGCAAATGCCACACCCATCACAATGGTACTGTTTTTTAGATGTATCATCATCAA
AAAGCTTGCAAGTCTTGCAGAAATATTCTCCCATGCATACACCACAATTAACACAGACTT
GTTGAACTTCTTGTTCAGTTCCACAAAGAGAGCATATCACCTGTTTGACTTCATGGCGTG
GGATATCATGTCTAAGCTTCTGATCAACATTAATATTGCTCTTAGCCTCATTATGACAAT
GACGACAATCAAAGATTTCATTGCAACAAGGAGCTCTAATGCGGCACCGTCTTCTATAGT
GCTGGCATCCATACTCCATCAGCCCCTTCTTTAGCAATTCAGTCATATCCATATGATCTG
TTGATACTACTCCAAACCGGCACTCCACATTTTCCAGCTGAGAGTCTGAGATCAGTGCCA
CACTTTCTTCCAATTTGCATATGTTCTTATGCTCAAACTGTTGATAGTCAGAATGATTGG
CTGTTACTTCTGCCATTGCAAAGTACTATTCTGAGATCAGATAGCTGAAGTTGGCAAAAA
GCCTAGGGCACTTTCAATCAGAATTCAGGAGATCGCCGATCGTAATGGCAAGATCCGACA
GGTCCCCGGAGCTCCAATCAAATCTGCTCTCAAGCGCAAATGCAGGGTTGTGACTATACG
TGTTGAATGAGAGATTTTGAATTGGATATATATGTATAGAGAGAGAGAGAGAGAGAGAGA
GATGTGGAAGTGACGGTTTCTGATTCCAGAGGCTTCGAAATCGGCTGCTTGAGTTGAGCA
CGAAAGCGAAGGCGGTGTCGTAACTAGTTGTCACCGGATTTTGACAGGTTGTATTATGAA
AATATGAATATACCTTCCATAGTTTAAAAAAATATCTTCACCGATTTTTATGGAAAAAGG
TTAGGATACAATGAAGGCCGG
>ntig_317_consensus
GGGTGGAAGGGCTGAGAACAGAGTCGCATGAGCCATCTTTTGACTTCAGCGTTTGGGTCC
TCTTCGATACAATTCGGACAAGGTTTTATATTAATAATTATCCATTCGGTAAATACGATA
ATTACAAATTAAAGCGGGAACAACAGGGGAAAAATGATAAAAAAGAAGCTGGAACAACAC
AAGCTGTAGAGTCCCGACGATCGCGCGTGGAGCAACCCCTTCAAGCCACCACGATCGTAT
CCCAGTAGACCATGGGAAGTTAACTGTGACAGTTGATATCATAGCCGAATTTGCAACCAG
CGGCGCCACACTTCCTCAGCCCCTAGTTACCCGAGTGTTATATGACTTGCAATTTGGGCA
CTTGTGAGCCACCACATGGAAACGTACGTTCAAGGTCTTACCACAATCGTTGCAGAGGAT
CCAAACCATTTTGTTTAGGTATGATTCCGGCATTGGCGTAGCGGCGATCTCCATGTCATA
TTTCTCCCAGACCTTGGACATGTCACAGATGGACTTCGAACAGAGCGGGCAAGCGTATTG
ATAATGTTCTCTCATCTCCTTCAAGCAGTTTTTGTGAATGGTGTGCCCACATGGCATGAC
GGTTACATCATTTCTTGAGTCAAATAAGTACTCACAGCAAACAGGACAGTCATGATGCAT
AGCACCTTCAACACATGGGTGGCTGTTCTTCAAAAGGCTCGAGTAGCAGCAACCACACTT

ACGGCAATGAAAGAAATTCTCACGGCCACCGATTCTGCAAATGCCACACCCATCACAATG
ATACTGTTTCTTAGATGTATCGTCATCAAACAGCTTGCAAGTCTTGCAAAAATATTCTCC
CATGCAAACACCACAATTAATACAAAATTGTTGAACTTCTTGTTCAGTCCCACAAAGAGA
GCATATCACCTGTTTGACTTGGTGGCGTGGGATATCATGTCTAAGCTTCTGATCCACATT
AATATTGTTCATAGCCTCATTATGACAATGACGGCAATCAAAGATCTCATTGCAACAAGG
AGCTCTAATGCGGCACCGTCTTCGATAGTGTTGGCATCCATACTCCATGTGTCCATACTT
TCGTAATTCAGTCATATCCACATGATCTTGTGATACTACTTCTATCCTGCACTCCACATT
TTCCAGCTCAGAGTCTGAGATCACTGGCTCAATTTCTTCCTTCTTCGGCTCAAACTGTTG
ATATTCAGAATAATTGGCTGCTACTTCTGCCATTGCAAAGTAGTATGCTGAGACCAAACA
GCTGAAATTGGCAAAAAGTCTAGGGCACTTTCAATCAGAATTCAGAAGAGCGCGGATCGA
AATTAGAGAACTCAATGGCAGGATTCGCAGGTCTCTGTAGCTCCAATCCAAATCTGCTCT
CAAACTCAAATAAAAGATTGAGATTTGAATCAAGAGGATTTCAACTGGAGTGACATATAT
ATAGGTGGCGAGGACATCGGTTTCTTATTCCAGAAGAGACTTCGAAATTTGCATTTTAAA
CACGAAAGCGGTTTTTACTGAACATTTGACAGGCTGTTTTCTGAAATTACCAATACG
>ntig_318_consensus
TCTGATCGGAACTTTACCGCTAAATAAAATACTAAAAAAGAATTATACAAATGATATGAC
CAAGAAATCTTAAAACCTCAAACTGCACTCAGGTCATCAAAACTAAAACTTCTATGAGTT
CCAAAAATCCATAACAAAAATCATCAATATCGAAATCGGCATACAAAAAGCACACACTTA
CTCCAAAATTTTGCTGCCTATTTTCGGACCTCCTTTTACTTCTTATCTTCCTTTTCAGCC
TCTGAAGCCTTTTTCAGCCTTGCACCAACATGGCGGCTGTTCATCCTCTCCACACGCAGC
TTGTTGTATGCCTTGAAGGACTTCATCTCTGGAGTAACTTTAACAAGCTCCACCGTAGGC
TTCTCAAGAGTGATTGGCATATAAGATCCTTGTACTTGGGTTGCTGTCGCCAGTTCCTCT
GGAGCAGAATCGCCAGCCTTGACCTTGCGAGCTCTTCTTGGGAAGACAACCAATTTGGCC
TTGTATGTCTTCAGCCTCTGAACATTGGCTTGGAGGCTCTCCAGAGATCGGTTCTTACGA
C
>ntig_319_consensus
ATCAGATCGGAACTTTACCTCTAAATAAAATACTAAAAAAAAAAACTGGCAAGGAATTAT
ACAAATGTTATGACCAAGAAATCTTAAACCTCAAACTGCACTCACGTCATCAAAACTAAA
ACTTCTATGAGTTCCAAAAATCTATAACAAAAATCATCAATATCGAAATCCACATGCAAA
AAGCGCACACACTTGGACTCCAAAATTTTGCTGCCTATTTTCGGACCTCGTTTTACTTCT
TATCTTCCTTTTCAGCCTCTGCAGCCTTTTTCAGCCTTGCACCAACATGGCGGCTGTTCA
TCCTCTCCACACGCAGCTTCTTGTATGCCTTGAAGGACTTCATCTCTGGAGTAACTTTAA
CAAGCTCCACCGTAGGCTTCTCAAGAGTGATAGGCATATACGGTCCTTGTACTTGGGTTG
CTGTCGCCAGTTCCTCTGGAGCAGAATCACCAGCCTTGACCTTGCGAGCTCTTCTTGGGA
AGACAACCAATTTGGCCTTGTATGTCTTCAGCCTCTGAACATTGGCTTGGAGGCCCTCCA
GAGATCGGTTCTTACGAC
>ntig_320_consensus
GCTGCCTTTATTGATAATAAAGCTTGCATCTACAGTACTTTAGTGCAAGTATGCAATTTT
CTTACAGGAAGGGGAAAGAAAGAAAGAGCAATCAGCTCTTTGTACTTCAGCAAAGGGAAC
TCAAATTACAACACATTGACAAAAAAATTCAAACAAACTTCTTTATGATGCAGCAACTGG
TTTTAAGAGTTGCAAGACAGCTTCTTTTGTAGGCAGTGCAGGAATTGCACCTCTCTCTGT
TACCGTGAGGGCGCCACAGGCATTTGCGAAAAGTAGAGCTTCTCTAAGCCGCTTCTCATC
CTTGAATAGGCTTAGATCAGAAGCCAGGCAACTAAGTATCCCACTGACAAATGCATCACC
GGCACCAGTGGTGTCCACAGATGTAGCTTTAACACCAGGAACAAAACCTCTAAATTCCT
>ntig_321_consensus
GTTTGATATGAAGATAATAAGATGACAGAAAAAAAAAAAAAAATTCTCACTGATAAAAAAT
CAAATGTTATTTATATACAGCAAAATGAGTGTTGCTTTGTCATCTCTTCCAGGTCTCTAA
TAAAAATCTAAACCACTTACTCAAATCACAACACAAATTTCCAAATTTTTCAAAGCAAAG
TGGCACTGCAGCTTGCAGCAGCAGCTGCTGCTTCTATAACTTACATAACCAACCTTCCTT
ATTCTGAATCACTTTCGACACTCCCTGTGCCCTTCAATCCTCTTGGTCTCTTCTTAGAGC
TCTTCTTCTCCCTAACCTCATACCTCTCCTGGCACATATCAGTCAACCAAGCACCAGGAC
TTACCAAAAGTAAGCTTCTTTGAATAACCTTGGCTCTCTTCTTAGGCCTTTGAGGTGGCT
TACACCCTTTCATAGCTAAGAAATCCTCCTCTTTTTCTTTGTTTGACAAAGTAATATAAA
GCTTCGGCCAAACAACCAATTTATTTTCCTCTCCATTGTTAGCATCTACAGGAACCTTGC
TGTTCTCATCACCAACCACTGATCCTCTTGTGGTGTAGTGCCTATCTTCCTTCTCAGGTG
ACAATGATTTTCGGTTCCTGTTTGAAGCCGACCGGAGTATCCCAGCCTCCGAATTCCTAG
CAAGACGACTTGTGGTTTCTTTGTCTGAGGCAGTAACCAAACATCGATCCATTCTTGTGA
TTCTCTTGCCAATTACGCCGTTTGGTTTCTGTGAAAGTTTTTTTGGGTCCTTGACCCTCA

CACATCTTAACCTCTTCTTGTTTCCCCACTGTAAGAAAAACTTCGGCTCCATTGATAATG
ATGATGATTTACAAGCTATCGGATTCCTATCTTCCGTCTCAATACCTACCAGCAAAACAA
ACTCACAAAATCTGGGCTTCGTCTGCACGTAAAAAGTTTGAAATTTTAATAACACATGAA
AATTAAGCACAAAAAAAATAATATTTTTGTTATACCCATTTCGGGTCTTTGAGCAATTCAG
TGGTTTATGGAGGAAAATAACTTTTATTTTTCACTTAATATCTAGAAAACCCAGAAGTGA
GACTCAAAGAAAT
>ntig_322_consensus
CTCATCCGCAAAGGCAAACATTAATGTATGATGAGCAAGCAATTACAAGTGGCGCAGCCA
TGACACAAAATTTCTAGTTTGGTTTGACTCGGTGCAAATAATCAACCAAGAACACAGCAA
TTCTTATTACAATGCTTTTATTAAATTCGTCATCTCATACACGACATAATAATGGCTACG
AAGTGCAGCAACCAACAAAGGTATTCTACCGAGCATCCTTTGATAAGAGACTATTGGTTT
CAACTTTCCTTGCATTACAAAGCTCATCCGCACAGTCACCTCTTTCAAAGACAGCAGCTG
CTCCCATCCCTGTGCCTATGCACATAGATACCACTCCAAAGCGACAGTCTCTGCCTCGCC
GCTTCATTTCATGCAAGAGAGTGGCGACACACCGCGCACCTGTTGCACCCAGAGGATGTC
CAATGGCCATTGCACCTCCATTAACATTGATCTTTTCTGGATCAAGTCCCAGCTTGTTAC
GGCAATATACAAATTGGGATGCAAATGCCTCATTTATCTCAAAAAGATCAATATCGTCAA
GTTCAAGACCAGCAGCCTTGACTGCAGCGGGAATTGCCACAGCTGGGCCGACACCCATGA
TGGCAGGATCAACACCAACAGCAGCAAAAGTCCTGAATACACCAAGAATTGGTAGGCCTT
TACGCATAGCAACACTTCTCTTCATGAGCAGTACAGCGCCAGCACCATCACTCACTTGGC
TAGAATTTCCAGCTGTGGTGCTCCCATCTTTCTTAAACACAGGCTTCAACTTTGCCAAAT
CTGATAATGATGCATTTGGCCGAAACCCATCATCAACTGTGATTGAAACAGGTCTCTCGT
CACCAGTTTTTGGGTCAACAATCTTGGTAGACACAGGGATGATTTCATCTTTAAATTTAC
CAGCAGCAGTAGCAGCTGCAGCCTTCCTATGCGACTCGAGCGCAGCCTGGTCTTGTTCCT
GCCTTGTCACACCAAATCGATGTGCAACATTTTCTGAAGTAATACCCATGGGAAGAAGAC
AATTCTGGGCTTGTTCAAATGTCTTCACTTTTGGATTGACTGATCCTTCCCAAGCCATTG
GATTGGCTGTCATGGATTCCAACCCAGCTCCAATACCAATGTCATAAAAGCCAGCTTTAA
TAGCAGCAGCTACATCAGCAACTGCCTGAAGACCAGATGAGCATTGCCTATTGACAGTTC
TAACGGGCACAGTATCAGGGAAACCAGCATAGAATGCAGCCATCCTGCATTCACTAGCTC
TCTGGGACCCTGGAGCCAAAACGGTTCCCACAACAATGTCCCCAACTTCACTCGGATCCA
AGTTTGTTTTCTCTATCAATGCCTTCAAAACAGGCGCCAGCAAATCGTCCGGGTAAGTAT
CCTTGAAACCGCCCCTTTTTGATTTACAAATCGCAGTACGATGAGCTGCCACAATCACCA
CATCGTCTCCATATACAGAAGCCCTCTGATATGCGGCGCTATCCCCAGCAGCGCATACTG
ATGCGTCGAGAGCGGACTGGTAATCGTGAGAAGAAGAAATAGAGGGGCGGAGATGCTGCA
GCAACACTTTCTGTCTGTTGATTGCTTTCTCCATTTTCAACGAGTCGGAACGAGTTTTGC
GATAATTGCAATTGTGAAGGAAAGGAAGAGGCCAATAATCTATTTAAATACATACCCTCT
AGAAGAT
>ntig_323_consensus
ATTTGATGAGTTCACTAGAAAGCATCCAAGATACTGTAAATCAATAGCCACAATAGAATA
AGGTTGTTGTCAAATTCGAATAGACTTGAATCAATATTATCCTATGGTTGTGAAGGGTTT
CTAAGACAAATACAAAACAAACAGAAGTTAGGCAGCTATGACTGGCAGTGACTATGGCCG
GCCAAGTGGACAAGAAATATTTTTCCTTCGGTTGAGAGCCACAAGCATGTGATGTGACCA
AATGGTCAATTAAAAATAAGTGGTCAAGGTTAGTCACCTCCTTTCTGACTTCACCACTAT
AGGGTCGTTTAGAAGCAGCTTCATAAATAAATTAAAACAATGGAGCCTGAATTTGTTTCA
AACACTTACCAGAAAGGCACTTATAAAGAGCATGGGATGTACAGCTTAAATTTCGATAAC
TAGTTGAAAAGGGAAAGCAAACTGAATAACATTCCATTATCATTCCACATTACAGAATCC
AGAGAACTAGAAAAGCACTAGTACCACAGACATCATATCAAACGAACTGGGAAATGACCA
TGATATAATTGCACCAACATATAAATAGTTTTTGATAGCATCTTCTGCACTGACATTAGA
AGCATCCAGTGTGCAGACAGTCAAGTTGCTCAGGCTTAGAAACCATGGATTTTAATGTGC
TCCTTCTTCACAATGCCAGCCTGGACAAGGAAGGTGGACACGTTCTTACGCTGGTCACCT
TGGAGTTGAATAACCTGTCCTAATTCAGGGTCCTGGACAACTGTACCATTGCAGCAGAAC
TCCTTCTTGAGGTCCTTGAGAATCTTGTTGTAGCTGAAGTCTTTCTTCAACCCCTGGACG
GTTGTCAGGCTTTTCCTACCATTCCTTTGCTGAATACGAATATGCACATACTCCTTTGTC
CCAGCGCCTGAGTCCTCAGCATTTGCATCAGCAAACGGATCAAAGGTAGAAGGAATCTGA
ATATCGAGATCAGACATGAACCTTGGCTGATGAAACGGGCCTAACAACCAGAACTCAGAC
CTGATCCGAATATTAGGGTTCAAGAATCGAGAGAGTCCGATCGATGAGATCAAAAAACCC
TAAACCGGATAAGTCCAATCTGGCGTTTGGTGAAAATGAGTGGAAGATCTAGAATTGGAG
TCGATGTAAAAGCGAAATTTCCA
>ntig_324_consensus

CACACATTTTCATTTACTCTCCGGTCAAATCAGCAGTCGACAAAACCGACAAAAGTTAAG
CAACTATGGCTGTTAGTAATTACAGTATGCGAAGTGGGCAAGTATTATTTGCGAGTTAAA
AGCATGCGCCAAAATGGCTACTAAGAATAGAAGAGGGTCAAAGTTAGCCATCTCCTTTTA
ACTTCGTCTATATAGACTATAGGCAAAGTTACTCCTGTACAAGCCTTTCACACATGAGCA
ACGATCATTTCCCTACGCAAGAAGCTAAAAATTGACCGATTAAGGTGCTGTTAGAACGTG
CAAGTAATGTACTCTTTAAATTTCGATAACAAGTTGAAGGGGAAAAGCAGACTACATAGC
ATCCCATTAGCATTCTACAATACAGAATCCAGTGAAATAGAAAACACAAGTAATACGAAA
TGCATACATCATATCACGACGATATAATTGCACCAACATATAAGTAGTTTTTTTATAGNN
NGTTGCTTAAAAACCATGGATTTTGATGTGCTCCTTCTTCACAATGCCAGCCTGGACAAG
GAAGGTGGACACGTTCTTACGCTGGTCACCCTGGAGTTGAATAACCTGCCCTAATTCAGG
GTCCTGGACGACTGTACCATTGCAGCAGAACTCCTTCTTGAGGTCCTTGAGAATCTTGTT
GTAACTGAATTCTTTCTTCAACCCCTGGACTGTTGTCAAGCTTTTCCTACCATTCCTTTG
CTGAATACGAATATGCACATACTCCTTTGTCCCAGCACCTGAGTCCTCAGCATTTGCATC
AGCAAACGGATCAAAGGTAGTTGGAATCTGGACATCGAGCTCAGACATGAACCTTGGCTG
ATGAATCGGGCTTAACCACCAGAACTCAGACCTGATCCGAATATTAGGGTTCGAGATTCG
GGAGAGTATGATAGATGAGATCAAAAGAAACCCTAAACCGGATAAGTCGAAGTTGGTTTT
TTGTGACTGAAATAATAAAAGGGAAAGACCT
>ntig_325_consensus
GAGCGCACCATCATATTCCAACAAAATGCTCTCTGTCAACATATTATAGCTAAACAAAAC
CACCAACTTAAACCATAATTCCTCAAAATCTATTCAAATCTTTTGAGAAATTAAAAACAT
TCAAATATTAAAGCCACAGATCAGGTTTTTTGCATCATCCTCCTATTTATGCGTAGCTTA
AGCTTCAGCAAATCCGAGCTCAGAGAGCTTGAGATGAGCATCTGCATAATCAGCAAAGAA
AGCATCCTCATCCGCAGCATATTTTTCAACCAATGGTAGAAAAACAGGGTCATTAAGAAG
GGTCTTGTCAGAAGGCAATTGGAGAAGCCCCTCCTTCTCTCCGGCCAAGAGTTCCTTAAA
GTAGGAGTTATCAAAGATGAGAGGGTTAGGAGTCCACGGTCCCTCAAATCCAGACCTCTC
CTTGTGACACCTTCCCAGGGTGTGGCCACCAGAGAGTGCAACAATATCCTGGTCACTGAG
ACCCATGGTGCCAAAGACATCCCTCAAGTGATCAGAACCCTTCGTGGCATCAGGAAGACG
TCCTTCTGGAGGTGGCTCAGGCTTGTCCTCTCTTCCAGGGTGGAAGGGGATTTCAGGTCC
ACCAGTGACCTCAACAGCAACAACACCGGCCAGCTGATAGAAGTCAGCATAGCTGAGGAT
TGGAAACTGCGCCTTGATCGGCTCCAAAAGCCTGACGGCAATATCAAGGCCATTGTTAGC
GCTGTGATCCAGCTCGGCACGATGCCTCATGGTCCCGAATGGACCGCCGGTCTTCGTCTT
CACATCAAAAGTACCAGCCGAGTGCCATGCCAGACGAAGCATGATAGGAGCACAGTTCTT
CTCAGCGATGAGACCTCTGAGCTTCCTCTTGCATTTCTCAACGGCCTTCTGGTATTCAGC
GCTAACAGTTGGGTAATTCTTGGTCATCTTGTCGAATGATTCTTGGAAGCAGTTACAGTA
AAGTGATAAGATACAAAGACAGATTAAAATCCCTAGCACACGCTATCACGAGCAGAGCAC
AAGCTCAGTCAAGC
>ntig_326_consensus
CCTGCTTCTTAAGATTGTTCAAGCTTGTTCACAAATCAATGCACTTGTATTCGTATAACA
CTCAAACAAGTTGTATTTGTATTGGTTAATGAAGCAAAACACTCAACATACGGAGGATCA
ACCTGCTAGTTTTATGTCATATCGATCATCATCATATACAAATACACACTTATCTTTACA
ACTCAACTTACCTAACCTTATCTGTTGAGTATCCAGAGCCGATTTTTAGGGCCCATCTTT
CAAGAGTTTGATCCCTCGTAAGCCTGCCCAATGAGGCCAGTTATGACTAAGTCCCATCCA
CAAGTTCTAGGCGAACAAATCCCACGAGAACTGTATGTTTCTTCTGTAATCCACCACTTC
CAACCAAGTGCCTAGAATTTAATTCCAAGACAAATCACATCCCAAATGTTCTGTGATATA
GATCTCCGGAACTAGGCATCCGAAACTTTGATTCTTCGTACCCAGTATAAAACTTGTTAA
AACCCAGTCTCTCATTTCTAAGGAGCTCTGCCATACGTAAACTGTTTCCACCCTGGACCT
CACTCCACCCATCCCAGTGGCCATTTGAAATCAGGGAATTTCTGGACTGTTGAATGGACA
GCTG
>ntig_327_consensus
TCTCTCTCCAACAATGGATTAATCCTCCAAATTTGGAGTTTCAATTTCGACCGAATTAGT
TTTGGAAGACACTCTGACTAGAATATTTCAAACCAGGATCCGGTGGACATGGAGGGGAGA
ATTGGTTGTACCCCAGACTGCCCGGATCCACCTTCCTCCACTGAATTGTAGAAAGACTTG
GAATAAAAATAGCGGCTTTTTCGTCTCACAAAGGCAAATGGCTTCCTACTTGTCTTCCAA
AAAGAGAGGAGAAACCAAGCAAACACCTCCCCTGAGGGGCCAACCAGGACACTTTTGGCG
TGTCAGGACTATCAGTCGGTTCTTTAAAAACCTACAAAATAGGAAGTTCAATCACAACCA
TGCCATTTGCAAATAACATGGCCAAGAGTCTAAATTGACATCTCTGTCAGGCTCTCTTCA
CTTTCACCAACTTGGACAAGAGGGCCAAAAATGGCCATTTTCTGCTTCAAGTTTTGAAGG
GCTTCTTTCTTGTTTGCCAATTCCATAAGAACCTTGATCCTCCGGAAAGAAATGTTGCTT

GGCTTTTGCCCTTTCTCTATCATCTCCAAGAAACACTTCTCTGCTTCCACCAACTTTCCC
ATATCACAAAGCAAATCAAACAACACATCAGATACCAATATATAAGACCCAAAACCCTTC
TCCACCATGTCATTCCACAACCGCAATGCCATCTTTACCTTTTCCTGCCTCCTAAATAAC
CGAATGAGAAACATACAAGACTGTGTGTTTGGGAGGCACCCACTATCCATCATTCTCTGA
TATAAATTCCATGCACTTTCCAAATTATTTGACCAATAAAAAACCCTAAAGAAGAGATTG
TAAGTAGTGGCATTTGGGTCTAATCCCTTGGGCACCATCTCATCCATCAAGTCAAATGCA
TCACCAAGTCTCTTTGCAATACAGTAATTCCTTATGGCAGAATTGTAAGCTGCAGTATCC
GGGTAACATCCATACTCCTTCATTTCTTTCAAAACATCTCTAGCTTTATCAGGCTGACCA
ACCAACCCCAAACCCCCAATCAAACTTGTATATGAGATAACATCAGGTGATATACCCTCA
TCTCTCATTTTCTCAAACACCTTATATGCCTTCTCTACTTGTCTACCTTTACAATAAACA
TCAATCAAACAATTATATGAAACGATATCCGGTTTTACTCCCATTTCCATCATCTCCTGA
AAGAAACCCTCAGCGTCCTCCACAGATTTCCAGCCAGACAAAAGTATATTAAAAGTCTGC
AAATTAGGCTTAAATTCATACTTTAAACTATGATACACATTT
>ntig_328_consensus
CTTCATCAGAACGCTGAAGTGTTTGCATTATAATATTAAGTATTCATACAACTGAAACAG
AAACTTCCAACGGCCATTTTTAGGCAATAGGCCATGGGGGATATTCAACTGTGGCTGAGG
GTGTATCTTAGAGAAGTTACAAAAGCCCAAAAGCATCCACACTACTACTGGACCATAAAA
GGACAAACCATCCACAAGGCAGACATCAAACTTACTCCCAAAACCAAAGACAAAAGAGTC
CAGACTTGAACTAAATCCAAGGTATATGTAAACGAAACAGATGACATCAAATGAGTAAGT
CAACAAGAAACGACCTTTTGCCATCAAATCTATAAACCAAAGCGCATTTCAATACAGAAA
TGCTTTGGTTATTACAGTGACTTCACCCTACTGAGAAAAAAGTGAAAAGATATGTGTGTG
ATAACTTCAAGCGGCGCCATTACAATGACCACCGAGCATAGATTAACCCAGTCGAACAGT
TCTCCCACACAACAAATCAAGGCATTGCCGCAACACACATAGTAGGTAGAGAAAAAGTGC
CTGCAGTCAGTTTATAACATTTCTTGCGATTTTCTCACACAGCGGCTCACCTTTTTCTTG
AAATCGTCTCTCCTCTCTCTCCATTCCTTAGCTGCTTCAACATTAGCAGGAGATTCATCA
TTAGGACTGGAAAGCATTGATATGATACTCAAAACAATACTTTCAACAGTATGGACGGGC
ATCCAGCGCTCACTTGCAAGCTCATAGCCATTAGGGTCATCACCCGGAGGATGAAGAATT
GATATGCAAACACGCCCATCAGGATATACATTAGGATGCCAAATCTCTGTAGTGAACTTC
ACTGTAGGAGGGCTATTAGGATAATTTGATGGAAAGCTCATAATGGCATTGAAAAACCCC
CCCTCATAAAGAGTATCCGGTGGTCCAATAATCGTAACGCTCCATTCAAATATATTAGTC
TCATCCACCAAGCCAGCCGAGAACCCAGCAACAGGATTCTTGCAAAGATCTTTAAGCTGT
TTTTGAAGGAGGAGGCTTGCCTGAGATGCCGCCATGGATTTTATTCAAAAAGAGAAGAGA
GAGCTGAAATGGCCCTGTAAATTTGTGAGTGGAGGAAAGAGACGTTGACAGGCGACAG
>ntig_329_consensus
GATACTAATTAATTAGTTTAGCTCATTTGACCTCCAGAAAAAATTAAAGCAGAAATACTC
CATAAACTAAACTAAAACCCCTCAAATATATGTTTAAAAAGCATAAATTTAACCCTTCTA
ACACAACAATCAAACATAGTCTCATCACCATCATCATCATAAAATAATATACACTCATTC
ATTATCATCAAAACTAAAAAGCCAACAACAACATGGAATCTAAATAGCCCACATCTTATT
CCGGCTCTCTTTCACTTCTTGAGGCTTCCCCGCACACCGGGCTTAGGTTTCGAAGATTCC
GTCACACGGCTTGCGTGAAGATTAACCAGCTTTTCTTCTTCAAAGGGGATCTTCGGGTGA
CGAGCATCGTGATGAATCTGCATAGACTTGAGATCAGGTGCCGTCGTTTTGCAGTGGGGA
CATTCGAACTTGGCGTGTCCCCCCTTCTCAATACCTGTCCTATCGGCCAGACCCGCTTTT
CCCCCACCGCGGTTGGTGGTTGCGGCGTCCACCTTCATCGCTATCTCCTTAGCCGTATGC
TTCTTTGGCTTCGCCTTGCCGGTCATGATGATGATGGGATGGGATTGGATTGGATTAACG
CCTGTGATTGAACGAATGAGAAGCGGAAAAGAAGTGAAATTTTGCAAGAAACCCTACTGC
CTTGATGTATACATATATATAGGATGCGAAGATGGGGAAGAATGAGATAAAAGAGAGAAC
AAATTTTGCAGAAAGGTGATAGAATCTCCAAC
>ntig_330_consensus
ATTCTGAAAATTGAAAAGTCTAAAAAAATTACAAAATAAAATAATACACAAATTATTAAT
AAATAATTAAATATAACACTTATTTATTTATTTTAAATAAAAGCACCATTTTATTCAATC
AACTAATTGATTACATTCGACTCACTCGGGAGGAGCCCATTGCCTTGGCCATGGCTCTCA
GCATATACTTTTTAATTTTCCCGGTAGACGTTTTAGGCAAGTCGTCTTTGAAAACGACCG
TTTTCGGCACCATATAGTGCGGCAACCTTGCCCTGCAAAACTCCATTATCTCCTTCTCAC
TTGGCCTTTTGTTCAACTCAGCCTTCAAGCTCACAAAAGCGCACGGTGTCTCACCCCAAT
AATCATCAGGTCTCGCTACAACGGACGCCTCATTAACGGCTGGGTGCATGTACAACACCG
ACTCCACCTCCACACTGCTCAAATTCTCTCCGCCGCTGATTATAACATCCTTCGATCGAT
CTTTAATCTCTATATAACCATCAGGATGCATTACGCCAACATCGCCTGTGTAAAACCATC
CATCGTTAGTCATACACTTGCCGGTGCCGACTGGATCTTTAAGATAACCGAGCATAACAC

ACCCTCCTCTCAAAACTATTTCACCAAGCGATGACCCGTCACGTTTCACACTCTCTCCAG
ATTCACCATTCACCACGTCAATCTCAGTAAAACCCACAATTTTTACTCCTTGTCTCGATT
TTAACCTCGCCCTCTCTGTCGCCGGCAGCACATTCCATTTACGTTTCCAAGCGCATGACA
CCACAAGTCCCGCCGTTTCTGTTAACCCATACCCGTGACTCACTATAAAACCCAGTGACT
CAGTCCTCGAGAGGATCGCTGCAGGCGGTGGAGCTCCAGCGGTGAGAATGTGTACCGGGT
TTTTAAGGGGTTCTGTATTAGGAATATTGGATAGCATGTTGAGAACCACAGGGGCACCAC
ACATGTGAGTGACACCGTGTCTTTTGATCAAACCGTAGATTACAGAAGCGTCGAATTTTC
TGAGGCAGATATTGGTCCCTCCAACCGCGGCCATGCCCCACGGGTAACTCCAGCCGTTGG
CGTGAAACATAGGTAGAGTCCACAAGTAGACAGCTTGTTTGGGTACGGACCAATCAATCA
GAGATCCAACAGTAATCGTGAAGATCCCACGGTGGCAATGGACTACACCTTTCGGAGCCG
AGGTTGTCCCCGATGTATAATTCAGTATCATCGGATCCCACTCACTTTGAGGCCTGACCC
ACTTGAAATTCGGATCACCTTTCTCCACCAGGGTCTCGTAAGTGGAACAAAAATTAGAAT
CCACCGTTGGGGCTGACGTGTTATCATCATCATCATCATCCGTGATGAGGATCAAGCGAG
GAGGTTTGGTATTAGACGGGAACAAAGAGACGGCTTCAAGAACAAGAGAACTGAACTGAT
GATCCACCAAAACCAGCTTTGATTCGCTGTGCCGCAGGAGAACAGAGACTGTGCGCGCGT
CCAGGCGCGTGTTGATGGTATTGAGAATGGCACCTGTAAAGGGTATGGCAAAGTGGACCT
CGTACATGGAAGGGATATTGGCGGCCACAACAGAGACAACGTGACCTGGGGAGACACCGT
AGGATGAGAGAGAAGAAGCTAACTGGAGACATCGACGGTGGGTTTGCGACCAACTGTAGG
TGGTGTTGTTGTAAATGATAGAGATGCAGTCACCGTAAACGGTGGCTGCTCTTTCCAAGA
AATCAAGTGGAGTAAGAGGGGAGGAATTTGCAGGGCGTGGCTTTAGATCATCCATCTGTG
GAATGGAGACAGCATTTTGGATTTGGTATGATTTTATATAATTATATATGAGATGCAGAG
TGGAGATAAGGATGCAAATTTGGG
>ntig_331_consensus
GGTTCATCACTATCCAACTTAACCCTTCTACACTCCGGCATATGCAAATTTTTGTTTTTT
TGCAAGAGCATGAAAAAACATACAAGGAAAGATTCAAAAAGGCATGGAATGTAGAGATAG
ACATTGGAAATGAACAAAATGATGCAAATGCAATAGCATTTCGTAATATAAATAGGTTTC
TGAATTCTATTAATCATAGGTTATAGAAGGCAAAAATTATACAATCATTGAGTTAGTCTC
TGCATCTCATTTTGGTCATTGAGGGTTTCCATGAGAAGGGGAGGTGCATATTGAGCTGTG
CCTTGGTGCTCCTCTGAGTCCTGGCAGAATGAAAGGATGATGGTATCAATGGACATCTCT
ACCACTCCAAAGAACAGAGTAGCGACAACATAGCCAAGACCCCAGCAAACCAACACAGGA
AACAACGGGGAGGATATTTTGTTATGAGCAGATCTGTATTTGTGGGTATCCAACATGAGG
AAAGCGAAAACTGCACTCGAAAGGCTGACACACAGTTTCCCAAGAAACAGAATAACATCT
CCAATCACATTCACCCGCCCTATGCGAAGAATGTTATTCATGATTAATTCTGTTGCGATT
GCAGAAGCCCTACAGAAGCTTTTTCCCATTATAGCAATC
>ntig_332_consensus
ATTGGGCTCGTGGTGAAACATCGCCAGAGATTCCATTTCTTCCTGTTATTTCCTCTATGA
AACGGCTGCTGCGTTACAGTCTTGGTTCTGTGGCTCTTGGATCCTTGATTGTGTCATTTG
TAGAATCTATTCGCTTTATTCTTGAGTCAATTCGCCGCAAAATGAAAATTGGTGGTACTA
CACCTGATAGCTGGTATGGAAAGGCAGCATTTTATTCTTCTCAGGGCTGTCTGAGGAGTA
TTGAGTGGACCATCAAATCTGTGAATCGCAATGCCTATATAATGATTGCTATAATGGGAA
AAAGCTTCTGTAGAGATCGGAAGCGCGTCGTGTAGGAAAAGACAGTAGTACTTACACCAA
TACGGATA
>ntig_333_consensus
GTGCTTGTAATATAAGAAGAATTTCTATTATTTTTCATTGTCTCTCTTAAAACTATTTAC
ATCAAGGCAAATTCTTTCTAAAAATCTAATAAATGAGTGGATAGAACTAAAAAACAGAAA
TCACAATGTTTTCCAACAACAGCTGGATTTGAATTTTGCTAAATTTCTGCGCTACTCATT
TCCATGGAGTCCCAAAGAAGCTAAATCCATTAGTTCACAAAGAGAAGAGGGGCAATGTAT
TTGAAGAAGTAGTGGAATAAGGATTTTCTAATAATATTTACAGATGAACTCTAAAACGTA
GTACAAACACTAGGTTCACTTATGAACATGCGAGGAGTCTTCATCAATCTACATCTCTGA
ATCCCCAAGCTAATACAAAATTGTTTAACAACTCAGCACATGTCATGTTGACATGTGTAT
AACGAAGACTACCTTTGAGCAAGGTGTAGATGGAGGAGGTGAATGATGATCACACTATCT
TGCTTTATTAGAATCTTCAAAGAAGTTGAACAAAACTTGTGGGAGGGAGGCAATCCTTGG
AAGACTGTGCAGCAGTTCTGCAACTGAGTCCTTTCTCAACATGGCCGGTTGCTGCTTGCG
GCCAGAAAGATTGCTCGACTCTCCCTCTGATGCACGAAAACCTTGCCCACCCTCGTTTTC
ACAAGCCGAGGCAAAGCCTGAGTTGGTTTCAGATATTTTTGTGACACCGTTTATGTCAAC
ACCTTCACCATTCTTGTCCTGCAAATTTTGTATTGCTGTAGAATCTTTCTGCAGAAGGCA
GCAAAGTGAATTGACCCTTGACATCAGAGACTTCTCATCAGAGGCTGATGTAAGTTGAGT
GTCACTAAACAGGTATTGAGTAATCTCCTCTAGAATGTCTCTACCCTGTAGGTCATCACC

GAAAATTAAGGAACCACCAGATGTCCTTTGTTCTGAAATGCAATGCCCAATGTGATTCAC
CAGATCACTCATAGACATAGACGCGTGAAGTCCCGGAACTTTGATCAAGTCACTATGACT
AAGCATCTTTGGTTCCTTGGCTCCACTGCTTCTGATTTCTCCAGTTGCACAGGTGTCTAT
TACTG
>ntig_334_consensus
GAGCAGGCGTTCGGATTCTCATCACTGAACGTCTGCAGAGCGTCCAAGCGAGGGTTGTGG
GGGTACATCCCATAGCCTTCGTTCACCTCCGGAATTGGATGATGCTCATGTTCCACGGGG
TAACTCTGATGCCCGTACACTGATGTGTCGTAGCAGCTAGTGGGAGGTGGTGGGTAGAGG
TTCGGGGCGTCACCGCCACCAGCCGCAG
>ntig_335_consensus
AGCGAGATAATTTAATGAAGAAACAACAGTGTGTTGAATCACCTAAATATTGTAATATAG
AACCAGAAAATGGTACCACTAAAAATAATTCCTTTGAAAAATCTAAATGAATGCAATACC
CAACTTCACGCTTTCTCTACAGAGAATAGAACCCTAGAATTCTTGCTATAATTCCTGCTT
CCCAAGCACAGGGCAGGATCTACAGGTGTAATAAAAGTAATAAAAAGAACAAACCCATCA
ATCTTCCTAGGTAAGTATGCGTGTTAGCAAAAACATGTGCATTCTTCAAAGCCACGCAAA
AGCAAACTAAAGACTAATTTGATAAATTTCCGCACCTCAATAATTGCCAGTAAGGAGGAT
GGCTTGAGACATGTGAATTATTTTAATGGTCATATACATACTCTGCAAGCAGGAAAATTG
CAACTGATAAAAGCAAAAGATCCTTGCTATTCATAAAGTAGCATGTAGGACCTTTTATTA
TCTTTACGTCTTTGCATGAGGTCTGAGTTCAATTTTTTATTGACACCTAGGCTGCATCAT
CATGCTCAGATGCACCAGGATCACTGCTAGTTGATTCTTTAACTGTTAGAAGGTAAGACC
ATGCTAAGCCTGCAGTGCCAAGGGAGATTGCAGCAGCAATTGAACCCCAAATCCATCTCT
GTTTTCTTTTCCTTCTTTGTCTATTACGCCGCGCCATCTCTACAGCTTCCTGGTTCCTCT
GCGACATTTCTTGATTCATTCCCTCATAATAATGAAGCCGATCCCACAATCGGGCAATCT
CAAAGTTCTTTGCTTCAATTTGAGCTTCCATTTTTGCCTCCATCTCAGCCTTTGCAATGC
CCCTCCCAATAGATTCTGATACAAATCGAGCAAGGTGAAGTTGTTGGCAAAGCTCTTCAG
AGATATTGATATCTGGTTGTCCATCCTCCCCTGCAACAGTGGGATCTGCAGGCAGAGTCA
ATCCTGCATAAGTCAACTGTTCCCTAACCCTCAGCCACTGACTTTTTAAATCCTTCAGAG
CTTCTTCTGCTTGCTTTCTCTTCTCTAATTCCATGAGTAAAGTCAATCTCATTTCACGCA
GTTCAGTCTCAATATCCTGAGGAGAAGACTGTGACCCACCCTCAGATGACAACTCTTCCC
AAGCATCATAAAACTCTCCCATTGGAGTAGCAAGCTTTGCAGAACTTTCCGTCCAGGCGT
TATCTTCTCCATCTGTGCTACTTACCACACTCATTGATTCCTTTGGATCAAAAAAATCTT
CAAACTCACTACTACTCTCTGTCTTGGATCCAATAAGCTTTGGCAAATCACTTTTCCCAG
GCAAACCACTACTTAAATTGCTGCTTTGAAGTCCACTGTTACTGCTCTTAAATTGCCCAT
TGCTGCTTCCAGGCTCACCATCATGCACACCATTCAGATGCTCCTTTTCTGTAGGACCAT
CACAGAAACCATTCACAAGTTTGTTTGCAGGACTATTCTGGACATCACTCCTGTGTTCCC
CTTCAACTGGTCCAGGTGTATTGAAGGAGACAGAAGCATAATTGGTTGAATCAACACTCC
TTGTTTCAGCATTTTTTGTAGCACTCTCAATGACCTTTCCTTCATCTTTTGGTTTTTCGC
ACAAGGCCTCATCAGCCTGTGAGAAACTCTTCACTAGCCGCGGCCCACGCCGCTTGTGAT
TGATGATGTAAGGTGAAGGAGGGAATGAAGTTGGTGAATCAGGAAGCGGAGTTGGCTTAG
GAGTTGTATAGAGTGCTGGTGTTATTGGAGGACGATTAGTTTTCCTCCCAATGACCACAG
CAGCACTATTTCTCCTCTCCGACTCCAAATTTGGATGAGCAGGTTTTGATTTAACCAAAG
GCTTTGAATTGGCAACTGGCATGTCAGCAGATTTGGACTCTCTAGGTTCTATCAGCCTGT
CAAGAGATAAAGCAGTAAATGTTGGCATGGCTCTCGAATACAAATGACAATAAGAAGGGG
AGAAAACCCTAGAAGCAAAAAGTTGCTGGAGAGATCAGAGACGAGTCGGCGACTAAGTGT
ATATATATATATAACGGCAATGTTTTAAATACAACAACGTAACGCGATTCTTTTTCTAGT
GGTTTCTTACGCTGTTTCCCCGACCTTCAAAG
>ntig_336_consensus
ATGATGCTAAGTGCAACAGAAAAACAAATATGCTATGCCTATAAAATAATCCAACCAATG
CACAAGCAACCAAAACCTAGTCACAGCCGGCCCATAACATTGGGGCTAAAAAATAAAATC
ATCGAAGCCTCTTTTGTGTGACCAAACATAAGCAGCCTCTAAAGGGCACAGAAGCGATAA
TTACTACTAATCCAATTCAAATTCCAGAACCTCTAAGAGTGACAGTAAGAGGCATAACTT
GAGATATTTGAATTATTTTAACGGTATATACATCCTCTGGAAGCAGGAAAATTGCAACTG
ATAAAAGAAAGAGAACCTTGCTATACACAAACTAGCATGCATGACCTTTTTATTCTCTTT
GCATGAGATCTAACATCAATTTTTGATTCACTACTTAGCTGCATCATCATGCTCAGGCAC
ACCAGGATAACGGTTAGTTGATTCTTTCTCTGTTGGGAGGTAAGACCATGCTAAGGCTGC
GCTGCCAAGGCTAATTGCAGCAGCGATTGAGCCCCAAATCCACCTCTGCTTTCTTTTCCT
TCTTTGTCTATTCTGCCGTGCCATTTCTACAGCTTCCTGGTTCCTCTGTGACATTTCTCG
ATTCATGGCCTCATAATAGCGAAGCCGATCCCACAATCGAGCAATTTCAAAGTTCTTTGC

TTCAATCTGAGCTTCCATTTCTGCTTCCATCTCAGCTGTGGCAATACCCCTCCCAATAGA
TTCTGATACAACTCGAGCAAGATGAAGTTGTTGGCAAAGCTCTTCTGCAAGATTGATATT
TGGTTGTCCATCCTCCCCTACTATAGTGGGATCTGCAGGCAGTATCAGTCCTGCATGAGC
TAACTGTTCCCTAACCCTCTGCCACTGACTTCGCACATCATTCAGAGCTTCTTCTGCTTG
CTTTCGCTTTTCTAATTCCATGAGTAAACTCAATTTTATTTCACGCAGTTCAGTCCCAAT
ATCCTTAGGTGAAGACTGGAGCCCACCCTCGGATGATAACTCTTCCCAAGCATCATAAAA
CTCTCCCTTTGGTGTAGCAAGCTTTGTAGAACTTTCTGCCCAGGTATTATCTTCTCCATC
TGTGTTACTTGCAACACTCATTGAATCCTGTGGATCAAAAAAATCTTCAAACTCACTATT
ACTCTCTGACTTTAATCCAGTAAGCTGTGGCAGTCCGCTTTTCCCAGGCAAAGCATTATT
CGAGCTGCTGCTTTGGAGTCCCCAGTTACTGCCCTCAAATTGCCAATATCTGCTTCCAGG
CTCACCATCATGCACACCATTCACATGCTCTTTTTCTGTAGGACCATCATGGAAACCATT
CAGAAGTTCCTTTGCAGGAATATTCTGGGCATCATTGTTTTCCCCTTCAACAGGTCCAAA
AATACTGACAGAGACAGAAGCACCATTGGTTGGATCAACACTCTCTGTTTCAGCATTTTT
TGCAGCATTCTCAATGACCTTTCCTTCATCTTTTGGTTTTCTACACAAGGCCACACCAAC
CTGCGAGAAACTCTTCAATAGCCGTGGCCCACGCCGCTTGTGATTGATGATGTAAGGTGA
AGGAGGAAATGAAGTTGGTGAATCAGGAAGAGGAGGCGGCTCAGGAGTTGTGTAGAGTGC
TGGTGTTATTGAAGGTCGATTGGCTTTCCTTCCAATTATCAATGAAGTGCTATTTCTCCT
CTCCAACATTGAGTTTGGCTGAGAAGCAGGTTTTGATTTAAAAGGAGGCTTTGAATTAGG
AACTGGCATGTCAACAGATTTGGACTCTTTAGGTTCTATCAGCGTATCAAAAGCTATGGC
AGTAAGCGTTGGCATGGCTCCTGAAGAAGAAGAAAACAGAAAAAAGGGAGAAAACCCTAG
AAGCAGTAAGTTGGTGGAGAGATCAGAGACAAGTCGGCGACCAAGTATATATAAAACCGC
ATGTTTTAAGGAAACGTAAAGCCAATCTTTTTCCTCGTGGTTTGCTTCGCTGTTTCTCTG
ACCTTCAAAAGTGTATGGGAACCGACTCCGCCAAGCTTTTATCGCAACGACCGCCTCCCT
ATCTATATCTCGCTTCTTTTCCCCCCGCAAATGTTTGAGTTCCTT
>ntig_337_consensus
ATTACCATAGACTCCTCGCCTCTTTGCCCATAATTTGACACATCGCAGCACTGATTGGAA
TTTCTCCAAGTCTGGAACAAGCTGAATAATATGCTGATTTGCACGTACTCCAGACAAGCT
TTTCCAACTAGTTTCATCAATATCCCTTACAAAGAAGGGATTGAGTACATCCACATCATC
AGGAACAGATGATACTTTAAGCTGCGCATATGGAAGATCAATCAAAACCCCATCAAATTT
AAACCGCATCAGGGGAACTTTTGCATCCTTCACGCAGTGGATCTCCGTAATTTCTGATCT
GCTCTTAAGCATATTGCGCAAAACAATGAAAAAATCCTCTGTCATTGTAGCAAATTGAGG
GCCAACACATAAAGCATCGATATCTGACTCTGAACCGTGAACACCAAGACCATAAGAACC
GTAAGTCAATATCGTTGCATGAGTCTCTGCAATTTGTTGTTTGGGAAGTCGACGTTGCCA
AGCAACCTTCTTAATCCATGACAACACTATCTGTTTAAGCTTTTTAATCACACTTTTTCT
TTTCTCTTCTTCCTCTCTTGCCGGCACGAGTCCTTCATTAGCCATCAACTGAAGAAGTGA
CATCGATCTTTCCTCGTCCATTTGAATCAAAAGAGAAGGATTCAACGGAACACCGAAAGG
AGGAACAACAAAGAGTAAAGGATTGTAGGGTAGAAGAACTTGATCAAAATTAGGATTTTG
CACCACGCCGCAACTGGAGTTCGGGTTTAAGGGCCCAAAATTCACGAAATTAATTTGATT
GTCGTTGTAAGCATAAGCTGCCATACAAAACTTTTTTTTTCTTCAAAACAAAAATGGAAA
TAAAATTTCTGCTTTCCGGTTTTTGTTTTCCGTCTTTCCGAAATTTTCCCTGTTTTCGAC
AAATGAAAACAGAAGATAGCAAGTACTGCGTATAGATCGTTTCCGTTTCTGCCTTTTTCT
GGACCCTCAAGAACGGAGA
>ntig_338_consensus
TCAGCTGCACTGAGGACCTTGGGGGTAGAACACCCTCCAATGCCTTACACTGATCTGGGG
TGAGCGATTCAGGGAAGTCGACGGTGAAATGAATGTACAGCTTACCCCTCATGAATGGTC
TTTGATACATAGGCATTCCTTCATCATTTATGGCCTTAAATTGGTCAGGCTTTACCACTT
CCCCAGGATTGGATTTGATTAGGAGCTGCCTTCCATCCAAATGAGTTAAAATAAATTGGA
AGCCACAGAGAGCCTCAGTTAGAGACAGGGTATGTTCAACAAATAGGTCATCACCCTTTC
GCTTGAACTTAGGATGCTCTTTTTGTTGTAGAACAAAAACAATGTCTCCTGTGATAGTTT
CAGGAGCTTCATCAGCTTCTCCAGGGAATGTAATCTTCTGTCCATTTTGCATACCCTTCT
CAACTACCACTTCCAGCACCTTCTTTTCTTGAACAACCTTCTCACCCTTACATGCTGTGC
AGCGATCCTTGTCACTGATAGTTTCTCCAGTACCTTTGCACTCATTGCAAGGATGCTGCA
TTTGTTGTATCATAGAGGGGCCAAGGTGCCTTATTGAGACTTTCATTCCAGATCCTTGGC
AGCCAGAACATTTCATTGATGCACCAGATTTAGATCCTTTACCTTTACACTTGGCACAGA
TTACGTTGCGAGAGAGAGAAAGCTTCTTAGATGTCCCATTGTAGAGGTCCTCCAGAGAAA
CCTTGAGGGGATGGATTACATCCTCTCCCCTCCTCTGCCTGCGGCCTCTGCTGCTTCCAC
CACCACCAAATGGGTTGCCACCAAAGAATGACTGGAAAATGTCAAACGGGTCATGGCAC
CACCACCTCCACCCATTCCCTCCTTGAGAGCATCCTCGCCATACTGATCATATATCTCTC

GCTTCTCTGGGTCACTAAGAACCTCATAAGCTTGTGCCAGTTCTTTAAACTTCTCAGGAT
CACCACCCTTATCCGGATGGTTCTTGATAGCGGCCTTCCTATACGCTTTCTTTAAGTCAT
CTTGAGAAGCGTTCTTCGACACTCCAAGAATCTCATAGTACTTCGTGTTGTCGCTTTTCT
TCGGCGGTCTTCCAAACATTTTCTCCTAACAACCAAACCGAGTGGCCCTTGAAGAGAGAA
GAGATACGGAGATTAGAGAAGCGAGACGGCAAGGGAAAGAGAGAGAGGTTGGAGTTGGAT
CAGGAAATTTATTTGGCAGAATGAACGGTCAGGATTCAACTAATGCCTTAAGGTTGGTTT
TAGACGTTGAG
>ntig_339_consensus
GTCGCACATTTCTTGAACAACTTCAACATGGAGAACCATTTTTTTGCCTGGAATTGCCTA
GGAAATTATGAGGATTGCTGCTTCCAAGAAGTTATAGAACTAGTAAACAGTTTCTTGGAC
CATCTCTGCTGCATTTAACCAATACAAAACATTCGAGAGAATTTTGGATTACTGTGCGTA
CACTAGAGAGAATTTTGGATCCCTGACCTAAGGGCACTCTGCTGCTGTCGCAACTGGTTA
AGACGAGGTAACATGTGACTTGAGGTCTCTTCGATGATGCCGCCAGAGCAACATTATCCA
ATTGCTTGATCAAAGCCGAGATGAAATTGGTGAGGCATCCATCGACATCGTTCAGTTGTG
GCTCCCTAATCTGTGTTATCACTAGCGCATTTAGAAATGAAATGAAAGAGCTCTGGAAAA
CATTCCATTGGACTACGGTCTTTGGCTGTCTTCCGCTTTGCCATTTTCTTTCTGTGACAA
TTTCAAAATGTTAGCTGCTCCAACATTGCCAAAACATAAGTTACTTGACTCAACTGGCTC
AATTTTTGTTTCCTCATCGACAATGGGCAGCTCAACATCTAAAGACTTCTAAATTGAAAA
ACTCAGGAAAAATTACTGAAGTTACGCAATAAAACAGTATGTTTTAACGTTATATGAAGA
AGCAAAATGCTAACAGTTTCATACATTCATCAGTTTTTTGAATATTAATAGAAAAATCAC
ATGCCAAAACTGACACCTCTAATCCTACAACAAAAGATAAGTTACTTGAGAATCTTATTA
TTCTCCGAAATCGTCGACAACCCTTTCTTCTCCATACCTCTCCCCAGTTAATCAGAACCA
ATACTGCCACAACAACCACTATCAATTACCGAGAGATATGGTCATCACTGCTGAGCACAT
TGCACTCTCTGGGCACCACCATGCATATCGTCATCTTCGTCGTAGGCCTCTTGGGCAGCT
TGCTGCTTCCTTCGCATCTCTTCCTCAATATTCACATCATGAAGAGTGGTTTCTTCACAC
TCATCCAGCTCCATATCAGTCAGCTGCACTGAGAACCTTGGGGGTAGCACAGCCTCCAAA
GCCTTATATTGGTCTGGGGAGAGCGAGTCAGGGAACTCAACTGTGAAATGAATGTACAAC
TTACCCCTCATGAATGGCCTTTGATACATAGGCATTCCTTCATCGTTTATGGCCTTGAAT
TGGTCAGGCTTTACCACTTCCCCAGGGTTGGATTTGATTAGAAGCTGCCTGCCATCCAAA
TGAGTTAAAATGAATTGGAAGCCACAGAGAGCCTCCGTAAGTGACAGAGTGTGTTCAACA
AATAGGTCATCACCCTTTCTCTTGAACTTAGGGTGCTCCTTTTGTTGAAGAAGAAAAACA
ATGTCTCCTGTGATAGTCTCAGGAGCTTCATCAGCTTCTCCAGGGAATGTAATTTTTTGT
CCATTTTGCATACCCTTCTCAACTACTACTTCCAGCACCTTCTTCTCTTGAACAACCTTC
TCACCTTTGCATGCTGAGCAGCGATCCTTATCACTGATAGTTTCTCCAGTACCTTTGCAC
TCATTGCAAGGATGCTGCATTTGCTGTATCATAGAGGGGCCAAGGTGCCTTATTGAGACT
TTCATTCCAGAACCTTGGCAGCCAGAACATTTCATTGATGCACCAGATTTAGATCCTTTA
CCTTTACACTTAGCACAGATTACATTACGTGAGAGGGAAAGCTTCTTAGATGTCCCATTG
TAGAGGTCTTCCAAAGAAACTTTGAGGGGATGGATCACATCCTCTCCCCTCCTCTGCCTG
CGACCTCTGCTGCTTCCGCCACCTCCAAATGGGCTGCCACCAAAGAATGACTGGAAAATG
TCAAATGGATCATGGCCACCACCACCTCCACCCATTCCCTCCTTGAGAGCATCCTCGCCA
TACTGATCATATATCTCACGCTTTTCTGGGTCACTCAAAACCTCGTACGCTTGTGCCAAC
TCTTTAAACTTCTCAGGATCGCCACCCTTATCCGGATGGTTCTTGATAGCTGCTTTCCTG
TACGCCTTCTTTAAATCATCCTGTGAAGCATTTTTTGACACTCCAAGAATCTCATAGTAC
TTCGTGTTATCGCTTTTCTTCGGCGCTCTCCCAAACATTTTCTCTCAACAAACCGAGCGG
ATCTTGAAGAGAGAAAGAATACAGAGAAGAGCCTCGAGACGGCAAGTGTAAGAGAGAGGA
AGAGCTTGTGGTTGGATCGGGAGCTTTAAGGGAGAGAATCAACGGTCAAGATTCGATAAT
GTTGTGAGTTACCCAG
>ntig_340_consensus
TCCATGGGTTTGCCTCTAAAACATCATAGCTACTGGTGTCGAAAGTCTGATGTAAATGCC
TGCGCATATCATAAGCACGGGAAGATACTTGCCTTACAAACTCGTTAGAACTCCCAGGAA
TGGTTCCGTCCAAGTGAAGGGCTCTCTCAATTTCTTGGCTGTCTTCATCATCTTTATTTC
CCGTGCTGGCTTTACAATAGAGTTGTGGAAAGCTACCAGTACGGCGTCCAGCCAAGGAAG
AAGAGAGAGCAAAAACACGATTTTGAACAGTGGGAGAAGAGAAACGAAGACGGAGGGGAT
TCCTGGGAGGGAGAACTATGGAAGGGGAGACAGATGAGAGTGAATCCATGGTATGGCGAT
TTGGTTCACCTGCCTAACTAATTATGATCACCCGCTGAGGACTTTTTTCGTTTAAACAAC
ACGTGGTTTGTGCGGAAATAATAAGAGAAATTCTCGGCGTGCAAGCAGCTGAGTAACCGC
CAAATGAAGGAGGAAGAAGGAAACTTGGATTTGGTGGCTACATCTGGATATTTTGTGCAG
TGGGGCCTTGGAAGAGTTATAGTTTGGCCAGTGAGAGGCGACGAGTCAAACTTGGAACCG

CAAAAATTTCAATGTTGGGAAGCGAATAATGCCAATACGATCAGGTGGTGCCTTGTCCAC
GTGTAGCTGCCCGGCCTGTTCCTAAGATCTTGCCCACAGCCTAACCCGTTAAGCTCACGA
GCTTACTTTGTACCATATCCGACGGCCTGACCCATTTGACATCTTGCCTTCTTTCCTTAC
AACCCCAAAAGTTAAGGGGCATCTTGAGATTGACCAACCCATCCCGTTCTGTAAGCTTTA
AACCCTTCAGTATGTTGATTCCCTTCTTTCACGCCCCATTTGCCTCAAGACGTCCCAGCA
AGTATTAATATTAATCATTTGGGATCCTTGCTGTAATCTAATGAATATCCAAGAATAGAG
GCACTGATGCACGGTATGCTATGTTAACCTCCTATTCTAGCACCTTAAGAAGCCAAGGTT
TCACTTGATGGAAAAGAAGTCATTTGATCGGATCCCAAACCTCCACCTGTTTCACTCTGC
CTATTTTATGACTGTTCTGAATCTTGATCAGAGAACACCTCAGATTGCAAAGATGGAACA
ATGTTATTGACGTGGAGCCATGTAAGTTCCCATAAGCTATCTCCTCCTAATGCACTTCTC
TGTACAAGC
>ntig_341_consensus
GCCAGATATTTAAGTTATTGAAACTAGGAAGAATTGTAGCGCCAGTCGTCTGACTCTAAA
CAAAGCGCCTGAAACCCTGATTCTTGAACAAGCACTTATCCTAAGGAGGTTTTCAAGCAA
CCATTAACAATTCAACAAGGTTGTACTCATACAACAGCAACATGTACATCAGACGCTTTC
TCTCACAAAACTGTTCAAGACTACAGTAAATGAAGCAATAATCATAAAACATACGACCGA
ACTGGAAACTATAAATAACATACACCAAACATGCTGTCACTCACATGCCAATTTTGTGCC
AAAGCTTGTAAGGCAAATGGCAAATGCCTGGAAGGCTGACAAAGGCTGTCTGAAATCCAT
AGTGAAAGTATCATCTGCCACCTTACCAAACTGGAGGACCGTTTCTTCATCTCCTTTACC
TCCTGGTTGGCTTTGGTCCACTGTTGCTACCAGCTGAAAGTTCTTTACTGATGCAACCGT
TACCCGTCCATGGAAATTCAAGCACCAGCACTGCAAATGCTCATGCCACCTAGGTGATTT
ATTCCTCAAAACTGTGTACCCAGAAAAAGCAGTCTCTGGTTTCTTCATCTTGGAATTGTC
CAGATACTTGTCACCAGCAGTTTCTCCTGACGTTGGGCACTTCAGCCTGCAAACCATTCT
CCTTGGACCTCTGGACTTTAGGAGGTTAAACTTATAGGAGACTTGTCCAATCTCAAAATT
GCCTGCTGGTAATTGGGGGCTTATTTGCTTGCTTGCAAATCTTCGACTAGACCTATTGCT
TGAAGGCTTTGCACCACAATGTGGTGGCTGACTATCATAGATTGTAAAGTTTGTGCCAAG
AAAATCCGAACTTAATTTTCCAACATAGGCATTACTTCCTTGTGATAGATCATCAGAATC
AAGTGATATAATATACTCGATGTGAGCACCACACCTATATCTCCGAGCTGCTAAAAGAAA
CTTTCCCTTATCGTTGAGTGATGGCGTAAGAGCAAGATAAAGGTAAAAGGTTGAGGTCTT
CTTATTCCTCCTTATCAGACAATGATGAGGAAGGTCACGTGGACCAGGCTGCTTAAGGCA
AGAAGGAAAAGTAATTTTTCCATTAAGAAGAGGAGACCCAACAATATCTTTCGCAATATC
TCTCCATCTCTTACAAACGCCGGCACAACCGACGACGCTTTGGCGCTGCGGCCACCGATC
CTCACTCGCGTCCACGCGCTTAATTATTTCTCCCAACAATTCCGGTAGCAACCCGGCCCA
GGAACCGGAGGCCGAGTCCAACTCAATCAAGTCGTCACCGGCGCCGCCTAGCCGCGTCTC
TTTCACCGACTTCTGCGTCTCCCTGAAGGATCTCGAGATCCTTCGAGAGAGCCACGACCT
TCTCAGAGACATTCGCCTGTCTAAATTTTTTTTTTTTTAAATTTT
>ntig_342_consensus
GGGTAGGTTGAACAAAGAAGAAAGTTGCAATAAAAGGTGCTAATTGAAACAGTTACCTAG
GAACCAAAGAGTCTAATCGAAGTAAGATTATCAGATAGAAAGGATTTTGAAATCGAAATC
AAGGAGATGATGAATTACAAGAATAAACAGAGATCTAACGTACTTGTGAATGGTCAAATC
AAAATTCTACCGTATATTACACATGGTATCTAACACTAAAGGTCCAAGAAATTTTGCTTA
AGTCCATCACTGAGAATCAACAGCTTTACCCTGAGAGATTTAGATTATACTGACAAGTTA
TCACTACAGATTCCAAATCTACAAAGATGTGATTCCTTGCAGCCCAGTAGCATAAATAGT
AGTTAATTGCTCCATTCCACGCCAAATAAGATTGCAGGAATGCATTCACCTCAAAATATA
CTCCCATATTCCTTCTGCTACCCATTCAAGTCAAATTCCATCCATGTAGCCACACCATGT
TACACTAACCTTGCCCAACTCCACATCAATCTCAAATGAAAATCTGGAGATTGTTGAACC
ATCAGGGCCATCCACCACACTAACACAAAACAATACACAGCCCCATACGACTCAAACTAC
CAACCCAAATCAAAAGCTAAAGAAACAACCCGATACCAACTACCACTACCAAGCTAACTC
TTTATTTTCTTCAACTGTATATACAGTCTAACACTACCCACTTCAAAAACTAAAGTTATT
TACAGGAGTACGCAAAGCGCCTTCTACTTCATTCATCAGCATTGTCAAGATTGAGAATGC
GCCGCAAAGTCTTTGTCGCCGAGTCAGCACTAAGAAATATATCCCTAGTACAGCCATTAA
GCTCCCTCGTTGGTGATGAGGGTGCAGATTTAGCCATTGGGCGAACCATTTCAACGGCAG
TCTTGTCAGCCATTACTGGCAAATCAAGTGAACTGGTGCGTTTGGGCCTCATCGAAAACT
TAGGAATCGGCAAAGAGCTCGGAGGCGGTGAGTTGGAGTAAGCTGGCCCAGCCCACAGCT
CAGAGAATGAGAAGGATTCAAATTCACCGAGGAGTTTCCCAGTGAAAGATCTCTCATTTC
TTGGAGTTTTCATGTTGATCGCAATAGG
>ntig_343_consensus
CTTTAATCACTAATCACATCCAATCAGGAAAATCCTGAATGACAGTCTATTTCATAATTA

CTAATCTCTTAAATCAAAATTTTAACTTGATATGAGAAAACTCCACCTCATACTTATGAC
ACCTTATTACATAAGAAACATCCTCTTATTGCCACTCAAGAGGACAGATTTGCCCCAAGT
TACTCTATCGCATTATCATATCTGCACATTGTGACATATACTTCATCATTACTTACGATT
CTATAAGCACAGCTAGACACCCAATGCCCAGACTTTAACTTCTACATATTCCAATCTACT
AAATTAAGCATTGCTCCCATCGGATTTTACGTGAGCCAAAACCCATTGCTGTCCATATCA
TTATTTGGGACAGAATCTTGCGCAAGAACAATTGCATGCACTACTATAAAACCGGTTATG
CAGTCAATAAAGAAACATCGCATATTATAGGTTCTCAAATAACAGACACAATAAAGATAC
TCATCTCATAGCCATCACCTTTAAACCTACTAAAAGCATCGTATATATCTCCTTGGACAA
CCAAGTATGACATCACATAAATGCGCAAATGTAAACAAAAAATATTACCTTCTAACCCAG
CAGAATAAGTAATGGACCAAATCGGCTTCAAACAATAAAAAAGCCTACGTCCCTCCCGCT
CCATAGCTTTCTTCATCATATTCTACCCCCATGAGAAAATATCATCCAGAGGCAGTGATC
GCCTGGCCCAAATAGAGCCAAGATACAACTGATGTAATAGTGTAATATAGTAGTCTTCTA
TCTGCTGCTTCAATCAGCCAGTCCAGCCTTGGCCTTGAGGTGCTGCTTGAAGTCCATAAT
GTCACCCTCCCAACGAGTAACAGTTTGATTTTCACAAACCCATATCTCATGGGCTACCTG
ATTTATTAGCCTGAAGTCATGGCTAACAAGAACCAGACCGCCATCCCACTCATTCAATGC
CTCAGCTAGGGAGTCAATTGTCTCAATATCCAAATGATTAGTTGGCTCATCCAAGAGAAG
CATGTGGGGCTGCCTATACGCTAACCAGGCAAAAATCACACGGCTCCTTTGCCCATCGGA
CAAATTCTTCATTGGCATCACCTGGGCTTTACCTGTCAATCCAAATTTCCCGATTGCTGC
CCTCATTCTCTCTTCCTCGTTGCCTGGGTACTCTCTCATCATATACTGAAGGGCAGACAG
TTCCAAATCGAGTTTCTCAGCTAGGTGCTGATGGAATTGTGCAATTCTCAGGTGATTATG
ACGCCGAACCATGCCATCAATAGGCACCAAATCTCCTGTCATCAGCTTCAGCAGCGTACT
CTTCCCAGCCCCATTTGGTCCCACCAAAGCTACCCTGGAGTCTAAGTCTACCCCAAAATC
AAGGTTCTTGTAAATCAGATTATCAGGTGTGTAGCCAAATGTGACTTCCACAAACTGCAG
CACTGGTGGCGGTAGCTTTCCGACATCAACAAAACGGAAGACAAGTATCTTGTCTCTCAC
AACCTTCTCTGTAAGCCCACCTCGCTCCATCTTTGCCAGTGTTTTCTCCTTGCTCTGGGC
CTGGCGAGCTAGTTTTGCTGACCCATGACCAAATCGGGCAATATACTCCTTCATTGAAGA
TATCTGCTCTTGCTCCCACTTATACTGTTTCATCTGGTTCTCTTCCAGTTCAGAACGGGT
CTGAACATACTGATCATAATTACCAGTGTAGAGTTTCAATTTCTTGTTTTGCATATGAAT
GATGTTTGTGCAAACACCATTCAGGAAATCCTGAGAGTGTGAAATCACAACCAAGATGCG
CTCAAAATTCTTCAGAGTCTCCTCCAGCCAGACACAAGCTTCTAAATCTAGATGATTGGT
GGGTTCATCAAGCAACAGGATTGTTGGGTTCATGAATAGAGCCCTTGCCAATGCAATTCT
CATTCTCCAACCACCAGAGAAATCTCGAGTTTTCTTTGCTTGCATCACCTTGTTGAAACC
AAGACCATACAAGATTTCAGCAGCACGTTTCTCTGCAGTTGATGCATCCATGGCTTCTAA
ACGCTCATATATGCGTTCAAGTTGCTCTCCACCTCCATCATCCTGCCCTGCCAAGGCTTC
AGCTTCTTTCTCCAATTGCAGCCTTTCCTCATCACAACTTATGACAGCTTCAAGAGCAGA
CATGTCAGAAGCTTCAATCTCCCTTGTAAGGTGATAAATATCCATGTGGTCTGGAATTGG
GAGCTCACGACACCCTATAGCAGTCAATAGAGTAGATTTCCCACACCCATTTAAGCCAAG
CAAACCATAACGTCTCCCATAGTTAAGCTCCAGCTCAGAATCAACTATGAGATCATGTCC
ATGAAAAGTGACTGATAAGGACTCGATCCGAATATCTCTGGAGAGAGGATGCGAGCAAAG
AACTCCAGTACAAGTTCGATCGGAAATTTGAATCGCTCCAATCCCGTCCGCAACTTTATC
ANNNNNNNNNNNNNNNNNGCCGCCTTCTTCTGAGCGGCCTTCTTCTTGCTTGCCTCCGAG
ACCATCTTTGTTAAGAGCAGAGAAATGAGATCTAGGGTTCAAGAGTATGGATTTTCGGTG
CTACTTGAGAGGAGAGAAGCAAAACCAGATTGTGAGGA
>ntig_344_consensus
CCACAATCTTATGTTATTTTGATAATCAAGCTGCTTATTTTTAAGAATTTTGGATGGGAA
ACTAAGTAAAAATTATGCCTCCGAAATAGGGATCAAATGCAAACTTCCATTATTTCAATC
ACAACAGGGCGACAAACCAAGACCATTCACTTAAATTTACAAGAACATAAATTCCATTAA
TGGATTGCAACCTCTTTCACATCCTTTTTCGGTTTTTCAGTCCTGGGAATGGTGATATTC
AGCACACCATCCTTCAATTCAGCTTTGATATCATCAACTTTTGCATCATCAGGCAACAAA
AGGCTTGTGTTATAGTAACCATAACTCTTTGCTGACCAGAATTCGTCATCTGAACCATTT
TCTTCCTCTTCCTTGTGCTCTCCCTTTATCGTTAAAACTCCGTCGTGTACGGTGATCTTC
ACATCATCTTTTCCCAGCCCCGGCACATCGTACCGAAGCTTGTAGCATTCATCCTGTTCT
TTAACTCTGCCCATCAGCTGTGAAGGCGATATGTTCAGGTTCTCGAATAGCCTATTGATG
TTTTCTGTCGCCTGCAATAGCGCACTTCCCAACCCTGACGGGAAAAGTTCATAAAGAGAA
GGAATGTCATTTTGCCTCCAGAGACTCTTCCTGCTGCTTCTCCTTGGAAAAAACCTGGAC
GTCTTATTCTTTTTAGTCACTTGGATCTCTTTCCCTTCAGTTTTCTCACCATCTCCGGAA
GTTGCAATAAACCTCTTCAACAACTCATTACTCCACCTTTGCCTTCCAACAGCACCAACA
TAATTATTATGACTCACCAAAGAAGAAGATGATGAAACCTTTCGCTGCAAATTCCTCAGG

GCCAAACGAGCCAATGCCATTGATATCAACTTCCAAGTTCAGATTTTTTATCTCTTTTTT
TTGGTTGTCTTGTTGATGAAAGTGAAGGGAGACACAGTTTTTAAAAGGGTGGAGAGTTAG
TTGGAAAGTTGGGTTTCTAAAGGAGTGAAGAAGTCTCTAAGATGTTCTGGGACACATCAT
TATCTTCATCTGGAGATGATTC
>ntig_345_consensus
CACAGTTTTCACAAGCCTAAGGTGACATAAGTACTTTTCTTAATTCATGACTCTTCCCAG
CGAGGCATCCTGAACTCGCCAAAGAGGTTATCCAGACTTCATCAGACTTCACTGGCTCAT
CGTCATCGTTTCGGTGCCAACTCCAAAATGCATGAGTTGAATTCACAATCTTGAGTTCCC
CATGTCCAAAGCTTGCTTCCACTCAGGCTGCGGATTTTTGTACTTGCGTGCTAGACCTTC
TTTGTTCCCTCCATCTCCAATGGTTATGTGGACAGCACCACAAGGATCAGATTTTCCATT
GTTAATCCGTTTCGATCTTTCATAAGCATGCACATGACCAGACAACACCATATCAACGCT
AGCAGCATGGAGCAATGGTTCCATGGCTGCCATCATCCCGTCCCCTTCACCTTGATGTGC
ATCGTTGCTGTTATACCATGGCACATGGAATAGAACAAGCAGCCATGGTGTTTTATGCCG
GTCAACCTTTGAAAGATCGTCCTTCAACCAGTTATATTGATCTGAGAACTTATCATACTC
TGTATATGAACCAAGCATGATAACATGAGTCCCTGCAACTTCAAAAGAGTAATAAAGATT
GGAGCTTGATCCGCTCTCTTGAAATGGCATCTTCCACCTGGCATTATAGGAATCAAATCC
ATCATTAAGAAGTGGTATTCTCTCCTTTTCATGGTTCCCTTCGGTTACCATCCACGGCCT
TGCACTTGCAAGTGGCTGGACAAGCTCACCAAATGTGTCCCACCTGTGCTGCATGTAATC
AGCATATGAAAGGTCCCCTGGAAGCAAGTGTACATCATATTTGCATAGGTCAATGTGGTC
TAGTGTTGATTTAGTCCAGCCAGTTTGACCCAAATCCCCTG
>ntig_346_consensus
ATGAATAATTGTCTTTCTGTTTCCAATTCTTATACAAGAAAAAGTTGTAAAACAAATCTA
TAATAACCAAACATTCGTCAGTAAAATGCTTTTAGAGAAGAAGAAATAATAGCACCAAAA
GAGCACGAATCACATTGCACAAAGATTTTAAGCAAACTAGTCATTAATTTAATAATGGAA
AGTCGATAAAAACAAAGACCAAAATTAAAAAGACTCGCAAAATAATAACTCAGACACGAT
GATCTCGCCACCGTCGGTCAAGAACCCAGTTCTGAGACCAGCATCCCCGCCGCCAAGGCA
ATGACTTTAAACTATAACCATCAAGATTAACACCCAAACATGCGACTTCTGGAGTCCAAA
CAAGAACCCAGTCTTTATTAAAACGACACTGATACCCCTCACTGGCAGAGGCTGCCACCT
GCTCGGTTCACTTCTTCTTGGCAGCAGATTTAGTCACCTTAGCTCCACTGGGATCCTTCT
TCTCCACGTTCTTGATGACTCCAACAGCAACAGTCTGGCGCATGTCCCTCACAGCAAATC
TACCAAGTGGGGGATACTCAGAGAAAGTTTCAACCACCATAGGCTTGGTGGGAACCATCT
TAATCATACCAGCATCACCATTCTTCAAGAATTTTGGTTCCTTCTCAAGCTCCTTACCAG
ATCGCCTGTCAATTTTCGTTAAGATCTCAGCAAACTTAACAGCAATGTGAGAGGTGTGGC
AATCAAGAACTGGCGCATATCCATTTCCAATCTGCCCAGGGTGGTTCATGATGATAACTT
GGGATGTGAAGTTGGCTGCCTCCTTGGCGGGATCATCCTTGGAGTTTGATGCAACAAAAC
CACGCTTGAGATCCTTAACAGCAACATTCTTAACATTGAATCCAACATTGTCACCAGGAA
GAGCCTCTTGGAGAGCCTCATGGTGCATCTCAACCGACTTAACTTCAGTGGTCAAACCAG
ATGGACCAAATGTGACAACCATACCAGGCTTGAGGACACCAGTTTCAACACGGCCAACAG
GTACAGTTCCAATACCACCAATCTTATAGACATCCTGAAGTGGAAGTCGAAGAGGCTTGT
CTGATGGCCTCTTGGGCTCGTTAATCTGGTCAAGGGCTTCAAGAAGGGTAGGTCCCTTGT
ACCAGTCAAGGTTGGTAGACCTTTCAATCATGTTGTCACCCTCAAAACCTGAGATGGGGA
CAAAAGGAATCTTCTCTGGGTTGTAACCAACCTTCTTCAAGTAGGATGAAACTTCCTTCA
CAATTTCGTCATACCTTGCCTTTGAGTATTTGGGAGTGGTGGCATCCATCTTGTTACAGC
AGCAAATCATTTGCTTCACGCCAAGAGTGAAGGCAAGAAGAGCATGCTCACGAGTCTGCC
CATCCTTAGAGATACCAGCCTCAAAACCACCAGTGGTGGAGTCAATAATAAGGACAGCAC
AGTCAGCCTGGGAAGTACCAGTAATCATGTTCTTAATAAAGTCACGATGTCCAGGGGCAT
CAATGACGGTGCAGTAGTACTTTGTGGTCTCAAACTTCCACAAGGCAATATCAATGGTAA
TACCACGCTCACGCTCAGCCTTAAGCTTGTCAAGCACCCAGGCATATTTGAATGATCTCT
TGTTCATCTCAGCAGCTTCTTTCTCAAACCTCTCAATCACACGCTTGTCAATACCACCAA
GCTTGTAGATCAAGTGTCCAGTCGTGGTGGACTTCCCAGAGTCGACATGGCCAATGAC
>ntig_347_consensus
CGGACGTGCAAATATTCATATGGTGGAGACTCTTCATAGTGGGGATGACCCTTTGAGAGG
TTATAAATAGCTAGCACAGTGCAAGTAGCAATGCCTAAATATGTGATCTTCTCCCACTTG
GCGGTCTCATAGGCATCGTCTTGATGACCAGAAGACGCCAATCCACGCTTGGGAGGAGCA
GCAGATGCTCTAGAGCCTCCACGAAGAGCCGTTCTCAGGACACCAGATCTCACCATCGCC
ATCGCCATTTTTTCTGTGATATTCCTTCTCCTTTGGTAGAAAATAACAAGACCAAGCCCT
AGCTTTAAAAACCCGGCGACCTATTGTACTCGACTGCCACGTGTGAATTGAT
>ntig_348_consensus

CCTCTTCAATAACAAAATAACATTTCCACATAAAAAAAGGGCTAATTTATATCATTCATC
AGCCTCATTATTTGCATGCGGAGTTTACTGACACAATTTGAAAAAGCTGAACAACTTGCA
GTAAGCCAGTCTTTAAATCCACGATTGAGATTTGCAAAGAGCAATCCTAACATTAAAAAG
CAAAGAAACATTCATGATTGTCGTTAAACCATTTGACAGAATATGAGAGCTGCAGAAGAG
TAGTCCGGGGAGGTATCTCCATGAAATGAGCCATCAATTGCCCTAATGACGCTTTGCTTC
AAAAAGGCCATCTGGACCCCATGGGAACTCTTTGTTGCGAATGTGCAGATATGGGTATGG
AGGAGGCTCTTCATGGTGGTGATGACCCTTTGAGAGGTTATAAATAGCTAGCACAGTACA
TGTAGCGATGCCTAAATATGTTATCTTCTCCCACTTGGCCGTCTCATAGGCGTCGTCGTG
ATGGCCAGAAGAAGCGAATCCACGCTTGGGAGAAGCAGCAGATGCTCTTGAACCTCCGCG
AAGAGCCGATCTCAGGAAACAAGACCTTACCGTTGCCATCGCCATTTTTTCCGATCTAAT
CCCTCTCTAACTGTTGCTTGTAAATAAGTAGATCAAACACTTGTTTTCAACTCCGATAGC
TGAAGTTTTCTACCGGCACGTGCGAAATGTATCTCGGTTGGGCTCTGAATTAACATCACT
TTGGGCTATAAAGTTTCGG
>ntig_349_consensus
CCTTCTTTTACAATTTTTCGTTTTAGGCAAATCTCCCATGCATTATCCATTTCCTTCCTA
TATATATATGGAACTATTTAAAAATCCCTTCATCCATTCAGGTGTCCTTTGATTGTGCTT
GCTCTTAGTTATCACATCACAAGGTTGTTCAATAGAGCCGGCCTAATTCTTTTTGGGTTC
AATACAAATTTCCCGAACCGAGAGCCCACAGAGTGGAAACCAGGAAAATAGCAAACAAAA
TGATGATATATAGCAATAAAGGAGGCCTACACGAGCCCAAAGAACCGAGTTCCAAAATAC
ATAACGAGAAACACAAACAAAACAAAAAAGAAGCAGGACGAACGTATTATTGGCTTTCTT
CATACAACTTTTGATCACAGTTCGCCATAGCCGTCTTCTTTCCGCTCTATGCGTATGTTG
CCTTCTTCATCTATGCGTCCAATATCGTTGAAAGTTTGTCCCGTAATCTGATCCGACGCT
TTCAATGTTTCCGCAATGGCGCCGGGACGCGTCTCTTCAATGGTTATCTCCACTCCTTCA
ACAGGCACAGTTCTTCCTGTGATCCCATGCTCACGTGCAGCCCTATTCTCTTCCTCTTTC
AGCTTCAACTCCTCCATTTGCTGCCTAGCTGCCTCTTCCGATTCACTCAACTTGTCCTCT
GCCGCTTTAGTCTCTTCAGCCTTCTGCTTAGTATCCTCCTTCTTACCAGTCAAGTAATCC
ATTGCTTTCTTGGCAGCACCAGCAGCAGAATCCTTGAGCTCGCCCAATTTACCCATAGTA
GTGTCCCTTCCTTCTTTTGCTATCTCAGCAGTATAATCCTTGTATTCCCCAGCCTTTTGC
GTTGCAGTGTCCTTGGCCCCCTTGGCCTTCTCCGCTGTATAATCTCTATACTCTCCAGCC
TTTCCGAAAGTCGTGTCCTTCGCCTCCTTTGCCTTATCTGCGGTATAATCTGTAGCTTCC
>ntig_350_consensus
GCAAGTGTTAAAATTTCTGTTCTTGGGTGCTCTAGAATTGAAACATCTGCATATACTGAA
TACAAATATGCACTAATAGTTATTAGTCCATTGATATTAACAGAAAATTAGGTTTTCAAT
ATATATATTAATTATACCATAAAACAGACTACTTCCTAGGATGAGTATCCTCGGGCCTGG
CGACGTTAGCAGACACCTTGGCGACATCACGGGCGTCAGCTTCAGGCTTTTTCTTAGCAT
ATAGCGTTAAGTATCCAACCGTGGCAACGATGAGGAAGCCGGCGACAGCCATAGTGGTGA
GGCTGAATGGTAGATTGCGTCTTTGGTGTAGTACTTCACCAGGGTGTTGGCCTGGTGGCC
TTCTGGATCCTTCCACGCCTGCTCTCTTCACTTGTTCAGGAGCCATCTTGTGAGTGTCTG
CATTTCTTCTCAAGTCTTCTTCTCCTCCTGCCATCTCTCAACTTCTCTTGTTTTCTGAAA
CTGGACGTACGTGGTTAGTTAAATCAGACTCCGGGTGAAGCTGTTTTGCCACGTTTGGGT
GGTGGTGTTGGCTCGAAGGTTTTATTTATCTCCACGTACCATTTGCGTGGCATTGTTTCA
TACTGACAAGTGTCATCAC
>ntig_351_consensus
TTTTGAAATATTGATCCACATCAACAGGTCATATAACAAAGAAACATTTCCTACACCAGA
TAAATAGCCAGCGCGAAAAATAACAACATACTCAAACGTCACCAAATTTGATTAACTAGA
TTCAAACAAACAAAATTAAAAACTCTAAAACAGAGATTCATGGGCAAGAAATCTGTGCAG
AACATCTTGCACCAAAAATTACAATCCAATGACATAACAAATGCAAAACATTAAGCAATA
AACTAACTCTCAGTCTGTTCCCGCAGCCTGCGGATGGTGCTCCTGACAGTTACGGCACTT
GCAGTGTTCAAGGTGTAAGCACCTCCTGCCTTAACCTTGCCACAGTCTTTGCAACCCCAG
ATTCCAACAGCCTTCCTCTTTACTGCATACTTCCCACAGAACTCACAGAAGTACTTGCTG
TGTTGACTGACCTCCATCTTTTTAATCTGCTTGCGCAAACTAGCACCGTAACGAGTACCA
TACTTCCCAACAATTCCAGCCTTCTTGGTTCTCTTCGTCATGTACAAGATACACAAGCAA
GAATACGAGTTAACAAAATTTACAATTTATCAGTGAACAAATGCTCCAGCCATCAGAAAG
AAAAAATAAATAAAAATGAATATCAATGAAATAAAATAAAGCTGCCATTCATGGGGTTTT
CGTTTCGAATAATATACATAAACCATCACGAACAAACTACTAGCCCTTAGTTGAATCTAA
TATACATAAACCATCAATAGCACTTAAATTTCTATTTTCTCTAATTCAAATATCACAGTT
GAATCTAATAGTATTATCAAGCTTAATATTGGTCCAGATGGGAGATGTCATAAGTCTTTC
AAACGCAATCAAGAATCATCGATCTCATTAATCTTCATCTTGGATGGATTTGGAGAA

>ntig_352_consensus
CAACTTTTAAAAGTAAGGAGTGAGCCACAGCCAATCCAAAAATTACAAAGTGACTATTAG
CTTAATTGATTGCTGCTCTGTCGGTGTATCTTACAGTACACTGATCATCATTATACTCAC
CTACTCTCTCAAAACACATTCAACAGTGAAGGAGAATCATAAAAAATTTGAGAAAAACCA
CTATCTAATTAGTAGCTCTAAACTATTGGGTTCATGACTGCCAGAATAAATCAGGGTCAT
TACTATAAAGTGAGTAATCTTGTATTTCTTGATTAGCCACATGGTAATCAATCAGTCTGG
CTTGAATTATTTTACACACCACCAATCCACAAGGAAGCATCCACCATTCACTTTCATCCA
TGCTAAAATTCCAGGAGTGAAAGTTTGTGTGTTTTTTACTTGAGTGGTCTCTATAATGAA
CAAATCCACATATGAATGCAGTTGCGATGTTGATCAAGGATGTTACATTCCACAAAGCAT
ATACACGAGCAAGGCCGGCTGACCGTCTAGGTCCATGACTGAACAAAGCATTAATTCCAG
CTGGGAATGGAAACAGTAGACCAAGAGGAAGGATAAACAAAACTAAGAAAAAGTTGAGCA
GTGATATAGAATACATCTGCAACAAAGTAAGCAAAACTAAGCTAAAATCGGCAAGCAGTA
GTATTGAGATAAGCAAACCAACAAGATCCTGATGGCCAACAGGCTTAGAA
>ntig_353_consensus
GTAAGATTTCACAGCTCATTTACCAACAGAACAAAAATATTATAAGTTATATTGTTCATA
AATTTAAGGCAAAGTTGACATTGATAATGAAAAGTCGATAAAAACATCTGAGGAGCTCAG
GCAAAACCTAAAACTCCTGAGCGGATTATGGGAATAGAAGACATCCAGTTTGACAAGACA
AAATGACCAAAATTAAAGACTAAAAATGACTCCAGACAATAATCTCGCCACCGTCGGTCA
AGAACCCAGTTCTGAGAACAGCATGCCCGCCACCGAGGTGATGACTTAAAAGTATAACCA
TCAAAATTAACATCTAAACATACAACTTTTGGAATCTTAACAAGCACCCAGTCTTTATTG
TATCGATATTGGAAACTATCATTTGCAGACACAGCAGCCGGCACGGTTCACTTCTTCTTG
GCAGCAGATTTAGTCACCTTAGCTCCAGTGGGATCCTTCTTCTCCACATTCTTGATGACT
CCAACGGCAACAGTCTGGCGCATGTCCCTCACAGCAAATCGACCAAGAGGAGGATATTCA
GAGAAAGTTTCAACCACCATGGGCTTGGTGGGAACCATCTTAATCATACCTGAGTCACCA
TTCTTCAAGAATTTGGGCTCCTTTTCAAGCTCCTTACCAGATCGCCTGTCTATCTTGGTT
AAGATCTCAGCAAACTTGACAGCAATGTGGGAGGTGTGGCAATCAAGAACTGGGGCATAT
CCATTTCCAATCTGCCCAGGGTGGTTCATGATGATAACTTGGGATGTGAAGTTGGCTGCC
TCCTTAGCAGGATCATCCTTGGAGTTTGATGCAACAAAACCACGCTTGAGATCCTTAACA
GCAACATTCTTGACATTGAATCCAACATTATCACCTGGAAGAGCCTCTTGGAGAGCCTCG
TGGTGCATCTCAACTGACTTGACTTCAGTGGTCAATCCAGATGGACCAAATGTGACAACC
ATACCAGGCTTGAGGACACCAGTTTCAACACGGCCCACAGGTACAGTTCCAATACCACCA
ATCTTGTAGACATCCTGAAGTGGAAGTCGAAGGGGCTTGTCTGATGGCCTCTTAGGCTCA
TTAATCTGGTCAAGGGCCTCAAGAAGGGTTGGACCCTTGTACCAGTCAAGGTTGGTGGAC
CTTTCAATCATGTTGTCACCCTCAAATCCAGAGATGGGGACAAAAGGAATCTTCTCAGGG
TTGTAACCAACTTTCTTCAAGTAGGACGAAACTTCCTTAACAATTTCATCATACCTTGCC
TTTGAATACTTCGGGGTGGTGGCATCCATCTTGTTACAGCAGCAAATCATTTGCCTCACA
CCAAGAGTGAAAGCAAGAAGAGCATGCTCACGTGTCTGACCATCCTTAGATATACCAGCC
TCAAAACCACCAGTGGTAGAATCGATAATAAGCACAGCACAGTCAGC
>ntig_354_consensus
ATCCAAAGGGAGAGATGAAAGGTATGCCCAAGACAAATTATTTCTGCTTTAAGCATTTAA
ACTCTTAAACAATTGAGTGTCTATTGAGCTGTTGCCTAAAATACGTTGATTCAAACTGAC
AAATATATTGATTTCAAATTAATTTTCAAAGTTTGCCTTAAATCTGAAATTATGAAATGT
GTAAAAAATACACTTCAAACATTTTAGTTGATGTAATGTTATTATTATATTTTAGTTATC
TAAAGTGTTACATTGACTGTTGTTGACAATTTCTACTGGTAGGTTCCGCAATCACTGGTC
CAA
>ntig_355_consensus
ATCAAAATCAAAATGCCTAATGTAAGCAATCAACCCACCAAAAATTTCCCGTCTTTACCA
ATTTTCTGCATTGATTCTCATACTCTTCTTTCTTATTTTCTGCTCATTCAGCAGGTCGTA
GAACTGAAAGTGGGCTTNNGTGACGGGTAATATCACACCAGAAGAAGTGATCAGAGTTCT
GCAAAAGATTGGCAAATCTGCAAGCCCTTGGGAGGAAGCTCAACCCAATGTTGATTACGA
TAGCTCCACAAATTATAACATAGCAAGTTAGTATTTTAGCACTTGATCTTTATCTTTATT
AATGGCTGCTGCCATTAAAGATTATAAATCTGTAACAGGGGTTTCAACCAAATCTACCCT
TCGAGGAATGCATCAAAATATCACCTCAATAATATTGAAACATTACTAAACAAAACCATT
GCTATATAGGATCACAAACTGCAAAGTCACACTCAAAATTTCTCATTTAACAACTCAAAT
TACATTTTATTATGCCCAGTGATAGCTATTATTACAGTATTTACAATCGCTATCGATTTT
CTCCAACTACAAAGTNNNNNNCTCGAATCGACTTAGCTCAGCCGTCACCAGCCGGGACAT
ATCACTCTACTTAGGCTGCTGAATCGGCCTTGGACTCAGCTTCACTTTCACGATCTTTGG
GACCCTCTCTGCATACCTCGGCTTCTCATTCAAAACTCGACCCTTTTTCCATTTCAAGGT

CTCAATTGAAATCAACTGCTTCCCAGACTCCATCTCCTCCGCCTGTTGGTGGCTGCGGTC
GCCGTCTTCGAAGAGATCGTCGTCGCTAGCGAAGGCAAGATCGTCAAAATCGGAGAAAAA
AGGGTCGAATTGGGGATCAAAGTAGCGTTCTTGGAGCCAGTAGTCACGGAACCATGGAGT
AGATTGGATAAGGGCCCACCATTGATCAGAGAAATCTTCGACCGTCCGATAGGCGAGTGG
GATGAACATTGGAGCGTTGGGATTTAAAGTTGAGGATGAAGATGAAATTACCTCCATTGC
CCCTTACGACAGTGTAGCAGTCCTTAAAAGAGATAAGGATAAGCAGACCAAACAGAGACC
TTTTTAAATCCCAAC
>ntig_356_consensus
CTGCAATTTCAGCCTCATTGCAACCTTGGTCATCAGTGACAACACCAGGTGTACCCTTGC
TTGTATTTTCAAAGTTATCAGATATTGCTCCTGAGCAGTTACAAACATTTACTTCAACCG
TAACTGGTGTTGGACTTACATCTGTCTGGTCACATAATGGCTTTTGATTTTTATCTGATA
GTTTAGAAAATAAAGATGGTCGGTTATGATCTTTTTCTGTATGAGTGATTGCTCCAAACA
TTCCTTGTGAAGACAAGTTCTTCTCTTTGGAGATTTTTTTCACACCCAGTCTAGGTTTAC
GAGAAATCCTATTA
>ntig_357_consensus
CGGCCGAAATTGCAGATATTTTGACAAATGGTCCCCTTTTAATTCCATTTACATTGCTTT
TGTGTGTTGGATGGATTGCATATGGTGCGTGATTTAACCTGTCAAAATAACCCTGTTTTG
GTTGGGTTGGGATGCCATTTTGCAAGAGGGCTGTAAAAATCTCCAAACATTTACATGTAA
TGAAGACTATACATAAAGGATATCAACCGATTGCCATTATGAAGAGAATTTTACACGTTC
CAAATCTTAAATTGTTTGTAAAAAAGAAAAAAAAAAAATCTGATAGATTAAGTATTTTCAT
CTGGTACAGTTGCTATAGGTCATAGAGCCATAACTTCAATGCCGGAATGTCTTTACCACC
TGGTCAATTCGTCTCCGGCATAGAGGGCAGCTGGTCAAGTGTGAGGAACAGGCCGTACAA
CAACACATATGACCACACGGGACAAAAACAGCATTATACTCCTGCTCAAGGCATATCACA
CATATATCTGGCATTACACGGTCTCTCTTAGTACTATCTGATCCATTCTCTGATGCTCCA
TTTGAACCTTCACTGTCTTGCCCTGCTCTCTTGGTTGCAGCAGCAAGAACCCTTTTCTGC
AGTTCCCAGCGGCGCTTTCTTTCCAAGAGATATTGGATAGAATGCTTGGCAATCAGGAAA
ATGCCAAAAACT
>ntig_358_consensus
GTCATAGAGCCATAACTTCAATGCCGGAATGTCTTTACCACCTGGTCAATTCGTCTCCGG
CATAGAGGGCAGCTGGTCAAGTGTGAGGAACAGGCCGTACAACAACACATATGACCACAC
CTGCAGACATGAACGATAGGGGACTCGTTCTATTTAGCAAACACAAGTCAAAACTGCAGA
GAGAAGGGATAATTGTTCCACAAAGTACGTAATAAAATGAGAAGACACGGTAGAACAAAT
GAACAAATAAATGAAGGCTCCGCTAATGCAATCTGTGATATTAAGGTATTCAAGAATATT
TGCAATGTTTATTGTGATACTATGATCAGTTTTTGTTAGTGATCAAAACTAAATTATTCC
GGCTGACACCAATACAAAAGCCATAACACTACTATAATTTATACGCTTGGGGAGAAAAAG
TTAATGGCATCTATCATTTATTCCCAAAGTGCATTTATATGAGGAAATGAGCCACTACTC
CCATTCTTGGTCTAAGGAAAATAGTAAGAATTTCGTAACAGGGCAGTGTAACACAAACAA
ATAGGCAATTCGGCAATCTATGTAAATCATGCAAAAGATAGTAGTGAACTTACGGGACAA
AAACAGCATTATACTCCTGCTCAAGGCATATCACACATATATCTGGCATTACACGGTCTC
TCTT
>ntig_359_consensus
TACATAAGGCAGATGCTTTAGACGCACCTCCATGCGACACAAATATTATACTTTATTCAG
TTGGCTCACACAACATATCCTACTTTTCCCAGGAAGATTCATACAACACAGCCTAGAACC
CAAGCTTGCGCTCTTTAGGAAAATCGGTGAAAAGCAGGCGGCTTACATCCACCACACAGA
CTACTACATTTGCATTTCCGGGTAATACATTTTACTCATGCAGTGCCCGCTTGTTCACAG
AGGTCTAACAGATCCAGAGTCTCAGGAATTGAGAGCTCAAGACGGAACTTCGGGCCATCG
TAATGATGCAAGACTGGCCTAAAAGCATCTTCTTCCAATGCCTTGCATAGTTTTCTAACA
CGGATCCTCACCTGAGCAGGAAACCTTGATTCTCCCTTGCACTTTTTATCTTCCCAGGCT
GTCGGATCAATGTTGGAACCTCCAAAACTTGCTGCCTCAAAAATTCCATGCAATTGGTGA
GTAGTATAATTATAAAGGAACAGAGGTAAGCCTGGCGTTATTGCCCGAACAGAATCCCTG
TATCTTGGAGGCAAACCAAATAGCTGTCGCTTTAAGTCTTCCTGCATTGTGTCATTGTTA
CAGACAAAGATATATCCTCCAAGCACCTCATTTCGTGGGAGTGTTTCTGTGGCAGGCAAA
GTCTTGAACCTTTTGTCAATTGCACTGTTGTTGTTATCATTGCTGTTATTGCTGTTGTTT
TCAGCCTCTTTGACAATCAAATTATTGCCACTATACTTGTTAACCATCAGGTTCCCCATA
TTGTGCCCACCATTTTTTTGGTACACTGCATTCATGTTGTAAATACCATTCCTCAAGGTG
CTTTTACTCACGTTTTCAGCAACTTTAGAGTCCAAATTCAGCATGTTGAAGTTAAAGTTA
AAGTTTCATAATTGTTATCTTCATGGTACAAAATTTTATCCCTTGGTCTTGATTCTGTC
GAGCCCTTTGAAAGATCAAGATTATTCATGCGTTCCCCTTTCAATCCTGTCTGCTCAGCC

AACCTAAGGGCAGCCCTTACCCATGATTGATCCTCTGAGGTTTTCGATTGTCCTCGAAGC
TCATCACCCAATTGCCAAAAACTGTGTGTGCTGTCCATCTTGCAAAACCCTTTACTACCT
CAGCCAAACTCTGACACCTGCGAGACACCACGACCCACACAAACCGCA
>ntig_360_consensus
GTTTAGATTTGAACGTAGCACCTTATGTGCATATACATCAAATTGAAGATTATATTAATA
TAATTTAGTATATTTAACACTTAAACCATATAAAATTGTTATCCTTCAATTTCCAGATGC
TAAAGCTTGTTGTTGCATTATTTCCGTTTCAGGCTAGTGCCCAGCCGTTTCTGGTGCTTG
GTTTGCTTGTCACCGCTTTAGCAGTAAGTCCTCCCTGATGTTCTCAAGCAGTTTCGTACG
GAGGGGAGATATAGAAGAAAGAGGAAACACGAAAACATTATGATTGTAGGAAAAGTGATC
TGAACATCGTTTTTGGCTCATCATCTATCTGTAAAACTCGGCTAAACTTCATTATTCCAG
CTGCCTTCTCCCACCTTGCCTCGCAAAATGTTGTGTAATGAAGAGCATGTTTTAAGAACT
TACGACGCACCAAGAGTTCCATTTTCTATTTTTGAGCCTGAATTTGGATTTATTTGCCCA
CATTAAATTATGTTGCTTGGCCTTCGTTTTAATGGGTGAACAGCATATTGGAAAATATTC
AGAAGTCACAAACATGTTACTGCAACAAAGAGAACATAAGCTGACGATGCAGCATGGTCT
CAGAAAATGCGGAACTTACGAACAGTTGTGTCAACTTCGCCTGCTTCTGCAGCCCCCGCC
CGCCCCATCTCTTTGCTGCTTTCAGCCCCATCCCGCTCCTTTGTCTGCTGTCCCTGATTT
TGATGGCCTCTTGAGTCCAGGTATCCTGGTACTCCAAGGTGCTCTCCCTTTGTTTGCCCT
CCTTGGTGCGCTTACATTGGCCTCATGTTCTGCCCTTTGCTGCTCTGAAGCCATATTTTT
TTAGTTCTTGATCTTAAAACGGAAACTCCGTCAACTTTAAGATGAAAGTGAAGGGCTTAT
TCTTATATGTGGAGGAAGGGCCTTTTTTGAGAAGATAAGAGATCGTTGAGACACAAGGGA
AGAAGGGGTCACGTAAGTGATGAAGCAGGAACACAGCAGCTATATCAGCCAGCCATGGCA
AGTTTCTACAAGTCAATCAATTGACACGAGTCCATTTAATTATCTCAGTCGGTTGCGATT
CTTTGCATCCTCCACGTGTCCTGAGGGATCATTAC
>ntig_361_consensus
TTTTTTTTTTATAGAAAAAACACATTTATTATAGATTAGAAACCAAAATGTCTGAAACAA
CAGGCGTTTCATAATACTCTATGCAATAATATAGAAATTCTCTTCGAGAGACAGAAAATT
CCAAATGCTTATTACTCAACAGAAATCTATTCCTCCATGCGAATGATGCACCTCAGGCTA
TCTCCTTTGAGCATGTAGTCAAAGGCCTTATTGATCTCGGAGAAAGGTATAGAGTGAGTG
ATAAATTTCTCAAGTTGCAGATCCAACTCCTTGTTCATGTATTGCTCCACAACTCTGGGA
AGATCAGTGCGAGGCTTGTAGTTGCCAAAGAAGGTACCCTTAAGAGTCCTCTCGTTAAGC
AGATTCATTGGATGAGTTTTGAATGCATCATCTTTGTTTGGAACACCAACAAGTACAGCA
ACACCCCAACCATCGTGGACACATTCAAATGCTGAAATCATGGCCTGGATACTTCCAGTA
CATTCAACACTCCGATCAACTCCGCCATCAGTCATGTCAGCAATAACCTGTTGAACGGGT
TTGTCATAATCTTTTGGGTTCACAAAATCTGTGCAGCCAAACTTCTTAGCTTCAGCAAAT
CTGCTGGGGTTCAAATCAACACCAATGATCCTTGAAGCTCCAGCTATCCTAGCCCCCTCA
ACAGCGGCAAGACCAACAGCACCCAGTCCAAAAACAGCAACAGATTGGCCCTTCTTGGGT
TTTGCAACATTCAAAGTAGCCCCAAGACCTGTGGATATTCCACAGCTAAGCACACAAACT
TTGTCAAGAGGAGCGGCAGGGTGGATCTTGGCAAGACAGCCAACATGGACAACAGTGTAT
TCACTGAATGTGGAAGTTCCAACAAAATGGTATATTGGCTTCCCATTGATGGAAAACCTA
GACTGACCATCATGTATCATAACACCCCTGTCAGTATTGATTCTGAGGAGATCACACATG
TTGCTTTCCTCTGACTGACAGTGTCGACACTCTCCACACTCTCCAGTGAACACAGGAAGA
ACATGGTCTCCTGGTTGTAGATCAGTTACTCCTTCCCCTACGCTCTCCACAATTCCTCCA
GCTTCATGACCTAAAATGCGAGGAAACACTGGTGTTTGTCCCTTGGCTTCCCAGAAGTAA
ACATCAGTGTGGCACAAAGAGGTGAAGAGGATCTTCAATCGAACTTCATTTTTCTGCGGT
GGTGCCACCTCCACTTCTTCAATAATCAGTGGCTTCCCAGCCTCCCATGCCACAGCAGCC
TTGCAACGAATAACTTGACCAGCTTTGTTAGACATCTTTTTTCAAGTTGAAGATAAACCA
AAAGTATCTGTTTGAAGTGAAGAAAAGCGATGTCTTTGAGTAGCTATTTATAGCGTGATT
GTTATTCTTTTATTTTTTCACCTTCC
>ntig_362_consensus
CTTCCTCTTCAGGATCATACAAAGTAACGAAGTTGCCTTCTGAATTGGATAATGCATCCA
CTTCCAGCTGTGTTTTGTGAACTTTTAACTGATGACTATCTTAATGCAACACCAGCATCA
ATACAACACAGCGATTTGTTGAAAGAAAAAAATAGAATTATGAGAAGTTTTATTCCACAA
ATCTAAATATATATACAGGTACCAAAACATGCCTTTGATTTCAGTTCGGCAGATATCCCC
AGTGCTCCAGGCTGTCTAATTTGTCAAGATTTTATATGTGTAGCACAACTAGTACAACAA
CC
>ntig_363_consensus
TAATTCTTCAATCATCTCAGGCATTTTATCACCCAAACCAAGCGAAACGGCGAGTTTTGG
CATCTGCTTACGCCAGGCGGAGCCAACCACAAGCTTCCGATACAAGTTCACATCCTCCTT

CTTAACAATCCCAAAAGTTACGAGATGCTGCAAAAAGGTATGCACGTCAGGCGTCTTCAC
GTTCTCAATCCCACCTCTCTCCTCCAGGCTCGCCTTCCAAGTCTCAGCGATCTCTTTGGC
TTTCGTCTTCACACTCGGCGTCACCAGCAACCTCGACTTCCCTATCACCGGGTCAACCAC
CACAGGTATCAAACTTTCCAGCGCCAACACACAAGCCCAGCCCAAATCATTACCACTCTT
GTCCTTTCTCTTGTCCACAGGAAAGACCTCCGATATAGCCTCCATAACAAACTTCGCAGG
ATCAACGCATTCAGTTAGCGCCACCGGCAAGACATTTCTCATCTCGTCTATTTCTTTCTT
TTTAGTAGTCACAAACTTCCAGAACTCTCTAGCCTTCATTTTAAGACAATAACACTTTAG
TTTCATCAATAACGCGTCGCCGTCGTCGACTTCGCCGTCAGGGTTTTCAAATTCCTTTTC
TTCCGCCGCAGAATTCAACTTACTCAGTGCAGCCTCCGTGCGTTCTTCCAGCTTCTCTAA
AGCGATCTCAACGCTGCCGTCGATGGTGACCTCCCGCTTCTTCAGAAAGTCCAAGGAAGC
TTTCGTCTCACTGTCTAGTGCTTGTATCTTGTGTCTCATCGCAGCAGATTTTTTCATGAT
GTTCTGTTCAAGAGACGTAAAGTGATCAGACAGCTCCTTCCAAAGAAGTGTACAGCTAGT
CATCAACGACGTTTGCCGCTGGAACTCATCAAAATTTGGCGGAGTCAACTCCGTCAACTC
GCCTGGATCGGGGATCGACCCCATGGTTAAAGCTGATAGATTCGATTCTCTCCGTTTGGT
GACTCAGTAACTGAGTTAACAACGACAAAGAAAACAAACAAAATACCAAACCCTAGAAAC
GTAAAGAGAGAGAGAGAGAGAGAGTCGAATCTATCAGCA
>ntig_364_consensus
GTGCCAAGTTCAGTGTGATTGATCAGATCTCTGGCATACAATGACATTGCTTACATTCAG
ATAACCAATGTTAGACAGTTCTACCAAAAAGTTTGATCCATATCTAAATGAAAGTAAAAT
TCATTGATAACCGATTAACATCAAATTGCAATGGTAGTGGCCATTCTTGACAAGCCAGCT
TAAATACAATGGGAAACATCAAAGCTTCTCCCTTATCCTACACAAGTTAACATAGTACAA
ACTAGACATCAAAATGAATCAGGCACATACTCATAAATACCTTCATAAGCGCTTGGGACA
GACATAAAGAAGTTGGCTTTTCTGGGTGACTAAAACCACCATGTGGCTTATCTACTTCAT
TCTTTACCGTCAGCAGCACGCAGTGAACCCAGTTGAACTGGTGCTTGAGTTCCAACCACC
TTAGATGACCCATAAATAGACAGGTCAATACCTTGCGCCTTCTCTTTCTCAATCTGGTCC
TTGATCCAGAAATATGTAATTCTCAGACCATCCTTCAACCTCATTGTAGGTGCCCAGCCA
AGCTTCTCTTTAATCAGGGTATTGTCTGAATTACGGCCCCGAACCCCCTCGGGGCCAGGA
ATGTGATGAATAGGGAGCTTCTTGTCCTCAAAGCCAAGAACAATCTCAGCCATCTCATTC
ATGCTGACCATCTCATCACTTCCAATGTTGACTGGCTCACGGAAGTCTGACTTTGTCAAC
CTCAGCACACCTTCTACACATTCATCAATGAAGGTGAAAGATCGGGTCTGAAGTCCATCA
CCCCACATCTCAAACTTGTCAACAGAAGTGATGGCCTTTCTGCAAAAAGCAGCAGGAGCC
TTTTCCCTTCCACCTTTCCATGTACCAAAAGGACCATAAATATTATGGAATCTTCCAATA
CGGCACTCAATTCCAAAGTCTTTGGTGTAGTGCTTGCACAGTTCCTCAGTTGCAAGCTTC
TCCAAGCCATAAGCATCTTGAGGCTCTGCAGGCCAGGCATCAGACTCTTTCAAGCTCACA
TTAGTATCCAGCTGCTTAAATTCAGGGTAAATGCATGCACTAGAGGCATAGAAAAACCTC
TTAACTCCGTTGATCCTAGAGGCCTCAAGCATGTTGAAGCTGATCATTGTATTGTTATAC
ATAATGACAGAATGGTTGGACTGAATGAAGCCCATCCCACCCATATCAGCAGCAAGGTTA
AACACATGGTCAACTCCCTGAGAAACTTTCAAGCAATTGTCCATGACCCTAAGGTCAACA
AGGTGAAATTCATGACAGAACATATCTTCAGTCATGTGCTCATTCTTCTTCCAGTCGGAA
GCAATAATATAATGGCCCTCACTCTTCAAACGACGAGCAATGTGGGAGGCAATAAAACCA
CCTGCTCCAGTAATGGAGATACGTAGTTTCTCAGATGGCCAGTAAGGTTCCCTCTCAAGT
GCCTCATAGGTGTAGGCACCATAGTTGGTTCCATCATTACTTCCCATTCCGACAGACTGA
AACTTGGAGTCACAGGAAATGAAGAGGACAAGGCACGAGGGAATGCAGAGAAGTGAATAC
AGCGCCCGCATATCATAACAAGTGCCCTGCGATCCATATATAGACCAAACACACCCTCAC
>ntig_365_consensus
ATACATGTCTGAATAAAAGTAGAGATTCTTATTGATAAACAATCAACATAAGATGGTGAT
CATAGTAACCGTTCTTGAAAAGCCAGATTAAAAACAATGGAAGAATCAAAGCCTCTCCCT
TCTCTTACACAGTACTAACTAGACACCGAAATGAATAATAATGCATACTCATAAATACCC
TTCTAGATCTCCACAACCATAAATACTTTCATAAGCCATTAGGATAGACACAATAAAACT
TGGCTTCTCTGGGACAACTACTACAACCATGAGGTTTATTCACTTCATTCTTTCCCATCA
GCAGCACGCAGTGAACCCAGCTGAACTGGGGCTTGAGTTCCCACCACCTTAGATGACCCA
TACATAGACAGGTCAACACCTTTAGCCTTCTCCTTCTCAACCTGGTCCTTGATCCAGAAA
TAAGTAATTCTCAGCCCGTCCTTCAACCTCATTGCAGGCGCCCAGCCAAGCTTCTCTTTA
ATCAGTGTATTGTCTGAATTACGGCCACGAACCCCCTCGGGGCCAGGAATATGATGAATA
GGGAGCTTCTTGTCCTCAAAGCTCAGAACAATCTCAGCCATCTCATTCATGCTGACCATC
TCATCACTCCCAATGTTCACAGGCTCACGGAAGTCTGACTTTGTCAACCTGAGCACACCC
TCTACACATTCATCAATGAAGGTGAAAGATCGGGTCTGAAGTCCATCACCCCACATCTCA
AACTTATCAGTAGAAGTGATAGCCTTTCTGCAAAAAGCAGCAGGTGCCTTTTCCCTTCCA

CCTTTCCATGTACCAAAAGGACCATATATATTGTGGAATCTTCCAATACGGCACTCAATT
CCAAAGTCTTTGGTGTAGTGCTTGCAAAGCTCCTCAGTTGCAAGCTTCTCCAAGCCATAA
GCATCTTGAGGCTCAGCAGGCCAGGCATCAGACTCCTTCAAGCTTACATTGGTATCCAAC
TGCTTAAATTCAGGATAGATGCACGCACTAGAGGCATAAAAAAACCTCCTAACTCCATTG
ATCCTAGAGGCCTCAAGCATGTTGAAGCTAATCATTGTATTGTTGTACATAATAACAGAG
TGGTTGGACTGAATAAAACCCATCCCACCCATATCAGCAGCAAGGTTAAACACATGATCA
ACTCCCTGAGTAACCTTCAAGCAATTGTCCATGACCCTAAGGTCGACAAGGTGAAACTCG
TGACAGAACATATCTTCAGTCATGTGCTCGTTCTTCTTCCAGTCAGAAGCAATAATGTAA
TGGCCCTCACTCTTCAACCGACGAGCAATGTGGGAAGCAATAAAACCACCTGCCCCCGTG
ATGGAGATCCGGAGTTTCTCATACGGCCAGTAAAGTTCCCTCTCAAGCTCTTCATAGGTG
TATGCACCATAGTTGGTTCCATCATTGCTTCCCATTCTGAAAAGACTGAAACTTGGGAGT
GACTGCGATGAAGAAGATAAGGCAAGAGAAAATGCAAAGAAGATAAAAGATTAGCACGTG
AATGAAGCGCCCTCAATAAATCTCCCGCGTGCTATATATAGACCCAACAGGCACACCCAG
CAGACAGAGTACACAGCACCAAAAGTTTA
>ntig_366_consensus
AGAGGGTATGGAAGTAAGAACAAAATTCTTATTTATTTCTCGCTAAAAGAAATAGTACTT
ACAAAATGTATATACATATACACATATATAAAAGCAAACCTTACATCTCTGATAAAAGAT
TGCTGATTTTCCGAAGCTCTTTGAGAGCTGTCATGGCTGTAGCTTGTCCTTTGCCAACTA
GAACATTTTTAGAGAAGTTTTCTAAAGGCCCTACCTGACCCCGATCTTGCTGGAAGACTG
CCTCAATTACCTGAATTTGTTCCAGCATAGAATGCCCAAGATTCTTCAAAGTTGCCATCA
TGTTCTGGTCCACAGTATCTAGTTTGCTGCAGGCAGATGACTTAATCGACCCTTCAGAAT
CTGAAGAGTCTGTATTGCTGCTCTGATGACCTTCAACATTCGCAGAATTCTTTGTCTCAA
TGGCACCTACTCTTTCAGACGACTCCTCACAACCACAAGCATTTCGTCCAATTTTCCAAA
TCCATGGGAATTTACCAAAAAGGAGCTTCCGCTCTTTGGGACCTTTCACTGATTTTTCTG
TAGAGTCATGGCCCATTTGAGAATCTTGACAAACATCATCAGTGTTATGAGAAACAGGAA
GAGGTGAACCATCTGGGACAGTCTGGGGATGTCCATCATTTTCATCAATAGATGTATTTG
ATGCAACACTACTTTTTTCTGAGTCATTTTCATTGTCATGAGCACCACTAACTGAACTTG
CTGGATCTGAGAATATAGAGGAGTTTTCCTCACTACCAACATTTCCATTCACACATCTGT
CATCAGATGTGTAAGTAAAGTCCTTAATGACACCATCTTGGCCCTCAACTTCAACCGAGT
GTTTTTCCTTTGTGGTTGAAACTTCACCATTATTGGCTTTCTCATAATCTCCCTCCAAAC
CTAGTTCCCGTGACAGATCTTCCAGTAAACTTCGCCTAACAGTTGACTTCAGGTCCTTTT
TGTCATTACCAAACTTGGCAGAAGATGGGTCAGATTCTGTTCTAGAGAGGTTCAGTTTTA
CTCTTTCCGACCATCCTCTTTTCTTTGCTGGCTTTTGTTTTCCGGGACCACCTAGTCTTT
TTTCTTCTTCCTTATGTAGAACTCTCCACTTTTCTTCCCAGTAGCTATCAGGTACAACAC
TTAAAGGAGTTCTTGGAGAAATAGAGTCAGATGGAAGGTGGGGTCCTCTCACCACTGTTG
ATTTACTCAAGTTATAAGTTCCAGTGGATGAAGAATATAATGATGAAATATCAGTACACA
GCACAAGAGCCTGTAAAGATTTTGCTTTGACTATAATTTTCCCTAGCTTTACATTCTCTG
GGAAATTCAACAATCTCTGAAGGCAGGAAGTTGCATTTTCAGCGGCAAGCAGAGATGATC
TCAAATAAAGCATCATGGAAACTGCAATAGCTGCAATTAATGCCCCCCGTGGCGAACCTA
GAACTTTAAAGCTGGAGTCTTCATCGTTTTCAACACTTTTGCTTAATTTAGCATTGTCTG
ATGCAAATATCTCATCCCATAATATCAACAAGTCTCCGAGTGAAAACTCCCGTCCAAATA
AGACCCGCAACCAGCGTAATCCAAAATACTGAGGTTCAACACCCAGCTCAACAAGATGGC
AGTGTAAAGATGAATCAACAATTGACAACAAATGGTACAATGCAGCAGAAGCTTCAATGA
TAGGAGGTAAGCTAGTGAGGGGTCCACCTGCAAGGGAGGGAGAAAAAAAATCTGCCATAG
AAACTGAACCATGGGCTCCACTCATTAAAGCCTCAAACATACAGTAAGCATCATGTTCCA
TAAATTTCTCAGACAAAACAATACCCAGTTCACCTTCAGCCCCATATGCATCATTTAGCA
GTATAATTGTCTGTATCTCAGGATCAAGCTCATCAAGGCTCTTAACTTTCGTTGAGTTTC
CATGGGATCCAAAATCTTCTTCTGTGGAATCTATAAACTTCTTGAAATCAAAGTTATATG
TAAGATCATTTTCATGAAATGATAAACCATCAAATCTGTCAGTGAAGTGGTCTTCATACT
CCTTTCGCACTTGACAAAGACGATCCACATCAACATGAAGAACATATAATAAAGGAGCCA
AAAGTTCATGCATTCCTTGCTTATAACCATATTCTGGATGTCTAAGGCACCACAATAACA
AGATCCGTCTTAACATGCCTTGGCATCCAGGTGCCTGAAAGTAACTCCCATGCTCTGGAT
ACAATCGTGATAGGTCCTGGTCAACCATTTTTTCCAGCTCAGCATTCCGAAAGAAGCGGC
CCCATGTGCTATCTGGATTTTGTGAAAGTGGATTGTCCATGACGGGATCGGGATAATTAC
TTCCTTCCTTGGGCAAATGTGGATCAACAAGTAGGCTCCTTCTTAAGCCAGCATATCTTC
TTCGAGAATCAGCCGTGACTCTACGGAGATCATCAATTGAAGAAGAAGAAGCGGGTAAAA
TCCCGAGATGAACACGCCATTGAACACCTCTAAGGTTTGCAAAACGACCAGATCTCCCTG
AAACGGCATCGTTAGTTGATGATTCTTCTAGATGCTGCTCAATTGCTGAAGCTGGCATCA

TCGCTTAAAAAAATCAACTCTCCCGATGCAAACACAAAGCCACAAAGCCACAAAAATTCA
ACACCAACCCAATTGCTCAATGGTTCTGGATTGCATAAAAAATTAAACAAATAAATAACC
CCCGAAATCAGTTCTTTCACAAATTAAAGACGAATCAAGTTGCTGAATGGTTAAAAAAAC
AAAATCAGTTATTTTACAAAGTAAACAGAAAACCGGTGGTTGAGATACAAAAAGAATCCA
CCAAAGAAGGGGAGGAGGATGAGATGAATTACATGGGGGTTGGGGAGAAAGAAA
>ntig_367_consensus
CCACTAAGTGCATTGATTAATAAAGAAAATATGATATATAGAGGAAAAATAACCTTACAG
AAATCATTCAAATATGATACAACAGTAGTAATTTTCCCGTTTGACTATAGATTCAACATA
AATGACAATGAATCAAATGCAGCTGATAATCATGATAAGTATGAAAGTAACTTCATTATG
TTGATGCGAGCTACACGACTACAATTAAATCTTATAGAAGAAGACAAACAAAACAGATAA
GTCATTTGGGTCCACGATGACCTCGCCCACCACATTCAGTGCACATAATATTACCAGTTC
CCCCACATCCTAGGCAGCGGACTTTGACAATGATGCCCTGGCTCTCTAATATTGAGTCCA
CATATAGGCCTGAACCACAGCAAGTTGTGCACAGGACAGCACCTTTGGCTTGGCAATCAG
TGCAGCGAAGATTATTTCCAATTACTTTTTCAGCAAGTGAAAGCCCAACTGGCTTCTCAA
TTCTTTCCTCTGGAATAGGCTTATCATAACAAAACAGAGATTCAAGACTGCTTCTTTGCC
TGTTCATATCTCCAACTGAACTTGACTCAGCCCCATTTAAAGAAGAGGGAGGAAGAGGGC
CGTTAGAAGAGATGGCATAAATTCTTCCGGTGGAGTGAAATGTATTGAATTGAAACCCAA
AACTGAAATCAATGTGTTTATTGTTATGGAGTAATCTTGATGAGGAGGAGATGGGGACGG
CAATGGATGAAGCTAATCTCAGAGTAGAATCCATTAGAGCCTAAAAACACATGAACAAAA
CTTTGAAGCGGAAAACTACAGGACGAACGAGTTAAGAAAAGAACTGGCTAAATTTGGATC
GGAAGAGAAGGTAGCTTTGGAGAAATC
>ntig_368_consensus
CCTGAACTATGATAGATGAAAGAAGGCCAAACTAGCAAACATACCAAATAAAAATGGCAA
TTCATCCTATAGCAACTGCATCCAAACTAGTCATTGAACAAGATTCAAATTCTTTAACAA
GGTATGTACACACTTTTTGAAAATCAAAGTATGGACTTACAGCATAAAGAGTCATACATT
CCTATTTTACAGCCTTCGGAGATTCAGACCCATGAACCTGAACATAGCCTTCTGCTTTTC
TAGCCTCTCCCTCTTCAGCCAACACCTTCTCTCGTGTCTTCTGACTCACTTCCCCAGCTA
CCTTTGAGACACGATTAAATGCACCAGTAACCCATGAAGCCCCAGTCAGAACATAGCGGT
TCTTCATGATAGCAGATCCTGCATTACTAACTGTCTGCTCAGCAGCTGACAAGGCAGACT
TGGTCTTTCCTGAAACCTGAAACTTTTCATCCATCTCCCTTACTTTGTCATTGACAAGAG
ATGTGCCAGCACTTATTTTCTCGCTGAGGCCAATTTTTTGATCAATAGAAACAACTGTTG
CTGTGGCAGTAGATGTGAATCGGTGCTTCTCATCGAAGGCCTTCGCTTTGTTGACTGCAT
CTTTACCAAGAACAAAGCCCTTGGCCAGCATGCTGCTGACAACATCCTCTGCCTTCTGGA
CAGTGGATTCAGCACCACCAGCATTTTTGCTCTGTGCTCCGTGAGATGAAGCAGAAGTAG
CAGCAGGCTGCTTAAAATCTGGATCCAAATCTATTGTTACAGACTGATCCACTATGGTTG
CCCCCGAAAGAAGAATTGCTGTCTCTGCCCCTTGGGGATCCTTGAAGACAACATATGCAA
TTTGAGACCGCTCATTGCCACTGTGCAGCTCAACATGTTCAATTTCCCCAGAAAAGGAGA
AGAACTCCTTTATATCTTGCTCAGATGCACCTAATGAAACATTGCTCACTTTCACTGTTC
TTACCGTCATCGAGCACACCAAAGACT
>ntig_369_consensus
TCTAAAAATAATATATAAATTAAAATTAAAATATAATTTATACAATCATTACATAGTTTT
CCCAATCAGATTTGGTTGAAATAAAGATCGACCCTAACGCTTTGACTTCTAGTGCCTAAT
ATTGCTCAACGGCCACATTTTCATGTGATGATACCAAAAAGTTAAATAAAAATATACTAT
TTCATACGAACGTTGGCCTTGTCGTTTTTAAGGTGCCTTCAACTAGAATTTTCGCGTACG
ATCGTAGTAATCAATTTCAAATTTAAGTGATAATCTGGCTGGGGGCTTTTCCAAAATGGG
ACTTGATAGACCTTTCAGAAAGTCAAATTTAATTGCAGACTCTGCAACTGAGTCTCTTTA
CAGAGAGACACCATTGTGATTGATCACTGAACATTGACATCCAGAATTTTACCACGACGA
CTAGACTTGGGGATGCTTATATACAGAACCCCATCTTTGACCTCAGCTTTAATCTTGTCA
AACTCAACATTCTCAGGCAGAGCAATCCTACAACTATACTTACCATAGCTTTTAGCTGAC
CATTCTTCTTCATTATTCTGCTGGTCTTTCAGAGTTTGGGCCACCTTTTCAGCCTTCACA
ACAAGCATCTTCTCCTCCACCCAAACCTTGACATCATGTTTAGTCATTCCAGGCATGTCA
AACCTCGTCTTGTACTCATTTTCACTTTCTTTTATCTCCCATGGCGTTCTTCCTCTGCTG
TAACCGCCTGTCTCGCTTGGCAGAGAAGAGGGCCACGACCCGGCATATGTAAATGGATCC
TCCATCATCGTCTCCATCGTCTCCATCATCTGCTGCACCGTTCTTGCAGTAGGAAATCGA
TCCCATAACCCTATTGGTGCAACAGGAGCAACACGTTTCCCGGGTTGGGGTTGCTGCTGT
TTGGTGCTGGCTCGCTGCAAATGATCAAGGTTGTCCCGGCCTTCTCCGGCCATGGCCCTG
ATGGTGACATAGCCGCGGCCATAGCCTCCTCCAATGTTAGGTTTCTTGAATTTGAAGTA
GTTTTAAAAGGTACAGGAAGGGAGATGCTTAGATTGGAGAGAGACTGAGACATATTCAGA

GTCTGTGTTCAATGTTACAGTCAAGTGTATCGGCAACAAAGGAGGAGGAGTTCTGTTGAT
TTATAAATGGAAGAGAAGAGAAGAGAAGACATTTCTAGAAAGAAATGGAATGGAATTAGA
ATTTGGATGAAGAATTTCTAGACTTCTCCGGAAAAAGAAGACCTTCCAGAGGACTTTCTG
GAGTTGAAACGACGTCGTTAGAATATGAGTATTTGGAGACAGATAAAGATAAACCTGCCT
CCAAACATATCCTTTCTTGCAGTCCACCACCTTTCTCAAAATTTACACGTGTCTTCATTC
AGTTCGAATGGCTTAAACACTATG
>ntig_370_consensus
GCTGGTGAAAGCAGAAACCCTGATACTCGAAGGAGATCCCAAAGACATGATTTGTCAAGC
CGCAGAACAAATGCACATGGATCTTCTTGTTCTGGGAAGCCGCGGCCTCGGCAAGATTAA
GAGGTGACTGAGATTACATTTGTATTATAAGTATGATCCATCATATTTGCAGAATCATTA
ACATGTAACACTGGAATGAGTTAATCAGTCCATACTGAACAAAACCATCTTCTGTAATTG
CAGAGCTCTTTTGGGCAGTGTAA
>ntig_371_consensus
TGACAATTGATTTTCTTCACACTACAAAAGATGGTGCCAGCGCAAGGGATGATGAATGTA
CATCACAACCAATCGAGCATCTTTTTTCACACTAAACAAATAAATTTCACTTCTATGAAA
AGGAATGAGGTCTTTCCAGCTCAACAATTTGCAACAGAAAGAGATAGGAATTCAAACAAG
TTTAATCTGTCCTATTTTGCAAAAACAGGCTTCATGCCAAATTCAACAGTCAAAATTTTA
AGTAGAAACCTATTCAAAGCAATTGTAGAGCTAAATTAAATAACAACGCAAAACATCTAA
ATCCCATCCCCAACTCTTCAGGCCTCTGTAATAGGATATTCAAAAACAACATCTTTCCCA
GTAAGCTTCCTGTAAACTACCGAAAATGTCTCCAACTTGTACTCAGTGTTGTTCCTCTCC
TTGGGATCCAAGAAAATCTTGATGATTTTGGAGCCATCAAGACGGTATCTGACACGTTTC
CCAACAATCTCCGCTGGATACACTACATCCTCAAGGATCGCATCATGGACAGCTGTGAGT
GTGCGGGAGCGAGGTCGCTTAACAGCAGAGCCTTTCTTTGGAGGCCTTGATATCCTTCGG
GTGGCAATCATAACAACATCCT
>ntig_372_consensus
CTAACAGATTTGTTTCTAGCTCTTCGGATGCAGAAACAGCAGGCTCATCTTCAATTACAC
CCAGAGGAAGTTCAGCTAACAGATTTGTTTCTAGCTCTTCGGATGCAGAAACAGCTGGCT
CATCTTCAATTGCACCCAGAGGAAGTTCAGCCAACATATTTATTTCCGACTCTTTGGTGG
CCACTGTCTTCTTGTCAACTTCAACTTTAGTCGCCATCATTTTATCAATATCAGTAGATA
AATTTGGATCTGATAACATGCTGGATTCCATTTCATGATCTTCAATCTTGTTTTCCCCTT
TCCCAGTTTGTTCAACTTCCTCCAACCTGGAATTGGATGCCTGCAGAATATCATCCACAT
GTATGGCAACCTTTGCTCTATCCTCCAGCCCATTTGGTTTATAACACAAGTCCATAGCTG
GTGCCTCCACTATTTCCTCAGAGATGGTCTTCTCTTCTGTGACTAAAGGAAATTGAAGAG
ACTGCTCAAGTTCAGGGTGTACACCATTTTTCTCTTCTAGCTGTGTCTTGTCAACTTCAA
TACCATCATGCGCTCCATTGACAGCCTTCTCTTCTAGTTGCAGATTTGAAGTTAATACTA
CCACCGGAGGGTCCATGTCACTTGCCACAAATAATTCCCGGATCTCATTTTCACAACTTC
CAAGCAACTTTTTATCAGTAAGGGAAGGCTCTGAGGTATCTTGACACTCGGTTGGTGCAC
TTTGGGAAAGCTGACTCTGGTCTTGCTCTTTCGTGTTTGAGCTACTATCACCACTGGTCT
CAACTGAATTTTCAATAATTCCTGCCTCCTTGTTTTGCATTTCTTGCATGCCTACAACAC
TATCAGTGCCAATCTTGGGATCAACAGCAAGTTTAGTAGCCTCTCTATTCTTTGTAACCA
GTGAATCAAGCTTTTCCTGCAGGGTTACAAGCTCCCTCACCAAAGCTTTTCTTACGTCTC
TTAGACTTGAATGCAAGCCCTGAACGGTATCTAATTTCAGAAGAAGCCGCATTATCATTT
CTCCAATCGCATCTCTTTCCCTATCATTTTTCTGTAAATCAGCGGTAGATTCAAGAGCTA
GTATATTGTTTCTAATCTCTGCCACCTGCTTACGAACTTCAGCCATTTGCTTCAGTTTCT
TTAGTGGTTCCATTTTTCTCACCTCAAACCCACGGTAGGCAGACTGAATAAGCTTGGCAG
CTTCCATATCTGACAAATTTTTTATCTTTCTTACCCCTTCACCATCAGCTACTTCAGCTG
AATGACATATTTTATTTTTAGTTGCCATCTCACCCACCATATCTCTCCCATCTCTTACCC
TTTCATCATCTTCAAACTTGTCAGTGTCCCTTTCAGTTCCCTTTGTAGAAGATTTATTGA
ACGTGTCTTCTTCAGAATCAATGTGCAACCTCATAGGCTTTTTCCTCTGAGATCCAGTCC
TCCAGTCTTCATTCTTGTTTTCGCAGTTTCTGTCATCTGCTAGGTGAACAACTTTCCTCT
CCATTTCCGCAGGCTCCACCTTCTCGCTCTTGTTCATGAAACAATCCTGTGTTTGTATCT
CCTGTTGCGTGTTCTCTTTCAAACTTGATGAAGCAGAAGACAGGGAAGGCTCTTTTGCTT
TTTCTAGTGATCCTTTGTTGGAGCCCGGAGGACTAGGAGATCTTGAACTGCAACTACCAT
TTTTCTTTTTTAGTAAAGGTTCAACTCTCAGACAAACGGGAGGTAACTTAGATGTCTTTT
GAGGGGATAACTGTTTGCTCAAAATGGCTCTCGTAGGACTATCTGTAGCAACAGTTTCAC
TGTACTTTGAAGTATCATCACTTACTTTCCTAGTTTCATTACAGCTGTCTTCTTTCTTCT
GTACTTCCACTCTCTTTGCCGAATTGAGTCTCTGGTTGGCAATGTTGTCCATTGTCTGTG
TGCTTTTAGCCTGATTACCAACATTATTCCTGTTTTCAGATTTGTTCAAGCTATCATCCT

CTATCTCTGAAGTCTCCACCGGAATGACCTTGAAGCTGGATAGGGGTTCTTCAGCCTTCA
AAGCAACATTTGAATCCCTTTGGTCTTCCTTGTCAGCCTGCCCTTGCTCACTGTGAGAAG
GGATCCAGATAATAGGATAAGGAAATTGCCTCCTTTTATCCTCAACTTGTTGATTCTGCT
TTCCCTTCTCAACTTCATCCTTCATCAAGGATTTGAGGCCACTAATGTCAATTGGAAACC
AGCCATTCATCTTCTTTTGGTCCTTTTGAGCATCCTCTTTTCCATCATTAGAAGGCATCC
AGATAATGGGATGTGGGAATTGCCTCAACTTATCCTCAGTCAGACAGTTGTCTATTCTCT
CCTTATCCTCTTCATGCATCAAAGGTTTAAGGCTGTTCAAGTCTAGTGGAAACCAGCCAT
TCCAAACTCTTGTCCCTTTTTCAGGAGACTTCGTATCCTCATGGGACTCTGTGTTACGTG
GAGCATTCTTCTGGTCTCCTACCTCTGATTCAAGGGGTCTTCCCTTCTCATATATTTTCT
TAATGTACTCAGGTGGTATCCACATAACAGGATAAGGGTAATTTTTTGTGTTAATAGGAA
CTAACGAATCATTTTTTCTCACAATTTCAGGGTCCTGCTCTTCGATGTTTACACCTCCAC
TTTTCTGATTGCATATGTGATTAGGGCAGCCACTGCAGTGATAGTCTCTTTGCGCTTCCT
TATCGTATTCATACCTACGCTGCACCGCTGAATGATGTGGAGTAAATTGGTAATTGACAT
GGGATGGTGCAGTAAATACAGGGTGATGTCCACAGCTATGAAGGAATGGTGACGATGTCA
AATGAGAAGCAAGAGGGTTGAAGCTGCAATAAACTGGGACGTTGACATGGTAGCAGCATG
CATAACACGGGATCATGTGGCCAGGGTTGCTGCCATTACTACAAGGTCCATACATCATAG
GATATCTAGCTTGATCCACTGTCATCTGAGGCGGTATACCGTCATAGCAAGGATAATAAC
ACTGACCAGAAGGCACTTGGCTTCTTAGGTGAGGATATGAGTCCATGTAGATGGGCATCA
TTTTAATTTAAGCAACACCCCAAACAGTTGTCGAAAAAGTTCCCTGCACCTCCCAACGTG
TTGATAATATTACCGGCAATATTTCAAGGTATCT
>ntig_373_consensus
CTAGGTTTCATTGATGAAAGGTATTTTAGACTCCTATTACAAAATACGATAAGCCATTGC
GGGAAGTTTACAATCACAAGATACAAAGATTGACAATACGAACAGTTGACATTGAATCTA
CAAGGGAGCATCATAATAATACAAAGCTGCGATGTGTTGACATCGTTGGCCCAATGTAAA
GCACCCTACAAAACCAAATCTTGAAGCTACACTGCAATCCATCACTCGCTACTTCTAACA
GCGTGATTCAAATAAAAACATGAGACCTGAAAAAAGATGCAATACATACTTTGACAACCT
CCAGATCTAGGTGGCAAGTTATTCACACTAAGGTTTTATTTTCACAACAAATACACCTAA
GAATAATAACAAATAATTTTAGACTATAGCATAATGAATAACATGCTACAGTTAGTCACT
AGAAAATTCACCACCAACTTGAAATGGTTGAGCACACGAGTTTAGCTTTAGCTGGAGGAA
ATCTAAGCGAATGGATTTCCTCCTACAGAAGCAACATGTCCTTGAGCTGGGACATTCGGT
AATTGCAAGGTTGGCGCCTGCCCTGACAGGTATGCAAAGCCACCCTGTGATGCTGCAGGG
ATAAAAGGAGTAACTGGATCTTGAGGAAACAACGACTGAGTTGCACCACCAATGAAGGTG
CATGGCAATTGTGCATTCGGAAGTGCAGCTTGCAAGGAGCTCATGTCCAAGAACATATTA
TTTTGCTCCAAATCAGTATCATAAGGAAAATCGAATGGGTTTATTGATTTAAGATCAGTG
GCATGTGCTTGTGTCTCTCCCACTAGAGGAGGCACTGATGGTGTACATAGAGGCCCTATG
CCAGCATGTAATGGTACAGCCACCAGCTGCGGTCCATGTTGAGACACAGCTCTCTGATTT
CCACCTTGATTACAATCACTGAAATGCTGATCTGTAATTGATGATGACTGATTAGCTGTT
AATTGCAGTTCAAAATTTTGCTGAATTTCCTCCATTGGAAGATTGCCAGTACAACCATCA
AAAGCATTCCATGACTGAGTGCTTGCAGCCATAGTGGGAGCTTGAACATTGTTTAAACCA
TCAAACCACAGATCAGATGATGGAGTAGGTGGGTCAGCATCAGAATTTAGATTTGCTGGC
CACTGAGAAGTTGGATTGAAGGATGAAAGTTGGTCAAATTTGACTGAAGAAACTCCATTA
ACAGGAACT
>ntig_374_consensus
TGATGCTTTCAACAATGTTAAAGCTCCCACTATAGCTACAAGCACTCAGTCATGGAAGGC
ATTTGATGATTCTACCACCAATCTTCCTTTGGAGGGGTTCGAGCAAAATTTTGAACCAAA
ATTGGCAGCTAATCAGCCTTCATCAACTGCAGATTGGCTAATCAGAGACTCTAATCATGG
TGGAAATCAGAGAGCTGTCTCTCAACATGGACCACAACCAATAACTGTACCACTGCATGT
TGACACGGGACCGCTATATACACCATCGGTGCTTCCTTTAGTGGTAGAGAAACAAGCACA
TGCCACTGATCTTAAATCAACAAACCCATTTGATTTTCCTTATGATTCTGATTTGGAGCA
GAATGATAAGTTCTTGGACATGAGCTCCTTGCATTCTGCACTTCCAAATGCACAGTTACC
ATCAACCTTCCTCGGTGGTGTAACTCAATCGTGGTTTTCTCAAGATCCAGTGACCCCTTT
TATCCCTGCAGCATCGCAGGGTGGCTTATCGTACATTTCTGTGCAGGCACCAACCTCTCA
ATTGCAGAATGTCCCAGCACAAGAACCTGTTGCTTCTGTCGGAGGAAATCCATTCGCTTA
GAATTTCTGCGGCGAAACTTGTGTTCAACGATTTCAAGTTGGTAGTGAAAATTTCTAGTG
CATAATCGATGCGTGTTCTTTATTACACTATAGTCTAAAAGTATTTATGTCATTATTCTT
GAGCCTATTTGATGTGAAAGCAAAATTTTAATGTGAATAACTTGGCACCAAATGTGGAAG
GGCTTGAAAGTATTTATTGCATCTTTTCGAGGCCTTATGTTTTTATTTGAATCACATTAG
AACTAGTGATACGCTAGAGATTGCAGCTACGGGAATTGGTTTTATAGTAAGGTTCTTTAT

GTTGGCTCAAAGATCTGGTTCTTATAATTGTGATGCG
>ntig_375_consensus
CTCCAATTTAACAAAATATCATTTAGTTACTCGTCTATTCTATCAACCAATTCATTTTTA
GCCAAAGCAAAACACCCTGTCATTTCACTCCAACTGAAGCCATCAGATCTTCATACTCCG
GATCATTCTCCTGTTTTCTGGTCACTGTTGCTGAATTAGCTCCCTGCTGTTGAGGTGGAA
TAGAGGCAGAAGTAGGTGAAAACGGATCATAAATCTGTGGTCCTCGAGGACCTGGTGCCA
ACTTATCAGATGTTAAG
>ntig_376_consensus
TGTCTAGAAATTTGATGCCAATTGGAGGGGAAATTGGGGGAGAAATAAATTACTTGAGAT
ACAAGATTGAAAAATAAATTTTGTTTGCTTAATGTTATAACCGCTTCACATTGCATAATC
AGCAAGCAAACCAAAAAAGAAGAGAGAAACTAATGAGAATCAGTAATCAAGGATGAAATA
TGCATTAATTTAATTCTACTTTCTGCAACAATCTCCAATTTAACAAAATATCATTCAGTT
ACTCGTCCATTCTATCAACCAATTCATTTTTGGCCAAAGCAAAACACCCTGTCATTTCAC
TCCAACTGAAGCCATCAGATCTTCATACTCCGGATCATTCTCCTGTTTTCTGGTCACTGT
TGCTGGATTAGCTCCCTGCTGTTGAGGTGGAATAGAGGCAGAAGTAGGTGAAAACGGATC
ATAAATCTGTGGTCCTCGAGGACCTTGTGCCAACTTATCAGATGTTAAGGGGCGATTGTT
TCCAAACTGCTGAATTAGACCTATGTGGTTTTCATGCCGATTTGCTGGTGCTGGATAATT
ATGCCGAAGCTGCAAAGGATTTCCTGACCTAGGAAGGAAAGGACCAGCTAGATTGGGCAC
TTGTGGACCCATACCAAAGTTCCTCGGTCTCATTTGAGAAACTACAGTTGTAGGAGCAAC
ATGACTTACATTTGCAAATGCTGCACTTGGGTTTCTGGCAAACATAGGAGCTTGAGGTTG
ACCCATCTGGTTGCCAACCTGTGGCTGAGGAAAACCTTGCATGACAGTTTGAGCAGTGGC
ACTAGGACCAGTCAGTCTAAAAGATGGAGCCTGAAGGTTTGGCAATAGCCCCATTGGTCT
TTGCGGTGGAGTACTCATCATTGCAGGCTGACTGCTTGGCCTGGATACAGTTAGAGGGGG
TAGATTCTGTTGCCGGTGAGGTTGAAAAGTAAAATCACCGGAACCTGGGAGTTGAGGTCT
CGAAGGTGTTCCAGTGGGTGGATTGGTAGTAGAAAAATTAGGTGGTACAAATGGAGAAGA
CTGCATAGGAGCAGAAACAGGAAATGCTGGAGAAGATCCCACTTGTGGCAGAGGACCAGA
AGGAAAAACAGGTCCTACAGAAAGATTTAGGGCTGTGCTAGATGGCCCAGATGGAAAAGC
AGAAACCATATTGCCTGCTGACATATTTGGAAGACCATTGGATGCAACACCTGGTTGTGA
AACCCCAGGACGAGGACCCTGGGGAGGAACCATTACCGGAGGCAATTGTCCCAGGTTGCT
TGAACCTAAAGATGCTCCTGACCATCCAGAAGAGCTCCCTGTAGGGGTCAATGGCCGGTC
AGGTAGTGGTCTAGGTGGAAAACTTGACACAGCTTGAGGCAATGAAGGGATCAAAGGTCT
TCCTTGCAGTTGAGCTGATGAAGTTTGTGGAGACGGCATTGGAAAATTACCTGATGAACC
AGTATGACCCAAAGGACGGATCTGCAGAATATATGGATGCGGAACTTGCATAAGAGGTTG
TGAATTTTGAGGAGCAATAGGTGAGTTTGGAAAAGTTGAAGTAAATCCAGCTGGCCGAGG
GCCAAATGGCGAAGGCATGTTCACAGGATTGGTGGGAACAGGTGCTGAAGTATTTAGAGG
ATGTATTGGAGTTTGAGGTGGACCTGAAGTAAACCATTGATTTTGGTATTGAAACTGAAC
TTGAACAGGAGTTTGCATGGATCCGACCACTGACACTACTCCCTGATTTACAGCAGCACT
ATGGATAAACGGGTTACCTTCATCCTGGCCTTGACTAAGAGTAGTGGCATTTTGCCCTGT
AACCAAAGTAGATGTGGAAATAGCAGCTAGATTTTCTGATACTGAATTAACTAGCAATTC
AATAAGGTCTGCTGCTGCATCAACTTTTTCATAGGTGTCAGCCGAGATATTAATATACAA
CTCTTCAAAAGAACTCTGGACAATTCCATCAGATGGAGATATTTCAA
>ntig_377_consensus
AAAAGAAAAGAAAAGAAAATTTGACATTAAATTCACACACGGATTCGATAATTTGGACAA
GCTTTTCTATATCTCTGTTTGTGACCAAACCCATGTCCTAATAGATTATAGCGGCTGCAA
CTCTTGCTCATGCCCAGCAGTATCAACTGGTTTGTAGGGATTTACATTTTGCTGTCTCTG
CCTCTGTATTAACCACCAAATTCCAGAAGCTGACAGTCCAGCAATCAGCGTAAGGGTAAT
GGCTAATAACACAACCAAATAATGTTGCTGCCACCATGTCCTATTTGCGGGGGGCTTGAT
TTTCCATTGAAGCAACTTATAATTTCCAAAGGTATTAGGAAGGTGCACACGATGTTCTGC
AAGCTGGGAAATTATGTTAATTGCAGTGTCATTAGAAATGTAGTTAGCAGGTCCAAAAGG
GAATATAGCCCATGTAATAAGACAACTATCTCCTTTGGATGTAACATTTAAAAGATGAAC
CTGAGAAGTATTAACATCTAACTCCTGAGCAAAGAACTCAGTAAGTTGCGGGATCTGGGG
TTCCAAATTTGAGTAGTTGATGCTCAAAGACATATCAAATGTTATTTGACCAATTCGGAG
CACCCCTGCAAGAGCATCATTCGGTGGTTCATTAGGAGGCAATTCTTCACTTGAGTTCTT
TCCACTTGAAGGAACCATTGGCGGAGTAACAGTTTCCTTCATTCGTTCCCATAACTCAGA
ACAATTGGTCTTCCAAAAACCAATCTTTGAATGTTCACGATCATACATAACAAGAGTATT
GCGAACAACGATCCCTCCAAGGAGTGTAGTTGGATCCTTTCCATTTTGGAAAATCCCCAG
ACAATATGCACCGCGAACCTTTGAATGCCGGAACAAGTAATTTTCTGGTGACAGCAACAG
CTTTTGCCCATTGCCAAATACCATCTCAACTGATGGAAAGGTATTTAAGAGTTGAGAGGC

ATCACTTGAAGCACCAGAAAAACAAATATCATGGTAATTAGGATCAGGACCACGGATTTG
CTTTAGGGACTGAAGTTCATTCATAATTGCATTCTTAAAAGCCACAAATGCTGCTTCTGG
CAGGTAGGCATATGTTGTGCCACTATCAAGAACAGTTCCATGTTTGCCATCAAAGACCCT
TGCGTTTAAAGGTAATGATTTTCCCGCAACATGTATTGCCTTCAAATTAATATTGTAGTA
TGGACTGTTGCCATCCCGAAACTGCGTACAGGGTCTGACTTAGCAAAAACCATATTTGCT
GGGGGAGATATATCACCAAGAACCATAGCACCCCCGCCAATATCCATCCCACCATAACAT
AATGAGAATGAATCACTAATCACACCTTTATCAACAAGTTGATCAACAACACTGAGATCT
CCACGGCCCAATCCCATTATTCCATCAGCATGCTGACTATAAAGGTCACCAGTTTCAACA
TTTTCACAACCAAAAACAGCGCGCTGAGGTAGAAGTTCACATTCATTGCCAAAGGATAAA
ATATCCTCACCAAGAACACCACTACTTGTACTCATCTCAGCATACTGTCTCTCATAAACA
CATTGTACCCTGTCACGGTCACAATTGCAGTCCAGATTGCATTTTACAGGTTGATATGTG
CTTGATAAGTCAGGCTCGAATTTAGGATCCTGATGCTGGCCGCACTGCCTGCAAGTAGCA
CAGGGAACATAGGTAACAGTGCTCCCTGTATCAACGATAAGAGCGAATTGTTGAGGTGGG
GTACCGATCCAGAGACGCGTAGTATAATACCCGTTGAGAAGTAGATCGTCGTAGAGTCGC
ATGCGAGCGCTAGGGTGAGAATCGGAGGTGGATCTCTGAAGATGACGACGAGAATTCGGG
AAACTGATTCTGGAGATATTAGGAGGGGAAAGGTAGAGTGGCAATACCATGGCGGGACGG
GTGCCGCCGTGGAGGAGACTGGCAGTTGAGGAGGCCGGATTTGATTGGAGGAGATACAAG
AAGATGAAAATGGTGGTGAGAAATCGGGTAAGTGCCCAAGCCATTAGTGAGGACGGCCAG
GGCAATTCAGGCGAGAACAAGACGGCGCGGATCGAGAAAAGGAATTGAATTTAAGAGTGA
AATAATATAAATAAATAAAAAAATAGTCCTCAGACTCTGAGAACGCTTCTCTATTCCTTC
GAGAGCGAGAGGGAACTCGAGGGCTCAGAATGAACCAAACAGCTTTTGTCCTCAAATCGA
TAAGATATTG
>ntig_378_consensus
AGCTGCTAGATTGGTGGATGATTTATAATTTTCATTGCATTTGTATTAGGTAATTGCTCA
GTATAGTTCCTTTTTGGAATAACGAGGTGGGAGAATAATATAGCGAGTGATTTGGTAATC
TGGGTTTGATAATGGGCTGCGATATGTATAGACATGTGAGACTATGTATAGTTTCGAGGC
ACAAGGTTTAACATGTAACTATAAGCTGCTAGATTGGTGGATGATTATTATGATTTATTG
CTTTTGTATTAGGTAATTCATAATTGTTATTTGTCCTAGTCGAAGATTTTGGACTTGATC
TGAGGTTGTGTGGGGGTATTATAGTTTAACTAAGTTAATGAGGGTTATGTTTAATCTAAA
CATGTAATTATGGTGATCAGAGTTGTTTTAATTATTTTGTAAGTTGGTCACTACAGAATT
GCAGGCT
>ntig_379_consensus
CTCAAGACTAAGGGTTGTTTTTTTATTTCAAAAAACACCATTTTGCTATTTTGTAAATTT
TTTTTACAAGAAGTTCAGCTGCGGGACTCTACAAGGAATGTAAACTTTGATTATAGTCTA
TAGCCATAAACAGGAAACAAATTATATTACTAAAAAAAATTTCATGTTTACAGGCCAAAA
CCAGGGGAAGTGTTCCAAAAAAAATGTTCAGTCCCGTGAAGCGTTTTGAGTTTTATTTCC
TGCTCACATTCCACAATGAAAAGTTCCCTCGGATGAAAGCAACGTCAATAATTAACAACG
TAGTTGCTCATTGCTGAATCAGGTTCCGAATAACATATGGAAGAATACCTCCATTGTTGA
AATAAGCCAATTCCACCTCAGTGTCAAAGCGAACAGTACAGGTAAAAGATTTTCCCGTGT
CTGTTGTGACAGTCACATCTTGGCCAGGCCTAATGTCACTGATCTTACTTGGGAGATCAA
TAGTATAACGCTCATGACCAGTCAAACCCAGAATGTCTGCGTCCTCTCCAGTCTTAAAGC
AGAGTGGAATGATACCCATTCCCACCAAATTGCTGCGATGAATTCTCTCAAAACTTTTGG
CAATCACTGCTTTGACTCCCAACAACATTGGACCCTTGGCAGCCCAATCTCGAGAACTTC
CACTTCCATACTCAGCTCCAGCCAAAATAATTGTGTCATGACCAGCAGTCTTGTATTCCA
TTGCAGCATCAAACACATAAAGTTTCTCCCCTGTTGGTACATGGATAGTCTTTGGGCCCA
CTTCCCCATTCAGAAGCTTGTTTACTAGGCGAATATTTGCAAAAGTTCCCCTAGCCATCA
CTTCATCATTGCCACGACGACTACCATAAGAGTTGAAGTCCTTGCGCTCTACCCCACGCT
CAAGGAGATACTTTGCAGCTGGACTGTCTTTGTGGATGCTTCCAGCGGGAGAAATGTGAT
CTGTTGTGATACTGTCACCAAAGTTTAGCAAGCAGTAGGCATCCTTGACTCCATGGGCTC
CAGGAGGATCCATAGTCATGCCCTTGAAGTATGGTGGCTCATGGATATAGGTAGAGCTGG
GTTCCCATGAGTACAACGTACCGCCGGGTACTGATAGCTGATTCCACATGGGGTTGCCCT
TTGTGATAGACTCATAAGTACTTCTAAACATATCAGGCAGCACACTGGATTGAACAACCT
CAGCAATTTCTTCAGTAGTCGGCCAGATATGCTTAAAATAAACACTCTTACCATCTTTGC
CCGTTCCAATTGGCTCCTTCTCAAAATCTATGTCAACTGTGCCAGCAAGAGCATAGGCAA
CAACTAGAGGAGGTGATGCAAGGTAATTAGCTCTCGTCAAGGCGTGAACACGACCCTCAA
AATTCCTGTTCCCAGACAGCACAGCAGCAGCAACAATATCATTCTCTGAAATAGCAGAGG
CAACAGATTCATCCAATTCTCCAGAATTTCCAATACATGTGGTGCAGCCATATCCAACAA
TATTGAATCCCTGCTGATTTAAATATTTTTGCAGTCCACTTGCAAGTAGATATTTAGTAA

CGACTCCTGAGCCTGGGGCAAGACTTGTCTTTACCCAAGGTTTGACTTTTAGACCAAGTT
CACAAGCTTTTTTTGCAACAAGACCAGCACCAAGCATCACACTTGGGTTTGATGTATTGG
TGCAACTTGTGATTGCAGCAATGACAACACTTCCATGCTTAAGCTCTGCTGGTTGTCCAT
GGAAAGAAAACTTAGCCACTTTTTCCTGTGCCTCTTTAGGAACTGCAAAGCCCTTGAACC
CAACTTTATTGCCAAGACATGAATGCCAATCTGCCTTCATTTCCCTTAAAGGAACACGAT
CATGAGGTCTCTTTGGCCCTGAAATACAGGGTTCAACATCAGCAAGGTTCAGTTCGAGGT
ATGAAGAGTACACCCTTTCTTGTTGGGGCTCATTGTAGTCAACAAACATTTTATTTGCNN
CCCATGGTTGCACCATATTCAGGAGACATATTAGCAATAGTAGCCCTATCAGCTAGTGAA
AGCTCACCCATACCATCCCCATAGAACTCGACAAATTTCCCAACAACACCATGTTTCCTC
AGCATTTGTGTCACAGTTAGAACCAGGTCAGTAGCCGTGACCCCATTGCGCAACTTTCCT
GATAGCTTGAACCCAACAACACCAGGCAACACCATGCTCATAGGCTGGCCAAGCATTGCC
GCCTCTGCTTCAATACCTCCAACACCCCAGCCAGCAACTCCTAACCCATCAATCATGGTT
GTGTGAGAATCAGTTCCAACCACACTGTCAGGGTATAGTATGCTATCAGTATTGAAGACA
ACCCGTCCCAGATATTCAAGATTGACCTGATGCACAATACCAGAGCCAGGAGGAACAACA
AGCATGTTGCGGAAAGCATTTGACCCCCATTTAAGGAAAGCAAATCTCTCCTTGTTCCTC
TGAAATTCAAGCTCCATATTTGCCTGAACTGCATTTTCGGACCTTGTTACATCAACTTGA
ACTGAGTGATCAATGACAAGATCTACAGGAACCAAAGGATTGATCTTGTTAGAATCACTG
CCAAGCTTGTTCATAGCATCACGCATACAAGCAAGGTCAACAACAGCTGGTACTCCAGTA
AAATCCTGTAAGAGAACTCGTGCAGGCTTAAACGGAATTTCAACTTGCTTTGGAGCTGAA
TTTTCCCAGTCAATAATTTTCTCAACATCTTCCTTTTTGACTTGAAAATTATCACAATTA
CGAATTGCAGATTCAAGAAGGATTCTAATAGAGTACGGTAGCTTGTCAATCCTTGGGTCA
TTAAGGGCAGGAAGGCTATAGAACTTTCCAAACTCGCCACCTCCAGGTTTTGGAAGACTG
GTCAAGATCCCTTTGAATGCATGATCAGTAGCAATAGTGGCAAAGGAGCGTTCAAGGCGG
TCGATGATAGGAGCAACGGTTCTGATCTGAGCGCGAAGACTTAGAGGTGAGCGCCAATCG
ACACCGTGGCTCCAGCGAGGGACAGAGCAGCGGAGAGATCGACAGGCAGAGGATAAGCTA
AGAGATCGGTGAGTGATGAAGGTAGAAGCAGAAACGTTGGAAGACGAAGATGATGAGGGC
TTCAAAGAGGATAAAGAAGAGATGAATCTTGATCTAGAAGCTCTAAGTAAAGAAGAAGCT
GAAGTATACATGATGTAGCAGTATGTCTATAAGGATCACGCAGTCGCATAAAACTGGTAA
GATAGGGTTTGGTGCAGAGCAGAGGTGAGCAAAATTAATAATTCAGCTATGGCGTACGGT
AGAGTATTCTGTTTGAGAGAAGAGGAGGAGCGGAGACGAGGCGTGTGGAGAGCAAAAGAA
GCCTAGGGCTGCGTGCCGTTCCTATAATTGAAAAGTATTTAACGGTGCTTCTTCACCGTT
CGCCTCCTTCAAAATTGGAAAGTTCAAATAGTTAG
>ntig_380_consensus
AGTGCCAGGCTAAACGAGCAGTCTTTAACTTCAAAGGAGTAAGAATAATGAGTTCCATAA
AACCTTCAATGGCGAAAATAGTAACAATTCTCCAATCATTTAAATTAAAGAACATTCCAG
TCTCTACATTACATTCCAACAGCAACTATGAATCAGAATTTGCTTTGGCCTATCCCTCAC
AGGGCCCCACTTGGTGTTGTTTCTTTGGNNNNNNNNNNNNNNNNNNNNATAGTTACAAAC
TATAAACTAGAGGAAAACGGGAGAGATAATTCCAACACAGAATTATGTATGATTATATAA
TCAACAGTTAAAAGAGGAGGCGTCCACGGATAAATTAGGGACATCAACATCTTGAAAAGT
TTGCTGCGAAGGCTCCACAGAATATGTAATCGGTATAAGACACTGTTAACCACAAAAGTA
TATTCACCCAACATGGAAGAGAAAAGTCAGAACAGAAGCAAAACTACAGAAAAGCAAGGT
GGAAATAGGAGAGGGACACTTGGATCAATCGTCAAACTCGTGCATATGATCTAAAAGATA
GTTGGCTGCCAACTCCTCATTCTTGTTGCATGCAAAGAAAACCTCCAACACTAGGGAGCG
ATCAAAGCCCATTGCTTCAAGACGCTCAATTGCTTCTCTCTCTTCAGGAGTCACAGTAAC
AGCCTGTTGCATTGCTGAGGCCAATGGACCTAGTATGTTCCCCTCTCCTCCTTCAACAGG
CTCATTTATCAATCGCAAGAAGTCAGCCTGATGCTCTTGGATGAGCCTCATAAGCCCTGG
ATTTTGCTTTCCAAGCTCCTGAAGCATAGGCTGCAAAATTTGAGGATTTGCTTGCACCAT
AGCTCGCAATGCTTGGAACTGCTGACTGTTGCGCAAGAAATCCAAGGTGCCTGCACTTGC
GTTTGAACCCATGTTCGGAAGACCCTGAGGAAAAAGATCCAGTGGGTTTGCATTGGGGCC
ACTTGCGGGTGCAGGTGCTGCTGGCTGTTGAACCTGGGCTGGTGGTTCAACAGGCTGCCC
ACCAGCAGGGACTCGAGAAACTGGTGGAACTCCAGTTTGTTCAGGAATGCCAGAATATAG
ATATTCAACAGCTCTTTCAGGGTTGTTATATGCAGCACGTAGAGCACGGAGAACAGTTTC
CCTGTCCCAACCACCTCCACCCATATCAAGTATCTGTTGCACAATAGTTTCTAAAGTACT
TCCGGCAACAAGATTTGATGCAGCTTGGCCATAAACATCTGATCCTGAAAAGACAGGTGC
AGCTGCAGCAGGGGTAGATTCAGGTACAGCCTGTGGCGTTGCTACCACTGGTGCAGGAAT
CTGAGATGTTGTGGGTGGTTGCATCGATACAGCAGGTGCAGTTGAAGTTGGTTGGGCCGG
ATTTGCTGGCACAGCTGAAACAGTTGAGGGTCCACTTGATGAGACCTTACTCTTGGTCAA
CATGACAACTACAAAACTGTTTTCAGTAACTTTGTTTTCATCCAGGGTTGTGTCATCCTT

GAGAACCTTTCCCTGGTGAATAAGCATCAGTTGCGCAGCTGGACAAACATCTGCACCTTG
TACACTTTCTATATTTTTCTTCACATCAGCAACCTTGTCTTCGGGCTTCACTTCGATCTC
GAAGTGAGTTCCCTTCAAAGTCTTAACGACCACCTTCATCCTCTCCTGAACTCACTGCTC
CGAACAGTCTAAGATTCTCCACCGAATTAACCGAGCGTCTCTCACCGCCGGCGATCAGTC
TCGAAGAGAGACAAAAGCGAAATTATTATTATTATTTAT
>ntig_381_consensus
GTCACTCAAACACATTAACCTTTTAATTATAAAAAGGAACAGACCCAGCTTTCCCCGGGA
GACCTTTATCGTTCACTTTGCCTTTGCCTAAATAAAATTAGTTAACCATAATAATAATTT
CGCTTTCATCTCTCTGTAAAAGTGATTGCCGGCGGTGACTGTAGATCGGATAATTCGCCG
GTGAGTCGAAACTGTTGGGATTAGGAAGTTTGGGAGAAGATGAAGGTTTTTGTTAAGACT
TTGAAGGGAACTCACTTCGAGATCGAGGTGAAGCCAGAAGATAAGGTGGTTGCTGATGTC
AAGAAAAAATATAGAGACTGTACAAGGTGCAGATGTTTATCCAGCTGCTCAACTGATGCTT
ATTCATCAGGGAAAAGTTCTTAAGGATGAAACAACTCTGGAAGAAAACAAAGTTGCTGAA
AACAGTTTTGTTGTCGTCATGCTGACCAAGGGTAAGGTTTCATCAAGTGGAGCCTCAACT
GCTTCAGCGGCACCAGCAAATCTGGCCCAACCAACTACTACTCCACCTCCTGTATCAACA
CAACCATCCACAACACATCAGATTCCTGCACCAGCTGTAGCAATGCCACAGGCTGTACCT
GAATCTACCCAGCCTGCAGCTCCTCCTGTCTCTTCAGAATCAGATGTCTATGGCCAAGCT
GCATCAAATCTTGTTGCAGGAAGTAATTTAGAAACTATTGTTCAACAGATACTTGATATG
GGTGGAGGTATTTGGGACAGGGAAACAGTTCTCCGTGCTCTACGAGCGGCATATAACAAC
CCTGAAAGAGCTGTTGAATATCTTTATTCTGGAATTCCTGAACAAACTGGAGTTCCACCT
GTTTCTCGAGCTCCTGCTGGCGGGCAACCTGTTAACCCACCAGCCCATGTTCAACAACCC
ACAGCACCTGCTGGTGGTCCCAATGCAAACCCACTGGATCTTTTTCCTCAGGGCCTTCCA
AACATGGGTTCGAATGCTAGTGCAGGTACTTTGGATTTCTTGCGTAACAGTCAACAGTTC
CAAGCCTTGCGAGCTATGGTGCAAGCAAATCCCCAAATTTTGCAGCCTATGCTTCAGGAG
CTTGGAAAGCAAAATCCAGGGCTTATGAGGCTCATCCAAGAGCATCAGGCTGACTTCTTG
CGTTTGATAAATGAGCCTGTTGAAGGTGGAGAGGGGAACATACTGAATCAGTTGGCCTCG
GCAATGCCACAAGCTGTGACTGTGACTCCTGAAGAGAGAGAAGCAATTGAGCGTCTTGAA
GCTATGGGCTTTGATCGCGCCCTTGTGCTGGAGGTGTTCTTTGCATGTAACAAGAATGAG
GAGTTGGCGGCCAACTATCTTTTAGATCATATCAACGAGTTTGAGGATTGACCAAGTGGC
TCTTACGTGTTTCCTCCTTTCTTTACGTTAGTTTTTATTTATGCATCTGACTTTTCTTTT
CCTTGTTGGGTGAATATACTTTTGTTGATAATAGTGTCTTATATCGATTTTATACTCTGT
GGAGTCTTTGGAGCAAACTTGTCTAAAGATGTTGATGTCCCCAATTTGTCCGTGGACGCC
ATAACATTTTCTCAGTTCCTCAGTTCCTTCTCTTTAATTATTGTTAATATAATTATACAT
AATTCTGTGTTGGAATTAGACCTCCCTCTTCTTTCATTTATAGTTTGTGACTAGTGATTT
CTTAATTTTTATGAACTTCATTGTATAAACACATTCCTTTCTTTCTCTTTATTATTTTTT
TTTTTCTGCCAAAGAAACAACACCTTGGGGGTGTGGGTGTATGAGGGATAGACTAATACA
GATTTTGATTTGTAGGACTCCAGGGATTCTAGCTGTTCTTGTTTGTATCGGAGAATCTTT
TCTTGGTCAAGGGAGAGAGAGGTTGAAGATTCGGTTTGGAGGGTTGATCTATTGAATGAC
TCGGCATTTCTTTAATCTTAATGATCTAGAGAGTGTGGGTCACATTTAGAATTAAAGATG
TTTTGTAAAAATTTCAGCTATTGAATCATAATTAAAAG
>ntig_382_consensus
CTCCTGAAAGAAATTACTATCTGGGTACAAATTGCTCAGAGCTGTCCTCAGAGATGTAGG
GGCAACTGCTTCTTTCCGCATATCGGTTGAAGCAACACTTAGGGCAGTGAAAATCTCATA
CAATGCACAGACAACACATGGATCTCCCACATGCACATGTTCTGAAGGTGATCTCCTCAA
GAACTCCTCTCGAAACTGTACTGTATAATAACATTCAGAAAGCAATTATATTCACCCACT
TCATTCTTCAACCCAGTTCCATACATGTCAGTTCCATTCACCTTTTCACTGATGACTTCA
TTTGATGAAACCCCGGAAGTATTTGCTTCCAGAGGTATCTTCTGTGGTATCCTCAAATTT
GAAGTCAAGGGCATTTTTTGAAGTGCTTGAAAGGCATCAAGACTTTGGCGTACAGCTTGC
TTTAGGTCAGCTTGGAACCTTTCTTCGTCATCCTCCTCTGCTTGTAGTTGTCTTAACGTC
TTTGTTACACTATCTCCCGGATTAGGACCAGCACCAGTTTGAAAGCTATCATGGGATCTA
TTAACTTCAATATCATCCTTTTCAGATAACAAACCCTGATTCTTCCCATCTTGAAATCTA
GTAGAACTCCTCTGCCGTCTACCTCTCCTTCCACTCCGTCTATCGCAGGCTAAAAACCCA
CCCTCGGCAGTACCTACATTAGGTATCCCTTGTTTAAAGCTGAGATTATGTGAAGTAATA
TTAGTCTGGAAACTGGAATGGGATGAATATTTGGCTGGCCCAACAGCACCATTGGCAGAA
CTCATAGGTACACCTTCTGTGTTGCTTGAAAACCCATTCTTTGACATCAAATGTATTGGT
GCCAGATTTTTAATTTGTTCATGCAAAGCACGATCATCAGCACCATGATCCAAGTAAACA
TCACAGACTCCATCCGTCAACTTTTCAGGAAATGTTTGAGCAGATCTTTTATTTGCTCA
GCAAGGTGTTTCCGTTTAGCCTCATTCTCCATTCGCCTTTGATACTCCAAGGTTTCTTCA

AGCTTTCTTTCCTCAGCTTCAAGCTCAATTTTACGTCTAAATTCCTCTTCCTGAAGTTTT
AAGTCATCACCATTCACAGAAATACCGGTCTCTGAATCTGGATGATCACCATCAGATTCT
GGATAGGAAACCCGCTCAGAAGTCTCATCATGAAGCATATCATGCTGATTACCACCAGCA
GACTTTGAATCCTTGGTTTTCCTGAAATCTTTGTTTCTTTTTTTATCTTTCGTCTTATCG
TGTGTATGCTTTGAGTTATCACCACCTCCATGGGCACTCTTCTTAGAATCAAGCGCAAGT
TCTGCTAAAAATGCTTCCCTAGCAGCATCAGACTTCTCTGTGGCATATTTCTCAGCAAGG
TCCTCCAAATGAGCCGTTAAGTATGACTGCACTAGAGGCAATAATATCAACCGATAGTCA
TAGACTGAGATAGGCTCAAGTTTGAGTTCCAATTGCTGCATCCCAGTAACATTGCGCATG
ATTCGAGCATCAATTTTGCTGAGCTCTACAGATAGATGTTCTTTCTGCCTCTGAATTGCA
ACTTCGATGCACGTGTCTACTTGATGTAAATAGTCCTTGGTTCTCCAATCATCATCTTCA
CCAGATTCCAAATCACACAACTGGGAAGTCATACCACCATAAGTATCCTCATATCCAGAT
TGATTGACATTAAGTGCTTCAGCTTCTTTTAAAACATTTGCTATTGCAT
>ntig_383_consensus
CCACAGGCTTGGCTCAGCCATATACGAGTAGCTAGTACCTCAGGGAACATGGAGGATTAA
AATGTTTTATAGAATAAATCTCTTTATACAACCACACTCCACACACAATTCCTTTAAAAC
AACAGTAACATTGATTACAGTAAAAATGTCAACCCAGTTCTAACATAAAGCTGCCAGCTG
CGTCAAATACATCATAAATTGTTTACCTAGTGAGATAGTATACAATTAAGAAAATGACCA
GAAATGATGCAATCAGGGTAAACATTCTCCGGCTTGATTTTGTTTCGAACACCATTTTAA
ACTTATCCATGGTTCCTGACAAGATTCCTCTTGAGGAATCCATATCATTGCCCATTCGAT
CCAGCATGCGGTTGTGGCTCTCCACCTCCTCGTTTATATCACCCGAAAGTCTCTTCAAAA
GATTGACCCTGTCTTGCAGTCCTTCCATAGCTCTTTCATTATCATGTTCGTCAATTTCAT
GGGAGTAAGAAGAAGAGGCTCTAATGCCACCCTCCTCAATGCCATCAAAAAGTGAAACTT
TATTATTACGGTAATCCCTTCTGGAATTCATGAGTCAAAGATCACAGAATCTTTCCTTTT
CAGTGCTCTACAGTGTATTCCCTTGGAGGGGAGATGAAATTCCGGTGCAGTAGTGGTAGA
GAAGCTCAAGAGATGAAAGAAAATAGGTGGGCTGTACATATATAAATCGAGGCAGCGCTA
TGAATCGGAACGTTATGG
>ntig_384_consensus
TTTTTTTTTTATCCATTTGAAAGAGTACTCTCCATCACTATTAGTCATGTTTTGTAATCT
ACAACTTACAATATAAAAAGAAGCGCAAACTGTTAAGTGCCGACACACCACTTCAGATGG
CACTTGATTCAAACTTGTCCTAAAAAGTTCACATAAAGATACAGTATCACAGCTATTTTC
TCTAGGTGTTTCAACACGTTAACTAAACAGCAATCACGGCCCAGGCCTCAAAATTTTGGA
AAAGAGCAACAATGAAATTAACCGAAAGCTCAAGAAATCCTTGGACAGCCAGCAAAATAA
TAAACCAGCATTTGTCAACTTCTATAACCGTCGAATCAGACAAACTGTTTAATAGTTTTA
GTCAGGAAGTATTTGCTCACCATCTGCGTCTGCTTCTGTCTCAATCGTGGGTTTTGAAGG
TGCTTGGCCCGCGCCAGGGGCCTGCTTCTTCTTAATCCCACTTACAATGTCATCAATCCT
CAAAAGCATGCAGGCAGCTTCAATTGCTGTCTTAAAGGTTTGTGCTTTCACATTGTATGC
ATCCCAGATCTTTCGCTCTTTCATGTCAGTAATTGCACCAGTGTTTCCATCTATGCCAAC
CCAGGCATTTTCAACATTTGCATGCTTTCCTTGCAAGGTAGTCATGGTCCGAATCACATT
AACCCCACAATTCTGTGCCAATGTCCGTGGTATAGCTTCAAAGGCAATTGCAGCAGCTTC
ATATGGCCACTTTTCAATTCCTTCAATTGACGAACTCTTTTGCTTCAAAGTGGCTGATAC
AGTTAACTCTGTAGCTCCACCACCAGGAACAAGTTTTGGATTCTTGATTATATTTCTTGC
AACAGACATAGCATCCTGCAGATTTCTTTCCACTTCATTCAGGAGGTCCTTACTGGCACC
TCTTAAAAGAACAGTACAAGCCTTTGGATCTTTGCAATCTACAATAAACGCAAAGAATTC
ATCTCCAATTTTCTTAACCTCAAATAAGCCAGCCCCAGTACCAACATCAGACTCCTGCAA
TTCATCTGGTCTGTTCACAATGACAGCACCACAAGCTTTGGCAATTCGGTTATTGTCCGT
CTTTCGTAGCCTCCTGATTGCACTAACACCAGCCTTGCTGAGATAATGGCATGCCAAATC
ACTTAGCCCCTTTTCAGTAATTACCAAGTCTGGTTTGAACTTCAGTATCTGCATGCAGAG
GTTTTCAATATATTCCTCTTCCATTTTCAAGAGGACTGCCCAATCTTCTTCCTTAACCAA
CTCAGCATTCGTCTGGTTTTCCCCTTTCTTGTATTCAAGAGGAGAATCAAGAAGTATAAT
ACGAGGGTTGACAATCTTTCTTCTCATTTTACCAGGGGCAACAACATCTTTATTAAACAT
AACTCCATTTAGAACCCTAGAATCTTCCAACTGACCACCAGGGACCTTCTCAACCTTAAT
GTACTTCTTAATATCCACCTCCCTCAACCCTTGGCCAAGATCAACACCAACTATGGTGGT
GGCATCGATAGCTAAGTCTGCAATTAAATCGCCAAATTGACTGGTGAACTTTGTGCCTAT
GCAACTCTTAACCAGTCCTAACATCGTTGCACGATCCTTAGTATCAATGGGCATTGCAAT
TTTATCAAGCACAGCAACAGCATCTTCCAAAGCTTTGTTATAAGCTCGGCAGATAACCGT
AGGATGATAATTCTTCTCAATGAACTCTTCAGCAACATGAAGCATTTCACCAGCAAGGAC
AATGACGGAAGTTGTTCCATCGCCTACTTCTTCATCCTGAGTGCGACTTAACTCAATCAT
CGACTTAGCTGCTGGATGGGCAAGATCAAGCTCACGAAGAATAGCATTTCCATCATTAGT

AACTACAATTCCTCCACTAGCATCAAGAAGCATCTTCAACATGGACCGGGGACCCAAGGT
TGTACGAATGATGTCAGCAACAGCCTTGGAAGCCTGAATATTAGCATGATGAACCTTAGT
TCCAGACTCGCGCTTCAACGAATCCTTGAGAACGAGTACTGGAGCGTGCATGATTGTTGG
TAGAAGAGTACCACAGTCTACCGCTCTGTCCTTCAAACGCTCCAAGAAAAC
>ntig_385_consensus
CACATTTAATTACCCAATAACATTAAAATAAAAACTTGTCAGAAAAAAGTGCACATAAAA
CTAAAATATCATAGCTTACAATAAAAAAAACAACTGCAATCACAGCCAAGGCCTCAAAAA
TTTGGAAAACAGAAACAATGCCGGCAAGACAATATATCAGAATTTTCTCAAATGCTTTAA
CCACCAAGTCAATAAAACTGTTCAGCAATTTTAGTCAGGAAGTATTTGCTCACCATCTGC
ATCTGCTTCTGTCTCAATCTTGGGTTTTGATGGCGCTTGGCCTGCGCCAGGAGCCTGCTG
CTTCTTAATCCCACTTACAATATCATCAATCCTCAAAAGCATGCAGGCAGCTTCAATTGC
TGTCTTGAAGGTTTGTGCTTTCACATTGTATGCATCCCAGATCTTTTGCTCTTTCATGTC
AGTGATTGCACCAGTGTTTCCATCTATGCCAACCCAGGCGTTTTCACCATTTGCATGCTT
TCCTTGCAGGGCAGTCATGGTCCGAATCACATTAACCCCACAATTCTGTGCCAATGTCCG
TGGAATAGCCTCAAAGGCAATAGCAGCAGCTTCAAAAGGCCACTTTTCAATTCCTTCAAT
CGATGAACTCTTTTGCTTCAAAGTAGCCGATACTGTTAACTCTGTAGCCCCACCACCAGG
AACAAGTTTTGGATTCTTGATTATATTTCTTGCCACAGACATAGCATCCTGCAGATTTCT
TTCCACTTCATTTAGGAGATCCTTACTGGCACCTCTCAATAGAACAGTGCAAGCCTTTGG
GTCTTTGCAATCTATAATAAATGCAAAGAATTCATCTCCAATTTCTTAACCTCAAATAA
GCCAGCCCCAGTACCAACATCAGACTCCTGCAATTCATCTGGTCTATTAACAATAACAGC
ACCACATGCTTTGGCAATTCGGTTATTGTCCGTCTTCCTTAACCTCCTGATTGCACTAAC
ACCAGCCTTGCTCAAATAATGGCATGCAAGATCACTTAGCCCCTTCTCTGTAATTACCAA
GTCAGGTTTGAACTTCAGTATCTGCATGCAGAGGTTTTCAATATATTCCTCTTCCATTTT
CAAGAGGACTGCCCAGTCTTCCTCCTTAACTAACTCAGCATTTGTTTGGTTTTCCCCTTT
CTTGTATTCAAGGGGAGAGTCAAGAAGTATAATACGAGGGTTGACAATCTTTCTCCTCAT
TTTTCCAGGGGCAACAACATCTTTGTTGAACATAACTCCTTTAAGAACCCTAGAATCTTC
CAACTGGCCACCAGGGACCTTCTCAACTTTAATGTACTTCTTAATATCCACCTCCCTCAA
CCCTTTGCCAAGATCAACACCAACTGTCGTGGTAGCATCGATAGCTAAATCTGCAATTAA
ATCACCAAATTGGCTGGTAAACTTTGTGCCTATGCAACTCTTAAC
>ntig_386_consensus
AACCAATCACAGATTGACTTAGACAAATCCACCACTATTGCCAACTAGTTCTGTCATCCT
TCGGTAATCATTTATTACGCTAATGTATCATAAAACTATCTGTAAGAAACTGATGAAAGC
ATCAAGGAGGAAACATCTCAAGGCTTGTATTTTCTGAAAGAAACATATCCTACACAAGAA
TGTCAAGTTCCTCGAGTATGAGCAAGAAAAAAATCACTGAAGCAAAATTTTTTAAGTTGTA
GTTGGATAAAACTAGTTAAGTTTATATTTAGACACTCTGTGAAACAGCTTCTTGGTTTTC
TGCAGCTGAAGCATCTGAATTGGAATCGAGTCTCCTGAAGACTAGCTTTTGCTGGGGAAG
TTGGCCATTGGAGAATGCTGTAACCTCTGTGTCAATCAAAACTGTGTCCTCATCTTTGAA
ATCTCCTCTCAAGATACCCTTTGCAAGTTCGTTTTCAACATTCTGCTGAATTACCCGCTT
GACTGGCCTTGCACCATAGTTAGGGTCATAACCAAGACTTCCTAGAAGTTCAACAGCGGC
ATCAGTCACCTTGATTTTCATCTTCCGATCTGCAATCCTCTTTTGCACGCGTTCCAACTG
TAACTTCACAATCTTGGTTATCTGTTCACGGTCAAGAGGCTGGAAGACAATATACTCATC
TACACGGTTCATAAACTCAGGGCGGAATACAGATCTTGCAGCATCCATTACCCGCTGTTT
GATAGTTTCATAAGCTACCTCCTTTGGTAAGGTCTCATCATCTGTATTGAGTATGTATTG
TGAGCCCACATTTGAAGTCATAATGATAACGGTATTGGTGAAACTCACAGTGCGGCCCTG
TGAATCAGTCACTCTTCCATCATCCAGAATTTGAAGGAACACATTAAATACGTCAGAATG
GGCCTTCTCTATCTCATCAAATAGAATGACTGCATATGGCCTGCGTCGTACAGTCTCTGT
CAACTGTCCTCCCTCCTCGTATCCAACATAACCAGGTGGAGCTCCAATCAACCTTGAAAC
TGCATGCTTCTCCATATACTCACTCATGTCAATTCGTACAAGAGCCTCTTCTGTGTTGAA
TAGGTAGGAAGCAAGTGCCTTAGCCAGTTCAGTCTTTCCAACACCAGTAGGACCCATGAA
CATGAAACTAGCTATTGGACGGTGAGGATCAGAGAGGCCTGCTCTAGATCGCTGAATAGC
CTCAGCTACTGCTTTCACAGCAGGATCTTGACCCACAACACGTTCATGCAGCACTTCTTC
CAAATGTAAAAGCTTTTCCCTCTCTGATTGTTGAAGCTTAGAAACAGGAATACCTGTCCA
TTTACTGACGATTTCAGCAATATCATTTCCTGTAACTTCTTCCCTCAACATGGATTTTCC
AGAACTCAGGTATTCATCTAACTCTTTTTCAGCACCCTCAAGTTGGCGCTGCAGAGAGTT
CAGGCTTCCATACTTCAATTCAGCTGCACGGTTAAGATCATACTCCCGCTCAGCTTGCTG
AATCTCAAGATTTACCCTGTCAATCTCTTCTTTAATGGACTGAATACGTGTCATGACAGA
TTTTTCATGCTCCCACTGCTCTGTTAACTGAGCTTGTTTCTCCTTTAAGAGTGACAATTC
AGCCTCAAGGCGGCTCAATCTATCTTTCGAAGCTTTATCAGTGTCATTTGTAAGCGATAG

CCTCTCCATCTCCAGTTTCAATACTGAACGATTAATCTCATCAAGGGCAGTGGGTTTCGA
AGTAATTTCCATTTTTAGTTTTGCGGCTGCTTCATCCACTAGATCAATAGCTTTGTCAGG
TAAAAACCGGCCACTAATATATCGATCAGAGAGAATTGCAGCTTCCACAAGTGCACTGTC
AGAAATGCGAACTCCATGATGCAGCTCATATCTTTCACGCAGCCCACGTAAAATAGAAAT
TGTATCTTCCACAGTAGGTTGGTCAACATAAACTTGCTGGAAACGACGCTCCAGTGCTGG
ATCTTTCTCGATATATTTTCGATATTCATCAAGTGTTGTTGCACCAATACATCGCAACTC
TCCCCGACCAAGCATAGGTTTCAAGAGATTACCCGCATCCATGGCACCGTTTGTGGCACC
TGCCCCAACGACTGTGTGGATCTCATCAATGAATAGGATAGTCTGGCCTTCTGACTCTGT
TACTTCCTTGAGAACAGCCTTCAGTCTGTCCTCAAATTCTCCCCGATATTTTGCCCCAGC
AATAAGAGCACCCATGTCAAGAGATATTAGCTTACGATTCATTAAAGCTTGCGGCACATC
ACCCTGCACAATTCTCTGAGCAAGCCCTTCAGAAATTGCAGTTTTTCCAACACCAGGTTC
ACCAATTAGCACAGGGTTGTTCTTTGTTCTCCTTGAAAGAATCTGGATACACCTCCGTAT
TTCATCGTCTCTTCCAATCACTGGATCAAGCTTTCCTGCTCTCGCCATAGCCGTTAAATC
TTTTCCATATTTCTCTAATGCTTCATACTTTCCCTCAGGATCTTGGTCAATAACTGACTG
GCGTCCCCTTATAGATTCAATTGCAGATTTCAAAGTCTGTGGAGATATTTGAAAATCCCT
AAACAATTGCTTCCCAAAGCGTTGATCCTTTGTAAATCCAAGAACCAGATGCTCAACAGA
AACAAATGAGTCCCCATACTCTTTTTTATATTCCCTAGCTCGCTGGAATAGGGCTTCCAA
ATCCCTTCCTAACATTGAACCAGCTGTATCACCAAGCACCTTTGGTTGGCGTTGGATGAA
CTTATCAGTAGCTTCAAGAAGGCGTGTGTTATCAACTCCAGCCTTTGAAAAGATTCGGCG
GGCAAGCCCATTCTTCTGCTCCAAGAGTGCCTTCATCAAATGCTCTGTCTCCACAATTTG
ATGCTTGTTCTCTTTTGCCACATCAGGCGAGGACACAATGGCTTGCCAAGCCATTTCTGT
AAATTCCTGTTGTGTAATCCTTCCACTTGCAGCCTCGCATCTAACACCAAACGACCTCGA
CTTCCTGGACGACCAGTCGTACCCATTACTGAACAACCCATTTCGCTTAAATTGGACCGA
CTTCAAGTTCAAACCTTTAAAAGAGTTTCCCTTTCTTGGAAAACTGAGCAAGCACTGGGG
CTGCGAAAACACCAACGACGACGCCGTGCCGTTCCTGTGACGTGGCACCGAAGTAGCAAT
ACCAACCCCACTAAAGGAGGCTGCAGAGGCCATTGTAACCGCTTTTCTAACGTGGTATTC
GGGCAATTGAAGAAAAAAGCGATGGGTTTGCGTGGGTAAGTAGGAAGAGAACAAATATAG
AAGTCGGGTCAATGTGTTTCTTGGAAGTGTTTTCCCTGTTGCGAATACAGGGGAACGAGC
CTCCTTCTGCTTACCCAC
>ntig_387_consensus
GTTTCGGGAATGGGCGAGAAGTTCGCAGGCGAAGGACACGTAATTGAAGAGAGAAAGCAT
CGAGTTTGGCCGTAGAGTGTGTACTGTACTGGTACACTCAGGTTCAGTCACATCGAAATC
CCATACCAGATTCGCCTGTTAATTTGTTCTGCTTCCCTCTATTCATTTACCATTTCTATT
CATGTAAACCCACAGTTTTGTACATCTCTTTGATCATGAAAGGGAAAAAATAAAACAAAT
ATTCCAACAGTTACAAAATTAAATTAAAAAAAGAAACAGAAAAAGAGACAAAGGGTCGAA
ATTCACATCTCTTCTACGGGAATTAACAGAGCCGCCAAGCTGCCAAAATTCTTAAGCTGA
ATAATCGAGCCCCAGCTCCACCTTACACGTCCCTCCCTGACACGCTTCCAGTTTTCTCTT
CTTCATTGGCACCACGGGGGTTGCATGTGAAACCACCGGCTCAAACCCCAGTGTCAGCTC
CAGTCCCAACCCCATATCATCATTACAAGAGCAATCCTTGATGACTCGCTCGCCACACTC
TGGCTCGGTTCGGAAGAAAAGCGAGGCCGTTTCGCTTGTCTCTGCAGACACCATGCTCTC
ACTCTCCTTGCTCTCATTCTCAACAACGGCCAACTCCGACTGGTGACTCAGTGACGACTC
GTGGCTCGTGGACCGGTTTGTTTCATCCACATAACCCTTCTTGGTCTCCGGGCACCGCGC
TGGTTCATCAACCGATTTGTTAAAGTCAATCTTACTAGCTTTCGGCTTGATGGCTCGCTT
AACCCGGCAACGAGTCTTGATCCGGTCAACTGACCCCTTGGAGTTCTCATCTTTCGAAAC
ATGACGAATATCGTACGCCTTGAGTGGGGGACCCAGGCTGATCGCCGCGGCTTCAGAATT
AATAGGCGTAATCGGAGCCCCCTTGAGCACGGCCTCAACAGCCGCTTGGCATAGCTGCCA
ACTACCCGACCATAACAGACCCACCGATCCGTAAATCGGGTTCACTATTCTCCCGCAAGC
CTCGTATAACAAAGACCTGAAAATCGCAGGACGGAGATGTTCAGGGCCAGCGTTTATGAG
GTTCATTAAACCAGCACGGCCATAGAACTTGGCGAGAAAGACAGTGGCGTTGGCTTGAGA
CTCGGGGGATTTGATCCATTGCAGACAAGGTCGTATACTGCAATTCTCACTACAGCCTTT
GCGAAGAACGCGACACCCATTACAGCTCATACGCATTTTCAAAGTAGTATAAGAAGAAAA
ATGATGATCAAGTGTTGTTCTGCCGAAAATGAAAGAGGAAGAGAGGAGGAGGATGGTGTC
TCCTTGAATGGAAGAAGAGATGAATGAAGCGATGGTATTTAAGAGAAAGAGAGACAAGGG
TGAAGAGCCGCTGGGTTTTTCAGGTTCCTTAGCGGACGCAGCTGTTTTAATCTCCACAAC
GCTGGTTTCCACATCCAAGGAGGTTAGGGCTTGTACCACTTTTGAACTTAACACAAAACG
GATCTCAA
>ntig_388_consensus
GTAGCATATTGATACATGAAATGTCCCTGAAGGTTGATTGTGTAGAATTCAATAAAGTAC

TCTTATTACTAAAGCGAACGAACCATACTAGAAGGCTCTTGCCCTGCACTCCGCCCACTC
ACCGGGGAGTGGGGTTCCTCTCTGGACAGGGAAAATCCGTCAGCATAACACCTTGAAGAC
GAAATGTAGCCTATTCTAACAACTTGTGAAATAGTAATATAACATAATGCGGCCTGTAGA
TATGAAAACACAAGAGACGGAAAGCTTTTATTTGTTTTACAAATCTGATGTACAAATCTC
AGACTATGAACAAACCATAGCCTATTTTAGTTTAAGTTACTTAGGTGAAGTCTCTTTCAG
CTTTCAGGAATCTTCGCGGCTCATGTTCCAGAATTCGACTTCATGTTCCAGGACAAGAAT
TAGTGCTACTTCAGCTTTGTCCAGGACCTCCTTTNNACCAAAACCATCGTTGCCCCATCT
TTGGCAAGTCCCCTGTAGTTCAGCTGGGGTCTTAGCGCCATCTTCTAGACAATGAGCAAA
ACTTGCTTGATAGACAGCTTCAATGGCCCAAAAGGCAGTGATAGCCACAGTATATCCCAC
TTCTGGGCTTATCAAGTACTCCAGAAATCTGCAGTAGTTCTCGTTAGCTTTCCGAGGAGC
GATGTTGGAGAGTGGAACATCCCACTTGGAGGCTTCTCTCTTGAACCATTCAACTTCATC
GTTTAGTGCAGCCAGGCCTCCCAATATAACTTCTGTATCACCATAGCTATCGTCTGATTC
TTTCCATGCTTTCATCAGAACGCCCGCTACAAAAGGAGCAAACTCCCTCACAAATAGGTA
ATCTTGTCCCAGCCATGTTCTGAATGAGGAGAGATCGATGGTGCCATCACGAATGCTGAG
GATGAAGGGATGCTTGGTGGCTCCAC
>ntig_389_consensus
AAAAAATTAAAAAAAAAAAAAAGAAAATCATAATCTAACTCCAAACACTTCATCATTCAA
CTGATCACAGCAACACCAACCTGAAGCTCTCAACGTGGACTGATCACTGCTGTGGTTGTT
GGTACGCCCCTGGCTGCTGGTAATTTCCATATCCGGGATAACCCCCATAATACATGTTCG
GATCTTGTGGAGGAGGCGCATATCCATACGCCTCGTATCCTTGTGCATATCCATAATATC
CACCATTCCATTGAGCTTGATCAGGCTGATCCTGTTTGTTTGAAGGACTCCGTCCCCATG
AAAGTCGAATGTTCTGTCCTCCTAATTGGGTTCCATTCAACATTGACAAGGCTTGCTCCG
CACAAGCT
>ntig_390_consensus
TGGCTGCTGGTAATTTCCATATCCAGGATAGCCACCGTAGTACATGTTAGGGTCTTGGGG
AGGAGGCGCATATCCATATGCTTCATATCCTTGTGCATATCCATAGTATCCACCATTCCA
CTGGGCTTGATCAGGCTGATCCTGTTTGTTCGAAGGACTACGTCCCCATGAAAGTCGAAT
GCTTTGTCCTCCTAATTGGGTTCCATTCAACATCGACAAGGCTTGCTCCGCACAAGCT
>ntig_391_consensus
TTTTTTTTTTGAAATTCAATGCAAACTAATTCATAAAATTCTATTTTACAAAAGACCAAA
GAGTCAGCACACTCAAAAGTCATACTATAATATGATCGAATAGCTATAACGTTCAAATTA
TGAAAACATGGCGTGAGAAATACTGGATAAATTCAACAACTCCAGACATAGAACAGATGA
AACAGGAATGTTGGTAACATGCCCTAAAACCTTCAGACTTGAATTACACCATCAACCCAC
AAGGATTAGCTAGTCTGCTGTCCAGCTCTGCTCCACTATTTCCCTTACTCTTCGGTTGTA
CTCACGTTTGTTCTCACTAAACATCCGAGCAGCTTCAGAATTTGCAGGGGAATTTGGATT
GGGATCACAGAGCAATGACTGGATAGATGTGAGTATAGCTGCCACGTCATATATAGGGCT
CCATTGATTTTGTAAAATATCCAAACAAATACTTCCATCAGCATAAATATTTGGATGAAA
CATTCGAGAAACAAATCTAACTGTTGGGGGTTTGTTTGGATAATCCTCTGTAAATTGAAG
TGTCAACTTGAATGTGCCTGAGGGACCAAATATCACAGCATTCCAGAGCATAATATTGTT
CTCTTGGGGTGCCCCGCTGATGCCTGCAGGCGGGTCTTGCTGTAACCTCTTGAAATCTCT
CATAAGCCTCTTCCTCGCAGGAGTCGACATTGGCACCACCTCAGAGCAGAAGCTAGGGTT
TCAGTTTTTGCTTCTAACTGATTGAAAAACAAGGATTGATGAAAATGGAGAGAAGAAAAG
AAAGAGTGAATGAAATTCTATTTTAGGTATTTGTTGGGATGCGTTTAGCTTCTTGAGCTG
CGCTGCAGCCGTCCTCAC
>ntig_392_consensus
ACCGAACGAGAAAGTCAGTTCCCTACAAAATGTGGCATCAATCAAATCGGACCATTTGTA
TGCAAAGCATGGCATTTCATAAAAACGAAACGAAATGCAAATTGAAAAGTAAAATGTTTT
TTTTTTTAATTTTATGCAGGCACAGATACACAACAGTCATACCATACTAGGGATAAAATA
GCTCCAACGTTCATATTATGAAAGGATGATATTTGAGATACAACAGGGCAAGGTAAGTTC
AACAGTTCCAGCCATCATAGGTGGCTTAAGCGTGCGTGCCTTAGAAAACTTCAAATTTGA
ACTGACTCACCAATTGTACATGAGGCTAGTCTGCTGTCCAGCTCTGCTCCACTATTTCCC
TTACTCTCCGGTTGTACTCACGCTTGTTCTCACTAAACATCCGAGCAGCTTCAGAATTTG
CCGGAGAGTTTGGATTGGGATCGTATAGCAATGACTGCCAATCACCAAATAGATATGCAG
CTTCAGAATTTGCAGGAGAGTTTGGATTGGGATCGCATAGCAATGACTGGATAGACGTGA
GTATAGCAGCTACATCGTATATAGGACTCCATTGGTTTTGTAAAATATCCAAACAAATAC
TTCCATCAGCATAAATATTTGGATGAAACATTCGGGAAACAAATCGCACAGTTGGAGGCT
TGTTTGGATAATCCTCTGTAAATTGAAGTGTCAACTTGAATGTGCCTCCGTCCCATGGAG
TATCATCAGGAC

>ntig_393_consensus
AAAAAAAAAAAAAAAAAGATACATAATGCATGATAATGAAACATTTAAGAAAAGTTAATT
CATGGAGCTGAGTTTGAAGAGTATTTCCTTTTACAGGCTAATCTTATATGCAGAGAGATA
AAAGGGATCGGAGCAAACAAATCTTTTGAGCACTTAACTATCGGTCACAGAGGATGCAAA
TCCAATATTGAAATCAAAGGCAATGCACGTTAACTCCTCCTTGTTTCCATCGTACATTTT
CATCTTCAGAGAGTATGAGCCAGGTGGAGTGAATCCAGGTAGAACTTGTGAGTGAGATAC
CACAAAATCGCCAATTGATACAGGGCAGGAAGTCTCTTCACAAAGATCATGTGTCTCACT
ATGGATGTGCCATCCAAAGTATGAAACATCAATCACCAGTTTTCCTCCAGAGATTGCTTT
ATCTGTAGTAGCAGCAATGCTGAAGGTAGCTGTCTTGCCTCTTGCCACTGGATCAGGAGA
TATCTCAACTTCTTTGACTTCTACACCATAGTCAGCCTTCTTATCGCAGTACTGAATGTC
CCTCGCTTGAGTCAAACGTCCAACCAAACAAAGCGACAGTACAATCATCAGCTTGAACTT
GAACGTCATCTCCATGATCGATTCACAAGAATCACTCCAACTAAGGATTTTGATTTTACA
AATTTTTTTATCTATTTTAATTTTTTTTAAGAGAGTGAGT
>ntig_394_consensus
CCGCCCGCCACCGAAACTTACACCCTTTCCCTACACGACGCCCTTCCGACACCCCACCAA
AAAAGAAAAAAAAAAAAAAAAAAAAAAAAAAAAAAGGGGAGTTTGGCGCGTTCGCTTTCCA
CTATTCTCGTCCACTGTTCGTTTTCATCTACAAATACAATTACGACGCCCTGACTGATTG
GTCTCGTAATGATATCACATCATGTAGATAATATCTGACTCTTCTTCAATACAATTTCAC
TGGCATACAGACAACAACCATTGACGGCCTAACACTCTACCCAAAATTTCTCTTTCAAAA
AATATTTAATCAAATAAACGAACTGATACTCCCTTCATCCTCCAATTATGCCCAGGCCCA
TTCTTGTAACTGACCCTAGCTTTTTCCAGGCTTCCAACTGAGCCCAACCCAAGTACTTGA
TTGGAATTACTTTTACCTTCAATCCACAACAACGCGATCAATTTTAGCCTTTAACCAGAA
TGACTCTGCGATGTCTCTCCCCTGTTTGATTCTTCTTTAACTCTCTGATAAAATAACACG
TAAGCAGCCGATGTCTTTATCTCAGCTTCATTAACAGGAGAAACATGGCTATCATCGAAA
TGGTACCATCTATTCTCATCAATTAACTTGGCATAAGCTGTGTAGTGCCCACCTCCTAGA
CCACCATAATGGTTGCTGATGGCAAACAGGTCATACACATAAGATTCACCACTCTTGCTT
TTTATGTATTTACTCAAGTCAAGATTATTAATGGGAAAATTCACGAAAGTGTCAAGTTTA
TTCTTCATGTATCTGCTGTAGGAGAACCTTTTTAAGTGAAAGACAAGTACATCAGGCAAT
GTCCACAAATCTAGCTTCTTGGTTGCTTGTCTATGCTCCTTGCAACGGGGGCAATACCAC
ATGTCATCAGGTCCCAGAGGCTCTTCAGTCAAGAATGCGTCCAGACATGAAAATAAGGAG
ATAGCTTCTTGCCGGGTTTTCTGCACAGTGAAGCCTGTCTTATGTACCTCTGGAAGATCC
TCCAAGAAGCTGGGATCATATAATTCACGTACATTTTCAGTCCAGTCCAGTAGCACCTTA
ATGTGCTGCCCAGGTTTTATGACAGTATCCTTCTTAATGGGCTTCCAGCTAGTAATATTA
TGATCGCTTTGAGAGAGCTGGAAGCATAACCCCCTGCTGGGTGAATCCTCTAGTTCTGCA
CTCTCCATCAACTGGTCACAATTATTTGATGGTTCATCATCAGCTTCAGAAAGAAAGCCA
TTTTCCTTGGCCTTATGACCTTTAACTGAAGAATATGTTCTTCTCAAAGGTGACAGCATT
CTAGAAACAGCACTAGCAATGTCAGTTCCACTTAAATGATCTTCTTCTAAATAAGTAACC
ATAGGTGCTCCAAAAAGTTTCCTCTCACCACCCTTCAGAAAAGCTGTTGAAGATTTCTGC
TAGCAGAAGGCTTTCAT
>ntig_395_consensus
TGCTCATAGATACACTACAGAAATTAGGCAACAACCCTTGTCACCCTAATATTACTCTAA
CCAGAATTACTATTTCAAAAAAAATATATAAATAAAATTAATGAAATAAACAAACTAATA
CTCCCCTCATCTTCCAATTATACCCCTTTCAAGTTCTTAAATGATGATATGGTATATAGA
AAAATCAAGCCAGCTTAACCTTCAATAAAGCAAGCTTCTATCCACAAAAGAACTAAATGC
AAACAATTCTTGCCTTCTAACAGGAATGACCTTGCGATGTCTCTCCCATCCCCACCTTCG
GTTCACCTTTAACTCTTTGATAGAATAACACATAAGCAGCAGAAGTCTTTATTTCAGTTT
CATTAACAGGAGAAACATGGCTGTCATCAAAATGGTACCATCTATTCTCATCAATTAACT
TGGCATAAGCAGTGTAGTGTCCACCACCTAGACCACCATAATGGTTGCTGATGGCGTATA
GGTCGTACACATAAGATTCACCACTCTTGCTTTTCATGTACTTACTCAGGTCAAGATTAT
GAATTGGAAAATTCACAAAAGTGTCAATTTTATTCTTCATGTATCTGCTGTATGAGAACC
TCTTTAAGTGAAAGACAAGAACATCAGGCAATGTCCACAAATCTAGCTTCTTGGTCGCTT
GTCTATGTTCCTTGCAACGGGGGCAATACCACATGTCATCAGGCCCTAGAGGTTCTTCAG
TCAAAAATGCATCCAGACATGAAAATAAGGAGATAGCTTCCTGCTGGGTTTTCTTCACAG
TGAAACCTGTCTTGTGTACCTCTGGAAGATCCCCCAAGAAGCTGGGATCATATAATTCAT
GTACATTATCAGTCCAGTCCAGCAGCACCTTTATATGTTGCCCAGGTTTTATAACAGCAT
CCTTCTTAATGGGCTTCCAGTTAGTAACCTTATGATCACTCTGAGAAAGCTGGAAGGATA
ACTCCCTGCTGGGTGAATCCTCTAGTTCTGCAGTCTCCATCAGCTGGTCACAGTTATTTG
ATGGTTCATCATCAGCTTCAGAAAGAAAGCCATTTCCCTTGCCCTTATGAGCTTTAACTG

AAGAATATGTTCTTCTCAAAGGTGACAGCATTCTCGAAACAGTATTATTAATATCAGCTC
CACTTAAATGATCTTCTTCTAAGTAGGTAACCAAAGGCGCTCCAAATAGTTTCTTCTCAC
CACCCTTCAGATAATCTGATGAAGATCTCTGCTAGCAGAAGGCTTTCATCACTCTTCAGG
CAGCACGCAGTACTCAAAGCGGAAATGAGATCCTTACAGCAGCCATGCTTGATTACTGTC
ACAGTGAATGGCATTGGGAGAGCACTTCCATCAGCATAAAACACAGTTACTGTCATTGAC
CGAGTGACAGTTGAAGGTAGCGGCAGTGTCAGGTACATAAATGGATCAAAAGTAATTGAA
ATCTTGTTGCAAAGTGGGCAAACCAGTGTCGACTTATATTGACCTTGAAAAACATCCACA
ATCAAAGAATCATTGCGAGCCTTATGATTTATCCAACACTCATTTGCCACCTCCTCATCT
GGCCGACCATCAGAGTCCTTCATTTCAATATAAGGCTTTTGTTTGACACGATTCAAATCT
TCATGCAGGCCATCCAATAAGAAGGCAAGAAGTTCCTGAGAATCATGCTGGTTATAACCA
CTGAATTGGGGAGCAAATCGAGCAAGTTTTCCCTTGAAAGCACGTGGTGCAATTGTTGTT
CGCCCAGAGGACCACAATTTCCTCAACAACTCACCAAATGCAATTGCAAGCTCACCCTTG
ATGGTTCTACGGGTACCAAAGTCAGTCCATTTCCAGCAGAATCCATGTCAAAGCGAGAAG
AATAACCTCCATCCACTTGAACCTCCAGAAGAATATGTTGGTCCATTTGCAGCATTGCAT
CATCCAGGGTTTGGCTCATGGCATCCAACAGTGATCCTGAATGTTTTTGCTTATTGAAGT
AATCCCAAATGCGAGCCTTTTCTTTCGGTACTCCTTTTAATTTACAGACATTTTCATAAA
GTTCACATATAGAAGCCAATGAAAAAGTTTTTCCCAAACTTGTTCAGGAACTAAAACATA
GTCCTGCCCTTCCACCAAATTTCTATGAAGCTCTAGAGCATCACCTTCACTACCATTACT
ATTTTCTAGTAAATCAGAATTATCAATGGGTCCAGGCCTCTCTGAAGAAACCCCATCGAA
ACGTTGAGAATCAAACGATTGCTTGTCAATCAATGGTTCATTACCACCAACGTGCCTCTC
CCAAATTTTATACCACCTCGTTGAAATTAGGTAGTACAAGTTGCCTTCTTTCAAATCTAT
CACAGATTGAGTAGTCAAGTCTTGTACGATCTGTCTCTCTTCGTTAGGCGTGCAAGGCAA
GCAACTCCCTGCGTTCTCCATCATCAAAACCGAAGAATCACGAATCGTCATCTTAGATTT
TGCTCCCATCGTACTTTCAGCGATTTCGTCTTTCTCCCGTTTGCTAAAACGCACCCTTTT
ACATCCTCCAACCCTTTGCAAAACAAATAGGTTGACCGCTGTCTTCAGGCGCCTCTTCAC
GAAAATATATTTTGCCTTCCTCTTGTTTTGCAGAAACATTGCCAAACTCATTGTCTACCA
ACA
>ntig_396_consensus
TTTTTTTTTTCAATTAAACCAAACAGCTTTCATCAATAAAATATGGTGCTTGAACATCCA
ATCAGATACAATGCTTCTTCATACTATCAGTATGAATCAAAGTACTAGTACTTAATAAGC
TACTGAACAAAATTCACAGAAAACATATAATCAATCCTCGAAAAAAGGGCCGAGCAAAAC
CTTCCCCCCAAAAATTATATATATTGAAAACTGTTGCATGTGACAAGGGATCATTTACCC
CCGTTATTGTTTCTAGAGCTTCGTAAACAATGCACTTAAAGCGCCATTATAAAAACAACT
GCGACCAAGTCAAAATAGCATGAAACTGACTTGCACACAACGCTAGTCAGACATTATCAT
CAACCACTTTATGGGAAATTTCCATGGTCTTGGGTGGATTAAACCAAATGGTGCAATCAA
AGGAAGAGGCTTTCAGAAAGACACGAACATTCAAATGGCTTACAAATCAACTGTAGGATA
GGAACTTGGCATGAACATACGACCACTTCCATTGGAATCAGTAGTAGCATAATGTGAGTG
TAGCTCACCTGAGTGAGATGACTTCCGGGACTTGAGATTTTTCGGAGAAGCCGAGTAGGA
ATCATTGGAACCACTGCTAGTGGTATACATTGAGGATGTCGGTAGCCCATGGATCTTTGG
CAACCCTGTTGGATCCGCACCCATATTGTATCCCCTCTCAACTGATTCAGCTTCCAGTGG
CAACTCATCCAAAGTCCGGAAAGCCTGTTGAAGAACCCGGAGTGTTTCTCGAGTGCGCTT
TCTCTTCGTTGCTACCTCGTCAGGTTCCTGCAACATCTCTTCGAACAGGTTTTCTCTGTA
GAGCTTTTTAATGAAGACATTATGCAGCTCACGTTTTGTATGGTTCACCNNTGCTTTAGG
TACAGAGTCCTCAATATTCTTCCTAACAATGTCATAGTACGATCTCAATAGTAATTTTGT
CACAGCAATTTCAATAGACTCCTGCTCCGAATGGTTTTCTGAAGGCCTCAACACAGTTGG
GGGCTCTCTCAGATGGATCATAGAGAAAGCTAGATCCATGCTCTGAATGGGTTCACTGTA
TTGCTTGTTTGTTGAGCTCTCTTTAGCAGCATGCGCACGACCATCACTTCCTCCAAAAAT
TGAAGAAATTCCCCAACTTGTACCACTTGTATTTCCAGACGAAGCAGCTTTTTCAACATC
TGCCGTTTGCCGAACTCCCTGATCGGGAACAAAGCCATTAGCTTGTCTTGCAAGAATAGC
TCGAGATTTTAAGCTTCTTTCAGATGTTGGTGCTTTATCAGAGTCCAAACCATCCTTCTG
CCTCACTAGGGGTAGAGGAATCTTAGTAGACTTAACCTGTTGCAGAGCAATCTCGACAGC
CTTACTTCCGCCAACAAAATTTGGATGGGAAGTATTTATATAGTCCATCTCCATTTCAAT
AATGTGTCCAATCATTGTTTCAGAGGGTTCAAGGCCTTCACGCAGAAAGTTCCCTATAAC
TTCATCCATACGCTTTCTGAGAACAGGAAAGCGTTGCAGTTCATTCACAAGACAACGGTG
GCTAATCTTTATCAACTCATCATATATAAACCTGGCACACTGAAGACTTGGATCTAACAA
ACGGGCAATTTGCCTTCGTACAAGAACTTCAAATGGCACTTCTGGTACAAATAGTGCAGA
CTTAGGACCAGTTGCATTCTGAATGGCAGTCCGAATATCATCATCAGTCAAGTCCTCACA
TGGATCAACTTCCTCTAAACTCTTCACAAAAATCGATTGAAATATATAGTGTATTCTTGC

TCCACCAGAAAGCTCAGATGTTGACATTTCTTCATTTTTTCCCTCTACCATTGAAGAAAA
TGCTTCAGAGTACTTAGAAAGAATGTTCAGGAGAAGAGCTCCCTGACCAGCCTTTGATTC
TGTGATGTCTCCATAGCTTGCATGCTCCTTTGCAACAGAAACCAGGGCAGAGCTTATGCG
TGACTTTAGCCCTGGAAGTATAGCCTTGATATGCTGTACTAGAATCTGGTTCAATTTTTT
TGCCAACTGAGGAACACCACAACGATCTGCTAAACCATTATATACTGGTCGACTGCGGAA
GAACTTTTCCTCAGCCGCTAGTGCATCCTTGATACTTCGATTGTGAATAATATCCTCCTG
ACTACGATTCACAACACCAACGTAACCAAGTCGAAGTGGGATTACTTTACCCAGCAACAA
ATTGCGGGCATCAGTACCTCTGTCCATAATATCCAACTTCGTAATTACACCTATAGTTCT
GTAACCATCTGGATCAGCAATGCCAGCAATTTGAAGAGCATCTGAGTTTGCTAAATCAGA
ATTTGCAGGTGTAACTGCTAGAATAAGACAGCTTGGTTGTTTGATGTATGACATAATCAT
TGTTCTGATTCGTGCTTCAATATCAGCAGGCTGCTCACCCACAGGAACCTTTGTTATACC
AGGCAGATCAACAAGTGTGATATCAAGAACATGAGGAGAAAAAATCTTCAACCGAATTTG
CTTGTCTGATACACCTTTATTTCCTCCTGCTTCCTTA
>ntig_397_consensus
TCTCTATGATCTATTTGAGAGAGGTGAACCATACAAAACGTGAACTGCATAATGTCTTCA
TTAAAAAGCTTTACAGAGAGAACCTATTTGAAGAGATGTTGCAGGAGCCTGATGAGGTAG
CAACAAAGAGAAAGCGCACTCGAGAAGCACTTCGGGTTCTTCAGCAAGCTTTCCGGACCC
TGGATGAATTGCCATTGGAGGCTGAATCAGTTGAGAGGGGATACAATATGGTTGCTGATC
C
>ntig_398_consensus
GGATACAATATGGTTGCTGATCCAACAGGGTTGCCAAAGATCCATGGGCTTCCGACATCA
TCAATGTATACTACTAGCAGTGTTTCCAATGATTCTTACTCAGCTTCTCCGAAGAACCCG
AAGTACAGGAAGTCATCTCACTCAGGGGAGCTACCATCACATTATCCTAGTGCTGACTCT
AATGGAAGTGGTCGCATGTACATGCCAGGTCTCTATCCAACAGTTGAAATGTAAGCCGTT
TGAATGACGGTGGTGTCTTCCT
>ntig_399_consensus
AACTCAGTTTAGCAAGTTTACGGAGGAAATGAGGGTGAGTTTAAAAGCCTATAGATTAGC
TAAATTAAATGCTAAACCAGTAGGGAGGGTTGTGTATTGAGTGAGATAGGGTTGACAAGA
TGATTCTGCTCCCTTGCAAATAAATCCTCTGGCTTTGGAGCAGTTAAGTATATTTCTCTA
TACAAGTATGTTTGTTGTCTTTATATTGCAACAATCAATAGAGGAAGCAACAAGATATAT
ACTTCTGAATGCATTATGCTAGTTTGATTGTTGATTGCAAAGGTTTTGGCTGAGAGTTTT
GGTTCTTTTTTGTATCATTCTTATTGTGAGAAGGGATATCTTGATAAGGAAACTTATGTT
ACATTATGAAAGTTTTTGAGAACACATTACATATTGCATCATCCACAGAACAGAACAGAG
TTCAAGTACATGCATTAGAAACCTTATTCTCCAACAAACGACACATCGACACACATCATC
AGCATCTGAAAATTTCTGAAGAAAAACCTCTAAAGCTCTTGCTTGGATTCACTCTTGAGT
TTGACAAGTTCATCCTCGCCTCCAGCAATGCTGACAGTCCTAGGTCCTTTAATTTTATTT
GGAGATAACTTTTTCAATGACAATGTCAACACACCATTCTCAAGCTTAGCCTGAACTGAA
TCCAAATCCACATTGTCTGGTAACTTAAACTGCCTCCAAAACTTGCCATAGGATCTCTCC
ACTCTATGCCAGTGATCACCCTTTTTCTCCTCCTCTTTCTTCCTCTCACCGCTCACTCTC
AACACCCGGTTCTCCTCCACTTCAATCTTTAACTCCTCCTTCTTCAACCCTGGAACATCC
AGCATTATCACATGACCCTCTGGTTTTTCCTTCCAGTCTACTCTGGCTAGTGAGACTGCC
GGGTTCTCATCCCTGTCAATCCCAAATGGGATTTGTTCCAGCACCCGAAAAGGGTCTGCC
AACCGGTCTGCCCAGAGATTAGACAGTAGGCTTCCTGGAGTGTCCACAAATGGCAGTAGC
AGAGAGCCATTTGATGAACCAAGAAGACAAAGCAAAGATACTAGCAATGAGGTGACGACT
AAGAGCTGTGGCCTCATATTCACCTAAAAATTTGGTGCGGTTTATGTGAAGTTTTTGCTT
GGAAGATATTGTGCTCAGATGATTTTGCTTGAACAATTTTAGAAGTGGGGTTTGGTATTT
ATATGTGTTAGGGTTCATAGGTGGATAAATGGGGAAG
>ntig_400_consensus
CTCAAACCGACACCTTTTCAACTACCAACCACATAGTAAGCATAATGTTTCAATTATGTT
TCACTAGATAAATTTAGAAATTTAATCTTGAAATAGCCCCCAAGGTGACTTGAACCCATG
TCCTGAAACATGGAAGCCAGCCAAAGGAGCCACCCAGGCTACTCCCTGGGGGCAATAAGT
TGGCATTGTTGCTAGGCTATGTTTGGGATGTGTTCTAGTGAAACAAAAATGAGTGTAAAT
TCATCATATGTGGTGGACTTCTCTGGAATTCTGAGGTTGGTACACACAATACAAGCATCA
AATATTTAAGGTTATTGTTAAAGTATCAAAGCATCTTTAATATGTAATACACTTCCATAA
ACCAAACATTATTCAATACTAGAAATTCATAACCAGATTACCAGCAGCATTCCCTCTAAG
ATTTAGATGCAGTAGCTCCAGACTCTGTAAGAAGCGGAAGCAGCTTCCCTTCCTTGTGCA
AACCAGTTGTCGCATCGCAGCCGCCAATGTGTTTTCCGCCAATGAACACATTTGGCACCG
TCTTCTGTCCGGTCCACTCCGCCAATGCTGCTTGAATTTCCTTTCCATCACTTTCAGTGT

CCAATTCAATGGCTTTGTAAGTGGCTCCCACATCATCCAAAAGCTTTTTCACAGTGACAC
AGAAGGGGCAGAAAGACTTGCTGAAAACGACGACGGAATTCGATTGAACCATCTCCTTGG
CCTTAGGCAGTGCCATCTCGACAGATCTCAGATTCTC
>ntig_401_consensus
GTCTACACTGTCCCATTAAATTTCATCAAAGTGTCACTCACAGATACGACTCCTACATGG
AAAATTTCTCCTTAGTTTTTATAATCTCAGGCGTAGGCAAGATTTAAGTGGCAATTTTAG
TGTTGTGACTCCATTACTTGCTATACTTTTGTGGGATGAAGAGTAAAACTCGATGGCCTT
GGTTGCTTTGTAGTATACCTTTCTCAGCGCATCCTTGACTCTATTTATTGTATATTTCTT
TGCAGAATACCATTCTTATTATCGAAACCGAATTTATACAGAGATTTTGTATGATAAAGG
TAACCAACAGGAGCTGAAGTTATTATTTTGTTTAAAAGCCAAGACAAGAACAAAACGCTC
TGAAAACATTACCCAAAGCCTCACATTTTCAGATCACTTTACTGAGCATTTTGGGATAAC
AGTGAGGCTCTTAGGACTCTGATCATGTCAGTGAGAGAAACAAGACCAAGAAGCAAGCCC
TGTTGATCCACGACCCAGACTCGGTGGACGTGATGTCTGAGAGCCTTGCTGATCACTTGG
GCGAACGGCGAATCCATGTGGCATGTTATTAGCTGTCTTGGTTCAGTTGTGTTGGAAGTT
GTATATAGAGGGCTTGTTGAGATGTTCAGTGTGAAGTGTTGAGCAGTTAGAGGTAACCAT
GTTTGAAGTGAAGCAAAATGACAACCCTTTAAATCTGTTGCCGAAAATGTTCCTATAAGC
TGCCTCCCTTTCCCGTTTATAAGCTGTTTGGGATCTTCTTCTTGCATGGTTTGAGAGGTT
TTAACTATGGGAACAGCTTGAAGCAAAGCAGCCTTCATGCATTTGATAGCATCAATAACT
GTTGTGCGTTCAGTTATGGCGAAAACAGTTTCATTCACCGCTGCCAATTCTCTCACAGAC
TGCGACATAATATCCTTGATTTCATTGGCATACTGCATGTTCATCAAAAACTTCAACAAA
TCCATTTGCGTCAACATTCGGTAGCTCGATGCAGACTCCACAAGTTCAACTCCTGAAATG
TTCTCCATCTGGCCCTCTGTTGGAACCAGAACTCGATGGATTCCCTTGCTCAACACTTCC
ATGCAGTCTATGATACTCGTATTGGGATTTAAAGTCCAAAGACTTAAACCTTCGAGGCAA
TGCCCTATGATGGAGGAAACAGGGACGGACGTCTTCTTATCAAGATCAGACCCATAATCA
CCACCGTTCATCTGATCATCACCGGCAATGTGTGCCAAAATATCCACCATGGTAAGCATA
CCTATGTAGTGTTTCCTGACGGCTCCCGTCTGTTTATCCGACTCCAAGATCATGGACCCA
CCGGCACCGATCCAATGCCCCGGCGGTGCAGCCACCGGCACCGCCACCACCTTGTTGGCC
ACTAAAGTGTTCATAGTGTGAGCTAAGGATGCAGTGTAGGGCACCTCCACTAGCCTCCTC
TTGTCCACCACTAAATCCTTCACTTTCTTGTCTCTCAGCCTTTGATTTTCAGTCACATGA
CTGGGGCTACTGTCTTGCTCCTTTGTCTGCATTTTGTAAATGAAATGAAAGTAGAAAC
>ntig_402_consensus
CAACCGTTTATACACAATGGAAAAGTTAAAAACATTGAACACTCAATGGATACTCCATGC
AGCTTGTAATTACATTGAAAACAAAAGTATAATAACAAAAATTCATGGCAAGACTTTGCT
CTTTCCAACACACAGAATGTACAGAAGCCAGAATTGGGGAAAAAACAGCCAAAACAAAAT
CAACTGCAAAACCAAGACGCAATTTACTCAGAAATTTAATTATACAACAAAAGCACAAAA
GATGGACATCAGCACAGTGAAATGCGACTATCTCATTTCATATCCTACTTTCTTTACACA
ATATATGCTGATAAATAAAATGAAAGTTTTCCTCCTTACCACGTTTATTTCCCGCTATCC
TCAAACACTCTACCTTTATCTTCCAGGCATTCCCAGTAAAGTCAAAATACAACAAACCCA
TTTGCAATTTTCACATTTGACACCTATAATCAAATTTTCAAATAGAAACTAGTCAGGACC
GTGCATCCTTTCCCAAGCAATCAAGCCCTTCGGTTCGGCTGCGCACAATCTTATGGAACA
CCTCTCTTACTTGTCTCTCCAACGGGTCCAATCCCTCCTTTACCGTTTCAGAAACCGTTG
ACAATTCCTTCACTCTTTGTCTCACCTCGCTCTCCTTCTCCTCAGCCACTGGATACTGAA
TCGAGTCCAACAGCTCATTCATCAACCTCGTGCTGTTCTCAATCTGATGAATCTCCCTCA
TCAACCCACAAGCATTTCTCCTGTCTCGTTTCTTCGACTCCTCCAAAATCTTCTCGTGCA
ATACCATCATTGGCCCCGCCCAAATAAACTGCCTGGACATCGGAAAATGCACTTGCAAAC
CACGATCCTGACACGGAATTGCCGCCACAAGCGCCCACATTACAAACAGCAACACAGAAC
TCATGGTAAACACCAACAGGGCAAGCCCATTTGTAGACATGATTTCATTACCCTTTGGGG
CATACAAATTATTCCCAATCGCCTGCAACTGCCGGGCAGCCGACCAAGACCTCGAAACAC
TCCAGGACAACGATCGAAAATGCCCCAAGGATCGATCCTTGATGTTATTCCTTCCAAAAG
ACCGGTTCCTATGAGCCAAAGCGGAGCCGGAATCTTTCTCATCAAGCATACTAATAGCCA
AATCAATCACAGCCTTCTTAGCTCGCCGAGCCTGTCCTTCACACAACATTCTCCGATCAT
CCAAAGCAACAAGAATGATTTCAATAAGCTTCTGCCACTGTCTGATCTGCTCAATCCCAT
CACGAATAGCATTACAAACGTCAAGCGCCTTCACACTCCGTTCAAAATACTCCGCTACAA
GTCGATCCATGGGCGGCTTATGGAGATGCGACTTATTATTAAACAAAACAACACGAAACT
CATCCTGCGAAACGAGGAACAAGTTTAGAAGCTTTTGAATCCAGGCAAGCGAGAGCAAGT
CGTCCGAACCGGAGGACAAGTCCAAAAAACGGTCCGAAATCTTTCGCTGGAAAGACTCTA
AGTCCACTTCGACGTCGGAAGCAGAGACGTTTCCGGAGTCCATGGAGTGTAGGGGTTGAT
CGCGGCGGAGACTGAGAATGGTACGGCCGATGTTAGAGAAGGTTGCCGGCGTCGAGCCCT

GACAATCGGTGGCGGGCATGATTCGAAGAAAGAAAGGAAATTTGAGATGGGCGAGACTTA
GGGAAGGGTGAGAATCTAGAGGGGGATTTTATTGAGCGAG
>ntig_403_consensus
GAATGGAAAAGTTTCTTGCCCAGCTGAATAATTTTGGAAAATACATTCAAGATTGAATGT
GAAAAGTACATCCAACCACTTCCATCAAAGGAACACTTTGGGTTATTCTGAACGTAATGG
TGAAATGGATGACACGGTAGCACCAAATACATATCCCAGCAGGGTGCAACTTCACTCTCC
AAGGATTTAGAGTTTGCTGAGCCTGTTCCTAAACAATAAAAATCACTCTCTATGCTACAA
GTTTTCCAATTGCTTTGTTGATGTAACAAGAGAAGGGGATCATGTTGACGTTTTCTCAAG
AATGATACACCAGTTCGATTTGTCATAAAATCGATCAACCACCACTGCATTGTTCATATC
AGACGTCAATCTTTCAAGTTCACCTGCACTAAAGACGTGGTAATATCTATTGTACACCAC
AGCACCCTTTTTATCATCTTTCTTCGCCAGGCCATTCGCAAGAGCACTAGCAGAAGCACC
ACTGACTTCTGCTCGGTGATAAGGTAAGTGCCAAGGGACAAAATATTCTTGTTGATTGTT
GGTGGTTTGTACATCTTTTGAAACTGCCAAATTACCTTCACTTTGGGAGGGTAAACACAG
AGATTCCCACAACTTGTCAGTAGATTTCTCTCTGGATTCTTTTACGCAATCTCCCATACC
ATGTTCCTCTGTTTCAGGAATACTTTCTAATGTTGAGGATGAAGGGCTACGGATACGAGG
ACTCCCTGGTCCTATCCACTCTTCCACATATTTTTCGGAAAGCGGTGTCCACTTGGTTAC
CAAAGATCTATCTTCTTGTTCCACAGCCCAGACTGTTATTAGAACTAGACCACCCTTTTT
GACAACTCGAACTAATTCTTCAATTGCTTTCTTTCTCCTATTTTCAGTGCTTAGGTGATG
TAATACAGCTATAGATATTGCTGCATCTCCATAACCAGTTCTGTAGGGAAGGTTCACAGC
GTCTGCAACCAAAACCTCATGCCCTCTATCTGCACAAATTTTTATTAGGGGAGGACTAAT
ATCACAACCCAGAAAGAAGCAATTTGGATTTAAACCCAAGTATTTCCCATTTCCACATCC
TGCATCCAAGACAAGTGAACCTGAAGGCAAGGAATTCAAAAACGTGGCAACTTTTGGCCA
CTTAGCAAAACGAGTGGAACTGAAATGGGGGGCAATAGCATCATAAACATGATGTACGTA
CTTCTTCTCCAACTCAGGGGTGGATTTTATGCTAGAGGATGAGCACCTTTGGTCCTTGCT
TACAGAAACAGATTCTTGAAAGTTTGGTTCTCCAGCAGCAGCCAGAGAACACAAACTAGA
GGCACCTTTAAGTTTAACTTCTCTCATGATTCCGAAAATTAATGGAAGACAAGCACACCT
GATTGAATGAAGATAGCATTGATGTGGGTTTTTCT
>ntig_404_consensus
ATGGCAAAGTTGTGTTATATTATTACTCGAAAGAAGTAGACAAAAGACATATGCAGAGGC
ACATTTTATTTTAGAGGGAGCTTTAGCTTGCTTGCCCATGGAAAGAACAACATTGCCAAC
GAGAAGAGCGACTTATCGGTACTGGGGTGCCAGTTCGCCTGCCTTGACTATGTAATTTTG
TTGAGGAAAATCTTCTCCCTTGAAGAACCTCTCGAGCATATCCTTGACTCCGCCAGCATA
TCGCAACTGTGCATCAATAGTTGTGCCCGAAACATGAGGAGTCATAGCCTGGTTTGGCAT
ATAACGCCAAGGGTGGTCCCTTGGAGCCGGCTGTGGGTTCCAGACATCACCACTGTAACC
TCCAATGTGCCCACTTGAGCATGCGTCAACCACTGCTTGAGTATCCATGACGGCTCCTCG
AGCATTGTTAACAATCATGACTCCCTTCTTCAGCTTCGCGATCCTATCTTTGTCAAACAT
TCCTCTTGTTTTCTCAGTAAGAGGCACGTTGATGACAATTACATCACATTTGGGAAGCAT
TCTGTCAAGATCCTCCTCAAAGGTTGCACCGGTTTGTGTCTCCAATTCTGGGTCCATTTT
CACCCGATCATGATAGAGAAGATTGCAATTGAAGGGTTTCAACCGTTGTAGTAAAAGCCT
GCCAATACGTCCACAACCAACAGTTCCCACTGTCTTCCCTTCAAGATCGTAGGATCTATG
GGCAACACCTGCAACATTCCACTCGCCATTGCAAATCTGATGATACCCAGGCAAAAAATT
ACGGAGAAGAATAAGGATTCTCATGAGCTCATCTTCTGCTACTGACACTACATTGCTTCC
GGTGACCTCTGCAACAGTTAAACCCGCAGCAGCGGCAGCTTGCAGGTCAATATGATCAGA
ACCAACTCCAGCTGTGAGAAGAAGTTTCAAATTTTTGGCTTTTCTGATCCTCTCTGCTGT
CACATAGGCAGGGTGGAAAGGAGTTGATATGAGGACATGCGCCTCAGCAAGATGTTTCTC
CAGCTCAGAGTCTGGTCCTTCTTTGTCATCAGTGACTATATACTGATGACCTTGCGATTC
CAGCCAGTCACGGATGCCCAAAGCATTCTCCGTACAGCCCAAAAAGTTGGGATTCGTTGA
AGCATATTCGTCGCCCTTGTAAAACACCCCAACAATCTTCATGGCCATGTCTGATATCTC
TTAATTTTGAATCTGATCTCTGCTCTGCCACCGCTTGTGCTTAAATAATGCCAAGAAGAG
TAGTTCATTTTGGCTTGCTCTAGATTTCTTTCTCTTTTTGGCGCATGGAGAAGGG
>ntig_405_consensus
GGTGGGTTTAGTTGATTTAGGTGGCTTTGTAAACATTTGTTATGATTTTGAGTTTTGGCA
AGAGAGGAGTTTATATTTTTGGTTTGTTTTGAGTAATATAATAGAGATTGTGTAGTTTGG
TGGTTGGTGTTCAAGGTGGCGAAAATGAGTTTGGGCACTGCCGAGGATGATTCGGCATCA
GAGATTCATATACCAGCGGATATAGACTGGCATATGCTTGATAAATCTAAATTCTTCCTT
TTGGGTGCTGCTTTGTTTTCTGGTGTTTCTGCTGCTCTTTATCCTGTCGTTGTGTTAAAA
ACCCGTCAGCAAGTAGCTGATACTCCAGTTTCGTGCTTCAAAATGTCATATACCATGATG
CGTTATGAAGGTTTTAGAGGTTTCTACCGAGGGTTCGGCACCTCTTTGATGGGAACAATA

CCAGCTCGTGCACTTTACATGACAGCACTTGAGGTTACCAAGAGTAATGTTGGGACTGCA
ACTGTAAGATTGGGATTTTCAGATACTACAGCCACTGCTATAGCTAATGCTGCAGCAGGG
TTGAGTTCAGCTATGGCTGCACAGCTGGTTTGGACTCCAATTGATGTTGTGAGTCAAAGA
TTAATGGTTCAAGGATGCCACAATAACAGTAGCAAAGACATCATCCCTAATGTAGGCTCT
TGTAGATATAGTAATGGTCTTGATGCATTTAGGAAAATTTTGGTTGCGGATGGTCCAAGA
GGATTATACAGGGGAATTGGGATATCAATACTGACATACGCGCCATCTAATGCAGTTTGG
TGGGCATCTTACTCTGTGTCACATAGGTTCATATGGGGTGGTGTTGGTTGCTATATGAGT
AGAAAAGATGAAAGTTCTTTGAGTAATGGGTGTTGTTACAGGCCTAGTTCAAAGGCAGTA
GTGGCAGTGCAGGGTTTAAGTGCAGCCATGGCAAGTGGTGTATCAGCTATAATCACTATG
CCACTTGATACAATCAAGACAAGATTACAGGTTTTGGATGGAGAAGAAAATGGGAAAAGA
AGGCCGCTGACTATCCTGCAGACGGTTCGGAATTTGGTGAAGGAAGGAGGATTGGCAGCT
TGTTACAGGGGATTGGGGCCTAGGTGGGCTTCAATGTCTCTGTCTGCCACCACCATGATC
ACTACGTACGAGTATTTGAAACGGCTTTCTGCTAAAAGCCAATAGCAGTGGACATCATAA
CTGGAGTAGCTGCAAGAAACATAAATTTTCCCATCGAAGAGCCCTGCACTTTTTCTCTGG
CTGATTATTGTAAATTCCCAGCTTTCTTTTCCCTTTCCTGTTTTCTTGATCATGTTGGAC
ATAACTGAAAATTTGTACAGTATTTGCAGGATATCTGTAATATTGTGCTTTGCCACTTTC
ATCTATGTCCATACTGTACAAGTATATGATGTTTATGGTTAACAAGTTTTAGTTTACGTG
GGTGACATGGTCTCTGAGTCACCCATCAATATAATTGAAGTTTATGGAATGAAAGGTGTA
GGGTGAAGTAAGACTGTTGAAAGGGCAAATCCGCTCCGCCTCTTCTCACTTGTG
>ntig_406_consensus
AGCACAAATACATACAGAAAGATGGCATCAATTACACACAAAAAGGTTCAGCCTTGGCCT
AAATAAAATTTTACAGTGCCCTACACTTTCTTTCCCCACAAATAATAGTCTCTGATTTGA
ATTTACCTAAAATGAAACTTTAACACAAAATAAAGAAGTGATGACATGCCATACAAGTAA
AACTGTAATTAGATGGAAGCCTTTAAACTGGGTATTAGGACAAGCATTTTACCCAGTCAG
TTTTTTACATGGTTCAACTAAGTACTTACAGTGCCGATTCCCATCCTCCACATTTCAACT
TCAGACTTCCATATACACATTCCACAAAAAGCACATATGCCACTCCATATTTTGTGACAC
CATTTTAAAATTTACAAGGTACAGATTCTGGGAGTTTGTCTGCTAATAGTGTATCCTCTC
ATCCTTATTGAAATTCAGGCTTTCAATGCCTCTAAGAGTGGAATCAAAGTCTCGTATGTT
TGAAGTGTGTCTGCCAGAACTTGTGTGATGGTTTTCTGATGATACAACAGGTAAACTTCT
TTGCCCACTAAAAATCTTTTGAAATGTTCCTGGGCTGGCATTTGTGATACGAGGGTCAGC
TTCACTTCCAAATGCAGCTGCATCACGTGACACATCACGACTAGAGATTGCAGCCCTCCT
TGATGACCCACCTGACCTCAAGAACCTATAACTGGATGTCATAGCACCTTTAGAATGAGA
AGGATCATTTAAAACTGGAATTTTTTGTTTTGTTAAGTTTGCAGAATTCACAAGTGGTCC
AGAGTTCCTCCTGCCAGTGGGATCTGATGCAGACCAACCAGTATGTCTACTTTCTTCCCC
AGCTGCTTGTCTATCAACAATGCCAGCACCAAGAGGCATGCCAGAGCTTGGTCCAGCACC
AGGTCCAAGTGCACGAGCAGGAGGATTGGCCATCTGTGATTGTTGATACTTCAAAATTGT
CCAATCAAATACATAGTCAAACTGGAAACCTTCACGGATGAAAAGGTCACGGAAAATTCT
CTTCAAATAAGGATAGTCAGGTTTATCATCAAAGCGCAGTGATCGGCAGTAGTGGAAGTA
GGATGCAAATTCCGTAGGATAACTCCGGCACAAGGCCTCAATAGAAGTCGATACTTTCTT
CTCACTGATCTTCTCATACTTCTGCTTCTTAGTGCCAGCTTTTAATCCTTGCCATGGAAG
ACTTCCTCTTAAAAAATACATCAGAACATAGCCAAGTGATTCTAAGTCATCCCTGCGACT
TTGCTCGATGCCAAGGTGAGTGTTCATGCTTGCATATCTTGCAGTTCCTGTCAAATTCTT
ATTTTCTCTGTAAGGAATATGCTGATGTGTTGCAGTGTCTCTATACCTCTTAGCCAGACC
GAAGTCAATGATATTAACCTGATTAGCACGCCTCCCCAATCCCATAAGGAAATTGTCAGG
CTTAATATCACGATGCAAAAATGATTTGGAATGAACAAATTCAACTCGATTGATCATTTG
ATCTGCAAGCATAAGAACAGTTTTGAGAGACAGCTTCCTACTGCAGAAGTTAAATAAATC
TTCAAGGCTTGGCCCCAATAAATCCATCACAAGAACATTATAGTCTCCTTCAACACCAAA
CCATTTCACATTGGGAATTCCAGTTCCTCCTTGTAATATTTTATACAACTTTGATTCATA
CTGTAGCTGAGGATGCTTTGTCTTGATATTTTCAAGCTTAATTGCAACTTCTTCATTAGT
CTGAATATTAGTACCCAGATAGATCTCCCCAAAAGAGCCGCTACCGATCTTTCTCCCCAA
CCGAAACTTTTTTCCAACTAGCGGCTCCATAATCAGAATTCAAAACCCGTGGAAAAAAGG
TCTTGGTTGTGATCAATTTTGGTCGCACAACATTGAAACCCCTCCACTTACGATCGGAGG
CTAAGTAAGTTTGGGACTTGGGTGGAATTAAAGTTGAAACTTCTCTCTCTAGAGTCTTAG
AGAGAGAGAAGTTTCAACTTTAA
>ntig_407_consensus
GGATTTTTATTTCTTTTTGGATAAGTACCTTCAAAAGGGTTGTCTGTGATTTTTTATAAA
CATATAATTAACCCATGAATTGTTTTTACATGGCTGAAGAAGTTAATCCAGTGAACAAAT
TCAATTAAACAAAAAACCAGGAAATTTTTTTTCCCAGTGATATGGGGATACTGACATGGA

AGTAAGTTTCGTTAGGATGAATCATCTTTCTGGCATTACAGAGCTTTTTTCTCATTGTGA
ACTGGCGAGAGGAAAACATGTATCTTCACCGCCGGTGCTCCAGAGAAAAGAAGCGTATTT
GGAAGCATGATAAGGGTTGCCTGGATCAGCTGACATTGCTTGTAAATATCTCTCTTCAGC
ACCCCAGAGGTCCTTCCTTATTGTCCAGAGGAAGTCGGCAAATCTACTGAATGCCTCAGC
ATCCGGCGGTTCTATTTGCACTGCTCTCTTGTAGCATGCTTCTGCCCTATTGTAGTCCCG
CGCGACAAGATGAAGCAACTGAGCATAATTTGCGAGCAGAAGGGAATTATTGGGTTCCTC
AGCTAAACCCATCTGGTATAGGAGATCTGTTCGGAAATAATCCACATAATGTTCTGGTTC
AAGCTTCACAGTTATTGGCGTCACAAAAAGCTTCTTGGTCTCAGAATCAAGAACCTTATG
TCCTGATTCCTCTTGCAATCTTGAGGCTTCTTCCACCATAGAGTTCCACAACTCTACCTC
TTGTTCCGTCATTTCATGATTTACCTCAGACTTTTCATCAGGAAGAAATCTAGGCTGTTC
AAATAAAAGTGACGGTCTGCGCATATGCCAATCCCCACCTTCAGTTCCATCAACAGTTGG
CCTCACTTTTTTGCCACCACCATTGATGCCCACATCAGAATTTGAGTCTTCCAATTGCTT
ATCGATCTCTGACTCTGAGATGGTCTCGCAAGGCACGATTGAAGTAGCAGCAACAATAGT
GTGATCACTCATTGAATGGACGGTAAAATTCGCCAGAAGAATCATTACATAGATCATCAA
AGTGGGCGTTCGTGAGAAAACTTGTTGGAAGAGCCAAACAAATGATGTATTCATTTCTCT
CTGGACTTTACTCATAATCTCCTTTAAGTCCTCAGAATAAAGGCTCTCCCTTATATGCAA
GGCATAGTTCTGAAGTTCTCTGATAATGAAAACTAGTGAAGAAAAAGCCTTCTTCACAGA
ACAATATGCAAAATCCCCAGCATGCCCACAACTCGGTTGCCATTTTTGTTTTCTCTTTAT
AATCTTCAGTGACAAAGGGATGCCAAAGCAGCTAGCTTTCTGTTCAATATTCACTCTATT
AATTTCTTCTCTCTCCGGCCAGTGCTCCTGCTCTGGATGAATGCCAAGCAAATTATGCTC
TAGAAATTCAAACTGTGAAGGCATAAATCGTGTTGAATTCTTCATTTTTTTATCAGTTTC
AGGCTGTTTTTTACTTGCGTTGCAAGAAATTTCTCTTCTCGCATCGCCTGCGTCTTTATC
ATTTTCTCCTCCTTCATCCTCAATAACATCAAACTTTTCTGCCAAACCTTCAATCATTTT
AAATAACTCCTCTTCAGGAAATTCATCAAACTCGATGCTGCAGGATCGCTTCAAGCTATT
TTTTCTCGAATTACACAACCCAAGATTCCAATTATTCGCTCTATTTTGAAAAATTAAAGG
TGGGTTCCTTCTAGTACGAGTTGTCAACAAAAAAGAAGAGATGAAAAAGGCTGGTTTTGG
AGAAGAAAAAGAAGAAGAGAGTGTATAAGGCAAAATAGGCTGCACGCCCTTGGATGCCAA
ATTTCGTACCATTTCTAACTATGGAACCTCAAGCAAAAATTAACAACAAAGAGAAAACAC
AGAACTTTTAAATGATATAGAAGAAGAAGAACAGAATAGAAA
>ntig_408_consensus
CTGGAAGTGATTCCGTGGGCAAAACAATTGAAAAAAAAATAATTTTAATACATCAATACC
AAGAGATAAACATCCAGGCCTGCAGCCTATGAAGGAATCCTAAACACCACAACCGACCAT
TAAACAATACTTAAATACTTGGCGGCGTGGCAACCACAATAGAATCCATTCCTACCTCAC
TTGATACTTTGTCTAAAGACTCAAAACTGCTATTATCCACCACTTTAGCAGACTTACATT
ACACAAACCACCATATTGGCGCATGAACTCACTTGACATTCCATAGTTTTGCGATGAATG
ATGCCAAATATAAATAAACCACTCATCCCAAAAAGTCACCCTTCCTTACAGAATTCTTTT
CTCAATCCAAGAGCCCCATCCTCCATCATGCTCATGTTCCACTTGTTGTCTGTTTAGCCA
CATAAATCTGCTTCATCTTGGAGGGAGCAAAGCCCTTTTCTGCACTGAAAGATTGGTTCC
GCCCATGGAACCCCATCCTTCGGTGGGACCATTTGCTCCCAGAATTCTTTTTTAAAGAAG
TCCCACCTCCTCTTTGATAACTGTACCCTGAGGAATTACTGTGCTGCTGGGAAGACATAT
TCTTTTGCTGTGAAGACTCAGGCTTAAAAGTAAAATTGTTTTGGTTAACACTCTCACTGC
TGCTGCTGCTGCTGCTGATGCTATTCTTAACATCATCAGTCTTGCCAGCATCTGTGATGG
TGCTTACGGTCAAGCTCTTAGCAACACCATTCTGTGTTGAAGTCCCATGATCAGTAGAGC
TTGATGAATCAAGTAACACCAGTTCATCAGGCAACTGGTTGACAGTTGCATCACTAGCTG
GATGAAAATTCTGGCTGCTATCAGAGGGTGTTGATGTTCGGGACTCACAAAACTTATTGG
CATTAGATGACTGGTCAACAGGAGGTCCACGGTTATACTGCAGAGGAAGCACACCATCTG
GCGGTTGTTGCAATGGCATTGATATGGAAACAGGCTGATGAGGAGCTGTAGTAACATTTG
ACCACTGAGCTTGAACTGACCTATCCGAAGAGGACTGAAACGGAGAAACGTCAAACATGG
TCAAAGTAGAAGCCATAGGCATGAGAGGCGACCCAGGGGCAAGATGCTGGATTGGAGCAG
TTAAATTGGTAGGATTGCGTTGGGCAGCAACCATGTTCAAATTTTTCACCTCTCCTTCGC
CAACACCCATTGCAGAAGAAACAGGATGGCGCTTCCAATCAGATTGCTTTGCAGATGGAA
TATAGGTGGTACCCATAAAACTCAAACCAACTTGTCCAAACTGTCCTACTGGTGCAAAAT
GGTTGTAGACAACCATGTGTGGTGGGCCCTGAACCCCTGGAATACCCCCAGTTGGGCTGA
TGAAAGGGCCAGCATATCCTGTGGGAGCGCCATAGAATGAATCTACACCAGAATTGCATT
GTTGCCAGGTCCCAAGGGGAGCTGAACTAGGAGTACTACTCTTTTGGGTCTGTGTGCGAG
CAGGTGCAGATTCATCATGAGGCCCAAAAGTGAAGATCGGACCCCCCAACATTGGATTTA
TCTCATAGAAAGGAAAATGAGATGGTGGACCTCCAGGGAAATGTGAGATCATCTGGCTAG
AAGAATTTGGTGGGCTTGGTAAAGGTGGCCACAAGGAAATTGGCGGGGTCTCAACAGACA

AATCTGCTGGCAAAGCTACAGAGAGAGACTCTTCAGCCTTAGATTGACTCACTGATTGCT
GATCACCTGCTGTTATACCATCAATTTCTGCACCTCCAAAATTTTTAGTCTCTGATACAG
AGGCACAACCAGTTCCCAGTCCATTCCCAACAATCTCATCACTACTTATAGCTGCAACTG
CAATGGCAGAGGCAGCCGCTTCAGCTTCAGAATCTTCCAGATTGACACAAGTTTCATTAG
AATGCTTCTCTTTGTCAAAAAAGAGAGTGCAATCATTACTAGTTGTGGAGATATTGTGGG
CAACTTGGATTTCTGAGAGGCACGGACCAGGAGGGCCTATTCCATGAGAAATGGCGCAAG
TGCTAGGTGGGAGAAATGTAGGAGATGTCACAGCACCAAATTGAATTTTCTCCCCAGCAA
GCAAAGAGTTTATTGGGCTTGCAATGGAAGAGAATGCCTTGTCTTTTCTCAAGATAGTTG
ATGATGGCATGCTGGGCTCACCAACTGGGCTAGTATGATCTTTACTGGAAACAAGTGAAT
CAAATTGCCCAGGATTCATGGCCTCATCAAGCTGGTTCTGGGTGAGGGCCATTACCTGTT
GATTAAGCCGAGAATTGCCCCACGAGCCCATAGGTGTTTGAACATTATCCAGAACCTTAT
TCTTGCTCTCAAATATGAAGCCTGATGAGAGATTCTTTTCACCACCAGATAAGACAGGTA
CAGGGCTCTTTTGGAGAGACTTGATTGTCTGGGGATTTTTATCAGCCTGGACATCAGTTT
TCAGAGCTGGAGTGCCAATTGGAGCCAAGGGTTGAGACACTTTGGTGGCACCAAATCCCG
TAGACACTTCCATGTTTGCCAAATTGTGTCCATCAGCGGCAACAAAATCAGAGCAAATAT
CATTTGCTGCTTCACTCATCGATGAAGAAATTTTGTTTGAACTTGCCCAGACAAGAGAAC
TTTGTGAAGTTGAATGGGATTTTCTTGGCATCTTGGTGACCCGAGACTTTGCCTTGATCT
CTTTTTCTCTCTGTTCACGCCGATCATTCAGCATTTGCCTCTTCGACCTCACCTCAATAA
AGTCATCTTCGTCACTAGGAGCTTCTATGCCAGGTTGCTCAAAAACACGCACAACACCAC
TTTGTAAAGGAGTATCAACTTCCTCTTCAGAGCGAATGGTTCTTTTAAGGTTTCCCTCTT
CATGATGTGGAATGTTCTGACTCCTAGTCAAAGATTCTCTTCCTACTACCTTTCCAGCCT
TGACTCCGGAATCTATTTCCCGTGAATTTATTCTACCTGAATCAAGCAATTCCAATTTAT
TCATCTGTTTAGACAATTTATTTGACACAAAGACTCTTCTAAACCCACTTTGAGTAGAAA
ATCCAGTACCCCTTCCATTCACATTTGATTTATCATCCAGAACTATTTGGTTAGCTGAAA
CAACACCACTAGATAGCCGCTTATCAGTATTTTCCCGAACTCGAAACTCAGTTCGCTGGC
GCCGAGGCCTCCTCTGAAATCCACCAGATTCCAACCGAGAAGTTTCAGGGGCCACAAAAG
ATGACTTTAAGCCAGAATTATTTCTAACTGATACAACATATTTTTTATTTCTGCCACCAG
ATATTAGACCTTGAGCTTTTGAAACAGTCAACATTTTTTCTTTCGAACCTGGCACAGAAG
ATGCTGCTCTAGTCTGAAGCAGGTTTTCTGATTTGTTAGATAAGGATTTGAAGTTTCTCA
TGACTGAAATATGGTGAGCTTGGTCCTCCACTGGAAAACCTGAATCAGACCTTGTTTTCT
TATCACCAAGGTGGGAAGCCTTGGCATGATCCTTCTGTGCCATAAAAGTAGTTTCCATAC
TTTCACTTGCTGGTAAGAAAGTTTCATTTAATGCATTCCTTTCAGAGAGATCCACATGCC
TTTGAGCAAGGCCTAGTTGGTTATCCTGCGACAGAGATACTACATCACTTTCCATCAAAT
CATGAGAAGTTTCTGGACTAGATTGAATGGGATCTGAAACTGCTGCATTCTGATTCAATG
AAAAGTTGGCTGACATATTAGGTTGAACAGAAGGCACTGACTGAGTTGCCAAAGGAAGTA
CTCCCTGGGAAATAGGTGAAGAATATCTCAACTGTCCAAACTGGGAGAGAGGAGATTGTG
CGGGGTGAATATGGGCAAGGGATGGACCCACTTGGGTATGCAGAAGAGGCATCTGAATAG
AGCCAATCTGTATGGCTGGAATAGGAGAAGGTATCAAAGAAGGCCCTGAAAACAGCCCAA
ACTGCAGCTTAACAGAAGCCTCGACCTGACTTGGACCACCACAAATTGCAGGTGTCACAG
AGTGATCAGATGATGAATTTGGAGTCATAGCAACAGAAGACAGTATTGGCTGCTGATTAG
ACACGACAGAACCACTGTTAGCATCAACATGCATCATCAGCTCAGAAGCTACTAATAATT
GTGTACAACTGGGCTTAGTAACCAAATCCTGCATTACATTCTCAGCCTCTGAGATAATTC
TTGAAGAATCACCAGCACTTGCTTGGGAAGGAGCATCAGCAGATTGAAGTGTTCCACATA
CACTATCAACAGATCCCCGGTCTTCAACAACATCGCCATCAGAGACGTGTGGTACTGGAA
ATGAAGATTCTTCATTTTGGGGACATTTTTCGAACTCTTCAATGGGCATTACTACCTCAA
CACCCTCATTGAAGCCTAAGACCAAGTTGTTCACCATGTCAGAGGAGCTTTTTTCCACTA
GATGCATATCTCCAAACTCCTGACTCAGATTAATATTAGCATCATCTCCTTCACGAATCT
CATCTTCTTCCTCATATCCATCTTCATCCTCATCATATTCTTCTTGCTCCTGCAGTCGTT
CATCATTCTCTACTGTCCATTCTTCATCATCACCAACTGAGACAGAACTAGAGTGAGTCA
TCATATTCTCTTTCCCAGATTCCATGGACAACACAACAGGATTGTTGTCTTGTCCTAATA
AGACATCTTTATCCTCATCGGTGGATAATGCCGGCGAATCTTCAGATTCATCCAAGTCAT
CATGAGATAGATGAACAGGGGAATCAGGAGGGCTAGAGACAGAGAGTGACGATTGTGAAT
CACATCTTGGTGTAGTGTTTGTATCAAGATTCTCCTCCAGATTTGCAGTATTCCCCGGCT
GGACATCAATTATTTCAGGTTGTACAAGATTACGTTGATTGCTGCTGTCAAAACCTGATT
TCCCGGTAGTTTCACTTCTTGCTGCACGATTGTACTCTATCTCATTTTCTAGAAAAGTTG
AAGGGCCAGGGAGCTCACTCTCACATCTATAGAAAGTTTGTTGCATTGCAGTCAGAGAAG
GAGGAGGGAGTACACGAGGGTGCCTCATGGAATACCTAGACCTCCCAATGGAAGAAAGCC
CATCTGTATCAGGACATTGAAATAGTCGTTCAGGGTATGAAGGATAAGGATTGCCACGAT

ACTGACTATGTCCCCATCCAACATCACTGAATCTCTCAGTGTAATTCTCATGGAAGTCAG
ATTCTGCCTCAGCATTTCTGACATAGCGATCACCATCACCAGATATACTCCACCTCTGAC
CGCTAGGCTGATTAATTTCATCTATATGTTGCTCTTGAATTCCTGTTTTATAATAAGCCC
TTGAAGACATAAAACCAGGTTCTCCAATAAACTCTTTGCGAGGAAATGCTTTCCCTCCAA
CAGTTGCATCTTGACTGGGGCTATAGTGACCATTATCTGAGTCCTGTGGAAAGAAGGTTG
AGCTGTTCCCATTCTCAAATGCATCCCTTCTCCATGAGTTCTTAGGCTTATCTCTTTCCA
TAATGGCAGCAGAATTATCTCTAGCAAAGTGAGGCCTACAACTCATGTCAAAGGCTCTAT
TGAGACCAGAAGAATCAGAAGATGCTGAAGTTGTAATTCTCTCCATCATCCTTTCACCAT
CTTCCAAGTCAGCCAAATCTGCCACTTTGGAAATATCTCTTTCTTTAGACATTCCTGAAA
GTTTCTCTTCAGCAACAATGCAAGAATTACTAGCACTCTTAGTTGCTTCAGCCTGCCTCT
TGGCCATTCTTTCTTCCAATTCTAAGAG
>ntig_409_consensus
CAACAGGGAATGCAGAGGAGAGTCATCTGAATCTGATTTTAAGTCTTTCTAATCCTTGAA
AATCTACAATAAAAGTGATGAATGAACAGAATTTGAGGGGCGTGTCATCCCCTGAGCTCA
CATTTATCATTAGTAACAAGAAAACAAAAAATCAGCAACATCATACATAATCGTGATACA
TCAACACCAAGAGACGAACAATCGCGTTTGCAGCCTAGAAAGGACAAATAACGGAATCAT
TAGACCACAACCGACTATTAAACAATACTGGTGAACAGCGCCGTGGCTACCACCCCAACT
AGCACACTAGAATCCATTCCTCCCACTAATTTTAGTCAGAAGACTCAAAACTGCTGTTAT
CAACCAGTTTTGCAAACTTACATTACAAAAACCAGTATAGCTTTAATTATATTGGCACCG
GAACCACACACTTGACATTCCATATTTATTCAATGAATCATGCCAAATGTAAAGGAAAAT
TCATTATCCCAAAAAGTTGGTGTTTGTTCCGGCACTCTTTTATGAAGCTTATCCTCCGTC
ATGCTCATGATGGTGCTGATGTCCCACTTGTGGTCTGTTTAGCCACATAGATCTGTTTCA
TCTTTGGCGGGGCAAAACCCTTTTCTGCATTTAAAGACTGGTTCCGCCCATGGAACACTA
TTCTTCGGTGGGACCATTCACCCCCAGAATTATTCTTCTGTGAAATCCCACTTCCTCTTT
GATAACCATACCCAGAAGAACTGCTGTACTGCTGGGAAGAAGTATTCTTTTGCTGCAAAG
ACTGAGGCTTAAAAGTGGTGATACTGTTTTGGCCACCACTGTCGATGCTGCTGCTACTAC
TGCTATTCTGAACAATATCACTCTCGCCAGCATCTGTAATGGTGCTTGGAGACAAGCTCT
TAGCAACAACATTCTGTGTCAAAGTCCCAGGACCACTAGAGCTTGATGAGTCAACTAACC
CCAGTTCATCAGGCAACTGGGTGACAGTTGCATCACTAGCTGCATGAAAATTTCCACTGC
CATCAGAGGGTGTTGATGTCCGGGATCTATTGGCATTAGCTGATTGGTCAACAGGAAGTC
CATAGTTATATTGTGGAGGACGTACACCATCTGGCTGTTGTTGCAATGGCATTGATACGG
AAACAGGCTGATGAGGAGCTGCAGTAACATTTGACCAATGAGCTTGCAGTGACATATCCG
AAGCGGACTGAAATGGGGAAACATCAAACATGGCCAAAGTAGAAGCCATAGGCATGAGTG
GTGACCCAGGGGCAAGATGCTGGATTGGAGCAGTTAAGTTGGTTGGATTGCGTTGCGCAG
CAACCATATTTAAATTGTTCACCTCCCCTTCACCAACACCCATAGCAGAAGATACAGGAT
GTCGCTTCCAATCAGGTTGCTTTGCAGATGGAATATAGGTGGTACCCATAAAACTCAAAC
CAACTTGTCCAAACTGTCCCACTGGTGCAAAATGGTTGTAGACAACCATGTGTGGTGGAC
CTTGAACCCCTGGTAGACCTCCACTCGGGTTGATGAAAGGGCCTGCATAACCCGTGGGAG
CACCATAGAATGAATCCACGGCAGAATGGCACTGTTGCCAGGTCCGAAGGGGAGTTGAAC
TTGGAGTATTACTCTTCTGGGTCTGTGATTGCGAAGGGGCAGATTCATCATGAGGTCCAA
AAGCGAAGATCGGACCCCCCAACATGGGATTCATCTCATAGAAAGGAAAATGAGATGTTG
GGCCTCCAGTGAAATGTGAAACCATCTGACTAGAAGAATTTGGTGGGCTTGGTAAAGGTG
GCCACAAGGAAATTGGAGGGGTCTCAACAGACAAATCTGCTGGTAAAGCTACAGTGAGAG
ACTCTTCAGCTTTAGATTGACTCACTAATTGTTTATCACCATCAATTTTTGCACCTCCAA
AACTTTTGGTCTCTGATACAGAGGCAGAGCAAGTCCCCAGTCCATTCCCAACAATCTCAT
CACTACTAATAGCAGCAACAGCAATGGCAGAGGCAGCTGCTTCTGCTTCAGCTTCAGCTT
CAGAGTCTTCCAGATTGACACAAGTTTCAGTAGAGTGCTTCTCTTTGTCAAAGAAGAGAG
TGCAATCATTCTTAGCCTCCGAAATACTGTGGGCAATTTGGATTTCTGTGCGGCATGGAC
CAGGAGGACCTATTCCATGAGAAATGGCACGAGTGCTAGGTGGGAGAATTGTGGGAGAAG
TCACAGCACCAAATTGAATTTTCTCCCCAGCAAGCAAAGAGTTTATTGGGCTTGCAGTGG
AAGAGAATGACTTGTCTTCTGTCAAAATAGCTGATGATGGTATGCTGGGCTCACCAACTG
AGCTATGATCTTTACTGGAAACACGAGAATCAAATTGCCCAGGATTCATAGCCTCATCAA
GCTGGTTCTGAGTTAGGGCCATTACCTGCTGGTCAAGTCGAGAATTGCCCCATAATCCCA
TAGGTGTTTGAACGTTATCCAGAACCTTATTCTTGCTGTCAAATATAAAGCCTGAAGCGA
GATTCTTTTCCCTACCAGTTAAGACTGGCACAGAGCTCTTTTGGAGAGACTTGATTGTCT
GTGGTCTTTTATCATCCTGGACTTCAGTTTTCACAGCTGGAGTGCCAATTGGAGCCAAGG
GTTGAGACACTTTGGTGGCACCGAATCCAGTAGACACTTCCATGTTTGCCGAATTCAGTC
CATCAGTGACAACAAAGTCAGGGGGAACATCATTTGCTTCATTCATTGAAGCAGAAATTT

TGTTTGAACTTGCTGAGACATGAGAATTTTGCGAAGTTGAACGAAATTTCCTTAGCATCT
TTGTGAACCGGGACTTTGCCTTGATCTCTTTTTCTCTCTGTTCACGCCGATTATTCACCA
TTTGCCTCTTTGACCTCACCTCAATAAAGTCATCTTCGTCGCTAGGGGCTTCTATGCCAG
GTTGCTCAAACACACGCACAACACCACTTTGTAAAGGAGTATCAACATCCTCTTCAGTGC
GAATGGTCCTTTTAAGGTTTCCCCCTTCAAGATTCGGGATGCTCTTACTCTTTGTCGATG
ATCCTCTTCTTACTCCCTTTCCAGTCTTGCCACCAGAATCTATTTCCCGTGAGCTTGCTA
CGCCTGAATTCATGGATTCTGATTCATTCATTTGTTTCAACGATTTATTTGACACAATGA
CTCTCCTATACCCACTTTGGGTAGAAAATCCAGGATCCCTGATATTTGATTTATTATCCC
GAGCTATTTGGTTAGTTGAAGCAACACCAGTATCTAGCCGCCTATCAATATTTTCCCTAA
CTCGAAACTCGGTTCGCTGGTGCCGTGGCCTCCTCTGAAATCCACTAGAATCTGAGTAAG
ACGCTTCAGGAGCTACAAAAGAAGACCTTGAGCCAGTATTATCCCTAACTGAAACAAAAT
ATTTTTTAATTCTGCCACCAGATAATAGTCCTTGAGCTTTTGACACATTTAAAGTTTTTT
CTTTTGAACCTGACAAAGAAGATGCTGCTCTAGTTTGAAGCAGGTTCTCAGATTCTTTGG
ACAAGGATTTGAAGTTCCTAGTGACTGAAATATGGTGATCCCGGTCCTGTACTTGAAGAC
CTGAATCAGACCCTGTTTTCTTGTCAGCCAGGTGGGAAACCTCTGCACAACCCTGTTGTG
CCATAGAGGTAGTTTCTGCACTTTCACTTGCTGGCAATGAAGTTGCCTCACTTAATGCAA
TCCCTTCAGCGAGATCCAAATGCCTTTGAGCAAGGCCTGGTTGATTATCCACGGAAAGAG
ACAATGCATCACTTTTCATCAAATTAAGGGAAGTTTCTGAACTAGACAGAATAGGCTGTG
AAACTGCTGCATTCTGATACGAAAAGTTAGCTGAAATATTAGATTGAACAAGAGGCACTG
GTTGAGGTGACAAAGGAAGTACTCCCTGGGAAATAGGTGAAGAATATCTCAACTGTCCAA
ATTGGAAAAAAGAAGATTGTGAGGGATGCATATGGGCAAGGGATGGACCTGCTTGGGGAT
GCAAAAGAGGCATCTGTATAGAACCAATTTGTATGGCCGGAATAGGAGAAGGTATCAAAG
AAGGCCCTGAAAACAGCCCAAACTGCAGCTTAACAGGTGCTTCCACCTGACTTGGACCAG
CAGTTATTTCAGACATGACAGTCTGATCAGATGATGAATGTGAGGTCATATCAACAGAAG
ACGGTAGTGGCTGCTGATTAGACACGACAGAACCACTGTTAGCATCTACATGAACAATTA
GCTCAGAAGCTGCTGAAAATTGAGTAATATTGGGCTGAATAGCTAAATCCTGAATTGCAT
TCTCAGCCTCCTGGAAGATTCTAGAAGAACCACCAGTACTTGATTGGGAAGGAATATCGG
CAGATTGAAGTGTTCCACAAACACCATCAAAAGATCCTAGGTCTCCAACAACAGTTGTTG
GCACTGAAAATGAAGATTCTTCATTTTGGGGACTTCTTTCAAACTCATCAGTGGGCATTG
CAACCTCAACACCCTCATTGAAGCCTAAGACCACATTGTTCACCATGTCGGGAGAAATTT
TCTCCACCAGATGCATGTCTTCAAACTCCTGTGTCAGGTTAATATTCTCATCATGAATTT
CATCTTCTTCCTCATATCCATCTTCATCCTCATCATATTCTTCTTGCTCCTGTAATCGTT
CATTCTCAATTGTCCATTCTTCATCATCACCAACCGAGACAGAACTAGAGTGAATCATCG
TATTCACTTTCCGGGATTCCATGGGCAACACAACAGGATCATTGCCCTGTCCTGGTGAGA
TATCTTTATCCTCATTGGCAGATAATGCTGGAGAATCTTCAGATTCATCCAAGTCATCAT
GAGATAGATGAACTGGGGAATCAGGAGGGCTAGAGACAGAAAGTGAAGACTGTGAATCAC
ATCTTGGTGTGGTGTTCACATCCAGATTCTGCTCCAGATTTGCAGTATTCTCCTGCTGGA
CATCAATTATTTCAGGTTGTGCAAGATTATGCTGATTGCTGCTGTCAAAAACTATCTTAC
CAGTAGCTTCACTTCTTGCTGCACGACTATAGTCTATCTCATTCTGTTGAAAAGTTGAAG
GGCCAGGTCGCTCATTCTCACGTCTATAGGAAGTTTTTTGCATTGCACTCAGAGATGGAG
GGGGGAGTACACGGGGGTGCCTCATGGAATACCTCGACCTCCCAAAGGAAGAAAGGCCAT
CTATATCTGGAATTTGATATGGACGTTCAGGGTATGAAGAATAAGGATTGCTACGATACC
GACTGTGGCCCAATCCAACTTCACTGTATCTCTCAGTGTGATTCTCATGAAAGTCAGATT
CAACCTCAATATTTCTGGCATAGTGATCACCATCCCCAGGTATATTCCATCTCTGACCTC
TTGGATGATTAACTTCATCTGTGTGTGACTCTTGAATCCCTGCTTTATAATAAGTCCTAG
ACGACATAAAACCAGGTCCTCCAGAATACTCTTTCCGTGGAAATGATTTCCCTCCAACAG
TTCCATCTCGCCTGGGGCTATAATGACCATTATCCGAATCATGTGGAAAGAAGGTTGAGT
TGTTCCCATTCTCAAAGGCATCCCTTCCCCATGTGTTAACAGGCTTACCTCTTTCCCGAA
CAGCAGGAGAATTATCTCTAACAAAGTGAGGCCTACAGCTCAAGTCAAAGGCTCTATTCA
GACCAGAGGAATCAGAAGATGCAGAAGTTGTAATCCTCTCCACCATCCTTTCACCTTCTT
CCCAATCAGCCAAATCTGCCACTTTGGGGGACATTCCTGAAAATTTATCTTCTGCAAGAA
TGGAAGTGTTACTACCACACTTAGTTCCCTCAACCTGCTTCTTGGCCATTCTTTCTTCCA
ATTCTTCAACCTGCCTCTTGGCAATTCTTTCTTCCAATTCTAAGAGTTTCTGCTTCGCGG
CCTGTTTCCTCCTCTCCTCCTCCATAATAATCCTCTGTTTCTCCTCCTCTCTAGCTATCC
TCTGTTCTTCAGCCTTGCGCAAGGCTTCCAATCGCTCTTGCTCAGCTCTCCATGCAGTTT
CTCTCGCTTCCTCTTCTATCCTTCTCCGTTGTTCTTCTTGTTCCCTAGCTACTCTTAGCC
TCTCCTCTTCTTCTCTGCGAGCTAGGTCTAAAGCTCTTTCCTGTTCCTCAATAATCCGTT
GCCTCTCCTGCTCCTGCATCTTTTGGACTCTCTCAAGCTCAGCCTCAAAAGATTCCCGGA

CAGGGTCATGAAAATCAGTCTGCTTAAGATCCTTCTTCTTTTTAACAACCCCAGCAAGAC
CCCCAGTAAATGGATCCCGTACATCAAAACCAGTAACCCCAAAATCCTTCATGAAAGGGT
CCTCCAGGTAAGGTTTCTCACTCTTCATTGCTGCACGTTTATCCCTACCGAAATTTAGAA
TGGGATCATTTACTGGAAGACCTCTGCCACCCGAGGAAAACAATGATTTTGACGCAGAAT
TATTCTGGAAAGAATCACCTCTGTATGTATTGTATTGTTCATTGCCATATCGGTCCCATG
GATTCCTCTCAGCCCTCTGACTGTAAGATGAGTGTCGTGTAGAGTTCCAAGGCTGCCTCC
CAGCCTGTCCATGCCCCAAATCCCTTTTTCCAAAATCATCAGGTGGCAAGTCTCGAAACA
ATGAAGATACTAACTTATTCTCCTTGTTTGCTTCTCTGTTCTGACTATATGGCGTGACAC
CAAAGCTATTTCTGCCATTTCCAATCTCTTGTCCACCAAATCCATCTTTTTGAAGAGGAG
GAGAAGCTCTCCACAAATTCCCTTCATGTACTTCTCTGCTAGATGTTCTTGTATCTCTAC
CAAAAGGGTCTACTTCAGTAACTTCACTGGAAGAAACCTTTCCAGTTTCATTATCACGCT
GAGACCACCTGTCAAAAACATTATGAGCTGGTTTGTGTGGTTTTTCACTAGGCCTTGGCA
TATCAAAATCTCTATCCAAGTAAGCTTCACTCCTTGAGAACCCATGATCTCTGTCCTGGA
CTGCAAAACCATGACCAGTATCACGTTCATCATCAGCCCAATCAGATCTGGGATTCAATT
TAACCAATGGCAATGGACCTGGAAAGTATTCATCCTGCTTTCTTACTTGTTCCAGTCCAC
GAGAACCACCCAAGTCACGATTTCTACCAGCATTCTCAATAAATCCATTATCAAAACCAC
CTGACTTCGACCGAGGATGCACGTCAACAAGGGAGTTCAATCTAGAACCATGGTTTAGGT
CACTAGTACCATACTCCTCACCCATTGCTTGCTTCTGTTTTTGATTCAAACCATCTCTAT
GTTTCTTTTCAGACACTGATATAGGTGGCAATGCAGCTCTTAAAGACGGGAAATCTTCAC
CCCTCAATACACTTACCTTCTCTACATGCGGAAAAGTAGAAGATGGAGGCCCAACAGGAC
CAGACCGCACAGAAGGCAGTATATAGGTGCCACTACTACCACTTTCCTTACTCACTCCTT
CAACACCATCATCTGTTATATGATTACCAACAACTACTCCTCCCTTCTCTTGCAAAGCAA
CTGCTGTGCCCGGCTTAGTCCAACCCATGCCAGAGGAACCAGGCCTCTGTCCACCACCTG
AAACCCCTCCCCCAGCAGACCCACCACTGGATCCCAACGAATCAAATCTCTCATGCTCTT
TCCGTAAGGATGGAAGATTCAAGGGGGGTGGAACAGAAAGCTTAGGTCCAGTGGCTTTCT
GGGAACTTCGGGGCCGTGACAGGACCACCATTCCTCCTCCTCCACCAGCACCACCACCAC
CAGGACGGGACCTATTGGACCCATGATAGCCTGAATGAGATAAGTTGTGCTGATGATGTG
ATTGCCCATAAGATTTGTTCAGATTCACAGACAAAAACTTTGTCCCCACCCCGGGATTAG
CCATCTTACGTTCTGACCCAAATACGTATCCCCTTCCACAGATTCCAATCCCCAGCAACG
CATCAAATAACTAGAATACCAAAATACAACCCTACAAATTGATTTATGTTCTCTTCGCGG
ATCAGATTAAGCACCGCACCCTACGTTCTCTCCTCACCTCCGGGGTATTCGATAGTGAGC
TGCTAGCTAAAGTGCGCCGCGAGGGTAAAAGAAAGTTAATATAGATACGAGACAGAGTAG
>ntig_410_consensus
GGCAGGTAAGTAAAATCTGATATTTTTTTTACTCTATCTTTACTCAATCAAATGATAGA
AAATTAAAAATTCAAATGATAAGCTATATGCTATATGCTCTCAAATGAGTCAGTCAGAAA
TGCATTACATGGCACTGGCCTATTGCTTTTCGGAGCTGATAGTGAGGATATTCCTTAATG
CATATCATGGTTCAAGGAGTGAGACTTGGTAATCTGAAAAACGTTTATCAACCAGAGAAT
CACCAACTGATCCCAACTGGTACAGCCTTAGGAAATCATGCTGCCCTATATGATAGCAAG
CTTTATCCTAGAATGAGTGCCTTCTCTGTCCAAGAAAGAATGCATCATCAATGGTCAATC
GCTTCTATCTTCATCACTTTCCCTCCTCCAATCTTGATCTTCAAACAAGTCTTCAGGCGG
ATCATATACAATCATGTCAGGAAAGAGCTCCAAGAAATCATCCTTGACCTTTTCTCTCAA
TTCTGGATATGGAATTAGACCTTTCAACATCACACGGAATGGCAATGATATCGGGGGATC
AGGAGATCTTCTCATTTCATCATATATATCCATTGCTTCTGAGGGTAGTCCACTATCTGA
GAAGGCCCTGACAAGGTCACCAAATGTATGCTGATCAAACAAAACTTCCTCTCTCTTGAG
ATCTTCCCAAACCAATTTTGCTTCATTCACCTTTTTGTTTCTTGCAAGCATCATAAGCAT
GTCTCTGTAAAAGAACATGTCTGGGTGGTACCATATTTCTCT
>ntig_411_consensus
ATTTGGAAGAACAAAGATGACGCTTAAACTGGAAGCTTCATGATTACAAGATAAACGAGC
ATGCCTGTCGACTTGAATGTCTCGAGTACTATGATACGATAAATACAAGCTTACTTAAAA
AGCAGAGGTATATTGCAATACAACAACCACACATGTTTATTATGTACTGAAATTACACAA
CATGATTACATGACTTAATTCACTGGAAAGTAATATCACACTCTGCAACTATAAGATTAA
ATCAGATTGAGTTTTCAACTGGGCGGGACTGGTAAGTGCGACGTCTAAGTTGAGAATCTG
GCGTGGGCTGCTGCAAAAAGATTCTTGTTGGATCATTTGGATCAACATATGCATGTCTCA
TCATTTCATCAACAGGGGTTTGTGCTGCTTGTAAAGGATCTACACGAGGTTCTGCTGCCA
TATTTGTTTGTTGATTATACTGTCTGACTGAAGAAATCAAGTTGAAAAAAAGTGGTACAA
AGGTACCACAACCCCCTTCCTTAAACAAAATCTTGAACGAGTGTGTGGAGTACAGAGCCC
TATGCTCATTTTCAGGGACCACAGGCTCCACAAGACCAGAGATGTTGTTGCACTGAAATA
TTGGCTGGTTAAATTTCTCATCGTGGACGTAGAGCAGGGGCATATCAAAAGCAACAAAGT

TTCCGACAGCTTTACTAGCAACAAAGACCATGCGAATATTTGACAAGTAAATTATCCCCT
TTGCCTTTACGTTGCCTCCATGAGCTCCGGGAATTTT
>ntig_412_consensus
CTGATTCTTTAACCTTCTTCTTCCTAGACTTCTCTTTGGAAGAAGCTCTTCGCTTCTTTT
TGGAAGTTTGTGCACTGTAATCTCCAAGGGGAAAGGATTCTTCATAATCTGACTGCCAAT
TGCAGAAAATCTCTTGTTGCTGATCCACATATTGTTTCTGTAATGGCTCCCCAGCTCCAG
GAAGCAAGTTGCTAAAAGTCATCTCACCATCAGATCCTCCTTTACGCTCCAGCAAGACTG
GATCAGCTCTAATTATGGTGCCAAGAATGCTTCTGTTAGGCCCTAAGCTAAAGATGGTCT
TCGGATTTGTCAAAGTCGCAAGCGCCATTATAAAAGCTGATTTAATCTGAAAGTAATTAA
AAGAGTTCTTCCCAATGTCGTTTTCTGGTTCCTGTGGGTCCTCAATAGAGATGAGAAATG
GTCTTCCTTTATTTGTAAACCCTTTGCTACTCTTTTTAAAGAAGGTGCCAGTCCCTTTGC
AAGATACGCCAACATCTAGGGTGTTCAATTTGCGTCCATACATATCAAAGAAGTGCACCA
AAAGGACTCCCAAATTATG
>ntig_413_consensus
CTTGCAATATTATTCATAAAAATTTTATTTCTGTGAGCCTTGTCATGATCTTATAATGAC
AAGAGGTTGGTTTGTATGTTACACAGACAAATTACAATTCGTCAACACAATAAAACAAAT
CATCACTCACAATTCACACACCAAAACCCAAAACAAGCAACAAAAAATTACCACATCAAT
TAATCACCCATCAAAACACCAAATAAACAAGCAATCCCACCACCAACCCCACCACATTAT
AACTCAAAACATTGATCTTTCTCAACCCCAAAGAACTTGCTCCACTGGTGGTAAAGTTCC
CGCTGGTGGTGTTCGTAGTCAGCACCAGCACCATCCAGTCATCGGCGCAGCCCAGTCCGG
CTCCTGTGTAATTGGAACTCTTCAAAAACTTTGTGTACTTGTAAGTATATGTAAAGTTTG
TGAACACGAGACTTGGCACCAGTTTGTGCACACAAACTGACAGTATAACTCCATCTTTGG
TGCTTTCCGGGAGAATTTTACACTTGCTTAGAATTTTGTCGGTGACAAACTTTTGCTGGG
TGGACACTGGTGTGTTTACACCGTTGGTGGCGTTGCATTTATAGTCCTCTAAATCTTCTG
CAATTCTTTCGGCCATGCACTTTGCTTTTTTGTTCTCTGTGAGCTGTGGTTCACCTAGTA
ATGTTCTATAACTGTTGAGTCCTTGGAGAAGATCGTCCTCGTCGCCGCAGTGCACTCCAT
GAGGAAGAAAGCAAATTGCTTGAAGGAGCACGAGGAAGAAGAGGCTAAGTTTGAGAGCTG
CCATATATATGTGCTTTATCAAACAAAGGGAGTGATATGTGTGTGCTAATTGATAAGAAC
GCTAAAGCGAAGACTACAGCACAATGAAGTACGTATTGGCTTCTTAAGGTTGTGATTTTC
CAAGGCAAATTAAAGAGAAAGCTTCCTGGAATCGAATTCTTAATCATTGCCTACGGTTTT
ATCCATGTATCTTTCTTTATCGACACAGGCTTCAACGTCAAGCTGCTTGCAAAAGCCGCT
CAGAATAATCTCACTTCTCTTTAAATCAATATTCAACTAATCATCACGGTGAGATGATAG
AGGTTGTACTAGAAGTTGTCTGGGCTTTTTCCGATCGTACAAAATATGATAAACATGCAA
ATATTTGAAGAGTCGCTGAGAATTAAACGAAGTCTACAAAATTATAGACTGAAAGATCCA
AAATCTACAGTTGATTCAAAAGGGCAAAAAGCACTTATACTAACACACCAAATAAACAAG
CAATCCCAAAAGCAAAGCCACCCAGCTGGAATATCCCAAACCCGCCTTCGAAACCAAAGA
ACTTGCTTCACTAGCAAAGCTTCCAGCACCAGTGTTGGTAGACAGAACCATAACCATCCA
ATCATCTTCCGAGCCCACTCCCACGCCGGTATACTTCGAGTTGTTTAGATACTTCGCGTA
ATTAGTGTGTGTATAGTTTGTGAGCACAAGCGTTGGAACCAATTTGGGTACACAGACCGG
CAGCACAACGCCGTCGGTTGTGGTGTTGGGATCAATTTTACACTTGCTTATGTAGTTGTT
GTAGTTGGCTGATTGAAGCGCCCTGGCGGGTACAGTGCTGCTGCAAGGCTGATCGTCAAG
GTCATCTGCAATTTCGTCGGCCAGACATCCAGCCTTGTCGTTCTTTTGGAGGGCTGGAAG
GTTTACTGATGTCCTGTAACTGTTAATGCCTTGAAGAAGATTGTCCTCGTCTTCGTCTTT
CGAGTAAACCACTTGAGGAAGCAAGAGAGCTGCAAGAAGGAGCGATGAGAGGAAGAAGAA
GGAGCCAAGGTTGAAACAGGCCATTGCTAGATATCAAATTGCAACAAGACAGAAATGAAA
TATCAAGAATGCACCAGAAGGTTTATATGAAGCAAATGGAGTTTTTAGAGATCTATATAT
GTGCTTCTTACTCTATATATGCTCTAAAAACTTTTCCTCTTTAGATATGTCTTCAACATG
TACAGGCGCATGAGGAGTCATTACAGGTGCCAATTTGTGAGCTCGCTAGCACCATAAAAG
CTGGTTTCCAAATGACAAGTATAAGTTAATAACTCCAGCACGTGGCTGCCATGCAAAGCG
TCAATTGCAATCCTTCTAACCTCTCGATTGTGTTTCCAC
>ntig_414_consensus
CAACCAGACTGAACTGAAGATAAGTTTTCAGAATAAAACTCCTGAGCAGTGTCATTAAGA
AACTTGTCGGAAAAGCTTGAGCAAACTTTGCTATCAATCAAACAGCTCTTGATCTTGTTC
CAGTTACTAGTACTATTAACTCTTTTCTGCAACCAGTTTGAGTAATCTCCCAATCTGTAC
TCTTTATATCCTCTGTTGGACAACACTTTACCGGCGCCTTTGTTGGTGACAACGAAGGCA
AAGATGGTGAAGCAAAACAAGACGACAATCAGCAAGAACATGACCAACAAGTAGAGCCAA
AGAAGCCAAGAGACTCTACAGCAGGCGCCAATCAAACCGGCCAAAGAAACCAACATGAGA
AACACTCCGAGAATAATCACCGGCGTGTCGAGGAACTTCTCGCACTCTGAAGCACCATGT

TTGGAAAGCCAAATACCGGCCCAGAGGATGGGAATCGAAAGAAGAAAAGTGATGATGTTG
AGGATGCCGATCAAGTTATTGCTCAAACGAAACATTGTAAAAAGAAAAACCCAGGTGCAG
AAGCGAAAAAGATGGAGAAAGATTCAAGCTTTGGGGAGAAAAAAAGAAGCCCGAGATAGG
GATCTGAGTATCGAAGAGGGGATTTAAATGGAAAGGTTGAGTTTTTCCCCGAGTAACTGC
AGGGAAGTTTCTTCTTAAAGAGAGAAGGGAGAGATACTGGTTTCTAACAGAGGAAGAGTT
ATTCTGGTTGTACACGTTTTCGGTGACCTTTCAATGGG
>ntig_415_consensus
AGTGTAACTCTTTATGAACCTTGTTACACAGTAAGTAACAAAATTAAGAGGCTAAATTTG
AGTTATTTGACATGCGCAACTCTTAATGCTGAAAAGAATTATGCACTATACATCAAAAAT
ACCATGCTATACCAATCATAAACCTTTTAGTAGAACCTGGGCAAGAGTGCAGAATTCATG
CCAGACTCAGACATACAAAAATGAATCGGTACACTTCCCAACTTAAAAGCATCCATGAAA
AGCAAAACTTCTCACCATGGGCAAACATGAAATACTAGATACCAGGCAATATCAGCAAAC
TACCAAGCACATATTATAAAGGATGGCAGATTGATTATCTCGATATCTACAATAAGTATC
AGGCTAGCGTCGCTTTTCAAAATCACTTAGCCATGCTTATGGCTCTGTTTTAATGCCTTA
CATATAACCGCTAAGATGAATCTACCACAGAAAATCACAATCTTGTGGGCGAAATGAATT
ATGTTGCATATTTTACTTCATAACTTCTGACAACCATTTAGTCTGGACATCCTGATGGCT
AACATAAGACTCGAGCAGTCCACCACTTTATAACCAGAAATCTGGTTTAGAGTCCATTAT
TGATCTGTCTGGAGAGACAAATATGTAAATAGCCCATTCCAGACTTGAAAATAGAAGAAT
CTTGCTCACAGGTTCTCTGATACGAAGTTAAGTATCTTCCTCCTAAATGATATTCCTACG
TGAAAACTTGAATGCCCTGATAATTTTCTCTCCTAATTTCAGGGTCAGAACCTTAAATGA
GGACAAATCTCAAAACTTGGAACTAGTCAATTAATCAAATAATCATCTTCACATGCCAAT
GCAAATAAGATATGCAGATGTTTTTGGTGACTAGAAAAAAAACTTCTGAAATTTTTTGACA
GCTAAACTTAGCAACAGTACCTTGTAAGACCACAAATTGCAATCAAACATGATGAGTTTG
AGTTATTGGAAAAATGTCACTGGTAAGAACAGAAAAAAAAAAAAAAAAAAAAAAAAAAAA
CCTGTTTTAAAAAAAAAAAAAAAAAAAAAAAAAAAAAAAAAAAAAAAAAATAAAAAAAAAA
TAAGGAAGGTTGAGATGCGTGAGGTTGGTCTTCGAAGGAGAGATGGTGGCAGTGAGACCT
CTCCAGGTTAGCCATAGATGAGTGACATCGTTTGCATGGGAAACATGAGAGACTGTTGAT
ATAGCTGGTTGGACGCTTGTTGAAAAGAGGGATACCGGGGAAGGCAGTTTTACAGAACCT
TTCTAGCTACATCTTTTATAGCTCTCTTCCGACGCTGGCAGTGAGAAGACCAATTCTATG
CAGGGGATGACGATGTACCGAAGACGTGAGACAAGGGGCTGTTTTCACAATTATTCTGAA
TACTATTTACACATGTTTTAATTACAAAGGGATATTTTCACAATTAACCAAATCAAATAA
TGTGCATTCTACATAAACTGTAACTAAAGCTTTACACACTACTGTTATGCACAAGTTCTT
CACAGATTCACAGTCCCATCACATGTTATCAGTTAGGTTTATATACCATGGCCCATGAAC
ACTTACAGAGACGGTGGCTGCTACCACCAAAACTTCAGCAGCTTCCACCACCATTTTAGT
AAGGCTTACGGCTCCAATCTGCCTGAGGGCCAGTTCAACCAACTTTCTTCCATTGCTTCC
ACTTCCCCCAAGCACAACTCCTCTTGCAGCCAAATAAACAGCTCGAGATACCTTTTCAAA
TACTGCATCTCCAGCTTGCAAACTTTTTGCCAGCATCCTAGTCATTACTACTTTTCTCAC
TTGATGCTTATCAGAATCTACTGTCTTGCCATCATCGCAAGACACTCCACCAATCACTTC
AACAATATCTTCAATTCCTGCATCTTCAATGCGGTCTAAGAGTGCCAAGAGTCTTGCAGT
ACGGTTAGATACTATTTTGTCCATGTCTGCAGGACTGGCTACTGCTTGCTCACCGAGGAG
GGTTTGACGGCAGATGAGAATGCTGGTGGAAACTACAATAATCTTCTGAATCTGGGCCTG
AAGAGCTCTCAACCTTAAGAGGTTAAGCATGAAAGTTTCAGGCAAAGATTCTTGTGTTAG
ACCAGAGATCCCACTTACTAACTTCAACAAGCCAAGCCTCACTGACAAGTCAAGTTTTTC
TCCCTTACATTCTGGTTGCTGATTACCTGATGTAGATGTATTCTGGCTTTCACCTGTTTT
GACCAGAAAGCTTCCACCAGTTCTCAGTGTGGCAGAAGACAGAGGCATTCCAGACAAGCT
TTCATGCCTTGTTAAAGCTGAAAGAGAATTCTTGTGTTCATCCCACTCCTGATCTTTGCA
AGTCCATACAGATGAAAGCCACTGAGAAGTCAATGGTAAAGAGCTGTAGGCATCAGACGG
GGGTCCATAACGGTTTGCAAAAGCTTTTCTAAGGTAGTCAAAACCGGCAGGCCCCTTCAA
CAAAGGCTCCATAAGTCTTATGCGTGCTTTGCTTATCTCTTGCTTAAGAGCCTGAATCTG
CTCTAGAACAAAGCGCAAACCCTTGACTATTGCAATAACAGGTAAATGATTTGATCCATC
TCTGGCCTGACATATCTCAGTTAACTCTTTCAGTAAATTCTGGTTCTTGGCCTTCATCTC
TTCATCCTTGGCTGGAGAAGAGAATTTCTGCAAAGTGCTCAACGCAAACTCCAGAATGCT
TCCAAGATAATTAATGTCCAGGTTACCCGAATTCAGAACCTGAGCAAGAATTTCCAGATC
AATAGCTTCAGTAATTTCTTCTTTCCAGCTCTGAGGAGCCATCTCACAAATTTCATCCCT
CACTTCCTTCACAAGTTGAGTAACCCGGTCATAGTTTGGCTCATCCAGTTTCAAGGATTC
CATGATGCCATCCCAGAAAACCTTTTCCATTGTCTCCCTTATCTTTGCCTTGATACTGTT
TTGATTGTCATCAGTGGCACTGGAGCTATCAACATAAGCACGGTGGTTATGGACAAATTC
ATTAACTATCAATTCATTCTCAGTGACTGGCTGTTTTTCAACAGATGAAACCAACTGACC

ATCTTTGTGGATTCCACTAGGTGCAGAGGAACCAATTCCTTTGTATGGAGACAAATTCTC
CTTAAATAAGGAGCGAACAACACTGCTTGGCCTTTCAACACCCTCAGTCAGATTACTTCT
CCTATCCACACCAGCAACAGATGGAGGAACAGCTGGTGAACTCAATGGACCAGGGGTTAA
AAAGTTTGTGATAGGAGAACCAACTGGGCTTCCATTTAATTTCGCTTCAAAGTACTTAGA
TCTTGTTTCAGATAATGCAGATTCCATGCGCTCAATCCCAGCTTCTCCACCAAGATGATG
TACTTTTTCCCTTATAAGTTTTTGGTCCTCTGTAACCTGTTTCTGAATAGCCTTCATATC
ATGAGTAAGAGCAGTGTTTTCTCCGTCTGCAGTCATATTACATGTTTTAATCATAGAGAG
TTCAAGCTGGCAAGCAGCTCTCACCAAATCATCTTCCAACAACTTAGCATCCTTAACTTT
CCACATTACAAAGCAATTCAAATAGGAGCACCATACTTGATCAAACGCAGCAAGCTGAGA
CCTGAAGGTCCAACGTTTTGGCAGCAAAGAGTCAGACTCTTCATCAGAACTCTGCATTGG
CCCCTCCAATATAATTTTTATCAAAATCTCAAACTGTCTAACAAATTCTTCTGCAGATTT
GGCCAGAGCAATCTCCAGCTCTCCTTGACCACTGAACACCGCATCTGGGTGACCCAAAAT
CATGTAAGCACAAAGCACTACTCTCACTGGATACCTTGACAATTTAGATGGACATCTGCC
TCCCTCCCGAGAGTAACTTACTTTCTTTGCCTCTCTGCCTTTCATAGGAGTTTGAGAATT
AGGTCTTCTCTTGGGAGTAACAACTCTTTTAAGAAGGTGATCAATGTTATCAAAAGTGAA
TGCATGACTGTCAACAGCACCAACAGCCCTAGAGATTTTAAGGCGGCTCTCAAGCCGGTC
AAGTAGAGCTTTCACAGTATGAAGGGCAGAAGTTGACTCAATCAGAATGGCCAACTGTTC
AAATGGCATTGCCTTGACAGAAGTCCCATTAATCTTTAAGGCATCATAAGCTTTTGCCAA
CTCCAAGGTAGTTCTCCTCTGATTACGAAATTGCCTCCAGAACCTCGCTAATTTTCTGGA
AAGGTGATCGGCCTGTGTGCACATCCTAAAAGAAACAGGATTATTCAGCCGTCCTCTCTT
CCTTAAATATTCCGCTCTTTGTAACTTCGCCCTTTGCAATCGATCTTCCAACTGATCACT
AATTCTCCTTCTTTCAACCTCTCGTTGGTGATTGACAATCTTAGCTACCCGCCTGACTTT
CAGCACCCGAGTATGTGCCCTCTTCTTCTCAGCTTCCAACAATTGCAGTCGATTCTTCTC
AGCAGCTGCTCGCTTCTGGTGGATTGCAGCACACACAAGCTCCTTATACTTGCTCTCACA
AGCCATCCTTCGCAGCAATGACTGAGATGACCGCTCCTTTAATTTTGCCCTTCTTTGGCT
GTAAGCCTTGAGGAGAAGCATTCTATTTGTCTCTGCCTGTTGAAACCTAGATTCCACTTT
TGAGCCAAGAATCTCACGTTCCCTCTCATAACGCATTTCTACCCCCGTTTTAGCTGCTTG
TCGCAACTCATCAAGCCTAGCCAGGCGCATTTGAGCATTTGCCAAAATGCTTAGCCTCTT
CTGCTGGGCTGCTTGAAGCTTCGCTTCAAGACGCTGACCAAGGTCCTCCTCATGAAATGA
GGATTGTGAAGGGCTCCTCCGCTTTGGTCTTGCCTTGTTTGATAACTTCTCATAGAATTG
CTGTCTGCGAAGATCAGCAAGTCGAAGCTTGGCTTCAATATCTTCAACAGTACAAGGACT
TTTATTCTCGTTCAAAAGTCTCTTCCGAATCCTCCCAGGGACTCTGATCGGCGACGAAAA
CGAAGACATTTCGGCGGTTTCATCACTGAGAGATAGTTCCACCGCAATACCCGCTACTCT
CTCCGTTTCAGCCATAACTAAAACATAAAGTGATCAAATTTTGATCAAATAGTTTGGACG
TATTGAACTGAAAAGGCGAGAACAGTGAAAACCGATTCTAGGTAATTGGGTTGATATAGT
TTTGAAGAAAATTTGAGAAGAACTTCGGAGCTTTTCTTTTTATGAAGATGATGGAAGGGT
GATGAAGCGTGTCGTGTTTGTGCT
>ntig_416_consensus
CTGGGGAGGTTAAAATTGCATAGAAATTCAATTTAAAGAAAGGTAGATAAGCTTCTATTA
CATTGACATAGGTAAACATCTCATTACAAATTCTTAGCTAATTAAGATCAAATATTCAGG
AACATTTTATTTACATGATATGTAACCTCAAGAAAATTCCTCTGCATGAGACCAACACAA
CAGGAACATTTAGCATCAAGACATAACATTTTTCTTATAGCTACGTTCAGGAGCATGGAC
GCATCAAACCCAGAAGAGAAGGGGTTTATCTGAGCAAAAGTGCTTTCAGAATGGCTTCAC
AGTTCTTTGTCTTGTAAATGAGCAGATGTCCAGCGTCTGCAACCTCATGATACTGCATCC
ATGGAACCTTCTCCGAGATATAACCATTTATTTCAGATGGAATCATTCTATCGTCAAGGC
CTTGCCAGATGTGAACTGACCCCTCATTGTCAGGAAAAGGATTGATAAGATCCATGGGAT
CAAATTTCCATTTGGCATAGCCTGTTATTATATCTTGATAAAGACACTCGTAAACACCTT
GCTGTCTAATCTTTTCCTGACCAACAATAGGGCATTCTGACATCTTCTTCAAGATCTCAT
GATCTTGGGGACTAAAAACTGACACATTACAATTCATCATGCTCCATTTCTGAGACATCC
ACCAGTTGATTAACCAAGGAAAATGATGAGCAACACGAAATGTCATTTGATACGGCACAG
GCAGCTTTTTGAAGGCTTCTTTTGCTAAATTGGCAGGAAGACAGGGCCACCAGTAGTGCA
CAAATGGAACCACCAGGGAAGCTCCTAATAACCTATTTGGTATGTATTTTAGGCAACCAT
AAACAGGATACGCTCCCATTGAGAGACCAATTACATAGAACCTAGATCCAATCTCCAACT
TGTCTGCTAGTTCTTGAATATCATTTGCTTCACTCTCGACTGACCGTGATGGATCTGGAT
CACTCTCGCCATAACCTGGTCGGTCGAAGAACAGGAAATATATCTTGAGCTCCTCTATAA
GCTCTTGAGGGACAGGTAAACTGAGATCTTTGGAGCTGTCAAAGCCATGAACAATGATGA
TTTTGTGTTTAGCCTCCTCCTTGGCAACCCCAGTTTCCCGATAAGCCAAATGCCTCCCAT
CACTCAGTTTTACTCTGGGTGAAGTAACAGGGGGACCATCTGGTGACCCGCATATCTTTG

CAGGAGGTGGCTTTATAACCTGATAAGCCCACCCCAAAAGACCCACTGCCAAGGCCACTG
CTATTGGCGCAAACATTT
>ntig_417_consensus
TGGCATAATTTCTAATAAAAGAATAATAAACAGTCTCGCAGTTCCACAAACTGAACACTA
AGCTATCATTAGTATGAACACAACCAATGAAATGTAGCCTGAAAAATCTGGGGAAGTACA
GTACATAATAACATCAAAGCTTCCCAACTCAACGACAGTAAGAATATGAGAAGTACAGCT
AATTACAACACTAAGGGAAGTTGATGCCAAGAGCCTCCACAAATCACTGAATGAGAACTC
TCACTCTCAAGTTGTAGTATGGTATGCTGCCTCAAGCTGTTTCCAGACCTCAAATTACAT
GTCACCCTTAGCTTTTTTTCCAGTAACAATCTGGAAGATCCTAAGGCCTCTGCTGAATGC
TTCATTGAATAGTATCCTTGTCTGCATAGACTGGAATCCAGGCATCCATGATAGAAATGC
CACAGGGGTCATGACAATCACTCCAAACATAATATCATACAATCTAGCAACAGAAACCAC
AGCGTGCCAGACCCTTGTGGAATCTAAAAAGGGTCGGAATACTTGGGCAATTAATATCAG
GCCCCATCCAGTAGGGACAAATGCCAAGAGACTGGTGAAAAGATCTATAAATTTGAACTG
TGTGAATTCCATCAGAGCAATTATCACAAGTATAGCAAAAATGACAATAAGGAATTGAAC
CAGTCGGTAGTAGATGTGTTCTTTCGCAGCATATTTGTCACGAGCATAGGATATTACTGC
ATAAATCCCAAATGCCACAGCAACATAGATCCATGAAAGCAAGTAAACGGAAATACTGGT
ACTTCCAGCAGTGATACCTAGCTGGTATACTATGGCATACTGAAAAAAGAAGAAGCGGAG
GTCTAAAATTATCTCCAGTATCTTTCCCCAGAAACCAGTTGTCCTTAAATGATCTTGTTC
CTCATACCACCATTGTTCCCAGCTCTCTTCTGCTTTTGCAAAAACACCACCATGGAACCA
AATCCAGTTCATGAATTCATCAAAGTCATATACAGTCTTCAACCAATCAAAACCAGAAGG
ATTAAAGACAAACGGAGCCATTATCCACGAAACAACCATGAACCAACTTGAGATGCTCAT
GGCTATGTAAACAAATGTGTCCTTAGCTATAGGGCTGTATGCTGCATAAACTGTAAGAAT
CAATCCAAGCTCAATGGCTTTTATAAAGTGGCTACGTGCATAAAGCCGATAATTTTCTGC
AAAGCCCTTGTGCTGCACAACAAAACCACGTCCAGTGGCTCGATACTTTGCACCACCATG
AAGGATAGTCCGGCCAAAAAAGTGAGTGCGAGTTCCCATGGAGAATGTGTAGAAAACAGA
TGATAACTGGAGCATCATAGTCAGGAAATCCCAAATAGCTTGAAGAAATCCATGCTCAAG
AGAGTTCTCCACAATCATCGGAAGGGCTGTAAACAAACCAAGTTGGATAATGAACTGTTG
GTTCAAGATGGCACCAAGTGCTGCATTGGTTGCGCTGTCGCTTTTAACAGAAGCCTCAAC
ACCACTAAGAGCCAGATAAAGTCGGCCCCATAAAAATGCATACACAGTCAACACAATCAT
CATTGTGTTGAAAAAGAATCCAACAGTAGTGTAAAAAAATGATAACATACGGAAGAAGTC
CAATCTATGACCCAACCTATACACATCTCTGCTGAGAACTTGTTCCCCATTTCCACTAGC
AACCTTGGCTTCAAACATAGATATCTGATTCAATCCAACATCCCTTCCCTTGCCAACTTG
GATGTATTCATGGTGTGTAACACTGCCTCCTCGTAATGTGCAGTTAAAGCCAGCAAAAAT
GTCCTCACTGATGTTAATCACCCTAGAAGCTTTGCTGAGGCCTCCCCGAGTCAAGAACCA
AAACCTGTCAAATACATCAGGATGGCCATAATGCATTCGGATTTTCAAAGGGTTTGCCAA
GACACGCTGACCCAAGGTGACAAAACTGGTTTCCTGAGCCGACATAAACCAAGCAAGTGA
CGATACTGAACCAGTAAAAATGTGCTCCCTAACACCTAAGATGGTAGGTTTCCTAATACC
ATGGTAGTGCCTGTATTCTTCCAACAAATTCCGCATTTTAAGTGCCTCCTCAAAGTAATT
GTCCTGATTCATATCAATAGTCTGAACTGCATCTCCTCTAGTGAAGATAAGGGCATGGTT
TTGGTTCTCTGGTTTTCCCTCACCAAGCTTCAAGGGACCGGGCAATTTAACCCTGTAGAT
TTCCACTTCCTTCTCTAACTGCTGATCATATTTTACAAGAACAGAATAATACTCCTTATC
ATCCCTCCCTGTGGAAACCTCATCAACATATGCAACTCGAAGGGCTTCATTGTTCTGCAT
CAGAATCAAAATTTCCTCTGCATGGGGGTCTTTCTTCTCCTTTTGCTGTCCATATATCTG
GCAGGCAATAACATATACGTATTTCATCAAGGCAGTTCCATACTCATGGCCCTTGAATAG
CATGCTAACTGAACTACCATTTCTACTTAAACTTTTCACAGAAGATGACCTTGCTGAGCC
AAATCGATCCAAACTGGCATCTCGCCTTATTGAACCAAGTTCCTGGGCCCCTTCTCTAAT
GTCCATCTCAGATGCAGAATCAAGAAAAGCCAGCAACTTAAGAGCCCGGTAATAATACAT
CATTCCCCGCACAGTGCGAGCAAGCGTCTGGCCTCTGTATGATGCCCAAAGCCTCAGATC
TTTCAACTTCTCTCCCCAGATTTCATTATCCTTCACCATTCCCTCTCGGCGCATCCTCTC
CATGAAATTCTTCCAGTCATCATGATAAATGGTCTGCAGATAGTACAGGATCGATATGCC
ATCCTCATTCTCACTCCATAGTTGCTCTTTACTGTAGAGAACGTCTTCATTGTAGTAAGG
CGTGAGAACACTGAAGGACATCATTTTCTCAACTTGGGGAGCATGGGGCATATTCATAAA
GAGAGAATTGCTAAAAAAGGCTATTCTGCGCCTTGCTTCGAGATTAACAGGAATATTATT
CATTGAATCCCTAGATGTTAGAATTGTGTATAGGCGCCTAACCTGCCGATAGAAGGTCTC
GTTATCATAGTCCGGCAAGTGAACAGCATTCTCAAACAGCAAGGCTGGATTCCTAGGAGC
TAACCCATCCTCAACAAGCTGGTCAGTGCCCCTCTTCTCCAAAAAAAAATCCCGAATAAC
GATCTCATAAAGGGCCTGTAGTGAATTCACCACCTTGTCAATGTCTTTACTGGGCTTGTT
CAATAAATCAACAAGTTT

>ntig_418_consensus
GACCAAAAGTATAGAACAAAAAATAAAAAAAGAAGGGTGGATCATGTAACCGAAACTGGC
AAAAACTCGAAAATGTTTTTATTATTACTGGATCAAACGTGGTTTACAAGTGTTACTTTT
GCTGTCACATCTGAAGAGGGTAGATTTCCCTAGACGATGTTGACAAAGAGTAGAAGCATT
GAGGCACTAACCCAAAAGTATTTTTCTTGCAACAGATGTAATCAACTCCTCTGTACTTGA
TGACACAAGCTATGCTCTGGTTGGGCGAAGTTTTGTCATCAACAGGACAGCCAAATACAT
TCTACTCATCCTTGAACCTTGAAGAGAGCACCACCATCTGAAACTTGTTGGCCAGCTTTT
ACTTGAAGCCCATTGACAATCCCACCACATGGTGCCTTTACCACATGCTCCATCTTCATT
GCTTCCAACACTAAAATAGGTTGTCCTTCTTCCACCTTAGTCCCATCTTTCACAAGAACC
TTTACTACTAAACCAGCCATGGGTGCTAACACAGTCCCTGGAGGGCCTTTTGCAGTCTCT
TGACTGCTCTTATGTTGGATTTCTTCATCATTAGACAACTCAAGCCCTAATTTTTGCTTG
AAATGGTGATGTTGTAAACCATTCCATACATGAATGTGCTTAGTCCGATCCTTAGAGTAA
ACAGCTAAACTAACATCTGTGCTTGTACCATCAAATTCAACTCTAAAATCATGCTCGCCT
AGGCATGTTGCTTTGACTTCTAAACCAGGTGAACCATCTTCTGGTATCTCAATTAGATAG
CTCCCATTTGGCTGGTAAATGACATTGAGTGTCAAAAGCTTTGAGCAAGTATTATCATAC
TCATTCTCCCACTCAAGTTCCATAATAGACCTAGCAAGATGGTGGACTCTGAAAGGAGGA
TGAGCATACCATATGGAAAGTACCCTGTGGCTTCCAAGGGGACTTTCCTTACAGGCAGAA
TGTTCTTGTTCACAAAAACATGCAGCAACCAGGATTGCATTGAGTCTTGCAGCACTATAA
GTAGCCTTTGCTAATGTGGAACTACTAGAATTAACAAAGAGGTCATCTTTATGGTTCTCA
ATAAAATGAGTTTCCACACTTCCACTTCGAAATGCCCTATGATTAGCAAGTTTTTGGAGA
AAGTCGATATTAGTGGGCACTCCTGCAACCTGAAAGTTTGATAAACAATTCTTCAGCTTG
ATCAGTGCTGCAGCACGGTTTTCACCCCGCACTACAAGCTTTGCAATCATAGGATCATAA
TGCATGCTAACAGTGTCTCCTTGTTCTACTCCAGTCTCAACCCGAACTTCAGAGGAGACT
GGGACAGGATGATAATGATGAAGAACTCCAGTTGCAGGAAGAAATCCTTTAGGGACATTT
TCAGCATATATCCGGGCTTCAAAAGCATGACCTAATAAGGGAACTTGTGATTGACTCAGA
GGAAGAGGTTCTCCATTTGCAACACGGATTTGCCACTCTACTAGATCTTGACCAACAATC
ATCTCAGTCACAGGATGTTCAACCTGAAGACGGGTGTTCATCTCCATAAAATAAAAGTGG
CCTGATTCAGTATCAACTATGAACTCTACAGTACCAGCATTGTGGTAATTAACTGCCTTG
GCAGCAGATACAGCAGCCTGCCCCAAATGTGAGCGAAAGTCATTGGTGACATTTGG
>ntig_419_consensus
GGAGCCAACCATGTTTCAAGAGAAACCACAAGGGATATTCATCAGTCATCCCAAGCGCTA
TCTGCTGAACCCTCCCTTTTCGTGTTTGAAGTAGCAATCATGAACCTATATTTGTCTTCG
ACGTTAATTGATGTAGGTCCCATTACTGACTTGGACATGGTAATTCTTGCACCTTTATGA
GCTGCTGCTGCTGCTTCTCTCTCCTTCCTTCTTGCAGCCTTCCCAGTTGCAAATGCCACT
GCATTTCCAGATTCAACATCTAATATTGCTTTCTTTATTTGTGCCACAAGGAACATATCA
CGATCAGTGTAAAATGAAGTTGCTTGGCCCATTGACCCAGCACGGCCTGTCCTTCCAATC
CGATGCACATAATCTTCCATGGTCCTTTGGAAGATCTAGATTAACAACATGAGCAACTCC
TGTAACATCCAAACCACGTGATGCAACATCTGTTGCTACCAAAATATTAGTAGAGCCATT
TCTAAAATCACGTAGAGCAGACTCCCTTTCACACTGACTACGGCCACCATGTAAAGCAAC
TGCAAGTAAACCTTGAACAACCAAAGCTTCAGCCACCTCATCACACCTTGTCTTCCTCTC
AACAAACACAATAGTTAAAGGGAATGGATGACCAGATCTTTCAGTCTGGGAAGCCTCTTC
TACGAGCAAAGCTAGAAGCCGATCAATCTTCTCACTCTCAGAAACCTTCAGTAATATCTG
AATAACATTTGCTGTTGGACTACTCACTTTACCCACCTTCACTCGGACAGGGTCAGCTAG
ATACTCCTGTGCTAATGCTTCTATCTCTACAGGCATGGTTGCACTAAACAGCAAAGTCTG
ATGCTTCTCTGGAAGATTGCACATCACCTCTCTTATTTGTGGTTCAAACCCCATATCAAG
CATTCTATCAGCTTCATCCAGAACAACAAATGAAACCCTAGAAAGAGATGTGTTTCCTTG
TTGTAAATGATCAAGAAATCTACCCGGAGTAGCAACAACTATGCTGACTCCACCACGTAA
CTCAGCCCTCTGTTCAGAGATGTTTGTTCCACCCACAACAATTGCAGTTTTGAAGGACTC
AAGAGATCTACTAAAAGCTTTAACCTCTTGTTCTATTTGTTGGGCAAGTTCTCTAGTAGG
AGCCAATACCAATGCCAATGGTCCATCACCATGCCTAGCTGGTGTTTGAGCCAGGCAGTG
CTGTATCATAGGAATGGTGAATGCAGCAGTTTTACCACTACCAGTTTCAGCACAACCCAA
CAGATCCCTTCCACTAAGTGCTACTGGCATTGCCTGAGCCTGAATAGAAGTTGGCCTAGA
ATATTCATGAAATTCAACATCCTCCATGATGCTTGGGTGTAAACACATGTCTGTAAAAGA
TTCAATTGGTGCAGGAGCAGGAAGAGCGCCTGAACCAACAGTAACATCAACATGAAGCCG
AGAACGTACCTCTTCAATTTGTTGAGGTTGCAAGCGGAGGACGCGGTTGGAGGGTTTCCA
GGAAGCGAAAACAGGTTCAGGGAGGGTCTGAGCTGCGGCGGTCCGACGAAAGGCATTAGG
GAAGGAGGCGACAGAACCTGAATTTGAATTTGAAGAAAATGAGAGCTTGGAGTGACCATC
GTTTAGGGTTACTGCAGGGGAGGAGATATGAGATGCTTTGCTGATTGAGTTTGAAGTGGA

GTTCCTGAGGTGTGGCGGCAGATACGACATCGTCTTAGTCTTTTTGCCTGAATTTTAGAG
GTGGGCGCTTGAGCGAAGAGGACTGACTGGCCCTTCGTGGTGAT
>ntig_420_consensus
CGAGATCTACACTCTTTCCCTACACGACGCTCTTCCGATCTGTGCAGCAAACTTCAATAT
GTCAACGTTACAACACACGGGGTAACAGCCCCAACCACTAGCCATGATTCTAAAACAAAA
GAAGACGAAGACCACACAGCCCTAGACCTAACCGAAAACTTGACATAAATAAAAAAGACT
TTCTCCGCTTGTATTAGATTTGCATTTTGATGTTACGACGAACTCCACTTAAGCTCTGTT
CGTCTTTACTTTCTTAGTCCACTTCCTCCATCTTGCTCTCCTCATTTCCTTCCTCCTCCA
ATGGTGGCATCTCAGTATCATCACCGGCGATCTCGTCCTCACCGATGCTGAGACCCAGTT
TAAGCATCCTGTGAATCCTTGCTGCAAAAGTGTTGGGATCATCCAGACTGAAACCGGAGG
TCAAGAGAGCGGTCTCGAAGAGCAGCAGCACAAGATCCTTCACTGACTTGTCATTCTTAT
CAGCCTCAGCCCTCTTCCTAAGCTCCTCCATTATACCGTTGTCAGGATTGATCTCCATAG
TCTTTTTGCTCGACATGTAGGCACTCATGCTGTTATCCCTCAATGCCTGAGCTTTCATAA
TTCTCTCCATGTTAGCTGTCCAACCATATTCGCCAGTGACCAAACAGCAGGGAGAGTCAA
CAATCCTGTCAGACACGACGACTTTCTCTACTCTGTCCCCCAGAATTTCCTTGATTGTCT
TGCACAGGTTCTCAAAGGACTTCTGCTTTTCCTCCTTTTTCTTCTTCTCTTCCTCATCGT
CATCAAGTCTTAGGCCTTCTTTGGTGGCCGAGACCAGCTTCTTGTCATCATACTCCTTCA
ATTGACCAACAGCATACTCATCAATGGCATCCACCATGAACAGCACTTCGTATCCCTTCT
TCTTGAGCTTCTCAAGGAAAGGAGAATTCTCAACGGCTTTCTTACTTTCACCAGTGATGT
AGTAGATATCCTTTTGGCCCTCCTTCATCCTTGTAACATAGTCTTTCAAGCTGGTCATCT
CATCACCACTCTTGGTCGAGTGGTATCTGAGCAGGTCAGCAAGTTTGGCTCTGTTCTGAC
CGTCTTCATGAATACCCAATTTCAGATTCTTTGAGAAGGCTTCATAAAATTTATTATAAT
CTTCCTTGTTCTCAGCTATCTCATTGAACATCTCAATGCACTTCTTCACCAAGTTCTTCC
TGATCACCTTGAGGATCTTGTTTTGTTGCAGCGTTTCACGAGAAATGTTAAGCGGGAGAT
CATCTGAATCAACAACACCCTTCACAAAGCTGAGATAGTCTGGAATTAGCTCTTCACAAT
TGTCCATGATGAACACCCTCCTCACATAAAGCTTAATGTTGTTCATCTTCTTCCTCGTGT
CAAAGAGATCAAATGGGGCTCTTTTAGGAACAAAAAGAATGGCCTTAAACTCAAGCTGCC
CTTCCACAGAGAAGTGTTTCACAGCAAGGTGATCTTCCCAATCATTGGTTAGGCTCTTGT
AGAAGGACGCATACTCTTCCTTGGTAATCTCCTCAGGTTTTCGCAACCAAATTGGTTTTT
GTTTTTTGATCAGTTGCCACTCATGCGTCACTTCCTTAACCTTCTTCTTTTTACCCTTCT
TCTCTTTCTCCTCATCAACTTCAACCACATCACCTTCCTCCTCCTTTTTGGTCTCCTCAT
CCTCATCATCACTGACCTCTTTCTCACTAGTTTTTCAGTCCAGAGATAGATGGGATAGC
TAATGAACTCAGAATGCTTCTTCACAAGATCCTTCAATTTCCTCTCCTCCAAGTACTCCA
ACTGATCCTCCTTGAGGAAGAGTGTGATCTTGGTTCCCCTGCCCAGTGGCTCACCGTTAA
CATCCCTAGTAACAGTGAAAGACCCTCCAGCCTGAGACTCCCAAACGTACTGTTCATCGT
CATTATGTTTACTTGTTACGATGACCTTCTCAGCCACCAAATATGCCGAGTAGAAACCAA
CACCAAACTGCCCAATCATGCTGACATCAGCGCCAGCTTGTAACGCCTCCATAAACTCCT
TGGTACCAGACCTCGCAATCGTTCCCAAATTATTCACCAAATCAGCTTTAGTCATGCCGA
CGCCACTATCGATAATTGAGAGCGTTTTGTTGGCTTTATCAGGAACAAGCCTGATAAAGA
GCTCGGGTTGCGCATCGAGCTTGCTCTTGTCAGTTAAACTCTCATATCGGATTTTATCCA
ATGCATCGGAGGCGTTACTGATCAGTTCACGAAGGAAAATCTCTTTGTTGCTGTAGAACG
TATTGATGATCAAGCTCAAGAGCTGATTGATCTCGGCTTGGAAAGCAAAAGTTTCAACAT
CTCCCATGTGCACTTCAGCCATCGACGATCAAATTGCGATCAGAATTAGAAAGTGAGCGA
TCAATTGACGATTGTAGAAGTTCAAGCGTGCGCAAAGTATTACCAGCTAGCTGAATTTCG
ATTAATTGTGAGAGAGAACGAGGGGATTGTGATTTTATAGTGTGGGGGAGAAGTTCTTCT
GGGACCTTCTTTGCGTTCCGGAATCGATTTTCGCGGGGACTCTAG
>ntig_421_consensus
TTTTTTTTCAAACACAATAATTATTTATACATAACAGTTTTACTAAAACAAACAATAATC
TTCCCAACACACCCTTCTCACAATATCAATCTCTTCCCTTCCTATTCACAGGTGTTATCT
CAAACTGGGGCAGCTAAACTATTTATTAAAAGATTGCACTCAGAGAATAAAAAAAAAATG
TCAATTTAAAGAAGAAAAGCAAAAATAGGGGTAAAATGTATAATCCTTTTTTACAAAAAT
CAAGCTCCCTGTTTCTCAATGCCTAAAAGGAAACCCATATAGGACCAGGAAAATTCATGT
GTCAAAACTTTCAACCGCAATAACCAAGTCAATCAGTTTTGGCAGCAGCAGCCTGTTTCG
CACCAACAGCAAAGAACTCTGTTATCACTTCGAGGGGCATGCCCTTTGTTTCTGGGACTT
TCAAGAAAACAAACACCCATGATATGACACAAACTACCGCATATATGCCAAAGACACCAG
CAAGGCCGACAGATTTAAGCATCACCGGGAGAGAATACGTGACTATGATGTCCCCTATCC
AAAATGTGAGTGCACATACGGCAATGCAAAGCCCACGTACTCTTGTGGGGAAGATTTCTG
AACAGAGTATGTTTGGGATTGGCCCGAACCCCATGACAAAGCAACAGAAGTATATAACAA

CACTGGCTGTCGAGATTGCTGCATGCACAACAGAGCCCATCTTTATGACACTTCCAAGTA
CCAGGATGATAAGCGATGCTATCAATACAGGAATTGTGCTGAGAAGCAAAGTCCTTCTGC
CAGAAAGATCCATGAGCCTCATGGCAACAGCTATGCATGGAATCATCAACAAGGTTGTGA
TGCCACTAATCATCAGAGAAGTAGAAGCTGCACTAAGGCCCATATTTGAAAGAAGAATTC
CAACACCTGCTTGCTCAAGAATTTGAGGAGTGTAGTAGAGAACCCCATTTATGCCAGAGA
ACTGCTGAAGAATTTGAATTCCCACCCCAACAATCAATGCATGTTTGACTCCTGGTTCAA
AGAGTTCACTCCAACTTGGCCCTTGTACTGCTTTTTCAGATGGATGAACCATAGCTGGCC
CAATTGGATGCTGTTTCATGAGTTCCTTGGAATAAAGTGCTGGCTGACTAACCAGAGCAG
CAGCCTGGATAACTTCATTTTCAGCAGGTATGTCTGCACCTGTGAGAGAAACTATAGATC
CACGCCTCGATCCTGGGGCACCCTCTTGATGCAAATAAATTCTTTTGAATCCCCCTTCCT
TCTTTCCATCTTGGCCTTCTTTCTCAGACCATTTCCATGCCAACTGCCAACCTCCACCTA
TTCCCATGCTACCTACTGGCTCTCCAGCTTGCACCTGACTGCCATGCCTCATGCTTGCAA
GGCTTCCATGGGCCTGTGGGACCAAGTCCTTTTCCATGCTTGTTGTCTGACGGGATATTA
ATGGACTATGCAAATTATCATCAGAATCAGCACCAGCAGCATCAGATGCATACTCTTCAC
CATCTCTAGCAAGGCTCTCCTCATCCCACTCCTCATTTCTAGCTTGATTCCCTCCCACAC
TGAACATGCTGCCAAAGTGAGGGAATAGAGTGCTTCGCATGCTTCCTGTGTCAGGAAGCT
TCTCATGCACACTACCAAACAGGGTGACAAGCGGATCCATTAGAGGCACAGTCTGGTTTG
CCATACTTCCATGCCTGGACACTAGACCAAGAGAACTTTGTCCAGTAACTGGTCTAGCAA
CCCAAGAGAGGCCTTCTTCAGGCCCATATAATTTGATTTGATCCTTATCAACAGAGATGT
CTTGGTCATCAGCGAGATCATTGGCTGGACCAATTATGTACTCCTCTAAAGATGTTTCAC
CCCCAACTTCCAGTCCCTCAACTAGTAAAGCCATCTCACCAGAGACATCCTCCCTGCCAC
GAATCTTCTGCAGAACCCTTTTAGCCTCAAGCATCTGCCCTTTACTCACAAGCCACCTTG
GTGACTCGGGCAAGTAAAATACAGTCAGCACAAAATAAATAAGAGAGGGAATACAAAGAA
CCCCAAGCATCAATCTCCAACTTGGAGAATTCTTCAAAGACATTCCAAAGACCATACAAT
ATGAGAAAAACATTCCAACACAACCAGTGAACTGTGGAAGTGTATTTAATAACCCCCTTA
TTTCAGGCGGCGCTGTTTCAGATATATATACTGGGACCAGAGTAACCGCCAAACCAATTC
CAAATCCATCCAGAAGCCTACCCAGAAGTAGGACATAAACATTGGGAGACCAGAGCATTA
CAAGACTACCAATGAAATAGAGGACAGATGAGATTATCAGCATTGGACGACGGCCTAGCC
AATCAGATATGGCTCCAGAGCATGTAGTTATTATAGTGGCCCCAATAAGTGACATAGCCA
CGATTAGCCCTTCAACAGTGGGTTCAGTTTCCAAATTGAACTCTCTCTTTATGTACAAGA
CAGCCCCTGCAATTGTTGCATTATCCCATCCTTGCAGTAAATTACCAATGGCCGCAGCAA
TAGCAACAAGCACAGCTCCACTCATTTTTCCCCAGAAGCAGTTATTAGAATCCCCTCAAA
ACTTCCACCAAGAAACCTATGGGAAGAAGCAGGAAGTTTTAGATGAGAGTTTCAGGTATC
ATCAGATGCACAAAGTCCATAACACTTGCTGCTAATTGCCAATTTGATCTTAAAACGTTG
GTCTTTCAGTGAC
>ntig_422_consensus
ATCCATTTTTTTTATTTCCACATACATATTTGCTGATTTATTTATTACATGGAGTTATTT
ATTGTTGTGGAATGCCGGATTTTCTACCGCAGAGCCAAAACTTAACAGAAGAAAACATTA
TAACTCAACACACCTTCCTTTTCCATGGCTTGACAACATCTATATACTTACTGAGACCTT
TATGTGATGACTGTAGGTTTCTCCCTCCCTGTGAAATCCTTCAGCTTTGATACGGACAAT
GCCAAATCTATCAATGGCTTTAAGGCTACTTGAGCATCCATATCGTGTTTAGAGACATCA
GGCTCAAACTGTTCAATGTACATTCTGACAGTTGCACCAGCTGAACCAGTTCCAGATAGG
CGGAATATTATCCTTGATCCATCTGTGAAAACAAACCGGACACCTTGCTTTTGTACTATA
CTTCCGTCAACTGGATCAGTGTATGTGAAGTCATCTGCAAATTGAAGAGTGTAACTTCCA
TACTTATCACCTGCCTTGCTCTTAGAGATCAAATCTCTAAGATATTCTACCATTTTATTG
GCTCCTTTGGATTCACATTCTTCATAATCATATCTTGAAAAGAAGTTTCTCCCATACGTT
GCCCAATGTGCTTTCACAACATCGGCGACAGATACTAATTTTTCCCCTGGTTTTTTGTCT
TTGTTTTGGTGTGCAACAATTGAAAGCCATGCCAGTACAGCCCATATGCCATCCTTCTCA
CGGATATGATCAGAGCCTGTACCAAAACTTTCTTCCCCACAAATTGACAACTTCCCAGCA
TCCATAAGATTTCCAAAAAAATTTCCATCCAGTAGGGACCTCAAAAAATGGAAGCTTTAAT
TTTTCAGCAACACGATCAAGAGCACCACTTGTTGGCATCGATCTGGCCAAGCCCTTAGGG
CCACTCTTAAAGTATGGAATGGCTTCTTGTGCATTTGCTGCAATAATTGCAACTGAGTCT
GATGGAGTAACAAAAAACCCCTTTCCTAAAATCATGTTTCTATCACCATCCCCATCACTT
GCTGCTCCAAAATCAGGTCCATTATTGCTATACATAACATCCACCAGATCTTTTGCATAC
GTAAGATTTGGGTCAGGATGACCATGCCCAAAATCCTCCAAAGGCACCCCATTTGAAATC
GAATCC
>ntig_423_consensus
CCCATTTCCAAATCTACACAATTCTACAACTTTCATTGCTACTCTAAACTTTTTGTCAAA

AAAAATTCATATAACAAAATATACTACAGCAACAATCCAAATATAACAAAAATAAACCCA
AACTTACAATCTCACTCTCTCACTATTGCCCACTTTCAGCCTCCAATTTCTTCACATAAG
CCTTCAACACCATCAACACATCTCTAGCTGGAGCCTGTAAAGTACTCACCAAACCCAAAG
ACGGTCCCTGCAATGCCCCCAACATCTTCGCATAAATCTCTGCTCTGGTGGGCATACTTT
CTAAAGCCTTAAAATCACCAGGCCCATAAAATTTTCCTTCAAAAACAGCACCTGTAAAGT
CATTCTCTTCCAATTTCTTCTCTTTCTGAAAAGTCCTGTACGGCTTTATCGCCGCGGGTA
TCTCTTCACTGTGGACAAAAAGCCAGGCATTCATGCCCTTCATGCACGGCTCTAAAGCTT
CCCATTTAGTGCCCTCAATTGCTTTATAAACCAATGTGTTTTTAGCCACAAGAAGCTTGG
TGTTTTCCGGCAATGATCGCCTAAGATCCTGGAATTGCTTGACGGTGAAGCCCTCGTACT
TGATGGCGGCGAGGAGGAAGCAGTTCTCGAGATTGGTTTTAACGGTTTCAACTGTTTGTT
CTTTCTTAGTACGCGAAATAGCGGCTCTGATGTGGGTGGGTCTAGAAGGAGTGCCTCTCG
AAGGGGAAAAATAGAAATGGTTATTGAAAAGTGGGTGGGGGTTCGGTTTTAGGGTTAGGG
TTCGGGGAGAAGGGGATGAGAAATTGGAAGAATAAAAAGAAAAGAGAGTGATTTCCATGG
TGAAAGAAAAGAGAGGCTGTGTTGGGAAGAAGATAAGAGTCCTTACTGTTTTCGTTGAAT
TCATGGG
>ntig_424_consensus
CGACATTATTTTTACTATTTGATATATTTATCAAACTGAGATCAATAAGCCCAAACTTCG
GCTTCGGCTTAGGCTCAATTCCTATAAAGTTAAATCAAATTCGAATTCAACTCAATTCGA
ATTCAACTTTAGGCATCAAATGAATAATTCATGTATTTAATAGCAGGCAAATAATGCACA
CATCAAATTACCCCCGGAACGACATGATGAAATACGGTAAAGTTCCACTTCTATCAAAGA
TCAGATTGGACAAGATCAAAGATCTCATAATAAGCTCTTAATAATCTACTTTAAAGCTTA
CAAAAGGCTGTAGAAAAATTCAACAATGGCATAAGATCTAATTTTAGACATGACACACAA
ATACTGCAAAAAAGATAAAGTAAATTAACAGGCATCACTTCAGGGAAAGCCTGAACCTGA
CGGAGCTGCACCATATCCTCCACCTCCACGACCAAAGCTAGGCCTTACACCAGCACCCCT
GAAGGATGGCTGGAAATCTGCTGGGGCTCCTCCTTTCTCACCACCAAAATCACCACCACG
GGGACCTCCACGGTATCCTTCACGATCACCAAACCTTGGACGGTCTCCCTCAAAGCGAGG
TGGGCCCCTTGGACGATCACCAGGAGGGCCACCCATAGGGCGACCAACAGGCTTTGCAGA
TTTCTTCAAGGTGGCAGGCACAATTTCTGACGGAAGATTGAGGTAAGTTCTCAAAAACTC
AATTCCATCATTGGTAAGATACCAGTAGTAGTGCATCCAAGCGAATGTCTCCCGCACATA
CTCCCTGGATTTGAAGCTCTGCATTAGCTTAATCACTTGCAGGTTGGGAACATCAATCTC
TGGATGCTTGGCGAGATTATAATCCTTCTTTGCACAACAGACTCCCTCTTGGAAGAGGTA
TTTGCAGATTTCTTTACGGTTCTTCTCTGGAATAATCATGGCTGCGAAGTGTGTAAATAT
GATAAACCCTTTCTGTCTGGCTGTTTTGTAGCGGCAGCCGCAAGATAAATGTCGAGCTAC
TTTCGGGGCCGCAGGAGGTGAACCCTAGCGTACCGATTGTCAATTTATACAGGTTACTTC
GGTCCCGAGAT
>ntig_425_consensus
ATGATTATGAAACTACCAAAACAGTCCACAAAACCAAACTCCTGCCTCCTTACGACCCCT
ACAACAATAAGCCTGACGACCCCAGAAACCTAGAAATATTTCATAAAATGAGACCAGAAA
GTAGATGAGGACTCGGAGCTTTCGTAGCAGATGAAGATTGTGGCTAATGAGAAGGCTGTG
AGGGCAGTTCTTAACAACAAAAGGGGCCCTGTTTTGGGGAAGTTATGGACCTTCTCGTTA
GCAACTAGACTGTATACGGAACAGTTTTTGAGCTTCTGCTTGTAATGTGCGTGTTACAAA
ATGGGTAATAGGCTTGACAACTAATTTAAGTACAAAAGAAACCACCACAATGTGGTGGGC
AAACAGTCAATAATACAAAGATCCCGATTCCTGTGACTATAATTATAGACCAAATGCGTG
GCCTACGGCTGCTATGAAATTAATACAAAGTAAAGCTTGGCAACGGTGGCCTCCTCCATC
TATTTATGAAATCCGAAGGAAAAGCTGCTGCCGAGCACATATACCAGACAAGTCTTAATT
ATCAGTATATTCGAGTGGTCTGCTCCCAGAACGCCAACTGTTGAAACAGGAACCTTCAAC
GTAGAAATTAGTAGTCGGGCTTGTTAGGGCATTCTGATGCTCTGTGCCCGAACTTCCCAC
AGTTGAAACAAGAACCTCCAAATCTGTCCTTGGATGATGGCCTGCTTGATCTTCTACCGC
CAATCAACCAATCATCTCCACTGCTTTTTGAAAACCTAGACCAGCTATTTTGACTTCTAG
AACTTCGACTACCACGCCTATATCCATCCTCATCATCGCTGTTCCAACCACGGCGAGATT
CTCTAAAACCTCTCTTGTCCCTGGAACCTCCCCGGGGAAACCTATCCCTGTTAGAGAATC
TGCCATAATTATCACTTGAAGGGCCATCATCTTGCAATGCAGGCAACTTGGTAATTTTGG
AAATGGTGTTCCCAGGTGGTATTTGCATATTCAGCAACTCCTTTGCAATCTCCTCCGGAA
GGTCAAAAACTGCTCCTCGAACCCTGTCATCAGCAATTATATTTATTTTTCCTAGCTCAT
CAGCAGCAGCAGGGTAAATAGCAGAAAGGAACCCAGTGACAGATCTAGCAGACAAATACC
CTCTGGAATAAACTGAATCCTGCGTCAATTGTAATGTCACCCATCCCTGCTCATGGGTAA
TAAGAGAGCGCGACGAAGGAGGCTGTGAGAAACCACTCAACTGTGCTAGTGCAGCAGCAA
GAGCATTTGTTCCTTGTTCTTCAATCAACTTTTGGGCAATAGGGACAAAGAACTCCACAG

ACTCAGGATGAACTCCATTAAGAGTAGCAACTACTTGCTCAGCTGATGATTCCAAAACCT
CTTCAATAGCCGGTGGGCTAACAAACTCAAACTTACACCCAACATCACGCTCCAGAGATC
TAACTGTTCTCCTTTGGCTACTAGTAAACATCAGAATAGCAGTACCTTCCTTTCCTGCTC
GCCCAGTACGACCGGAGCGATGAACAAAAGTCTCTGGATCATTTGGCAGTTCATAGTGGA
TAATTAAATCAACATTGGGGATATCAAGCCCACGAGCTGCAACATCAGTGGCAACAAGAA
CAGTGAATTTTCCTTGTCGAAAACCATTTAATGTTCTCTCCCTCTGGTGCTGAGACATAT
CTCCATGCAATGCCTCAGAACTTATACTGTTTGTCAACGCCATTGATACTTCATCAGCAT
CTCGTTTTGTCTGTGTAAATACAATGGTCTTCCCACCCTTTGCATATACCGTTATAAGAT
CACTAAGAATGGTTCGCTTTGAGGTTGCAGTTGTTGATATAGCATAAAGTTTGATCCCTT
CTGCAAGCTTTTCATCTTGGTCACCAACCAAATCTATATTGAGAGGACTGTCCAAATATT
TTCTTGATAATTTCTGGACCCAAGGAGGCATGGTTGCAGAGAAAAGCATACTCTGTCTCT
TGGGTGGAAGATTTTCCAAAATAACTTCGACATCCTCCTCAAATCCAACAGCAAGCATTT
GATCAGCTTCATCAAGAACCAAAAACTCTATTTCGCCCAATCTGAGGGAGCCACTTTTAA
TCAGGTCAATGATTCGACCAGGAGTTCCAACAACCACATCCACCCCACGGGCAAGAGCAT
TTTGTTGAATGTTATATGAAACCCCTCCATAAACACATACAGTGTTCAAATAAGAAGCAG
ACTCTTTGAGTTCTTTCTCTACTTGCTTGGCTAACTCACGAGTAGGTGCAAGAACCAAAA
CTTTTGGAGGTCGACCCCGCCTGAGAGCACGAGCTTGATCAGCCTCTTCAGTAAGACGCT
TAAGTATCGGGATTCCAAAGGCTAATGTCTTCCCAGTTCCCGTCTTTGCACGAGCAATCA
AATCTTTACCTTGTAGAGCCGGAATCAAAACAGCTCTCTGAATGGGGAAAAGGTGTGTAA
TGCCACGCTTCTCAAGAGTATCAACGAGACGGTCAGGTAAACCGAGCTTAGAAATGGCGA
GTTCATTTTTGTCTGTACTAGCGTCCTCGGACTCAAATTCCTCCTCAAGAGAGCTGGAAT
TGTCAGAGTAGTCACCGAGTCCTTTGAAAGCTTCTTCACTGAGGAAGTTGTTAGGAGTAG
CTATGGCCGAAGCAACAAAGGTGAGGCCATTGCTCCTAAGAACCAGAGGCTTATTGCTCG
AAGCAATAAAACCATTGAAGTGAGACTTGTCAGCCAACGGCAAAAACGACGTCGTGGGTC
GTTTAGCCAAGACGGGAGTGGAAGATACACCGAGTATAGAGGACATTTTGAAGTATAAAG
AATGGAAAGAAACGTAGAAGGCGGTGTTAAGGTGAAAAAGAAAAGGAAGTTGAGGGATAA
GCAGGCCACTCTTTTGCTAATGAATGGCCTCGCACACCTCCACCTCCTCCGAACCAGCGA
CATAAATT
>ntig_426_consensus
TCTTTCTTGAGCCCTGGAAGATCGGCCTTGAACACGTGAGCCTCCGGGGTTTCCCTCCAG
TCTATCCGGGTATTGACAAAAGTAGATGTTTCTTGAGGGAAACGAGTGGAGAGTGAAGAA
AAATCCTTGAATGGATCCCAAACATCGAGGGAGAAAGGATCGAAACTACTGCTGCGCTGG
TTGCCGAAGAAGCTTGGAATTAGCGACATATTGTCTCAAAGAATGCGAGGGAATTGTATA
GATTGCTCAATCTGATGCTTTAACTTTGAAGAGTTGTCTCTGATGGTCTAAAACGGAGGT
GCTGGGGCCTTTATAAGGAGATGGAG
>ntig_427_consensus
AACTGAGGATTCCTCCCTCTTCACAGCTTCCTCAGAATGTGGAAACCTGAGGTCTCACCT
TGCCAATGATGCAAACATATTACAAGAGCAGTGAAGAGAAGCCATTGTTTCACCAAATAG
GATTTCTACTTTTGAAGATTAATAATCCCCAAGAATAAAATTGACCCTGACACTGTTGCG
ATGTTTATAATACACAAAGCTCCATTGGTTAGTTTCAACAACTCATGATAAATCAAAAGA
TCCCTGCCCTTCGCTGATTGCATAAAGCAGTTTTTTGTACATAATTTCCTTGCTAGAGTA
CGGGGGTAGCTTCAGGTAATTGGCACAAGTCATCACGCTTGGCAAATCTTCATCTGCTGA
TTCCGATGTCCCAGTTCCATTTGTTGCAGCATTACTTGCAGTTGAAGAATGCTTTCTCAC
TATAGTCAGCTTGGGATTAAGCACCGCCAGGCCACCTGGTGGAAGCCTAGGTGCACCAGT
AACAAACTGGCAGAAGGCTCGTTGCTGCTCTGGTGTAAACTCACCCATGATCTCAAGTAA
GTTAACAATTGCAGGGCTCTTGGCAGTATAACCGTGATCAAACTTTATATGCTCAGCAAG
CGTCTCAGCCTCCCACAACTCTCTGCGACCACAAAGCAGATGGTCCAATTCATGAGGCGT
AAATATTTGCAGTGACGAGATGTCAAAGACCTGATTGAAGCCTGCTCTAAATGCTTCCAT
TTGCCGCATAATTCCAGTCTTCACAGTTGCATCAACTACGAGAGATATATACTCCTCTAA
GTTATCGATGCCAACGTTTTCATCTCCTTGTTTCAAAATGTAGTCTGGGTAACCAGGAAC
AGTAAAATCCAAGCAGAGATCCTCAATTGGGGCCCCACGGAATCGCAAGTCAGAAATTGC
ATTACAATTATCACAACTTGCTGACTCTAAATATTGTTTCCGGCAAACAATGAGATGCAG
CTCTTGCAAAATCTTCCCAAATTCAGAATCAAAAGAAAGAATATCATGCAAATCAAGGTC
TTGATCAAGAACAAGTTTATAAAATGCTGTTGACAATGGCAGATCTAAAAGTCTCCCATC
TTGAAGAGCTTTAGCCATCACACGCCCAACCAGCCGGAAGTACTCAATGACTTTAGAAAA
CTGGCTACCTTCAGAAGTATCAGCATTTGGAGGCCAAGGTCGAGGGAAAAAACCTAGTGG
AGCTTGAACTAGATCTCCTGTTACATTATTCGATTTCCCCTTTTTTTCTTCACCCACATC
AGTTTCCATAGATGATTTCTCTGATGAAGAATTTGACCTCCACATTTCAAGTACAACTTT

CTGTAAGTCACGACTTAGAAGGGTATAAAATTCAAGGGTGGGACCCAAACCTGTGCCAAC
TTCCCCAAAATATTCTACTTCAAGGACAGCCTTTTGGCTAGAATACATCTCCATTACTTT
TACAGCAGAATCCAAAATACGGTTCCTAGAGACACGAACTTTCTGCCGCTCTAATCTCCC
AACTCTTACCTCTCTTTCACTTACTGATCCATGACCATCAGCACCCTGCTGTTGCTGAAG
GCGATATAAAGCACGAGATAACCCAAAAGCAGTTGAATAAAAATACTGACGCCTGGTCTC
AAAAGGAAACAAGAATGGGCATGCTTTGGTCAACTGATAACACCATGAGGGAAGACTACC
ACTGCACAAAGCAAGTGCATCTTGAATTTGTCGAGCTAATTTAGGAGTGAGTTTACCGTT
TACAAAATCCTCTGAAGGTACCCTAGCACCCATTGTGCTCTGTTCATCTAGACTTGAAAT
TTTCCCCTCAGCGTAACTATCAGAAATCATCTGGGCTCTCAAACGTGGTGCCAGCTGATT
CAAACCCTCTAGTACACGCAACAGTGCCAATATATTGTAAGTAGGATTAGATTTCTCCAA
ATCACAAGGAAGTTCCCCCTGCAAGATGCTGTCTAAAAGTGGCATACGATGAGATTGCGC
ATCAGAGTTGGAATTGGAAGCAGAACCAGATTTTGTAGATTTTGATGGACCCGTAGAACT
TGATCCTCCAATGGGTATTCTATCAGATTGGCTGTTTGCCCTCTGGTATGTTATAGTGTA
AATATCACTCCACAGTCTGCTTCCATCACTTGATATGAAGTCACAGCCACCAAACCTATC
ATCATCATCCTCATCCAGCACAAGCTGTTGTTGAATGGCTTGATAAATAGTCAAATGCCT
GTTAAGCTGTTTACCACCAGCAGTGAATATCAACTTTGGAGGTTCATTGGAACTGCCAAA
CAGAGGACGTCCATGTCGATCTCTGCCCCCCCTTACACCTCTTCCATTAGCGGAACCAAG
CCCAGCCATAGCAGCAGCAGCAAACGCTCCCCTTGAACCGTAAGTATTGCCACTCCTAAA
GTCAGCAGAATCAGAACCCCTGACTGTAGCAGCTTTGCCACTAGATCCGGATTCTGGGTT
AGTCTGACCATCACTAGCTGCTGAAGCAATGGCACTATCCTCAGCCGAATCACCCAATTT
TACATCATGTACTTTGTCGGGCATGCAAAAAGGAAGAGAATCATCTTTCAACACATCTTC
ATGATCTTCATCATCATCATCATCATCAGAGATATCATCATCTTCAATCACCAATGCATC
ATCAATCTCTACTGGAGATATATCCAATTCCTCATCCTCAGAACTTGTATCTGCAGTCAC
TTGCTTCATTTGTGCGTCTTTGTCTAGAGCAGCTCTTCTACGAGCAGCATTTCTTGTTTG
AGGTCCTCTTACCTCCTCCTGAGCAGACTTCAAAACAGCTTTACCCTTCCCCTTGGATGA
GCTTGAGCTCCTCTCTTGGGAAGGTTCTTTTTTTGTGGCACCTCCAATATTAACAGATAA
TCTGGATCTTGAAGAATGACGACGAGCTGTAGAAGTAGGAGTTGAGGTAGAAGGAGACGC
CGCACCAGCTCCAGAGGGTGTGGTCCCAGACTCTGAATTCCCCACAGATGCTGCAGGCTT
TTGGCCAGACTCACCTCGCTGAACTCGAGGCCAAAGAAATTCTTCAACAGCTGCTAAACT
TGCTAATGGATCAATCAGTACAACTTTAGAAGAATAATCCCGGATAGATTTTTCACCTTG
AGCTCGACAGAGGCGCAACTTAAAAGGCTGAGATAATGCACTAAGTCCAGAAGAGAGACG
AGCACTTCCACCAGATGACCTAGATGAATGGCTCAGTACGACAGGAAAACGTTCTAGTGA
GGACAAAGCATTTTGAAGCTTCTGAACTAAGACGGTCAAAGGAGCCACATTTCCTGCATC
CACACTAGATGGGAGTGAAACAGAAAGAAATGACTTAAATCTTTTAAGAGCTTGTTGGCG
AAGCTTGAGCAGAGTGTATTCTGAAATTTTTTCCTTAGAGAAATAACCACACGAAAAATA
ATTCAACAAAGCTGCAACAACACCACTGCCAATAAACTCAAAAGTGGATACACCATCCCC
TTTGCTTAGTTCAGTTAACATCTCAGATACCACCCCAAACAAATACTCTTCCTTACTAGC
AGATATATCAGCTATTTGAGATCCAGAGGCTTTAGATTTTCCCTTTGTTTTAGTCCTTTG
GTCATCAACACCAATATTCAACTTCAAACAAAGATTCTTCAGAAGTAATAGATGATCTGT
AACTCCAGCTTCGGCAGCCCCAGGATCAGAAGGAAAGTACTTGTCCTTAAACGCTTTAGC
ACTTGCACTGACAGAAGTTCGAAGATTGGAATTTACTGTTGGAATTTCTACTGAACTTGG
AGGCGAACCAACATTTGGTGAAAGAGGATTCTTTGATTCTTCTGAATAATTTCCTTCAGG
ATTTGAGTTACAACTGCGTCGTCTATAACGCCTAGAACGTAACGATGTACCAGATCCAGA
ATCATTGTCCTTATCACCTGCTTGGGCAGGAACAGTATTTGGATTACCAACTAAAATAAG
TTGATCTACAGCATGAACCACACCTTCTCTAACAAACATCTTAGAGAATGTGGCAGGAAG
CTTTTCCATAAGAATCTCAGCAATTTGGAGGGCAGGAACCAAGACATGTGGATCTTTCCA
TGCCAAAACACCAGCTAAAAAGCTTGATATGTTTGTCACACTTAATAGAGACTGAATCAT
CTCTGCACTGCTGAAATACATTAATTTCCCAATAACAGAAAGACATTTGTGGCGAACTGG
ACTGTTGACACTGGAACCATAGATCTGTATTAGAACAGGAAGAAGATCCATTCCAAACTG
CTGGAGGAGTTCAGGCTGATCATTTAATAATTTCTCTCGAGTAGAAACCTCAGGAGCACT
AACATTTGCACCATCCTGTTTCCCAGAACCACTAGCAGGAGACTTCCTCACCAGAGGACC
TTTCATAAATATATTGGAGCTGGCTGGGAGGGCAATAGTTCCTTGCGGCAGTGGGGGGAG
GAGCTCGTTTGCTAGGTTGACTATCTCGAAAATCTGCTCTGATGGTCTACTTAAAGAGGG
TGGAACAGATGTATTAGCAGAAACACCAGATCCCGATAGTATATCTTTCAGAATTCCACT
GATCCCAAGGAGAAGCAAAGTTTTGGCACAAAGAGGTGATCCACTAGCACAAGTGGACAG
CAATCGAATTAAACCCGTATAGGTAGGTGTGCTGAGAGATGCCTGCCCACCTCCAGAATT
ACTGGTTGAAATAAGAGAGGCAGCCTGTGTAACCAGTCCATGATTACATAGTTCATCTAA
CTTGTCTGGAGATGATGCGAATGCTTCAGCAATTCGAGTCAAACAAACAGAAGCATGTTC

CAACACCTTTGAATCATGGTACTGAAGAAGGTTGGTCAACAGAGGCACAGCTTCCATCAC
AAAGTCAGCAGCATCAGAAGGGAGTTTCTTACACATATTTGCAGCAGTAGACAATGCTAC
CCTCTGCACTCCTGTGGAGAAAAAATCCAGATACGAAAGAACTGCCATTAGAGCACCTGC
TCTCAAGCAGACAGTTGGGTGCTCCTGCGATATCTTCTTTAAAGCTTGCAGAGACTGTTC
AGCCAAATCCATGTATTCAATTGTGAGCAACCGAGCTACAAAACATGTAACCGCCCCATA
ATGCACAACAGCAGCACAAGAAGACGGCAAAACATCACACAAATGTGTGAGTGCCCTAGC
CGCAAGCAACATAATATCAGGATTATTTTCATGATTCAGTAACCCAACCAACACTGGCAC
AAATAAATCCACCGAGAAAGTACTCAAACTCTCCTCCGTCCCAATGGACAACATCTCGCA
AAGCTGTGTCAACGCCTCTACTTGCTTCCCCTCCTCACCGTCAGCCCTCAGCCCCGATAA
AATCTTCTTTAATCTTCCGCTCTGATGGGATGACGACGTCGACCCCATAGCTGCGGACGG
TAAAAGATCATCTAACCCAGCGCCGAGCTTCCTCAACAGCCCTTGCAAGGCACTGCTAGC
TGAGGCTAGATTCTGATGCAAAATTCCAACTCCACCCTCGCTGTCATTGTCATCATCGTC
CATACCCGTATCCATATTCAACCCAATGGCCCGACCCGTGTTAGCCAAATCCGGATTGTT
ATTGTTGTTATTACTATTGTGTTCTTTTTCTTTACCTTTGTCGGCGGAATTGTCGGCGTT
AGATGAGGAGATTTTGGAGCGGCGAGTGTGGCGAGAACCGGAGGATTCAGTGGTGGGGTC
CATGGAGAGAGGGCGTGAGTGACGTGAGCGCGTGGTAGCGGAAGCGGAGGAAGCGGGGGC
GGTTGTTATTGTCGAGCTAGTGGGAGCTACACGTGGTCGTTTATTGCTGCAAGAACGAGT
GATGGGCCCAGACGAAGATGATGAAATCGAAGGGGCAGTTGAGGAGGCCTCCGCCCGCTT
CCGGCTCCGAGTTTCCATACAAAAACCACTCCAAAATCGGAACGGCTTCGCTCGCTCGAA
ACCACGAGCGAAGCGATTACACGGTTTCCGATCCAATTCCCAC
>ntig_428_consensus
TTTTTTTTTTTGAAGCAGAATTATGAATTTCTTTCCATATATTTACATCCAATTCATAAC
ATCAAAATGTTACATAGGCTTTACACCTCTCGTACAGTTAAAACAACTTTCATACAAAGA
TTTTCTATAACTAAAAAATGGCACTGCCTCACTGCCCAAGTTGCCGAGTAGAATCTACTT
TAAGTTGAAAAGTTTGCGAGTAGGTACTACAATTGATTCACACTAAAGGATAACCTCAAT
GGCACCGCTAGATTTAAGAGAATGTATCCTCCCGATCACCCTCTGCAGTTTCACCCTCCT
CCAGGTTGCTGAACTCCAAGAAGTGGGTTGGTCTGTGATCTTCATGGTAGTATTCTCTAG
GTGTGCCAAGCTTCACCCCATATTTCATGCTCACAGTATGTTTTACTCCCATGAAGTTAT
AGTTCCATGGCCCATTGTCGGGAACCATGTAGAAACCAAGGAACCGGTCACTTAAAAGCA
TCTGGACTTTCTCATAATGAGTTGGAAGATAGCCATGGGGATTGCTTCCTGTGTCCTTAT
TGACTCGTCCCCACTCATATCCGCTTGGGGTAAGTTTATATGCAGTTAATGAGCAGGAAC
CTGGAGTGAAACTGCAGGTCAAGATAATGCACTTCTCTCCATCCCATTGCTTGTTGTTTT
CTAAAATCCGAGCATGACTAGTGAGATCCTGAGGCGATAACTGGGGAAGCTCATTTGGTT
GTGTGTGCATCCATCCCAAAGGCTCAAGATCATTAAGGAAATCATGCTCAGGAAGAGCTG
AGGGAAGATGAACTTGCTGATGAGTCCCCCACTGCGGTGGCATGGCAATACAACGAATTT
CCTTAACTTGAGGATTATCCGGTGGGCTTATACCATACAAGTATCCAGCAATCTGAGTTC
GTAGATCTGCTATGCAGATGAACTTCTTCAATATATTCTTCGGCATAATGTAAGTATATC
CAGTCTCCTTTATGTCCTCTGAGTTGACATATATATGATTCACTCGAAGATAGAGATTTG
TGGCTGATATTGCCCTTACACGCCAATCTGTTTTGGACCCAAATGCAGCTTGCTCATAGG
GACTAGTGGTGGTGACAATAAGCTCATCCCCATGCACATTTGTAGTCCTTGTTGTGACTG
CCGTCAGCTGGCTTGCTTCCTTTGCCTGTTTCTCAATCTCAGCTATCTGTTGCCTCTGTT
GGGAAGGTGGCGTAATCTCCGCTCCAAGTATAATATCACGGATCTCAGACTGTGTTAGAG
CTGATGTATTCACATTGTTCTTCTTGGCATAGTCTGACAGTATGAGATCTCTTAGAGCGA
CCTCAACCTTCATCCATTGATCATCAGACAGAGAAGGCCAGATGTGATGTGGCTCTGTGA
TGATTGTTTTGTCAGGCTTTAAAAGCATCTTTGCTTTCTCATTGTTTACATGAAGTGCAC
GAAGAATCAGAATGAGTCGGGAGAATGCTGTGTATGATGAGATACTCTTCAGCCAATCAT
CATAGATATTGAACAAAACCATTTGAGGCTCCGTTGCTTTCAAAATTAGATCACCAAATT
TCTCAATTTTCAAGCAGGCCTGGAAAGGAAGCTGGAGTTCACTTCCTTTGATAACTATGT
TGGGGAAATCCAACAAGTGAACCTCCAATGGATCCAACATTCCCTTACGAGTAACAATGA
TTTGTTTTGGTTGTTCTTCAACAGGTAGAGACCGAACAAGAGCAGCAACTTCTTCTGCTG
TTTTCCACTTGGCTAACTGACCAAGACGCTTCTGACCTGCCCACACACTTGTGTGGATGA
CCTTAAGGAACAGCTGTCCAGTCCTTGGATTGAATATAAAGATAGCCCCATTGATTGGTT
TAGTTGTGAGATTTCCTTCAAATGTTTTGTGGATTGTAACACGATACACATTGGTGTCAT
CAACAAACCATATTATCTGATTGCTGAAAATCTCTCCATAGTTTTGAGATGATAAATATG
GTTCAGTAGGTTCGGAAGAATACAACTGCAAACCTTTCCTTATACGCTCTCTCAAGACAT
ACAAAGCTGGATTTGACTTCATAATCTTATTCATTGCTTGAGCAAGCAATGGTTTTGATC
CAGGGAACCAGTTACCAAATGCAGAATGCAAATTATACGCCAGATCAAGCCCAATCATGA
CACCAGTTGGAGATGGATAGATAGACATGTTGTCAGTCGTATAATCCATAAATTTGGCCC

GTGTGTACCGTTCAATGTCGTGAGAATCATAATCTCCCCAACGGAGTTGTACATCTATCC
AGTATTTATTGCTTGCTTTCTGGTCAAACATATCCTTTGACTCGGCAACAAGACTAGGCT
TTGACATAGGCCATCGATGAGCAGCAAATAGGAGAATATCTGCACAAGAGCTGTTCATTT
TGTAACTCTTCCTCGGATGGATTGTTTCTTTCTGCACAGTTTCAATTTCCAAAGCATCCA
ACTCCTGATCCAAGACCTGACAGAGATCCATGACCACACTCTCATGAATCTTTTGCCACA
GATGAGCACGGAAGATCTGAATCAGGGAAATCTTCAGTGTAGGAATCTTTCCATGCATGA
AAATTCCAGTTAAATCCAACTGCACTTGGAAACCAACATAGACATTGGCCCTATTTATTG
TAGGAGACCACCAGAGAGTGAACCGACGGTTAGGAATTTGGTTCAGACCAGACCTCTGTG
CATTAGTAAGCTTCTTATATTTCATTGATTCCTCAAAACCTGATGCCTTCTCCCAGAAGA
GACCTTCCCAAGTTGGAAAGTATGTTCCTTTGAACAAGGTGTGCTCAAGAATACCCTCGA
CACCTCCAAGAGCTTGGATGACATCAGTACGGTAGTTGTTCAAGTTCCACAATTTCCCAT
CATGCCTTTGGTGGGTCCACCAGAAGGGATTCTGTTTTAATACTTGATACTGCTTAAAGT
CTGTGCGCACTCTCCAACCTTTATCATAGGCTAAAGTATGTCTGTCCTTCTGGAACAAAG
TATTTATACGTGGTATTCCCCTATCCCATGAATCTTCCAGATCCTCAAGGGTTAGCCGCC
TATTCTGAGCCTGAGCTTCCTGCCTCTTCAGTGCATACTCTGCCCAAACACGCTGCGAAT
CTATGAATTCACTTTCCCATGGCTGTATGTACCGATAAAGATTTGGAATCAACTGGTCCT
CCTCATGGCTCATTCCACTCCTGAAGTGGGTGACACCAACATCTGTCTGCTGACTATATC
GGAGATCACTTTGAGGAATCAATATGTGACCCATGGATAACATTCCAAGTCCTCCAATTT
CTTTGGGGGTATAAAAAATGACAGGAGGAAATCTGCTAGGCATTTTAGAGTTCAACCCAA
TCTTAATACGTGTTTGGATTTTGTTTTCACATTTGACCAGCAAATCTAAAAGTTCTTGGG
TATGTACAGTTGCTTCACGGAAATAAGTCATAAGACCTATCAGGGCGGTATTCCACTTGT
TGACAATTTTGGTAAACGTTGTTGAACCTGAAGACATAAGAATCTGCCTGACACGGTTCT
CAAATACCTTCATGTGTTCATCATCAACTCGTAAGAACGCAACTGCAGTCCGCTCTTTGG
TCTGTTCATTCTGCAAATTCCATACCCCATCCCTTGTATTGCTGAATGCTTCTTGGGTCA
TTCTTATTTTTGGAAGTATTCGAACTTCAAATCCACACATGCTGAAAAGCAAATTTGGAT
TGTCCTTGCTGTAAACAGAAACAAAGCTGTTCTCCCACTCCAAAGTTGTAATGCTTCTGG
GCAGACGGTTCTTCATATCCCAGAAAACACTCCTACCAAGATTGACATCATGTTTCATGA
GCCTCATTCTCGCATCTCTTGGCCAACACTTCTTGTTGTTGTATCCAACCATATTCTCGT
TGTTGGGATCTGGATGCTCAGTAAGATAACGCTGGATAAGATCGCGAGCCTCTTCATGAG
TGAAGCGGAACAAGATGTGAACCCTATCAATATACCTAGAATACAACCTTATTGGATGCC
TTGTCTCAACTTTTGTGTCCCAGTAAGTGATGAACTCATTTGGCATCTGTGGGGGCCCAG
CAATCTCACTAGCACGTGTCAAACCAAGAAGCAAGAGATCCAGCACAAGTCCATAATACT
GTACAACAAATGAAGCAAACTGAAGACCACGTATGAGGCCATAAGAGTTTGTATGGCTCA
TATCTTTGTAGGACAAGACAACATTGTTCTTTGCAGTGACATAATCTGCAATATTGTGGT
CAAGGACCAAACGCAAAAGCCTGTTTAGCATGGTCAAGTCAATCTTTTCAAAGAACCTCT
CAAACTTAGTTTGAAGCATTACCACACACTGCCCATCACTTGTATCCCATATCCCTTGCA
GATTGTTTATACCCTGACACCATTTATATACCAAAAGTGGTGGAGGCTCTGAATCTGCAG
GCTTAATCCAGTTTGGAAAAAGGTGTCGCTTGTCACCTTCATACCATAAATACTGGTCAA
GATATGCATCTGTAATTTTCTCCAGTGGCTCAATCTCATAAACTGGAATCAAATAGCTGT
ACAAGTCCATGAACTCAATGCCAACTTCTTTAAAGGCACGCTGAGTGAGCAGATGACGCT
TAATCCTAGACAAAGCCTCATGAGGATTATCATATGCTTGTTCTATGAGACCCAGCTCTT
CTCTCTGCAACTGATTTAATCTAACAGCCACACTGTATGACTCTTTTAGCCTCTCCAGAG
CAAGGATAAGTAGCTTCGTGTCATGCTTGTATGACAATGGTGGAAATGGAATGGGAGAAA
ACTTTCTTGATTCCAACCAATGCACAGTTGTTGTATAAATAGCAACAGCTTCTTCTGGGG
TGACATATGGACCATCTTTCAAATAGTTGTGTTGTCTTTCCTGCTCAGCCTTCAGCCAAA
GACGAGTCAATCTTCCAAGGTTCTTCCTACAAACAGTCTTATCTACTGTTGCACCTCGTC
TAATACGTTCCCGATTGTAGTGAGCAACATTTGTCCACCAGTCGGCTTTTGACTTGACAT
ACCGAAGGATCATATTCTCAATGGGTACAGGCAAGCCAGGTACCTTCCAAGGTATATTTG
CTTTCCAACAGCGCCATGCCTCACTGAGATGCTGCAAAATAGTTCTAGCCTTGTTTTGCT
TAATACCCTCCGGCATGGCATCTAGAACATCATGCATGACAGCAGCTCGAAGCTCCAAGT
CAAAGTGACTTTCAACACGCTGCTTAGTGACAGTCTTGGCAACTCCTTTTGAATGGCGAC
CTTCAAATTGTCGAGCCAGTAAATTTCCTAACCATCTTTCCAAGAGAGGCACTATGCCAC
GAAGGAAAAACAGCCATACCCTCCACATTGGAGCCCAGAATCCACATCCAGGTCCCTTAC
CCACTGGCCCAGTATTGAAGCGGTAGTAGATCAAGTGCTTCAAGTCCTTGCACATCCTAA
TCTGGCGCATCAGCCTGTACTTGTAACGATACATACCAGTCAACTGACCCACATGAGAGA
AAGTATATTGCAACCCATCAGCCAATTGGAAGGCATCTACATTACCCAGGCGGAACTGAA
TATTGGCATCAACTACAAGCTTTGTCAATCGAAGAATTTCTCGGCAAAGATGAAAAGCAT
TTCCAAACCTAGACTTCTTACGCTCTTTTGTAGTGAGAGTTTTCACAGGCTTCAAATTGA

AATTGTAATCAAGATGAAGGTAATTCAAATTTTTTCTATGAATCAACAGATTCAGCATGT
TGTACCCCTGCTTACAAACTTGAAGACCTGCTTCAGCCCAATCAAGCTCTGTGGTTTGAA
AGAACTTCGTTGCCTGAAGAGACCGGAACAAGTGCTTCTTCTTTTGAGCCTTGGGTGGTC
GATGATGCAGCTCATTCAAGACAAAACATTTCAACAACTTCTGGTAGCTGACTCGAACTT
TTACTGGATAAGATGGAGGGCAGTGCTCCTTATACCACTCTGACACAAGGGGTATATCTT
CTGCTCGTCGCATACGACCAGATCTCATATTGAAAGGACGTGGAGCAAATAATAGGGAAA
TTCCAGCAGCTGTAGTATCAGTGTAAAGTTGAGTATCTTTTAGCAATGGCTCCACCTGCT
CCGGCAGATAAAAATCATCATCCTCATCATCACCATCATCATAAACCTTCTTTTCTCGGC
GATCCTTGTTAGTGGAAGTTATGGGGTGTATCAAAGGATCATAATAAAATGCAGGCAAAT
CAGGATCTTCAGTTTTTATGTACATGACCATGGGAGTATGATATACCCCAAGCCTTACTT
TACGAGGCCTGTTGTTATAAAGGTGAGGGAAGGCAATTCTGTACTCGGTCCTCAAAGGTG
ACCGAATAATGAGTTTGTTGATATCGTTGAATTCATTCCAGTCCTCATCACCTTTCTCCA
TATCACGGTACAAAGGCTCAAATTTAGGGCCACCAGGAATGCACATATTCAATGCCTTGG
CAGTAAAGAATGACTCCATGTCAAACAAGTAGAAATAGTTGTGGTCAATTAAATCGGAAA
GTAGCTGCCCTGCAAGCCGGTGAAGAGTAGCCATGATGGGAAGAGAAAGGTGCCATTTGC
GGTAACTTGGACCATTTATAAGCTTTGTTTTCACAAGAGGCTTATGGTCATAAAACCAAG
TATATACCGCAGAATCTTCTTCTTCATCCAACTCCAGTTGAATTGGCTCCAGAGGATCAA
CATCTAACAGATTATCAGCATAGTCTAGTGGAGGTTCCTCATCATCAAATGGTGGAAAAC
GCATTCTCTTGAAATGTCTGCGATCTCTCTTCTCTCTTCGCATCATGATCCACATTGTAC
CCCACTGAGCTAAATAAATAGGTTCAACAACCCATGGAATTTCATTCACAAAAGTGATGG
CTCCAGTAATATGATATAAAACCTTCACATCACGAACTTGCTCCCATGGCATTGGCATAT
TCTCAAGGATCTTGTAAACTGCATGTGGAATGAATTTAAGAGCTCCTAGATACACGCGTT
TATCGTGACGATATTTCTTTGAGGACATATCCCCATGGTCTCTAATAATCTTCCTCACGT
GCTCAGGAGGCATGTCCTCTTTCTGGGCCTCAACGAACCCAAACTTCCTCTTATCCCCAT
ATCTCTTCGAGTTAAGTTGCATCCATTTTCTAGCTTTCTCCTCCAACCTCGCTTCCGCTT
CTTGAGGCGACGGCGTCGTTGTAAGCACGGTATAAGACGGCTGCGACGGGGGCGGGATGG
GCACAGCTCCACTCGTTCCCGGCGGGGCTATCGGGCCCCCGTTGTTGCTGTTGTTGTTAT
ACATGGCGGCTGATAGTGACGACCCGGTGTTTAAGATCCAATGTCAAAAAGAAAATTAAT
CCTAAAAACCCCCAAAACAAAACCCACGAGCGAGTTTATATACTTGTTGCGTTTTTTCTT
GTGACTGAATTTGGGTTTTAAATTGACAATAATGTTTAGGGTTCCGGGATTTCGCTTCCC
TCCCTCCCGGAGTTTGCGTCGAGAGAGTGAAGTGGAAATTTTAAAACCCAAC
>ntig_429_consensus
CAACGGTTGGATTAAGCACAAAAGCAGACTCCTAAAATATTGGACATTGAGGCGCGTGAT
TAACTAACTTCGGAAAAGATATTTAAAAATGGAGAAAAATATTTCGGACAATGTGCACTA
TAACGTTATGTCATCAAGTTCAATATAGTTATCTTCGCATTGTGGCGAATAACACCTATT
CTCCACTTAGATCGGCGTCTGTATCACCTGTCTGCCCAATGTATGACAAGTGTCCCTCAC
CACCTCGACCGAGCAAGCCACGTGTCCACCTCCTTCAACCGGAGCTGGTCTGCCACCCAT
GGCAATTAAGCAGAAACATGATGGGAAAAATTCTAACAGGCATGTTCTCGCCCCCGGCGA
TGAGAATTACCAATTATAGTAACGAAGTAAAGAAGCTAATTAAAAACAAAAATACGAAAA
GAAATCACCGCTGGACATGAAATTAGCAACGGTAAGAACAGTACATTGACACAAAATAAA
ACTCACACAGATTCCAGTACAACGCGCTGTTTATTAACGACATACACATACAACCAATAA
CTTGTCAACTACAACACTTGATTTTATCATAGTAGCCGTTTGATCTCCACCCAATTATCT
ATTACCATAGTCCATCACATGGCACAGATCACATTAACGTAACGCGTTTAAGAGCTCTGA
TGCTGCTGCGACCCCGTGTGCTGCTGTCCATATTGATCAACCTTGTCTTTCCATTCTCGG
GCTTTACCAGTCAGCTTATTGCGCGCATAATCAAGTTGATCGGTCCCAGGTGGGTGCTTC
CCAGTAACAAACCTGTAGATCCATGACAAAACAGTGATAGCTGCTACACCGAAACCACCG
GAGGCGAGGAAGCCCGTTGTCAAAAGAGCCACTGTAATGACCGCAGGGACAAGAACAGGA
CTGAATATGACGAAGAGTGGTGTGGCAATGGTGAGTGCAATGACCGTACCAGCAAGAGTC
AAACCAGAGAGAAGCAAAAGAGAGCCACCAGCGGTGACAGCAGTGGCGGTCTTCACAACC
TGGAAGGGCCGAGGTTGTTGTTGGTTGTAGTGCTGCGTTTGGTAGTGCTGATTTTGGTAA
TGCTGTTGGTAATGTTCAGCCATTGACGAAATATATGACGGTTACAAAGAGGTCCTGAAG
ATATTTGAGAAAAGGGAATGGATGATTCTGATGAGTTGAGG
>ntig_430_consensus
AGGTCCTTTTATTCTATTGCTCATCAAAGTTATAATACTATGTATTATACACAAACCTAT
GTTATATATAAACCAAGTCACCATCCACAGGGAAGGAAGGAGGGGAATTTACATCACATA
ATTACAGAAAATCAAACACATCACTCAGTTTTCAAAGGAAAACAAGGTTTTGTCTATGTC
TATCTGTTTCCCAGACAAAGAGCTTTTTGCAGACCGGTTTGCCACTTCGACAGAATCACA
AGCACTAAACCTTGTCAGGCAGGTGTATTTTTACTCCATATCTATCTGAAGCCTACCCAA

AGATTAAAAGAGTTATGTTCCTACTATCATCTCCGGCGTCGTTTTGTATCCACCTTAGTA
TCTGAGTGTCTGTCTGTGGTAAGAGAATTCTGCAACTTCTTTTGGCCTTCAGTGGAACAG
TTTTCACAGAAGAAGCGGTCAAGTCTTTTAGCCTCCTCAGCAGCCATGTTTATACAATTA
GGATGAAACCAGTCGTTGCATCCTTCGCACTGAACCATTAGATCATCAGGGTTGTAAGGC
ATTTCACATTTACAATAGACGGCGATGCGATCGGGATTGAAAGCTCCCGTGGTGGAGTCA
TACTCGAAACGACAGAAAAAATCGTCGTTTCCGACGGCGTCAAGCTTCGTGTAACTCTTG
AAACTGTGGACCGTACACTTGCCTTCGATTGTGTCGGCGCTCTGAAGGTCGTAATGATCG
GAGAGGAAAACCTCCTTAGAGCCGTGAAATTGACGGCGGCCTCCGATCGACTCCTCAGGT
CGGTAGTACCAGCGCACACGCACCTTCACGTTGGCGCGACGCGCGTCAGATTCGATCCCG
TCGATCTTCGCCACGTAGGAAGGCTTCGAAGGATCCGACGGACGCAGTAGAACGCAGTCG
CCCGGCTTAATTGTCTTGTTGATGTGTTTGACTGTGTAAGACTCGAGAGTTCGCTTAGGA
GCTTTCGGTTTGGCCATTGACGAGAACAAGCAGTGACGGAGCCCGCTGATTCTGCCAAGC
CCG
>ntig_431_consensus
GGTGTCTGCTTGAAGCCTACGCAAAGATTAACAGGGGATAATTCTTGACTTGTTTATATT
CCTACTATCACCTCCGGCGTCGTTTCATATCCACCTTAGTATCTGAGTGTCTGGCTGTGG
TATGAGAATTCTGCAACTTCTTTTGGCCTTCAGTAGAACAATTTTCACAGAAGAAGTGGT
CAAGTCTTTTGGCCTCCTCGACAGTCATGTTTATACAAGTAGGATGAAACCAATCGCTAC
ATCCTTCACACTGAACCATTAGATCGTCGGGATTGTATGGCATTTCACATTTGCAATACA
CGGCGACACGATCGGGATTGAAAGCTCCGGTGGAGGAGTTATACTCGAAACGGCAGAAGA
AATCGTCGTTTCCGACGGCGTCAAGCTTCGTATAACTCTTGAAACTGTGGACGGTACACT
TTCCTTCGATTGTGTCGGCGCTCTGAAGGTCATAATGATCAGAGAGAAAGACCTCCTTAG
AGCCGTGAAATTGGCGGCGGCCTCCGATCGACTCCTCCGGCCGGTAATACCATCGCACAC
GCACCTTCACGTTGGCGCCACGCGCGTCGGATTCGATCCCCTCGAGCTTCGCCACGTACG
AAGGCTTCGACGGATCCGATGGGCGCATCAAAACGCAGTCGCCGGGTTTAATAGTTTTGT
TGATGTGTTTGACTGTGTAAGACTCGAGAGTTCGCTTAGGAGCTCTGGGTTTGGCCATTG
ATCAGCACAAGTAGTGATGGAGAATACACAAGACAACAGAGATCGTAAAAAAATTCATAT
TTTTATTTTTTTAATTTTTATTTTATTGAAGGCCGTGTTTGTTTTAACATCTGACTGCG
TAAGAACTTC
>ntig_432_consensus
CCAAATTTGTGAAAAAAAAATCTTGGACACCTTCATCATCTTACGTTCTGATGAGCAGTT
TCCCCGAGGAAAATAGAAAAGGAAGAATTATTACGTATAAACCACACTAACCAAATAGAT
CAGGCTGTTGACTCTTTCAAATATGCAATTAGATCGGCACGCTCCTGTGGCTTCTTCAAT
CCAGGGAAGACCATCTTCGTTCCAGGAATGTACTTCTTTGGGTTAAGCAAGTACTCGTAC
AATGTGTTCTCTCCCCAAACAACAGCCATATTCTTGTTAGCAGCAGAATAGGAATACCCT
GGGGTTGTCCCAGATTGTCTCCCAAAAAGCCCATTCAAATTAGGTCCTTGCTTATGTTCG
GCTCCTTTCTCAACGGTGTGACACTGAGAGCACCTGGTTTTGAAGATGTTG
>ntig_433_consensus
ATGCAGCTTAAAATTTAATTATTAAGAGCTGAGAGATCATGAAAGTTTGAGAAGTGCAAC
AAAATTTTCAACTCCAAATTTGTGAAAAAGATCTTGGACACCTTCATCATCTTACTTTCT
GATGAGCAGTTTCCCCGAGGAAAATAAAAAAGGAAGAATTATTACGTATAAACCACACTA
ACCAAATAGATCAGGCTGTTGACTCTTTCAAATACACAATTACATCGGCACGCTCCTGTG
GCTTCTTCAATCCAGGGAAGACCATCTTCGTTCCAGGAATATACTTCTTTGGGTCAAGCA
AGTACTCGTCCAATGTGTTCTCTCCCCAAATAACAGCCATATTCTTGTTAGCAGCAGAAT
AGGAATACCCTGGGGTTGTCCCAGATTGTCTCCCAAAAAGCCCATTCAAATTAGGTCCCG
AAAATTTACAACATTCACAGAGCACAAATCAACTTGCTTATGTTCGGCTCCTT
>ntig_434_consensus
GGGGGGAGAAAATTTCTATACACAAAGCATTAAACCACCAAAAGCCATACGAAATAATCA
AAATGAACAACATTTTCAAACCACCCTAAAAAACATCCAGCAGCAAAACTGAAAAATCTC
ATCATTTCCAGATATATAACAGTACCAAACATGCAGCTTAAAATTTAATTATTGAGAGCC
GAGAGATCATGAAAGTTTGAGAAGTGCAACAAAATTTTCAACTCCAAATTTGTGAAAAAG
ATCTTGGACACCTTCATCATCTTATTTTCTGATGAGCAGTTCCCCTAAGGAAAATAGAAA
AGGAAGATTTATTATGTATAAACCACACTAACCAAATAGATCAGGCTGTTGACTCTTTCA
AATATGCAATTAGATCGGCACGCTCCTGTGGCTTCTTCAATCCAGGGAAGACCATCTTCG
TTCCAGGAATGTACT
>ntig_435_consensus
AAGACCATCTTCGTTCCAGGAATGTACTTCTTTGGGTTAAGCAAGTACTCGTACAATGTG
TTCTCTTCCCAAATAACAGCCATATTCTTGTTAGCAGCAGAATAGGAATACCCTGGGGTT

GTCCCAGATTGTCTCCCAAAAAGCCCATTCAAATTAGGTCCTTGCTTATGTCCAGCGCCT
TTCTCAACGGTGTGGCACTGAGCGCACTTGGTTTTGAAGATCTTCTCACCGGCCTTGGCA
TTGCCAGGTGGCGCTTCTTGGAACGACGCCATTTCGCTCTGTGATGATCGAATTAATTGA
ACGAAGTCGATCGAT
>ntig_436_consensus
CATCGTTATAAAACAAATTAGACTCACACTAAACATGGTTTATTGATCTTAAAAAGACAG
CATAATCCCCAAGGGCAGACACATTAACTGATAAAGTATGGACATGGGTCAATGTGATCG
AACTAGACTAAGTAATCTTCATGTCTAGTTTATAAAGCAGCCATCGCATCTGTTGGCTAT
CTTAGTACAAAATGAGAACCCCCACCAACCAAAGGTATTTTAGTACAACATCACAAACCA
AAGAACTAAATTCAAAACATCGTAGGTGAATAAACTTATAAATGCTTTTCTATGGACACG
ATATACAAAAGCATTCACAATTCATCTAATGTTGGATTTGGGCCGAGCCAAGCCCAATGT
CAAGCTGGCCACCTTGAACCAAATTCAAGCTGAATTTGAACTGGAAAAGATCGTCGATAG
AATAAATAGGGTTACTAACAAGAGTATCGCTGACAGGATGACAAGTATCATTAGAGGAAG
ATTCAAACCGAGCTGCTGAATTGAAACTTTAGCTCGGATTTGGCTCAAAACAAGCTGACT
CACTGCAAATCCACTCCTAAATTCATCCCACTTCTTCTTTGGGTTAAGCAAGTACTCGTA
CAATGTGTTCTCTTCCCAAATAACAGCCATATTCTTGTTAGCAGCAGAATAGGAATACCC
TGGGGTTGTCCCAGATTGTCTCCCAAAAAGCCCATTCAAATTAGG
>ntig_437_consensus
GCTCTAATAACTAACATGTTCTTTTATTTTACAAATGCTACTCTTCATGTTTACCTCGGT
GCCATCACTACATAGACAATTAAAAACAAAGATTAAATAGAACAGGGCAACAAACAAAGA
TTTGGCAATTATTCTGACTACATTACAGAAAATTTCCTCGAAGTGTAACTGCCACTCCAT
TAAAACAAATCCATCATCCTGTTTAAGTTAACCTACTGTTGCTTGATTAAAACAAAAACA
TCGTCCTGGACAAGTTAACCTACATTTTAGCGCTTCTTCTTGCCCCCTTTTGAAGCACTT
GATGGACTCGTGATCAGATACACGAACAGGGCAATGATGGAGATCACAGATATTTGAGAA
CCATATTTCACAAGCAACCCCTTAGCCATGTCAAGCTTATTCACTACAGGCTTCTCAGCA
AGGATATCTAAAGGCAAAATTGGGGTTGAATATGCCTCCTGGAGAGCAGCCTTTGTAGGG
ATGCGGAAGGTCACGATCGCAGGTGCACCATAAAACATTCCTTTTACTTTGGCATCCAAT
TCAAATGAATGGGAGAGAATACCACCAGCATCAAGTCTTTCCCAGGACTGTGAAGTATTA
CCGCTTACTACATCAAACACTTCTTTAACCCAACTCTCATCCGTAAGACTCACATCATAA
GCCGTCGAGGTCCCTTGATTGTAGATGTCGATGGAGACAGAGATACGCTCGATGCCGGAT
TTGAGTCGTTTCATTGTGGCCTTCTTGTGAGCCACGATGAAGGGAACGTCTGAGCTGGCG
AAAGAAGATGAGATCAAGAAGAGAGCAATCAAAGCTGAGATCAGAGACCTGGTTATCGGA
TTCGCCATTGCCGAAGAGATTGGTTCGCTTCAGAGCTATGGAGGATCCTGGGAGATTCCT
GTATGATTTAGAGGACAAAGCCAAATTGAACTAGGGTTGATAGTTAAATTGCTGTAGTAA
TCTCGGGTCAAGGTCAAGGTCATCTGAAACGGCAGCGTTTTACGCTATCTGCAAATATCT
AAATTGTTGG
>ntig_438_consensus
TATTATTATATTACAACTAATACCAGAGTTCTAAGTCCCATGCCATAATAAGAACACTAT
TTAGATGACAGAGAAGCAGCTTGTGGATCAGTCCTCTAATTTATTTAACAACTTTTGGCA
TCTGTATATTGCCCTACACTCAATTTGCACAAGGCACATACATAGCAACTTCTAATTTAC
ATAAACGCGCAGCTTCCTTCCGCTGGGGCCAAAATGCTGCCAACGAAACCTTGATAACTT
CTTCAGTAGCCACTGAAGTTACAGGTTTACAATGTTCTCTTGATCCCATTTTGAAGCCAA
TCCATGCATGGAGTGGTTGAGATTCTTCTATATGATCGTCCCATCTTCTATTGTTGCTTT
CTCGAGTATAACTGTAATTCCTGATCGAATGTAGAATCCAGCTTCTGGTCTATCTGCTTC
TTCAACACCATCTTTGTTCATAATGACCACATCTTTTCCTATCTTTGTATTCTTGTCAAT
GATGCAGTTCCTGATTTTCGTATTCCTTCCAATCCCAATAGGGACCTTTCCTTCTGCCAG
TAGAGATGCAATTTCAGATTCAGTTTGATAATAGTCTGCACCCATCATCACAGAATCCTT
CAGCTCAACACCATAGTCTAAACGTGAGCGCTCACCAACTATGGAGTGTTCCACAAAGCA
TTCTCGCAAGAAACATCCATGTGAAATGATAGCATCCTTAATCCGGCAGTTATCAATCTT
GGTTGGTGGCAAGAAGCGAGGAGAAGTGTAGAAAGGAGTCTTTGGGTCATAAAATTCAAA
ATTTGGAACCTCTTCAGTGAGGCCCAAGTTAGCTTCATAAAAGGATTTTATTGTTCCAAT
GTCGTCCCAGTAGTCTCTGAAGATGTACCCTTGGACATTGTGCTCCATAACAGCTGCAGG
AATGATCTCAGAACCAAAGTCATTCGATGTAGGATATCTCCACCTCAGAAGCTTTAACAA
GACATCTGTTGTAAATACATAGACCCCCATGGATGCGATGTACGGGGATTTAATAGCTTC
TTGGGGTGATAATCCTAAAAGAGTGGTATCTGCTTGCATAGCTTTCAGATCAGCGCCCCT
TGGTTTTTCAGCAAACTGCACAATGCGACCTCTATTGTCCATCTTCACCAATCCATAATC
CGATGCACGGCTTTGACCTACTGCGGCACATGATATTGTAATATCAGCATTTCTGTCAAT
GTGACTCTGCAAAAAATCCATATAATCCATTCGGTAGAGATGATCCCCTGACAAGATCAA

TATATTGTCAATGTTCCTGTTCCTGGCATCCTCAAATACCCAAATAAATTGCCTCACAGC
ATCAGCTGTTCCTTGAAACCAATTCTTTCCTGTTTCCCCTGGTGTTTGAGTGGCAGCCAG
GACCTCCACAAATCCATCACCAAAGTTGGTACCATTGCCAAAATATGTCCGTGCAAGGTG
CCGATTGAGAGAAGCTGAGTTGAACTGGGTCAACACAAAAATCTTGTTTATGCCACTGTT
GATGCAGTTACTCATAGGGATGTCTATGAGCCGATAACATCCACCAACTGGAACAGCTGG
TGTTGCTGCTGTTTTTGTAAGAGGAAAGAGCTGAGTCCCTGCACCTCCTCCCAGTATGAT
GGCAGCAACATTTTTTGGGTCTGCCCTCCATCTGCCAAACCTCGGAGTTGGAGGGGATAA
GGTCTCTTTGTGAAGATCGGAAGAGACACGTCTGAACTCCAGTNNCTCTTTGTGATTGTT
GGACGTGATCACAGCAAAAGCAACACCTGGTTTGACCTTCTTGTCTCTCTTCTCAGCTTT
CAAGCCTTTTGCCAACTGATTAAACTCACCACTGTTGCTTAAGCTACCTCTAATCCTCTC
TCCCCAAAATGCATTATCTCCATTCTTGAAACCTTGTTTGGTAGCTTTTGCCACATGAGT
AGTGGCCGTCAAGCCCACACAGCGAGAATCCATTGCAGCCTTTTCT
>ntig_439_consensus
TGACAAACGATCTCTAAATTAAACAACAAAATAATTAAGATATATTTTAAAGCAAACAAC
AGATTCTATAGTACAATCCAAAAAAAATTGCAAGTTTAACGAGGACAGCAGCAATGGACT
ATCTTGTTGCAAGTTACAACTCGTTCTCAACATCAACTAGCTTAAGACCCCAACTCATGT
CTTCACAATGCCTCACATGAGGAACTACTAAGCCTGTATCAATCCCTCTCTTAGCCTTGC
AATCATAAAAGGGAGGCGCATGAAAACAAGGCTCCATTGACATGACTCGGCGACAAGGTG
GATTAGGAACCGTCCTATTCTCAGGCTTATGGAGTACCCATGACTTCAGACCTCCAAGAC
CTTGAGCCACATACCCAAACGTTGACCATGAACTTGTGAC
>ntig_440_consensus
TGACAAACGATCTCTAAATTAAATAACAAAATAATTAAGATATATTTTAAAGCAAACAAC
AGATTCTATAGTACAATCCAAAAAAAATTGCAAGTTTAACGAGGACAGCAGCAATGGACT
ATCTTGTTGCAACTTACAACTCGTTCTCAATATCAACTAGCTTAAGACCCCAACTCATGT
CTTCACAATGCCTCACGTGAGGAACTACTAAGCCTGTATCAATCCCTCTCTTAGCCTTGC
AATCATAGAAGGGAGGTGCATGAAAACAAGGCTCCATTGACATGACTCGGCGACAAGGTG
GATTAGGAACTGTCCTATTCTCAGGCTTATGGAGTACCCATGACTTCAGACCTCCAAGAC
CTTGAGCCACATACCCGAACGTTGACCATGAACTTGTGAC
>ntig_441_consensus
ACTGATAAAGCCAGTGGAAAAGACTATAAAAAGCATAAGATATAAGGTTACAAACAGAGA
ACATAATTTGGAAATAAATAATTAGAACTTTTGTTTCATATGTTAAGGTTGTGTACTCAC
AGCTATAGCCTCAAGGCCTCCTTTTTGGGATGAAGGAGCCACCTCTCGACTCATCGACAC
AATGTAGTGTCCTTGCACAAGATCAATCGCATCCTTCGAATAGTGCCAAGTATCTGTTTT
GTTCCATCACAGAAG
>ntig_442_consensus
GTCTACTTATGTCACATGAGTTTGAACATACAATTTCTCTTGATTGAATATCAAACATAA
ATAGATTTTGCATTAATTTCCATAAGGCCTTCAGAGACAACAAACAGGATAATAAAGGGC
TGTGGAAGGATAAAGCAAGTTCGACGCCATGAATGCCTATAATATCGAATGACTACCGTT
TTGCACCATCGGATAACCTATCACTTAATGAAATCTCACAATATTCCGCTCGTATGAAAA
AAGAAAAAGAAAAAAGAAAAATAAAACCATAATGTACCAATTTTGATCAAATGAGGCATG
CTGCCATCCTGGTGGAAGAAATGCATTATTCATAACTGCTCAAAATCTCAATCATTACTT
CCACAAACAAGGGTGCCATTGTTTTTGAATCCTCTGTTGTATCATTACTCACTCCCTCTT
CTGCCTTTTGGAGTTTGGTGCACTCTGTTCAGAATCACTTGGTCGATGTGATGTCCTAAC
ATTAGAACTCTCAGCATGGGTGGAAGAATCTTGCTCCATTCTAAGTTCAGAAGTATCTGC
TTCCTCAGAAGACGGTACCATTTGAGATGCACTAGATTGTTGAGATATGATGGGCATGAT
CTGCTGAAGCAAGTTAGATAAAAATAACCCTTCATCAGTAACACTTGCTTCTGCTTCTGC
TTCTGCTTCTGAAGGTCCACTGGCTGTTACAGCACGATCTGGGGGAGACACGGTTGCTGG
TTCAGGAACAGAACCTGNNNNNNNNNNNNNNNNNNNACCTGGAATTTGCCGCTCAGGTTCTT
GGCTGTTTTGGGTCCCACTGGCAGAAAGAACACTGATATTCAAACTGCGTCCACTAGAAG
GTTCTTGTTGTCTTGAGTTCAGACTTGGTAACAATCCATTTCTTGCAGGATCCTCACTGT
TCTGTGGCTGCTCCACAGACACAGGAGCAGATTGCCGCTCATTTTGCCCACCAGATGCTA
GAAATCCTCGTTCACCAGTCACGTGTCCAGAAGCTACATGTTGAAATCTTCCAACAACTG
GATAGTATAATCCCACAGGATTACCAGATAAATCTGACGGTAGCCGACTGAAAGGAGCAG
GCACTGCTGCTACCATGGTCCTAATTGGCACCACCCGTACTCCAGATTCTCCAGTGAAAG
ATGAACCCTCCGAGATCCTTGAAGTTGTTTGGTTGTCAGGATTTTCAATACCAAGTCCAG
TTGCTGAGTTACCTTGCCCTGACGGCTGCTGAGTATTACCATGTTCTTCTCGATTAGCGT
TAGAAGTTGCCATTGGTGAACCTCTTCGTATTTGTATATCAATACGCCTTGGAAGAAATC
CAGTACCACGCCCATTAACCAAACCCGTGCCAGCCTGTACCGACCCCATGGGGATGGCGC

CAAAGCTTGTTCCCGGTTGGAAAGGAAGAGGTTGAACCATGAGAGGGTTAGGACCAGAAG
GAGATACAAAAACAGCAGGACCAGCATTGACCACAGCTTCAGACGGAGTTAGACCTAATC
GCACAGTCATGGTTGTACGACCAAGTTCAAGCAAAAAAGCACCCAGATTATGAAGTAGAA
CTCCAAAGCTCAATGCAGTAGATTGTGTGCTCATCCGTACAGACGAATCAGTCACATTTG
CCTGATTCTCCAGTTGCCCGGCAAGTTGATGCAGACACTCTCCAACTTGTTCAATGAGCA
TCTGTCTAGTGCACAGCATGACTTCTGCCAAAGATGCAGGCGTTGGAAGACCTTCTTGTA
CAGTCCCTGACTGTGGTGCAGAACTAGAATCTCTCTCTCCAGTCATATCCATGGTGGTTG
CTTGTGAATTGTTACCCACTCCCCTGCCAATTCCGTCTAATTCGCGCCTTATATTACTCA
GATATTGGGACAAGGTGGTCAATGAATCAGGAATCACTTGAGGCTGCAAAGACCCCAAAG
AGACTGCTGTTGGTATTCCAACGGCGCTTTGCAGTCTATTTGATATTCTTATGCCAGCTT
GTTCAGATTGCTGCTGAGTTGAATCTGACATACCAGTAGCAACTGGTGTTCTTTCAAGTC
TTTGCAATGCAGATTCCCTGACATCAATCCCATTACCACCACCTCCCATATTTGAAAGTC
CAAACGTACCGAGAACAGCCGAGACAATCTGACTGATTTCTGGAGGAACTCCATCCCCTG
GATCAGGAACATTGAAAGTTTCAATTACAACACTAGGGGTTACCTGGCTTCCATGACTGC
GACTTGTACCTGGATGGTTGTGGCCACCATCGGATGAGGGGAGGACTGGTTGCCTGACCA
CCATATGCAAGGTGTGACCATCCTCAACATGATAAGCAGAGAGGAGCTGATCATCCTTAA
GAACTTTTCCACGACATATTAGACGTTGTTGCTCAGATAAAACACCAGTTATAGAGGCAA
TCTGCTCTTTCAAAGCAGGAACCGGTACCTGTTTATCCACTCTCAAAGTATAGGTTTGTG
AGTCTAGAGTTTTTATTTTGATTTCAATGGTGGTTTCAGAGGCTTCAGCCTTGTTAATTC
CTGGAATTTTTTCAGCACCACTGCTTTCCATTTTTTGCAGAATGAACAGGTACCAGAGAT
CAAAGAGTGAAACTTTCGAGATTTACGAGGAGAGAAGAGGAAAAACACAACCAAAAGACG
CAGTAGCAGAAGTGCCGGCGGTGATGATGTGTTGTACGGTGAACTTAAAGAAGAGGGTGA
GGTTCTGTTCTGGCTTCTCTCTGGAGCCTTGAGCGTGCCAGCCAAATGAAGGCGGAGGAG
GGAAATCTTAC
>ntig_443_consensus
CAGCCTTGCACCAACATGGCGAGCATTCATCCGCTCCACGCGGAGCTTGTTGTATGCCTT
GACAGACTTCATCTCTGAAGTCACCTTAACAAGCTCCACAGTTGGCTTCTCACGGGTGAT
CGGCATGTAGGGTCCTTGTACTTGGGTTGCTGTTGCCAGTTCCTCTGGAGCAGAATCACC
AGCCTTGACCTTGCGAGCACGTCTTGGGAAGACAACCAATTTGGCCTTGTATGTCTTCAG
CCTCTGAACATTGGCTTGAAGGCTCTCAAGAGATCTGTTCTTGCGACGATGGTCCACTGA
TATGCCAATAGTTGGAGCAAGCTTCTTGGGAATTCCTGCAGCTTTAAGTTCTTCCAGNNN
NNCGAAGAGGGCCCGCGGTGGGACGAGGAAAGATCTTCACGGCCTTCTTTTGACGAGCAA
TGCGACGCCTTTCCTTTCTAGCGGGCTGGTTAAACCAGGTCTTGACATAGTTTTGCCAGT
GCTTTTTGAAGTGCCCATTTGGCACAACGTTGTTATGCTTCACCATTTCTCACTTACCTT
TG
>ntig_444_consensus
CCTTCAGTGTATATTTCTTTAAACAAAACCACGGTCAGTGCTTCATACAAAGTTAATGAA
TAATCAGAGAATTTACAAAGAACTTATAAAGCTTAAGTGCGTAAGTTTAAGAAGGAACAG
TAGAGTTTGTATTATGCCAATCTTGAATGAGGGAACGAAGGATAAGATTAGGGGTGAGAA
ATTTGTGTTTAAGTTTCAGATTTGTCATTGGAGAAGTGTCATGCCCCATTCCTAGCCATT
CCTCCATTGCTCCTAGCTCGTAAGAAAATCCGTCTGCAGCTACATGTGGATTTTTCATTA
CCTCCTGATATATGGGGCAGAGAAAAACACTGGGAACATCACTTGAGTCCTCTTTAGTTG
CAAATTCATCAATCACCACCTCACTGGCTCCTCTTGCTGCTAAACCATCCGCCTTTTTCC
TCAATTCATCAAGCTCTTTCATAACACTTGCAATGCTCAAATCTTTAAGGGCCTCTTGGC
TTTCTGACAAACACCTCATTGTTATCCCAGCCATTTCCTCTGCCAAATCCAAAGGCCAGT
TTCCAGCCATCTCATCCAAAACTCGGACCAGAGCCGTCCGATCCAACACCATCGCTTCCT
CAACAAGCCCAGTCCAGTTCCTCCCTGTTAAAAGATTCAACAACACCACCCCAAATGCTC
TAACATCCAATCTTTCATCACATTGATCAGAGAATCTTCCCAACCCAACTCCATTAATCT
TTGCAACAAGATTGCGGTCAAGGAGGATGTTTGATGGTGTGAG
>ntig_445_consensus
AGCCAAAATTACATCTGATCTGACATTTCCCCGTAAGGAATAATATGAATTATAGAGAGA
GGTATCTGAAAATATACAACCTTATAATGCACTCTACATAAATAAGAGAGATCATCTCCC
TGTAAAAGCACCAAAAAGAAAAAGCCTGGAACTTTACATGGTTTTGCCGACTACACCACA
ACGGCTGACTCGATCTTTTCTAGTGTTTCCAACCTGGTTTTCCTCTTCCAACCTTTCATT
GAAGCTACCAGGTTGATCAGCCCCACAATTTGGCTTTTGCTATATTCAGGGTGAGCTGCT
GCCCAGTGAACATATTCTGTTTCCGGTAAGTAGTAAGCCTTGATGAAAGCTTCAACTGTT
TGTAACTTCGGCTTGACATTTATGGAGACAAAATGTTGGAGGCCATTGATTAAAACCTGA
AGATCTAACGACATGAGAGCTCGTCCTTCATCAGTACATCTCTTAACTCTTGATAAACCT

TCTATAAGAGTTTCAGCAACAATGTCGACTCCATATTCTAATAGGTGGTCTTGAACCTCT
TTGCGAATACCTCCATGGGTAAGCCTTGTTTTATAGTGCTTAAATTCTCCCAACAACAAA
TCAACATATCCATTATGTTCAAGTCCAAGCTCCTTTACTTCCCATTTAGCATTAGAAATT
CGATCAACGTAGCCATCGATATGGAGCAGTAATCTTGCAGTTGTCCTATGTATGTGCTCC
GTAAGATCTGGCACAGAATCCACCAGGTGCACATAGAAATCTTCAACTGCATTACTTTGC
AGAAGCATTGACTGGAGATGAGTTCTTGATCTATACAAAATCTGAGCAACGAGAGATACA
GTGTCAGCGCCTACACATCTTTCCTTGAGACCAAAAGATGTGCCTGATTGTTGACTTGGA
GGACTAGTAGGTGCAACATCTACATGCATCGAAAGTGCAGGTGAAGAAGGTATATATGAT
GTCAAATCGGGTTTTAACCACTCGTCACAATCTTGTGCTATCCTGGTTAAAGCTGTCTTC
AGCCGATAATTAAGTGGGTTGTTGGAGCCTTTTGCATATGAATTATTGTTTTGCTGACCA
AATGTTTCATATACATAATGGAAAAAAACCTCAAACAATTGGCACATTCCCTTAAAAAAC
TCAACATTAACAATTTCTAATTTCTGCATAAGCCTTGCATATTTATCCATCGACCTGAGA
AGGCAAAGAGATGATC
>ntig_446_consensus
GTCGGTCAAACAGAATATATATAGGTTCTATTGCTAATGTCTAGAAATCCAGAAAAGCCA
GTAACCAAACATACAAAGACTTCCGGTAAATGATATCTCAAACCAAGAAAATGCCAATTG
TACAAAAGACTATTTATAACTACCAAGAGTAGCTAGAAAACAACATGGCACACGCACTGT
TCTTCCCCAGGAGGCGTATTTTTCACGATCTTGACCTTCTAACTGGTGAGCGGCTGCGGT
ATCTACCATACTCAGGACTTCTATTCCGCCCATAATCAGGACTTCTGCGCTTATCGTACA
TTGGACCAGGGCTCCTGTTCCTCCCATAATCTGGACTTCGGCGCCTGTCATACACTGGCC
CATTGTATCGATCATAAACTGGGCTTCGGGCTCTGCCATAATCAGGACTTGGTCTCCTAC
GATAAGCAGGACTTGGTGACCTCCCATATGGACTATCCCCACGCCTTCCATAACTACCCC
TTCTTGGACTATCATATCTGTCATCCCTTTCACTATCATCTTTTAAGGCATACTCAACAG
AAATAACCCTGTCCAATAACTTGCTTCTATTTGTAGCATCAAGGGCCTTGGTTGCATCGT
CTTGTGTCTCAAACTGCACAAATGCAAAGTTTCGTCGAATCCGAACATGAAGGACATTTC
CATAGGGCTCAAAGTGCCTTTTTATATCCCGCTCCTTTGTTCGAATTGGATCAAAGTTAA
TCACAAACAAGGTTTTAGTGGGCCTCTGATTTGCCATTGACCTAGACCCTTCACGATGCC
GACCACGTTCGCCCCTAGCCCACTCCACTGACAACCTGCGTCGGTCATAACCAAATGGCT
GATTATCAAGTCCACGGATGGCATCAGCAGCATCTCGATCATCCTCAAAATAAACAAAAG
CAAAGCCAGATTTCATGTCTACTCGCTCCACCCTTCCATATTTGGAGAAAACACGCTCTA
GCTCGGATTGGCGAGTCTCGTACTCAAAGTTTCCAACGAAAATCGGTCTCGACATCGTGA
TTTCGGCAACAACAGCTTCAACCGAGCAAAAATCTGCCTGGAGTGAAGAAAACCCTAACT
TCGAAGCGAGAATGGCACTCGATCAGGCAGGCAAGAATGTGGTAC
>ntig_447_consensus
CTAAATTTTTGTCCAGGACAACATCTAAATTTTTGTCCTGGACAACATTTCCATTACAAC
AGAAGTCTTTTTTGAGGTCTTTGAGGATCTTGGAGAACCTGAAATTCTGGTTCAGTCCTT
CAACAGTGGTTATGCTTTTCTTTCCATTCCTCTGCTGAACCCGGAGATGAACATAACCAC
CTTTGGAATTGTCAGAATTCTTGGTCTCGGCGAATGGGTCAAAGGATGATGGTATCTCAA
CGTCTAGGTCAACCATGAACTTTGCTTGCTCTCATCGTGCATTTTAAGTTGCAATGATTC
ACAGGCACAGCGGGAATGGGAGAATGTACTTCTGTACTTCTTTCTAGATCTTATTATAAA
CTTTGAGGGTAAAATGAAAGTAAAAGGCTGCTGAACCATAGCTCACTTATGTACTACTGC
TGCTTGCTTAGACTTCGAACAACAAATGGGAGAATTCCTCCATGGTCGAAATATGCCAAT
TCCACCTCGGTATCAAAGCGTACGATACATGTGAAGGATTTCCCGGTGTCAGTTGTGACA
GTGACATCTTGGCCAGGCCTGATCTCGCTAACTTTAGTTGGTAGGTCTATTGTGTATCGC
TCATGACCGGTTAATCCTAGCATATCTGCATCCTCGCCTGGCTTGAAACACAGTGGAATG
ATCCCCATACCAACAAGGTTACTACGATGAATTCGCTCAAAGCTCTTGGCAATCACTGCT
TTCACTCCCAGAAGCTTGGGACCCTTGGCAGCCCAGTCTCTAGAGCTTCCGCTTCCATAC
TCTGCTCCAGCTAGGACAATTGTATCCTGCCCAGCAGACTTGTACCTCATTGCTACATCA
AAAACAGAAAGCTTTTCCCCAGTGGGAATATGAATTGTTTTTGGACCAACTTCTCCATTC
AAGAGCTTGTTGACAAGACGGATATTGGCAAAGGTTCCTCTTGTCATCACTTCATCATTG
CCTCGACGGCTACCATAGGAATTGAAGTCCTTGAGATCCACCCCACGCTCAAGCAGATAC
TTTGCAGCGGGGCTGTCTTTGTGGATACTTCCAGCGGGAGATATGTGATCAGTGGTTATG
CTGTCACCAAAGTTCAGCAAGCAGTAGGCATCCTTCACTCTATGAGGGCCTTGTGGACCT
GTAGTCATGTTCTTGAAATATGGAGGCTCGTGAATGTAGGTAGAGTTGGGATCCCAAGAG
TAAAGAGTGGCAGTGGGAATAGAAAGCTCATTCCACATAGGATTTCCCTTTGTAACTGCC
TCATAAGTGCTCTCGAACATATCAGGCAAGACACTAGATTGAACAACCTCTGCAATTTCT
TCATTGGTAGGCCATATATCCTTGAAGTACACATCCTTTCCGCCCTTCCCGGTTCCAATT
GGCTCCTTCTCAAAGTCTATGTCAACAGTGCCAGCAAGAGCATAAGCAACAACTAGAGGA

GGAGAAGCAAGATAGTTAGCTCTTGTTAGAGGATGCACACGGCCTTCAAAGTTTCGATTA
CCAGAAAGCACAGCTGCTGCAACAATGTCATTTTCTGATATTGCAGCACTAACTGATTCA
TCAAGTTCTCCAGAATTTCCAATACATGTTGTGCAGCCATAGCCAACAATGTGAAAGCCC
TGCTGATTCAAGTACTGTTGCAGTCCACTCTGGAGCAAATACTTTGTAACAACACCAGAG
CCTGGTGCAAGACTGGTTTTCACCCATGGCTTAACCTCCAGACCCAGTTCACAGGCCTTT
TTAGCGACAAGACCAGCGCCTAGCATGACACTAGGGTTTGATGTGTTCGTACAGCTGGTT
ATGGCTGCAATAACGACACTACCATGCTTAAGTTCAGCAGGCTGTCCATGGAATGAAAAT
TTTGCCACTTTATCCTGCTCTTCCTTGGGTACAGCAAAACCCTTGAATCCAACTGGATTG
TCAAGACATGCATGCCAATCAACCTTCATGTCTTTTAAAGCTACTCGGTCATGAGGCCGT
TTTGGTCCTGAAATAGAGGTTTCAACTTCTGCTAGGTCCAACTGCAGATAAGATGAGTAT
GCTCTTTCTTGTTGAGGCTCATTGTAATCAACAAACATTTTGTTCGCACGCAGGTATTCC
TCTATCATTGTGACTGTCTCATCGCTTCTTCCAGTTAACCTCAGATACTGCAATGTAACA
TGGTCTACAGGGAAGAAACCCATGGTTGCTCCATATTCAGGAGACATGTTCGCAATGGTG
GCCCTATCAGCCAGAGATAGTTCACCCATACCTTCCCCATAAAACTCAACAAACTTGCCA
ACAACACCATGCTTCCTCAACATTTGTGTAACAGTCAAAACCAAGTCAGTAGCTGTCACA
CCATTACGCAACTTCCCACTCAACTTGAATCCAACAACACCAGGCAATACCATACTCATG
GGCTGGCCAAGCATTGCAGCCTCTGCTTCAATTCCTCCAACTCCCCAACCAGCAACTCCC
AACCCATCAATCATAGTTGTGTGGGAATCAGTACCAACCACACTATCAGGGTAGAGAATA
TTATCAGTCTTAAAGACAACCCGTCCAAGGTACTCCAAGTTGACCTGGTGAACAATTCCA
GAACCGGGAGGAACAACGAGCATATTATGGAAAGCAGATGAACCCCACTTAAGGAAAGCA
AATCTCTCTTTGTTCCTCTGAAATTCAAGTTCCATATTTCTTTGTACTGCATTTTCTGAT
CGTGCTACATCAACCTGAACTGAATGATCAATGACAAGATCAACCGGAACCAATGGATTG
ATCTTGTTGGGATCGCTGCCCAGACTCTTCATGGCATCCCTCATACAAGCAAGGTCAACA
ACAG
>ntig_448_consensus
CTCTCGCTTGAAAACAGAGCAGGCTCTCCCACTTGTTTTAATTAAAAATCCGTTTTCACC
TCCCCGGGTCTCCATTATTTTATTCAAAATTTTATTTCAAATTCTCCACCGTTTAATGTA
AACGCTTCCATCAACTTCGCAATAGCGGCGCTCTTTCTTCTTATCCTTATATAAACTCTT
TCAACTCCACATTTTCTTTCTTCAGAACCAAGAATTTCAGCCACCATTTGGCGTAAGTTT
TCCACTTCTCTAGAGAAATATCTTTGAGCTTTGTCATGTGCTGTTACCTTTTAAAGGTGG
CGAACAAGAACGAGAACACTTTTGATCATCATCGTCCTCTGGTTGATAATGAGTTTCAAT
ATCAACCCTTGGATGATTATCAATTATCTAGCGGTGGCGGCGCTAGGGGTGATGAGACGT
GGATGTTTTCGAGGGCGCGCAGGGAGAGGGAGATGTCGGTTATGGTGTCCGCTCTGAAGC
ACGTGGTGGCAGGAGATGATTGCGTGCAGCTGCTGCAGCACGACGGTTTTGATGAATTAA
TGGATAATATGAACGTTGCTTCGATTGAAAGCTCCCTGCATGGCCATGGCTCCTTTTCTT
CATCTTCTTCATGGAGTGGCCACAAAAGAGGAAGAGAGGAAAAAACAATGCAGAGTTCTC
AAGATTTTTCTTCTCTTAATGAAAACTCAACAACAGCAATGCAAGCAGAAATTACATCAT
ACAACACAAAATATGAATACAGAAGCAATAATGGCAATCAAAATAATAACCCCACCGCCA
ATAATGTTCAAGAGCCAAGTAGAAGATACAGAGGGGTGAGGCAGCGGCCGTGGGGGAAAT
GGGCGGCGGAGATAAGAGATCCATTCAAGGCTGCAAGAGTGTGGCTCGGCACATTTGACA
CGGCGGAGGATGCAGCCAGAGCGTATGATGAAGCCGCTTTACGGTTCAGGGGCAGCAAGG
CGAAGCTAAATTTCCCTGAAAATGTCAAGCTCCGATCACCACCCCACGCTGCGGCAACCG
CTGAGGCGGCGTCAACCCAGTTCATTATTTCTGATTCTGCACCAACCCTTTTGACCATTC
CCTTATCCACCGGCCCTAATCTTCATTCTCAAGAGAGGGCTCCAGCCCCTATCGTTTTGG
ACTTTGCTGAATATCGGAGGCAGCCTGTGAGTTTGTCTGAGCCGCTTGTGTTTTCTTCTC
CATTGGCTTCTAATTTATCGCCGCCGCAGTTGGTGCCACCGCATTTCATGTTCCGGCCTC
GGCCTCAGCATCAGCCTCAGTCGCAGTCTCATCATTTCATGCCAGCCTCCACTAGCGGTG
GTGAAAATTTTCAAATTCCTTCATGGATGGGCTCCCATCATGATCCTCCCAGTTCACGGT
AAATTTTTGGTTTCTATATTATATAATATATATATAAAAAAAATAACTAGTTAGT
>ntig_449_consensus
ATATAGAAACCATACCTTAACCCCTCACAAACTTATAATTAATACAAGAATATGGTATCC
GCAAGATAATGTTCTCGCTGATTGCCTTAACCCAACATAATAAGTGCAAACCCCCGTATG
TCATTAACCATCCTCTGACAGAGGATAAACACACATCATAGAGAAACNNCACAGCAACAA
CTGTGATGTAGGGAACTGCTGTTTACTAGCAGACACTACTGTGGAAGATCCAAAAGCCTG
TTGATTGAGAACACCATTACCCCCTAGTTTTAGCTCTCTTCATGCCAAGGCTGTTGTTGA
AGGGAGACACTGAACCTGACATGGGAGAACCTTCNNNNNNNNNNNNNNNNNNCCACAGGC
TTCAGCAAGTCTTCTAGTATCTCTCGTGCTTGCTTTAGGCGAGCATCAACTATTTCGACT
GGTAGTTCTGCTTCAACCAGGATATGAAGGGGTTCATTCAGATGCTCGTAACC

>ntig_450_consensus
AAAGCCTGTTGCTGCTTGTCTGATATACAAGTGTCTCCTTCATTGGAGGTCATTTGAGGT
TGACAGAACCAGTGTCTTCGACCGTATCATTCAAACAATAAGCTCAGCAATAGATGTCCA
TGATAACAATGATAAGTTGGCCTATTGGTTATCAAATTCATCCACACTGCTGTTACTACT
TCAACGCACATTGAAGGCAAGTGGAGCAGCAAGCTTAACTTCACAGCGAAGGAGATCAAC
ATCAACTTCTCTACTTGGAAGAATGTCACAAGGACTTCGTGCCTCTCCACAAAGTGCTGG
TTTCTCATTCCTTCAAAGCCGTATGCTTGGTGGAGTAGATGGCTTGCGACAAGTTGAAGC
AAAATATCCAGCTTTGCTTTTTAAACAACAGCTCACAGCCTTCCTTGAGAAGATATATGG
AATGATAAGAGACAATTTGAAAAAAGAAATATCCCCGTTGCTTGGGTTGTGTATCCAGGC
ACCTAGGACATCACGGGCTAGTTTAGTTAAGGGACGTACCCAAGCTAATGCTGTAGCTCA
ACAAGCGCTGATTGCACATTGGCAAAGCATTGTGAAAAGTCTAAATAATTACTTGAAGAC
ACTGAGAGCAAACTATGTTCCTTCATTCCTAGCACGCAAGGTCTTTACACAGATATTCTC
ATTTATCAATGTCCAATTGTTTAACAGTCTTCTTCTACGACGTGAATGTTGCTCATTTAG
CAACGGGGAGTATGTCAAAGCTGGGCTGGCTGAGCTGGAACAGTGGTACCATGAAGCAAC
CGAGGAGTTTGCAGGCTCAGCTTGGGACGAGCTAAGGCATATTAGACAAGCAGTCGGCTT
TCTGGTCATTCATCAGAAGCCCAAGAAGTCATTGAAAGAAATAACAAATGATCTTTGCCC
GGTGCTTAGCATACAGCAACTTTACAGGATCAGTACAATGTACTGGGATGACAAATATGG
TACACACAGCGTATCTTCGGACGTAATTTCAAGTATGAGAGTGATGATGACCGAGTACTC
AAACAATACTGTCAGCAGTTCGTTTTTACTAGACGACGACTCAAGCATTCCATTCACTGT
TGATGACATCTCCAAGTCCTTGCAACACGTGGACATAGCTGAAATTGAACCTCCAGCTTT
AATTCGTGAAATCTCAGGTTTTGGGTTCTTACTTCCACGGTCAGAGTGAAAGTCACCAGC
ATGAATTAAAATATTTGAATCTCAGCTACACGTCTGTGCATATAACCTATAACTATTCAT
GTGTATAACGCATTTTCCATAGACTCAGGCTACCTTTGTTGAAGCCTGGGCCGATTCATT
CTTTCCCTCACGTTTC
>ntig_451_consensus
GGATCAGTACAATGTACTGGGATGACAAATATGGCACACACACTGTGTCTTCAGAGGTTA
TTTCAAGTATGAGAATCCTCATGACCGAGGACTCAAACAATGCTGTAAGCAGTTCTTTCC
TATTAGATGATGACTCGAGCATCCCATTCACGGTTGACGACATTTCCAAGTCGTTGCAAC
AAGTGGACATAGCTGAAGTTGAAACTCCAGCATTAATTCGTGAAAACTCAGG
>ntig_452_consensus
CTGTGGTCCAACACTTTCCAAGCAAAATTATACAAAGTACCTTACTAATTCAGAATCACT
TGTAGAAGTTCTTCCCTTTTTTTTTTTTTTTTTCACAATTTATGTAGCAAGTTTCAAACT
CTTTTAACTGAACTACAACGTGACCATTCCAAATCACCCATCTCAAAACATACATAAATA
TATGTTTCCAAACTAGGTTTCCAAGGATACCATACGGTTCATTCCTGGGAATAAGCACAC
AGCAGGTTCAAAATTGGACCCCAAGGGCTACGGAAAGAACCAGGCCTTGATGAAGACCGC
CAGAGCCATTTGCAAAAAAAACGAAGCAACCACCAGTATTGTCAGATCACTCCCCTCTAT
ATAAGATTTCAGATTTAATGCGACAAAGCAAGATGAGACTGTCCCAGCCAGAAGTACGAT
GATCCAACGAAGAAACTCAACGGGAATAAGAAGTAAAAAAGAGCTCAAAATAAAGACAAA
GAGTGAATATCCCCACAGGCACCAAAATCGAACAAGGCTAGCATTTGAACCCATATACCG
AACCAAAAAGTAATAGGCCAGGGGCACCAAAATTGCATAACCGTAGATAACGCTTGCCGC
CACATTTACATAGCTGACGTCAAAGCTCCAAGAAGTACTGCTGCTNNNNNAAAAAATCTC
CAGTAAAGGGGTACAAAGAACTCATCAATCTGTTTACGACAATATCTGTATCCACATTAA
AATATTGCTGGTATGATGCGACATTAAAAACTCCCCTCCAATTGTTTGCTGGTTGTTGCT
CAAAGCCTTCAGTTGGACTTCCAAGGGTTTGATAACCTTGCCTCCTTCCTCCTCCATTAT
TTGGGGGAAATGTCTGCATATTTGCTTCAGGGACCTCATAGTTTGTGTTTTTATTTTCTT
CGGCAGCAACCGCGGGAACTGAACCAAGCAAGTGGCTTGAAGACATATTATTATAGGAGT
CGTCCATGGAGGAAGAGATCGAAAACTAAGAACAAGAATTTGTTAGTGTTGTTGATAGGA
GTTTAGCTCAGAGAAGCATTGTGGATTTGAGAAATTGAAGTGGATCGAGATTCCAGTTAT
GTTGAGGTATTTCCGAGAGGCGTTAGGTTTTTCGTAGTGTGGAAGTTAATGGAGTAGACG
AGAGAGAAGCTGACTTGATGCCGACCGGTTTTGGCTTCGAATCCGACGAAGTAAAGAAAA
TGAGAAGCTTTTATTTCCACAG
>ntig_453_consensus
GCTGCTATTAGCTTCCCCGATAGAATCTTCACAGAACATCTCAATTGTCAATTTTTTGTT
AAAAAGAAAAATCTGGGTTAAGAATGGATTCCTGCTGTGCTGCATTGAAGGCAAATGCTC
ATCCTATTGTTAGTAAAGGAAGTAGTGGGATTTGGGGTGAAAGTGTCAGGGGCAGTCTTA
ATAGCAGGGTTTTGAGTTGTCATTTATGGAAGAGCTTGAAAACAGAAAAGCTGGTTAAAG
AGGCTAATAAGCCTGGAGTTGCTTTCTCCCTTCTTACACCTGATATTGACAAGGCGAGAG
TGTCTTTTGAGGCTCCGCAGTTTCAGACTCAAGAAGCAGATCCAAAAAAGGTGGCTTCGA

TCATTTTGGGTGGAGGTGCAGGGACACGGTTGTTTCCTCTTACTGGGAAAAGGGCGAAGC
CAGCGGTCCCAATTGGAGGATGTTACCGGTTAATAGACATCCCAATGAGTAACTGCATTA
ACAGTGGCATAAAAAAGATATTTATTATGACACAGTTTAACTCCTTCTCCCTCAATCGCC
ACTTAGCCCGGACTTATAATTTTGGAAATGGTGTGAATTTTGGAGATGGGTTTGTGGAGG
TTCTAGCTGCCACTCAAACACCTGGAGAAGCAGGGAAGAAGTGGTTCCAAGGAACAGCAG
ATGCTGTGAGGCAATTTGTATGGTTATTCGAGGATACCAAGAATAAGAATCTGGAGCATG
TACTGATTTTGTCTGGTGATCATCTTTATCGGATGAACTATAGGGATTTTCTGCAGAGGC
ATATTGACAGTGGTGCTGATATTACTGTATCTTGTGTGCCAATGGATGACAGCCGTGCAT
CAGATTATGGACTGATGAAGGTTGACGGCACAGGGCGAATTATCCAATTCTCGGAGAAAC
CTAAGGGCCCTGACCTGAAAGCAATGCAAGTTGATACTACTCTCCTTGGGTTGCCCATGC
AAGAAGCTGTCAAGTTACCATACATTGCATCAATGGGTGTTTATGTGTTTAGAACTGATG
TCCTACTAAAGCTTCTCAAATGGAGCTACCCCTCATGCAATGACTTTGGCTCTGAGATAA
TTCCTTCTGCTGTTAAGCACCATAATGTCCAGGCATATTTATTCAATGACTACTGGGAAG
ACATTGGAACCATAGAGTCCTTTTTTAATGCCAACTTGGCCCTCACAGAACAGCCCCCGA
AGTTTGAATTTTATGACCCCAAGACGCCGTTCTACACTTCTCCTAGATTTTTACCACCAA
CAAAAGTTGATAAATGCAGGATTGTGGACGCCATAATTTCACACGGTTGCTTCTTGCGAG
AATGCAGTGTCCAGCACTCCATAGTGGGCGTGCGCTCACGTTTAGAAACTGGAGTGGAGC
TTCAGGATACCATGATGATGGGTGCAGACTACTACCAAACTGAATCTGAAATTGCATCTC
TGCTTGCGGAAGGAAAGGTTCCGATTGGTGTCGGGCAGAATACAAAAATCAAGAATTGCA
TCATTGACAAGAATGCCAAGATTGGAAAAAATGTCATAATTGCAAATGGTGATGGTGTCA
AGGAAGCCGAGAGACCGAAAGAAGGATTTTACATAAGGTCTGGGATCACAGTCATTCTGA
AGAATGCAACAATCAACGATGGAACAGTTATTTGAAATCTATGAAGGCTTCCTAGAGCTG
CTTGTGCAGGCGAAGCACGCAATGGTAACTGAGAGGATTTGGTCTGGTGCTGCCCCTCAC
TAGTTTACAGTGAAAAAGTAGTACTCAGGCTTCTGTATGTAAATTGCATGTTCCTACAGT
GTAAAAGAGCTTTTGGAAAAAGTATTTAATTGTAGTTCCTAAATAAATGCCAGTGCTATT
ATGACTCACAAGACCATTAATAAATCACAGAACTCCATTAGATCTTAAAAAAAAA
>ntig_454_consensus
GTTCTTCCATAAACAAATGGAAGGAACCAAACCCACAAATGGAGAGAATCATTCTATACT
TTTCCATAATCCTTCCTAAAAATCTATAAACGTGAAAAACCTAAATATAATTCTAAAATA
CTCGAATATCATGATCTAAAATTAATCTTTTGTAAATCAACTCAAGTTGACAAGAACCCT
TTTTTATTTTATCTCTTTCATATTATTCATATAATAAACCAAGCAGAAATAAAATGGCAC
ATCTCAATAAGAAAACAAACATATAATATTAAACGAAACAACAAACTTAAAAGTTCGAAA
ACAAACACTTTGATTGATAATATGCAAGAACATCAACTTCAGTAACAATCTTATCATTAT
CATTAATAAAACACTCTAAATATTATCATTACCATACTTTTCCAGAGCCGGACCAGTCAA
CCGACAGATGCGCCATTCATCAAGGACTTTAGCTCCCATCTCCTCATAAAACTTAATAGC
ATTAACATTCCAATCAAGCACAACCCACTCAACTCTTCCGTACCCCATTTTCACAGCTTG
TTTCGCAACTGCACTTAACAACATCCTTCCAAAACCCTTCCTTCTGTAACACTCTCTCAC
GAACAAGTCCTCAATATAGAACCCCGGTTTCGCCAGAAAAGTGGAATAATTCGGAAAAAA
TAAAACGAAACCAGCAATAGTAACATCGTTAACTTGATCGGAGATGAAATTTTCGCGCTC
GGGATCGACGATGGGGTGATCTAAATAAATGACGCGGTCGGGGGATGGGAAGGGTTTTGG
GGAGACTTCGAGGAGGAAAATGGTGAAGGAGGTGAAAGGAGCAGAGTTGAAGAGGGTAGA
AGAGAGGGAGGATTCCGTGGCAGAGAAGAGGTGGGTAAGGCGTTCGAAGACGGCCATTTG
GTGAATGAGTTTGTGGATGTGGGGCACGTCGGCGGGAGTGGCAAGACGGATGCGGGTGAA
AAGAGGGTTGTCTCGGCAAGAACAGTCTTGTAAAGCTAGCGAGGGCTCCGGCGCTGCGGA
TGGTGGCGGCGGAGGTGCTGCAGCGGCCATTTTCCTCTTTTTGGTGGGTTGAAGATGAAG
AGCTGTAGAGGCAGTTTCCAGAGAAGTGAGCTCGGTAAGGTTAAGAACCGCCAATTTGAG
GTAAATGGCC
>ntig_455_consensus
AGAAGCACAAGACCAAGAAGAAAGAAAGGGACGACATAATTATAGAACAATAAACAATAA
TAAAAAAGAAAACAAACGGATTGTTTTCTCTCTTTATGGTTTTTTATTATGATGAACGAT
ACATTGGGTTTATATAATATATGTATATCACTGCTGCACCATTCGTAGCTGCTGCGGCTG
CATTATTTGGAGGTTCGCCCGGGAACATGAGCGAGCCATTTCGCCGGCGAGCGGAATCAC
CGGAAGATTATCGCAGTCGCATATTTCGATCATGAAGCCATCCGGGTCATGGAAAAACAT
CTGTTCTACGTCGATCCCACCTTCCTGCACTAACGATCTTACGTACTTTATTTCCATTTC
TTTCAGCTTCTTCTCCACCGCTCCCATGCTCTCGCACTGGAAAGAGATATGATTATCCTT
GGGATTATTCTC
>ntig_456_consensus
AGAAGCACAAGACCAAGAAGAAAAAAAGGGACGACATAATTATAGAACAATAAACAATAA

TAAAAAAGAAAACGAACGGGTTGTTTTCTCTCTTTATGGTTTTTTATTATGATGAACGAT
ACATTGGGTTTATATAATATATATATATCACTGCTGCACCATTCGTAGCTGCTGCGGCTG
CATTATTTGGAGGTTCGCCCGGGAACATGAGCGAGCCATTTCGCCGACGAGCGGAATCAC
CGGAAGATTATCGCAGTCGCATATTTCGATCATGAAGCCATCCGGGTCATGGAAAAACAT
CTGTTCTACGTCGATCCCACCTTCCTGCACCAACGATCTTACGTACTTTATTTCCATTTC
TTTCAGCTTCTTCTCCACCGCTCCCATGCTCTCGCACTGGCAAGAGATATGATTATCCTT
GGGATTAATCTCACTTTTCTT
>ntig_457_consensus
GTAGAATGAGAAGGAACACATCCTAATGAAATTCTACTCTGCAACCACAAATGTGTACAA
CTCAAACCGGGATTTGGTATGACATCCAATACCATAGAAGACTGTTTATTAAACAACATA
GACTGAACTCTACCATCACCAGTCTGTTTCTGATTCTCTGATACTAGAGGTGCAGAGCTC
AGCTTGTGCAGTGCATTACCAAGACGCTCATCATTTACTTTTCCTGCAAACTCAGAAAAT
CCAACAAAGGGACTCAAAGGTGATGATGGTTTAGAATTCAAGGACACCACTGTTGGAGAA
GTAAATGACGATGGAGCATGAACTGTCAAAGTCAGATCTTTGGATGTCAGGTCTGATGCC
TGAAGAGTTAAGACCTTGGCTGGAAGCTGCGTGATTCTATCATTCAGCTCAGAATAATGT
CATGACATTTCAGATGCAACAGAAATCATGAGATCCCTTGAGTTGTGTGGTTGCCACATT
GTTGGTTGCTTAAAAAATAATCTTGACTCCATAATTGCAAAATGATTAGCAGTTGAAGCA
CTCATCTTTCCTTCCAAAGTCTTGGAAGGAGGGTGCAAACTTGACGATCAAGAACTTTTT
TCACCATAAGCTTTTTGATCCATCCACAATGAAGATGCTGGCTTTAGAACAAAAGAGTGT
TCTTCACCCCTCCTGAGTGATAAATTTGGCAAGCTGTGGTCATTTCCACCTTCAATACAT
GCAATTGGCAATGATGAGGATGATCCACCTTCTAACGCCTCCTCAAAGACAATTGTTATA
GTATCTTCTTTATCTGAACACAAAGAAGGTCATCTGTATTGCAGTCAGCAACAAAAGAAT
GAATTTCTACCAGTTGAATTATTTCAAGTTTCCTCCTCCACCTTCTTTCTGAAGCATATA
TGCCTTCCAGTCCACAACGAACAGAGAATCTTTCTTGCTCCATTGATACACCCCGAAAAG
ATAAAAGCCTTTCAGTTTTATTTCTTTGATCCTTCATCATTTTCTGAGACAAGAACTTAT
CCCAATCATGATGATCAAAGGATCGCTTAGATCTTTGGGCTTTAACAGGTACAGGAGCTG
TGGTATTTTGAGGAAGTGATGACATAGAATAACTTCTAAGATGGGATAACAGCTCAGAGC
CAGAAGAGGTAGACTTGTGATGACTGCTGCCAATTCCGGTAACAGGACCCATAGCAGGAC
GAGATAAAGAACGAGCTGGAGAAGGAACAGTTTTATCCAGTGGAAGCAACCATTTTAATA
ATTCTCCACATGGATCAGAATTTGCATTCATTAAGTTGTCCTGGTTCTCATAATAAACTT
TTCGTTCCTTACATTTCTCAAATTGAAGAATTTCAATGATAGGGTCTCTCAATAAATCAA
CACTAACACTCACTTGTAAAAGCACCTGCATCCACTATATCCCCATTAGAAAGCGAGCAG
CACTCCACTGAATTTCTTGCCACTCCACCACAGATATTGCGTCAAAATTTCCTCTATCAA
TAACTGACTCTGCAATTCTAACAAAAAAATGTGAGTCCTTGAATTTTTGCAACAAAGTTT
CAGTTTGTCTTTTGTGATCTTCCATTCTAAGCAATGATTTGCTGGCTGAAATATCTTTTT
GTAAGTCCATCTTCTCTTCTGCTGAGTTTTCATGATTGCAATTCGTAACTTGCCCATCAG
GACTCGCTTCCCTATCTACAGCCACTGAATTCATTATGTATTCATTGTTTTCATTTTTTG
TCTCAGGCCCCAGTCCTCTACCTCTACTC
>ntig_458_consensus
CAATCATCAAATGGGGATACATTTTGCAAACAGTGTATCATAAAAATAGCTTTACATCGA
TGGTCAGTCTCCAGGTTCATCATTACAGCAGACAATGTACTGAAAGCTCTGCGGTGAAAT
ACAATATTCTGCTTGATTGAATGAGTAAAGTAAATTCCAGGAAAAAGATCGACTACTACA
GAGGAAGAAAGAGCAACATCGCAAAATCACAAGCCACCATCAATCACTTCCATTATCATT
TTCCCGTATTCATCATCAGAGTAATATTGTCTCCCTTCAAGAGTATCCTCCCCAGTGACT
TCCTAGTCTTCTTCTTCACACTAACTTCTTCAGCATCATCCAAAACCAAATTCATGTACT
CATCAAATCCAATGATACGGCCTTCGATTCTCAAATCTTTCTGCTCAAACAGCCAAATCT
GAATGCGTGCTTTGCTTTGGAGAAACCTGAAAATCAGGTTAATGGGTTGGGTCATAATCC
TCTGAACCTTGGTGCTGGCCATGGTTGCTGCCGCGCCTTCACTCAACAAACTTGTTCTTC
TGTTCTTTTCTGTTCGCAAATGGTCAAGGCCAAGTATGGAGCGAAACCGGCACCCCCAAA
ACCCTAAACCCAGTTCATTGACTAATTGACCATTTTACCCTGGGAAATTTGTAGGATCT
>ntig_459_consensus
TCACCTCACTTATCACTTTCCCCTGAACATAAACAAAATATTATTTCATGAGAGACAGTA
AATTCTGGGGAAAGATTGTCTAGGCAAGAAAATGAATTTAAAAATAAAACTTGAGCGCAT
TGTGTATGAGTTTTAGAGAGAGACAGGGAGCGAGCTTGGTCTTACGTATTCATCATAAGA
GTTATATTGTCTCCCTTCAAGAGTATCCTCCC
>ntig_460_consensus
GCGCAGGAAAAAATAGGACTGGAAGTTGTGTTGAGGGCATGTTCCCCCCACCATCCATTC
AAAAAAAAAAAAAAACACTAGATTACTGATTTTTCCCACTTGTACAAGGCGTAACATTTT

AAGTTTAGGGAAACCGTGGAAAAAAGATCATATATAATCATAAACTGGGAATAGATTGTC
CAGACAGAATGTCATACAAATACCTATACATCTATATCAGTCACAAGGAGGATTCATCAT
TACAACAGGCATGTAATTGAAAGCTTAGCAGCGAATTTCCAATAATCTGCTTGATTCATT
GACTGGAGAAAATTGCAGGAAGAAAGAAAAACTTTGCTATGTCACAAGCCACCATCACCT
CACTTATCACTTTCCCGTATTCATCATAAGAGTTATATTGTCTCCCTTCAAGAGTATCCT
CCCCAGTGACTTCCTACTCTTCTTCTTTACATTAACTTCTTCAGCATCATCCAAAACTAA
ATTCATGTACTCATCAAATCCAATGATACGGCCTTCAATTCTCAAATCTTTCTGCTCAAA
CAGCCAAATCTGAATGCGAGCTTTACTTTGGAGAAACCTGAAAATCAGGTTAATGGGTTG
GGTCATGATCCTCTGAACTTTGGTGCTGGCCATGGTTGCTGCTGCGCCTTCACTCAACAA
ACTTGTTCTTGCTGCTATTTTCT
>ntig_461_consensus
GACTTGCCGAATACTCTTCTTATGTTTTAAATAGGCAGTGCAGGATGTCGACTTAGGGGG
AGTTGATGAATTCGACAGCAACAGCTTCCAAATCTAAGAGCATGGGATCAGGCTGCTGGA
GACAGTCGGCGTGTGAAACAAGACTAGAGTCGCCTCCATCCTCCAGGCAGACAAGGTTAC
AGGCCTTGCAGCTGAGGAATCCCATTGCGGGAGAGCTGGCGTATCTGAAAGCGGCAACTG
TCAGATCTAAAGGCATCGATCACAGGCCTAGCAGC
>ntig_462_consensus
GACTTGCCGAATACTCTTCTTATATTTTAAATAGGCAGTGCAGGATGTCGACTTAGGGGG
AGTTGCTGAATTCGAAAGCAACAGCTTCCAAATCTAAGGGCATGGGTCACGGAGGGGATC
AGGCTGTTGGAGACAGTCGGCGTGTGCAACAAGACTAGAGTCGCCTCCATCCTCCAGGCA
GACAAGGTTACAGGCCTTGCAGCTCAGGAATCCCATTGCGGGAGAGCTGGCGTATCTGAA
AGCGGCAACTTTCAGATCTAAAAGCATCGATCACAGGCCTAGCAGC
>ntig_463_consensus
TTATCGCCGTCGCATATTTCGATCATGAAGCCAGCCGGGTCATGGAAAAACATCTGTTCT
ACGTCGATCCCACCTTCCTGCACCAACGATCTTACGTACTTTATTTCCATTTCTTTCAGC
TTCTTCTCCACCGCTCCCATGCTCTCGCACTGGAAAGAGATATGATTATCCTTGGGATTA
ATCTCACTTTTCTTCGGCAGGCTCTCAGGATCCTCTGATTGCAATAAATGGATGCCAATT
CCGTAGCCAAACAGCCATGCTCCATCAAAGTCGAAAGAACCGGGCCTCCTTATGGGGAAA
AAACCGAGAACATTTTGGTAGAAATCAATGGACTCTTCAACCGATCGACAAACGAGTGAG
ACGTGGTTCAAAGACTTGAGGTGCAAAGGGTTTCCCACGGATTCCTTCATTTTCCAGGAA
ACGGAGAATAAATGGAGATTAAGAAAAAGTGGTTGAATTAGTCACGGAATTAAAATGAAT
GAATCCGAGGG
>ntig_464_consensus
GGTAACAATATGTTTATAAAATATAAACGATTTAGAAATGATAAACTATAAATTAACAGG
AAAGATTCATGAACACAAAAGAAGCAAAGTGGGGTCCATATGATCACACCTACATGATAT
ATATTTTATTTATGAGCGACATCCATAAATCGCGAGATAATATACTTATGGGTATGGAGC
CGTTGTTTTCATCTCAGTCCGCTCAATATCGATGGCAAAGCCCTTGCCATGCTGCTTCCA
GTAGTCAGCCTGCGGGTTGATCCACTCGGGCTCTTCCATCTCCACCCTTGAATTCTTCAA
TGCTTCGGTTGATATGTTGTACTTGCCGTCCCTTTTCTGAACCAGCTCTGGAGTCACCAT
CCCCGACTTCACTCCTTGGGGAGACATCATCAGTGCTGGAGCTCCCAGTGGCAGCCCATC
ACCTCTGTCCACCTGCCATGTGCACCAAAACTTGCCATAAGTTTTGGTCAAATTTTCGAG
CTCTGCTTTCATTATCATTTCTGGGACTCGAGGATTAACCCAGAGGCCTGATTTGATCTC
ATAAGCATGTGAGTGCCAAAGCTTCTGCTCTTCTGGTGGCAAAGCGTCAAAGATCTTATC
CGAAACTATGTACTCCACTCCAATGAGTCGGCCGTTGGAATCATCAGAGTCATAAACGGC
GCACTGGAGGAAGTCCTGGTTGAGTCTNNNNNNNNNNNNNNNNNNNNNNNNNNTTTATGGG
TTTCAAAGACTGCATCATCTGAGCGCCTTTGTCAAGTAAGTGCTGCTGCATCGTCATTGG
CGCCCCCGGCGGCTGCTTCTCTCCGTAGGGAGGTGTTGGATCCGGAGACTTGTCACTGGA
GGACATTGAAACCGCACAGCTTAGTTGTTTGCGCTCACAGTCAATTGTAGATTTGC
>ntig_465_consensus
AATGCCAACAAAATGGCTACTGATAGGCAAAATCTTCTACAAAAACTAAATTAGTAAAAT
GTAGCAAACATAAAAGCAAGAAGATTTCAGAAACAGAAGTGGTTAGAGCAGAACCACTTT
GGAATTCCAGGAGTTTAAAATTTCATGTCTAAACAGCACAAATATGTAACAAAATATGTC
ACAGATGCAGCTTTTACATGTTTGAATTGCTAATCGGATTTAGTATCCCCGTTGTGCTTT
CTACTCCCAAGGATCATCATCCCTAAACCCTTCTCTTTTGACCATTTGCTTTCATTTCTG
TATTCTGGCTCTAACTGTCGTAGAAGCGGGATCGACGTCTCCGTGAACTGCGAAGTCCAG
AGCTACCCCTAGAGGGGCCACTTGTACTCACAAATTCATCATCTGTCTCATCTATTCTAT
ACTCTGCAAATTCTACAGATGTCACAGAACCTTCATCAGAGTTCGAAGTTTCATAACTCA
GGCGGCGACTTCCTCTTCTGGTATCACTGACTCTTGATCTTCTTCTGTTTCTACCATTCC

TGAAATTATCAAATACCTGATACAAGATGCAGGAGGTCCACCAGTTACCCTCATCGCCAG
GGAAGTCTTCAAATTCATCTCCAGTGTCATCATCCCCATATTCAATAACATAGTCTCCCA
AAACCACCCCTCGCGGTACTTCTGAATGGATTGTGCTCAAAACATCAATGATCTCAGATG
ACTGCTGGAAATTTTCCCAATCAAGCTGCCGAGCAGGATCAATTTTTGAAGGACGAGAAT
GAGGGTGCTCTAGTTGAGCATGTTTTCTTAGTTCCATGTAGGTCCCAGTAAAAGAACATC
GGTCCTCCTCACAACAACGCTTCTTTTCATTCAGAATTGTACGGACCTTATCAACAACCA
CCCACCCTGTGACTTCCCCTCTGCATAAGGGACAAGCTGATTTGCAACTACCCTCTGATG
CCACTGGTTGAATGTCTGAAGTGGAATTTACGTCAGAAATTGACGGGGGTGACATACCAT
AGGCATTTTTGAAACGGTCCAAACAATTTGAGTGCAAATGATCTGTGTCACATACAAAAG
CACGACATCCTTTCTCATATGATGAACATTGAAGGAGCACACCATTGTGAGGGGAATCCA
AGCATATTGGGCAAATAACATCATCCCAATTTGTATTCAACTGAACATCCTCAATGCCAG
AGACACTGAGTTGGCACCCCCTCCCATGAAAATGTTCAAAGGCCATACTATTAATTACAA
AAAGACCTAAAAGAACAAGATATGCACTCTCCTCCCAGATCAGAAATGAAGATCCAGTTA
ACAGAAGCTTATTAAAAATTCAAAGCATAGTCCACTGATCTCCAGATATTAACAACAGAA
AAATCCTCGGTCTATCATACCATGCCATCCAAAGTAACTGCATATAACAAAAAACAAGCA
GAAATAAAACATTGAGCATAGTGCACAAAAAATTCACACATCTATGAAAAACAGACAACA
TACAACAAAAGCTAACAGAAGAGCATTTTCTTTGCCTGGCATCAAGTTATTCCAGCTATG
AATATTTTAAGTCAGTAAATGCATCTTTACAACAGCTTGAACAAATTAGAACTTGAAACT
CAGGAAAAGAACCGGAAACCTAACAACAATCATCGCACAGAAAATAAAATTAC
>ntig_466_consensus
ATTGTTCATTCTCCTATCAATCCTGCAAAGGACCACATGAACCAATGACATAGCCTGACT
TAACTCACCACAGTGAAAACAGCCCCAGGAAATTGCAGTGAAAGTCCACTGGATTTCCAG
GTGCCTTCACCATAAGTTCAATTCAAAGGAGGACAACAAGCCAGCCAGCACTCAGTGGAA
TCCAATCATATCATCAATTTCATTAAAATTCAATCAAAATGGAGGAATTGCATATTGGTT
TTGTTATAAAACAACAATAACAATCCAATTATGTCGAAACAGTTGAATATCAAGATCAAA
ATTTAAATCTAATCCACCCCGACTAGGGTTTCAAGATTCCTACCCAGAAAAATAAAGAGA
AAATTGTAACTGGTAATGATCCAGGCATTCTTTGGCTGCAAGCCTGCAATCTTTTATTCC
TATACCATTGTATTACAAATAATCTGCTGAAATTTAAACTTCTTCAATCAATCCATTCAA
AACTTGAAAAAACCATGCTTCTGTTTCCTCTAAAGAATCAAAAAAGTGCAGGCGATTTAA
ACTACTATTATGATCTTGACATCGGGTTCATAAAAGATTCAAGACATTACAAGATTAATT
AGTTTTTAAAGGGAAACACTACATGGACATCAATAGGGTATCAAGACATGACGACAAAGC
TTGTGTTGTACATATGCATTAATTATAAACACACTCATTTAGGAGCGCATTCCAGTTGAG
CCGAATCTGTAAGGCTAGTTTCAGTTCGGCTCAAATCTTAAGCATCGAGGTCGAGCCACC
TCAAATAGTGAACAGTGGTGCTAGAAAAGACAATGGATCACCGTAGGTGAATAAAAATTG
CCAGATATAAAAAAAAAGCTCCGGTAAAGAATTGCCTATCAGCCCTTAAACTCAAGCTGA
GATGGAGCTTCAACTCAAGTTTGGCTCGTTTGAGCCAAACATAGTTCCAAGCTGAAGCCA
GCTCGGCTCAAATACAACCCTACTCAAATCCACCCCTTGGCTCATTGGATGGTTATACAT
GCATCCGATACTCGATGAAATCACTCGTAACTATAGTCGAGATTTTAGTTTCTAAATATG
TAACCTTCAATCAAAAATAGTTTGCAAATAATTGAATCAATGTTAAACAAGATAAACCCA
CGTCCAGAACAAAAGAAATAGCCAAAATTGAAGCATTTGAACGATGGAAAATCCATATTG
GTAAACAGAAAACAAAAATACATTCACAATTTAACATGCATAGGATGAGTATAATGCCTT
TTGAAGAAAATCCAAACGACAATATTCACGTCAATAGATCACGCAAACAAATGGCAACAA
CCATAATAACAGAATAAACAGAAAAGTACAAAAATCAGCAGAAAAAAGGGAAAATGTCTA
AATCTGATTTCAGATAAGTAAGGATCTTATCGTATGCCACGGAAAGGAAAGAAACCCAGA
TCTTATATCATAAAAAAACCCTAATCCATTGACGAGAACAAGAAAAAGAAGTTCATTTCA
TCATCATCATCATCATCATCATCATCATCATCATCATCATCATAATCATATATTAAAATT
TTTTGAAGACATCATGCCATGATCATCACACTCATAGAATTAAGATAATTTCATCTTAAT
CTTTTTTCTTCTTTTTTTTTCTTTTTTTTGATCCGCATAATAGAGAAAGAAAGACAAAAA
AAATTAATAATGATAATAATAATAGAGATGAGATAAGCACCACCATCAGAGACATAGAGC
GGTGCACTGCAGATCATCACCGTTGAAATCAACATCATCCGACGGTGGCAAGTTTAGATC
AAACGGCAACAGCTTTCGGCAGGAAGATAAGGCAATGTTTTCATCGTCGTCAATCACCGA
TGAGGAGGAATCACAGTCGCTATGACAATCTTCCGGCATCACCGGCGGCGTCCGTGGATA
TCTCCGTGAACTTGCGGCCGCCGCAGTCAGATTCGTCGATCTCGACTGCTGTGGTGGCCG
TGGACCGCTAAACGACTCAACAGTACTGCTCATACTGCTCGTCGTGGGTCTCTGTGGATT
TACAATCTGCTGATCTTGAAAATCTTGGAATCCATTAACGGTACCGTATAATCTCTGATC
CAAAAACGGATCATTACTGGGGTAAAACGAAGGCATGTTTGTCGTTGAAGTGATTGGAAA
GTTTGTCTTCGCTTTAGGTCCACGGAGAGTACGCGCCGCCGCATCATAGGCACGGGCCGC
GTCCTCAGCTGAGTCAAATGTTCCGAGCCATACACGTGTCTTCTTCCACGGATCTCTGAT

CTCGGCAGCAAATCTCCCCCATGGTCTCTTCCGAACCCCTCTGTATCTCGGCTCTTTCGA
CGCGTTTCTCGCCGCAGATCCTCCATTAGCCTCTATCACTTTGCTGGCAGGCGCCGCTGC
TGCCGTCGTCACAACTCCTCTCCCTCTCCTCATAGATGAAACCACNNNNNNNNNNNNNNAC
GGATGAATATGTAAATTTTGAGAGCTCCTCGGGACGAAAGAAGAAATGGGTATTGGTAAA
AAGACGAAGAGAAGGAGAAACGAGAAAGAAGGGACAGGTGGGTGAGGTGGGAACTGGATG
AACCCAGCAGGCAGGCACCTTCTCTTCTACACAAAAATGATTTTTTTTTTCAATTTTTAA
TTTTTTTTAAATAAATAAACTTTTCGATTTC
>ntig_467_consensus
GAACGGTTTGGTTTTTTACCCTTTAAAAGTCACGCGCAAAAGAGTATGTTACGAGATCGA
AAGCAGAAAAGATGGAGTTTTTGCAGCAGAAAAGATAAGAAAAAAACAACCTCCATGTCA
TAAAATTGATTCATTCATTCTTCCAAGTATATATATTCCATTTGAGGCCACTTTAACACT
CACAAAAAGTCACCATTTTGTATTTATTTTGCCACCCATTTTCATGTTCTTGGTTTCCTG
GGGGTGTTGGTCTAAAATTCATTCAGTACAATCAAATGAAAGAAAGTTCAAAACATAAGC
AAAAGGAGCAGAAAGAATGATCGGTACCCTGTTAAGTAGTGGAAAGGCAAACAATAGGGG
ACTCTTATGAAGTGAACAGTTTTAACAATAAACTAGATCACCCAGTCAAGATAGTGAAAA
ATGATTGAAAAAACTAATCAAATACAAAACAACTGGACCCCTAAAAAGGGCCACTCTTCT
TGAGCGGTCAGGCACAATATTAGGCCAGCATACTTGAGAACAAATTCCATGGCGTTTATA
GTTTCATTTCTTTCCTCAATAAGACATATAGCTCTGACCCCGGAAAAATATACAAAACTC
GATAAAATTACCTATTTGTTTTTGGCAGCTCCACTTATCGAATAATACAAATTGCTTACC
TTGGAGATTAGACGGCCTAGAGAGCAAGCACAGCAGCTCTTTGTGGATACTGACACTGAA
GTGCTCATTCATAAAAATATTAGACTGCTAGAACAAACTCCGGCAGGACATGATATCAGA
TGCTTGGGAATCCAAAATATTTTTCCCAGAGGGACTTGAACTTATTGTCTTCATGACTGC
ATCAAAAATAGTTTTCTGGAACCTATCATCTTCAATTTTCATAAGGATCTCCTTTACAAC
TTTAAAATCCAATCCCTCCACCAAAACAAGTTTCCCAACAATCTCGCCAAAATTATTAGG
TGCTTTGGGCAAATCAATAGCAATATCATCCATCAACATGCCATAGTTCAAGCATCCAGT
TCCTATGTCTCTAGGTGTAATTACCTTCTTTGCAAGCAAGTATTCCAAGAGTTTTGCTAC
TGCTTCAACACAAGGTGGACTTTTCTCAAGTGCAAGGGAGATGGATTCCTTGACAAACTC
AGGGTGGTAACCCGGAGACTTCAGTTCCTCCACGCATGTTAGTGCTTCACCCAAATCTTG
AATATCAAAATATTCTTCCAGAAGTGCAACTGTTTTTTTACGAAGTTCAGCTGTGTTCAA
CCTTGCAGCTGGTGCCTGTGATTTCTCTACTGGAGGCACATATGGTGCAGGACCAGGTTT
AGCAGGTACAGGAGCATGGAATGAAGGTTCTTTTGCTGAATAAAAGTTGGAATGCCGGCC
AGGAGGCAGACCACTCCCCTGCAACAAGGCACTTCTGCCACTCATTAGGCTACCAGTACC
CTGCGGTAATAGCCTTGTGTTAAGTGATGCTGACTTATTCATCGCCAGTGATTGTATGCG
TCCCATCTGCTGCATTCCTACTCCATCACCCCTAGGCATACTGCGTTGCCTAGGTATCTC
CCAATTATCATTATCAATGCCAGGCATCCCAGGCATCTTTCTAGTTCCTGGCATGCCTGG
CATCATACCACCCAAGCCAGGACGTGTGATGGGTGGCATGCCACCAGGACTGGTAACCCC
TTGAGCCCCAGAAATACGATTATTTCTAATACTTGCAGTAGCACCTGGACGTAACCCAAG
ATTTTTTTCTGCCTCTGAATGAATTTCAGTGATTGTTTTTGCTTTCACCTCTTCTCTCCT
GGGAACCCAGTTGTTTGAACGCAAATCTAAAACATTACGAACCATGAATCTCAGTCGGGG
AGCAAGCTGAGGGTTTGAAGTCAAGTCCTTCAATCTACCGAAGTACATATCATTTATACG
CCGTGATTTTGGGCTCTCGTCAAGCTGCTTACCAATGGTGTTGAAAAACTGACATATGGC
CTCAACATTTTCTTCTGCAGGACAAGATGCACTATCATGTCCTAAAAGTTCCTGAACAAT
GTGATGGACAATCTTTTCAGGCACCATCTTCTGCTTCAAAAGCTCACCAATTAAGCGAAT
ATTTCCAAGAGTACGAAGCTTGACCATTCTTTCTTTATCCCTGCGCTCCATGTCTTGCTC
TGGTGAAGTCATCTGCCTTACTTCTTCCCTTAGTTTGTCAGCACCCTCAAATGCCTCNNN
NNNNNNNNNNNNNATGGGGCAGAATGTGGGCTCAAGAACAGCCTTATCAAATATAAGAGA
TATAACATCCCTTAAGATATCAGCTGATGTGATGCCAGAATCTATGAGTTGACCCTTAAG
TACATCAAATTTCTCCGGGGTTAGCTTATTCAAAATACCTTTTACAGTCTTCAAGACACG
TTCCTTGTCAGAGAGATTTCCCCTTCTAGCCGACCATGGAACCTCAGCCTTGACTAAAAC
AGGAGTTGATCCCTCTTGGTTAGATGAGATTTGTGCCCTTGGAAACTGGTAGTTGAGGTC
TTGGCGATTAAATTGATTTCCATCCTGTCTGGACTCATATCGTCCACCATACTCTCTATC
GTCTTGCGATCTCTCTTCTCCGGAGTGGCCAAACTGTGCAGGACGGCGCCAGTCACGATT
ATCAGGTTCAGAATAACGACTCTGAGGTTGATTTGGAGGATTACTTTCAACATGCCCAGC
ATGACCCCAGTTTTGCTCTTCCCCAAAAACTTCAGCATCAAATTCCCGTTTGATTTTGAG
AATTTCATCAGAAATAACAACAGCCTCTCTAAGCTGTAATAACTGGTCTCGAGTGTAATG
CACACGCTCATGATTCTCAAACCTTGCATCTCCAGACGTTAGCGGGAAAGCAGAGACAGG
CGCGGCGCCATGGGGACGTAAAACAGGGAGATCAGAAGAGAAAGAACCAAACGACAAGGA
CGGGGGCGCAGACGACGATGAAGAGGAAGACGAAGCACCAAAGAGTCGGTTGCTGCCTCC

ACGACCACCACCAGGCCTCAAACTAAGCAGAGTGTGATCACCTTGATGCATAACGATATC
AAATCTCGACCAAAAACGACAAGGCAACAACCCAAAGAAAACTCGAGAAAAGAGGGCGCA
AATAAGGAATAAAGAGAATTGTTCGAGAGGAAAGTTGGTACGTATAGGCGTATATATAGA
TTGTATTCTTGTACGATACTTTTCCTCCCTCTGTGCGGAGAAGCGGAGAGAGGAGAAGAG
AGAGCTTACGAGAAGACGGTCTTACAGAGAGAAGAGGACTAGAAACCCTCCTCTGATTAG
AGTTTTCTCGTAAGC
>ntig_468_consensus
ATCCAATTCAAGTAGGCTAGCCGAAAAGGTGATGGTTGAATCAAAATAATTCCTTTTAAA
TTTCTGTTTCTGTCAGAGGGCAATATGAATGTTATATCAGGATGGAAGAAGAATCAGCGG
CAAGACTAATTCTTTGATCTAAGCTTGAGCAGCTCGGAATCAACCTCTAGCTATGAGTTT
CCCATTGAAATAATATTCTCAAACTTAACATAGTTTTTTTTTTTTTTTTTTTTTTTTTAA
ATCAAATACTAAATTCATTCATACACTGCAACGGTTCAGGGAACTCAGTGGTTAAAGGCT
AACAGTTAAACAAGGATCACAGTCACCTTTTTGCTTTCTGCCTTCTTTACCGAATTAGTG
AAGATAAGGGGTCTGGTACTGGTAGCCATTTCCAAAGTAGTTTCCATGATTGGGAGAGAA
GCTGTAAGTAGCAGAACCCAGAAGAGACGGGCAATGGTTGTCAGCAGGTCCAGTGTAAAC
GTAGGGTCTGTCATACACATACTGTGGATACCTATCTGTGACTCTAGTTGGATAGAATGT
CTTGTCAGCAGCATTGTTAGTGACTCGAGGCCCATATCCTGCACCATTGGCACGAGGTCT
CTTTGATTGAGGCTTTGCAGCTTCCATTGCCCTTTTTGTTTCTGCCTTTGCCTTCTCTAG
CTGGACAACACGTTTCTGGAGAGGATCCACAGGATAATGCTCTTCAAGGTTATGCTCTTC
AATGCATTTGATCACAGCCTTAAGAGCAGTTAGCTCTCGTTCACTGACCTCAGTCTCTAC
AGTGGGACAGACATTTCCAACCTTGCCTGGTGAAGAAGCCTTTCTAGCCTCCTTCAAGTA
GGATTTCAATAAAGGCACAGGTGAGAACTGCTCAGTAATCTCAAATGCAAAAGCCAGGTT
AACTGCATCAATTTGCCTTCCACTATTAATTAGAACTTCAATGACACCTGGCATCTTTTC
TGATAACCCAAGAGAGCGACATAGATCAGCTGTCTGGCGGCGACGAGAGACCATTGGTAT
GAGCCTTGACAATTCTTCCTCATTAAAATCAGAAGCAATACCAAAAGTGGCCAAAAGTTG
CAGGAATGCATGAGCCTCCAAGGAGTTCCCATTGCTAGCATCAACATCAAGAGCAACCAA
CTTAGGTTTCCACTCTTCAGCAATTGCCTTTGCCTGATCCTTCACATTTTCTGAGATCAC
AGCTGAAAGAGAAACCATATCCAGATTTGCTAGCAAAATGCTAAGGCATTCCATCAACAT
AATGCAGGTTCTGCGGAGACCCAACAAGCTTGAATCCTTCTTTCCATCCCCATTTGGAAC
CTCCACATGATAAAAACCTTCCAAAGAGTCCAACACCAAGTGGGCTGGATCTACTGCTGA
CTTTAATGCAAGTGGAATCTCTTTCTTCAGGGCAGCAAGATTCTTACGGTTGTCTGATAC
AAATTTGTGCAAGCCTTCTGAGTCCATCTCTTCACAGAGTTTGAATAACTGTGGATAAGC
CTTCAGCTCCATGTTTTCACTCTCGGAAGAGGTTTTGCTACCTTCCAAGTTACTTTCAAC
AGACCCAGAATCTGGTAATTTGTCTTCCACAGTTACTGCCCTCATTTCCCCATCATCGCG
AACAACAGGCTCTGCAGATGATAGTTTCCTCTGCTTCTCTAGAGCAGTAGTAATAGCAAA
GACAGCAGCATCTCTCTTCTTTTGGAGCTTCTCCAAGGAAGCATGTTCCTTGGCCACAAC
AGCAGCTTCCCGCTTCTGCAACATTTCTTGTGCTTTCCTAGTTTTGGTTTCAAACTCCTT
TTCTTGGTCTTCCAGCTCATGAAAGCGTCTTTTTAAGGACTTTTCAAGCCCATGAAAATG
TTCTTCAAGTTCTTTCCATTTCAAATTAAGGGTTATGGCCCTGTGACTTTCCAGTTCAGC
AAACGCTTTCTGAAGTTGTTGTATCTTAGAGGTTGTAGAGTCCATAAGCATAGCAACTGA
CTGGCTGTCTTCCATTGCAGAAATCACGTAGAACAGATGGAGATGGTAAAAATATCAGCG
CAAATAGAAATTTATCAATCCATTTCGTTTTCTGTGGCTGCGAAGTCTGCTGAAATCTAA
ATCCCTAAGAGAGAGGAAAGTTTTG
>ntig_469_consensus
AGTGGGTTTCACAGAATCATGAAACTGCAGAGAATCAACTAAAGGCCCTTGCATCAGAAG
TTGTAGCACTGCAAAGGCGGATTCTTCCCGGTAAATATAAAGAGGAAGAGAAATGTAGCT
ATTGTTTGGCACCTGTCCCTTTTGAATCCCCAGAAGTAGCTTTTTGCCAAGGTCTGGAAG
GCACTGGTGGAGCTAGCCAGAACCACAAACTAGTAAGGTGTGCTGTTTCTATGCAAGCTT
GCCCCACTACTCCTCTATGGTTTTGCAAGTGTTGTCATAGACGGGCTTCCAAGTTAGCAT
CAGAGTCTCTTTTTAAAATGCCTAAATATCCAGTAGATTTCAGGAAGATAACAGAATCTT
CTGTTCAAGAAGAAATCTCAAAACCATTGTGTCCCTTCTGTGGGATACTTCTACAGAGAT
TACAGCCAGAATTTTTACTCTCAGCATCTCCAGTGTAAGCTACATATATTTTTTTTTTTT
TCAGTTTTTTTTTAATCAAATACTAAATTCATTCATACACTGCAAGGGTGAAGGGAACTC
AGTGGTTAAAGGCTAACAGTCAAATGAGGATCATAGTCACCTTTAGCTTTCTGCCATCTT
TACCGAATTAGTGAAGATAAGGGGGTTTGGTACTGGTAGCCGTTTCCAAAGTAGTTGCCAT
GATTGGGAGAGAAGCTGTAAGCAGCAGAACCCAGAAGAGCCGGACCATGGTTGTCAGCAT
GTCCAGTGTAAACGTATGGTCTATCATACACATACTGTGGATATCTATCTGTGACTCTAG
TAGGATAGAATGTCTTGTCAGCACCAACATTACTGACTCGAGGCCCATATCCTACACCGT

TGGCCCGGGGTCTCTTTGGTTGAGGCTTTGCAGCTTCAGTTGCCCCTTTCTTATCTGCCT
TTGCTTTCTCTATCTGGATAACACGTTTCTGGAGTGGGTCCACAGGATAATGCTCTTCAA
GGTTATGCTCTTCAATGCATTTGATCACAGCTTTAAGAGCGGTTAGCTCTCTTTCACTGA
CCTCGGTCTCTGCAGTGGGAGAGACATGTCCAACCTTGCCTGGGGAAGAAGCTTTTCTAG
CCTCCTTCAAGTAGGACTTTAGTAAAGGCACAGGTGAAAACCGCTCAGTAAGCTGAAATG
CAAAAGCCAGGTTAACCGCATCAATTTGCCTTCCACTATTAACCAGAACTTCAATAACAC
CTGGCATCTTTTCTGATAACCCGAGGAAGCGACATAAATCAGCTGTCTGGCGGCGACGAG
AGACCATTGGTATGAGCCTTGATAATTCTTCCCCATTAAAACTAGAAGCAATACCAAAAG
TGGCCAGAAGTTGCAGGAATGCATGAGCCTCCAAGGAGTTCCCATTGCTAGCATCAACAT
CAAGAGCATCCAACTTAGGTTTCCACTCTTCAGCAATTGCCTTTGCCTGCTGCTTCACAT
TTTCTGAGATTACAGCTGAAAGAGCCACCATATCCAGATTTGCTAGCAAAATGCTAAGGC
ATTCCATCAACATAATGCAGGTTCTGCGGAGACCCAATAAGCTTGAATCTTTTTTTCCGT
CCACATTTGACACCTCCACATGATAAAAACCTTCCAAAGAGTCCAACACCAAACAGGCTG
GATCTGCTGCAGCCTTTAATGCAAATGGAATCTCTTCCTTTAGGACAGCAAGGTTCTTAC
GATTGTCTGATACGAATTTGTGCAAGCCTTCTGAATCCATCTCTTCACACAGTTTGAATA
GCTGTGGATAAGCCTTCAACTCCATATTTCTCTCTCAGAAGAGGATTTGATAATTTCCA
CATTACTTTCAACATACATAGAATCCAGTGTTTTGTCTTCCACAGTTGCTGCCCTCATTC
CACCATCATCGCTAACAACAAAAGGCTCTGCAGATGATACTTTCCTCTGCTTCTCTAGAG
CAGTAGTAATGGCAAAGACAGCAGCATCTCTCTTCTTTTGGAGCTTCTCCAAAGAGACAT
GCTCCTTGGCCACAACAGCAGCCTCCCGCTTCTGCAACATTTCTCGTGCTTTCCTTGTTT
TGGTTTTGAACTCCCTTTCTTGGTCTTCCAGCTCATGAAAGCGTCTCTTTAAGGATTTCT
CAAGCCCATGGAAATGTTCTTCAAGTTCTTTCCACTTCAAGTTAAGGGTTATGGCTCGGT
GACTTTCCAATTCAGCAAATGCCTTCTGCAGCTGTTGTATCTTAGACGTTGTAGAGTCCA
TGAGCATAGCAACTGATTGGCTGTCTTCCATTGCAGAAACNNNNNNNNNNNNNNNNNCGAC
GTAGAACGGGTAAGGAATAGCAAGAATTTTGGGCGGAAATCCAAAGTATCAATCCATTTT
GTTTTGTGTGGCTGCTACGAATCTGCCTGCCTGCCTGCAATCTAAACCCCTAAGAGAGAG
GAAAGTTTTGTCAAGGGCGACAAGGAGAGATTTTATACTGTCACTGGCGAGTCACGCTAC
TCACAGTCACAATCACAGTTACAGTCACAGTCACAGAGGTCATGTCCTCT
>ntig_470_consensus
GTGTGTGTGTGTGTGCGTGCGTGTGCGTGTGTGCTTGCTGAATCAATTGCTAGCCTTCTT
CCTTCAATGGGAAAAAAAAGTTAAAAAAGTTTGGCAGCCTTAACAAGAAACATCCATAAG
TAGTTGCATTTAGTTTCCATCCAAGGAAATTAAGGGAGAGACAAAACTGTACATTATTCC
TTCGGAACAAGCCCACGATAATACCCAAACAAAAGCTGCAACAGAAACACTATAAACATC
CTGTAGATAGTGAACACCACTGTATCCCTCTTATTAACCGCTTCATTGAAGCGACTCCCA
TGCCTTTTCTGTTCAACCTTTTCCCCTAACATCCATAACCCTAACCCAGGAGATTGATCC
TGTAGGTCTTCGCTATGTAGTTCATTCATTCTTTCATTGATGGTCCCAAATATTTGATTT
GGAACCACCACTTGTTTATGCTGTGTATCAGTTGTACCGGTTTTGGGCAGTGTGTGAAGA
AGAACAGAATCCCCTTAAGTCATAACCTTGTGTTTGTCTTCTTGTTGCCTCTTGGTGAGG
TCCTTTTGCCTCTCGTTATCAACATCATTGTCCTGACTGACAGATTTGGAGTCTTCTCTT
GTGAGGTATTTATCCACTTGTGCAGCAGCTTGGCGACCCTCTGAGATTGCCCACACCACG
AGGGACTGGCCTCGCCTGCAATCCCCAGCTGCAAAAATCCCTTCCACACTAGTTGCAAAG
CGGCCATAATCTGCCTTGAAGTTTGACCGATTGTCTCGCTCCAGTCCCAGCTTATCTGAC
AAAGTCTGCTCAGGACCAAGGAAACCCATGGCTAATAAAACGAGGTCAGCCTCGATAGTT
TCTTCAGAGCCTTCAACCTCCTTAAACTGAAACTTCCCACTAGCATCCTTCTCCCATTGG
ACACGAACCACCTCAAGTCCCTTCACAACACCATTTTCATCTCCTACAAACCGCTTAGTC
AATACCTCGTATGATCTTGGGTCTTGGCCAAACTTGGCAGCAGCTTCCTGGTGCCCATAA
TCTACACGGAAGATTCTAGGCCACTGGGGCCAAGGGTTGCCTGGAGCCCTAGTTTTTGGG
GGCTGAGGTAGAAGCTCAAGATTAACAACGCTACTACAACCATGTCGGACTGAGGTCCCT
ATGCAGTCTGTACCAGTGTCACCTCCACCAATGACGATTACTTTCTTGCCCTTAGCAGAG
ATGTAGTTACCATCCTGAAGATCACTGTCAAGCAAGCTCTTAGTGTTTTTATGAAGGAAC
TCCATGGCAAAATGAACGCCTGACAGTTCCCGTCCAGGTACAGGAAGGTCCCTAGGCTTT
GTGGCACCTACTGCCAAAACAATGGCATCATTCTCCTCTCGTAGCCGATCAAGAGAAAAC
GAAGGATCAACACCAACATTAGCATTGACAACAAAGTTAACACCTTCTTCAGCCATGAGG
TTAACCCGCCTCTGAACTACATCCACTTTGTCAGTCTTCATGTTGGGAACTCCATACATC
ATAAGCCCTCCAATTCGATCTGCGCGCTCATAGACAGTTACCAAATGACCCATTCTGTTA
AGCTGATCAGCAGCAGCCAAGCCAGATGGTCCACTTCCAATGATAGCGATCCTTCTCCCA
GTTCTCCTCAGGGGAGGTCGTGGCACCATCCAACCCTCCTCAAACGCCTTGTCAATGATG
GTGCACTCAATGTTTTTTATGGATACAGGATTCTCAATAATACCAAGCACACAAGAACCT

TCACAAGGTGCAGGACACACTCTGCCAGTGAATTCTGGGAAGTTATTCGTCTCAAGGAGT
CGATCTAGTGCTTCACGCCACCTATTTTGGTACACTAATTCATTGAATTCTGGTATTTTA
TTTCCAAGTGGACAACCAGAGTGCTCCTGGTGGCAGAAAGGGGTACCACAATCCATACAA
CGGGCAGATTGAGTCTTTAAAAGTGGGCCAGGTGTTGATTCCTCCATAACTTCCTTCCAG
TCATTCATCCGAACATTAGGATCCCTGTATTGAACACCTTCACGCTCATAAGCAATAAAA
CCTCGATGTTTAACAGCATCAGCAACCCGACTAGGCCTTGTGGTTTCTCTAGCATGATCA
ACCTTCTCACTGGATTTCTCTTTCAAAGATGCAGCAGCCAACTTCTTGAGCTCTTCAAAA
GCATCTTTTTCCATTAATTCTGTCTCATCTTGTTCCTCAACTTCTTTGTCAGCATGATTA
ATTGCCTCGTTTGAGGCAGCTTCTTCTTTCATGTTTGCAAGAACTCGTTTGTAATCCCTA
GGGAATACCTTTATAAATTTAGGCAGAAGATTCTCAAAGTCAGCAAGAACTTCTTTGGCC
AACTGGCTGTTTGTGTATCGCTGATGTTGCTGTATCATCATTCTCAGAGTCATGATATCC
TCTTCCTCTTCAACCTTATCAAGATCTACCAGCTCAGGATTGCAGCGAGAGTGAAATTTG
CTGTCCACATCAAGAACATAAGCAATACCCCCACTCATACCCGCTGCAAAATTTCTTCCA
GTTTTTCCAAGCACAACAACTGTCCCACCAGTCATATATTCGCATCCATGATCACCAACA
CCTTCTACAACAGCTTTTGCTCCTGAATTACGGACACAGAATCTTTCAGCTGCCATTCCA
TTAAAATATGCCTCCCCACTGATGGCACCATAAAGAGCCACATTTCCAATGACAATATTT
TCTTTTGGATCAAAACGACTTCCGTTTGGTGGATAAACTACAATCTTGCCACCAGACAAT
CCTTTACCAACATAGTCATTGC
>ntig_471_consensus
ATCATTGACTATGGCCTTGCAAAAAAGTATAGGGATCTTCAGACTCACAGGCACATCCCA
TACAGGGAAAATAAGAATCTCACCGGCACAGCTCGCTATGCAAGTGTCAACACTCACCTT
GGTGTTGAGCAAAGCCGAAGAGATGATCTGGAATCTCTTGGTTATGTGCTTATGTATTTT
CTGCGGGGAAGCCTTCCCTGGCAGGGACTAAAAGCAGGTACCAAAAAGCAGAAATATGAT
AAGATTAGCGAGAAAAAGATGCATACTCCCATTGAGGTCCTTTGCAAATCCTATCCATCA
GAGTTCACATCATACTTCCATTATTGCCGATCATTGCGGTTTGAAGACAAACCGGATTAC
TCATACTTGAAGAGGCTTTTTCGGGATTTGTTTATTCGAGAAGGTTATCAGTTTGATTAC
GTTTTTGATTGGACTATTTTGAAGTATCCACAGACTGGCTCTAGTTCAAGGGCACGACCA
AGTGGCAAACAAGCCTTGAATGCTGCAGGACCATCGGCTGAAAGATTAGAGAAACCTTCA
GTAGGACAAGAGGTTCGAGATAGATTTTCTGGTGCAGTTGAAGCCTTTGTTAGACGGAAT
GCTTCTGGGCATGGTTTGCATGGTGATCACTCTAGGCACAGATCTTCTGAGGATGTGCCA
TCGTCGAAGGATGTGCAACTTGATTCGGAGAGGGCTCACTCCTCATCTCGAAATGGAACT
TCTTCAAAGAGGGCTGTCCTAGGAAACAGCCGACCAAGCTCCTCTGGTGAACCCAGTGAG
AATCGATCAAGCCGACTGGTTTCTAGCAGTGGTCGGCTGTCTACAACTCAAAGGATCCAA
CCTGGATTTGAATCCAAATCATCGTCTTTTGTTCGTACTGCAAACACAAGAAGTGGTCGC
GATGATGCACTTCGGAGCTTTGATCTCCTGACTATTGGCACAGGAAAGAGGAAATGAAGA
ACATATCCCATCTGGTAAGAAAATGTGTCAACGGTGAAGTTTTCCTTTATGGCTTCTATC
AAGATCCCATGTTCTTACGCTACTTTGTATATGTGTATCCTTTACTGGATTCCCATCCAT
CAACAGCCATAGCTTCACAAAGCATATCCCATTAAAGAAAAAAAAAAAAATAAAATGAAAA
AACATACAAAGTCAGTGTAACTTTCAGCACATAAACATACAAATTAATGCCGGGGAAACA
TAGACAGAAAATATAAAACCAACATAACATTGATAGGGATCTATGTGGTAAATTTCCTTA
ACAAAAAAATCTCAGCACAAGAGCCATGTCAGTAAATATTTCCAGTACACAGCATATACC
AGAGGGCAAACTGAGCAAAAATAGAAAACAGCCTGACATTCAACATAAGGGATCTTTCTC
ATGGCCTCACTAAACACAATTGGGAAGAAAAAAGTTTCCTGATTACAGAAGCAAAAATCC
ATTCATTACGTAAGAGGCATTTCATATCTATAACAAAACACCATTCGTATTTAAAGCCTG
CGAACTGCAGCAATTATAATTTTGTAAAGCATCATGTAAGTATCAATGTTCCTCAAAATC
ACCAAAGATGATTGTAGAAATAATAATCCTAGACAAAGAGAAACATGGGAAAAAGAAAAC
ATGGATGGTCAACTGGATACCTGCCCCTAAACCAGTCTGGCCAGTGTCCTGGGGAGACTG
TGACCGACCAAGTTGAGCCAACAGGGATTGCTGCAAAGCCAACATTTGCTGCATTGTCTC
AGGAGAAGTCAACTGACGAAGAAATTCAGGATTTTGCATCATTTCTCTGAGTTGAAGTAC
ACTACGCGTCTGGGGATTAGGCCCAAGAATCTGGTTCATGTACTGTGGGTTGGAGAGAAG
GCTTTGCATCATTTGTGAAATAGCTGGATTCTGCATCATCTGATTCAGTGAATTAGTATC
CTGCATGCCACCCAATATGCTTTCAAACCCTGGGAGACCAAGTCCACCTAAGCCAGCAGG
TGTCTGTGGCCTAGCATCCCCATGCGGATTTGACCTAGTGGTGTTAGATGGAACACTTCC
AGTGCCAGCAGATGCCCAAGGATTTGGAAGGGGGTTAGTATTGGGAGCAGGAGAACCAGT
AGCATCAGAATTTGTGGTTGATGGGTTAGTTGATCCATCTCTGGCTTGATTCCCAGCTTG
ATTACCCAATAGAGCAGCAAAAGGATTTGCAGTTTCATTTCCACCATTTCCAGTCATGGA
GGTTGCATTAAGAAATGGTTCTTGAACAGTTTCATACATGCGGCGAAGCATATTGAATCC
CTCAGGAGAAGATTCAATGTTGCTCATTGCTCTATCAGTATTGCGCATCATTTCCCGCAT

TAGCTCAGGGTTTCTAGCTGCTTCAAGTGTCTGACGTAGTGTGCTAGGATCATTAAGTAT
GTGTGCTAGCTCAGGGTTTCGATCAATGATTTCACGCATTTGAGGATTATTCATGATCAA
GTTTCGCATGATCTCCGGGTTATTCATGAGGTTCTGCACAACAGGCATGTTCATTATTTC
TCTCATGATGTTGGGGTTCCGGGTCAACTGTTGCTGCACTTGTTCAAACTCAGGAAGTTC
AGCTCCAAATAAACCACCAGGTGCACCACTGCCACCTAAACCATTCATGCCAAGGCCAGG
AAACAGTGAAGCTCCAA
>ntig_472_consensus
AGACCCCTCAGGCAAAACAACCTGTAAAGCAAAAATACTCAATCAGATGACCACAGAAGA
CACTTTTGAGCCACCAGGCACAATCCATGCTTCCAATAATTCTATGGGTGCTTTATCAAC
AAAGCCCATTACATCTGATTTTAAGAAGCATAGAGAAAGTTAATCCCACCCAATATCCAA
CCCACTGCAAATACAACAATGAAAAAAATTTATATCAACAATCATCAAATCACCAACCTT
AACAATGAGATTATCA
>ntig_473_consensus
TGCTTGTTGCGTGCGTGCGTCACTTGGCTGCTCACTTAGCTGTCTGCTTATGCTGCGAGC
TGCTTATGCTTGTGCACGTGTGCGTCTAAGCTGCTTCCGCATATGGCCTGCGTGCACTGC
TAGCTGCTCACGCACCTGTNNNNNNTGCGTGTGCGCGCAACCTGCTTGTTGGCACACCTG
TTTGCGTCCGCATGTGGGCTTGCATAACCTGCTTACACACGTGGGCTCTTGAGCTGTTTC
TGCACCTGCGTTCACACTTGATCGGCCTGTGTTCACNNNNNNNNNNNNNNNNNNGGAATT
TGCTGGTTGTGACAATAAGCTCTGCAGACGTTTTCGAGAATTTCTATTTTGGGTGCGACC
AATATAACCGTGGCCTATCTGGTAAAACAGCTGGTTTGCTTGGCCTTGGCCGAAAGGCAA
TCTCTATGGTCTCTCAAACAGCCACAAAATACAAACAATTCTTCTCTTACTGCCTGCCGT
CGCCAG
>ntig_474_consensus
GCATGAAGAAAGGAAAGATAAGGAAAATAAACTACAGTGAACTCTATTTCTATATTTTCC
TTGGTTGTTTCGGTGGGTCAATCGATTAGACCTATAAAAAGGAGCTCTGGCTAGTGGTGG
TTTCTTTCAAAACTACTGCAGAGTCACAAACGTGTTCACGTTTTTCAACACTTCAATGGG
AAGCAAAAGTTTTTTACTGAAAAGCACTTTAACAAAGTCCACAAACCGCACTTTCTTCCA
GTCTACCAGGAAGCTCCATCTCTCCCATCACATAAAAAGATGCAATAATTTACCCCACAA
AAGAAACTAGCAGTAAACAATACACTGTGAACACCCAGAAGGTTCCCGATTATTTTTCAA
ACATATTCATCTGCTACATTTCAACAATCAACAAGCTAAAAGATGAACAAAACAGCCACA
ACTTGCAATCAAGATTTCTTTGGTATATTTCATTTTTTTTTTTTCTTAATTTCTTTTGCA
CAAAGCATGGTTCAGGGAACAAACAAAAAGGTTAATTCTCAGGGAAAAAAACAGAGCCAT
CCTCCTCGCTGGGTACATCAAAGCCAAACACACTCCTTATATATGTATACACAGTTAAAT
TACAAAGGTTAATAAGATTTTCAACATGGGTGATGGGTTGGATTGTGATTCTTCTTCTTT
ATCTTCGGCCCTTGCCTGTGGTTCCGATCCTCCATGTTTTCACTATTTATCTCTTCTTTT
TTTTTCTCTCTTTATGTTTTTATGAGACAAAGTAATTTATATTATCGGTGATTACGCAGC
TGCAACAGTACTGGTACGGCACGAGTACTCCTGATTTTCGGTTGGTGATTTCCTCTGCTC
TTTCTTCCTTAATGCCTTCTTGGATGAAGAGAGGTTCCGATTTTTGTCTTTAGCTTCATC
CATATCTAACTGCTCTTGCCTACACTCTTCACTGCAAAATGGAGTGTCTCCTCTGTACAT
GTAGATGTCTCTATTTCCAAGACGTTTCTTGCAAAGAAAACATGCTTCCAAGAAATAGGG
TTGCTGCTCCTCGAATCTTGCATCATAAAACCTAGGTCCAGATCTCGGAGAAAAAGAAGC
CGCGGAAGAAGATGGAGGAAGATTCACGAAACTACTCTTCCTCGCATAAGAAAGATGCCT
AGAAAAGAAAGTATAATTATTCTGGTGATGGTTCCCGGAGAACCCAGCTTCCATATCGGC
CAAAGAAGCCAACCCATAATCTTCTTCGGTGAAGCAAGACCTTCTTGAAACCACAGGATC
CATTTCTGTCAATTTCTTGGAAGTTCCACAAATGCGGGCCGGTTTAGGTCTGAGAAAGGG
GAGGAGAGACAACAAACAGGA
>ntig_475_consensus
CTGGTGTATCTGCCAATATTGGAACAATATTTAATACTCATTATTTATTAGTGTTAAACC
TTGGAATGCTATATATTAAAGGTGCTTTCTTGGGAGTTTTTTACCAGGGCACTGCTTATC
TCTATTCGCGGACACAGACACTAGGAAACATTCATCAAGAACAATGTCTACCATTGTTGC
TCCTCTCGAGCATTCTCCTGCTGAAGATGCTGAAGTTATTTACCAGGCTTGCAAAGGATG
GGGGACTGATGAGAAAAAACTAATTTCAATACTGGGTCATAGAACTGCATATCAAAGGCA
GCAAATCAGGAGAGCTTATCATGATCTTCACCAGGAAGACCTTGTTAAACGCCTCGAGTC
TGAACTCAGCGGAGAGTTCGAGAGGGCTGTCTATCGATGGATTTTGGAACCAGCCGATCG
TGATGCTGTCCTAGCCCACGTAGCCCTCAAGAAACTGAACCCTAATGTGATCATCGAAAT
CGCTTGCGTTCTATGTCCCGAAGAGCTCTTGGCCGTGAGGAGGGCTTACCAGGCTCGCTA
CCAGCGTTCCCTGGAAGAAGATACGGCTGCTCATACCACTGGTGATTTGCGCAAGCTCTT
GGTTGGCTTAGTGAGTGCATATAGGTATGCTGGTTGTGAGATCTCTGAGAGAACGGCTAA

GTCTGAGGCTGAAGCTCTTTATGATGCTATCAGGCATAAGGATCATGATGAAATCATTAG
GATTTTAACCACAAGGAGCAAGGCACAGCTCACGGCAACTTTCAATCGTTACAAAGATGA
ACGCGGCACTTCCATTACTAAGAATCTGTCAGGCTCTAAGCATGATGAGTTTTCAGCTGC
TCTGCGTACAGTCGTTCGCTGCATCGACGACTCAACTAAGTACTATGAAAAGGTTATCCG
CAATGCACTCAAAGGGATTGGGACTGATGAGGGTGCACTCACTCGCGTGATCGTTACCCG
GGCAGAGAAGGACTTGAGGCAGATCAAAGAGGCTTATTACAACAGGAACAGTGTCTCTCT
TGAACATGCAGTGGCCAAGGAGACTTCAGGGGATTACGAAGCCTTCCTTTTGACTCTGCT
GGGCAGAGAAAATTAAGATGCTCCTTGTGGGTAGTAGTCAGATGGTGCAAATTTGCAATA
ATGGGGGATTTAGTTAAACTTTGTTGGTCAGTCTTATTTGGCACTCTGGTCTTTCAATTT
TTTTGTGAGTTTCTTCATGTCGGTTGTGTTGCTTAATTATGTCCCTATCTAATGTGTTTC
CAGTTTGCGTTACTGAAATGAGAATCAGTCTTTTCTAAGAGCTATCGATAAAATTTTAGG
AGTAACTTGCTATGGAAATATTTATGGTGGTCTGGATTTCTTAGCCAGAATATAAGATTT
GCATGAAAAAACTGATAGGGAAGTTTTCACTAAGGAGGAAGATTACCAAAGGAGAGAGTA
TTCATCACTCTAATTGCACCATTGTTTTTAATATATTTTCACATACACTTTTGCACAACT
ACTTTTATACGAAGGCCTTAATTAGTACACGTTCGACATATTATCCAGAAGTTGCATGTT
TACAGTAAGTCGCGCTTATTTACCTGTTGATCACTGTATCTACTTAAGTGTAATGATAAA
GTTATTCGTTAAGTCATTTTGATGAACATTTTAAATCTGTACTTTCTGCTTTCAGATAAA
CTTTTTAAGATTTTGGATCACCACTAT
>ntig_476_consensus
CACAAATACTAACGATAACATATGGTAAAAAACCAATGACTTTTAACGAAAATACAAACA
AATGTATTCACAGAAACAAAAAGTGAAAATACAGTTTCTCCCAAGTATATGCGTAACAAA
TAGTCTAACACCAAGATTTTGAGGGAAAAAAATACATAAAAGATAACCTCTCCCCTCTCC
ATGATTGAATTTGACAAATCACAGTGTAATATCTAGACAAGAACCTCCTCTTCATCCTCA
AAACTCAAATTCAATGTCTGCAACTTAAATCCTGAACTCTGTATGGTCTCAATCAAAGTG
GCAGCCACGCCTGTCGCTTGTACTGTTGTTTGTTTCTTCAACTCAATGGTGGCAATACCC
TCAAGGACTTGAACATTTAAATCTGTAATCCCCTCAGTTCCCTCCAAGGCCTTCGTCACA
TTAGGAATAGACGCTTCATTCATAGTTCCATCTGCCTGGAAGTGCATGGTAAGCTTATCC
GAAGGAGAAACAGGAACAACAAAAGGCTGCTGTTCCTCTGAAGCAACAACTGTCTCTTCT
CCTTGTTCTTGTTCTGGCACTGCTTCACTATCTTGTTCCGTAAGTAAAACTCCTTCCTCT
TCAGCTGACCTCAGCCTTCTCATTCCATTAAAACCCCACTTCCTGTTGGCACTCTTCAAG
GTCAACTTGGTCTGAAACTGGCGGATTCTTATGGGAATTGTGAAAGAATGAGAGGAGACT
GGTCGGTAAGTGCGGGGTTTGGCTATGAAATATGAATGAAGAGCTGAAGAAACTGAGACG
GTGGCCATTTTGGTTTTGAATTTTCGAGTGGAGAGAGAAAAGCTCCGTGAAACCCTCAAC
TTATCGTTAAAGACTCGCAACTTCAAGTGAGCGCTATTTCGGTCCCCTATCCACTTCACA
GAGATTATCTGGTGTCTGAGTACTCACATGTATCTTAAATTACCATTCTTTTGAGAGACA
TATATTGATCTCTTTTG
>ntig_477_consensus
TGGCGTTCTAAGAATGGTGTAATTTATTAGAGCAAACAATAACAGTAAGTTAATTATTCA
TGGCGAATCCTTGCACCTGAAGGATAGTGTTATCAGAGCAAACAATTGCACTCAGCCTTT
CGCACATGGTGAATCCTGGATCAATCATTGATAGCAGATTCTTGATCATGGAAAAATGGT
GTTATCACAACAAATAATAACAGTCAGGTTGAAAAGGCTTGACAATATATATTGATCAGA
TTCAAGAGTGTTGGCCAATTTTGACCAATTCTAACTTGTTTGTGAAAACAATCGTTCTGA
ATATAGATATCAAAGGCTTTTAACATTCAAATCTTAAACAGGATTCAACCATTCTCTGAT
CACTTATTTCGTCCATTAAGCTGAAAAAGCTTTTATATAGATCAACCCAGCCGATCATTA
TCATCCATTACAATCGAACTTTCAAACCATCTTCTGAGAAAGCCTCTTTGAAGTAGGCAA
CCTGCTCACCAGAATAATATCTTCCAAAGTGCAAGACACTGAAGTATTTTCAGCCCAATG
GCTCATGCTGATAGTACTGTTATTTGCTTATGATGCACACTTTTTCCCAGCTCACAAGTA
TTTGAGCATGCCGCGACCCTCCACGCTAAGTTTGGTGGCTTGCATCTGCTTCTTTTTGGG
TGCCTTCAACTGCTGCAACTTCTTATTCATCTTCATTCGGCGTTGATCTTGCATAACTTG
TTTAGCTCGGGCTCTAATTTCCTCTGGATCAATTTTAGATTGGGGACGCACCACAAAATC
TAATGGGGTTGCTTCTGGTCTTGATGCATGTTGCCTTGTGGCTGAGTCTTGCCTTGAAGC
CTGTTGTGCAGAAGAGGATTGACCAGACTTGCGGTCCCTAGATATAACCAAATCATCAAA
ATCACCATCTCTAGATTCCATTGCTGCAGATTTATTTGTAGGCCTTTTAACCGATGGCCG
CCGAAAAGGAACCCTGTCATCATCATTATTTTTCATGTCCTCAAACCTCGTGCTCTTATT
AAATATGGGTCGACTCCATTTATCAACGAGATCCTTGGCAAGCTTTCTGTTTGACGTGGT
TTCCTCATCAGATTTTGATAAAAACATGATAACCTTTCCAAGACCACTTTTCTTCAATTG
CTCTCTTCTATCATACTGCTCCAGATCAATTGGAAAATCAGTCAAAATCTTTAGAATAGC
TGCACGGATGTTTATATTTGGTAAACTCCCATCTGGAAGGGGTTCAAGCCAATTCTTTAA

CAGAGTCAGCACTCCATGGTCTAAAAATTCTTGTTGAAGCTGCTTCTTTGAGAGAACCTC
AATAAGAAGAGACAACTTCTTGAGTTTATTAATAGCAGGTTTATTCTGTCTATTAAGTTC
AGCATCTTCTTCAGCAGTGACCTCAAGCTCAGCCATGACATTCTCAACTAGCAATGCAAT
TTCTGCAGGACTTTTCTCGTTCTTTTTCTTCTTTTTGCCCATCTTGAAGAGCTCATTGAT
TTCCTCATCCTCATCAGCCTCCTCTGCCTGAGGAGCATCATGAGCAAAGCGCATTTCATT
GTCACTTCCATACCTGTCACTAGGGTCAACACCACTGTCATCTATGAAGTTGTCATCATC
CACTGTCCTAACACCTTCCTGATCGTCCTCAGAATCGCCAGGCGGTGCAACAGCGTTCCA
CATCTCATTAAGCATTTTATCACTATCTTTCATATTTCCCTTTTTCCCAGACTTGGACCC
ACTGGCCTTCCCCAACCTCCTATCCCTCTCCAACTTTACTCTCTTATTACTCTCCTTCCC
TTCCTTCGCCCACTTCTTCCTCTTCCTCTCCTCCATTTCCCTCCCAATATCATCCCCAAT
AAAGTTATCCTCATCATCGTCATTTTCATTAACCAAATTAGGCGTACCACTCCTCGGAAT
TGATCCCCCCTTTTTCACCAGCCTCTTCCTAGGTTTCGATTTTTCGTTATTGTAAACAGG
AGTCTGCGACCGCTCCCTCTCGGGGAAGTCCCCAATGTCGTCTTCCAAATCATCAAGGAT
ATCGTTTTGACCAGGCTCGGGAGATGGCTCCCGGTCCGATTGCATGTCATCATAGTCCAT
CAATGGCTCTCCATCCTCGTCACGATAAGGATCGTCTTCGTAACCCATTGCGGTGATGGA
TTGCTTGATAAGATGGCAGTAGGGATTGATCGATATTTGTAGATTTATAGAAGCGCTAAG
CTTTGGCGGTCTTGAACTACTTCTGTTTGTTTGTGGAATGGGATCGGGAGAGGGGGATTG
AGCCTTGATCCTATACGGGAAGGATCACC
>ntig_478_consensus
CCTTTTTCGATTTAATTTGAAAAAAAAAAAATTAAATAACCCCAAAGTTATTACGTGTTA
TTTACCACAGAAATTATTCATATCACAGTCAGATTCGAAGGAGGAAGAGGTAGCCTAACT
AAGGTCTCATATGATGGCAGAAAAACAGAAATTTTCAGTCTAATGGAGAGGACAAACATG
GCAAACGACTCTACGGGAAAGAAAAAAAGGGAAACTTTATGAATATGTGAGAAAGGCAAC
AGACCATAAATCCCTTGTCTTGAAATATGGTCTACTCTTAAAATATGTATGCTCTTTACT
CCTTCACCTGCAAAGCATCAACTGGTCTATGCAGAGCCATGAATGCACCTCATCTCCAGC
TCTACACCAACACAGCAAGTTCATCTTCAAAGCATAGGTCCAATTCAATGAAGCTCCACT
AGCTCAGTTACATATTCAGGCCAACTACACGGCTTCTCAATCAAACCTTCCAACTCATGA
AATATTTGAGCGTCGTTAACAGCTAGTTCTTTATCAGCAACATTTTCAGGAACTGAACTG
TCCCCAAGTAGGCTTCCCACCAACATGCGATATACAGATGATGCATCTTCGTCGCTTGTT
TCACTCATCACAGTGCTCTTAATATTCTCAGTATGATCGCACAATTCATTTTCACCGTCC
ATAGCACCTGGGAATGGAGCCCTTTCAATTGTTTCAGTAATTATAGTACTACTGTTTGGT
AACATATCAATAAGCTCAGATTGCATTGAAGCCAATTTTTCCTGAGCTTGGTTTCTACGA
TTGACACTTTCAGGAAAGTTTTCAGCCTCTTGGGCTGCAAGCAATCTTCTTCTCTTACTA
ATTTCACCTCCATCAAGCTCTCTTTGTTGCTTTCTCTTCGCAATTAATTGCTGAACAAAG
TTGGGGCATTTGGCTATTTTGGCAAAGAAACTCAACATCTGATGCTGCTTGCAATCTGCA
CGTCTTATACGTTCTTCAGTAGAAGAAATTTGATTCTGTGAATCCTCTTGCTGTTGTCTC
AGTTTCAAGATTTCCAATCTCAGTGTACTCAGATCTTCTCTTAAACTCTCAACCTCAGCT
TCCAACCCGGATTTGGTCAAATCAACGCAAGATATTAGCCCATCTTGTGGCTTGTTAAAT
CTACTTCTTCTTTTGATGCCTTTAAGTAAATGCTTCTTCCCTCCCTGAAACATTTCATTT
GCAAATTCCCATCTGTCCGAATGAATCTTCCTAAATCCGTAAGTATTGAGCTGGCGAATG
AAGGTAGAGAAATTCTTGTGCTTGAAATACTTTGGAAGAAGATTTTCAGAAAAGTCATGA
GAATCCCAAACAATAAAGCTACTACGATTCGCATCCCAGGAAACCACCGAGTCGGTCTCC
GGATCCTCCACCATCTCATATAGCTTCTTCAAAAACGGCGGGGGAGCTAACTCGTTCAGC
CCTTGCATTGGCTGAGGCAAACTCACCGATGAATTCGAAGAAGACGACGATCCATTTGAA
TTAATATCACCATCATAACTGCAGTCCGCCACGTCACCTTCTTCTTCTTCCTCTTCTTTG
ATCATCCCAGCTTTCTCAGCTTCCACATCTCTTGTAATTTTAAAAGCTATTTGGTCCAGC
TCTTCTTTCACCGCAATATTTTCTGCTTCTACTCTCAGTTCAGTAGAAGGAAATGACGAC
CGAGTCCCGCCACCATCGCCACCGCTAACACTAGTCACCGCCACCATCTTTTGTGAAGTT
GGCCACCGCCTTCACAGTGTGTTAATTCAACTGAATAAGGGTCGACACGTTCCTACACTT
ATACCCACTCGCAGCCATGTGTCCCTGCCTAAAATTAATTTCTTTGGAACGCCAGATGTC
ATTCTGGGAAATTGGATGGTGGCGATGAGGCGGTGTATTCCATGTTCAACTGGTGACCAT
TTGCCGAAAATCTTGATCTGGCTATATCTACA
>ntig_479_consensus
TGCACATATTTTGTCTAGACAGTTGTTTTTAGTTTTCTCAACTGGAAAAAAACAGCTTTAT
TCTTGTTCATAATCATGCTATAGGCTACCTTCCATTCCACCCTCAAATTTTATCACCTTC
GCCCCAAATAGCATAAGCTTCCTCCCCATGAACAGCTTCATCACCAATTCTCATATCTTC
TTCAGACATTCTCAGAGGTACAAATAATTTAATTAAAAGACAGATTAAGCTCGTACTAAT
TATGTTGAGAGTGGCGATAAACACAACCCCAAGAAGTTGAATTCCTATTTGCCGAAATCC

AGTACTGATTTGTCCCATATCAAAGCCATAGAACAGACCAACATAATCACCATATGTAGT
ATAGAACAAATAGTTGAGTCTTGGTTCTGCGAAGAGACCAGAAAGGATTCCCCCAAGAGC
ACCAGCGATGGCATGAGTGTGTAGAACTGCCATTGTGTCATCAACTCTTTGAAGAAGCTC
GGATCTCTTATGGACTACCATCATGGTGAACCATGGAATTGAGCCTGAGCACATCCCCAT
TACAATTGCTGCCCATCCTTGAACAACGCCAGCTGCTGGGGTGATGCAGACTAAACCAGT
GATCATGCCTTGAACTGCGCCAATGACAGAAGATTTGCCGAAAACTACAAGATCTAGCAT
GACCCAAACAACCAAGCTGGTTGCAGTACATACATGTGTGTTTAAAACAGCCAAAGAAGC
GTCACTGTTTACAACATAAGGGTCTCCTCCGTTGAACCCTGTCCAACCCATCCAGAGAAG
CCCCCCAAAATGCAGCTGTGTAACCAGCAACTCCTGCAGACAGATGAATCACATATCCAC
CAGAGTAATCTATTAGGCCTTTCTTGAAGAGGAAACCATTAGGACTCCAAATAATGATCT
GAGCTTGGATCGCTTATCCCCACGTCTTTTGCCTCTTGTCATTGTTTTCCACGTCCATTT
GCGTTCCATTTAGAAGATCTCTTTCATTTAATGTCTGTCATTACTTGAAGAACTAATGTC
AGACAAGGAGGAAAAATATGAATCAGAATGAGATGATTCCATCTCAGAACCTGAGGAATT
CTCTGAATCAGAGTAATAGTGTCTTTTCTGCTTATATTAATAATACATTATATTAATAAA
TCAGAGGAATACATTATATTAATAATTCACACTCAAAAAGAAAAAAAAACAAAGGACTTT
CCAACAAAGAGGTAAGAATAATTTCTCACCATCACCAAATTCAACACAATTGATTATCTT
CAATGTTACAGTTGGCCTCCCTTCTTCATGGCCAGCATTTTCAGTTTTTTTTTTTTTTTT
TTCCGAAAACCTCTTCCCCCTGCACAAGCTCCCCACAGACTACATATTTCCTGTTAATAA
TCGACGAAGACCCCACACTAACTATATGGAAATTCAGTGAAATATACCGCATAAGCAAAA
GAAAATGTCAGCGATAAACTAATAGATAGAAATATTTATTTATACCTGGAAACTGTTAAA
ATAGAATTCTACTTGATTAAACTAACATACCTATCAAGATTATGATGAACCTCAAAGGTG
ATCATAAATGAGATCCCAGTGCGTGGCGGTCAGCAACGGACATGGATAAGAAGCCAAGTC
CATCATGTTTTAATCTGGGTCACAAGATGCAAGTGCTGATATATTGGGGCTGTTACTTCC
ATTGCATGTTGAAATATGAGCCACTTGAGAATACTGTGAGGAAACCAGAAAGATAATGAA
AGGGATAAAAGCTACGCAAGCAACTCCCATGGAATAAAATTGAGGTAAAAGTAACTAAAT
ACAGTTCAAATAAATTGTAAATCCTGACAAGGGAAAATTACAAAAGGGTACTTATGCATA
GAGGATGTAGCCCAAGCACAAGCTTAAACAAAAGGTTGAAACCGATTAAACAGGAAGCAG
ATGGTCTAGTATGCGAGCCCAGAATCAATTGTAGACCATGTATTTGGGGGTTGCACTGAA
CTTTTGGTATCCCTTAATAACTTTATTCACTGCATCGAGGAAGTCTTTCTCTGTCACTGT
TTTCCTTCTTGCCCGAATAGCGTACATTCCAGCTTCTGTGCATACACTCCTTATATCAGC
TCCAGTCGAATTTGGGCAAAGCCGAGCCAAAAGTTCAAAACGGATATCTCTTTCACAGTT
CATTGTTCTTGTATGAATCTTAAATATCTGTGTTCGGCTCTCCAAATCAGGAAGGCCGAA
CTCAACCTTACGATCTAATCGTCCAGGTCGTAACAATGCAGGATCAAGCGTGTCAGGCCT
GTTTGTTGCCATAAGGACTTTGATATTTCCACGAGCATCAAAACCATCAAGTTGATTCAC
AATTTCAAGCATTGTACGCTGGACCTCATTATCTCCGCCCACACCATCATCAAAACGGGC
ACCTCCTATTGCATCAACTTCATCAAAAAACACAATACAAGCCTTCTTTGAACGTGCCAT
CTGAAACAACTCACGAACCATACGAGCTCCCTCACCAACGTATTTTTGAACAAGCTCACT
TCCAATAACACGGATGAAACAAGCATCAGTTCTATTAGCCACAGCTCTGGCTAGAAGTGT
TTTACCAGTTCCAGGAGGGCCATAGCAGAGGACACCCTTTGGGGGGTCAATACCAAGCTT
TACAAATTTCTCAGGATGAAGCATGGGTAGCTCAACAACTTCTCGCATTTTTTCAATTTG
CTCCTTGCATCCCCCAACGTCATTATATGTCACATCTGGCTTCTCCTCTACTGTCATCAT
GGTCACACTTGGATCTATTTTTGGAGGCAAGGGAATTTGAATTTGATATTTATTTCGATC
AACCCCCACCCGCATGCCTTCTTCAATATCAGTAGGGGAAACTTTGTCACCAAGCCCAAC
AACAAACTTAGCGATTTGCTTAACATTAATGACATATTTGGCATCCTCAGAGTTAGGATT
GATTATCTTGGTGCACCTTGCCACCTGAAGAGGCTGCTCCTCCTGCATCATCTGCTTATC
GGAAACAAGATCCCACTGGCTGGGCGCAGCTAAGCCAGTGTCAGACTCTTTAATACCACA
TAGGTCATTCACCTTCTTAGCCATATCCTTAATTTCTTTCTCAACTTTCTTAATGCCTGT
CGAGTAAGGTCCTAAACCATAGGTTTTGAGAAGAGCGATATCGTCCTCATCGAGAGGTCT
AGGGTTCTTCTCGTCCTT
>ntig_480_consensus
TATCGCTCTTCTCAAAACCTATGGTTTAGGACCTTACTCGACAAGCATTAAGAAAGTTGA
GAAAGAAATTAAAGATATGGCTAAAAAGGTGAATGACTTATGTGGTATCAAGGAGTCTGA
CACTGGATTAGCTGCACCAAGTCAGTGGGATCTTGTTTCTGATAAGCAAATGATGCAGGA
GGAGCAACCTCTTCAGGTGGCAAGGTGCACGAAGATAATCAATCCTAACTCTGAGGATGC
CAAATATGTTATTAATGTTAAGCAAATTGCTAAGTTTGTTGTTGGGCTTGGTGACAAAGT
TTCCCCTACTGATATTGAAGAAGGCATGCGTGTGGGGGTTGATCGGAACAAGTATCAGAT
TCAAATTCCATTGCCTCCAAAAATTGATCCAAGTGTGACCATGATGACAGTGGAGGAGAA
GCCAGATGTGACATATAATGATGTTGGGGGTTGCAAGGAGCAAATTGAAAAGATGCGAGA

AGTTGTTGAGCTACCCATGCTTCATCCTGAGAAATTTGTGAAGCTCGGAATTGATCCACC
AAAGGGTGTTCTCTGCTATGGGCCACCTGGAACGGGTAAAACACTTCTAGCTAGAGCTGT
GGCTAATAGAACTGATGCATGTTTCATCCGTGTTATTGGAAGTGAGCTTGTTCAGAAATA
TGTTGGTGAGGGAGCTCGTATGGTTCGTGAGTTGTTTCAGATGGCTCGTTCAAAAAAGGC
CTGTATTGTGTTTTTCGACGAAGTTGATGCCATAGGAGGTGCACGTTTTGATGATGGTGT
TGGGGGAGATAATGAGGTCCAGCGTACAATGCTTGAAATTGTGAATCAACTTGATGGGTT
TGATGCTCGTGGAAATATCAAAGTCCTAATGGCAACAAACAGGCCTGACACACTTGATCC
TGCATTGTTACGACCGGGACGGTTAGATCGGAAGGTTGAGTTTGGCCTTCCTGATCTGGA
GAGTCGAACACAGATATTTAAGATCCACACACGAACAATGAACTGTGAAAGAGATATTCG
ATTTGAACTCTTGGCTCGGCTTTGCCCAAATTCAACTGGAGCTGATATAAGGAGTGTATG
CACAGAAGCTGGAATGTACGCCATTCGGGCAAGAAGGAAAACAGTGACAGAGAAAGACTT
CCTTGATGCAGTGAATAAAGTCATTAAGGGATACCAAAAGTTTAGTGCAACCCCCAAATA
CATGGTCTACAATTAATTCAGGGGCACACATCCTAGACCATCTGCTTCCTGTTTAATCGA
TTTCAATCTCTGTTGTTTAAATTTTTTGTTCTTGGGGCTGCATCCTTT
>ntig_481_consensus
GTGTAATCTGTCTCAATCTTCCCATGGGCCATTGCACCAACCACAAAAACAAGGTCCACA
CCATTGCTGATAGGAGCCACATGATTCCTCATTTTAACCAATTTGTCCGCACTATATGAG
AAACCTACCTGCAGTAGTTTCTCACGCTTACCAACAGCAGTTATACTTAGCTTTTGTAGC
AGCTGCAACAAAATACCAGAGAAACGTTTATATGTCCTTGGTAGCCGAACATGCGGTTTA
ACTTCAAAAAGAACACCTTTCTCCGTTTTAACAAACACAGCTCACAACCTCCCAGCCTTA
GTTAGTGGGCTGTCTGATGAACGATGTCAGGCCTGTAATCACCCGGATTTTTGTTATTCC
TTTTCAAGAAGTTATCATGATCATCTGAATTTAATATCTGGTAACTCTTTCCAACTTTTG
CAACTTCAAGAGAAGCTTTCTCTAGAACAAATATAACTCCGGGTTTCTTCGTACTTTGAT
CGGA
>ntig_482_consensus
GTGTAATCTGTCTCAATCTTCCCGTGGGCCATTGCACCAACCACAAAAACAAGGTCCACA
TCATTGCTGATAGGAGCCACATGATTCCTCATTTTAACCAATTTGTCTGCACTATATGAG
AAACCTACCTGCAGTAGTTTCTCACGCTTACCAGCAGCAGTTATACTTAGCTTTTGTAGC
AGCTGCAACATAATACCAGAGAAACGTTTATATGTCCTTGGTAGCCGAACATGCGGTTTA
ACTTCAAAAAGAACACCTTTCTCCGTTTTAACAAACACAGCTCGCAACCTCCCAGCCTTA
GTTAGTGGGCTGTCTAAAATTGATAAGAGAGCCTGATGAACGATGTCAGGCCTGTAATCA
CCCGGATTTTTGTTATTCCTTTTCAAGAAATTTGCATGATCATCTGAATTTAATATCTGG
TAACTCTTTCCAACTTTTGCAACTTCAAGAGAAGCTTTCTCCAGAACAAATATAACTCCA
GGTTTCTTCGTACTTTGATCGGA
>ntig_483_consensus
TCTGCCAAAAAATACATGATTTATTAAACTAAGTCCTGCAGCTGCTCTCAAAATTTATCC
TGATAACTTTCGACTCCTAACTTGTGCAAGAAGAGAGTAAGCAAATGTAATCACAAACAT
CATTGATGATCAAAGCTCTACTAATGAAGTTAAACATAATATTTATGCAGATTTTGACTA
AGACAACCAACCAAAAAACTTATAAGATACCAGTAAAAATCAATCACCTTCATTCTCAAA
CACAATGACCTGTTCTTCCACCCCAAATACAAAGGCTTCAACATTTATTTCAAGCAAGGC
TCACGCAATGGCCATACTCTTATCATAAGATATCAAAAATTGCTCAGAGATACTAACAAA
AGCAACCTATGCTAGAAAAAACACACACACACACACTCAAGACAAGCCAATCATGAACTG
TAACTAGTGGACAAAATGGCAGAAACAAAAACTTTGAAAGGATGGGACTTCCAAACAAAA
GTTGGCCGAAACAGAACACCTACACACAATATCCCTTCAAGACAATCACTGCTTGAATGT
TTAAACCCTCAACAAAATAGCGCAAAGTAGCAAAAACAACTGAATATAAATAACCTTACC
CTGATGCTTCCACTTATGAGAATGTCACCTTAGACAAAATGATAGAAGAGATGTAATCAT
TTTCAATAAGGACTTTTAAAAGCATGCACACTTGTTGAATTGAATTATCTTCCATATGTA
AATTATCAAGAAATGCTCACACAAAACCCACAAATTATTTGAACATATTTATAGGACATT
AGAATTCAAGTATTATTTCCATACATAGAACAACACTTTGAATATGAGAATGGAATTTAC
AGCACCCAAATGCTTTAAAGTTATAGGAACAAAGATTGCATATATATAAACCACAAGCCA
TTATTATTAATTAATTGCAGGTACAAAAAGGGGAACGATCGTGGGACTTGAATTTTTAAG
AAACCTCCTGGAAAACCCTCTATCTCTTGAAAAAGTCCTCTCTTTCCGACTCTTCTCCAT
ATACAATGCTCGTTCCCTCTCCTTCTCCTTCTTTTCTCTTTTCAACCGTTCCTCTCTCAA
TTCTTGTAACGTCTTCTTCCCTCCCTTCTTTTGCTCCTCCTTTTGCACTCTCACTAACCC
ATCATCCATATGATTCTCAGCATCTACATCAACATTGCTCCTGCGCTCAAGATACCAAGG
CAACTTAACCCCTTTTCCCGCAACCCCATAACCCAGTCTATACTTCTCATCATCTGGTGT
CACAACCTTTTTCAATGTCTCCTCCTCTTTCCTCATCTTCTTCCTCTTAAGCCCATCTTC
CTCTCCACCACCCTCTTTCTTTGACCCGTTACTTGGGTCAAAAATCTTAATCCCTTCAAA

GAGATTAATATGATTCGACTTGGGTTCAGATTCAACATGCTCTGCAGTAGCCTCTTTGGC
TGACTCTTTAACTTGTTTCAACGGAGCCAAACCACGGGCAGCTCGAAGCTGCTCTAAACG
AAACTCAGCATCTCTTTTTCTGGCCTGTTCACGCTTGATCTGCTCTTCTTTTGCTGCAGC
CTCTTCATCTTTACGGACTTTTTCTCTGTTATCATAATTGTACACATTCCATCGCTTCTG
CGGAAGAATATTTAAACCACCATGTCCTCCCATAATTCGAAATTGTACGTATAACTTGAG
AATCAC
>ntig_484_consensus
ATCTTTCCACTAAAAATTTTATATATCTAGGACATGTAAAATTAGTCAATAATAAGATGA
ACATATGTCCGAGCACCGACATGTTTACTGTTCCTATTGCCATGGCCACATGCCACAAAC
AAATTCAATGGTAGTCAAGCCAGGCCAAAGTTGAAATCAATATGATTCTTTTAAAACAAT
AAATATCAAAGCCTTCACCACAAACATTAAATCTGAAATAGATTCAATGCAAAGGATTCA
ACCATGCTCTGATCACTTCTTTTATCGCAAAGGGGCCATTAGGCTGAAAAGGCTCATATT
ATAGGTCAACCCCGCCGATCATCCATTACAAAATCGAGCTCTTAAGCAATCTTCTTATTT
GAAGAAGGCAATCTGTACGGCAGGAATATCTTCCAAAGTGCAAGACGCTACTCGGTAATT
TCAGCCCAACAGCTCGTGTTGGTAGTACATGTTATGTGCTTATGATGCAGACTTTTTTCC
CAGCAACCGAGTTCAACAGCTCACAAGTATTTGAGCATGCCACGACCCTCTACGCTAAGT
CTGGTAGCTTGCATCTGCTTCTTCTTGGGCGCCTTCAACTGCTGCAACTTCTTATTCATC
TTCATTCGACGTTGATCTTGCATAACTTGTTTAGCACGCGCTCTAATTTCTTCTGGATCA
ATTTTAGATTGAGGGCGCACCACAAAATCCAAGGGAGTTGCTTCTGGTCTCGAAGCATGT
TGCCTCATGGCTGAGTCCTGCCTTGAAGTCTGTTGGCCAAAAGATGATAGACCAGATTTG
TGGTCCCTAGATATAACCAAATCATCAAAATCGCCATCTCTGGATTCCATTGCTGCAGAC
TTATTTGTGGGCTTTTTAGTGGATGGCCTTCGAAAGGGAACTCTGTCATCATCAATAGTT
TTCATGTCCTCGAATCTGGTGCTCTTATTAAATATAGGTCGGCTCCATTTGTCAACTAGA
TCCTTGGCAATTTTTCTGTTAGATGTAGTTTCCTCATCAGATTTTGATAAAAACATAATA
ACCTTTCCAAGGCCACTCTTCTTCAATTGTTCCCTTCTATCATACTGCTCCAGATCAATT
GGAAAGTCAGTCAGAATTTTCAGAATAGCTGCACGGATGTTTATATTTGGTAAACTCCCA
TCTGGAAGGGGTTCAAGCCAATTCTTTAACAGAGTCAATACTCCATGGTCTAAAAACTCT
TGCTGAAGCTGCTTCTTTGAGAGAACCTCTATAAGAAGAGACAACTTCTTGAGTTTATTA
ATAGCAGGTTTATTCTGTCTATTGAGTTCAGCATCTTCTTCAGCAGTGACCTCAAGCTCA
GCCAT
>ntig_485_consensus
GCTTTATATGAATTTGAATCATAGATGTATTTGTAATATAAGAGCGAATGCAGATGAAAT
ATTTAATGAAGATTTTAAAACAAACAGTAAACCTTAAACAATTAAAACCAGGAGAAGGAA
ACCAGAAACCTCTTTTTACTTATTAAGAAGATTAAATTCTTGACATGGCTCAGAGAATCA
GATTCAAAATGCTAAAGCCTCTCGCTTTTTAGTCGACGTTAACATGCAACACTTCACTCC
TCTCTTCTTCCTTTACCTTGGGCACCGTCACCTTCAATACGCCGTTCTTCATCTCCGCCT
TGATCTCGTTGGTGTTGTACATCTTCTCTGGCAAGTCAATTCTGCTCGTGAATCTTCGAA
CGCTCTCTTCATCATCACCTTCCTTTGCTCCTTCACCTTTGATCACTAGAGTGTTCTGTT
CAACTGTCACCTTCACATCTTCCTTTCCCAGCCCCGGCATGTCCATTCGGAGATTCAGGG
CATTTTCGTCTTCTTTCGCATCCCAGCCTCGGCGTAGCGCGCCACGTGTCCCGGAGAAGA
ACGGGTTCTCTGTCATCTGGTCCATCAGGTTCAAGACCTGGCTCAAGGTCCTCGTTGGAG
AGAATGGATCAAACACATCTGAGAAGAAATCCCGACGGCGAGCAGCAGAACGATGGTCAA
GATCAACCTCACGGTCATCGGACTCGTTATTGGATTGACGAACAGCATTGGTGTTGAAAA
AGCGAGAGGCTGAGGTGACAGTCGGAGCGATGGTATGACTTAGAGACCGGGTGAAAAGCG
TTGAGGAAGAAAGTCTCTTGAGAACTAAAGAGGAAGGCATTGATGGAACTGAGTGAACTC
TCGACAAATTCGAAATAGCTGCAGGGATGGAGAAAAT
>ntig_486_consensus
CCTTAAACAATTAAAACCAGGAGGAGGAAACCAGAAACCTCTTTTTACTCATTAAGAAGA
TTAAATTCTTGACATGGCTCAGAGCATCAGATTCAAAATGCTAAAACCTCTCGCCTTTTA
TTCGACGTTAACATGCAACACTTCACTCCTCTCTTCTTCCTTTACCTTGGGCACCGTCAC
CTTCAATACGCCGTTCTTCATCTCTGCCTTGATCTCGTTGGTGTTGTACATCTTCTCTGG
CAAGTCAATTCTGCTCGTGTATCTTCGAACGCTCTCTTCATCGTCACCTTCCTTTGCTCC
TTCAC
>ntig_487_consensus
TCCCAGAACCATTGACACCATTTGAACAATCTTCTTCCATTGCATCACTCGGTTTTGATA
GTGGAGTAACTGCAGCTTCAACATCCCATACAAAAACCTCCCCCCCAAGACCACCAGAAG
CAACAATATTGCTATTTTTTTCTGCTGCTGCTAGACAAGTAACATAGTCAGAGTGCTGAC
GCAGAGTCCTGGTACAAGTTCCATCAGAAAAGCAATTCCATGTCTTGAGGGTGGTATCAG

AAGAGCAGGAAACAAGTGTACTATCACCTGCAAGTACAGCATCATTAACCCAATCAACAT
GTGACTCAAAAGTAGCAGAGCAGCTAGCAGTGTCTTCAGCCAGTGTCCATCTCTTTAATG
TGCCATCTCGGCTCCCAGTGAATAGGTAATCACAGCCATCAGATGCTGACGACTTTGATA
AAGCCAAACAGTTTATCCCAGCACAATGCTTCGTGTCATTGGCATCATTTAACACATATG
TCAATCTCTTCTCTTTACGGGGCCGAGTAGAATTGGAGTTATTACCCGCACTACCCACTC
GGTGCATTGCAACTCCAGTGTTGTGATGTATCAATTAGACGACAAAACTGAAGATAAACC
CTAGAAAGTGATTTGTACTGACACGCTTGTGGCCTGTTGGCCTGTTCGTTCAG
>ntig_488_consensus
CAAGCTTCTTAATTCCATGCACACAAAAAACAACACATAAAATTTGGAAAGTAAAATTGA
GTGGCATTTAACTAACACTGAAATCAACACAAATTAAACCATTATATCAGTAAGTAATTC
ACCATAACAAGTCTTAACTAATCTAAACAACTAAAAAAACTGATAAAAAAAAAAACCAAGC
AGTGGTTGGACCTATCTAACTCTTCATCGTTTCAAGCTTTTACTTCTCTGATGAGTTAGG
ACCCCTCTTCTTTCCAAAACCAACAACTGCAGTGACGAAACGGCGGTTGTATTGCATACG
CTTGTAGGCACGGCCGCGTGGCTTCTTCTTCTTGTCTTGCTTAGCAACTTTTGGAGTTTG
TCCTCTCACCTTACCAGCACGAGCTAAAGAACCGTGCACCTTACCCATTGCTGATTACTA
TAAACGTACTGCTGCTGCAAGTTGCTGTCCTTCTCTTTCCTTTCCCGCAAAAGACTAGGA
AGCTTAGGGTTAAGGTTAGGGTTTTGGCTGATTATTTATCGTAGGTTTGATTCTCAAACA
GATAAGGCCGGGTTACAGCCCGATATGCGAATTCTCGGACCTGGAGTCTGCGCCATTACT
CTTCAAAGACCATTTTTTAATTGTTTTGTTTTTTATTTTAATATAACCAAAAATTGTGGG
ATATTATTTAAAAGAACA
>ntig_489_consensus
ACCTTTTTTAATGGAGGTAGGACTCTTCCTCCCCCCTGGGCGATATTTTGTTTGCTTGGG
AATGCTGCTTGTGTATTTCTCATTCCTAGTAGCAGCCTGATTTTGATTAGCCATTTCCAG
CTTGCGTTGCACACCTGTAATCATAGAAGCATTTAACTTTGGAGAAGGAAAACTGCTAAA
CAGCTTCAAATTAGATATTGCCCCAGAAGACCTCGGAGCACCTTGCTGTTCCAATGCTCC
CCTTGTTCCACCAGACGGAGGTGTTCTCAAGACAGTTGCTCTGGGGCGAAGTGATGGTGG
AACATAAAAGCAACTCTGGAAGAATGGGTGTTGCAAGGCCTCCGCAGCAGTAGGCCTCTT
GCAGGGATCCCAAGAACAAAGTGACCTGATCAGACTAAGTGCATCCTCACTCGCCGATGG
CATTAATGTAGAGAGATGGATACCAGCAAACTGTGGGAACTGATAGTTTGTAGCCCTTGC
AAGTAGAAGTCCATCAGCCCAAGAATCCTTAGTTGGTGTTCCAATTATGTTGCATATTTT
GTAGATTTCATCCGCTTCACTAGCACCCGGAAAGAGAGGACGGAGCGTGATCAACTCAGC
CATGATTGCACCCATAGCCCACATGTCAACTTTAGAACTATACAGATACGACTGAAGCAG
CACTTCAGGAGCCCGATACCACCGTGTCGAGACATACGCCGTATAAGGTGGACGAGAATT
GATCTCCCGTGCAAGACCAAAATCAGCAATTTTGATTATGTCTTTTGAAACAAGCAAGTT
CTCAGGTTTAAGGTCACGATGAAAATATCCCTGCTGGTGCATATAAGCAAGGCCTTGGAA
AACTTGGAAACACCAATTTCTGATTTCAGTTTCAGAGAAAAGCTTTTCTCTATCTTTCAT
AAGTTGGTAAAGGTTGCATTCCATATATTCAAACACGAAGTACAAAATGTCATTTTCTCT
GATAACTTCCTTAAGCTTCACAATATTCGGATGATTCATCTTCCGCAATGACTTAACCTC
TCTAAGATTTATGCACTCGTCCCATGAATAATATTTCTTCTTCATTTTTTTAATTGCAAC
GACTTCACCAGATTGCTTATTAATAGCTCGCCAGACACTCCCAAACGTTCCATCACCTAC
TTCCTTGATTAACTTGTACCTCTCCATTCTTAGCAGCTCTATCTTTATTCAAGAGAAGTA
TAAGAACCAACAAAGATATATGACATTTATTGTCACAAATTGATTACTAGTAAAAACTAT
AAATTTATAAATCGCCTTTCTGGTCCAGCTGTGTTCACCATTAATTTTCTGTGCCAAAAC
GAAAGAGAACAAGACCAGAATTTTTCTGATGGAGAAGCATTGCAGCCTTATTTTCATATA
AAGTCACAGCTTCACAACAGTACCAAGCAACAGATTGAAGGAAAAGAAGAATTCCACAAA
ACATATTGATTCCAACAACAAATCAAAGTAGAATGGTCGTTCCAGCCAATAGATGTATCC
AAAGTGCAAAACAAGGACAACCAGGGTCTAGAAACAAAAGAAAAGGTAGCTCAACTATAC
TGTAAGTTGAAAAATCTTCTGCATACTGTTATCTCTTGTCCTTCTAATCAGTGTCCATCA
GGAGTAATAACAGAAGAATATCTAAGAACTATAGTCTGCAGGGTTTCGATCGGAAATGCT
TAACTAACGCTACAAAGCAACCTTATCACGCACTTGCAGACC
>ntig_490_consensus
ACCAGATTCATTATATCAGCAGCACTCTTTATCATATTAGAAAGAAACATAAGAAATATG
AGTAAATGACGGGGCACAGTACATATTATACTCCAACGTTTTGAAACTAAAACACATAAA
ATCCACAAAACACTCAATTCATCATTAATTCGTACTAGTAGTACTCCACACATTCCACAC
GCAATCCCATTATTCAAGCCCCCTCTAAAAAGCCCCTTCTCCTTTAGCCCGCAACTTTCC
TGGTGTAAGTTCTCCCAGATTGCAGTGATTGAGTAGATCTCAGAAACATATCACCTCCTG
ACTCAGCATTCCATTGTACCCCAGTCTTCATAGGAGGTGGCTTCAGCGGCTGCCCCACAG
ACCGCCTGCCGCTACCTATTGTCATGCTCGCCAACTTTTCAACTGCATCTGAAACTCCTC

GGGCAGTTCTACCTTTGCTTAGTGAGGTTGGACTCTTCCTAAACACTGGGCGATACTTCA
TTTGCTTAGGAATGCTGCTTGTGTATTTATCATTACTAGTAGCAGCCTGATTCTGATTAA
CCATTTCCAGCTTGCGTTGCACACCTGTAATCATAGGAGCATTTAACTTTGGAGAAGAAA
AATTACTAACGAGCTTTGAATTAGATATTGCCCCAGAATACCTCCTAGCACCCTGCTGGT
CCAACACTCCACCTGTTCCAGCAGAAGGAGGAGTTCTCGTGATAGCTGCTCTGGAGCGAA
GCGATGGCGGAACATAAAAGCAACTCTGGAAGAATGGATGTTGCAAGGCCTCTGCAGCAG
TAGGCCTCTTGCAGGGATCCCAAGAACAAAGCAACCTAATCAGATTAAGCGCATCCTCAC
TCGCTGATGGTATTAATGTAGAGAGATGGACACCAGCTAACTGTGGGAATTGATAGTTTA
TAGCCCTTGCAAGTAGAAGTCCATCAGCCCAAGAATCCTTAGTTGGAGTTCCTATAACAT
TGCATATTTTGTAGATTTCATCGGCTTCGCTAGAACCAGGAAAGAGAGGACGGAGTGTGA
TCAATTCAGCCATGATAGCGCCCATAGCCCACATGTCAACTTTAGAACTATACAGGTACG
ACTGAAGAAGCACTTCAGGGGCTCGATACCACCGTGTTGAGACATACTCAGTATACGGTG
GACTTGAATTGATCTCCCGTGCAAGACCAAAATCAGCAATTTTGATTATGTCTTTTGAAA
CAAGCAAGTTCTCAGGTTTAAGGTCACGATGAAAATATCCCCGCTGGTGCATATAAGCCA
GGCCTTGAAAAACTTGGAAACACCAATTTCTGATTTCAGTTTCAGAGAAAAGCTTTTCTC
TATCTTTCATAAGTTGGTAAAGGTTGCATTCCATGTATTCAAACACAAAGTACAAGATGT
CATTTTCTCTGATAACTTCCTTAAGCTTCACAATATTCAGATGATTCATTTTCCGCAATG
ACTTAACTTCTCTAAGATTTATGCACTCATCCCATGAATAATATTTCTTCTTCATTTTCT
TAATTGCAACAACTTCACCAGACTGCTTATTAATAGCTCGCCAAACACTCCCAAATGTTC
CATCACCTACTTCCTTGATTAACTTGTACCTCTCCATTCTTATTGTTGAACAAAAAGCAG
CGCTTTCTTTATTCAAGATAAGTATACGAACCCACAAGGAGATATTACATTTATTGTCAC
AAACTGATTGCTAGTAAAAACTATAAATTTATTAAAAAAAGGCCTTTTTGGTCCAGCTGA
ATCCACCATTAATTTTCTGTAGCAAAATGAAAGAAAACAAAACCAGAATTTTTCTGATGC
AGAAGCTTTGTAGCCTTATTTTCTTCGAAATTCACATTTTTGAAACAGTACCAAGCAACA
GATTGAAGGAAAGGAAGAATTCCACAAAACATATTTGTCCAGCAATGAACCAAAGTAGAA
TGGATGTTCCAGCCAAGGGATATATCCAATGTGCAAAGCACAGATAGCCAAGGGTTCAGA
AACTAAAAAACCGGTAGCTAAACGATACTCTAAACTGGAAGTTGAAGAATCTTCTGTGAA
CTGATATCTCTTATCCCTCTAGTGGGTACCCATCAGGAGAAATAACATTATAATATCTGC
AGAGTTTCAATCAGAAATGCTTGTCTAACACTACAGAAGCAACCTTATCATGCACTTGTA
GACCTCAACCCCCAGAAGATGTTTTCTTAAAATTAGAGGCTGGATGTTCAATCAGTGATG
CATTGTAAAAGTGAGCTAAATATCCAACACCTATCTCAGCAAACGTTTGCATTACGCCTC
CTACACTGATCATTAAGGTGATTCAGACCACTAGGTGCAATTGACAAAGGACACCAAAAG
AAAACTTCCAAGTATGAGACACCGCCAGTCCAGCACATCCTGGAATGAAAAAACTCGATA
CTGATAGCAATTTAAGCAGATGCGGGCTTGCTCCATGTCTTAAAAGAGACTATTAATCTG
TGTAGATCCAAGTCCGGCACAACCTGAGAGCCAACCGGCAAAAAAACCGTCGCGGAGGAC
GGGAAAAGGTGGAATCCAGAGCTTCTCCCAATTTGCGTCGAAACCAAACCAAAAGAACAC
CACCACAACTCTCTC
>ntig_491_consensus
CATCTATTGGAACTCAGTTGACCAAACTTTATAAGTTACACGATCTGTCCCATGTTTCCT
CATAATTTACCAATAAGAATGAACACAAGCCCATCAAAAGTTGCCATCTCACTGATGCAA
TTAGGGTACATGAGTGAAGATTTTGAACTAAAAACACTGTTCAAAGTGTATCTAACCATG
AATCAAAATCATCCAACACTTTAGACTTCGTAACAGAGTGAGATGAACGTGTATCACGGG
TAACAACCTTCACACCCTCCGACTGGCTCATCAAATTTGATGACTCCTGTAATAAGTCAT
CAAGTTCATCATCAAAACTGGTAGTCACTGGATTCCCTGAAAGTTGCGATGGTTTAGACT
TTGAAACAGAGTGAGGAGGAGGAGTATCATGAGGAACAGCCTTAACATCCTGAGACTGGC
CCATCAAATTTGATGATTGCTGTAATAAGTCATCGAGTACATCATCAAAACTGGCAGTAA
GTGGATGTCCTGAAGGTTGTGGTGGAGCTGTGGAACATGCTTGTGGGGACATGGTGAAGC
TATGATTGTGTTTGTAGCCTGAAGAATCGAGATACTTGGTATCACCACATGAATCAAGAA
GCAAATCTAGTTCTGCTTCAGCAGCTGCTGCTTCAAATTTTACAGTGGACTCCAGCATTC
TGCTCTGCGCAGATTTATCAAAATGACTGGAAGCCAAACCATCTTTATCTTGATTGGCCA
AATCAGGCCCTTCACTATGAAATTTTGCATCTTTATGATACATGCCATAACTAAATGAAG
TAAATTCAGTATCTGTAACTTCAACATTCTCAGATAAATCATTGAAACCCACATCTTCAG
ACATATCTGCAGCAGATTCACTTTCATGAGTTGTTTGAATCTGGCCATGATCATCAATGA
TTCTCACCAATGATCGCTCTGCTCGCAGCTCTGATGGCAGCAAATCTGCTTCAATAAACA
GCCTCTCTGATAAGTCTACTTTTGCTAGCTGTTCTGCAAGAGCATTCAAATTTAGGGAGA
GAAACGATGCCTCTTGAACTGAAGTTTTTTTATCGTCCACAACGAAGTTATCCTCCCCAA
TCCATGATAAAATGCCCTCCCCCTTAACTGAAAGCATAGAGCCGACTCCCTCAATGAAGC
CTCCGGGCATAACATCTTCTAAAGAAGGAAAGCTATGTGAATCACAATATGATTGCGATT

GAGCCTGAGATCGAGCCTCTGCAATGAGGTAAGTATAGTCTGCCCCCTTGCTTTTTGGCA
CAACAACATCAGAAGATTGACTTGTGCTATCACTTGAAAGATCTTCACCATCCAAATCAA
AATTCTGTTCATACCGATCCCAGTTGGAAGGGAGTCTAGATATACGCTGGACCTGGGGGA
TTTTCTCCCCAATCTGCTTTCCTTTTGGCTTAGTTGTGCTTCCAGCATTGTCCGAACTGA
CGGATGGAGCTTTAGATTTCTGATTTGGATGAGACTTATTTTTATGGTGTTGAGAGTGAG
CTCTCTTGGATTTTGCCAAGGCCTTAGCATCCATTATACGTATCTAATTAACAGTTCATA
ACGCAATTAACTAGAGTTTCCATTCAGCACCCTGACATGATGATCATCTGAGGCGACCAC
TGAAGATTTCGCTTTGCCGGTTTCCCGGAGGTGACTGGTTACGTCTTGCGGCGACAAAGA
GCCGGCAGTTTCCGTGGTCGGCAGTTACAAGGATTGAAGAGATGACCGGTGGGCGGTGAC
ACGAATTAGGGATTGGGGATTTAAGAAAATTAAGGATTTTG
>ntig_492_consensus
GGCATCGTTTCTCTCCCTAAATTTGAATGCTCTTGCAGAACAGCTAGCAAAAGTAGACTT
ATCAGAGAGGCTGTTTATTGAAGCAGATTTGCTGCCATCAGAGCTGGTATTTTTAGTGGT
TTACCTAATTTGCTTTTTTATTTTGTTGCATTTAGAGTTTGTGACACTATTTTAAGGTCA
GATGGGGCCATAGCCCTTCTGTAGCTCCATCTATCACCTTTTGCCCTAGAGCTGCACACA
GTATTAAAACAAAAAGAAACATTCTGCCTTGAATTGGTCGTTCAAACAGTACACAGTGAT
TATAATAGCACTGGTTATTTGAACTATTTGAGCTTTTCGGTTTATCAAATTAGACGTTCT
CTAAACCTTCCAGA
>ntig_493_consensus
GTCACTTTCTAAACCTAGTGTACTCGCCAAAGCATTCATCATAGTTACGACTATACATAA
CAACCCCAATGTTCAGATTAAGAACAGTCCACTTCCACTTGTTTTAGAGACTGAAAAATC
ACCCAATCCTACTCTGACACTTCGGGAAACCCTAAAACAACATGGTTCTAGACTCTTCTT
CCGTTCATTCCTTATTGTTTGGAGCTCAGATTTTCCAGACCCTTCTATTTCTCTTCTTCC
TCGCCTTTTAACCCTCTTTTCTCATTCATTTCTCTCACATTCATAATCGAATTCTCACCA
CGTTCAATTACAACTCACAAACTAATTCACTTCAATGGCGAAGTACGGCGAGGGAGACAA
GCGTTGGATCGTTGAAGACAGACCAGACGGCGCCAATGTCCACAACTGGCACTGGGCAGA
AACGGATTGCATAGAATGGTCGAGAAATTTCTTCAACAAACACTTTACAGACGTCATCGT
ATTGAATGGCGAAGGAAATATTTATATCAAGATCACGAAGCTTGAAAAGCTCGAGGGTGA
AGCTTACGTTAATGTCCGGAAGGGGAAAATTATCCCGGGATATGAATTGAATGTCACCCT
TCTGTGGGAGGGCGAAGCAAGGGATTCTGAAGGTGGGGTTTTGTTGAAAGCTGAAGGTTC
GGTGGAGATCCCTTATATTGCGGACGAGAATGCGGACGAGAACCCGGAAATTAGGGTTTT
AGTGAAGGATGAGGGGCCAATTGGGAAGAGGTTGAGAGAGGCGATGTGGGAGAAAGGGAA
AGTTTTGATTTTTGAGAAGGTGAGAGGTTATGTCGAGAGTATGGCGAAAGGCGGGCCGTG
TAAGGAGGAGTTAGAGGCGAAGAAAGTGGCATCGAAGCCAGGGAAGAAGGAGGAAGCTGG
AGGGAGTGTGGTGGTGGAGAAGAAGGAGGTAGCTAAGAAAGAAGTGAAGAAAGAGGAGAA
GAGAAAAGAAGGGTTTAAGACGATAACTATGACCGAAAAATTTAGCTGTAGAGCTAGGGA
TCTGTATGAGATAATGATGGATGAGAATAGGTGGAAGGGGTTTACAATGAGTAATGCTAA
AATAAGTAAGGAGGTCGGTGGGGAGATTAGTATCTTTGATGGGTCGGTTACGGGGAAGAA
TTTGGAGTTGCAAGAGGGGAAATTAATTGTGCAGAAATGGAGGTTTGGTAGTTGGCCTGA
TGGGATAGATTCGACGGTACGTTTAGTTGGCTTTCCGTTGATTGGTTTGGTTGGAGAACA
ATTTGGATTGATTATAATGGGATTTTGTGTGTGTGTGTGTGTGTGTGTGTGTGTGTTGTA
GCAGCAGTGGCGGTGGCGGTGGCGATGGTGGTTGGGTGTTCCAGTTTTCCATTATGTACA
AGTTAGAATTCGGAGGGAAGTGAACTGACCAAGTTCAGAAAAATACAGGCACCTCTCCAA
GCTTGTAGACAACTCGAAAATAAAAATCACATGGAACCAGAAATGCTCAAGCAATTGAAC
CATACCAAAACCTCGGAAATGAGCAACCCTCACAAGTATGCCAGCCCCCCAATATACAAT
ATGTGAAAACAAAAAAATCCACGGGCATTGATACAAGTATCGGCCACTGTCTCCGACTGG
CTTTGTTAAGTTGTCAGGATCCATTTTCAAAATTGGGAATGTCATCATCTCAATGCCACC
AGTAAACAAGTAATTACTTGAGGCTAATGGCTGTCTTCCTTCACACTATTACTTCTTTCT
TTGGCTCGAAAGTCAGCTATGCTACGATATTCTTCCCTTAGTAACCTTTCCAGCTCTGGA
TGTTTCTTTAGTGGCTCTTTCTGCTCGCCTGACCCTCGATAATGATGGAATGCCCAATAT
TTTTCAAGGCCATAAAGATTGCCTCTGTGGTAGAATTCAAGTGTCAGCCATTCAAAGTCG
TTATAAAGGTCCTCTCTGAACTCTTTTTCCAAACCATAACTATAAAACCTGAAAAGACAC
TCTATCCCATAATTATAATTAGCAGCGGCATCTTCGAGAGCAAGCTTCTTAAACTCATTA
TACATTGTAGGGATGAACATGTCTCTAATGAAATATGACCAAAACCTGTACAGTGTATTC
ATTTCCT
>ntig_494_consensus
AAATCCCAGCGAGCCAGAAAAGCTCAAGCAATAGAACCATATCAAATGAGCAGCCCTCAC
AAGTAAAAGATCCCCCCCAATATACATTATACAATATACAAAAAAAAAAAACCACCAAAA

ATGGGCCATTATCTCAACTCAAACTCCCTTTGTAAATTCGCCAGGATTCACTTTCAAAAC
TGGGATGGGATGTCCCCAGTAGCCAAAGTAATTACTTGAGGCTAACGGCTATCTTCTTAA
GTAGTATTCCTTCTTTCTTTCCTGCGGAAGTCCTCTATGCTTTGATATTCTTCCCTAAGC
AACCTCGCCAACTCTGGATGTTTCTTAAGGGGCTCCTTCTGCTCATGTTGCCCTCGATAA
TGATGGAATGCCCTGTCATATTAATTAGAAGAACAAATTTAAGAAATTTTTTTTAAAAT
ATATGGGATTCAAACTGCATCCTAGTTTCAAAGTG
>ntig_495_consensus
AATATATTAATTTTCATACTTATTGATTATTTTTTAATTTTTAATTGAGAAAATGAGAT
AAGATTTTAAAATTATACAAAAGTTGATAGACATAACCATAGACTTAACTCAGGATAAGC
ATAGTACATTGTTATTTATTAGCGAAATCCATTACAAGCACACAACATACGTAAACAAGA
CAATCCGTGGCTTTGTTCTCAAACAGAAGCATATGCAGTGGTTTCAACCGGAGATTTGAA
TGGCCTTAACCTCAGGTTTCTTAACCTCAACTTTTGGAACAGTGACAGTCAGAACTCCGT
TTTCCATAGAAGCCTTAATCTCCTCCAATTTCACGTTCTCCGGTAGCTTAAACCTCCTGG
AGAATTTCCCACTACTACGTTCCACACGATGCCAAGTGTCGTTCTTGTCTTCCTTCTCTA
TATTCCTCTCTCCGCTAATTTGAAGCGTACTGTTGTCTTCAATCTCCACCTT
>ntig_496_consensus
ACCCAAATGTAAGCCTCAAGAAACTTTCAAGCTTCTTTCCTGGTAATCCTGAAGCAATTT
ATACCCAAAGCCGTTGTGATTTCGTGAATAACAGATGTGGCAAAGTGGAGATAGAGGGCC
ACTGTAAATGCACAGTACCCAAGCAAAACCCAGAATTCATCAACTAATGGAAATCCATGC
ACATGTTAGTTTTCAATCCCTTCGGTTCATCGCACAAATGAGAAAGAATCATCCTTCCCA
CAAGGAACCCAAACGCAAGTCCAGTTCCCAATACAACTAAATGTGGATAGTTCCTCATTA
TGTCAGATGGAGACAAATAATCCCATGCAAGCACTCCTCCCATAAGTACAACAAAAGGGT
AAAGCATTGCCAAAGCCAGTAACATGCTTCCTTTTCGAGCATGAACGACCCTGTAGACAT
TTGACACATTGGAGTAAACCGTAGGTATAACAGCAAAGAATATCATCAAATATAATACAG
CTTTGTAAGTTGGGATCCCACTAATCAGTGGCACCCAACTCAAGAAAGGGATAGAATTCC
CAACATGCTGATACCACCATTCAGCACCAACTAAGGCTGTAAAAAAGTGCGCTACATAGA
TCAGCATAAGACCCTCTGTTGGTCCATTGACCACTGGAAGAATCAGGGTATTGGTAAAAT
AGTGTTCCCATGTTGCTCCATAAAATGGAACAGCTGAAATTACCCAGTACCAGAAAGTAT
CTCTTCCACACATGGCAGTGCTTCCGAAAGCCATGGACTCAAACGCACATGCAAGAGCAT
CACATCCATGGTCAAAAAGCTCGCCCAATGGACTAGATGAATTTGTCCGTCTTGCCTGCT
TCCCATCAACAGCATCAAAAGTCTGATACAAAAAAAGAAGTAAGCCATGTGCAAAATGAA
CCCATCTTGGCGGAGGTGAGTCCAAATGGGGTGAATATATATATCCAAGCAGAGCAGATG
TGAGTAAGAACAAGAATCCCGTAAGTGTTATCATGTTCGGTGGCATCCATAAGGGGAAGA
TTTTGACGAAACGACTCCAAAAAGGTTGCAATAGATATTTGGCAACGTAAGAATTATCAA
CTCCACTGTATTTGTATCTGTGCAACGCTGCTACTCCATGTGCTCCTATATAACCCATTT
TTTCCTCAAGTGTCAAGTCTCTTCGCTAATTCTCGAAGAAAGGAGAGATGGAAAGAGACG
AAGACTTGCAAACTGAAAGGAAAGTGCATGAAACGCAACCGTTTTATGCCTCTCCCGCCC
TCCACTAAAGCCGGAAGAAAGTGAAG
>ntig_497_consensus
GTTGAATTTTATCCTTTGAAGGAACCTTAGTGGGAATAAGTCTTCTAGAACAAGGAAAAT
TAATACACGGGCTGACGATAAGCACCAGCAGGCTGTCATGGATGGAATGAATAACATGAG
CATGTTACCACCAACACCATCATCCATGAGCCACAGTATGGTACCCACCTGACTTTCTTT
TGGGGGTAAAAACGCTGATTGGCCTTGAACAAGCCGTGGCGTGTATGCAATGGCCTGATA
TTTGTCCTGGTGCCTGTATCTGGATTGAGTAGCTGTCGCATCGCAAGTTGACCGAACCTT
ATTCCATCCATTTCGCCGCTGGTTTGATTCAGACCTTCTTGTACGCCTTGTGGATAGGAT
ATGCCTTGGGCTGTAAAATTCTTTAGTAAATAAACTGCTGCTCCTAAAATCTCTGATCTA
CCTCTCAAAGAGTCGTTGATTATGATGGAAGAAGCTAAACGAAACTCTCTTTACAGCTAC
CTCTCAAACATCTTATTTTGTAAAATTTATAGCCTTTGTAATATGAATTCATAAACTAAT
AAATGAAGAAACTTACTACTTCACTTTCATTAATTCCATCGTATAAATAACTGAAGAGCA
AATTTTGGATGATATAATTACATACTTAATTCAGAACAATGTTATTACACTGTTATTTAT
TAGCAAAATCTATTACAGGGATTACAATATGCGTAGATGACAATCTATAGCGTTGTTCTC
ATACACAACTATATGCAGCGGTTTCAGCCAGAGATTTCAATGGCCTTAACCTCAGGTTTC
TTAATCTCAACTTTAGGAACAGTGACAGTCAAAACTCCGTTTTCCATAGAAGCCTTAATC
TGCT
>ntig_498_consensus
GAATTTTTGAATTTTAAAAAATTAAAAGGTAAGACTTTGTTTCTAGCCCAAACTATGGTC
CTTTGGCGCAACAAATATTCAACTGCTAAATTCATTCTTGTGACGATGTGAATTAATATT
TAACGTCAATGTTATTTAATTAGTAACGAAAGACAACAGACGAGGAGACACCGACAAAAG

ACACCAAGGACCTACTTATGCCCTTGAGTTTGACCAGACACATCTCCAATGACTAATCGG
ACCCAAATGCAGAAGAATAACACTATTGCACACACACTAAGAAAGACTAGTCAACCTCCT
CCATCTTGCTCTCCTCGGCACCGTCTTCCTCCAGAGCCGGCATGTCAGTGTCCTCATCGG
CAGTCTCATCCTCATCGATGCTGAGACCCAATTTCAACATCCTATGAATCCTAGAGGCGA
AAGTGTTTGGGTCATCAAGGCTGAACCCCGAGGTCAACAGAGCAGTCTCGAACAGCAGCA
TCACTAGATCCTTAACGGACTTGTCATTCTTGTCAGCCTCGGCTCTCTTTCTGAGTTCCT
CCATGATCCCGTTGTCAGGGTTAATCTCCATTGTCTTCTTGCTTGACATGTACGAACCCA
TGCTGTTGTCCCTCAAGGCTTGAGCCTTCATGATCCTCTCCATGTTAGCACTCCAACCAT
ACTCTCCGGTCACCAAACAGCAAGGGGAGTCGACTATTCTGTCAGACACAACCACCTTCT
CAACTTTATCGCCCAAGATATCCTTGATTGTCTTGCAGAGGTTTTCAAAAGACGACTTCT
TTTCCTCTTTCTTCTTTTTCTCTTCCTCGGTCTCATCCTCAAGTTTCAATCCTTCCTTCG
TAGCTGAAACCAGCTTCTTGCCGTCGTACTCCTTTAGCTGACCAACTGCATATTCATCAA
TGGCGTCGACCATAAAGAGGACTTCATAGCCCTTCTTCTTGAGTCGCTCCAAGAATGGGG
AATTCTCCACGGCCTTCTTGCTTTCACCAGTAATGTAGTAGATGTCCTTCTGACCTTCTT
TCATCCTGGTGACATAATCCTTCAAACTTGTCAACTCATCACCACTCTTGGTTGAGTGGT
ACCTGAGCAGGTCAGCCAACTTGGCTCTATTTTGGCTGTCTTCATGGATACCCAACTTCA
AATTCTTTGAGAATGCCTCATAGAACTTGTTATAATCTTCCTTATTCTCCGCAATCTCGT
TAAACATTTCAATGCATTTCTTCACAAGGTTCTTCCTGATTACCTTGAGAATTTTGTTCT
GTTGCAGCGTCTCACGAGAGATGTTCAGGGGCAAGTCATCAGAGTCAACAACACCCTTGA
CAAACCCAAGAAACTCAGGGATAAGCTCCTCACAGTTGTCCATAATAAACACCCTCCTGA
CATAGAGCTTGATGTTGTTCATCTTCTTGCGGGTGTCAAAGAGATCAAATGGTGCCCTTT
TAGGGACAAAGAGAATGGCCTTAAATTCAAGCTGACCCTCAACAGAGAAATGCTTGACAG
CAAGGTGCTCTTCCCAGTCATTGGTCAAGCTCTTGTAGAAGGCAGCATATTCCTCCTTGG
TGATCTCTTCTGGCTTCCGCAACCAGATGGGTTTCTGCTTGTTGATTAGCTGCCACTCAT
GAGAAACCTCCTTAATCTTTTTCTTCTTTGATTTGGTGTCTTTCTCTTCATCAACGTCTT
CAACAGCACCTTCTTCCTCCTTCTTAGGTTCATCATCTTCGTCGTCACTGATTTCTTTCT
CGGTTGTTTTCTCAGTCCAGAGGTAGATGGGGTAGCTAATAAATTCAGAGTGCTTCTTCA
CAAGATCCTTAATTCTCCGCTCTTCCAAGTATTCAAGCTGATCCTCTTTGAGAAAGAGAG
TCATCTTGGTACCCCTGCCAAGTGGCTCTCCACTAACGTCCCTTGTAACAGTGAAGGAGC
CACCAGCTTGTGATTCCCAAATATACTGCTCATCGTCATTGTGCTTTGTTGTGACAATAA
CCTTCTCTGCAACAAGGTAAGCGGAGTAGAAACCGACGCCAAATTGACCGATCATGGACA
CGTCCGCCCCAGCCTGTAAAGCTTCCATAAACTCCTTGGTTCCAGATCTCGCAATTGTAC
CCAAATTATTCACCAAGTCTGCTTTGGTCATCCCGATGCCGCTGTCAATTATAGAGAGCG
TCTTGTTAGCCTTGTCAGGCACGAGTCTAATGAACAACTCTGGTTGAGCATCGAGCTTGC
TCTTATCAGTCAAGCTCTCAAATCTGATCTTGTCCAAGGCATCAGAAGAATTACTGATGA
GTTCACGAAGAAAGATCTCCTTGTTGCTGTAGAACGTATTGATGATCAAACTCAACAGCT
GATTGATCTCTGCCTGGAACGCGAAGGTCTCAGCGTCCGCCATTGCTCCGCACGAAACAA
AGAATAATAATAAGATTTCAGAAACAAGAAAATTGATTAATCGAAGAGAAAGATCTTGCA
CAACGAGAAAC
>ntig_499_consensus
CGAGATGTGTCCGGAATTTTGAATTTATAATGAAAACATGCAAATTAAATCAAAATAAAC
CACCCCCCTCTTGCGCCTTTCGTCAGATTATTCCCGTCTGACTTTAATGGCCACAAAGAA
TCCAACATAAAAAGGTGCGGAGATTTCAAAGAAACCAGCTTACAATTGACATAGACACAA
GACACTGGCAAAAAGACACTTAAGGACTCCCAAACGCTCAAAGAATAGAATACAACTATT
GCCCAACCTAGATCAGAACTTAGTCGACCTCCTCCATCTTGCTCTCCTCAGCGCCGTCTT
CCTCCAATGCAGGCATGTCAGTGTCATCCTCCGCAGTTTCTTCCTCATCAATGCTAAGAC
CCAATTTCAACATCCTGTGAATCCTAGACCCAAACGTGTTCGGATCATCAAGGCTGAATC
CAGAAGTCAAAAGAGCAGTCTCGAACAGCAGCATCACTAGGTCCTTGACGGACTTGTCAT
TTTTGTCGGCCTCAGCTCTCTTTCTGAGCTCCTCCATGATTCCATTGTCTGGGTTAATCT
CCATTGTCTTCTTGCTTGACATGTATGAACCCATGCTATTGTCCCTCAAGGCTTGCGCCT
TCATGATCCGCTCCATGTTGGCACTCCAACCATACTCTCCAGTCACCAAGCAGCAAGGAG
AATCCACGATTCTGTCAGAAACAACCACCTTCTCAACTTTATCGCCCAAGATATCTTTAA
TCGTCTTGCAGAGGTTCTCAAAAGAAGTTTTCTTTTCTTCTCTCTTCTTCTTCTCTTCCT
CCGTCTCGTCCTCCAGTTTCAATCCTTCCTTTGTGGCAGAAACCAGCTTCTTTCCGTCGT
ATTCCTTCAACTGACCAACAGCATATTCGTCAATAGCGTCAACCATGAACAGGACTTCAT
AGCCTTTCTTCTTGAGTCGCTCCAAGAATGGAGAGTTCTCCACAGCCTTCTTGCTTTCAC
CAGTAATGTAGTAAATGTCCTTCTGACCCTCCTTCATCCTGGTGACATAGTCCTTTAAGC
TTGTCAACTCATCGCCACTCTTGGTTGAATGGTACCTGAGCAGGTCAGCCAACTTGGCTC

TGTTCTGGCCGTCTTCATGGATGCCAAGCTTCAAATTTTTTGAGAAAGCCTCATAGAACT
TGTTATAGTCTTCCTTATTCTCCGCGATTTCATTGAACATTTCAATGCATTTCTTCACAA
GGTTCTTCCTGATTACCTTCAGGATTTTGTTCTGCTGCAACATCTCACGAGAGATGTTGA
GTGGCAAGTCATCAGAGTCAACAACTCCCTTGACAAATCCAAGGTACTCGGGGATGAGCT
CCTCGCAGTTGTCCATAATGAACACCCTTCTGACATAGAGTTTGATATTGTTCATCTTCT
TGCGAGTGTCAAAGAGATCAAAGGGTGCCCTTTTAGGGACAAAGAGAATAGCCTTAAATT
CAAGCTGGCCTTCGACAGAGAAATGCTTGACAGCAAGGTGCTCCTCCCAGTCATTGGTCA
AGCTCTTGTAAAAGGCAGCATATTCCTCCTTGGTGATTTCCTCCGGCTTGCGCAACCAGA
TGGGTTTCTGCTTATTTATCAGCTGCCACTCATAAGAAACCTCCTTAATCTTCTTCTTCT
TTGATTTGGTCTCTTTCTCTTCATCAACGTCTTCAACTGCACCTTCCTCCTCCTTGGGTT
CATCATCTTCGTCGTCACTGATCTCCTTCTCAGTTGTTTTCTCAGTCCAGAGGTTGATGG
GGTAGCTGATGAATTCAGAATGCTTCTTTACAAGGTCCTTAATTCTCCGTTCTTCCAGGT
ATTCAAGCTGATCCTCCTTGAGGAAAAGAGTCATCTTGGTACCCCTTCCAAGTGGTTCTC
CACTAACGTCCCTTGTAACAGTGAAGGAACCACCAGCCTGTGATTCCCAGATATACTGCT
CATCGTCATTGTGCTTTGTGGTGACAACCACCTTCTCTGCAACAAGGTAAGCAGAGTAGA
AACCGACACCGAATTGACCAATCATGGACACATCAGCACCAGCCTGCAAAGCTTCCATAA
ACTCTTTGGTTCCAGATCTTGCAATTGTGCCCAAATTATTCACCAAATCTGCTTTGGTCA
TCCCGACGCCGCTATCAATGATCGATAGAGTCTTGTTCGCCTTATCAGGCACGATTCTGA
TGAACAACTCAGGTTGAGCATCGAGCTTGCTCTTGTCCGTCAAGCTCTCAAATCTGATCT
TGTCCAAGGCATCAGAAGAATTACTGATGAGT
>ntig_500_consensus
GTCCACGACTTTTCTAGTTTGGAACAACCCATTCTCCAAAATGGTTTTCTTGTACGGAAT
AACCATCAGTCTCATTTAGGTAGATGGGTAATCTATCAGTTTGACACTTTGTAAAGGCAT
GTTACAGAATACAAAGCCAACGCACTAAAGCCTATATATCAAGGACCACGTAAGATACTT
TTCCCAACTCTCAACAGGTGTCATGGGGGCCAAATTTGAAACCATTGCAATATAGCGGAA
AAGCAACCCCGTGTGATTTGCTTAATTAGCATCATCTCGAGCACTAGTCTTATGTGCATC
AACCTTGTAATTTAAACTAATAAAGAACAAAAAAGACATTGATTACTCATAAGAGAAATA
ACCCTAACTTCTGACTAGTTTATGTAAAATAAACCTAAAATTCTTTAATCAATGTTGTCC
ATCTGACTTACATTGGGCACATCCCGTCATGGAGTACGCCGATCGCCAACGACAACACAC
TCACGAAAAATAGCATTCACACACACACACAATCCAAATCCAACCATACATCGTCACTTC
CTGTGTCTTTGCTATGTTTACTAGCCTTGAGTTGTGACAAACCTTCCCCCATCTTAAATG
TGCAGATCCTCATGCACACCTCATTTTTGCAACACCTTCAAACATCGTAAGATCGCACTC
TTGCGCTAACAATACATGCTTGTGCTATCAAACAAGTGCAACTAAATCATCATATCTGCA
AGCACGACATCCTTGGAAAAAAATATTTCCACAACGGCTGCAAGATCATCTTTAACATGC
ACCAAGCTCACACGCATGTACATGTGACAAACCCTTGTTCCCAATGGACATTACTACTTG
CCTGTTGGCTCAGCGACCTTCTGCCATGGCTAGGAAAAACGATGTGCTTGTTAATTTTCA
GCATTACTTACGTTTGAACTTTTGAGAAGATTCAGGGACTTTCGTGCATTCGATGTTGAT
TATGTTGGATTCACGCGAAAATAGACTCGCCCAACCTAGGATTTGATACCAATTGTCACG
GGCACTATCTCAAAAACCAACTTTATATTCGTCAATCCCGCAATTTAAATTGATAAAGTA
CAAAAAAAACACTGATTTCGTTCTTCTTTGAATTCATTAGGAAATTGACTCCAACCCCCG
ATCAATTTGTATAGGATAAGACTAAAAACTTTTAACCAATGCTGTCCGCATGCCCGCATT
GTCCACAGCACTCACGCTCACTAGCCTTTGAGCCGTGACAACAAAGGTATCCTCATTTGT
ACAGCTTCGTAATATGAATCCAAGAACTGACACTCTACTTTTTCATTTTAGTTAACTACA
TCATAACAAATTTTGGATAATACAATTATATACTTAATTCAGAACAAGGATATTGAACTG
TTATTTATTAGCGAAATCCATTAAGGGCATTACAAATGACTTAGACGACAATCCATTGCA
GTGTTCTCAAATACAACTATAAGCAGTAGTTTCAACCGGAGATTTGAATGGCCTTAACCT
CAGGTTTCTTAACCTCAACTTTAGGAACAGTGACAGTCAGAACTCCGTTTTCCATAGAAG
CCTTAATCTCCTCCAATTTCACGTTCTCCGGTAGCTTAAACCTCCTGGAGAATTTCCCAC
TACTACGTTCCACACGATGCCAAGTGTCGTTCTTGTCTTCCTTCTCTATATTCCTCTCTC
CGCTAATTTGAAGCATACTGTTGTCTTCAATCTCCACCTT
>ntig_501_consensus
CCGAAATCCGTTACAGTCACACAATCTACGTAGACAAGACAATGCATTGCATTGTTCTCA
TACACAACTATATGCAGTGGTTTCAGCCAGAGATTTCAATGGCCTTAACCTCAGGTTTCT
TAATCTCAACTTTAGGAACAGTGACAGTCAAAACTCCGTTTTCCATGGAAGCCTTAATCT
GCTCCAATTTCACGTTCTCCGGTAACTTAAACCTCCTGGAGAATTTCCCACTACTACGTT
CCACACGATGCCAGGTGTCGTTCTTATCTTCTTTCTCTACATTCCTCTCTCCGCTAATTT
GAAGCACGCTGTTATCTTCAATCTCGACCTTCACTTCCTCTTTCTTGAGCCCTGGAAGAT
CGGCCTTGAACACGTGAGCCTCCGGGGTTTCCCTCCAGTCTATCCGGGTATTGACAAAAG

TAGATGTTTCTTGAGGGAAACGAGTGGAGAGTGAAGAAAAGTCCTTGAATGGATCCCAAA
CATCGAGGCAGAAGGGATCTAAAACACTGCTGCGCTGGTTGCCGAAGAAGCTTGGAATTA
GTGACATACCGTTTCTAAGAATGCGAGAGCGTTATATCGTATTGATTGCTGAATCTAATG
CTTGAACTTCGAAAATTTGTCTCCGATGATCTGAAAGAGTTGTGTTGGGGCATTTATAAG
GAGAACGGAAAATTCTGGTGCTTTGGGGATCAACCTTCTGGTTAGGTGATGAAGTCTGTC
GGGCTTTCTGGTAAATTCTGGAAATCCTTATGGTCCTGGGCTCGCTAGAAAGTTCTGGGC
CCTATTCTCTGATTGCCGGCGTCAACCATTCGTTAAAGGCCCAAACAGTAATTTTGATTC
TTTGATTTTTCAAATACGTGGTTAATAATCTACCAGGCCACAAATTAAGAGCAAGATCCA
GTAACCAACTCATTGAAAGCTAAAACTTTGCCACGTTCATTTCAAGATGTTTTTATATCG
TGGGGGCCGCTTTTTTTTAATCTTATCATCAGC
>ntig_502_consensus
CCGAAATCCGTTACAGTCACACAATCTACGTAGACAAGACAATGCATTGCATTGTTCTCA
TACACAACTGTATGCAGTGGTTTCAGCCAGAGATTTCAATGGCCTTAACCTCAGGTTTCT
TAATCTCAACTTTAGGAACAGTGACAGTCAAAACTCCGTTTTCCATAGAAGCTTTAATCT
GCTCCAATTTCACGTTCTCCGGTAGCTTAAACCTCCTGGAGAATTTCCCACTACTACGTT
CCACACGATGCCAGGTGTCGTTCTTGTCTTCCTTCTCTACCTCCCTCTCTCCGCTAATTT
GAAGCACATTGCTATCTTCGATCTCCACCTTCACTTCCTCTTTCTTGAGTCCTGGAAGAT
CGGCCTTGAACACGTGAGCCTCCGGAGTTTCCCTCCAGTCAATCCGGGCATTGACAAAAG
TAGACGTCTCTTGAGGGAAACGAGTGGAAAGTGAAGAAAAGTCTTTGATTGGATCCCAAA
CATCGAGAGAGAAGGGATCGAAAATACTGCTGCGCTGGTTGCCGAAGAAGCTTGGAATTA
ACGACATATTGATTCAAGGAATGCGAGAGTATTGATTGCCGAATCTAATGCTTAAACTTC
GAAGAATTGTCTTTGACGGTCTACAACGGTTGTGTGATGGGGCATTTATAAGGGGAAAGG
AGAATTCTCGTACGTTGGTGAGCAATCTTCTGGTTGGTTGTTGAAGTTTGTCGTGGTTTC
TGGTAAATTCTGGA
>ntig_503_consensus
ATATTCCTCAAGAGGGCATAATTGGAGATACCCATTTGAAATCATACAATAAACTCTAAC
CTGTGCATGCAAATAAGGCAATAGAATCTACATAACAAGTCAATTTGGAAACAGTATCTT
AGAGAATTGATACACATCTTGGTAACCGCCAGCCAAAACTTCAATGGTTGCACCCATCTG
TAGCAGAGATGAATCTAAGCTTGTGGTGTAAGTGAAGAAGTTTTCAGCCATAAGGTTCGG
CCTAATATGGCACTTCCATGAATGGAAAGGTAAGCGGCATTTAACCTGTCAACATCTGGT
GTAGCCAAGGGATCATACAATTCCTCCTTTTGCTCAACACCTGGAACAGCACCAGCCATA
GCCGCCATATCTATAACACCTTCAACCTGTGGAGCATATTGTGGGAAGCCAGCATATGCA
CCATATGTGTACAAAGATGGATCTTGAGTGGTCCCATATGCATAAGCATCATAGCCCTGT
CCATAACCATAGTAGGCACTCCATTGACTCTGATCTACTTGTGCTCCCAAGCTACCAGTT
ACATCCTGTTTTCTGCCCCAAGAAATGCGGATTTGTTGTTGACCAATCACATGACCTTGC
ATCCTTAACATGCTTTCTTCAGCAGATGTCCTGGCTGCAAACTGTACAAAACCACATCCT
CTGCCCACAGGAATCTTTACGTTGACTATCTCCCCAAAGTGTAAGAATGTTTGCCTAAGT
TCTTCTTCGGTAACATTTGGATCCAAGTTGCCAACAAATATTGTTGTATTATTGATATCA
TTATCTGCAGGAAGCACCTGCACTGGAGTAGTGTATGCTGGCAAGGGATATACGGCTTTT
GTTGCAGCATACTGTTGTTGAAAACCAGTTGCCTTCCTAGGTGTTGCTGCACTAATACGC
ATTGGTCTAGTTGAGCAAAAGACACCATTCATTTCAGTCATTGCACGATTTCTTTCATTC
TCATCCAAAAATTTGACAAACCCATATCCCTTTGAGCGTCCAGTATTTGGATCTGTCACT
ACCTTAGCACCTCTAACAGATGGATATTGAGCACGAAAGGTCTCTTGCAGCAAATAATCT
GTAACATCAGGTGCCAAATCCCCTACAAAAATTGAATGCTCAGGACCAGCATCAGTACGT
TTTTCTCCCATACCAAAAGAAGCCCAATTCAACCTGAAAGTTTGCTCAGTCCCAGGCATT
GGTATCCCATTATAAGTCTGCAAAATCCTCTCAGCTGCAGCATGAGAAACAAATTCCACA
AACCCATAACCCTCTGGCTGACCAGTAATCTTATTACGAATAATCTTTATTGACAATACC
TCGCCGGTGTGAGCGAAGCAAGAATTGAGATAGTTCTCGTCGACCCAGTACTGTAAGTCT
CCAATCCACAGAGTTCTTACTTCTTCTAGCGTCATCGGATGATGATGATATCCTGCTCCT
TGTGCCGTCGCCATTGTTGTTGTCGTTCTAATTTATCTTTCTTCTTCGCGGTCTCGCTTA
CAC
>ntig_504_consensus
TTTTTTTTTTTTTAATGAACATAATAAAATAACACCAGGGCAAATGGTAAGGACATCTCA
ATCACTAGTATTCCCTTCAAATGGTTTCCAAAAAGGAAAAAAAAATGAAAATTATTCAAA
AGCCTTGTTGTTATGCATTCTTCCTTGAGAGTTAAGTTCAGAACGCAAACTTGAACTCAT
TCATTGTGAATCCCTCTCACACAGACTCCCAAAAAATCTTAAAACAATAATACAAACCTAA
ACACAGCTCCACATTATTAAATATATCCCAGCTACAAAATTCGGAAAACTACAGATTTAG
CTACGTTCTGAGGCAGCTAAATTGACCGTACTTCAATCACGTTTGTAGGCTACATTTTAG

GACATTCATAAGAGTTCAGAGTACATGATTTTGGAAATATCCATACCCCTCCTAAGAAGC
ACCAGGAACCACAGTTGCAGCAGCATACATGCTTGGATCTTGATTAGGTGGCAAGGCATA
TCCATTCCCACTATAGTAGTGACCTCCATAATGTGCCCCATTATTCCAGTGGTTATGAGA
ATCTGATCTCCACTGCTTGTTCCCAGGATTGCGACCCCAAGAAAGACGTACTGCTTGCTT
GCCTATTACAGTCCCTTGTAAATTTTGCAATGCATCCTCAGCATCCTTTCTATTTGCAAA
TTGAACAAATCCACATCCTTTTCCGACTGGTATTTTCACAGAGACAATTTCACCGAACTG
AGAAAATGACTCCCTCAGTTCCTCATCACCGACATCAGAATCAAGTGCCCCCACAAATAT
>ntig_505_consensus
TAGTGGCCTCCATGATGTGCCCCATTATTCCAGTGGTTATAAGAATCTGATCTCCACTGT
TTGTTCCCAGGATTGCGACCCCAAGAAAGACGAACGGTTTGCTTGCCTATAATCATCCCT
TGTAACTTTTCCAATGCATCCTCAGCATCCTTTCTGTTTGCAAACTGAACAAATCCACAT
CCTTTACCAACTGGTATTTTCACAGAGACAATTTCACCGAACTGAGAAAATGACTCCCTC
AGTTCCTCATCACCGACATCAGAATCAAGTGCCCCCACAAATATAGTTGCATTATTTGAC
TCGCCATCAGATTGAGACCCCTGGACCCTATTAGCATTTGATGCATGTCCACCAGCCAAT
ACCAGAGCTTGTGAGGAATACTGTTGTTGA
>ntig_506_consensus
CAACCCAACACTAATAACATCCTTAAAGTTTTCAGTTGAGTGAGGAAATCAGAAACATTG
ATTTCAAACATTTTATTTCTATTAAAAAAAAAAAAAAAATCTCACCTTGGAAGGATGAAT
CTTCCATATATCTCGCAATTTGAGCTTTGAGATCAATTTCCTCGGTTAAAGTTGGTTGAG
CTTCAGCTGAATCTTTTTTGACCTCAGGGCAGCAGTTAGCCACTAATGATTGGAAGCTAG
GAGGCATTGCCAGAGAAGTAACTTCAAAGTTATGATTAACCTTGATTTCCTGGGTGCAAT
GGGGTTGCCCAACTTCATTACTCAAGTTTGGTGGCATTTCAGGAGTATAAGTGTCAACAG
GATCTATACTACTCAATTCCTCACAAACTTGAGGTCTATTAGAAGACACGAAGTCAGAAC
GGGCCGAAATTTCAGACCGAAAGTCACTGCTTAGAGGTTCTGTTTCTTCCTCGCTCTTCA
ATATTTCCTTCAAAGCATTGATGAATGTCTCAGCTTCATGACTTGTTGGAAATCGCAAGG
CAAACTTCTGGATCT
>ntig_507_consensus
GGTAGCTCACTGTATGCTTCATGCAACCTCACTAAAATAGGGAACTCACTCATATCAAGA
TTGAACCTTTTAAATCCCGCATCAAGCTGTGGAGCTAGAAAACAGTCTGCCAGGTATACT
TCATTTCCAGTTGCATATTTTCCAGCATAGTCTTTTAACAGCTTCTCAAGTGCTGCAAAA
CCTTTCCCGAGGAACTGTTTAACCCAAGGAAGTTTCTCATCAGGGCCAAATTTCTCACCA
ATGTAATTCAATACAACAAGATTTTGCAGAGGCTGAATGCTTGAGGACACAATATTTGCA
ACCTCAAAATTGATAGCTTTCATCGTAAAATCAGAAGGCAACAATGGATGCTGAGGATAC
TTCTCTTCCAGATACATTAAAATAGCAAAAGAATCGGAGAGAACAACATCCCCGTCCACC
AAAACCGGCACATAACCAAGAGGATTGAGCTTTCTAAACTCAGGACTGTTCTGCTCTTCC
TTCACCAAGTTCACGGCTTTATATTCATAATTTAACCCTTTCAAATTAAGGCCAATTCGG
ACACGGCAAGAGCAAGAACTCCTCCAGTAAGAATAGAGTTGGAGCTTATTGTTACCGCTG
TCATTTCCGCTTGCCATGTTTGAGTCCCTGACCAAGCCAGACAAGACAAGATCAAGAAAA
TAAGTTTAGGTCTAACCGAAAAAACTAAAAGTGCTCTACT
>ntig_508_consensus
ATATATATATATATATATATAGAATTAATATATAAATACAAATTTGATCCATGTAATATTTT
GATTATAATGCTATATGCCTACCCAATTGTCCCAAACAGATGGGATACCTTTATTTAAAC
ATGAAGACCACAGCATAAATATTTACATGCAATCAACCAAACCTATTTTAGATATTTCAG
AAAGTGAAATCTGCAAAGTCACACCCAAACACAACCTTAGAAATAAGGATTCTTTAGGAA
AGGAAGGGAGCATAATTCCCAACCGTCCAGTATGTCTAGGTAATACAGCACGCTAATTGT
TAACAAAATCATGCACGTAGACCATCAGAAACGCATCCAACAAGCAGATTATTTAAGATA
AGTAGTGATTCATCCATGATTGGAAGCGTGACCATGCCAGCTCAACTGCAGCATCATCCT
CATCAGGCATTCCCATACTCTTCCTCCTCTGCACTCCCTCTGGTGACCTATTCATGAAAG
CATGTGCATTGCCAGGATAGATGTGAACCTCAAATGGAACACCAGAAAGTTTCAGCTTTT
CCTCCAAAGCTTTTGCAGCTGTCACATCTGAAAAGCCAACAAAGTTGTCAAGCTCCCCAA
AATGAGCCTGAACGGGTGCCTTGGCCTTGACAGGATCTGCTAGCTCTGATGACGGAACCC
CATAAAATGCAACAACAGCATCAACCTCAGGAATTAAAACAGAACTTGCAATAGAGAGAG
CACCCCCCATGCAATATCCAGTCACGCCAACCTTTTTAGAACCATTTTTCTTAAGCCAAT
TAACTGAAGCACTGATGTCCTTGACAGCACCTTGCCAGTCAAGGCCGTCCATCAAGTGTT
GTGCTTCCGCAACATCCAATCCAACTTTTCCTCTATACAAATCAGGGATAAGTGCCTTAA
ATCCAGGATTGAGCTGGGAGATTTTGACAGCATGATTCTTGATCTCATAGTCAATACCCC
ACCACTCCTGAATCACAACAATCCCGGGACCATCTTCTTTCCCAACAACATACGCATCGA
ATGTAGTATCGTCTCTCTGAATCTGGACTTTCTTGAAGGGAGAAGCTGCAGAATCAGCCA

TTGCCCGGACGGTAAAACGATAACGGGAGGGAGAGAGAGTGCGAGCGAGAGAAGGTTTAA
AGAGAGGAGTTTGGCTGAAGATTCTGGAAGCTGTTGCTAACATTCTTCGCTGAAATCTTA
CTCAAAGTCAGTATTTAC
>ntig_509_consensus
CTGGCACTGCTAATACAACATAAATATGAGAACCATAAGCTACAAAATTGTAATCAACCA
AGACCAAAACTAGAATGATTTCTTCCCTCACAGAAATTGTAGAGACAAGTTTTTTTTTTT
TTTCTAATTTTATATAAAATCTGGATCAAATAACCATAACGTATACAAAGAGACCCTAAT
CTTATCTCCTCTTATTCTTTGACAGTTTCTTGTTGATTCCCAGAGTTCTTTCGATATCAT
CATCTTCATCTCCGCCACCCCTTCTTCTCTTCTTGCCTCCGGATTCTTTGATTGTCATGA
TGGATAATCTTTTGGCTTCCATGACACGCTCCAACAATAGCAGGACCTCCTCTTCTTGAG
CAGGAAACTCTGGTAACTTTTTGCCAAGAAGCTTCTCTATCTGTATATACCATTCCAACT
CGTACTGATTCACTAGTGAGATTGCAACACCAGATCGTCCTGCACGAGCAGTTCTTCCCA
CTCGATGGATGTAATCCTTAGAGTTCGTTGGGATATCATAATTGATAACCATGTCAACAG
ACGGAATATCAAGCCCTCTACTTGCCACATCGGTGCAAATAAGAATATTGCACTCTCCAG
CCTTAAACTTATTCAAGGCACCAAGTCTTTTTGACTGGGTCATCTGACCACTAATTGGGA
TGGCTCTTTGACCAAGATTTCTGAGCGTCAAAGCTAAAAGGCGAGTTGCATCACATGTTC
GCGTGAAAACCATCGTTGAAGATCCAGGCATCTCAGTCAGGATAAACACCAGGTAGCAAT
CTTTGTACTTAGCAGGAATGAAGCAGTACTGCTGCTTTAGTGTGTCAACAGTGGAATATT
TAGATGCTGCTTCAATCTTAACAGGATTCTTCAAACAAGCTCTCTGGAGCTTTCTCACCT
TCTTGGTCATTGTGGCCGAAAATAAGAATGTTCTCCGTGAGTGAGGAATAACATTCAAAA
TTTCGTCAATTGATTTTTCAAATTCCTCATTCAGTAGCCTGTCNNAACCTTTGGTGTTAG
TTAAATGATCCATAAGGCGTCCAGGCGTAGCAACAACAACATGTGGCCGCTTTCCAAGGG
CAAGGGTCTGTTGCATCATGTCTATCCCTCCAACAAGCACTGCACATCTAAGGCTAATTC
CAGATCCTAAAGCTTCAAACTGCTCAGCTATCTGAATTGCAAGCTCTCTTGTTGGAGAGA
GCACACAAGCAAAAAACGCGGGAACTGTGCGTTGAGTCTCGGCAATATCTAGAAGGGCTT
GCAATATGGGAAGGGCAAACGCTCCAGTTTTACCAGACCCAGTTTGCGCAAGCCCAATCA
AATCTTTACCTTCAAGAGCATGAGGTATAGCTTCAGCTTGAATCTTAGAAGGATTTTTCC
ACCCTAAATTATCGCAGGCTTCAACCAGTTCATCGCGCAATCCTAACTCCTTAAAGCTCT
TTACTTCCTCCGTCTCTTCCGCCATAAAGATAACAAATAAACTTACAGACTTCAGATGAA
CTGCTGCTGCAGCAGCGACGGCGCCTGTATTATAATCACGTG
>ntig_510_consensus
CAACTCATACAAACCATTAGCTACAAAACTGGCGAATCAGGTAAAACCCTAACAAAACTA
TAATCACAAGTTTTTTCTGTTCTATACAAAATTTGAATCAAATTATCATGAGACACATGC
AAAGAAACACTATTCTTATCTCCTCTTAGTCTTTGACAATTTCTTGTTGATTCCTAGAGT
TCTTTCAACATCATCGCCCTCATCTCCACCACCCCTTCTTCTCTTCTTGCCTCCAGATTC
CTTGATTGTCATGATGGATAATCTTTTGGCTTCCATGACACGCTCCAACAATAGCAGGAC
TTCCTCTTCTTGAGCAGGAAACTCAGGCAACTTGTTGCCTGCCAATAAGCTTCTCTATCT
GTATATACCATTCCAACTCGTACTGATTCACTAGTGAGATTGCAACACCCGATTGTCCTG
CCCGAGCAGTTCTTCCCACTCAATGGATGTAATCCTTAGAGTTTGTTGGGATATCATAAT
TGATAACCATGTCGACAGAGGGAATATCAAGCCCTCTACTTGCCACATCAGTGCAACTAA
GAATATTGCACTCCCCAGCCTTAAACTTATTCAAGGCACCAAGTCTCTTTGACTGTCACA
CATATTCAACTGGCTCATTTGACCACTAATTGGGATGGCTCTTTGACCAAGATTTCTAAG
CGTCAAAGCTAAAAGGCAAGTTGCATCACATGTTCAAGTAAAAACCATCGTTGAAGATCC
TGGCATCTCAGTCAGGATATATACAAGATAGCATTCCTTGTACTTAGCAGGGATAAAGCA
GTACTGCTGCTTCAGCGTCTCAACAGTGGAATATTTAGATGCTGCTTCAATCTTCACAGG
ATTCTTCAAACATGCTCTCTGGAGCTTTCTCACCTTCTTGGTCATCGTGGCCGAAAATAA
GTATGTTCTCCGAGAGTGAGGAATAACATTCAAGATTTCATCTATTGATTTTTCAAATTC
CTCATTTAGCAGCCTGTCTGCCTCATCTAGGACCAAATATTTCAATGTGGCCAGCGAAAA
GCCTTTGGTGTTTGTTAGATGATCCATAAGGCGTCCAGGGGTAGCGACAACAACATGTGG
CCGCTTTCCAAGGGCAAGGGTCTGTTGCATCATGTCTACCCCACTGCACATCTAAGGCTA
ATTCCAGATCCTAAAGCTTCAAACTGCTCAGCTATCTGAATTGCAAGCTCCCTCGTCGGA
GAGAAAACACAAGCGAAAAATGCAGGAACTGTGCGTTGAGTCTCCGCAATATCTAGAAGT
GCTTGCAATATGGGAAGGGCAAACGCTCCAGTTTTACCAGACCCAGTTTGCGCAAGCCCA
ATCAAATCTTTTCCTTCTAGAGCATGAGGTATAGCTTCAGCTTGAATCTTTGAAGGACTT
TTCCACCCCAAATTATCGCACGCCTCCACCAGTTCATCGCGCAATCCTAATTCTTTAAAG
CTCTTCACTTCCTCCGTCTCTTCCGCCATTGTTAATCAATTGACTTCTGACGAGATACTG
CCGCTGCCGCGGTGTCGCGTACTCCGAAGTCTTGCGTCTGAACAGAGGTTTATGCTCGGG
GCTTTAGAGAT

>ntig_511_consensus
CTATTGGATATTTAATAGAAGATAAATATCAGCAGGGAATCAATAACTACCATGTATAAA
AAGAGGAAAACTTACGTTATACAAAGTTACAAAATCCATATGAGAGCATCATAAGGTTAA
TGTTTTCCTCTATAAAAAAAAAAAGCCAGCAGGTCTAGCTTAAGAGAACGTCAGCACGAC
AAAAATTCAACCATTATGTCCGGGTCATTGTCTTCCCATTTCAATTGACATCTTCCTCTT
CCTCTTCCTCTTCTTCTTCTTCTTCTTCTTCTTCTTCGTCCACCTTTCCTTCAGCCTCAT
CTTCCTCTTCATCATTCACTTCAGACTTTGGCTTGTCTGTCTTCTCATCACTATCATCAG
ATATGTTTTCCTGCTTCTTATTGTAAGCATTCATAAGCTTTTCATACTCTCCCTTCCCTT
TTGCTGCTCTGGCTTCGTATGGAGCTTTTTCGTCAGGAGACATTGATTTCCACTTCTCTC
CTGCAGCTTTCCCAACAGCTGACACAGCAGTTGCATGAGGGTTGTCTTTCTTGAAGGTTT
TCCTGAACTCCTCAAGGAACACAAAGAAAGCACTCGCAGGTCTCTTTGGTTTGTTTGGGT
CCTTCTTCACCTTCTTAGTTTTCCATGTACCACTTTTATCAACCTTTGCAGTAGTCTTTC
TCTTTCCAACTTTTC
>ntig_512_consensus
AGGACATAGGCAACAAAAAGTTATTGACTTCAGCAGCAGAGAACCCAAGTTAGAGAAATA
GTAAAGGCAGCAGACAGATAGTTCTTGAGAATCCCTGGAAAGCCTTACAAAAGGACCATA
AATTATGTCTCCGTCTTGACATGAGCTATAGAACTTATGAAGTGTGCATTGCATGTTCTC
AAATTCCCAGGCAAAGGTAGAATTCAAGTAAGAACATTTTCCCAGGCAAAGTAGAATGTT
CAACACAACCTGGGAGGAGGATGGGTGCTCATCATCTAGCAGGATTAGCAACTGGGGAAG
AGCTCTTATCCATGTAATCTGATGTTCAAATAAGGGATCACTGGCATCTAGGAACACCAT
ATCATCTCTAGGAACAAGCATTTCTTCAATTGTGGAAAGACAAGCCAACTTAAACGAAGA
CTTTGGGTTGCTATCCTCAAATGTCTTGGTAAATGCCTGAAGAAGACGATGCTTCCAACC
ACTTGTTATTCTCAGTAAAAGTTTTGAAAGAAAAGGCAGCAATAAAAGTAAATGTTTTTC
CCAGACAGCTTTACCAGACCGTGAGTCACTGCAGCTCTTGCCAAGAAGCGCATTTTCTAT
AAATTCTAGAAATTCCTCCAACAAGACTGCAGGAGGGCAGATCCACTCACTATAATGCAT
AAATATTTTTGTAATAACAATGTTCAAGATAAAATATCTATCATCGACCTTATCAGAAAG
GTGATGCATTGGATTTAGGGGAAATACACTGAATAACTTTTTCAACAATACCGACGATAT
AGTTTGATCCAATATTGCTGCATCAAGTCCTCCAAAAGAAGGTTGCGATGCTGGTTTGCT
CTGGTGGGTTCCATAAACAAAAAATCCAACTGCAAGATCTATGCTTTGAAGTATATAATG
CATACAATCAAATGATCGTGCATCCAGTGATGTCACGTCATGAACCAATGGAATAAATTC
TTGGAAACAGTTGACTAAAACTGGGACAAGGTCCTTCAGTTTCCTTGCGATGGAAGAAAA
CCCAGAAGCTTCTGTAGGTACTTCAGGCTCAAAAGGATGCAATATTCTTTCTCCAGCAAT
ATTCTTTTCACATGAATCTTGACTTGTTTTACATGGCAAGAGTGACAAGCAATACACCAA
CCCGGCAAGAGCACTTTTGAGCTTGCCTTTATCTTGTAAGTATAATTGGTTTTTCCGAAG
AATATCCTCATAATTTTGGAGAATCTTTTCAGCATACAAAGAAAAGGAAGGTGGATAGTA
CTGCACAACAAGGTCAGAAAACTTGAAAGCCATCAACCGA
>ntig_513_consensus
CTTGGACGTTAACAAATGGTTGAAACGTATAATAATAATTTTATATTGGAAGGCTTCGTA
CATTTACATAATCATATTATGGGCATTAACCCTTATAACATCCGTTTGGAAGCCATAAGA
CAAGCAATAACCAATATAATATTATCTTCATAAAATAACACAACAAACAAGGAATGCAGA
GGTACTGCCTTATTTATGCCTTCTACAGATGGATTTGGGTGGGTTCCTTCTGTGGATAAT
ACTTGATAACAATTATACAAACATATATTTTACTATCATAAAATTATGGTACACGGCATT
CTTTGATGTTCCAGATTTCCTTAGCATATTGATTGATTGTGCGGTCACTGGAGAACTTGC
CGCTTCCAGCAGTGCTTAATATGGATCTCTTAAGCCAATTCTTCCGATCCTTATAAGCTT
TGTCAACTCTTTCTTGAGCATCCATGTAGCTTGGGAAGTCATAGCCGACCAGGAAATAAT
CACCGCGGCCATAACCTGTGTTCCCTTCCAGGGAATCAAGCAGAGGGTTGTAGTCATAAC
TTCCGAATGCTCCACTTCTTATGAAGTTTTTGGCCTCCTCAAATCGGGGATCTGGTTTGA
ACAATCCATCCTCTCTTTCCTTGCGCAGTACAGCGACTTGATCAGCAGTGGCACCAAAGA
GAAAGAAATTCTCATCTCCAATTTCCTCCCTGATTTCCACATTAGCCCCATCCAATGTTC
CTATTATGAGGCAGCCATTAAGTGAAAATTTCATGTTGCTTGTGCCACTTGCCTCCATGC
CAGCAGTGCTTATATGCTGAGAAAGCTCACTTCCTGGAATAAGCACCTCCGCCACAGATA
CATTGTAGTTTGGAACAAATACTACCTTCAAATGATTATTGACCTCAGGATCACTATTGA
TGACATCACCAACATCATTCACCAACTTCACTATTCTCTTAGCATTTGTATATGTTGCAA
ATGCTTTCCCTCCAATCATGATGGTGCGTGGAGTAGTATTCTTCCGCTCCTCAGGACTCA
TCTCCTTTAACTTCTTGTATCTATAGACTGCGCCCAAAATATTCAATAGCTGTCTCTTGT
ATTCATGGATGCGCTTGACTTGTATGTCAAATAAGCTATTTGGATCAATACTGACACCCG
TTACGCGCTGTATGTACTGTCCCAAACGCTTCTTACTGGCCATCTTGGCAGACGCCCATT
CAGCTTGGAGTTCTGCATTGTCAGAAAATTGTTGAAGACCTACAAGTAGGTCAAGGTTGG

TAATCCACTGGTCAGTATCTAACCATTTTGTGATTATGTTACTAAGCTCGGGATTGCAAA
ACCGGAGCCATCGACGAGGAGTAATGCCATTAGTCTTATTTTGGAGCTTGTTCGGCCACA
AGGAGACATAGTCTGCAAACAATTCAGCCTTTAAGATGTCACTGTGTAGTTGGGCAACAC
CATTCACCGCGTGTGATGCCACTACACATAAATTTGCCATCCTAACAACTGGCTTTCGGG
GATTATCATCCAAGATGCGCATACTGGATATCTTGCTCTCAAGGTCACTCCGTGTGGCAC
GAACCATCTCAATGAACCTCTTGTCAATCTCTTGAATAATCTCCATGTGCCGAGGAAGAA
GTTTCCACATAATTGATTGTGACCACTTCTCAAGTGCTTCAGGAAGTACTGTGTGATTGG
TATAAGCTATTGTCCTTGTTGTCACCTCCCAAGCTTCATCCCATCCAAGTCCTTCCTCAT
CCATCAATAATCTCATCAACTCTGGTACCGCAAGTGTAGGATGAGTGTCATTCAGTTGTA
CAGCAACTTTGTTAGGAAATTCAGACCATTGGCATGAGCCCTTTTCACTTCTCCTCTGCT
TGAATCTAAGAATAATGTCCTGAAGTGAAGCACTGCAGAGAAAGAATTGTTGTTTTAGCC
GCAAAAGCTTCCCACTTTCATTGGTGTCTCCAGGATATAGAACAGCACAAATCTGCTGAG
CTCGTGAATGAAGCTGTGCAGCAGCTTCATATTGTCCATCATTGAACTGAAACAAATTAA
AATCCTGTGAACCAGCTTTTGCTTCCCAAAGACGAAGACTGTTAGTGTTCTTAGTTTTGT
ATCCTGGGATTGGCACATCATAGGCTAGCGCTTGGACAACTTCCCCCCCAACCCATTTTC
GCGATCCATTTGGATTAACTTCAACCTGCCCAAAGAATCTAACCAGGTATACCAAATCAT
GTCTTACGACTTCCCAAGGACTAAACTTCTCAAGCCAGTCCTCAGCAACTTCTTCTTGAC
CCTCCTTCGTGATCTTCTGTTTGAACAGCCCATACTTGTACCTCAAACCATACCCCCATG
CAGGCAAATTTAATGTTGCCATGGAATCCAAAAAGCAAGAAGCTAGCCTTCCCAGACCAC
CATTTCCAAGTGCTGCATCTTTCTCCTGCTCAGTTATTTCCTCAAGGTCATGGCCCAACT
TATTTAAAGCATCAGCATATGCATTTTGAGTGTTCAGGTTCCCAATTGCATTGGTCAAGG
TCCGTCCTTGAAGATATTCCATAGATAAGTAGTATGTCTGCTTTGGATCAACTTTGTGAT
AATGAAGGTAAGTCTCGTTCCATTGTTGAACAAGACGATCGCGGACACTTCCCGCTGTGG
CAAAGAAAGCTTGCTCCGGCTCAAACTTGAAAGGAGAAAAATGCGGACTATACTGCGCAT
GGTATTTAATATTTGACGCAATTTCCACCGGCTCATTCGATAACGGTTGCGCAGTTATCG
GCACTTTGGCTGACTCGGCCGATGCACTTGTACTTTTGGCACTTTTGGCCTCTGTCGCCA
TTACGTATCAAAAATC
>ntig_514_consensus
ATATGCAATAGTTTAATAAAATTAATCATTCGACCTTATTTTTTAGTTGAAAGCCAGCTA
ACTGGTCCCACCACCTCGCTCCGTTCACTTCCCTTATCCAAACACGTGCCAGTATGTCAT
ACGTTATCAAAAGATGAGGACAATTGATTGATTAAAGAGAAAACGACATTCACTAAGGCA
AAATTATTTTATTCTAATAACAACAAAGGCGAAGCAGCTGATCAAAACCCCACATCACAT
TAACAACACTGGACCTTATTTCACTTAGCAACTCCATAGAGTTGACTCAGTCAGCTTTTA
AGTCTGAGACAGGACACGTCACACACACCCGAAAATAAAATAAAAATTAAAAATAAAACC
ATATTTACATAAGGAGGTAGCCCATCACCATCATTGTCACCACCACCATCATCGTCATCA
TCGTCGGCGTCTCAGTGATGGTTCTTGCTCCTGGTCTCGCCACTGTAGAGCCAGTCATAC
ACAGTGGGCGAGTTGGCATGAGCAGGCTTGTCAAACATCTGAGCACCGATGCCCTTGGTG
GCAAGGTTGCTACCTGGGTGGAATACGCTCCTCCACACGTTATCCTTACGGGCCGACGTC
GGTGTCGTTGGTGTCACTGGAGTCGTCGGTGTCGCTGGAGTTGCCGGTATTGACAGAGAC
CTCTGGTACTTGCTCGACTCTCCTTCACCATCTTTGACGTTCAAGGGCTGAGTGGTGATC
TTCCTGAGCTTGCCGAGACCCCGTTCAGGGTGGGGGCCTCCCAAGCTGTCGTCCCATAGC
TTATCGAGAAGCATCTTGATAGTTGCTTTTAAATTTTCTTCTCTCCTTGTAGACGCTGGT
AATGTGGCTAGCTCGGCTCCCTGGCTTATATAGAAACATGGATCCCCAAATATCT
>ntig_515_consensus
CTAACAATACTTGATTTGCCAGTGGTCCTACTGTTACATAGGCATTTGTCGAATAAGCTG
TATGCTGAAATTCTTCATTCAAAACACGATCTACGATAAGCTTTAACACAAGTTGTTCTA
TTTCCTCTTTCTTCAAGTCAGAACCTAGTTCCTTATGCTTGGTTTTCATTTTGTCCACTA
GTTGCAACAGTGTCAATCTCTGATCATTCTCTTTCATGTCTTGCAGCAAAGAAACAATAA
ATTTTGCATGACGAGATACATCCACTTCTTTAACCTCACTTGAGTATGCACAGTTGTCAC
ACATCCCATTGCAGTCCTGTAATGGCTCAGCAAAATGCCGAAAAAAAGTACTTCGTCGGC
ATTGCTTTTTAGACTGACAATATCGTACAATGTCATAGAGATTCTGCAATCCAGAATTCT
CATAGAAGACCATTGAACTCTGCCTAGGAACATCAGCAGGCCTAAAAAAGAGCAGGCACT
CAGAAGGAAGTCCATCTCGTCCAGCACGGCCACTTTCCTGATAATATGTTTCCACTGATT
TACTCAGACTGTGATGGATGACAAACCTGACATCTGGTTTGTTGATTCCCATACCAAATG
CCACCGTACCAACAATCACTTGCAACTTATTTTTGCTCCACCTCATATGAACCTTTTCAC
GAACATTCACATCCATGTCTGCATGATAATAATCGGCTGAAATTCCTCTCTCACGTAACT
CATAAGC
>ntig_516_consensus

CGACAATTAAAAATTTTATTATTTTTTAAAAATGTGTATCATGGACGGTACATTACTAAA
CATTGAAATCAAAATACAAGACGACTACCAGGATAGTAACTAACAAAATCACCTTGTTCT
TTAAATAAAATCTTAAACTCCTAGCGACGACAAATGATGATACTAAGAACGACCTTACTC
TTAAATTATTCTTAAACACCTACCGACAAATAATGATAACAATTTTTCAACTGCGTAGAG
GGGTACCTTTCATTGGACTATCTGACTACCATAATCTTCATTGAACTTCTCATATCGATG
TGCGTAAAGATGAGCTGATGGTCTTGTGTTCTTCAAAGCAGTCTGTATGTCTTCTGCTCT
AATTGGCCCAACTTTTGGTAGTTCATCCTCAGGTACTACATCCTG
>ntig_517_consensus
TGAAAACTTATAAAATATAAAATGAAGACATCACCCAAATGCGAAGAGACAGCAGATAAA
TGCCTCTACAAATAGTTTATAGCCCCCAGTAGAGGAGTAAGAAGAAAGAACTTCAGGAGA
GCTACCATTTCCCTGTGATATAACCATTACCGTATCTCGTATGAAAATGCCAGCAGCACC
AAGCCAAACTCCCCTCAATGTTTGGCAAACATTCCTTGTGTTAGCGACGAAAAATCATAA
TCCTTTTCGGCTTGTGAAGCTGGTGGTGAAGAGACTGGAGATGCTGTTGGCCGTTGTTTA
CTAGTTCCAGTTCCTGGAGTTGACACACCATTAGCCGAGGTAGCTTGCCCCATAGCATAA
AAGCTGGATGTTGGATATGGAACAGCACCGCCCACCTGCTGTGTTGGTGCCATGCTCATA
GCCTGCATAAGCTTCTGCAGCTCAGCCTGTCCTACAACTGGCATCACCATTCCAGGTATT
TGGTTGCCAAAGTTTGGCAAATTCTGAGTGGGTAAGTTGGGCCCATTGGCTGCAGCTGCC
ATCAAAAGTGACTGTTGCTGTGCCAACA
>ntig_518_consensus
AAACATTTATTATTTAACGTTATCTAATAATCAATTTAATAAAAGCGACTTTTTAGCCAG
GTGAAAACCCCAACTGAGACTAAAAACGCTGTTATGGTCTATGTATAATCTAACATTTTA
AAAATTCCAAAGCAGGTGGTGGAAGACGTGCCCAGTACACATTAATTACCCTGTTCAACA
TAAGTCCAAGGGCTGCTTCTTTGTGGGAGAAAAAGAATTTTGGTGGTTTTGGCGTGCAGT
GTTGAGTAGAGACTTCTGTCGATTTAAAGCGCTCTCAATGTATTCTTGCAGAGGTGGAAG
GCAATCTTCGTCACCGCCTTTGATTACTTGTCCTCGTATTATCTTTGATGCCCATGAGCG
CTCTGCTTGCTGACACACATCCCTTATATCCCTTCCAGACATTTCTTCTGTAGCAGTGGC
AAATGCAAGTAAATCAGACTTTGATAAGTGCTTTGCATATGAAGCTGCTATTTCCTCTCG
AGTCTGATAATCGGGTAGGCCAAAGGTAATGCAAGAATCAAATCGACTCATTAAAGCAGG
ATCAAGGTCTTGCTTTCTATTTGTTGCAGCAATGACAATCACTTTTTTATCCTGTTCAAA
TCCATCGATCTGTCGCAAAAGCACTGACAAAATTCTACGTGTAGCTTCGTGCATTTCACT
ATCACGTGCAGTTGCTAAGGAATCAACCTCATCCAGGAAAATAATAGCACCATTAGGCAG
CTTATCTGCAAGTGTGAAAACTTTGCCCAACAAACGTTCACTTTCACCGTAATATTTTGA
CAGGATAACCTCCAGTGGCAGATATACTAATGGAGCACCCGCCTGATTTGCAATAACACG
AGCACAAGATGTCTTTCCGGTACCAGGTGGGCCTTCAAAGAGCACAGCTCGAGGTCTATT
TGATTCAAACTTGCGTCGAGTCCCACGAGCAATATCATCAAACACTTCAGGACTCTGGAG
AGACAATAATACAGTATCTTCTATTTCCCTCTTTTGCTGATCATACCCAGCAATATTGTC
CCATGATATCTCAGTATTTGAGGAATTCACTTGGGGCTTGCCAAGTCCATATATTCTCAC
TCCCATAGACTCAAGAGTAGATATTGTATTTTCTACTGGTGTCTCTTGTGTAAATCCATC
TCTAGGTTTTCTATCCAAGTTTCTATTTTGGCCCAACCTTCCACTGGCTAATTGCAAAGC
AGACACAAAAGCATCAAGCTCTGCAGAACTCAAGCTCCCTTTTTTTATGAACTCAATTTC
TGCTTTATCTGAGGTAATAAGTGAACGGAATATGAGGATGCATAAGTCATCTTCGTGTGT
AGTCATATCACTTGAATGGGTTTGATCCCCTCCATGTTTATTCAGCTTTCCTGGATGCCT
AAGCGTTAATTGCCAAGCAACTGCACTGTCCCAAGCACGCAATGACATGTCTGAACCACC
ACCATGTTCTTCAACCTTCAGTCCAAGATTTGAGACAAGGTTTGCAATCAATTGTGGAAC
TTCACATCCGGGTGGTATTTGGAACTTAATGGTGATCTTTTGGCCCTTAACAGCAACGGT
AAACCTGGGATAAAATCCGTTTGGCTGTCCCTTCTTTTTGATTGACTCTTCAATTCGCTG
CCGCTCCTTCTTTGCAATTTCCTCCAAGTCCCCATAACTTGAAGAAGATTCTGATGGCAA
TGGAGGTTTAGCATTACCTGCCTGCTCCCCAGCGTCAGAATATGCAATATTGATCACCCC
AGCACCAAATAAACTAGCTAAGATAGCCGGAAACACGGAACGCAGACCTGAAACTTCAGA
GAATAATATCGGCCTTCCTATTTCTGAAGCATTGGAATGAATTTGACGCTCGAGAAATCG
GTTAGGAAGACGGCGAGGCAAGTCACGTGAAGCAGCGCAGGAAGGGAGCAGGATACGCCC
TGCCGGAACTGTCTTCGCTAGCCTCCGCAGAGCCGCCATGCGATGGATAAAGCAGTAGTA
AGCTTTTGATCTTTCTCCGACTTTCAGAGCATACATGCCCTCAGAG
>ntig_519_consensus
TCATAAAAAAGTCCGAGAAGACCAGGCATAGAACCCAAAATTCCCAAAATTCTATGCCAC
CACCTGCCACATCAATATTCCCAAAATTCACGCGCGGATTTCTTTTTTTCCTCTCTTTCT
CCTGCATACCTCTGTTTGTTTCCCAGGAAAATTTCTTGCATCCGGAAAAAAAAACATTGA
AATTTTCGGGCAAAAAAAATGGTTTCCCCGGAAAATACGAATTGGCTGTATGATTTCACT

ATGATCGATGACATGCCTGTCCACGATGCCAATTTCTCTGTCTCTGCTTCGGGCTTTACT
TGGCCTGTTCAACGTATGAATGGGTCCACTAATGTCAGTGTGGATCCTATTGCTGATGCC
ATATTTGGGGATTCAGATGGCTTAAAAGAAAGTGGCTCAAAAAAGAGGGGTAGATCTGAA
TCGTGTACTGCTGCATCTAGCTCCAAGGCATGTAGGGAGAAGTTGCGTAGGGATAGGCTG
AATGATAAGTTTGTGGAGTTAGGCTCTATCTTGGAGCCTGGGAGGCCTCCTAAAACAGAT
AAGGCAGCTATTTTGATTGATGCTGTACGATTGGTTACGCAACTGCGGGGTGAGGCCCAG
AAGTTGAAGGATTCAAATTCAAGTCTCCAAGAGAAGATTAAAGAGTTGAAGGCTGAGAAG
AATGAGCTTCGTGATGAGAAGCAAAGGTTAAAGGCAGAGAAAGAGAAGTTGGAGCAGCAA
CTCAAGGCCATGAACACACAGCCCAGCTTCTTGGCCCCTCCTGCAATTCCTGCTGCATTT
TCAGCCCAAGGCCAAGTTCCTGGGAACAAGTTGGTGCCTTTCATTAGTTACCCGGGAGTT
GCGATGTGGCAATTCATGCCACCAGCTGCCGTTGATACCTCACAGGATCATGTGCTCCGC
CCTCCAGTTGCATAAGCCTGCAACTTATGTTTTGGGTTGGCAGGTTGCAACTAACAAATG
TATCCGTTTTGCTATTATTCTCGACTTCATTAGTTTTCTGTAGTCTTTCAAGTGGATTGG
GTGGATGGCTGTACTTGAATTTCTGTACTTTTATGAATTCTTGCTGGTGTCACGTGTAAA
TTTTAATAAGCTATGTGGCAACCCATATTTTTCAAAATTTGAGTTGAAGCCC
>ntig_520_consensus
TCGCACTACTACATCGAAGTGATGAGAATTGTTGTGAAAGATTTACCAGAGCACGAACAA
AGTAGGCTGAGTCCAAGCATGAAACTATAATTGACTGTCTGTTGACAACAATATTTTTTA
GCAGAACATCTATGCCAACATTCTCTACCCTATAGTTAAAAAGTAGCCCAAAACATTATA
CCACTTACCAAACAGGGTGGAGAGTTCGCCTCTCGCAGATTCAGCATCCCATTAATACAT
GCTAACTACCATAAAGATTGATCCCTTTAACAGTGTTCAATTGATTCCAATATGCAATTG
ATTTTGAATATGACAACATAACAGATTTCCACAGCAGAATGAGAACCGAATCAGTCAACT
ATGATCCACACGGCCTCACCTGCAATAGGTCTTTCACATGCAGAGTAACCCGGCAAATTT
AGGAAGACTCAACTACCCACTCGTTAGGCAATGAAGATCCAAATATTTGAAGCAGCAGCA
GTCTCTTTTTTCTAACCAAATTCTTTTTGCAGTACTTATGAACACCAAGTCTAAGTGCTT
CTTTTCCTTCCTGTAAAGTTCTCTTTCTAATGGCTGTCAGCAGACCTGACCCTTGTGGAT
CAAAGCCAGTCACCTTTTTCACACTTCCGGATATCTCATTGCTCACTATTGAAGTGGCAC
TGCCATCTGCGTTCTCTTCAGACTGATTTTTGTCTACTTCATCAGTTTCAGCTTCCTCCA
CATGAGTACTCAAGTTACCTTCAGAACTGCTGCCACCCCACCATTTATATGTAGCAGCAG
ATAAGCCATCTGATGGAAATCCATATTTAAATTCATCTGGAGCCATGCTTCCTTTCGATG
AGCTTGGGTTGAGCAAGATCTGCAACTGTCCAACTACAGTGTTCTTGCCTCCTTGTTGCC
GCAAATTAGTAGGTGATGAGACACTGTGCAATTTAGACATATTATTGTTTTGGGATTGCA
CAGAGCTTTGCCTAGCAAGCTTTGAAACCTCAGCAATTTGTTTGTATTTTTCTCTAGCAG
CATACTTTTTAATTTCTTCAATGCTCTTGCCACATAGAAACTTGCGATAGGGCCACCGAT
CAAGGCCGTTCTCGCGGCAAATTTTCTTGAGAACACTCACGCAAACGCCGAGATGAGTAG
CGGCGTCAGAGAGGGGCAGAGAGAAATAGTTAGCGACGTCATCGAACGAGGGAGATTCAG
TTGGGTTGGAGCCTAACTTTGAAATATTTTGTACAGAAATCATTACATATATTTATTAAA
ATACCTTTGAATTAAATAAATAAATAAATATTAACACAGATTTGAAAATGAAAGGGTTTT
TGTTCTCCCTGCTCTTCAAGTTGTTTCGCACAGTCACAGAGCTGACCCAGATCCAAACCC
GAAAACACGTTAAAGGGTTTAGTTTTAGACTTTTTTATTTCTTTTTTCTCTAATTTTGAA
CGGCCTCTGATTTTTGCCACCCCTCCGCTAAAGAATCATCCATTGAATTTGGAACTTGCT
GGAACTCGACTGTTCTTG
>ntig_521_consensus
ATTTCCTTGCTCATCCTATCAGCAATGCCAGGGAACATGGTTGAACCACCACTGAGAACA
ATGTTTCCATACAAATCCTTCCTAATATCGACATCACACTTCATAATGGAGTTGTATGTG
GTTTCATGAATGCCTGCAGATTCCATTCCAATCATGGATGGCTGGAAAAGGACTTCAGGG
CATCGGAAACGCTCAGCACCAATGGTGATCACTTGCCCATCAGGCAATTCATAGCTCTTC
TCGACTGCAGAGCTGGTCTTGGCAGTCTCCAACTCTTGTTCATAGTCAAGGGCAATGTAT
GCCAACTTCTCCTTCATATCCCTAACAATTTCACGCTCGGCTGTGGTGGTAAAGGAATAA
CCACGCTCTGTCAAGATTTTCATGAGGGCATCAGTAAGGTCACGACCTGCCAGGTCAAGA
CGGAGGATGGCATGAGGAAGGGCATAGCCCTCATAAATGGGGACAGTGTGGCTGACACCA
TCTCCAGAGTCCAGAACAATACCAGTTGTACGACCACTGGCATAAAGAGAGAGAACAGCC
TGGATGGCAACATACATTGCAGGAGTATTGAAAGTCTCAAACATGATTTGGGTCATCTTC
TCACGATTAGCCTTTGGATTGAGAGGTGCTTCAGTAAGCAGAACTGGGTGTTCTTCAGGG
GCCACACGAAGTTCATTGTAGAAGGTGTGATGCCAGATCTTCTCCATATCATCCCAGTTG
TTTACAATACCATGCTCAATTGGATATTTCAGAGTTAAAATACCCCGCTTGGACTGAGCC
TCATCCCCAACATATGCATCTTTTTGGCCCATGCCAACCATTACCCCAGTGTGACGTGGG
CGGCCCACAATGCTTGGAAACACTGCCCTTGGAGCATCATCACCAGCAAATCCAGCCTTA

ACCATTCCTGTCCCATTATCACAGACGAGTGGCTGTATATCCTCAGCATCTGCCATCTTC
TATAACTAGCTTAAAAACGAAGATTGAAAGAAAGAGGAGGCTGCGATCTCAGCAGCGCGT
CCTTGGCTTCAAAAAAGGTAATGAAAATTGAAACTACCCTCTAGTGAAACTCTATGGC
>ntig_522_consensus
CAGATATATTCTAATCACAATAGATTTGTAATATGCCGATTTATTTTCTATTAACTTTTA
GTTTTTACATTACAAGGAAGAAAATACAACTATACCTGCTGATATTTTCTACAAGAGGCT
GCTCGACAAGCAACACCAAACAGGTTTTGAAAAATACTTAATGCCTGATGGTAAGCTCAG
CCCTTAGATGAAGGACGCCATTGATAAAATAAAGGCTGTCATCCGCCATAAATGCTGTCC
AGGATACACCAAAAAGATTTCTATACCCAACAGCCTTCCCTCCTGTGAAAGTATAATTCC
CTTTATACTTGCTCAGGTAGTCTTCAGATGGCTTTGTTCTTGCAGCAAACTCATAGTCGA
CAGCAAATGACACTGATCCTTTCTCTTGCATCCCCAGAAACAAACCAAAACAATGGAATG
AACTTTGTTGGTCCATATTGCAGTGTGCTGACAGAAAAAAGCCCTGCCCACCAAGGTGAA
ACGCCTGTGAATAAACCCGTCCAGCTGGAAAGAGATGAGCACACTCTTCCTGCTTAAGGT
CCAAGTACACAACACACTGCTGACGAGGCAATTCGAACTCCACCACCTTGACAGGGCGAT
ACTTGTAAGCCCGCTCCACAAAGCGACGATGAATTGCGTTAGCCTCCTCTGCTGCAAGAG
AACGCTGCCGGTATGGTGTCTCAGCCTTAAAAAAGAGAGACTCAAGCACAAGTTTAGACG
CAAAATCAGGATCAAAGTCATTGCAGGTTAAAACTTTCTTCAGCTTGCGACAAGTCATGT
ATGGAAAACGAATTAGTCGGCCAAGTCGAGAACCAAGGACTTCCCGTCGTTCCTCTAATT
TTGGATAATGAGTCCGAGCCCACTTCAGCACAAAATCATAAACAGCATCCTCTGAAGCTA
TCTGGAGTTCATCACTGGACAATACTGCCTCAATACCAGCCAAGGGCAAGTTAAGAACCT
CTTCCTGAAATTTAGTTATGTCCTTGTATCGCGCAGCCAGAAATTGTTTCGCTGCATCTG
TCAGCGGCTGCACAGCTTCAGCCATTAAAACAGTAGAAGGCAGGTCCAGATACAGTAAAG
CAGACTCACAGGTCATTGGCAAGTCCCTCAATAACCTGCTGCAGTATCTCATGCATGACG
CAACCTCAAATTTGTCAGCAGCCATTAGCACGTCCAGCAAAGCTGTTGGAGTGCTTGTTG
ATAAGGTTCTAGTATACATGAAATTGAGGAGGTCCATAAGGGCTGCTTCTTCAGAGGCAT
GTATTCGCAAAGTTACATGCCGCTGCTCTGACTCCCTCATTCCATTAGAAAACAACTTAT
AGAAAAAGGGACTCTTTGCTGCCAAAATAGGAGAGCTAATATGTACAGTCTTGACCCTAA
GAACAGTTGAACAATCCATAGTCCACGATGAATCATTGCTATGACTGGCTTCCTCACCAT
CACAATTCAAGCACAACTTCAATCCAACATCAACAGGTGATTCCTCAATCATTGCAACAG
GTTCGTCATCCTGATTCTCATAGGCTACACCATCCTCGCTGTCTGGCATGTTACAATGTA
AAATCTGTTCCTCCCGTTGCATGAAAACATCCACGCCAGTGTCTTTCTTCATCTCCTCTC
TCCGTCGTTTACGGTTGCGGGCCCAATCGGCGATGGAGGAACAACCGTCCCCGTCGGATT
TAGA
>ntig_523_consensus
GCTTATCATATCATCACTTACCAAAAAAAAAAGTTCAGCAAATAGAGTAGAATCATTTAT
GAACAGTAGTTTCTCCATGATCCCTTAGCCCTCTGGAATCTTAATAGGTTTAGCTGTAGT
AGAGCTCGAGGCCCATGCCATCATGGATCTCGTAGTCCTTAAGAGTGATATGATCCTTAT
AGATGGTATACCACTTCTGAATCCGAATCTTGTCGGCTCGAGTACCGGTTTGAGCCGCCA
CCAGCTTCTTCAAGTCGCCGATCGTATCGTCTTCGTTGCACTTCACGCGCACCTTCTTCC
CTAGCCGATCGTTTAACACCACCTCAATCATCTTCTCTCCCCAAGGTTCTGTGTCTTTTC
CGCGTGGCTTTCTTCTGATTCTCCCCTTTTTGCTTGCCAAGTTCTTCCTTCAAATAATAT
ATAAAATTATCTCAACCGCCCTCGCGCCGAACGGAATTGACCCAGTAAAATCTAAACGAC
GTCGTTCAAGGGTTAAAGTGGCAAAATCGTACTCGTAAACA
>ntig_524_consensus
CTTTTCTTTTCCCTGTTTCTGTTATTCGTCCCAGAGGCAGTGGTATGTAGAGTAGATGTA
ACTGTCCTGGAGGAATGATTTTTGTAAATAACTGAGAGAGACCTGATGGCTTCTGTTTTG
TTTGCTTTCTTCCTCGTTATCTAAATTTCACAATAAATTTCTGCAGATAGTTTGTACTTT
TAACATTAATAAAATTCGGTTTACTCTTTCTTTTCAAGGCTGCTTACTTTCAAACATCCT
CCCGTCAGTCCCATCGCTTTCTCGGGTAGCTTAGAGTATTTTCTAAGATAAACATACAGT
CTTCTGCATGTACAATACAAGAGATAAAAAATCAAATATAAACAGAAGAATGTACTAACA
AATAAAAGAGATGAAAATCTCTTAATAACCTTTATTTACAAGATGGGTTTTAATTTTTTT
CCTCAAAGAAAAAGAAAAATCTGTTAATTATGGAAAACAATTTGTGAATCCTGCACGCTT
CCAAGGACAATAACAAAAATTGCGAGTGAACACTGTTACTATCTCTGCATGCCAAGGGGA
GGATATATTGTATTTAACCAATTTTCACCAGGGTGGTCTGAGTGGTAGCTTGCAGTTCTA
TTTTCCATAGATTCATCATAATTCCATAATGTATAAATTCGTACCTCTGCCACTCTTCTC
CTCCTGA
>ntig_525_consensus
TAGGGACTAGTGGTGGTGACAATGAGCTCATCCCCATGAACATTTGTAGTCCTTGTTGTG

ACTGCTGTCAACTGGCTTGCTTCCTTCGCCTGTTTCTCAATCTCAGCTATCTGCTGCCTC
TGTTGGGAAGGTGGAGTAATCTCAGCTCCAAGGATAATATCACGGATCTCAGACTGAGTC
AGAGCTGAGGTATTCACATTGTTCTTCTTGGCATAGTCAGAGAGTATGAGATCTCTTAGG
GCAACCTCAACCTTCATCCATTGATCATCGGACAAAGAAGGCCAGATGTGGTGTGGCTCT
GTTATAACTGTTTTGTCAGGCTTTAGTAGCATCTTTGCTTTCTCATTATTCACATGAAGT
GCACGAAGAATGAGAATGAGACGCGAGAATGCAGTGTATGATGAGATACTCTTCAGCCAA
TCATCATAGATATTGAACAAAACCATTTGAGGCTCTGTCGCCTTCAAAATTAGATCACCA
AATTTCTCAATCTTCAAGCAAGCCTGGAAAGGAAGCTGCAGCTCACTTCCTTTGATAACT
ATGTTAGGAAAATCCAACAAGTGAACCTCCAATGGATCCAACATTCCTTTACGAGTAACA
ATGATTTGTTTTGGTTGCTCCTCAACAGGCAGAGACCGAACAAGAGCAGCAACTTCTTCT
GCTGTTTTCCACTTCGCTAACTGGCCAAGACGCTTCTGACCTGCCCATACACTTGTATGG
ATGACCTTAAGGAACAGCTGGCCAGTCCTCGGATTAAATATAAAGATAGCGCCATTGATG
GGTTTAGTTGTGAGATTTCCTTCAAATGTTTTGTGGATTGTAACACGATACACATTGGTG
TCATCCACAAACCATATTATCTGATTGCTGAAAATTTCTCCATAGTTTTGCGATGATAAA
TATGGTTCAGTAGGTTCGGAAGAGTACAACTGCAAACCTTTCCTTATACGCTCTCTCAAG
ACATACAAAGCTGGATTTGACTTCATTATCTTATTCATTGCCTGAGCAAGCAATGGTTTT
GATCCAGGGAACCAGTTACCAAATGCAGAATGCAAATTATATGCCAGATCAAGCCCAATC
ATCACCCCAGTTGGAGATGGATAGATAGACATGTTGTCTGTTGTATAATCCATAAATTTG
GCCCGAGTGTACCGCTCAATATCATGAGAGTCATAATCTCCCCAACGAAGTTGTACATCT
ATCCAGTATTTATTACTTGCTTTCTGGTCAAACACATCCTTTGACTCGGCGACAAGACTA
GGCTTTGACATTGGCCATCGATGAGCAGCAAATAGAAGAACATCTGCACAAGAGCTGTTC
ATTTTGTAACTCTTCCTTGGATGGATTGTTTCTTTCTGCACAGTTTCAATTTCCAAAGCA
TCCAACTCTTGATCCAAGACCTGACAGAGATCCATGACCACACTCTCATGAATCTTTTGC
CATAGATGTGCACGGAAGATCTGAATCAGAGATATCTTCAAGGTAGGAATCTTCCCATGC
ATGAAAATTCCAGTTAAATCCAACTGCACTTGGAAACCCACATAGACATTGGCCCTATTT
ATTGTTGGAGACCACCAGAGCGTAAATCGACGGTTAGGAATTTGATTCAGACCAGATCTT
TGTGCATTAGTAAGCTTCTTATATTTCATTGATTCCTCAAAACCTGATGCCTTTTCCCAG
AAGAGACCTTCCCAGGTTGGAAAGTATGTTCCTTTGAACAAAGTGTGCTCAAGAATGCCC
TCAACACCTCCAAGAGCTTGAATGACATCAGTTCGATAGTTGTTCAAGTTCCACAATTTA
CCATCATGCCTTTGGTGGGTCCACCAGAAAGGGTTCTGTTTTAATACTTGATACTGCTTA
AAGTCTGTGCGCACTCTCCAACCTTTATCATAGGCTAAAGTATGCCTGTCCTTCTGGAAC
AAAGTATTTATGCGAGGTATTCCCCTGTCCCATGAATCTTCCAGATCCTCAAGGGTTAAA
CGCCTATTCTGGGCCTGAGCTTCCTGCCTCTTCAGTGCATACTCTGCCCAAACACGCTGT
GAATCTATGAACTCACTCTCCCACGGCTGTATGTAACGATAAAGATTTGGAATCAGTTGG
TCCTCCTCATGGCTCATTCCACTCCTGAAATGGGTAACACCAACATCTGTCTGCTGACTA
TACCGGAGGTCACTTTGAGGAATCAATATGTGACCCATGGATAGCATTCCAAGTCCTCCA
ATTTCTTTTGGGGTATAAAAAATGACCGGAGGAAATCTGCTAGGCATTTTTGAGTTCAAC
CCAATCTTAATACGTGTTTGGATTTTGTTTTCACATTTGACCAGCAAATCTAAAAGTTCT
TGGGTATGTACAGTTGCTTCACGGAAGTAAGTCATGAGACCTATCAGGGCTGTATTCCAT
TTGTTGACAATTTTGGTGAATGTCGTTGACCCTGAAGACATAAGAATTTGCCTAACGCGG
TTCTCAAATACCTTCATGTGTTCATCATCAACTCGTAAGAAAGCAACTGCAGTTCTCTCT
TTGGTCTGTTCATTCTGCAAATTCCAGACCCCATCCCTTGTATTGCTGAATGCTTCTTGG
GTCATTCTTATTTTAGGAAGTATTCGAACTTCAAATCCACACATGCTGAAAAGCAGATTT
GGATTGTCCTTGCTGTAAACAGAAACAAAGCTGTTCTCCCACTCCAAAGTTGTAATGCTT
CTGGGAAGACGGTTCTTCATATCCCAGAAAACACTCCTGCCAAGATTGACATCATGTTTC
ATTAGCCTCATTCTCGCATCTCGTGGCCAACACTTCTTGTTATTGTATCCAACCATATTC
TCATTGTTGGGATCAGGATGCTCAGTAAGATAACGCTGGATAAGATCTCGAGCCTCTTCG
TGAGTGAAACGGAACAAGATGTGAACCCTATCAATGTACCTAGAATACAGCCTTATTGGA
TGCCTTGTCTCAACTTTTGTGTCCCAGTAAGTGATGAACTCATTTGGCATCTGTGGTGGT
CCAGCAATCTCACTAGCACGTGTCAAACCAAGAAGCAAGAGATCCAGCACAAGTCCATAA
TACTGTACAACAAATGAAGCAAACTGAAGACCCCGTATAAGGCCATAAGAGTTTGTATGG
CTCATATCTTTATAGGACAAGACAACATTGTTCTTTGCAGTGACATAATCGGCAATATTG
TGGTCAAGGACCAAACGCAGAAGCCTGTTTAGCATGGTCAAGTCAATCTTTTCAAAGAAC
TTCTCAAACTTAGTTTGAAGCATCACCACACACTGCCCATCACTTGTGTCCCATATTCCT
TGCAGATTGTTTATCCCCTGACACCATTTATAAACCAGAAGAGGAGGAGGCTCTGAATCT
GCAGGCTTAATCCAGTTTGGAAAGAGGTGTCGCTTGTCACCTTCATACCATAAATACTGG
TCAAGATAAGCATCTGTTATTTTCTCCAGTGGCTCAATTTCATAAACTGGAATCAAGTAG
CTGTACAAGTCCATGAACTCAATGCCAACTTCTTTAAAGGCACGCTGGGTGAGCAGATGA

CGCTTAATTCTAGACAAAGCCTCATGAGGATTATCATATGCTTGTTCAATGAGACCCAGC
TCTTCTCTCTGCAACTGATTTAATCTGACAGCCACACTGTACGACTCTTTTAACCTCTCC
AGAGCAAGAATAAGTAGCTTTGTGTCATGCTTGTATGACAATGGTGGAAATGGAATGGGA
GAAAACTTTCTTGATTCCAACCAATGCACAGTTGTTGTGTAAATAGCAACAGCTTCTTCT
GGGGTAACATATGGACCATCTTTCAAATAGTTGTGTTGTCGTTCCTGTTCGGCTTTCAGC
CAAAGGCGAGTCAATCTTCCAAGATTCTTCCGACAAACGGTCTTATCTACTGTTGCACCT
CGTCTAATACGCTCACGATTGTAGTGGGCAACATTTGTCCACCAGTCAGCTTTTGACTTA
ACATACCGAAGGATCATGTTCTCAATGGGCACAGGCAAACCAGGTACCTTCCAAGGTATG
TTTGCTTTCCAACAGCGCCATGCCTCACTAAGATGCTGCAAAATAGTCCTGGCCTTGTTT
TGCTTGATACCCTCTGGCATGGCATCAAGAACATCATGCATGACAGCAGCTCGAAGCTCC
AAGTCAAAATGACTTTCAACACGTTGCTTAGTGACAGTCTTCGCAACTCCTTTTGAATGG
CGCCCTTCAAATTGTCGAGCCAGTAAGTTTCCTAACCATCTTTCCAAGAGAGGCACTATA
CCACGAAGGAAAAATAGCCACACCCTCCACATTGGAGCCCAAAATCCACATCCAGGTCCC
TTCCCCACTGGCCCAGTGTTGAAGCGGTAGTAGATCAAGTGCTTCAAGTCCTTGCACATC
CTAATCTGGCGCATTAGCCTGTACTTGTAACGATACATACCAGTCAACTGACCCACATGG
GAAAAAGTATATTGCAACCCATCAGCCAATTGGAAGGCATCGACATTACCCAAGCGGAAC
TGGATATTGGCATCAACTACAAGCTTTGTCAAACGCAGAATTTCACGGCAAAGATGAAAA
GCATTTCCAAACCTAGACTTCTTACGCTCTTTAGTAGTGAGAGTTTTCACAGGCTTCAAA
TTGAAGTTGTAATCAAGATGAAGGTAATTCAAATTTTTTCTATGAATCAACAGATTCAGC
ATGTTGTACCCCTGCTTGCAAACTTGAAGACCTGCTTCAGCCCAATCAAGCTCTGTGGTT
TGGAAGAACTTTGTTGCCTGGAGAGACCGGAACAAGTGCTTTTTCTTCTGAGCCTTCGGC
GGTCGATGATGCAGCTCATTCAACACAAAACATTTCAACAACTTTTGATAGCTAACTCGA
ACTTTTACAGGATAAGATGGAGGACAGTGCTCCTTATACCACTCTGAAACAAGGGGTATA
TCCTCAGCTCGTCGCATTCGACCCGATCTCATGTTGAAGGGACGTGGAGCAAATAACAGA
GAAATTCCAGCAGCTGTAGTATCAGTGTAAAGTTGAGTATCTTTCAGCAATGGCTCCACC
TGCTCTGGCAGAAAAAAATCATCATCCTCATCATCCCCATCATCATAAGCCTTCTTTTCT
CGCCGATCCTTGCTTGTGGAAGTTATGGGGTGTATCAAAGGATCATAATAAAATGCAGGT
AAATCAGGATCCTCAGTTTTTATGTACATGACCATGGGAGTATGGTATACCCCAAGCCTT
ACTTTACGAGGCCTGTTGTTATAAAGATGTGGAAAGGCGATTCTATACTCAGTCCTCAGA
GGTGAACGAATAATGAGTTTGTTGATATCATTGAATTCATTCCAGTCCTCATCACCCTTC
TCCATGTCACGATACAAAGGCTCAAATTTAGGGCCACCAGGAATGCACATATTTAATGCC
TTGGCTGTAAAGAATGACTCCATGTCAAACAAGTAGAAATAGTTGCGGTCAATTAAATCG
GAGAGTAGCTGCCCTGCAAGCCGGTGAAGAGTAGCCATGATTGGAAGAGAAAGGTGCCAT
TTGCGGTAACTTGGACCATTAATAAGCTTTGTTTTCACAAGAGGCTTATGATCATAAAAC
CAAGTATATACAGCAGAATCTTCTTCTTCATCCATCTCTAGTTGAATCGGTTCCAGAGGA
TCAACATCTAACAGATTATCAGCATAGTCTAATGGAGGTTCCTCATCATCAAATGGTGGA
AAACGCATTCTCTTGAAATGCCGACGATCTCTCTTCTCTCTTCGCATCATGATCCACATT
GTACCCCACTGAGCTAAATAAATTGGTTCGACTACCCATGGAATTTCATTCACAAATGTG
ATGGCTCCAGTAATATGATATAAAACCTTCACATCACGAACTTGTTCCCATGGCATTGGC
ATATTCTCAAGGAGCTTGTAAACTGCATGTGGAATGAATTTAAGCGCCCCTAGATACACG
CGTTTATCATGACGAAATTTCTTCGAGGACATATCCCCATGGTCTCTAATAATCTTCCTC
ACGTGCTCAGGAGGCATGTCCTCCTTCTGGGCCTCAACGAACCCGAACTTCCTTTTATCT
CCATACCTCTTCGAGTTAAGTTGCATCCATTTTCTAGCTTTTTCCTCCAGCCTTGCCTCC
GCCTCTTGAGGCGACGGCGTTGTTGTCAGCACGGTATACGAAGGCTGCGACGGGGGCGGT
ATTGGCACGGCTCCACTCGTGCCCGGCGGAACTATGGTCCCCCCGTTGTTGTTGTTATAC
ATGGCGGCAGATTAAGACTATCCCGTCTTTAAGATCTGATGCCAAAATGAAAATAAACCC
GAGAAACCCCGAAGCAAACCCTGCCAACGTGTTGATATGATTGTTGCTTTTATTCTTGTG
ATTGAATTTGGGTTTCGCTTTGAAAATAATACCTAGGGTTTAGGGGCTCTCGTCTGTCCC
TTTGAGGTTGTGTTACGCTCACTGAAGTGGGAAATTTGACAAGAGCGATGTGGGGCTTTG
TTTGAAGATGGGTGCGAAATTCCCTTACG
>ntig_526_consensus
CGCATAAATAATTAAAATCCAATATTCCTCAAGATAATACCCAAAAAGCCTTATTATTAT
TTACACCCAGTTACATCTATCGCTGGGAAAGAACTCATACACTAAAAAAATAAAATCAAA
GTCCTATAATTTAGCTGAGGAAAGACACAAATTAGTGCCAGAACTACCACGAAAGAAGAT
ACAACGAACCATTACCAAAGGGATTTCTGGGTTTCTGATTCTGATTGATAAATGTATGTA
TAATCAGTGCTAATGGCCCAGCTTACGCTTCTTCGCCAACTTTCTCATTCGCTCGCGCTT
CTCCTCTTCCTCTATAAGACGTAGTTGTTCTTCATCCTCTTTTTGAGCAATTCTTGCACT
TCGTTTCTCCTCTCTCATGATATCCTCAAAATTTGCCTCCATGTCACTATCATCATCATC

ATAAGTGGCAAACTTACGGGGTCCCAACATACTCCTAAGCATACTGAAAGCCTCAGCATC
TTCATCATCAGAATAACTTGGTCTCTTCTTGGGACGAGTATCTGGAACACGTGATGAAAT
TTGCTTCAAAGGCTTGATTACCTGAGACTTTGACGAAGCAACCATTTGTTTCGGTATTGT
TTTACTCCTATCGAGTTCCTCAGTGCCCCTTCTTGGTTCTACGTGTTTCTTCTGAATAGA
TGGCAGCATCTTTGCAGGTGGTACCTTTGCACTGGGTAAGTGATTCTTTGCAACTGGGAT
AGAAGCCCTCTTCTGCAGGGAGGCCACGGGCATTTTGGAAGGCTGGGGTTTTGACCCTGC
AGGTCTGCCAGGACCAATTCCATTGCTACTATAAAGCTGCCTTCTAGAATCAATTGGTGT
CAAATTGGGTTTACTTGCAGAAGTAGGCCTGTTAGTTCCTGATTTAGAATGCATTTGGCC
ATTTAGAGAAACTATTTTTCTTCCACCAAAAGCCAAATTCATTTGGCTATTAGAAGAAAC
TGATTTCCTGTCATCACGAGCTAAATGCATTTGCCCATTAGAAGAAATTACTTTCCTTTC
TTCATGGGCAGCATGAATGCTTCTGCCATTAGTGCTCAACGATTGTTTGCTCCTTGGTGG
TACTTGAGCTGATTGCGCCTCAGAATTTGGAGCCGAGACATTTTGAGATACAGGCTTCTT
TTTAGGTGCTGGAAGCACTGCATCATCAGATAGAAAGGAGTAATCTCTTGAGACTTTAAT
CCTTTCAACTTTCCTTTGCATCTCATTGACTTTAGGCTTCCAACTGTGTACTCCATTCTT
TGATACTGTACTATTTGAAGCACAGATCTTTCCATTCTTGTGCTGGGAATTTGAAAGCCT
GGAAGCCAAATGCTGGGTTTCTAATATTGACTTGCTTTCTCGGAGTACTCTCTGAGAAAT
AGCTGGCTGAGAAGGACCAAAGAAGGATCCAAAATTGTCATATGGAAATCTGTTCCTTTT
TTCTTGAGAACTAGCCCGATCAGAGCCATTTTCTCGTTTTAAATTTCTTCTGTGAAGTTC
TTTTAGCTTTTGCCTGTATGCAAGAATTTTCAATTCTTCTTCTGTTGGTTTATGTTCTTC
CTCTTCATATTCCTCCTCCGCCCCCTCTTCACCATCTTCATATTGCTCTTCTCCTTCATC
CTCATATTCATCATCTTCTTCAGCTTCCTGAATCAAGCGATTTTGAAGAGAGAAACGCAG
GGCAGATCGAGATCAAGATTTAGATGAAACGAAACGACAACATAGAAGAAGGCCTTCTTT
GCGGTCTGCGGAGTCTTATTGCGTTAATGCGTGTGGCGTGGGGGTACGGGGGGATAATAG
ATTAAAGAGAGTGCGAACCGAAGGACCCAGAACGTGAAGGGGGTTGTGCTATAATACGCC
TCTAAGCGTAGTGCGCACCCCCAAAGTTTCAGCC
>ntig_527_consensus
GTATCAAAGTCCCTTACATATAAAAATTATGTATGATACTAAAATTTTTTATGTTCACTG
AAATTACAAACCAACAAAATCCACATGTATAAAAAAAGGAAAAAAAAAAAATCAATGAAAG
CTTTGGCAAAAGAAATATCCATCTCAAACCACCCCAATCCTTTTTGTATCTACAAACAAC
TCTCCACCATCTTCTTCTGTGGCTTATTGTGTCTTCACCTTCAGCTGAAAACTGTAGCCT
GCTTATGCAAATATCAAGATGCTTTTTGTACATATCTCAGTTGCAATCCAACCCACAAAC
TTCTAACACAACACCGCCTTTTGACACAAATGGGTCGACTCTTGCCCAGAGAAGGGAGAA
TATTGAAGCTAAAAGTATTGACCACACAACAATGATGGTTGGAATCCTGTCTTGTTTTCC
CATGAACCCTTTGAGAAATGGGTAAAGATGGACAATGACCCATAAGGCAAAAAAGAGCCT
ACCGAAGAGAGGACCCCATGAATCATAGCCATTACTGATGGCATCTGCTACTCCTACTAT
GACTCCGATGATGTTTATGATCAACAAAGTCAAGGGAGGGACCAATAAAGACGTCCATTT
GAAGAGGTAGAGCTCTGAAAATTCTCCATCGTCAGCTGCTTTGGAGGTAACAGTGAAATT
TGTGTTAACTCCAGCCAACACCTTGAGCAGACCTTGGATGAGGGCAAACAAGTGTGATGA
GACACCACCAATCACCCAGAACTGCTCGTTTCTCCACCAGTCATGTATGCCAACACCTCC
CCATTGCATTTCAAGTATGCTAGTTGCAGCAATGGATATGAACAGGGCCATGAAAATGAT
ACTGGCATAGTTGCTAATCTCAGGGACAATGAACTTCCCAGTAAGGAGGCAGACAGCTGG
CAATGTACAATATGTGATCAAGGGAATGGATGTCAAGGGGTAAACAACAGAGTTTATATA
CGAGAAACGCTCCAATGGTTTCAAGCCGCACCCGAATCCATACCATATTGGACAGTGCCT
GCTAAAAAAGATCTCAACAGATCCCAGAGCCCATCGGAGAACCTGATGTAGACGATCCGA
CAGGTTAATAGGAGCTGAACCCTTGAAAGCAGGCCTTTTGGGTATGCAATAAACCGATCG
CCAACCATGGCAGTGCATTTTGAAACCAGTTAATATATCTTCTGTAACAGATCCATATAT
CCAGCCAACCTCTTTACCCCACTCCGTTTTGTCCTCGTAGCCACAGCTAATCACATGTAT
GGCTTCTTTCAACAGTGATGCCGATGAAGCTCCTCTAGGAACTCCGCCTTCTTCCATAAG
TGTTGAAGATATGAAAACAGGGGATTGTCCAAATTTCTTCTCTAACTTTATTTGGGACAT
CAGAGCTGACTTCTCATTATCCACTCCTTCAATTCCCTCTATGTTTTCTAGAGCATGTAT
CTGCTTTAAAGTCTCCTTGTCATTCTTTTTCTCATTGGACTTCATTTTTCTACTTCTTCT
TGATTTGCAACAACAGCAACAGCAACAGTAGCACCATTTCGGGAAACAATTGCAGGTCTT
CCTTGGAGGTTTTTTCTTGATGGGGGCATCATATCCATAGAGTGCCTGCCTCCTGAAAAC
ACACCCTGTTCCAACATAAATGGGCCCTTGGATCCCATCCAATCCTTTCATGTTGATATC
AAAAAATACAACATTGCGGTTAGAGTATCTATCATTACGATCAATTCCATCAAATCTTTG
AGGAAATTGCACATAACAGATTTTCTTTCCTGATGTGGGGTCCATCATAAAGCACATGGC
TTCACGAAGTGCCTTACTGTTGTTTATATAATGATCACAATCAACATTCAGCAGGTAAGG
AGCATTCGATATGATTGCTGAAACTCGGATCAAAGCATTCATTGCTCCAGCCTTTTTGTG

GTGATCAAATCCTGGTCTCTTTTCACGAGACACATAAACCAGACGAGGTAATTCATTTCC
TTCAATATCATGAACCCCATTATGGCCAAGGAAGACCTGAATCATTCCAGGATGATCCCT
CACATTGTTCCCTGGCCAGGGAGTTCCATCCTGCATTGTCCAACCTTCCTCAGGAACTTT
CTGTGCCATGGCAACCAACCCATTTATCCGAACTTTAAACTCTTCATATTCCCTCTTTAT
AGCACGACGCTCCCTCACAAATGTTGGGTCTACTTTATTTTTTAAATAGTCAACCTTCTG
GGCAAAATACCACTCTGGAGCCCGGGGCTCAATACT
>ntig_528_consensus
GTACATCTCGGGTTGAGAAGGAGCTGAGGATGGGAATATTATCAGTAAGGCATGCCTTGG
GTTGTTAAACAACATAAAGCCATTCAATTCTCTCCCAAATGTTACCCGCTTATCAAGGTT
TTGAACAACTCATTCCATTATTTTTAACCAACTTACTGTCTTATTAGTCGTTCAACAGAG
TTGCCAGCACAAGATCTTGCACAAACCCAGCTCTCCCAGCAGCTTGTCTCATCCTTTTTT
CCCGCTCTTCATGACTCAAAGAAGGATCAAAACTTGATTTCCAGATATGTGTTGGTGCTG
GATTCTTTGCAGCAGTGTCCTCAGCACTTGTTACAACAGACTTTGAAATTTCCTCTACTT
CAGATGAAGGAAAATTCAAAAACAGAGATGAAAGGAAAGGATCAGTTGCTGCATTAGATA
CATTCGCATTGTTTGACCGATATCCACCACCGCCTAAAAGCACTTGAAGATGAGCTTCCC
TGACATCTCGGCCCAAAAGAGACAATGCTTGACTGCTAGGAATTGCAACTCTACGTAATC
TACGTCGTCTCTGTAACTTAAATAGGTGTCCATGTTGCAGAGTAATATGACTTAACATGT
CCCTTGCAACTTTAACAGAGCAAATAGGGCATACCGTAACCTTTGACTCGCACGAGTGTT
CATCTTCGAGATGCGAACAGAGCGACGCAATATCGAAATCCTCGTAACAATATGGACATG
GGAAATCAGGCCGTACCTCATCTTCTACCTCAAAATCATCTATGCTCAATCGATCCAGGT
GCGAGCTCTGATGATGATGATGATGCAACGTGTATTGACGCTTGGCAGCCGCCAGACGAG
AGGTCCAGAAATCAGAGTCCATTGAAAGATCAGAAACCGAGCTAAGCCGATATTGAATAC
GATTTATTTAATACTAATTACGATTTTATATATTCACCTAAAAACATCAAAAAATGATAA
AAAGCTGTTTCGAACTTCTCTTCTCGGTTCTCTTTGACTTATTCTCAGAGTTCACAGATC
GATCTAAGTAAGCTTCAGAAACGAGAAGAGAGAGAGAAAGACTAGGTGGGGTAGTTTTTA
TTATTAAAAAAAACTTTTTCCAGAAATATAGAAGGAGAAGAACGTTCAAATATGGAGGAT
TGAATCAAGGAGACTGAAAGAGAGAGGGCTGTTGTTTGTGGCTATGTGAACGTGACGTAC
AGCCAAGGCAATCAAGAGGAAATGTCGGCCCCCTTGCTTCGTTATCAACACACGTTCTTG
TTTGTCTGTTTCTTAATTTCCTTACTATTATTAATGATTATTTGTTTATGTGGACA
>ntig_529_consensus
CACCAGCATAATTAAATAAAGTTTTCAATATGTTAGAAGAGCATTGTATCCCCAGAATGT
TGTACATCTCAGGTTGAGAAGGAGCTGAAGATGGGAATATTAGCAGTAAGGCATGCATTG
GGTTGTTAGACAACATAAAACCATCATAATCTTATATCACGATTTTCAACAACTTGTTCC
ATTATTTTTAAAACCAACTTACTGTCTCCCCTTAGTTGTTTAAAAGAGTTGTGAGCACAA
GATCTTGCAGAAAACCAGCTCTTCCAGCAGCTTGCCTCATCCTTTTCTCTCTCTCTTCGT
GGCTCAAAGAGGGATCAAAACTCAATTTCCAGATATGTGTTGGTGCTGGATTCTTTGCAG
CAGTGTCCTCAGCAGTTGATTAGCATTGTTTGACCGATATCCTCCACCCCCTAAAAGCAC
TTGAAGATGAGCTTCCCTGAGATCTCGGCCTAGAAGAGACAATGCTTGACTGCTAGGAAT
TGCAACTCTACGTAATCTACAACGTCTCTGTAACTTGATTAGGTGTCCATGTTGCAGAGT
AATATGACTTAACATGTCCCTTGCAACTTTAACCGAGCAAATAGGACATACCGTAACTTT
TGACTCGCACGAGTGTTCATCTTCGAGATGCGAACAGAGCGACGCGATATCGAAATCCTC
GTAACAATATGGACACGGAAAATCAGGCCGTACCTCATCTTCTACCTCAAAATCATCCAG
GTGCGAACTCTGATGATGCTGCTGCTGCAACGTGTATTGAAGCTTGGCAGCCGCCAGATG
AGAGGTCCAGAAATCGGAGTCCATTGAAAGAACAGAAACCGAGCTACGCTGGTACTGAAT
ATTATTTATTAAATAACAATTACAATTTTTATATATTCACCCAAAAAACAAACAAAATAA
CGAAAAACTTTTTCGAAAATCTCTTTTCGGCAGTGTTTGACTTATTCTTAGGATTTGCAG
GACAATCTAAGCAAGCTTCAGACATGAGAGAGAGAGAGAGAGAAGGTG
>ntig_530_consensus
TCCTGCTCTGGATTTACATTTCACAAGAAAACAATTAAAGATTATTATTGATCATCTCAA
GGCATCAGAGAAAATTTTGTTAGCAAAACTTGGAAATGCAAGAAAAGTTGAACATGTTCC
AACAGAAAAAACACACAGTAATTCTCAGCTTTTTACCCGCAGCACTTGCTTTACGTATTT
CCAATATGTATGTTGCCGCATTGCTGTATTTAGACATTTGGTATTCCAGAAATCTGCAGT
ATAGTCGTTTCCTTCCAACAAGACCCCGACCAGCAATTGCAACCTCTGGGAGTATGAAAA
AACTCTGCTCAAACATTCATCAGCTATGTACTTTATTGTATTTTGAAAAGGTTCTTCAGC
TTCACAACCAACTTCCATTCCTCAATAGACAGTTCATCCCGATAATTGGCTTTAAGTATA
TCAATTGACTTCCTTGAAATGTGGCTGACAAGCTCATCGTCGATTTCAAAATGCCTTCCG
TACATTTTTTGCTGCTCCTCAGAATTAGACCCAACAATCTTGATGGCTACCTTATGCCCT
TTCTTGGCAACATCAACAGGCTTATGGTTATTTTCAATGGATGCAATTCGACCAATATCA

ATAAAGTCCCTTTGAGGAATACAAATAGGAGTTCCAACCTTAAGTATGCCTTCAAGGACA
TCAACCCCCAACACAATTGGATCTTTCTTGTTAAACACACAATTCGGTAAAATCTTAAGA
ACGCATGGGAAGACTGCTTCATCAGCAGCTTCCCTCTTCTTCTCCTCCCTGAGATTGTCA
ATATAAGCCTTGAATTGATCGAATAGGTGGTAAATGATATCAGCAATGAAAATTTTCACA
CCCAGTTCATCCGCCAGTTCCCGAGCCTCTGGTGTCACTTTGACATCAAATGCCAAGATA
GTGGCATACTCCTGCTTCTTCTCAAGCATTACACTGGCCTTCATGACATCCTTTTTGTGT
ACAGGGCCTATGTTTATACCACTGACAGGTATATTCACTTCAGGGGTCTTTAAAAATTCC
AGCAAAGCTTCCAAAGAACCCAAGGTAGATGCTTGTACACAAACTCCCTCACCGCTTTTA
TCAATCCTGCTCATGACTGATTTCATATCTTCCATAGCAGCTTCCTTGATATCATCCAAA
TCATCATCAGGCCCAACCACATATAGACCTGTGCCAGCAATAGCATGCTCCAGGCCCTGA
GCAGTGATCTTAATACCCTGTGCAGCCTTGATTTCTTTATGATGCAGATAAGTTCCCTTA
ACTCGGAGTTCCTTCATTGGGTGGGGTGTCAATAAAGCTCGAATTGTGGTAACAATAGGT
CCCTGCAAGCCGCAAACAACAATTTGATCTCCCTCATGAAGCACACCATTGACCAACACA
ACATCAATTGTTGTTCCATGGCCTTCAATTACCTTGACCTCCAACACTGTACACTGCACT
TCATTACTGAAAGTAAGCTTCTCAACCATCGTTTTCTGAGTCCACTGAACCAGTAATAAT
AGCAGATCTGGAATACCTTCTCCAGTAATAGCACTTGTTGGTACAATACTGAAAGTCTCC
CCCATTTCTTTATTTTTGTAGTATAATTCAGTATTCAATCCTTGCTCTTTAAACTGTGTT
ATAATCTGCACAAGCCTCATGTTAAATTCATTTTGTACGTCTTTTGATTGTTGTTTCATG
GATTTTATAATAGGAGCATTGCGAGTAGTTTTCCACCCATAGAGTCTGTCAACCTTGTTT
AGTGCAATGATAAACTCTGTATTTCTCATTTTCAAAAGATTAAGTGACTCTATAGTTTGT
GGCTCTAAGCCATGCATTATGTCAACCACCAAAATTGCAATATCACATAGCCCTGAACCA
CGCGATCGCAGATTTGTAAAAGATTCATGACCAGGGGTATCAATAACTAATAAACCAGGG
ACCTTCAATTTTGCATCAGCTTTCAATTCCTTGGTTCTCTCACGTATATTCATAGCAGGG
AAATACGTTGCACCAATCTGTTGAGTAATGCCCCCAGCTTCGCCTTCCTGAACATTAGTA
CCTCGGATACAGTCCAGCAGCTTTGTTTTACCAGTATCAACGTGGCCCATAATACAGCAG
ATTGGGGAACGAAGATTTTCTTCAGCCTCTTTAGGGGTAGCATCTGCACTTGATGCTTTA
TTCTTAGCTCCAGACTCTTTCCTCTTAGTCATGTCTTCAACTTCAACCACAGGCTGGCTC
TTCTTATTCTCAGCATCCTGAGATTTCATCAATTGCACAGGCATGACACTCTTGCCTGCA
ACTTGGGTCTCTTTCTTTATTTCAGGTTCAGGCCCAGAATCAACCTCTTCATCATCAACT
GCGCCTTTAACATTAAGATTAACATCATCCCAGCTTTTTGCATCCCAATCCATATCATCA
TCTTCCTCATCATTTCCATTTTGCTTGGACAAATCAACAACTTCTGGCTTCTCTTCCACA
GTCAATGATTCCCCTTCTTCAACCTTCTCTGTTTCAAAGAATCCAACTCACCAATGGTT
TCTAGGGGCTCTTGCTCTTTTTCTTTTGTTTGTACAATCTCCTCCACCTTAGAAGGAGCA
GCACCATTTGCTTGATGGTGAGCTGGTTTTGACTTCTTTGTCTGGTATTTTGGCCTTTTT
GGTGGTGCACTTTCCTTGTCACCAGCAGGGAGAGCAATTCCTGCATTAGCCAGTATCTGG
TTTCTCATAGCCTCTAACCGACGAGCTTCTTCCTTCTGCTTCCCTGTTAAAAGCTTGCCT
TCCTGTTTCTTCTTCAAGAGCTTCTCCTTTTCCTTTTCCTTCTTCCTACGTCTAGCCTCT
TCTGCTTGCCTCTCAAGTTCTTCTTGCCTTTTTCGCTCCTCTTCTTCCTTCCTCATCCTT
TCCTCTTCTTCCCTTTTCTTCCTCTCCTCTGCTTCTTTTCGTCTAGCAAGCGCTTCTTGC
ATCTCCCTCACTTGCTTCGAAAGTTTGGGCCCTTTATTCTTCCCATCAACCTTTTTGGGT
TCTGCTTCTTGTGTTTCTTCCACTGCAGCAGCAGCAGCTGCTGCCTTCTTCTCTTTTTCC
TTCTCTTTCTTCTTTTTTTTCTTCTTTGCAGCAGCAGACTCCTCTTTTTTTTCCTCCCCT
TCCTTCTCATCAGCAGCATCTGCCGCTTGCACTGATTCAACCAGTTCAAGAGGCTGAACT
TGTACTTTCTCGTCTTGTGGAGTAGTGGTGGGTTTCGAAGTGGCAGGCGCTTCACCAAGC
TCTGCAAGGATCTTATCCAAATCATCCTCTTCTTGAGCTGTCCTTCCACTCTTCTTTTTT
TTCTTCTTACTCTTTGATGACTCTGCCGCCTCTTCCTTCTTACCAACCTTAGAATCATTA
GAGTCAACACAACTAATTCCCGTGATCTGTTGCTCTGGTTCAACTGCATCCGTAACATCA
CTCCCAAGTCCAGATTCTTCACCTAATGAAGCAAAAGCACTATTACTCTTCTTCTTAGAA
GACTTCTTCTTACCTGTAAATGAAATTACAGGTTCATCTTCATCCTCAACTTTATTAAGC
TCAGCATCATCATCATTTTCCCCATCAAGCAATGCTGCACTAAATGAAACACCACCTTTC
TTTGAAGACTTTGACGACTTCTTTTTCTTCCCAGAGAAACTAATTGGTCCAACATCATCA
TCCTCATCTTTCCGCTCCTCCTCATCAACACCTTCAACATCATCATCAAGCCCTGCAAAT
GCAGAAGCTGCAAACAAACTCCCACCACCCTTTTTGCCACTCTTCTTTGACTTCTTATTC
CCTTTAAAAGCAATTTCCACCAAATCATCTTTGTCATCTTTACTCTCCACAACATTATCA
TCATCAATGACCTCACTTTCGCCTTCACCAGTAGCATCAATTGCATCAAAAACTGATGCT
GTCAACAAGCTACCACTCGTTTTAGCACCTTTCTTTGATGGCTTCTTTTTACCAGTGAAA
CTAACTACAGTATCATCCTCTGCCTCACTATCTTTATCGCCATTATCATCTTCATCTTCA
AGTAACCCAAAAGCTGACGATGAAAAAACACTATTCCCACCTTTCTTGCCCTTAGACTTC

TTCTCAGTTCTAGTCTCAATTGTCTCAACATCGTCCTTTTCATCCCCCTCCAAATCATCC
TCATCATCCTCATCATCCTTTCTAGAACCTTTTTTATTTGCCTTTTTACCCTTCTTTTTC
CCAGCAATCACCACCTTCTCTTCTTGAGCTGTAGGTTCCTCAGACAGTTCAGCACCAATA
GAGTACTCATCATCATCAATAACAACTGCGTTCTTCTTAGATTTTCCGCCACCACCCAGA
GCGGCAACAGGCGCGTTGTTCTCGTCATCTCTCGCTGTCGATTTCTTTCTACCCATTGAA
ATTCAGGGTTTAACTCGACAAATTTCAGTTCACTTCGATAGTCTCTAAAATTGGAAGCGA
TAGCGACGAAAGGAAGAGCGAAAACAGATAGAAGATTGAAGAGAGACGAGAAGCATAAAG
TACATATGCGAGTAATTAAGGGTTTTATTTTTT
>ntig_531_consensus
CCCCGCTACTGAGGGATTCAACCAACTGACTATTCTCATCTTCATCACCCTCAGTCACTA
TCTCCAATATTCCCCCACCATCATCATAATCTACATCCTCCATAAGAAAATCTCCATTTT
CTTCGTCATCGTCATCATCAAGCTCACTCACACTATCCTCGTCATCACTATTACTCAAAT
CATCTCCCTCGCTATCACCATCCCCATCTAAATCTGGGCCAAGTAGAGGGACCACATCGC
CATCATCATCGTCATCCTCCTCATCTTCCTCATCACTCTCAGCATCATCAGGATCTGAGT
CATCGTCAGTTGGTCTTCGGCGACCAATTTCATAAATTCTACCTGATGAAAACATCTCTT
CTTGGTCATCCATGGTTATCAATCCAACATTTGAATCAGTTGGTTCTGTCGCAAAGTCAA
GGACACAACGATCAACTGGTATGGTGGCAATGTCCGAGTAGTTGATGGCATCCACAGTGC
GAAACGCAGCAAACAGTGGGTGCTTGACTCGCCGGGTGTGAACTGCCGACATGACGTCCT
CGAGATTTCTCCTCAGTATGGCGTAAATTACATCCCCACGTGCATTGAAGGTTATTGTTG
TTTGGTCCAAGGATGGTACACTTCGGAGGAGCCTAAATTTACGGAGATCCCAGACCTCGG
AGTTTATAATAACCTCGTTCCCAGCAGGATGAAAACCACCACCCCCATAATCTGTGAACT
GATCAAAACGATGAACAGGGACAGAATTCCGGCGGTCCCACAACACTCCATTCCAGAGAA
GCATTGTGTCTGAAGGACTGAAGTGTATCTGTGAATATGCATTGCCCCGTCCGGTGGAAT
TGGCAGTTGTGTCAGACAGTTTCAATTCCAAATGGCAGGTTTGAATATCATAGAGGAGGA
TTCCTCGTTGGGAAGTCTCCGTAGGTAGGGCTGCAAAAATGCCTCCAGAATTGCTAAATC
TTGCTGCCTTGCACCCCTCGAATGAATGCATTGGCCCACCTGAGATTGAAGAGGCATCCC
ACAGCCGTACATCCTGCGAGCTCGAAGAAAGCAAAAGATGGATCTCAGCTGAAAGATGAG
ATTGGACAAGCGTCAAAGGACCCTGGTGGCTTATGCAGCTCTCCAGTACATTGCTGCTGT
TGGTGTCAAAAATTTTAAGCTCATTTGTGTGGCTACCGACTGCAATATGAGATGAGTCTC
CAAGAAATGTTATGCATGTCAAAAGGGCACCAGCATCATCCCGGCATGTCCGCCACGGCC
TGAATCTGCTATAAACAAACTGGCGATCTCTACGATTTCTGTGGATTCCACTGTACATAC
TCTTATGCTCACGCATACCAAGCCGAGCTGTTACATTTGATGGAGCATCAAGGCTCCGCT
TTGGTTCAGGACAAACATGAGGATGTAGAAGTGAGAGTGGTGGAAGAGTGGTTATAGGGG
CTGGACACTGCCGATGCTGTTGCTTCAGATACTGAACAACAAGAGAGTCTAGGGTTAACC
GCTCTGAATTGCCAGACTGAGAATCATTTAGGAGTCCAAGTTGGGAGGAAGGTACTGATT
GTCCCATTTGGTAATTGCCATATTGGTTGTCATCCAAATAATCTGCCAAACTGCTCGGTG
TTGATCGTCCATGTTGATCCCTTAGGTTAGAAGATGGTGTACCATGAACATCATTAAGAA
GACAGCTTTTACGGAGAGAATTAGGTGTTGGACAAACTGGAGACCTAAGTCCACAATCAC
TGGTGTTGCATCGCTTTCCATACAACGTCAATCCAGTATCCTTTAACTCTGGAAGTTTTC
GTTTCATAGGCAGCACAATCGGAGTCTTACATTGAGAGTCTGTATCAAGACAGGTTTTTG
TCATGGGTTCAGATGGAATTTCTAATACACTTGGAGGTGCAGAAGACTGTTTACAACTAC
TGCACAATTTTCTAATTGATGGTGCTTGGCAATCATGAGACTCGGGCTGATTTCTTGACT
GTGAACCAAAACTAAGTGAGAAAACCAGCTGCTTCTTCTTTGAAGACACACTTGAATCAC
ACTTCAAACTAATATCCTCAACCCCTGCAGTAAATTTCAATTTATCAGCCAGAAATCCAC
CAGGCGTACGACCAGAAGGCCACTGAAATTGTGTTGAGGGGGTCTCTTGCATTGAGGCTT
GTTGTGCAAGAGATGAAGGAGCTGCCAATGAAGGCAAAGGGGTCAACTGAGCCTCTTTCA
GCAGGGAAGCTGCTGTTGTGCCCAAACCAGATTCCTGCTGGAATGGTAAGTGATGGGGGT
GGCAGCAGCTATGGCAGCTCTTTCGATGCGCCTTAAAGTAGGGGTAGCGGCATCAGTAGC
TGCTAATGTACTTGCACGCCCTGAATTTGTAACAACCGCAATAAGCTCGATCGCCACCTG
AGAAAGTTCAGCTTGCCATCTGCCTTGGTCAGTTCCAGGTATGTGGCTACCTGAATCTCG
AATCAGTTCTGACAACTTTTTCCCCACCTGAAGCTTAGTCAATATGTGTGCAATTGTATC
ATCTCTGGCCAAGCCAAGAAGGACTCGGCATGCGAGTGCTCGAAGACAATCCAGAGCAGC
AGGTGGAGAATAAATGCGTGGTTGCAGGAGATGTAGAAGTACCTTTATGCCATTATTGGC
GCGAAATGCCTCCCTTGCTTGACGATATCCTTGTTCCAGTTGTGCAGCAAGACCAGCACA
ACCTGCTCCAGCACCTAAAGATATTCTACGATCCCCAACTAATCCTGATGTTGGTGTGGG
AAAAGGTGTCTGAGAACTGCCACTGGTACACAGTGTGTTTGCCACACCGCCTCTATCTAG
CACACTAGATTCCCCATTACGCTCTCTTAAGTCACTCTGACTAGGAATGTTAACAGCTCT
GTCTGAAATATTACGTTCTGCATTCCTATCTCTATTTTCCATAGACAAACAGTTTGAGGG

TTGACCAGAAGCAGACTGCTGGCCTTGAGCAAGTAGAAGTGGCTTGTTGCTGATTGAAGG
TGGAGGACAGACAAGATTGATCAACACATTAAGTGCTGGCTGTATGATCTCGGGGTCCAC
ATAACTACTTGTAACATTTGCAGCATCCAAAATGACTGCTATACCAGAATGATTATTACT
GAGTGTGGCAGTGACTATCATCTTGCGGCTGTTAGGTACCAATGTAACAATGTGCAGAAC
ACCCAATGCATACTGAAGCAAGTCGTGCAAATAACGCTCAACAGGAGGGGCCTGACACAA
TTCCAACATAGTAATATGCCCATTAGAACTTAAGAAACTGTCAACAGCAGGCCACCGAGT
TCTAACAAATGCAGGGCCTAGCTTCCTATCTTTCTGTAATTGTAGAAAAACTGCATCAAT
AGCCTCATTACTAATATCAAGTGGCTTGTAGACAGCCCTTACATTCGGAATATTCCGAGG
TGCACTTCGATTACTTTTATTAGGACGAATGGAATCAACAAGCAAAAGAAAATGTGCTCT
GAAATACTGTCGCAAAGCAACACAGGCGTGATAAGCTATCTGCTTCTCTGAGGATGTTAG
TACTTCTGGAGGTGATCGATCATTTCGAAGCGATGTTGAACTAGAAAGCCCTAAAGCCCC
AGAATTACCTCCAGATCTCACAGAGGCAGCATCATTTAAAAGGCCCAGCAATTTCTGTAA
GCCATCCTGAGCATCAAAAGCATCAATAACTGCTCTGAACACAAATGCAGCAGCAAAAAA
TAAGGCTGCATTTTTTTCTGGCTAGATCCTGGGAGCACTCAAGAAGGTAAATAGCTAACTC
GACCACCTGGTAGACAACATCAGAAGGAAGTGCACATACACGTTCCATTATGCCCTGAAG
AGATCCTATGGTAAATAAACAAGAAGAAAGACCATAAAAAGTCTGATTGATTCTTGGCAC
AGCAAGCAACCTTTGCATGCCACCTCGATCCACAAACAATGCAGCAAATTTTCGGTGAGC
AGCCAAAGCGCAAATTAGCCTCATAACATCAGGCAAAATCTTTGTAACCTTTGATGCTTC
TTCAGATTTGCAACTCTGTTGCAATAGTGCAAGACAGACATCTACTCCCTTTTCATGTAA
CACAGGACCCAGAACTTCCATGTATTCACCTAATGTCTCAAGGCATTGAATGCAGTATTT
TTCTCTCAATTGTGCAAGCGTTTCAATATCAGGGATGAAATATTCTTCAATATCCTCAAG
CACTTCGGGTTCTGTGACACTTAAACTCACTGACTCAGCATTAGTGCAGTTACAGCTCCT
TGAAACTTCAACCGCATTAGCAGCATCAATAACAGTAGATGCGGCTCTGGAAGCAGCCAA
AACAGTAGCTTCTTCATCGTTGGTAGTTTTAAATTCCTCTAAAGCTGCACTTTTGACAAC
TTCAGCAGCTGCATCACCAGCTGCTTTGATGGCTTCTACAGGAGCATTTGCTGCTCTAGC
TTCAGCCTCAGCAGCTATAACTGCTTTCTTTACCAAATCAAAGATGTCTTTGCTTCCAAT
TCTGCATCCCTGGAAGCAGTCATCGCCATCTTCTCTCTCCACAATCAAACTATCAGAACT
GTTTTTTCCTGAATATTTCCTAGAATCTGGTGCTTTTTTTCAAATCTAAAGTTCTTGAGGA
ACTCTTATCTCTTACACTCCGTCCCTGTCCTAGACGACTACCAGATCCTGGAGATGTCAA
AATTTGCTCACTTTCCATAGGCCCTTCATAAATCCTGCCCTTCCCTCTAGATCTTGCCCA
TCCACGATTTGTCCTACGCCTTGAGGAGTCATCTCTTCCACCTTCATCAAGATCACTAAA
TTTTGTCTTACCCTCACGAGCATGCCACCTTTCCTGACAATCAGTATTTACCTCACGTAT
ATCAACCCCCTCTGCTAACCCATCAGGTGGTTCTCCATCATCAATCCTGCACTCTTCTCC
ACGTGCATGCCTACCAATACCTCTGTCCTGATCCCTTTCAATGTCTTGATCATCTAAAGA
TCGCTCATCAGCTGTTCTTTGGTCATCAGAATGAGTTGTCTCAAAAACCTGTCGAACTCT
ACCCCTACCTTCTTCTCTGCCTCTCAAGGAAGTAGCACCAGAGGTATTCCTACTGTCAGT
TAAATGGCTAGCATCTTTTTGACTTGTACTTGTCTCCCCAAGGACACGTATACGCAGATA
ACGCATAAGCTTTGCAGACAATCCAGATGTCAACACATCTTCAACGACCACTTGACCGCC
ACCTGACAAACACAGAACAAGAAGTCCGGTGGAATAAGTTCTCAACATTTCAGAATCTGT
AGCTTCCCTTTTCCCAACTTCATGCCTCGAATTACGATGTTCCCCAGAAAATCGGGCAGA
CTCATCCATTACCCAATTCTTTATATTCTCCACAACGGGTTCTTCAAAAACGTGAGGATA
TATCCAAGTAACAGAACAGCTTGATAGAAGGCGTGCAGCAGCTGCTTGGACGGAGGTGGA
ATATCTACTTTCACATAAAAACTTGGATGATATCAGGTCAAAAAAATCATCGTTCTCCCG
AAGTAAGTTGCCAAGGCGTCCGATAGCATGAGAAGCTCGCGCGGAATGAGCGTTACTGGT
GGAATAACTATTCTCTTGCAAAAATAGTGATTCTTGGATCTCCAACAAAGAGGCGAGGGC
ATGGAGAAGTGTAGGATTAGGGTTATCAGATGAGGAAGTAATCTTTTCCATAAGCCTCTG
TGCCTTTGCCATCAACTCTTCCTCTTCTTTCTTCGTTGTCTCTTCTTCATGAGCAGGAGG
ACTTTCACCATGCCCGTGCCCTTGGCTCGATGATCCTTCCATAATCAATAAAATCACCGA
ACAACAATCAATCGTTCATCAGACCTCACAGTACGTAATTTTAGGGAAAACTACCAAAAG
AATCCTTGTGTGGGCTTGTGGTTGTTTTATAGCATCAGAGGGACAGTTCTTTCTTCTTCA
ATAAAATGTAAATGGATCCGAGGCTTCCCCAATGACCC
>ntig_532_consensus
GTCGGGGAACTTGAGAGGATAAGGTGACAGAAAACAAAAAGAAGCTGAAGACAACAGCTC
ATTATCCAATTGGCTCTCGCATAACGGCCAGGTGACAAAAACATGGTACGCATGAATCCA
GAGTGTAAAGAACAGTATCACAAAGATCCTATTCATCTCTAGCATCCCAGGGAATCTAAT
CCCGTAGCAATCGAGAGAATAAAATACATTATGTAATGTGAAACAATGAAATTGTAATAT
ATACAACTCAATTAAATTAAGGCTGCTTTCTGGTCCATTGATTATAAAATGTAATTATAG
AGGACAAAATTCTGGGAAATATAGGAAGTATGCAACTGTTGTGCACCCTGGTTACAGGAT

ACTTTAACTTCTAATGNNACAGTTCTTCCCCAGAAGGGCTGGAACTAATGCTCCTCATAG
CAGCATCAAAGATTGTTTTCCGGAAATAATCATCTTCCACCTTCTTCAGAACTTCTTTTA
CAACCTTAAAGTCCAAACACTGAGCTACAACCAACTTCCCAACCACCTCACCATAGTTAT
TTGGTGCTTTTGGTAAATCTATGCCAATATCATCTAACAAAGAGCCATATAGAAGACACC
CAGTTCCAATGTCTCTGGTTGTCAAGACATTCCTACTTAGCAAGAACTTGAGAAGGTTTA
TGGTGGGTTCCACACATGGGGGGATTTTTTCAAGAGCAAGGGCAATGGCTTGTTTAACGA
CCTCAGGATGATATGCTGGGGCTTTCAGTTCCTCCACACACTGTAGTGCTTCATCAAGAA
TTCGAACACTAAAATATTCTTCAAGAAGAGAGATGGTTCTCTTTTGAAGATATACAATAT
CTAATTTTGTAGCAGGTGCC
>ntig_533_consensus
TTCTAATCGAACATTTAGCCACTGTTTCTGTTTTTTACATAATTTCTAATCCAAAACCGA
CCTCGAATAAGAACATGTACTACACGGATGCATAATGAGAGTGGTTCCGAATTTCCTGAT
TGCCCTTCAGCCGCTCTCTGGTTTTCCCTATAAAATTACGCCATTCCTCGCCAACGGGGA
CTCATCCAACTCGCGTTCTTCGTCTTCCTTGTATGTTGAGAAACCCTAACTTCTCTCTTG
AAAATCTTTTCTTTCTTTAGACCCTTGAGTCTTCCGCCTTACCTTTTCATTTTGAAATCG
AAATCGATTAGTCTGAAATTGATTTTAACTTTCTTCTTTGTCGAATTTCTGATTATTAGG
TTTTAAATTTTTGATTTTTTTATATTTATATTGAGGATGACTTTAGCAGTTCTAAATCCT
GAAGACATTCTGAAAGAGAATCGAATCTCTCAACGACCTTTGATTTCTTCTTCTTCTCCT
TCTGAGGTGAAATACCAAAAAAATCCTTGCCCTAATTTGAAACGGTCGAACCGGATGCAA
TCTACCCGGAGGAAGCGGAGTCCGCCTCGGAATCAAAACACGTCGCTTCCTTCACGCGGC
GTGGGGGCGGCGGCGGCTGCGGAGGCCCCTAAACTCCCCGTTAAAAACAATCTCGTGATT
GGGCAAGTCAAGATCCTAAAACGCGGAGAGGAACTCACGAAAACGACGACGGTTCCGTCA
ACAGAGCCTGTGAAAAAGATCGTCGAGAAGCTTGAAGTTCCAGATCTGGGATCCACTGAG
AGGTTGGGACCTGATCCCGAGTCGGTCCCTCTTCTAATTGAGTCCAAGGGCCGGGTCGTA
CCTTTCTACGCCGGGTCAGCCTTCATTGCTTCCCCTCCTCCTAGTTCGCTTCCTTTACCT
GCTTTTTTCACGAAGAAGACAGTGACAGTCAGTCAGAACGACCCAACGAGTGATTTAAGA
AGGATTTTGAAGCTAGATGTACTGTGAATTAAATTTGAAATTTGTTTGGGTTTTGGGTTC
TCTGATCATCGATCTGATCCCTTATTTTTTCAAGGGGATAAAGATGGAATTGGCTGACTC
AACTTGGCAATTAGTATTGGTAATGGTAAATTAAGTAGTTTTTTTCTTGAAGGTTTGTTA
GGAACGTTAGCGGGTTCATCAAGAGTTTAATGTATAGTTTAAAATCTAGCTGTAGTTTAA
AGGGCTCATGTAAGATTTGGGGTTGTGGGTTTGTCGTCGTTTCCTTTTGGTTCGAGTAGA
ATATGTTAAGGTTTATTTGTGAAGTTACAGATTCTATGTATCTGCTTGTGGTTATGGAAT
CTGTATCCCATTATGTAGTTGCTTTCGTTTCTGTAATGACGAAGTTTAGTTATATTATGT
TTTGTGATGATGTTCTTTGGTTGTCTCGTGATTATTTTGGCTTGGGAGCGATTGATTTTT
GTGATTCAGATGTGGTATTATTGGACCCTTTCCAATGCTTTTTAGGGTAGAATTTTATTG
ATCGTGGTGATGCTCGAGAGATTTGTGATGCTATAAGTGGATTTTAATGGAGGTGTTATG
AACCATTGAGATGTTGTTGAAGACATGTCTGTGTGGAAC
>ntig_534_consensus
CTGGCCAGGATCCACCATTATGGTAACTCCAGCTTCTGTTCTTTGGATTATATCCATTGC
CAATCCTCCATTCATGTCCCTCCAAGACTGGTTAAGAGATCTTCAAAGGCATCTCTTCTA
TCAAATCCTCCCAATTGTCTTCAATCAAATCCATAATTGCTGTTGCCTGCACAGGCATGG
CAAGAGAATTCAAAATGGCAATGCAGTTTCCTACTAAGAACCACCGGAAGTCCATCCAAG
CCGGACAAACATTGCCAATCAAATATCCTCCTCTCAAGGGCATGAAATCAAAAACCTACT
CAGGGATAGAATCCAGAATGTCTTTGAACTTGTTAACAGCTGTGTGGGAGTACTCGGTCT
TATAAAGGTATGTATTATTTAGCTGTGTAAAAGCAAGCCAGTAGTACTTCTGGGTAGCTG
AGAGCTGTGATCCTCTTGTCAATTCTTTCAATCAATCCCTTGACATCTTGTTCTGGGTTT
AACATTTGTTTTGGCTCACCTCAATGCAAAATAGAAGAGAGCCTGGATCTCAATTGGATA
TTAATATATTCCCTTTTGCAAAGTTTCAATCATATATATGGAAAGAACAACAATGCTGGC
TCCAACAGGGATGAGCTGCTCCCTATGCCAAATTCCTTGTTTAACAACTTCAGTCAGCAC
CAAAGAAGTCGGACTCAGCTCACTTGTGCTCAAGACAGCAAACCCAAGGGCCATAAATAT
TTGAACAGTTAGCATCTAACTTCAAACCCATGGGCCTATCGCCATTCTCCTGTCAATCAT
GCTACATCCATCTGCCCACAAAAGTGTAGCGAAAGTGTCAAAGCCATCTGAGAGACAATC
GTTGAGAATCAACTTCATTTCCTCCCTGCACCTCACACTGTTCTGCTAGTGTATAATCAC
GAGTACACTTGGTATATGACCCGAGCAGTATGATCCACCAAAAGCCAGAATCCACAGGCG
CTACTCTTCCTATGGCACTACCGCTGAAATCTGCAATCAAGGTCTCCTGTTGCTGATATG
AATTAAAGAGTACCTTAAAACTTGCAGGCATCACACCTTTTCTGAGGGTAAATTTATCAA
TCTTTCTCCCAACCTTGAAGGTGAAGGATTTTCGGTAGAAAGTTTTTTACTATCTCAGGT
TCTGTAGGATCCCTCATCAAGCAAGCTAAGCCAGTTGAACAAAGTCTCTCACAAAAACCT

GATTGTAGTTCAAGGCTTCGGGCATAGGATCCATTCAGCGAACGTCTCCACAGGCTTTCC
TTTGAAGTAGACGTAAGATTTCTTCAGTCTCTTCCACACCTCTTCCATCATCGCGGCACC
GTTAGGCTCTCCACCTCCACCGTTCGATATTGTTGAGGCTTAAATAAAACGGTGCGTTTT
TGATTTAATCAAAACGCATCGTTTCAGCTGTTGTAAATAAAGGGTGGATAAAAAGTTTGG
CTAATTTCTCATTCCAGAAGTTTCATTTGGGAGCTAGGGCTTCGATGTGCTGCTGCTCTG
CTGATCCTCTGCTGGAGTTTCGCTGCGATTCATCTTCAATTAGAGATAAGCTTTGTTTTT
CTTTTCAGTTAATTGTTAGTTAGTAGTTAGTTTAGTTTGTTTTTCAGTTATTCTTGTAAT
TGAATATAATTGGCTACTGTAAATCTTTCCAGTGCTTTCTTTTGTTGTAATCTTTGTCTA
TAAATTCAGATGTACTCTTCTGTATTTTGTGTGAGTTTAATAAAGAGTTTCAGAGATTTA
TTCCAGCTTGGGAAGATTTGAGGAGGCGGTTCCGGTTTTAGAGCGGTCCATGGAGATCTC
AGATGTTGGAAACGAGTTGGATCATGCTCTGGCCAAGTTTTCGGGATACATACAAGCTTG
GGGACATGCATTCGTCGATGGGTTTGTTGG
>ntig_535_consensus
CAGCTCGCTAGGCTATAATATTCCCATTCATAAATTGGATACAACTAATTTCAAATATGT
ACAAAACAAAATCAGGATATAAAGAAGTATTGTTCAGATCATCTTATACCTCAATTATCA
CCAAAATGAAGCCTTGATAAGTGATTTTCAAAAAAAAAAAAAAAAAAAAGTCTGGTAACTA
ATAGAAGTCCGATATTCTGCCATTTATTAGTATGTCACAGTAAACACATTCCAGCTACTG
CAAATATGTTCACCTTGCTCTTTGTACTGTCAGGCTTTGCTATGCAATCATGTTGTTAAA
ATGAGGCTGAGCGAGTGAGCTTTGGCTTGGCTATCTTCTTGTCCTCCTCAAGAGAAATCA
TCAGGAGATTGGAAGGGTTCTCAACCATCATTTTTGCAACCAAATATCCAGCAATGCTCC
ATGTCTGATATTTTCTTGCCTGTTTCCCAACATAACGTCCAGTCTTACCATCGTAGTACT
CAGGCCAACCATCTTTAGAGAGCCGCTGTTCCGCCAACTCTATTGCTCGTTTTGCAATTT
GAGGCCTTCCGGTCTTGATGGAAGCTGCAGCAAGTAGCCACAAGAGAACTGGCCAAGATC
CGCCATTATGGTAACTCCAGCGTACGTTCTTTGGATCATATCCAGTGCCAATCCTCCATT
CATGTCCCTCCAAGGCTGGGTAGGAAATCTTTAAAGGCATCTCTCCTATCAAGTCCTCCC
AGCGCTCCTCAATCAAATCCATAATTGCTGTTGCCTGTGCAGGCGTGGCAAGAGAACTCA
AAATGGCAATGCAGTTCCCTACTAAGAACCACCGGAAGTCCATGCGAGCGGGGCTAACAT
TGCCAATCAAATATCCTCCTTTCAAGGGCATGAAATCAAAAACCCATTCGGGGATAGAAT
CCGGGATGACATTAAATTTGTTGACAGCTGTATGGGAGTACTCCTCCGTCTTATAACGGT
ATATGTTATTTAACTGCGTAAAATCAAGCCAGTAGTATTTCTGTATATGATAACTGAGAG
CTGTGATCCTCTTATCAATTCGTTCAATCAATTCCTTGCCGTCGCGCTCTGGTTTTAACA
TTTGTCTGGCACACCTCAGTGCAAAATAGAAGAGGGCCTGGATCTCAATTGGATACCCAT
ATATTCCCATCCTCCGGTCAACCATGCTGCAACCATCTGCACACAAAAGTGTAGGGAAAG
TGTCAAAGCCATCTGAGAGACAAAGGTTGAGAATCAACTTCATTCCTCTCTGCACCTCAG
GCCGTTCTGCTAGTGTATAATCACGAGTACACTTGGTATATGACCTTAGCAGTATAATCC
ACCAAAAGCCAGAATCCACAGGCGCTACTCTTCCTATGGCACTACCTCCAAAATCTGCAA
TCAAGGTCTCCTTCTGCTGATGTGAGTCAAAGAGTACCTTAAAACTCGCAGGCATCACAC
CTTCTCCAAGGGTAAAGTTATCAATTCTTTTCTCCCAACCTTGGAGATGAAGGGTCTTCA
ATAAGAAGTTCTTTACAATCTCAGGTTCTGCAGGATCCCTCATCAGGCAAGCTAAGCCAG
TTGGGACAAAATCTCTCACAAAAACCTGATTGTAGTTCAAAGCCTCGGCCATAGGATCCA
TAGCAGCGAATGTTCCCACAGGCTTTCCTTTGAAGTAGACGTAAGATTTCTTCAGTCTTT
CCCACGCCTCTTCCATCATCGCGGCACCATTATTCTCTCCACCACCACCGTTCGATCTCA
TCACATAATCATATATTTCAAGATTCGTGCCAACACTTGGACACGGCTTTAACGACATTG
CCTTGGGAAGATTCACGTTATCCGACATGTTCTCGTTGCTCTGCAATCTCACCGTCTTCT
TCACTTTCTCCGGCTTCACTATCGCTCCAGATTCAAAGCTTTCTTTCTTTACAGATTCTC
TATCTTCCAAAGATACTGAATCATTTTTTTCTTTGGATTCCAAATCGCATTTTGAAGTCT
CAGTCGCTATAAAATCCATCGAAGATTTCTCAGAAATCGTCGAGTCCTCCGAAGTGACTT
CCGATAGATCATCCGTCTTGGATTCAGAAATCGATGAGTCTTTCTTGCTGCCAGATAAAG
ACACCACTAATTTTTGTTCCTTGTCTGATAAACTCTTTGTTTCTGGACAATCTCCGTTGA
CAGCGGAGAAAGAGTTCGTCGGTGCCGGTGGAGGAGCAACCTCTGCTTCCTGTGCATCAT
TTAGTCTCTTCGACATTCTGATTAGCAGCAAGAGATTACCTTCGAAGTGTCTTTTGGCGT
TTGGCTATAAAGAGAGGAGGCGAGAGAGAAATTCAGGTACCACAATAAAGGGAGTGACTG
ATTCACTCTCTCTACTTGAAAGAAACAGAGAGCACGGAGACTCTGTTTTCTGTTTTGGCA
G
>ntig_536_consensus
CTTAAATGATCACAATATGCAGCAATAACATATCCCTCTTACTGTCAAATGTTACAAATT
TTAACACGCTAGAAGGCTGATATGTAATGCGTCGAACATATACTTCTCAGGCAAAGAACA
TAAAAATTTTAAGAAGTAAGCGAATCAATAATGGTTGTGTGAAGAGGGTCACTCAAGTGA

GTGGCCCAGTTGTTGAGAGGGCCTTTTCCAGTAACAGCAGCTTGAACCGCAAAGCCAAGA
AATGCAACCATGGCAAGGCGGGCGTGCTTGATCTCTGCCAATTGAAGTGTGGCTTTCTTT
TCAGGATCAGCAGCCAATCCCAGAGGGTCAAAAAACTTTCCACCAGGGTAAAGCCTCTTC
TCCGGATCAAGCTCCGCGTTCCTCTGAAACTCAATGTAACCAATTACCAAAACCTCGATC
CATATCAATGTTGTTATGGAGAACGGCAGTGGCTGCCCAAGGTACGATGACCCTTCAACT
AATTCCACCTTGCCGGCGTCTTGCCAGGTAACACCTGTGAGCCACTCGACGGAGAGGGCA
CCAAGAGTGGCTAACATAGCCCATCGTCCATGAATGAGCTCGCACTCACGGAACCTCTGA
AGTCCAAACACTTCAGTGTATGGCTGAAACGGAGTCGACTTCACTTCAGAGTTCTCAAAA
CGGGTCCCAATAATTTCACCAGCCATGTTCTTCGCCAAGTTCTGGTCCAATGAATCCAAA
TCAAACTGCAAGTACTCCGCGGGCTTCCCAAGCCCGAATGGGTCAAATCCATAGTCACCA
ACCAAGCTCCCGTCTAGCCATTCTGGGGCCTTGGCTCCAGGGTACCACAGTGGACGATCA
GAACTGGGTTTCGAAATCTTCTTCGGGCTAGCCTTCTTGGTACCAAACCCAAACCGGGCT
AGGACCCGGCCCGAGTTAGAAGACACATCGGGGAGGCGGGTTCCTATAAAAGAAGATGTA
GCAGCTGCTGCTGTGGTGGTGGCCATGGCTCGTCGCTAACCTAGCGTACAGTGCTCAGAG
AGAGATTTGAGGCTTATGAGAGTCCACTGAGATGATACTTGGAGTTATAAAGTTTTGATG
CTTTATCCTTGTTGGTGTGTTGCTGGGCGTAGGATTGAACTGCGTATCCATCTGACGGCT
TGTAAAGAATCTGCCTTATCTTTCCTTATCTGATAGTAGAGGATATACCCTGCTAGAAGG
TTGTGGCTGCTGTTGCCAGGAGAAAATCTTTCCGACAGAAAGATTTTTTTAGAAAAAAAC
AAAATCTGACTGAAGGCCCAAACTTACGAGTTTTTCAGAGATAAGTTTAAATC
>ntig_537_consensus
CACCCAGCATTCTTTAAGCTTTGGTTAATTAACAAAGAATTTCAGTCTAGCTTTCTATTT
GTTTCGTAAAGGTAGGAATCAGAATAAGAACCGAAACCGAACAGGTAGGAACCTATCCCT
ACAAGTTCCAGTTCCAGCAACAAGGTACCCAGACCTGTTCATCTCTATGTATAAATGGGA
TCAGACCTACCAAGAAATGTATTAGAAGGATATTGCACCTGCAGCAGCCTTAATATACAA
TCAAATAGTTAATTACATCAGCCAAAGCCGAAAAAGAAAAATATGAACAAAAAGGACGAA
TCCTCCTTTTACTTCACTTCACCTAGCTGGTTTGCCATCCCTATTTAGGTCCTCCCATCG
TATTTCCTGAGCGCATTCTCCACATACTGTGCTGCAACCTGCTTTTGTGCCTTGACTTCG
AAGCGTCAGCAAATGCGAAAGATTAGTGCATTTACGCAACACCATACGACTCCTCAGAAT
GCCTCTGGGTTGTGTCATAGTATTGCTGGTCTCTGGTGCAGGAGGAACCACATTTGACAT
GGCAGTAGGCTTTCGCTCTGATGCTGATTCATCAAGTGGTACACGTTGCACTTCCTCCCA
TTGTAATGATCTCCGGTACACTTCCCAAGCCAAGTCTTGTTCCTCCTTTGAAAGCCTTTC
CTCTTCATTCTCTTGCAAAAGAGTCTCATGCTCATGGTAATTGGCAATCCACCTTGGATG
ATGCTTGCCAAGTAAAGTTTCCATTAACTTGTCAGAGGGGCAGCTTCCATGAGAAAGAGG
CAACTTATGCTTCAGAGAACTTCCTATTTTGCAAGTTGGAGTCCGACAGTTTTCTTGTCC
ATTTTCTTGGCAGACTTCAGGTAGAGGATCAGGGTTTTCATCGTCACCGAATTCAAAAAG
ATGTAACATTTCTTCTTTAGAGATAAACCTATGTACTTGTTGTCTATCAACCACCCTTGC
AGCAAGCCCCTCCTTCGTCACCTGACGCTTATAAATCTTCTCCTCCATGGTTCCATGCGC
CATCAATCGGTAAGCAAAAACTGGCTTTTTCTGCCCATACCTCCAAGCACGAAATATGGC
CTGAAGATCATACGTTGGATTCCAGGAACCATCAACAATAATTACACGGTTGGCAGCATG
AAGATTTATCCCCAGAGATCCAGCTCTAGTTGAAATTAATGTACACTTCACCCTTTTATT
GAAGGGCTCATTAAATCTCTCTACCAACTTTTGCCTTTCAGAACTTTCTGTTCTCCCGTC
TAGCCTATACCAGTCCTTTCCTTTTTTCCAAAACTTCCCTCGTTTGCCTGATCGAGGTAG
TTTTGAAAGATACAGCTCTATGAGGTCTAATGTTGGTATACTCTGGCTAAAAACCAATGC
TTTATCACCTATGTCAGAACACAGGGTTAAAATATCAAGCAGCAAAACCATTTTGCCACT
GTAATCAAGCTCTTTATAAGTATGTTCACGCAGAAGATCTTTCCACCAGTCCTGTTGAAA
AAAGCCATCATCG
>ntig_538_consensus
CCGAAGCCTTAAATTGTTGAACAAGAACATTCGAGCTTGCTGTCATAAATGGAAATTTGG
CTATAATGAACCACCAATAGATGTGCTTGATCATTATGAAACTGGACTTTAGTTTCTACG
ACCTAGGGGGATTAACGGCCAGCTGGTGCTTGTATGAACCTTGATTTCAGCTTCTCCCAC
TTATCAATGCTTCACATACATAGCTGCGCATCAGCCCCTGACAACACTAGTGCATTTAAA
GTTTGGGAAAATGCCAGCCCTGTGATTCAACACAAGTCAAACCATTAATAGAAAAAAAAT
TACAGCAAGGATATATCAATTATGTTTTTCCTAGAGAAGCAG
>ntig_539_consensus
GTGGGAGAGGGACATGTGCAGTATAAACTAAGCATCTTTACAGTGGAACAGCCAAAAATA
AATAATCCAAAAGGCTGTTTTGTGGGTACATAAACAGACAGGCATGTCAACAATAGCCAT
TTTTCATTCCAAAAACCATCAATGTATACATCATCATAAAAGGCAAGTATCATCAAATCA
GCTTTGGGGGAGGGTGTGTTCTCAGTTTCTGATAAAATGTCCTATAGAATTTCACGACAC

ATAACAGCCGAGGGGAAAGAAGATTTACAACAGATAGAGAATTCCATGATTTAGAGGAAG
TTATTAGAGAAAAAAGAAGTGAGGGTGTAGTTTAAAATTAAGAACAAAAGAAAAATGTGC
AGCTCAATATGTCTATACTCCTTATGAGCTAACCTTCTCATTTATATGCCATGGCGTTTA
CCTGGGAGGGAGTTCTGACAGTTGACCACTCAACGAAGGATTTGATGAATTAGAAGGGAG
GGAGCCATTGTCTCGGGAGGGTGTGCCACCCCACTTCAGGTCAGCATCAGAAGGCTCGAC
TACGTATACTGCTCCATCACTCATGCCAAGAGCAATCTGATAAGGCTCAGATGGATGAGC
CGCTATAACAAAAGGATGGGTGGCAGTGTTGCTGCTGCCAGCAAAAGAAGGTATGTAAGC
AGAAGGTGCTACTCTACATCTGAGCCTTAAATTTTCCGCATCAAAAACTCCAACAGCACC
ATCACAGAAACCAGCATATACTAGTAAGCCATCACATGAATATGTTGCACTTGAAATGGG
AGCAGGGAGTGTATCTTTTGGATACCATGAATGGGAACATTCAAGCTTGCTGTCATAAAT
AGAAATTTGGCTATCATGAGCGACCAACAGATGTGTTTGATCGTTATGAAACTGTACTTT
AGTTTCTCCAACCAAAGGGGATTGCCGGCCAGCTGGTGCTTGTATGAACCTTGATTTCAA
CTTCTCCCATTTATCAATGCTCCACATACATAGCTGCGCATCAGCCCCAGAAGACACCAG
TGCATTTAAAGTTTGAGAAAATGCAAGCCCCGTGATTCCATTTTGGTGACCCTTAAGTTT
GGTCTTGACCTCATCCACTCTAACATTGTATATCTGAATGGTAGAATCCTCCATCCCAAT
GGCAATAATATTATTATCTTGAGGATGGAATGCCAAAAAGGTGGCTGCCGGAGGTGGGGG
CATGAACATTGTCATGACCTTGAATGTCATCATGTTGAACAAGGAAACCTTTCCACCAGA
GGCAGACATAACATAAGAATCATTTTTAGATAATGCGATGCATGCAGCAGATTCTTCAGC
TGGTTTACCGTCATTTATGTCATTGGTCATAATTGTTCCACTGGGAGGCTGCCACAACTG
GGGCACCACGTATGCAGTAGCCTTCCCCAAGGGATTCCTTTCACTATGCTGCCATTTCCA
CAGCTTGTGAACAGCACTGGCAGTAAGGGCCAATAATGCTAGGCCAGAGTTGGTATAGAT
CAACCGAACAACCTTGCTTGGTGCTATAGAATCAGGTAAGCGCAGAGCTTTCATATGTGA
TGGATCTGAAATATCAGGAATTTTCCAGCTCTTAACCTTGTCTCCATCATCCACAATACG
TGGTTTAACATCAACTAGTCTGCTGCTGTCCATAGTACCAATACTGTTGATAGATACAGC
TGGAGGAACTCTATCTAGGCGATCCAGAGTAGGAGCAATGGCACCAGAAACATTTCCCGC
TGGACCGAGAGCATTAACAATCAATGGCTTACTGTTGACGGGTTCAGTGGGGACCCGATT
TTTGTCAATAGCCCTGCTTTCTAACATTCTTATCAATCGCAAACCATCACTGTTGGCTAA
AATTTTGATACCATTGTCACTTGTTGTAACTGCCAGCAGAGATCCTTCCTTATTGAATCT
CAGTCTAGGACTAGCCGGCAATCCACCGTCTGCATCAACAGCGGTCAACATATTTGGATT
GTCCATATCCCAGAATTTAATTTGAAATTCATC
>ntig_540_consensus
ATCGATTTACTAATCATGTATGATGCACGTGGTATGCAAAACTTGGTATACATACAATTT
TAAATATATTTATAAAGATTGATTTTAATTGAATATATTTTTTTTAATTTATAATTTGAT
TAAAAAAATATTTTCCCGTTAGGGAAGTCATTAGAATAGTGTTGGAGCCTGGATGCCAGT
GGTGGGAAGCCACCCAAAGGCATTTAAAACATGAGAGCCTTGTCAAAAACAAAAGAACGT
CACCAAAAATCCAAGATGTTCTTATTAAAAGATAAAAATTCTCTATTATTCTTATATTAT
AGATGGGTTATAAAGACGTGAACGTAAAAATGAAGATTAGGATACTGTATCAAGTGTTAG
AAATTATAAAAAAATTATTTTTAACCTTCTCATAATTTTTGTTTTTACTTATGAGGATAT
GATTGTAATAAAAATTATTTTCCTTCATTATTTAATTTCATAACCTTACGTTGATAATAT
TGTAACACGAGACAGACAAAAACTGAGAACGAAAATGAAAATGGCATGGTTAAGTCCTGT
TTAGAGCCTAGATGATCCCTTGGGTTGAAGAATGACGATATGATTGTAGGCGTCCAGAGT
CAGAAAATCATCAAGTTTCGCCGCCGTGCATTGCCTGGTAAACAACTTACAGGCCTTTTT
GTATATTTCCTTCTTGAACTTTTCTTTCCCAACTTCCCTTTCAATCCTAGCCATTTCTTC
ATCCACAACTCTACCAAAGAGGTCACGGGTCACTCTCACTCCAAGTCCATCTCCATCCAA
TTCCACTACATATTTCAGCCATTGCCAGTTCTGAACTCTGCTAATCTCAGCAGTGGCTGC
ATCTTCCATCAGGTTATAAAGAGGCACTGATCCTGATCCAGTGAGCCATGCTGCCAAATA
CTGGATTCCAACTCTGGTATTTAGACGAAGACCTTCCAGGGTACGAACTCCTCTAGGCCT
CTGTAAGAGGTCTTCTTCTGTTATTTTCACAGCATCTTCTCGCTTCATGGTTTGTATCTG
ATTAGGGGCATCGCCCATGTTGTTGTTGAAGACTTCCATGCAGGCTGGGATTAGTCCAGG
ATGCGCCGCCCAAGTTCCATCGTGCCCAGCCCTGACCTCTCTTAGCTTGTCCTTCCTAAC
AAGATGTAGTGCTGCCTCATTTGCGGCCGTATCATCTCTAATTGGAATCTGAGCTGCCAT
GCCGCCCATCGCGTGAACACCGCGTCTATGGCATGTCCTGATGAGGAGATCGGAGTAACT
TTTCATGAAGTGTTGAGACATGTCAACCTGGACCCTGTCGGGTAGCAGCCGATCTGGGTG
ACCTTGGAATGTCTTCACATAGCTGAAGATGTAATCCCATCGTCCACAATTCAAACCAAC
TGAATGATCCCTAAGCTCAGACAGGATTTCATCCATTTGGAAGACAGCAGGAAGGGTTTC
GATTAGAACAGTGGCTCTGATGCTTCCTCTTTCTATTCCTGCCATCGCCTCTGCCCTTTC
AAACACAGAGTTCCATATTTTTGCTTCCCTTGAATGTTCCATCTTAGGGAGATAGAAGAA
AGGCCCAAATCCTTCACCGTGATTTCGCCGGAAGGCATCATAGTTGTGGTAAAAATATAG

GCCGAAGTCTACAAGGCAGCCAGTTGCAGGCTCTCCGTCAATAAGGATATGGGCCTCAGG
CAGATGCCAACCTCGTGGGCGGACGAATAGCTTAGCCGGCTGATTATTGAGCTTGTAAAC
TCTGTTCCTGGCCTTGTCATGGAAGCTTATGGTCCCTTCAACAGCATCCTTCAAATTCAC
TTGTCCACTAATAAGGTTCTCCCAACTGGGCGAGAGTGCATCTTCGAAATCAGCCATGAA
AACTTTAGCTCCAGAGTTGAGTGCATTGATGATCATCTTCCTCTCCACCGGGCCCGTAAT
TTCCACCCTCCGATCAGCAACAACAGCTGGAACAGGCCCACATGTCCACTCCTCTTCCCT
TATATATCTTGTTGCAGGATCAAACCCCGGTAACGCCCCTTCGTTGTACCTCCTTTTGGC
CTCTTTACGGCACTCCATTGCATACTTGATATGGTTTCTAAATTCTCGCTGCAAATCTGC
GACAAACTGCAAAGCATCGTTGGTGAGAATTTTTGAATACTCTTTACTGTATCGGCCTCT
AATCTCCACTCCCTGAGGGACATCATCGTATGCATTGATGTTTTCTGCAACCGTTGCTCG
GTAACCATTTGAACCATAGCCCATCATCTCGTTCTTGCTGCTTTATTTTTTCATAAAGCA
ATGCTTGTTAGAAG
>ntig_541_consensus
GCGAGATCATGTTCCAAATATCCGAAGCCTTCTCCATCATTTTATGCTGCACATATTCCT
TGGTGGGTCCTTCACGGGAGAAAGCAACAACCAACTCTGAAAGGGCACCACTTTGAACAA
AGTTGTTGAGCTCGTCCTCATAAATGTAATCCATTTGGCGATTGCGACATCCAAAAAACA
ATATGGAGGCTCCCAGCTCAATTCCATCTTCTTTCAGAGCAAATCTTTCCTGCAGGAAAC
CCCGGAAGGGAGCCAATCCAGTGCCAGGGCCAATCATGATAACAGGCACTTTAGGATCAG
TTGGAAGTTTGAAGTTCGATTGCCTNNNNNNNNNCTTCATCCAAGTAGAGCACACTCCTTT
GTGAATGCGTCCTGTGGGTGTTTTCTCATAAACCAGAGCACAAGTAACATGAATTCTGGA
AGGTGCCATCCTTGGTGATGAAGAAATTGAATAGTATCTAGGTTGCAAGTGTGGAACAAC
TGCTGCAAAGAAAACACCAAGTGGGGGCTTGGCAGAGGGAAATTCTGCCATGACCTCCAG
GAGACTTCTCTGACTTGCAACTACCCATTGTGCATATTCATCCTTTCCAGCAGGTGATGC
AAGATGTCTTAACCGATCAGCTTCAGTAGGGTCAGATGCATGAGCTGCTAAAGCAAGTAA
TGCAGACTTTTTAGGAGAACTCAAAAGATCAGCATATTTGGTTAATGCTGTTCTTAAAGT
GCAAGGTG
>ntig_542_consensus
CCCAAATATATTGAAAAACTATCTGCATTCAACTATAACTATAGTTACACACAATCAATT
TGATTGCATCCAAGTTAAGAAAAGCCATAAGCATCTTTTAACAATTATATAAATAGGTAA
CAAAGAAAGGAAAGTTCAAGCCCGTTAAATAATTTTTCCAGGGGACTGAAGCCACTGCAC
AGAACAAAAGGTCCAACTCATGAATTATTGGAGTAAAATCACACAAAAGGTTCCTAGGAT
GATTGCTGTATATTCAAAAACAGCTTAGCATATCTCTCCCTTCACATGACATTGCGCCAG
TGAACAAAGAAATTAGTTGGATTTATTATACCATAAGAATGTAGCAGAACTGCCAAGAAG
CAACTTGAGATGTCCTCATTTAGTCCACCAGTGGTAAAAATCCTTGCGTCGGTTGATTTT
CTTCTGCAACTGGAGAAGGCCATAGAGCAGTGCCTCAGCAGTGGGAGGGCAACCTGGAAC
GTAGATGTCCACAGGGACAATCCTATCACAACCCCTAACAACAGAGTAAGAGTAATGGTA
GTATCCACCACCATTTGCACAGCTTCCCATTGAGATAACCCACCTAGGTTCTGGCATTTG
GTCATAGACCCTGCGAAGAGCAGGGGCCATCTTGTTGGTGAGAGTGCCAGCGACGATCAT
ACAATCGGACTGGCGAGGGCTGGGCCTGAAAATGATACCGAAACGGTCCAGATCATAACG
AGCGGCTCCAGTGTGCATCATCTCCACGGCGCAGCAAGCGAGCCCAAAAGTCATGGGCCA
GATAGAGCCACGGCGCACATAGTTGATGACATCATCGACTTTAGAGATGACATATTCGGC
CGTCTTGGACAAAGCAGGCGGCTGGGCCGAGCGTGACAGCGAGGGAGGTGAGGGATGAGA
GAACGATGAGGGTGAGGAGGCAGATGAAAAAGATGGAAGAGAAGTGTGGAGAGAAGCGGC
GCATCGTGGGAGGAGGTAAGGGAGGCGCGTGGCGGTATTTCTGGAGATCATAGCCATGGC
TGATATTCTCTTGAGAAAGGGGAATCGCGCGGCTGAAGAGATCTGAGGAGGTGGGCAATC
GATCTCCCTCAAAAATGAGACAGTTGGGACAATTTTAATGGGAG
>ntig_543_consensus
AAAAATTTGTAAGACTTCTATGATGAAATCAAAGACCTGAAAAGCAATCAACACGATAAC
AAACAGGGAAAATGCAGCTCCGACTCTGAGTCCACCAAAGAGCAGTGAAGTCTACAAGTT
CCTAGCCAGTTAAATTGCTGTCCTAGAACTAAAAATTAAAACAAATGAATACCAGAAACT
ATTTGTATTGAATCCAAGTTAGACCAAAACATTAAGCATCTTCTTTAAATTACATAAATA
GATAATAAACAAAGGAAAGTTCACGCCTGTCACATACTTTTTACAGGGACTGTAGCCACT
GCAGATAACAAATGGTCCAATTCATGAATTATTGGTGTAATACCACAAAAGGTGTCTTCC
TAGGACGATTGATGTATTCTCAAAAACAGGTTTCATTGCACCAGTGAACATATGAAATGG
TGGAGTTATTATTCCATAAGAGTGTAGCAACTACTTGAGATATCCTCATTTGGTCCACCA
GTGGTAAAAATCCTTGCGTCGGTTGATCTTCTTCTGCAGCTGAAGGATGCCATAGAGTAG
AGCCTCAGCGGTGGGAGGGCAACCTGGAACATAGATGTCCACAGGGACAATCCTATCACA
ACCTCTAACAACAGAGTAAGAGTAATGGTAGTATCCACCACCATTTGCACAGCTTCCCAT

GGAAATGACCCACCTAGGTTCCGGCATTTGGTCATAAACCCTTCGAAGAGCAGGGGCCAT
CTTGTTGGTGAGAGTGCCAGCGACGATCATACAATCGGATTGGCGAGGGCTGGGCCTGAA
AATGATACCGAAACGATCGAGATCATAACGAGCAGCTCCTGTGTGCATCATCTCGACAGC
GCAGCAGGCGAGCCCGAAGGTCATAGGCCAGATAGAGCCACGGCGGACATAGTTGATGAC
ATCATCCACTTTAGAGATGACGTATTCCGCGGTCTTGGACAGGGCAGGGGGCTGGCCCGA
GGGCGAGGGAGGTGAAGGACGAGAGTATGAAGCAGGAGAAGAGGAAGATGAAAGAGAGGG
GAGAGTAGTGTGGAGAGAAGCAACGCGTTGAGAGAGGAGATAAGGGAGGCGCGTGGCGGT
GCTTCTGGAGATCATTGCCATGGTGATCCTCTACAGGAATTCGGCCCCTGCCTCAACGGA
TTTGAAGAGGAAGGCGGTTGCTCTCCACACTAC
>ntig_544_consensus
TTTGACGTATTTAGAATGAGATGATTGCAAAGCACTTGATGTTTGCTGTGGCAATGTCAT
ATTAGCTGCTGAACTGCTGTTCACACCAAGAGTCTGATTTCCAGATTGTGATTGTTGAGA
CATTCCAGGAGTAGGAATCATGGTGCCTGTTCCAGAAGAGACTCCAGACGGACCAAGACC
AGTCATCATATTCTGATTCATGCTGATTCCACTTTGTACCATTTGTCCTCCTGAATGATT
TCCCTGGCTCATACCAGGTACAGATTGACCCATACTAACACCTCCTTGAAGAGAACCCAT
CGGCTGTGAGATCCCAAGGTTTGAATTTCCAGACATATTAGAAGTTGCAGACGTGAATGA
ATGAAGACTTGAATTTTGTGGAACCTGTGAAGTCCCGGTAAGAGTCCCTGATCCAGTTAT
AGATGCAATGCTTGGTTGTCCAGATGAATAAACAGTTTGAGCAGCAGGAACTGATGAAGC
CATGCCACTGGATATCATGTTTGACATATGGATAGCCATAGGAGTTTGACTCATTGACTG
GAGTCCAATAGAAGTTCCTCCCGTCAGGGCTGCAGAGTTCATCACCTGTCGTGCTTGAGA
GAGATTATTCAGTATACTCACATTTGCAGCAGCTGGAGGAGGCACAGGACGCAAGGGCTG
TGACATGCCACTGACAATAGGTTTTACTTCTGGCACACCATCACCATTTGTGATCATCTC
TTGGGATGTAGAAGGTGGTGAAGAAGTCTGCAAGCTAGAGACTCCTTGAGAAGCAGGACG
GGCAACAGAAGAAATATGCAGAAAAGCAGATCCATTTACCATGGGAGTTACAGTGGTTGG
CTCAACTTTTACAGTAGCAGTAGGAACACTTCCAACAGAAATTGGTTGCCGGCTCATCAT
GGATCCATTCACTGATGGAATAGATGTTGGAGCTGGCCCTGTTACTGAAGTAACAGGAGC
CATGTCCATTTTTACAGGATTTTGATTGGCAGCCAAATTTGTAACTCCTGAACGACTTAG
AGAAGCACGAGCCTCCATAAAATTCTCTGAGATCAGGATAAGAAATTGAGGGGTTTTACC
ATTATCAACTGGTGGATCTGCTGCTCTTGGATTGCGCTTTCCCGCATTGTAGATTGCTCT
GAGTTTTGGAAGCTGTTTTGGACATATGACAGATAAAGAAACAGAGCACTGAGCAAATGA
TTTAGCCACTGTCTCTGCATCAGATAGGCGACTTTCAGCCTGCATTTCAATGTTTTCACT
CTGCTCCATATTCTGCATCTGTGGTCGATAAACTGGTGTGGGCAAGGGGTAAGGATTACT
TGCAGCGACAAGAATGCAATGCCTTTGCCCATCCACATTTTGTTGATTTTGGCTTCCATT
TGAGGCAACAGAAAACATCATTAGAGCTTCAGAAAGCCCTTCTGCTATTGCAGCATCATT
GAAACCACCACCAGCAAAAGGTATGGTTGATAGCCGTTGTAAAAAGATGTCAACATCTTT
TGTCCATCCACTCCGCTGTACCAAACATGCACAATATGATCCATGGGTATTAAAGGTGAC
CACCGACAGTTCAACATTTGAGGTAGAGCCCTTCTGCCCAACCAACTCATTACCACAAAA
AGACCTGATTATTTTGTCGAGGTAATCAGAGACGATAGTATGCCAGAAAGGGCCCATTGC
GGCGGTGCCTTCTACGGCCACGATCAACTGCTTCTCCGACATGGTGGATTACGCAGGAGA
CGATCAGAGTGGGCCGATGTTAGAGGTCGAGCTTCCCTGTGTCTAATTGTTGGGGTGTGT
GGGGAAAATTTTTTAAAATTTGTTTCCCACCCGAGGTAGAGTGTTGTTGTGGGAGCTGAG
GTTTTGGAGCTCGGTTTGCCGTGGGGATTCGTACCCGTTCTTTTGATGGGCGATATCCAT
AAACGGTAGGTCAGTTTGGACTGTTGATGATGAAGGGGCACAAAACGCTCCGTTCAAGTG
TTTCCT
>ntig_545_consensus
CCATGTATATACATAAACATTACAAAAGAGAAGGAAAGAAAAGAACCAAATCCTACTAGC
ATGTCAAGTAGTGGATTAGATAGCTATTCAATTTCCACATTTCACTGCTTGTAGAACATG
ACAATAACTTTTGATGTTGATACAAAATAATTCCAAAAATCTGAGAGTTATATTTCACAA
ATTTCCCTTGAAGATAAATGAGAAGTTTACAAGACAAGACTTAGTAAAGTCTTTCCCGCA
ACACATTGCACATGTTAAAACTTCCCCTGTATTGTAGAAATCCTCACCTTTTCTAATAAA
CTTAAGTCCAAAAATTTTACTTAAAGAACAAAAAAACCCAAAAGGGTAAAGTGGACACAG
AAATAGTTCAAGACACTGTCACAAAGGTAACACAGATCTACCATCTTTCCCAAGGAGCTA
ACAATAAATATTTCAACTACTTCTTAAGAGGATTCATCCGGTCCCTGAGATAGTAAAGTT
TGGCCCTCCTCACTTTTTTCTTGTCTAACACTTTTATCTCCTTTATGTTGGGAGAGTACA
GCATGAAGAGATTTTCAACTCCAACCCCAGCAACCAGCCTCCTTAATCTAAATGTAGTAT
TTAGACCAGCATTACGCCTTGCTATAACAATACCCTTCAAGATCGAAACACGTCTTTTAT
TCTCAGGGACTTCCACTTTAAGTTGCACAATGTAACCAGGCCTTATATCTGGTATTTCTC
TCTGTCCCTTCACCTCCTCCACAGCTTCCTTATCTACAATCTGCATTATGTGCCTAGCAG

TTTTATCAAGCCTCTTGAATTTAATGCGGGGAGCTACATCAGTGATAGCAGCAGAAGGAT
CTTGTGAAGCTGATGCAACAGAATGTCCAATCGTCGTGAGGCACCTACTGGCAAAAGGTT
GAAACATGCCCGCCCTTGTACTTCTACCATAATAGTTGACTGATGTCAATGACTCAGTTT
GGGAGCCAAAATTTACCTGAGAGCGAGAAGCGAGCTCTTGTGGTATTCGCTTAAATAGAT
GTCCTGCCGACTTGGACGAAAGGAATCCTGTTGTAAGTGAAGAAGTGTTCATGGTAAAGT
AATGTCTTTGAACCAACCGGATACTCCCACGAAAAGACAACATTTTTTCCCAAACACTGA
CTGTATTTGAGATGCTGAGAAAACAACAGGATTAATTTGATTACAATAATACCTCTGAAG
ATGGTTTTTCTCCTG
>ntig_546_consensus
CAAGAAATAAATGTTTTTTGATAGGTTAGGTTTTAAGGTTGTTCGTGTGAGAAGAGTTAA
AAATGTATGATTCTGTTATTGTCGTCGTTGTAGTATAAGAGTAAACCAGGAGTTTGGAAG
CAAATGGAAAATTTACTATTCCCAAGTTACCTCGTTTGGGGGTGGTGAAATAATAAATGG
AAAGCTTAAAGAAAAAATGGATATTTTTAATTTGTAAGACATGGGGAGCTGAAGAGGGCA
AATAGACAATTGAGTTTTGATTAACATTAAACAGGATAAACACATCGGACACAAAAATAC
AGGAACATCAAGGACAACAACACTTGAAAACACAATCATGTGTAAGCGCGGAGTAGGACT
GTAACAATTTGCCAGGCTCCTCGGGGCTTCTCTCCACAGTAATGCCGATTTCCTACCTTT
CCCCTCTCATTACCAGTTCCTCTCCTTGGAAGAATCATTTTCATGAGTGCCCAATCGCCT
GGCCACCTCCTCGGACTCGGTTACTTTTCCCATAGGCCTATCTGTTTCATCAGGTTCTTC
CTTTCGCTTGTGTAGAGCGTCTGAGATTACATCACTGACAGGCCTAGGGGTTGATGCAGG
TTTCTCCTGATTCACTTTCTTCTTTTGCAAAGCCTCAGATATCACCTCTGATAGAGCCCT
ATCTTCTTCTCCCGGATTCAATTTCTCTGCAAAATAGTCCTTCGCTGATATTCCTTTATC
TTGCTCTTCAACCCCACGTGCCTGGGTGTTGCCAGTACCAGTACGTATGGTGCAGCCCTG
CATTTTTGACATGACTGTACATCCTGCTCCTGCAACACTCTCATAAACTGGAGTTAACTT
CTCTGTCACAGCAGTAGCCATTTTCTTTCCATAGTCCGTTACCGATTCTGGCATGGAACT
TTTGTCTCCTACATGAGTTTCATATCCTTCTCCGTGGTGTTTNNNNNNNNNCGGATGAAA
TTTTCTCACTGTAGCTACCCTGGTTCGAGGGCCTATCAACTATGTTTTCTTGCGGGTAGT
CTTCTGCGCTTCTACGTCGTGGGGGAGGTATTTCTGGCAAGAACTGATCGCGCTCATTCG
AGGACCTGCCAAAAGTGTTCTCTTGTGGGTGGTTTTCTGTGGTCATACGTTCTGGGGGAG
CTAATTCTGGTAAGAACTGGTCATGGCTTCCAGTTGGCACACTGGTAGGAAAATCTCCCA
TTCCAGAAGGTGAGCTCAGTTTCTCTCCTGAATTTAATTTTGGTTCGTCATAGATTTCCA
TTTTGTCGAAAGACTGAATAGCAGGGGAAACTCCTCCTTCTTCACCACCTTTGCCAATGT
GGTAACTGTCTTTGGTATGACAATTCCCAGGAACATAAGCACCAGCTGTGTCCTTTGGGG
CGCCAGGATCCTCCACCAGGCCATTTGGCCTTTGCAGGTTGACTTTCGGTTGCCCTGAAT
GTTCCTCTTCAGGGTAGAGAAAGGTTCTGGAAGGGTCACTGTCTCGGGTGTCTTGATTTC
CAGGATCAGTATGAGATGCAGGTGTATTCTGTGGTGCTTCAGAAATTTCACCCAGGACTG
TTGTCAAACCCAGATTGACCCTTTGATTTCCCTTACCTACGTCTTTCCCACGATCATCAG
GATTCAGGTTTGGCAACCGAGGTTCAACCTCCATGCCCGTTGACCTCCCAGTGTCGACTC
CAGAGTCCACTACCCCAATGGTCTTAATGGCAGTTGCCTCGTATATTGGTGCACCAGGAA
CTTCAGGATCTTCAACCAGTTCTTCCTCATCATCGTCTTCTTCATCAACATCATGATCAT
CAGGTATATGATCACGATGACCATGACCATGTTTGTGAGTGTGTCTTTTATCTTCCTTG
CCTTTGCCTTGACCTTCTTCAACACCGAGTTCTTCTCATGGTGATGCTCATCTTCACCTT
CTGTCACGCTACTGGCGTGATGCCCAACAGTGGGTCCTTCATGCTCATGAGTTTTGCCAT
GACCATGAGCATCTGCCGTCTGGAATTCCATCGGAACAGCGTTGACACAGAAGAGAACTT
TATAGAATTGGTAGTTACAGATAGAAGTTGAGAATGTTTGGATGTCGAAGACGATG
>ntig_547_consensus
ATCGATAGATTCAGATGTTAACTGCAGACAAGATAATGTAAGGAGAAACTTTTGGTGACT
TTATTGCTTCTCCTAGTGTTCACACCTCAATAAACAAAGTACATCATCCAATCAAATCCA
TCAGCACAGACGTCATAATCACAGCCATGGCATCATCTGAATAAGACATCATTTTTTTTT
TTTTGCGGTTTCTGCTCCTAAATACAATCATCTTTCTGTTATTTCTCTTTGTTTTTCTAT
CTACAAATCCCGTTCACTTCCCTGTCTACCTATCTATCGCAAGGGAAGTCCATGTCATTC
TCAGTTACTTTAAAGTGCTGGGGCTTCTGCCCTGAGCAGTGGAGATTCCACATTTTGAAG
TATGACCTCTCCAAATTCTTAACCCAGCGTGCTGTATCAAATAGAGGACATGTAAGGCGA
ATTGACTTGAGTTTATTTGCAAGTGCCTGGAGCTTTTGCCTATTCAGTGCCAAAGATACT
GCCCTTTCTTCATATTCCTTCATACTATTAACAATCATCTCATCTCCAAGTCCAGTAGCA
CGGCAAAGCGATCCAGCAACCCTCGTAGCCATTTTCTCAAGAGGTAGAGTTATCATTGGT
AGACCTGCCCATAAAATATCTGTACCTGTAGTATGGGCATTGCACAAAGGCGTGTCAAGA
AATAGATCTGCTAAAGCACTGCGCCTGATATGTTCATGTTTCATGGCAACATCAGTGAAA
ATAATTTGATCTGGCTGCAGACCTTGGGCCATAGCATATGAACGAAGTCTCATCTCCCCT

GCAGCAGGGAACCTAAGGAGCCAGAGTGCACTGTTGGGTACTCGTTTAAGAATATTGCAC
CAAGTATTGAAGATTTCGGGATCCATCTTGTACAATTGATTGAAGCATGCAAATATGAAC
TTGTCTACAGGGAGACCATAATCTGATCGTTTGTGCTGGCAATTTGGATCCAACACATCA
AGATTTTTCTGTTTATAATCATTAACAAAGTAGCAATGTGGGAGATGCACCAGCTTTTCT
GAATATATATGTGCATAGCGTAGAGGTGAAACAAACTCATCAGTGACCAAATAATCAATG
TATGATGCACCAGTGGTCCCAGGAAACCCCATGTATGAAACCTGTATAGGTGCAGGTTGC
ATAGCAAATATTTCATTCCTAGCTCCCTTGGTATAGCCATTGAGATTCACAAGGATCTGT
ATCTTATCCTCATTGATAAGTTTGGCAATCATATCTGATGACATAGCAGAGACATCAATG
AAATGCTCTGCTTCAGATTGTGTACGCTTTCTCCATTCAGTACCATCATTTGGACTTAAT
GCATAACAGAACACCTCAACATTTTCTCTGTTGTGCATGCCAAACACAGATCCCATAAGA
TGTGACAGAGGGTGATTACCAAAATCACTGCTCACATACCCAACCTTCAGCCTCTTAAGT
CCAGCCTCATGCCTAATAGGAACTGGAGCAGGGTGGTTAAATGGAGGAAGTGCAAAGCGG
GATGCAATAATAGAGCAATGTGCTGCATATTTACGGCTTATTTCAAGTGCAAGCATTGGA
TCAATGGGATATGCTATAGCATGAAACGGTTGGACACTGGGAAGAACTGACAAATTGATC
TGCCGCCTGATAATCCCTTCAACTTCATTGAACATTCTGTCACGATCTTCCCAACTGCAG
ACACACTGTAGGGTATGTAGAAGGTTGCAAGTGGCTTCTGGAAAGTCAGACCGAAAAGCC
AGTGCCTGTTTGTAGCTCTTGATAGCAGCCTCCACATGTCCACTATCCTTGTATGCTGAA
GCCAAATTTGCATGGGCTTCAGCCATGGTTGGCCGGATAGTAATAGCACGTATATAGTCC
TGGATTGCATCACTCACTCTACCAATCTCTTTGTAAGTGTTTCCCCGATTGACTAGTCCA
TCAGCAGCCAAGGGATCAATGCGGAGAACCTCATTGTAACAAGATATAGCATCAGCATAA
TTTCCCTGTTGTTTATTTATTACAGCAAGATTGTTATAGGGCGCAGACAATCCTGTTGTT
ACTGTCAATGTTGCCTTATAATATGAAGCAGCAGCAGCTAACATGTTCCATTCCATGTAT
ATATTCCCAAGGTTGGTAAGAGCTTGTGGATGGTTTGGTTGTAAGGCCAGACATTGATTA
TAGCACTGAATTGCTTCCTCGACTCGACCCACATCTTTCAATGCATTACCCATATTATTG
TAAGCCTCCAAAAATCTAGGATCAAAAGATATAGCTTGCTTGTAATAAAGGATTGCCATA
TCAGTTTGACCTCTCTCATAATATGTACTAGCCAAGTTACCAAAAGCTATTGCATTGGGA
CGAGTCTGAACAGCACGCTGATAACACACGATAGCTTCTTGAGGCATTCCCAAAGCCTTA
TAAACATTTCCAAGATTTAGGTAGGCATCAGGAAATGTAGGTTTAAGTTTCACGGCTTCC
TTGTAGTATTGGAGAGCTCGATTAAGATCTCCAGACTCCATAAAGAGACCCGCAAGATTA
GACCATGCAATAGCAAAAGTAGGTTGTATGCGTAAAGCCTCAAGGTAGCAGCTGTATGCN
NNNNNNNNNNNNNNNNNNNNNGCAAGCGCCTGGCGACAGCACTGTGCTGCCTCATTAAG
TCTTCCCTTTCGCATGTATGCACTAGCCAAGTTCGACCATGCATCAGCAAACTTGGGGCG
AAGCTCAATGGCAACCAGATAATAGCGAATGGCAAGATCAATATCACCTTTCTCCTTCCA
AGCATTTGCCATGTTTCCATAACACTCAGCAAATCGTGGTTCAAGTCGAAGGGCTTCTTC
ATTTCTGGCAATGCACATATCATACTCATGCAACTGATAATAAATCGCACCCAAGAGAAG
AAGATTATCTGTTCGTAGAGGACTTCTCTCATAAACGCTATTGCTATGCTCTAGAGCTTG
TTTAAAATTGCCAGCCTTGTACATTTTATGAGCAAGGGCCATTTGAATATCTTCACCGAC
TTCATGAGAATCGTGGCCCTTGAACGACGACAGACTCAGCGCTGATGCCGAGTCCTGCTT
CACAGTCGGAAATCCAACCTCACTGGCCCGATCCGAGCCCACCAACGCCCGCGAAACTCC
GTTTTGTAACGAAATCATAATGATCTCTTTTTCCCTCAAATCAAACTCAAACTTCCAAAA
TAAAATCCGATCATCAAACAAAACCCAGATCCACCAGTGCAAGATCAAATAAACAAAAAC
GGCGAAGAAGATGACTCGATAAAAAACCCCAGGATGGTGGTTTAGCGAGCTTCCTTCTTC
AAAACGACAGAGTCTTAGCGTTCTAATAGCGTTGTTTAAGG
>ntig_548_consensus
CTCTAAGATTCACTCAAAAGAAAATATAAAGGATCGACCCCTGCGTATGGAAAAATGATT
TATAACAAAAACTGGTTTTCATCACTTAAATCACAAATGCAAAAATGTATATTAATTACG
TCAATCAAACAGAGTTTTGACAAATTATACCAAGTTTCTCCAGTGTTTCATTTAGAACCA
AATGCAAACGACAACTTCGGCCGAGATTTGTTCTTACCCAACAGCTTCATCTGATTCAAT
CGTTCTTCCTCCTCTTGCTGTTTCTTCCTTCTCAAAGCCTCTTCTTTTTCTCTTTGGATG
CGCTCCAGTTCACGATAACGCTCCTCCTCTCTTCGCTGCTGCTCTTCCGCTTCTTTTCTC
TGAGCTTCTTCCACCCTCCTCCGGTTTTCTTCAAGCATTTTCTCCAATTCTTCTTTCTCT
TTTCGGACTTGTTCCTCCTTTCTTCTAGCCTCGATTAGAGCATCTTCTTTTTCTTTCTCG
AGTTGAGCTGTGACTTCATCTAGAAGTCTCTTCCTCCCTTCCTCCAACCGCTTCTGAATT
TCCAGCTTTATCTCCACAGAGCTCAAGCTCTCTTCAACTTTCTTCCTGATAGCTTCTTCC
ACCCTCCTCATTGTCTCTTCTTCTATCAGTTTCAATTCAGCTTCCTGCTGACGCC
>ntig_549_consensus
AGGCGGAAAAGTCATTCAATTTCCCCACGACGCCGCAAAGCTCATTCTCCGTCTCCAAGG
CGCCGTAAAAGTCGTTCTCCAACTCCAAGGCGCTATAAGAGACAAAAAAGTAGGAGTTCT

TCACTGTCTCCTACACGTAAATCCTCAAGTCCAAGTCTTGGGTCATTAGAGCAGAAAAAT
GCAATTGAAAAATTAAGAAAAGAAGAGGAAGAGAAAAAAAGGCGTCAACAGGAAGCTGAA
TTGAAATTGATAGAAGAAGAGACGGCCAGGAGGGTCGAAGAAGCTATTAGGAGGAAAGTT
GAAGAGAGCTTGAACTCTGAGGAGATTAAGTGGGAAATTCAGAAGCGGGTAGAGGAGGGA
CGGAAGAGACTTCTAGATGAAGTCACAGCTAAACTCGAGAAAGAAAAGGAAGATGCTCTA
ATGGAGGCTAGAAGGAAGGAGGAACAAATCCAGAAGGAGAAAGAAGAATTGGAACAAATG
CTTGAAGAAAACCAAAGGAGGGTGGAAGAATCTCAGAGAAAGGAAGCGGAGGAGCAGCAG
CGAAGAGAGGAGGAGCGTTACCGTGAACTGGAACGCATCCAAAGGGAAAAGGAAGAGGTA
TTGAGAAGGAAGAAACAACAGGAGGAGGAAGATCGTAAGAATCAGATGAAGTTGTTGGGT
AAGAACAAATCTCGACCTAAGTTGTCGTTTGCGATTGGATCTAAATGAACACCAGAGAAA
CTTCATATATATTTGTTAAAATGTCTCTGCTTGATTGGCTTAATTAATATGCTGGTTTGG
ATTTGTGATTTAAATGATGAAAT
>ntig_550_consensus
CACTAGGAAAATGGTAAGCTCCAGTCCAGTGGCCAACCATTTTTAACTTTTTAACGCTAG
AGAAAAAAAAAAAAACTCTTCCCTTTATGTACATAGCCAACAGTTCAATCTACCTTAAAA
AACCTATTAAATCGAACATCTCGCTCCCTTCCCTCAACCTTTCCTTCAGAAATTTATAAA
GGAACTTTGCGATACACCCAACATCTCCAGAGTCACTCTTCTGAGCAGCGACCCATTTGA
TGGGCAACCAAACATCTCTCCAACGAATCAGAAGTCAAATTCTCCCGTTGCCTTATTAAA
GAGAATGTCCTTGTTGTTTATATGCATAATTCCATCCTTCAATGCACATTTCCATTTGCT
TTTGGTTCGAGTCACCTTGTCAAACTGAGCCAACACTAAATGTTGAGTGTTCAGCTCCTC
TCCTTGATCCACATCATCCAAATCATCTTCATCATCATTTTCATTCAATGGCTCATCATC
ATCATCATCATCCACAGCCTCATTTTGTGCAACAATTGCAGGAGTACTAGCCTGTAGGTC
AGTCGGAGCAGGTGTGTTCACTACGTTGTAATCTTCACTAACAACTCCTTGGTAGTTGTA
TATATTGGGAGTAGAAAGCACATCGTCATATGGATCAGGAATTGGTCCATCCAGTTGAGG
AATCTTCACAGATGACATGGATGAATGCACTAGGGACTCCCTATTTGAAGTGGTAACAGT
GTTACCTTTACCAGTGAAATCACTGCCTGGGCTTACCTCAAGCTCAAAAATCTTCAGTAT
AGCATCTCCAGCTCCATCTTGCTGGGGTATATTATATCCACCATTCTGGTACTGTGTAGG
GAAATCTTCACGTTTACGTTTTCCAGATGACATCGTGAAGAAGTCCTGTGTAAGGGGTTG
ATGAGAGGTTCCTCTATCTGCCTCATCACGCCCTTCCACATAAGCAACATTAACATCAAC
ACTTAGAGGGGTCCTCTGATTCATCCATGGAGAAGGAGGAGGTGGCATGTATGGGCTTGG
CCTTCCATTTCCACTTCCACCTTTAGCCTCAGTACTAGCACCAGAATCATTTACTGGCGT
GGGATAATCACTAGAACCAGTAGGGATGTTATACATTGAGCTATCCGCAGTTCCTGGCAA
TGGGGTACTTGTGGGTAATGGTGTCTGAATGGGAGTTTGCAAAGGAGTCGGGGGAAAGAG
TATCTCAGCAGTAGGTGTTTCATACTCCTCCGTCCCTTCGTAAGGTACATTAAGATCGTG
AACTGGAGTTACACCAGATGCCAGCTTTGGCGCCGACAACCTATCTATCGGACCACTCAC
AACTCCTGCTTGCACCATCTTCATTTCCCAAATTCCTTGAAGATCGTTGAGAACACTCTC
GCCAGGACCGCCCTTGACGAACTCCTCACGGACCTTGCTGACAACATCTTCGATGACGCG
GATGTAGACATTGCTGGTCGTCGATGTCGTCATTTCCTTTTCAATCCGAATTCAATGCAA
AATCTCCCCAACTCCTCCAATAAAATGTTCTCGGGAAGAAAAAAAGATAATCGAAATAGA
AAGATGATTCTCTCGAGAAATTCAGGGGAGCATCAGGAAAATCTTAGGGTTTCATTTAAT
TATGAGATGTTTCCGGAGATGAGAGCCAGCTCAACCGAGAGAGATCGGAGAGAGGAGATT
AAGAACGAGAGGGGGCGGACGAG
>ntig_551_consensus
GCAGGAGTACTAGCCTGTAGATCAGCTGGAGCAGGGGTGTTCACTATGTTGTAGTCTTCA
CTGACAACTCCTTGGTAGTTGTATATATTGGGAGTAGAAAGCACATCGTCATATGGATCA
GGAATTGGCCCATCCAATTGAGGAATCTTCACAGTTGACCTAGACGAATGCAGTAGGGAC
TCCCTATTTGCAGTGGAGAAAGTGTTACCTTTACCAGTGAAACCACTCCCAGGCCTTACC
TCAAGCTCGAAAATCTCCGGGACAGCATCTCCAGCTCCATCTTGCTGGGGTATGTTATAT
CCACCATTCTGGTACTGTGTAGGGAAATCTTCACGTTTACGCTTTCCAGATGACATCATG
AAGAAGTCCTGTGTAAGGGGTTGACGAGAGGCTCCTCTATCTGCCTCATCGCGCCCTTCC
ACATAAGCAACATT
>ntig_552_consensus
AGGAGGCCAGATCTGGCCGCCTCCTCCAAAACTGGAGGCGGCCGATCTGGCCGCCTCCAC
CAAACGGGGAGGCCAGATCTGGCCTCCCCTGGAACCAGGTGGCCGGAAATGGCCACCTAG
TTCTATTATGGGTGGCCACTGCCGCCGGTGCAGGCGGCGGTGACTACCGGATTTTTTTAA
TTTTTTAAAAATTTTTTTAAACAATAAAATAAAAATTACTTAACTATATAGGTCCATCAA
AACAATTTGTTCTAGGGGATATTCTCACCCTGAGCTTAAGGAAAACTAGCAACATAAATG
ATAAACAAACCTTGCTAACAGAACATAATATCCCATGAAAGAGGACACCAAACTCAGCGC

TTTTTGGAGACACCAACAGTCTTTCCACGCCGACCAGTTGTCTTTGTGTGCTGTCCACGG
ACACGGAGACCCCAATAGTGTCGAAGACCACGGTGGTTTCTGATCTTCTTCAGACGTTCC
AAATCATCTCTCAGCTTCATGTCCAGTGCATTTGATACAACCTGAGAATACTTGCCATCC
TTGTAATCCTTTTGTCTATTCAAAAACCAGTCTGGGATTTTAAACTGACGGGGATTTGCA
ACAATCGTCATGAGATTGTCCAGCTCAGCTCTCTTGTTCATGTCAACATCAGCCTTCTTG
CAGACGATGTTAGCGAAACGGCGACCGATACCTTTAATGGAAGTGAGAGCGAACATTATC
TTCTGTTTGCCATCCACGTTCGTGTTCAATACACGAAGAATGTGTTGAAAGTCCTCGTTC
GCCACCAAAGACATGGTGTTGTGGGTCTGCCTTGGTTGCTTGCCAAAGAGCCCTTGCAAA
ACCTTTGAAGGTTGCCTCAATAATATGGTGAGAAATTCTTCCAGCATGCTGCCGAATATG
GAGTGTCACACCTGAAGCATTCACCCAAGACTGAAAAAAGTGCTCCACCAACAGTTTAAT
TGTACTTCGATTTCCAAATTCTCAAAGATGCAGAGAGTATCATACTAAGTCAAATCTCTA
CAGGACAAAGAGTTCCACTGGCAAGCGCAACATCTTCGTTGGGATGGTGATCAATGATCA
TCAATGTGAATGTCTCCAGTTGCCCCTTCTCGCACCTAACATGATTAGCTGAATCGGTGG
CAATTTGCATGGAAGAAGTTGCATCAACT
>ntig_553_consensus
GGAGTTTGAGATTTCATTTGATTAAAACCATATATGTACCATCAACACCAATAACGAGGG
CAGACATTGCACCAATCTTTTGATTAAATGTGATATATATTGTCTACTTGACTTGATCAC
TTTTCTTTTAATATATTATTAAAAAATTTCTTAAGTAAAAAGAAAATCTTATCCTTCTAG
AGTCCGTCCACTACTCCTTGAATTTAGAAGACAAGAAAGGCATGGAAGGCCTTCCACATA
GAATGTTGTTGGGCAATCTCCCTTTAGTGCAGGTGACTGCACCCCACCTTATATAACATC
TTATTGTAAGTTCGTAAAACACCATTAAGGTGTCAAATAAAGGTAGATGATAATCAGATA
GTCACCAAACATTATTCTTCCACATTTGGGAAATATCACCTAATTGTAAAACAAGAACAA
AATCAAACAACAGACACTCAGCATTGGATAGGAAATCACTAGATGTTCAATATAACTGAT
GCGAATTAAGTAAACCAACTGTAGATAAACACTTAAAATATATAAACGGAAAATTATTAG
ATAGTACTACTCAAAAAGCTCAAACAGTCTTAATACTGGTGGATGCTAAACCTAGAACTA
AAAGATGAAACAGGTGAAGAAGCTTCCCCAAAAGAGTATCCCCTCCAGTCTTGCAGCCTC
ACCAGTGTAGTCATATGCTGCAAAGCCAAAGAACGAAGATACTTGATGAGAAACCCCATC
CATGTTCCCGAGCCTACTTAGATCTCTGCCTCATCTTTCGGCGCTTCCTCTTCAACCTTC
TCATACGCTTCTTCTTCCACTTAGCTCTCATCTTGGATTACGATCTCTCTTTCTTGGCCT
ACTTCTGTAAGACGATGAAGAACAATCCTTTGGTTCGCCGAGAATGCTGTGGATGTGAAA
ATATGAAATCAATTATCAGGTTAATATTTATAGGGTTAGGGTTTTCTATCGCTGGAGCCC
TAATTCCCCCGGAATTGTTTAAAGGTTTCTAGACCGGGTCGGCCCGGGCGGGAGCGAAAA
CTGAGGC
>ntig_554_consensus
AGCTCATCAAGTAGAAAAAGAAGGAACAAAAGACCAGAGATAATATGGCACACAAAGTCA
AAAAAAAGATGCCAATTGCTGCTACCTCCAACCATCTCATCCTCTCCAAAAATAGCAACT
CTAAAACAAATCTGGAAAACAAAACACCACCAAACCAGAATATGATGGGTATGAACAATT
AAGGCAGCTAGCAATATATGCAGCTTGTTCACCGTAGGCAGTCTTTTGGGATCTATTTTT
TTACACAATTTGAAA
>ntig_555_consensus
GCTGAAAAACGGATGCGCGCTAGTAGCGTGGCTCGCTTCGCTTTTGTTGCGGAGCTCGCT
GCTTTTCATTTTGTTAGGAGTAGGTGCTTGCCCTTAGTTGGTTGGGGGAAGAGGGCATTT
CCATCGCGAAGGATTCAATCCAGCCACAGGTTCCCCTACGGCTACCTTGTTACGACTTCA
CCCCAGTCGAAGACCCCACCGTGGTATGCGCCAATAAGACCACCAAAAGCCTTTGTGGCA
CTAGTGGTACACAGAAGTCATGGGTGATCATTGGTCCGATGCTTCGGGCGAAACCAATTC
CCAGGGTGTGACGGGCGGTGTGTACAGGGCCCGGGTACATATTCACCGCGGCATGCTGAT
CCGCGATTACTAGCGATTCCAACTTCATGTTCCCGAGTTGCAGAGAACAATCCGAACTGA
GGCAATCTTTCCGGATTCGCTCCGCCTTACAGCCTTGCTTCCCATTGTAATTGCCATTGT
AGCACGTGTGTGGCCCAGCCCATAAGGGCCATGCGGACTTGACGTCATCCCCACCTTCCT
CCAGTATCTCACTGGCAGTCCCTCGTGAGTGCGGCACGCACCTTTTTGTTTGTTTCGGAG
CCAGGGGCGTACTAAACCCACTACGTACCACACCACCGGGCGGCTCGCCTGAATGCCGAG
TCTTTCTCCGCCGCCAACTCGACGTCGTCGTCACCTGCAAGATAAGGCCAAAAACTTGAC
TTTACTAAACAAGCGAGAAAAGCCCTTTCTATCTTATTAGTAAAGCGCGCTAGCTGCAAT
CAAACTAAAGCGCACAGCACACTAGAAAGTGCTTCGAAAGGCGCCGGCTCCCTTCTTACT
GACAGCACACAGCTGTGTGCTGGCACTCAATTCGAAGCGCTGGCACGTCACTCGGCTCAC
TTCGGTTGCAAAGACTTTCTCCTTAGGCGCATGTCTCAGCAACACAAAACGAGGGTTTCG
CTCGTTATAGGACTTGACCAAACATCTCACGACACGAGCTGACGACAGCCATGCAGCACC
TGTATGAAAGTAAGTACCATCCCGTTAAGGACAGGTTTTGTTGTTCATATGTCAAGGGCT

GGTAAGGTTTTGCGCGTTGTATCGAATTAAACCACATGCTCCACCGCTTGTGCAGGCCCC
CGTCAATTCCTTTGAGTTTCGGTCTCTAGACCGTACTCCCCAGGCGGAGTGTTTCACGCG
TTAGCTGGGCCCCTGATCCGCGTAGACCAAGGGCGAACACTCATCGTTTACGGCATGGAC
TACCAGGGTATCTAATCCCGTTCGCTCCCCATGCTTTCGCACTCCAGCGTCGGTAGGGAC
CCAGAGAGCTGCCTTCGCTTTTGGCGTTCCTTCGTAGATCTCCGGATTTAACCCCTACAC
ACGAAATTCCACTCTCCTCTGTCTCACTCAAGTGAATTGGTTTCGAGAGTCTTCCGCCAG
TTTTTGGCGACTTTCACTTTCAACCCGATTCACCGCCTACGTGCCCTTTACGCCCAGTCA
TTCCGAAGAACACTTGCCCCCCCCGTCTTACCGCGGCTGCTGGCACGGAGTTAGCCGGGG
CTTCTTCCTCGAGTCCTGTCATGATCGCGCACTCGACGAAAGAGCTTTACAAGCGGCATT
GCCCTTCTTCACTCACGCGATATTGCTGGATCGGGCTTTCGCCCATTGTCCAAGATTCCC
CACTGCTGCCCCCCGTGGGAGTCCGGGCCGTGTCTCAGTCCCAGTGTGGCTGATCATCCG
AAAAGACCAGCTAAGCATCATTGGCTTGGTCAGCCTTTACCTGACCAACTACCTAATACT
ACGCAGGCTCATCAAACAGCGCTTTTTAGCTTTCTTCAGGATTTGGCCCGAACTGTTCGG
CAGATTCCCACGCGTTACGCACCCGTTCGCCACTTTGTTCTCAACTGTTCCCACCTCCTG
GGCGAGACAACTTTAGCTAGGAGCCTCTTTTCCTTCTGCCCCGAAAACAACGTTCGACTT
GCATGTGTTAAGCATATAGCTAGCGTTCCTTCTGAGCCAGGATCAAACTCTTCTTTTGAC
TATGATTGGGCCCTGCAGTGGTAGAACCTGGTGAACCGGGCGTACCATTTCCCAACCTTC
TGTGGACCTTGCTTCTGGATCTTCCGCGGGAACCGTATG
>ntig_556_consensus
CCTCTCTGATTTTAGTTCACAACCATCAAAATTTCAACACAGCACAAAAGGGGTTCCAGA
TAAAGCAAAACAAGCTTAATGTTTTCCATAATCAATCAACTAAAATAAACTTGAATCAGA
AATATATACAGTACGATGGCATTTTGCCTGAACGTTTAACATTGGTGAACAATCGCAAGG
GGTTCTTTTTATGACACTGAGATTTAGATGCCCATTACTGTAACAAAAGTGATAACACAT
TACATTACTGAAATTTTATCAGACAGAAGTTGAACCTAATCTTATATCTCACCCAAATCT
TACAACAACAACGGAAATGTCATCCGAGCTGTTTCTAGCAAGTGCCTCTTCAGTAAGATG
CTTTGCGGCAGATCGAGCATCCTTTACATTCTTGATGGCATCTGCTGCTTCTTGGTTTGA
CATTACCTTCCATAACCCATCACTTGCCAAGATAATGAACTCCGTATCATCATCAATCAT
CTCTACTTCAATGTCTGGTTCCGAACTGAGATGTTTTTTCAAGCTCTTGTCACCAAATGC
TCTTGCCACTGCCAACTGTCCATCAACTCGTGGAACATCCCCTGGAAAGTTCGACACAAA
CCCGCCTCTGCCTTCAATCTTATCCTTTTCTGAGCTTGGCTCATGATCAACTGAAAGCTG
CTTGGCCACACCATTTTTGCATATAACTGCCCGAGAATCACCAATATTAGCTACTACCAG
CTTGTGACAGTTGATCAATATTGCTGTAACCGCAGTTGAACCTCCCTTACCCAAATCAAC
TGCTTTCTCCAAAATAGTATCATCAGTAATACGATAAGCTCTCTGGATTGCAGTCTCTGG
CTCTGTCCAGAAATTAGGCTCATTTAAGATTGTCTCAAACAAATGGTTCCGCAGATAGTC
AGGAATTTCGTGGCTCAAATGCCCATCAAATATTGCAAATAAACCAAGTTCATTGTCCTC
AACCTGCTTGAAGTGTGCAACAACATAGTCTTCCATA
>ntig_557_consensus
TTTTTTTTTTTAATTTTTTTTAATTTTTTAATTAATTTTAAATTTTTGACAATTTCAAC
CCAATTACTTGATATTAATTCCAATAAGGGGAGCTTATAGCAAGCAAGACACTAATAAGA
GGTTTTTCATACAATAGCTATTAACATTAATTGTTCTCGAGGAGGAAGAAAAAATCAAGA
TAAAAATCCTTCAAATAAGAAAAATTTAGAAAATGATAGGAAAAGCATCACAACCACTCC
TCATCTCCAGCTACAAACATTCCTAATAAAAAAAACAAACGTGGTTGAACCCCAGATTAT
GTTCAAGGCAAGGGTAAGGTGGATTGGTCGACATCTAGAGGACCATTAGAATTAAAAGCC
TCTCAATGACAATGACGATGATGGCATGAGAGGCTTCATATGATACAACAATTATACGAA
TTATACTCTTTCCCTTCGGTGATGACCCTGACCTCGACACTTCTCCGCATCAACCAGCAT
GAGTCCACATGACACAGACACAAAAACCAACTAAATGCAAAAAGAACTCACCATCAGAGA
ACTTTGATAACTTAGAAGCACTTCCTGTGGACAATGGATGGACCCGACTCATCATACTCA
CCCTTTGAAATCCACATCTGCTGGAAGGTGCTCAGGGATGCCAGGATTGATCCTCCAATC
CAGACACTGTACTTTCTCTCTGGTGGAGCAACAACCTTGATCTTCATGCTGCTTGGAGCA
AGTGCAGTAATTTCCTTGCTCATTCGGTCAGCAATACCAGGGAACATGGTTGAACCACCA
CTAAGCACGATGTTACCATAGAGATCCTTTCTGATATCAACATCACACTTCATGATCGAG
TTGTAGGTGGTCTCATGGATACCTGCAGCTTCCATACCGATGAGTGATGGCTGGAAAAGG
ACTTCTGGGCAGCGGAATCTCTCAGCACCAATGGTGATTACTTGGCCATCAGGCAGCTCA
TAGTTCTTCTCAACAGAAGAGCTGCTCTTAGCAGTCTCAAGTTCCTGCTCATAGTCCAGA
GCAACATAAGCGAGCTTCTCCTTCATGTCGCGGACAATTTCCCGTTCAGCAGTGGTGGTG
AACATGTAACCTCTCTCAGTAAGAATCTTCATCAATGCATCAGTAAGATCACGACCAGCA
AGATCCAAACGAAGAATTGCATGAGGGAGAGCATAACCCTCATAAATTGGCACAGTGTGA
GAGACACCATCACCAGAATCCAGCACAATACCAGTTGTACGCCCACTGGCATAGAGGGAC

AGAACAGCCTGGATAGCAACATACATGGCAGGGACATTGAATGTTTCAAACATAATTTGC
GTCATCTTCTCTCTGTTAGCCTTGGGATTTAAGGGTGCCTCAGTGAGAAGCACTGGGTGC
TCTTCAGGGGCAACTCGAAGCTCATTGTAGAAGGTGTGATGCCAGATCTTTTCCATATCA
TCCCAGTTGCTTACTATACCATGCTCAATGGGGTATTTCAAAGTAAGAATACCTCTTTTG
GATTGGGCCTCATCACCAACATAGGCATCCTTCTGCCCCATCCCAACCATGACGCCGGTG
TGTCTTGGCCTACCCACAATACTGGGAAACACTGCCCTGGGAGCATCATCACCAGCAAAC
CCAGCCTTAACCATTCCAGTTCCATTGTCACAGACAAGGGGCTGAATATCCTCAGCATCG
GCCATTTTTAATAAATTTAGAAGAGAAGAGACGGCTGCAGCAGCGAGTGAAGGAGTGAGG
AAGTGAGATTCCTTCTCGCTCTGATGAAGGAGGAGAAAGAGAAGTGTGTAGATACAGAGT
GAGAGAAGGCGAAGAGGACGCGAGAGGGTATATATATTAAGGGCGTGTGAATTTTTAAAA
TTCACATGAAATTTCCACTCGCC
>ntig_558_consensus
GTCTGAGTTAATTGAATCTTGCCATTTGGAGTTGGTTGGGTTAAAGTGAACTAACTGGAC
TACTTATTGGCACCTTTGTCCAACAGCAAATCCAACAAAATTTGTAGGTTCAAAAATTTT
CGACTAGATAGACTTCTACGCTTTAAACTTGCTATTGCCATTGCTACAGAACTCCTACAG
CTTTTAGATAATGGCGCTTTCAGTTAATCGAAATCAGACTATACAAAAAGAATGGATGGC
TATATATATTTGTATCTATGATGATACATCATCACCTGGACCGAGAGTTTCAGTTTCACC
TGTTTGTCTAGCAGATATCACCCTGTCTAATGTCTGGTCAGGAACTGTAATTCCACTCGT
CTCCATGTCTGCAACTATCGCAATGGCTGCATCCAGTTCTCCCAGATCGAGACATAATGA
GATTATTTCATCATAAAGGGGACTGCCAAATACATAACCAAGACTGTGTGTCGTCCGTAA
GATCTTCAAGAAAGCTGAAGGCAGCGGAGCCCTTATGGCTGCTCTTATTATCATGGCACA
GTCTCCGATTGTAGGCTTTCCACCCAATTCTATAACCTTCTGCATAACTTGGATTGCCAA
CTCAACTTCCCATTCCTGTGACCATTTTTGAGGAGGTTTATTCTTCAGTTCTCTCTCCCA
CGTCCAACCCCAAACATCAGCTATTGTATACATGTCAGATACATCAAACACCTTCCTTTT
CCTCATCTCCTTAAATGCTTCAGATAAATCCTCTGGAAACCAGTCATCATCATCATCATC
CTCCACCATTTTGGACATTTTTTTGCTTGCACGTTTTGATGTGGTAGTTGATTCATCAGA
ATCTTTCAACAGCTGGACTCCGATCATTTGAAGAGGGTTCTTGGCTTCAACTTCTTTCTC
CTTAACCCTGTCTACATCTTGGGACTCAGTCTCAGGTTGTTCTACTTCCTCATCGTCATC
GGCCTCATCATCTTCAACATCCTTTGCAGCAATTTCTACATCTATACCATCATTGTCCAA
CTCATCTGGAAGTTCTGATTCATCCATGTCCGTAGTCTTATCATGATCATTATCCAAGCC
TTCCCCAAGAAATCGACGTTTCCAGAACTCAGTGTTTCCTTCTTCCAGTTTGATCCGTGA
TATCAGCTCATCTAGCTCTTCATCAACCTCCTCCTCCTCCTCCTCTACAAGTGGAACCCA
AAGAGGTCGACCCCGAGAACGGTTAATTCTTCTTGCTTTTTGGACACGCTGATAAAGAAC
ATTTCTAGTTCCATCAATCGGTAGCCCTTGTGCTTCTAGCTCTTCCTTTAATTCAGACAC
TAGCATTTTACTAGCAGCCTTGGGCTTTAGAACGTTCTGCTGTGGACCCTTTATTATTTT
CCTTAGTCTCTCTTGAACTCTTATATAATCAGATTCCGACACATCCCCCAGAACTGCACG
TCCTTCATCTTGCAGTTTTTTCAAAAGCTTTTTATGGTACATCCTTGAATCCTCCCGACA
TCTTTGCTTGAAGGAAGTTTCAATCGGACTTGAATATACGAATCCAACAGTATCAGGATC
TAAAGGAGTTTTTCCTCTTCGAGGCACCCATCGTTTTCGTTCACCAGTAAGTCCACCTTC
TGCTATGTACCTAGCAATGTAGTCAATCTCGTATCCCAGTTCCGCTTCTTCTTGCAAAGG
TTCAATCCATGAGCTAACTAATGTCCGATATTTTCTGCTCAAGATCATGGCTCTTGGTGG
AATGGATTGATTATCTTTGGCCATTGCTTCTAAAGCTTCAAGTAAATCAACTATCCTTCC
TTCTCTGCATAAAGCCCGCAGATATAAAGACAGTGGATCCCCAAAGTTTCCTTCATGATG
CAAATATGTTGTTCCCCCCTCATAGTTTTTTAGTGCACGGAAGTGTCGAATAGCTTCCTT
TACTACACAGTACTTGGTAAAACACTCAACTAAGAGCGCATAGGTTTTCATATTAGGTTG
TAGACGTTTGTGACTTTCAACCATCATGCCAAGTAACTCAGCAACATCTTGCACCCTGTC
ATAAGATTCAGCTCGGGTATATGCTTGAATCACCCAATTATAAGTCTCAGTATCAGGTTT
CATAAAATCTTCTCCATATTCCATGTTCTCAAATGTTGCAAAAGCTATTTCAGGTATTCC
ACAAGTAGCTTGCACACTAAGAAGATGATTGAAATGACAAGTAGTTGCCATCCGCCCAGC
TGCCTCCATTTCATAGGCAATTTCTAAGGCATTAGAATGGTCCCCAACTTTACAATCTTC
CTCAATCATGAGATCATAGAGTTCATCGGTAGCTTTCAATCCCCCCTTGGCACCCCTCAA
GAACACTTTGTTGGCATCTTCCAAAAATTTGTTCCTAACAAGCTCCTC
>ntig_559_consensus
CGTTTATGTAAGAACCTATATGTATGTTTGTGAATATACACACGAATATATGTGTAAATA
TATATACCATTGCGTGTGAATCATGAATGTTATGTATGTGTTGCAACAGCCCCAAACCTC
GGCCTAGACTGCTACGGGTCTATTGTTCCAGATCTTGACCCACTGTTGATCAGTTATGGC
GGGCCCTTTCACCCATTTTCTGTCTATGATAGGTATTGTCCTCCCCCTCAGAACTTCTTC
GCCTCCACACCACTCGAACTTAGGGGACTAAAGGCAAGTGAAAGTATGGTGTGAAACACG

CCTTTTATCAGATAATCTCTGGTGATGCAGATGACATGGCACTGGACTGGTCCCTGAGCT
TCATCACCTCAGACTGAAATCGTTCCATAGCCAGTGTTGAAAACTTAAGATGCTTTATTG
CAGCTCACAGCAAGTGCACGCGACATGAGAACCTCATTTGCCATGATTTTGAGTACCATG
GAATGCCTTGAACAACCATCATTGATACAGCTCAAGAATAAATGCAGCCTAACAATATTC
ACAGTAGGCCCTTTCACAAAACTGCTCCAGCTCTCTCCTGCAGCTTAGTAAAAGAGGGAG
GCATGGCTTCACTAGCCAACTCTCAACTCACATAGCTTTCATGGACGAGAAAGCGCCAAA
AGAAATGGTATGGGGCCAAGGCAACAAATAGCAAGCCTTTTTGTGGGTTCTTAAGATTGG
ACCTCCTGGTTCGTTTCATGAATCAGACTCTACTGATATGGACTCAGAAAAACCATTGGA
ATCGGGGAATGTATGGTTAAATCCTTTGGGATCGAAGGGTGTACACTGAGGTACGTTAAC
CATATATGAAGAGACGACTTTGAATTGAAAAATGAACTGGGGACAGATTCACACACAGAT
TAGTCTTTCCCACAACAAACACAAACATCATATTACCGTTAACAAATATACAAGAACATT
AAATCCAATATCTCACACACATTATTCACATGCAGTGACCACTTACAACAACAAACCCAA
AACACAAAAACACTCCATCGCATAGATTAAGATGCCATTTTTAATTTCAGCCGGAAATAT
CAATGGCCTTCACATTGGGTTTCTTCATCTCCACTTTAGGAACAGTCACAGTCAGAACTC
CATTCTCCATGGAAGCCTTAATCTGCTCCATTTTCGCGTTCTCAGGCAACCTAAACCTCC
TGGAGAATTGGCCACTGCTACGTTCCACACGGTGCCACGTGTCATTCTTGTCCTCCTTCT
CTATATTCCTCTGCCCACTGATTTTAAGCACCCTGTCGTCTTCGATCTCCACCTTCACTT
CCTCTTTCTTCAGCCCTGGAAGATCAGCCTTGAAGACGTGAGCTTCAGGGGTTTCCCTCC
AGTCCACACGAGTGTTGACGAAAGCAGACGTTTCTCGAGGCCAATAAGAAGAGGACAGTG
AAGAAGGGAAAGGGAAGTCCTTGAAAGGATCCCAAACATCGAGGGAAAGTGGATCGAACC
AGTTTCCTCTTGGAACCAGAGACATTTTTGAGCTTCTGAAGAAATTGAACAAAGCTCAGA
GAGAACCGAGACTAGCTAATAACAGAGCCAGAAAACCTTCGGTAAAGTTGTAAGGTGAAT
TGGTCAATTTATATTGCGTGGCAAGAGTATTCTAGTGCTTTCAAGATTGTAAAGATAAAA
TATCCTCAACAGATAGGCTTTTGGCTGACGAATACCAGTGATTGGAGAATGCTCTGGAAG
GACGATGCTAGGATTTCGTTACTTGGGCTTTCTGGAAGAGGTTTTACATCCATGGGCCTT
GTTTATGGGCTTCTTCAAACAAATTCTTCAGCTGGAACATTACTTCATATGTAAAATCAG
AAGGCAAGGAGAT
>ntig_560_consensus
GCTGGATTAAATTAAATTACAATTGCCCCGAGAAAAGAAGATAGCTGCCGAACACCAGCT
CTAAAATACGATGCCTGCATTTCGAATTATTACAAAATGACAAACAGGGGGACAGCTGCT
TCAGCCGTCTCTTTTCACAACTAAGACTGCCATTGTCTCGCCTTTTTGTAATTCATTCAC
CAGCATGCCAAGACTCTCTTACAAAACAACTATCTAATTTGTTAATAATTCATGACTACA
AGAAAATTTCAATTCTAAGCTCCATTTACCAAACATCTCGGGGGCGATACCAGTTCTACT
GTAAGAGGCACCTACTATCAACAACCACAGCCGACCATATATGGGGACCATGTCACTTTG
CCTCCTCTCCTTCACCAACACCATGTCCACCAGCTGAGCTGGCCTGACCATCAGCACCAG
TTGTCCCAGCATCAGAAGCATCTTGCTTGCTTCCAATACGGACATCATTTGGGCCTGTGC
CTGACAGAGCCCCTGCACCGCTACCCACTCCAAGAGTGCTCTCAGCATGCATGGTATGCA
TGCCATTGTTGGCAGCACCAGGTCTCATTCCCAATTGTCCTAGCATAGCTTGATGTTGCT
GATGTAGCTGTTGCTGTGGATCCTGAATGTGGTGAGAGTTATTAAATTGTAATGGCATCT
TTGGTGAAAATATTCCGGGTTGTTGAGCCATTGCTGCAGCCTGAGGATGTTGCATGTAAA
ATCCCCCTGCTTGCATTGCAGGATGTGGGGTCATCTGTGGAGGCATAGCTGGTGCTTGAG
GCTGTGCATCAGCAATTGCAGCTAAATACATTAAATTCTTTTGAAGCTGAGCTTGGTACT
GTGCACACTCTGCAAGTTTTCCAAGATTTTGATTGTCCAAAATTGCCAAAATCAACTTTT
TGTTCTCATCAAGGTACTTTTGAATCTGCTCGGTGGTAATGTTTGTGGGTGGAAAAGAAG
GCATGACAGGCATCATTTGTGGGGGCTGCTGCATCTTGTAACAGAAACCCTAACCCTGTG
TCTCTGCGTAACTAGGTGTAAAAGAAGATGTTTTAGATGTGGACGAGGTTTCTGGAATTT
GAGACAAAGTCTGAGCAGAGCAATCTAAATCAAAACTACTTAAAAAAAAAAAAAAAAAAC
TCTTGACAAACAAAACAAAG
>ntig_561_consensus
ATTAAAATAAATTACTACCTGCAAAACAAGTTAAACTGCCGAACTCAGTTCTAGAACACG
ATGCATGCATTTCAAGTTATTACAAAATGACAAACATGGAGGTAGAGCCCACATGCTTCA
GGCTTCTCTTTTCACAAGTAAGACCGCCATTATCTCACCTTTGCAACTCAATTCACTGGC
ATGCCAAGACTTTCTTACAACACAACTTACCTAATCTGTTAAACACTCAAGAGTACATGA
AAATTTCAATCCTAAACTCCCAACTACTAAACATCTCTGGAGCGATACGGGTTCTACTGT
GAGAGGCACCTACTATCAACAACCACAGTGAGCCACTCATGGGACCATGTCACTTTGCCT
CGTCTGCGTCACCTGCACCATGTCCACCAGCTGAGCTGCCCTGACCATCAGCACCTGTTG
TCCCAGCATCAGAAGCATCTTGCTTGTTTCCACTACGGACATCATTTGGGCCTGCGGCTG
AAGGAGCACCGCCACTGCTACCACCTCCAAGAGTGCTCTCAGCATGCATGGCATGCATGC

CATTGTTGGAAGCACCAAGTCTCATTCCCATGTGCCCTTGCATAGCTTGATGTAACTGTT
GCTGTGGATCCTGAATTTGGTGAGGGTTATTGAATTGTAATGGCATCTTTGGAGGAAAAA
TTCCTTGTTGAGCCATTGCTGCTGCCTGAGGATGTTGCATGTAAAATCCCCCTGCTTGCA
TCGCAGGATGTGGGGCCATCTGGGGAGGCATTGCTGGTGCTTGAGGTTGTGCATCAGCAA
TTGCAGCTAAATACATCAAATTCTTTTGAAGTTGAGCCTGGTACTGTGCACATTCTGCAA
GTTTTCCAAGATTTTGATTGTCTAAAATTGCCAAAATCAACTTCTTGTTCTCATCAAGGT
ACTTTTGAATCTGCTCGGTGGTAATGTTTGTGGGTGGAAAAGAAGGCATCATAGGAATCA
TTTGCGGTGGCTGCTGCATCTTGTGACGAAAACCCTAACCCCGTGTCTGTGCGTGGGTAG
GTGAAAAAGAAGATCTTTTTGAAGTCGATGAGGTTTTGGGAATTTAAAACAAAGTTGAGC
TGAGCAATCCAATCCAAACTATTAAAAAACTCCTACCAAACTAATAATTTTCTAGATTTG
GTCTGTCCTTTGAAGACAAAAC
>ntig_562_consensus
GGGAGGATCCATAGTCATGCCCTTGAAGTATGGGGGCTCATGAATGTAGGTAGAGTTGGG
TTCCCATGAGTAAAAGGTACCACCTGGTACTGATAGCTGATTCCACATGGGATTGCCCTT
TGTAATAGACTCATAAGTACTTCTAAACATATCAGGCAGCACACTAGATTGAACAACCTC
TGCAATTTCTTCGGTTGTTGGCCAGATATCCTTGAAATAAACACTCTTACCATCCTTGTC
AGTTCCAATCGGCTCCTTCTCAAAATCTATGTCAACCGTGCCAGCAAGGGCATAGGCAAC
AACTAGAGGAGGTGATGCAAGGTAATTTGCTCTTGTCAAGGCATGAACACGGCCTTCGAA
ATTCCTGTTCCCAGAAAGCACAGCTGCAGCTACAATGTCATTCTCTGAAATAGCAGAAGC
AACAGATTCATNNNNNNNNNNACGACTCCTGAGCCTGGGGCAAGACTTGTCTTTACCCAAG
GTTTGACTTTTAGACCAAGTTCGCAAGCCTTTTTCGCAACAAGACCAGCACCAAGCATCA
CACTGGGGTTTGATGTATTAGTGCAACTTGTGATTGCAGCAATTACGACACTTCCGTGCT
TAAGCTCAGCTGGTTGTCCATGGAAAGAAAACTTGGCCACTTTTTCCTGTGCCTCTTTGG
GTACAGCAAAGCCCTTGAATCCAACTTTATTGTCAAGACATGAATGCCAATCTGCTTTCA
TTTCCTTTAAAGGAACACGATCATGAGGTCTCTTTGGTCCTGAAATACAAGGTTCAACAT
CAGCAAGGTTCAGTTCTAGGTAAGAAGAGTACACCCTTTCTTGCTGGGGCTCATTATAGT
CAACAAACATTTTATTTGCTCGGAGATAACCTTCTATCATTGCCACTGTTTCATCACTCC
TTCCAGTCAACTTGAGATATTGTAAAGTAACATGATCTACAGGGAAGAACCCCATGGTTG
CACCATATTCAGGCGACATATTAGCAATAGTGGCCCTATCAGCTAGTGAAAGTTCACCCA
TACCATCCCCATAGAACTCAACAAATTTGCCAACAACACCATGTTTCCTTAGCATTTGTG
TCACGGTTAGAACCAGGTCGGTAGCTGTAACCCCATTGCACAACTTTCCAGAGAGCTTGA
ACCCAACAACACCAGGCAACACCATACTCATAGGCTGTCCAAGCATTGCTGCCTCTGCTT
CAATACCTCCAACACCCCAGCCAGCAACTCCTAGCCCATCAATCATGGTTGTATGAGAAT
CAGTTCCAACCACACTATCAGGGTATAGTATGCCATCAGTATTGAAGACAACCCGTCCCA
AATATTCAAGATTGACCTGATGTACGATACCAGAACCAGGGGGAACAACAAGCATGTTAC
GGAAAGCATTAGACCCCCACTTCAAGAAAGCAAATCTCTCTTTGTTCCTCTGGAATTCAA
GTTCCATATTAGCCTGAACTGCATTTTCTGACCTTGTTACATCAACTTGAACTGAGTGAT
CAATGACAAGATCTACAGGAACCAGAGGATTGATCTTGTTAGAATCACTGCCAAGCTTGT
TCATAGCATCACGCATACAAGCAAGGTCAACAACAGCTGGTACTCCAGTAAAATCCTGCA
AGAGAACTCGAGCAGGCTTGAATGGAATTTCAACTTGCTTCGGAGCTGTATTTTCCCAAT
CAATAATTTTCTCAACATCTTCCTTTTTAACTTGAAAATTGTCACAATTACGAATTGCAG
ATTCTAGAAGGATTCTGATAGAGTAAGGTAGTTTATCAATCCTAGGGTCGTTAAGGGCAG
GAAGGCTATAGAACTTTCCAAACTCGCCACCTCCAGGTTTTGGAAGACTGGTCAAGATCC
CTTTGAATGCATGTTCCGTAGCCATAGTAGCAAAGGTGCGTTCAAGGCGGTCGATGATAG
GAGCAACAGTTCTGATCTGAGCGCGAAGACTAAGAGGCGAGCGCCAATCGACACCGTGGC
TCCAGCGAGGGACAGAGCAGCGGAGGGATCGAGCGGCAGAGGATAGGCTAAGAGATCGGT
GAGTGATGAAAGAAGAAGGAGAAACTTTGGGAGACGAAGATGATGAGGTCTTGAAGGAGG
ATAAAGAGGAAATGAATCTTGACCTAGAAGCCCTAAGTAAAAAAGAAGCCGAAGTAGACG
CAGTTATATACATGACGAAACAGTATGTCAATATGGATCACGCATTCACATAAAACAAGT
TAGATTGGAGGATAAGGATTTGGCGCAGAGCAAGGCAAGCAGAAACAATACTTCAGTTCA
TGGCGTGTAGCACAGAGAGCATTCTGTTTGACAGAAGTGGAGAAGAGGCG
>ntig_563_consensus
CCCATACTCAGAGCCATGGCACATTTATAATAGAAATGATGCACAACTAATAATTCAAAA
AAATTACTTCAACTACACCATGACAAGCCCATTAAATCACAGCAGAAACACAATTGTAAT
ATTTATGACCAAAAACAATAGTGCTACACAAGATAAGCAACACAACAAAGGGAAGGGCTA
TAGACGGACTGTGGACTGTGAACAAACTCCCTCCCATATAAATGCCACTCAAAAGTACTT
TTTCTAGTTGAAGGTGCGTCAATAGTCTTCACCTTCCTCTTCGTCATCAACACCTTCTGC
ACCCACTTCTTCATAATCTTTCTCAAGTGCAGCCAAATCCTCTCGTGCTTCTGAGAATTC

CCCTTCTTCCATGCCTTCACCAACATACCAGTGCACAAATGCCCTCTTGGCATACATGAG
ATCGAATTTGTGATCAATGCGGGAGAACACTTCAGCCACAGCTGTGTTGTTGCTGATCAT
GCAAACAGCTCGCTGCACCTTAGCCAGGTCGCCCCCAGGGACAACAGTTGGAGGCTCATA
GTTAATACCACATTTGAAGCCAGTTGGACACCAGTCAACAAACTGTACAGTCCGTTTGGT
TTTTATTGTGGCAACTGCAGCATTTACATCCTTGGGAACAACATCTCCCCGGTACATCAA
GCAGCAAGCCATGTACTTCCCATGCCTGGGATCGCACTTGGCCATCATACTTGATGGCTC
AAACACTGCATTTGTGATCTCAGGAATTGATAACTGTTCATGGAATGCTTTTGCAGCTGA
GATAACAGGGGCATATGACGATAGCATGAAATGGATACGGGGATATGGCACAAGGTTGGT
CTGGAACTCTGTAATATCAACATTGATTGCTCCATCAAACCTCAATGAAGTTGTCAAAGA
TGATATGATTTGAGAAATCAATCGATTCAAATTAGTGTATGTTGGCCTCTCAATATCGAG
AGATCTGCGGCATATGTCGTAAATAGCTTCATTGTCCAAGAGCACAGCAACATCTGTGTG
CTCAAGAAGGGAATGAGTAGACAGAACACTGTTATAAGGCTCGACAACTGCAGTTGACAC
CTGAGGAGAAGGGTAGATAGTGAAGCCAAGCTTTGACTTCTTCCCATAATCTACTGACAG
GCGTTCTAACAGCAAAGACCCCAGACCAGATCCAGTGCCGCCACCAACAGCATTAAAAAC
CAAAAATCCTTGCAGCCCTGTACAGTTATCAGCCAACTTCCTCACTCTATCAAGGCAAAG
ATCAACAATTTCCTTCCCAACAGTATAGTGTCCCCTCGCAAAGTTGTTAGCTGCATCTTC
CTTCCCAGAAATAAGCTGCTCTGGGTGGAAAAGTTGGCGGTAAGTACCAGTTCTAACTTC
ATCAATGACTGTTGGTTCAAGATCAACAAACACGGCTCTTGGCACGTGCTTGCCAGCACC
AGTTTCACTGAAGAATGTGTTGAAGGCATCATGGCAAGAGCCCACAGTAGTATCACTGGG
CATCATCCCATCAGGCTGAATGCCATGTTCCAGGCAATAGAGCTCCCAACATGAATTTCC
CACCTGAATTCCAGCTTGTCCAATGTGTATACTGATTATCTCTCTCATTTCTTCGTCTCT
GCGCTCACTTCAAAGTACCCAAAACCCTAATCTCCAGCAATATAAATATATATATATATA
GATTTTTTTGGTAAGTGGGTATGCC
>ntig_564_consensus
TACCTCCCAATTTAATATCACTACTCTAATAAATTGCAACACATAAAATGGATCACTTTT
TCGAGTTAAACAGTCATGAAAAATATTCTTGGTTCAACAATCACTTCAAATCAATAAAAA
ACATAAGCTCATCTTGGTGTTCCTCGATATTTTAAACCACAACTGGCACAATACTTGCAC
CAAAACCAAAATCACTACCATACCACTCGAACAAAAGTCACGACTCTCCTTGGTAACACT
CTTTAAATCCTTTAGCGGTACTTAATGAATCCAATTTCCTTGGCGTTGCTTCGGAAGCAC
TGTCTGCAGCACATGAGCCCATACTTCCTGATTAAACCATGTGGGTTCCCACATACTCGG
CAAGTACGAGAACCGGGCCCGTAGTTCTTCGGGTGAGAGTTCCAAACGTTGGAGTGACCC
ATGATTAGCTCTTAATTCTTCAGAAGAGACGAGAGCTGCTGCACAGAGAGCTAATGAAAG
TGAAAACCCTAGAAAATTGCTGAGGAAATTGAAGGCGGAACTAAAGTGGGTTTTTATAGT
GATGTCAAAAACCCTAGATTTCGATTGTGAACATGGTCCGTGTTAAGCATACTGATAAGG
CGACGTGGCTAACAATTATGATTGGGCCGGACTAGAAATATAGAAAAAACTGGACCGCCC
TTTTGTCATCCAAGCCAGCCCAAATAGTTCAGCCCGTTGGCTGACATGACACAGC
>ntig_565_consensus
TTTATCCTGACAGGCTCATTAACAATTACTAGTACATCTGTACCAAATTCCTTTAGACGT
AGATTTGCCAAATAGACCCTCACAATATTTTGTGCTTCCTTTCCTTGCCTCCCTTTAGAA
ATGGCCATTTGGCCAACTGCAGTTGTAAAGACGGCAGGGATATTTCCAAAGGGCAATCCA
GGGGCTTCTATCACTCCTGAGCCCTCCAACATCACACATCCTTCAGCATCTTGTTCAGTA
GCAAGGTCTTTAAGGAACCAATTAGCACTTTCACTATCAGCAACTTCATGCTTGAAATCT
AACAGCTCAAATATCAAGCTTTCATCACGTACAGGGTCCACAAATACCTCCTGATGATCA
GGGACTTGTCGAATATCGCTCACATCCTGGAACCTCTGGGGAAAAGTACTAGTAATTGCA
CCGCCAAACAGAGGACGTTCCAGAAAAGATTCCTGAGACATGTTAAAATAAAAAAGGGAA
ACTCC
>ntig_566_consensus
TTCCACGCCCACTGATTTCTACACTTTTCCCTACACGACGCTCTTCCGATCTGTTCGCAT
GCTTAATGTCAGATTTCATTGTTAGATCATATATGACGCTTAATATTTTATCTAAAATTT
CATCCAAATAGATCGCGTTTGGAATCTGCCATGATTAATTTGGCAATTATTGTGAGGAAA
TTAAAGGGCAACAAAAAATCAAGAAGAAAAAATTGTTAAAATAGTTTCTTTTCTACTATTT
CCAGCATCGTGGATAGTTACAACACATCCGTATTCTTAGAACAAACATCTTTCATTACCC
AACAGAAGATTAAACAGAATCGATGAAAATATTTGAAACAGCGGAGAACACTTGATCATT
CTACATTGCAGCCTTGGTGGCCTTGACATCTTCACCAGAAGAACTCCCTTCAGCAATATT
GATAACCTTCGTCTGCCTCTTCTTCTCCACGGCAAGCTTCGGCACAGTGATCCTCAACAC
CCCATCTTCAAGATGAGCTTTAACATGCTCCAAATCCGCATTCACCGGCAGCCTAAATTG
CCTCCAGAACTTCCCATTAGTCCTCTCAGCTCGGTGCCATTTCTCGCCCTCAACTTCCCC
TTCACTCTTCCTCTCCCCGCTCACTCTCAGCACCCTGTTCTCTTCCACTTCGATCTTGAT

GTCCTCCTTCTTCATCCCTGGAATATCAAGAGTAATCACGTGGGTTGACGCCGTCTCCTT
CCAGTCAGCTCTTGCCAATGCCAGAGTCTCCATGCCTTTTGGGATGGTTAATGGGGTTTG
TTCCAGGATCCTGAAGGGGTCTTCTGTTGGGAGCATCATGTCCAGGAAAGATGGTCTTGT
GTATGGGACGAGTGCAATGGCTTGTGTTGCCATGAAGATGATTGCTAGCATGACAAGGCT
TCTTGGATTCGCCATTTCGGCTTGCATTGAGATTGTTAGCTGATTGTTGATATAATCCGT
TTGCTGTTTCTGATAATACGGAATCCAACAGAGGTGAAGTGGGCATTTATAGGTTTGATT
GGGGATTCTCTGGATTGTTCTCCACAAGTCTTCAGGTAGAAAGTCAGAGAAACTCTAGGA
GAGTCCAGAGATTTCTTAGATTGATGGGATCAATGACATGTCATGTGGGCTTCACATAAC
AGAGATCAGCATAGTTGATGGGCCAAAATCCAGTTTAGTCTTAGCCCATATTACATTCAA
AAAGGGCATTAAAA
>ntig_567_consensus
CTGAAAACTGATACCGATACATAAAGTACGGCTAAACTCACGAACAGAAGCTAGTACAAA
ACGGGAAACTGAAACTTAAATACAACTTCTTCAACTATAACCTTTCATCAACTACTAAAA
CACAGCTTAACTCTTGGTCCTGAACTTGGATTCGTCAATCGAGACTCCCTCCTGAGCTGC
ACGTTCCCCGCCGGACTGGTCACCCGTGCTGAGCCCGCCCTTGCGGCCCATTTCCTGGTA
CTCTTCTGTCCCCGGTTGCTCCTTCCTTGCCTGCCCTCCACGGCTCCTCCCTTCTGCAAG
GTGTTCTTGGGCTTCCAGGCTTTTGCCACCAGTTCCACCAGGCACAACAGTTTCTCCCTT
CCTGGCCCTGGCGTCTAGTTCAGCGCGTTGTTGATCTGAAGACATGTTTTCGTCCGGGCT
GAAGTTAAAACCTGTAAAGAAACTATTAGAATTTCTCCAACTTAAACTGGAAACTGAGAC
CGGCGCCTTTGTTGGTGACAACGAAGG
>ntig_568_consensus
TTTTTTTTTTAATAATAATAATAATAATAATAATAATAATAATCACCTTAATTATTAACT
AATATAACATCTCTAGAAAAATACACAATAGATTTTCGCTCAATGTCATCCTCATCGAAC
GGTCTAAAAACAAGATCCACGTTCATATTACATATATCACATGGCAGAAGACAAAGACTT
AAATTCTCGTTTACCGCCGTCGTGTTGCGGCAGGGGAGCATAATATGGACAATAGTTTCT
GATAGCCGTCAACTCATAAGGCGGCGCATCGAGTGCCAACCACTGCTCCACTGTGAACTG
TTGTTGAGCGCAAGGTGTGTGGACCCCGGTGGCTGTGACCTCGACGTAGTTGACATACCA
TCCATGATGCGGTCCTGAGCCGTCAGATGTAAGATTCATGGCGCATATGGGTGCACTCAA
GCACCACCCTCTTCCGCTGAAAATGTCCAAATTACCCCTCTCGAAATAGTTGTGACCGGG
ATCCATCAAGCCTCCCCATGCCTCCAGATTCGTCAGCTCCAGCATCTCGCCGTATACGTC
ATACAAACGCAAGCTGATGATCGAATCAGTGCCCCCCTTCAAGATCGAGCCGGTTCTGAT
GTACACCGTATACACACAATCATCTCCATCAGAGAAGGCGATCGTCGCGTAGGAGATGAG
GAGGAGGAGAAGGAAGTAGAGAGTGTTGGAAGCCATGGCTAAATACGGGCGAGAG
>ntig_569_consensus
CCGGTTATTCGTTCTATATGGTATCGGAAAAAAATCGCGAGCTGGCAAGCAGACGCAGAC
GCAGACTTCATCCTTGTCTGTCACATACAGTTCTAACCTCATACAAAAGGCCTAAACAAG
AAAACTGAAATATAATGGGCCAAATCTTAATTTTGTATCAAATTTAAAGATTTTTTTTTT
GGGTAGATTCATGGCGCCAGACCGCCGTTGCTCACAATCAAGCCACCTTGGTCTATAGTA
ATAGTTACAACACCATAAGATACCTTCAAATCGAAGTTCCTGTTGAAATATAACTAATTT
CTTCATATAAAAACGGACAGCAACATAACTAGATTAATTCCAGAAACAAAGCTTCCTGTC
TACTCGTCAGACCTAGTTCATTCAGTGTTAAACTGCTCTCCCCATTACTGAAAGCTCGCC
TTGGGTATGGCCTCACCAGTCTGTATGTGCCAGCCTTGACCCCTCTACCAATATCTATGA
AGTCAAAAAGAGACTGTAGCTTGTTGGATTTCAGAAATCGGCGTCCACGACGACTACCGT
CTGGCATTTTCACCAGAAGGGTCACAGCATTTTCATCATCTGCTGATGGCTCCTGAGGCA
GAGAAGCTTCTTTGGCTGCTAATTGTCTTTCATATTCCTGCTCCTCCTCCATTTTCCTGC
GTGTTTCTTCCTTGTTCCTTTTTTCTTCTTCAAGTGCAGCTTTTCTAGCTTCTTCCTCCT
TTAAACGACGAGCTTCAGCTTCCTCCCTAGCCTTCAATTCTTTCTCTCTGTCAGCTTGCA
ATGATGCAAGATATTCATCATCCTGCTGTTCACGTATCAAACGTTGAGCTTCTAAAGATG
GTGAAGGAGGACGTGGTGTCCGCCATGTAAAAGATCCATCAGGTTGCATAAACTGATGAG
GTGCATATGGATAATTATATCTACTTTCAGGAATCCCACCAAACATAGCAGCCTCAAGCA
TAACTGCTTCATCATGCTCCTCAGAAGAAATGCCACCCCACTCATCTGGGAATGCATTTC
CATTGTGCTGTGGAAGATTGCTGATAATATGTTGTCCAGGACTTGAAGGTGAGCTAGCCT
CAGCAACCGGAGCTTCTTTAGACAAATCCACAGAGCCAGAAGATATGTGTCTAGACCTGT
GCCTAACCAGAGGCTGCTCCTCCACTTCTTCAGCTTCATCTCGGATGGATGAACTTCCAG
CTTCTAACCTTCCATTTGATGTTGGTTCTTTCCCCAGCTCCCCCTCAGCTGGCTCAGAAG
ATCCTGATTTTGCTGCTTCAACAATTCCTCCTAGCTGATGTAGAGCTTTTTCTTGCTCCG
CTGTCTTCAATGACAACGAAACTGCATGTGCCAGATCGGCATCTTCCAAATGTGTTTGCC
TATGCTCAGTCCTGGAACCACTTATGTCCTCAACCTCTCTTTTTGAAGCCTCAATGGCAG

CCCGAACCATTTCTTCTTCTATGTCATTGCTATAGTCTGGCAAATTCTCAAAAGTGGGAG
ATGTGAGGCTGCTATGTCTTTCACGAGAACCATCATTCTGCAGTGTGGCTGGTGCAGCCG
TGGCAAATGGGGTATCCTCATCCACTTCATCAATTATGACCGTCTCATGGCTTTCATGGC
CATAAGCATGTGTGGATTCAGTGATATCCTCAATGGTAGGAGCATGACCAGATGTATCAC
TGCCATCCTTAACCTCAATGGGGATCTCTCTAACCTCTCTTGGTTGGGTAACAAATGGTT
TACGATTAATAAAGTCAGAACCACCCTCGAAAAAAGTTCTAGGAATGTTTGGATCAAGCA
GTGAGAATGGATTTAAAGTTCTTGGCACGGATAGGAGAGGAAAAGGAGGAGCCTGAGCTC
CAGTTTCAATTGGATCATCTATTTCCATTACGTCATCTTGGGAAGGAATAGGTGTCTGAT
GAACAGTGTTCATAGATGTGTTTCTATCTCCTTCACTGAAAAATGCATTCACAGCTGCAT
TGAGATCACCACCATGCTCCTCTAGCTTTTGCAGCGCGACCGATTCAGTGACGCCCGTGA
TGCTAATAAAGGTGTCGATTGCTTCTTGATTAGGCCTCACCATTTTCTCCGATAATATAA
TTGTTTTGAAGTTTAGGTTTTCAATTGGATCACCCTTGTGTGTTTTCTTTTTAAGAAAGA
AAAGAAGTGAAAAGGAG
>ntig_570_consensus
CTCGCCTTGGGTATGGCCTCACCAGTCTATATGTGCCAGTCTTAAACTGCCGACCAATGT
CTATGAAATCAAAAAGAGACTGCAGCTTGTTGAATTTGAGAAATCGGCGTCCACGGCGAC
TACCATCTGGCATCTTCACCAGAAGGGTCACAGCATTTTCATCATCTGCTGATGGCTCCT
GAGGCAGAGAATCTTCCTTGGCTACTAATTGTCT
>ntig_571_consensus
CTTTCCCTACCGACGCTCTTCCGATCTGGCAGCCACCCTGAAATTCGATTATTTACCAAA
AACAATCCATAATGACTTTACACAAGAAAAAATTATTGGGACTCAATTCTACACCACCGC
CATCCACCACTCTATGTACATCAGCAGCCACCCTGAAATTCGATTATTTACCAAAAACAA
TCCATAATGACTTTACACAAGAAAAAATTATTGGGACTCAATTCTACACCACCGCCATCC
GCCACTCTATGTACATCAGCCAGGAACTCACCCATTACAGCTGGCAAATCGACACCTACA
AAACTGGTAGCTGCTCTTTCTAGAATCTGACTGAAGCAGCCAGAAACAGTAAGAATTCCC
TCCTAATCAACAGATTTTATTTTGAAAAAATATTTACATTCAACGGCCTGCACTGGCATC
CAATAAGCTCCTCAAGCTGCACAAGGCCTCTGCAGACAAGCTGCAACACCTTATTCTCCG
ATGCCGAGGTGGCGATAACTCTTGGTGATCAAGGGAACTGAAGCATATAATTATTACAGT
GAGATTGTCAATTGTGTTGAGGCGCAAGGCCTCCATGACAAGATCTCGGGCACACTGTTC
AGGATCATCGTGACGTCGCAATCCTCGATGTACAAGGCTTACTGCATGCTGACTTGTCAT
CACATCCCAGATTCCGTCACATGCAATGATGAGAAACTCATCATCTTCTGTTAAAACCAT
CTGCTGGAATTCCGGCTCTGCTATGAGAGGTGAAGGAGAACCTTGAGGGAACTTCATGTC
CCAATCACCCAAGGCTCTGGAAACTGATAAATCACCATTTAGATACCCATCATCAACATA
ACCACCCAACTGTTCAACTCGCCTCTGTTCAGATGGGTATATTGGTCTGTGGTCTTGAGA
CATGTCAATTGCTTCTCCCCTCCGGCTGAGAACTGCACGACAATCACCAGCATTAGCCAC
CATCAGAAGCCTTCCAAATATCAAGGCAGTAAGTGCTGTTGTCCCAGAGGAGCTGCTCAC
ACTGGAGTCATTGGCCAAAGCCATGTCGGCAAGTAGATATGCTTTTTGAAGGCAGTTCTC
AACCGCCTCCAGGAAAATATCATCATCTTCAGATGACTGGGGGAAGTTGACATCTTCAAA
AAGAAATCTAATTACATTTTTCCTGATATACGCCGCTGCTTCAGGTCCTCCATGACCATC
AAACACCCCATAAAATGCACTTGGCTTGGGAAATTTAAAGAGGGATCCCAAATGAGATGA
CAAGTCATCTATTCGTATATGCTCATCCTCCATGTATCTTCGTGGACCAATGTCAGCAAA
ACTACCAGAACGAATGCTGGGAACAAATTGCAGGGCAGCAGATTCAAGACTAGCATCTGG
AATCTTTTCTGCAGACTTTATGTCCACTTGAGAGGTGGAGAAGTCGGTTGAAACCGACTC
CGATAAACGAACTTGATTCACAGCTCGCGGAGAAACCGCCGATGTAATTTTAACAATATC
TTCAATCTCCTCAATTTTGCTTCCTTTACCGAGATAATTATTAACATCCAACACAGCCAC
GCTCTGTTGCAAACAAACAACCTCTGATCCAGCTACCATCTTCAACAAGCATTTACTTCT
TCAAATCTTTCAAGATCTAAACCTCCCAAACTTAATGAAATTTCTAAATTTTCTTTTTTC
TTTTTTCTCCTGTCGCAGCGCTTTAAATTT
>ntig_572_consensus
AACTACAGCCTCTCGCTACGTTATCTAATATATAATCATAAGCACAAAAAAATCTTCCGT
ATGAAATGTGTCTCTTACAAGGTTATAATCGTAAAGCTCAAAAGTTCTTGCCTATGAAAT
GACCTTGACACCAAGAAGCAACACCGCAACAAATATGACAACCATACCGCCAATCCACAA
AGATTCAAACCCAATATGTATAGAACAAAACATATCACCAAGCAAGAAGTATGCGACTGC
CATTAAACTAAGTCATGGCTACTTATCCGACTTTTCAACTTCCTCAGCTTCCAAACCTTC
TTTGGAAGGACTAGTACTTTGGTGCGTACTATCACCCAAAGGAACACTCAGATTAATGTT
CGAATCTTTGGTCAAGGAGCTCATTATAGCTGAATTGGTAACAGAACTTGAAACATGCTC
TTCTGATTCTTTTGGCCTATTAAGGGCACTACAGTGTGGGCAGTAATAAGTAATAAACGA
GTAATCCTCCTTCCTTGCAAGTCCATTGTGCATATGGCAATTGCCACATATGAGGGCATA

AGATTGTGTAGGATCTTCCCCGACAAGCAATGCAGCAATTCGAGCAATCCATCCTCCATC
ATTTATGGTAGAACCCTGAGGATTCTGATGATGGACAAGAAGTTTATCATGCTCAGAAGT
TTGTGGATGCTCATTCACAACAGAATGAGAGGATTCTTCATCAGAATGATGCAATGGGTT
GCTTCCGGCACTACTGGATCTGGTGTGCACTGGCTTACGATTTCGAAGACCACTAGACTG
CACAACCTCAACATCATTGCTCTTCCCTTTAGCAACATTAAGTTTAGATTCATCCCCTAC
AAACACTTTCAAGCCTGAATCAGCACCCAGCTTAGATGCCAGAACACTTGCAGCAGCAGC
CTTAGCTGCCGGGTCAGGATCATATCTCTGAATGAGCTGTTGAGTAGTGTAATAATTTGT
CTTCTCTTTAAGTTCATCAATTTTGGCTTGCCTCTCAGCCCGAAGCCGTTCCAGAGTTTT
CTGGTCCCTGTGATCACAGATCCTTGCGAAATTCCTGATGGCTGAATAAGCAAGAGCAGA
TAAACCAGGCAACACGAACATTGGCAAAACTCGAAAAGCCCTCCTTTTCCAATCTAAGTC
CATTGATCTTGTAGTCATGATAGCATAACCAACCGCAATGACCTCAAAAATGACACTGAA
TATAATAAGATGCCGAGTCATCCTCCTCCAAGTTTGAGATCTCCGCTTCATTCTAGAAAG
AACAGCGGCTTCTTCTTTAGAAATGTATTGCAATCTCTTCTCAAAATCATCCCCCTTTAA
TCTGAAGATACCATTCCAAATACGAGAAATGATTCCCCTGCTCTTCTTTTTTCCTATCTC
AGGCGCAGAAGCAGCAGGGTCTTTCTTATCACTTTCAGAAATACCTTTGCTATCAGACAT
TCCTTCTTTTGCTTCACTTCCTCCTTGAGAGACGATCCTCAAGATTTTGCAACACTAAAT
GGAAGATGGTACAATCGATGGCCAGCGCCAAAAAAGTACGACGACTAACGATTTCGTTTC
GAAGAAGAAAGCTCCAGAACCAGAAGATTAATAATTCACCGCGT
>ntig_573_consensus
GCCTTTAAAGGAAAGACATTCCAAAGTTATGCTGTCAGTTCATATTTAGGTTACAATCAA
AACCCTCTAAACATCCATACCAGAAGTTATCTCAATTAAGAAAATTAGTTGACTACACAA
TTATTTGGTAATGAAGCGGAATCACCAAAAAATCGTGATTCTGCACTATTCATTGTAATC
TGCATCATATGTTCTTAGTCAATTTTCAGGATTCAGTCTCTGTATAACCTATGAGCTCGT
TTTGACAATCTGCACAAACAAAACTGTGCAGTCACCTCAGAAAACTTTAGCTAATCATAG
AGGCCTTCTTCATGCCAGATGTCAACTAAGAAATCCACCATCTCAGCTAGTGGGATTGGC
TGTCGAAAAGGTTCATTAGAACAAAGCAGGTCCTCATAAGTTGTGGTCAAGCTCATAACT
GGTTCTCTAGGTAATCTCTTTGACTTTTGAGGCTGAACACCAACCCACTTCTTCCTCCCC
TCATGCCAAACTATCTCAGCTTGATTTAAAAAAACCACTTTCTCAGTTGAACAGTCTTTG
CTAGCATCATTGGGAACCTCTGAAGTCTGGTTTTCATTACTACCATTGGTTTCCATTGTC
TTCAGAATGAACAAAAATCACCTTCTAAACTATGGTTCTGTGAAAAAGCAAACAGTATAG
AGGCAGAGCCCATCTGACAGTCAATTCATAAACCCACAAAAGAAAAAAAAA
>ntig_574_consensus
AGATGGTTCGTCCTTGTCAAGATTCAAACATAATTGCCACGAATGCTAAACATAATGTCA
AGTCTTTTTCACAATCCTGGGCATAATATATCATTCTGAGTGCAAAGAATGGATAGGCTG
CTTACAGAACCATTTCGCTCACACAAAAGTCAGCAAAGCCAACAAATAATACCTCATGGG
CGACCATTTAGGTCCTTAGAAAGTAGCAGAAGAGCCCATATTGAAGGGAGGGTTGGCACT
TCCGGTCAAATATCTAACTGCTTCTTGGTAACAAGTTTGCCAGAGAGGGAACCCCTTGGT
TCCTCATTTCCTCTTGCGCACGTCTCATCTCTTCAGGATCCATATTCTCCATTAATTTGG
GCATTAAAAACACAACCACCAGCATGAATCCCACCATAAGTCCCATTGGACTTTTCACAA
CAGACATTATGGAGAATGGTTCTCTAATCTCATAATATTGCTCCTCTCTCAATGGCTCTA
AAACCAATTCATTCAGGCCCCGCCTATTCTCTGTCAACGCTGCCTGAACCTTGCCGGGGT
TTCTTGCACTAACATCGACTCGGACTGGGGAAAAGAAATAGCCAATTGCAGCCACTTCAA
TCAAATGAGTCCCTGCTGACATGTTATGGAATGAAAAGTATCCATCAGGCCTCAAAAATG
TTACACTTTGGCCACCATTGAGTATAACCTTGGCATTTGATATTTTTCCACCAGGAGAAC
CAAATGCTTTTATGCCTACACCTGGAATCTTCACCCTACCACTGATACTGTAGCCATCGC
CAGACCGAGGTGAAAGTGCAAGGGCAGAAGAAATAAGAGATAGGCAAAAGTGCATGAAAA
GGATCGCAAGAACCAGTTTAGATCTGAAGGTGGACGCCATATTGCTATTAGAAGAGGCTT
GCAGGGTAATCGAGTTCAACAACGAACGATGAAAACTAAAAGAAGACGGATTGTGTAATA
CAGAGTCTATTTTGGTTCTGTTTTATCATGAAATCTGGAACCTCGACATCTTTTTAGGGC
CAAACCTTAATTAAATCTTGTTGATAGAGCTGGGCCCGATCCACGCTCAGCAGTCGGAAT
GATTTGAGTTTGCGTTAGAGGTTT
>ntig_575_consensus
ACCTCCAGGTCGCCCTCCATCCAGATGCTCTTTGAATATCCGGTCAAATGCTCGGCATAG
TTCCAAGATAGTGTATAGCTGAGCCCCAGCATCAACAGCAATAGGCCGACCAAGATGGTC
CATCTCCGATTCAAGTTCTTCCATGCTTTTATTTATTAAGGAAGTGATACTTGGTATTCT
AGCCCTAATAACAGACTCCAAATGCTTTGAGAGAAGCTTTGCAAGATACTCTGAACCCAT
TTTGCTAGCTAAGTGTCCATAGTCTGGACTTGTAGCAAAGTACTCACGTTCCTTGCGCCT
AGCAACAATCATATCAACATTTCTATTTATATCTGCTTGTGAGCGATTAACAATTCCAAC

CCAAGGATGTTGCAGTCGATAAGATCTTCCTTCAAGCACATCTAATGCATTTGTTCCTTT
GTCCATCAAATCTAGCTTTGTCAGCACCCCAAACGTACGTTCACCAGTTGGATCAACTTC
TCTAGCGAGTTTGATAGCATCAGAAGTTGCTATATCTTGATTAGCTGGAGATATAGCTAG
TATGATGCAATTAGGCTTCTCAACATATGTCCTCACCATACTCTCAATGTCTTGTACGAT
ACTTTCAGGCTGCCCCTCAACAGCAACTTTAGTGAGACCGGGTAAATCAATCAGGGTTAA
GTTGACAACATTTGGAGAATAGATACTAAGATGGATGGGAACGGGAGAAATTTGTTTGGT
CCTCCCTGTCATTCTATCAGTTTCATCCTGAATTTCTTTACGAACCGTAGAGAAATCCGT
AAATCTCCTCTTAGGCAAGTGAAGAAATTCAGCGTACTCCTGTGAACCATCTTCTGTCTT
GTGAAGCTGTAGAACAAGAGGCCGTCTAGTAACAATTCCGGATCCTCTAGGAAGGAAGTC
TCGGCCTACTATGCTTTCAAGCACCGATGATTTTCCAGAACTCTGGCCACCAACGACGGC
AACAGAAGGAAGAGCTTCCCAAAGAGAAGAAAAAGTGTTATCAGCAGCTCCGTAATCTCC
AAGCATAGTACATGCCCTCTGAATTCTATTAACCAGTCCAATCAAGCTCTCCATCGTCGT
CATTTTTCAATTCCAACAAAATCTAAGTCAGATGAGCTTTGGGATAATAGAATTACAGAT
TTTTGCGACAAAAACCCTAAAAGAAGAAATGGTTT
>ntig_576_consensus
TTGGAGTTGAAAATGACGACGATGGAGAGCTTGATTGGCCTGGTTAATAGAATTCAGAGA
GCATGTACTATGCTCGGAGATTATGGAGGAGGTGGTGATAACGCTTTTTCTTCTCTTTGG
GAAGCTCTTCCTTCTGTCGCCGTCGTTGGCGGCCAGAGTTCTGGAAAATCATCGGTGCTT
GAAAGCATAGTGGGCCGAGACTTTCTTCCCAGAGGATCCGGAATTGTTACTAGACGGCCT
CTTGTTCTGCAGCTTCACAAGACAGAAGATGGTTCTCAGGAGTACGCTGAGTTTCTTCAC
TTGCCTAAGAGGAGATTTATGGATTTCTCTACGGTTCGAAATGAAATTCAGGATGAAACA
GATAGAATGACAGGGAGGACCAAACAGATATCTCCCGTTCCTATCCATCTTAGTATCTAC
TCTCCCAATGTTGTCAACTTAACCCTGATTGATTTACCTGGTCTCACTAAAGTTGCAGTT
GAGGGGCAGCCTGAAAGTATCGTTCAAGAAATTGAGAGTATGGTGCGAACATATGTTGAG
AAGCCTAACTGCATCATACTAGCTATATCTCCAGCTAATCAAGATATAGCAACATCTGAT
GCTATCAAACTGGCAAGGGACGTTGATCCAACTGGTGAGCGAACTTTTGGGGTGTTGACA
AAGCTAGATTTGATGGACAAAGGAACAAATGCATTGGATGTGCTTGAAGGAAGATCTTAT
CGACTGCAACATCCTTGGGTTGGAATTGTTAATCGCTCGCAAGCAGATATAAATAGAAAT
GTCGATATGATTGTTGCTAGACGCAAGGAGCGTGAGTACTTTGCTACAAGTCCAGATTAT
GGACACTTAGCCAGCAAAATGGGATCAGAGTATCTTGCAAAGCTTCTCTCAAAGCATTTG
GAATCTGTAATTAAGGCTCGAATACCGAATATCACTTCCTTGATAAATAAAACCATTGAA
GAACTTGAATTGGAGATGGACCATCTTGGTCGGCCTATTTCTGTTGATGCTGGAGCTCAA
CTATACACTATCTTGGAACTATGCCGAGCATTTGATCGTATATTTAAGGAGCATTTGGAT
GGAGGGCGACCTGGAGGT
>ntig_577_consensus
GTTAGTACAGAATTGATCAGTTGAACCAGGAGCATGGAGAAAGACAAATCCAGACCCAGC
TTCCCCTTTAACAGGGTTAAAAGAAAAAATCATAGCAACAAAATATTATATATTTTTTTT
CTCCAGCAAACAAAGATTCGTACAGAAACAGAAACTGCCTACGATGCATGATTCCAACTA
GATGTTTTCATCAAATTTATCCTGCCCGAATACAATTGGCTTTGAAACCAGAGCTGTTAT
CAAGTTTCTGGGAATTAAAAGGGATTATGCTTGGAAAAACCACAGAAAAGGATAAAACCT
AAAAACCCCTTTCCCTGTAGATTCTTAACCAATATAAACAACCCATAAGCATCTGTCAGA
GGCTAGAAACTTAACTTAAGATCACATGATATAAAGTAGATATATAATTATTACATTAGC
TACAATGTGAGGAGATCCCACAAACTGGCAGTCACTTCCAAAGCTTGACACATTTGTCAT
GACTTGCTGAGGCTACCAAACCAGTAACATTTGATACTGCTAGTGAAGATATAAGCTTGT
CATGTGCATTCAGGGTCAATGTCTTGTTCTCAGTCATGATCCAAAGCTCCAGAGTCTCAT
AACAGCCAATGACTAACAGAGAAGGGTGTGTGGGATGGAACACACAAGTACGAAATTTGT
TGCCAGTACAGCTTAGCTCCTGAACATACTCCCCTTTGCTGCCGGAGCCAACATTCCACA
CTCTAACCAGTTCATCACTCACTGACACCAGAAACTCGCCAGTAGGATCCCAGCATACAG
AATGAACTTGATTTTTATGACCCTGCAATTTAAGTCTGCAAACTTGGGTCTCTACATCAA
ATATGGATACATAGTTATCTGCAGCAGCTGCGAGAATCCTTCCAAGACGTGGTTGAAATC
TCATCTGGGTTGCACCACCCTTGAAAACACCAGCACAATTTCCATTGTTGATACTCCAGC
ATCGTATCTCGCTATTATTGTCACAAGAATAGATAAGGTCCTCTTTACTAGGGTGGAAAT
CCAGAGACATAACAGTTGTAGAATGCCCAGTAAAGGTACGAAGAGAATAACCAGGATTAT
CAGTGTCCCAAACCCTGACAGTTCTATCAGCAGAAGATGTAGCAAGCCGTGACATGCTTG
GACTGAAACGAACGTCAGTAATCCATTGAGTATGCTCTTCAAGAGTGGACTTCAATGTAT
AAGACTCTGTGCACCATAATACAGCCTTTCTATCATGTCCACCAGTGGCAAGGAGTTTTC
CATCTGATGAGAAGTGACAAGATTCAAGCTTATTGTTACTTGCAGAAATAAGCTGGATTT
CCGAAAATGTGAAGCCCTTGCCGACTTCAGCAAGCCGACCAACTCTATCTCTAGGATCTG

CATCGTCAGGTGACAAGAAAGACTCCACATTATCATCCAGGGATCCGTCATCCACAAAAC
GTTCAATATCAGCCAACTGATTTGGTGCTGATGTAAGACCCAAACCATCAGAGCTAAACA
TAAGCATAGACTTAGAGGAGCCACCATTATGTTGCAAAGTAGGCCTCGAAACCACATCTC
CAGGTGTGTGAGTAGAAGGCGATGAAGGTGAACTTGGGGATGGTCCAGTGGTGTTTGCAG
TTCCAGAGCTATTGGCAGGACCAGAAGATGATACTGGCTGCTTTCTCTTTCGCCCAATTT
GACTTTTTGATGCCTGATCATTTCCTTGAAAGGTATTTGACATGCTACCATCCATTGTCA
TGCTGCCAGCGCCTATCATTTTATCTTGCTGCAGCAGATGGGAGTTTGAATTCTGAGACT
GCTGGCTTGAAAGGGGATGCTGTGGATATTGTTGCATCTGTTGGTTGTTCTGCATCTGTT
GTTGCTGTAACTTCATTAGCATATCAACTTCTCCACGAGGCAGCACAGGGCAACTAGCTT
GCATAGTTGATCCAACATTGGACACTACATTGCCAACAGAATTCACAGGGCTTTCTTTAC
CAAGACCAATATTTCGACTGCTGAGAAGCATTCTAAGTTTTCTGCATTCCATATCATTGA
CAGATGGAGATGTCAAATTCTGCTGTGCTTGAAGCATAAGTTGTTGTTGCAACTGAATTT
GATTGAAGGGCTGTGGGGACTGCATCGAAGACTTCTGTGTTAAAGGCCATCCTTTCAAAG
TCACATTGCTGCCACCTGGATTTGAACCATGAACACCAATTAATGATCCATCTGAACCAG
CAGGTCTAGGACTCATCATAGGGTTCATCTCACTCTTTATCTCCTGTGAAGACACTTGAG
GTTGCTGATTCCGACTTTGAACCAGTTGACTACCTCCAGGTGTACCATGCAGTGTTTGCC
CTGAAGGCTGGCCTGACATTGCAGTTGCTTTCAACAAAGAGGTCTGATTTGGATCCAAAA
GCTGGCTCATATTCTCACCCAATCTTGGCTTCATGGTTGCATCACCCATGGAATCCCCCT
GAAGTGGAAGCTTTAACCGATCCTCGTAAATTTTTGCTGTCAAGGTATTTGCAGCTCCAG
GGCTCTGCCTCATTAGAGGGTCATTGCTACTACCACTCAAAAGCTGGGCACTATCTCTTC
TCTGCAGCTGTTGCTGCTGCTGCTGTTGT
>ntig_578_consensus
CTTCCACTTCTTTTAAATTTAATATTCTATTTATTTTTCTATCTATACAATAGAATACAT
CCCTGTAGAAAAGAAGAAAAAACATAAAAAGATTCATACAAGGCAATGATAATTAAAGGC
CTTAAAATATAAGGACCAGCTAGCTATAGTGGGTAGGGTTTCTATCATTCTTGTCGAGGG
CGAGCCACCCAGAGAGAGCTGAGAGCTCGGAGACGATGGAAGTACTCTGCAATCGCCAGC
AGACATCTTGCAGCTTGACGGGTAGTTAGAAGTTGATGCAAACGGTGTAATGTTTGATGC
CTCAGATTATCTGCCTGCCTAACAAAACCTTCGAGCGACGAGAGTTTGTTCATGGCTACG
GCCATTTGGCCCATGTAATTACCCATGTTAGGTGGGCCACTCAGGGAATCAGAAGCTATG
ATATCTGAAATGGACTGGTTGAGAGCTTCAAGTCCTTGAGAGAGAGCTTCTTCAGCTTCC
TGGGTAGATTGTTGCAGTGCACAGGTGCCCAAGATCTGTTGCTCAGTTAATGGCTCAATT
TGGCTCAGTATAACCTTAATGAGCTCCGATGGCCGGAAGCCACCCAACCACAAGAAGCAT
CTCTCGGCCGGTGTCTTCCACAAGCCAGAGACAAGATGAAACACGTCCGTTTTTGCCAAC
ATGCATTTGAGGTTCATGATCTCATCGTAGTGCGCTAAGCAGTTGTCTACGAGGAGCCGA
AGTTCGTTCTCTGGTAAATGCTCTTGTACTGCTGCTCGAAGCTCACACATTAGGCGATGA
TGCACCTCGAGCCATCGTGCATACTCCATGTCAAATACAGCAGAATCCGAGTTAATACTG
TTGATGCCAACAGGAAGCCCTTGGTCTCCACCTAAAAGCCCACCGAGAAACATGCCTTGA
GTTCTGGCTCGTTGTAGCTCTTGTTCCAGTTGGGTAAGTTTGATCCTGCTTGATTCTAGC
TGCTGGACATAGGCCTTTTTACGCAACCTGCTTTTCCTTGCCGCCTCTCTATTCTGAGCA
AGTCTTCTCAGTGTCTTAGCGTCTGGTGTTTTGGGTCCTTCTTGTTCTGAGCTTGATGTT
GGACCTTTTCGGTTTCCTTCACG
>ntig_579_consensus
ATTCCAAATAGTCTTTTTTTTTTTCTAAGCTCCATAGTACAAGTATATAGACAAAGAAGAG
GGGGAAAATACAGTACAAAAAAGAAGATCCAGTAAACTTCTGTACCCAGATAGACTAGAC
TGACCTAATTGACTGAAACCTTACACTAACCAACTTCCCCTTTTCTTCTCGACATGATAT
GTTAGACAAAGGACTTTGATTTGTATATGTTGTGTAACTCATAACCCCTGTTATGTTACA
GACCATTAGTCTACACATATCTTTGACAGGCCTACTTCTTTCTTTACAGGTAAAATAAGG
ATGATGGAAAATGGAAATAACACCCATCAACGTTAGATCTAAAACTAATTCCTGCTACTT
CATTATTTCTAAGCTCAAGATCAGCAAGAATGTGCAGGAAAAGTCCTGATGAACATTAGT
CCTCTAAGGGTTGACTTTCTCCCATTCAAAACGTCCTTGGAGTGGTATATCATTAAAGGG
ATCAAAGTTATACTTCTCTACAAATTGTCTTAGTTGCTCTTCTTCTGCATCGGCAAAGAA
CTGATTTATTTCATCCGTTGTTGGGATTTGTCTCTGAGTTGAATTCCGTACTCCCCTGCC
CTCCATTGAACCAGTAGGCCTAGCAGTAGAAGGCCTAGTAGTAGAACCAGGGGTTCTTAC
AGACTCTGGCTGCCTTATCAAACTGCATGGAGTGGATTCCCTAGTGCCTCTCTCTCTTCC
TTCAATCTCCAAAGCATTTTCCCCAAAAGAAGCTTCAACACCCAAATCCTTGTTGGTCTC
TTCAACCTCAATCCCCTCGTTTTG
>ntig_580_consensus
TTTTTTTTTTAGGAAGAGGAAGAAAAGGGTCATTTATTCCAGATAGTTTTTTTTAGGTTC

CATCAGTACAAGTATATAGACAAAGGAGGGGAGGAAAATACAGTACAAAAAAGAAAAAAG
ATCTAGTAAATTTCTGTACCCAGATAGACAACTGACCTAATTGACTGAAACCTAATCCTA
AAGCCCCCTTTCCCTTCTCTTCTTCCCAACATGATATGTTAGACAAAGGACCTTGGTTTG
TATTTGTTGTGTAACCCATAACCTCTGTTATGTTACAGACCATTAGTCTACACATATCTT
TGGCAGCCCTACTCTTTCTTTACAGGTAAAATAAGGATGATGGAAAATGGAAATAACATC
ATCGATGTTAGATCTAAATCTAATTCCTGCTACTTTAATAAACCTAAGCCCAAGACAAGA
AGAACTAGCAGGAAAACAAGTCTTGATGGAACATCAGTCTTCTAAGGGTCGACCTTCTCC
CATTCAAAGCGTCCTGGGAGAGGCTTCTCATTCACGGGATCAAAGTTGTACTTCTCTGAA
AATTGTCTTTGCTGCTCTTTTTCTGCACCCATAAAGAATTGATTCATTTCATGTGCTGTT
GGGATTTGTCTCCGAGTTGAATTCCCTATTCCCCTCCCCTCCATTGAGCTAGTAGGCCGA
GCACTAGAAGGCCTAGTAGTAGAACCAGGGGTTCTTATGGAATCTGGTTGCCTTATCAAA
CTGCATGGAGTGGATTCCCTAGTGCCCCTCTCTCTTCCTTCAATATCCAAAGCATTTTCC
CCAAAAGACGCTTCAACCCCCAAATCCTTGTTGGTATCTTCTTTGAACCCAACCCCGACT
CCCACGTTTTCATTTCCATGACTATTACTCTCGTTCTTTTGACCAGACCCATTCGACCCA
GAATCCGCCTGACTCACCCTCACCCTAGCCCTCGAATTACTAGAATTGGGGTTCTGATTT
TGAGTCTCCAAAGCACAGTTCTCCTTCTCCTTAGCCGGTCCCTGCTTCGCCCGCTTAGCA
GAATCATTTACCAAAATCGGCGGCTTCTCGAGACGGCGGCTCCTGAGCTGGAGGTAAGAA
CCACCAGCGACGGCAGAGGAGGGCGAAGGAGAAGGGGTAATAGAAGACTTTTGAAGAGCT
AGGGTTCGAGCGCGTGTACGGACGCCGAGGGTGAGGTCCATGATGGCAACCTCGCCAGCC
GTTTTGGCCTTCCTCCGCATATACTTACCCATTCGTTGGTATTGTAACTTTGTGTCCAAG
TTGAGCTTTCAAGCGAAGGCGAAGCTCACATGGAAACAAAAATGTGACCGAAGACGGGAA
AACCAAAGTAGGGTTTGGGTGGGGTCTTCAGTTGGCTCCC
>ntig_581_consensus
CCAAATCCTTCTGTCTTTCTTGTTGTATTAACTCCCTAACAGGCCTATAGGCAGCTGTTG
TGCACAAATTCTTAAGGTCACTTCCAGAATATCCATCTGTCATGCTTGCAACCGCCTCAA
AGTCAACATCAGGAGACAAATCCTCTTTGGCTAGTATAACTTTTAATATCTTCGCTCTGT
TTGGAGCATCTGGTAAATTTACCATCAATCTGCGGGGAAGCCTTCTAATGACAGCCTCAT
CAAGGTCAAAAGGCCTATTTGTGGCTGCAAGCACGAGAACTCTTTCTGTATCTTTTGTCC
GTAACCCATCCCAGTTCACCATAAATTCATTTTTCATCTTACGCATGGCCTCATGCTCTC
CTGGATTTTCTCTCCGGCCCAACATGCTGTCAACCTCATCAACAAATATGACACTAGGAG
CAATTTTACTTGCTAGGGAGAAAACTGCTTTGACATACTTCTCACCCTCACCAAACCACT
TTGATGTAATGCTTGACATCGAAATGTTAATGAAGTTTGCACCAGCTTCAGTTGCAACAG
CCTTTGCAAGCATGGTCTTCCCAGTCCCAGGGGGTCCAAACAGAAGTATGCCCTTACAAG
GCTTAGTCAATTGCCCCTTGCAGAATAATTCTGGCCTTTGTAAAGGAAGCATCACCAACT
CCTTCAATGTATCCTTGACATTCTCAAGAGCTCCAATATCATCAAATGTAACTCCAATGT
CACTGGGCGGAATCACATCAGATAACAGCCTTTTCTCAAATTCATTTTCTGTCACAACAT
CCTTAAGTGACTTCTTCAAGCTCTTAGATTCATTCTGGATAGCCTGTAGGATTCCAATCC
CATACTGGATGCTATCACTCAATAACACAAGTCTAGCATCAGGATCCGCTGCAGGGTTCT
GCATTAGATGGTGGCTTAAAGCCCATCCAACCACCTTTTCTGCACTTTCGTTTGTGAGTG
ATTGGTCCTTGATGCATAAGGTCTCAAACCCTTCACATTCCACCCCACTCCGACTAAGAA
CAGAGCGTAGATGATTGAGGTTTCCCTTCATTTTTAGTGTTTCGGCATCACGATCCAACT
GATGCTTCCAAGAAACTAGAAGTGCCTCATCCTGTGGCATGTGAATGGTTACTTTATTTG
GGAAAAGCTTAGTTAGAAGTTTTGTTGCCTTTGGGACTTCCTTCCCTCTCTCATGCAGTC
TTCCAAAACTATCTGGAAAAGCCAAGTCAAGTAGAGCAGTTTGATTGCTGCCAAATTTTG
TGAAAAGCAAGCCACCAGGATGTGACTTCTCCTTACGATTGTCAGTATGAGTGTGTGAAC
CAATTACAATCACTTTATCAGGAAGCTTTTCCAGTTTGCTTTTAAATGTGGAATATGAAT
CAGAATTTCCGGCAACAGACTTCTCAGCATCTTTCATAAACAAAATGAATGGAGAACTTC
GACTTTCACTGTCTACCACCTTGCAGGTGGTGATGTTGGATATATACCACCAGAAGCAGA
ACCAATGTATCTTATCCTATCACCT
>ntig_582_consensus
GTTCATAATATCATGCATATCATATATACCAAACCCAAAGGCTTATGGTAGTGTAAGAAA
CAAAACCCCTCAACTGCTGCAGGACCAATTTGAAGATTTGTAACTCTAAATTTATACACC
ATCTGCATATATACACCAGTTCCAGTTTATAGAAAAACGTTTGCTAGAAGTACAGGCTAC
AGAAGCCCGGCATCATGATTACAGAAACAAATATGTTATTGCAAAACCATAACATTATTC
AAAGACAGACCTCTGTGGCAAACACAGATAGTTTTGTGATCTTCCTGATGATTAAGAGGC
TACAACTGGTGAATCCGCTGAAAACTGCTGTTTAGCTCCCATGGCAGAACAAATCGGTGA
ATAATGTAATGTCATGGCTAATCCGAGTAAATCTTTTTGGATTGAGAGTGTGCAAAGATG
ATCACGCAGCACTCAAGCAGCTCTCCCAAGTCCCATATTTTGAGCTAAAACTGCCTCCTC

CGCTCTGTTGCGCATATTCTGCTATCACAGCACGGGCACACACATCCCAAGTCCTTGCTA
TCACCCCAAGAGTCATGGGTTGTGGAAGGCTTCTCAGAGATATGCTAAACCTTAACTTTG
CTTTCACCGAAAGATCTTCATATTCCTTGCTGACTTCAACTGGAAACTTGTCTAACAATA
TTGGAGAGCAATTTGGGTAATTAGTTGGAACAAGCAAACGCAATGGCTGAATAGGTGACA
TTTGTGCTGAAGCATACTGTGATTTCAAGCTTGGACTAAAAGCCACTGCACTGAAAGAGC
ATCTGACAATGATTCCTTCACCTCCTTCAGCAGTAGCACCAGCAGCAGTCGGATCAACAT
CTTCCTCACTGATATCAACCACTGTGTCTATCAGTAGCTGATTTATTTCCCTTATTTCCT
CCAAAAGAGCATGATTAGCCTCCATTCTTGGCCTTTTCATACTGGATGTGGCAGTTGACT
CCAGATCAGATGTCTCTGAACCATTTAACTGCTTGAAACTATCATTCATACTGCCAGCTG
AAGAAACAACATTTAAAGGCATGGCACTTGTGTAGCGCCTCATTTTCCTAGGTCCAGCCA
TTCCATCTTGTGCGATGAAATTTCTAGCTTGCAGACGACACTTCGTCATGGCAACCAAAT
CCTCACCAACTGCAGCTCTAGACCCATTACCTGGCGCTGATCCAGCTATCCTATCCATCA
TACTCACAACTGAGCCAATGTCACTGACAGATGCACTCAATGCTTTGGGTGACATTGATT
TCACCACTTTGATTAAGCGCTCAAGAGGCTGCTCTGTAACACCAGACATGCCTGAAACAG
TAGTCAAGGCATTACCATGAGTGCCATCTGGACCAGTAAACTCAGCAAGCAAAGGTGATG
CTGATATCCCAGGAGTGCCAATTGCAAGAGACGGTGCTGTTGCTTGTGTATTAGAATTCT
GTTGATGGGTGCTATTCCCAGCATTTGCTAGGGATGAAATACCAGATACTGGTTTATCAG
AATCTCCTGGTATGGGTGATGGAGCCAAAGATGTTGAAGGAGATGGTTCAACAAAAGGAG
AGCTTGCAGATTGCAAGGGGGTTCCAGGTTTTGTGAGTGATGAAAGCAGATTCTGCTGGT
CAACCTGTGGAGAAGAATGTTGTGGCACTTGAGGGGATGCAGCCTGTACCATTTGGGGAG
AACGAATAGCAAATGTAGCTCCTGGTTTCAACTGTTGATGGGAATAGGCTGAGCGTTGGC
TTGATGGCATATGTTGCTGAAAGACCCCTGGTTTAACACTCATCCCCTGTCGCATCTTTA
AGTCATTCACATCATTCATCTGATGCAGCTGTGGCATTTGATGCATCTGCATCTGCATCT
GCATCTGCCCCGGCAGCTGCTGCTTTGCTTGCTGTTGCAACTGCTGGTGCTGCTGTTGCA
ATACTTGTTGCTTCTGCATAAATTGATGCGGTATCTGGCGCTGTTGCAATTGTTGTTTGA
TCTGTTGGGACTGCAGTATTTGTTGCTGCTCCTGTTGTTTTATATGCTGGTGTTGTAGCA
TATTTGAGTTTGACTGGAGGGGATTGATATTTGTTGGCAGGATATTAACTCCACTTTGTG
ATGACAAACCGTTCATGTTTGCTTGTTGAGCACTCACAGAATTTTGTTGTAGAGATCCAA
CAGAAACCTGATGCAATGGGTTCAGTGAATTTCCTTGTCCTGAATCCAAATTTGAACATG
GCTGCAAGGTATGTAACATGTTCTGTGATGCTGTTGAGACCCCCGAAATAGAAGGCAGAG
AATTATGCTGCATATTTGTCACATTATTTTGTGGCATTGTTCCAACAGAACCTTGTAAGT
TCATTGATTGCATCTGTGGATTCATTTGATTGTCATGGGGCTGAACTTGAGTAAGTTGTG
ATTGTGGCTGCTGCATGGAATGCAAATGAGGCGGGAGATGTTGCCCTGGCTGCATTGAAG
AAACAGGCTTCCTGGGCGGGCGATTTGTATTAATATAATTGATAATCAGCTTCTCATAGG
AACCCAGCTTTTCCCCAAAATTAGGTGAGACATTACTTTTGGACACCTGTAAGAACAATA
TAATACGCTCCAACATAGTCTTAAACTTTTCCAGCCTCTCAAGCTGGTCCGACTTTGGCG
GTTGAGGAAGAGAATCATGCTGCTGCAATTTAGCAGCGATTTTCTGATACATTTCAGTTA
ATTCGGGCAAATACATCTCCTTCATGGCTTTAATCTTTTGGTAGACCTCCTCTTGCCAAT
CACCCCCATTTACTTGTCCAGTCTGGGCTGTGGAATCTACAGATGTTGATGATGTTTCTG
GAAGGGGTCTTTGTGATTGATATAACTGCTTTTGCTGATCCATTACATTCTGCGGTTGAT
GCAAGGAACCGGATGCTTGATTTGATGCCTGAAGCCTTTGCTGTATATTTTGTTGTAATT
GATTAGGTTGTTGCAATCCCAACTGCTGTTGCAGCTGTGCAGGCTGTGATTGGATCTGTG
ACATAAATTGCTGCTGTGGCTGTTGCGACTGTGACTGCTGCCCCTGATTTGGTAATAAAG
GTGCACTCTGCTGGCTTTGTTGATGCAATGGAACCTTGGATTGTTGTAGCATATGCACAG
AGTGTTGATTAGTTTGCATGCTTGAGTTTCCAGGATGGTTTCCAAGTAACTGTTGCTGCT
TCTGTGGCTGTAATCCTGAAACATTGCTTTGTGGGCCCAATTGTTGATGATGCATACTTG
AGAGGCTTTGCTGAGCCAACATCTGTTGCTGCTGCATATTTGAAACATTACTCTGCTGCC
CTAACAACCTTGGTTGTTGTTGCTGCTGCATATCACTAACACCATTTTGTTGGCTAATCA
ATTGATTCTGCTGCATGTTTGAAGCATTTGAAGGTTGGCCAATTAGCTGCTGCTGCTGTG
GCATCATTGATGACTGTGTCATTGGAGGTTGCTGTTGATGAATACCAACAGGCTGTTGTT
TCTGTTGCTGCGGCCTAAGGACTGATTGCTGGTGAGGCTGAATCATGGATTGTGTTGACG
GTTGAACAGATGACTGCTGGTTCTGCTGAATACTAGAGAGAGGGGCTGATTGGATCACTG
ATGGTTGCATAACAGATGATGATTGCATGCCAGATTGCTGAGACGGTTGTAACTGATTAG
GCTGTAGTATGTTTGGCTGCTGCTGTTGCAGTTGAGGTTGCATTAGTGAGTGTGAGAGAT
TTCCCTGCTGCAGTTTCTGCTTCATTAATTGATGCTGCTGATACATATACTGCGGATTCT
GGGATTGCTGTTGTTGCTGTGGGGGTAGAACCTGTTGCCTTCCTTGCATTTGCCTCTGGG
AATTGGCAAAGAGATTGGAGGGCACCCCTTGCCCCATTGAAGGCACTGTGTTCTGTGAAA
CAGCGGATATACTTTGCATGCTGGAATTTTGGTTTACAACACTGGGGATTGGAGCCTGTG

ATAAACCAGAAACAGAAGGAAGAGCAGATGGCAATCCAGAAGAACCCTGAACTCCGGTTG
ATGTCATGTTACTCTGAATGTTCTGTGATAACAGCTGTTGGCGTGGTTGAGACTGATTAG
CTGAGATAGGGATAGGAAGTGATTGCCCTTGGTTCTGAACTGGGTTTTGCATGACAGCTC
CTTGATCCTCTGGTATGTTGCTACTGCTAGAGGGATTGGATGGCAAAGAACTAGGCATAG
GATTTTGATATTTTGACTCCAGTGAAAGCATCTTCAAAGATATTTCCCGCAAATAATCTG
ACTGACTTGTTGCAGCAGCATATATCATATCCTCAAACCTTCCAGCAATTTTCTGGAGTT
CATTCAATCCCTCTTGAGCAGAAAAAGGAAGATGCCTCTTCAGTGTCTCCGTAATCTTGT
TGACAATTCTTTGTCGTGAATCAGGCTGCAACTGATTTCTCCAATCGCCAGTGTCTATGA
CAGGTTCTCTGCCTTGAGAAGTAGGCCCCCAATTATTGGTATCCATCTAATTCTAACCTA
AAAACAAACAAAAAC
>ntig_583_consensus
GAAACCAAATCCATGGCACCAAAAATCATGGCTATCAGTAGAACATACAAAACAGCAAAG
ACAGTACAAATTATGATAACAAAAAAAATAGCCAATGAACTGGTAGACAAGACAATCTCA
ACATCCGTTTGATGAAGAAGCAAACTAGACAATAAATATGAACAAGTTTACCCTAAACTA
GAATTTGCATGCATTTTGAGACTGAGTATCAGGTAAAAACAAGGTCAAGACTCACTTGTC
TGCTCTAGTAACTGATCACCTACAAAGTAATCAGATAAAAGACATTACAGGAAGAGCTGC
TACACGAATTGTTTTTTCATCACCTATCAAGCCACCATCATAATAGAGAAACAACACTAT
CAATAATATTATTATTCAAATCAAGAAAAACAACTCAAGAACTTAGTATATTTGAAACAA
ACAAAACATATAAAAAAAATAACAATAAATCCAAAAAGTTCCCTTTGAAGCCTCACTCAG
AGCGATTAGCATACCTCTCAACCTTATCATCATTCAAATTGATACCCACCATAGCCTCAC
CCAGTCCACAGCTCACTTCTGCCAAAATGTCGGGGTCACTATAATTCGTAACCGCTTGCA
CAATTGCCCTCGCTCTCCTAACCGGGTCCCCGCTTTTAAACACACCCGACCCCACAAACA
CTCCATCACACCCTAACTGCATCATCAAAGCCGCATCCGCAGGCGTCGCCACTCCGCCTG
CGGCGAAATGCACCACTGGCAGCCGCCCCAACTGCTTCGTCTGCATCACCAAATCGTAGG
GAGCCGCGATTTTTTTGGCAAACGTAAACACCTCGTCGTCATCCATGTTTCGTAAAACCC
TAATTTCGCCCATCACCGACCTTACGTGCCTCACCGCCTCCACGATGTTCCCCGTCCCCG
CCTCTCCCTTCGTCCGGATCATCGCCGCGCCCTCTCGGATCCGCCGCAAGGCCTCCCCCA
AATTTCGGCACCCGCAGACAAAAGGAATCCTGAAATTATGTTTGTTGATGTGGTTCTCTT
CATCGGCCGGAGTCAATACTTCGCTCTCGTCAACGTAATCAACTCCGATAGCTTCCAGAA
TTTGCGCTTCAACGAAATGGCCGATACGCGCCTTGGCCATGACAGGGATGGTGACGCTGT
TTTTGATTTGTTTAATCAACTGTGGGTCTGACATGCGGGCCACGCCTCCCTGAGCACGGA
TATCTGCTGGAACGCGTTCGAGAGCCATTACGGCACAGGCGCCGGCTTCTTCGGCGATAC
GGGCCTGTTCAGGAGTGACGACGTCCATAATGACGCCGCCACGAAGCATTTGGGCGAGGC
CTACTTTGACGGAAAACGGAGACTTTTTCGTGGTTTCATAGATGGCACCGTTACCGTAGA
CGGTGACAACTCCGGTGTCCGCCATTTGTTGGAAGAGAAACGCGGGAGAGCAGAGATAAG
AGAGAGATGTGGCGTGCTAGCGGCCGGAGTATTTTAAATGAATGTTCGGATTCGTTTTGT
GGAGAGTCGGTGAAGGAGGGACACGTGTGTGGCTTGGATTCATTCGTTAGGT
>ntig_584_consensus
CTGTTTAAAAGGGTCAACAGAAGGTTGCCACAAACACGGTGAACATTTGAAAAAAAGACG
CAAGCTTGACGCGCAGAACCCAAAAAGCCGACATCAAAACACTGCCAAAGCCTTGCTGAG
AACACAATTAGCCATTCACTATAACCCCACCATTGGGGTGGAGGACCTGCCCAGTTATGT
AAGACGAGCAGTGGTTACAAGCTAGGAAGACAAAGCAGGGTGCAACTTCAATGGGCTGCC
CAGCTCTCTTCATGGGAACTTGGGACCCGAAATTTGCAGTCTCGTCTTCATCGAAAGATG
AAGGAATTAAAGGTGTCCAGATCGGTCCTGGAGCCACTGCATTCACTCGTATTCCTTTTT
CCACAATTTGGAGAGCAAGTCCCCTAGTGAAAGCCACAATCGCTCCTTTGGTAGATGTGT
AGTCAAGGAGCTTGGCGTTTCCTTTGTACGCATTGACTGATGTGGTGTTGATTATCGAGC
TTCCCTCCTTCATATGCTTCAAGGCGTGCCTGGTTACGAAGAAGTAAGAGAAAATGTTAG
TCCTGAACACTCTCTCAAGCCTTTCTTCGTCGATCTCCTCGACAGATTTTGCTTTGTGCT
GCTCAGCTGCATTGTTGACGAGAATGTCAATCTTTCCAAAGGCCTTCACCACTTCATCAA
CAACCCTTTTGCAATTCTCATCAAATCCTAAATCCGCTGCCAGCGCCATCGGGTCCTTGG
CGTCGGAGGTTTTGGCCTTTCTGAGCATCTCAAGAGTATCCTTCGCATCTTTGTCCTCTT
GAGCCTTTACATACGTGAAAGCCACAGTTGCACCTTCCTGCGCAAAGCAGTGACACACAG
CTCGCCCTATCCCGGAGTCGCCTCCGGTCACCAGCGCCACCTTTCCCTTCAGCTTATTTG
ATGGCGAGTATTCATGGCTAGCATATTGAGGAATTGGGTCCATGACGTGCTCTTTTCCAG
GCTGTGCTTTCTGCTTCTGGGGAGGAAACTGCTGATTATTACCAGAAGCCATTGTTCTTA
CACCTACACTACTGATGAGTCTCTGACTGTTCGGGCAAAATATTGCCGGAATAAAACTCT
TCCGGTGAGTTTTTTTCAGACGGATTTCGTCTAGAGTAAAAGAAGCTTTAGTTGATGACA
GTCTTGATGGAGAGAGTGGCTCAGGTGTGAGCATATATAGAGTGCACGAAGCTGAAGAAG

CATGGTGAATAAAAAATTGTGATGACTTTATATAGAGTTAGCAGGAGGACACGTAGGAAA
ACGTGGCGGAAGAAGGAG
>ntig_585_consensus
ACTTTAGCCAGCTTAGCTTCATTCTCTTCAATCACAGCTGGATCCGCAGTCTGGGCATGT
CAACTGAGTGCCCTTGTCAGTATGCTCCTCAGCGATGTATTGGGTAATTGCCCTCGATTC
GAATAGCTTCTGGTCTCCAACTTCTAATGCTGGGACTTGACCAAATGGATTGAGGGAAAG
AAATGCTTCAGATTTGTGGTCACCAGCTTTCATGTTTACAGGAACAAACTGGAACTCAAG
TCCCTTCTCGTAAAGGCAGGCAAAAACACGCATCGAAGCTGTCGAGAAAACAGCACCGTG
GACCTTCAAGGCTGCCATGACGTTGTGATTACACCGAGAGGAAATAGCTAAGTGGGCGTA
GATGGATGAGGTTAATTGCAGAGGCAGGAGGCGTTTATAAAGAGGGAT
>ntig_586_consensus
CTGGGCAGGTCAACTGAGTGCCCTTGTCATTATGCTCCTCAGCGATGTACTGGGTAATTG
CCCTCGATTCGAATAGCTTCTGGTCTCCAACTTCAAATGCTGGGACTTGACCAAATGGAT
TGAGGGAAAGAAATGCTTCAGATTTGTGGTCACCAGCTTTCATGTTTACAGGAATAAACT
GGAACTCAAGTCCCTTCTCGTAAAGGCAAGCAAAAACACGCATCGAAGCTGTCGAGAAAA
CAGCACCGTGGACCTTCAAGGCTGCCATGACGTTGTGATTACACCGAGAGGAGATAGCTA
AATGGGTGTAGATGGATGAGGTTAAAAGCAGAGGCAGGAGGCGTTT
>ntig_587_consensus
GATTAACCAAACCAAATCAATTTCTAATGAAGAGATCAACACTCGACAATAAAAAAAACT
CCTCACTCTTGGTCTCTTCTCTCATCACCAGATTCAGTTTGCACATCAACAAAATCTCTC
TTTGTCAAAGTATAGAATTGGATCCAGTTGATCAGAATTGTCAACATAACCAAATCAAAA
ACCACAGATGTCCCAAACAAAAGCGGAAAATCAAAAGCATTCTTGCATCTAATCAATGGG
AATGAAAAATCCCTGTAACTACAGCAGCAAATTAAACAACTGAGCAAAGAATCACAACCT
TCTCCCTCGTCTGCCACCCTTTCTGCGGGTACTGTCAGTGGGAATTGGTGTCACATCCTC
GATGCGACCAATTTTCATGCCTGAACGGGCAAGGGCTCTAAGCGCTGACTGTGCACCTGG
ACCAGGTGTTTTAGTCTTGTTCCCTCCAGTAGCCCGGAGCTTGATATGAAGAGCAGTTAT
GCCAAGCTCCTTGCATCGTTGAGAAACATCTTGTGCTGCAAGCATGGCTGCATATGGAGA
GGATTCATCCCTATCAGCTTTCACCTTCATACCACCAGTGATGCGAACAAGCGTTTCTCT
GCCCGACAAGTCAGTAACATGAATGAATGTATCATTAAACGAGGCAAAAATATGGGCAAC
ACCAAAGACATGCTCCCCATCTCGTACAGCAGGTCCAAGTGTAATGTTTTCCTCCTTAGG
CTCTCTGGCTTTCTTCTTTGACATGGCCGTTAGTGAAACAGAG
>ntig_588_consensus
TACCATTACAAAAATAATTGGCTTCTGAACATAAATTCAGAACCTATAAACGAAGTAAAC
AGTTCTTTAACATTGAACAGATCCAATTTATCAAAATTGTAAACATCACCATAGCCAAAA
CTACAGATGTCTCAAACAAAAAAGTTGGAAAATCAAAGAAAGTATTGCATCTAATCAATG
AGAAGGAAAAATCCCTGTAACAGCAGCAGCAGCTTAAACAACAAAGCAAAGAATCACAAC
CTTCTTCCCCTTCTGCCACCCTTTCTGCGGGTACTGTCAGTGGGAATTGGTGTCACGTCC
TCTATTCGACCAATTTTCATGCCGGAACGAGCAAGAGCTCTAAGTGCTGACTGAGCACCT
GGACCAGGTGTTTTAGTCTTGTTCCCTCCAGT
>ntig_589_consensus
GTCTTGTTCCCTCCAGTAGCCCGGAGCTTGATATGAAGAGCAGTTATGCCAAGCTCCTGC
AGGTTACCAGTTCTATAAGTTGATATTTTTTTGGAAACAATAAACAGTTCCTTCTATAAT
AAGAAAAGCATCAAATTCTAGTTTATATGAAATTACAGTAACTATTAGATTACTATAGTG
AAGATCACATGGATATAAAACAAATATCCATTGGGGGAACTGAAAAGTAAAACCTTGCAT
CGTTGAGAAACATCT
>ntig_590_consensus
AACTTTGCTTCGACTAGGTACCATAGGACCTGTTGATTGGTTCTTCTGATTAAACTCATT
AGCTAAATTTGACTGCAAGCCAGAAGATTTGTAGTCAGCTTGTGGTTGCACTGCTGCTGA
AAGAACTGTTGGCCTCTGAGTTGGTGTACGTTCTTTGAGAGTCAGGTAAGCAACTTTTGC
CGCACTCATCTCTGCCTGCTTCTTCGTCTTTGCTTCCTGCCCAGTAAAAACCTCTTCTTC
TACCTCAACAGTTGAAACAAAAGTTGGCATATGAGCTTCACCAGATCGATTTGTGTGATA
AACAGGCAAAGTATAACCTTCTTTCTGAGCCAATTCTTGTAAAAGGTTCTTGTAAAAACC
AGAGTCATCCCCTTGAACTCCATCTGAAGACAATGACATTAGAGCAACTTTTGCTGCTCC
ATGCTCAGCTTCCTTTAATGTGGGGAAAAACTCCTGACACTCGTAAGTTTGTCCACCAAC
TGTGACCTTACTCTTAAAACGACTAGCATGAGGAGGACCTTCCCGCTCACAAGAATAAAT
AGGAAAAGCAAGATTTTGCTTTTGTGCATAGGATTGCAGCTGGTTCTTGTACAGGTGTAA
TGTATCTCTTGATCTGGAAACATCTTTAACAACTGAGGAGGTTGCATTGACCATAGGAGT
TTGATCTCTTGATCTGTCAACATCTTTCCCACTTGAGGTGGTTGCATTGACTTGAGGAGT

TTGATCTCTTAATCTGCTATCATTTTTCATGACCGAGATTGTTGCACTGACCTCAGGAGT
CTGATCTCTTCGTTGTATATTCATTTGCAATGTACTTGTGCTTGAAGAACCTCTTGATCT
GCAAAAATCTTTAACGACAGACGAGGTGGCATTGACCAGAGGAGTTTGATCTCTTGATCT
GTCAACATCTTTCCCAATTGAGGTGGTTGCATTGACTTGAGAAGTTTGATCTCTTAATCT
GTTATCATCTTTGTTAACTGAAACTGCTACACCAACCCGAGGAGTCTGATCTCTTCGTTG
TATATTCATTTGCAATGTACTTG
>ntig_591_consensus
CTTTCTTCTTCTTCTTCTCTCTTTTTAGCTTCTTCCTCTTCCCTTTCCCTACTTTCCCTT
TCTTCTTCTTCTTCTCTCTTTTTAGCTTCTTCCTCTTCCCTTTCCCTTCTTTCCCTTTCT
TCCTCTTCTTCTTCTCTCTTTTTCTTCTCCTCTCTCTTCCTGGCTTCTTCTGCTCTTTTT
TCTTCTTCCTCTTTTCTTTTTTTCTCTTCTTCCTCTCTCTTCTTGGCTCCCTCTTCCTCC
TTTTCCCTTTTAATTTCTTCCTTCATTATGCTTTCCTCTTCCTCAGCACAAGACGTGCAA
TCTAATATGACTGACTCTGCCTGCTGTGCCAGCAGGTGATCAATTGTTGTGTTAGAGACA
TTAAAGGATACAGCCAAAATTCTGTTGTCTAACGTTTGAATGACTGAGCTCTTTCCTGCT
AAAAATTGAGGATAGTTTCTCCTCCTTGTTGCGCTGAACCCCATGAAAACAAATGAGTCA
TTGTTAAAAGACATCTGAGCCATCGGATGGAACCTGGGTACAACAAAGACATCTCCTTCC
TCCGCCCTAAACCTCACGTTTTTGCAGCTTGACTTTTTTGCACTGCTTGGACAAACCACA
CGAATCATCCCTTGTCCATGTAGCACTACCGCTATCTCCATGGCCATTGGATTCCAATGC
GGCCCCATCATCGAACCCTTTGTCAAGTTCACCATGAAAATTCCAATGTTTGACTCCTTC
AATAATTTCAAGTCTTTCCTATCTACTGTTAGGCTCCAGCCGTTACAGTTGCTGAAATCT
GGATCAACATCAAAGACATTATATGTTCTAGTTTTCTTCCTCTTGCCGTTCATGGCATGC
ATGTTGAAGCCTCTGTCACCTACAATGGCTTTCAGGAATCGACTTTCAAGTTCCCAAAAG
GTTGATTCCTTCTCCCTTGGCAATGCATGAATGATGGGTGGTGGCCTTGTTGTATTTCTT
AGTGCTTCGATCAAATCTTCAGACACCTTGAAAGCTGATTGCAGGACTTTGTCATCAAAT
CCTAAGACCAGATCACCAATGCTAGTGTATGCGCCAATTTCTGGGTCGAATGATTCTTCA
TCTGTACTAGAAAAGACTGCATGGATCCTAAGCTTCTCTCGCTCTGGCTCCAAATCGCTC
TCTATGAAGAAAATGGACCCTGGTTGCAGTCTATACACATCTCCTCTCTTAATATTCAAG
CTTTTTGTGTCCTTTTCATCAGTCCAACTCAGTCTCCCACTTCCTGTGTGGACATAGAAC
ACCATGCCTGCACGGAGGAGGACAGGAAGAAAGAGAGAACTTGGCTCCAATGTGATGAAC
TGAAGAAAATAAGGGCCTCTTGTTCCATCACTGATCCTAACTTGAGAAATCTCTCCATAT
TCTGTCTCAACAACAGATTTTCTGTCTCCTCTCTTCACCATAGACGCCACCTGTGAAGCA
TCCTCATGATGATCTTTAAAAGCGTCTACATTGATTAACGAAACAGAAAGAACAAGAAAT
ACGCAGAATAGTGAGAGAGATCGAAGCAACGAAACCAAAGATTTGTTCAACATCTTTGAT
TTGTGTCG
>ntig_592_consensus
TTCCAATTCATTAAACACAAACAATTTTACTCAAAAAAAGTTCGGTACAAAACATAAATG
TATTTATCAGAAAACAACATAAATAAATATCATACCACAGACCCATACGAGCACATATTC
ACAGTATGTGGGTCTATTTCCCAAACATAACAGGCTGGCCACACACCATCCATATCTAAT
GTGGGACCCACATGCAAATTTTTAAACCATGGAAACCTCATGATAATAAACTAATTGCAT
TACCCACATCTCAACTCTTTTTGTAGATGAAACACAAGCCAAAGGTGAATAAGATGCAAA
TAAATTATTTCCAAACAATGAACATAGGATGATTATTTCAAGAAAATGAAACAATACTAG
GTAACATCATGCATCAAGTGATCACAGCTGTAATTACTACAACTAAGACACATTCAATCC
CCCTAAGAACGAATCAATCACTCCAGAAAAAAAGGGATTGTTTGTGTTGGACTTGGGACT
ATAAAATTGGTTTGAAATATGTCGCAATCAGACTGCTGCTGAAACAGAGGATAGACAAAA
GCATAAGGTTGTTTGTTATCACAGCCTTCTGCTTACAAAAGTAAAACTCATCTAACACAC
TGAAATATGCCCATAGCATTCACCAAAATCCAACATAAAAGTGTGTATGTTACTGAGTCC
TGTTGTTTACAGGAAAGCATCCTTCTCCAACAGCTTTAGTACCTCATGCTGGTTGTTGAG
CTTGGCAACATCAATGGGAGTCTTGCCATCCATGTTTTGAAGGGTTACTGCAGCGCCATT
CTCCAACAAGAGGGAAACACATTCCTTCCTGCCATAACCGGCTGCATAATGAAGTGCAGT
GTTCTTATTCTTGTCCAAAGCATCTACATTGGCTCCAGCCTCAAGAAGGATCTGAGCGCA
CGTCAACTCACCATATCCGCAGGCAAAATGCAATGCTGTCCTTCCTTCTGAATCTTCTTC
ATCCTTGTCAGCACCAGAGGCTAGTGCAGCTTTCAAACCTTCAACATCACCAACACTAGC
AGTATTATGAACAATAGACTCATCGTCATTTCCAGCTTCTTCTGTTTCATCTTGCCGAGC
ATTGTCAGCAGAAGTGACTGGATCTCCAGCAACTGCAAGACCCATTGCTTCCCCCAACTT
CTGAAGCACATCTTTATCATTCCAGTACTTCATCATGGCAGTTGGTCCGCCCCTCTCTAT
CTCTTCTAAAATAGGCTTCAAAGATGGATCTTCCTTTATTCGTGCCATTCGTTCCTCGAG
CTGGTCATTGTGTGACGGGTTTGATAAACTCTCCAACATAGTAGACATGGATGGGTCCTC
CATCAATGCATTACCAAGGCGCTCAGCCATAGCCATGAATTGAGGATTCTGCATAACCTG

TTGCATGGTGGAGTAATACTGCTGTGTATCAAACTGTGGGATACCATCATCAGGGGATAC
ACCTTGAAAAGTTGTCTGAAGTTGCTCTGCCATCTGATTGAATGCAGGGTCTTTTGATAT
CTGTTCAGCTAGTTCCTTAATGCTAGGGTCATTAAGGAGACCACTCATGGCAGAGAAATC
AAAAGGATTGGGTGGAAATCCAGGTCCTGGTGCAGAAGGTGCAGCTTCTCTTCCTTGTGA
TTCTCTAATAGAACCAGTGGATGTTTCCGATGTTGGTGTTGAACCTCCTGCCTTCTCATC
ACCAGCAGGAGCGTCTTTGGTGGGATTAGAAGCCATGAGATAAAGCTGAAACCGAGTGTG
AAAGAGAGAGAGAGAGAGAGA
>ntig_593_consensus
GTGTGTCAGTGATCAAGGTTTAGTTTCTAATTTCCCAAAGCGAGATGTAATGAAGGAGCC
AATCCGAATCCAAAGCGAGAACCTGAACCCTCGGAATTGTACTCTTACTGATTTTTTGGA
TCCTCCTCTAACGAATTCCAAGTCCTTGGCAATGCTATACTGAATCTGAATGCTTCCTAT
GTCATCAAGCAGAGGTAATGCAACTGAATCCTGGGACTCCAGGTTTTTTATCCACCCACG
CAGTAAAAAAATTCACAATTTCAGGCACTTCATCATGAGGGCAGTGACCAGCTGGACTGAG
CTCATAGTATGGAACTTCAGGTAGCTGCCTTTTCACCTCAAGGCCCCAGATAGGCTTAAC
CCAGGGGTCTTCTTTTCCATACATGAGACAGATTGGAACATCATTCA
>ntig_594_consensus
CTCAATATGTAAGAATGGTTGAGAGACAAAAAGACAATCACACCTAGAAAAATACTTTAC
TAGTTTCTTGCAATACACCAAAATAAGAGGTTCTTTTTGAGTTAACTATAATATATACTC
ACTCACCATGCAGAAAAACAAATGCCTAATTATAGAATCTAGTATTACAACAACGACTCC
AAAGGATAAATTGAAAGACTCAACACTGGCCCAACTACCACAACCCCTTCAAGGACGTCC
CTGCTTGTGCCTCAGCATCAAATACATCGCATTCTCATAGTTTTCCTTGCCTAAAAAGAT
TCGGATGTCCACATGCAATAATTAAAACCCAACTGGCCTTGCTTTATTAGACACTGTAGT
ATAGACTGACCTCTGTTGGCAACTCTTCAACCAAACAAGACACGGGACCAACCAATAACA
TAACACTTACAGTTGCCCTGACCCGACCCGACCAGACCAGACCTGACTTGAACCTGACAT
ACACCTTCGTCAACCCCAACATGAACTTGGGTTCCATTTGACAAACTCCATATCCCCTCC
CTCGTGCACATGAACTGCAATATGCAACTTCAACTGAAGTTGAATCCTCCAGATGGAACT
TGAACCTCATTCCCTCCAAATTGGAAACCTGGCTGGGCATCATCAGCCCGAGGTAAAGCC
TCATCCTCCTCTTCCAACCAGAATGTTTCAAGAATCTTAACTGCCTTTTCATAGATCTCA
TTATTGTCATGACTTTGTAGGTTTTCAATCTTTTCTAAACCCTCGGCCTCCTCAACTAAT
TGAGCATAGTAATTGACTTCTCCACTGTTGCCCATACTCTTCTCAGCTTCTCCGACCTTC
AAAATGTTCTCTAACCCTTCCAGACAAACAGTGACAATGCGTGGATCAGGGCATGCAAAA
AGATCACAGAGTGGTTTTATACATCCCTGAGCCACAAGGTACTTGATCTGCTCATGAGTA
CCGCCAGAGGTGGCATTTGAAATTGCCCAAGCTGCCTCTTTCTTTATCTCAAACTCAGCA
TTTTGAAGTAGATTGACAAGGGGACCGATTAAACCAGCATCAATTACAGCCTGAATTTGT
TCCCTGTTTCCAGCTGTAATGTTTGAGATAGTCCAGCAAGCTTCTTTCTTTATGCTCTTT
TTATGATTATGGGTCAACAGGCTCAAAAGGCATGGCAAAGCGCCATGGTGAATTATACAC
TGAGTCTGCATATCATCTCCTGTGACAATATTTCCCACTGTCCGCAGGGCAGGAATGAGC
ACTGAGGGAGATGGATGAAGGAGGAGCTCAACAAGTCGTGGGCAAACACCTGCCTCAATC
ACAGCTTGAATTTTATCATTTGTACCATCAGAAAGATAGGAGAGCGCCCAGCAGGCATCA
GTCAAGACTTCTTCATCATTGGAATGAACAAGTTGTGCAAGTGCTGGAAGAGCAGGCTTC
ACCTGATCAAAAGGTGGCTGTGGTTTGCCCCTACAGAAGTTTGATAGTGTCCACGTGGCA
TTTCTTAGCATTGACAGCTTTGCATTCTCATTCAATTGTGCCAGCAATGGCATCAAAGCT
CCTTGGCTAAGCACAAGATCACGGCACCTAGGTGAATCACCAGCAACATTTCCCAGTGCC
CAAACAGCCTGTTCACGAACATCATCACTTGGAGAAGCAAGCAGCTTGACAAATATAGGA
ACAGCACCATGGTCAATCACTACCTTAGTATTCTCTGATGTTCCAGAAGCAATGTTCGTA
AGAGCCCATGCAGCCTCAAACTGAAGTTGCGGGAAATCCTCTCTCATAAGAAACTCAACA
AAGCGTGGAACAACCCCAGATTGTATCACTTCCTCAATTGGAGGACTCCTCTCAATGGAA
AGCAGTTTCCGAAACTGAGTAGTTGCTTCGAGCTGCAGGCTGTTATCGTTCGACCAAACA
CCGGCCACCATCGAAGGAAGACTTTCCAACTTGTTTTGGAGGCTGAGATTGGGAAGAGAA
GCAGCCGGTACTTGGAGCCCTTCACGGCGCTTCTTAAGCAAGCTTTCTTCACGCTTACTT
TTGCGGATCTCCACCATATTGTCCTCTCTTCTTCGTCGACCTTCATCGGCGTCCACGGCA
ACCTTGTAACGGTTCCGGCGAACTTCGGTTCTGGCGCTCGGTCTAAGCGACATCGTTTTT
TTGGGTAAAAGTTGAGAGCTTTGAAAGGCCCTAACAGATGATTGAAGAAGTATAAAAAAA
AAGGCTAGGGATTTGGAGCGACTATGAAGGCCTTCGAAGAGGGAAAGGGGAAGAGTAACT
TATATCCAAAAG
>ntig_595_consensus
CAACCAAATGCACAAAATTGATAATTGTAAAGTATCAGCGTCAATTTCTGCCATTGTTTC
TCTACCTGTGGAATTTCATGCTGGTAGTTATGTGGGCGAAAAGATAATCCATTTTAGGAG

TGAATTGTTCAGCATCCCCCAAGTCTTGCGCTAGATCAGCTTATATGTGATAAGTGTAAC
ATCGGGGGTGCAACCTTTTGCTACTCACATCTGACTATGAAAAGTTCCCTATTTTTTCTT
GAGCACTGCAAATATCCTGAAAATTGCATCTCTCCTTTATTGACATACATAAATATAATG
CTCTGTTGGGGTCACCATTCCTATTGAATTGCCTCATCTCGTTCTTATCCTTCTTTCAAA
CCCACTCACGAACGAACCTGTGTTCAACATTACCCTTTGAACTTCATTATATGGATAATT
AAATTGACATAAATTCACCAATCTTATCCACTTGGAAACAGAAATTTATAAAGAGAGTAA
GTGATTCTTACACACAGAACAACCTAAGCATGTAAACAAGCGGTACAGAGAGAAAACCTT
TGCATTCAACAGCGTCCTTATTACATTTTTTGCCACACTACCATTACAAACTTCACCTCT
AAAACAACATTAAAAGATAATGGCCCGACTCCGCCCACTGGACCTTAAGGCCTTGCGTTT
CATTTTGTAAGCTACTTCTCATGTTTTCACTCCTCCCTTGGTTTCCTTATTGGTTTCCAT
TTCCCTTCCAGTTTCCTGGGCCTTAGCATAGATTGTCTGGCCCGTTTCTCTAGTCTTCTG
GTCTACTTGGCCCGCAGTTTCCTGAGCGAGTCGCTTCAGATGCTCCATCTGTTCGGGGAC
TCGCATTCTCCGCAGAGCATTAGCCATCCAAGACAAGGCAGAAAGAGCGCTGATTCCAAA
GGCTCCCGATGTTAAGAAACCAGTGATAGCCAAACCAATGACGAGCACCGCAGGGACCAA
AACCGGGCTGAAGATTACAAAAAGCGGTGTGGTGATAGCTAGGCCGATGAGTGTTCCCGC
CAGAGTGAGTCCAGATAGGAGGAGAAGAGTGCCACCAACGGGTAGAAGAGTGACAAGTGC
AAGAACTTGAGTTTTTGATGGAGCCCATTCATGAAACATGCCCTTGAAGGCTTCATTTGG
ATGGTACTGGTCGTAGTGGCGTGGATAATGCTGTTGCTGGTGAGTTTGGCGGTGGTCAGC
CATTAAAAATGGTTTCTGACTATGAGTGACAGAAAGAGTGGAGGTGATAAGCTTGAGGTG
TGCATTTTATGGAGGAGGTGACGGTTTACACGTGGAAAATGGCAACACAGCTGGGTGAAA
AATAGAAGCGGATGTTTGCATGGGAATTTCTCCTTGTCAGTCTCACCCTAAATTAATAAT
GGCATCCCATAAAATTATTGGAAACTTCAGAAGACTGAGCAAAAAAGTT
>ntig_596_consensus
GAAGGAGAAGAAACAAACTGTCTCGGTAAGCTGAAAAGATCTTTCTCTTCAAATGGTGAA
GTCCACTCGCAAGAAACAGCAGCTGATGAATCACTATTACATACTACACCATCCTTCAAT
CCTGGAATGGGAACTCCATTGCTGCTGGCTGGAGTCAATCTTAATATTGGCTTAACACTC
ACAGGATTAGCATTTGAATCATATATATGCGAGTTCTTAACCAATGATGTTCCTAGCCGC
AATCCGCTAGGTCTTGGGGAAGACAAGCCTCGATTCAGACTCCAATCAATATTTGTATAA
TCAAATTGTGCCAACGAGCCAGCAGTGCTCCAGTCAACTGGAGAAGACCCAGCTGTGCCA
GTTGAGCCCAACCCTGAGAAAAGTCCAAGGGAGGAAGCAGCAGAAATTGTAGGTGCAGAA
CCCGTCCTACTCCATGAAGTATTTCCATAAATATTTCCCATAGGCTCCTTTGGACTGCCA
TGAGGTATAAAGTACTGCCACTGTTGGTGATAGTGAAGTGGGTGATACATCTGACTTGAG
TTAATGGTGATCGCACTGCTACTTGTACTTGGGATATGGCTCATAAACCCATTGGACTTC
ATATTTTCAACACTACTCGTAGGATAATAATTAGCCGCAGTTGTTGGAGATGAGCTCACA
CTTGTAGCTGGAACATGCTTTGCACTCATAGATTGGGGTCGCTGGATCATAATAACTGGT
TCCCTGACTGTTGATGAAAGATTCTTGATTTCACTTCCAAGAAGTATGTACCGAGTTTCA
GCCTTGGCCGGTGGAGGTGATGCTTGCAAAGGTGATGTCAAGGAATAGGAACCTGAAGAA
TTAGATCCTGTAGTTGTCCATCTTTTCTCAGAAGGTACCACAGTTTTTTGCGGTTTTGCC
CACTCAAGCTTTGGTTCAATTCTCTTCAAGGGTGGAACACTTTTAATATTAGATTGGGAA
GTTACTCCAACTGTAACAGCTGCTTTTGTGTAGTTGAAATCCTTGTCATCACTCTGCTGT
TGTAGAGTAGGTGGATTGGATTTTAATTGTGACCTTACTTCCGAGTTGCGACTAGTACTA
ACAGAAAGCACACCACTATCTCCAGTAGGAGGATCACCAGTCTCATCTGGTTTTGATGGT
GCAGAGGGTGGTTCCTCCTTCATTGCCCTCAAGGTTTCCTCTGCTTTCTCTAGATCTCCC
TCTGCACCAACAACAGCTCTTTGAACTTCCTGCTTTGTACACCTGTACATCATTTCCATA
TCTTCAATCCGAGCAAGTTCTTCCGATATGTCAATTTTCAAATTGCCCCCACCAGGATTC
AGATCCTTGTTCATATCTGCTTCTTCACCCCCTTCGAAAAGCCAAGCAACAGACTGTTCT
ACTTTGCCTTCATTCATGATAAGAGCCATTGTTGCACTGTCATGAGAAAATCCCATTGCC
ACAAGCTGCTGAGACAATGCTTCAAGTTTTCTTGACATGAGATAGCCACTAAATCGCTCC
TGTAATTCCTGAGCTCGCCTCTCTTTCTGACGTTGATGCTTCTTCTCATTTTTCTGACG
>ntig_597_consensus
GGCAATTCATTCCATTCCAAACAATTCCATTCCATGGCTGACTTTGATTCTAAGACCATC
ATTATCTTTTCCAACACTCCCAGAAAATTACGTAGCTATTCTACATAAATAATACGTACA
AATTCAAATACAACTTTCCGAACAAAAACAACCATTACTAAAATTCAAAACTAGAAAACT
GCCTATTCACTTTGGTTCCCCCACGTTTCCAGTAAAAACATAAACTGGAAAGAGAGAGCC
TTATTAGACGCCCAAAAATGACCGACACTGGAATTCCCAATGCTCCTTTGCCTGCCTAAT
CCTGTCCACTCTTTCTGTTCGAAGCCTCTGTTCCCAGTAGTCACGGCAGCTTCTGCTTAT
TAGCATACTAAG
>ntig_598_consensus

TCGACTGTCACCAAATAATACATTAACACTAGTGTATTACACCAAATCCAAATACATTGC
CAAGTCCAAATACATTAAAGAGGCACACGCTAACAATAAATGAATTTCAAATCCCAGCAT
ACATCAACTTGACTAAACAAACAGTGGCAGCATACACTATATATCTTACCGGAGTCTGAA
TGAATAGATGTCTTTTATCTTGTCTTGATCACATTACATACAGTGTACAACATGGTGAAA
AGTTTTGCCACGGCAAGTCATTAGCCTTGAGGCTGCAATGCTGTCTGAAGATTTTTCACA
GCACTGTCGTAGTTCACAAAACCCATGAACCAAAACTCGTGGTTGTCAATAGAGATAACC
TGGATGTACTTCTCTCCTTGTTTAACTTTGCTTGTTGAGGGATTGACTGCCTTCAACTGA
TTTATGGGAATAACTACCTTATAATAGCTCCATTGAGTCTGCTCACCAACTTTATAAGAC
AAAGGATTGTCACTACAAAATGCAAGCTTCGCAGTGGACAAATATAAAACTCCCATTACT
GGACCAGCAGATGTTGAGAGGTAGCATGCATATGTCTTTAGAAGTTGTTCCTCAGGAACA
GTCTCAAAACTTTGCCGAAAAATCTTCTCGTATCCACCTTCTGCAAGAACTTTTGTTCCC
TGTGCAATTCTTCCCATTGCAGCATCAGTAAAACTTGGACCTGTTTTCAAGTGTTGCCAG
AAATTCCCAGCAAGATCCTCAGCCTTCTTCGTGGACTCCACAGCCTTCTTCCCAAATTTT
CCCAAGGCACTCTTCACCGAGTCCATCTTATTCTTGGTAGAGGACTGAGGAACCGGAGAA
GGAGAGACGTATGGATTGGACTCCGGCGGCATAGTGGTGGCGGCACTCTCGTTGATAGGT
GCGGGGCCTACGGAAACGTTCGTCCAATTCTGTGGAGGTGGAGAGACGTCGTTAGGATCA
AGTTTAGGGTATGGTGCATAATCAGATGAGTGGAGCTGCTGGTTGTTTGACTGTGACGAG
GGAGCTTTACTTTGATCGCCTTGATGCTCATTCATTCTCAATTCCTTTTCTTTCTCTCCT
GTATAAACCTTCCTCCCTTTTCTCGGCGAATCTCGGAAAAAATTGGGGTTCTAGCGTGAT
CTCATCCAGTGCTGATGTGCCCCTGTGAAGGACGTTTCTGGAATTCAGTTTTGAAATTAG
CAATTTCTGCCGGCCTCAGTTGTCT
>ntig_599_consensus
TTTTTTTTCAACGACAAACGACTAGAAATTTCATGCATACAACACTTCAAAAGATAATGA
GAAGAGGGCTCCATGGTCTAGGCCTTGGAGAATTTAAGCTATCCAGGCACAATCCTTGTA
TGAATAATGATATCTTCAAACGAAAGGAATAAAATCTTGAGATTAAAAGAGGACAAGAAT
AGAACAACACGTGGCTGTTAAAATATGTAACGTAAACAATCATGTAGCCGCGGTTGGCTC
GGGTTCCGTGACAGGCTGCTCATTAGCAACTGGAGCATCTTCTTTAGGCGTCTCGGGAGA
ACCATTTGAGGTGGTATCTTCGGCCGGAGACTCAGGCTTCCCTTTTGCCGATGCCACAAC
CTTCCCATCCTTTCCATCTTTGGCTTCCTTTGCTGGAGGTGGTGGGGGTGGCAACATGTG
AGGAGGAGGGTTGTGTTTCAGCTTGACCAAAATATGCCGCAATCTTGACAAATCTGTGGG
TTTCCCCGGCTTAGGACCTTGGATGACCGTGATTGACGGTTTCTTCTCTTGATCCTTCTT
TCCTCTCTTTTTCTCAATGTTGGGAACCTCATTAGGAATGAGTTCTGCTATCGCTTTCCA
GTAATGTTTGTCAGCCTCTTTATGAAACTTCTCTTGATTGGTCAAATATAACTTCTCCCT
GTCTCTGTTATTAGTCTTGTTAGTCTCTATATTAAGCTTTCTTTTCTCATAGAAACCACG
TTTATACTCTTCAGCTTCTTCAATTATCTGAATTCTCATTTCCTTTTCCCTTTTCTCCTT
CTCCTCAAGTTGAATTGCATTTTGACGGCGCCATTCACGTAGAGCAA
>ntig_600_consensus
AGACTTTTTCCCTTTCCCTACCCGACGCTCTTCCGATCTGGGATATCAACCATACGCAAC
TGAGAAGCATTTTTTAGATGATAAAACGTATAAGCCTCTTTTCCAAGTTGACCCGACTCT
GAGGACTGCACAGAAGGTCCACCACTGGCAAAATGATGTCGAGTAACCAGACGTTGATAA
TCTCGAAGCAATGGACTAAGTTCCTTGATTGGTATTGTCTTTGGTGTACACTTCTTCCAA
TGCTCACGAAGCACCTGAGATGCCTGAGCTTGTGATTGTATACTACTGGAAACTACTCCT
CCAGTTAGCCTTGATGCTAGGCCAAGGAATTCTTTATCTTCTGCCTCATTCACAATTAAA
ACTCTAGGACTTGAGAGTGAAACTGAGACTTCTAATTTTTTTTGCAGTTCTGCAACGGCA
GAACCAACCTTGTGGCTTTCCTTGAAAGCAGATAAGGAATTATCATTTTCCATTGCAACA
TCCTTAATTTCAGAGAGAACTTCAGGTTGAGATTCAGCAAACCGGTTTGCAATTGGGTCC
TCTTCAGGAACCAGCCACCACATATCCCCAAATGTTGATTTCCCATCAAAGCCACCAAAT
AAGAGATAGCGTGAGCCTATAAGTGTCATAGAATGATATGCTCGAGCAGGAGGTGGTTCA
TTAGCGATAGGTAAGCGCTTCCACTGTGCTGATACTCTATCTAAGATAATAGAGTCATTA
TAATAAATGTCATAACGACTTAACCAGCCACCTGTACTATGTCCTCCAAATAGCAAGAGA
TGGTGCCCTCCAGATGTAATGGTATGGCCACAGCGGGAAGAGGGTGATTGACCAGGAAGC
TTCAATTGTGTCCATCCAGGACTTTCATTCTCTTTCATCTTCAATAAGCCCCTTTAAAGC
CCATAAATCACCCATGATTGGTCCACCACCACCTCTGCCACCATACACAAGCAATCGCTT
CTCCAGCATTGTAGCTGTATGGCCACATCTAGGTGGTGGCACTGATCCAGAAATAGGCAA
TTCCATCCACTCAAGTGATAGTGTGTCCAAGATATATACATCTGACAACCACTTTTTACC
ATCCCAGCCACCATACATGACTATTTTATGGTTTCCAATGGCTGATGCAGCAGCAAAATC
TCGAGGAGAAGGTAAATCACCAAAAGTGGTCAGTTCTGACCATTGCCATATATCTGTGTC
TAGAACCCAGAAGTCACCTAACCTTTTATTTCCACACCGTCCACCAAAGACAAACATATG

ACAATCAATTGCAATGGCAATGTGAAATGCTCGTGGGCTGGGACCCACC
>ntig_601_consensus
CATTTATAAGCTACGTTGAAACTGGTACAATTGCTTACAGTAAACAGATTTTTAGATAAA
ATCTCACAACTCCAGAAACAGTTTACATCCCATAAAAGATAAACAATCAAATATGCATAA
AAGAAGCTTTTGTACATGAAATCTGAGGTCCAAGTCACTTCTAGTATTTTGCCTTCATCT
TAGTTCTCTCCAGTTTCACTTTCTCCAGACGTGGGCTACTAGGAATAAAACACAACCAAT
AAGAAAAAGTTCTGTACTAGAAAAGTAAAAATTGCAGATAAAAACTATATCCCACTACCG
ATTTGTAATGTTAAGACTAATTTACAGGACTACAACTTCTTGTCAACAGTCAAACATTTC
TGCTACATACCCGAAATTTACAGTGCAAACTAAAACTACATCCATTACTCTCTAATTCTG
TACAATTAAAACTCAGACACTTGCCATATTTACAGACAGAGAGCTTCATAACTTTGATCC
CATTGCGGAACTTTATGGACAAAAATGTCACCATAAGCTTCATCCTGCCTCACACCCACA
AACCCTTTACTTTTCAGCACTAGTATTTCATAGAAAAGCCTTGCACTTTCTTTCTTGGTT
TTTCCTCCCAAAAGCCCAAACAAATTCACTGCTTCATCCCCACTTCTGGTCTTGTGATAC
TGAAAACTTCTATGCAGGTACTCGGCAACCATTCTGGTTCTTGCAGAGAATTCATGCAGT
TCCTGGTCATCATCGTCAAAAAAATTTGCTTCCTCAGTCATCAAAATGGGGCCGAATCCT
TGATCCCTGCTTAACGATAGCTCTTTCTCCATAATCTGAAACGACTGAGCAAATTTTACT
GTATCTGAGTGAGAATCTTTGAGGCTCTCTGGAGTGTCAGATGGTTTTGAAGCTACTAAC
TGTAACAAGGGTGTTTCTGAACCAACAGCCATTTGTTCAGGTCTGCTCGGTGTTTCAGGA
TCAACTATGATTTGCTTTAAAGATCTAGCTGTAGGTGTTCCTGGAGCAATTTGGTCTAAA
AATCTAGCTGTAGGTGTTACTGGAGCAATTTGCTCCAAAGATCTAGCTATAGGTGTTTCT
GGGGCAATTTGCTCTAAAGATCTAGATATAGGTGTTTCTGAAGCAACAACCATCTTCTCT
AAAGATCGGGCTGTAGGTGTTTCTAGGGCAACAGCCATTTGCTTTAAAGATTTATCCTTA
GGTGTTTCTGGAGCAATAGCCAGTCGCTCCATTGATATACCAACAGCAGGAGATTCTGGT
TCTAGAGCCTCCACTGTTTCGGCAGCCCCTGCAATCTTCAGTCTCTCTTGGCAAAAAAGG
GATGTAAGCTCTGAAGACATACAAGGTAGCAAAGGAACAGAGAAACCCTGTGAGAGATTG
GATATCTGCAAGGCTCTCCAGGCAGTGAGAGCAGTATGAACTTTTCTCCGTTTTGAGACC
AAGTCACTTGCATCTTCTATACGTTTCCTAATTAGATGATTGGGAAACACTATAACATTG
TCAAAGCAACATTTTCTTCTTTTCGAAATCTGAGCACGCTCCTTAGTTGCTGGGGTGTGA
ACAATCATAGACCCTGGTGTACTATCTCCTGGAGTATTAAGGAACTTGGATTGATCTGCA
TCCACTTTAACTGACAGTGGATCCTTTCTCTTCATTAACTGACATTTATCATTTTCTGGC
AATGACATTTCCTGGAACTTCATTGGCTCTGCATTAGAGTGATGCTCTTTACCAGATGAT
TTAACTAATTCTGAAGGTTCCTTAGACCTACTAAACATTTCAACATCTACAGATACTTCT
TGATGCAACCTATGGTCTGGAAAGTTCTCCGTGCTTGCTTGTAAGTACCTTAAATTATTG
TCTTCACTCATAACCAAGCACGTATCTTTTTCCAACTGTGCTGAACCTGGAAGATTTTTC
AGCTCTCCCTCCAATGGATGGTCTTCAGCAGATGATTTGATTAATTCTGAAGGTTTCTTA
GACCTACTAAACATTTCAATATCTACAGATACTTCAATACACAATGTGTGGTCTCGAAGC
TTCTCCGTGCTTGCTTGTAAGTTGCTTAAATTATTATCTTCATTTCTAGCCAAGGACATC
TCTTTTTCCAACT
>ntig_602_consensus
ACCATTATTAGTCCAAGACACGAACATCTTTCTGCAGTCAATAATATATACCCTCCTATT
ACAAGTTCTTCATTGTGGTATTACATCACAGTTTCCAGCATATGAGATTTCATAAATAAA
AAAACAAATGGTACCTTCCAGGTAAATCCTTTGCAATTTCAGCCCATTTGTTCCCATATC
TCCGGTGGGCCTTCATTAGAGCTAATTCCTCCTCTAGAGTCCAGGCATCCTTCTTTATAT
CAGGATTCAAATGATTGTGCCACCTCTCTCGGCATTGTTTCCCTATGCGACCAGGTAAAG
ACTTTGCAATGACAGACCATTTTGTAGGCCCATATCTTTCCACAAGTTCAATTATTGTAG
CATCCTCCTCTTGAGTCCAAGGCCCTTTGACAAGGTCTGGATTGAGAACTTTCTGCCACC
GATGCAGACATTGCACTTCTGATCTATCAGGAAAAAACTCAGCAATTTTCTTCCAACTCT
TCCCTTTAAAAGTTGCCACAGCATTTCTTAATGTCTCATCCTCCTCTGGTGTCCAACCTC
CTTTAGCTCGCCTAATTGGACCCGTGGTCCTCCTGTGAGTTGGTGATTTAGTTGCAGGGC
TTGATACACCAGGGGACTTGAGTATAGCACTGCCACTACCTTCAGATACAGAAGAGCTTG
AAGCGGCCGTTAATTGTTTATTCTCAAGGCAACACTCCTCTACTATCAACTCTGCCATGT
CCTTCTACACATCATCTCCAAAACCCAAAATCCT
>ntig_603_consensus
TTTTTTTTTTCAACAGAAACAAGGATCATTCATTAGCATTAACAGTACTTTATTTATGTC
AACACATGTAGATGTAATACATAAAAATTAAACAGTCATCGGGTTTCAACCCTGTGCCCC
AAGAAAAAAGGCAAAAGATAGGAGTTTCATCCAACAGCAAGACACATAAACAATGCTGAG
TTCATTATTATTTGGGAGGTCTATACCCAAATGCCATCTCCATGCGGGTCAATCAGTGAA
GGTTCAAAAGTAATTGCTCTTATAGAAGTTTTTCAGAAAACTGGACATGCCCTCATCCTT

AGAATCCCGATCCTGCACAAGCCCTTCACGACCCACAACATATTGGCCAATTATATGATG
CCGCTGCTTGGCATGCTCCAAAATGTTATTCAGTTCTTCTGTTCCCTTGAAAAGCAGCAG
ATCAATCACCTTAGGGCTTGTGATATGAGCGTTCTTGCGGATCTCGGAGGCGATGGCGGA
GCGGAGCTGTGACTTGGAAACGATATCGTCCAGGTTGTAGATTTCCATAATTGAAGGTAT
TGATCTGCACGCTGTCCTGAAGAAGTCGAACACGCGGCTGCGAGCTTCCTCCAGGCTCGC
TGAGTTTGGCGGCACCTTCAGTGCTCTGGCTGTGAACGCCATTTCGAGTGTAAATGTACT
AACTCACAGAGTTATTGACTCGTTCTGTGATTTTGCTTCTGGAGAGAGAGAGAGAGAGAG
AG
>ntig_604_consensus
GTTCCTACTAATGAATACTACTTTCTATTTGTATCCATTTTATCTCTAATTTCTAATTTG
AAAGTACCCAAATAGTAACATATCTTCATTCATACATTGTTAGTATACATGGTCTTACCA
ATAATAAAAATTGTTTAAGAAAGATCCAATAGACAAGAAAGCATACTAGATTGTGCCCAT
TACACAAGCATGCCACCATCTTCCCAGCCAAACTGAATTTCCATCTGAATTTAAAATAAT
ACAGCATATACCTCGTGTCTTTCTCTCAGAAGCAGCATATCATTGTTGACCAGGCGCTTG
TCCACGTCCACCGCGCCCACCACGAACACCTCGACCACGTGCAGCTGTCCTTCCACGGCC
CCTACTCACTGATGGTGGAACAACTCCAGCTTGTCTTTGCATAGCCCTCTCCCTTGCTCG
CTCTCGTAAGTTAACTATATTAGGAGCATTATTTACAGGGGAAGCTTGTTTTACTTCAGC
TGCTGTTGTTGTACTGGAACCAACTAGCTTTGACGAAGCATCAGGTTCTGCAACTGAACT
TGTTGAAGCCGTTTCACTGATAACTGAAGCGGCTTCAGGCACCTGATCTGTCTCTACTGC
AGCTTGGTCATTACCATCATTTGACTTGTCAGAACCCTCAGCATTCTCCTCAGTCACATC
ACCTTCTTCATTTTTCTCATCATTTGCTGCCTCGGGTGAAGTGACATCACCTGTCTCTGC
AGACACTGCAACCATACCTTCATTCTCTACCCTCGTGGGCGATGCCACTGGGGTTCCAAT
AACCTCCATCTGCACTTCCTCAGCCTCAGGGCTTCCTGCCACATTTGAAATATCATGGAC
TCCCTCAGGATCAGTTACATCCGGTAATAGCTCTCCCTCTTCTCTCTCACTCTCGGATTC
TAGTGTCAACTGTTGGTTCTCTTGCTCTGTTTGATCCTTCGGACCCTCATCAGTTGCCAT
CTCTGCACTAGTTGCCTTGTCTAAATTCTCTTCTGAAGCATAGTTCTTATCATTCTGCAT
TTCAGCTTCAGTTGTCCCATCTACATGTTGTGATTCTTTTAGCTCCTCAGTCTTCTCTCC
AATAGTGTCCACCTCTTCCTTCTCAGCATCAACAGCTTCTTCTTGAGGTGTATCACCCAC
AGCATCAGTAGATTCTTCAATAACTGGTTGAGCATCAATATTTTCCACTGGGGCGGCCAC
AGCAGATTGGCCAGCAGCATCCTCAGGTAATGAATCTATTCCCTTGGACTTTTTTAGCAC
AGGCGCAGCAATATCAGTGCTGGGCTCCCCTTGCAGGGGAGACTCCTCACGTAGCTCAGG
AGTGGATGAAGCCAGGCGTTTACGAACCAATGAATGACTCTGTTGAGTCAGACTGACTTG
AGTTTCCACATCCTGAGACGATGCAACCTTTCCGATAACATTAGATACTTCCGCCTCTGA
CATCTCAACATCACCTTGTGCTTCTTCAGGTTTACCAAGTCGGGGGCGGACCAACTTGCG
CCCTTGCTTACGAGTTTCAACATTGGTTTGGGAAGAACTAGCTTCTCTTTTCCATCTGT
TGCTTTTGGTGGTATATGACTTGGTGCAGTTCCTGCTGAAGAACTGGAAATCAGTGCTTG
GGTAGCAACAGCTGGACCTGTTGTAGCTGCTGCGGGAGCATCCAAGACTAAAGAAGTCTC
TGGAGGAGGAGGGGCACCAGATCCAAGCTCGCCAAAAACTGTGCGTGCCACCCTTTCAAA
ATTTTCGACAGCCAATAAATAGGAGGAAGCATGATCATCCAAAATCGTACCAGAAAGATG
CTGGACCACCAATGTACCCTCTGGAAAATTGGCTTCAGCATTTTTTATCTTCTCTAATTC
ATCTGAAAGGCGCTTC
>ntig_605_consensus
TCTTACCAATAATAAAAATTGTTCAAGAAAGATCCAATAGACAAGAAAGCATACTAGATT
GTGCCCATTACACAAGCATGCCACCATCTTCCCAGCCAAACTGAATTTCCATCTGAATTT
AAAATAATACATCATATACCTCATGTCTTTCTCTCAGAAGCCGCATATCATTGTTGACCA
GGCGCTTGTCCACGTCCACCACGCCCACCACGAACACCTCGACCACGTACAGCTGTCCTT
CCACGGCCCCTA
>ntig_606_consensus
GTTGGAAAAGAGTTGTAATTGGCTATATATAGAAGACTACAAAAATTTTCGGAATTTCTC
AATCACTAATTCTGATCAAACAGCACAACCTGACATGACTCGAGTCCGATCATAGTATTC
GTTACAAGCTGGGATTTCAGAAACAGTGATTTGTCCAATGCGGAACAAAATCCCGTGATA
GATTTAGTGGACTAGAATATACACCACGGCTACGAAGTTTCAACAAGATAAAGAAGGGCC
ATAGAAGAACTACAGAATGGTTCAAAGGGAAATATACACTAAAACAAGTAAACAGTTTCA
ACATGGCTAATCAGTGGCGCCGGCATCAACTTTAATGTTCAGCTTACGTGCTTAAGATCG
AAGTGATTTGGAATATGTCCATTTCAACCAAAAAAAAAAAAATACCAGCTATTTTAATATA
CTCATCCTCCTCGCATATGTGCTCCAAGTTCACCGAACTGTTCATCAGCTTCAACAGTAT
CCTCAGACAGGCGAACTGCATCCAGCTTTTGTTTAAGAACTTCTATGGCATTTAATACCA
TCTGAGAAGCTTTAATTGCACCGGTGGATTCCACAGTGAATATAAAGCTGTCTTCTTTGC

CATAAATCTCTACAAGTCCCGGCTTCCCCATGGCTTCTGCCTTCTTAATCACTTCATCAT
CATAAGTGTATCCCTCAGGATCAAGCACTACAACCTGTCCAGTAGCTTCATCAAGGTCAA
AGACTTTGGTTGGACTACTATCAATCCAAGCTTGTTTCTCGGCAAGGGTCAGAGTTTCCA
TTAAATCTTCATTGATGTGGATCTCTGGTTCATACATGAAAGTGACAGTCGCAGCAGGTG
ACCATTTTGCATGATCTTTACCAATCCCTTTTCTGGCAATTGCTCTAAGCCTCAATTCTT
GCCCACGACGCAGCTTCACAATGAGAATGCCCCTTTGCTGATCAGTTGAATCGTAGGCTG
AAGAATCGGTGAAATCAACAGGAACAACGTTATGGTCAGAACTGATCAAGTCCTTACTCG
TGACATCCAAAGTCTGGTCGCCCATACACTTGGCTCTCAGATGGAACTCCACGGAGCAAT
ACTCGCACTGGCCGTCTCCATCACACGCGTCGCAATCACGTGAGAAGCGCATGCTCATGG
CTTTCTCGCTGGTGAGAGGGATAAGACCGAGACGATGGGCTATGAACTCGTCGTTGAGAA
CGGAAGAGTTGACTTCGATTTCGACGAGGTCGATAGCAATGGTCGGAACCTCCGCTATCA
TCACGCGCCGAAGAGCATTGGCCATCGACGCGTCCGTGTCGTGGAGCTCGAACTTCACGT
AGTCGTCCTTGAGCTCGCGAATTTTGACTCTGGGAAATCGCTGGTATGAAACGCCCTCCA
TTGTGCTATCCCGCTCTCTGGACTAGGGTTTTGTAAGCTCTCTGCCGTTGAAACTCGAAA
TCAAAATAACGGAGGTTTTGAGTCG
>ntig_607_consensus
CTTTGTTGATCGCTTGAATCGGATGCGGAAGAATCGGCGAAGTCTACGGGAACAACGTTA
TGGTCGGAACTGATCAGGTCCTTACTAGTGACATCCAAAGTTTGGTCGCTCATGCACTTG
GCTCTCAGATGGAATTCCACGGAGCAGTACTCGCATTGACCATCTCCGTCACACGCATCG
CAGTCACGTGAGAAGCGCATGCTCATGGCTTTCTCGCTGGTGAGAGGGATAAGGCCAAGA
CGGTGGGCGATGAACTCGTCGTTGAGGACGGAAGAGTTGACTTCGATTTCGACCAGGTCG
ATAGCAATGGTGGGTACCTCGGCAATCATTACACGCCGAAGAGCGTTGGCCATCGACGCA
TCGGTGTCGCGGAGCTCGAACTTCACGTAGTCGTCTTTGAGCTCACGAATTTTGACTCTG
GGAAATCGCTGGTATGATACGCTCTCCATAGTGCGATCCCTCGCTCCGGACCAGGGTTCT
GTCAGCTGTGTTAAAACCCTAGATCAAAATGACGGGGTTATGAC
>ntig_608_consensus
GTGGTGTCTGTGGAATCTGGTCATTTAAAGTTACAATCTTTTCATAATTTATTACGATAG
AAACTATTAAAAAAGCAAATTTCATAACATCATGTAAAATAACAAATTTAAACATGCATA
TGTACAGACGGTAAAAATTTTCAGTTAGAAGAATAGAACGGGCAATGAGATCACAAGGTT
GAGACCTTACTTCCATATGTACAGCGGCGGATCTTCCAGGGGTTCCAAGCCGTAGAAAAG
GCAAGAGGAAGCACTTTGAATAGTCATCGAATCCATCTTAGAGCCCCGAGGTATNNACCA
ATTCATAGCAAGATTCCTCAGCAGAAAGACCAGGGAGGGTCCTGAAGCCAAGAGATCATC
TTTTTGTAGAAGACTTGCTCCCCGAGACATGGCAGGTTTCGGACACAAGATCAAGTCCAT
GAGACAAGTAATTGAAATGGCTGTGACATGCTCGACAATGCTTCCATATATGACTTAGGA
CCACTGCTTCTATCAAGAATGCACAGGCTTCATATGTTCAAATCCGCACATGTTCAAATC
CATGGTGTCCATCCTTCGTACATATTGCAAAGATTATCCACACTCGATGCTTGCTTGAGT
AGATAATCTACTTGCTCGGCAATGCTGATCTCCCTGTTCTCAGCAATATCTCTACCATCA
AGTTTCATCTCAACACGCCTTAAGACTGATAATGCATAAGCATTTTTACCCCTGGCAACT
CGGATAAAAGAACGTGCATTAGGAATAGGATCTTCATTACTTTCCTCAGTCATCTTAACT
TTATTTAATGAGCATAGTTTATCTTTAACTCCAAGAGTCACCTCTGAATCCTTATTCAAT
GGTTGTGAGCTGCCTACAGGCACAATTGCAGCTTCATAACCTTGTGATTTTACTTCTTCC
AGATTTACATTTGCTTCATTAATATTTGACTTGGCAGAACCATTATCACAATGGTCCACA
ACCAATGGCCAAGAGTGTGATTGGAGATCAGTATGGGAAGATGGAATAAAACTTGGCTGA
AACTCATCCTCCCCATGTCCCACCAATTCTTCTGGGTTGTTGGATTCTGGAAGACTTGCT
CCACCTGAGGTGACACCAGACCCGGAGCTACTGCTATAGATGCTATCTGGAGGAGATATC
CATCCTTTATCTTGGTAAGAGAGTCCAGTAACACCAAGAAGATCATCTTTGGTGCTCTCA
CTATCAGACCTGGTGAGATCCTCTCTCTCATTGTCATCAAATTGGGTAGCATCAGCAGCA
GCCAGGTCTGACCTTGACAGACTAATCCCCTGTGATTTTACCTCTTGGCTTTCTCCTAGT
CCTTCAAGAGCTTTATGAAGATTGCCTGCATGGAGACTTGCAGTTCGTGCAAGCCTTGTA
AGCATTGAATATAGTCCCTTTACTGAAAGCGCAAGCGATGGAAGCAAGGGCTTAAAGATC
TGAAATGCCTCCCTCACTCTAAGACCATAAGACTGATATATAGCTTGTCCATGAATAGGA
GAAACTTCCATCTTGGTTTCTTTTTCACTAGCCATAGCATCAGTCATGGCAGAGACGTCT
TTGGACAAAACAGATTCAAGTGGGAGAAGGATGGAATTTGCCTTGGAAAGGTCCTCCATA
AGAGAATGGTGTACTGCAGCAGCCTCCTTCCCCAAACTGTGAACATCATGCAGATCTTCC
GTGATTGCCAGAACTTCTTCAAGCATGGAACCAGATTCGGAGGTCAAAGCAGTATGGCTC
CTTGATACACGACCAAAAGCAGATAGGGCAACACTGGCTTCATATGCACAGTCCCTCATT
GCAAGAACAGTGTTTTGTAAATCACCTGAAGCTGACTTTGCTTTGAGGGAGGCAATGCAA
AGTTCGTTTGTAGCAGAATACAAGCCATTAGAAGCAGATTCCATGCTGCTTTGTGCAAGC

TTCCACTCTTGTTCAGTACATGTGAATGAATGATAAGCAACCTCCAGTTTTGTTAGTGCA
TTCAAGTAAGCCTGCTGCCATGTCCTTCCATCACCACCACCTCTCGAAGGATAAATCTCT
CCTGGAATCTGAAAAATACCATCTCTCGACAATTCAAAATCCAACACTGACATACATATT
TTGACAATATCAGATGCCTTTTCTCTAGCTTCCCATAGTAGTTGCCGACGCCTCATTAAA
TGGTCATGGAATTCATGTGGAATTCCTGAAGGCTTGGTTGAAGAATTTCCAGCTGTACTG
GAATTTGGTCCACCACAAGCCCACCCCATTGCTCTCTCAAGCTGCCCTTCTGCAGTGAGA
GAAGTTCGTTCACAAGCTTGCAGGCACTCAATTGATCTAGCTGTTTTTGACACAGCAGAC
TGTAAAGTTTCCAGCAATTTTGGTCTGCTCAGGTTTAAAATAATTGGATATATATTATCA
TCATTCGGCAAAAATCTCTGATATATTATCCTGCTATTATATGAGGGTGTCAATACAACA
TCATGCATCCACTCCATTTGGACGATCTCTAGGCTAGTCTTCAGAAGAGCCTCCCTGAGC
TCATTCACCTGCCTTTTCATCAGGGAAGCAGCTGTTCTTGCTGCTCTAGCTGTTTCATGT
GCATTACAATATTCCTCAAGCATTAGTTGGACCCTTCTCCCAGCCTCAGATTCCTTTTTG
ACTTCCTCAGCTGTAAGTTCCTTTGTTCCCTCAGCCAATTTCTTCAAATTCTCAATTTCT
TTATCTAACGAAGCCAATAAACATGGAGCAACTCGTTCTTTTAAGTATAGACCAAC
>ntig_609_consensus
GTCCTCTTTAACGAACAATGCTAATATGAAAACACACAAACATACTATTATACCAATAGC
CTAATGGAACACTTACGTAGAGTTACAATTCCACTCAAAACCCAGTTAATGTCTCAAATG
CCTTAGAAGTTCACTAGTAAATCAACATGCAACACAGCAGACGCAGTACTTTGGACTGAA
AAACTGGGGCGACACAGAAATGTGATCACCTAGGATAGCCAGGAGGTGGATAAGCAGGAG
GATAGCCTTGTGCCGGAGGTGGGTAGCTTTGAGCAGGAGGAGGATAGGGCTGTCCATATG
CAGCTTGCTGTGGTGGATATCCAACCGATGGAGGATACTGCTGATCAATGCGAGACATCT
GTTGAACAGGGGGCACTGCCATAGGTTGTTGACCAAACACGCCATCTCTTTTAT
>ntig_610_consensus
CTCAAATCAGAATTCATTGTAAATGCACATAGCATATAAAAATTATGTCCTCTTTAACCA
ACAATGCTAATATGAAAACACACAAACATAGTAGTACACCAATAGCCTAATGGAACACTT
ACGTAGAGTTACAGTTCCACTCAAAACCCAGTTAATATCTCAAATGCCTTAGAAGTTCAC
TAGTAAATCAACATGCAACACGGCAGAAGCAGTACTTTGGACTGAAAAACTGGGGCGACA
CAGAAATGTGATCACCTAGGATAGCCAGGAGGTGGATAAGCAGGAGGATAGCCTTGTGCC
GGGGGTGGGTAGCTTTGAGCAGGAGGAGGATAGGGCTGTCCATATGCAGCTTGCTGTGGT
GGATATCCAACCGAGGGAGGATACTGCTGATCAATGCGAGACATCTGTTGAACAGGGGGC
ACTGCCATAGGTTGTGTACCAAACACGCCATCTCTTTTAT
>ntig_611_consensus
CACCGCAAATAGCATTATGCCTAATTCGAATCAGTAATATCCAATTCAGACTCGTTCAAA
ATAGTATACAATATCGTAGAAAAATTACTGATAGGCTAAACAATTTTGTCGATAAAGCAA
ACAATGTCATTAAAGTGATAAACAATATTATCACTAGGATGAATAATGTCACCAAAGAGG
CAAATAAACCAAACTTGAACCGAGCTGGACTTCCAACTCAAGTTTGGATCATTTGAGCCG
AACAATAGACTTCAGTCAAATCGACTCAACTCGAAGCTACCCCTAATACCATTCAGTGGC
AATACAAGTTAGAGAAAAAAATTGCAGAGAAGAGGAAAAACTAGAAGACAAATATTGACAA
CGGACCAGAAAACAATTATGTATGTTTTATTCAACAATGGTGACATGAAAACACAAATAT
GCATATATGACAATAGTCTATTACACTTTGCGTAGAGTTGCAGCTCCATTCAAATTTGAG
TTAACAACTCAATAGGCTTGGAAGTTCAGTGGAAAATCAACAAGCAACACAACAAAAACA
TTACCTTGGACTGGAAGGCCGTGGGCAGGTTGTAACACAGAAAATGCGATCACCTATAAT
AGCCAGGAGGTGGGTAAGCGGAAGGATAGCCTTGTGCCGGAGGCGGATAACCTTGACCCT
GGGGAGGTGGATAAACATACGGCTGTCCATAAGCACCTTGCTGTTGTGGATATCCAACTG
AGGGAGGATATTGCTGATCAATGCGAGACATCTGTTGAACAGGGGGCACTGCCATAGGTT
GTGGACCAAAAACACCATCTCTTTTGTCCAATTCAACCTTGTGTTGAGTCTGCATGCATG
CACAAACCGAGCAATAAACTAGATCAGCCAACAAATTCAAAATCTGAGAAGCCTCCTGAA
GCTCATCACCTCCAACAATGCATGCGATTATGGAAACTACGCAAGCAAGTTGTTGAAGGC
ATATCATAAACCCAATAATAAAATTGTCGCACTTAGTCGTCTGGATGTTGAATTCATCTT
GCAACAGAAAACGAGTAGAGGCCACCGAATTTCCAAAGCAGCAGAAAACCTCAGTGCAAA
GGCAAAATTCAGGACAAGAACTTTCGCGACACTTGCCGCTGCAGGGCATATAACCAGCAC
AACAAACATACCTGGACATGTCATTGTAAAGTGCTCGTTTCCGAAGCAGGTAAGATACAC
ACGGGGGACAGAAAACCGCAAAGCAGCAATAAGGCGTGTCTTGAGTAATGGACCCAAGGA
GATCGGTGTGCCACATGTTTGGGTAGGTTTGACGAAGCTGCATCTTCGCCACAGCCTCGT
TGGACACCATCTTCACCGACTGATCTGGCGAGTTTGAAATCGAAAAATCAGAAAGTGGGT
GTCGTCGTCCGGACGAAAAGTTTTAGTCTTTGGCAGAACCGAGACGGAGTTCCTGGATTT
AAAACTCCCTATTGAGAAGACTTTGGAAGAAGACTAAGGGTAGG
>ntig_612_consensus

ACCAAACACGCCATCTCTTTTATCCAATTCAATCTTGTGTTGTGTCTGCATGCAAGCACA
GACTGAGCAATAAACTAGATCAGCCAACAAATTCAAAATCTGAGAAGCCTCCCGAAGTTC
ATCACTTCCAACAATGCAAGCAATTAGGGAAAATATGCATGCAACTTGTTGAAGGCATAA
CATAAACCCAATAATAAAATTGTCGCACTTAGTCGTCTGGATGTTGAATTCATCTTGCAA
CAGAAAACCTCAGTGCAAAGGCAAAATTCAGGACAAGAACTTTCGCGACACTTGCCGCTG
CAGGGCATATAACCAGCACAACAAACATACCTGGACATGTCATCGTAAAGTGCTCTTTTC
CGAAGCAAGTAAGATACGCATGGGGGACAGAAAACCGCAAAGCAACAATAAGGAGTGTCT
TTAGTAATGGAGCCGAGGAGGTCACTATGCCATACGTTTCGGTAGGATTGACGGAGCTGC
ATCTTCTCAATAGCCGCACCGGACGACGCCATCTTCACCGTCTGATCTGGCGAGATTCAA
GTT
>ntig_613_consensus
GGAGGCTTCTCAGATTTTGAATTTGTTGGCTGATCTAGTTTATTGCTCGTAAGCCTTTTT
GTCTGCTCCCCACTAAAAGCTTTTAGTCAAAAAAATTGCTACAAAATATCATACTGATTC
ATTGTGTGTTTTCCTTTGCAGAGTCTGTGCTTGCATGCAGGTATGCAACTAATTGCAAGC
AACTCACATACATGCGAATGTGATGGTTTCAATGTAATATTTCTTTACGTTTTGTTTTGA
TTATGTGCAGACACAACACAAGATTGAATTGGA
>ntig_614_consensus
AAAAAAAAAAAAAAAAGGGCCAACATTCTTTCATTGCTTAGGTTTTTACTCAGTACACAA
CCTCAAAAAATCCATTAATAAAAATTATATGATCACTTTTACAACCACAAAATTTGTAAA
TGAGACTTCAAAATCTCAAATCTCAATGTAATATTTGCCGAATAGATGTAATCTTCTCGC
TGACTCTGCCAATTTGAGTTGGAAAAAATTTCTGGCAACCCTTCACCCCGCCGTGCCGCA
AAACTCTAGTATCTGTACAGAATTTGATAACTTTCTTTCAACTGTACAGCACAGACTGCT
AGGCCATCTCCAGGAAAATCGTACAACTGAAGACCACAAGGAGAACCCCAGTAACTATCA
CGCTAGTGTGCTTTCAACATATTCCATAGCTTTCTGCTTCACATATTCTATTCGTGATTC
ATCGCCAATGGATTTCTCGTACTCCAGATACTTCTTAAATAAGAATTTCATTTTTTTAGG
CGGGAGACTCAAGCTAGTAGCTCTCTCGAATAGTGAACGAATCAAATCAACATCTCCAAG
TCGAATCTCTTGATCAAGATAAACACTCCACAGATCTGTTCTCTTAGGGTACTCACGCAA
AATTCCTTCAAACATGGATCTACCTCTATCCGGGACCCCAGTTTTAAACTCAAGTATAGC
TGTTTGTGAGATAAATTTAATATGCTTGTGGCGGGGAAGACTTAATAAAGCACGTTGTAC
CAAAGACTGAACTCCATCTTGTTGATGCTTCAAAATCCTTTGTACCCGCCGTAACCAAAC
CTTGCATGAATGTTTGAACTTCTTTATCATTTTGTCAAGAAGTTCATCAGCCAATTTGTG
TTGATCAGTCCTTTCGTACATGGCCAAGAGTGCTAGATGGACCTTTTTGGGATCAGAATA
CTGCAATGCTCTTTGAAATAGTTTCACAACAGCTTCCTCTGGAGGATTTCCATATTCATT
TTCCAAATTAAAATAAGCCACCCAAATATTTAGCTTCTCATTCTCTTCTCGGATGTTTAT
TGTTCTCAAAGCCCTTTCAGCGATAGAGCGGGCTTTCTCAACATCAGCCAAAGCAAGCAT
AAAAGCCATATACTTCAACCAAACAAAGCTGCTATTCGGGGAGCTTCTAACCAATCTCTC
AAATTCATCAGGAGTTTTTGGCGCATCATTCACTAATAGCCTTTCCTCAGCAGCCCTAAT
TTCTACCTCCCTCTCTTCCTTAGCTTTCTTCTTGGCCTGTCGTTTATTCTTCTCATCCTT
GTTATCTGTCACATCAATCTGTTCCGTGTTCTGACCAACTACATTATTCATGTCAGGCTG
TTCTATGTCTTCAAGAGCGACCTCAAGTGGAGGAACAGAAGCCCTAGATTCTGTCTGAGC
AAGAAGCATAGCTTCTCCATCTTCAGACTCAATGTCACTATATTCAACAGCAGGTAAACT
GCCATCCAAGATAAATGACTGGTCATGAGAACCATTTTCTTCAGTATCTTCATCAGCCTC
TTCTTCAGAAAGCATTTGAAGGGCATTATCACTACTAAAATATGAATTCTTCATTCCAAG
AGAAATTCTGCCCTTCTCCTTGTCCACCTTCAATATCTTAACCATAACTTTCTCCCCAGC
TCTGTATTTAGTCTCAATATTTTCAACGCGGTCATCTGAAATCTCGGACACGTGGCATAA
TCCAACCAAGGTTGTTTGGTCAATTGTAATAAACAAACCATATGTCTCCACCCGCTTAAC
CCTTCCAGGAACTACATCTCCAACATGCAGACTACTTAAATCATTCATTTCTGGTTTTGT
AGCATTGCTGGAATTCAATGGTTTCAAGGTAACTTCAACACGCTTCGACAAAGGCTCCAC
AGATAAAACCCTGCCAACCACAAGTTTTCCAATAGGAAATTCTTCATCAGGTCTTTCAAC
AAATCCATCTGACAAATTTGAGAGGAGAATTTTGGCATCAAGCTTCCGTGATAACAAAAT
AAAGCATCCTTTCGATGTAACATTCTTAACATAACCCTTCACAGCCATATTAGGAGAAAG
ATCCTCAATCTTTTCCAATCGATTGCCCGGAGTATCTACATCAATATCAGAAGAACTTTG
ACCGTGCACGCCATCTAATGATAAACGTAATGAAAGATCAACATGAATAGTTCCCTTAAT
AGAACGACTGGTTTCCAAGACCTTGCACTTGACAAATTGGCCTTCATGATATCCAGACAA
TGGATCAGAGACCCATGAATCGTTGAGTTCCGTAAAATGAACCCTACCAAACATATGAGG
ACCAATTTGCACAACCAATCCACCAACACCCGGAAGTAGTTGGGAAATCCTGCCACCAAC
AACATCTCCTTCATGTATATGAGCTGCAATATTATCATCTGAAATTTTAATATCATCCAT
CTTATTGCTTCCTTGATGACTAGAAGGCAGCCTCACCAAGCGCAACAACTTCTTCTCCTT

GTTGATGCTCAAAACATAACCAGTAACAGCTTTCCCCACATGAAAGGATTTTTGGAATTC
TTGAAGTTCACTAGGTTCACAGGCGCTGTCAAGGATAAAAAGCCTAGCCTTTACTTCCCG
AGATACTGCTAACCAAGCCCATTCACCATCCACTTTATATACATAACCAGTGAGACATTG
TCCAGTAGAAAAATCAAATTCTTCATTCAAAAGCTTGCTCTCCAATTCAAAGGAACCTGT
GACCAGGGTGGGTTTGATGGAAAGTTCCCACTGGTGGCTTTTCTTGTTATCCGGTTTATT
AGGCTTGGCTACAATCCTTGCAGTCACTGTTTGCCCAACTCTAAAGTTGCTTAATGGATT
TTCTGGAATATTATCCTTACCATCGTTCACCTCTGTAATGTGAATCCTCCCACGGAAACC
AATTCCAAATTTCAATCTCAGTTCCAGAGGCTTAATCTCAGTAATCTCTGCCTGAACCAA
AGATCCAATGCTGTAAGCAGATTTCCTTTTGGACCTCTTGGAACTGGAAGTCTCATTGAC
TTCACAAATAGATTTAAGAAGCAAAAGCAACCTCCCAGCAGTTGAAGGACTTGGAAGAGC
CATAATGGTAGCAATAACACTTTGTCCATTCAAAAATTGTTTGTGTGGAAAATTTTGTAT
GTTGTAGTCGCATAACGATGCATATCCTATAGCATAATCACACTCTGGCAAAGAGAGAAC
CAAGTAATTCTCTTTCACAATCTCCACAACTGCATTTACTGTCTGATGCACCTGCAAGTC
CTTGGTTGCTTTTCTTTTGCGTTTCTTCTTGTGAGTCTGGCTGCTTGAACTTTGCTCTCT
GGCTCTGTCAATGAACACTGTTTTTAGCGATAAATCAACAAGGCGTTCTGTCTTGGCAAC
ATCAAGAACCACAGCTTGAATGGTGGAGCCTTCTTCCACAATAGCTCCATCTAACTGATA
ATGAGTAATAAAGCCAAAAACATCACCATGTTTCTCAAAACTAACAACCACTCCAACATC
CTTTGCCTCCTGAATTTTTCCTTCAATGGTACTGCCAACGGTAAATCCTTCCACCCACTC
TAACTCTGATCCCTTAGGGTCTGATGATTGCAATATAGCAATCTTCTCTTCTAATATGAA
GTATTCTTGCATGAACGATGCATCTGTCGAGGCACAGCATGACTGCTTCAAAGACACTGT
AATTCTACCTGTATCATTATTGACATCAACTATATTGCTGCGAACAGATTGCCCAACATA
AAAAGCTTTCAAAGGGTCGGCTCTCTGATCGTCCATTGCCTTGCTTCTAGGAGCAAACCC
AGTTAGACGCCCAAGATAGCGGACAAAGCAACCAGTTTCAATTAAGTTGCATATGTAACC
ATGGACAACTGTGTGGGGATGGATATGACCAACATCTGAAGGAAGTTGTTGAGCAGAGTT
GATCAAAGAATATTTAGCAGAAAGTACCATGGTGTTGCCCTCAATATCTAGGACCACCAG
CCGATCAAACTCATATCCAGGCTTTATGACTGACTTCATCAAAATAGCATGCCCATGGTG
ATCTGCCAAATGTTCAGTAGAAATTGTACCCTTAGAGTAACCCCTAGCATTAACATAGAC
AATAACTGCATTGGAGGTTACTACATCAACCACTCCAGAAACTATGCTACCCAGTTTGAC
TAAATCGTCCTCAGAAACCCTTGTAGGCTTCATTAAGAAACTAAGGTTGATGCGTCGTGA
GGCAGGAATAGAACTCACTACTCTGCATTTGACAACTTGTCCAACATGATAAATGGAACT
TGGTTCGCTTCCAGGATCTAATCCAAGCTCAGATCTAGGAGCAAAGCCTTGGACTCCATT
GTAAAACCGAACAAAGCAACCGTGCTTCTCGATCTTCACTATCCATCCATGTGTTATC
>ntig_615_consensus
GTGTGATTGAATGAAGAGGAAAAATGACTTGAGCTGTAGTGTTTTTTCTTTTTATCTGTC
ATTTCCCTTCTGTTCTCTATGCGTAAGTTATTGGCTGTGGCTCATTCATAGATGACTGCA
ATTCATTTGGTTTTCAGCCTCCATTCTGCAGATGTCTCGGAAACGTGTACAATATCTTGC
AAGCTATTAGATTCTAAACATCACGCAAACTCTGCTTATATTATTTCAACTGCATGGAAA
CATATGGCATGGTAAGATACTATGTGGATGTCAACAAGCTCACAAGTAACAAAGCAGCAC
AATCTTTATTCAGGGTTAAATTTAGAAAGCATCTTTATGTAAGGATTTTTAATTGGCTGC
AGCTTGAAAAGCACTCAGAAAAACCTCAGGAGGCTGGCCACCACTCAGCTGATGCTTTCC
ATTAATCACATAATGTGGAACTCCTGAAATTCGTGATGAGTATTTCTCAAGGTCCTCCTT
GACCTCCTTCAGTCCATTATTGGGATCTTCAAGAAACTCCGCTGCGCCTTCTACACCCAC
TTTCTTAGCAGACTCAACAAGAAATTCCCTGTCACCAATGTATTTACCCTGTGTGAAGTA
GCCAAGGAACAGTTCCTCCACAAGTTTATGTTGCTTATCAAGATCCTGCTGCCCAGCAAA
ATATATAAGCCTGTGGCTATCAAGGGTACTTCCCGTGAGTCCGGACATGTTGTATTCCAA
CCCAAGACCCCTAAAAATCTCTGTCATTCGATCTATGATCCCTTGAGCTCGAGCTCCAAA
CTTGTTCTCATAATAGCTTTTCTTATCAACACCATCCTTAGAGGCGGTAGGATTGAGAAA
AAAAGGATGCCATTTAATCTCAAAATTATATTGGTCCTTAGATGTAGCTATAGCCTTGTC
CAAATTTCTTTTTCCTACAAAGCACCATGGGCATACAGAGTCTGAACTTATATCAACTTT
GATAATCTTCTTCTCATTGCTGCCATTAACTGACTGGGCCATGATTCTTCTGTATCTGTG
GTCTTTCTAGCATATAGATGAGAATATCAGGCGCAAACTCAAAAGAGTTAGAATAGTCTT
TCTCCAGTTATATTTCTAAAGTATCAATGCGAAATGGCTAAACAAATTTATCAGCCAAAA
CAGCGTGAAAAAAGAACAAATATCCTCTTCAGTATACTATCCAATAGGAAAATTTTGTGA
CACAATCCAAGAACAATTCAGAAAGTTAGAGATTAAATAAAGACCAATACACCAAAACAC
AAAACACTTTATAATGATTTGAATTGTACCAAAATATGATGACTTGTACCAAGGCCAGTT
ATCAAATTCTACCCATTTAGTTCTAACAAAGATTTTACCCGTTGTCAAAGAAGAGGCAAA
CTAAAGTCTTTGCAAGATTCTTACCAAACAAACTTTTCCCTTTCTTTATATTGAAAATCA
CTGTATCCAACAAAATTATGAAGATTAACTTGGATATTTTTGTCAACCACATATCTATTC

CATATAATTTCAAGCAGTGAAGATAATGAATAAACTAAAACCACATCAATTAAGAGTTGT
CCCAGCAACCAGAGTCAGCAAATTTCCCACTTCAACAAAGTTTACTTTTTTACACCATGT
TCATTGTTTGAGTCCAACCCTCATAACAATTATTAAAGTAATCTCAAAAGGGCATCCTTA
TTTTGTCAAATAAATTGATGTTCTAGAAACAAATAACCACAAAAGACTTGCAAAATGAAT
AACAGTGAAGAGCAGAAACTAATGAAACATAAGAAGAAAACAATAACGATGAAATATAGA
GCGGAAAGAAGGCTTGTTATGCAAAGAAAAGATGTTACCTGGAAGTACAAGTAGAGACC
>ntig_616_consensus
GAAGGCAAAACCTTTCCCACAGCCTTTGCAGCCCCAGTACTGCTAGGAATAATGTTGAAT
GAAGCAGCCCTGCCACCTCTCCAGTCCTTCATTGATGATCCATCAACAGTTTTTTGTGTA
GCTGTAGTTGCATGCACAGTTGTCATTAAACCTTCAAGAATACCAAATTCCTCATGGACC
ACCTTGGCCAGAGGGGCAAGACAATTAGTAGTGCAGCTGGCATTAGAAACAATGTCCATG
CTTGGCTTGTAAGTCTTCTCATTAACTCCTACCACAAACATAGGTGCATCAGCTGATGGT
GCTGATATTACCACTTTCTTTGCACCACCCTTCATGTGAGCTGAAGCCTTGTCCCTTGTT
GTGAATACCCCAGAAGATTCAACAACATACTCAGCCCCATAATCACCCCAAGGGATCTCA
GCCGGGTCCCTTTTGCTGAAAATTTTAATCTGCTTCCCATTTATTTCCAAGGTGGATTCA
TCCACAACCCTGAGAGTTCCCCTAAAAACTCCATGGGTAGAATCATACTTGAGCATGTAA
GCCATGTACTTGGCATCAACAAAAGGATCGTTTACTGCTACCACGTGAACATCATCCCTG
GATGTTGCTATTCTCAAAACCAGTCTTCCAATGCGACCAAAACCATTGATTCCAATCTTT
GTGCACCCACTGCTCCGAGTTTTTTGAACCATTGGCGGCAACTCTGTGGCAGTGGCTTTG
ACGGGTTGAATGCTCTTGGCAACACATTTCTGTAAAGAAGAAGACCGTCTTGTGACCGAA
GAACCAAAAATGCTTGAAGTGCTGAGATTGCGATTAATCCCAACGCAGGCCCCCTTGAGA
CGATCGGAGGAGGAGGAAGCGAGGTCGGCGGAGCGTGAGAGAGGAACGGTGGCAGTAGAT
CTGACGAGAGAAGAGAAAGCCATTCGTTGAAAAACAAGCTGTGTCAGGTTCCCGATGTTT
CTCTTTCAGCTAGCCCAAAAAACCCTTGAGACACAGGCAGTGACAGAAAAATGAAAATGA
AACGGCGTTTTTAAAAGCAGGAAAAGCAACTGTTTTCGAAAAAGGGAAATTGAAAATTGA
GCGGAGTTTCTTATGTCAACAACGCTACACTACAAC
>ntig_617_consensus
AAAAATTTATTGACAGCTTCTCAGTGGCTCACTGAACTCAAATTGTCTAACTCGTTCAAA
TTAAACACCAATCAAAGACTGAACCGGGTCAACTCAGATCCAGCCTGAGATTGATGGGCT
TAAATGTTCACATCACGAGTTTCTCATTTGTCTCAGCATTTGCCCTATAAATCATGCTTT
AAGCCAGATGGGACACTTCTAGTCTGTAAAGCTCATATAATTGAAGAGTTGCGTGTCCAG
GGTTCTCAATGGAATTGGACCCCAGCAACTTCACTAAGGCAGATAAAGGGCAGACAACAT
GACAGCCTCTGCCACATCAGCAACCATGAAAGCCGATAAATCTTGCCTTACTGCTGGGCA
CACTG
>ntig_618_consensus
GTCCAACACCAACCGAATATCATGGATAACATAGCATCCAAATTTATGTATTACCAGAAA
CATTATCTATATCACCAACTACTGTAGATCAATAACCTGTTTTATGAATCAAAACACAAA
AATTCAGGGACGACAGGATCATAAAATAAAATTCTACATAGCAACTTCACTAAGGCAGAT
AAAGGGCAGACAACATGACAGCCTCTGCCATATCAGCAACCATGAAAGCCGATAAATCTT
GCCCTACTGCTGGGCACACTGAACTCG
>ntig_619_consensus
GTCCAACACCAACCGAATATCATAGATAACATAGCATCCGAATTTATGTATTACCAGAAA
CATTATCTATATCACCAACTACTGTAGATCAATAACCTGTTTTATGAATTAAAACACAAA
AATTCAGGGACGACAGGATCATAAAATAAAATTCTACATACCAACTTCACTAAGGCAGAT
AAAGGGCAGACAACATGACAGCCTCTGCCACATCAGCAACCATGAAAGCCGATAAATCTT
GCCTTACTGCTGGGCACACTGAACTCG
>ntig_620_consensus
TGGGCAATTTTGGGATTTTCATTTATTGTTCTAATGTACATTTGATGATTCTCATCCCAA
ATTTCTAACACCATTATAAAACACTTTCAATCATATATAGGATACAGTGAAGTTGATCTA
GTATGGTATTATTCCATTAGTGCCTAGTTGCTCCAAGCTTACACTACTAACATTGACAAA
TTTACAAACTTGGACTAGTACATGACCTACAATTTGATGATGACAATCCATCCTCATCAC
TGGAAAGATTTAGGATGTCTACGTTGCACAAAGCCGATCACTTTGATCAGGAGTATCGAT
TTGTTATAGTAGAACAAAACTTTGAGCTGCAGAGTTGTCACTCTGAAATTCATTGAGTTA
ACAACATACAAGAAGGTTGAGATAGACATTAATTCACTCTGATTCGTTCCCTAGGGGTTT
CAAAGAAACCGCTGGTAAATATAGGCAAGCTACATAGTTATTTGGTTAACACAAGCACAC
CGTGCCTGTGGGAGGGTTTTAGAGTCATTGTGGTTGGAATTCAACACATGAAAATAGAGT
GAGGAATTCCAACGAGAGGTACATTATCCATGTCTGGAAAATTGGAAAATGACTGGAAGA
GAGAAGATTGAAATACTTGCATAAAAAACTGATGATGCCAAAAAAAGGAAGTATGAGAC

AAAGGGCTCGAATTATGTATCTTTGCCGATTTTAATATTTCCTTTTTTCGGGACGGACTT
AACACCAAAAAGCCAGCCAAAGGCTGACAAGGAGTTAAAACTCAAGAAAGTTTTATTCAC
AACAGATTAAGCTTTCACAAGACAAAAACAAACATCAAATTATACTACGTTATAACAAAC
ATGTTATTCATACGCAATTCTCGATTACCCAAACGCATTCACAGGACAGAGACACGTATG
CCCCTTTTAATTTCAACCAGAAATATGGATGGTCCTCACATCGGTTTTCTTCATCTCCAC
TTTAGGAACAGTAACAGTAAGAACTCCATTCTCCATAGAAGCTTTAACCTGATCCATCTT
CGCATTATCCGGCAATCTAAACCTCCTCGAGAACTTGCC
>ntig_621_consensus
TAATCTCATCCCCAGCATCATCAGTGATATCAGAAGTCCAAAGTGTAAGATTATCTCGGA
GAAGTTGCATGATCAATGTACTGTCCTTGTATGATTCTTCACCCAATGTATCCAACTCAG
AGATAGCCTCATCGAATGCCTGCTTGGCGAGATTGCAAGCACGATCAGGTGAGTTAAGGA
TTTCATAGTAAAACACAGAGAAGTTAAGAGCCAGTCCCAGCCTTATTGGGTGAGTAGGAG
CAAGTTCAGCCAAAGCAATATCCTGAGCAGACTTGTAGGCCAACAAAGTGCTCTCAGCAG
CTTCCTTTCTCTCAGCCCCAGTCTTGAACTCGGCCAAGTACCTGTGGTAATCGCCCTTCA
TCTTAAGGTAAAACACCTTGGACTCGGCAGAAGAGGCCGAGGGAATGAGATGCGACTCGA
GGAGGCTCAAGATTCCATCACAAATCTTGCTCAACTCCGCCTCAATTTTGCCTCTGTACT
CCTTAATGACTGATACGTGGTCCTCATTTCCTCTGCTCTCTTCCTTTTGCTCAATGGATG
AGATGATCCTCCATGAAGCCCTCCTGGCCCCAATCACATTCTTGTAAGCAACAGAGAGAA
GGTTCCTTTCTTCCACAGTTAACTCCTCAACATCAACTGTCTTTGCAACTTTCTCCATGA
ACTCCACCATTTCTTCATAACGTTCAGCTTGCTCAGCCAACTTGGCCATGAAAACATTTT
CCTCACGAGTTGATTCAGTAGGTGACATCTTTGCTATATCAATCTGAAATGCAATTCGTA
TAAAGGTGTTGTGTTGTGTTGTGCGAGGGCGTGAGCAGATGGTTGAGAACTAAATAAAAG
AGAGTGAAGAGAGGAAGGGTCCAATGACAAAGCGACAACTGTTAGGCAGCAGCGGAGAAC
CCGTGAAGGTTGATGCCCTCGGACGTCAGCCACGCTGGCGTTGACCGTCAACTCTGAAAA
CCCATTCACACACACTGACGCTTC
>ntig_622_consensus
TACAAACTTGATAGAAACAGGACACGTTCTTTGATAATTAGAGGACAAGACTCTCTATCC
AATAAATAAATTGATTCCAACAAACCATCAATCTAATATAAAGCCACACAATTATAATTT
GCTCATACCAGGCAAAGCAAATTATAATTGGTGCTTAGGTTAAGGCTTTTCCCACCACAA
TCATAAATATTATTATTAATGCAATTATGATATCACTGTACAGTATACAAGCAACTAACA
AATAAACCCATGTAAGGTATATATATCTCAAGGACTGAAGCTGCCAGCATTCCCGAAATC
TTTAGGCCATTAATTAACAAAGACGACCATATTGACATTGTCTCCTCCACTGCACTGCGG
CGGAGTTTTCATTTCCTTTCCTCCTCGCCCTTGTAAATTTGTCTTTCATGTCCTAATCGC
AAGCAGGCAGGATATCCACATATATACAAACAACCAATAAGCTTCCACCACAACCATCAA
CTGGCGCCATAAAATTAACAACTAGAATTAGTTTGTGACAAGTCCTGTATCAGCGTGATT
TCTAAACAGCTTCACTTCAGGAACCCCTTATGGCTGCACTTTCTTTCAGTATCTGAGATG
CAAGTATTACTAACTCCACCATTTCAAATCCAGCAGCACAAACTGAATCTATCTTTTCAC
TGCTAGAGTCCCCATATATATCTGGTATACTGCCGATAGTTTCACCCATTGATCCATCAT
CTGCATCAGCTGACCAACTTAAGGGTAGGGAGCTGCCCAGGGCAGTCTTTACAAGTTTTG
CGTCTGTAACGATTTCATCAAGAGCTTCAGACATGCTAGTAGTAACCGTGCCTTCAGAAA
TGACTGATTCTTTGACCTTAGAAAGTGAGACACGTAGTCTATGAATTGAAGAGGTCAGAT
GCTCCATACTTTCCATGCTCTCCTTAAGGGTTGCATTTTCAATCCTCATTCTGGCAAAAT
CCATACTACTGGACCTTTTGGCCGTCATGTCCCCTGCATTATTTAGGTGAGTTTGAGTAT
TCTGTCCACTGAAATCTTGTGCACCGATCCAACGTCTTAGATATGTTTTCTTCAACAAAT
TCCTTAACCTTCCATCTCTTTCAGGAGTGACAAGTCTATTTTCCCCCATTTCAGCCCAAT
CTTTCCCTTGTTTCATTCCATCACTGATTTTC
>ntig_623_consensus
TTTCATTCCATCACTGATTTTCTCTTGCAGCAGGTGGTATTCCTGCTTTAACTGTGCCAA
CTCCACGAGAGCTTTTACCTTTTCATTGGTAACTTGTAACAAGTTTCGCTGAAGTTCCTC
AATCTTTTTCTGAAGAGCTACATTCACATTTTTTTCCAATAAATGTCTTTCTTCCTGCTG
CGATAAAAGCAATAATGCTGCAACCTTCTCTTGCAAAGCCAGTGCAAGGGCTTCAGAGGT
ATCAGCCTTTCTTTCAGCTACAACATTAGATAAACCAGGGCTATTCCTCTTAGAAACTAA
ACTGGCATTAGCATCAGAATTTATATGTACATCTCGACACTCATATTCCTGTGACATTTC
ATCTCTCTGATCAGTCCCCATATTCTGTCCTCTGCCCACATCAAAATGTTGCATATTTTC
TTGGATCACATCAGCAACTGATTTAATATGGGATTTTCCCTCTTCAAGCAAATTCGTGAT
ATCAACTCTAAGCTGAGAATGGAAGTGCCGTAATTCTACAATCCCATTATTAAAGAATTT
GTTTGAATGGATTATCTTCTTTTCCAATTCACGAACACTCTTCTTTAGATGGTTTTCAAT
TTCCAGACCCATGCGTAATTTACTTTGAAGATTCTCTACAGATCTCTTCATCTTCTCCAT

CTCATCTTCCAATGCACTCTGTTACGTATGTAGAGGCGGAAGTATCATTGAAATTCCACA
TTTCCACAGGATCCT
>ntig_624_consensus
CTGCCATATCAGCAACCATGAAAGCCGATAAATCTTGCCTTACTGCTGGGCACACTGAAC
TCGTGGCATTGATGGCTCATCATCATCTTCGTCATATGCCTCTTGTTGATGCTGATGCTG
TTTCCGTCTCATCTCATATTCAATGTTGACATCGTGCAAGGTGGTCTCTTCACAATCATC
CACCTCCATATCCGACANNNNNNNNNNNNAAATGGATGTAAAGTCTGCCTTTAATGAATGG
CCTTTGGTGATGCGGCATTCCCTCATCATTAATTGCTTTGTATTGACCAGGCTTAATGAT
CTCGCCAGGATTTGATTTGATAAGTAGCTGCCTGCCATCAAGATGGATCAAAGCAAACTG
AAACCCACAGAGAGCCTCAGTGAGACTAAGGGTGCGTTCTACATAAAGATCATCATACTT
TCGCTTGAACCTGGGGTGCTCCTTCTGTTGCAATACAAACACTATATCCCCTGTAATTGT
ATCAGGAGCTTCATCGGCTTGCCCCTCAAAGACAATTTTTTGGCCATGCTGCATCCCTTT
TTCCACATGCACTTCCAGAACCTTCTTTTCCTGAGAAATCTTGTTTCCTTTGCAGTGCTG
GCATTTATCTCTTTCACTGATGACCTCACCTGAGCCTCTGCATTCTGGACAGACATGTTG
CATCTGTTGAATCATGCCCAGTCCAATCTGTCGAGTTGTTATTTTCATTCCAGTACCCTG
GCATCCATAACATTTCCCAGAGGTCCCACTTTTTGAACCTTTCCCTTTACATTTCAGGCA
CAGAATATTCCTAGAAAGAGAAAGTTTCTTTGTTGCTCCGTTATACAATTCCTCCAGAGA
GCATTTCAGAGTATGCACAACATCTTCACCACGCTTTTGCCTTCTCACTCTTGAGCTACC
ACCACCACCAAAGGCTCCTCCACCAAAAAATGATTCAAATATATCAAATGGATTATGCGA
AGGCCCAGCTCCTCCCATTCCTTCTTTAAGAGCATCTTCACCATATTGATCATAAATCTC
TCTTTTATCAGGGTCACTAAGAACCTCATAAGCTTGAGTCAGCTCCTTGAACTTCTCAGG
ATCTCCACCCTT
>ntig_625_consensus
CCTATTTCAGCAACAAAAAGACCCATAGTTTGATTCTCTAATCACTCCGACAATTCCTGG
ATATTGACGCAACCTTCGAACAGATGTGATGGGCAGGTATCTTTGCAAAACTTCAAAATC
TACCATATCATCACAAAAATAGTAGACAGATTCAAGGAAAATTTAAATTCTATGATCATA
TGCTCATACATTCATTCAATACATACTTGCCTCTTCCGCTTCTCTCTCGGTGAATTCCAC
GTAATCTGAGCTCTCGATTAACATATGAGTGTTCTTATAGGCATTTTGAAATGTAAGCTC
TAACAGGACTCATACAGGTTAACTAATATCACAGCTGCCACCCTTGCGATCGCTATAGTT
AACTGTCACCCGTGACAATTGTGGAAATTAAGATCACAGTCCTCGCATTCCACATCATTC
TTTTTTAACAGGTTTATCAATTGCCGCCAGTCTTTCAACAAGTGGTGGATGCGAATAGTG
ATAAGCTGAGTACCAAGGATCTGTGTTCATAGCTGACAAATTTTCTTCCTGTAATTTCAC
AAGAGCAGCACGAAGAGCAGATGCATAACCAAGCTTCTTAGCAAAGGCATCGGCCTGAAA
TTCAAAAGATCGGCTGACAAGATTCAGACCAAAGCTTACTAGGTGTTGCAGTGGGATGAC
AGTGTGCTGAAAAATGATGAGACCAATGAGTACTGGCTGTGTATCAAATCCAAAACTTTG
GAAGAGATCATCTGAGTTTCTCACAAGGGTATAACCTCCAAATTGCAAAAATGTAAGAAT
CTGAACGGCAATGAATGAGTACATTGTATGATTAAGTTTCCAGTGACCAAGTTCATGGGC
AATAACTGCTACAATTTCCTCCTCATTTTTGCACTGCTGAATCAAGGTGTCATAAAGGAC
AATTCGCTTGTTCTTAAAGAATCCATACATATAAGCATTGCTATGGCTTGACCTTGTGGA
TCCATCAACAACAAACAACTTCTTCAAAGGAAACTTAAGGGATGAAGCAAGGCCCTCAAT
TTTATGCCTGAGCTCTCCTTCTGGAAGAGGTGTGAACTTGTTAAACAGAGGGGCTATCAA
AATGGGGTAAAGTGTCATCATCACAAGACAAAGAACAAACATGAATCCCCAAAGATAGAT
GGCCAGATAAGGACCTCCTTTCTGTACTATTATGATGATTGCAGAAACAATAGGTGGGCC
AAGAATAATAGCAAGACACATTCCTTTGAGCATATCCCTAAAAAATAACCATATAGTTTG
TTTATTAAAACCATGATGAGCCTCGATCACAAAAGTTGAATAGAGAGAGAATGGCAAATC
GGTAAGCTGTGACCATAAAGTAACACCAGCCAAAAATGCTAGGGTGTGTATTATTTCATT
CTCTGCATCAAGACCAGCTAAAACCAAAATGTTCCCCGATTTCTTCCAAAGCCAAGGCAA
TATACCAAAGAACAAAATTGCTGAGTCCATCAGTATGGTCACAAACTCATGAACAAAATG
GAAGTTGCTTTTATCAAGACTATAGGCTCGAGACTTCTCAAATTTTTCTTGGCTGATTAC
TCCTTCCAAAGCTTTGGGAAGTGTTGGCAGTTTGAGAGCAGCATGTTGCCGCAAATCCAG
GTAAGTTTCAAAAAAATACATCAAGATCATAAAACCGACGACGGATTCCATGTAGGGAAA
CACCATGGCGAAAAAATTTAAGCACGCAGTGGAGAGAGAATT
>ntig_626_consensus
AGGAAAAAGAAACCAACTACAACCGGCTCATCAAATCCACCGGTCCATTCATCACAAACT
GATTTCATGGCTGATTCGCCTTCGTCTACGGTGAACACAGAACACAACAGTAAGCAAGCC
GAGTCCGACAATCCGGTTATTCAACCGGATCAATCATCCACTTCCGTACCGCCGCCAGAG
CAACAATCAGTGGCGCCAAACCCTAATAGCAACCATCCGCCACTGCCTCCACCGATCCAA
TCTTATGTTCCTCCACAGCCAATATCGGTGCCTCCGACGGCACCGTCGTATCGTCCGGTA

GCGATGCCGGCTGTGCCGCAGTTCTCTCCGGTTCAAAATTTCCAAAATAATGCTGTGCAG
CCGCCGGGCGTGGGCGTTAGTGGTGGCGTTGCGGTTACCATGGCGCCGCCAATGATGCCG
TATCATGTTGTCCCAGGTCAGCATCCGAATCCAGCGCTTAGACCATATGCACCGGTGCCG
AACGGATATGGTGTCGTTCATGCTCCCGCTCCGGGTCTTCCTCGGTATCCTGCTCCATAT
CCAGCTATGGTTCGGCCTGTGTTTCCTCCACGTCCTCCGGGACCTATCGGAGTGCTTCCA
GCAGTTGCACGTCCTCCTGTTCCAGGTGTTGCTGGTATTCGCCCAATTATTCCACCAATT
GTTAGGCCAGCTGGTGTTCCTGTTGGTACACCAGCAGAGAAACTTGAAACCACTGTTTAT
GTTGGCAAGATAGCATCAACTGCAGATAATGATTTTATGCTTTCTCTTCTTCAGGTATGT
GGACCTGTCAAGAGCTGGAAACGTCCTCAGCATCCCTCAAATGGGACACTTAAAGGCTTT
GGATTCTGTGAATTTGACTCTGCTGAAGGGGTTCTTCGTGCGTTGCGACTGCTGAGTAAA
TTTAATGTTGATGGGCAAGAACTAATGTTAAATGCTACCCAAGCAACAAAAGATTATCTG
GAGCGTTATGTTGGGAAGAAAACTGAAAACACAAAGAAACTCAAGGACGCTCAAGATACT
GGGGCTGAAAAAGAGGATGCTAGTGTGCAAGGTACTGAGAAAAAAGAGCCACCCAAAGCT
TCTGAAGACTTGAAGGACAGTGAAACTGGAGACAAAGAGAACCATGACTATTGCAACTTT
GGGATTGTGACTGATGAGGATAGGGAAACTGACCGAGAAGCTTTGGAGAAGCTTAAAAGC
ATGATTGAAGAGAGGTTAAAGGCCAAGCCTCTGCCTCCACCCCCTAGTCAATCAGTCGTT
GATGATTCTGGGATTTCATGCTCAGAAGTGCTAGCTAAAAACAGGGATGGAGACTCTGAT
GTGGACATATTGAGAAATGATGTAGCTGCAGATAAAATTGACGATGAGACAGCCAGTGAT
TCCAAGGCAACTAGTGATCATGAGAGGCCTGAGACAAGCTCACCTGATAGGAGGACATAT
GATAGGAGAAGTAGAGATAGAGACATAGAGCGAGATCTAAAACGAGAAAAGGAACGCGAA
ATTGATAGATATGAAAGGGAAGCGGAGCGAGAACGTGTTAGGAAAGAGAGAGAGCTAAGA
AGGAAGATTGAGGAGGCTGAGCGTGAGTATGAAAGGTGCCTTAAAGATTGGGAGTATAGG
GAAAGAGAGAGAGAGAGAAAGAGAGGCAGTATGAGAAGGAGAGGGTAAAAGAGAGGGAACAC
AAGCGGAAAAAAGAAATCCTTTATGATGAGGAAGAGGAGGATGATGATTCTCGTAAGAGA
TGGCGTAGGAGTGTGTTAGAAGACAAGAGAAGGAAGAGGCAAAGAGAAAAGGAGGATGAT
TTCAATGACAAAATTAAAGAAGAGGAAGAAATTGCTGAGGCTAAGAAGAGGGCTGAGGAG
GAACAGCTGCAGCAGCACCAAAGAGATGCATTGAAGCTTTTGTCTGGGCATGAAGCAAAT
GGAACCAAAAAACCTGCGTTGGCTGAAGAATCTTGCATTGAAACCAAAGATAAGACTGCT
GTTGAGCAGGATTATGAGGTTGATTCCATCAATGAAAAGCATTTAGCTGGTGATGAAGTT
TCACAAAATGGTACTGGTGATGAATCAATGAATGCATCCGTTTCTGCATTGGATATGCGG
CAGAACAATAATACCCCAGCAAGAAAGCTGGGTTTTGGTCTTGTTGGCTCTGGCAAACGA
ACTGCTGTCCCTTCTGTCTTCCATGAGGAGGAGGATGATGATGCACAGAAGGAGAAAAAG
ATGAGACCCCTGGTTCCCATTGATTACTCCACTGAGGAACTGCAGGCAGCCCAACCTATT
GTCTCTGGAGCAAACCAGCCAAATTTGGCAGCAGCAGCAGAATTTGCAAAGCGCATTTCA
AATGTTAGTTCCAAAGAAGAGAAGCTTGATTCTGAAAGAGAAAGAAGCAGGCGCTCTCAT
GATAGGTCAGGCCAGCGTGATAAGGACCGGAATGGTGAGGATAATAGGCGCACAAGAGAT
GAGAACAAAGAGAAAATCCTTGATCGTGATAGAGATCGGGAACATGGGTTAGATAAG
>ntig_627_consensus
ATTGTGTTAAAGAATGTGTGTCTGGAGAGTAGACGATCGAGGCCAACGTAGCTGACCTCC
AAGGAGATCATCCCTCACGGAACCTCCGCTGCCTTACAGCTGTCGCGTTTTCATTGGACC
ACTTCCTCCCCTCTCTCGCTTTAACTATTTAAACAACTCACTTCACGTTTGAATTCTCGA
CAAAAACAATCAGATCCCCTCCGTCTTCACCGAACACACTCCCTTTATACGCATTACATT
TTAGACAGATATAGCAAAGATGTTGCCTCCTGAATTAACTCGTGAGGAAAATGTGTTCAT
GGCCAAGTTGGCTGAGCAAGCTGAACGTTATGAAGAAATGGTGGAGTTCATGGAGAAGGT
TGCAAAGACAGTTGATGTTGAGGAGTTAACTGTGGAAGAAAGGAACCTTCTCTCTGTTGC
TTACAAGAATGTGATTGGGGCCAGGAGGGCTTCATGGAGGATCATCTCTTCCATTGAGCA
AAAGGAAGAGAGCAGAGGAAATGAAGACCATGTGTCAGTCATTAAGGAGTACAGAGGCAA
TATTGAGGCGGAGTTGAGCAAGATCTGTGATGGAATTCTGAGCCTCCTTGAGTCGCATCT
CATTCCTTCAGCATCTTCTGCTGAGTCCAAGGTGTTTTACCTTAAGATGAAGGGTGATTA
TCACAGGTATTTGGCCGAGTTCAAGACTGGTGCTGAGAGGAAGGAAGCTGCTGAGAGCAC
TTTGTTGGCCTACAAGTCTGCTCAGGATATTGCTTTGGCTGAACTTGCTCCTACCCACCC
AATAAGGCTGGGACTGGCTCTTAACTTCTCTGTGTTTTACTATGAAATTCTTAACTCACC
TGATCGTGCATGCAATCTTGCCAAGCAGGCTTTTGATGAGGCTATCTCTGAGCTGGATAC
ATTAGGAGAAGAGTCATACAAGGACAGTACATTGATCATGCAACTTCTCCGAGATAACCT
TACACTCTGGACTTCTGATATTAATGATGATGCTGGGGATGAGATTAAGGAAGCATCAAA
ACGTGAATAAGGCGACATGCTGCAGTGACGAGTTCAGTTGATAGGATATGTTCTCTGTAT
CTTTATCTTGTGGCTTTAAATCTACATGGACGCCATGTGGGTAGTTGTAGCAGGATTTCG
TGAACTGATAGATGGTCTTTGAGTGTCGACAACACTTGGGATTGAAATGTTCCAACAGAT

TGTGAACTAGCTTAAATTATTGATGAGAAGGATCTGGTTTGGCTTTCATGTAGTTTTTTG
AGTGCATGTTTTTGAGTTTATGGATGTGGATAATTTTCTTTCTTCATCCTGGTAATTAGG
GTTTTAAGCAAGGGGAGGCAAATCTGGAACGGTATATCTTGTGCCATGTCTGTGGTTCTT
AATTGTCTTCTGAGAGCATCGCATTTGGTGAGAAAGCTTGTGGTGTGGCGTGTACTACGT
AGTCCTTGGCATGTTACCCACAGGTAACAGCGCACTCTTGCAGAGACACCTTCGAATCAA
ATTCGGACAGCCCAAGTAGCCGGCGCCGCTTAAAGCGCTTAACGCTCTCTCTTGGGGCTC
GGGAGAAGACGGGCAGTTGGGAATAGGAAACAAGGAAGAGAAAGAATGGGTCTGCGTTGT
CAAAGCTCTTGAGCCTTACAAAGTCCGTTCTGTTGTTGCTGGTAGCCGTAACTCCCTCGC
CATTTGTGACGACGGCAAGTTGTTTACTTGGGGGTGGAACCAGAGAGGAACCTTAGGTCA
CCCACCTGAGACCAAGACTGAAAATGTTCCCAGTCAGGTTAAGGCTCTTGCTAATGTTAA
AATTGTTCAGGCGGCTATTGGTGGATGGCATTGTTTGGCCGTTGATGACCAAGGCCGTGC
CTATGCTTGGGGTGGGAATGAGTATGGTCAATGTGGTGAAGAACCTGAGCGAAAAGATGA
TACTGGAAGGCCTTTGAGGAGGGACATTGTGATTCCTCAGCCATGTGTCCCGAAGCTTGT
TGTTCGCCAGGTTGCTGCAGGGGGAACCCATTCCGTGGTGCTTACACGCGAGGGACATGT
TTGGACCTGGGGTCAGCCATGGCCTCCTGGAGACATAAAACAAATTTTTATTCCAGTGCG
AGTACAAGGGCTCGAAAAGGTGAGGCTCATTGCCGTCGGGGCATTTCATAATTTGGCTCT
CCAGGAGGATGGAACATTGTGGGCATGGGGTAACAATGAATATGGGCAACTTGGTACTGG
AGATACCCAACCAAGATCACAACCTATTCCTGTCCAAGGACTCTCTGGTCTCACTTTGGT
TGATATTGCTGCTGGAGGATGGCATTCTACTGCATTGACTGATGATGGGGAGGTGTATGG
TTGGGGAAGAGGTGAACATGGGAGACTTGGATTTGGGGATAATGACAAGAGCAGTAAAAT
GCTGCCCCAACGGGTTCAACTTTTAGCTGGGGAGGACATTGTTCAGTTTGGACGAGGTGA
CCATGGACGTCTTGGATATGGGAGGAAGGTGACAACAGGCCAACCAATGGAAGTTCCGAT
AAACATCCCTCCAAGAAATACAAGCGAAAGCGGAGCTGAGGGACATTGGTTTGCTAAACT
CGTTGCTTCTGGGGGACGCCACACTCTGGCTTTAGTAGAATGGCAGGCTGAAAAATCCAA
GCAGTTATGAAATCATGAACCTAATAATATTGGCTATGTACTTTACAAATGCATAAACTC
ATAATATCAGAACACATAGAACAGTTTAATTTTCCTTTGGTTAATCACCAATTTACTTTT
CCCCTATTATGTGGAGTTTTACATGAGTTTAGGGGTCAAACCAAATCAAACAAGAATAAG
AGTCC
>ntig_628_consensus
CAACAAGTTGCCATGTCATTCATAAAAAGAATGGCCTTAAATTTGATTAGACTTAATCTG
TGCTAGTCATCGAACACATTCAGCACAGAAAAAAAAAAGCTTTCTAACAAGGTGCATAAT
TGCTTATCCATCTTCACAAGAAACAGGAAATAAAGTAGTTGCATCAGACAAAAACCGGCT
AGTATTTGCGAGTACAGGACAAGCACCATAACCAAAAAAAAAAATTATTATCTAATTCAA
TTAGTAGTTTTATGGTTGAAAGCTACATCACAAAATGAATGCACTACAATCTAAACACTC
AAAATTATCACCCAACATTAGCTGTGCCAATTATTAATGTATCCATAACCCACAAACACA
GGATGTGAAAAACCACCCACAACTTCATATTTCAAAGTCTGCTGCAAGAAAGTTGCTAGA
GATAGACACCTTCTCTCCCAGTTTGAATGGTGGAAGGCCCTTGTATGGGCATGTACCGCA
TCGAAAAGCATCCCCTAATCCACAATTTCCACATGCTGACTGAGGATTGTCCAACTGATC
TGTAGTCAATCCTAACTTCTCTACTTTCTGCTCTGCCTCAGCCCTTCCGCAAGTGCAGTT
TTTGCAAGCTTTCCTTGTACTTCCAACTTCACATTCACCAACACGTGGTAACTGTGGTTT
CTTCAAATCATCTTCAGTTAAGAGAGAATCTTCATCGATCAAATCAGAATCATCATCCAA
CTGAACTTTCAATAAACTTTTAGGGGCCTTCTTCAAAGCAAAGGAAGACCCAAGTTTCCA
GGAAGGCTTCTTACCCTTCACCCCAAACGAGCAAGCATTTTCCGCAGCCACAACTGATTT
TAGTTCAAGACGAACTGCATCTAGAAATCCGGCTAACAATAACTTGCGCTCAAGAGCCAA
GAATGCCTTAGCATCATTCCCTTCACCAGACTTCAAACTTTTGTAAAGCAGGATAGTTCC
ACCGGGCTTCAGAACCCTAGAAACTTCACCAAGCATCTGATCAGCCGGAAATTCAAGTGA
ACTACTGATAGATACGACAATGTCAACAGAGGAGGATTCCATTCGCAGCTGACTTATTGA
AGACGCGTGAGTGATAATGAGCGGATCGCAGTTTTCCACTGATTCATCCCCGAGCTCTCT
TATTGCATTTAAAACCGAACTCACAGAAAGAACTTCATCGTCAGTAAAAGAAAGCACAAC
ACTCTTCTTCACCGTATCCATGTGGTTGAATCTTCAGAAATCCGGCAGAGAGTTTTGGTT
TTGGTTTCTGGGGTTGGTTTCTGGAATGTGTCAAAAGACAAATTGCAGCGGTTTTAAGTT
TCCTCTTGCTTGTTATTAAGCAGCGCCCCTACCCGAGAAAGGGTTTAAGAGAAAGAAGGT
GGGCAACTGTTGCGTGGCTGACCGACATATGGTGATGACCTGGTGAAAAGTTGTTGACAG
CCCAGTGAAATGTCGTCCGAAGTTCAAAAAGAAATTTGG
>ntig_629_consensus
TGGATCAATCTTACATTCAATATTAAAAATGTAGTCCTACTTATAACGAAATGATATGAC
CTTCTAGCCGACGATGCTAACACCAATAGCATAAAAGCGAGATCGTTTATCCATTTTCCC
CAAGAATTGGGAAATTAAGGTGTCGCATCAGCCATACTCAAGACAGGCACCATATAAACA

AAAATTGAAATCATAATTAGTTGTCTTACAGTCAAAACTACAATCGATCAGCAAAGATGA
TGCAAAATAATCGTAACCTTACATTATTCATGCTTATTATTAATCCATAATCCTCAAACA
TAAGATGCGAAACATCACCCACGACTTTGTACTTAAAAGTCTACCGCAAGAAAGTTGCTA
GAGAGTGATACCTTTTCTCCCGGTTTGAATGGTGGAAGACCTTTGTATGGGCATGTACTG
CATCGAAAAGCATCCCCTAACCCACAATTTCCACATGCTGACTGAGGATTGTCTAGCTGA
TCCTTAGTGAGTCCTAACTTCTCTACTTTCTCCTCTGACTCAGCCCTTCCGCAAGTGCAG
TTTTTACAAGCTTTCCTTGTACTTCCAACTTCACATTCACCAACAGTTGGTAATTGTGGT
TTCTTCAAATCCTCTTCAGTTAAGAGAGAGTCTTCATCGATCAAATGAGAGTCATCATCG
AACTGAACTTTCAATAAACCTTTGGGGGCCTTCTTCAAAGGGAAGGAAGACCCAACATTC
CAGGAAGGCTTCTTACCCTTCACCCCGAACAAACAAAAAGTTTCAGCTGCCACAACTGAT
TTTACTTCAAGACAAACTGCTTCTAGAAATCCGGCTAACAATAACTTGCGCTCAAGAGCC
GAGAATGCCTTAAGGGCATTTCCTTCGTCAGATTTGAAATTCTTGTAAAGAAGAATCGTT
CCACCAGGCTTCAGAACTCTCAAAGTTTCGCCAAGCATCCTATCATCAGGAAATTCAAGT
GAAGCACTGATAGATATAGCAACGTCAACAGAGGAGGTCTCCATTGGCAATTGACATATT
GAAGATGCTTGAGTGATAATGTGAGGGTCACAGTTTTCCACAACTTCATCCCCGAATTCC
CTTATTGCATTTATAACTGAACTCACGGCAAGAACTTTTTCGTCTGTAAAAGAAAGCACA
ACGCTCTTCTTCGCCGTATCCATGTGATTGAATACTCAGAAACCCAGAAGAGATTTTGTT
TTTGTTTTTGGAGTTTGAGTTGGCTTCTGGAATGAGCCTTGAACAAAAGAGGCTTTTTGT
TTCATAATTTGTACGCTGAG
>ntig_630_consensus
TTTTTTTCAAATAACAATAAATTTCATTTCAGTTTCACACAAATTTCATACTACAATTGA
AAGAATTCATTTACTATAACAACAAACATCAGAAGTAGAAACAAAAACTATAATCACAAA
TTTATTTAATATTTAGTAACATCATATACAAAGTACAAACAAACAAAACTTATTTAACCG
ACTATAGACACGTAATCAAACCAATCTAAAACCTCACCTATCGTTTCTTGCTATCGTAAT
CAAGGATCTCCGAATCCGTTACCGATCTCGAGTGCTGAACTATCGACCGTCCGATCGAGT
TAATCCAGTCCTCCTTCTCCTTCTCCGAATCGGCGATGAAGTACATCGTCTCGTTCCTCG
TACTCAGCTCAAATGCGTACTGTTTGTTAAGAACATCTTCGGCTCCTTTGACTGTGAGAC
ACGAGGCCACGGGGATGACGCCACGTGGCCGAGAAGTGCGCATAACAGTTGAATCTTTGA
ACCAGAAAAGTTTGCCTTGTTTTAAAACGAACCATCGGCGACGCCACGTTTTAATGTACT
CGCCTTGTTTGGTCAACCAACCAGTTCGCTCAGGGTTTGACCAGAACTCGACGCCGTCGT
AGGCAGTGAGCTGATAAGTTTGGCCCGACACAGCCCGCCAGAGGCTAGCCATGGGTTGAT
TGCTGCGTTGGTTTCGGGTAGAATCGTTGGTATTTTTGATAGGAATATAAGTAGCTAAAT
TCTCGGCCCTCTCTTTTTAGCAAATTGTATTATTCTACTTTTTATTAAATTACATTTTTG
>ntig_631_consensus
GCTGTTATAGGCCAATGTAATTGTCACTAATAGCATAGAAAATAATAGATTAAAGTTGAA
GTTATATAAAACTTATATAACTTTGGGATAATAACTGTAACATAATAAAATATTCATCAT
TTCAGAAAACATCATCATATTAATAATAAACCTAACACTGAGTGTCTAAAACTAAACTGT
GCCAAAAAAACCAGGTTCTCTGCCAAAACATAAAATCAACAAACCAAACTAGGCCGTACT
TATAAAAGGGTCAAGACAAAACGCACAATGCTGCCCTTACTCCCAGCCAGTGGCTTGAGG
GGCAGGGACGTCAGGAACAACTCCAGCCATTTGTGGAGCAGGCACCGCCTCCCATCCATC
TCCAGCAGCTGCGGGAACATCTGGGAAATAGGTAGCAGGGACTGCAGCAATGGGTTTCTG
AGCTTCACCCGACCACTGTACATCAGAGATTTGGCTAGGCCATTGATCATTAGTCAAGTT
CATAGGGAAGTCAGGAGCTGCACTTCCATATTCGATGGGAGCAATAAGCTCTTCTTCTTC
AGCTTGCTTGGCCTCCTCAGGTTCTCTGTAGAAGAACAGATCCACCATGACGTCCCACTT
ATGGCCAGGACGAATTGTGCCACGCATCTGTAGAACCATTCTTGCCAACAGCCAGAAGAG
GCACCCAATGCTATGCTTGCCCTTGTTATTAGCAGGGATACCAATGTCAACATATCGCAT
TGGTGAGTCAGTATCACAGAAGGCAATGGTGGGGATGTTTCCAAGCGCAGCTTCCTTAAT
AGGCTGGTGATCAGTTCTTGGATCAGTCAGGATAAGGAGACGGGGCTCACTGAAAGATGT
TTGGAGCTGATTGGTGAATGTACCAGGAGTGTGCCTCCCAGCAATAGCATGAGCACCAGT
GTACTGAGCAAATTTCAGAACAGCTCTCTGACCATAGGGCCTGGCAGACTGCACAATAAT
GTCCTGAGGGTTCTCAACAGCAACAATGACTCTAGCAGCCATCTGAAGCTTCTCCCATGT
CTTGCCAAGATTGATGATATAAATACCATCGTTCCTGCG
>ntig_632_consensus
TTTTTTTTGTAATCAACAAGTTTATTTTTATTTCTAATTTAAAAGCCCCTAATCAGATAA
TAACACGACTATCATACATCCAAAGTTTACACAGGACTCGGAATGCTTATTCTTTTATTA
TCGTGTTACAAGATTTATTCAGGGTACATTTTTTTGCCACCAGCTTTCCCTAGAGAGTTC
ATTTACATTGAAAAACATATCAAACAGGATGCCATGTTCATCACAAACAAAAGCTTGTGG
CCAGACCAACTACCTTCTTGGTTCTGCAGGCATTCTCGGAATTACGGTAATTCCACAGGC

TCTATGTTCCATATGTCTCGAGCGTATTCATGAATAGTTCTGTCACTACTGAACTTGTAT
GAACCCGCTGTATTCAGGATTGACATTCGTGTCCATCGCTTTTGGTCTCGATATGCTTCG
TCAACCTTCTCTTGGCACTCTAAGTAACTAGGGAAGTCTTTGCCCACAAGGAAATAATCT
GCACGACCAAAACCTTCAGTTCCTTCCAGCGACCCAATCAGTTCATTATAGTTATTTGAA
CCGAAAACACCACTTCTAATGAATTCCTTTACTTCTTCAAAACGTGGATCTGGAACAAAC
TTGCCTTCAGATCTCTCTTTTCTGAACCCGGCTATCTCATGAGCTTTAGCTCCGAAGAGG
AAAAAGTTTTCTTCTCCAACTTCTTGCCTTATTTCAACATTGGCACCGTCCAAGGTCCCA
ATTAAAATGCAACCATTCATTGCAAACTTCATGTTACTGGTACCACTAGCCTCCATGCCA
GCAGTACTGATATGCTGTGAGAGCTCACTAGCAGGGATAAGCAATTCAGCCACACTGACA
TTGTAATCAGGGACAAAAATAACCTTCAGCAAGTCACCTATTTCAGCATCATGATTGACA
GTAGCCCCAACATCAGTGATAAATTTTACAATTCTCTTGGCTTGCACATAAGTAGCAAAA
GCTTTTCCCCCAAATATACAAACTCGTGGAACAAACTTCGCTTTCCTTTCTGCAGTGCTC
ATTTCTTTCATCTTTTTGTAACGGTAAACAATACCCAAAATATTCATCAGTTGCCGCTTA
TATTCATGGATGCGCTTCACCTGGATGTCAAACATGGCATCAGGGCTGACCAAATAGCCC
GTTTTTCTTTAATGAGTGACACAACTTTCATTTTGTTGTTCCTTTTCGCCAACCTGAAC
TGCTTTTGGAGATCCTCATTTTCTGCAAACCTTCTTAACTCGGCCAGCTTCTCAGTATTT
GTGACCCAGTCTTCTGTGCCAATCCACTTGCTGATAATTTCACTTATATCTGGATTGCAG
AAAAGCATCCACCTTCTTGGTGTAACCCCATTAGTTTTGTTTTGAAATTTATCAGGCCAT
AACTCATAAAACTCATTAAACACTTCATTTTTTACTATGTCACTATGTATCTCAGCCACT
CCATTTACTGCATGACCACCCACAACACAAAGATTAGCCATACGAACCAACTTAGGTGGT
TCAGGTACCACTTCTGCCTCCCTTTTTCCCTCTGCCGGAGGACTAATTTCTTTTTCAGCC
TCGGATTCCTCATCAGTTGGTTCAACTTCTTCAAAAATTGTAACTTTATCACTAGGATCA
ACAACAGCACTTTCCTTGGGTTTTACAAGCAAATCTGCAAAGGGGGCAGGCAAATCAACA
TTCTCTAGAATTCTCATCTCCTTCAGCTTTTTCTCCAGTAAATCGACATCTGCTGTACCA
TATTCTGAAACTATGGCATGAATCAGCTCTTCATCAATCATTTCTATGATCTCCACATGC
CTAGGTAACAATTTTTGCATAAGTTCTAAACTCCATTTTTCCAATGCCTCAGGTAGAACA
GTATGGTTTGTGTATGCCACCGTTCTCTGTGTGATATTCCAGGCTTCTTTCCAGCTTAAA
CCTTTCTCATCTACCAAAATTCTCATCAGCTCTGGAATGCAGAGAGTTGGGTGCGTATCA
TTCATTTGAACGGCAACCTTCTCAGGGAATTCTTCCCATCGTACATTTGTTCCAGATCTT
CTCTCAAATCGTGCAATGATATCTTGCAGAGAAGCAGAGCATAAGGTGTATTGTTGCTTC
AAACGAAGGATCTTGCCCTCATCAGATTCATCCCCAGGGTAGAGTATATAGCAGATCTTC
TCAGCATTTGATAGGGCATCAGCTGCTTTGGTATGGTCTCCAGAGTTAAAAGCATATAAA
TCAAAATCTTCAGAAGGAGCTTTAGTTGACCAAAGTCTCAGGTTGATTGTGGTTTTAGTT
CTATACCCTGGTATTGGGACATCATATGCAACAGCCTGTATATCTTCTCCACCTATCCAG
TGTCTTTTTCCATCTGATCCTGAAACAACCTTGCCATAAAATTTTATGGGATATGAGACA
TCATTCCTCACCATTTCCCATGGGTTTCCCAGCTCAAGCCAATTTTCAGCAACTTCTTCT
TGACCATCCTTTGTGATCCTCTGTTTGAATAACCCGTACTTGTATCTAAGTCCGTAACCC
CATGCTGGGTAATTTAGTGTTGCCAAAGAGTCTAGAAAACAGGAGGCAAGTCGGCCAAGA
CCCCCATTACCAAGTGCAGCATCAGGCTCCTGGCTAACCACATTCTCCAGATTGTGTCCA
AGCTTGCTCAAGGCCTCTGCATAAGCACCAGTAAGCCCTAAGTTTCCAATTGCATTCAGC
AGTGCCCGACCCTGTAAAAACTCCATTGATAAATAATATGCCTGTTTCACATTCAACTTG
TCATAATAATTATATGTCTCATTCCAGTTTATAATAAGATTATCACGAACGCTTTGTGCA
GTGGCAAAGAAAGCCTTTGGAAGCTCAAATTTCTCTGGGGAGAACAATGGGGTGAACTCC
GCATGATACTTGATATTTAAGGCAATAGAGGCAGCATCTCGAGAGTCTGGATTACAACCG
GTAGTTGCTTCTTTAACAAAGGACTCCTTAACTTTAGCCGTAACCTCAGTGGACACATTC
TTCACCGAAAACGACCTCGTCACACGCCACGACCGCCAGTTCAACTTCCCCCCGCTCGTC
GCACTAAAGCCGATGACCCTCGACAAAGAACAGTACTGCACGAACGACGTCGACGACGAA
GACGGACACGAAGCTGCAGAGAATTGCGAAAATGCCATTCCCTTCTTCAGATCAAATTTG
CTATGAATGCAAGAGTGTGATCCTGGTTTCTTTTTCACGGAGTCCTTGACCTCGA
>ntig_633_consensus
TCGTGTCTGTGCCTTTTAGAACAGTTATGCCAAGTCCTACCATGAAAGAGCTCAATTATG
TCTGCCATGTCACAGAAGTGGCATTCTTCTCTTGATTATTCAAATCATCAAAATCTAGCA
TTGATGTAACCAGGCAAGTATATCCAATACAGCAACAAAGAAATAAAAGCATAGCCATCT
CTAAATACAGTGCTTTATTTAGGAGGTCATCTCACCATATACATAATCCCCATTTTTCTA
AAATATTCCCTCACTCGGTTCAAAATTATCCTTCACAAGTTCAATACAAACACGAAAGCT
GAAATCCAGAAAACATAGCTTCAAGTTCTCAGATAACCAGATTACTGTGAATCAAAGCCA
GAAGTGTGTGCCGCCATTCAGTGCTTGCTGGAGCTGAGATCCTTCACGACCGTCACTGGG
CAAGGTGCGTGTGTCATCACATAGTTGCTCACACTCCCCAAAAGAATCCTTTGGACGGTA

CCAAGGCCTCTGCTCCCCATAACAATAGAGTCCAGTTTCAGGTCCTCGATAGCTTCAAGT
AGCTTGTCTCTGGCGTCTCCCCAGTAAATCTTGGCGACAACTTTCACCTCTTTCTGTCTG
AAAGCAGTGTCAAGCATGTCAAGCACATCGATGTCAGTTTGAACAGAGTACTTCTTTACA
ACCTCCGGCTCTCTGAACTCAATCAAAGGAATAAGAGGAGAACCAGATTGAGCCCAGAGT
TGATTGGAAGATTCATCTAGATGATGGGGGTTGACATGGATGATATACAGCGTATCGGCC
TTATCAACCAAATTATCAATAGCCCATTTGAGAGCATTCTTGCTGCTCTTCGAGAAGTCC
ATAGCCACCCCAACCGTTTTATCTCCTGACATTGTCGCTCTCTCGCTTCAGCTTCTTCTG
CTTCTGTAGATGATGCTGATGGTACTGGATCTAGTTCTGGCTTTGTGACTAAGAAGCAAA
TGGGTTTGTTATAAGCCGTATAGCCTCA
>ntig_634_consensus
CTGGTGTTGATGAAGACTTGAGATGATTATCAACACTTGTAGGCACAAAAGCATTGTTTA
GGAACACCAGGTGCAGTATATAAATCACACAGCCCCTTGCCATTGTAGCGGTTTTTACAG
TCTTCCCAGCAACGTGTATCTCCTTTACAGTTCCACACCTTGTTGCAGTCTTTTGCTTCT
GCCACAGGCACCGATATCTCACCATCTCAAAGGATCTCTCTCACATCCAAGTTGCAGAAA
TCCTCTTCCATTTAATCTTCCTAGATCTTAATCTTTTTCACAGCACCTTGCAGTGGGCAT
TGCGCACA
>ntig_635_consensus
GGTTGTTATAATTTTAAACTTTTATCATGTCATTGTTCAATTTACAGACTGATTCTTTGA
TCGGATTAAGGCCCAACAGGGGCTTAAATCCTAGAAGAAAGGAAGGAAGACACACACATG
CTTATACTGTCAGCACTAAACCACAATAATCGAATCATCCATGTATCTTTCACACCTTCT
GTCTTTCCAAGATCAAAGCCTTCAAACTTACAATATGAACTCTCAAAGACAAAGAAAAAA
AAAAAGAATCAAGTTCAAAGAATGTTAGAACTCAGGAAAAGTTGTTTCAATTATTCTGAA
TTTAAATAACCAAATCGCAATGATGAACTTACTCAGAATGCCTGCCAGGGACATAATATC
AATCCATGCAAAGATGAACTCACCTCAGCTTAATATGAAAATTAAGAAGCAATACAAATT
TTGTACCCACAAATAATGCAACTTTCATGCATAACATTCATCATGTAATGTCAATATAAT
GGAGTACACAAACAGGCCATGAAAATTGTGAGCTGCCTCATATTAAGATTAAGATTTAAT
CACATATATTGTCCAAGCCCCCATTAGCGTATGGGAAAGGAAAACACAGAAGCATCAACC
TTGCAATGTGTGACGGATTAATTGTCTTAACTTTTGAAGGGGTAATTCAGATGCATTATT
CCATATAAAGGAGTCTGAAAACCATTATTCAGAACATTCAACTCAACAGAATTAAAATTA
AGACTGAGCATAACGTACAGGAATTCGCCGCTGGATAGGACTCCTACAAGAAGGACAATC
CTTCATCCCCTGTTTCTCATGGAGCTCATTGCACACTGTGCAAACTACCTGATGGGCACA
TGGAAGAAAAACTACTGACATCTCCTCTGAAAGGCACATCACACACTCGCGTTCACGTTT
CACACCTCCTGTCCCAGAGAAGTTACATAGCTTTGTCATTACTTCTGAGATAATAGGAGT
CTGGGACTTCTTGTTTATTGAGTCATTTCCAATGTCGCTAGGTCTGCCAGCATAGCTCCC
ATCAATGCCCCTCCGAAGTGCAGCAATTTTTGAAGAGTCAGTCTTCAGTCTCAATTGAGA
GATTTCTTTCTCGAGCTTTTGGATATCATCCTTATACCTCATCAAATTTGTTTCTGCCTT
TGATTTGATCATATCTTCCTTTGATTTTGCTGTAGCCTCAATCTGCTCTCTTTCCTTTCT
TATTGAACTGGCCTGCATAAGTAATTCTTCCTTCACCTTTTGCTCTTGTTGCCATCGAGC
CTCAAACTGTTCCTGTAGAACTTTCTCTCGCTCTAGTTCTTGTAGCAGCTGTGCAACCTT
CCTTTTTTCAGTCACAAGGTCCTCTTGGAACAATGTTTTTTGCTTCTCCCATGACTGGAA
CTTCATTTGTGTCTTCTTCTCCCTCTTTGATAC
>ntig_636_consensus
ATCAAAAGATAAACATCTCAGCTTGATATCAACATTAGGAAGCAACAAAAATTTGCAATC
GTAAATGATGCAACTTTATCATTTACTTTGATGCATAACATTCTTTGTATAGTGTAAGTA
TAATGGAGAACATATGAAGCCTCATATTATGATTCAAATTTAGGCATATATACTGCCAAG
CCCCAGTAAACATATGGGATAACTACACAGAAGCATCAACCTTGCAATGTATGACAGATT
CGTTGTCTTAACTCTTGAAAGGGGTAACTCAGATGCTTTATTCCATATAAGGAGTCCGAC
AACGATTATTCAGAACACTCCACTCAACACAACTAAAATTAAGAGTGAGCATAACGTACA
GGAATACGCCGTTGGATAGGACTCCTACAAGAAGGACAATCCTTCATCCCCTGTTTCTCA
TGGAGCTCATTGCATGTTGTGCATACTACCTGATGGGCACACGGAAGAAAAACAACTGAC
ATCTCCTCTGAAAGGCACATCACACACTCACGTTCACGTTTCACACCTCCTGTCCCAGAG
AACTCATAGAGGTTAGTCACTACTTCTGAGATAACAGAAGTCTGGGGTTCCTTGTTTATT
GGGCCATTTCTGACGTTGCTAAGTCTGCCAGCATAGCTTCCATCAATGCCCCTTCGAAGT
GCAGCAATTTTTGAAGAGTCTGTCTTCAATCTCAATTGTGAGATATGTTTCTCGAGCTTT
TGGATATCATCTTTACACCTCATCAAATTCTGTTCTGCTTTTGATTTGATCATATCTTCC
TTGGATTTTGCTGTAGCCTCAATCTGCTCTCTTTCTTTTCTTATTGAACTGGCCTGCCTA
AGTAATTCTTCCCTTGCCTTTTGTTCCTGTTGCCACCGAGCCTCAAATTGTTCCTGGAGA
ACTTTCTTTTGCTCTAGTTCCTGTAGCTGCTGTGCAACCTTGCGTTTTTCAGTCACCAGA

TCCTCTTGTAATAAAGTTTTTTGCTTCTCCCATGACTGAAACTTTATTTGTGTCTTCTTC
TCTCTCTTTGATACTTCCTGACAGCTTGCAGCTGACTCTGCAGCACGTAATTTAGCCGCT
TCCATCTCTTGTCTCAGAGCAGCATTCTCTACCTCAAGCCTACGCACAGCAGTATTAGCT
CGCTCCACTTGCCCACTGGCCTTACACAAAGCATTCTCCATTTCAGACAGCTTCTTCATG
GTGTTTTCCTCCAAAATCTGTTTCTCTTTCTTAAGTCGTTCCACTTCCTCTTTCTCTTGC
CTCAGTGTCTTAAGTTCAGCCTTGTCCTTACTAAGCCTTCGAGCAGCCTGCATAACCTTC
TGATTAACCCACTCATTCCATTCATGGAGTTGATTCTGCAGTTCCCGCACCCGTGGAACA
AGCTTCACAATCAGCTCATCCTTCTTGTCCTGAGGAATCCACTGTCCCAGAGACATGTCA
CACTGTAAACCAGCATAACTACCATTAGGTCTGTTGGCATTAGCACTAACAGGCACTTGA
GCAAAGTTGCCTTTGGTAGGCAACGAAAGAGAAAGCTCTGTATCGGTAGTTGATAATGCG
GACAGGTTATTGGCTAAAGGTAAAGTAGATGGAGTACCAACTGGAGGGAGTGTGGATAGT
ATACTGGCCTTTGGCAATGCAGAAATAGCACTAGAACTTTCTGAAGTAAACACTACAGGC
AAAGAGGATCCAGAAGTGGTAGATGGATTATGGTTTCCATTTTCCTGATGTGCTTCAATG
GCCTTGTTTATTTTTAAGGAAGTATTCTTTATGTTAAGGGATGCATTCTCAGTCACTGAT
TTTAGTTTCTTATCTAAGATTAAGCCAGCCAAACCATTCAGTTTCCCAGCTCTTGAAGAT
CCCTTAGATCCATATGTCCGGTAGTGTTTCTCCAAATGAAGAGACTTCTGACGAAGTACA
TATTCTCTC
>ntig_637_consensus
GCAGCAGCCTTCTGGTTCCTTCTCTTCACTCTACCTGGGCGTCCACCACCAAATGGACTT
GTGATGGAGAAATCAATGTGCTTCTGCGAGTCCATTCTCACCATGAATGACGGGATATTG
ACAACCTGCCTGCCAACTCTAATGTGCCTCTGCCTGATTAGAACTCGGGCATGGTGGATT
GATTTAGCCATTCCATTCTTGAACACAAGAGTTTGGAGACGGCGCTCAAGGAAGTTCTCC
ACAGTGAGGGCCAACACATAATCCAGCTTATTCTGACTCTCATCAAGAAGCCCATACCTA
TTCATCCTCCTCAAAAGGGCCTCACCCTCAAAAATGCGACGAGGATTCTTCTCATCAAGG
GTGAGAAGCTCCCTTGCTGCATTTCGAATGCGGCTCAGTGCATATTGAACTCTCCAGAGT
TCTCTTTTGCAGCGAAGACCATATTCTCCAACAAGCCTCAGTTCAGCATCCAATCGCTCC
TTCTCATAGGGACGTCTGGGCTTCTTAAAAGTTTTACCATAGTTGCGGTAGAAGGAAACG
TGAACCATGTTCGTGACGCGCTAATCCTCTCTTCGCCGCTACGAACCCGCAAAAGTGAAG
GAACCAAATGCGATATGTATCACTCTTTATATAGGCTTTCCGAAACC
>ntig_638_consensus
CCCGTCGCTCTTCCGATTTTTCGTAAAGTCCTTCACCAGATGTGGCGCATGTGCTCTGGA
TGTACCAGTGACGTTGGCGCAGGGAGTGAAGACCGAGCTTATCAGTAATCTCAGCAGCAT
TCATAGCATTTGGAAGATCTTGCTTGTTTGCAAAAACAAGCAGTACAGCATCCCTCAACT
CATCCTCATTCAGCATCCGGTGCAGCTCATCCCTGGCTTCAACCACACGATCACGATCAT
TGCTATCAACCACGAAGATAAGCCCTTGGGTATTTTGGAAATAATGTCTCCACAATGGAC
GAATCTTGTCCTGACCCCCAACATCCCAAACAGTGAAACTAATGTTCTTATACTCAACAG
TCTCCACGTTGAATCCAATTGTAGGAATGGTTGTCACAATCTCGCCCAGCTTGAGCTTGT
ACAAAATGGTGGTCTTACCAGCTGCATCAAGACCCACCATCAGAATTCTAGTTTTACAGT
GGGGAAAGAACCACTTTGCAACTCAGAGATATATGTTTACCCATATCACAACTCCAGCCA
C
>ntig_639_consensus
CCTTCCTGTATACACACATCATCAAGAAGGTATAAGACATGAAATCACAAAAGAATATTT
GGCTCAATTTTCACGTAACTGCAATTACCAGAATATATCATTCACAACCTAGTCACATCC
AACAGATCTGATAAATCACAAAAAGATGATCAGGTAGAATCGACAGAAACTTGACTGGGA
AAAAGGCCAAACAGAAACCCAATTTTTCTATTTCAAATCCGCACTACATGGCAGACTGAA
AATCCAACAGCATGAAACTCAAAGGCTCTAGTGGCCATACTTCTGGAACTCCCACTCGCT
ATTATCCTGACGTGTCTTAAGCCATCGACTGATGCAGTGAAAGTGAAAGGCGTGATTGCA
AACCCCCCAAGCTACAGTGCATTCCTCGCTCGTGGCGCTAGCCTGGTTCGCTTGACACTC
GATGCAGAGGTCCATGATATGGTTTCGGCAAATCGCACAGTTATCGACAACAATATCCCA
AGCCCAAAGGGCAACGGCGCTCCACTTCTTGATCTCGAAACGCTTAGGCTTCTTGGAGGA
TGAGGAAGGAGCAGAACTGCTTGATGGCTCGGTGACAGGAACCATAGTAACGTCGGAGTC
CAAAGAAGCCATTAATTTCTGAACCTCTGAATCAATTGCAACAAAAGCTTGTCTGAATTT
CTAAAGACCAAAACAAGAAAATCCGTGAAGCCCTGGGAATTTTAGTATTCGGGTTTGGGT
TTCAAATTTTTACAAAAAGCAGAATTGGGTTCTGTTAGGC
>ntig_640_consensus
AAGGATTATAACAGTGTGAGTAAATGTGGATACCAGAAACGTTGATATATCTGCAACTAC
TTCAACTATTTGGTATTACATTGACTACTACTCGCACCATGGGACAATATAGTGGCGTCT
TCCTAATTAGAAAACCAAAAGCACAATCATGGAGATGCATGCATTATATTCCACAGTGTC

GGGGCTCAACTGTTCATTGCTAGAAATAAAAGATCCATTTGGTTTTGAGCTAAAACAGAA
GGTACTTAACTAACTAAACAACATCACGGGTTTCATAAGCAGAAATTATGATTTCACTTC
AGCACGGTCGTCATTAGAGGCGGCGGTTCAAATGGGAAATTACCATCCGTTGATGAGGCA
ATGAAAGATCATCTTCAGTCATCAGCTGGGGGTGACCGACTGCGGTATCTGTCATTGTCG
GGGCTCTCAGCTCCACCATAATTGGGGCTATGTCTAGCTTCAGCTATAGAGTTTGGGCTG
GGACGACGACCATTGTCAGGGCTACCTTCTCCGTCGTAGGGACTTCCACTGGCTTGCCGA
TCATCGTCAGGACTAGACCTCTCTCTTCCATATGGATCGCGGCTGGGACCATGACCATTG
TCTGGGCTTGACTTCTCTCTCTTATATGGATTGCGGCTAGATCCATGGCCATTTTCAGGA
CTAGGTCTCTCTCTTCGGTAGGGACTACGGCTAGGGCCATGACCGTGGTCAGCACCAGCC
CTCTCTTTTCTGTAAGGACTATGGCTAGTACCCCGTCCATAATCAGGACTGCCACGATCT
CTCCTATATGGACTTGGGCTACGAACACGGCCATAACTGGGGCTTGCTCTTTCTCTCCGA
TAAGGACTGCGGCTAGATCCACGGCCGTAATCAGGGCTTCCCCTCTCTCTTCGGTAAGGG
CTTGGTGATCTCCTTCTACCCCTATCAGGTGACCTGTCATGACTCCTATCAGGACTATAA
CCATTCCTTCGGTCATCATCATCACGAACTGCATACTCAACTGAAATAACTCGATCCATC
AACTTGCTCATATATGTAGCATCCAATGCTCTAGTGGCATCCTCCTGTGCTTCATATTGA
ACAAATGCAAAATTCCTTCTGATCCTTACACTCACTATCTTTCCATATGGCTCAAAGTGC
CTTTCTATGTCCCTAGTCCGGGTATGGAATGGATCAAAATTGATGACAAACAAGGTCTTC
GAAGGTCTGGTATTTGAAGAAGGTCTTCTTGAGCCACCTGGTCTTCTAATAGCACGTTCA
TGCTTTGTCCATTCAACACGAAGTCTGCGACCCTTGCGTCCAAACTCAGTCCGATCAAGC
CCTCGAATTGCATCTTCAGCATCTCTCTCATCTTCCATATAAATAAAAGCAAACCC
>ntig_641_consensus
CCCATTTTATTTAGTAACTGTCTCCAATCTCCAGATTTGTGACTTTGTTCAGCCTAGAGA
CTAGCAACTCAAACAATTTAAAGTGAGAGACAAGTCTGAGAACAATAAAGCATTTCTCTG
TTAAGGAGTTGCAAACATTAATTGTTTCCAAATCCGATATACAAAGATGCAAACTGCAGA
TATTGTACTGTTGGGGGATGTACTTCTCCCTAGAACAGCTTCTTGGTAACTTAAATAACA
AAAGCCATAGAAATAGAGTCGTTGGCAGCCTCAGAAAAGAACACTTACATATAAAGTCCT
TCTGGTACCCCTTCTTTCTTCTTAAACATAACCTTCTCCTTCGCTTTCTTGAAGTCTGCA
TGTGTCACCTTCATGCGGCGCTCTCTTAATGCAAGCAAGCCAGCTTCTGTACAAATTGCT
TTGATATCGGCACCAGAAAACTCATCCTTGGTCATCACAAACTCTTCCAAATTGACATCA
TCAGCTAAGGTCATCCTTGAAGTATGTATCTGAAAAATTCGTCTTCTAGTTTTGATGTCA
GGAAGAGGAAACTCAATCTTCCTGTCTATTCGCCCAGGACGCAGCAAAGCAGGATCCAAA
CTCTCAATTCTATTTGTTGCAAGAATCACTTTGACATCTCCTCTTGAATCAAAACCATCT
AACTGATTGAGCAACTCCAACATTGTCCTCTGAATCTCACGCTCACCGCCTGAATGTGCA
TCATATCTCTTTGTGCCAACTGCATCAATTTCATCAATAAAGACAATTGATGGTGAAAGA
TCATCCGCAACTCTAAAGAGCTCCCTGACCAATTTTGGGCCATCTCCCAAGTATTTCTGA
ATCAACTCGCTTCCAACAACACGCAAGAAAGTTGCTGATGTTGAGTTTGCCACCGCCTTA
GCAAGCAAAGTTTTTCCAGTTCCTGGCTCCCCATAGAGTATGACTCCTTTAGGAGGTTTG
ATACCAATGTCTTCATACAACTCAGGATGAGTAAGCGGAAGCTCCACAGCTTCTTTAATT
TCCTGTATTTGGGCATCCAACCCACCAATGTCAGCATATGATTCCAAGGGGGCCTTCTCA
ACCTTCATCACAGACACCATGGGATCAACATCATCTTGAAGAAGCCCAACAACAGAAAGG
ACCTTGTTATGCATAAGAATAGCACATCCAGGCTCCAGCTGATCCTTATCAACGAACGAT
AAAATCCCCACATAATACTCAGGCCCCACAGACGACGAGACAATCGCATGATTCTCATCA
ATCAACTCCTCCAAATTGCCCACACTCATGGGTGTCCCACGCAGATCATCAACCTTCGAT
CGGTCCTCCTCATTCTTCTCCTCCTGCGGCTTCAACCGCTCTTGATTCGCCACGAACTCC
TCCTCCATTAACAGATAATCCTTGATGCGCTCCAGCTTCAGGAGCCGAAGCTTGCATTTC
GTCAGTGGGGTCACGGTGGGGAGGCGTGACGCAGCCTCTGAGCCCTTCTGCTTCCGCTGT
TTGCGACCGACGCGTGACGGCGGAGCTGCCGGCTCGAATTTCTTGTCCTTCTTGTTGTTT
GAAGGGTCTTTCCGGTCACCACCCTGCCGGTTTAGACCGCCTGAGGGGGCTTGTCCCATT
GGCTTGGTTGTGTTTTGGGGTTCAAAGGAGTGAAATTAAAGTGCCCTTAAGAAGTTTGAG
GAAAGGTTGGTTTTTGGTTTAAAATAAGACGAAGAGAGTAGAAGATTGTTTGGTCGAGAA
GAAAGGCAC
>ntig_642_consensus
GTTCAATGTAGTAAGACTGCATTAAAATTATACTTGTTCACAAGAATACAGAGATAGTTA
ATGAGCAATTCTGTCATTATGAGAATACAAATTTGCAGCTACAAGGCATCTTCAAGAAGC
AGTAATGGGAGTCCACAAGACAGATTTGACTCCATTGGAAGGCTTCTTAATGCCAACTCC
AACTTTTCAAAATCAATCAGGCTAAAACATAATCCTGAAATTCTACTCTCATTACTGTAC
AACTACAAAGGCCACTAAGTCAAACAAAAAAACATGACTTTCCACATTGCCCGAGCCCAC
TATTTACAAACATCCTCACCACCAAGCTACAAACCCCCATCAAATTAGACAGCAAATAAT

ACACCTTAACCAATCTTACTTAAGGCATTACAAACTTCTGTATTACATTTCATGTTGGGA
CATCAACAACTTCTGTAGTTTTACAAATAGGCTCAGAGGTCTCAAAATCGTCTGCATCTG
ACCTGCTTCCACTGTCATCACTGTCTTCCTTTGTCTGATCGTTATCTTTCGCATCATTAC
ATTGGACACCATGTACTTCATCACCACCTTCTCCATTTATTTGTTCTTGTGATGCAGACC
TCTCCTTGTTGAAACAATCCATGTCTTCTTGAAGGTTATCACAACTGGCTAAAGTGCCGA
ACTTTGGTTTTTTCTCTTCTGCAATATCGAGTTTAGTACTACTAGGAATATCAGTATATG
ACACATCCAAACCATCAGGCTTGACATCCTTGGCAACAGTTGAAGACAAGGAAATTTTAT
TCTCTGGGTTTGTAAGTGCAGATATCACAGAGAGTATGTCAGGTTTTGCCTCAATATCAA
CCCACTGGTTCCCCACCCAATCTTTAGAGATTCTGATGTCCTTTTTGTGCACGTTCAAGA
AGTGGCTCTCCCAAGGAAGATAAACTTGAAGATTGTCATTGCTATCACTGTCAAATCCAG
TTACAACCCCTTCCCACCAGCCATCACTCCACCATGCATCAACAGCAGCCCCAATATCAA
GTGAGAGGGGCACCAGATCATTAGAAGGAGATGGTCGGATTGTTGGGCGGTCAGAACACC
TCATACCAAGTTTATCAGGCAAAGCTACTTTATACACAGGGACCCACTCCTCAAGATTGC
CATATCCATCTTCATCCAGTAGATCATCATATTGGACTCTAATATGTTTCCGGGATACCC
GTAAAACTACACACCTGAACCAGCAACCTCGAATGCCACTGTCTTGGCACAGCAACTCTA
TCTTTTCGTCAACCTTATACACTGGACTAGACCGAGGCTGACACTCAATGTGCTTTAGGG
AAACCAACCTTATGCCCAACAAACCGTATTTCAAGTTCTTGTATGATGGCTCATACCTCA
TCACATTGTAACCCCCACCAGATCTGACTCCCAAATTATTGGATATTTGACTCCCCCTAC
CACATCTTGTCCTCTTGGTTCCCAGCTTTACGTTATCACCAGGAGCTAGTTCATCTTCTT
CCATGTCAGTCTCCTG
>ntig_643_consensus
GGGGAGAGCTTTTTCTTGAATGTGCACAAAAAGGATATCCGAACTTCTAAAGATTGGGTC
GGGAACCAGTGGGTTGCTATTGAGGCAAAACTTGACATACTCACAGCAATATCTGCAGTT
ATTAACCCAGATAATAAAATTTCCTTGTCCTCAACTGTTGCCAAGCATGTCAAGCCTGAT
GGTTTGGATGTGTCATATACTGATGTTCCTAGTAGTACTAAACTTGATGTCGCTGAAGAT
GAGAAACCAAAGTTAGGCACTTTAGCCAGTTGCCATAACCTTCAAGATGAAGACATGGGT
TGTTTCAACGAGGATAAGCCTGCATCACAAGTAGAAGTTAACGGAGAAGGTGGTGATGGT
GTAAATGTGGGTTATGATGAACTACATGGTTCCCAAAGGAACAATGTAAAAGATATAGAT
GAGACAGAGGATGATAGTGATGAAAATGGAAGCGAGGCCGATGCAGAGGATTTAGATACC
TC
>ntig_644_consensus
AATGCCACAGCAGATTAAACGTTTTAATACTTCCAATATATCATTTCTAGCTCAAAACAA
AACAAGTTCTTCAGTAATAGAAAAACTGCATAAGCTAAACAAACTTATCTATCTATAATG
AAGTCAGAAGTCAAGTTCACTTCTTCTTATCTCCACCTCCAGTGGCCCTCATCTTGCGGA
GAACACTCGACATCTCTTCACGCTTCTTCTTTGCTCTCTTGTGGGTGCCCAGTTTTCTTT
TAGCAACCTTCAATGCTCGCTTGTCCTTTCCAACTTTCAAAAGCTCAGTGATCCTCTTCT
CGTATGGTGCAAAACCAGCAACTTCCCTGATTACATTCCTCACAAAGTGGACCCTTTTGC
TTGTTTTCCCTTTACGATCAGATGGGCGTGGAGGCAATTCTTTCTTGGTTACAATATGAC
CTTTGTTTAACCCTACAAAGAGACCAGTATTTGGCTGTTTTGGAGCCATTACC
>ntig_645_consensus
AAGCACTATAACATAATTGAAAACAAATAACTAAATAATTCTCCATACAAATCAAAAACA
CATGCCAGAATAATCGTTTGAAAGGCATCATCATATCATTACTTCCAATATCTCATCTTC
AATTCAAACAAACAAAGTTCTTGATTCAGCTAAACCAACTCATATAACTATCAAATCAAA
AGTCAAGCTCACTTCTTCTTGTCTCCACCTCCAGTGGCCCTCATCTTGCGGAGAACACTA
GACATTTCCTCTCGCTTCTTCTTTGCCCTCTTGTGGGTACCCAACTTTCTTTTGGCAACC
TTCAGGGCTCGCTTGTCCTTTCCTACTTTCAGAAGCTCAGTGATCCTCTTCTCATATGGT
GCAAAACCAGCAACTTCTCTGATTACATTCCTCACAAAGTGTACCCTTTTGCTTGTTTTC
CCTTTACGATCAGATGGGCGTGGAGCCAATTCTTTCTTGGTCACAACATGACCTTTGTTT
AACCCTACAAAGAGACCGGTATTAGGCTGTTTGGTAGCCATAACCTCTGGACGAGCCTGA
GCTGCCAATATCTGATCTGACGGAAGGAAAATTAGGGTTTTGGCCTTTTGG
>ntig_646_consensus
AGCATCAACTGCTACAATAGCCATACCAACATTGTCTCCAGAGGCCCCAGTCACTCTCTC
CCCGAACATAAATGAGTTGTTTCCATGAATTCCCATGTACACCACCAGATCAATCATCAA
CATCTGAATTTCTTGGAGATGGACTTCGGGAATCAGCACGACGACCTCGTGGTGAAGCAC
TACGAGGAGTTGGAGATCGCTCATCATGACTGCGTCTATCCAGGCTCTCACCCCTAGGGG
AAATCTTGCGAGGAGAAATACTCCTGCGAGGACTAGGACTACGCTGAGGGGATGCACTTT
CGACTGATCGACTAGCACTCCGGCGATCATCATCATATCGACCTCTGCCTCGGCCCCTAG
AGTATTCAGGACTGGCACTACGACTTCTACTTCTGCGACGATAATCTCTCTGCCTCCCAC

GATGTCTATCACGTTCATACCTATCCAAGCTTCTACTGCGACTTCTCCTCTTATAATCCC
TATCTCTATAATCATCCCGATACTTCCTTCGAGGACTGCGGCTTCTTGACCTGTGTCGAG
TTCTAGGAAATGATTCACTAATCCTTCCTTTATGACTGAAAAAGTAAGTTAATGAGTTGA
TAAGTGACTGGAGTTCAAACTAAATTATGGCTAGAACATAGAACAATTTGGTTATAGTAT
CGCATTCCCATCAAAAATGATCAGTAAAACCCTCACAAATTTCAAACTAGAAAACCAAGA
AATCCAAACTCTCTATCTATAAATAAAAATATTATATGCTATACACACACATAAAGCAAT
GCCAGGAAATTTCACCAGACTGGGTCTCAACTGTTGGTAAAGCAACCATGCTTCACCTAA
CTGAGAACTTAATTTAACGGCACTCCAAAGGTCATAAAATACAAATAAAAAACATCTCCT
GGATAAGCTTCGCGACCGGGAGGTCTTCGTAATAGAAGAGACATTTGTCGATAAGCCTGC
GCTTGTTTAGAGAGATCATCATAAATGATTAAAGTGTGTCGTTCACGGTACATAAAATAT
TCAGCCAGAGCTGCTCCCGTATAAGGAGCGAGATATTGTAACGTAGCGGGGGAATCCGCC
GTTTCGGCTACCACAATAGTGTATTCCAGCGCCCCCCTTTCCTGGAAAGTAGTCACTACC
TGAGCCACAGAAGATGCTTTTTGACCAATAGCTACATAAACACATATTACATTTTGCCCT
TGTTGATTGAGAATCGTATCCGTGGCTACTGCCGTTTTACCGGTCTGTCTGTCCCCAATA
ATTAATTCTCGTTGACCGCGTCCTATAGGGATCATCGAATCAATAGCAATAAGTCCTGTT
TGAAGAGGCTCATATACGGAACGTCTCGAAATAATACCTGGGGCGGGGGATTCAATTAAT
CGAGATTCAGAAGCTGAAATTTCACCTCGACCGTCAATAGGTTTAGCCAGGGCATTTATA
ACACGACCCAAATAAGCCTCGCTTACTGGTATCTGAGCAATTTTTCCTGTTGCTTTTACA
GAACTTCCCTCTTGTATCATTAAACCGTCACCCATTAATACAACACCAACATTATTTGAT
TCCAAATTGAGAGCAATGCCTATTGTACCCTCTTCAAATTCTACTAATTCACCTGCCATT
ACTTCATCAAGACCATAAATACGAGCGATGCCATCCCCTACTTGAAGTACGGTACCGATA
TTTACAATCTTTACTTCTCTATTATATTGCTCAATACGTTCACGGATAATATTACTAATT
TCGTCGGCTCTAATTGTTACCATGAGTCAATTTTTTTTGGAAGAAAAAAAAAAATGCCTAC
TGCAAAAGGACTAATCAGTTATTTCTTTCAGTGCCCCAAACATGCGAATATTAGCACTAA
TGGTACGTAAATGTAACTCCTTGTTCAAACAACTATTTAGAGTTCCTAGAGCTCCTTGTA
AAGCTTGTTGGAAAACCCGTTGTCGGACTTGATTAATTGCTCTTTGTTGTTCAAAATGGA
TGGTTTCATTTTTGTAATTTTCTAATTGTTCCAAAGTTTTAGAAGTTGAATTGATCAAAT
TCCATTTTTCTCGTTCTATCTCAGAGTACCCATTCACTCGAAACTGATCTGCCTCCATTT
CAACTTTCCGTAACCGGGCCCGGGCTTTTTCCAGCCGTTCAATGGCCCCTTCCTGCAGTT
CTTCTGAATTTCGAATAGTAGTTAAGATCCTCAGTTTTCGATTATCTAATAAATCACTTA
ATGAAAGTAGATTATCGTTCCATTCATCTCAAAATTTCCATAATCCCTTCCCGAACCAAA
CATGAATTTTTCGATTCATTTGGCTCTCACACTCAATTACTTGAACTATTTATGTATGGG
GGGCATTCCCCTATCTTTTTTTTACTGTAATGAGCCTATCCTCTTCCTCTCTTCTCTATT
CATATTCCAAAATGTCGTGACTGATCAATAAAATCAAGACAAGAATATTCGGAGGACTCT
TCTGACCAAAGAAAAATAGATAATTGTCAGCAAAGTTGTTTCTTTTTTCTTAAAATCCAA
AGAATTCTTCTTACTTTAATATATAGGTCATCGATTCAGCATAAAAAGGGGTTGATTGAA
ATACCCATCTTTCCAATATTTTCCAATAAAAAATAAAAGTCTCAAATCATTTTATCGACA
CGAGTGTTTTATATCGAAAAAAAAAAAAAAAAAATTACATCCCATTAAAAATAAAAAAAA
AAAAAAAAAAAAAAATTCAAAAAAAAAAAAAAAAAAATAAATAAACCTTTAAAAAAAAAA
AAAAAACCCCCCAAAAATTTTCTTTTTTAAAAAAAAAACCTTAATATCCATGCGCGAAAC
CCCGTGGAACAACCC
>ntig_647_consensus
CCAGGAATCTGTGAACATATTATAGAAATACTATGTTTTTGAGAAAATTTAAAGACATCT
CAGTATAGAGGAATCTGTTCTATCGTTGTATATTCTTCCACCCAGTTAGTTCAGGTGGGA
TTATATGTTCATTACAAGAAAGCACAGCCTGTGCCATTAATTTGCCAAGCCAACCATGGT
ACAATAAACCTCTTGAACCAAGTCCTCCGAATAGCCAGTATTTACAGCTCTGAGTTTTAC
CTATAAGATCTTCAATACAACCCAGAAGGGGAATTGATCCGTTGACTGTAAGTGGTGGCA
TTGCTCTCAAGCCAGCCCTAGCTTCGGTGAAGGACCAATCTCTTATGTCTGGATATATGG
AAGACACCTTTGGCAGAAGCTCATTTAGAGCTTTTGAAGCTTCATACACTGAGACTTTTG
GTGATGAATTTCTTGAATTCCATTCCCATGTTGAGCCCATATATAGACTGCGAGTCCCCT
GTACAGCTAGCCATGCATCTGCCAATATTGATGGGCCATCATCTGGATAATCTTGCCAGA
GAGCTCTGGAAGCATGTCTGCTTGGGCACCCATGCATACAATCACAGCATCATATTCCCC
TTTCAACTCCAGGAGATTACTAATGGATTTTTGGTGGAGATAGAACTCTTTCCTTCCAAA
ACCAGAAGTAGACAATTCCTTCACTAGATTTTTACATGCTAAGAAGAGCCCCCCAAGATA
GCGCAGGGGATGAATGTTGACAGCATCAGGCATATAAAAGGCTGTGTTCAAAGGAACGCA
TAAATTGGGAACAAGACTTTGGGCAGAATCCTTACCAATAGTCTCTATTCTGCAACTGGG
AAGGCAGTTCTGAGCATTATCATTTAATGTATTCAAAGACTTCGTATTTGTTGCAGGCCG
CAAGATGCCCCTTCTCCTTACAACAAATTCACTCAAATATTGACCAGATTCCTCCAATTC

TTCTGAACCAATTGCTGCTTCTGCAATGCCTAAAAGCTTTAAACTTTCTTTCCAACATTC
AGCACCCCGCCAAAGAAGCTTAGCTTTAGGAGAATAAGGATGGAGAAGTCCTCCTGATAC
TCCAGAAGCACCACCTCCTATGCCAATCTCATCATATATATCAATACATACATTCAACTC
CTTAGCACTCTCTTTCAACAAATGCCAAGCGACTGAGAGACCAGCAAAGCCAGCACCGAG
CACGGCGTATCTAAGAGGGCGTTGGGGTAGAGAAGAGACTACTGGTGAAGCGGTTCTTTT
ACTATGTAAGCGACAGTGATGGCGGCGGTCGTAGCCGCTGATATAAGGCCAGCTCCAATC
GACCAGACGACTCTTGCCTGGCGGCGGCGGCATGGCTGGCTTCGAGTTCTATCTAATATA
GTTAATTCTGCCAGAT
>ntig_648_consensus
GGGATACAATAATAACAATAGCAATAAAGAGGTAAATAAGCTAGAAATAATCCCATTCCT
GAGTTAACAATGCCATTCACTTAATTCAACAAAATGTATCGAAACATAAAATCAGATACC
AATACCACTACTTAATCGGCTAAATAACCAAACGACGCCACAACTGAGAAGACTACTTAG
ATCTCTGTCGCATCTTTCGGCGCTTCCTCTTCAACCTTCTCATGCGCTTCTT
>ntig_649_consensus
TGGAAATTTTTATTATAGTATCATCAATAGTCGAGTTTTGCATCCCAAATAATGTCAATA
TATTTTAGACGTCCAATGAAATTGTTTTAGAGTTACAGGTCTTGCAATCAAATTATCAAC
AATATATTCCGTTGTCAACTTTTTAGTACCTCCATGGGGGAGCCTGAAACTCAACCAGCA
AAGGTTCTGAGACCAAACTATATCAGAAAAAGAGCTTTTGATGACTTAGAAATATAACCA
AGAAGGGCATAGAATACAAAGACGTACAGCAACAGACTACTGCAACAAAACTTTTAATAT
TATAATCCGACCACAGCTCAACTTGACAGTTTCCACCTTGCAAATGTGGATGACCTCATA
TGATTAATTGGTCAGGTATGTTTGAGAACAAGATTAACTTAAGACACTAGACGAAAAAAG
GAAACAAAAAAGAAAAAAGCAAGAAGTTAAAATAGTACTTGCAAAATAAGAGAAACTCCT
CAGCCTCAAAAAACTTACAAAAAAGAACCTCATCAAAGGAGATCAGCAACATTTGAAGGC
AGCTCCTCGATCACCACGTTATAGAATTTCTGGATATCAAAAAGCATTCTGTCGTCATCC
TTTGTCACAAAGTTAATGGCAACACCTTTTCGTCCAAATCGACCACTTCGTCCAATTCGA
TGAAGGTAGTTTTCTGGTTGAGTCGGCAGATCGTAGTTTATGACAAGGGAGACTTGCTGC
ACATCAATACCACGAGCCAAGAGATCAGTGGTGATTAGAACACGAGATGAGCCAGAGCGG
AATTCCCGCATAATGATATCACGAGTGTTCTGGTCCATGTCTCCATGGGTAGCAGACACT
GTGTGGTCACGACTTCTCATCTTGTCAGTAAGCCAGTCGACCTTACGCCGGGTATTCACA
AAGATGACACTCTGGGTGATGGCCAAAGTCTCATACAGATCGCAGAGTGTCTCTAGTTTC
CACTCTTCCTTCTCAACATTTACATAAAACTGTTTGATACCCTCAAGAGTGAGCTCATCA
CGTTTAACCAAGATTCTCACAGGCTTGTTCATGAACTTCCTTGTAATTTCAAGAGCTTCT
GGAGGCATTGTGGCAGAAAAAACCCCAACCTGAACTTTTGCGGGAAGCAGCTGGAAGATA
TCATAGATCTGATCTTTAAAACCACGGGAAAGCATCTCATCGGCCTCATCAAGTACAAAC
ATCTTAATATAATCTGGGCGAAGAGATTGCCTACGCAGCATATCAAACACACGACCAGGA
GTGCCAACAACAACATGGACACCAGCTTGAAGAATGCGTTGATCCTCACGGACACTTGTC
CCTCCAACACAAGCATGTACCTTAACACCAAGATAATCACCCAGGGCTCGCATGACCTTC
TCAATTTGTTGAGCAAGTTCCCTAGTGGGTGCCAAAACCAAAGCCTGACATTGTACAAGA
CCATAATCAAGTTGCTGCAAAATGCCAGAGCAGAAAGTAGCTGTCTTTCCAGTTCCAGAC
TGAGCCTGTTGAATCACATCAAGACCTTTGCAGAAGGGAACAATTCCCCTTTGCTGAATC
GCAGAAGGCTTCTCAAAACCATAAGCATAGATACCCCTCAAAAGGTTTTCCTGCAATCCC
ATTGCATCAAAACTGTCATATGACTCATCATATGAAGTGAAGAAGTCTTGTCCATCAGAC
TGGAGCAACTCAGACATCTTGGCATCATACTGACGAGCATCAAATTGAGTTCCTTCCGGT
GCCAAACCTGCCATGACTGCTAGTGTTGTTGATCAATAAAGAGAGAAACTTTGAGAGTTT
TAGGTGCCTTAAGAGAGCTTTTTATTAGGGTTTCTTCAAAACTCCTCGGATCTGCGCCGT
GGAGTTAAAAGAGGAAGAGGCTACCAGATGCGGAAGATTTATATACCAAGCCCACAGTGT
TC
>ntig_650_consensus
GCACAACCAGCAAATGTTGAGACCAAACCAAATCAGAAAATAAGCTTCAGATGACTAAGA
ATAGAAGTAGAATTGATGACATAAAATACAAACTAGAGCAACAAAACTTGCAATAATGTA
AACCGCCCAGTGCAATTTGACAGTCTTAATGTTGCAAATGTGCATGACCCCATATGATTA
AATGGTCAGATATGTTTGAGAACATTAATTTAAGACGCTAGAAGGAAAAAGATAAACAAG
TTAAATAGCGCTTGCAAATCAGCAAAACTCCTCGCCCTCAACAAACTTACAAAAAAAGAA
TCTCATCAAAGGAGATCAGCGACATTTGATGGCAGCTCCTCAATCACCACATTATAGAAT
TTCTGGATATCAAAAAGCATTCTGTCATCATCTTTTGTCACAAAATTAATGGCAACACCT
TTTCTTCCAAATCGACCACTTCGTCCAATACGATGAAGGTAGTTTTCTGGTTGAGTCGGC
AGATCATAGTTTATAACAAGGGAGACTTGCTGCACATCAATACCACGAGCCAATAGATCA
GTAGTGATGAGAACACGAGAGGAGCCAGAGCGGAATTCCCGCATAATGATATCACGAGTG

TTCTGGTCCATGTCTCCATGCGTAGCGGAAACTGTGTGGTCACGACTTCTCATCTTGTCA
GTAAGCCAATCAACCTTGCGCCGAGTATTCACAAAGATGACACTCTGGGTGATGGCCAAA
GTCTCATACAGATCACACAGTGTTTCAAGTTTCCACTCTTCCTTCTCAACATTAACATAA
AACTGCTTGATACCCTCAAGGGTGAGCTCATCACGTTTAACCAATATTCTCACGGGCTTG
TTCATGAACTTCCTTGTAATTTCGAGAGCTTCTGGAGGCATTGTAGCAGAAAACACCCCA
ACCTGAACTTTTGAGGGTAGCAGCTGGAAGATATCATAGATCTGATCTTTAAAACCACGG
GAAAGCATCTCATCAGCCTCATCCAGTACAAACATCCTAATATAATCTGGGCGAAGAGAC
TGCCTCCGCAGCATATCAAAAACACGACCAGGAGTACCAACAACAACATGTACACCAGCT
TGAAGAATGCGTTGATCCTCACGGACACTTGTCCCACCAACACAAGCATGAACCTTAACA
CCAAGATAATCACCCAGAGCCCGCATGACCTTCTCAATCTGTTGAGCAAGTTCCCTAGTG
GGTGCCAAAACCAGAGCCTGACATTGGACAAGACCATAATCAAGTTGCTGCAAAATGCCA
GAGCAGAAAGTAGCTGTCTTTCCAGTTCCAGACTGAGCCTGTTGAATCACATCAAGCCCC
TTGCAGAATGGAACAATTCCCCTCTGTTGTATTGCAGAAGGCTTCTCAAAACCATAAGCA
TAGATACCCCTCAAAAGGTTTCCTGCAACCCCATTGCATCAAAACTGTCATATGACTCA
TCATATGACGTGAAAAAGTCTTGTCCATCAGACTGGAGCAACTCAGACATCTTCGCATCA
TACTGACGAGCATCAAATTGAGTTCCTTCTGGTGCCACACCTGCCATAACTGCTAATGTT
GATGATCAACAACGAGAGAAAGTTCGTAGTTTTAGGTACGTTTGGAGAGCAAAAAAGAGA
GCTTTTTATTAGGGTTTCTTCCAAACTCCTCGGATCTGCGCCGTGGAGTTGAAGGGAAAG
AGGCGGCAAGCGAAGCGTGCAAGATTTATAT
>ntig_651_consensus
TCCAATACATGCTTCTGATATTGACTGATTAGATGATTAATTTGCTATAATAGAACACAT
AAAAGCGATCATATTTTGTAGAGATTTACAAACCGCCTCAAGGGATGCCTCAATGCAGAT
ACTTTTAGTTTAAGGATGTGCAAAATTATTATGTTCATGGTGATGAGAAGGCCAATATAT
TCCTTCTCTTTGGCATGGTAGAGGTCCAAGAGGAGCAGGCTGAGCTATGTAGGTGGGATA
CTGTTGCCCCGGCATCAATACCGAGAAATCCAATGCATACGAGGCTGACACAGTTTGTGC
ATGGCCAAGCTTTGCCTTGGGTCCTGAATCTGAAGGAGCATTGTTCATCACATCTGGTGA
AGATAAGGAAGTTGTAGGCCTAAAGAGATGCTCGAAAATCGCCATAATTAGAAACATCGA
TATCAAGATAGCTGTGGCAACAAATCCGAAAGAAATAGCATTCATTGATGTTCCAAAGCT
TTTCCATGGAGCTTCCTGATCGACAGCTGCCTGAGATGGACTTGGATCTGACGTCGTGTA
TATGTTCCAGGGCTTCGATCTTTCGGAAGCAAACTCATTCGAACTCATTATATGCTGCTG
CACAGGCCTTCACACGCTGGAACCTAGTTTAAACCGGGGAGCTATTCTTGATAACCACAA
CTAAACAAATCTCTTAACAGCTTGTTGTTCC
>ntig_652_consensus
ATCGACTTGGCCAGTTTCAGAAACGCCTGGTAGAATGGAGTCCTTGGATGTCCACCCCCC
ATTACAAAATTCCGCTGATCTAAATTTCCAAAGAATGAGGCTTCCATCTTGGAATGAACA
ACCACTAGGTACTTGCTACTGCAAAATTTCCCAAATATGGAATCAGGGTTTTGACCCAGC
ATGTCCAGTGGATCCATCTCCCTCAGAGCAAGAAACTGCTGGAAGAAACTCTCTTTGTTA
ACTATAAGCTTATCTGATTTAATGCCAAAGCTTTCCTGCTGAAAGCCACTGAACATTTTT
TGGCATATATGAGACTCAAATGCATATTTCTTGTGTGCCCTCTTTGCATAAACAACATTG
GGTTCTATAGAATTAGCTGCAGCATCAAGGTCCCAGCCAGCTGCTTTCATCATGTTGATC
AATGGTTTAGAAAAATCATGAATAGCTTTGAAAGCAGCTTCAACAGTCGCTGTGAATAGA
TCAGGAGTTAGATCCACTGGAAAATTTCCATTTTCATCTGCAGAACCTTCTGACTCTTTG
GTCGACAAACCCCTAAGCTTGAGATTCTTTTCCAGCTTAACTCGCTTCTGATCTGCCTCC
TCAATCAGCTGTTGCAACTGAACAATCTCAGTGTCTTTATTCTGAATTTCAGATTGAAAC
TTCTTCACCATAACTTCATATGTTTTCAGAAGGCTTTGTTGTTCTTGGATCTCCGCAGCT
AGGCGGGAGTCCTGAGGAGAAGCACATACTGGTTTAGGGTTGTTCTCCCTGTAAAAATGC
TTGAGTTCAGATAGATTCTTTAACTCTGAGATTACAAGTTTATCAGCAGCTTGGATTTTA
TCAGGGTCATAGGGAGTATGAGCAGATTGGAGCTGGATATAAGCTGCCTTTAGGGAGGAG
ATGTTTGCAAAAATCTTGGACACCAAAGCTTCAACAGCTTCTGGATTCTGATTCATGGCC
TCTTCCATGGGTTGAGGATGGACCTTTTGGCTGTTATTCTCGCGGTGTGGGTTATCTTTC
ACCCCAGTAGGTAGCATAGCTACTACCAACAGAGGGGTAGTTTAATTGAATTCCACCAGA
AGAAACTACAATGCAACGAAAC
>ntig_653_consensus
CTGGACGTCATGTAAACAGTAAACGAGTTCTCTAAAACAATATGCTTTTATTTCGTCCTG
ACTCTTGAAGAAGCTGGTTAAAAACAAGATCCCCTCTTCTCCATCAATAGAACAGAGAAT
TAAACCATACAATGTGGTATAATGCCTTAACTGGTAACAAAAAGACGACAGACTACGAAA
TCAATCTCATTCTCCATCTAGATGAAGCACCTTTGACCATATACGTCAAGAAATGTTGGA
ATTTCAGATATCTATCTCTAAAGGGTTCTCCCTCCCAGAAAGTATTCAGCTATTATACCG

GCACGGAAGCATCCAAAAGCCAGAAATATGGTGACACACTCCAGGAACAAGAATATCTCT
CTTTTGGCATATAATAATACTTCATGAGATCGCTGCTTTGAACAAGATAAGAGTTAAATT
TAAATGAGATAACTGAAAAAGACTGCATGCACGTTTACCACTTTCTCATTCCAAGCAGCA
GAAGAATGCAACTGAAAAAACTAAACTGAGCTTTTCTTGTTTCTAAAGTTCTCCTTCAGC
AGTGCAAACTTTTTCTTACACTGATTTACAGTCTTTCCAGGAACAGCCGCTGCAACTCGC
TCCCAGCGCTGACTGGTCTCCTTAGGGAAGGTTTTAGAGCCTGAACCAATGCTCTTTCT
TGTACAGCAGACCACACGTCTTGATCAGAACTTGAGGAAACCCCATTTGCAGCAGACACA
TCCACAGGACTGTTTGAACTTGAAGACTCTTCTGGGATTTCCACCTTTGCAGCTGTTTTC
TGAGGCCCCTGGGGGATAGATAAACCTTCCACATCTTCCCTAGTTGTAAGAGGAGAAGCA
ATGGACTGTGCAGGTTTCCTCTTTTCAAGAAAAGAATCAAAAGCTTTTGCACCATCAGGC
TTCTGTAGGAGGACTGTTTTGGTTGCCTTCAGAATTTCTTCCACAGTTCTTCCTGTACCA
ATGTACTCTGAAATAACCTCCCACCTTCTAGATGTTCCTTTGGTATATTTTTGGATTCCT
TTTCTCAAGAGCTCAATCTCTTCCTTTCCCCAAGGCTTCTCCTTCTTCTCAAAGCTTTTT
AAATGGATACTTCCATTAGCCTCCGCAGAACCATTCTGCTGAAAATTCTTTTGTTCATCT
TGTTTCTTTCCCTCACATTCATCAACGTGCCCTAGTGCATTCCTTACAAATTTTGCTTGT
TCAAGGCCATCCTTGTTTTCCAGCTTATCACACAAACTTTTCAGCTTCTCAATATCAAGT
GACATACAGAGACTTTCCACATCCTCCTCAGAGAGATCAAGCAAATGCTGGGATACAACA
GATGCTGAAAGTGTTCTAAGACGAGTCCGCTCTTTGCGCAAGAGCTTCTTCTCTTTTTCC
TTCAGCTTCTTCTGTTGCAATGCAGCTTCAGCAGCTAGTTTTTCCTCCTCCTCTTTTCGG
CGCTTCTCCTCTTCTACAGCCCTAGCAGCTTCTTCCTCCTGCAACTTCTTCGCCAGAAAC
TTAGCTTCCTTTTTCCTTTGCTTCTCAGCTTTCTCCTCTTCCTTTCTCTTCAGTATTCTT
GGGTCTCGTCTGTATGCATTGTCAACAAGAGAACGCACTCGTGCATATTCTTCCTTCCTA
GCTTTTTCTGAAAGTTTAGCATTCTGCCTCTCCATCCATCTCCTATGGTCACGAGACTCA
GCCTGTTCAAGGTCAAACTCGTCTGCATGTGGGAACTCTCTCCAGCTTTTAAAGCTATAC
CAAAAATTGTAAAAACTATCAACTTCCTTCAATGGAGTACTCTCATCACCTAAAGATGGT
ATTGGTTGATTAACTGACCACCTTCCATTCCTCATAAAAGCTGGACCAAAGACCTTGAAA
AAATCTTGTGGGGAACAGTCAGATGGGATTTCATCATCAAACTCATCTGTGGAGTCATAT
ATCCTTCTCTTCAGAGGGTCAATCAAAACCTCATACGCTTCTTGGACAGCCTTAAAGTGA
CTCTCTATTTCATCCTTCTTTGCCTGTTTTGCAGCTTCAGTTTCCTCGGCAAGAAGCAGT
GCAGCCTGCTTGTCAGGATGGTATTTCAAAGCAGTCTCACGATAAGCTTTTCTTATCTGA
TCCTCTGTAGCAAGGTATCTCAAATGACTCAATCCCAGCAGTGCATAATGATCTTGCTGA
TTGCTTCCTGCACCAGACTTCTTTTTACCTTTGCCACTATAAGACTCATATTGCGGCAAA
GATGTCTGTTCCTTATCATTGGAGACTTTCTGATCATCAGCATCAGTATCTTCCTCACAT
CCGAGCAGTTTAAGTGCAGCAGCATGAAAAGAATGACCAG
>ntig_654_consensus
ATGGCTTAAAACACGATCCCCTCTTCTCTATCAATAAAACATAGAATTAAACCAATACAA
TAATATATTACGCCTTCTGATAACAAAAAATAAAAGATTACAAAATTAATCTCAATCTCC
ATCAATATGGAGTACCTTCTTCGGCCATATATCAAGAAAATTTGGATATCCTAATACCTG
TATCTAAAAGGTTCTCCATCACAAAAAGTTTCAGCTATAATACCTGTATGGGTAACAACA
CCAGACAGATTCCAGACGCTCTCTTTTACCATATAATAATCCTTCAAGAGATCACCGCCT
TAAACCAGATAATAATCAAACTTAAAAGCGATAACTGGAAAGATCTCTCTCATGTCTAAC
ACTTTCGTTTTCTAAGCAGCAGTAGGAGGCAACTGAAAAAACTAAGCCGCAGTTTTCTTA
TTTCTAAAGTTCTCCTTAAGCAGTGCAAACTTTTTCTTACACTGATTCACAGTCTTTCCA
GGAACAGCAGCTGCAACTCGCTCCCAGCGCTGACTGGTTTCTTTGGGGAAGGTTTTTAAA
GCCTGAACCAATGCCCTTTCTTGTACAGAAGACCACACATCTTGGTCGGAATTTGAAGAA
ACTCCATTTTGAGCAGACACATCCACAGGACTATTTGAACTTGAAGACTCTTCTGGGATA
ACCACCTTTGCAGCTGTATTTTGAGTCCCCTGAGGTGTCAATACCCCTTCCACATTTTCC
CTAGTTGTCAGGGGAGAAGTAATGGACTGTGCAGGTTTCCTCTTCTCAAGAAAAGAATCG
AAAGCTTTTAAATCGTCAGGCTTCTGGAGGAGGACTGTCTTGGTTGCCTTCAGAATTTCT
TCCACAGTTCTTCCTGTGCCAATGTACTCTGAAATAACCTCCCACCTTCTAGACGTTCCT
TTGGGATATTTCAGCATTCCTTTTCTCAACAGCTCAATCTCTTCCTTTCCCCAAGGCTTC
TCCTTCTTTTCAAAACGTTTTAAAGGGATACTTCCATTAGCCTCCACAGAACCATTCTGC
TGTAAATTCTTCCTCTCATCTTGTTTCTTACACTCACATTCATCAGCAATTCCATGTGCA
TTCCTAATAACTTTTGCTTGTTCCAGGCCTTCCTTGTTTTCCATCTTATCACACAAACTT
CTCAGCTTCTCAATATCAAGTGACATACACAGACTTTCCACATCCTCCTCAGAGAGATCA
AGCAACTGCTGGGATACAATGGATGCTGAAAGTGTTCTAAGACGAGTCCGCTCTTTGCGC
AAGAGCTTCTTCTCTTTTTCCTTCAGTTTCTTCTGTTGTAATGCAGCTTCAGCTGCTTGT
TTTTCCTCCTCCTCTTTTCGGCGTTTCTCCTCTTCAGCAGCCCTGGCAGCTTCTTCCTCC

TGCAACTTCTTTGCCAGAAATTTAGCTTCCTTTTTCCTTTGCTTCTCAGCTTTCTCCTCC
TCTTTTCTCTTTAGAATTCTAGGGTCTCGTTTGTATGCATTGTCAACCAGAGTGCGCACT
CGTGCATGTTCTTCCTTCCTAGCTTTTTCTGAAAGTTTAGCATTTTGCCTTTCCATCCAT
CTCTTATGATCACGAGACTCCGCCTGTTCAAGATCGAACTCGTCTGCATGTGGGAACTCT
CTCCAACTTTTAAAGCTATACCAAAAATTATAAAAATTATCGACCTCTTTTAGAGGAGTA
CTCTCATCACCTAAGGAGGGTACTGGTTGATTAACTGACCACCTTCCGTTCCTCATAAAA
GCTGGACCAAAGACCTTGAAAAAATCTTGTAGGGAACAGTCAGATGGGATTTCATCATCA
AACTCATCCGTGGAGTCATATATCCTTCTCTTCACAGGGTCAATCAAAACCTCATATGCC
TCTTGGACAGCCTTAAAGTGGCTTTCTATTTCATCTTTCTTTGTCTGTTTTGCAGCTTCA
GAGTCCTCAGCAAGAAGAAGTGCAGCCTGCTTGTCAGGATGGTATTTCAAAGCAGTCTCA
CGGTAAGCTTTTCTTATCTGATCCTCTGTGGCAAGGTATCTCAAATGACTCAATCCCAGC
AATGCATAATGATCTTGCTGATTGCTTCCTGTACCTGACTTCTTTTTACCTTTGCTACTA
TAAGACTCATATTGTGGCAAGGATGTCTGCTCCTTATCGTTGGAGACTTTCTGATCATCA
ACATCAGTATCTTCCTCACATCCAAGGAGTTTAAGTGCAGCAGCATGAAAAGCATGACCA
GCAGGTTCATATTTTGAAGCCTTTACAGGAAGACAATTTGAAGAAACAAAAATTGGCCGT
CCATCTACAATCTCTTCTGAGTAGGAAATAAGATGATAACTCGCATGGATTACCATAATG
CCTCAAAGCCCTCAGAGAACAGAAAGGAGGGCTATGCCAGTACCAAAATCCGAAAGGGTT
TTATGCTGCTTCAGAATCCTTATTTACAATATGAACCTTATGGACGTCAAATCTGCACAA
ATAAACGAAAGTGAAGCAAAGACGGTAGATAGATTTCTTCTTCTTCGACTTGTCCGTAC
AGCAACTTAGACTGAGAGTGAAGGAGAGAGACAGAAACTCAGGGAGAAGGAGTAACAGAG
GCAGATCGGAAGAGCACAACG
>ntig_655_consensus
CTGAGGAACATCTAGTGGCCCATGTATGGGGCGCCACCATACTGGTTGACGCCATGCTGG
CCCCTTCCAGACCCACCTTGTGCCATCTGCTGATTTGGATAACCACCCTGGTTCCCATAC
CCTGGTATCACCCCAGGAGTCACAGCCTGATTTGTATAAGCACCCTGCATTCCAGCACCC
GCACCAGCAGCAGAGCCTAGTCCTAGCAAAGCCTGCCCAAGTGCAGGGTTGATCCCCAAT
CCAGCACCTTGAGTCGCCAGTATAGCTGTCAACGCCTGCCCAATAGCCGGGTTCAATGCC
GGAGCTCCAGCTCCAACCCCAGGTCCAGAGGGTGCCATCAAGTGGCCAGGTCGTGCAGCC
GCGCCAACATAACCTGCATTGTCATTTCTCTGAAAATGTGAATTCTGGGCATTCTGGTGA
TGGCGGTGCAGAGATTTTCCTGGTTTCGGTCCATCAACAGCCTTCTGGCAATGCAAAACA
TGACCTTCAAAATTTTTGTGCGGCTCTTCCAAAGCCTTCTTTGCAGCTTCAAAGGTCCTA
TAAACAAACAAACAAAACCCCTTAGGCTTTCCTGTGACCTTATCCAGACCCAGCGGTCCT
TCCTCAATCTCCCCAAACTTCGAGAAAAACTCAAGCAACTTCTGTGGATCCAACTCCGCC
GAAACATTGCTAACAAAAATCTTCCTTTGAGTATACTCCGACACTGGCTGTGGCTGCTGC
TGCTGCTGTTGATGATGATGCTGCGTCATATTGGCAGCCGGAGTCGCCACAGGGCCAACA
GAGGCCAGCTGGCAAGACGTCATCCGACTCCCAATCTTCTTTTGTGGCTCCTTAAGTGCC
TTACGCGCCCCACTTCGCGTCTTGAAAAGAATGAAACCATAACCCTTGGATTTCCCAGAG
ACCTTATCACAAACAGCTTTGCAATCCTCAATCTCGCCGTACTGTTTGAACGCATCAATT
AAAGTCTCGGCCTTTGTGTCCCAACCAAGACCGTGCACAAATATCTTCCGGTGAACAGGA
TCTTCATTGGCTACTTCGCGAATCCGATTCGCCAAGTCAATATTCTTTTCAGCTGCTTCA
CGGAGCAGATTTACAAGCTGATCCTTGCTAAAAGGTTCTAGAAGCTTCTCTATCGGCTCA
TCATC
>ntig_656_consensus
CCCCTGCCAACCAAACTTTTTATTTGGTTCCTAAATTTTAAAACAATACAAATTCTCTGG
CCGTGAAAAGACAAAACCCCAGAGATATTCATTAAAGTATCCAATCTTACAATCACCATT
GATAACATGGAATTTCAGGAAACTAGATGAGAGATTTCACACCAAGTTGTTGCATTTCTC
TAAGCTTCCATACTGGGTAAAAAGCATCCTCTCCAAGATCTTCTGGATCTACTTGCTGCA
TTGCCCATGCATAGTACACTGTATGTATAGTATCTTTTCCTCCTTCTGTTATTGCACCTT
GTCTGTCGCGTACACAATAAATTTGCTGTGTTTGAAACGCTACAATGATTATAGGGGAAG
CTCCCATCATTTTAGTCTCTCTTACTTCCACATCTGATATATGCAGAATCCTGTTAACAA
AAAAAATGCCATGGCTCTGATAAGCAGTATGCTCTGCTTTACATCGTTCAATCACTTCAG
AGCTACAGTATTTCTTCAAAGATTCAATATCTCCCTTCATGTAAGCATTAAGGACTGGTC
TGATAGCTTCCTGAACTTCTGCCACAAAATCTGGTAAGGAGAAAGATGGATCTCGGCGCC
GTATTTCCTTAATCGATGCTGCAGCATCTGTTTCTTGGAAGATAGTCTCATTGATATCCT
GAATTTTGTGAACAATAGCACTATCACTAGTCTCCCATCTTTCCCGCATGTCCTCTGCAA
TCTCTTGACCTTTGGTCACAACTGGTTCACTTAGGCCACTTATGTGCTTGAACACAGGAT
AGCTTTGCATCTTCTGTTTGACTTTATTCCACATAGACTGCCTTCTAGGTGTAATAACGA
GATCAGTTCTTGCACTTCTTTCACCTTTCCATGAGGGAGGAGCAGTGTATTCCAGATGTT

TTCTCTTACTAGGATTACCACTTAACTCATCCTTTACAATGTCATATCCCTTCTTTGCTA
AGTCAACCACTTTTGCTTCCTTCAATTTTTGGAAGACCGTGGTAACTTTAGGGGAAGAGA
AACTCAACTTAAATTTCCCATAAAATGTTTCTGCATTGTTACTAGACCCTGAATTTTCAT
GTTTTTCTTCTCCAGATGGAGTGTTAAATCCATCTTTTACATCATCTCTGGGTCTAGAAG
TGTGTATGGAACCCTTCCAAAAAGTCTCTTTGACCTCCTCCGTTGCCGCTGAAATCTTCT
CCTTCACGTTGGCAGAAACCTTTTTGACACTAGATTCAGCCTCCGTCCACACACCATCCA
CATGTTTATAAAGCTGCTCAGTCGTCTGCTTTGTTCTGCTGACTTTCAGCTCATCTTTCA
CCCCTTTTAGTTCCTCTGCTTTGTCCTTCAGTTCCTTCACTGACTGATGAAATTCTGGGT
GTCTCTCAGCTTCGCCTTTAACTTTCTTCGAAAACTCATTAAACACACTGAATCCACGAT
TACTCGAACACCTATTTGAATAAAGTCGCCAGTGTCTCGTACTGGAACCCTGTTTAGACA
TTAACTGACAAAACAGAGGCTGCCTAGAAAGAAGCAAGTCTCTAACCAGCTTTCTACTCG
CCATTATTGACAGAAGACACAATTTTTGCGAGAAAGCAGAGTGATATTATTTTATTTGAT
GGAAATTTATCATTCGTTTTCTCCCAAAGAGGGAAGGGAATGAAGAAGGGGAGGAGGAGA
TTATGTAGTTTTCTTGAAAAACAGACGATGGAGAGACTGAGTGAGGATAGCCGCGAAC
>ntig_657_consensus
GCTTGCGGCTGCGTTTAGTCGGTCTCCGTTGTTTGTTTTCCAAGAGAATTATAGGCTTTC
TTCCTTTCCTCCTCTCTTCGGAAGGAAACGAATTATACATTTTCATAAAATAGAATAATT
CTAATTTCCTCCAGAAAATTTGTGGCTTTTGCTTACAATGGCTAGTAGAAAGCTGGTTCG
AGACTTTCTTCTCCCTAGATACCCTCTGTTTTGTCAGTTAACCGCTAAACAGGGTTCGAG
TACGAGACAGTTGTTGCTTTCTTCTATTAGGTATTCGAGTAATCGTGGATTCAATGTGTT
TAGCGAGTTTTCAAAGAAGGTTAAAGGCGAAGCTGAAAGAAACCCAGAATTTCATCAGTC
GGTTAAGGAGCTGAAGGAGAAAGCAGAAGAATTGAAGGGGGTGAAAGATGAGCTGAAAGT
GAGAGAACAAAGCAGACAACTGAGCAGCTATACAAACATATGGATGGTGTGTGGACGGAG
GCTGAATCTACTGTGAAAAAGGTTTCTGCCAACATGAAGGAGAAGATATCAGCTGCAACA
GAGGAGGTCAAAGAGACTTTTGGAAAGGGATCCACTGGCACTTCATCTAAATCCAGTGAT
GATGTTAAAGATGGATTTAAGGCTGCATCTGCGGAAGAAAAACATGAAAATTCAGGGTCT
GGCGATAATGCAGAAACATTTTATGGAAAATTTAAGTCAAGTTTCTCTTCCCCAAAAGTT
ACCATGGCCTTTCAAAAATTGAAGGAAGCAAAAGTAGTTGACTTAGCAAAGAAGGGATAT
GATGTTGTAAAGGATGAGCTAAGTGGTAATCCAAGTAAGAGAAAACATCTGGAATATACT
GCTCCTCCCTCATGGACAGGTGAAAGAAGTACAAGAACTGATCTGGTTACTGCACCTATG
AAGCAGTCTATGTGGAGTAAAGTGAAAGAGAAGTTGCAAAGGAATCCTGTATTTAAGCGC
ATAAGTGGTCTAAGTGAACCTGTTGTGACCAAAGGCCAAGAGATTGCAGAGGACATGTGG
GAAAGATGGGAGACTAGTGATAGTACCATTGTTCACAAAATCCAGGATATCAATGAGAGT
ATCTTCCAAGAAACAGATGCTGCAGCATCAATTAAGGAAATAAGACGACGAGATCCATCT
TTCTCTTTACCAGATTTTGTGGCAGAAGTTCAGGAGGCTATCAGACCAGTCCTTAACGCT
TACATGAAGGGGGATATTGAATCTTTGAAGAAGTATTGTAGCTCTGAAGTGATTGAAAGA
TGTAAAGCTGAGCATACTGCTTATCAGAGCCATGGCATATTTTTTGATAACAGGATTCTG
CACATATCAGATGTGGAGGTAAGAGAGACTAAAATGATGGGAGCTTCTCCTATAATTATT
GTAGCGTTTCAAACACAGCAAATTTATTGTGTACGTGATAGACAGGGTGCAATAACAGAA
GGAGGAAAGGATACGATACATACTGTGTACTATGCATGGGCAATGCAGCAAGTAGATCCA
GAAGAACTTGGAGAAGATGCCCTCTACCCAATATGGAAGCTTAGAGAAATGCAACAACTT
GGTGTCCAATCCCTGATCTAGTTTCCTGAAATTCCATCTTATAAATGGTGGTTGTCAGAT
TGGGTATTTTTCATTTTTCTGAATTTCTCTGGGTTTTTGTCTTTTCACTGCCAGGGAAAT
TATATTGACATTATTTTTCAAAATTATAAGAACCAAATAAAGAAATTGGTTAGCGGGCAA
ATTCATTTATCTTTTTTATTTATGTGTGACGGAAGGCCCCTGTCGAATTGGTTGAAAAAC
TTTAATCTGTTGACAAATGACAATGTGCTGCCCATTCAAG
>ntig_658_consensus
AAGAAATTTCAGTTCAGTTGTGAAAGAGTATTATGGTCTATTTGTTTGCAATTGAATGTT
AAGTCAGTTTCAGACCAAAAGGGATAAAGCAAGCCAAGTGCACATCCCACAAAAGTACTT
CCTATAAGTTTTTCTGGCATTGGGGGTATAAATTTTGTTTTGAAGAATAGTGGAGATATT
TTTCCTTTTTTTTTTTTAACAGATCCTCCCTGCCCCAAATCAAACATTAGAAAATTTCAA
AACAATGCAATTCCAAACCTTCATCTGAAAGGAAAAACATTTTCTTCTACTGCATTTTAC
CTTACACAGCATCCTGATAAAACATGTTAAATCTACATCTTTGCATCTTGTTTTTCGAGT
AAATTACACGTCACAAAGCTCAAACTAAATTAGGAAAGGGCCAGCAATCCATGCATGCAA
AAGGACTTGTGCTCTGCCAGTCGTTGCATCATCTTTGGGCTTGGTCTGAACCTGAAGCCC
CAGCCGGAGGAGCAGCTGGTACACTGCTTGTTGACCCATTCTCAACAGGTGGAGTTGTGC
CCCATTTTCCTTCAGATTCAAGGGGCTCAAAGACATGAACTCCACCATCCGATAGTCCCA
AAGCAAACTGATTTGCTTCTTGTGGATGTGCAGCAATCACAAGCGGGTTGACTTTGGAAC

TACTGACATTAGCAGGAAGATATGCAGATGGATTGATACGACAACGTAATCTGAGATTTG
AAGCGCTAAAAACACAGACAGTTGCATCCAAGAAGCAAGCATATACCAGCAGACTATCAC
ATGAGAATGTTGCATGACTGATTGGGGCAGAAGACTCCCGTGGGACCCACTGCTTCACAC
ATTCTAGTTTTGTTGTTTCAAAAATTGCAAGCTGAGTCTCATGTACAACCAGGAAGTGAA
TCTGGTCTTGGTGAAATTGTACACGTGTATCAGATAGTGCTGTTGGCGTTCTCCCAGCTG
GAATTTGCAAGAATCTGTTCTTTTGCCTTTCCCATCCATCGGAACTCCATACACAAAGCT
GAGCATCAGCTCCAGATGAAACTAGCACATTCAGTACATGAGAAAAGGCAAGGCCAGTTA
CTTTTTTGGAATGGCCTTTAAGCTTGCTTTTAACCTCATCTACACGAACATTATATATCT
GAATTGATGAATCATCCATGCCAATAGCAATAATATTGTTATCTTGAGGATGAAAGGCAA
GAAATGTTGCTGCAGGTGGTGGTGGCATGAAAGTTGCCATTGTCTTAAAAGTCATCATAT
TGAAGAGGGAAATTTTTCCACCAGAAGCAGACATTACATAAGAATCATTCTTGGACAAAG
CAAAGCAACAAACAGCCTCTTCAGGGTTTGTGTCCGAAACTTCATTGGTCATCAAAATGC
CACTTGGTGGTTGCCATAGTTGAGGTGGCACACTGGCAGTTGCCTTCCCAGTTGAATTTC
GGTCACTTCGCTGCCACTTCCAGAGCAGGTGAATAGCATTTGATGCTAATGCCAAAATAG
CATTCCCTGAATTCGTGAAGATTAACCTTGATATCTTGGTTGCCCTCCCATTTTCAGGAA
GTCTTAAAGTTCGACACTGAGATGGTTCACTGATTTCAGTTAGCTTCCAAATCTTTGATT
TGTCATTAGATTCTTCAGTTATTCTAGGCTTCACGTCTGCCAAGCTCCGGGCATCCCCAT
TCATTCCAGGAATGGCAACCACTGGCGCCCCTCTGTCTGCAAGTCCAGCACTAGTGGCGG
CTGCTGCTGCTGCAGCTGAGATTGGATTTACCACAGGCTTCAAGGCTTCAGATGTTCTTG
TCGCATCATAAGAAAGATTCTCAAATGTACGCAGTAACCGCATGCCATCTGAATTTGCCA
AGATCTTAATCCCATTCTCATTGGCAGAAACAGCCAAGAGACTGCCATCCTTGTTAAAGC
GGATACGTGGCCTAGCTGGAAGGCCTCCATCAGCATCGATGCCCATCAAAAGTTGTACAT
TGTCCATTTCCCAGAATTTAATGGAGAAATCATCACCAGCAGCCAAAAACCGATTTTTAG
TGGTGTCAAATTGCACAACACCCAGAGACCGTTTGCGGAATCCTAGATAGGTCCTTCTCA
CAGCTCCTTCACTTTCATTCCATTCAACAATATATGACTCCCCATCTTTACTTGTCCCAC
ATGAAAAGAGCCTTGTGCCATCAGCACTGTAAGCCATTGTTGTGCACCAACGGCCAGGAG
CATCATAATCAACACGAGATCCCAAATTGTCATACAACCAAGCTTTTATCTTTCCATCAA
GAGCTGTTGAAAAGATGAACTGAATATTTTCCTTATGATGAGGGCAGACAGAATATACAG
GAGCCTCGTGACCTTCAAATATATATTGTTTTGCACCAGTAGCAGCATCCCACACCTTTA
TGGTCTTGTCATCACCACAGGTAATCACACAAAGTTGCTTATTTGGGTGAGAGAAAGCAA
GATCATTAACTCCACCAACATGAGCATCAATCTCCAAGTGTTGTCGAACGTCATCTCCCC
CATGATATGAATATATTTGAACAATATGCCTTGAGTATGCAACTCCAAATAAAGAACCAT
CAGGGCTCCATATAACACGGTTAACAGACACACCAGGATATTTGACAAGAGCAGCCTGGA
GAGGCATTGAACATGCACTAAGATCCCAAACTTTAAAGTTTCTCAAAACCAGCCGTTCCC
TAGAACCGACATCCCACAATCCTATGTCCCCCACATTGGTTCCAACTAAAAGAAGTGTCT
GTTGAACAGGATGAAAATCCATGCTCATTGGAGATGATCCCTGATTCAACGTTCTTGTGA
CTGTCTTTGGCAAGTCTTCAGGTGCATTAAAAACCTGACTATGACTGTGACCTGGAAATG
TTACTGGCAAAACATTTACAGGTAGATTTACCTCATCAGATATTCCCATTGGCCGTGTTC
TTTTGGAAAGATGATCAGCTTCCCCCGGTGGGTAATCCACAGAAGGGTTAGTTGGAGGAG
TTCTGGGATGCTTTAAAGCAGCTGGGATAGATGAAGCACCAAGACCAATACCTCCTCCAG
AAACTGCTGGATGAGTTACTGTGGGGGGATTAGACGTCCAACCTGCAAGGGTTGTTGGAA
CTGCTGCTGGTGTAGGTTGAAATGGCCCATGTGC
>ntig_659_consensus
TACTCAAATCCAAAACATAATTAAATTTCTAAATGAATATGTTTAAAGAAACTTTCTAAA
ATGTTAATCCAATCAGCTTACATATATTGGGAAAAAGAACTCGAATCTTTGAAGATGCAT
AAATCATGCCACAGTAATTTTACTGCAAACATGACTTTGTATACCGAAGCTAACAGCCAA
AATCCTGATAGAAATGGAGGATCCTTGGCACTGAAGGACATAGCTGTCATTTAAAGACTC
AAAATTTCTACCCATACTCAACTGCTGTACAGACATAACCTACATAACCGAAGGCCAACG
AAAGCCCCTCGAAAAACTGATACCATAACCAGAAGAGCACGGCCACAATCACTGAACCGA
GTTATGCACACCATTACCATTGCTATACACACCATTTAATGATCCATTCGACGACTTAAT
ACCATTTCCATTTGGTTGGATTTTCCCCATTGGTTTTCCGTTACTGAATCCATTAGGCAA
TACCTTACTTGCATGGCCACACTTTAGGACTGTATCCAACCGCCCACATGCTGCTGCTAT
AAGTCCTAAGAAAACTGCCGAAGGTTTTCCTGGCCTGGACTTGAATTCAGGATGGAACTG
AACGCCAACAAAGTATGGATGACTAGGCAGCTCAAGGATTTCCATGCGCTGTCCACTTTC
ATCTCTGCCAACAAATGATAGACCGGCACTTTCAAGTTGTGACACCATATCAGGATTGAC
CTCATATCTATGTCGATGCCGCTCATCAACATATCTTGCATTGCCATACAATTTTGCAGA
GTTGCAGTCTGCAACCTTAAAATATGTCCTCCTTGATCCCAATCGCATAGTTCCACCCAT
ATGAGTTTTTGAACCCTCTGGCATAAATATGACACAAGGATTTGAAGTTTCAGGATCAAA

CTCTGTGCTGTTTGCGTCGTGCAAACCAAGGACAGAACGAGCAAACTCAATAACAGCAAT
TTGCATCCCCAGGCAAATGCCCAGGTATGGCACTTTTTTCTCACGAGCATACTTTGCAGC
AAGAATTTTTCCTTGGACTCCTCTATCACCAAACCCTCCAGGAACTAGCACGCCATCAGC
ACTCTTCAATAGATTCCAAGCAGCTTTATAAACATCAGGTGCCTCTTCAGCAGTAGCATC
TTCTAGGTCAGCAGCTGCAATCCATTCTACAATAAGTTTCCGATGGCAAGCAACAGAAGC
ATGCAATAGAGCCTTCAAAACAGACAGGTAAGCATCTGAAAGGCCAGTGTACTTCCCAAC
CATTGCAATTTTAACAGGATCATGCAACATATCATAAACTTTAGTCCTGGAAGTCCATTC
AACCAAATTCGGCTTCCTGGCAAAGCCTGAAAGGTTCAATCCCTTCAAGATAGCTTCATG
AGCCTTTTGATCTCTCAGTAATAATGGTACATGCCAGATGTTTGGAACATCATATAAAGT
AACAATGTTTTCCGCCGCTACATGGCAAAATTGAGAGAGTTTTGCCTTAACATTCTCTTC
AAGTGCCTTTGTACTGCGACAAGCTAGAAGATTTGGTGTCAACCCAAGTCCTCTAAGTAA
CTTGACACTATGCTGGGTAGGCTTTGTCTTTTGTTCACCAACAACATTGAGGACAGGGAC
AAGGCTGACATGAACCAAGCAGAAATTGCCAGGACCTACACGGTATGAGAACTGGCCAAG
TGCCTCAATAAATGGCATAGACTCAATGTCCCCTATGGTTCCACCCAATTCAATAACGCA
AACATCAGCTGGACCATCTTTTCCATCCACTGGAATCATTGATACATTCTCTATCCATTC
TTGAATTGCATCTGTAATGTGCGGAACTACCTGAACAGTTTTTCCTAGATAATCTCCCTT
TCTTTCCTTGTCAATAACAGACTGATATATTTTTCCCGTGGTTATATTATTGTCACGGGT
TAACTTGATGTTTAGAAACCGCTCATAATTACCAAGGTCCAGGTCCACCTCGCCACCATC
ATCTAATACAAAAACCTCCCCATGCTCAAAAGGGGACATTGTCCCTGCATCCATGTTCAA
GTAAGGATCGATTTTAATAGAAGTAACACGAAGACCACATTCTTTAAGAAGAAGACCGAT
GCTACTGGCTGTAACACCTTTGCCAAGCCCACTAACAACACCACCAGTCACTAAAACGTA
CTTCATTATAAATAATGGGGAAGTGTCTTGTACACAATGACGGTCCTTACAAAGTTCAAA
TCCCCTCTGCTTCTTCTCCCCCCTTGCTTGCTGGTGGTTTTTCTTAGTAATGCCACACAC
ACAAAAGCAAAGGCTTCCTTACT
>ntig_660_consensus
TTTTTTTTTTGTTAAAATTCTAGTGCTTATATATATATATATATATTAGAAAAAAAAGTTCC
TGTGAAACGGCCCAAAATAAAAATGAAAAATAATACCATACATCTAACACATGCACAAGC
TGAACTCTCTGTTACGTACCAAATATATAACACAAATATTAAAAATTCTCAAGCCAGAGG
GACACAATGTACCACAATACAAGTGTGAAGAAGTCAATGCAGACAGAAGCTAAAGCCAAG
GAAAAGAAGACAAGTCGTGGAAGCTGTAAGATAGAACACCAATTTCACTTCAAGACATTG
TACAAATAGGCAGCAATGCCTAAGATAACACACAAGAGGATGATATCAATGCAAAAGTTG
CGGCTGGACCTCAGCTGGTTTACAGTATCCTTGAGCCTCACATTTGTACTTTTCAGGTCA
GAGGTAGCCCTGTCAACCTTTTCATCTATTTCATCCATCAGAGGCACTTGTCTATCCACT
TCCTCATTCATGTCATGGGCCATGTTTTTCAACGTGTCCAATCCTTCTGCTATTACTTCT
AAACCTTGATCCTGTTTCATCCTTCGCATTTCGTACTCTTTTCTGAACTGGTTAGACTCT
TCCGTTTCTTGAAAGTACTCGTTATCGAATTTCCCATCTGAATATGAATCAATTTTGATT
CCTGTATACGAGGCCGAAGTTGCCCCACCATCACTTTGTTTAGCTGCAGTTGTTGATCCA
TCAGGTATCGCTTCAATCCTATCTTTCAGTGCAGCAACTAAATCACTTCTAGCCTCTAGC
TCTTCCTTTGAAAGCCCTTTGACCTTTTTAAAAGCAAGTCTCTGTAACTTCGGGACTTCT
TCAAGCAATTTAGCTTTCGTTCGTCGAATTTCCGCGTTCATGGCTACGGCTGTCGCCCGA
TTTTTCTCCGTTGCAACAGCCTCTGATTTCTGAAGAGCAGCATCAAGGTCAGCCTCTACA
ACACCGTATAAGCGAGCGAAAGCGTCATCGCCGGCTACGTTGTTGTCTTTCTGCTTATCG
ACATCGTATTTTTCATATTTCTTGCAAATCGCATCGACTCTGGTGATCAAATCGATCACA
GTCATCTTTTGCTTTTGAGTATAGGCGTGAGTTAGGGTTTCTGAGTTCACTAAGTTGAAG
AGAAAGCAGAAGACGACGACGACGACGACGA
>ntig_661_consensus
CTCTCACTCTGAGATTATATTTACTCAATTATAGAAATAGAACATACACCGATCATATCG
TATGTATTAATTTATTTATGCAGACAATTTTCTCCGTTATGAATATATTAGAGGTGCAAT
TATCATTATACTTTTGTGTTTGGCTAGACATCTGTCCCTTTCTTCTATCAACAAACAAGG
GAAACATCATATAGAACAGCCGGACAACTGGTTGGTATTTAATTCTAACCGAACAAAAAT
ATATAAAACAATCCCCAAGTTTTCAATTCAAGCTTTCTCGTGAACAGACGAAGGGATACT
CGGAAAAACTATTTAACAGAACAAGACAACGATACATTACCCTGCCAAACTGCAGAAGCA
GGTCAAACTCTTAATATTGTCTTCAAAACAACTTTATTTCAACTCAGCTGTCTCTACATA
AGATGCAAGCAACACACTCCCATCAAGCAGGCATATAAGATTGCCAATCCGCTTAGTGGC
TCTTTGACATGTGAACAGCCAGATCAACCACACGTGTGCTGTAGCCCCACTCATTGTCAT
ACCATGAGACAAGCTTCACAAAGTTCTTGCTCAGAGCGATACCAGCCTTGGCATCAAAGA
TGCTTGACCTGCTATCACCAACAAAGTCAGTGGAAACCACATCCTCTTCAGTGTATCCCA
AGATTCCCTTTAATTTTCCCTCAGAAGCCGCCTTGATAACAGCTTTAATTTCATCGTAAG

AAGCTTCCTTCTCCAATCTCACTGTGAGATCAACCACCGAGACATCGACAGTGGGCACAC
GGAAAGCCATTCCAGTCAATTTTCCATTCAGCGAAGGCAAAACCTTTCCCACAGCCTTTG
CAGCCCCAGTACTGCTAGGAATAATGTTGAATGAAGCAGCCCTGCCACCTCTCCAGTCCT
TCATTGATGGTCCATCAACAGTTTTTTGTGTAGCTGTGATGGAGTGGACAGTGGTCATCA
AACCCTCAATGATGCCAAAATTGTCATGAATAACCTTTGCTAGGGGAGCAAGGCAGTTGG
TCGTGCAGCTAGCATTGGACACAATGTCAAGCTCTGGCTTGTATTCATGCTCATTAACAC
CCACAACAAACATAGGAGCATCCTTGCTTGGGGCTGAGATGATAACTTTCTTAGCACCAC
CCTTCAAATGGGCAGCAGCCTTGTCCTTGTCAGTAAAGACTCCAGTTGACTCCACAATGT
AGTCGGCTCCAGTCTCACCCCAAGGGATGTCTTCGGGGTTCCTAATCCCGAACACAGTGA
CGGGCTTCTCACCAAAGAGAAGAGTCTTGTCATCCTTCACCTTCAGTTCATGATGCTTCC
ATTGACCGTGAACAGTGTCATACTTGAACATATATGTCATGTAATCAGTGCTGATGAATG
GATCATTAACAGCAACGAGCTCAACATCATCCCTCTGCAAGATAACTCTTGCAACCAGCC
TTCCTATCCTTCCAAAACCGTTGATTCCGATCTTGACCTTTCCCATGATTCTAGAGAGCT
GAAGAAAGATGCACTAGTGACAAAGAGGAAATCGTTCAGTAGTAGTGGGATATGGGAAAC
TGAGTTTTCATTATAAAGGAAGAGAATGGAGACCGGGACGACCGGTCAGGTGTAAAAATA
TACAATGCCGCGCTTGTTGTCCACCGTCTGATGT
>ntig_662_consensus
CTGTAAAGAGATCCCCAAGTCTTGTTAGCCTTTTTCCAAACACTTTGCCCCTTCAACTGA
TGATCGCATTTGATTTTCCTGTTGACCGCCTGTAGTTTCTAGTAAGGCCAAATTCATCAT
TAACTTCAAACTCTCTCATTTCCCATTTCAAAATTCAATCCCACTTTACCATTTCCCATT
CAACACAATGTAACCCACAATGATTCTTTCATTTATAAACCCAGAAACGAGCTCATCCCC
CACAAAGAAGTGAAAAAATTCAAGAGTAATTCTAGGTGTACAAACAATTCTACTAACTGA
TTCGACCGTGCCTGAATAAATATGATTGAATAATATTGAAGTGATAATAATAGACAATCA
CCCCAGTCAATCACATATTGTCTAGACATTTTGTATGTGTAATCCCTCCAAAATTAATCA
ACAGAAAGAAATAATCAAATCAGTCACCAAGCAAGCTGTTAGATACCTGCAAATTATACT
GGGTATATATATTTTACACGTGAAATCGTCAAATGGAAAAAAAGAAAAAAGAAAAAAAAG
ATTGTATAATTTTATTTTCAAAGCGTACAGCCACAGAACAAGAAGAAGTAACAAAGCATG
GAGACTAAAAGAAAAGACTCGAGCACAGTAACAAAGGTCCACAACAAGTCGTCAATGGAG
AAATCAAACCATCTGAAACTCTGCTGCAAATTCCACCCACTTCTGAAATAAGAAAAATCG
AACTCGGGCCAGCGGAAAAACGACGCTGAAAAGGGAAATTCGAACTCCGGCAACCGGAAA
TATGAAGTCGACCACCTTGC
>ntig_663_consensus
AGGGGAAGAAGTGAAAAAATTTAGATTGAGCCCAGAAGGTTTTCTTATGTGGTACCTAGT
GTAATATCATATACAAAGTGTTGGGAGAAAATTACAAGGAGAAGACAATCTATGCAATGT
AATCATTACCATGATCCCTATCTGCTGATACATTTACAGTTTCATCTGTATCTTCCCAGT
CCCCAATTCCAAATCCGATAGAGCTAGTTCGGTCATTTGTGAAGTGAAAAGTTCCTCCAA
TATGATTAGCCGATGCAGCTGCCTGCATTGCGTCTATGTACAATATTTTTACCAAATTTC
TGAACCTACGCCGAAACGTTGGTACCCTGGACATGGAACCTCTTCTAACAATGGCTTGCA
ACTGTGCTTTCCGGACATTGTCACTGGATGGGAATCCACAGGTATCCCAGTCCTCAGGTT
TATCATTTTTGCATTCCATAATAATTTTTCTAAGTGAAATTTCTTCATTCACATTTAACT
GGAGATTCTTCATTTGCTCTTTCATTATCAAGAGTGGTGCAATGAACCAGTCAAACAACT
TATCTCTTGGACTGTTCAACCTTGTCAATTCCAAATCATCAGTGATCACCAACCCAGAAG
AGTCAGATTTTGCGGATGCTAGCAGACACTGCAATATGGACCAAGCAGGTAGTTTAATGC
CCAAC
>ntig_664_consensus
GTTCAACCTTGTCAATTCCAAATCATCAGTGATCACCAACCCAGAAGAGTCAGATTTTGC
GGATGCTAGCAGACACTGCAATATGGACCAAGCAGGTAGTTTAATGCCCAACTTCTTACA
ATTTCCCTTCACAATGCATTCCTCAATATCCTTAATTTTTATAAGACCTTCACGAAGGAT
TATCCTACCATTCACTTCACACGATTTAAACAGCCAGTCCCACATCTGCACAGGTTTATA
TTGTTGGACTGCCCGCTTCAGAGTTCTTGATCTTTCTGAGATGAGTTTTGCATTGAGAGA
ACCTTCCCTTCGAATGCTTAGATCAGTGTCACTGTCACTAAGCTTTTTCATCCCACAGCT
AGTGCGCATGTTCCTACGATATCTGGGCCTGGGCAAGCATGATCCTTCACTCAGGTAGAG
TAAATCATTTACGTATTCATCGAATAGGGAAACCACTGCTGTTGGACAATAACTCCAGCA
TAAAGTCCAAGAAAGAAGCTGGAGATTATAGCAGCTATCACAGCTCCCACAACAGCCAGA
GGCCATAAGAGAATTGCAAGACCAGCAAATGGGACACAAACGGTCTCCAAGAATGGTCCT
TCTCTGCCAATCAAGTCCTCCAATAGCCGCTTCCATCCTTTAAACAACATGTAAGGACTT
TTCCACAAAGCAACAACTGTAATTAAAGGTCCATCCACCAGCACTCCAACTAGGCTTACC
AAAATACAACTTGGCAATTTCTTTAACTTTATGTCCATGGGCATTTCATCCTCAGGCACT

TTCTCATTTAGCTCGTCCATGTAGGAGAAGTATGAATGGAAGCAAAAGTCTATAAAATCT
TGCACTGCTGTGCAGCTTGCTTTGATAGTGGGTAAACAACCATCAACAAAGCAGTGGTAG
AATTTGTCAGTAACATTCTCTCCAACAACCTCAAAAGTTGCAAACAGGGGAGTGAAAAAT
CCAAAGCTTATTCCACCTAATAGACTTCCAATAATTCCAATAATTGGCCAAAGAATCAGA
GGCAGCGGCAACACTATGAGCACCACAATCTTTAGAACAGGTCCAAGCCTCTTGGTTCT
>ntig_665_consensus
ATTTAATCTGATCAAAACGTGCATCAGCTGGGGAGAAAATGGAGTTGTTAATTAGCAAGT
TTGGCTGCTCAGGTATGTTGCCTCCCGTCATAACCACTTGGCTGTAGAAGCTATCATCCA
TCTGAAATTGAAATTTCTGCTCCTGTTGATGCTGATGCTGAGTTTGTTCATCAATAACAT
TAAGCCCCACAGGCACGTTAAGACCCCCAGTATTCAGGTTCTTGTTCCGCNNATCATAGA
AAGACATCAGATCAGAAATCATTTTTTGCCCATCATCTGAGAGTCCAAGTCCGGACACAT
TAAAACGTGAATGAGTCTGGTTCACCAGTGGAGTCTGGAGCTTCTGTTGAGTTGGTTGAG
GTAATTGAGCTTGCTGAGCAACAGGTAAAGAGAACACAGAAGGTTTGTCGTCGTCCATTT
GAAAATTTGTCACTCCAAACCCTTCAGAAGAATTGCCACGATATGGGCAAGTCATCTGGT
GGTTGTTCCTCGAAAGCCTGTCGAGGAATCCAAACCGGTAGTCACTATTCGGGCACTGTG
GGTTCTCACAGGTATACCTCTTCTGATCCATCATCATGTGAGACTCCTCTGCCAGCTGCT
TCCTCTTCTGTAAGAAATCTGAGTTGGTTTCAACAAGCTCGCCCTTAATTTGAGGAGCCA
AAGGTTGTGTTATCATAAGCCTCTCCCTCGGTCCCACCATCCCGATATTGAAGAAATTCA
CATCACGAGGCTTGACCTCCTCCACTTCAACATTTGGATCATCATCCACCCCCCCTTCAA
CATCATAGTCACTGGTATCACTGATAACAAATGAACCACTCCCACCAGCAGATGCAGGAG
GGCAGCTATCAGGATAAAGTTTTCTTGACAACAACTCTTCTTGATTAATGATTGCCAACC
AAGTGGCGCTCTCCTTGGCAGTCATCTTATCTTGCAAGCACTTAGATTGACGAACAAGCT
TTCTGATCTTAGCAATATCTGGTGATATGTGTTTGATGACTGCCGTGAGAACACTTACTT
TCCAAGCCTTCTTCAGATCATGAGGCTTCTTGTATGGGGGAGCACCTTGATCCTTGGTTA
GACCCACTTGAGGCCACCACTCTTCATTTCCAGT
>ntig_666_consensus
GCCCAGAAATTTAATTTACTTTCTTTCAACTTTGCTGTTACTAATCAAACTATAGTTTTG
GAAAGTTAAATACATGCTTCGGAAATATAAGCATCAAGAACCTGACCTGAATGTATTAGG
TACAGACTTTACAACAGAGAAACGATATTCTATCAATGCGCTGAAATTGTTTAGTCCATT
GTCACACCACTGCATTCACAAATTACCTGGAGTTGTCGGCTGTGCTAGCACTTATAATGC
TCACATTTTGACATACTTATCAACATAGCCCGTGACAAGTTTGGTGAGTTTCCGCTGTGA
TGAGTCAATGTATTGATCTATTTTTGCCTGCGACTTGGGTATCTGATGACTCTTCATGGC
TCCCGAGACTTTATCAACAGTCTTCTTGACAATGGTCTTGAATGCTTCCTTACTCATATT
ACCCTGGCGCCAAGACGGTTTCAAAACCTCCTTTACAAAATCTGCAAGGGCAACCTTGAA
AAGCTTCATCGACCGGGAGTCTTTACTCTTCTT
>ntig_667_consensus
AAGAAGAGTAAAGACTCCCGGTCAATGAAGCTTTTCAAGGTTGCCCTTGCAGATTTTGTA
AAGGAGGTTTTGAAACCATCTTGGCGCCAGGGTAATATGAGTAAGGAAGCATTCAAGACC
ATTGTCAAGAAGACTGTTGATAAAGTCTCGGGAGCCATGAAGAGTCATCAGATACCCAAG
TCACAGGCAAAAATAGATCAATACATTGACTCATCACAGCGGAAACTCACCAAACTTGTC
ACGGGCTATGTTGATAAGTATGTCAAAATGTGAGTATTATAAGTGCTATCACAGCCGACA
ACTCCGGGTAATTTGTGAATGCAGTGGTG
>ntig_668_consensus
GATCAAAAGTGTTACAGTTCCAAATTTGGTTCCATTAGCTCGGTTCACAAATCACAATAA
AGATCTGAGATTCTGCCACTTCTAAACTCACTGAAAACTATAATTAAAAGAAAAGGCAGA
GAAGCTTCGCTAAAATGGGCTCAGGTGTAACCAGTTAGTAAAGATTGACAATCATCTTTA
ACAGATTTGTGCTGCAAATTGTGTACTAAGGAAAACTAAACAAATTCAAGTACATAAAAC
AAGCAACAATATTAAGTTTTCACCAAATGGGTGCAAGTAAAATTTTCAATAACAGTTCTA
GTACCAACTTGCAGCGCTGAAAACAGTAACACAGACAACCTTGGAGCAATTTCTTTACAC
AATCCTCTCCAACTCTCTTGCATAACTGAGCGTCTGGTTAATGAGTTGCAGTGACGGGAT
CTCCTTGCAAGGTGCAGATTCTTTTGCCTTCTGGAAGAATACGGATCCATTCTTTACCTC
CCATTCAGGATGCTC
>ntig_669_consensus
TTACCTGTGCTGCTGATGGGCAGATTCGTCATGCTCAAATTCTGGAAGGCGGTGGAGTGG
AGACTACTTTGCTTGCCAAGCATGAAGGACGGGCACATAAATTGGCTATTGAACCTGGAA
GTCCTCACATTCTTTATACTTGTGGTGAAGATGGACTGGTGCAACATATTGATTTGAGAA
CTGGGGTTGCGACTGAACTTTTCACTTGCTGTCCAATTGATGGTAGAAGAAGTTACATGC
CAGTAGTAAATCTAAATGCAATTGCAATTGACCCAAGAAATCCAAATCTCTTTGCGGTTG

CTGGATCGGATGAGTATACTCGAATTTATGATATTCGCAAGTATAAGTGGGATGGTTCTA
CAGATTTTGGTCAACCTTCTGATTACTTTTGCCCTCCAAATTTGGTTGGATATGGGATTG
GGGGCTAACCTGGTACGTTCCTCTCCATTGTCGACTGGGAGTGATGCTAGTGAAATAGCA
TCCGGTCATCCCTCATCTCAAACTATGGATGTTGGTGATATAGTTACACCACAAGTTTAT
AAGGGCCACAGAAATTGTGTGACAGTAAAAGGTGTGAACTTCTTTGGACCAAAATGCGAA
TATGTGGTCAGTGGGTCCGACTGTGGTCGAATATTCATTTGGAAGAAAAAGGGTGGAGAG
CTCATTCGTGTCATGGAAGCAGATAAGCATGTGGTAAACTGTATTGAGCCACATCCTTAC
TCCACAGTGCTTGCTAGTAGTGGAATTGAAAGTGACGTCAAGATATGGACCCCAGAGGCT
ATTGAGAGAGCTATTTTACCCACAAAGATTGAACAGGTATGCTTTGTCTATAAATGTTAT
TATTGCTGGTCAAAATTTCATTAATATTTTCATTATACTTGTTTGAGGGTTAACTAAAAT
GGTTAAAGTTATTTCAAGACACCAAATCTCTATTGCTTCAAGGGCTCATATACCTGACAT
TGAATCTTAATGCATATCCTCTTTCATCCTTCCCTCTTCCTGCCATAAGCATAATTACCA
AAACCAATAGGAATTAGTAGTTTTGTAGCTGGCAAAGGACATTAACTACGTAAAAATTGT
CATTTTGTTGTTTAAGATCTTTGATATTTAGTAATAGAGTAAATTGCACTGATGTAGGAT
TAAAGTTCAAAATTTTGACATTTGGTGTAGGACTAAAAAATAATCACGATGGCTCTTTAG
AACTTGAGCTGAAGCCTTAGCTTTTAGTGCTTAAGATCTTTGATATGTAGAAAGATTTGA
CCCATCTGTTCAACTTGTGTCCCTGTTATTGGAAAAAAAGAAAAAAAAGAAAAAAAAAAAA
AAAAAAAAAGAAACTCTAAAATACTTGAATGGGTGAATAGGTGGAAAACTAATAGACTAT
AGGACTTGAATGGTTCATTTAAGCAGGTAATGTGCTGTTTATGAGTAAGTATCTGTGGAA
TGGACTACAACACTGCTTTTATAACTGCAGCAGAGGAGCTAATTTCTCTGCTGTGACTTG
TTTGAAAGGGTCTGTTACCTGGTCGTACTCATTGGATTGCACTTGGTGATGATGACGACG
ACGATGATTATTATTTTTATGATATTGATGATGACGACGATGACAATAATGACGAGGATG
GAGATGATGATGATGAAGACGATGACGGGGATCACAGTGATGATGATCATGTTAATGATG
ATGATGATGATGATGATGATGATAATAATTATTATGATGATGATTACAGCATGGAAGATG
TCAATGATGTAGATGATGATGATGAATGGTGTGATGACGATGATGATGGAAGTGCTAAAG
TTGATGGCTAAAAAGATTCTGATAATTTTGCTGGAGGTTATGATAATGCTAATAATGATA
ATTTGTGATTTTGCACTTTGTGTCCAGTTCAAACCGAAGGCTAGGGGCTGGATGCACCGT
GTATTTTCACCGGAGGACCTGATGATGCAATTATTTTCACTGCAAAGGCGAAGGACCGAT
CCAGATCCAGAGCACATTGGCGAAAACACAGCTTCAGGTCAGGAACTTTTGGAGCTTATA
TTGACATTCAGCGCCAACAGTGATGGATCTTCAGAGGATGGAGGAAATGAGCCCGAGCCA
TGAAGATTTG
>ntig_670_consensus
ATCCATAATTTCAAAACACTCAATCATATAATAACATATCATCACTAGTAAACAAATTAA
TACTCTTTGATAGTATACATAGTTTTATTGAATGAATATCCATGTGCAAAAATCTTATCA
GGTGGGCATACAAATTATATAATCTTCATATTCTTCCCAAGACATTTTCACGTTTCCACA
AGCATTAGGTAGCCGACGGCATTACCAGGGCCATTGCGGTTGACTCAAAAAAACTCAGCA
GTGCCTGCACTAGAACCAATAGGTACAGAAAAGCGAGGACCTAATGTTTGCACTGTGAAT
GTTCAATATTATTTTCGACCAAGAATAGACCACCAACCCACAATAGTTTTTATGATCTTG
TTGTCACAAGCTTTGTTTGTCACAAAAGGGAACTTGATCACATCTAATTGACTAGAATTT
CTCTGAATCTTGGCGAGGTTTTGTGACATCCTCACCACAAACAGGAAACATATTAGAATT
TACCCTTACAATAAAATTTTATAAACTTACATATCTGGAAGAAAATTAAAAAGTGGTGTA
CTGATATAAACTGATCTCAGTAGGACATTTTTCTTCCTCGAACAAGTATCATTCATCATA
ATTGTGTTCATGGAAACATATTCAGCCAAAACTTATTGTACAAGTTTGTAAATTACTGTG
CTATTCTAGTTAACATGTAAGTAACCCCAGGAACAAACCTCACTTCTTAAGGAACATTTC
TCTGAAATCATTATTTGACTTCGGCTTCTCATCTTGCCCTTCCCCAGGTTTTGGATTTGT
CAAAGGCACACCAAGTGGTCTGACATTCCTTGGCAATGCAAACGTGTTCCTCCCCTTCAG
CTGAATGTTATCACTACCTCTGTTAGCAGCAGGGTGCAGGCCTTTAGACTCCCTGAAAGT
CTGATTGCTACTTTGCGCATGTTCACCAGAGGCGCCATGATCAGCAGCACCCTTTCTCTT
TTGCTTCGGATTTGACCGTGCAATGCTTACCTTTTTCCCAAGAAACATTTGCTTGTTCTT
TTGTAGAGCTGCAGTGAGGTGTGCATCATCGGCAAAATCTACATATGCCAAGCCCCTTGA
CTTTCCAGTGAACTTGTCCTGCAGAACTCGAACAGAGACAACCCCACCAACATCGCTTAA
GAATTTTTTCAGATCTTCAGAAGTTGCCCTGAGGTTAAGGTTTGATACAAATGCAGTACA
CTCGTCGGTATATACTTTGGCTCTTCCACTGGTGGAGTCTTTGATGTGCTGGTCATCTGG
ATGCTCTTCAAAGTTGGGTTTTGGTTCTTCTACTTCATTTTCTTCAGCTAAGTTCTGTGC
TTGTCCCTTTTCTTTCTCATGCACTTTCTTTGGCTTTTGGGCATTATTTTTCTGTCGTTT
AGCTGGANNNNNNNNNNNNNNNNNNNNNNNNNNNNNNNTTCAGGAACAGATTTGGCTTCCTG
CTGTAACCTAAATAATTGCAGCTCTTCCAAGCGAGGTGTAACCTTCTGTGTAGCATGGTC
AAAATCTTCAAGTGTTCCATATTCCCTCTCAAAACGCAACCAGTTGTGGCATACATCCTC

TGAACCTGTCCCACTAAATCTTTTACTGTAACATCTCCTGTAGATGGACCTAGCTTCATT
TAAATGACCCAATTCAATTTCCATATTTATATAACCTTGCCAAGCTTCCAACAATGACCC
ACAGATCTTAACCAGGCTCTCCCAAACAGCACGAGCTGCAGCTTGATCTTTTCCCAAATT
TTGCTCCAAACGAGCCCAATAAGCATACAAATGCAGCAAACCATCCGTATTCTTCATCTG
TTGAGATAGGTAATCTGATGCACGCTGAAAGGTTTCCCTTATCAATGAAAAATTCAATGC
ATCATCTTCTACTCCAGGCAAAAGAATTCTTCGTCTCAACCCATCCACTCGAGTGAGGAA
TAAGTTTAGGTACTCCTCAATGGTTGAAAAAGTGCATAGAAGGGACTTCTCAAAGACAAT
AGATATCTCTTTCTCAGGAGCACGACAACGTTCCAAAGCAAGCAAATATTGAACCCACAG
ATCACCAACCCAGGGGCAATTTTTCGTAGCCCTGGAATGAACATCCCGCACAATATTTCC
CATCTTCAAGGTTTTATCCAAATAGTGAGTATAACCAAGCCAGAGATCGCTTGATACAGG
AAACTCAGTAATAGCCCGTTCATAGAGAAGTTGAACACGAGCTGGATCTCCAGAAGATTG
TTCAAACTTCAAGTAGATCATTAATTGTTGATATTTTTCAGAGTCAGATACATCCTGCTT
TGCAATCTGTTCTTCAAAATCAGCTCGAGCATTATACATCTCCAATGCCTTTTGGTATGC
TGAGGCAACAGAAGAAGGAATCCCACCTAAATCACTTGATTCAACATCTAGAGCAGTTCC
AAGTTCCACCTCCCAAGACTTATATGCAAGAAGGGTTGACTGTAAGTTAGCAAGGGGAAC
AGACAGCTGGCGGTGAAATATACTTCGGATGCGCTGAACCTGTTTTTCTTTCTCCTTAAT
GTTGGTCTCATCAATGGTATGGAGAATGGCTTGCTCAAATTCCCTGTATGCTTCCCATAT
TTTACTGCCTTCAGCAACATGCAAACCAGCAGCAGTTATAGCACGCTCAAACAGATGTCT
TGCCTTTGCAATGCCATCAAGTGCAAATTCACGTACAGATGGATCATGCTCTTGCACAAA
TCTCAAGTAGTCACACCAAAGTGAGACAGACAGATAATCAGAAACTCCTCTTTCATAAAT
CTTCTCAATTCCCAAAAGAGCCTCATGTCCAGTTGATATAGAAGCTTCATCTCGAGCCCA
CTCTTGCCACATGACAGGAGTCAGTGGAAAAATCTCACTCATAGCTTCTCTTGCTTGTCT
CAGCTTTTCAATCTCACCCATCTTCCTCAAAACCCTAATGTATTGAACATGGGCTTCATA
GTTGGAGGGATTACTAACGAGCTGGGATTCGATATTTTGAAGCTCAATTTTTTGCTGTGC
TTCATCTTCTGAGTCCGATTCCGAGTCACTTGACTCAATCGAGTCTGGAGAGTTAGGCTC
TGTTGCTTCTGGGTTTTCATTAGTATCCACCATATCCGTGTCTGTGTCTTCTTCTTCTTC
TCGGTTTCTTGAAGCTAGGATTTGATCTTCTGGTTCCATTATTTTGCTCTTGCCCTTGCT
CTATGTTCCTCTCAAGCACACCGGGAAGGATCGCGTTTATTGCACTGCCGCTTCCACTTT
TTGAAGTCCCAAGTTTCCGAAGTTTCCTGTCACCCCCAAAATACT
>ntig_671_consensus
GGGATATATTTAGTAAGCAGATGTTCTACACATGGCTTTGCAATATTCATACATCATTTT
TAACTGCTATTATGTATTACAATAAGAAGTACACAACACAAATTATCAGCTGACTAATGA
TAAATCCACATATATATACAAGCATTATTCTTACAAGTTTGAATTGGTTGCCAGGACTTC
TTGATGTGAGCAGCAGTTGGACATAGTGTACTTGTCTCTTTATTACTTCTTCCACTGGGG
TACCCTTTGAACCACAACCTTGCCAGAGTTCTCTTTATTTCCCACAATAGTTTGACAAGC
CAGTCTCCAAGATTCAGGCTTCTTTTTCAGATAGCGGAGTTCAGTATTTGTTCTCTCATT
CAAAAGATCTTTCCCATCAATTATCTCCACAATGCAAGTTCCACAGCTTCCACTACCTCC
ACAGTTCATTACTTTCCCATACGTGGCGTAAAGCTCGATCTTGTTATCCAACATGATGTT
CCTCATCAGATTCTCTCCACTTATCGCTTTAGCTCTGTCCACCGGGTACGATCCATCACT
TCCCGCTTTCGGACCAATGAATTCTAGCTCGATTTCAGGCTTTTCCGGTAAACTCGGCGA
TGATTCCGGTGAGTTCACTGCAAATGACACAGAATTGGCTCTCCTTTTCACCGTAAAAGT
AGTCGTAATGTTGCCTCTCAAGTACAATTCTGGTGGCTGTTGAGATACTGATATGGCGGT
GAAGTTGAGCAGAGAAGCCATCGTAGTTCCGTCAGGATTTCCTTTTATTTTTTATTCAAC
GGGTGGTCACGGCATGGTCCGGTTCGATTCTTCTATAGGATTCTACTAAGGCCAAAGAAC
TTATT
>ntig_672_consensus
CGAAAACAAACTAAATTGTAATTTCAGTTATATTTGATTAAATATTTGATATTAAAAAGC
TTCTTTAAAACTCAAAACAAGCAGCTGAGCTGAGCTGAATATACATGCTTCTGCCAACTT
TATTGAAAACTAAACAGGCATCCTCATGATAAGTTGGCCGTCCTTTAAACTTCGGATGTA
ATAGTAACAAAAATCAGCTAAGATGAAGGTTTGAATCATTTCAGCAAGGAAAGCCATTGG
GAACCATAGGTATCCATATCCAATCAAATATAGATAACGCCCTCTTGTCTCAATAACCTG
TAAAATCCAGTGAGCGCAAGAAAAGAATCGTGCAACACCCAACGCAAATACATAATGGGA
TGTAAACGGTTCAATCATCTTTGCGTTCTGCATCATTCGAAGCTGAGGAAACACGGAGAC
TGATTCCAAGTACACACAAAATGCCCAAAGAACTCCACTAACTCTTATAGTATACCTATA
AGGATGGATTATTAAAGCAAGGATCGCACAAGGCACCACTATAAAGCGTAAGGGGAAGTT
GTCTAACTCCTTGACGTAGGTTGACTTCAGCTTGAAGCGGATCATATATATAACCCATGC
TGTGGATACGAGGGTTGCAAAATCTAGCAATGTGTGAATATCACCCTCCATTATAAAGCT
GCACAACAATCTTACAGCTAGGAACAGAGCTGTGAGTTCTTGAGACCTTAGTGAGAGACC

AGAACAGGACTTCTTGGTTGTGAGCTTATAGATCAAGACGATGATCCCAGCAGCATGGGT
AGCCTCCGATGCAATGAAGAAATAATCATGATTTTTAACCCAAAGCTTCATCGCCATCAA
AGAACACACCACAAACGTTACTGCAAGAAAGCTCTTGACCTTCATTGACTGCCGTTTCAG
CCATTCAAGCACCACATCCAAACCAGATCCTTCCCTCTTCGAGA
>ntig_673_consensus
GTAATAAAGTAACAAAATTAACTCAAAATGGGAGCAAATACCAGTGACCGATTGGTAAGA
GAGAAAAGTAGTAACCAAAATGGTAACGCATCAAAACTACCGGATAGTCGAGATCATCAA
CAAGGCAGTTAATGGGGCTTTCATCACATAAGAAAACAGACAGCAAATCTCCCCCTCCCC
CAACCCCAAAAAACGTAAACAAAGAAACGATGTTGATGCTGAATTGTACCCTTTTCATAA
TTGCAGTTAATACTATGAAGAAATAATAAGAGGCATGGAGAAGGAAAGCCCCATATTGCC
ATGCTGCCATACATATATCCCACCAACCCTATCCCCGCCCACCAAGAAAAAATATTTTCGC
AAAATTATTGAGTACCCTAACAGCACTGTGGATGAATTTCTTCTACCAAGATACAATCAG
CATCACTATTCTCTAAATAAAATTTTCTAATATCCTTGATCTATTGGGATCTCGAGGCCG
GAAATCCTCCAATCTCAATTTAGAGCTCATTCGAATCTCATTTAATGCAGTTTCTCCTTT
AATTGATTTAATATTCTCTAATATGCTACCAAGAACATCACCAGCAAGCCCAATTTCAAG
AAGTCGTCCTGGCTCCTCTCCTCCACACAACATTCGCCCTACGTCTCTCAGGGATATCAC
GTTCTCAACAATGACTTTCCCAAAAATGCGGCCAAGAAATTCTGCTGCTTTTGGGGCATC
ATTTACCGCATCCTCCAAACTACTCACAACAGATTCAAACCCTTCTGTGAGCTGAGCTGC
ACTTAACATGCTGTCCCGAGATTTTGCAAGATTAACAAGAAGCTTAGCCAAAAGATCCCT
TTCCATATCCTTCCTCTCAAAAGAGTCTGTAACCCAGAGAGAGACCATTGAAGGATGGAA
GCCTGGGGAATTCAAATCTTTAATGCACAAAGCAACTTCTTTCTCATCCCTGGCACTGTA
AAATTCTTTAATTGCTTCCAAGGACATATTTCGCAGGTTTTCTTCAGACAAAACATTTTG
ACTGGAAGCTTGTCCCTGGCCTCGTGC
>ntig_674_consensus
GGTTCAACAGATGTTGAAAGTTTGCATGGTAATATGATTTCATATGTGGATACCTTGTCA
AGCAGAAGTAGTGCAATAGACCAAAACTCCACTCACATTTCAACCAAAGAAACTCCAGAA
GAAATTATGAGATATGAAAGGGAAGGTCTTGAGAACAGGGGGGCTGGATTGGTGTCAGTT
CCAGGATCTGGTTCCAAAGATAAGCCTGTACTGGAATTAACTAGGTCAAAGAGCACTACT
TCTGGAGGAAAGAAGAGAAGAGAAATTCTTCAGAAAGCAGATGCTGCTGGGACAACTTCT
GATCTCTATATGGCATATAAGGGTCCCGAAGAGAAGAAAGAGGTTGCCATTCTTTCAGAA
ACTGCCAACAATCCATCTACCATTGTTAATTTACAGCAGTCGGCAGTGGATGCTATAGCA
AGTGAGAAGATTATGCAGAGTAAAATGGAGCCTGATGATTGGGAAGATGCTGCTGACATC
TCCACACCTAAATTAGAACCTACAGGTGCTGGGGAACAGTCTTCTAGACCATTGGTGCAA
AACGAGGACAATGGAGATGGTAACCTGGGAAAAAAGTACTCCAGAGATTTCCTCCTGAAA
TTTTCCCAGCATTTTACTGAGCTTCCAGAGGGCTTTGAGATTGCATCTGATTTTGCAGAG
GTCTTGATGGGTACCAATGTCAGTGTCTCTCATCTTGCTGATCGTGATTCATACCCTAGT
CCTGGAAGAATAATGGATAGGCAGACTGGGGGACCTCGAATGGATCGTCGTGGTAGTGTT
ATTATTGATGACGACAGGTGGGGTAGATTGACTGGTCCAAGTTTGGTGAGGGATATGCGT
CTGGATGTTGGTTATGGAGGTAATGCAGGATATCGACCTGGTCAAGGAGGCAACTTTGGT
GTTCTAAGGAATCCACGGGCACAGATGCCTGTGCAGTATGCTGGAGGGATCTTATCTGGA
CCTATGCAATCCATGGGTCCTCAGGGTGGGATGCAAAGAAATAGTCCTGATGCTGACAGG
TGGCAACGTGCTACTAATTTCCAGCAGAAGGGTTTAATTCCTTCTCCAACTCCATTGCAG
GTCATGCACAAAGCAGAGAAGAAGTATGAAGTGGGTAAAGTGACAGACGAGGAAGAAGCT
AAACAGCGGATGTTAAAATCTATTCTGAACAAACTAACCCCACAGAACTTTGATAGACTT
TTTGATCAAGTAAAAGCTGTAAACATTGACAATGCTGTCACTCTTACTGGTGTCATCTCT
CAAATTTTTGATAAAGCTTTAACGGAGCCTACATTCTGTGAGATGTATGCTAACTTTTGC
TTTCATCTTGCTGGAGAGTTGCCTGATTTTTGTCAAGACAATGAAAAGATTACTTTTAAG
AGAATGTTGCTGAACAAATGCCAAGAGGAATTTGAGAGAGGGGAGAGAGAGCAAGAAGAA
GCTAACAAAGCTGACAAAGAAGGTGAGATTCAGCAGTCTGATGAGGAAAGAGAAGAAAAG
AGAATCAAGGCTCGAAGACGGATGCTTGGTAACATTAGACTGATTGGTGAATTGTACAAG
AAGAAAATGTTGACTGAGAGAATAATGCATGAATGTATCAAGAAGTTGCTGGGTCAGCAT
GAGAATCCTGATGAGGAAGATGTTGAAGCTTTGTGTAAATTGATGAGCACAATTGGGGAG
ATGATAGACCATCCTAGAGCCAAAGAGCATATGGATGCATACTTTGAAAGGATGGTGCAG
TTGTCGAACAATTTGAAGTTTTCTTCTAGAGTTAGGTTTATGTTGAAGGATGCAATTGAG
TTGAGGACTAATAAGTGGCAGCAAAGGAGGAAGGTTGAAGGGCCTAAAAAGATCGAGGAA
GTGCACAGAGATGCTGCTCAAGAAAGGCAAGCTCAAACTAGTAGGCTTTCCAGAGGTTCC
AGCATGAATTCTTCATCAAGAAGGACACCTACAGATTTTGGTCCTAGATCAGCTATGTTG
TCTTCCCCAGCTTCTCAGATGGGTGGTTTTCGTGGATTGTCCTCACAAAGTCGTGGATAT

GGCATTCAAGATGTACGGATGGATGATAGACAATCTTATGAGGCAAGGGTGTTAGCTCCC
TCACCTCAAAGACCTATAGGTGATGATTCAATTACTTTGGGGCCCCAAGGTGGTCTTGCC
AGACGAATGTCTAGTGTTGGCGAACCTAGAATAATGACTGCTGGTTTGAATGGTTTCAGT
TCTTTATCAGAGCGACCTGCTTTTGGTTCTAGGGAGGATCTAATGCCAAGATATCATCCA
AATAGATATGCTGGCCCAACTACTTCTGATCAAATGAGTGCTCAAGAGCATAACATGACG
TATGTTAATCGTGATACAAGGAACCCAGATCAAAGTTTTGAAAGGCCTCTTGCAACTATG
GCACCCACATTAGGGCAGGGACAAGCTTCCACTCAAAATGTTTCATGTGAAGTGGCGCCA
GAAGAATGCCTGAGAAATATGTCCTTGTCAGCAATTAAAGAATTTTACAGTGCCAGGGAT
GAGAAAGAAGTTGCTTTGTGCATTAAAGATTTGAATTCCCCAAGCTTTCATCCTTCAATG
GTCTCTCTCTGGGTTACAGACTCTTTTGAGAGGAAGGACATGGAACGGGATCTTTTGGCT
AAGCTTCTAGTTAATCTTGCAAAATCTCGGGACGGCATGTTAAGTCCAGTTCAGCTTGTG
AAAGGGTTTGAATCTGTTCTGTGTAATTTGGAGGATGCAGTAAATGATGCCCCAAAGGCA
GCAGAATTTCTAGGCCATATTTTAGGGAAAGTCATTCTTGAGAATGTGATACCCTTGAGA
GACATAGGGCGGTTGTTGCATGAAGGAGGAGAGGAGCCAGGACGCCTTCTTCAAATTGGG
CTTGCAGGTGATGTTCTTGGTAGCATATTAGAGATTATTAAATCAGATAAAGGAGAAACT
GTGTTAAATGAGATTCGAATGAGCTCCAATTTGCAGTTGGAGGATTTCCAACGTCCTGAC
CCTAACAGATCAAAGATATTAGAAAAATTTATTTAGAGATAGTGATGCTGATTGTATCTT
GTTAGAAGAAATTAATCCACATTACTTCTAGGGTACTTGTTAATTTGCGAAAATATCTTT
CTTATTGGTTGGGTTGGGGTGGGGTCGGGTGGATGGATATGAATGTCTGCAGTATAGGGT
TTTCCTTCTCCATGCCTTAAATATAATTTCTTCAAAATATTCACTGCAATTATGAAAAGG
TTTCAAATTCAGCATCAAAATCGTATCTTCTTGTGGCTGTCTATTTTCAACTTTTCACGT
ATATTGTAAAGTTGCATGGAATTGGCTGACAAGTTTTGATTAGGATAGACTTGTGTCAAG
TTTGATCTATTTGAGTAGTATTTGGTTTGCGGAATTCAGGAGAAGAATCTGCAGGAGAGT
AGCATAACGCTGAGGAAGACTATAGCATTAAAAGGTGTGGTTGGTCGATATCATGCTGAT
AATACCACAAGTATTGAATTATTCAGCTTTGCGTC
>ntig_675_consensus
GGGGGATTAAATAAGCTTTTATTTCTTGATGTACAAATAGGGCCTCGTTATTATCAAGCA
GCTTGAGCTCGGTTCAGTGAACCTTTGCTAGAAATGGAAATATTGGGTTGTAGTCCACAA
CCATAATGCAAAACCAAAACCAATCACCAACAGGAAATAAATTACATTTTACTAAAATAA
TTTACTTGTGCACAGGCCAGGTATTCCGACCCGAAAGTGTTCCAAAAATTGCCTCAGTTC
CCTTCTTTCTATTATTTGGAACTTTATTCACTTCTCACATTCCACAATGAAAAGTTTCCT
TGGATGAAAGCAAAAACCTTAATTANNNNNNNNNNNNNNNNNNTGCAGGTAAAAGATTTTCC
CGTGTCAGTGGTAACAGTCACATCTTGGCCAGGCCTTATATCACTGATTGTACTTGGGAG
ATCAATAGTATAGCGCTCATGACCAGTCAAACCCAGCGTGTCTGCATCCTCACCAGTCTT
AAAGCAGAGTGGAATGATACCCATTCCCACCAAATTGCTCCGATGAATTCTCTCGAAACT
TTTGGCTATCACAGCTTTAACTCCCAACAACATTGGACCCTTCGCAGCCCAATCTCGGGA
ACTTCCACTTCCATACTCAGCTCCAGCCAAAATAATTGTGTCATGACCAGCAGTCTTGTA
TTTCATTGCAGCATCAAACACATACAGTTTCTCCCCTGTTGGAACATGGATAGTCTTTGG
GCCCACTTCCCCATTCAGGAGCTTGTTAACAAGGCGAATATTTGCAAAAGTTCCCCTAGC
CATCACTTCATCATTGCCACGACGACTACCATAAGAGTTGAAGTCCTTGCGCTCTACCCC
A
>ntig_676_consensus
ACCACCGAGATCTACACTCTTTCCCTACACCGACGCTCTTCCGATCTGCCAAAAACGCCG
TCCAAGTCAAACACGGCTTTACCAAATCACTGGCTATTGGATTGACCTCCCTGTAGACCC
GACAACAGCCCGCTAAAGCCCCAAATGTAGCTAAACACAAAATCAACCCANNNNNNNNNN
NNNNNNNNNNNNNNNNNNNNNNNNNNNNNNNNNNTTGGGTTCTTGTGGAAACCGCCTTCA
GCCCACACAAGCCACGACGAGAGACCCATTAAGACGTTCGAAGCCATGCAAGCTGTGTGT
ATTACCCATAATGGAGGGAACCAGAATGGAGTCTTCATCGCGACATACCTACGACTTGAA
CCAAAAAAATAGATGTCAAAAAGGATAAGAGCGAGAGGAAGAGCAACGGCTATGGACAAT
GATCTAAGGCCACGCTTGGCAGATCGGAAAAACACACCTCTG
>ntig_677_consensus
AAAAACGCCGTCCAAGTCAAACATGGCTTTACTAAATCACTGGCTATTGGATTGACCTCC
CTGTAGACCCGACAACAGCCCGCTAAAGCCCCAAATATAGCTAAACACAAAATCAACCCA
NNNNNNNNNNNNNNNNNNNNNNNNNNNNNNNNNNNNNNNNNNNNNNNNTTGGGTTCTTGTGGA
AACCGCCTGCAGCCCACACAAGCCACGACGAGAGACCCATTAAGACGTTCGAAGCCATGC
AAGCTGTGTGTATTACCCATAATGGAGGGAACCAGAATGGAGTCTTCATTGCGACATAGC
TACGACTTGAACCAAAAAAATA
>ntig_678_consensus

ACCCACGTGTTAAGTCTTACGTCCTCTTTAACCAGAGAAACTCACGCTTTCCTCTATTTA
ACATCCACCTGAAATTCCTCTCGTGACACATATTTCTCTTCCGCTCTCTCTCTTTCTGTA
TTGACATACACGTAACACAAACTTACATTCATGGCTTCCCAGACCCTGAAGCAACGTATC
AAGGATGACCCGGCCGACACGCCGACTCCGGACAACGACAACAACAACTACACCAAAAAC
AAACGGGATAAGAGAGCGGACATGGCCAAGCGTGGCCTTAGATCATTGTCCATAGCCGTT
GCTCTTCCTCTCGCTCTTATCCTTTTTGACATCTATTTTTTTGGTTCAAGTCGTAGGTAT
GTCGCGATGAAGACTCCATTCTGGTTCCCTCCATTATGGGTA
>ntig_679_consensus
GCAAGGATCGCACAAGGCACCACCTACCATGGAGTTTGAAGACAAGTAAAATATTTTGCA
GGTTCTGTTATTTAAATAAAACCCAGCTATCCATCTGCTTAAGTATCTGCATAATAACAT
ATACTTTCTATCCACTTTATCAAGCAACTTGAGTATACAGATGATGTAGAACAATATACT
TACTATAAAGCGTAAGGGGAAGTTGTCTAACTCCTTGACGTAGGTTGACTTCA
>ntig_680_consensus
GCCAAATTTAATTTTACAACTATACTCACTGGAAAGAAGATATGTTCATCAATTAATTAC
ATGATCAGAGCCACCCGAAAAAGGGCAGTTTCCAAATTCCGACCCCAATTGGCAGGCTCT
GGAAATTAAACAAACAATAAACAAAGATGGCCTAAGATGAGGAATCCCTTTTCTCAATTC
AACAATGCAATGGATTCTTTGGCAACATCAAAGCTACAACCAATTCAAATCTTACAAACA
TTACTATGTGCAATTCTAAATTAGTTTGTATAATAACAGACACAAACATGAGGGGTTGTA
GTGCCNNNNNNNNNNAGCAAAATCTAACAGCCCACGTCCTACTGTCGAGAAATTGGTGCC
GTTGGCATTGATGATGGTTGGCCATAGAAATTACCATCAGAGCCTTGGAAACTCTGATAA
GGATTCGTTGAAGGGGCTGCGAAAGGAGGCATGCCTGTCATCGGAGTTGCAACTCCTGAA
TAACCAGGCACGGTAGGAGGTGGTTGTGGTGGTTGGCTCATGTTATAGCTGTATGGGGCA
CTTGTCATCGCTGCAGTGTTTTGGATGTACAGGGGCATCTGATATGGTGGCAGAGGTGGT
AGTGGAGGCATTGGTGGAGGAGAAGACGGTGGAGGAGGTGGTGGGTCATTTGTAGTTAAA
TGTTGAGTTGTGGTCGTTACATTATGTTGCAGAGATTCAGGGTGTGGGAAAGAAGGAACT
GGAGGCTGCTGGGGGTTCTGTGATGGCAAGAATGATTGCTCATTCTCAATCTTAGGCCTC
TTCTCTGAAGGAAAATCACCTGAGGGTTCATTACTTGAATTACCAATGACACCCTCAGAT
GCCAAAGAGGAAAGGACGTAAGAGAGCATCTGGGCAGAGGATGTCGATGCAGTTAGCTTT
GCTGCAACTGCAGCAGCTGCAGATTTCCGAGGGTCCTCCTCAATATGACCAGATTTTTCA
GGAAAAGATACCTGTCGAGTGTACATTACAGGAGCTGTCTGTTCTCTGTCTCCAGACATC
AAGTTTGGAGGCGCCATTGAAGGATGAGCTTCCTTTGAGTTCTGCACAGTGCCGTAATTT
AGTAACTGCCGGTAGACCTGCTCTGACTGGGATTGTGCAGCCTGTA
>ntig_681_consensus
CATGAACAAAATGGAAACATGTATTGTAGAGTTGAAAGACTACTTTTATTCTTTAACAGA
AAGAAGTGTGAAAGCAACCAATTTCCTATCCTTTCTTATCTCAAAGCCCTTTTTAAGTGA
AGCTCAAAACTCTTTCCTGATTCATATTCTCTTTCTCCCCTGATAGACCCTGAAACCCAT
TATGTCCCATATAGATATAGTTAATCTTTATTGGGCCTCCATAAATGCCAAAATTGATGC
CCATCGCAAGCAGCTGGCTTCATCAACCACCACTTTGTCTGGGACCCCATGCACCATGTG
CTCCGTTCAGCCTGCTACACGAAGGTCCATGACCCCAGGTCACTTGCAGCCAGCGCCCCC
TGCCACTTGAGTCAACAGGCAACTTATCTGAATAGGAGAACGATCAAGACATGTGGAATT
GGCACCCAAGCAGTTGAGAAACACACTTTCAATTCCAACCCTTATTGATTCCTTGACTCT
TTCAACCTGTGTCCACATGCCATAATGCAGTTGATCCATGGGGCTGTGGTGATCGAATTA
CAGCATCAGAATAAACAAAGCCTCAAACATTGACTAACAATTATTGCCAGAATTACAGAA
ATGGGCACAATGAGATTTGGTAAATCTTCTCCAGTATATGGCAAATATCCAACTTGAAAG
TGTAATTGATACGGTTGTCAAGAGCAGTCCTTTGAGATCCGGGCCACAAAGTCTTCCATG
GAAGAAAAAAACTTGCATATGCAGTCGCATTATATGGTAACCACTCTTTCTTTCTTAATT
TTCCCCACGAACGTACTGCAACCAAAGATCCTGAATGAACCTTCTGACCACAAGCATTCT
TCCCATTAATGCAGCAACTCCAGCTTCAAAATGTTTTACAGAATCAGCAACTGGGACTTG
TGATCAATAC
>ntig_682_consensus
TGCATTAATGTGATAATAATAATGTAGCAAAGGTCAAGTGAATTGACATACGAAGCCTCT
ACACTTTTACTACAGGACAGCGGGCTATGAAAATTATTTCCCCTCCCCAATTAATTAATT
AATTAATTATTTATTATATAATTAAAGCTTTAGATATTTACTAGGCAATTTGATCACCAG
AAAGGGTCAAAAAAGGCAGAAAAGGCAGATGAAATCTGGCAATTACAAAAGAACTGGGAA
TTAAATCATACAGGCATTAATCACACGATACAACAATTCAGAGCAAAACAACAGTGATGG
TGATGGTGATGGTGATGAAGATGATAATCATGATGATTGAAAAACAGATCAACGCTCATA
AATTAACACCAAGTAACAAATTAAATACACGAGGAACAGAAAAGCCGCACGTGTCACTGG
GTTTGCATTCATCGCTTAGTACTCAGTAGTTGGCAGCGGTTCGTGTGAGGTGCTGATAAA

GAAGAAGTCGTATATGAGGCCAGCAAGCCCACCACCGATCAATGGACCGGCCCAGTAGAC
CCAGTGGTTCTCCCAGGTCCAGCTCACCAAAGCTGGCCCAAAAGACACGGCTGGGTTCAT
TGAAGCTCCATCAAAAGCTCCGCCGGCCAAGATGTTGGCGCCCACAATGAAACCGATTGC
AATGGGGGCTATAATTCCCAAACTGCCCTTCTTTGGGTCAAGCGCTGTCGCATAGACGGT
GTAAACCAGACCAAAAGTCATCACGATCTCGAAAACAAATGCGTTCCATACACCAACTCC
TGATGATAGAGAGAAAGCTGACGTGGTCTGGCCGTTGGTGGAGAATTTAAGAAGCAGGCA
AGCGACGGTGGATCCGAGAAGTTGAGCGATCCAGTAGAGGATTCCACGGAGGAGGGTGAT
GTTGCCACCAACGAAAGCACCGAAAGTGACAGCGGGGTTGACGTGACCACCGGAAATGTT
GGCTCCGACGGCAACGGCAACGAATAGGCCGAAACCGTGAGCAAGGGAGGCGGCAACAAG
GCCAGCGGGGGTAGTGGAACCGTTATCAGTGAGCTTGTTGAATGCCATGCCTGATCCTTC
ACCGGCGAAAACAAAGATTAGAGTTGATATGAACTCTGCCAAGGCAGCTTTTAAGGCGTC
GGGATGAGCTGCCTCATGTGGATGGCCAACGGCAATATTTCTGATCGGCATGATGATCAA
GATCGACGGTGGAGATCGTGAGAGAGAGACTTACAGAGCTCACTGGCGTGTTATGTGTAC
TAACAGGTTTCCACTTCTTACACTGGAGGTGGTACTTATACTGGAACCGTGTGCATGTTG
AAAACCAGTGGTAGCCCGTTTCCGGTAAACAAAAAATCGAAGTAAATAAAAAGATTTCTT
AATGTTGTGATTGGTGG
>ntig_683_consensus
TGGCACCAAATAACAACATATGTTACTGTAGAAAGCTTTCAGGCTGAGATTAACAATTTG
TACAAATCAACAGTAGATGCTCAACACCATAAAAGAAGAAGCAGAGCAGGTTGAAAAGCT
TTGCCAGCAAACTTCAGGCTATACAATCATGTACACACGCATGCAAAATACAAATTTTCA
TGAACCTTTTCCTAAATAAACTATCTCGAACTTCACCCACCNNNNNNNNNNNNNNNNNNN
NTGTAACTAAATGTAATGGGAGGAGATTACTCGTTCAAGGGCAAGAGAATGTACTTATTA
TCGTTCACTTATCTTAGGTGGGAATCGTATGCGTCAAAAAGGTGATCAGGAACTATCAGT
GGCAACTTATCTATATACATGAAGAGCCTTCTAAGATTCTCCCTGGTCACAGTTGCCATA
TCGATTATATCCCTGCTATCTGTATATACCCAGAGATCACGAGAGTTCACTCTCTTCAGA
TTGTCACGGCAAAAGGGGCATGACTGTGACCTCACTCGCCATTCACGGTAGCATTTCAAG
C
>ntig_684_consensus
CGTACCATGACATATATATGATGCAATCGAAACCCAAGTCTCAACTGTCGTTTTGAGTTA
CAAAATATTGCTTCAGTGTATATAATTCATGATGACGTTTATCGACCTTTAAATGTTACA
TTTCTCCAAGAAATTTCAACACTTGTATGAGAAGAAAAGAAGGGAAAAAAAAATACGCTT
TCTCAGCTTTACGCACAAAAAATGCAAAGCGGATAGCCTAATCACTGGGTTAAAATTTGT
GGCCAACAACAATACTTAACTGGGTTAAATTTGTAGTCAAAATGGAGCAGAAATTTGGAT
CAGGTCATGAAGCTGAATACTTGTCCTTCACAGCTAAGGCTGCTGCTCGGTACCATGAGG
CTGCAGCCATGGCACCAGGGTCCGGAACCGATGCAAGAATCTCTCCAGAGACATAGGTTG
AACGACCAGCCTGTGCCTGCATGTGTTTGGTCGATTCTGCTCCGTCCAAAGCTGCTTCAG
ATGATAAAAGAAAGGCAGTACAAGGATCGATCCCAGCATTTAACCTCTCCTTAAGAACTG
CAGCAGCTGGAATCAGGGCATCTAACAATGTCCGATACCCAGCAACTGCTCCACCATATT
TGCTAACTGCAGCAATTGAAGCTTCAAATGCCTCAGCCCATTGTTTTGCTGTGACAACCA
ACTCAGAATGCGCATTGGCTTTTAACCGTGCATATGCTGCCTTACAAAATATATTATATA
TGATTCCACTTGTTCCACCCATGACTCTTCTAATAGATGCTCCGATTTCATTCACAGTTT
CTGCAGCATCATTCAAAGGATAATACTTTTTCATGTCCTCTAGAATTGCTGTTGCACCTC
TATACATTGTCGATCCACAGTCACCGTCACCAACTTTGCTATCCCATTCATTCAAACTGT
CTCGCAGATTGATCACTTCATTAGCTGCTGCTTCAATAGCCACCTCAAGGATGCGGCCTT
GTTGACTTAGCTGTAGTGGCCGATTTAAAGACTCCTCACTTTTTGTTGAATGAGAAGGTG
GCATCGGAACAGGAATCTTGGCAGGTGGATGATTACCATCAGCACCAACAGGCCAATAAG
GAGCTTTGGTTGCAGCATCAAGACGTTGCAGAATTCCCTGATCTGCCTTCATGATAGAAA
TTGAAAATCCTGCCATATCAAGAGAAGTCATGAAAGATCCCGTGTACACTCTATCAACAG
CCAACCCATGCTCCAATTGCAAGTTAGGGAC
>ntig_685_consensus
GTTGATGTTAAGCCATATTGTTCTTTTACTTCCGTCTCTTAGAAAAACTGTCTTGCTTGT
CAAAGACAAATATAACCTAACTATTCATGTTACTCATTTTTGCTAACCATCATTTCTTTG
CATTTTATATGTTCCTCGGCTAAGTAGACTTCACAGAGATAGGGAACATCCTGTGCTTAT
AAATTTGGGACAGTACATATTTTTGTTAAGCATCAAAATGCATCCTGTGCTATCATGGAA
AGTAGTACTTTATTGTCTTACAGGTTCAACAAGATTATCACCACGTATGAACATGTACAA
GCCAAAAGTCGTACAAATTGAAAAGACAGCTTCATAAGAACTAGGGTAGAAGTTGCATTA
CATACAGAACTCCCGGATCTATTTTATCCTGACAGATCAAACTGTTCATTTGGCCAGAGA
CTTGCCAGCTATCTTAGTGTCAAGAGCAAACCGCCTCAATTGCCTTGCAACTGGGCATCA

CGACTGGTCAAAGGAACATAACGAACAGAAGTTTCGCTCCGAACACTAATTGAACCATCT
GAGTTCTTGTCTATTACCTTCAGATCCTGAAATATGTTACCAACTGGTATTACCATCCTG
CCTCCAGGCTTCAACTGATCAATAAGTGGTTGTGGGATTTCTGATGCTGCCGCTCCAACA
TGAATGGCATCATATTGAGCAAACTCTGGCCAGCCTAGTCTTCCATCACCAACATGCACT
GAGAGAGAACCTTCTTTCAGTAATGGAGCTGCTGCACTTTTTTCAATATTCTTGATTGAG
GAATTCACCAACTGAGGAATGTGTTCCACACCTACTGCGCGCCCTTGTGGTCCAACCATA
ACTGCAAAGCATGCTGTCAAGTATCCAGTTCCTGAACCAACGTCTAAAGCGCGCATGCCA
GGTTTCAAATTCTCCTCCAACAATTGAAGGCACATTGCATGCATATGAGGTGCAGAAATG
GTGGCATTGTAACCTATAGCCATTGGGCTATCAACATAGGGTGGAGTCCCATCTGGCACA
AATAGAGCCCTATCAACAGTTTCCATTACTTCAGCTGTCCTCTTTGATGTAATGACTCCA
TAACGCTGCAGATGCTCTACCATTCCTTTATTCTTGCTGATGTTATTACCACTCCAGAAT
CGCCGTGAGGAGACGGTTGAGGTTAGCGGGGAGATGAAAATTAGACGACAAAGGGAGAGG
CGCTAAGTAAGGACGGGGATGATGATGAGGAGGAATGACAGTCGTAAAGGTTATCAACCG
CTTTATACAGGGCATATAATAGCGGCAACTATACGCTACTGCTGAATACTGATGCATATC
AAACACATCAATTTTTTTTTGTTTCACCTTTTAAAAATATTCTCTTTCTTTTTCGAGTT
CCTTCTGCTACATTCTCTGGCACACTTGTCACCTCTCA
>ntig_686_consensus
CTTGAATTTCAGCGTCTTTGAGTTTTTCCAATGGAGCGTTTTCCAGAGAACAAATGCTTT
GGTTTCAATGTAGGAATAACTCTATCAGCCTCTCCACGACGAGCATCCTTGTTCCTCTTC
TTAACAGATTTCTTACTGAGTTTAATTGCCTTGACCTTTTGAGCAGAGTCCTTGAAGCCC
TCTCCAGGAACAACTTCACCAGGAGGCCGTGATTTTGACCTTGACCGGGATGTGGACCTC
AGACGCAGCTTCTTGTTTGGTTGGTCCACATCCATATCCATAGCATCACCATTATCGATG
TCTCCTCTGTCAGGTGACCTCTCCCTTTTCCGGCCTCTAGACCTGCTGCGGGCACGATTC
ACTGCCAAAGAAGGATCCAGTCCCAAAGAAGATAGCTGCCTCCCCATTCTCTGTACCGTG
AACCTCCTTTCCTTGTCAAACTTTCTAGGTACAGTAGGCCGACTCTCAGCATTGCTTTTC
TTGATCCTATGTTGTTGAATAAGCAAGCTTTTCTTTTTCCTGATCTCTGCTAATGCTTCT
TGTTCTTCTGGGGTCAGTTCCTCACCATCCATTTCAAAATCATCATCTTCCTCGTCTGCC
TCCCTAATACCTTCTTCCTGTTCCAACTCTTCAAGCCTTACTATGATATCAGGGTCAACA
AAGTCATAAACATTATGGCCATCAAGAATTTCAGGCATAATATCTTCTTTCCATTCATCA
TTGGACAAGATGTAATTCTTTTTCAGACTGGCAGAATATACACCAGCACCACCATTCTCA
TTCTCCAAATCCTTCTCAGTCTTCCTCTTTTCCTGTTCAGCTGCTTGCTTGGCTTTAGCA
TCCAAAACTGCCTGTGGTATACATGGTGGCCTTTCCTTCTGGTCACGTGGCTTTGGCATT
GCAACATGAAACCGATTCATGCAGTCATTTATCTTTTTGGTTTTCATCTTCAATTCCACC
CTTTGATTCAATAACCGCTCACAAGCTGCATTTTTCACAGCAATTACCCCATCTTCAGTT
AAAGTGCTCATAGTTAACAGCACCCCCTCATCAGTCTCAGGTTCTCCTCCTGGACCCATC
ACAGTTTTCAATGCTTCAGCTTTCATCTCCATGACCAGTTTCATATCTTCCTCAGATATG
TTTTCCATTGGCTGCAAATCAGTCTTGTTGCAGACAATAATCAAAGGCTTGTTCATAAAC
AGAGACTTAATGCTGTGAAAAAGAGCTGCCTGCTGGGCAATGCTGTATCCACATGATCCT
GAGATGTCCAAGAAGAAGAGTACAGCAGCTCGCAAATGTGCCAGAGCCGTTATACTGCAC
ATCTCAATAATGTTACGATCTTCAAATGGCCGGTCCAAAATCCCTGGGGTATCAATCACC
TGATACCTCAGATATTTATAATCTGTATGACCTACAAAGAGTGACTTTGTGGTAAAAGCA
TAAGGCTGGACATCCACATCGGCCCTAGTAATCTTGTTCATAAATGAGCTCTTTCCCACA
TTAGGGTACCCACAAATCAAAATAGTCCTAGTATTTGGATCAATTGAAGGAAGCCTTGCC
ATGTGTTGTCTGATTTGTTCTAAATATGCCAAACTAGGGCTAATTCTCTTTATCACAGTA
CACATTCGACCGAGAGCAGCAACCTTCAGAGACTTGCAACGATAAAGTGAGTCACCATAC
TTCAACAACTTCACATAATCTTTAGCAATCTTGCTGATGAGGTTCCTTGCAGTGTTTATT
TGGCCAAGGGCAAGCTTGTAATGATCTTTATTATACAGCACATGAAGAAGGTCTCCATAA
AAAGGGTGAATATCGTCTAAGCGGGGAAACTCTTCAATAATCGTAGACAGCTTCTCATGA
AAATTTTGTTGCGTATATTTTACCTTACGCATGTAAAACTGACGGAGACGAGAAATGGCA
TATCCTTTATGAACAACAGTTGGTGTCTGCCGCTGGGTACGGGATAGAATGATGTCGATG
AAATCCTTTCCATTAGGCACTACAGTAATCTTCTTGAAATTATATTGCACCATCTTTAAG
TGCTTGACTGCCGCTTGTGACGGCGTGCGCTAGAATCAGTGCAGCCAGTGATGGTGCCTC
TGCCGTTAGTGATGGCTTCGGATGAAAGTAAAGCTGCCGCCTGTGACGGTGAAGATGGAC
AGTAGAGCTGCCGCTTCAGTGCAGAATTGTAATGCAAAGCAGAACAAAATG
>ntig_687_consensus
CTTTAGATGATCAGCGTCAACTCCCAAAGATGGCACAAATGCAGCAGCAAAATTAGGTGA
TTACATATCAAAACATACTCGCCATCAATGTAAATGAAGTTCAAACCTAAAATCTTCCAG
ACAAGTGCCAAAAAATAATAAATCCTGAAACACTGAACTCCACTTCTGATTATAATAAAA

CTTTCTCCACCTAGATATTTAGGAAAAATACTTGGTGGAAGGAACCACGTACCAATCTTT
TCTCTCATTTTCCTTCCATATATTCTAGTCCATGACTCAATGCAATGCAAACCAATATTG
TTCCGACAGCAAAACAAAAAGGATTAACAAAATAGTCTTACATTAAACTTTAAAACCAAA
AAAAGCCACTACTGTGACACCCCAAGCCACTTTGAGAAGTTCTCGAATTCAGTATAATCA
CTGTCTGATATCATTGTTTGGTACCATGCCTTGCCAGCAGCCTTGATTGCGGTAGCCTCC
TCCTTGGTAATGGGCTTCCTCTTCTTGTCAAGCTTCTCCTTCTTAGCCTTAACTCGTTGA
GCCTCAGCCAAAAGCGACATTCTCCTTGCAGTCTTAGCATACGGGTTGAGCCTGAGCATA
GTGTTGAGATTCTTCAGAGGATTCTTCTTCAATGGTGGTCTCTTCACTTCCTTCTTAATC
GGTCTCACTACAGATTGAACCTCATCAGAGTTAATGATCCTAGCCAAATCGGCATTCACC
ATCTTGGCACGTGGCAACACGTACCCCTTCTTCTTCTCAGACCCCTTATCGAAGGTTCCA
TAGATTGAATCCAACTTCTCAATGGCCGACTTGGTCCAGATAACAAATCGTCCAAGATGT
CCACCTGGAGCAAGCTTCAACAGATTCAACCTCTCAACATTGAGCACCTCGACACCAGGA
ATGTTCCTAAAGGCCTTCACGAGCTTAGCGCCTTCAGTTGAGTACACAACCAATGGGCCC
TTTCTGGAAATGTAACGGCGGTTTCTCATTTTACCCTTTCCAGGACGAATGGACTGGCTG
TCCTTGGCCTTCTCGGCATCATCATAAGCACCAATCTGTTTCAAAACCTTCAAAGCGGCG
GATGTTTTCTCAACACCTTCGACTGAGTCACTGACAACCAGGGGCAACTCGGGAACTTTT
TCAATCTTGTGGCCACGTGCCATCACAAGGGAAGGGATGGCAGAGGCGGCGATAGCGGAG
ACGATAGCATACCTCTTTTGGTTCACGTTTATCTTACGGTGCCAGCGGCGCCAGATCTTG
GTAGGAGCAAACATGCGTCCACCACGACACATGTTTCCAAAAGCTCCCTGACCAGCTCGG
TGAGTACCACCACCGGGAACACGGGGGATACGGGAGACAGCGCGACCGGTACCCCATGAT
TCGGCGGAGGTCTGGTGACCTGCCTTCTT
>ntig_688_consensus
AGGCCCTTCTTGTTATGACTCATTTTCTGCTCTCTATTTTTCTCACCTTCCATATCTTCT
TCCAACTCCAGACATAACTTTAGTAGACGGGACTCCACATGCTCAAGTCTCTCAATGACG
GTTCCCTTGACTCCAGTCTCCATGACCACACGATTAATCGGACGGCAATGCTTCTCTAGA
CTCTTAGGGGAGTAATCAACGGATGAGGGAAGCCCATCGCTATTTAGGATCCCACTTGAT
GGAGTAGAAGAAGCACATGAACTCTTTCCTGACCTACTGCAGCATCTGAGTTCTTCTAGC
TGTCTCAACATGTTGTCTAAACG
>ntig_689_consensus
CCTTGGGTGGGAATATGTTATTTAGCTTTGAGGCATGATCCACAACACGATAACCTATGC
CTTAATAGGTTTTTTTATGCAGGTCACGGACGTAGACCCACACAAATTATTATTATTATT
TTAATAAATTTATCAATTTTTTATATTATTATTTGTTGCTTTTATTACAATCCATCAAAA
ATTCCCGTGAAAAGTTAAGAACCAAAATATACACAAAATTTTAAAAAATAAAAATCAAAC
CAGCCTTTTATCTTTATGCTTGCGCCTGATAATTTGTTTGACAAATTGCTTCAGGCCCTT
CTTGTTATGACTCATTTTCTGCTCTCTATTTTTCTCACCTTCCANNCTTTAGTAGACGGG
ACTCCACATGCTCAAGTCTCTCAATGACGGTTCCCTTGACTCCAGTCTCCATGACCACAC
GATTAATCGGACGGCAATGCTTCTCTAGATTCTTTGGGGAGTAATCAACGGATGAGGGAA
GCCCATCGCTATTTAGGATCCCACTTGATGGAGTAGAAGAAGCACATGAACTCTTTCCTG
ACCTACTGCAGCATCTGAGTTCTTCTAGCTGTCTCAAC
>ntig_690_consensus
TTTTTTTATATGAACCATAAGATTTAAATACAATTTCTCAAGTTGGGTTCGAATCAATAA
TTTCTATTTCGATCTCAAGTTTGAGCCTGCAACTCTTAAACTTGAGCCATGCATCTACCC
TATCAAACCGAGCTCAAGGCCAACAATACTTGTTTCAGCGTATTTGCAGCACTAGTATAG
ATATCTCTACAAAATCACAGCGTTAGCTTTGAATCTAGAGAGTTCAGACATCTCCATCCA
ATCACAACATACCTGGCGTTTCCTCATACTTGAAGGAAATAGTGAATGCCTGCTGGGGAA
ATGGAGCTTGGTTTATAAGTGGATTATATCAGGACAACGTGCTTCAGTACGACTTGATTT
TTTACAGAGTGGCTGTCGCAATAAAAAACTAGTAGTTCCGAGCAGGATCTGATGTATATG
ATCAACGTGCCTTAGTTTGTTTGAGAAGTGCAAGCACACAATATGCTTACCTTTCGCAGA
TTTTCTTGCAGAATGGGTGGTGTTAAAATCACTGAAATCCTCATCTCATGACAAAAAATG
GGCTTGTTGAAAGAAACAATAATAGTAGCTATTAAGTTGCATTAAAGCAAAGACAATTTA
CAGGAGTTCCGATTCCAAACCTCATTTAGTGTACTCGTTGCAAAAAAGTGATTACATTCA
AGGGAAAGATTTTCAGGGATAGAAGTAGCTCGAAAGTAAAATAGGGCCCAGGTCTGATTT
CATCTTAGATTTGTTCATGTGAAGGATGGCATCTAAAGTTGCAACAGCTTCCACAATATA
TTTCCCTTTGTTCAACATTACACAGCTTGCCCTNNTCCAGAACCTGAGTTGCCCAAATGA
CTGGTACATGGGCAGCAGCACAAATAGCAAGAATTTCTTCCTGTATTTCAGCCAACCTTT
CCCACCCACATTCCACTGCAAGATCTCCTCTTGCAATCATAACCCCAAGGGGATTTGAAG
ATTTCATAGCTTCCAGTAGTAAGTGAGGCAGGTTATGAAACCCACTCCTTGTCTCAATTT
TCAAAACTATACCCAGGCTCTGAAGTTTCTGTTTCTGTAATTCTTTTTGAAGCACAACAA

TATCATGAATATCTCGAACAAAAGAAACGCCCACCATGTCAGCATGAGTGGCAACAAATT
CAAGATCCATGAGATCCTTTGAGGTAAGACCTTCAAACCGGATATTGCTCTCTGGGATGT
TTATGGATTTTTCTGATCCAAGTTTAGTGCCTCTAAGACCGGCACGAGTGATTGAGACAA
CAATCTCTGAGTTACTTGCTCCCTGGATTACACCCCAAATCTTTCCATCATCAAAAGCTA
TAGGCTCTCCGGGCATAACTGAATCAAATAGATAGCCACAAGAGCAAGTTATCCTATGAG
CACCACTTGGTGGACAAGATAATCCTTCCTGATCCAATGAACTATCTCGTGATATGGTTA
GCAAGTCTCCAACTTTTAATCTGATAAATCGCTTTTTGGCAGGAACGTTTACCACCTTCC
CAGTTAATAGCCTACCCTTCTTTCCTTTAGTGCACAATTCTGTCCCAGACTGAATATAAG
CAGTCTTTGTACATTCAGCCGCAAACCCAGTACCGTTAAAAACATGAAACTCCCTAGTAA
TCTTGAGCATCCTTTTTTTCCCTCTAGCGTCATAAAACTTCAAAGTGTCACCTACCTTGA
GCTCATTGAGAAATATTTTGTCATCTATGAATAGAGCTGCATCAGGAGACAAGTGAGGAG
GAGGAGGACCAGATCCTTTGTACGAAAGCCAAACTTGAGCTGGCAATATCACATTACCAG
TAGCATTTTTCTTGGGGGATATTTAATTACACATGGACCAGGCTTCAATTTCCCTGTAC
GGAATTTTGGTCCAGCTAAATCCATAAGAATTCGACATGGCATCTCTAGCATTTGCGAAC
TTCTTTTTACTCTTCTGATTATTTCACTCCATACACTAGGGTTTCCATGAGCACAATTGA
TCCTAATAACGGAGGCCCCAGCCCTAAGAATGTCACTTACAAGCGTGTCACTTTCAGTAG
CTTCTTGGCCAATTGTCACCATAACATAAGTAGTTCTGTTACCCCGAGGTGAGCCCAACA
ATAACTCTTTATTGAACAACTGTTTCTTCCTCAATGCTCTCGATGTGAGCTTCTCATTTT
GCCTTTGGTACAATGTTTCCTTAGTGCAACTTCGCACACTAATATTCTCTTTTATATCCA
GAGAAGTAGAATTCGGGTTCTCCAATAGTTGGATTCCAGCATTTAGACATGCTAGAACAT
ATGGATTGATAGTCTTCAAATTCAAAGACCAATAGAAGACAGTTCTTCCCTCAGCTGCT
CAATGTCAAGACCTTTCAGAGTCAGATAATGAATCAAGTTTGTTGCACTCTCCAAGTAAT
GCCCATGACACAATCTGAGTTCAGAAGTGTTCCATTGTTCAGAAGCTAATACATGTAAGC
GAACAGCCTTTAGTTTTTCAAGAAGGCTTCCTGGCTTTCTCGAATCCTCCACACAGTGAG
AAAGTTTATCTTCCGAAGATATAACGGAGGCAACGACATCTACATCTTGGTGCTCAGAAT
TTTCATCATTCCCGGAACAAACCAGGCCATCACTGGTGCAATCATGAGATATTTCTGTTT
CCAAAACCAGGCCATCATCAATGCAATCATCGGATGTGTCTCTTTCAGCTTCATCATTCT
CATGTGGCACAGAAAAAGCTAAGGTCTTAGGCACAAGTTCCCTCCTCTTTACTGCTAATA
AGTGAACAACATGTACAGGCTGTCTCACTTGATGTCTACATGACAAGTGTGAGAGCTTCC
CCCTTGGGTTGGACAATGCAAACTCTACATGATGAGCNNNNNNNNNNNNNNNNNNNNNNN
NNNNNCTTTAACAGGTTTTACAATATAAGAATGGTTTTCCCGAGCAAATATACTTAGTGT
TTTTGTGGCGAGAAAGTGATGGAAATTTTGGAAGGATGGAGGGAGTGTGTTTTACAATAT
AAGAATGGTTTT
>ntig_691_consensus
AATCACCACAAAGCTTCTCCCCTTAATAAAAGCAATGTTAATCACCACAAAGCTTCTCCC
CTTAAGAGAGGCAGTTTCTGCAAAATACAGAAATAGACATCACTCATGGATGTTCCTCAT
CAGACCTTGACTCTGTCAAACGCCGCTGCCTTCTTGGGACTGGAGATCCATCTTCACCTG
CATCACTCAATATGTGTAGATTGTCATCATCATCATCCTGTAGACCCAAAAGATTCTCCC
CCCAGAGGAACCGGCGACGCTCAGATAAAGCTCCCGTAGAACGTCGATGTCTTCTCCAAG
CCCTTGACCTAGCCCAGTGCCACTTTCTCTTCCAGCTGATAATCTCTCTCCGTTCTCAAT
TACATAGTCCCCAACAACAACAGCACCTGGCATGGATGAGCGAATGGCACTGACAATATC
ACCGTATTCCCGTTGGTGCTCAAGGCATCTCCAAGCTCGTTCTCTAGATGGGTCAATATC
TGAGGGTCGTACAGTTGGGTGAACCCTCCTAGCATGTCGACGCAATTCCTGGTAACTACC
AACAAAGGAGCATGATTCCCCCAAGCACTCTACACAGAATT
>ntig_692_consensus
ACATCTGTGTAAAGTGCTTGTGGGGAAAATAACATCCAAAATCTAGGTAGGAATTTAGAA
ATTCAAGATGGTAATCTGAATTTGGAAATGGAGGACTCAGAGTCTGTGCATGAGAGAATT
GAAGTTGAAGAGGTAGATGTGGATAATTTGTTAGGATCAGGTTTGAATTTGACATGCCCC
ATGTGCCGAGGAGCCATACAAGGCTGGGAGGTTGTAGAAGAGGCTAGAAAATATCTTAAC
TTGAAGAAGCGATCTTGTTCCCGAGAATCTTGCTCCTTTGTTGGTAATTATCANNTACCT
GGCGACGCCTTGAGCACCAACGAGAATATGGTGATATTGTCAGTGCCATTCGCTCATCCA
TGCCTGGTGCTGTTGTAGTTGGGGACTATGTAATTGAGAATGGTGAGAGATTATCAGCTG
GAAGAGAAAGTGACACTGGTGAAGTTAATGCGCCGTGGTGGACTACTTTCTTTTTGTTTC
AGATGATGGGTTCAATTGACAGTGCTGGTGAATCAAGGGCTAGGTCAAGGGCTTGGACAA
GACATCGACGTTCTGCAGGTGCTTTATCTGAGCGCCGCCGGTTCCTGTGGGGTGAAAATC
TTTTGGGTTTACAAGATGATGATGAAGATGATGATGATAACGACGACGATGATGACGACC
TGCATATATTGAGTGATGTAGGTGAAGATGCATCTTCCGTCCCAAGAAGACGACGGCGTT
TGACACAGTCGAGGTCTGACTAGGACCAGCCATGAGTGATCTCTATTTCTGGTAAATTCT

GCTTCTTGACAAGCCTGTGATATCCACTGAAGTTGTATATAGGATTTCTTTTGGCTGACC
CAAATAGTATTTTTACCATCAATGTTCCAGTATTATGCAGAAACTGCTCTTTACAAGGGG
AGAAGCCTTGCGGTGATTAACATATTTTTTACAAACCTTCTGCTTGGAATGAAGGCTAAG
CAGGGTCTTTCATACGGAGATATTGATTGGGATTGTTTTTAATCTTGACTGGT
>ntig_693_consensus
GCCTCAGTTTTATTCATATGTTGAAATTAATTACGGGTCGATTAACAAGAACATCCGTTC
CGTCCCATGGCAGCCACACAAAGGCAGTTTACATGTGTTCTGGACCTAAAGTCTTCTTCG
TGCTCCACAAATAATATTCTAGGAAAACAGTTACATAGACATTCACAAAGAGGAATTTGT
TACAATTTCTTCATAAACCCATCATCACAGGAGCAGAGGCCAACATCGGATAATTTAGGG
GTTAGCCGAACCCACAAAGGTCACGTATGCACAATTAAACCAACAGAAAAGAGAAATAAT
ATTTCAAAAGCGCCATAATATTTTTCACTTGCAGATCCCAGATAGATATTGAGTGCCTAC
ACCTTCATCGAAAACTATGTGCCGCTATATTTCTGTATCATCTTCCATCGATTCATTTTT
TAGTCTCTATGCTCCCCAGTTTGGAATGTTTGCTGTCGCTTCATTCACCATTTTCACCAG
AGTCACCACACTTTCCGGGACACCTGCTGGAGGCAGGGGCAGGTTCTTTGGTGGTGCAAC
ACTATCATAAGATCTTTTGTCAAACCTCCATTCGCCAATTTTACCATAAGTCAATTTACC
CTTCATATCATAGTCACATCCGTAAGTGAAATGGATTATGTACTTGTTACCAACTTCAGT
ATCCCAAGGAGGCTGAATCATGAAGTCTTTATGTAAAATGTTGGCGACATCATGCAGAGC
CGAAGCAACAGCATATGCATACATTTCAAGCACCCAACCAAAAGCCTTATCTGCTTCAGG
ATCCTTCTTCATTGCAATGGACACATTCATCCAAGTGGGAGCAATCTTCTTAAGAGAATC
CTTTGCAACAATAACAGGAGAATTCCCAATAGGGTCAATATTTGTTATCGGTCCCTTCTC
CTCAGGGAAGTACATTCGCAAAATAGACTCATACTTTTTCGGCTCAATATAGAAGAAAGG
AAATGCTGCTCCATACCCATCTCTAGACAAATTTGGTATGGGTTTGACAATGATATGATC
TGGTTCTGCCATCAATATGTAATCTTCTTTTATGTTCGCTTGTTGAAGCCATTGTACAAA
TGCCCACGGTCTGTTCAGAACTATATAACCCTGATCCGTGCCAGGTGGCAAAGGCTGGGC
AATAAAAGTTGGGATCTCATCCATGAACTTATCAGCTTTACCAGAGTGCAAGATCCTGGT
GAAGCCACCCATCTCGGAATCAGGGCCGTGCCTGAACTTCTTGAACCAGTAGTACATAAT
TCTACACTGCCACGTGTTGTACACAGAATCCGACGCTGTAACCGCCGTGTGAAACAGTCT
CTTCTTACCGAATTTTCGTGATCTATCGAGTGGCATCTTGATCACAGGGTCCACAGAGAT
TGACGAGGATTTTATCCGGCCAGGCAGATCTTGCTTCAGAGGAGCATTAGCTGAGATTAT
TACATTGTATGTAATCAGAGCCACAGAGAATATTATAAGTAAGTTGTAGAACAAATTCCC
ACAACCCATTTTCTCACTTTTTCTCTCACTTTCTCTCCC
>ntig_694_consensus
ACCCCAATTAAAGGTCCCCCCTTCAGACCTCTTGGCTGAAGTAAATTGACCCTTGTTCCT
CTGCATCCTAAGAGCAACTTCTTGGCGGACACTATATCTAACTTTCTTGTCAAAACAGCG
TTCTTTCCTCTTCAGCCGGAACCTGTTTAACGAAGCAACTCTCTGTGGTTGCGTACACCT
TCCTGGAAAGTCAGCAACACCCCCTCCATTCCTGGTGGGGCCGACGACATCTCACATCCT
CCCAGCAAGAGTAGCACAGCTTGAACCTTTTCAGGAGTGACGGAGTCAAAGACATAGACC
TGGCCGCGAAAGGAGAGGGTAAGCTGGCTCGAATTACCCAATTCGGAGCCAGAGGATGGA
CCGTACACAGAATCAGGAGCAGCGTCTTCTACAACAACGCCGTTTTCGAGGGAGTGAGTG
TCGTAGGAAAGGTGGTGATTGTCGGCGCCGACGGTGACGTCATCTTCTTCGGCACTGGGG
GACATTTGAGTGGGGATGTTCATTGGCTGACAGTGACCGTACATTGCTGTTTCTGAGCTA
AAATAAGGGGTTGCGCAGCATTACAAAAAAAAGAAAGAAAAGAAAAAGGGAGAAAAACAA
ATTGAGAGATTAAAATTTCAAAGGTAACCCTAACCCTAAGTATAAGAGGGTGAAGAGTGA
GATAAATAAATTAAAAAAGGGAAGAACAAGAAGAGAGCTCTAACAGAGAAATAAAGAGAG
CCTATGTTTTTGTGTTGCGTAGGAAGCTTGTTTGTGAGAAGAACGAACGACAAATAGGGA
GAGAGAGAGAGAGAGAGAGAGAACAGTTTTGTTATAAATTTAAGTATTGCCGCCGCGGTC
CTGCTTGCAGTAACTAACAATCGTAATTATTTTCCTATATTTCCTTGTTTACCATTGCGT
TTGAGGACTTAAATCACAAGTTGATAATAGCAATTTCATATTCTTGTGGAAACGACACTG
GAAAATCTTTTGGCTTATCTCAACTTCATCGCAGCAAATGCTGTCGCCAAATCCATGGCC
TTGCTCACTTGTGCTGCGTCTGTTCCCTGGCCAGAAGCAAGACCACCTTTTCCTTTACCA
CACCTCCCTTTCAAAGGTTCCATTGCTGCCGTTAACCACTCTGATACCTCCAACTGCTTT
GCCTCGTCTGACTTCTCTGGTACGCCGGCACACACCCACAGCCTTATTTGTGGTTTGATCT
GTACTGAAGACCATAACTGATATGCCCTTCTGCTCAATGACTTTCAAAACAGCCTCGCGA
ACCGCGGCTGCATCCAAGCCAACCTCAACGTGAGAGATGCAGAAGGCTTTCCCATCTGAA
GCAGCAACTTCAGCTGTCTGAGTTGCAACCTTGACAGCTTTCTGAATATTTTCTTCAGCA
ATCCTCTTTTGTGCCTTTCTCACTTGATTCTGAAGCCAAGCAATCTTGGCCCTGATATCA
GCTTTCTTGGCTGCTGGAATTGCTGCTGCATCTACACTACTTTTCAAAGAAGCCACTTTC
TTCTCTAGCAAGCTTCCCTCTATCTTTGATGCCTCATTTACTTCCTGCTCAAGCAAGGAT

GCCGATTCCATTGCCTTAAAAGCAACATCAGTTGTGACAGCAGTTATTCTACGGATTCCT
TTAGCAATGCCCTCCTCAGATAGAAGAGCAAATGCTTTAGCTTCCCGGGTATTTGATATA
TGTGTCCCTCCACATAATTCTGCTGAAATTGATAACCACTCTTGATTTTCTGGATCAGCC
AAAAGGTCCTCAACTTTTCGACCAATTGCCACAACTCTAACAGGATCAGGATAAACCTCT
CCAAAAACAGCTCGCAAACCATTGATACGCTTGGCCTCAGCAAGGGTTGCGTCCTTTGCA
AATACATCCAATTCAGCTTTGATTTGCTCGTTCACAATTGACTCAATTCTTCTCAAATTT
TCAGGATCAACTGGCTTACCGTGAGAAAAGTCGAATCTTAATTTTTCAGGAAGAACAATG
GATCCTTTTTGATCAACATGGTCACCAAGTACTTCCCTAAGTGCAAAGTTTAGCATATGT
GTGCAAGTATGATTAGGGGCAATGAGCTTTCTCCTGTCATAGTCAACTTTGCATGTTACT
TTATCGCCCACAGAGAATCTGCCACTGTCTCCACTAAAAGAACCAATATGAAGAATAAAA
CCTCCAAAAAATTTGAACATTACATACTTGAAATGAGCCAAAGGGACCTTCCAATGATCCA
GTGTCAAAAATCTGACCACCTTGCTCAGCATAGAAACTTGTGGATTCCAAAATAATACCA
ATTTCATTGCCAGAAGAAGCACTTCCTATGAACTCTGAACCAGTATAGATGCCTTTTATC
ACAGTGGAGTGGTCCTGGAACCAAATAAACTTGTAGGAATCATCTGTTGCAGAAATTCCT
CTCTTGTGCAATGCAGAGGTAGCATCAGCATCCATGACAATGGCCCCACCAGCTTGCTTG
TTCTGAGCACTCCTTGATCTCTCACGTGCCTCATCCATAGCTTGATCAAAACCATCCTTG
TCAACCAGTAAACCCCTTTCTTCTGCCATCAACTGAGTCAAATCTAATGGGAAACCATAT
GTGTCCCACAGTATAAATGCATCCTGCCCACTCAAAACTTTCCCCTGAACATCCTGAGCA
GCCTTCTTAAATTTCTCAATACCTTTAAGCAAAGTCTTGCCAAAACTAGCTTCTTCAGCA
GCAATAATATCCCTGATGTGAGCTTCATGTTGCTTCAGTTCTGGAAAAACATCTCCCATC
ACTCTTACAACAACATTAACAAGCCCATTGAAAAATCCTTCTTGAGCTTTTAGAACCTCA
CTTCCATATCGAACAGCTCGACGAAGAATACGCCTCAGAACATATTCACGACCCTCATTG
CCTGGACAAGAACCATCAGCAATAGCAAATGAGAGAGTTCTAATGTGGTCAGCTACAACC
CGATATGCCATATCAACCTTGTCTTCATCATCTGGTCCAACTTTTCCTGAATACGGACGA
GCTCCAGTTGCTTGTTGAATAGCTTCAAAGATGGGCAAGAATACATCAGTGTCATAGTTG
CTCATCTTGTTCTGAAGTATGGATGTCAATCTTTCAAAACCCATTCCAGTGTCAACATGC
TTAGCAGGCAAAGGTCTTAACGAGCCATCACTTTCTCTGTTGAACTGAATAAAGACAAGG
TTCCAGATCTCAATGCATGTAGGATCATCATTATTAACCAATGATGCAGCATCGCGGTTA
CCAATTCTATCAAAGTGAATTTCAGTGCAAGGTCCACAAGGACCAGTATCACCCATCTCC
CAGAAATTATCCTTACAACCAAAAGGCAGAACACGACTGGGAGGTAGAAACTTGAGCCAA
ATATCTTTGGCTTCATTATCAGGCACAAGACCCGCGCTCTCATCTCCACCAAAATAGGTT
GCATAAATTCGATCACTAGGAAGCCCATAAACTTTCGTAAGAAGCTCCCAAGCCCATTCA
ATAGCTTCTTGCTTAAAATAGTCACCAAATGACCAGTTTCCCAACATTTCAAAGAAAGTA
TGATGATAAGTATCCTTCCCTACATCATCCAAATCATTATGCTTTCCACCCGCGCGTATA
CACTTCTGAGTATTACACGCGCGAGTTAGCTTACTCAATTGAGTGTTCGGATCCGCCGTC
CCGAGAAAGATAGGTTTGAACTGATTCATACCGGCATTGGCAAACAAAAGAGTCGGATCA
TTGAGAGGCACGACAGGGCTGGATTTCCAGTTGACGTGGTTCTTGCCTTCGAAGTATTTG
ATGTAGTTATCGCGGACTTTAGCGGCCGGCCACTCAAGCTGTTGAGGATCGACGCTGGGC
ATGGCTAAAGTAGAAACTGAACAAGTAAAAGAACGGAATGTGAATAAGCGAGATCTGAGT
GACGGATGGTTGAAGAAAACCCTAGCCGCCACCAAAGAACTCGGAAGAAGAAGAGACGCT
GATTTTGAGGTTTTAAGGAATAGACTTCTCATTCATTTTTTTTAGTTTT
>ntig_695_consensus
GGATTCACCTCCCTGAGGAGGTGGTGGGGTTGAGGGAGCCTCAGGCGTGGCTGCTTTAGC
TGGCTTTGGTTTTGTCATTATTGGCCTGCAGAACCTGTCAAGTGCTTCAGCCTTCCTTCT
CACATCAGCTAATAAGAGAACAGGAGTAGCATATTTGGGAAGAGAATCCTGATGCTGCTT
CTTCTCTCTCAACCAGGCCTCTGCTTCGACACACTCATTCAAGACCTTCTGCTTTTCGGC
CAAATCAATGTGATCGAATTTGGGATCACTAGACATAGCTGCTTCTCTGTAACTGTTGAT
ACAGTAAGCAAGTTGATCAATCACAGAACTCCTTTCTGTGAATTCCTTGTAACGCTCTTC
AATGGGGTCACCTTGCTATACTTTTTCAGCTCCTCAAGCTTGGCAATATAAACACCTTTG
GTTTCATCCTCACCATCTTCATACAACCAATCTTCTACTTCCTGTAGTCTGGCAGTGAAC
TGCTCCCTTTCAGAATCAGTGACAAATTCCTGGTATTTGTCGCAGAGCTTATTCCTCATG
TCATAGACATAAGCCTCAACAGCATTTTTCCTATCTTTAGTTTCTTCCATTACTCGATCT
TGTAGAGCCATTTCAAACTCCTTCTCCACAGCCTTTTGCACATCCACTGGTACCATTGCA
CCATATACCAATTCTGCTACAGGGATGTTTGTTTTCTTCACCTTCTTCTTCAGAGGCTTA
GCATCAATCTCCATTTCGGCAGTCTTTTCACCTGACTCAGGAACACCATTTTCAGAACCA
GGGGCATCAGAACTGGCTTTAGCATCTTGAATATTTATATCAATCTCATTGGAACTAGGA
GGAGCAGAATCACCAGGAGCCTCATCAGTCTCCATCTTAGCAGCTTCCTTTTCTTGCTCT
TTTGTGACTGGAACCTCAACTTCTTCTTCCTCCAAGAGTGTTGCAGACTCAATAGATACA

ATTCCATGCAAATTCAACCGAACTTTGACTTTCACTTTGGCCCGTTCACTTTTAGTAGAT
TGGAAGGGACCAATCGTATAAGTACTGATCTTAGCGGGTGCCTGTAACTCACTCACATCA
GCATACAGGACATCCACATTGAAAGTACCTGACCTATAGAAAGTCAAAGCTTTAACACTA
GGCATTGGATTTCCCTTTGGAAACACAATAGTGCTTTGCTGATTATCAGTTGCCTCATTT
TGAGCTTCTGGTCCAGAGCCTTTCCATGACAAAGCAATTGAAAATGGGAAGCTCTCATTT
ACCTGAAACTCTCTCACTTTAAAAGTTGGACTAAGGATGGCACATTGCAAAGCACAGCCC
CTTGCAACACACTCACTTGCATTCATTGTTCGCCTAGGCTCCTTCCCAAAGAAATCTGTT
AAGATTTTGATTATGGAAGGAACACGAGAACCTGATCCAACAACCTCAACCACATGAATA
CTCTCAACTGAAAGCCCAGTTTCAGCGAGAGCCTTCTCTAAAGGCCTCTTCACACGTTCC
AGAATGGAAGCACTGATTTGTTCAAACTCATCCCTCTTAATGAAGCCCCTCACATCCTTC
TCATCCATCAAGCATTCAATATTCAAAGGTGCCTCAGGATTGGCACTAAGAATTTTCTTT
AGTTTCTCACAGGCAGCTCTAAGACGAAGGCAGGCTCGGGCATTCTGGAAAACATCAATC
TTGTATTCGTCCTTGAACTTGGCTGCAAAATGCTGGAATAGAGCTTCATCAAAATCTCTA
CCACCCAATGACCGATCAAAAGAGTGACCCAATATCTTAAGTTGACCCTTCTTGAAACCA
GCTATGCATACTTGGAAACTTGAATTACCAACATCCACAAAGGCAACATTCAATTGGTCA
TTTTCTGGCAAATCTGTCTTATAAATGCCATAAGCCAATGTTGTGGCTGTAGTTTCATGA
ATCAATCGGAGGGGATGCAACCCTGCAATTTGAGCTGCATCCAGCACAGCTCTTCTTTGA
AGCTCGGTGAAATAAACGGGAACTCCAATGCAGCAATCTACCACAACTGCATTCAGATTC
TTCTCAGCTATGCTTTTCAAATCTGACAACAACATTCCCAGAACTTGGGTAGGGGTAAAA
GCCCTCATTTCACCCAGGTATCGTGCATGTATCAAAGGGAATCCATCAGGACCTTCAGTG
ACAGCAAATGGCAATGACTTCAGATCCCTTTGCAATTCAGGGTCAGAAAACTGTCTCCCA
ATCAAACGTTTAATCTGGGAGATTGTGTTTTTGGGGTTCATCATAGATGATGCAGCCCCT
GCAGTCCCAATGAACCTCTGCTTGTCACCAAAGCATACAACGGCTGGTGTCTCACGCTTC
GATTCATCATTAAGCACAACATCAATGCCCCTTTGCCGAGCAACAGCAACAATGCAGCTC
TCATTGCCAACATCAAAACCTACAACGCTCATCTTGTCGAAAAACTAAGCAATTTATTAG
AATTGGTCTGCTCCTTATAAAGACAGGAGGGGTTTGCAGAGGCTGTGAGCTCTGGAAACT
TCTATATTGTGTTTGGGAGAGGAGGAATACCGCAAACACAAACGAGAGAGTGGGCTTTAT
TGGGAGGATCTGGACACCTAAGG
>ntig_696_consensus
GGGGGGACTGGAGTTTCAACGTGTGCGCTTCCCAAAAAAAAAGGAAAAAAAAAAAAAAAA
AAAATAAAAAAAAAAAAAAAAAAGAAGTGCACAAACATAGGGCCTCGTTCTTTTCATCAT
CCCACCGAAAAGTTTCTGTTCCAGGCTCACGGAAATAGAGATTCTTATACATAGGAACCA
AGATCTTGCCCGACATAAATCATTAAAGGCCAACCCAACTCTAAATTTATGCAGTATTTG
ATGTGTTTTCTGACCTGTCAGTCTCCATAGGTTCTCCAGATGTTGCTGGTGCCTCACCTG
TCGCATTTCCTGCATTTTCATTTGGATGGGCATCCTCACCATGTGGTTGGGATTCACCTC
CCTGAGGAGGTGGGGTTGCCGGGGCTTCGGGAGTGGCCGCTTTAGCTGGCTTTGGTTTCG
TCATTATTGGCCTACAGAACCTGTCTAGAGCTTCAGCTTTCCTTCTCACATCAGCTAACA
AAAGAACAGGAGCAGCATACTTGGGAAGCGAGTCTTGATGCTGCTGCTTCTCTCTCAACC
AGGCCTCTGCTTCAACACATTCATTCAGTACCTTCTGCTTTTCAGCCATGTCAATGTGAT
CAAATTTGGGATCACTAGACATAGCTGCTTCTCTGTAACTGTTGATACAATAAGCAAGTT
GATCAATCACAGAACTCCTTTCTGTGAATTCCTTGTAACGTTCTTCAATGGGGTCACCTT
GCTTTTTCAGCTCCTCAAGCTTGGCAATATAAACACCTTTGGTTTCATCCTCACCATCTT
CATACAACCAATCTTCTACTTCCTGAAGTCTGGCAGAAAACTGCTCCCTTTCAGAATCAG
TCACAAAATCCTGATATTTGTCACAAAGCTTATTCCTCATGTCATAGACATAAGCCTCAA
CAGCATTTTTCCTATCTTTTGTTTCTTCCATTACTCGATCTTGCAAAGCCATTTCGAACT
CCTTCTCCACCGCCTTCTGCAAATCAGCTGGTACCATTCCACCATATACCAATTCTGACA
CAGGGATATTTGTTTTCTTCACCTTTTTCTTTGGGGCCTTAGCATCAGTCTCCATTTCAG
CAGTCTTTTCACCTGACTCAGGAACACCATTTTCAGAACCAGAGGCATTAGCACTAGCCT
TAGCATCTTGCATATTCACATCAGTCCCATCAGCATTTGAGGGAGGAGCATCAGCGGGAG
CCTCATCAGTCTCCATCTTAGAAGCTTCCTTTTCTGGCTCTTTTGTGACCGGTACTTCAA
TTTCTTCTTCTTCCAGGAGTGTTGCCGACTCTATGGATACAATTCCATGCAAATTCAATC
GAACTTTCACTTTGACTTTGGCTCTTTCACCTTTAGTAGATTGGAAGGGACCAATTGTAT
AAGTACTGATCTTGGCAGGTGCCTGCATGTCACTCACATCAGCATACTGGACATCTACAG
TGAATGTACCTGACCTATAGAAAGTCAAAGCCTTAACACTGGGTATTGGATTTCCCTTTG
GAAACACAATAGTGCTTTGCTGGTTCTCAGCTCCTTCATTTTGAGCCTCTGGTGCAGAGC
CTTTCCATGACAAAGCAACTGAAAATGGAAAGCTCTCATTTACCTGAAACTCTCTCACTT
TGAACGTAGGACTAAGGATTGCACATTGTAAAGCACAGCCCCTGGCAACACACTCACTTG
CATTCATTGTACGTCTAGGCTCCTTCCCAAAAAATTCTGTTAAGATCTTGATTATAGCAG

GAACACGAGAGCCTGAACCAACAACCTCAACCATGTGAATATTCTCAACAGAAAGCCCAG
TTTCAGCAAGAGCCTTTTCCAAAGGCCTCTTCACACGTTCCAAAATTGGAGCACTAATTT
GTTCAAACTCATCCCTCTTAATGAAGCCCCTCACATCCTTCTCATCCATCAAACATTCAA
TATTCAAAGGTGCCTCAGGATTGGCACTAAGAACTTTCTTCAATTTCTCACAGGCAGCTC
TAAGACGAAGGCAGGCCCGGGCATTCTGGTAAACATCAATCTTGTATTCATCCTTGAACT
TTGCTGCGAAATGCTGTAACAGAACTTCATCAAAATCTCTACCACCAAGTGACCGATCAA
AAGAGTGACCCAATATCTTAAGTTGACCCTTCTTGAAACCAGCTATGCATACTTGCAAGC
TTGAATTCCCAACATCCACAAAGGCGACATTCAATTGGTCATTTTCCGGCAAATCTGTCT
TATAAATACCATATGCCAAGGCTGTGGCTGTAGTTTCATGAATCAGGCGGAGAGGATGCA
AGCCTGCAATTTGGGCTGCATCCAGCACAGCTCTTCTTTGAAGGTCAGTGAAATAAACAG
GAATTCCAATGCAGCAGTCCGCCACAGCTGCATTCAGATTCTTCTCAGCTATGCTTTTCA
AATCTGACAACAACATTCCCAGAACTTGGGTAGGAGTGAATGTCCTTATTTCACCCAAGT
ATCGTGCATGTATCAAGGGAAATCCATCAGGACCTTCAGTGACAGCAAATGGCAATGACT
TCAGATCCCTTTGCAACTCAGGGTCAGAAAATTGTCGCCCAATCAAACGTTTTATTTGCG
AGATTGTGTTTTTGGGGTTCATCATAGACGAGGCAGCCCCTGCAGTCCCAATGAACCGCT
GCTTGTCACCAAAACAAACAACAGCTGGAGTCTCACGCTTTGATTCATCATTAAGCACAA
CATCAATACCCCTTTGCCTCGCCACAGCAACAATGCAGCTCTCATTGCCAAAATCAAAAC
CTACCACGCTCATCTTGTCCAAAAACTAACTGATTTTATTGGCTCAAATTGGACTGCTC
>ntig_697_consensus
ACGAAGAATGAATAGCTGCTAATTGGTATCAAATGAGTCAACAGAAAGAAAACAAGCGAA
TATAAATTCAAAGCATAACCTCGTGGTGTGTCGTTAACAGAAAGCTAGAAAAAAGGGCAT
AATCTTTAAAATTCTTTAGATTTCATACACAACCAACATTCATTTCAGAAAGAAAGGGAG
GCTCTGTGTCTGTCGCAATATATTATTTATTTCCAGCTCGACAGCTCAAACCCATCTTAA
ACAAACTGCCCCTTGCTCAAGGGAATTCTTTCTCGTACACTTCATTGGCATACTTCCAGT
TTATGACTTCCCAAATGTTTTTCAAATAATCCGGTCTCACATTCTTGTACTGTAAATAGT
ATGCATGCTCCCAAACATCTATGCCAAGCAAAGGAACCAAACTTTGGCCTTTAGTTACCA
GTGGATCCTGATTTGCAGTGGTTTCAACCACAAGCTTCTTCAACTCCTTGTCCACACCAA
GCCACACCCATCCAGAGCCCTGTAAAGCTGAACCCTCAGCACTCATCTTTTGTATCAATG
CTTCCAAAGAGCCAAATTGTGTGTCAATAGCCCAACCAAGGGAACCCTTTGGTGGCTCTC
CACCTCCTTCATGAACAGGAGTCAGATTCTTCCAGAAAATTGAGTGGTTGACATGACCTC
CGCCGTTGAACTTGATGGCACTTTGCAACTTGACAACAGTAGAAGCGTCGCCTTTGTTTA
TGGCTTGGTCGAGCTGCTCAAGTGCCTTGTTGAAATTAGTAATGTAAGTCTGGTGGTGCT
TCTGGTGATGCAACTGCATAATCTCGCCGCTAATCGCTGGTTCCAAAGCGCCATAATCAT
AGGGAAGATCGGGCAACGTAAAGGTCTGAAGTCCACGGACGTGCGACTTCAATCCAAGGC
GTAGAGATTCAGTTAGGGATTTCCTGGTCAGGTGAGAGAGAAATGCCATCGCGCTGTAGA
GAAGAGGAGTGGAGACGAGCTCTAGAGATTATCTGAAGGGTACCAAATGAAGTGTAAATT
TTTTGTTAATTTATAGTGGATGATGTGGCGGAAATCAGGGTGCCACGGCTGCACCGTGAC
GCGTGGCGGTACGAAATTCTGT
>ntig_698_consensus
CTAGCAGCGGTTCAAAAACAAACCTACATATTTTGACAGTCACAAATGCATTAGCCTCTC
CCACCTTAGCAACTCAGTACTATATTTTGTAAGTTCTAAGTTATGCATGATCCCTTTCCT
TGGAAAGTACAAAGATTATCCCCCTGATGATCTCCTTTAATAAAAATCCATGAAATCATC
AGTTCTTGATCAACAGCTCCTATGTTAACCTTGGGAAGTCTTCAGAGGCAACAAAACAAT
GGCATCGAGGCCAATCATAAGCCATAACTTCACCTGTTGCTTCAGGGTTACAAAGAAACA
AAATCCAAAAGGACCATGCTGAAGAGCCCTAAGCTTTGGACCACCTAAACTCAAACCAGA
TCTCGTAGTGATTAAAAAGCAAGCTATCATGTGTATACTTCCCAACATATTCAAGTCCCA
AACTTGTGAGCCTCTTTGTACACTCTTCCCCTCCTAATCGTGAACAAAACTTTATGTTAT
GCGACACAAAGATTTGCCCATCGTAAGGTTTCAACTTCTTTGCTAACCTCTCAAGCCCAA
GCCACACAAGTTTGTCAGGCATATGAAGGAACACAGCACTAGCATATATCAAATCATACA
CAATTTCAGAACCAAACTTTGTAAAATCCATATCCCCACCTCGCACAATCAGAGGCCGCT
TATTTAAAAGACCTTGAGAAGGGAGCTCATATCTGAAAGCAGCCATTAAAGATAGCTCAT
CTTGCTCAAGGCAATGAAAGTGTTCGGGACTTAGATACCTAATAAAATGCAGGCCTACGC
GAAGTGTACCGCATCCAATCTCAAGGACATGTGAATTGGGCGTCAAGTTACTTGCCTCAG
CAAGGAACTCAAATACATCTCGACCACCGGCCCAGGGCTCCCCATAGTTGCTGTGATGCT
CCTCAACAAGGAGCTCACCAGGGCATGGAAGGGCCGTGTGATTGTTCGCTTCAGGCTCTT
CATAATGCGATATGCCTTTGAATTGGATAAGATCTTGAAGTTGCTGAGCTCTAGTGCGAG
GGTTGATCCCTTTCCTCAACACATTGTCTTGCATATGAAGTCCGTTTCGAGAGATCTGAT
CCTTTACCCACTCAATGTCGGCTTCCGTCGGCGGAATCCGATCTCCAGAGCGGTACTCTT

TCGGCGGCAGGTGGGCGACAGAGGAAACGGCAGCTTCTGAGGCGGAGGAAATAGGGCAGT
TGCAGATCGAGAGAGAGGAGAGGAGGAAGAAAAGGAAAATGGCAGCGACGGAGAGCGTGA
GAACGAAAACCGGTACGGTTATTGTGATTCCTTTTTGTTGTGTTAGCGCCATTTTAGGGA
TGTGTATGTTGAGTTTTGTTTTGGTTTCGATTAGAGGCGAAGGCGGGGGCGAGGCTTTGC
TTGGAGATGAACATGGAAGATGGTGATCTGCTCCAAGAGGGCGTTTGCTGCTCGAGGCAT
ACGTCGTCGCTTTCCGCTGGGTTTCACTCAAATACAATTCTCAGCCTCAACACTTAAAAC
CCAAACTATC
>ntig_699_consensus
CTGTAAATGGTAAAGTATCTCGTGATTCAATGGAAGCTTCTCATCAATAACATGATCAAG
GTAACCACGGATCTCTCTTAGTCGTGCATCCAGACCTTTAAGGGCTGTAAGTTTTCCAGT
GACCTCTGTTGCTAAAGTACTGATGGTGGTATCTTTCACATCCCTCAGCAAGTGTTCCAC
TCCAATTTCCTCGACTTCATGAGCTGCAATTTCTGAAGGCACGTGTACAAAAACCTTTTG
GCTTTTTTGAGTAGCATTCTCTTTAACCTCCTCAACATCATAGTAGGCCTTGGTGGGTAT
TCCCAGCTCCTTTGGTTGCACATCAATGATAACTAATACAGGATTTGGAACATAGTTGTG
GAATAATTGGTGAATATCAAGATCATTCTCCCGCAGCTTCGGGCCTGTACTATACCACCC
TACAACATGCTCCTTGGCATTAATTCTTTTGAACATCGAAAACATGGATTCATGGTAGTT
GTGGTCAAGAAACCAGATGCTAGGGTCCTTGTCATCTTCCTCAAAAGGCACTGCATAGCT
GTTGGAGATATCGACGGTGCCTTTGAAGGAACTACCGAGGAGGACACCGACGACACGCTT
GCGGGTGTCCTTAGCAACACGGTTATAGTTGTCGACTATACTCAGTAACACGAGTGGGTG
TACGATCACTTTCTCTATTGGTCTGGTTGATATTTGCTGTGTCTTTATCACGTCCATCTT
CTTCAACACCCTCTCGGTCTCTTTTTCTGCCCTTATTGTTTCGCACAGCTTCTTTCAGTT
TCGTGCTTCTTATTTGGGTCTCACTGTGTCTGTGTTGTACTTGG
>ntig_700_consensus
ATATAGTTCAACTAAGTTATACAATGATAAAAAGTCTTGCCACTTCTATATTTTACAACT
TCATACAATATGAAGCATGTAAACATTAACATGAATTTCATATCAAGAGTTCATAAATTA
GCCTGCAGTCCATTCCTACGATGCATCAACAAGTTGATATTATCCCCACAGCTAAAGTAA
ATCAGTTTTTGAAATAAATGTCAACAAAGTAGGAATAATTGATATTCATTCACCAGCATT
GATTTCACCCAATGCTCAGCATTTTCATGATGACCGCTGCACCTAAATATAACAATCGGG
GAGAGATCACACTATAACTGCCAAAATTTTCCATTGTTTAACATGGCAAATCTTTAGCCA
AAATTCTTATTGGTCTTAGTGTACACAGAAAAAATTGTAGAACCTTCTGTCTTGGTCAATC
TACCTCTCCAAGAGCATCACCAGCTCACAGTGTGAAGTATGTGGGAAAAGATCTACAGCC
ATGGCTTTCACAGGTTGGAAAGGCTCTGACACTGGCATAGATTTGGCTCGATGCCGAGCC
AATCCTGCACTGCTCATGTTCCTCCATCTCCTGTTGTCTTTATTTCCTTTCTCAACCTTT
TTGGGAGATGGTGTGCAGAGTTCAATAGCATTTGCCACTAAGGTTTCAGGATTGCAGGAT
ATGTAAACAAGTCGTCGCAGATGTGGATGAGTCCGCAAAGCTTTAATCACAGTTGGATGA
AGTCCACCACGGGGAGGATCAACAATAACAACAACATTTTTAAACTGCTGCACAGAGGAA
TTTTCATCTTCCATACAACCCTTGTGGACTTGATTTTCAGGTTCTTTTCTACTACTTCCT
GAACACTCAGAAGTGCCAACACCATTTTCAGACTCATGGACTGAGCTCTCATCACTGTCT
AGAATGTTATCCAAGGAGGAATCTTTCTCTTCTGTACTAGCTATTTCTTTGTCATTACTC
TCCAATACATGGTCATCTTCTTTTTGAGGCAAATTTAGATACTCCCTTAATAAAGATCCC
ATCACATCCTCTGCC
>ntig_701_consensus
GCAATTTGTAGATTCTCCAGCTCTTTCATAGCAAACTTATTATGGACGAACAACCCACTG
AACAGATCAAAACTATCCTTTAAGGTATTATCTTTCAATTGTAGAAGTGTCTCAAATTGC
TTTGTGTTCATGGGTGTTGATTGTATCTCCATTTCTGATAAAACCCTGCCGATTGTATGC
GTAAGAAGTTGGCTTTTGGCTGCTGCTTTATCGTGTTCTTCGCAGGACATTTCAACCATT
TTGCAACCCTCAACTTCAAAGATTTGAAGGAAGGTGGAGCAGGTTTCCTCATCTCTTATC
CGAACTCTGTCGTACACGAAAGGGAGATCTTTCCAGATATCTTTCCCACTTTCTGGACCA
AACATAGGGTGAGTGCAAAGCACATCCATCTCTTCCGGCAACACTTGCAATAGAACGTTT
CTTGGGTGCACTTTGACAGAAAGAACATCGGCAATGAGCGTTGGCCGTTTTAGTTTCTTC
AGTGGCAGTGACTTGAGGACCTCGGAGAGAGATAAAATCGATGTGCATATCAATATGACA
TCGTTATCTGCTTCAAGGAATGCACCTGGGTCC
>ntig_702_consensus
CTGAGCAGCTAGCTATAACTGAGCAAAATACACCCATATTGACACACCTAAATTTTCAAT
GAAGTGGAAAACTAAAAAGCGCCATCATCTATTGCTCATGGTTCTATGGTTCTTGTTTGG
AAATTTGCACATACACCATCTGGGTATATCCATTAAATACAGAGTTCTCAAGTCTACCTA
AACCAAGTTGCTGAAACAAGCTGAAGCGGCCATCTTCAACAATTGGACTGGATGCACGAG
CAGGATCATATCGTGTTTGATGAGTCAGGGAAGGACCAGCCACCGGAGGGAGCTCCCAAT

AAACATCTAGAATTGATTCGATAAAACGACTGTTCTGAAAATGTTCCATATATGAGATTA
GCTTGTTAGTGTGATTGAAGTTATCTCGCATCTCTCCTCTAACACCCCACCAGGTTTCAT
CTGGTCCACATAACTCTTCTACGTCCACCGCCTTCTCCCAGAAATTCTTCCTTCTTGATC
TAACCCTGGCTTCATCACTATCTCTAAAGCAGCTATGGCCACCTCCTTTTATATAAAACT
GATCACATATTTGCCTCAAAGAATAATGATCTCCAGTTCCAGTATCATAATTATCCTCAA
AAACAAGATGATGAAAGCCAGCCTTCAGCGCCTGCTTAACTCTTTTCAATTCATTTTGAT
GGTCATCAAAAAAGATAAGGACTCGGCTGAGATTCGTAATCCCATGTTGCTTCATCACAC
TCCCCCAATTAACACTACCAAAATCCACAAAATCTTTTCCAGCAAAATATGTGCAGTTTC
CATCAACATAAGCAGGTCCCTTTTGTAGGTACTTACCTGGATGCCGGGGTGAAAGTGAAA
TGATTTTAGTCTCTGGCATTGCTTGCCGTAAAACCCAAGTTGAATGCCCCTTGAAAGCAC
CACTCTCAATCATTAGATCTGGTTTCAGCCACTGTGCAATGAACCAAAGCCCATAGCTGT
GGTCGAAACCCATCCCATAAATATTGTTCTTAATCGGACGAGTTTCATATATTGGTACAA
ACTCTTCCAAACCCTTGAGTAAATCCTTGGATGTCCATGCAACCATTCTTCCATGTTTCG
ATCTGCACCAGGGAACACCAAGATATCCGAAATCAGATTCCAATGCGTCGGGCCGAAAGA
GTCCGGGTCCGCCCCGAGAACGCGGATCGTATGGCGAAACACAGACGAGGCCACGAAAGT
AGTGATAGACCAAGGAGGCGACTACGAGGAAGACAAAGAGAAAAATAACGAGAGATGGCA
ACACGCGGCCGATGCGTGAGAGGTACTTGGAAGCTAATAAGGAAATGTGTTGGCGTTTGG
CCTTGGAGTCATCCACGGCGTCGGATGCGGCGTCAGAGGGTGGAGAGGATAGGGGCTCGG
TTTCAGGGGGGTAAGGAGAGGGTCGCCGCGAGGAGACCGAGCGGTCGAGCATTGTGGCGG
GGATGGATTTCGGCGAGTGAGGTGAGGTTGACAGAGTGTGA
>ntig_703_consensus
GCGTCAATAAATGATACATTCAGTTTTTAGGCGCTATATTAAAGTTAAAATCAGTGATAT
GATACATATATTGAATGAACAATAGAAACGATACTTGATGGCAATAGTTCTGCGCGGCTA
GCTATGACTATGCGTGACCAAAAACACTGATATCTACACACCTGAAGTGCAAAACTATAA
AAAACATCCTCATCTATTGCTCTTTATTCTATGATTCTTGTTCAGAAATTTGAAGATACA
CCATCTGGGTATATCCATTAAATACAGAGTTCTCAAGCCTACCTAAGCCAAGTCGCTGAA
ACAAACGAAAGCGGCCATCTTCAACGATTGGGCAGGAAGCACGAGCAGGATCATATCGTG
TTTGGTGAGTAAGGGAAGGACCAGCCACCGGAGGGAGCTCCCAGTAAACATCTAAGATTG
ATTCGATAAAACGGCTGTTCTGAAAATGCTCCATATAGGAGATTGGCTTGTTACTGTGGT
TGAAGTTATCTCGCATCTCCCCTCTGACACCCCCACCAAGTTTCATCTGGTCCGCATAATT
CTTCTACATCCACTGCCTTCTCCCAGAAGTTCTTCCTTCTTGATCTAATCCTTGATTCGT
CACTATCTCTAAAGCAGCTATGGCCACCTCCTTTTATATAAAACTGATCACATATCTGCC
TCAAAGAATAATGGTCTCCAGTTCCAGTATCATAATTATCCTCAAAAACAAGATGCTGAA
AGCCAGCTTTCAGTGCCTGCTTTACTCTTTTCAATTCGTTTTGATGGTCATCAAAAAATA
CTAGAACTCGGCTGAGATTGGTAATACCATGCTGCTTCATCACATTCCCCCAGTTTACAC
TACCAAAATCCACAAAATCTTTTCCAACAAAATATGTGCAGTTTCCATCGACATAAGCAG
GTCCCTTTTGTAGGTACTTTGCAGGATGCCGGGGTGAAAGTGAAATGATTTTAGATTCTG
GCATTGCTTGCCGCAAAACCCAAGTTGAATGCCCCTTGAAAGCACCACTCTCAATCATTA
GATCAGGTTTCAGCCATCGAGTAATGAACCAAAGCCCGAAGCTATGGTCAAAACCCATCC
CATACATGTTGTTCTTAATTGGACGAGTTTCATATATTGGTACAAACTCTTCCAAACCCT
TGAGTAAATCCTTGGATGTCCATTCAACTGTTTTTCCATGTTTCGATCTGCACCAGGGAA
CGCCAAGATATCCGAAATCAGATTCCAACTCATCGGGTCGAAAGAGCCCGGGTCCGCCCC
GGGAACGCGGATCGTACGGCGAAACACAAACAAGGCCACGAGAGTACTGAAAGATCAAGG
AGGCAACAAAGAGGAAGCCAAAGAGAGAGAGGATGAGAGCGGGCGATACGTTACCGATGC
GCGAGAGGTACTTGGAGGCTGACAAGGAAATGTGATGGCGTTTGGTCTTGGAGTCGTCGA
TAGCATCGAACGCAGTGTCGTAGGGTGGAGCAGATAGAGTCTCGGTGTCAGCGGAGGGAC
GGCGGGAGGAGACGGAGCGGTCGACCATCGCGGCTGTGGTCGTGGAATTGGGAGAGTGAG
GGTGACGGAGTGCAGTGGTGCTG
>ntig_704_consensus
ATTGCTGCATTCAGATTATTCAAAAGATCAATCATTTGCGTATTCGTTATAGTTTGACGC
AATTCAATATGGAACATGTAGTTTTAAAGAAGTGAAAACTGGAAATGGGTGAAGAAGCTG
CCGCAGCATCTACAGTGTAAGCGGTTTTGTCCCCAGCAAAATTAACATGGAAACTTGTAA
TGAAAAACAAAACTACTTTTCAGTCCTAAGCTGTCCTCTACCCTTTAATAACTTTTCCAT
CCATGTTAATATGCATCTCCAATTCGGTCACGCCCTCCACCACGTCGATGATCATCTCGA
CCCCGTTCGATGTACATATCTCGACGATACTCTTTTGGTGGAGGTGCACCTCTAATTGGA
GATCTGCTCCTCTCGCGCATGTCTCGTGCCCATCTCCCGTGCTGAGGAGGAATTGGTGCA
ACCGGAGGGGGAGAGGGTACCCGTCTGTCGTACCCTTTCCTTTCATTGAAGAAGTTGTCC
CTTCCACGATCTCTATGGCCCCAACCCATAGGTAGCATTGGCTTGTTCCTCCCTCGGTAA

CCATCATCTGGAAAATCAAGACGATCCCTCCGCATGTGATCAGAAAACCTGCCTTCATGC
CGAGTAGGTTGTGGTCCACCAGCTCCAAACTCTCTAGGACCATGGCCATCCCGGCTCCAA
CCACGAGGAGGCGACCTATAGCCATTCATGGGTCTGCCAGGGGAAATATCCACAGGAGGA
ACAGGAAATCGCCTGGGAGGAGGGAGAGGCGGTGGTGGAGGGCCAGGATAACCCCTTCTA
GGACTCCCACCAGGTGCATAGCGAAATCTCTTGCAATTGTTACAGTACTCTCGCCTGGCA
AAGTTCAGGTTGCCGCAAACAGGATCAGGGCAAATCCAGTCTCCTTCCCGTGGTCGCACA
TTTGGATTATTTCGATTCATGCCTTCACCTCTTAAAGGCCCAGGACCAAGTGCAGGTCCA
TCAAAACCTCTGCCAAAAGCCCTGCCACCACCCCTTCCTCGAGAATAAGGTGGTGAAGGG
TCTCGATATCTACCATGATCCCTCCCAC
>ntig_705_consensus
GGTGCCGCATTCCTCGACAACCTGCAAGGCCTCGAAACCCTGAAAAACTTCGACGCCGAC
GACCACGCCGCCACCGAAATGGACAAGAAAGCCGAAGGCTTCCGCGTCATGACCATGCGC
GTGCTGCAGATCCAGCTGCGCTCGCTGACCGCCATGGATATCGTGGCCTACGGCGGTGCG
GCGGCCGGCATTGGTGTGGCTCTATGGCAGTATGCGCATACCGCCGCGGCCGCCGCAGGA
GCCAGCGCCGCCGGCTGGTCTCCGATTATGCTCGCCGCCCACCTGCCCGGTGCGTTCGCC
TACCTGGCCTACGGCCTGCAATACCTGATGCCGTTCGGTGCGGGCTTCCCGCTCACCCTC
GCCGGACTGTTGCTGATCGTGCTGCTGTCCGCTGAATTCTTCATCCCCATGCGCCAGCTC
GGCTCCTACTTCCACGTGGCGATGAACGGCATGACCTCCACCAAGCGCATCTTCGCCCTG
CTGGACACCCCCGAGCCCACGAGACATCTCAGGATCTCGTATGCCGTCTTCTGCTTGAAA
AAAAAAAAAAACGGCAAGCGTTCTAGGGATCAACTGCAATTAGTTTCTTGCCATACCAT
>ntig_706_consensus
ACCATTTGTACCAAACTTTTAAGCACAGAGAAGTACAATATGAACCTAACAATTCATCAG
CCAGTTACTAACTAGTAGTTTGAATTACATCCTTCACTGTAACCAAACCAAAAAAATACC
ATGAAATTCGGATGAAACATTTCCACATCACATCTCAGTAGCTGAATGAACATGGGTTTC
TAGTCATGTGTTAAATCTAANNGTCCAAAATCAGAGCACAACCCAGGTACACAGCCTTAA
GATAACATCATCAAGGAGATTTCACAAAATGCGGGGATAGCAGTTTAGCAATCTCCAGAA
AGCCAACAGTGTCCCTTCCATTGAAAATACTCTTCAACTTCTCATCACAACGAATCTCCT
TCTTATTTGCAGGATTCTGGAGGTCGTGGAGCTTGATATGTTGCCATATTTTCTTGGTAA
CTTCAGCACGAGAAGCTTCGTTGACTCCAAGAAATTTTCCCAGTTGTGGAGATACAGGAA
CAGGTTTCGCAATCCCTTTTTTCTCTTTCTCCGAGACAATAGCTGCTGCTGATGAAGATG
ATTTGGCTGCTGCCATCAGTGCCCTGCATCCTCCAAATGCCCTGTTGCAC
>ntig_707_consensus
GCTTATACTTTGACTCACCCAGTAATAACCATTACATGCATTAACAAAACCATATACTTA
CTTGATAATGTCAGAAGCGCCCATTGCATACTTGGTCGACAATCATTACATTCTGACATT
TTATCTGCCCATTGCACAGAACTAATACGTCCTCCATTGTATTGTACTATTAAAGTGAAA
AATGCTATAAAAAAAAAGGCTCGGTACATTACAGTTATATTGTGCTGTCCTTAGATGTCC
CCAAAACTGAATTAGAATATGAAGTGTACATCTTGTCACTCGACAAAGACAAAGCAAAAT
TTTTATCTACGAGACGTGTACAACAACTCCAGAAGGCAAGGCAAGATCAGTCGTTAGCAA
CCATGAGAGACGTGCTGTCCAATTTCTTGATGCACAATCTGTTTTGGGCAGGTCGGTCTT
TGGCCAATGGTGAATCATCAACATCGACAATAACCGAATCCTCCTCCTTAAAATCTCCTC
TCAACACCCCCATCGCAATTTCATTCTCAACCAACTGTTGTATCACCCTTTTAACCGGCC
TCGCTCCGAAGTTCGGGTCAAAGCCTAAAGTACCCAGAACCTCAACAGCTTCTTTTGTGT
AGTGAAGATCAATGTTCTTTTGTTTCAGCCTATCTCTCAATCGGTTCATCTGTATCTCAA
CAATCTTACTGATTTCCTTGGAGTCCAGAGGCTGGAAAACTATATACTCATCAATTCGAT
TCATAAACTCTGGACGGAATGTTTGTCTAGCCAATTCCACGACTTGTCTTTTCATGATAT
CATAAACAACTTCCTTGCTATCCTCCGTATTACGTAGAGTTTCAAGTATATAGTGGGAGC
CAATGTTTGAAGTCATTATCACAACGCAGTTTGTAAAACTGACAGTCCGTCCTTGAGAAT
CAGTTATCCTTCCATCATCCAACAACTGTAATAATATGTTGAAGACATCATGATGCGCTT
TCTCAATTTCATCAAAGAGGACTACAGAATATGGCCTTCGACGAACCACCTCAGTGAGCT
GCCCACCTTCCTCATAACCAACATAACCAGGAGGGGCCCCAACCAATCGCGAAACTGCAT
GCTTTTCCATGTACTCACTCATATCAATTCTGACAAGAGCATTTTCAGTGTTAAAGAGGT
AGCTGGCTAAAGCTTTTGCAAGCTCAGTTTTGCCAACACCAGTAGGACCCATAAACATAA
AGCTAGCTATAGGTCGATTGGGATCAGACAATCCTGCCCTTGAACGGCGGATTGCATCAG
CCACCGATTTAACTGCCATATCTTGACCAACCACCCTTTTGTGGAGCACATCTTCCAGCA
TGACTAGCTTGTCGCGCTCTGATTGTTGAAGATTTGACAAGGGTATACCTGTCCATTTAC
TCACAATTTCAGCAATATCTAGATCAGTCACCTCCTCTCGAAGAAAAGACTGCCCAGACT
TTTGGAAGTCAGCAAGGTTCTTTTCAGCCTCTTCTAATTGACGCTGAAGGACATCAAAG
TTCCATATTTGAGCTCAGCAGCACGATTTAGGTCATACTCACGCTCAGCAGCTTCCATCT

CCAAGTTAACTCTATCAATCTCTTCTTTAATTGACCGTATCTTTGTCATGAGATTCTTCT
CCCGGCCCCACTGTTCATTTAGTTCTCTCTGTTTTTGTTTTAGCTCATTCAGATCATGTT
CCAGTTTGCTTAACCTTTCTTTGGAGGCTTTATCAGTGCCATTTTTCACAGAAAGCTTCT
CCATCTCCAACTTAAGTACAGCTCTATCTATCTCATCCAACTCTGTAGGTTTAGATGTGA
TCTCCATTTTCAGCTTTGCAGCAGCTTCATCGACAAGATCAATGGCTTTGTCAGGCAAAA
AACGCTCTGTGATGTATCGATCTGCAAGAACTGCTGCTGAAACAAGGGCACCATCTGATA
TTTTAACACCATGATGCAACTCATAGCGTTCACGCAGCCCACGAAGAATGGATATCGTAT
CTTCAACAGATGGTTGACCACAAAAAACTTGTTGAAATCTGCGTTCAAGTGCAGGATCCT
TTTCAATGTATTTCCTGTACTCATTCAATGTAGTTGCTCCTATACACCGGAGTTCACCAC
GGCCAAGCATCGGTTTCAACAGATTCCCAGCATCCATTGCACCACTTGTAGCCCCTGCGC
CAACTACTGTATGGATCTCATCAATGAATAAGATAATCTGCCCATTTGAAGCAGTAACCT
CCTTTAAGACAGCTTTCAACCTTTCCTCAAAATCTCCACGAAACTTAGCACCAGCAACCA
ATGAGCCCATATCTAAGGATATCAACTTTCTGTTCAGCAATGGCTCTGGAACATCCCCTC
TAACAATACGTTGGGCTAATCCCTCAGCAATTGCAGTTTTCCCCACACCAGGCTCACCAA
TAATAACAGGATTATTTTTAGTTCTTCGAGATAATATCTGGATACACCGCCGAATTTCAT
CATCTCGACCTATAACTGGATCAAGTTTGCCACGCCTAGCAAGCTCTGTCAAATCATTTC
CATATTTGTCCAGCACCTCATATTTTCCTTCAGGATTTTGATCTGTTACTCTCTGATTTC
CACAAACAGCTTTGATGGCCTCCTTCAAGTCCTTCTTGCTAAGCTGAAGGTTCTTGAATA
ACCGTTGCCCAAACCTCTTATCAGAATGAAATGCCAACAAAAGATGCTCAACAGACACAA
AA
>ntig_708_consensus
ATAGAAAAGAATTTGATTCACACTTGTAAGTTGGAATATACTGGAAGTAACTGTGCCTAA
AATCATCAATACCTGTTACACTTCACCTGAAAAAAATCCTGCATTATAGCATTTCAATAA
AAATGGAATTCGGGAAGCAATAATTACCCATTCCCGTTTGAAGACATCAAATGAAATTGC
CTCAAAAAGTTTCTACAAAATTTTATAGAACAAACTTCCCTGGAGCCTCTATTTGGGTTA
AATACAGTTCAAGGGCAGACTTTCTTTCACAAAGCCTCCTTAAGACGAAAGGTCCTAGCT
ATGTTCCTTGCATTGTCATAATCATTGAGCTCAACCAACATTTTTTTCAAACGACGAGCA
AGTCCGCCTGTCTCATTCTCACTCACGCCAGCCAAATGCTTCTTCTCAAGTGTCTTTCCT
AATGATAGCATGTAATCAACCAATGGTTGCAACTTGCAGCTGCGACTGTGGCTTCCAGTG
ACATTTGAACCAGCCCAGACCCCAGATAAAGAAGCAACAATTTTTTCAGTTCTCATGATT
GCATCATGAACATTTGATGTGATGCTTCGAACATGTGGCAAAACTTTTCTACACAAAAGC
TCATCAAGGACAAGCTCTTCTAAAATTGGCAATGCTAGGATTTCTTTCCATGAGCAAATA
TTTCTCATCAAACGAACAGACATCCCAAACCGGTATGCTGCAATCCGTGCTGCATTTGGA
ACAGCATTCATTAAAAGAGGGTTCCATGTAGGGACTGTAAGGTTTGAAACAGCTTCAGCC
AGACGGGTACGAATAGCAAGTAGTAATTCTGCTAGTGCCTCACTGGAAGTAGGAACATAG
GTGACAACCAAGCTTGTAGCAGAAACAGCATTCCTTGTTTCTCGGGTACTAAGCATGTCC
CAACAATGAGCAATTTCATGGTGCAGAATAGGAAGTGCAACCTTTTCCACCAGTGTAGGA
ACCAGGTTAGCATCAGCATCATCAGGGGCAAAATCTCTCCCATCTTCTGGCAAACCATAA
TTGAATAGTAAAGAATGCCATTTCATATTGGAGAAATCAGCATCCTCATTTAACGGGTCC
CACTTTAAGAGCTCCAACCTCACATATGGAGACAAAATAGTAGGGGCACTTAAGGACATG
TATGCATCACGATAGCTCGATGAATACTCCCTCTTCCATTTTTCAAACCTTTCTTTAACC
AATGAGAGTTTGGAATATTCCTCAGATGCATCACTGAAAATCTCATTAGCAGTTTGGAGC
AACAATTCACGGTTTGACTTGTATGCAGAACTCTCACTATCACTTTCATCAGTGCTTGAT
TCTCCTTCAATTTTCTGATTGGAACTGTCAATATCCATGGATGACAAGCACTTTGACTCA
AATCGAGCTCTCCTACATTGGCGTGCTTCAGCCCTCCGTGCCATATCCATCCGTTTCTGC
AGGTTCACATCTCTACCAAATTCATCTAGCTTCACTGGTAAATTTGTTTGTTCTCTAATG
GCAGAAGATGCCGCCTGAGCTGCACTTTTGGCAGCTGCAATCATTGCAGCACTGTTGCCC
CGTTCGTTGAATACTGTCATAGCTGCTTTCACACCTGCTTCTACCTCAATCATTTCATCA
TCATTATCGGCAGCCCTTCTTTCAAATACAGCAGATGCACGTTCTTCACGGAGCTTTTGC
ATTTGCTCTTCAAGTTCCTCTATGTAATGAGCTTTATGCAGCAGCAGACAGAGATCTTTC
GAGATCAGTAATTTTCAAGAGTGAAGCAGACAAATCTTCATCAGCCTTTGTGAGCGATGA
CATAACCCTACCATGAGTTTCCTTAAGCCTGTTGATATTATCGTGCACAGCTTTTAAGGC
ATTCTCTCCTTTCTGAGATATTGACATTGTATCAAATCCTTGTAATGCTCCAATTGCCCC
TCCAATATTCGGAGGAGCTGCTGTTGGGTACAAGTTCTGGGGCTGCACCTGTGGCTGCTG
AGGCATAGGAGCACTTGCACTAGCACTAGAAACAACCATAGCAGTAACAGAAGTATCATC
TATTCTCTTCCCTAAACCCTTCCTCACTTGCTCCTCTTCCCAAATTTTAGCAACTTCATC
TTCATCTTCATCATCCTCCACGTTTCTCTTCCCAGCAATACCAACCACCACAGGATTCTC
ATTTTCCTCATCATCCTCGAAAACACCCTTTTTCTTCACATTCTTCTTCCCACCCTCTAC

CTTCTCTCCGAACATAGCCACTCGCCTGGGAAACTCCGGTTCTTCATCACTCAAATCCTC
TCCACCCAAACGATGGTCACTCCCTCCTTCCAATGATATATAATCCGGTGCCGCCCGCCC
GCGTGATTGCCTCATTCTCTCCCTCTTTGCTCTAATTGCTTCTATTGTAGCCTTATCAGG
AATAAAGTCTTTCCCTTTGGCAATCCCCATCGAAGCAAATCTCTTCTCTGTCTCATCTTT
ATTATCAGAATCTGAATCCGAAGAAACCCTAGGAGGTTTGTTTTCAACAAAGTTGGAATT
TTGAGGCTTGAGCAGGCCCTTGAGGACAATCGTGGGTTCAGAAGAAGGCGGGGGCTTAGA
GGAAGGAGCGGCTAGGGTTTTGGTGTTCTTTTGAAGTTCAAGGAGGGCTTCTTTAGTGTA
AGTTCCAGCTTGGGGTTGAACATTGGAGAGAAGAGGAAGGCGGTCTTTAGAGGAAGTAAT
TTTGTGAGATGAAGAGGGTTTGTTGAGACGTGAAGAGGGTTTGTTTTTCACTCTATTCGA
AGATGGTTGGTCTTCTTCTTCATCGTCAGCGAAGCTCAGCAGTTTTTTAGGTTTTGATGA
TAAGGGTTTCTTAATGCTGCTGGTGGTGGTGGCCGCGGCGGGGGACACGGTGGAGGGAGC
GGTCTCCGTGTTGTCTTCATCCTCGTCGCTGCGACGGCGGAAATTTCTAGCTCTAGAAGT
GCTCATGGTGGCGG
>ntig_709_consensus
CTCGCTAGAAAGACAAATTAATTGAATTCAAAAATCGAGTTACAAAACTAAAGCTTTAAC
TCATTCAGATCAAATGATGGATTCAAACCGAACCAAACCAATTTGAATCCACCCCTAAAT
CAGTCAAACAGAAGACCATCCTTTTGACTGATTCAACGGCATGTCCAAGTCCAAAAACAC
TGACAAACAGATTGAGACGCAGTACACACAGAATTCTTTTGTTGTTTTTTGAAAGTAAAT
AAAAACTGAAGCTTTCCCCATGTTTTGAGGTTCGGGCTCTCAGATCACGAATATTACAGA
AGATAAAGTTGAGAATAAAGCACCTAGAACTAATATGCACAAGAGTTGAAATGACACATA
AATTTAAATACAATGCAGGGGCACCAGCAGACACCATAACCAGTGCCAGAGACCATCTAA
ATACCCAAATGCTGGGACTAGAACTAGTCTATAAAAGAATCATTTCTTGATTCTATGCAT
GTTGACCTTTTTTTTTTCTTTTTTCCAGAGAAAAAAAAAATAATAAAGCATTGTTTTATT
CCTTCCTACATGGTTGCCAAAATCCCATTTCAGCATCCATCAGTCTACACCACTGCTGGT
GGCTCTCCCTGAGCACCAGCAGGCATATAAAGGTTTTAGATACAAGATGGTAAGGGAAGC
GCAGGCTGCATCTTCCATTATCCTTCTCCACCATAAGGAGCGACTCAGACGACAGACTTC
TCTGGTGAGCATGAACACTATGTATGACCTCTAGAATGCAAGAAAATTAATTTGCAAGGC
TAGATCCGAAGCCCGCCACTCAAGTAGCAGTTCCGCTGCTCTTAGTCCTGCTGCAATCTA
AGGATACCTTGATCATCCCCTCACCCAGTTGTTTGGTCAGAACCGCTACCTTGCATAAAA
GGAGCAGGAACAGCAGGATATGGTGCCTGAACTGTGGGCATAGGTGAAGTTGTTATTGCA
TTTACGCTTGGTCCACTGAACTGCACAATCTGATGACCAGGCACTCCATATCCCTGAAAT
GGCGTATAACCATGACCTCCAGGAACAGTTTGACCCAATTGTCCATAAGGATAGAGAGCT
GTGTTGACTGTCCCAGGTAATCCATATATCTGAAGGTACTGCTGACCCATGTATGGGTTG
TAAACACCCTGTGGGTAAACATATTCAGGACCATATGTTGAATACCCATAAGGAGGATAC
ACCAATCCTTGTTGGTAGCTGTAAGAAAGCGGTTGCTGATAGCCAAAACTTCCAATATAA
GGGCCCCGGGTGGCTTGCAAGCTCCCACTATATAGGGTAGTTGGCCTTGGACGCCCATAG
AATATTGGAGGTCGAGGTCGCCCAAGTGAAGCCAAATTACAGTTTGCTCGCCTACCATCA
ATTACTGGAGTTGGATCAGCACATGCTCTCTTAGCAGCCTCTGGTTCCCGAAAAGTCACC
TACACCCCGAATTGCTCAAAATAGCGACGCATGGTCTCACTTTGAGTCTCCCAAGCGAGT
CCCCCCACAAATACCTTGGTGTATGTGGTATCTCCAAAAGGAGAATTCATGTATTGAAAC
CCAGAACTGGAACTCGATCCCGAGCTAGGACCCGGAATCGGCTGATAGGCCATCAAAAAT
AGAAGAATAAAATTATATACTGATTCTAACTAAGACTAGGATTAAAAGACAGGATCGAAG
AATCTTCTAGAAAATAAAAAAGCGGAGTCGATCAATGAAATTGAAGATAATCAAGAGAT
>ntig_710_consensus
GAGCTTGAACTATGTATGACCTCTAGAATGCAAGAAAATTAATTTGCAAGGCTAGATCTG
TAGCCTGCTGCTCAAGTAGCAGTTACACTGTTCTTAGTCCTGCTGCAATCTAAGGATCCC
TTGATCAACCTCTCACCCAGTTGTTTGGTCAGAACCGCTACCTTGCATAAACTGAGAAGG
AGCAGGAACTAAAAATTGAGGTTGTGCTGGAACAGGGGCAGCAATACCAGCAGGATATGG
TGCCTGAACTGTGGGCATAGGTGAAGTTGTTATTGCATTTACGCTTGGTCCACTGAACTG
CACAATCTGATGACCAGGCACTCCATATCCCGGCAATGGGGTATGACCATGACCTCCA
>ntig_711_consensus
CTTCAATCTAATGACAACACATGCAATGTCAAAATTCTTATGCAAGATTACAGGGAGACA
ACAAACAATAATCAAATTTATTGCAACACAAAAACCTTTGTTTGGTTCACAACATCTATT
AAAGAAACCACCAGATTTAATGATATTGTTGCCCCATCAGCATAACATAGTTCATAAAAT
TGTACAACTTAAACAAAGTTGCCAACCAATTCAAAGTTCTTCCAGTGAGATGCAGGAAAA
TCCACTCCACTCAATACCTATGTTCCAACTTCTCTTTCTCCTGGTATCTCTGGACATACG
TGTATGTGCCGACGAGAGGGCCAAGGAGGAGGGCAGCGTTGATCCAATTCTCGGAGATCT
TGTGATGGATCTTGCCCGGTAGATCCTTCCATAAGCCAGTCATTATCTTCTGCTGAAACG

GCGACAGCGCGTAAACCACCGCCTTCATTCTCACTTGATTCTTCCCCATGTCTCTCTCGG
TGCTAACAGTATTCCAATTGACGATTGCTTGTATCTCAGCCACAGCTACAGCCGCTCTTC
TTTTATTTATACAAAAGCGGAGTTGAACTTGGACCGGTATAAACTTATTCCTCCATCCAA
ACACTCTATTTTAATTCCAG
>ntig_712_consensus
AAGACAACACAAATTTGCCACTCAACTAAATATTATCAGGGTGGCACTCCAAAAATCCGA
AAATTTCATTTTTACATATCCCATCATGAAAAAGAAAGAAAGAAATAAGAAAAAAATTATA
TTGGAAAAAACAAAGTTCAGCAATTGCTAGAACAAATCTTCTGAAAATTAGTATTCAACA
GATTGAACATTTTACCTCACTGTACAAATCCAGCAATCGGCCCTTCCTCATCAAACATTG
TCCTTGTGAGTCTCTTCCTCGACAGACATTGCGGTTGGACTCAGCTGGGAAACTTCATGT
GATCCTGCAAGCTCAACATTACTTCTTCCCTCGGAATTTTCAGATTCTACATTTCCTGAT
GCAGCACCTTCCTGAGAAACAATGCACACCAAGTTCATACCTTCCTGAAAATCTGCATCA
CAGGATCCCTTAGGAGCAGATGTCGAATTCTCTGTCTCTGCAGTACGTTCAGGGGAAAGA
ATGCAGGCTGATTCTTCTTGCTGAACATCAGCATCTGAAGATCCCTTAAGAGCAGACAAT
GAATTTTCTCTGTTACCTAGGTCATTGCCAGAGCCGCTAGACATCAGCCACATAAACTGC
AAAACGTGGCCTTGCCAGCATTTACCATTTTGAAATGCCCTCTCAGCTGAATGACGTGTT
GTGAATGCTATACGAGCTGAGCAATTTTTCTCTGAATCTAATCCATTAGCACCCTCATGG
AGTTCGCCACCTTCCAACTCAACAGCAGAAAGATCACCATACGATGAAAAGTGCTCCTTC
AAGANNTCCTACAGGTGTTAATGGCCGAATGTGCTGCTTGAAGCTTGTAGATTCATGCAA
CACCGTGGCTGTGCTTGATTTGGGACTATGGGATACAACAGTGTCCACAATTTTGTTCTT
GTCAGCCACCGTCTCAGCACTCAGTGATGCCACTGCTGCACCAGAATCAGATGACCTTCC
AGCATCTTTTGCAAGATCAGCTGGTACCCCTACTTTGGGTCTCTTAGCAGTTTGCTCCAT
CCCCAACTCACCCTTGACTCCTCCAGATTGTTTCTCTAATTTGTCCAGCTTACGGCGGAA
GTCATTCCGCTTTTGGTCCAGCAATTCCTGTTTCTTTCGAAGTTCCTCCTTCAACTGCTC
CAGACTATCAAACTTCTTTTGCAAAAGGGGTGGAACTTTCAGACTATTTGAGATGACAGG
TGAAGAATGATCAGAGGCAGGAAGAAAATCATCAGAAGCAGGGGTAGTACTACCCTTCAC
AGCAGCAGATTGAAGATTATTATCCTTTATTTTGTTTGCAACAGATAGATGGGCTGGGAC
TGAAGCTGCTGTCACACCACGAGGAGTCAGAGATGTGCTAGTGCCACTGCTTATGCCATC
ATCTGGTATGCTATCACGGTTAGCCCACCAGAGCTTGATAAAGCGGTTACCCATAACAGC
ATCAGGTGCCTTTAGAGCAGCCTCTGCCTCTTCTCTTTTGGAAAACTGCACAAAAGCCCG
CTCACTATTTAATGGAATATAAATGTCAACAACCTCTCCAAACTTGCGGAAATGTGAAAG
AAGTGCCTCTCTTCTGTTATTTTTCTGGGGAATTCCATTGACAAATAGGGTGCGCAATGC
CTTTTGTGATGGTTTCCGGATATTATGCACAGTATCAATCTGTAACTTGGCAGATGCATC
CATAGCTTTTGAGCCAGCATCCTCGCCAATACTCCGCTTTCCTCGTCGGAAAGCATTATG
AAGGCTGGATAACACATCTTTATCTTCCTTGGTCTCATTCTCAAGACAATCTGAGGAACT
TAATGTTACATCAGTTTTCTCTTTCACATTTGATCTATTTTTACCACCAATTCTACCCCA
AACAGATGGATTTGTGCTCTGTGAAACACCAGCAGACCCAGAATTTCTCATTGGGCATTC
ATTATCAACACCATCACCAAACCTGACATGATGACGATCAGAAGAATCGTCATTTAACAA
AGGTTCAGTTTCATCCTTAGATGACTGTAGTGCGGTGAGTGTAGGTGTTCCAAGACCATT
ACTATTCCACAGGGGTTGATCTGGATCATACAAATCAGCTCCATCTGCAGTAGCATCTGA
ATATGCACCATTTAAGCCCAAACCATCATCAGGCTTGTTACCTTTATTATGAGCTCCTTT
GGTACCCATCAGTGTAGTTGACGGAGCACCAACTAGAGGTGGTCCAAGTCCAGGTGCTGT
TGCCAATAGTGGTGCATTTGGAAGTGGGACAGTGAGATTAAACTGTGAAAGGCTCTGAAC
ATCTTCAACAACAATACGATTGACACCATGCTCCATCGGGCACATGTCTCCCCTCAGACA
AAATCCACGCTCCTCAAAGTCTCTACACCTTTGACGAGTAATGCCCAGATTCAATGAAGA
ACTCAAAGGCGGTCTAAGTGTCCCTTGCAAACCAATGGAATGGAGTGTATCAAAGCCACC
ATTTGGTAAACTTGGAATTAATCCATATGCATTCCATGATGCACTTTGTGAATTTGAAAC
ATTAGGCAAGCCCC
>ntig_713_consensus
CTACCATTAATAACCAACAAATTTGAGTTGATACAGAAATATAATACTAGAGCAGACCAT
TGCTTTAACCATTGAGGTTTGTGCCCAAGACACAAAAATCCTCCACTAAAAGTTAGATTA
AGGCGCAATTTGAGTCATGATAACTTAAACACAGAACTCATGCAGGAGCCACATTTGAAT
AAACACTCATATTTTCATTCATTCAGATAGTAGTATCTACATCTTCAACTAAATGCCAGA
TCTACTGCGCTGCAGTAAATACTTTACTGACCAATAATTGATTTTTATGGCTTTGTCGGG
TACATGTGATCAGATGCCTTGTTCACGTTTTCTTGTTCAATTTTTACACTTGCTTCATCG
ATTTGCATTGAAGTTGTATCTGATACCTTTGTGTTTGCAGTTTCGTCAACAGTTGTGTCA
AACCCTCTAGCATGATCAGCAATTCCTTTATGACTCTC
>ntig_714_consensus

TCGATTTGCATTGAAGTTGTATCTGATACCTGAAGAGTAACAACGGAAAAACAATAAGAT
ATATATGTTAGTGTGAAGGACCTTCTCCACAAATGTGACTGACTACACATTCAAGCATGT
GCTAGGCTTTTCATATTTGTTCTTGCATGCTTTTAACTTCACCTTTGTGTTTGCAGTTTC
GTCAACAGTTGTGTCAAACCCTCTAGCATGATCAGCAATTCCTTTATGACTCTC
>ntig_715_consensus
GCCAAAGACAATAAAAAACCAGCAATGCCGTAGTATAACCAAATTGTATCACATTCTAGT
TTTTGTTTGTAACGGAAAATCAGACACAAAATTCCACATGTTTTCATTAGCCGGCTACAT
ACCCTACATTACAGTCATGGACATAATCTGTTGTGCAAGACCTGGATCTTGCTGTAGGAG
CTCTGTCAAGTTACTTTCCACTTCTGCCTTTTGCTTCTCTAAGTACTCCTTTGAGGCCTG
CAACGCTGCAATTGCAGTTTCACTATCCTTAAATTTCTGCTCTTGTTCATTCATTAAAAC
TGACTTGGGCTCCAAAACAAACGTTCTCCCTATGGATTTGTATGTATTTGTATCATTGGG
CAACAGGCGCAGCTCCTCCAAGGTTAGGTAAGCACGCTTCTTCTCCCCTTCTTTATTTCG
CATCTGGTTCTGCACCTGCTTCAATTTGCCAGTATTTTCTATCATGCGACCTTTAATCTC
CAGAAACGCGGTTCTGTTGGCTTCGTCGGACATTGTTGAAGATTCTTCAGTGCCGATATG
ATGGATTCTATTTACCTTCAAAGCCCTACTCTGTTGCAACCATGAGAT
>ntig_716_consensus
ATTACAGTCATGGACATAATCTGTTGTGCAAGACCTGGATCTTGCTGTAGGAGCTCTGTC
AAGTTACTTTCCACTTCTGCCTTTTGCTTCTCTAAGTACTCCTTTGAGGCCTGTTCAATG
AAAAGATATCAGCCTATTCCAATCCTTAGCCTTTTTAGTAGGAGCACAGTCAAAAGAAAA
ATAATAGATTAGAAAATTCTGTATTTGGTTGGCAAGAAAAAGAGGAAAGGATGCACAATT
CCTCAAAGCTCAAAGAACTTGACCCTTCAAGAAGTGAAATGAATAGATATAATTGTTT
>ntig_717_consensus
CCTCTATAACAACATTGCGACCAGGATCTCCAGCCTTACCGAAAGCATAAAAAAAGATTT
GAAGAGGTGTAGTGTATATTTGCAGTCTTGCAAAAAGTAAACTACAACGTGATTTTTCTT
GTCGGTTTCAAGTTTGAAGAAATTTAATCAAAATGGCTCAGACTGTGGGAAGAAATATAG
CGGCTCCATTGTTGTTTCTCAACATGATTATGTATCTTATCGTTCTGGGGTTCGCTAGCT
GGTGTCTTAACAGGTTTATCAATGGCCAAACTAATCACCCCAGTTTTGGAGGAAATGGTG
CAACAATGTTCTTTCTGATATTTTCTATATTAGCCGCCGTTATTGGGGTGGCATCAAAGC
TCGTGGGAGCGAACCACATCAGAACTTGGAGAAGTGATAGTCTTGCTTCGGCAGGATCAT
CTTCATTAGTGGCATGGGCCTTGACTGCACTAGCTTTTGGGTTGGCTTGCAAGGAGATAA
ACATAGGAGGGCACAGAGGGTGGAGACTGAGGGTGCTGGAGGCATTCATCATAATACTAA
CATTCACTCAGCTTCTGTATCTCATATTGCTTCATGCTGGCTTGTTTAGCAGCAGGTATG
GACCTGGTTACAGAGACACCGACTACGGCATGGGGGCGGCCGGTGTGGATCCAGCAGGCC
ACAAGGGAGGTGGTGCAGTGGCTGGATCTAGGGTTTGAATTGAGAATGAAATGTAGGAGT
TGATGATTATAAGTGTCATCATCTGCATTATCTTCTTTTTTTTTAATTTCTATTCCACTT
GTGGTGTCTATAAGTAGTGTTGCTATTTTGGCTTTTGTCTGGATTACTGGACTCCAGTTG
TATCATCTGTAACTTCAAAAGACTTTTTGCTGCCTTTGTTTAATTGGTGTTCTTGTGGTG
ATTTTGCAATGTTAGATCGATATGGCAACTTTCTACACGATCTTAAATTTGATCTGTTTG
AAGTTGGTTTTTGAATGGAATTAAATTTACGTAC
>ntig_718_consensus
ATCGTAACATATTCTCATAACCAAATTTTTAATACTTTATCTGTACTTAGATCACTCAAC
TAGAATAAGAACAACTCCATCATCACATTGGATGGGATATGCAGACTAATTTACATAACT
GTGCTTCTCTACAATGTTTTTTAGGTCTTGCTAGGTTTAAGAAATTCAAATTTTCTTTTA
TACAACAAAGCCAACCATACATAGAACAGAGCCAAAGACAGTTTACCTATTGCTCTTCAG
CTGTAAAATCAGGCTCCGCTGGCTTCTGAAGCAGCAACGGTGCTACCCTCTCCTGTACTC
TTGCCTTACGAGTACCATTACGCCGAATTGCACGAAGGGCATTGGCAGCAAATCTAGAAG
CATAAATGGTAGCTCCTAAACTTGGTGAATTTCGACTAGCCTTGGACAACACATCTTGCA
ATCGATTCTCTTCTGCACGCAAAGACTCTTCAAGCTTCTTTTTGTTATAGCAGCGCCAAG
CTGTTTGTATGAAACAAGCTGCCCAAGTCCTCCACTGTTGTGAATAGAATCTAAAAGTAT
GACGCAACTGCTTGCTGTGAAGCCGCCTGAATTGAGAGGCGACAAACTTCAAGTCATCAG
CCTTTAGAGCAAAGGCTTCAACTTCTGTAAGAGTTCTAACAGTTCTAGTTGAGATGGGGA
GATTGGAAGAGGATCGAGGATCCAGAGCCCATGTGAGAAGTTCTTCACCACAGAAATCGC
CAGCCCCAAGATATTCAGAGTTAAAGAAACCAGTTCTTCCACCATTGGTAGTTATAGTCA
ATAGTTTGCCTCGCATGATGAAGAGCATTTCGTCTACAGGGTCACCCTCTCGGACAATGT
AGCTTTCTTCTGTGTACAGCACTGGCTTGAGACGATCACACATTGCATCCAAAAGTTGTT
CATCCATTTTTTCAAACATTGGCACCCTCATGAGCAAAGCCAAGCAGAGATGTCGCTTTA
TGTCCCTTTTAAGATCCTTGGGAAGATTACAGAGAAGATTCTCTTCATCAACACCTCTGG
TTTCTTGCCACTTATATTGCTCATAACGTCTTATTCGCTCCCTAAGAGTATCAGGGAGCA

ACCGGTGGGCCATCCACTGTTCTGCATCACGTCTTTTCACTCTCATCTCCTCCAATCTTG
TAGTTGTGGACTGCAAGTATGTCATTCAGGAACCACTGTATTGCTCCAAATGCACCAAAC
CTCTGAATGAGAATTTGATGAGCCCTAACCAACTTGTTTCAAGGTTTTGACCAAGAGAAC
TCAGATTTTGCAGACCCCACCAGAAACAGTAAAAGAA
>ntig_719_consensus
TATTTCTGGGTCCAGCTCCTGGCAGTTGTCTCATACTTGCTCCTGTCAGTCTTGTACATG
TGGGCAATCTCCGGCACCAATGGGTCATCAGGATTTGGGTCTGTCAATAATGAGCAGATC
GATAGCAAAACCTTGGAAATGGTTAAAGCAGGGCTCCATTGCTCTTTCAAGATGTCAAGA
CAAATGCTGCCGTTGCTATTTATATTTGGATGGAAAACCTTTGTCCTGAATGCAACCTTG
GGAGGCTTAAATGGATAATCTGGGGGGAAATGAATAGTAACTAGGAAAACACCCCCTGCG
TAAGGACTATCTGGAGGACCCAT
>ntig_720_consensus
GCATCTTTGACAGGCAAGTAGCTCTTGACCAACGTTTTAATTCCATAAATCTTCAATAAA
CAAAGTTCACTTCTGTCATCCCAAGATACTTCATTGTCTTCTTTTTTATTGCCGCACTGA
AGAATCTTGCTTTTTATGAATTCCACAATTTCCCTCTCTCTAGTTTCAAAAACTGCTGTT
GCAGTCTCAGCAATACATCCCAGAGATTGTAGAACAGCGGGCAAATGAGTCTTCTCCTCC
AACATGTCTACAAGCCTCTTGTATAAAACAGAGAGAGACTTGAGCCCGTCATCCTTTGTT
ATTGCTGCTAGTGCATGTACAGCATACTTGGCCTGCCTTCGACTACCTTCTAAACAAAGC
CTTTCCAGTACCAGGTCAATTGAACTTGATGACAATGCAAGTTGTTCTCGGATGGTCCCA
CCAGCTTTTGCTAAAACATGCAAGACACCTTCTTTTATAATTTCATTGTCATCTTTCAGT
AAATTTACCAGTTCTTCTTCGGTGCCACCCAGCAAAAATGGACTGAAGCGTGCAAGAATC
CCTAGAATATCCATGCAAGATTGTATATATTGTGCATTTCCCGAAGACTTTTGTATGGCA
ACCTCCAGAAATATTTCTTTTACATGTTCCTGATTAAAAAGTAAATAAGAACATTTCATA
GAAAGAGTGCTCAGGAAATCATAGAGTCGGTGTTTTTCACCAAGTATTTTAAGCAAATCA
TCCCGACTAGAACAAGCTTGATGAAAGCTAATGTTTGGATCAAGAACATTCGTCAATATC
TCCCAGATATTAGCATCTTTCAATTGATCAAGAATCTGAAAATTCTCCTCAGCTTTTGTA
GGCTCAGCAAATGAACGAGACATGACTCTGAAGCAAAACAATACTTTTTTCTGGATCTCA
GGGGCATCACCGTCCTGATGCATCTGCCTAAGGGAGAGATACCTCTGCATCTCTTGCTGC
AACCGTTGTTTTTGCTCAAGTATCTTCTCAAGAGCCTTCATCTCAACTCTATCAAAGCCT
GAGAAAATTCTCACCCAAAGTCTAACTCTATCTTTTACAAAGAACTCGGTTGGGAACAAG
GAACCAGATAAAATAGATTCAATCGTATCAGATCTGAAGTCTCTGTCATAGAAACATTTC
AAAATCTTCCCAGGAATCCAATCAAAGTCATTCTGATCAATTAAACCCTCAGAGGACCTC
AGGCAATATACTCTGAAAACCTCAGCCAGCCTCTCCAGAGTATATATTTTAACAAGTAGA
GATTTGTCTCGAATACGCTCTGCAACTAGTTTCAGAGTCTCAACAGGAATAGAATTCAAA
GCTTGGCATGCCACATCACAAATTACAGCAACAACTAGCTTTCTTACATTTTCATCGTAG
TCCAAGAGCCGATCACAAAGGACAGAAAGAATTTGAGGAGCCTCAGCCCTTAAAGGATCT
GACAGTAGGCAGCTCTTGACATGATTAAGGACAGACATACGAACTGCAACTACTCTGTCA
GTCGACCTTTTCAAAAACTCTGAAAAGATAGGCTGAAATACTTCTGCATTAGAAGACCCA
GGCAGAGCAAACAGATCTCCAACTAATTGCACTGCTTTTAGACGGGTATCTAGTTGCTCA
GTCAACAACTCTCCTGTGAGGTACGGAACAACTCCTGACAAGATTTGAGGAGCACAACGA
TAAATATCATAAATAACTTCATGGTAGTCAATATGACTATTTTCTGGCCTGTTATCGCCT
GACATTGATGATACAAGGAATTGTTTTATGCCAACTTCAAGTTTTCCAGCACATTGCTTT
ATAACATTCATAGCAAGCCTCCTTGGGGTATCACTTCTACTTCGACCTAATGAAGACAAT
AAAACAAGTAAAAGATCTTCTTGAATATCCTCACTTTCTTCTAATAGAACAATCATGATT
GTTTGCATTGACGATAGAACATTTTCTGGATGATCATCACAGGCAACATTAAAAAATGTA
CTGAACATTTGATTCACTAGATCATCACATTCAAGATCCAGCATCACAACACATGACCTA
TATTTTGCAAGAGTCTCCAGAATGACAACTCTCCTCCCAAATGACGGACCACTAATGTCC
TTTAACCCACTAAATGTACCCACAATCAACTGAAAAGTATCCTTGAGAATGTCATCACTG
TAGGGAGCTTCAGGTGCAGTAATCCTGGTTATCTCACAAATACAGGTAGCTACTAGAAGC
TTGACATCCTTATCTTGATGCTTCAGAAATACTGGCTTGACAATTGCATTAAGAAAAGGT
TGCATTGCCTCTAACATTGAAGCCGGTGGAGATTGGTCCAACTCAGAAAGGCACGTTGCA
CCTTGCTTTAAGAGCTTGACGAGAGCGTCTTTGTTGGAAGGAGGCGTCTCGAGCTTGGAT
CCAACCTCCTTCAGCTGCTGTTCGAGCTCTCCAGGCATTTTTTTGGGGAACTCTAGCAAC
GTCGTTTAGCTGAGAAACTGAAGTTAAGTGTAATGAACCGAACTTGAACCCCTCTCTCAC
ATGGAATTTTCGAGATAAATTCGACAGAGAGCGAGAGGGCTTCGACTTTTTTTTTTTTTT
TTCCTTTTTCTTTTTTTTTTCTTCTTCTGTCCTTTTTTTTTTTTTAACTAGACCAGGCCC
ACGTGATGTTATGGACCTTTCTTTAAGTCATGGGATTTGACAGACAGAACATTCATTCAT
TACAGACACCGACAGACAGACAAAAAATACAAAAGAAAAGCGTCCCCTCCCTCACCTGCG

CCACAACTTCTTTCAGCCCATGGCATATTTCTGGGTCCAGCTCCTTGCTGTTGTCTCGTA
CTTATTCCTGTCCGTCTTGTACATATGAGCAATCTCTGGCACCAATGGATCATCCGGATT
TGGATCCGTCAACAGCGAACAGATCGACAGTAACACCTTGGAAATGGTTAAAGCAGGGCT
CCATTGCTCCTTCAAGATGTCAAGACAAATGCTGCCGTTGCTATTTATATTTGGATGGAA
AACCTTTGTCCTGAATGCAACCTTAGGTGGCTTAAATGGATAATCTGGAGGAAAATGAAT
AGTAACTAGGAAGACACCCCCCG
>ntig_721_consensus
CTCAGAAATAGTGCTGTGATCTATCTTTCTGGCGGTGACGGGCAGTACATTCTCCTGTAG
AAGAAACTTAAGGGCTTTCCAAACATTTAATTCAGTCACTAACATAAGATAAAGCCAGTT
TACAACTCTACAAATTAGGTTCCTGCACAATACAACATAAGGTCGCTGCATCATCTACCG
TCTAATCTAATATAAAGATACAGATTGATTCTTCCTACATACAATTAAATCTACCAACAC
CATCAACAAGTTCAATGTTACAGTCATCCACGACATCTCAAAAAGTTCCAGGACAAATGA
CAGGATAATATAGGAGTATTATACGGGCTAAAGTTGATGGATAAATAGTACAGCACATTT
AGCAGCTCACTGGAGCTGGCTGTTGTGAGGTGTCAGTTCGCCCCATTTGGTCGACTTTCC
CATTTTCAATAACCTCTGCTTCAATTTCTTTACCTTTCTCATTGACCTGCACAGGTGCTG
ATCTATTGGCCTGTTCTTTTTCCGCTCCTCCATTTGCATCTGTGGTCACACTAGCAGGGT
TGTCCACTTTGTTAGCGGTCTCTTTAGCTTCTGGCTTTTGCACTTCTGTTACACTTGCTT
CTGGCTTTTGCACTTCTTTTGCGCTTGCATTTTCTTTAGCGTCAAGAACTCCTTGTTCTT
CAATCTCCTTGGTTTCTTCAGCATGTTTTTGGTTCTCTGCACTAGATTTCTCCAAATCAA
CATCTTTGTTCATGATGAGACCTTCAGCATTTTCCTTCTGAGTGACTTCTGTATTTTGCA
TTTCAACATCTTTCCCTTCCTCAGTTTTCTCTGGAGTAGCTTCGTCTTCTTTGCTACCCT
TCTTTGAGCCTGATGATGATTTTCGGCTTCGCTTCTTCGGAGGTTCCTTTTCTGGTGTTG
GAGTTGCCTTTGCCCTCTCACTGATCCTCGCTGACCTCCTTGGTGTCTCACCAGTGCTCC
AGTCGAACTCTGAGATGGCAGGATTACCAGGGTGAGATTTTAGGTATTGCTCCAGTTGTT
TTCTGTTATTAATCTCCTCCCCAGTAGGTGCAATGAACATAATCTCACTTTTCCTAGGGG
TACCTGCTTTCTTGGGCAAGTACATTTTCTTCCAAGCAGCAGGTGCTGGCAGCTCAACAG
AAATAACTTCCTCTTTGCTCTCCATTTTTCACCTGTAAAACCAACAAACCAGAACCTTTT
CTCTCTCTCTTCTCTCGTCTTGTCTCCCCTTAAAGTTGTGTCACAAATACCGTTGAGTGG
ATTTTTTAGATATGTTTTGTTTTGCTGGTTGGCTTGGTTTTTTCATTTTTATTTTTATTT
TTATTTGTGTAACTTTCATTAATAATTTCTTTATGCCATGGCTGCCATCACCACCATGGT
TCGCCACAACTGCCACCTCGACTCCCTCCCCCTATTTGACGTCTGCCTTCTCTATCAATT
TGATCTTCTTTCTCTTTCTCAAAATTTTTTCTTGCACCATCAACACCACTCAAATCAATT
ATGAAGATGAAATTTTGACTCCCAAAATTGACTGCTTCATCTTTAATGAATTTGTTGGTA
GTCGCAAACAAACTTTTTCACGGTTCAGTCGGTCACTAATCTTAAAATTATTTCTCTTCT
AACCACCCAAAATAACGAAG
>ntig_722_consensus
ACTCTCACAAGTTCCATATCAGAAATTCTATAGACCAGCATCAGACATGAAAATTGTTCC
TGTGCACTTGAGCACTATCCCTTCCTCTAAAATCGTGTAAATGGGAATTGTTGCCTCTCC
ATCAATAACAACACTTTTAAGAAGTGAATTATGCCGACGACCGTATGCAAGTAATAAGTG
CTCTGATGTGGGTGAGAACTGAATTGAAGTTAAACAATGAGCTGCTCTAATTGCCCGAGA
TGCAAGCACCAAACCAAATGTTGCCTCCTCCAAGGAATATATCCGAAGCTCATATATAAC
TTGGTGAGCTGAAATTGGGTGTCGAGTCGGGGAGGTTCCAGCCCCTGAGACATCACGATG
CACTTGACTTTGTAATCCAGGATCAGCTTCTAAATGAGGCAACACACATGCAACACAGGC
TGCTAAATATCTTCCACAAGGTGAAAAATGAGCACCCATTTCACTCTTCAAGATGAGCAC
ATGGATCTTTTATATCATGTGGCCAGATTCTTAGCTTCACTGTGCAAGGCAATTCAGCAG
CAGCTGCTGAAGCCAGGGATGTGGAGAACGCGGATTGAAATCTGCTAACAACAGGTTGTG
TACCACTTTCACTATGTGGGATGCTAACAGAAGCAGTCCTTTGACCAGATCCAGATCCAG
ATCCAGTTCCAGATGTTATCCGGTAACGTGAAAATTGATGGCCTGAACCAGATCTTCCAC
TGACCCTAGATTGACCAGAATTGTTTGATATCACTGGGGAAGTCGGTGGATTTTCCAGTT
CACCAGATGCCGCAAAGTTATCATGAGAAGAACCATTAAGCGGCCCAGTTGTGTGCTGTG
CCGCTTGGCTATGTCCAAATAACGAACCTTGCAAGAAAGGAAGCTCCCAATATGTTGGAT
CACCCCATGGGAACATATGATTACTTCTTCCTTCAGGCTGCACCTCCACAGTTTCCACAG
CAGTTTCAGGAACTGCATTAACCATTTCATTCACCAAATAATTTGTCACTGCTTCTTCTG
GAATGGGAGATGAGCTATTAGGTTCAATAGGAGACAACACAAGTTCATACTGCCCTGAAG
GAGTTGAAAAAGTCAAAAGGCACACTGAAGTAGAAGAATCCACTCTCTCTTGTGTTCGAC
TTGCAGCAACATCCCCATCAATATGTTGCAAAGACATTCTATCATAGTCTCTAGCAAATG
AAGGCCAGAGCAAGAAGGGCAAGGTCATGAAAGGTAGTTCATCAGCAGAGGTAGGACGAC
CACCCAAAAAAGCATCAGCCATATATACAGCAGGTGGAGGATAGTGCAAATAACCTGGAC

AGGTTGCAAGTGTCATTGACGACTCTGATGGGTCTAGGTCATTCACCTCAGCTGTTAAGA
GAAACGGAGCACCATGAGGGTGAAAGTGCACAGCACGCAGTGAACGGCGTGTCTTCAGTA
CAACTTTTGGTGAAGATGTCTCCCCTCTTTTGTTATAATGCCATATATATAGCTTGTGAC
CAGATGCTACAGCAAGATGCTCCCCATGGGCATGAAAAGCTATAGAAGCAATAGGACGGT
AAAAATCACGTGATCCTATACACTCTGTAGTATTTGCATCCCACAACCGGACTTCATGAT
CCAAACTTCCACTTGCAAGTATTACAGGATTTAATGGATGAAACCGAACCACCCAGGGTG
TCCTCCGATGACCACGCAACACTCTTAAGCATCTTCCAGTTTGGCAATCAATAATCTTTA
CTGTATGGTCCCCATGTGTAGAGGCCAGTGTCTTTCCATCAGGGCTGAATGCTGCTGCAA
TAGTTGATCTCGGGGGAGGCAACAATGCACAATAATTTGCTGATAAACGCTGCAATGATT
CTGACTCTACCCATGAGATAAGACCTCGTCTTGCATCTTGCTCTTTTTCAAATCTTGATG
CCAATGAATTA
>ntig_723_consensus
CTCGTTTAGAAGCTTATATCTACCAAACTCATTAAGCTCAAATTCGATTTTTTATTAGCC
AAACCAAATTCGTGCTCGGCTCAGATCCAACCCTAGCCTCTTTCAATGACCAAATTTGTC
ACTCCAATCCTTAATGTACTTACCTGAGAGCTACCAGAAATATAGTAACTTTGTAGCAAA
TGATCCAAACACATGATAGAAGAAACTTAACAATTCAATACCGAAGTGCCAAAACTTTTA
AAACAGACAACTATCCGAAAATAAAGAAAAACATTTATAACTAACGAGAGGAAGTAGCTT
AAACATCAACTCCATGATCCTCAGCAATGAGTAGCTTCCCAGTAGGCTTTGCCAAGAGAT
CTGTGTACTCCTGGAATGGAGATTTCAGGAAGCGGGTCTCTCTCCAAAAGTCGGGNNAGA
GTCTTTGTAGACCCTCGTGAGGAAGTGAAAACATCCTCAATTCCGGCGAACTGAAGAACC
TTCTTTGGAACCCTAGCAGCCACGATTCCAGCGCCACGGGGTGCCGGCACCATTCTCACG
GTAACACTACCACACTTCCCGGTAACCTTGCAGGGAACAGTGTGTGGCTTACCGATCTTG
TTACCCCAGTACCCTCTTCTCACCGGGATAACAGATAGCTTGGCCAATATGATAGCTCCA
CGAATAGCAGTAGCCACTTCCTTTGAGCATTTGACACCGAGGCCGACGTGACCGTTACCG
TCACCGACTACCACAAAAGCCTTGAACCTGGTCCTCTGACCTGCACGTGTCTGCTTCTGA
ACAGGCATGATTTTCATGACCTCGTCCTTCAGCGAGGGCCCGACGAGTTGATCCACAATT
TGATACTCCTTAACCGGCAAGGAGTGGAGGTAAATCTGTTCAAGGCTCCTAATCTTGCCC
TCTTTGACCAAGCGCCCAAGTTTAGTCACTGGAACCCATTTCTCTTCTTCCTCTCTACGG
CCAGCGCGGCGACGGCCACGAGGACCGCCACGATCACCACGGCCACCTCGGCCAAATCCA
CGCCCGAAACCACGTTCGCCACCTCGTTCTGCCATGCTTGTAATTTGG
>ntig_724_consensus
TTTTCCTGATTTTCCAACTGTGATTGGTTACAATATCAAAATAAGAAACCCTCCAGAAAA
ATGTAACTTTCAAAAACAGAACAGTTTGTTAATCTAAGATAAGAAACCCTAAGATGAAGC
ACGCACGGAGTAGAAGAACCTCACTTCTTAAGTGCCTATTAATAAAAAGCCTCAGCACAC
TAACTTGGTAAAAGCACCTCTTTCACTTCACATAGAAAACAATAGTTTCTAAAATGGAAA
AGGAATCTGCATCGAACAACTATCCCGTCTTCAAAACATCTGTAGGTCTCACATGTCAAT
CACTCTATTGACGCCAGCTTCTCCTCATCAACCTCAAAAGCTTTCCTCGCTTGTTTTAGC
TCCTCTTGCATTGACAGCAGTTCTTTACAGCGGTTAAGTTTTGGCATATCCTTTCTGATT
AAATACAGAAAATCCTCAACTGATAACTTTCCCCTCTTTGATCCGATATCCTGTGCTTTA
TGTGCCAAATCTGTGACATATTCCACAACTATGTCCTCCACAAGTGCCACAGTTTCTGGA
AGCGGAATCTTTTTGAAAGACTCCTCGCTTGCGCTTAAATGAAGTTGTTTCATTAGGCTG
CGAGGATCCTGCTTTTGATTTTGAGGATTGTCCAGCAGCAGAGTTGCTCATTCTGTATGC
ACAATATCTCTCACTTCACTAC
>ntig_725_consensus
GGTATATTCCCCAGAAACTAACATGAATATATGGTACAGCAAAAAAACATTAGCCTCGTA
GCTCTTTCCAACATTCAGATCGAGCACTCAAATTTGGTTATAAAAACAAAAGTATCTGGA
TACAAAACTGATTTATAATATTATCATTATATTACAAAAGCAGACCTTTAAGATCATATA
TATTACAGCACCCTGTAATGTTACCAAGACTCCAGAAACAAACGAAAATCAAATCTCTCA
CAACTCACAAGTAGCATTCTCGAACTTACATTATTATATACGTGTGTATCTGCAGGAATT
GGAAACAAAACTCATGTTCCATGAAAGCCCTTATTCATCATAATACTCTTACATATATCT
AACAGCAAGCAGCACCGCTGATCCTCTATGCAACTCCCAGGAGTTTCTTTATATCATTCT
CAAAGCTGGAAGGAGTAGTAGTTGGCTCATAATGATTGACAACATTCCCATCTTTGTCAA
TCAGGAACTTGGAAAAATTCCACTTGATGTCGTCTCCACCCTTGCTCGACTTCAGGAACT
TGTATACAGGAGCAGTATTCTCACCATTTACATCGACCTTGTCAAATATGGGATACTCAG
CCTTAAAGCGACTGCAAGCAAATTCTATGATCTCTTCATTGCTTCCAGGTTCCTGACCTC
CAAACTGGTTACATGGAAATGCTAGAATTTCCAGGCCTTGATCTTTGTATTTCTGGTATA
ACTGACTCAGCTCAGTGTAGTTGGAATTGGTCAAGCCACACTGTGATGCAACATTGACAA
TCAAGAGGACCTTTCCTTTGTAAATGCTAAGATCAACAGCATTTCCTTTGGCATCCGTAA

CAGTGAAGTCATGGACTGATGTCTTGGATTCGCCGGCCATTATGTGATCAGATTTCAAAC
TGCTGATGGAGAAATCAAAAGGGTTTTCTTAGAATGTGAAAAGAAGCTTTTTAGGAAGAA
GAGAAGCGGATAAAGTTCTTGCTTCAAGATTATTTCAAGAGCAGTCTACGAGTCGGAGAA
GAGAGGCAAAGAGGATTTTATAGCTAATGTGGATGGTTGGCATGGCTACACGTGGATGAA
ATTTGGATCAGAGATTACGTCACGGTCTTTTATACAGTTACAATTCTGGACATCTTGTCC
TATGGTGAAGGCGACGCTTAGAGTTTGAGGCTTTTGCTTCAATCCAACCGGACGGCAGCT
TTTTTCTGAT
>ntig_726_consensus
CCACGATCTTAATTAGAAGGCTTTGATAAAGCAACAAATTGACAGGCTTATGAATGAATC
TTATGCAACAGCCTTAAATACCAAGAAACAAGCAAATAGTTACTTTTCTAATGGCTGAGC
AACAAACATCAGATACCATAGCTATAATGATCAACACTGCATTTATTGGATAAGGGACCA
GTGTCCATTCCGGCTATTCAAAGCCCCAGTCTCGCCTACATTTCATAACTAAGGTTTAGA
GACATTATAATCTAGGACTAGTTCATGCATTGACCTTCGGCTCCCATCCTGCAATCACAT
ATTGCGGTGCAGCCTTGGTTTTCTGAAATGATTCCTGACTGAACAGTTTCTTCATGTAGT
TGTGGACATGAGGCAAGCTCTCAGGGATACTCCACTTCTTGAAATGCCCTAGAGTTACCT
CAAGGTGGTACAGCTTTGGTGCCAAGCTCAAATCAACTGCAGTGATCTTTTCGCCAGCAA
TAAACGGACCCCCATGTTTCTTCAAATGCTCATCCAACGCCTTCAATTCCTCAAGCAAAG
CCTGCTCTGTCCCATCATTTGGATCCTTGCTTTTCAGAAACTGGAAGAAAGTTGAGAAAA
TCTTTGATCCCACAGAGGCAAATTCAGGAGGAGTAGCGAGAGGAGGCTCAGGGTACTTCT
CCTCAAGAATTCCAACAATCACGTCAGAGTCAGGCACCCATTTATCATCAAACTTCGCCA
CTGGCACCTTCCCTTCTGGGTTTACCTCCAAAAACCATTTGGGTTTGTCACTGGTGTTGA
TGTAGTGTGTCTTGTAAGGAACTTTTTTCTCCTCCAACGTTAGCAGAACTCTCTGCGTGA
ACGGACAGTCTCCAAGACGATCGGGAGCACCAACGGCAGCTTTCACACAGATTTCCAGAG
CCATTTGCTGACTGTGAATGAGAGTAATTTCTCCTTCGTTCACAATGGAGATGTTGCAAA
TACCAGGACACGCTAGTACCTTACAAGACACACTACATC
>ntig_727_consensus
TCTTTCTATTAAATTTTTTGCTGTCTTGGAGCTAGTAAAACTTACACAACAAGCAAGAAG
AAGCAAAGAGTTAGTCTACTGCATTGGCATTTACAAGTTCTTCTGTTTTACAATTTAAGA
CCAGTACTGACAAACAATGCAACCTTTAAAATACTCCCTGGGTTGATTCATAGAAATTGA
TCAACATGCTCAAACGTATCCGAACAAATGATTAAGTGAATACAGGATATGTTTTTATTC
ATTGAAAAGAAAACTGAATGCATAGCCAATAATCCTATACACCAAAGCCTAAAAGAAAGA
AAAGGACTAGTCAATGAAGTCAAATTTTGCGTCAGTATTTGCTCTATAGATATTTTGCAG
CTCTATGTTTGCATTGTATTTTTCACAGATAAAGCAAGTGATTCAAGCCTCTTTCTGGTA
TCCAGCTGGGTTGTTCTTTGCCCAGTTCCATTGGTCCCTGCACATCTCTTCAATACCATA
TTTCGGCGTCCAGCCGAGTTCCTTCTGAGCTTTGTCGATAGCAGCATAAACTGCAGTTGC
ATCCCCAGGTCTTCTTGGACATAATTTGGTAGGGATTTTCTTGCCACAAGCCTTTTCAAA
TGCGGCAACCATTTCAAGCACAGATGTACCTCGTCCAGTTCCCAAGTTATAAGCTGCAAA
ACCTAAATCTTCCTCTGTAAAGAGCTTCTTAAGGGCAGCAATATGACCATCAGCTACGTC
CATAACATGGATGTAGTCTCGCACACTGCAGTGCCGTCCTTTGTCGGATAATCATGACCA
TAAACATTAAGCTCAGGCAATCTACCAACAGCTACTTGGTGTAAGTAGGGCATCAGATTG
TTTGGGATACCCTTGGGGTCTTCACCAAGTTTACCGCTCTCATGAGCCCCAACAGGATTG
AAGTACCTCAGTGAAATGATTCTCCATTCTGGATCTGCCTTCTGGACATCTTTACCCATT
TCTTCACAGTAAACCTTGCTTCGTCCATAAGGATTCATAGGCATATAAGGGTAGTCCTCA
GCACAAGGTATTTTCTCAGGTTGTCCATAAACAGTTGCAGATGATGAGAACACCATCTTC
TTAACGTTGTACTTGGCCATAACCTGATAGAGGTTAATAGTCCCAATCAAATTATTGTCA
AAGTATCGAAAAGGATATTGCATACTCTCCCCAACAGCCTTAAGGGCACCAAAGTGGATC
ACAGCATCATATTTATTCAGAGAAAAAACCTTCTC
>ntig_728_consensus
TATCATAGCCTTACGTACATACATACAAAAAAGTGTCCGAAATTTAAAGAAGCATATCAT
ATCCATACATGCATGTTGGTCACTTTCAACACTAAGACCCAACTTATTCATACGAGTAAT
ATTTGACCTACTTGATGCTTCCTGAAATCATTTATTCCTCACTTAAGTTCCTTGAGAAAA
TCTTCATTGTCGACCAGGACAGTCTTCCAGCCATCTGGGTCCAGGAAAGAGGTAATTTTG
GTGTTGATTCCAGGAATTGGTCCTGGTTGTCGAGTTATCTTTCCTCCGAGCTCTTGTGTG
ACCAGGTTGACAACCTCAGCACTTTTGTATACATCATCTGTACCAATAGCAATCTGAGCA
TATGCATTTCCCTTGGTATATTCAGTCACACCATAATTGTAAGTTAATTCCAGAACAGTT
GTCTCATATTCATCNNNNNNNNNNNNNNNNNNNNNNNNNNNNNNTCTAGATCACCAACACGA
AGCATTACTTGGCATAATGGTTCAGGTGTTGGACCTCTTTGGATGAGTTCAAAAATATAA
CCATCAGGATCCTTTATAAAGGCAATGTGAGTTGTTCCACCTTTGACTGGACCAGGCTCT

CTGGTGATATTGCCACCCTTGGCACGAATGTTTTCAACCATTTTGTAGACATCTTGAGTT
GCAATTCCAAAATGCCCAAAACCAGTACCAATATCATATGATGATACACCATAATTGTAA
GTTAACTCCACAACAAAGTTTGACTCTTCTGGGCCAAACCCAAGAAAGGCATTAGAATAT
TTCTCTTCTGGGATGTCTCTTTGCCTCAACAGTTTCATTCCTAAGCATTCAGTATAGAAC
TTAATGGTGCGATCAAGATCACCAACACGATATACAACATGAAGCATGCGCCGCTTATCT
TTCTTTGGCCACTCCAACAGCTCAGTATTAGCCTCGGCCATTTTCAAATGAGATAAATTT
CAGAGAGAGAGAGAGAGAGAGAGAGAGAGAGAGGAGGATTTCGAAGCTCAGTGAAGCCTGAA
GAGTTGTGACGAAATGCTCATGACCGATCTGAGAATAGAGAT
>ntig_729_consensus
GTACTCACTTAGTTCTTAGGAATTCTTGTATGATTAGTTTTAGCATATTGATGGAATAA
TAACTGCAAAGAGGTCAATCGGAATGGGAGTTGAATGGCTTTCTTTTGTTTGCAAACAGA
AGAAAGTGCATCCAACTATGCCCACCATTGTAAATCTGAAGTTAAAATAAATTATGGTCT
TCGAGATTTCTCTAAAGTAGGGTCCCACAAGGATTTACCTCTCCATAGCCGGTCCCAGAA
ACATTTTTCATTCGGTTCGGTCAGGTTCCAGGAAAGCCCCGTCTCGAACCAACTTATCAC
ACACCGATGCATCCTGCCATTGTCCATTCTGACCGCATCACAGCAAATCACTATTTAAGT
CAATTTATGATACATTGCACGATCATAGTTAATAGACAACATCAGCCTCAAATTGATACA
CACGAAACAAGTGTCTGAAATTTAAGCTGTATCATAGTTCAAACCTAACTTATACATAAG
CGGTAATACTAGCTGCTTCCTGAAAATTCTTCATTCCTTAAGTTCCTTTAGAAAATCTTC
GTTGTCCACCAGGACTGTCTTCCAGCCATCTGGGTCAAGGAAAGAGGTAATTTTGGTGTT
GATTCCAGGCATTGGTCCAGGTTGTCGCGTTATCTTTCCTCCAAGCTCTTGTGTGACCAG
GTTCACAACATCGGCACTTTTGTACACATCATCAGTACCGATAGCAATCTGAGCATATGC
ATTTCCCTTGGTATATTCAGTCACACCATAATTGTAAGTTAATTCCAGAACAGTTGTCTC
ATATTCATCTGCATATCCAATCATAGCTAGGGTGTACTTATAGTCAGGCTTATCAACCTT
CCTTAACAGCTTCATCCCCAACGCCTTTTCATAGAACTTAATGGAGCGGTCTAGATCACC
AACACGAAGCATTACTTGGCATAATGGTTCAGGTGTTGGACCTCTTTGGATGAGTTCAAA
AATATAACCATCAGGATCCTTTATAAAGGCAATGTGAGTTGTTCCACCTTTGACTGGACC
AGGCTCTCTAGTGATATTGCCGCCCTTGGCACGAATATTTTCAACCATTTTGTAAACATC
TTGAGTTGCAATTCCAAAATGCCCAAAACCAGTTCCAATATCATATGATGATACACCATA
ATTATAAGTTAATTCCACAACAAAGTGAGACTCTTCTGGGCCAAACCCAAGAAAGGCATT
GGAGTATTTCTCTTCTAGGATGTCTCTTTGCCTCAA
>ntig_730_consensus
ACACAGACAATCAACTACATGTGCATATTGAGAGTTATCTTTCCATGTATTGCTTTTGTT
TAGTTAATGCGGAAACATTTGTAATGACACCTAATTTTCCATTAGTAAAGTACATTTTAA
ATGGGTTGTCATCTTAATCACAAATTCCAACCAATACTATAACGACAAAGAGTACCAGGT
CACTTAACCTCGCTTTACAGGCCTCTGAACGTAAGATTTGTTGGGATCCTCTGTCTTCAG
CTTTGGAGGTCGAATTTCTCCTTTCTTACCCTTCTTTCCTGTCTTCATGAAGTCTCTCCA
GCTTGAAACCCGTTGTTCTCTTGTTCCTTCCCACTGCTCCTCATGCTCACGCTTTCTCTT
CCACATTTCTTTTTGTTCCTCTTCATCCTTTTTCAATCTACCTTCCTCTTCTGATATCCT
CATTCGCATTTTCCTCCTCCGCCATTCTTGTTCTGTTAATATGTCTCGAACCTTCAGTTT
CAGCTCCTTCTTAAACTCCTCTGACTGTTCGTACTGTTGCTCATATTTGCCCTCATCAAC
TAATGACTTTATTTTAGAGGCTGCATCTTTCTTCAACTGCCTCTTTCTCTTTGCCCTAAG
CTCTTCTTTTGCTGCATTAACTTGAGTAAGAATATAATCCCTTTCTTGATCATCACCCAG
TAGTTGCTGTGCTTTTGCCAGTGCTGACAACTTCATTATCTCTTTCAACTTCGCTAACTT
CGGCGAAGAAGCTTTTAAGAAGCAAATCCTCATCGACGGTGTCTCCCATGGTGCCGTTGC
TGTTCGGATGTCTGTCTTCTCTCTCTAGTCGAGTAACGTCACAGACGAGTCGTTTTGGGA
GTTGGCTT
>ntig_731_consensus
GAAATGTGTAGAAATTTATGTAAAATGGTGTTTAAATATTTTAAGTAATCAAAGGCTAGA
GGTAAGGGTATTTTAGCCATAAATATATGCATTATACATTGTCCTTCAATTGTTCCACCT
AATTAAGAATAAAGTCCCAGCCAGGAATTTAGCTTCTTCACCTTTACTATTAAGGCTTCT
TTCTAACTGTAATAAGCTCTTCCACAGACAAACCNNNNNNNNNNNNNNNNGGCCTTTGAAC
ATATGATTTATTGGGATCCTCTGTCTTGAGCTTTGGAGGTCGAATTTCTCCTTTCTTACC
CTTCTTTCCTGTCTTCATGAAGTCCCTCCAGCTTGAAACCCGTTGTTCTCTTGTTCCTTC
CCACTGCTCCTCATGCTCACGCTTTCTCTTCCACAATTCCTTTTGTTCCTCTTCATCCTT
TTTTAATCTACCTTCCTCTTCTGATATCCTCATCTGCATTTTCCTCCTCCGCCATTCTTG
TTCTGTTAATATTTCTTGAACCTTCAATTTCAGCTCCCGCTTAAACTCCTCTGATTGTTC
ATACTGCTGCTCATATTTCCCTCGTCAACTAATGACTTTATTTAGAGGCAGCATCTTT
CTTCAACTGCCTCTTTCTCTTTGCCCTAAGTTCTTCTTTTGCTGCATTAACTTGAGTAAG

AATATAATCCCTTTCTTGTTCATCACCCAATAGTTGCTGTGCTTTTGCCAGTGCTGCAAA
AGCTTCCTTTGCTTGTGGATGCTTGCATTTGTCAGGATGAACCAACAAAGACAACTTACG
ATACTGCTTCTTTATATCATCTGGAGTTGCATCAAATGGTAGGTTTAAGTACTCAAATGG
ATTCAGCTTGAAACACGATAGAATCCTGATGACTTCGTTATCTCTTTCGACTTCGCTGAC
TTCGGCGAAGAAGCTCTTGAGAAGCAATTCGTCATGGATGGCGTCTCCCATTGCGCCTTT
GCTTTTACGGTATCGGTCTTCCTCTTTCTCCCTACGCGAGTAGCGTTACAGATGGCTCGT
TTTGGGTGTTCACTTGACTGTCCAGATTAAAAGAAAATCACTC
>ntig_732_consensus
TTGCTTTCTATATAGAAAGCAAAGTCAAAATCGGTGATTCATATCTTCAAAATCAAGGCC
AATATGGAAGGGAACGTTTTTCGAACACTAGAGCACGTCAGTTCAGTAGCTAGATATGGC
TTTTACGTGTTAGTCAGAAGATACAATCCCACTACCACTTAAAACCATTACGAAAGAAGA
TAGAACTAATCTCAACAGCAGAAAAAAGAATAGCACCTCCAGCAAAAAGGTCTTCATTAC
AAGTCTAGGTTCAGAGATAAATCAATTTCACGAAAATTCGTCATGCCTAGCTAGACATCA
TCACATGATCGATCGACTGCAGGGCTTGATTGGCAAAAAAACGGACATCAACATCTGGAT
CCTCACTAAGCTCAACCAAGCTTGGACGGATTGTCTTTTCCACCACCGACTGGTCTACAA
TCGGGATCAGAGACTGCAGCACTTTTGCAACATTAAACTTTATGTTGGGTACTCTGTCTT
TTGATGCATGGACTACCACTGGCAACAATTTAGTACAAGTGATTTCTGATCCCATAACAG
GGGCAAGTAGAGAGATAGCACGCAAAATGGTCATCCGATACAAATAGTGGGCATTGTTGA
TCATATCCAAAACCTGTGGAATTATGTGCTGCATTGCCCACTCTGGACCAAATTCTTCAG
CAAGGCGCTTTAAATTATTAGCAGCAGCATCACGAATAGAATAAACCTTATCCTGTAACC
ACTGCATACAAAGAGAACCAAGTTTGTCATCAAAAAATCCTACTCCCAACTGGCTGGCCA
ATAAAGGTATATACTCTATTATTGCAAGACGAACCCTCCAATGTCTATCCTCAGCAAGTT
CAACAATGGCAGGTAGTAAGGATTGAGACAATAGATCAATTCCAATAACCTGGTTCACTT
GATCAAGCTTGCTGATAATATTGAGTCGCACATCTGGAAATTCATCCTTCAAAAGAGAGA
GAAATATTGGAAGAAGATGCTCGATTGTTGCATCCTTTCCTAGAACAGGGGCCATCCCCA
TTATTACAGAAGCCAGTGCAGAGCGGACATGCTGGGAAGAATCTGATGACAATTCCTTCA
CACAGGGAAGGATATGTTGAATCGCAAGCTCTGGATTTAGAATCCGGCAAAACTTGGTTA
CTTTGCCAGCAGCTGCTATACGTACTTCAGCTTCATTATCACGAAGTAATCGCACATATG
CAGGGACCAAGTCCGTCCTAGTAGGCTCAGGACCCACAGCTTCACAAAGTTCATATAATT
GATTTGCAACCATGTAGCGCACACGCCAAGACTTATCCTGGGAGAAATTTACTATAACAG
GGAGAATATGTGCAACACAATCCTGAGGCTCCAACAACTTTCCAAGAGCTGCACAACCCT
CGACAGCCAACAGTCGTACAGAATCTTGCTCATCTTGTGTAAGATCCTCAAATATTGTCA
TGATGTCAGTCTTCAGATGAGCAGGTTCAACTGTTGCAGCAAATTTCCCAAGATTTGATG
CAGCAGATCTCCGAACCATGGGCGTGTCATCTTGGCACAACTGGGTATATATTGATCGCA
ACTCTGTCTTCAACATATCTGGGGCACTTGGATATGCAATATGGAACAATCCACAAGAAG
ACACTCGAGCTGTAAACCATTCACCAGCCGCCAGTCTCTTAACTAAAGGAATAAACCAAT
CAACCAAGTCACTCTCCCTCATCTGGGACCCAATCCTACACAGCGACTCCACAGCTTTGT
CCCTTACACAGGTCTCTTCGACAGTGCAAAGTGTCTCCAGTGGTGGAAGCAAAACATGGG
CATGTTCAACTCCACCAACGTATGGAATAAACACCCCCAACTCTTCTGCCATTGCAAGAA
GCACCTCGTCATCATCATCATTGTTTTCACTCAAAAAGGGAATTAATTCCTTCCTTGTCC
GCTCCTCTCCGAGAGCACGTGCAATTGTTGACAATCTACGGATCGAGTTCAACCTAAGCT
GAATGTCATCATTTTTCAGCTCATCTATTAGGACAGCTATTGGGTATAGTGGTTCATCAA
TCATAGACATTGTGCTTCAAATCTTTTTCACAAATTTCTTACAATCACTTATCGTCACAC
AAGCACCGATCTCAATGTCTTCCCGCCGGCGGTCTCTGATTAGAGAGAAAAGAGACTCTT
AATGATGGATTTGTTACTTTTGTA
>ntig_733_consensus
AGGGCATATTACATAAATGATTGCTTCTATACAGACTTCTAAGGCAAAATCTGTTATTAA
ACATCTGGTAGAGCAACGCCAGAAAACCAAAAAATAAACTAAAAATAGAAGAGAAATACA
ACATGATCTTCAAGGCCAATACGGGATGCTTCTCAAACACTACACATTAGTTCAGTAGCC
GAAAAATGGCTTGTACATGTTAGTGGACACAATCGCACTCCCACTGAAAACCATTTGGAA
AGAAGATAGGACTGTCCTGAACAACAGAAAATAATAGCACCTCCGGCAAGAAGGTCTTCA
TTAAAAGTGAAAGTTCAGAGATAGGTCGATTTTACAGATACTGGTGATGCCTAGCTAGAC
ATCATCACATGATCAACCGACTGTAAGGCTTGATTGGCAAAGAAACGGACATCAACATCT
GGATCCTCACTAAGCTCAAACAAGCTTGGACGGATTGTCTTTTCCAACACAGACTGATCA
ACAATTGGGATGAGAGACTGCAACACTTTTGCAACATTGAACTTTATGTTGGGTACCCTG
TCTTTTGATGCATGGATGACCACTGGCAACAATTTAGAACAAGTGATTTCCGATCCCATA
ACAGGGGCAACCAGAGAGACAGCACGCAAAATGGTCATCCGATACAAATAGTGGGGATTG
CTAACCATAGCCAACACCTGAGGAATTATGTGCTGCATTGCCCACTCTGGACCAAATTCT

TCAGCAAGGCGTTTCAAATTGCTGGCAGCAGCATCACGAATAGAATAAACCTTATCCTGT
AACCACTGCATACAAAGTGCACCAAGTTTATCATCAAAAAAGCCTACACCCAACTGGCTG
GCCAATAAAGGTATATACTCTATTATTGCAAGACGAACCCTCCAATGTCTATCCTCAGCA
AGTTCAACAATTGCAGGTAGTAAGGATTGAGATAATAGATCAATTCCAATAACCTGGTTC
ACTTGATCAAGCTTGCTGATAATATTGAGTCTCACATCAGGAAATTCATCCTTCAAAAGA
GAGAGAAATATTGGAAGAAGATGCTCGATTGTTGCATCCTTTCCTAGAACAGGGGCCATC
CCCATTATTACAGAAGCCAGAGCAGAGCGAACATGCTGGGAAGAATCTGACGACAATTCC
TTCACGCAAGGAAGAACGTGTTGAATTGCCAGTTCTGGGTTTAGAATCCGGCAAAACTTA
GTTACTTTGCCAGCAGCTGCTATACGTACTTCAGCTTCATTATCTCGAAGTAATCGCACA
TATGCAGGGACCAAGTCCATCCTAGTTGGCTCAGGCCCCACAGCTTCACAAAGTTCATAT
AGTTGATTTGCAACCATGTAGCGCACACGCCAAGACTTGTCCTGAGAGAAGTTTACTATA
ACAGGAAGAATATGTGCAACACAATCCTGAGGCTCCAACAATTTTCCAAGAGCTGCACAA
CCCTCAACAGCCAATAGTCGCACAGAATCTTGTTCATCCTGTGTAAGATCCTCAAATATT
GACATGATGTCAGTCTTCAAATGTGCAGGTTCAACAGTTGCAGCAAATTTCCCTAGATTT
GATGCAGCAGATCTTCTAACCATGGGCATGTCATCTTGGCACAACTGGGCATATATTGAC
TGCAACTCTGTCTTCAACATATCTGGGGCACTTGGGTAGGCAATGTGGAACAGCCCACAA
GCAGATACTCGAGCTGTGTACCATTCACCAGCTGCTAGCCTCTTAACCAGAGGAATAAAC
CAGTCAACCAAGTCACTCTCTCTCATCTGAGACCCAATCCTACACAGAGACTCCACAGCT
TTCTCCCTCACACAGGTCTCCTCGACAGTGCAAAGTGTCTCCAAAGGCTGAAGCAAAACA
TGGGCATGCTCAACTCCACCAACGTATGGAATAAACACCCCCAACTCTTCGGCCATTGCA
AGAAGCACCTCATCGTCATCATCATTGTTTTCACTCAGAAAGGGAATTAATTCCTTCCTT
GTCCGCTCCTCCCCAAGAGCACGTGCAATTGTTGACAATCTCCGGATGGAGTTCAACCTA
AGCTGGATGTCATCATTTTTCAGTTCATCTATGAGGACAGCTATTGGGTATAGTGGTTCA
TCGACCATAGACATTATGTTTCAAATCTTTTTCACGAATTTCTTACAATCCCTTATCGTC
ACACAAGCACCGATCTCAATGTCTTCCCGTCGGCAGACTCTGATTAGAAAGAAAGAAGAC
ACTCAAAGATTTCTAGCAATTATTTGTAAACATCAGAAAACTCGGCTATGATAATTAGCC
CGCGGGCCTAATTTGGACTGGATTTGAATATCTGTGCCCTGAAAATCGAATCCTCGAGTT
CAACTCTCAATTTGACTCCG
>ntig_734_consensus
CAAAAATCTATCTCAGAGAGCTAGCCTAAATGATGATTTCACTGGAAAAACAATATTTCA
AAGACAAATAACAAAGAACTAAGACAAACACTATGAAAACATAAAGCATACGTAATGTGT
CATAATATATCATGTAAAAAAGAGGAAAGAAAAAAGCTAACTCTGTTTTTGATATGAAGG
TAAAGATAACAACTTAGCGCTTTTGTGCTTTCCTTCGTCGTGATTGTGCCTTCTGCCATT
TCTTTCTTGAAACCAGTTTGCTCTTTGGTTTCAATTTTATCTCAGGTGAGAACTTATAAT
TTGGATCTCTCATCCGTGCTTGTATGTCCTTGAAGAACTCCATATTCAATCTCTTAGGCC
CTCCCTGTCTAAGTTCCTCATATTCCGGTCCTGAGACAATGTTTTCAAATGCTCCTATAA
GTATATCTATGTCACTCATGACTTCCCACACATTTATATAGCGCCCATCTGGCCCTTTCT
TTAATTTTTTCAAGGCATTTTCAACTGCAAGTTTCCGAGCTTGCTTTCCAGGAGACAGCT
CTTCTGGCTCATTTTCAGCTTTCAAAAGTTCTGAGATGGATCGATATTTAGGTTTCTCCT
CAAACTCGAACAATTTATCAATATGCTTATCACTAGCTTCATTGGGATAGGGCTCAGTTG
GTACATCATCATCATCATCATCTTC
>ntig_735_consensus
GAAACATTGAATTTTATTAAAGTGATGTCCAGACACTACAAATTTTTCAATCAAATAAAA
ATTTGAACACACTTTAAATCACCAAATCAACAGCCGCCAGCTAGTCCCGCGAATTTCTAC
CTCCCTCTACACGGAAACAAAGTCAAAAGTCCCAAAGCCCACGTCACCGATCCGCGTAAA
TCTCAACCGAAAATCTGAACCCTTCATTTCAAACTTATCCGCGGTTCACAACTACTCTTA
CAACACCACCGCGGATCGAAACCCAAACACCCGACACCAGACGTTTCTATAAATACGACC
CCAGATGATTCAGATTCAGCCGCAACAACACTCTTAGAATCTTCTTGTCTCTTCATCAAC
TCTCAAAATCAGAAAATGTCGGGCCGTGGAAAAGGGGGCAAGGGTTTGGGCAAGGGAGGG
GCGAAGCGACACCGTAAGGTTCTCCGAGATAATATCCAGGGGATCACAAAGCCTGCGATT
CGGCGTTTGGCTCGTAGGGGTGGAGTGAAGCGTATCAGTGGGTTGATCTATGAGGAGACA
CGTGGTGTTCTGAAGATATTCTTGGAGAATGTGATCCGAGATGCCGTGACATACACTGAG
CACGCTAGGAGGAAGACGGTAACTGCTATGGACGTGGTGTATGCGCTCAAGAGGCAGGGC
AGGACTCTCTATGGGTTTGGTGGTTAAAGTTGTTTTATTTGATTTTATGTAGAGAGACAA
GAAGTCTTGCTGTCAAGAAGATTATGCTTGGTTTTTAGGGTTACGTGTTTGAAATTCATT
CTCTGAATATGGTTTCTATTTGTTTCCTGTAGAATTGGTGGTAATTATGAAACAATGGTA
GATTGTAGTTCAAGTTTAAGTCTTTGGCTTCCATATTGTTCTAACAGGCTGTTAATATTG
ATAGTTAGCAACAAGATTCCAGGATTGAATACAAATCAAAAAGATTTTCCAGTACTTGCA

GATTTGGAAGCTTTGAATTAAGAAATATTATGATTTAAATTGTGCCATTGCAATTCTATA
TAATCAATGACGAAGTCATTCTGAGTCCAAGGTTGTAGCATTTTCTATGTCTCAGACTCA
GACAAGGATCCTTTATTGGGTTGGTTAGTACTCACTCCTCTTTGTGTTTTGCGATGTTGC
GAGCGTGAGAATATCTTAACCCGCTGGTACACCTTCCAATGAACTTAAGAACCTCATCGA
TTAGAATCACTGGGAAAGCAACAGCCACGACCAAGAGCCATTCATTCAGACTGAGAGGGA
CGATGCCAAATACTTGAGCAAGGAATGGCACATAAAGGATCAGAAAGTGTAGGCCAAATG
AAGTAGACATAGCCAAAAGAAGATACGGGTTGACCCATGGGGGCATTGTCACCAGGCTGG
AGTCCTCAGACAAAGCATTAAGGGAATTGAACATCTCAATTGCAACCAGTACAGAGAGGG
AGAGAGTGGTGGCTTTGATTTTCCCAGAGTGGAAATACTCACACGGATTTGCATCAAATG
TGAATTTTTGGCTTCCAGCTGTGAAGGGCGACACTGAAAAATTCTGCCAGGAGTGGCACT
GTCCCCAATTAGCAAGCTGAGAGTAGGTCACAAGACTATGGCCGTCTCCACTCAGGTCAA
TGCCCAAGAACGAGTCGTGTGTATACCATATAATGAATACCCCCACAGTTGCTAACCCAA
CATAGAGTCCAATCACCAGATATCGGAATAAAATCCAAGGAGTGATCAATGAGTCATCAC
TTCTTCTGGGGGGTTTCTTCATTATATCCTTATCTGGAGGGTTAAATCCTAAGGCTGTTG
CAGGAGGACCGTCAGTAACAAGATTTACCCAAAGAAGCTGGACCGGGATCATGCCTTCTG
GAATGCCCAAAGCAGCTGTCAAAAATATAGAGGCAACCTCACCGATATTTGATGATATCA
TGTACCTGATAAATGCCTTCATATTGTCGTAGATAGATCTGCCTTCACCAACAGCAGCTA
CTATAGTGCTAAAATTATCATCTGCTAACACCATATCAGATGCTTCCTTAGCAACCTCTG
TCCCAGTAATGCCCATTGCTACACCTATATCAGCCAACTTCAATGCAGGTGCATCGTTCA
CTCCATCTCCTGTCATTGCAACAACTTCACCATCCTCTTTAAGTAATCTTACTATTTCTT
GTTTATGCCTTGGTTCCGCCCTGGAGAACAGGAGGCCTCCACTTTGCCGCAGATGATTTT
TCTGATTGGGATGAGTCATAAAATCCTTTCCGGTTAGACTTCTTGAACTAATATCTTCTT
GAGGTCCAAATACACCAATGTCACGACAAATAGCTTCAGCTGTGTGCTTGTTGTCCCCAG
TAATAACCATGACACGAATTCCAGCAGTTCTACAGTCCTCAATTGCTTGACGAACCTCTG
GCCGAGGAGGATCCTTCAGCCCAACCAAGCCAACAAAAATGAGTCTACTTTCAATTGAAT
AATAATTGCCAGGATTAAGCAAGATCTGATGAGCTGGATGATCTTCATCACCATTATACG
TTGCAAACTCAGAGAGGTCATCCTTGTATGCAAAACCCAAACAGCGTAATGCACTGGTTG
ACATTTCATGAAGACTTTGTAAAATATGATCCTTTGAATATTCATCCAATTCGACAACAG
AACCATCAAGTAATTGAATGAATGAGCTCCTCTCCAATAAATTTTCAACAGCACCCTTCA
CTAACAATGACTTTTTCCCTAAGCTGGAATTTACAATGACCCCCATGGATTTCCGATCAT
GATCAAACTCAAGAGTTGCAATCCGCTGATCTAGTTTATTCCACAGTTGACAACAACGTA
GGACATCCTTGGGACTTGGAGAAGAATCATCATTAAATCCCCCAGGGAATCCCATTTTCT
CAACC
>ntig_736_consensus
AAAAAAAAAAAAAAAAAACAAGCAACCAATGCAATGCACGAACTCTCTCAAAGTGGCTCTT
CACATATGAGAGAGCCTTAAACAAAACATATCAATTTTGACCTTGCCAGTGCTCAGGTAT
CCTCAACTCGCGAATGGAATACAAAACTTGGAAACGTTGTGGTGATGTCCTTGAGATATT
GAAGGGTCATATTCTTCAAAAGGGATGGGTGTCGGTAAACAAGGCTTCTCCACTCTTCTT
TGTCAATCTTCCCATCATGTTTTGTATCTGCTTCCTCAAAGGTCTTGTCGATAATACTTT
CTATAACATCGTCTGAAAGGTTCATACCAGATTCAGCAAGTGTAGCTACTACCATTTGCT
TCACCTCCTGCCTCTCAATAAAACCTTGCTGCTTGAGATCATACAGTTGAAAAGAAAAAT
CGATTTTATCATCAATAGGGGCATTTGGATGGAAAACAGACAAAGCACGAGCAAACTCTT
CAAAACCTAGTATTCCGTTGTGCTTTGTATCGAACAAGTCAAAAACCCGATCAGCAAACA
AACTCTCCTTTTTGTTTGTCTTAAATAATGCCAACTGAAACTCCTCCTTGTTAATCAACC
CGTCATCAATCACAGCACTGCTGATCTTCTTAAACAGCTCATAAAGTGCCTCGATTTCAC
TTACACTAAACACTGTCTCTCTAGCTAGAACTTCAGGATCTTCAAGGCCTCTTGACTGTC
TGTACAAATCAAGATCACAACACTGCAATAGGGAAGCACATAAATGCTTAACTCCCTCTA
TGCACTGCAACATGATAGTTTCTGGACATCAAAACACTCCATACCTTTTAACCATTTCCT
GCTTCTCCAAACTCAAAACCAAGCCCCTAAATTCCTTTAAACCTGCAACTCTCAACACTT
TATNNNNNNNNNNNNNNNNNNNNCGAGAAGAACGTGGCGATCCGATTGGTAGCCGAGAAAC
GGAGGAGGAATTGAGGTTTAGAGAGAATTTTCTTTTGTTTTCTCGGGAAACATACAGATT
ATGTGGAGTTATTCGTTACCTTCTTATAGAGAAAGGCAAAGGATCAGGAGAGTGGAAATT
ATGGAGAAAAATAAATTAAAATTTAAAAATAACCTAAATTTTATTTGAATTTGGATTTTA
ATTTAT
>ntig_737_consensus
TAATAGACATTAATAACCATAACACAATCTACGGTTCAATCCCATCTGGAAACAAAAAAA
TATACTGTACATGCGTTTGTTGCATCAGAGCCCTTTTTCCCAGCCAAAAATGAAGTTGAC
TATCGGATCCAACTTTCAGGAGCCGCCAACCCATATAGTAACCAACCCACCATCACTCCA

AAACAGCTACCTCTTGATCAACCCTCCAGTAGTTTGCATCATTGAACTCCTTCATACCAG
CTCCACTCTCGTCCGAATTTTCCATTTCCGACACCTTTTGGATAATGTTCCTCTTCAAAG
GCCCAGCTTCTCCAACTATTCCCTCCTTCAGAGCCGGTTCCATTGCCTTCTGAGTACTCT
CTAATTCCATACCCACAAATTCAACATCTTCAAACAATGAGGGCACTGCTGCAGATCTCT
GACTCCTGTCACTTGCAGATCCCTCAATAGAATCCATGGATGTAACAGTACCATTTGGGA
CTATAGAATCCGCACTTGATGAGTTTGCAACTTCGGTGTGATTAGCAATGTTAACACCAG
AATTGTCATTATCAATAAAAGGATTCAAGCTTGACCCACCAGCAGCTTGCAAATCCGTTG
ATTCTCTCCATCTTTCCCAATCTGGTAAAGGCCTATCCACAAAGAAGTCTTGATTGTCTG
GTGTGTCAAACCTGAAAAATCCCATATCATTGGAAGCATTCGCATTCTCATTTTGGGGAT
CCAAATTTCCACTATTTGTAGTATCACTCCCAGAAAATGGATTCATATAGTTTGTGTTGG
AAGTGGAAGTGCCATTGACAGAATCTTTACTTTCAATCAATTCATCATCTTCCCCAACCA
CCACCTCATCATCACTACTGCTGTTACCACCATTAGCAGTTCCATTCAAGTTAATATCAT
CCATCATCTCACATGGTGATGTGCCCACAGGCGCATTACCAATTCTGTCATCTTGGAAGG
CAAACCAGTTGGAATTCGTGAAGAGACTCCCTTGGTCATCACCAAGCCTCAGGGATGATA
TCACGACTTCAGTAGATTCATCATCAAAGTACACATCCTCATTATCCCGATCCAGAGCTC
CATGGTCCTCTTCAGCATCCTCATTCCCATATATTTTGTATCTGAAAGCCTGAGTTAAGT
TATTTGCAAGACCAGCAACATCATAATCCCTGTCATGAATGTCATCATCATCACTATCCC
TTGTCCTATCTTGTAAAGCTGCTGGACGCCCACAAGCCCATTGGTAGACATTTTCAACTG
CATTACGCTCTTGCAGGACATTAACTTGCCATCTGCTCCACTCATCATTTTCCTGAAGAT
ATGCATGAATGTAGCTGTCATTGCTGGCTAATTGAACAAGCTTATTAGAAATCCGGGTAA
TGTGTCCAAGGTTTCCTGCTCGTGGTGCCTGTTTCCCAGAAGCGGGTAAAGTTGGCTTAC
CAGACTCGCCAGAAAGAACAGGATCTTTATCAGTTTGGAGAATCTTCCCAACCATATCAC
ACTCTCGAAGAAGATGATCCACCATGTTATTACTCTTGCTCTCCAGACAAGAATATATAA
TACTCTCCACATGGTGATGCAATGCATTATTGTATGGATACTCAAAGAAAAGATCAAGAA
CTCTTTGAACGGTTCCAGAGCTGACCAATTCCTTCTCTGCAGCTTCATTGCCAGTTTTCA
GTAGCACAGCGATGAACTCGACAATCTTTAAGCGATGCTTCCCTAGTGGTGGTCCCAATT
CTCCAAATGTTGTTGGCAAAATCTTCTCATCAGATGCGACATTAAGAAGCATAAGTAAGT
CATGAAGTTTTGGCAGCATCGCACTAATAGTCTCTGGATTAACAGGGATTGGAGATTCAT
ACATATGTTGGCTTCGAAAAGAATGCATTAGAGGAGAAGAAGTTGCTGATCTTTTTGGAT
CTAACAAGGAGATACACACAGAAAGGGAGTGAAAAAGGCCAGACTTTGAATGTGAATCTT
CCAATGCATGACCAAATATTCTTGCAATGAAACTTGGGCTTGCAAGTCTTGTGGCCAAGG
CTGATGGAGCATTTCGAGTTATTGCACATAGTGTCTCTGCTGCATTAGCATGAACTTCAG
GAGGGCAAGATGGACTTAGTTTATCCACAATCATTTCAAGCAAATTGCTGTCAGCCAGCC
ATTGCATCACATCCATAAAATTGGGATATACATGATCGTCAGCACCAACAAGCCTAACCA
AAACCTCCATGATAGAGGTAATCCCAATCAAATCAACCAACTGGCGGAAAACATCTGGAT
GGACTTGAACATAGTTCATAAGTGAGACTGTCTTTCGCAACATAAGGCACACAACAACCT
TGCTGAAATACCCAGCCAATAAAGCACTATGAGGTCGGTTTGGTTCCAAGAAGGAGAAAA
GCAAGTCCATTAACTCCTCTTCCTCCACCAGTGTCTTGAGAATTACATCAATTTCGCAGG
TAAATATCTCACATGCAATAAAGGGAAACTTGAATGCAACTTTGCTGTCAGCATCCTCAG
GGGCTTCTTCAATTATATACCGCAATAACTGCTCCACTTGTGCTCTGTCTCGTAAAAAAT
TGATGAGTCGGCTGTTTAAGGCTTTGCATTCTTGGATTATTTCTTCCTCATCCAGCAGTT
CTTCCAACGTAAAATTTTCCTTGTCCAAAACTGACTCCACCGGAGAGGATAGAGCTGTGA
GCTTCCAAAACATGATGGAGAAGCAAAGGCAGGTAAGAGATTTGAGAGAGGTGAGCAGAT
CTAACTTTGGGAGACTAAGAGATCGAAAAAAGTTGAGCGCCGTGAAGATCGAGACGTAGA
AAGACAGAAAAA
>ntig_738_consensus
GAGATACCTCCTCCTCGGCCTAAGAGGAAACCTATGCATCCATATCCCCGTAAGTCTACA
GATTTACACCAAGGAACACCTGCTTTGAGTCACTCAGAAAGGTCTCTATCAGCAAATGTA
CTGGCTTTGGATAAAGATAATCAATCTCCCGCCTCAGTTTTATCTCCATTTGCTTCTGAT
ACATTGGGGTCTGCAGTCTCCAACCAGCAAAATGGATGTTCCTCGCCGACTTCATGTACC
ACTGAGATGCAGTTAACTAATGTGTTAATTGCCAAGAAAGAGCATGAATATGTGACATCC
AACTCTTCTCCCGAAGAAGAGAAAGTTTCTATGTCATCAGCCAGTCCAAACGGCGGTGAC
CTACTGTCTGTGAAACTTGAACCTCAGTCCAAGGACTCACCGTGCATAAAAGGAGATCCA
GCACCGGAAACATGTGCAAGTATAAAGCTTTTTGGGAAGACAGTTTTGATAACAGCTTCT
CATGAGACAAGTCTACCAGATGCAAAGAGTCACAATTCAATGATAATCCAAAGATGTCAA
GAGAATCTGAACATTGACATTGGAAATTCTATTGACGTATTCGCATCTCAGAAACTGAAC
AGACATTCATCACTTGGAATGGTTACAGGGAAGTCTTTCCCATTGGAGGCTCCTTGTAGG
GAGCCACAGAAACAGAACAGCATCGCAGAAAACAGCCCCAATAGTTCTTTTAAACTGCGG

CCTATTATGCATCAAAGCCTACCCTTTTTATATGCCATGCCA
>ntig_739_consensus
GAGATACCTCCTCCTCGGCCTAAAAGGAAACCTATGCATCCATATCCCCGTAAGTCTACA
GATTTACACCAAGGAACACCTGCTTTGAGTCACTCAGAAAGGTCTCTATCAACAAATGTA
CTGGCTTTGGATAAAGATAATCAATCTCCCGCCTCAGTTTTATCTCCATTTGCTTCTGAT
ACATTAGGGTCTGCAGTCTCCGACCAGCAAAATGGATGTTCCTCG
>ntig_740_consensus
CCTCTGATCTCCAAATCTTAAGTACTATGCCATAAATGAATGCATGTCCATGAACTGGGC
AACTTAAGCTTATTACCCTTTCTAGTTTCTCCACAACTACAAGCAGGGGGAACTACAGAC
ATTACATCACTGATCCTTCCCAAGAAAAGTCACTGTTCCCTTCCATAGAAAAGGGAACAT
GCTGAACTTAGCAGATAAATCAACATGGGAAAGTGAAGTTCAAAAATGAGTTATGTTACA
TAAGTTATACAGAATCTCAACTTCATAACTTATACAAGGCTCAGGAGAACAAGGACAGTG
AAAATGGTTCATGTCCCTGCATCCCAAAGTCTTCCGACCCTTGTAACAAAACCAGCTTTT
ATAGGAGCCACAGACTGGATAGAACCACACTGCTCACAACCAAGAAAGAAAAGTTGATTC
TCCTTTGTAAGAATTGTATCTGGACTTCTACAGTCATTACATATTACATATTCATTTATG
TATCTATGCAGAATCCCTTCAACGTTTTTAGGAGGAAATCTACCCTTTACTACCAATTGC
TGCTGCCTGTCAAGGGATCCACTTGCCCCCATTTCAGCTAGTAAAAAGGTCATGACATCC
TCTGCCTGTCTATGCATC
>ntig_741_consensus
CATCCTCTGCCTGTCTATGCATCCTGAGTCAAACAAAATAATTTTACAAATGGAAGATAA
GTAACAAACTATTATCAGAGATCACAGAAAAATATAAATCCCATATTAAGATGAAGATGC
TTAAACTCTTAAGAGTACAGAAGAATAAGATAAAACTATGAAAGCAAGTTTTAAAAAAATA
AAAACCAAATCATTAATCATACGTTTCACAAAGATCAATAAA
>ntig_742_consensus
TCCAAATTCAAATTCCATTTCTTGTACACCAAAATATTACTGGGTCTATATATGATCGTT
AATAACAAAGCAATCGCGCAACAAAAACAACACATAAAAGTGATATCTTTTGCGAAACAA
AATGCTGAGTCAATAAAAACATAGCAAGGAAACTACAGCAAGAAATTCAAAAACATAATT
ATAAATGTCATTATCCTAGATTTTATCATAACAAAGTAGGTGTTCTTAGTCGAACAAACT
GAAGCCCATGTCATCATCAGACTCTTCTGCTGGTTCTTCCTTCTTCTCTTCCTCGGCAGC
AGCAGCAGCAGCTGGTGGAGCACCTCCAGATGCGGCAGCTGGTGCAGCAGCAACAGCGAA
CTTACTGGGATCCTTGAGGTACTCCTTCACTTTGTCTGCCTGAGGGAAGGAATACTCAGT
TGCTAATGCCACAGATAGAACATTCTTGTAAGCATTGATGAACATGTGTGGTGCCGCTGC
CAAAGTAGGGTAAGAGATGGCCAGTGCCAATGAGGTAACCATTGAGACACCAGCAGCAAA
CTTCTCAACCAGGTCATCCTCAGTCAGGTCAAGCACCTCTGGGCTGAAGACAGACCCATT
GTCATAGACAGACTGGACAACAAGGCCATAAGAGAAGGGCCTAATGCCAAGCTTAGCCAG
CAAAGCAGCCTCAGAAGATCCCACCTTGTCACCCTTCTTGATAAGCTCCACAGGGGTGAT
AATTTCAACGGTACCCTTGTTAATCTTGGTGGGAATGTTGAGCACCTGGAAGAAAGAGGT
TTGAGATGGGTCGAGCCCGGTGTTGCCCGGCGGAACAACAACATCAATTGGGGCAATCAA
ACCGACACGAGCAGGAGCTCCAACCTTGTACTTGGCAACCTCCTCACTGACTTCCTTCAA
ATCACCCTTGGTGAAAATCAACCCCACATTACCCTGAAGAAGTGGAATCAAGTTGAGGAA
GGCCTTATTCCCCGTCTTCTCAGAGTGAATCCTAATGGAGCGTTTCATCATGGTATTCTT
TCCCATCAGTACAATAGAGTCGCCACGTAGACCTTTCCTGATGTTCTGGAGCTGATTGGA
TCCCACGTTGTCCGCGGCAGCTACCAGGACCTGGTGGTACTCATCCAACAATTTGCATAG
CTTTGCGTCATAAGCGATCTTCTTGTCGGCCTTTGATGGTTTCACCGCCATTGGATATTG
TTCTGTTTCTAAGCTATAACTTACTAAATAAACGAACAAAAATGAGGGCCAAGACGACAC
TACAGCTCAGTCAAGGCGGACAGCAGGACGATGCCGTCTCTTTAACCTCGTCGGAATTTA
TAGATTATTAACACTGACTTGTGTATACTTCGGAACGCGAGGACTGCACGTTTCTTAGCT
GCTCT
>ntig_743_consensus
TTTTTTTTCGGAGCAAAAGAATCAATTAAAATCTATATAAAATAAGGGATATGATAGCAG
AAAGATGCCTACTTAGCTAAACTCCATATTATATCAGGTAAGACGACTGTGAATAAAACA
AGATCAAGATCAACTCGTAGGAAGCTGTTAACACGAACAACCAAACATTATTACTAGATA
TATTGGCTCTTTCATCTCTTACGGTTACCAGTTACTTAAGCATCCCCATGTCCTGTCAAT
GCCAGAAGCATTTTTTCGTAGTCCCCATGGGTGTCCTTGGCAATGGCATGATCCAGAGGG
ATACTATTTCTTTTCTGGTACTCTTCTTTTATTACCTTCATGTCAATCTCTGCCCTTGTG
GTCACAACTCTAGTGAGAGCTCCTTCATCTGTTCCTCGTCTGTTTATTGCTAGACGAAGA
ACCTTTTCGAAGTACTTTTGAGGTTGAATCAAACATTTAACTGTGGCTCTAAGCAGGGAA
AGGTACTCTTCCTTAGGATCAGCCTTCAAGTCCTTGTTTATATCATTCCCGAATTCATTT

TTATACTGATTTAAAGTTGCATTAATCTGTGCTTTGCTTCTTGTAGCCAAGATCCTGATG
AGTTCCTCATCATTGTAAGCTTTGTTGGAGATCTTCTCATGGAGTATTTTAGCTTCTGAT
TTTGCTAGTGTCATGTTCACCTCATCTCCCTCGTATCGGTATGAGCTCACAAGAGGTTGC
AAAAGCT
>ntig_744_consensus
GTGCGATAGAATATATTGCAATACAAGTTCCAACTTACAACCAATCAAACTTCTCATATA
GTGTTTGATTCTTGAATACACAATAAAATAAAATAAAAAACTGAAGAAAAGAAGTACTTT
GATGATCACATGATGGCGCAGGCTTGAGGATTCTCATCACTGAAAGCGGTGGTGTACCGG
ACTTCAGTGGAACTGGCACGTGCCAGATGGGACGTCTGTGGGTCGTCTGCTCTTCGTACA
TAACCGCTTGGAGCCTTCTTGTCATACACTCCCGGGGCATACGGATGAGCCACAACATCG
TATGGAACATAGGGCCAGAGCTCTGCTTTCTTCCCCGTTCGATGTGCGACGCGTGCCACT
ACCTTAGACGGATCCACATATCCGACGACGGTCACCTTGTTGGCTTTGCGCTCCACGTCC
ACTTGCTTCACTCCTTTCATCCCCTCCACCGCCCGTTTCACCTTGCGCTCGCATCCTTCG
CAGTCTATACGTACTTTGATCTCCACCGTCTGCAACTGCTTGCGTTTCTTGAGCTTGGAG
CTGCCATGTGAGCAGTCAAAGAGATGAGAAAAATGATCTAGAGCACCCATTTTTTTTTCT
TTCTTCTCTGGTGTCGATACAAAACAAAGATTGCTGCTACAAAGATGTAGAATTATGAGC
TTCTTTCTGTAAAGATCAGAATGATAAGAAAACAAAATATCCCAGAAGTGCCTTCG
>ntig_745_consensus
CTTTCCTTTCCTTTCCTGCCATACACGATTACAGAAACTTGAAGCAATGATGTGACATTT
ATTTTGGTGATTGAATTGAATGGACGTTTATGGCAAAAGGGGATTGCCCATGATCAAGCT
GTTGAGTGCACATGGGTGATGAATTTCAGATTGCATTCAGTTCACACATATTTTCGTAGA
TTTAACACAGCGAGAGACAGCTTCAGTTCTTAGAAGAAATAAAAATAAAAATAAAAATAT
TGCAGCACACAAAATCAACAAGCTAATAATACATTATCATGAAGATGGTGATGATCATCA
CATGATGGCGCACGCTTGAGGGTTTTCGTCACTGAAAGCTGTGGTGTACCTAACTTCGGT
GGAACTCGCCCGTGCAAGATGTGACACTTGTGGGTCGTCCACTCTGCGCACGTACCCACT
TGGGGCCTTCTTGTCATACACCCCTGGGGCGTATGGGTGGGCCACCACATCATATGGCAC
ATAGGGCCAGAGCTCTGCTTTCTTCCCCGTTCGATGTGCGACACGAGCCACCACCTTTGA
GGGTTCCACATATCCCACGACGGTGACTTTGTTGGCTTTGCGCTCCACGTCCACTTGTTT
CACCCCTTTCATTCCCTCCAGCGCCCGTTTCACCTTGCGCTCGCATCCCTCACAGTCTAT
GCGAACTTTGACCTCTACCGTCTGCAACTGCTTGCGTTTCTTGAGCTTGGAGCTTCCATG
GGAGCAGTCAAAGAGATGTGAAAAATGATCCAGAGCGCCCATTTTTTTTCTTCTCTTTCG
ATGATACTTACAAGACAAAAGCTGCTGTTACAAAGAAGTAGAATTATAAGCTTCTTTCTG
TAAAAGATAAGAATGATAATCCAGAATATCTCAGAGAAGTGCTATTATTACTTCAAAGAG
TAGCAATGATGGGC
>ntig_746_consensus
TTTTTTTTCTTTCTTCTCTGGTGTCGATACAAAACAAAGATTGCTGCTACAAAGATGTAG
AATTATGAGCTTCTTTCTGTAAAGATCAGAATGATAAGAAAACAAAATATCCCAGAAGTG
CCTTCG
>ntig_747_consensus
GGAGAATTTTTTTTTTTTTTTTAACCTGAATTTTAGGCGTTATTAAGTTAGTTAAGCCAC
CTTGACATCACAGATAATATATTACAACAACCTTTGCAAGAGATTGAATAGCCCTAACCC
AAATTCGTTCTAATGCTGCTACATGTTTATCAGAGAAGTTGGAAGTTGAAGATGACAACA
GCACCCTATTCAATCATTTATCTTACAGTCACCAGCGAAGTGCTTCTCTGCAGTTTGATC
CTTCCATCCACAATGCATTATATATTTCTTATTTTTACCAGAGTTCAAGGAGAGTACATT
CTTCATTCTTACAAGAAGGAAGTGCTTTTCATGCATGTCTTGATGCATCATCATGCAGCT
GCGCTGGTCTCACGCTGGGATCATCACGAATACCATAGGAAAAACCAGTTTGTGCCCGGA
AGAAATCCATTTGTCCCAGCCCATGCATGCCTTGCTGTGCGCTGTAGTAACCATCATGGC
TTGGTGCAATGGAGTTCAACTGTCCCAGCCCCTGAATGGTCTGTTGACTCCCATAGTAAT
TATCACGAGTTGGTGCCATTAGGTTAAGTTGTACCATTCCAGGCACACTCTGTTGCGCAC
CATAATAGCCATCCAGGGTTACCGCTCTTGCACTTAATTTGTCCATATGCTGCAAGCTGT
CTTGTGCCCCTACAGCCATAACCTCTTGATCTGAATTCACCTTTCTTTTCTTTGTTGGAT
TCTTTCTTTTATTTGTCTTGTTCATACTTCTACTTTGGTTATCTTCTTCAATGCATATCA
GACCGTGAGTTGGTGATGCAACAGCTTCCACGAGACTCTTAGTAGAAGTATTTACACTAA
CACAGCTTCCAAGAGCTTCTTCTAGTGCACGAAATGCAATGCTGTAACTCTCTTGAGACA
ATGATGCCTCTTCACTCAATTTCATTGCACGCAGACATAAATCATTGTACCTTTGAAGTC
TAGATTGCACCTGATCACATTCCTCTGTCAAAAATTGCCTGCTCTTAGCCTCTTTTGTCC
ATCGCTTTAAGATATATTGAGATGGGATGGAAGAAAGGCCACGAATTTGAAGAACAATCA
ATGCATGTCTACACAGATAACCTTTATATTCAAATAATCGGCAGAGACAAAAAACTTCTG

ACTTAATTTGATTCCACATAACAACAAAGTCCTGATTCTTTTCAAAATCTTGAACTCTAA
AAACAATGCTCGTTTCATCTTGATTTTCCTGTTTAGGATGACAAGCCACAGCCCCCAAAA
CCTCAACTTGGAATTTCTTGAAAACTGCATGAGTATACACCCCGGAAACACTCTTCTCAA
AAGGTGATGGAGATTTTAATGTGGGTTGTTTGTTCCATGTATCAGTATCTGCTTTGGCTT
CCTCTTCATACCTATCTTGCAAAATAGTTTCATAAAGTTTCACAAACTCTTGCACAGTGG
TCTTCTTATGCACATACTTATCAAAGAAGGAGTTTACACTCTCAGACCGTTGAACATTGG
ACATCCCAGCCAAAAAGGTATCTCTCATGTAGGTTGGCACCCATTGTTTACGGTCTTCAT
ATAATGACTGCATCCATTCATCTCCCCTGAGTTCAAATCTCTCAACAAGTTTCCACCACC
TTCTCCCAAAGTCATCTTCTGTCCATGACCTATAGATACATTTTTCAAATTTTGCCATAA
AGTTTTCATGTTGTTTAATTACATGACCCAAATTTTCAGAAACCTTTGCCAATACTTGCC
ACAAGCTAAAGCAATGACAAGCATCAGGAAAGACCTCTGAAACAACAGATTTCAAGGCCT
TATCTTGATCAGTAA
>ntig_748_consensus
ACCAAAATAGCATAGGCCTCAAAGCCATGTGACAACCAAATAAAAAAGGAAAATCAAAAT
ATCCTTGGAAACATTTTTGAACAAAATCTAAATTAGGTGTTCGAAACTGCGACTAGTTTG
TCATAAACAGAAAAAAGAAAAAGCATTATAGCAATCACTCTCAGTCTTCTTATTCCTTCC
TAGTAACGCCCGATTCCCCACACTCTGATCACACCATCTGTATATCCACTAAACAAGGTG
CTTCCATCCGCGCTCCAGTTCAAGCTTGTGCAGTAGATGTTCTTCTTTTTTATGGTGGTA
GTAGCGGTGGTGCCCTCACTCTTCTCAGCCTCGGTCTTTAAATCAACTTTAAGATCCTCC
ACAATGCTCTTGCTCTCGAGATCCCAGATCTTAATACTCTGCTCAGTTGCAGCACACAGC
CAATACCTGTTCGGACTGAAGCACAGAGCGTGAATCACAGCCCCTGCATCCAATGAGTAC
AGCCTCTTGCCTTCCGCCAAATCCCACAACAATATCACTCCATCTTTCCCTCCACTGGCG
CACAAGGAACCGTCAGGTGACACCGCCACAGTATTAACATATCCAGTGTGCCCTGACAGA
GTGGATCTCAGCTTACAATTGCTCAAGTTCCATACTTTCACAGTCCTATCCCATGAGGCA
GAGACGATTGTAGGCTGGAGAGTATTAGGACTGAACCTAACACAAGAAACCCACTCGGTG
TGGGCGTCACCGTCTTGGATGGTGTACTTGCATTCACCGAGAGTGTTCCACAGCTTGATC
GTGCGGTCACGGGAGGCTGAAACGATCTGACGATTATCAATGGAGAAGGCGACAGAGAGA
ACATCCTTGGTGTGGCCAACAAAACGGCGGGCGGAGACACCGGCAGCCAGGTCCCACAGC
CGGAGCTCACCGTCCCATGAGCCGGAGAGAGCGAACTGTCCATCGGATGAAAGAACAACG
TCCTGGACGAAGTGAGAGTGCCCCGTCAAACGACGTCGTGGGACACCGTAAGTTTTCTCT
TCCTTAGTGAGATGCCACAGAATGATGGACTTGTCACGTGAGGCGGAGACAATCATGTCG
GAGTTGTCGATGGGGGTGGCGATGGCAGTGACCATGTCGGTATGGGCACGCATGGTTCCA
CGAAGAACGAGACCTTCAGCCATTGTTGGTTTGTTTGTAAGGTCTCTTCTAGTTAGGGTT
TGCAGAAATTAAAGAGGCGAGAGGAGCTAACTGAGAAGGAGGGCAAGAAATGGTAACGTT
TATATAGTGAGAGTGGCGGAGATTAGGGTTTTGGGGTGACCGGAAGAGTCAGTTGCAAGT
CA
>ntig_749_consensus
CTCATTGTGAAAAATTGTAGACAATTTCATCTAATGAATCAACAATCAAATAGAATTACA
GATTGGAAACTGCAGGGAATATTCAGCAACTCAAAATCAGTCACAAAATGTTCTGTTAAT
AATATGTATGCAACCTAGAAGGAATGACAAACATGCTATATGTTCAATATGAGCATTGTA
TCGTGTGTACTTGAGAGCACCTCTATCATATAATGGGTTGTATAATATAGCATAATAAGA
CCCTTATAACAACAAAATAATAATAATTAACTTGTGCAATACTAATAACCAATATTTAAC
CGCAGCATTGGCTCTTTGATTCTGGATGAGAGCAACAAAATAAACTTACTTCTCAGATTT
TGCCTTGTTAAACTCCTAATTCCCTGCAGCTTTAGTGGCAGGTTCATCCTGAGGTTTCAT
GGCTGGGAAGAGTAGAACCTCCTTGATGTTTTGTGAGTCAGTAAACAACATGGTCAGCCG
ATCAATTCCCAAACCCCATCCACCTGTTGGAGGCAATCCATATTCTAGAGCCATACAAAA
TGTCTCATCCAGAGCCATTGCTTCATCATCACCTGATTGTCGGTCCTTTAGTTGTTCAGC
AAATCGTTGACGTTGAACAATGGGATCATTCAGCTCAGTGTATGCATTGCAAAGTTCATG
CTTGTTAATGAACAATTCAAAACGTTCAGTCAAGCCTGGCTTTGATCTGTGCCACTTTGC
TAAAGGACTCATTATCTCAGGATGGTTAATGATGAAAGCAGGATTAACACATGTCTCTTC
CAAAAAGTGACCCACAAGCTTGTCTAACAGGCGGGCAGTTGTTTGAGGAGGGGGACATTT
AATATCATACTTCAAGCAAGCATCCTGCAGATATTTGTTAGCCTCATCACTTGATAGGTC
CTTCGGAATATTGAGGTTAGCCATCCTTTCCAACTCATCTATCATGTCAATCCTTCTGAA
AGGAGGTGTGAAATCAATCTCTATTGGATCCTTATCAAGCCCATTAGCATGATATTTGAT
TTTATAGCCACCAGTAATTTCCTTGACCATCCCCCTCGTTTCTAAATTGTTTTCCAATCT
CATAAACTCTATCCAGTCCACCAACAACGAGCTCCTTAAGATACAGTTCAGGTGCAATGC
GCATAAACAACTTCATATTAAGCTCATTGTGATGGGTAACAAAAGGACGTGCAGCTGCTC
CACCAGCAATCATGTTCATCATGGGTGTTTCAACCTCCAAGAAATCAAGATTGTCAAGGA

ATCTCCTAATATAAGAAATAATCTTTGCTCTAGTCTTGAATATTTGCCGAACTTCTGCAT
TCAACATCAAGTCCAAGTAACGTTGACGATACCGAGTTTCCTGATCTTTTAATATATAAT
CGTCAGGGTTCCTAGTACTACCTGGACCCATATGTCAGCTTTCTTAGAATTATCTGCAG
CAGGATCAGGTTTAACTTTGTTCCCCTTAGTGTTATCCCCAGGACCAGGTTTCTTACTTG
GCATCATATGGAGACAATGAGAAAGCACCACGAATATTTTAGGAAAGATACTCAATTCCC
CCCTCTTAGTTTTTCCTGGAAATCCAGTGACACCAACAATGTCACCACGCTTGACACTAG
AATGGAACTTTGAAAATTCAACTTCATCCATGTCTGACTTACTAGCATCAGCCATAACTT
GGACTTTTACACCACCACCGTGCAAATCATAGAAGAAGAGCTTTGATGATGAAGATCGTT
TGCTCATTATTCTTCCTGCCAATGATACATTCACATCCTCAAGATGCTCCCCATTGCCTA
AGCTTCCATATTTCTCAGTATACTCAAGTATAGACATGTCAACAAAGAATTTATGGGGAT
ATGGATTTCTTCCATCAGCCTTCTGGGCAGAAAGATATTTCAATCGATTTTCAAAGTATT
GTGTTGGATCCATATCTTCATCATCAGCTGCAGGTTTCTGGTTCTGCAGGCTTGCCTTAG
CAGCCGCCTGCTTCGCCTTTTCCTCTTCTTTGCGCTTCCTCTCCTCCTCTCTCTGCTTGT
TCTTCAGCTCCTTCTTGCGCGCATTTTTGCTCATGGTTTCTTCGAGTGCTGCAGGCTCAG
AAGAAGATGAATCCACGGCCAAGCTCGATACAGCTTTCGCTGTTTTGTCAACTAAGCCCT
CCATCACTATGGCTCGTTCGTTTGTGCGTGGGTTTGCCTGTGAGATGTGAGCTTGAGTGA
ACTGTGAAACTAGAATGAGACAGGGAACTCGAAGAATAAGCAGAGAGAGGAGG
>ntig_750_consensus
GTATGCAACCTAGAAGGAACGACAAATATGCTATATGTTCAATATGAGCATTGTATCGTG
TGTACTTGAGAGCACCTCTATCATATAATGGGTTGTATAATATAGCATAATAAGACCATT
CTAACAACAAAATAATAATAATTAACTTGTGCAATACTAATAACCAATATTTAACCGCCG
CATTGGCTCTTTGATTCTGGATGAGAGCAACAAAATAAACTTACTTCTCAGATTTTGCCT
AGTTAAACTTCTAATTCCCTGCAGCT
>ntig_751_consensus
GATTGGTCAAGATGTTCACCACCAAAAAGAAAATCCAAAAAGACAATGAAGCTGAGCCCA
CTGAATTTGAAGAGACAGTCGCCCAGGCTTTGTTTGATTTGGAGAATACCAACCAGGAGT
TGAAAAGTGACTTGAAAGATCTTTTCATTAACCAAGCAATTCAATTGGATGTTTCTGGAA
ACCGCAAAGCCATTGTTGTTTATGTTCCTTATAGATTGAGGAAGGCTTACCGCAAGATTC
ATCTTCGCCTTGTCAGAGAGCTGGAGAAGAAGTTCAGTGGAAAGGATGTTATTGTGATTG
CCACCCGAAGGATATCAAGGCCACCAAAGAAAGGCTCTGCTGTTCAGCGACCTCGCTCTC
GCACGCTCACAGCTGTGCATGATGCCATACTTGAAGATGTAGTTTATCCAGCAGAGATTG
TTGGAAAACGTATCAGATACCGTCTCGATGGCTCCAAAATCATCAAGATTTTCTTGGATC
CCAAGGAGAGAAATAACACTGAGTACAAGCTGGAGACATTTTCAGGAGTTTACAGGAAGC
TTACTGGGAAAGATGTGGTTTTTGAATATCCAGTTACCGAGGCGTGAAGAGTTGAGGATG
GGGCTTTTATGTTTTGCCTTGTTATTTAATTGAGCTTTACAATTGCTTTATACGGTGCTT
AAAATTTTGGCTGTTAAATTTGGTATGATGCCTCTTTTTGGCAAAATGGGGCAGATTCAA
CTTCTTTGAATTTCAATCTCTCTGTTACAAATTGTTGAGCTGAAGAGACCTCATGCCTTT
GCGTTGAAGTGAATTTATTTGTTTACTGTGAAACAAAATGCC
>ntig_752_consensus
CTGAATCTACATCAACCACAAAACTCCTTCTGTAATTCCTTCTAGAATTGTTTTTAGCTT
CAGCAAAAGCCCTGGGAGACGACTCAATAACATCATCTTCAATTGCCTCAACATCAATTG
GAGCAGGTGGTATTGACAACCCTGGTTGCCTAGCTTGCACTTCCTGTAAACCGATATTAT
TTGACGTCCCCTCTCGATCTCTGATTTCACATGGTGGTGCATTAAGATCCAGAGAAGCCT
TCCTTCGGCGATATCCTCTTAAAGGAGGTCCTCTCACAGACTGAGTGCTCATGCTCTGAG
GAGCTTATATTCAAGTTTTCCACAGTTGAAAAAGTTTAAAACTCCTCAGAGGATAAAGTC
AACGCCGAGGATTATCACCAAAATGTCGAAGGTACAGAGAGAGACGGAATGATACTTTAC
AATTCTTGGGTTTGAGTTACAAGGAAAAAGGATTAGGGGAAGGCAAAATGAATTTTTGGA
ACACAAGACTGAGAGATTAAAAAAGTATCATTCCGTCT
>ntig_753_consensus
CTGAATCTACATCAACCACAAAATTCCTTCCATGGTTCCTTCTAGAATTATTTTTAGCTT
CAGCAAAAGCCCTGGGAGACGACTCAATAACATCGTCATCAATAGTCTCAACATCAATCG
GAGCAGGTGGTATTGACAACCCTTGTTGGCCAGCTTGCACTTCCTGTAAACTAATATCAT
TTGAAGTCCCCTCTCGATCTCTGCTTTCACATGGTGGTGCATTAAGATCTAGCATTCCCT
TCCTCCGGCGATATCCCCTTAAAGGAGGGCCCTTCACTGCCTGGGTACTCATGCTCTACG
GAGCTACTATTCAAGTTCTCTGTAGCCTTAAAACTCCTCAGATAGTAAATTCAACAGTGA
AGATTATAGTCAAAACGTCGAAGTTACAGAGAAAGACGTAAGGATTTTGTTCAAGTCTGT
CTAGGGCTTCAGCTTGAAGAAAGAAGGATGAGGGGAGGGTGAATTAATTCTTGGGAAGAC
GGGCTGAGAAGTAAGAAATGGGCTTTCGCACTGTGGGCCGAATATATGACACGTAGGTCG

GTGAGGTCTAG
>ntig_754_consensus
GGGTCAAGGAGTGTTTCAAAAATTTGAGACCTAGCTTCCCACAAAGCATTTGTGACGGGA
GAATGGTACATTCCGGGCCACAAACTTAAAGGCTTTTTGTTGGCTTTGGGGTCAGGAGAG
GACTTATCAAATGGTGAAGCAAATGTTGAGAAAACAGTGATGGTTTTTGGAGACGAGGAA
TTGTTGAACTCCATTATGGTGGGTTTGTAGCAATGGATCTGCTATGCGTTGACTTCGATT
GATCAAAGTTGCGTTCTTACACGACGAAAGGAGGAAATTTTAGTTCAAAAAACGCTGCTT
GGTCTTGGGAATGGGGCAAAGCGTTTTCTTGTTGGGGGGTTCAAGGATGTCTGAGAGATC
AGACACAGG
>ntig_755_consensus
CCAGCAGTAATAAGAGTCGGATAATTTAGTAAAACATTCCAGTACTGTTTAAAACTACTT
TATCGGCAGTTCAAGCAAGAACACGGTAGTGTAATGTGCTCGAAACCAGTTGCTCATCCA
AAATACACAGCATAAAAGAAGCAATTCAGGTATGAAAACCACACACTCAAGCAGCAGTGG
TTTCTTTGACGGATTCACGCCAGGCATACTCCCTTGCTCCATCTTTGTCAACTATCTTTA
TCACGAAGTTTGGTGGTGCCACAACCAGCCTGGACCGGATCTCTATTATGCACTTATCAA
CCAAATCAATTGCTTCTTCCACTGACATGCCACTGTGGTAGTGTCTGTCCATCATGGACA
ATGAAAAGTAGGAGCCATAACCAAATGCGCCCTTATCAACCTTGTGAAGGCTGGCAATGT
AGTCAATATAGTATAGAGATGGTCCTGTCTCCTTGTCATAGCCAGCTAAAAGGATGTTGA
CCATGTATGGGCTCTTTCTCAAAGCAGTAGCGAGCTCTCCTCGCGTAAAGTTGGCCGCTG
CCGCAGTCGTCAACGGAATCCCGTTACGAAATTGGTACAATGCCACGTTCTTCTGTATAA
ACTCCGTAAACTGAACTCTGTCACCGGGTTCGCCGCTTGCAGCGATGAGTTTATGAGAGT
CCAGGACCATGATCTTGTCTTCGTTAGATTTGTGAACAAGTATACTGTGAACGGCCGACG
TGTCCGCCACTAAAATTGCGAAACCGTTGCCGACTAATCCGAACACGCACTCCATGTTTC
AGCGGATCTGATGGCGATGGCGGTGGTGGTGGTACTGTCGATCTGTTACTGTGTTTCGTA
CCTTTGTTTTAGTATTGGCGTCTTGAGGAACAAGAAAGAATCGGATCTCCGGAGGGTAGT
TTGGTCATTGGAATTTTTCCCCAGAGTTTAGACTCCCACTGTTTGGACGCAGGAAGAAAC
AGAATCTCG
>ntig_756_consensus
ACGAGAATTCCACTTCAGAATTAAATTTCCAAAACATTAGCTATTCTTAAATATCATGCA
AGAAGAACAAAGTACTGTTTGAAACTAGTTTGGGCAGTTAAATATAACATTCTACCGCCT
CATCCAAATTACAAATCTTAATCAAAGCAATTCAAGTATGAAACCCCTCACGCTCAAGCT
GGGGTTTCTTTGACAGATTCACGCCAGGCATACTCCCTTGCTCCATCCTTGTCAACAATC
TTTATCACGAAGTTTGGAGGTGCCACGACCAGCCTGGACCGTATCTCCATTATGCACTTA
TCAACCAAATCTATGGCTTCTTCCACTGACATGCCACTGTGGTAGTGTCTGTCCATCATA
GAGAGTGAAAAATAGGAGCCATAACCAAATGCGCCCTTATCAACCTTGTGAAGAGTGGCA
ATGTAGTCAATGTAGTATAGAGATGGTCCTGTCTCCTTGTCGTAGCCAGCCAAAAGGATG
TTGACCATGTATGGGCTCTTCCTCAAAGCAGTGGCGAGCTCTCCTCGCGTAAAGTTCGCC
GCTGCCGCAGTCGTCAACGGAATTCCGTTACGAAACTGGTACAACGCCACGTTCTTCTGT
ATAAACTCCGTAAACTGAACTCTGTCACCGGGTTCACCGCTGGCGGCGATGAGTTTGTGA
GAGTCGAGGATCATGATCTTATCTTCGTTAGATTTGTGAACCAGTATGCTGTGAACAGCG
GACGTGTCGGCCGCTAAGATTGCGAAACCGTTTCCTACTAAACCGAAAACGCACTCCATG
TTTCTGCGTATCTGGTGGTGGAACTTTCGATCTGTTTCTGAGTGTCGCTGGTACCTGGAG
AGATGCAACTGGGCTTTTATTTGTTTTACTTTTTGAAGAAATAGCTGTCTTGAGAAACAA
GAAAGAATCGGGCTTTGAAAGGGTAGTTTGGTCA
>ntig_757_consensus
CCCCCCCCCCCAAAAAAAAAAAAAAAAAACCATTTGCTTATTTACAAAATTACACATGCTG
TCACATGCTATATCGTTAAAATGCAGATTTTCAGATCCGACAACTGTATTTCCATAACTA
CCCCTCCGATCAGCTCGTCGAGCCGATATTACCCACACTCGCGGACTTTCCGTGTCGTGT
CACGGCACAAGGCTTTCTACACTATCAATTCAAATCACTTTAAAAGGGCTTCTGAAGGTA
CATGAACATATCCTGCAGCGGCGACAGCTGATTACTGCTTTGAAACTGAGAATGGAAGCT
CAAGTCCATATTAGGATCCAAGTAATTGTACGGAAACTCGTGGTTACTGTTATCCCAAGG
TTGTAACTTGGGCTCGCTCTGCACCTCACAGGTGAATTCCGGTGATACCACTTGCTCCGA
ACAGCTCGAGTCCGTGTGCAACTTCGGCACTGAATCCGACGTGTCAAAGTACACGTAATC
ATTAACCATTCCAGCCGCCGGATTCGGAGGAAGCGCTGAGCCGTTAACTATATCTGGTTT
CTTATCTTCCTCCATCACCGGTGAATCCATTTTCCGATTGACCGCTTGATTTTGGTGCTT
CTCGAGGCTTCCCTTCTTGTTGTATATGCGACATAGGACCCAGTCATCAAG
>ntig_758_consensus
CTTGGCGGGGATAGAACCAGCGCTTAGTAAGCGGCAGCGCCAGCCCGGTTGGAGCTGACC

TTATTATTATGAAAAAGAGAAAGGGCTTTTGAAGCTTGCTGACTAGGGTTGGCAGCTAAA
TTCAGTCTTCGAGACCCCATAACATAAGAAAGTCGTGGAGTGCATAAGCCCCCTTAGAGA
TAGGGGCGACTTTCTTGTGGTAGTAATTGCGAATGAGCAAGTAGGGGGCGGCAACTAAAG
AGGTAGCGAGACTGACCGCAATTGCAGGAATAGGTTGACTTTCTTTCATTTTGGTTATGG
AAAGTGAAAGTGGTTTCGTTTAGTACGAGATCGCTTCACACCTCGCGGTGCTTACACCTC
TCGCCTATCGAAGTTCTGTTCAAAAACCTCGTCCGAGAACTTGTATAGAGAAGGATTTCC
CGCGGCGCAGCAGTTCTTCCATACCAACTTAGCTGCCCGGCGCTGCTATTGGCATAACAA
CCGGTACACCATAGGTTGGCCCAACCCAGTCCTCTCGTACTAGGGTTGGCTTCTCGCAGT
TCTCCCTTTAACACCAACGGTAGATAGGAACCGAACTGTCTCACGACGTTCTAAACCCAA
CTCACGTACCACTTGAATCGGCGAACAACCGAACCCTTGGGACCTTCTTCAACCCCAGGA
TGTGATGAGTCGACATCGAGGTGCCAAACGACTCCGTCGATAAGAGCTCTTGGGAGTCAT
CAGCCTGTTATCCCCGGCGTACCTTTGATCCGTTGAGCGAGAGCCCTTCCACACGGGACT
CCCGGATCACTATGGCCGACTTTCGTCTCTGTTCGACCAGTCGGTCTCACAGTCAGGCAG
GCTTATACCATTACGCTCACGAGCAGAATCTTAGCTTGAGCCTACCTTCGCACACCTCCG
TTACTCTTTAGGAGGCATCCGCCCCAGATAAACTACCCACCTCGCAGTGTCCCGCCCCCC
CCCGAATTCTCGGTGCGGCGGTTAGGCATCCTTAGACGAAAGAGTGGTCTTTCAGGATTG
GTCGTTGTGTGTCACCACCTCCCACCTATCCTACACATTCGATCAAGGTTGTCACTGCGA
AGCTATAGTTAAGGTGCACGGGGTCTTACCGTCTAGCCGTTGGTACTCCGCATCTTCACG
GAGAATTCAATTTCACCGGGTCCATGTCGGAGACAGCGGGGCAGTCGTTACACCATTCGT
GCAGGTCGCTACTTATGCGACAAGGAATTTCGCTACCTTAGGACAGTTAGAGTTACTGCC
GCCGTTTACCGGGGCTTCCATTCAAAGCTTATAACACTTCTCCTTTTGACTTTCCAGCAC
CGGGCAGGTGTCAGACTCTATACATCGTGTTACCACTTAGCAGAGTCCTGTGTTTTTAAT
AAACAGTCGCTACCCCCTGGTATGTGCCGCTTTCCTAATCAAAAGATAGGAGAGCACCCC
TTCTCCCGAGGTTACGGGGTCATTTTGCCGAGTTCCTTCGACATGGTTCTCTCAAGCGCC
CTAGTATACTCTACTTGTTCACCTGTGTCGGTTTGGGGTACGGTCAGTTCACCGGGAGGA
TCGCCCTCCCAATTCGCAGTTTTTTCCTGGAAGTTTCAACCTTGTTGACTATGACAACAG
TCGCGACTATGGCAGGGCGGTACGCTCCGCTCTCTCGCGACCCCTACTCTAATCAAAAGA
CTAAGAGTTTCGCCTTTTGAAGCGCCAGTCGCGTAGGGCGACCGGGCCAGGCCGAGTCAG
AAAGGCTTTGATGAGTCAAGTACTATATAGGGGGAAAGGAGAGTGAGGGGAAGAGGGGGC
TGCCCTCGGCCCGATCATCCAATTCGCTCCAACAGACAGGCATGGTTCTGTAGTCAAAGC
AACTTCGTCACTTTCGTGTACCCATCGGACGGCAGCCCTTTCGGGGGTTCCTTAGGGACC
GATTCACTCTGCGTAGATTGACTGAACGCAGAAAGCCTTCCACTGGCAGGCGATCGTGTT
TTTCACAGGATTTCTCGTTACTCATGTCAGCATTCTCACTTCTGATATCTCCAGGTGTTG
TCACCAAAAACCTTCCACGATTGACAGAACGTCCCGCTACTGACACTTGAAAAAGCTGCT
TTCAAGGTCTCGTCGCTTCGGTGAATCACTTGAGCCCTGATACATTTTCGGTGCCATGGA
GCTAGACCAGTGAGCTATTACGCTTTCTTCAAAGGATGGCTGCTTCCAAGCCCACCTCCT
GGTTGTCATCGCTCGATCACTTCCTTTTCCACTAAGTTCTTGCTTAGGGACCTTAGCGTA
CGATCTGGGCTGTTTCCCTCTCGACTTTGGATCTTAGCACCCCTTCAGTCTGTCTGTACA
AACGAGAACGGCCTGTATTCGGAGTTTCCCTGGGGTTGGTAAGGCGAAATGGGGCCACCC
TAGCCCATTGAGTGCTCTACCTCGGGCCATCGACATCATACGCTCTACTGAAATAGATTT
CGCGGAAAACCAGCTATATCCGATCTTGGTTGGCCTTTCACCCCTAGCCACAAGTCATCC
CCGTATTTTGCCACATACGTGGGTTCGGTCCTCCAAGGCCTGTTAGAGCTCTCTTCAACC
TGCTCATGGCTAGATCGATCGGTTTCGGGTCAAATAGGAAGAACTATAAGATTCCACCTC
TGGAAAGCGCCTACACCTAATGGCTTAAGCCGCTGTTCCCATTTCCTCGCTGACCCATCA
TGCAAAAGGTACGCCGTTAGAGTGAGTGCGCTTTACTACTCCTTCGACTGATTGTTCGCA
TCGGATCTCAGGTTCTCTATTGCACTCCCTAAATAGGGTTCTTTTCACCTTTCCCTCACG
GTACTACTTCGCTATCGGTCATTGAGGAAGACTTAGGCTTAGAGGGTGGTCCCCCTTTCT
CGCGTAAAAGCGATCAGAATTCGAACACGCCGCGTTTTACTGGGAAGGATCGAACCATGG
GAACGAATCTACAGGGCTATCACCTTCTTTGGCCAGATCTTCCAACCTTTTCACAATTAC
AGTTCACTGCGCCCTTGACTAATAGAATCTAAAGTCAAGGGGCTTGCTGGTTTTTCCATC
ATCCAATCCACAACAAATCGAATGAAACCTGGCGAAAAAGAAGTGAACACTTTGCTGAGG
AAGACGTCTTTGTTTTTCTTCAAACCCCCAATCCGCTCTCGCTCGCCGCTACTAACGGGG
TCTCGGTTGATTTCCCTTCCTTTAGCTACTCAGATGTTTCAGTTCGCTAAGTTTGAAAAG
TCCAAAGAGCGCAGACTAGCCACGGAGCTTGGATACGGTTTCCCGATCGGAGATCCATGG
ATCACAGACGGTATCTCCCCATGGCCTTTCGCCTCATAAAGCGTCCTTCCTTCTCAATGC
CCGGGCATCCATCCAATGCATGATCTCGACCTCGGGCCATCGACATCATACGCTCTACTG
AAATAGATTTC
>ntig_759_consensus

CTTGTTCTCATTTCTACAACCCGTCTCCATTTTCAACACATTCTCTTAAAGACTGTCAGG
TGGAAAAGAGTTACTGAGGTTTTTCAACCATAGCGCAAGCTCTGTAATATACCCAGATGG
TTTATTCTTAACAGAAAGAAAGTACCATTATGTTAAAAATATATACATATTACATTGCAT
GATCAGAAAAGGGAACAAGTGCATACAAAGTCTAGAAATTTTTACATGGATATCTTTTAT
ACGATTTCATGATAATAATACCAGCATCAATAAACAGAAATGAAATAAAATAAGGGATCA
TCCATGTCCCATACCTATACAAAAGTTGGCTGTTTCTTTTTCTTCTTCAAATTAGTCTTC
TCAAACTTCCCAGAAAATTTAATCAGAACGAGATTCTGATCTTCAACCATAGCTTGATAA
TATATAGAGATATATTTTTCTAATATTTACACCATCTTCAATCTGTTGCTTCCAAAATTA
AACTGCATCTTTCTATTCCTCTCTGCTTCCCCATCAGATAGTGCTCACAGGGAGAGGAAA
CAAATAGTCCGCCCAAGTAGAACCCAAAAAATCGATTTGAGGAAATAATTCATTACCAAT
ACCAAACCCTTCCAAGAACACTCGCCAGCTATGATATGATGGGCTCAGTTCGAACTGTTT
ACCAAATCCGAAACCCAATCTACATCTGGAGCCCCACTAGATGAAAACGGCTCAACCCCT
TTCAGAGAGTTCTCTTTGCTTAATTTCTCAAACATTCTTGCGCGCAAGTCCCTCCCTGAC
TCAACCCTTTCCATTATAGGTTCTTCCTCGCTAATATTTTCGAACGGATCCAAGTAACGT
ACACCGGGTCGGGTCGGAGTGCCCGTTGGGGTGGAAGGAAGGCTTAAAAATCCGGACCGG
ATCATGGACCCACGGGGAGATCCGTATCCGGGAGAACCACCCATATGAATATTCCATGAA
GATGGAAGAGATTTCACCTTCCCCAACTGCAGATTCCTCAAAGAAGCCACAATTTCATTG
ATAGAACTCGAACCAAGAGATCGACTCAGAGATAGACTCAAAGGCGACTCAGCTGTTGGA
CTCATAGGAGGAGAATTCGACTCCGGGGAGGATAAAAACGACAACGTCTTAGCACAGGAA
GTCGTATCGACACTCCTCGGGCTCTGCTGAGGTAAGATTCTAAGCTGCTCCGGTGTGTGG
GCGAAAAAACACACTCGTCTCCTACAACCGGACCCATCTTTACAAGGCTGAGTGCGGTAA
CGAGCCGGATGAAGCCAACACTCAAACACACCATGAGCGAACTCACAAGAGTCGCCTTTC
TTACAGCTTCCCTTTCGGAACTCGGGGCAAGCCGTCCCCGAATAGTGATACTTTCTCGGG
TCACGCCGACGTGCCTTCTCCCCTGGGTGAGCGTACGGACATTCGGTCCAGTCATGAGAC
CTTCCACGTGCACACTTTCTTACCTTGAACTCGAACATGCGAAAATGGTCACATGAGTAC
GCGTCCACCGGAGACTCCGAATCTATACCCGAGATTTCAGCTTCCGAGTCCAAATCCTGT
TCATTAGACGGCAGAAAAGGTTGAAGTGCCGCTAGGGCTTCCTGGAGATAGTACGGACAG
TTAGAATCAGGGCTTCCGTTTGCATTACCATGAAAGGAGTGGGGAGAGATAACGCCAGCC
TTCGGATCTTCATGGAGAGGATGCCACGGAGGGATGTTAACCGTCGGATTCGGACGGTGG
CGCTCACCGAGCATCATTTTCAGTGTGTTTATCTTGGTCGTGAATGCTGTTATTTAAGGG
AACACCATGAACCTTTGCTTCTGTTTATAAACTGCCAAGAAAGGTATAACTGTGGTTAAT
TTCAAAAGCAACCATGGTTAAGGGCT
>ntig_760_consensus
GTTAATTTAAGCACTACTCATTAATGCATACAAGATTTAAGGGAAAAGTTGGAATGTTGG
CAGCATTTTTATTTTGAAGACTTCTTATAGTAACAAGGTTCCTGACAGTGATCTTTAAAT
GTTAAAAATGTAAGCAGCGACAATGTCAGACATGCAGACACCGACATGGTTTCCCTACAT
TTCCAATCTGTGTTTCGGTCTAACCCAGATCCAGTCCTACTTATTCAGCGGCTTTGCCTT
TTCTTCTGCTGCTGCTACCGGTTTCTAGCAGGTAACACACCATAGAATCGACGTCGTCAC
CAAATATGCTGTAAGCTTCTATCACTTGCAAATAGCTGAACCCCATCGATAATACCATCT
GCATACCACTGCTCAGAACTGTATCCTGTATAGTGCTGTCTCTCCCACCACCTTCCACCT
TGCTCTCACTACATGATGACCTAGCTCCAGAGGATGAAGGTTCAGCCCCTGAAGTAGATG
ACTCTTTACGGGCAAGGTGCTGCTTGAATTTGTCATTAGCAAGATACTCAGCTCGAGAAA
CTTCCATCACCATACACTCAATTTCCTTTTCAGTAAGCTCCAGATCAGAGTAAAACCGCC
CCTCAGCTAGGAGTGCATTATCAATCTGCTGGTCTTGTTGAGCCTTGATGGCAGCCTTGA
CTTGATCTCTGTCAACATTTGTCCCTCGAAGACAACTGAACCCAAGTCCTGCACCAATTG
TCAAGCGCCGTGGGTCAACAAGGGAGTTGTAGTGATTCCCATGATGATAACTCAGCCGTA
TGGGAGGCGTATCAGTTTTGTAGCTCCCATGAAATATGTTGATGGGTTCTGTACTGTAGG
AATAAATATGAATAGGCCGATTATACATTTCAGATAAGGCTTGAATCTCAACATTGTTTC
CGTAGACCTTGTCTCTTCTCTTCCTCTTGCAGTAAGATGTAAAACCTTCGGTTATAAATT
GAGAAAAATGATCCCTCTCTTGTTCCATATAATCTATGCACATCTGTCTCGCCAAATCAT
ATAATTCAGAGTCCCCATACACCTGATCTGCAACAGCACGAAAGAGACAATTCCCATCCT
CTAGCATCCGTTTCACTTCAAAATCTTTTGCCCGCCTGATATCAATCTCAAATTGACGTT
CTCTTTCCAGGTCATCATAGCAGGATACAAAACAAGGATTGTGCTCATCAGCGCTATTAT
ACCCCTCACTTTCAACATGGGACCTGGGAGATGAAGGCCTTGACCCAGATGGTGAAGTCC
TTGTGGAAACAACAGGCCAGGCAACTGCTCTTCTAGATGATCCAATACGCCCAGCATTTG
AGCTTCCTGAGAAAGATCTTCTTGAATTTGAGCTTGATGCCAAAGGTTTTGGTGGTGGAA
CAGGAGGTGGAGGTGGTTGTGGACTTCCACCACTCAGCTGTGCTGACTCACCACTTGAAC
CTTCAATCTCGGCTGGAACTCTCTCTGAAATTTTTAACACACCCAGATTCCTAACTAACT

CCCCTGAACCCACATTCTCCTCATTAATCAATTTATCGCCACAAATTTCATCACTCAAAC
ACTCACTCTCATCGTTATTGATTTTTCCCACCAAAAAATCATCACTTTTCACAATTTTAT
TATCACTAGTTCCAGAACACACCAAAAGCTCATCGACAACTTGTTCCGGCACTTGCTCTC
CAATTTCCTCATCTTTCACAGAAGAAAGAACCTGAGGAGAACTACAAACCTGTGGCTCTC
CACGAGCCGAAGAAGACCCAGCACGGCTAAGATTTGATGACGAGCCCCCAGCAGAACCTC
GCTGAACCAAAATTCGAGTCATTGAATTCAATGTTTATAATCTCCAACTATTTAGCCCAA
ATACGCCCTTTAAGAACCCACGTTTAGAGAACCAAAACCCTAGTCGTCATGGGTTTGTCA
CGCGCTCTGGCCTTGGGCACAGTGATGTCAATTCCTTGGCTGAAGGATTTTTTCCAGCCC
AGCAGTGTGTTCTTGTTCTGCTCCTCAGGAAACGATCTCCTTCAAGAAGCCCACGCTGAA
ATAAGCACAGAGACAGGAAGAGAAGAAACCTCGAAACCCTGGCGAAGAGATCTGCAAAAG
CAGATGATATGAATTTTTAGTTTTTTTTATTTTTTTATTTTTTTT
>ntig_761_consensus
AACAGAACTTGAAATCTTGGATTGAGCTCAGGTTATATCCTTTATCCCAGACTTGCCATC
TTTCATTTGACATGAGTGAAGTTGCCTAGTGAGCTCGCATCATCTTTACCCTAGGCATCG
CTTTCTTCATTTGACGAGCACGGTCTTTTGCATCCTTCCTGCGAATGGCCTCAGCACTGA
GCTTTGCCTCAGCTTCCTCCATCTTTCTCCTTTCTGCCTTCTTTTTCTGCAGGGCTTCTT
GCCTAGCGTTTTGAAGTTCTTTGTATGCCTCTTGAGCAGCCCTGTGTCTGGCTGCTTCTG
TTTTGGATCGAGCCCCGTCCAATTAGATCAATGTAGTAGGGTACGAGAGCCACCAAACGG
CTCATGTCACCCATGTTATTGGCATCAGGAAGAGCAAATTTAAATAGCAGCATCTTCTTA
TGTGTGCCAGGGTGTTGATCAGAAAAATGCATTGAAATGAAAC
>ntig_762_consensus
CAACTACTACAAAGACCATACATATTATATTTGAAGCAAATTCAAACACAAAGTTATAGG
GTGTAATTACTTATATTAAATCAAATTTACAGATAATTATTCCTACTGGTTTCAAGTCAA
AAGCAGTTTAATATAGTGGTAGTGCATAGCATTAAGTATAATCCTTGCCTCAATATCAAT
AATGTACCTATGACTCACAATTCGTCGTGCAGGCTTCCCTTCAACAAGGGAGCCTTGGGT
TGATTTTCCAAGAGGTATCTTGAAGCCAATGATGCATGTAGGGCAGCTCCGTACGGAAGT
GCATCTTCGTTGATCCTGAAGTGAGATGAATGAGCTGGTTCAAACTGCCCTTTCATCTCA
TCGTTCATACCAAGGTAGAAGAAGTATCCTGAAACTGCCTCTGCGAAAAATGAAAAGTCT
TCCGAACCCATCGCCGGTTGCTTTTCTTTGACGTTTTGAAGACCCAGCATGTCCTCTGCA
ACATTTAGGAAGTACTTATGCAACTCCTTGTTATTTACAGTCACTGGGTAATTGATTTGA
TTATTTGCATTAAAGTTGACTGTGGCGTTGCACCTATGCACACTAGCTTGTTTCATGATA
ACCTCTTCAATTCTTTCCTTTAGTTGAACAAAGCTTTCTTTTGAAAATGCCCTGAATGTG
CCACCTATTGTAACAGAATCAGGAATAACATTGAAGGCACCACCTCCTTGAAACTTTGCC
ACAGTAACCACCTGTGAATCCAAGGGATCAGTTTCACGAGAAATTAGATGTTGCAAACTA
ACAATCACATTAGACGCTGCTAAGATTGGATCTATTGTATGCTGAGGAATGGCAGCATGC
CCCCCTTTTCCACTAATTATTGCCTCAAAGAAACCACTTCCAGCTAAAATAGGGCCAGGC
CTGGAGGCTACTGTGCCAACTGGGAATTTCGAAGAGACATGAAGCCCAAAGATGGCATCA
ACGTTCTCCAGCACGCCAGCATCTAACATCTGCTTTGCTCCACCTAATCCCTCCTCGGCT
GGTTGGAAAATTAGCACAACTGTTCCCTTCAATTCATCGCGATGCTTTTGAAGTATCTTT
GCAGCACCAAGAAGCATAGCAACATGAGCATCATGCCCACAAGCATGCATCTTACCAGGA
ACTTTACTCTTGTACTCCCACTCTACAGCTTCCTCCATAGCTAGAGCATCCATATCAGCT
CTGATTGCAACAAAAGGCGGTTCACCAGTCCCAATATAACCAACAATACCAGTTACAGCA
ACTGGGTATCTATAAGGGATGCCTATCTCATCAAGCTCAGCTCTTATAAGTTTACTAGTC
TCAAATTCCTCAAAACCAAGTTCAGGATTCTCATGTAGTCTCCTTCTAATGCTCACCATC
CAATCAAAAATCTCAGGTTTCCTGGCAAAGTCGAGGAATTTGACAGGCAATTTAGCGATG
TCATCTGCGCTTAAAGATAAAGTGGGGGAAGTGGGTGTTGGCATAAACAAGTAAAGAGTA
AAAAAGAAAGACAGGATATTGGAGAAAGCCATGGATTATCGATGGGTTGATGGAGACTAT
GAGGAGTTAGAGAGGTAGAACGCTGTTTCTAACTTTGGG
>ntig_763_consensus
TGACCCAAGGTAAATGACACATTAGAAAATATAACTTCATTCTTGACAGTAAACTCCCGA
TAGAGATACTGATGTTTATGCATCAAAATTTGAGAGTACACTTGATTGAATATGCAAACA
ACCACCAAACCTATATATTACATCACAGACCCCAACAACTTCAAACTGTTTTATGTAAAT
ATTAGTGCATTGCCTCATCCAAGCTAAGTTCAACAAGAACCAACATACAATACTTCTTAT
GGAGATGTCAAAGGCATATATCATTTACCGTCACCTGCAGGCCCACTGCCAAAGAGGTAA
CCCAGAGAAGATCCACCACCAGGGGCTGCATGGACCTTGGTTGATGGTCGGTCTGTGATG
AAGTTGCCAGTGTTCTGACCATCAGCACGTACATAGTTGTTTGATGAGTTACTATTGATA
CCAGCCGGAACCTGTTTGGTAATGTCTACTGGTTGAGGAGCAGCAACAGGCTTGCAAGGC
ACATTATTTACAGATTGCCCTTCATTCGCAGCAGGTTTAGGAGCCTCTCCGGTACCAAAC

AGATATCCCAATGAGCTCTGTCCCCCACCAGCACTAACTCCACGACCCATGCTCTCTCAA
TTCTAATGCAATGAATTTCCAAACCTAGCCGATTGGATCAGATCTGAGAGAGAGTGCCGA
AGAGCGAGTGAGCGAAAACGATGCCTGCTAGGTGGATTTTATGGAGGTAGATAAACTGTA
GACAGTGACGCCGCACAGTAAAAAGAATTATGA
>ntig_764_consensus
GATAATGATCCTGGACCAGGGCCTTCAGGTCTCCTGCCCCACCGATCAGATTCTGATAGG
GCTTGACCAAAGCCTTCAGTTCGCCTGCTGCTGCCACCCCACCTATCAGGTTGAGTAGGT
GGTGACTGCCCTGAACCCTCAGTTCTCTCTCGCATGAATTTGGGAACATATCTTCCAGTA
GATGGTGCAGCAGCTGCTGCTACAGCTGGAGCAACAGTTCCAGGCTCTGATGCACGGGCG
GGAAGCTCAAAAGGCTTGGCACCTATATCAGTTGGTTTCCCCAACAGAGCTTCTCTTCTC
AGCCTTTCCTTCTCTTCCAGTTCCAGCTCCCTTTGCCTCTGTTTTTCAGCAATCTCGTCT
AATTTTGCCTTGCGTTCAGCTTCTTCTTTTCTTCGTTTCTCCGCCTCTTCCCGCTTACGA
GCTTCTTCCTCCTCATGTTGCTTTTTCAGTCTTTCTTCTTCACATTTAACATAGTATATT
TTCTTTCTCATTGCCTCCCTCTCCCATTTGCGTGCTTGAACAATTTGGTTGATTCTCTCT
TCCCTCTCCACTCTGCACCTGTCAAACTCAGCTTTGCGGCGGTTCAATACTCTTTCCTGG
AATTTTTCCTTATTGTCCAACATTCTAGAAAGTCTAGTCTTTTCTCTGAGATCTCCATCA
TGACGTTGCCTGCTGAGTTCAGTCTCTAGCTGTTGTTCACGTTCATGAAGCACCTTCTCT
TCCACCAAATGTCGCTGAAAAGCAGCTTCAATCAAGGGAGCAGCCTCTTCTCTCTTTGCC
CTTTCAAGATAATCCATAGTTTTGGCTAATTTTTGTAACTTTTTCTCCATTTCCTGCCTC
TCCCTAAGTTGCTCAGTCAAAGCCCGCTCCATCAAAGTTTGTTTTGTTACTTTCTCTCCC
TCCAAAATTGGCTTTTTGCCACCCTTCTTTTTACTGCGCTTCTCAGCTTCCTCAAGCAGT
GCCTGGGCCTCTTCAAGTTCACGCTCCTCTATTTCCCTTAGAATTCTTTGATTTTTCCTT
TGCTCATATTCAGCAGCAAGCCTCTTCTGCTCAGCTTCTTCTGTTATTTTCTGTTGCTTT
AGCCTCCTTGACTCCTCCTCACGTTCCATTTCAATAAGCTGACGTTCATGTTCTTCCTTC
CTTTTCTCAATGATTGATTTTCTAGCAAGAAGTCTCTTATGCTCCGTATCAACAGTCTCC
CCTAAAACAGGCAACAATTCACCTAGTTTTGATGCTTTATTGGCAGGAGGATATATCATG
GCCCTCACTTTATTTAAAGACTCAGCAAGAACAGCCAAGTGATCCCTCAGACCATCAGAT
TCAAGACCCAAATTGCCAAACAAAACAACACCCCTCATATGGTCAACTTTCATGGCTATA
AAATTATGTTTTACAGCTTCAACAGAAATCTTCTCCACCGCTTTGAAGTCACAAAAGGAA
ATCATCTGGGACAACCTCTCAATTTTCATCATCTGATAAACCTGAGATACCTGCTGAAGC
AACCTCAGGATAACAAGCTTTTCAAGGGCTGGAACATAACGAGACAGCTGCACCTCTGGT
ACAGAAGAAGCTGAAGCAAGCTTACCACCTAACTTCGAAATTTTTGACAAAAGCGGCTGT
ACCTTTGAGGCAAGATCTAAAGGGAAAAACTCATGCTCCAAAAGGTTGTACAGATCTTTT
ACTTCCTGAGTTGCACAACTCAATACACCCTTGGCAACCAATTCTGAGAGAAGGGCTGTC
CTTGAAAGCACTTCTCTCTTTTCAAGTTTTGGGTCAAGCTCAAATCCTATCAGATTGGCC
ATCCTTGAGTTCTTCTCCTTAGCATTCTCAAGTTCCAAATGAGATGCACCATGGGTGTGA
TCATAAGGGAGCACTAAAAGAGCAGCCAAAACGACAGATGATGCTATCAGTTGCAAATCC
TTTAGGCTCAGGTTCTTATTGTAGGATTTTTGAAGCGTAAAAAGCTTAAACCAGGCATAA
GCATGATAAAGATGGCTTGAAGAAATCCAGAAAATCTCTGTCAACTTAGCATAATAAACA
ACCAGCAAAGATGGCTTGGGTGTTTTCTTAACCATGCACATCAATCCATAAATATCTTCA
ATGGAATAAAAAGCTTCCTGCCAGAGCTGGAGGTCAGTGGCGATCTTTAGCTGCTCAAAT
CTCGTATCAAGATAGAGTTGCAAACTCTCTGGAGCTGAAAGATCAGGTCGGTCCCTTTGG
TCTCTGTATTTGCTAAGATTCAGCAAATGGTTCCTGATAATTTCACAAAGTCTACGAAAT
TCAGTTGTCCGCTTATATTGCTTGCAAAACTGGAAGGCTCGATGTGCAGTCATCGCATAG
AGTGCCTCAAGCTTTGAGTTATTCCTTAATATTTCAAGAACTGTTCTGTAAGTCTCCCAT
AAGAATTTGAACCAAGGAGTAACAAGTTCACGATCAGACCTATCCTTTCCCTTCTCTCCA
CTGACATAACTAAGCATTAGGTCTTCTGGCCTTCTATCTGCTTCAAGGTCATCCACATCC
AGTGCTTCTTCGAGGGCTAGTGCCTGACTGCGAGCCTGCTCAGCCTTCTCAGTGGAGAGG
TGCATAAAGTGCTTGATAACCTCCTCCAAGGAAGTGACATTCACCTGTTGACAAACTATA
CGGTATTGAATCAGACCATCCTTGGCAAACTTACCTCTCCTCATATCAACACAAAGCTCT
ACATATTTGAACATAATCTTTTCAAGTATCTTTTGCCATGCTCGGTGCCTCTTTGAAGTA
ATCAGATCATGAAGCACTTGCAATGCATCTTGTTTCTGCCCAACATTGATCAAGGCTTCG
GCTTGATTCAATGCAGCCTCAGGTTTGGCGTAATTGGACATGGCGGGTTAAAATTCACAG
CCCACTCAATAAATATCTCCAATTATCTCGACCTGAAGGAACAGCGACAGCGACAGAGAG
TGGCTGCTCCTCGCCCTACGAAGAGTGTCGTGTGTAACGAAGAAAAAGAATAGCCGAG
>ntig_765_consensus
GATACTCTTGCTAAACATCTTTTTCACTGGGAGAATCAAACCATTGAATGGGCTATGCGT
CTGAGGGTTGCTCTGTATATTGCTGAAGCTTTAGATTATTGTAATAGTGAAGGACGTCCT

TTATACCATGATTTGAATGCATATAGGGTTCTCTTTGATGAGAATGGTGATCCTCGGCTT
TCCTGCTTTGGATTCATGAAAAATAGTAGAGACGGAAAAAGTTACAGCACGAATCTTGCT
TATACACCTCCAGAGTATCTTCGAAATGGAAGGGTTACTCCTGAAAGTGTTATTTTCAGC
TTTGGGACTGTCCTTTTGGATCTTCTTAGTGGAAAACATATTCCTCCTAGTCATGCTCTA
GATATGATACGGGGAAAAAATATTCTTCTCCTAATGGATTCACATCTGGAGGGAAACTTT
TCAACTGAAGAGGCTACAGTGGTCTTTGATCTTGCTTCACGGTGCTTACAGTATGAACCC
AGGGAGCGACCAAATACCAAAGATCTTGTTTCTACACTTGCTCCCCTGCAAAATAGACCT
GATGTTCCTTCTTATGTGATGCTAGGAATTACAAGGCATGAGGAAGCACCTCCTACTCCA
CAACACCCTCTCTCCCCCATGGGTGACGCTTGTTCACGGAAGGACCTCACAGCCATCCAT
CAGATTTTGGTGATGACACACTACAAAGATGATGAAGGAACCAACGAGTTATCTTTTCAA
GAATGGACCCAACAAATGAGGGATATGCTGGAGGCCAGAAAGCGAGGTGATGTGGCATTC
AGGGATAAAGATTTTAAAACTGCTATAGATTGCTATTCTCAGTTCATAGATGTGGGAACC
ATGATTTCTCCAACTGTGTATGCAAGGCGAAGTCTTTGCCATCTGCTAAGTGATCAGCCT
GATGCTGCCCTCCGAGATGCAATGCAAGCGCAATGTGTTTACCCGGATTGGTCCACAGCA
TTTTACATGCAAGCAGTTGCTCTTGCAAAGTTAGACATGCACAAGGATGCAGCTGACATG
TTGAAAGAGGCTGCTTCATTAGAAGAGAAAAGGCAACGGGGTGGGAGAGGTTCTTGAGTG
ACTATAACATTCTCTGTAACTATGCTCAATCAATTGTTATGGTTCCTGTTGTGAGAAAAT
ATTTGGGTTTATGTATGATTAAAGTAGGAAAATGCTTTTCATGCAAGATGTACCATAGAG
GGAGGGAGAGACACTTGTTTTGCTCGACTGTGCTATTTTTTTACAATTTCAGTGAAATAA
TCCGTCTGTCTTCATTTTTGTACATGCGAAGTTGGCTCAGGGAATGTAAACTTGATTTAT
GAGGATGCAACTTACTACAAGATTTATCAAAATAAGTTGTCTTTAAGAGTTGGAGCAAGC
TCATGAAGGAATGTCAATGGCGGCTACTATTAATCTCTGCCGGTTCTTCTCACAGGTTAC
AGTACCGGCTGGGCTCATGACTCTGATGCCTTCATATGGCAGCAATATTTGTAATATTTC
CCCGCAAGTTATATCTGTTTTTGTAATGTGTCAAGTTACTAGTAAAACCTGTTGGAATAC
CGTGATTTGTTAGTTGACACAGCTTGAGTAGTGATAGATTCAATTTGAATATGTTCGAG
>ntig_766_consensus
CTCAACTCGATTTGATTTCACCCCTAATCACCTGGAGACGGTGACGGTGGAACCGAAGAG
GACAAAGATGGGAAGGTTCAAATGGCGGTAACTAAGGGTTTTGGGGCATTTTAAAAAGGT
TTTCAGCTCAAAAAAGTCAGAGAGGACAATTACATAATTATTTCTATTTCTATTAATAAT
ACATCACTCTTAAGTGCTATATAACCAGCAATAATTTTTTACGAATGTTTTCTTTCCCAC
TTGCCTTCTCGTCTGCCATCTCTTTCTCTCCGCTCCATGACTTGCCTTGAAGATTTGCTC
CCTCTGGATCCATGCCTCTGACCCAAACCAAAACCACTCGACTGTCCATCAAATGCAGAT
ATTGATTCTGGGTCGCCCATACTCCCGGTGTCCCCTGAGTCCTTGCTCCCATCCAAATTT
TCTTCATAACTGCTGCCACAGTCGTCTGAACGGTCTCTAGATTGTAGAATAGTCGGAGGC
TGGCCTGAAAATGATCTAGAGCCTGAGAGAACAATGGTCGGCATTCCTCCAATGGATGAA
TTGCTGCCGGAAATTTCAGCCTGCAAACCTCCACTACTTGTCCTTGCACTTCCTTCAGTA
CCTGACCTTCCATTTTCCCTTTCACCACGATACCTATCCCGAGGGCGACTACTATGAGAG
GATGAAGAAAAGTCACGTCGTGAAGATCGTAGTCCAGATTCAGCCTGCTCTAGTTCTCGC
TGAAGTTCTCTCTGTGTTTGCCTCTCCTTCTCCATATCAAGGATGTGGCGCAGCTCCCTC
GATTTTGCACGCTCAAGTTCTAGCAATCGTTGCCTTTCTATCTCTTTTTCTAACTCAGCA
GTCCTTTCCCTATCAAGTTCCTGCAGAAGTTCAGCCTCCCGCAAAGCTGATTCCTCTAGA
TATTTTTGACGGGCACGTCTCATCAGAAGTTTCTTTTGCCTATGAGCTACTATTTCTTCC
CGGAGTTTTGACTTCTCTAGGGAAAGGATGCTATTTTTGATTTGCTGAAGACGGTTATCA
TCCTTCACAAATGCAGCTAGAGTAGGAACCTGATGCCCTTCTGTGGAATTTTTTGGTGAT
TCATGTGTTGCACATTCCTTTTGGATTATGTCCAATTCCATTTCTGCTTCCTCTAGTTCC
ATAGCCTCTTTATCAACTTTGCTTGAGAACAAATCAAAGTAAGACTCAACAGAAGTTAGA
TTGGCAAGGACTTGAAGAATAGATTCACGAGTAGTCCCAAATTCGGGGTCAACTTGATTT
AAAGAATTTGAAGTGAATGCTTCTTTCAAGACTTCTTTGGCTCCATCTCCTTCAATTCTC
AATCTACACAATACTTGGCAAGCTTTTTTCTCCAGATCCCCAAGCCTGCCTTCAGTTTGG
GACAATCCTAAGGTC
>ntig_767_consensus
ACAATAAGGACCTTTTTGTCCTCAAAAACACGTGTTGTTAGGAGAAACCCTCACGATGAA
ACAACTGCAATGACCATCTTGAATCATAATTTCCTCCGAGTAATTTATAAATTAATTCCA
CAAGAGAACATTGTAAATGACAGAACGACCATATTGTCATGATCCACACATCTCTGCCTT
TCTTTCATTAAAAACCCAATACAAACAGTCTAATAAAAGATTCACAGAGCCCTAAACCAT
GTCCCGAGCCTTGCTCTTCGGCTGATTTGACCTAATCCGAAAAACCGCCAATCGACAGGC
TTAAAAACAATCTGAAAAAACACGATAAATACCACGTAACAACAACCCGCCCGCTATCTT
GTCTATGTAGTAATAATTATTCTTTTTTAAATGATCCGACTTTCCAGCGACTCGCGTGTG

GTGATATAACAGGCATGTTTTCCCACAGGACACCTGATTGTTTCAATTCGAATGAAATAA
CATGTACGGAATCATTCTTTACTAGCCATATTCATATAATCTTAATGGCCTCGCATAGAG
GTTACAATGGCCATTAGTAAATGACCGTGAAAATCAGTCGAAAAACACAAAAGAAAAAGG
TTAAAAAAACAAAAAAGAAAAAGAAAAAAGGGTAAAAGTAGCTATCATAAAATAAATTTT
CAATAGCTTAGTAAGGAGATCAAACGGGGCCAAAAAGAGAGAAACCTATCTTCTAACTGG
AAAACCCGAAAAAGCCAGAATACTTGAGCTGCTGCCGCTTCTAAGTCAAATGAATTGGGG
CTGCAAATATTAAATTACGAGTCATTACCCACAAAAGAGTCTTCAGAATTATTATACCTT
GATCATCGCATCACGCTGTCCGTATCGTAACGCCTGAGTCTGACTCGAGGCATCATCTCG
TACCGGTTCCCCTGTTTTCTTCTATTGTCTTCGCCACCTACGTACTTATATTCCCTACCA
CCACCTCCCAGATCGGTCAATCTTCTGAGATGTACCGTGGGTCTGATAGACCGTGCTGAA
TCAAACTTTTGGCTTGGTCGGAACATCCTCATAGGTGGTGATTTCCCATCCCTGAAATTA
TCTAACACAGAAATCCGGTCATCAAACCTCGAGAAGCGACTTCTAGGAGGGGATACAAAC
CCTTCTTCGTACTCCGCTGCAAAACGTTTTTGAAAGGGCAACCTTACCCTATCCATTCTA
CCAGATCTATAATTCGGTGACCGACTCTGACGTCTTGATCCCTCATTCCGTTCCCTTGAC
AATAACCATGCACCAGGTGAGCGACTTCTGGATCTTGATCGAGTTTTGTTGTAAGTTTGA
GCATAACGCAGTCTTTCTCCGCTTGGCGAAATACTTCTTTCTCTCCTGCTCATTCGATAA
GGCATATGATCAGCATATTCAGAGGAATAATTAGTGATGGGCCTCCGAAATTCCTCCCTG
ATGCCTCTACTAACCCCTTGTGGGTATTTTCTATACCTACTCCTATCAGGATTGACATCA
TCTCTAGCAAGAGCGGTTCCAACATGAGTTCCATAAAAATCATCCCTGTAGGGCACATAT
CTCCTATTAAGAGGCCGATAGACACTGTTTGAAGAGGAGCTCAAATACTGCCTGCGAACA
CGGCTATCAGATCCTTCAACCGCTTCTGCATCTGAAATTGTACGACCAGAGGTCATTACA
AATCCACGGCTTTCAATAACATTTCTTGATCTAGGACGTGTGGAAGCATATGAGCTATGG
TAGGTAGGCGAGTGATGGCTCACAACAATGTGACCACTGGCAGGACTTCTGCAGCGTATT
TCTGAAGCATATCTATCCCTTCCAACAACTTTGTTTAAACCAGCTCCCCTTACAGGTCGA
GGACATACATCATCAAAATCATCAGATCTGTTTCTTTGTGCAGAATCCGAGGCATCAGTG
GACAAAGATCCCTGAATACTTGATGGCAAATCCCTCTTCAATACCCGAAGTCCAACCCCT
CTAGAGGTGAGGTCTTTACTACTGGGCCCATCCTCACAATCAACAGTCTGATGCTTTATC
TTGCTTCCATCAATGGTTTCCAAAGTTGCCGTCTTTGAGTTAGTTAGATGATCTCTAAAA
GCATTTCCTGGGGAACATTGTTCAATTTTTTCAGCTGCAACACTGGGCCCAGCCTTTTTA
TTCCCAGTATACTCCACTTCTAAACTAAATAATTTTTCTCCATCACAATCAGACCCATAG
TCCACTTGTTCAATTTCAGCATCCTCACCTTCATTATCCTCCCAGCAACGAACGTCTGAT
TCCCTCAATTCCCCATCCTCAAACTGAGAATCATAACCAGCTTCCTGGGGTACACCAGTA
TTATCTATGGCATCTATGTGAACCTTATCATCTTGATAAGAGTTGGACTCACAGGTTTCA
TCAGAAGGAAGTTCACTATTAATCTTATCCGAGCTATTAGTAGGATTGTTACCACAAGTA
TTCAGACATCCTGTTTCCATAGAATCAGTAAAGTTTTCAGAAGACATGTCAGTTAGTTTC
TCGGGCCCGATATCCACAGAAGAAATGATCTCTACATCATTGCCATGAAGAACTTCATCA
GCTACTCCCATTACATGTCCAGTTTGCACAGAAACGACTTTTCCAACAACCTTACTGGAA
GTAAGTACTTCGTCACATCTGCGTAAAGGATGGTCAAAACCCACTTCCTCTGCTTCACAT
GCAGGCTCCATTCCAAGGTATACGGGTTTCACCGCTTGACAACTATCCATACTCAACATC
TCTTGAGAAGAACAACTAGATGCACAGCCAGAACCTTCAGCATTTTCATCTAGGGTTGAC
CTCTTAACCTCACAAGTAACTCCATCTAATGCAGGTCCCAAAGTAGAGGCAACATCACAC
TTCTCAACTTCAACATTTTCTGCACACACAATTTCTTCCTCTGGGACACCAGAAGCACAA
CCTTCTGTCTTCTCCTCAAGGCAATCGCGCAGCACTATGTTGTCAACTGTATTACTAGGG
GAGGCAACATTTTCGAGGCATGCATCTATGCTGATAACCTGAGTATAAGCTTTTGCACTG
TCACTATACTCGTCATGGCAGCATTCAAGGCCAGAGCCAGTTTCCAATTGATGGTCCATT
CTGTTTGACTCATGAGGCAGATCTCCAGGATCAGAAAATGTGTGTGTCCCCCCATATACT
TCAGATGAATCTACTGTGACCACCTCACTATGTTTGATTTCTTTGGTTTCCTCCTCTGTC
TTGCAACCTTCTAACATGTGCAGATTTCCATTCCCAACTGCAGAAGTAATCAAAGCAGGG
TTTACTTGAGAACCAACGTTCTCAATATCACAAGGTTGCTCCCAGGCATCTATCATAACA
TTTAAATCCCAGTGTAACCTATCATCTCGCGTGGAAAAAGACTTTTCTGCTCTCTTGTTA
TCCTCATCATTAGGGGATTTCTGTGCAGACGTAGTGGTAGAATCCTGAAAAGACGACTCA
TCCTTTTTATCTTTAAGTACTAGATCTTTCATGGAGAGATCAGATGTTTCCATTGCAGTA
ACTGTTTCCTTAACTGGCTTCAAATCATCTTCATATACGGATTCTTCCACCATTTTATTC
TCCAATTCCTGATCAACACAAGCTGCAGCTGCAAGGATTTCAATACCAGAGAAATCTTCT
CTATAATCAAGCTTCCCATTCAGTTCCTCGGAAATCTCCCTTTTAACACCAGGACTACCA
GCTTCTATGGAAGCGTCAATCATCCTATGTTGTCTCTTGCTGTTCGAACTTGGATAATAT
CCTTGACTTGAAGAATCATACACCACTTCAATGCATGAAGGGGAACGAGGTGACGGCGAT
GGAGAACGTAGATTAAATTTTCTTTTCTTAATAGGTACATGTTCTATTTTGTCACTGAAT

CGACGTCCAAGAACAGCCACACCAAGCTTCTCAGATTCTGAAATGGTTCCATTCTCTGCC
CTTTCAGAATTCTTATCTGCTAGCTCCACTCCTGCTGTAAATTTTCTAACAAAAGGTTTC
TTTGTCCGCCTTGTAGCACTTCTATTTCCTTTCGCAACGGTCTTCCAAGTCAAGTTAGCA
CCTAAAAAGTTTGTAAGTTCTAGGCCTACAGTGCTCATCGCTGTTGTTGCTTCTACAGAT
CCTATGCCATCAACTAATCGGCCACCAGAACAAGTCTTGCAGTAAGCCGTTGCTCCTTGC
TTTCTGATTGACACCAGGGTTGATGAGACCCCAGCGCAGGTGCAAGACCTAGT
>ntig_768_consensus
ACACATTTGCAGAGGGAAGGAGCATCAAATGGGAAAAATCCCAATTATGTATCTATTTAC
CACACTTAGGAAAGTTTGCGAGGACAATAACACAAGGGTATATGCAATATCCTCAATACG
ATAGTTCATACTTCAGTTTCAAATTTAAATGAACACATAAACTGACCACCATATGGTGTG
TTTTCAAGCCGAATCCAGGCTGGTTGTAGACCACTACATGCCTAACTGTCAGCTGACCTC
AGCACCCGTAAAATGGAAATGAACAGGTTCAGTATATCCAAATAAAGAGCGACGGAGGCC
AAAATATATTCGTCATACGTGAAGCGCTTGATCAGATTATCGGTGTCATAAACTATGTAT
CCGCAGAAGACCAGAGCACTAATTCCACCATATACAGCAACAGAAGTTGACCCGAGAGGA
AAGAACATCTGCATAAAGCTAGTCAGGACAAGGATCATGAGGCTGGTGAATAAAATTGGT
CCAAGGAAGCTGAAGTCCTTGCCCTTCTTAGAGGCCCAGAAAGTGTAGCCAGTTAAAGAG
CACACTACAGCTGAGGTTAGAATTAGTGCTTCAAGCACAATCCTACCCGAGGTATTTGCG
CAGGTCACGGCAACAGGGAGACTTATTGACACGGTGAAGAGACCAAGAACAATCAGGTTC
ACAGGGTGCTTCTGGTGATACACATGCAATGGCCACAACAGGATGAAGGGGAGGAGGGCG
AGGAAAAGGATCAGGCCGGAATTGCCCTTGAGGAGATCGTTGATGGGAGAGTAGAGGACC
GTAACG
>ntig_769_consensus
CACATCGTATTTTCTCTCAAGCCCAAGTTTGATTTTATTGTTATTACACGGAAAACGAGA
CAAAATTATCTAATTTATGATAATTTATCCCCACATGAGCCGACTGCCTTAGCCAGTGTT
TTGCATACCTGCAGCGATACCTTTCATGGTCAAAATGACAGTATCTTCCAGACCAGGAGC
ATACTCACTTGTAGGGTTGAGCCTCACAAGCTCTGCAGCTGGCTTTCTTGATTCCATGTA
CTCTTTTGACAAGTGTGGCCTCACCTTCACATGGAAGTTGGAGTCACGAATCTGCTTCAG
AGTATAGGCTTGGCATACGTTAAGTGCAGTGATGTATGCATCGCGAAGTCGGAGCCTCTG
CTTCAAGTATGGATCTCCTTCAAGAAGATCTCTATGCCCAGCAACCTGGAGAAGAAGGTG
CTTTGTTTCTTTATAGTTGTGTCTCAATTTCTCTCCAAATGGCCTCAGTTCTTCAGATAC
AAGGAGTTTGTCATACAGAGCAGCAATGCCTGGGTCTCCCTTGGCAAACACCATTTCAAC
CAAGTCAATTGTGACCCTGAAGAAAGGCCACATGTTGTACATCTCCTGAAGCATGTGGAG
ATTCTTTATATCCTTCTCGATCACATGCTTAAATGCTGCCCCAAAACCAAGCCAAACAGG
TAGATGAAACCTCGTTTGAGTCCATGCAAAGATCCACGGGATGGCACGAAGTGATTCAAT
GCCTCCACTTGGCTTTCGTTTTGATGGACGACTACCAATGTTCATTCGACCATACTCCAA
CTCTGGTGTTGCAAGGCGAAAATATTCAACAAAACGTGGCTCCTGGAACACTATGGAACG
ATATTCCTTTGTAGCTATAACAGCCATCTCATCCATGAGTGCTCGCCATTCTGGCCTCGG
TGAGACTGGGGGATGCATTCCATGCTCCAGTGTAGCAGCTGTAAAACGCTGAAGTGTTCT
GAAGCATAAATGCTCCTCCCCAAAAGATTGCTCAATTACTTCACCTTGAACTGTCACACG
AAGTGATCCATGGATTGTATCTGGTGGCTGAGACAGTATAGCAAGATGAGTGGGCCCTCC
TCCTCTTCCAACAGTTCCTCCTCGTCCATGAAACATTGTGAGCTTAATGCCATAATGCTT
TGCAACCTTTACAAGCTCTTCTTGAGTCTTGTATAACTGCCAAGCTGCTGAAAAACGGCC
AGCATCCTTTCCAGAATCAGAATAACCAATCATGACCTCTTGTTTCCCATTGATCCGGTT
TCTGTACCAATCTATTGAGAAGAGACGAGCCACAGCAGCAGGAGCAGCTTCAAGATCAGC
AAGCTTTTCGAACAATGGAACAACCCTTAATGGATGCTTCACATGACATTCGCGTTGTAA
AAGCTCCACAGCAAGTACATCAGATGGGGCTGTAGCCATTGAAATTATATAAGCACCAAA
GTTATCAGGGGGAACTTCTGAAATGACATGAAGCGTGTCCAACACATCAGCAATTTCTTC
TGTTTTTGGAAGATCAGAGCCAAACAGAGGGCGTTTGCCTCTGAGCTCAGACAGAAGCCA
TTCCTGTCTTTTCTCCTCTGACCATTCTCGATAAGATCCTATGCCCAAGTACCGTGTAAT
AGCATCAATGACATCAGTATGCCTGTCCGATTCTTGACGGATATCAAGTCTCACAAGGCA
CAGCCCAAAGGTAGAAACTTGCCGTAAGAAATCAAGAAGGCTTCCATCAGCAATTGGCCG
GTCACCACAATCACAGAGTGATCTGTAACACAGTTCCAGGGGTTCCAAAAACTGCTCCAC
ATTAGTAAAAGCTGAATCCTCAGGAATGTCAGAAATCCCATTGGCTAACAGCTGACGAGA
ACGTTCCCTTGTGTTATACAGTTTGTCTCTGACATCACCAAGAATAACACGATAGGGTTC
ACTTGGAGGAATTTGTTTCCAAAATTCTATATAGTGTTTAGAATCTTTCTTTGAGGATCT
ATGAAGTTCATCTGCACGTGCACGAAGTTCGTCATTGCAGCGCCACAT
>ntig_770_consensus
GCTTCTTGCATTTGAATCCATAAAGATTTGAATGGTTAAGAACACAGTACTTGGAATTCA

AAATGCCAAATTACAAAGTACAACCAACTAGGTAAGTCTCTAATCCGGAAAGATGAAAAT
ATACTTGGTATAACACTATCACGCCATGAGCTCAATGTAAACAAAAATAAAGGAAAACTG
GACCAAATTATTGATATTACAATTTAAAGTTCCTAGAGAAGTGAATTTCAAAAATAAAAA
GTAAAGCATAATAAAATTTGGTAAAGGTTGCACTCTTTTATCTGCTGATAAACTGCAACT
GCTCTTCAGAATGGTAATCTTTGAAAAGGGGGGAGTTCTTCAGTCCAGCTTCACTGAAGA
GAACAAGTAATGCACAAACCAGAATGAAGAAAGGAATCCAACTGTGCCCGTTGCCAGCAT
AATTGCTATGACCATAAAGAGAGAGTATCCCAAATAAAGAGTGGCTGAGACAGGTCCACC
CAGATTTCGAAGATCAAAGACGAGATAGTTTATGGAGTACAAGAATATGTAGATTGCAAC
TGATCCCGAAGCAAAGAAGGATTTCCACCACCATTTCCAGTCCTCAACGCAAAGATGCAT
GTATGTGAGAACAAGAGACACTTCGGCACAAACCACCACAAGCAGTATCAAAACAATCAA
GAGGAAACCAAAAACATAGTAAACACGACCCATCCAGATGCTAGACATGATAAAGAAGAG
CTCAATGAAGAGGGTACCAAATGGAAGAGTACCAGCACCAAGAACTAAAAGCCAAGATGG
ATATCTCTGGGCCGGGATTTCACGCGGAATCTGGTTGGTTCTAACAGGGTATTCAATATG
AGGTGCCTTTGCCCCAAGGTATCCACCAAATAGGGTAAGGGGTACAGAGATGCAGAACCA
TAGCAGAAGCAAAATAACAAATAAAGAGAACGGAATGGCTCCTGTGCTATGACTACCCCA
CAACAAGAAATTCAAAGTCGTTAGAATCAAAAAGGCAATACCAGGGAAGAAACAAGCAAC
CCTCCATGCCACAGAAGCCCAACTTTGAAGATCAGCACAGCCAATTGTCCTCCATAGACG
AACTGCGACATAGCCACCTGGAACACCAAGAATCATGTAGAAAAAAAGCATACCTATGAT
GAGAGTACCACGGGAAGCCGGTGACATGAATCCAAGAGCAGCAAACAAAATAGTCACGAC
AGCCATCCCAAGAATCTGTACTCCATCTCCGACAACAATACACAAAAGTCCGGGATTGCT
TGGAGCACGGAAAACATCCCCCACAACAAGCTTCCACCCAGACAACTCCTCATTCATTTG
TGACTGCGCCTCTTTGTCAAGTTCCTCATACCGAGTCAGATCCCGCCGAACAGTCCTCAA
AAAGATCACAAGAACAATACCGGCAAGGAAAGTGATGACCATAAGAGAATTCAAGATTGA
GAACCAATGGACCTTTGATCCCTCCATCCTCAAATAGGCATCCCAACGTGATGGCCACTT
AATATCACTCAAATCAAAGTTAACCTCATAAGTAAACACAATTGGTTGACCCTCCTTAAT
TGGCATAGACACAACGTTTGATTCACATCTAATCTGACTTGGGTACTTGCCATACATCTT
CAAATCCTTCACTGAGTCAACATTATGCATCACACTACATGGAACAACCTCAAATCCAAC
AACCATATAACCGGGATTATCAGATCCCTCACTATCCTTGGTTGGAATCATCTCAGCACC
ATCAGCAGTACCCATAACACGTGCAACATTAGTTTCCTCATACTTGTGGACAAGAACCTT
AAACTTCAAATGATTAAACACATAATATGCATCTTGATACTTAACCCCAACCGGAAAACC
GGTCCACCTCAAAAGATAGCCATCCTTCTTGGTGTACCGTATTGCAGGCAAATTAT
>ntig_771_consensus
GCCTTGATGAACACAAATACAAAACTTCTTCATTTGAGAGTTTGTCAAATCTAAACTGAA
TACAAACATTCATTACCAATCACTGTACCGAACACAAACTATATACTGTTTAAATTCAGT
TAATATGCATCTATACGTCTGTTTACAAAGATGAAAAGACTAAATATTGAAAAAAAGAAA
AAAGGATATCACATACTCTTGTAATATCATAGAGATTTTATGAAGTGTTTTCATTATAAT
CCAGTATCAAGAAATAGTTGGTTAAAGTGGATTAAGCCATATATGTATTTAGCAATGTAG
TTTATACTTGGATCTCGATTCTTGGTTGTTAACGAGGGTGTTCAAAGAAAATATTTGAAG
GGTATACAAAACATGCACCTGGGTTTGTAAAGAAGAAAAGGCACCCATATTATGTTTGAA
GAAAGGAAGATAAAAATCAACCCCCGAAATACTTAAAGTATACAGGGCATCAAGTTCTTA
AAATATCAAAAAAGACTTCACTTCAGAGGTATAAGTCAAAAATCGAAAGACAATTGTCCC
ATATCATGTCAACAAAATATAAACAAACCCAACAAAACTTATAACTCCACAACAAACTTT
AAGGATAGCAACTACATAATCATATTGTGCAATAATATTACCTAACATATAGTTCCCACC
TGCTGTATTTATGAAAACTTGGCCCTTGAGAAATCCATCTCCGGATGCTCGGCCATGAAC
TTCTTTAGAATCTCTTGCTTCTGCATCTCGTCGCTAGTAGGAAGACCCATGGACTTCTGC
CTTTGATCAAACATCATCTTTTCAACTGTCTTCCGAGTCTCTGGATCTAGGTCAGATAAT
TTACTGTTCTCAGGTTCAACTTTCTGAGTGTCAATTTCAGGACCTCCCTTCAGCAATGAT
TTCCACCATTCCATCTGGTTCTGCTTGGTCAAAAGAACAGAGATGGAATTCTGATCCTCT
ATACTCCAATAACAATCATCAGGCTTAACATTCTGACTAAGTTCCCCATCAATAATTGGA
GGCTGACCCTTGAGTCCAACTTTTAG
>ntig_772_consensus
AAAACTTTATAATTCACTAACAAACCTTAATGGTAGCTACTATACAATCAGCATGATATT
GTATTAATTATATAGATAGTTCCCCGCTGCTGTATTTATGAGATCTTTGCCCTTGAGAAG
TCCATCTCCGGATGCTCAGCCATGAACTTCTTTAGAATCTCTTGTTTCTGCATCTCATCN
NNNNNNNCGAGTCTCTGGATCTAGGTCAGATAATTTACTGTTCTCAGGCTCAACTTTCTG
AGTGTCAATTTCAGGACCTCCTTTCAGCAATGATTTCCACCATTCCATCTGGTTCTGCTT
GGTCAAAAGAATAGAGATGGAGTTCTGATCCTCTATGCTCCAGTAACANNNNNNAGTCCA
ACTTTTAGATAGTTCTTCTTTATCTCACACACAACAAACCTTGATTTAGTTCCATTAGGG

ACTGGAACAATAATAGACAACTCTTGCAGGGTCTGCGTCCAAGAGTAATTGTCCAGATCA
AGACCATTTCCTTTATTTGCAGCTCTAGCTTGGTTTCCATTAGCATCGAGCTTCGGCTCC
GGCGGAGGAGCTTTCTTGACGTCACTCGTCGGCAAGTTCTCCAAATCTTGATCCCCCTTC
TTAGCTTTCTTGGGTTGAGTATCCTCCAACGGTTTCTTTCTCTTCTCTTTGGCCGCTCGA
AATACCGCCGTGATCTGACTCCC
>ntig_773_consensus
TTACATTTTAAACACAATTTTATATCTCAAATGAAATGTGATGCGCTTTTGACATCTTAA
AAGTCTTAATGGGGATCATTACAAGATTATTTCTTTCTGACACTAACCTATTTACATTAG
TGATTAACAGGAAAATAAATATGATAGTTTGGGTTTAGAAGGGCCACAAATCACCCCAGA
AAGATTTTCGAGCCAATCGATGTAATGTTGGATTCTTGCTTCGTGTCATTACAACATGCT
CAAATGAGCCCTCTGAGCCATCCCTTTTGATCGATCGACAGACATCCATTGCAACCTGCC
TGCCCCTTCGAACTGGACCAAAGATAATCCTTCCCCAGCCACCACCGACATCAGCACGGC
CTGTTTCATCTTCTTTCTCTTCCACCTCAAAATCTTTGTCATTATCATCAGCACCATCAG
AGTCGTAATAAACTGGCTTTACTAATTGAAGTTGCCTATCTTCAGCTTCAGCTTGTAATT
TAAGTAGATCCTCATAGTCAATTTCAAGATCTTCTGGATTTCCTTTGGCATTTTGCTCCT
TTAAAGTATCAAATTTCATACCATCAAGAGGCCAAGGTTCCCTGGGCCTTTGACCACGTC
GGAAAGCAACAAAGCAAAATTTCTCATCTTCAAATCCCCGTAAAGGCTCACCCTTCGAAC
GCTTATAAGCACGCTGTGATGTTGTCCTCTGCAAACGCTGAACAAAATGACAATATTTTC
CAGAATTCTCCAGTGGACAACGCCCATCATGAGGGCACCTTTTCTCCATCCATAGTATAT
GAGACCGCATTTGAGATATGATGGTAGATCCCTGTGGTGTACCTGGTTCCACTAAAACCA
GAACATCCCCTGTAAGATCCCAAAGTTGGCGAACTACAGTAATTCGGTCCTTCAGTGATG
GTATCTCTCCAAGCACGTACGAAGCAATTACAAGGTCATGTTCCCTCTCAGATTTTTTGA
TATCTTTAGACAGTGCTTGAATGCTACTGTAACTATGAATAAGGGGCAAATTCTTCAGGC
CTTGTCTAAGGCTCTGACCTGCACGCTGCATTGACTGGGATGGTTCAATGATATTCACTT
TTTCCAAAGACTTAGGCCAAACTTCTTGTAATGCCCAGAAAGCGGAACCAGTGCCAGCCC
CAAAATCCAGTACTCTTGCAGGAGAGAAACCTGGTAACCTTCTTCGAACCTCAGAGAGAA
CCCTATAACAAGCAGAGTAAACGGCAGGCATTCTAGAAGCAACATAAGCAATACTCTCAT
CGTCCCTGTACTTGAGACCAATGTCTCCATAAGCGCTTTTGATCTTCCACCGCTTGGATT
GTTCCATAGACTTCAAAGGGTCTTCAAAAAGCTCCCTCGAAGTGGACTCTGTCAGCATCA
GATTCACATTCTTAATTCCGCTTAACGATTCTGACAACCTTAACACTTTCCTCTTCATGT
GTGGCTCATCTTGCTCTCGGAGGTATCTTTTGATTGCGCGGCGGAGACGGACAGGGACGA
TAAGGCAGCGCTCCGACTGTTTGGCGGCGGATCGAAGGGTTTCCGGTGTGAAGAGTTTCT
GGGTTGTTTCAGGGATTAGACTAGCCATATTCTACTGCCCGTGGTGGAGAAGATCCGAGT
CAAAGAGAGCCAAGGTTTTGATGAAGCTCTGCTCTC
>ntig_774_consensus
TTTTTTTTTAAAAAGCAAAATCATCTTTATATCACATAATACCAATTTAATACAGGTAAT
CTAGCTATACATATAGCTTTAAGGGATTTAAACTGATATTTTCATCACTCAAATAAAAAA
CTAAAAAATATTTACAACAACAAAGAGCTAGCCTCCAACAAGAGCAAACCAACAAAGACA
TGAAATTTGCTCTTCAAATAACCTTGAGATTAGCAGGCACGTATCCAAAAAAATAAATCC
AAGTGCCTGCAATTCCTTTGGAACACAAATACATTACGATATTACATGAAAGAAAGAACA
CAAGATGGAGCTGTACAAGGATAATGGAAGAAGGTGAGGGTTGGCAAAAATTAACCACAT
CATACATCATCTCGCGGAAGCAACCAATGGTTTATCTTTACTGTCTTGAGTTGAGTTTAA
GGTTGTTGAAGGAAAAGGCAAGGGTAGATCAGAGATTGTACGAGGTGTGAATAAATTTGG
AACTGGTAATTCTTCTTCAGCAGGGACAGGTGTTGGCTGTGGTTTTGGTGTGTAGTTGAA
GGAGAGATCTTTGTTAGCAGCCAATGCAATGAGTTCTTTCTCCTCCAACCCTTTTGTTGG
CTCAAGGCTGCATCGGTCAGTGATGTGCTTAGCCAAAGCATCTTTAAATTTTTTGATCGT
GGCATTAGTGCAGCTAAAGCTACACAGTCGACCGTGAGCTCCTCTGTAAAATCGAAAGAA
CGGCAGGACATGAACATTAAGACTATAACACATGGATTTATGCTCCTCATAATTCACCTG
AAGAAACTGCACATATGGGTGCATCTCTGCCAATTGACATATCTTGGGGTGGAGAGCTTT
GCAGCCACCACAACCAGGAGAGAAAAAATCAACAACAACAAGTTTGTCCCCAGCATTCAA
AAGGGAGTCTACAAGATCTTGTGCAGAGGCTGCCTCTTTCATGTTTGGTTGCAGCCCCTT
TTCCCACCACTTCTGAGATTTTCCTATGATGCCGATATGCCGTGCTTTTATGGAGACCTG
TAAAGAATTTCCTCTTCCGGGAAAGCCTTTGTTCTCTTTGACAACAAGTCCCTTTCCATT
AAAATCACTGTTCAAAGATGAAGAAAGTCTCAAAGCATGTGATTTCAATTTCACTGGAAA
ACCTCTCATAGAGCATGAACCACTACAGCTTTTTGGGATCAAAGAGAGGCCATTACTGTG
TTGATTTGGATTAATGTTTGAGAACAGATTTGATTTGTTCAACACATCAACCATCTTTTC
TCACAATAGAAAAAAAAAACACTTCCCCAAAACAAAGAAAGCAGTTTCACTGAAACCCAG
ATGGAAGCAGCCAAGCAGGAATAGATTCTGTTGAAAGAAACAATTATTATTGAGCAAATT

AGGTTCTTGAAAGAGAAACCAAAAAATGGAAACGAGAGAGTATGTAAAAGGATTGGATTT
CAGTTGCTTAAAGAACAAAAACACAGCTTTTCTTGATTTATAAGGAACGGTTTCGAAGAA
ATCCCTGAAACAAAAGGACAGCGAAGAAATTCTGCTCTCGAAAGTTATTTTGGCAGCAAT
AATCTGCGTTCTGAAAACAAAAGAGAAGAGTTTCAAAGAAGAAAATGCCAACAGCT
>ntig_775_consensus
CAACCAGTCAGCATGTGAGCGTTTCAAGAGCTGTTAGAAATATTTACCAAACATAAGCTA
TAATCCATTATCTTCTGAACTCCAACTGAATTAGACTACAAAAAAACGTTTAAGAATGAA
AAGCAGGATCTCTGATAAAAATTGTTAGAATGCTATCTCCTCAAGAACTTTAAGCGTTTT
CTGATGAACCCATTCAAGATGTTGTGCTGTAATACCAGGGACATTTCCAGAATATATACC
GCCTAAGACCTTGGTGACAGTCTCAAGAAACATGCCAGGGCGCACAGGAAGAGGTTTCCG
TCCCGTAATACTTTCCCTTCCAATAACTCCATTGAATTTTCTACCATTAGTATCAACCTC
ATCACTTCCATAACTTTCTGCACCTTCGTCTTTCCCAAGCATTACATTTTTTATCATCTC
TTCAACAGTTTTCCCAGGTTTCAAAGCAACCAGTTTGGTTAAGAAGTTCACCTCTGATTC
AGGAAGAGTAGTAAAGCCACGGTCATTTCTTTCATCCAATATTCCACCTCCCATCATATT
TCGAGCTTCCTCTCTGATCAATACAAGCCTTGCAAGTAGTTTTCGATCTGGAACAACAGG
TCTAGTTTCCATTGTCTCCAATAAATCATCTGCCTGGTTAGCTATATCGTTTAAATTTGC
TCCAGGAATATGAACTTTGATCCCATTCTCACTTTTAAAAACACCACCACCAGCAGCCAC
TCGACGCAGCAATTCATGTCTGCTTTCTTTCAGGGGAGTTCTACAAAGCAGGCCAATCAC
TTGAACNNNNNNNNNNNNNNNNNNNNNNNNNNNNNNNNNNNNNNNNNNCTTGTCATCTTGGT
CCTTCTCAGCAAGTTGTATCATGTAATCAAGGGCCATCATAAAACCTGATTCCATCTGAT
CAACTCTTTTACCTATAACTCCTTGTGCAGCCAATTCCACAGCCACCCCCTCAGATGCTT
CGTTCATATTCATATCTTCCAAAAGAGTGATCATTTCCTGAAAAACAGAAATGACTTGGT
CACCCCCTTTGAGCAAAGCTTCATACTGAGCATTGTCCATTTTCTGGTCCCCATCAAAGA
GGGTTTTATTTTGGTGTGAATTGAAATCTTCAGCTGGGTAACCGCCACCTACTTCATAAG
AGGAACACAACCTCAATTGGTAAGTGTTGTTAGCGGCGATGGCGGGTTGTTGTAAAAGTG
GGAAGTGGGATTTGTGGTGGTGGTGGTGTAATGTGAAGGCCATCAATGAAGGATTTAGAG
TTGTACAAAGTGAAGTTGAGGAAGATAATGCAATCAAACCCAACATTTGGTTTTGTTTGC
TCTTTGG
>ntig_776_consensus
TGGCACAATATGATTAACAGTTACGTAACCAAAAGCATTCTCAATATCACCTATTCAAAA
CTTCAAACAAATCGATCCCAATTGTCAACATAAAAATCGAAATCAACAAAGCTTTCACAA
TATGTCCTTGAGACAAAAAGTAATCTCGTAAAGAACTCGTCAAAACCATTAAATTTGTTT
TCTGATTCTCTAATAAGGCTTTAACGGTTGGCCTTGGCAACTCTCTCAATTTCATCTTTC
TTCTTGATGGCATAGCTGTTAGATGAACCCTTTGCAGCATTGATGAGTTCATCAGCTAGG
CATTCTGCAATTGTCTTGATGTTCCTGAATGCGGATTCACGAGCACCAGTAGTAAGAAGA
TAGATGGCCTGGTTCACACGTCTTAATGGAGAAATATCAACAGCCTGCCGTCTAACAACA
CCAGCTGAACCAATTCTGGTAGCATCTTCACGAGGACCACTGTTGATGACAGCATCAACA
ATGACTTGAATTGGGTTCTGGTCGGTCAAAAGGTGAATAATTTCCATGGCATGCTTCACA
ATCCTAACAGCCATCAATTTCTTACCATTGTTCCTTCCGTGCATCATGAGAGAGTTTGTA
AGCCTCTCTACAATTGGGCACTGAGCCTTCCTGAATCGTTTCACAGAGTATCTACCAGCT
GTGTGGGGCACATAGGTTGCATGCTTAGCAGCTTGGACACCAATGTAGTCATTCAATGAA
ATATCAACAACCTGAACGTCGTCGAAGGTCCAGCGGTTGAAGAGCTTCACATCGTAGTGA
GGCTGTTCCTGAGACGGTAGAGCAGACGCCACAGCGGTCGTTGCCATCTTACCTTAGACC
GAGAGCTCTCGGGAGAGAGTGAGAGGAAGAGAAATAGGGTTTTGGAAGGTGCGGCTGCTC
ACAATGAAGATCTCTCAGGTT
>ntig_777_consensus
ATCCCCTTGAAATCAAAATTTTACTTTCTTCGCCTGGGATAAAGGGTTATTCATTTCATT
GAGAGTCGATAATTACACGAGCACCCGGCCCTTCTAACATTTATTTTATTCATAGAAAGA
TCCAACTAATAACCGACAGCCTTACATCCACCACCGTTCAACCACAACATTTAGGAATGT
TACCCCTCAAGCTCAACATCCATTCTATGCTCCATAGTTCACATTATGAGTTCCAAAATA
TTCTGGCATCATAAGAACCACATGAGACTTTCAGATTACAGCCACTAGATAAGTAGAAAA
TTAAAATTTAGCTCTGGATATTTTCATCAACAAACACATAATTTAAGCATCCGCTGCCTT
CACAAACTGAGCTGCCTCTGCACTGGTACTTGATTCGGCACGTTTAGCTTCATAATCTTT
TACAGCTGCCTTAATAGCATCTTCAGCCAGCATGCTGCAGTGAAGCTTAACAGGAGGTAG
AGAAAGATGTTTTGCTATTTCAGTGTTTTTAATGGTCAAGACCTCTTGCATTTGCTTCCC
TTTCACCCACTCAGTAGCAACAGATGACGAAGCAATTGCGGACCCACAACCGAAGGTCTT
GAAACAAGCGTCAACGATTTGACCAGTCTTGTCATCGACCCTAATCTGAAGCTTCATCAC
ATCGCCACACGCAGGCGCACCTACAAGGCCAGTGCCTACCGTCGGATCGTTCTTGTCAAA

CGAACCGACGTTTCGCGGATTGTTGTAGTGATCAATCACGTTCTCATGATAAAATCTAGG
CAGCGCCACCTGGACCGACCGCGAGATCAACTGGGCGTCGCCAGCCGCTAGGCCTAGTAG
CCTCTTTGAACCGAATCTCAGCATTTCTTTCTTCTTTGGCCAACGAATCGCAAGAGGTTC
GTTTTGAATTTTATTTGGTGAGTTTACAAGAATTTTGGGTTTTGAGG
>ntig_778_consensus
CTATTTGAGCACAATTTTTTATTACCACATTTCTATAATGCCGAGTACAAAGTTCGTATA
AGTTTAGACACAACTTCGTGGCTACATAACCGCCTGCTAAATGAACAAAATGTTAACATC
CTCCGACCATATTACATCTCCCGCATTACGCTTCACTTCCATTTCTTGGACGAGGCATGT
GTTTAAGGAACACAAGCATGTAAAACTCGGGTCCCGAGATTGCTCCAGAGTGTCTTCATC
CTCCAAACAAAATTAAAACTGAGATCATTTCGTAGCGCTTCTAAACACACATTTTGAAGT
AGCCAATATCAATTTGTCGCTCCTGGCAATAAAGATTAAGGGCTAGCACACTCCTGACTA
TCTGACAAGCAACACTTCATGAAGTGGTGGATAGTGTCAGGCCTACATAATAATAGCTCT
CCAGTGAGGGTTTTCCGAGCCATGCTTGCAAAACCTTTCAGCATTGATTTTGTGCCCGAG
AATTGCTTTCAGCATTTGGATAGCACCTTCAAGTGAGAAAAATGAAAACTCCCTCCCCTT
TAACTTGGCATCATAACAACCAGTTGCAGTTTGCCTAGCTGGCAAAGTTTTAATAGATTT
TTGTATACTTTAACTTTAGCTTGATCATCACTAAATAAAACAACATGCGCAATGCTGAAA
GATTCACCTTTCTCAACAGCAAATTGCCACTCTCTCATTGTTATATTGAACCAGTCCTTT
CTTGCAGATCTGGTTGATTTAACTTCAATGTACTCTAGACTATTTTCATTCTCTCTTATC
ACAATGTCATAGGGCAACCCAGTTTCATTGTTTTCATTCACCCAATTCACAGCTGTCTTA
CCAAAGAGTTCTGTGAAATGCTTAAAAGCAGCAAGCTCGCCCATTCTCCCAGTTCGCAAA
GCTTGCACTACATCAGGTGCACCAGTATTAAGCCGATCTCTGTTGCAAAACTTAGATAAC
TCGCAGCCTTTGGATACAAGATCTAGATCAACAGGATCACATGCAGAATTCATATTAGAA
GGATTCTGACAAAATGCAAGGCCAAACTGATCTCCCATATTTTCAGGAGCTGGCAAGAAC
AAGGCTGCTGAAGTTATTGCAAAATCATTTCCAAGAGTCACATTAGAATCAATCTCCAAG
GGAGTAACATCATTTGTTTGTGTGACATCTTTGTTATCATCCCTGTTTCTAGTTTGTGAG
CTGCCGCTTGGCTGTGCGGTTGCTGCCTGTGTTTTAAAACCATTTGCACGAGCATAGTTG
AAATCTGGGGCAGTTTTCCAATCAGCAGGTAGCCAGCTTGAGCTATTTCCTGCTTTCCTT
TTGGATTTTGAAAAAATTTGATCCTTAATATCTAGTGATGTTGAAGTTTTTTGAAGTGAT
GCATCATTCTGAGTCAAAATATTTGCAAATGAGAGTGACCAGACAGATTCTTCAACAGGA
AGCTTTGGCACTTTTTGGCTATTTAAGATGAAAAACTCTGTCTGCTCCTCTGTAGAACCT
GATTCTGCCATGGTTGTGATCATATGGAGAAAATTTGCAAAGTGCACCTCTGGATTTCCA
TCAAAGAATAGGCAAGAAAGCTCCATGTATAAGGCATGAGAGTCAGAGTCCTGTCTTGTG
TACAAAATGTTACCCTGCAGAAGACAGCTGCATTCAAAACGCTTTTTAGATGCACCACCA
TTGCTTTTAATAACATTTCTGTAGAACAACTTGTCAACAACTACAACTCTTAGATGATTC
AGTGTGTCAAACCCAGACTTCTTCAATTTTAAATATTTATCAGGGTGTAAACTAAAGATG
TAGCGCTGAGCATATGGAAGGGCCCAGTTCACCAATGAAGCCTTAAAACTACAATCAGCC
AAACCACAGTATATTGCTTCACGAGCTACAACCTCTGAAAGAGCAGGAATCCCTAGAGTT
CGCATGAGAGTGGTCACTTTAGTTTGAAGCATCTCTTGTTCATTGTCACCAAGTTTGCCA
AAGTCTAGAAAATCAATATTCTCTAAATGCTTAAATCTCTTCTTCAACTTCTTATCATCA
CACCAACATACAAGACCAAATGAAGGATGTAGGGAAGCCCACTTATCTTGAACAGTGGGA
AGCACTTTATACTCCAATTTTGTAAGACATTCTTTCAAGTAAAGAGTATCTTCGGAACTC
ATTACAGCAGATTTTAGTCCATCAGCCCACTTCAGGAAAACTTGGAAAACAAAGTTGGCG
ACTTGTGAAGGCAATGCAACAGTTGACAACTGCAGCAAAATCTCAAGGTAGCTACGGAGA
GAAGGACTCTCATGTATTCCACACCTATTAACAAAAAAAATCGTGAAGGCCTGGGTATACA
TCCGACAACATCTTAGCTGACTGTTGCACAGAAACACCAACAGAGGTACACTGAGGATAC
ATCTTCTTCATTAGGTCCACAGTGCCAGTTGAATCATGCCAATACACTTCTTCATAAGAC
AAAAAGGTTCCCAATACAACATCCTCATACCCTTCTCCAGATGTGCATGGAACAAATATG
AATGGCTCTGAATGTAATTCCTCAGTAATTTTGCACTTTGAAGCTGGCTTTAAATGGATT
ATCAAATCTTCTCCATACTTTGAGGATTTCCATGATGTCCTCAAGGGTAACTTTAGACTT
TAATCCAATATCGTGTACCAACTTTTCACTTTTAACCTTTGGAAAAACATACGGAGCCGA
AGCACCCAGGATAGATCGCACTGCATCACAGTCGTGGAATAAATCTTCAAGATAATGAAG
CTCATGATCCATGCTTGATACTGCCCACTGAACACTACATATGCTGTTTACAAATGAAGA
TTTGAAGGGTCTCCCATCTCCATTGGACTTAAAATTGTAGAGACCTGTTGTTTTGTCACT
GTAGCAGTCATCCCATAGTGTATCAAGAATCTCTAATAAATATATACAACTTTGGTGGTT
GGCACTTATAGTAAGTAGGGATAACAAACAAACCAACTCATCAGATTCCCAATCTTTCGC
AGTTGATCCAGGTGAGATTACCTCCCCATCCCATATCAAGTTTTTGGAAACCAAATGAGA
TATATCACCAACATTCTTATTAACTTGAACGACTTGCACAAAATCAGTTACACCAATTTC
TTGGAAAAATTTTCTCCACTTTATCAGCCCACCTGAGAGTGACTTGTTTGCTGGATGCTT

CAAATAGGTGGTGTCAACTTCATGCCATTTAGCATCCATGGCATCAATCAACTTATTAAC
ATTAGCAGGATTTCCAAATTCTTTACTGAAGTGAATTGGCACCTCAGTCAGTCGTTTGAA
CCCATGATTTGTTAATATAAAAGCTTTAATTCGTAACTCTGAAACAATGTACTCCCTTTC
ATCATGACAATTGGAGCAGTAAGATTGTAAGTGGATCATCACAAAACAGAGATAATCCAT
CATCAACATTTTGTCTCTATTTACAATTTTTTCATCACATATGGCTGGCAAAATATGCAC
CTTCACTATATCATGAGCAGATAGCTGTTGGACACCAATTTTGAGAAGCATCCTAGTAAG
GTTGTCTACTAAGATCCNNNNNNNNNNCCACATAGATTTGGGAAAGCTTCAAGTCCAAGCC
CACCATCAAACATAGAGTAATCAGAATGTAACCAAATTGTCTCTTCATCCACTGCACCGA
ATGTACCATTGGAAAGGGGAATAAATGGAATTTTTCGGAGGCTATCAATAAGATCAATTT
CTATTCCTGAGTTTAATGAAGATTTCCCAAAAGAAATAAAAGAAATTGTATAAAGCTCAT
TTAGCCAAGAAGATAGCCAACTGATTCCCATAGATTTCAGGCCATTCTCTGCTTGACATA
CAGAGGATATGATTTGAACTAGAATTTTAGGTCCATACTCCTCAATACCCAGTGCCCTTG
CCAATGAATCTGACAAAACTATATCCTTATTCAAGAACCCTAGGCCCAGATGCTCATGAA
GCAAGCTATCAGGAAAAATAGCATGTGCCTGATCAGTCCAACCTCTGAGAACCTTGCAGG
GAGGAACCCACTGATTATTGTCTCCTTCCAGAAGCAAGCAGTTAGCCATTCGTAATTTAG
AAATAATCATTCGAGGAAGAGTAGAAAAAAAACCATGCACTTCGCCTAAAAGTGGGACAA
AACTCATATAAGCTGCCACAGCTTTTCCTTGATTATCCCTAAAACTAGGAAGAGAACAAA
AAGCTCTTTGTGCAGTGACAAATAAGTGAGGAACTTCTGACAATAACCACTGATTCCAGG
GACTATTCCCATCTACTTCTTCTCTAGATGATGGGAGAACAAAATCACCTTGCAGAATAA
ATTTTAAACCATAAGTTCTAAGAGGAAGAAAAGCGAAAACAGGTTGTTGGTACAGAAGTG
GACTGTAGTTCCCCTCATTCAACTCC
>ntig_779_consensus
TTCCAAATACCACCAACAGTGAAGGCAACTACAATTCCACTGCATATCTCCTTGACAACA
CTTGGTCCCTTCAACGTGGCATGAGCAATCTTGTGGGCAGCCATTTCACTTATAGAACCT
GAGATGGTCAGATAGAAAGCGCAAAATCCGATCGTGCGAATACGCTCTCTCGAGACTCGA
TTGGCTTATACATTCCAACTTCCGTTTACTTGCAATTTATTAAATCTCTATTTTACCCCT
ATCACATAGTTTGGGCTAGAAAGGTTTAGATGGCGGCTTTATGC
>ntig_780_consensus
CCTTAACCAATAAACTGACCAAAAATTAACATACACTATAATGACCTAGCTTCCTGGATT
TCTTGTTTCAGTCATGCATATACGTTGAATTGAAAACAACTAACATCTCGGGGATCACCA
AAATCCATAAACCAATAATGTCGTGTCCACATCTCCATAAAACAATTTATTCATTAAAGA
ACCACAACTAAGTATGCCCATCTTAGCTTATGAGCCAAGTAAAATCACAAAGTGGAACAA
GAAAGGAAAGTTTAATCTTCTGCGACAACACTAATTTCACCTTTCTCGAGCATATCGTAA
AATGCCCTCACTTTCCTCTGTTCATTCCAGTGATGCATTTTCCAAATACCACCAACAGTG
AAGGCAACTACAATTCCACCGCATATCTCCTTGACAACACTTGGTCCTTTCAATGTGGCA
TGAGCAATCTTGTGGGCAGCCATTTCACTAATAGTTAAAGGCAATAAACCAGAGCGACTC
CGAGAAGCGAATGTGCAAAATCCAAC
>ntig_781_consensus
CCTTCTCTATATTCCTCTCTCCACTAATTTGAAGCACACTGTTCTCTTCGATCTCGACCT
TCACTTCCTCTTTCTTGAGCCCTGGAAGATCGGCCTTGAACACGTGAGCCTCGGGGGTTT
CCCCCCAGTCTATCCGGGTGTTGACGAAAGTAGATGTTTCTTGAGGGAAACAAACGGAAG
GTGAAGAAAATTCCTTGAATGGATCCCAAACGTCTAGGGAGAAGGGATCGAAAATACTGC
TGCGCTTGTTGCCGAAGAAGCTCGGAATTAGTGACATATTGTTTCACAGGATGCAAGAGT
ACTGTATTGATATGTATTGATTGCTAAATCTAATACCCAAACTTTGAAGAATTGT
>ntig_782_consensus
TACGGTAAGAGGGAATTGGACCTTTGCTGAATCGCTTGAACAGCGTGATTTGCTGCATCC
CGAACAACAGGATCATGAGCTGGTACTTCTTGCCATCCAGGTCCATGGCAACCGAGACCA
GAAGAGGTAAATGCTGGAGGGGAATCACCAACGTGCTTAAATTCCTGTAAATCTTTGAAA
TTCATCCACGGCTTCACCCAAACCTTTGCTTCGCAAAGCTTCTTCTTCCCTGCTTCAACA
GCCCCGATAGTAAGATGATACAAAGTACCAGCCACCACCTGCTCTTGTGCCTTCACCACT
CTTGCAAACTCTAGCACTGTATTCTGTTTCTTATTATGTTCTTGGACAGCAAAATGGCCG
AGTTCGTCGATGTCGACGCTGTTCTCAGCGCCCCGTGATTCCTGCAGAC
>ntig_783_consensus
GCCATCCAGGTCCATGGCAACCTAATTTACAAAGCACAAATCAGCAAAAGCAAAATGCAT
TTGACCATAAAAAAATGCATTCAATCTTCGTTCTGAACAAACTCACCCACAATATTGCAT
TGAGGATATATGCATTAGAACTAAAATTTCCCAGCAAAAACGGCAGCATAAATTATAAGT
TAAAAAGTAAAAGTGGATCACTTTTCGTCCTACCGAGACCAGAAGAGGTAAATGCTGGAG
GG

>ntig_784_consensus
CGAGAGCAGAAGAGGTAAATGCTGGAGGGGAATCACCAACGTGCTTAAATTCCTGTAAAT
CTTTGAAATTCATCAACGGCTTCACCCAAACCTTTGCTTCGCAAAGCTTCTTCTTCCCTG
CTTCAATAGCCTCGATAGTAAGATGATACAAAGTACCAGCCACCGCCTGCTCTTGTGCCT
TCACCACTCTTGCAAACTCTAGCACTGTATTCTGTTTCTTATTATGTTCTTGGACAGCAA
AATGGCCGAGATCGTCGACGTCGACGCTGTTCTAAGCGCCCCGTGATTCCTGCAGAC
>ntig_785_consensus
ATCAAACATATTCAAGTTCAATGTAATTATAAACAATCAAATGCCATGCCTAAACATCCA
ACAATTGATTTCCCCTCTAAAAGAATGTTTCCCATTAAAATACAGCTGAAAATAACCACG
GAGAAATTCTCAGGTGATCATGTTTAACACATGATAGGGAATGAGCTATAATTACTAGCT
AAGAAGCAAACCTGTGCCTCTTAACCATGCAAGAGGATAGGCACGATATTCAGCTTCTGA
TGAACCCACACATATAGTGTCAATTCTATTTCCCTGTGCCTCCTTTTACTTTAACCACCA
AACAGCTGAATCAACAAGCCTGGATTCACTGGTCCATATTGTACCACTATTTCTCTAAAA
AACAAAGCCCAACTACTGAACAGGCAAGCCTAGAATCCAACAAAAAGTGGACCATGTGAG
CAAATAGCAGCCTCTACCCCACCATGTCCTTGGCTAAGAGACCTCATACCCTTCTCATAA
GATATCTGCCTGACCAGCCAAAAGCATAAATTTCCTCCAGTGGGAGTCACTTTTCCTATT
TAAGGCTTCATCTTTACCAGCATTTCCTACACTTGGAGCTCATGTTCATGGCAAATGCTT
CTCCAAAAGAGTTGATATCATGAAGTCAAATGCTTAGTTGGCATGGTCTTATTAATGGCC
TGTAAAACATAACAGGGAACTTCTATGCTTCATGGTTTCAAAATATGCTGTATTTGTTGC
CACTCTCCAGGCATGCACAGCTTCAACTGCAGCTGAACAGCTCTTTCAATTTTTGATGTG
GTATGGTTTCTGTGGATATGGGAGTGAAAATTGGTGCCGTATTGAAGCACTTGGATGTTG
GCATTATTTTATGTGCCAATTGAACAATCAAAATGGCAACCAAGGCAAGGGCATTGGGCT
TTTATTTTTCAATAAGCAAGTGGTGCATGCTGTGGGCGTGTGGCAAGCTATTATGGGTGG
TGCTTGCTAAGTCAACTTCTCGGGCAGCAGATTTAATGTCCCAAGTGAAAGTCAGATTTA
TTGTGGCTTTGCTATATGGCGGCAGCGCAAATACATCTCTTTTGTGCATGCAGGACAACT
AAGCTTGTTGGGTTGCATGGACGCCTAAAGGCTGGAGCTGCCAGCCAAACCAATTGTGTG
TGTCTCAGCAATGCCATCGACAACTTATGGAAAGCATAAGTTGATGTAGAAAATTGCAAA
GACAGCAGAATGCCACACCTTAGAGGGATCAAAACCAAACCCACTCATTTGCATCATCCG
CTGTGTATCATCAGTAGCATGATCGACTGCTGACATAGCTAACATCAACAGTGTTCAAGT
CAATCCCATTCTGTAACATTGACCTGAACCTCTGAGTCAGTGGGAATGGTATCTTTGCTG
CAACAAACCCGGAGAAGAAAAAGTTAACCCAAGCAAAAGTAAGTGTCTGGGGTATGATCA
TAGACAGATTTTTCTTCATCATATCCATTGCCATGTTAGGATCAGAAAACATTTGTGCCT
GTGCATTTTGAGCCTGCCCTTTGGGAACATGCAATAACCCATTTTCCTCGTTATTGAAAT
AAATCCTACGAGCACGAAACGACTTGGGAGGGATGAAATTGGCGCCACCACGTAAATTGC
GTGCCCTAAGAATCACCTGACCTTCTTTGACGATTTTGAGATCGGGAACTTGAGTGGAGC
GCATGAGTTTAGAGACGAAGTAGCGGAGGATACCGATGAGTACCATAACTACTGAGAGAG
GAATTAGAACCCAATCTCTGATCGCCGTGTCGAGTACCAGATCCTCCGCCATTAATTCTT
GATTGATGATCACGATCTACCGTGGAGTTCTGATACGGAGAAGATGCCAATTACTAAAAC
GGCACACTCGACACAAAGCGGAGGGAATGCGGCCTTTTTGCAAGATAAATCGTTTGGAAA
CGGGGCGTTTTCTAACTCTAGTGTTG
>ntig_786_consensus
TTTTTTTTTTTTGGAGTTTTGTTTTTGAATTTATTTATCGGATAAAACTGGTTTATTACA
GACGCATCACTATCTTCGGTTAATCAAATTGTATTACAAGAAACAAATTTTGGCCCACCC
GCACCTCACAGCGGTCCGATTACTTCGCCTTCTTCCCGTTCGAATGCATCAAACTGTCCA
CAAATTCATTGATATTCTTATCAGAGCTTCCTCCCTCGCCAATGGCTTCTTCTGCCGACT
TCTTCCACTGTCTAGTATTCCTCTTAATTGCTTCACTTCTCTCCCCTTCCATTACTAACT
TCAAACACTTGAGAAATTCTTCTTTCTTCACTATCCCCATTTCATCCTCCTTAGCTCTGA
CTCCTACCTCCCAAATTTCCTCCACATACATGGCGTCAGGCAGCTGATCAGCCCACTGTG
GCATGCCCACCATTGGAATGCCAAGGCTCAGCCCTTCAAGCGTCGAGTTCCACCCGCAAT
GTGTCACGAAGCAACCCACTGCTTCGTGTGCCAACACTTCCAGTTGGTTGCACCATGTCA
CGACTAAACCCTTTTCTTTTGTGGACTCTGTGAACCCATCTGGCATTTTGTCAAGTTGAG
ATTCTCTGATAACCCAAAGGAAATGGAAATTGCTGTCCTTCAACCCACAGGCTATCTCTT
TCATTTGTTTGGCTGTTAGTGAGACCATGCTCCCAAAAGAGATATACACCACCGATTGCT
TCGGCTTTGTTTCAAGCCATTCAATGCACTCTACGCTGAGTGGCTTCCACAGACTCGATC
CATATCCTCTGTCGTCTTTGATTCGCCCGTCCAAGTATGCTGATGGCACCATGGGACCTA
TCAACTTCGCTGGCCATAATTCTGATACGCTTCTCGCCTCCTGGGGTTCTAATTCTTCAA
AGGTATTTCCGAATATCCAATCAGCCTTCTCCAAGTTCGAAAACTGACTGAGTTTCATAG
CCAAGTATGCCGGGTAACTTTCCGGAAACCTTACAAAAGTTGGCAAGTCAGAAAAGTTAA

ACGGAGGCAACCCGGGTATGGACAACGGTGTGTTTCCCGGCTTTACAGGAAGAGTCAGCA
AGCCATGGTGTATACGACAGAAAATGTTACACACAGCAGCTGAATTGGTAAAGAATGCCG
CTCCATACAAGCCATGTTGCTTGGCAACATCAAGAGCCCAAGGTAGAAATGAGTCATACA
CGATGCAAGTGACAGGGGCGACAGAATTTTCATATTTTCTAATGAGTTGAGACAAAGTTT
TGGAACCATTTGTTGCAAAAGACTTGAGGAAAAAGTCCTCGTTTTGGGCTTCACTAAACC
CACCTTCATCGAACCCATCGGAGATGGGCTCAACTTGAACGTGTGGTGTACAAATGGAGT
GAACGGTGTACTGGGTGGTGGCTAATGTAGCCTTGACGCCTTTAGAAACCAAACGCTTTG
CGAATTGAAGAAGAGGATTAATATGGCCTTGGCTGGGATAGGGAACCAGAATTACGTGGC
TTCTGTACTCCTTCATGACTAAATTTTTTTAGTGCCTTTCAATCTCAAAGATGCTGAGCT
CCATTGCTACCATGCACGCCAAGCCCTATTTATAGTCGAAACATTTACAAGTTCC
>ntig_787_consensus
CATCGGTGAAAATGTCATTTCCTTGATTGAACTAAATGAAAATGTCAGTCGCAACTGATA
CAGCAATAATGAAGCAGTGACCAACTAACACACCAAGTTCCATGGCTATGACGATTGTCC
CCTACATACAAACCCAATGCTTTGACAGATATACAGTGTTTAGTTAGTTATTCACTAACT
GGTATGCACCACGCCATGATAGTGAGTTCAAAATTAAAACGAGAAATATCATCTTCAACC
TACCTCAGCCTCATAGTTAAGAAGTTACTCATAATTAATCCACCAGTGTTTGCAGTATGA
AAACAGATAATATATACCCGTTATTACTAAGTCTGCATGGTGAATAAACACTTGGGTCAA
TGAATCTGAGCTAGCTCCAACAGAAATCCTTCTCAGGATGCATTCAAAAACCACTGGAAT
AAGCATTTAAAATCTCGTTCCACTTCTTCACTTCATAAGCATCCCTACGCTTTGCCACAT
GACGGTGACCTGTTAATATAATTACAGGATGTGGAACTTGCAAAACTGAACCATAAGAGA
AAGTAGAAGGGGCCAAATCAACAATGGCATTTATGGTAATCTGATATCTTATAGGGTCAA
CTGTCTCAATCATCCAAAGCGGTGAGACAGGAGACTCTCCTTCACTTCTTGCCAGGCCTT
TTCAGCATCTGTTCTGCCCGATTCTTAGCCCTTCTCTTAGAGGATTTGGCATCAAAACCA
AATACACCCTTCATCAATGAGCTTACATCAACAGAGCGTTCCCCCATGATCTTATCATGG
TAAATCTTACTCCCAATCTCAGTAATTTGTTCCTCCACTTCTTCTAGTGGTTGGTCAATG
TTGAGCTGTAGCTCTCCTCGCCTGATGACAGTTACATTAAAACCATTTGCTTTGGCTTCT
CGAAAAGCATCCTTAGGAGGCCTTGCAACAACAAAGGCTTGGCCACTTCCGAGCATCTCC
AAATGGCTCTGAATGATATAACAAAGGTTTTTGCAATCACCAGCATCTTCAAAAGCAATC
ACATGGTGCGGTTTGGGTTCCAACTCAAGATCTGCAGCCATCTCCAGTGAATAGAATCCT
ATATTTTGTTCCCCGTCTTGAATTGTGTATAGTTCGATGCACAGCACATGCGGTAGATCT
AGCCACCATAGGTAATCCTCTTCTTTCTTTTCCGCATATTCACGGTATTTGCTCACTACA
GCTTGTGGTCCAAACAACTCCATCTCTCTTTTGAGCTTGTTGATTTTCTTCTCCATGAAT
TTCTTATTCCTCTCAGGTAATTTATTCATCAGGTCAGCAGCTTCCTGTATTTGTTCTTCA
GTGATCCATCGATCTAAATCCTTTCCCATATTTTTCATAATGGATTTAACTTCTGGGTCT
GTCTCTGCATTATAAGATTCCAAGAATCCACGTGACCATTTCTTTGTGTGTTGCCAATAC
TCCTTCCCCAATTTTTTACCAGATCTGACAGAACCATCGCTGCCTTCTATAACTGTCTTG
GAATGTTCTGGATCCTTATTTTGTGGCTTCTTCAAATCATTAGGAGCCAAAGAGGAAGAG
GCATGCTCATGGGAGGGAAACCTAGTTGATTTTTGACAAAATTTGTCCTCATCCTCCGTC
ACAGGAGCTTTTCCAGCAAAAAGAGCCTCCCTTTGTTTAAGGAAGTCATCCAGGAACTCA
GGACTTTTCTCCAATAGAGGCCCCTTTCCAGCGCGGTATGATTTTCTCTGGTGGATCATAC
AAGCTGATACCATCTGCTCCATAATCAAGATTTTCAATGTTTGAATGGAGCCATTCATGC
ACATGGGTTAACCTCTTATTCTCTTCCTCAACTTTCTTTTCAAGACCCTGAAAGAATGCA
AGTTTGTCTTCAGCATCCATCAAATAAAATGGATCTCGCCCCGCCAATTCATTTTCTCGA
ACTTGAAAAACAATTTCTCTCAAATTATCGTCTTTCATCCACTCAAGTTCACTGTCAGCT
>ntig_788_consensus
TGAGCTGCTGCTTGTCTTGGTCTTCTTTGCTCAGGTGGAGGGCCATCTCGTCGTCTCTCA
TATCTTTTGCTAACATATTTTGATTCCTTTCGTTGCTTTGGCTGATACGTAGGGTATGTG
CATGGAATTATCTCCCCATTTATATATTTATCACCTCCATAATCTTTATTTTTGACATCT
ATGTATGAGTCAGGCAACACCCAGAGAACACCCGGCAGTCCCTTGAATTTCTCGGAAGTT
TCTTCAGATACAGTGCACTGGAATCCAGTGTAAGTGGTCGTGCTGAAAGCATACATATTC
TTCTTTGCCTCTTCCATGCTGCCAAGAACGGTAGCAAGGGTGTTGAGATAAGTCTCAATC
ATTTGCTCCCTAGTTGGAGCTGGGTCTTTGGGAAACTCCATAACAATGAGCCAGTGGTTG
TAGTCACATCCCGGTAACAGTATCGTCTCCCTCGG
>ntig_789_consensus
CAGTAGATAAAACACATGAAATAATTAAAAATTAAGAAAGAATCCTGCATTCAGATGGTC
ATAACCTTAATACAGCAGAGTAGTAGATAATAATTTACACACATTTCTTAACACAGCCCA
ATACACAAAACACACAACAGCAGAAAATGAAGCTGACAAAATACATGGACGAAAAACTT
TTAAGGCCTGCTCTTGAAAGGAATGGCTCTGATGATTATCTGGTGGTACACGGCCGCCAG

GGCAGCTCCAATGAAAGGTCCAACCCAGAAGATCCACTGGTCATCCCATGCATGGTCTTT
GTCGAAGATGATGGCAGCTCCAAGACTCCTGGCTGGGTTGATGCCAGTGCCAGTGATTGG
GATTGTGGCCAGGTGGACCAAGAACACTGCGAACCCGATGGGGAGAGGAGCCAGAATAGG
GACATGAGAGTCTCTAGCATTTCTCTTGGCATCAGTTGCAGAGAAGACAGTGTAGACAAG
AACAAAGGTGCCAACAATCTCAGCACCAAGACCATCACCCTTGGTGTAGCCATGGTTCAC
AACGTTGGCTCCACCTCCCAACCTTCCATAGTTTCCTGAACCTTCAAAACCCTTCACAAC
ACCAGCACCACAGATGGCACCAAGGCACTGCATGATCATGTAGGAAACAGCCCTAGTGAG
GGACAGCTTCCTAGCCAAAAACAGGCCGAAAGTCACTGCTGGGTTGATGTGTCCTCCTGA
GATACCAGCAGTGCAGTAAACAAGGGCAAAGATCATACCACCAAAGGCCCAAGCAATACC
TTGGATACCAACTGTGGCACACTTGGTGTTGGACTTAAGGACGCCCATTACAGTCAAGAT
AGTGATGTAAAGGAACAAAAAGGTGGCCACAAACTCAGCAATACAAGCTCTATAGAAAGA
CCATGACTTGAGCTCACCAGGCTCAAAGAGTGGAGCTGGTGGTGGCTCCTTATAGTCTTT
GTCAGTCTGAGCTGAGGTTCCAATGGGTTGCCTCTCTGGGAACTTGTTAGCTCCGAGCTT
AACATCCTCCTCTTTGCCCTCCATAGCAGCCTCTCCCAAAACTCAAAAGATTATCTTTCT
ACAAAGAAACAAGCCCAACTCTAGGCTGTGCAAGCTGTTGCAGTAGCAGTAGTAGTGAAA
AAATTAAGGATAAAAGGTGGTTTTCAGGAGAGGCA
>ntig_790_consensus
GTGGGAAAATGTCCTTTGGCCATTAAAAAAAGTGGGTTCCCTTCTGTGATTCTTAAACGT
ATGAAAGCAACCTCATCATCCCCCTGACAGAAGTCAAATATGGCAGTATTTCTTACTTAG
AATTTCAACTTAAATTTTACAATGCAAAAACAAAGACCATACCTAACCTAACCTAACCTA
AACTTTGAGGTCGATCAAAATGGTGGATCATGGGTTCATTTTAGGGCAGATCTCTTCACG
GCCGTGCTGCAGTCTGTCCTGAGTGCTTCGTATAAATACAATGTTGTATTGACAGAAGCA
GTAGGATTTATCCGTTCATTATCCAGATAACAAAGACTTTGGTCAAGCTTCATCCAGCTT
GCTGCCTGCTGACCATCCACTAGCCTCATCAAACTCTGTTCTGCAGAAGCGTGCCATGTT
TTGCTTCTTCACCGCT
>ntig_791_consensus
AGACAATTTCGGATCAGTTTATGTCAGATCAATTCAGATGTAATTCAGATTAGTTTAGAA
TTAAAAATTTTCAATAAGACTCTAAATTAGATCGAGTTATGAATTGACTATGAATTGACA
GTTTCTAACCCAAAACCCGAGCTTCTGGTCAAAATGTTGCAATGCATCGAGGTGATGGGA
ATTGAATCTTCTTCCACTCTCAGCAACTTTGTTTCTTATCTTTTGCTGGGCAGATGGTCA
AGAAGATTACTGGTACAAGAGGTGCCTTCAGCTTGCTTCTGTTTCTTGGAACTACAGCTC
TCTTCTTCCAATGCTCTCAATTTGTTCTTGATTGCGGCTATTTTAGCACTGCGGTTCATC
TGTCCACCACTACCACCAATAATGCCTAATTTGCTTGCATCGCCCACCGCTTTGGAAGAA
TTTTGTGTTGTTTCTTCCAAGTAGTACTTCTCACTTGCTAGACGAACAACCAAAGGGCGC
CCACAAGCTAGTCTCCCATGCATCTTCTCCTTGGCCAATTCTGCTTCCTGCATGCTCACT
CTTTGGTGCTAAACTGAATAAATGCAAACCCTCGTGGTTCTCCACGCTTTGGCCCACGAG
TATGCCAGAGAAAATCCTCAGCTACAATCTTCCCATACGGAGAAAACATCTTAATCAGTG
CAGCCTCTGTTATCTTCAGGTCAAGATTTCCAATATAAAGTCTGCTTTCACTCTTCTCAT
CAACGAGGCCATTAGGATCATGAGTGATGAATTCCTCGGGAAAGACCATTTAGTCCTGAA
CTTCATCATCTAGCTTGTCAGTTCAATGAATAGAAATAATCAAATCTCTTTTTTAAGGAA
AACTTACAAGCATAGGGTTCAAAATAATAAAAGTTGATAGACTAACTAAAATGACGTCGT
TTCGATATATATTAATCAAATAACATTATTTTAATCAATTTGAACGACTCGGGTTACAAG
CTCGAGCTCGAACTCGAGCTC
>ntig_792_consensus
TAGCCAGAGAGACTGATGGTTCCTCCTTGATTATTCCACACTTGGAAAATACCTCCACCA
CTTCCTTCTCAGTGACATCATCAGGCAATCCGGTCACATAAACATGCGTATTGACTTTCA
ACTCAAACCAAGTATCAGGAGGTTTGTTAGCTTCCTTCTTCTCCATTTCCTTGTCTAACT
GTTTTCTCTTGCCATTATGTTTTGTTTCTGCAACCTTATTGGCACCATTATCTTGATCCT
CCACAGCGTCTTCCTTTACAGATGTATCAGCAACATGCACACTTGAAAAGACCTCTTCTT
CTTCCAAGAAAGTCATCTCTTCTACCCTATATTGCTCATTTCCACCTGACATGTTTTCCT
GAGGAACCCATGCTCTAAGACTCCTATCCCACTTGTATGTAGTACCATCGTCATCAGTGA
ATTCTTCCTCCCCTTCTGGTGATGCTGGAACAACATCGTGATCACCAACCCCCAAGTCAT
CACCAATGTTGCTGGAACCAGATCCGTTTTTCAATTTTTTTGCCTCTAACTCTGCCTCTC
TAACCTCTTGCTTCCATTTCTCAAACTCATCATCAACATTAGCAGGTACTTCCTGTTCAT
AGCTGGGAAAAGATCCCCTTTCCAAGCCGGTGGATTCTATTGCCTCCTGTCTCCCAACCT
GTGCATGATGCTCGAGATCCTTGCTGGGTTCTGCAGAGGAATAAGCAGCATCCGGTTGAG
AAATTCCAGACAACAATAGTGGGATTGAGGACAGTGGCTGCCATTCACTTCTTCCTTGAC
ACCAGACGAGGGTCGTTTCTAAGAGGTAGCCATTTAAGAAATGTTCACGCAACTCTGAAA

GTGCATAAGGACCAACATGTTGTTGGTTTTCGTCAAGTAAATACCACCCTTCCTCACCAA
CTATCTCGCGATGCTCATTGCCAGCTGGCTCTGCACCAGAAACTTGTTGATTATTGACGC
CGTTTGAAGAATTCATGTCTTCCCTTCCTCCCATGCTTCTCCGAAT
>ntig_793_consensus
TCCTGCAACATGGCATCAGTAACATCTGGAGCCAAATCTCCTACAAATATGGACAGATCA
GAACCGGAATCTACTCGCCTGTCACCCGCAAAGGTTGCCCAATTAAGACGGAAGGGCTGC
TCAGTGTTTGGCATCACAGAACCGCTATAGCTCTGCAAAACGTTTTCAGCTGCTGTACGT
GTAAAGAATTCAACAAACCCATACCCCTCTGACAGGCCAGTATGCTTGTTGCGTATAACT
TTAACATTTGAAACCTGGCCAGTGTGAGAGAAACAGGTATGAAGATAAGTCTCGTCCATC
CAATGGAACAAGTCACCGACCCAGATAGTCTTAGTCTCATCATTTGTATTACTACCGTTA
ATAGATCCATTTTGTTTCTGATTATTATTATTATTGTTGTTGTTGTTGTTGTTGTTATTA
CTATGCTTCCCGTTTTGATGGTGATGCTGTTGTTGCTGATAATACTGCTGCCCACCGCCA
TAAGCCGCCATGTAGTGTTGCGGATACATCATCATCTGTTGTTGCATCATGGCCATTGCC
GCCGCCGGATACTGCATCATACCCATTCCCATCCATTGCTGAGGATGTTGATGATGCTGT
GGGACCCACTGGTAAGACTGAGCCTGCTGCTGCTGTGCCGGCCCCGCTGTTGGTGCGCCA
GCGGCAGCTCCACCACCAGCTGCTGGTGGTGGAGCTTCCGCTGGGGCACCAACAACGGGG
CCGGCGC
>ntig_794_consensus
TGACACAATTTTCTCTTTTATGAATATATTAGGGGTGCATTTATCATTATACTTTGTGTT
TGGCTAGACATCTGTCCATTTCTTCTACCCACAAATAAGGGAAAGATCATATAGAACAGC
CGGACAACTGGTTGGTATTTAATTCTAACCGAACAAAAACATACAAAACAATCCCCAAGT
TTTCAATTCAAGCTTTCTCGTGAACAGACGAAGGGATACTCGGAAAAACTATTTAACAGA
ACAAGACAACGATACATTACCCTGCCCAACTGCAGAAGCAGGTCAAACTCTTAATATTGT
CTTCAAAACAACTTTATTTCAACTCAGCTGTCTCTACATAAGATGCAAGCAACACACTCC
CATCAAGCAGGCATATAAGATTGCCATTCCACTTAGTGGCTCTTTGACATGTGAACAGCC
AGATCAACCACACGTGTGCTGTAGCCCCACTCATTGTCGTACCATGAGACAAGCTTCACA
AA
>ntig_795_consensus
CTTCATTTTATTTTTATCTTGCTCTTACCAAGCCAAACCAATCTCTTTGTTTTGATCCCA
GATTGGCTTTTATCTGATCTGATTCATCTTCACCTCACATTCCCACCTCTCGTCTTGATC
CCTTAAATGGCAACGCTGCAGAGCCTGATTGGCCTCGTCAATAAAATTCAACGGGCCTGT
ACTGTTCTCGGCGATTATGGCGGCGAAGGCTTGTCGCTATGGGAAGCTCTCCCGTCTGTT
GCTGTTGTCGGTGGCCAGAGTTCTGGGAAATCTTCAGTTTTGGAAAGCATTGTAGGGAGG
GATTTCTTGCCCCGTGGATCTGGTATTGTCACAAGGAGGCCATTAGTGTTGCAACTTCAT
AAGACAGAGGGGGGAGCTGACTATGCTGAGTTTCTTCATGCACCAAAGAAGAGGCTTACT
GATTTTGCTGCTGTGCGTAAGGAGATACAAGACGAAACAGATCGAATAACTGGAAAGACT
AAGCACATCTCTAACGTTCCAATTCATCTGAGCATATATTCTCCAAATGTTGTTAACCTA
ACCCTCATAGATCTTCCTGGGTTGACAAAGGTTGCTGTAGAGGGGCAGGCGGATAGTATT
GTTCAAGATATTGAGAATATGGTGCGCACTTATGTTGAGAAGCCTAACTGCATTATATTG
GCCATCTCTCCTGCTAATCAAGATATTGCCACCTCAGATGCTATAAAGCTTGCCAGAGAA
GTTGATCCAACAGGTGAAAGAACTTTTGGTGTGCTGACAAAACTTGATCTGATGGACAAG
GGAACCAATGCTGTGGATGTTCTCGAAGGAAGGCAATACAGGCTGCAACATCCCTGGGTT
GGAATTGTAAATCGTTCACAAGCTGATATTAACAAGAATGTTGACATGATTTTTGCTCGC
CGAAAGGAGCGTGAATATTTTGAAAGTAGCCCTGACTATGGACATTTGGCAAGTAAAATG
GGATCTGAATATCTTGCAAAACTATTGTCACAGCATTTGGAAACTGTTATCAGGCAACGT
ATACCCAGTATCATTGCATTAATAAATAAGACCATCGATGAGATTAATGCAGAGCTGGAC
CGGATAGGCAGGCCTATTGGAGGTGATTCAGGGGCCCAGCTGTATACGATCTTGGAAATG
TGTCGTGCATTTGAGCGTGTATTCAAGGAACACCTGGATGGAGGGCGTCCTGGTGGGGAT
CGGATATATGGAGTTTTTGACCACCAACTGCCAGCTGCTTTAAAAAAGCTCCCCTTTGAT
CGGCATCTCTCCACAAGAAATGTCCAAAAAGTTGTTTCAGAGGCTGATGGTTACCAGCCA
CATCTAATTGCTCCTGAACAAGGTTACAGAAGACTCATTGATGGATCTATCAGCTTTTTC
AGAGGCCCTGCTGAAGCCTCTGTGGATGCTGTACACTTTGTATTGAAAGAACTCGTACGG
AAGTCTATAGCCGAAACTGAGGAATTAAGGCGGTTTCCAACTCTTCAGTCTGACATAGCT
GCTGCTGCAAATGAAGCTTTGGAAAGATTCCGTGAAGAAGGCCGTAGAACAGTTTTAAGG
CTGGTGGATATGGAATCTAGCTACCTGACAGTTGAATTTTTCCGAAAGCTTCATCTTGAA
CCCGAGAAGAGTCTTAATCAACAAGGCCCAAATGCACCAAATGGACCAAATGCTCCAAAT
CAGGATCGATATTCTGACAATCATTTTAGGAAAATTGGATCAAATGTCAGTGCTTATATC
GGCATGGTTTGTGATACACTGAGAAATTCAATTCCCAAAGCTGTGGTGTATTGCCAAGTC

CTCTCGGCCAAGAAATCACTACTCAACAACTTCTATGCTCAAGTCGGAAGGAAGGAGAAG
GAGCGGCTTGGTGCAATGTTGGATGAAGATCCACAACTCATGGAGAGGAGAGCAACCTTA
GCTAAAAGGCTTGAGTTGTACAAGCAAGCTCGAGAAGACATTGACTCGGTAGCATGGAAA
TGATTGATTTCCAGTTGGGTGAGCCTGCGTTCCAGTTGGTGACCTCCTCTTCAAATCATC
ATCAAGTACAGGATCTTGGCTTTCTGCAGAATACAAAAATCTTTGGAAGAATTCCCAGAA
CCAGAAGCAACCTGCAATGAAGAAACTGGAATACAGCACTCTGGTTGCCCACACTGGTGA
ATACAGTAATATACATAAATACAGGGTAGTCCAATAAAATCCACATGTGTTATGGCCTTT
TTACAGAAAATACATATCTGAACAACAGACAACACATCATGAGAACAAATCAAAATATGG
AACGAGAAAAAAAATATGGAAGAAGACACCGCAACTGGCACACATCGTAGAGCAAAACCA
TATTCTTGAGCAGTCAGTCAAGAAACTGACTATGGTCACTTGTTATTTAATCATGGGTCT
GGGTCTGGATCTGAGTCTTGCCTGCTGGCTAAGCGCAACTCATGCAATTCGTTAAGTAAC
CACTCCTCTCCAGTGTGCCCACCTAACACCAGAACCCTAGTCCCTCCAACCACACAGGTG
CTGTGACCCCAAGCAAATTTAGGTGGCTGACCAGGAACATTGAGGATCCTCCAAGATGGT
TTCGCCTCGGAAGGATCTAGAAGGAATAGCTGAGAAGGAGAGTGCAGCCCGGCAATGGAA
CCACCAAAGATGATAATCCTTCCGCATGGCATGCTGACGGCAACATGGTCAAGTCTAGGT
GGGGGAACCACAGCACTCTGGCTGCCCACTCCTGTGAATGCGCTACACTCCAGTTGCTTC
CACTGTGGTTCCTCATCTCCCAAATCAATGGTATAGGAATCACCTGATCGCAGTCGCAAA
TGCCCACTGTTAGCAAGCCCACCAAACATCAGCACTTTTGTCCGACCATAAACCGAGAGA
GAATGCCCCAATCTGGGAGGAGGAGTAAATGAAGTGAGTATTTCTCTCCACGTTGGCTTC
TCTTTAGCAAGATCCAATAAGTACGTGTCACTAAGAAGTACCCCAGCATCAGTACACCCG
CCTGTTACCACCAATTTGGAACCTTCTATTGTGCAAGAGCTATGCCAAGATCTCGGAAGC
GGGGGAGTCCCACCAGAAACTTCTTTCCATGTTGGGTGCTTGGCATCCAAATCGAGAACA
TATACATCGTTGAGCAATCCTTGCCTTCCACATCCACCAAATACCACCAACCAGGAACCA
TTCAAACAGGAGAGAGTGTGGCCCCAGCGTCCTGGTGGAGATGATTTAACACCAACCCGC
TGCCATTCTGGGTTTGCTGCATCAAGATTTAGAACAAATGTGTCATCCATAGGCTGCATA
TTAACTCCTTCTCCTCCAAACAATACTAGTCGATTCCCAGCAGCACAGGCACTAAAATTG
CAACGTGAAGGCTGCACTGCTCCTCCGACTGTAAGTTTTCTCCAGCAAACTGCCTCAAGG
GTAGTCAATTCCCTAGCCAGACGTCCCCACCCTAACTTCTTGGTCATCAGTTCTAACGCA
CCTGTAACGTCCCTTCCCCATGCATTTTGACATACCATCTTCTTTACATGTTCATTCTTT
GTCAACTGGCGTATCCTCCTACAGACGGATCCAATGGATGCAACATCCCTTGGTGTCAGG
CGTGATAAAATGTTGTGAGCCAGAACTTCATCTGAGAGCTGAAGTATTCCACATATTTCT
TGATGTTGAATGAATGATGATTGCCCACTCAGGGAACTATATTGAGCTGACTGGTCATAC
TGCTGATTGCAGGTCTCTTTGAATACTGGATAAGATACATGTTTCAAATCTATTTTTGCT
TCAGAAAACACCTTGGAATTCGATACATTCCTCAAGACATCTTCTGATCTCAGAAACAAC
AACAGGATCTACCAAAGGATGCCTTCTTTGAGCACGAGGATCCCTGTATTGTAAGAACCG
ACAGTTTCGACCAAGGACTTCATCTGCGCGGTAACCGGTGGAGATCTCGAACACTTTATT
GACGTAAATAATAGGAAAATCATGCTCCAGAGCATCGGAAACCACAAATGACGTCGGAGT
TGTCGGATAGAAGAAGAAACCTTTCTCCAACGCAAGTTCACTCTCCTCTCCATCGCGCTC
CTCCGCTTTGTTAGTAGTACATTTCTGACGCTTTACGGAGCTCCTAATCTCGTCGTCGTC
GTCTTCTTCCTTGTTACCTGTCATGGAGTTTCGGCTTTGAGCTGAGAGAGAATTGTTTTG
GTGCTCGCTTAGTTTCCTTTGTCTCTGAAAGCGAAGATATTTTTATTGTGTCTATATGGG
TTGTCATAAATCGGGC
>ntig_796_consensus
TTTAATCATGGGTCTGGGTCTGGGTCGGGGTCTGGGTCTTGTCTGCTCGCTAGGCACAAC
TCATGCAATTCATTGAGGAGCCACTCCTCACCGGTGTGCCCACCTAATACCAGAACCCTA
GTCCCTCCAACCACGCAGGTGCTATGACCCCAAGCAAATTTAGGTGGCTGACCTGGAACA
TTGAGAATTCTCCAAGATGGTTTCTCCTCAGAAGGATCAAGAAGGAAGAGCTGAGAAGGA
GAGTGCAGCCCGGCAATGGAACCACCGAAGATGATAATCCTTCCACAAGGCATGCTGACT
GCAACATGGTCAAGTCTAGGAGGGGGCACCACAGCACTCTGGCTACCCACACCAGTGAAT
GCATTACACATCAGTTGCTTCCACACTGGTTCTTCATCACCCAAATCAATGGTATAGGAA
TCACCTGATCGCAGCCGCAAATGCCCACTGCTAGCAAGTCCACCAAACATCAAAATTTTT
GTCCGACCATAAACTGAGAGAGAATGCCCCAATCTGGAAGGAGGGGTAAATGAAGTGGGT
ATTTCTCTCCATGCTGGCTTGTCTGTCGTTAGATCCAGTAAGTATGTGTCGCTAAGAAGT
ACCCCAGCATCTGTGCAGCCGCCTGTTACAACCAATTTAGAACCTTCTATTGTGCAAGAG
CTATGCCAAGATCTAGGAAGAGGCGGAGTTCCACCAGAAACTTCTTTCCATGTTGGTTGT
TTGGCATCCAAATCCAGAACAAATACATCATTGAGCAACCCTTGCCTTCCACATCCTCCA
AATACCACTAACCAGGAACCATTCAAACAGGAGAGAGTGTGGCCCCAGCGTCCTGGTGGA
GATGATTTAACACTCACTCGCTGCCATTCCGGATTTGCTGCATCAAGATTTAGAACAAAT

GTGTCATCCATTGGCTGCATATTAACTCCTTCCCCTCCAAAGAGTACTAGACGATTTCCA
GCTGCACATGCACTAAAATTGCAACGTGAAGGCTCCACTGCTCCTCCTACTGTAAGTTTT
CTCCAGCAAACTGCCTCAAGGGTAGTCAGTTCCCTAGCTAGACGTCCCCATCCTAATTTC
TTAGTCATCAGTTCTAACGCACCTGTCACCTCTCTTCCCCATGCATTTTGACATACCATC
TTCCTTACATGTTCATTCTTTGTCAATTGGCGAATCCTTCTACAGACAGATCCAATGGAT
GCAACATCTCTTGGTGTCAAGCGTGATAGAATGTTGTGAGCCAAAACTTCATCTGAGAGC
TGAAGTATTCCACATATTTCTTGATGTCGAATGAACAAAGATTTCCCACCCTGAGAACCA
TATTGAGCTGACTGATCATAATGTTGATTGCAGGTCTCTTTGAATACCGGGTATGATACA
TGATTCAAATCTATTTTTGCTTCAGTAAACACTTGAATACCGATAATGTGTGTGACAGTT
CCATCATCATCATGAATGGGTGCAAGTCTTAGCCTGTTCACCAGAGTAGTACCATCTTTC
CGAAAATTGAGTAACTCACCTTGGAATTCAATACCTTGCTCAAGACATCTTCTGATCTCA
GAAACAACAACAGGATCTACCAAAGGGTGTCTTCTTTGAGCATGAGGATCCCTGTACTGT
AAGAACCGACAGTTACGACCGAGGACTTCATCGGCGCGATAACCGGTGGAGATCTCGAAC
ACTTTGTTGACGTAAATGATAGGAAAATCAAGCTCCAGAGCATCGGAAACCACAAACGAC
GTCGGTGTGGTAGGATAGAAGAAGAAACCTGTCTCCAGTGGAAGCTCACTCTCCTCTCCG
T
>ntig_797_consensus
ATTCAGTTAGGGATTTCCTGGTCAGGTGAGAGAGAAATGCCATCGCGCTGTAGAGAAGAC
ACCTGACCATTATGTGGGTAAAGGCCTTGTTTTTGGTTAGCAAAAGATGAGTACTTTCCC
ATTGGGTATCCCTTCAAAAGGCCTGCCGCAAAATCAGAACTCAACTGAGGTACCTTGCTT
AAAGGTTTTGTAGGTTGTGCATATGAAGGCTTGGAAATTTTGTTAGTAACATTTCCATTA
GGTTTCATATAGTTCAAGCCATTTGACTTTCCAAAAGCAGTAGGGCCACCATATTTTGCA
TTTCCAGAACTAGTGAAATTTCCGTTTTGAGCATCTCCAACATATGTTGAATCCCATGTA
TAACATGGCACAGCTTCTGTTCCATAGGAAACAGGGTGCTGGAGATAACCAGAGGACAAA
AAATAAGGTTGCTGACCAACACGTTGCCCGTCAACACCAACAAGAGTTCCCGTGCCATAT
GGATCATAGCCAGGCAGATAATAGATAAGTGAACTATTGTCAGATTGCATACCCATATGA
GAGGCATCTGTCTGATAATATCCATTGTTGTCCACTTGAGCAAAGGGTCCATTATCTGCT
GGGTACATATAGTTGTCAAAGACAGTAGGCTGATTGCCAGGACCATGCTCTCCAGCTGAC
TCTTGATTAGTCTCAACTTTCATCCTAGAACATGCATCCCCCAAGGACACCATGGTAGAT
GTTAAATCAGATGGTATTCCATTGTTTCCAGAAGCCACATCATGCTCGGCTGATTTACCG
ATGGCACCTGACTTTAATTCCATTGCCAATGGCTGATCTTTTTCAATGCTCTTTTCTCCA
GCCATCACTGTCTTCTTCAAATACCCAAGAAAGAATTAGCTCCCCTAAAATCGACGGATC
GAACCGCCGGAAGCGATCTGAGAGGATAAAGAATCAGCACGAGAAACTGCTCAACCGCTT
CCGACCGGCCGACGGAACGAACGGCGGCTTTCGACGTGGAGAGATCGGACGCAAGTGGCG
TTTATGTGAATTTCAGTGGGGAAAGAGAGAGGGAGAGAGGAGAGGAGTCATGAGAGTGAA
GAGTGGGATGATAAAGGAGAGAAGGGACGGCTGAGATTAGTTCCTGCTTTTGGGAGACGG
TACTGGTGGC
>ntig_798_consensus
CTACGTGTAACTGACATTGAAATTCTCCCCATAAACATCATAACTCATAAGCCCAAGAAA
ATTTAATAAAACCGGTCAAGAAACATAAGGTACAAATTAAGCGAATACCAAGAAATCTCC
TTTCAACTACAAATTTTGAGTAACAAGATCCAAAATAAACAAAAAGGTACATAGATTCCA
CAAAACCACTCTGCCATAGTAACCGAGTACACATTCTATCAATGGATTTATCCTTCAATA
CTTTACTAGCAGTCTCACTTCTTGGATTTTTTTGATGCCTCGGTAGAAGCAGCAGGTGGT
GATGTTGTTGGCTTATCTACTCCAGGTCCCTGGGCTAGTCTTTCCTCCCTCCTAGCTATT
TTTCTCTCCCTGCTAGCCTTATTCTTTGCCCTTTTAGCCTCAAACTGGTCAGACAAAGTC
TTTTCTCTTGCCTTCTCAGCCTTCGACTTGTGGATACTCTCCATCAAAACTCTCTTATTC
TTGAAGACATTACCCTTTACCTTCATGTACATGTCATGATACATGTGTTTGTCAATCTTC
TTAGATTCCCGGTACTTGCGAAGCAAGCGCCTGAGAACTCGCATCCTCCTCATCCACAGA
ACTTTAGTTGGTAATCTAGCTTCTCTTGTACCCTTACGCTTACCATA
>ntig_799_consensus
TGGCTGTTCTCTTATATATTATCAACAAGGATAGGATTCTATATACAATATACTCTCTCA
TGAATACAAATGCTTATATCATGTATGATCAGACATCCCAAAGCATACTTCTATCAATGC
TACAACATAAATATTACAGAATTAAACCTGAAAAACATCAATAGCTTCCATTTTCCAGTT
GCCATGCCCCAATGCAGTTTCCTACCATCTTCGACGCCTCCTCCCATACCAATATGCAAA
TGCAGCTACAGATGCAGCAAGAACAACACCAGCTGTTGCATGCCAAGGATATAGGTTTCT
CTCTAGAACAAAGCCAGAACTCTGTTTTGCCTCAGCAGGAGGTGGCAGCTGCACTGCTTC
CCGACTAACTGCCAACGCAAAATCCAAACCTTGCCTCTCCAGCTCTGCTATATCTTCAAT
TGCTGGCTTCAGCCTTGTGGCTTTGGCTAAAGCTTCATACTCTTCTTTTGTTCCACCTTC

AAGAAAAGATCCAACGAGATGACCCTTACTCACCCAGTAAGCCCCAAATGTATTTTGTGA
GAAATCCCCATAGTGGACTACTTCGCCTGCGTTGTCCCCATAAAATTGCCAAGAGAAAGC
AAAGACTCTAGAGTAGAAGAATGGCAGGTAGTCAAATTCGCCTGTTTTGTCGGGTTCCAT
TATTGCAGCTACAGCATGTCTTGCAGATTTTCGAGCAGAGTCAACATGTTCAAGCCTTCG
GATTTCACCAAATATTTTGACTGGAAATGCCGCAACATCGCCAACTGCATAAATTGAGCC
ATTGCTTGATTGTAATCGTCCATTTACTTTAATCCCACCCTTTTCCAAAGTTAGTTGACC
TTCAAAAAGGCTTGTGTTTGGACGTATTCCAATCCCTACTATAACCATGTCTACAGGCAA
TCGACTTCCATCCCTAAGATTAACAGCTACAACCTACCTTTAATGAATTTCACCCCTTTA
GTTGTGTAATAATCTTCATAATAACTTGCAATCTTGGGTGTAAATAGCCGGGCCATGAGA
TGTGCTTCGGGAAAAACCATGGTTACATTTATTTTATTGATCACCAAAGACGCAGCGCAC
TCCATTCCTATGTAGCCACCACCAATGACAACAGCATTCCCACCAACAGAAGATTGCATA
ACATCTACAATCCTATTTGCATCAGCCAAATCCCGTAAATAACACACGTTTTCAGCATCT
GATCCACTCACACCAAATTCCTCAAGCTTCAGAGCCCCGAGCACCTGTTGCAACAATGAG
GATCTTATAACTTATAGTCTCTCCAGCTGCAGTTAATAATGTCTGACGCCTCACATCAGC
AGACTTAACTCTAGTTCCAAGAACCAGCTCAATTCCATGTTCCTTGTACCATTTTGGAGT
TAACCTTTCCTCATTAGCACCAACACAAGTGTGAAATGATGGAAGGCGTGCAGCAGACTC
TGGAAGTAAATAACCCTTGCTTAACGCAGGTCTCTCATAAGGTGCAACTGTTTCCTCAGA
AATGATGCAGAGTTCACCCGGAGCAACTCCTCGCTTCGTGAACTCAAGAGCTGCATATCC
AGCTACTACTCCTCCTCCAAGAATTACATACACAAATGCTCTTCCCATTCTCTGAATAGC
CTCT
>ntig_800_consensus
GCCATCAGTAGTCTGCAATGGAAATTCCATTAAATCCAACATCCCAAAAACCTAACAAAA
TTCATCTTTTAACATGTACAAGAAGTTCACTCACATCAACTAAACTTTCCTGCGCAATAA
TGTGAACTCCTCCCTTTATTGTTCCGTTTCAGACAAGCAGTAAAAGGTAATCTTAAGCAT
CAGGAATCTGATTGACTAGATTTATACATCGACTGAAATGTTGGCCTTTCTGGGAGCAAG
CACAATGTTTTTGTTGCCAGGGAAAGCCTCTCGAGAGACGTCTACAAGGGCCTTGAAACT
ATAAGGTTCGTGCATTGCCAGTTCTGACAGAACTTTCCTGTTCAGTTGGATGTTCTCCTT
CAGTAGCCCATGCATGAAGTTACCATAGTTAACGCCATGTTGACGAGTGCCAGCATTGAT
GCGTTGGATCCAAAGGGAACGCATGTCTCGCTTCTTGTTGCGTCTGTCTCTATATGAATA
CTCCAAGGCCTTCTCAACTCTTTCTCTTGCGATTCTTATGCAGTTTTTGGCTCTTCCCCT
GAACCCTTTTGCCAGTTTGAATATCTCCTTCTTATTCATCATTTTTTTCTCTGTTTCTGA
ATTCTCCTCTCTCGCTTTTATTTTTGTTGCTTCTGCTCTGCCCTTGTATTCTGTCCTGTT
GAGACCCCGAGGCTTAAACTCAAACCCTAGCTTAGATATACATTCTTT
>ntig_801_consensus
CAGGAGTTCAAAAACTTGATAGCAAAAGCAAACATTGCAAATATGTCTTGACTATCCAAG
CTCCCACCTTAAACTAACTTTAACTAAGCCAAACCAGAAGGGTCCATAACTTGCAATTCA
CTTATCCTCCCCCCCTTTTTTATAAAGCCAAGGTTTTGAATACAATTTCATATCATGATC
TCTTCTCTATAGCCTATTTTTACCTGAAATCAGGTTGGTTTTACAGTTGAACTCTCAAAT
AGTGCTTGTGGATCAAGTAGTGCAGCTCGAGAATACAAAGGATAAGTGATTTTACTTAAC
AAAGGAAGGAACCTTACCGGTGACTCATAAAAACCAACTGGTGTTAAGTTCATTCTAGAA
AGACCTGAATCCTGTCACCCATCGCATCGTTGAAATCTAACAGAAGCTTTCCTCCTACTT
CATCGGCTGCCTCGTCCAAAAGTTCTTTGAGGTTTACACCCCCCAATGCTGGAACTATAT
TTGTGACTCCTTCCAAGAACCCAGCAGGAATTCTAACGTCGAGTCTCTCAAATCCATCTC
TACCAGTTATTTTGATTCTCAAAGTCCGAGACCAAATCCCTGTAAAGGGGCTCCTTGAAC
TATCTGCACCATAGTTATCCATTACTTCAGGAGTTATAACTTCCACTACAGGTTCTGCAA
GACTTCGGCCACTCCTTGCTGTATTATCTTGAATGCTTGGTTGTCTATTGTATGAGTAAC
TGCTCACTCTTTCTCATAAGCTGAGAGACGAGTAGTGGCATACTGTTTCTCTTCTTCATA
ATAACTGTTGAAACCTAAAATTTCTTTCAGTTCTTCAAAAGATGCCATACTTCCTGGAGG
AGGAATACGACCTCCCTTAATTGCAGCCAGTGCATCCTGCATTGCTTGAATAGATACTCC
TATCAAGGAAAGAGGATAAGCCACAAGTTTAAATCCAACCTCCTCAAGTTCAAGAGGATT
AAGAATCGGTGTTTTTCCTCCACCTTCAAGCATGTTGGCCATTTTTGGAACTAGAGGAGA
AATTTCACAGAATGCTTTCATCTCTTCTTTTGAAGCTAGTGCATCAACAAAAAGAACATC
AGCCCCAGCATCAGCAAAAGCCCTTGACCTCCAGAGTGCTTCCTCCAAAGACAATGCTTG
ACGAGAATCAGTTCGTGCAACAATAACGATGTCAGAGCCACTTTCTTTCCGAGCATCAAC
TGCCGCTTTTATATGCATCACTGCTTCATCTCTAGACACCACTTTCCTGCCACGTGTATG
ACCACATGCTTTTGGAGATACCTGATCTTCAAGAATGATTCCTGCAAAGCCAGCTCTGAT
AAATCCCTTGACAGTTCTCTTAACATTCATTGCATTACCATACCCATTATCACCATCCCC
TATAACAGGAATCGTCACGGCTTGGGTAATCTGCTGGCCCTGATCCACCATCTCTCCATA

GGACAAAAAATCCAGTGTCTGGCAAGGCCAGTCGAGCAGCAGATATAGAAAATCCACTTGT
AAAACAATAAAGAAAGCCAGCATCTTGAACAAGCTTAGCGCTGAGAGCATCGAAACAAGC
GGGGCCCTGATGGACCCCAGGCAACTGAAGAATCTGCCGGAGAGATTTAGACGGAGAAAC
TCTCTCCGGCAAAGAAGCTACGATGGGTCTGTGATGGGTTCTTGTTCTGATATTGTTAAG
CTTTTGAAACGTGGCGTTGGAGGGCAAGAATTGTGACTGTTTGAGAGAAAAAGAAGGAGC
TTTGAATTCCAGAAACGGTGATGGGTTAGAGTGAGGAGGACACATATTACTGGAAAGTGC
AAGCGAAAGTGAAGAATTCATTTGTTTTGAGTTTGCAAATGATGAGTTTAGCTTAAGAGT
GAGTCAAGATGAGTTGAGGG
>ntig_802_consensus
TTTTTTTTTTTTTTAACAAGAACCATTAAGAGATAATTTTTCATTAACACCAACATAAAA
AGATTTAAAATACCCAACTATCTATAACTGAGAAATTGACAATCTCCACGTAAAGATCAT
AACTATGAGCCAAGAAAAACTAATAAAACTGGTCAAGAAACATAACGTACAAATTAAGCA
AATACCAAGAAATCTCCTTTCAACTACCATGTTTAAGTAACAAGGTCCACAAGAAACTAG
AAAGAACATAGCTTCAACAAAACTANNNNNNNNNNNGTAGAGGCTGTAGGTGGTGCTGTT
GTTGGCTTATCTACTCCGGGTCCCTGGGCTAGTCGTTCCTCCCTTCTGGCAATTTTTCTT
TCCCTGCTTGCCTTATTCTTTGCCCTTTTAGCCTCAAATTGGTCAGACAAAGTCTTTTCT
CTTGCCTTCTCAGCCTTAGACTTGTGGATACTCTCCATCAAAACCCTCTTATTCTTGAAG
ACATTACCCTTCACCTTCATATACATGTCGTGATACATGTGTTTGTCAATCTTCTTTGCT
TCCCGGTACTTGCGTAGCAAGCGCCTGAGAACACGCATCCTCCTCATCCACAGTACCTTA
GTTGGCAGCCTAGCTTCCCTTGTACCCTTACGCTTACCAT
>ntig_803_consensus
GGAATCATAAATCAAAAACTTGACCACCTAACTGACCCCAGCCCGTAGTGTTCTTTAAAG
CAGGAAGTTCTGAAGGGGATCCAAGAACAAGCCCGGAATCTGCTGGTTCATTACGTATTT
GAGATAAAAGAAAGATGATATAAAAACCCTTTCTCCAAGTTTATAACCCATTTCTAGAAT
TCACTATCAAAGTTAAATCCAAGTTAAGATTGAAAAAATCGATTGGAGGCTGCTTTAATT
TGCCTCTGATTTCCATTTCAATAAGAAAGAATTACAAAGAACATAAAACTACAAATATAT
ATAGATATATTTTTATTAATTCCAAAATCTGACTCACATGGCAGCATGAGGCGGGTGAAT
TGTGGAAGTTGAGTACACATCACAGCTCACAGCTACGCCTTGGATGCTTTCTCTTTTACA
GCATCGTATGCTTCTGTTGCCTTATTTTTGGCTTCATCGTAAACATATACTGCGTCTTCT
TTTCCATGTTCCATTGCTTCTGCCATGCTCTCTTTAGCCTTTTCTGAACCACTCTTCACT
TTTTCTTCAGCTTCAGCTGCGCCTTCGGATACCGAGCTCAATAACCTGCTTCCTTTCTGT
TCCAATGTACTTCTGGCCTCAGCCTTCCCTTCAGCATATTCTTTTGCTTTGCTTAATTCG
TCACTCGTTGCGCTGGCTGCCTTCCCAGCCTTATCGGAAATATAACCGGCTGCTTCAGAT
GTAGCATATTCAGCATTTTTCATGAGTTCTTGAGCTTTCTCTTTTGTTTTATGTTGGTCC
ATTCCAAGATTCTTGTGGATCTTTTCTTTGGCCCAATCAGACCATGACTCATGCTTCTCT
TCGTCGTCATCGTCTCCAAACGCCACCCTTGTCGATACCATAAACAACATCACACATATC
AACAACATTGCTGATCCCTTTCCCATCTTTTTCTACCTCAAGATATTCAACCTGTTATGC
ATCTGAATCCCAATCTTGATGCTTATATTGAGGACAAGACAAACTGTTTGATTTGAAACG
TGGCAGTATACTGATAGAATT
>ntig_804_consensus
TGATGTCCTACATCTCAAGTTTTTCTTCTTCTAAGAAATCTCCAACAGGAACAAAATTAT
AAAACCATATCTTTTTAATCCATAAAAGAAACTATCAATATTATAGAACACACTGCCATA
ACATATCGTAGTTCTTATATAACATAACATAGTTCTTATAGATAGCAACAGTGACTACTT
CTCTAGCATTATACTGTTCCCTAGTATTCTGTTTCTAGCTATTCAACCGTCAGCATCTCC
TCATAAAAAGGGACTCAATATGGTCACTCACTACTGAAGGAAGCGTCACGCCAAAACTAT
AAGAAGATCATTTCTCATTTTCATCTTTGTCCTTGAAGCTTTGCATCTCCGAGATTTTAA
TATAGAGAATATCAAAACCTTACTTCCTGCTCCTCTTGGAGCCAGATTTCCTGGAGCTTT
CTCTTCCACGTTTTCCAGTGGAACCAACTCCAGATTTGTTTGGATTAGAGTGGGCACGAT
GAACAGGCACATGATGCCCAGGAACCAAAGCAGGGAACCCAACAGCAGGGATTCCAACAA
GAGCACGGCAGACTCCTGCTTGGCCGAGGATGCACCAAATGCATTGCCCTTTTGTAAAGT
AAGACGAAGAACTTGATTTTCTGCAACAACACACTGAAGCAACCGCCCCAGTCTCCTGCA
TTCACCCTCTAAGTACCGACTCTTCATTTCAAGATCCTTTACATGCATCTTCTTCCTCTC
CCTCGATCTAACTGCTGCATCCCTATTTCTTAACTGTCTTCTCCGTTTCTTGGAGATGGG
ATCGTCAGCGCAGCCAGCGTCGCCATCCTTATCATTGTTACTGGTATTATTATTTTCGCT
GCTATTGTTATTGTCATTGGCTTCTATTTCGTGGACGTGCTTCTCTTCCTCCTCTGGAGT
AACAGGAACAGATTCAGCGGAAACATTAGAGTCTTTATGAGTAGCCGCTGCCACGTCATC
GACCTCCGCAGAAGCAGCAGCAGGTGAATCAACAAGTATATCTGAAAAGAAATCGTCCAG
CGATTGCTGATTCAGCGGCTCTAATTCGTGGTCGTCATCATTGTCCTTCATTAACAAGCA

CTCAATCTCCGCCGCCCATGAAGACCCCGAAACGTCTGACGAAGGATTCGAGAAAGTCTC
CGATGATAATGGATCCGACAGTTCCGTTAACAGATCGTTCCAATCGATATCGTATTCGAT
AAACACGTTATCTTCCATAACTATTTACTATAAAAAATTAAACAAAAAAATGAAAAAAAT
AAAGGAAGAAAGAGGACTATGTTTCATGGATGCTCAATGG
>ntig_805_consensus
CTACCAATATTTCAAAAATAACTTAAAAAGAAAGCACAGAGAGCAGAGTTTGATAACATA
TCATAATTATTATTGATAGCAGCACAAGTGACTTTCTGCAGCATTAGAAACTGTTCCCTA
GTACTTTTTTCTAGTCATGCAACCTTCAACACCATCCTGCCAGATACAAAGGACTCAATA
TGGGATCTACTGAAGGAAGTCTCATACCAAAACTCTAAGAATATCGTTTCTCATTTTCAT
TTTTGTCCTTGAAGCTTTGCATCTCCGAGATTTTAACAGAGAGAATATCAAAACCTTATT
TCCTGCTCCTCTTGAAGCCGGATTTCCTGGAGCTTTCTCTTCCATGTTTTCCATTGGAAA
CAACTCCAGATTTGCCTGGATTAGTGTGGGCATGGTCAACCGGCACATGATGCCCAGGAA
CCAAAGCAGGGAACCCAACAGCAGGGATTCCAACAAGAGCACAGCAGACTCCTGCTTGGC
CAAGGATGCACCAAGTGCATTGCCATTTCGTAAACTAAGACGAAGAGCTTGATTTTCTGC
TACAACAAACTGAAGAAACCACCTCAGTTTCCTGCATTCGCCTTCTAAATACCTACTCTT
CATTTCAAGATCCTTCACATACATCTTCTTCCTCTCCCTCGATCTAACTGCTGCATCCCA
TTTCTCAACTTCCTTCTCTGTTTGTTGGAGATGGGGTCATCAGCCCCAGCGTCATTGTTA
CTGGTGTTCTTTTGACTGTTATGGTTCTTGTCGTTGGTTTTTTCTCCTTCATGGACCCGC
TTCTCTTCCTCCCCCAGTGCACCAGGCACAGATTCATCGGAAACATTAGAATCTTTATCA
TTTGCCACGTCGTCAACAACCTCCGTATAAGCTGCAGGAGGCGGCGAACCCGCGAGTATA
TCGGAAAAAAAAATCGTCGAGCGATAGTTGATTCAGCGGCTCTATATCGTCATGATTATCC
TTCATCAACAAGCTCTCGATATTCGCCGCCCATGAAGACACGGAAAAAATCGCGTAATGAG
GGGTTCGAGAAAGTCTCCAACGGTGATGGATCCGACAATTCCGTGAAAAGATCGTTCCAG
TCGATCTCATATTCGACAAATACGTTGTCTTCCTCCATCACTATTTACCTTAAAAACTAA
T
>ntig_806_consensus
CTTGCCAAGATTTCCCAACCAAATGAGAATAAACACAGAATAACAGACATAGAGAACAGA
AGCACGGTGGTGGGCATCTTCATGTTGACTGGGTCAAAAAAACATAAACACATAAATATA
CATGGATCTGGAATGGATAGACGAGAGAGGAAAACCCAGCTATGCTTCCCATCACTTAAT
TCAGTTGTACAGTCAGATCCCACTTTGATGATAAGCTGCTTTTACTATCGTGCCACAAAG
AATACCAACAAAGAAGGCAAATAGATTTCATTTCCAATCCAAATTATAAGAGAGAGAAAC
ATACAAGACAGTTGGTGGACTTGGCCTCTTTATAACGCAAACGCAGGGGAAGTGACAAAT
CATCAAATTATTGGACCCACCTCAACTACAGAAAAATCCATGCTATAAAGTACCAAATAG
TCAGGCTCGGAGTCAGAGACACTTCACGAAGAATGCAGCTTACTTCAACAACTGGTACAA
CTTCAACAAGTCTAACATCAAAGGTAAGCCCTTTCATGAACTTCAACTTCAGTCACCTAG
CTCAATTAGGATTGAGCCAAGGACCCAAGAAATGATCAAGACCATCCATCCGTTCAGAAT
TGGGATTCTCTTCTCATGGAAAGAAACCCCACATGGAGAGAGGGTTCTGCAACTTGTACA
ACACTTTCCACACATAATTGATGGGCCTCACTGTCGATTTATCACTCCATAGAGAGAAAG
AGAAGCCGATCACACAGTTCTACTATACTACATGGTCCATCAATATGCATATTTGACATT
TATTACCCATCTCACATTGCTAACAGCAATAATAAATTCAAAAGTCAGGTGCTACAACAC
ATCTGAAAAAAGAAAACATAAAAAAAAAAACAGAACGAGAGAGAGAGAGAGAGAGAGAAA
GAGAGATGGTTCAGTCTTGGGCATCTAGTTTTCACCAGCTACACCTTATCTAACTACAAT
TTAATCAAGATAACAGACTAAAATCATTCCAGAGTAAGTGCTTGCTTTGGCTTCTGATTC
TCTACTGCTGGTGTCAGAAGAATGATTTTATCAATTTCAGCATTTAATATCACCAATAGC
TCGAGATCCGTATCTATTTCTTCTTCATCAAGGACAAGCAATTTGTTCAGGTGAAAACAC
AGATATACACTCACTTCTTGATGTTGATCTACAACCCCCTCCCTTCTCTAGCAGCCTTCT
TTATGATAAAATGAATATGAAAACAAAAAGTAACATAGAATTTAGAAGTGTACCAATACA
AGAATATGAAACGGTCCAATACCTTGACAAAAACATCCACCTTGTGCATTAGCCCCCAAT
CCTCTGTGATCATTGAATGAACAGACAAAGAAGATATGAAGTCCTTTATGTTGCTTCCGC
CTTGATTGCCACTGCAACAGTCCACACTAAGAACAGAAGCTCTTTACTTGACTTTATTTG
ACAAGTACGCAGGACCTTCTTTGCGGGCTATATAAAAGGCACCCGCCGGTTCTGGTGCAT
CATTCATC
>ntig_807_consensus
AGAGACACAAAATAAACATATGGATCTGGGCAACTTGGTTGGCATGGATAGACAAGAAAG
AGAACCAGAGGGTTTGCTTCCCATCACTTATTCAATTGTACAGTCAGATCCCACTTAGAT
GATAAGTGAACTCAAGCTTACCTGCTTTACAACGCAAACGCAGGGGGTGTGACAAATCAT
CAAATTATTGGACCCACGTCAACTACACAAAAATCCGTGATACAAAGGGTGTAAATAAGT
CAAGCCAGGCCAAGGAATGCAACTTTCTTAACAACTGACACCAACTTCAACTAAAATCAA

GCTAAGGTAAACCCTTCCATGAACTTGAATTGGCTGGCTCTGGTTCTGGTAATTTACTTC
TATATTAACCCATCCATGCCTTGTGAATTGGGATTCTCTTCTCATGTAAAGAAAGGCTGT
GAGGGGGTCCCGCAACGTCTACTTGTACAACACTTTCCACACATAATTAACGGGCCTTAT
TCATCGTTCTGCATACAGAAAGTGAAGGCAACGAATACTGTATGGTCCATGAACATGCAT
ATTTGACATTTATTACCC
>ntig_808_consensus
ATGCTTGAGATCTTACATACCTTAAACAATGCTAGGTAGCATGAGAATGTGGATGGCATG
GAACTCACAAGGAAAGAGCACAAATTGCTTGACAGATGGTTGGGAAATCTGCTGAGCTAG
GTGAGCGGCTACAACATTAAAAGGGCGATGCTGACAGATTCAATCAGTCTTCCTAGCTTC
TCAGGATTCAGTAGCAATGTGATCCCGCCACATCTTTCTCCAAACCATCTTTCTCAAGAT
TGGCTCTCATCTTGAATGAATTGCACTATTTGC
>ntig_809_consensus
CCTTGTTCATACAAAATTATGTTTACTAATTTACATTATTCAACATCATCAAATGATATT
ATTGTCAAATAATTCATCTAAACTAGTCAACCAAAATATATATTGGCAGCATCTCTTCCT
TCCTTAAGAGTATACCAAACTTCACAAAATTCTTTCCTCCTTTAAAGTATAACAAGCTTC
ACAAAATTCTTTCCCCTGCAATCTCCAGATAGGGTAAAGAGATGTAGGTTTTACCGAACT
GTATATATGATCATTCTTTTTTCTTGCTTTTATCTTGTCCAATCGTGTGCAAAGTATTCT
GCATTCATTCTGCAACAGGAGCATCCTCATTCTGAACCTGAGAAGAGACTGTGGAATCAG
AGGCAGCATCGCTGTTGGTCAAGCTTGAAACTTCACCACCCAGAGAGGAGGCAGTATCAT
GAATGGTCACATTTGACACTTCACCACCCAGGGAGGAGGTCGCATCGTCGATGGTCAAGT
TTGAAACTTCACCACCCAGAGATGATGTTTCATCTTCGCCACTGATAGATGGTGCTGGGG
ATGCCTCAGGAGTAGCTATACAGTGGCCATTAGCTGGAGATGCTGGAGATGCCTGAGTAG
CCACACTGTTAGCTGGAGATGCCTCAGGAGTAACCACACTGTGGCCATTAGCTGTGGTTG
GGGAAGGCGCAGGTGAGGAGAAGGGAGATGTATCTGCCTCTTCCACTAGTTCTTGTAGGC
TCCAATACATTATGCTGGCAGTTAATGTAAGGATCATCTTCTGGTTTACCTCCATAATGT
CCTCAGGTAACAAGAAATTGGAACAGCCAAGTTTTCGAGCAACGCTAATGATGTATGTAG
CATTCAACCTCTTTTCCTCATCACTTTCACCCTTGGTTACAAGGTTCCAGTTCACCACTC
GCGGCTCCACAGAGCTTAGAAGCTCAAGGAAGAATAGTCCACTAGACAGGCTTTTATCCT
TGAAGCTCTCCATTTGAGAAGTTCTGCCTGTACTTTTGACCTTGCTGTTTGCCCAATTAA
GGATATCAGCATCAGTGATCTCCTTCCCTTGGGAGCGGGATCGCAAGTTTCTCAACAGTT
GAAGCATGTTGAATCTCATCAATTGCCATAGGAAAGCAAGTATGAGCTTCTTATTTCCTT
GTACGATATCATTTCCAGCAACATTCACAAGAGAAAATTTCAATTGCTTTCCAATCTTTA
CAACTTGATTGCAATTCTCTACTTTTCTAAACGGCATCTTAATTGGCGGCTTTGATGCAT
GCTTCCAGTTAACCGATCCAGGGGAAACTTTGTCCAGCACTTCAAGAAGAATCCATCCAG
TCCTGACATCCTCGAACACATTATTACAGTAAGTAGCAACTCCAAGACTGTTAATCCACA
GTCTAAAGCATCTCTCTTCTCTAGATGTAAGCACATCATCTGTCATCATCTCCGCAAACG
AAACCTTTTTGCTGTCTGTAGTCAAGCCACTCCTTTGATGAAATATTTGTGCTACAAATG
CAAGGTTCAAGTTTGCTGATCCCTCGACTATGTCTTTTGGGGTTAAATATCGTTTGCAGT
CCATTTTCTCAGCATGGTCAAGTACCAGCTTAGCCCTTTCGCTGGAATCCTTGGCATCTA
ATGTGGCTGGATTACAGTGTTCCGGTGCAAGAACATTAAGCAGATAAGCATAAGCCTCAC
CATCCTTCAAATCAGAAGAAAAATTTGTGACAGGCTTCTTGTATCCTGCCTTTTTCAAAT
GAAAATTCATCCATTTTAATAAGACTTTCTCAGGGGCTAATCCCATAAGCTCCTCAACAT
CAGTGCTGTCTTCCACCAATTCCACAAGTTGAGGTGTCTTCTTAAGGTTGAGATCTGCTA
GCAGTTGGATCTTTATAATTTGGGAAATCAATCCAAGTACCAGATGAGGTCTTCCTTCAA
CCAGATCCTGTGTACCTATGTTAACAACCGTACAGCCAATAGCCTTTGCAGAGTTGAGGC
ACAGAGTGTGATTCTCATTCCTCTCCCATGGATTGAGTATCCGTTTTGTGTTGATAGCTC
GTTCATCTATTGTTCCAGGCACAGCAACATTGATGAGCTTACATAGTAGAACTCCATCTT
TTGCAAGCTCAAATAAATCATTCGTAGCTGGGTCTAAGGGAAGAAACTGCTTCAGAAAAG
GATCATCCCCAAGATAGCTGTTTATGTGAGCAACATAAGATGCTTTCTCTGATTCACTTA
TCGTGTGAAGAAGTGTGGTCATGGCAGCCTTAAGAAACGATGAAGAGTTCTTTGCATTGC
CCACTTTTGTGCTAGCTCGACCTTGCAAATCAAGATATGCCCTCAGAAAAGCTTCAAAAT
CAATCTCATTGCTAGAGTCGGAATATGACTCTGCCAAGATCCCCTTGATATCCTCCTCGG
TGAACATCTTACTAAAAACCTTCAATTTCATCATTAAAGAT
>ntig_810_consensus
GTAACACGCCTTGAGAGTTGTGGGACTTGTGATGGTTCCGGAGCAAAACCAGGTACAAAG
CCATCTAAATGTACAACCTGTGGTGGGCAAGGGCAAGTTGTCTCATCAGCAAGGACTCCT
TTAGGTGTCTTCCAGCAGGTAATGACCTGCTCCTCATGTAATGGGACTGGGGAAGTGTTT
ACTCCGTGCAACACATGTTCTGGGGATGGACGTGTGAGGAGAACCAAACGCATCAGTCTG

AAAGTTCCAGCTGGTGTGGACACTGGTAGCCGCTTAAGGGTCCGTGGTGAAGGGAATGCT
GGAAGAAGAGGTGGATCTCCTGGTGACCTCTTTGTTGTGATTGATGTTATTCCAGACCCT
GTACTAAAACGTGACGACACCAACGTTCTTTACACCTGCAAAGTTTCATATATAGATGCC
ATTTTGGGGACTACAATCAAGGTCCCGACAGTGGATGGCATGGTTGATTTGAAGATCCCA
GCTGGGACTCAGCCGAACACAACTCTTGTTATGGCAAGGAAAGGGGTTCCAGTATTAAAT
AAAAGCAACATGAGAGGAGATCAGTTGGTACGCGTGCAAGTTGAAATCCCAAAAAGATTG
AGCAGTGAAGAGAGAAAGCTTGTTGAGGAGCTAGCTGATCTTAGGAAGGCAAAGACAGTG
AGCAGGAGATAGTTGAATGTACATTTTTGTATTTTCGATTCTTAATTTGCTTATCTTTGA
AATCATATCCCTGGTTTGATAGAAGAAACCTGGGAGCTATGGAAATCTCTATGAATCTGT
GGAAGTAACCAGGTGTTCTTAGTGGTACTAGGTAAAATTATATTATAATACTTTTGTCCC
ATAAAATGAAAGATATGTTCAGGGAAAGTTAGATGGCGAAATGGGAGATACATCCCTCTT
TAATTTTGGTTTTATACTTATACAGAATATGCCTATAATTTATTTTTTTGGGACTTGGAC
>ntig_811_consensus
CTCTTTTAATGTTGAAGCTTATGATTTTCAGGTTAAAATATAATGAAACTCGAGATTCAA
GATTGGGAAAATCCAGTGAACTGATTCCGGATAAGCCCAATTTATGATGCCACAATAAGT
GATATGGGCTACATTTGCTTGTACAATCTCATTAATAGTGTACAAAAAGGCGCGCCTTCA
AATCAATAAGGTAAACTCTTCAAAACCCTTCTTTACCTATAAAACCCTAACCTCGTGCTG
ATAGTCCTGGCGATGAGGTCATATGGTCAGGCCTTGTGGGGGAGCCCCCGCTGGAGTTTT
GGGCTTTGGCTTTTCCACAACAATAGCCTGGGTAGTGAGGACCATTCCTGACACTGATGC
TGCATTCTGTAAGGCACATCTTGCCACCTTTGCCGGGTCAATAACTCCAGCTTCAAGCAG
GTTCTCATACTTGTCTGTCATGGCATTGTAGCCAATCTCCCATTCACTGCTTTTCAACTT
CTCCACTACAACCTCACCCTCAACTCCGGCATTCTCCGCTATCAGTGCTGCAGGTGCTAC
CAGTGCCTTCTGCACAATGTCAGCACCTAACCGCTCCTCAGCATCCTCGAGCTTATCCTT
AATTGCAGGAACATGAGAAGAGAGGTGAACCAGTGCAGCACCACCACCAGGCACAATCCC
TTCCTCTATGGCAGCAAAAGTTGCATTCTTAGCATCTTCAATACGGAGCTTGCGGTCCTC
AAGCTCAGTTTCTGTTGCAGCTCCAACTTTAACAACAGCAACTCCACCAGACAATTTGGC
AATTCTCTCAGCTAGTTTCTCCGTGTCATATACAGAATCTGTCTCAGCCAACTCTCTCTT
CAGCTGAGAAATTCTGGCTTGTAACTCATCCTTTGATGCAGCATCAGCAATAATGGTGGT
TGAATCCTTGGAAATGGTCACCTTTCTAGCTATGCCTAGCTGCTCAACTGAGGTGTTCTC
AATTGACAAGCCCAAATCACTAGCTTGAAATTCAGCTCCAGTCAAAATGGCAATATCCTG
AAGAAGAGCTTTTCTTCGCTCACCAAAACCTGGAGCTTTAATGGCAGCGACATTAAGAAT
ACCTCGCAGCTTGTTCACAACTAGGGTGGCCAGGGCCTCACCAGTAACGTCCTCAGCAAT
TATGAGCAAAGGAGCTCTTAATTGTGTGGTCTTCTCTAACAAGGGAATAATATCCTTTAT
AGCCGAAATTTTTTGATCTGTGACCAACACTCTAGCATTTTCAAATTCAACAATTAATTT
CTCTGGGTTGGTGACAAACTGGGGAGAAATGTAACCTCTGTCAATCTCCATTCCTTCCTC
AACATCCACAGTAGTCTCAAACGAGGATGACGATTCAATAGACAAGACACCGTCAGGCCC
AACCTTGTCAATTGCGTCAGCTATCATTGTGCCAATCAATTCATCATTCCCAGCCGAAAT
TGTAGCAACAGCTTTAATATCTTCACGACCTTTTACAGGCCTAGCCTTCCTCTCAAGCTC
TTCAACTAACCCTTGTACGGTTTTATCTATGCCTCTCTTCACTGACACTGGATTTGCACC
AGAAGTGACACTCAAAAGTCCCAGTTTAATAATTTCTCGTGCAAGTACAGAGGCAGTAGT
TGTCCCATCACCAGCAGAGTCGTTTGTTTTACTTGCAACCTCCCTGATAAGAGCAGCTCC
AGCATTTTCCATGGGATCAGCCAACTCAATTGCTCGAGCTATTGTTACTCCATCATTAAC
TACCTTAGGACTCCCAAACTCATCCAGAACAACATTCCTCCCTCTGGGACCGAGAGTCAA
AGCAACCGCATCTGAAAGCTTATCAATCCCGGCCTGAATTGCAGTTCTAGAGTTCTGATC
AAAGGCAATCTCCTTTGCAGCTGCTCGAACAGATAACCTTCTAATATTTACTCTCTGGTT
ACTACTCTGCGGTCTTCTCCTCAAACTCGCCTCTTTGGGAGAACAGAAAATAGAAGCTGA
AGAAAGACCACTAGCAGAAGCCATTTTCTTCGCGTGTGGATAAGGAGGAAACTACGAGAA
AGCTGGTAGGAGTTTTGGTTAGGAGTTGGTTTGCTTTAGTTTTATTGACAGAGTCTGGAG
GTTCCAGAAGGCTCTTTTAAATGGTGGAAGCCTTCGCTCACCCGGCCCAACCAAATTTTG
CTTTCAAGATACTTGTTTTCGGTTTCGAGTAAAAGTTTGGTTGAAATTATATGTTTTTCA
ATTTAATAATAAATTGAGGATAATTTATTTACGACAAATACATATTTCCCCT
>ntig_812_consensus
CTCCTGAACTCAAACTCATAATTCATTTATTAAATTTCTCAGAGTCAATTGTCACAATAA
TTCAAATTCCATTCATTATACTTCAAAATGAACAAGACATTGCCAACTCACTCAAGCTTT
CTTATAGATATAACCTAATTTCAACACTAATCTGAATTATGATGCCACAATACAGGATAT
CAAGTAATGGAGACTACATTTGCAACAACAATCTCGTTACAAAAAAGGCATGTCTTCATA
CCAATAAAGGTAAAATCTTGTTCACCGCAATAAACTGATAAAACCCTAACCTGCTTCTGA
TAAGTACCGGCAATGAGATCATATAGTAAGGCCTTGTGGAGGAGCTGCTGCTGGAGTTTT

GGGCTTTGGTTTTTCCACAACAATAGCCTGGGTGGTGAGGACCATTCCTGCCACTGATGC
TGCATTCTGTAATGCACATCTTGTCACCTTTGCTGGATCAATAACTCCAGCTTCAAGTAG
GTTCTCATACTTGTCTGTCATGGCATTGTAACCAATCTCCCATTCACTGCTTTTCAACTT
CTCCACTACAACCTCACCCTCAACTCCGGCATTCTGTGCTATCAGTGATGCAGGTGCCAC
CAGAGCCTTCTGCACAATGTCAGCACCTAACCTCTCCTCAGCATCCTCAAGCTTATCCTT
AATTGCAGCAACATGAGAAGAGAGGTGAACGAGTGCAGCGCCACCACCAGGCACAATCCC
TTCTTCTATGGCAGCAAAAGTCGCATTCTTAGCATCTTCAATGCGGAGCTTACGGTCCTC
AAGCTCAGTCTCTGTTGCAGCTCCAACTTTAACAACTGCAACTCCACCAGACAATTTGGC
AATTCTCTCAGCTAATTTCTCTGTGTCATATACAGAATCTGTCTCGGCCAACTCTTTCTT
CAGCTGAGATATCCTAGCCTGTAACTCATCCTTCGATGCAGCATCTGCAATAATGGTGGT
CGAATCCTTGCTAATGGTCAACTTTCTAGCTATACCTAGCTGTTCAACTGATGCGTTCTC
AATGAGCAAGCCCAAATCACTAGCCTGAAACTCAGCTCCAGTCAAAATGGCAATATCTTG
AAGAAGAGCTTTTCTTCGCTCACCGAAACCAGGAGCCTTAATGGCAGCCACATTAAGGAT
ACCACGTAGCTTGTTCACAACCAGCGTGGCCAGAGCCTCGCCAGTGACATCCTCAGCAAT
TATGAGCAAAGGAGCCCTTAATTGTGTGGTCTTCTCTAACAAGGGGATTATATCCTTTAT
AGCTGAAATCTTTTGATCTGTGACCAACACTCTAGCATTCTCAAATTCAACAATTAATTT
CTCTGGGTTGGTGACAAACTGGGGGGAAATGTAACCTCTGTCAATCTCCATTCCTTCCTC
AACTTCCACAGTAGTCTCAAATGAGGATGATGATTCGATAGACAACACACCATCAGGCCC
AACCTTGTCAATTGCATCAGCAATCATTGTCCCAATAAGGTCATCGTTCCCAGCAGAAAT
GGAAGCAACAGCTTTAATATCTTCACGACCTTTTACAGGCCTAGCCTTCCTCTCAAGCTC
TTCTACTAACCCTTGGACGGTTTTATCAATACCTCTCTTCACTGACACTGGATTTGCACC
AGAGGTGACACTCAAAAGCCCAAGTTTAATGATCTCTCGTGCAAGTACTGAGGCAGTTGT
TGTCCCATCACCTGCAGAGTCATTTGTTTTACTTGCAACCTCCCTAATAAGAGCAGCTCC
GGCATTTTCCATTGGATCAGCTAACTCAATTGCTCGGGCTATTGTCACTCCGTCATTAAC
AACCTTGGGACTACCAAACTCATCCAGAACAACATTCCTCCCTCTGGGACCGAGAGTCAA
ACCAACAGCATCTGATAGCTTATCAATGCCGGCCTGCATTGCAGTCCTGGAGTTCTGGTC
AAAGGCAATCTCCTTTGCAGCTGCTCGAACAGAAAACCTTCTATAATTTACTCTCTGGTT
ATTACTCTGCTGTCTTCTTCTCAGACTCCCCTGTTTGGGAGAGCAGAAAATAGAAGCAGA
CGAAAGAGCATTAGCAGAAGCCATTTTCTGTTGTGTGTGGATAAGGAGGCAACAATGAGA
AAGCTGTTAAGAGAT
>ntig_813_consensus
TTGAGTAAAACTTCATCATTTATTGAATCCTTAAGTGGCCATTTCTTTATCATATATTTA
CATCTTGTTCAAAACATCAAACATCGAATAGGAGTATGTTCTACAACCAAAAAAGGGCAC
CGCCTCATTGCCCAAGTTGCCCGTGTAGAATCTACTTTAAAAGTTAAGCTTGCAAGCAAA
TACTACAATTATTTCACACTAGACTATAACCTCAATTGCGCCACTATGTTTAAGAGAATG
>ntig_814_consensus
TTGAGTAAAACTTCATCATTTATCGAATCCTTGGGTGGCCATTTCTTTATCATATATTTA
CATCTTGTTCAAAACATCAAAACGTTATGTAGGCTTACATCTCTCATCCTATTAAAACAA
ACATCGAATACGAGTATGTTCTACAACCAAAAAAAGGGCACTGCCTCATTGCCCAAGTTG
CTTCTACTTTAAAAGTTAAGCTTGCAAGCAAATACTACAATTATTTCACACTAGACTATA
ACCTCAATGGCGCCACTATGTTTAAGAGAATG
>ntig_815_consensus
TGCCCGTGTAGAATCTACTTTAAAAGTTAAGCTTGCAAGCAAATACTACAATTATTTCAC
ACTAGACTATAACCTCAATTGCGCCACTATGTTTAAGAGAATGTATCCTCACGATCCCCC
TCTGCTGTTTCCCCCTCTTCCAAGTTGCTGAACTCCAGGAAATGAGTTGGTCTGTGGTCT
TCATGGTAGTATTCTCTGGGTGTGCCAAGCTTCACCCCATATTTCATGCTCACAGTATGC
TTCACTCCCATAAAGTTATAGTTCCATGGACCATTGTCGGGAACCATGTAGAAACCGAGG
AACCGGTCACTTAAAAGCATCTGTACTTTCTCATAATGAGTTGGAAGATAGCCATGGGGA
TTGCTTCCAGTGTCCTTGTTGACTCGTCCCCACTCATAACCGCTTGGGGTAAGCTTATAT
GCAGTTAATGAGCANNNNNNNNNNNNNNNNNAATCCGGGCATGGCTAGTAAGATCCTGAGG
CGATAATTGGGGAAGTTCATTTGGTTGTGTGTGCATCCATCCCAAAGGCTCAAGATCATT
AAGGAAATCATGTTCAGGAAGAGCTGAAGGAAGATGAACTTGCTGATGAGTCCCCCACTG
TGGAGGCATGGCAATACATCGAATTTCCTTAACCTGAGGATTATCTGGTGGACTAATGCC
ATACAAGTATCCAGCAATTTGAGTTCGTAGATCTGCTACGCAGATGAACTTCTTCAAAAT
ATTCTTTGGCATGATATAAGTATATCCAGTTTCCTTTATATCCTCTGAGTTGACGTATAT
ATGATTAACTCGAAGATAGAGATTTGTAGCTGATATTGCCCTTACACGCCAATCTGTTTT
GGACCCAAATGCAGCTTGCTCATAGGGACTAGTGGTGGTGACAAT
>ntig_816_consensus

AATGCGTCATGTATTAAGCTACAAACATGAGTATGATCACTGATGTATCATCATATGATC
AAACATTTTTGAAGTAGAAATAAAATATTTTTCAATCACATGATAACACGTCAATATTTA
TACTCGTATTTATATATAAGACTACCCATAGGAGCCTTGAAGGAGCTAACTTCAGTATGA
AAACAATGGAAGCTAGAGAAAGAAGAAAATGTTCATAAAATTGCAAGCAAACATGTAATT
TATTTGGATATTAACAAAGGAATTCAAGAACCTAGTTGAAACGTTCAGATAAACATCAAT
AAAGAAGCAAGTGGCACAAACACATTTCATTTAAGAGAAAGACTCACCCACCTTTTAAGC
ACAAGGCCAGTTACAA
>ntig_817_consensus
CAGAAATTTATTAATATCATACTTAAATTTAGTCGTGAGCTAGCAAACTCACTGAATCAA
ACAAGAAGAAGCCCACAATTAAACTTGAGTTCCACCCATTAGCAGCGTTGGTATATACGT
TAACAAGCTCTTTCGCAATCTTATGTTCATAAAGATTATATAAAACAGGTATTAAATTGA
ATCTAAACCTAGATGCAGGATCTAGAATTGCAATGAGGAATTCAGATGCAAATTGGAAGA
TTGTGATTGGGCATCATACTATAAGAAGCATTGGGCATCATGCTGACACCAAGGAACTTG
TACAGCACCTTCTACCAATACTTGAGGTAACCTTAAATTTCCCTAGGCTTTTCAGACATC
AAAATGCTATAGAAATAAAATCATCAGAACTTATATATGTTATATGCTATACTGTTAGTT
TTCTCTCATGAATATGAGCTCTAATTTCAGGCCAACAATGCTGATATGTACATCAATGGC
CATGACCATTGCTTGCAACATATAAGTAGCACCAGAAGGTAATTTACAACCTAGAAAAAG
TAGTGGGAAAGTCACATGTTTGCTTGCTGAGCTAGGCTCAACTTGATTAATGTTGAGCCG
AATTCAACTCGCTCAAGTTCAGCTCTCAAATTGAACACAAGCTTTATGTTGACTAGCTCA
ATGAGCTCACAAGCTCACAGTTCGACTCAAAAGATTCTCCATTCAAATATTGGCAATAAA
CAATATTAGCATAGTTTGATTTTGAGTAACAAACATAAATTCTTTCTGGATATATGCAGC
CCAATTCAATTTTTGACTAGTGGAGGAGGATCAAAGGCCTGGAAAGGTGATATTGATAAA
AAGAACCAAAATGGAACAAGATTTTATTACGATGGTCAAGGTTTTATGTCTATTCAACTT
AGGCAAGCTGAAGCAAGGATCCTGTTCTATGATGTTTTTGGTAGGGTTCTGCACAATCTA
AATCTATCCAAGCAACTCAATGCTGCCATGTAGTATTTGATTATGAATGTATGTAATGAT
GGTACTTTCACTTAATAAAATAATTTAGATCAGACCCTCATCTAAACTTACTGAAAATGC
ATACAATCTATGATGTTATGCAAATTTCATATAAAACTATA
>ntig_818_consensus
TGTATATGTATGTATGTATGTATGTATGTATGTATGTATATGTACACACACCAGAAACTG
AAGCTAAAATGGTGTAACAAACACTCTTTAACTTCAGGACCGCTGATTCTTAAGAGCTTG
AAAAACTCTGGAACTCTTATCAAAGATGTGGGCTCTCCTGTTATAATCAGTTGGTTCTTT
TCCTTCATCGTCTCCCATTATTGCTTCTCTTTCGAGGCATTCTAACAACTCCACCATTCC
GTTTTTCTCCTTGCACTTGTCTCGTAACTTTCCCTTTTGCAGCCACTCTTCTTCTTCTTC
TTCTTCTTCTTCATCCTTCATCTTATTAATCTCACCCTTATCATCCGATCCCAAACCCAC
ACACCCATTCTTCCACCGCTGCTGCTTTAGCCTTTGCCTCACTTTGAAGCTGTAAACTGG
ATGATTGTGATGATTATGAAGGTTTGGACATAGAGTTGCGGGTTGTTTTATTCTGGTCGA
TAGAGAAAAGTTGAATATTCTAGCTGATTCTATGCTCATTGTGATAGCTTAAGCTTAAGG
TATCACTTGTGAGGTTTATACAGAAAGCACTGTTAGTAGTAGCCCTCATAGTTCCACGTG
TATGTATGATGTTCAAAAAGTAGACGTGTAAGAAAACGGTGGAGCATTCTGGATAATGTT
TATCTTTTGTTGAAAACTTGACCCAAAATACACCACAAAGATACTGCATATGGTCAAACC
AGGATCATCAGGTCATAAAAAGGGAAGGTTTTTATTTGATAGGACAAAGAAAAGCTTCA
TTCTCGGCTC
>ntig_819_consensus
AGCCCAGAAAGGCAATTTTAACATTGCTTCAAATATCAGAACAAAATTGAGTACAATTAG
CAATGTCAAGCAATTGGGAGACACACCTGTCTAAACAAAGCAAACGCCTAGAGGGTTCTT
AAAAATATACCACAAGTAAAAAAATTGCTGACAATAATTTAAGTTATCCAATTAAAACTA
TGTCGATATGAAAAAGTAAAATAATGTATCAATACAAGTACATGTCCCCTACTTATTGAA
TCCTATAGTGATGTGACAGATTGTGTTCTGAAAATCTTTCTGATGAAGGTAATCGGTATA
GGCAAGTCAACCATCCACATTTTGTGGGGAAGTTGTGGCTGAAATCAATGGTTATTCACG
TTATTGAAGAAATGTTCGGATCGCTTTCTCACTGAAGCACAGTAGAAGCCACCGACAGCA
ATTTCTTTGTACTATGGGTTTTGCTGTACTTGCCGATGTATCTGCACAACCCAGTCAACA
TAACCACTGCTACCACTGATCTGGTCCTCAACAAGGTTTGCAAGAAGAAAGAAGCCTTCT
CTGGGTTGTTCACCTTGTCTTACAAGATGACACAGCTGATAATACGAAAGATCAATCCTT
CTAAGTCCCTCTAATATCTTTGCTAACTTTCTTGACATTTTATTATCACGCTCAGTAAGA
GCAACCTTGGATAACTCGGCCGCAAAGTCTTGGCCAAGCAGATTCATAGCTACATCAGGT
GGGAGCATTTTACCAAACCATATAATGAAACGGAAACCGTCATCATATATATAGAGTGCT
CTAGAATCTAGGCTCTCTGCAGCCAGTGGTAACCTCTTCATGTTATCTAAAAACTCCTCA
ACATCACCTGAAGGCATGACAAGAAAATCATCAACTCGTATCAAGCTGGGGTACAAAAGT

TTCAACAAGCTCTTAACAGGCAATGCCATCATAGTGTAACCTGCTGCACAGCGTTCATCA
AGTGAAACATCAGCATATCCTCCTCGTAGTGGTGTTGATTTACAAAGTGCTAAACCATAC
AGAGGCAAATACTTCAGAGATTCAGGATATATCATCCTGGCTCCCAGGCGATGCTGCACA
GCATGGAGATTCCGATAGTCCTTCAGAGCTTTGACAATCCTTTGCTGCAAAGAATTCCGT
GCATCTTCCAGCTTATGTGACAATGTTTTCTCAATTGCTAACCTAGAAAATAAAGAAACA
ATGGCACCAGTGTCTGCCTGGCGAAACATCTCACTTAGATCTGCAACAACAGGAGCTGCT
AAAGTATGTACCCGGATACGCCTCTCTCCACA
>ntig_820_consensus
CATGGAAAATGGTCCTTTGGCCGCTAACAACGGTCATAATTCCTCGTCAGGACGACCAAT
TTTACTTGATTATTGCTTTACATTGCGTTTTAATGATAATTTTGTATTTACGTAGTTTTT
TAACTTTTTGGTAACCCAAGGACCACCAATCCCCCGTCGGCTTGTAGGAAGATGGTTAAC
CTCCTCGGATCAACCTCTCCGTCTTGCGGGACACTGGGATGATGGTGGAAACCCATACAA
CATGTTCCACTACCCTGATGGAATCAGTAACGAGACAGCTGACAGAGGAAGTCAAGACAC
TGATGTTTAACTTCTCCAGAAGAGTTCCGTCACTAACGTCACATGATCATAGATTTCGAC
CAAATATTACCATAGCCTTGTTAGATGTTACTTCATGCCAACATTCCACTTATGTTAACA
CCACAAAAACGCAACAATTTTTAATGTGACTTGGAAACGCAGATCTTATCAGGTTTTTGC
AAATACATAGAAAGATCACATACCAAAACTAACACCTATAATGCCTATAACAGAAGGTAA
GTGAAAGTCGCCAATAATCTTAATTCTCTCAAGTAATGTGAACAACCACAACCCTTCCTT
CGTTCCTCTTCCTCACCATTACCACAGTCATCACTGCTGAGCACATTGCACCCTCTGGGC
ACCACCATGCATCTCCTCATCTTCATCGTACGCCTCCTGGCTGGCTTGCTGCTTCCTTCG
CATCTCTTCCTCAATGTTCACATCATGAAGAGTGGTTTCCTCACACTCATCCAGCTCCAT
ATCGGTCAGCTGCACTGAGGACCTTGGGGGTAGAACACCCTCCAATGCCTTACACTGATC
TGGGGTGAGCGATTCAGGGAAGTCGACGGTGAAATGAATGTACAGCTTACC
>ntig_821_consensus
GGCACGGGTATAAAATGTACATATCTATATATATATATTTATCAAGTTATAATTAGGTCC
AAAATACCAATCACCCATTAACAAACTAAAGTCTTGACAAGTTCAATCGGAATTCTATCA
TCTAAAACACATAAAATGTTCAGTCTCTTATTGGTAATATATACTACTTAGCAACTTGAA
AAATAAGGGTCTTCTTCAGCTAAGCCTTCTCCCCAGTCAGAAGATCCATGACAACACGCT
TTATACTCCCATTGTTCTTCTTGAGCAGCCTCTTGTTCATTTCTTCATCACAGAATCCCA
TCTCCTGTAGCTCAAACATTGGATCCCAATCAGAAACACCACAGAGATCATCCACAGATT
GCTCCAAGTCATACTCATTCTTCCTCAAGATCTCCTTGTTTAAATCAACCTGCTTGAAGC
CCATCTCCTCAAGCTCCTTGAGGAGAGTCTGCTCAACAGCATCTTTATCACTGATCTCCT
CAGGTGATATTGGCACAGTTGTAAGAGGGGTTGATGAATGAGATGCCTTGGCCGCGTCCA
CATCAGTGAGATCAAGGATGGGATATGGCACAGTTGGTCTAGACGGTGGCAGCAGCTGAG
CAGCAGAAGCTAAGCCACCATTACCAACCAGGAAGCTAACATTTGTAAGGGAATTCCCCT
CCTTTGGCTGTTCATCAGCCACTGGTTTTACTGGTTCAATAACTGGAGAAGGGATGCAAG
GCCCTTGCCAGCTTCCATCCACAATGGGTTGAACATTCATATCTATAATTTCACAGCTTT
TGGGAAAATTGCTCTCAGGCGGCAAATTCAAGTTCAAGCCTTCAAAGCTAACACCATAGC
CATCCTTCACAGACCCATCAACCTGGATAAGAACCCACACACGCTGTCCAAATTTCAAAC
CGGAAGGTGACGCCACCCTCCAGTAAGAGATATAACGACCAGGCAACTCAGGTGCAGTAA
AATCAACTGATATATCAACCTCATCTTCCACAGGCAAGCCAACAGCAGGAACCTTAATAT
CAACTGAAACTGCGTTACTAAACCTATCTCCCCCAATCCACATGAGTTGTGTTCCCTGGG
ACCACACTTGAGTGCCGCTATTGCACATCCGCCATATCTTAGTGAATGGGGTGGATGGAG
CCATTATAGTCCCATCTGCAACATTGACATCCAAAATAAACCGACTGTCTAACTTTGGCC
TGCCATCCTTGATTCCAATGCGTCTCATCATAGGAGGAGGTGTTGGGAAACCATTATGTG
GTGTTAAGGGAAAAAAACGCTTATAATTCTGTCTATGATCATATAACCCTTTGAATGGCC
TTGGATTTTGGTAATATACGGGCTTATCCAATCGAATGTAATCATTCTCATTGCCCATTG
CAGCAAAGCAGATACTGCACAGATCATAATCATCTTTCACTTTCGACTTGAATCGAGGTC
CAGTAATGGGATGAACACCACAGCCATCACATTGGATGCCCCTGTGGAACGTTCCAACCA
TGGCATTGCAATTCATGTTTGGCTGACCAATGCGGAGGTGCTGATGAATGCCAGCTGAGT
CATCTGACACAGGAATTCCACTAAATGGACACTCATTAGAAGCATTACCACCCAAACCCA
TGGCATGGGGTTGATCGGAATTAGAAGGCCTAGTTGGAGCATGAGAACTAGCAAACTTGC
CAATGTGTTGACCACCAATATTCTTTGCCTCATTCCTATCAAAGGTAGCAAGAATATTAT
AATCATTTGGTGCACAGCTCACATTTGAGCTTCCAGAAGGAACTGAACCAATCTGTGGGA
TTGTATTCAGGTTAACAGCAGGTGCTGAAACTCCTATCGAATCTCCAGCATCACAATCTT
GGCTACTCATAGTATTTAGGTCCATAGGTTTTGAAATTAAAGATACTTTTCTCAGGCTAC
CATCCCTTGGTGCATTTGGATCTTTAAGAACTGAAGAAGCCAAAAAGCTTTCAGATGCCT
CAGTGCGTTTACTTGACTCTGCATCTGCACCAGACTGGGAAACTTGATTCGGCTGAGAAA

TACCCAACTTCAAAAAGCACTCAGCAAGATCAGAAAAAATCGGACTTGAGGATGCTGTCT
TTGAAGCCAAGTCAAGAGATAGCTTTGACAGTATTTCAAACAGTGGCTCAGGCACCAATT
TCAATGCCTCAGCAAACTTATTGTTAATGTCTGGCAATGGATGCTGGACTCGAGGGGATC
TCAAGGGTGTAGAGCTTCCACTTGATCTTGTAAAAGGTTTCCCAGATTTTTCATTCTTTA
GCCGTACGTCAATTCTCAAGAATTTGAGACGCTGCTTCATCACATCAAGTAGATCATTGT
CATCAACAAGAGTTACCATGTCACCATCTTCATCAATGTATGTCAGCCCAAGATCAGCAT
CATGAGGAAAATTAAAGAGACCTATGATCCTCGCCCTTAATCCATCAATATCAAGATCCA
ACTTCTCATTTTCATCAATGCGAACGTTAAAGCGCCTAAGTGTATCTCCATATTTAACCT
TGATAACCATAGAAGACTCCATCGCCACAGACAGTTAGAAGTCTGTTCCCGCGAACCAGA
CAGTGAATAAAACCAACTTTCTAAAATAAATACAGAAAAAGAAAGACCCAAACCCTAATG
AACCTTAACCAGAGTTGGATCAAAAGTTAAAGAGCTGAAAAATTCAAACACCAAATCGGA
ATAGATTACGACGGTATATTAGATCGCTTAACCCGAATTCAACGCTCCCTTTCACACACT
AGAACGATATATGAAAGGGAGCGTTGAATTCGGG
>ntig_822_consensus
ACGACTATCTCTGTGGACTGCATAGCCCCGTTAGATTTTTTCTCTACATCTTTCTCTTTC
TCTTCCTCCTCCTCCTCTTCCTCTTCATCTTCCTCTTCCTCTTCCTCTTCCTCTTCTTGT
TGGTTTCTTTCATTACCAATCCAATCCCTCTCAGCCACACCTAACCTCTGCTGTTCCTCC
CTAATCTTTGCCACCAAAATCGCTTCCTCAGATCGCCTAATTAAAGTCTCTGACCACCTC
TCTCCTGGCCTGGCCATATTCCTCCCAGTCAAATCCTGAAACTTCCCGACATACTCATGA
TGCAGATACGGCTCCCTCTCCCTCATTGCATCCTCCGAAAAGTACTGACCATCCCGTATT
AATTGATTAAGATATGCCAGCCTCCTGTTCTTCACAGTCACCGACCTAGACCGCAACTCC
TCCGAAGTAGGGTTCATCTTGCTCCTAAGATTTTTTAAGTGCCAATTAATCTCATAGTCA
TTGTTGAGCGCATCAAACTTGCCAAGCTCATCACAAGTCAACTGCGATCCATATCTTTCT
AGGAAAACGGCGGCGTCCCGGGAGAGGAGGTCGAGGAGAATAGATTTGCGCTGAGAGGGA
GTGATAGCGTTGGATTGAAGAGCGCGAGGAAAGTAGAGATCGTCCAAACAAGAAAGTCTC
TCACTGATGCTTTCTTTCACTTCTCCTTCCATTTTTTCTCTTCCTTCACTTTCCCTTCCT
CCGACTTAATTATCTCTCTTATACATGTCTCCGTTAGTCGTTGGCGGTGGTGTGTCGCCT
GCTTCTGTACCCAGGTGCCGTTATAGTAATCCATTCTGGGTTATGCTACTAGCTGTCACG
TGACATGCAGGTGCCTTAGTTTCGATTCATTTA
>ntig_823_consensus
AATAATACTATAAACATGCACATATTTTAAACATTTTTTTGTTGATTTAAATTACAGAGA
TGCTGCATGAATGCAAGGGAGTGAGCATAACAAGAAACTAAACCCTAAAATATAAGTAGC
CTAAGCTTTCTAGAAATACAAACTAAGCTACCAACAAAAAGAGAGTCCATGTCCAACACT
GTGAGCAGACCAGTGATGAAAATGGGCAGCAGCAGTTTCACACAACCTTACAAAAACAAA
AACGTGGTTTTCATCAATTCACTCATGGAATAAATTTTTCATCTCCCACCTACAACAACC
TCGTACAAAAATATCCATTACAAAATTCCCCTCTCACTATATCGTTATCTTGCCTGTCAA
AAGAGGTTTTTCAGTAACTGACCGGCATAGCATGGTGTTTGCCCTCCACTTGTAGTTTGT
GGTTCATGACGCTTTGTTATTCCTTGGCAGCAAGCAGCTTTTCGATTTTTTTTGTTGGTT
CACTCTCAGTTGACAGAACTTACTAATGATAGCTCGTGGAGTTCACTTAACATCCATTCT
TCTCCCGTTTGGCCACCTAGGACTATAGTCCTTGTGCCGCCTACAACACAAGTACTGTGA
CCCCAGGCAAATCTTGGAGGTCGGCCAGGTACATTTAATAATCTCCATGTTGGTTTTTCA
TCGGTAGGGTCCAGAAGATAAAGCTGAGTCGCAGAGTGAAGCCCGGCTACTGACCCACCA
AAGATAAGAACTCTCCCACCTGGGAGGCTCACAGCCACGTGATCGAGCCTAGGAGGAGGT
GCTACGCCACCAGGGTTTCCAGCGCCAGGCATTCCACATCCAGTTACAGATCTCCAGCAA
GGTTCCTCCTCACTAAGATCCATTGTAAAGACATCACTAGAACGAAACCGCAGTGGACCA
CTCTTCGCTAAACCCCCAAACATTAATATTTTCCTACCACCATAAACTGACAATGTATGG
CCCAACCGAGAAGGGGGAGTCCAGGCCGCAGGTATCTCTCTCCAAACAGGTTTCTCCATT
GACAGATCAAGAAGAAATGTGTCACTTAAAAGTACCCCAGAGTCTGCACAACCACCAGAA
ACTATCAATTTGGTGCCATCAAGAGTGCAGGAGCTATGCCATGATCTCGGAAGTGGAGGT
GCTAATCCGGAGATTTCACGCCAAGTTGGAGGCTTAGCATCCAAATCCAGAACAAAAACA
TCATTCAGTAAGCCCTGCCTTCCACAGCCACCGAATACCACCAAATTAGACCCGTTTACA
CATGAAAGAGTATGACCCCAACGGCCCGGAGGAGGAGAGCTCACTTGGACATGTTGCCAT
TCCGGTTTACTAGAGTTCAGGTCCAATACAAAAGTGTCATTCATTGGTTGCATATTAACC
CCTTCCCCACCAAAGAGAACAACCCGATTCCCAACTGCACAGGCACTAAAGTTGCACCGT
GAGGGTTCAACAGCACCTCCAACTGTTAGCTTCCTCCATGCTGCAGCTTCAAGAGTAGTC
AACTCCCTTGCCAGGCGACCGCAGCCCAATCTCTTTGCACCAGGCACAGTCTCTAAAACA
CGAGTTGTTTCACTACCCCAGGCATTCTGGCAGACCATTCTCCAAAGGTCCTCATTTTTT
GTCAGCTCGTAAAGTCGTCTACAGACCGAACCAACTGATGCAATATCTCTTGGAGTCAAC

CCTGAAAGTATCTTAAGAGACAGCACCTCATCGCTCAATTGCAATATCCCACAAACTCCA
CGGGAAACATTTCGGTCTCCCACTGGGACAGGATGTGAAGTGGAAAAAGCAGGAAAAAAC
CGGTCAGATGCTTTTCCAGACTGCTTCACTGAATATCCTGGAACTGGACCCAGATCAACA
TTTGCTTCTGTGAAGAACTGGATTCCAATAACATGAGTTATTGTGTCATCATTTTCATAT
ATAGGAGTCAGCCGCAATCTGTTCATCAGAGGAGATCCATCTTTCCGGAAGTTCAACAAC
TCACCTTGGAATTCAATACCCTCCTTGAGGCATCTCCTAATTTCTGAAACAACTGATGAG
TCTACTAATGGATGTCTTCTCTTAGCAAATGGTCCTCTACATTGCAAGAAACGGCAATTT
CGACCGAGAACCTCCTCGGCACGATACCCAGTAACAGTCTCGAACACAGAATTAACATAA
ATAATAGGGTGGTCAGGCTCAAGTGCATCGGTGACAACGAACCCACACGGTGCCGCTTGA
AACAAATTCTCCACGGGGAAAGGGAGGGGACCAGCGGGCTCATCATCTTCGTCGTCGTTA
AGAATGAACTCCTTGTCCTCATCCTCACTGCCATCAGAATTACTATCCCACTCCATGAAA
GACGGCGAAACCGGACGCGGGTTCAACCCATATGAATAGGGGGAAGAATAAGCGGAGTGA
AATGGCGAAAAGGAGGATACTTTGGGTGCTAAAATGAAAGATAACGTGGGGTGACTAGTG
TCTGGGAGGAATATATTTTGGGCTAACTAAGCTAAAGGTAAGGAAAGGACGAGCTAGAAG
TGACAGCGAGGGAGAGGGAGATG
>ntig_824_consensus
TTTTTTTTTTTAAATAATAAATAGCATTAGCCAATAGAGATAAAAAGATTTGATCTGTGT
TACAATAATACTATAAACATGCACATTTTTTAACACTTTTTAGTTGGTTTAAATTACAGA
TGCTGAATGAATGCAAGAGAGTGAGCATAACAAGAAATTAAGCCCCTAAAATACATAGTA
GCCTAAGCTTTTTCTAGCAAATACAACTAACCTACCAACAAAGAGTAGTCCAAGTCCATT
GTGTGCAGGGAGAGCAGTGATGACGATGGGCAGTCGCAGTTTCACACAAACCTTACACAA
ACATAAACATGGTTTTCATCAATTCACTCATGGAATAAGTGTTTTCATCTCCTACCCTCA
AAACCTCGTACAAGAATATCCATTACAATAGTCCCTCTCATTAGACCATTATCTTATCTA
TCAATAAAATTTCAGTAATTGACCATCATGGCATGGTGCTCATATTCAACCTCCCACTTG
TGTCATGGTTATGATATTCTGTGGTTCATGACGTTTTGTCATTCTTTTTGCAGCAGCTTT
CAATTTTTTTTTCGTTGGTATATCTCAAATGATGGAAGAACTCACTAATGATAGCTCATG
GAGCTCACTTAGCATCCACTCTTCTCCTGTTTGGCCACCTAGGACTATAGCCCTTGTGCC
ACCTACTACACATGTACTGTGTCCCCATGCAAATCTTGGAGGCCGTCCGGGTACATTCAG
TACTCTCCATGTAGGCTTTTCATCAGTGGGATCCAGAAGATAAATCTGGGTCGCAGAGTG
AAGACCAGCTACTGATCCACCAAAGATAAGAACTCTCCCACCCGTGAGGCTCACAGCCAC
ATGATCAAGTCTAGGAGGAGGAGCTACTCCACCTGGGTTTCCAGCACCAGGCATTCCACT
TCCAGTTACAGATCTCCAGCAAGGTTCCTCCTCACTAAGATCCATGGTGAAGACATCACT
AGAACGAAAACGTAGATGACCACTCTTGGCTAAACCCCCAAACATCAATATTTTCCGACC
ACCGTAAACTGATAACGTATGGCCCAACCGAGAAGGGGGAGTCCAGGCTGCAGGTATCTC
CCTCCAAACAGGTTTCTCCATTGACAGATCAAGAAGAAATGTATCACTTAAAAGCACTCC
AGAGTCTGCACAACCACCAGAAACAATCAATTTGGTACCATCAAGAGTGCAGGAGCTGTG
CCATGATCTTGGAAGTGGAGGAGCCAATCCAGAGATTTCACGCCAAGTTGGAGGCTTGGC
ATCTAAGTCCAAAACAAAAACATCATTGAGTAGGCCTTGCCTTCCACAGCCACCAAATAC
CACCAAATTAGACCCATTTACACATGAAAGAGTATGACCCCAACGACCAGGAGGAGGAGA
ACTCACTTGGACATGCTCCCACTCTGGTTTACTAGAATTCAGGTCCAATACGAAGGTGTC
ATTCATTGGTTGCATGTTAACCCCTTCTCCACCAAAAAGAACCACCCGATTCCCAACAGC
ACAGGCACTAAAGTTGCACCTTGAGGGTTCAACGGCACCTCCAACTGTTAGCTTTCTCCA
TGCTGCAGCTTCAAGAGTAGTCAACTCCCTAGCCAGGCGACCCCAGCCCAGTCTCTTTGC
ACCAGGCACAGTCTCTAAAACACGAGTTGTTTCAGTACCCCAGGCGTTCTGGCAGACCAT
TCTCCAAAGGTCCTCATTTTTCGTCAACTCATAAAGTCGTCTACAGACAGAACCAACTGA
TGCAATATCTCTGGGAGTCAACCATGATAGTATCTTAAGAGACAGCACCTCATCACTCAA
TTGCAATATCCCACAAACTCCACGAGAAACATTTCGGTCTCCCACTGGGACAGGATGCAA
AGTGGAAAAAGCAGAACGGAACCGGTCAGATGCTTTTCCAGACTCCTTCAGTGAGTATCC
AGGCACTGGACCCAAGTCAACATTTGCTTCTGTGAAGAACTGGATTCCAATAACATGAGT
TATTGTGTCATCATCTTCATATATAGGAGTTAGCCGCAATCTGTTCATCAGAGGAGATCC
ATCTTTACGGAAGTTCAGCAATTCACCTTGGAATTCAATACCCTCCTCAAGGCATCTCCT
AATTTCTGTAACAACTGTTGAATCCACTAATGGATGTCTTCTCTTAGCAAATGGCCCTCT
GAATTGCAAGAAACGGCAATTTCGACCGAGAACCTCTTCGGCACGATACCCAGTAACAGT
CTCGAACACGGAATTGACATAAATAATGGGGTGGTCGGGCTCAAGCGCATCAGTGACGAC
GAACCCACACGGCGCCGCTTGTAACAAATTCGCCACGGGAAAAGGGAGGGGCCCAGCGGG
ATCATCATCTACTTCGTCGTTAAGAATAAACTCCTCCTCATCCTCATCCTCACTGTGATC
GGAATTACTATCCCACTCCATGAAAGACAGCGAAAGCGGAGGAGGGTTCAACCCATTTGA
GGTAGGGAAAAAAAAGACGAGGTTAAGTTGAGAGAAGGAGAAAAACTATACGGAGTGTAA

AAAAGAAAGAAAACGTGGGGTGAGTAATGTTTCAGGAGGATTTTGTTTTGGGTTAAGCTA
TAGGTCAGGAAGGGAGGAGAAGAGCTAGCACTGAGAGAGATGGATTCTTGAGAAATAAGA
TTTTTG
>ntig_825_consensus
ATAAAACCATATTTACATAAGGAGGTAGCCCATCACCATCATTGTCACCACCACCATCAT
CGTCATCATCGTCGGCGTCTCAGTGATGGTTCTTGCTCCTGGTCTCGCCACTGTAGAGCT
GGAAAGATCTATTTCAACGCGCTCATCAGGCAGACCGCCACGTTTTCCTCCCTGCTTCTG
GCCTTCGTTTGGTTGCTGAGAAAACGGTGAAAGGGAAAAACAAATACCAAAACACGTATT
TGAGCCTCATGTTTATTTTATTTATCACAACGATAAAAACATATTTTTTCAACTTGAATC
TTGTCACAACAAACAGATACAACATGAGAAACTTAAAAAGTTGTTAACTGGAACTACAAA
TAATCTTCTTAGCAACCAAACTGGGCACCGCCTGTTCCATCATAACATTTTATAGGGACA
ATATAGGAGGGCGGGGGCTAACATGCATGTAAGATGAAACATGGGTTATGTGCGTACCAG
TCATACACAGTGGGCGA
>ntig_826_consensus
ATGTCTCTCTGAATAATCCGTCTGTCAACGAAAATGATAGTGTTGATTAAATCAACCTTA
GAGTTTTACAACAAGAAGCCTAACCAGATAACTTATAATCAACTGTAATTGAAATATATT
GTTTTTTGACTAACAAAGGTCTTAACATGAACAGATCTTTCTTCCTCCTTTCTCACGCAG
ACCAATTCGGCAACACATTAATTATTTGTCGATTTGACGACTTCAAATTCTGGGCCTTCC
AAACCAGAAACAATACAAGATTTTAATATTGCAGTAACATAAAGAAATTTTTATTTTTAT
TTATAAGAATATGAAGCTATTACATTGTACATTATTTTACCTCATTACGGATTCATGTTT
CCTGTAAATACATACAACCCCGTATTATTAATTAATTATTTAACCAGAAATTTCAATGGC
CTTGACATCAGGTTTCTTAACCTCCACTTTAGGAATGGTAACAGTAAGAACTCCATTCTC
CATAGAAGCCTTAACCTCATCTATTTTCACATTCTCCGGCAGCCTGAACCTCCTTGAGAA
CTTCCCACTGCTACGTTCCACACGGTGCCATGTGTTGTTCTTGTCTTCCTTCTCTATTTT
CCTCTCTCCACTTATTTGAAGCACCCTGTCATCTGCAATCTCCACTTTTACTTCCTCTTT
CTTCACCCCTGAAAGATCCGCCTTGAACACGTGAGCCTCTGGGGTTTCCTTCCAGTCCAT
ACGCGTGTTAACAAAAGCTGAATTTTCTTGAGGTGATCGAGAAGAGAGTGAAGTTAAAGA
AGGTGGGAAACTAAAGTCCTCGAAAGGGTCCCACGAAGGATCAAAGATGCTGCTTCGAGA
GTTGCCAAAGAAGCTAGGAATCAGGGACATTTTTCTACAGGAATTTGTGTTTTTAAGGAA
GGACTTAATGTTGGAGTTCAGAACTGAAAGTCGTTTGTACGCTGGAAAAGTTCTCTGCTG
GTTTCTTGGCAAATAACAGTTGGGTTAAGTTGGGTATTTATCAGATGCAAG
>ntig_827_consensus
GCAATCTACGCATCACACCGACAAGCCTGTTCGACGAAGAAACCGACCATGAGGCTGCGG
GGAGGGTCAAGGTTTGCAGTGAGTAGGACGGGTGGGAGGATACTAAGGGGTCGAGTAAAG
AGGAATTGTTGGGGGTGACTACGACGGTGACCTTTAGGCCGCGGCTGAGTAAGCGGTGGG
TGAGATCAAGGAGAGGGATGATGTGGCCGGAGGTTGGGAATGGATAGACGAGAATATGCG
CTGCAGCGCGTGGCATTTTCGAAATTCTAGACTCTTTTTGTTCTGAGTGGTGTTGTTTTG
AGTTTGTAATTGCCCCTTTATAGGGCG
>ntig_828_consensus
CAAAAGAAAATATAAAGACAAACATCCCACGAGGCCGACCACATATGTGGATTGCTCTTT
TAGCTGATGGACGTCCACGAATCACAGGTGTAAGAGGAGGCCTCATCTTGGCAGCTAGAT
GTGGAGAGAATTGTCTCACAGTTCTTGTAACCTTCTCACTGAAAGTGCCTGCAAAGCTGT
CGTTCAGAAATGCAATGCTAAGCCCTGTCAAAATAGCAGCAACAATAGCAAGTGGCTTTC
TGAGGAAACCCAGTCCCAGAATAAGAGTAATCATGATGAAGTAGTTTGTTCGGTAGTAGT
AGAGATTGCACTTCAGTCGGCTGTTCCATTTGGCGTATGATCGGGGGAGGGTGAATCTGG
AGAAGAATTCGGAGAGAGGACGGGGTGCATTTGACCAGTCAACTTCACGAAGCGCGTCGA
TCAGATCTTCTGCAGTTACGTTTCCCCAATCCATTTGATTATGAACTTCAGTTGTATCGC
AACCAAGAGTGAAGAAAAGGAGGAGCAAAGTAGTCGATTTAGAGAGACGAAGAATTTGGA
ATCAGATTTGAG
>ntig_829_consensus
CTTCTTAAGGAACATTTATCATTTGTTGAAACTAAATCAAGAACTTGCAACGTACAAAGT
AGTTGTCTTGATTTTTGAATGCTCCTTGTCACAAACAGGTATGTGGCACCAGTCTCAGTT
GATACCCTCACAACCTGAAGTCCTAGACCAAATAACACCTCTTTAACATCTTCAATCTTG
TGGCAACCCAGTAAACTTAAGCTAGTGCCTTTCTCTGTACATGGAATCTTGCTCAAGCAT
ACAGCAAAACATATACTGCCTACAAGTTTCGTGACCTCTGGAATCAGCAACACTTGTATT
AAAGAAGTTTTCAACAAAATCATCTCCCATTAGAGATGAGCATTTAGCCACGGATGATCT
TCCAATACTATCAAATGAAGAGGTTGAAATTGCAATTTCCTGAGCCTGTTTCTCATAGGT
AGCTTTCCTAACATCATTCTGACCAAAGATTAGCGTTCCTTGTTCATTTTCAAAACCAAC

TCCACTAATATCCAAATTTCCATTCGATTTTTGTGATTCTTCCACCTCACCAACCATAGT
TTGTTCCTCTAATAGAGAAATAACTCTCTTTCTATGTTTCTTTCTTTTTTTCCTTTCCAA
CCAATCAGCCTCTTGGTTGACTAAATGAGCAACTTCATCATCGTTAGCAACAGCAGACAA
ATTATTGCTTACGCCATTTTCTCTCACATGATCAATGTCATTTAGATGTCCATAATCATG
ACCCTCAAACAGATTAGATGAAGAATGCCCTTTGTCAGTCCTACACACATCTTCAAGGTT
AAGAGACTGATCAACTTCAAGCATTGATGGCCCATATAAGCAGGAATCATCATCACTGCA
GCTAGTATTACTATCAGATGAAGCCACAGAATAGGAATCTGCAGATAGTTGCAAGATTTC
CTCAACCAAATTGTGCCGACGATGCAGAATATCCTCCTGGTAATGAGGTGGAGATCCAGG
ACAAGCAGATGAGGAATGAGCATCTGTGATGTCATCTATTGCAGTCAAGGGAGACTCACT
GACATTTTCCACCATCATACGACCTTGAATAGTAAGTATATCAGAATAGGAGCTTTTAGA
TTGCTTGGAAACAGTGCCAACCCCTTCATGTAGAAGAGATTTGTGATGCTCCTTTCTCAA
GTCCATTCTCCCCATCCCTGGTAAAGAAACCCCACCAGTAATCCCAAGGGAACCAATATG
GTCATATTGATGAGTGTGCAAACCCATGGACATATCTACAAGGGAATTGTCAAACTCTAA
AATGTTTGTACTACTCTCGTCTCCTGAAGCTTGAACAGAGTCTGAGACATATCTTGAACT
TTCAACAAAATGCATCTGGCTCTTTTTATTCTTCAAATGATTTTCTTTTCTTGAATTTAA
AGTACCTGTATCCAGAAAATCTCCAGAAGTATGATCCATCCAGTTTTTCAATTCACGTAA
CCATAGAATAGAACGCTCTTTCTTCATATGCTCCACCCTATTCATCAAATCAACTATTTC
AATATCATCATCAGACATGATAGTATCCTCTTTGTTTTGAATCTCATTATCACATGAAAT
AGAGCCTTGGTCAGAACCGACATATGTGCTCTCTTCTTCACATTCAATAGCAGCAAGACG
AGAGACCTTTTTCATTTTTCTATTGTGAATCCCTTCGGCATCAGTATCTTCTTTTGCAGG
AGAATAGAATCCAAAGGCCGCTGGTCGCTTCATCCTGCTTGCAATTATAATTTGCCTCTT
CCAGAATTCTCTTGTGCTGATTTCCTTGTCATCTAATTTCAA
>ntig_830_consensus
GCACCAGCAATGCCTTCAGGATCAAAAAACTGTGTGAAAATCCTTGTGAAGAAATTCAAG
TTGCGGGAAACATTGTAAGCCATGGCAGGTGGAAGAGGCTTAACGATGGTGTCTATGCTG
AATACTCTTTGTAGAAACTGAAAGTTACGCTGGAAGCTGTAACGAAGCTGGGGGTCGATT
TGGAGGGACTCGTCGTCGTCATCGAGATCATCGTAATTGGAGGTCTTTTTCTTGCCAGGC
ACGTGCATCGTGGAAGTGCCACCAGAGTCACTCGTGACTTTGAGCTTGGCGTCCTTGAAT
TTGTTCGATTCGTCGTCGTCTTCTCCTCTCCATGGAGCTTTGGTTAGCAGGTCCTTCTTG
GCTTTGCCCATTGGTTTTGCCTTGCCTTTGCTTCTCTCGCTTGAGCGCGATCGAATACTT
GTGTTTTGTTGATAGGGTTTCTGTTTGGTTGCTCGGATTCGCTCCCCCTGTTTGGTCGCG
CCGATTGCTTTACCC
>ntig_831_consensus
GGGCAAGTCTTAAAAGAATATTATCATTTATTACAGTTTGTGCACAAAGCAGAAACTAAT
GAATTTCCTGTCATAGCACTTAAACATTGAGAGAAGAATATGAATGAAGTAATTGTAAAT
TTTCTATTGGGATTTTTTTCCTGGTGAACCTGTAAAAGTAAGTAGTATTTCCCTGTACAA
TGCTACCTGTATTCAATTGCCCTAAACCCAATTGTATTGTGTTTCCCTATAGTCATTTTA
AAGTAAGTCATTTTTCCTGTGATTCCTGATGAGAAGTTTCAGACTTGTTATGTACAACCT
CTGCAGAAAGGCTTTTAAGCAAGTTTATGACTTCTGAAGTATCATCAAGATAATATTTCG
CCATGCTTGGTTTCTGACCAACTGTGCAGGCAAATACTTCCGCAATTGCTGGCAAAGATG
GATTCGCAACAGCACGTCCAATGCTCTCAAACATATCCTCATCCGAACGATCGTCACCGA
TGCACAGCACGAAATCTGGAGATTTACCCTTGCTTCGCATAGTTGAAATCAGATTTTCTA
CCACTATACCTTTGCTTACTCCCTGTGGCTTCACTTCAACAATGTGTTGGCCTCTTTTGA
CAACCACTGGCTCATTAGCTAGGACACTTTCCAGATGATCGAGAAGCTCCTTCGCCTGCC
ATGTGCCAAAATGAGGGTCAGCATCCTGGTGGTGCCACACCAGTGCAGTTTCTTTATGTT
CAATGAAGGAGCCATCGGTTGTTTCGGTATAAAGCTTCATCACAGGCTCTGCAATCTTCT
TCCAGTCAAAATCCATCGACAACGAGCAGGACTCCCATGGAACATCCTTTGTCCACCTAA
GAAAGTAACCATGCTCAGCTGAAATCCCCAATTTCTCACAAGGCGAAAACCATTTGCTCA
AGGAATCTTTTCCTCTGCCACTAACTATAAACACAATATTTCTGGGATCACTGCATAACT
TATTCAAGATTGAGATCACCTCATCACTTGGTCTTTTGTCAACTGAAGC
>ntig_832_consensus
GCTCCACAGTGAGACGAGAATATGAATAAAGTAATTTGTAAATTTCTATTGGATTTTGTT
CGAGATGAACCTGTGAAAATAAGTAATATTTCCCTGTACATTGCTACCTGTATCAGTTGC
CCTAAACCCCATTGTTTGTGTTTCTCTATAGGTATTTCAAAGTTATAAGTCATTCCTGAG
ATTCCCGATGAGAAGTTTCAGACTTATTATGTACAACCTCTGCAGAAAGAACTTTAATCA
AGTTTATGACTTCTGAAGTATCATCAAGATAATATTTTGCCATGCTCGGTTTCTGACCAA
CAGTGCAGGCAAACACTTCTGCAATGGCTGGTAAGGATGGATTCGAGACAGCACGTGCGA
TGCTCTCAAACATATCTTCATCCGAACGATCATCACCAATGCATAGCACGAAATCTGGAG

ATTTCCCCTCACTTCGCATAGTTGAAATCAGTTTTTCTACCACTATACCTTTGCTTACAC
CCTGTGGCTTGACTTCAACAATGTGTTGGCCTCTTTTGACAACCACAGGCTCATTTGCAA
GGACACTTTCCAAATGATCAAGAAGCTCCTTGGCCTGCCATGTGCCAAAATGAGGGTCAG
CATCCTGATGGTGCCAGACCAGTGCAGTTTCTTTATGTTCAATGAAGGAGCCATCAGTTG
TCTCAGTATAAAGCTTCATCACAGGTTCTGCAATCTTCTTCCAGTCGAAATCCATCGCTA
ACGAGCACATTTCCCATGGGGAATTGCTAGTCCACCTAAGGAAGTAACCATGTTCAGCCG
AAATTCCCAATTTCTCACATGGAGAAAACCAATTGCTGAGAGAATCCTTTCCTCTCCCAC
TAACTATAAACACTACATTTCTGGGGTCATTGCATAAATTACTCAAGATTGAGATCACCT
CACTGCTTGGTCTTTTGTCAACTGAAGCCTGTGGCATCACTGTCCCATCATAGTCCAAAA
GGATCAACCGGCTATTAGTCCTCTTGTATGCATCACTAATATGATGCACACCGAGTTTCC
TGAACTCCGGACCCAAGGCAACAATCCTGAATCCCAATCCCAACCCAACGCCCCAACACC
GCTTGAACGAATGATCTCTGCAAGCCCTTTCAAGGTCTTGATCGAAACTCCTCGCCCAGT
AGGCAACATCATGAGAGCTAATGTACTTGTAATGCTTCTCATGGCGCAATGTTTTCTCTG
TCTCTGGCATTCTCAGGGCTGAATCCATGGCATCGGCAATTGCATCAATATTCCACGGAT
TGACACGAATAGCGCCACTAAGAGAAGGTGAGCAGCCAATGAATTCAGACACAATTATCA
CACTCTTCTTTGGAGTTGGATTCTTCTCATCAAAACCCAAGGCCTTGTCCAAAACATGGC
TGCCCTGCCTACTCACGGTATACTTATAAGGCACCAAATTCATCCCATCTCTCACACAAT
TCACCACACAACACTCTGCAATTGCATAATAAGTAACCTTTTCCAGTGTACTAACAGGCC
CTTGAATCATAACAATCGGCTCATAGCCCGATTTGCCAAACTTTTTATTAATCTCATCAG
CAATTGTATTGATTTCATCAACAACATCTTGCACGTCTTTCCCACAGCTTCTAGCAGGGT
TGGTAATCTGAATGAAAACAACTTTTCCTCTCAATTCAGGATGCAGCTCCAAAAGTTGCC
CCATTGCAGAAAACTTCAAACTAATCCCCTTAAACAAATCCATATCATCCACACCTAACA
AGACAATTTTCTCTTTATATTTTTCCCTCAAATCTTTCGCCCTCTTTGCAGTCTCTTCAA
GAGACAAAACAGATTCAAGCTGCCCCATATGAATCCCAACAGGCAAAATCTTTATACTCA
CAGTTCTACCATAATAATCCAAACAAATGTAACCC
>ntig_833_consensus
TGGGTCAATAATTATTAGGACTAAACAACTCAATCTAATATGAAACCAATACGTAAATAA
TTAAAAGTTAAAACCATTTCTGACATCCAGAATCTAGTTTAACTTAAAAACTTATTTTAA
TGCATTTTAGAAAATCAAATAAAAAACAGAAAATCCCGACATCCATAACACCAGATTGAG
CACCCAGATAACATCCAACTGCTCAACTGTCTAGATACTGTAATGGAAGCATATAAATAA
ACACTGTCCACAAAAACAACAAAAATGAAATAAAACAAAACAGGTACAACAAAATTCAGA
GCATAAGCCTCACTACAGTAGACCTTCAAAACACCTCATTTCTTCTTGGCTGCAGATTTG
GTGACCTTGGCACCGGTGGGGTCTTTCTTCTCAACGTTCTTGATGACACCAACAGCCACA
GTCTGCCTCATGTCCCTCACAGCAAAACGACCAAGAGGTGGGTATTCTGAGAAAGTCTCA
ACAACCATGGGCTTGGTGGGAACCATCTTAATCATCCCGGCATCACCATTCTTCAAGAAC
TTGGGCTCCTTCTCAAGCTCCTTGCCAGATCGTCTGTCAATCTTGGTCAAGATCTCAGCG
AACTTGACAGCAATGTGGGATGTGTGGCAGTCAAGCACTGGAGCATATCCGTTACCAATC
TGGCCAGGGTGGTTCATAATGATGACCTGAGAGGTGAAGTTAGCAGCCTCCTTGGCAGGA
TCATCCTTCGAGTTAGATGCAACAAAACCACGCTTGAGATCCTTGACTGCAACATTCTTC
ACATTGAAACCAACATTGTCACCAGGAAGTGCCTCCTGGAGAGCTTCATGGTGCATCTCA
ACAGACTTAACTTCAGTGGTCAGTCCAGATGGGCCAAATGTAACAACCATACCGGGCTTG
AGGACACCAGTTTCCACTCGACCCACAGGGACAGTTCCAATACCACCAATCTTGTACACA
TCCTGAAGTGGCAGACGGAGAGGCTTGTCTGAGGGCCTCTTGGGCTCATTGATTTGATCA
AGAGCCTCAAGCAGGGTTGGTCCCTTGTACCAGTCGAGGTTGGTAGACCTCTCAATCATG
TTGTCACCCTCAAAACCAGAGATGGGGACAAAGGGGATCTTCTCAGGATTGTATCCAACC
TTCTTGAGGTAGGAAGACACTTCCTTCACAATTTCATCATACCTTGCCTTGGAGTACTTG
GGGGTTGTGGCATCCATCTTGTTACAGCAGCAAATCATCTGCTTGACCCCAAGGGTAAAA
GCAAGCAACGCATGCTCACGAGTCTGACCATCCTTGGAAATACCAGCTTCAAAACCACCA
GT
>ntig_834_consensus
GGTTACCTTAGCACCTGTTGGGTCCTTCTTCTCTACGTTCTTGATGACACCAACAGCCAC
AGTCTGCCTCATGTCCCTAACAGCAAAACGACCAAGAGGTGGGTACTCTGAGAATGTCTC
CACAACCATGGGCTTGGTGGGAACCATCTTAATCATACCAGCATCACCATTCTTCAAAAA
CTTGGGCTCCTTCTCAAGCTCCTTGCCAGATCGTCTGTCAATCTTGGTCAAGATCTCAGC
AAACTTCACAGCAATGTGGGAGGTGTGGCAATCCAGCACTGGGGCATATCCGTTCCCAAT
CTGCCCTGGGTGGTTCATGATGATGACCTGAGAGGTGAAGTTAGCAGCCTCCTTCGCAGG
ATCATCCTTGGAGTTAGATGCAACGAAACCACGCTTGAGATCCTTGACCGCAACGTTCTT
CACATTAAAACCAACATTGTCACCGGGAAGAGCCTCCTGGAGAGCTTCATGGTGCATTTC

AACAGACTTAACTTCAGTAGTCAGTCCAGAGGGACCAAACGTAACAACCATACCAGGCTT
CAGAACACCAGTTTCCACACGACCCACTGGGACAGTTCCAATACCACCAATCTTGTACAC
ATCCTGAAGTGGAAGTCGAAGGGGCTTGTCTGAGGGCCTCTTCGGCTCATTGATCTGATC
AAGAGCCTCAAGGAGGGTTGGACCCTTGTACCAGTCAAGGTTGGTAGATCTCTCAATCAT
GTTGTCACCCTCAAAACCGGAGATGGGAACAAAAGGGATCTTCTCGGGGTTGTATCCAAC
CTTCTTGAGGTAGGAAGAGACTTCCTTGACGATTTCATCATACCTTGCCTTGGAGTACTT
GGGGGTTGTGGCATCCATCTTGTTACAGCAGCAAATCATCTGCTTGACACCAAGGGTAAA
AGCAAGCAAGGCATGCTCCCGAGTCTGACCGTCCTTGGAAATACCAGCTTCAAAACCACC
AGTGGTGGAGTCAATGATAAGGACAGCACAGTCAGCCTGTGAAGT
>ntig_835_consensus
AGGAGGTGGCTATGGCAAGAAAAAAAAATCTAATAAAAACACAATAATTATAGCAAACTTG
TATCCAGTTAATTAGGCCATGTATGAGCTTGGCCTTCCATTTCCACTTCCACCTTTAGCC
TCAGTACTGCCAACAGAATCATTTACTGGAGTCGGATAATCACTAGATCCAGTAGGGATG
TTATACATTGAGTTATCTGCAGTTCCTGATAATGGCGTACTCCCTGGTAAAGGAGTACTT
CCTGGTAATGGTGTCTGAATAGGAGTTTGCAAAGGCGTCGGGGGGAAGAGTATCTCAGCA
GTAGGTGTTTCGTACTCCTCAGTCCCTTCATAAGGTACATTAAGATCGTGAACTGGAGTT
ATGCCTGGCACCGGCTTTGGTGCCGATGACCTATCGGCACACACAACTCCTGCGTGCATC
ATCTTCATTTCCCAGATCCCTTGAAGCTCGTTGAGAACACTTTCTCCAGTACCACTATTG
ACGAACTCGTCACGGACCTTGCTGATGACATCCTCGATGACGCGGATGTACACAGTGCTG
GTCGTCGATGCCGTCATTTCTTTTTCTCAATTAAATTCAAAATCTCCCTAGCTCCTCGGA
TAAAATTTTCTCGGAAAAGAAAAAAGGAATAAGTGAAAAGAAAATTCTCTTGGGAAAAAT
CAGGAAAGCATCGGGAAAATCTTAGGGTTTAAT
>ntig_836_consensus
GTGAGCTAATTAATCATAATATATGGTTATATTTTATCTATTTTATTAATATTCTACATT
ATATCAAATCTAAAATAATATCAATATCCAAGGATGCTAATTTCTGGTAACCCCTTCACT
ATATCTAGGCATTTGTCAAGCCAAAATAACAACTTCATTGACACAAAAACAAACAGTTCC
AATGCACCATTACTAGAGCTACAATACTTTGTTCGACTACATAAACCTTGTATTGACATC
ACAAACTACTTGGTCTGTCACACCATGGAACATAAAAGTTGATTCCATGGTCTGTGTGGA
TCACGTTCATAGTAATAAACTTCACTTCATGCATAGCCATATACATATAGATTATCTAAA
ATATGGATAGGAAGTGAGTTCGGAATCTGTAAAATAGTAACGATTCCGAAATTCTACTCA
CCTTAACAAAAACCTTGTGTTGTCGAATATCTTTAAACTAGAATACTGTTTTCTGAGTTA
TCATGAAGTTTAGGGGTCATTTCCATGCTCCAGGAGGCTCTCTGTGCTAAGACTAGTGCC
CCAAGCTTTCTCTTTCCAAATCTGTTCCACTTCAGGAAATGTCAATCCCTGAGTCTCAGG
CACAAACACAATCACAAACACGATTGCAAGTACAGCAATGCCAGCAAGAATCAAGAAAGT
GGATCCAGTACCGACAGCATCAGCTACTGAAAGAAAGCTTTCGGCTACAATCAAGTTTGA
GACCCAATTTACAGTTGCAGACATTCCACCACATATCCCTCGATATTGCTCAGGATACAC
CTCAGAGTTCACAGTCCATGGCACAGGTCCCATTCCAGGTGAGAAGAAAGCTATATATAG
GGCTAACCCTAGAACTGCAATCCACCCATAGAGTCCACTAGAAGAACCAGATGATTCAGC
AAAGAATGCCCAAGCAAGGAGGCCAAGGGATACAACTACACCAGCTAAACTTGTGATGGC
CAACTTTTTCCTTCCAACATGGTCTATAAGGTAAATCCCAACAATTGTGCCAGCAGCGTT
TACAGCAGCAACAGCAAGAGATAGAAGAAGTGCAAGCTGATTTGACTCGAAGCCAGCCAT
TTGAACAATTGTTGGGCTGTAATACATGACTGTATTTATACCAGTGAACTGTTGAAATGC
CTGAAGCCCAGCCCCAGCCAGAAAAGCAAGTCTAATCTCTTTCGATTTAAAAACATCAAT
GTATCTGACAGTATTCTTTTTCTGACGTTCTTCCTCT
>ntig_837_consensus
CACCTGTTTTTCTTCATAACACCGTCCTGAAATCAACAATATACTAGTAGTTCATAACAC
ACACTTGGAATATTAAGTAATAAAAGCCAAACTAATGACTTCTATTAACATTTACAGGAA
ATACAAAATTGAAAGAAGAGATGCCTTCAAACTGCATATGAGCTAATTAATACATTATAA
ACATTTTGAGTTCAGGAGGTTCAACAAAATACTTACAAAAAATTTTTAAAATTCACGTGA
TGCAATAACTGGAACAGTCAAGTGCCCTCTTCCAGATCGTCAAGCTCCTCCAAGATAACA
TATTTTGTAGTATCGTTTGCGGGGGGTGAACTCAAAATGTCATTGGCAGTTGTATGACCC
TTCTGTAGCCTCTGGTATCAATATGGTTACAGCTTCCTTAACAACTCCTGCTATTGTTAC
TGTGACAGCACTTGTTACTGAGACAAGTACGTACTCCGTCAAAACCATGAAAAATGCTAG
AGTTCCACCGAAAAGCATCAGCAAGCAACTTCTAGTGACATGCCATGAATTATTGAAGTA
ATTGTTCTCTCTGAATTCATGCCATGGATCCAACAAAAGGGATAGAAAAGCAGTCACTAC
TGCCATCACGGGAGTCAAGTAGCTCATCAAAGTAAGTGGATTTTTAAGACCGTAAGATTC
TTTCTGTAGAAGAATTTGAGTCATGCACCAACGAAACCCAGACATGACAGCAGCAAGCAT
AACAAAAACAAAACCCCAAAACTCAAAGTCTGTCTCCTTTGCAACTGTTAATAGGATTCC

AACTGAGATAACCACTATGATACTGAAAAGTTTAAAGCTTGGAGACTCCAACCTGAATGC
AAAAGCAAATAAAAGTAGAAATATAGGAGCAGCAGATTTACACATTGTGGCAAAAGTAAC
AGAGATGAAAACAAGGGATGCATTGCTTAAGTTTACATCCAGTGCTGTTGCTAGAGCCGT
TGGTACAACT
>ntig_838_consensus
CTTGGCCCTTACAAAAGCAGCCTGTACATAGCCCGTTTCACCAGACCAATTCCCATCAGG
TTGGGACAATACAGGCACAATCTTAACGCCAGAAGCTTCCCACTCTTTAAACCTATCCTG
ATAAGCCATTCTCTTAAGGTTTCTAGCTCCGTAATAGAGTCTGACATCAGGTCTCTCTTT
CGCACTAAATCCCGCTTCTATAAGTGACCTGATTGGACTAATTCCCGATCCGGTAGCGAA
AATCACAACCGTGGGATACGCATCAGCCGGCTGGATTCTATCGATATCGAAACCTCTTCC
CATGACTTGACTAATCTCAACAACATCACCCTTCTTTAAACCACACAGCGCCTCCGCGGT
CGAACCGGCTATACTCTTCACCAGGAACTCAAACGCACCACTCGTGGACGCAAAAGACGG
CGGAGAGGCGATCGCAAGGAACGTCGGCTTCGCTACGTCGGCGAGACGGAGCTGCAGGTA
CTGTCCCGCGCGCGTGTGGCTTTCCGATAGATCCGGCGCGCTGGAGACGTCGATGGATAC
GTGAAATAGAGACTCGGCAGCGGGGGAGACTTCGGAGAGCGGAACCGGAGTCCAGATGGT
GGTATCCTGGCGTACAGCTGCGGCAGCTACGGTGGTGAAGTTGCGACGGCGAAATTTAAG
GTGAAACGGTTTTAGCTGGTGAGGGAAGGGAGGGAAGCGGCGCAGGATAGACATGGCATG
GAGGGAGGGGCTAAAGTGCGCATGGGGTAGGCACGGTGATGGGGAGACGGTTAAGGACAT
TGTTTAGAAGACAGATTATATGGAGGCAGTATTGGGTGGCGGCGGCGGCAAAGGTG
>ntig_839_consensus
ATGCAATTATAAAACAAGCACGCTTGAAATACGACCATGTAACTAAGATTGAATTCTTTG
GACATTTCCGTCATTCGTTATTGATAGTCACAGTCGTATCCCACCATCCACTTTACACTT
TTGCCTGTGTAAAATAACAATGTCTTCTCTTTTAATGCTATCTCAGTTTGTTTCCTCAAA
GCTCTTCCAGAGGTTTCGGATAGCAGGATGAAACTGCGCCTGCGACTGTGAGAAAGATTT
TGTCCTCGCCGACCATGTCTGCAAACTGGGATGCGTGGAGCTTGTCAATCACCACTGAAC
CCGGGTTTGCCACAACAAGCTGAACGTCTCTCTTTTCTAGACTCTTGTATAACTCTTCCA
AGGCATGGATACCACTGGTGTCTATGTCAGTAACAGGTGACATGTCAACTATCAGATATT
GGATTTTATGTTCACCGACTGCTTTATTCTGTTCTTCTTCATCTGTCAACCATCTTAAGA
TCCTCTCCCTAATATAATTAGAGTTTGAAAAGTAAATTGCAGAATCGACTCTCACAATCA
AGACACCAGGAACCTTGGTTGCATTTGGATATTGTTGGATGTTTCGGTACACAGTAGTCC
CAGGAACCTTTCCCAGAAGAGCTGTCCGGGGCCTGGTTACTTGCAAGAGGATTTTAGCAA
AGGATATAGAGACCGCAATTAAGAGGCCTATCTCAACAGAGGCAAAAACCACTCCAAAGA
AGGCTCCAATGCATGCAACAAAATCGAATTTGTCAACCTTCCATATCAAACATGCTGCCT
CAATGTCGATTAGGCCAAGCACAGCTGATATAATGATGGCAGCAAGAATTGCATTTGGAG
TATACTTGAAAAGAGGTGTGATGAACTCTAAGGTTAGAAATACGACACAAGACATGACAA
TGTTGGAGACTGCAGTCTGGCAGCCTGCCATGTAGTTTACTGCCGATCGAGAAAATGAAC
CTGTGGCCACATAGCACGAAGTCATTGAACCGACAATGTTCATCGCTCCTAGTGCTACCA
TTTCTTTGTTTCCATCCAATTGATAGTCCTTCATGGAAGCAAAAGTTCTACCGATTGCTA
TAGCTTCTGTCAATGCTATCATTGCAGCAACCACACCAATTTTGACACCTTTAAGGAGAT
ATTCACCACTGAAATATATTTGATTCACAGATGAGGGGTTGACTCCTTTCTCTATATGTT
TTACTATTTGAACACCTTCCTTATCTGCATGGGTTATATATACAAAAAATGTTGACAGAA
TTACAGAGATTAACGGGGCAATTGCTGGCACCCAGAAAAATTTATTGTTCTTCTTTCCAA
TGTACTTTGCAAAAAGAAGGAAACCTAGAAAGCTTGCTCCAATAACAATGGTCTGCCAGT
TCCAACCGTGATGAGCTGACTTGATCACTGAGTTCATCACCGAGACAATATCTGATTTCT
TAGTGAAATTCTTGATGCCCAGAAAACCCTTAAGCTGTTGGAGAGCAATAGTTATTGCAG
CTCCACCCATAAAACCCACAACAGCAGCATGAGATAGGAAGTCAATCAGGAATCCCAACC
TGAAAATTCCGAGGGCAGCTTGTGTAATTCCTGCAAAAAAAGTGGCAGTGAAAGCAAGAC
GCCGATACTCCACCACATGTGTTTTATAATCAATTTCATCCTGAAGCAGAGTCCCCAACA
GGAGAGATACCACTGCAACTGGTCCTATGGCGATATCTCTTGAACTACCCATAAATGCAT
AAATCAAAGGTGGAACAAAACTGGAGTACAACCCATATTGTGGATCCAAGTTCGCTAGCT
TTGCATAGCCAATATCCTGAGGAATGCAGAGACTTGCAATGGTCAGACCGGCAATGACAT
CCCCTTTAAATTTAGTAAAATTATAATACCTTCCCCATTCAAGAATGGGGAAGATGGCCT
GGATGCCAAGGATGAACTTACGTGACCTAGGTTGATTCTTAAAGTGTCGCAGTGGATCAT
CAGCTAAGAATGTTTCTTTAACAGTTGTTTTGAATTCCTTGAATAGATTTTGTTTAGGGG
GAATCCCCACCTTGTGCACATAAGGCTCATTTTGTGAGTAACGTTGAGAAGATAACTCGT
GTTTAATATCAATCTCTTTTGTTTCAACCTCTCTGGTTGAAGGATCCATGGAAAGTTACG
AGTACTTATTCAATAGCAACTTTCACTTCTCGGTGAGCCTCTTTTTCTTTGTGTTTTCCT
CCTGGTAAAACTCGCCTCCTTTATATGCTTCTCACCAAAGCCATTGGTAGTGGATCCAAA

AAAGTCTTTGCCTCCCAAAATCAGTTTCTCTCTCAACCCACCTATAACTTT
>ntig_840_consensus
TTTTTTTTTTTTAAAGTAATTTAGATTTGCATTACATGAAACCTCAACTGTAGCGAAACA
CAAGTCATAAAATTTGCAGTTTACAAGAAACTCTTGGGTGCAAATGTTAACCCTTGTGTC
CATAAGAAAAATTAAGGCCACATTCCAATCATTTACCCATCTAAGGTGGCTGCTGAGATG
ATGCATTGTTGAAGTTGGCAACCTTTCAGCAGCTCAGGAACATCATAATCTCAAGCAGCA
GTTTGGGGAAGCGTGCTCTGTATGTTGGAAGGCATCAGGATGTTAAAACCATCTGATGCA
GTGGATACAAGCATTCCAGGAATCTGATTATGCCAATGAAGTTCTTTCAAGTCTTTTTGT
CCCTGATGCACAAACAAAAGTTGAGGTGGCAAATCTTCTGGGACATTTACTTGCTCTTTT
GTTTTGGCTTTGAATTCTGCTTCCTCTTCCTCATCTTTTTCCAAAGAAAGGTCCCATATC
GTCAGCTGATTGTCAGATGATGAAACAGCCAGAGTGGAGCCTTCATGTGGACTCCACTCA
ATGGATGTAATTGGGTGTTTGTGATACTCAAAATGTGCTACCACAGAATCTCCTCCCTTG
AGCAATCTCAGATCGTGAATGGAAAATGTTCCATCATCACTTCCAGATGCCAACAAACAA
CTAGCCAACCTGTTCCATGAGATAACATTCACATCTGCATTATGTGCCTTAAAAGAAGTT
AATGGTGACTTCCCTGCACGGGTATCCCAAATTGCTATGTTTCCATCCACAGAACAAGAG
GCAAATACATCTGATTCTGTAGGGCTCCATTGTAGATCTTCAACACTGGCTGAATGTCCA
ACAAAAGGATTTGGATCAACATTCCATGTAGAATCAGATGCAGGCTCCCATAAATGAATG
CAACTCTTGCAGTCCCCAGTTACAAGTCTTCCGGGAGTTAAAGGACTCCAATCTATCGCA
TAGCCCTCGTCTTTGTGCCCACCAGACTTCATTAATGGAGTCTGATTGAATACCTGAGAA
GCTCCCTGGCTAGCTTCTGTTTCTGATTCAGCTAACGCATTTAGATGAGAACGGAAATCC
CATACCTGCACATGGCCAGTGTCTCCCCAAGATGCGCATATATGGGGATTTTGCGCCATT
GCACGTATACGATTGACACATCCTTGATGAGCTACCTTGCGTAACTGCAAAGTTGGGGAT
CCTGAACCACCTTCCCCCTCATCTTCACTATCTTCATCACTATCACTACTTTCACTACCC
ATATCATCATCACCAGCAGCAGGTTTAGGTACTAAGTCACGTCTTTTCCCAGAAATATTA
GATACTTTAAACACTCCAATAGAATTCCAAGCAGCTTTCTCTGCCTGAGTCCCAGCGACA
AAATACACAGTGTGTGGAAATTCATTCCGAACCAAACCTAACGTATCACGTACAACATCA
AAGCTTAAACAAGGCCGACCCATGTGAAAGGCGTGAAGGGAATTGTAGGCAGTTGGGTCA
CACTGAAGCTCCTCTCCAAACTTATCAATTCCTGGTTGCCAAACTTTTGTAGGTAATGAC
GGTGCTGAAGAAGACGATGATGATCCATCTCCTCCCTTTACACAATAAATATGATTCCAA
AATCTAGTAAGGAAGAAACTTCAGCCCACGTTCCAAACTGGTGGTTATGGCTCTCACCGA
TTTGCCGGCAGAGTATCCCCTTTGTACACCTTCCAGGGTCCTTCATAGAATTTCGGCTTC
ATGAGTGGCACTGCTTCCCCAGGGTGATCTTCCCAGTACTTTTCCTGATCAACAAAACAA
ACACAATTCTCAGATCAAAGCCTCAAATTGCAGCTCTGAGATAATTG
>ntig_841_consensus
ATATATATATATATATATATCAGTAATCATTTGTTCATGAAAAAGAAAAATAATTCCTTG
CACAATGCAATGCCTAACACAATGCCAAAATTAAGATGTTTGATACCAATGAAACTCAGA
GAGTACAACTGCTAAACTCATGTCCAAAAACAGCATAACAAGTAGAAAATGCCAAAGAAT
CCTAAAACTCCCCAACAAGTCTCTAAAACACAAGTTCATGAGGTTTCATGCCAGCCTTGA
GGGAAAACCTTGCAGCCAGATAAGACTTCAAGTCCGGAACACTCTCGATGAATTTGATCA
ATGTGGTATCAACCAACTTCTGATCATCCTTTTTCTCCTGTGGAAGAACCTTTTTCTCCT
CTTTCTCTGCCTCAAAGAACTCATTCTCTCCCTTCTTTTTCTTCTTTTCAACCTCCTTGG
CAAAGTATTTGTCATCAAACTTGTCTAAATTCAGTCCAGAAATGTCAACCTTGGTAGAAG
TTCCAATCACATAGGCTTGATTTACTCGTCTTAAAGGCACACCATTGATCTTAAATGGTC
CAGTAACCAAAAGCAACCCAGAGGGAAGCTGCTTCAAGAACACAACTCTCTTCCCCTTGA
ACCTTCCAGCAAGTATAATCAACACTGTGCCGGGAGTAATACTCGCTCTAAGCTTGGTGG
GTTTTGGCTTGCGCTTATTAAAGAGCGGTTTCTTGATGTCATCGGCGGGATAAAACTTGG
GGGGCTTCTCAGCCGGAGAGGCAACCTTGGGTTTAGGGTCATGGCTGGGGAAAACGCCGC
CGTTTTTGGCTTTGATGGCCCAGAGACCTCTCTTATGGTACATCTTGGAACGAGAGAATT
TTCCAATTCCTCGGATCAGCTCCGGATTACGGCTTACCCTCGGCTTCCCCTGCTTCGGCG
CCATTGCTGGATTCGACTCACTTGTCTCTCTCCTTGAGTGAGTGACTGTGCTGAAACGGT
AGAGCAAACCCTAAGTACTGAAGATATTTATACGCTCGAAGTGGAAACCCTAGAAAAGTA
GGAACCCAATTAC
>ntig_842_consensus
ACTCACATTAACCAGAAGTTAACCAAGATCATATGAAAAAGAACTTGTCACATATAATGT
ATTAGCAATCATTTGTTCATGAAAGAAAAAAGTAATTCCTTGCACAATGCAATGGCTAAC
ACAATAACAAAATTTAGATGTTTGATACCAATGAAACTCTGAGAGTACAACTGCTAAACT
CAAAGTCCAAATAAAACAAGTAGGAAATGCCGATGAATCCTGAACCTCCCCCAAATGTTT
CTCTAAAACACAAGCTCGTGAGGTTTCATGCCAGCCTTGAGGGAAAACCTTGCAGCCAGA

TAAGACTTCAAGTCCGGAACACTCTCAATGGATTTGATCAACACTGTATCAACTGACTTC
TGATCATCCTTTTTCTCCTGCGGAAGAGCCTTTTTCTCCTCTTTCTCTGCCTCAAAGAAC
TCATTCTCTCCCTTCTTTTTCTTCTTTTCAACCTCCTTGGCAAAGTATTTGTCATCAAAC
TTATCCATATTCACCCCAGAGACGTCAACCTTGGTGGAAGTTCCAACCACATAGGATTGA
TTTACACGTCTCAAAGGCACACCATTGATCTTAAACGGTCCAGTAACCAAAAGCAACCCA
GATGGAAGCTGCTTCAAGAACACAACTCTCTTCCCCTTGAACCTTCCAGCAAGTATAATC
AACACGGTACCGGGAGTAATACTCGCTCTGAGCTTGGTGGGTTTTGGCTTGCGCTTATTA
ACAAGTGGTTTCTTGACGTCATCTGCGGGGTAGAACTTGGGAGGCTTCTCAGCTGGAGAG
ACAGCTTTGGGCTGGGGGTCATGGCGTGGGAAAACGCCGCCGTTTTTAGCTTTGATGGCC
CAGAGACCCCTCTTGTGGTACATCTTGGAACGAGAGAATTTTCCGATTCCCCGGATCAGC
TCCGGGTTACGGCTTACCTTCGGCTTCCTCTGTTTCGGCGCCATTGATAGATTCTAGCCG
TCTCTCTCGTTGAGTTAGTAAGCAAACTCGAAGAACTCAAGCTATTTATATCCTCAAAAT
GAAAACCCTAAAAATTAGAAACCCTAATATATTTCGGCTTTTG
>ntig_843_consensus
AACTTCTACTTCAGCATGTCCATTCTCAGTTTTCTCAGCACTTGTTGAATCAGCCGAGTC
AGTGTCATTCTTCCTCCAACTCCTGCTCTCATTAAACCCGCCTCGCCCATTACCGAGTCC
CCTTCTCCCAAATGCGGGCATATCAGCCTTCTCCTCTTTAATCTTCAGCGCCTCAAGCTT
TTCATCAATCTCCTTCGGATCCATCCCTTTCTCCGCCAACACCTGCTCTCTCGGCCTTGC
CTCTCCAAAAGGATTGGCCCCCTTAGGTTTCACTATGTCAGCACCCCCCTCACTCACAGG
CAACGAACGTGGCTGCAAAACCAGCCTTGGCCTCGGCCTCTCACTCTCACTACTATCCTC
TCTTTTCTTCCCCCAATTATCTCTATCCCTCGACAAAGAATCAAAACTGCTCTTCCTCTC
AGCACCACTGTTGAACCCGAATCGGCTCCGAGTTTCAGTCTTGTTCGAAACCCAGCTGTC
ACTTTCATCAGCCTTGGATGACTGCGAATCGAAGAACCCCCCACTCCTCGCGATGGGGGC
AGGCTTCTTAGCTGCAGCCCAATTATCGATCTCGTCTGCACGCGACGGTTGAGATTCTCT
ATTCACATCCCTATTAGAAACCCTAGAAGAGCCCCAGCGAGAGCCAGACGAATCTTCACT
GTTATTAGAACCAAACGAATTGTTACTATATCTATTATTGGACCCGTAATTTCTAAATCC
ACCGCCTAGACGAGAGCGTTCGAGTTCTTC
>ntig_844_consensus
CTTAGCCCGAATTCAGAGTTTCTGGAGAAACAAGCATACAATTCTTCATTAAGATAAACT
CAAAGAGAAGTACATAAAATTAAAAACATGTAAACTAATTGACCATATAGATAGCAGACT
CCCTGCAATTGTTGTTACTATGCATACAGTGATGCATTATGATCAAGTTGCTGCTATATG
TGCTCCCAATTAGGAAATAATTTAGCAGTGATATGACATCCACTAAGAGGAAGGAATATT
CAATTATAGCCTACAACATAATTTACGTAGTTGACAAAAACCATTTTCCTTCCATCTTCT
TATGCCTCATCAGATCCAGATTGTTTATTCCACTAGCACAACCCAGTTCACACCCAAGA
AAGAACCTTGAGCACAGGTCCAGTCGGAGCACATATGACAAAGCTGATAACAGCTGTGGA
AGAACAGACCTCTTAAGCAAATTAAGATTCTACCCACCTCCAAAAGCAAAAACCATCAAT
AATCTCTTGACCTTATTAATTCTAGCAATTATACATGGGTTCCACAGAAGCCCTGGGTCC
CATATTGATGCAGAATATATATTATTTCTATACTATCATGGGAGATCATTAGACAATGTA
GTAGTAAACCAGTAATAAACATAGGAGAATCACGATTTTTGCCGTTAAACAAACTGAACA
TCAAATCAACTGGACTACAACAGTTAATCACTTGAACTAGGTAGATTAAAAAATAAATGA
ACTCTCCAGAAACCCTATAGGGGATATAGAATTTCTTTACGCAGTCAATGCTTATAGCTT
AAGCCAACAAACTGATATTAAAGTAACTGATTCATTCAGGATAAAACTATGACAACACGC
AGATTCTTCAACAAGAATATATGAACATTAAGAGAATCAAATGTGGCAACCAGAATCCTA
ATCCCTACACAAGAAAAAAAAAAAAAATGGGAACTAGATGCTAGTTCCTAAAACAAGATAA
GTGATTGCTTACAGAACGCTTCCTACTTGTAAAACCAATTAACAGGATTGAAGTTACCAT
TTCAATACAATAGTTTTATGTCAATTAATTCAAGCATCATTGCCATAGTCATCATATATA
GAGTTCTCCACCTTTCTAAGTATTTGTACCTTAGCCCGATAAGATTTGATGACCGCAATC
AACAACTGATCTCCAGCAATTTGTCTCACTCGTTGTATGAGCTCATGACGTGATATCTTA
TTTTGTCTATGATCTCTATGGTGTTTGGTAATCAAAGCAATAGTGGGTGAAGGTAAAAAC
TTTGACAGCGCAGAAATGAGAATTGGAAATGGCATCCAAGGTGAAGTCGGCATTCTCAAC
GATTCCCCAGCTCTCCCAAAACCCCTCAAGGCAGAGGGAGCCTTTAAACTTATGATATAC
TCTGGCAAGATATGAGTATTCATGTTGCTTGTCCAGACAATATACCTCTTTGGCACCTTT
GAGTTGTCAACACCAGAATCAAACTCTGATGAACTCGGATGAGACTGATCAGAACCAGGA
TGCACAACCTCTACCTTGCCCAACATGACCCGGCACAACAACATATATCGTAGTCCATCT
TTGTCAACAACAGAGTTCTTCAAACATTCCATAGGTGAATCATCAGGAAAGAGATAGATG
CCGCTACCGTACAATCCATTGTTATTAGGCTTCCCATGATAGCTAAAACCATGCTGTATA
ATCTTGCAAATCTCATCTTTTGTACTAGAGTACCAAGCATATTTCACATTTGCATCACCG
CCACACTTTGTAGCCAATGCTTTGGCGAAAATCTGAAACGAGTGGATCCTAGCTTGTCCT

ATAATCCCAGGAAACGAGTTTTTTAGAATGGCCACTAAATCAGCTTGTGTCCCGAGCGCA
CCCAACCCCAAAATCAATCTTCGTTTTATAATATCATAAACTTTGTCTCCTTCGTTCAAG
CTTATCAACCCCTCACCAAATGGAGGACTTGGTTCAAAAGCGGGACGAGAATTGGCACTT
TCACAATCAGAAATTATTGAATCATCGTCATCGTCATGTGTAGTATCTAAACCCGAATTC
TTAGTACATGTTTGTCGGGTAAACTGGCTCTCTAATGTGTTTTTTGTTGGACTGTTAGAT
GCTAAAACGCTATAATTCATTGTCAAGAGATCAAACAAACAAAAGAGACTTTACAATCAA
ATTACACAGAAAATCTACAAGAAACAGATATCTACACAGAAAACGAAAAATGCAAGGAGA
AATTCAAAAGCAAGAAATCTGTAAGCTACACATCTCTAACGAAAAGGAACTTAAAATAAG
AACCTGCAAAAAAAAAAAAAAAA
>ntig_845_consensus
CTATATGATTCACTTGTGGCATACGCATCAGACAAAATGTTGTATGAGAGTACGGTAAAA
GTCCCAGAAGATGAGATACGGCCATCTGAATCTAGATGCCCCATCATGTCAGCTCCATTG
ACTGGGATCAAGCGACGAGGACTAGGAGACGGGGCAGGAATTACACGCGAAGTNNGTTGG
AGTGTATGTCTTAGCTCGTCCTACTTCAAACCAAGTTTCGCCACTCCTCTGAGTAACTGT
TGCAGGATATAAAGTTCCTGAACCATTGGTCAAACTAGTACTTGATGCAGAACCAGATAA
GCCAGCATTGATAACTCCGGAGCTGGAGCCATTGAACCGCCCAAATAACTC
>ntig_846_consensus
CTATATGATTCACTTGTGGCATATGCATCAGACAAAATGTTGTATGAGAGTACAGTAAAA
GTCCCAGAAGATGAGATACGGCCATCTGAATCTAGATGCCCCATCATGTCAGCTCCATTG
ATTGGGATCAAGCGACGAGGACTAGGAGATGGGGCAGGAATTACACGCGAAGTTAATAGA
GTGCTGCCAGGTCCCACAACTAATTTAGTCTCTGCATCTACCACAACACACTCAAACTTG
AGAACATGACCAATATCATCAGCTGTTGGAGTGTATGTCTTAGCTCGACCCACTTCAAAC
CAAGTTTCGCCACTCCTCTGAGTAACTGTTGCAGGATATAAAGTTCCTGAACCATTGGTC
AAACTAGTACTTGATGCAGAACCAGATAAGCCAGCATTGATAACTCCAGAGCCGGATCCA
TTGAACCGCCCAAATAACTC
>ntig_847_consensus
TTTTATTTTTTTAAAATAAAATCTTTTTATTTAATATATAAATTTGATTTTTAATTTTAA
TTTTAATTTTAATTTTTCATCATCAAATACTCCACTTTTATTTCCAAACTTCTCTGTAAA
ACCCTAATTATCGGTCTTCTTCTTTCTATCTGATCCATTCAAAAACCCTAACTTCTTCCC
TCTCTAAACGACTCGTATTCCTTTAGTTTACCGTCGATTTCAGTCTTCGTTCAAGATCTG
TAACCGTTGTTGACGCACGGTCTTTTTTTTAGGGCTTTTATTTTTGGGGTTTCCGGTAAA
TGCTGGCGGGTAATTGGGTCGATTAAATGGCGGATCCGCCGCGTGTCCGCCAGGTTTTTA
ACAGTGACGGCAATAATAGGAATAGAATAGCTGTAGCAGCGAAGAGAGGTGTTGTTAGAT
TGGAGGATTTGATAGGAAGACATAGCGGTGACGAAAGAGAAGAGTGAAAGCATGCTGAGC
GTGCTACGTGTGCACCTTCCTTCAGATATTCCAATTGTTGGCTGTGAGCTCACGCCATAC
GTGCTTTTGCGGCGCCCCGACAAGACCGTCACCACCGAGGACGTGTCGGAATCCGCCCCT
GTTGACGGCCATTTCTTGAGATACAAGTGGTACCGTATACAGAGTGATAAAAAAGTTGCT
ATATGTAGTGTACATCCATCTGAGCAGGCCACACTGCAATGTCTAGCTTGTGTTAAGGCT
AAAATACCCGTTGCCAAGAGTTACCATTGTTCTCCCAAGTGCTTCTCTGATGCATGGCAA
CACCATCGTGTTCTTCATGACCGTGCTACAAGTGCCATAAATGAAAATGGAAATGAAGAG
GAAGAGTTATTTGGGCGCTTCAATAGCACAGGCTCTGGAGTTATTAATGCTGGCTTATCT
GGCTCTGCATCAAGTACTAGTTTGACTAATGGTTCAACACCTTTATATCCTGCAACAGTT
ACACAGAGGAATGGTGGTGAAACTTGGTTTGAAGTAGGACGATCTAAGACATACACGCCA
ATAGGCGACGATATTGGTCATGTTCTCAAGTTTGAGTGTGTTGTGGTAGACGCTGAGACT
AAACTAGTTGTGGGACCTGGCAGCACTCTGTTGACTTCACGTGTTATTCCTGCCCCCTCT
CCTAGTCCTCGTCACTTGATCCCAGTCAATGCAGCAGATGTAATGGGGCATCTGGATTCA
GATGGCCGCATTTCATCTTCTGGGACTTTCACTGTACTCTCATACAACATTTTGTCTGAT
GCACATGCCACAAGTGAATCATATAGTTATTGCCCATCATGGGCTCTTTCCTGGCCTTAT
CGCAGACAGAATTTGTTGCGGGAAATTGTTGGATATCATGCTGATATTGTATGTCTTCAG
GAGGTTCAAAATGACCACTTTGAGGAATTCTTTGCCCCTGAGCTGGATAAGCATGGATAT
CAAGCTCTCTATAAGAGAAAAACAAACGAGGTTTACAATGGAAATACCCATACTATTGAT
GGTTGTGCAACATTCTTTTGTAGAGATAGATTTTCACATGTCAAAAAATATGAGGTTGAG
TTTAATAAGGCTGCTCAGTCATTGACTGATGCTATACTTCCAAGTGCTCAGAAGAAGAAT
GCTTTGAATCGATTGGTTAAGGATAATGTTGCATTAATAGTGGTTTTGGAAGCGAAATTC
AATAACCAAGGAGTTGATAATCCTGGGAAACGGCAACTTCTCTGTGTGGCAAATACACAT
GTAAATGTTCACCAAGATCTAA
>ntig_848_consensus
CATAGAAGGCATCTTGCATGGGCCAGCCACCACGTAGTCTAGATATGTCCTTTGTTACTA

TATCCCTTCCAGCAGTAGCCAATGCTTCATTGAAAACAATTTTTGAATTTGTGTTCTCCG
CTAGCGAAGAAATAGCCCCAAGAGTTTCCAACCGCAAATGTTTTTCAGTCACATGAACTG
CAGCGGCAAGCAAAGATTGAGTAGTCCTTGAAACATCAGTTTCACTCAGTTCAGCTCCCC
TTTTTGTGACAAACTCAATTACGGCTTGGATAGCCCCTTCAGCAGACTGCTTAATTCTAT
CGCATATAGCTGTGGTACAACCATGCAAGGTGCCTACAAGCTCGTCCTTCGTCAATAGAG
TACAGACAGAAGAAACAATTCTGTTGAAAACCTCTGATGGATCAATAGAAGCATCACTTC
TCAAAATGGCAATAACATCCTCCAAGGAGGATAAAGCACTGTAGGACAACTCTATATCCA
CACCAGAATTTGAACCAACAGACCTTGGAAGTGAAAGAGAGATGCTGAAGAGCTGATCAA
GAATCTGAGCAGAAAGTTTTCTAACTTCAGAATTAGTGTCTGCACAACGTGGAAGATACA
TTATCACCCTATCTCCTAAACACAGGGCTTCACGACTGGGTAATACATATGCTGATGGTA
AGTTTGAAAAATTCCCATGCCCAGTACGGTCAATCTGCTTGCTGTGAGTGCAACTCCCAT
GGCAGCCTAGTGCACAATAGCCGCTAGCACAAAGTGTCCGAAACTTCAGAAGCATCTCAT
GCACTGCAAGAGAACCCCTTCTCCTTTGATATTCCACAGGTGAAGAAACATATTGATCAA
TTTGTCGCAAAATATGCAATAGTTGGTCTGCTCGACTTCTCCCATCCTCTCCACTTGTCA
GAAGAATCGCACATAACAGAGTGATAAGGTTGTCAATAAGTGGATTGACAACTTCTATAG
GGTCATTTGGTAACGCAAAGAACCCTAAGGTGGCCTTCATAACATGATTTCTTGTTTCAA
TTGTCAGCTTTGGCTCCACAGAAACCAATGTAGTACATGCACTAAGAGCAAGGGCCTGGG
TGCGTAGAAGTTCAAGACTTGAATCAGCAAAATCATCATTTTCTTCTATGCCCATCAAAG
TTAATATATAGTCGAGCAACACGTCTCTCCTTTTCAGTGGGAAAGATGTCCCATTTTCCG
CAGCATTAATGACAGCACGACCTAGTAAATCAATAGCAGTTATAACAGCCTGCTTTGCTG
TTGGGTGGCGTACATGAAGAAGCCGTGAAAGCATATTAGTACCAACAAGTGCATCAATTC
TGGCTTCAATAACTGTTGATGGGGCATATCTTGCAGCATATCCGTACATTAGAGCCAAAG
CAGCATGTATATCATCAGATTCTTCCATTCGACGACCATCTGAGAAGAACCATAATAATC
TCTGAAAAATACTTTGCCCAACATTATCCAGAATCACTTTTAGCTTATCCAGTACAGTAT
CCAAGTGAGATGCTGCAACCAATCCCATAGCTTTTGCCAGACCAAGCCTGTTACTTGGAA
TAGCAATATTGGCTTGTTTATACATCCAATCAATCTTATCACGAACATAAGCCCTATCAT
CAACTTTCTGAAGAAGAATGCCCAGGCAACAGTGAAGAAGTGCCGAATGCTCATCATCAG
GCGTATAAAGCACATATTGTTCAGTAAATGCATATCCAAGAGAAATCACCCATGCAGTGT
CTTGAATTACATCCAAAGATTCTGAAAGAAAATTGATGATCATGTCATCCCAGGTCTCCT
GGTATGAAGGATCGAGCTTTAGGTCTTCTGTATCACTAACATATGCCTTCATCTTTGGAA
TCTCATCTTGCCAAAATAAGTTAATGTTCTTTGGAAAGAGAGGTGCCAAATAATAGAGAA
CCATCAAAATATGAGTTGCTTGTTGCTCCCTAGCCAGAGGATCATGCAAAAGCACCACCA
ATCGGGCAAACAATTCCTCAGGATTTGGAATATCAGCATGACCTTTGCACTCATCTAGCA
TGGCAGTGCTGTAAGAAGATCTATGTCTACATAATTCAGAGATGCATCTGCAAACAGTGG
CAGATGCACCAGTATAAACCCGTGGAATGATCATCTTTAAAAGAAAAGGCCAAAGAATGT
GCTCCATCTCAGGAATTGTGAAAGTTATTAAAAGAAGGCCCCTTTTCACATATTCCTCTTA
GCTCTGTCGGACCAAATATCCCTAACTTCACCTTGGAGCTCTCATGGTCACATTGATCCT
GATCTGACAATGCACAATGTCGTACAAGATATTCAACACACAACTCCCCAGAAGGACCAG
TCAAATAGCAATGTGAAGCCATGACCACAATTAACTCAGAAACAGCCTTTCGAACACCTA
AATTTTGTTCATCCAGTAAGGACTTTACTGTGTCAACAAGCAAAGGTTGTTTACCATGCC
AAGCTTCAGAAGACCTCGGCAAGAGGTGCTTTAGAACACAAAGTGCACCAAACGTCAAAG
GTTCTTCCTTTAGCCGGCATTT
>ntig_849_consensus
ATCTGTTCTCAAAATCCAAAAGAAAGAAAGCAAACCATGTACAAGATTCTGTTCATGTGC
AAGAAAAAGAGTATTTGAACAAATTATGATCCAATGGTATCAGTAAACAGAGAGCCATAA
AAAGAAAACTTGAGCTCTAATAGATGCGTTTGCTATAAAGCAATGTGAAAAAGTAACTTT
TATTTTCCAAACGATGCATTCCATCAGATGAAAAGGCAGAGCATGATCAAACTGAATCAA
ACAAGATTATAAATACTTGGGTGACTGGCACCTTAGATGATGTCAAAGTTAACAGGTTCT
CGCAGGATCCTCTCCATTTGCTGTGCCCTAGTCTTAATGGACATGTCGAACACCTTTTGG
TCAAATTCAACCACAAGTCCACCAAGAATGCTTGGATCAATCTTTTGTTCAACCTTAACC
TTCTTTCCTTGTCCAAGTATATCTAGAAGTGTTTCTTTCAAATCTTTCTCCTCTTCCGGG
GGGAGAGGAATGACAGTTGTGACAATTACTTTCACTTCTCCCTTATGTGCCATAGTCAAC
TCCACAAATCTCTTCACAATGCTGTCTAAGTATCTCAGCCTCTCATTCTCAGCCAATACA
ACCAGAAAGTTCTTCGTAACCTCTGAAAATTTAGCTTGGGCGCAAATATCGTTAATGGCC
TTAACCTTTGTATCTGCAGACACAGCCAAGTCCTTCGTGAACTGAAAAAATATAGGTGCT
CTCTTTGAAGCCTCAATAAGTTCAAGAAGCTCAGTCTCAACTTTCTCAAGAGAATTAGCT
TTCACCGCTGCAAGGAATAACGCAGAGGCATAGTTTCCAGATCCCCCAAACACGGTCACA
GGCACCTTAACTTTCTCCTCCTTTGGCCCTGAGGTCGTTGCAAAGTTCTTGGAAAACTGT

GGGTGAAGGATGGATCGCCGAGAAACGACAGAGTCGGATCTGATAAGTTTGCTGAACAGA
GACACGCTCGATCTGAGACGCCCGGCCGTTGCCATTTTCCCAAGTTGAAAAAAAAAACCC
TAATCTCCTCTGTTTAGTCTTTTTGTTTTGCGAATACGAAAGAGCTGAGGCAGGGCCAGA
AGACG
>ntig_850_consensus
ATGCAAAAATGTTTTATTTGGCCAACTACAAAACGGATGTTTAAACTGTACTAGGCACAT
GAATCGTAAATCAAAACAACATTTTTATAATCAAAATTGTAACAAAGAATTATTTCTATA
AAAGAACAACGGTATAGGCCTAATGCTACCGAAATACAACCCACCAAATGCCAGCCATTG
AATATTTTACACCAAAGCAACACTTAGCGCCAAGCAGATGCCGAGATCAAAGCCCTGTCT
TTCCACCCCAACAACAAAGCTCCATCAGTCTCCACCAGCTTATAATGCTCTGAGTAACAC
CTCAACAAGCCCTTAATCACAGAGTTGACATACAGGCTTAACGGGCACTGCCGGAATCCT
GCCATTGTAAACCTAGACTTCCATTTGCCAAAGAGTTCGTGTCGCTCCACCCTCTCCATA
CCCTCACAAGCAATAACATTCACGATATCCCTGGCCAAACAATGTTGTTCGACATTGATT
CGCTGCTTTTGGTCCCTTGGCATGGTCGTATCAATTGACTCAAACATTGCCAAGTAATAG
TTTAGTGCTTCAATGAACCTAGGAAGGAAAGCAGCAGTGTTTGTGTTCGATTCTTGCTCC
ACCAAGGTGACCACTTTCGGATTAAGGGACTTTATCTTCCTCAAAAGCCCATCCCTTGGG
TTGTTCACATCAATGCTTTCATCTGGGGTGTGGTGGAGCTGCAGAGGGAAGTTGACAGCG
AGAGCTTCCCCCGGCCTGACATCAAGCATTTCCAGGGTCACTTGAGGAGCAAAAACAGAT
ATAGGATGAAACTCAACAGGGATATTGAATTTCTGAGAGATTGCAGCCAAATGTTTACCA
ACTGCCTCCAATCCATTACC
>ntig_851_consensus
CTTGTTGATAGATTACCATTCAGCTAGAGCTAGAAAGAATCGACAAAACAGTCTTTCAAT
ATGATAGATACTTCACTGATTACTCAAACCAGTCAAAGTTACACATCTAGGTTAGAGAAG
CCTAATCCCTAAACCCCTACTTGATGATATAAATTTAAGACAGCTCATTTTACACAAACT
CTTAAATCTATACCTCTTCTTCAAGAAATGGTAACTTCAACAGTCTTAGACTTGGGCAGT
GGAGGAAGTTTCTCAACAACAATGGTCAAGACACCATTTTCACATTTGGCTGTGATAGCT
GACCAATTGGCATTTTCAGGCAACCTAAACTTCCTTATCAACTTTTGCGGTACTTTCCTC
TCAAGCCTGATGTACTTGCAGCTTTCCCCTTCACCATCTTCGCGCTTCCTCTTCCCACTG
CTTCGGATCACCAGTGTTTTCTCATCCTCGACAATCACCTGAATGTCGGATTTAGAGAGG
CCAGGAACATCCATGTAAAAAATGTACTCCTTGGGAGAGTCCAATATGTCCACAGGAATG
CTACTAACTCCCTTGTTCTCATTTGCCTCAAAATTCGAACCTGAATGGAACACCAATTTC
TCCATTGTTTCCGGAAAACCAAACAAATGGTTCACTGCTGCCGCTACATCGTCAGCCACT
CTGCTCATTCTCAACCGATCT
>ntig_852_consensus
AGAGAGTAAAACAGGATTTGTAATTTCTGCTTCTGAGTGCATTAACAAAAGTATTGTAAG
AGCATTTCATCTAAAAAGTATGAAAAGAAAACCCATATCATTCTCAAAGTAAAATCACAT
GACATTTATCTAACACCAAATTCAACAATGTTATCAATAACAAAATCACCATGACAAGTC
TTACAAGAACAATGAAAACACCAGGACCGATCTAATCCTTCCCCCTTTCAAGTTTTACTT
CTCAGATGAGTTGGGTCCTCTCTTCTTTCCAAAACCAACAACTGCAGTGACGAAACGGCG
GTTGTATTGCATGCGCTTGTAGGCACGGCCGCGTGGCTTCTTCTTCTTGTCTTGCTTAGC
TACTTTTGGAGTTTGTCCCCTCACCTTACCGGCACGAGCAAGAGAACCGTGAACCTTACC
CATGGCTGATTGCAAAACCTGCTGCTGCTGTGTTTCTGTTTCTCGCTTTGGGCAGCGGCT
AAGTGGAGCAGGAGAATAGGTAAAACGGTACTAGGGTTTCGGCTGAATATATAAATTATG
TTAAATACATTGGGCCGGGTAATGGCCCGATATGATTCTCAAAGAAAGGCGGTAACTCAT
TTAAGTCCATAAGA
>ntig_853_consensus
TCTACCACCACCTCGGCCACCTCCACGGCCTCTACCACCAAACCGACCACCACCATCTCT
ACCACCACTTCTACCACCACTTCTACCACCACTGCGGCCGCCACCTCGAGCACTGCCTGA
GCCATCACCCCTTGGTCTTGCCTCGTCCACAATCAAAGAATATCCACCAAGTTCAGATCC
ATTCATTTCTAGAGCTTTGTTGAAACTACTGCTATCTGTGAAGTCCATGTAAGCAATCCC
CTTAACACCACCAGTATTATAATCTTGAGGAATGGAAATCCTTGACACCTCTCCACATGA
GCCAAAATGTTTTTCCAGGGCACTCCTAATCTCATCTTCTCCAAGTGATGAATCAAAACC
CCGGACAAATATTGTTTGGGACTGACCTCTCCCTCCCTTGTTGAATGAGTTGGCTTCTTT
ACCGCTATAAGGTGTAGTGTACAAGCCCCTTTCGCGAGCCAAATCGAGCCTAACTTCACG
ATTACCCAACAATTTACCATTCAACTTAAGAGCTTTATTTGCAGCTTCTGCCGTAGAAAA
TTCAACATGTCCAAAGCCTTTAAATCTCCCATCAACATCTGATGCCAAACGGACGTCAAC
TACCTCCCCAGCATCCTTAAAGAAATGTTCTATATCAGCTTGTTCAACTTGGAAAGGTAG
ATTGCCCACAAACAATGTTTTCGAACCAGTTGATTGAACTTGAGGAGTGATGGGTGTCTG

GGGAGCTTTCTTTCTAGATTTCAAATCAGCATCTACCATTTCTACATCAGTATCCTTTTT
CTTTGGAGTCTTGGCTTCTTCCTCCTCACTTTCATCTGAATCATCTGAGTCATCCTCATC
ACTACTGCTTTCCTTTTTAGCAGTTTTAACATTATTTACAGGCATAGTGGGCTTTTTCTT
CTTAGGCTCATCATCACTTTCCTCCTCAGAGCTACTATCAATATCCATCTTTTTCTCTTC
CTCACTATCCTCAGAGGAGCTACTGGTTGACTCCACCTTTTTCTTGGGAGCTGCAACAGA
TCCTTTCTTCACAACAGCTGCTTTCTCGTCTTCTTCGCTACTATCATCAGTATCCTCGTC
AGAGCTATCACTGGATTCAGCTATTTTCTTGGGTGCAGCAGCAGAAACATTATTAGTGAA
GGTAACCTTCTTAGCTTCTGAAGCCTTTGGAGGGGCTCCATTCTCCTCATCAGAATCCGA
AGAATCTTCAGAGCCTGAAGTATCATCATCTGAACTATCACTTGATTTCTTAGAAGCAAG
AGACCCTTTCTTGGCAGCAGATACTGGCAATTTTTTTGGTTGGGTAGTCACTTTGGTGGA
TTTCTCATCCTCATCTGAGCTATCATCTGATTCCTCATCTGAACTGTCACTTGATGAATC
TTTCTTCTTTGGAGGAGCAGAACCAACATTTTTAGCCTTAGCCAATGATGCTGTTACCTT
GGCTTTAGCATCCTCGTCAGAATCAGAATCATCTGAGGAATCTGAACTGCTAACAGGCTT
GCTTTTCTTGGCAAGGGCAGCAACTGCCTGCTTCTTTGGAGGCTCCTCATCAGACTCAGA
ATCAGAGGAATCTGAACTGCTGCTAGCCACCTTGCTTTTCTTAGTAGGGGCAAAAACAGA
GCCATTTTTGACAGCTACTGGCTTCTTCGGGGGCTCTTCATCAGAAGATGAATCATCCTC
TTCACTACTAGAGTCTTTGACAGGGGGTTTTGCAACATTAACTCCCTTCTTAGCCACAAC
TTTAACCTTCTGCTCTTCCTCAGAATCTGAGGAATCCTCATCCGAGCTACTGGTTTCCTC
TTTTTTTATTTTCTTAGTTGTCTTAGCTTGAGCTTTCTCCTTCTGAACAGCCTGTGCTAC
TCCATCATTTCCTTTCTGCTTCTTCGAAATCACTGGCTTCTCCTCAATAGCATCTTCAGC
TTCTCTCTTGACACCTTTCTTGGCAGACTTTGTAGCTGCTACAACAGGAGCAACATCAAC
TTTGGTAGCAGATTTCTTGCTCGACTTTCCCATGTTAGAGTTGAAGAGAGAGGTGTAGAT
ATTTTAAAGATTGAAAAGAGGAAAACACAACACAAAACCCTAGTTTTAAGATATTTTGAA
GGTCGAAGGGTTTTGATTAGGAGAGTTTGAAGAATCGAACGGCAAAGCTACCTTGTTTAT
ATAGCGCGGTACCTCGT
>ntig_854_consensus
CCTGAATGCCATTATATATCATACTTTTGTAGTGGTACAAGTAACATAGCAAGAACCCCT
CACAATTTAGAGAGGTCATATCCAACTTCTTCCTATACTATAGACATCTGTCTCAATTTC
AAACACTCTATAAGGCTTTATCAACTTTTACATTATCAGCCTGGTGACTCACCAATGATT
CCATTCTGTAGTTTGGTTTCCCGAACTTACCAGGAAATGTAGTCGTTCCATCAACTCTGA
GAGCATAATCTTGCTCCCTTGTATTCTCACACCAACATCTGGGGGATTCTCAAGAACACA
AAAGAGAGGGAATACAAAACAGTGGCAATTATAACTGCTGTAACCAAGTTGTAGAGAAGG
CGCTTGTCATCTTTACTGGGCACAAATGCTATTTTTAATGTATTGGTCAGCTCAAATAAT
CTGTAAGAAACTGCAACATATATGGCACTGGTGAGCATAAAGTTCAACATTGGGTAATCA
GGGATCAGTGAAAGCAACAATTTAGGTTGTCCATCAGGAACTGCCGATCTTAACCAGATA
TGGAATTGTGAGATGTAGGTCTCCAGTGTTACCTTTCCAAGCCATGCAAAGAGAGTCAAA
CTGTAGCTACGGAAAGACTGTGTGATGTTTCGCAAACAAATATAGACAGTTATGGGAATC
CACGAGGTATAAGGATGATACTTGTTGTAAGTAATCTTATCCAGTTTGTATACATACTCG
AACCAGATGAATCCAACCATTAGGGCTACAAGTCCAACAGCTGTTTTAATTAACAGTCGG
CGCTTCACTTCTGTTTCCTCTAGTTTCTCCATCCACCGTTCGACCGTTGGATGATAGTAA
GCATATGTCATCCCAACTATCCATATGTAACGATCAAGCCCTGAGCGGAAATGCCACTCA
TGCAAGATGGGCAATGTTGGCTTGGCTGGGTCAGTATAACCAACAAGGAAAGTAAATGGG
CTCCACAGCAATTCAAACACTCCAGGTACTTCCCAGACTAGAATAACCACCAAGAAGCAG
GCAATAATTTTTACAGCAATGACTGATCCAATCTCATTATACTTGTTCAGTATACCAAGT
GCACCATAAACCATGAGAGTAAACAAAGTGTGCATTGGGCAAATGTAATACAGCATGTAA
CTATTGTTAAGGACGACACAGCAAAACAAGACCAGGAAATTAAGCCTCCACATCATCTGT
GCAAACCTTGCACGACTGAAATCTTTGCGTACATAATAATATGAGAAGTTACCAAATCCA
GTCATCCACACATAAGCAGCAATAAATAGTCGGATTGCATTGTAAATTTCTGTTGCAGCA
AAGTAATGGTACATCAAAAACAACACCTGCATCCAACCTTTCCACTCTTCAGTTTGATGT
CTATTCAAATAGAGCATGGATTTCCCCGAGAATGGTGACTTATCATGATGTATCTTGAAG
GAAGTTATTGCAGAAACTATGATAAGAAGGGAGTAGAGAAATAAGAAAAGATCCCGATTG
TAACTCTTTTTTGCTGACCCAAAGACATCCGTCCGATCACAGATGTAAAAGTAAGCCAAA
AGCACACCAAATTCAGATATAGCTCTAAGCGTGAGACGATTTTCGATCAGGAATGCCTCT
TCCATCATAAGAAATCTGAAGATAGGTGAAGATGCAGACGAACTCCGAGCTTTCGGAGAC
TCTAATTGCAATCCTCCGCCTTCTAGAAGAACTGCTTTGTCATCTTCCTTCACCGTCCCG
TCACCAGCTCTCAATTCATCCAAACTGACATCAGAGTGTACTTTGTTCGGCAATAAATTC
TTTCTGTACTCCAAATATTCTGAATAAATCCATGATGCAAACACGGCAACTATTCCGATG
AAAAACGAGACCTGGCCGGGAGTGAGAGGACTGGAGATCGTCATTTTTAGCAAATCAAAA

GAACACTAGTAAACCGACGCGCATTTCACTGACACTTCAAATTCTGATCGGATCTGATCC
AAGTCCGAGCAAAATGAATCTTTGATGGAAGTGACGCTGCT
>ntig_855_consensus
CCTTTATCATGTTGTTTCAAAGCCACAGAGCAGCAGCATGCAGCATACAGCCAAAAACGG
CGCCGGACCTTATGCGAGAGTCTTGACTTGTTACACGTGATTCAAAACCCCGCTCTACGC
AACAGAAGTGTTGGAATTTTATTGGAATTGAGGACATCCTACCACTTACCAAACTTAATG
TATCAGATAGCATAAATCTCTCTTGAGTTCCTCAGATATTTAACCGCGGCTTACACGAAA
CATTAATATATCTCTACACCATAACAAAAATATAACTATGTTTTTTCCCTTCTCGTTCGA
CAACCTGCATCTGCATTTTGCAAAGTAATATTAGACAACTGCAGAGGAAAATCAACACAA
CGGCCAATTTACAGGGGCCAAAAAGTTGTAGACCTGTGAGTTTCAGACATAAAAAATCAA
AGCTTAAACTTAACAGCCCACAACAGAACTTTTGGAGAAAAAAAGGTAGCCGTGAAAATA
CACCCAGCTTTATGGACAAAGCCTTTACTATTTTGTGCCATTAAAAATCAGCATTTCAAA
CTCGTTTTCCAAATCATAAACTCCCCCCTTCAACCGTGCAATTCCAATCCCTCATTTCCC
TAACTTTCTTCGGCACTCCACCAGCTTAATAACCTTCACTTTTGCTGCACCAAACACTTC
ATGATTTACAATGCACTGTCATGAACATCTAATAATGAACCCTCTCATCACCATCAAAAT
GCAGGCCCTCAATGCCCTTTAGGGTAGTTTCATAATTTTTAATGTGACCAGTATTTCTCC
CCGATGATGTGCGCCTTGGATCTGAAGATCCAACAGGGGAACTTCTTTGTGCACTTAAAA
CTCTGTGCACTGCTCCAGGGCTTGGTTCGGTAGTGCGAGAGTGTTGATGATCAGCCTCAC
TTCCAGCAAATGCATCCCGACTGCTTGAAACAGCAGCAGCTCGCCTAGAGGATCCACTTG
GTCGTCCCACAATATTGGAGCCTGATAACATAGCATCTTTACTAATTCCAGATTCATTTA
CAACTGGACTCTTTTGCCTAGAGAAATTTCCAGAATTTATGATGGGCCCTGACATCATTC
TCCGTCGTGAGGACTCCATTGAAGACAAACCCGATGGTCGACTATCTTCCCCACCTGTTT
TTCTGTCAGCACTGGCTATAACAGGAGGTATTGCAGAACTGGTGCCAGCACCGGGGACAA
GGGCACGAGTTGGTGGTGTGGCCAGCTGTGATTGCTGATACTTCAAAATGGTCCAATCAA
ATACATAATCAAATTGGAAGCCTTCACGAATGAACAGGTCACGGAAAATTCTTTTAAGAT
AAGCATAATCTGGCTTATCATCAAATCTCAGTGAACGACAATAATGGAAGTATGAAGCAA
ATTCTGTTGGATAGCCTCGACACAAGGCCTCTATGGAAGTAGAAACTTTCTTTTCACTAA
TTTTCTCATATTTCTGTTTCTTAGTTCCTGCTTTCAATCCCTGCCAGGGAAGGCTTCCTC
TCAAGAAGTACATAAGAACATAACCCAGAGACTCTAGATCATCCCTTCGGCTTTGCTCAA
TCCCGAGATGAGTGTTCATGCTGGCATATCTTGCAGTACCAGTCAAATTTTTATTCTCCC
TGTAAGGAATATGTTGATGGGTTGAACTATCTCTGTATTTCTTAGCCAGACCAAAGTCAA
TGATGTAGACCTGATTTGCACGTCTTCCTAAGCCCATAAGAAAATTATCAGGCTTAATAT
CTCGATGTAGGAATGATTTTGAATGAACAAACTCAACACGATTAATCATCTGATCTGCAA
GCATAAGAACTGTCTTCAGGGAGAGTTTCCTACTACAGAAGTTAAATAGGTCTTCAAGAC
TCGGTCCAAGTAAATCCATCACCAGTACATTATAGTCTCCCTCAACACCAAACCATCTCA
CATTTGGAATCCCAGTTCCTCCCTGCAGGATTCTGTATAATTTGGATTCATAGAGCAATT
GTGGATGCTTTGTCTTGACGTTTTCAAGCTTAATGGCGACCTCTTCATTAGTCTGAATAT
TAGTACCAAGATAGATCTCTCCAAAAGACCCGCTACCGATTTTTCGACCCAACCGAAACT
TATTCCCCACACGAGGCTCCATGAACACGAGTTACAATTGAAACCCTAGCTCTATTCACC
AACAAACCCTAACTAATAGCTCTAGATTAAACCAAATATATTCTCAAAAATAAACAAATA
AAAATATCTCAAAAACACGACACTCCCCGCCGAATCGACTGCTCCGACAACCTNNNNNNN
NNNNNNNNNNNNNNNNNNNNNNNNNNNNNNNNCCTTTTCCCCCAAAAGAAAAAGAAAATAG
CGTGGAGTTAGGTCAGTGAAACGCAAACGGGCTTTCAGTTTTCGGCATCTCTATCTCTCT
ATCTGTAAGGACTTTCTTTTTATTAATCAAATTATCCAATCCAAGCATTTACTTTGAACA
GCGGTC
>ntig_856_consensus
AACTTAAGATGGGTCCTGACGTCGTTCTCCGCCGTGAGGACTCCATTGAAGACAGACCAG
CTGGTCGATTCTCTTCTCCACCTGTTTGTCTGTCAGCACTGGCTATAACAGGAGGTGTCG
CAGAACTAGTTCCGGCACCAGGGACAAGGGCACGAGCTGGTGGTGTAGCCAGCTGTGATT
GCTGATACTTCAAAATGGTCCAATCAAACACATAATCAAATTGGAAGCCTTCACGAATGA
AGAGATCCCGGAAAATTCTTTTCAGATAGGCATAATCTGGCTTATCATCAAATCGCAGTG
AACGACAATAATGGAAGTATGAAGCAAATTCTGTTGGATAGCCCCGACACAAGGCCTCTA
TGGAAGTAGAAACTTTCTTTTCACTAATTTTCTCATATTTCTGTTTCTTAGTTCCTGCTT
TCAATCCCTGCCACGGAAGGCTTCCTCTCAAGAAGTACATAAGGACATAACCAAGAGACT
CTAGATCATCCCTCCGGCTTTGCTCAATCCCAAGATGAGTGTTCATGCTGGCATATCTTG
CAGTACCAGTCAAATTTTTATTCTCCCTGTAAGGAATATGTTGATGAGTTGAACTATCTC
TATATTTCTTAGCCAGACCAAAATCAATGGCGTAGACCTGATTAGCCCGTCTTCCTAAAC
CCATCAGAAAATTGTCAGGTTTAATATCTCGATGTAGGAATGATTTTGAATGAATGAACT

CGACACGATTAATCATCTGATCGGCAAGCATAAGAACTGTCTTCAAAGAGAGTTTCCTAC
TACAGAAGTTAAACAGATCTTCAAGACTAGGTCCAAGCAAATCCATCACTAGAACATTAT
AGTCTCCCTCAACACCAAACCATCTCACATTTGGAATCCCAGAAGCTTAATGGCGACCTC
TTCATTAGTCTGAATATTAGTACCAAGATAGATCTCTCCAAAGGACCCGCTACCGATTTT
CCTACCCAGCCGAAACTTGTTCCCCACACGAGGCTCCATCAACACCAATTACAATTAAAA
GTCCTAGCTCTATTCACAAACAAACCCTAACTAATAACTCTAGATTAAAAAGAATCTCAA
AAATAAACAGATATCAATTTCTCAAAACACGACACTCCCTGCCGAATCGACCACACCAAC
AACCTATTAAAGCCTTAAAAAATTTCCCACTAATTAAAAAAAAAAAAAAGGAAAGAA
>ntig_857_consensus
AATAATAATAATAATAATAAATGGTGAAAGCTTGAGCTCTAAAACCTTGTCAAGACCCTA
ACAAACTTGAAAATGGTTTCTGCTCAAACTATCAGCTTGCATTCCTTGATTACAAGTATC
TTTTAAATTTCATGTGTATAACACTCATAACTTCTTAAGGGTAGATTTCAATAGGGATAA
AAAGCGCTATATGGTGAAGTTATAATATCCTTAACATATTTGGAAGAAAACCATTAAAGA
CTAGTAAGACAGAAATCCATAATTCTCTAATACAAAAAACTCCACAAGAACAAAATAATG
AGGTAGAAGAAAGAACATAAAACTTTCATATGACATTAACTTGAAAATTAATCATTCTTC
ATCCACCATCGTGCCCTTTTCACTGACATAAATACCATCAAGGAATTTCCTGATGTCCTT
GTTCTTAACATGACATTTCTGATTAATCAAAGCACAAGACCGAGACACCAGCTCAATGTC
ATTTCCATCCAATATGAGCTCATCCTTTACCTTTTCAGATCGAACAATGGTCACACCTCC
AAGCATATCCACCTTTCTAACCTTCTTCTCTCCAAGAAAGTTACGTATCTCAATGGACTT
GTTTCCATTGGTAATGGAAGCATTAATGGGAAAGTGAGCGTAGACGAATCTCATCTTATA
ACGGTAACCTTTAGTGACACCTGTGATAAGATTCTCCACGTGGCTGAGGGCGGTTCGAAT
GGCGGCGCTGGTCTTGCGGGAGCCGAACCAGGCGTCAATCTTNNNNNNNNNTTGAAGTTGC
GGCTGAGCTTCCCCCTGGGCCCCTCCACTTCGATAATCTTGGCGTTAATCTTGATCTTAA
CGCCGTCAGGGATGTCCATCGTCTCTGAGGAGAGAATCGTCTTCATTTTTTAGTAGGGAT
CGTACTCTGTCGAGTCTTCTTCTTCTCCTTGCAGCGGCAAAAATGCAGAGTAAAGCCATC
TCTAAACCCTACAACAAGTTCGTTTATACGAGTAGTTGGACGGGTTTCAC
>ntig_858_consensus
GTCCATCCACTGAAAAGTAAGTTTCAATAAAAATCAACAGAACAGTACAGCAAGGTTTTA
ATATATCCATAACATATTAAAGCAAAACCATTAAAAGCCTAGCAAATCCAAAATTCTTTA
ATATAAAAAATTCCAGAAAAACAATCATTATAAGAAAACAAAGAACTCCCGAAACATTAA
TATGAAATAAAATTGACGATTAATCATTCTTCTTCAAGAATTGTGCCCTTTTCACTGACA
TAGATACCGTCAAGGAATTTCCTAATATCCTTGTTCTTGACATGGCATTTCTGATTAATC
AAAGCACAAGACCGGGACACAAGCTCAATGTCATTCCCATCCAATATGAGCTCATCCTTA
ACCTTTTCAGACCGCTCAATTCTTACACCCTCAAGCATATCCACTTTACGAACCTTCTTC
TCTCCAAGAAAGTTACGGATCTCAATCGACTTGTTTCCGTTGGTGATGGAAGCGTTAATA
GGAAAGTGAGCGTAGACGAATCTCATCTTGTACCGATAACCTTTGGTAACACCGGTAATG
AGATTCTCCACGTGGCTGAGAGCGGTACGAATGGCGGCACTGGTCTTGCGGGAGCCAAAC
CAGGCGTCGATCTTGAGCTTTCGCTTTCCGGTTTCCTCGTCGGTCATGAGGTGGAAGTCG
AGGTTGAGGTGCTTGAAGTTGCGGCTGAGCTTGCCTCGGGGACCCTCCACTTCGATGATT
TTGGCGTTGACCTTGATCTTCACGCCGTCAGGAATATCCATTGTCTCTGAAGAAAGAATC
GTCTTCATTTTCTTCCAGCTGCGGGTGCGAAGCGGAACCAGCTAGGGCACAGGGGTCGTT
CAGAGACTGAAATGAAAAACCCTAAAGTGTCTTCATA
>ntig_859_consensus
CCTTGAACCACACACGAATTTACAAAAAAACTGCTACTCATTGTTAAAGCAAAATCAATA
TGAATATTAAGTTATTTGAAAGAAAAAAAAAAAAAAAACTCTTCCAATGATCTTTGATCT
TTTTTTTCACAGCCGCCACCAACCATTCTACTCTCCAGTAATGTTTCTAATATTAAATAC
AGTTGATTCTACATACCTAGCAGTTCAGAGTCGATTCAGCGGCCGGAGCAATTTAATATG
CAATCCTGAAAACCAGCTTTGTGACCGATAATCCATTATTATCAAAACTGTTCTTTTCTT
TTCCGGACAGCAAAGAGGCCTTGGTAAGAATGCTACTCTGCAGTCATACCTTGGTTTCAA
AATTTGTCTTCGATCACCTGTCTTCCACCTGAACTGCAAAAAGCAATAAAAAATCTTCAG
CGGATACCTTACCAGACCAAATGTCCTTTGACATGGTTTATGAAATGGTCTAAATTGCTT
GCTGTCAAAAATTCGATATTCCACGTCATCTGCAAATGCCTTTGTTTTTTTTTTCTCTCC
ATTCCAATGTTTTTATGTACAGTGTGAAGTTTCTGAAGGACAGTAAAACATCTTGGCTAG
AGCTGCAAAATTTCTGAAGGGGACAGGAGGGCAAAAAATCTCCTCAGAGAGGAAACAGGT
TGAGCCAACAACTGTTTAATCTGAAAGCATTTCTTCCAACTCAGGCTCCTCAGAGGTGGC
TACATCATAATCTAAAGCAGTTCCGTTGTAGAGAACCTTCTCTGGAAAGCCTCTTTGATC
TTCATCCTCATCCATGTCAGCCAATTCCAGTTCATCATCTTCATTCTGATCGTCACTTAA
AATAACCTCTTTTTTAGATTCACCATTCTCCATCCCCTTTCCAACTTTTCTAGGTAAAGA

ATCACTCTTTGCCTTGAGCACACTATCTTCACAAATGCAGCAGGCTGGATTCTCATGAAA
CTCACCCCTGCCTGGCCTTACCTCATTTGGCTCTCCCATGAAGCCTCTCTGATAACTACG
AACTTTGGAGGCAAATACTTCCCATGTTGTGTTGGTCTGGTTTAAGAACAACCGATAGAG
TTTTTTAGCATTTGGCCGAATGGTGGGTTTGTGCCCAAAATATTTCCTCCTTCCAGCTAA
TATTCTAGCCATATTACCATTGTTGTTGGTGACAAATACATCACTTTCATCACAAACAAT
AAAATCCAAAGCAGCCATCCGAGATGAGAATCGAGAAAATGGTGCCAACTCCTCGTTTGT
TGCAATAGTCTCTTTTGAATGGAAGTGTGGAAAAAGTGCTTTTAAAGGCCCCAATGTCTC
TTCCCCTCCATATACTTCGCCAGAAGCCACATAGATATGAACATCGCTGCCATATCCCAG
TGCTCTCAGCATTAGACCAACTTCTAGTGGAGTAAGT
>ntig_860_consensus
CCACAATTCCTTCAATTTCTCAAAATTAAATATACACTGCAACTCATAGATAAAGCAAAA
ATCAATTTTGGAAGAAATTTAAAGAAAAACACAATGAATGATCTTTGAAAATTTCGTTTC
CTATCATTCTAGTCTCCATAATGTTTCTAACATATAATTCAGTTGACCTTACATTAGTTC
GGACTGGATACCATGACAAAGGGGCAGACACGATGCAATCCCAAAACCAGCTTTGCAATC
AATCCATTATTATAAAAGCTCTTCTTTTCATTGCAGAAAGCAAGAGGCCTTGGTAAAATC
CTATTCTGCAGTTCATACCTTGGCTCCAAAATTTTTCTACCATCAACATCTTTGATCTGA
GCTGCAAAAGGGAATATAAGACAGTAGATTCCATTCCAGACCAAATGTCCATTGACATGA
CTTATAAAATGATCCTTCAAGACTGATTAAATTACTTGCTGTCAAATATCCAATATTCCA
CATATCATCTGCAAATGCCTTTGTTTTCTTTCCATTCCAATGCTTTCATTCCTGAAGGAC
AGTTAAAACATTTTGGCTTGGGCTGCAAAATTTCAGAAGGGGACAGAAGGATGACAAGAA
TTCTCCTGAAAGAAACAGATTGAGCCAAAAACTGTTTAGTCGGAAAGCATTTCTTCCAAC
TCGGGCTCCTCAGAGGTGACTGCATCATAATCCAAAGCTGTTTCGTTATAGAGACCCTTC
TCTGGAATGCCTCTTTGATCTTCATTGTCATCCATATCAGAGAATTCCAGTTCATCATCA
TCATCATTCTGATCATCACTTATACTAACATCTTTTTTAGGTTCAGCATGCTCCATCCCC
TTTCCAACTTTTCTAGGTAAAGAATCATTCTTTGCCTTGAGCACGGTATCTTCACAAATG
CAGCAGGCTGGATTCTCATGAAACTCACCCCTGCCTGGTCTTACCTCATTTGGCTCCCCC
ATGAAGCCTCTCTGGTAACTACGAACTTTGGAGGCAAATACTTCCCATGTTGTGTTGGTC
TGGTTTAAGAACAACCGATAGAGTTTTTTAGCATTTGGACGAATGGTGCGTTTGTGCCCA
AAATATTTCCTTCTTCCAGCTAATATTCTAGCCATATTACCGTTGTTATTAGTAACAAAT
ACATCACTTTCATCACAAACAATGAAATCCAATGCAGCCATTCGAGATGAGAATCG
>ntig_861_consensus
GTGCCTGACAAGAGCAAAGTGAAAGAGATATTCAACTGTTTTTTTTTTTTTTTTTGGGTA
AATAAGAGATATTCAACTTACATCAGTGAAGTTAGAGACAATGTCATTAAGAAGGAGCTC
CATGTCCTTATCATAATAATCTTTCAGAGCTGTTAGAATGATCTCCCTATCCCAGACATG
GTGTAGATTAGATTTGTGCGTAAACCAGCGTAAGTCGATGGTGTTTCCTCCTTGATCACT
CGTAAACCCCACATGCATTGGCTGGTGGACATCTCCCATGAAGTGGGATAAGAACAGCAA
CGCCTCTGTCATGTTATGTTTGCGATCAGAGGTTCCTTCTCTGTAGTGCAAAAGCTGAGA
GGTGAAATTCTGGATGGCACCAACAACACAC
>ntig_862_consensus
CTTATAAGGATTCTCAAATAACAACAACAAACGGTGCTTAACCTCACTAGGAAGGGGCAT
TTGAGTCTGAGAATCTTTGCGTCCACGATTTCCTGGAGCTTACCGTCCATTTGCCCTCGG
GGTTAGGGTTTTTAATTCTAGTAATAAGTGAACCTTACGCGAGCCTTTTTGAAAGCTGCC
ATGATAATCGATTCCATCCCAATCTGATTAACTCGTATTTCTAGTTTCCATCAAGCGTCT
CTCTTCCACAAACAACACCACACCCCCACCCCCCCTGCATTGAAATCACAAAACTTTACT
GGGCTTTCTTTATTAATCAATTACAATGGACAGCCCGCTCAAAGTGAAGGGAGGTGTTTT
GGTTGTTGATGTGTTGAAAGTAATGGAATTCGGTCTGTTAAATGCTATCGAGGGTTAGAT
ATTCTTTTAGAGAGTAATTGAATCAAGGGTATTTTGCGTTAGGGCCTAAAATGCCATCAG
CTGCTCTAACGAAACTGAACCCTACCGTTTGTTCTGGCTTTTGGAGAGTTTGTAATTCCA
CAAGTTTATTTGGGCGAAGAAAGCTCTTGTTGGGACAATCTAGGTCATCTGAGTTTTTGC
CATATTCAATTCGGTTGTTATCTGGGACCTCACGGTTTAATTTAAGTGGTCCGTGTGACA
GGAGTTCAATGGCTGCCTCTGGTACTAAAGCTGTTTTTGGAGAGGTTTACATTGATGATT
TAGTTTCAAGTTGTTGTAATGGCTTAGGCTTTGCAAAACCTAGTAGTGTTTATTTCAATG
ATAAAAGTCAGAGCCGATGTCAAAAAGCTAGTATGAGTTTGAGAAAACAGGGGTCACCAA
GGAATGGGCTTCTTTGCGGGTATTATGTTTTTGATTCTGTAAGAAAGAATTGGAACTCTA
ATTTTCTTGTTGGCCCATGGTTGAAAAATATTCATACCTCCTCTTCTGTGTGCTTCTCGA
CTGGGGCTGCTCATGATGTGTCATTTGATGGTGGTTCTCATGATGAACAGCTTGGGAGTT
CCTCTATTTCATCTGACCAAAATATTCTAGGAGAGAGACACTTGAAGCTACTATCAGGAT
CATGCTACCTGCCACATCCTGCTAAAGAAGAAACAGGTGGAGAGGATGCTCACTTTATTT

GTGTGGATGAGCAAGCCATTGGTGTAGCAGATGGTGTAGGGGGATGGGCAGATGTTGGTG
TTGATGCAGGAGAATTTGCCCGTGAACTTATGTCTCATTCAGTGACAGCAATTCAAGATG
AGCCCAAGGGTGAAATTGACCCTGTGAGGGTGTTGGAGAAGGCCCACTCAAGCACAAAAG
CCAAGGGTTCATCAACAGCCTGCATCATTGCCCTTACAAGCAAGGGAATACATGCAATTA
ATCTGGGAGATAGTGGATTCATTGTGGTTAGAGATGGATGTACCATCTTCCAGTCTCCAG
TGCAGCAGCATGGCTTCAATTTTACGTATCAACTAGAGAGTGGCAATACTGGTGATCTAC
CCAGCTCTGGTCAGTACGTGCTGGCTTAGGACCCCAAGTGACCGCTCAGAAGATAGCTGC
TTTGGCTCGTCAACGAGCACAGGATAGGAACAGGCAGACTCCGTTTTCAACTGCTGCTCA
AGATGCTGGATTCCGCTATTACGGTGGCAAGCTTGATGACATAACTGTCGTTGTGTCTTA
CATAAGTAGCTCCAATGATGTCTGAGTCTGTTCAACAAAATCATCGCGTCTATCATCAAC
CTTATATGTTCTCATATTCTTTTTCGTTGACCAGTGGCGTTTGGAAGGCCATTAATCCTG
TTCTGCTTATGCTGAAACGTTGAGCAAATTGTATTTGACAGTGCTTCTTCTATTACATCC
TTTGATTGGCGTCACTGAATTTCTACTCTCCTCCTAATCCTTGTTTCTGTGTCTGATCAG
GTTCGATCATCACAAATCATATAAATTATTTCAGCAATAGTCCCTTTGGGAGTTTTCACT
GAAGAGTTCTCACCCAATCATTGTAGAGGAAAATATGAAATATATTAAAAAGCTTATTTA
TTTACCGTGAGAAAACATGGGGTCACTCAGTTCTTACATCAACAACCTCAAAACTAAGAA
GAAAAACACGGCGTTACAGATTTTTATTAAACACACAGCTATTCAAACGGAATGAGGCTG
CACGTTGGAGTTTAGGGAACAGGTATGTATCCTGCTAAGGACACATGTTTTAGAACTCTT
CTCTGCCTATGGCCACCTTCAGTATCTTGTTCTCTTCTCCATGGTTCTTCTTCTTGTTCC
TCTCTCCTTTGCCTTTCTTCTTCTTGTCTCCTGGCCTCTTCTTCCTCTTCCTTTGCTGCT
GCTTCTCGCTTTCTCCTTTCTTCTCCTTCTTCTTCTTCTTCTTCTTCTTCTTGTCTTTCT
TTCTCTTTCTGTCTTCTCCTTTCTTCTTCTTCCTTCTCTATCTTTCTTTGTTCTTCCCTT
TCCCTTCTTTCCCTTTCTTCTTCTTCTTTTTCTCTCTTTTTAGCTTCTGCCTCTTCCCTT
TCCCTTCTTTCCCTTTCTTCTTCTTCTTCTTCTCTCTTTTTAGCTTCTTCCTCTTGCCTT
TCCCTTCTTTCCCTTTCTTCTTCTTCTCTCTTTTTAGCTTCTTCCTCTTGCCT
>ntig_863_consensus
CATAATAGAAGCCTTCTGACACCTGAAAAAGATAATCACATTTAACAGACAATAGCATAC
ACAAACTCTGGCTGAAAAAAAGGTTCCAGACCTAAAATAACTATTGGTATTTGTTTCCAC
TCACTCTTGAGATGCCCTACTTCTCTACATTATATTGTACTTATTCATGAAATGGTTAAA
TGCTGCTCAAAAGTGCTTTAACCTCCCTGTATATGTTCTAGGGTATATTTGTACAGAATC
TAAACAGAGACCGCCTTTAGTGTGTGTGCAATCAATTTGAGTCATAGAGAACTTAATCGT
TGTTAATGCATTGGAATTCTCAACAACGAAGTCCCCAACGTGGTATTGAATCCATCTTCC
AGATTCAATCAACATACATTGAGATGAAGCATGCTGACCGTCTGAAGTCCAAAGTTGAAA
CTGGACAGGCTTTCTATCCCAGCCATGGACGTGCTCTGTGTTGCAGATGCGTCGACCAAA
CCTCTTTGCCGTCCGACCCAACTGCAGTCTGAAGAACACACTATAAGTTCCCAGGGGAAA
TGGGAACTCTATCTCGCCATCAACTTCAAACCACCAGATTTGCTGAAGATAAGCAATTGA
AGCAAAGCTAGATTCCTCGGTCGGAATATGATTCCAATATCTCCGATCATCAATACCAGT
GATTGCTAAGCCCTTTGCAGAAATTGATAAACACACACCACCGCTACTCTTATCAAGCCA
TACTTTCTTTGTGCCACCATCAAGGGTATTAGCACCACAAAGCCTGGCATAAATCTCTCT
TTTACCCAAGTTTTCTTTGAGATCACCAAGTACTTTGACAATCAAAGTTTGATAATTTGA
AGGCAATTTCGATTCCCAAACAAAATCAGCCAAAGAAGCGCCACGAAAAGCCCAATTTAG
CTTAGCGAATTTACAAATCTCCGGCGGATCCAAATAGGCGATAATTGACGCCACACAACT
CTCCGGCAAATCACCAAGACCCGGCTTCGCTGGTACCGAAACAGAAGAGCCATTTGCATC
AAAGAACAAAGCAGAGAAAACAGAACCCATCAAGAGAAAAAGCAAGCAAGGAAATGAAAG
CGACAATGAGGAATAATAAAATGGGGAAGAAGGCGTACAGAGATTAAAG
>ntig_864_consensus
TCTTAAATAGATGCCTATCTTGTTAGATACCTGAGCATGGAACACTGATAAATGAGAGCA
CAATTCTAAGTAAAAGACATATGAGTCTGAAAATCGAGAGAGAGAGATTGAAGAAAGATG
AAAAGAACATGGTAGAATGAAGATGGGAAAGACATATGAAGATGATAGTATAGAGATGAG
GCAGAAAGATTTGTATTTTTAAAGATTTTCTACTTAGAACAATTGTGATTTCTCAAGGCA
ATAAAATGGCATTTTAGAAGAGAAAAAAAAATGTTTAGGAAAGTTGCAAGCCATGTGTAA
CACATGTTGATATAAACATACTACATAATATTTTCGTGTACAAAGGAAATGATGTAAATG
GCAGGACAAATGAGGCTTCGTGGTCTTGACACAAACATATGTATATATAGAGGTTTCACA
TTTTTTATCGGGTGGACACTTGAGTTTATTTACGCAGGATCTATGTAGATATATAATATA
AAAGCATGGCAGTAGCTTAGATGTCTCCTTTCCACAGAGTCCAGTTTCAAACTTTCACAT
GCACCATGGAGTCTGCAACACATCCCATCCTGCTCCATCTAGCTGGTGGCTACCAAAGAG
CCCTGGCTCTTCCAGCATAGAATAATCAATGGACATCGCCGCTTCTGCCCATATGGATTC
ATCGAACACTGGTAAGTTCACGTTCAAATTCTCCCAGAATCCATCACTTCCCTGATCATT

CTGCCCAACTTTGGCTTCATTTTCTGCCAAATTCGACTGAGACTCAATGCTCTCGTTGGG
AAAGGAAATGATCGAGTTCTTGTTGATGTCGATGTCGATGTTGTACATCTGTGTTGCATC
GTTCACCGAGGGAAGCGGCGTATTTGGTGAGCATTTGATACCTGAATCGCGAATGATTTG
AGGTGGCTTTGGCATTTGGGGAACCTGGGCAATGTCAGAAACAGTTGGATATTGAGAATC
AACGCTCAACTCTGGCAAATTGAGCTTGGCATTTGGGCCATAAAGTTTGCGAGCAGCAGC
ATCATAAGCCATAGCAGCTTCATGCGACGTGTCGAATGTCCCGAGCCAAAGACGAGCGCC
GCGATTGGGTTCACGAATTTCGGCAACCCATTTGCCCCAAGTTCTCTGACGAACACCTTT
ATAAGTGCAAAGCGCATTCTCTGGACCTCCCTTGCCTCTCATGCATCCTTTCCGCGAGCT
CGCCTGTGCAGGCTTTTTGAGCTGTTTTTCCCTGCAATACTCCCCATGAGTGCTTTTGA
CATTATTACAACAGAGGACCCTGAAAACTAGGAAGGTTTGGTGATGATAAGTACATCTCT
TCCAAGAGGTGGGCGGTATTTATACATGAAAAGGGGAGATAGGAGCTTGAATGCAGTAAT
AAGGCACACAAGAATTGATCTGAAACGTGTCATTACATTGTGGGTTTGGGCTGATACCCG
TCATGAAGAACAAGTTTAA
>ntig_865_consensus
ATATTAACATCATCTCAACTCTCAAGCTTAACTAGTAAAAAAAAAACCCAAATTATAAAG
CTGGCATGCTACCTATACATGGAAGTTCCGATATCACTTTTGCCCATGTCAAAAGTTCTT
CTGTAGTGACGGCCTGCAAGCACTCACTGAATTACTTCTACCTATCGATGACAAAACAGA
TCCTCTGCTAGTCGAAGACTTCTTTGAGATCGTGGCCTTGGGACTGTATGACTTCCTGTG
GCAATCTAACCCAGCTGCATAGCTCTTGCATGACTTCATTTTTAATCTACAAGGAGCTCC
CTTCTTTACAAGATCTTCTAGGTTCTTCACACTTGCTAAAGATGTGAAAGACTGAGACTT
CCCCTGATAAAACTTTGAAAGACCCCTC
>ntig_866_consensus
TTTTTTTTTTTTGGCTTCCCTTTCCCAGTGGTTGGGTTTTTTTTTTTTTTTTTTTTTTTT
TTTTTTTTTTTGCAAGCAAATGACAAAGTTTTGTCAGTGTAATTGAACTTATAGAATGTT
TAAAAAAAAAAAAAAAGAAATATTCTATCCCACCCTTTTGGATGACCATTCACTGGAAGT
ATCTATCAAGAAAAAGACAAAGGTCAACAAGAAATTAATTCATTTCTCTAGGTGCATTGA
TAAAAGTAAAGCATTTGAAACACGAGCATGCTGAAATTCAAATTCAAAAGATCTAAAAAC
CTACCACAAAACCAAAACTGATGCCACTATTCAGTTTGAGATATTATAATGACCCATATG
CCCTAATAGTTTAAACTGAAAATTGGGTATGCATGCATGTCAAAAGACTTAAAACATGGG
CAAATATCTAAAATTTACACTCAAGACGACTCAGCATTAAAGATGGTTTTTTTTTATGGA
GGAAACATCATTCCTGTTAAATAAACTTTGACTTGACGAGACTCAAATGGATAACAAGAT
TCCAAACAGGCGAACATGAGAGTGTGCTGAAGATGAGATAATGTTTCACTTGCCGCCCAA
TGAGGGAAAATGCCCAGGATCCTCAATAGCAGGGGCTGCTACACTATTGGATGAGTAGCC
TCCATAGCCACCTCTTGAGCCACGGCCACGACCTCGACCCCGGCCACCAGGGTTATAGTA
CCTCTCTCCTTCAGCAGGTTTCAAAAACTCATTAATGCTAACAGACTTCTTAGCTTTCTC
TTCTTTATCAGCAGCATCTTTGCGTTTGTCCTTATCAGAACCCAGTTTGATAAAGATGTC
ATCATTGCTTTTCTTATTTGAAAGTTGTTGCATGGACTTGAACTCTTTGTCCACAACAAC
CTTCCTTTCCTCAGACTTCAAGGCAAGCAAAGCTTTCCTCTTCTCTTCAAAAACCTTTTC
ATACTCCTCCAAAGTCATTTCCTTTTCCTCAGGCTCCTTATTTTCTTGCTCATTTACGGG
ACTCTCCTTATTGGCACCCCCAACATCTTCTTCACCAGCAGGCTTGTCAGCACTCACATT
CTTCTCATTTTCAACAACAGGCTCTTCAGTCTCCTGCGCAATTTCATCGACTGGAGTTCC
CCAATTGCCACGACCAGCGCCATCACGTTTAATTTCATGCCCACGCCCTGTCCCACTACG
ACGTTCAAATTGTCTCCTTGGACGATCTCCTTCCCCAGATTCTCCATTGCCAGAGCCACT
GCGGCGACCCCCACGGAAAGAACCACGAGGAGCACCATAACTACGTCTTTCAGATGGCTT
TCCTCCTTCTCCCTCTTCTGAGGGTCTGTATCCTCCAGAAAAACCATTATTGCCACCGAA
TGTGTCATTGTTGCTTAAATCGCGGTCAAAGCTACGACCTCCTCCACGCCCAAAACCACG
ACCACCACGGCCCCCACCACGTCCAACTTCAGTCTTTGAATCCCTCATGGCTTGAGTGGG
AGGGGCTGGCTTGGTGGGAAATTTAGCCGCCGGCTTAGCCGCTGACTGAGCTTGAGGATT
TAACGCGGATTTCTTAGGCTTGTCAGCGAGCTGGGGCTGCTGGACAGAGACGAGCTGACT
AAGGTCCTCGTTATCGTTGTCACCCAGTAGATCAAAGGGGTTCATTGTGGCCATTGGTTT
GGATAAGTGTGGATACTACCGAGACAAAATCAGGAATTCGATAGAAGGAGATGAAGTCTT
ATTACTTAGAAAAAGCCATTGAAAAATCGATCTAAGGAAAATAGATTGCTGAAGCAGAGA
AAGAGAAAGTGACAGAAACCCTAGCCTATGCGGCCACAATGAACCCCAAC
>ntig_867_consensus
GTCCCGGCTCTCAATGCCCGCATGGCTGTATTGGCAGTTTGCAAACTCTTGGTGAAAACT
CCGGCAGCTAGACCATATCTTGTTGTGTTTGCCCTTCTTATCGCATCATTAATATCCTTG
AACTTCAATATGGATTGCACTGGGCCAAATATCTCATCCTGTGCTATTAACATATCGTCC
TGAACATTTGAGAAGACGGTTGGCTGAACGTAGTATCCTCTGGTGCCAAATCTGNNNNNN

NNNNNNNGAACTGCTCTGAGTCAATCTGAGGACCTTGTTCAATACCCTTCTTGAAGGGAT
CACCAACAATACGGTTAATGGCACGTGCCTTTGACTTCTCTACAAACTCATCATATACAC
GTTCATGTACATAAGTACGAGACCCAGCACAGCAACATTGTCCCTGATTAAAGAACAAAG
CAAAGTGTGCTAGCTCCACAGCCTGGTCAATGTCAGCATCTTCACATATTATGAAAGGTG
ATTTCCC
>ntig_868_consensus
CCGGCAGCTAGACCATATCTTGTCGTGTTTGCCCTTCTTATCGCATCATTAATATCCTTG
AACTTCAATATGGATTGCACTGGGCCAAATATCTCATCCTGTGCTATTAACATATCGTCC
TGAACATTTGAGAAGACGGTTGGCTGAACGTAGTATCCTCTGGTGCCAAATCTGTCACCT
CCACATTCGAGGGTTGCACTGCTTTCAATGCCTGACTTTATGTACCTAAGGACCTTCTGG
AACTGCTCTGAGTCAATCTGAGAATTTGATAGGGCTCATTAGAGGTACCACTTTGCAACT
CAAGCACTTCATGTAAATTTGTTGTTAAATTTGAATGGGATGAAGATGACACATAGGTAT
AGTTTCAGAAAACTTTCACAAAACTGCAGATCAGAATTAGTATTCAGTTTAAGGGAAAGC
ATAATTTCCATCTGACTGAAAGCAAAGGCATTTTAGTAGTCTTAGTGTTATGCATGGTAA
GAAACTTAAGTCAATCTGGAGACACAATGAATGTACCCTCTCAGTCCTGACTTTTGTTGA
AACCCAAAATTAAGTTGAAACTGAGCCCTACATAAACTTCTACGAGACTATTTCAGAAAA
CCTACCACACAGCATATGAATATACTATAATTTATTGAAATAAGTCAAACGTTTAAGACT
TATCACATTGCAGGAAGAGAAGAAGATAAAGGGGCTGAGGGCAGAGGAGGACCTGAGGAC
CTTGTTCAATACCCTTC
>ntig_869_consensus
AGGTCAAGAGTAGAGTAGAATAGTAAGAGTTTGAGAGCCCGGAGAGATAGACAGGCGCTG
TTCCGTTTTGTTTTTGTGCAAGCTTTTGCATATCTCAGAGTGAAGAAAAGTTAGGTGTTC
TGATTTTCAGGGTTCGGCCATGGCTGCTCGCAGAATCTCTTCCCTGCTGTCTCGCTCACT
CTCTGCTTCTTCTCTTTCTCTGCTTCGCTCTCTTGGAAAAAAACTCTAGCAGGGGAAGAGG
AATCAGCCGGTTTAGCACTGCTGCGGTACTTGAGGAGCCGATCATTCCACCTGTTCAAAT
CAATTACACTCAGCATCTAATCAATGGGCAATTTGTAGATGCAGCGTCAGGAAAAACATT
CCCAACCTATGATCCTCGTACTGGAGAGGTGATTGCCAATGTTGCTGAAGGTGATGTGGA
AGATATCAACCACGCTGTTGCCGCTGCTCGCAAGGCATTTGATGAAGGACCGTGGCCAAA
GATGACTGCTTATGAAAGGTCGCGGATATTGTTGCGCTTTGCTGATTTGGTTGAGAAACA
CAGTGATGAGCTGGCAGCTCTAGAGACATGGAACTGTGGAAAGCCCNNNNNNNNNNNNNN
NNNNGGTTGGGCTGATAAGATCCATGGACTCACGGTTCCTGGCGATGGAAACTACCATGT
TCAAACATTGCATGAGCCAATTGGTGTTGCAGGCCAAATTATTCCATGGAACTTTCCTCT
TATCATGTTTGCTTGGAAAGTTGGACCTGCTCTAGCATGTGGTAATACTATTGTTCTAAA
GACTGCAGAGCAAACACCTCTGACTGCTCTTTATGCGGCAAAGCTATTCCATGAGGCTGG
TCTTCCTCCAGGTGTTCTGAATATAGTTTCTGGATATGGTCCAACTGCTGGTGCGGCTCT
TGCTAGCCATAGGGATGTGGACAAGATTGCTTTCACAGGATCTACTAAAACTGGTCAGAT
TGTTCTTGAATTGGCTGCAAAAAGCAATCTGAAGCCAGTGACCTTAGAGCTTGGAGGGAA
ATCACCTTTCATAATATG
>ntig_870_consensus
GGATGGCTAATATATTATGACGCATGATACATATCTCACAAAATACAAATGAAAAACAAA
TCATATCATAAATTGTATAGAAAATTCGATAAGTGGCTGCTCTTTAATAATAAGAGACCA
AAAACACCCTCCCCGCAATGGTAATAAATCAGTAATATCCAGGGCTTAACGTCAACTACT
AGAAAAATAACAGCACGGAAATATTTTATAGCAATCCAGTAAACAGGTCTTTCCCAACCA
AACTTAATCTGAGATAACATAAAATCTGTCCATCAAAATAACTAGTTTGGGACTAGTACG
GCACATTCTTGGGCAGGTTTCGGATGACATTACTCTTCATACCAATCTTTGTCCGCATGG
CCTCGACCCCTGATCTATTCATCTCCTTCATCACCTTCATTTTCACCATGTGCTCTCTTG
ATTGGACGGTTGCTAGCCCCATGCTCCTATACCTTGAGGCCAGTGCAGCTGTGATGGCCG
TACTATTTGCAGGTGTTGGGCGTGGCTTCTGGCGATAATAGCGCAAATATTCCCGGGAAC
CAAATGTTTTAGTTGCCACTCCATTGTCAGTTCTTCTGCTGATGATAAGTTCAGAACCAC
CACCAAGTTCTATAGTGTTGTCCATGTCACTAGATGAGACCAACTGTTTACCGTCCTCAT
CCATGTAACTGCTGCTATAATCATAGAACTCTTCTAACTCTGCTTCCTCATCATCACCAC
CATCTCCATAATGTACTTTGCAATGACTTTTTGCTTCCATATGCTTCCTAACTGCTTCCA
AGCTGTTAAAAGGATGGCACCTATCATTGCAGTACAAACACATGAAGTCCTTCTTTACCT
TAAGACCAAGATAAGTGAGCAGGCCTCTAGGATCCTTCAAATATTCAACATCAGGAATGA
AAAAACCATGGTGCTTGTGCATGTGAACCATGCAGCTCTCGATTGTCTCATGCTCAATAT
CACACATGAAGCAGCAAGATGGATCAAACTCCTCAAAGTCCTCATCCTCATCCTCCATGA
CAACATCAGAGGTGGATCCCACATTCAAATTAGTCAAGGACTTTGCAGCCTCACCTACCA
ATTCTTCATCTGAATCAACCTCCTCCCATTCATCTTCACTCTCTTCACTGTAAGCCTCTC

TTCGAGGGGGAGGCTTATTTACAGTACGACGTGGAAGAGGCTTAATTATCACTGTCTCCC
TGTCTTCATCATTCATTCCTTGAGAAGCCCGCATGATGTGGCTTTTTGATTGAAGATGCT
GTGAATGAGCCTTGGAACTTCTGTACCCCTTACCACAAAGCCCACAACTATAAAGCATTG
GCGTTTCACTCAGCTTATTCTTCTCTTGAGCAAGGGCTGATTGTCTAGCCAGAAATAACG
CTTCTGTCACCCCTCCTTGCGTTTGAGATTGTAGCGATGCCAATCGGACTTGTAATGAAG
CTTCTGCTCGGCGTCATCGCTGAACTCTTTGTTACAAGCATTACAGGTTAGTCCCGGCAT
TTCTATCTCCGCCCAATAT
>ntig_871_consensus
GATTCCCTTTCTGCTTCATCCCAATTGATTCAAAAATATGATTAATTAAATTAATATGAT
ATTTAAAGATCATTTAGATTGCAAATTTTAAAAACAAAAAACAATTTATTTAAATTCTAA
TACATGAATTACATGCTAATTAATAATTTGAGATTAAATTAAAATTAATTGTACAATGAC
TTTGTGCTTGTTATGCTGACCATTGCCATTCCGCCCCGTGGAAATATCTAACCCAAAGAA
TTCCCTTATTTACAAATTTACCCTTATCAATTTGTTAGCCCGACCCGGTCACCACCTCTT
TATGACACGATCAAAATACGTATAATTATCATCATAATGCACAGATTATACGTATTGAGT
AAATGTGAATGTGCTTTTATACTTTGGAAACAGTCCAACACTCGGGAGAGGTTTCACTGT
CTAAACCCGAATGCACCTTGATGGATCGGGTATCTGAATCCAAACGGGTCAACCGAGTTA
GAGAAGTTTTGAGTCATTGCGCCCGAGTTTTGCGGGAAAAGGACTGAGTTGTTAACTCGT
TGATTTCTAAGACTACTTTCAACCTCGTCATCCGTCACGTCACCAATCTTTCCAGAATCC
ACATGGCAAAGCTGGGGCACTGAAGGAATATACACGTCATTGTTCGCGTAATTGACGATC
CCCTGACTCTGAGTTTGCCCACTCGGCACGAGTTCAGGCACCGAGTTCAGTCCCGCAAGA
CTTGCCCAGTCGAAATTCCCCGAACCCAAATTGTTTATGTTTATCTTCTCATCAGGCTCT
GCAGTTTTCAATGAGTTCATCCGTGGCAAAGCAAAAAGCCTGTCGTCAAACTCTGGTAAA
GACTCCAACACGTCATCAAGGTGTGACGATGCAGACGAACAGGTGCCATTACTGTGTTCT
TTGCTTGAAACACTCGTCGTCGAAATTTCTTTCTGAGCACCCGAATTCTTCTTATAAATT
CTGCAGAGAACCCACTCATCGAGCTTTGAGCTTCCATTTTTGTGAGAAGATCCGCAGAGG
CGATACTCATGCATGATCCAATTCGTTTTGGTGCCTTTCGGAGCCTTGCCAACGTAAAAG
ACTAGAGCTTTCTTGATACCAACTTTACGGCCTTCTGTTGTGATCATTTTATCAGTTCCC
GTAGCTTTCCAGTACCCGGAACCGGCAACTCTATTTGGTCTTGACCCGTTCGGATACTTC
CGCTCTCTAGGACTGAAAAAATACCATTCTTTTTCACCAAATATTGCCTTACTTGGTAAA
ACCCATGGATCAAACTTGTACAAATCAATTTCACCAATAATTGGCAGAGAAAAATTCTGC
CCTGCAACTTTGCGACATAGATACTGAACAAGGAGCTCCTCATCAGTCGGGTAAAACCGA
AAACCCGGCGGCAAGTTCAATTGAGAAAGAGGGTCTGTCTGTTGTACTCCCATTTTTTTA
GGCTAACAGAGTTCCAGAAAATATAAGAAGAGGCTAAGGCTGAGAGAGGCTCTGAGAGAG
AGAGAGAGAGAGAGAGAGAGAGAGAGAGAGAGAGAGGAGAGC
>ntig_872_consensus
TACGGTTAACGGTTACCTCAACGTTTTTAGCGGTAAATTTAATGTCCGAATATCTCCTTC
CATAGCAAATGAGTATGTACAAAGGATTTTTCTCTTCAATCTATCCGTCCCAACCAAAAT
CTGGGGTTTAAATCTCGCAGCATACACAAACTCTTCCTGGTGAACTCACTCCAAGTTGAT
TCAGCCACCGCTTCAAGTTTTCCGTCGGCTCCACGCCACATTGACTTGAATCGACTGAAC
TAGATATGAAAGAAAACAGAAACCGGAACCAACCTGTGATGAAAATCAAAGGTCTTCAGC
TTTGGACATGATCGAAAGCCGACTCCGCCTGGGCTGGAACGCCTCGCGTCTCTTCTGATG
ATGCGAAGAAGGACGAATCAAATCTCTCAACGATCTCTTCATCCACTCTCCTTCCACACC
ACCGATCCNNNNNNNNNNNNNNNNCCGTGACTGTGGGGGTTGACGCCAGAGTTCTTATGCA
GACTACAGCGGAACGAGCCAGGGTGCGTCGTAGGTGAACACATACAAGTTCTTTTCTGGA
AGGAAATAGTATTGTTTTTCTTCGTAACAGAAACCGATCTGTTAGGAGAAATAGATCTGT
CAATTGAGAAGCGAACAGCTGACGAAGAACGAGAAGCATTTGTGTAGAGATTGACACGCG
TAGGAGACGCGGATCTGTGATGACTGTGATGGTGGTGATGGTCGTTGTCTTGTCGGTGAA
AGAAGGTAGACGAAGAGGAAAAGCTTGAGCTCGTTGAAGAAGCGAAAGACGAAGAGGAAG
ACGAAAGATAAGAGTTAGCTGTATCCGATGTGCAGAATCTACCGGACGGTGACAGTGATC
TCAGAACTGGACCGCTGGATCTTCTGGACGACGAAATGGGCATTTTTTTTT
>ntig_873_consensus
CTCACTGTAATTATTCCTTTATTGTATGGTGCCCCTACAAAAAATTCCCTGATCCATGTA
CACGAATACCTCACTGCCTCACTTACACCATGCGTGGTGAACGGCAAGGAGGTAGCCCAG
ACTAAATCAATTGCTCCTTCTCAAAANNNNNNNNNNNNNNNNNNNNNNNTTGTTTTGGTCA
ATCTTCAAGCCGCTGGATCTCACCCATCATGATCCGGTTGCTAGAAACTTTGAAGCCAAG
GAGAGCAGGATCAATCTGTCTTCCTTTCTTCCCTTTCTTTTTGCCACTTCGCCCAGTTGG
TGCACCCGCATCAGATGTCTCCAAAGCAGCTCCATTTAATGGATCAGCTTCTGGAAGAAC
AGGCTTCTTAAGCATGTCTATGAATGATGTCTCTGAAACAGCAGCATCATTGCAAGATGA

GGTCCTCCTAAATCGCAGATCTTTTTTGAGAGATGCTTGAATTTCAGGAACTCGGGTCAC
TACAGGATTACCAACTGACTCCGGCTTCCCTTCATCTGATGTAAGGGATATTGAACTTTT
CTGCTTGACGAATGTCACAGAAGATGACTCAGGCAAAAGATCCTGGGGAGATGAAGCACT
TAATAGAAGCTGTTGTTTGCCTAAATCATTGTCAAGCGCTTTTGGTAAATTAGGGGGAAG
CCTTTCATTTGAAATTTCTCCAAGGGACTTGTCTAGCCCAATTTCATAATTGTATAAGCC
TCCATTTCCGCCAACACTACTTAGAGAACTATGCCTACTGTGGGCACAAATTGGTAGCTC
TCCATAATCCAGAGAGTTTTCTGCTTGCTCCACCAAATTGTCTTTGATCTCTGAAACCAA
CATGCTCGTGCCTTTGGACCCATGCCCCTTCCCTTTAGGTCCTTCCAGCTCTAAGTCCCT
ATCCAAAGTTGATTTACCAATGGAGCTTGTTCTGGACAAGAATGGCTGTTCTAGTAGTAC
TCCAGAATTAGATCTGAGGATTAATCTTTCACCATTCCCTGAATGCTTGAATTGTGCAAT
TGAATTGAAATTTTGAGGCCCTTCCACAAAGGAGCTGTTCCCAATAACTTCTTGATCTGG
GAGAAGATTAAAAGGGAGATTTGACAACTGTGTCTCAGAAACTGGCCAAAATGTATCTTG
GGTTTTAGCAGACAATTTATTATGTTGGTAATCAACATCTGAGGATTGCACCGACTGAAG
ACCCGAGTTTTGACGGAGAAGATCCATTAGAACTTGCTCCGAGTTCTCCCCATCACCTCC
AGTGGATGTCCACAAACTTGAATCTATAGAAGTCATATTAACATC
>ntig_874_consensus
GTTAGTATTATTTATCCTTTATTTCCTTCAAAGTCTTGGTAACATATCTCAGACTGTTAT
TTTATGCCGAATCCCCATATAATAAGTTGGCAAAATCCCCATCTTCATTCAAGATGCTCA
TCATTAGTTATCATTCCATCTGGTATTTCATGTCACAATATGGTACATCGTTGAAGTCCA
AGAGCCGAAAAGAGTCTTCCACTGGTTGGCCAGAATCGGTAAAACCAGCCCATTCGCGGT
GCCTCAGCAGTGGGTTCAAGGAATGCCCGTGTTGTCTCCTCTCTATCTGAATCCTCCCTA
CTGTCATCTTTCTCATTTTCTCTGGCGGCTTCATCATCAGAGATATTCTCAGTTCCAGCA
TCAGAAATTTCTCCTGGCTTCTTGTCAAACAATCCTTTAAATTGTTTCTTAGCCTTCCTC
TCCAGTTCCTGCATTGTCTTCTTTAGTTTTGAAAGTGCTGCAGTCGCATCCAGTTCGGAT
GACTTGTCAACCTTCATCATCATTTCAAAATCATCCCTAGCTTCCTCAAAATCTCCAACA
GCCATGTAGGCCATTCCACGGCGATATAGAGCCTTCACGTGAGCAGGATTCACATCTAAA
ACCTTATTGCATGCCTCAATGGACTTTCTGCATTCTCCCATTTTAAGGAAGCACGCAGCT
ACATTCAGGTGTAGTGAATTCCTTGTGTTTACAAACACCTTCCCTTCCTCATCGTCTTGT
GGATTAACATGATTAAACTCCCGAAGCACCTTCTCATATTTTGCCTTCGCAAGTTCAAAC
TTCCCTTCCTTAAAGAGCCTGTTACCCGTGACTCTAATCTTCTCCGCTTCATCCATTATA
TTTTCAAAGGTCAAACCAGTCCAGTCCTTTGGCATCTCGAATCCAAGAAGTTCAATCTCC
CACTGAATATGAGCACCTTCAGGAACATTTTCTGGTCTAGGGAAATTATCATAAGCATAA
TCAGGAGGGCATGTGACGAGGGCTAACTCTCCAGGCAACATCAAGCGAATACACATTTCA
AATCCCTCAGGCACACGCCCCTCCCCAGAACTGAACTCCAAAGGCTGACCATCATTATCA
ACTTGAGTGTCATAGAAGACCCTCTTTTCTTCATTAAGAAGCATGCCCTTGTAATGGACG
CGAAGTAGACTGTCGTGAAGTGGACAATCCATTGGAAATTCACCTTTTCCATCACGAAGT
CGACGTTTTATTAGACGTCCATCACCAAGCATGTCTCGTACCTGAATGATATGGACGAGT
TCCACTTCAAAATGAATTTCCTCATAACCATCTATCACAGGTAGGAGGGGCGATTGAGTT
AGGTACTGACTTGTAACATATATTACAGCCTTCTCTTTGCGTGTCATTGTTCCTATTCCC
ATCTCAAGACCTTTAGGGACCTCTGATTTTCCAAAAGTAAAGAAATATGGTTCTTCTGTG
TGAGAGAAAATCAATCTCCCATTGCCAGTCTTTGCAGAAATCCAAGCTTTTACTTCATAA
GGTTCCCTTGGTGACTCCCATCCCTGCCCTTCATTTATTACCTTTTTTAAGACACCCAAA
TCATCCGCAATGACC
>ntig_875_consensus
GCTTCTCCAATTGTAATTGCACCTTGCTGGACAGCCAAAGCTGCAACTGTCTGTGGCACT
GGCCTAGCCTGCGTAAATTGCCCAACAATCTACACAAATACGACAGAACTGACCACTATA
TAACTATTAACACTATGAACACAGTCGTTGTATATTGAATTGAACTTGTACAATCGTGCC
CCTGCCCTCTAGCTTAACGTCAAGCATAAACATTAGGTCGCAGTGCCATTTTAAGTAAGT
TCTTAATGTTATTGAACTTAATTAAATACTTCTTAATTGTAATCCAATAGCCCAAAAAAT
TTATCGTACCCGCCTGCCATTTAGACAGAGAGATAATACCTGTCCACCAACTGATTCAGT
GACCACCCGAGCACCAGGCACATCAGTTTCAATAACA
>ntig_876_consensus
TCTTTTTCTTTTTTTTTAATTCAAACCCTTAACAGAACAACCACCAATATCTCAAAATCA
TCTTACCAAACACAGTTCAGAATCAACAACAAAGTAGTAGATAGGTCTTGCTTCACTCTT
ACATCTCATCCTGTTCCTCAACGCCCTCCTCATACTCCTCATCATCGGCAGTGGCATCCT
GGTATTGCTGATACTCTGCAACCAAGTCATTCATATTGCTCTCTGCCTCTGTAAACTCCA
TCTCATCCATCCCTTCCCCAGTATACCAATGCAAGAAGGCCTTGCGGCGAAACATAGCAG
TAAATTGCTCACTAACCCTCCTGAACATCTCTTGGATGGATGTTGAGTTACCCACAAAAG

TAGATGCCATCTTCAGACCCCTGGGTGGAATATCACACACACTGGACTTCACGTTGTTGG
GAATCCACTCCACAAAGTAGGATGAATTCTTGTTCTGCACATTAATCATCTGCTCATCCA
CCTCTTTAGTGCTCATCTTACCCCTAAACATGGCTGAGGCTGTCAGGTACCGACCATGGC
GGGGATCAGCAGCACACATCATGTTCTTAGAATCCCACATTTGCTGGGTAAGCTCTGGGA
CAGTTAGGGAGATGTACTGCTGCGATCCACGTGAAGTGAGTGGTGCAAAACCCACCATGA
AGAAATGAAGACGTGGGAATGGGATCAGATTTACAGCCAGCTTCCGCAGATCAGAGTTCA
GCTGCCCAGGGAATCTCAGGCAGCAAGTCACACCACTCATGGTTGCAGAGATTAAATGAT
TCAAGTCTCCAAAGCTTGGAGTGCTAAGCTTTAGAGTCCGGAAGCAAATATCATAAAGTG
CTTCATTGTCAAGGACCATGCATTCATCAGCATTCTCTACCAATTGGTGCACAGAGAGGG
TTGCATTGTAGGGCTCCACAACTGTGTCAGAGACCTTAGGTGAAGGGAAAACAGAGAATG
TCATCATCATCCTATCTGGGTATTCCTCTCTAATCTTTGATATCAGGAGAGTTCCCATGC
CAGAACCTGTACCTCCTCCAAGCGAGTGACATACCTGAAAACCTTGCAAGCAGTCGCAAT
TCTCAGCCTCCTTGCGAACAACATCGAGAACAGCATCGATCAATTCTGCTCCCTCAGTGT
AATGACCTTTGGCCCAATTATTACCGGCACCAGACTGCCCGAATACAAAGTTATCGGGAC
GGAAGATCTGCCCATAGGGGCCAGATCTGATACTGTCCATAGTACCAGGTTCAAGATCCA
TCAGAACGGCCCTGGGCACGTACCTTCCACCAGAGGCCTCATTGTAGTAAACATTAATTC
GCTCCAGCTGAAGATCAGAGGACCCATCTCCTTTGTATCTGCCGGTGGGGTCGACTCCAT
GCTCGTCACATATAACCTCCCAAAACTTAGAGCCGATTTGGTTGCCGCATTGGCCTCCTT
GAATGTGCAAGATTTCTCTCATATTTGATTGAAATGAAAAGGGATGGAGCCAAGCAAGCA
ATGAGGAAGGGCCGGGAAGGAGGATATTTTTGAAGGTTGTGGCGTGGTGAGAGGGCCTTA
TATTGTAAAATATAAAGAG
>ntig_877_consensus
GGTTTTATTAAACAACAAATGATACAACAATTACAAGTGGAAACAGGCATCTGCATTCTT
CTGCAGACTCATCAGTAAGCCCCCTAACCAGAAAAATGGCATAGAAAAATTGGTGCAAAA
TTACAATACAATTTACAAGTTGAATTAACTACATTTAAGAACCCTACCTACACGGCAGCC
ACGGACAATACAGCCTGAGAAGCATTAATTACAATGGGCTCACCTTCTACAAATAACTAA
CTATGTTAATGAACATGGTCTTATCAAGACGCTAGCATGAAGGAGGCTGGAGTTGCTTAA
GCCGCCGTACAACTTGTTTCATCGTTGGCCTAGAAGAAAGAGAGTCAACGGTGCATACGA
CTGCTAAGCGTAAAACCTCAACCAGATCATCATGACAGCCTGCATCCCATAATCCTGCAG
TGAAAAACTCCTTGGCACGACCTTGTCGCAAGAGCATATACCCCCATGCAACTATGTTGA
AACCATTGCCGAATGAGGAAAATGAGGGATCTAGAGCTTTCTTGTCTGAAAGTAGCTCAA
GAAGTACAACCCCGTAACTGTAGACATCAGCCTTATCAGAAACCCGGCAAGTCATTGCAT
ATTCTGGAGCAACATAACCAAAAGTCCCAGCTACTCCAGTAGTGGCATGGGTTTCAGAGG
TGCCTAGAAGCCTTGCCAAGCCAAAGTCGGATAAATAAGCATTGAAATCATCATCTAACA
GAATGTTGCTGGGCTTGACATCACGATGGAGGACACGTGGTACACACTGATCATGAAGGT
AGGCAAGAGCACGGGCAACATCCAGAGCAATCTTGTGAAGTATCCTCCAATCCACAGCCC
TTGTGGATCTTTTCTGGATAAAATTTTCCAAATTACCCCCAGGCAAATAATTGTAAATCA
GGAACATTTCAGTTTCACTCGCATGATAGCCAATTAACGTGACCAAATTCGGATGCCGAA
GCCTTCCAAGGGTTCTAATTTCTGCATGGAACTGTTGAATCCCTTGAAAGCGTCCAACAG
CAAGCCGTTTTATGGCCACTAATACTCCCGGTGAGATCTCAGCCTTATAAGTTGCCCCAA
AACCTCCATTCCCAATACAGTTGCTTGCGTTGAAATTTCCTGCAGCTTGTACAACATTCT
CAAATGTTACTGGAACTCCAATGCCTGTGAAAATTGTTACTTCCCTTCCGGTAGAACCTA
TTTTGGATTGTGGATTCAATCTTCTGGTGTAGAAGAATAACACAATGAGAGCTAAAAGAA
CTGACACAATAGCCGATGCAGATGCTATGGATGCTATCTCAATTGAATTGAAGCCAGGGC
TCTCACTACTTACTGGACTTTCCACTCGACCTGCAGCATAATTCAGTGGATCCCTAACCC
CCTGCATACCCGAAGATGGAACTGCCAGGGAAAGTGGCTTGCAAGGATATAAATAAGGGT
TTCCAAGAACACCACCGCACTTCATCAAATTATTGCTTGAGGGCAACGGCCCAGACAAGT
TATTGAAGGACACATTGAATGATGAGAGTGTGGACACATTTGCCAAACCAGAGGGTATCG
ATCCAGAGAGGTCATTATTGTTGAGTAGAAGAGTAGTCAAACTTCTCAAGTTCTCAAGAT
CATTTGGGATCACACCAGTGAGAGAATTTGAAGAAAGATCCAGCACTTCCAATAACTGCA
ATTGACCCAAACTGGAAGGTATAGAGCCAGTGAGGTTATTACCAGCCAAAGAAAGATACT
TTAGACTCTTTATCTGCCCAAGACTAGTTGGAATCTGGTCATGCATAAGGTTCCAACTCA
GATTAAGAGCAACAAGAGACGTCAATTCCCCAACACCAAGGGGAAGGAGACCAACAATCT
GATTCCAAGATGCATCCAGAAATTTAAGAGAATTGCACCTGGTACCAATGTCAGGTGGAA
CCTGACCAGACATTCTGTTGTTGCTAACATTGACCATTATTGCACCCAATCTATCACATT
GCTCAAACANNNNNNNNNNNNNNNNNNNNNNNNNNNNNNNNNNNNNGGAAGGTAGAATGCCA
GTAAAATTATTCACTCCAAAATTGTGAAATACTGCAAGAATATTATCTCCTTCAAGTAAT
GGCAAAGGGGTTCCAACCTGAGCTTTGTTAGTTAAAAATAATAAATATGCTGTAGGTGGG

TCATAGGTTTCAAAATGGTTCCCCTTTAAGGAAGAAACAGGAGGACAAGCCCTACTACAA
AATGTTGGGATTAAACCTGACAGAGAGTTTCGACTGACATCAAAGAGAGTCATACAAGGC
ACTGGGAGTTCCTCAGCAAGCTCTCCAGTAAGCTGATTTGAGCTCAAGTCAAGAAAATGA
AGTTTGTTGCAGCGACCAAGTGCACCAGGAATCTCTCCTTTGAAAAAATTGTGGCCCAAA
TTAAGCATCTCCAAGTTGTCACAAGTACCCCAATCACTTGGTAACTTGCCCTCAAGTGTT
GCTCGTGGTGCCCACAATATCCTCAAATTTGGAAGAGAGGCGACTGCCTCAGGAAATCCA
CCTTGATAAAAGTTCAAATCATCATTCATGGAACTTGACTGCTCAGCTGGGGAATCCCCT
CTTGCACTCCGAACATTTTCAAACGTATCAAATAGATTTGAAAGCACAAGAACAGTCAAT
TTGGTGCAGTTTCCTATCTCAACAGGTATTGAGCCACTAAGACTATTCCTTGACACGTCT
AACACTTCAAGATTCTGAAGCATACCCAATTCTGCAGGAATAACTTCCTCCAACATATTG
GAATTCAACAACAAGGATCGCAACTGAAAACACTTCCCCAAACTCGGAGGAATTTCACCA
ACGAAGTAATTCCCTGACAAATCCAAATGCTGAAGGTTCTCACAATTCTCTCCAATTTCA
CTCGGCAGAGACCAACCAAGATTATTAAATGACAAATACACCCCTCTCAACCTCCCAAGA
AAAGCAGGTACTGTTCCATTCACCTGATTACCGGCCAAGTTCAACACTTCCAAACTCACA
AAATCTGAGAAAGAAGCGGGTATCTCTCCAGCTATTCTATTAAACCCCAAGTTAAGGGCC
CTCAACTTTTTAACCCGGAAAATGAATAAGGAAGATATCCA
>ntig_878_consensus
GAGATTACTTTGATTCCATCACTGTAAGCTTTTTCCCTCTTTGGAATCTATTTTGCATTT
ATGGCTTCCCCGTTTCTATTTTTATCCTTCATGGATTTATGGACTAGTTGCCTTAACTTT
CATGTTTGTTTGCTTGTGTAGAAAATAGTGAGAAATTTGATCAACTTAAGAAAGCAGTGT
GATTATTTAGGAAGGAAACTTTTTCTTCTTTACAAATTACATAATACACTAGCAAACTGA
TTTATATGAGGACTCAAAATCTCGCACATTATTTATTGAATTTGAACATCAATGACCTTG
CGTTCAACTTTTGTTTTAGGAATGATGATGTAAAGAACACCATTCTTGAGCTCAGCCTTG
ATGTTATCCTTGTCACAATTATCAGGAAGTTGAAGACGAGTATCGTAACTACTGTAGCTT
CTGTTCGACCAAGAATCATCTCCATCGCCGCCTTCCTCTTTTTTGTGCTCTCCTTTTATT
ATAAGGACATCATCCTCCACCGAGATTTTCACATCTTCCTTAGCGAGTCCTGGCATATCG
AAACGCATTTTGATATCGTTTTCCTCGTTCTTAATGTCCCAAGGAGCCCGCACTTCTCCT
GCCGTCCGATTCCCGCTTCCGGGGAACGTCATGGCTTCGTCGAAGAGACGGTCCATTGTG
TCCAACATCTGACGCATTGTCCTCATTGGAGAAAAAGGATCCATCAAACCAAAAGGTGAA
ATGTCCCCAGCAAGTCTTCTCGGCCGCCTCTCTACAGAGGACGTCCCTTGCTGTCCTTGA
TTAACATGCACTTCCACGGCATTTTCCTTGCCGTCCCTGGCAGCCTCAGCTCTAACAACC
GGCAGCGGAGAGCTCCTCCTCGACGGTAAGACAACTCGGCAAGGCCTTAAATTATTTCTT
GCTGAAGTTCTGGGATTGGTTGAGAGGAAAGGCGAGGCTGAAATGGACAGTGCTGAAGCC
ATTGACCAATACAAACTGAAACTGGAGTGGTGAAATTCGGATAATCGAAATTTGAAAAGG
GTAGAAGCGTATTTATAGACATGTTGAAAGACAGTTGGGGAAGTTTGCAGAGAAATCTTG
AAGGTTATGATTCGAGAGTGAAGTTTCTGGACCAACGTTTTGCCTTTCTACTATTTTCTC
GCGCTTTCGGGAAAACTCCCTACTCTGGGAACAAATAACAGATAAATGATTTTGCAAGTA
GGTCCTTGTATTTTACTTTTTTTTTTTTTACGTTTATGCCATCCGATTTTGCTACATTCA
CTTGAATTGACATCATCTCTCATTTAGCTACCCTTTCAACACCAAA
>ntig_879_consensus
TGACTGAAATGTTCAACCATATTGTCCTAAACTATAACTATACAAAGAAAATAACTTCGT
ATTCCCAGTTCAGCCAGGACATTTATACTACTGAAAAGGAGGTAGAAAAGCTCATCAACA
AAAAATAAAATAAAGAACCATCTTCATTTCCATTGCCATTATTGCAACAAAAACAACATG
TCCGATTCATTGAAGCAAAATTAACCATAAAAAGCCCTCAACAACTGAACTTACGGGCTA
CAGTTCAACATTAAAAAGTAACATAAACATAATTAAAGCATAAAAGAGATCACTATATAT
CACTGTACAAGCACAAGCCTATTGTTTCCCATACCTCCGCTAAAATCCCCTTTTTCACCA
CCGTCAACACTGCCACGACCACTGCCTTCTCCCTCCTCCAGACCCTCCAAGTCCCGACCT
TTAGGCACTGTCACTATCAGCTCCCCATCCACATAAACCGCACTCGCCAATTCGGGTCGG
GTCGTCTCGGGTAGCCGAAATCGCCACGTATCCAGCTCCAGCTCAAGCTCATCCAATGAA
GACAACTCCACAGACCCATTTTGCCTAACAACGATTTTAGTGATCCCAGGATGGATTTGG
ATCATATGGGCCCTTACTTCTCCGATACCGGTGGTTTCAACAACGAACTTGAAGCAATCC
GGGTTCTCTTCGACCGCCACGTCAGCATCGGACCGGAAAGGCAGCTCAAGAACCCGAGCA
AAGATATGGGGCAAGCGACGGAGCTTCTTATGAGACCCGGTTGAAAGATTCTGTGGGTTT
CTCAAATTGATATTATTAGCATAATATTGAATGGTGATGTTGTTTCTCTTCTTTGTGGTG
GGCACAGGGTGAACCTTCATGGCTGTTGCTGCAGCCGAGCTTTGCGGTGGCTTGTTGTTA
TCTGTAATAATAGTCGAAGAAAACAAGAACCCAGAAGAGAGAAAGATAGATATAAACAAG
AAGGAGAAGAAGAAAGATGCTATTTTATAGAAGAAAAACGAACAATTATAGTAGGAAGGA
ATCAAAATCTTGATGTGATTTTGATGGCAAGAAAGTAAAGGCAAAATCATAAATTGAGTA

AGATTGTTAACAATTTATTCTCTGTTTTGGTTATTGAGTTTTTGGTTCTGTTAATGTTTG
CCATGGCCCACCG
>ntig_880_consensus
TTTTTTTCTCAGAACAAAAACATATGTAAATAATCTCCAAAAGCACCCCAAGAAAAAGTG
ATAATCTTTACAAGCAAACCAGCACAGATCACTAAACAGACAAAAACCAATAATATTCAA
TCCTGTTTCTTCAGTACATGCCTGATTACAATAGGCAGATAACAGTATATTCAAGCCCTA
ACTCAAAAACCGCAAGATGCTGCCACGCCAAGCAGTGGAAGCTCTTGAAGCAGACCAGAG
AAGATAACCAAGTGTATGAGATAGGAGATATGTGTCCACTTCATATACCCTTGGAACCGG
GAAGGTCAATCCATTGTAGTTGAATCTCCACCTCACCACANNTGGACAACTTTGCCATCA
TTCCAAGAAATGCAACTCTCCTCGGCGAGACAATTGTGCCTGCTTGGTACAACTCTTGAG
ATTATGGTGCCACTAGGGAGGCCCTCTAAATTCATCTTTAAGGCCTCTATATATCCTTTG
ATGTCGAACTCTGCATCTCCCATTTTGTCATCTTTGCTAAACATGTCGTGATCATACACA
GTCTGATTGCCCATCTTAACAA
>ntig_881_consensus
CAGACCTAAAATTAAATCTAAATAATCTTCAAAAGCACAAATAAGAAACGAAAAACATAA
ATGTAACAGACTTTGCAAGCATGCGACACAAATCACTCCGTATTGGTTAACCAAAAATAT
GCAATACTACAATGTTAAGTACAAGCCTGATTACAATAGGAAGAAACTGTAAAAGATAAC
AGTACCCAAACCAATAACCAAACTAAAAACAGCAAGGCTCGGCTGCGCCAGGTGGCAGCA
GAAGCCCTGAAAGCAGACCAGAGAGACAGACATTAACTAAGCTTATGATATAAGGAGTGG
TCATACACTTTTTACAAGCCCTTGGAGCCGGGAAGGTCAATCCATTGTAGTTGAATCTCC
ACTTCACCACATTCCACATTTCTCAGTCTGAGAGAGAGATCTTGGACAACTTTGCCATCA
TTCCACCAAATGCAGCTCTCCTCGGCAAGATCATTGTGTCCGCTTGGTGTAATTCTTGAG
ATTATGGTGCCAGATGGGAGGTCGTTCAAATTCTTCTTTAAGGCTTCTATGAAAGGTCTA
ATGTCGAACTCTGCATCTCCCATTTTGTCATCTTTGCTAAATGTGTCATGGTCATACACG
GTCCTGATTGCCCATCTTAACAACAACATAAGGATCGCTACTGCGAACATCACGAACGGC
GAGATTATTGCCCCGCTTGACACAAATCCTTAGAAGCCCCAGTAGACCCTCCATGATAGA
CGTTTTTCCCGTGTCTCCGTTCTGTGAGTCCGCCATCACAGTCGTCTTCTGGGTCTTTCT
GGTGCGGAAAAGTAAACGGCTTAGCACGAAAGCGCAGCCGTCTCTTATGTATCTTTCAAT
TTTAATGAATCAGAAATCGACCACTCCTTCTTTCTCTTTCTTCTTAAGGCAACACGCACA
CACGCGCAAAATCTCTCTCAATCGAAGTTTCTAGTTTCCTCTGCCTCTCCAATTCTTTCT
TTTCAGGTTACAGACAATTCCGGTATCCGGTAACACTTATCCCCTGTTATTATTATTAAT
AATTTTTTGGATCTATG
>ntig_882_consensus
GGCCTATCAAAAATTATATGAAATTAAAAGTCAGCATATGATTCAAGACACTTGAATATT
GTCTCAGCTGTTTAAAGCCAATAATTCATCCAAAACAGTACTAAGTCAAATGAAACACAA
TAGATAAAACTGTCACTTTTTTGCAAGCAGTTTCTTTGAAAGCTTCTCCACCGGGGTTGA
TTTCAGTAGGAAAAGCCTGGCAGCACGCTCCTGCAAGGTGCCCCCACATTTCAGCCCACG
TGCTTGGAGTTCAGATTTCAATCTTTCCATGCCAAGAGCCTCCATCTCCACTGCTGAATT
GAATTCATTAAAATCCAAGGGCCTTTCTGATTCTCCAACAGTTGTACAGGCAACAACTGC
TTCAGCATCCATGGGTTTGTTTTCGTTAACTTCCTCATTTCTTGAACTCAAAATATTTGG
GGATTGAGTAGCTATTTCCTCTACATCTCCAGATGCAGGTCCATCACTGACCTTGTGATC
AGCTTCAGTTGCAGAAACCAATTTCAGCTCCACGCTAGAGGCTTCAGTACTCTGCACCTT
TTCTTCATTAATCTTGGGTTCAGCCACCCCATTCTCCTCAGGGTGCACATCTTCACCGGA
ATATCCACTGGATTCAAATTTTTCCTGCACAAGAGTCCCTCTCTCTTCTTCAGAAACACT
CTCAGATGAACCTTCATCAGAAAGTTCTCCATCAGGTTTCCCACCCGTAACTGAACCTGT
ACTCCCCTCAGTTTCCTCATTTGAATCTGATTGATTCCCAGCATTCAAAACTATAGATTT
CTCATTTTCTTCACCATCATCATCGTCACTATCTTCATCTTCACTTTCACCCAATTTTCT
CTTCCCCATCCAAATCTTCAACCTTTTGAACTCCGGTGCTGCTGTTCCAACAGTCTTACG
TTTACCATTGCCAAACGCCTGTCTCACCGATTCCTCAACCTTAGCCACACACTTCGCCGA
CTCCTCTCTATACCTAGACACATACTTCTCGGCCTCCCCATCTCCTACTCCTTTTTTCCC
TTTTTTCGAAGCCTTCTTAATAAATTCCTCAGCAATTTTCTCCAACCTCCTCTCCTCTTC
CTCCACCTTCCACTCTTCCAACTTCTTCTCCGCATTCACGTGCCTCAACCTCCGTCCACT
CATATCTCGACACGCGTCAAAGTTGTTTGTCTTCTTTTGGCCAGCCTTCGTAGCCGCCCC
ACGAAGTAAAGACCCGAACCCACCTTTACCTCCCAAAAGAGGAAGCACGAGGTGGAAGGT
GATGTTGTCATCGGAAATTACGGATCCTTCCTTCAATTGGAAGCCAGAGTAGATGAGTCG
CTGAAGATGGGTGGGTATTTTGGTGGTTTCGTAAATGCGATTCTTGATCTCGTGGCCATA
CACGCTTGGAGTAGAGAAATTTAGGGTTGCGGTTTTTCCGTTCAGAAGCTTCACGAAGAG
CCGTAATGGCTTTGGACTGATATTGTGATCTTCCATTCCTTTGATTGTGAAGAAGACGAT

CAATCTGTTAATTATGATAGCACGAATACAAATGATGTTTGATTTGG
>ntig_883_consensus
GTCAGCTTATGATATAAAGCACTTGAATATAACCTCTGTTGTTTACAGCCGATAATTCAT
CCATAACAGCACCAGATCAGATAAGGTCAGATATAATGTCACTTTTTAGCAAGCAGCTTC
TTTGGAAGCTTTTCCACTGGGGTTGATTTTAGCAGGAAAAGCCTGGCAGCACGCTCCTGC
AAGGTGCCCCCGCATTTTAGTCCACGTGCTTGGAGTTCAAACTTCAATCTTTCCATGCCA
AGGGCCTCCATCTCCACTGCTGAACTAAATTCATTAAAATCTAAGGGCCTCTCCGATTCT
CCAACATTTATACAAG
>ntig_884_consensus
CACAAATTCAAACTATTGATTCAAATAAAATCTAAATTGAATCCAATCAAACTATCTTTA
ATTCATAAAAAAAAAATTCACAGAAATTAGAATCCTAATTCTGAAACTAGCGAGGGCAAG
AGTCACAATTGCCAATATTATTTGATTCTTAAACCAAAAAAAGACAGAACAAAACAAAAG
GGAAAATACCTACTACTTGACTCCAATAATAGACATGAGAAGCCTGAGCTTCTCATGAAC
TTCAACAAGATCAGAAGGGTCCTTAAGCAAGGCAGGAGGCTCAACTCCAGTGAACAAATA
CAGAGCCTTCAACTCCTCCATACATGTGCCCTGTGGTGCTGCTTTGTATCCAACTATCCG
CCGCATTGCCTCCATAAGTTCCCAGATTTTCTCCTCACTGTACACCTCCAGTGTCTTCCT
GTGTGAAAATTGGAGCATCGTTGCACTTGTGTTGTCCTGGGAAAACAGGAAGCGCTATAT
AATCAACTGTCTCTGGACTGCCATGAGTACTTGAAGAGTGCTCATTTTCTGCATATGAAT
CGGCAGCTGCATTTTTATTATCAAATACGTCAAACCCACTTTCATCAGCAATTTCTCTTA
CATCTGCATTTGACGAGGTCAGATTTCCCTTCTCCTTATGCGATTGAATATCTTGTACTA
CATTCTTTGCAACATTATTATCTCCTCCTCCCTGCAACATAAGAGGCTTGTTTAAAACTG
GATTTTGCTTCTCTTCTTTGAGGCGACAGACATCATTTGTTCCAATCATTTTGTCCACTG
GTTTTAACAAACCTCCGTCGGTTTTTCTTAAGTCTGTCTCTTTTGGAATCACATCCGCCT
TTGCCGCATTGTCATCTTCTTTCTTTTCTACCTCTGTAAGAGGCTTACTGCCAATTCTAC
CTTCCATTGACTTTTTTGTTTCTTCGACATTCTCTCCATTATCAGTCATACCCTCCTCCC
TGAGCTTTTCAAGTAATCCCCTTGTCTCTTCCTCCAATTCCCCGTCTTCTTTGACTTCAT
TACTCTGACATAGTTCCACAACAATGTCTGTCACTTCAAATGGGGTATCTTCAATGGTTC
CGCCACTTTTCTCATCTCCTTCAATGTCAACAACAGCAGCAAGGACATCTTCTACTAGCA
TATGATCCGAGCTTTCTACAATTTTAGGCTCTTCACCTTCCTCAATAGTTCCGCCGCTTT
TCTCATCTCCTTCGATATCAACAACAGCAGCAAGTACATCATCTACTGGCATATGATCCG
AGCTTTCTACAATCGCAGGCTTTTCCTCTTCCTCAATGGTTCTGCCGCTTTTCTCATCTC
CTTCAATATCAACAACAACAACAGCAAGCACATCATCTACTGGCATATGATCCGAGCTTT
CTACAATCGCAGGCTCTTCCTCTTCCTCAATGGTTCTGCTGCCTTTCTCATCTCCTTCAA
TATCAACAACAACAGCAAGCACATCATCTACCGGCATTTGATCCGAGCTTTCTTCAATCG
CAGTCTCTTTCTCTTGTTCTTCTCCAACTCCTTCAATTCTCACCTCCACTTCTAATTTCA
TTGGTTCTTCCTCATTTTCATCTTCATACTCATCAATATCTTCTTCATATCCAATTTCGG
CCACTATCTCAATTTCATCTTTTTTTATACGTCCGTGCTTTGCGTCATCAATATAAGGAC
CATTGTCATTCTCGACTAATTCAATCCTCATTTCAACTGTTTCCCGCATATCCTCCAACA
TCTCTTCTTCTTCCTTCTCCGTATCACCACCTCCTTTACGCCACTCATCTTCCTCTTCAC
CATAGTCATCATCCTCGACAACTCCATCCATGTACGACGCTTCTCCATAATCCAAAAGAA
ATGGAGGAGGTCTAGACCCCGGAAGCAACATACTCATGAGCTTATCAGTGCAAGCATAGC
TCGCCAACAGAAGCCCAACCGGAACTGAACAAGCAAACCCAAGAAAGGAGATAACCGCAA
GAGGAGGAACTACCACAGGAGCAGAAGACACCACAAAACCTGTCACCAAAATCTTCTTTC
CCACTGTTAATCCCTTGCACAAGATCCAGCTCACTTTACCTCTGCGTTCCTCTGATGAGT
ATTCTTCAATACTCCCCTCCATGGCCGAGTGTTGATCTTCCATATTTAATCGTTTAAAAA
AAATACTTGTGATCAGATACAAACACAAAGGATTATTATTTTAACATTTATCTTCAAAAA
TATTCTTCGCACAAGAGCTTTAGAAAGAACATCTAGGATCAACAATGGGGAAGATTATTT
TAATTATTCAGTAGCATATTTTCTTCGAGGCACTCGAAATAAAGATCGACACTGGCAAAA
AATTTTAAATTTCTACAGGCTAACAGTGACACATCTGGAATCAATAATACAGGGGGAAAC
ATTTCTCAAATCCCGGATTAAAATCCAAAACAATATTCATTGAAAAAAAAAAAATGAACA
GCTACACCAAAGGAACATACTAATCAAAGGGGTTTCTAAGTGAATAGGAACTTTCTCTGC
AAATTAAAGTATTTTCGTAGAAAAGAACTGAGAAAAAATAAAATCAAGAGGAAGAAATTA
CATTTTCCATGAAGAACCTCAAGTTTAACCATGCATATCTAAATAAAATTTACCTGAATA
TATATACGGCTTAATG
>ntig_885_consensus
TCCTTTTATCTCCAATCCTGGACCCTGATCTTCCTTCACCAATGAATTATTCAAATCATT
ACCAAGTTTTGACAGGATGGCATGATCACATGCAGTTTCAGAAAGCACTTACTCCTTATT
TGTAACTGATGTGACGAAGCGATGGACTGGTTGCCACTGGAAAGACTAGGGGTCTTCTCT

CTCTTCCGATGCGGAAGATCCATCCTCTGCTTTGGTGTCAATATTGACATCAACCGGGGC
CTCAGACTCTGTAGTCATAAATCGCTGCTGACCTTCAGCTTCTGCCATGACCCTATCTTT
GATCCACTCTTCTGATTCGGCCATTGATCCTGGAGCCTGAGGGTCACATTGATGTTCTGT
TTCCTGACTTGATGCTGGGGCTGGTGCTGATGCCGGTGCCTGTGGCAGCATCGCTGTAGC
TGCTACACCTGATACTTGAGCAGCCAGTAGTCCCGGCAACTGTGCAGCAAGCACTCCTGG
TGCTTGAGCTTGAGAAAGTAAATATGTCAGTAGCTGGGCCAGAACAACCTGTGGATCACT
AATACCATGTTGGACTTGAGGAATGACAGAAGATGAGGAGGCATTAAAGTTAGGCGAGTA
GAGTGCTTGTAGCCCTGCTTCTCCATACAAAGCCCGGTGACCTTGAAACCGCTTAACATT
CATCAGGTGCTTTTTACCAGCCAAATGACTGTCAAAAACAACTTGTGACTCACACTTGAC
ATTACACAATTCACATATAAAAGGAATCCCCTCTTTAAGTTTGGCAGGCTCAACAGGTTG
TCTAGGCCCTTCATTAGGTCTGATATGCTTAACCCCTCGGCGCCCTCTCCTTTTGCGCTT
GAAACCACAATTACGACCATCAAATTGATCCCTTGACTTTCTTTGAGGTTCGGCTGCAGA
ATCCTCTGTGTGATCTTTAGACTTCTTAACTGCATCTTTATGCAGCTCAGTCCTGATATT
GTTATCATCAGTAAGAGCTTGGGAGAGTAAACTCCGCTCTGAGGGTTGTTTTTCCTTGGA
TCTCTCGACTGTCTGAGGCTGAGGAAGTTCTGGTTGACTTTCAGAATTTGGAATTTGAAC
ATTCTGCTGTTGTGTTACAACTTTATTAAATAAGTCTGCATGTGTTTGCAAATTTTTCTT
ATGTCGCTTTCCATTCTTATGTTGCTCCAGGATTTCTGGTGTGTTGCAGTCAACCCTACA
AAGTTCACACCATGCCATACGTGGAGGTGGCCAGGTAGCTGGAGGTACTTGGGCAGGTAC
AGTTGCTGTAGAACTTGATGCTGCACCATGTGATGGAACGTAGTTACCACCACCCTGGCC
ACGTCCCCGATTATCTGATCTAGGGCCTCGACCACGACCCCCTCTAAAAGATCTGCCACC
ATTTCTACCACTACCTCTATGTGCAGTTTGACTAATCCGAGGGTACATGCTCCGCTGTGG
CGCATCCGTACATGCCGTCCCTGTAAATTGTATGAGATTTGATCGATTATTAGTAACTGC
AAGAGAATAATAGGCATCCTGGTGGTTCTGGAAATGGGTTGGGTCAGGCGAGATTGGAAC
GCCCGGAGGATGAACGGAGACGGGTTCTTGATGAAATTGTTGAGAAGGGTAATATTGTTG
ATGATGATGATGATCGTAATCGTAAGCGTAATAGGGTTGCGATTGAGATGTGCCGTAAAC
TTCAGCAGTTTGCCGTTGATGTTGATATTGCTGGTGGTAGTATTGTTGGTAGTGTTGGTG
TTGGTGTTGGTGTTCGGTGGTGTAATCCATGGTGCACTTCTTCTGTATAAATTTTTAACC
GACGATTGTGATTATGCGTGACTGTTTCTCATGCTTGCCTGTTGTTGAAGAAAAA
>ntig_886_consensus
TCTCTCTCTCTCTCTCTCTACAGCTTCATTCATCGGTCTCTAATTTCTCAAAAAATATTT
ATCGAGACTGTGACTTCATTGGTCCACCAATACACACAGACAACATATGATTGCAAATGA
TTCGCACCAAAAATACATTATGCCCTAACATTAAAACCTTTACTGAATTTAAGGCTCTCA
AACACAATTCAATTCATCCACATTTGATCTGAGTGCCATATGAATATATATTATGCCGCC
ATGGATCATAAGAAATTAAGCCATGCCTATCAGCCCCAAGAAACTCTACGTTATTGTTAT
GTCTCATTGGAATCTCATTACAAAAATATAGAACATTTCAAGTGACGAGCAAAAAAAAAA
AAACTACCGATCTAAGCAGCAACTAGAGACACCTTTTCCAGAGATCGCATTGAGACTTGG
CGGCAACAAAGCTCCAGAGGCGGTTCATCTCAATGCATAGGGACTGGATTACATAAACAA
ATGCATTCATCTCCAGAATTATTACTATATGTAGAGCACCAGTCGATTTGCAGCCCATAC
ATCGAAGGCCAATGATGTAAAATTACCAGCTACCAATTTTCTGAAAACAAAATTTATGGG
AAAGCTTATAGCAGCAAGAACCAGAATCACAAGCCAGCCAGAATAACTAAACACTCATCC
AACCCAAAAAGATTGGAAGTGTCTGCAATTGAATGGAAAGGATGGAAGTGCCAGAGACAG
TAACATTTGCAGTACTGGTAAACTGTTATTGGCAGCAACAAAAATTTAGTGTACTGTTAC
AGAAAGGAATTTACTAATGACCTATTTTTATTAAAGGGAAAAAGAAGTACTAATGTGCAC
TACAAGCATGGGGTCAGCGGTAGGACAAAGATGGACCAGCAAAGTAACGTGATGAAACTG
GTGCGGCTGCTGAGTTAGGCTTGGCCACCTCATAGTGGTGCTGCTGGTTATGGTTCGATA
AAGCACCAGCAGCATATTTCGAGCATAATCTATCCACTGCATCTGTTTCTCTTCTCCCTA
AGGGATCAGTTCCAACCACGTCAGTCTCTCTCCTTCCATCCACAAGATAGGAACCTGACA
AAAGCTCTTCTCTCCATGGAGGTGGAAGATATGGGTCAACAGATGCAGTGTAATAGTGAC
TTTTATATGGATCCCTAATATAGGAGTCTGATTTTGTGGCAGCTGCAGTAATAGAGGCAG
CTGCCGGAGAAATTACAGATTGCAGTTCATGTCTAGCACCAACATTATAAGTTTGATACT
CCCTCGCACTCAAGTAGAGAGGAGCAGCAGACAAGGAATCTCTGGATGCAGGAATATTGT
CTCTAAAAGGACGATCCACGTGGCTTGGAAGGCGCTGGTGCTCTCTTTCATGAATCCTCT
GATAAGGGTCTAAGGGAGCAATATGATGAGTGCTAGTAAGGTTTCTCCTCTCTCCCTGAA
GACCATAAGTTCGATATTCCTTTTCACTCAAGTACAAATCTTGGGGGTATTCCTCTCTGT
AAGAAGACCCCACCTTTTGATATGCAAAGTGCTGATCCCTTTCACGAGACAATCCATTAT
AACTCCTAGAATTAGCATTGGAATAAGAATCTCTAGCAAATGTTTCCTGCTTTAATTCAC
TTAATGCATCATAACCTTTCCCATCCTGAACTTTAGGCATTGATGAGCTGCGGACATGCG
GCGAAGTTAAATGGACTGAAGCTGGCCGGAAAAGTTTAGAGAGCTTCCAGACCTGTTTGG

GGGTAAGCTCAAATTTGAACTTGTTCTTTTCATTATAATTTTCCTTAATTGCCTTCTTGA
AAATGCTCTCAGGAAGTGGAAGGCAAGCGATGTGAACACTGAACCGCACCTGAGCAGGAA
AGGCCCCATCAAAAGCATTTGGCTCAAGTTTCATGCCACCAAAAGAGGATGCCTCGTAAA
TCCCATACATAAGCTTGAAATCATAATCATAAAGAAAAAGTTTAAGTCCAGGTTTGATAC
GCAACACAAAGTCTCTTTTAGTGGCTGATACACCCATGACACGATAATGGAAACAATCAG
GTTTGGTTTTCTTGCTGCACATAAAAATCAAACCACCAAGCTTTTCTTTCTTCTTTTCCT
TATCAAGGCCCTTTCCTGAGTCACTATGTTTTTCTTTGTTCTTCTGATATTTCAAACTCC
CGCCTGACTCACCAGTCTTCCCAGCATCACTGTGTTTTTCTCTGTTCTTTCGATTCCTTG
AACTCTTGTCTGACCCTCTCACTTGTAATTCACTATGTTTTTCTTTGTTCTT
>ntig_887_consensus
CCCAACAACTTTTTAGTTTTCAATTTATTTTTTTAATATTTTTATTTTTTTTCATAAAATT
TATTTTATTATTTATAATTTTATTTAAAAAATAAAAATACTCTTTTAACTATTACTTTTA
AACCATTAACCATTAAACTTACTTTAATTGAAAGTGGGCGTTTGGGCTTTCAAAGAGTCA
AGGGTGAAATTTTGTCAATTTGGCAAACCCAAGTAGAAATATGTCCTTTGGCCATAAAAT
ATTGAGAAAAAAGACATCACCACCACCCATAAAAAATTGCCCTACCCTTGTTATATAATC
CATGTACATGCTTCGAGTTACAGAATGGGAATTTAAGTTGCTTGACACGCATAACAGCTA
AAAAGATCACCACCTAAAATTAACAATTCATGATAAGATTGAAATGGATTCTTGACATAT
GTCCGGGAATCCGATTCCATTAATTTCAAAGGAATGCTCATTCCTTGTATTTAGCATAAT
GATCAAAGGAATATTGCCAATTCCTTTCAACCGCCAGAACTTTGTAAATTATTGGCCAAA
ATAAAAGTGCAAACAACTATGATAAAAAACTGTAAGAAACAAAACAAAACAAAAATAAGA
AATAAAAATATATAATACATCCTAAAACAGAAATTAGAAACATCAATGGATGCTGAATAT
TGAAATAAGTTAAACATATACTAATTTGAATGAAATTAGAATGCATCACCTCAAAGCCTT
AGTTTTAGATTAGACTGCCATCACCTTAATGACCTCATTACTAAGCCTTACAAGTTTAAT
CGAAAAGAGTGAGTGGCTGCGAAAGAATAACGACATAGAAAGCTCTAGCCTTATCATCTT
CTTGATTTATTCCAAAACTTCGTACAGCAGTGTAAGCATCTGGAATCTTCAGATGCTTCC
AAGCTAATAAATTCTTTTATAGTGTTGAAATGCTCTCCTGTAGGGACTTCAATGATTCCA
CCACACAAAAACTATGAGAAACCAAATATCATCAAACCCTACAACATCAATCTCCTTGAT
AACCGAAATAGGCCAAAAGAACTGCCAGTTCCCATCAAAAACTACAAGCAACCATAACCG
TCCAATACAAAGGAAACAACAAACAGAGCAGCGAGAGACCATTTGTAAACCAGGAAAGTC
GGAAAATGTAAGTGTAGTTGTAACAATAATTGTGCAATAGAACAAATTAAATAGTGAGAT
TATATTGCACATTAAATTTCTTAACTATAATGGACTTCAAAACAAATACGATTTCGTTTT
GGCTTTGAACTATACATCAATGGTGAATAACTTATAGCCTGCAGGTAACTTTAACAAGTA
AAAGTAAATTCCATTGTACCTTTGAAAATCACTGTGTTGACCTCCTTGCAATTCTAAGCG
TAACTGTGAATGACAAATATTAATATATGGTCTGTCAGATATGTTATTAATATAATTTTA
AGGCATATCAGGAATACCCAGCAGTTGAGAGGAATGGTATAAAGAT
>ntig_888_consensus
TATTGCCCACAAAACCCCAAATAAGAGGCTATGTACAATATATAAATTTAGGAGCTTGGA
ATGACAGAGAACCAAGCAATTCCATGTTTCTATTCCCATAATCTGAACTGATGAAGGAAC
ATAAACATAAATCCCGGACAAGAAGATATCAAGTCAGGCACTTGGGTGCTTAACCCACTT
ACAATTAATCCCACAAAATATAAAGACGATCCTGAGTTAGAGCTGTACACCTATGGTTTT
TTACTTTTTAATGAAAACCTGCAATATGATGATCAGTTCCTGTCAACAACTTCAGCCGGC
AAGTTCAAGTCAAAAAAGCCACCACCAAAACTGTCCTTTCTAGGACGCTTCCGAGAAGAG
CATCCACTATCATCTAAACTCCCGGAGCCTGAAGGCGGATTATGTTCTGATGACCCTCCC
CCACTTGTGCCACAGTCCAATGGTAGACAATTATCAGTAGTATTCGATTTGGTTGTCCGA
CAAGTAGAAAGTTCTGATGCCCTCTCAATGCCCTTTGACTTGTAAGGAGCTATATTGTGA
GTACCAAGTTGAAGACAACCCAACAGATCATTGTCAATGCACTCATACATTTTCTTACAG
AGGTTATCAATGATCACAGACAAATTGATGACTTGCATTCTTATCTCCATGACCTTGTCA
TCAGAATATTGGTCAAATGGCTCCTCTAAGTTCTTGGAGAGTTTCTCAACATTAGCCTCA
AGCTGCTGTTGCTGCTCCTCAAATAGATGCTGTTTTATTTCCCTTTCTTGCAAGTTCATC
TCATCCCTGAAGAGCTCATCCCCAAACATGTAAAATGCAAATGGGTATGAATAGGAAAGC
ACACGCCTTGATCTAAAAAGTCTGTACAACCCGTTAGTTACCCAGCTAAAATCTCTAAGC
CGTGATTCCCTCTCCTCTGATATTGACACCTTCTCCAGAATTGTCTCCTTAAGTTTACTT
TCTAGCTTAAAGGAATCTGTATGAGCTTTATAACGATTATGATAGTGCATATACCGATAT
AGATCCCGCTTGGCCCGTTCAGTTTTGTTTGCTTTGTCTTCTTTGTAGCGATTACAACTA
TGCCCCACAATTCTTGACCAAGTATGGTCCCGGCCAGTAGCTCCACCACATAGCCAACAA
AATGCCTGTCCGCAAATACAGCTCACGAGGTTACAGCCACCATTCTTCTCCACAGGTTTG
TGGCACTTTGGACAGGGCTTTGTATGAACAGTGATCCAATTAACTGTTTCAGATTCATCT
CGGCACTTCTTGGCCCAAAGTTCCCACATCAAACATGAACATGGCGAGTGTGCTTCTGAC

AAACAACTAAAACAGAATTGTAAGCCACAAGCACATTCTACCTCACAAAATTCATCTTCC
TCAACACGTATTGCATTCCCACAATGAGGAGTGCTTGGGCACCACTTAACCATTTTATTA
TCCTCAATATACGACTCAAGAAGAAATCGATCAAATTTCTCTGCCAAATCAGGATGCTTT
TCACTGACCAGATTCCTGACAACTGCTTCATCACAAATAGCATTGCATTTGTGCGCCATG
CACCTAATGCGCCTGCTCTGCCCCTCATTTATTTTGACAATAAAATGTCCTCTCCAACAT
TCATTGCAAAAGCAATGGCCACAGTCCATTTTGGTTGCAGCTGCAGCAGAAACTTCTTCC
ATGCATATATCACACATTACTTCAGAAGGAAAATACAGTGATGAACCAGTATCTTCATTC
ACAGTGACACCTGCATCAGCAAACATGCTTGCTTTCCCTTTCTCCACAAGAACCGCTAAT
AACTTCTCAACATCCCAACGATGATGAATAAGTAAAGTGCGTGCGTGATGTTCCCTCAGA
GAAAGCAAGTCCATCACTGTCCGCAAATCTTCCCTCTGCGCAACCAAAAGTGATTCTTTT
GTTATAACCTTGGTGGAGGAATCCTTGGGCGCGGCCCACTGCAGCGCCTCATCATTCTCC
ATGATATCATGCGAGTCAAGATCGGACTCAGAGTGATCCGCGTCATTATAATCCGAGTCT
CCACTCATACAATCGTCCATCGATCAAAATTCCCCCAGACAAAAACAATTCAATTTCCCT
GCTAAATAAATACTCTGTAACCACAAATTGAAGAACCTAACAGGCCGATCGTAATACAAA
GGCAGAGAAAAAAAAATCCGACGGTTCTGTCGCCGGCGATAATAGTGGAAACACTGCTG
>ntig_889_consensus
CATAGAATGTAATATTAGCTCAAAAGTTATATATATTATATGAGTATTGCTCAAATCTTA
GGTTCAAAATTTACATATCTTATTGCCCACAAAACCCCCAAATAAGATGCTATGTACAAT
ATATTAATTTAGGATCTCAGAATAACAGAGAACCAAGCAATTCCACGTTCCTCTTCTGGT
ATATCTCCCGAAGGAACATAAACATATTCCCACGACAAGAAGATACCAAGTTAGTCACTT
AGGTGCTTAATCCACTTACGATTAATCCCACAAAAGATAAAGAGAATCCTGCGTTAGAGC
TGCTGTACACCTATTTTTTTTTTTTTTTTTATGAAAACCTGCAATATAAGAGATCAATTC
CTGTCAATAACTTCAGCCGGCAAGTTCAAGTCAAACAAGCCACCACCAAAACCCTCCCTT
CTAGGACGCTTCCGAGAAGTGCATCCACTATCACCTGAACTCCCAGAGCCTGAAGGCCGA
TCATGTTCTGAAGATCCTCCACTTGTGCCACAGTCCAATGGTAGACAATTATAAGTAGTA
TTAGGTTTGTTTGTCCAGCAAGTAGAAAGTTCTGATGCCCTTTCAATTCCCTTTGACTTG
TAAGGAGATATATTATGGGTACCAAGTTGAAGACAGCCCAACAAATCATTCTCAATGCAC
TCATACATTTTCTTACAGAGTTTATCAATGATCGCAGACAGGTTGATGACTTGCATCCTT
ATCTCCATGACTTTGTCATCAGGATATTGGTCAAATGGCTCCTCTAAGTTCTTGGAGAGT
TTCTCAACATTTTCCTCAAGCTGCTGTTGCAGGTTCTCAAAAAGATGCTGTTTTATTTCC
CTTTCTTGAGAGTTCATCTCATCTCTGAAGAGCTCATCCCCAAACATGTAAAATGCAAAT
GGGTATGAATAGGAAAGGACACGCCTTGATCTAAAAAGTCTGTACAACCCATTAGTTACC
CAGCTAAAATCTCTAAGCCTTGAATCCCTCTCTTCTGATAATGACACCTTCTCTAGAATC
GTCTCCTTTAGTTTGGTTTCTAGCTTAAAGGAATCTGTATGAGCTTTATAACGGTTATGG
TAATGCATATACCGATATAGATCCCGCTTAGCCCGTTCAGTTTTGTTCGCATTATCTTCT
TTGTAACGACCACAACTGTGCCCAGCAATTCTTGACCAAGTATGATCTCGGCCAGTAGCT
CCACCACATAG
>ntig_890_consensus
GAACTCAAAAGTTTCAGTCCCCTCTAGAATCAAGAATAGGTTGAATGAAACATACAGAGA
GAGAGAGAGAGATATTGACAAATGATTTTAAACCATATAAAACTCTGATTATTCAACCTG
TAATCTTCCTCAAACTCAGAGCAAAGACTAGACACACCTGTATAAAATTTCTTACCACAT
TCACCAAAGTTTTTCACACGAGGACTCAATCAAATCCTCCATTATCTATAGTGTTTTTTC
TCTACAAATCATGAAAAATCTAGACATCAAACTCTTCATAACAATTATTTGCAACATCAA
ATATACACACATACATTGTATAGAAGACTAAAATCTTAGCAATGTATTGAAGGTTGTGCT
TGTAATATCAGGACCTCCCAGCATTACCATGCCGATGGTTTCTGAAGTAACTAAGGAGTG
ATTGTGCCTTTTCTTTGGCCCTTGGTGTGCCTGACTGTGACAATGCCACTAGCGGTGGGA
CAGCTCCTTCTTGAAGCACCATATTGCAATACCTGCCACTGCTTGTGCAAAGTTGTAACA
GGGCAGCTGCTGCGTTTTCCTTCCCTCTAGCAGACCCCAACTCAACAACCTCAACCAGAA
CAGGGATCCCATTTCCCTGACCGATTGCAGTCCTTCCCTCAGGAATTGTAGCAAGATTTG
CTAAAACAGCAACTGCCTTATCAACCATCCCAGCTGCCGGATCCATCAAATCCACAAGGT
GCTTCACAGCACCAGCTTGCACAATCCGGTCCTTGTTTTCATGATGTATTGACAAATTAA
ACAGTGCTGTGGCTGCATCTTTCTTACCCCTAGGAGTCCCATTCCCCAGCAGATCAACAA
GAGGTCCAATTGCCCCAGACCTCCCTATCTTGATCTTATTATCCTCGATCACAGATAGGC
TAAAGAGTGTGGCAGCCGAATTTTCTCTAGCTTCTGGGCTTCCTGTCTGAAGGACATGGA
TTAGAGGTTCGATGGCATTGGCATTAGCAATTGCACTTTTGTTGTTATCATTAATTGATA
AGTTAAGAAGTGCTGTAACAGCATTTTCCTGAATCTTTGCATCTGTTGAGTGAAGCAAAT
CAACTAATAAGCTGATGGCTCCACAGTTTGCTATTACCATCCGATTATCCATATTGTGCT
TAGCAAGAAACCTGAGTTCTTCAGTTGCATTCCTTTGAGTATCAAGTGATGTACTCTTCA

AGTCCTCAATCAACTTCCGTACTTGGGTTTCAATACCAGAGAGATCAGCTCTGGTTTCAA
TAGCCGAATTAGAAACTATTCTAGGAACCAACCTCTCAGATGGGCGGCGCCTGAGCACTT
GGCTCCGAGACCTTGTTTCAACCATACGAGATGGGAATTCGGTTTCCCTCTGTGTGGTGG
TCAAAGTAGCAGCTGTATGAGGTTCTGATTTTACCTCTCCAGAAGGATCAGTTTGATGGT
TTGATCCCTCTGAA
>ntig_891_consensus
TATGGAAACAAACTCAGTCTTTGATATAAACTCAAACAATTCGGATCAAATTCAATTATT
TTCTCTTTTCTATGGCTGCTTTTACAGCGATTCTTTATATTTTTACATTGTAACCATCGG
CATCTACAATATAAAAAGATAATAATAATAATAATAAGGTTCATTTATATGCACAAAGAA
AGTGCCTAATTCAATCAAAATGAGACCATGTTAACAAGGCAAAAAAATATGGAATAATAA
AAGAGACAAAGATGTAACAGTTCACCACGGCATGATGACATGAGAAGTGAAATCAAATAA
TACATACATGTCACTCATCTTTCATTGCAAGTTCATGCCTCTTTTTCTGCCCAACTTCGA
CAAGCAAAGTCAAAAGTGCCTCACAAACCTGGGAAGCATCTGCCTCGCAAGGATCATCAT
TCAATGAAGAATAAACCATCCAAGCATGGTCTAGTTTACGTTTCGGCCGCACTACAATGG
ATCCTGGTACTTCTGCATCGCGACAGGTTACAAGCCCGTCACTCTTCTCACCATATCTCA
CCTGTAAAAGCTGGGCGCATGCTGCCATCACTGCACCAAGGGGCATTACCACTGGGACTT
TTGCTGGTTCACCTGCTGATAGAGAGGGAAGCTCTGCATGAGCAACATGAGATAATGTAG
CAAGAACTGCAGGGGTGATGCCAGCTTCAGTATGGAATGAGACAACAGGAAGTTCTCTTG
GCAGTTGGTGTTTCTTCAAAAATTCCTTCCTCCTCTCGTAAGTCAAGTCTTCTAAAGATT
GCAAGTCACCCTTGATCACTTTACAGATCAGAATCTCCATAAGTTTCCGTAAATAAACAT
AATCACCAAGTTGTCCTTCTCGTAAAATATCTGAAGCTATAGGGCTCCCACCGTATGGGC
TCTGAGCTAATGCCAGCCCAGCAACCTTATCTTTCAAGTCTGGCCAATACAGTGATAACG
CAGCTGCTGCATCTACACCCCCCTTTGCTATGCCCT
>ntig_892_consensus
AATTAGTTTTTTAAATTAAATTCCAGGTTAAACGGACCAATAGATCAAGACTCAGTGATC
AACCTATTCCAGATTAATATACCAAGTCTAGGTATGAAAAAATTGCTTGAAAGTAAACCT
CAAACATGTTAATGTTGACATAGGAATGATGCTATTCCTAGATCAATAACTAAGAGTAAA
AGCTACAACTGACTCCATAACAAAACCAAAATTTTACTTTATCTGCGTTCATCAGATTCA
GCTTGAGCTGGCCAACTCAATTCCCGGGATCTCTTCACCCATAAGGTCACTAGACATTGC
AGACGGTATTCTGTGAAGTTTCAATATTCTAGCTGAGGCAATTTTCGCCCTCTGATAGTC
CAATGCAATCACCCTGTCATCGTCCAAATCAAACCTCAAGCTTTCCGTGCCACAGTTTTC
AACTTGTTCGGATAAATGCTTCAAGTTTTCAATTTCCACCCCATTGACTCTCTTAATCTG
TAATTCAGCAAAACGCTCATACCCGGCATTGATATCATCCATCAGAAC
>ntig_893_consensus
CACCATTAAACTAATTTTAAAAAATTTATTTGTCATCAACCCAACATTTGATAAATGAAT
CTAGATACATACTTTTGATGGCAGTGTCTACCATTTTGTCTGTAATACGAAAAATAAAAG
TCCATCCCTGAAATCAACAAAATGCCGAAAACCAAAAAACTAATAGAACTTTTCTTACTG
TACCAGCTTCAATATACTCAAGACACTCGATTACCAACAGTTTGATACAGAGACCTTAGC
TGTGTGTTCCATTTGTCTATAGCAGTATACTTCTTCATTCCCTTTGACCTGTTGCCACGC
TCTAAGAGCCGGTTCACTTGATCAATATGTCCATCAATACGATTATCCAAGATTAGTGAA
ACCAACAATTGTTCAACGTCTTTTTCTGGCACATTGAGCTCCTTTGATATGAAAGGGATT
CGGATCCTTGTATACGGCTTGATAAGCTTAAGCAGCACTTGAGTGCGGACGTTCTTCAAT
AGATCTTCAATGTAATTACGGATAAAGGGATCATCCATTATTGTTCTTCTGTTACTCTTA
AGGATTTTCTCAAATTCTAATATCTCATTTCGTTGATACGCCGCTATCAAGTTTGTCATT
GCTAGAATTTCAGGGTCATTTTTATATGGCTTTGCCTCTTGTCCGTCAAATGGATTAACT
TCTGATTCCATCAACATATTCGCTAGAACCAGATATTTTAAACATTGTATACGCCTCTGA
TTCCCAGCTTCATCATAGTTCTTGAAAGCTTCAAAGAAATCCGTGGCTGCATCTGCCCAC
TGACGTTCAGCCATATGCATCTTCCCACCACATTCACGTATTATTCCCATGATTCTCGGA
TGGGGAATAGCTGATTTAATAGCAAGTGCTTTCTGGTACAATTGCTTTAGCTTCTTGTTA
TTCTTAGTCTCGGTGTACATTTGAATCTCAATTGCATAGACCTCCAAAAGTTGACTTCCT
TTCTTTTGGTCATCTGTACCATCTTCTCTTTGACAAGATTTATGAAGTTCCTTCAAAATC
TTGCTCATTCTCCCATATTCACCCATATCAAACCATATCTTGCAAAGCTTAAGATTTGTC
TTGAACCAAAGTCTCTCATTCTTTGCTTCTTCAAGGGCCTTCAGAGTGGTCTGATAAAAT
TCTTGCAGGAGACTAAAATTCTGACTAGCAGATCCAGAAACAAAATCCATTATGTTGTTT
ATGCATTTTTCACTGTAATTTCGTGTCACTGCTGATTTAATATATGTAAGCATTTCCCTG
TAAGCATCCATCATTTCTTTATACTTCCCTAATCGATAATAGAGCTTTACGGTTTGCTTC
AGAGCCTTGAATCCCCATTCAGCCTTCTCAGGTTCCATGGCAACAACTTCAGCAAAGCCA
GCAAGTGCCCCTTCTGGATCCGTTTCAACCAGACCTTTAGAGTTGTAATATTGATTCTCG

ATATCAACGTCCTGTTCCTCAGGCTCCTCATCTGAGTACTCAAACCCATAATCCTCCATA
TCCGCATCTGAACCCATGACTGAGTTAGAAAGAAGAAAATCGGATGACAAAATTAATAGA
GATATCTGATTCTGGTAATTTCTTGTAATTTCTGTTAGGG
>ntig_894_consensus
GGTCGGTGTTTAAACTCATGTCCGAACCTCCAAATTCCACTTATTGTGGTAATTTTTAAT
TTCATTATTGTAAGATAATCACTCTGGGAACTTCATAAATACATAATTACAATAATATAA
TAAGCTTGTCCCACCAAAGACTTCAACTCTATCTTTTCATGCCACCTTCCACTGTAGGAG
CGGAAGGAGCAACAACTCCCATGGCCACTCCCCGATTTTGCCTCTCCTGACCCTGCCATC
CAATAGACATGTCAAATCCATGGCTTTGGAGCTCGCGATATTCTTGACAAAGGGCACAGG
GCTCGCAGCAAAAATGAACCAAGCAGTCGCAGCAAGGTTCCTCTTCCAGCTTGAACTGCT
GCCGGATCTTTGTGCGATAAAAGCAAGAGTAACAAAATGTCAAACCAGTCACGGCACCAA
GTAGACAATAAAGCACTCCTGCTGTAGCACAAGAGGTATATCCTTGGTCTGCAATCTCGG
CGACTCGTCCAAAAGTGATGCATGGACACCAACACGTCAAGCAGCAGCTTGAGCAGTCAG
AGCAGCAGTCACAAAGTCCAGAAGTCCAAGCTCCGAATCTGGGTTGATGATCGAAGCCTT
GAGCTACCGGGACTCCTGTTGCTGTCATGCTGATGAAGGTTTGGAATTCTATTGGGAATG
CTATGAGACGAAGGTTGATATGCTGGCTAAGCGTCCTTGGTGGGCTTATATAATGAAG
>ntig_895_consensus
GGTGAAAGCCCTATCTGCATAAATATTTCGGAAATTATTTGTCCCACTTCTTTCTGCACT
GAGGAGGAAGCTACAAATAAATGTAAATGTGAAGCCACTTGGTAACAGTATCTCCTCAAG
ACAAGAAAACTCTTGTTACATAATTGACCAGTCTTTGAGCTAGAATTTTCATGAGCTTTT
TCATTTTGGTTACTTCATCAAAACTCAATGTCATTGGACGTATCCATGGACCTTGTAATG
AGTCCACAAAGGCTCATTAGTCCATTACTATCACCAGGACTACGAAATCCAGTAAGCTAT
TATTGATGGATCAAACAAACCACAAAGTACACCAAGCAGAAAAAAACAGACAGTACATATT
ACATGGATCCGCTCATAGTGCTAGACCTTCCAGCCTCTTACAGTTTTGAAGCTGCATCTT
TGTCTAAAAACCAGGCTAATTCAACTTCAGCTGACACCATTTGCACGGGTAGCTTATCCG
AATTTTGACTACTTCCCAATGCAGCCTGCACTGGACCAGCTTTCCCAGCACCACACACCA
CAAGTGCGTTGTACGCAGATGAATTGATTATAGGGAAAGTAAAAGTGATTCTCTCTGGAG
GAGGCTTTGGTGAGTCTTTGATGAAGGTAACCCATTTCTCTTTCTCGTTTACAAGAGGAT
GACCTGGAAATAAAGAAGCTACATGTCCATCTGGACCCATACCCAGAAGCATGAGATCAA
ACTTCGGCAACCCGGTTGTAGATGACACATTTACTATATTACCTTGAACCAAATGTTTGA
GACAGATCTCATAATCTTCAGCTGCACCCTCAGCTGACAGTGCATCATTGATAGCATAGA
CGTTACCGGGGGTAATGGGTACCTTGGAGAGAAAACCATCATACGCTAGTTTATAATTAC
TATCTTCATGATCCTTCGGAACAACTCTCTCATCCACCCAAAAAACATGCCATTTCTCCC
ATTCAATTGAATCAATATACGGCGGTTCCACCAATTTCCTGAGGGACTTGATGAGAGAAC
CACCAGACAAAACGACAGAGAAACTACCCCTCTCTTTGCCAAACTTCTCCGACAGATCGG
CCGTGTATTTGGCCAATGAAACAGCAAGATCTTCCTCCGAATCAAATACCTGCACCTTCC
TTCTATTGTCCGTGGAAGTGGCGGCCATTGATGCCTTGGTTCTTAAACACCTGAACTTTT
TCGGATTCTTGGCGATCACAGAAACGCTTGGAAAAACGACGGTCCGGTTTGATCTGATTT
GTTGAAGAGTGTGGAGTTGAGTGTTAAATATGGCTCGTAGAGAACTACAGAAAGTGAGAG
AAGAGGAGGCCATTTTGTAGATCGGAAGAGCCACACGTC
>ntig_896_consensus
CTGAATGGCCTTTGCAAGTTGTTCATCCTCCTCTAGTTGAGCCTTGGCCTTTGCAAGTCG
TTCATCCTCCTCTAGTTGAGCTTTGGCTCGACGTTCATATTCCTCTTGTTGAGCTCGACA
TTCATATTCCTCTTGTTTAGCTCGTCGGTCATATTCCTCTTGTTGAGCTTTAGCTAGTCG
TTCATCTTCCTCTATCCGAGCTAGAGCAAATGGTTCATCCTCATCTTCATCCGAGTCAGA
TTTAATACATTGTATGTCCTCCTCCGAGTCAGATTCATTTTCAATCACTTTTTTTCCTTT
ATGATCCACATCTGAAAGAGAAATTGCAATAGCATAATCAAGTTCTTCTCTGTCAAAACC
GGATGAATCATCCACTGAATCACTAGGCTCATTCCAAATTCTATCACGTGCATATTTCCC
ATGATATCGACCTTTATTACTGGAACCTTTCAGAATTTTGGTCAGCCATCCCATGATACA
AGGTTTTTTGCCAGCAGGATGGAAATTTTTTGCC
>ntig_897_consensus
ATTGCAGCCTGCACAGGTCCTGTAACCAAAAGGATAAAAGAACGGAAAAGGTTGAAATAT
ATTTGAAGTATCATATTGAGAAGAAGGCTCGACACTGAAACTTTCCTGAATAGCCTTCGC
AAGTTGTTCATCCTCCTCTAGTTGAGCTTTGGCTTGACGTTCATATTCCTCTTGTTGAGC
TTTAGCTTGTTGTTCATATTCCTCTATCTGAGCTTTAGCATATAGTTCATCTTCTTCTTC
ATCAGAGTCAGATTTAATACATTGTATATCTTCCTCCGAGTCAGATTCATATTCAATCAC
TTTTTTTCCTTTTTGATCCACTTCTGAAAGAGACATTGCAAGAGCACAATCAAGTTCATC
TCTGTCAAAACCGGATGAATCATCCACTGAATCGCTAGGCTCATTCCAAATTCTATCATC

TCCATATTTCCCATGATATTGACCTTTATGACTGGAACCTTTCAGAATTTTGGTCAGCCA
TCCCATGATGAGAGATAATTTGCCGGTAGGATAGCAAATTTTTGCCCTGTAGTAAGTAAA
GAATCTGCAAAAGCAATAGAATCCGGCGAATCTCCTATCAGACACAGCACCCAATTCTTA
ATGCATAGTCATTGAAGAAGCATAAGTGGGTGCAAAGAGATGCCATCCTTTATTAATTGC
AATGAATCCAGCAAAATCACTCCACCTTTTTTTTTTCTCACTGACTTGTTGTCTTTGTTG
CCCGCGTACTCATCTTCTTCTCACCCCACGTCATCTTACCCCACATCATAGACATATGAG
CAAGTGACCTTCCCCTTTTTCTCCCTCCGTTCCTTTCCTGGTTGAATGGAAACCGTGTAT
TTGCCTATGGCATTTATGGGCTGGTTAGTCATAT
>ntig_898_consensus
TAAATTATTACCCACAAACCAAAAATTTTCCAACATTGTAAATGGATAGAAATTCCCAAT
AACAAACTTACAAGGGAACTACCTACAACTACATCTGTACAATCATTGAGAGAAGAGACA
CCATAAATTTATATAAAAAAGCAAAATTGTATGTGTGTCTGAGCGTGGGAGGCACTTTTA
GATCCATATACAGCCTGATAAACAATGTGACTCATGACACGATAACTTAAAATAGAAGTG
TACCAACCATACCAAGTTGAAATTATCATTAGTGTCCTTTGTCGAGGTTTGCTGATCACT
CTGTAAGTCGCTGCTTGCAACTGCTGAAATTGAGGCTAAAATCATTTGTTTTCATTCTAA
CTTGATCATATCCCCTTGTTGCTTCTTTGGTTCAGCGGGGAAGTATTTAATGCCCACAAG
TTCCCCAACCTGACTGATGACTTCCAAGGCTCTAATTAGATCTTCCCTGGTATGAGAAGC
AGATATGCATATTCGAGCTCTTGCCAACAGCAGTGGGGTTGCTGGAAAAGCAACAGTGAC
AACAGCCACATTCCTCTTAAGGCACTCCCGTGAAAAGGCAGGGATTTTCGCAGGGTTGTA
AAGCATAATTGGCATCACTGGAGAATCATTATCCCCAAGAACCTCAAAACCCATTTTCTG
CAGTTCTGACCTAAAAAAGTTGCTATTCTCACGAATTCGTGCAAGTTTCCGGGCACCTCT
ACTGGAACCATCCTCCCCAAGAATAACCTTTATTGAAGACACTATTTGCTCTGCTGCTGG
TGGTGAAATTGAAGTCGCATAAAGATGAGCAGGGCAAGTGTATTTCAAGTATTGGATCAA
TTCCTTAGATCCTGCAATGTAGCCACCACATGATCCAAATGATTTAGTAAAAGTTCCCAT
CATGATATCTACATCAGCGGTATCTACTCCTAAGAGCTCACAAACCCCTCTTCCTGTTTT
CCCCACTGCTCCAATGCTGTGGGCTTCATCCAAGTATATATATGCCTTATACTTTTTGCA
GATTGATATGATTTCTGGAAGTTTGCAAAGTTCCCCTTCCATGCTGTAAATCCCCTCCAC
TATGACAATAATCTTTTTCCATGGTCTGTGGGTCCTAGGTTGTCCCTCTGCAATTTGTTC
TCTCAAAACTTCCTCCAAATGAGTCGGTGTATTGTGTTGGAAAACACGAATGGTTGCTCC
TGATCCTCGTGCACCATTGACAATGGAGTTATGGTTCAAAGAATCACTAATAATCAAGCC
TCCCTTGCCAATCAAGACCGGAAGGATAGAAGAGTTTGTCAAATAGCCCATTCCAAACAC
TATAGCAGCTGGTTTTCCAACAAACCTCGCAACATACTCCTCCAATTTGTTATGCAATAC
CGTTGTGCCCCCATCCACTCGGCTACTACAGGTACTAGGAGAATATTTTTTCAATGATTC
TATTACCCGGGGTGTGCAGTATTCATCTGCTGCAGCAAACCCCAGGTAGTTGTATGATCC
CAAATTTAGGCATCTACTCACATTATTGGTTCGTATCAGCGTCTTATTACAGTCATTGGA
GTATCGTTCGACCACATCAAACCACCCATCAGGGGCACTAGCAATAGGTCGGCCAAAACA
GTCCTGTATTCGAAGATACAAACGTCGAAGATAAAAATCTTCGAGTCCTAAACAGATAGG
CGCATATCCCTGAAGATTATTGGTGCTCCACCAGTCAATAGTTTTGCGAAAGAAATCTCT
GATTTGGCCAAAAGCAAACAACAAGCCGTAGCTGAAGTAGGTCGTTAAGGCAGTTAGATA
GGGAATTGCAATCATTTCTAAGCAAAAAAACCGGTGAAATTCAAGAAAGGAAGAGCAGAT
CTAAGTGTAAGAGAGAATGATGAAATGGGATCCCGCTGGAAGATCCGAGCGAACGAAAGA
TCCCAAAAAAGGACCCAAGAGAAGGGGACGGCAGCGATCGGGAATTAAGAGAAT
>ntig_899_consensus
AAGCTCATAGACTTCTTTGTGGAAGTTTTATTACTCAGGACAACGTCGTACCAGACTACA
TGTGATTGCAAAGCGAGCACTTTGCCTTATTTAATTAGTTAAGTTTATGTAACTATAAGG
TGATGCTGCAGAAATGAGAGGTACTTCAGGGACAGAACACAACCTTATAGTTAGTACCAC
CTGGGCAAGTAAATACGCTTGTTGCGTCATCTTTAGGGTAACTATAAGCATCTGGGCATC
TATCCTTGAAAAACCTGGAGTATTTTGTAGGTCCACAATTACCAGAATTACAGCAATATT
GATCTGTCTTGAAAACGGGACATGCCCCGTTACATCCACCAGGGACTCGCAGCTCCTTCG
GACACTGCCCAATAATATCCCCGACACATTTGATCACACGGTTGCACCCTCCGGACACCG
AGCTGAATTCCATTGGAACATTAAACCCATCGATATTAGAAATATCAATAAAATCAAGGT
TGTTGAACTGGTTCAGTGCGTATTCAGCTAAGGTGTTAGGGGGTGAACCATAGCCTTGAC
ATTGGAGAAGCCCCCCACAGTCACCAGTCTGGCAACGCCCACGCCCAGCTGCGTCAAATT
GGCAACCCGTCCGAGCCCATATGCGGGCTGCTTTTGTGCCAGGGTTCACGTTAAGGACCC
AATTTTGACCACGATTAAGTTGTCTGCCTCCACCCGGAGAGGCAGCCGCCCAGACCGTGT
AGGGACAATTGTTTATGACATTGAAAGTGGCTGCATTGGCCACAGCGAAACAGAAAAAGA
GTACTAGAAGCAAAGAAGACATTAATTGAGAGCTTATTCAAAAAGACTATGTGATGAGTG
TTGGTGTGGCC

>ntig_900_consensus
TCCAAGAACAATCAATTTACTACTTGAAAAAAAATTGATTCATAATCAAAAGTACTAAAA
TTATATCTCAAACCAGCCATCCATAAGCACAGAACTTAAACCAACAGGAAGAATATGTTT
AGTAAAGACCCACTCACAGATGCCTAAAAGTCTAGCAAAATTTTTGATTGCAACAGTAAG
AAATAGCAACTCTCCAGAACAATTAAACACTGAATTCAACTTAAACTCTGAATCCCCTGC
TGTTTCTCCTTCCTCTAGCCCTCTCAAACTTCCTTCCCTTGGACCGCACATAGGGTTTGG
TATGGCTGTGCGGCACACCAGGAGCTGGACCAAAGTGCTTCACTGCCTCACGAGCATTCT
TTGGACCTCTGAGAAGAACCGTGTTCTGTCCCAACGGAGCCCTGAGAGCAAGTTGATCAA
AAGTCAAGCACTCTCCACCAGCCTTCTCAATCCTTGCCCTAGCCGTCTCAGTGAACCTCA
AAGCAGTAACCTTCAAAGCTGGCACCTCATAAACCCTAATGTCATCGGTCACTGTTCCCA
CTACCACTGCTATCTTCTCTTCCTTTCCTTTCATGAAGCGAATGAGTCTAGAGAGAGATA
GTGGGGGCTTGTTGACCTTGCTCATGAACAGCCTCTTCAATATCACTTTATTGAAGTTAC
TATCTGTCCTCCTCACGAGAAATCGGTACAGCTTGACGAGGAGCTTAAGATAAATATCAT
CGGACTTGGGGGCAGTCCTCTTGGACTTCTTGCTCTTACCTCCTGCGATCAGATCGATAC
CCATGGCAACAGCTCTTCTGCTTGATGGCGCCGCTGGCAAGCGTGAGTTGTGAGAGGACG
AAAATGAGGTAGGGTTTTG
>ntig_901_consensus
TGCACTTGAAACACTTTATTGCAAAAATCCGTAGCTGTGCACTTTACTACATGAGAGGCT
TATGCCAATTTCCATTCTATTTTCATGGCCTCCGCAACATGACAGTATACAACCAATAAA
CCAAACTCTGGAACCAGGAAACCACTAAATTATAGTCACTTCCGGGTTTCAATGTTCAGG
GTGAAGAAAGTGAGATGAAAATTTTAGGAAACAACCAGAGGCAATAATTCTTTTATTGAC
CCTATCAACCATAATAGATCATGGACATGACCCGCACCAATAAATGGATAACAGATGCCA
ACAAATGTCAAATACCCCAAAAAATAAAAAACAAAAGACATGAAACTATCCCACGGAACA
ACTAAAAAATGTGGGTCTATGTAAAAATAACAGGACACCAANNTCTTCTGCCTCCTTTCC
CAATCAAATTGCAAAAAGGAAAGTTGAAAACAGCCCTGCAAAGCCCTTAGAGAGACAACC
TTCAACAGCACAAGGAGATTTGGAGGAACCCAATGGACCATTTTGTTTCACTATTTCCAT
GCAGTCATCACCAGCAGTTTCTGGTGGATTATCTTCCTGGTCCAGAGGTCTCTGAGCAAC
CAACTTGCTTCTCTTAGGAGTTAACATGTAATTTAGGTCTGTTAAGTGAGGGAAGCGATC
CTCCGATGGCTCCATTTCATC
>ntig_902_consensus
ACTCCTGTGACCTTTAATGATTGCCTCCCCAGGTCTCTTCACTTCTCTCACAAGTTTTTT
TTGACGCCGTTTCTCTCTTCTAGAGGATTGTGAAAAACTGTTATTTAAAACAAGTTCACT
GATCAAAACACCTTGCATATATTCACGTTCCAAAATTGGTGGTACATTTGAATCACTCTC
TTTCTCCAGGCATTCTGAAAAAGATTCAGACAAGTCCAATGATGAATCGTGTCCAATCAC
CTTTTCGATAGATGCCTCAAGGCTCCAACGCCTTTCCAGGGAAACATTTCTGCTATGGGA
CTGCTCCAAATTTATTAAATTCCCCTTGGAGAGTTTATCAGCTGAAATGCGACGAGACCC
TCTGTTCCCACCAACCTTCACATTTGCTATATCCACTGGAGAAGTATGATGAGCAT
>ntig_903_consensus
TGTTGACTTGACCTTAGCTTGCACTGTCAAAGCCAGGGTCTACAAAATTGGATAAAGCCA
TAAGAAAATCTTCTCCATTAGAATCTTCCATCGGCTGAAAATCCGCCAACTTTCCAACAA
ACCTAGAATAGGGATAAGTAGAATCTAGCTGATTGTTGGATAGTGTCAGTTGTCCCTCTT
TACAATTCGGTGAATCAATACAATTTGTCCCTAAATGATTAAGGGAATGTCTAGAACCAA
GAAACGGCATAGACAAGCAACTACTGACATTGTTCGGATCATAGCCATAGCTTTCAAATT
TTGGTTGAAAAGATATCAAGGATTGCATCGTGGGAACTTGATACAAGTCTCCTTGTAAAT
CCACTGTAGATGAATTGTATGCTGCAGGATGTGATTCACCATAGTCCCTGT
>ntig_904_consensus
TCTACACAGAGAGGCTGCCATTCACACTTAGAGTTCATCTTTCGCAGATGATTCATCTTT
GACAGATTCTTGAGGGGCAGATTTATCCCTGTTCTTCTCAATGAATTCGATAATGTCTTC
CTTGGTCCTATCACCCTCATAGGCCAATATATTTCCACTACTTGACCTGAAGTACACGGT
TGGGAAACCTTGGACATCAAAAGTTCCGCTTGGGATATCATTTGCAGTTGCATCAAATTT
TGCGATAACAACATCGGCATCATTCTGATAAGAGACTGCTACTTCATCCAATATTGGAGC
CAATTTTTTGCAATGTCCACACCAAGGAGCATAAAACTCCAG
>ntig_905_consensus
TCTACACAGAGAGGCTGCCATTCTTAGAGTTCATCTTTTGCAGATGATTCATCTTTGACA
GATTCTTGAGGGGCAGATTTATCCCTGTTCTTCTCAATGAATTCAATAATGTCTTCCTTG
GTCCTATCACCCTCATAGGCCAATATATTTCCACTACTTGACCTGAAGTACACGGTTGGG
AAACCTTGAACATCAAAAGTTCCGCTTGGGATATCATTTGCAGTTGCATCAAATTTTGCA
ATAACAACATCGGCATCATTCTGATAAGAGACTGCTACTTCATCCAATATTGGAGCCAAT

TTTTTGCAATGTCCGCACCAAGGAGCATAAAACTCCAG
>ntig_906_consensus
CTTCATTCTAAAATTCCTACCAATATCCAACCAATTAGGAAACCTGATTATATTGTTTGT
AATTTGAAACGTGAATATACATAATCCATTTATACATCTGCCCGATGTGCACAAACCGGA
TTCAAACTAAGCTGAACTCAAACTCACAGGGTGTGAAATTGGCAGAAAAATGTACAACTC
TGTTCAGTTTCTCCAAGGTCCGATTCCAGCTCTCAAGAGGGAAGCATATAACAATTCATT
CCAAGTGTCTGGTACTCCAGGCAAGCACCAATGACTGCAATCTTGAACTTGCTCAGCGGC
AGCTTGTTCTTCGGCAGTCTTGTATTCCATTCTATATGTTGCAGGGTGTCCATCTTTTCT
GAAAGCAGTAAGCCTACTTATGTTCAAATATAACACAGGAGATCTCATTTCTGGAAGCAC
ATGCTCCAGTACTTTCATTTTTGAAGGATACTTAGGTAAGTAAGTTCCATTGAAGAATGG
CTCAGTTTCTTTGTGGCACTGTCCTCCTGAGTTCCATGGGCCTCCTCTAAAATGGGTGAC
TGAATATCCTCTGAAGAAAATCTGAGTTCGATTGCTATCAATGTTATTGTCAACCCATCT
GGCCCAAGTGGTGAGTGCTCTCCTGTAAGCTTCTAGCACCTTAAGTCTTGGGTGCACATA
GTTACCTTCTTGGTAATAGTCTTCTCCTCTAGAAGTTTTCTCATGGGTCCACCAATGACC
TGTATTGAAGATAATGATGTCAGCATCATGGTACATTGAAGTTGTACGGTCCATCAAATC
TAATCTTAGTGTTTCCATGGATCCATTTTTCCCCCTACATGATGATTCTCTAACAAGAAA
TGGAGAGCCAACGAAATCCACAGTACAATTATAGTCCTCAAATAGGAAAGCATAAAAACC
TTTCTTTTTAAACTCTGTCTTTCCAGAAATCTCATAGACTCTTCTCTTGTTTCGAACACT
ATGGCGGAGTATACACACTAGAGATTCCCACATATTCCTATTCAGAGAATCTCCAACAAA
AACTAGCTTCTTGTCTCTCAATCTCTCAAGAAAATCAGTTGCATTCAAACTAGGGATGTC
ACACCCGTATGGCTGCCATTTCCATTTAACAAAAACATCATCTGGCCTTCCATTAATGTG
ACAATCAAAATCCCTTTCTCGTCGGAGCTAGTCGAAGCTGTTCAGGGAAGGAAAGTTTCT
TCAAATCCATTACAGAGACAAAAAATGGCGT
>ntig_907_consensus
ATTACCCAGAATGTATGAGCCACACAGTAACTTTGGAGATGACAGATGGAAAGAACCATC
AACAAATTTGACCAATTACAAAAACCATCTACTTTGGTCCACCAAATTTCGACATCATCT
TAGCAATTATAGGGGCCACTTTAGGATTTGCTTGATGCTTTGCAAGGTTAGCAGGATTCT
TCATGACATCCTGAAGGGCAGCCATAACTTCTGGATCACTAAATGCTGCCATCAGTTCAG
GGTCATTCAATATCTTGCTGAAATCAACATTTCCCGGCATCCCTCCAGGCATGCTACCAG
GGAAGCCACCAGGCATACCCCCTGGAAAGCCACCAGGCATGCCTTCCGGTTTTCTACTCG
AGGAAGAGTGCTCTTGCTGCTTAGCCTTCTCATAAGCAGCCTGTGCTTCAGCACGACGAC
GTAGTCTTTCACGCTCAATCTTTCTTTCCTCCCTCTCTTTCCGCAGTCTGTCATACTTCC
TACGATGTTCTTCAATCCTGTGTGCATTAGGTTCAACCTTCTTAAGCACAGCACTGATTT
CTTCATCATAATCTAACTTTGATGCCAAATGCAAATCCTTTGCAGCGTCCTCCCACTGAC
CAAGCAATGCCCTTGCCATACCTCGCGACTTATAGCCCTTCGCAGAATCTGGGTTAATCT
CTAAAGCTGCATTAGCATCCCTGATGGCAGCATTAGGCTTCTTCATTTTGACGTATACAC
TAGCTCTAGTCCCATACATAATTGCTGATGTCGGATTGAGCAAAATTGCATCAGTCAGAT
GCCCAATGGCCTCCTCGAAACTTCCTTCAGAGATGGCTTCCATGGCCTTTGCCTTGGCCT
CCTGCGAGGCGACACGGCTCTCTTCAGTAACCTCAATCGAATGGTCTCCCATCTTTTGTG
GAGCATCATCGTCAGGTTCAACTGTTTCTCCTTCAAGCTCAATATCAGATTCAATTATCT
CACCCTCTTCCTCTTCTTCTTCAACAGTCGTTTCTGCTCCTTCATTCTCTTCCATTTCAT
CTTCGCTCCCCTCCACAACGTAACTCTTCGATTTGGAGTCGCCTTTATCGTATGAAGCAG
GAGGCAGCTTTGCTCCAAGACTTTCAAGGTAGTCGCGGAAGAAGGAGAGGGAAGGATCAC
CGAGAATAGAAGGATTGGATTTGCACTGATTGATGAACAACTCGAGCTCTCTCAATTTTG
AAGCGTCCATCATTGTGATTTCAGAGAGATTGTTGTTACGTTACTGAGAGTAACGAGAGT
TCTTCTTTCGGGTCTTGTGTTTTGTCTTTCGTCG
>ntig_908_consensus
TATCTACAAAGGGGAAACTGATCTGCACCTCCCAGAATAAATTCAACCAACAAACAGCTT
GGAAACCCAAATAGATCTTTATGATAAAGTTGATCCAGCAGAAGGACGGGGGGTCGGAGA
NNNNNNNNNNNNNNNNNNNNNNNNNNNNNNNNNNNNNNNNNNNNNNNNNNNNNNNNNNNN
NGTTGAAGAAAGGATTTCATGGGTCAGCAAATGGTGAAACATGCAATTCCTATGGCCATG
AAGGTCGCTTGTAGCTATCCCATCCTCCTTCAGGTTCCTTCCTCTTCAGACCGCCACCAG
CCATCTGATATATTCTTGCTTGCTCGTCTGCCAGGGCCTGTGAACTAGTAACAGATAGCT
GCCTTGGCACAAGAGGTGATATCTCATCTCTACCTTCTATATCTGGGAGTCCAGGCCCTG
CATTTAAGTCTAGACCCTGTCTTCCCCATTTCCAACCACTCTCAGTATTAGAACTATTGC
TACCATCAGGAAGGCTAACTACAAAAGACTTTGGGTAATGTGGTCCCAATATTTGTGAAT
TGACTGCAGGGAGACATAGCCTCCCACCAGAAGATGAATCCATATATGTTGTTGAACCAC
CAGAGAAAGTGGCTGATGGAAGAGGAAAGGTGGTTCCAAAAGGGAAGACAGGATACTGGA

AGTGAGGAGATGGAAAGGGAACTGCAGAAGATGACAAAACTGGCCCCCTGAAAACATCAT
GACCAAATGGGGTGCCAACAGAAGGGGGGTTCAACATCCTTTGGTGTGCACCCGGTGCAA
CAATTGAAAAAG
>ntig_909_consensus
CACTAGTGAAGAAAGCAAAGGTAAACTTACGTTACACATTTACAAGGTGAAGCAAAAAAT
GAACATAAAGACCAAAAAATCACATACCAGGCAGAACACTTTGAGGATATCACAAAAAAT
CAGACACCCCAGCTAGCTTTGCCTGACGATGAAGTCCTGTCCTATCTGCTAAACATATGC
AAGAGGCACTCTGTTCTCGTCACAAACAAGAAACTGAACAGCCTTTGAATACACAAAAAA
CTCGAGGTTTTTTATTATTCGAGGATGGCCACAAAAAAAGAGCCATACTAACATCCCTCA
AAAATTACACAGCTAATACAAGGTTAATAGCAAATCTGATTCTGCACAAGTTAAGGGTTT
TCCCATAGTTGCCCAGAATGTCTTCTGTCAAAATAATGCACACTGATAACTTTAGATACG
TGAAATGCCTTGATCTGCAATGTATAGTAGCATGACATGGAAGCTAACCAATTACTCCAA
AAAGTTTGGAGATGTCTCCTACACTGGCTAACCTTTAAATAACAAGAATTCAACTTGAAA
ACTTCAAATGAAAAGCCAAACTTGATATCTTTCAACAAATAAAAGAGCATTACTTCACTG
TAATAGGGACGAGGGAAGCAAACATCACGTCCATTATCCTGGCCCAACTATTGAGAGGCA
CTGACAGCTAATTTGCTGCACCTATGGCGCACACAGAACTTTGCAGGCATCACCATAAAC
TCACCTCATCTTACAACTTCGGCCTCTCTAGAAGATGGGGATCTGAACCACACACTTCTG
CATCTGAACCACCTGTCTTATGCATGCTTGCAAAATTAAGCATATTCCACAAAGGTTTAT
CAAAATCAGGCAAATGCTGCCAGCGATGGGATAGCTCGCAAGTGTCATGGAGTTATGGAC
TTAACTTCCCATTGCAGGTCTCCACATAAAGAACGAAGTACTAACACACAGTTGAGTCAG
CAGATTGCAAATCCAGTTCAGTTATCATTTGACAGGTGAAGTATGCCACGCATTTGAAGG
TAAGGATTCTCTAACAGTTTTTCGTTGTTGCTCTGCTGAGCTTTAAAGACAAGATTCTGT
AGCATGTGCTGGTACTTCTCAGGTTTCTCTGTCATCATTCTCTTTAATAGAAAAGCTGAA
GCATAATATGCAACCCTTGAATCAGTATCATCTAAAAGTTCCCTAAAGAATTCTTCTCCA
CCAACTTCTTGAAAAGCAGCAGGATCTGCAGTGCATTTACCAATTAGAAGCAATAGAAGA
GTAGCTCGAATATCGGATGTGGCACCAGGAAGGTTCCCCCTTCCTTTGCTTCCAACGGCA
ACACCCAAAGCAATATTATCTGTTGCTGCACCAGCAAGTTGAATCAATGGCCAATAAAAT
AAAGCAGCAGGTACCCGTGCAACTAATTGCATAGGAACAACTGCCTGCCCGTGCAAGAGC
TGTGCTGCCATTGATGCTGTTTCAAAAATTGGATGATTCATATTATAATGGGATCTGCTC
TCAGTTTCCTCATGGAATTCATCCCAGCAGCCAGTCTCTTTTTGAGAGCCATGCTCAGCA
ACATCATGTTTTTTATTCTCATCTAGGGAACTAAGAACTTTACTCTGATGCAAGCCATCT
CCAAATGACTTTGCAGTTGCAGGCAAAACTTTGAGGCACAATTGAGAGAACAGGATGTCA
CACATCTTCAAGATGTTGATACGGTCTGTTTCATTAATTTGAGCAACCAAAGACAAAGCA
ATACTCATGATATCAATCATAGTATTTGCTTTTTCAAGTCGGCTATCTTCATGTTCATTA
GCAACAACACCAATTGGATGTTGCTCCTT
>ntig_910_consensus
TACAAAAATTCCATCTTCACTTACGCCGATGGACAAAGAATCTATCAACGGTTCTCATTC
ATTCTCTGTACACATGTTTAACGGAGAGTCTACCACAGTTGCACAGAATTATCTCCAATA
ATTACCATTGGCTGATGGGAAATTGGGAGAATCTTTGATCAGGCATGCGATGGAAAGGCA
ACATTACACAGGGTGCATAGAGTTTGTAGAGTAATGAAAGTGA
>ntig_911_consensus
TACAAAAATTCCATCTTCACTTATGCCGATGGACAAAGAATCTATCAACGGTTCTCATTC
ATTCTCTGTACACATGTTTAACAGAGAGTCTACCACAGTTGCACAGAATTCTCTCCAATA
ATTACCATTGGCTGATGGGAAATTGGGAGAATCTTTGATCAGGCATGCGATGGAAAGGCA
ACATTACACAGGGTGCATAGTTTGTAGAGTAATGAAAGTGATGTTATCATGATTATTGAA
TGCACCATCAATTCCGTTGGCTTGGCTGAGTAGGACCCAAATATTTTTCCATGGATTCCT
TCTTATGGTCAGGATCAAGGCTATTCCTGAGGCCAAGAAGAGCATAGTGTGCAGCACTTG
CACAAGCAGAAGCTGGAGCTATTGCAGCTGCAGGAACTCCCCGTACAGCAGTTGCCAAAG
CAGATCCAGCACTGGCTCCTCGCTGGTACTTTTTGAATGGTTGTTGAATTAAAGCAGAAG
CAGATCTCCCCAGGCCATCAGTGAAACTCTCATAAGCCTGTTGGAATCCTTGGTGGAAAT
CTTTTGGCTGATTACATCTTACATTTGTCTTTGTCTTCTCCTGCACAGGGCATGATACAG
GAGGAATGCTTGTAAGAATACATTCAGTTTGAAGCAAAATGTCATGAGCCCCAGCTGCCA
AATGAACCCCCAGTCCAACAGCTTCAAGTGATATACTTCTAAGAAAAGCAATAGTACCTC
TTTGCATTCCTTTGAGTAC
>ntig_912_consensus
CGACCCATGGTCAAGACCTCTGCCCTCCACGACGCTCTTCCGATCTTTTTTTTTTTTTTT
TTAAAGATGATGGTTCAGTCGGAGTCAATCCATTGAGCCATGGCGAGCTCAGGTGGCACC
GGCCAGTTATCCTTCCACTGCCAAGAGCGGGAGGTAGACGCTGCTCTGTGAAGCCAGCCT

CACGCGGTGTCCTCCACTCTAGTAGCCATGAAACTCCAGCCTTCGCCAGAGACCCATGGC
TCATCTCCTAGCTTGGAACGGGCTAGCCAGTTTAAAGCCAGCCCAAGCCCAACTCAATTG
GTTGACCCAACTGTTCTAGCATCTTAAAGCCCCCACTTGGGTTACAAGCTCACGCAACCA
AGCTCGGCC
>ntig_913_consensus
CCCATATAAGTTATCTATTTCCCTACACGACGCTCTTCCGACCTGTCAACCTACTGAGCC
ACGGTGACCCCAGGAGGCACCGGCCAGTGATCCTTCCACTGCCAAGAGCGGGAGGTTGTC
GCTGCTCTGTGAAGCCAGCCTCACGCGGTATCCCCCACTCTAGTAGCCATGAACCTCCAG
CTTTCGCTGGGGATCCAAGGCTCAACTCTTGGCTTGGAATGGGCTAGCCAGGTCAGTGGC
AATCCTAGGACCGACTCAGTCCGATCGAACCAAAGTCCCACCATCTCAAAGCCCCCACTT
GGGTCACAAGCTCACGCTACCAAG
>ntig_914_consensus
TAACCAAACAAATAAAGCAAATACAAAGAGAGAGAGAGAGTTTCAGAATGTAACACCTCGGA
TCCATAATTACAAACATACTTTCTTCTGGGGAAACTCAAAAACTAAATCCATGCTGGATT
TTTCAGAGGGGAAACAACTGCCTTCACTTGCAAGTAATTGTGAAGGCTATAAATTCCCTT
TTCCCTGCCTATGCCGCTCATCTTGTATCCACCGAAAGGAATAGCAGCATCAAACACATC
GTAGCAGTTAATCCATACCGTCCCAGCTCTCAATGCCCGCATGGCTGTATTGGCAGTTTG
CAAACTCTTGGTGAAAACTCCGGCAGCTAGACCATATCTTGTTGTGTTTGCCCTTCTTAT
CGCATCATTAATATCCTTGAACTTCAATATGGATTGCACTGGGCCAAATATCTCATCC
>ntig_915_consensus
GAATGGCTTCCTCCAAGACTTTCCTTCTTCTTGGCTTTGCCTTGTTAGTTGTTCTTCTCA
TCTCCTCCGAGGTGTCTGCTCGTGAGCTTGCTGAGACCACCCAGGAAACCAAGCATGATG
GTGCTGATGGCCACCACCACCACCACCACCACCACCACCACCATCATCACCATCATGGAA
AACACCATGGAGGGCACCCTGCCGATGAGACAAAGGAGGTCGAAACTGAAGCTGGAGGGA
AGTAGAGAAAGTTTGATTTGTACCTGCTCAAATAAGACTGTACGTGTGTGTCAATAATAA
GTGTGCGAGGATGTGAGCTTGAATGTTTCATATAACCAAAAAAAGCTTTATATAAATAAG
TATACAGTCATGTATAATTACTCTGTCATGGGATGTGTTCTCCTACGAGCAATTATGATT
AGATCCAATCTATGCTAACTTGGTT
>ntig_916_consensus
TCCAAGACTTTCGTTCTTCTTGGCTTTGCCTTGTTAGTTGTTCTTCTCATCTCCTCCGAG
GTGTCTGCTCGTGAGCTTGCTGAGACCACCCAGGAAACCAAGCATGATGGTGGTGATGGC
CACCACCACCACCACCACCATCATCACCATCATGGAAAACACCATGGAGGGCACCCTGCC
GATGAGACAAAGGAGGTCGAAACTGAAGCTGGGGGGAACTAGAGAAAGTTTGATTTGTAC
CTGCTCAAATAAGACTGTGCGTGTGTGTCAATAATAAGTGTGCGAGGCTGTGAGCTTGAA
TGTTTCATATAACAAAAAAAAAGCTTTATATAAATAAGTATACAGTCCTGTATAATTACT
CTGTTATGAGATGTGTTCTCCTACGAGCG
>ntig_917_consensus
GCTGAGACCAGCCAGGAAACCAAACTGGATCAGTCCAATTATGCTGACCACGGTCATGGC
CACCACCACCACCACCACCACCACCATCATGGAAAACACCATGGAGGGCACCCTGCCGAT
GAGACAAAGGAGGTTGAAACTGAAGATGGAGGGAACTAGAGAAAGTTTGGTTTGTTACCT
GATCAAATAAGATTGTACGTGTGTC
>ntig_918_consensus
AGTGGAAAATTTCCGTTTATTGACATGATCGTGCCATTGTCATGCGGAATAGGACCTTAT
GATTATGCTGTCAATAGAATCCTACGCAACCGTAAGTCATATTCAGTGCTTACAAGACCC
TTAGTCCATGCTAAATTTACCCTACGCTCACCGAACCAACGGGTCTTGATCTTTAGAGCT
TTAGTTTAGCGGGGTTTTCATGCGGCATCCAACTCCTGCTCAATGGTTGGTTAATAAGCA
CCTATAAAACTATGTATGTTAGGATATATATATATGTTAACATGCTCTCCCATAATTTAG
GCAGGCAAAGTACTTGAGTTATTTGCGCTAAACACCTAAAAAATTACATTGACATATCAT
CCACATTTTAACATAATCATGAGGGTTGAAATTGGATGTGGGGGCATGGCCTTTCTGGTT
CTCCTCTTGTCCACAGTATTTCAAGTTCACCTCTTCTCTTCCCCAATGTCAAAAAAGTCC
CAAGTGTAAATGGAACAATATAAAGCAGAGCCGGTTGGCCATGTCCATCCATCAGATTCA
GAGCCACATATGTGATCAGGAGACCTAGACCATAGGCAGTCATTGCCCACACAAAGTATC
CCGTACGAAGATTCCTCTTGGCCAGCCAATCATATCTCAGTGAAAATGCCACAAGCAGTC
CTGGTAAGATAATGTCACCAAAACCAATAACACTGTAGCCACCCCAAGGATCGAACATCC
GTGGGATTTTCAATAACATGGGGATACCATCCTCCCCACTTTTATCACCGCGAGCTACTA
CTATCATTACACTCTCATGGAACCACCATTTAGAAATGAACACCCAGAAGATGTCGTACA
GGAAGGCACAACTGAGCAGAACAGTTCCAACCTTAAGGTTTGGTACACGGACAATCTGAA
GAACTGTGATCATCAGTGCAATACCAAGGATATCTTGACCTATCCAAGCAAAGGATATTC

GACGAAAAACAGCCCATATAACAGAAAATGCTATGCAGAACGGACAAACTGCCAACGTCA
GATATGAGATAGCTCCAACGAAGGGTACTTTAACAAACGATTCTCCAGCATGTTGAAACC
ATCTGAAACATGACAATAATGCTACCAAGCAAGTTTGCAGACCCTCTACCCCACCAATGC
AAAATAGAATCACCAGAACCTCAAGAAACCAGAATGACATCAGTTTGTAGAGCATAACCA
AGAAACATGAAGCAACCACAACAAAGAGAACTGCTGATTTAGTGCTGATATCCATTACAC
CACTGGAACTAACATTTTCCGTGTTTAAAAGTTCCTCTGAAGCATCCTTTAACAGCTTTT
CCTGCTCAATGGCTGCCTCTCTGGCAGTCCAAGCAGACCAGTAGGATGCACATAAAATAG
TACTAACAGCCATAAGCCATAAGAACACTTCTGCAACATCAACCATTGGACGCTTTGGAG
AGTACAGCTGCACGAAGACCGAAGAGTTGTTCTTTATATGACTTTCCAAGCTTGCACCAG
CGTCTTGGGGAAGCATCACAACAGGTATTTGTATATTTACATCAGTTTCGTTGGCTTCAC
AAACCATCTTTAAAAGTTCTGTTTTGTAGTTTATTATGAGGATGGCTGAAGCATTTACCT
CTTCAGCAAAATCTGCCTTAGTTGTAAAACTGCAACCACCTCTATGCACTAGAAGTACTT
CTCGATTAAGCTTATTCTTTGGTTTGCTACAACAATCAGGAGGGTCTGCCAGTACAAGTC
TCGTTTCATTAGCATGTTTTTCCTTGGACTCCAAAGGAGTGCCAAAACGAGCACCAACAC
CAACATATTCAACATCCAGCACACCATCAACCCAAGTTGGAACTTTAACCAAAACAAAGT
TGTTATCACAACCAGGCCTCTTGGGAGGATTGTCGTCTTCGTGAACTATATCGCCGGCTG
ACCCCAACCACGGACACAGCCATAATACTATTATTAATATATGTGCAGCTCTCTGTATAT
CCATCTAAATATACATAGAACGATAACCTAGGTTTCTATTTTACGAGAGAAACGGAGTCT
AGAGAAGAGAGAAGAGTTTTGAAAGAGGAAATTGCCAAGGGGCC
>ntig_919_consensus
GCATTGAGAAAAAAAAATTTTCTTTGATTAAAAAAAAAAAATTTAATGTATAGGTATATACT
AAAAGAATGAATACCTATATCTTGTAATTATTAGTTTAAAAGATTATATGTAAAAAGTTA
TTATAATAGTTTATAAAGAAACCGGTGTAACTTCAGGAATTGGTCCAGGTTCAAATTAGA
GCTAAATGGGCTGCACAATCTAGACCAGCGTGAAATGTGTCTCCACTGGAAGAGAGCAAT
GCACAGATTGTGTCCACTGGACCTATCAATTGCAGAAACGTATTAATGCTATTATGTGTA
GCTATTACTTTTTCATTATTTCCGGACCGAGCAACTTTACATCTAGGAATCTACTTAGAA
TCCAATGTTCAAAAATCCCAGTTTCGGACTGAAATAACTAAAATTCATTACAGTAGACAC
AGATAACAACAAGGGGACCAATTGTTCTATAATTTATTGGCGATGAGCTGAAAAGGCTGT
AGTGGGACTTACAATAAGTTTCCTTGGAGGTGTGCCCTCCAAAAGAGGCCCGCTTGTGCC
CTCCAGAAAAAGGGGCCTCCCAGTGTCAAGGAGCGACGTTCTTCTTTTACCGTGTGTCGA
AATCAGGAGTCGGAAAGTCCAGTAAAGGATGCTCGGTGCAAACACCCTGTAGAGGAGGAA
TATATTGTACCAGTTTGGAAACTTAACATATGCATCGCCTCGACAAGCCCCGGCTACAAT
TAACCTAGCGAAATCCTCCACAGGGCCGCCGGCCACATGGACTTCTCTTTCTTCTTTCCA
TTGCATCTCGGCGCCCTCTTCCACCATGAATTTGCCTCTGGTCATTTCGCTTCCAAGCCA
CCCGTGAGTTGCAATTGTTATTCCAACATCGTCAGTTAATTCGAATCTCAATGTGTCGTA
GAAGTTTACAAGGGCTGCTTTTGCGGCAGAGTAAAGGCTCATTCTTGGCAATGGTAACCA
GTTCTCCACTGATGCATTCACAACGATACGGCCGTTGCTCTCATGTAGGTAAGGAAGAGC
AACAAGGGTTGGATAAACGTTTCCCCAAAAATTTATGTCCATCAAATGAGGAAACACAGA
GGTATCCGCGACTTCCTCAAAGTAAAATGTGTGTCCCAAGCTTGCTGTATTTACGAGATG
ATCCACGCGTCCGAAGAAGTTTATGGTTTCATTAACAAATCTTCTACATTCCTCTTCCTT
GACAACATCTGCAGCTATGGCTATAACGTGCCTTGCACCCATCTGTCTTGCATTCTCACT
GATACCTTGGAGTCGGTGATCTCTGCGTGCCACCAACACAAGATTCGCCCTCCTCTTTGC
ATACTCGTATGCAATTTGCTCTCCGATACCAGAAGAAGCTCCGGTGATAATGACCACTTT
TTCTTCCATGTCCAAACCGTACAAACTATTATAAAGCCATTCGCAAGAGTTTATAAAACA
CATGGCTGGCCATGCAAGGGCCAGCATCACTGAACTCGCTGGAGGCACCACCAAATTCAG
AAAAGTGTTTAGCAAATCCATCCCCAGAAACCCAGAAAGGCTCACATATATTTGAGGGTA
AGTTTGTGGGTTTATATATATATAAAAA
>ntig_920_consensus
TTTTTTTTTAAACTGCAACTACATCCCTATCTTTATTAAACATGGATGGCACATTTTACA
TATACACACACACACACACACATACATATGTATGTATCTATGTATCTATGTATGTATATA
AGTACAAGCAATAGAGCCTGTCCGGCACTTACACAACCCCAATCCAAAAATCTCCGTTTT
TAGGTCTCTATCGTCTATAAAAGCCTATGAAAGATCGAAAGGGCAAGAGACAATACAAAA
AACTCTGCAATTCATTACCACAACGCACAATACCCAAAGAAAACAAAGACCAGAAGATAC
TAGACAAAACCGAAAAACAGAATACATGTATGACAAACAAAAATTATCGACTTTTAGCCG
CTGTAAAGACTCGAATTTGATTTTATGTCCAGTTCGTCTTTTTCTTTTTAATCCACTTCC
TCCATCTTGCTCTCCTCATTTCCCTCCTCCTCCAGTGGTGGCATCTCCGTATCATCATCA
CCGGTCTCATCCTCGTCGATGCTGAGACCCAATTTAAGCATCCTGTGAATCCTTGCTGCA
AAAGTGTTGGGATCATCCAGGCTGAAACCGGAGGTCAAGAGAGCGGTCTCGTAGAGTAGC

AGTACCAGATCCTTCACTGACTTGTCATTCTTATCTACTTCCGCCCTCTTCCTCAGCTCC
TCCATTATACCATTGTCAGGATTAATCTCCATAGTCTTTTTGCTTGACATGTAGGAACTC
ATGCTGTTATCCCTCAATGCCTGAGCTTTCATAATCCTCTCCATGTTTGCTGTCCAACCG
TATTCGCCGGTGACCAAGCAGCACGGAGAGTCTACAATCCTATCAGACACCACAACCTTC
TCAACTTTGTCTCCCAGTATTTCCTTGATTATCTTGCAGAGGTTCTCAAAGGATTTCTTC
TTTTCCTCCTTTTTCTTCTTCTCTTCTTCGTCTTCATCAAGTTTTAGTCCTTCTTTGGTG
GCCGAGACCAGCTTCTTTCCATCATACTCCTTCAATTGACCAACGGCATACTCATCGATG
GCATCAACCATGTACAGCACTTCATACCCCTTCTTCTTTAGCCTCTCTAGGAAGGGAGAG
TTCTCCACAGCTTTCTTACTCTCACCAGTGATGTAGTATATGTCCTTCTGGCCCTCCTTC
ATCCTTGTTACATAATCTTTCAAGCTTGTCATTTCATCACCACTCTTGGTTGAGTAGAAT
CTGAGCAAGTCAGCAAGTTTGGCCCTGTTCTGACTGTCTTCATGAATACCCAATTTCAGA
TTCTTGGAGAAGGCTTCATAGAATTTGTTGTAATCTTCTTTGTTCTCGGCTATTTCATTG
AACATCTCAATGCACTTCTTCACCAAGTTCTTCCTGATCACCTTGAGTATCTTGTTTTGT
TGCAACATTTCACGAGAAATGTTAAGTGGGAGATCGTCTGAATCAACAACACCCTTTACA
AAACCAAGATACTCTGGAATGAGCTCTTCACAGTTGTCCATGATGAACACCCTCCTCACG
TAAAGCTTGATGTTGTTCATTTTCTTCCTCGTGTCAAAAATATCAAATGGGGCTCTCTTA
GGAACAAAAAGAATGGCCTTAAACTCAAGTTGCCCTTCCACAGAGAAGTGTTTCACAGCA
AGGTGATCTTCCCAGTCATTGGTGATGCTCTTGTAGAAGGAGGCATACTCTTCCTTGGTA
ATCTCCTCAGGTTTTCGCAGCCAAATTGGTTTCTGTTTGTTGATCTGTTGCCATTCATGG
GTCACTTCCTTAACCTTCTTCTTCTTCCCCTTCTTCTCTTTCTCCTCATCCACTTCCTCC
ACATCACCTTCCTCTTCCTTCTTGGTTTCCTCATCTTCGTCATCACTGACCTCTTTCTCA
GTGGTCTTTTCAGTCCAGAGATAGACGGGGTAGCTAATGAACTCTGAGTGCTTCTTCACA
AGGTCCTTCAGTCTCCTCTCCTCCAAGTACTCCAATTGATCCTCCTTGAGGAAGAGTGTG
ATCTTGGTTCCTCTACCTAGTGGCTCACCGTTAACATCCCTAGTGACGGTGAAAGAACCC
CCAGCCTGAGACTCCCAAATGTACTGTTCATCATCGTTATGTTTAGTTGTCACAATAACC
TTCTCAGCCACTAAATATGCAGAATAGAAACCAACGCCAAATTGCCCAATCATGCTAACA
TCAGCGCCTGCCTGTAATGCCTCCATAAACTCCTTCGTACCAGACCTCGCAATAGTTCCC
AAATTATTCACCAAATCAGCTTTAGTCATGCCGACACCACTATCGATAATTGAGAGTGTT
TTGTTGGCTTTATCAGGAACAAGCCGGATAAAAAGCTCTGGTTGAGCATCAAGCTTGCTC
TTGTCAGTTAAGCTTTCAAATCGGATTTTATCCAACGCATCGGATGCGTTACTGATGAGC
TCACGAAGGAAGATCTCTTTGTTGCTGTAGAACGTATTGATGATCAAGCTCAAGAGCTGA
TTGATTTCGGCTTGAAAAGCGAAGGTCTCAACGTCTCCCATCTGAACCTCAGCCATTGAC
AATCCAATTGACGGTGGCAGAAGATCTTGCGCGCAATGAAGTGAATGCGAAGTGCTTTCT
GAGCGAGTTAAACAACGCAGCTTTCAATTGATCGTGGTGAGGCCGAGGACGGTTGTGATT
ATGTAGTGAAAGGAGAACTTCTGGGAGCTTCTTTCGCCAACGAGTTACTGGACGAGTGTT
TTGG
>ntig_921_consensus
ACTTCACACCAACCGTCTCTTGCAAACACAATGTGAATATTAAAAATGACAGAAATGATA
GCGGGTAGTAAATTCCATAAATTTCTAATTTAAAACCAAATGTCAAGATAATCGTTTACA
ATGTGCCAATGAGATTATGTTACATACACAAGTTACAGCCGAAACTGAAATTTCAAAGAA
GAAAAGCTGTTATGCCACACCT
>ntig_922_consensus
CCCTAAATCAGCGATAAGGAACAAAAATAGAGAGAAAGGTATGATATTAAGCCGAAAGTT
GCCGGAGCATCCAAATTTATTTTTTTTTTTCTTGTGAATTTTTGATGCGACTCGGACTC
TTAGGGTTGAAATAGAAACAATTTGGTTGGAAATTTTAGGGTAACTTTTATTCATATGAA
AAAAACGTGGGCAACCCCGAACCCACGTTTCTCAAAATTATGGTGACACCTCAAGTGAAA
AAATAAAAAATAAAAACAATTAACCCCGACAAGTTCTGGTAAATTACCAAAGGGGGTGGA
TGTGTGAATTATTATGAACAATTCAAAAAGATATTCTTTTCGAATCAGGAAGCGTGGTCG
ACAAAATGGGCTTTGACTCTGCCGATCAGAAACTATTGGAATAGTAGCTGTACCTGCCTT
AAATGCCACTTGAATCTCTTCCAAAGACAAAACGCCTCATCCAGTCGGTGATCACCAATG
TCCGTGTGCGCCAGCTCACAAGCTTACT
>ntig_923_consensus
ACATTTTGCCTCCCAGCAGAGAGGTCCTTCTCACGATCCCAAGGCTTCCATGGATGTTGG
CCAACCCACTCTTCTTTCTGTGCTGGCTGTTTGGACTTTTTCTTTGAACGACTTTGAGCT
TCTTCTTGGTGCTTTTGAACCAAAGATTTTGAGCGCTTCTCTTTGTTGTATTTATCCACT
ATATCTGCATCTGCACTTGACCTTTTCTTTTCTTGTTCATTTGAAGCAAGTTCAGAAGCT
GCATTATACGCTTCCAAATAGTTCATCTTCGCTTTCTGGGCTCTGTCAGAAGGGGTATCA
GTCCAAACACTGGTGTCACCACGACCTTCTTTAGTGCCCTTGCTGAATTTTGTTGATTGC

ATAGTCATACCAGGTTTTCTCTCAGGTGGCAGGGTTGTCATCCATTCATCCCTTTTTGGT
GCTGTTTTAACTTCTACTTCCGCCAAGAGCTCATCATCACCCTCCATTGATATGCCTTGC
AATCTTCTCCACTTAGCAGCTTCACGCATTGCTTTCAACTCAGCCTTAAATGCCTCCTGC
TCTTCCTTGAGTTTAATTTTCTCGTCCAACACTTTACGCTTTTCTGGATCTTGTATTTCT
TTAAAAGCTTTATTCAATATAATGAATGCTTGGTGTGCTTGAGGATGGGGGCATTTGTCT
GGGTGAACCAAAAGAGACAGCTTCCAGTACTTTTTCTTTATGTTATTAATTGGTGTATTG
TGGTTTGCTCCTAGCACCTCATATGGGGAATCAGCATCAACTCCCATAATTCTTGCTACC
TCTTCAAAACGCTCTGCCTCATTTGCTGATTCAGCTTCAGCAACCACTGCAGGTGGGGGT
GGTCCTACAAACAGTTCATTATCTTCCTCCAACTCCGCTTCTCTAAGTTCAGCTTGGGCT
TCAGTTAATTTGGCTGCTGCAGCAAGTAACTCAGCTGATGGCAGTTCAGGGCCAAGCACC
CTTCTTTTTGGACCACAGGAGTCAACCCTGGGACTTGGCATCACCGAAGGACTATGATTA
TCATCGTTTGCTTGTCTATTTTCAGCCTGAGGTAATTCTGAATGTGCTTGTTCCACAGAC
ACAGAAGGATCAACTTGCTGCTCTTTTGAATCCATATGAGCTTGAATGATGAATCCAACG
ACATCCAAAACAGGACGAGCATTTTCCGGCCTTAAAAAAACTCCTTCTCCACTCTCCTTC
AATTTCAATGAAACAAACAGCGTCTTCAAACGCTTACTCAAAGACCTCTCAGATATCCCG
CGTATGTCAACAGCTTGTCCATCATCAATCATTTGCAGAAGCTGTTTCAAATCGCGTCCC
ACGTTAGCAAACTCACTTAACATATTACGAACAACTTCTTCGGGATCCATCCTAGACCTC
CCTGGTTCAGAGTCCGACCCCGACACGTCATCACTCAACGAATAATCCCTCTTCCTCTTC
TCCTCTCTCCTCATTTTTCTTCTGCTTCTCTCCTTTTCACGCTCCCTCTTCTTCCTCTTA
TCTCTCTTACTCTCCCTCTCCCTCCTCGAGCTCCCATACCCTCTGTGCCGCTCTTTCCGG
TGTCTCCGCCGCCTTCTTGACGAAGAATCAGAATCGTCAGAGCCGGACAGAGCGGACGAC
GATGAGGACGCATCATCGTCGCGCTTCTTCCTGCCTTGCTTACCCATATTTGATTTGGCG
AACACCGGAATAGAAAGCTAATACTTTTGAAAAAATTTGACTACACTAGGGTTTCTGAGT
TCTAATCTCATTTGATGCTGAGTTGACTCGGAATTCTTAGCTAGAGTTCGAAGACGGTGA
CATTGAATTTTAGCGACGGAGG
>ntig_924_consensus
TAGGCATTTACTCCTTGTCCACACATCAAATTTTTTGAATATATACACAAGAGAACTTTT
ATATAATTAATATTGATAAAGACTCCAGAGAAATGAAAGATCTAGTGAGGAGGTTTACAG
TGGCCCGGGCTGCCAACAGGGATGTAGGTGCAAGGATTCGAACCGGAAGGCGGCGAACTC
CCATGATCTCGAGGGTAATATATAGGAGATGCTTTCTTCCTTCCATTGCCAATGTCGTCC
ACTGGCATAGGCCTTGCAGCAG
>ntig_925_consensus
GCATCTTATTTATATTGCTGAATTCTTTATTCCTGAGGTGAATATACTGCTCACAGTGTC
TACATGGGAAAAGAAAGCATATCAATTCGGATAATAATAGTATTTTCACCAAACAGTGCA
ACTAAAGGAACAGGACCACACATTACTCCTAAAAGAAAATACAAATATTAAACGAGAGAA
AAAAAAATAGAACAAACTTACTCGATATACAAACAAACTAATAATTATAGAGAAGCCAGA
ATAGTTTGCCTGGTTTGTCCATAAAACAAACTTTAGTCTTTTAATACTAACATGCAAGAT
GAAAAGGCTGCTGCTAATGCTAAGCCTGCAATTACTCAAAACGTGATACATTGCCAAAAA
TACAGTTTGGCGCTTCTGTGATCAATACTTTGTTCTTCCATCACCTTCTGTGATCACATC
TTTAAAAGCTCCACCACTGGGAATCATAGGAAGGACATGCTCTTGATGGGGAACAATGAC
ATCCAGAAGGTATGGCCCAGGTGTGTCCAACATCGTCTGAATTGCAGCTCTAAGATCTTC
CTTCCTTGTGACACGTGCAGCAGGGATGCCACAAGCTTCTGCAAACTTCAACATATTTGG
AAATATTTCAGACTCCCTTGATGGATCTCCCAGATATGTATGAGCCCTGTTTGCCTTGTA
AAATCTATCCTCCCATTGCACCACCATGCCCAAGTGCTGATTATTCAAAAGCAATATCTT
AACGGGGAGATTCTCTACCTTGATAGTTGCCAGTTCTTGGACATTCATGATAAAACTCCC
ATCACCATCAATGTCTACCACAACTGCACCAGGGTTTGCAACAGCAGCTCCAATAGCAGC
AGGCAATCCAAAACCCATAGCTCCTAGTCCTCCAGACGTTAACCACTGGCGAGGTCTCTT
ATACTTGTAAAACTGAGCAGCCCACATCTGATGCTGCCCGACACCAGTACTAATAATTGC
ATTTCCATCGGTTAACTCATCAAGAACCTGAATTGCATACTGTGGAGGAATCGCTTCCTC
AAATGTCTTGAAACTCAATGGGTATTTTGCTTTCTGCTCATCAAGCTCCTCCCTCCAAGC
CGAAAAATCAAACTTAACACTACCAGCTTTACTCTCCAACAACTTGTTTATTACCTTCAT
TGCCAGCTTCACATCTCCACAGACTGACACATGAGGCTGCTTATTCTTTCCAATCTCAGC
TGAATCAATATCAATGTGAACAATCTTCGCTCGGCTAGCAAAAGCCTCAAGCTTCCCCGT
CACCCGGTCATCAAACCTGACCCCAAATGCAAGCAACAGATCACTTTTATCCACAGCATA
GTTAGCATAAACAGTCCCATGCATTCCAAGCATTTGCAATGATAACTTATCAGAACTAGG
ATACGAGCCCAAACCCATCAAAGTACTAGCTACAGGGATCCCCGTAAGTTCAACAAACCG
CCTTAACTCCTGACTAGCATTCAAACACCCACCACCAATATACAACACCGGTTTTTTTGA
CTCTGATACCAACCTCACAATCTGCTCTAATTGTTCCTCATTGGATTCTTTAGGTAATCT

AGACATATAACCCGGCAATCTAATTTGTTGGTTCCAATCAGGAACCGCTAGCTGTTGTTG
AATATCTTTAGGTATGTCAATCAAAACCGGGCCGGGCCGACCAGAGGTTGCTAAGAAAAA
TGCCTCACTAACAATCGTAGGAATGTCATCAACATCAAGCACAAGATAATTATGTTTTGT
AATAGACCGAGTTACCTCCACAATTGGGGTCTCTTGAAAAGCGTCCGTACCGATCATCCT
ACGTGGCACCTGACCAGTGATTGCCACGACTGGAACGCTATCGAGGAGCGCATCAGCGAG
TCCGCTTACCAAATTGGTGGCACCGGGTCCAGACGTTGCAATACAAACTCCGGGTTTTCC
GGAGGAACGCGCGTATCCCTCAGCAGCAAAGATTCCACCTTGTTCGTGGCGAGGGAGAAC
GTTGCGTATGATATTAGAGCGCGTGAGGGCTTGGTGGATCTCCAATGAAGCCCCACCAGG
GTAAGCGAAGACATCGGTGACACCCTGGCGTTCGAGAGCTTCAACAAGGATGTCAGCACC
CTTACGGGGCTCATCAGGGGAAAATCTGGAAGGAACTTTGGTTAATGGGATTGAAGTTGT
CGTTGTTGGGGTTGTGGCGGAAGCAGAAGCAGAAGCGGCGGACGGTTTGGGGAGAGAGTT
TGTGACGTGGAGAGCGGGGTTTTGGCGGGTATAAGAAACGGGAAGAATAGATCTTGAAAC
GGGTAATGAAGATTTGTTATTATTAATGGTGGTGGATGTAATGAAAGATGAAGGCTTGAT
GAAAGCGGCGCGTGTAGCGGAAGAAGAGGCCGCCATTGTAGAACACATAGACAAGGGAGG
CTCCTGCAACGACAGGGGCTTTGTGTGTGTGGCTAGAGTGGGAGCCT
>ntig_926_consensus
ATTTGTCAATGTGAATCATGATGGCCAAAACTCCCATCTCATCTATCTTTTGCTGAATCC
TTTATCTATGAGACGAGTAAGCCGGTTATAGTGTCAAAATGGGAAAAGAAAGCATCTCAA
TTTGAGAATAGTGTTTTCACAAACAGTGCAACTAAACGAAACAAACGAAACAAACTAGTT
GATACATTAACAAGCTAATAATTAGAGAGAAGCAAGTACAGCTTTCGTCCTTCGACACCC
ACATGCAAGATAAAAAGATTGCCACTCATACTACGACTGCCATTCCTAATTCGTCGTCAA
AAATGCACATTTGCTGCTTCCGTTGTCAATACTTGGTTCTTCCATCACCTTCTGTGATCA
CATCTTTAAAAGCTCCACCACTGGGAATCATAGGAAGGACATGCTCTTGATGGGGAACAA
TAACATCCAGAAGGTATGGTCCAGGAGCATCCAGCATTGTCTTAATTGCAGCTCTAAGAT
CTTCCTTCCTTGTGCAACGTGCTGCAGGGATTCCACAGGCTTCTGCAAACTTCAACATAT
TTGGGAATATTTCAGATTCTCTTGATGGATCTCCCAGATATGTGTGAGCCCTATTTGCCT
TATAAAACCGATCCTCCCATTGCACCACCATGCCCAAATGCTGATTATTCAACAGCAATA
TCTTAATGGGGAGATTCTCTACCTTTATAGTTGCCAGTTCCTGGACATTCATGATGAAAC
TCCCATCACCATCAATGTCTACCACAATTGCACCAGGGTTAGCAACAGCAGCTCCAATAG
CAGCAGGCAATCCGAAACCCATAGCCCCTAGTCCTCCAGATGTTAACCACTGGCGAGGTC
TCTTGTACTTGTAAAACTGAGCAGCCCACATCTGATGCTGCCCAACACCTGTACTAATAA
TTGCGTTTCCATCAGTCAACTCATCAAGAACCTGAATTGCATACTGTGGAGGAATCGCTT
CTTCAAACGTCTTGAAACTCAATGGGTATTT
>ntig_927_consensus
GCTAGCTCTGGCTAAATTAAAATGAACATTAATGCTGCAAACTGACTGACGTTCTAATCA
ATGTGTTCCATAATAGAAACTTTACAATGAAAAATCAAAAGTAGAAAAAAAATGTTAATG
CTGAGGTAAAGCTGGTCTCTTATCCACCATTTCGTAATACACAACAAAATTATCCTTGCG
GATTATTTCAAAAGTGCTTGGATCAAAACAGTGATAAGACCCTCGCTCGTATGTGTTTGT
CTTAACTAGTGGCTCCACATTAGGCACTCTGATGAACCAATACCGATGATCCTTGTGATA
CATCCAATTCCTATTATATAGTTCATTTGCAGCATGCAATTGGGCCTCATCTTTTGGCAT
GCTGTAAAATATATAGAACAATGTTTCCACCGAAAACTTTGAGAAGTAACCTTGATGTAG
CACAGGTGGTTGTTTAGCATAATAACATTGTGGCACAGTGAATTCTGGATCACCCTTGAC
TGGTTCATCAGACCATGGTGAACCAAAAGTCTTATGAAGATTTTCTGCTGAATTCAAGTT
CAACCCAAGTGTTGTCAGGTCAATTCCAAGTGCCAGAGAAGTCAGATCAGGATCACTCAT
CTTTATCACACTTAACAAGCCAAGCAAGCCAAACGGGTCGGGAGAAGAATGTGGTGACTG
CATGGATTTTATATCCTGATTCCTAAAGGACTGATTGACAGCAGACATCTGTTGTAGGCG
AAATTGAGATTGGTTCTGATGTTGCTGATATTGCTGAATAAGCTGGTCATAGGAGCCAAG
ACCAGAAACTGGATTGGCAGAATTCAAAGGTCTTAGACCTATGCCGGGTGGTCCACTGGT
CTGTGAGTGGTAGCTTGAATTTGAAGATGGGAACATATCAGAACCATGTAAATGGAGAAG
ATCCTGGTTGTTAACGGATGAAAAGGAGACACCACTACTACTAACAGATGGAGCATGTTG
CTGTTGCTGCTGTGGACGATGAGAAGAGTAGGTACTACCTAAATTAAATCCAGCAGATCG
GCCCATAGAGAAGTGCTGAGATTGCATCATTGACATGTTGTTTTCATGAAGTTGTTCTTT
CTGATGTAAATCCATAGCATAGTCAGCATTACCACCTTTAAAACCCGGTAAAGCAGGA
>ntig_928_consensus
TCCTGCTTTACCGGGGTTTAAAGGTGGTAATGCTGATTATGCTATGGATTTGCATCAGAA
AGAACAACTTCATGAAAACACCTTGTCAATGATGCAATCTCAACACTTCTCTATGGGCAG
ATCTGCTGGGTTTAATTTAGGTGGAACCTATTCTTCTCATCGTCCACAGCAGCAACAACA
ACATGCTTCATCAGTCAGTAGTGGTGCTGCCTCCTTTTCGTCTGTTAACAACCAGGATCT

TCTTCATTTACATGGTTCTGATATGTTCCCATCTTCGCATTCAAGCTACCACTCGCAGAC
CAGTGGACCACCTGGCATTGGACTAAGAACTTTGAATTCTGCTAATCCAGTTTCTGGTAT
TGGCTCGTATGACCAACTTATTCAGCAGTATCAGCAACATCAGAACCAATCTCAATTTCG
CCTACAACAGATGTCTGCTGTCAATCAGTCATTTAGGAATCAGGATATAAAAACCATACA
ATCAGCACTTTCTACTCCTGATCCATTTGGCTTGCTTGGCTTGTTAAGTGTGATAAAGAT
GAGTGATCCTGATCTGACTTCGCTTGCACTTGGAATTGACCTGACTACACTGGGGTTGAA
CTTGAATTCAGCTGAAAATCTTCATAAGACTTTTGGTTCACCATGGTCTGATGAACCAGC
TAAGGGTGATACGGAGTTCACTGTACCTCAATGTTATTATGCTAAACAACCACCTGCCCT
ACATCATGCCAAAAGATGAGGCCCAATTATATGCTGCAAATGAACTTTATAATAGAAATT
GGATGTATCACAAAGATCATCGGTATTGGTTCATCAGAGTCCCTAATCTGGAGCCACTAG
TTAAGACGAACACGTATGAGCGCGGGTCCTACCACTGTTTTGATCCTAGCACTTTTGAAA
TAATCCGCAAGGATAATTTTGTTGTATATTACGAGATAGTGGATAAGACACCAGCTTTAC
CGCAACATCAACATTAACGTTATTTCTTCTCTCGATTTTATCATGTAAAGTTGATATTAT
GAAACACATTGATTAGAACGGTAGCCAGTTTGCAGCATTAATG
>ntig_929_consensus
CACCCACCTCGAAGATACCATGTTGTCAAAAGAATTTTAAGCATTATTTGTCTCCACAAA
GTACATGTTTGTAGCAAACAGGCATCTGTGAAAGCCTGTAATATGTTTGATATCATCAAA
TAAAAAAAAGAAAAGAAAAGCAAATACAAAGTTTTCCTAGTTCTTACTGTAAACCTGTTG
ATTGCAACATATAGAAAGAAGAAAATATGACAAAGGAAGAATCAGATTTTCAAGTAAAAT
ACCCAGAACTTTTACCCATCATCTTTCCATAATCACATATGGGGTTTAAGCCTTCTATGC
GGCATTATCAATCATAAAATGCTTCAACTCATTGTTATCATGCTGCACCCTTCCAGTTAT
ATCCAAGCGTAAGCCGTTGGGAAGAGACTGGTTTGTTTGTTTTCCCTTATCAGGTTTCTT
TCTAAGTCCTGGATTTATAAAGACTGGTTGTACTGTAAGAGTTGGATTTGGTTTAATTCG
ATTCACTTCTCTGTCATCTGGTGGCCGTAACAGTATTCTGTTCTCTGTTTCCAGCTCAGA
AATTTCATCTTTATTCAACAAAACCTTGCGACCAACCAAATCAAAAGTCACAACTACTTT
ATTTCGATTGGCCTGTTCAGCTTCCTCAATCTCCTCCTGTTTCTTCCTTAAAAGTTCCTT
TTCCTCCTTAGAGAGCCAGCTATTTCCCTCAATCTCATAGTAGTCACTTTGGTCATCAAT
AACCGTTGTACGAGCTGCAGAGTTTCGGTCATATTCAACAAGCCTTTTTGCATAAGCCTC
AGCTGCTGCTTCAGAATCTGACAGGGGAGCTAAACTTTCTTCCAGACCAGCATACGTGCT
TCCTTCCTTTAGCACAAGAGCACCACAAAATTGGCAAGGTCCCTCCCCCTCTTGTTCACA
AACTATCTTGCCACATGACAAACAATTGCTGACCAATCTGTGCTGACGTGCTTGACAAGA
GCATGGCCTTCCCTGTTGGAAAACAATTGACCCTTCAGCAACCTCAGCAAGAGAAATAAC
TTTTCCAGCTTTCTTCTTTTTGTTATTATTTTGGTTCCCTTTATTCACAGTTCTTGATTC
ACTGCTCTCAGTTGAGACCTGGTTCTCACGATAACTAGGGATAGCACCTTTTTTAGGTTC
AACCTGATAACTTGAAGCAATAACCGTTTTAGGTTGAGCCTGATGACTGGGGCCTGTAAC
CTCTTTAGGAGTATTAGATGGTTTCTTGGATCCACCAGCAGATCCTTCATCCGAGCGTGG
TTTAACATACGCATGCAAATTTGTATTTGAAACCTCAGAAGTGCTGCTGCTGAGCTCTGA
GTGACCTCTCCGTCGAAGATACTCCTCAATCACACTTTTGCCCACTTCTTGGCCAATAAT
ATTGTCGAGGTACTCTTTGGCGTCGAGTGGTTGAGCGAATTCACAATAGGAGACTAAGCC
CGAAATTATCTCCCGGTCCAGATCCAATCCCGTCTTTACTCGCTTGCACAGATCCACTAG
TGCTTCCTCCAGCCATTGTCCGGCGGTCTCCATCCTTTCTAAGTCTCTGCGAGAACGCAA
AGGGTTGTATACTGTGCCTCTCCAGGTATCAG
>ntig_930_consensus
TTTTTTCTACAAAAGATGAAACATCTTAACTAAATCATTAAGCTTTCAAAGCAAAGTACA
TATAGTAGCGAATCTCCCATCTACAACTATTAAATTTGAAAAGGAGAAAGAGAAAATGAC
AAAAGGAGAAACATAATTGCCTTACAAGACAACAGTATCTAAAATTTAACATGAAGCACA
AACATAAACATATGTTGTTCAAACACTGTCACAACCTAAATGACTATGTTATCTGCCTTC
CCAATTCCTACTCATCATCATCCTCCTCGCCACTGCCCTCTTCATCTTCATCATCATCAT
TCACCTCGGACATTGACTTCTCAGACCCTTCTTCATCTTCAGCCTTAGGGGCTTCAGCCT
GTTTCTTATTATAGTTTTTCAATTCTTTCTCATAATCAACCTTGCGTTTCTCTGCCTTAG
CAACATATGGTGCTTTGTCAGCATCAGACATGGACTTCCATTTCTCTCCACCAGCTTTAC
CAACAGCCGCTACTGATTTGTTTTTAGGATGGTCCTTCTTGTACTGCTCGCGGAACTCCT
CCATGAAAACGAAGAAGGCACTGGCCGGTCTCTTGGGCTTGTTTGGATCTTTAGCTGCCT
TTCCCGCTTTCTTTCCGGCTGCCTTTTTAGGCTTTTTGTTCACAGCAAGCCTTGAGCTCT
TGGCATCTGACTTGGATTTAGCTCCTTTCATGGCGAATCAGGTTAAATAGCGTTAGGTTA
GGGTTTAAGATGAGAGAGAAAGTGAGACGACCAAGAAAGAGAAGTAGCGAGGCGCCTTAT
TTGTTAAGCGTTGTGAGCTAAGTCATCAAAGGGCGAAGATAATAAGTGAGTCGTCCAAGA
AGGACC

>ntig_931_consensus
TTTTTTCTACAAAAGATGAAACACCTCAACTAATTCATTAAAGCTTTCAAAGAGAAGTAC
ATATAGAGTAAATCTCCCATCTACAACTGTTAATTTGGAAAAGGAACGGGAGAAAATGAC
AGAGGGAGGGACATAATCCACTTACATGACAAAAGTATCTAAAAAAATTAACATGAAGCA
CATAACATTAACATATGTGGTTGAAACACTGTCACAGCCTAAATGATTAAGCTATCTTCC
TTGCCAATTCCTACTCGTCATCATCCTCCTCGCCACTGCCCTCCTCCTCATCATCCTCGT
CGTTCACCTCTGACATTGACTTCTCAGACTCCTCTTCGTCCTCAGCCTTGGGGGCTTCAG
CCTGTCTCTTGTTGTAATTTTTCAATTCTTTCTCATAATCAACCTTGCGTTTCTCAGCCT
TGGCAACATATGGTGCTTTGTCAGCATCAGACATGGACTTCCATTTCTCTCCGCCAGCTT
TACCAACAGCGGCTACTGATTTGTTTTTCGGGTGGTCCTTCTTGTACTGCTCACGGAACT
CCTCCATGAAAACGAAGAAGGCACTGGCCGGCCTCTTGGGCTTGTTTGGATCTTTAACTG
CCTTTCCCGCTTTCTTTCCAGCTGCTTTTGTAGGCTTCTTGTTTACAGCAAGCCTTGAGC
TCTTGGCTTCGGATTTGGATTTACCTCCTTTCATTGCGAATCGGGCGAATAAGGGTTAGG
TTAGGGCTTGAAAACGAGAGAAAAGAGAAGAACAGAAGAGAGAGAGAGAGAGAAGCGTAT
TATGTGTTGCGCTTTGTTGAGAGACGAG
>ntig_932_consensus
CCATTGGACAACAATTTTGTGATAGATTAATGATAAATTATAATAGCTCAGTAAGAAACA
TACATGCCACTTAATACTTGCAACTGACACAACAAACTTAATCCAAGCCTACCACCTTAA
CTCGAAATTCTTGCAGCTCAAAATCTTCTACCAAAAACAATGATAAATATTAGTACTTCA
TAAAAGTACAAAACTAGCCAAAAACAAAAAACCGTCCTCAATGCTTTACTAATCCTCGTC
ATTGNNNNNNNNNNNNNNNNNNNNNNNNNNNNTCCACGCCCTCTACCACCACCTCGGCCAC
CTCCACGCCCTCTACCACCACCTCGCCCACCACCATCTCTACTGCCAAACCGACCACCAC
CATCTCTACTGCCAAACCGACCACCACTTCTCCCGCCACTTCTCCCACCACTGCGGCCAC
CACCTCGAGCACTGCCTGAGCCATCACCCCTTGGTCTAGCCTCTTCCACAACCAAAGAAT
ACCCACCAAGTTCAGATCCATTCAATTCTAGAGCTTTGTTGACACTGCTGTTATCCATGA
AGTCCATGTAAGCAAACCCCTTGAGGCCACCAGTGTTATAATCTTGAGGAATAGCAATCC
TTGTCGCCTCTCCACAAGAACCAAAATGTTCTTCCAGGGCCCTCTTAATCTCATCTTGTC
CAAGTGATGAATCAAAACCCCTGATAAATACTGTTTGAGATGAACTTTTCCCTTCCTTCT
GGAATGAATTGGTTTCTTTGCCGCTATAAGGTGTTGTGTATGAGCCCCTTTCACGTGCCA
AATCAAGCCTAACTTCACGATTACCCAACATTTGACCATTCAACTCCAGAGCTTTATTTG
CAGCTTCTACTGTAGAAAATTCAACATGTCCGAAGCCTTTAAATCTTCCATCAACATCTG
ATGCCAAACGGATATCAACTACCTCCCCAGCATCCTTAAAGAAATTTATTATATCAGCTT
CTTCAACTTGGAAAGGTAAATTGCCAACAAACAATGTTTTTGATCCTGTTGATTGAGCTT
GAGGAGTGTGGGGAGTTTGGGGAGCTTTCTTTCCGGATTTCGAATCAACATCTACCATTT
CTACATCAGTCTCCTTTTTCTTTGGAGTCTTGGCAAATTTTTCATCCTCACTTTCATCTT
CTTCATCCTCATCACTACTGCTTTCCTTTTTAACAGTTTTAGCATTTTTTACAGATACAT
TGGGCTTTTTTTTCTGAGGCTCATCATCACTTTCCTCCTCAGAACTACTATCAACATCCA
TCTTTTTCTCGTCTTCATCACTACTATCATCAGTATCCTCATCAGAGCTATCACTTGATT
TAGCTTTTTTCTTGGGTGCAGCAGCAGTAACATTCTTAGCAGCTGGGGCCTTCTTGGCTA
CCAAATCTGTTGTAGGAGCTCCATTCTCCTCATCAGAATCTGAGGAATCTTCTGAGCTTG
AAGTATCATCATCTGAACTATCACTTGCTTTCTTAGCAGCAGCAGATACTGGCAATTGTT
TTGGTTGGGAAGTCACTTTGGCAGATTTATCATCCTCATCAGAACTATCATCTGATTCCT
CATCTGAACTGTCACGTGATGTATCTTTCTTTTGTGGAGGAGCAGAAGAACCATTTTTAG
CTCCAGCCAATGTTGTTGTTACCTTGGTTTTAGGATCCTCATCAGAATCAGATTCAGAAT
CATCTGAGGAATCCGAGCTACTATCAGGCTTGCTATTCTTAGTGGGAGCAGCAGCTGCCT
GCTTCTTTGGAGGTTCCTCATCAGAACCAGAATTGGAATCATCATCAGAAGAATCTGAAC
TGCTACTAGCTGCCTTGTTTATCTTAGCAGGAGCAATGATAGAGCCATTTTTTGCAGCTA
TGGGCTTCTTCCGGGGTTCTTCATCAGAAGATGAATCGTCCTCTTCACTACTAGAGTCTT
TGACAGAGGGTTTTGCAACATTAACTCCCTTCTTGGCCACAGCTTTAACCTTCTGTTCTT
CATCGGAATCTGAATCTGAACTACTGATTTCCTCTTTCTTTATTTTCTTACGTGTCTTGG
GTTGAGCTTTCTCCTTCTGAACAGACTGCGCTACCCCTTCATTTCCTTTCTGCTTCTTCA
AAACAACTGGCTTCTCCTCAATAGCATCTTCAGCTTCTCTCTTGACACCTTTCTTGGCAG
ACTTCGTAGGAGCAGATACAACTGGAGCAACATCAACTTTGGCAGCAGATTTCTTGCTAG
ACTTTCCCATGCTAGAATTGAAGACAGACGCTGAGAAATATAAAAGATTGACAAAAGAAA
TACACAGCACAAAACCCTAGTCCTCAGATTTTTTGGGGGCTGTAGCGTTTTGAGTACGAG
GGTGAAAAGAG
>ntig_933_consensus
TCCTCAACATGCAAACATGACCCACGGCTCGACCAATTGTTGACAGACTCAAACCGGACA

TAGATCCAAACCACTAAAACCTATAAATAGGTCCAACTATTTTCACCAGCTTTAAGCGCC
AAGACATGGGCAAAACGAAGTCAACCAACTGACTTAATCATACCCAAACCGCTTAAGTTG
AGGAAGCAGACTCGTCAACTTTCTTCACAGCGTCTTTACTTTTTGATTCTTTCTCCTTAG
CTTCAGGGGCCTCTTCCTTAGCTTTGTCCTCAGTTTTATCTTCTTCGACACTCAACTTCT
CTAGAAGACCAGCAGTTGACGTTGCTTCTTTATTTTCCTCTTTAGATTTTTGGGATT
>ntig_934_consensus
GCCTGGGCGATGAAAACATTACCGATAAAGAAAAAAATTTATAATACATCAAAGTTTTCC
TCGTTTTCTTTAAAAAATAAAATATGAAGCATGCATTATTTTCAAAGACAATACAAGTGC
CTGAGCTACATTGTTTATCAGAGGGAAATTTTCTATCAACCAGAATCTGCTTCACCACTT
GAGGGTACTTGATTATTTGTAGCCGATGGACCCCCTCCTAAAAGCAGGGGAGCTTCCTTC
TTTTTGGTACTGGACGTAGTATTGGCATCAGGATTTGCTGCCCAAGCTCTAAGGGGAGGC
TCAAAATGCTGACTGGCAACATCAGGTGGAGGAGCATCCTCCCCAGGAAGAACAAGAAGG
CCATTCTCTCGAAGAGAAGACGGCTCCTTGTAGCATGTATTCCAGAGGGAGTCTACAAGC
TGCCTTTCCTCCCTAGCAGCACCTAGATCTTCTGTTGACATTAAGCTGATCCCTTTAATC
CGATCTTCAAGCAGCTGCTTTAGTTTCTCATCTTCTTCACCGAATTTACCAGTGTTGCCA
TCTGGCTTCTCACGGGCAATCTCAAGAAGGCCCCGAAGAGCAGCTTCACGTACATCTCCA
TCTTCACTAGAGGCAAGATGCATCATAATGCGGGGAAATCCTAGCTCACTTACCACATTG
CAGTCTGCACTATTCTCATGAAGTAGATACTGAATCAAGTTCAGAGCTTTCCTTTGAAAC
CTAACACTCTCAGATCCCAAAGCATCTCTCAAGCCAGCATAACCATTGGCTAGACGAAAA
GCTGCAATACCAGGTTTGTTGTGCCGGATTAATGAGGATATGGCACCAAGTGCTTTCGTC
CGAACAGTCACATCAGGGTCTGAAGTAAAATTAGAAAGAAGAGGTTCTAATCCATTTGCT
TCCATAACCAGTTGTTGACTCCGTGGATTGTTCTGCACAATGGTGGTAACCACATCTGCA
GCTTTTGCCCGAATATTGGCATGTGAGTTCCTTAAATAACTCAGAAGAGGAGCTAAACCA
CCAATGGAATGTAGATCATTGGCCATATCAATGGACTCCACATGCTCTTGTAGTTCATCC
AACATATCTTCAATATCTGCGGGAGTAACTCCTTGAGCCTCCAAAATTTGCTCAGGAGTT
TGCATGACAAGAGTTATCTCTTTCATGCGCTGAATTACATCCACAGTCTGCGATTGCATC
GCTTCCATAAACCACCTCCGATCTTCCTCGCTCAAATTGCTCTGTGGGCGGGTGCCATCA
GAGTGCGCGATGCTCCATTTTAGCAATCCGTCCCAATTGGGTCCTTTACCGGCCATTTTT
TTCTGAATAAAACCCTAGTTCTTAACCTATATGTAAACAAGCTGTAGTTTAAGATAGAAA
TTGAAAATGAAGTTCAAAGCAGGAGAAACTCTACGATATAAATAGCAGCAGCTACCGGAA
AGCTGGAATCGTCTCCACGATTCCAGAAGCCGTAGCTTTATTTTGACTGCTCTGACAATA
G
>ntig_935_consensus
ATCGGATTGGTAAGTGATTCAAAATGTTCTTCGACAAAATTCTCTTGAATATTACAATCA
TTTCAAAATCCTAAACACGTTTCAGGCATTTTATGCTCTGCTTATTGATATAATCTGCAG
ATTTTGACAGCTTAGGCAGATATATATATGAGGGCATATTAGGAGCTATTCCCATTGCAT
ATGCCGTTCAACATCCATGTTTGAAACATTAACGGTAGCCTTTTCATCAGGGCCTATGGA
ATCATACAACGAATCCTCTGAAAATTCAGCTGTCATTGATTTCCAGTGTAGTCTCGGTTC
CCAATCTGCTTCTCTTAGAACCTTCAGAATTTCTTTTACCACGATCTTGCTCCCCTCTGG
CGAAAAGTGAATCCCATCC
>ntig_936_consensus
CTGGTGGAGGAAATTCTCTAGCATGGCAAGTTTGCCCATTGCAATTGCCATATGCTCCAT
GTAATCAGCAACATTTCCAGAACCAGCAGGGCCAAGAGATGCCCTTGTAAGTGTGTCTAC
AAGAGACTGTTGCAAAGCTTCCATTCCTTGGGATAAGGCATCTTCAGCCTGCTGGGAGGA
CTGCTGTAGATTGCAAATGCCCATCAACTGTTGATCTGTCAAGGGTTCTAGATGGTTGCC
AAGGATCTTGACAAGGTCAGATGATCGAAATCCACCCAACCACATGAAACACCTTTCAGC
AGGTGTTTTCCACATGCCAGAGAGCATGTGAAATACATCAGCCTTTGCACCAATGCTCTT
TAGTTGGAATACTTCATCATAATGTGCCATAACACTGTCAACATGGTGGCGTAATTCATT
ATCACCCATATGAGAATTTACAGCTAATCTTAGGTCATTGATCAGCCTCTGGTGTTCATC
AAGCCAGCGTGCATAATCCAGGTCAAATGCCAAAGTCCCACTTCCAGTCCCAGAATGGGC
CGGCTCCCCTGAAACTCCAGTTGGAATAAAAAAACCCTGCTGGTGAGCTCCTTGAAGCTC
CTGCTCTAGTTGTGTAAGCCTGAGACGACTACTCTCTAGCTGCTGGACATATGCCTTCTT
CCTCAGTCGACTCTTTCTTGCAGCTTCCCGATTCTGAGCCAATCTACGCAGCACCTTTTG
GTCACCGGTTTTAACCTTGGATTGATCCATGGAATCCACAACTACAAGTGCTCCACGCTG
AAGTCCTTGAACCTGGTTTTTTTCATCAGTGTCAACATCTGTAGAAGTGTCAGTCTGTTG
GCTGTTATCAGCCACGCCTGAGCCTGACTCGCCCCAGTTCTCGATATGCCCGTTTCCCAA
CAAGGAACCAACCGCTGTCCCCTTCTGGTATATAAATTGACCTGTATCCAAACACCCCCC
AGTCCCAGTTGAAGCAACTATTTCTGTAACACACCCGATGCTCGTGGTCAAGGTGTTATA

GTGGAAGTTATTGGAAACAACAGAAGCATTATTGGCTTTTAAACAAAACATGGCGCTTGA
GCTTAAATCAACAGCATCGTTCTGAAAAGGTTGTTCAAGCTCTCCAAGATCCGCAAAACG
CGTCTGGTTTTGGCTACTTTCATCTACTCCCCTGAGAAAGAAGGAAGAGTGGCAATATAT
ATCAGGGTCAGTAGCAGGAGCTGGTTTGAAGCTTTGCATAGTTAAACAGAGAGAGCCAAA
AAGAGGGTGGGTGTTGGAGAAGGACTATATGGCCAGAAATGGAGAAAATCTTCAAGGATC
TGTATTGAAGAAGATGGTATATCCATAAAAAACCATCAACCTCTTTATTACTGCCTTCTT
AAGTTGGCATTTTAGTTTCCCAACACTCCTACACATCTCTATATCCTCTTTCAATCTCTT
CCTACAGAGCGTTGTTT
>ntig_937_consensus
ATATATCTGATAAGTATGACACCACCCCATTAAAAAAAAAAAAAAAAAAACAGAAAAAAGA
AATTGAATAGGGCACATGTATGAGTTTAAAAACATTAGGGATCACTTAGGCTCATATAAA
AGCAGCAAGAACACATTCAAATTATTCATCTACTACCCAATAACGCAGAGCTTGAGTGGT
AGGAATTAGTAGAATCCAACTCAATCTCAAGGTTGAGGTTTCTCCCACTTGAGAGGTTTC
ACCACCTCCCATGTGAAGTCAGGGTCCTCCCTTCCAAAATGCCCATATGCAGCGGTCTTA
AGAAACCTGCCATTACCCCCTCTCTTGAGATCCAAGTTGATGGTGATCATTCCAGGCCTA
AAGTCAAAGTTTTCCTTTACAATCTTCAGGATCTCCTTGTCAGGAAGCTTGCCAGTTCCA
TATGTATCAACAAAAACAGATAAGGGGTCAGGCACACCAATGGCATATGAGACTTGTACA
ATGCACCTTCGAGCTAGACCATTTGCTACAATACTCTTGGCAGCCTGCCTAACAATGTAT
GCACCACTCCTATCCACCTTGGTAGGGTCTTTTCCAGAGAAGGCACCACCACCATGGGCT
CCCCAGCCACCATAAGTGTCTATGATGATCTTACGTCCAGTGAGACCAGCATCACCGTGA
GGGCCACCAATGACAAAACGGCCTGATGGGTTCAGGTGGAAGATTGTCTTCTCATCTAGG
TACTTCTCGGGGATGACCGGCTTGATTACATGTTCCTTAAGATCAGCAGCAATTTCGTCA
TTTGTAACTGTCTCATCATGTTGGGTGGAGATGAGAACTGTGTGAACTCGGACTGGAACC
ATAGCACCATTCTCGTTGTAGTACTCCACCGTGACCTGGGTCTTACCATCAGGCCTAAGC
CAAGGGCAAGTAGCATTTTTCCTAACCTCTGTGAGGCGAGCACCAAGCTTGGTTGCCAGG
ACATGGCTTAGAGGCATTAATTCAGGGGTCTCATCAGTGGCATAACCAAACATGTGACCC
TGGTCACCAGCACCTATCTCCTCTGGGCGCTTGGTGAAGTGACCATGAACACCCTGAGCA
ATATCAGGGCTCTGTTGCTCAATGTTGACCAGGACTTTGCAGTTTTCAGCATCAAGACCC
ACATCCTCAGAAACAAATCCAACGGAGCGGCATGTATCACGAACAATCTTCTCATAGTCT
ATGTCGGCCTTGGTAGTAATTTCCCCAAAGACCATGACCATGTTGGTCTTGGTGCATGTC
TCGCAGGCAACCTTGCTGTCAGGATCCTTCTCGAGGCAGGCGTCAAGTACCGCATCAGAG
ATCTGGTCACACAACTTATCTGGGTGGCCCTCGTTCACAGATTCAGAGGTGAATAGAAAG
GTCTCCATCTTATCAAACTGAGGCTACTGTTTCGGTTCAGATCTAGAGTGTTAAGTTTCC
AGAACCCCAGCTAAAGCGGAACTCAACGATTTAGTACGCCTCTTTTACCCGTCAAAACCC
TCTATTTATAGATAAATGGCCCTAATGTCCGGTTACCTGGCACAACAGTTATAGTTCTCG
CTGGAATGTGGAATCTACGTCAGCTTCCTTACTTAATCGTTTGCTCATAAATGCTAGCCG
TTGGATACAAGACTCACCAGGGACAATATCCGGTGGGGTTGGTGGGTGAGAAAGACATTT
CGCTGAATGTCCACGTACGTTGTCACCATTAGTTTCACCGGAAATAGCCGGTTTCCCATT
CTCACCA
>ntig_938_consensus
AACTCGATTTATTTCTAGTTGCACTTTCTCCACATCTTACAAAATCATATTCCAAGCTAA
TTAAAAGCAACTCTGCAGTACCAATTAACTCAGGAAACTACAACTGAATCGACCCTCCGA
CAGAGACAATTTCTCTGCTTTTGCAACTGCATCTAACGGGAGAGGAGCGAAACTTTTGGA
TGGGCCATAAAAATGAGGGAAGCTATTTGAGCCATCCACAAGTTCATAGATCAATCCATC
CCCAAGCTTTTCAGCATCAATTTGGAGAAGATACAGATCCACGTTAGTGTTTAAGAAAAA
GTTCTGTAATGTCAATTGCACCTGATCTAGTTTACTGAGGTGGATGCAACCGGTAGACTT
GTCGAGGTCTCCACCAAAAATAGATCCATTCTTCTGGAGCTCTTCCCACTCTTGCGCCGT
ACTGATTCTGTAAACAAACTCTTTACCTTCTCCTTCTTTTGCAGCCATTCTCTTACCCAA
CACAAACTACAACTGCTCTGACAATTGTTTTACACACACAGGATATGCTCAGATCCCTCG
AAACTCCAATTCAATCCCCGTCGACGCCAATTTAAATGGGG
>ntig_939_consensus
TGTAAGGTCATCAGCAAAACTTTCATTGGTGGATACTTAGAAAAGGGAGCATGGCCATGA
GCAAGTTCCAGTGCGGTTATTCCAAATGACCAGATATCTGCCTTAAAGTCATATCCATGT
AATTGCTGCATTACTTCAGGAGCCATCCAGCATGGAGTTCCAACAAAAGTATTTCTGGTA
CGTTGCCTATCTCCAGTATCAAACATACATGCTGACACGCCAAAGTCAGCTAACTTAACC
ACACCATTAGAATTGATCAATATATTCCCTGACTTCACATCCCTATGGATGTGCCCATGA
GCATGAAGATAAACAAGAGCTTTGAGAGTCTCCTTCAATAAAGTAGCAATAACAGTCTCT
TCAAAACCTTCTGGATAAGATGATTTCATGATATGAAGACATGATCCCCCTGCCATGTAT

GGCATCACAACCCAAAGGCTCTGACCAGCAGTGAAAGAGCAATGGGCCTCTAGTATATTT
GGATGATCAATCAAAGTCATAGTTTGCACCTCACGCCTGATACCATCCTGCCAATCCAAA
GGCAGGAAAACGGCGTCGGACTGAGCTGGGCAATAAGTGTCAGTAGTTCATCGAAACAAG
AAGACACTGAAAAACTAGTTTGTTTCAGGTGTTCAACAAACCCGATGAAAAGACGGTAAG
AGTTTGAGATTTCTACGTCTCTGTTTGGAAGACCCCTGAGATCGAATACCTCACCGTCT
>ntig_940_consensus
CCTCATTTCTTCCAGTTGCTTGGTCCAAGGCCCCTGGTGAGGGATTCTGAAGAAACCACT
GCTCCATATCTAATTGGGCAGTATCCTTCCAACCAGGGCTTGTTGCACCTTCATCATCCT
CTCCGATATCAAAACAACCTTTAAGTGCTTGCAATGGTTTGAAAGGAAATGAAGCAAGTG
AACTCTCCAAATCATGAAGATCTTTGAAGCCATCCTGACTTGGTGCAGCACTTGAATGGT
TAGCTATCTCTGGAGACAGTCTTTCTGCTGGAACCTCGACAGCATCATAGCTGTCCCATT
GCTTCCCACTCCCACTTGGATCTTCATTTCTTGTAAAATCATCATCCTGAATAAGCGCAG
CCTGACTTTTCAAATCCTCAAGGTTGAAATTCCAGGCACTTATTCCTCTTATGTACTCTT
GCTGTGATAGCTGCTCTTTGTCACCGTAGAGAGCTTTATTCTGCAATAGATCTGCCTCTT
TCGCCTCCTAATGGAGCAAGATCATCAAGAATAGTGCGATCCAAATAATCACATGAACGT
GCATGTTTAAAGAAAGAGTGTTTCAAAAGCTTTTCTGATGTTGGGCGTTTTTTTGGATCC
TTAACTAAGCATGCAGCAACCATCTCTTTGAAAGACTTTGAGAACCTCTTATCCCTTTCA
TAATCAAGACCTGGAGGTGCATTTTGTAATGTCATCAGCAAAACTTTCATTGGTGGATAC
TTTGAAAAGGGAGCATGGCCATGAGCAAGTTCCAGAGCCGTTATTCCAAAAGACCAGATA
TCTGCTTTAAAATCATATCCATGCAATTGCTGCATCACTTCAGGAGCCATCCAGCATGGA
GTTCCAACAAAAGTATTTCTAGCACGTTGTCTATCTCCGGTATCAAACATGCATGCTGAT
ACACCAAAGTCCGCTAACTTGACCACACCGTTAGAATCAATTAATATATTACCTGACTTC
ACATCTCTATGGATGTGCCCGTGAGCATGAAGATAAACAAGCGCTTTGAGGGTCTCTTTC
AACAAAGTAGCAATAACAGGCTCTTCAAAACCTTCTGGATAAGATGATTTCATGATATGA
AGGCATGATCCCCCGGCCATGTATGGCATCACAACCCAAAGGCTATGACCAGTAGTGAAA
GAGCAATGGGCTCTTAGTACATTTGGATGATCAATCAAAGTCATTGTTTGTACCTCACGT
CTGATACCATCCAAGTCATTATTGCACTTTTCCAGATCAAGGACCTTGATAGCAACTATT
TCATTTAGTGGAATGCAGAGAGCCCGATAAACAGTAGCACTGACGCCTTCACCAACTTCT
TCGTATAATTTATAATCTTTGGCATTGAGAGGAAATCGTTTCTCCAAAACAGAATCCATG
TTCTCTACTTACAGTACACTTTTAACATCATCTTTACTCCCAAATGATCCAGAAAAGATT
GATTTTATGCACATGGGAGTCAAGTCAATGTGGGGCTTCTTCCAATTGGGAAGCATTTTC
CCTTTCCTCAGCATAAAACAGAATGTGAATCAAGTGACCTTGTCTCCCAAAAACTTGGAT
ATCACAAGAAGACTTGAATACTATCAAATTGTGTTAGTGCCTGACTAAACAAAGTAACAA
AAACGGCGTCGGACTGAGCTGGATGCCAAGTCTCAAATCAATAGTTCATCCAAATAACAA
GCAGTTGGACTCAGATGTTCGACAAAACCGATGAAAATACGGTGAGAGCTTCAGATTTCT
CCGCCTCTATCGAGCGTTTGGAGGTTCTCTGAGATCGAATCTCTCACCGTTTACATCAAA
TGAAATAAAC
>ntig_941_consensus
TTATCTAGCATTCTTGAATCTGGCCATAGAAGTGAAATTGCTTGGATGTAGAGGTTGTCC
TCTTTAGAATATTTAGAAGTTGGTATATCTGGTTCTACTTGATTCCAATATTTGATATTT
ATTTTTATTCCATCGCACTCATTTATGTAAGCTATTTTCTCCTTGTTTCATGTCTTTCTG
GCAAGATTGGAGAATTCATTTTTATATGTTGACTTCTTTATGATAGTTTTGGGGGTCTAT
GAGTAATTTGATTTTATCGTGAAGTAGGGTTACAGGGAAGATGATGGATTGAATCTGGAA
AGTGATGCAGATGAGCAACTTCTGATCTATATTCCTTTCACTCAAGTCATTAAACTTCAT
TCTTTTGTCATTAAAGGAACTGAAGAAGAAGGTCCC
>ntig_942_consensus
CTGTAATGAACAAATTCACATTCCAAAGCCCCCTTGATATTATATTAGTCATCTAACAGC
AAGACTGAGAAAAAGAAGGGCTTGGATTAATCATCAATGTCGATTGATATTTGCATATTA
GGAACTATTTAACACTTCTATTTATTGTTCAGCCATCGATTCCTTCAATCTTGTCTTCAA
CTTCTGAGTAGAGCTGCCTCAACTTCTCAATATTCTCCTCTGAAGTTTCCCAGTACCCGC
GTCCATTAGCCTCCAGGAATGTCTGCACCAACTTCCTGAATGAGTTGGGATTAGTATTCA
TGAGCCTGTTTAGCATCTCCTCATCCTGGATAAAGGTGGAGTTAGCCTCTTCATAAACCC
AGTTGTCAACTTGGCCTGAAGTTGCACTCCACCCAACTGTATTGGTAAGGCGCTTCTCAA
TTTCACGAACACCTTCATAGCCAGTTGACATCATTCCCTCATACCACTTGGGGTTTAACA
ATTTGGTTCTAGCATCAAGCCGCATGGTCTCAGCAAGAGTGCGTACCTGAGCATTGGCTG
TGGTAGTATCAGCAATGAATGCACTAGGTTTCTTTCCATCTTTCCTCAAGTTCTGGACTA
AATTTGTTGGATCTGAATCGAAGTAATGACTGACATCAGTAAGCGAGATTTCTGAGGAGT
CAAGATTTTGGAATGTGGCATCTGCTGTACTCAGAGCCATCTCAAAAATTTTTCTTTTCT

CAGTCATGCCTGCACCAGGGGCATCACAGTCAAAGGCAAAAGACTTCCTGCTCAAGTACA
TGTCTTGGAGCTGTTTCTCATCATTCCATGAGGAATTCTCGACAGCAAGGTTTATATTGG
AGGAGTAGGAGCCTGAAGCATTTGAGAAGATACGAGTTGCAGCTTCTCGAACTTCAATAC
CCAGGGCTGTTGCTTGTTCTAGTGCATGTTTCCTGACAAAGTTCTGTTCTGATGGTTCAT
CTAGTTCGGCAACCATCTTCACTGCCCGGTCAAGGAGGTTCATCTGATTTATGAAGAGAT
CTCTGAAAACTCCTGAGCAATTAACAACAACATCAATCCTAGGCCTTCCAAGTTCTTCAA
GACTAACTGGCTCAACTCTGTTGACTCTACCAAATGTGTCAGCCACAGGCCTAACCCCAA
TCATCCACAAAACCTGGGCTAGTGATTCACCATAAGTTTTTATATTGTCAGTTCCCCACA
ACACGAGTGCAACTGTCTCAGGGTACACGCCTCCATTG
>ntig_943_consensus
CTCAAGCAAAAATTAGGGCTGTATTGTAATAATCAGAAACCAGTTTGACGCTCAATTTCA
ATCACGCTAATTTACAACCAACCACGCCACTCTTAAAAAAAGAAAAAAAGGGGTTTCAAC
TTAACCGCTAATATCTTTTACAATCATTTTTACGTATCACCGCATGTTGGATCCTAGCAT
TGGTTGCATTGCATAATTTAATATCATACATGGGAATTCCTACCTAAAATCATGCGCCAC
AATACAAGAAAGCAATCAAGCCTCGACATTGGCTTTGTTTCCCGCAAGAATGAGGGAGAT
CTCGAGTCCCCGGCTAAATGCTTGATTAAAGAGAAGACGAGACTGGAAGGTAGAAATAAA
TGGGAACCAGGACAGAAAAGCCACTGGAGCAAAGATTATCATACCCATCCCAGCATCATA
CATTCTGGCAAATTCCCGTACTGAATCCCACAAGCCCAGGCTCCTGATCAACTTCTTCCA
TGTAATGGCTAGACATATAATCATCCAACCCGTAGGTATAAATGCAAGAATGCTTGCCAA
AAGGTCTGTAATCGATAATTGAGTGAATATAACAAAAAGGGCAATTGCTATTACAAGTCC
TATAGATGTCACTCCCTGCACAAATCGCATCATTAGCTGGAAGTTGCCCGACCTTTTAGG
ACTAAGGGTAAATATCTTAAATATCATGACAATGAACACCAATATAACCCAGGAGAAGCC
ATAAATC
>ntig_944_consensus
TTCTCATTTAAGGCACATACATCCATGATTCTTACATCATAACATTAGCTAAATCCCAGA
GAACAATGTTCGTATTGATTAGTAGTACTCATCATTTTAGATATATATAACATGTGTTTC
TGCAAGCACTGAGACATATTTAACAGAACCATTAAGACTATTTTACGAGACCAAGTAATG
TAACTTCTGCCTCAATGATGACAGTGTCATCCACCAAGTAACCCTTGTCTGGATCCTTCA
ATTTAGCCAA
>ntig_945_consensus
ACTCAGATGTTCTCGAGAACTTGGCTCCTAAAGTCAGGAAGCGTGTGGAGGTTCTCAGAG
AGATCCAGAGTCAGCATGATGAATTAGAGGCAAAATTTTTTGAGGAGCGAGCAGCACTTG
AAGCCAAATATCAGAAACTGTACCAGCCTTTATACACCAAGCGTTTTGATATTGTGAATG
GTGTTGTTGAAGTTGAGGGAGCTTCAAATGAAGCTGCAATGGACCAAGAAGAGGATAAGG
CTGCTGAAGAGAAAGGAGTACCTGATTTCTGGCTCGCTGCAATGAAAAATAATGAAGTGC
TGGCTGAGGAGATAACTGAACGTGATGAAGGAGCTCTCAAGTATCTTAAAGACATTAAGT
GGTATAGGATAGAGGAACCTAAAGGATTCAAGCTTGAATTTTACTTTGATACCAATCCTT
TTTTCAAGAACTCTATATTGACGAAGACTTATCACATGATTGACGAAGATGAACCTATTC
TTGAGAAAGCCATTGGGACTGAGATTGAGTGGTATCCAGGAAAATGCTTGACACAGAAGC
TCCTTAAGAAGAAGCCTAAGAAGGGATCAAAGAATGCTAAGCCTATCACGAAAACTGAAG
ATTGTGAAAGTTTTTTCAATTTTTTTAATCCACCTCAAGTCCCTGAAGATGATGAAGACA
TCGATGAAGATACTGCTGAGGAGCTTCAAAATCAAATGGAACAAGATTATGATATTGGGT
CAACCATTAGAGACAAGATTATTCCCCATGCCGTATCATGGTTTACAGGCGAGGCTATTC
AAGGAGATGAGCTTGAGATAGAAGATGATGATGATGAAGATGAGGATATTGATGAGGATG
ATGATGAAGGAGAGGATGATGATGAGGAAGACGAAGATGGAGATGATGAAGATGATGAAG
AGGAAGTCAAGACCAAAAAGAAGTCAGCTGGAAACAAGAAGAGTGGAAGACCACAAAGTG
GAGATGGTCAGCAGG
>ntig_946_consensus
GTGGAATGTTTGGATTGCCATTGTAACACTTATAATTTTGTCTGTATAGTTTACAGATCA
TGAATTATTGACTGATTTAGAATTCCAGATCTGCGCAAACAAAGGAATTGCTAGAAAAGA
CACACATACAAAACAACTTAGCACCCTACTACATACACACCAGAAAGTAAAATCCTAGTA
CTAACACACAAATTAGTGAAGCCTAATTACGATCACTTCAAGATTTGGAAACTTAGTCAT
CACGAGACTCATAGTTAGTTACCGCCTTGTTCCCTTCAGCAACGGCATGCCTCCCAAGAT
CTCCTGGCAAAACCAGCTTCACTGCTCCTTGAATCTCCCTGGACGAGAGTGTAGTCCGTT
TGTTATACTTGGAAAGCTTCATGGCCTCGTTTGTAATCCTCTCAAACATGTCGGCCATAA
AATTGTTAATGACTGTCATTGCCTTGGACGATATGGCCATACCTGGTTGAACCTGCTTCA
GGAC
>ntig_947_consensus

CTGGTGACAAAAGGTTAAAAACAAAACTACATTAACAATCCCAGATCGTAAATAACAACT
GAAAATTTGTGAAGCTGGAAACTTAGCTTCACATTATCTGATCTTTCTATGATGTGGAAA
ACATGTCTATAGATTATAATATGGTGGTGCCACTGCCACCTTGTCATTTTATTGTTTCAA
TCTTCTAGTTGAGAGATGATGTTGACAAAAACCTGGTATCCTTGGCACATATCAGATAGA
AGACAGTACTCGTTCTTGGCATGGTATGTGGCCATTAAACCATAGCCTGCAGTCTGAACA
TCAAAGCCTTCGTCTTGCAGTTCTCGAATGAGTGGCAAACTCCCAGTGATTGAATAAGGT
TTTACATGCCCAACTGCCTCTTCGGTTGCCTTGCAGAGAACTTGAAAGCCCCGAGAATCA
AGATTACAAGCAACTCCAGCATTCGCCTCATCAAAAGTTAATGTAAGGCTTCCTCTCATG
TTTTCATCATGAAGAACATATTTTGAAACTGGACCCCTTGTATCCAGCTTCTCTATGTTC
TCATTTATGTCATCCACGTATTCTTGTAGTTTCTCCATCACATCTTTTACATTGTAGAAG
GGAGTTAACCTGACATCTCCTGATACTGTACACTCAAATGGAATTTGATTGATACCACCT
CCTGGATAATGCCATTGGGTTGGTTTCATGGTTGATGGTGTTGCAAATCCATAGACCTGC
TCCTTTGGGTGAGGTGGAAAGTCTCTGTAAAATCGTGTTTGGATTTCTTTAAGAGCCTCC
ATGGCCAGCTCCAAAGGATTAATAGCCTTATGCGCTAGACCACTGTGAAAAAGCTTCCCA
GTAACATGAAGTTTCCAAGGTATCATACCTCCTGTACCGATGCAAGGTTGTTTATCTGCT
GTGTCAATCCAGTAAAGAGGACCTCCCTTCAGCTTATCGAGAAGCCCATCTTTCACAAGT
GCATCCACACCAACCCCTGTTATGGCAGAGTTCTCTTCATTAGCTATGAACACTGCCACC
ACAGTTGATTTCAATTTTGGCTTGGTCTCTCCCAACGTCCTCATAAGCTCAGTTACTAGG
GCAACATGTCCCAAACAATCAGTAGTACCGCGACCGCGAAGCTTTTCCCCATCAATGCTC
AATGTGAAAGGATCAAATTCCCAATCATTAGGATTAGCAGTGACAACATCCATGTGCATC
CCAACAAAAGACAAGATTTTTCCCGGCTCGGTTCCTGGATACTCCACAATAAGATTTCCT
CGACCGGGAAAATATGTGGCATGGTTAATAAGCAAGGGGCCTCGTCCACTGGTAGTGCTG
TAGGGAAGAAGAACATCGAGCACGTGCTTGACCACTCTGTCCTCTTCTGGGATCAACTCT
GGAGGGTTGTTTTGGACGAACTTGGACTCTCCAATGAGGTTAGTGAGGAGAGCAACGAAA
GACTCCTTACTCAGAGTGCCTAGACTTTCTTTGACTTGTTGAGAAGCCATCGGCAATCGC
AGAGATTTGAACAAGATTTACACTACAAGAGACTGGTTTAGTCGATGGGGACGAG
>ntig_948_consensus
AACCATCAAAATTATAGCCATCATATAAATCAATCATTTAATCAAAACTGTCTTAAACTA
TGTATACAGAGGAAGAACAAAGTCATTATACAAGAACACTGGAAAGAGGACAATATAAAT
GGAAGGGAAACCATCATATATATATATATGGTGCTTCATAATTTATTTCCATAGTGGATC
ATCATCTCCATCTCTTAACATAGTAGGAAAGGACATCGATACTTCATAGTAGCTAGCTAT
GGCGGCAACCTAAGGCTTACACAAGCTAACACACACCACACAATATTATTAAACTCTACA
TGCTTGAAGCCGGTAAAACATTACGGGTGATGGTGATGGTGATGCGGTTTGTGTGGGTCG
TGGGGGCCTGCAGGTGGAGGCGGTCCATGAGGTCCTGGTGGAGGAGGGCCTGGCTGGTGA
GGAGGCGGTG
>ntig_949_consensus
TCATCTAAATTTTTTATGTACAAGTTTGCACCCTGAAATTTATCTGCTGCTTCCTTCATA
TTCTGCTCAAATCGATATTTTAGTTCAAGTTCCCTTTCAGATTTCCTCTGGGCTTTCCCA
ACATACCATTCCTTATCATCAAATTTCTTTCCATTGAGGGCCTCGACAGCGTTTGCAGCA
TCATCAGCATCCTCAAAATTGACAAAACCAAAGCACTTTGATTTTCCATCCGCATCCCTC
ATCACCACAGCACTAGTAATTGTGCCAAATTCACCAAAAATGTTCTGCAAATCTTCTTCC
GTTATTGCTTCTGAGAAATTCTTTACATAGACATTGTTGAATTTGTTCTTGTTGATTTCA
ATTTCTCTCTCCTGTTTGCGGAGGAATGGCCCAACATAAACTTGCTTATCATTTAAAAGC
ATACCATTCAGCTTCTCTATAGCTTTTTGGGCAGATTCCTCATTCTCAAATTGTACAAAG
CCATAGCCTCTTGACAGACCATTCAAATCTGTTGCCACCTTGCAAGAGAGGATATTCCCA
AATGCAGAAAATGTATCATGTAATGCCTTGTGATCGATGGCCTTATCCAGATTCTTGATA
AATATATTCCCTGCCCCACTTTTCCGAAGAGTAGGGTCACGATGAGAGTACATAATCCTA
ATAGGCTTTCCATTCACAGAAGTAAAGTTCAGCACCTCCAGTGCTCTTGCCGCATCCTGT
GGATTGCTGTAATTAACGTAACCGTAACCGAGTGATCGCCGGGTCGACAAGTCCCGACAA
ACCCGAACCGAAACCACCTGACCCATCTGGTTAAAGAGATCGTAGAGCTGGGAATCCGTG
ACGTTCGCCTCCAGATCACCCACGTAAAGAGACGTCGTCACGAATTGATTGGCCCCGCCA
TTCACGTTCTGTCCCTGAACAGCCACCTGAGCTTGACCCTGAGCCATCTGCTAAACCAAC
AACAATAAACAACGCAAACAGAAACCCTAGTTTTCTTTTTTTTTCCCCCCTCAAATTTTC
TTCTTTTTTTCTTTTTTTGGTTTTCTCTTCCCTCTCCTCTTGTTATTCTTCAAGAGCAAA
CCAAACCCTAAACAACATAGACAAACAAGAGACAGAACCAAAGAGAAACACAACACCACT
CGTCACAGAACCCCAAAGTGCTTTCGCTTTCCGTTCAGTGTTTTGCTTTTTTTTTGTTTT
TGTTTTTCCTAAATAGATAGATAGATAGATAGATA
>ntig_950_consensus

ATAGATAATTTATAGAAAATTTATATAGCCTTTTATCCAATCTAATAATGAAAAGGAAAA
AGGCAATAACATGCAAAATCAGACACCAAACATAAAGAAACAACCGTCACAACCTTCAAA
TTTGATCCTGAAACTCTCCCAATCAGTTGAAAACAAAAGGCCAACATTCGTACGTAGTGG
TGCCAATTCTATCAAATTGTTGGCTTTTTGTAAGTCTTAACAAGCCAAAGTATATATAAA
AAAAAAAAAAAAAAATGTATTTCCGAGACCAACTTAGGAAACCAAATTATCACTCAATGAC
AATGAGGCCAGTTGATCAGCTGCGTTGCCCGCCTGCTGTTGCTGAACAACATTCCTCAAA
ACCTCCATTGCCTCGGCCACCTTTGCTTTCAGAGCCTCAGGTGACTCAAGCAAGTGCAGA
ACCTCCGTCTGGTCCATCTCCAAGAGCATTCCTGTAACCTTAGCTGGTGCTTCAGGCTCC
AGCTGCTCCACAAGAGGATAAAGATTCTCCCCCAGCATTGTCCTCTGCTGCTCTGGAGAA
GCATTTGCAAGGGCAGACGCCAAAGCCCCAATAGGAACTTGCTGAGAAATTGCTGCATCC
CGCAAAGGCATACCACCCATGTCATATGGAAGCATGCCTCCAACACCAGGCATGGGAACA
TCATGCAGTCCACGGCCAGGCGGGTAGCGGTAGACTCGTCCCCTAGGCAGAAGCATCTGT
GGCTGCATTACAGGAACATGTTGTTGGGTTTGCTGAATGGCTCCTGCCCGTCTGCCACTA
GGTCTCTGACTCTGTTGTCCTGGAGGAACCACTGGAACATAGAAATTCTGCATTGGGGCA
CCACCGGGTCTTATCCCAGGAACAAGCTGCTGCTGATACCCAAATCCAGGCTGAGGAATC
ATGGCAGGTGGAGCTTGACCATAAAATAGCTGTTGCCCAAGACCAGGACCACCAGGAGGG
TAAATTGGTAATCGAGGAGCAACAGAAGGTGCTATTGCAACCGGCCGCATTTGAGAAAAC
TGAGCCTGCAGCCTAGCTTTTCTATCTTCCTTACGTTGTGCAAGTGCAACATACAGCGGC
TTGCTAACTACCATTTTTCCATTCATCTCCAGGAGAGCTCTAGATGCCTCTTCAGGACTG
GAGAATGCAACAAATCCTGATCCTCTACTAATTCCATTAGGGTCACGCATCACCTTGCAT
GATGTGATGGAACCAAATTGAGAGAACAACTGCTTAAGTTTATCATCATCAATGCTATCA
TCTAAGTTTTTGAGGTACAAGTTTGCCCCCTGAAACTTATCTGCTGCTTCCTTCATATTC
TGCTCAAATCGATATTTTAGTTCAAGTTCCCTTTCAGATTTTCTCTGCGCTTTTCCAACA
TACCATTCCTTATCATCAACTTTCTTTCCATTCAGGGCCTCAACAGCATTTGCAGCATCA
TCAGCATTCTCAAAATTGACAAAACCAAAACACTTTGATTTGCCATCTGCGTCCCTCATC
ACCACGACACTAGTGATTGTACCAAATTCACCAAAAGTTTTCTGCAAATCTTCTTCAGTT
GTTGCTTCAGAGAGATTCTTTACATAAACATTGTTGAATTTGTTCTTGTTGATTTCAGTT
TCTCTCTCCTGTTTGCGAAGGAATGGCCCAACATAAACTTGCTTATCATTCAATAGCATA
CCATTCAGCTTCTCTATAGCTTTTTGGGCAGACTCCTCATTTTCAAATTGCACAAAGCCA
TAACCTTTTGACTGACCATTCAAATCTGTCGCCACCTTGCAAGAGAGGATGTTCCCAAAT
GCAGAAAATGTATCATGTAATGCCTTGTGATCAATAGCCTTATCCAGATTCTTGATAAAT
ATATTCCCTGCCCCACTTTTGCGAAGAGTTGGATCACGATGAGAGTACATAATCCTGATG
GGCTTTCCATTCACAGGAGTGAAGTTCAGCACATCCAATGCCCTCGCAGCATCCTGTGGA
TTGCCGTAATTAACGTAACCATAACCGAGAGATCTCCGAGTTGACAAGTCCCGACAAACC
CGAACCGAAACCACCTGACCCATCTGGTTGAACAGATCGTAGAGCTGCGAATCCGTGATG
TTCGCCTCCAGATCGCCCACATAAAGCGACGTTGTCACGAATTGATTGGCCCCGCCATTC
ACGTTCTGTCCCTGGACAGCAACCTGAGCTTGCACCTGAGCCATCTCCTAAACCAAAAAC
AACGAAAACAGAAACCCTAGTTTTTTTCCGAGGTTTTTT
>ntig_951_consensus
CTGGGTATAAAAACGAAAATTTTTCTGTGACAGAGGAAACCTTGAACGGCAGTAAATTAT
TAATTTTTACATGTCATTTGTTTTGGTTAAAGTTAGCTGCTGCAGCCATGGAAGACGCCA
CTCCACCAGGTGTGGTTCTCATATCAGCTTTATTTCTTATCTCTGCACTAATTACCCTTT
CAGCATCTTCACGCGTCACTGGTTTGTCACCAGGCAGCTTAGTAGTTGCATCCGCCAAAA
CGTCAGAAATGGTGGTCTTATCAGCGAAGAACGTTGTTCGCTGATTATGAGCGGCTGCGG
ACTGTGCTGTGGCACCAACCCCACCAGGTTTANNNNNTAGCCGTTGCCTCTAAAGCTTCA
CCGATTGTGATTGAGTCAGGATCAAAAACAGCTCCCGGAGATCTCGTTGGGACATCCGGT
TTTTCATATTGCCCCACAATTTGGTCACCAAGGGCTTCAACGATAACACGTGTGCCGTCT
ATGACAGTTTCAGTGACTGTAACGCCTTGGTCTGTCGCTATATCAGTAACAGCGCGGCGG
TTGATAAGACCAGCCCTCTGATTCACCGTGGCTGCTGATTGCATGACGGCTGCTGGACCG
CCTTTTTGGGTCTGTCCGAGGACCACGCTCTCGGCTGCCTGCATGGTAGCGGCGTCTCGC
GCTGCAATAGGCTTGCCTGCTAGCTTGCCAGAGACGTCGAAAACATCACCATACTTGATG
GGTTCTTGGTTGGGTGACTGGTCGATCTGGGACCTTCCTGGTTGTTGCTGGCTCATCTTC
GGCTTAACCTCAACGGAGTTTGGACAACAGAGATCGTGTTATACTCAGTTTGAATACAAA
AGAAGGAAGAAAACTGTGGTTGTTTAGCAGTCGCAGGGAGGTGTCGTATATAAGCAGTAC
CAGTCCGAGGTTGGAAGCTTGGACGTTTCTTTATAATTTGACTCGTGTACAAAAG
>ntig_952_consensus
AAAAATTAAAATGGTCTGTGATAATGATAAAGAATGGCTCAATTTCCGAAGTGCCTGACT
CATCAATCTAGCTTGCATTGCCATGTGAGCATCACCCATCTCACCATCAAGTTCACTTTT

AGGCACTAAAGCTGCTACACTGTCAACAACAACTACATCAACAGAACCACTTCGGATCAG
GGTATCCACAAGACTCAGAGCTTGTTCACCACAGTCAGGTTGTGAAAGAAGCAAATTTTC
TGTGTTTACTCCAATAGCCTCAGCTAACGATGAATCAAGAGCATGCTCAGCATCAATAAA
AACACAATAACCTCCTTGTTTTTGTGCCTCAGCAATTACATGTAGTGCAAGAGTTGTTTT
TCCAGAAGCCTCAGGACCATAAATCTCCACAACACGACCCTTTGGGAGCCCGCCAACTCC
TAGAGCTACATCCAATGCAAAAGAACCGGTGGACACCACTGGAACATGTCTGGAAGATAC
AGACCGACCAAGCCACATAATAGATCCTTTCCCAAACGAGCTAGTAATCTGATCCAAAGC
>ntig_953_consensus
GCCAGACGCCGAAAGAAAGGTCGGTTGCATTTCAGATGCTTTCACGGGCCTGAAAGAGCC
TAGCCCTTCGAACTAGAAATTGAGGTAGATAGGGGCATCACTTCCGCTGCAAGAGTGTCT
GCAGTCTGGGAATAAAATGCATAGTGGGATTAAGCTCGAAATTACGTATTAGCTAATCTC
GGGGCTCGAGGGAAATTCTTTCATTTACGTCCATTCGTCACAAATTGGATCCTAAGAGTA
TTTCAATCCCCTGAACTAAAATAATCCATGGCCGGGGTAGTAATCCGACCATCTTACGTG
GTAACGGACAAGACAACGAATAGAAAATGTAACCAAGAATTAAAGAGAGACTAACTTCCT
TCCATACAAAAAGAAAGCTTACTTATTAAAAACAAGTGCAAAACAAACAAGCTTCGCCTC
ATCCAAGACCATTTTCAAGTTTGATTAATAAAGTTCCACGCTCACAAACCATACAACCAC
AAGCCCTAACATAACAAGAGACCCATGCATAGGAAACCATATAATGCCTAGAAACCACCG
CGGAGGCGAAGGACAAGATGCAGAGTAGATTCCTTCTGGATATTGTAATCAGCCAAGGTG
CGCCCATCCTCCAGTTGCTTCCCAGCAAAGATCAACCTCTGCTGATCAGGAGGAATTCCC
TCCTTATCCTGAATTTTAGCTTTAACATTATCAATAGTGTCCGAGCTCTCAACCTCCAGG
GTGATGGTTTTTCCGGTCAGGGTCTTCACAAAGATCTGCATTCCGCCACGCAGCCTCAAC
ACCAAGTGCAGGGTGGATTCCTTCTGAATATTGTAATCTGCAAGGGTCCTTCCATCTTCA
AGCTGCTTGCCAGCAAAGATCAACCTCTGCTGGTCTGGTGGGATGCCTTCCTTGTCCTGA
ATTTTTGTCTTTACATTGTCAATCGTGTCCGAACTTTCAACCTCCAGAGTAATTGTCTTT
CCAGTAAGAGTCTTAACAAATATTTGCATGCCACCACGAAGTCGGAGCACAAGGTGGAGG
GTAGACTCCTTCTGAATATTATAATCAGCGAGGGTACGCCCATCCTCCAGCTGCTTCCCA
GCGAAGATAAGCCTCTGCTGATCCGGTGGAATCCCTTCCTTGTCTTGAATTTTAGCCTTC
ACATTGTCTATGGTGTCTGAACTCTCCACTTCCAAAGTGATGGTTTTTCCCGTCAAAGTC
TTCACAAAGATTTGCATCCCACCCCTAAGTCGGAGCACCAAATGGAGGGTGGACTCTTTC
TGTATATTGTAGTCAGCAAGGGTGCGGCCATCCTCAAGTTGTTTACCAGCAAAGATAAGT
CTCTGTTGGTCTGGTGGAATCCCTTCCTTATCCTGAATTTTTGCCTTCACATTATCAATA
GTATCAGAACTCTCAACCTCCAGCGTGATCGTCTTTCCAGTAAGAGTCTTCACAAAGATC
TGCATACCACCTCGGAGACGG
>ntig_954_consensus
TTGACACCATCGGGCACAATTCGGTTCTCTGACTTGAACCTCAAATAATCATTGCGGTTG
AATTTAGTAAAGCCCATTGACTACTGACAGAAGTTATAAAAGCAGGATCTAAATTTTGCC
ACGGAGTAGAGGCCTTAAAACAGTCTGCATATGTATTAAAAGAAACTTCAATCGCAATAC
AGAAACAAAGTTTATGTTTTAAATGGAAAAAGACTAGCAGTACTTGACATTAAAACAGAA
TAAAGATAACATAGAAAGGGTACTTGAAACACAATAGAATGCCTAAATTCTATCATGTTT
TAGAACAATCAAACAAAGATTCCTAAGCTGATGCAGCAGTATCCAGAAACGCTTGTCCTG
GTTGACGATTCGCCAGTGGTCCATGACATCCAAGTAGCTTAGCATTGACACCATCGGGCA
CAATTCGGTTCTCTGACTTGAACCTCAAATAATCATTGCGGTTGAATTTAGTAAAGCCCC
ACTTCCTGCT
>ntig_955_consensus
GAAATGAAAAAAAAAAAAAAGAAAAAAGAAAAACGGAGTACAATTACAGCAACAAAAAAA
TAAAAAAAATCCAAAGTTTTGACTGAGTAAAGGATAGGAGAGAGCTGCATGATTTATATC
CCATAATGAAGAAAATCGCTCAGCACATATGGGTATTCACAACGCTTCGGGGAACTTTCC
CAGCCAGAGGCTGCGACTTGTTCCCAGAAAATTGTTGCTCTAATCAGTGTAGAGAGATTC
ATCCTTGGCAAGCAGAACAGAACAGAGCTCCAAGAATATAATCAGCTGATAAATCAGTTA
AAATTCTCGTCCTTTATACGGCATTTCAGGCTGCTGGTAGCTTGACATGTAAAAGAGTGG
CCGTGGCTGGCCAACAACCATCTGCTGTCCATACTGAGGTCCATGTGGGTGATAATAGAC
TTGGGGTGAATGCATGGGAACCTGCAGATTATACATGACAGGCTGGTGCCCGGAAAATGC
AGGTCCAATCCCAAAACCTACAGGCATGCCAGATATATGTGGTACTGCAGATACATTAGG
TGGATGATAGAAGGGACTATCAGTGGCAGGGGATTCGGGCCTAGCAGCCACTTGAGATGG
GATAAAACTCTTTGCATAAGGATTGAGTTTAAATTCCTTGGCATGGGGATTCAATGTTGA
TCTTTCTGAAGATAAAGAGCCCACTGATGAGCTTGGTGATAAACCAGGACCACTAGAAGC
TGAGGCAGCAAATGCACTTTCAGAATTGGATGACGGAGAACTACCAGATCTTCCACTAGA
ATTTAAAGATTGTGGTTCTCTGGGTGCTTTACTAGGCACTGGCACCTCCAATTGTTCACA

GGGTGAATTTGTCTTTTCATTGCCCTTTGATGGAGCACAGGACGGAGCAAATGAAGAGGC
ATTTGCAGATAGTCCCACTCCATCAGAACCATCTATATTGCCCTCTAATGAGTCTGAATC
CTCTGATTTTGACACTTGAGCCTCTTCTGCTAGCAGATACTTATCCAAAAACTCCTTTAA
TTTATTGTTTGCTCCATGTTCACCAAGCAAATTCTCCTGGACCCTTTTTTCACTGTCCAA
AGTAGAAATACTTTCATATGGAATTCCAGATACCATCTGTCTAGCATGATCATAAGGAAC
TGAGTGCTGTGGATCCGCAGCAGTGCTAGAGTGGGAAGATTGTACCTCATCCACTAGTGA
AGAGCTTGAAGACAATTGAGCTGCATCATTTCTTTTTCCACTGGTCAAATCAGCAGGTCT
CTGGCTTACAGAACCAGATGAACCACCAAAGGTTTCATCATTGCATGAGTCCAACATTAT
ATCCTCATCTTCCTCATATCCACTATCATCAAGGGCACTACCCCTGTAGACTGAAGAGAA
TCTGGTTTCCTCATCGATATCAAAATTTTCATGAAGCTTCATGCCTCTTTCCTCTGCTAA
ATGAAGATCTTGAGTATCCTCACCCTCAATTTCTCTGGCTATCCTCATAGCTTCTCTTTC
CAACTCTCTCGTTTGGGGGCCCTTCAGAAGTTTTGTTGTATAAAGTTCCTCATTGAAAGT
TGTCTTCACACCAAATAATGTTTCATTTGTTTCAAACTGATCCCAGTTCCTATTCCAAGA
GCCATCGAATATATTTTCCAATTCGGGGAGATGTGGATCATCTTCATCTGGGATCCATGG
TTCTAGCTCTCTTTCTACATCTGCATGACGAGATTGTGATATGAATGAATCTATCATAAT
TTCATGCTGTTTTTCTCGCTGTAGCTCACTTGCCAATCCATCACTAGTTACAGCCACTTC
CTTTGCTATAACTTGCACAAGTTCACTGGCAGGTATAACAAAAACACTTGAAGGTGCCTT
GCTAACAATCTCCACTTTCTGTCCTCGTGGATTATTACCATCTTTTGTCAAACGAGCCAT
TTTCAATATGACTCCAAACTCTTTTTCATCACTTGTTGAGTGAAATATTCCAGTGTATAT
TGATCCATTTTTCATCTGGATGTCCACAGGAAGTCCTATAAGACTTGTTGTCACATATAA
TAACCGATCGTGGGAAGGGCTCTCAAAACCTCCAAATTTGCCACCACTCAGTGCACCTGT
ACTCGTCACTCTAGCAGAGTTTGATTTTCCCGATGGCAATTTATTCTCCAGCCTATTCCC
CCCCTCTCTTTCACCTCTTCTACGGCCAAATCCATTAGCAGTGGACTTGGGGGGCACAAG
TTGTTGCAAATTCATCATCAGCACAATATGCCTCTGGAATCTTAATAAGCAAGGAAAAGA
AGGGACGGAAATAAGCGGTGACTTGGCAGTAAGAGAGAGGAGTTTTGAGTCGGGGGGTTA
GGGTTAGACGGAAGCGACGATCACGTGCGAATGACGAGGCAACAAGAGGGAAGGGACTAG
CTGTGATCCAGAGGAAAACAGAAAAGAGGGAAATGGGAGAGGGACAGAGTGGGAAAC
>ntig_956_consensus
AGGAGGCGACATCCAATGTCTTGATGCAAAAGATAGATTGTGGACTCATTGGATGTAGTA
GGCAACTCGCCACATTTTTGCTCATAGCAACATTTATGCTCTTTGGAGGAATACTTCTTT
TGCTAGTGAATTTATCATTATTGCTTGACGGTGATATCTGAAAACGGTTAGCCAGTGTTG
AACCAAATTCTTATTTCTGTTGACTTAGCAAGCAAGAGCAAGACCGGATATGTTGGTGCG
GCTAAAAGTTAGTTAGATACTCAGCTATGTAATTTACTTTATTCCGCATGTTGTTGCACT
TGATGCTACTTTTGTGATAAAAATGGTTGTGTCTATAATAGTATAGCATATGCTTCCTGC
AATTTACCGTTCTAATATTGGTGG
>ntig_957_consensus
CTACTGTTTTTGGAGTACTCTCGTTTGAACTACTTTGGTTATTTGCTACTGGTGGCCCTT
TTGTCACATGTGATGAACCAACTGTTTGTACATTGTTTGTCCAGGAAGATGCAGCAGCGC
TAGCCATTGGAACCATAAGCATTGAAGTGGAATCAGGATTCAGAAAATGTCCAGGTTCAA
GTTGAGCAGAAGATGACTGAAAGGTACCCGGGACTTGTGAATGCACTGGAGCATTTTTTG
AGGCTTTTTGAGGATAAGGATGAGCAGCTTTCCTTTTAGGTCTCGGAGGAGGTAGATGTT
CAGCTGTTCCACTCTTTTGAACCTTTAGAAAGTATTTCTGCGCATGACTTCGAATCTGAA
TGACTGTCTTTGATCCAACAAATGCTTCAATTTTTTTCCAGTCCCGGTCAAAGAGCTGAA
GCGCTTCAAGGAACTTGTCATGTTCAGGTTCGGTCCAGCTCTCTCTAGACTTGGTAATAG
TGTAGGGTTTTCGGATCTTCTTTGACGGATCCTCTGACGAAGACACCATAGAAGGCACGA
CGGCTGTACTCGCCGTCGGGACAAAAGGTCCGAGTCCGGGTAACTCCATTCCATTTGGGT
CCAAATAGAATCCTTCAGCCGGGTTCGGGTTTTTGGACACCATGATAAAAAATTTGGGCA
GCGGAGGATTAGCTTCGAAACTACGATGTAGT
>ntig_958_consensus
GTCTGCAGCATACATACACATATATTCAAATTCTGACCAAATACAGCAAAGTTAACGGAC
GTTGAAGAATTTATCATAAACATCAGAGAAAGAACAATAGGGCACACAAACAAGAAGAAG
AAGGTATGAGTTCAAAACTCCAAGAAATACATCTAGATCAACGGGACAGAGACAGATCAA
CACAAAACAGCACAAAGTCATCCTATTTCACACAATACATAGAGAAATAAAGAGTGATTT
TGGTTGCGTTGTTTGTTTAGTGATGCAGCTAGATATCATTTGCAAATGAAATATTGATTC
AAAATTTATATGTCAGAATCTGCAATCCCCAGCTCATGAACTCGGAGAATAATCATATAA
ACCAGATTACCTAAACAAAATAAGCTTAATGAGATTCCATATGAATTATTATACAATGCT
ACAACTATATATTAGAAAATCATTCAAGAAACAAAGACACACACACGACCATTCATTAAT
GACCTAAATGATCAGGATGACAAGACAGTACAAATTGCAGATAAATTTGGCTAGATCAAA

CTCTTTGATTGAACACTCACTTTTTCACTTGTACTCAAAATTGGGCACCGGAGGCGCATC
GAAACTCTCCAAAATCTCGGTCTTACTTCCACTGGCCGCCGGGGTTTCTTTCTTCTTTCC
CCCACTAAAGAACCCTCCAAACCAACCTGAATCCGACTCAGAAGAATTCGATTCCACTCC
TTGACCAGGCTGACCAGGTAAGCCAGCAGAAAACCCTGGCCCACCAACCATAGCAGGGAC
CGGCTCCTCAATAATAATCGGCATGTTTTGCGGGCCACTAAGAACTTTATTAAGCATGAT
CCCAGCTCCTTCGATTAGCGCCAAGAGAACACCGCCGAAAACAGCGGATCGCGCTGACGA
GCCAAGCCCCTGGCGCATGGAGAGGAAACCGCCAGTGGCGGCGCCGGCAAAGATGGAGTT
CCAAGGGTCTTCTTTCTGACGGAGGTAGACCATGGAGCAATCGAAGGTTGAGAAAAGCCC
GCCCCAGACAGCAAAGCTGCCGCCGACACGAGGCGCGTTCATGCGAACGGCTTGGGTACC
GCCGATGAGACGGGAGCCGCTTGGGGAGTTATAGACGCCTTTGAGGAAGTGGAAGGCGGA
GCCGCCAACAGCACCCATCCCGAAGGCTCCACCGATGTCGTCTAAGATGCGATCTGGACA
AGGTTCTCTGGAAGTCTCTGGGGTTCCCATCCCAGCTCTCTGTTTCCTGTTTCGGAGAAG
GGTCAAGATGTTTCAGGGGGTTTAACTAATTTGATTCCTCGGGCTTAAGGAATTTAGACG
CGTTCTAGGGTTTTGGGTGATTTGACGACAACGTCCTTGGAAAAAACAAGGCTTCCCTTA
TTTACAAATGAAAACA
>ntig_959_consensus
TAGTGGTATATTAGATTTTCAACATTTTGAAACTAGAAAATTAATAACCTAATGGCAGAG
TTTGCCCTCTCATCTATCATGACTTGCACCAATACAATTCAAATTCAACAAATGCTCATT
CTCAAGACCAGTGATCATTCATGGTCCGCTTACAATTTCTTTTTGGCTAGGCACATGGTT
ATCTACAGCTTAGATCATGATTGACATCCCTGACTAACATCAACAACTTGGAATAACATT
ACCCTTTTCCGAGGGTTAAAGAGGATCACCCTGTGGCCAAGATTCTAAAATCAAGATCAA
CAACCTTGGTCCAAGGCTGCACAGTATTACAATATGAAAGAGAGAAGATTTCCCCTTCCG
CCTGCGACTCTTAGACTAACCCCTGAAACTTAATTAGATGGTAGCACAATATACATTGAA
AAGCAAAGTAGACAGGTAGCAATACAGTTGTCTCGGAATGATGAATAAGCCCCAAGGTCA
CAGTGATCAATACAATGAGATCGAATTAGACGAAGAAGCCTAATTTTCTTTCACTCAACA
AAGTTTAATGACATTAAAAGTGTACATTAAATTGCAAGCCGATTCCCTAGCAAGCTATCC
TCATCAACATCCATGAATTATACTGATACTAACTGTTAAAATTTACAATACAAAAAGGGG
CTCTCACTAGCACTTGTTCCTACCATGGCGTATGGACCCAATTCGCTCTCTTCCTCGTGT
CTTCTGCTTAATTGAACTTAGTAGCCGTATCCTGAATTGTCCATTGGGTTCCCCGCAGGC
ACAGCAGGTTCAGGCTCCTTGATCTCAACCACCACACAATCGGACATCAGGAAAGTTTTT
GCAACTGAGGAAGCATGCTCCAGGCAACACCTGACCACCTTAGTTGGATCAATAATTCCT
GCAGCCATCAAATCTTCATATTTTCCAGTGGCAGCATTGTATCCGTATTTGTGGTTGTCA
CTAGAGAGCACCTTCTCACTGACAACACTCCCATTGACACCGGCATTTTTGGCAATTAAT
TTCAGAGGGTAACTCAGAGCCCTCTTAACAATTTCTGCCCCAACCTTTTCTTCATCATTA
TCCAGGCTTTCTTTAATGGCATCCACTTTCGATGATAGTCTCAACAGTGTACATCCACCA
CCAACTACAATACCTTCCTCCACAGCTGCCTTTGTTGCGTTAAGAGCATCTTCAACCCTC
AGTTTCTTCTCTTTGAGCTCTGTTTCAGTTTGTGCACCAACCTGAATAACAGCAACACCA
CCAGATAGTTTTGCAATTCTTTCATTAAGTTTCTCCCTTTCATAGTCTTGCTCAGCATTC
TCTATAAGATTTTTAATTTGTGCAACTCGTTTGTTTACTGCTTCCTGGGTGCTTCCATCA
CCCACAATGGTGGTAGTGTCCTTTGTGAGGACCACTTTAGAAGCATTACCAAGGACTTCC
TTGCCTACTTTGTCCAAGGCAAGTCCCACTTCATCCCTGATTACAGTTCCTCCAGTCAGG
ATAGCAATATCATCGAGGTACTGACTCTTACGCTCACCAAATCCAGGAGCTTTCAGAGCT
GCAATCTTCAATGTCCCTCTAAGCTTGTTCACAACAAGTGTTGCTAAAGCTTCCTGTTCA
ATGTCTTCTGCAATTATCACGATCGGGTATCCACTCCTAATAGCATCTTCCAAAATGTTG
ACAAGATCTCTTGCATTTGTTATTTTCTTATCTACGAGAAGCAACTTGCAGTTCTCATAT
TCAACTGCCATTTTCTCACTATCAGTGACAAAATAAGGAGAGATATAACCACGATCAAAT
TGCATTCCCTCAACAACATAGAGGGTGTTCTCAGCACTTTTTCCCTCTTCAAGAGTCACA
ACACCCTTCCGTCCCACTTTACTCAGTGCTTCAGCTATCATATTCCCTATTTCATAGTTG
TTACCAGCACTAACTGCCGCTACATCGGCAAGTTCACTATCTTCAACCTCTTTTGACATT
GACTTGAGCTCTGAAACCAAAGCCTTGGTTGTCTTCTCAATGCCTCTGGTGATTAAAACA
GGGTTAGCTCCAGCCGCCACCACCTTGACACCTTCGGCAATAAGACCTTGTGCAAGGACA
ACAGATGTTGTGGTTCCATCTCCAGCCAAGTCATTGGTCTTAGCAGCCGCCTGTCGCACC
AACTTAGCACCAATGTTCTCAACAGGGTCCTCCAACTCAACCTCTTTGGCCACAGTCACA
CCATCATTTACAATTTTTGGGGCACCATACTTGCTCTCTAGAACAACATTCCTGCCTTTG
GGACCAAGGGTAACCCCAACAAGATCTGCAAGCTTGTTCACACCAGTTTGTAGTTTCTTA
ATAGCCATTCCATCCTTGTTGAAATTTAAATCCTTTGCAGCATAAATTTTAGGAGAGCAA
GTCCTGCGTAGAGCAACAATTTGTTTTCTACCAAAAGAGGTTGAAGAAATGGAAGCGAAT
GATGACAGCTTATTTGAAGAAGATGCAAGTTTCTTATCAGTCATGCGTCCATTAGGAGCA

ACCATAGAGCCAATTGGAGATATGGTAGTGAATGTCGCCATTGTAAATTTAAAGAATGAA
ACGGCTGTAGAGTGGTA
>ntig_960_consensus
GGTAATATTGGTTGTCGTAAATTAATTACTGAGAGCATAATCCAATTATGAGACTTGGTA
ATTAGCTTTTTCTGTTCGTCATCTTTCATAATCAGTGGCAATTTCAATTTTTGTGATTAA
TGGAAAATCTGCTGCTTTTACATTACAAAGAAACCAAAATGTTCTTCTAAAACAAGGATT
ATTGTAAAGCAGAAACTAAAACGCCATTAAAGGAACAGAATCAATCAGTTTTTTTGTCCG
CTGGTTGGGATTTGTTTGCAGAGGTTGAATGTTTATCCTCTGCATACTTTCCCTGAATCT
CGTCTCTCTCCGTGAAGTACTTCTCAAAAGCCAGAGGAAGGAACCTTGGAATCAGGAAGC
AGAAGAGATTGGCAGAGAAATACACAAGATTGGCAACAGCAAGAGTCCTCCCAAACCAAA
ACCAGGCCATGTCCTTAACTTCCGCAGTGACAGGCAGAGTTTTGTTGAACCAAACATCAG
ATATCCAGTCAATTATCAGAAAGAGTCGCCTGACAGTATAAAGCAATGGAACTAGTGCCC
TCACTGGTGCTGAAAATAATGATAACCCACTTATGATATTCTCAGTCAGTATTTGAAACG
AGAGCAAGAACAATTGAGGTGTGGTTGAGCGAACTGCTTCGTCGTCGCCTCTGGCGAATC
CTCCCAGCACATATGCCAGTGGCAAGAACAGCCCGATTGTGGTTCCTACAAGAACATAAA
ACCTGAAGAGTTTGCTCCCTTGGAAGATCTCCAGCGAGCTTGAGGAAGCCTTGCCGTGAG
CGGGGAAGACAAAACGTGAAAGTGATAGTAGATAACCCGATGTGACGGCGGGGAAGACTA
AATCTAGGAGTGGGACGAGTCCACTGGCTGAGAAAACCATAATGAAGGCCACTAGTTGGA
GTTCAATTACGCGCAATGATCCCATCACACTCCCCACTGTACTTAACTGCCCCTGCCGCT
GATTCCTCAGGGGCTCTTGGGTGGCTTTGTCAACCTCATGTTGAGGCTCAACAGCAAGAG
ATACACCGGACATTGCCCAGCAAGC
>ntig_961_consensus
AGAAGAAGAAAAAAACCAAGACTGTTGTTGAGAAATACTGGGACTGGCAGCTCACTAATG
AGACTCAACCTATATGGCTCCGCAACCCAAAGGAAGTCACCACTGAAGAATACAATGAGT
TTTACAGGAAAACCTTTAATGAATATTTGGATCCATTGGCATCATCTCATTTCACAACAG
AGGGTGAGGTAGAGTTCAGATCCATTCTCTATGTTCCAGCAGTTGCTCCCACAGGGAAGG
ATGACATTGTCAATCCCAAGACTAAAAATATTAGGCTCTACGTTAAAAGGGTGTTTATTT
CAGATGATTTTGATGGAGAATTGTTCCCTCGATATCTAAGCTTTGTCAAAGGTGTGGTGG
ACTCAAATGACCTTCCCCTCAATGTTTCACGGGAGATTCTTCAGGAGAGTCGAATTGTAC
GCATTATGAGGAAACGGCTGGTCCGGAAAGCGTTTGACATGATTCTGGGAATATCCATGA
GTGAGAACAGAGAGGATTATGAGAAATTCTGGGAGAACTTTGGCAAACATTTGAAATTGG
GTTGCATAGAAGACCGTGAAAATCACAAGCGCCTTGCACCATTGCTTCGATTTTTCTCAT
CTCAAAGTGAAGATGAGATGATTAGCTTGGATGAATATGTTGAAAATATGAAACCTGAAC
AGAAAAATATTTATTATATTGCTTCTGACAGTGTCACCAGTGCTAAAAACACACCTTTCC
TTGAGAAACTTATGGAGAAGGATCTTGAAGTGTTGTATTTGGTTGATCCCATTGATGAAG
TTGCCATCCAGAGCCTGAAATCTTACAAGGAAAAGGATTTTGTTGACATTAGCAAGGAAG
ATTTGGATTTAGGTGATAAGAACGAGGAGAAAGAAAAAGTGATGAAAGAGCAGTTCAGCC
AAACTTGTGATTGGATTAAAAAGCGATTGGGTGACAAAGTTGCCAGTGTTCAAATCTCAA
ATCGATTGAGCTCGTCACCCTGTGTTCTGGTCTCTGGAAAGTTTGGTTGGTCGGCTAACA
TGGAGAGGTTAATGAAGGCACAACAAGTTGGTGATACATCCAGCCTGGAATTCATGAGGG
GAAGAAGGGTGTTTGAGATTAATCCTGAACATCCTATTATTCAGAACTTGAATGCTGCAT
ACAGGAGTAACCCTGATGACAATGATGCACTGAGGGCTGTAGATCTTCTGTATGATGCAG
CTCTTGTTTCTAGTGGTTTTACTCCCGAAAATCCAGCACAGCTTGGTGGCAAGATATACG
AGATGTTGAGTGTGGCTCTTTCAGGCAAGTGGTCAGT
>ntig_962_consensus
CCCTGATCATGTAATGTAAATTAATTATTCTTGATTTTCTCTGAGGTTGTTTTCCCTCGG
ATTAAGCCGGCTGCCATGTGCTTTGAAAGGATTGCTATTAATATACATGAAAGAAAAATT
ATACATAGAATAATATAAAATTTATTACATTTTATATTGTACCTTGCATGGATATGGCAG
AACATTATTACAAGGAAAAACCCAGAAAGCAGGAAATCACACTTTAGAACAAACACAGCA
CAAACATTTATAATATCAATCAATCACACACAATCCTTGAACAATTTCTCAGCCCTTGAT
TTCTTCAATCTCGATCACTTTCTTCTCAGGTTTCTTCACTTCTTGTTTAGGAATCGTCAC
CACAAGCACTCCATTCTCCATCGACGCCTTCACTTCATCCGTCTTGGCATTTCCAGGCAA
CCTGAACCTTCTAAGAAACTTGCCACGGAAACGTTCAACCCTATGCCACTTATCATTTTT
ATCATCTTTTTCCACGTTCCTCTCTCCGCTTATTTGCAGCACTCTGTCTTCTTCGACCTC
AACTTTCACCTCCTCTTTCTTTAGGCCTGGGAGATCAGCCGTGAACACATGGGCGTCTGG
GGTCTCCTTCCAGTCCGTTGGAGTGTTGAGGACTGGACATAGGTTAACCATTGAGAGAAA
CGGGTCAAATGGTTCATCACCAAGCAGTTTTGGTATAAGCGACATGATGAATACTGTCAG
AGATCAATGAATATTGTCAAGGATATATGTTTCGGTGATTAAAGGCATGTATTTATAAGA

AGAGAGAGGTGATTCCTGAAATTTCTTG
>ntig_963_consensus
GCTTCCAGAATGCAAATTGAATAATCTATTTTTCTACAGTAAATGATTGTATGTGAAAAA
TATCCGACTGGCATAAAACATCAAAGGCTCCTGATGATATCTATGAATTTTGAACAAAGA
GCAGCTAAGCTCCTGAACAAAATGCTTGAGCTTTATGTATACTAGGCAACAATCCAGACT
CATCACTAACTCTTCATACACTCTGAAACTATATCTCCAACAACAATTACATTTACCAAA
AGTGAGAAGATTAAAAGAAAGAAACTGCAAGACTTCAATTTGAAGAATATCATACATCCC
AAAATGTGGCATCTGACTCCGGTAAGATCAACATGACCTTAGTAAAACAGGACCCACAGA
TGAGCTTAGCATATCGCTGAGAGCTGCTGGAACCTCATTTGATCCATGTTCACAGCTTAT
CAAGACTGAAAGAAGAGGAAAAAACTGAGACAAATTCTTCTCGAAGCTAGTCTCTCCTAA
AGTACAGATCGCCTGGAGTGTAGACACAATAAGAGAGGCCCGTGCAGCCAATTCCCTCCG
TCTCCCAGAACCTAAAGGTATTGCCCACTGCAATTTCGTATTGACATATGCCTCGGATGT
TTGTCCTGAGAGAGAAGTGTCAATATAGAACTGTAACACCTCGCGACAAAGGTCAATCAG
ATAGGACTCCACTTCAGCCTCATCATAAGTAGGAGGCCTGTCCAGTATTAGATTCTGTAA
AAATGACAGACAAATCTGAAAAGACTCATTTTCTAGGCGTAGTAGGGGTGGATCTTGCAT
TTGTGTCATAGAGCCAAATTCTTGTAGCTTTGTGCGTAACATGTTATTGCTATTGATCTT
ATGAGCATGGGTTGCCACGTCATGCAAAGCCTCAAAGAGGACTAAGACGTTTTTAGCAGA
AAGGTGAGGCCGATACATGTTGTAGATCTCCATCACTGCCTGAATCAATAAAAGTTGTAC
AGCAGCCCTGCATTTTGCATCAGCTATAGAAGCATATAGACGGCGAGTCTTCTGGTCCTC
ATCATCATCAGCCATACCAGAACCAGAAGCCTCCCCATTAGTTTGTCCATTTGACACATT
CTCATGGTTCCTGACCATGCTCTCTTCACTAAAAATGTAAGAAAAATCAGGAGCAGTGGA
CTTGGCTGCTTCTTTTAATGAAATGACCACTTCCAGCCACTTTTCATCAGAAAATAGATC
ACCAGCATTGCTCATTAAACGGACAAAAGCAGCAATTCCAATACCCGCAAGGCTCTGATG
AGGACGTTTGATAAAACTTACCAGCAGCATCAGCACCTTCTTCAAAAGTGGATTGACAGT
GTTGTAAAATTTGACAAAAAGATCCACAACTAATTGGAGGGCCAACGTGCACGTCTCATA
GAGCCATGCATCTTGATCAAGTTCACCCATATCACCATCAATCCCTTGCCCAGGCGAGTT
CTCCCCTGAGGGATCAATGGCATGCCGCACATAGTCAAATATTGGAAATAGGACTGATTC
AAACACCCGCTCCCACAGAGGCACTGAGAAAAGATGACCATGATTACGCAAAGTTTCAAA
CAGTACTTGTAAGGCACTCTCCCTGATTTCTGGCCTAGGGTCAAAACTAAGTTCTGATAA
ACCAGCCAACAAAGGAAACCAGAAATAGAGATGATCATCTTTATCAATTATCTCTCCATT
ATCTAGTTTTCCTTCCTTCAGAGGTTGAGACTTAGGAGAAATCTTTCCAGACACTTCCTT
GTCTTTTTTCCTTGACGAGGCACTAAGATCTCCTTCTGCAAGTTTAGTGGCACAGAACCG
AAGAAATGCAATAGCATTCAGACTGATATCTTTGTTGAATCTACTGTTTGTGAATGCTAT
TAAGCAATTCACACAATCTGTAAAGGTGGTTGTTTCAGTCTCAGTAATGTAGGGGAAGTA
GTCTCGGATAATCTTCTCAATAATTTCAAAGGCCAAAAGTACAATATTTTTGTGATCATC
ATATGCTGCTGTTGTGAAAACCATGAACATGCTTTTCCATCCTGATTTGACATTATTGAC
ACGAGATAACACCATCTGTGAGACGCATCTGATGATTAATTCTCTGATTTCAACAGCACT
ACTCTTCCGCATTACAACAACAAAAGGCTTCATAAATTCATTCTGAAAATTATAATTAGC
CAACTCTTCTCGCTCTAAGAATTTCATCGATAACTGGCGTAAAGAATCCATTGCAAAAAT
TGCTATTGAGAGGTTCTCAGAGCAGCCAATGGTTACAAAGAAATCAGAGAGGACATGCCA
GATGCTTGACCACACAAGCCTGATGCGATTCATGTTATAGTGCGCAATCTCAACAATTTT
TGTGAGGCTGAAAACCCGTGGATCTGTTGCAGATCGGAGTTCCTCCATTGAGACCTTGCA
AAGAGCCTTAACAAAGTCTATTATTGCCTCACTGTTCAATTTCTGACTTCGTGTAAATAT
ACGATTCATTTCAGAGCTTCCAACTTGTTCTAACATGTTTAAATTGGTGACTAAATTGTT
CATCAGATCAGATGTAAGCACCCCAGAAGCATTGCCACCAATGCCTGCACTATCATATGC
ACCCCTCATCACAGCAGAAGCTGCATACTGAATCCTCCCAGGTCCTTTCTTCTTTAAAAC
TGGAAGGATGGTTGACTTAGCTTGTTTGGATTTTTCTGATTCATTCTGTGGAAAAGCAAA
GAATGTGGCATCTGGAGGAGCACCTTCTCCCAAGAGATGCAAATGCTCAAATCTGGACAC
ACATGTCAAAATATGCTCCCATCCCTCCTGTAAATAATTTCCATCTTCATCTGCAACTGT
GACTATCGCCTTGATGGCAGCTATGTTTTTCTGCTTTATGTCAGCAGGAGAGTGAAGAGA
AGTAAATTTTGCTAATGAAGTCACAAAAGCATCCCTATGAGTCTTCATAGACATTACAGC
GGTTACATGGATAGCATAACGAAAGCCTTCCAGACAAAGAGCTAAAATCACTTCATCATC
ACTCTGGTCAAGGGGAACACTAAAGGCAGCCAACATGGGTGCCCAGCACACTTCAATCAT
GAATCTTAGAATTACCACATCAGTTGCTGCATAGTATACTGATTCAGATTTGCGAGCTTG
TTCCTTAAATTGTTCTTGCATGTGCCTGATGAGATCATCACTAGTCTCCATATGCCTTTC
TTCCCCCCGCTTTCGAATCACAATATTCAAGATACTGTCCAAGCCTAATATTTTGTTTGA
GTTCATAGACTGCTTCTGTTGAAGAGCCAAATCATCTTCTTTCATTTTGATCTCATTTCT
TGATATCCGATCAAACAATGACCTCAGATACTCCTCAGGTAAATCTTTTCCATCGTCTAT

GCCACGATTGTTTCTTACAAAATCTTCAGCCGACATCTTGTTCTTCACCATAGGATTATG
GGCATCAGTGTTGAGCATTATTACAGAATATGCAAGGACATAAGCTGTGTCAGCACTAAT
GAATGCCTTTGGATTACATTTACAATAACGTTCAGCAAACTTTTCCATGATTCGATCAAT
CTTTTGTGCCTCACCAGGCAACCTAAAGCCTTGCAAAAAGGCCCTGATTGCCTCATCAAA
CTCCATGCCTTGAAATTCAAAAGAATCCACATAGGCATGCATTACTTTAAGGGATAACTC
CTCCCTTTCTCCTAGATAATCACCAATCAGAGTCTTGTTCAAATCAGAGGCATTTTTTAG
AAAAGCTGCTATCTCCTCCGGTGAGTTGCCCACCTTATTTGCATTAATAAGAAATTCAAT
TCCTTTCTTGGGTTTCCGATTAAAAAGAGATATACCTTCTTGAAGTTCAAGCTTGTAAGC
TCGACGTTGTTCAATGGTCAAAACTTCAGAAACCTCACTGGACACCTCAGATTGAGAATC
TGATCCCTCAACAGACTCATCCCCATTCCCATTTGCCACTGAAACAGTTCCAAGCTCAGG
AAAGTTCTCCACTGCCTCATATTTCTTAGTAGAATGAGGATCTGGAATACGCAATTGTTT
GTTCATCCAGTCTCCCATTGATCTCAATATCGCTACAAGGCACTTCATGGCTTCAAGCTT
CATTGTCACCTCTTGAGGGGGCAACAGTGTGGTGGTCATGCCAGGAGGAACACCTTGAGC
AGTTTTAAGAAGTCCATTGACCATTCTCTCAAATATGTTTGATGAGTTGACATCACAATC
ATAGTTGATAAAGATATCTACTAAAATCTGCGAATCAACACAGAGCTTCTCTAAAAAACG
AAGCACTATCATCTTCTGTTGAAAATTTGGTTGAGCAACATTTTCCAAAACTCTGAGCAC
AATCATAGGAAAGAACACCCCAATCTCTGCTTTCAATCCAGCTCTAAATCTTGATACCAG
ACTAATGAAAATGGAGCAAGAAAGCTGGAAAACAATCATAAGAGTTGAAGCACTATTCTT
CAACAATGACAGACACAAATACTGCTTAATGGCACCCAAAAACCTTTCACTCGTCCGAAA
CACAGCTCCAGCATTCTCCAAGAGGATCTTCAACAACTCAAGAGCCACAATCTTCCCTCT
CATCAACTGGGGATCCGCCAATGCCTCCTTAGGAGGAGTCTTCATAGACAACTTACAAAG
TGCTCTAAATACCAAAAATGCATCTCGTCTCAACTTATTCCCAATTTGAACCTCCAAATC
TTCATCTCTTTCCCCTTCTCCGTCAGCCAATTCCCCTTTCCTCCCCTCCAACGCCGTCTT
ATACATACTAATCTCCCAATACTTAGCGTCCAACATGTCCTTATCAGTTGAATCCAGTAA
ATCAGCTGGATTAGTTGTCTCCACAGTAGTAGTTTCAAATGCACCATCGTGACCTGCGGC
TGAAATCTTACTGGGCGTAACTGGATTCAAAACTCCGTCGATATCTTGCATAATTTTGGT
TATAAAACCTTGCACAAACATTGTCATGGACCCATCCGCATCAGATTTTTCTACTGGTTC
CATCAATTCAGCAACCACAATTGGTTGAATTGGAACTGTTGATGAATCTGCTTCCATCCT
TCTAAAAACGATCACTAACATTTGGATCAGTGAAGCCTTAGCGGTCGTCTGATTAACCAC
ATTTTTACTTCCTAGATAAATGTCATAACAAGTCCTCACAATTTGCAACAAACAATCTCC
GTGAATCCTTAACGACATCGACGTGACGGCCGATAGAAGCGTTTTTAAAACTAGTAACTC
AACAGCGTCATCACCTAAATCATGGCATTTACAGACCGATTCAATTAATTTGGAAAGAAA
TATCGCTTCGGGACCTCCGGATGGGTCCGCCTCGCCGCGAAGATAACCATGGGCGATCAA
TTTTTGAATACAATCAACGGCAGGATCAACGATCTTAACGAAGCCAGTACCGCAAGCGTT
GATCAGAGGGCTAAGAATGGATTCAGATTCTGAGAGCGAGTATTCTAGCGTCCCGCTGTC
GTGGAGAGGGCCAGGGACGGAGCTGTCGGGTTCCGAATCTGTAGGCGTTGAGGGCGACTG
TTTCTGCGCGGAAGTGAGTCTCTCGAGAACTGATTTACATTCATGAGCGAGTTTCGAATG
TTTGCGCCACGACGCGTTTTTTATGATCTTTTCCAGAGCTGGAGCTAGGGCTTGACTCAA
GCGGGAATCGGCTTCCGAAGAAGCCATTCCCGAGTTGGGACTAGCTGTGACTCAGTGAGT
CAGATCTGGAAGTGACGAGCAGAGTCGAAGAGTGAGCGTCGTTGCTCTTGGTTTTGATCG
CATAAAAATCGCACGGAATGAACAATTTTACCACAAAAAGAAGAGAGAGCGTGAAA
>ntig_964_consensus
GACAAGCAGTCAACTCGTTCAATTATTGAAAAGAAAATCACGCACAACGCCAGCAAATAT
TAATATCAAAATGCTATGAATCGGTAAACATAAAAAAATTACATGCGACAGATTTATATA
TTTACATGCTCAAATGCCATTTTTCAGCCAAATGCAAATAGCAGCAGCACATAACCCGTA
ACGGAAACCCAAACAAGTCCTTAACTCCAGACTATTTCTAGCTTAGAAACGTCTAGCTGA
AACTGAAGTGCCCCTACGTCTGCTTGATGGCATAGAACTTTGTGACCAGGATGACGGTTT
AAAAGCTCCTGTCAGCAAGTTGGATGCTGCACTTGCACATATTTTCCTTCGCTTCGCTTC
AAGCTCCATTCGTTTATTACTTGACGAGGAAGACCCAGATGACTCGGGAAATATTGATGC
AGCTTTAATCAATGCTCGCTTATCCTTCCTATTTTTTTCAAACTTTGAAGAAAACCTCAC
ATTTGCAGCAGCAGCAGTGTCTTCTTCACTTTTTGGGAGCAGGCGTAAGCCAAGACCCAG
TTTCCTCGAAGAAGCCTCTTCTTCAGCAACTCTCCTCTTTTGAGATCTAAGTTTAGCCCT
GAGAGCTTTGTTCAGGGCATAGTCATCTGAATGCCTAGCATTAGATACTCTCTGAAGGCT
CACTAGTACTGGCTCCGCCTGTTTTTTCTTCTGCAGGTCCTGTTCCTGGTGCTCAAGACG
ATAAAAGGGATCTGAAAGCTTACCCCGTTCTTCATCTGCAGGGAGTTCAAGAGTCTCTGC
ATCCTCAACATCGAATTCTTCAACCTTCCGCTGGGCACCGCTAATAATAACATATTCACA
GTTCTTTGGATCTGTCTGGATAACAATCTGGTGTTTGCAGCATGCAGACTTCATGGAAAA
GCTCCATATCTTTGTAGAATAATAATTTCCCACTTGTTTCTTTTCTGCATTGAACCGAAC

ACCCTTTGCTATCATAGAATTGCAACCACCACACCATATATTGAAAGGCATTTCGAACCT
TATAATCAAAATGCCCTGATCTATTTTTCTTGCTCTCTCTCTTAAAGCATGTTGACCATG
AAACTTGTTCAAAGAACCCTGATTTGGTGTCCATTCTGGAGGATAGTAAAAATTGTCTGC
TCTAGCAGCTGCAAGTGAAGACATATTTTCTTCAAATTCTCAAGACTAGGATCGATGTTG
GGAGATTTCTGATACAGAGGAAATTGTTGCCTTTGGCACTAAATTT
>ntig_965_consensus
GTTGTTGGTTCTGATCTCCTCTTAGCTGATGGTTGCCATATCCTCTGGGAATGCTTTGGT
TGGCCTGTGGCCCACCCCATTGATGTGCACTACCACCACCAGGTGGTCTGTAGCTCTGAA
AGCTATTGTAACCACCTTGATGTGAATTAGGAGTTGCCCCAGACGCCGAGGCCATAGGCC
TGGGTGGGTACCTAGGTCCACCATTCAGGTGAATTCCTGAAGGGTTATATAAATCAAACG
GCTGTTCTCCACTGTTAGCTGACCAACTATTTCTATCATATTGAGAATGAGACCTTGTGT
GCGGAGTTGATATCGAATGCCGTTGACCATACCCAACATCGTAGGAATGGGGAGGAACCG
AAGACCGAGAGTGGCCAACAGGAGAATAATCTGTTCTAGGTTGTGATATTCTAGGATGTC
CCTGATCATGACCCCTGTAATCATGATAAGAAGGAACCGTTGGGATATAGGGAGTGGCAG
CATAAGGTACTGGTGGACCCCGCATATGATTACCATATCCATTATGATCCACCTTCAACT
GTAAGCTATTAACAACAAGTCGATGTGATGCCTCGCCAAGCTCACGGCCAGAAATGGTTC
CACGGGGATTTTGCCTTCCATTTTCATAAGGTCTTCTCCCGGAATCCTCATGCCACAATG
TAGGCTCAGGTTTTATATCATCCAGTTGCACCATCTTTTTAGGAAATATGACCCCAACAG
GAGGTCTGGTGACGTGTTTATGTGCATCTGGGAGTTTGTATATAGCACACACGACTTGGT
TGCCCATGATATCTTCCATCCCGTCAACTGGAGACCTAAATATAGGAGGATGAGGATCAC
CAGCACATGGTGATATATAACCATTCATCCCACCACGTTCCTTGACCTCAGCTCGCTCTT
TGCTTTTTAATTGTTTACAATGCTCATCAAGTGTATAGATGCTTGCAGAGAGAGGATGGG
ATAATAGCACAAAAAGCATGTCAGACATCATACTGTTTCTCCACACTTCCTCCTCCGTTA
AAGTATGTTCAACTTTCTTAACCTCAGCAAGAAGGCGATCTTCATCAATGAAAGGCAGCT
TGGCAATACCCTGCCAAGCATACCGCTTCCCATTCATGTCCACTTCAAAATCAGTTGGGT
AAAAATCAATAATAGGTGAATTTGGATCTGTCATCAATTTTCTGTAATGCTCGGGGAGTG
CGTGAGCACTTGCAGCAGGGAAGACACCCAAGAGTTGATTAAATGGTTTGAAAGGTTGAC
CCAATTCAAACCTGATATCCAGCTCAGCAAGATATTTGAGATCAGAAGCAAAGGGTGCAT
AATGATAAGGATAATACCACTGCCAAGAGCAGACACCTTCATAGTAATAATGCATGACCC
AGCATAACCCTTCTGTGTACCGCAAAACAACATCTTTTCGTACATCTTCACGTTCCTCGG
GAGTTATTGCATTAAACTTCTCCTCATAATACCTTTCTTTCCAGCCTGGCTCTCCCAATT
TAACCTTATCTTCTTCTGGATTTTCCGAGTTGAAGGCATCAGATTTCCCACGAAGTAACT
CCTTCAACTTTGCTTTCAATTCTTCTTTATTTTCCTGCATATCTGTTTCAAGACTGTCCT
CTG
>ntig_966_consensus
ATTGCGAAACCATACCTTCCGTTTTATATTAAATCGTTACAACCAATTCAATTGGCCATT
GGATCAATTATTTCACTAACAAAATACAACACCCACTTAACAATCATCCATTCAGAGCTT
AGCAAGCAGAGCATTCTGGTTAGGCTTTTCGGCCATTCTATTGCTAACAATTATCCACTC
ACAGCTTAGCAAGCAGAGCATTCTGGATAGGCTTTTCAGCCATTCCATTGCTGTCTTTGA
TATCCGAATCGCTGGCACAGGGAGGAACATCCACTGCTGGTGAGCAATTGAAGAACCCAT
GTGGCATGAGCATAAAACCAATGCGATCCACTGGCATTACAGGCCAATCTTCGAGTCGAG
GAATGTAAGTCACTCCAAATACATACCATAGAACTATATCAGTTTCTTCCAGAGATCGAT
TTTGCTTAACCCATGTTGCCAATCCTTCTCCATCTCGTGGATTCTGATTAGGAAACTCTC
CCCCAGGGTACATTTCATCATGTGCATAACGAGTGACCCAAAGATTATGCTTCAAGAAAG
CAGCTCTTCTCAAAAATTTGGCCTCAGAACCAGCTAATGGTAAACAGTTTGAACCAGGCA
CTAGCTTATATCCAGTTAACTGCCCAGTACGGTTAACATTTCTTGTGTTTCTTATAATCC
AATGGCGAGCAGATAAAGGATTACAATCACGCATTGCTTGCAATTCTGATCTTAGTAGTT
CCTCTTCAGCATAGAATGCATTATTATGAACATTATTCTTTCC
>ntig_967_consensus
GGGTTTGGGTTTGGGTTTGGGTTTAGGCTCCACTCTTGCCTTCTTCCCACCAAAAATAAT
CTTCAAAAGAACAGAGAAGATAACCACCACAACTGAAACAAGAATGCCAATTGTGAGCTT
CGGTTGTTTCTCTCCCTTCTCAATAATATCAAGAATCTGAAGTTTGTATGCATCCAAGAA
AGGAATGTCTGCAATCTTGTAAAGCAGATCAAATACCTTTTTCTGAAAGCCTGCAAGACC
ATCTGAACCAGCAGCAGCTTCCTCGGCCTCCTGTTTCTCTTTCTCAATATCATACTTTGG
CTTCCATGTGCTCCTCCGATAAGACTCTGCAACCTTCTCATCCTTGGCTATGAGAATATT
GTCAAATAGTATACCATCTTGCATGGTCCAAATCTCAATGCCGACAGCAGCGATAGGCTC
AAAGTCAGGTTTATTGAGCTCAACGTAATTGGGATTTGGAATCTCTTGAGGCTTCCAAAT
ACCCTTGTAATTGGGGTTTTCAATAAGTGGGGCATGCCATTTACCCTTGTAAGCAGGATT

CTTCTTCATTGGCCTCTTCCATACCCCACATCCAGGGGCTGTTTCACACTTCGGGTTATC
AATCTTCGGGGCCTCCCATTCACCATCCTCCTCATCATCCCAATCTTCTGGTTTTGTTGC
GTCTGGATCATCAATCTCCTCAGGCTCATCATCTAACCATCCTTCAGGCTTCACAGCATC
CTCATCTTCGATTTCCA
>ntig_968_consensus
GGGCATTCAAGATCGTGAAGCAATCTAAAAAGTGAAAAATATGAGTAGTCTGATAAAATT
TGATACATTATGTAACTTCAAAAACAGAGAACTTACAAGGCCAAGAATATCTAAGAGTGA
ACATTGAAACAATAAGAAAACAAAGAGATGTAAATTTTGAGACACGTATACAAAGGCCCC
CCAATATTGACAAGGGAATAAATATAATTCAATGCTCTCTTTGAAATACAGAAAAAAAAA
CTCAGAATACAAAAATAGAAGAGAATGAACCCTAACACTCATAGACGAATATAATAAAAG
AATTAATGAAAAACCATGTAAACAAATGACAATGTGTCTGTCCCCAGGATCTTCTATCTA
CAACACCCCCAAAAGCTTCTAGCTTTCTTTCACAAAATTTTCATCTTGTCTTCGACAAAT
CCTAACCAACCAACAAAGCACCGTGCAAAATATTTTGCATCAGGTGTTCAGAGTTGGAAG
AGCTATCTCTTCAGCAACTCCACAATTATGATTATGATCTTAAGGCCTTGTTTTTATTCA
TGTATCTTAGTCACAATTCAATCCACATATCTCTAACACAGGGCCATCACGGTTGACAAA
TGGGTTTATTCTGACCCACAAGAGGGTCAGGATTGAGGCCAGAAGAATAGACCAGACTAA
AATGATGGTAGGCATCCTGTCTTGTTTTCCTAGTAACCCTTTGAGGAAGGGGTAGAGATG
AATGATGACCCAAAGGGCAAAGAACAGCCTACCAAAAAGTGGGCCCCAGGAATCATACCC
ATTGTTGATGGCATCTGAGACACCGACAATGACCCCTACTATGTTTATAATGAGCAAAGT
TGTGGGAGGTATTAGCAATGATGTCCACTTGAAGAGGTAGAGCTCAGAGAATTCACCATC
ATCTGCTCCTTTTGAGGTCACAGTGAAATTTGTGTTGACACCAGCTAAAACCTTGAGTAA
ACCCTGGAAGAGAGCAAAAAGATGAGATGAAACACCTCCGATCACCCAGAACTGTTCATT
TCTCCACCAGTCATCTATGCCAACACCACCCCACTGCATCTCAATGATACCAGTTGCAGC
AATAGATATGAAGAGAGCCATAAAAATAAGACTGGCATAGTTGCTAATCTCAGGAACAAT
GAATTTCCCAGTAAGAAGACAGATAGCTGGCAGGGTACAGTAAACAAGCAACGGAATTGA
TGTCCAAGGATATACCACCGAGTTTATGTACGAGAACCTTTCCAACCATTTCAAACCACC
CCCATAACCATACCAGATTGGACAATGTCTGCTGAAGAAAATCTCAACAGATCCAAGAGC
CCACCGAAGAACCTGGTGTAGACGATCTGAAAGATTGATAGGTGCCGAACCCTTGAAAGC
TGGTCGTTTAGGAATGCAGTACACTGATCTCCAGCCATGGCAGTGCATCTTGAATCCAGT
CAGGATATCCTCCGTCACAGAACCATAAATCCAGCCAACTTCCTTTCCCCATTCTGTTTT
ATCTTCATAGCCACAGCTGATCACTTGGATGGCTTCCCTCAACAGTGATGCTGGACTGGG
ATCACGAGGAACGCCACCATTCTCCAGAAGAGTAGATGCGACAAAAACCGGAGACTGCCC
AAACTTCTTCTCCAATTTCATTTGTGAAATACCAGTATATTTCTCAATAGCTAATTCTTC
CACTCCCTCTTCAATATTCTCAAGAGCATGTATTTGCTTTGATGTCTCCTTGTTCTTTGA
CTTCTTTTTCTTATCCTTTGCTGTCTTTGCCTTTTTGTTCTTCCTAGATCCACAACACAA
ACAGCACCATCTTGGCCAGCAATTGCAGGTCTTTCCAGGAGGCTTCTTCTTGACTGGTGC
ATCATAACCGTAAAGTGCTTGCCTTCTGAAAACACACCCAGTTCCAACATATATTGGTCC
TTGGATGCCATCTAATCCTTTCATGTTGATATCAAAGAACACAACATTTCGATTTGAGTA
TCTATCATGGCGATCAATT
>ntig_969_consensus
ACCACCTGAAGTTTTCTTCTTATCGGCAGAGTCACGTGGGTTCTTTTGGTTTCCATCAAT
CCTCACTGATTCTTCCTTGGGAGCTTCTCCAAATATGATCTGTTTCCTTGCAGCTTCAAA
ATCAAATGGCTTTATCTGCAAAAAGCCACTAGGTTTTTCAGATTTCTGGAGTTTTCCAGG
TTTCCTATTTTTGTTCACTGATTGCAGGCATT
>ntig_970_consensus
ACCACCTGAAGTTTTCTTCTTATTGGCAGAGTCACGTGGGTTCTTTTGGTTTCCATCAAT
CCTCACTGATTCTTCCTTGGGAGCTTCTCCAAATATGATCTGTTTCCTTGCGGCTTCAAA
ATCAAATGGCTTTATCTGCAAAAAGCCACTAGGTTTTTCAGATTTCTGGAGTTTTCCAGG
TTTCCTATCTTTGTTCACTGATTGCAGGCATT
>ntig_971_consensus
AACAGAAGGAAGCTCATGAAGTGGTAGCCCTAGCCCTAGGTCAATTCTGGTCTGGTCCAT
TAGGGTTTTCTTTTCTCCAAAGCTCTAAACCTTCAAGCTCAGCCCATTAACTCCTCCTTC
TACCTCCAATTTTCTCCTGTAACCATAATTCAAAATTTTTATTTTCATTTTTATTTTTAT
CCAGTTTCCTGGAAAATTTTCAGTTCTGAATAGATTCAAAATTTTCTGAGCGTTTCTCGG
TCTTCCTCTGGCCCTTTTCTTCTTTAGATCCGTCAATAAAGGACTATTCTTTCTCCTTTT
TAGATGGATTCCCCTGTTGCAGCTGTGATGCCTACGACTCCTATTGATAACAGTGAACCA
CCTGGTTCAGAAACACAACCTAATAAGCGTAGGAGAAAGAAGTCTATCGTGTGGGAATAT
TTCACTGTAGAAACTGTAGGAGACGGATGTACTAGGGCCTGCTGCAAGCAGTGCAAGAAA

TCATTTGCCTATATTACTGGTTCAAAGCTGGCTGGAACCAGCCACCTTAAACGACATATT
ACCATGGGCATATGCCCTGTAAGCCGCCAGAAAAATCAACAATCTCCGTACACTCCAAAT
TCCATCCCAGGGAATGCTACTGATCCACCCAAAAGACGCTACAGATCACATTCTGGATTT
GCTAGCATCCCTTTCAATCAGGATCGTTGCAGCCATGAAATTGCTAAGATGATCATTATG
CATGATTATCCTCCCCACATTGTTGAACATTCTGGTTTCGTTGATTTTGTTCATACTCTG
CAACCCCAGTTTAATGTGGTCAGCTTCAACACCATCCAAGGGGATTGTGTGGCAATGTAC
CTGAAAGAGAAGCAAAGCCTTCTGAATTTTATTAGTAATATTCCTGGACGTGTGAACCTT
ACATTGGATTTGTGGAATTCAAATCAAACCGTTGGCTATGCAGTCTTTACGGGGTACTTC
ATTGATGGTGATTGGAACTTGCATCGTCGGATTCTTAATGTTGTTATGGTTCCTTCACCA
GATTCAGATTTTGCGTTTAATCAAGCTCTTGTGACTTGCCTATCTGACTGGCATCTGAAG
AGCAAGTTGCTGACCCTCACAGTTGATCACTCCTTTTCAAATGAAACCATAAATCAATAT
CTCAGAGGCTTCCTTCCTGTTAAGAATCCGCTTATGTTGAATGGTCAGTTGTTAATAGGA
AATTGCTATGCTCGTGTTATAAGTCGTTTAGCACAAGATGCAATCGGAGCAACTGGGGCA
GCTGTCAAGAAAATTCGTGATAGTGTAAAGTATGTCAAAACTTCAGATGCTCATGAAGAG
AAGTTTCTTGAGCTGAAGGAACAACTTCGAGTGCCCAGCAAAATAGAATTGTTCATTGAC
GACCAAACTAAATGGAACACAACTTTCCATATGCTGGGGGCTGCCAATGAGCTAAAGCAA
GTCTTTTCCTGTTTAGAAACGTATGATCCTGATTACAAAATAGTCCCCCCATCGATGGAT
GAGTGGAAGCAGGTAGAAATTCTCTGCATATACTTGAAGTCTTTCTTTGATGCGGCTAAC
ATCTTAACTTCTCCAACATACCCAACTGCTAGTGCCTTTTACCATGAAGTGTCAAAAATT
CAATTAGAGTTGACACAAGCAGCCATGAGTGAAGACCCCTTTGTCAGTTACTTGACCAGA
CCTTTGAAAGAAAAGTTCAATGAATATTGGAGAGAATGCTTTCTTGTTTTGGCGGTTGCT
GTTATTATGGATCCAAGGTTCAAAATGAAGCTCATTGAGTTCAGTTTCTCCAGGATATAT
GGTGAGGATGCTGATACCTGGATCAAGGTTGTTGATGATGGCATTCATGAACTCTTTGAT
GAATATCTTCTGCTTGCGCTTCCTCCTCCTGATGCTTTTATGGAAGAAGGGAATGAGGTC
CTCCCAAAAAATGAAACTCTTCAAGAACAAATCCCCCAAGAAGACACCACCATAGAAGAG
ATCCCTCAAGAAGGAACCTATCAGGACGGGCATCCTCAAGAAGGTAACTATCAAGAAAAT
CCCCAAGAGGGGCACTATGAAGGAGGAAATCCTCAAGAAGGGCACTATCAAGGAGGAAAT
CCTCAAGAAGCGACCTATCAAGGAGGAAATCCTCAAGAAGGGACATATCAAGGAGGAAAT
CCTCAAGAAGGGATCTATCAGGGAGGAAATACTCAAGAAGGGATCTATCAAGAAGGAAAT
ACTCAAGACAGGATCTATCAAGAAAGAAATACTCAAGAAGGGATCTATCAAGAAGGAAAT
ACTCAAGAAGGGATCTATCAAGAAGGAAATACTCAAGATAGGATCTATCAAGAAGGGAAT
ACTCGAGAAGGAATCTATCAAGAGGGAAATACTCAGGAAGGGATCTATCAAGAAGGAAAT
GCTCAAGAACGAACCTATCAAGAAGGAAATGCTCAAGAGGGAAATACTCAGGAAGGGAAT
ACTCGAGAAGGA
>ntig_972_consensus
ATTCAGCATTAATTCAATTTCAGCATTCAGCAAGTCTCTTCAACAGAGAGCACACCAAGT
AGTCATTATGACACTTGGCTTCCCATGCAGGCAAAAGCTACAACTAACTGCCTAGACTAG
AATAAAGATCTTCAAGGGCAGCATACAAAGGCTCCGCAGCTTTGGGCCTGTCCACCATCC
CAAGTAGAATGTCGGCAGTTGTAAGATAAGGATCACCATAAAACTGTAAATTCATATCCA
TCCTCGTCGCAACAATGTTCCCTTCTAGTGACACTCCCACAAATGCACCTTTGCTACAAC
TATAAGTATAGCACATGCCAGAACCTCTGTCTCCAGCCCGAATATCTGCTTCCAGCACTC
TCCCGATCGGCCCTGCTGCAGCACTGCAACCAGCACCAAGAGAAAAATGCACACGACTAC
AAAATGTCTTCACAGCTTTTGAACCATGAAGAACAATTATGAAGTCCATGAGTTCACCTC
CAATCTGAGCACCCCATCCTAAACCAGCTGAAAATATGGCTGATGGAGCAGACCAGGAGC
CATCTGACCTTCTAGCAACAACCAAACCAGTGCCAAGTTTGTAAGAAACTAGCACACCGG
CTTTAGCAACTGTTAAGATTGCAAGGCCTTTGGCTCCTTTCAGAACAGCTAAGGGTATGG
ACCTCTCAGAACTCAATGCAGCAGCCTGGCAGTAGCTTCTTAATGTATTACAAGCTTTGT
ATATCTCCTGTTCCATTGACAAACCAACTGGAAGGTTAAGCCATCCCC
>ntig_973_consensus
CCCGTCTCAAAATAAACCATTGAGACCACCTATCAAATCAAATGTCCTAAACAATAAGTT
AAACAAGCAAATCAAGAACACTGTAATTTCTTAAATTGACTGGTCTTAAAATAACTCCAT
CATCATTTAAATAAGCTAAAAACTAACCATCATAACTGAAATGTTTGATCTCAAATTAGC
TAAATGAAACTTCCAAGAATTCCAACAAGCTCTATAATAGTAAAACCATATCCAACTACA
AACCAAAAACTTGCAATAATTGAATAAAAGCACTAATGACCAAAGCTAAAAAGCCTTCAT
ACAAAAAAACATATATTCTGATCTTAGTCGAAGAGGCTAAAACCCATATCATCATCAGAC
TCTTCTTTCTCTTCCACCTTCTCTTCTTTCTTTGCCTCGGCAGCAGGGGCTGCAGCAGTA
GCACCTCCCACTGCACCTGCTGAGGCAGCAACTGCAACACCACCACCAGAAGGCACTGAT
GCCAACTTCTCCCTTCCGGATGCAATTAGCTCAGTGATATCCTTGCCCCGTACTTCAGAC

AATAGCAACTCTATCCTGTCATCGTCAGCATCGGCTCCAACTGATCCGAGAATACCCTTC
AAATCACTAGCTGATGGGCTTGTGTTGCCTCCCAAAACGGCAAGCAAATATGCGGCGACG
ACCTTCATTTTTGGAATTGGAGAAACTAGTTGAGAAGATTGGCGTTGAAGGCGAGCGCTT
CGCGTTGCTCTAGTCTAGGGTTTGTTAAGGACTCACAGGAAGCAGGGCTTATATATAGAG
CTAGGGTTTTTGGTTTGTTAAGA
>ntig_974_consensus
TTCAGCCTTTGCCGTCGATACCAGTCTTCTCATCTCGCCCCACCACTCTCAAAAATGAAG
GTCATCGCCGCATATTTGCTTGCTGTTTTGGGAGGCAACACCTGCCCAACGGCTAGTGAT
TTGAAGCATATTCTTGGATCAGTTGGAGCCGATGCTGATGATGACAAGCTAGAGTTGCTG
TTGTCTGAAGTTAAGGGCAAGGATATCACTGAGCTAATTGCATCCGGAAGGGAGAAGTTG
GCATCTGTGCCTTCTGGTGGTGGTGTNNNNNNNNNNNNNNNNNNNNNNNNNNNNACTAAGA
TCAGAATACTGGTTTTTGCTGTATGAAGGCTTTTTATCTTTGGTCATTAATGCTTTTATT
TAAATGTTACAAGTTTTTGGTCTGTAGTTGGATATGGTTTTACCATTTTAGAGGTTGTTG
TAACTCTTGGAAGTTTGGTTTAGCTGATGAGTTATGGTCAAACATTTCAGTTATAATTGT
TACTTTTAAGCATATCTAAATGATGATGGAATTTTTTAAGCCAGCCAGTGTTGATAATTG
CTTTCACAGTGGC
>ntig_975_consensus
CCACCAAACAGTTCAACTAGTATATAGTACATTCACTGAACATAGAGGCTACACATTTAA
TGCAGAAGAAATGCTAAGAAGCCCACTTAACCTGAAATTCTGCTCATTGTCGCAACCTCA
ACTCATCCACCTAAAGCAGAAGAAAGAGCTGTTCTTTGGATTCCAGCTGCTTTCTCATTG
CCACTCCCTCGACGATTGCCAGGGTTGTGAAGAGCAAGTAGTTCCAACTCATCTTCAAGC
CATTTCACAGGACTGACAACAAGGGGAGGAGTTCCTTGAGCATCTGTTTAACAGCAGAAG
ACCACAGTTAATTCCACAAGTAAGGTCCACCAACTGCTTGAAGGGGTAATGCACCCATG
>ntig_976_consensus
CCCAAAACAGGATAAAAATGGAAGATGGAAACCCTGGGATTTTGCTCAGAGGGGCGCTGG
GAAGAAACACTAGAGTTGTAATATATAAACTCGAAGTCCTTGCTTGCTTAGCTATGAGCC
AACCACTGCGAACATGCAAAGTAGAACAAAGCTTAAAAACACACTGGATTCTATGTGTAA
TCAGTATTACAATCACAACCATGTACAAAATCAACAAACAAATAAGGCTTTCATGGCAGC
AACCAGTTGATCCCCGTGTGACAACCTTATGAGGCTGTTCTCCATTGCAACTCGATCTGG
ATTCGCCCATTCTTTGAGTCGATAAGATGGTACCTCTCATTGATCCGTTTGTTGCTAACA
ACATCTGAAAGATTGATGTCCACGTAACCTAGAGTTTCCTTTGGATGCAGCAGTCCAATC
CTTGATGAGGTGCTGACAACTTCCACATGTAGTCTATCATTAGTTGGAGGCTCCTGTAGC
ATGAATGTGAACTCTTCCTCCCATCTTGGATCTCTGTTCTTCTTCAGATACTTAGTTTTT
CTCTCCTCCCCTCGAAAAAGAAGTCGTACATAAGGATTAGTGTGGTGCTTTCCTTCAACA
TCTTGAGCTTCGTGGACTATAACTACAAGCAAACCTCCACCAGGAGGAGTATTATCAGGA
GCTCTTCCCACTGTATGTGATTCTTCAAAACTTGTAGGCATTTCCTCCTCTTTAAATGGT
TTATATGCCAATTCCACTACAAGCTGTCCACGTGACTTCTCATTTTGTGCATCATTTAAG
TTCATGTTTTTGAGAAGATCAAGAGTCATAACTTTTGGCTCTTCAGGAGTAAGTTCTTTT
AGAGGAATGACATTCATACCCATCTTTTCATGTTTGCCGATCTGCTCCCAATCATACACA
ACAAGCTCTAAAGCCTGAGACTCTGGGTCTTTGATGACAAAATTAAATTCCTGATTCCAT
TCAGGGTTCAAGTTCTTGTGCTTCACAGCAGATTTTTTTGATGGAAGCTTATCCTCAGTG
AGCTTTAGTTTCACATAAGGGTCTGATGCACCCAGAAGATCTTTCTTCCTTAGATTCATT
GCCCTTATAAGCTTCACATGGAGAATTCCAACAGGTCTCCTGTCGGCTTTAGCTGGATCC
AAGATTGGAATTTGTAGGGTTTTGGGCCACAGATACATGTTGGCCACTTGATCTTTAATA
CGCTCCTGGACAAACTTGTAGAGACCAGGTATAGACATAAGATCAGCCCCTATAAGCTTT
AGTCCAAAGTCAACATGAGGCTTCTCCATGAGAGAAACATAGATTTTTGCAAAGCAAGGA
AAGCTAGGAACCAAAGGCTTCAGAGTGATACGTGGTGCAACAAAAACTTGTAAATCAACC
ACCTGAACAGTTGCCTTCAACCCAAATGCTTTAACAGCAACAATGACATTGGGATTTCCA
GCCCATTTTAGACATGGTTCCATGATCAATTCCTTCTCATCAGTGAGATAAACTTTCATA
CACACGATCATAGTCTGGATTTTTCACCCAAAGTGGTATCTCTGGAAGCATCCGCTGCAA
AGAATCTGTATCTTGCTCAACCAACGGGCGAATTTTAGGATTCTTGACATCAGTAGGCTG
AAAGTAAATGAAAAAGAAGTAACCAATCACAAGTCCAGCGGAAACGCCGACGCCAAAGCC
GCAAAAACCTAACACTGTACTTACAATTCCCATCCTTCACTTTCTCTCTTAATTTTCTCG
GCAAGCAAACGGCCCCTTCTCAATTTTTATCTTCTCCAATCAC
>ntig_977_consensus
CGCCAGTATAAGAATGTAGTGAACAAATACCTGTTCACCCTCTAACTTAAAAAACTTGAT
GAAACCAAAAAGAAAGATCTATCAACATAAAAATATTATCTCAATAAGAATTTTTCAAAA
GTTGATATCTTATTAATTTGCTTGCAAGAGTAAAACTCAAATACCCCAACCACAAATTCA

CCTGTATGCTGATATTGGAAACTGTTTTATCCTTGCCAAACATTTAATATGGGAAAAGGC
AGCCTTGCTCCAGAGTATTAAATTTCAAGTTAAATGAAAAAGGGAATTATAGTATCAAGA
GTGTAAAAAGAAGAAACTAATGTTATTATGTGTAAACCGATTTTTCACTACATTTTTATT
TCGTTCAGAAGGCTTTGCACGAAGAGATGATAGACCAACTCTGAAACTTTGGTCGAAAAA
TCATTACCATACCTCAGGACGGAGTTCACCGCTTGCTGGGGGCATCCATTTACCTGAGTG
GTAGACACTTTCACCAACCTTCCAGCCAGGCACATCCTTCATTACTTCAGCTTCATACTC
AAGGTATTTCTTCCACTCTTTGACAAATCTTTCATCTTCTTCAGCTTGAAGCATGGGCAA
TATTGCCCTGCGAGCAGCATACTTTTCTTCCTTAAGTGCCCTTCGGATCTTGTTGCCCTG
GCCCACCTGATACATGCCATAAGAGAAAGCGCCAAAAGCAGTAAGAAACATGGCCATGGC
GCTGGGACCCTTACTGGGTATACGACGGGCGTATCGGGCCGGAGGGAACCCGCCTGGAGG
CGGCCCATCTTGAAGGATAGGCATGTCTTTCACGGTAGCCATTCC
>ntig_978_consensus
AATTCCATACTAGTTTGGTTTCCTGTACATAAAACGTAGAACAAAACCACAAATTCCAAA
TAAATAAAAACAAATTAACGTTTTTCTACGATGGTTGTTATCATGTATATATTAAATGCC
GTCTACATGTAATGCTCTAAACTTGTAGAAAAAGGACTACCAAAAGAACTCCCTGCTCAA
ACATAAATCACAAATAACATTTTCCTACTTGCCACGCCAAATTTTGTCTTTGCAAAAGAT
AAACAAAATATCCATGACCAATCACAAAATCACAGGTGTCAGAATTACCAAGCTTAGGAT
CAGTTATCATGCAAAGCCTTCATCAAATATAGTCTGTTGAAGCTCTGTCCAGTGACCCCA
TTGATTTGGTTGCAGTAGTTTGCAATTCGATTTGTTACAAGAAAGCTTTCTAAGCGGGAT
GGCTCAGGGATTGGCTTAAAGATGGGATTTGAAGGATCTTCCTCAGGTAAAGGTTCTTCT
CCAGCAGCTTTACGTGCCTTATTCTCATCCCTTCTCTTTTGAAGCCATGATTGCTGCTGG
GCTTGTTGGCGTGTCAGGTTCCGGTAGTAATATTGAAACTTCTGTTGTTCCGCTGATAAA
TCATCCATGCATTCAATCAAAAATTCCACATTCCTTGCCAAAAAAGGATTGCTTGATAAT
CGCAGGCGGTCATAATCACACTGGGTAACTGGTGTATCAGCTTCCAACTCAGTCATAAAA
GCACTGACAAGTGCAGAATTTGAGACTTTTATAGGTATTTCCTCAAAAATATCCACCCAT
GACAAGTTTTTCTCTCTCAATTTCTCTCCTGTAAAATTGTTATTGCGGTAAAGATCCATG
AAAGAATCAGAAAGCTTCAAAGCCTTGAGAGCTAAGGCACCTTGATTTGACCTTGAAGGA
TCATAAATTATGCACACACAACGTCTAATATTCTCCTGGTAATTCATAAATGTCTCAATC
AGTTCCACTGTCTGAAAACAGCCAAGCAGTGTCGATTGGTACCATCCGACAGTGTTGTTA
TCAACGTTAACCTCCCTCAAACACCTCATCATATCAAGCTGGTAATTAGCACCATCAGCC
TCAATCTCCTCATCCTCTTCCTGGATCGGGAAGGGGAAACAGTTAGTAACTTCAAGAACG
CTACCAACATCCAATCCTAGAAGCTGACCAGTCACCAAAGCTGGAGAAAACTCCTTGCAG
TGTTTAATTATCTTCAATACAACCAGTCCCTCGATTTGAACAACTCTTAGAGGAGTAGCA
ACTTCTTCCGTTGCCGCTACTTGAAGAAACGACCTTGCCATGGAATTAGCCATTATTATG
AAAATTTAATACTATTCTGGAAACTCTCTGCTCCTGTGTAGTGCCTGCGTCTGGAAAAAC
CCTAGTATTTTTGTAGGTCTTTCCGACTATCACTTTTTTTTTTTTTTTCTCCCTGATGTT
TATTATTAAATTTTTT
>ntig_979_consensus
CAGGGAGAAAGCAGAATTAGAAAGGGGAAAGCCCTATGATTTTGAATAGAGATTTCCAAA
AAGAAAAACATTTCGTAATCAAAGGGTAAGATCAAGTTGTCCTAGTAATAATAGAGGCAT
CCCAACAGTAACAGGGTAACTTCAGTTCAGTAGTCCTTTGCGAGTCATTAGCAGCTCCTC
CCAATGACATTAATATACACAAAAAGCAGAGCTGTTCTCAAATTTACATGCTTGTTTGCT
TCCTTCCAAAAAAGGCCCAACTCAAACAAGGACAACAGCTTCAGCTATCATCGCTAAAAC
TTCACAAATATTAGGTGTGTGTGAAAGAAAGAGAGAGAAAATGCTAGCCTCAAAGTATTG
CATCATGACTCCTGCGCTATCTGATCAATAAGCTCTTTATACCCTTAATTTGGTTCATGC
AGTATACAGATTCCCACCTCAGGATGATGACCTAGACGATGCAGATTCACCTAGGGAGAC
AGAAGCCAGGGAAGCCCAGACACGTGCATTCTTTGCACCTTCATGGAAGTTCCTGGTCTT
GCAGAGTCTTAATTTGGTCAAGACCCTCCTCACATCGATCATGTGCAGGGCACGGCCAAT
TATTTGGCTTGTCTTGCCAAATATTTCCAAGTTTAAGTGAGGAACATATGATTCCCAGAA
CGCTTTTAGATGAGATAAGACTTCTGGGAGTATGGATAAAAGCAACCTCAAATAGAGGAA
GAATACTGCAAATGCCAAGTAGGGTTCATTCTGGATCAATTTCCTCAGTGATGACCAGTT
CCAGGACTTCATGATTAATATGTCGCTACCACCCAAGCTGGTTGATTCATCTTCAGCAAA
ATATTCCACCGGTTCAAGTCCTGTTGTTTTCTGATAAAATTGTACGAGGGAGTGGAGATC
TTTTGGACCATGATATTGCAACCTAGATGTTTGATTCACCAATAAAATTGAGGGAAGACT
ATGGATTCCATATCTTGAAAATACGCTTGGCAGAGCTGAAGATTGTTCAACGGCAAAATG
TTCCATCTGTGGGAACATGGAACTAAGAACTTCAAATGTAGGATGCATACTGCGAGAAAA
TGGGCACCAGGAAGCATAAAAAAGCACTGAAGCGTAAGTGTTTCTCTGTTTGGAGCTCAA
AGCTCTGTCCAGGAACTCTCCATCCACCCGAATCGGAGGATTTGGCGAGATCCCGAGAGA

GCACTGCAATTGAAGGCTATGTCTAAACACATCTAATTGTAGGGGGCACAGAGGCGCCGA
TGACACGCAGCGTATTGTAAAAACAACACCACTAACTAAGAAAAGAAGACGGAAAAAACC
AGAGCAAGGAGAAACCATGGAAAAGGGTACAAAAAGTGAATTCACAACACGACCAAGGAC
AAAATGGGTAACAAAAAAAATAGAAGATCAGAATCACAAAAATTGATGAGGAACGAAAAA
ACCCAGGAACAATTGAGATCTTATTTAATGGGTTTGATTGTTTAGAGCGCTCTATCTCTC
TCTCTCTCTCTCTCTCTCTCTCTCTCTCTCTCTCTCTCCCTTGTTGTGCCGATGATAGATTA
ATTATCAGATATCAAT
>ntig_980_consensus
AGGATTAGGAAGATTTATGGAAGGCAAGAAAAACACGTTCAACATTCCTAAACCAGACAA
GCAAATATCACACTTGAACTAAAGCATTGAGTGAAAAAGAAAATAGTAGAACACATCAAA
TGCAACATTATAATTCACACATCTCAGTTATATACTGCATTAATCATGCCCAATGAAATG
CTAAGTGAGTCTGGCACTGCACTTCATTCATAGCTACATAATGCTTTTTCTTTGTATAAG
CTGTATAGCCAGGCACACAGGCCAGGCAGTTAAATAAAAAGGCAACTAGGGAATGAGTAC
TCAGGATTAGCTTTTTGAATGAACAGCAATTTGGTGGATGTCCATGTCCTTCAACATAAC
AGCACGCCCACAAGAATCACAAGGAGCTGTTCTTGATCCACATATGCTCTCATGTTCAGA
TAGTCCTCTTAGTCGGTCCCGCGCATCCAAAGGCAAGCTTCCAGCCTGGACCATATCCCC
ACAAAACCGGCAAGTAATTAAGCGTAGGGGGCAAGTTGAGGCTTGGTGTTGTACCATTGC
AGCATTCTCAAGGACTTTTCCACAGGGGCAAGTACGTGGCTCATGAAACACTTTCATGTG
CTTCTCCAATTCTCCTTGCTGAAGACCTTCCCCACATTTTTCACAATGAACATGGGTTTT
GGCCTCCTCAATTCTCAGTACAACCCCACAACCAGCATGCTGACAAGCCACACTATGTCT
ACTACAATAGGCTTCATGCAACACAATACTCTGGGTGGGTATAAAATGGTTGCAGTTCCT
ACACTTCACAGTATCCATCTCCATAGATGACAAAGACGATGTTGCTTGTTGACCCACTTT
ACGGCCACTATTATCCTCAATTGTCATCAATACGTGGAACTTTGTTGTATCCTTGAAGCC
ACATACACCAATGCTATATGTACCAACCCCAAAGTTCTTATCCTTAGAGCTGAGAATCAG
AACTTTAGAACTCACATCATGAGAAGACCATTCATGCTGGTGAAGGTTTGGGTATATCAG
CGGATGCTTAGATACATAAAGATTAATGTCTCCTCGATCTTTCTCTGTGTCAATTCTTAC
CTCAACTCTTTTCTCTCCAGAAACTATTTTTTCCCAGGTATCCTCGCCAATTGAGAACTT
GTAATACACATAGTTTCCTTCATCAACCACTCCAGATTCTGACTTTCCAAATACAAGTGG
CTTCAGTACAAATCGATTATCCCCCTCTGATTTCACATCAGGATTTTCTATATCAACCTC
AATATCAGTTTCTAGAACAGATATGCTTGAAGAGGGCTTCAACTCAAGAACTTGTAACTT
ATATTCCAGAGCTCCATAATTGACAGTAAGGACATCACCCTGAGAAAGAGTGGCATGTTG
GCGAAGGCTTGTTTCCAGGACAGCCTTTTGGTTGGGTAATTCTGAAAACCCAAGCCCATC
AGCCTGAAGTTTGGCATAAGTTCCTTTTGGTAGTCGCACATAACGAACTTCAACTAATGA
AGCCTTTGGAGTCATAGTGGGGAATAAGTTGCTCCATACATGAGAAGGAAGCCCAACAAA
ACCTTCATCTGCAGTGAACTCCAAAACACCAGAATGGGTGCTTCTCTGAATTTCACCATC
CTTCGTGTTTGATGTTTCCTCTTGATGAACTACTGATAATGTGAAATGCAGAGGACCCTT
ATCGAAAGCACCTTGACCAGACAATTCAGTGAAACATGAGGGTGGTAATTTGATCTTATC
TCCACTACCTTGGTAAGGGACAGCTTCTAAAATACGATAAAATGCAATTCCATTTCCAGC
AAGCAAATTTTCTTGCAGCTCTTGTTCGACCTTCTGTTGGGCATGAATGGCATCGAGTCT
TCGAGATCTCTGAACCGCTTCAATTGCATCACGTTGTTTCTTAGCCTCTTCTTTAGCCTT
GCGTTCTCTTTCAAGTCTCAATCTTGCTTTTTCTTTCCTCTCCTTCTGTTCTTTCTCAAG
CTTTTCTCTTGCTCTTCTCAGCTCAAAATCCATCTTTTCTCTATGAAAAAAAGATATATC
AGGGTATAAATTTTCGATGAGATGATAGGGTTGGTGTTGTTTGAAATGGGATTTTAACCC
TGTTTTTGGAGTTTGAAAGGTGATGAAGAAGACTCAAACTTCGCGTTGGCAATCCAACTC
GTTTACCGAGATCGGAAGAGCACCACGTCTGAAC
>ntig_981_consensus
CCAAAGCACTGAAAGGAATTTGTTGCCGTCTTGTATTTTTATGTTCATTTCCTGATGGTA
AAGAAGCATCTATGGAAATATCAGTAAACGCCCTTGTATCATCATCAGCCTGACAGGCTT
CTTCATCAGAAGCAAAAGAAGGTGCCCCATCTCCCTTGTCAACTCCAAGAATTGTTTCAT
TCTTTTCCTTGGGGCTATGATCAAACTGA
>ntig_982_consensus
GCATCTATGGAAATCTCAGTAAACGCCCTTGTATCATCATCAGCCTGACAGCCTTCTTCA
TCAGAAGCAAAAGAAGGTGCCCCATCTCCCTTGTCAACTCCAAGAATTGTGTCATTCTTT
CCCTTGGGGCTATGATCAAACTGATCATGCTTCTCTGAGAGCACAGGAGAAGATGCTCTG
CTGTTTGTGACCGCAGCATCTTCAAGAGATCCTGCACTGGCACTTTGGGCTGGACTGCGA
CCTCGATTTGGGATCTTAACTATTAGCTTGTGATTATTCCCCTCAATAACAGATACATCA
ATTGCTTTTTCACTCGTTAAACTCAACTGAGATGGTCTTTCTGAAGCTTGATTCCTGTGC
TGAGAAGTTCTGTTTGATCCAGTTTCTCTTTGAACCCCGGAAAGATTTGAACAAGGGAAA

CCATTAACAGATTTTCGAGACCGCAAAGAACCAGGTGATGCCTTATTTGCAGTCACTGAA
ACAGAGGTAGAGCTCCTAGTATCCTCTTTCCCGCAGAATGCCCCAATTTTGGCATGGTCA
ACAGTACAAGAATGACTATTGTTGTGGGAATGACCAGCACTGCTGCTTTTCTCATCCTTT
GCTGGAGTTGCAGGTAGGTCAGTTGCACCAACACCAGCAGAATTGCCAGATTGTCCATCT
TTTAGGTTAGTGCCCACTGATGTAGGTGATGGTGCTGACTTCACAGACACTGGAGACGCA
AATGCTGACTTTGTAGCGGTCTCCCCTGGTACAAGCTTGACTGAAGAAGTTTTAGAAGCA
GTAGGCTGTATCACCAAGCTCTTTATAGCAACCTCAGAAGATACACCTGACTGTCTGTTC
CCACCAGGAGTAACCTCGGGAAGCCTGGGTCTAGCTGGCCAGGAGACAGCCTGATTTGGG
TTTGCATCCATTTCAGCCTCAACACCTTTCTTCCAGGTGTCAACTAAACTCCTTGCTTTC
TTCTGAATTTCCAAATTCTTGTGCGTACGCAAATGATTCACAGACTTACCAATGTTACAC
ATTTTCAGTGCATGAAGATTTATAGGCAGCTTATCAAGAGCGTGAAGTAAAATTAAGAGA
AACTCCTCAATTGATTTATCACCATCCTTAGGGCTGTTACCATCACCAATCTTTCCTTTG
TGGACCTCTTGGAGCCATTCATCATACACAGGCAAACCTTCAAGCTGCACAAACCAAT
>ntig_983_consensus
CTTCTCAAGCTTGTACTTGATTCTCTCCTCAGCATTTGCAAACTTCTGCTTCTTCTTCCC
TTTAACATTAAGCCGACGTGGGTCCTTCTTGTTGGCTGCTTTTCGCTTCTCTTTCTCCCT
CTTTCTCTTTAACTTCAACCTCTTTTTAGTCATCCACTTCACCTTCTTCGGAGGGTCAAT
TTCATGAGTTTCAAATTTAGGTTTTCCTTCTGAAATCACAAGACTCACTGTTCCATGACT
CAAATTTCTTATTACACATGGAGGGTTAGTTTGTCTTAGCGGAATTAAGTGAGTATGTGA
ACACAAAATAGACTCTTTAGCTGGAAAAACCGACCTGAGGGAGAAAGGGTCCAGCAAGGA
TCGGAAAAGTGAGGAGGCTCTTCGAAAGTTGCAAAATAACGTTTTTGCCATTGGAACAAA
AGATAATAACGGAGAAATTAAACGCTGCCTGAGAAAGGTGTGAG
>ntig_984_consensus
TTGACAGCATAGAGCTTGTGCTAATGAAGAGAAAACTTGGGACAATTGAACCATGCTGAG
TTATTCATCTCTAGTTGAAACTTCTCGCACACCTTCTGTGTCAGGATTTTCCAGAATCTT
TCCCATTTGTCCATTGTGTTCTTTAAGCGGACACTCTTTTTTCCTTTAAAGAGATACCTT
ACGAAATCGTTTGTTAACCGAACCAGCTCAACACCATCAATGAAGCAAATGTACACGAGT
ATGACGAAAATTGTTGCTAATATCTGTTTTCCAGTTTCATCACCTGAATCTCCAGAACCA
TTTCCACCACCGTTGCCGCCACTGCCACCACCACCACGTGGTGGACTCTTGCCACCACTT
CCATTATAGTATTCTTTCTTCTCAATCTGCTGCCGAAGCAAATCCTCAATCGACTCTGCC
TTCCAATTTCCCATATTTTTTCCAAAAGGATCCCATGAATGTTTCTCGTCTTCTGACTTG
TCTTTGCCACCCAGAACGCAAACAACCCCTCCTGGTTGCCGCGCTTTCAACCGAGGTACG
AATAGTGGGTACGACGTCGTCCAGTTCAGCTTCACCAACGCCCTACTTGGCGTGCACGGC
ACGCTCTTTAAACCAGCCCTGTTCGCAAAAACCCCACCAGACTTGCAAAACCCAGTTGCC
TGAATGCAACTCATCTTTCAACAATAAAAATAAAAAATGAAATGGAATCTATGTGTTGAA
ATTGATGAGCGTAATCATCAAAGTTATTCTCTTTATTTTTTGTTTTGATTTTAATTTGAA
ACCCTAGAAATTTCTCGTTTTCTTTTTTGATCGAGCGGGGAGGAAGTGAAGGGCAAAAGT
TAGGAGTCTTAATTCAAAATCAAAACAAAAAATAAAGAG
>ntig_985_consensus
CCAATCCAAAATTTCTAGTCTTTCTCAAAATAACACTAAAAAGTTCCCGACAAATCAAGA
TCCTTTCCAATCCAAACTAAAACTTGAACAGCATTTCCTTCTCAAAAGCCAAATCTGCAA
TGGTTTTGCGAGTACGGTTTCCAGGATCTGGATAAGATAGTGCACCACGCGTTATGAGGT
ACCAAACTGTCTCCTGTCTGCAACAATATGCTGCCCAATGTAATGCAGTCCATCCATTCA
CATCAGGGTAATTGATGTCTACTCCTGCCACAACCATGGCTTTTAGGGCCCAATGATAAC
CAAGAGCAGCTGCAAAATGTATCACACCTTGACCTCCTTCATCTAACATAGTAAGCCCAT
TTCCACCTTCAACTACCTTCTGTAGGAGCCACAAATGTAACTTCTCCTTCAAAATCTTCT
CCTTCACCATGTCTGATGAAATTTTCTCCTCCAAACTTGGTATTGATATCTTTCCCCAAT
CATGACCTTCGTCGTCTAGTGACTCTTTTATTTTATGGTACAATACTGGTAATTTTCTTA
TATTGTGTTCAGAATAATGTGGACACACATAACTCAGAGACAACAGATTTTCAAATCTTC
TAAGGAAACTTTCAGTTGTAATGTTGCCAAAATCAGATGCACGATCCTGCCGCTCTGTAT
TCTGTTGGGAAATCGTATCACATTCACCAATGCTACTAGCAGGATTCTCTCCCGATATGT
CCTTTTCTCGTTCACATCTTTTCCTACTTCTCTGATAATTCCGGAAATGAAAGAATTAGA
TCCAAAA
>ntig_986_consensus
CTAGTTGGAGGTGCTGAGTCCTCGTCATCTGACTGGACATCACCATATTGCCACATCCCC
AGCCTTCGAGTCTTGGCTTCTTCCTGGAACTTTTCCAGATTTTCAAGAGCAGCCTGCCGA
TCCTTGGGCTCCCATTTCTTCCTTCTTTCTACCCGAGCAAGTCCTTCCTGTAGCATAAGA
GCATTTATGCTTATCTCAGCATCCACAGCAACAAGAGTTACGATAAGACTCGTCCCAGTT

CCCTGTCCCTTAATTTTTCCTCCAGAAGTATCCCTCTCCTCTACTTTGGCTCTAAATTCT
TTTGAACTGCTTAATGTGTGCTCACTCAAAAATTCTGCAGCTTCAGGGCCATACTCATCC
TCCAAGCCAGGGACCTTAACGTAAGCAAGGCTGCATAGCTGAGCCAGACCAGGTGCCACA
GATAGCGAAGGATCAATATGCCTTAGGTTACTGTATGGAACAACTTCTTGATTTCCATAA
TCGATGTAGAACACTTCAAACTTATCATTTGAAGATTCAACATTTCCTCGAGGCGCATTG
ACAATCATAGCTCGATTCCAGGAATTATCTGCACTAAATTGAGCAAGGACAATGTCACCC
TTCTTAGGATTAAAAGCACCGATAACAGGAGCTTCTTGAAGATTAAATGAAGCAAGTTGT
TGCTGAATTGATGCAACTTTCTGATCCCCAACTTGCTGGACATAAAATTTACCACCACCC
AGAACTTCTGTAACTACTACCTTCAGTACTTCTTTCTGTTTACTTTCAACAGCTGAACCA
TTGGTGGAGGCCACTTCCTCCCCTTCAACATAATTCTCCCAAATTTTCAGTTTCTGAGAT
TTTGCAGATCGCTCTGCCTGTTCAAGAAGATGAGCCTCTGGAATTCTATCACTGCCAAAA
GAAGTTTGCAGCTTTGCCAGGCCAGCTTCAAGAAGTGTTGCTGCCATATTGGTCCTTGAC
TCCCACAAGGATCCCAAGAAAGTTCCAGTTCTATCAACTGTTTCCACTTCAATCTCCACA
TCTCTCTGTAAAATCTTGCGTCTCATAAGAGCAATTGCTTCTTCAGAATAGGGCTCACCA
CGACCAGGGCAACGGACACCAGAAAATGAAAAGGCAATGCTGCATGTTTCCTTGGGAATC
AACAACTTAAATCGATGACCACTAAGAACATATTCAACAATGGCAGGAATTCTCCTGCTC
CGGTGGAGGAATGGCAAGAAATCTCTAGCTTTCTTGACTGATGCCACTGTCAAGTCTGTT
ATGTGCATGACTGGGGGATCCTTGGAAGAGTGTACCCCTTTCTTCCCAGCACTAGCACGT
GACTCAGCAGCAAGAAGGGCATCATAGTAATTTGATCTTTCCTCAAAATCTCGATGCTTA
ATTACGTTCCCAAAGCCACGAGATACCACAAGCTCAGCTACATTGATACCAGTTGGCTGG
CCAGCAGACTTTGATGGAGCCACTGAAGATGTATCATCACCCTCTCCCTTAACAGAAGAT
TGGAGAAAGACTGATCCAAAATCCATTATTCTTGAATCTGAAGTTGTACTTCCATCTGCC
ATGGGGACCTTCCTGGAATACTCCATTTGAACATTCACTTGTTTGCCGATGAGTCGTGTT
CTTAAAAAATCTCTTGCCTCCTTGGCATAGGGGGCTGGCTTTTCATCTTTACGTGGATTA
CCAATCTTTGGACACCTAATACTTGAAAGATTGACTCGTCGCTCGGCCAATGGACTACCA
TATGGGACAGAATCATCAGCCACAATAATGCAGTCCCCACTGACAACCTCCACAACCTTC
CCAGTGAAGTTCTGGCCATGAATTGCTTTTGAATTTGTTGCTGGTGGTACATAGTTCGTC
CAGATCCTCAACCGGCTTTTCTTGGCTTGAAGCTCTGCATTCTTTAGTCTACGCTTAGCA
TCTTCTTCCATCAAATTCGCACTCCATTCAACATATTTAGCTAAGCCATTTTCTACAAGC
TCCAGCGCTAGGTCCTTAGCTGTTTCTCCGTCAGAATAATACACTGAGCCAATCAAATTA
TTGAATTTGTCAACGCCTTCGAGGACAATGCGGACCTCTCTGTTCAAAACACGAAGCTCC
GTAAAATGTTTGGCATCCACTGTATATGGCTCATCAGTAGTTGCTCCTGTTGAGGCTGCG
AGTCTCTGAGCAGAATTCAGTGGACCAAGAGATCCACTTGAAATATCTTCATTGATCTCA
TCGGAGGTGGTATCTACTTCACTAGCAGCTGCAGGCTTCCGACCAACTGCTGGGGCCTGG
ATTCCCGCTACAAGGACACGGACAAATTGAAATTCTGGAAGCAGGTAAACCTGTATAGTG
CTGCCATCACGAACCTGTTCAACCATACATTCCATGGGTCTACCCTTATTTGCAGCTAAC
AGACCCATTGCATCAAAATTACCAGAATCACCAATGGCAGAGGGTGGTAATTTCCTTATG
GAAGCCTCAGCAGCACCTGGAACCTTGGTCCAGCGGCCAACACCTTGTTGTTTTGCTTGC
TCTTCTAGACGTAGCAATTCAGCAAGGAAAGGACTTGCTTCACCTTTCTGGGAACCCTGC
TCCTTGACCTTTGCCCAGCCTTCAGCAACAACCAACATTGCCACATTTTTATCATCAAGG
AAAACAGTGCCAAACTCTCTGCCTATACTTGCTACAGTATAATCGACTCTAAAAGTCACC
TCCTTTCCTATGCAAAGCTTCCTCAAAAATTCTCTGCTGTCCCAAGCAAATGGCTCATCT
ATACCTCCTCTACGAGCCAGTCGAGGAGCAATAATAGATGATAACGTGAGGGTTTTATCA
CGCGGAGGTCCAGCCTTGAGGGCAGACAAGGCCGTAACTACCAAACTGTCCCCCGATGTA
ACCGCTTTCACTTTTCCTCTGTACCATTGTCCCCCTGCTGCTGGCGCCGCCATCTCACAC
TTATAGAGAACGGGCGGCGGA
>ntig_987_consensus
AAATAAAATTAAGACAAAATTTAAACAAGTTTCAGAGGAGAATTCCGTCATACCTATAAC
TTATCTTACGCCTACACCTCAACGCAATTTAGGCCACTACTTGACTTATATCTCTAGTTT
ATCAAGGCCTCGTCTGGTCTCTACTCATCGTCGACCACCAGCCTTTCTAGCTGGAGGTGC
TGAGTCCTCATCATCTGACTGGATATCTCCATACTGCCAAATCCCGAGCCTTCCAGTCTT
GGCTTCTTCCTGGAACTTCTCCAAATTTTCCAGGACGGCCTGTCTATCCCTGGGCTCCCA
TTTCTTCCTCCTCTCTACCCTAGCCAGTCCTTCCTGCAACATAAGAGCATTTATGCTGAT
CTCAGCATCCACAGCAACCAAAGTCACGACAAGAATTGTCCCAGTTCCCTGTCCCTTAAC
TTTTCCTCCAGATGTATCCTTCTCCTCAACTTTGGCTCTAAATTCTTTTGAACTGATTAA
CGTGTGCTCACTCAAAAATTCAGCAGCTTCAGGGCCATACTCATCCTCCAAGCCGGGAAC
CTTAATGTAAGCAAGGCTGCATAGCTGAGCCAGACCAGGTGCCGCAGACAAAGAAGGTTC
AATAGGCCTTAGGTTACTGTATGGAATAACTTCTTGATTTCCATAATCGATATAGAACAC

TTCAAATTTCTCATTTGAAGATTCAACATTTCCCCGAGGTGCATTGACAATCATAGCTCG
ATTCCAGGAATTATCTGCACTAAATTGAGCAAGGACAACGTCACCCTTCTTTGGATTGAA
GGCACCAATAACAGGAGTATCTTGAAGATTTAATGAAGCAAGTTGCTGCTGAATTGAAGC
AACAGTCTGATCGCCAACCTGCTGGACATAAAATTTACCACCACCCAGAACTTCTGTAAC
CACCACCTTCAGTACTTCTTTCTGTTTGCTGTCATTGACTGAACCATTGGTGGAAGCCAC
TTCCTCCCCTTCAACATAATTCTCCCAAATTTTCAATTTCTGAGATTTTGCAGATCTCTC
AGCCTGTTCAAGAAGATGAATATCTGGAATTCTATCACTGCCAAAAGAAGTCTGCAGCTT
TGCCAGGCCAGCTTCAAGAATTGTTGCTGCCATATTGGTCCTTGACTCCCACAAAGATCC
CAAGAAAGTTCCAGTTCTATCAACTGTTTCCACTTCAATCTCCACATCTCTCTGTAAAAT
CTTGCGTCTCATTAATGCAATTGCTTCATCAGAATAGGGCTCACCACGACCAGGGCAACG
GACACCAGAGAATGAGAAAGCAATGCTGCATGTTTCCTTGGGAATCAACAACTTAAATCG
ATGGCCACTAAGGACATATTCAACAATTGCAGGAATTCTCCTGTTCCTCTGGAGGAATGG
CAAAAAATCTCTGGCTTTCTTAACTGATGCCGTAGTCAAGTCTGTTATGTGCATGACTGG
GGGATCCTTGGAAGAGTGAACCCCTTTCTTCCCAGCACTAGCACGTGACTCAGCAGCAAG
AAGGGCATCATAATAGTTTGATCTTTCCTCAAAATCTCGATGCTTGATTACGTTGCCAAA
GCCACGTGATACCACAAGCTCGGCAACATTAATTCCAGCTGGCTGACCAGCAGAATTGGA
TGGAGCCACTGAAGATGTATCATCACCCTCAGCCTTAGCAGAAGATTGGAGGAAGACTGA
GCCAAAATCCATTATTCTGGCATCTGAAGCTACAGTTCCATCCGCATTGGGGACCTTCCT
GGAATATTCCATTTGAACAGTCACTTGTTTGCCTATGAGTCGTGTTCTTAAAAACTCTCT
CACTTCCTTGGCATAGGGGGCTGGCTTTTCATCTTTACGTGGGTTGCCAATTTTTGGACA
CCTAATACTTGAAAGATTGACTCGTCGCTCTGCCAATGGACTACCATATGGGATAGAATC
ATCAGCCACAATTATGCAGTCCCCACTGACAACCTCAACAACCTTCCCAGTGAAATTCTG
ATCATGAATTGCTTTTGAATTTGTTACTGGAGGCACATAGTTCATCCAGATCCTCAACCG
GCTTTTTTTGGCTTGAAGCTCTGCATTCTTCAGTCTCCGCTTAGCATCTTCTTCCATCAA
ATTTGCACTCCATTCAACATATTTAGCCAAGCCATTTTCCACAAGCTCCAATGCTAGGTC
CTTGGCTGTTTCCCCATCAGGATAATACACTGATCCAATCAAATTGTTGAATTTGTCAAC
ACCTTCTAGAACAACGCGGACCTCTCTGTTCAAAACACGAAGCTCCGTAAAATGCTTGGC
ATCCACTGCATATGGATCATCAGTAGTTGCTCCAGTTGAGGATGCAAGTCTCTGAGCAGA
ATTCAAAGGACCTTGAGATCCAGATGAAATTTCTCCATTGATCTCATCAGAGGGGGTATC
TACTTCACTAACAGCTGTGGCCTTCCGACCGATTGCAGGGGCCTGGATTCCAGCAACCAA
GACTCGGACAAACTGAAATTCTGGAAGCAGGTAGACCCGTACAGTGCTGCCATCCTGAAC
CTGTTCTACCATACATTCCATTGGTCTCCCCTTGTTTGCAGTTAATAGACCCATTGCATC
AAAATTACCAGAATCACCAATGGCAGAGGGTGGCAGTTTCCTTATGGCAGCCTCAGCAGC
CCCTGGAACCTTGCTCCAGCGGCCAAGACCTTGTTGCTTTGCTTGCTCTTCTAGTCGCAG
CAATTCATCAAGGAAAGGACTAGCTTCACCTTTCTGGGAGCCCTGCTCCTTGACCTTTGC
CCAGCCTTCAGCAACAACCAACATTGCAACATTCTTATCATCAAGGATAACAGTTCCAAA
TTCTCTGCCTATGCTGGCTACAGTATAATCCACTCTAAATGTGACCTCCTTTCCTATGCA
GAACTTCCTCAAAAATTCTCTGCTGTCCCAAGCAAATGGCTCATCTATACCTCCCCTGCG
GGCCTGTCGAGGAGCAATAATAGATGATAAAATGAGGGTTTTATCACGTGGGGGTCCAGC
CTTCAGGGCAGACAAGGCCGTCACTACCAAACTGTCCCCTGATGGAACTGCTTTCACTTT
TCCCCTGTACCATTGTCCCCCTGCTGCCGGCGCCGCCATCTCACACCAAAGATCTGGATC
AGTTAAAACGGTGCGTTACGGATCTAAAAACGAAGTAAAAGAAGAGATAAATCTGTGAGT
GATAATTTAGAATAATTAAGAATAGAGAAGAGGCGACAGATTTACAGAGAAC
>ntig_988_consensus
TGAAAGTTGTAATAAATTACCATTCTCAAATGGATTAAACTCTTGACACGAGAATCAGTA
ATTGGTAGGGGAGAAGGGTTTCGGTAAGTATAGATGCTTCTATCATTAGGTAAATAATAA
GATTACAAGATTCTATATATGCTTTGTACTGCAATACAGGATGCTTCATCTTAGACACAG
AATAATTTCAGGCAGACCCTTTTCTGTTTAGCTCTACTAAATAATCAGATGGTATCAGTC
CCAGTAAACGTTTCCGGATTTTCTAGATCCGCCTGGCGGCGATAAACCCTCCGTGGTGAA
TATGGGAATTGGCCTGGTCTATCCAAAACTTGGGAACTATGATGTTGCTGCTGAGACTGA
ACTCGCCAGCGTCTCCTCCATCTCAACAACTCAACAAGAATAGAACTCCCACTCATGGCA
ACTCCAAAGCCAGCAAATGTTGCGAGGAGAATCGACAGAACTGCTTGCTCCTGAACCAAG
GAATAAAATATGTGAGCAAACAGAACAACCAGAGCGAACTGTATTGATGCGTAGAACCAG
ACAAATCTTCTCTTTACCATAATTGATGAGGCCATGGTCGAGAGGAGACCCAATACAAAA
GAAAATGGAATGGATATTGCAATTGCCCCAGTACCCATCTTTGCAAACAGAAGCTGCTCG
ATAAAACAAAAATGAGCAAGCATGCTGACAATTACGAGCACAGGAACTTCCCGACAAACC
CAGCATCCATTGAGATCTACCACCTGTCCTCTGTCTGCTCCACCATTTACAGTTCTCGTG
CTTTGAATTCGTAATAGAGTGACAGGTAAATTCTGAACCTCTTGCTTGCACACATCACAT

GTCTTATTACCTTTAATGGTAAACCACTTTATGGCACATTCTTTGTGTGCTAGAGCAAGT
TCTCCCTTGCAGCTGCATTCCATCTTGAGGGTTTCACCCCCTTCACAAAGTTCAACCATA
CAAATTCTACAAACGGCTTCTTCTTCAGGTATGTCTTCACCATCAGCATCACTATTGTTT
TCTGCTTCTACTATCTGAGATGTGTCTGACACTATCTCTCCTTCCTTTACTCGAGGAGTT
GTGGGAATTACTCGGAAAATTGAATCCATTCTCCTTACGCTCCCTTTTTTGTTATTGACA
GGAACTGAAAGCGAACGATTTATCTGCTTCTGCTTCTGGGTTTCCTTTCTACTTGAATTT
AGAGAATCAACAACATTTCCACTGCATGAGGATTCCAGGTTTGTATGCATGATTGGAGTC
ACAGGCAGTGATGACGTTCTTTTCATCCTGGGAGTAAAAATCTTTGTTAGTGACAATGAC
CTTGAGACGGAAGGCTTCTCCCGTGGTGCCATATTTGAAGTTTCTGGAGCTAGATTGGCA
GCCTTCTCAATATCTGAGTAAGAAATTCGATACTTGAAACTTATTTTTGGTAAGAGACTT
TTTAAGGAAGATTTGGCTCTGGATAAGGAAGGACTAGGGGAACCACTGGTTCTTGCATCT
GCATGATTAAAGTTGAAATTCACCCTCCTGGGAGTTGGAGTGGGACTAGGAGTTGCGGGC
ATCTTTATTATGACAGATTCTTGTGAAGAGTCTTCCACTGTTCTTGAGGGTATTTCTAAA
AGGAGGTTTCTTCGCTTCCATTTTTGAGAATGAGAGTTTTCTTCAATTATTCCCATTGAA
TCTTTGACCTTGTAAACTGGACTAACAGTTAAAGTTTCCGTTGCTGCAGCAGCAGCAGCA
GCCTCATGAGAGTCCTCAGACAGCGGCTTGTCCTCTCCAGTTCCCATTTGTTCCATAAAA
GTGAGAAAGGAGAACCCAGAAGCAGAAACCCCAAGAAAAAACCAAGAAGAAGAAGAATAA
AAGCTTTGAAGCAAAAAAACGACAGAAATTAAAGAGTGATGGACAGGGAGTTAATGGTAG
TTTCTTGGTAAGTGTTGGGGCGAAGGATTAAAAGGGAAGAGGGGACAAGTGGAAAAGAAA
GATAGAAGAGTTTGGAAAAATGATTCTCTCTCAACTCAATCTCATTTATGTCAGAAAGCG
G
>ntig_989_consensus
GCTCAGACCAACTCAATATCAGATGTATTTTTAAATCTTGACATATAAATCCCAACATTT
TATAACTGGTCCTAAAATTCAAACGGAAGGTGGTGCAGGCATGAGAAAACATAAAAATAA
AAGGGGCGAGGGTAAAGAAATTTACTGGGTAAGAAAAAAACTGGTCCCAGGGCAACAGAC
AAGCATATCGACAACAAAGCATCACAATCAGTAACATTTAATTTAATAGCTTCAGAATGT
TTAGACAAATATAGAACTCATATTACTTGCGATCACAATAGCATAATCTTTTTCCTTGTA
AAGAACACAGCTCTTGAGGTGGAGATATTCGACACAAAAATGAACAAGCCAAGGCCGTCA
GCAAAGCTTGTTATGATATGTTGCTACTCCTCCATCTATTTGCCAGCACTGTGCTTTGCC
TTACTGTGAGATTGTAGAGCACCATCTGACCCAAATGACTTGCTACACGATTTGCAAGTA
AATTGACCACCACCAGATTTGGGAGTTTTGTCATTAGGTGTTTTGCTGGCCTGCTTTGAT
GGGTGAGGAGTTGCAACATGACCCCCCTTCTTACCATCAGTCTGAGGAGTAGCTGCTCCC
TTAGATTTCTTAGCAGGAACAGGAGTCTTTGTAGCTGATTCTGTTGGTCTCTTCTTTCCA
GCTACAGCCTTCTTAGGTGTCTCTTCATCTTCATCAGAGTCATCATCTTCTGTATCATCT
TCATCGGAATCTTCTTCATCAGAACCTTCCTCATCACCAGAAATCTCATCAGAATCATCT
TCCCTCACATCATCCATTGACTCATCTTCATCATCTGAGTCATTGTCATCATTCTCCTCA
GGTTTAGTTGTTACCTTGCTTGGCTTGGCAGAAATTTCAGGCTTACCAGAATTTGCCTTC
TCAGGAGCTTTTGATTTCTCAGCCTTTAATCCAGTTTTCCCATTCTCAGCTGCAACCGCT
GGGATATCCTCTTCCTCATCTTCATCTGACCCCTCCATATCAAACTCATCAGAACTATCT
TGTGGCAAGGGGGTTTGATAACCACAAAAGTAGACACTACCATTCTTCCAGTTGTGAGAA
AGCTCAAACTCTTTCTCAAACACTAGATCAAAAGATAGTTGTGGGATGGTATCAGTAACA
AGAGTTCCAAGAACAAGTTTCTGGTCACCAAATTTCACAAAGAGGGGCACAGAGTCATGT
CCTTTATCCTTCTTTGACTCACCAAGTGAAGCCTGTGAGAGGTGAATGATATTGTCAAAG
GTTGGTTTTACTTTAAGTGGCTGTCCGGCCTTAACTTCAACACCCCAGAACTCCATGGAA
GAAGAACAAGTGACTAAGCGTGAGAAATGGTGAGAGAAAGTTAGGGTTTTAGAGAGAATT
AATGGGAGAGGCGTAAGTGAATAGGCTTTGGAGGGAATTTATAAGAGAATTGCCGCCGAT
TTTAGGCTCTAGGGTCTTGGTTTTGGGTTTTTTATATTTTCTTTTGGCCAAAGCACTTAT
GGCCA
>ntig_990_consensus
TAGTCTATTTGCCAGCACTGTGCTTTGCCTTTGTGTGAGATTCTAGAGCTTTTTCAGATC
CAAATGTCTTGCTACATGATTTGCAAGAGACTTGACCACCAGATTTGGGACTTTTGTCAT
TAGACGTTTTTCCAGCCTGCTTTGATGGGTGAGGAGTTGCAACACGGCCTCCCTTATTGC
CATCAGTCTGAGGAGTAGCTTCTCCCTTTGCCTTCTTAGCAGGAAGAGGAGTTTTTGTGG
CTGATTCTGTTAGCCTCTTCTTTCCAATGACAACCTTCTTAGGTGTCTCCTCATCTTCAG
AGTCTTCATCTTCTGAATCCTCC
>ntig_991_consensus
TGCCATGCCATGAATGCCTAAAATTTTCAACATTTGCTCTCCAATGGTTTTCTATACATT
TTCAATTAAATCTCTGCAGCTTCATAAGAAGCAGCAGGTCAACATGTCCCACCAACTTGA

TTTCCCCGTTAAAGAGGGGCAAATATTATTCAACCCGGCCTCAATAAATAAAGCAATCCA
GATGCCCACCAATCTTGTCAAGGAAAATACAATGTTATCATTATGAATAAAAATTACATG
AGAAAATTTCACCAAAGGGTTAAGTAGAATACAGATATACCCCCAAGTTTCTATACGGGG
GAAACCTGAGGTTCCAGAGGTGCAGATCCATCCTCCACACCTCCACTCTCTGATGCCTGT
GAACTCTGCTCTTCCTCTTGAAAACCTAGCTTGAATCTATCATAATTTGTCAGCTCACTG
GATTTGAATGGCCGCTCACCCAGAACTCGAACCAAATCAGCTTGGTGGAGAACTTCCTTT
TCTAGCAACAACTCAGCAATCTGAGCCACTTGCTCCTTGTGTTCCTGTAGAAGGTGAACC
GTCCGAGCATATGCCTTGCCCACCCAATCTCGCACTTCACTGTCTATAATTGCCCCAGTC
TTGCTGCTGTAGGGCTTTGACATCTCAAACGTGTCTTCCCTTTGTGGGAAAGAGAGCAGA
CCCACCTTATCACTAAAGCCATACACTGCAACTTGGGCATATGTCATTTTTGTAACCTTC
TCCAAATCATTTTGTGCTCCGGTCGAGATTTTCCCCAACAAAACCTCTTCAGCAGCTCGA
CCACCTAGTGTCATGCAAGTCATATCAAAAAGCTGCTCCTTTGTCAGAAGAAGGTTTTCA
TTTGGAACATACTGAGCAAATCCAAGCGCTGCCGTACCACGAGGAACAATTGTCACTTTC
AACAAAGGTTCTGCATGTTCCAAGAACCAGCCAGCAACAGCATGGCCTGATTCATGGTAA
GCAACAGTCCGGCGCTCCAGCTTGCTTATCACCTTATTTTTCTTCTCAAGACCACCAATG
ACCCTATCTATAGCAGACTCAAAATGATCCATTGTGACCTGTTCACTTTCACCCCTTGCA
GCAATCAATGCAGCTTCGTTACACACATTTGCAATGTCTGCCCCAGCAAATCCAGGAGTG
AGTGCAGCAAGTCTTTGAGAGAAATATGATGGCTCATGGTCGAGTTTGATCTTTTTCAAG
TAGATCTGAAATATCTGCTCACGGCCTTTAATATCAGGTTTATCAATGGAAATTTGGCGA
TCAAAACGACCAGGCCTCAGTAGAGCTTTGTCTAAAATATCTGGCCTGTTAGTGCCAGCA
AGCACAACAACTCCAGAAGTAGTCCCAAAGCCATCCATCTCTACCAGCAATTGATTGAGG
GTGCTTTCACGCTCATCATTGGAACCTGAGAAGCCTCCACGGCCCCTTGCTCGACCAATA
GCATCAATCTCATCAATAAAAATTATACTAGGAGCACATTGCCTTGCTTCTTGAAACAAG
TTTCTAACCCTAGACGGTCCAACACCAACAAACATCTCCATGAAATCTGAACCAGATATT
GACAGAAAAGGCACACCAGACTCCCCTGCAGTTGCTTTTGCAAGAAGGGTTTTACCTGTC
CCTGGAGGGCCTACCAAAAGTGCACCTTTTGGAATTTTTGCTCCAAGTTCCTCATACTTC
TTGGGGTTCTTCAGGAAGTGCACGAACTCCATGATTTCTTGCTTCGCCTCATCACAGCCC
GCAACATCTTTGAAATAGACCTTGTTCTTGGAATTTTTATCTACTTTTGTTACATGGGCT
TTCCCTATGTTAAATATTCCACGGGCACCCTTTCCACCACCACCACCACCAACACCAAAT
CCAGCTTGCATTCTCCGCCCCATGAACATGAGGGTTCCCAAAAGCAAGAGTGTTGGTGCA
AACCGCATCAATTCTTGGTACCAAACCATTTCAGAGACATAAGTCACAGGTACATAATCA
TGAGGGTCTATCCCCAGAGCTTCCTGGGCTTCCTCCAGCTTCTCCTCGAAAGACTCAACA
CTGCCAATATTAAAGTAGTATTTATACTGACCACTATGTCCTTTTGCAGGGGTACCACTG
ACAGGACCTTGGATATCATCTACACTAGTTTGATTGTGCGGGGAGCTCCTCACATATACT
TTTGCAACTGACTTGTTAGAAACAACTATATGGTCCACTAAACCTGGTTCCAGAAGTTTG
TTTTTAAACTCCTGGAAACTAACCTGCTGCTGTTCCTGGGAACCAAAAGAAAAGGAAGAA
AGGAATAGAGCAATCATCAGTAAAGGAGTGATCAAACTTTGAAACTGCTTCATGAAAGTT
TCTTGAAAATTTCCATGATCATCTGTATTTGAATCTTCTTTGGATTCAGATTTATGCTCA
TTTCCTTTTGGTATTTCCTTCTTCTCCTTTGGGTAGAAATTCTCATAATTCTTCTTATTC
TTCGGCGCTTCACTGGAGAAAAACCTGGTAATTCCAGGTTTTGCAAGAGCTTGATTCAAA
TTTGACAAATACGACTCGTTTCGACCTCCTATTGCAGCTAAATATCCCCTCCAAAATCCT
AATCTTCCATCTACTCCATCCGCAACACATGGCACCAGTAGAATCCTCTCGCCCTCAATC
AGCGATCGCCGTCCACCGGTTCCGTAAAGCGAACTTCTAGAACGAGAAGATCGCGTGAGA
CCACGACTGAGCTTGGAAAAAATCATTATTAATCAGAACGAATGAAGATAGGGT
>ntig_992_consensus
AAAAAGTAATAATTTACTCTATGCAAAACTTTAAATTATCCCGAGCATATAAATACTTAA
AAATCCTACCCCTGTTTCACACTGGAGCCAACTGAGCTTGAACCAATTGATTTTGCCTAC
AACTACAAAACTTATATTAATTTACACTTTCCCCTCAAAAATCTTGGAAGCTAAATACAT
TGGCTAGTTCTGCTATGGGATTTAGATGACAAAATTGAATGTGTACAGACAAACTATATC
ACAGGTATTGTCGAAGGCTTTCGCCGCTCATAGACTCCATTTCCTTGATAATATCTTGAG
CTAGAGATTTCACAGGTAACTCTATATCCTCTAGCAATTCACCAAGGAAGGGCATTGTTT
CAGCCAGCAAAACCAGATATTCTTCCTTTAAGTTATCAACAAAGTACTTCACAATTCTCA
GTCCCAGAATTCTAGACCGAACCTTCTCAGTACGAGTTTGCATTAATACCTCATGGTTCA
GGGGTTTCCACAGAAGGTCACTTCCTGCAGTAACAGCCATTTGACCAATACAAACAACCA
ACAAGTCATCAACTTCATCCACAGATGGTACATTCACATGCTCTGCCAGATCAGCCGGTG
GTTCTGCAATAAGCTGCGAGACAATAGGTTTTAATAGAACCTGAAAATTTGAGGAGTCAA
GAAACTTCAGGCTTCCAGTATCATAGAGAAAACATTTATGCAATGACGATATGACCAAAG
CCCTGAGGTGCCAGTTATTAATTGATAATAAACTGTTTTGCTCTTTTACATTGCCTGCTT

CCTGAGTCCTGGCCTTCTTTTTTTTATGAGTCAAATTAGCAATGTTTACATCTCCAGTGT
CAGTAAGATGCTGAACACAACCCTCGAGTAAGTACTTGAAATAAGGGACAAACAAAGACC
GGTGATTCTCAGCAAGTTTGTTTACAAGGCCATAGAAAGTTATCGCCCTATCAATACTTT
TGCTTCCCATAGAGACAATGTCTTCCACTTCTGACTCTGCCCATTCTACACTTCTGATAA
AAAGAGGCCTAAACATAGTCTCCGTTAATTTCATTGTAAGAGCAATCATTGTAGTTATGA
CACTCTTTTCTACAATATCAATGTTCTGAACTGAGACAGGGTGTTGGCGGCGAAGATCAA
GAGCAATCAAGCACTGGTCAAAAA
>ntig_993_consensus
AGCAATCAAGCACTGGTCAAAAATCTTTCCATGGAAACCACCAACAGATGATCTATCCAT
TCTCCCAACAACATTTCCGAGCATCTCAAAAGCAATTACCAAACTTGCATCACCAGCATC
AACGGCATCAACATATATATTCAGCAAAGGTGGCAGAACAAGTCGAACAGGGATTTTCTC
AGTAAGGAGCTTTCGAACTGCATCAGCTTTCACTTTCAGTTTTGGATCTGAACCCAGCAG
ATATTCAGGATGAAGCACCAAAATTTTTGTAATATCTCCAACATATGGATTTAGAAAACC
ACCGAGCTTGTCTATAACTGCCTCCAAGGTGATAAGAATAGATGCCAGAGATTCTCTCAG
AGCTGCATTATCATCAGTTTCATTTTTAAAGTCAACAGAGGTAGAGATTTCATGTGCCCT
CTTTATCACATTCTCCATTACACAAGGCAGCTCCACCAATGCCTTTGGCCCAAGCACATT
GATCAAAGCACCAGTTGTTCGGAGACAGCTAGAAGAGACAGCCAGGTTCTGTGATCTAAT
GCTGTTTGTAACAGATGCAAGGCACATGTTGAAGACAGAATGATGGGAAGGAAACTTATT
GGCTAAAACCTCTAAAGTTGTAACTGCAGCCAGTTTTAATGAAATGTTTGATTCTCCAGC
AGAGTCATCAACTATTTGAACAATTTCCAAGCACATCCTATTGAAAGATTCAAAAGCATT
CTCATCCAGATGAAGCCAATGACTACTTGAATTTGGATTAAGTTCTCTTCTTGCCTTGTG
CTTTGGTTTAACTGACTCATGACCCTTCACTGTTTCACATAAAAGCCCAAGAGCCTTCTT
TCTCACATTGCCATCTGCATTCTTAAGCAAATTAATGATGCTTTTAAAGTATGCAGATGG
AATCATAACCATTGTAATAGTCCTCAAGACACAATGCATACACTCCTTCAATTCTTTTCT
AATAGCAACAGAAACACTTAATTGTTTTCTTCTTCTGTCAACATATTCTAAGAGGAAAAC
GACCTGCTCCATAAGTTCTTCAAGTGTTCCCTGAATGCTCTCTGAATATTCTGTTGATTC
ACGCTTAAATGCAAATTCTGGATCTTGCATCTTGTGTAAAATGAATTGCATTGCAAGCAG
CAATTCCATAAAAATTACTTCCAAATTACCAATCTCTATTTGTTTAAGCATCATAACAAG
CGAAGGAAGCCATATGACACATGAATATTGCTCACATATTTGCACAGCAAAGGTATACAC
CCACTCTCTTTGTGCAAAAGATACAAATTTATCAGCACTGTGTACATCATCAAGGCAAGA
TGACCCTTTCCTTGAAAGCAAGGAGCGGAAAAGGAGGACAAGCAATGAAGCCAAACTATC
ACATTCTCCCAAAGTCCT
>ntig_994_consensus
TTCAAACCATTCTGAAGCTCAGAGGCTTCAATATCCTGCTAGATTTACAAACTAGCATAC
GCTTATAGACACACACATGTAGCAGCGTAGAACTTTCAACACTACAGTCGACAGGAGTAG
TAGGTAGGTCCCCTGCAAACTCCATCCTCCTGATCGCCTCCTCTTGTCCCTCTCTCTTGT
TAAGTATCACACATCGTATGGATTAGTAGGTCTCCGAAGTTCATCCGTGTTTGCCTTCTC
GTATGCATCCGATGTAGACTTTCTACGAAACCGAAAACTCTCTCTATTTCCNNNNNNAAC
TTTTGTCGTCTCCAACGATGATTCGTCAACGTCTGATCTCACGTTCCCACCTTTAGATTC
CTTGCCGGCTTTCGGTCGGAAACTGAAGGTGGAGTGCTTTCGGAGCTTGCCAAGCCCAGA
CTCCGGCCGAGGCCCGGCCAAGGTGTCGTCCCACAACTGGTCAAGTAAGCTCATGGCGCA
AGATAGCAAGACCTGAAGATGTTGACGAGATTATCCTCTC
>ntig_995_consensus
TGCTTCTCTTCTCTTCTCTTCTTTTCTCCTCTTCTCTCCCGTTGTGCCGTCTCGTTTTTT
CTGCTTCTGAAAATACAAGGTAAAATGACTATCCCTAGTTTTTCCGAACAAGCAGACGAT
GCCATGTCGGTCAATAGGAATGATGCAGATGTTACAGAAATAATGAAACAAGCGCAACAC
CGATTTTTAAGTCCAGTTGAAATAGCTGTAATAATCAAGAAATATGAAGGGCTTGGCATT
TTTCTCTCTCAAAGACCAAAAAAACCTACAAGTGGTTCATTCTTTGTAATTAAGCGTCAC
ATGGTGGGAGAGTTCAAAGCAGATGACCATAACTGGACTGTTCAGGAAGCTCAAGAGAAA
CTCAAGGTGGAAAATGTCGAGGTGTTACAGTGCTTGTATGCCCACGGAGAAGAAAATGAA
AATTTTCAAAGAC
>ntig_996_consensus
ATTCATATAGATTTTGGCGAGGAAAGATACCTAAAAGACTTATTGGTCTTAGCAAACAAA
ATTCGATCTTTAACAAAATTCCCATAGCAACAGTCTCAAACAAGCGCAATTGAACAAAAA
TTGCCACTAACTCGTTTAATTCAGCTTGTAATAAAAAATGCTAGAACTCTCTTGCGACAA
AAATCATCTCTCAAGGAAGATTTGAGCTTGTTGGCCCTGCACCAAAACCACCACCACCAC
GACCAAAGCCAGGCCTTCCACCAGGGCCCCTGAAAGATGGCTGGTAGTCAGCAGGAGCTC
CACCCTTGTCCCCAAAATCACCACTTGGTCCACGGGGACCTCCACGGTAGCCATCCCTAT

CACCAAACCTCCGCTCTCCCCCCTCAAAGCGAGGTGGACCAC
>ntig_997_consensus
CCTGATATGATAAATCATAAGAAACTCAATCCAAGTTTTGCTTTGACTGAAAAATCCCTT
GAAATACCATGACCTAACCCAGAATCACATTATCGGTGAGAGCGAGTGCCCAGCTTTACC
ACTTAAAGCAACCTTACAGCTAAAAAATATTCAAAAACTGCTTGAACTGAGCCAACTAAA
TTTAATATTCAACAGTCTCGGTAGCGAAATAGAACAAAAATTGCCATGAAATAAAGTAAC
CGTATTTTTTCAGCTTGTAAATAAAAAAAAAAAGGCCAAAACTATCATGATGTAGAAATCA
TCTCTCAAGGAAGATTTGAGCTTGTTGGTCCTGCACCAAAACCACCACCACCGCGGCCAA
AGCCAGGCCTTCCACCAGGACCCCTGAAGGAAGGCTGGTAGTCAGCTGGAGCTCCACCCT
TGTCACCAAAATCACCACCTGGTCCTCGGGGACCTCCACGGTAGCCATCCCTATCACCGA
ACCTCCGCTCTCCTCCCTCAAAGCGAGGTGGACCACGTGGACGATCGCCTGGGCCACCAA
AGGGCCTACCACCAGCTGGCTTGGCCTGCTTCTTCAAAGTAGCAGGGACAATTTCAGAGG
GAAGATTGAGGTAACCTCGAAGGAATTCAATGCCTTCATTGGTGAGGTACCAGTAGTAAT
GCATCCAAGCAAAGGTCTCTCGCACATACTCCTTTGACTTGAAGCTCTGCATCAACTTAA
TCACCTGTAGATTTGGAACATCGATATCTGGGTGCTTTGCCAAATTGTAGTCCTTCTTTG
CGTAACACACACCCTCCTGAAAGAGATACTTGGAAATCTCACGACGGTTCTTCTCTGGAA
TGATCATGATTGAAAAAAGGAGGAGATTGGTGGGGAATGACTAGGAGGCGGCAAAAATGC
GCCAATATCAGACTCACTGTTATGGCTCCCGCAGCAGAAAACCCT
>ntig_998_consensus
ATAACAGCATCAGCCACAGAGGATTCAGGTCCAGTAGTATCCATGGACATTGACAAAGCC
TGTTGTAATAAAGCATTCTCCTCATCCATTGTTTCAGTAGTTTTCTCGTTTGCTTCAGCA
GCTTTATCAACATTCTCCGTCATTGTTGCATCCTGTGAACTGGATGGTGGCTCCTCACTT
TTTCCCTCTTTCACAGCTTCATCAGCAGCCTTTTTAGCAGCAGCTTCTTGCCTTGCTCGC
TCCTCTTCCATCGAAACTCTAAGGGCAAGAGCCAG
>ntig_999_consensus
CCGGAACGAAGCCTCTACTCCAAGAAACTCTCACAATCCAAAGGGCATAGAAAGAAAGCA
TTCAAGCAGAGTTAAGGTTTCCAAAACACTTCTCTTCCTTGTCTGCTAAACCCCTAAAAA
CATCATGGTTCTCGAGGCGACTATGATATGTATTGACAATTCAGAATGGATGAGAAATGG
GGATTACTCCCCGTCTCGATTGCGGCCCAATCAGACGCTGTTAGTCTCATTTGTGGAGC
CAAAACACAATCTAATCCGGAGAATACAGTGGGAGTATTGACGATGGCGGGAAAAGGTGT
TCGTGTATTAACNNNNNNNNNNNNNNNNNNNNNNNNNAGGTTGCTCAATTGGCCCTTAAGCAT
CGCCAGAACAAAAACCAGCGCCAAAGGATAATTGTTTTTGCTGGAAGTCCTTTGAAAAAT
GATAAGAAGGTAATGGAGATGATAGGTAAGAAATTGAAAAAGAACAGTGTAGCTCTTGAT
ATTGTTAATTTTGGTGAAGAAGATGATGGGAAGCCAGAGAAGTTGGAAGCGCTNNNNNNN
NGATGTACTTATCAGCACACCCATCTTCAGTGTTGATGGTGAAGGAGGAAGTGGCTTCGC
AGCAGCCGCTGCAGCAGCTGCAGCTGGCAGTGTTTCTGACTTTGAATTTGGGGTGGATCC
CAATATAGATCCTGAGCTGGCTCTTGCCCTTAGAGTTTCGATGGAAGAGGAGCGAGCAAG
GCAAGAAGCTGCTGCTAAAAAGGCTGCTGATGAAGCTGT
>tig_1000_consensus
CTGCAGAAGTCCAGAAGCATCTTCCTCCATTGTTGTTTCAACTGAATCCATGGCGGTCTT
CTTTCTTTTCTGTCAATAATTCAATCTTGTATGTTCAGCAGATTCTGAGAGTCTAAGATT
CTCCTGAAGGGCTATAATACTCGAAGAATTAAAACCGGATTTCTCTCCTGATATCCTCAG
CTTCAACCAATCTTGGAGTTGCACATGATCAGATCCTGCCATAGGATGTTGT
>tig_1001_consensus
TTCACTAAGCGTATATAATAAAATAGAGTGAAAGGGTCCACCCCGAAACCAAGGGGGTAC
TAACTAACAGCTGAGTCCTCTCCGAACCGCAGGAGATAGTTGCCCATCATACGGCTCACC
AACTTGACTTGCCTCTATGGGAGGCTCGCTCCGGGCAGGTTCGGATCACTTACAATACAC
AGCTCTACGAAGGGGGTTAAGAACATTTTCAATATGATTCTTTTCCCGTATTTGTTCGAT
GAGAAATGCGAGACGAAAAAGGAACCCATCTTTTCGACTGGGAAATGCGAGTATGTTTTG
TATACTTTCTCCATCCCCCTCTATCAAAATGATCAAAAAGGAAGGCAAGCTTGCTTCTTA
TTCCCGTGTTCGATCTCTTCCATCTCTGACCCGCTCGTTGTCACCTATGTTGAAGAAAGG
TTTGGAACCCTAACCCTGTAGAGAATGAAGAGGGGCCAAGGATCTTCCTCTCAACAGTGC
TAGTGCTTCTTGAGGCTCTCCCCCCTGAAAAAAAGTAAGGCCCCGTTAGCCTGGGCGAAG
ATGGGGATAAGGAATAAGGATTGAAGCCCCCTTAGCTCTGCCAGGCACTGGACAGGGGTT
AGCTCGGTAAATGTGTAGAGCCAAGTGTAGTGTGGTGTAGTAGTAGGCACTTCTAGGCCC
CTTCCCGGCTACTGGATCACTCCAGTGCTTTGGGTACTACGGACCCTCTGCCATCCATTG
CAGCAGAGCCGTTTCATGAGCGGGGGGCTAAGCGCAGTTCTTTGAATCAAAGGTGGAATG
AAATCGATTCTTTTTTAGATATCAAAATGGAAATCAGATAGGATAGATGGATGGATCTAT

CTTTCTATTTATATATATTACTCAAAAAAAATGGATTTTTATAGATAGTTAAGTAGCGTA
GCAAGAAGCCCCAAATCCTTGATTTGGCCAGGAAAGACTGCACTGCTTTGGGCCCAGGAA
GCGAAGGGAATGAGCTCGGCTGCTTCTCCTCCACACTGATTTTTCTCCGTGCCCGTTCCG
TATGCGCTTCGCGCGCCATTGGCGCTTTGCTCTCCTCTTATTCTTCATTGGACGGTTCGG
ATGGACTTCGCCGTTCTTTCCCAACTCAAATCGAAAAGGCTGTCTCACATCGAGATGTCG
ATTCGTTTTCCGCCCCCAATGAGATGGGGGATTTGTAACCCCCTATTTAGACTTTTTGGC
CCTTCATCTTTCTGAATCGAGGCCCGGCCCGGCTCGCGTCGTTTCAACAACCGGCGGGGA
GCACCTCAGTATACGATCGCCCGCAGTAACAGGGAGTCCTATTACACCTAAGGCCAACTT
CAATTCACCAAACCAAGGTTCATCTCGTGTAGTGATTGTGGACTCGTACAAGGATATTGA
GTAGACGGTTGATGTATCAGACTCGACCCTATCTTTCGTAGCATGTATTCCCATCCGTGT
CGCAACTGATTCGGTAAACTACGTGTCCGGTGCACGGAGAACTGCCTTCGGTCCCCCAAA
CTGACTTACTCGTGGCAACCTTCCGGCCGCCCAACAACCTATAACGCTAGTCACTACTCC
CACTGAGGCTAGGAAGTCAGCCCCACAACCCAAAGCGGCGAAGAACAAATAGAATTTGCT
ACAAAAGCCGGCTAACGGGGGTATTCCCGCGTATGAGAACATAGTAATGGAGAAGGTAAT
AGCCAAAATAGGATTCGTTTTGGCTAGAGCGCCCAAATCCGCTATATATTTGACACGGGT
TTGCCGTAATGCTAAAACTATGGCGAATGCATCTATCGTCATTAATGCATAAATAAAGAT
ACCAATTAGTAGTGATTGAATTCCTTCTATGGTTCCACATGAGAAACCAATACAAATATA
ACCTACATGTCCAATTGAACTATAAGCTAGAAGTCTTTTTACTTTCGTTTGGGCCATGGC
GGCCAGTGCTCCTAAGATCATAGAAGCAATGCTGCAGAAAAAGAAGATTTGTTGCAATGT
AGCTCCATAGGAACCATAAATAAAAACACGTAAAATATTAGCAAAAATCGAGATTTTAGG
CGCAATAGAAAGGAATGCTGTAACCGGGGTGGGTGAACCCTCATAGATATCAGGTGCCCA
CATATGAAAAGGAACTGCAGTGATCTTGAATAGGAATCCTACAGCGATAAATAGAATCCC
CATAAAAATACCACTAGATCGAGCACCTGTGATTTCGTATCCGGTCAAAATCTTGGCTAA
TTGATCGAAGTGGGTAGCTCCAGTAGACCCATAGATCATGGAACAACCAAACAATAATAT
TCCAGAGGAAAATGCACCTAAGATCAAATATTTCAAGCCGGCTTCCGTGGAAAATTCAGA
CTTTCTTTTTGATGCTGCGATTACATAAAAACATAAACTTTGAAGCTCAATAGCTAAATA
CATGGCAATTAAATCATAAGCCGAGATCATAAAGAGCATACTGCGAGTAGAAAGTAGAAT
TAATACAATGAATTCAAAAGCATCAAACCTCTCTTGTTCGAAAAAATCGAAACACATCGA
AATGGTACCAGCCGTACTTAATAATAGAAAGATTTGGCAAAAATATGTAAAATTGTCCCT
CCTAAAAAAATTATTCCAGAATAAATGGGCAATAGTTAGGAGAGGTGCGCCAGCGGCGAG
CAGAAGCAAGGTTATTAGAACACTAAGTAATCCAAGCCAACCCACATTACTGACTAACGG
CGGATAATCATATTTCTTAGAGGTACTAAATACAACTCCATGAATGAGCAAAATGAAGGT
TGCATTAATGATAAAGATCTCTGGGAAAACCGCTAAAAAAAGATTGAACATGTGGGAGGA
TTCGAACGAATTCTGCTTTCATTTCCCCCGTATGGAGCACTGAGTTACTGACGTTACGGT
CTTCAGCAGGCAGGCTGCACTCTAGGCGGCGGCTAGGAAGGATCGCTAGACCAGCAGCCA
AACCAACAATGAATGTGTAATGATGGCCCCGGGCTCAATAAAGAATTGAATGTAGAAAGG
TAAACTAAAAAGGAGTCCATGACCCCTTTTTAGAGCGTATAAGGAGAGGTCGAATTCCAA
ACTTTTCTCTCGAGTATACCCATTACCAAAAAATGGACGTCACTTTGGGACACATGCGGA
TAGCCGCCCCGCGTGGCTAAAAGTAAACCAAAAGAGCTAAAACTCTTTTTTTTAGTTTCA
CTGAGAAACTCGAGTCTTATAAAAGACGCCACTATACGAGGGAAGTCCTCGGGTCGAGTC
CTTGCGAGTTCATCCCAAACAAAGCGAACCCCTTCCGGGTCTTGTTCGGGAAGATGCAGA
TAAAACGCCAATAGCCTGATGTCAGCTTCTACGGCATAAGCTTTAAAGAAGAGAGCTATC
AACGCAATAATAAACATTTCATAAGAAAATCCAATTTTTTTTTATCTCAAAGGTATAGGT
GAAATTTGGAACAGTCATTTCAATCTTTTTCTTCCGAAAGAGAAATCCCGAAAAATCCGT
CAATAAATGACGAACCCCATTATACAGATGATAGGACAGAGCTAAGGCAGTAATCTCGAC
GGAGATTAGGATAAGCTTTGATGAATCAAAGAAAAATTGGTAGAAATTCTCATAGGTGAA
GCAGAAGGATTGAGATTTTCGTAAGTAATGTTCGAAAGCACTGTTCCCAACAAACTATAA
GTCATGGGACATTCCTCAGGCATTCGCTTGCAGCGGATTCGGAAAATGTTCGGAAGTGAG
AGTTCGATTCCAAAGCCCCTTCTTTCCCGCTTCCTGGCTTTTCCTAAGAAAGTGGATTCG
GGATACACGCCAAACCCCCATGCCCCAGCTTCACGCTCTACCAGTCAGTCCAGATATCCA
TCTTTCCTCTCCTCCTCCCCCAGTCTGGTCCCCTTCTTTGTATCGGTATCAATGCGCAAA
CGCTCATCTCTATCTGTATACGTTGTTTGATTAATTGAATCTTCCTTCTAACGCTCGAAC
TGGAGTCCGAGCTCCTGCAAGAGCAAGAAAAGAAGGATGTCTCATCTTCCTTCCTCTCTT
CACTCTCCGGATTTCGATCTGCACTCTTCTGGTACAAAGGCCTATGCTTCCACTTTCAGA
CAGTTCTAAAGGAAGAAGTCGAAAAGAAGGCACTCTCCTGCTAACCTCCTGGCTTTCAGA
TTATACTGAGCTTGCTGCACTCTGATCTAATATATTCGACTTCGCTTGCTTGCACATTAT
AGGCTGTCTTCCTTCCACTTCTCCGAGGGACAGGGACTACGGCTTGACCTTCGGGGAAGA
ACTCCGTGGGACCCCTCCTTCTAGGGCGCCTAGATAGTGAAAGGTTATTCCTTTGCAACG

CTGGAAGCTAAGCAAAGTCACGAAGCTGGCTGACTACGCTCGAGCAGAGCTCAGGCCCGG
AGGTGGTGGCTGAACTCGCCGCAGAGTTCAGGAGCGAGCAAGACTATCTTGGTGAATGCA
ATAAGAATCGGCTGCTTGCCGCAGTTGCCCATGCTGCGATCCGCCGCCGAAGCGCTCAAG
GGATTCGTGCTTGGATGTCGAGATCAGGGGACTCTATCCAACGGCGAAATTTGTGGTGGA
ACAAAGAAAAATATATATATTTTTCTTTGATTTGCTTCGATACAAGAGATCGGCCCCGAA
GCAAGGTATGGTGGGTGCCAGCAACTTTCACTTGTTAGGAGTAGGGGCTGAAAAGAGCTC
TTTGTATAGGTTGGATTTTCTGGGCTTTGCTTTGATGCTTACCTTATAGGGAAGGAAGGT
TTGATTGATAAAGGAGCTTTTAAGCCCTTTTGCTGGATTGTTGGTCCGCAGCCCCGCCCT
ATAGAATGAATAAGGAGAGGCCTATCGATGACCGAGCCACTCTTATACTACTCCTTACCT
TACCCCCTTGTCACTTAAAGGGCGAAGTCGCTGAAGCGAGTCTAAAGGCCAGGCCAAAGA
GTGATCTTTGAACGCTACTACGCATCCTTACAAATAGTATAGTGTAAGGTGGGTTTGGGT
ATAGCAGGACTTGAACCCGCGACCATTAGGTTAAAAGCCCAATGCTCTACCAACTGAGCT
ATACACCCAAAAAAAAAAGATGTAGTAGTAAATAAGGATTGGTGCGGAAAAGAAAAGAGG
GCGGCCCCTCTTCTTCTCATCATATTAAAGGGGCGCGGCTCTGTGCTCGTACTGCAGAGC
AAGCGAAGAAAACGTAAGGGCGCGCGTTAGCACTCCTGCAAGAAAACGGCCTAGCTAACG
CGCCCCTGATTTCAAACTCAAGGATCGATATGAGAAAGATGCGCTCAAGCACCCAACCTA
TACAAGGGGCTTGGGCTCTAAAGCCGGCCTTACTCAACAAGCACCTTTATTAGTAAGGTT
GCTTGTTTCCACTCATTGAAGATTCTATCTACAAAAGAAGGTCAACGGGGAATACTAAAG
TTGCTCTCACTGACACCATCTACGAGAAGGTGAGGTCGTCTTTCTTTCCAGCCGC
>tig_1002_consensus
CTCCAATCAAATATACCCTAATTAAATAACAAAAATATAATCCTAAATTAAACAAAATAA
AATTCATATTTCATTAAATTGAATAAAATATTACCTTATATTTAGTTGTTACTTGAGAAA
GAAAATTGTATGGAACCTTTGGCTTGGAATGTTGGGCAGATAGCCTACTTGGGACAATAT
TGACATGGTATTGGCTTTTAATCTTGACGAAGATAACCTCTTCGGGGAAGAATTGCAACA
GTTAATTTTCATAACGTCAAAATTTATACTATACATGCTTATGTGTCATACAAGGCTGAG
GCAGAGTTGAATGACTAAATTTACATATAAATTCAAGCCAAAACAATGAGAAAGTGACTA
CTGTTTAAATTCAAATCGACTTAATTTAAACACGTTTCACTCATTAAATAGTAGCTAAAT
AATAAAAACAACTTAATAATAATCCTAAAAAAGAAAACAGACAGATTGTTTTCCTCTTCT
TGAACCACACGCCGTGTTCATACATATATATATATATATCACTGCTACTGCACCACTCAT
AGCTGCTGCATTTCTTGGAGGTTCACCTCGGAACACGAGCGACCCATCTCACCTACCAGC
GGAATCACTGGAAGATTGTCGCAGTCGCATATTTCGATCATAAATCCATCAGGATCATGG
AAAAACATCTGTTCCACATGGATCCCCCCTTCCTCCACCTTTGCCCTCACGTACTTAATC
CTCATTTCCTTCAGCTTTTTCTCCATTGCCCCCATGCTCTCACACTGGAAAGAGATATGA
TTATCCTTGGGATTAATCTCACTTTTCTTCGGCAAACTCTCAGGATCCTCCGATTGCAAC
AAGTGAATCCCAATTCCGTAGCCAAACAGCCATGCACCATCAAAGTCGAAAGAACCAGGC
CTCCTTATGGGGACAAAACCGAGAACACCTTGGTAGAAGTCAATGGACTCTTCAACCGAT
CGACAAACGAGTGAGACATGGTTCAAAGACTTAAGATGCAAAGGGTTTCCCACGGGTTCC
TTCATTTTCCCGGAAAGTAAGCCAGAAAATTTTGCTATCTGAAGTTAGGAAAATTGAGCG
AGGCGATTAAGAAAAAGTGGGTGAAGTAGACGGCAGCATACGAACTAGTGGATGGAGAGA
GATAGAGGAAGGGGGTGGCTGGCCGTAAGCATATACCGCACAAAGGAATGTTCATGCACG
GCACGTGTCCAGTTTAAGCATCGTGTCGGAAATCGATAAC
>tig_1003_consensus
AAAATTGATCTCTTCCATGTCATCACTAAGATACACGAGATGTAGGGTCATCAATTAATC
ATCGCCACAAACCTCCTCGCCCATGGAGCTCGTACTTCCATCTTTAACCGTCTGATTGTC
ATCATCATCAGAATCATCACTAACTTTGTCGTCGTCCTCATCATCGCAACGATCTTCATC
AGACGATAAGGGCAGAAGCCAACAGGTCCCGTGTAAGTGCCCATTCAAACAAACAAAATA
CTCTAACCCACCATCATGCGTGCCCAGCCGCCGAGCCGCGCTCTTAGGAATAAGTCGAGC
CGCTGTCATGCTCCAAACACGGGCCCCACAGTAAGGACATCTCACGCTCTCCTCCAACCC
CACCTGGCGGCGGATCAAACATGCTCTAGTCCTGGATCTCGTGAATCCCCGAAATACCCC
TCGAAAAATTCCAACGTCATCATCCTTGTCCACCAGTGGATGCTCACACGGGTCACTTAC
ATACAACAAATCGCCCCGACATTTC
>tig_1004_consensus
ACAACAAATCGCCCCGACATTTCTTCGTTAAAAAGCTCCGACCCGAAGTTTTTGAAAACC
GAGAGGATTTAACAAAATGTCCTGACTGACGTTGATTCAGTTGGAAATTACGAGTTGACT
CACAACCGCAACAGAAAAACATTAGCTTAGCGAGCGCGTTCCATCCACCGTCAATACGAC
TGTTTGGGGCTCCATTAGCCAACGCCTTCACCATGCGTGGTGCCCTATGCGTGCAGAGCT
TCCGCCAGAGAAGCCGCTCTGCAATAGCCCGGAGCTTCTGGTTGAGGGAGCCAGTCAAGC
AAAGGACGTGAATGTCCCATTTTACTGACTCAAAGATGAGTAAAAGTATGCTCTCGTTGA

GTAAAGGTGACTCGCCCGAGTCAGAAGTCGACGCGTTTGGTTTGAGCCTCTTTCTCTCAC
TCATCGAGACACTGCCTGAGACAGTGATGGACTCATTAGAAATATTCACAGGCTTAT
>tig_1005_consensus
TTAACATGAATATTGGTCTAACCTGTTACAGAGGGTTCTTTAGCAACAGCTACAAATTGA
GACTAACCAAGAATTTGATTTTAATTATATGTTGATGTGCGAGGTTTTGGTGGAATGTAA
AAAGATGTACCACAATCGATTGGAGTATGCATAAGTGGATTCCATCTATCAAAAACTATA
CGACCCCCTCGGCCAATTCTACCATGGAACTTATAAGTTGGTGCCGACATGCTATTCTTG
GCTGAAGCATCTGATGGTGGGACAATGCCTGCAGCAACTAACTTTTCCGGGACCAGAGGT
TTTGTGAACAGCAGAACTGGCTCAAGAGGATCCAATTTATGGAGCCATCCAAGT
>tig_1006_consensus
CCAATTTATGGAGCCATCCAAGTGGTGTATGGCGCCTGAACTCTTGTTTAATGTTAGCTG
TTGGAATTGTAACAAGGTTGGACTCAGGGTATAGCTGATTCTGAACTGCAGCAGGTCGCA
TACGTGGGCGGCTACTTGCAAGGTCATCTGAGTCCACCAATTCATCCTCACTTGAAGCAA
ACTTGAAAGAAACAGGAGGAAACCCAGGAAGTGCCAAGCTATCCTCAAGAAGCTCAATTT
CATGCTTGTACTTCATTTGCATCCTTTGGAGATTAACTTCACTCTCCAAGACATCTCTCT
TTTTCTCTTCTCTCTTAATCAAAGCCTCAATTATAGTCTTTGCTTGGTCAAGGTTGCGTC
TGACCTGGCGAAGCTTTTCAAATGACTGTACATTATTTTCTCTCCTTTGCATCCTTCTTG
TATGGAGTCTGTGGGCTTTCTCTCTTGGCCTAAAGACATTGTATGGGTTGGTATCATTAA
CTGGCGGAGGGGGCTGCAAACGTCGCAATATAGGTTTTTGCCATCTTTCACGCTTGTCTT
TCCAATAATTATAAACAGACTGGAAGACTACATATCTGATGGTCTGAGCTTGTAGTGCCT
CAATGGCAACATCAAGTTGCAGAAGTACAGGAATGGGTGAGCCAAGAGTAGGTGTTATAA
GTCCAGCTCTTTCTCGAGCTTTATGATCCAACACCTCCAACTTAAAAAGAAGAATCTCAA
AT
>tig_1007_consensus
CTTTCATCAAAACCATTACATAAATTTACATCCAATAAAGGTGAAGCCCTGAAACCATAG
AAATGACACTGATTTTCTCATACATTCCATTTTCTGATTGCAATATACAAATCCCATTTA
CATTCAACAAACCTTCCCATTCAAATGTGATATACTATAACCAGACTGGAAGATTTAAAA
TTAAAAATAAAAAATAAAACCGAAGTTTTTAACAGAACTTCCAATAAACTAGAGCAACTA
AGCCACTACTGGTACACAACATATAATCATGAAGAAGTCATGCAGCGCTAAGAGGCAGGA
TTTCAGAGCCCACTTGTTTTATTTTGATCACAATGAACAAATTCATAAGCACTTGCTTAG
TGGCTTGCCAACTTCAGAGAGTTTGCCTTCTCTTGCAAGGTGTCAAGTAGACTATTAAAG
CTCTTCTTGAAGGAGTCCACTCCTTCAAGTTCAAGTTGAGTGCCAACATAGTTCCAGTCG
ATTCCCAACTTCTCAAGTGCACTATAAACACCTTCAGCTTCAGAAACATTTACATCGATC
GTCCTTAAAACAGCACCATGATCAACAAACGCTTGGAGAGCTTGGTCTGGCATGGTTGAA
ACCGTATCAGGTCCAATGAGAGGAGCAACATACAAAGTATCGGGGTATGCAGGGTTCTTA
ACGCTGGTTGAGGCCCACAATAACCTCTGCTTCTTTGCACCTTTCTTCACAAGAGCTTCC
CATCTTGGACCAGAAAACTTCTTCTGGTAGAGCTGGTATGCCAAGGCAGCTTGAGCCACG
GCAGCCTTTCCCCGGAGATCAAGACCTTCTGGTGTTCCAATCCTTTCAAGCATTTTGTCA
ATAAGGGTGTCAACTCGACTAACAAAAAAGGATGCAACACTTGTGACTCTGGAGAGGTCA
TCTAGACCAGAAGCCTCTAGGCCATCCAAGTAAGCATCAATGACTGCTTCATATCTTGCA
AGGGAGAAAATGAGAGTCACGTTGACGCTAATGCCATTTGCTATAACTTCCTTGATAGAA
GGAATGCATGGAGCTGTGGCAGGAATCTTAATGTAGACATTGGGGCGGTCAACCACTTTA
TGAAGCCATTTTGCAGCTTCCACAGTCCCATTAGTATCATCAGCAAGTCTAGGCGAAACT
TCAACCGAAACATAGCCATCACCACCATTAGTTTCATCATAGATATGCTCAAAAAGTTTG
CACGCATCTTGAATGTCCTTAATCACAAGTTCCCAGTATGCAGCTTCAATGTCTTTTCCA
GATTGTACCAGTTCCCTGAATTGATCGTTGTAAGCATTTGAAGATGAGATAGCTTTCTGG
AAAATCGCTGGATTGCTAGTTACACCTCTCACACCACTTGCAATCAGTGGAAGCAAATCT
GTAACTGGACGGCACAGATTATCATACCAAGGGCTCTGACCTTCCTTTTCATAAAGATCA
TGAAGAGATGTTCTTTTTGCTGCACTTCCATTTCCACCTGCTTGTGAACACCTGACAACC
AAGGAGCCTCTTAAAGCATGGCTACTTTTAAGGTTCAACTGAGAGCACAGAACTTTGGAG
TTCAACCCAGTTTTGTCTTTGAGAGAAACGAAAAAAACCAACCCAGAAGAGGATCTGGGT
TTGATCGGTGGAGAAAAAGAGGAGAGAGCAGCAGGTGGTGAATTGCAAAGTTTTGAAACA
GTAGCCATACCAAGGGTTTGGTGAAACAGAAGT
>tig_1008_consensus
AGGCCTTCTATCCTTTGTCTCCACCAGTCATGATGCAGAATACAAGACACTATACACTGA
AATGCTTGTCAATGAAGCTAACACACCAAATAACATTACAAAATCTTGAGCAACTGAAAA
AACAAATGAAATTGGCATGCAGAATTGTAACTGATTTCACCCTTATTGTTGACTTTACAC
AACTTTTTTACAATGCAATCTCATTCACTCACTCCCAACCTTTCCACTGAAGAATATAGT

CCACTTCACACTAAAAATTGTACAACACAATCACTTGCTCACCCCCAAAATCAAGGGGAA
GAAGAAAAATATATAAATTACAACGTGGGAAAAATAATACCTCACCAACTGCTTGATGCT
TGAATGTTAGTGGTAGTATAATAAACATGTTGCCTACTGAAAGATATCCCAACAAAAATG
CACACCTTATGAAGTCAAAAACCTGTTGTATCTCTTTGTATATGAAAAAAGCAAACCTGT
GATGCTGTACATTTAGTTACCGGTCTTCATCACCGTGATTGTAGTGCAGCTGACATTCTT
TCTGGATCATCAATACATTGCAAGAATCTTGGCGATGAGTATGCATATACCCTCAAGCAA
AGGTTTCATCTCTTCTTAACCCAATCCTTCGTAATTCATGTAGGTGAGAAGCTATCTCCT
GAATGAATTTAGTTCCTTCTTCTCCATTTTTGAGTGATTTAATTGTGAGCTCAAGAAAGT
TCTGGTCTGCCTCAAGTGCTTCCAATCTCTCATTCAGATCTGCAAACTCACTACGGAGAG
AAGCTAAATCGGTCCCTCCACAAAGGACGAGAGTTTGCTCACTGCAATTCAATTCAGTGT
CTTCCTTGCTACAGAGCCGAGGTTCCTTGTGCGAGGAAACATGTCCATCAGCAAGTGGAC
TTCCTCTTGATGTAGAATCATCATTGAGCATAGACAAGTCACTCCCTTCTGTTCTGCTGT
TTGCTTTAGGATCCACTTTACAGTTGACATGCTGTGAATCACTTATGTTAACTCCATTTC
CAGGATATTCACCATTATCCAAGCCTGAGCAAAGCCCATTATATGAGAACATATAAAGCT
TCTTCTCCAATTTTTTCAAACTTTGTAAAATATACGACCTTTCATCTTCAAACTCTAAGA
GTGAATTCTTCACAGTAGCTGTACGCATATCTTCAGATGGTGTATCTGTTTCAAGATTTT
TGATGACTGACTTTGTGAGAGTATCTGCCTTATCTTCACCACATGTATCTTTTGAATGAT
CCATTCTGATCTCTCTTTCCTTGCAATTATGAGTTGGCTCCAATATGTTCTCCATCATAT
ATACATTTGGGAACTTCTTTCGGCAAAACTCAAGTTCTTCTTCAAGATCTTGAATCTCCT
TCTCCTTCTCTGCTAGAAGGTCATTTGTCTTTTGCAGTGCTTCCATGTCATACTCAGCTT
GTTCCTCCATCATTCTCAAGTTCTGCAAGGCTTCCATGTGGAGTGATGCCTTCTCCTCCT
GCAGTCTAGTAATCATTGCCATTGATTGATTTACAGCTACAGCTGAAGCATTTCTTTCTT
CTTCCAATTCCTTATACAAAGCACTCAATAATTTCTTATCATGCTCAACCTGCCGTTTCA
ACCGATCAACCACACTTTCCCCTTCAATTTCGCTGACACTGCTTCCATCCAGAGATTCAA
AACCAGATTCATTTCTCTCAAGTGAAATCCTCTTTTGAAGAAGTTGCATCCCGTTAGAGC
TAGAATCATCGCTATTCAAAGACACCTTTGGACTCATATCAGGCAGTACCTGTTCAAAGC
CTCGAGTGGCAGATAGTTGTGACAACAAGAGCTTCAGATCTTCACTAAGTCTAGAAGAAT
CCTTCCCAATCCACTGTTCAGCAAGCAGTCCAGACAGTTGTCTTCCTCTGTTGCCAACAG
CTACCTTATAAGCATCACTGAGGTCCAATAAGTTGGATGCTTGAAGACCAGTATCGCTTG
ATAGAGGATTTTTTTCCATAACAGTTTCATTTGATGTATTTGGCTCACTGCCGGCCTTGC
AGTTAGATGTCTTCTCATTTTCATCACTTCTTGAAACACGGCTCAGCTTTGGTGCTTCAA
TTGGCGATTCAGTGTCGTTAGATAACGGTGGGACATCATCAGGGAGAATAACTTTGCTGT
CATTTGACACTTCAGGAGTATCCCTAGCATTTGATGGTGAAGGGACACCATCAACAGAAA
TAATCTTGCTCTCCTCTGATACATTAACAGCAAATTCAGTAGCATTAGACATTGGTTGGA
TGTTATCTAGGAGAACAACTTTGCTATCTTTTGACACTTCAGAAGGGGTATCCATGCCAG
TTGAAGAGATCTTACTGACAGAAATAAGCTCGATCTCCTCTGACTCTGATACATCAATAG
GAGACAGAGCAGCATTGGACATGGGTGGGACATTATCAGGGAGAATAAGTTTGCTATCTT
TTGACACTTCAGGAGGAGTATCCCTGATCTTTGGGGACAAAGGAACATCATTGGCAGAAA
TATGCTTGCTGTCCTCTAACACATCAACAGGACTTTCAACATCCGATGAGAAAGGAACCC
GATCAAGGACAATAAGCTTGCTCTCTTCAGTTGCATCAACAAGAGATTCCCTTGAATTTG
ATGATGGGGCAACGTCCTGATTATTAAGCCTACTCTCCTCTGATACATTTGTATGAGCTC
CAGCATTGGATGATGGAGGAGAGACCTCATTAAGACAAATAAGCTTCGTCTCTTCGGTTA
CATCAACGAGAGATTCCCTAGAATTTGATGGTGGAGAGACATCGTGGTTGATTAGTTCAC
TGTCTTTTGAGATATCAATAGAAGTTTTTCCTGCATTTGATGATGGAGGAACATCATTGA
GGGAAATCAGCTGAGTCCGTGCAGAGGGATCAGCCTTGACATCAATCAGTTCCCAGTTAA
GCTCCTCCAAGCCATGTCTAACTGCAGTAGTAGATTCTGTAGATCTGTCATTATCTGGAT
GGGTGATATCTGATTGTAACTCTGAGGCTGGAAGTAAAGGCTCAGGTGCAATGGCAGGAT
CTATTAATTTTTCTCGAACTAATTCATGTGACAGGGTAAAAACTTGCGGCTCCAACTGCA
CACATATATGTTCAAGATCATCCTTGGGACCACTAGTTTCATGGATTACAACATCTGCAT
CATCATCATCAGATAAAAGCAATTCAGATTCAGTATCTGAAGTAATCTTCAGCTCCGCGT
ATCCTACATGAGACAGTGGATCTGGCCCACGATTCTCCACATGAGTGGCTCTTGTAGGAA
TTGATCGTTTTTCCTTTCTCTTCTTCAAGTAATGCAGGCTATGTTCACCAGCAACAGATA
CAGGCAAATCAAGTTCAGACGTCTCAGACTCGAATGATTTAGTCTGTATAAGTTTCTGGG
CATACCCTCTTGGGATCCATGGTTCATTGCAGCAAGAACAATGCTTAATACTTGATTGAT
TATTGTTATGATGACCCTGAGGCAGTGGTTCCTTATCAAATCCAAACCAAGAATCCTCCC
CCAACTGGCCGACCAACAACCTGTATGTTTCAGTGTTGGATTTGTTGACTGTGGCAAATG
AGAACAGACAATTTTCACACATTCCATGGACATCAACAAGCTTGTTGTGCGCATGGCAGA
AAACTAAGGATGAGATCTCCAACTTATGACTGCTGCAGAACAAATCCCAATAATGTTTTA

ATCTTTTATTGCCCAAAACATGATCAAGTCTAGAGCAAAGCAGACAGGGCGTCTGCAATT
CACAATACCACGCAAATCTAGTAATTAGGTATGAAAATATAGCATCGATGAACAAGAAAA
GTATCAGCAACCATTCAAGAATAGCTGCCACCAATACTCTGCGAAAACTCAGGGAAGTTC
TCTGTTGTTTAACAGCTGAATTTCCCCTGGCAGCCATGCCCTTGCAAAACCAAGAGGCTT
TGAAGAGGAATTAATTCCTTGAAAGTTGAAATGCACGAAAATCCAATCCTCGCAAAAGTA
AATTAAAATCCCAGACAAGATCAACACTGTTCGGGTGAGGTGAAAGAACATCAAGAACTC
AGAAGGAGAACAAAAAATCACAGCGACCCAGATTGAAATTGACTACAAAGATGAGAACCA
TGTAGTAGTTGCTAAAGATACAAAGATTAAAGCTAAGAAAAACAAAATATATAGAAACGA
GAGTGCACGTAGTTTTCACTTTATTAAAAGTAAATAAATAGAAAGAAATACACAATCAGC
CGACACAGCCAAAAAGTCGGCCGCTGACAAGAGAAAAACGACATGTACCGAAAGTTGAAC
ATCAAAAGTACAGCCCCCCCG
>tig_1009_consensus
ATTGCTATTATGCACATTCTCAAGTTAGGATGAACAATGACAAGAATTAAACCGATTAAA
ATCTATTCAATAAAGGACACTTCCATTTGCCAAAAAAAAAAGGACGCTTCCAATATGAAA
GCTTTACAAATGGCCAACCTCAGAGATTTTCGGGCTCCTGCAAAAATTTTCAAACTCCGA
GTACATTCCTTTGCAAATATCATCTTGTCGGCACCTAATATTGAAAACCATACTTCGCCC
ATTTACGGCCCTGGAGATCAAACCACATGAACCTCTAGTTCAATGGCTACATGTTCATAG
ATTACAGGTACACGAAACACCATAACTACAAGAAGCAACACCCAAACCTTACCATATAAA
AGCTTATTTACCAACGTTACCATATATAATAATATTAAGACTTATGTTTTTACTGAAACA
AAGATCCCCGCAACAAACTCAACAGGGTTTGCAGTCTATCTCCCTTAGTGTAGTCTTCAG
TCCCTTCCCCGCAGCATTAGCCAGTGGATGTATCCCATGCTCCGGTCCCGACATGTACGC
TCTGTTCTCGCTCTCTTTGGCAAACGATACACCATATCGTTTCTCAACATCTGATGCAAG
GTTTTGATAAAGCTGGCCATCGCGAGTAAAGGACATCATAATCTGGGGTAGTCCAAGAGG
CAAATTATCACCCCTATCAACCTGCCAAAAATGTATGGTCTTACCATATGTCTTGCAAAC
CTTATCAAGGTCTTGCCTCTGAATGGGACCAGGAATCCCAGGCATGAACAACATTCCACT
TTTCACTTCGAACTGGTGAGAATGCCACAGCGGCTTCTCAGAGTCCGGCAACGTCATGAA
CAGTTTCTCCGATACAATATATTCAATCCCAATAAGCTTCGCATCGGCTTCTGGGCTATT
GTATATCAGGCACTGCCGCATCTCCTCGTTTTGATGTCCGCAAAAGTGGTGCGCCTCCAC
TTGCCGAGTCATGTCATCAGAATAAAAATGAAACGCGCAGAGGTGTTGGTGAATCTTGTT
GGTGGGGCCAAAGCCTTGAATGGCGGCTGTTGTGGTCTCTAGGAGAGCAGTGCCGGTGGC
AATTTGCTCGCCAGGAACTAGAGTGCGTTGCGTTGATGCCATTATTAAATTAACCTACAG
ATTGGTGTATTGCAGCTCTCG
>tig_1010_consensus
TAGCAAAAATTAACAAGGAATAAGCAATTATATCAGCTGAGAAACAACAAGATGTTAAGA
AGAAGCCATGACTAATTAAACTTAAGTTGTAGATGATGAAACCCCAAATGAGCATATCAT
AAATATTAAAACATACAAAGCTATAGGAAATTAAACGACTCTCCATTAAAGAGAATGATA
TAATCACAAATACCACATAACTCCACAATTAGCCAACACTAAACACGAAACATTTATAGC
TTAATTGTCATTCTCAAAACAACCACCACAAAAAGCATAAATTATACCTTGAAACAACCA
ACAACCTAATGACCCGAAGATTTCATGGCCTCCAACATCAACTTAGTCTGCTCCTTGATT
AAATCATTACGCTCCACAAACAACTGTAACTGAGCAATCTGCAACTTCTTCCACCTTTCC
TCCAATTCCACTTTTTTGGGTCCTCCAATAATTTCTAAACCCTGGTTGATTACAAACTCC
TCCAAACCAGCCATATCAACACTCTTATCAAACAAAAGTTTCTCTTTTGCACTGCCCAAA
CTCCCTTCTTTATAATTGTCAATCTCCATCTTCTCCCCCTCCTTAGCTCCATCATTCAAC
TCAGCTTTCAGAAGGGCTAAGGACTTACTATTCCCTCTCTGGTTTTGATTCTTTCTTGCT
TTTCCGTTAGACTTAGCAGCGGATTCAAGAACACCACTAGTACTTTCACTGCCCCATAGC
TTTTTAGACAAATCATAAGCTTGTCGTTCATGTAGCTTTGAAAAAGTTCTATCTTCGCCT
TTCTTCCCTCTCCCAAGATTGTTCTTATATTTTTTCTTCAATCTCCTAATTTTGTCCATC
ACTTGAGCTCTTGTAACATCCACATGAAGCGATTTCTTGATAAAATCATGAAAAGCATTC
ATATCTTGATTTGGGTCCATCCCCTTTTTCGTGGTGAAGTCAATCATCCCCTTCAAAACA
GCAATTTCATCCTCCTCGCTCCATAGCCTTTGGAAAAGCTGCTTCTTTGTATCCTCTCCC
GGCTTCTTCTGATCCTCATCATCCAGATCCGCATCCGTTTCCTTCTTTTTCACCCGTTTC
GAGTCTTTCAGAGAATCCTCCGTCTCATTGAGGCGTTTTGCCGGAGCTTTCGACCTGGGT
TTTGTCGTGGTAGCTTTTGGGGCTTCTTCCATGGGTTTGGTGGCAATAGGCTGAGGAGTT
GATTCGGGGTCGGAGTCGGATTCAGATTCGGAATCTTCTTCGGAACTAAATGATTTAGAT
TGAGGGGTTGACGCAACAGAGTGGGTTGGATTATGGGTTTGTTGAGGAAGAGGAGAAGAA
AGTTTCGTGGGTTCTTCAGTTTCTTCATCGTCAGAGGTAGTAAGTTCTTCAGAAGAGCTA
TCCTCATCTTCTGAGGAGGAAGCAGCTGGTGGGTCTTCCATTGGATTTGGTTTCTTCGGT
GCCATTATGAGACGGAGATCAGGAGATTTAAGAGGAACTGAATTGAGAAGAGAAAGGCTA

GGGTTTGTGCGGAGGTTTATAGGGAAAAACAATGGCCACGCG
>tig_1011_consensus
TAGAACTTAGAAAAATAAACAAACACATAAGCAACATAATAAGTCATGCGAGCATATAAA
GTAGAAAAATGCTACTTTCTCTGAGAGTATTACATAAGCTAATTTGCATATTTCATATTG
GTTTTACTGCAAGTCATCTCTATCTAGAACATGCCAAAAAAGTTGACCTTCTTGGATATT
GCAAATTATTTCCATCTAAAAGAAAATGGAAGGGATATATTTTCTCAAGCAGAAGAACTT
ACAGCACTGTATGAACAATTGGACATGAGATACATGAAAAAGAATGACAGAGTGGCGCAA
ACAGAGAAAACCATTGCTAGTACCTCCACGACACAACTAATACAAAAATCAAACATCTAT
CAAATATTACTTACTACGTATTTGCAGAAGCCCAAGCCAATACAAGAATAATGAAATCAG
TCAGTCCTACTTAACTAAAATAATTAACAAGTACAAAGATTTTATACGAATGGGGGTCAA
ATAGCCTCACTATTTATCAAAACCACCAAGAGATGATATTAACCTTCATACATTAGCCAT
ACCAAGAAGAATCACCAGCAACATGTAAACAATAAGAGATTATATTAGGTAAGAACCGAC
AAAACTTTAAGAAAAGGTATCAACTATCTAAAGAAGGCAAGTTCCAGATAAATAAACTCA
AATGCGCAAGTCAAAATCATTAAACATATTTATGAGGCTATAAGAAGTTTAAAGTACTCT
ACATTCAAGATTAATGATATAATCACAAATACCACACAAGTCCACAATTAGTCAACACTA
AACACACAACCTTAATAGCTTAATAGTTATTCTCAAAACAACAACCACAAAAAGCATAAA
TCATACCTCCAAACAACCAACAACCTATTGACCTGATGATTTCATGGCTTCCAACATTAA
CTTAGTCTGCTCGTTAATCAAATCATTCCGCTCGGCAAACAACTGCAACTGAGCAATCTG
TAACTTCTTCCACCTTTCCTCCAATTCCGCTTTTTTGGCTCCTCCAATTATATCTAAACC
CTGGTTGATTACAAACTCCTCCAAACCAGTCATCCTAATACTCTTATTAAACAAAAGCTT
CTCTTTTGCAGTGCTCAAACAGCAACCTTCTTTGTCATTATCAATCTCCATCTTCTCCCC
TTCCTTAGCTCCATCATTCAACTCAGCTTTCAAAGTAGCTAAGGACTTACTATTCCCCCT
CTGGTTTTGATTCTTTTTTGCTTTCACATCAGACTTTGCAGCTGATTCAAGAAGACCACT
AGTATTTTCACTTCCCCATAGCTTTTTAGACAGTTCATAAGCTTGTCGTTCATGCAGTTT
CGAAAAAGTCCGATCGGTACCTTTCTTTTCTTTGCCAAGATTGTTCTCATACTTCTTCTT
CAATCTCCTAATCTTGTCCATCAATTGAACTCTTGTAACATCCACATGAAGCGATTTTTT
GATAAAATCATGAAAACCATTCATATCTTGATTTGGGTCCATCCCTTTTTTTGCGGTGAA
TTCAATCAACCCCTTCAGCACAACAATTTCATCCTCCTCGCTCCATAGTCTTTGAAAAAG
CTGCTTCTTTGTATCCTCTCCAGGCTTCTTCTGATCCTCGTCATCAGGCTCCGCATCTGT
TTCCTTCTTCTTCGCCCGTTTGGAGTCCTTCACAGTCTCCTCCGTCTCATTGGGGCGTTT
TGCAGGAGCTTTCGACCTGGGTTTTGTAGTTGCAGCTGCTTTTGGGGTTTCTTCCATGGG
TTTGGTGGCAATAGGCTTCGGAGTTGAAGCCGGGACGGGTTCCGAATCTGACTCAGATTC
GGATCCGGAATCTTCTTCAGAGCTAAACGATTTCTTCTGCAGGGTGGAGGCAACGGAGTG
AGTTGGTCTCTGGGTTTGTTGCGGAGGAGGCGAAGTAAGTTTTGTGGGTTCTTCAGTCTC
TTCATCGTCGGAGGAAGTGACTTCCTCAGAAGAGCTCTCTTCGTCATCACCGGAGGAAGC
AGCTGGAGGTTGTTCCATGGGGTTTGGCTTCTTGGGTGCCATTCTGACTGATGACTTGTG
AGAGAGAGGAATGGAATTGAGACGGCTCCTGCTTGCTGAGGTTTTCAGGAAGAAAC
>tig_1012_consensus
ATCCAAGTACAGACCTTATATTCGAGGTGGCTTAAAAATGAATTACATGAGCAAACAAAT
TCGCAGAGGTTTTACAGGAACGAAACAGCAAAAGCAACACAATATGCATCAAAAGAACAT
AATGTAATATCCAAATCATACTCTTCTTGATGTCACCTACCTCGCTCAGCCAAATTTAAG
TATAAAGCACAAGATTAACAAATTTTATTTTTAAGAAAGAAAGCCTCTCAAATTGAATAA
TGTAACTAGGTCTAAGCATGTAACAGGTATTCAAGGCTCTCAATATTTTGTTCAAAAACC
TACAGGCCTGACTGTACTAAGCAAAATATGAAATCTAATCGCTTTCAATATCTCTCCCCA
AACCGGAAGAATATTGCACCAGTCCCTGTATTATGATCAACAGCATACTCAGCTCGCACA
AGACCCAGCTTGACACCAACACCATATGATGAGCCATGACCCATGCGCCTATAGACCTCA
GTGGGGTTCCCCTTGACATCCTTTGAACTTCCTAGATCATTGCCATGCTCTGCAAATGCA
TACATGTGTGTATTTCTCACAGGTATCCGGATTTCAGCTCCAAGCTCAAGAATGTTCCTG
GCTGCGCCTAACTCACCCATATTATAACCCCTCACAGAGTATGGGCCCCCAAGTGTAAAT
GCATCGTAACTTGGAAGATCTCCCACACAACCTCCATAGTGGCCATGAAGTACTAGAACA
GGNNNNNNNNNNNNNNNNNNNNNNNNNNNNNGGAAACTTGCTGCCAATACCAAGGCCTTGGT
CCACCTGAAAAACATTCCTCTCACCAACAATAGCTCCATTCACAAACTTGGTGTTGTCAC
GGGTAATATTAGCCTGTACAAAGGCCATTCGGTCAATGCCAGTACCACTAAGGGTAGTTG
GAGGTCCATCTGCGCTAATTCCACCACTTGGCAACACTCTCTGACCATTTGGAGAGATAT
GACTGCTTTCATCACGTGTTGTTATCTCTTCCATGACAAGACCATAAGTGAATTTGCTTT
GACGGGTGAAATTCTCTGTAATGTTAGCTTTAACACCAGCACGATCAACCCAGATAGGTG
GGACTTCATCCAGTCCTGGTCCACCAGTGAACACTGGACTCAGTTTCCGACTGTTGAAGC
AGCTTGCACGGAATGTACGGTTACGAGGGTTATTCACACCATCCAAATATGGATGTACAT

ACTCCAGCTTAAAAGCAAGATCATCCTGAGGATTTAAGAAGTTGCTGGTGGTCACTGAAC
CGAGAAGGGATCTATTCAGCCCTTTCAGATTGCGGTGTTCAAAAGAAACAGTTCCACCCG
GCTGAAGTGAAGCAAATGTGGGGCGTCCACCTCGTCCAGGAACAAGACTCCATTCAGCAC
TAACTTCAGCTGATTTTTGATCCAGTTCTTTAAGCTTAATCTCAACAATGATTCCTCCTT
CATTCTTTTCATCAGGGCGTGGGTTCACCTCAATATTGGAAAACAAAGCCAGCGAGTTTA
TATTTCTCAGAGCTTGTTTACCAGCTTCTATGTTAAAAACATAGCCTGGTCGAAGCTGTT
TGGGAAACTCTCTCTTAACAACAGGCAATTGGGTGTTCCCTTCAACCACATTTCCAAGCT
TATCCTGAAACTGCACCACCAACTGAGTTATATCCCCTTCCACAACCTCACAAACCACTT
CCCTCGTATTCAAGTTGCCAAAATTCACCACTTGTGCACAAGCATACCCTTCATCATGGT
ACCACTTCTGTACCCTGTCCCTTATCTTCTGCAACAACCTCGCACTCACTTTTCCATGGT
CCTTCAACATATGCAAAATCTCCCGGTGCACCGAATGGGGCAACAAACACGGCCTCGCCT
TTTCAATCCTCCTCCTATAGTCCTTCTCCTGACTCCTATAATACTCCATTTTCTCCTTAT
CAGTCATATCAGGATCCATTTCAATAGGTTTCGATTGGGCCATCAAACCCACGTTTATGC
ACCTAATTCTGTCAGCTGACTGCCACGTGCTCTCCAAAAATGATATTGTCAATGCTAATG
TACCATCGGGTTTCGTTTTACTCTCCATATCAACCTTCTCAAACATCCCACAAGTGGCTA
AAGATTCGAGCTCTTTCACAAGCTGGGCTTTAGTGTAAACTCCTCCAGGACGTAAAGAAA
CCATTTCAAAGAAAGAATCTTCAGTCCCCACAGTGACGCCACGTCGCCGATCAAAGAACA
ATATTTCTGAAAGCTTATATTTTTTGAATCCGCTGAGCTTGTTGAGTTGAACAATGATAT
TCGCTGGGAGACCATGAGAGTCCCACTCATTTGACTCGTTATTTTCATCTGCAATAGCTG
AATCTTGACAAACTAGGTTTCTCCAAAACCCATTATCACCATTACCACCAAAGTTTTCTC
CTCCACCGCCGTCACCACCACCAAATCCGCCATTTCCACTACCTCCACCGTAACCGCCAT
CGAGAATTGGAGTGAGACGGAGGAAGAGAGTGGCAGTGGCGGCGGATGCAACAGCTAGGG
GTTTAGAGAGGGATTTAAGGACATTAGAGTAATTGGGAGATTTGTTGGTAGACTTGTCAC
GGTGAGAGGAGAGATTACATTGTATAGAAGTGGCGCGTGCGGTAGTGGAAGATGAGGAGC
GAGTGAAGAGTAATTTAGGACGAGAGAAAGTGGGGGTGGATGAGGAGGTAGGGAGGAGGT
GGGTAGGAGCGGCGAAGGAGGACATGGTGTTTGTTTTAGGGATAAAGGAAGAATAGACAA
GAGAAGAGGATTTAAGGGTTTAGAGGACTT
>tig_1013_consensus
ACATGGGTCTTTGAATTTGGTTTTCACTTGGGGTGGCATACTAATCAATTTATCTTTTAC
AGAGAAGAATGTAACAAACATAGACGGTCGCCCTTTCTAGCATTTAAATTGGTATCGACC
TACCTCTCCCAGACTCTAAACTTGTATCCCAACTCCACGAATTTATATAATGAAATTAAA
AGAAGACCTGAACCAATACAATACATGAATAACAGACAGTAAGCTTAAGCAGAACTCAAG
AAGACCATAAGCAAGTAATACCTTACACAGAATATAATGGACTCAGCAGATCCAAATTTA
TGAAGTGCAAGCACATAATTTTTGGCTTTCTAATACCAGCACATTGGCATTAACCATATC
CTTTTAACATTATAGGTAAATCCCGACCGGATCCAATTTCTTTAATGCCTAGATCTTATC
AATATCTTCATCTTCAAACTCAATGTAATCGTCACCAGCACCTTCATCCTCCTCGTCAAG
ACCACCGGCAATACCCTCATTGAGACGGGTGCTCTCTGGAAGCTCACCATATGCCTTCAG
AAGCCTCGCCTCATCAGGCATGTACTTGAGGATGACATCAGCTTTGTCATCTTGATAGTC
ACGAAGCCCAACAAGTATTATATCACCAGCCGCAATCCAAACCTTCTTGTGCATCTTACC
ACGGATGTGGCAAAGACGTTTGGTGCCATCAATGCACATTGCTTCACACCGCCCATTCCC
GAGCATGCGAAGAACTTGGGCGTACTCTTGTCCATCTTCCTTGAACAAAAGCTCAC
>tig_1014_consensus
CAAGAAAGAAGTCATGTGCAAAGATGCTTCTTCTTAGAGATTGCATATTTAGCCTATCTC
CAAGTTGCTTTCTATCTTTCAAGAAAACATGAATCTCTTTCTTTCTACCTAGAATGTGCT
CATGAGTTTACGGCTTGTATATATAATAAATCCCTTGTACATGGTATGCTCTAAATGACT
TAAAAATATATTTTACAAAGGCTGTTGTTCCCCTATCCATTCAATGTCACAACCTCAAGA
AGCTGCCCAGTATATTACACAAGGCCATCAATGACTTGCTGGCATCCTAATCATATCAGT
TTACACAGAAGAAAGTAACAGGCCCTTCCCAGCCGTTGAATTGCTATCTCCTCCTAGCTT
TCAAACTAGTATCTAACTCTAAACCTTGTAGTGAAACAGAAGAAGGCCTGAACCAATACA
TATACCAAAACAAAGCAAGATTCGAGAAGCCATAAGCAAGTAATACCTCACACCAAATAT
TATAAAAATCACCACAGCCAAATTTATAAAAGAGCAAGACAAATAACTTTTGGCTTTCAA
ATACCAGTACATTGGCAGCAAGCATATGTTTTTTTAACATTAAACATAAACCCCAAATAG
AGTAGATTTCTATAAAGTCTAGATCTTATCAATATCTTCATCTTCAAACTCAATGTAATC
ATCACCAGCCCCTTCATCCTCCTCGTCCAGACCACCGGCAATGCCCTCATTGAGTCGGGT
GCTCTCTGGAAGCTCACCATATGCCTTCAGAAGCCTAGCCTCATCAGGCATGTACTTGAG
GATGACATCAGCCTTGTCATCTTGATAGTCACGGAGCCCAACAAGAATTATATCACCAGC
TGCAATCCAAACCTTCTTGTGCATCTTACCACGGATGTGGCACAGACGTTTGGTGCCATC
AATGCACATAGCTTCACACCGCCCATTACCAAGCATGCGTAGAACTTGGGCATACTCTTG

CCCATCTTCCTTGAACAAAAGCTCACGTTTCTCATCATCTGCTTCATTTTTTCCCCTCTT
CCTGTTCTTTCCTCCCTTTCCCTTGTTCTTCGGCATGTTTTCTGATCCTTCGGCGAAGAA
GAGGTGGAGAGTAAAAAAAGTAGCTAAATGTTCTGAGACTAACCCTAGCGGTTTTGTACA
GAAGATCAGGTCAAGTTGAACTTTATGGGCTTCCCACCGACCTGACCTAGCCCGGCCCAA
TTGACTTCCTTAACCGCCCCTCCCTGCTCCCTGGCTACTTCTCGTATACGATAACTTGGA
GAATTCAAAGTTGTTTTTGATTTGT
>tig_1015_consensus
GTAAGTTTTGCTCAAACTATTAAATCACATTAAAAATTCTCTCAGATTCACGGGAACGTC
AGATGGATATGAGCTAGTATCTCATATAAATAGAATAATAGCAAAATGGGAGAAATTTGA
GGCCTGCAATGCATAAGGCTTCAACAATGCCAGAATCAGATGCTTGACGAGTTCTATGTA
TACGAACTTTACTAAGCACAAACCGATAAAATTGAAACAATCCCTCACCTTAAACTTATT
CCTGTCAACATCACAAGATTGGCAACGTAATAGCAATATAAATAATATAATCATCCTACT
ATTATAATGCAAGGCTTCACATTGTATATAATTATCCGCACTTAAAACCAAACCAGAGAA
AATTCATCATCCTACTAGCTACTACATTAATAGATCCAACTGCTGAATATAACTTTTACA
CAAACAGTTAAGCACACAAGTATAAACAATTTCTTAGTTGTCTACAACCAATTACAGATC
TGTGTTCCAAAACTAAATGCATCATGCAATGTTATTACAGCAGATATAAGAACTCAAGTA
TGATAATAACAGACATGTATAAAAGCAACCATTATCTTGGAACAAGATGTCTCACTAACC
CAAGGTTTGCATGCTAAGAAATGGGACAACTTAGCCGTAGCATCTGATAGGATCATTGCA
GCCTTTTTCAGCAGATTGCCTTAATCCAAAACGTCCCCCCTTTGCAAGGTACTGCTGGTG
GTACTCCTCAGCTCGGTAGAATTTCTTGGCAGGCAGAATCTCAGTAACAATTTTCCTGTT
CAAAAGCTTCTGCTGTCTCTCCAATGACTCCTGAGCTGCCTTTTCTTGCTCAGGGCTGTA
GAAGTATATTCCAGATCTGTACTGAGTTCCCACATCATTAGCCTGACGATTTAGCGTGGT
GGGATCATGCCTAGCCCAAAAAACATCAAGCAAAGTCTCAAAACTACACTCTTTAGGGTC
ATATTGAACCCTCACAACTTCATTATGATTAGTTTTCCCAGTACACACATCTTCATAGCT
AGGATTATGCAAAAACCCTTGACTATATCCGACCTCAGTTTTGGTAACACCAGGCACTCT
CTGAAACGCCAATTCAACACCCCAAAAACACCCAGCGCCGAACTGAGCGAACAGCTGACC
TGGCGCCGGCACATCATCATCCGGACCTTGAGCAATTGAAGAATTCTCCATGGAGGCAGC
GTCGGGCGTGGGCTTGAAACCAAAGCCAAGCTTGTTAAGAATGTTCATGGGAGGTTTGTA
AAGAGAAATGGGTTTTCTAGTATTAGGGAGAGTGATGGGATTAGGTTTAGAGAGAGAAAA
GGAAAGTTTAGAACAGAGAGAAGAAGAGAAGAGGAGGAGTGGGGACGTGGAGGAGGAGGA
GGAGGAGGCGACGACGGAGGACGCGGTGGTTGTTTTGGCTAGGCCTAAACACTTATTTAG
GACCATACTTTTTGGTTAGAAACGAGTTCGGTTATGTTCTATCTAGTCGAGATTGGATCA
TCCAAGTTACCATACAACTTTTTCG
>tig_1016_consensus
GTTTGTGGCATCCAGCACAAGTAAAGTGTTGTGCTTCTAGCATCTGTTTCATGGATTTTT
GTGGCTGAATTGCAACAATAAGAGATATGGTCTTCCCTAAAGGAGAAACATTTTGTGCAT
CTAACCCTCTAGTAAAAGATGTGGACTGAGAAACCAGCGTATTTGAAAAAGGGAAACTGA
CTAATGATGCCTGTGGAAGAAAGAACCAAATCAATGCACCTGGAGGGCTGGGAAAGAAAC
TGGAATTGATGACAGATTGTAAACACTCTTGAATGAGAACACTTCTCTCAGCAACAACAA
CAGGAGATGCATTTCCAAATATCTTGCGAGATTCTTTTTCAACAGATGACCAGGGAGAAG
GGAGAATCCAGCCCTGGTCAGCATAAAATGATTTCAA
>tig_1017_consensus
GGGAGAGAACCCATTAAAATAACATTGTTGCTGTCTTTATGCTCGCTTTTTTCTGGCTTT
GCCCTAGAAAATAGTCCCCAGAGCAGTCCCGTAGATGGTCCAGAATCTGATCCCTTCCCT
AGTGTGATCAATGTTCCATTAGTCTCCCCACTGTCATTCTGGCTTACCCTTCTGGCAGAA
TTGGCAGCTTCATCCATCTCAAGGCTTCCACCGCAGGGGCAGTGTGCCAGCTTTCTGAAG
CAAGGCTTATGGAATACTGCTTCACAAGATTTACACTTTTCAATGTCATCTTCCTGAAAA
GTGAAAATGAGAGAAGATGGGTCATTACAAGCTTGTCGAGCACTGCAGGGGACTCCAACA
TCGCAACATATAAGGCACTGCTCTGTAATATGCTCCAGGATTTTTCTTGAAACATTTTCC
ATCATTACAGGTAGTGCTGCAAATGGCCCTTTGGAAAGGTCAATGAGGTCTCTGAGGGCA
AAAAAGTCATTGCATTCAAGAAGATATCTTCGAGATCCAAGTCCTTCGTTGATGGATCTA
CGAAACGGGCAGCGAACAAATGAAAGCATAGCTCCGATTTTTTTCCTTACACCCAGTACA
TGCTGTAGGGCTGGGACCTTTGAGTACAGGAAAGGATTAACTGCACTCAAGCAAAGCATG
GGCTGATCATACACAGAATCCAAATATGATTTAGCCAATTGAGAAACTGGATATCGTGTG
AAGTCCCAATGATGCAATACTCTTGCTGGCAAGACTGCCGTGTCACTTGTATGGCAAGAA
GAACAGAACAACTGACCAGTATATTCACAAAGTCGAGGTTTTCCCCACCCAAAAGTCTGA
ACAAAGTCTTGTATTAGAGTCATTCCATCATCAAAATGTTTGTGGCATCCAGCACAAGTA
TAATGTTGTGCTTCTAGCATCTGTTTCATGGATTTTTGTGGTTGAATTGCCACAATAAGA

GATATGGTCTTCCCTAAAGGAGAAATGTTTTGTACATCTGCCCCTCCAGTAAAAGATGTG
GACCGCGAAACCGGTGAATTTGAAACAGGGGAACTAGGTAATGATTCATGCGGAGACAAG
AACCAAATCAACGCACCTGGAGGGCTGGAAAAAGAACTAGAACGGATGACAGCTTGTAAA
CACTCTTGAATGAGAACACTTCTCTCAGCAACAACATCAGGAGATGCATTTCCAAATATC
TTGCGAGATTCTTTTTCTACAGATGACCAGGGAGAAGGGAGAATCCAGCCCTGGTCAGCA
TAAAATGATTTCAA
>tig_1018_consensus
CCCCCATTTACAATCACCAAACTTTCTCATTCAAATGTTATATCTTACAATCAGATTGGA
AGATTTGAAATTAAAACTAAAAATTAAAAAATAAAAGCCAAGTTTTTGGTAGAGCTTCCA
ATGAAATAGAGCAACTAAGCCACTACTTGTACACAACACATAATATCATTAACAAGTCTG
ACAAAGCAAAGAGGCAGGATTTGAGAACCCACTTGTTTTATTTTGATCACAATGACAAAA
TGGCAACAAATTCTTAAGCACTTAGTGGCTTACCAGCTTCAGAGAGTTTGCCTTCTCTTG
CAGGGTGTCCAGCAGACTGTCGAAGCTCTTTTTGAAGGAGTCGACTCCTTCAAGTTCAAG
TTGAGTGCCAACATAGTTCCAGTCAATGCCCAACTTCTCAAGTGCACTGTAAACACCTTC
AGCTTCGGATACATTTAGGTCAATTGTTCTTGAAACAGCACCATGATCAACAAATGCTTG
GAGAGCTTGGTCTGGCATTGTCGAAACCGTATCAGGTCCAATGAGAGGAGCAACATACAA
AGTATCAGGGTAGGCGGGATTCTTAACGCTGGTTGAAGCCCACAACAACCTCTGCTTCTT
AGCACCTTTCTTCACAAGAGCCTCCCATCTTGGACCAGAGAATTTCTTCTGGTAGAGCTG
GAATGCCAAAGCAGCTTGAGCTACGGCAGCCTTTCCCCGAAGATCAAGAGCTTCTGGTGT
TCCAATCTTTTCAAGCATTTTGTCAATAAGGGTGTCAACTCGACTAACAAAAAAGGAAGC
AACACTTGTAACTTTAGAGAGGTCATCTAGACCAGAAGCCTCAAGGCCATCCAAGTAAGC
ATCAATGACTGCTTCATATCTAGCAAGGGAGAAAATGAG
>tig_1019_consensus
CTTAGTTTCAACGTTCTCTTAAGTTTTTAGATCATCTGACATAGCAGAAGGTATGCAATG
TGTTGAAAGAATGTCAGAGGTAGCTGCCTTGGCTGTCTTAGTCTGTAGAACCACTATCTG
TTGGTACTCCAGATCAAATTTTAAATATTCATCTTCACAACTCTCAACCATGTCAGCTAG
GCTTTTAAGATTCCTCACGGGCTTACCATTGAAAGCAAGAACCTGTGTATTGACAATTTC
CTCATATCCAATATTGATATCAGCCACAAGCACCTGAGAAATGACAACAACCTGTTCATC
GATCGATTGTGCCATTGCATGCAAAAGCTTATCCAGCAGTTTGACTGGAGCATCAAATTC
ATAATCCTTTCCATACTCAGAACGCAGATATGGAACAGTTACAGCTGTGAAGACAAATCC
CGCAATTATGTAATAAGAAGGAGGTTTGCCATTGATGTGTGCTGGGATAAGCCGCTTATG
AGTCGCAAGCTTTATGTTGAATTCTAAAACCTTTGAATTTCGAAGAACTTTTACATTAGC
AGTATCCCCAGTGTACTTTTGAGAAACTAAGTAACTAAAACTTATGCGCTCCCCATGCCT
GAATGGAACTGTTCCATCATTAGCAATATCGATGCCATCAAAGCTAATGATGATATCAGA
TGGCTTCAGAACCTGAGACTCTGGAGCTGTTGGTTCAATTCTTCTAATGCGAACACCCTT
TTGTTCACGTCTCATCCCCATTGCCATGCGCAAATCAGGATTTTCCATCTTCTGCCACTC
AACTCCAAGAATTGGAAAACCTGTGTAAGCACCATTTCTCTCATAATCTTGAATAAAGTG
CATTATAACAGGTGTGGGTATCACATAACCTATATTTTCTACATCTTCATGTTTTAGAGA
TTGAAATGCAATGCCAACACATTTGCCCTTGTCACTAAATGCAGGTCCACCAGAATTTCC
AGAATTTATAGCTGCGTCTATCTGCAGTCCCAGCAGTTCAGTTGAGCCATGGGCATAAGA
TAGTATCTCCATGCGTGAAACAACCCCACTTGTCACAGAGATAGTGTCACCCCCAATCGG
ATACCCCACAACAGTGACAGCATCTTGAAGAGCTGGTAAATCTCCAAACTCCACTGGTGA
AACTCCTTCCCAGAACTCATCATCATTAACAGTCAACATTGCAATATCACACTCTGTCCC
AACAGACAAAACGGTGGCCAAATATTTCGTATCGGAGCCCCGCTTCTTCAATTTGACTTG
CGTATGATGATCAACCGAATGAGCATTAGTTAAAACTCTTCTTCCCCCCACAATAAACCC
ACTACTACTCGAACTATACTGCCGTTTCCTTTGCCACGCCAGCGAGAAATTCGGTTCCGT
ATGCACACAAAACACTTTCACGACAGCATCCATCGACGGCACAGCTTTAACTGCCACGCT
GTCCCACCTCGGGGGCAATATAGTTATTTCATTCGCATCCCCGTGTCTGCTCCGTTCGGG
CAAAGCCAAATGGCCAACGTTAGAATTTGAATTGTTAACGTTGCCGTTGTTGTTTTCGTC
ATCGGTGAGGGTTCGTCTCGTGCGGCCGCGTGTTCGACGAGGGGAAGAAGGGTTATGATG
CGTATCGGCGACTGCGGTGGCGGCAGCGTCGTTTTGGAAAGTGGGGTTCGTATATTGATA
ATCCATGGTTTGGGTTTCGGCTTCGGGGGGTTTGCGTCCACGTTTTCTTGGGCGCGGCAT
TTCCAGGCGGTTAGGGTTTTAGCGCGTTTGGGTTCTGCGTATATTTTCTTCTCGGAGGCG
GCAGTTAAACGGGAGAGAGTAAGGTCAGAATTGAGTCTATTGGGCTTTCTAAATGGGCCC
TTATCATTTTCTCAGTGGGAGATTATCCCTTTCCTCCCTAGTTTTTACGGTAC
>tig_1020_consensus
TTTTTTTTTTCTAAAATAAAAGGCAAAAGGGTATATCATCATCATCATCATCCTTGAACC
ATTTCAAAACTTAATGAGCTGAATGAATGGAACCTGTTCTGCATTCTGCTCTATGTATGT

ATTGTTTTGAATAATGGGGCCAAAAAGTTGTGAATCCAAAATTGAAAAAAAAAAATCAACA
CTGTGTTAAAATTTCCGCACCAGTTCTAGTAATTAAAATTGTATGCTCAAACTGTGCAGC
AGGGCTACCATCGGCTGTCAAAGTTGTCCAATTGTCTGGCCAGGTTATACACTCGATGCT
TCCCATAGTAAGAATTGGTTCAATAGTAAATGTTTGACCTTCA
>tig_1021_consensus
CATCGGCTGTCAAAGTTGTCCAATTGTCTGGCCAGGTTATACACTCGATGCTTCCCATAG
TAAGAATTGGTTCTGCAAAAAGAGCTCAAATGAATTACTGTATCTGTTTCATAGTGTCAA
TGTAAGAACATTTATGGTATTTGATATGAAGACAGTCCAAAACTTGATTGTTATCAACTC
CATAACTTTCAGAAACCCCAAGCTGGGTTCAATACTCATCTTAAAAATGAAAAGTACTAG
TTTTGCCGACTCATAGCTCACTTTTCTGGGAAGTTCTCAACACCCAGTAAATCCTACTTT
GCCCTGCAGTTAGCAAGGGTAAAGTGACAAGGAGCATCCCTACTGCCATAAAATCTAACA
TAATTGCCACCCAGGCCAATCTATTTCAAGTGTGTCTGGGTTGACAATGTGCTTTGCCAG
GCAATTTATTTGGTCCCACCTGAACTAAACTTGGAAGTGAAAATACCTCCTGGATTCCCA
GGAAAGAAAGAAATGCACTCACCAATAGTAAATGTTTGACCTTCA
>tig_1022_consensus
TCTCTCTTGAATTAAATCATGTAATTACGTCTAAGCATTTAACATATATTCCTGGTTCTC
AGTATTTGTTCACAAACCTAAAAGCCTGACTGTACTACGCAAACTATGTAATATAATGGC
TTTCAATATCTCTCTCCAAATCGAAAGAATATTGCACCAGTACCAGTATTATGGTCAACT
GCATACTCAGCCCGCACTAGACCCAGCTTGACACCAACACCATATGATGAACCATGGCCC
ATGCGCCTATAGACCTCAGTGGGGTTTCCCTTGACATCCTTCGAACTTCCTAGATCATTG
CCATGCTCTGCAAAAGCATACACGTGTGTATTTCTCACAGGTATCCGGATTTCAGCTCCA
AGCTCAAGAATGTTCCTGGCTGCGCCTAACTCACCCATATTATAACCTCTCACAGAGTAT
GGACCCCCAAGAGTAAATGCATCATAACTTGGAAGGTCTCCCACACAACCTCCATAGTGG
CCATGAAGTACTAGAACAGGCGGTGGTGGTTGATTAGCACCTTCTTCAACTGACTTTAAT
TGGATAAATCGTGTCAATGTCAATTGGTGGCGGTTAAAGAATGGAAACTTGCTGCCAATA
CCAAGGCCTTGGTCCACCTGAAAAACATTCCTCTCACCAAGAATCGCTCCATTCACAAAC
TTGGTGTTGTCACGGGTAATATTTGCCTGTAAAAAAGCCATTCGGTCAATGCCAGTACCA
CTGAGAGTGGTTGGAGGTCCATCTGCACTAATTCCTCCACTTGGCAACACTCTTTGACCA
TTTGGAGAGATATGACTGCTTTCATCACGTGTTGTTATCTCTTCCATGACAAGACCATAA
GTGAATTTGCTTTGACGGGTGAAATTCTCTGTAATATTAGCTTTGACACCAGCTCGATCA
ACCCAAATAGGGGCGACTTCATCCACTCCTGGTCCACCAGTGAACACAGGACTTAGTTTC
CGAATGTTGAAGCAGCTTGCACGGAAAGTACGGTTACGAGGGTTATATACACCATCCAAA
TATGGATGTATGTACTCCAGCTTAAAAGCAAGATCATCCTGAGGATTTAAGAAGTTGCTG
GTTGTCACTGAACCGAGAATGGATCTATTTAGTCCTTTCAGATTCCGGTGTTCGAAAGAA
ACAGTTCCACCAGGCTGTAGTGAAGCAACTGTGGGGCGTCCACCTCGACCAGGAACAAGA
CTCCATTCAGCACTGACTTCAGCTGATTTTTGATCCAGTTCTTTAAGCTTGATCTCAACA
ATGATTCCTCCTTCATTCTTTTCATCAGGACGAGGATTCACCTCGATATTGGAAAACAAA
GCCAACGAGTTTATATTTCTCAGAGCTTGCTTCCCAGCTTCTATGTTAAAGACATAGCCT
TTTCGAAGCTGTTTGGGCAACTCTCTCTGGACAACAGGCAATTGAGTGTTCCCTTCAACC
ACATTTCCCAGCTTGTCCAGAAACTGCACAACCAACTGAGTTATATCCCCTTCCACAACC
TCACAAACTACCTCTCTCGTATTCAAGTTCCCAAAATTCACCACTTGTGCACAAGCATAC
CCTTCATCATGGTACCACTTCTGCACCCTGTCCCTTATCTTCTGCAACAACCTCGCACTC
ACCTTTCCATGGTCCTTCAACATGTGCAAAATCTCCCTGTGCACCGTAAGAGGCAACAAA
CACGGCCTCGCCTTATCAATTCTCCTCCTATAATCCTTCTCCTGACTCCTATAATACTCC
AATTTCTCTTTATCGGTCATATCAGGATCCATTTC
>tig_1023_consensus
GACAGAATTCTTTAAAATATTATCTTTAAAGAAGAGAGGAGGAAGGCAGTTGCATACTGA
CAGAGCTCCCAGACAGCCAGAAAATAAACGCTCCCTAGTTTTACAGAAGACTAAGTATAT
ATTTGAACAAGAAAGCACAGGCTCCACCACTACCAGAAACAAAGTTGGCCCTCTTTCTTG
AATTCCCATCCCAAAAATAGTTAGTGGAATTATGTACCATATTGAATCATAAATCAGGAA
AAATAATTAGAAGAAAGGAAGCGACTCAGGATGACTAAACATTTCATTGATAAAACACTA
TGCTATATCTTTTACTTTTGGAATTAGTACAAAGCGGGCGGGAATGGATGCCCTTCACTA
TTGTGTCATGTACTAATGTCGTGATCTGCAGAGGGCCACCAGGCAAGCAAACTTGCCGAT
TCTGATTCTCTTTCTCATGCAAGGCTGCTGATGCGTAAGCAGAGCCATAAGCAACAATCC
CTAAGCATCATTTGTTCGGAAGATCCTTTTATGAGGATGGTTTCCAATCTAATTGAAGAT
TATTAAGACCACTAACAGCTCCAAGACCAAGTAGCTGGTTCCCCAAATCCTGCTGTTGAT
GATTCATGCTTTGATCTCGAGGAGGCACTGTTGTCATGCCTACCCTTCCGTTCATCATGG
AATTATTACCCATTGCATTTCTGATTCCATTAACCATTGCAGACTGGCCAAGTCCGCCAC

CCATGTTGCCAAATCCTGCACTTCCAATGCCTGAATTATTGTTGACCGTCCCATTTCCCA
CCAGACCATTACCAGTGTTGAGAACTGTATTGGTACCTGTCGATAAAATCCCATTAACAT
TTTTAACATCATTTCCCATTGAACCAACACCAAGCATGCCACCCCCACCATTAAGTTGGC
TGCACATTATCATTTCATGTATGATTTTCTGGAAAGCGTTTTGAGAGTCACTTGAGTCAG
TCTCAGTAGAAAGAGATGGTTGTGGAACCAAGATATTGGCTGGTGAATTTGTAGAACTCA
AGTGATTGGCAGCTGTTAAGGCACTGTGAGATGTTTGCAGGGGATTGTTAGTTGAGGGTG
TTGGTGATTGAAATGGTGAAGGGCTTTGAGCCTGTGCAATAGTATTAGAAGATCCCGGCG
ACGGAATCTGTACTGAACCTCCACCATAAGGACTGCTTGCATTATTCATAGAACTCTGCT
GTCTAGAGTTCATTGAATTTTGGTGAAGAAGTCCAACAATAGTGCTCGCCGAGGTGGATG
CAGATCCAGGATTGAGTGAGTTATTTACACTAGCCACACCATTGCTAGCAGCAAGTTGTA
CTGCATTGGCCTGGACAGAGCTCTGATCACCATTTGAGTTTGGGGCCACTGATTGCTGTT
GCTGATGTTGTTGTAACTGATCCTCGGGCCGTTGAGTTTGGCCTTGCAACGCAGAAGAAG
CACTAGTTCGTCGGGGGAACTTAGCCAAGCTCTCCATAGGTCCAGTCCCTGTCTCTCGAC
TGTAGTCAATCAAGTCCTTCATACTATTAACCACTTCTGATATCTGAAGACATCGGACGT
ATCTCTTCGTATACCCTAAATCATTAACCAATGGCACCTCCAATGCTTTTGCCAATTGTC
GAGCCGATGCAACAAACGTATTACAATTATTTTGTAATTCTGAGACAGACAAACTCGATG
ATGCATTTTGAGTTGTAGCCTGGTATTTTTGGGCTGCAGACCCAAGCTGAGTAACCTGTG
GTATCAGCAATCTTCTCGGGATAAGCTCTTCATGACGCCGAGCACAAAACTCCCAAGAAC
GTATCTTCAGATCTGGAGAGAAAACTATCCGAAGTTGACCATCACGAACAACACGAAGTT
GCTCGAAAACACTTTCCTGTATTGCTTTTGCATAGTCCAGGACAATTTCACCAGATGAAT
TCTGATATTCGTAGGGCATATCAACATAAAGAAGCTCTTCTAAAGTGCCACTTTCATACT
TGATTTTAAAAAGCCTAGGAAGAACCTCAACGGTTGCTTCAAAACCACGGCCTGGTTTAC
GATTACATATCTCACAATGCCATACATCCTGAGGAAAAACTCCAGTCGTTTGCCGGCCAC
TTCCATACATAGACACACACCACTTTTTCTTGGCGTTCGGAGCAAAGTACTCAGCAACAA
ATTTTCTCCAAAAATCAATGTTGTTGTCTTCAGGTCTATGCTGTTGCTGATACATGTAAT
GTGTCAAACGCCGGGCACACATCCCAGGTTCATATACAGGTTTCACAGAAGATCTCACAG
GCAAATTCTGCTGTTGAACTGACTGTGGCAACTGAGGACGTTGTTGAGGCATCACCTTCA
ATAGTTGTTGTTGCTGGTGATGTTGCAGTTGCAACAATCTTTGTCGCTGTAAAAGGTTAA
TTTGGGCAACAGCCTGCTGAGAGGACTGCCTTGACATGTGAAGGAACTGCTGTTGCTGTT
GTTGGTGCTGCTGCTGTTGCTGTTGCTGTTGTTGATGATGCAAAAACAGAGACTGCTCTG
AATGTTGAGGCTCCATTTTTACAGGTGCCAGATTTCTCATTGTCGGAATTTGTTGTGGTT
CCAATTTCACTGGACCAAGGTTTCTCGACGGTTGCAACTGTTGCTGCTGCTGTTGCGGTC
CATGTTGATCATTACCCACCTGCTCCAACTTAACTGGTCCACCAGCTAATCCTCGAATTG
ATTGATATTGTTGTTGCTGCGGCACCTGTTGGGTATTGTGAGCAGCAGAAAATAGCTGCA
TTGGTTGCTGGCCATGCTGAAAATTTTGAGTTTCAAGCTGCTGTGACTGCTGCTGATCAG
GCAACTGATTACCAGAAGGGTTTGAGAACTGCTGACCCTGAACTTGACCAGATGAACCAG
GACTCACCAAGTTGGATGGTACAAAAGATGTCGAAGGAGCATTAAACCCCATTCCATTAC
CAACACCTGAAAGTGGATCAGACTCAGCCCCAGTATCCATACCTCCATGCTGATTATTCC
CAGGGCCAGAAAGACCTGGATTTGGACCACCATTTCCAAATGTCTGATTTAGTAGAGAAG
TTACATTGGGAACATTTCCAAGCATATTCATGTTATTGTACTGTGTTCGTGGCGACATAA
GTGATGGGAATGCTGCCTGGGACGGTAAAGGACCACCCTGACCTCCCAGCATTCCAGAGT
TAGAACGCAAAAGAGAAGGTGAAACAGACTGCGCACCACCAATTGGAGCCTGGCCTGGTG
CTACCATTTTTTGGGAATAACAATAATCTATACTCAGTGAAAGCTACAACTATACTTAAA
ATAACATTAATTCAAATAAAAATTAAAATTACACAACCAAACACGAAAAACTGGTCAAAG
AGTTCAACTCAATTAGCATTTCACCCCCGCATCCAGGACTATAGGCTTCCAAGTCTTTGT
TGTGCACCAAAAGAAAAGGGTATGTTTTGTGCCTAAGCATATGGTTTTGAGTTTTACCAT
AGACAAGCCACAAATTAAAAAGAACGAAAACCAAGGAACAAGCTTTGATTCTGAGCATTC
TAGTGTTGTACTATCTCCGCTAGGCGAGCAACACTTGAAAGGCTTTGGCATGAATCT
>tig_1024_consensus
AACAACAACAGCAGCAGCAGCAATTCCTTCATATGTCGAGGCAGTCCTCTCATCAGGCTG
TTGCCCAAATTAACCTTTTACAGCAACAAAGATTCTTGCAACTACAACAGCAGCAGCAAC
TACAACAGCAGCAGCAACTCTTAAAGGCAATGCCTCAACAGCGTCCCCAATTGACACAGC
CATTTCAACCGCAGAATTTGCCTATGAGATCTCCTGTGAAACCTGTATATGAACCTGGGA
TGTGTGCCCGGCGTTTGACACATTACATGTATCAGCAACAACATAGACCTGAAGACAACA
ACATTGATTTTTGGAGAAAATTTGTGGCTGAGTACTTTGCTCCTAATGCCAAGAAAAAGT
GGTGTGTGTCTATGTATGGAAGTGGTCGGCAAACGACTGGAGTATTTCCTCAGGATGTTT
GGCATTGTGAGATATGTAATCGCAAGCCAGGCCGTGGTTTTGAAGCAACTGTAGAGGTTC
TTCCGAGGCTTTTTAAAATCAAGTACGAAAGTGGTACCTTGGAGGAGCTTCTTTATGTTG

ATATGCCCCGCGAATACCAGAATTCATCTGGTCAAATTGTCCTGGACTATGCAAAAGCAA
TACAGGAAAGTGTTTTTGAGCATCTTCGTGTTGTTCGTGATGGTCAGCTTCGGATAGTTT
TTTCTCTGGATCTGAAGATATGTTCTTGGGAGTTTTGTGCTCGGCGTCATGAAGAGCTTA
TCCCACGAAGATTACTGATACCACAGGTTAGTCAGCTTGGTTATGCAGCTCAAAAATACC
AGGCTGCTACTCAAAATGTATCATCTAGTTTATCTGTCCCAGAATTACAGAGTAATTGTA
ATATGTTTGTTGCATCGGCTCGGCAATTGGCAAAAGCATTAGAAGTGCCATTGGTTAATG
ATTTAGGATATACAAAGAGATATGTTCGATGTCTTCAGATATCTGAAGTGGTTAATAGTA
TGAAAGATTTGATTGACTACAGTCGAGAGACGGGGACGGGACCGATGGAGAGCTTGGCCA
AGTACCCCCGACGGACAAGTGCTACTTCTGGGTTTCACAGTCAAACACAACAGGCTGAGG
ATCAGTTACACCAACAGCAACCAGTGGCTCCCAACTCAAACGATGATCAGAGCTCTGCCC
AGGCCAATGCAATGCAACTGGCTGCTAGCAATAGTGTGGCTAGTGTAAATAACTCACTCA
ACCCTGCATCTGCATCTGTATCTGTATCCACCTCAGCCAGCACTATTGTTGGACTTCTTC
ACCAGAATTCCATGAACTCTAGACAGCAAAATACTATGAATAACGCAAGCAGTCCTTATG
GTGGAAGTTCAGTACGGATTCCGTCTCCAGGATCTTCTAATGCTATTCCTCAGGCACAAA
ACCCTTCACCAATTCAGTCAACAACACCCTCATCATCTAACAATATTCCACAAACATCTC
ATAATGCTTTAACGGCTGCCAATCACATGAGTTCAACAAGTTCACCAGCCAATATCTCAG
TGCAACAACCATCTCTATCTACTGAGACTGACCCGAGTGACTCTCAAAGCGCTGTCCAGA
AAATCATGCATGAAATTATGATGTGCGGCCAACTTAATGGTGGGGGTGGCATGGTTGGTG
TTGGTTCAATGGGAAATGATGTCAAAAATGTTAATGGGATTTTACCAACAGGTAATAATA
CAGTCCTCAACAGTGGTAATGGCCTGCTGGGAAGTGGGGCAGTCAACAATAATTCAGGTA
TTGGAAATGTAGGCTTTGGTAACATGGGTGGTGGACTTGGCCAGGCTGCAATGGTTAATG
GAATCAGAAATGCAATGGGTAATAACTCCATGATGAGCGGAAGGGTAGGCATGACAACAA
TGCCTCCTCGAGATCAAAGCATGAATCATCAGCAGGATTTGGGGAATCAGCTACTCAGTG
GTCTTAGTGCTGTCAATGGTTATAATAATCTTCAATTTGGAAACCATCCCCATGAAAGGA
TATAGCAGTCAAATGAAGTTTTGGGGTTTTTGATTATGGCTGCTTATGCTGCAGCAGTTG
GCATGACAAAGACAATTCAGAATCGGCAAATTTGTTCGGTTGGTAGCCCTGCACAGATCT
CAATGAGGGTACATGACATAATAGTTAAGGGCATCCATCCCCTCCCACTCTGTACTAATT
CCAGAAAGTAAAACATATAGCATAGTGTTTTATCAATGAAATGTTAAGTCATCCTGTGTT
GCTTCATTTCTTCTAATTGTTTTTCCTGATTTATAATTCAATATAGTCACATAATTCTAC
TAACAATCCTTGGGATGGGAATTCAGGAAAGAAGGTCGACTGTTTTTTGGTAGTGGTGGA
GCTTGTGCTTGCCTTGTTCAATTATTCCTCTTAGTCTTCTGTAAAATAGGGAGTGTATTT
TCTGGCTTTGTCTGGGATCCTTTTTTCATCTCTTCTTAAGTACTATAATATTTTTTAGAT
TTCTCCCCTTCTCCCTGTATCACCACTCTTTTCAATCCCATTATTAATATTCATTCAGTT
GCTTCATAAATGTCTCTAAACTGTTAATTCATGGTGCCTATGCTTGATGGAGCTGTAGCC
CC
>tig_1025_consensus
CTTTGCTGCAAAGCTACCTCCATAAGCTCTCACCAAGTCCTTCATCCTTAATAATGGTGC
AATATGAGGATTTGCCTGACTCACTATAAAATGGTTGACATTAAAGAGCTCCTTCAATTG
GATCATTGGTAAATCAATCTCTAAGCTGCCATCCCTCCACCTACGCACTTGTGCGCCCTC
CTCAGGATCAACATTAAAGGGTGGATGATAAGGAACAATGTCTCCGCTTCTATCCTTGGC
CATTAGCTCCTGGGCCTCAAAAAGGCCAGGAAAGGCACAAGATGCAGTCACAGCACTCCA
TATAACGACATGAGGCGAAGTTAAGTAGTTAAGGCACCTAGGCGGTTCGTGCTTCCTCGG
GGAGCAGACCGTTATTCCAAGAATTCGACCTGTCATGTCATAGGCTTCTTGAAATGTAAG
ATTACTTGTAAGATTCCTTAGCATCCACTGCAGTTGCCTAATATCATGAACAGCACCTTG
TGTCATGACCCTCCTTACAACCTTAAAAATTCCGCCCAACTGATCAAAAAACTGCAACGA
GTGCCAAGAATCCTCAAAAAAGCTTTGCAGCTCGGGCCATGACCTAGTGGCAACAACAGA
ACACATAATTGATCCCACACTAGAACCAGCAATAATACGGGGCAAAAGCTTATGCTCTAC
CAATGTTTTGACAACACCCACATGAAACGCTCCAAGGGAAGCACCCCCACTCAAAAGCAA
TGCTGTTCTCCCGAAGGCATGTCTTGTTTCATGCATAAAAGCAAGCTTTTCTTCTAAAGA
AAGCTCCTCTGAATCTGAGTCACAGACCATCCTAAGTTGAGTTGAGACCTCATCTATATA
TTCTTTTATAAGTTTGGGCACTTGAAGCCGACCCTTGTGAAGTTCAGGATTGCACATATT
TCCGAGATTTCTGATGAGATCAGCTCTCATGCAAAAGATTATATCTCTAAGAGAACCTTC
TTGACGGCGGTGGTGGAGTTCTTGAAGCTTATTTCGCACCAGTTCTTCATCATAAAGGTC
TGCTTCATTCATCTTTGGGGTTTCCTTATCAAGCATCTTAGCAGCGTGAGCCCATTCCTC
GTACGTTAATGCCGTTCTCATCGTGTTCCTCCAAAATTTTCTCCGATACGCCATTTCAGC
CCTCACTTTCACGTTTGTATACCGTTTCAACAAAAAAGCAATTATCGTTACCATAGCTAA
TATCCCTTGAGGATTTCGGGGATGCAGCATTGATATCATAGGCCCCAAGAAATCCCTAAA
TCTAAATATATAATCCAACAAGACACGAAAGATCTCATGCCTTAATTGTGAAATTGACTT

GCAGAATAAGACTCTGAAAGCAATTATCCGACCAACTATGGATGATGGCCCAATCGAAAA
ACGATCAACACTAGCCTCGTTACTAATATCCATATCTTTGCTTAAACAAAATTCATATGT
TAGGATAATTTCACAATCGCATAAAGATGTTGAAACTTATCGAGGCTTTCTGGCTAATGA
AATGACAAACCCAACAAGGAATTCTCAATTAACGTAAAATTATTACACCAACTATAACCT
TTATAGCAATATGTTTAATCTTTTAAAGAACGAAACAGAAACAAATAAACTTTTCTAGTA
ATCTGCTAAATTAATCGAAAGCAATTATCAAGAAATGGAAGCCAGTAAAAGATTAAGACT
TCTTGATCCAGTTTAGAATTTGACCTGAAAACACCGACAGAAATTATTTTTCATAAATCA
AAATGTTTACCCTAAAAAGAAGAAGAAGAAACCCAAAGAAGAAATCTAAGGAACCAAATT
TGAAGGAAGAAAATAAAAATAAATTCTGAAAGAAACCAAAACACCTGAAATACAAATAAG
ATCAGAAAACCCAGATGGGGTTTCTCAAGGTCATGTCCAACCAGGAACCTCAAAATAATA
ATCATAAACTAAGCCAAACTAAAATAACTACCAGAAAACTAATCTAAGCACAAAAAGGTT
ATAGCTAAAGTTAGAAGCAACTAGAAATAACGAAAAGATGAAGTTCTATGATGAAACGGG
GATGCATTGGGAGGCGGGAAGAGATAAAGATACCGA
>tig_1026_consensus
TTCTGGTATAAGACAAGGCAGACCCACAAGGCTGGGTGGCCTATATTGAACTCAAAGCTC
TAGGCTTGGAAGTACTCTCCAAACGCAAGTAAGCCTCGAGCTGCCTCGCGAGTTGTTAGG
ATACGAGACATCTGCTGTAGTGTTTCTTGGCGAGGATAATCAGCCTGTTCGGGCAAGAGC
TTATAAAATTGGTAACATGTCCAGATGTTACAAGTCAAAACTGACTTAGTTCAGATGGCA
A
>tig_1027_consensus
AACTGACATAGTTCGGATGGCAATTTCTCCATGCCTTGAGTCAGAGCATCTTCTGCCTGC
TGGCATGACAGTCTTAGTGAAATGTTAGTACAAGGATACTTTTCATCGATAAAAAAGACA
GTATATACAAGAATACAGCTTGACTTTGGCCTAAATTTTCTCCTAAAATTCTTAACAAAT
ATTGCTAAGGATACAATACAGCTCATATGATACAGCTCACCAGAATAATGTACAAATATG
GCAACAGAATAAACAAAATCAATGATAATCAAACAATAGCCCACTTCAAGTTGGAGCATG
GATGTCAAGAATGCCCAACTTGCGAAGTAAAAATTGAAACTGATCACGACCCGAGGCCTT
GGTGAAAATGTCAGCAAAGTGGAGTTAGGTAGGCGCATGGGACATAACAATATCACCGGC
TTGAAGCCTTTCTCGAATAAAATTGACAATCAGTTTTAATGTGTTTTGTCCGTTC
>tig_1028_consensus
ATCAAGCTCAAGGTGCTGATGTTTTGGCTGAAAATTCTAAAAGCTCCTTCTCAGATCCTA
TTAATTTGTCGAGGTTTTCTGGGCTTGATGAAGGTGTAAGTTCTGTTGTCAGAAATCAAG
TCCAAAGGACGGAAAATACTGAAAGTTCTTCCCCTATGCCAATTTGTTTGCCAAGATTTT
CTGGGCTAGATCAAGACAAAGGAGATATTTCTGTTGCTAGTAATGATGGCCAAGATGCTG
ATGATGTGACCATGTACCATAATTATGGCTCTGTTAATAACGCATTTGTGAGGGAGCAGG
ACCCAGTGCTTGAGGAGGCTCCCTTGAAGATGGGGTTGGGCACTATTGATTCCAGATTGG
AAATTATTGATCAAAATTTGAGTAACAAGGAGCTCAGTATCAATAACAGTGGAACTTTAG
AGAAGAAAGAATTTGGGTATTTTAAGGCACAATTGGCCCCTCTTGCTGATATTCCCATAG
ATCAACTTTGTGTTGATTCAATTGGATCTGCTAACATGTCAAATTCAGAATCCTTTGAAG
ACTGTGAGACGAAACCATCCCCATGTACATTTGTAAATATCTTGAATCCATTAAAGAATG
CTCCAGCCTCAGCAGATCTTTCTGACGACCATCTGGCCATGACCAAGAAAGAGAATCTTG
AGTTACGTTATTTTTATGACGAGATTGTTCATGAGATGGAAGAAATTTTACTTGACTACA
ATGCATCCCCTGGGGCTATGTTCCCTCAGGGTAACCAAATCTTTAAATCTCAAGTTTCTC
TACCTCTGAGAGATGGTGGCTCCACAGCTTCTACTTCTGGTACAGATGATGTTTACCCTC
TGACTCAACTTCCCCTGAAAATCGATGGGGTTGAAGTGGTAGGTGCCAAGCAGAAGAAAG
GTGATGTTTCACTAAGTGAGAGATTGGTTGGAGTGAAGGAATACACTGTTTATAAAATAA
GAGTGTGGAGCGGAAAGGACCAGTGGGAAGTTGAACGCAGATATCGTGATTTCTTTACTC
TCTATCGTCGCTTGAAATCATTTTATGCTGACCA
>tig_1029_consensus
CTCGAGAAGACTGGAATTAAGTCCATTCTTTCCTAACCTTTCTTGATCCTTGTTTTGTCA
TTAAGGAAATCGACAACTTTGTGCGGGCTGAAGAGCCTTTACTGTCCACAACATAGGCCG
CAGGCACATGAATATGAACAGACAATACATTTCAATTTAGGCCAATTTCACAGAGCAAAC
AAAATTCAAACAACGCAAATTTCTATGACTGTGTTGCCTGCCGAAGCCAGAGAAAGAGCA
ATTATAATTGGCTACCTTCAAAACTAAGGAAACAGAAATGGTGATGGGTTGTTGCAATTT
GTGGGGTTTGGTCCAGTTGATATGTGACTTCTGATGTGTCTTACTTATGGTTTATATCCA
ACAGAAAACTTGTTCTTGATAAACTTTACTGAAATAAAGAACAGATACAGATGTACAACA
AGAAAAACAAAAGACGATAAAATCTCAGAACTCTAACCCAAAAAAAAACGAGAAAGGAAAA
ACTAAGCCCCTTCACACATGCATGAAGCAGCAGTTTAGAGCTCATCATCACGAACTTTAC
TAGCTACATTATCTTTCAACTCTTTGGACTTGTGCTTTGTAGCTTCATAGTTTGAAGCAA

TGGTATCTTTAGCAGACTCTAGTGTCTCTTGTGCTTTATTCTTGGCTGTTTCATAACCAT
GCTTGGCTTTCTCCTTGACCCACCCTAATTGCTCCCCTGTTTCCTCTTGCTTTCTCTTGC
TTTTTTCCTCAGCCCTCTTACCCCTTTCCTTCGCCGCTTCTTTTGCTTCCTCTGCATTTC
TGGATGCATGATGTTTTGCTTCCTCTGCCGTTTTTGATACCCTTTCCTCTGCTTCTTTGG
CCTTATCTACAGCTGCATCCTTCGCATTGCTAGCCTTCTCTTTTGCTGTCTCCTTTGCAG
TACATACTTTATCTTTCGTCGAGTCTTTCACATCACACGTCTTCTCTTTTGTCGCCGAAC
TGAGCTTTTCCTTAGTTGTACCTGCTATGTCACTAGCTTTCTCAGTTGCTGCATCCTTCA
TCTCACGGGCCTTCTCCTTAGCTTTGTTGGCTATGTCACTGGCCTTCTCCGAAGATACAT
ACATAGCATCAGTAGTTTTTTCCTTGGCTGCGTTCGTCATGTCACCTGCTACATTCTTGG
CATCTCCGGCTTTCTCAACTGTGTATTTAGCGGCTCCAGGAGCCATATCTTCAACCGTGT
CTTTAGTCTTCTTAGCAGTTTCAGTGGCTGAATCAGAAGCTTTCTTGGCAGTCTCCTTTG
CGTCCTCCGTTTTCAGGCCAAGTCCTTCTGAGATCTTCTCCTTTGCCCATACTGCCCATG
ACTCCGATGTCTCTTTGGTCTCTTTACCCGTCTCTGCCGCCTTATCTTTGACTTCTTTTG
TCTTGCCTTTCAACTCCCCATAATCTTTCCATTCATCTTTACTTGAGGGCATATGTCCGA
CGCTGGTGCAGCTACAACTATTGGCAAAGGCCATCACCATCAACATTACCAAACCCAATG
AAACATAAAGCCTAGAAGTTTGCTTCAAACCCATGATATTATATCGAAAATGTTAATGGT
TTAAGCATAAGCTGTATGGAAGTTGGAATCCGATTTGGAGGTCAGG
>tig_1030_consensus
CCCCATAAACAACAATGAAAAAGCTGATTACAATTTTTTCCAGACGAAACAAGCTATGAT
AAATTATTATGTACTCATGCACAAAGTTCTAGTAGCAACATAATGCAAATACAACAATTG
GCAAACTTAGCTCTATACTAAATCGGGTAGGCGTAGGCTTGGCACTTCATCTTCTAGTTA
TATACAGACAAGTGAATAAATCTAAGTCTTATTCTGGAGAAGAATTGGCCAAGACGATTT
TAAGTCTAAATGAGCATACCTTAAAGATGTAGAATTATCGTTTTATTATTTTTTGCCTGG
ACGAAAGGATGTTGAAAGTTTTTGGTCGAGTTGGCATGAAATTTTTTATAGGCTACTTCC
AGATAGCATCATGATCAATCAACTACTTGGGACATAGTCTGATTGGACAGACAGAGAATG
CGATACAACGAGCTGCTTTTGCCAGCATAAAGCGCTCACTTTCTGTCAAAGGCCTCTCAA
TCACCTTCACCACATTAGCATCCTTCTTATGTTCTCGCATATAAGGCCTCACACTGGCAA
TATCACCAACCATGATTACGTTATTGACATTGCCACGTTCATCCCTTTCTATAGGTGAAG
CTGTTGAATGCCGCCATTGCCCATTAATAATGAACTTATAGTAGCGC
>tig_1031_consensus
CTTCACTTGCCGGCCCATTTTGCTCATAACAAGTAACCAAACTATTCCAAGAAACTATAT
TCCTCTCTCTCATCCCATCAAACACCATCTGGGCACAACCCACACACCCACATTTACCAT
ACATGTCTATAAGAGCTGACCCCATATACACATCAGACAAGTACCTTGATTTTTCTAACA
AACCATGTATTTGGGTACCCAATTTCAAGTCCATTAACCCCGCACAGGTGCTAAGAGCAC
TACCAAATGAATACTCA
>tig_1032_consensus
TTCATACCGGCTCCTCCAAGATTACAATTTTCATATAGTTCCAAACAACCATACCATATC
TGCCAAAATTTCTAAAGATGTTTCTTATTTTGTTTATAAATACAGCGTAGGTACGTAAAA
ACAAGCAAAAAGGAAAAAGAATATTATTTCCATTGCACAGTGGAGCCTAACATCCAAGCG
CATGCCGAGATCAGGCTCAGGTAATAACAAAATGCCGAGCTAGCAATGACTAGAGCAAAA
ATTCAAAACATGGGAGAATTGAGTCATATCTTTACAAACCGAACATCGTTCTGTCCGAAA
GCTGTTCCACACCCAGGGCACTTCCGGTGCCTTATCTCTAAATTTCTTTGGATACATGGA
TTGCAGAAGAGGTGATAGCATTTCACAATCACAACCTCCTTTGGGCGGTCAAAGCACACG
CCACACTTGAGAATAGCTTTGCAATCCTTTATTTCATCCCTGAGCTTCTGTATCGCTGCC
TCTCCACTTTCAGAAGTCAACTCGGCAACCTTGTTATTCAATTCCATGAGATCTTCCTCG
AGCTTCATCCTCTCTCTTCTTTCTTCGTCCAGTTCTTTTTGGATGTCATCCAACTTCCGC
TCAATCTGCTCATGTTCCTTCTCAGAAGAAGCAACAGCAGATTTCAACCACTTCAATTCC
TTCTCAGCATCAGCTAATTCCCACTTTGCTGTTTCAAGGTTGACCATCAGATGTCTGTCT
TCTTGAGTTGATTTAAGAGCCTCAGTCAAACAGACTTTCATCTGCTCTTCACAGTGGAGA
ATCCGTGATTTTAAAGATTCGACCAGTGCATTAATTTGTTGAAGCTGCTTCACTGATGCC
TGCTTCTCAGAGATTAGAAAGCTTTGTGCCTGCTTTGTTTTGACACTCTCAGAAACAAGC
TTGATATTGTAGTCATCCCTCTCAGTCACCTGCAGCAGCAAATGCTGGTTCTGGGTCTGC
ATATCTTCATATGCTTGACCGATTGTCTCAATTTCAGAAATATATGCCTCTGCTTCCTTA
TCTTTGCTTTTAATAGCCTCTGTAAGCTCCATAACATCCCTCTCAGAAGCATCTAGTTTA
GCCCTTAGTTCAGCAATTTCAGCTTCAGCAACTGAAAGCCTTTGTTGACAAGCAGCCTCG
GTCTCATTAGCTGCTTTCACCCTCAATTCCAAACTATGTTCATCCAAAGCACTTTTTAAG
ACTTCAGCTTGTGAATGAGCCCGACGTTCAGATTCTTTTATCTCAGCCACATCTCCAGAG
CCTTCAGACTTTTGATCTCCACCATCTGCTCTGCACATTTATCTTCCAAACACCTTTGTT

CATGCGTCTTAGTACTTAAGGAAGCTTTTAGTGATTGTGCTTCATCACGCAAGGAAACAG
CTTCATCAGCAGTTTCCTTCCACCGATTCAGCTGAGCTTCCATCATGCCCATTTCCTCTG
AATCCTGAACAGCTTCTTCCATTTTCATCTCAAGATCATTTTTTTCAATTATACACTTCT
GAAGCTGATGCTCTAGCTGTTCAATCCTAGATTCAGAATCATCAATGATGTTCCTAGCAG
CATCTGCTGACTCAGATCTTACACTCAGTTCTTTCTCCCTTCTTACAACAAAAGACCTGT
CAATCTGCAATGAATCAGTCAAAGCTTTGTAACGTTCCAACTCAGCATTCCAATGTAGGA
TTTGATCATTGACTAAAGTATATTGTCGAGATGAGTACACATACTTGTCATCATTCAAGT
CATTCTGAAGGTTTTGCAATTGTTTTGACAAGTTAATGTTGTCCTGCTGTGCATCTTCAA
GCTCGGCAAGACGATCAGCAGCCACTATCTTTGTTTCCTCAATGGAATCCTTCAATTCCT
GCATGTCCACTGTCCTATCAGCAGACTTCACAGGAGAAACATTTCCATTAGCAATTAACA
CACCTGGAGTCTGCAAATGCATGCCAGATGCCCCATCTTTTTGCATCTTCAGATTGACCA
GCTTTCTTCTACTTTCTTCAAGTTCAGCCATGCTCTCTTCAAGCTCACCTGCAATGCGTT
TAATCTCAGACTGATCAACTGAATGGTTGCATATATAAGTTTGAATCCAATCAGCATACT
CTTTATGCTTCAAGTGGAGAATTTCAATCACCTCATGCAGACTTTTAGCCTCTTCTTTCA
TCATATCATCCATCTTGGACAACTGGATGATGGCATCTTCTGCAGATAGCTTTCCTTGCA
AAGCCTGAGCTACACTCTCAGTTTTCACCCTCTGAGCATCAATGGCACCTTCCAGAGACT
TCATCAACTCCTTAGTGGATGAATGACGTGAAGCAAGGGTTTCTTCAACGTATTTGGCAA
TCCCCTCATTACCATTATTCTCAATGGCATTTACTTGTAACAGTCTACAAAGAAACATAT
CCTCCGGAGGACAAGATGGAACTGAATCTTCTGGCTGGTCTTTGCCATCCAAGTTAATTA
ACATATCAGGTCCTCCACCAGCTCGTACCCCAAGCAAGATCAAATCATCAACCAACTGAT
TCCAAAGTTGATTCACTGTAATTAACATGTCATCATATGAAGTTTGCTTATCTCTTAATT
CCCTAATTTTCACTTCAAGATCATGCAACTGATGCTTCTGAGCATCTAGTTGCTGCACAA
GTTTTTGATTCTGATACTGAAGAACTGCTGCATCAACTGTTTTGTTATTCGAAGGAGAAG
TGGCGGAGTTGCGAGCCATGGAGGGTGGAAAAGGGTGAGAATTCACGTGCGGCTTCTTCT
TGTCTGGCTCTTTCGGCTCTTCGTTTTCCATTTACAACATCTAGACGCCTGAGCAAGCCC
TAGAATAGTCAGTGTGCGCGTCGCGAGAGTCCGAGAG
>tig_1033_consensus
TGGTCCATTGCTAAGAGACCGAAGGACTGTGCTTGCCCTAGCTTCCACCCCGGCACCATG
TTCTTCATCAAGCAGGTCATTGATAATATCAAGGTGAGGAAATTCATCTGCCAATACACC
TTGGGCTTGTCTTCCAGATGTACAGGCAGAAAACTCATTACTAAAGTGCTCCCTGGATCC
GGAGGGCAGAGACTTGTATAAATCAAGGTTTTGCATATCATTGACCAGGGAGGAAGGATC
ACGGTGCATCATTCTGTTGCCATCTCTTTGGGAACTCTCAACCCACTGCTGGCCATTCTG
CAAGACATCCTGAGAGACCACGCCAAAAGGAAAGCCTGACTGGACTGGATTTGGTTCCAT
CCTTTCTGAGCTCTGGGATACATACATAGGTGCAGATACCAACCCAGGCTGTGGTGAGTA
TGCCTGTGATGGATTCACCCCCAAACTTGGAGAGCTAGGATGAGTTAAACCAGCTGAACT
TGAACTAACAAGGTTTCCCAATTTGGCATTTCTATATGACTGGGGAATGTAACTATGAGT
AGCAGGTGAGAGATCAGGACCTAACCGCCCAGCTGCACTAACTGAACGGGCAAGTAGTGG
GGAAGTATGGACCATGGAAACAACAGGAGCACTTGGCCGAGGACCAGGCCCAAGAGGGGC
ACTAGATGGCCTTGACATCATAGGCAGTTNNNACCACAGCAGTGGATTGGGATGAAGTAT
TCAATGCTGCTATTTGATCAGTCTGCTGCTGGCCATTTGGAGTCAATTTGATATTTGGAA
CAGGATCTGCAGTAGCAGCACTCTTTGGATCTAACTTTAATTGGGCACTGGACTGCATAT
TTCTAGGTGGACTTCTTGAAGAGGTTGATACAGCTGTTGTGTTCACTTTAGCTCTTTCTT
TATCCACTGGATCTTTGATAATCGATTTCTTTTGTACTGGAACAACTTCTTCCTTCTTGG
CAACATGCTGTTCAAACCACTTCGGCTGATCTTGCAAGGATGAAACAACTGACTCAGACT
CAGATTCAACAGCCTTATTGCTTGGAGAGACATCATTATGCTCCCCCGCATCTGTGCCAC
ACCTACAAGGCTGTTCATCTGTTTCATTTGCCCAATTACCTCCATCACGTGTCACCTTAC
CTTGCTGGCTCTTCCCTCTGCTAGGTGACTTCTGATTTTGGTAGTTTAAGGAGTTCCCTT
TAGAAGGCCCATTCATAACAACTGACGGGACAGAATCAGTAGAACATGTTGATGAACTAT
CATCCATTAAACTCCTTTTTTCAGCCATACCATTTGGTACAGATGACAGACCACAAATTC
CACTGCTAGTGGCTTCCATGGGAGGAGTAACTTCGGATGTATCAGTATCCCTGTCTTCTG
AATCATGCTGAAGCACTTCAGCAACACCATCAACTGAATCAGAAACGTCTGAAGTATCTT
CCAGCACATCAGGCTTATCAAGCACAGGCCGTGCTTCTTCTGGAATAGATTCCTTTTTCT
CATCGCTAGGGTTTTCTTCCTCATGCTTGTCCTGATTACCAGCACCGGGCCTTTCATCCC
TCTTTTTTTCTTTCCCTTTGCGATTGTTCCGTTTCTGCTTGGCCTGCTTTTTCTTTGTTT
TCTTCTCCTTCTCAGCAGCTCCACGTTTCGCCTTCCGCTCACTTTCAGCCAGCCATGCGG
CTTCCTCTTCACGAATTAGCTCTTCTTGCCTCCTCAATGCAACAGCTTCCTGATAGGCAA
CTTCAATTTTATTGCTGAATATATG
>tig_1034_consensus

TGGTCCATTGCTAAGAGACCGAAAGGCTGTGCTTGCCCTAGCTGCCACCCCGGCACCATG
TTCTTCATCAAGTAGGTCATTGATAATATCAAGGTGAGGAAATTCATCTGCCAATACACC
TTGGGCTTGTCTTCCAGATGTACAGGCAAAAAACTCATTACTAAAGTGCTCCCTGGATCC
GGAGGGCACAGACTTGCATAAATCAAGGTTTTGCATATCATTGCCCAGGGAGGAAGGATC
ACGGTGCATCATTATGTTGCCATCTCTTTGGGAACTC
>tig_1035_consensus
GGACCCTCTCATTTAATACCCTCCCAGTAGCCCCTTGCCTACTTCTATTATTAACTAAAT
TTCAGTCTATTGAGTTCAGTTGTCAAATGAATTCTAGCACGCACTCAACTAAAGTACCTA
AGCTTCAAGATAATTCCGAAGAGAAAACCATCAGAACACTTATCTTAAGAGACAATCCAT
CAACCAGGCGACAAGTACTCTGCAGACTTCCAACAATCAACACAATTCTATGGTACGACA
AAGAACTGCTTTTTAAATCCTTAATTCAAACTTTAACACACTAACTGACAATGGAAAGCA
ACATATCACCATTTTGTCACTGAATGATAATATGATGAATGCTAATTTCTAAAATTCAAT
CTGTCATTGTTCTTCATTCAAATGTGTCTGATCTAACCTCTAATGGGCAAACACAACAAA
GCTGAGAAGATAATATTACAAACTACTCTTTGGAGGATTTGTTGATGAGGGATTTGTGAA
TGTGTGGGATAACTCCTCCACCAGCTATCGTTCCTTTGATGAGGGTATCAAGTTCCTCAT
CTCCTCGAATTGCAAGCTGCAGATGCCTAGGCGTAATACGTTTCACCTTTAAATCCTTGC
TAGCATTCCCCGCCAACTCCAGAACTTCAGCTGTCAGATATTCCAAAATGGCTGCTGAAT
AAACGGCAGCTGTGGCTCCAACCCTTCCATTTGCAGCAACTCTTTCCTTCAATAAACGGT
GGACCCGACCCACTGGGAACTGGAGACCAGCGCGAGAAGAGCGAGAGATAGGCTTCTTCT
TATCCTTGTCTTTGTTAACCGTGGTTTTTCCAGTAATCAAACCCTTAGCTCCTTTTCCTG
ACATTTTCCCGCCAAATTTTTAGTGATTTTATATTAAATATTTGATTATTTAATTTTCTA
AACCAGGCAGAAAAAGGAAGCAAGAGCGGAGAAAGCTAAGGGCGAGTGCGAGGCAGGAAC
AGGGGACACCGGGTGAGAAGAAGGATCTAGACCTTC
>tig_1036_consensus
TTTGGCACAAACTATGAGACCTGTAATGGCACAAGAGGGATCAGATATTCATAGAAATCA
AGTTTCTTATTTAGGTGCTTTTATGGCTCTACTCATCCAAAGGCAGCTGCCAACGTTTTA
ATGTTTAAAACTAACAACTTAAATTGTCAGACTTTCCAACACCACAGTCAAATGCAACGC
TGCAGCTACAAAACTTAACCACGCGTTACTATTATAAAGAGATCAAAGATCACAGAGTTC
TCTGAATTTGAGAAAACACCGACGCATGGTCTAGAATCAATGCTGAACTAGAGTACATCT
TTAATCCTGAAGTTTTCGCTAAGTTGATTTTCTAGAGAAAATAAGTTTCTTTAAGTAAAT
CCAGCGCCCTTCGTGGTCTGATTTAGCAAATTAAACAGGTGCCAAGTAGTTAAGTTATAA
CGCTGAAAGAAGTGGACATAAGGGAAAAGAAAGCTAGCAAACGGCCTCATGAATATAACA
AAGTATAAGCTCTACACCTTGTTATAATGATGCATTACTTCAATGGTTTTCTATCTAATA
AACTCTTCTCTACTGCAACATTTAGTCTTGGAATATAAATTATGCATTAAAACCCTACAA
GTGTAAGGAAACAAGTCAAACCTTGAAAGTCAGCTTCTCATATAACATATGAAACCCATA
AAGCAGTTTCTTTCAGCGAAAGTAACCCGTAACTGGCAAATTTCAACAGAGCATGAATAA
AAAGAGTCCCTTCAAAAATAAACTTTACCCACCTGCTGGCCTCTGGATTATGCACTGCAT
TGATTAGCTCTGAGCACACTCAAATGGATGCAGGGATCCCTCAGAAAATTCATGCATTCG
AAACCGTTCAAGTAGTTCAAAGGGCAGGATAGGTTCACGACTACGACATAATTTCCGTTG
ATTGCTTTCCATGGCCTCCAATACCTCGGCAGATTCTGGTCCTGCTGCTTCTCCAGTAAC
AATTGAGGGAACAGAAACACTAGGAGTCCATATCTTTATTGTGTTATCAATCCCACTGGT
TGCCACAACAGAATCAAAGGGATGGCACTGTACACAGTTCACAACAGCATCATCTCCAAC
AAGCATTTTGATCAATCTGCCAGTTTGCTTTTCCCAAATAAACCATCTACCATCATCACT
CCCGCTTGCAACATAGTCACCCCGTTGACCAAGAAAACTGGCTTGCTTAATGTCAGTTCC
AACATTACAATGGCCAACAAATCGACGTTTCATATCAACAACTGTCTCAGGCTGGTAAGG
TGTCCAATCATTTTGAGATGATGAGGCAGTTGAACCACAGGAACCATTTCCACCAATTTC
TTTAGTGGAATCACCTCTCCGATGGATTCTCACATTCAAGCTTCCATGTAGAACATTGGT
TTCAACATCTCGCCCTTCATCACCTGATATTGATGTTTCAAAGTCCACCTCCAATTCTTC
ATCAAAATCAGAATCTTCTCTTTCAGACCTTGGACCATCTTGAGAAGCATCACTATTAGC
CTCTGATCGGTACAGTATATCACTCAATGAGAGTACTCTTCCAGATCGAGGTTCGGGCCT
TGCCACACCATCATTTGCCTTACTACTTTTTTCTGATTCAGCTGCGGCAATCAGCTTCTT
TAAGTTCTCCACCTTTTCTACAACACTAGAATTGGATGGATCCCGACATTGAGCAGCAAG
AGCAAATTCTAAAGCTTCCTTATATCTGGACAACTGCTCTAGAGCTTCTGACATATAGTA
GTGAGCTCTAAAGGAAGTGTTATCAATTCTCCGAGCATTATAGCAATCTCTTATGGCAAT
ATAGGCATCATTTTTCCACTTTCGCTTAAGCAATAAGGCAGCATGTGTGCAAAGACAATC
ATGCCTCAGCCTGGATCCAATTTCACTACCATGCCCATCCAAAACTTCATTGCATGCCTC
AACTCCATAAAAAGGATTCGCCCCTTCATCTAAGCAATTTCCCGCAATTTCAATTAACAT
CCCCTGGCAGCAAGAACATTGCAGGAAACATCAGATACTTGATGATGCAGTTCAACTCCG

TCAAGTACAGGCATGAAACTCGTCATTTTTGAAGCAGCTCCTGCAGTATATCGCATAGCA
CTTCCACGAGCATGATTTACATCCATAAGATATACATGCTCCCCACTATAACTGAGGAGA
ACCTCTTCTCCATTTGGACTAAAAGTAACATGTGTCAGGTGTAAGTTTGAACAACCATGT
TCAGAAAGATGCATAGGGCAGAAATAGTTAACACAAGGTGGTGGTGGCATTCTTTTCCGA
CAGGAAGTCAATGGAGGGAGCATCCTTCTATCATATAAACGAGCAAATGCATCGCTCCCA
CCAACCAGGAGTAAATGGGGCCTAGTGGAACTAATATCACAAGACTTCAAAGCAAGAGTT
TGCTTAGGTGGATCAACTAGAGACCTCTTTGCCCCACAACGTAAATCAAGCAGAATATTA
CGGCATTCTTGATGAGAAGATCCAGCGGGAGGACAAGAAGCACCCTCCCGAAAATCATGC
TGTCTCAAAGTCCCATCTTCACTTGCACTCCAAACCACATGAGGATTTCCAACTTCAACA
GCTATTTTTTTACTCTTCTGGTGTGACACTGGTACAGAGCTGATGGATGAATAGCATTA
TCATCAAGGCCTCGCCCACTAAATCGAGAAAGATTAAACAGCCGAACTTCTGCATCACCA
GCCCCAGAAACCACAAGCTCATCAGAAGTTTCAGGGACAAATTTTGTACAGAATATGTTG
GCAGAATGCCCAGTCTCTATGGAATGCAAAAGCTTTCGACTAGAATAGCTCCAAATGTTG
ATACGTGTATCATCTGACCCAGATATCAGAAGTGAACCTGTGGAATTCCAAGAAATAGCA
TTCACGCAACCCTGATGCCCCTCCAATTCTCTTTCCTTCGAAAGCCTCTGCAGCAATGAT
GAGTGCATTTGCATACTGTGATTAACATCATGGCGAGTGTCGGTGAGTCTGCTATCGAGG
AAATTATAGATATTGCCATCGTGAAACGGCCAACTCTCCATTGTTGAAACGACGTCAAAT
CAAAAGGCACGAATCCTCAACTGAAGTGATACTGATAGTGAGCCTGAATCTTCAGGATCG
CTCCGCTTGGCACTTACCCTTTGTATTGAAACAACGACGCCACGATCTGCTCTGTTGCTT
GCCTCGTGCGTGCTCTAGTTCTTAATTT
>tig_1037_consensus
CTTTTGCAATTATAGAACAAAAGTTACATTAAGTCTTCTCTTGGGAACAATTTATTACTC
ACCTTATATTTGGCTATAGTTGCTACATCATTTGACTAAACTCATGCTGAAATCCCAGAG
CTTCTTTGCCAAATCAGCATCCCTGCCCTGCGCAGTTGTCTTTGCCAGGTTACTGTCCGA
GAAGTACTCCCCGCTTATCCCCTGGACTCCGGGATGTAGCGCCACATAGCATGTTGTCGC
AGCTCCCTGCTTAACATCTTTAAGCACAAATTTGCCAACCGTGTTACAAAGGCCTCTTAT
TACTCCATTATACCGGAACAGATTTGTAGCAATTGATCCTGGATGAAGCGAATTCGCAGT
TATATTCACTCCTTCTTCCTTCAATTGTTTTGCAAGCTCATTAGCAT
>tig_1038_consensus
CGTTTAAGACGATAATTCATCAATATAAGATGAAGATACCTAATACATGATGGCTGCCTA
CAAGCCCATAGTTTTTGTCCACGTCCCCTTGAAAGGAGAGATGGACCCAACGACAATACC
GAAGCCAACAAATTCGAAACAGTGCTACCAGCCGGTTATTTTTAAGAGTCATTAGGGGCA
TTCACTACAGTACGAATGTGAAATCCATAATATGCAGTACGCAAGATCTCCAGGTCCACA
ATCCCCAAGAGTCGACAACATTTAAAAAGCTTTACGATGAAGGGCTGACAGATCTTGAAA
TGGATCTGGTGCTCGCGCTACCGTTACTCCTAG
>tig_1039_consensus
CGTTTAAGACGATAATTCATCAAATATAAGATGAAGATACGTAATACATGATGGCTGCCT
ACAAGCCCATAGTTTTTGTCCACGTCCCCTTGAACGGAGAGATGGACCCAACGACAATAC
CGAAGCCAACAAATTCGAAACAGTGCTACCAGCCGGTTATTTTTAACAGTCATTAGGGGC
ATTCACTACAGTACGAATGTGAAATCCATAATATGCAGTACGCAAGATCTCCAGGTCCAC
AATCCCCAAGAGTCGACAACATTTAAAGAGCTTTACGATGAAGGGCTGACAGATCTTGAA
ATGGATCTGGAGCTCGCGCTACCGTTACTCCTAG
>tig_1040_consensus
CCCCAACCGACGCTCTTCCGATCTGGGGAATCAAAATGATAAAAAAAAAAAAAAAAAAAT
GACAAGAAAAAACAGAAAAGTCCAACACCACATAAAAGGGCAAGAGGTTTGGGGCCAGTG
ATCACTGGTCATACTAAGAAGAACACAAAATTGTTATCAATGAAATGCCTCATGATACAA
CCTGCCTCACCCTCCTTCCTCTAACCCTTCCAAATGTTTGAGTTCTGTCATGGCTGAAAC
CAAAATGCTCAAAAAAACTAGTTATTTAGCTTCCACTGGCAGCTGGGACTGCTGCTGGCT
TTGCATCCTCAGCCTTCTCATGTTCCTTGTTCAGCATCTTGTTATTGATCAAGTTGTGCA
GTGCAATGACACTGCGGATGAGTGACGAAAGATATATGACCAACATCATATCATTTGTTT
TTACTG
>tig_1041_consensus
TATTTAGGGGACTGCTACCACTTCCACTAGTACTACTACATTTTCATTACTTTATACAAG
CTTTCCATAAAAAAGCACATACCCACAATCACTCCCAAGTCATAAATGGCAACATACCCA
CAATCACTATGGCCACAAAAAGGAGNNGCCAGCCACCATTTCAGTATGTTCTAACTTTCA
AGTAACTAAATCTACTACTTAGCCCTCCATAGCATAATGAAATTACGTCCAGGAGGTAAA
TATTTACCCTGCTAGTGCTTGTTACTGGAACCAAGTGATGGGTGTGGAAGTGCTTTGAGG
AAGCTGGGGGATCAGGCAGTGCTGTTGGGGCCATTGTTGTAAGCCCTCACCCTCCCTCAT

TCCACCAAATTCATTTGCGTGACCTTGCCCTATGCTAGAGTTACTGTTATCCTCAATATC
CATTTCAGCATCCTCCATATTATCAGCTTCCATCAAATCTGCTGGCTCTGTTTGAGAAGT
ATCCACACCTGGGTCAGAGGCAATGGGAAACTGGGAAGGAACCCAAGGAACAACGGCTAA
ACACTTGTCTGTTGTTGCATTGTTCTTGTCATGCAAATAATCTTCGTCTTCAACCAATTT
CATGTGCCCGGTTTGGCTTCCCCTAAGAAATTGATTCTTAAACCCAGAAACCAAGTTCAA
AGAATCCAAATTCACACTAACACTAGGAGAAGACGGCATTCGAGGTTTGTACAAAACAAT
AGCTCTCTCTTCATTAACAGGGTCGCCAAAAAACCCCATCTGCGAATCGACGCCGTCTTC
AACAATCGGCGACGAACCCACATGCAGACTCTCCGGTTCTCTCTCCTCCTCAAAAATCGG
AGGCAACTGCGCATTCAGGCGCCGAATCTTCCGAGCCGGGGAAGAAAGAGAGAAGTCATG
AAAGTCATCACTGACTTCATCAAGATCAATCTCCTTTCTCTTCATCTTCATGGGGATGTA
ATTACCACCATTTCCCACCATTTTTTCTTCTTTTTCTTGCGCTTTTT
>tig_1042_consensus
TGTCTGATGTTGCATCGTTCTTGTCATGCATATGTTCTTCCTCCTCAACCAATTTCTTGG
GACCAGTTTGGCTTCCTGGAAGAATTTGATTCTTAAACCCAGAAACCAAGTTCAAAGAAT
CCAAATTCACAACCACACTAGGAGAAGATGGCATTCGAGGCTTGTACAGAACAATAGCCC
TCTCTTCATTAACAGGCTCACACAAAGACCCCATCTCCGAAACCACGCCGTCTTCAATAA
TCAGCAATGAACCCACATGCAGATTATCTTGCTCCCTCTCCTCCATAATGGGCGGCAACT
GCACATTCAGGCGGCGAATCTTTCGAGCTGGGGAAGAAAGAGGGAAGTCGTGGAATTCAT
CACTGACTTCATCGAGATCAAATTTTTTTCTCTTCATCTTCATGGGCATGTAATTACCAC
CATTTCCCACCATTTTTTTCTTCTTTTTCTTGCGC
>tig_1043_consensus
TGGGGCAAATGAAATTGATATTCTTAATTGCTCATTAATGATAAAAGTCTCTACAATCAA
CAACTCTGAAAACTGGATATGAAACGGTTACCTAACAATCCATATTCTCCATTAAGAGGC
ACAGCATGACCAATACAAATTTGTACAGATGATAATTTCATTGCTAGGCTAAATCAGTCT
GCCTCTGGTACCATCTTCTGCACACTGCCTATACCATGAGCTCGTCTTCAAGATAGTTGT
ACTCGAAAGTGAACTCTGTCTTAATTCTTTGACACCATCCGTGTTGGAGATCATCATTAG
CAATATGAAAATCGAAGTATACATAAGTCCCTTGTTCATATTCATCAGTAGCAACTTTTG
GCTCCTCGTGCCGTTGGAACCATGTATTGTATTTTCTGTGGTACCTCCAAGATTGCTTTT
TTAGCTCTTTTGCAGCCAAATATTGTTGGTATGTGTTCTGTTGGTAGTAGAATGCAAAGA
ACAGTGTATCAGTACCAAAACTATCGAGTGAAAATCGCTCCCAAAACGCTGGATTATTCA
CAATAGGTGCCTGAACTTGCGGATAGTTATGAGGCGTCACTGCAGGGTGCCTAGGAATGT
AGCTCCTGGGACGCTCTGAGTCCTTTGGTTGTGGAAGTTTATAAAATGCAGACTCAAGCA
TCTTCAAATTATACATCTGATCATGCATTGCTCCAGAATTCACAGATGAACCACTCAAAT
TATCGCCAATGGCACCAAGATCAGAAACACTTCTCCGACCAATAACACCAAGGCCACCAG
AAGGCTGGCTGGACTGTAAGGGTTGCCCAGGAGATAATTCAATCTCTCTGACTACTTGAG
CAGGCTCTGTCAAAGAGCCAGACACTCCTGCAGGAGAATCCATTGCATATGAAACTTTGA
GATCATCCTCATTCATAAGATTCTTGCCAACACCAGGACTAGGAGCAGATTCGACAGTTG
ATTCCTCGGGTAAATTCTGCTGTTGTTGCTGCTCGTCAACTTTCAAGTGGCCAATATCTG
CATCTTTCTGACCACTAGGCATCAATGCCTGTTGACTAGATTGTTGATGGATAGAATTTG
ACTGCTGTTGCAAGCCAGCAGATGTAACAGGATTGAGTATTGGTGCCTGAATCCCAAGTC
CCAGGCCAGGTTGAGACATGGGTGAGTTTGGTGTAAACTGCTGCAGCAGTGGGTTTTGTT
GTTGGGAAGAGAACTGCTTATTATTTCCACCACCAATACCAAGAAGGGTACTGTGATTTT
GTTGCTGCACTTGTTGGAGCCGTTGCAAGAACTTCTCCCTCTGATCAGGTGTTATTTCAG
TTCTTCCACGAAACTGCCCCGGTTCATTCTGGTTTTGAAAGGAACTTCCAGGCCTCCATT
GCATGCTTACTGAAGGAGAGAAAACTCTGCTACCAATTGCCATACCTTCTCCAGCATTAT
TAGAATCAACCGATCCAGTTCCATCATTACCCTTGGCAGCCTGAGGCAACATCATTCTGT
TACTAAGCGGGGAAACCAGAGATTGTGCCATGCCAGTACTGCCAAGTCTTTCATCAGCTC
CCAATATATTTCTCTTTGCCATATCTGAGACTGAAGGAACAGAGCCAAGGTTTCCATTGC
TAGGAACTGCAGTGGCAGAACTAACAGGTATGCTAGATGAAGGTTGGCTGGAGAGGCTGC
CTCTTCCAATGCCCCTTACATCTGCAAGGGATGGAGATGATCTACGGCCTGGGAAGCTCA
CAATATCTTCTTCCTTAGTAGGATTTGTCAGATTTACTGGAGGTGAAGAACTGATGGCAC
TTGTACTATCCAATACACCTCGAACAGAACTTGAAAGTGCAGCTGGAGCATTTGACACAC
TTGACAAATTATGAGCTGGGACACTGATGGCAATGGGAGCTGTTTGACCAACAGCTGGGG
TTGATGCAGTTGAAGAACTAATTCCACTACTTTTTGAAGGCGGTGTCCTTGGAACCATAT
CTGAATTACTATCCTGGGAAGCCGTATCTTCAGCTAGTTCTGGGACAGAAGTACCCTGCT
GATGCGTGGAAGTGACAGTAGCAGGCATTTGAGATGCTGATGCAGCCAAGGAAGTCTTCA
AGCTAAGAGCTGGAGCACCCTTAACAAGACCTGGAGGACCAATTGTAACCAGATCTTCAA
GGGATTCCACCTTGTCTAGTGGTAATGTGTTGTAGAGCTCATCAACGTCGCTAAATTCTT

CAAAATCCTCCTGATTACGTTCCACATAGTCATCCAAGAAGTCTTTCACGTCATTGACCT
GCTCTGGACTCAGTTCATCATTATCTAGTAGCCTCAAGATCAATTCTAACTTCATAATAT
GAGCCTTGTGCCGTGTAATAGAAGTCTCTAAATGAGTCAATCTGGGTGGCCTTGTCTTCC
CTTTCTTCACAGATAGCCCTTCAATTTCTGCTTCAAAATTATCGATCTGGGATTCCAACT
CCCCAACCACATTATTCAACCAATCCCTTGTCTCTGCCTTGGCCTTCTCCTTTGGATCAG
TTTTTGGTTGTTGTCCCAAACCTTCTTTAGAAAATGCTTTTGTTTTAGTTTCCTTTTCAC
ATATCTTAAATCTTTCCATTTCACGCTCAATCTGCTTGCGCGCATCCACCAGAGCTTGCT
CATACGAGGCACTGACTTTCTTATCCTTGATTTCACTAGACTGAATCCATGTCTTGATCT
GATCACGGTACCTCTGCAGCTTCTTGATTTCCTTCTTCAAGTCAGCCTCAAACTTCTCCT
TCTGGTTTGCATTGTCTGTATCATATACCTTGTTCCAGATACTATCGAAGACATCAACAC
CTTCTTGGACCTTCTTCAGCACTCGATCGATCTCGCCTTGTAGCTTGCGACTGGCGCCCA
TTTTTTCGACTGAATTGAGTGACAGTGAAGAAGAGAACTTCTGTTGAGGCAATTTGATTT
ATCGCAAGGTTTTGACTCAATGCTGATGGGTTTGAGTAACTGGTTTGGCGTTGTTCTAGG
GTTCTTTATTGATTGACTGAGAGAGATGAGACGAGGACGCGGAGAGTAGATCAATGCTTG
GATTGGGCCGTAAT
>tig_1044_consensus
CAGAAAAATATAGCCATTCAATTCGCCTATGAAACCAAACTTCCCTCAACATCAGGAAAG
CAAGTTTTGAGTCACTAAGCCAACCGCAAAACCTAATGTAATCTAAAAATAAACTAAATT
TTGTTGCTTTATGATTATGTCATCTGAAATTAACTAAACCGCTTAGTCAGTTTCATCACG
GTACAACCGGAATTTTACATGATCTAAAATTGAGAGTAAGAATTGCAAAACTGAGCACAT
CAAAACTATACATCGATTGTGCCTCGTTTCAAGATTGGTGGTCCTAACTTTGACTTGATG
TTTTCCCGTCACTTGTGACTTCTTCTTTTATCTCTTTAATCATAGCAATGATGATATTAC
TCATGACTTCAATAGCTCGAGAAGTTTCTGCAGCATCAAGATCTCGTTGCACAGCAATAG
TCACTAGAGAGGCATCTGATTCGAGGCCCTGTGACCCATCAGGCATTATTGCAAAACCTG
ATGGTTTTAAGGATTTAGTGATCAAAGATGACGACAAAGCAGCATTGATGTGTGATTGGG
TTACAGGTGATGAAATAACAAAGGAACAAAATTCCTCTTTTGAAGCCTCTTGTAACATGT
ATTCACAACACTCCGTGGACTCATTTTGAGGAACTACTTTCCTATGTAAAGTAATACAGT
TACTATCATCTTCTGCCACAAGCCTAATAACTTCTTCAGGTTCTTTGGCATTTAACAAAC
TAGGCCACTGCAATTCAAGATTCCTTTTGACCAGAAAATCGAAAACCCTGTGGGGTTTTA
CTGGCACACGAAAGCTAGTCAATCCAACATAACTGTTGTAGCCCTGCGTATGTGAATTGA
ATATGGCTTTATCCCACAAAATCCTCACCCCTTGGTCTGATATAATATCCCATTTATATC
CATTTGGAGCTTCACTAACACAACTAAAATAGATTATCTCCATGCAGTCAGTCAATTTCA
GCATAATAGCTTGAGCTACACAACCCACAATTTTAGCATGTGTTAATTTCAAAAGTCTTG
AAACTTTCAAAATAAATACAGCACAATTCTAAAATATAAAGGACCTAATGTATCCCAACA
GTCCAAGACAATATGATAACCACAAAAAGAGTCATACTTGTGCCTTTTAACATTTGATAC
AGCCAACATTACTTCTTAATTCTAGCTCATGTTAAAGATTATGCAATTCTCAGAAATACT
TCCATAACTTATTAACTAGAATGTGAATAATGCTGAGAAAGAAAATGAACTCTGCAGCCA
TGCTTAGTACTTCAATAACCTTACGTTAATAAAGTTATTAATCAGTAAACTCTTGTCAAG
CATTAAAAATAGTAAGTAATAAATATGTGCAATTAAGATAAGTGAAACACAAGTAAATTT
CAAAATAAGTAAACAAATGCAAACCATAAATCAGGTAGGAAGAACATAGTAAAAGGTTTA
AAAATCTTGACCATGATATCTGATACAAAAAATGACATGAAAAACTAGATGCGAAGAGAA
TGCAATCCAATCACTGAAACTTAACGGATTACTTAGTTACTGACACCCAATTAGTACCTG
ATCCGCTAAGGCAATACCAGATCATTACCCAATTTAAGAGCAAAAAGTAGACTGTGAGTT
ATAGAAAAAAGTGGGATTCTCAATGTACTTTGAGTTAATTTATGCAGAATACCACCATTT
GTTTTCAATAAAAATGAAGAATTGGGAATTATATTATTTCCACCCTTTAGTAAATAATTC
ATCAACCAAATTTGACCTTCTATAACATATATTGAAGTAACATATATGAAAGTGAGTAAA
TTGTATATTACTGTCTCCTTCCAGATATCTAAAACCGACATGGACTGAAAAATCAAATAC
CTTGGTTGGCCACTTGCATCTT
>tig_1045_consensus
TTTTTTTTTTTCAAACACACTTAACGCCTATTATTAATGTTTTCAAAAATTACTAATTGA
ACAATCCAAAATAAAGTCTCCAAGCGTGAGATAAAATTTATAAGAAAATGTTAACAACAA
GCTCTTGGAAACCATTAGGAACAACCCAGCCATTTCAGTAGTAAACTAACCAACAATCTC
AAACAAAGTAATGGCGAAGAAGTAATCATAAAGTGTCCGCCACATCAAATATAGAATTTA
AAACTGAGAATCTGGCAAGTAATCTCAATATCAAACAAACTAAAAACTAGAACATTTAAG
GTTTGACAACAATGCGAAGCACTGTCATCATAATCTCACAACAAAATTTCTCTCATCCAT
TTCTTGAAGCCCAAAAAGTTGGCTTTCAACCTCCCTTTTTTTAGCTCCTTGCTGCCTGCT
TTTCCTTTGCTTTCTGGATCTTCAAGACCTTCTTCACAATCTTTTGCTCTTCCACCAAGA
AAGCTCGAATTATCCTCTCCCTAACAGCAGCTCCAGACAATACACCACCATAAGGACGAT

TCACAGTCCTGCGGGTCCTAGACAATCTTGACCTCTTATACTCAGCAGGTCTCAAGTGAG
GGATTCCCTGAATTCTCTTGCCAGTAACAGGACACTTGGGCCCGCTTGCTCTCTTCTTCG
TGGTCTGATACACCAATTTCCCACCAGGGGTTTTCACTACCCGGTGCTGGTTCGATTTGG
TGGCATAACTGTGCCGAGTACGATACGTTAATCGCTGAACCATTTTTGGGTGTGTTATTT
ATTTTTTCTTTCAGGGTTTTCAGCAAGGAAGGAGAGAGCAAGCG
>tig_1046_consensus
CTACAGGATACTGGCAAAGAACCTGTTAAATTCTGGTTAAGATGCACATCTAGAATAAAC
GGAATATTCAACTGCATAACAATGACCAGTTTATGACTCAACTGACAAGAAATTACAATA
CAAAGAGCAAAATGCAGACCAGTGTGGGATAAATTTTCGTCACTGGGACAATCCTTGTGG
TGCAGGTGCTGGTGGAGCAGATCCTGAAGGGGTTGATGTTGAGGGTAAAGATCCTGACGG
TGCGGGTGCAGACACTGGGGGAGCAGGCCTCGGGGGAGCAGATTGCTGATACTGCTGTGT
CATTGGTAGGGCATTTGGGTGGGGCATTGAAGACTGATATGGAGGGTAATGTTGATATGG
TGCCATTGAAGGCATGTATCCATATGGAGGATAAAGATGTTGTTGGTATTGCCCATGAGG
AGGGGGAATGGGTGGATAATTATAATGCTGTCCTTGTTGCTGGTTCTCTAGACCAGGCTT
GCTCTCACCTTCCCCGGATGGAACAAGTGCACCCATTCTTTGAGGATCCATGGATGGATA
ATAGGCCCTTTCCTGCGGAGGTGGAGGAGGAATATTAAAGTAAGGCGTTGGTGCAGCTTG
GTCCTGAGTGCCAGGCTGTTGATATTGATTTTGCTGCTGAGATATAAGTGAACGGGGCAA
CATCCCGCTATGAGCCACAGCCTGCTGCCTAGCTTCATCTGAACCTTCTGATTCAGGTCT
TTGTGTCTGGGGCTTTCCCCACATAAGCTTCAGCCTCAGACCCTTTATAACTAGTTTGTT
TGACAGCTCTTCAGCAGCCTTCTCTGCACCTTCTCGTGTTGTGTAGGTTACAAAGGCAAA
TGCTTTCTGTGGATGCATCTTTATGGATTCAATTTCACCATGGGCATAAAAGTTATCCCT
CAGATCTTGTTCAGTGACCCTTGCATCAACTCCACCCACATAAAGAGTTCTAATGCTTTC
ATCCTCAGGGGGTTCCAGAGATGGCATTTCACCAGCCTTATTAAGTAGCTTTAGTGCAAC
TGGGTCATTCACTCCATAATACCGATCTTTAATATTTTGCTGTGACAACTCTCCAGTTAT
AGGCATTTCATGCCTATAAGGGCACTCAGCACCTCTTGTGCATTCACCACGAACATAAAA
ACTGCATACATGTGCTCTGTTGCGCTTGTAGTAAGGTTGTGTTCTTTGAAGCTTCAGGAT
GGTATCATTTGGACGTGCCTTCCCATAGGAGGATTCATAGTCAATACCAGCTCTAGCCCT
CCGATCATGCTCCTCCGCAAAGTATTCCCTGTTCACATCACTCTTTGGAATGGCATCATT
CGAATTGATGCTGAGAGCAGTATCACGAACCTGAACAGGCAACCCATATTCTAGATCCAG
AAGACATACTTGACAAACATTTTTCAACTTACTGCATGTCTGGCAAATCTCAGTCTTCTT
AAACCTTGCATCACGACCAGGCCTCCACCTAAAAACTGTGAATGGTCGCGTGCAAATCTT
ACACTCCTTATCATAATCTGCTTTTGTCATTCGCACATACGGATTGTCACCGAGACATGA
CTCACAGATTATAGGAAAATCAGAACGTTCCCAACCATCAGCTTGGTGATCTCTCAAAAA
TCGGTGCGCCATCGAAATATTCCCAAGCTTCCCTCACTGCTCTCTCCGTGTGCGCTTCCA
AAATTGTCTTAGGAACTCAGGGTTTGACGCGAACCTTCTTACAGAAATCGAGTTCTGCCA
ATTAGCTAGGTCATGGTTCTGTTCTTTCTGTAAATTGATTAAGGCTCTAAGCCACCGCCC
GCCAGAAGATAGGCTTAGAT
>tig_1047_consensus
GCTCATACAACTAAATATTTGAATGATTCAACAATTTATTGAACAGAAAATGGTACAACA
AACAAAAGATCAGGGCTTTGAATTCTACAAGCACACAAGAATTCTTCCACTAGGTTGCCT
ACATATAATAATTTGATGTCCGTATTCCTAATTCCTTTCAACCTAAGGCTGTCTTTCATG
CTACAACTCCGGTCCAGATAACCAGTTCAGTGGAAAGATCTTCACTATTCCACCAAATGT
AAACCTTCCCAAGAGCACAAAAACATTGCAGATGCAGCAAACTCTAATCTAAAAGAAGCT
GCAATATGTATATCAATAAAAAAAGGGAAGG
>tig_1048_consensus
CACGCATGATGTATATTTTAATCTGTGTCATTAACATAGAGTGAATGCTTAGTCTAAACT
ATTTATATACCAGCTTGATGAAATCCACCATAATATATCCATAAAGACTTTTATTCTAAA
AACAACAAACACCACAAGAAAAACACATACACAACAACTTCTCTATATACAAACAAACAC
AAAACTTCTACTCCATGCCATCCCTTCTCATCTCTTCAAACCATTCTGAAGCTCAGAAGC
TTCAATATCCTGCTAGATTTACAAACTAGCATACGCTTATAGACACACATGTAGCAGCGT
AGAAACTTTCAAAACTACAGTCGACAGGACTAGTGGGTAGGTCCCCTGCTAACTCCATCC
TCCTAATCGCCTCCTCTTGTCCCTCTCTCTGGTTAAGTATCACACATCTAATGGAGTAGT
TGGTCTCCGAAATTCATCCGTGTTTGCCTTCTCGTATGCATCCGATGTAGACTTTCTACG
AAATCGAAAACTCTCTCTATTTCCAGAGAAAGGAGACACCGGAGGTGTAGAACCGGCCGG
AGAAACAGGCGGCGATCCGTTCTGGTAACCAGGTGGTTTTACAATCATGATGCTCCGTGT
AACTTTTGTCGTCTCCAACGATGGTTCGTCAACGTCTGATCTCACGTTCCCACCTTTAGA
TTCCTTGCCGGCTTTCGGTCCGAAACTGAAGGAGGAGTGCTTTCGGAGCTTGCCAAGCCC
AGACTCCGGCCGAGGCCCGGCCAAGGTGTCGTCCCACAACTGGTCAAGTAAGCTCATGAC

GCAAGTTAACAAGACCTGAGGATGTTGACGAGATTATCCTCTC
>tig_1049_consensus
CTGGGGAGGCTTCATCAGCTCCATCGAGACATACTGAGAAACAACAAGAAGGACAGGTAA
AACAAGGTATGCAGCAGTGTCTTGCCAACCAAGCGGTGGATGGCCATCCACAAATGGAAA
GAGCCAAGAAATGCCAGATCCACTTTGTCGAGCAGCAATTGTTGTTGGCCCACCCAATGA
AGGTATCCAAAAGAAACCTTCAGTTAACAATCCCTCATTTGCAACATTTGAAAGTGCTTG
ATACAGACCTATCCAGACAGGTATAGTAACCAATGTTGGCAAACAACCTGCTAATGGGTT
AACCCCTGCCTGCTTATATAAACGAGATGTCTCAAGTTGTATTCTCTCCTGATTGCCAGC
GTACCTTTGTTGAATAGCCTTGATCTTTGGTTGAAGGTTTTGCATAGCTAATGTTGATTC
CACCTGTTGCTTTGTTAAGGGCAATGTGACAATTTTAACAATAATTGTCAACAAAATAAT
AGCAAAGCCATAGGCATAAGGCACATGAACAGCTGAAAGCCCGTCCTTCAACACCTTGAG
AACAAACTCCATGGATTCGGAGATAAAGCCGAACCAACCGCCATTTTTCTGCGCAGCAGC
ATCTGCCGACGTGGAGGCCGAGCCACTGGAAGCGCTATCAAGTGAAACAGCAGCATCAGC
GAGCGTGTAGAGTAAGCTCTCGGCTCGGCTATAAATTAAACGGACGTCAACTGAAGAGTC
GAAGGAATGAATTGGAGGAATTTCTTGGAAGCTGAGTTTCACTCTGGTGGAGGCGAGTCT
ACGGTGAGGGAGCGGGTGGAGACCATGGCGAGAGAGTGATAATGATGGGAGAGGTACGCC
GATGAACGGTGGTGATGAAATCAGAGTTTTCGCCATGGGTTGTGGAACCTTCTGCTCAGC
TGCTCATGAGTGGTAGAGATGAGGGAAGGCTTTGAAGCAGAGGTAAATTGATTTTTCGAA
TTATCCGTTATCTTTATTATGCCAACTAAACTATAAAGATCTCCGAGACCGAGAGCCTTT
TGTCTATGGTGCTTCTTTAATGAATACATGAACCTCACCTCACCACTCTG
>tig_1050_consensus
TCTCTGGGTTTTTGAGAGCTTTCAATAACGTATTGGATAAAGCAATTCCTTCAAGAGTCT
CTCTTATCATCTCTGCACCTGCACTGGCAAGAATAACCCTAGCCACGTTCTCTACTGTCA
TACGCTTTATCCCCATTCAATAACCCAATACACCAAATGAATGCTATGGACCAGAATCAA
CATTATCTTCTTCACCCACCCCTCCAGCCCCAGCCCCATATTCAAATTAGCACTGACAAA
GGTTCAGTTTCAAACCTGAAAGGCGACAGCCTGATCATTGCCACTTCATAAACGTAAACA
AAATCTAACCAAATATCTAATGGTTAATAATCCAAAGGCAATGATTTTGACGTAAGATTA
CCACCATTGAAGACACCAGCTAGCGTATCAGTTTTCTTTTAATTCAACATACAAAAATAT
ACTAGATCATTAACAGTAACAACATGAAAGTCGGAAATTGAACAAAGAGGAATTGATCAT
CTTCACAAACAAGAAGCTACTGACCACCAAAATGTTTGATTAATTCACAGAAAAAGTAGG
AGAAGAAACACTCAATAAGGCGGTGGCAGTGCTCTAGCAGTCGGTGCCCATATCACGTCA
CCACCAGGCAAATGACAACTATACATGGACATACCACATGACTCTCCTCCCGCCACTTGT
TCACTCTCAGCCCCACCGCCGCCACCACCACCACTTGAACCGACTGGCGATTGCCCTTCA
GCACCACCACCTGAAACCGAAGTTCTTGGCTCATCCTGAACAGGCAGTCGATGATAGGAT
GGATTATTAAACGTAGCTGCGACCACGAACACAGTCCCAGCCGCCATTAGTGGGCCCACC
ACGGAGCCTCCCACAATCTGCCCCTGCGGCCCCGCCAAAGAAATTGCGAATATGTTTGGC
ACCGGAAGACACGCTGCATTCTGAGGCAAGAATGTCGCCGAGATGGAGAGAATGTCGAAT
CTTCCGTGAAAGGTTATCGTAGCGCCAGGGGTTGCAGAAGGCTGACGGAGAGTAACGTTA
GCTACAGTTCCAGAGCCGGTGAGGACACAGATTCCAAGGCTCTTGCGGTGGCAGAAATTT
GAAATAGCTTCAACGATGTCGTTCCCACCAAGGACTTCGAGAATGTGCGGGCTCATGATA
GATTCGTGTTCCCGTGTTATAAAAACAGGGGGTTTGGGCTTATTTTTGGAGCCCGGTGGT
CTACCTCTTGGGCGCCGAACAACTTCAATAGTAGCTCCATCACCAGGCTCATGTTGCTTG
AGTTTATGCCCGGTGGTGGCCGCAGAGTTGTGGCTGTTGGTTTCTTCTTCAGAGGTTTGA
GAATCACGTGAAAGTTGAAAGTGATGAGAGAAAGGATGCTGATTGTGTTGATGCTGGTGT
TGTTGCTGATGAAGCTTTGAGAACATGTTGGGGGAGTTTGAAGTTTTGGGTTCCACATAT
TCACCTTTCATGTTTTGGTTTTTAAAAAAAATATTTGTTTGTAAAAGATCTCTCTAATTT
AAAACTTGGGGTTTTCTTATATTTTATCGAGAGGGAAGGGAAAGAACAAGGTGGAATGGG
AAGAGATGGGTCAAGGTTGAGAGAGATTATAAATATGGAGGGAAGTAGAGGTGAAAGAGA
AGAAGGGGGGATGGTGAAAGTGGTGGATGAGCTTGGAAACCACGTAAGGACTAGCTTGCG
CTGCTGGATGGAGACTTAAAGTAGGGTCCATTAATGAGGGCAGATAGGTCAATGTCCTTG
GTTAGCTCACCTTACACCGCCTTTGTCCTTTTTTCAAAAAGAGAAAGAGAGTGAGAAGAG
TTTTCTGTGCCAACTCTCTCTCTCTTACCGGACCTAGTAGATTGATTATGACTAGTGATG
TTTACATTTTTGGGCAATGGGGGATGCCCTTTTTATGTCCTTCCTCAAATTCTCTGTATT
TG
>tig_1051_consensus
GGAAAAGGAGCTCACTGAGGTTTCATCTGTAGATCTTTCTATCACCTAGCTTCCTCCTAT
TTTGAAGGATCAACATGTTGCTCAAAAAGGCAACAAGAATTAACTGCTATGGATCCCGTG
AAGGTTTATTTTCATTTGTGATTTAGGAAGGTGGTGCTATGTGTCGTGATGCCCAGCCTG

CTTCCCTCTCACCTGATTTCAGATGTCACACTGAGATTCTAAGTTTGAAAGTGATCACTT
TGACTGAGGAACTCCTTGCAACTGCAAAGCAAAATATGGTTTCTGGATCTGATACTGGGA
CAAGTGCTACTGCATATCCTAGTTTGCTCCAGTCTGATGGGAATAAAATGGAATCAGGTG
GCATCTCTACCCATCACAGGGATTTTACGGTGGGCACGAAGGTTCAAGCTGTTTGGAGTG
AAGATGGGGAATGGTACGATGCAACTGTTGAGGCCATTACACCAAATGGATATTATGTAG
CATTTGATGGTTGGGGTAATAAGGAAGAGGTTGATCCTGCCAATGTAAGGCCCATTCAGT
TTAATGCTTTGTTGGAAGCTGAGAAGGTAGCTGAAGCTACAAAACAAGCAATAAAACGGA
AAATTGAGCAAGCTGCTGCTATTGACTTTCAATCACGAAGTTTACCTGCGAAGCTCCGTA
TAAATCCTGAAGACCCAGAGGATGTGAAAGCAGCCAAACGGAAGAAAATACATGCTTTTA
AGTCAAAAGTGAGGTTTGAGCAACTTGAAGTCTCCCAAAACAAGCGACAGAATGCTTGGC
AGCAGTTCCAGACAACAAAAGGCAAGACTAAGAAGGTTGGTTTCTTCTCAGGCCGCAAAC
GAGAGAGCATCTTCAAGTCTCCTGATGACCCCCAAGGTAAGGTTGGTGTAACAGGAAGCG
GAAAGGGCTTGACGGAGTTTCAAAAAAGGGAAAAGCACCTGCATCTCAAGGGAGGAGGAG
ATGCCGAGATCGAAGAGTAGACTCTCAAGCTCATAATATTTCTAGTGCTTTCTCTATATC
CATGCCATGATTCTGACCTCTGGCAAACCAGCTCAGTCTTAAGTCGACCCTAAAGTACTG
GGCATCTCCTGTTAGTAGTAATGACTAGGAAGAATATGGTTAAACACTGATATTAACAGC
TGCTTTGTTAAGAACATTTGTTTTTGTTGAATAATGGTAGTATTTTCTTAATCAGTTGTT
TCCTGTTAACCTGCTTTTGCCTCTTATTTATTTCTGTAAGGGGGAGGGTCGAGGAAGAC
AAAAGGATTAAGTGGGTAGTAAC
>tig_1052_consensus
TATCTTGTTCCTCCTGTTTTTCTTACTTGAGTGCTGCGCAAAAGTAAGCTCTTTGCAGAA
AGCTTCATAACGCCTAGGATAGTGTATCACAAAACAAACTAGACAAACTTCACTCCAACA
TCCTCCACCACCAAAATACTATGCCGCTCTCCTCTCTGATCGTTGGCATCGTTTAATTCA
ACACGATCAAGACTGAGAGATATCAAAGAAGAAGACAGCTTCACCAAGGAAGCACGCGCA
TTTGAAGAGATTTCCGCTCCATCAGGAAAAAGATATACACTGCAGACTTAACAAACAGAT
TTGCGCTGGAAGGAGTTCGAAGAAGTTGAACAAGATACACATTGAATCGAGTCCGCCACA
CGTAATAATTCGGTATCCTTTTGAGTTTGCTTTTTTTACAAATTGCAAATTGGGATGTAT
GATACGATGTCGTTTTAAGGCGTTTCCAATGTAAACCTTATACAACGTCCAAGCGGTCTG
AAAAGAAAATTGGAGGGTAACGAAAACTCATTTGGATTGCCAAGTGGTTATCGTTTCTCT
CTTGGTTGGTTTTCAAATTTCTCTCATTGTTCAACGTGCGAGACCTTGAACAATGACAGA
AGAAGGGATACAAGTTTAAGAGCTGCGGCAAAAGATCATGTAAATGGTTTCTCAGTTGGT
CTTGGAGTTCGAATGACAACAGAGTTCATAGAAGGAAGTCATAGTCGGTTAATTGTATGG
ACGGAGCAAATTTGAAGACAGCTCTTGAAGCCTCGATCATAGCGGGGATGGCCAACCAGG
ATGAAAATTTTGACAACAGGATATTAGAGCGGAATACTTTTATGAAGTCAACAGAGTTCT
CAATGAAAATCGAATAAGAATAGCAACCCTGCATTTATAAAATGACGGTATTCGATGGCA
CCAAGATCTCCTCAAATCCAGGAATGAAACATTGGTCTTGTGGAAGGAAGCAATTCTATA
AATTGTTATCTCTCAAAGCAAACAAATCTTTTCAAATGCTTTGGTAGTTTGTTGAGAAAT
TGATTTTGCGTCACCTTGTTCTGAATGCATTCAATAACTCAGCTGAGAGTTCTCGGTCCC
TCTCAACCAAATAAAAGCATTATTCCACATGACAACCCTTGGTTGTAACAGTTTAATACC
AAGTTGCCACATATGTCTAACTCTGGGGTGACTTGGATACTCCACTCTGTTGAACAGGCT
GAGAAGCTGGTGCTGCATTGCCCTCAGCAGAATACTGTGGGTACCCACCACCATAAGAGT
TGTAAGATGGTGGCCCCTGACCATAACCAGGTTGACTGACTGGCGGTGCACCATATCCAT
ACCCTTGCTGAGCAGCTGCACCATAACTGGATGGTGGGGCACGTTGAGAACCTGAATCTG
GTTGAGAATAGCCAGATGATGGTGGCTGTGAATGAGGATAACCCGGCTGAACAGGCGCAG
GCTGCCCATAGCCCCCTGCAGCATTAGGCGACTGTTGAGTCTGTCCATAAGCAGGGGGGT
TGGGTTTCTGTCCTTGGGGTGGCCCATAGTTGGAATACCCAGACTGAACAGGTGCTTGAT
TCCCATAGGCACCCTGAGAAGTTGGAGGCACTCCATAACCTGGCTGTGCAGCTCCTTGAG
GTGGATAACTGGCAGGATTTGGGCTAGGCTGTTGACTGGTACCATAGCCCTGTGCAGGTG
GAGGTACCTGTGTTGAATCACCTGCTCCATATGACGGAGTATGCCCCTCTTGAGTTGAGT
TGGTAACATTACCATAGCCAGATGCAGAGTTGTAACCTTGCTGTTGATCATATGATGCAG
GCTGCCCATACCCTGACTGAGGCGCATGGTATCCACCATAGCCATCTTGAGAATAACCTT
GTCCCTGTTGGCTATAGCCAGATGCTGCTTGCTGGTTGTAACTGTAACCAGAGTTATCAG
CTGGAGCTGCTGAACCACCAGGGGCTGGTGGTTGCTGAGAAGATGGATGTTGACTATAGT
AATCGTAACTACTCCCCTGGCTGGTTTGCTGGGATGGTGGTACAGAGGACTGGTCCCAAT
TGGTGGAATATCCACCAGAAGTTGATTGGGAAGGATAGCCACCGTATGGAGGCTGAGACA
TGTTATACTGTTGTGATGGACCAGAATATGCTCCAGGCTGCATGTAACCGTATCCAGGTT
GTTGCATTGGAGGAGCCCCAGGTGTGCCCCAGTTTGTAGGTGGACGTGCTTGATAACCTT
GCTGAGAATATCCTCCAGACATTGCAGGATTTCTAAGACGATT

>tig_1053_consensus
TGTCTCTCTGCCTATCTTCATTATTCGTCTTCTCAAAGACATGGATGACAAAGCGGTGTC
GTTTTTGGTGTTCCTTTCTCTCCTCCTTGTCTCGGCAATTTCGGCCGACACCTTCTCCGA
CGATCAGGTGATCAAGCAAGTAACGGACGGTTCCGATGAGCTCCTGGGAGCCGAGCATCA
CTTCATGCTCTTCAAGAGAAAGTTCGGAAAATCTTACGGTTCTCAGGAGGAGCACGACTA
CAGGTTCAGAGTGTTCAAGAAAAACTTGAGACGTGCCGCGCGTCACCAGAAGCTCGACCC
TACCGCCAGTCACGGTGTGACTCAGTTCTCCGATTTGACTCCGGCCGAATTTCGAAGGAC
CTATCTCGGTTTGAGGAAGTTGAGGCTGCCTAAGGACGCGAATCAAGCGCCGATTTTGCC
GACTAATGATTTGCCTGAGGATTTTGATTGGAGAGAAAAAGGAGCTGTGACGCCTGTGAA
AAATCAGGGTTCATGTGGATCATGCTGGAGTTTCAGCACTACAGGAGCTTTGGAAGGTGC
TAACTTCCTTGCAACTGGAAACCTTGTTAGCCTAAGTGAGCAACAGCTTGTAGATTGCGA
TCATGAGTGTGATCCAGAGGAGGAAGGTGCTTGTGATTCTGGATGCAATGGTGGGCTCAT
GAATAGTGCCTTTGAGTACACACTCAAAGCTGGTGGACTTATGCGAGAGGAAGACTATCC
TTACACTGGTACTGACGGTAGCACCTGCAAATTTGACAAGACAAAACTTGCTGCCAAAGT
GGCCAATTTCAGTGTTGTCTCCCTTGACGAGGACCAGATTGCTGCCAATCTCGTGAAAAA
TGGCCCCCTTGCAGTGGCCATTAATGCTGTGTTTATGCAAACATACATAGGAGGTGTTTC
ATGCCCATACATTTGCTCAAAGCGGTTGGATCACGGGGTGTTGCTTGTGGGTTATGGCTC
AGCTGGCTATGCTCCAGTCAGGATGAAGGAGAAGCCATACTGGATCATTAAGAATTCATG
GGGAGAAAACTGGGGAGAGAATGGTTTCTACAAAATCTGCAGAGGTCGTAACATTTGTGG
AGTTGACTCCATGGTATCGACTGTTGCTGCTGCTGTTCGTAGTGACTCTGATTAGGCGGT
GAACATTGCTGATTTGCATATAAGTTGTTTTAGTTTTATGTGGGAGAAAAGAAAACTTGA
TTCTACTTTTAGCTTGTCATATGTCCCCACCGATCAAAGCATAAGATTGAAGCTTATGCC
TGTGGGTTGCTCAAACTACTCGGTGTTCTATTTAGTACTCTAGTGCATCTTAACCTGTCT
TTATTGTAAGAATGCTGTATTATCCAAATCAAACATAAACATCCATAAATACAAAAATCA
AAAGACAATCCTCCATGGCTGACCAATCACCATACAACTATCAGATTATCTCTCACATGC
AAAATCAAACAGAAATGAGTGAAAGATAAATGTTCGATAAATAAGAACACTTATCCTACA
TTAATCACATTTCATCTTCTATTGTATCGGCATTCATCCT
>tig_1054_consensus
ATGTGATTCACAATATAAACAATTAAGTTTTGGATACATATCATACATAATATGTATTTG
CACAACCATCAAATTGAATAAGATACAGTACTAATAATATATCAGGCACACTAGAATCTC
TTTTCTTTTGCAATTATAGAACAAAAGGTTACATTAAGTCTTCTCTTGGGAACAATTTAT
TACTCACCTTATATTTGGCTATAGTTGCTACATCATTTGACTGAACTCATGCTGAAATCC
CAGAGCTTCTTTGCCAAATCAGCATCCCTGCCCTGTGCAGTTGTCTTTGCCAGGTTACTG
TCCGAGAAGTACTCCCCGCTTATCCCCTGGACTCCGGTATGTAGCGCCACATAGCATGTT
GTCGCAGCTCCCTGCTTAACATCTTTAAGCACAAATTTGCCAAACGTGTTACAAAGGCCT
CTTATTACTCCATTATACCGGAACAGATTTGTAGCAATTGATCCTGGATGAAGCGAATTC
GCAGTTATATTCACTCCTTCTTCCTTCATTTGTTTTGCAAGCTCATTAGCAT
>tig_1055_consensus
CTCGTTCATTTTCTCTCTGATTTTTATAGATAAAACAGATTGTAATCGGAGGTGACACCA
AATGTTTTGGAAGATACTGCTCGACCAAGTGATTACACAAACAATAACCAGTCGTCCATA
ACATATGATCATTGTGCAACAAAAATCATAATATCAATATATAAAAACCGCTAAGCTTGC
ATCAACCAGCAGTGATGCTGACAAATTGTTTGACAGGCTGCTTCTCAATGATGAACTCCT
GTCAGCTTCCGTGCAAGAGATTGCTGTCATACAAGTCAGTGAAAGAATATTCAAGTGAAA
TTCTCTAATTCCCTAGTTGTTATTGCTCTTCATCATCCTCGCCAACCAATCAGTAATGTA
TTAAAGGAAGAGCAATCAACAAAGTAAACAGTTTCAGTGTGCAATATATTAAGCAATTCC
AATATTTCAAGCGACCCATGTTTGCTGTCAGAAGTCCAATGATCCGCACTGTTTGCTCTC
TAAACTCTTTGTTCCCCTAGTCTGTTTCATATCCTTTTTACCATCATTATTTAGGCCCTT
ATTCCGTCCAATGCCCAACTTAGGCAAACCCCGATTCAGTCCCTCCAGCAGGACACAAGC
TTTACACCATTTCTGGCTAGAAATGTAGCCACAGCGTTCACAGGTACCCTGCTCTGGCAT
TTTTGTTGAAGTGGCAATTCTGAAATTCTCCCCTGATTTAATGATGTCAAGTATGGCCCT
GGGTCTTATTCTCTCCAAATCTTTAATGAACTCACGAACAAAACCACGATAAGCATTTGG
AGAATATATGCATTCGGTAGAGAAGTAGTCTAGCCTCTTGAAATAAGCATACATTACAAT
CTCCTTCTCGTAAGTGTACTTAAAAGGTTTGCATCTTGGAATTGGTCCATCTTCACCAGT
TGTTATTGAGGTACATCTACTCAATCTAGCAATATCACCACGTAAGATGTTCAAGAGAAC
CGTTTCTGCAATATCATCTGCATTATGTCCAGTAGCGAGCTTGTCCACTTTCAACAAAGA
AGCACCTCGATCGAGGGCCTGACGGCGGAAAACACCACAGAACGTGCAATTATTCTTTAA
ACCAATCATCTTCACTATTTCATCCATTGTCCATCCATATAGATCCTTGTAAGAAATAAT
CTTCAGTGGGAGGCCATACTGCATTTCATTTCTTTTAACAGTTTCCAGTGAGTCATCCCT

ATAACCTGTAATGCCCTCATCAATTGACAAGAGGAAGAGATCCAAGCCGTAGTTGTGGCG
CCGGTTTAGCTCCGACAGTACATAAGCAAGAACAGTAGAATCTTTTCCACCAGATGCGCC
AATAGCAATACGCTCACCAGGCCTGAATAACTGGTTATCCACAATTACTTGGTGAATCTC
CTCTTCAAAAACCTCATAGAAACACTCCCTGCATATCTGTTCTAGGGTTTTAGGTCTTTT
GAGAGCAGCCCTTCTCTGGCTACAGAAACAACAAAGGCGGCCCCCAGCCTTCTTCAGCTT
CCCGTCGGCTTCTTCCATTCAAGTGCTTCTGGAGAACCGAGACCAAAGTCCTTAGCAGTC
AAACTGACGACAAAGTGATACGAAAACGTTCCTTTGAAAGCCCGAAATTGTTGTTGCCCA
GGGGAGCTTCAGCGGCTGGCAAT
>tig_1056_consensus
CGAGCAATAAAACAAGCAAACCAAGTACATTATGAGTTCATGCTAAAACTGCAACTCTCT
TGCTTCCAATGAATGCTTTTCGAAGCCTAACATAACCATTTCTATAATGCTCTAGAGAGA
AACACAGCTGCCTTATTGCTTCTCTTTTCTCTTCTGCTCCTTCCATTATGACAACTCTTT
GTCTCTCTAACTCCTTCTCCATTTCTTTGCCTCTTTCTCTCAACTCCCTTTCAACCTTAC
GACCTTCTTCCAACCCTGCATTTAGCTTCACATGTTCCATGTGTAATTCCTCCAAATGCT
TTTGCATTTTACTAATCTCATCATCTTTACAACTCATTTCAACTTTAAGTCTATTCATTC
CTGAATTAAGTTCATCTCTCTCTGACTTAAATGCGTCAATCTGTTCATTCAACACATTTA
TATGATCCTCCCTCGCTGCAACTTCTGACTTCAACTCGCTAGTTTTATCACGGAGTCTTT
GCTCCGTCTCAGTTTTCTCAGCCTTGATCTTTCTGATATCGTCTTCTAGGCACCTGCCTC
GTGCTTCCCATTCTCTTAGCTGCTCCTGCAAGCAAGCCTTTTCATCTAACAAACCTGATA
TTTCAGCCTTAATATGTGCCTTTTCGGGGAAAATCTTTTGTTCAGCATCGGATACTGCCA
TTTTCAAATCTCTGATCTCATGATCACGGTCTGATAAACTAGTTTTCAACCTTGCGATTC
TTTCTTGCAGCTTGGAAACTTCTCTTTTCTCTGAATTCAGCTTAGCTTTCCATGTTGTGG
TATCTTTCTGAGACAATTCAAGTTGCTTCTGCAAATTCTGGATTTTTTCAGACCTGATAG
CATCAAATTCATGTTTTAAAGCAGCAATTTCTTTTTCTGAACTCTGAAGCTTCTCTTTTG
AAGTTCTTAGCTCTTCCAAAAGTGCCTGAATCTTAGCATCTGGATCCAATGTATCTGCTT
CTTTAAGTTGTAATGATCCAGATTCAGCCTGCAAACGGCTGGAGATTTCCGATGATTGGT
ATTTTTCAAGCTCAATCTTCAACCTAGAAATTTCTTCTCCTGAAAGCTTTATCTTCTCAT
TAGCATGTTTCAGTTCTTCCTCATATTCTGCTATTCTCACAAGAAGGTCTTCAGACTTGG
CAATTTCAGGATCCTTTACTGATCCATCAGCATTTGATTGCTGCAACCAGAGTTTTTCTT
TAACTTCACGAAGTTCAATCTCCAATTCAATGACTTTTGATCGAAGCCCTTGATCACCAC
TATTCCCAACTATACTAGAGTAGTTATTCACTGAAGAATCATCGGATTCTGACTCAGAAT
CTGTTAACGAAGATGAGTCATCTCCTTCTTTATGGTAAAGATCAGAGCTGCTTCCGGAAG
AGCCAAGAAAAACATTGAAACCAGCAGCTCGGGGGCCAGATTTTTGAGGGCTTGGCTTTC
GTTCGGGAGAGGGACATGGAGAGGGCAACTCAGTGCTAATGTCAGAAATGCCTGAGCCCT
GGGATTGCAGGTCAGATGGAATATTTTTCTTCAATTCTCCTGTCACATGATCATAGCGCT
CAGCCAATGACCTGTACTTTCTGTAGAACTCTTCAACATGAGATATTAATTCTGGCCTCT
TTTGGTAATACATCTCAGCTTTCTTCGCAAATGAATCTCCTTCCTCTTCAATCAACTTCA
ACATTGTCTTCACGTGTTGGTCCATCTCCTCAAGATTCTCTGCTAGCCACTTAGAATTTT
TGGGACTGATGTGACTATCCCACCACCAGGAATGTGACTTTCTCGAATTCATCCTTCTCA
GATTTTTATTTGAGCGAATCAAAGATGTAGCCATTACTTGGTGAAAACACAAAGCTGATA
CTAATCCTTGTCACAGACAAAAGGGTTGGTTGAGCTTCAGATCCCACCCCAAGGTGTTGA
AGAAAACCTGAAAGGACGAGAAGTTTTGGAGACACAGTGACTCAAGAGAGATGAAGTTGG
GTAGACGAGCTTTTTAATACGAA
>tig_1057_consensus
ACAAGGTGGCAAAAAAGAGAAGAACCCCTTTTGGGATCAATGACCCATGAAGAAGAACGCT
TGAAACTATTTCTTCTCATAGTGTATCTGCTACTTTCAGGGGTGGTGGTGACAGTGATTT
GATGATGAAGTTGGGGGCTGATTGAATAATCCATGGTCCCTGCTGCCACCGCCACCATTG
GCTTTTCTAGCATGCTTGACCCTCTTGGTGATGAAGAAATTATGCTATCATTAGCATAAT
TACTATTAGAATTCATCTTCTTCTCGCCACGCTGAATCCCTTTTTTGGAATGGCTTCTCT
GCAAAGTGATTCTTGAATTTGCATTGATATCTTTGGCTCCCTCATTGATGTTATCCATGT
CAACTACAAAAGTATCAGATTTCTTTGCGGTACCGTACTGAACCCTGTTTAGAGTATCAG
AAAGCGAGGTTTTTTCTGGTTCCGCACTCTCCTCCATGTCCTTCATAGAC
>tig_1058_consensus
GGACATCAAAGCAATAACAAAAAATTAACTTATTTTACGAATAACTTTGATTTCAAACAA
ACTGGCTTACAAGAAAGCAAAAGATAACGTGAAAAGAGGGTAAAAGTAAAAGACCATCTC
CTTATATCATCAAAATTACAAGTAGGAATCAAGATACCCATAGGTGTGCCTCTTGCTATA
CTGAAGCAAACTAGCATAAGTCCTGTCCCATATTCCTATAAATAAAGACTCATCATCTGG
ACTAAGAGATACTCCAGATATCTCACCGAAGAAATCTATCTCTTGCTGCATTTTGAAATC

TCCCTTTGTACTATACACATGCACGAAATCTGCAGGTTCAGCGACAACCATAAACTGTCC
ATCGGATGAAAAGCGAACTGACCGAATGGCCCCTAGGTTTCCTTTAAGAGTTGCTACTGG
CACAGAAAGCTTCCTTATATCCCAGACTCGGCAGGTCTTATCCTGATTCCCTGTGGCAAA
GATGCGCCCATCTGGGTGCCATGAAGACGCAAAAGAGTAATCCTGGTGACCAGCCACTGT
GGCAACAGTCTTCCCATTTTGTGAATCAACAAGCAATGCATGTTGGTGATCTCCAACAGC
AACAAAAAGCTTGCAATCGGGACTCATTGAGGTATGATTCACTGGCCAAGGGAAACGGTA
GTGATTTAAAAGCTGAAATGCTGCCATATCATATTCTCTTACACCACAGTCATTGTTGGA
TGCCATTAAATGTGTTCCACCCCTCAAGCTGTCATAGATCTCTACAGCATTCGTTATGGA
ATTATCATCGCAAGTCATCCGGGTGCAAAAGCTTACTCCTTGCTTATCCAAACACTTGCA
AATTAGCTCTCCTTGGAAGCCACCTGCAACCATAAAATTATCTTTCACTGCTAGAGTGCT
TATCTGCGTTTGTGTAAAACCTTCTAATAAACTTCCTGCATGTTTCT
>tig_1059_consensus
CTTGCAAATTAGCTCTCCTTGGAAGCCACCTGCAACCATAAAATTATCTTTCACTGCTAG
AGTGCTTATCTGCGTTTGTGTAAAACCTTCTAATAAACTTCCTGCATGTTTCTACATTAT
CAAACAGTTCAAGTTAGCAAATACTTGAGAAAAATTGTATGTTAGAGAGATAGGATCCAC
CAAAAAAGCACATCCACTGGTAAGTATCGTTTACTCAATTTGCCTCCTAGATTATAGACA
TATTTTGATTTAAAGAACTATCCAACTCAACCATTCATCACAAGCATTCAAGTAAATACT
TTATAAATTATACCTTTGAATGAAATCTTCTTCAAAGAACTTATTCAGTTCCATAAATTA
ACTTTATAGCAAAACTAAAGGTGCACCCTTGTGCCATACATATAGTAACTTATAGCGGCT
AACGGAATAAACTATATAACAAAGTAACTTATATACCTCACTGGGGGCTATGTGTCCTGC
AAAATTGAGGATCTCCGACAAATTGGAAGATAGTGTTGACCAATGCATGACTGAATAGTG
GGACAT
>tig_1060_consensus
CTTTACGATGAAGGGCTGACAGATCTTGAAATGGATCTGGTGCTCGCGCTACCGTTACTC
CTAGAAGAGCCCCTAGGGTAACTTTCACGTGAGTCTTCCTTCTTAAGAGATCGAGAAACA
CTCTTCCTTGGACTTGGGGAGATGCTCCTGGAATAGCTCCTTCTTGAGGTCCTTAGTCTA
GGCGACAGACTGCGTGAATAACTTCGCCTCGACTTTGTCCTCTTTGGAGAGACGCTTCGT
GAATAGCTTCTCCTAGAGCTCCTCCTCCTAGGTGGTGTGGGGCTTCGAGAAACTGAACCA
TACCGATGCCTTTTATTTGGCGAATCATACCTGTCATCATACGGTGTGTAGGATTGATCA
CGCCTTCTATAATAGCGGTCATCATATCTAGTGTCATATGGAGAATACGACCGATCCCGC
CTTCCATAGTAGTGATCATCCGGCGACCTACTGTAGGAGAAACGGGTGCGGGAGTAGGAT
CTCTGCCGACTATAATAAGGAGAATAAGACCTCCGGCTGCTATGGTATGGATAGGGAGAA
TAAGATCTGCTCCTGCTTCTTTCTGAGGAATACCGAGGAGACCGACTAAGGCTCCTGCGG
TAAGGAGTGTAGCTAGGAGATCGTCGACGAGGTGAATAGCTAGGTGTTCGACGCCGAACA
CGTATTGTTCTCAGTCCCAAATATCTTCCTGGAGTAGGAGTTCTTCCTCTTCGTCGCCGT
GC
>tig_1061_consensus
CACCGAGATCTACACCTTTCCTACACGACGCTCTTCCGATCTCACTGATTTTGCTTGGTC
TACCTTAATCATCTTTGCAAATATGTTGCCATAAAATTGTGCATCCTTCTTATTGTACTC
TCTCACTTTCTCCCTCAATAACTTGTACTCATGTTTCACATCCCTATTGTCAGGTTCAAT
CTCAAGAGCTTTTTTGATATCCAGTTCAGCCAGATCCAAATCAACAAGTTGAATATATGC
TTGGGCCCTCCTATAAAGAGCTTTAACATTCTTACTGTCAAGTTCTAACACCTTAGTGCA
TAACTTCTCTGCCTGCTTGTACTCTTTAAGTTTGAGCTGGCAAGCAGCATTGTTCAGGTT
GCAGGTAATTTTCAGCACCTTGGATTTCTGCTTCTCCTCATCAGTGAAAGAAGAATCATA
CTCTATAAATTTGACAGCCTTTTCATATCTCTTGGATGCTCTTTCATATTTTCCAGCCTT
GAACAATGCATTTCCTTCTTCCTTTTTCTT
>tig_1062_consensus
CATAATTTCTACACCCTCTTAAATATGATAAATAGCTGCTTTCTAGATCTAGTAGTACAA
ATCATGAAAAAGAAGTGGAAAAGACAAACTCCACAAGCTTAAAGGACTAAAGAGCTGATC
CTCTTAGCCAAGTAGAAGGGGTAGAAGAGAAGAAGAAAAACTTTACTAAAATATGTCTTG
ATAATTAACAATAGAAACACATGCACACCCACATATGCACATTAAAATATGTCTTCCTGG
ATTCTTTTCTTTCAACCCCACGAGAGGATTATTATGCCTGAAAAGATAAGGATAGCTAAA
GAGAACAAGGTAGAGTTTGCAAGTCTCAACCCACCATGATTGTAGTCTCTGTCAAAGCCA
CCTCCAGAAGGGCCTAAACCTGAGCCAACTCCTATTATTCCATTTGAAGGTGTTGTTGTG
TACGGATTGGCAGTGGTGGTGGTGGTGGGGGTTGAATTTCCAGTAGTCGGGGTATTTGAT
GTAACGGGTGTTTTCGGGGTCACTGTGGTGGTGCCCGCAGCGCTGGCGCTAGCGGGGTAT
TGGCA
>tig_1063_consensus

TGCCTGTACATAATGTGCTACCTTCAAATTTACACACTCTCAAGCATTCTAGTTATGTAG
CATATGATACACACAAACAGAAACAGTTACACAAAGACAAAGATGGACTCAAACCAGAAA
AACAACTGCCTGTACATAATGTGCTACCTTCAAATTTACACACTCTCAAGCATTCTAGTT
ATGTAGCATATGATACACACAAACAGAAACAGTTACACAAAGACAAAGATAATAGCAATT
AATAGCTACTGATTCCAGCATCTGTGTCTGATTTGGTGGAAGCTGCAAGATCGGCAATGT
GGAACTGAGATGTATAAGTGTTGGAACCGCCTTCAAGCACAGGAGGTTCATTTAGAAATG
CCCAGTTGCTCCTAGTCCACCAGCAGTTATTCTTTATGAAATTCTCCAGGCTTGTTTCTT
TATCCGTGTTAATGAAACCATCCTTGGTATTGGCCAGAGGCAGGGAGGCTGATCTCTGGA
AATTGATTTTAGATTCCTCATCCAAACTTCTCTGAGCCTGACTCTTTCCTGATGGACTGG
CAATAGCCTTGAGATGTTCCTCCTCAATTTTCTTCTTCTGTCTCCAGTAAGCATCGATCT
CCTCCTTGGTCAGTGACCGATTCCTCCTCAATTTCACCAATTTAGGATCTGGTATATGTG
AATCCCAGCCTGCCATTAAAGAACCCATTTTACTCAAAATCTTTGAATTATTAGACTTTG
ACAGAATATATCTTTCTACTTTGAAACTATATGCAGTTGTATTTATAGTCTTGACACTGC
TAATGGACTATCCCATCTTTTTCTTTGTTCTTATCCTTACAGATATCTTTTCTACAAAGA
TCGGAAAAAAAGATGTTAATCATGTAGTGCCCAC
>tig_1064_consensus
TACCTGTGAGATGGAACACGCCACAAAGACTCCCATACTTTAATCACTTAGGAATACTTT
TTGTGGATCAAGTTGTCCGCTACAGTAACTAGAAGGAACAGTAGATATTTCCTGTCGTCC
TCTCCACTCTTCACCAGGTTNNNNNNNNNNNNNNNNNNNNNNNNNNNNNNNNNNNNCAGC
ATCTGGAAGTCCATCCTTCCGCAATACATAAGTACGCTTCCTCTCATGGTCCAGAATAGC
AATTTTTGTAGGTGTACTCAGATATATCTTGTCTACTTCTGATTCAAATGTTATTGCATC
CCCTTGTTCAGTAAATCGCTCTTTACCCTTTAAGTTGTCCAGATAATCCAGTGTCTCTAA
TCCTTCTACCCGCACTTCACTGATATCTGAAACAGCAAAATAGGTGTGATACGCAAATGT
AAATGCAAATGATTTTCCATTTGTATTTGTATTTCGAATGCGAGAAGTCAACATCAAATC
TCCCCCAGGTCCAAGAGTTATCCTTAGGCGAAACTCATATCTATGAGGCCAGATTTTCAC
ATCCTCTTCAGAGTGCTTAAGGATTAAATCAATAAAGGCCCTGTTTGAGGTGTTTGTTGG
AAATGGTGGAGGATCATGATCAATGCTCCACATCCTGTTCCTTGCAAATCCGTGTTGCTC
AAGAGAGCCATGATTTCCAAACTGAGGAAAGCAGATTGGAATGCCTCCACGAATAGCCTT
TGGAAGTTTAAAAGTAGCCTTGCTACTAACAAAAAGCAATTCTTCCCCATATTCATTCTT
CCAAGATGTTACTTGTCCTCCATAGAGATATACCTCAACAGATGAGCCACGGACTTCGCG
TAAAATAACTCTCTCGAGGCCGTTAATGCCCTTACTATGCTCGACGAATAGGTTATCATC
GCCGGAGGAGGTCGAGCGATCCATGGATCGGTCTTTTTGTGTGTCTTTGGATCGAACCGA
ATCCATTGGAATCGTGTGGGGGAATTTATAGGAGATTGAGACTGCAAAGAAAGTTTAATA
TTTTTT
>tig_1065_consensus
GCCTGTAGTACTTGGCTAAAAGATTATTGAACTACCAGGGCTATACATTTTACAGCTTTC
AGCTAAAAAATAAAACACTACCTACACAGCTACTGTCAGCATCAGATTCATGTTTCATCA
AGTCCGCAAATGATACATTTTGGTGCAATCCCACAGAACTTTGATTACCCCAAGCATTTC
TTCAGTCTCACCGAATGTGGAGCCTGTGTCTTCCAATGGGTATGGAAGCTTAGTCCATTT
ATCAGCCTTTAGCTCAACCACATCCACAACAGACGTCCCTGAGTCCTCCAACTCTTTTCC
GTACCATTTACCATAAACCTGATTTACTATTCCCTTACCAATCTCTTCTCCCTTCCCATC
TAAAAGCACAACATACTGACCTA
>tig_1066_consensus
CTGCAGTAAAAACTTCAGCTGATTTCTTTTCTATTTATTTGCATTTATAGATATGTAGAG
AAAATTAAGAAACAGTAACAAGCATGTACACAATCCCGAGCATATATTCAAATATTATCC
CTCTGACTACATTGACAACATATTTTATACGATACCCACAGATGGAAATAAAATTGGCCT
ACTCATTGCTCTTACTGGCGACTTCCAATCCATTACCATCTGGATCAAAGAAGAAGATTT
GTTTGACCTTCCCGTTAGGGAGGGACCTTCGGAAAGTCTGTATTCCCTTGTCCTGAAGAG
TGTGCACAAGGGATTCAAAATTGTCGACAGTGAAGCAAATATGATGACCTCTGGGAAGAT
GGCTGGGGTCAAGGACGGGTGACGTGGCACTGTACGGACCTTCTGGAAGCTTGGTGACGG
GGCTTCTCTCGATAAGATGAAGAGCAAAAGCTCCGGGCAGATTCAGCCATATCACTTTGA
ATTCCCCGAAATCTGGGGCTTCAATTTCCTCAAAACCAAAAATCTCCTTGTAGAAGTTGG
CGAGGCGCCTTATGTCCGAGGATTCTCTAGAGATGTGGTTCAGCGTTGCTCCTTGAGCCA
CTGGCATTTCGGTGAGTATATTTTCAGACTTGAAGAGAATCTGCCACGGCCAG
>tig_1067_consensus
ATGGATTCAAATTAAGTCGGTCTGGGTTTGGTTTAGTGTTAGGCTTAAACAGTATTCGGA
GAAAATAAGTATTTTGAGCAAGAAAACAAATTGGAGCTCTCAATTCTCAAATTTAAAGAA
ACAAGCCCTTCGGAACAGGATTTGTTGTACTAGTAATAATACAACTTGATGGCTTTGTAC

AGGCAAATATAACATGAAAGACAAGGCAAGGGAAGTGCAGGAAGAGCTTACTGGACTCTA
TGAGACACCTTTCTGTGGGAGAGTCTCGAAGGCGGAGACTGTGTTTTCAAATTTAATCCG
ATGGAGAGGAGCACCGCTCTGCTTTATCGCACAAGCTATCTTGTCTTCCAGCGGGCAACG
TGCTTTCAGCATGGAGAACACATGATTGTTCAACCTCCTTGCTTTCTTCATTGCTTCTAG
TACAGCTTCCCATCCCTGGTTAAAATGAGGGTTCTTGTGTGTTTGTGCAACCGATATAGC
TTCCACTTCAGCAACATTAAGGACTTGATATGAAAAATCAAAAAGCTCAAAATGCAGCTG
CTGCCCAAGTAAGTATATTATGGTACACCCACCCCAAGCAACTGAATCTCCCAGCACTTC
ACGATTGCTAGATGGTGCTTGATCAGATTCCTCCAGATATCCAATTTGAAGGCCAGAATA
TATACGATAAAAATCCTTTGAGGTTGTAATGTCAATGAAACCTGTCTTAGGAACAGCACT
CCATTTACTACAATACTTATCCAGTGCTGCGCTTGTAAATGCAAGGGCATATTCAAGCAC
ACTTCCTGTATTCATGTTGGCCTTGTACAATAGATCTGCAGCTTCTGCCTGTTTGGACAT
GGTGTAGAACGATGTTGGATTTGGGCACCCGGGGTTTGACACAATTGCAGAAGTAGCTGA
TTTGAAGAGATTGACGACAGGGCTATCTCCACCATCTTGATGATGCGAAATTTGTCCATC
TGCAGCTGGAAGCAAGCCCAACCAAGGGGCTGTTTGCATGAAATGGGTTGTATCTACTTC
TCTCAATACAATATCAAGCAGCCCCATCCAATATAATACACTTCCAATCTCTTTTATCCC
TCGAAGAACCTCTGCTTTAAGCTCTGCCTTTGACCCCCAATTAAGATTTTCTTTAACAAG
CCTCATACAACCTGTTACACCACTATCAAAAGGAAGCAGACCAATAGATTTTGGCAGTGC
TTCTTGCAGTCCTGTAATCATTGGTTCAAGTGTAGTTATCTTATTTGATATATGATCAAG
TAGTGCTCGGATAAGCCAAGGCAAAGATCTGGATCCAAGCAACTTAACAATGGAGAACAT
GTGGGGTATCCCAAAGAATGAACTATGCAAACGTGCAAAACTTTGATGTGCGGAATTCAA
ATCTTGAGTACCACAGTAGAAGTTAGGCTTGGCATACGGAACAGATGGCTTTTGAACAGG
AACAAGAGGAACTTTAGATGATCGGATGAAACGCTGAGTAGTATTACAGAGGATAAAGTT
CGGCAAGAAATCACTTTGCATCTCTGACCAAATCTGAGAGGCAATTCGACTAGAAAATGA
CACGAGCGATATGTTTTCTTGCATCTCATTCAACATGAGACTGAATGAATCTATTGTAAG
ATCTTTGGAAAGTAATTCATGTGCATGCTTTAAAATATCCACCAGCTTTTCTAATTCTAC
TATGGCACATAGATCTTGAGACTCAAAGCGATCAAAAAGAAACTCGAGATTCTCTCTAAA
AACTTTATTCATTCGTTCAGCAAT
>tig_1068_consensus
GCTCGGTAACTTTGGAATAATATTTGTAACACAAAAAAATATTCATCGCTTGGAATACTA
CCATAATCTTGAACAACAAAACCTAACTCTCAATGTCTAGAACAAAAATGTGCCAAAAGA
ACCAAGTTCTTTGCCCAAACATAAATAATAACAGTTCAAAACTAAGTTGTAAATTATAAA
GGAATAGTAAGGGAATGCGGTCACCACAAAACTCATTGTGCACTGCCCTTAGTCCCAGCC
AGTAGTTTGAGGGGCAGGAACGTCCGGAGCAACTCCAGCCATTTGTGGAGCAGGCACAGC
ATCCCATCCATCTCCAGCAGCCAAGGGAACTTCTGGGATGTAGGTAACCCCAGGAACAGC
AGCAATGGGTTTTTGAACTTCACCAGCCCACTGTGCATCAGAGATTTGTGTAGGCCATTG
ATCGCCAGAAAGGTTCATGCCAGCATAATCAGGAGCTGCACTTCCATATTCAATGGGAGC
AGCAAGCTCTTCGTCTTCAGCTTGCTTGGCCTCCTCAGGTTCTCTGTAGAAGAACAAGTC
CACCATGACATCCCACTTATGGCCAGGACGAATTGTGCCACGCATCTGCAAAACCATTCT
TGCCAATAGCCAGAAAAGACACCCAATGCTGTGCTTGCCCTTGTTATTAGCAGGGATACC
AATATCAACATACCGCATTGGTGAGTCTGTATCACAGAATGCAATGGTGGGTATATTCCC
AAGAGCTGCTTCCTTAATAGGCTGGTGATCAGTTCTTGGATCAGTCAGGATAAGGAGACG
AGGCTCACTGAATGATGTCTGGAGCTGATTAGTGAATGTACCAGGAGTGTGTCTCCCAGC
GATAGCATGGGCACCAGTGTACTGGGCAAATTTCAGAACAGCTCTTTGACCATAGGGTCT
GGCTGACTGGACAATAATGTCCTGAGGGTTCTCGATGGCAACAATAACTCTGGCTGCCAT
CTGAAGCTTCTCCCATGTCTTGCCAAGGTTGATGATATAAATACCATCATTCCTGCGCTT
GAAGACATAACGTTCCATCTGGAAGTCACAGTTCTTGGTTCCAAGGTGGACTTCGGCGGC
CAACATCATCTGAATGTCAGCCTCCTTTTGAGACGGTTGCCTCGGGGCAGTTGCATTGGA
CGACGCCATTTTTTCCCTGTATCTGCTTCTCTGTAGGAAACCCAATGTGGGTTTAGAGTG
TCGTGAGAGAATAAGAGCTGGAGAGTGGAGAAAAAAATGTGAAAAGAGAAAGGAC
>tig_1069_consensus
AAGCCACATTGCCAGAGTGACCTGGTCCAATACTTCATCAGCAAGTAAAGAATAAACATG
AAAATGATGGGAAGATTTCAAGAAGCTGAATAACAGTATCTATTAAGAATTTTATTTAAT
GCAATACTAAGCTTTGCTTAAGAGTTAAGATGAATTCTAATGAACCCCTGATCAAGTTCA
ACCAAACATCCAACATACCATCAAAGTATTGTTACAATGGGCTAGAAACTAGTATATTCT
TCTTTTCTTCTGGCATTAATTGCCCTAGACATCAGTATCCACAATAAGAAGACTGACCCA
CATACTGTAAGATGCTATTCGAAGCCAGTAATAGGTTAGATGAATTCAAAATATATGGAT
TCAAAATATAAATTTTCAAAGAGATAAGAGATTAGACCAAAATTGTAGACCACATTGATC
TCATACGGGGTATGAGGGCAAAGCCCCCGCATGTCATATATTTTTTAGCTAAGGTTTGTT

AAAAAGGGTGTATGCGCCACAAGCATAGGGGAGAGCTAAGATTAAGAACTCAAACAGACA
GTACAAACAATATCTTCTTCATCTTAGAAGTTGCCCCTACTTATGCATTTTTGCTATTTG
TTTTCATTGGATTTCATAACAGAGTAGAAACATCAAAATTGTAGTTGCTGTTGTCACATA
TGTTTGACAACTTAGCAGAGAAGCGAAAAGGAAGAACCCCCAATTGGAAGTACATAAAGG
GGTTCACATTTGTTGAAATTCTCAACTATATATGAGAGTGGGGAAACTTCCTTAATAAAG
CAGCCACAATGAATCAATCTAATGATTATCTGGGAACTTTGGAAAACACTTGAAGAGAAT
TCAAAAGTTATCCTATTGGCATGTATAACATCGTAAACCAGAGTACCATGAACAATTCAG
CTAAATCTGACATCAAACAGCCCATTTGAAATTATCTACCTACACACGCAAAGAAACCGA
AGCAACAGTACCCACACTATATTTCTCTATCCCAATATCACAAGCCTATCGCTCTATGCA
AGTGACAGATATGGCACAGTTTAGCATTCTAAATTCCGCCGAATAATACATAACAAACAG
CATGAAAAGAATAAAAAATTGCACAGGTCTACTTCTTTAGTTACGAAGTAAAACAAGCGA
CAAGCTCAGAAACAAGCTGTAGAAACAGAGTTATCACACAGAAAATGAACAAAATAAAAC
CTCACTCATTAAATAAAAGCAAATTCGGTGAGCGTATCTGAAACCGTAACATAATTTTCA
AGCAAAGATTAAAAAACAACCTCAAATGATCGATCAATCCTTGTTCTTGAGCCTCTCGAT
CCTCTGATTCAGCTGCTCGATCCTCACCACGAGATGTGTGACAGAATACAGCAGCCAATA
GAACAGCAAAGCGGACGCGATCAAGAGCGCGTTGCGCTGACTCTTCATGATCGACTTCTG
ATGACGCAAGTGCTCCGACGGGCTACACGCTTCCGCCTCGCATCTCGGGCGCGTCTCGTA
CTTCCAATAGATATCCATCAGCAGAAACAGACAGAACGGGACCACCGAGAGGAACGGCTT
GAGGAGATTACGCGTCACTGCGACGAGGCCCCTGCGTATACCGTCGAGGCCGGGAATAGT
GAGGAGGAGCACCATTATCGCTTCTGCCGCCGCGGCGTAGCCGAGCACAACCCATTCGAG
CGCCATGGAGAAAACCTAGATCTGCAAACGGTGCTTTTCTTCGTCGGGAAGGGAAGAATT
TTTTGCAGATCTAGG
>tig_1070_consensus
ACTCGCTTGATTAGCAATGAATTACTGAAACGTGGTTCAAAAGGATGAATTAGCTACCAT
CTTTTCTGTGACTCAATCTGGGCTACCCCCGAGCTTAATTAGTTCAAACCAACATCCGTA
CCAGAAAAAGCAGAAAACTTAGAGAATTACATTATTAATAAAGCCATAAGTAGCATCGTA
CATAAGATGATAACGTTCTTATCACTGTTCAATTTATTCTAAGTCCTTCGTAATATTCTT
TATGATGGCCTTCTCAACCAGATTGCTGGCTCCACTCCATAGTAATTCAGTGAATGGTTT
CACAGTTGGTTTTAGAGGAGACTGCGAAACCAACGTTGATGCTGCAAAGGCCGGGAATCT
TGCTCAGGTATGCATCGTTTACCTTCATAGACTTGGCAGCAGTCTTTAAGCAGCGGCAAA
TATCCTTCTTATCATCAACACTTTTCACCGACTGAGCAAGCTGTTTCAGTCCGCTGCAGC
ATGCACCAGAGGGGCTTGGTTCGTTGCCTGTGGCATACCCCGCACATGCAGCGGCTTTAT
TAACCACAAAGTCGCATGTAATCGCCGCCTCGTCGACATTGGCAAGGAGGTAAAGGACTG
AGAGGAGAAGCAGCACGGATAAAAAGAGGTTCTTCTTCATGGCTAAAAAAGGTAATGTAA
CAATGTTTGCAATATGATCCTGGAAGTAATTTGATGGTGAACTTGTTTGGTTGATGGGAG
AACAAGGAGGGGTTAGGGGGTTTATATAGTGAAAGCTGATTCATTATTGAGACACTGGAA
ATGGAAGTTTTGAG
>tig_1071_consensus
CACGGATCTTGAATTTAAAAACAAATGACACTTCCATGAAGGTACGATTGATAAAAAGAA
AGAAATTTATCACCTGCGTGCTAGCCCATAACAAATAACCATAAACATTTACATCCAGTA
ATTTACAAACATTAAATGTTTAATAAGAAAAGGGGAAGGGGAAATTGGGNNGGATAATTC
TGGAGTAGCTTCAGATTCGAAGTGAAATCCCCTGCTAGCAACCGCCTTCTGATCAGAATT
AGCATTGCACAGCATATCCGCAGCAGAGTCTCCAGTGGACCTTCAGGGTCACTCAGAAGC
GTGTCCCAAATGTGAAGGCTGTCTGCGAAATTGAACTCCTGAGTCAAGAGGAGAGTAATC
CACCTAAATGCATAAAATTGGGGGTTCACTTTGGTTGTGACCTCTAGGTGACGCCAAAGC
TCTTCATCATGTTCCTTCAGGAGCTGCGATAATTTTGTGATTGTGGATCTGATTCCAACC
ACACTGTTGTCAAGTTGCTGACAAAAATGATCCCGAAATCCACTCAACAACTCAACAAAA
CAAAAGAATGTGTCTGCTTCTGCAGAGGCCGCAAATTCCTCGTCAGGATCATTTCTGAAT
ACATAGAACAGAGGTGCCAAAATTTCATTCATCCCTTGGACATATCTAATACCAGGATTT
AACTTCGCAAAGACAGTTAATATACTCTTCAAAGCCTCCTGATTAGATTTTGCAAAAGGT
GAGTCTCCAGAGAAAAAATGCAGGTCTGGATGAGTGCGCTTTACATCTCGATCAATCTGT
TCCATGATTTCTGTGTCCTGAAAAAATTTATTCCAAATGCTGGTCTTTCCAAGGCTCAAA
GGATGCTCTCCATGAGTAATATTTGATCTTGAGAGCATACCACTACTTTCACTTTTGGGG
TCATCATTGTCAGACTTTTCCAACTTCCTCGCAATTTCTGAAGGATTCATCAAAAGCTCC
TCCTTGAAGTGCTTGTACTGACACCTCTTCTTAGCTAATTCAGATGACCAAAGCCCACGA
TCT
>tig_1072_consensus
ATCAGAATTAGCATTGCACAGCATATCCGCAGCAGAGTCTCCTGTAGTATGTATCTATCA

ATGTCACAACTCTCTCTCTTTTTCTTAACCTCATAATTAACAAACCATAAACACACACAT
ATATGTATGTTTCTTTATATACGGTAAAATTATCCCGAGAATCAGGATCTTTGTGACAAT
ATAAAATGAAAAATCTTAACACAAGAACAGAAAATATATGAATTTTAAACCTACCAGTGG
ACCTTCAGGGTCACTCAGAAGCGTGTCCCAAATGTGAAGGCTGTCTGCGAAATTGAACTC
CTGAGTCAAGAGGAGAGTAATCCACCTAAATGCATAAAATTGGGGGTTCACTTTGGTTGT
GACCTCTAGGTGACGCCAA
>tig_1073_consensus
TCTCCACCACACAATAACCAAATCATTTAAAATCGATAATCTCATACAGAAATAAGACAA
TTCAAGTGCCATTAACTCTTGAGAAAAACTCATAGCATCCAAAATTAAATATTTAATAAC
AAGGTTGCAAAACAAGTTTAATAACTCATGTCATCTTAAGTTCAAAGTTTGATCTAAGAA
GGGGAAACACAAGGGTAAAACTAGAAAAATTGATCTGCAAGATGTGGAGCTCGGCAAAAA
ATTTTTTAAGCAGCACCAGCTCCCTTGCCCTTCTTCCTCTGCAATTTCTTCATGTAGAAC
TCCAGCTCTTTGCCTTCCAAGATGTATCCATCAGCACGACCACACTGGCCAGGTCGTGAT
GAGATGCAAGCCAACAAACGACCACCACCAAATTGCTCTTCAATATGTGAGTCAAGCTTG
CGTTCCTTTTGGCGCTTCTCCAGCTTTCGTACAACATGGTTGCTCTTCTTTGTGTCTTCA
GTAGCAGCTGCAGCCTCACCTTCCTCTGTTTCTTTCTTGGCTGAAGCTGCAGCCTTCTTC
TTGCGTCCAATCTCAACACCATAATGCTGGAGATACCACTGCTTGAAGGGAGCAGCATCA
ACCTGAACAATGGCACTCTTCACCAGAGTTTGAGTACGGACCAACTCATTGTTAGAGGCA
TTGTACACCACATCCAAAATTCTAGTTTTACGAGTCACTGCCTCGCTTCCCCATGAGTAG
TTTCCTGTGTCAAGCCTCAAGGCACGCCACTTGACATTTCCACCTCTAACCCTAACCCTC
CTAACGGTCTTGTTGCTTGACAGCTTGGTGTTAGCAGGTTGCCTTCCAAGCTCATACTTT
CTCTTCTTCCTCCAAGCCTTCTTCTTGCCTCCAGTGGCACG
>tig_1074_consensus
AGGTATCACTAACTCTAGGAAAAACTGGAAATATCCAAAATTAACAACATTAAACAATAA
CAAGGTTTTAAAAATAGGTTTAATAACTCATCATCTCAAGTTCAAGGTTGATCTAAAAAG
AGAAAACACAAAGGTGGAACTAGAAGAATTGATCTGGAAGATGTGGAGTTCAGCAATCAA
AATTTTAAGCAGCACCAGCTCCCTTGCCCTTCTTCCTCTGTAATTTCTTCATGTAGAACT
CCAGCTCTTTGCCTTCCAAGATGTATCCATCAGCACGACCACACTGACCAGGCCGTGATG
AGATGCATGCTAACAACCGACCACCACCGAATTGCTCTTCAATATGTGGGTCAAGCTTGC
GTTCCTTTTGACGCTTCTCCAGCTTTCTTACAACATGGTTACTCTTCTTTGCCTCTTCAG
TTGTAACCGGAGCCTCACCTTCCTCTGATTCTTTCTTAGCAGAAGCTGCAGCCTTCTTCT
TGCGACCGATGTCAACACCATAATGCTGGAGATACCACTGCTTGAATGGAGCAGCATCAA
CCTGAACAATGGCACTCTTCACCAGAGTTTGAGTACGGACCAACTCATTGTTAGAGGCAT
TATACACCACATCTAGAATTCTAGTTTTACGGGTCACCGCCTCACTTCCCCATGAGTAGT
TTCCTGTGTCAAGTCTCAAGGCTCGCCACTTGACGTTGCCACCTCTAACCCTAACCCTCC
TAACTGTCTTGTTGCTTGACAGCTTGGTGTTAGCAGGTTGCCTCCCGAGCTCATACTTTC
TCTTCTTCCTCCAAGNNNNNNNNNNNNNNNNNNCTCTTAGAGAGATTTCAAGCTTCTTCT
CGGACTCAGTAAAGCTGTGAAGCTTAGGGTTTGCCACAGAGAGAAACAGAAGGCTGGTGG
TGGCTGAATTTATACACACTCTATATAGCTAAACCCTAGTTTCTATTGGATACACGACGT
CGTCTTACCAGAATCAG
>tig_1075_consensus
GGACGCATCCCTTATATAAAGCTGCATTACAGGTCTTTGGTCAATCTTCTTGGGCAATAA
TTCAAGCTTTTTGTTATCCAACAAAAGTATAGCAGCTTGCGGAAAGTAACGACATATATG
GTCACCAATGTTCTTAGCCACACCGTTAAGATCTGAGTCATCATACCTCTCATTAGCATG
AAAATATCCCACAATGCCAAGGCCTTGAGCACTATAATACTCCTCTACCAAGATAAGGGA
GATCTCGAGATTGGGAAGGAGCCCAAGGTGGGAGTGAAAGAGAGGGACGGAATCAGTGAT
TTCCACAACGTCATCGTTTCCAGGCGACACTCGTCCCAGTAAGACACCGTTGACCGCCGC
TGTCTTGTGCTTCTGAGCGTGTAGCACGAGCTTAATGTACGCGTCTTGAGAAATTTCATA
TCTCAATTCCCCCATGTTTCTCACTCACAGAATGAGTCAATGATGGGCGTAAATCGCAAC
TTCAACTTGAA
>tig_1076_consensus
TTTTTTTTTTTTTTTTTTTTCAAGCTTTGTTATCCATAAACAAGCATGGGAGCTGCTGAC
GCGTAATTATTCGGAGTCTGTTAGCCAGCCATTACTACAAAACATTTGTAAAGAGTTTAG
AGAAGGAAAAAATGTGAATGAATACCTCCTTTCTGTTAAATTCTTCTCTTATGTTGTTCC
TCTCTCTACTATATACCTTTTCTATCTAATTTGTTTTCCTCATAATCTCTCTCTCACAAG
TCCAGAGCGCACCAGATTAGTCAGCATTGACGATTTCAGATGCTCTATCATCGTTTGCTG
TTCTTCCACTTCTTGGTGATTCTTTTACTTGATCATCGTTGGCTGTTCTTGGGCTGCCTG
GTAATCCCATTCCTGAACCTCGGTGATCTTCTCTTTTCTCATCAATTCTGGTACTTCTTT

GGCCAGGATGTGTTGATGAGATGTTTCCTTCTGAACCTGGAATGAAGGGAACTGTCTTGG
TCAGTGAATTATGTCTAACTGCCTGACAGAGACTCGTGTATTTTGATACTACATCCTTGT
ACACTCTCAGCAATCTCTCCACATCCCCAACAGTTAATTCATCAGCTTCAGCTTCCATAA
AAGGATAATTATATCCACCACTTGAATGTATTCCATCATCTTGAATTATTCCTGACAGAC
CAGGACCTCTCAGATCTCCCAAGGCTTTAAAGTCGTTTAAAGAGGATGAGACTCCTACAT
GTCCGACTTTGTTGGCTAATTTAGCAGCCTGCATACTTTCCTCGAATTCAATTTCCTCAA
TAGAAAGAGATTTGGCATTTAAGTCGACAACAAAGGTCTTGGCTGACATAAGATTCGTGA
AATAATAAGCTGCTTCTGAAACAAGCTTTGTCTGCCTCCGGTGCAACTGGATGAACTTAA
GATTAGAATGCAACTGAGGAGGATTGGCC
>tig_1077_consensus
AATTAAATGATAAAACAAGAGTTACATCACTATAGTTATACCTACCTCAATCCTTTTCTC
CTCTAATAATAACTGATGAAAGGAAGATTGCATAGTCTCAACAAAAGCTCATTTAATTTC
AAAGCTCAGAGACCTATCTACACAATCCACTAGTATGGGCAAACTGGACTTCTAGGTGAC
CTCCTGTGACCATGCCTAACAGGGGATCTTGATCGGCTACAATCACGATCCCTCCTTTCA
TCTCTTTCTTTCTTGTGTCTCCTATTCATCCCCCTCACTTCTACCATTCAACTCCCGAGA
CCTTTCATGATCCCGATCACGTTCTCTATATCGCTCTCTCTCCATGTTACGGTCTCGATC
ACGATACCTGTCCCTATCACCTGAATCACTTCT
>tig_1078_consensus
ATTGCATAGTCTCAACAAAAGCTGTGCGATGGCAAAAAAAGTTTCATCCATGATGATTAA
TGACAATGAAATGCAAGCCTCTAAAAGCACACATTTATAAAACTCTGAGATATTATAAAA
AAAACTACCTCATTTAATTTCAAAGCTCAGAGACCTATCTACACAATCCACTAGTATGGG
CAAACTGGACTTCTAGGTGACCTCCTGTGACCATGCCTAACAGGGGATCTTGATCGGCTA
CGATCACGATCCCTGCTTTCGTCTCTTTCTTTCTTGTGTCTCCTACTCATCCCCCTCCTT
CCATCCCGACCGCTTCTACCATTCAACTCCCGAGACCTTTCATGATCCCGATCACGTTCT
CTATATCGCTCTTGCTCCATGTTACGGTCTCTATCACGATACCTTTCCCTATCACCTGAA
TCACTTCTTCTCTTTCTACTCTCGTATTCTTTCTCCCTCTGCTTCTGCCTTTCTTCTGCT
TCTTTTTCTCTTGCTAATCTTTCCTCTTCCCTTGCCTTCTCCTTTGCAGTCTTGTATTCA
GTAATGAAATCACGAACCATGCCATAACCAATATGCTGCTTTCCAGTAATATGAGACTGA
GTCCTCTCAGCCGCATCATTTGCAACCAGAAATGAACCACAGATTTCACAAAGAGCCATC
TTCTTCTCCTGAGCCATCATCAACAACTTCTCATTCTGGGGTTGTAGGGTCAAGGCTGTC
TTCTCAATATTAAGTAGCTCCACCTTTTTCATAAGCGCTTCTGCTTCATCCACCTTACCA
GCTTCACCAAGGGCCTCTACTTGTTCCAGAAGATTCTTTATCTTCTCCTCCAACACAGAG
AGTTGTTCAGATTTTTCCTCCGGTACTGGTGCAGGTGGTACAGGTTCCACCTCTTGAGCA
AGACGTTCTCGCCCACGTCGAACTCTTCTATCCAGGTCCATGACCAATTTCTCACAAAAC
TGAGCAAGTTCAGCTTCAAATTTGGGCACATAAGCATCATGTCTTGTGGACTTCTCAAAA
CTTTCCTTCAACTTTGGATCATGAATTCTAGAGCATGGTCCAAGATCGCTTCGAGTATTG
ACAAAGAGGTCATGAGGACAAAATCGAACCATATAAAATGCGCAAACCTCC
>tig_1079_consensus
GGCCTTCAACTCCTATGCATAAATTTGCATCAGATGTACAGAGAGCTCAGCAATCCCAGA
TTACTGTTACAATACCAACTAGTATTATGTTACAAATGGCATAAAAAACTGTGTTACAAA
GGCATCCAATGCCTCGATGTTAAACATCAGAGGTGATTATTACATCAAAGCCAATTACTT
CGGGGCAGAAAATGGGACAATAATTGATATGAAAAAGATTAATTCTGGTCTCGCATTCAA
ATTATAAGCAGTTGTGAAGCCCATCTTTGGTGAAAGTTCATTATAAAGTCTCTGGCTTTG
GCATTCTTCAAACTTGTAGTTTCAGCCCTGTGCCACCTCCAATATCATCTTTACACTTCA
AGTCCCTGATGGGTTATTCTTCCACATCCACTCCTGAGAAGCCTGAAGCCCCTGCTGTTG
GAAGCGTGTTGGATCATTGACTGAACCAGCTGCAGCAGGTAATTGAGGATATCGATGTTC
CTGAGTACTATGTTGACCTCGAATTCCAGAAGCTGGATACGATGAAGGAAATGGCTGTTG
CAACTGCTGCTGTTGTTGACGAAGCTGTGGTTGAAGTTGCATTAGTTGAGCTTGTGGAAC
TTGCTGTGCAGGCAAAACTTGGGGGTTTGACTGGGTTTGAGGCTGTTGAAGTGGCAGTTT
TTGTTGTTGCTGCTGCTCAAATAATATTAACTCTTCAGGTTTCTCCCACTTGCTTTCACC
AGTTATGCCATTATAATAGTACTTGTATCCATCCGGAGAGGTATGTTCTGTCCAATTACA
TTTTACAGAAGGTCCAGTCTGGGTTGTTACAGATAAAGCAGGGGCAGCTGACGTAGATGG
AGGTGCATCTGTGGCTGGTATGGAGCTAGAGGCGGTGGCAACAGTCTGTGGTGCAATCCC
AGCCCACTGAGACTGTTGTTTTGTAGACTGCGAACCCTGCTGCGATGATAGATTTTGATT
AGATGGCTGCATCAACTGCAATTGCTGCTGCAGTTGAGAGAATGCTTGCTGGGATGATTG
AAAGGTCGCTTGCAGAGTTTGAGTCTGCTGTGACAGCATCTGAGCTAACTGAGAAGGGGA
TTGCTGAAAAGGTTGAAGCAATTGCTGCTGAACAGGAGCAGTCAACTGCTGTTGATTTGT
AGCATTGGACAGTGAATTGGATTGAACATTCAGATTTTGAGGAGTTTGTAGTGGAGCAGA

TGAAGCTATCTGCTGAACCTGAGGTTGTGAACCAGTCAACTGTCCACTTAAGCCATGTAA
ATGCTGTGATGGCATTGCTTGGCTGAAAGGTGTCTGACTCGGAACCTGCAGCTGACCAAC
ATTTGAGGTCTGAGTCTGTGGGTAAGAAGATTGAGCCGGAGGATGTAGTTGAAGTGAAGG
AGGCATATGCTGAGGGGATTGAAGAGGCTTCTGTAATGGTGATATTTGCTGACCAAGTGA
TGGTACTCGTGATAAAGGTTGGTTAAAACTCTGTTGAGAAGCAGATGAAGTTGATGGAAC
AGGAAGTCCTGGAAGAGGACCATCAGTAAGGCCACCCGTATTTAAAGGCATTCCTAGATC
ACCAGACCTAGGAAGCAACTGATTCCCAAAACCTCGGATGCTAGGATTTGGAGATGGTCC
CATGTTTTGGGGACTCATTGGACGCCAAGCATTCTGAGGAGCTCGATCAGCCATAGGGTC
GCCAAAATTGGGTGCTGGTCTAGGCCCTGTTGGTGGTAACCGAGGACCGAAACCGGGACC
TCCAAATGCTGGAGAATTCCTGAAGTCTCCAGGCCTTGGCCTCTTAGGATCAGCAAACCG
GACTGTTAATGGTTGATCACAACCTCTCATGGTATAAATTCCATTCAGAGCATTAATGGC
GGCCATAGCCATCTCTCTGTTAGAGTATTTAACAAATCCACATCCACGACTCTGCTTAAA
TTCATCACGCATGAGGTAAACATCTTCAACTCTACCATATGGTGAAAAAATTTCTTCAAC
TTCTTTTTCGATAGCTTGTTTGTTCAGTGAGCCCACAAACAATTTATATTCAACTGCACC
AAGACGTTCTCGCTCCCCATCTGCGTATCGAACTTGGATAGGACCCGTTCCCCCAGACAA
AGTATGTTGATTATGCAAGGCTCTAATGGCCCTATCAGCTTCTTCTGAAGTTGCATATTT
TATAAAACAACAACCTTGTTGTTGTCCAGTCCTCTTGTCTTTGATCAAAGCAACCTCTAT
CACGCGTCCATGTTCTTCAAACAAAGGGCGAATATCCTCTTCAGTGGCTGTTTTTGGAAC
TGAGCCAACAAAAAGTTTGGCAAAACTGCCACCATCAAATCGATCGGGAGAAGACCCTCG
GCCAGAGAAACCTCTTTTCTGTCCCAAAACTTGCGGCAAAGGTGGTGGCGCCACAGGATA
GTTCGAGCCAAACCCTCCGCCCGTTGGCCGGAAACCTCCTCCTGCCCCGCTGCCACCACC
ACCCATGGGCCTGAATCCTCCTCCACTTCCACCAACCGACGGATAACGCGGAGGGCTTTC
GAAAGCACGGTGACCCCCTCCTCCTCCTCCTCCTCCTACTCCTCCTCCACCACTACCGCC
ACTTCCTCGAAAATCGTTAGGGCTACGGCGGCGGCTGCTATATTGATAAGATTCTTCATT
ATTAATGCTATTATTATCGTTGTAGCGACTCATAGGACCGTCGGAAAAGCGAGAGGGACG
ACTGTGACGATTGTAGTGAGAGTCCTGATAATCATTATTATTATAGTTGTCGTAATTGTA
GCTGCTACCGCTGTTTCCATTGTTGTTGTTGCCGCCATAATAACGATCTCCTCTGTGTCT
ATCCAGCTTTCCTGACTGATTGATGTTAAACCCTAACCCTAGATTTTTGAAAGAGAAATA
AGGAAATTATGCAGTGCAGTGTTGCAAGGGATGCGAACCGGAGAG
>tig_1080_consensus
TAATTAATTATTTATAAACGACAAGTTTATTCTTACGAGGAACGACATGTTTATTAGGAC
ACATACATAGCATACAAAATTATTCCAAATCCATATTCGAGACCCGAAATCCTAGAGCTT
GCTGCTTATGTTGATGTTTCTAACTTTATTGGCCTTTGGGCTTGCATCTTTTGGGACAAC
AACAGTGAGAACGCCATTCTCTACATGGGCCTTGATCTGTTCCAACTTCACATTTTCCGG
CAGTTCTATTTCCCGAGAAAAATCTCGGTTTCCAATCCCTCTTTCAGCTACGTGCCAGAC
CGTATCTTTCCCATTTTGCTCCTCTTTGCTTCCTTCTCCCGTTATCCGCAGTAAGTTCCC
GTCTTCTATTTGCACTTTTATGTTCTCTTTGCTCAATCCTGGGACGTTGATTTTGAAGAT
GTGGGCGTTGGGAGATTCAAGCCAATCCATGAGGGCGGGTGATCCGGACCGTTCACGGAA
GACGGGAGTGCTCAAAAAGAAACGATGAAAAGGGTGTCCCAACAAGTCGGCCATTGGAAC
GATCAATAGAGAAGACGAGGTGTTGATTGAGAATCTGAAGAGGAATGGGTTTTAGTTTAA
TTTTATTTGTTGGAAGACTCATCCAGAATCTTCGATGCTTCTTATCCTTTATTTTCCAGG
AAATTTGAGAAGATG
>tig_1081_consensus
GCTTAACAACAACAGCCATATGACTATGACCTTTCTGGAACTCATTCAATATATCATATA
ATGGCAAAGTTTCTTGAACCCTTGGAATCCTGCGTATAGTGACACTCTTAACAGGAACTT
CATCCTCTGGATGAACTGTCAGCAAATTCTTGACCAAAATGAGCCCAATTATGTTTGTGG
GTTGCTCATAATAAACCGGCACCCTGCTATGTCCTTTCTCCAGTATTAGACNNNNNNNNN
NNNNNNNNNNNNNNNAGAGAGCTCCAGTGCCCCAGCAATAATTGTTGTCTCATCATGTGTC
AGTTCTCCACCTTTTCCAGCCTCATTACCATGCAAACCTACAAGTGTTTTCAGCTCAGCT
CTGCGAAAGAGTGCTACATGTCCATGGCCCAACAAATAGTCCAATAGCTTGCTTATTGGA
AAAGCAACAGGATAGCAGACCCCAACAAGAATACGGACAAATGGAGCCACAGTCGCACCT
ATTGCCAAACCATGTCGAGAACAAACAGACTGTGGAATTATCTCACCAAACAGAAGAATC
AGAGTAACTGAAATCAGAATAGCACCCCAAGCTGTTACCAGACCATCAAGAAAAATTGGA
AGCGCCTCCATGGCTGCCGCATTGCATATAAGTAGAGTGCANNTGGCGAGAACTTCAAGA
TCAACAAGACTCATAGACATAAGTCCCAAAGTAAGGCCAGACATCAACCCAGCAAACATC
ACAAGCAACACTATTATCACAATATGCATAAAGAAATTAATCCCACAACAACTATACTCC
ACCGCCATTCTTTCTTCAAATCAAATGAACACTCAACCTTCACCCTAACCGAAACTCTTT
TATCTCCCAACAACTAACTAAAAACGACTGCGTTTTCAGCAACACTAAGAAAAAGTGTTT

ACACAGAATGTTAAAGAAATTTATTATTTTCCTTTTCTGAGTCGGTTTATTATTTTCCCA
GCCGTGGCAATGAAACGAGAGAAATCCACGTGTATGTACAACAAAAGTGCCGAAAGATAT
GGAGAGAGAAAAAGCGTAGGCGTGTGTGCGGCGGACTGTAAGGTGTTAGCCGACAAGCCC
GCGGAGAGAAAAAGCTTCAGAGAGCTTGCCGGTGGTGTTGTCTGAAAGAGGCGTTGCGTG
CAATGGTTCTCCTGCGTGCGGTCAATACTTACAGCGATGTCGTTTTAATGACCTTTTTCT
TTTATAAGAAAATAATTAAATAAAAGTGATTGAGAGGGTGGCAAGTCGTTGGCATGTGGA
TTTGGAT
>tig_1082_consensus
CTGAAAAAGAACAAACGACAAAACGAAAAGTCAAATCAACCCAATCCAACTTGTGAACCG
CTTACCATGGTTACTGCTTTGATACTATCCCTTGTTAGCTGAGATTGGCGGCTTGATTGA
CTTTGATGATCCCTCTCAATTTTCAACAGCACATCAATAATGTCCTCGTGCTCATGTTTT
CTCCCTCCACCCTCAGGTTTAAGATGATCGTTGATGATTTGCTCGTAGAAATTGTCCAAC
GCGTGGCAAACTTTCTCAAGCTTTGAATAATAACCAGTTACTCTATCAATCAACCAACCC
ACATAAGGGAAGCATTCTTCTGCAGTAAAGCCTCCCGCCACAGCCATAGCTGCATGAATG
AACTCTTGAAATCTATAGTCATTATCATCGAAATTACTCCCCCGGAATCTCTTTCCAAAG
GCCATCCTAAATATTATACTTCCAGTTAGAGCATACATCTTCTTAGAGAGCTCCACAGGA
GAGGCAGTAGATGCCGACTGAGATATGGAATCCATCAGCGATGCCACTTCTTCTTCCCTA
ATAAACCG
>tig_1083_consensus
CATGGTTTAAACTAAATTCATGTTCAAGTCAAACTCATTAAAGGACAAAAATGGCAACTG
AGCAACTTAAATAAAGTACAAAATAGCTACTCAACAAACTCTAAATACTTGGAGTCCACA
TAGATTGAACGTGGGAGTGGAAAGAGACCATCACCAACAGCACCAGCACCAGCACCATCA
AGCTCCAAGGCAAGTTACTAAATTTGCTTCCAGCATCAGCAGGGCGCCGGTATGTTCTTG
GCCTAATAAACACGGTTAATTGCACCGCGAAAATCAGCAACACAGGCAAAAACAAGAGGA
ACACCTTTAAGTTGGTCATAGTGTGCTGCATCTGTAACTTGTAGTTGACGTAAGATGAAA
GCCAAAAAAGAAGAAAAATGATTCCGAAAATGGCAACTAAAGGTAGAGGAGGTGCAGAAA
TGGAGGATAGATTTCGCTCTGCCCACCCTTGCTTCCATGCAGGCCCTCTTCTTTCTGTGA
AAAACCAACCCATGTCTCTGATTGTTGTGTAGTCGTAGATGGAGATATAGATGCTAAGAA
GCTTCTAGTATGCTTTGGCATGAATGATGGGATTTTGATGCTATCCCCAACCGACCCAAC
TTTAGGAGGAATTGATACCTCCATCGATCCCTGGTAAATTCCTACTAGAGAAACGCAGAA
ACCAGAGAC
>tig_1084_consensus
CAATGGGCCCCAAGTTGACATACCTTTCTAATACTTCCACATATGAACCTTTTGCATCAG
GTTGTAGATTTAGCTTTATAAGCTGTGAATCTCCATAGCTTGAGCCAATATACACAAATG
CATTATCCAAATATGATATAGTAGATGCAATAGATGTTTCCCCCAACAGCTCAATTTTTA
GTCCAGTGACCTTCTCTTTCTCATGGGTTATAACCAGCAGGTGAAGGAGGCCAGCATGGT
CACCAAGTAGATACTGGTCTTATTGGAATTGCTTTAAAAGTGTTAGCACTACAATAGACA
ATTGTTTCTTCTCCAATTATTAGAACACCACAGAGAGGTGGAGGTACTGGTATCAATAAA
TCAGCTCCATTGTCCAAATTATTCTGCGACCAAGGGCCCTCAGCAAAATCTTTATCTTTC
AGAGCAACCTCATATGTTTTGACATGACGAGCATCTTTATTATCCTGGTAGAGGACTACA
ATCGTTGGCTTTGAACAACCATAAAGAAACTTGATATCCAAAACCTGAAGCTCCTCAAGC
CTAATGTTAAAAGCTTCCTTCAGCTGTCCTTTACTATCGAAAGGAATAACCTTAAACAAC
CCATCATACAGATGGAGTCCAATCAATCTACAATCAGGATCAATTATGCCAATCTGTCCA
TTATCTGTAGGACGGCCAATACGATCAGAGACATCTCCCATTGCTCTTGTAATAAGCTCA
GATGACTCAGCATCCCATTGGAGAACACAGAATTTGTATCTTTCAGTAGCAATAAACAGA
AAATCCTGTGCTTCACCATG
>tig_1085_consensus
CCCGTGTCCCCTGTTTTCATTCTCAATCATCAATCGAATTGTCTATCTCGGATAAATGTG
TCAAGAGATTTCCAGAGCCTCTAAATAAGCAACGACTTCTGCTTGTCAATCCAGGACATG
ATATTTTTATAAACATTTTCTGGATTAGGAAAGCAGCTAACATTGTCAATGCTTAAACGA
AGAGTTGAACTAAAAAATCAGGACATTTAATTTTGTAGAATGCAGAAAAACCTAATTTGA
AACGACCATGTGAGCTCTGTAAGCATTGTTATCGTGCTTTCATGTATTGCATGCAATAGA
AATGCCTTGTCCAGATGACTACAAAAGGGTAGACGGGTTAACGACTATATGCTTCAGGGG
GTGTGCAACATCGCCACCACCAGCGTGCTTCACGAATTCAGCCGGCGAGAGAAAGCTACC
ATGGCAAACACATATTATTCTTACCTCTTCACCCTTCCTATACCTGTAAAGAAACCCTTC
TATTCTTTTCCCATTGGGGCCATCACCTTTGGCGGACACACAAGGCATATCTTCTAGCAC
ATTCCTCACCAGTTCTTTCACTCCCTTTTCAGCAGCTGTCGTCTTATTTGATTCATCCTT
TGTTGGAACCTCTGCAATTTTACTTGATTTTTCTGCGATTGGTGCTTTAGGATTGGTCGA

TGATTTGTGCTCACTCTTTAGTATAGACTGAACACTTGAAGGACTTATTGCTTCAGTACA
TTTGTTCAATCCTGGAAATTGTTGATTCTCATATTCAGAAATCCTAGAGGAGCCACTCCC
TTGTGCTGATCCAATTGAGCCTTGTGACGCCGGCGGCGGCGGTGATGCACGTGGTGGTAA
TACTAAACTTCCGTCACCAATACCACCACCCTTGACTGCAATGTTACAATTGGCTTCTTT
GCTGCCATTAACCCAACTTTGCAGGCAATTTTCGGCCTCAAAAATCCCATCGACGCTAGC
CTCTTCTTCTTCCCCAAAAACTATACTTCTCTCCCTCGAAATCTTCCAATTCCTTTGCTT
CTCTGACCTCTTCCTTTTGGCTTCCATTCTCCTCAGAGTTTGCACCTCCTTCCTCTTCCT
CCAATCCTCCTCAGTCTCCGTAGGCAAGGAACATGTCCTTGAAATATTATCACCACAATT
TCCAACTGGGTTCAAAAAGTCCGGGATTGAAGATGATCTTTTAAGCTTATTTGCTCTTTC
TGGGTCTACCCCAAAACGTCCGTTCAACGACAGCCCCAAACTCAGCTCAATATCCTCCGT
TTCTTCCCCACCACCGCCGCCGCCCTGTTGAAAATCACTGTAATCAAACTGGTTTGTGCC
ATTCAAGAATCTATGTAACAGATCTCTAGAAAAACCACTCACTTGAGCTTCTTTCGCCAT
TAAACCCGGATCATAATAAAAACTATAAACAAAAAACAATCATTCCTTTTCTTCATCGTT
GTCAGCTTCATCTACTTTTCAATGAAAGGGAATCAAATTACTGTAAACACCGCATAAAGG
CAATGGGAAAGTGACACGTGGAGGGCATGAAGGAGGATGAACAGAAGAAAATATCACACG
TGGTGTTAAAAACCAGGATC
>tig_1086_consensus
TGCTTCAGTACATTTGTTCAATCAAACAATCACCAACTTATCATGGACTACTTCCATCCC
TTCGTTCAGGTGAAATCAAGATAAAGAAACATCTGTCGGTCTCTTTCCTTGTCTTCCCTT
TTCTGGCTAGTCATATTATAACCGCTTGAATTTGTCCGAAGGGAGTCAAAATGTAACCGG
AGTACTGGAAATTGTTGATTCTCATA
>tig_1087_consensus
CCTGAACAGAAAAAAAAATGGAATAAAAGGGTAAAAAGGTAAATTTGTATGGAATAACAAA
ATTGATATCTCATTAGCGCAAAATCCATTACTTTCTCTAGTGCGACATCTTTCAAACTCC
TTTCTCTTGTTTTTCTCTTTTTCAAACTCTCTTTCGCTCAACCGTTAACGAAAAAACAGG
ATCCACCTACCAGTTCTCTTGCCAGATTTTGGAGAGGGCTATTTCAATTAACAAAGTTAT
AATTTGGGTAGGAAAATTTTATTAATACCAACTTTTGCACCAAACCTTGGGTGGGAAAAT
GTTAATTAAAGTAATGGAATATCCCACAGATCATCTCCTTTTACAGTTGCATTGGGAACG
GAATAAAGATATTTTAAATACTTCAAAACAAAAAAAGAATATGAAGAACAATAAAATAAC
CTAAATGAAGTTGATGCACCATGGTATAACCATTATATAGAAATATCTTATTACACCTAC
ATATCATATAGAAAAGGGAGGGGGCGGCCTTTTCCTATCCAATACAGAGAGACTGCTTAT
ATAGTTAAAATCCGTGCTACATATAGAAATGCATACAAGAAAATAAACAAGTTTTCCATG
GTTTAGATGACATGATTCGTTTACCTGATTCGCTTCCTACTCCTTTCTCCTCCATGCCAA
GGATGACCGAAATTAAGAGGCTGATTTCTGGCCGTCACACTCAGGACTCTGGTCAAAATT
TTTGCTGGGAAACTTTGCGGAGGCCATTCCAACTTCAAACACCAAATTATCCCCACTGGA
TCGGATCCCCGTGATGGACCACCACCTAAAGAAAGCCCTGACCCGAATACCATCCAGCTC
CGCGATCCGACCTTGCTCGATCTTTCCCTTGATGTTACTAGAGTATGTAGCCAAATAGTT
GTCCGGAGGGAGCGTTAGTTTACATGCACCACCGAGCTGCACGGAGAACTCGCCAGAGGT
CTGGTTCACAGAGTAGGTTTGAACCGATGAGGGAAGCAGCCCGATCG
>tig_1088_consensus
ACCAGCTATCTTGCATCTTGCAGGAGTTTTGCAATAACGTAATAAACTAATTCATAGACA
TATGATTCATTCATAAACTAAGAGGAACTCATGGTCTACAATGTTTTAAAAGCATATGAT
CTATAATCAGAACTAGCAAGATTACAACTCTGCAAACAAGGATGAGAATATGCATGCAAA
TTGGTAAAGAGTCAAATATAAGAAAAGAAAATTGAACAGATAAATGCAACGTAGAGTGAC
AGAGAGAGAGTCGGAGCTCACTGGTGGGCAAGCCATGTAGTAACACGAGTACCACCTCAC
AATGCGATGTCCTTCAAAGCATATGTGAGCTCTTGCTTTGGTAAAACAATCCTCACCTCC
CAAACAATTGAAAGGAGATCCATTTGGCATGAAATAAATTGTTGATAGCTTGTGTCTTAC
AGCTGCTTAAATTCTTCCCCAAAGATCACCACTTGAATATTCTCAGGTTCATCAGCATGA
GCCTTGTAAACTTCAGTTCATTCTTAAACACTTGTCGATGCTCAGAAACGATAATTCAGT
TTAGAATGTAACCAGTCAATGAATTCTATTGACATCACAACAAATAATCCAGAACTCAAC
ACACCGTCTGTTCACCAGCCTGCAATCTCCCACTTCTGTCAGTTTCTTCACTATCTTCTA
AAATTTCTACTTCCAAGTAATCTTTGGAATTAGCATTCTCTACTTCCCAAGTTTTTTCCA
AATCCTTTACTGTCTCTTCTTTTGTAGCTTTGTCAGCACAATCCACAGCATATACTTCCT
TAACTTTTTCCTCATTCTCCAAAACCTCTTCTTCCTTAACATCGATTATCACCTGAAGAA
GGTCTACTGGTGTAGCTGCTGGATCCAACTCCCTGCAACCCTTCGGAGCATTTGCAGCCT
CCTGACCCGTAAGCAAGTATGAGGGGACATGATGAGAGAACTGAAACATCTCTTCTTTTG
GAATCCTCCTAACTTCTCTAGGGTCCAGATGCTGATGAAACACTGTCTTAAAACCAGCAA
CTTTTAGGAGTGGAGTAACAGTCACGCCAAGCTCTTCATCATAGTCTTCAAGTACTTCCA

CCATGTCATACTTGTGTATAACTTCGTCAGCTGTCAGCTCATTCCAGTCCGGGGACCAAT
TCCTGTAGAGAGCCCAGACATCCCCCTTCCTAGGAAATATACGAATGGCCCCACGTGGAC
CTTTTGACCACTTGACCTTGTGAGAAAAGGAATTGAGTGAGTTACTGATCTCATATTTGC
CTACCCTGAAATCTCCACATGTTTTGGAGAAACCAGAAGCCACCCAGTTTAGTGGACCCA
GCTCACTGTTAGTTCTTGAATTAAGCCAACTGATCCGCATCCTAAACGGATTCAAAGAAA
TCACATTATGAATCATAGCATAGTACCTCGGCATCCCATCATCACCATCATATGCAGCCC
ATACCTGGTTATCTCCAAATGACCTTTCTGTTCTGTCCTTGTCAAAATCATGGAAATCAG
GATCTGGGACATTTATGGACAATGTTTCCACGGTCTCTGCATTGGCATTGTCATAAGATC
TTTTAGGGATTCCATTTTTTTCATCAACTGATTCATGACTCTTGTTTCGTTCATTTGCTC
CATTGTTTACCAAAGGTTTCTCTACTTTCTCAGTTCCATTGACCTCATCTTTCACTGCAA
TTTTAGCAGTTGTACCAGACTTCCATTCATTTAGCTTCTTGCAAATTTCTGTCCTAGCTT
TCTCCATCAGCAGATTTTGAATATCAAGTTGGAATACTTCCTTTATGTTGTTGGGCCTAG
TGATTCCATTAACCCTAATTGTTTGAAAATTACCTTGTTTTGTTCCAGATAAATTAGCTG
CACCAGCTCCTCCATTTCCTATTCCCATTTGATTAGCAATATTAGTCCCATTGTTGCTAG
CACCAAGATCCTCCATGCCTCTTTTTCTCTTTGCAGAAGTGGATAGACCATGATGAGCAC
TGCTTGTCCTTTTAGAAGCATTTTGCTTTCTTTGCAGGAACTCCTCCTTTTTTGTGGATG
CCTGTGCTTCTCCACGTTCTCTCCTCACTTTTTCATAAGCTTGTTGAACCACAGTAGCAG
CCTGAGCAGCAGTTGAAGCACCACCGGTCCCAGAGAATGGACCCCACTGAAAGTTCGTGT
GATTATGGGAATCGGGACTATTAAATCCCCCTACTCCCACATTTGGGGATGTAGCCTTAG
TCTTTGCTGTATTAACAGTATTTTTCCCACCTGCCTGATGATTTGTGCTCTGCCGCTGCT
GTGTAAAGTTCCATGGGGTTTAGAACTGTTTGAAGAAGGTGGAGCTGTTTCTACTGCCA
AAAATGGCTCATGGCAATTAGGGCATAAGAGATTATGATTCAGATAAACTCTGAGATACT
CGTACTGCATTTTGCATCGATGGCACACAGTCCAGAAGGTATTAGGTTTGGACTTATAAG
ATGGAGCAGGAGTTGAAGAATGACCTGACCGAGGGGTACTCTTTTGAGCCTTTGTACTAG
GTTTTGTAAAATTGTAAAAGCCATTTGGCCTGGGTGGTGTTGGTGTACCCCCACTAGTAG
TTGAAACCTTTTGAAACACCTTTCCATTCCTCTTACTATCATATGCTACTCTCTTAGCCT
TATCAGACAACAAGCTCCA
>tig_1089_consensus
CACATATTATTGAAAATCCACAAAAGTTCATACGTAATATTTTAAATTACAAAAGCTGCC
TAGTAAAATTGCCTCAAAATTTTCCAACAATTGAACTTGTAAATCAATATAGAAAGAAAA
ACATCAATTAATCACTTTCTCATTGCTAAATATTTCAACTGATGAACTTGAAAGGTGACA
TGCATGTTTAGAGCATGCTTCGTCCCTAAATCTGAATTACTTCCTTCCCCTGCTAAGCCT
CTGCAGCTGATAATTAATACTTCCCCCAGGCTTTCTCTTTTTACTAGTCTGTGCATCCAT
ACAGGCACTCAAAAGTTCCTTCTGCCGAACACACTTCTCATCATTCATCTGGTTAAGAGA
TAGGCAGTTAAGAAATGATATCATTTCCTTCATGCAAGGTCTGGTGACTGCCCCAAATTT
CTTTGGGTTTATATACAGCGAACCAGCTTTTCGGCCCATTACTGTATTGGTTCAAGCTTG
ATTCGGCGGTTATTGGAGCAGCTGGATGGAATAAAAGGCCGTAAAAGTTCCTGGCGTCGT
GTCTGTGGTTCGAAATCTACCGGCGTGCTTGGTCCACCTCGGCCCGGTGGTGAGCGTCGG
AGAAGAGCGGCTTTGTGTTATATTCTGTTGATATAGAGTTTATCAGTTAATTCTACAGTA
CTCG
>tig_1090_consensus
ACATGATATGAGAACAAATAAGAAGAAACAATAGTGATTTCTAGTAAATTTTTTATTCAC
AGAAGAAAGAACCACCCTGGCTAATATTACCTCCTTGATGCGTTATTCTTATAAACAATG
TTATATACACTTAAAATGAGTATCCAATGAGTGTTAATAAAGATGTATCCCTGTATGATT
GAGTGATTTTGAATTAAAGATAAAGTAAATACTCTATCACATAGTAACACATTATCATTA
GTATTTAATTTAGCTTCTTATTTACATTACCTTATAATTTTTCCCTTGCAGTTACCAAAA
CAGTAGTAGTTTTATCCCCCAAAGATAGAGTATTGGGCCACAAGGTCTGCACAACAAAGC
AAACAAACACACGCACTGGAAGCATATAACCTGGCAAAGTGACTCTCCAACAGTCCGTTT
AGGCAATAGCAAAATCCCCATAGGATCATGAATAATTCCTCAGCCCTTGAAACCTAAGAA
ACCGCGCTTAGTTTGTCTTCGAGACGAACTTCAGACCCAAGGCTTGTGTTATCCTTAGAA
CCATGATCA
>tig_1091_consensus
ATTTAGCTTCTTATTTACATTACCTTATAATTTTTCCCTTTCAGTTACCAAAACAGTAGT
ATTTTTATCCCCCAAAGATAGAGTATTGGGCCACAAGGTCTGTACAACAAAGCAAACAAA
CACAAGCACTGGAAGCATATAACCTGGCAAAGTGACTCTCCAACAGTCCGTTTAGGCAAT
AGCAAAACCCCCATAGGATCATGAATAATTCCTCAGCCCTTGAAACCTACGAAACCGCGC
TTAGTTTGTCTTCGAGACGAACTTCAGACCCAAGGCTTGTGTTGTTATCCTTAGAACCAT
GATCA

>tig_1092_consensus
GTTGCATTCCAATTGCTTCGGGACCCTTAGTTCGGTCCAGCTTATAAATATTGCAATTGT
AACTAAAAAAGAATACAAAATGGTCTAAATCAAAATCAGAAACAACAAAGCAGCCCATTG
CCCACCTTGTCGATCTTGCTCTGGGCCGTGGATTTCTCATAGGGCAGGAAAGCAGAAGCT
TTTTCCATCCTTGTTTCTTCTCCAATTATGCTAAAATGAAAGGGACAAGCACTATGCATT
TATTGTTTACAAGCTTATAATCAAAGAGATGAATGCCCATGGTTTGAGAAATAACCTAAT
TAAGGCAGGAAAGCCAGCAGGGGCCAATTAATCGAACTCAGGTCGTTTTCTGTTCATAAG
ATTGGCAAGAACTAGCTGACAGACACATTACCCAATATACACATAGCTTTTAGTAGGAGC
CACTTACAAATCAAATCAATTGCATGGAAAAGTAAGTTTTGTGGTTAGTTTTCATCAACT
GCCATTTACAGTAATCATAGAACTGTTTACAGCCTGTGGGTATTAGCCAAAAAAACACAT
CAATCACAAGTCACAGAGACATGACATTTTAGAGTTTCAGCTTTTATAATACAATTTCTA
GTGTAGCAGCAAGAGTGGGGAGGGGGGAAAAAAAAAAAGGCTAAAAAAGAATCAGTGGTC
TGTTCGAAGCCTACCTTTGAGCTGCAGAAGGGACAGGAAGTCCAAAAAAAATTCAGCGATC
GGCCACTAAATCTGCTGTCATCAAGGACAGTTGTTATATCAATTCACTTTCAAGAGGTTT
AAAAATTAGACTAGGTCGTAGCAATGATCAAATTTAGAAACAATATGGAGGAATTCAACG
ACCAGCACAAGCCACATGGATTTTAAATTCTGCCTTTTCTTTTTTACAAGTTGTCATACT
TTTAGGCACTTGTAAGACCAACAAAATACAGCTACAGAAGAACCTCGATAAAGAAGACAA
TAAAGTTAATACTTCTTCTAATCTAGTGGTTTTAATATCGTGGTTTGCGTCAGAAAACCC
ACCGACCATCAATCAACGCCACGATGAAACAGTGAGAAGAGGCACCTTGTTCCATGCCAG
AGATAGAAACTTGGGTTGAGACTTAATTAAAGAGTAGCAAGTAGTGCAATTATAGTAGTG
CCCCAACAGTATCTCGCCCTGACTGACTGCATAATGGCTCAATGGAACCGGCTCGAAACC
AGCACTCTCCATCAGAACTTTCCATTGTTCCTTACTCTCCAAACATTCTCTTCTACTTCC
CACACCTTCACCTCCAATCAAAGCAGCAATTTTCCGACCAAGTATTAGTCTTTCTATTTG
TAGTCTCTCCGTTGAATCACGACTCATGTTTGGCTCTAGCGACTCAAACACCGCTAAGTA
ATATTTCAAAGCATTCTTCACACGGGTTACAAACCCTACTCGGTTCAAACTCGATTCATA
CTCACTGAGAGTCACCACTTTCGGATTTAAGGATTTCGCCATTCTCAGAGCAGTCTCTAC
AGCAACTGGGTTATCATCTAATAGATTGCATAACTGAAGCATGAAGTTCACCGCCAACAC
CTCATCCGGCTCAATTTTGAAACATGAAGCATTGAGTTGCTGAATTGGAGTTAGAATGGG
CTCGAACTCAAATTTTAGTTTCAAAACTCTAGCAAACTCAAGGAGACGATTACCCGTAGC
AATGAGAGAAGCAGTAGGTGATTTACCGAGAGCCGGAGCGGGTATACCAGAGATCCGAAT
TTTGAGTAAGTTTGGTTTTGGGCGTGACGCGAGAGCTTGTAACAGTGATGCCCATTGAAC
ACCATGAACGATTCCAAAATCAACTATGTGAATCTTGTTTGCTTTAGCAGTAGCTTCAAG
TATGGCTTGATTAGCCGTTAAATGCGCAAACTTTGAATAGGGACACGCATCGTTTAAGGC
CTCGTAAGAGAGAATTAAATCCCGACAGTTTGTCCCCAAGATGGATACTGTTTCCTGTGC
TGGGGGAGACACTTTGTTGTAGAGAGCTTCAGTGAAGTAAAACGCCATTCTTTCAGTTGG
ATCTCCGCGTTCAGATACTGACTCTCTGAGCTTACTCAGCAATTTAATAGCTCGGTCCGG
CTCAGACTCTGCGAGTCGAGCGCAGTCCAATAAAGCCTTAATAATTGGTAGTGCTGTGTC
ATTTTCGGGTGAGCTAAACAAGGCTAACGCACCTTCGACTTGGTTTTTGAGTACACTTAG
CTGAAAAGGAGGACTTGGTGCCTTGGTTTCTTTGGCCGGCGACGCGGAATGCGGGGGAGA
TGAAGGTGACGTTGTCAGTGTAATTGTTGGTGTTGTTGGTGCCCATGCTGCAGGGATAAC
GTTCTTTTGAGGTTCCGTAAAGTTAACTTGGTTAAGATCAGACGGCGAAGAATTACCGCC
GCTGAGTCCACTCGGGCAGCTGACAAAGGGATCGGTGGAGTAAAGACCAAAATCAGCATT
GTTTTGCCACGCGTCACAGCCAGATGGAAGATTAGAGCTATCTGTAGAGTGGCCACCGTC
CATTAAAGTCACCATCCATTCATCGGAGTCGAACTCAGCACCTGGCCCACCGCCGAAATT
AGGAAATCTAAAGCCACTGGAGTGGTTGTCAATCTGAGGAAATTGAAACGCAGCTTCTCC
AGTATCTAACGGGTCAGAGAAGGAGGAGCCAGCAAGATTATATCCCATATTTGGAGAATT
AGACATAGGGTTGGTGGACCAAGTGTTTAAAGAGAAAGGATTGAGACCTAAAAGTTGCTG
TTGCTGCTGGCTATGTTGCTGTTGTTGCTCTTGCTCTTGCTGTTGCTTTTGCTTGATCAC
TTGTTGGGCAACCGCCATTAGATTGCCACTATCTGCACACATATATGCCATTCCCCAGCC
AAATAGAACAGAAGTAGAGGCCCAGAAATAACGGGATCACAAGTTGAACTTGGCTGAGCT
AGAGGAGAGAGAGATGCTCAGCAAGTATAGAAGACGGCTTTAAAGAACGAACGCTCAACCCC
AGGAGCTGGACACAGTGGGTCCACCTTTCTCCATTTACGTCAAA
>tig_1093_consensus
GACATTTCTCAAGAAAGTGTATTTTATTTTAAATGATTTTCATTAAGAAACTAAACAAAT
ACTATTACAAAGTCTTCTAGGAGACTAAATCATGTCCATAACCACTTTAATGCCCCATAA
TTTATTGTATAACAAGGTTCATTTATTGGAGATATGCATGAAGCATAATTTTTCTGCAAT
GTCAAACTTTCTCTTTCGTCATCATCAGAGAATCACACTCACAGCTTACTTCTTTTAACA
GGTCCCATCTCTTTCGCCTTGGCCCTTAGAATATGCTTCTGAATCTTCCCAGTAGCCGTC

TTTGGTAATGGTCCAAATATTACTGACTTTGGAACCCAGTAAGCAGGCATCTTGGATCGG
CAGAACTTCATTATATCTTCTGCCAGCCGCGCCTCATCTTTGTTGTCTGCCCCTTGCTTC
AATGTTATGAAAGCACAAGGAGACTCTCCCCAACGCTCATCTGGCCTAGCCACCACAGAC
GCCTCAAGAACTGCAGGGTGCAAGTAAAGTATATTTTCTACCTCCAAACTACTGATGTTT
TCACCTCCAGAGATGATAATGTCCTTCGATCTGTCCTTGATTTCTATGTAGTTGTCAGGA
TGCTTTACGGCGAGATCCCCGGAATGAAACCACCCATCTGCAAAAGCTTCCTCATTGGCT
TTTGGATTCTTTAAATAGCCCTTCATCACAGAATTACCACCAAACACTATCTCTCCTATG
GTTTTTCCATCAGCTGGAACAGGCTTCTTTGTTCGAGTGTCAATGACGTCTAAAAATTCT
AAACCAATGTACCGCACCCCTTGGCGGGCATTAAGGCGAGCTTGGGTTTCAGGTGGCAGT
GAGTTCCACTCAGGCTTCCATGCACAAACAGTGGAAGGACCATAGGTTTCTGAGAGACCG
TATGTATGGGTGACACGGAAGCCCTTCTGGGACATTGCAAAGAGAACAGATTGCGGTGGA
GCAGCACCTGCAGTCATCACATTTACAACATGGGGGAGGGGAAGGATAGTGTCCTCCGGT
GAGGCATTGACTATTGTGTTAAGAACCACAGGTGCAGCACAGAAGTGAGTCACACCATAG
TTGGCTATGGCAGAGTAAACTGCCTTGGCAGCCACCTGCCGAAGGCATATGCTTGTACCA
CAAAGAGCTGCAAGTGACCAAGTGTAGCACCAGCCATTGCAATGAAACATAGGTAAAGTC
CACAGGTATACAGCTCCTTCATTTATCCCCCAAATAAGACAATTACTCATAGACATTACA
TATGCCCCACGGTGACTCAAAACCACACCCTTTGGACTGGCCGTTGTACCTGAAGTATAT
CCTAAAGCAATGCTTTGCCACTCATCCTGTGGTGGCTTCCAAGCAAACTCAGGGTCACCT
GTTTCCAGGAATTTCTCATACTCAAGGGCACCTCTTCCCAAAGCATATTGCAGTAACTTA
GGATCACAGCTTTCATCACCTATGACAATCAACATTGGAGGCTTATAGTTGCTTTTGCTT
TTCTCCTCCAAGATTTTCAAAGCTTCTTCTGCCAGAGAAAAATACTCTTGGTCCACCATA
ACAACAGCAGATGATGAATGACCAAGAAGGAAGGCAATTGTTTGTGCATTTAGACGAATA
TTGACACTATTTAACACAGCTCCAGCCATTGGAACTCCAAAATGAGCTTCATAGAGAGCT
GGGACATTTGGTGCAATCACGGCTACCGTGCTACCGAGTCCAACAGACCGTTGAGAAAGA
GCAGAGGCCAATCTACAGCAACGCTGGTAAGTCTGACTCCAACTATAGCGTACGGATCCG
TGCACCAGGGATGCTCTTTCAGGGTGAACCGTAGCTGCTCTTTCTAGAAACCAGAGCGGC
GTCAACGCCGTATAATTAGCGGCGTTTTTCGGAAGATCGTCGATGTCGTTCCTCGCCGCC
ATCTCTAAGCAAATTCACCACCACTTATCACTGTGTGTGTGAGTGTTAGTTTTTTCAGCT
TGCTTGAAAGGCACCAAACAAAGACGCCTGGAAGAAGCTATTTATAGTTGGGACTCGGCC
ATGATTGAAGCATACGTGTCAAGCCTTCAGGACTACAGGATAGAGGG
>tig_1094_consensus
AGGGCTTGGAATATGCGTTGGTTAAAAGGACATGAATGATCTTGACTTGGGATTACATTT
AGGACTGAAGGCTGAAAAACAATTCAGAAAGATGACTTTGTCATCTTGTTCCCAATATGC
GTGTTCTTGGGCAATTCAATTTAACTTTTTCAACACCACCGGGTCAGTGGGTCTAGTACA
AAGCTGGACGAGAGTCAGAATATCCATTTTTCAACACAACCATTTATATAATTGCTTCCA
TCTAAACGCCATTGGACTTAGTTGCAGTCATATCATCTTTGAGCACTGGAGCTTGGAACA
TACACCAATCATATTTGTGGGATGGAGGAAAGAACATAACAGTGTGATAGTAGAATGCAG
CAACACCGTGATGTCTTTGAAGAAGAACAATATCTGGACAGTCATACCAAGAGCCATTCA
CTCTGTTCCGAATTGGTGCCGATGTATTCCATTAGAAACAGCTCAAGATCTTCTCTCCAG
CTTCCTTTCACTAGATCTTCTTTCTTCTGCCTCTCGCCGTCTTTGTTGAGCAAAAGCGGT
GTATCCAGATCCAGCATTAGCAGCAGATAAGATTGCTGCATGAGCCCCAGCTGCAGATAG
AGAATCCTGCCCCACCATAGATAGAGGATTCCTTCGATCACCATCCACCT
>tig_1095_consensus
TTTCCCTACACGACCGCTCTTCCGATCTCAGCAATTTTTGCAAGTAAATATGGAACAACA
AAGGAAGCCTCCAGAGTCCAGGCCCCTAAAATCAGACGTTGCGCAGGGCTATGGGAGTTA
CAACACGAAGTTAACAATAGCACAATGTCCGTATATTAGACCCAAATCCAATGATGTAAT
AATGCCAGATCCGGTAGATAATACAAATAGACAAGGAAAGAAAGTTCTAAACTTGGGACA
TCAACCCATAGGAAGGGTTGACCAAGTAAGCTCATTCATTGCCCATCTAAACACACAAAG
CTAGAATTTACCGCTGCATTTCTCTATATCCTACATGTAGTTTTCCCTTTTTGAAAAATA
AAAGCCTAAATTGAGGCACACAAATCCACAGCAACTTTATGTACAATTATTTTAAGTTCG
CCTATATGATAGTTCCATCACTGATATGTAGAATCGTGGAGGAAATCAGCGCAATCCACT
TTTCTCCATGCCTTCTGCCCTTCCTTTCATATCGTCCACTAGAATCTTCAGACACATCAG
AATTAGAATCGTCTGAATGTCGATGACTTCTCCTCTTTTCCCTCCTACTCTTCCTCTTAT
GGTATCGGTGCTTATCCTCTCCATCCGAGGACCAATCTCTCTTACTCCTCTTTTCCCTCC
TCCTCTTCCTTCTACTTTCATCCTCATCCTCTGAGTCACTATTTTCCCTCTTCCGACTTC
TCCTCTTCTTGGACCTCCCTCTCTTCCTCTTTCCTGAATCAGAACTGGAGTTTGTTCCAT
CTTCATATGAAAAATAGGTTTTTCTCTTCCTGGATGATCTGCCTCTGTTACTAACTTTCT
TCCCATATCTCTCACCAATAATCCTCTCAATCTCAGAATCTGCATCTGAATCCGAGCTCT

CACTCTCCTCCTCCTCTTCTTCCTCACTTTCCTCCTCATTTTCAACCTTTCCCTTCCCAT
TCAATTTCCCATCTTTTCCCTTCAATTTATCCAACTCAGCCATAACTAAAGCCTGAACTG
CTTCTGTATTTTTTTCTTATTATCATCCTTGACAAAATTTCTACACTGAAAGGTTAAGTG
CCCAACACGACCACACTTCTTGCAACCACCACGGGCCTCATCAGCATTCATATTAGTTAT
GCGGGCAAGAGCTAAAAAGCCGTGGTAGCTATTATATGGATTCTCAGCATCAGGCTCGTT
ATTTGATGACTTGGGCTATGAGTTCTCAGAGTCATCTTTGTTGGGTGCATATGGATCATA
CCCAATTGCACTCTGCCATATACCGTGAGTTTGCAGGGCTGCACTGCTATGGACCCTATT
ATTAGCTGGCATGCGAACTCGTCCAGCGGTGGCCGGCATCTT
>tig_1096_consensus
GAGACCATAGAAGACTGATGACTTGACTTTAAAGATCAGATTGCTCAAGAAAAGCTTGAA
GAGCTTAGTCTAAGCCTGCAAGCTAATCAAGTCGAGTAATAACTTATTCAAACTTAGACT
CTACAATAACCAAGCCAAGCCACAAATAGTTGACATGCGGCTCCATAAGAACACATTCAA
TTGCTAATGCTAAGAGCTGATGCAAGCAACTCTTTTAACTTAATTCAAAACTGGGTCAAG
AGCATTCTAATACATGCCACTTTGATTTGTCAGCAACCAAATTAAGTATACAAAAAATGA
AAATAGAAAACAGAACTTCTGGAAATGGATCTATGCTGTTGCCCCTGGTGTCCATCTGAA
AAAATAACACTGTCCCTCATAGGATGTGAAATGATCCAAGGCCTTCACAAGTGAAACAAC
CATCTATTTCCTTTTCCTCTTGAGTGGCACAACTAAGCCTCTCTTCTCAAGACTTTTGTA
GCGATCCTTTACGAGGGTGCAACAACCCTTTAGTTTTCTAAGGGATCCAGTGATTTCTTC
AGATAATAAGACTTGAGTAGGAGCGGGCACAAATTTGTGCTTTCCCAAACGTGGTGGACG
AGCCTTTAGTCTTTCTTGCTTAGCTACAACTCTGCGCAGATGTCTTTTCTGCTTCTCCTC
ATCCTCTTTGGCTATTTCTTGAATGATATCATCCAAGCTGTCAATTTGTTTGGAAAGTCC
TTCCACCTTTTTTGCTTCAGATTCTTTTCTCTCCAGTTCTTTACGTTTAACTCTCTTATT
CAACTCAACTTTAGTCACTGGCTTTGTTTTTGTGGAT
>tig_1097_consensus
CCCAAATCAAACCTACCCAGTTCCTCGTATTTTTCTATTTATTCATAGTCATCGTTTTCA
TGGATTCAAACTGCAATTCACCCAAAAGACAAGACTTGACATCTTAAATCATGCTGCTAT
ACATGCAAGGCATGCTCTTAGAAATAGTTTCAGAAACAATCTTTTCAAAATCTCAGACAC
AACTCCTTTCACAATTATTAAGAAAAAGATCTTGGGTCATGTAAAGACTAAGCTCCAACC
CGTTCTACACAAGAGATTTATTAGGTTAAAGCAAAACTCTCACATATCAAAATAGTATAT
CATCGATTGCTAGAACTCTAGTATGGTTCAACGGGTGTGCGGAAGTTTGCTCCAGCATAA
ACAGCTTCGGCACCAAGCTCTTCCTCAATTCTCAGAAGCTGATTGTACTTTGCAAGACGC
TCTGACCTACACGGTGCTCCAGTCTTAATTTGACCCGTGGCCAAGCCAACTGAGAGATCA
GCAATGAAAGTATCTTCAGTCTCACCACTGCGGTGGCTAGCCATCACACCCCACCCAGCT
CTCTTGGACATTTTGACAGCTTCAATGCTCTCAGTTACAGATCCAATTTGGTTGACCTTG
AGGAGAAGGGCATTGCAAGTCTTCTCTTTTATAGCCTTCTCTACCCTCTTGGGATTGGTA
ACCAATAGATCATCTCCCACAATTTGTACATTTGCTCCAATTTCATCAGTCAGCTTGGCA
TAGTGCTCCCAGTCATCCTGGTCAAATGGATCTTCAATTGAAACAATAGGATACTCAGAG
ACAAATGACTTGTAAAGATCTTTCAATGCATCCCCTGAGATCTTTTGTGAACCATCATTT
TTCTCTTCTTTGAAGTTCAGATCATAGGTCTTATCTGAACCATAGAACTCAGATGCAGCA
ACATCCATTCCAATGACAACTTTGCCAGTGTAACCAGCTTTGGCAATGGCAGTCTTGAGC
AACTCAAGTCCTTCCTTGTTTTCCTGAATGTTAGGTGCAAAACCACCTTCGTCACCAACA
TTTGTTGCATCCTGACCATACTTCTTCTTAATGACAGCCTTCAAATGGTGGTATACTTCA
ACACCCATCTTCATGGCCTCCTTGAAAGTAGAAGCTCCAACAGGAAGAATCATAAACTCC
TGCATTGCAAGTTTGTTGCCAGCATGTGATCCACCATTGATGACATTGAAAGCAGGTACC
GGCAACACCAACTTCTTGTTACCAGCAAGTTCGGCAATATGCTTGTACAGAGGAATCTTC
TTGACATGGGCACCAGCTTTGCACACTGCAAGAGACACTGCCAAGATGGCGTTTGCTCCA
AGCTTCTGTTTGCACCAACCCCACTCATTCACGGTTCCGTCAAGACGTTGAACCATGATA
TTGTCAATTTCAGTCTGCTTGGTTGGGTCCTTGCCAATGAGAGCTGGGCCAATGATTGTA
TTGACATTCTCAACGGCCTTCAATACACCTTTTCCAAGGTAGTCTGAGCCTCCATCTCTC
AATTCAAGAGCCTCATAAATTCCAGTTGAAGCACCACTAGGAACAGCAGCTCTCGATAAG
TAATCGTCTGATGTTTTCACGTCAACCTCGACGGTCGGATTGCCACGGCTGTCGAAGATC
TGCCTGGCCTTGACGGAGACAATAACGATGGTCATATCTAAACCTTACAGATGGATGTAG
AAAGAGAGAAAATTGAGACACTGAACAGAGTGGCTGCAAGAGTGAACGACAGTTTGGTGC
AGGGCAGGTATTTTATAGATGGGAGGTCAATCAGAGCCGTCTGATTTATGATTAAAATTA
GG
>tig_1098_consensus
CCCCTTGGCCTTCTCCGCTGTATAATCTCTATACTCTCCAGCCTTTCCGAAAGTCGTGTC
CTTCGCCTCCTTTGCCTTATCTGCGGTATAATCTGTAGCTTCCTTCGCCTTCTGTGCGGT

ATAATCNNNNNNCTTTTGCTTCCTTGGCTTTCAGTGCAGCATAGTCTGCAGTCTCCTTTG
CTTTCTCCCCGTACTCTCCCGCCTTTGCTTTAGTGGATTCTTTGGTTTCTTCAGCCTTCT
GCGCCGCGTTATTTTCATATTCCTCCATCTTCTGACTGGCCTTGTTGGTAGTATCCCTCG
CCTTTTCAGTCGTGTAGTCCCTATTCTCTTTCGCTTTCTCCACCGCCGTTTGATCTCCTT
CCTTTGTTGTCTCCGCATCCTCGTAGCTCTTCCCGACAACAGCGTCTTTGGCATGCCCAA
TGGTCCCCGTCACAGCCTTCAAAACCGATCCAATCACCCCTGGTCTATCCTGTTGTTGCT
GAGGCTGATCCGTGGGCGTATCAGCCATATAAACTCCCTCTTCAAACTCTCTTTCTCGGT
TCACGTCTCTCAGGTCAGCTGCAGCAATTCTCGCTGCCGCCTCGGCTCTCTCTTCCTTTT
GTTGCCTTGTCGCCATTGCTCTTCACTTGTAAATCACACCCAAAAACAATTTTTTTCCTT
AGAATTTCTTTGTTCACGTCTCTCAGGTCAGCTG
>tig_1099_consensus
CCCCTTGGCCTTCTCCGCTGTATGATCTCTATACTCTCCAGCCTTTCCCAAAGTCGTGCC
CTTCGCCTCCTTTGCCTTCTCCGCGGCATAATCTTTATACTCCCCAGCCTTCCCCAACGT
CGTGTCCTTCGCCTCCTTTGCCTTATCTGCGTTATAATCTGTAGCTTCTTTCGCCTTCTG
TGCGGTATAATCNNNNNNNNNNNNNNNNNNNNNNNNNNNNNNNNNNNNCTTTTGCTTCCTT
GGCTTTCAGTCCAGCATAGTCTGCAGTCTCCTTTGCTTTCTCCCCGTACTCTCCCGCCTT
TGCTTTAGTGGATTCTTTAGCTTCTTCAGCCTTCTGCGCCGCGTTATTTTCATATTCCTC
CATCTTCTGACTGGCCTTGTTGGTAGTATCCCTCGCCTTTTCAGTCGTGTAGTCCCTATT
CTCTTTCGCTTTCTGCACCGCCGTTTGATCTCCTTCCT
>tig_1100_consensus
GCAAAAGCAGTGTATCCAGAGCCAGCATTAGCAGAGGATAAAATTGCAGCATGAGCCCCA
ACTGCAGATAGAGAATCCTGCCCCAATGTAGATAGAGTATTCCTTCGATCACCATCCACC
TTGTCCCACTTTGATTTCTTGTTTTGTCTACCATCCCTTGCAACAGCATCATTTGCATTC
AGCACTGGATTCAATCCAGAAACTGCAGCTGGAACAGGTATATTAGGTTTTGGGGCAGCA
GAAACTATTCCAGGTTGCACTCCTCCTGCAACAAATTCAACCTTCTGATTCTTTTTACTT
TCTTGATCCTTTCTTTCCCTTTCACGCCTCATGTCAGCCTCTATTTCATCATAGTAATCG
CTTTTGTCATATCCATGTGGGTCAAATACATCTTTACTGAAGCAAGATCCTATCTGATCA
ATATCTTGATACCTCACTGCATGAAGTAAGAAGTCTGGATTTCTATATTCCTTCCTATTC
CGAACTTCTGCATTGAAGCTTTTCCCAATTCTCTTTAAACCTAGAAATTTGTCTATCTTC
CTCTGCAGCTCCTCTGAGCACTTGGCTTTCGGAGGTGGAGGGAGAAACTTATCTAACAGA
TCAACTTCTTCTTCTGTATTCTCCAGAGTGTCCTGAACTACCACAGCTTCTGATTCCGTC
GGAGAAAAACTTATTGGATCAGGTTGTGATTGATCAAAATGCTCTGTTGATTGAGGATTT
CTTTGAACACCAGGTGTCAAAACCAGAAGAGTTCCCATAGGTGTCCTTTCTTGGAAATCA
TCATTAGCATTTTGAAAGTCAATTTCCCCTTCCTCAGGTTCAGGCGACATGGCAACCTCG
TCATGCCCATAGTCTACAATAGTCAACCTCCCTTTTCCACTTCTCCTCGAAGTAGGAGAA
GCCCTGTGTTGCTTGGGTGATATCAACGAGATCTTAGGCTGTTGCGGTGTTGAAGTCTCG
TCACCAGCGACGTTATCATCACCAAGGTTCATTCTCGAATCTTCCTCTAAATTATTTGCC
TCTCGATTTTCATCGTCACGTTGTTGTTGTTGTTGTTGTTCTTCTTCTTCTTCTTCTTCT
AATTTCCCTCTTCCTGTAGTGTCTTGCAACCTTCTCTTCGAATTCTATTCACAGAGGCAG
ACAAGAACACTATGAGAGAAGGCAAAAAGAGCTCTGTTAGAGCAATCTCACAGAAGAAGA
AAGATAAGCTGAAGAAGACCAAGATGAATAAAAAAGACAAACAACACAACAAAAACACAA
TAACAAAGAAGCTACTAGGCGAGAATAAAGTTCCAAATAAAGAAGAAAAAGAACCTCAAA
AGAAGAAATAAGTACAGCAAAATTTACCTGTTGATTCCGAGTCTGAGTCCGGCT
>tig_1101_consensus
CCGGGATCTGTTATGTCTTTTAAACTCATCTCTGTATGTCATTGGTTTAAACTCTGAATC
ATTTGCAGACTCACTTTCAGACATGTTACTTGAACTCTCATCCCCTGCATATCCTTTAAA
TCCGGAAACTGAAAATTTTATGGAACCATTGCCAAAACTGTTTTTCATCTTCTTAGTGAT
TTCCTTGGCAATTTCTCTTGATTCTTTAGAATTATGTCTCGAAAATCCTACGTCATTAAT
ATTTCTCTTTGCCCATGACTCTGCCTCCTTATTATTGGTGCCTGGAAATTCTGCATGCTT
TCCACAATCTGATGGATAAGCATGCGAAGAATGAGGTGATGAAAC
>tig_1102_consensus
ACCCTCCATAACGGAATATACGCGACTCATCGGTGATTTGATGGTCTCCCAAGGAAGATT
CATAAAACTTGACAGGTGCTCAAAGAATGTTCACATGCATTTCCTCTCCAACTACTGTGA
ATATTTCGTTGACCCTCTGAGTATCTATGTTCACCTCTGCAAGCCAAGTTGTTAAAGCAA
CCTGCAATATCTCCTGGCTTGGCTGAAGGTTCAATTGTATAGCAGCCTTATCAGAGTTCC
GGTCCATCAATCTTGGGCTAACTGGCTCACAAATTTCCTTTGAATACAAATCACACAATC
TCAAATCATCAAATTCCCCTAAATAAAGCTCATTGGACCGGCAAAGCTCATTGACTCGCG
AAGTTTCTGAACCGATAGAGAGTAACTCTACCACCAGCAGTCGCTTCAAATTCAGNNNNC

ATGTCCTTGTAAGATGATAGAAGTTTGCTCCGATAGTTCTCCTGCTGCTTAAACAATTTA
CAGGAAGCCTTAATTCGTATATCTGGCATATCACTCAATGCACCTAATTCCATTGAAGAC
ATGTACCTTTGAGATAAATACGAGTTGA
>tig_1103_consensus
GAGGCAGGCAACCATGCTGCTGTGAGTTATGCATTTTCTGTCTCTTTGCTTCTTCATGTT
GGCATTGCTGCTGCCTTCTTTTCTTTGTCTGGTACTCATGCGATGATTCATATTGAGGCA
AGTTGAATAAATAAAGAAGAACAATATATCCTCCACTCTGGATTCCTCCTGGGTACCTGT
ATGGACCTAAGAGAGGCCCTCGGGCTTTCCATAGTTATGGTGACCAGGACCAGCAGGAAC
TGAAGGTGCTGCTGTGTTGTATTGGAGGCAGGCAACCACACTGCTGCGAGTAATGCGTTT
TTTGTCTCTTTGCTTCTTCATGTTGGCATTGCTGCTGCCTTCTTCGCTTTGTCTCATGCG
ATGATTCGTATTGAGGCAAGCTAAGCTCATTTTTTTCTGGCAAGATAGGCTTTCCATTCA
ATAGCTCTGCAAAGATGCAGCCAACAGACTACATGTCTACAGCTGGTCCATACTTTGTGG
CTTCAAGTAGCAACTCTGGAGGCCAATAGCACAAAGTAATAACACGATTTGTAAGAGTGT
CATTGTGGTGACAAGAAAATGGCCTTGCAAGACCAAAATCTGCTAGCTTCAAATTTTCTT
CATCGACTATCAAAAGATTTGAGC
>tig_1104_consensus
CGACTATCAAAAGATTTGAGCCTTTATCTGGGGGATTGCAAATCGAAGTCCAGGACGATC
AACAGGGCCTGTCAAGTCATGGTCCATGTACTCAAAAACCATATAGGTGCCGCCCTTATA
CTTGTTACCATCTGGCCTCCATCCCTCTCTGGACCTGGAGAAGTAACAATCTCTTTCAGC
TTAATCACATTTTCATGGTGAAGCTTCTTCAGAATCTTAATTTTACATATGGCTGTGATA
GGGAACCCTTCTTTCTCATTATCCATGCGTATCTTTTTCAAAGCAATAATCTACCCAGTC
TTAATTTGTCTGGCCATGAACACTTGCCCACATGTTCCTTCACCAATCTCTCGAAGCAGT
CAACACTACGAGAACCCCACGCCGGCGGAGGCTCCTCCACGTTGAGTTGTTCAAGTGCCG
CCACTGCCATATCTCACAACAACGGTCGCTTCCGTCGATTTCTTAGAGACGTTTGCTTTA
TTCCCTTGTGATTTTTAATGATGGTTGGTTATGTTTCCACGTCTCACAAGGAGTCGAAAG
ATTTTTACATATTCGCTTTATCCGGATACGAGTGGTT
>tig_1105_consensus
ACTCCCCTTTCCGCAGGCAAGCAATGCATGAAATAAGCTTGCGGGCCTGCTAATTTCATG
AGTGTTTCATCCACCTGCAAAATTTTGCCAGTAAAAATAAGCAGGACATAGACTTGAACC
AACAATACCTTCTGGTTAATATTCATGGCTTATTACCATAGAGCAGATGGTTAGAATTTA
TCCAATACATTAGGTATTACCAATATTCTGTAGTAATACAAATGCTTCCAAATAGCACCT
GAAATCCTTGAAACACTTGCCGGCGATATGCAGCCTCTTCCTTCTGCCCCATGCTGGCCC
ACACATCAGTGTACACAACATTGGCACCTCGAACAGCTTCTTTTGG
>tig_1106_consensus
CGAACTAAACTCTTGAATCTAAACCTAAACCCAAACTTTGCTACAAAAAATTCAACAAAT
ATATATGTCCGCATGCGCGCCAATTCATTCAGTTTAACTCAAATTCAGCCCTAATCCATA
GATAAACACCACACCTAATCATGTTTTACTACAGAAAATCAAAAATTTAATTCTGATATA
ACCCATCGACTATTATAACTCACGTCTAACATTTTTGCAGGTGTTGCCGGTGGCATAGGA
GTGGCATTGGCTTGGTCAATTTGCAAGCCAGGCTACTCCCTCCGAGCTTATTTTTTACAC
AAGCTTTGTGGAGCTCTTACAATAAACGTTTCTCCCTAAAGCATTGGCAGCAAGGACCAC
CATTCAACCAAAGTTTTCCTGCCCCATAAAGGGCCACTGTTAAGAACGGAAGTACATCCG
CTCGGCCAGCTGCTCAGCCCTTCTAATTATTCCTGATGGTGATGCCTGAATACTTGAACT
AATCCAATCTAAACCACAGCACTATAGAACTCAATGAAACATACCATAAAACAAATCCAA
CCCGTTTTTTACCCCTGTTCTACTGAAATATGAAACTGTCAATTTAAAGGGTTTGAAATG
GCTGCAACTTCAGAAGCCTTCTATAAAGCAGGCACTAATCATGAACATGAACCAGGTTGG
CATCATTACAAGCCCAGTACATGAAGCATTATAGCATTCTGTGCATGCATCCGATTCTCA
GCCTGTGGGAAGACAATGGAATTTGGTGCTTCAATAACACCATCAGTCACCTCTACTCCC
CTTTCCGCAGGCAAGCAATGCATGAAATAAGCTTGCGGGCCTGCTAATTTCATGAGTGTT
TCATCCACCTGAAATCCTTGAAACACTTGCCGGCGATATGCAGCCTCTTCCTTCTGCCCC
ATGCTGGCCCACACATCAGTGTACACAACATTGGCACCTCGAACAGCTTCTTTTGGGTCA
TTTGTAATCTCGATCTTGCTAATTCCTGCATGCTGAGCCTTCTCAACTGTTTCCCTATCT
GGTTCAAAACCTTTTGGGCAAGCACAAACAAAGTGGAAAGGAATCACTGATGCCAACAAC
AACCAAGAATGCACAATATTATTTCCATCTCCAACATAAACAACCTTGGATCCTTCCAAC
TGACCAATGTGTTCAATAATGGTAAGGGCATCAGCCATTATTTGGCAAGGATGGTTGTAG
TCTGTTAGGCCATTGACGACAGGTACAGTAGCATATTTAGCTAGATCAAGAATGTCCTGA
TGAGCAAAGACACGTGCCATAATAATGTCATTGTAGCCACTTAAAACACGAGCAACATCA
CGTGTCTCCTCTCTCTTACCCATCTGGATATCATTTGGTCCTAAATAAATAGCATGACCT
CCCAACAGGGAAAACCCAGTCTCAAATGATACCCTAGTTCTCATGGATGGCTTGGCAAAG

ATCATAGACATTGTCTTTCCTTTAAACGGTAGAAATGTCCTCTCTCCTGATTTAATCAGT
GCTTTGACCTCAGCAGCCCGGTCTAAAATCTTCAATATGGTACTTTTGTCAAAATCGCTA
ATGTGCAAAAAATCCTTCAGCTCCGCTTTTGCTTTTCCATTGACGGAAGATTGAGGTGAA
GTGGCAGAAGCGGCCTGGCACGATATAGTTCTTCTGAGAACCGGAAAAGTCGGGGGAGGA
GACACAGAGGCGGCAAAGAATCGCGAGTATTTAGAGTGAATATTTCCCGAGAAAGAGGAA
GATAAACCGGAGAGAGCCACTGTGTCCGATCTAAGTGAACAATTACCAGAAATCGCCGCC
ATCGTTGTCGGTAGGAAGAGCTTCGGGAGAGTGAGAGTATAAAATGTACATGGACCGT
>tig_1107_consensus
TGATTGTTTTGTGTCATTGACCCCTTGTTTCGGCTTACAAATTTTACTTATCAAAAAAAA
ATTATCAGTCCTCATCCTCTTAGGCATCTAACATAACCATTCGCTATAGCAAGCCAAAAA
GATTTAGATGAACTTTTCCTGCGTACTACGAAACAATGCAGCCACACCCTCCCCTTTGGC
AACATTGGATAGATCACAGCAAAATCTACCCAACATTAACAAGTGTCACAAGACAAGTCG
ACTGCCACCAAGTTCTTAAAACTTATTAATGAAAAACACTAATTTTAGTCACTTTACATC
TACCAAATTTACTATTGTAAAAGATAAAAGCAATTTAAAATTAGCTTAAGCTTTGTATTG
CATGAGTGATCAAAACTGAAACTACATCAAACCCAAATTGATCCTAAAACTGATTGTAAC
AAAAAATTTTATAATAACTCACGCAATTTCCAGGGTGCTTAGCATCCAAGAACACAAAGA
TGAGAAAATCAGAACTACTTTTGTCACATCATTTCCACTTAGCTATCTTAAACATGACTA
GCACATAATATCTGAACAATGTAAGATAAACCAAAACCGTAGAACTAATTTTTTTCAGTT
ACTTTACACACTACATTCCAAGAGTGATTCTCTTACCTTTAATCAGATGGCAACTAGTCC
AAACAAGGACGGTGATGATTCTTCCACTGTCCACTCAAAGCCGATAACTACCGTAGCCAC
CGATGCCAGAAGAACCATAGCCACCATACAGTTGCGACGATAATGATGATGATGAGTACA
ATGAACTCCCCAATTGAGAATATGAAGATCTATAACCGGTGTCTATCTTTCCATAGCTGC
TTGTAACTGTTTCTTTGTGATTGTTCATAAATTCCTGAATCAGTTGTTGAGCCATCTGAA
CCTGTGATGAGGAGCCTTTAATTTCTACAGTGATTTCATCAGGTAATCCCCTGCTCTCTT
GGACAGTTATGATGGCCCCACTAGTTCGACGGATGTGTTCAATACTATTACCTCCAATAC
CAATGATGTCCTCAGCATAAGATATTGGTATTTGCATAGTTTGTGCAACCTGTGTAACAA
TAGGACCACTAGCACAAGCAACTCCTGATGAACGTATTGCTGAAATTGCAGGGTCTTGCC
CATAGAGTGAAACTCCAGGGTATCTAAAACGTGACTCCAATTGTGGTTCACGTTCCAAGT
ACAAAGATTCCCTCCTTGTTGAGGGAGTATACTCAGAGCTAATTCCACTTTGAGGTGCAT
TACGCAGTGATGACTTGTCAGTCCAAGTCTCCACTTGACGCTCTTGAGAGATTGCAGCGT
TGTATGTTTTCTCAAAAAGAGGAAGAACGCTATGATCAACCAAGAACTTCCTCAGATGTC
CAACCACTGCTTCTAGAGCTTTGAGAACCTTCATAGCTTCTCCATGCATCTCCACAACCC
TCTCATCTGTGGCAGCATAAAATGGCACTTCTTCTCCCGAAAGTACACGCACAGAAGCGC
CAGTGCTTTCTTGAATAGACTTAATCAATGAACCCTGCTTTCCTATTAAATTAATTGCTT
GTGTAGATGCCACCAACAACCGCACTGAACAACTTGCAGATCCAGCTCCTTTAACATCAC
CATCATTTTCAGGCAACCCCAATACTCGTTTGAAAACTCTAATTGTCGCATTCATAGCAG
GAGAAACAGGTGCCTCCAGCTCTTCCTTTCCTGATACCATCACAATGCGGTCAGGATTTC
CAACAGGGCCGTCAAGTACTTTAATGCGAGCTCGAGTCTCCTCACACATCTTCTTAATGA
GATCGCCCTTGCGTCCAATAATGCTTCCAACTTTTAAAACAGGTACAATAAGCCGAAACA
CGCAATACCCTGGCCATCCGGGCCACCGCTTCTCCACTAGTGGAGTGGTGGTGGTGGGTT
CGATGGCCTCGGCTGAGTCGGGGTCGGAGCTGTCTGGGTTTTGGTTTTGAGGGGATCCAA
CAGAGCCTTCTATAGGGTTTATAGTGGGTTCCGGGTCAAATTCTGCGGCATATGTGGAGC
CGTTCTCGCTGGGCAGCTCCTCGTCGGCGGTGTTGGTGGTCGTGGCGGTCGCCATGTGAG
AAGAGTGCTTGGATAAGGGCAAGGTTTTACAGTGGAGCGCGTGGTGGGATTTTCCAGAAT
TGGGAAAGATAGATTACCTGGTTCAATGTTGGGCTTGAACTTCAAGAAACTTGAAACGTA
ATAGCCGATTCAATAACTTTGAAAAGCCCAAACAACAAAGTCCCACGCAAGGTTAACTAA
CAAAGTACAATAAAAGGTTTTAAAAACTAAATACGCACC
>tig_1108_consensus
TTGGGCAAGTGCAACAGCATCCTCATTGGGTACTACATCCCATAGTCCATCACTTGCAAG
AACAAGCAAGTCAAATTCTTTTCCTATCTCTTGCTCCTGGATATCAGGTTCAGCAACAAC
AAATTGCTTCAACATACGGTTACCAAATGCTCGTGACATAGCAAGAACACCACCAACTCT
CCAAGTGCCTGCCCACATTACAACACCACCAGCATTTTCAATTCTCTTCCGCTCGTCACT
TCTGTTGGGTTTATGGTCCTCAGAGAGAGGGATAGCTTTACCAGCCTTTGATATCACAGT
CCTTGAATCTCCAACATTGGCAACATATAGGTGGTTACCAACCAAAACTGCTGTCGAAGC
AGTAGAACCATCATCCCTTACAGTATCTCTTTCAGACTCCAAAAAGGCGGTATCAGTCTG
TTGATATGTTTCACNNNNNNNACCATCAAAGATTCCAAACATGTAGACCGGTTGCCCATC
AATCGTGGAAGCCTTAATGTCAAAGAAATCCTCCATGGTCGCCCTCTTTCCCCTAAAGCT
TGAATAACCACATCTCAGTTTTCCATCTTCACTTTTCCATCCTCCACTAACATATCCACC

ATCGTCTTCTTTCTCCGGCAATATATCAACAATTGGCCCGCGTTTCGCCGCCGCACCTGA
TGAATCAACCATCATCTTCAAGCTCCTTCTAAATTCGCAATTCCACGTAAAATTTACACC
AACAGACGCATGAAAAATCCTCCAGGTGTTGCTTATTTGAACCCTCCTTTTCGTGAATAT
CCCTACTCTCCCAGCTCGTAAAATCGCACCCCCTAAATAACTGTTGCATACCATCTAAAA
TCTACCGCTCCAACCTGCGATGCCAAATGGATCTTCGATTGAATGAATCCGCGGAATTGG
AGGTCCTAAATTGAAGCGGATCGAATCAGAAGGAGCGCAATGGAAGGTGGAGGTGATCGG
GTTCGGGTCGGATAAAATGGATGATCCGAATCTGACAAAACACTTGGTTTGAACTTCGAA
TTGGATTTAGATTTGTTTGAGAAGGTTTCTCTCCCTGAAGGATAGTCTGGAAACCCTAGA
GAGAGGGATTGAAGAAGCAGCGGTGAG
>tig_1109_consensus
ACCCAACATGCATGTGCAAGATTTGGAATAATGATCTATACCTGAGTGAGCTGCCAATAA
ATATACACATGCAACATAAAATTAGGATGTCTTATTAGGCTGGGGATCAGCTGGACCTGC
ATCATCACCATGGTGGAATCTCACCACAATGCAGGTTATATTGTCAGCACTGCCACAGGT
AAAAGCAGTCTCTGTTAATTTCCGAGCTGCAGCCTCGGGTTTTTCTTCTGTTCGGGCAAG
TGCAATAGCATCCTTATTGGGCAATACATCCCATAGTCCATCACTTGCAAGCACAAGCAA
CTCAAATTCTTCATCTATCTCTTGATCCTGAATATCAGGTTCAGCAACAACAAATTGCTT
CAACATACGGTTACCAAATGCCCGTGACATAGCTAGAACACCTCCAACTCTCCAGGTGCC
TGCCCACATTACAACCCCACCAGCATTTTCAATTCTTTTCCGCTCATCACTTCTGTTGGG
TTTATGATCCTCAGAGAGAGGAATGGCTTTACCAGCTTTTGATATCACAGTCCTTGAATC
TCCAACATTGGCAACATATAGGTGGTCACCAACCAATACTGCTGTTGAAGCAGTAGAACC
ATCATCTCGGAAAGTATCTCTTTCAGACTCCAAAAAGTTGGCATCAGTCTGTTTATATGT
TTCACTTATAGCCAATTTAGTATCTTTCAAGAATTCTGGATGCTTCATGAGATTATCGAA
CAGATGTTGCTTCAAATACTCAGCAGCCCGAGAACCACCATGACCATCAAAGATTCCGAA
CATGCAGACTGCTTGCCCATCAATTGTGGAAGTCTTAACATCGAAGAAATCCTCCATCGT
TGCCCTCTTTCCCCTAAAACTTGAATAACCACAGCTCAGTTTTCCATCTTCACTTTTCCA
TCCTCCACTAATATATCCACCGCCATCTTCTTTATCTGGCAATATATCAACAATCGGCCC
ACGTCTGGCTGCTGCACCCGATGAATCGACCATCATCTTTAAGCTCCTTCTAAATTCGCA
GTTCCGCGAATAACTTACACCGACGCACGCATGCAAACTCCTCCCAGTGTCGCTTACTTG
CACTTTCCTTTTCGTCAATATTCCTATTCTCGCAGCCCGTACAACAAGACTCCTTGAATA
ACTGTTGCATAACATCTAAAATCAACCACCAAATGGATCCTCGATTGAATGAATTATGAA
TCTGCCGAATCGGAGGTCCGAAAGGGAAACGGATGGAATCTAAGGGAGCGTAGCGGAGGT
GGAGGCAATTGAGTTCGGATTGGATATAGTGGATGATCCGAATCCGATGAAAGACATAAT
TTGAATTGGAATAAGATTTGTTTGAGAAGCTTTCTCTCTCTGAAAAGGAATC
>tig_1110_consensus
GTATGAAATTCCAGTAACTTTGCAACAAATCTGGAATTAGCATAGAGTGTTCAGGTTCAT
CCTGTCATTTTAGTCAAGTCGTATTATATCAATTCCACACATTTCCTCTTCCATTTGAAT
TCTGTCCCCTAGAATCAGATGATGAGCTGTGAAAGACAAAACTTGATCTCTTCTTCCTGT
GGGTAGTCCATGAAAATAAATGCCCATTCAAACTACTGCCTTGAACACCTTGATGTTCAG
GCTTTTCAAAACTCTGATATTCAGCTTTTTCTGAATTTCCCCTCTCAAACTTCAGTCTCT
TTGGGGCTGAACCATAGGTATCCTCACTTGTTCCCTCACTGGCAATAGGCAGATTCCCAT
TCTTCACTTCTAATACCTTTTTCTCTGCTCCTGTACTATTACTAGCTGTTTCATTATCCT
CCAATGGAAGATAGTCTCTTCTCTTTTTAGTTGAAAGCTTCAATATGTCTTGTCCGTTTT
CTACAGCTTTCATCCATTGCAAATCACTTAATGTATCAGCGTAAACCACTTCCTTCCTTT
TACGTTTTCCTGTAATGTTGCTGCTATCATGTTCAAAACCTTTGTTCTGACCTTCCTTAT
TATCAGGTGCAGAATATGCCCACTCAGGAACTTCATGCTCTTCCATAAGACGAGATCTGT
AGTTCTCTTTTTGCCTTCTCTCCTCATCCATCTTCTCAAACAGCCAAAATTCCTCATCTG
AGCGGGCTACAAGATGGTTAATTTCTCTCTCACTTGGTACATCTGTTCCAAGGGAGCTTG
TACCTTTACGCATGATCTCCTTTAACATCTCTCTTCTATCCTGAGCAGTGGAAGTTGTAT
TGAAAAGTCCAGCCTGGATAACCTTGGCATCAATGCCCATCTTCTGCTTAGCACGTTCCA
AGATTACCTCTTCAATTGATCCAACACTAACCAACACAAAAACCCTGACTTCTTTCTTCT
GTCCAATTCGATGGGCCCGATCCTCTGCCTGTTGGTCCATTTGTGGATTCCAATCACTGT
CAAAGATTATTACAGTATCTGCTGTTTGCAAGTTCAAACCAAGTCCTCCAGCACGTGTAC
TCAAGAGAAACATAAAATAGGGAGAGTCTGGAGCATTAAATTGCTTCAGCAATGCCCCTC
TTTCCTCTGTTTTTGTTGAGCCATCAAGTCTAAGAAACTTAAAATCATTTAGTTTCAAAT
ATATTTCGAGAATGTCCATGAGACGAGTCATCTGTGAGAAAAGGAGGACTCTATGCCCAG
ATCTACGGAGTTTCGGGAGTAAACGATCAAGTAGCTCAAATTTTCCAGATGCTCTGATGA
TCTCCTCCTTGCGCCACATATTGTATTCTCCAACAAAAAGGTATGGGTGGTTACAACACT
TTCTGAGCTGCATAGACAAGTTCTGCAGACTCTTTGATTTCCCAGAACCATTATCTAGTC

CAACTCTACCCAACTCTGTAACTTGGTGGTAATACACTTTTTGCCATGCTGACATGTCGC
ATTTAAGTATGACCTGGGATTTCCCAGGAAGGTACTTCTCTACCTCATCTTTTTTCCTCC
TCAAAATGAATGGCCTTATTACATGATGCAAACGACGGATAATCAACAATTGCTCTTCAT
CTGTAAGAGCAACCTGTCCCCGATCTTTGAATGGAGCATTGAACCACTCTTCAAAGTTCT
CAACAGAATTGAAAATGGTTGGGAGAAGAAAATTGAGGAGGGACCATAGTTCCTGCAAAC
TATTCTGTATTGGTGTCCCAGTTAACAAAAGTCTTCGTTGAATCTGATACCCGGCAATAG
TCTTTGCAAGAGCACACTCATGATTCTTTAAACGATGCCCTTCATCGACAATCATATAGG
TCCAGAAAATTTTCTTCAAATATTGCCTATCTCTCATTATGAGATCATAATGTGTGATCA
AAACATTATATCGTCCTCTCTCTGAAAAGAACTCCTCCCTTAGTGCCTTTCGCTCATCTG
GTCGTCCATCATAAACAACTGCTGCAATACTGGGAGCCCACGTTGAAAATTCATTTATCC
AATTGGGTAGCACAGCCTTTGGAGCTACTATAAGGTGGGGGCCTGTAACACCCTTGTTCT
CCATGAGATATGCAACCAAAGATATTGTTTGAATTGTCTTTCCCAGTCCCATCTCATCAG
CTAAAATTCCATTTAGGTTGTTATTGAACAATGAAAGCATCCACTGGAGCCCCTCTATCT
GGTATGGTCTCAATTCTCCACCTTGAAGTAAGGTCGGTTGCTCAGTTACCTTCTCTTCAA
TTGAGTGAATTGCAGAGTTATACTGCCTCTGGCCTTCGAGTAAATCAGCAGATTCATCAT
TATGATCAGAATCAATAATGTCATCTTCCTCAGAAGGTGAATCCCGAGGAGTTCCATTCT
CTGAAGCATCCAAGTCAAGCAAATCATCTTCTGACTCTTTCAAGGGTTCAATGCCATCTA
AATGTTTAGCATCTTTCTGACGTTGAACAGCAGCTCCCAAATTAACAAGGAGCTTATTTG
TTTCTTCAAGAAGTGTGGTCAACCGCTCATTCTTGCTCTCTTTGACTAATCTCATGTATG
CTTCTTGATCATCAGCCTTTAGAGCTTGGAACCTCAATTTCTCAGCCCGTGTAGCACGTT
GCCTTTGCCTTCCATGCCATGCCTGGACTCCATCATTCCTTTGTTTTCGGCGTTTTAAAG
AAGCTTGAATTTGCACTTGGAATTCGCGGACAGCATTAAGTATTTCTGCAAAAAATTTTC
TTTTTCTAGTCTCAATTTGGTTCCTTGCCTCCTCTTCCAACCTTGATAGCCTCTCAGCAT
CCCGCTTCTTCCTGAATTGATCATCAGCTTCCATGGCAAAAGCATCTCCGACACCATACA
GAGGACGGCGCAATCGCATCATACCCCAATCAAACAGTTGCTTTTCAGGACATGCACAGT
TCAAGCGAAGCCAGTATTCTGAACTCACATCTGACCGAACTTTGTTTTGTAACCCTGCTA
GCTTTAGTCCATAGAGTTCTAGTAAGCACTTTGACTGCAGCTCCTCCCCTCTACTTGAAG
GCAATTCTTCAAGTTCATTTAATCGGTGCTGAATGTGGCTCTTATAGCGATTTTCCCTTG
ACTCTTCTAATCCAGTGCCTGACATGCATTTTGGCCGTTGCTTTGACAATGAATTCTCAA
ACTCACTCATCAAATCTGCTCCATTAGGAATCTCAAGACCATCCAAACCCTTGTCCGCCA
CGGTTGTCTGGTCAGATCCATCGGCACCAGGGACCTCATCATCAACAACCTCGGTAACGC
CCCCTTGGTGGCCATAATAGATGGATGAAACGGTGTCGAATAACTCAGAAGGTAGCGGGA
GGTCTCGAGATATGAAGTTCAATGCACAGATCAGAGACTTTGCCCTCTCAACATGGTCTA
AAGAGGGAGTCACACTCTCCTCAAGCTGAGTCACCAT
>tig_1111_consensus
TTTTTTTTTTCAAATATTCAATCTCATAGCATTACGAAAATTTCTTAGTACAGACTTTTT
CCTGTTGAAATCGACATTGAAATAGTAAATTAAAGTACAAGACAAATTCGGGTTCTTTCT
CCACTACCAAAACCCTACAACACAATGCCGCACAAAAGTTTTCATCAGCTTGACTACCCA
GTATCTGATAATAGAAAATGGAAAATGTAATAGCAGAAGCAGCTTTATGATGAGATGAGA
CTATGAAGCTTAAAAATGCCAAGTTATAGACTAACCAACATTAATATTGAGACTAGAAGT
ATGATATTCAATAATGCGGCAGTTCTACTAACAGCTTATATGGGCAGAAAACACTTTGGC
AATGGTGGAATAACTGGGAAGTGGAGAATAAGCCATGACTACACCAGTAAAAAAATCATG
TAATCTTAATCACTTGAGAACCCGGACTTCTGTGCCAAAGGAACGATGATCATGCTGAAA
GAAATTCTGGCAATGAGCAGAGACCATTCAGGTTCATCCTGTCATTTTCACCGAGTTATT
TTGGGTTAACTCCACATATTTCCTCTTCCATTTGAATTATGCCCTCTAGAATCAGATGAT
GAGCTCTGGATGATGTAACTTGATCTCTTCTTCTTGTGGGTACTCCATGTAAATAGATGC
CCATTCAAACTAATTCCTTGAACACCCAGATATTCAGGCTTCTCAACGCTCTGATATTCA
GATTTTTCTGAATTTCCCCTCTCCAATCTCAGTCTCTTTGGGGCTGAACCATAGGTATCC
TCACTTGTTCCCTCACTGGCGATAGGCAGATTATCATTCTTCGCTTCTAATACCTTTTTC
TCTGCTCCTGTACCATTACTACCTGTTTCATTGCCCTCCAATGGAAGATGCTCCCTTCTC
TTTTTAGTTGAAAGCTTTGATATGTCTTGTCCGTTTTCCACAGCTTTCATCCATTGTAAA
TCACTTAATGTATCAGTGTAAACCACTTCCTTTCTTTTCCGTTTTCCTGTAATATTGCTG
CTATCATGCTCAAAACCTCTGTTCTGATCTTCCTTGTCCTCAGGTGCAGAATACACCCAC
TCAGGAACTTCATGTTCTTCCATGAGACGAGACCGATAGTTCTCTTTTTGTCTTCTCTCC
TCATCCATCTTCTCAAACAGCCAAAATTCTTCATCTGTGCGGGCTGCAAGACGGTTGATT
TCTCTCTCACTTGGTACATCATTTCCAAGGGAGCTTGTACCTTTACACATTATCTCCTTC
AACATCTCTCTTCTGTCCTGAGCAGTGGAAGTTGTATTGAAGAGTCCAGCCTGGATAACC
TTGGCATCAATGCCCATCTTCTGCTTAGCACGTTCCAAGATTACCTCTTCAATTGATCCA

ACGCTTACCAACACAAAAACCCTGACTTCTTTCTTCTGTCCAATTCGATGGGCACGATCC
TCCGCCTGTTGGTCCATTTGTGGATTCCAATCACTGTCAAATATTATTACAGTATCTGCT
GTTTGCAAGTTCAAACCAAGTCCTCCAGCACGAGTACTCAAGAGAAACATAAAGTAGGGA
GAGTCTGGAGCATTAAATTGCTTTAGTAATGCCCCTCTTTCTTCTGTTTTTGTTGAGCCA
TCAAGTCTAAGAAACTTAAAATCATTTAATTTCAAATATATTTCGAGAATGTCCATGAGA
CGAGTCATCTGTGAGAAAAGTAGGACTCGATGCCCAGATCTCCGGAGTTTTGGGAGTAAA
CGATCAAGTAACTCAAATTTTCCAGATGCTCTGATGATCTCCTCCTTGCGCCACATATTG
TAATCTCCAACAAAAAGGTACGGGTGGTTACAACACTTCCTGAGCTGCATTGACAAGTTC
TGCAGACTCTTTGATTTCCCAGATCCGTTATCTAGCCCAACTCTGCCCAACTCAGTCACT
TGATGGTAATATACTTTTTGCCATGCTGACAGGTCGCACTTAAGTATGACCTGAGACTTC
CCAGGAAGGTACTTCTCTACCTCATCTTTTTTCCTCCTTAAAATGAATGGCCTTATAACA
TGATGCAAACGACGAATAATCAACAATTGCTCTTCATCTGTAAGAGCAACCTGACCCCGA
TCTTTGAAAGGTGCATTGAACCACTCTTCAAAGTTCTCAACAGAATTGAAAATGGTTGGG
AGGAGAAAATTGAGGAGAGACCATAATTCCTGTAAGCTGTTTTGTATCGGTGTCCCAGTT
AACAAAAGTCTACGATGAATCTGATATCCGGCTATAGTCTTTGCAAGAGCACACTCATGA
TTCTTTAATCGATGCCCTTCATCAACGATCATGTAGTTCCAATGAACTTTCTTCAAATAT
TGTTTATCTCTCATTATGAGATCATAATGTGTGATCAACACATTAAATCTTCCTCTCTCT
GAAAAGTACTCCTCCCTTAGTGCCTTCCGCTCATCTGGTCGTCCATCATAAACAACTGCC
AAAATACTCGGAGCCCATGTTGAAAATTCATTGATCCAATTGGGTAGCACAGCCTTTGGA
GCCACTATTATATGGGGCCCTGTAACACCCTTGTTTTCCATTAGATATGCAACCAAAGAA
ATTGTTTGAATTGTCTTTCCCAGTCCCATCTCATCAGCTAAAATTCCATTTAAGTTGTTA
TTGAACAATGAGAGCATCCATTGAAGCCCTTCTATCTGGTATGACCTCAATTCTCCACCT
TGAAGCAAGGACGGTTGCTCAGTTACCTTCTCTTCAATTGAGTGAATTGCAGAGTTATAC
TGCCTCTGGCCTTCAAGTAAATCAGCAGAATCATCATTATGATCAGAGTCAATAATTTCA
TCTTCCTCATAAGGTGAATCCCGAGGAGTTCCATTCTCTGAAGTATCTAACTCAAGCAAA
TCATCTTCTGAGTCTTTCAAGGGTTCAATACCATCTAGATGTTTAGTATCTTTCTGACGT
TGAACAGCAGCTCCCAAATTAACAAGCAGCTTATTTGTTTCTTCAAGAAGTGTGGTTAAC
CGCTCATTCTTGCTCTCTTTGACTAATCTCATGTATGCTTCTTGATCATCAGCCTTTAGA
GCTTGGAACCTCAATTTCTCAGCCCGTGTAGCACGTTGTCTTTGCCTTCCATGCCATGCC
TGGATTCCATCATTCCTTTGCTTTCGACGTTTTACAGAAGCTTGAATTTGCACTTGGAAT
TCACGGACAGCATTAAGTATTTCTGCAAAAAATTTTCTTTTTCTATTTTCAATTTGGTTC
CTTGCCTCTTCTTCCAACCTTGACAGCCTCTCAGCATCCCGCTTCTTCCTGAACTGATCA
TCAGCTTCCATGGCAAAAGCATCTCCAACACCATACAGAGGACGGTGCAATCGCATCATG
CCCCAATCAAACAGTTGCTGTTGAGGGAATGCACAGTTCAAGCGAAGCCAGTATTCTGAG
CTCACATCAGATCGAACCTTGTTTTGCAACCCCACTAGCTTTAGTCCGTAGAGTTCAATT
AAGCACTTTGTCTGCAGCTCCTCACCTCTACTTGAAGGCAATTCTTCAAGTTCATTTAAC
CGGTGCTGAATATGGCTCTGATAACGATATTCCCTTGACTCTTTCAATCCAGTCCCTGAC
ATGCATTTTGGCCGTTGATTTGACAATACATTCTCAAACTCACTCATCAAATCTGCTCCA
TTAGGAATCTCTGGACCATCCAAACTCTTCCCAGCCACGGCTCCCCGGTCATATCCATCG
GCATCACGGACCCCATCATCAAGAAGCTCGGTAAAGCCCTCTTGGTGGCCATAATAGATG
GATGAAACGGTGTCGAATAACTCAGGAGGTAGAGGGAGGCCTCGAGATATGAAGTTCAAG
GCACAGATCAAAGACTTTGTCCTCTCAACATGGTCTAGAGAGGGAGTCACACTCACACTC
TCCTCAAGCTGAGTCTGAGCCACCATGG
>tig_1112_consensus
TCCAAAACACAGTGCCCATTACTTGTATACATAGTAACCGGAGAGCATAGAGCTTGATTA
GAATTTCTGGAGAACCACAGAATTGGCAAGGCCAGCCTGCTTTATTGTCTGAGATCGGTC
CATAAGAGTTTTAGGAGGGAATCCCATCATCTGCAGTTGATAATTTCTTGCATTCCCAGG
TGCAAATGCATCGATGTGAGCATGGATGTCATTGATTGTATGATGGAGATTGAATTGTGC
TACCATGCGGGTTCCATCAGCCAACCTGATCTGAATTGATGTTGATGGTAGCTTGTCATC
CACAATCAGACCTGCAGAAGGGGTTTGAGCAGCAACAGCAGAAGTGGAACCGACAGTTGG
TTCGGGTGCTGCTGGAGCACTGGCACTACCTAAAGTTCTTCCCACACCCTGAAATGGAAC
ATGACGCGTCTCTTGTTCCGGGCACTTCACATCCTTCTTTATCAGATTGACATGAACTGA
GGACCTTCTATCAGCAGGTTCTAGCTCTTTTGGACACTCAGACTTTCTGATGCTCTCTAA
GAAAGATGCATTTTCGGGATCATTCAACCTCCTCAAAGGGCCATCATTTACAGTAAAACC
ATTTGTCCAAAAAACAATGTTGTGAATGACAGGCTCAGGCTGCTGAGGTGCAGATGGTAC
GTTTTCCCCTGAGAGTAATCTACCAGTTCCAGTAAAGCTCCTTGAGCTTGAAGATGGATG
AAGATGTTCAGGAGGCCCTTCCACAGCTCCCAGTTCTCTAGCTTGATTGAAAATTGCATC
AACATCATTAGGATTACCCTTTGAAGGATCTTGGACAAGCATACCACTCTTTTCACCGCC

AGTGTAGAATTCTTGAGGACCATCAGAGTCGGAATCTGAATCAGGTCCGGTCCTCCGGTT
CAGATCGGAGAGGGTTCGTATGCCGCTGGTACGGCTACTCGATGGCTTTGCAGGTTTCTT
GTCACGTGATGCCATGGATTGGTTTCTCTGATTGAAAGTTGAAAGTCGAACTCGAATAGA
GAAATGATTCTTAAATTTTCAAGTTAATATTTTATACGTGGTTTTTTTTTTTTTTTTTTT
TTAAATATGTACCCTTTTTTTTTTTTTTTTTGTGGTATTAGAGTCAACAACTAAATGGTG
CTTGATGCGACATCAGATTTATTCTTCAAACGTACCACACCGATACATCCTGCTTTGGGT
GAGGATCCATGGTGAAATCAGCTGGTGCCATGTTGAATGGTGAGCTAAACCTGAAGTCAG
CAATATTATCACCTTGATTGGTTTCAAATGGTGAGTCCAATGGTTTGATCTTATTGAAAC
GAGTCTCTGCTGGGGAGAAGTTGTTAACTGGCAAGTTGGGCTGCTCTGGTACATTGCCAC
CCATCATAACACCTTGGTTGTAGAAGCTATCATCCAGCTGAAATCGAAATTTTGGCTGCT
GCTGCTGTTGTTGCTGACACGGTTGATGCAGAGTTTGACTGTTTACAACATTAAGCCCCA
CAGGCACGTTAAGACCCCCAGTATTCAGGTTCTTGTTCCGCTGATGATTAGAATCATAGA
AAGACATCAGATCAGAAATCATTTTTTGCCCATCATCTGAGAGTCCAAGTCCGGACACAT
TAAATTGTGAATGCGCCAGATTCAACAGTGAAGTTGGAGGCTTCTGTTGAGGTGGCTGAG
GTAATTGAGCCGGCTGAGCAACAGGCAAAGAGAACACTGCAGGTTTGTCATTGTTCATTT
GAAAGTTTGTCACTCCAAACCCTTGAGAAGAATTGCTACGATATGGGCAATTCATCTGGT
GATTGTTCCTTGATGACCTATCAAGAAATCCAAGGCGATAGTCACTATAGGGGCACTGTG
GGTACTCACAGGTATACATCTTTTGATCCATCATGATGTGAGGCTCATCTGCCAGCTGCT
TCCTCTTCTGAAAGAAATCTGAGTTAGTTTCAACAAGTTCCCCCTTAATTTGAGGAACCA
AAGGTTGTGTCATGGTAAGCCTCTCCCTAGGTCCTACTGCCCCAATGCCGAAGAGATTCA
CATCATGAGGCTTGACCTCCTCTACTTCAACATTTTGATCATCATCGCCCCCTTCAACAT
CATAATCACTGATATCACTGATAACAAAAGAACCACTTCCACCGGCAGATGCAGGAGGGC
AGCTATCAGGATAAAGTTTTCGTGACAAAGCTTCTTCTTGATTAATTATTGCCAACCAAG
TGGCACTCTCTTTGGCAGTCATCTTATCCTGCAAGCACTTAGATTGCCGAACAAGCTTTC
TGATCTTAGCAATATCCGGTGACATGTGCTTGATGACTGCTGTGAGAACGCTCACTTTCC
AAGCTTTCTTCAGATCATGAGGTTTCTTGTACGGGGGAGCACCTTGATCCTTGGTTAGAC
CCACTTGAGGCCACCACTCTTCATTTCCAGTTGGCCACCAGGGTGGTGCAACACCTCTCT
CTAATGGAAATCGCC
>tig_1113_consensus
GTGCTGATTAATAATATAATAACAGAAGGAGAACCAGAGTTACAAGGTGCTACTATTATT
TACATATTACAGCTGACTAGGTAATGTATTTCCTCATTCTGTTATTTACAGATTCAACAG
AAAGATGAAGATGAAACCCGACATTGTATCCACAATTCCGGATAATCATAAAAGTCAAGC
CCCCCTATCCCTCAATATTTCTCTACATTTAACCCACAATATTAACACCTGTTAATTAGC
CTTCCTGGAGAATAAAAGAATTTACTGCACACCTCTAATCAAACTCATCACCTTCATCTT
CATAATGATTCTCCATTTTCAGGCCTTCAACGTTATTATTTACAAACTTGAGTTGAGCCT
CCATTCTTAAGCCCTCACTATTGTTTACAGTCCCAAGCCACGTATTGGTTTTTAGGCTGT
CACTATTATTTACATAGCCCTTGCTTTCAAATGGCATCCGAGGACCATCCCAACCATTTT
CTCCAGCATAACTGTTATGATCAGGTACTGACGCCACAGTATCAGTCACTGATTCAAGAG
AATCAGAGCTGCTTACGCATAAAAGCCTGCCCTTTCCAGAAAGCCCAGACCTCTGATGCA
GCACCCGTCCATTGGTATTGTTATCTACAGCAGGAACCCTTGCTTGTTGAGGTTTGGGAC
TCTGAAGATGGTCAAAAACTGGGATCAAGTGTTTTGACCTAGCTTCAATCACATGAGTAG
ATATCCTTTCAATTTCAACTTCTCCTTCAAATCCATCCTCCACTTCAATGCCCTCCATCA
TCATCAACCGAAAGCATATCTCTGGTTTTGCCCACAATGGAATATGAGGTGGATGCATCT
GCAGTTCAGCTTCAGCAACATACATGTGATGTTTATCCTCAAGACTGGCCTTTTTCTCAA
CTGCACCCCTAGAATCAGTAAGAAGACAATTTCCCTCCTTCATTTCTTCAGGGTATATCT
TATTGCTGTCTGATGCTCCATTCTCACCATATATGTCTAGATTATCTTCTCGCTCTCTTC
GAATCTGCTTCTGACATATACTCCACTTCTGGATAGCATCAAGGACTAACCCAGCATCAC
CATCCGGAACTGAATCAAAAGCAGAGACCAATCCAGACACACCTGTCATCCCATCATGAC
CAGCTGATACTCGCAATGCATATTGTATCATACACCCAGAGGGAGAGAAAACCAGCAGAT
GGTACTTCATCTTCGAATATACATAAGAATATACATCCCCATTTCCTTTACAGTTGTGAA
AAGATGACGCAATAGCACCAGAAAGGGAACTCATCCTTCCTGTTGCAGCTGCTGCCATAC
CACTTACATTACCTCTCCAACCATTATTTCCATTTCTTATTCTACTAATAACAGAGAGTG
TAACTGGAGGACCAGATGCATAAAGACTATGTTGATTACGCAATTGAATATTAGAATTAG
GTGGCCAACGAACAACAGGCTTAGTCCTGAAACCCGATCCATTATGTTTTGCAGCAAAAC
CAGTATCAGCAGACTGAAAATAAACAGATCCCCCTGAAGGATTTATAGCAAACAAATGGC
TTGTACCCCTGGAGGAACTAATCATAATCCAGTTGCTGTCATTACTGAAACTAATGTCCT
GTATAACAGCATTTGTGATACCACGTTGCAGCTGGTAAAGATGTACATAAGGTTTATCAG
CCTCACATGCAGAGGAGCTTCCTTCTAGTCCAGGCATTATTTTGAAGACATTTATGTTAT

GACCCTGAACTGAAGCAGTCACTAAAAGAACCCCACTAGGATCAAAGCATAATGCTGCAA
TAGGACTCTGGTGCGCCCTAAACTGGGCAATAACAACTTTACTGACAATGTCTCTCACAA
TGACCATTCCAACATTATCTGCATCTGGCAGAAGACCATTAACAGTTCCATTGCCCTTCC
CACCAGGACTCGCTGATTGTAGAGAATTCTGGGAATCAGGTAGAAGCTCAGAATAGTATC
TTGACAACTTCTTATAACCTATATCTCCTAGTGTCACTATCCCAGTAGCAAGGTGCTTGC
TTGATACTTTTGCATATTCTTTTGCATAGTGAGCAACCCGGCTTCCATTTGATGCAAGGC
CAGAAAAGCTTGGAGAAGGACCTAAATGTTGTGGACTGACACGCCCAGAATTGGAGACTA
CAACTGAACTTCCACTATAAGCCAACCACCTGGGACCAACTGCAAGGGGTCCAATGCCTA
CACCTATGCCTCCAGTAGTAGGATATCCAGCACCTATAGGATTTGTCAGAATGGCATATT
CTCTCTCCAATGTTGCAGCATCAAAGCAGTG
>tig_1114_consensus
CTACCGTCTTTTAACCAATGGTGCAAAATTCACTTCACATAGTCCAACAAGTAATTGAAA
CTTGCAAGAGAGCTACAAGGAATAAAAACAAAATTACATTTGAAAGGGGAAAGGAAAATC
ACACAATTTATTCAAGGCGAATACATGCCAGCTACTTCTTGTCTTTTGCATAACATTATT
CATGGGGACATGTACAATAGCACTATAAGATATGAAGTACTTGAGGCAGAGACTCCAATG
CTGAATGCTCATGCAGCAGCAGTAGGTGAAATTTTGTAGTCAAGATCCCAGAGCTCGAAA
CTTCCTTCAACAAAATCATAATAAGCTCCCTTCAAAGCCAGTCTCTTTTTCACCACACTG
TCTCTCACAAATGGATATGTCAACAGGTTCCCTAACGATACGTTCACAGCCTCCT
>tig_1115_consensus
TACGAGCAAAGTAGGCAAAAGGATTTTAACAACTATGCATACAGGTATTTGCACAATCCT
ACAACGAAGAAACTGAAGGATATTACATTTTGCATTACCTGTCCACTCCAGACACTCCAT
TGTTGTAAAGTCCATACTTAATTAACAAATGACGTCAAGGAGACAGGAAAAAGGAAAAAG
AGCAAAGGCAAAAATAAATAAAAGAAAAATGTATGATTTCATAGACAGAAAGGAGCGACC
ACCGTCGTCACCTTACTTCAATCCTCAAACTCATGCATGTGATCCAGAAGATAGTTAGCA
GCCAGTTCCTCGTTCTTGTTGCAGGCAAAGAACACCTCCAATACAGTTGCCCGATCGAAG
CCCATTGCTTCAAGCCTTTCTATTGCTTCACGCTCTTCAGGTGTGACAGTTATTGCCTGA
GGAATTGCACCAGAAAGCGATCCCCCAATATTCCCCTCACCACCTTCGACAGGTTCATTG
ATAAGCCGAAGGAAGTCAGCCTGATGCTCCTGAATGAGCCTCACTAATGCAGGATTTTGT
TTCCCCAACTCTTGAAGCATGGGCTGCAATAAATGTGGGTTGGCCTGCACCATAGCTCGC
AAGACTTGAAACTGTGGACTGTTGCGTAAAAAATTCAAGAGTTCCTGTCCCAGCTGCACCT
GAGCCCATCTCTGGAAGGCCCTGCGGAAAGAGGTCTAAAGGGTTTGCATTGGGTCCACTA
GTAGGAGCAGGTGTTGGTTGTGTGGGCTGGGTAGCTTGTGCACTAACAGGAACTCGGGCG
ACAGGCGGAGCTTCTGCTTGCTCAGGGATACCAGAATACAAATATTCAACAGCTCTCTCC
GGGTTATTGTAAGCAGCACGGAGAGCACGGACAACGGTATCTCTATCCCATGTACCTCCA
CCCATATCAAGAATTTGCTGGATTGCTCCCTCCAGGTTATTCCCTGCAACCAGATTAGAT
GCAGCTTGGCCATAGACATTGGAATCCAACGTAGCAGTAACTGATGCAACAGGGGCAGGA
GCAACAGCAGGACTAGGAGTAGGAGCAGGAGTGCATACAGGCGCTGCTGATGTTGGTGCA
GAAGCCTCAGGAACTGTTGCTGGGGCTGGGGCTGGTGTACTTGTTTGGGCAGGCTTAGTT
GTAGGTGCAGTTGATGTAGTTGAACTCTCGCCAGAAGGTTTCTTAGTCTTAGTTACCATA
ATCACAACAAAACTATTTTCCGTTACTTTGTTCTCTTCCAGTGTAGTGTCATCTTTGAGA
ACTTTCCCCTGAAAAATAAGCATCTGCTGTGCCGCAGGATAAGCATCCTGTCCTTGAACT
GTTTCTATCTTCTTTTTCACGTCAACAACCGTGTCTTCAGGTTTCACTTCGATGTCGAAG
TTAGTGCCTTTGAGTGTTTTAACGAAAATCTTCATCTTCTACGTCTGTGTCCGTGATATG
AGAATTCAGTCAAGATCCGCAATTAATTTCTATCGTTTATCTATTTATATAGTGAGAAAG
AAAACCAAAGCACAAGTTAAGAACTTCGGAATATCGTCT
>tig_1116_consensus
TGACCAGCTGCCTCCTCCATGTTCAGATCTTCCTCCTTCATTCCATTCGGCAATTTCCAA
TCGAAGCAATACAGGAGATTTGCGAGTGCTAACTCCGAGGTTATTAATCCCAGATGAATT
CCAGGACAACTTCTTCGACCAGCTCCAAATGGTAAATACTCAAAATTTTGCCCTTTATAA
TCAAAAGAACTGTCAATAAACCTTTCCGGGAAGAATTCTTCCGGTTGCCTCCAACAGTTA
GGGTCTCTTCCAATTGCCCAAGCATTGACTTGTATCATCGTTTTAGGATATATGTCGTAA
TTCTTAACCCTAAAGTGGGATATAGTTTCTCTTGGGAGTAGAAGTGGGGCAGGAGGGTGC
AGTCTAAGGGTTTCTTTCATTATTAGTTTTAGGTATTCGAGTTTATCTATGTCGGCTTCT
GTAACTCTTCCTTTATTTCCAACGCAGTTTCTCACTTCGTCCTGTGCTTTTTTCATGGCT
CTTGGATTCCTGGCTAGCTCCGCCATTGCCCAGACTATGGTAATTGCACTGGTGTCAACT
CCGGCCAAAAATATATCCATGGTTACTGCTTTGATACTA
>tig_1117_consensus
GACTCATCATTTGAAACTCCAATGTCATCCATGCATTTCCGCAAAAGAGATTCAGGCAAA

ATTTTTCTCTCAAGCCACAAGCGTAAAACCTTGAGACACTGACGACGATTTTCACGAGCC
CCATTTCCAGGTGGAGCAGCAGCACCTAAAAGACGTGGTAATGCAGATTGAACGGCAGGA
ATATATGAAGCTCCAGCAATTCCTTTTTGATTATGGGAACACTGAGTGATTGAATCAACA
AGAAAGAACAGGTCCACTTTACGATGATAACTAGGCTCATTCTCCAACTTGCGGATTAGA
AGTTCCACAACCTCAATAGCAATACCATACTTGGCACAATCAATGGCAAGACGAGTTGCC
CGTCCAATACTTTCTTTGGTCCTCGACAAAGTTTCTATCATTCCTTCAAACGCATCACGA
GATACAGCTGCCTCAGTACCACCACTCAATGAGCCTCCACCAGCTCTGTGTCCAGAGCTA
ACTCTTCTCTCCTCAATTTCATCAGTATCCAGTTGACTAAAAGTCACTGACTGATGACCA
TGAGTTGACGGAGAGGCTAAACTAGAGCAATTATTAGATCCCTGCATATCTGCCTGTAGC
ACATTGTTGGTGCCAGACAAAGAAGGCTGAACAGAAGAGGGGCTAGGGCTCTTCCCCTGG
ACCTCAGTAACTGAAGTAAAAGCAGTATTAGGATTTCCAAGAGAAAATTGTTGCGAGTGA
GCTTGTTTCCTTTTTGCCTGAGCAGCTGCAATAAGATGTTTCATGGACATAACAGAGTCT
GGAGTCTTAGTATCAAAGAATGAACTGGCTCTATCTTCTCTACTAGACTCCATTATTTCC
AAGGGCAAAGAACTTTGATCTATTACAGTTTCTGTCAAAACAGCACGGCCATTCATATGT
GAAGCTGCTTTGGGAGTGGTTTTTGGTCGTTCAGCAGAAGAGGCTGTCCTATTTCTTTGA
TTGGCAACCTGATTTTGAAGAGAGCCTATGCTATCAGAAGCAAGAATTGAACCCTTACCA
GACATTGCCTGAACCTTTTTCTGAGTACCAGTAGAAGTAACAGAACCTTTAGTAACCACA
GGTCTTGAGGTTTTATGTTGTTCTGCAGTTGTCCTGGTAGTGGAAACCAAATGAGGGGAC
CTTTTAGGGGAATCTGATTTTACTTTAGCATCCTTTAACAGTAATTGCTCAGTTTCTACT
TTCCCAAGACCGCATGCAACATGATAATCCAACGTCCTTTCTGAATTTTGGGAAGGTGAC
AATGACTCATTATGTTTTTGCACTGAGGATTCCTTTGGCAAACTCTCCACTCTAACAGAA
TCTTTAGCACTAGCGTTCTGCTGATCATTAACATAACTCTCATGATGTGCAGCTGTTCTA
CAATTATCAGAAACAGATGAAGAAGCTTTCACATTTCTGGAAGATCCTCCGTGAACTGGA
GTTTTGGGTTCTTCATCATCATCCTCATCAAATATGCAAACAGCTCTCCATCTTTTGCGC
GACTGTAGCACCGGAGGTTTAACATTATTAGAACATGACACATCATTTTTCATTTCATTA
AAATTCTTCCCCAATTTATCATCAGAACTATGAGTAGGAGAATTAAACATAGCCTCCAAA
GCCCGCCTACGTCGTTTTGTTAATGGCAGCACAGCTTCATCACCATCAGATTCAACTTTA
TTTTTACAAGTCTGAGCTTTCAACGCCAAAACCAATTCAGTTTTCTCATGGGATGAGGAC
CGTTTCAAATCGACAG
>tig_1118_consensus
CCAATATTTTCAGGTCAGCACTTAAAATAGGAAACTATCAATTCCAGCCAGGTAACAGAT
TATTTTAGAGAGAGGAAAGCTGCACAATGGCTTGGTTGTATTGAAAAACAAAGCAAATGA
AGAGAACAAGTTCCAGCCACAAACAGCATTATGAAAGAAATTGCAGACAACGACGAAAAG
GCCAATGGATAGTTGAATACTATATACCTAGACAGTCATCCTGAGGCCTTATGGTGCAAG
TTCATCAAGGTAACGATCCATGATGACTTCAACATATTCAAGAAACACTTTATCACTTGA
TCCTGGGAGCACAGCTTCCTGTGCTTCTTGAACAATCCGCTCAATCACGGACTCATCCTT
CAGTGTTTTCCTGCACATGATGCTTCCGATGGTGAAGGGAGTAAATCCTCTCTTTGTACC
TTTCTTTAGAAGCATTGTGGAGATTCGAAGGATCCAGGCACACTCAGGTGGTATGTCCCA
GCCGTGAAACTTCAGAAGTTCAATATCCCGTTCAGCATCCAGTGAGTTTATATAGTCAAT
GGTTTCAGGGGAGTAAGGTTGGTGAGCTTGTGGCCAGTAGAGCCAGTCAAATGTACAATC
TTCAAAATTCTCAGGCAAGCAGTAGCCATGATCAATTGGAATAAGCCTAATTCGACCCTC
TTCATCTTTGCTGACCAATATATTCCCAGCATGCCTATCTGCATTTGCCAACCTTATGTC
CAGAACAGAGATCTTGTGTACCTCATCCACTGGAAAAGCACGAGGTCCCATATCCTCACA
ACTTCCTATATTCTTCATGAACATTTGCAGTGATCCTATCTTCACATTCTTGGCAGAATG
TTCATAACCCTCTGGATGATTGAATCCTTTGTGCAAGCACTTAACCATAACTGTGGGGGG
AACCCCGGCAAAGCCTCTCTCCTTGCCATCAGAAGAGTCCAATGCACCCTTTGGGTAATC
CAATATGTATGCTGCAACTTCCCTCAATGCTCCTTCTCCGACTCTTGTGCCTTTCTTCAA
TCCTTCACCATCTTCTGACAGGGGTAAGCCTCGAGGATTATTAACAGCCATTGGTTCCTC
ATCAATTGGTTTAAAAACAGAAATATACTTCTGACCTGATGAATCTTGCATAAAATAAGC
TCCTCCTGATCCCTCAGAGGAGCGAACAGGTTCATATCCCCTGTCTAACCCATCAAAAGT
AGATTCAATTAGCTTTTTAATTTCTATTGGCAAGTCAATATTGGAATCAACAACTAAAGG
TTCCAAAAGGAGATCTCTCTGCAACAATTTCCTATCTGTGTCCTCATGTCCCTTGAATAG
TGTTTCCAACTGGTATTCTCCTTCAACATCAGCCACATTCGCATTCAAATTATCAGCATC
AATGGACACTTCAAAATCTTTCTCAATAGGCTTAGCCCTTACTTTAGCAGACTTTCGAAC
CAGAAAGCGGACAACAGAATCATTGTTTTTACATAAATCAGTAATCAGTCTCTTATCTTC
AAGCTCCTCACCATCGCATATGAGTTCTTGAACCTTGAGATCAATGAGCCCTTTTCCCTT
TTTAGCAACTTGCTGCTTCACATAACCTACATTTCTACTCCTTTCAACATGAAATTCAAA
TACCTTTCCACATACAGTTGTCACAGTGATGGCCTGGAGATCTGAAAGTCTAAGCACCAA

ATGCAAAACATTCCCATCAGCAAGCCCATAGTCTTTGACAAGAGAATTGGTCCTAGCCAG
TTCCCTCCCTCCATACACCAGTTTCTGCTTGTTCACAAAAAGCCCTTTGAAACTCCGAAT
CCTCATTTTCACAGAAGCAATTGAATCTGACTCCATAACACGCATGGGGATCACAGAACC
CCCAACACTCAGAAAAATCAGGATCGAGTCATCCGCAGGCAGACCAAGTCGATGGGTGAA
ATAGCCCGGAAAGTTCAAAGACTCTTCAAGAACCGGACTAAGAGCTACACTGGCAATAGA
CATTTTTATCTTGGAAATCTAGAACCCTACGTACCTTATATCAACTTAAAACTTCTATGT
ACAACTCCT
>tig_1119_consensus
GGAGGGAGCGTTAGTTTACATGCGCCACCGAGCTGCACGGAGAACTCACCAGAGGTCTGG
TTCACAGAGTAGGTTTGAACCGATGAGGGAAGCAGCCCGATCGGGAAGCCGTAGTTTGTT
AATTCCGCATGGGCAAGGGTTGGAGGATGGGGATTCGAATACGAGAGACGCGTTGAGAGG
AAGAGGAGGAGGAGAGAGAAGAGCAAGAGAGCTTTGGCCATTGTCGTGTT
>tig_1120_consensus
GGAGGGAGCGTTAGTTTACATGCACCACCGAGCTGCACGGAGAACTCGCCAGAGGTCTGG
TTCACAGAGTAGGTTTGAACCGATGAGGGAAGCAGCCCGATCGGGAAGCCGTAGTTTGTT
AATTCCGCGTGGGCAAGGGTTGGAGGATCGGGATTCGAATACGAGAGACGCGTTGAGAGG
AAGAGGAGGAGGAGAGAGAAGAGCAACAGAGCTTTGGCCATTGTCGTGTTATCTCGAAGG
AAACGCTGTTCAATTACGTGACAATATTGTACAACTGTGACGGTTTGACAATTGACTTAG
GAAGAAGAGAAATA
>tig_1121_consensus
CCCATTTCAGAAATTGATATCCTTAACGTGAAACATATTAAGTAATTCTAATTCTTGCCT
CGGTTCTATCCTCATTGCTAGTAATAATCAAAAGAGCAAACTATTCATCGATATTCCATG
CCACACAATTGTCTAGTCCACAACCAAGGTTTACGTCATTTACAACACCCAAAATCTCAC
AGTAAATAGCTAAAAACTCAATACATATCTATTAAGAAGATTTCACTACTGAATACAAAC
TCACAGGCTGAAACCAACTCCAAAACAGAGAACCTATGCGGCATATTATACAGGTTTCCA
AACTGTAAGCACTTCTTGGAACATCTCCAGCGTCAGTTCACGGTCTGCTTGCCTGAAAAA
ATTATTGATTTCATCTCTTACATCGTATATCTTGCCAAAGTCAAACAAATACATCATGCA
TCCCTTCTTCAATTTGTTCAACTGGTATAGCCAGGCTTCCTGCAGCCTCTCAAGGACATT
CGAAGAGTTGATGTCTTCCATGAGACTATCCTCACAAGCATCTTTTAAGTCAGTAATGTC
ATATTTGTTCGCTGCCCCCAGAAGTGACAGCCTGTGCTTCCAGAAATCCTCTTGTTTTAT
GGTTCCATACAAGTAACTGAGAAGGGCCATACAAGATTCTACAGACATGTCTTCTATGTT
AATTATGGAGGATTCTTTTTCCTTGAGATCATGATGGAACATGCTCTGAAAAACAGGAGA
ACTCGCAGCAAGAACTGCCTTGTGAGCTCTTAGAGTGCCATCAGCAACGTGGATGGCAAC
ATCTGCATGGATGCCTTCATCAAGCATGCGTGAGAGACATCGAAGAGTGCTTTGGGCTGA
GACCGACTGCATCAGTCCATCACAAGGCCATATAGAACTGGGCAACCCACCATTCAGAGG
ACAGACTTTCAGGTCCAGAAACTCAACATCAATGATGAAGCGACCCTGGAAAGTAGTATC
AACAGGCCAGACAAAGTCTTCACAAGTCCGAAGCAATCTCTCATGAACTGGAGAGATGTA
TGGTTTGCGGTTAGCCCCAGCTATAGAAACTCGAAGAACGAAACTAGCAATAGGAGGCTG
TTCTTTCGACACACGGGATGGTTCTGGAAATAGTCGTACATACAGATAACGATTTTTCTC
CACGGATAAGTACCAGTTCCAAATACCGACCTTGAAAGCTTCGGATTTTTTGTAAGAGCA
AGGTCCGAAGTTTTCGATTCTCCATTGTGCTAATCTCGAGATTGTCTCCACTCGAGAATC
CCCCATTTGTTTGCGTTTTTTTCTGAGTTGCTCTTCTTGTATATCTGCGGAGTTCTCTAC
TGTGGGAAGAGAGGAAGGGGCAGGAAGGGGAGAGCTTGGCGTTGAAGTTGTTTGACGCAT
TATTGACGTAAAAGCGCC
>tig_1122_consensus
CGGGCTTTTAATAGAGATTTGAGGGAAATAAAAGGCAAGGGAGAAGCAACACAACACTTA
TTTTGGGAGATCTGTATGTATATTAATTGACTACATACACTATACAGAGTATGTAAACTG
AAAATGCTTCCACCTAAAATATTTTGAATGGCAAAACCGGAAAACAACAGCATATACAAA
CTTTTCCTTTTTCATCGGATTTACAGTCCATTGAACCGCTTGTGCAAGACTTGGCCTAGC
TGTATGTACAGGTCTTGCTGCTCCTCAAAAGTCAGATTTGTCAAAATCAGCTCCAAGATC
ATTTCAACAGAGAGGGTTTCAGGCACAACAAATGACTGATGATAGATGTATGCAAATACG
GCCATCACCATCTGGCAGTCCATTCTATCTGTGATTCCACCATGTCCAGGAATACTATCA
CCAAAATCCTTGATCTTAAAAGCTCGTTTAAAACCACTTGCAAAGAAGCCCCCAAAAGGC
GCTATTATAGATGCAAACAAACCAAGCCATAAGGCATGCCATTGAACTGGCAGAATAGAA
ATTTCTTTACCAGGAAACCATTGAGGAACCCATCCAGATAAACTAAAAGTCTGTGGCTTA
AACAATGGGCCTGGATCACAGTGCAGCCAACCCATTAACAAATCCTTTCGAGGACATGTT
AGCCATTGGAAATGACCAAGGATATTTGCAAG
>tig_1123_consensus

GCATATACAAACTTTTCCTTTTTCATCGGATTTACAGTCCATTGAACCGCTTGTGCAAGA
CTTGGCCTAGCTGTATGTACAGGTCTTGCTGCTCCTCAAAAGTCAGATTTGTCAAAATCT
GCCAAAGGAAAATCCATTAATAAGTCTTCTTTTAATTTATTTATGCACTAAAATTGAAAT
AACACAATTGAAGCTCAAGAGGAGAAATCACCAGCTCCAAGATCATTTCAACAGAGAGGG
TTTCAGGCACAACAAATGACTGATGATAGATGTATGCAAATACGGCCATCACCAT
>tig_1124_consensus
ATTTCATGGTTTGCGTACGGTCACACCAACACTAAAGACAAAGAAAATGGTAAACGTAAA
TCTTCTTCAAATAAGAAACTGATTTTTGGTTGCCGCGTTTGTTGTTCATTTAAGACATGA
ACGTAGAGGTGGAGCCTCAAAAATTGTGTCGGTTGATTGGTTGGTATAGGGACACCGCGA
CAAAACACACATACCAATATCATCGACCAACTCTTGATTCTTGACGCTCTTTCTCAAGAC
CGGTCATAGTGCCCCCCTTCATCATGCTTCAGAAACAGAATCCGGAATGATCTTACCAAC
CATGTTAAAGTGACATACTCTGATTGTTTTGTAGGTAGATTTTCGGATGCTGAAGTTACA
TTTTGCA
>tig_1125_consensus
GTTGTTGATGTGAAAAAGAAGATTGAAACAGTTCAGGGACCAGATGTTTATCCTGCTGCA
CAGCAGATGCTTATTTTTCAGGGGAAAGTCCTCAAAGATGACACTACACTGGAAGGGAAC
AAAGTGACTGAAAATAGTTTTGTTGTGATTATGGTAACTAAGACTAAGAAAACATCTGGT
GAGGGTTCGACTACATCAACTGAGCCTACAGCTAAGTCTCCTCAAACAAGTACTCCAACA
CCAGCACCAGCGACAGCAGTAGCTCCTGAGACTCCTGCACCAACATTAGAACCGCCTCTA
TCTGCTCCTGCCCCAGTCACATCAGTTACTGCTACGGAATAACCTTGAGGGAGCAATCCA
GCAAATTCTTGATATGGGTGGAGGTACTTGGGATAGAGATACTGTTGTCCGTGCTCTGCG
TGCTGCTTACAATAACCCGGAGAGAGCTGTTGAATATTTGTATTCTGGTATTCCTGAACA
AGCTGAAGCTCCAGCTGTGGCCCGTGTCCCTGTTAGTGGACAAGCTACAAACTCGCCAAC
ACAGCCAACACAACCAGCAACTGCTCTTGCTAGTGGACCCAATGCAAACCCTTTAGACCT
CTTTCCACAGGGCCTTCCCAACATGGGCTCAGGTGCAGCTGGTGTAGGAACTCTTGAATT
TTTACGTAATAGTCCACAGTTTCAAGTCTTGCGAGCTATGGTGCAGGCCAACCCACATTT
ATTGCAGCCCATGCTTCAAGAGTTGGGGAAACAAAATCCTGCATTAGTGAGACTTATTCA
GGAGCATCAGGCTGACTTCCTACGGCTTGTCAATGAACCTGTAGAAGGTGGTGAGGGGAA
TATTGGGGGACAGCTTTCTGGGGCAATTCCCCAGGCAGTAACCGTCACGCCTGAAGAGCG
TGAAGCAATAGAACGGCTTGAGGCAATGGGCTTTGATCGGGCAACAGTGTTGGAAGTGTT
CTTTGCCTGCAACAAGAACGAGGAACTGGCGGCCAATTATCTTCTTGATCACATGCACGA
GTTTGAGGATTGATTTAAGGTGATGAGGATTGTCACTCCCTTCTGTGTGCTCCTTTGGAA
TCTTTTACCTTTACATTTATTTCTTTCCACATTTTTTCTGTTTCCTCTCTCCTTGACTTC
ATTTGCTAATCAAGTTTGGCGTTTACAAAAATGGAGTGGACAAGTAATGCAAAATGTAAT
ATTCTTCAGTTTCTTCCTGTAGGGGACTATAAAAATGCCTGTA
>tig_1126_consensus
AAAAAAAAAAAAAAAAACATTATGGACAACTGAGACTGGCACCAGTAAGTGCGTAATCCG
TGTCAACATAACATAATAGAACAAGAATTCTCATATCTACCTCGTTGGACAAAAGTCACC
ACAAATTGCATTCTTCAAACCATGTCAAAGTCTCTAGGAACAGGAGCTCAACGAAAATCC
CAACTGCTTCACCAAAGTTTCATTATATTTCACTAAAATAATAGACCAAAAATTTCCTCA
GTACAGTAGGCAGAACCCCTTTAAGCAATTAAATGATTCCTGTATATGGATATCGCAACT
GATAAATTTCTTGTATGTACCATGCAGTTCACAACTCAAACAGCACTCTCAAAAGACACC
TGGTTTTGTTCAAGATACCTAGGCTTTGAATTTGTGGCATCTGAAAGACCCTTAAGCATT
CTAACAACATCAACAGCATCATCAAGATAATACTTGGCCTTGCTCGGTTTTCGCCCAATT
GTGCACGCAAAGATCTCAGGAGCTACAGGCAACGATTGCGTAGAGAGGGTATTCAGTATG
CTCTCAAACATATCTTCATCAGATCTATCATCTCCAACACACATCACAAAATCTGGTGGC
CTCCCACTATTGACCATTTGCAGAAGAACCTTTTCAGCAACCAACCCTTTGTTCACACCC
TGTGGCTTAACTTCAACAATATGTTGACCCCTCTTAACAACTGCAGGTTCATTCGCCAGA
ACATTTTCCAGGTGATCCAGCATTTCCTTCGCTTGGCAGGACCCAAAGTCGGGGTCTGCA
TCTTGATATTGCCAAACCACAGCACTCTCTTTAGTCTCTATACTAGAGCCATCAGTTGCC
TCTGTATACAATTTCATGATAGGTTCCACAATCCCAGTCCATTCAAGGTCAGCACACGGG
TGACTGGTTTCCCAATCAGATGCTTTATTCCACCTTATGAAGTATCCATGTTCAGCGGCT
ATTCCCAGCTTCTCACATGGTGCAAGCCAATCACTGAGAGTTTTTCTCCCTCTCCCACTA
ACAATAAACACAGTGTTCTTTGGATCACTGCAAAGATTTTTCAGGATATCTATGACTTCA
GGACTGGGGGACTTAATGATAGAAGTTTCAGGAACAACGGTACCATCATAGTCCAGAAAT
ATTGTCCTTCTATTTGTCCTTTTATATGCTGACACTATATGATCAGTAGACAACCTCCTA
AAACTAGGAGATAAAGATACCACTCTGAACCTCAGGCCCCAACCAATCCCCCAGCACCGT
TTACTATAATGATCACGGCATGCTCTCTCCAAATCTTGCGCAAAGCTGCGTGCCCAATAA

GCCACATCGTGAGTACTGACATACCTATAGTGCTTCTCATGCCGCAATTGCTTCTCAGAA
TCAGACAAGCACACAGCCAGATGTAAGGCATCACCCACAGCCTCAATGTCCCATGGGTTG
ACCCTAATAGCTCCACTTAAAGAAGGTGAGCAACCAATGAACTCAGACACAACAAGCATG
CTTGTACGAAGAGAGTCTGAATTTCTACCAATAGCTGCATCCAAACTTGGAGTTCCCTCC
CTGCAGACAATGTACTTATATGGAACTAAGTTCATCCCATCCCTTACGGCATTTACTATG
CAGCAGTCAGCTATAGCATAATAGGCAGACTTTTCGTAACGAGGAACATGGCGATCAATC
AGAATCACTGGTTGATAATCAGGTGAACCATAAACCTCATTGATTCTTTTAGCTGTTAAA
TAGGTTTCTCTCCTTGCCTCCTGGACATCTTTCCCTGAACCCCTCGCAGGATTAACAATT
TGAACCAATACTATATTGCCTTGCATTTTGGTATGTAGCTGTAATAGATGTTCAAGAGCA
AGCAGTTTCAGACTAATACCCTTAAATATATCCATATCATCAATACCAACAATCACGCTT
TTCCCATCAAATTGTTTTTGAAGCTCTTGAACTTTAGCAGAAGTGGAAGGAAGATCCAAA
ACAGATTCCAGCCGTCCCATATGAACACCTACCGGCAGTATTTTGATATATACTGTCCGG
CCAAAATAATCAAGTCCAATATGTCCACGCTTAGATTCATAGTCCAGGCCCAGCATCCTA
CTGCAGCAGGATAAGAAATGGCGAGCATAATCAAATGTATGAAAACCAATAAGATCACAA
TTCAGTAGGCCCCTAAGGATTTCATCTCGAACAGGTAAGGTTCTATATATTTCTGAAGAA
GGAAATGGGCTGTGGAGGAAGAATCCAAGCTTAATTCTGTTGAACCGTTTTCTCAAAAAT
GTAGGGAGAACCATCAAGTGATAATCATGAACCCACACATAATCATCATCTGGATTAATT
ACTTCCATTACCTTGTCAGCAAATAATTTGTTAGCGGAAACATAGGCCTGCCAAAGTATG
CGGTCAAAACGATCACCATGGTCTGGGCACATGGGCAGCATGTAGTGAAAAAGTGGCCAC
AACTGCTGTTTACAGAATCCAAGATAGAACTTCTTCTGCATTTCAGGAGGCAAAAACGTT
GGCACACAATTGAAATCCTCCAGCAGCTTCTGAGCAACTTCTTCCTGTTCCCTCTGATCT
ATGTCAACCTTGAGAGACCCCACATAAATAAACTCAGTCTCCGAGGAAAATCCATCCTTC
AGGTGTAACAAAAGTGAATCCTCGTCTAAGCTGAAGCACCATCTGCCAGATTCTGTATCC
CTTTTAGCATGCAATGGTAACATATTTGCCACTATAATCTTCCTCTCATGACTATTAGAT
GAGCTAACATCTGAATCCTCATCATTATTGCCATAACCATCCACGTCAGAGATAATTCCA
GGAACAGTCATCACCCGAGGAAGACCTCTTGGAGTTTGAGGAAAATCCAAGAGATCTCCA
GAAGCCAAGTCCAAAAAATTTACAGATGCTCTTGATGCCATTACCGCGTAAAGAGATTAA
TCCTACTAAATGATACAGAAGAGTACCTTCCCTAGTGGAAAATCTTCAAATGGTGAAGCC
CACAGATCTGAACAACCAAAATGAATTTCAACAATATGATTAGATCATCAGAATTCAATC
AGAACACTATAACCCCATGCTTCCAGTAGCACTGCCTTTAGAGGCTCCGGAGGGCAGAGA
TGGCGGTTAAATCGAAAGCAGTCAAGTTCTGTTAAGTCGCGGCGACAGAGAAGAGAAGAG
AAGAGAAGGAGAGAGATACACAGAGCCGCCTGTCAGATTTTTTATTTTCT
>tig_1127_consensus
ACCAAGGCGAACTCCACTCTTTCACTACACGCCGCTCTTCCGATATCCACAAAGTCGGCG
TATCGAACTGTTTCTTGAAACCAGAGGAGAAAATCAAACCCTTCATCTGCCACAGATGGG
TCCACATCTTCCAGGCTATTAGCATTGGCAGTCGCCTTACTTCCAGCATCGGCAGTCGTC
TTACTTCCAGCATCGGCAGAAGCCTTACTTCCAGCCAGGCCTGGATGATAAACAAAGGTG
ATACCAGGCTTCTTGACATAGGACAACAGAACAGAAGTGTTATGGTCAACAAAATAGACA
TTGAAATCCTTGCGATCCACAGGATACGATGGCAAGAACCAATTTGTAGCTCGGGAGTTC
AGGTGCTCCCCTGCCCCAATATCAATATACACTAATCGCTTCCTAGAGGCAACATCCATA
AACTTGGGCAAATAAACAATCCTTTTCTCAAACCCCATTGGTTTCTCCTCCGTGAGAGGC
TCAATTAACTCCATAAATGGTTTGTTGTTCATAAGAGATGGGCAATCTGATGGAAGGCCA
TACTGCTCAAAGTAAGAGCAATTTTCAATTTCCTTCTTGAAAACAACCAGAGTAAACTGA
TTCACATAACCAACATGGACAATAGTGGAAGTTTTTAGAATTGAGGAAATTGGTGTTGCA
GACCTAATTAAACCATTTGGGCTCGAACTACTAACACCCACAAGCATCGCACCAGTTCCA
CCTGGCCTAAGAACACGCTCAATCTCGAGCACTAAAAGAGCAGGGACAGAAACCTTATCT
AAATCCCTGGAGTAAACAAAATCAAAGGAATTGTCCTCATAATCAAGCTCATACACGAGC
TTCTTATGCTTAAGCGAGAAAAAATGTGCGTCTGTAAACCCCATAAGTATGGCTAAACCCC
ATTTCTCGCAATGCATGCACTGACGAGGACGAACCTTCACCAACACAGAGAGTTTTTGCA
TCATTGTTCAACATTTGCATACCCACCAACTCTCTAGCTACAATGGTAGTCAGATTTGCA
TTTTCCTTGCACTGCATTGACCCAAGGGAACCCAGGATGGGATAGATAAACCGACCCAGA
AACACATGCGGGTTTGAATAATCATAAATGGAAGAACAATCATTAAAGCCCACCACATCG
AATATAATGGGATCGGAGCCAGATAAGATTTGCAGGAAAGGAACGACTGACATAGCCGTG
CAGAACAAAACGGCGCGCAACAGCACACGCCTCGTAACAGACCCATTACGGATCTTGAGT
TTCAAGGATTTCAAATCCATCAAGTTTCAATTTTTGACTCAACAAACAGGAGCAATAACA
GATCTTTAATTAAGAATGAGTAACAAAGAGCAATGTGATTAATAATATTCAAGAGAGAAT
GAAAAGCCAGATTGAGATTGAGGAAGAAACTTAGCGAGAACAAAACCTGGAGGCAGTGTG
ATTCCCACCCACGAGGACTCCCATTGATTTTTGCACCTTTAACCAGACCCCTCTCGGAAC

CCTCTTTGCCCTTAATTTGAACCACATTTCATCCATTCTTTCCCAAACCATGAACCAAAA
CTTNNTACATCTTCGGTATCGAATTTCAGTCAATCAAAATGGGTTGTGATTCAAACAAAC
AGAAAACAAAAACCCCAAATGTGATACACCAGAAGATAGGGTTTCAGTCAAGTGAAGTGA
AGTGAAGATGAGGAGTAGGGAC
>tig_1128_consensus
CTCCACAATCTCATATATCTTCCTCAAAAAAGGCGGGGGACCTACCTCATTCAGCCCTTG
CATTGGCTTTGCCAAACTCATCGATGAAGTCGAAGAAAACGACGATCCATTTGAATCAAT
GTCACCGCCGTCAATATCATCACAAACGCCGTTTGGAAAATCATGATCTTCCTCCTCTTC
TTTGACTGTCACCGTCTTTTCATCTCCATCATCAATTAGAATTTTAAAAGACGAGAAAGG
GTTGTCGCCGTTAGGCACTACCATTTG
>tig_1129_consensus
AACAATCGAGTCGGTCTCCGGATTCTCCACAAACTCATATATCTTCCTCAAAAAAGGCGG
AGACCTACCTCATTCAGCCCTTGCATTGGCTTTGGCAAACTCATCGATGAAGTCGAAGAA
AACGACGATCCATTTGAATCAATGTCACCGCCGTCAACATCATCACAAACGCCGTTTGGA
AAATCATGATCTTCCTTCTCTTCTTTGACTGTCACCGTCTTTTCATCTCCATCATCATTT
AGAATTTTAAAAGACGAGAAAGGGTTCTCGCCGTTAGGCACTACCATTTG
>tig_1130_consensus
ACCTCGTGATATTGACTACAGAAATTCATTATTTTTGTATCATAGTGAAGACAATGGATA
AGAAAGAATTGGACCATGTTCAACGCCAATTTCAATCGAGTCTCCACCTTGTTAAAATTT
TCTCTTAACACGCACACATATACACGGCTAAACCTGAGGTACTGCAAGCCCCAGCGTGGG
CATCTAGTGTGCACAAATCACCCCTCCTGCCACGTGCTGCTGTAAATAACACACAAAGAA
ATAGCTGGATATGACAAGGTCAATTCAGCTCTTTCTACACCAGAGGAGGGGAAATAAATA
TCAAGCAGCATGATAGCTACTTTCTTTACCACCACATTCAAGTCCACACAAATTCCATAA
ACACAAATGCCAAACTACATTCCCTTTTTTCCTTGGTTTTCTTTTCTCTACAAACTACAA
AGGATTCTCAGCTTAAAAATTTTTAAGTCAAATAGAAATCCACAACTGGGACGGATAAC
TTCAACTTGTCATCATCAAGAATCCTTTGAATTTGATTAATGATCATCACATTGCCCTTT
TTTCGTTCCTTGGGATCACCTTTAAGGGCAACCCCCGTTTGGGTACAGGAAATGGCCCAT
ATTGTATCCCATCTTCGCCTCCCACAACTTGCCACCTGTAATGAGTAGTGAGATGGTGAA
GAAGAATGTGCATTTCAAGCTTAGCCAGCTCACTGCCTGGACAAGAGTGCACTCCATTGC
CAAATGGCAGGTATGTGTTTGGTCTGGGTGGCACCTCGAATCTTGAAGGGTCAAATTTTT
CGGGTTGAGGGAAGAGCTCAGCGCAATGATGAATGGTTCTGAAGAGAGGGAGAACTTTCC
AACCTCGAGGGATAAAGTATCCTTCAAACTCAACGTCTTCCACTGCTTCTCTGAATGTGA
ATGAGAGTATGCTTGCTGTTCTTAAAGTCTCTTGAATCACCCGGGTAGTCAAAGGCATGT
GCCTAGTATCCTCCCATGTAAGCCCGCGATTTGCTTCTATTATTTTGCGTCGAATAGCTT
CTTGTTCCCTCGTAACAGCTTCTAGTAAATCTCCATTATCATGCAGGTACTTTAAGATCC
ATGTTAAGACACTTGCAGTTGTGTCATGAGCAGCAAAGATAACTCCAATAATATTGTCAG
CAAT
>tig_1131_consensus
AACTAGAAAGTATAAACTATGCATAAGACTGCTTTCCCTTCAGACAACAATGCCATTATT
AAATCAATTGTAATGACACTTGGGACAGTATTTCTTATTAGTCATTTCTTTTGTGACCAC
CAAAATTTGTTGACTCGAAACATGAGATTGAAACATTCTTGAAAAATTTAAAGGCTGGAT
ACAATTTCATTAACCAAATTTCGGATTTCCATTGCCGGGTATTGAAGGCCATAGAACCTT
GTGAATCTAAAGATAATAGTTTTTGACCAACTATCCATCATAAATAAGAAATTGCTTTAA
AATTACTTGTTCTCATCTTGTGAAAGAGAACAACCAAAACTCACCATATATCCACATGAA
CCATCAATAAATGAAGCACCCAATGAATTACACAGTTGAGCCAAAGTTCACAGAAAGTTA
AGATGGCAATGCAACAACAACATCTGAAACACAAACTCTCACCCTAATTGTGGTAAAATC
CCCTAAATTTCTCATGAAAACACAATTCTATAAATATGACTCCTTGCTTAGCGACCTAGC
TGTCTGGGAAGACATGATTTTCAGTGACCTAGTTGTCCCGGAAGAGACACAAATGTGAAC
TAACTAGCTCTCTTCATGCTGTTGTACAGTTCTTGGTTCTATAGACTTAACACACTAAGC
TCCATTTGCTCAATCAAAGCTAGCATTCAAGAATTTCAGCAACTCATTTCTTTTATTTTC
TGCAGCTTTATCCTCCTTTGATTTATCAAGTTTTAATAGAATCTTTTTTCCAGAGACAGC
ACTTCGGCGGACTCCATCATCTTTGTCACGCCCTAAAAACTTTGATAGCTGTACAGGGCC
ACTATTTCCCTCATCCTGCTGCTTCTATATATGGCTCCCATTGCCAAGACAAACCAGACC
ACCCAGAAACAAAATCCAGGCAAGTCAATAGCACCCAGTCATCATGCAATAGACCCCAGC
TCCTCTTTAACTTTATCCTTATGCCGTTTGTGGTGATGACTCTTGCTCTTGCCCTTTTCT
TTAGTCTTCTTTGCTCTGTCCCTCTTCTTCCGCCTCCTTGTGTGACTTTCATGCTCACTA
TCAGAACTCTCACTCCTGGAGGAATTAGATTGGGAGGAATCTGACTCTGAAGAGTAATTG
TGGTGTCTGCGACGTCGTTTTGCGTGTGATCGAGACTTTTTACGGATCTTAAGAGATTCC

CTATCTCTATCTTTTTCGCGGCCGATACTTCGGCGGCGGCGACGACGGTGGGATGAGGAT
GACGTGTCAGAGGACGCGTAATCGGAGGATTCGGAGGACACAGAGGTCGACGAAGCCGCC
ATTCTATAAGACAAAACAGAGGAGGGTTTGGGGAGAGGCAAGACAATACGAGAGAGTTTG
TGTTTTACAAGGAATGGCAGAAAAACGCTGAATAAATCC
>tig_1132_consensus
GGAATTACTCCAACAGTAGAAACAGCACCAATTGAAGGCCGAAACCCGTAGATTCCACAA
TATGCTGCAGGTACTCTTACACTTCCTCCAGTGTCAGTTCCCAAGGAAAAATCTACAAGC
TTTGCACCAACTGCAACAGCTGATCCACTAGAAGATCCTCCAGGTATCCGGTCACGTGCA
CAAGGATTTCTCGGCGTTCCATAATGTATATTTTCTCC
>tig_1133_consensus
GGAGAAAATATACATTATGGAACACCGAGAAATCCTTGTGCACCTGACCGGATACCTGGA
GGATCTTCTAGTGGATCAGCTGTTGCGGTTGGTGCAAAGCTTGTAGATTTTTCCTTGGGA
ACTGACACTGGAGGAAGTGTAAGAGTACCTGCAGCATATTGTGGAATCTACGGGTTTCGG
CCTTCAATTGGTGCCGTTTCTACTGTTGGAGTAATTCC
>tig_1134_consensus
GCTAACGATGCAGTGAAATATCACCACTGCTCACATTTCATTTGAAACGATAAAATGGTT
TTTACAATTGGTTGGTGTAATCACATAATTTTTGTGCACCTAACTAAGCAAGACAAGCTC
CCTAGAGGTAATTAATAGGGTTTAATTGGCGCTTCGATTTCAAGCACATTCTAAAATTGC
TTAAGATCAATTAGTACGCCAATTTGCAGAAAAAAAAAAAAAAAAAAGGTTAAATAATGTT
TTTTCCCAACATGGTGAAAAAAAAAAAAAAAAAATCAGACAAGAAAACCATAAGAGCCGTG
CACATAACACCTAAATAAAATGACCTGCCATTCTAGTTAACTACAGGGAAGAAAATAGTT
CTCCAGTAGGTTTCTCTGCTGGGCATAATATTTGGATTCCTATCATGAGCTCAAAATCAA
GGCAGCCAAAGAACCTTCTTTAGGCTAAGGAAAATTAATGATAGAAGCCAATTGCACTCC
TAACATCAACTCAGCCCAAGAAGGGAGGAATGAGCATGACAAACCAAGTTAATCAAGGAC
AGCCCAATTCGCCGAAGCCATTCACCTCTCTCATTCTCCCCTCCAATGTATCAATTCACT
ACGAGGTGCATATAACTTTGCCACCTGCTCTTCCGCTTCAGTATCATTAACTATGCCCCG
ACACCTTGGAAAGCCGCAATAGCATGATAGTTGTTCATCGATTGAAAAGAACCTATAATC
ATAAGTCAGCTCTTCCCATTGCTTGATATCTCTTTTAGCAAATATAATAATGTGCTCATC
ACCATTAACACTTATAACTCTGGAATAGCAATTTGGTTCACACGAGTGATTAATTAGATG
TGCAATGCTCCCAGCCCTTGTAGCATCAATGACTCGTTCATCATCAATCCGAAACATGTA
AGTGCCAGCACCCACCAACGAATTATAAATGAAGTGTTCTCTCCGATCGGCAATAGGAGG
TCGAACAAGTTCTCCAGTGTATTCAATCACCATGTCTCCTGCTCTATGTGGATGCTTTGC
AAATATGCCAAACCCATGAATAGCTGATTTCCCAAACGCCAATCTCTTTCTGAAAGTCTC
CCTCATGTATTTGTATTTCTCAGCCATGGAAAGAATATTGTTATGAGCATCCCTATGAAT
GCTGAAAGAGAATTTGGAGCCAATGACTCCAATAGAAGGCAATATGTTTTCTGACAATCC
ATGTTGGCAGTAGCGACCAACCAGGTATGGCTGATTCTCTACAAACAAACGCTTTAATGA
TGCAGCGGCAAGAGCTTCAGGTTCTTTCCGCCCTCTTCTTCCAACATTATTGTAAGGCTC
TGTCAGTATCTCAC
>tig_1135_consensus
GGCCAAAAAGAGGGATATGCATTCATCAATTTCAATATAAAATATAAATGCCAATTCCCA
TATATCAATTACAGTAGTATACTTCCGTAAATCCCTTACTATCAAAATTTAATTTACAGG
TGCTGCAACAGAAAGGAAAATTCAGTAAATCCCACCTCCTCCCTTTGACCTCTTAAATTT
CTATAAGATCTTGAGGTATTAATAAGTCAAGCCAAAAAGGCAAAGAAAGTAAAATAGTTC
ACTACCTATGAGAAATCACAGGGAAAATGAATTTAAAACAACATGAGTAACAATAACGGC
ACCTGTCACATTCATAACTTTTAAAGGTCAGCAACACGTACTCCCACCAAGGACTTCTAA
GATGCATACTATATTTACACTAATATCTGGCCAGAGCTTCAGAGCAACCAAGTTTCGTCA
TGAGTTGTTTAGCTAGATCAGTAACTAGGATTCCAATTGACCTGTGCAACCTGTGAATGC
TGATAAGCACCAGATGGGAGTGCCATAGTTTCAAAAGCTGCAGCCACAGCTTGAGATTGC
TGCAGTAATGGATTAACATTTGAAGGGGCAGCTAATGTCTGTGCTGGTTGATAAATTCCA
GGAAAGGCATTATGACTAGTCTGTGCTGGGGCAAATGCAATGTGCTGTCCCTGGAGGGAA
AGATTGTATAGAGGATTGATCTGCATGTTGGATAAATCATGTCCTGCCGTAGGTATCCAC
ATACCTGAACCTTCACTCAGTGGTCTTGTTGTGTAGACTTGATTATCTTTCAATTGAGAG
GTAAGATCCTCATTATCGGTAGACTTTACAGGGGCTGCCACAGTAATGGGATTGAAACCA
ACAGGAGAAGAGCCATATGAACCATAAGCAGCTGCAATTCCCATATGAGCTGAGTTTCCT
GCATTACTTCCTGGCTTGAACTGTGAAAGAGGAAACTTGACTCCAGGAGCAGTGGCAGGA
GCTGCTGCTGACGATAAATATACGTTACCAGCAGAAGGCTGCTGTGGAAGCCCGTTGGGG
CTCAAGAACTGATGTATTGGTGGTATATAATAAGGTGTTAGGTAGTGACCATATGGGAGG
AAGCTTGGATAAGGATGCCTGAAAAGATGAACAGGTTGTGGAGCAGCAACAGAGCTTTGC

ATGGACAGGGCTGGACTTGAGCTAGGGGGCACAGAAACTCCATTCTGAGTCTCAGGTACT
TCCACTTGTATAGGCTGGCTCACAGCTGAGGGGAACATTAAACCAAGACCATAGTTTGAA
ACGCTTTGAATAGGAGGTTTCTCTTGGCCTTCTGGAAGCAAGGGCATCTCCTGCTTCAAC
TCATGCTTCAGGTTTGCAACACTTGGGATATTGCCAACTGTTGGAGGTACATTATCCACA
TACAGAGTAGACTGTGTATGATCAAGAGTTTCCCCTTGAGTAGTTGGGGATATGCTTTGA
CTTCTGGGAGAAGGCTCCCCAGCAGCTTCAGCACCGGCAGAAGATGATTCAGAGGGAGAC
ATAGAGTTAATCTCACCATCAGTGTCATTAACTTCCTTCGATATCGATCCAGAACAAGTA
TCAAAGCTTCCAAAAGTCAACCAATTTTTCACTGCTGCTGAGACTTTGAAGTGACTAGGA
AAAGTAACATGTTGACCATCAGAAGGAGCTGACTCAGGATGCACCTGAGAATCGGCTTTA
ACTTCAGAAATTGTATGAGACTCTAGCAGTGCTTGACTATCATCATCAGAAGAACAGATA
GCCAAAGAAGTGTCATGGACTAACAAGGAAGAAGACTCTAAAGGCTGAGACAGCTGATTC
TTTTCCACTTCTTCTGATTGGCTTTCATCCTTCTTTTTGTGGATAGCATCTGAAATGTTC
GAGGCTGTCTTTTCACTCTTGGATGATTCCGAATCCACATTAGAGGAAACATTATTGTCT
TCCTTAATATAGTTGGATTCAGCAGCTTTATAATGGCTCCCCACTTCATGCATAATTGTT
CCTACAGCAGCTGGATTCTGAGGCACTGGCGACACCAAAACAAGATCTGAGGCCAAGGAA
TGGAGTCCAGAAACAGATGCTTGACTGGCAGAGGTAGGAAGGTGATCAGAAGTCAATGAA
GACTTTTCGTGGTGAGCACTAATCAAGGAGGCAAAGGTGAGAGTAGGTAGAAGTGGTGCA
GTCTCCAGCTTTTTTGCGTCTATAGTAGTATTATCTTTACTTT
>tig_1136_consensus
ATATTTCCCAAACTTTCATCTCTTATGCCAATTTTCATCCCAAACCGTTCCACTCCACTC
CTAGCCGATCTTTGAAGCCAAAGTTGCAGCTGCTTATAAAGACTTACCGTGGTAAGTCTT
TAAAAAAGACAAGAAAGAACACATGTAGAGGAGATAGATCGAATTTACATTATTGACAAA
GAAAACTACACACAGGCAACTCCTACTTGGAAAGATCGTACTACAATTTATACAATTTAT
GCTAGTAAGCTGCAACTTTATGGGTCTCTAAACAGCCATTTTCCGGGCCTTCCTTGCCAC
TGGAGTCCTGATAGACTTCAGTTGCTTAGGCTTTCCCGGGGATTTGGTCTTCTTCAGGGA
AGCCTTCACTGCAACCTCAG
>tig_1137_consensus
CGTCCTTATAAATGGAAGCAAGGATAGAATCATTACAAACTGAACCAATCCTAGGATTAA
CTCAAGCTCAAATAATCAATCGATCGTTAAATTAACATGACCCTAATTTCAAAGCTCGTG
TCTACATCTAGCATCATGGAATAAAAGGCTCATTATTAACTCAAAGTTGTATGTTACAAT
AGTATCTTATATTTCCCAAACTTTCATCTCTCATGCCAATTTTCATCCCAAACCGTTCCA
CTCCACTCCTAGCCGATCTTTGAAGCCAAAGTTGCAGCAGCTTAGAAAGACTTCCCGTGG
TAAGTCTTTAAAAAAAACAAGAAAGAACACATGTAGAGGAGATAGATCGAATTTACATTA
TTGACAAAGAAACCTACACACAGGCAACTCCTACTTCGAAAGATCGTACTACAATTTATA
CAATTTATGCTAGTAAGCTGCAACTTTATGGGTCTCTAAACAGCCATTTTCCGGGCCTTC
CTTGCTACTGGAGTCCTGATAGACTTCAGTTGCTTAGGCTTTCCCGGGGATTTGGTCTTC
TTCATGGAAGCCTTCACTGCAACCTCAGCTTTTTTCACAGTTGCAGATCTTGTCTTCTCC
ATGATGATGATGGGTACTTTCTCCTTCACACGCACCTTCTCCTTTGTAACTACTCCTTCA
GCTAACATGTAAGACGCCCTGATCTTAATGAGCTTCCCCTTAGCGGCGGAGTTCTTCAAC
TGCAAACCCAGCATCTTCTTGAAGTTCGCCGGGAGCACTGCCTTGTGCTTCTCTTCAATC
CACTTGGCTATGGCGCGAGGGCTCGAACCACTCTTATCCTTCAGAGCCAAAAGAGCCTCC
TTTATCATCTCAAAGTAAGGAGGATGAGCAGCAGCTTTTGGTTTCTTTTCTTTAGGAGTC
TTGGGCTTATTCTCCTCCGGAGTTTTGGGCTTCTTCTCTTTGGGAGTCTTAGGCCTCCTC
TCCCTTGTAGCCATCTTTTCCGGCGGCTTGGCTTCGACTTCTTCAGCAGTCATGAAAGCT
TGACAAATGCTTCTTCTCCACTCTTGATATTTTACAGAAGTGGT
>tig_1138_consensus
ATCTATAATAAACTCATATTGAACTATTTTTTATTCAAACTAAATTGATATCATAAAGTT
TGAACCATGCCTGATTCATATAGTTTTTACATCAAATCCCCCAAACCTTAAACTAAAACT
CAGTACTCCTAACAGCATTGATGTTGGCCTATCAATTTAGTTCTATTTTAGTTCACAGCC
AGCCTTAAATAAAAAATAATCCCAAAAAACCATTTCTCACTGATATTTTAAGACATTACA
CAAACATTTTTAAGAAGGTGCAACCAGTTACCCACCCATCATAAATGCACTACTACATAT
TCCAAGCAGGCTATGATTTTCCCACCACAAAACACAAACATTTAACATATAAAATGCAAA
ATCTAGATGATTAAAATTTCTTTCTTTCTTTCTTCCCCGCAAAAGTAGAAGAAGAGACAT
AATGACACAGACAGTACAACTAACTCTTAGGACCAATGTCCTTCAGGGCACAGATCTGCT
CCTCACCCATGGCAGACATCACACTCACAACCAAATCCTTCCCTGCTTCGAACCCTTCCT
TGATCTGGCTCAGCAGGCTCTCATCCGTCGGGAGCCTAAGATCATCCTT
>tig_1139_consensus
GCTTTTTAGTCAAACATAAATGAAATAATCAAGAGGAAGAAACTTGATAATATACATCTG

TTAATAAGACAGATTCCAAAAATTAAAATGATTTTTTCCACTGGATTTTTGAAATCCTTT
ATAATCTAGCAGTTCCTGATTAACTTTCATACATAATTGAAAGTCAGGAAACTTGGTCCG
GTGAGGCCATAAAGATAGGCGAAACAAGAAAAGAGCAGGATTCGGATCTTACGTTTGACA
TGCAGAAAGACCAAAACAGAATCAGGGATTTATTGTATCTAATGAAGCACATCATGGGAA
AATTCTGCGTCGATGGCGGGGCGTCTCTCGCTTCACGTACATTTTTTCCATTTCATTCAA
AGGTCGGCTAGACCCATCTGGCATGCTAACAAACTTCCAGCCACCACCCCCTCCACGCTT
GCCTCGTGGCCCCAATTTCTCGACTGTGAATTGCCAACCTTCAGAAGGACGACGCCTGCT
ATATTTGTGGTGTTTCACCTTCCCTTCTATTTTCATTTTGTTGAAGTTCATTCTCTGGAC
AAAATCTTGATATAACATTTCATCCTCTTTCTGGGAGATCTCGTAATGAACTTCATCTAG
GTCTTTGATAGTACCATCCCAACCCTCTGGCATAACCTTAAAGTCAGGTTTATATGGAAG
GGATGCCACATGGAATTCTCGGTCGTGAGATATAAAGAATTCAGGACAGGTGTCAAGCAG
AAGCTCAACAGAATCATCCAATGGATGGCCAAGCTTTTTCTCCTCTTCCTCCTCAAGCTC
CTTTAGCCACAAAATTGCATGCACTCTCTGCCTCATAATCCTATAATCCTTGGCAAGCCT
CTCAACAGTATAAATCTCAGGATTCTGTTTGTGCAAGCGGTACATCTCTGCCTTCTCTGA
GTCCTTGAGATAAGACCCTCTTGCACCAGGCTCTCGTACTTGCTTCAGCAACATACATGT
CTCACGCATTCTCTCACCCCATCCCTCAACGAAAGCCTTGCTCTTTCTGTTCTCTGCCTC
CATTTCCCTTGTTGCATCTTCAATCTCTTGCATATTAGCGACCACTTTAAATTGATGATA
TGTATCCTGTTCACTACTGCTGGAACTAGCTTGATGTCCAACAGCCTCTCCATTAAAATG
CTCCTTGGTCAATCCAGTTGACCACATTGAACTTGTTTCCCAACTCAGATCATCAGAATT
GGTCCCACCAATTGCCTTCTCCCATTCATTTTTACCTTCACTCCCATCCTCACCACCACC
TGATTTCACAGAAAATGTTCGGGTTACAATACCCCGATTAGCCTCATATCCACCCTTTGA
AGCGATCTTCGCATCATTTCCCAATGTGGGCAAAGTTTTCTCCAAAATTCTAGTGGTGTT
TGTAACAGTAAAAAGGGAAGTAGATGACAAGCGAGATAAGACCCGGGTAAGATTATGCAT
ATTAGCAGTACTCAAAATCAGCCTTTTTTTCCAAAACCACACAAAAAGTACACAGTCCGG
AAAAAGAGGCGGTGACTGGCACAAAGAGCTTGGCGGAGGCTGACTTCGACCAGGGAGAGA
GTCGGCGGCCGCTCAAATGTTGTGATATTAGAAGAGCCAGTGACACCGGTTTAGGGGGTT
TAAGGGATTTTTTTC
>tig_1140_consensus
GGGAAAATTTATAACCGTAACATTAGCTGCTTAAGATAAGTGTACAAATGGTGAATCAAA
TCATTCTACTGGGACTAGTTTGATAAGTTTACAGATCCAATAGTCAAAAGTGCTTTTGTA
CAGGAGCTGGAGGAGGCCTACTGCTAGCTAGAAAAAATGCTTCTTCTACCAGGAATTGATA
AAGAAGAAAAAGAACTTCTTGAAGACCTCTTAGATATGGTGGCCTTTGGATCAAGAGTAT
ATGATTTATGACTATCCAATCCACCTCCATAACTTTTACTTGTCTTCATTTTCTTCCTGT
AAGGACTCTCCTTCTTTGGCAAGTCTTCTATGCTGTTCACTCTTGCTAGAGATGTAAAAG
ACTGAGACTTACCTTGGAAAAAATTTAGAAAGCCCTCTCTTGATGGGGAGTTGAGCCATGA
GGTCTGAGAACTCAAACAAAGGACCATTAGAAGCTGGAGAAGAGTTCGAAGAAGGTGGTG
AAGAAGAAGAAGCATCATCAACCAAGTCAGAGGAAGAGGTTGTTGATCCATGGGAATTAG
TTGTACTTGAATCTCCAAGGGAAGAAGAAGAAGAAGAAGAAGAAGAAGATGACATTTTCT
TGTAGGCATCTTGATGATTATCTTTGTCTCTGTCCATTATCATATCCCAATGACCTTCAT
GTTTTGCAACTGCATGCTTCTTCTTCTTGTTATCACTATTATCATTATCTTGCAGCCCTA
TGAAGCATGGAGCTTGCAGAAAATTTTGATCACCTTTGGAAGCCATCAATGGTGCATCAA
ATCAAAAGATACCTTGAGAAGAAATATATTTGTTTGAGGATCATTAACTTCTCTCGG
>tig_1141_consensus
AGAGATGTAAAAGACTGAGACTTCCCTTGGAAAAAATTTAGAAAGCCCTCTCTTGATGGGG
AGTTGAGCCATGAGGTCTGAGAACTCAAACAAAGGACCATTAGAAGTTGGAGAAGAGTTT
GAAGAAGGTGGTGAAGAAGAAGAAGCATCATCAACCAAGTCAGAGGAAGAGGATGTTGAT
CCATGGGAATTAGTTGTACTTGAATCTCCAAGGGAAGAAGAAGAAGAAGAAGAAGAAGAA
GATGACATTTTCTTGTAGGCATCTTGATGATTATCTTTATCTCTGTCCATTATCATATCC
CA
>tig_1142_consensus
GCTTGGAGAATCATAATATAAATAAGAAATTCCTTGAGCACAAAATCGGATGCCTTGAAC
TTTAGAGTATTAAAACATTCTAAGAACAACTAACAGATTTTGCACATGATGATTACATAT
TTAAATTGGAAAACATAGTGGACTATAATCAATACTCAAGCTTTAGCTAGCCTGCATAAG
TTCAGAGATTTAAAGCCATTAAATGTATGTTTAGAATCTTATCTTTTCCTCTTCATAACG
GGATATGCTAATAAACCATCTAAGACTTTATCATAAAATTTGTAACTCAATTAATTGCTC
TCAAATCATTCAAGTGGGATTAGTTTGATAAGTTTACAGATCAAATAACCTGAAGTGCTT
TTGTAAAGGAGCTGGAGGAGGCCTACTGCTAGGTAGAGAACTGCTTCTTCTACCAGGAAA
AGATAAAGAAAAAGAACTTCTTGAAGACTTCTTTGATATGGTCGCCTTTGGATAAAGAGC

ATATGATTTGTAACAATCCCGTCCACCCTCAGAACTTCTACATGTCTTCATTTTCTTTCT
GTAAGGACTCTCTTTCTTTGCCAAGTCTTGTATGCTCTTCACTCTTGACAGAGATGTGAA
AGACTGAGACTTCCCTTGGAAAAATTTAGAAAGCCCTCTC
>tig_1143_consensus
GGAAAAATTTAGAAAGCCCTCTCCTGCATATATATAAATACAATCACTATTTAGCACAAA
TAATTATATATGAAACTATTATTTCTTCTAAAACATTTAAAAGCTTCAAAATTTAAATTA
AATCCTTTGGTATTTTGATATATCCTTCTTGTATAATTCTCTAGAGATCTAACCCGCGTC
AACGAAAAGGGTTTATATATATAATTCAAGAACTTATAAATGAGCTAGAGAACTGATTAG
ATTACTTACTTGATGGGGAGTTGAGCCATGAGGTCTGAGAACTCAAACAAAGGACCATTA
GAAG
>tig_1144_consensus
GTCAAAATGTACTGAAATTGCATAAAAAACTGGATACAGTAGATGAAAAAACAAGCTAAT
GTCAAATAAAGTTACACAAACAACATAGAATTCACAAACAATGAGAGTTAGATTTGCAAC
ATCATTTCATCCTATTACTTTTACAGATTTTCTCTTTCCTCCTGTGTTTCAGTTAGAATA
ACTTCTACAACTCTCTCTGATTCAGGGCAATGAGGCTCAGGTTCCTAAAACATCACAACA
AATTCACAAGCTTTTGCACAGTTTCTACTTATCTTAAGTCTGAACCATTGGCAGTTGAGG
CTGCGGCTGCACAGTTTGTTGTTGTTGTCGAGCAGGTGACGCTGGCGGTTGGGCTACTTG
AGTGTCATCTTTCAGCTGGGTGCTAATGTCCATCACTGCCAGCGCTTCTAGCTCTTGGAT
TGCTGCCAATGCACTGATCTGGCTTTCACTGCTTAACTGAAGCTTCAATACGCTGACAGC
TTGCTTGACTTTCGTTTGAAAGAGTTCAAGCTTTCTTTTAAGGTCTCCAATGGCTGTAGA
GCCTGCTGTTTCAGTTGATGCTCCTGATGGGTTCTTCTTGGGCCGACCAACGGGCCTCCC
GGTACTGCTGACACCATTCTTCGGCGGCCGGCCACGCTGCTTGACGGCATCATCCCCAGC
CATAGTTGTGCCGCCGATCTTCTTTGGTCGCCCTCTACGCCTCGCTTCATTGGTCATTGC
TATATTTGCATCATGCTCACTTGCAACATAAGTAACCGCCGCAGGATTAAGCGTCTGCTC
TTGGCCTTGAGCTTGAGCTTGAACTTGAACTTTAGGAGGACGGCCAGGACCCAACTTACC
AGTCAAACTCTTCGGCTTTTTAGGACGTCCCGGACCCCTCTTCTTTGCCTTTGGTGGTCT
GCCTCTCCCCTTATTAGGCCCACGTGGAGCCGCCGCTGCCGCCGCCACCACCACTACATC
ACCCTTCTTCGGACGACCTGGCCCTCGCTTGGGCTGATCAACCAGCCCGAGCGAAGCCAA
GACACTATCACTGGTAGTAGTAGCCTTGGGCGGACGGCCCCGTCCCCGACCCGGTTTAGG
CCCATGGGTTGAACTAATAGCAGTGGCATTTTCGGAAACAACATTTAAACTTTCCTGAGG
AATAATTGAAGCGACAACATCATCAGATCTAGGAAGCTTGTAAGATTTCTTCACCATAAC
AAGCAGACCCGTGTTCTTCAACCGCTTGAGGTGGTGAGTCAACAAGGCCGAGTGATTCGG
CGGGAGAAGATTCCCGTAAACTTTCTCTATGTACTTGGCGATAGCTCGCTTGCTGGACCC
GTCTTTCTCTTTCAAAGCAGTGATGGCGGAGCTTATCATCTCCGTGTAAGGAGGGTGATT
GTTATTGTGGTTAACAGAAGGAGGAGTAGGGTTGGCGATGGTGGCGGCAGAGGAAGGTGG
TGGTGGCGGTGGTGGAGGGGGTGCAGGAAAGAGGGCGTGGAGGTGGGCAAGAGACGGAGG
AGCAGGCGGCGGCGGTTGATAAGGCGGAGGCGGATGAGAGTCGGTAGAAAGGTGTAGAAA
GGAGGAAGGGTCCATGGAGATAGAGGAGGTATTTTGGTAATTTCTCAAAGTTTGATTTTT
GGATTTCGAAAAGCGAGAGATATAGTGGTAGGTTAGATCACGAAGCAGAAGAAGATGAAG
ATGAAAGGATTAGGAGATGAGGTGGACGGTTGAGATTCATTATTATGTGCGTTTTGGGTG
GTTTTCGATTGCTG
>tig_1145_consensus
TCTGGCTTCGAGAGTGGTCCCGCCTAGAATCATACTCCCGCACACGAACATAGGACCGAG
AAAATGCATTCCGAAACTCTGAGTCATTAAGTTTTTAATTGCATGCTTCATGTCATCAT
AGTTTGTGTAATCCACAATTCCAGTTGTGCCACTACCATCACGGAAGACTTGGGAAAAAC
AGACATCTCCCGCTCGACGCATATGATCCTTAAGGTCTTGCCACGAAGCAGAAGAGGGCA
ATCCACTGACTAACACGCGATATTCAGATCGCCTAGATACTCCACGTCCACGACCACTGC
TATAACTACTATGACGATCAGATGATGAACGTCCTCGCCCACCATGTGCAAGTTCCACCC
GTAATCGGTGCCCATCAAAGTCATAGCCATCCCGACCACGAATTGCATCCTCAGCGTCTC
GAGCCTCTTCAAACTCAATAAATGCGTAGCCAGGAGGCCTAGGTGGGATCTTCAGATCAA
TATGGGCTATAGGTCCATACTTGTAAAACAAATCTTCAACTTCTCGCTCACGAATATCCC
CAGGGAGATTTCCAACATAAAGGGTTCTGCTTGCACGGCTGCTCATTTTTCTGTGAGACG
ATACCACCAGAGAGCCTCGTAGAATATAGGGTTTTGGAGTCGGGGTG
>tig_1146_consensus
AAAAAAAAAAAAAAAAAGCAAAGAGCAATGCCATCTGACTTTGATTGCAATTTGAAGCCAG
TGAACAACTTATTTTGCGGGGGTTGGTTGAAATGAGAAACGTGGGAGTGGGCCCTGGACA
AGTTAGTGAGATTTTAGGATGGGACCATGGGAAGGAAGGGGGAATACCCTTTTGCTTAAA
ATCTACGGAGGAGGATTAGTCTCTGGCTTGACTATAGATGAGGTATCAGGAGGTTGAGTA

TGGTAAATGGCCGTTGCCAAAGAGATTGGCATTATGCAGAGAGCTTTTGACTGAAGAAAC
TGCATTGCAGCTCCAACATTTTCTTCCATCAGCTTTGCTACCTGCCTTTCAGTTCCATCA
TTTGACCATTTCTCCCAGGCTGGTTGGTGTCTTCCACCTTCCCCAGACTCATCCTCAACT
GATGATAGTGGTATATCTGTTATTAGTGGAGCCACTGCACCAGCTGCGCCCAATCTACTC
ATGCTCAAAACCTTAACTTGGAGCCTCAGAAACTTCACATAATCCACAATTTCATCAAGC
ATAGCTGCTCGGTCCGTCTTGTTAACACTAGGAACTAATTCCTGCAGTGCCCTGATTCGT
TCTGCTATTCTTTCTCGCCGCAGTCGTTCAGCAATGCTGTGGGGGTCTGTGGCCTGCCCT
CTCCTAGCTCGCACCCTAGGGCGTATTGCTGGAGGATGTGGAGCTGCAGGAACAGTTGTG
GGCATTGGTTGACCATGGTACAC
>tig_1147_consensus
GCATAGACTTCAACTTATCACTTTACATAGAATTAAGAGCATGGCAAACATTATAACGCC
ATCAAATTCAGTTCTAACAAACTTGACAGTCCATTTTCAAATGATGGCAAAAAGAAAAGA
ACTACCATGATGATCATCCAAAGAAAAAGCACCAAAGATGACGTACAGATACAATGGAAC
CATTTAAAAAAAAAGGCTTTATCAGGCAAGTACAACTAATATTTGGGGCAAAACTTTCAC
TGCAACATGCAACACACTTCCTTACATTTGGACAAAGCTGTAAATACGAAATATTTGGTA
AATATATCGTATTTACCAAAAAACTAAAACTACTCTTCCATGGCGTAATTCTCCTCTGCT
TGTGCTTGGGTGACCTTGACTTTGACCTTTGGAAGAGTGAAAGATTGATCCTGTGAGAGA
ACAGATGGCGCTTTGACCAGGTGGATTCCTTGCTCCAATTCGATTGCTGCCTCTCTCTGC
TCCTTGGCTTTCTTCCCCTTCATCCTATGTTTATGGTGTTTTTCCTCCCTGGCAACTCTA
ATTTTATCAATCTTCTTGATAGCCTTTAGAGTGTTTTCTGCAAGATTTCTGTCATATCTC
TCTGGCCTATTACGTTTCCTCTCAAATTCAAAGGTTGAGTCCTGTGTCATGTCCTTCCCA
TGCAACCGCCTATAGGCCTTGGTCCATTTTACTTTACGAGGGTTTCTCTTCATCTTGAAG
TTTTTGTGACATTTGGATCTACAAAATCGGAATATCTTTGCATCATTCCGAACAAACTGA
ATACCATGTCCTGGATATACAGTGGAAGAACAGAACCAACACTTCTCTAATCTCATCTTT
TCTTCAACTTAACCCCAAACC
>tig_1148_consensus
ATAATGTTTACCTTTTTGCAGCACATTTAAGAAAATGGGAGAGAAAACTGCGGAAGTTGT
AGTCTTCTCCTTATTGAAGATTGGCAAGAAATACGACAATATCCAAACAAGTACAAGATG
TATTGTTCATGAAATGATGTGTAATAATAAAGATTTTGCACGAGCACAGAAAGTAGACAA
AGAAATTACTGGGTCTTGGTGGGTTGCCAGGATTGGTACCTGGACCAGTCTCTCTGCCCA
GGGTTGAGGGCATGTCCTTTGGAATGATAAATATACTCCTCACCCTCTCCGGATAAATTT
TCCTGGTGCTCAACACCATATCTCGCTGGTTTCAGCATCAGAAGCTCATCTCCTGTGTGA
TCAGTTATGTAGTGGAGCCAACCGTGCCATTCTGGTGGCACCTGAGATGCATTGTAGCGA
TTCTTCTCAGCATATTCGACCCACCTGTGTCTTC
>tig_1149_consensus
TCCTTTCTGGGCGAAGAAGGTGGTGGCATAGCGTAACTAATTATGGATTTACCCAAAACC
GATATGGTAGAAACAATCCCAGCAACATAAAACACCATAATCAATCCAAGAACCCAAGGC
ATTAACATAAACCCTATAAAGAACGTTACTGATCCACACAACATCAAAGCCAACGAAATC
CCTAGCATCAAAGACGCAAATCCCGCCGGTGAAATCTGCGTCGAGGACGAAGACCTCGTT
GACGACACAACTGCCGTCGATGACTGATCGGAGTACGGTATCGGCGATGGCGGCGATCGC
AGGATGTTCAACACCAGAGCTGATA
>tig_1150_consensus
CCCGATTTGTTATTAGTCAAGCTCAACTCAAACTCCGGTACAGGAAATATAAATTTACCT
ACTGAACAAAATAGAAAGAAAAGAAACCCAACACAGCCAAATCAGAACAAAGTCTGTTTC
TTGCTTAAATGGTGACAGATGAACCGTACAGACACTGTTAAAAAAGGAAAATGTTCCAAA
AGACTAAATAGGACAAAGACTGAAAACAGTCATACCATCACATCGAATTCCCTGAAATGT
AAAGACTGAACATTTTCGCCATCATTGTAAACCATCTCATTTATCATGATTATCCCCGCT
TAACCAAGCGACTACGTGTGCATGATAACCATCTTCCGCAATCTATTTTTGGGCTATCCT
CTCCTTAGAAGATCTTGAAAAGCAGGCCACCGTGAGTGGCAAAGAAAGGAGCAAATACCA
GTGATTCAACAGCCATAAGCTTGATGAGGATGTTTAATGATGGGCCAGATGTATCCTTGA
GTGGATCTCCAATGGTGTCGCCGATCACAGCTGCCTTATGTGGCTCCGACCCCTTAGGTC
CAAGAGTCCTTGCATGCTCAGAAGCACCCGCCTCGATGTACTTCTTGGCATTGTCCCATG
CACCACCAGTGTTAGATGCGGAAATTGCAATCTGGACACCAGAAACAAGGGCGCCTGCAA
GCACACCAGACAAGGTTTCCACGCCAAAGAAGATTCCCACAATGAGGGGTGTAAGCATGA
CAAGAGCACCAGGAGGAATCATCTCCCTGATGGATGCATCTGTTGAGATCTTGACACAGG
TAGCATAATCAGGTTTGGTGTGGCCTTCCATGAGACCTTCAATGGAGTTGAACTGCCTGC
GAACCTCCTCAACCATCTTTAATGCAGCACTTCCCACACTCTTCATAGTCATGGCAGAGA
ACCAGTATGGAAGCATAGCACCAACAATCAAACCAATGAAGACTTTTGGGGTCAAAACAT

CAACGGTAGAGATTGCTGCACGGCTGACGAAGGCACCAAATAGTGCCAAAGACACCAGTG
CAGCTGATCCAATAGCAAATCCCTTTCCAATGGCAGCAGTGGTATTCCCAGCAGCATCAA
GGGCATCAGTTCTCTCACGAATGCGGTGGCTCATTCCAGCCATCTCAGCAATACCTCCAG
CATTGTCACTTATGGGACCATAAGCATCAATAGCCAAACCAGTGGCGATAGTGCTGAGCA
TTCCAAGGGCAGCCACTGCAATACCATACATGGCAGCAAAGCTAAAACTAACAAAAATGC
TGACTGCAATGGCAAAAATTGGAATGATTACAGATTTGTATCCTAAGGCAAGGCCAAAGA
TAACATTGGTGGCAGCTCCTGTCCTGCAGGAATCAGCAACATCTTGCACAGGGCTGTATG
CATTGCTAGTATAATACTCGGTGACAAACCCAATAATAAGTCCAGCCCAAAGACCAACAG
CCACACATAAGAACAGTTGCCAGTTTTTCACAACCTTCTGAACCCCAAAATTGTAGATTG
TGAAAGAAGAAGGCAAGCCTATCCAGGAGACAATTGCAATTGCAATAGTCATAATAACAG
TGGAGATTATAAGTTGTTGCTTCAATTTTGGTTCAATTTCTTTGACAGCCTTGATTTCAG
ACCAATCAGTGGCAACCATTGTAGTGAACAAACAGACAATGATACCCGCCGAACTAATTA
GCAGAGGATATAGCATAGAAGTGAAATCATGGTTGATCCCAAAGGATGATATGGAAGCAA
CAACAAGAGCAGCACAGGATGATTCAGCATATGAACCAAAAAGATCTGAACCCATTCCTG
CAATATCCCCCACATTATCACCAACATTGTCTGCTATGACAGCTGGGTTTCTGGGATCAT
CTTCTGGAATGTTCCTTTCAACCTTTCCAACAAGATCAGCACCTACATCAGCAGCCTTGG
TGTAGATACCACCACCAACTCTCCCAAAGAGAGCCATGGAAGATCCACCAAGACCATAAC
CAGTAATAGCCTCAAAAAGGCCTTCCCAATCATCACCATAGTACAACTTGAAAAGATTGA
TAGCAATGAACAGCACCAAAAGACCATTTGCTGCAAGGAGGAAACCCATCACTGCACCTG
ATCTAAATGCAGTAATAAAAGCCTTCCCAACACCCTTCCTTGCTTCTAAGGTTGTCCTTG
CATTGGCATAAGTTGCAATTTTCATCCCAAGGAAACCCGAAAGCAAAGAGGTGATAGCAC
CAAGCAAAAATGACAGGGTGCTGAAAAGTGCAGTGGCAAGAGCAGGCTTGCATGTCTTCT
GCTTATCATAAGTGCACGGTTGGCTCTTGGTGCTAAATCCCTCCACAGAACCCAGAAAGA
GGAAGATCAAGATTGCGAAAGCAACCATGAAGACTCCAACATACTGATATTCAGTAAAAA
GAAAGGATGTCGCACCTTCAGAGATAGCGCTCTGAATTTCAGCACACTTAACCACAACAC
TCTGGTCGTTAATCCCCTCCTCTTGCTCAATCAAATAATCATTGAACCCATTCTTCTTGT
TTTTGCTACTCGGAGGGTGCTCGTGCGTAAGTTTCACGCGCGAAACGAGAACCCACTGAA
TGAGCGAGAAAATGATGCCAACGACGGCGCAGACTGGCACCAAAATCTCCGTCCCCAACT
CCGGAAGAATTGTCGGCGACCCCATAACGCAACAACAACAAAAGCAGCCCAAAAGAAACG
AAATCGATATTTCGTGAAGGAGAAAGAAACCGAAGTGAAC
>tig_1151_consensus
ACCGTTCCCGACTTCGCTTTCTTTTCGCTTCATTTCTCTCGTCACGATCCCTTCGTTTCA
TTTCTTTTGCGTTCTTTTTGTTGATGCGTTATGACGTCGCCGACGATTCTTCCGGAGTTA
GGAACGGAGATTTTGGTGCCGGTCTGCGCCGTCGTCGGCATCATCTTCTCGCTCATTCAG
TGGGGTCTCGTTTCGCGCGTGAAGCTAACGCACGAGCGGACTCCGAGTGACAACAACAAA
AAGAATGGGTTCAATGATTGTTTGATCGAGGAAGAGGAAGGGACTAGTGACCACAGTGTT
GTTACTAAGTGTGCTGAAATTCAAAACGCTATCTCTGAAGGTGCAACATCCTTTCTTTTT
ACCGAATATCAGTACGTTGGAGTCTTCATGGTTGCTTTTGCAATCTTGATCTTCCTCTTT
CTTGGTTCTGTGGAGGGCTTCAGCACCAAGAGCCAACCTTGCACTTATGATAAGCAGAAA
TCTTGCAAGCCTGCTCTTGCCACTGCACTTTTTAGCACTGTGTCATTCTTGCTTGGTGCT
GTAACCTCCTTGCTTTCTGGTTTCCTTGGGATGAAAATTGCCACTTTTGCCAATGCAAGG
ACAACCTTAGAAGCAAGGAAGAGTGTTGGGAAGGCTTTTATTGCTGCATTCAGGTCTGGT
GCAGTGATGGGTTTCCTGCTTGCAGCAAATGGTCTTTTGGTGCTGTTCATTGCTATCAAT
CTTTTCAAGCTGTACTATGGTGATGACTGGGAAGGCCTTTTTGAGGCTATTACTGGTTAT
GGTCTTGGTGGATCTTCCATGGCTCTCTTTGGGAGAGTTGGTGGTGGTATCTACACTAAG
GCTGCTGATGTTGGTGCTGATCTTGTTGGAAAGGTTGAAAGAAACATTCCAGAAGATGAC
CCCAGAAACCCAGCTGTCATAGCAGACAATGTTGGTGATAACGTTGGGGACATTGCTGGA
ATGGGTTCTGATCTTTTTGGCTCATATGCTGAATCATCCTGTGCAGCTCTTGTTGTTGCT
TCCATATCATCTTTTGGGATCAACCATGATTTCACTTCTATGCTATATCCTCTTCTCATC
AGTTCTGCAGGTATCATTGTCTGTTTGTTCACAACTATGGTTGCGACTGATTGGTCTGAA
ATCAAGAATGTCAAAGAAATTGAACCAAAATTGAAGCAGCAGCTTATAATCTCCACTGTT
ATTATGACTATTGCAATTGCAATTGTTTCCTGGATTGGCTTGCCGCCTTCTTTCACAATC
TACAATTTTGGGGTTCAGAAGGATGTGAAAAACTGGCAACTGTTCTTGTGTGTGGCTGTT
GGTCTTTGGGCTGGACTTATTATTGGGTTTGTCACTGAGTATTACACCAGCAATGCATAC
AGCCCTGTGCAAGACGTTGCTGATTCCTGCAGGACAGGAGCTGCCACCAATGTTATCTTT
GGCCTTGCTTTGGGATACAAATCCGTAATTATTCCAATTTTTGCCATTGCAGTCAGCATC
TTTGTTAGTTTTAGCTTTGCTGCAATGTATGGTATTGCAATGGCTGCACTTGGAATGCTC
AGCACTATAGCCACTGGTTTGGCCATTGATGCTTATGGTCCCATAAGTGACAATGCTGGG

GGTATTGCGGAGATGGCTGGAATGAGCCACCGCATTCGTGAGAGAACTGATGCCCTTGAT
GCTGCTGGGAATACCACTGCTGCCATTGGAAAGGGATTTGCCATTGGATCAGCTGCACTT
GTGTCCTTGGCTCTATTTGGTGCTTTTGTCAGCCGTGCAGCAATTTCTAATGTTGATGTT
TTGACCCCAAAGGTCTTCATTGGTTTGATTGTTGGTGCTATGCTTCCTTACTGGTTCTCA
GCCATGACTATGAAGAGTGTGGGAAGTGCTGCTTTGAAGATGGTTGAGGAGGTTCGCAGG
CAGTTCAACAGCATTGCTGGTCTCATGGAGGGCCACACCAAACCTGATTATGCCACCTGT
GTCAAAATCTCCACTGATGCATCCATCAAGGAGATGATTCCCCCTGGTGCTCTTGTAATG
TTAACACCCCTTATTGTGGGAACCTTCTTTGGCGTGGAAACATTGTCTGGCGTGCTTGCA
GGTGCTCTTGTATCTGGTGTCCAGATTGCAATTTCTGCATCTAATACTGGGGGTGCATGG
GACAATGCCAAGAAGTACATTGAGGCTGGTGCCTCTGAGCACGCAAGGAGTCTTGGACCA
AAGGGATCAGAGCCACACAAGGCAGCTGTGATTGGCGACACCATTGGAGATCCACTCAAG
GATACCTCAGGCCCATCACTAAACATCCTGATCAAGCTCATGGCTGTTGAATCACTGGTG
TTTGCTCCTTTTTTTGCCACTCATGGCGGCCTGCTTTTCAAGATCTTCTAAGGGGAACCT
AAAAAATACAATTGGGAAGAGGGTCTTCAACCCGTGGAGACTTTGCTTTCACTTATAATA
GTAGTATCCTCACCAACCCTTTGATAACTTAAGTCAATTTTTGTACTTCACAGTAATGGG
GTTTCTAAGTTAGTTTGGTTAGTAGTGAACACTTTTTTTTCCAAAAGCGGGGATAATGAC
GATAATCGAGATGATTTTTGAATGATGGCAAAAACTGTTCAGTCTTCACATTCCATGGGA
GTCGTGGTGGTGGCATCTCTTTCGGTCTTTTTGTCCTGTTATTGTCATTTACAACAACTT
CCATTTTTAACTTTCCTGTAAGGTTCATCTGTCATCATTTAAGGAAGGAAAAGAGCATTT
AGACTCCATTTGAGCGAGAATGAGCTTTTGTACTGGTTTGCTAGTTTTATCTCTGGCTGA
GTTGCTGGCCACCAAAATTTGAGGCATGTATTTCTGTTTAAATAAGGGGACTGATTATAC
TTATTGTGATCCTATCATTCCAG
>tig_1152_consensus
GGCCCGTTGGTCGGCCCAAGAAGGTATAAGCTAGAGTGTTTTATAGCATGGAAAGTGTTT
TTTCCTATTTATCACACATCATTTTCGTATCCACATTCGGATTTCAAAGAAAAGTACATA
ATTTTACTGCTATAACCATGACTTATATTGGCAAGGACGTGACATTAAATCAAATTCATG
TGAATATTGAGAAAATCACCGTCTAAAAACTAGTTGTGAGATTGAAGTTTGCTAGTGTTC
TTCGGCGTCCGAATTGCATTTTAGATGATTGTTTTGTTGAATTTGCTTAAATCTGGCTAG
TGGTTTAAACATTCGTTTGTAAATTTTGTGAACTCTTGTTCAATTGTTGTTGCCTATGAA
GAACCCATCAGGAGCATCAA
>tig_1153_consensus
GCAATATCAAAATTCAATTAGTATAAAATATAATAGGAAGAAACCCAAAAACAATGAAGC
TAAAACAAATCCAGGTCCCATTAAAATCAAGAATTTTGAGACAAATAAGACAACGGACAA
ATAATCTCTAAGTGATAACAATCTATCACTTTGAGAAGCCCAAGTGCGATAGCAATCTAT
CACTTATGTGAATGCTAACATGAAATAACAATCTAACACTTATGGAGAGAACAGCAGCAA
TTTTCAGTTCCTATATCTGCCGCAATGCAAGGAGCCTTAGCTTTCCCCTCCGCGTACCAA
GTTGAATGGTGCTCAACCAACTCATTCAAGAAATCATCCAGTTTGTCAATGCTGACATTT
GGCATAACCACAACATGAGCAATATTACCTTGGCAAGCAAGCTGCCACTTTCGAACAAAC
TCTTCATCTTGAGGACGCTCAAACACAACTGTGCTGCTAAGTTCATTCAACATGGCACTG
ATACCAGCATCACGAAGGCGGTCCTTCAAGTAGTGAGCATTTCTAAGACACTTTTGGACT
TCTTTTTGGAACCCCTTGTATCCCTTTCTATTGAGGGTGTACCAGAGGAAGATAGGAGCA
TGGCCATTTCGGCTTCCCATGATTGTGGCATCCCTCGAAGCAAGGTACTCGACATTTCTT
GAGAGAGCATTGATGTGATCCATCCTTGTTATCTGAACACCACAAGGCATGGGGCATCCC
ACAAACTTGTGGCCAGAGACAGACACACTTCCAATGGGTTTCTTGAAAGTAACCTTCGGT
GCACGTTTAACAAAGGGCATCATGAGTCCAAACAGAGCTCCATCACAATGAATATAGAAC
TGATCATGCTTAAATCCACTTTCTTCAAGCGTTTGTATCACAAGATCAAGGTCATCGACA
GCTCCTTTGACAGTTGTTCCTATATTAACATTGATAATAGCTGGTTTGTCCTTGTGAGCA
AGAAGTTTAACTTTGAAATCAGCACAATCAATTTCACCAGACAACAGAGTGTTAACTTTA
ACACATTCCATTCTATACATCCGTGCAGCTTTAAAGACAGAATAATGGGACTCTTTTGAT
GCATAAAGAATGCCATCTGGAAACACCTCCCTCCCAACCAAAATGCCGTGAAGATTGCCT
TCTGTACCACAATTTGTCATGTATCCCCAGTATTCATCTTTCTCTATTTCCCACAGACGA
GCAAACCAATCCAAAACACCAACTTCAAACTGTCTTGAATGGACACCATAGTTGCTCTCA
ATAAATGGATCCCCAAGATTGTTGATGGAGAAATGTTGCAACTGACTAAGAGCACCATAA
TCGAAGTCAAGATTATAAGGATAACCTAAATGATGCTTAGTCCGTTCGATCAAGGTCTTC
CTATAGCGAGCCAAGACTCCAGCCATGTAAGCCTCCTTATCACCAGTGAACTCATCGTTA
GCTTCCGGCTCTGTGACAGCAAGGCAAGAGGTGTGTACGTTCTTCCCGAGAACAATCTCT
CTCTCAGTCTCCGTCTCAGACTCAGCAATAATCTTCATAGTCCCGCCGTTCTCCTCCGCC
ACAACCGGAGGCAGCGGTTCAGGCACCACAGCGGACGGATCGAAATCTCTAGAAAAGATC

TCCGCCATCGCTCCTTTCACAAGGTTCAAACCAGCCTTCCCTTCCGCTTCACCACCTGAT
TCGAGATGATTGAGGCGG
>tig_1154_consensus
AATTGGTGTGTTTGGTGGCTGAGTCTAGAATCCCTTTTGTTGTTTGATGTCTGACTTCTC
CTCAGTCCCAGAAAAACATTTACTGCCTGATATTGGTATGGAAACCATACTAAGAACAAA
CACAAAAGCAAGACAGATTTGTAGAAGTACAAGAAAAGCAACATACCCAAAAAAAGAAGA
AAAAGGCCCAATTCACCACCACATAGACAGGCTTGCTAACTCTCCACCCCTAACATTATT
TTCTGACTTACATTTAATTAAAGCATTTTAGTGCATACTGCGCATGATGCCATGTAGATA
ACCGCCTGTCATCACAAGGGACACAAGAGAGACAACACCGGCTGGAAATACCTTGCCTGA
ATTCTTGAAACGCGAACCCATCACCACCAAAAGCACAGCAGACAAGCCAAGCCCTATGGA
TGATGCAAGAACTGGTTTTGTGGGAAGCTCTGTATAAACATAATATAGCAGTGCAGCCGA
CACTCCCCCTGCCAAGAGTGACTTCTGGCTGCCGCTTTTCAAGTAGCCCATAAGACCACC
CACTCCAACAAGTGCAGCATAACCCAGAGTCAGTTTTTGAGACATAGACATCCCCATCTT
TTCCTTCCCACTAGATTCATCTTCTCCTCCTTCGTTCTTACCCTCATCTCCACCGCCGCC
GCCACCGCCACCGCCGCTGCCAAACTTCTCTCCACCACCGCTGCCGCTTCCGCCTCCGAT
ATCGGGCACAATCTCGCTCCCATCTTTTACGGTGTCAACAATATTGGTGAGTATAGTTGA
GGATTTGGAGTCAGATGAGAGCACTCCGGCAATAGTGGCGATCCTCTTAGAAGAAATGTT
AAGACTTAGATAGCTGATGTTGTTAGAGTGTCT
>tig_1155_consensus
AACAACTTGCCGGACTCCTTGAAACGATAACCCATCACTGCTGAAAGCACACCCGACACA
CCAAGCCCTATGGATGATGCCAGAACTGGTTTCGAGGGAAGCTCTGTATAAACATAATAT
AGCACTGACGCTGACACTCCTCCTGCAAGGAGTGACTTCTGGCTGCCGCGTTTCAAATAG
CCCATAAGGCCACCCACTCCAACAAGTGCAGCATAACCCAGAGTCAGATTCTGAGACAAA
GACATGCCCATCTTTTTCTTTCCACTCTCGTCACTCGATTCACTTTCTCCACCGCCGCCA
AACTTCTCTCCCCCACCACTGCCACCGCCAATATTGGGCACGGTCTGCCTCCCGTCTTTT
CCGGTGTCAACACTGATGACGATTACAGTTGAGGATTTGGAGTCTGATGAGACCAGCCCA
GCAGCAGTGACGATTCTCTCGGAAGAAATGTCAAGTCTTTGACAGCTGATGTGGTTAGAA
GAAAAGATGACTGTCTGTGATACACCTAACAATTTGTTTGACTCGCTCCCTTTGTTTTGC
CCTTCTACGTTCTGCCCCAAACCCAAACTCAGGGACAGTTTCTTG
>tig_1156_consensus
CCTGAATCAAACACCCTCCAAATTCAAGTTTGACAATATAAAACTCTTAGACTTAATTTC
AAATTAAAGCTCATCAAACCCAAACTGGGAATCAAGTTCAAACCACCTTTATTCGAATTC
ACTCCTATTCCCTGGAGAAACCACCAAAGGAACCAAAGAAAAACGATCATAAAAATTGGA
AAAACAGACCTACAACAAAATATCGTGGTAGAAAATAAAAAATCAACAGAAAATATAAAT
CTGCAGGCAACTTTCAATTACTCGGTCGAAAGGAGGTTGCCCCAACTTGAATTTGGCTAG
TCTTAGCCAAACACTGAGCCAAGCCCGATTATCCATAATGGTAAAGTTGTTACCCTATAT
AGAACATGTACACAAATGATTACGGGAAAACATGGAGTCCAAGATTGTTGATAAACTATG
ACTCTTCACCACTGGAAATTTCCTGGTATTTCTTGGGACGAAAACAAGAAACACCAGCTG
CCAATCAGTTTTTTCCCTCGGAAATAGCTTCAATAGTCAAATGAAAGGTGTTCTGTAATA
TGAAAATGGTAATTGTATCGTGTCAATATGCAATTATTTATCTCTCAATACCACGTAGTT
GTTTGGTCCATTGGTCTTCTGTATCTTTGAAATTCTCCTAAGAATATCGGCAAAGGCCCT
CTTATCCTCAGAGGATTTCAGTCTTGCTTTAAAATTAGCAACAAGATCCTGGGTTGTCAC
AGGCGCCTTCTGCAGTAAAACAGCCCTGATTTCTTCTTCAGTGACTGGACCAGAAGCAGC
AGTTGATCCAGTTTTCGTTGACGATGATGATGATGGTGTAACTCTTGGAGGCCCAGCACT
TCTTGAACTGTTTTCCTCCTTTACAGAAGTTTTAGCATCATTTTCCGTCTTCACCTTCTT
TAGAGGTGCACTGTTAGATGTTTTTGCATCATCACCATTAGATTTCCTCTTACCCTTTGT
AGATTTGGATGCAGGAGTTCCCTTGGTTCCAGAAGGAGCTGGTTTTGATGGACTGTTATC
AACAGGCTCCTCCTTCTTAGGTGTTTCTTTCTGCTTTGGAGCCGGTACAGAGGAAAAGCC
AATGTCATCATCCATATCATCATCATCATCGTCATCATCCTCAGCTTCTGATTCATTTAG
CCCACTAGTTCGCCCAAGCAGCTTCTTCAACTCTTTTCCAGATTTGCTCAGTCCCCCACC
CTCTTCCTCTTCCTCTTCATCCTCTTCATCCTGCTTTATTTCTGGCGGAGCAGGAACTTC
AGGGGCTAAATCTTCCCGCTCCTCAGGATCATTACCAACAGCTTCATCATCATCAGTAAA
AATTTCCTCATGTTCCCAATCATCACCCTTCTCAACATCATCATCATCCAAATCAAGATC
ACCTCCTCTCGGACCTTCTTCATCATCATCACCACTTCTTTTATTGAGTCCAAGCCTATT
CTTTCTGGCTGCCTCCTCTTCTTCATCTTCTTCTCCTCTATCCGAGACATTACCTTCATC
ATCCTCACCACTTTTTTTGCGTCCTCTCCCACCAGCCCCAGCACTCTCCTTGTCATCCAA
ATTTTTTTCCACTTCACCAAATGCAGCAGCTCCATTATTTGCAGCTTTCATCATCCATCT
TTCATATCCATCTGCAGTCTTCCTCCTGTTCTTCATTTTTTCTTCAGCTTCCTCCAAGGT

AAGTTGCTTATAATGAGCAACCTTGTTAAAATTAAACCAAGAACCAGCAGGAATAGCAAC
AAATTCTTTATTATTCAGTACTAGCAGATAGTAAGTTGCTGACTGAGCGCCTTCAAGGTG
ACCATGGTATTGGAACTGGCCTGTTTCATCCTCCAAGAGCCAAGGCTTATTCTTGTACTT
CTCCCGCAAAGTGTCTGTAACCTGGCGCCCTTGTAATCCATCCTTCTGAAGCGTCCACTC
TGAATTTTTCTTCTTTGAAAAAGGCGGTACTCCGGACACAAATCTTCCGATAAAGTAATG
CTTGTCAACGCTAGCACTCGGTCGAACATTATACTCTCGAATCAAGCGAGTGACAACGGC
GCCACAATCGAAACATTTGGCTTTGTTCTGAGCCATGGTTTGGCCACACGTGAGGCAAAG
AGTCATGTGCTTGCAGTTGCTTCCGTACAAATCCACACTGGATCCACATCTGCTACACGC
CGACTTCAACGATAGATCCAACGACATCTCTCTTCGCCTTCTTCTTCTTCTACTTCTTCA
AAGCCACCGATAATTTTAGGGTTTCAAATTATAGACATGATAATAATCGGTTCCATCTGT
TTATTTATGTTTACTTTGATCAAGATTCTGAATTGCGTTTCAAGTTCGGTGGCTGACTGT
TTAAAGTTTAGGGTCGCTTCAAT
>tig_1157_consensus
CCTCACCCATGGCAGACATCACACTCACAACCAAATCCTTCCCTGCTTCGAACCCTTCCT
TGATCTGGCTCAGCAGGCTCTCATCCGTCGGGAGCCTAAGATCATCCTTAGTGCTCCCAC
TTTCAGTAAGCAAACTCACAAACCCGTCCTCGGAAATATCAATCAGCTGATAGTCAGTAC
GATTGACATGGGGAACATCACAATTGTGGGAAGAGGGGACAATATCTTCCAGCTTCTTTC
CATTGAAGATATCAATACCAACAAAGTGGCACTTAGCATGACCATGCTTACCAGTCTTGG
AGGTGGAAACTTCCACAACCTTGCAAGGACGATTCTTAATAACAATGTAACCGTTCTTAC
GGATGGTACCGGCTTGTTGAGGGAAAGTTTTGGAGGCTCCGGCATCCGCCTTAGACTCGA
AATGGTGCTCCTCGTCCGACATCTTTTCAGTAACAACTTAAATCGGGCCTTTCAGGGAAA
GATCGATAAGAGAGAGGCTGAGGAGACGAAGAAATTTATATGCATATAGTGTAGGCGTTC
TATATTGTGTCGGCTTTGTCGTTTTTCGTTTTTAGGGTTTCGTCATGAAAG
>tig_1158_consensus
TCTGCTCTGGTTCTGTTGATTTGAGGAAGAACATGTGCATGTCCAATTGCTTACACCTGA
TGTGCCAGAAAGCTCATCACAAAGGCTTTGCTGAATCCAGGCCAAGCCAGTGAGACAAGC
AAGGGCAACTTTCACTTGAAGCTTCATTTATGCTAGTCCAAACACTTCATTGGGGGCCAG
AGGTTTTTCCAACTTTTTTGTTCTCAGACTGCCCAATCTAAAAAGATAGTGAAGATTATG
ATTTTATTTTATATTACCAGCACGTCTCTTCTCAGCTACAGAAGCATTTTGAAAGACACC
GAACAAGCACTTTTTCTTAACTTAATAAACTTATAATAAAAAGTTCAAGCACAAGCACTG
TG
>tig_1159_consensus
GAACCCATTTCATCTACATATACAAACTCCATGTCGGCCAAACTAAAAACCGAACCACCA
ATTTGATGTGTTCAAAATGGACACCTGTGTCCCACTTAGACATATTTTAGATTTAAACCA
ATAAAGACACAACACGTAATTTGAAATAAAATAGAATCGAGTGCATATAATAAATCACTC
TCCTTGCCTCCTTGCCTTAAAATGAGCTGTTGAGCGACTGACTAGACATGAAATTTGTCT
ATCGTTGTCCTCTCGCTTTGGCTTTGGCGACATCTTCGAAGCTAAACTAAACGAAACAAA
AACACTGGGTCAACTCCTTCGTTATCTCCGCCTCTCAGCCATAAAATCCTATAGAATTAT
TCCTTTCTTCTCTTCATTTCTCTCATTTTCAAACCCTAGCTGGTAGAAGGTTCCGTCGTT
TCTCGCCATGTCGCAATCTCTTCAACTCTTCACTCATGCCTCCAACATTACTCTCTTTAA
ACCCAAACTCAATCACGCGCTTTTAACTGTCTCCCCTCGCCGTTTACACGTCACCACTAT
CGAACAACGCTCTGTCACAATCAAAGCATCCGCGTCATCGTCTCCTGATCCGAAAGTTTT
ACTCGCTCACAATGGAACGCCTGAGTCTGGGCCACACGTGGTCAAATCACAATCGGTTTC
TGCTGATTCCTTATCGATCGATGTCGATGCTGTGACGGAAGCGGAATTGAAGGAGAATGG
TTTCAGAAGCACGAGGCGGACGAAACTGGTGTGCACGATCGGGCCGGCCTCTTGCGGTTT
TGAGCAGTTGGAGGCGTTGGCCGTTGGGGGGATGAACGTGGCGAGAATCAATATGTGCCA
TGGCACGCGCGAGTGGCACAAGCGAGTGATCGAACGCGTTAGGAGGTTGAATGAAGAGAA
AGGTTATGCGGTGGCGATTATGATGGACACTGAAGGTAGTGAGATTCATATGGGTGACCT
TGGCGGTGCTCCTTCTGCTAAAGCTGAGGATGGTGAGATCTGGACTTTTAGTGTCAGAGC
TTTTGATGCACCTCTTCCAGAACGCACCATCAATGTCAATTATGATGGATTTGCTGAGGA
TGTGAGGGTTGGAGATGAACTCCTTGTGGATGGTGGAATGGTGAGGTTTGAGGTGACTGA
GAAGATTGGTCCAGATGTCAAATGCCGTTGCACTGATCCTGGATTGTTGCTACCACGAGC
TAATTTAACATTCTGGAGGAATGGGAGTGTTGTACGAGAGCATAATGCAATGCTTCCCAC
AATTTCTTCTAAGGATTGGTTGGACATTGATTTTGGAATTTCAGAGGGTGTTGACTTCAT
TGCCATTTCATTTGTCAAGTCAGCTGAAGTGATTAATCATCTTAAGAGTTACATTGCTGC
ACGATCTCATGATAGTGATATTGCTGTAATTGCGAAGATTGAAAGTATTGATTCATTGAA
GAACCTAGAACAGATTATTCAAGCATCTGATGGAGCTATGGTTGCAAGAGGAGATCTGGG
TGCCCAAATACCTTTGGAACAGGTTCCATCAGCACAGCAAAGGATTGTTCAGGTGTGCAG

GCAGCTGAATAAGCCAGTTATTGTTGCCTCTCAGTTACTTGAATCTATGATTGAGTATCC
TACACCAACCAGAGCTGAAGTAGCTGATGTTTCTGAAGCAGTTCGGCAGAGGGCAGATGC
TCTGATGCTCTCTGAAGAGTCAGCTATGGGCCAGTTCGCTGATAAGGCATTGACTGTTCT
TAGAAGTGTCAGTCTCAGAATTGAGAAATGGTGGAGAGAAGAGAAACGTCATGAAGCTAT
GGAACTCCCTGATATTGGATCCTCATTTGCAGACAGTATTTCAGAAGAAATTTGTAAGTC
TGCTGCTGAGATGG
>tig_1160_consensus
GACTTGTAGAAATTTTCTAATAAGCAAGGATTAAAAAGGGGCACTTAGCAAGTAGCAATC
TTATAGTACAATAAAAATCGGCAACCAACCAAAATTTGACACAGCTAATCAAGAAATCTC
AGCAGCTGCAATTGATAGACTCTATATTCATCTCGACAGCAGAACATTTCAATCCAATAA
ACTCAAAGATATGTTCCAAGGCAAAAACGGCTACCGCCTTTTCTGGTTCTTTCTCTTACC
TTTCTTTTTGTCTCTTTTATTTTGTGGCTTTGACTGTGATTTGTGTCTTGCAACTTTCTT
GAATGATTTACTTTTATGCACAGGTATATTGTGCTTTTTATTGACTCCAATAGAGCTGCG
AAAAGTAGTTGGAGTGTTTCCACTAGGAAAGTTTTCTTCTTCACCGGTTATCTCACTAGT
TATCACAGTGACTTTCATGTTGTCATTGTCATATAGTGTTGTTCCTGTAATTGGTGCCAC
CGGCTCTGCTTCTTCATCCTCCTCAAAGTATTCCTCAGTTTCGTCAGGCACAGAATCAGT
GGCTGGAGGATTTCCACCATATAAGGCAAGCTCTTTTTCCAATCTCCTCTTTTTTCGTTC
CTCAAGACGCTTAAGCCTTTTAGCCTCCTCTTGTTTCCTTTCCGCTTCTTTTCTCCTCTT
CTTCTTCCTCTTGTGAAACCCAGTCACGTAGTCCTTGAGGCCTTTCTCGTCGAATGAAAC
GGAAAGAGCTTTATTCTTGAGAGCCCTTTTCTTTATGTGTCGGCCTCGGACTTTTGGGAC
CTCTGGTCCTTCCTGTTCAATCTCTTCTTCCATTACAACTCCTCAACAGAGATGGATTGG
GTGTCCTGAAAACTATTGTTTCACAAAAGGGTTCCATTTAGTTGGTCGGGTCATCAAACG
GGACATTTTTT
>tig_1161_consensus
CTTTGATCTTTGTTTGTTTCCTCCTTAGACAAAGAATCAGCTCAAGTACACGATTGCTCT
TCAGGGGGTCCTCCAGTTTTCTTTGCTACTTTCTGTGTACCAACAAGATGATATAAACAC
ACTTTCCCTTGGCTTGCGTACGATAAGAATTCGTGTATCACTCAAAATAGGTAAGCGGTT
TAGGAGAGAATCGTTTTCCTATGTCCTTCGTGTATCCAGTTTTGTGCTGTACAAGTTTTA
TTTCAATCCCAAACCCCAAGTTAAATGAGCCGTATCCCACTCCTCTACTTGAAGTAACAA
AATAAACACACCCTAACGCGCTTTATCCAATT
>tig_1162_consensus
CTCCAAACCGAACACACTCTAAAAATAATTTCGCTTTCCTGCAGGTTTAACTAAGCTAAG
GCCTGGACAATAATGGGGAGACCACAAAACAAAAAAGAAATCTGCATCAGCAACAGTAAG
TGAGTGATAAGTTGAGAGAGTATCAACACACTATGATCATGGTCTTCAAGTCACCTTTTC
CGTACTTCTAATACTACTACTAAAGAAACATGACAATGTAGTTGTGTTTCTAAACTACCA
AGGATCAACCACAAAAATTTACTTAGTTTGAAGTCAGTACTGTAGACCAATTAACTGCAA
TACCGTCAATTTTTATCACCTTCGTTTATTTCAGATGAAGAATCAAATGTAGGTTTCTTC
GTGGTACTTGATTCTCCCGAAACACTGCTCATTGATACTCGCTTAACTCCTCTTCCTACA
ATCCCAAGATGTGTTACTCCAGCACTAGCAGCCCCATTTGTGTGAGCAGTGGAAGCAGTT
GGAGAATCAAATTCACCGCCACTGCTAGCAGTAGCCATCTGTGAAGAGCTCAGACCAGAT
GCAGATGTGGCCTTCTCATTGCCTTTTCCCTTGGCAGATGCCATTGCAAGCAATTCTGAG
AGAAGGGACTTGGGGTTCAAGACCAGCTGTTGCAAATCTTCATGCTTCTTTTCTAGGTCT
CCTAAGAGTCCCAGAAGTGTCTCAATCTCTGCTTTCATATCTGACACAGTTTGATCATTT
TGGGGCTCATTTGAGGACTGTTGAGAAGAAGCTGACAAAGAAGTTGCCTGTGAGAGACTC
TTACATTCATTCATGAGCCGCTGTACTCTTGACTTGCAAACTGATATAGCCTTCTCACAA
TATGGAATTGCTTCCTGAGCCTTGGAGCCAATCTCCAAACATAAGCATATGCGAAAATTT
AGTTCAGCAATTTGTCGACTGTCAGGCTCAACCAGCTGAACCAAAATGGATAATGCATTT
TGATAGTCACTAAGAGATGTTTCGAAGTCCTCTCTTTCCAATGACACCTCGGCCAGTGTT
GATAATATGTCAACTTTTTCCAAAGTGTTACCGAGGTTCTTTTCAGCAATTGCTCTTGCA
ACATCTAGTGTTTTCCATGCCAAATCCAGGTCAGATTCATCTTCATCAGCTTCATCCATG
TCCTCTTGATCACTACCCTCTTCAACCTCTTCCTTGCCTTTCCGACCAGAAACATCATTT
TCTGTTACTTCCAGTTTATTTGAGCTCCTATCAAGCTGAGTATTAGTTGAAGCAGAAGCT
GTGGAAGATTCTCCATCCACAAGATTCTTAACAGATCCATCATTATCAGAACCTACTTCA
AATTCATCCTCCTTTTTAGGCACTGAAACCAATGGATCTGCCTCCTCTTGGGCTCTGTAC
AGCAATGCACGTCCATATTGATAGTAAGCATTCAGACACTCTGGTGCAAGTTCGCCATAC
ACTTCAACCCTGATCTCTAAAGCACGGCTAAAACACTCAGCAGCTTCGCCGAAGTCTTTT
TCTTGCAAAGCTTTGGTCCCTTTCAGCAATAACTCGTTGGAGAACTCCAGCTTCTTTTCT
TGATCTCCATCATCAGGCACACCAGAAATTTCACCGTCCTCGTTGCCATTTGAGTCTATA

CCTCCCTCGAACAAAGATTGTATGGTAGCTTCAACTGAGGCTCGAGTTGTTTCTAGGGTT
TCCGTGGATTTCGGAACATCTTCTGTCATTGTCACTGAATCTTCTTCTTCCGACGCCATT
GCCATTGCCATTGCCTTTCAGTTGCTTCAACAATATATAAAACGCGAAAATAGCGCTTTT
AAATATATCGCCACCACTCCAAAACGGTGCCGTTGTCATTGTTTGACAGAATGACTATA
>tig_1163_consensus
GGCAAATAATTTACATTTGTATATTGGTGTGAGCGATCGAAACCATGGACAATAAATCCT
TTGGATGAACAAAAGATCAACAACAATTAATGCATAGAAAATGGCAGAATTGATTCATGA
AATTAATTGTCCTGTTAACCAGGATGCCAAAGTTCTGTCTGTCAATTGTTATAATTCAAA
TGGAACAGTGCTTGCATACTTTCTAGCCTTCTGTACATCCCCTCCCCTTTACTATGATCA
TCATCATCACCACCACCATCAACTCAACTCCACCAGTAGTGGACTAAAACTAAAACAGTA
GACCTCTAAATTGCACATCATATTACATTACAACTAGAATTTATCTCCCTACTAAAATAT
GCATGTGTATGATACTAAGTTTCTCAACGGAACCAAAGTACACACTCCACTTATTTGCAG
TTTACACATCCATATCAAATCAAGGAATCCAGGTATGAGAAGTTACGGCACCGACCGTAT
TCCAGAAGGCTGCCATATGTTCGATCCCATACACCAATAAACAAAGCCTCTGTGTCTGGA
CTGTATGATATTCCAGATATCTCACCAAAGAAGTCGATTTCCTGCTCCCTCTCATACCCA
CTTTTAACATCATACACATGCACAAAGTCAGCAGGCTCAGCTGCTGCCATGTAACGACCA
TCAGATGAATAGCGAATGGAACGGATGGCTCCAAGGTTGCCCTTCAAAACAGCAACAGAC
TTGGATAAGTTCCGTATATCCCAAACTCTGCATGTTTTGTCCTGGTTCCCTGTAGCAAAT
GTGACACCATCAGGCTGCCACGCTGATGCAAAAGAGTAGTCCAAGTGCCCACACAATGGC
TCAATAGTCTTTCCGGTATTAGAATCCACCAGCATACCCTCTGGGTTGTCTCCAACAATT
ATTAGAAGTTTACCATCAGGACTAAGCGAAGTATGATTCACTGGCCAAGAAAATCGAAAA
TGCTTAGAGAGTTGATACTTTTCCATATCAAAATCTCTGACACCACAGTCATTATTTGAG
GCAGTGAAATGGACTGCACCACTGGGACTGACATATATATCAACAGCGTTGGTAATAGCA
TTATCATCATATGTTGTACGAGAACAAAAACTCACTCCTGGTCTATCTAGATGCTTGCAA
ATTAATTCTCCCTGAAATCCACCTGCAACTAGCAACTTATCTTTTACAGCAAGTGTGCTA
ACTTGAGTCTGCGTAAATCCTTCCAATAGACTGCCAGGGTGTTTTTCGGTTGGTGCCACA
TGGCCAGAAACATTGAGAACCTGAGACCTGCTGCAAGTTAATGAAGACCAATGAACGACT
GAAAAATGTGACATAAGGTAGACATCGTGCTTTGATGTAGCCCAAACCAAGTTCCTTAGC
TGAAAATGAAGAATCGTTGATTTCACAGATCTAGAATTTCTTCTGAACTCATAAAACATA
GCCCCTTTTTTAGTACCCTTGCAATCCTTCCCTGACCCTTCTCCAGACTGTGGGATGTTT
TCATAGTTCTTGTACTGTTCTAGCCTAGTTTGCCGGTATTTTTCCCTAGTGATACTAAGC
CTGTCCCAAGGTATACCCTGGATATCTTTACCTCTTCTAGCTTGAGCAGCAGAGGTATCA
GCTATCTTATTATTTGAGTACTCATATTCCTCAAATTCAGAATCTGATCCACCCATTTCT
CTGCCGCGAAATTGTTCATCCACATCATCATCTATGTCTTCCATGTCATATTCATCTGCC
ATATACTCAGCATCATCCCCTTGGTAATTAGACATATTTAGCCTCTTATTGAGAATTAGG
GTTTACAGGGAACAGCCCCGATCTAAAGATCAAAAAATCAGAAGCCGAAGAGCATATGGA
AGATTTATGTGACCAACTTGTTCATACGAAAAAATCAAAATAAAAAAGGAAAAAAATAAA
AATTGACGGTGGAATACTTGATCGCAAGAATTCGAGAAAATGACGAGCAAACGCAAGAAG
GGTTTGTGTTTATCCAGTGGCTAGA
>tig_1164_consensus
GGTGGGAGTCATTTAAATTTGGAAATCAATTTTTCATTATAATATTTATAAATAGAATAA
CGAACATTGCCCCAAAACAAGGGATGTCTACTCTCAAATTGAATTGTTCATCATTATTCT
CACGACCAATCATTTCTCAAAATGTCACTCCACTCTTAACCTGAGTCGCTCATCGTTATT
CCGGCAGCTAGTCATTTCTCAAAATGTCACTCCCTAAGTTCAGTCTTGGAGAATATTTAC
CGTATATAATAACAAGATTTTAGTAGACATTACTCGGGCAACCTAGGCCTTTGTCTTCCG
AGCACTTACAACATTCAAAAGACAAAGACCCAGCGGCCCAATTCATATCAGAAAAGTAAG
AGACAAACCGACGAAGTTCATCAAGAACAAATATTCATACTGAATGCCAATTACAGCAAA
AGACTGTTCAAAAGAAACAACAACATTGACAATTTAAAGCTTAATTGAAAAAAACCACCC
AAACCAAAGCTCCTCGGTCAAGCTTTTATTACTGCAACCTAATAATATGAAGACAAAAAA
TGCCCATAAACTACGCCTGAAAATGGGACAAAGCAAATGTTATAATGCCATTACCTCTCA
TAATTCATAAAACACATGACCAGAAGTGTCAAACTTCTTGATTTGGTTTAGTATTTGTAG
TCCTTGACATGGAGAGACTCAGCAGCACCCTCACCAAGCTTAGCATCGCCCTTGTAAGTA
CCAAGGGTGGCCTCTGAGTTGGCCTTGGCCCTTGTAAGGAATGCACACTGCGCCTTCTGC
ACATTCTCTTTCTTTCCTCCCCATGCCTTGAGTGTGCTCTGCTGAAGTGCACGTCCAAAG
GAGAATGAAAGTGACCATGGCTTCTTTCCCTTGAGTTTGTTCATGGCATTCAGGTTGAGG
GTAGCTTCTTCCTCACTCTGTCCACCAGACAAGAACACAATTGCAGGAACTGCAGCAGGC
ACAGTGCGCTGCAAGGCACGCACGGTGTACTCAGCAATCACCTCTGGTGCAACCTTGGGA
GCATCAGATCCAGGGGTAACCATGTTGGGCTTCAAGAGGGTGCCTTCAAGCATAACATGG

TGGTCATTCAAAGCCTTATAACAGGCAGCAAGAACACGTTCAGTCACAGCAGCACACTTC
TCAATATCATGGGAGCCATCAACGAGGATCTCAGGCTCCACAATGGGGACAAGACCATTC
TCCTGGCAGATGCTGGCATATCTAGCCAAACCATAGGCATTCTCATGGATGGCCAGTTCA
GAAGGCTCATTTGGACCAATCTTCAAAACAGCACGCCATTTAGCAAAGCGAGCACCAGCT
TCATAGTACTTAGCACAGCGCTTACCAAGGTCATCAAAACCCTGGGTGGTGGTTTCACCA
TTGGTTCCAGCAAGTTCAACGGTACCCTTATCAACCTTGATCCCTGGGAGGACTCCACCT
TCC
>tig_1165_consensus
AGAAAAAATATTCAGATTGATTGTCAATTTTAACAAAAAGACTGTTCAAGAGAAACGACA
ACATTAGCAGTCTAAAAACTTAATTGACAAAACCATTCAAACCGAAGCTCCTCGCTCAAG
CTTTTTATTACCCTAACCTAATAATATGAGAAGAAAAAAAAGCCCCTAAATTAAGGCTGA
GAACGGGGCAAAGCTAAAGTTTTCCGCCATTCACACTCTTAATTTATAAAACAAATGACC
AGAAGTCTCATCATCTTCGTTTAGATTAGTATTTGTAGTCCTTGACATGGAGAGACTCCG
CAGCGCCCTCGCCAAGCTTAGCATCGCCCCTGTAAATACCAAGGGTGGCCTCTGAGTTGG
CCTTGGCCCTTACAAGGAATGCAGCCTGGGCCTTCTGCACATTCTCCTTCTTTCCTCCCC
ATGCCTTGAGAGTGCTCTGCTGAAGTGCCCGTCCGAATGAGAATGAAAGTGACCAAGGCT
TCTTTCCCTGGAGTTTGTTCATGGCGTTAAGGTTGAGGGTAGCTTCCTCCTCACTCTGAC
CACCAGACAAGAACACAATTGCAGGAACTGCTGCAGGCACAGTGCGCTGCAAGGCTCGCA
CGGTGTACTCAGCAATCACCTCTGGTGCAACCTTGGGAGCATCAGATCCAGGGGTAACCA
TGTTGGGCTTCAACAGGGTACCTTCAAGCATAACATGGTGGTCATTCAAAGCCTTGTAGC
ATGCAGCAAGAACACGCTCAGTCACAGCTGCACACTTGTTAATATCGTGGGAGCCATCAA
CAAGGATCTCCGGCTCAACAATGGGCACAAGACCATTCTCCTGGCAGATGCTGGCATATC
TAGCCAAACCATAGGCATTCTCATGGATGGCAAGTTCAGAAGGCTCATTTGGACCAATCT
TCAGAACAGCACGCCATTTAGCAAAGCGGGCACCAGCTTCATAGTACTTAGCACAACGCT
TGCCAAGGTCATCAAAACCCTGAGTGGTGGTTTCACCATTCGTTCCAGCAAGTTCAACCG
TACCCTTATCAACCTTGATACCTGGGAGGACTCCACCTTCCTT
>tig_1166_consensus
CTTGCCTTCTTGTTACAGTGCTATTGTGACCAGCAACATTGCTGTTTATAGCATTTAATC
TAACTCTGCTAAAGCTAGACATTCGCTCAAGTGCCCGGTTCACTAAAGGACGTTCTGATG
CTGATGCAGGAGTGGTAGGTATTGACTCCACAAAAGCCCACAAAGAAGAAGAAAACCAAC
CAGATGCAACTCCACCACCCTCTTGGCCATTTTGAGTTTGATTTGCTTCAGCCAGAGGTT
GCGTAAATTCTTGTACAGTACCTGAAGTAGCAGAGTACAGAATTTCCTGATTGGATTCTG
CTGTTCTAAAAATATTACTTTGTTGCTCTGGCATTGATGGTTGAGTGCTGGTGTGATCTA
CGCCAGATGAACCAGACAGTAACTGGTAATCCACCTGGTTTACCATGAAATGTGGTTGCA
CTTGATGCTCCAGAAGGCTCACTACTGAATTGGACTGGAATTGGTAATCCACCTGGTTTA
CTGCAAGATTTTGTTGAAACTGCTGATCCACTTGACTTACCAAAGGAGGAGGTTGAAACT
GATGATCCACCTGGTTTAACACATCATCCCCCAGCACACTCATTTCCTGATGAATAGGCC
CCATGTCATTAAGAAACATGGCTGCATCATGGTACAGTTCAAATTCACTCAATCCATCGA
GCTCATCAAACTGTAAGTTCTCAAACGGTTTCTTGGCAGCAGAAACAGTAGGTCCAGGAC
AACTCAGATCATCCATTTCCAGGAAATCCTCAGCACATAGCTCCAGTAACTGCTCAGATT
TCTGGGAAGGAGCAGATGTGACCTCAGGTGCCTCATTCAGTTGCAAATTAGATGAAGCTG
ACCAGACGAAATCAAAGCTCACCGGCGTATTATCACACACTCTATCCTGCTTAGGAGAGA
CAACTTCCCTAGATGATGTGTTTAGCAAAGTACTTTGGGCCTCTTCCTCAACAAAAACCT
CAGGCAATTCATAACCATAATTGATTTGTGGTTCAGGATCATCAGCAAGTTGTCTCATAA
ACTGATCAAGATCATCCAACATAGATGGTACTTGATAATTGGCTCCCCCATTATCAACAA
GATCAACTTCTCCATTCAGATGCTTTGCAGGAATATCTGGATCAACATTTTTGGTAACAT
CTGGTACATCATCAAACCAGTCTTCCTCTCTAAAAGGTGCTCCATATTGCTCACCATTTT
TAGGACCAGGCCCACTTTTTTTGAAGACCTTATAAAGTGCATAATAATCCTGTATATTTT
GGCACCTCTTGAGTTCCTCTTCATCCAAGGTATACTCATGCATCACCCAGTCAGTACGCT
CACCACTTGGTGCACGGCCTCTATAGAAAACGAGGGTTTTCTTCACTCCAACATTACGAG
CACTACAAGTTATGATGCGATCCTTTCCGGTGGCTTTCCAATACCCAAGTCGAGTTGCCC
TATTTGACCTTGCTCCATTAGGGTACTTTCTATCCCTAGGACTGAAGAAGAACCACTGCC
GATCCCCCGTCTTCAACAATGATAGCCTAGGAAGTTCTTCAGGATCCCACTTATAAACAT
CAGTCTCAGCGATGATATCGAGCTTGGTCTTCCGGTTACAGATCTTCCTCTTGAGCCAGT
ACACAACCAGCTCCTCATCGGTGGGGTGAAACCTGAAGCCGGGAGGCCAAACGTCATCCG
CGTTCACTTTCATCATCAATTCTGAGACAATTCAAGAAAAAAACCAAAAAAAACCCACAA
CGGAAATAGAGAAATTGAAACGAAAAACAACAAAAAAATAAAGAGGGGGATCGGGTAGAG
TTTGTTGGGAGAAGAAAAACCAAAATCGAAAAACAAACCCTAGAAGACGAGAGGACTAC

>tig_1167_consensus
AGATTCATTCACAAATAGCAATTATGGGAGGAATTTCAAAGTGGTTTTGAGATCAGGATT
CAAATCGTCAAATATTACGGCAACCTAAAGTAAAGGCCACCATGACTACAAACCCTCAAG
CAAGCTTTTATTGAACTACAAACCATGGCTGTACATTACAGGGGACATAAAAGGGCTGAC
GCGGAGTTTTTGAAACAAAACAGAAGCAAACACTAGGATTAAAGAGCTAGCTAGATAGCC
ACATTGGGTTACTTCCGGCAGGTTTTTTTTTTTTTAACTTGTTCATTTTACAATTCTCAT
TTTGGTAACCCTCTTTTACTCCTCTAATCCACAACCACTTGAATTCTCGCACATCGATTT
TGACATTTCAGTGTAAGCAGGGATGAAAAGATCTCCTACTGTGTGGCTGCAAGAACTGGT
GGCCCTTCCACCTTAAACGTGGCACCCCAGATCTCTTCCTCCTCAGCAGGAACTGCAGTG
ATCTTATTCTTTGGATTCTCATAGCTTCTAAGACCTCCAATAGTGGTGGAGTAGAAACAC
CCTTGAGGGAAAGAAGCAAGTGACTTGCCACAAGCACCTTTAAGCAGGTCAGCATCATCA
GCAAAGGCAACGTATCCATCAGCAGTGATTCCCCAATAGAGAGGAACCTTACCAAATTGG
TCTGAAGCCACAAACAAGGTGGATGTAGACTTGTCAAAGACAATGAAAGCGAAATTCCCA
CTCAGGTGACCAACAACATGGTTGGGAGGGTAGGGTGCTCGATCTCGAAGAGCCTTGTAA
GCCTCAATAACCAAGATCACCTCATTTGCTGACTTAGCTAGCCCATATTGCTGCCTCAGA
CTTCCCAAGTTGTCAAGTACTCCCTCAAACAAGCAGAAAATCTCGTCCTTGACGGCAAAT
GATCTTGGTTGTAAAATAGACTCGTTCTGATGAGTGTAAGCCAGTTGAGCATTGTCTCCG
ACCTGCACGGACACGGCAGAGGAGTTTTTCTGGAGGAAGCGGCTGATCAGTGCTGTCGAT
GTGATCTTCGGTGAAGGAGTCCGGCTTCCGGCCGCCACTAGTTCCTCCGGAGGCGACACG
ATCGCGCTGCTAAATACTCCCAACATTTTTGCCAGGAAACTTCTCCTCTCACTTTCTCAC
GCTAGAAAACTCTCTCTCCTGCAAGAAAAACTAATATATTTTTATAATTATAATAACTAC
TCGGTAGCTCTAGCTAGAAGGCTCAGAGCTCGGAAGAATGAGAGAGGTGA
>tig_1168_consensus
CTTCCACTCAGGAAATTCATCACAATTCAAAATTTGTAAGTTCAGCATTCAGGCGGATTA
CAGAATCAGAATTAGTACGAACTCAAAACCTGCAATATATTCCATAATCCTGATTTTCAT
CAACCAACCTTGAATCACTTGGACCCTAGAAACAATTTGCATATCCAAAAGCTTCGGATA
AAGCTTAATTTGTAAGGTCTTCATGACTGCTAAAGACTAAAAATTATCACAAATTCATCA
TAAAACCTCATTCTAAAAAGATATATTCAATTTCTGACAATAAACCTAAATAACAGCCCA
ACTAATTTCCATCATTTTTAAGAAGTCAACCCAACAGCTGAATTACAATGATACCTACAC
GTCATTTTTCCAGACTAGTTGTGACAATCAAAATCTCAGGACTTCGATGAGCATGAGATT
ACATGAGACTTAAATTTCTCGAGCTCAGCTAAAACCTCCTCTTCTGGCCTATAGATCAAA
TCGCTCATCCGCCATATCTGCAATGTGCCCCCTCCACCTGTCGTCTCACAGTCATCAGAG
ACACTAACAACAGTCCATGGATCAGATGCATTCCAATGGAAATCAACAATTTTATCCCTG
TGCCCCGCATGCTGGAAAAATAAGCCTGCAGGAGAACTTGGAGATCTTGACCCATGCTCC
ACCTTTTTACCAACCTTCTCATAATCCCAAATGTTTAAGAGACCATCCTCAGCAGAACTT
CCAAATACAGATGATTTGTCTGGAGACCACTGCACACAAAGAACAGCAGCTCCATGACCC
TCAAATTTATGGATAGGTGACCCAACCCCATTGGAAGTCAGATTCCGGCGATCAAACATG
CGAACAGAATTATCAGCTGAACCGGTAAGGATAAGATTATCATCGTGGGGATTCCAATCA
ACACAGTGAAGATCAGCATTATGCGCTTTCTCAACCTTTATAACTGGGTTTGAACCAACT
CGGGCATCCCATAATATGAGGCAAGAATCATCACCTACGCTACAAAATTCCTGTGCACTG
GACGGACAGAATGTCACATCTTCAACTGTATCCTCATGCCCATAGTAGATACCTCGTGGG
CCAACAGAAGGACCATCAGCAGCTTTATCATTACCTTCCCCAGGCTTGGGATTCTGTTTA
ATGATTGATCCACCTGATCCAGCAGATTTAGCTGTGGATGGATCTGTGGTTGATGTTGTT
ATATGGTCCTGGATACTCCATAAAACCACCGATTTGTCCTTACCTCCAGAGAGCACATAG
GGTTCTGTAGGGCACATAGCAAGAGCAAATTCTGCATTATCTTGATGTCCAGTCAAAATC
AAATCTGGCCGAGAATTAGTAGCTCCAAGAACGGCATGGCGGTTAGGTTGAGTTTCAACA
TCCCAAATGAGAACATCAGGACCGTCAGTGTGTGTGGCCACTATCTTAGTATTTTGTGGG
AGCTCCCTAATTCTGTTGACCTCTCCAGGATGTATAATGGTCTTGTGCTTCTTGACAAAT
GGAGATCGAGCCTCTTCATTAAACTGAGAAATGTGCTCCGCAGCTGCAACCCTAGGTTTG
ACAACCTCACAATTCGCGATAACCAATGTATTCGGAACGCTACCATCAGTCTGTTCAGAA
AGGTAAAGTCGTTGCCGATTCTTGTAAGTAGCTTGCTCAAGCTGCGGGCCCCAACGGCAA
GAAAGAGAAGGCCAAACGAGGTTGTGATTAGCGAGCCAGTCATAAAGAACAGGAACGAGA
GATTTCCACTGAGTGTACTTGTCGTCAACAGAGTGATGTTGAGTCCTCTTGGCCGTGTCT
TTCATCTTAGGTGTTTGTTGTTGTTGTTCGTCTTTCTTGTCTTCCTTAGGTTTGGGTTTA
CGGCCTCGCTTCTTGGGTGCCTGTTGGGGATCCATTGTAAGCGATTAAAAGTCGCCGCCA
CACTCAAAGTTACAATCAAAACCACCGGGGGGGAGGGGCCGGGGACCTACTCATACTGAT
GGCGACATGATCCCTTGCCCTCTGTTACCTCTCCGTGGTCTTTTCACTTGGTAAAATTCT
TC

>tig_1169_consensus
CACACTCTTACCGGGTTCGGATATGGCGTCAGTGGGACGCACGGTGCCGGTCCTGGTGCT
GGCGCAGGGGCTGGTGCTGGTGCTGGTGCTGGTGCTGGCGCAGGGGCTGGTGCGGGTGCT
GGTGCTGGTGCTGGTGCTGGCGCAGGGGCTGGTGCGGGTGGTTTCTCTTTAGGTTTCTCC
GGTTCTTTTGGTTTTTCAGGCTCCTTTGGCTTCTCAGGCTCTTTAGGCTTCGCAGGTTCT
TTTGGTTTCTCAGGCTCTTTCGGCTTCTCAGGTTCCTTTGGTTTTTCAGGCTCTTTGGGC
TTCTCGGGTTCTTTGGGTTTGGCGGGTTCTTTAGGCTTTTCAGGTGGCTTCGGTGGAGGT
ACAATATCAATACTCTTGATGGATTCGCCACCTTTGCAGATAATTTTTTCCTTGACCTTC
TCAGGACTGCAACACACCACTTTGATCGTCACTGTGTTCT
>tig_1170_consensus
GTTCGCTATGTAGGGTTCAAGATTTAGTAGTAAGTGTACATAGAGAAAGGTTGAGACTGA
AATAACTTCTACACAAGTTTATAAATGCAATTTTCACCTCAAACTTTTTCTAAAGTGGCC
ATAATCTCTGTTTTCTGCCAATATTATTACAACATAGGCATGCATTTTCCAAGCAAATCT
GCATACACCTTTACAATTATGATTGGAGAGGTAGAGCCCCAGGGATTTCGTGAATTAGTC
AACACTAACAATGATAAATCAGAATTATACCAAAATCAATGAGATGGTGGAGGTATCAAC
AATGATGATGGTATCACAATTCTCACTAGGCTTGAAAATATAGAGACAAATTTTTCACAT
AAATTTAGCAAAGGCTTAAAGAAAAACCATAAATTAAAATCCAGTGTGATCAAAGTATGG
TAAAATATGAGCGAACATTAAATTTCCATAGAAACTTTTAAGCTATTGACCCTTCAATTT
GCATTACCTTTTGATAATGAGGCAATATGGAGACAAGTACAGACATCAAGCATCATGAAA
TTCTTCTCAATTCCAATTTCTTCTTGCCAACTTACTGCATTATATGCTGAAAGAAGGTAA
CAATTAATGCATCACCTTAATTCTACAGGCCATAGAGTCTGCTACTTCCCATTAATCCAC
CATTAATCCATAAGGCAACCAACAGATAATCATTCCCACTCAATAGTAGATGGAGGCTTT
GATGTGATGTCTTGAACAACTCGGTTTACACCCCGAACATTGTTACAAATTTTTCTTGCT
ACATCATCAAGAAATTTGTGCTCAAAATAGTACCAATCTGCTGTCATTCCATCTTGGCTT
GTAACAGCTCTAAGAGCAACAACATGTGAATGTGTTCTTTGATCACCTTGAACTCCGACT
GATCTTACAGGCAAAAACACTGCAAAAGCTTGCCAAATTGAATCATAAAGCCCAGCATCC
TTGATTGATTGAATAAAAATTTCATCCACCAGGCGGAGGATATCTAATGCATTGCCATCA
GTTACATCACCTAGAACTCGTACTGCAAGCCCAGGCCCAGGGAATGGGTGACGCTTTAAA
AACGGTTCAGGGACATTTAAGATTTTACCAAGTTGACGAACCTCGTCTTTAAATAAAAGT
TTAAGTGGTTCAATGAGCTTCAACTTCATGTCCTTAGGAAGGCCCCCAACATTATGATGA
CTTTTGATTGTGTGAGAGTGGGCTCTTCCAGTTCCCGGTGGCGGGCATGATTCAATTACA
TCTGGATACAAGGTTCCTTGGACCAAATAAGCAGATTTCTTTCCAAGTTTCTGCTCCAAC
TCATGGGCAAAAGCATCAAAGATGCAGATGAATTCCTTTCCAATTATCTTTCTTTTCATC
TCAGGATCTACCACACCTTTCAGCTTACTAAGAAACTGATTTGTCGCATCAACACAAGTT
ACTGGTAGATGTAGATCCTTTTCAAAGGTTTCCATAACACGTTCTCTCTCTTTGTACCTT
AACAAACCATTGTCAACAAAAACACAGTGAAGCCTATCCCCAATTGCCTTATGAACAAGA
GTTGCAGCAACTGTAGAATCTACACCCCCAGATAGAGCACAAATGACATGTTCTTCAGGT
CCCACTTTTTTGTTAATTATTCTAATTTCCTCATCCAATACATTTTCCATCTTCCAGCCA
GCACTAACTCCACAAACATCAAACAGAAAATACCTTAGTGTATCCATCCCTTCAGGTGAG
TGTGTCACCTCCGGATGATATTGAAGCC
>tig_1171_consensus
ACGCAGCTAAATTTAGCTGGAAAATTGGGTTTTGCTTGCATAAACAAAATCAGCAGTTTC
TGATCAGATCCAATTTCTGCATTATTTCCCTAATCCTATAAAGTCAGTGGCTTGCATCAT
GTTGACAGCTATGAGCTGTAATGATGTTGATATTTCATTACTCATAAGTCGTTTGTCCTT
TCTCACTATAGATTTTGGTTCCCAGATCTCTCTATCTCCGCTTGACATCTGTTAATTTGT
CTAAGCACTGAAATACTGCACGTATTTTGATTGATTTTGGGGTCTGAGTACTGATTCTGA
TTCTGGGTTTGGCTCACTTTTTACTAAAAGTAGCACAGGAACTGGCACCACACCAACCGC
AACACCAACAACATCAACCACACCTTCAACTACAACGGGCAGTCCAAATGTGTTCGGTGG
CACCACAGGTCTTGGCCCGACAGGAAGCGGAACTGGCGGCATTACGGACTCTGGTG
>tig_1172_consensus
ATCCACAACAGTGTCACATTTCTACATCCAAAATTTACAATATTTAAGGACCCAAAACAT
CCTATAAAAGAGACAAAACATGCTCACTCTCAGTAGGCAAAGGCTTGTGAAAACAATACA
CACTTAAAAGAAATCAATACCATAAACTCATTCTTGAAGGAATCAATAGAAGGAAAAAAC
AAAAAAAAGTAGAACCAAATGCATCTCCCACCAAACACAATGTTATACATAACTCAACCA
AGCCCATCACTAAGGAACAAACCATCAGTGTATTTCTTCCTCAACTTGTTTTTGGATTCT
CACTGTCACTCTGAATCACGGGCCTGAGCAACAACAGGTGATGGAACTACATTTCCACTT
ATGTGATTTTCCTTCTCACTGTTGGATATACTCAAATTTGAACTTTTAGATTTTTCCTCA
GTCACCTGCTGTCACACTATTTTCCAGCTTAACAGATTCAACAATACTTGATTCAATTTG

CTCACATCATTTTCTTCTAATTCTGCTGATCATTTCTCTGACTCCAAAGGTTTCGATAAC
TCAACCTGATTACTTTGTGAAACCTGCTTCAAAGCAGCATGAATATCCTCCAGAGAACTT
GCTTCCAGAACAGGTAAGTCTGAGTTGGTGCCTACTGAATCTACATTTTTACCAACAACT
ACCTGATCCTCAAGCATACTGGGTAGGATGACTTCCTCCACATCTACACCTTCATCAAGT
TGCTTAAATGCCAAGACAATATCTTGAATTGATTTTGCTTCAAGAGTTGGCAGCTCTGCA
TAGGTTGCTGCCAGTATGGAATCATTGGGCAATGACTCAGCATTTGAGATATTGGTCTCC
TCCAATGTCGGGTCAATATGAATGGACTCACCTACTTCTTTGACACTAAAGTCACCAACA
GTGTCCAGTTCAGACAGCAACCCTTCATCAATTTCTCTTAATTCATCTGCTTCTTCGTTG
AAGTTTTCCTCATCAATGCAAGCAGCATATGATTCTACTGAATTACTGATATGTTCTTCA
ACCTGATCACGATCCCTATAAACAACTCTATCATGGATTCCATCTTTCACGTCCTCTCTA
CCAGGAGATCTATCTCCAGTACTCCCAAATAAGGTTGAATTAGAGGTTAGGGATGAAGAA
GCTTTCAAAGTTTTTTCCTCATGATCACGAACTTCAGAGACATTTGCAATGGTGAGAATG
CTTACTTTCTCCCCATCCGGAAATCTACTTCCAAGACTCTTGAATGTGGTTGAAGAGGAA
TTGAAAGTAATGGATGATGAACTTTTCACCTCATGATCATGATCAAGAACTTCAGATACG
TTTGCATCATTGACAGTGTTTACTTCCTCAGCAGATCTTGTCAGAAGGATTGGTGCTTCC
AGCGTTTCTTCATCATCATCATAAGATGGTGATTTATCTGACCAAGATAATTGGTTCTCC
AATTCAGATGTAGATATTTTTTCAGAACTAACATCTTCGTAACTGTACTTGGCAGAAATC
GCCTTTGTGTCCATATCCTCGTGGAGACCAGAATCAATCTTCGACTCAGAAGATGATTGG
ACTTGATCCTTTTCAGGCTGATGAGTTTCTCCCTTATCTAATATATGCTCCTGTAAATGA
TCCGTCTCTGGAGAATGATGAACTGTTGGAACTAGCTTGGTCAACAAGGGAGACTGCCAT
TCCACTGGTGTAGACAACGTTAAATTATTGGAATCCATATGCTGGGAACTCAATGGCACA
GAATACAAATCCTCAGAAACATCTTGGTGGCCATCAATATGGATAACTCCGTCAATACTT
AATGTGTGGACTTGGCCTAGTTCCTCCAGCAAATGTCTGTCCTTGTCTGTCATGCCTTCC
TCTACTGACTTCTTGTCTAATGAACTTGACAAATGGACTTCATCCAGTTCATGTTGCATG
GTTTCTTCCTTATCATTTGCTTTTTCCTCCACAGGCTCGGACTCACTAGTGCGCAGAATA
ACATGTTCAACCACCATTGAAGGTTGTTGCCTCTCTGGAGCTGACAAACTTGAATAGGAG
TCAGATATCTGATCACCAGGAGCTGCAGACGCTAACTTCTCAATCACCTCTTCATGATTC
TCAGAATTATGTCTTGATGAGACAACTTGATCCTTTTCATGGAGAAATTGCTCTCCCCTA
TACACCGTATACTCCTCTACAGGAATAATGTCCGAAGAGAACAATCTCACAGTGGCTCCC
TTTTGATTGTCAGTGCTTGGATTAAGATCATGCTCACTTGTCTCTGTGAATGCAACTTCC
CTTAATGTTGATTTATCTTCATCTGCTGCATGACCTTCTGAGGAGGCTGTATCCACCTCT
TCAAAATCATTAGAAGTATCCTTCTCATTGTCCTCAATG
>tig_1173_consensus
TTTCGGGTATTAAAAACTATTAAAATATTTTTGAAAATTATTTCTGGACTATTTTTGATT
CTCAGACTAATCTCCACTAAATATTACACAAAATTAGATATGAAGAACTATATTTACTTA
AAACAGTCCAAACCCAAAAGAGGAAATTGCTTGAGGCCCCTGCCATGCCCATGCCATCTT
CGGAAATCAGGAAAAAAATGAATTCATTCAGATCATTTGCTGAGCTCTCACACAAATCTT
CTAACTCACTTCCAGTCAGTCAAAACGTTTTCTGCATTATTTAAGCTGCCGCTTCTTTTC
ACCCATATTATAGCCTTGGTACCTCGGAAAGAATAGGGGCTGCTGGGGTTAAAAGTTTTC
CTCAACTCTCTACCAACACACACATTCATGAAGAGTGAAAATTTTTACTACCAAAATCTT
CCAAGTTCCAAGTAGACAATCATATACAGGAAAATTTAACAATAGCAACAATCCAGTGTC
TAATCACGATGTCCACAAGGTATGCATAGCATGTAATTCAGTGATATTAGTGGACTTCAA
AGACATTTTGTAAAGCGCCCTGAGCTTTCCAACATCCCCAGACTGAGTGGATGGAGTTGA
CTGGGTAATGGGAGAAGATTTGTTTGTACTTGCAGAAAGTGACATNNTCAGATATATGGC
GTCCTTCGAGTAGGCGAGATAATGCTGATTCCAGAATTGCATTTGAGTGTAAAGAGCAGG
AAAGTCTGAATGAAAGAGCAGATCTTTCTATTAGAATAGTCAGGTACATAGTGGGATGTG
TCGTGGGACCATAAAAGTCCATTGCTTTCTGCCAATTTCTCTCAGCTAGGGAAGCATATT
GTTTTACCCTCTGGACAATGCTATTTTCCCCTTTAGGCAAGTTATTTTTCTCATGATCGG
ATTCCAAAAACTTCAAACAGCAATCCCTTTGGTAGCAAGCAAGTTGAAAATAGGTATATG
CAGCCTCTTGTCTGCGTAAATCTCCCATTGATTCATAGACAGATAGTGCCTCTCTNNAAG
GAACAGATAACTCTTTCAAGGCTCTATTTTCATAAACTTCAGCAGTTGTATCTTCTCTAG
CCAAAAGCATGCCAAGCCTTAAATAGGTATGAGCAAACTGTGTATATACTTCATTCCTCA
AGCTGTCTGACACTGAACTAGCCTCTTTCACAATAGTATTCAGATGTGACTTTGCTGCCC
CATAATATCTGAGTGATTCACTGTATTCCAATTTTGCATTCTCCAGTGATTGTTTGTATG
CACTATGGAAGATTGCATGCATTTTGAAACCTTCAACCTTTGATACCATCTCTTCGGCCA
ATGCTCGTCTGCCATGAGCCAAGTTACAGTTAATCAAAATAATGTTGGTGTGGTCAGAAA
CTTCTTTAAACACATTTATAGCCTCAGCAAAAGCATGTTCAGCTTTGTCCAATTCTTTCC
TTTCAAGCCTGATTCGACCCAGTTCATTGCAAACCCACCCTTTCTTTTTCAGAACAGATT

TCAGTTCTTCTGAAACTGCAGGAAGTCCACCCAATGCCTTCCTAGCTTCTTCATAACAAC
TTAGTGCGGCTAAGAGGCTGTGTTCTACATCTCCAAAAATGGGACCCTCAAGATATTTAA
ATATCCCACCATTTTTCATTTTGGGAGCTTCTCTTGAAGCAATTTCAGCTTGTTCAGGCA
CTGTGAACTCTGCATTATGTGTTTCTGACGACTCTTGTGCTCCTACCGGTGCATCACCAC
CTTTGTTGAGTTGAAAATTTTTGTTTTCAGGACTCTTCCCAGTCTCATCCTTTTGATTAC
CATATGCTTTGTTAGGGTCTTCCAAAAGTGAGTATGTTGCATTCTTAACATGTGATCTCT
TACCGTGTTTTCTGCCATACGCAACTGACCATTTATCTGCACTGCTACTACTTGCACTGC
TTCCACTGCTGGCCCTATCACTCTGGCAGCAGCAATTTACCAAAGAACATGAACTGCAGT
TCTGGTTAGACTGACCCAGCTTCCTCTTCAGCCTTTGGAATTCTTTGACTACCTCTGAAG
ACATTCTTAACTCCCTCGTAGAAGGTTTGCTGCCTGCTTGTATTGATATCTCGGTACCCT
TTATCCTATGGAATTCAACATAAACATCTCCAACTAGTGTCCAAGCCTTAGCCCAGAAAA
GATACGTGAACGATAATTTCTGTAAAGTATGACAATCATCATTGGTACTTGATTTCAGTT
CTTTCAAATCACCAACAAAAGATCTAGTCCTTTTACTTCTATCATTGAATGCTATTTGTG
GTAAGAAGCTTTTAGTCATTGAGGAAATAAATTTTGTGTCCTCAAGATGCTGAGGCATGG
AACCATAAACAGAACAAGCTAATTCTACAATCTTCAAAGCTTGATGCAATTGCCCATCCT
CCCTATGAGCTTGCCCAAGTGCCAAATAAGATTCTCCAAGCAGAAGAACAAGTTTCCACA
ACTTATGGTCCAATTTCGATGTTGGAAGCCATTCCCGAATGTCACAGACTTCAATACAGT
CAGCATCACCACATGCACAAACGGAGAAGTCCACAGGTGGAGGCCCATCATCAATTCCAA
TGCCCTGGTGGAAAATCTCTGGTTCAGAGCTCTGTAGTTTACGCATCCATCTGAGAGACT
TAATGGCCTGTGAAACATGATGTACTGCAGCTAACTTGGAGGAGATCGGATCAACTACAG
TTTGGACCACATTTGTTGACAGTAGAGGTGTTCCACACACCACAGAACCTTCATCATTGC
TAGAACATGACAGATATCTTTCCTGATCTCTCAACTCCAAGTCACCAGATTCTATCAGTT
TCTGGGTAGATGACACATTTGCCTCCAAAGTCATCTTCACAGAAGCTTCTGGCACCAAAC
CTTGCAAAGTCTTTTCATCTTCACTCAATTCTCCATCCATAACTAATGAGTCCATTTCAA
GGACAACAGATTCAGAAGAACTACCAATCAGGTCCATGGATTCTTCATTAGCATCATTAA
CCGTAATTTCACATTCAACGGGCAGAGATTCAGATGTTAATTCCAGGTCTTCTTCATAAC
TCAAAATGAGTCTAGCAAACTGTTCATGAGCAAATGCCCGCACAACCAGATGATCTGGCT
CATCCAGAAATTCCAAACATTTTTGAAAGAACCTGACACACTTAGCCCTATTATCAGGTG
CCATTGAAAGCGAAAGTCTGTGAGCAATACGGTACAGGAGTGTTCCCAAAGAAAATAATG
CATCACTTCTTCCTCTGTAAATTAGGGGAATAGAGCTTGACCTGTCTTCACAAGCACTAG
ATGTATGGTTCTTGGGAATAACAGAAAGATCAAAAAGTTGAATTACATCTTCGCCAGCAC
TTTTATATAGCCAGTAAGCACCAGGATCTTGCTTGCAGTTTTCTTGAAGGAACCTCAAAA
CTGAAAGCCCGTTTTGCTGAACAACATGAGGATGAAAAGCAGGAGAACCATCTTCAGAGA
TGCCTTTTAACAAAAATATGTCATCTGTTTTCAGAAGCTCATAACCTTGGACAACACCAT
TTTCATGATAACAGATTGCCAACTCGGGCACACTTGCCATGACATTGTCAAGCCAGGCTT
CAAGCCATGTCAAAGGAGTGACCTGTCGAGTGACATCCCATAAATGCAAGCTCACAGCAG
CATATTTTTCATTACTGAACAAAAGCAAATCACTACCCAGTAGCATGTGGAAATTGTGAA
ATTGCCAAAACAAAACCCTTAAAAATCCATCATTTCCAAGTCTCCTATGTTTTTCAGACT
CTTGAATTGAGCATCTAGGCTTTTCACCAACATGTGAGGCCTTTTTAATAGGATCATGGC
CATTATTTCTCCTATTCTTCACACTTTCCCAAATGTAGTTATCCTGTTTGACCCGTGGAG
ACTCTGTACAACGTTCAAAGTCATGCTTTTGAGCAACATCATCACTTTGTCTCACAAAAT
GGGATGCATCATCCCCAGGAAGAACTGATGAAGTTGATTGACTCTCGCGTGGAGAATGAT
GAGCTGGCGGACAATCACAAGCCTCCATTCTAACTGAATGCATTGCAAAGTTTAAAAATA
AAGATTGATCTGCACATTTTGATTGACTGCTATGTCTTCTGCGCAGCTTCTCTCCTTCTT
CAACATCAGACCCAGTATTCAGAACAAGAGTCTTGCCAACACGGTGAACTGCAATTGATA
ATCGAGCCTTGGAATATGGTATTTTAAAGATTTGTTTCAAAATGTCTGTTGGAGCAATCA
CATCAATTTTATCTCCATATTCTACCAAACCAGTTACAGCAAGGGCTTCACATCTCCTGC
TAAGATTTGGTGGGACAGCCCCTTCTCTACTAATATCTCCTGTGGATTTCGACTGTGCTG
CACCAATAGGGAGAACCTTTTCCGGTAAATTGGGCACAACCGGGAGCCTATTAAGATCGG
TTTCAGTGGGCAGCATCCTATAGCGAGGAGCGCTAACTGTTTGCGGAGCGGGACGGGGGA
CGAGAGCTGAATTGAAGGCGTCATGGAAGGACTTGTCGGTGGGAACTGGAATTGAACCGC
AGAGAAAACCCACCGGTTTGGGTTTTACGATTTCTAAAGTTCCTACGCACTGAAGCTCCT
TGGAGCTTGATCCTGGTGCTGGTTTTTCCATGCTTCTTCACTTGTTTCTAGTCCCC
>tig_1174_consensus
CTCACATAATAATTTTATAAAATAAAATATGACACTAGAATGTCCCAGGGGTTGTACATT
ACAGCGGTGCCACATTAATTTCCATGACACCAAAATGTGACATGAACTAAGAAGCAGGGT
AACAAGTCCTAAGCCCTTGTGCACCAACCTAATAAAAACCTTCAGTTGGCTCATCATTAC
AGAGTAAGTTACCAGCGAAACACTTGTTACTATGCACAAATAGATTTTTCTATCTTACAC

CTTTCCGAATGCCAGATACCCGAGCTACAGGTTTAGTTCTTCCTTTCCATAAATTCGTTG
AAGCTCCCGAGTTGCTGCTTGAAATGACTCT
>tig_1175_consensus
CAAAGGTCCTTCCTAGTCAAGATCTTCTTGTCTGGGCTCTGAACCTCTAGTCCCTGGGTT
CGAATCTCCATCATTGTTCCCACCCCCATTGCTAGTCCCGGCAGATGAACTAAAGGCCGT
AAATGGCCATGGCAAGGAGATACTAAACCTGCGCTCCACCGTCCTTGGAGTCTCAGGATT
CCCTCTACCATTCCCACCATCACCACCAACAGACATCGCACCTCCAATCGCAACACTAAA
ACCCTGATTATTATTGTTATTAACAGTACTATCATTGCTACTTATATTATTGTCATTATT
ATTCCCATTACGCCTTTGCTCGTAATCTGCATCATCCGTGGGCAATTCGTACCGACAAAC
AGGGCAAGAATTATGCAACTCAAGCCAAGGCATAATACAATCCGAATGATATATATGTTT
ACAAGGCATCTGTTTCACAACTTCGTTCAACTCAAAACTATCCTTACACACAGCACACTG
AGATGAATCCGAGCCCATCAATTCCTCCGATATCTTGATATTTGGCAGCCCCTCAATGGC
TGATTTCGAAGCCGGAGGCGTCCCATATCGATTCGGATCGTTCTCCGCCAATTGTTGGAT
CAACTGCTCGAGGCCCGGCCCAATGAAGTAATCCCCTAGATTCGCTGGGAACCGAAACGC
CGCCCCTCCACCGGTAGGGTCCATCGAGGGGCTGTTCTCTATGACGAACTGGATATTTGC
ACCCCGTGCCGTCAGGGTTTGAAGATAATTTTGAAGAAAAACGAGGGGGTTGAACTCGGC
CGGGTCTGTGACCGTGAAAGAGGAGCGAGTCGGAGTGGGTCCCATGCCCAACAGCGTATT
GAGATCGTCTAAGAAGAAGGCATTGAAAGGATGATTAGAGTTGAAATTAAGGTTAGGGAA
AGGGTCAGGATTAGGGTTAGGTTCGAGTTCTTCAAGGAAGCCACCGTTGCAAGTTGGGCA
AGCCAGTTCGATGGAAAAAGACGGAGCGACCCTGACCGTGCGCTCGCACTGGTAACAGTA
ATAAATCTGTGGCTGAGTAGGTGGCGATGGCGCCGCGGCGGCAGCGCCACCATTTCCGCC
GGAGGAAGACATGTCAACCAGATCGGAAGAGCCACGTCTGAACTCCAGTCACGCCAAT
>tig_1176_consensus
TCTTGATCCTTTCTTTCCCTTTCACGCCTCATGTCAGCCTCCTTCAATGGCTAAAGTATG
AGGATTGCCAGTTAACCTTCAAAATAAAATCAACAAGCAAGACAATTTATCTATTTCATC
ATAGTAGTCGCTTTTGTCATATCCATGTGGGTCAAACACATCTTTACAGAAGCAAGACCC
TATCTGATCAATATCTTGATA
>tig_1177_consensus
ATGCTAGTCGGAGCTACCAGCAACACCAACAAGAGAGGCTTGCAACTAAATTTCAAAGCC
AAATTTTTTCTAGCACTTAAGAACTTTCAGTTTTTCCAAAGAACTACGGAGGTCAGAAGT
TGCCCAATCCTCAGGAGTCTGAATAATATTGGGGTTGTCAAATGAGATGACATCTGAGAG
AGGGTAACTCCGGTTGGCATGAACTTGATTACTTGCACTGCTACAAGCTCCCTTCATTAC
TACAGTTTTGTGCAAGCCACCAAGAAGTCCTTCATAATCTGTGTCCCCAGAATCTCCAAC
AAAAACCACCATCTTAGACAAC
>tig_1178_consensus
AGCATAGATTAATGGGTTTAATTTACCTTTTCCAGTTTTAAACAGCACTGTGTGTTCTTT
TGTTACACTGTGCTACCTGTCTCCTGGTATACTTGCCTTGGTAGCCTCATGAAAAATACT
TACCAAAAAAAAAAAAAAAGGAGATGGGACCATGTATGGAATTCCATGAATAAAAGTCCC
AGATCTTAGCCACCTTTTACGATGACCAAGAAGAATAAACTACACAAGAATGGGCTATAC
ACAATGATAACATCTCTACATGAAGTAACTGGGAGATGTCTTCTTTTTGGATCCACCTTC
TCCATACAAATCATTCCACTGGAGGAGCTCATTCATATTTGTAGACTCAGATGACACACT
GGCACATACCTGCTCATGTGCATATCTAAAATCCTCCATCTTCAGAGGACGAATGTCAGC
ACTGCTATGCAATGCAGGTGAAGGTCTATTCTCAGCCAATGCCAAAGCTCTCTCCTTCTT
TTCCCTCCCCAAAATTTCTCTTATGGGGCAATGAGCAGCAGTCACACATAGGTTCTTTAG
ATCGCTTCCCGAGTACCCATCAGTCATATTTGCAACCACTTCCAAATCAATATCCAGTGC
CAATTCTTCTTTGGCTAAAATAACTTTTAATATTTTCTCTCTGTTTGGCGCATCCGGCAA
GTTAACCATCAATCTCCGGGGAAGCCTCCTAACTACAGCCTCATCAAGGTCAAAAGGCCT
ATTGGTAGCAGCAAGTACCAATACACGTTCCTTATCCTTTGTACGTAGACCATCCCAATT
TACCATAAATTCATTCTTCATTTTACGCATAGCCTCATGCTCCCCCGGATTTTCCCGTCT
TCCTAACATGCTATCAACCTCATCAACAAAAACAACACTAGGAGCAATTTTACTGGCCAA
AGAGAAGACCGCTTTAACATACTTCTCTCCTTCACCAAACCACTTTGAGGTAATGCTTGA
CATGGATATGTTGATAAAGTTTGCACCAGCTTCAGTTGCAACAGCCTTCGCTAGCATTGT
TTTCCCAGTACCAGGAGGTCCAAACAATAAAATTCCCTTGCAAGGCTTTGTTAGCTGTCC
TTTGCAAAACAACTCAGGCCGTTGAAGAGGTAGCATCACCAATTCCTTCAAGGTATCTTT
AACATTTTCCAAGGCACCAATGTTGTCAAAAGTAACCCCAATATCACTAGGGGGAATAAC
ATCAGCAAGGAGTTTCTTCTCAAATTCATTCTCAGTAACAACATCCTTCAGAGACTTTTT
CAAGCTTTTGCTTTCATTTTGAATGCCCTGCAGAATGTTCAGCCCATACTTAATGCTTTC
AGTAGATATCTTAAGTTTAGCATCTTTTTCTGGAGATTCAGAACAATGCATAAAATGGTA

GCTCAAAGCCCAGCCAACTAACTTTTCCACACTTTCAGTTGTAAGGCTTTGATCTTTGAT
GCAGAGAGTTTCAAGATCAGGGCACTCCAAGCCATTTCGGCAAGTATTGAGCGTATGCT
AACTATGTTTGACTGTATTTTTAAAGTTTCAATATCACGCTCCAACTGCTGCTTCCAATC
CAAAAGCAAAGCTTCATCCTGAGGCAGTTGAATAATCACTTTGTTTGGAAAAAGGCGAGA
AACTTGCTTTGCAGCTTTGGGTGTCTCTTTGCTTCTATCATGCAGTCTACTAAAGTTATC
CGGAAAAGCAAGATCAAGTAAAGCTGTTTGATTGCTTCCAAACTTCGTAAATAAGAGACC
ACCTGAATGAGACTTCTCTTTGCGATTGTCCATTTGGCTATGGGATCCAATAACAACAAC
ATTGCTGGGTAAATTTTCAAGTTTACTCTTTAGGACACTATATGCATCACCATTCCCTAC
CAGAGACTTCTCCACATCTTTCACAAACAATATTAAGGGACTACCTTTGCTTTCATTCAA
TGCAACCTCAAAGAGTTCGTTAATAGCAAGCTTGTCAATGTCATCACCCACAGAAGGATC
TAAGCGCAGTGAACTAGCTGGGCAAAAGAAACCATGAAGATCTTCACAAAAGCCACCTAG
ATTATTGCCTTCTACGACCGTCTTATCAAACCTAACGCCAATTTTAGAAGAATCGTTTTC
TTCAAAAGGTAGAACTACTCTTCCTTTAAGCCCAATGGTCGGTCCTCTTGATTTAGGTTG
CATAGGAGAAATTCCAGAAGGTAGATTACCCACAAATCTTACTCTATCACCTTTTTTGAA
AGTGTAGTTTTTGGATGATGCAGTTGACGCCTCTGGCGTTGTCTGTGCCTGTGAGCCTAC
TGCAGACCCACCTGTAATATCCGCCTCCACACTAGATGTTGGTTTCTTGTGCTGTACAAG
AGCAGCTCTTTTAGCAAATACAGATACTCTTTCAGACCTTGAACTTTCCTTTAAATGATC
AGCTTCTTTTGCTGCCTCTCCACAGGGCAAGAGAAGAGAATCAACAATAAGCAACCTAAC
ACCAAAGTGTTTGGCAAGTGCCTTTGCCAAAGTCACCTGATATATTTCTGAACCTGCAGG
ACCAGATAACAA
>tig_1179_consensus
AAGAATGGGCTATACACAATGATACCTCCTCTACATAAAGTAGCTTAGAGATTTCTTCTT
TCTTGATCCACCTTCTCCGTACAAGTCATTCCACTGAAGCAGCTCATTCATATTACTAGA
CTCAGATGATACACTGGCACATACCTGCTCATGTGCATATCTAAAATCCTCCATCTTCAG
AGGACGAATGTCCACACTGCTATACAATGCAGGTGAAGGTCTATTCTCAGCCACTGCTAA
AGCTCTCTCCTTCTTCTCCTTTTCCAAAATTTCTCTAATGGGGCAATGAGCAGCAGTCAC
ACAAAGGTTCTTTAAATCACTTCCAGAGTACCCATCAGTCATATTTGCAACCACTTCCAA
ATCAANNNNNNNNCCGGCAAGTTAACCATCAATCTCCGGGGAAGCCTCCTAACCACAGCC
TCATCAAGGTCAAAAGGCCTATTGGTAGCAGCAAGTACCAACACACGTTCTTTGTCCTTT
GTACGTAGACCATCCCAATTTACCATGAATTCATTCTTCATTTTACGCATAGCCTCATGC
TCTCCAGGATTTTCTCGTCTTCCCAACATGCTATCAACCTCATCAACAAAAACAACACTA
GGAGCAATTTTACTTGCCAAAGAGAAGACTGCTTTAACATACTTCTCTCCCTCACCAAAC
CACTTTGAGGTGATGCTTGACATGGATATGTTAATAAAGTTGGCACCAGCTTCAGTTGCA
ACTGCCTTCGCAAGCATTGTTTTCCCAGTACCAGGAGGCCCGAACAGCAAAATCCCCTTG
CAAGGCTTTGTTAGCTGTCCTTTGCAAAACAACTCAGGCCTCTGAAGAGGAAGCATCACT
AATTCCTTTAAGGTATCTTTCACGTTCTCCAAGGCACCAATGTCATCAAAAGTAACCCCA
ATATCACTGGGAGGAATAACATCAGCAAGGAGTTTCTTCTCAAATTCATTCTCAGTAACA
ACATCCTTCAGAGACTTTTTCAAGCTTTTGCTTTCACTTTGAATGCCCTGTAGAATGTTC
AGCCCATACTTTATGCTGTCAGTAGATATCTTAAGTTTGGCATCTTTTTCAGGGGCTGCT
GAACAATGCATAAAATGGTAGCTTAATGCCCAGCCAACTAACTTTTCCACACTTTCAATT
GTAAGGCTTTGATCTTTGATGCACAGAGTTTCAAGATCAGGGCAATCCAAACCATTTCGA
GCAAGGACTGATCGTATGCTAACAATATTTGACTGCACTTTCAGAGTTTCAATATCACGC
TCCAACTGCTGCTTCCAGTCCAAAAGTAAAGCTTCATCCTGAGGCAGTTGAATAATCACT
TTGTTTGGAAGAAGACGAGAAACTTGCTTTGCTGCTTTGGGGGTCTCTTTACTCCTATCA
TGCAGTCTACTTAAGTTATCCGGAAAAGCAAGATCAAGTAAAGCCGTTTGATTGCTTCCA
AACTTCGTAAATAAGAGGCCACCAGGATGTGACTTTTCTTTGCGATTGTCCATTTGTGTA
TGGGATCCAATCACAACAACATTGCTGGGTAAATTTTCAAGTTTACCCTTTAATACACTA
TATGCATCACTGTTCCCCACCATAGACTTCTCCATGTCTTTCACAAACAATATTAGAGGA
CTACTTTTGCTTTCCCTTAATGCAACCTCAAAGAGTTCATTAATAGCAAGCTTGTCAACA
TCATCACCTCTGGGCAAAAGAAACCATAATGATCTTCACAAAAGCCACCTAGATTATTGC
CATCTATGACCGTTTTATCAAACCTAACACCAATCTTCGAAGAGTCATTTTCTTCAAAAG
GAAGGACTACTCTTCCTTTAAGCCCAACAGTTGGTCCCCTTAAATTAGGTTGCATGGGAG
AAGTTCCAGAAGGTAGATTACCCACATACCTCACTCTATCACCTTTTTTAAAAGTGTAAT
TTTTGGATGATGCAGTCGACGCCTCTGGCTTTGGCAGTGCCTGTGAGCTTACTGCAGATC
CACCGGTAATATCTGCCTCCACACTAGAAGTTGGTTTCTTGTGCTGCACAAGTGCAGCTC
TTTTAGCAAATACAGATGCTCTTTCAGGCCTCGAACTTTCTTTTACATAATCAGTTTCTT
TTGCTGTTACCCCACCAGGCAAGAGAAGAGAATCAACAATAAGCAACCTAGCACCAAAGT
GCTTCGCAAGTGCCTTGGCCAAAGTTTCCTGATATATTTCCGAACCTGCAGGACCAGATA

ACAATATGCGAGGACACATGGTAGGAAGATCTGCAGCATACTTCACAAATTTGTCACACT
TCAAATGAATGTATGTGGATGCTATCAGTACATTCTTTGTTGTATCACTTAAATAATACG
GAAAATTCTCGAACGACACATCTATATTATCAGGACTCAGAATTCCTTCCTGTAAACTAT
CCTTAAATGCTTGCCGTCT
>tig_1180_consensus
TCCCCTGGTTTTGCCACTTCTAGGTCCATTGAATCAGAAAGTTAGGATTCATAACCAACT
AGAATAATGCAACCCATGCCTATCCTTCCATATCCTAGGTGAATGATATGATTTGCAAAA
TTACAGCATATCCTAGTGTGCAGACATATCTATCCTTGTTAACCTTTTACACCTTTAGCA
ATTGTAGTTGAAACTCTTACGTCTTCCAAAAACAATCAAAATAACACAGAACAATATGAA
CATGAAAGGCCTCAGTAATTGTAAATGTCCTTCAAGAAACACAAACCAAACACAGTTTTG
GCTGAGTATGTAACATACTTTCAACAATCTATTTATGTTGTTTGTATCTAACCCACATGA
TCTACGACAGAACAAGTACCAATAAGACATCC
>tig_1181_consensus
ATCCGAGTAAAAGAGAAGAAAAGGAAAACAGGTTAATTGTACATATATTAACATAAATAC
CAAGATTCAAAAGCACATCACAATAGGAAACATAAAGGGGTAGCCAAATAACAATTGTTT
AACATTTTACTACGCATGTTTAAGTAAGCACATGTCCATCGAAAGGAAAACAAAAGACAG
AATTACATATTTTTCACTTCAGAATTTTCACTCAAAAGCCCACTGATTCTCCCTACGAAC
CTCCTCCTCCTCCTCTGGAGTAAAGTCATTCTTAATGCTGAATTCTTTGCGAATCTCCTC
TGGTGTTTTGCCCTTAATCATGTCTGCCACCGTCTGGCACATCAGGTCCAGCAAGCTCTT
GATGTTGAGATAATTTGCAGCGAGGATGAGTTCAAAAAGGGTGGCCTGATCAACTTTGGC
GAACTCGGCATCCCATGTCTTGAGATCTTCATCAGCAGCACGGTCGTCGGCCTTGGTGGG
CTCGACGTGCTTCTTGCAGTACTCGATGACCTTGGAGAGGATCCTGCTGGTTACATTAGG
AAGCGGAATTCCGTTGTCGGCGCAATCGTCCTCTATCATATGCTTAATCGTCTGCGATTC
CAAAGCCACCACTTCATCAACCTCAAAAGCCTCACCATCCGAGCTTTTCAGAATGATTTT
CTTGCTCGTTGCCATTCTTCCGTAAGGATCAATCGACCGATGGATCCAAGATCTCAGAAA
CCCTAAATGGGTATTTCCCCCGCTACTTCAATATAGAATATTAATACTGCATAAATGGCT
AAAGGACTTATTCCCACACATAGTTTTATGCTTACGCAAGGTCACAGCTTCCCGGTTTGT
AAAAGGCCAAAGGGCATTTTCC
>tig_1182_consensus
ATGTAAAAGCAAGGTTTGAACAGGGTTGAATGCTATGCATTCCAGGTCAGATGCGGCGAA
GTGAGGCCAGTCTCTTCTCAAGATCCTCAACATCAGTAGATTCAGAACTAGTGGTCGTTT
TAGGAGCAGCTGTAACAATGTTTGGAGCATTCCTTGAAGCGATCCGGCCTTTTGGAGCTG
AAGATAGCTGAGATGCAATATCCACACCAATTTCATCAAGAACCTGGTTAGTGAGCTCTT
CCGTCTCCTCTTCAGCCTCATCTTTGTCTAATGTCTCATCAATAGACTCTGACATCATCT
CAATCGTCATGTCCATTTGTGCTGACTGCTTCTGGAATTCTTTGATCACTTTAGCTTGCT
TTGAGGGTTCCATTTGCTTGTTCATTGCTACCATTGCCTTAGTTGCACCTTTCATTCCAG
TAGAAATTGAAGTACTTGCATATAGAGCCTGAGTATGAGTTGCCACTCCTCTGATCTGGG
CACGACTCCCCTGCAAATTAGTTATTTGTTGGCGTAGCCGAACAAGTTGACGAGCTAATA
TTCTTGTGGCAGCCTCATTTCCAGTTTTAGCTGTTTTCTTGATCTCCGCAACCAATTTTT
TCTCCTCCAATTGAAGAGAGGCAATCTCACGCTCAATTCCTCTAGTTGCAGTGGCCATGT
CCCTCTTGCTCTCCCTTAAAGCCTCTTTGGGGGAGGTTTTCTTCCTGAAAATGTTGATGG
TTTTCTTCAAGATCTTCATCGTTCCAAATTTTTCCCAACCCTAACTTCCCTGCAATGCAG
TGTTGGTGTTGGAGGGAGAGAAAGTGTGAGAAGTCCGAAGAGTGTTTTAAGGGAC
>tig_1183_consensus
GGTCATTGAATCCGGAGGCTTCTGTCTGACTATACATAGCTTCAGTTATAGATCGGTGCT
TGTTGCGTTGGAAATGGCACATTTTCAAAACCATTAGGGAGCAGTTTCACACAGAGACGG
ACATGGCAACCCGATTCGTCAAGTTGATGAATATGGGGACCCAGTTTATCAGTCTAGCAC
CACCACTGGCGACAACAGAACGCCGGTGGCTGGTAGCCACCTAGGTACTGGATACAATGA
CCAAAGGCATCATGGGCTTCAACGTTCTGGAAGCTCCAGTAGCTCTAGCTCCTCCGAGGA
CGATGGACATGGTGGGAGAAGGAAGAAGGGTCTGAAGGAGAAGATAAAGGAAAAGTTGCC
GGGCGTTGGGCACAAAGATGAAAGAAATCATGCCTCCGCAACCACCACTCCGGGCGGTTG
CTATATGAGTGGGGCGGGGCAGCACCATTATCATGAGAAGAAAGGAATGATGGATAAGAT
CAAGGATAAGCTTTCTGGACACCATCACTAGCTCCAGTTGAGCGGCATTGTAAAGCATTA
TTTAGTTGGCCATCACTGTATTATTAGCCGGCAAGCGATCCACTCTAAGCCTCATAAATG
TATGAGTTTGAATACATATAGATGTGGTTCAATCCTATTTATTACAAGAAAAAAATTAGTA
AAACTTTTTTTGAGTCAAGCTAAGACTGGACAGAATCCATAGTACCCTTTTGGTAAACCG
ATCTGAAAAAAAAATAAATTTTTATTTTTTCAAATATTAATTTCATTAAAATTTTAAAATA
AATAAGGACATTAATTAGAAAAAATTAAGTAGAA

>tig_1184_consensus
AGCTGCGGGTTTCCAATTTGGTGCTTGACCATGGGAAACAATGAAGACGTGAAGTCTTCA
AAGTCTGATAAATCATCTTCACCTGCACCACCGGATCAAGGCCATATTCCTGTATATCCT
GATTGGGCAGCTGTGCAGGCATATTATGGCCCTAGAGTTGCGATTCCACCATATTACAGC
TCTGCTGTGGCATCTGGACATGCTCCTCACCCCTATATGTGGGGTCCACCGCAGCCTGTG
ATGCCACCTTATGGAGCACCATATGCTGCAATTTACTCTCCGGGAGGAGTTTATGCACAT
CCTGCGGTTCCTCTGGCATCACATTCGCATGGTCACGGAGTTCCATCATCACCTGCTGCT
GTAGTCCCTTTGAGCACTGAAGCACCAACAAAATCATCAGGAAACTCAGAGCGAGGCTTA
ATAAAGAAGCTGAAAGGATTAGATGGTCTTGCAATGTCTATAGGCAGTGGCAATGCTGAG
AGTGCTAATGATGGAGCTGAACATAGGCTCTCACAGAGTGAGACTGAAGGTTCAACTGAG
GGGAGTGATGGGAATACTTCTAAGGAAGATCAAGCTGGAAGAAAAAGAACGCGGGAGGGA
ACACCTAACATAGGTGGAGATGCGAAAACTGAGACCCAGCCAAATCCTGTTCCTGGTGGG
GATGTGAATGCATCTCCTAATTTGGTGTTGTGTACAAGTGTTGCTGCTGCTGGTGTTCCA
GGAAAACCAGGTGGCCCTGTACTTTCTCCTGGCATGACAACTGCATTGGAGCTTAGGAAC
CCTACAAGCATGAATGCTAAGACAAGTCCAACTAGTGCTCTGCAAACATGTGCGGTTTTG
CCTACTGAAGGCTGGTTGCAGAATGTGCGGGAGCTCAAGAGGGAGAGGAGAAAGCAGTCT
AATCGAGAATCTGCTAGAAGGTCTAGGTTGAGGAAGCAGGCTGAGACTGAAGAACTGGCA
CGGAAAGTGGATACATTGAATGTTGAGAATATGACACTCAAATCTGAAATAGATCAATTA
ACTGAGAAGTCAGAGAAACTAAAGCTAGAAAACGCAACATTAATGGAGAAACTGAAAAAT
GCACATGCGCAAGAGATTATTTGAACAACATTGACGACAAGAGGGTCATGCCTATTAGT
ACTGAGAACCTTCTGTCTAGAGTTAAAAATTCAGGTTCTGTCGATAGAAACATGGGGGAA
GAGG
>tig_1185_consensus
TATGTGCCTTAAAAGAAGTTAATGGTGACTTCCCTGCACGGGTATCCCAAATTGCTATGT
TTCCATCCACAGAACAAGAGGCAAATACATCTGATTCTGTAGGGCTCCACTACATTGACA
AAAGTTGAAACGGTTACAAGATTGAAGTAGTAATTGATTAGCCATTAATGTTGCATTATA
AATTTGGATTCAAATACTAGATATCATATGGCTTCAAGTATTGATTACTAGGTAGAATGT
GTACTTGTAGATCTTCAACACTGGCTGAATGTCCAACAAAAGGATTTGGATCAACATTCC
ATGTAGAATCAGATGCAGGCTCCCATAAATGAATGCAACTCTTGCAGTCCCC
>tig_1186_consensus
CTGTTGCCCACCAACCAGATCTATCTTGCAACACAAATCTAAGTTACTAAAACCCTTTGG
ATATCTAATTCTTTTGATTTCTCTCCTCTCTTCCTTTTACTTTTTCCTTTCTCTTCTGTT
TTTCTTTAGAAGGAATAAGGATCGCGTGCGTGTTGTTGGCGGAGTTGGGGCGCGTGGCTG
GTTTTTTTTGGTGGGCTGAGAATTCTTCGTGTGGAGACATCTAACCGTTGGATTAAGTGA
TTGTGCAGCTAGGATTCTTTTGCTTTATGCTTCGCCACGCGGGCTCGGAAGTCACTTACA
GGTCCACCTTTAATCGGTCCTCCGAAGTTTTCGAACTTATCCCCAACAAGTTGAAAAGCG
TCTCTCACTCCCCAACAAAATGAAATTTGCTCACCTTCCCCGCCACCAG
>tig_1187_consensus
ATGAAATTTAGTACAAAATTATGTCCTTCCATCAGACATGACTGGTTACTCCACTCATAT
TTTTTTCAAATCTCTCAAAACAATACAAAATTTCACCTGTGTTGAACAGCAACTGAATGG
TAATATAAGCCAAAAGAAAGAATGCTTCTGTACGTGCAACTAGGGAAACCTATTCGAAAG
TTAACTGAATTCATCTTTTGGTAAAATCTGGGTGGAACACTGCTCAGTTGCGAAATAAGC
CCAAAGCACTACATTAACGTTGAGAGCCAATCAAAGGTTCCGTGCACCAACAAAGACGAC
TGTTGCAGGAACTGTTTCAAGCAAATGCCTTTTCACCCTTTCTTTTGAACAAATAAAGAA
ACCCTTCTATCTTTCTTTTGTGATTTCCAGACCCATAAAAGTAATTTTTAGACTTCACTG
AGCTCACTAACCAATAAAAAGATCGCTGGTCACGGATGTATTCTCCATCCCCGCTCAGCA
ATTGCTGTATTAACCTGACTAGCAGCCTCTGCAAGGTCGTTCAGTGGAGAATAGCTCCAG
TTCTTAGATATATCCTCTTTACCAGTGAATGGTCGGACATATCCTCTTTCCAGAAGAGAC
TTCTGGAAGTCAGCAAATTTCCATGTACACTGATCAGCTCCAACAACATATACAGGCTTC
CTTCACCATCCCAAATATTAACCTTAGGGTTATTACGAAATTCTTTGATTATAATCTCAG
CAACCTTCTCTGGTGTTCTTCTGGAAAAGGATATTCTGACACTTCCACAACTCCAGAGAA
CATCCTGCAGCATTGCAGCCAACTGCTTTGCAAGATCAGCACCATACCGACAGTTGCTTG
TAGGACCTCCAACATTGACCACAAGCAAAGGCTTTGGGAGCATGGCCAACTCATCATGCC
AAACAGAAGCAGCATGCCTCAGTGCTGCAGAGTCAGCCTGATGAAGAGCCCCAACTGTGA
GAATCACATGTCTATCAGGTCGTTCATGTGGAGTTATCCACCTCCTTAAAAACCAAGGAA
TTTGTTCTTGGGCATGAGGTGTCAATGGATAATAATCGTGACGAGGAGTAATCACCAGAT
CAAACCTGTTCAGCTGTGACCTTGGATGTTGTATCTGAATAACAAAAACATTTTCTGGAG
CTAACTTTTTTATGGAGCTTGCAACAGAAATGGTATCCCGACCTGATGCAACCACCAACA

ATGGGCCTTCT
>tig_1188_consensus
GCAGCTGCCATACTCCGTAGCTTCTGAGAGACTTCTGCAAATGAAGGTCTCTCTGCAGGA
TCGGAAGCCCAACAACTTTCCATCAGAGACTTCCACTCAGGATCACACCATGTTGGAATT
TTTGGGCGTAATGAGTTGTTCACAATTCCTCCAATAATAGAAGCAACATGCATATCTGTA
TAAGGTTCATCCCCTCTTTTCTGTTACCATGTGACTTTTTCCACTCAGAAGCTCAGGTGC
CATCCACGGTAAAGTTCCACGAACACCTCCTGACACTAGAGTCTGTTGCTTTACCTTTGA
CAAGCCTAAATCACCAATCTTGCATACTGGTCGCTGAGGATCTCTCATATTCACCAACAG
ATTTTCACATTTTAAATCAAAATGAACTATATTCTTTCCATGCAAATACTCCATTCCAAA
AGCTG
>tig_1189_consensus
TTTTTTTAAAAAAAAAAAAAAAAATAAAATTTTTTTTTTTTTTTTTTTCAATAAGACGAG
CAAAGTGAAATATACTGGCTTTAATATTTTGAGTTATTTCACATTGATCGCAGCTGCCAT
ACTCCGTAGCTTCTGTGAGACTTCTGAAAATGATGGTCTCTCTGTTGGATCAGAAGCCCA
ACAACTTTGCATCAGAGACTTCCATTCAGGATCACACCATGTTGGAATTTTTGGACGTAA
TGTGTTGTTCACAATTCCTCCAATAATAGAAGCACAATGCATGTCTGTATAAGGTTCATC
TCCCGTTAGTAGCTCCCACATAACTATCCCAAATGAGTATACATCAATCTTTTCTGATAC
CATATGACTTTTTCCACTCAGAAGCTCAGGTGCCATCCAGGGTAAAGTTCCTCGAACACC
TCCAGACACTAAAGTCTGTTGCTTTACCTTTGACAAGCCTAAATCACCAATCTTGCATAC
TGGTCGCTGTGGATCCCTCATATTCACTAACAGATTTTCACATTTTAAATCAAAATGTAC
AATGTTCTTTCCATGCAAATACTCCATTCCAAATGCTGCATCCATAGCTATGATGAGTCT
CTTACGCCGGTCAATAGTTCTATCCTTCTTCTGCAAAAACTGTTTCAAAGATCCGTTAAT
CATGAATTCCGTCACAGTTGCTAGAGATCCATCAGGACCATCACGTACTATACCATAAAA
AGACACAACATTTGGATGGTGCAAAGAACTCAATAACAAAGCCTCCTTCCAAAAATCCGC
AATCAAGCGCTCTCTTTCAGAAGGTCTCCCAGCAAAGCAGCTCGCTTTGATTCTTTTTAT
TGCTACATCAGAACCTTTCCACTTGCCATGGTACACAGCTCCATAGGTTCCTGAGCCTAA
TTCACGTATCTCCTCAAGATCATCATTTCTTATTGTCTGTAACCCCCTGGAAATAGCTTC
AGCCTCAGCCTTAGTTGGCTCAATTTTGGAATGCACATTGTCAATGTCAGAATCCATCTC
CAATTCCCCATGAACATTCGTTAACTCAGATTCATTTGTAAGTGCTTCCTTTGGCACTGA
ATCAGGCTTTGTAGAATCAGCATCCTCATCAACTTTAGCTTTCACCTCCTCTGCACCTTC
TAGTGCTGCCCTTTTTACAGAAGCAATTAAATCAGGTAAGAAACTCAAACAGTTATTTGA
GAGTTGTGGATCTTCCAGAGGAGCTGGAGGACCATTTTCACCCTGCTTCACAACATTGCC
ACTAACTTCCCCAATCCCCAAGCTATTTGGACCATTAGATTTATCAGCAATCACATTCTG
CTCAGGAGAAGATATGCTTAGCACATCAGTGTTTTCAACCTTTTTTACAGGACTTGCTTC
ATTTTCTTGTCCAAGATTAGTTTGTTCCTCCATCTGAACTACTTCAACCACATTATTTCT
CAATCCCATATTCGAAACTGAAGATACAGAAGACACCAATTTATCATGAATCATAACTGA
AGATGAATCTGATGGCTGCACAGGATCTGAAGATTGGTTTTCATGATTATGGTTTTGAGG
ATTTGACTGTTGAGCCTGGCCATAATAACATTCTGCAGAGGTGCCTGTGCCATCAAAACC
CTGTACTTTTGGTGGGGACTCAACCCAAGAGTTTTGATTATCGAACCCAACACCAACCCT
TGGACTATCATCCACTGTAGCCCTAAGAAATGCAGGATTTATGGAACCATTAACAATTGG
AGTAGATGCTTCATAAGAGGGACCACCTTGCATTGGGTTCTGAACATTTCTCCACAAAGT
TTGCATCTGCACATGTCCATGAGATACTGGGTGAAGATTATCAGCAACGTAAGGAAAATT
CCCATACAGCACACGTTCTTCAGTTGGAGGAACACGCATATGGGGTACTGCAGCCACAGC
TTGATCATGAAACATTTCATTTCCCATTTCAGGTCCAGCTCGATGGTGGATGTGATTTTT
GGACAAATGATGGCCCTCTGCCAGATTCCCATGCACATGATGAAAATTCATGCCAAGACT
ACCAGCCATGCAGGGATCACTCAATCTCCCAGCTCCGGAGATATTTGTTTTTATTTCATC
AACTCGAGGATTCAACTGTTGCTGCAGAACCCATCCTCTTTCATGACTCTGACTCTCATT
GTACAAAGGTCGAGTATCATTCTGATTTTTAGGATACATCCCATGTGGCAACTTTGCTTC
AGTGTTCAAAAGAATATTCTCCCTGTTTGGACGACATTCAGCTAATGGATTAGATACCTG
GGAAAATCCATTACCCATGCTGGGCTGATCCAAGTGCAGATGATTTCTGTGAATGGTAGG
CCGGCAGTGCTCACAGACTCCATTAGCATCATAAACATGATGGCCATGAAATAAATTACC
AGGGAAACCAGTCTTATCTCCAGATATAGCTCCTGGTGGGATCCTTACTAGATTCTCAGA
AATTTGTGGCTGGGCTTCGTAAGTGGGATGATGATTTACATGTTGCCGAGTATATTCATC
AGGCATTTTGTCCTCGCCAAATGGCATGCAGTACCAGGCAGAAGAAGGTGAAGAAGGGAA
CTCAGGTAATGGTCTTGGATCATGATGCCCATAGTGCCTCGGGGAATAATATGAAGGACT
CCAGGTACCTTCCAATTCATTATACCTCTGAGCAACAGGCTGCTGGAGTTGCCCCAATCC
CATATGAGGAATTGTAAGTTGATGCATATTAAACTGTGGGTGATTATGTAAACCACCCTC
AAGACTCATAGTGTTAAAAAACCTGTCAGCCAAATGAAGATCATCAACAGGACCAAACAC

GGGAGAATCTGGTTCCATCTTCCTAAAATCATTACCCTCATTCATATTATTCAGTGCATC
CACATACCTCCTCTCAGCCTCCCTATCATCTCCATCAACAAAAGGCCCTGAACCATCCTG
GTCTGGGTGCGAAAAAAGAAAAATCCTCAATCTTGTAAAGCCATCACCAGAACCCAGCTT
CTCATACTCCTCCATCATGTTAGTGACATCATCATCATTCACAACCGAAACCAAAGCATC
AAGATCCTCATCCGGTTGTTGATACTTCAATACCACAGCTCCCTCATAAAGCTCTCTCAT
CCTATTCATCAACTCCTCATAACTCACGTCCCTCGGCAAGCTCACAATCCGAGTCTCCCC
ACCAACATACCTCAATTTTCCATCTTGGGGTCGGGGCAAAATGCTACCTAAGAAGCTACA
CAAGAATTTTACACGACGGGCCTCATCATTTGAACCCGGAGTTGAAACGGAGGCAGAAGG
TGAATCCATGGAAAAAACAGTTTCTTGATTACCAGAGTGAACTAATGATTTGTTCAAGCA
TTCGATTTGTTCAAGCATTCATTTTCTTGATTATCCATGGAGGAGTTCTAAAGACTCACA
CCATGTAGCCCACAACTCAAAGGTCACTATCTATATCTTCAAGAAAGTTAATCAATAAAC
CCAAAAAAAAACCCTAAAACAAAGCAAAACTAGCCCTAAAATCAAGATTCGATCTTCATA
AAAGGGGAGGAAAAAAAGTACCTGATTGAGACAAACCCAATTCAATACACACGCTCTTTC
TATATAAATTCTTGGCTATTATATAGAATTATGAAGGTGATCTAGAGTTAAAATTTTCAA
AAACAAATGGTTAATCAATGAAAACAATGGTTAAAAAAAAAATTGAAGAACAAC
>tig_1190_consensus
CTCTCTTGCTTCCTCAAGTGGTTTACTCGAAAGGTTTATACTCTCTCTCTCTCTAACTAT
ATAACAGGTTTTGTTTATTGGGTTTTTATGTTTCCTTGTTCTTGCACTTGTATTTTTGTT
CATAAAGAGCTGCATAAAAAGCCAATTCCTCTGAATTTAATAATAGAGAAAATCAAACAG
AGCGAGCCTGAAATCCTCATTATTATTAGCTCTTTTATCATGTTCCAAATCAAGATTTTC
ATATATATAAAAAGATATCGTCGGTGCAGCCCCTATACTTTGTTGAGAGAATTTGACAAG
AAAGGGGAAAAAAGTGAAGAAGAATCCCCTACCAAGAAGTTATGTTGATCCCATGTAAAG
TAAAATCGATTAATTGGTCAAGTCTAGCTATTAAAATTATTTATTATTCTTTGAACAATT
TAAGAATCAAAGACTATTTTGGACGACCAGGGTTGGTCCACATGTGTTTTTAGACTTGGA
ATATTTTATGTTTGAAATATTTTTGGAATATTTTATTTCTGGGTCTGTTACCCAAATTAC
GGTCATTTAGTACAAACTAAATGACCAAGTAGTATTTCAATACTTTCAACCAAATTTGAG
TTGTTCACTGACCTTTTTGAAGAATGAAATACCGATGCAATTGCCTATGATTAACTAATT
GATACTTTCCTATTCACAATCAAAATTAGACACAAGAACTAAATTGATTAATTATGTTGT
TCCACTAAGTATATCAAAGTTGTCGGCATTTCCTATGCAATCGAAACGTGTAAAACTCTT
GATTTATTGTTATTTCCATGCATGCCTGCCTGCAGACGAAGACGAGGACAATCTTC
>tig_1191_consensus
GCACATAACAAAAAAAATGAATCCTCTCAATGGTAATACTTCCTTGATAACTTGATATAA
TAACAAAAAGGATTGTTATAACCAATACAAAACCCTTGCTATCCAATCTAAAAATTCAAC
GTCCAACAATCCATAAATTAGGGACATGTATTAAAGATGACGGATATAAGAATGATCAAA
ACCGTTCTGCTAGTCTAGCACTAACTGCTCTAAGTTTTGAATACACTTTTCCAGCAGGTG
ACCCTAAAATGAGGCTGCAAAGTGAATCCCTAGCAATTTTAATGCTGGCAAAAGATCCAA
GTATGTGAATTTTTGTGTCTGCAATCACAATCCTTGTCTTTGTAGCATTTTCAATGGTAA
ACTTTGTTTTGCCACCTTTACCAGACAGCCTTCCAATGGCACGGGACAAATGCTCACCAC
GAAGAGTTTTGACATCCTTGATCTCAAAAGATTCCACATACAGCTCATCAATACGCAAAA
GGGCAATAGCATCTATAACATCAAAACCTAGCATGAATGCATGCACAAAATCTGCACACT
TTTGTAGGTTACTTATGTCTGGTGTGTCTTGTCTAGTCTTCAATTCAACTCTACGAGCCT
TGAGATTCATACGGATGTCAATCTTCATCTGCTCATATACTGGTTCATAGATGTCCATCC
ATGCTTTCTTGAGAGGTGAATACCTATGAGGTGGGACAGAGACCTTCCGAAACTGCACTC
GACCATCAGACATCTCATGTGCCTTCAAAGGCTCAAACTGTGGCTTTGGTGGCAAATTGC
CAGAAGCCTTTTGGGTTTGACTTGAAACTGTTTCAACTTCCATGGTAACAGGGATTTCTC
TGTACTGAGTGTGCAAAAGCAAGTTCTCCCCCTTCAAATTCTGAAACGAACCTTCAGCTC
CGATCGATGTCGGCTACCGAGGACGGCGCAGTGCAAAGTGGACAAGGAAGCCGCCAAGG
>tig_1192_consensus
TCAGCTAAGACGCCAATGATGTCGAAGGCCTCCTGCAAATCACTGGCGGAATTCATGTCA
TCCAACCACGACAGCAACTCTTTGGACAAACTCACCATTTCCTTCATGTTCAATAGCCGA
TCTTTCATTACCACTTCCCAACCTTCCAAGCTTCCACTCTCCTCGGAGTCACCTTCGGCC
CCCTTTGACGTTCCTTCTACTTTGATTGGTGTTGCATTTGTTGATTTTTTGACCTTGAAA
AACTGCTGCAGCTGCTCGATCAAGCCAGTGTAGACGAGAATTGGTCTAACAATAGAAAGT
AGGTCATCAGTACCACCAGATCCTGCAGTAGAGCTTTTGCTACTACTTCCTGATTCTAAC
CTCACAACATTTGTTGAAGTGGATGATGCCCTAGCCATGGACCGACCATGGATGTGCTGC
ATATACGTGTGGTATATCCCACGGCTCAAAAAGGTGCGGCGGTGCTGCAACCAGGATTCA
TAAGACTCAGATAATAGTGAGTTAACCATCATAAACTGGACAGTTTCTTCGGTTCCCACT
GAAGGCGTTCCTGGAGTGGAAGGTCTAGCTTCTAACATGGAAGTGTTGATGTATGTTGAG

ACAGTTTTTGCCAAGCTGCAGCGCTGGGAGGGGCTTCCGTGTTCAAGAAGGTGACGAGCC
ATTTGGATCATGAACGGCAGAAATTTAGAGTTGCTTTCTCTTCCGCCACCTCGGCTCTCT
GCACTAAATGATGCCCCAGTTGCAAATCTAGCTAGCATCAGAACAATGTCATAAGTCAAT
AGCCGAAGCCGACTCCCATCAGCACGACCAAGAGCATTGAGATTATCCCAGTACTGGTCA
ACATATCGGACATACTGGGCAATTGGGACAGATGGACCTCTTACTGGAAATAAGGAGTTG
CAGAGAGATTCATTATTCCTGAGTGTAGCCCCCTCCCATTCTTTCTTTGGATTCCTCAGA
GCAGTATCTGCTCTCTTGGCCTCCTGATGACATTGGAAATGAATGATATTGAAATAACTG
ACAGTAGTATATACACACTCCCCACGAGCACTTCCTGAAGTACCAACACCAAGGTTCACC
CGCTTGCTGTAAGAGTAAACTCCCAGCAAGTCAGCAGGCCTCAAACTGTAGCCTTCCCGA
CATACCATGCATGCCAATCCATCTTCTTCTTCCTCTACATCTTCTAACCCTTCCAAAACT
GGCCTAGCAACAACAATTCGCTCACCACCATCTGAAGCTGGTTCTTGACGCATTCCAAGT
CCCTGAAGCAATTCCTCCCTCCTCCTTAAAGCACGGCGTCTCTTTTCATCTCTGGTTGCA
TCCCGTAACCTACGAATCTTTTCTTCCAAGAATCCATCTCCTTTACCCTCTTTATCAGAA
AGTGTGTCTAGTAAGTTTTCAGCCCTAGCCCCAATCTCATTTTCTACAGAGACTCCTTCC
AAAGCATGAAGCAAGGGTAAGATGCCACCCTCATCAATACACATCTGAGTGCCCAAGTGC
CCCATTGACAAGCCCCTCAGCATAGACAATATATGTGGAACAGATGGCAGTTTTAGACCC
ACTGCCCCTTCTGTACAGGATTTATAACCAGCCTGGCCTG
>tig_1193_consensus
AGTGGTCAAATTTGATTGATTAAGATTACTGGGTTCAGTCTTTTTTATTTCAGCAGCAAT
CATTGTTCTGATACTAAGAAAGATTGAAAAACTTTTTTTGGTATTGAAGGCAAGATTGTG
AAAATAAAATAACTCTTCAGAAACTAAGAAAGAAAAATTGAAATAACTAGGCGCTAAACA
TCTACAGTTAAGAGCTCTTGTTTTTACAAGGAGGAGAACTCTGCAATTAGGATCAAAAAT
TTCATACAGCCTGAATTACTCCATTTGTTCTAACTCCATCGAATCCACCTTACCAGCTGC
TATTTTGCTTATATTG
>tig_1194_consensus
TTTTTTTTTTTTAACGCAGAAGTTGGATGATTTTATTGCCTTGAGCAGAAACTGAAACAA
ATCTTTCATCTCAGTTTGCCAGAAACTCAAACTCCGTGAAATACTGTTTGATCTTCCAGT
TAACAAGAAACTATCAAAGGTCTTGGTATTGAAAGCTCTATATTGATGAAAAAAATAGAAA
AAGAAAGTAAAAGAGTTGAAACAAATCTATCTGGACTTCTGTTGACTATATCGTAGAATT
CCATGGACGTCGATAATGCCAGGGGGAAACACAGGATCGGTTGCAGCTCTCACAGCAACC
TTGGCTACGGCAGTGACATTGACAGGAGGAGTGAACAGAGGTCCAACAAGCGGCAACTGG
TTCAATGGTTTCGCATGTTGAAGAACCATCTCCACCGGAGAACCAACCATGCCTAGAGGA
ACTTTCATGCCCCCAATGCTTCGAGTTCCATAAATAAACCCAGGCCTCAAAATCACTCCT
CCGTAAGGGAATTTGGTCAGTAACTCTGTTTCAGCAGCTCTCTTTCCCTCGTAATATCCT
TGCAACAAGTAATTTGCTATGCCAAAATCAGCTGCAGATATATAAACAAATCTTTTTACA
CCTTTTTCTGAAGCTGCTCTAATTGCATTGATGTTTGCAGTCCCATTGATCTTGTACATG
TAAGAGTTGGATCCAAAACCACCAACACAGGAGATAACAGAAGTCACCCCATTCAAAGCT
TCCTTCAATGAATCAGGCGAAACAAGATTTCCTTGATGCCAAATTACATTGTTAGCCCAG
GAATCATGTAAAGATGACTGACCGGAC
>tig_1195_consensus
AAAAAAAAATGCAAGTTATCATTGAGTGAAAGCATACAGGGTTATAAGAATTATATCCCA
TGATGAAAGAAAAATGGCGCAGCACATATGGGATTCACAATTTTTAGGCAAACATTCGCA
GCCAGGGGGCCACAATTTGTTCCCAGAATATTGATGTTCCAATCAGCCTCAAGAGNNNNN
NNNNTTATACGGCATTTCAGGCTGCTGGTAGCTTTGCATGTAAAAGAGTGGCCGGCTCTG
GCCAACAATCATCTGCTGTCCATACTGAGGTCCATGCGGATGATAATAGGTGTGGGGTGA
CTGCATTGGAAACTGATGATTATATATGACAGGCTGGTGCCCAGCAAATGCAGGTCCAAT
TCCAAAACCTACAGGCATGCCAGGTATATGTGGTACTGCAGATACATTAGGTGGATGATA
GAAGGGACTATCAGTGACAGGGGATTGGGGCCTAGCACCCACTTGAGATGGGATGAAACT
CTTCGCATTAGGATTGGGTTTAAATTCCTTGGCATGGGGATTCAATGTTGATCTTTCCAA
AGACAAAGAACCCACTGATGAGCTTGGGGATAAACCAGGACCACTAGAAGCTGAGGCAGC
TAATGCACATTCTGAAGTGAATGACGCAGAACCACCAGATCTTCCATGAGAATTTACAGA
CTCTAGTTCTCCAGGAGCTTTACTAGGTGCTGCTGCCTCCAAGTGTTCAGCAGGTGAACT
TGTCTTTTCATTGCTCTTTAATGGAGCATGGGATGGAGCATATGCAGAGGCATTTGCAGA
TAGTCCCACTTTGTCAGAACTATCTATCTTGCTCTGTAAGGACTCTGAATCTTCTGATTT
TGACAGTTGATCATCCTCCGGTAGCAGATGCTCATCTAAAAAATCCTTAGCATCATTATT
TCCTCCATGTTCACCAAGCAAATTCTCCTGGACCCTTTTTTCACAGTCCAAAATAGAAAT
ACTTTTATGTGGAATTTCAGATGCTATCTGTGCAGCATGATCGTATGAAACTGAGTGTTG
TGGTTCTGTAGCAGCACAAGAGTGGGAACACTGTGCTTCATCCACTAACGAAGTGCTTGA

AGACAATCGAGCTACATCATTTCTTTTGCCACTAGTCAAATCAGCAGGTCTCTGGCTCAC
AGAACCAGACAAACCACCAAAGGTTTCATCATTGCGTGAATCCAACATTATATCCTCATC
TTCTTCGTATCCACTATCATCAAGGGCACCACCCCTATAGACTGAAGAGAATCTGGTCTC
CTCATCAATGTCATCATTTTCATGAAGGTTCATGCCTCTTTCCTCAGCCAAATGAAGATC
TTGAGTATCCTCACCCTCAATTTCTCTGGCTATCCTCATAGCTTCTCTTTCCATCTCTCT
TGTTTGGGGGCCCCTCTGAAGTTTTGTTGTATAAAGTTCCTCATCAAAAGTGGTCTTCAC
ACCAAATAATGTTTCATTTATTTGAAACTGGTCCCAGTTCCTATTCCAAGGGCCATCAAA
TATATTTTCCAATTCAGGCAGATGTGGATCGTCTTCATCTGGGATCCATGGTTCTAGCTC
TCTTTCTAAATCTACATGACGAGGTTGTGATATGTGTGAATCTATCATAATGTCATGTTG
CTTTTCTAGCTGTACCTCACTTGCCAATCCATCCCTGGTAACAGCCACTTCCTTCGCTAT
AACTTGTACCAGTTCACTTCCAGGTATAATAAAAATACTTGATGGTGCCTTGCTAACAGT
TTCCACTTTCTGTCCTCGGAGATTACCATCTTTTGTCAAGCGAGCCATTTTCAAGATGAC
TCC
>tig_1196_consensus
CGGGTAGCCATAAAAAATACTCTAATCTTCTCTACTATGCTTGCGAAAATGTAAAAGCAA
ATCAACATACATGTATAAGACAAAAACTTGAAAATGTACATTTCTTTCTTACTTACAAAA
CTTGCCTTCCTATTTGATCTTTGATTTTCTTGGTTCAAACAACTATACACTGTGATCAGT
ATATTTGCCTCTCATATGTGCAAGCAATGCCCTCTGCTATATTCCCTTTTATCCTTCTAA
CAAATTTATAGTAATATTCTTTCAAACCCGACATCACAAGCAAAAAATTGATGAAAGTTG
CCAACCATGCATCCATAACCTTAAACGGCCAAACAGATCGAACATCTGAACATTCTTCAT
GATGTAACTCAACCATCGACAGATAATTCAGTCACTCCCTAGTGATCCTTTTTAATTTCT
TCCTTTGCTAGAGGCTGTCTTGGATACAGAATAACTGCCAATAATAAAGAGAACGCAACA
CCAAATGCTCCACTAAACGGTTGGAATGGGTGAGTTCCAGTTATCCAAACAGGGAAATCC
GAATTGTAAGTTAGGAAGCCACCATTTTGTAAAACGAAACTCGAGGCCATAATCCCTGCT
CGCAAACCAATTGGAACAGAAAGGCTGCCTTGACTTCTTTCACGAACGCCTGCCAGGGCC
AAAGATAATAGCCATAGTCCTGGGATTACCTGTGGAGACCTCTGAAATATTGAAAAAGCA
AGTCCTGAGATTATAATTCCATGATGATAACCAAGATCTGTGGCAATTTCTTCAGGCAAC
CATGACCTGAAAAGCAATTCTTCAACTAGCACAATGGCAGTTGCTGTTATGATACCACGA
CATGCCAGCATAAAGGATTTCCCACACACTCTGAGCCATGTCGCAGCATTTAAAGGTGAT
GGAATACTTGATGGCCAACAGAAACTTGCACAACCAAGTAAAGCCTTCACTAACTGTAGT
AATAACACAAGCATGAGACCTCCAATAAACCCCTTCAAAAAATTTTGGACCTTGGATAAC
GATGTCATATCAAGCCCATACCGCTCAAGTGGATTTTCATATCCACGTATTCTTCTGCCC
CATAGCACCACCAGTATCATAACAGCAGTATAGAGCCCAATGATGCAAGCAAGCTCTGCA
ATTCTGGAGGGATTATTTGTTGTCCAGCTCTGCACAAGAGTTGGCAACAGTGGAACAACA
ATAGGGGACCATAGAACAAGCACCATGCTGACAAACCCAAGAATCCTTTGGAACAATGGA
CGATCAGCTATATGCAGAAACAAGATAAGCCTGTCAGTCAGACTCATTGCACCACGTAGA
CCACCCCAAAGCAAAGCAATTTTGCCTACTAGCTTCAACATGCCACCCCTTTGTCCTAAA
TCGGCCAACATTGTGACCAATCTTTCTTGATCAACTTCACCATCTTCCTTTGTTGGTACT
ACTGGACCAGCAACTGACATGGCTTTCTCAGCAAGGCTTGTCACAATGTTATTCTTTTCT
TTTTCAGACATTGCCCCCACAAGCTTTGCAGGTTCTTTTTGATGAATTTCTTTCTCATTT
AAGGGCCTGGACATATTTTCAGCAATTTTCTTGTCTTCATACAAATCCGATTGCTTTACA
AGTAATGCAGATGCTCCCAGGGCAGCTGTAACAGCACCAACCACGACACCTCCATTGTTT
ATGTTTTTCAATTCATTCCTTACCACTTCTCCATTTACTGAATCATTTGAATTCGTAGTT
TTGTCAAAAGGCATCTGACTAAACTCTGTTAAACGGACCTTGTCTTGTTTTCTCGCCCCA
GATTTCACGTTGAGATCATAGACTACTGGTTCATTTTTACTATTATCATTCTCGGTAGAC
ACATCAAAATGTTTTCGCAGAGCAGCTAAACTAGAGCCCACTATAACACCAACTGGAAGT
ACTTTTCTCAGATAATTTGTGCTCTGGACAGCAGAGGTAATGGCTCTGATAATATTTTCA
CCTTGAAGTGTGCCAACTTTTTTGGAAGTATGGTCAATAGTGCACTGTTTATCATCCAAA
TACCATATTTTTTCCCTGTCACTCCAAACAGCCAAAGAGACAGCATTTGCAACTTTCTTT
ATATCTCTAGCAACATCTGCTTCCATTTCTTTCCAATCAGATGGACTTAGCTTACGTTCA
ACTTCGACCTTCAAAGAATTAAACATGATATTTTCCACAAATTGCATTAACTCCGTCAGT
GCAACCTCGGTACTTTCAACATTTTCATTCATATGATCCTTCATTTTGTATTCTCTAACA
GGTTCTTGCTGTCTATCTGTATCTAATATTACATATGCTGGTTCAATAAATTTGTCTGCA
GCATCTACCTCTGCAAATGAGTGGGCTTCCACTTCTCTGTCATCATCCTCACAAGTTCTA
AAATCACCAATGGAATCTCCGAAATTTCCAGTTTGTTCCAAGAGCTTCCATTGACCTTCT
TCTGGAAAGTAGTCAAGCAATAGAGCAGTAGTTGTATCCAGATCCAATGATTTAGCATTT
GAAATTTTTGATATAGGATATCTGCTTAAGTATTCATTTTGTATTGAGTCCCCATAAAGA
TTCACAGGATTGAATAACGGGAAGTTATTTCCATTTACTTCATCTGAATGGTCAGACAAA

CGTTTGCTACCAACAAATGCCCTCTTGTTATCCGTATCTAAACTTTTAGCTATCTTATTT
CTGTGGAAATTATGAGGACTGCTGCTATCACATAAAATGGAATTTTGATTGTGTTCACCC
TCCACCCTCTCAGTTCTTACTTCATGCTGAATGTCCATACCACTCTCACGACTATTACAT
GCAGAAGGATCAGATGAAGCAGGAGCCTGTAAATTCTGATCGTTCCCAGTGCCTTGTTCT
TCTGATCTTTCAAGATTTCCAATTCTTTGCTTCTCAGAGAAATGATCAATTTTCTCATCC
TTAACCTCATTTCTCTCCTTGACATCATTTTCATCACCGTTTTCCTCTTCCAACTGAGTA
ATAATATTTTCTATTACACCGAAAACATTATTAACAGCCATTTGGGTGGAATCATCCATT
CCTGTCAATGCATCCAGTGCTTGAGAAACACTAAAGGTAGTAGGATTAAGATCAGTTGCA
GGCTGCAAAATTTTATTTTCTCTCTTCTGATTATCATGTCCTTCATTCTCCACTGGTTGC
ACTTCAGAAGAGGATCCCAGAGGCAAAACTGCCTCCTCTGAAACATTGGCAGATGTTGTC
TTATTTTGATCAGAGGAAGGATCAAGGGCCTTATCTCCAACTTTCTGGGTACTATTCTCA
GGTTTTTCTTCTACTTGGGATACTCCACCATCCTGTTCCACTTTGTGCTCGCATTCCCCA
GGGCCTACATCCTTTTCAGGCTGGCTATTATTGTTAGTATTAGCACTTGTCTCTGATCCA
TTTTCACCAATGCTAGAATATGGAACAGCCTTTTCTTTAGTGATCCCATCATTTTCATGA
TTATTTCCAGACTCATTAGTGTTCTTACTGACTGAACTAGATATATCAGCTTGCAGACCA
CTCATTGACTGAGATTGATCTACATCATCAGATTTATGCAATTTTTCTGAAGGTAATCCA
CCATCAGGCTTGGCCACGTTAGGTTGCACATTGTCAGAGCCCTCCACCAAATTGTCAACC
CTTTTCACCTGATCTGCCCTGTGAGGATATTTAGACTCTTCAGCACTTGATATTCCTCCG
ACCTTCTCTTGGATCTTTGACTTAAACTCTGATGTTACTTTAGGTATTTTACCAATACCC
AGAAGTCCATCCAATTTTAAATCTGTGCCCTCAGCATGCAAGAACCCAGATACTGCAGCC
AAAAGATTTCCTCGCGCATCTTCAGGCACCGCATCTTGCAGAGCTTTCATAAGTGTCTCT
CCTTGACCAACAGCAGTCAAGACCTTTTTCTTTTCTTCTTCTGTCAATGTGCCAGGCATA
GTTACATCGAGCATATTCATGACTACTTGTGCAGACTGCAACACTTGGCTCCTTTCACTA
TCTACTAGACTGGCTTCCTCCTCTTTGACCAGTTCTGCATCAAGAGGACCAGTCTCCT
>tig_1197_consensus
GGATGTATGATATTTATAAATTCATATGTGAGCATGTGAAAAGTTTTTAAGCTCATGTCG
GTTTACGAACTCAGATAACTGGCACAGCCTTCAAACAAATTACTAGAAAATCAGGCAACA
GATGCTTCAAATAAATTATAGTCACGAATGTCAAGAATTAGACACTAGGTCCCTTTTCAT
ACAATCCTCACTAATGCATATGAATTG
>tig_1198_consensus
GTCCGGGTGGGTGTAATCATCCCAATCATACGACCTGAGCATGCATTATTAAACAAATGA
CTACCCTCAACAATCACTGCTGGGTTTTAGTTGAATTGACAACTTAAGCTGAAAAACGTG
CCAAAAGCAAAACATACAAGGTACCCACATTCCCAGATAACATGGGTTTAGAAGTCCTAT
AAATATTTATTTGGAACAACAACAACCTTCAAATTTGATCCCCACCAAGTAAAAATGAGA
TTCATAATAACATCAATCTGCAAACTCTTAACTGACACTTAAGCAGAAACACCACCCTAT
ACCTGAAGCTGCTTGATATTCATAATCTGCTTCCCAGGATTCAAACCTTTGGGGCAAGCT
CGGGCACAGTTCAAAATGGTATGGCAGCGGTACAGCTTAAACTCATCGTTGATTGCTTCC
AATCGTTCCTTGGTGAATTCATCACGACTATCACTTATCCACCTATTAGCATGGAGCAAA
GCAGCCGGGCCCAGATAAGTCTCGGGGTTCCACCAGTAACTGGGACACGACGTGCTACAG
CAAGCGCAAAGTATACACTCGTACATCCCGTCCAACTTCGCCCTATCTTTCTTACTCTGC
AAAATCTCTTTCCCTGGAACCTCCGGTGGGTTCTTCCTCTTCAGCCAAGGCTCAATACTC
TTGTACTGGTTATAGAAGTTCGTCATATCCACCACCAAATCTTTAATCACATACATGTGC
GGTAACGGCGTGATGGTTGACTCGCCTCCCGATGAGATCTTCGTCAAGCACGCGAGACCA
TTACAGCCGTCGATGTTCATCGCGCAGGAGCCACAGATACCTTCACGACAAGAGCGGCGG
AAAGTAAGCGAAGGATCGATCTCATTCTTGATTTTGATGAGCGCATCCAGGACCATCGGG
CCGCAATCCTTGAGATTAATCTCGTACTCCTGAAGTTGAGGTTTCGAAGGCTGGTCCGGG
TTCCAACGATAGATTTGGAACTTCTTCAGATTCTTCTCCCCACTGGCCTTCGGCTCAACC
TTCTGCGCCTCCACTTCAGACGCGTGAAGCCGAGCGGCAACGTAGCGAGCCGACGGCGCT
ACTCTGGAAACCGATACCGCCCTCCGAATCAAGCCAGTCGCCATTGTCTTAGGCAGCAAC
TCCAATACTTCGATAACTGGGGTTTATTGGAAGAGTAAAATGACGAAAATGGGTAATACT
TATATGGTATCGGGTGCCGCAGTTGGTGGGACAGTTCTTCGAGGGGATGACGACGTGGCG
AATAGTAAACCGTTGATTAAGGATGGAAATATGATTTGATTTTTGTGGGTAGTTTTTGGT
GTTTCCAAGAGTTATAGGCGTGTGGTGAGCACGGTTTGAGATTAATTTGATAATAATGCG
>tig_1199_consensus
GGATGTCTTATTGGTACTTGTTCCGTCGTAGATCATGTGGGTTAGATACAAACAACATAA
ATAGATTGTTGAAAGTATGTTACATACTCAGCCAAAACTGTATTTGGTTTGTGTTTCTTG
AAGGACATTTACAATTACTGAGGCCTTTCATGTTCATATTGTTCCGTGTTATGTTGATTG
TTTTTGGAAGATGTAAGAGTTTCAACTACAATTGCTAAAGGTGTAAAAGGTTAACAACTA

TAGATATGTCTGCACACTAGGA
>tig_1200_consensus
AATTGAATCATGTGCTGACATCATTATTACAGGAATTTTCTTGCAAATATCATGCTCCAT
GACTAAAGTTAGAAGTGCAAATCCAGATATCGAAGGCAGCTCCACCTCAGTTAGTACAAG
GTCAATATTATGGGGTTGGCCCTTCAAAGTCTCCCATGCCATTAACCCATCAGAAACAGC
AGCAACTCTGTAACTGCATTTTCTAAGGAGAGCAGAAATTATATGACGCGTAGAATCATC
TGCTTCTACCAATAGAACTCTAAGAACCATCCTCGGCAGAAATCTTTCCCACCTAACAAC
CTCAGACGATCCATCCTTCTTAGCCTCGTTCTTATCTGTCTCCATCTCCATTTCAATTGC
CTCTTCACTGCTCAATACCACTTCACCCATTCTCATAAACCCTGCTTTTCAACACAAGAC
GACACAAATACTAAAACAATAACCCTAATTCTTAGTAAACATAACCATAGAAGCAAATAG
ATAAAGGGAACGTGTTTTGTAGATAAAACCATCCAATTAACCTCATCCTTGGCAAATATC
TGATACCGTATCAGCCTGAGAGCAAAAGTCAGACTGTAAACCGCTCAGAATAGATATCGT
CTTTCGCTTACAGCCGCGAAAATAGCTAAATATATCTTCTTGGCAGAAGACCTCCTCTCT
CACAGAGTCGGATTAAAAGGTGGGCCGCCATCGCTTTATGGTTCGACG
>tig_1201_consensus
GATCCTTGTTAATACTCCAGCCTTGATCCTTCATTAAGGAATAGTGTTCATTGTAAACAG
AATAAATTGGGGGCATCATAAAATAGGGGGGCCTTGGATTTTTGCATTGAAAGAAAGACA
TTCCACCTGATACTTCTTATAGCTTACAATCGAAATACAATTTTGATATATGAAAATACA
CATTGGTGCGAGAGGAAAAACAAGAATATAAAAATCACAAACAAACTTCAACTAGGTCAT
GGACTTTTCGCATAATAAGATGATGGAGCTACGTGGTCCTTTGACCAACTGAACCACATT
ATGAAACTAATAACACTTTCTCCACCACCGGTTCACAATCTATAGTTGTCTTCTTCATGC
CTGGCTCCATCAACAAAAATTTACAAGCATTGCAGGTCCTCCTAAGTAGTCACCAAAAGA
AGGCACTCATTGTGGCTGTGGCTGAGACTGAGGCTGTTTCGTATGCCCTATTTCCACCAT
CAGCTGGCCATCTTTCTCAACAGAAATAGTCACTAGTTCATGTTTTACCAAGGCCACATC
ATGCCAAGTACCTGAACGTATTGGGATTATTTTTATCCCTCTCAAGATAATCCCGCGTGA
TGAGGTCTTCAATCCTCTTTTTAATTGCCTTGAAATCAGGCTTGAACATGCGGCCTAACT
GCTCAACACATTCCAGCACCAACTGTTGGTGCCCCAAAACCTTCCTACTCTTCATGATGC
GAACAATTGACGCATCGATCGCATACCGTCTGTCTTTGTCAACATCTTCAATCACTTTCT
TTTTCTCGTCTACAGGGGGAAGAGGAATCTTGATCCTCCTCATTTTTTCAGTAAACTTGG
AGTTGAACACAAAGTGATCTGTGGGGGAGATGCTTTTCGTGTTTGGCTCTTTGTTAAGAA
TTTTATACTTTGCACATGACAAGGAATGTAGCAACCTAATAACATCATCATCAGTCAAGT
TCAACTGAGTCATGATCTCTGAATAACTCAGTCTATCTGAGGAATTGAATAGTAGCAATG
CGGAAGCTTGGTAGGTTGTCACAATCAGCTCTGTGGTTCTTGTTTCGAATTTCCCAATAA
GGTTACACGTGCCCAGTGAATATATCCAAGTAAGTTTTCTGTGCTTCGTTTTTGTCTGAT
AGAATTCTCTAAATACTTCAACACATTTCACCATCTCTGCTGGAAGGTTAAGATCAAATG
ATTTGTAACTTGGCCAAAAGCCTGTGGTCAGAACAGTAACTGTCAAATCAATCCCTGGAT
TTGCATTTGGGTTACCATTTAGATATTCCTCGAAATTGTTCTGATTTTCCTTTGCCAATG
TCAGGTCTGTAACCATCCCCTCCATCTTGGAGGTGAACTGACCACCACACTGCTGCTTCA
GCTTTGTCAAAATACTTCTTTCATGGTCATCATTAGCACTCTTATCAAAAAGAAGCCGCC
GAGCAAGCTTTTTCCTGTAAAATTCAGCAAATAAATCCTTATCACTAATATATGCGAGCA
GCTTCACTACCTTCTCAAGTGTTTCTTCAATTGCTTCATCACTAAGTTTCTCACTCCCAC
CTTTCTTAAGAATATTATCACAGAAAGTGGCTAGAAGTTCTGCACTTGAGCTTCCAGCAA
CACCCTTGTTGCAGAAGACTTCAAAAGCCTCCTTGAGAGACTTGTGGAAGAGGGTGTGAT
TTTGGAAACAATCATTAACATATGCTAGGTACTTGTCATGTAGGTCAATAACTTTCCTAA
CAAAAACCTGTTCTTGCAAACCAACCACGTCTCTTTTTTCAGCCTTCTTGTTACTTGCTG
CATCTTCAGCCTGTTTAACCAAAGCTGTACCCTCAGCTGTAACATGCTGCTTAAATATGT
TGGAAACTGGATCTAATCCTCGAGGAATTTTAGAAAAGAGCCTGAACATTCTTGACAAAT
CCTCCACCTTATCATCTCTAAGCAATGCATGGCATCCTGAGTGCTCTTTTTCAAACAGTT
GATTTGCAAATACGGTCAACAGCTCATGTTGAACTTTCTCAAGTAACTTTGGCTCACTAC
TAGAATGCAAGTAATGAGTGACCCTATCTTTCTCCCGTTTCAGACACTCCTCAGCTTTCA
ACATATAATCTGGACAAGAATCTTCTAAAATCCAACTTGAAGCCTTCCAAGAATAATATC
TAGATGTATCAAGGAGCATGGCTGCTTCAAAGTCATTTTCATAGCAATCCATTTGCCCCA
TCCCAATTTCAACGAATATATCTAAAACATTCTTCAATAAAGCCCGATCGATCTGTTCAC
CCTCACGTTCTTGATCAATCAAAGATATTACAGCATCTCTTACTTTTCCATTCAACTCCT
GATAGACCAGATCACGAAAACAGGAAAGCCCCACTTCATTAAGGGGTGGCAGAGACCTCC
TTGCTATAAAGTAACGATCAAGATAATGGAAGAATCGAGAAAGCCACCTGACATTAACTT
TATGGTTTGCCCACCGTTTTACAAGCTCTCTCAACATAAACTCATCATGCTTCTCCCTTA
TAGATGGCAATACCGTGGAGGTAATGTATTCTTCAAAAGACTCTCTGTACTTGTCATACA

GCTGTTGGGAGTAGTCATGGGGAGGCTTTTGTGTACACATGTTGTAGATGGTTGTATAGA
GCATCATGTAGTCCTCAGAACTGAACTGCGGTTCAGGAAGCCCTTCCAGAATGTTCTTCA
ACTTTGTAATTCCCTTCTGCATAAACTCCCATCCTTGTTCCAAGTCAATCGTCTTCCGTT
CATTCATTGTCATATTGCTAAACCCTAATTCTCAATCCCCAGAAAACCCAGAATCTGAAT
TGAAAGCGAAATCGGAACAAGGGAGAAGAAGCGGTGGACCGGTGGAAGCCAAGTTCGCTC
TCCAGCCAGACAGAGAAACAGAGAAATATCGGGCTCCTTCCGGTCTCGGATCCT
>tig_1202_consensus
ATACCAGCTTCAAAACCACCAGTAGTAGAATCAATGATGAGGACAGCACAGTCAGCCTGT
GAGGTACCAGTGATCATGTTCTTGATAAAATCACGATGTCCAGGAGCATCAATGACAGTG
CAGTAGTACTTGGTGGTCTCAAACTTCCACAGGGCAATATCAATGGTAATACCACGCTCA
CGCTCAGCCTTGAGCTTATCAAGCACCCAGGCATACTTGAATGACCTCTTGTTCATCTCA
GCAGCCTCCTTCTCAAACCTTTCAATGACACGCTTGTCAATACCTCCAAGCTTGTAGATC
AAGTGTCCAGTGGTGGTTGAC
>tig_1203_consensus
ACAGCACAGTCAGCCTGTGAAGTACCAGTGATCATGTTCTTGATAAAGTCACGATGTCCA
GGAGCATCAATAACAGTGCAGTAGTACTTGGTGGTCTCAAACTTCCATAGGGCAATATCA
ATGGTAATACCACGCTCTCGTTCAGCCTTGAGTTTATCAAGCACCCAGGCATACTTGAAT
GATCTCTTGTTCATTTCAGCAGCCTCCTTCTCGAACCTCTCAATCACACGCTTGTCAATA
CCTCCAAGCTTGTAGATCAAATGTCCAGTGGT
>tig_1204_consensus
AAAGATATGGAACTTTACAAATGTATGATGTCAGCCAATATCTACATGCAACTGCACATA
CAAAAAGCAAAGAAAGAGACAGGAAAAAAAAAACCCTAGTGACCAAAATTAAAATGAATA
ACCTGAGAGATGATTATACAAGAAGCTCATCAGGCTGCAAAACCTTGTAAGGCCTTTCAT
TCAGTTACTAAAGCACTCCAATGAACTCAACGACACCAGCAATATACCTTCTCATAAAAC
AATAGAAAGCTAAACAGAGTAGAATTGAAGTTTGCCACATAAAGAACAGCAACCCCACCC
CTAGATTTTTTTCCTAACCAGTTTGAGCAGATGAGGCAGCAGCTGGAAGGGCATCTTCTA
GTGTAATTCTGGCACCCATGGAGCCCTCAGAGGCAGAGATAGGTCTTTCAGTACGAGTGC
CTTCTTTGTTACCCTCATCCAAGCGAACAACTTGAGCAAAGCTCTTAGACATCTGCTTGA
TGAAATCACTGGAATATGTAACAGCATTTCTATGATCATTTTCTCCAACAACTCCTACAG
TCATCAGGCTAGCTTGCTGTCTTGACTTCCTTTCTTGCATGGCTTTCTGCCTATCTTCAT
AGAAGTCAAAATCATCTAGGATTGACATATCAGTTTCATAATTTTTGAAAATGTTCAACA
TCTCAATTCCCTGATCCAATTTCACCTCCTGAGTATCTCGACTGTTGGTCACAGGCTTAT
TGTCATTATTTTCAAGTACAATATGACGAAATTGGCTGTTTGGGACATCTTTAATAATAT
GCCACCTGACTGAGAACTGTCCACTCCATTTATCTTGCTGCCAGTAATCCACACTCTTTT
CAAAATCAACAGGCCCAACCATTTCAGCCACCCCACAGAACTGAGCACTAGCATTCACCG
AAAAGAAGAGAAAAAATTGGACTTGACTCTTGCTTCTCTTTAGCTTCAAGATAAGCAGAAT
CTAACTTTTTATTCCCATTTGGTGTGCTGGCCCAGACACCGTACTTGATGCTCTTGTGAA
CATTATCTTCACTATATGACTTGATGATGAAAAACTTAGCATCTTTGTATTCAGTGACAA
AATCTGGCCGGTTGTATGCCTCATCAAAAATCTTGACAGTAGCCTTGCTAAGTTTGTTAG
TATCCACAGAAGATCCCTGTTCAGTCATTTGACTCCTAGGCTTTGATGCCCTTGGTCCCC
GGTTTTGCTCACTAAGTATATCAAGATTACCATTGCAACCACATAGAGAGCCATTACCCC
TCCCACGTCGTTTATTGTCAAGAGAAATCCATCCTCGATTGTTCATTCCCAATGTAGAAA
TAGAAGCACCACCAAAACTGGAGCCCTGGTTAAAACCTCTGTGCATATAGCCTCTGTTGT
AGGAGTCCAAACCAGATCCAAACTCATAGAATGATCTTTGTTGCTGGGATGCCATTGGAA
CATTCTGCCCAAAGGACCCAAACGTGCCAATTGGTTGTGGAGATACAGCAGGTGATACTG
GAGTCAGAGATGGTTGTCTGTCAGAGGGTTTTGACCAATCTGACCAAAGTCCTCCAGATC
TCAGTCCATCAAAACCTTGCTGTGAACCATGGAAGCCAAGTGGACCAGGATTTCCAGGTA
TGCTGCCTCTACTGAAAGATCCCACAGGAGGAGGATAATCAGGCCGTGGTCGAAAGAGCA
TGTTGTCATTTTGTTGGTCAGCACCCAGTAACATGGTAAGCTCGGGTTGTGACACAGGAG
TTGGTGAAGTAATATATGG
>tig_1205_consensus
GGCAGCATATGAAGCAGCTAATCAATTGATCTAACAATCTGAGATGTGTGAGTGGTACAA
ACCAAGGAACTTTCTATTGACTTCAAAAATAAAATTACAACACAAGTAGGATTAATAGTG
AAAAGACTGATGGGTGTAAATTTCAAATGATGAGTTGTTCTAGGGACGTTTTGCGTCCAA
CTTCTTAAAGTCATCACTTTAAAGGTTGAACTTGAATTGAAACTGAGCACCCCTATCATT
GATAGGCCTCTGTTTTGTCCTTGAATAGCTTGAGTAGGCTCTCCATGCTCATGACTGAAA
TGTCTTTCTGATCTTTCACATCTCTCTCCCGTACACTGACCTGTCCGGTTTTGGCTTCCT
CTTCACCAACAACCAATATATAGTTATACTGTGCCAACTGAGCTTCCCGTATCTTTTTCT

GGATCTTTCTATCTGTTGTATCTGCATCAACGTAATAACCCACTTGATGAATTTGATCCC
GTACCTGCAATGCATATGGCTGGGATTTCTCTGAGACAGGGCAAACAATTGCTTGACGGG
GACTGAGCCAAAAGGGCCACTTTCCCTTGTAGTGTTCCAACAGTATAGCAAACATACGCT
CAACAGAACCTAATATGGCCCTGTGTATCATAACAGGTCTTTCTGTCTTCGCTTCATCTT
CGGCTGAGTAACCCAAATTAAATCGATCAGGTAGTTGGAAGTCAAGCTGTAATGTTGCAC
ACTGAAATTTCCTTTTCAATGCATCAGATACACTGATATCTATTTTTGGTCCATAGAATG
CTCCATCTCCTTCATCTATCTGCCATGGCTTACCAAACTCATTCAATGCTTCAGCAAGAG
CGCCTTCTGCTTTCTCCCATGTTGCCAAGTCACCTAGATACTTTTCTGGTCTAGTTGATA
GCTTCAGCTCATATGTGAACCCGAATATTGTGTATGCATAATTGATGAATTCCAGGACAC
CCTTTACTTCGTCTTTTAT
>tig_1206_consensus
GGAATAATTTTAACCAATCTAATGTGGCAAATCATAGAATAATTTACTTGCAGCAAAATT
CCAGCCAGGAAACTTTCTATTGACTTCCAAAATTGAAATAAAATCACATTACAAGGAGTA
ATAAAATTGAAAAGATTGTAAATTTCAAATGATAATCTTGTCCCTTGTTGGGGTGTTCTA
ATCAAGTTTTGCATCCAACACTTTAATGGTGAAGCACCCTTATTGATAGGCCTCCGTCTT
GTCCTTGAATAGCTTGAGTAAGCTTTCCATGCTCATTACTGAAATGTCTTTCTGATCTTT
CACATCCCTCTCCCGCACACTCACCTGTCCAGTTTTGGCTTCCTCTTCACCAACAACCAA
TATATAGTTGTATTGTGCCAACTGAGCTTCTCGAATCTTTTTCTGAATCTTTCTATCTGT
TGTATCTGCATCAACATAATAACCCACTTGCTGAATTTGATCCTTTACCTGCAATGCATA
TGGCTGGGATTTCTCTGAGACAGGGCAAACAATTGCTTGACGGGGACTAAGCCAAAAGGG
CCACTTCCCCTTGTAATGTTCCAACAATATAGCAAACATACGCTCAACAGAACCCAATAT
GGCCCTGTGTATCATAACAGGTCTTTCTGTCTTTGCTTCATCTTCGGCTGAGTAACCCAA
GTTGAATCGATTAGGTAGCTGGAAATCAAGCTGTAATGTTGCACACTGAAATTTCCTCTT
CAATGCATCAGATACACTTATATCTATTTTTGGTCCATAAAATGCTCCATCTCCTTCATT
TATCTGCCATGGTTTACCAAATTCATTCAACGCTTCAGTAAGAGCAACTTCTGCTTTCTC
CCATGTTGCCAAGTCACCTAGATACTTTTCTGGCCTAGTTGATAGCTTCAGTTCATATGT
GAACCCAAATATTGTGTATGCATAATTGATGAATTCCAACACACCCTTTACTTCGTCTTT
TAT
>tig_1207_consensus
TGTTGGGTTGGATAGTCAAAACCCTCGTGTTGAGTCCTCACCAAAAGTACAGGGTTTTGA
TGGGACAGGCAACCCTACAGAATATTATTATGGCCCCGCTTTAAAGTTTCATCCACAGAA
CCATAATCATGGAAAGCAATCTTCGGACCCTGTGCAGATATCAGATTCTTCTTCAGTTTT
GATTCATGATAGATTGGTGTCTTCTATATCTTCAGTTTCCAATATGGGATTGAGAAATAA
AAATGTGGTTGAAGTAGTTCAGACGGAGGATATAATTAACCTTGAACAAGAAAATGAAGC
AAATCATGTAAAAGAGGCCGAAAACATCGATGTGCTAACGATATCTTCTCCAGAACAGGA
TACAATTGCTGATAAATCTAGTAGCCCAAATAGCTTGGGGACTGGGGAAGTGAGTGGTAA
TGTTGTGAAGCAAGGTGAAAATGGTCCTCTAGCTCCTCCAAAAGATTCACAGCTGTTAGC
TAACTGTTTGAGTTTTTTACCTGATTTGATTGCTTCTGTAAAAAGAGCAGCACTAGAAGG
TGCAGAGGAGGTGAAAACTAAAGTTGACGAAGATGTTGATTCTAGAAAGCCCGATTCAGT
GCCAAAAGAAGTGCATGCAAGTGAATCTGAATTAATGAGCGTTCATGGGGAGTTGGAGAT
GGATTCTGATGTTGACAATGTAAATTCCAAAATTGAGCCAACAAAGGCAGAGGCTGAAGC
TATTGCCAGAGGGTTACAGACCATAAGAAATGCTGATCTTGAGGAGATATGCGAATTAGG
TTCAGGAACCTATGGAGCTGTTTACCATGGCAAGTGGAAGGGTTCTGATGTAGCAATAAA
AAGAATCAAAGCCAGCTGCTTTGCAGGGAGGCCTTCTGAAAGAGAACGCTTGATCGCTGA
TTTTTGGAAGGAGGCTTTGTTATTAAGTTCTTTGCACCATCCAAATGTTGTGTCATTTTA
TGGTATAGTACGTGATGGTCCTGATGGATCTCTAGCAACTGTGACCGAATTCATGGTTAA
TGGATCATTGAAACAGTTTTTGCGGAAGAAGGACAG
>tig_1208_consensus
ACCTCCTTCAATTTCTTTCTTACTTTCCTCTTGACACACATATAAACGTCGTCTCGCTTG
AAAATTTCCTCTTTACGAAATATAAAAAATGTCTGATTTTAAAGATGTATCATCTGATAC
TACAATAAGTACTTAAAAGGGTATGCTCATAGTATTGAGCAAACCAAAATCTAACCAAGA
CACTAGAAGAGGAACACTGGAACTGCTCTGCAATGGGAGGGAAATCCATGACTGCTGTCT
CAATCTTTTAGTTAGCAATTCGCCCAAGGCACCGAACGCAGCTATCCAGTTTAATACTCA
AATCCTCTGGTGCAGACTGAAACCAGACCTCTTAACTAAAACCTGGGAGAACTCTTATTC
ATATTTCGATGAAGGATCCGACCCACATTCGTATCGATACTATCAATCAGTTCTCTCAAC
CCCAAATTCCCTACTTTCCCTTCCTCTTCTTCTTCTTCTTCTTCTTCTTCCTCCTTTTTG
ACAACGTCACTTCCACGTGTCACTTTAACCTGCAACCTCTCAAAATAATTCCCATATATA
TACTCAGGTGAGTATCCATTCTCCTCGTCAAAAAGCATTATATGTCCGTGCCACTCCCAG

ACCCGATAATCCGAACCCGTTTCCTCGTGATATCCGACACAGATGTGGTGTTCCCCAACC
GTAACAGCCTCGCCCGAGTTGGGCTTAAGCGATTCGATGTTGCCGGATAGGATGATCTTG
ATTATCTGTTTTTCGAGCCTTGATTCTTCTTTGGAGAGTTGGGCTTTGTTTGAGTCATCA
TCGGATGAAGAATCTGAGAGATTAACTGCGTCAAAGAGGAGGTCTTCAAGGGACTGCGGC
CTGATTTGAAGGCGACCCATCAGGGTCTGAAGCAAGAGAAGATCAAAATTGCTGGTACTG
CTCGTGAAGGAGCTCATGTCGGTTTAGGCCTTGGGGTTTTTGTAAGTTGGAGATTGAGTT
GAGGGAGAAGGAAGAAAGAGGAAATATG
>tig_1209_consensus
GCACAAAATTGTTCTATATTAACCACAACTGTGACTGAACAAAAAGATCAATCACACTGA
TGCATTTGGTATAACACAACCATATGGAAGAGTTGACTCAAAAATGTGTTTTTATATTCA
AAAGCACAGTACTAAGAAAGCACAAGAAAAAACTAGCAAAAGTGATTTGGCAGTTCAATT
TAAAACAATATTCCAAGTGAAACTTCAAAAGCTGATGCTAGGCGACCACATAGAACCATT
TAACCAAAAACATTAAATCAACAGAATAATATATTAAGACTGCAAAATTCCTAGGTCATC
CTCTGTGCCGCTTCCTTTGCAAGAATTCTTTCTTTCGCCTTGGTTTTTGCCTGTGATTTG
GTGCCCATGACACCACCTCCCCATTTCTTCCTCAACTCATCATATTTGTCGTTGAAGTTT
GCCTTAATAGCTTCCAGGATCTTGCTGAACTCCAGCTTGTCTTCATTCTTCACTGTGGTC
AAACACAAGACAGAAGCTGTCTTCTTGTGGACAATCGATCCTAGACGTGATTTCCCTTTC
ACAATGCAGTAGGGGATCTCCATTTTTCTGCACAAGGCAGGAAGCCACACAACCAACTCT
ATTGGGTCAACATCATGCGCAATGACAACCAATTGTGCCTTGTTCTGCTCAATGAGATAG
GTGACATGGTTAAGACCGTATTTCACAACTATAGGCTTCTTAGCCTCTACAGTTTTTCCT
TCTGCCTCCGCCTGTGCTCTCTTCAAAAGACGTTCCTTCTTGGCAGCTTTGTCTTCTGGC
CTGTACTTCAGAAGCAACTTGAAAAGACTTGTGGCAAGATTCTTGTCCAAGGTCTTGGTG
AACTGGTTCAATGCCGGTGGCACCTTCAACCTCTGTCTGAGGATCCTTCTCTGCCTCTGA
ATTCGGATTGCCTTGGGCCACTTCACATAACGGTGAAGATCTTTCTTTGGAGGCAATGCA
CCTCCAATACCAAATTGCTTTGGCCGCTTCTCAAACAAAGGATTCACAACCTTCTCCGGC
TTCTTCTTTGCTGGTGCAGCAACTTTTCCTCCTCTCTTTGGCGCCATTCCGTGCTGCTGC
AGATGCTAGTCTTGGCTGGTGGTTTTTAGGAACGGCGAATGAAGAACCCTAGCCAGGCCA
CTCACAACGAAATAGCCAGAAGAGGCCTTTTATAATTAGGTTAGG
>tig_1210_consensus
AACCAGTGCTGAATAGATCCATCAAACTACTGCATTTAATAATGTTTTGATTAAAATGCA
CAAAACCAAGGGTGCAAGCATATAAAAATAACTGAGGTTAATTTAGCAGTTCAATAAAAA
CCATATTCCATGTGAAACTTAAAAGAGTAGATTCTAGACGACTACATCAAACCGTTTCAT
CCAAAAAACAGGGCATTAAATCAACAGATTGAAAGACTAAAGACTCAGCAAAAATTCCTA
GGTCATCCTTTGTGCAGCTTCCTTTGCAAGAAGTCTTTCATTGGCCTTGGTTTTTGCCTG
GGATTTGGAGCCCATAACACCACCTCCCCATTTCTTCCTCAACTCATCATATTTGTCATT
GAAGTTCGCCTTGATGGCTTCTAGGATCTTGCTAAACTCCAGCTTGTCCTCATTCTTGAC
TGTGGTCAAACACAGGACGGAAGCTGTCTTCTTGTGGACAATCGATCCTAGACGTGATTT
CCCTTTCACAATACAGTAAGGGATCTCCATCTTTCTGCACAAGGCAGGAAGCCAGACAAC
CAACTCTATCGGGTCCACATCATGTGCGATGACAACCAATTGTGCCTTGTTCTGCTCAAT
AAGATAGGTTACGTGGTTAAGACCATATTTCACAACAATAGGCTTCTTAGCCTCAACAGT
TTTTCCTTCAGCCTCTGCCTGAGCCCTTTTCAAAAGACGTTCCTTCTTGGCAGCTTTGTC
CTCAGGCCTATACTTCAGAAGCAACTTGAAGAGACTAGTAGCAAGATTTTTGTCCAGGGT
CTTGGTAAACTGGTTCAAAGCAGGTGGCACCTTCAGCCTTTGTCTAAGGATCCTTCTCTG
CCTCTGGATACGAATTGCCTTAGGCCACTTCACATAGCGGTGAAGATCTTTCTTTGGAGG
CAAGGCCCCTCCAATACCAAACTGCTTTGGTCGCTTCTCAAACAAAGGATTCACGACCTT
CTCCTGCTTCTTCTTTGCTGGTGCAGCAACTTTTCCTCCTCTCTTTGGAGCCATTTCGTG
CCGATTCTAGCCTTGGGTTGTGGTTTTTAAGAACGGCGAATGAAAAACCCTAGCCAGGCC
ACTCTTAGTGAAATAGCTGGAAAGGGTTTTTTATAGTTAGGTAAAGGTCCACAACCC
>tig_1211_consensus
ATTGTCACCATTCACATCAACCTTATCAAATATGGGATACTCAGCCTTAAAGCGTGTGCA
AGCAAATTCTACGATCTGTTCATTGGTCCCTGGCTCCTGAGCTCCAAATTGATTACAGGG
AAATGCAAGAATTTCCAAGCCTTGATCTTTGTATTTCTGATACAACTGGCTCAGCTCTGT
GTAGTTGGAATTAGTCAAGCCACATTGTGATGCAACATTGACAATCAAGAGCACTTTTCC
CTTGTAAATGCTGAGATCAACATCATTTCCCGTAGCATCCTTGACGGTGAAGTCATGAAC
TAATGTTTGGGATGGGCTTGCCATAGTATGATCCGATCTCAAACTGAACGACACGGACTT
AGAAATCGCAGATTGGTTTG
>tig_1212_consensus
ATAGTTACTTCTTCCACCATCTCCAAGGAAGGTGGGATTACATCTTCTAACACATTTTCA

GGCACAGCTCCCCCACCCTGAGATACATTTAATTGATAATAAGCTTCAGCACAGGATCAT
TCTATCTACTGTAAGACTGAGCTGCCAATTGGATCCTTCTTAGACAAAGCCTCTGTCTTT
TCTTGCTCCACCGCCACGAAAGGTGCAGGCAGACCCAAATGTTCTGAAACAGGTTCTTGC
TCAACCCCAGCATCCGATGTAACATCTATTTGATTACCTGCTT
>tig_1213_consensus
ATAGTTACTTCTTCCACCATCTCTAAGGAAGGTGGGATTACATCTTCTAACACATTTTCA
GGCACAGCTCCCCCACCCTGAGATACATTTAATTGATAATAAGCTTCAGCACAGGATCAT
TCTGTCTACTTTAAGACTGAGCTGCCAATTGGATCCTTCTCAGACAGAGCCTCTGTCTTT
TCTTGCTCCACCGCCACGAAAGGTGCAGGCAGACCCAAATGTTCTGAAACAGGTTCTTGC
TCAACCCCAGCATCCGATGCAACATCTATTTGATTACCTGCTTCAGCTACAGACCCTTTT
GGATCCTCCAGTGACACTAAAAATGCATCCTTCTCAAGAGGAATAATCTATCTTTTCTTC
CTCAACCACAAAGGAAGATAAAATCGACCCGGATCCACAGACAAGCTCTCTTGCACAATC
CC
>tig_1214_consensus
ACACAAATGGTCAATCAATAAAGGTAAAAGAAAATCGTCTAAATTCAGAAAGGTCGCCAA
CACAGAGTAGTAGTATCCATGAACACAAACACAAAGCTTTGCCCCTACATCATCTCAATA
AGAACTACATTTTTGGATCAAAATCAACTTAAAAAACAACCCACAAGATGCATTGCAAAC
TCAGATCTAACAAAAAATCTTAAGCTTTTTAATTACTAAGAAGCTAAGAGAGCTTCCACT
TTTGCAATCTGATCTTCTTCATCCTTGGGGCTGGGACTCCTGTTAGCATCCTGAAACAGG
TCATGCTTCCACTTTTCACCTCGAGTACCATTTGGGGGAAACCCTTGCCTTCCTACAAAT
GTATCTCTTCCCCTATAATCACCACGACCACCACCACTACCATACCTCTGTCTCGATAGA
TAGCCAACCCTTTGTGTTTCGCTCCTGTTTGCCAACCTATCTCCTGTAAGTGGCTTCTCA
ACTCTATCTGAATGGTGGGAGTCACGGCCTCTCTGCTCTCTTCTTTCACTTCCCAGTACA
GGACGGTCAGGACGACTAACCTTGGCTGATTCTTCTACTGTTTTATCAACATTTCCAGAG
TCCAATGTGATTTTCTCTTCCCTAAATGCTCGTCTTTTGAGGGCAGGTGGTGGCAGATCA
GTCTCCAATTTGAAGAATTCATCATGGCGCCAAGCACTGCTGTCAACCACCTTACCTTGT
GATTTCTCATCTCTTGGCCGTGTATCATATGCTTCTTTCTTGTTAGCAGCTCTTTCATTT
TTTTGACTCCTGGAATCCCTCTGCCATTTTCGATCAGAAGCAGCTCCACGACCAAAGCTT
CGACCAACTTGNNNNNNNNNNNNNNNNNNNNNNATCAGTTGGATCCTTGCTTTTCTCATGAA
GTCCATTGGGTTTCTTGTCAGCTTCTTTGGTCCAGGGCACAGTTCCAACCTCAGAATCAG
GAGCAGATGGAGCCTCAAGTGGTAATACATCCTGCAGCCGGCGTCGGCGAGTTTGATCAT
GGTCTACAAGGTCCTCAACATCCAAATTATTTTTGCTGGGTACTCTCTCTGTAGCAGGTT
TTCCATCTCTCCTAGACTTCTTAGGACTGGGTTCGCGGTCAAATCTAGAACGGTGTCGTT
TGGATTCAGAATCCCGACTCTCGCGTCGTGACATTGGCTTGGCTTGAGAATTAGGGTTTT
TTCAGGGCGACAGCAGTGGCTAATAGGATTTCCG
>tig_1215_consensus
GCTAGCTTGTAATAAATAAAATATCATTCATCCCAAGACATGTGAAGATTGGGGCTCAAA
AGGTCCCGTTAGATAATATCTAGTAGATCTAACTAAGCAAAGAGAGGTTCAGAGCTAGTA
AAATTCCAATCAAAACCTACAAAATTACAAATCAACTATAATTGATTACCATAACTCAAG
TTTCAAACATAAATCCTATAAGAACCACGAATCCACAAGATCTACCACCTTCAGCGGAAG
GTCACACTTAAAGAGAGTAAACAACCTCTTCTTTCTGCAAATCCTCACTCACTTGACAAT
GAAGGGGAATACTAAATGTCATACAACTAAAATGCGGCTGCAGATGTATCTGGCCTTCCT
CATAGCATGCAATCAGTTCGAGCTGGCAACCTCCTGAAGAAATTAAGTGGCACTGAGGGA
AGCTTATGACGAAACTTTGGATGTCTTAACACATAAAATCCTGTTAGGAGAGCCACAACT
TGGAATGGTGTAACATAAAGAACAACGGCAGCAACCAAACAGAAAATCACAAAAAGAGCA
GTGGCTCTTGGGTCTCTCCAGCTCAACAAAGATTGCAACCTCTCCCCTTGCGTAGCCAAA
TCACCAACCACAGTCTGAATTCTACCAGCAATACTTCTCAAACGATCATATCTCATCCTT
ACAATATCAGCAGGCCTAGAGGTTGGAAATGTATCAAATTCTTCATCCAATTCATCGGGA
TGGGGAGAATCTGCATGAGAGAGACGGGTGTCCATGTGAGGAGGATGCCTCGGCCTCCAC
CTATAATACCAAACTCCAATCAAGAATAGGTACAGGAAAATTGTGGGCAAAATAAGCTCT
GGGTACAACACCAGTATGACAAACAAAATGTGAATCAACACAGTGGTAATGGGGTTTTTC
CAATTGCAGATCTGATCAAACCATTTTCCCACAGATATTAAACCACCCAACACTCCCATA
ATCCTGAAAAAATTAGCTTTGCTACGTCTCATGCTCCACATGTGAGAACCCACATCCAGC
ATAAACTCCACAACCTCCTTCCTCAATGGTGGCTCAGCTCGGCTCAGCCTCATGGACACA
ATCTGAGTAGCTTGATGCCTCAGGTTATCAAGTTGGCTTACAGTTAACGGATGCAAATAG
TGCATCTTTGGTAACAACGGTTGTGAGTACATATGCATCATGTTAAGCAATGACGAGCAT
GTGAATCTCACAGCTAAATGAATTTCACCCATCTTCTTGACACCATTTTGATATAGAACC
AGAAGTGGATATGAATGAGTGTAGACACGGTCTGTTTCAAGCGTAGAGAGACGAATCCTT

ACTTTCCCAATTCTTGAATCCTTTGACCCTCCAGATTTATCTCCTCCATGCAAGTGCCAA
TTATCAAACACCCCAATTGTTATAACAGTACAAGGATCGAAAACTTCCCATGTGTATTGC
TCATTCCACCTGGGAGTAAAGCTATCTATGATTGTTCTAGTTCGAACCCATTTCTGCCCA
TACTTAGCCACACAATAAGCATCTGTAGTTCCCCGCCCATCTTTAGTCTTCATTGGCATC
AAACCTTGAGCATTCAAAATACCCAATTCCAGTACCCCTATGCTGGACTTCCATAACTGT
TTCGCTGTTGGCCTAAGATCACTACTGTAGTGTGTCGACTCATCCAGAACATGATAACCT
CCTTCCAAACAGATCCTCAGGTGAATCCTGCTAGCAAACTTTGTCTCTTTCTTCTTCTCC
CCTTCCGCAATCACATGCTTTTCTAGATTAAACCACCTGCTATTCACAGGTTTATGATCA
AGCCTCCTGTCAACATACTGCAGAGGAATCACACACCCTCCCAGAACCTCATCCTTGTTA
GGTGCAACTCTATCTTCCACACTCAAAATCAAGTGCTCCTCAAATGGTTCGGCTGCCACA
AACATCAAATCCTCATTCCACATAGGATTAATAGTCCTGCTAGCAGAACTTCTAGTTCTC
AAAGCCTGATTTCCAAGTCTGGCCTTGACAAAAACATCCGGGTATCTACCCTGGTCAGTG
GGTTGCAGGTCCTGTGCTTCAATGACATTAACCCTCAAATACCAAAGCTTAGGAGAGAGA
TACACCTTGGACCGGATATTAGCCAGACCATCCATGCCACTAACAGTGGCTGCATCCGAA
TGCCATGCCTCAGGAAATGCCTCATCTGCTTGAGTACCCATCCAAACAGCCAACATTAAT
TCTCCCTTCACTTTATCCCCCTTCCTATCCTCCAACCTATACC
>tig_1216_consensus
ATTTCTTTTCCTACCAACTGCAAATGAAGAACGATGTAGTTACAATTGACCAAAAAATGT
CAAATAAACTGAATTCTAAATTATATAGACAAATTGATAAATGGACTATGACAAAGTCAA
GATACAGAATCATATTTCACTTCTGTCATACAACAGTGGACCCTAATCCAAGTATCAAGA
TGATCCTGATGCCGAGAAAGAAAATTTTCAAGCATAACAGATCAGGCAGCCAACGTTTTC
ATTAGGATACCGAAATTTCTATCTCACATGGCTACAGAAAGGTCACTGGTATTCACTCCC
ACAAACACCCCGGCTGGTGCTTGCATCACCAATTGAAACTGTGGCAAGGGACTTGGAAGC
CCATGCCCTGGCATTCAACGCTCCTTCCTGCAAGTTACTTCTCTTGCAAGGCTCCTTCAG
AGAATTAAATATCTGCATTGCCCGATTCAAATAGGCCCAAGGATGCTCAAACAAGTTTCC
CAGTTTAACAATTCGAATGAGCCTTACCCAAGCAAATTGAGCAAATACAAGCAGAGTTGG
AAAGAATGCGTAAAGCAATCTCAAGAGAACAAATGCAGTTGCAAGAGCCAAATATGTTTC
CTGCGGAAGCAAATTCTCCGGTGATCTTGCCCATGAGAATGGGCAGCTTTCGTCATCCCC
ATTATTGTGCTTCTCTTGACTTGATATGTGCCCAACTTTGTCCAGGGATATTTTATCCAA
TGATGCAGAATTAATGCCTGTGACATCACTGTAGAAAGCAACAAGTGAACCAAGAGCCCG
AGAGCCATGATAACGAACACGCATCGAGGAATTTAAAAGAAAAAGGGTGGGAAATCCATG
AACCCCATACTTGGAAAGTATACTTGGTCTGATGGCTGATTCTTCGATGGCTAAATGGGG
AATTGATGAATATAAAGAAGAAAGGATAGAGAAATTCGGTCTAAAATTACGAGAGAAAGG
ACACCAAGACGCATAGAAAAGAACAGCCACAAAGTCATGACTATTCTTGTGAACCATATT
CAGAACCCTCTGCAATGAAAGCTCGCCGCCCTCGATAACATCGTGGGATTGAAAACTGGG
GTTAACTGGACAGCTGAATTTCCGGAACCCTAGAATCAAATCAGTAATGGATTCCGTAGG
ACATAACGGAAACCTAACTGTCTCGACGGCCGACGTTAATCTCCCCAACAGCAACAAAAC
CAAGATCCCCGTCTCCCAGACGCTCATCCCCATCACGACTCAACAGAGAATAAGAAAACT
TTTTACTTGGAAAAAAACCCAGAAAAAAATAAATCAAAAATCGAAGAATAATAAGAGGAC
AAATTTGGGAGAGCGGCGCGTTTTAGGAAAAATAAATTATGATTGAATTAAATGTGCCTA
ACTGTTCGACTTACAGACGGCTTTCGGACATGACATGAGAGAGAGGACTGGGTTGAAGAT
AACGACAGGGTGGCCTGTCAACGGTGTCTTCTTTAGGTTTGATGGTGGGGTGGGAAGGGA
TTCTCACAACTTCAGACTCGTTAGTCTTTGTGATTTATAGGAAAAAGAGAATGATAAAAT
AAAATAAAAAATCAGTCTATTATTCCCTAGTTAAATGTGTGTTGGGCGAGGGCAGGACCG
GTAAAACAATGCTCGGTCAAAATAACAAAGATTAATTTGACCCTAAATTTTTTTTTATA
AGAGTCTCTAGATATAAGGCTTTATAGTCTATGCCGGCCTTCACAAAACTCATATCTATA
AGCC
>tig_1217_consensus
GCCCAGATTATGAAATATAATTCTACAAAATCTGAGTTTGATGTAAGCATGCAACAAATA
AATATACAAATTTACATGCTCCAAGTAAGTTCAGTACCCAACTTTAACCAAAAACAATTA
TTATAGTTTCCTTCTCTAACAAACAGGACCATTTGCAACTCATCTGTTGAAACCTCCTCC
ACTGTCACCCATCCCACTCTGAAAACAAAACCTAAAAAGAAAACTTGCTTGTGCAAAAAT
CTATTATGACTGTCGAAGACTATTCATGGAGATAAGTTGTAACTAGTCTCTGTAAAATTG
TGGGTTTGCATTGTATTTTCCATTCCCGTAGAGTCATCAGCTTTTATGGTATTTTTCCTT
GGCGATTGGGGATTGATCAAGCTGATCAGATTGTAGTGGTTGCGTCTCTGTTTTCTTTTT
CTCCTTCAGAATCACCTGTCTAGGCATCACTTCTAGCAAAAAGCTTCCCCCATTCCATAT
AGAAGCAGAAACCTTTAGAACTTGGAATATTACATGCAATCTATATGACAAAAAGATGGG
GACAGTGAGTGCCATGGTTGCCACGGTAAATATAGCTTGGAAAGCAATGTACATGAACAT

GCGATTTTGATCCCCAAGCAAACCACTTAAACGCCACCATACGTTGTTTGCTTTCTGTGC
CTTTTTGGACAGTTCCCTATAAGAGGTCATGACTTCTGGATCTTGTAAAAGCCTCTGTCG
ACGAAGGACATTAACAATGAGAAAATAAAGAACTTGCCAAAGGGTGTAAGCAGCTAAAGG
AACCCAAAACAGCCAAGTTAAAAGGTAGGATTTATCTTCCACGTAAGGCCATGATGCTCT
TTGAGATGATCCTTCTGGTCGCATGGCTGCAAAGGTTTCTGGATTCCACCACCGAATGGT
GAAAAACACTAACCCAGGCAAAAGATGTATTAGGACACTAACAATTTTGTCCACAGAATT
GAAAACCAAGCTACAACGCCAAACAATCAATGCCCATGCTAAAGGCCCCTCAGCAAATGA
GAAGCAAACCATGAAAAACTTTTCGTTTCTTTGGTAGAGAAGAAGATCAACCAAGAAGAT
TGTATTGGCATAATAGCAAAAATCCAGAAGATAGTAATGCCATTTCTTATACCGATAATA
TATCCACCGAAGAGGAACAAAGGTGACATAGAACAAACAATATACGTAAGGAATATCTTG
GGGCCTTGCTCCCAACAGGAAACAAAAACCCCCGAACCCAAGAACGCCCAGAAGATGTGT
GACCTTATTAATAAATCTTTCATGTTCCTCTGCTTGTTTAGCGATCTTAACAGCCTGTTT
AGACAACATCTCTTTCGTCTCAACTGCCTTTTTCGACATCATCTCCTTTGTTTGAACCAC
TTTCAGAGATCGATCCTGGAAACTCTTCTTCACCATTCCAACTGAATGCCCGTTGCCATT
TCCGTTCATCTCCTCCGAAAGCTCTTCCATGTTGGCCATGCTTATATTCCTCTTCTGCCA
AGCAAATACAAGAAATCTGAAAATACGAATGATAGAGAACCGGTGGTGCTTCGTCTGCGT
GGCTGTGGCGGTATTACACAATAATTATGCATCCAAAATTCTAAATTGTAGCCAGAATTG
TTTGTTTATTTTGCGTTTGTGCCGTGTGT
>tig_1218_consensus
TAACAGTCACATACCAAGAGAAGTACATTTCAACTAAATTTAATGCTAAATTAGTCTCCC
AGTTTCTAGAACAGGACTCCCAAAAATGAAAATTTCAAACATAATTCAACAAAATGAACT
TGCTTGTTCTAAACTCATTGATATAACAGTGCAAGGTTTATAAGCTTTTACCAACTAAAC
AAGATTTCTACTCTCATAAACTTTCCTTTGAGCTTGGGAGCTTGTCATCTTGTTTCTCCT
CTATATTGACTGCTGTAGGATTCTCCACAGGCAGGTTTGTTTCAGAATCAGGATCCTTTT
TGAGCTCTGACTCAAACTCCTTTGCAGCCTGTTGGAAGCTCTTGATGGTCTTGCCAATAC
TCCTGCCCACTTCAGGCAACTTTTTGGGACCAAAAACTAAAGCAGCGACTCCAGCGATTA
CAACAAGTTCAGGCACACCGAGGCCAAACAAAGCATTACAAGAAAAGCCCTTCTTAGGGT
TTTGAGTTTTTGCTCTCCGTTGAATAAGAACCAGGGAGCTAGTTTTGAAGTTAGTTCTGC
TGAAGATAGAACTGGCGGTGAAGAAAGAGGAGTATGAAGAAGAGATGGGAAGAGAAGGTG
GTGGTAAAGGTCTCGTTGAAGAAGAGAGAGGGAGAGTTAAAGAAGAAGAGATCTCCATGG
TGATGCTCAGATTTCTTGTGCTTTCAAGCTGCCGAAGTTAGATAACATCTTGGCTTTGGT
GGGGAGCAGAGCCTTATTTTGCCTTAACAGTTCGATGGAGATTCTATTATTGGGAATCTA
TCATGCACCTCCCCTGGGGTTTCTGAAATTTTCAATACCTCCTGGACATATCAGAAAGAT
TAGCCTTCTTCC
>tig_1219_consensus
AAAAAAAAAAAAAAAAAAGAACAGGCGGTAATCGTGATGATAACAAAGGTTCTGTAAACTA
GGTTGGGAGGTCATCCTTAGAGGTGCAACCTGAAGATATTTACCAAGTTCAATTTAAATA
GCCCAATGGTTGCTTCGAAAGCGGTTCTACATGTTTTCTCTGGAGTTTCACCTTGTGTTA
ATTCATATGATTGGGGTATCTATAGTTCAAATTCAGTTCTGTCCATTAAGCCTAGTGTAA
TCTGTACAAAGAGTAAAGGAACTGGATGTACCAAATGGCGTGATGGTTTAAAAAACTGCT
TGAGTGTGTATGCTATTCACTTTGGACATGACAAATCTCAGGGAAAGACGAATGGTAATA
GGTTGAAGTTTTTGAGTTACAAATGCCGAGCTGTTGAAAGTCCAGGTGGTTTAACTGCAG
ATGATGGAAGAGAAACATTGCTTCCTGTTAGTGGTAAATCTAGTGTGCCAAGTAGTCAAA
ATTTTGAGTTTAGGCAACAATCAAAAAATGTAAATGGAGTTTTTTCGTCAAATGATGCAT
TAGAAACTTGTGGAGCAATTATAGATACAGTCAATAAGGCTGGGTCAACATCGATTGAGG
AAGAGGCATGGGATTTACTTAGAGCATCCATGGTTTATTATTGTGGGAATCCTATTGGAA
CAATTGCTGCTAACGACCCAAGTAGCTCTAGCATCCTGAATTATGATCAAGTCTTTATTC
GTGATTTCATACCATCTGGGATAGCATTCCTCTTGAAAGGAGAGTATGATATCGTACGAA
ATTTCATTCTTCACACCCTTCAGTTACAGAGCTGGGAGAAAACCATGGATTGTTATAGTC
CTGGTCAAGGATTAATGCCTGCAAGTTTCAAGGTGCGCACCATTCCTCTAGATGGTGATG
ATTCTGCAACTGAAGATGTATTAGATCCTGATTTTGGTGAAGCTGCAATTGGTCGTGTTG
CACCTGTTGATTCTGGATTGTGGTGGATCATTTTGTTACGAGCATATGGAAAATGCTCTG
GGGATCTTTCAGTGCAGGAGAGAGTTGATGTGCAGACTGGGATTAAAATGATTTTGAAGC
TGTGTCTTGCTGATGGTTTTGATATGTTCCCAACTCTATTGGTAACCGATGGCTCATGCA
TGATAGATCGTCGCATGGGAATTCATGGCCATCCTCTGGAGATTCAGGCACTTTTTTATT
CAGCATTGCTGTGTGGACGTGAAATGCTCGCTCAAGAGGATGGATCTGCTGACCTTACTA
GAGCTCTGAACAATCGTCTAGTTGCATTGTCATTCCACATCCGGGAATATTACTGGATTG
ATATGAAAAAACTTAATGAAATATACCGATACAAGACAGAGGAATACTCTTATGATGCAG

TTAATAAGTTCAACATCTACCCAGACCAAATTCCTCCCTGGCTGGTGGAATTTATGCCCA
ATAGAGGAGGCTATTTGATTGGAAATCTTCAGCCAGCTCACATGGATTTCCGTTTCTTTT
CTCTTGGAAATTTATGGTCTATTATAAGCAGTCTTGCTACAGTGGATCAATCACATGCCA
TATTGGATCTCGTTGAAGCAAAGTGGGCAGAATTAGTAGCAGATATGCCCTTCAAAATAT
GTTATCCTGCTCTTGAGGGCCAGGAATGGCGAATTATTACAGGCAGTGATCCCAAGAACA
CTCCTTGGTCTTATCACAATGGAGGTTCTTGGCCTACTTTGCTGTGGCAGCTTACTGTTG
CATGCATAAAGATGAATAGACCAGAGATTGCTGAAAAAGCTGTCAAACTTGCCGAGCGGC
GTATATGTGCTGATAAGTGGCCTGAATATTATGATACTAAGCGAGGAAGATTTATTGGAA
AGCAGTCGCGGCTATTTCAAACGTGGTCAATCGCTGGGTATCTTGTGGCGAAGCTGCTCC
TGGCCAACCCAAATGCAGCAAAAATTGTTGTAAATGAAGAAGATTCAGAACTTGTAAACA
GCTTTTCGTCGATGATTAGTTCAAACCCACGTAGGAAGCGTGGCCGAAAAGGATTGAACC
GCTCCTTCATCGTATGAGTTTAGACTATGAAGCAATGAGAGGTAAAAGCAAGGTAATACC
ATTGTGTTGTGCAATATTTTGTTTTAGCTAATACAATTTCTACAAAATATGATATAGCTC
CACAGTATTATTAGGATTGATAGTCACAATCTTCAATCTCTTCCTTCTGGTTTTGCATTG
TTGACAATGTTTCTTTTTTGGCCCAATGGAGGACTTGAGGTAAATATAAAGGCTTTGGAC
ACATGTAGCCAGCTCAGGAATCATAATTTTTCAAAATGTGCAG
>tig_1220_consensus
GAACCCTTTGCTGCTCTGATCAAAATTGCCTCTAAGAGTAATAATACAAAAGAATATACA
GTGGTGAAAGAATACAATCTATAATTTGCTAGATGTGGAAAAAGAATCACAATTACTATT
TGATGATCTTCTCTTCTCAACAACCTCGAATCCTAGTTCTATAATATGCCTCAAAATTTG
CGTTTCCTAGCAGTCAACAGAAGTAAATATGTTTACTGTAAGTGTCCATGTTAGAAGGAA
AGCTTCTCCACCTGAACAAGCCTTCTCCGCTTCCCAGTTGAAAAATGAATTTTGGTTCAC
GTTTGCTATGATCCAAGAAATAAACAACCCAGTTTTAAGCTATAACCATCGTCATAGGAG
ATTTTATCTTATTGTATGTACTTCTTTCGTCCAAGTGACGACTGTCTGTATCCGTGCCTC
AACCTTTCAAACAATTTCTCCTTTGTTTTCCTCTTCTTGTCCATCTTTAGCCTGTATCTT
TCTTCTCTCTCTCTTTCATAAATCCCTTGCCAATGCACTTCCCCTGTTAGTGGCCACAAC
CACTTCTCCCTTGGGTGGTCACCATCATCTGCAGCCTCGGCAAAATCTTCATCCATACTC
TTCAAAATTGTAAAATTAAAATACTTTCCCCATCTAGATCTATTGCGTTCATCTACAGGA
TGCTGCTCAGTTAGAGAACCAGAGTGCGGCTCCAAGTACACCATCTTTCGTCCGCTGTGG
TAAGCCCATACATTGACAAGAAGTTCCAACAACCGACAATAGCAGTGCTTTTTCTCTAGC
TCAGAGGAACTCAACAAGCACATATTTGCTTTACTAGAATTGGTATGCAGGGCGTCAAGA
GAATCTACAAACATCCTTGAAAACATCATGAACTCTAAAAAGGAAGGGGTTGGCATCACC
CAGCTATGAAGGGTGGACCAGAGACCACCATCCGCTGGCATCGCAGGGAGAGCATCAATG
TGTGATGGCAAGTCATACATCTTCCGGAAGGCATCTTCAAACGCAGCTCT
>tig_1221_consensus
TTCCCTTCTTTTCTTACGCCAGCACCGTCTCAAATCTCCGTTGAATCCGGCTCCTTTTTT
TTCCAGTCACACTTCTCCTCTGTCTACTACTTGATCAAAATAACTTATGTCAACTTACTT
TAAGAACTTACAAGAAAGACACACCCCAATGTCGTATTATTCTTCAACAGAATGGATAAA
GAGTTCGTGCAACCGAATGAATAAGAGAAAACAGTAAAGGAGAAAGAGTAGATCTTAATT
CTCCAAAGAGCCAAAATCGTCAATGCTCATTAACCGCGCGAGTTGTCTCAGTGCAGATCC
AACAAATGAAGCCAAAATCTATAAGAAAAACTAACTCCCATAAAGTAAACATAAGGTCTC
AATAACACTTGAACCACCATTACCAGTAACCAAATTACATTACAATACAATGAGATCCAT
TCTTGTGAGGCAGACCCATAAAATTTGACCTAACATCAGTAATCGGAAGGTGAGATGACA
TCATCAATTTTTAATATCATCTTGACAACTTGTGTTGCAAGAAGGATCTGCTGTTGTTTC
CCAATCAGCGTCTCAAACACATTCTGCTCACGCATATCTGTGGTGCCCACATCATTGCAA
TCTATTCCGCAATATGGATTGTTTTCCTTAATTTGCTGAGATTTCACAGCAGATAGCGTC
TCAATAGGTTGGAGACCACTATTTTCTGCAAGTGCCATTGGGACAGAATCCAAAGCATCT
GCGAATGCTCTTATAGCATATTGTTCAACTCCTGGGTATCTATCTGCAGCAGCCTCTACA
GCAACTGAGCAAGATATCTCAGCTGAACCGCCACCATAGACAATGGAATTGTTACGAATC
AAATTTCTAGCCACACACAATGCATCATGGAGGCTGCGCTTAGTCTCCTCAATCATCATT
TTATTACCTCCCCGAATAAATATGGTTACTGCCCTTGAATTTGCACAGTGTTCAATGTAC
AGCATTCGATCTTTTGTTGTACCAAAAGACTTCTCTCGAACCAAACCAGCCTTTCCCAGC
TTTTCAGGTGTCAACTCCTGAAACCTTGGAACAATTCTTCCACCTGTGGCTATTGCTATA
AGTTCCAATTCTACACCACCAACCCAACGTACTGCAGGCAAGTTCCTATGCATTAATAAG
TGATTTGCCTCATCATCAAAACCCCATTGACAGATCACCAAGGTAGCACCAACATCCTTG
CACTTCTGAACCATGTCATCAAAGTATTTCTGCTCCTGCAGACGCAAAGTCTGGAACTTT
TCCACTGTATCAATGTCCACCTTATGTTTAGTCTTTGGCTTTGGTGGTTCAAAGGGGCAA
GTCAAGATAGCAATTTTAGCATCTTCAATACGTTTTGGCATCTGGGGATGGCTCATATCC

TTATCAACAGTAATCCCATATATTAGCTCAGTGTCCTCCAGCTTTCCCCCTACTTTGCCT
TCAACTTTGATCAAATCTAAATTAACATCTTTCCTCTCTAAATCTGCCACAGCAAGAACT
GCTTTCACAGCAATTTCCGCCAGACTGCGCTTGCACCGGTTCACTATCTTAGAATTTAAA
GTAGTCATGCAAGTTTGAACCAAAGGCTCTATATTAGAAGCCCCAAATTCAAACTTCTGA
GCAATATGATCCAAATGTTCAACAGCTATTCTAGAAGCCATTTCATATCCCTCTGCAATT
CGAATAGGATGAATTCCACGTTCCAAAAGCTGCTCAGCCTGCTCCAAAAGTGCACCAGCC
AAAACAACAACACCAGTTGTCCCATCACCAATTTCATAATCCTGACTACGAGACAATTCA
ACCATCAATTTTGCAATCTGATTGTCAACATCCATCTGCTCAAGAATCGTGGCACCATCA
TTTGTAATGATAACGTCACCGTCAGGGCTCTGGAGCATTTTATCCATGCCTTTGGGTCCA
AGCGAGGTTCGGAGGATCCGAGCCACAGCTTTGCCAGCAGAAATGTTGGCTTTCTGAGCA
TCGAGTCCACGCAACCTGGACTTCTCATCTTGCTGCCTTATTAGTATAAATGGACGACCG
AACTCATCGAACGCTAATGCCATTTTTTTTCTTTACCTCCTTTGTCTTCTGGATTTTCAG
CTTTGCATCCTCGCACTCAAATCAGACTTCAAGAAGAATG
>tig_1222_consensus
TTTTTTTTTTTTTGAATCCTAGTACTAAAACTAAAAAACCAACGAGTCACACACTAAGCA
TAGCAATTATATAAAATGATTAATCGATTTTTATTCAACCCTATAGAATTTAGAATTGC
TCATTTTTGTTTTGATTGAACATCAAAAAAATGAAAGAGTTTGGCCTTTTTTGGTCTATT
TCCATCGCTGGGTAGAATGTAAAGACACATAAAGTCTTATTATTCTGCAACTACAAATAT
CCAAATTTTGATTCCTAATACCCCGTATATAGTTCGAACTGTATAGGAACAATAATCAAT
TTTAGCTCCAATGGTTTGTAGAGGAACCCTACCTTCTCTGATCCATTCGACGCGTGCAAT
TTCTTTTCCGTCAATACGACCTGCAATTTGTACTTGAATTCCTTTTGTATCCGCCTGTTC
CGTTAATTCAATAGCTTTTTTCATTGCTTTGCGAAAAGAAACTCTATTCTTTAATTGTGC
CGCTATAAATTCTGCAAGAATATTAGGGTGTCCATACGGATTTGAAATTTTTGTAATAGC
AATGTTTAGTTTTCGATTCACACAATTAAGTTTTTTTTGTACATTCAGTTGTAATTCTTC
GATTCTTCGCGATCGATTTTCTATTAATAATTTTGGAAATCCTATATAGATTATGACTTG
AATTAGATCGATTCTTTTTTGAATCTCTATCCGTGCAATTCCCTCAACACCGGAGGATAT
TCTCCTATTTTTTTGTACATAATTTTGAATACAATCTCTTATTTTTTGATCTTCTTGTAG
ACCTTCAGAATAATTTTTTGGCTGTGCAAACCAAAGAGAATGATGACTTTGTGTTGTACC
AAGTCGGAAACCGAGCGGATTTATTTTTTGTCCCATAAGCCCCCACTACTATATGTATGA
TGACATGTCATATTTGTGTTCTTATTTTTATTTATATTTGTCAATCCAGGTTTTTTTGAG
CACATGAAAAATTCTTCATATTCTTCTCAGAGAAGGAGATATCTTTCAAAACAATAGTTA
TATGACAAGTGGGTCTTTTTATCAGATAACTCCGCCCTCGAGCTCGAGGTTTTAATCTTT
TCGAAGCAGTTCCCTCGTTGACTTGGGCTTTATTAATGACTAAATTTGCTTCCTTAAAAC
CCCTATTGTGACTAGCATTCGCTGCTGCCGAATAAACCAATTTAAAAATGGGATAACATG
CTCGATAAGGCATGAGTTCTAGTATCATAAGGGTTTCTTCATAAGAACGCCCACGAATCT
GATCAATTACCCTACGAGCTTTGTGAGCAGACATACGTATATGTTGACCTAAAGTGTATA
CTTCATGTAAGTTCTTTTCTATCCTCTTTATCATAAGGTTCACCTCTCACCAATGAATCT
ATATTCACTTTATTAACGATTAACGACGGGATCTATTATCATTTTTTGCATGTACACGGA
AATTTAGAGTGGGCGCAAATTCTCCCAATTTATGTCCTACCATGCGATCTGTTATATAAA
TAGGCAAATGCTCCTTTCCATTATGGATAGCAATAGTATGGCCGACCATTGTGGGTATAA
TGCTAGATGGTCGGGACCAAGTTATTATTATTTCTTTTTCCGCTTTTGTATTAAGCTTAT
CGATTTTTCTTAATAAATGATTCGCTACAAAAGGATTTTTTTTTAGTGAACGTGTCACAG
TTAATTAACTCCTATTTTTTTTTTCTTTTGTAAATTGTAAAAACGAAGAAAGAAATTCGAT
TTTCTCTCCTATTTACTACGGCGGCGAAGAATCAAATTATCACTATATTTATTCCTTTTT
CTACTTCTTCTTCCAAGTGCAGGATAACCCCAAGGGGTTGCGGGTCTTTTTCTACCAATT
GGAGCCCTTCCTTCACCACCCCCATGGGGGTGGTCTACAGGGTTCATAACTACTCCTCTT
ACTACAGGACGCTTACCTAGCCAACATTTCGATCCGGCTCTACCCAAACTTTTCTGGTTC
ACCCCAACATTCCCCACTTGTCCGACTGTTGCTGAGCAGTTTTTGGATATCAAACGGACC
TCCCCAGAAGGTAATTTTAATGTGGCCGATTTCCCCTCTTTTGCAATCAGTTTCGCTACA
GCACCCGCTGCTCTAGCTAATTGTCCACCCTTTCCTAGTGTGATTTCTATGTTATGTATG
GCCGTGCCTAAGGGCATATCGGTTGAAGTAGATTCGTCTTTTTGATCAATCAAAACCCCT
TCCCAAACTGTACAAGCTTCTTCCAAAGCATACGGCTTTCTGGATGTAGATGATGATATC
TATACAGATGGATCTTATAGATATCATATAATGAAGTACCACATGAGTGGATATATAGGA
ATCCAAATCTGCCGAATCACTCATGTTATGATCTTCTACATCCTAGGTCTTCCCGTTCCG
TCATCTGGCTTATGTTCTTCATGTAGCATTCAGACCGAATGACTCTATGAAATTACGTCG
ATACTTCCACATATTATGGGTAACGTAGGAGACATCTCTATTTTTCCCCGGGGAAATCTT
TAGAATTACCACTGCTTAGCTTTCAATTCGCCTCTGACCATCAAATCAAATGTGAATAAC
CCGTCCTCCTCTCTTTGAAACAAGGGGTGCTTCTGGTTCTGTTGGTGCTTGAAACAATTT

GATCTTCTCCATATTACTATATCTCTAGAGTCAATAATTTTATATGAGGAACTACTGAAC
TCAATCACTTGCTGCCGTTACTCTTCATTTTTCTGTTGAGGTCTATCCTGTAAAGATACT
CAAATTGGATCAGTGATCGATTTCTAGGTTTCGTCGTAAACCTAATTGGTTACTTCCAAT
TACGTAAATCAATAGTTCAAACCGCACTCAAAGGTAGCTTCCAGATTTCAGGAAAGTGAG
GCACTACAGGTATTGGATTCAGCTTGAATTCAACTTTTTTCCTTTCATTTTGTTGGGGCT
GTTGGGGTCATTTCATGTACTGGAAGGATAATAGTCTTCTGCCACTCTGTAGAAATAAGA
CTTTCGTCGAGCTGGGATCACGTCCTAACACACCCGAGGCTTGGGCCCCGGAGCCTTCAC
GCCAGCTTCAGCAAACCTGGTTAGAAGCATAAGCTCTTCTAGGAGTACTGTCCGTGTGTT
CTAATCCAAGATACTGCTCCCAAGCACCATAAACATCCCTTCGCTGAAAACGACTGGAGA
CAACATCCGAATCCTGAAGATACTCTGGGCGACCAAGGGAAAGGAATGCAAACTTCCACT
TAGCAAATTCATCATCAGGAACCTGTAGTTTCTTCTGTATTCGCACTTTTATTTCTTCCA
AAGTTTCACCTTCATGTATGACTAAGAAAAAAGGTTCACCAAAGTTCTGAATTTGCATCT
GATTCTGTGTAGTGTCTTTTGTAAAATGATAAACATGAATTAGACGATCATGTGGACCAA
GATTCTTTTCTTCTTCTGGAATCTCTTCTGCCCTTAACGTCCAGTATTGATCATTTATAT
TCTCAATCTTTTCATTCAGCGGAAAAATCTTGTAAATCTTATGATAAAAAACTTCTAGCA
ACCGAAGTTCTGCATCAGGATGAGACAACTCCACCTTTGTCTTAAGATCATTAATAACAT
CGCCTACAGTACTCTGTTTTGGTAGTCTAATAGTGTGAACACTAACTTCATCCTTGATAG
CATGATGAAATGCTACTTTGAGAGTTTTCAAACATTGTAATTCTGGCAGAGGTATGTCCA
ATACTTCATAGTACAATATATCAGAAGTCTGGTTGTAGTGAATCAGCATATCCACCAAAT
GATCAATGCCACGGTACTTAATAGGTTGAGGTTTGGGTTGCTGAGAGTAGCAGTTATGTG
ACGTAAGCCTAATTTTGGATGGATCATCCAAGCCCAATTGTTGAGCTACTCTTTCTACGA
CATCATCATACGTAAAAAGCTTTGACATCTCTAAACAGAAATCATCTTCCTTCAGCTTTT
CAAGAGATCGGAAGTGAACAACCTGTCGATTACGCACATAGTCCAAAAATGAAGGAACAT
GTGGATAGCGATATTGTTCAGCACTTTCAATCGTATGAGATTTTTGAATGCAAACAATAT
CTCCATCTTCAAGCTGGCTAGCTCGAAA
>tig_1223_consensus
CTCACAACAAAATGATCTGCAAATTTCTTGTGGCATTTCATACTGTGCTTGGCTTAAATT
CTCTGTATATTTACAGACAGCCTTTAACTATGCACTCAAAAAACCAGAAATTTTAAACGA
AAAAATAATTATAAGTCCTGTTTGGTTATTAACAGATGGAAAAGTAGATATCCTATATAG
CCCACATCGATCATTGCACATGATCTTTGCATACATAAACATTCAAAATTCCAAAGGGGC
ACAATAATGTACAAACTATGAGATTTTACTGTTGGTCTATTTGTCCAAGAAAACCCCCAA
AGAGGAACAGCGAAGTGTCCACATGATGCCTTTCAGGCAGGGGCTGCTCTGTTTTTGGAA
CAGGTTTGACAGTTTGTTGTTCCTTGTCTGGAAATGGAAATGAAAGAGAAAGGGAAAGGG
ACGCAGACACATCCTCCTCCCCCCTATCAGTCATCTTATCTGGAGGCTTGTCCTGTTTAT
TATTTTTGTCTAATGATCCAACAAAGAAAGGCAACATTCCCTTATTTGGTGGTTTCTTCT
CAGCCCCTAAAGCAAGTTCAAGATTTGGAAACCCATCACGAAATTGATCATCATCTTTTG
ATGAGAGTGCTTTCAATGGCATGGGGTTAAACCCCAACCGAAAATCCTTTACATGTGAAT
CCATAGGAAAGAAGTACCTCTCTGTAGTTCCCAAGTCCTCAGGAATAACTTTTTCATCAC
ATCGCACATCACATCTCTTATCCTCAACTGAAGAACCGGGATGCAGATCATCTCTCTGTG
AAGAAAATCCATCACTAATAGAATCTCTATCTCTAGAACTACTACATCCATATGATCCAT
TAAAACATGTCTTTGGCTTCTTACTAGCACTTCCTCCATCTACAAATGCTTCTTTCCAAG
TCATCTTTTGGCTTGTGCTACTAGAATTTTCNNNNNNNNNNNNNNNNNNNNNNNNNNNCA
AGCCAGTTTCTGGGTCTATGTAACCATCATCATCCTTCAAGCACCTTTCAACGTCAAATT
GATTTCTGCTCCTTATCATTAAAACAGGCATCTTCTCTGAAACAACAGTCACCTCATTAC
CTGCAGGGAGAACATCTAATTTTGACCTCTCTTCTCCAACAAATGGAGCAACTACATGCA
CTTCTGTCTTCTCAATTTTGCCAACATCAGTATTAATTTCTTCAAGACAAGGTTTGGACT
CACTGCCCTGCTGGCAAACAGGAAGTTTACACTCTTCCAGTTGAAGAGTGGCGCATCTCA
TTTCCTTGGACAATTGAGTAGTTGCTGCAGTCCTTGAAGGAAATTTGGAGTCAAGTCCAT
CCTGTTGCTGGGAGTGGGTTTGTGAACCCAAAGATGTCTGCTCATCACAAGATAGCTTGT
TATTGTAATCCCCATTTACTATGATGCCTGTCTGGTCAGGACAACTGAATGTTGGGGCTA
TGTTATGAGAACTATCAGAAGCAGATGAATATTCATCTACATGCTTGGGCAAACTTAGAT
TCTGAGGCGAGGTTATGGCATCAGCAGAAATCGCTCTCTCCAAGACCACCATACATGAAC
CACCGATAATACCTGAATTCTTTGAGGCATTGGAGCAACTCACTTTCCTTGCCCTGAATA
CACCCCATAGAAAAAATAGCAAATTCCAACGCTGACAGTTCTCAGGAAGCTGGCTGGATG
GGAAAATCAGGAGTTCCACACCATCTAGGTTTCCCTTCAGTGCTAAATCATTCTTCATCA
TGCTATCCACCAGACACTTGTAATTTCTCTCATAGCTCTCATGATCTTTCGCAAAGAAGT
AAAGGGCAATATTATGTTCGTTTGCACCACTTTCATGAAACTTCATTGGCCATGTGCTCA
AACGAGGTACTTCATTCAATTGAATTTTCTGTGGCAATTTGTTCACCATTTCAAGAACTT

TAGGTGATGCATATGATGATAAATGAGCTTGAATTCCACTGCATAAATCTGGAAATTTTC
CTCCCCTATGTACCTCAAATCCCCCCTGCCAAATGTATTCATGCTCTGGGATGGCTGACA
TCTTCAAAAGAATTAATGATGCTGCCAAAGCGTGACCAGACAAGTCCCTCATTGGTGCCT
CTCCAAGAGGAGCAGTTGAACTTGGATCCCCAACTCTCAAATCAGTGGAACGGGCATCAA
ACTGTTTGCCTTCTTTGATAGTTGATTGTTTGTAAGAGTCAGAGGAGGAACAGTTTTGTA
TATTAGTTTGTGCTTCTTCAAAGGAAATCATATTCTTTGTCTTATTTGAGACACATAATT
GCTCTTGAGAAGCTGCTTCACAATCAAAATCCACATTTGTCATGCATAACCCATCTGATT
GATCATTCACTCTATTCCTTCCATATGATAGAGGCAACTTATGCATGGCGGCCTCAATTG
CAGCTTTCAACTTATTACCTTTGATCATCTCCTCCCTAGAATTCTTTCCAGCAGGTATAT
CGAAACCAGAAACATGTGGACTAGCGATGGGGCAACTTTCCACATCGTGACCCATTTCTT
TACACTTTTTACATGGAACACCTTTTGAACCATTAGTAGTAGAAGGCCTTGGATAACTAA
CATTGGGTTTTTCACCCTGAGTCGTTGATTCCAGTGATCGAGGTAGACTATCTTGCAGAA
TTTCATTAACATTATTTGATGGCCTATCAGCAGTCCAAGAAGAACTGGATGAGGGCTCAT
CCTTGGGGACAACAAGGAGAGGCTTCTGTTCAACAGAAGAACTGGACATTCCATTCATTG
GCAAAGCTCTAACCAGAGAAGCTGAGACTTCTGCATTCCTACGAGATAGATTGATGTTTG
ACTTTGACAATGTGCTTGCTTTTCCTTCAGATTTCAAAATCTTTGACTCCTGGCTGTCAC
CTGCAAATGAAAGAGGCACTATTTCGCCATGAGATGTGAGCTTTTGATCAACCTTAGCTG
TTGAGATAGCAGCACTAGCAGTTGCTGAACCAGGATGAGACTTATCCAACTTTGACAAAT
TTTTCCTTTCAAATACATTACGCTCTTTCACTTGTTTTAGTCCTTTCAGATCCTGAGACA
ATCGAGGTGAAAGGGCTCTAAATTTTGATTCGCTAGTACTTGTACGCCCAAAATTTGTAG
ATTTAAATGACTGAGATTTGCTCATTGCTTTAGAAAACCCTTCTTTCATATCAAGGGGTG
CTTGTTCTCTGCTCCCTTTCTGTTTTTGAGGAACAACTTCCGCAACAAGTTTCACTTTTG
GCTTGGAATTCAAAGTGCTGAATGAATTGGATTTTAATAAAGTACCCTTGGAGGTTTGAA
GCAGCAGACGACTAGGAGAACGTGCAGTTTCTGAGACATCAGTGCTGGAAATATTACCAA
AAGATGCTGGCTTTACTTTCCCCTTATCTATGCTTTTGAATGAATTATCCCGTGATAAAG
CAGCTGTTCTGGTAGGGCTGGATGATTTTGCTGGTCCTAGACTTGTTTCAACAGCCAGCC
TTTTAGCTGCCACAGCAGGCTCTAGATTTTCATGTTGTCTCTTACCAAAGCTTTGTGCAC
TTAAATTGTTTGCTCTTTTAGCCTCTGTATCTAAAACTTGCTTCTGGTTTTCAGTTTCCT
CTGCAAACTTGCATTCTTCGCATAGCCATTCACCTTCAGGAAGTTTTTGAAGCATTTCTT
TCATGCAATAGATGTGCTCTGCTCCATCACTGCATTTACTACAAATAGCAAGCAAATCCT
CTCGTCCTGCATCCCCACAAATATCACAGACTTTTACATCATGTTCTACAATGTCTGACT
CATCACTCTCATCCCCAGACACTGCTTGTGGAGGCTGCATATCAGGTAACTCAGCCAACT
CATTGAACTTCAAATCATGTTCATCACAACCTGAACTTTTAAAAGTTTCATCAGGTGGTT
CCCCACCATCCTTCTCAGCTTCTTCTGACTTTGGAGATATTTTTGTCCAGGCTTCAGGGA
AGCCTCCAACAAGTGACTTATTACTGTGAGAACGAGAAAATGGACTTTGATCCTTTGGAG
AACCACAACCAGTATCAGCCTCTTTTGCAGAAGGAATCTCTGAGGAGCCTAATTTCTGGG
AAAATGGTGCTTTCTTAGATCCTTCTGGACCTAAACTACAAACTAAAGCTGAAGTACGAG
ATAAATTCTTAATGTCTACATATCTGTCAGCATGAATGACTGCTGTATTGGCATCATGAC
CTCTAATACCACTTGACATATTATCATCATGTCCTTCCACACCCTTGGCATCATCATATT
TAGGAGAGAAGATTCTCTTATCCAAAACACATTCAGGTTTCGAAGATACTTTATCCTCTC
CACCAGCTCCACTAGACAACAACTTTGGATGAGTATCAAAATCTTCTGAAGTAACAGATG
TATCACAAGACCTAACAGTTGCTTTGCTTTCAGCATTCACCAAGAAAAAATCATGACTTG
AATTGGCACTGAGCAAGTTACTTGCTTCACTGACAGCATGTTGAAGGCTGCCACATGGTC
TACCTTTAGGGCCAACATTGTCATTGCCA
>tig_1224_consensus
GGCCAGACAAAAAAACCTCTAAATTTTATTTACAGATTCAATTTTAGTACAAATTACAGA
AGTAAATTTATCCTTTATCCTTTCTTTTCAAAGCCAATAACTCACACTGTGGAAGAGTGT
GACTTTTAAATCAGATACAAGATGGGAAGGAGAATGCCCCAGGAAAGAATGTGGATTTGC
CGGTTCTATGAAATGCTATAATCCCAGCTTCTTTGAGAACCAATCTGAAAGATTTGCTAT
TACAAATCCTGCAAAAACCTGAGCTGAGCCAAGAATGTATATTGCAGAGTAGTGACGGCC
ATGAATCACCATGTCTGCCACCATGGCAAGCGGAATGGTGAGTGACATCCCCAGTGTGGC
CACTAGCGGAGTGGTCCATACAACACATAATGCCCAAAAGTAATCTGAAAGGACACTACC
AATAAGGCCATTGGCAAGGACAACTTCATCCATTTTAGCTGAATGAGGAATTGAAAACTT
GGGCTCAATTCCCAAGGCTGACAACGGCCAAACCAGCCACCATAGAGCAACAAGTGTGAA
CAATCCTATGTATCCAAACAGCTTTTGTACATCAACTCTTTCTCCTTCCTCACCACAAAA
TTTCTTAAGAAGCACTGTGAATAATCCATATGACATAGCTGAGAGAAGGCCGAAAAGATC
TCCAACTAAAGAACGTTTCCCGTTGGCAGCAGCACTTAATTTGGACTCATCAGCCGCCCA
AGTCTTTCCAAGAGTAGTCATGGCAACACCAGCCATGCTAACAAAGACTGCAACTACTTT

TGACACATTTAATTTATCTTCCCCCAGGAAAGCACCAATGAAAAGTGTAAAAAGTCCTGA
AGTTGATGATAACACTGTTGTACTAGCAACACTTGTACGTGCAAGTGCTGCATTTGATAA
ATACTCTGTTAAAAACCAGATAGGAGCAATATAAAATCCAAACTTCACAACCTCCCTGCT
TGTAAGCTCTTTATGTGTGTCATCTTTGAGATTCAAAACCAGTGGCAGTCCCTCCTCATG
AGATGATAAGTCCAGTTCACTGTCCTTTCTAGTGTAAGTTCCTTGAAGCTCCATTTCAAC
AATTGTGTGCCTTGAAGGAGAATCAAGTGCAGAAGAAGACCCATTCAGACTTTCATGAGA
GAAGCTCCCAGATATGTCACTGCAAACGGCTGCTTGTAGTCATTAAAGATACCCTGGGTA
ACTTCTGCAGAGGTGACCCATATAATAACAACAGCAGCTATCAAGAACAAACCAGCTCTA
TATCTCCAACTCATAATAAAATCAAATCAAATCACAAACAAGAAAAGAGATCTGTTATTC
CTTTCAGATCTTTAAGCTTGTAAAATTTATTTACTTTTCACTCCGACCCCTCTGCCGACA
TGTAGCGTTATGTAACTAAACTTTGGTAGGATCTCAACTCCAGCTAGCGTGTGCTTCAAT
GCCTCAACAACATATAATTTGCTTGGATGGCTGGGGGCTGGTGGCTGCTGGTTGAAGAAT
AAAAAGGGCCCGAAAAGGGTAAAGGAAAAATGTAGGAAGAGAT
>tig_1225_consensus
GTTGGAAGTCAAAGGTCTATTTCCTTTGAAGAATAATTAAAAGTTTGTTTATGTATATGG
CTATTTCTGAGACACCACATGCTGTCTGAAATACAAAGACGACTCCACAATAGGCAAAGT
AACTATCAAATATAAGTATGAGAGGACAAAACAGAATTCAGTTGCACGAATTATATTAAA
CAAAGAGTCGAGACGGCAGAAAGATTCATCAAAAGTGTAATGATAACACCTGGATATGTC
CATGATGATGCAATTATTCAAATTATCCAGAGGGCTCTGAAGCCCATTTTTTGACTCCTA
TCTCGGAGTCACTGCAGGCTCAAGACTTCAATCTCAAACACCAGGACTGAATTGGGTTGA
ATTCCCCATGCTGGAAATCCACCAGCACCATAAGCATAATCTGGAGAGCACCGTAGACGA
GCAACCTCTCCCA
>tig_1226_consensus
AAAACAGAATTCAGTTGCACGAATTATATTAAATAAAGAGTCGAGACGGCAGAAAGATTC
ATCAAAAGTGTAATGATAACACCTGGATATGTCTATGATGATGCAAATTATTCAAATTAT
CTAGAGGGCTCTGAAGCCGATTTTTTGACTCCTATCTCGAAGTCACTGCAGGCTCAAGAC
TTCAATCTCAAACACCAGGACTGAATTGGGTTGAATTCCCCATGCTGGAAATCCACCAGC
ACCATAAGCATAATCTGGAGAGCACCGTAGACGAGCAACCTCTCCCACTTGCATT
>tig_1227_consensus
GTGCTATTAAATTCACCCCTAGAGGATCACTGAATGTGTCAGTGTCCATGGATTTCAGAA
GATTTCACTATCCCCCTTTTAAACAGGCATAAAAGGTGGTAAACCTCGGAATTCACGGAT
TTTCATTTATCTATTTGTGCAAAAAAGAGTATCAAAAATCCAAAACCCTAGAACACCGAT
GGGAGTGGAGAAGCAAATTTTGACCCCCGGAACCGGCCCCAAACCTACTGCTGGACAGAA
GGTCACTGTTCACTGCACCGGCTACGGGAAAAAATGGTGATCTCTCTCAAAAGTTCTGGAG
CACCAAGGATCCTGGGCAGCAGCCTTTTAGTTTCCAAATTGGCCAAGGTTCTGTTATAAA
AGGATGGGATGAAGGTGTGATGGGAATGCAAGTGGGAGAGGTTGCTCGTCTACGG
>tig_1228_consensus
GGCACTTAATAATTTACAAGCCAACTATTCTCTATACTCAAGCGGGGGAAAATGCTATTT
GTATACCTAAATTTAAGCTCTTCCAATCCACAATATTACTTGCACAGATAAAAATCAAAT
TGTGGAGTACTCATGAAGCATTCTTTTCCCAAAAAATCTGATCCAATGCAAAATAACAAC
TTGGTTGCCACACTGTTTCCGTTGCGCTCTATTCAACTAGAACCCACAATAACTATACTT
CAGTAAGCTGCATCTGTATGAATACAATCCCATGACCAGTCAAGAGTAAATCCAAAGTTC
AACTCAGCTTGCCTGTGTTAATGGTCTGATTCATTTCAACTCCATTTTCTTCAAAATTAG
TGGTGATTAAAATTGACGGTGCAGCCAGGTTTAAACCATTGGCCTTGACAGATCCAGGAG
TTGTGTCACTTGAGAATGTCTTCCCACTTGGCTCTTCAGATACCAATTGCTGTTGTAGCA
TTTTCAAGCGACTTAAAGATGGCACGTCGCCCTTACAGTACCTGTGCCACATGTTCCGGA
ATGTAGACTCCAGGCCAAGTGTGAAGTTTTGCCCATCACATACAGGGGACTTGGACATCA
ATTCTCGAAGGCTCATCCTCAAGTTTGAGAGAGCAGTAATGTCAGAGGCCAATTGCAGTG
CCAACCGGACATACTCATCTTCATTTTTGGCAATCAGATGAGGTAAGCCTACTTTACTGA
GAAGACTCACACCAACATTGTGAGCATGTACAGAACCAGCCATTGTAATACATGGAACTC
CCATGAATAAAGATTCACAGGTGGTTGTTGTTCCAGCATATGGGAATGTATCCAAGCTGA
TGTCCATAAGAGAATACGCTTGCATATGATCATGGTTCAGAAGAATTAGAGGCAAAAGAT
CAACTCGTAGTGATTCCAATCCCAGCTGCTCTAATGTTGAAAGAAATCTCAGCCTTACAC
TATCACAACAAAAAGGCTTACATTTTACTACGAGGCGAGAGTTGGGAACGGCACAAAGTA
TCCTTGCCCAAACTTGCAAAACCTTAGGAGTTATCTTTGCTAAATTATTAAAGCTACCAA
ACGTAACAAAGCCATTGGAAAGGGCTGGACTAGGGGAAACAGGCCCAGCCTCTGGCGAAG
GTGTATAACAAAGAAAGCATTCAGGTAATCTAACTAGCTCCTCAACATGCTTCTGTTTTG
TACCAGGAGGGTCTGCCAGTGAATCAGTGATTCTATAATCAATTGCAGGCAGACCTGTTG

TGTTTGGGTAGCCAATCCAAGTCACCTGAACAGGTGCTGGTCTGCATGCCATCATTCCCA
ACTTATTATTAGCTGTGTGACCAGTGAGTTCTACCAAAATGTCAACTTTATCTTCTCTAA
TCATGCTAGCAACCTTCTTTTCATCAATTCCATATATATCTCTCCATATTCCACCCTTTT
TCAGGACTTTCTCCCTAAACCTAACAGTTTTTGCATCAGCCTTCACAACTGCTGAATATA
CCACCACCTTGTAATTTTCATAGTCATGATAGACAAGGGGGGCTTCAATGAAATATGATA
CAGAGTGAGTAAAATAATCTGGAGATACGTATCCAATCACAAGCGGCCTCTCCGGATCTT
TTGGATTGTCCCATGAAGCATATTGAGGAAATAACCTCATAAAGCGCCTTCCCCAATCCC
TGTGTGCCTCAAAAAGCTTATCATCATGCCCCTCATTTATGTAATTCATAGCAAGCAATC
GATTCTGGCCTGCATTACGAGAATCAGGGTCTATCTTGAGACACTGCTCATATGCTCCAA
TAGCTAGAGAAATGTTGCCAGCATCTCTATAGAGAACCCCTAAATTGTTATATGCTTCAG
CATATGTTGGATTAGCAACAATGGCTTTTTCAATCATGCTTGCCGCAGCATCCATTTTAC
CCTGGACAGTGTAGACAACGCCAAGGTTGTTCAATGACTGGGAAAAGTTTGGTTTAATTG
ATAAAGCCATCTGATAACACTCTACAGCTTTATCAAGATTGTCTCGATCTTTGTATATCA
CTCCCAAATTGTTGCATGCTTCAGCACAATGGGGATTGAAATGAAAAGCAAGCTCATAGA
ACACAACCGCCATGTCAAATTTAAGCATTTCACCATAAGCAACCCCAAGATTATACATAG
CATCAGCATAGTGCCAATTATAATACAAAGCCTTCTTGTAATATGCTACACCTTGATGGA
TGTCACCCTCTAATTTAACCTTTGTTCCTAAATCTGTCAAGGCTATAGCCATATTGTTCT
TTGCAATCTCAAAATTTGGTGACACTGCAAGACACCTCTCATAACAAGCGATGGCTGACT
CCAAGTCACCACGATTTTTATAGATGACACCCATATTGCAGTATGCTTCAGCATACAGAG
GCCTCTCCAAAGCAGCCTTCTCATAGCAACTAAGCGCTGTATCATATTGCAGCATTTCAG
AGTAGACAACACCAAGGTTGTAATATGCTGGAGCATAATGTGGATCTATTCTTAAGGCTT
CATAGTACTTCTGAATTCCCTCTTGAGTGTTGCCAGCAAGCTTTAAGCCAGTTCCAAGAT
CTGTTAGAACAATTGCTAGGTACTCTGCTGCTGGTTTGTATGAGGGGTCTGCTCTCAACG
CCTTTTGGTATGACT
>tig_1229_consensus
CATACCACTTTCCTTCAGTATTCTCTTTCCCTGATCAACATTGGTGCCTTCAAGTCGAAC
CACCACTGGTACCTTTAGGGCAACCTGTTTGGCAGCATTGACAATTCCACTGGCAATTAC
ATCACATTTCATTATTCCTCCAAAAATGTTAACCAAAATTGCTTTGACTTTCTCATCAGA
AGTTAATATTTTAAATGCCTCAACCACCTGCCCTTCGGAAGCATTCCCACCTACATCTAG
AAAATTGGCAGGAGTTCCCCCATGTAATTTGATTATATCCATGGTGGCCATAGCTAATCC
AGCACCATTCACCATGCAACCAATTTCACCATCCAAGCCAATGTAATTTAAATCTGCTTT
GGCAGCAGCCACCTCTCGAGGATCCTCCTGTGATGGATCACGCATAGCAAATATCTCTTT
CTGCCGAAAGGCAGCATTATCATCAAAGTTCAACTTAGCATCAGCAGCTACCAACTGGTT
GCCAGAAGTCTCTGCAAGGGGATTAATTTCCAGTAATGTGCAGTCACACTTGCAAAATAG
ATCATACAATTTCTTCACTTGTTCAATTGCATCCTTTCTATCAGCAACCTTGGGTGCCAA
ACCATCCACAACCTTACCAGCATCTTCATCTGTGATTCCTTTAAAAACATCAATAGGTAC
CTTAACAATCATGTCAGGATATTTCTCTGCAAGATCTTCAATGCTAGTTCCTCCCTTCCT
ACAGCCAATTATTAGTGGACCAGCTGTTTTACGATCCAAAGTGATAGCGAAGTACATCTC
ATTGACAAGAGACAACTTTTCGCAAAGATAAACCTTGCTGACAATTTTGCCCTGTGGACC
AGTTTGTTTAGTAACAAGTATCTGCCCAAGCATCTTTCCAGCTATATCTTCAACCTGATC
AGCCTTAACAATGTGAACTCCACCCTTAAGACCGGATTTGAATGTTCCCAGACCTCGTCC
ACCAGCCAAAATTTGGCTCTTAACCACTAGCTCACTTTCATTAGGGAAAACATCCTTAAT
AGCCTTTCTGACTTCATCAACAGAAGAAACTGCCACACCTGTCGGGACATTGACTCCATA
TTTGCCCATCAACTCAGCTCCCTGATACTCGTGAATATTGAGGCGGCGTAATTGGTGGCG
CTGCCATTTTCCGGCGACAGAAAGAGAGCGAGAAACGAGCTTGTTCAACAACCCTCTCAC
CATGACGCTATGATTGATAAGATTTCGCAAACTGATATCTAGGCCAAATGAAACCCTAAC
GAAGAGAAACTAGAGT
>tig_1230_consensus
GAGCAAAATAAATTAGAGAAGACAAAAGTGTAACAGGCAAATGAATAATGTTTTATTACA
GCAAGTAGACGGCCCTGGCGTTTGACAGGCTGGATGGTAGAAATTGTAAACAGAGAGTAC
AGTGTCAAGATACTGACAACAAAAATGCTTGGCATGAGTAAACATCAATGCCAAAAAAGA
ACATTGTATCCATGCTAGGTACTATTCTGCATTATCTGCTGGAAATATTTTTCCTTTTCG
GCCACCGCAAAACATTCAGTTTTGCCCCAAATTACAAATTACAGAAAAAGGAAATTCCAT
CCACATCCAACAATTTTTTTACTTGAGCCAAGCAAAATTTAACAAAAGCTGCCAACTACC
AGTACAAAATTTTATTTCTCCCCTAATTCTATCCCCAAAAAAACCATTTCATATCTGCTG
CCTCTT
>tig_1231_consensus
ATCATCTGCTGGGCAAGGGCACATAATTTTTTGATTGCTCAACCATGTCTGAGTAATACC

TCAATGTTTCATAAAGGACAGCATTTACTTGCAAAGCTCTTATTGCTAATCACATGAGCA
GATACAGTACTGGAGATTCTAACCTAGGAATGTTGCTAAAATTTCCACCATAAAGCACAT
GAAATTCGGTCTTACCTATGACAACTTATGGAGAAACCGGAATTTACAGCAGTTGAGAAA
ATCCCGTGATTAGTGCCTTATTATGTGTACAATAACACAGGATGCGGTCCTCACGTGTCC
CGTCTGACTGCCACTTCCACTTCTACATCTCTCATTTTAGTTTGCGCCATCAGACTCATC
ATCTTCTATGGTTGGTAATGTTTCAATCATTTCCACTGTTTTGCAGAACATCTGCTGCTA
ATGTCTTATTGGCACTGGCCAATGAACTATTGTTGTGCTCTTCTTCTCTTTCTTCCACCC
GATGGTAAACCACACAGGTTCGAGCTGGCGAGAGTACTTTGAATGAGTTGGGACGGTTGT
TGAAATTTGTCTCAGGCACACCTGGTATCCCTTCTGGAGATGTAAAATGGTCTACATCAT
GGCCCACCCTTCCATGCCCACCAAATTCCCAAGCATCACTATCCAGTGCAACTCTATACT
TCCCTGGCAAGTCACACCCAACTTTATACCCTTCATATGTATTTTCTGGATGAAAATTGA
ACACAAAAACCAGATCTCCTCGCTCAAAGACTATAACCTTGTCGTCTTCATTTGTGCTGC
TCACAATCTGTTTTGATGACGTGAGGAATGAGAATTTATCATCAAGCATGTTCATAGCTC
TGTCGAATTCATTCAAGAACTTGTAGCGTAGGTGGTCAGTATCCACTAAGTTCCATTGGC
GTCTGCATTTTTCATAACTCCACCCATTGCCTTCTCTTGGGAANNNNNNNNNNNNTGCCA
TCGTTAAGAAATGTATCATCTTATGAAGAGCTATTCCTCGCTCAACAGTAGGGGAGGCCT
CTGTTAAACTAGACATACCAGAATACATCTCTTTATCCATTAAAAGAAATGCAATAGTCT
TGTCACCTACAATAGCCTGGTCATGACTTTCAGCATAAGATACACACTTCTCAGTGTATC
TCCTATTTGTCAAATTCCATGATATTTCCTTCATCGACCATTCTTCATCCTTCTTATTCT
TCAGATAATCAATCCACTGGTCAGGGATGGCCATCGCTAGACGGTAGTCAAAACCAATAC
CTCCCTCAGAGACAGGCAAACAAAGTCCAGGCGTACCAGAAACATCTTCAGCAATTATGG
TAGCATCTGGCAATATATTGTGAACCAGACTATTTGCCAGCATTATATAAACCACAGCAT
CAACATCTGTTGCTTCACTGAAATACTCATTATAATTCCCAGTAAAAGCCATGTTAATCC
CATGGTGATGATACAACATTGAAGTCACCCCATCAAATCGAAATCCATCAAATTTGTACT
CCTCAAGCCACCACCTCAAGTTGGACAAAAGGAAGCGAAGTACTTCCCAATTTGCATAGT
TAAAGAGCCTACTATCCCACAAACAATGGTAACCCTGACTGCCAGTGTGGAAATACGAAT
CTTGTGAGCTTTGGCCAACATCAAAGCCATTAAGGCCATCAGTGACGTTGTTACTTGCAT
GGCTGTGAACAACATCCATCAAGACCCGCAGACCTAAGCTATGGGCTTTATCAATCAAAT
ACTTAAGGTCATCAGGAGTTCCAGATCTGCTACTTACAGCAAAAAAGTTTGTTACATGAT
AACCAAATGATGCATAGTAAGAATGCTCCATCACAGCCATTAGTTGGACTGTGTTATAGT
TATTTGCTTGTATACGAGGCAAAACATCATCAGCAAATTCCCTATATGAATTCACACGGG
GTTCAGAGCTGCTCATTCCAACATGAGCTTCATATATCCTTGGGGCCATAGGCTTTGGGG
GCCGAGGGTGCTTAAACTCATACCTTTCTGAAGCTGGTGGATCCCAGTACACACCATCGT
ATGGGGCCGCAAATCTTGTAGGGTCTACAGTAGCATATTTAATCCATGCAGGGATTCGAT
CAACCCAAACCCCATTACCATGTTTGAATCGAAATTTAACTCGTGAACCATGAGGAATGG
CTGGAGTTCCACCAGAATCTGGAATATTGACACTCCAAACACCATATTGATCCATTTCCA
TCTTGTGGTTGGAACCATCCCATTGATTGAAGTCCCCGATAACTTGTGCTTCCTGAGCTG
CCGGGGCCCACTCACGGTACACAATGCTATCTTCTTCCCTGTTAAATCCAAATTTCAAAT
AGCCTTTTGCATATTCCTCAAGGCCTCCTTCATACTTCTCAAAAAGGTTTTTCTGATCTA
CATATTTCCTCACTCTGTATTTAAAGTGATCTTTAAATGGTTCTAAGCCTGGATCAATGC
GCAAGATACCAATATTTTCTGTATCATCCTCCACGCTTGTCATTGTCAAGGGGTCATTTG
TCAAAATGCAGGAGATAGGATAATTGTGCTCCAGCCGATGATGCACCGGAATTCTTGTTG
AGAGTATGTGATGCTTAAGCAATTTTTGACACCCAGATGTTCCCTTGTTTAGTAGTTGAT
CAAGAAGCTTCTTGTTTCTTGCAGTCCTTGTCGTCCTTGTGTAAATTTTGCTTGACGAAA
GCGAAAAAGACCCAGCAACAGCAGGCGAGAAAGCTAGACCCGAAGAATTCAACATTTTTT
TCTTCCGATTCAGCTTGTCTGGTTGATCTGAGAACGAAGCCTTCTTAAGCTTAGTAAAGC
AACTCTGTGAAAACACGTGAACATGGCGGCAATCCCATCAGAAAAATGTGAACCGTTGGA
TCTCTACGATGGACC
>tig_1232_consensus
TTGATGCTAGATCAACAGAACCTGCAATAGAACTTGTCACTTCAAATGTCAAATCTTAAG
CTTCCCAAGGAGGCAAGGCAACAAGCTGGCAACATGATGGACAGATAAAAGATTAAAATG
AACAGAGAAAGCCCTCTCAATAATTCATCGACTCCATGTTTTTAAATATAAAGCAAGATT
TAAAGATTAAAGTGCTGACTCATGAGAGATTGACTTTGCTTCCTCTGATACAGTGGATTC
ACATGCTTTTCCCAACCTCTTGAAACCATGAAAATCACAGACTAATGAAATTTCAAATTG
GTACATAAAACAATGGAAAACAATCAAAACTTTACTCATTTTACATTTTAAGAGACAAAT
TAAGATTACTTTTTATCGAATTACAAGACTTGCATTACACCAAAATCTTCCCACACAAAT
GAAACTTCAAAGAGATGAAAAAGAATTTGTAAGAATCTATCAACATGACCAGATAGTAAC
TTTAAATCAGTGCTACTATACATACACACATAGCTATAAAGATGAAAAAGGAGCAAGTGG

GGTAAAGGAAGAAAGAAAAAAATAAGGATACTAAAGTCGGGAGAGAGGAAAGACATTGCCT
CCATTACTATCTTCCTTGGACGAGGCCGCTAAAAACTTGGCTGTTCCCTGCAAGAGTTGC
TTCCCACTCCACAGAGGACAAGAGGATCTGTGACAGAGAAATTACAACTTGCTTCATGTC
CGGACGAAGGATAGGATCCTCATCGATGCATTGCTTCGCAAGCATGGCTACTGATGCTAA
TGACCGTCTCTCGTGATTTTTGGTCACCACACCTTCGGTTCGTATAATGGCCTCCTTCCC
TGATATTATCTCAAAAAGAACAACACCAAACGCATATACATCACTCTTGGTGGTGGCCAG
ACCATCACTTAAGTA
>tig_1233_consensus
TAAACAGGTTACAGATTATTTGCGCACCAAATTTAAAAACAGAACCAACTATATAAACAG
TTGTACTAACAAAACCAAAGCAAGATCTCTCCTTTACTCTGGTCCAATAACTTTATTGTT
TGTCTCCTCGCTCATACTATGCCTCCTCATAAATTCGCCATTCAATTTGACTGTCCACAT
CGGGCCTCTCCTAGCCTAGCTGCTGCTGCTGCTGCTCGTATTGGCTTGACTCAACTCCGC
TACCAATGACGAGGTCTTTTGTCGTTTGAGCAATTTCAATTACAGTTTCGCCAATGGCCT
TGAGTACTCCTCTTTGGATCTCTCCTTGTCCGTACTCTGCGCCCGAATTTTCTCGTTTTC
CTTGTTCTTGGCCTCCTTGAAAGATCTTTTAAACCCAACTTCAAATCAGGCATTCCAGAG
AG
>tig_1234_consensus
TAAACAGGTTACAGATTATTTGCACACCAAATTTAAAAACAGAACCAACTATATAAACAG
TTGTACTAACAAAACCAAAGCAAGATCTCTCCTTTACTCTGGTCCAATAACTTTATTTTT
TGTCTCCCTGCTCATACTATGCCTCCTCATAAATTCGCCATTCAATTTGACTGTCCACAT
CGAGCCTCTCCTAGCCTAGCTGCTGCTGCTGCTCGTATTGGCTTGACTCAACTCCGCTAC
CAATGACGAGGTCTTTTGTCGTTTGGGCAATTTCAATTACAGTTTCGCCAATGGCCTTGA
GTACTCCTCCTTGGATCTTTCCTTGTCCGTACTCTGCGCCCGAATTTTCTCGTTTTCCTT
GTCCTTGGCCTCCTTGAAAGATCTCTTCTTGTTCGTATTCTGAGCCTTCCTTTTCTGGCT
GTCCTCGTTCTTGGCCTCCTTGAAAGGTTTCCTTTCGACCGCATATCGAGCCTTCGTTTC
CTCGTTTTCCTTGTCCTTGGCCTTCTTGAAAGGTTTCCTGGATGGTGATTCCAGTTTTCT
CTTGTCCTTCTTCTGTGTGGGACTTTGGTTCTCCCTCGTGGTCGCCTCCCTGCTGCTGTC
TGCCAGAAGTCCTCTTAGCCTCAACTTCTCTCTCTGCCTCTTCCTTCTTCCTGGCAGTAT
ATTCTTTCATAGTCTCACCAGTAGAGACAGCCACTTCTTTAGCAGCACCCAGCGGTTTGG
TCACTATCTCAGCTGNNNCTTTCTCAGTCGTGTACTGTGCTACCTTTGCAGCTGCAGCAG
TCCCCTCCGCTGCCTTCTCAGTTGTGTAATGCGCTGTGCTCCAGCCTGCCACTGCAGCCT
TGTCCTTCACATCTGCTGCCACTTTCCCTGCATATTCAACGGCCCCTTTGCCACTCTCTA
CCGTCGCATATTTTGCAGCCGCAGCCTTCTCTCCAGTGTAACTTGCAGCCTTCTTTGTAG
TATCAACTGCTGTTTCTTTTGCCTCTGTAACCTTACCTATTGCATAATCTTTGGCTTGTT
CCGCTTTTGGAACGGTATATTCCTTGGCAGATTTGCCTGCGTTGTACAGTGTCTCTTTGG
AAGCCTCATAACCTTGTTGGCCCTTCTCTCCAGTGTAACATGCAGCCTTATTGGTAGTAT
CAACTGCCGTATCTTTTGCTTCCGTAACCTTTTCTATTGCATAATCTTTGGCTTGTTCCG
CTTTCGGAACGGTGTTTTCCTTGGCAGATTTTCCTGCATTGTACACTGTCTCTTTGGCAG
CCTCATAACCTCGTTGACCCTTCTCTAGAGTTGTGTCCTTGGCCTGACTGCCTTTCTCTG
CAACGAGCTGTGAAGTTTTCTGAGCACCCTGAAGCACGGTGTCCTTTGCTTGAGATCCCT
TTTCGGCGGCATATTCCGTTGCAGAACCAAGTGTCCGCCTGGCCTCTCCTTTCGCCTGAT
TGTATCGCTCTTCCGCAGCCCTTAAAGCCTCAGTTGAGTTCTGCTGCGCTCTTGCTCTGG
CCCTGGATATTTGTTCGAGAGAAGGTTGCTGTTGCTTCTCGTGACCCTCAGATTGGTCTT
GCAATTGTCCGCCCCTCTCCTTCTCCTCTCGGTCATGGGCCTGGCCCTGCATTTGTCTGC
CCATTTCCTCGCCCTGAAATTGTGTCACCATTTCTTTCTCCTGGCGGCCTCCCGCTGGGC
CCTCCATTTGCGTGCCCATTTGTCTCTCCTGGTGATCGTCCCTGGCCTGGCCTTCCGTTT
CCGTCCCCATTTGTCTCTCCTGGAGATCGCCTTCATCTTGGCCTTCCATTTGAGTGCCCA
TGTCTTTCTCCTGCCGATGGCCCCCGGCTTGCCCTTGTATTTGTCTTCCCCTTTCTTCAC
CGCTCGCGTCAAATTTTCCTACAGCACGAAGCCCTGTCGTCTTCTCCTGCTCTCCTGTAT
CTGATCTTTTATCTTCTGCCTGTTTCTCACCAGCCATATTTGCTCTCCTTGGTTGATCAG
CAAACGACTCTTTCTCTCGATCTTTTTCACGGTCTCTGGCCTTTTCAGCATTAACATTCT
TATTTATAGACTCGATGTCATGAGAATCCAACTCGTGGGATTCTCTGGCTTTCCCACTTG
CACCTACTTCGGCCGTTGTATTACGCTCATGAGGAGTTTCTGAATCCTTTCCTGTCGCAG
TCAAAGACTCGAAGTGACTTGTGATTTTAGGAACTGTGTCTTTCTCTATGTGAAACACGC
GTTCGTGAGTGGTATTGCCTCTGCTAACTTGCTCAGACGACATTTTTAACTCTCAGATAT
TATGTTGATTTCTCTCACAGATATTTTCTAACGGCACTTCTTTTTGTTGTTTCTTCG
>tig_1235_consensus
GATCCACTAAATACTGAAGTAATTTATCAAGGTGATTCTGATGAGGAATATCCTGAACCT

TTGATGCTTGAAGATGGAGATGATTTGGAAAGTGCTATATCCAGAACCAGGTCTTTCTAT
GGTGACAAGGCTCAGCATCATCTAAAAAATAAAGATAAAGTGGATGATTCCACCAGAGCT
GTTCAACAATCGGGTATCTCCACAGATGTTGATATCCTTGTGAACCTATCTGCATCTGAC
ATTCCCCAAGTGCACAGTTCATTGGAAGTGCAGGCGAAAGGAAAATATCCTTCATCTCAG
CCAGAAATTTTCATACAAGCAAAAGATGTCTCTGGTTTATCAGTATCTACTGTTTGTCAA
GAGATTTCCATGGAAGAAGAAATTAAGAACAATGCTTCATCTTTCATTCAACAAGTGGAG
ACAACTTCAACTTCTGGCCAATTGCTTCTTGATGGTGGTGATGCTAATAGGAATGACAAA
TCTGACACATTTGATGGCAAGCAGGGTTTGCTAAAATCTCGCCCTCATATAATAACTTCA
CATTCGCCTGGTTCTGTGAAGAAAGGGAAGATTAGTAATGGTCAACTAAACAGGAACAAA
GTTAAGATGGCAGCCAACAAATCTCAGCTTATCAAACCAAAGAAAGTAGTAGAAGGTTCT
AGTGGCCGGTTTGAAGCAGTTCAGGAGAAATTGAATGAGTTGCTGGATGCTGATGGAGGC
ATAAGTAAACGGAAAGATGCTCCTAGAGGATATTTGAAGCTTCTGCTACTGACTGCTGCT
TCTGGTGATAATGTCAATGGTGAAGCAATTCAGAGTAATCGAGACCTATCAATGATTCTT
GATGCTCTTTTGAAAACAAAATCGCGAGTAGTGTTGTTAGATATAATCAACAAAAAATGGT
TTACAGATGCTGCACAACATGATGAAGCAGTATAGAAAGAACTTTAAAAAGATTCCAATT
CTCCGGAAGCTTCTGAAGGTTTTAGAATATTTAGCAGTGAGGGAGATACTTTCACGGGAT
CATATAATCGGTGGTCCTCCTTGTGCTGGAATGGAGAGCTTTAGGGAGTCTATACTGTCA
TTCACTGAGCATGAGGATAAGCAGGTTCATCAAATTGCGCGTAATTTTAGAGATAAATGG
ATTCCCAAACCTTTCAGAAAATCTAGCTACATGGACAGGGATGAAGGCAGGATGGATTTT
CACAGGGGTGTAAGCGGCAATAGGTTCTCAGCTTCTCACAATCATTGGCACGATCAAGAT
TCAAGACCTTCAGAAGCAATTGATTGTGCCAAGCAGTCGGTCACAATCACTTCAGTGGAT
ATGATGGCTAATAAGACGGGTTCTGCACCTTGCACAGATGGTTGTCAGATAACTGGCTCC
GCAACTCGCAAACGTAAAAGTCGATGGGATCAGCCAGCAGAACCAGACCTAGATTCAAGA
TCTGTTACTCGTAAGGAACAGAAGCATGAATCTAAGTTACAACAGCAGTGTGACAATTCT
AGCTCACTTTCTATTAGAGGTCAATTAATGCCCAATCATGCTGATAAGCTGAGCAGAGAG
GACAACTGCCCTAACTGTGTCCACAATTACTATAACCGAGATGAAGCTGTTAGCTCTAAA
GATGGAAAGCAAACTACCCAAGAAGATGTTCCACCAGGTTTTTCATCTCCCTTTAACCCA
CCTCCCAGTTCATCAGATGCCTCATCAACTACTGATCTTACTCAGCAAAGTGTTTTACAT
CGAAAATGTATATGTGATGTGGCTATTGGGCATCCTCAGCAGAAATTCATTTCCCGCTTG
CCTGTTTCATATGGGATTCCATTACATATTTTGCAGCAGTTTGGATCACCCCAAGCTGAA
ACTCTAGATCGCTGGGTTATTGCTCCAGGCATGATTTTCCAACCTTTTCCACCTTTACCC
CCACTTCCTCGTGACAAGAGAGGCCCCTTACTTGGTTGTGCTGAAAGCTGTAAAACTGTC
AATGAATCTGGGGAAGAATTGCAACAAGATAGTCACCATTTACCCAGTTGCCATCCTGAG
GAAGACAATCCAAGTGTAACGGGAGCTAACCAATCAGATTTAGATATACAACAAACATGT
AAACGAATGAGGGGAACCTCAAATGATTTGGGAAGAAGGTACTTTAGGCAACAGAAAAGG
AAGGGCCCCCCATGGCTTTGGAGACGAAATGAGCATTCTTATTGTTCATAGGATAGTAGA
GCGGATAAACCAAGCAGTAATTTTTATCAGCGTCCATCCCAACAAAATCATCATTGAATT
GATTTATAGGAATCACTCTTGAGAGACAACTGGATTTTTCATCCAACACATTAATGTATT
CTCAATTCTTTACTTGTGGGTTCCATTTTTCTATGAAATTTTTTTTCTATTTCAGAATTC
CAGTCCAAATAATAATGTTACGGCAGAGGGAAAATGATGCTTCAATTTTTTGATGGCTCG
CTTTGGATATCGACCCATCAGTACACACCTCATAGAGGTAGACATTCTTTTTGAAGGTTT
ATGTATTATTTTATTATATAGAAATCAAAGTGTTTTTGAATGTTTTAACTAGTAGAATGA
GAAATTCACGCATTTGTTCATCTCTGCCGTTGAAATATGCTTAACAATGAAGATATCAAA
CTGTGTCTATTTGGTAATTATCCTGCAAATGGAAATTAGGTTATCTAATTTCTTTTCAGA
AACTAGAAATAATGGCATTCAGTTGAGGAAGCTGCATTGTTTGGGTAATTTCAACAAGAA
TCCAAGTTTTTCTTTCCATAATCTTGAAGCATCTAACACTAGATGGCAGCACATCGTACT
GTAGTTGAAGAGAAATGATTGTTGTAGTACAC
>tig_1236_consensus
CGTTATCTAATTCGGTATTTTGTATTAGAGAAAATTTAAACAATTAAATCAAAGGCTATC
TAGTGTGAATTCACAAGAAATTCCAGCACAATTGGCAGCCCCACACAACAGAAATGAATA
CATTGAGACAACCAGAGGCAGAAATTATGATCCCACTTGTCAAGGGAAAAAAAAAAAGGA
GTTCTGAAATCAATCAAACCATGTGATATGTTTCTAGATTCAGACCTGACTTCCTGGACT
CCTAGCACCACATTTATCGATGGCCCGTATGGAATAGGAACAAAACCGTAATTGCCACTT
AAAACTTACGATGAAAACTTTCCTATCAAAATAAGTGATTAGGGAACTGGCTTGGATGGG
ATTTCTTGTTATTATAAAAAAAAAAATAAAAATCAATCACAGGTTTCTAGATGGTAAAAAG
GCAGCGGTAACAGCCTATGCTAAAGAAGTTAAGCTTGTAAAGTCACGATGTTGCATGTAA
TAGGACATGTTATTCAAAGATACGAATGTGATCAATCAGGCAAAGCCCTACTGGATCTAG
AGTTTCACAGTATCGACACACATAAAATTGAAATATATGCAGCTGTGCGAAGATAAACGA

AAACGAAGAAACATGAATATCATCATTGTAATGCAAGTTAATAAAAACATGCTCCAATGC
GACAAGCATGGCAGCTAATAAATCCAAAGGGAAACATACAAAACTATCCATAACTTTATA
AAAGGTTTGAACAACAGATGCTAATGCTCAACTGATGTCATTAGGTAAATCTTATATCCC
TACCGTGTTCAACCCAACGTAATTGCAATCACTTTGCAAGCATCATCCTAACGAACTCAT
CATAATTCACCTGACCATCACCGTCAACATCTGCTTCTCGGATCATCTCCTCAACTTCTT
CATCTGTCAGCTTCTCGCCAAGATTTGTCATGACATGTCGAAGCTCCGCAGCAGAAATGA
AACCATTTTGATCTTTATCGAAAACCTTGAAGGCTTCTTTCAGTTCCTCCTCAGAATCAG
TATCCTTCATTTTGCGTGCCATCAAATTCAAGAATTCTGAGAAATCAATTGTGCCATTCT
GATCAGCATCCACTTCACTAATCATATCTTGAAGCTCAGCTTCACTTGGATTCTGTCCCA
GAGATCTCATCACCGTCCCCAACTCCTTGACAGTGATACAACCATCGCCGTCTTTGTCGA
AGAGGCTGAAAGCTTCTTTGAACTCGGCGATCTGTTCCTCAGTGAGTTGCTCGGTCATCT
TCCTCTTCTTCTTCTACTTCGTTTAAATTTCTTCAGAGACTTTGAAAGAAAATTGGAAAT
ATCTGTTGGCTGAGCTTAGAGCGGAAAAAATCTGTTGCCGTTCCCACGACTCTAATACAT
TTTACTCGCTTCGCCTTCGAGCAAGAATCAGATTCTTTTCTGACATCTGACGGTGGTTAC
GTGTTGACCCAAATATTAAACACTGGGTCCCTTGTCCTTCAGAGCTAAG
>tig_1237_consensus
ATTAGTGTACAAAAGTTCGAGTAACCAATACTTCAAAGAACTACACGACCAGCAGGGTAA
AATATTACTAGAGCACTAGAGCCTCCGAACACACACACAAGTTCTATTATAACAGGATAA
TTAACTACTACAGAAACATGATGTTAAGACTTTATATAAAGGAACACCCCAACTATTTAC
ATTGTGCACTCCAGCGCCCTAGAGAAACACAGAAAAGCTAGCCATATTTATGCGGCTTCT
GCAAATCCAACTCTCATATTGCCGTAGTCAAAAACTGTATGGTAGGCGCCCATAAAAACA
TCCCCCAAGATCCAGAGAGGTCCACGTGGAGGGGGTACATCCAGAGCTGTGAATCCGCTA
ATGCATTGGGCTGCTTCTCCCTCACCAACTTTCAGGATGTACTGCTCAGGACTGAGGTTG
AATGTCTTTTCACCAATTGTAAAGGCAACATTAGGCAAGGAAGACACAGTGCTACAATCA
ACAGCTGATTCTCCCATAGGACTTGGCAGCCGATCGCAGAGCTCATTGACATAGCTAATA
ATGCGCTCCTGTGTTTGGTTCTGCTTGAGCTGGTTCTGCATCCATACAACTGTCATCTCA
CATGTTGAGCACATTGCATCATGCACACCACCGGATGATCTTTTTGCATTTTCATCAACG
ACACTCTCAATGCCCATACTTACACCTCGGGTCCCATCAAATGTGCACAAACCAATTTGA
GAGCAAATTTTTTCTGGCCCATCCTTAGCTAAAAGCATATCAATTATGGTTTCTCCATAT
TGTGAAATTACAGCCTTGCATTCCTGACTAACCAACCCACTGGCTCCAATGGCATGGTTG
ACTTCAGCTATGATCGTCGTTGGACCAGCCAACAAAGAGGTTCCTGAATCAGCAATTGCT
GCACATCCCCTAGCACAGAACCCAGTTGTTTTACCATCAATCAAAACATCACCCATATCA
AACTGCCAATACCCCTTCTGTGACACAGGAACATATGTGTGCTCACCCTTGTAGTGGTTA
GGATCCATCCCACCAAAAACTATTTCTCCCCCTTCTTCTTCATCAGCTTTTCGGTTAAAC
CAAAATGAAAAAACTGGTTCACTAACAAGACCTTGATTCACCATATTGTACCACACAGGA
ACAGCATTTTTTACAGAAATCTCTTGAAATCCAAGTCCAAGGATGCCATCAAACTTAGCC
ATCAAGAATGTGAGGCCAGGCTCTTTGGTCGCTTCAATAAATTCCTGATCTTTAACCACA
AGATCACCAATCCTGACATCGTCTTCACTAAAGAAACCTGAAATTGCTCCAGTCCCATAA
TGGATATCCGCTGATTTACCATTCTTCTTGTAAGTACTTGAGCTGCTTGCTTTGTACTTG
GAATGGAAATAGCAGGCAACCGAGAAATAACATTTAGATGAGGGCACCCCACAAGTTTGAA
CTACCAGTATCAAATATAACAGTGAAATTTTGTGGAGGGGTACCAACACCAATCTCACCA
AAGTACTGAGCATCCATGTAGTTCTTCAACGCTACAATATCAGCATCCCCTGGCTCTCCT
AAGTTCCCACGAAGATGACATTTTCTAATAGAAGCTCTGAGTGACTCTCCCTCCTTGGAG
TCAAGCCGAGCAGCAAGCCGGTTGTTTTGATCGAACTTCCTCTTTTTGAGTCCAATTCTA
TATAACCCGCCATTGGGAGTAGAGAAAACCACGGGAAAAAGAAGCAAACAAAGGAAGAAG
CTGACCGTAATGGATTTCAATTTTGTTCCCATGTTGACAAAATCAGTGAGTCGCGATGGG
GAATCGATATAGAGAGTCGTCCAAACGAGCAACAATTGTAAGCTAAAAAGAATCAGTGCA
CGTCTGGTGCAGTAAATAAAGCGTTGGGTGGTCCGTT
>tig_1238_consensus
TAATATAATCAATTTATTTTACGTCTACTTAATTCTGATTTATTATTTTATTTTCCCTTG
GGGGCGCTTGTACATATTTTGTCTCAGCCAAAATACTAATTCAAGTAAAAGATTGGAATT
GCAAATAAATATGTTTAACATAATTTCTATATCAATTAAAAACAACTAGGGAATATTACA
ATCGTAGTTTCTTCTTGTAAATAGAGGAAACCATCAAACCCATGGCATCCAATAGACAAT
CCTTCGCGGAAACAGATTTTTTTATTATTTAAATTATTATTTGTTAATTTAGGTCTTGTT
CTTCAAGAGCAAGGCCCGAAGGTCGGCCACGTCGAGATCGTTGGCACCATTCTCAGGCCT
GTAGTAGCCGGTGACGGGGTCTGGGACCCATGACACCTTCTCATTGGATCCCATCATCTC
TTCTCCCGACTTCTTCACCATTGGCCCAGTTCTTGCTCCTGTTCCTCTAGTCACGTTCGA
CACAACTCCTTGTGATGCCGCCGCGTAACCTCGTCTATAGATAGCGTTAGAGATTCCGTC

GAAGGCAAAAGCAGAGAAAAGCTTAGCGTTGGAGAAAGAGCGAGCCATTTTTTATTTATC
TTCAACTTCGAGAGATTAAATAAACCACTGCTGCAGAAATTTAATCTTTTGTTCTCTTTT
CTTCACTGCAGAATACCCTCTGCAGCAGCCGGATAAACCTCTCACGCCAGCTGACTCTCT
CTCTCTCTTCAAAGGTCTATTATGTGTTAAAGAAAGAGGAGGCTTGGTCCTTATATAGAG
AAGAAACAGAGGAGCCTAAGTCTCTTCGGGGAATACCATCCACTTGTGTAAGACCGACCC
AGTCGAAGGATGCTTCTCCTCACATGCGAAGGACTTGTCGTTGTTTGCCACCTGGCAGAG
ACGCCCAAACTTTGATGTTACATAAGTACCCTTTTCTTTGCCTGTAAATTACCCGATCAA
TAGTTGGCTTGGCTTCAAAATCTGAAATTGATCGACGGTTGAGATTTGCTGGAACATGTG
ATCGAGACAGTCAACGTGTGGGGAGACCATTGAATAAAAC
>tig_1239_consensus
CTCAATTTCAAATTCATCTTTACATTAGGATATTCTAACCCTTCACAACTCAGTTGCTAT
CCCTATCAGCTACATACCTTAATATTTTTGAAATAGAAAAGGCCTAAACTAAAAGGGTAA
AACATTCATAAACCAATAAAAAGTACAGTACTGACCTTTTGGCAAAATAATCCAACAAAT
TTCTGAGGAATTAATGTGATAAAATATTACTAGTTCAAGAAACACAATACTAAATTGCCT
CATGGCAACAAAATTAACCATGTTCTCCAGTAAAACTAACGCATGTCACTTTTGTCATCG
AGTACCAACTTTTTAAACCAAAATTAAGCCAGGGCAATAATAAAAACACCAAATAACAAC
ACAAAATTTAGCGGTAGCGCCTGAGTGAGAGTGTATTGTTCCTCTTCCAGTTGGCTCTTC
TTGAAGGTCGAGCCTTGTGGTGAAGGTGTCCCTTTCCGCGAAGACCTCTGTACTTCTTCC
CAGCAGATGTGAGTCCACGAAGCTCCCTGTGTTTGTGAACAGGTTTGCAGATCCAGTTGA
TCCTGGGGTCATTACGGATCGCATTGTGAGCAGCATCAACCAAGATTACCTCATAGTATT
TGTATGTTGAATCCTCATTGATCCAGTAAGAGTTGAGAACCTTAAGGCCACCGAGTTTCC
TACCAGCTCGTTCCTCAGCAACTGAACGCTTGCTGCGTTGGAATTTCAGCTGAGTGACAC
CCTGATTTGTTGGCTTCCCATATACTATACCCTTGGGAACTGGTCTCTTCCGTCCACCAC
GTCTTACACGCACACGATAGACCACATAACCCTGTTTGGCCTTGTACCCCAATCGGCGAG
CCTTGTCAGGGCGAGTGGGATGGGTGACACGCACAATAGAAGGATGCTGGCGGTACTCCC
AGCACCTCACCCTCTGCAAAAACCTCATAACATCGGACTGCTTCTTCCTCCATAGCTCCG
AAACATAAGTGTAAGCACCCATTGTTGTTGAGCTGCTCTGCTCCCGAGTTTCGGCTGCAG
ACTGCGATGGGAATGATATATGAGTGCCTCACTATCGCTAGGGTTTTTCCAGAG
>tig_1240_consensus
TTTTTTTTTTTTTTTTTTGCATTTATTATAAGCTTGAAAGGCCTGGAACTGTTAACTTACA
AAGTAGCCCCACAAGCATCAAAGAATAAGATGGGTGGTGACACTTCCTTCTCTTACCCCT
CTCATGGTCATTTCCTTATACTAAAATGGAACAGGACCATGAAGGTGCTCTCTCTAAGGG
TCTGCCTTTCCCTCTCTTTCACTCACTCTCTTATGTTCAGTGATATATCATTCGCTATAC
ATGATAAAAATACAGAAAAAGATTTTCATTCTGGTTTCTCTTACTCTCGTTTTGTCATCC
TCCCGAGTTAGGAGAGACTCCAAATCGCCTCCTAAAATGTAGCACTCCATAGAGCGTTCT
CACCTTCTCCACACCGCCGATGGACGTCCTTAATCCTATCAGCAGATCTGCAAATCCCAC
TGCAACTCCAATCGAATGAAGCAACACAGATGTTGCCTGCTTGGGCCTTCCACTCGCAGT
CAGGTGGGGTGCCACAACATAGTCTCCGATCATCAATATGCTCAACATCAAGTCCAATAA
ACCAAGATCCCAATGAAACATCTTCATTAGCATATCTGTGTAGAACATGCTGATTTATTA
ATATATACGTAGCTAAACCTTTTGAAATTGCATAAAGCTGTCCAGTTGCATGACGGAAAT
ACTTGTTTCCTGCTTCACCGAATTTCCAATGCTCAGGTTCATGGTATCTCACTCCC
>tig_1241_consensus
CACAGAAGTGCCCATTAACCGAGGGACGGCTTATCAGCCCATTTACTGTGAAAGGGTAAA
ATAGTCATTGTACGACATGCTAAAACCCTAGTTATAGCGAGGGGAGGGCTCTCGTTTTTC
TCAGCAGTGGCCATCTCCTCAGCCGAAACTCGGGAAAACCGGAGCTCAACAAGCTATGGG
TGCTTACACTTACGTATCGGAGCTATGGAGGAAGAAGCAGTCCGATGTTATGAGGTTTCT
GCAGAGGGTGAGGTGCTGGGAGTACCGCCAACATCCTTCTATTGTGCGTGTCACTCACCC
GACTCGCCCTGACAAGGCTCGCCGCTTGGGGTACAAGGCTAAGCAGGGTTATGTGATCTA
CCGTGTGCGTGTAAGACGTGGTGGACGTAAGAGACCAGTTCCTAAGGGTATCGTATATGG
TAAGCCAACAAACCAGGGTGTCACGCAGCTGAAATTCCAACGCAGCAAGCGTTCAGTGGC
TGAGGAACGTGCTGGTAGGAAACTCGGTGGCCTTAAGGTTCTCAACTCTTACTGGATCAA
TGAGGATTCAACTTATAAATATTATGAAGTAATCTTGGTTGATGCTGCCCACAATGCCAT
CCGAAATGACCCCAGGATCAACTGGATCTGCAAACCTGTTCACAAACACAGGGAGCTTCG
GGGACTCACTTCTGCTGGAAAGAAGTACAGAGGTCTCCGTGGAAAGGGACACCTGCACCA
CAAGGCTCGGCCTTCACGAAGGGCCAACTGGAAGAGGAACAATACACTCTCACTCAGGCG
ATACCGCTAAATTTTGTGTTGTTATTTGGTGTTTCTATTGTTGCCTAGTTTAATTTGTGT
TCACCAAGTTTAAACTCAGACAAAGTGTTTGTTGATTAGTTTGTTGAACGTGGTTAATTT
TGTTGCTTTGAG

>tig_1242_consensus
CTGGGAAAGATAACTATAGCTGAAGAGACACACAAGTTTGGTTATATATATTGATCGCTG
TAAACAGTCCCTGTACTAACAATTCACTTTCTCAATACACACAGCATCATATAGCTCCTC
AGTTATAACTAAATTGAAAGCCCCGTCCAACAATCACTCTCATACAGACAAAACAAGATG
ATAATAGTTCACTGTTGGGTATTTGCTCACTCACATATTATTTTTGCCATCAGATTGTGA
CACACTTTGTTCAATTGACTCTAACTGGTTACGAAGACTTTGATTTTCTTCTAGCAAGCG
ATCATACTCGAGAAGGAATTCTTCCGATTGCTTTTTGAGCGCCTCTGCTTCAGATTTACA
GGCACTGCATTCCTTTTCTTTAGTCTCGTGTTCAGATTCCAACTCTTTAATCTTCGTCTT
CAAATTGGTAATTTCATTCTTTAAGCTATTAAGCTCATCCGCATTTGCATTTTTCCCTTC
CTCAAAACTCCGGTTTTGCTTTTTCGCAGCTTCCATGGTTTTCCTGAGTAGTCGAAGTTC
TCTTATGTAATGGTGTAATCTGTCTATCATCAGTGAGAGGAATAGCACAAATCCCATAAG
AGAAGCTTCGAGCATGTGCCTGGACATGAGGACCTGGTCGGTTGGATTAACGGCCCCATC
CTCGATGGTACGCTTCTGGATCTTGATCATGCTGTAGACGCTAGACATAAGCACAACGAA
AATAGTACTACCCACCGTCTTCACCATCACCGGACCCTGCCCTCGTTTGACCCGATCCAG
GCTCATTATCACCAGCTTTCTCAACGGCGTCTTGAAGAGCAGCGTCATGATCAACGCCAT
TTCCGAAAAGATTACCGTGTACAAGAGCTGTATCATGTTTGCTTTTCTGTTTGTTCAGTA
GAAGAAAGGCTCTCGGCAAGTCAGTTAGAATGGTGGGTTTGATTTTGTGGTTCTGTCCCG
TGTAATTTATGATGCGAGCATGGCCG
>tig_1243_consensus
TTTTTTGCTTGAACAGAATAAGATTTTGATTTACTACTACTGGAGAAAAATGTTATTAGG
GCTTCAAAAAGGGACAACATTTTAACAAGATCCAAGTCATTACAAGACAAACAAAAGAAC
ACCAAATTTGACGTACTTTGCTGAACCAAATATTCAAAACAATGTTTGAGCTAATCAAGC
CTTCAGTCTTCCATAGAACTTGGCCTTTTCCTGTGTAGTCTGGAACCGGCCATGACCAAA
CTTGGAGGATGTATCAATGAACTTGAGCTTAATCTCCTCCATTGCAACTCTGGAAGTCTG
CTTCAGCAATGACTGTCTCAATGTCACCACTCGCTTTTTAGGTCCAACACAGCAGCCCTT
GATCAGCAGGTAATCTTCTTTCACGATACCATAGTGGGGGAATCCTCCCATCGGTGTAAT
ATCCTTCTCAGTCCTGTCATATTCGGTCATGGCAGAGTGAGACTCCTCGCTGGTCTTTCC
AAGCTTGTAGATTTTCTTGTTCATCTCTGTGCGGTGATGGTAACCATTTTGTCCAGCCCT
CGCAACAGTGAAGGAGACCCTAGCTGGATGCCAGGCACCAATACAAGCAACCTTGCGAAG
ACCTCGGTGAGTCTTACGAGGAAGACGAGTGACACCCCATCGAGTCACCACACCCTCATA
ACCCTTACCTTTGGTCACACCGATGATGTCGAACATTTCATCTTTCTGGAACACAGCATC
AACAGGAATTTGCTTTTCAAAGAAACCATATGCAAAGTCAACCTTCTGGGCAATGGTTCC
ACCATTAACCTGGATCTCCATCAGATGAGCTTTCTTCTGCTTCAGTCCCTTCATTTTTCT
TATCTGGGTGTGAGCCAAAACCCGAATCACAGTGCAGTACTTCTTCATTTTCTCCAACTG
AGCCTCGATATCTTTTTTCCCAATTTCACTTTCAAACTTCTTTGAATACTTGGTGAAGGC
CTTCTTCTTAGACTTGCACCAATTCTTGTAGAACCTCCTCTTAATCTCTTCACTGAGATG
CTGAGCCCAAACAGTGCAAAGAGACCGAAGCCCCCGAGGTGTCTTGACATAACCAACAAC
ACCAACAACCACCATGGGAGGTGTTTCTATAATAGTTACTGCTTCACATGCTTCCTTCTT
GTGGAGCTTTGATCCTGGTTTCTCCACATCTCGGACAATGTGGGTCATTCCAGCTTTGTA
ACCAAGGAAAGCAGTCAGTCTGCATGGCTTCGTAGGATCATCTTTCGGGAAAGCCTTCAC
TTTTCCTCTGTGACGGGCTGCTCTCTTTCTTGGGAGAAACCCCAAAGATCCATGTCGAGG
GTGTTCAAACTTTCGATGAGACATCCTTCCAGTAACAGAGATGAATATGCGAAGTGGCTT
TGGGCAGGAAATGAAGTTTGATCGATAATACCTCCTGGATTTAACGAGAAAAA
>tig_1244_consensus
CCACAATATTATTGTCTGAATCTAAACATTCAACTTAATTTTTTATAGAGAAAATGAAAG
TGAGTAACGAGCACATTTGCGACAGTAAATCAGTCATGACACCAGTGTATGCCAAGAGTC
GATTAAACATCCAGGGACAGTAATATAAAGTGCAGAAAATAACTTCTTCATAGATTACGA
ACAGCAAACAACAACACAGCCATGTCTATGACTCTGATCTGAGAGAAGTTATTGGCATGA
CCACCATGCCAAACAAAGACCCAATCTCACCAAAAGTCATACATTTATTTCTGATTATTG
CTTCCATAGCAAGTGGGTTAGATTTCGATTCAGCTCTTATGTGGCTCCTCAGTTTTCTTA
TCAGAACCCTGTTTATCTTTCTCTAACTTTTCTTTCTCAGCCTTCTGCTTCAGCTTCTCC
AGCCTCTCCCTCTCCATTTTCTGAATGTAGACGTTCTCAGCAGCGCGTTCCTCCTCACTC
AAGACACGGCCTTTGTCATCGCTGAAGTAGCGAGTTGC
>tig_1245_consensus
GTGATACTGTTTTATCAAATTAAAACTTTTATTAATTCAAATGGATCTACTTGGGCTCAG
TCCAGCCCTTCAGAGCGTTGGACACACTATTCCTGATTGCTTGCGTAGGAGTGCTGCAAT
AGCGTGGATACACAGAAGAAGCTTATTCACATCGTTATATTCATAAGATTTGCGGCCCAT
TTCAGGTTTCCTGTACTTCGCAATAAGATTTCCGGTCTTGAAATTACAAAGCACGTAAAT

CATGTAATATAGAGTTTACATGGAATATGATCCACTGGTGGAAGTACAGACGATCATGTC
TCTGAAATGTTCAGACCCCAAGTATTATTTTAGATATCCAACCTCTCCTTTATAAGGAGA
GCCGAATATATATTAAGAGAGTTTTTTTGGTGGGTTCACGGCGTCAAGAGAGAGCTTCCT
TCCTTGATGAGCACAAACACACTGCCTGCATCTCCCCTTGAAATGCGAAGCTCTTCATTG
AGGAATGTTGTTAGTAACCAGGACTGTGCATTGCTATTTGAGATTGGGAATTTGAATGGC
GGCTGGCTAGAAATGGTCTTTGCAACGGATGAAGCTGTGTCTTGGACCGAAGTGAATAAG
CCTTTTAGGGAGGAGAGGTCAATCTTTTGTCCCAGAAATTCCAAGTTGTCTGGTAAAACT
ATTGAGTCCGTTATCTGAGGAGTCCCGATGATTCCTTCTTCAAACTTGATCTGCACACGT
TGGGGACTTCGGACTTCAAATTTGGCATTGGTACTGATAGATGTTGTAGCCAATGGCCCA
GAAAACCGGATAGAGTTCTGGACAGTGAAGGTCTCTGAGTCAATTGTCTGTGATATTTCC
TCCACTCTCGCCAACGACAATGAACCCCTAGACAATAAAGGGAACAAACCAGGGAAGGAT
GTGTACGCAAGAATCCATTTGCCGTTAAGCAAAGTTAAAGCCTCAGTTGGTGCAGGAGTC
GGATTCTTGGCTTCAAGCTGTGTGATAAGCTCCACGATCTCCGCTCTCGTTTCGCTAGTC
GCCGTCAGTCCACGATCGGTCCCATAGAACGAATCCACTAAAGCCTTCATTAAGTTATCA
ATCTCCGTCACTTCTTTTGGCTTCTCCTCCTCTACCACGGCCACGGAGCCACCTTCTTCC
TCCTTATCAGGGCCCCACTCGTCGTCCTCCGCGGCCCTTGTCACGAACGCAGGCCTTGAA
TTCAGAGACTTGCTAGTAATTCCTCCCGAAAGCTTGGCTGAGTTCAGAGGAAGAATCGAA
GGTTTTACACTAGTGGGCTGGTGATGAAGTGGAGTTGCAGAAAGCATCCTGCATGAGAAT
TGAGTGAGTTGACTAAGAGAAGCCATTGGATCTTCAGCTTCTCGATCACAAAGAGAAATC
TGAAGAAGCCAATTCACCCGATCTGGAGGGTATTAATG
>tig_1246_consensus
GCAAAGAAACCAATAACTGATATCTCACTTCAGAGGAATGAACCTTGAAGAATGGGTAGC
ACCAATACCAGGTCTGCCATGCTTGACAGGCTTGTATGAAATAGAAAATTCAGCAAGGTA
ATGACCAATCATTTCTGGCTTGATTTCAACCTGATTGAAGGTCTTGCCATTGTACACACC
AATAATGCTCCCAATCATTTCTGGCACAATGATCATGTTGCGGAGGTGAGTACGAACTGG
CTCTGGTTTCTCACCAGGTGGAGCCTC
>tig_1247_consensus
CCAATAACTGATATATCTCACTTCAGAGGAATGAACCTTGAAGAATGGGTAGCACCAATA
CCAGGTCTGCCATGCTTGACAGGCTTGTATGAAATAGAAAATTCAGCAAGGTAATGGCCA
ATCATTTCTGGCTTGATTTCAACCTGATTGAAGGTCTTGCCATTGTAAACACCAATAATG
CTCCCAATCATTTCTGGCACAATGATCATGTTGCGGAGGTGAGTACGAACTGGCTCCGGT
TTCTCACCAGGTGGAGCCTC
>tig_1248_consensus
TTCACAATGGTGTAATGTGCAGGGTTTAAGAAGTATGGAATGGAAAATCTTTCCCTCTCT
GAGTTCACCATTACCCTGTGCTCCACGCTCTCATAAGCTTCATTGCTCCACACCTGAATT
ATGTCACCAACGTTTATGATATAAGCATCGGGGGTGGGCTTGACACGAACCCACTCACCG
TCCGATTTTCTCTTCACTTCAAGCCCTCCAACATCATCCTGAGCCAGTACTGTTAAGGCC
CCGCCATCCTTGTGTCGACCAACGCCAAGTGTGAGGTGAGGGGCTGGGCAAGGCGGGTAG
TGATTGAGTCTAATGAAGGTGGTTTGTTCCTTGAAGAAGCCCTGGAATATATTTCCAGGC
AACCCCAGACTCAAAGCAATCAGCTCCAACAATTTGAAAGCCAGTTTCTCCATCTCTTT
>tig_1249_consensus
ATACATACATATATATATATATATATATATATATACATGTGCCTAGGTAAGTCTCCGGCAAA
AACACATCAAATGATCACAGAGCTGGATCGAAATCAAGATGCAAAGAATGCTGCCCGGCT
TCAAATGTTTACGACTCTGCATCGTAAAATCTCTCTTCAACTGAATCATGTCTGTCCTCA
CTTTGTTTTAAGAGTTCGACGAAACAGGAAACAGGTAAATAATCAAATGCTATATTCAAG
AGGCTGAAGAACCAATTCAGCATCATCTTTCTTAGGTTTTCTTTTCCCTGTGAGGGAAAA
TGGCAGGAGAATGAATCTCAATATGCGGCTCAATGGATCTTGCAGACCTTCCATCTCATA
GTGTCCAAACCGCCAGTCCCGTTCAACATAAGGCCGCTGCATTGGCTCATCAAGGCCTCC
TGCGTACATTAAACCACGTTTGTCAACTGCCCTGTAGAAAGGTCGTGTCTTTTTCACTTT
AGCCACAACTTGGAGATCATTCCATGCATGTGCTCGGTCTGCACGAGTAACTTTACCAGT
GTTCACAACCTTGGTAAAAAGATAATATGAAGGACGAGGAATCCGGGTGAGATTATTGGC
ACGGTCAACTGCAACAAGACCAAACTTGGGACCATAACCATCAGCCCACTCCCAATTATC
CGAAATAGTCCAAAACAGGTAACCAAGCACAGGAACACCTGTCATCATTGCCGCATAAAC
AGCAAGTAGATGTTCTATAAGATATGGTCGACGAATGATATCGGTCTCATCAGAAACTCC
GTTTTCGGTAATGATAAAGGGTACGTTTAGATGCTTGTATCTTTCATGGAACTGAATCAG
CATGCGGTATAGACCATCAGGATATACACCTCGCCCAGATTCACTATATTCATCAGTTTC
TACTAACTTCAAGCCAGCACCAGATATCACCTCCTGTCCATAGTAATTAATGCCCATGAA
ATCCAGCCTATTAGAGATACTATCCACATACGGGAAGAGGGTAAGAGAATTAGCCAGTGT

AACTGCAGCAACATCGAAGAGACCATATGGTCGTGTAAATGATACATGGTGTGCCACTCC
AACTTTTGACTTTGATAACGTGCTGCTTTTTTCATGGATATAATCGTAGGCCTTGGCGTG
TGCAATGGCTATCCAATGCATGGCCTGATTAAAAACACCAGTTGGCAGAGCAGACGTAGC
AACCTCCAGCATATCAGGATTTCCACCAGGCCAAGCCCCCGCACAATATGTAAGCATACA
GAAGACATGTGGCTCATTAAAGGTTACCCAATACTCTATCATATCTGATACAGTATCAAC
AACAAGCCTGGTAAAATCCACGAAATAGTCAACAGTTTTCTCCAGTTTCCAACCACCATA
GTCACCAGCCCATGGTGGCAATGAGTGATGAAATAGAGTGAGCATCACCTTCATTCCATA
AGAGCGAACCCTGTTGATGATCCACTTATAACGCTCCAGTGCTGCATAATTAACAGTTCC
CTTAAGACCATTGACTGGCTCCTCCGGCATGATCCTTGCCCAATCTATTCCCATACGGAA
CACAGATATACCAGTATCTTTAGCAAGCTTCAGTTCTCCGTCAGGATCAGACCAAAATCG
TAGCCTCTCCTCGGGGTGTGGAACATTGTGCCATGCAGCAACTTTATGATGCAATTCTTC
TTTTGGTGCATTTACTTCTTCTTCTCCTTCCATGTACTTCTCAAACCCCCTAATCATAGC
TTCCATGGCTATCTTAAGAGGCTTCTTTTTCTTCACCATTTGATTAACTGCTGTACCTGA
CATTGGAGCTTGCTGTGATCCACCATCACCCGTAGCAGAACCCATTATGGCATCCACAGG
TTTCAGGCCTTGCTGATCTGATGCGTGGCTGCGTTCTTCTGCTGCAAACTGGAGCCAGGC
ATCATTAAGCCGGTCTTCAACATGGGCTGGCGCAGTTGCCAACCCAAAGAAGAACTCATT
TTCCCCTTCACCTTCAATATTGAAGGACGCCAGAATATCAGCAGACTCGTCGATCGGAGA
CCGAATCCGCCGGAGATTCTTTCTTCGGTAACGAGAAAACGAAAAGGCATTAGCTGCTAC
TATAACAGTCGCTAAAATTCCAGCGAGTTTCGCCGCCGATACAAAGAGTGTTATGAAAGC
CATTTCGTCCGATTTTCGTTCATCAAAGGCCGAAAAAAGCCGAAAGGTACAGCTGCCCCT
ATCCTGACCGGTAAATTTTAAAGTCCATCAGTGAGT
>tig_1250_consensus
CATTGAAAGAGAGTGCCACAGCTTTCTGCTCCCTATGAAAATGGCAGTCTAGATTATAAA
TACACCAACTAGTAAAAACGACTTCTGAAATTTGAAACACCGCGATTATGATTCACAAGC
TGCAAAGCCTATCACAGAATTTATGGCTCTTAATAGCATTTGAATCCCCAAAATTTGTAG
TGACTGATCCCCAAAATCTATAGCTCTTACTGCAAAATTTGTAGTAACACAAAATTTTAA
ACCACAATAAAAATCCAGCAATGGATAATGGTACCAAAAACAAAAAAGGCATTAGAGAGG
CCAAACACAGCATATATATAAGTTTAGCAATCCTAGTATACTGATGTAGAAAGTTCCAAA
ACTTGTCTAAACAAAATTACACATAAAAATTAAAATTTAAACCCAATTCCAAAANNATCT
ACACTCTTTCCCTACACGCACGCTCTTCCGATCTCCCAATTCCAAAACAAAAAAAAAAAA
AAAAAAGTGATGCCAATTCCTTGGTTGAGGCCATGACCATGATTACTACGATGATGATGA
TGATATCCCACAATTCCGCCGTGACTACTATCGCAAAATTAATTACCAATCAACACAATA
GCTGAGGAACCTTAGCCTCAGTCACCCTTTCTTCTGAATGAGCACCCCTCTGTAAGCCTC
GCACGCCCACCTGTTGGCTGGCACAAAACTGTCTGGCAGTTCCCACACACCACCACTGTC
TGTGAATGACTAAACACAGTTGTTATGTTGAAGCAACCCTGGCACTTGACATCCATGAAG
AACGAGTTGGGCGACTGAACAAGGCGCTTCAACTTGTGTTTCCTCTTCTCCAACTCAGCT
GGAGGATTAAGCAAGTCGATGTCGTTTTGAAGAACCATTTTGATCTATATGAAAGAGATG
CTCAGAAACCCTAGAAACTCTTGGAGAAGACGCAAGAAAGAGAAGTGCCAGGGG
>tig_1251_consensus
GCCAATTTCCATGAATGAAATACCCGGAAAACCCAGAAGTTTTTTTGGGTAAAATACACT
CCCAACTGCCACTGAAGAATTTAATTAAAGACAAACAAAATTGCTTAGTTTACATGAACA
TTATCTCACAGCTGATACAAAAGTTACCTTCAAATCCACGGTATGATGCTGTAGACTTAA
TATTCAGCTTCCAAATGACACCAATCCCAGGTATAACAATTTACAGCAAAAATGCAAAGC
TGAAATTTTTATGCTCTGATTGAGCCTCCAAGCACAGATGATCCTACATATTTCAGATCT
CCTCGACTGTAAAAACCAACATTTGAAGAACTGATCCAACTCCAGCAAATTAACACAAAC
CCTACCAGCGGAGTCATTTTTGAAATGCAAATATTGGTCTGTTAAATCCGCTCTTGCTGT
CTGATAAGCCATTAGAAGTTATGACTTCGACCCTTCTAAACCCAATCTCATCCAGAAGAA
TTTCTTGAAATTGCTCTGGACGGAAGACAATACTTTTATAATTAGTTGCAGTGGTCTCAG
ATACACGACGATTATTTTCGTAAGATCGCCAAGGTTGAGGTTCCAACACAAAAATACCAC
CCCACATAATTGTATCATATTGCATCTGTAATGGGCGCCGACTCTGAACAAAATTTTCTT
GTTGGAAAGAAACTATGTCATGTAAATCTCTTTCTACAGGAGGAGAACAATCTTTTGAAA
CTTCCTCCTTCTCCGTTTCGGACATAACAGTTACACCAAGCTGAGAACCATCTGCATTCC
CTGGAACATCAGCTCTGGAAGCCTGAGCACGTTTTCTTTCAATTTTTTCCATTTTCACAA
TTTTCCTAAGGTGCCAATATGCATGCTCAATTAGATTAGAATCAATGTCAATTCCGAGAA
TCCTCCGGCACTGAAACTTTTTTGCAATTTGGATAGTTATAACGCCGCTGTTACAGCCAA
TGTCGAGACAGTTTTTTCCATGAAACCATTCCTTCTTGAAAACCTTGAGACGAGGGTCTT
CATCTAAGCCCTCACCAATACGATAGCCGTAGTAATTTTTGTAATTTCCGAAGGGAAAAA
CTTCTTTGCGTTTCCTCTTCTTCACTTGTTGAGCGTCATCATCAGCTTTCTTTTCTTCAA

CTTTATGTTGCATCTTCTCCATGTTTTCTCTGGTCGTAAAGGATGTTTAGGGTTTATTAA
GGTTTTAAATTTGCACGTATAATTAGCCCCTTTATGTCCTTGCCCATTAGGGTTTGGATT
TGGGTGGAATGGAATTTAAATTTGGATCAAT
>tig_1252_consensus
CCCTATCGTAAGCATTATAATCTATGTTCATAAATCTGTCAAAAAGGCACAGCTCTGAAA
CTCAACTGCTTTATTGCATGTTTGATAGTATTACACTCAATATTACGCCAAAAACCAAAA
AAGGGACAACAATGCTCTCTGTGTGCTAGGCGTCTGCGAGTATTTATCATATATCATCAT
GATGTTGCTGTTGTTGATGATCATAGTCTTGTGTGTTTCCAGTTTCTTCATTCTCATGTT
GCTCTAAAATGGTTTCTACACCGGTATCAGCAGGGGCCATCTTCTCTGAGCTTGTTTGGC
ACCCATCTGAAAAATTTTTGTTTGGCTCTGATGCGTTAGCCACATCTGGCTGCTTTTCCA
TCATCCCAGACAGGGCCAATGTTGGACTCCACTCAAGAACATCATCAATTATGTTTGACA
TCCGCTTATGATACCTGGCTCTAGCCGCTTCATCAGGGGCATGGTGCCTCAGCAATAATA
TTACCAGCACAGCTACTACAGCAAAGAAAAGTCTTGCTGTCCACTTAGGCCGTTCTTCTT
CATCTTTCTTGGGCCAGACACGGAATAGTTCCCCTAATGTTGCTTCCTCGGCAAGGATGT
TCGGGAAAAACCAAACACGCTTTCCAAGAATAATCCATATGGCACCAAATATTGCAGCTC
TCACCAGGAGTAAAGACAGGATAACCAGCAAAACCCCAGCACAAGAATAAAGTATTAATA
GCTTGCATCGATGTGGATAAACAGGAAACAAGCAAATTGCTAGGGTCAACAAAGGCCAGC
AAAATGAGAGAAGTGTTTGCCACAAAGGTCGTCGTTTCACAAATGTCCATGCAAAAAAGG
CATCATTATCAGAAAATATTTGCTCAGGGAAAACCTCTAGATGTGCCGGCCAGGTGGACA
ACTTCCTTTTCCCAGGACGTAAAGTTTTCACCACACGATCACAGCGCACCAAGAGATTCT
TTCTTAATAGATCATTGGCAATATCTTCCGTTTCAAGATTCTTGTCTGATTCTAGTATTT
CTTTAAGCTCTGGATGATTTCTCAAAAAGCTTACAAAATCCTTACCTCTAAAATATTCCA
CTCGAGTTTCCTGCAACACAGCCCACCTAGATTCCAATTCTTTATGGTCTCTCACCTTCT
CAGCAAAGAACTGGTAAACATCCTTCTTGCCAGATTGTTTCCTAGGTGGGGTATCATTGC
TGGGATCTCTGGTGCCATTTTGAGAGGCTGCTGATGATCTCCGGACCCTTTTCTTCTCTG
CTGCCCCTGCTTTCTTCATCTCTCTCTGTTCTCTTTTGTGGTTTCT
>tig_1253_consensus
GATATGAGATGATCCATTATTATTACAGTATGATACGTTGATTTTCAACAGCTATAATAT
TCCTCTAATAATAAACATCCTATCCTATACTTAGCATCGTAATCTATATTCATAAATCTG
ACACAAAGGCACAGTTTTCAAACTCAACTGCTTTATTGTCACGTTTGATATAGTGTTACA
CTCAAGATTATACCAAAAAAACTCACAATAANNNNNNNNNNNNNNNNNNNNNNNNNNNNN
TTTCTTCATTCTCATGTTGCTCTAAAATGGTTCCTGCACTTGTATCAGGAGGGGCTGTCC
TCTCTGAGTTTGTTTGGCTCCTGTCCGGAAAACTTTTGTTTGGCTCTGTATTGGTAGCCA
CATCTGGCTGCTTCTCCATCATCCCAGACAGGGCCAACCTTGGATTCCACTCAAGAACAT
CATCGATTATGTTTGACATCCGCTTATGATACCTGGCTCTAGCAGCTTCATCAGGGGCAT
GGTGCCTCAGCAATAATATTACCAGTACAGCTACTACAGCAAAGAAAACTCTTGCTGTCC
ATTTAGGCCGTTCCTCTTCATCTTTCTTGGGCCAGAAACGAAATAATTCCCCTAATGTTG
CCTCCTCAGCTAGAATGTTAGGGAAAAACCACACACGTTTTCCAAGGATAACCCATATGG
CACCAAATATAGCAGCTCTAACCAAGAGTAAAGAGAGAATAACCAGCAAGACCCCAGCAC
AAGAATAAAGTATCAACAGCTTGCACCGATGTGGATAAACAGGAAACAAGCAAATTGCTA
AGGTCAACACAGGCCAGCAAAATGAGAGAAATGTTTGCCACAAAGGCCGCTGTTTCACAA
ATGCCCATGCAAAAAAGGCACCATTATCAGAAAATATTTGCTCCTGCTCAGGTAAGATCT
CTAAATGTGCTGGCCAGGTGGACAACTTCTTTTTCCCAGGACGTACAGTTTTTACCACGC
GATCACAGCGCACCACGAGATTCTTTCTTAATAGAGTATTGGCAATAACTTCAGTTTCTA
GATTCTTGTCTGAATCTAGTATATCTTTAAGCTCTGGATGATTTCTCAAAAAGCTTACAA
AATCCTTACCTCTAAAATATTCTACTCGAGTTTCCTGTAATACGGCCCACCTAGATTCCA
ACTCTTTATGGTCTCTCACCTTCTCAGCAAACAACTGGTAAACATCCTTCTTGCCAGCTT
GTTTCCTAGGTGGGTTATCGTTGTTGGGATCTCTTGTGCCATTCTGATAGGCTGCTGATG
ATGATCTTCGGACCCTTTTTTTCTCTGCTGCACCTGCTTTCTTCATCTCTGTGTTAAGTT
TCGTTTCGTTTTCTCTCTTTCTTTCCTCCGAGTTTTCGTT
>tig_1254_consensus
CCCACCTACATCTCTACTCTTTCCCTACGCGACGCTCTTCCAATCTCTATGATACCACAT
CCGACTCTAGCTCCGGCATTCCCAGTCGTCTTGCTAAGTTCATGTCCACCTGTGTTTTGG
TTGATAATTGGTTAAAATTTCAAAAGATGTGAAAATAATTATGACAAGAAAAGAAATTAC
ATAAGCAACTGAACCATAAATCGATGAAAAAAAGGCATGCAAGAGGCTTTCACACAATAA
GGCAAGCTTATGTGGGTTTATATTTGCAGGTCTTCATTACACTCAACTATCTTAAGCCTA
AATTCTATATGGCTCAAAGAATGTAATTCATGCGAAGGCAGGTTTATATAAGCAAAC
>tig_1255_consensus

CGGACAGGTTGTTCACTTTACAGTATCGATAAATCAATAGAAAGATCATCAAACAGAACA
AATAAACGCCATAGATTAAGTGCTGACTCCTATTTACAGAGTTGTACAAAATGGCAAGTT
CAGCAAACTGAATTTATTTTTGAGTGATCATATATGTTCAAGTAAAATAGAAAAAAAAAA
AAAAACTTGTCAAGAATTTGATCACCATGAGATTTCATGCAGTTTATGGCTAGGAAATAA
CTGTAGAAGTATTTTAATCGGCACACAAGCAGTGTCTCTAAACAGAGGATTGAAGCCCTA
TGATACCACATCCGACTCTAGCTCCGGCATTCCCAGTCGTCTTGCTAAGTTCATGTCCAC
CTTGTCCAAGATCATCAGGATCAGCATGTACAACAACAGCCCTCCCTAGAATGGAGTGCT
GTCCACTCAAAGGAATCTGTCTGTCTGCAATTGAAACCTCAGCTACTCCATCTGGGCCTG
CAATGATGTTACCCAAATCACCAGCATGCCGCTCCTCATGACTGGGAGCTCCATGATTCT
TCTTTAGTGGATTAAAATGAGGTCCGGTAGAATTGCAGCCATTGGTGGTGTCACCAAGAG
CGTGAATATGGAACCCATGAAGGCCTGGTTTCAATCCCGTAATTCTCCCTTTAACATGAG
TAACTCCATTGGGGCCTTGAACAAAGTGGAGCGAGCCTCTAACGTTGGTATCTCCGGAGA
TGATAGCCACAGCTTTCACAGTGGGTTTTGCACCGACCGTCTGCTCCATTTTCTGTTCCT
TGCTAGTCTTTGATTTCGATAGAATATAGCAATATGCTACATATTTTTCCTTGGCTTCTT
TTTTTTTTTTTT
>tig_1256_consensus
GCTAAGGCACTCTTTACTCTGAAAGCTTATCACTTTCATTGTACAGTGGAGCACTTTCTC
TTGTGGTAGGGGAAATCCTTCGTGAATAGTATACCAAATTTTTTTTTTTTGCTTCTCAT
GCCATTAACCTCTGCAATCCACGATGAAGAGTATATTCATCAAGTCTCATGCCAATGGAC
TATGGAGTGATCATGAGAGGTTTTTTGGCTGACCCAGTGCTTTCTGTCTCCTTCATGAA
CCTTGATAGCAAGAAAACCT
>tig_1257_consensus
CAACTTATTTATTTAGCCAAAAAAAATAAATAAATTACAAAGTAATTAAAAATAAACATA
TTATCCCCACATGTTACAGCCCCATGTGCGTGCCCCCCTCCCTCACTAAAATTAAATCCA
AATTTCACCACCACTGTCGGCCGCCGCAACCCGACCGGAATTCATAACTAGAATTACCTG
TTCGTTGCATCTACTGCTGAAACTTGTTTGTTTGGTACGATTTCAAAGCCACTCCCATCA
TCTTCTTCATCATCATCCGCTGTATCTTTGTTGTCTTCCTCTCCATTTTGATCGCTGTCA
ATGTCGTCCATGACGGAGTCTTGATGTCTAAGTTGTTCAGGCTCAGGCGGCCATTGCTGC
TCCGGTCTTACCATTAATGGCACTGTGTTTTCGGGCTTCATTTGATTCGAAGAGCTATCA
AGTTTGTTCTTTTCTTTATATAAAGCTTCAAGTTGCTCGAAATATGGACAAGTTTTGGAG
TCCTCGGGCCTTCTCTTGTTATTTTCTTTCACTTTCTTGAAGTACTTGTTTATATTTTCC
CATTTCTCTTTGCATCTTTTGGCACTGCGATTGTAACCTAGCTTTCTCATCCCTCCTGAG
ATCTCTTCCCATAGTGGCCCTTTAGGTCCATTCCCTTGATACTTTGAGTCGAGATTAGTC
CTCAGACTAATCAATGCTAGAACCTCAACTTTTGGCCACCTTGAAGAGCTTGTTGCCATA
GAATTCTGGTCCCCATTTGACCCGTTTGACATCTCTAAATTTAATACCTGTCGTGTCTGT
GTCTGTGATTGTGGTCCTGGTGTCGATGGTTGTGCCTGCGGTGCTGGAGGCAATGATACT
GGCTGTAGTGGAGCCGGTGCTGCTGCTGCTGCTGCTACGGGAGGAGTCGCTGCCGGTGGT
GGCTGAGATACATTTATTACTGGAGGAACATTTATATTTTGTCCTTGCCCAGCAACGTGG
TTTTTCTGATCGGATAGTTTTTGCAAGAAAGCCATTACTGCTGCATCTTTAGCTGCAGCT
ATCGATCTTTCTTGAGCTAAAATTTCTCGCTCTCTATTGATTCTTGTCATTTCTTGCAAT
CTCCAAGCTTCTTCTCTAATCATTCTTTCATGCTCACGTTTCTCTATTGCTTCCAGGAAT
TTCTTCTGCAACTCTTCCTGCTTGTCCACCACCTCCTTTACCAACCTCTCAAAGAAGGCC
CTCCATTTTCTTTTTCTTTTCCTTGGCGCTCCGAATTCCAGGTCCGAAGAGGTCGACGAC
GACGTCGAATTCGACAGTAGATCAGTGGAAATATTCTGAAAAGAAGGATAATTCACAGGG
TTTGTTACTTGTACCGAAGGTAAATTTGTAGAAGGAAAGGAAGGAATTGCCAGGGTTGTT
GCTGGTATTGTGACAATGTTTTCAGCGACATTTGTTGTTGTTGTTGATGGAACAGTGGAA
GTAATAGTGATGTTATGAGCCAACGGCATTGATGCTGATAATACTGCAGCGACCACCGAA
GTTGGCTGAGACTTGGGTGGTTGTGGTGGTGAAGGTGTCACTGACGACAACGAAGGAGCT
TGATGATGTCCAAAAGCCTCTAATTGATTAAAAAATCTATAAGTTTTACCATGAGATTTA
CTGTTTCGGCCATCTTTGGTTCTCCTGTGATACTTGAAAACATTCTCAAATTTCTCTTTG
CATTTCTTGGCACTTCGATGATAACCAAGCTCGGCTAGCTTCCTGGAAACCTCTTCCCAC
AATGGACCTTTAACACTAGCATCTCGAAAGGCGACGTCCATATCCGACCTAATTTCAAGA
AGTGCAACAGTTTCTTGTCTCGGCCACCGGTTTCCTCCAAAGCTGCGGTCCCCTTTATCG
TCTTCGCCTCGAGAATTTGAGCCAACATCAACAGCACCACCAGCGGAAGTGTCATGAGCT
CCAATTGGGGGCGCAACGTCGCTGCCGGAGGAGGTTCCGAGCAAGGCTGATGCATCTTCT
GCAGCTAGCATAGCACATAGGAGACTTCTAGCAAATTACAGCTAGCGGCAGATCGGAAGA
GCACAC
>tig_1258_consensus

AGATGGAGACAACCGTAAGATTGGCGTGGCTATGGGATTGTTTGACCAGAGATAAATGAC
CTTCGTGGAGATAACCCATGGTGGGTACAAAGCCTATGGTTTTGCCTTGTGATCTCATGG
CTCTTGACCAGCTCCTCATCTGGTTCTTGTCTGTAATTATCTCTGGTTCTTTGGTTGCCA
TGGGTAACAACAGAAGGCACTGAGACTAACTAATCCTGTTTGGAGTTCAAAAGAAGGACT
TGTAGTGTGCGGAGTCAAAACTAAAGTTGGAGCTGCGTGGATTTGGGGAAGGACAAGGAT
GTGATGTTTTGTGTTTAAAAGGAGGAATTATAGTGTACGGAGTTTCAGTAGGCAGATGGG
AATGACGACGATGATGTGTGTGATTTTAGGATTTGGAATACTGGTAAGCCCAAGAGACAA
AATCTCAGAAAATGATTAATCTCAAGAGAGGAGGCGGCGGCCTGGGGGAGCTGCGATGTG
ACTCTGATTCTCCGCCCCTACGTGGTTGCGTTTCTGATTTGGCTTTATGAAGAGGCCAAA
CGCCTTACTC
>tig_1259_consensus
AAAAAAAAAAAAAATTATATATAGTAGTATAAAATGAAGGGGAAAATGGGTAACCACTTG
ATCTCGTCAAAACTCAAAAAAGGGAAATCACAGTAGAAAATTCAAACCCATCAAGTGATG
AAGGTCAGAGGTGAGCCCGCCTTCTGAAAATGAAAACAGTACTTCAAGTCCAGGTTATGA
TATTTCTTCTTACCTGCCGTTGCACCCAATTTGAAGGAACAAAAGAACGGGCACTTATTA
AGAGTCAAGAAGGCCTTCTCCTTGAAGCTATGTCATCATCATTTTCCCGCATATCAAAAG
ATTTTGGATTAACATTGTATTTTCCGTCCCTTTGATATGTGTATGAGCCTCTCTCTTCCC
TAAATCTTCTTGGTGCCTCCCCAATCCGACTCTCAATGCTCCTGCTAAGTTCCTTAGGTC
TTACTCTGCCATGAAAATCAGAAACATCTCCATAACAAGAAATACGAGCCTTCGCAAATC
CATCATCCAAATTATTACGGAATGGTTTTGCAATGCCATTCATACCATATCGCTTTGCAG
GGGGAAATGGCCCAAATCTACAACTATGTTTCTCCCTAGTATCATCATTGCCCTCATACC
TTACCCGTTCCCTGCCCACTCCATTGATTTCTGTAAATCTTCCAGGATGCACGTATCTGT
AGTTTCGCAGAGAATCGACATCAAACCTGTCATCACATGGGCCAAACTTTCCTGGTGATC
TCCTGTCTAAAATGGAAGTACGCTGACTTAAACCTTGCTCCCTGAGATGAACCACACCAT
CTTTCCAATCACCAATCCATCTGGAGTTGTGAGGTGAACCATGACTTCTGTGCATAGTCC
TAAATCCTACCACTCGGTCTCCCAAAAAACCAGGCCGCTGATGAGGTGACTTTCCTCTTG
GCACCCTTATCTCAGATCTGAAATTAGGCGACTTGCTTCGGTGTTTTAAATTTGGATTAC
CTGTCTCAGGAAAGCAAGTTCTAGATCTTGAAGGAGACTTAGATAGGGATTTACGTGCAC
GGTCTTCACCTCTTCTTTCATTGGGTGAAAAGCTTCTCTCTCTCTTAGCTGAATGATGGG
AATGGTTCAATGAAGACTCAGTGCAACCATGGTATCTTCCCCTGAGGCCTCTCTCATCCA
CTCGAGGACCATATCTTAGGGACCTGCCTCTGCCAAGCGTAATACACCGGCTAGGACTCA
GCTCTCTAGATGGTCTAAAACCCAATCGCAAACGAAATGCCTCATCCCTGTCAATTGGCA
GGTGCCGATGTACACCAAGTGAAGATGCATCAATCTTTTGTCTGCCAAAGGAAACATTTT
CAGGCCCTAAACGGCGAATAGATGTTGGGCCATGATAGTTAGGTGAATGATAGCGTTTAA
GACTCCTGTGACTCTCAGTAGAATTGACCCAATGATCACTCTCCTGACCTCTTGCATGAA
CATGCTGATTATATCTGCCCCTAACCAGAGATTCTCCATATCCAGGATCCCTTTCAGATC
TTAGATTAGTGATGTTATCAGTATCACTCCTTTCCTGCACATAAGGTTTGTCATCGTCTA
AATATGCATCATGAGAAACTCGTCTTTCTATATATGATAGCAATTCCCTCCTCAACTCTC
TTGGTCTGACCACCCTCGCCTCGGTACCATCTGTGTCTTGGACATCTATGCAATCATCAA
TCATGCTCTTCCTACTGCTGCCATCAATCTCAGTTGTCAACGAATCTGAAAGATTTTTAT
GATCCCACCTAGATGCTTTCTCAGCTGAATCAGCCGATAGACTTGGATTCTTTGTATCAT
CTACCCTCGAAACAACGACATCCCCCATAACCTTGTGAGTGCCTACTTCATCACCTGACT
CGATCTCAATCCTTCCATCTTTCCCTTTCAATGGTTTTTCCTTACCGGATCCAGATGAGC
CACTTAGGCAAGGTTGGAAGTCAGTTTCTTGTGTATGCTTCACAATTTTTTCAGCGTCAC
AACCCAGATTATTGGCATTGTCAGATCCATAGTCAATATGTTCAGCTTCCATGTCTTCCC
CATCATATTCTTCCCACGTATGAACAATTGATTCCCTGACCTCACCATCTTCATACTGTG
AATCATAATCTAAGACCTCAGTCGACTTCACATGAGCATGATCATGAGAAACATCAGAA
>tig_1260_consensus
CATAATATACATGGCATAACTTACGCCGGTGATAAAAAATTTTACCCTGCTATACCTAAC
AATTACATGAAACATTATACTAGTACAATCATCTACTCCTTTCTCATCCATTCAAAAAGG
ATGAAAATCCCAGCTCTCTTTCTAAGGAATATATATATATATATAGATAACTCTTGGAGT
CCAAGAACAAATAAGTAATTTAACCAACAGTGCTATATTATCTTCAGAAACCAAAGGGTG
ATTTTCTCCTGCTTCCACCTTCTCCATACTGTTCATTCCATTTTCTCAACTCATTCATGC
TTGCAGCATCATATGCGATGGATGGACCAACCTTGGTTTTTGATTTAATAAAATCCTCCA
AAGTAAGGGGCCTCAGTACCGGAGATGCAACATTTTTGCCCATCGTCCTTTCTTCCTCTA
AAAGTTCTTGAACAGGTATATATGCTGCGGCAATACAGAGGTTCTTCAAATCACTGCCAG
AATATCCCTCAGTTGCAAGTGCAAGTTCCTGCAATTGGAAATTAGGACCCAAATTTTCTT
GTGCAAGAAAAATGCTTAGAATCTTCATACGATTTTTAACATCTGGTAGATCCACATATA

TCCTTCTAGGTAAGCGGCGAATGACAGCATCATCAAGATCAAACGGACGATTTGTGGCAG
CAAGGATCAGGATCTTTTGGCTTTCTTTGGACCTCAATCCATCCCATGCAGACATGAACT
CATTTCTCATCCTTCTAGTTGCCTCATGCTCAAAGGCACCGCCTCGAGCACCAAGCAAAC
TGTCAACCTCATCAACAAATATTATTACAGGTGCCAGCTTGCCAGCAAAAGAGAAGAGAG
CCTTGGTAAGCTTTTCGGCATCACCAAACCACTTTGAAGTAAGAGTCGAGCCAGTTATGC
TAATAAAGTTAGCTCCTGCTTCTGTTGCAAGTGCCTTGGCAACAAGGGTTTTACCAGTTC
CAGGAGGTCCAAAAAGTAATATTCCTTTACATGGCCTTAACAAATTTCCATGAGAAAAGA
GCTCAGGTCTCCTCATTGGAAGAATGACAAGTTCATTCAGGGCCTTCTTCACATCTTCAA
GAGCACCAACATCATCAAATTTCACACCAATTTCACCGGGAGGTACCACAGCTGAGACAA
AATTACTCTCATACTCATCCTTTGCAAGGTTCTTGAAGTTCTGGGCAGGCTTTCTATTTG
CAGTTTCTTGCTCCTTCAACCTCGAAATTGCAATCTCAAGGCTTTCCCGAGGCAGGTAAA
GTCGCTCTCCTTTTACGAAAGGAAGCTCACAAGATGACAAGTAGTGATTTTTTGCCCAAC
CAACAACTTTCTCAGCTTTCTGCGTTGTCAGTATCACACCATCGGTGTTTACATGCAAAA
GATCATCACATGACAGTTCATGTTCCTCAAGAACCTTATGTAACTCATTTAAATTACTCC
TATATATTACAATTCTTCTGTCATCTTCAACCTGCTTATTAAATGTTCTGAGTAGATCTT
CCTCCTTGGGTGGATATATAGATAAAACATTGGTGAAGAGCTTAAAAATTTCATTATCAT
CAGATCTCTTTGTTGCTTTAAGTCCCTCAGTAAGGCGCCTAAGGGGGAGAGGCAGCTTAG
CAAGCCGACCAAAATTTGGGAGTATCATTGTAAATTTCTCCTTTTCCTTGGACCCAGTTT
CAACTTTGTTCTGCCCACAAATCAAAACTACTGGGCCTGATAATTGGTCAAACATCTCCT
GCACCTTATGGACAAACTCCTCACGATTAGACTTGGGAGCTGCTCTGGACAACCACTGAG
AAGAATCTGGGAAATAGACAATAAGAGGTTGCATGGTGTTCAAAACCTCACATAATGCCT
CCATTGCAATATAGCAATCCTCTGCCTGGGTATCTAGATCCTGCTCAATGTTCTTCACAT
CTATCCAATAAACCGGTGGTTTTGTAGACTGCTCTGCCACTTTGCCATCCTTCTCTCCTT
CATTAACTTTACCATCATCGCCAACATCCAAAATAACTGCAACTCGGTCTCCATTCACCT
CATACACCTCTCCACGTTGCCCATTAGTTAGTGGCCTGTTGTCAGCTTCAATGCGTATTG
AAGGCCCAGTGTATTTCACACGATCCCCTTTCTTCAGTGGCTGTTTCAATTTATCAGAAG
GTTCACCAACGTCAGACTTTGAGGACTTAGAAGAAGTCTCAACTTCTCCAGCGAATGTCT
TCTCAAAATCTTCCAGGTTGTAAGGAACAAGATTTTTAAAAGCTTTCTCAGCAGTAACCT
CCACATCAGCATCAGCATCACTACAATCTGTTTTGGCCTCATTATTGCTTGCCCAATCAT
CTTCATTACTAGCATCATTCTCATCCTCAACCTCTGACTCTGAAGTACAATCCTCACAGG
ATTCCACATTATCTTCATCTGACGAGTAATCATCGCCAAAATCATAAGGAGCAAGGATGT
TACTGTCAAGCACCAAGAGTGGAACTCGTAAATCTTGTGCAAGAGCTTTAACTAATCTCT
CCCGATAAAGCTCAGTGCCTGGAACACTTCGAAGCAATATTCTCCCACTTGAAGATGACA
GACGAGCACCGTATGAAGCAGCGAACTTCTTATGTTTCAAATGTGAAGCAACACATTCCA
CCAAAAGATTTTTGGTGTACTCATGAATGTGGTAAGGAAATGTCTCCCAAGAAATGTTTA
TTTTCTCCCATGGAACAACCCTCCGCGAGAACTCAATTTTGAACCTTTCACGTCTAGTCA
AAAATGGTGATTCTTTCTTTTTATGCTCAATGGTGAGTTTCTCATTTTGTAGCCATTCTT
TTTGTTCTTGTTCCCCAAGTAGAGCATGGGCATCACAGTGCTTCACATCTTCCCTGACTT
TTTCAGGCCACGTCTTTCCCTTCTCCCTCTTATCCAAGTTAGCACCGTCAGGAACGGGTT
TCTGATTATTCTCACTTGCATTCCTTCCATCACCTTCAGAACTGTAGGCACGCAATTGAC
TACTTCTCAAACCAATACTAGACCTCACATGGAAACCTGTATGACAGTTGCCAAGAGCAA
CAGCTGAAGACAAAATCGATTCTAACACATGTCTTCTTACTATAGGACTGCAAGGTGATT
CGTTTCTCAATGCAGCAGTATG
>tig_1261_consensus
CCAATATTATCATTAATTGTAAAAGTTACAATGTCTCATACAACAAAATAAATCTCTCAG
GGAATTAAATTTATTATCCTTGAATTTTTACTAGTAGTAAAATCATTGGTGCAGTTCAGA
AATACAGAAGACCGACTATGACAAAATTTTTCAAAATTTATGAATACTTAGAAATATTAT
TGTACCTGGCCGGCAAAACTTATGCCAGTAAAAAAATTCCTCTAAAATACCTGAAGATTT
CATGAGAACTATGCTATTGCCATCAGCAGCTCCTTCCTTCTCTTCCATTCAAAAAGGACG
AAATCCCGAGCTTTTCTTTATTAGCTTCAGAAACCGAAAGGTGATTTTCTCCTGCTTCCA
CCTTCTCCATACTGCTCATTCCATTTCCTCAATTCGTTCATGCTCGCCGCATCATATGCG
ACAGACGGTCCAACCTTGGTTTTTGATTGAATAAAGTCCTCCAATGTAAGAGGCCTCAGT
GCTGGAGAAACATGTTTTTCGCCCATCTTCATTTCTTCCTCTAAAAGTTCTTGAACAGGT
CTATATGCTGCAGCAATACAGAGGTTCTTCAAATCACTGCCAGAATATCCCTCAGTTACT
CGTGCAAGTTCCAGAAATTGGAAATTAGGTTCCAGATTTTCTTGTGCAAGAAAAATGCTC
AGAATCTTCATACGATTTTCGACATCTGGTAGGTCAACATATATCCTTCTAGGTAAGCGA
CGAATGACAGCATCATCAAGATCAAATGGACGATTTGTGGCACCAAGGATGAGGATTTTT
TGGCTTTCTTTGGACCTCAAACCATCCCATGCTGACATGAACTCATTCCTCATTCTTCTA

GTTGCTTCATGCTCAAAAGCACCACCACGAGCACCAAGCAAACTGTCAACCTCATCAACA
AATATTATTACAGGTGCCAACTTGCTGGCAAAAGAAAAGAGAGCCTTAGTAAGCTTTTCA
GCATCACCAAACCACTTTGAAGTAAGACTTGAGCCAGTTATGCTGATAAAGTTTGCTCCT
GCCTCCGTTGCAAGTGCCTTGGCAACAAGGGTTTTACCAGTTCCAGGAGGTCCAAAAAGT
AATATTCCTTTGCATGGCCTTAACAAATTTCCATGATTAAAGAGCTCAGGTCTCCTCATT
GGAAGAATGACAAGTTCATTCAAGGCCTTCTTCACATCTTCCAGAGCACCTACATCATCA
AATTTCACACCAATTTCACTGGGAGGAACCACAGCTGAGACAAAATGACTCTCATAGTCA
TCCTTTGCAAGGTTCTTCAAGTTCTGGGAAGGTTTTCTATTTGCAGCTTCTTGCTCCCTC
AACCTCAAAATTGCAATCTCAAGGCTTTCACGAGGCAGGTACAATCTTTCCCCTTTTATG
GAAGGAAGCTCACAAGAAGATAAGTAATGATTTCTAGCCCATCCAACAACTTTCTCAGCT
TTCTGCTTCGTCAGTATCACACTGTCAGTGTTTACATGCAATAGATCAATGCATGACAGC
TCATGCTCCCCAAGAACCTTATGTAATTCATTTAAATTACTCCTATATATTACAATTCTT
CTGTCCTCTTCAACCTGTTTATTAAATGTTCTCAGGAGATCTTCCTCCTTAGGTGGATGT
AAACATAAAACATTAGTGAAGAGCTTATAAATTTCACTATCATCAGATTTCTTTGTTGCT
TTAAGTCCCTCAGTAAGGCGCCTCAGCGGGAGAGGCAGCTTAGCAAGCCGACCAAAATTT
GGGAGTATCATTGTAAATTTCTCTTTATCCTTGGACCCAGTTTCAACTTTGTTCTGCCCA
CAAATCAAAACTGCTGGGCCTGATAATTGGTCAAACATTGCCTGCAGCTTCTGGACAAAC
TCCTTACGATTAGATCTGGGAGCTGCTCTTGACAACCACTGAGAAGAATCTGGAAAATAG
ACGATAAGAGGTTGCATGGAGACCAAAACCTCAGATAAAGCCTCGATTGCAATATAGCAA
TCCTCTGCCTGGGTATCCAGGTCCTGTTCAATGTTCTTCACATCAATCCAATAAATTGGT
GGTTTTGTAGACTGCTCTGCCACTTTGTCATCCTTCTGTCCTTCGCTCACTTTACTGTCA
CCAACAATATCCAAAATAACTGCAACTTGGTTTCCATTCACCTCATATACCTCCCCACGT
TGCCCACTGGTTAAAGGCCTGTTGTCAGCTTCAATGCGTATAGAAGGCCCAGTGTATTTC
ACTCGATCCCCTTTCTTTAGCGGTCTTTTAGATTTATCAAAAGGCTCACCACCCTCAGAC
TTTGAGGACTCAGAAGAACTCTCAACTTCTCCAGTGAATGTCTTTTCAAAATCTTCGAGG
TTGTAAGGAATAAGCTTCTTAAAGGCTTTCTCAGCAGTAGCTTGCACATCAACATCATCA
TCACTACAATCTGATTTTGCCTCATTATTGCTTGTCCAGTCATCTTCATTACTTGCATTA
TTCTCATCCTCAACCTCTGACT
>tig_1262_consensus
GCATTCAAGCACTAAAGAAATATTAGGTAAAGGCCATTGAGAAACTACCAGTACTGGTGG
AGTTTATAATTAAATATATATAATTTTGTGGAAAATCAATCACAATCTCCAGCTTTAACA
GCTTATAAAGCTACATGATCTCTAACCAGCGGTAGTGTCCTTATCGTAATCAGTGAAAGA
GTATTGTATTGAGCAAGGTTCCTGGCTACTACCGTTTTCTTTGTTTTTGTTAGGCTTCTT
CTCTATGCTTATCTTCTTTCATTATGCCTTCTTTGCTCCTTGAAGAACGTCTGTGCTTTT
GAAGAAAAAGAATCCATAGGCATGCAAGATGTACATGATCATCGTCATCATACACATACT
ATACAGGTCACCTCTCTTTTGTAGTCACCCATTTGCGATCCTTCCACCAAAGGTTGGCTT
TCACTTTAAATTCTGTGTCTGCAGTCTGCTAGATTAAGCTGTCTATAGTTATTTTAATAC
TTTGTCTCCTTAATCAATGTTGCATGTTTCAGCGACTGAAAGGAAGTCTTCTTTGAAGGT
AGTCCACAAGCATGGTTCCTCATTGGGATCAGGCAATTCATTGTTGCCGTGGGACTCGGC
ACTCCTAAGAAATCGTTGTCACTGATATTTGACACAGGCAGCGACCTCACTTGGACACAA
TGCAAGCCTTGCGTCAGATATTGCTGTCAGCAGAATGAGCCAGTAAATGACCCTTCTGCG
TCCAAAACATATACCAATGTCCTTGCTCATCAGACATATGCGGTCAGGTCAAGTCTGCCA
CAGGTAAATATTTACATATCTTAACAGTTTCATAATCCATACTAATTTATATAGAGGTAT
TAATCTGTTGCTATAAGTAGGTAATTCTCCGGGTTGTGCATCTTCAACGTGTGTTTATGA
AATCAGATACCGAGACGGTTCTTTCTCCATTGGATTTTTTAGCACGGACACTCTGACTAT
AATGTCACAACCCGCAAATTCCCGGTTGCTCTTTTCCTGGCTGTCTTTATTGCTGCCTCC
TCGGTGGTCTCTATTGCTATTGAACCGACTGTCCCGGCTGGACCTTTCGCGGATGTCCCT
TCGCGGAAAGTTGGTCTTTGGCTTATCGCGGTTACTTGGGATGTGGCGATCTCCTCCGCT
TTTTCCTTTGCTGAACTGGTCCCTACTCTTTTCAGGCTTGCAAAACTCCATGAGCGATTC
CGCGATTGTTATTGCGGTATTTAGATCCTGAGCGCCCCTACGTTGGATCTCCATTTTCGC
CCATGGTTGGAGCCCGTCCATGAAGTAAAAGAGGGCTTCCTTCTCTGGAAAGTCAGCGAT
CTCTAGCATCAATTCTGAAAATTCCTTCACGTAGTCTCTTATACTACTACGGTGCGTAAG
ACGTCGTAATCTGGCCCGTGCTTCGTGTTCGGCATTTTCAAGGTAGAACTGGCGCTTGAG
CTCTTCCTTGAACTCTTCCCAGGTCTTAATGCTGTTGTTGCCTCTCGCCCTTTCGCCATG
CCTACGGTGCCACCAACTCATGGCAGTGTCCTCGAGATATAGGTTGGCATAATCAATTTG
ACTGGTGTCATCCTCGACTCCAGCTGTTGGAAAATATTGTTTGAGACTCCAAAGGAAATT
ATCAATTTGCTTGGCATTCCTTGCTCCGCGGAAATATCTTGGCTTGGGGACTTCGAGTTT
GGAATGAGCTTGGACAGATGAGGTGGTAGCCATCCTTCCTTGTGTGACTGCCATTTTACA

GAGCGCCCAATCGCTCCTGACTTGTTCCAGCTCATTTCTCCATCTCGTGGCTTCCTCCCG
TAGTGTGGAAACCTCCTGTTGAAGGGTTTGAATATCAACAACAAGGGAGTCTTCGAGTGA
CTTACCGGTATTTTGGCATGCTTCCGAGACGACATTCAGTGCTCCCAGCATCTCCTCTCG
CAGCTCTTCTTGGCTGGTTTCGAGAGCTGTGGTGCGTTCGGCCAAATCGCCAAACTGGTC
GAATTTGTCACCTGTTGCAAGCTCCATCCTTGTGAGTCTTGCGTTCATGTCTACCAGGGT
GTCCCTCAATTTGTCCTTGCATTGCTTGGTATGTTTTGGTGCTTTATGTTCACGTTCACG
CCCTTTGTGTATGGCGGGAGTTTCACTTGAGCCTGCCATCTTTGTCCCAAATCACGAACT
TGCTCTGATACCACTTGTCACGGGATGAACAACTGACGATCACCCTGTGCGGCACTTGGC
CCTGTCCATTGAGTTAAGTAAGCCTTTCCTGATAATAGATGTGTGAAACAGAATAACGTT
ATGTGATAATCGAACACAGAAAGGTAGAATGAGAGAAATGAATATGTGCGGGAAATACCA
CTCTATTTCTCTTTA
>tig_1263_consensus
CCACATCTCACACTTTTTCCCTACCCCACCCCCCTCCCATCCCCCCCCCAAAAAAAAAA
AAAAAAAAAAAAAAAAAAATTATACACAGCCTCCCTCCTGGCCTCCGTTCAAACCCGAAA
AAAAAAGGGGGGGTTAGTGAGTGATCTATCATCATCAAAGTTGAAAATGAAAAAAATAAA
ATAAAAACCACTTTAGTCTAAATTGGTCTCCTGATCTCGACCCCACCCCACAGATGCTAG
GAATCCTTCATCAGACATAAGTCTCTCATTCTCCCCATTAACTTGGAGTAAACATGATGA
ACTTGCCCCATTTGGCTGCTGCTGCTGTATATAATTCTCTAATGGCCGGCTACTTTGTAC
AAGCATCAAAAGGCTAACTCCTGTTTCTCCAATGGGATCACAATTCAAATCTATTGGTCC
ACTGCTGCTCTCACCCACCTCTCCTTGAATTCTTTTCTGGCTCATTTCAGTCTGCGAAGG
ACTCATTTGCATCATCAATTCATTTCTAGTTGTACCGTTGTTCGTTTCTGATTCACCCTT
GTCATCGTTATGGTCCTTCTGAGCAAGTTCTGCTGCTTCATGTTCTGACTGACGTCTTTT
TTTGCGCAGCATTAGGGTTTTAAATCGGCGCTTCACAGTCATGCAAACATTGCATGTGCA
AGTGGGTTTGTGCCTTCCTTTGCCACTTGGGGGCTGGATGCACACTATGCAACTGCAGCC
AGGTCGGTGTCGGGGATGTCTAGTAGTGACTCCAACTGAAGGCTCACCTAAGTCTCCCGC
ATTGTCTCCTAGAACAGCAGCACTTGCTAGTGCGTCTAAGCCAGAAGGCTCGCGTTCTTG
GGCTGGGTTGCTCTCTACAATCTTTCGCTTCTTAGAGTCTCTACCAACCCTAAGAAGATT
CTCCAATTCCTTCTGACTCATCTCCTCAAGCGCAGAACATGAACACCTTATTAAATCCCA
AACATTATCTGAACATGTCCACTTTGGAGGGAGCAGAACATCCATTGGCAATTTTCGCCA
TTTAGAGCAATCATCACACTGTGCCCATTGCTCTTGTCCCCCAGATGGCCGGGCAGTGAA
TATAGTCCTCTTTCCGAAAACTGGAGGCTCATCATATTCTTCAAATTCATGGTCCTCAAT
AGTGACAATGCTCGGTTTGACACTTGGGGGTGGCCGCAGCAAGTCCTGTGCTTCTTCCCA
TGTAAGTCTTAGCTCCAGTGCTTCTTCACTGTGCATTAACAATCTCCTACTTTTGGAACC
AATATTTCTAGTTCTTTTCTTGTCTGAGTTTGGAACGGATTGCTGTGGTGACTCCTCTTT
TGTCTTGCTTTCGCGATTATCACTTTTATGCCAACCAATATTGCCATCAGCCAAACTCAA
ATGTTCTGGCAGTACATTCATGTGAGGTTCCTTGCTTCCTTTTGGTGTTTGGAAAAGACC
AGAATAACCGCTTATGGCAGGGAGATTCTCAGTAACACCAGAAACACAACTTTCCCCAGA
AGGAGCACCATTTAGAAGGGAAGATGTGTGAACATCCTGCATTTCTTCAGAGTTTGTTGC
CTTCCGAAACCCCATTATAAGCTTGCCACCAGGATCTATCCGACTAAATGTAATAGTATC
GCCTGCTCGTAATTGCATAGACTGTATGCAAGGGGTTACACCCTCTAAAACATACATCCT
GCTATTGTTGTTGGGCCAAAATCTGAACTGAAAAACCCACTCTTTCCCTTTAACATCTTG
AATTCTTAATGGAAGCCCTTCTGATTGAGATATATGAGGAAAATATGCTTCAGCACATGC
TTTAGGGAGAACCAAACGACCAATTCGACCAGCATCACTAGCACTTAGAACCTTTTCAAA
CAGTGGCACAATGGTGGAATTCAAATCTCCAGATAGCTGCTGCAGCTCTTGATCAGTAAT
CCTTGGCCAGTACCGAGGAAGTAAATGGTTTTTGCCCCGGCCCTCAACAGGCGGCCTTGC
AATCCGCAGATGAGAGGCTGCACCTTTACTTGTATCTGGACTTATGCTAAGGCCAGTTTT
AGGGGGTTTTGGTAATATAGGACGAGACCTTTGACCTTGTTGAAAGAGAGAAGGTACTTT
GCTTTGTTCCCTTCCATCAGCAGTTCCATTAGAAACGGGTAGCGCATAATTTGTACTCCC
AGATGGCACACCAAAGCTCATACTCAGAGATGGTTGAGCATGTGATTCATGCATATCTCT
AATGTCTAACATAGATCTACTGTCTGGCTTGGCAAGTTTAGATAATCCAGCAGATGGTTG
ATTGGCACTTGAAAAGCCTGTGCTAACTTCACCAACAGGTGTCCTGATCTCCTCCTGTTT
GTCTTGCCCAACAGACAAACTTGAATCGCCTATCTGAGATTTGAGGAAATTGGGTTCATT
TGCCTCCATAATTCTGCACAACTGCGTCAGCTTTCCCTCATCAGCAACTTTAGCAACCAC
CATCTTTTCAACGGCTTTGGACCGTAGACTATCAGCATCATTTATTGTTAATGCACCGAA
TTCATTACGAATTTCATCTCTCTGCATTGCATGGAGTCGAGAGCTCATTGAACAGCTGGT
ACACCCAACTCCCCCGAAATCCAATAGCTCAAGCAAAGATATTGAGGCTATACACCCACA
GTGCAGACGCTTGCAGCATAAGTAGCATTCCCTCCAACCAGGTTCCTCTAAGTGGAATGT
ATTACAAAACACAAAATTCTCATATGCAGATCCGCAATTGTAGCATAGATCAGCATATCC

ACCAGATCTCAAAGGCCAGCCTCTTTTCCATTCATGGGTAATGGCTGTTCCACATGATGC
ATTCATGCAAATTCTCGACCCCATTATTAACTAGTTGAAATCAAAAAACCCAGATCTTGA
AAATGCAACGGAAACCCATAATGATAAGTTGGTGAAGAAGAGGGTCAACTCCGGACTTGG
AAGGGAAAAAGAAGAACGGCGGGGTGGTTGTTGTATATGGGTCTCGACCCAAAAACCGTG
TTGAGTCAAGTTGAGTTGTATCTCAATTG
>tig_1264_consensus
CTCGGATTACTGGATTCCAAATTACACGTCCGCTTCATCTGAACAAGTTTCAAACCTAAC
ATATTCTGAAGTAACAAATCTACAAATAAAAGTCTTCAGCAAGACAACTGAACCATACAA
ATGAATGGAAAGACAATGAAGTGGAAAACAAATAATAATGGAACAGAGTGATATTTCTCA
TACTAAACTATTATAATAACCCCATTTATTTCTGGTGAACTGAGAAACAGATTAAAGGGG
GTCCTAACAGAATCTAAATACAGAAAAGAATGAAGAACAGATGACAAACCAACTTGAGAA
ACAAACCAAAACACATCAACACACGGAAAAACAAATACGAGGCATGGACCTTCCTTAGTC
AAAGAATTCCCAAGGGAACTTAGGTGGGGCTGAATTATTATGGGTGACGGGAGTAGTAGA
TGTGGATGTTGAGGGGATTGGCAGCAATGCAGGGCTGGAATCAAAGGTCTCCCATTTTGT
GGTAACGACAACCAATCCTGCCGTCGAATGCCTTGATTGTCGAAGTTGTTGATGCTGGTG
TTGCTCATTTATTGACAAATGTTCAAAAGAATGCCTAGCTTCCTGCTCTTGAATTGGCGC
AAGTCTATGCCCATTGGAATGCCTTGGTGGCTGCTCACGAATTGATGTTGATCTTTGGCC
ATTTGAATGCTTTTGTTGATTCTGCATTAATCTTTGAACTCCATTGCTGCTTGGCATAGA
GTTTTTTATCTTCAACATATCAAGAGTTTCAACATACTTCTGCACTCTTCTCACCTGCAT
TTTCCTCTGCAATTTTACATCTCCATCAGCCATAATACCATCCAATTTGAGCAACTGATT
CATCAACAACTCAATTAGACCAAGAACATCCGTTTCGGCCACTTGCCCTCCTTTAGTAAT
TATCGATTCAAGAGCTGACACCTGGCCTGCAAGCTGGTCAACTTCCAGGCTAATTTCGGA
GATGGATTTTGAAGCTTTCTCCATCTTGGCATTCTTTCTCATCTCAAGAAAACGTTTCTC
TTGACTAATTGGGTCCTCAACAAGCACAATTTTTGATCGGTCCTTCACACCCGCCGTGTC
CAGAAACGTCTTTGAATCTCTCTCTTTATCTTTATATATCAGCTTTTGATCCTGATGGTG
CAACCCCGTTGGTCCAGTCAACATCTTCTTCAACTCCCCAAATGTAGCTTGAGAACTTAT
ATGCATTTCATGGTAAACGGACCCGTATTTGACCCGAACTCTAATTGTCGGTGGTGGGGC
AGAGGTCCGATCAGTATCCGGGTTTCGTTTCTGTACAAGCATTCCACCTGGTCTCAGTTC
CCAATCATGAGCCAGAGGCTCAGGAGCTCCACCAGCTGAGCTACCATTCGTCATCGAGGA
AGGTGACAGCCCTGTGGTTTTAGTCTTCATCCTCATCATCTTAAAACTCTCACTTTTCAA
TATTTTATTTTCGTTGTTGTTCCGTTACAGAACAGAGTTTGACTGGAAGAAAAGAGTCTT
TAGATCTTCTATAACAGTGATTCCAGGCGATGCGTAACGAAAATCATAGATACCCAGTTC
AGAAAAAGCTCCTTAGAGTTTTAAGACCACAGCATTACACGAGCCCAAGTCCTTAAGCCA
TGCCCACGCGTACACAGACAGAGAAAACAACCTATGCGAACACAACCTTCCTTCCTTTCT
TCTTTTACCTTCCTCGCGGCACCAAATAGTGAAAAAAACAACAATCTCTGAACCGTATTT
ACCAAGAAAACCCAGCTAGAGATCGCCTCCACTGAACTTGCTAAAGCCAAAAACCGATCA
AAAGAATACTTCAGATCTCTTCAA
>tig_1265_consensus
ATTGGAAGACAATATAGTAAAAAAAAGTGATAATGAAACAGTGTAATATTTCTCATACTA
ATCACTATTGTAGTAACCCCATTTATTTCTGGAGAACTGAATAACAGATTGAAGTGGGGT
GCTAACAGAATCTAAATACAGAAAAGAATGAAGAACAGATGACAAACCAACTTGAGAGAC
AAACCCAAAGCACATCCCCACGCGACAAGACAAATACGTGGCATAGATCTTAGTCGAAGA
ATTCCCAAGGGAACTTAGGTTGGGCTGAATTATTATTGGTGACAGGAGTAGCTGTGGATG
TTGAGGGAACCGGCAGCAATGCTGGGCTAGCATCGAAGGTCTCCCATTTCGTGGTTATGA
CAACTGGTCCTGATGTTGAATGCCTTGACTGTTGATGTTGATTAGGGTATTGCTCATTGT
CATTTATTAACAAATGCTCAAAAGAATGCCTAGCTTGCTGCCCTTGAATTGGCACTAGTC
TCTGCTCATTGGAATGCCTTGGGGCTTCCTCTTGAATTAATGTTGATCTTTGACCATTTG
AATGGTTCTGTTGAATCGGCATTGATGTTCGAACTCCATTGCTACTTGGCATAGAGTTTT
TAATCTTCAACATATCCAGAGTTTCAACATACTTTTGCACTCTTCTCACCTGCATTTTTC
TCTGCAATTTTACATCTCCATCAGCCATAATACCATCCAATTTAAGCAATTGATTCATCA
ACAACTCAATTAGACCAAGAACATCTTTTTCGGCCACTTTCCCTCCTTTACTAATTATTG
ATTCAAGAGCCGACACCTGTCCAGCAAGCCGGTCAACTTCCAGACTGATTTCGGATATAG
ATTTGGAATCCTTCTCCATCTTGACATTCTTTCTCATCTCAATTAGACGTTTCTCTTGAC
TAATAGGGTCCTCAACAAGCACAATTTTTGATCGGTCTTTCACACCCGCCACGTCAAGAA
ACGCTTTTGAATCTCTCTCTTTATCTTTGTATATCAGCTTTTGATCCTGATGGTGCAATC
CTGTTGGTCCACTCAACAGCTTCTTCAACTCCCCGAATGTAGCTTGAGAGCTGATATGCA
TTTCATGGTAAATAGACCCGTATTTGACCCGAACTCTAATTGTCGGTGGCGGCGCAGAAG
TCCGATCAGAATCCGGATTTCGTTTCTGTACAAGCATTCCACCAGGTCTTAGTTCCCAGT

CACGAGCGAGAGGCTCAGGTGCTCCACCAGCTGAGCTACCATTCATCGTCGGAGAAGGTG
ACAGCCCTGTGGTTTTAGTCTTCGTCCTCATCATCTTAAACCTCAACTTTTGTATATTCT
ATTTTTGTTCTTGTCCCGTTACAGAAATGAGTTTGACTTGCAGAGAAGAGCCTTTAGATC
TTCTGCAACAGTGATTCCAGGCGATCCCTAACGAAAATCATAGATACCCAGTTCAGACAA
AGCTCGTTAGACTTGAAAGACCCCAGCATTACCCGAGTCCAAGTCCAAACAGAACCTTCT
TTACTTTCTTCTTCTCGGTACCAAATGGCGTCTCGTAAAAGAACAAAAAAAAAATCTTTG
AATCTTATTTACCAAGAAAACCCAGATAGAGATCTCCTCCCCTGAACTAGCTAAAGCCAA
AACCGATGAAAAGAATATTTTAGATCCCTTCAATGAATGCAGTCAACGTAATAAAACCGC
AAGAAAACTGAACCAAACAACGTTGGACCAGCGATTTTTCCCAAGAAAGTAACCGAAAAA
TCAATGAAAAATACAAATTTAAAAACAAAATCTCCAAGTATAACTTTTAC
>tig_1266_consensus
GTGCCAAGAATCTCGGTCCTTTGACGAGGAATGTCTATTTCTGTCCCTGCTGTGGTGTGA
AGATCGATGAGAATGATGTTTCTTTTTAGATCGCTTGTGACCATTTTCTTCCCTTTCTTC
TTCTTCTTCGTCGTCCTCTTCTTCATCCCTATTAGATCTTGATCGATACTTCCTCTTTGA
ACGTCTTTCAATATCTTCACCAAAATCTTTCATTTCAATCTTCTCATTTTTGCCAGATGT
ACGAACGTCAACTGGAGCCGAACTGCCCTCTCTTTCTTTGCCTAGGAAATTTGCAACCTC
TGCACCTGACCCAGAAAGCCCAGAATCCGGAGGTCCAACAGATAAGTTGTGCAATGTTTC
ATCTTTTGAAGGTGCTGCTTTACTTTTAATCATGGCAGCAAAAACCTTTGCCCGCTTTAA
TCTCTCAGCCTTCAACTTCTGCTCTCCTGTCAAGCTGGCTTCGGATGAGTCAGCCTCACT
TGCAGCTGCAATAGCAGCTGACTTTGCGATAGCATTTGCAACAGGGACAGGAATACTTGG
CTCCCCCTTCCGATCCAGCACCTGTTTAGAACTCTCGGGCTGAGAAGCCTGGCCACCCTC
ACTGCTAGGACCTTGACTGGAACCATTCAAAGACTTCTTCGATAGAATGTCTATATTGGG
ATTTCTAATGCCTCGTGTAGCTGCCTGAAGAATGGCAGCGGCTGCAGCTGCGTCCATGGT
GACACTGTTATTAGGTGCAGTGACTTTTGCAGGTTGTTCTTGTCCATCCTTCTTTGACTT
GCTTATTACCATCTTAAACTTCTCCTTCTTATCAGAATCATAAGGTATATCAGATCCTAA
GGATGGCATATCACTTTCTCTGGATACAGCCTTCTTCTTGTCCACCCCCAGCCCTGTTGA
ATCACGCCTATCAGAACTGGAACCCTTGCTAGGTAACTTTGACTCTTCAGATCTATTAAG
AACTTTTAGATAGTAGGGATAATACAAATTTGATGGCAGAAGGAATGGAAATCTTCCATA
TGTACGATCCTGTTCGACAAGAACGGTCTCAAACTCTTTCCCATTTCTGAGGATAAATTC
AACAATCTTATCAACCGCTCTCTTCATATCAGATGGAGGCTCCACAATAGGTAATTCAAC
CTTGGGCTTGCTTGATGAAGCAGAACTTTGCAGCTTATCAGCAGCAGGGCAAAGTGAAGC
CAAGTCACCATCTTTTTTCGTCCCACTTGCAGTCCTAACCTTTACCGCACTAACAGATTG
GTTTCTTTTTATCAAAGGAGCTTTTTCTTTTGAAGAAGGAAGTGAATGCTTAGAAACTTT
CTCGTCTCTCCTAGACACATTTCCAGAAGACTCTGTCTGTTCGGAACCCTTTGAACTGGT
TGCATTAACAGAATCAGAAGCACCCCCAGCTTCATTTCTTTTAAGTTCCAGGGTATCCTC
AGCTCCACCATCCTCATCCTCTCCAGAACCATACACAGAACCAAGCAAAGACAATGCACC
ACCTCCTCCAACATCACTGGTTTGATCAAGCCCACTTTCATTTTTGTTTTCTTCACTCAG
AAGATCTGAGTTATCAACAAGAAACCTATAGTATGAATGCAGATGATGATCAGGCATCAA
GAACCCAAATGTTGGGTTGTCTCCCTGTTTGACTCTCAAAACTATTTCAGATTGCCCACC
ATGCTTGCTTACAAACATAGCAGTCCTAGCAATGATTTGATGTACCTTCTCTGTCGGTGG
C
>tig_1267_consensus
AAATTACGAATGATGACAAATATTGTCGCTCTAGTTTGACCTTTTGACTTAAAACTTTTT
CCATTCATGGCTTCTAATTTTATGGTAATAGATTTCCAATTTGGCACGAAAAACATCCAT
CTAAAATGGAAGGGTAGGTAAAGTGATTCCTTCAGTCAGTTGGTAGCTAGTGAAACAGCT
TCATCACCATCTCCAATATTTTCAGTCATCTTTTTTGCAAGTTTATTGAGATTTGTTTGT
TTGGAGTCACTATCACTACAACAATGGTCCCATTGATATCTCTGATAATTTTAACAGACA
CATTTTCTTGGGTTGACAACGAAAGGACAAGAGTGTGAGAGCTAAAAGTAACAAATTTTA
CCTTTAAGATGCCTATAGAGACTACATTTCAAGATGTCCAAGCCATCCTCTTAACATCTT
TGAATAACTTTATTATCTCAACATTATTTGGCTCCTCTAGAGCTATATAATACAGCATCA
GAGTTGTAACGGAGCACCCACCATNNCCATACAGTTTTGACCATTCCTCCAATCTTGAAT
AAATGTACTTGGATCCCAGAGAGTCCGCCCAGAACATGATACCTCCCCTGTAAGGTGGAA
AGCCCATGCCCATAACAGCAGAAACATCGAGATCAGCAGCTTTGACTGCAATTCTTTCGG
CATATACTCGACAGGCCTCATTAACAACTGGAAAGAATATCATCTCCACAATGTCCTTCT
CTGATAATTTAGAAAACTTTGGGTCAATGATAACACCAGAAATGCTCCGAGCCTTCTCAA
TGTATTTCTTTAGTTCAGGATCTGGTCTAGCTTTGCGTTTATCATCATACAAGTAAAATC
CTTTGCGGGTACTTTCACCTGCTCTCTTATCCTCTTGCATGATTGGGATAATCATTGATC
TATAGGTCCTTTCAGGGAAATTCTCAATAAATTGCGTGCCAGTTGCAATTGCTACTCCAA

AACCAACAAGGTCAGCCAATCTGAAAGGACCCATTGGCATTCCAAATTTGGTAATCACCC
TATCAATCAGATACGGATCGGTTCCGCGTTCAACAAGCAACAAAGCAGCTTGTGTGTAAG
GGAAGAACATCCTGTTGACAGCAAAGCCTGTGCAATTACCAACCACAACTGGAGTCTTCT
TTATCTTTTTACCAACATCTAGTAAGTCCACAATCACTTGGGGAGAAGTCTTATTGGTAC
GAACGATTTCCAAAAGTGGCATGACATGAGCTGGACTAAAGAAATGGGCACCAATGATCC
TATCTTGGGACTTGGTCCGTTCACCAATCAAATTCAAGTCAATTGTTGAAGTGTTACTTG
CAAGTATGCAATGTGGTGGACAATATTTTTCAAGATCAGCAAAAATTTGTTGCTTCAAAG
AGACATTTTCAATAACGGCCTCAATGACCATATCAACATCTTTGAAGTTCTTGTAGTCCA
AAGTACCCTTAAGGAGAGACATGGTCTTGTCAAACTTTTCCTGAGTCATTTTTCCTTTTT
TGAGACGGCTTTGCAGATTGGCTCTGACTCTATTGATCCCAGCCTCCAAGAACTTCTCAT
TTACTTCTTTCAGAATCACAGGATAATTACTAAGAATCAATGCAGTGGCTATTCCAGAGC
CCATTAATCCTCCACCAAGAATTGCGACCTTATTCACTTGTCGTGGCCTCAAGCCAAGAT
CAGTTACCCCAGGTACCTTTGAAGTTCCACGTTGAGCAAAGAAGATATGAACCAAACTTT
TGCATGTGTCAGAATGTACAAGTTCTTGGAAAGCTTCAGCTTCCTTCAAAAGTCCAGCCC
GAGGACCAGAAACCACACCCTCTTCAACAACATTAATGCAGACTATTGGGTGTTTGAGAT
TTGGAGCCTGCTTCCTGGCCTGAGCCCTTGCAAACTTGAATATTTCCCTTGCCTCCCCAA
GGGGCTCTAACTTATCAGTCTTGTAAAGAGTGGCAACCCATGGCCTTCTAAGTTCCAAGA
TATCCAGGGCCCATTGGCGTGCCGTGCTTATCAACTGATTGGGTGCAACAATGGCATCTA
CAAGGCCCAAAGTATGAGCTTCCTCCCCTTTAACTGGTTTTGAAGTCAACATCATTTCAA
GGGACTTTGTAATACCAACAAGACGGGGAAGTCGCTGTGTTCCTCCAAATCCAGGAATGA
TTCCTAGCTGAAGTTCAGGAAGGCCTAACTGTGCAGTAGGGGTCGATATTCGTGCATGGC
AGACCATAGCAACCTCTAAACCTCCACCCAAGGCAAGTCCCTCAATGGCAGCAACTGAGG
GTTTTCTTGCAGCTTCCAGAGTGTCAGTTAGAACATCTTTTGATATAGAACCACCGGACT
GCTCCGCTTTTCCTTCTTGTACTCCAGAAAAACCAGAAATGTCAAAGCCACCAGAAAACT
TACCCATAGCTCCTGTAACAACAATTGCCTTCACGTCATCTCTGCTTAAGGCTTGTTCAT
AACTCTCTTTCAGGCTCCTTAACACGTCAATAGAGAGAGAATTGACAGGAGGATTGATAA
TGGTGATGAGAGCAACCCCATCAGCTCCAACCTCAATCGTTGTTCTTCCCTTACCCATCT
GGATTTCTCTTTGCTGTAATTTTTACTCTTCAAGAATTTCTTCTGTTGGGTCCAAATCGA
CGAGATTCAGAAGCCTCAACCCTTGTTGGTGTAAACTATGCACCAGTTTCGTTCGCCAGA
AGAGAAACAGCTACCGGGCTTAAGGAGGTTCC
>tig_1268_consensus
CTCGAATAAATATTCAGTTTGAACAAACCAAATTTGAACAAAGATAGCCTCAACTCAAAA
TCCAATCAAGTCAGTTCGACTCAACTGAGTTTGTCAAAATATCGCAATAACTTTTCATCC
TGTTTGACAATTTTTTGTTCTTCTCTAACAATTACTCACCTCCACCTTGCCAGCTCTTCT
TCACAAACGGCTCCTCTAGCACACTAAATCTTAAATTATCCATTCCAGATTTGGAGCCAA
GTTTGAGCTAACCTTGTTTATGACTTAAAGCTTTCTCTATCCTAATGGTATATGATTGCT
CACTATCACACTTCTCTGCAAGAAAAACCCGATATTAGTTATTCTTTAGAATCCTGCAGA
TTGACAGTATGGCCATGACCAAATCTTCGGAATACAATTCAAATTTCCAGACAAAGACAT
TCCAGTTCACTACACAACGTAAGTCTAGAAACAGAGCATATTAAGACACACCAACACAAA
AGTTCCGGATTTTATTAAGTAGCACAAACACTGGCAACGCATATGAGCACAGGCCGCACA
GCACAAACACAAGCA
>tig_1269_consensus
GCTATTTCCATATTCAATTAATTCACAGCCAAAAATGCTGCATTTTACTTAAGTCATTTC
CCGTAATTTTACCAATATTGGAAAAAGAGAAAGTCAAAAATTTTAGCTAGATTTTCGCCA
TTAAAAATAATTGTAACTTCGTACCCATTTTTTCCTAGGCATTTATGAACATACTTTCAT
AACTCTTGTCAGCCTGAGTTGGAGATTAATCAGCATCAAAGCCTCCGTCTCCATCCCCGT
CTCCGTCTCCAAAGTCATTATCATCTGCCTGCCCTTGGTCCGCGTCTCCCATATTAGCAT
AGAGTGCATTAGTTTCATTATGGTGTTGCCCATCAACATCCATTGGTTGGGCTCCCCTAT
CTTCAACCTTCGGCCTTGGCTTCATAGACTCCAAATTTTCATCGCTGAAAAACAAAGAAT
ATGGCTTCGACACCTTAAATAGACAGTCTCGCTTCTCAGCACTCGTCATTAGGTTCTTCC
AGTGCTCATCATGCTTCAACGTCGACTTAGAACCTACCACCAGTATAGAAGACTTTGCTC
TGGTAATCCCAACATTCATCCGACGAAAATCAGAGACAAATCCTATACTTTTATTTTCAT
TTGCCCTAACACATGAGAAGATTGCTACATCTTTCTCCCGTCCCTGGCAACCATCAACTG
TAGTTATATCAACTACCTTCTGTGACTCCATCCCAAAAGTTTCCTTGAATCGTTCTTGGA
ATTGCTTCACTTGATATCGATAGGGGGATATAATGGCCAGCTGAGAACTTGACTTAAGCT
GAGGAAACATACTCACCAACTTATGATAAAGAAGGAGAACAAAATCAACCTCATCATAAT
TTACCCAAGATCCACTTCCAGAGGGCTGAGACTCTTTCCCTTCATGTATGTCAAAGAAGC
AAAAAGGCCCAAAGCAGCGGTATTTGTGCCAGTCACGAGATGTATAATCCTCAATACCAG

ACCCATCTTCCAAAGCCTCATCATAGAACTCCCTTGAAGGAAAGCTTCGTATCTCTGGAT
GCATTCTGTACTGGGTCTTCAGCATCTTTACAGGATAACCAGCCCTTTGTAGTCTCTTGA
ACAAACTTGTGCCATATCCCAAACTCTCTGCTATAGGAGAAATTACAGTGGCCGGCAATT
GAACTGGATCACCAACCAGAAATACTTGTTTGCATCCGGTTGTCAAAGGAACTAGAGTTG
CAGGTTCCACAGCTTGGGCAGCTTCATCAATTATAACAACATCAAAACCATGATTCAATT
TGCTGAGAAGAGCTGAACCACTGAAGCTAAGGGTAGAGCAGACGATCACAGCCTCATTCA
GAATTGCAAAACGGATACTATCCCTATCCTTTCTTGTGGATCCATGTTTCTGTTTATCAG
CAGCAGAGTCATCACGTTTTTGTTCCACCAGGTGATCAATGGATACTGAATTGACAGAAT
GATGTGCTTTAAGACCAATCCGCACAATTTTAGGAGTATATGAGCGAACATTTTCATCTC
GAATCCCAGTATTTAGAAGGCGTATTACAATTTCATCAAGTGCAGAGTTCGATGGGGCAC
ATACCAGCACCCGTACACGATATTTGCGACTAGAATTAACTACTTCTGGTTTTAGTTCAT
TTCCAGTGGTAGGCAAAAAACCATCATCACCATCAATAGGCATAATATTATCTCTGGGAT
TAACACCAACTAACCATGGGGACGCCTGTCCCCAGTGATAGAATTTTTCCTGAACGGTTA
GTTCTGGACCACGCTGTACTTCACGCAAAACACCTCTGGACTGCATTCTAGCTGGTGTTG
CATGCAAAATGGCACTGAGAAGTCCAAGTATGGTCTGTGTTTTTCCTGTTCCTGGGGGGC
CCTGTATCAAGACAAAGGCTTTTCGAGATAGACCTTCATATATCGCCCCTTGTTGGGAGA
CATTATGATTTTCTTTAATATACTCATCCAGAGGTCCAGGAATTCTCCAACTCTGGGACT
GAGCAGAAGTTTTCTCAGTAGCTGCTAATATCAAATCCTTAAAAGGGAGAGAACCAATTG
ACCTTAGAGCAATATATTCCCGGGCAACAGTTGATAAGCTGCAAATCTTCAGACTGAACA
AACATTTCTCTACTGCTTTGACAGATGAAGTGATAAGAGAGCACATATTCAATAGCCTTT
CAGATTTAACAGCATCTTTATTTATATGTGTGACCTCTCCAGCTAAGTACATTCTAAGCC
TTAGAAGATTTGGTTGGGTATGCTCCACCAGTGCAAATGCATAGGCTGTTGGCAATTTTG
AACCTTCCTTGAACTCTCCTCTTTTGAAAGCAGTAAGAGATCATTTGGTGATATCGGCTC
TTCTTCCTCAGCATTGTAAGTAACAGCAGGTAAGTGAAACCCATCAGCCTCACCACATTC
CATGACCAGCCTAAGTTTCCAATCTCGCACTTCTTCGTCATCTTTCTTTTGAATAATCTG
AGCTTTAACTTCTTCAAACAAAAGGGGCTCATATGTAGCCAAATAATCATCCACATCCCT
GTAAATGTCCTTCACTTCTTTCAAACCCAATGCAGATGCATCAACCTTTTTACTATTTTT
GTTATTTTTCAAGAACAGTTTTGCAGAAACAGTATATTGAAGCTTCATCCTGAGGCTTGT
CTTTGTCAGCTGCCATCTCTGCAGACTTCAAAGGCTTGTCAATATTCTCATGATCCTTTC
TAATTGCATGTGTTCTAATCTGAAAAAGACGAGAAGAAAGAGAGACGTCGAGAAGAAGAA
GAGATGAGCAACGACGAGAGGAAGAAGAGATGGGTGAGCAGTGACGACACTAGGATTAGA
TTTTTATTTTTAATTTTTTTCTCTGCGTTTTTTGGGTGATGTGCGAATCGGTGGTCGGCC
GGTAGCTGGCTCGCTAGCACGGTGGCCTTGCTTTGCTTCTGTAATTTGG
>tig_1270_consensus
GGAGAAAATTTTTTCTTGTAAAAACAGACATTAATATATTTTACTCACTCTTACCCATTA
CCATCTCTATTGGTAATGGTTTACCAGTGTCAACAAATATTAAGCAACAAATATTATAAA
AGACATTTTCTTATATAGACAATTATAAGAATTTTGTATACAAGGTCGGATAAAATTGCA
ATGCCAAGGCTTTGACAGTCGACTTTCACTTGGTCTGAGCCTAGCTTTTTAAGTTCAACT
TTCGAGTAAGGTGATAATCAATGCAGAATTGACGCACAAGTAAACCTGAAAAAACTCAGC
TCTGACCAAGTGAGGTGATATGCAGATGAGATCATTTTCGTCATAGAACTTTTACAAATA
TTATTTAAAGGATGATAAGCAATGTAATAATAAAATCCATAATCAATTGAACTAACCAAA
ATAAACTCAATGAAACAGTTCAACATCCCACAATTAAAATTTAAAAAAATTCTGAAATGT
TTAATAGCAGAATGTCAGCCTGTCACATGCCAGTAGCATCCAAGTACTCTCAAAATCAAC
CAAAACTAAAAATTACCTCAACCTATAAAAGATATCCAGCACTGACATTACTTAATTCAC
CAAAAGCCAACACCTGTGAACTGTATCTAAGCAGCAATTGATGGCTTTGAGGCAGCAGAG
AGCCTTGATCTTTTCTTTGCCAGACTCTCACTGCGCCGCTCCCGTTGCTCCTTCAACCTG
GTAGCAAGGAGCTTCTGATACTCAGCTGCGTCAGTCTTAGCCTTTGCGATTCTCTTCTTC
TTTTCAGCAATTCTTGCACGCTTCCTTTGCAGGGTCAAGGGAGTTACCAGCCTCTGAATT
TTAGGAGCCTTGCTAACCTTCTTGCCAGTTTTTGTTGTAAAGGTCCTACGATAAGTGTTA
ACATACTTCCTGACATCATCCTCCTTGGACAAGTTGAACAACTTTCTGATCTTTGATGCT
CTCTTTGGACCTCTCATTCTAGGCTTCTCAGTATCGGTGAGTCCAGGTAAGTCCTGCTCA
CCCTTCTTCACAATGACCAAATTCAACACTGACAGATCTTGACTGACAATGCATCCACGC
ACAGACTTTCTCCTACGCTCTCCATCACGCCTGCCATATCCCCGGAAGCAAGGGGTACCT
CTGTGGAGCAAAAGACGAACTCGGCCAGGAGTGAGAACTCCCTGCTTCATAGGGAAGCCT
TGCTTGTCACAACCTCCCATGATCTTGAATACATAGCCCTTAAACTCCTCACCAAGAGCA
TCTCCGCTAACTTCCTGAGATATTCTCTTGTCAAAGAAAGCTCGGAGTTTCTGGTCATCG
TCGATTTCGAGCTTCTTCTGGCATCCAGTAGTAGGATTTGCAATATTGAACTTCATATTG
TTGATGGAGTCTGTCGCCCGCGGCTAGACGAGAATGCTAACGCTCTGCTGCTCAAACGAC

TCCTTGAGGAGGAACTAGGGTTTTTGGGAGTCCCTTATATA
>tig_1271_consensus
CGTACAGGTTCATAACCTTTGTCTAATCCATCAAAAGTATATTCAATTAGCTTTTCAATT
TCTAATGGTAAATCAATATTGGAATCAACAATTAAAGGTTCCAAAAGGAAATCTTTTTGT
AACAATTTCGTGTCCATGGCCTTATGTCCCATGAATAGGGTTTCCAACTGGTATTCTCCT
GCAACATCACCTATCTTCTCATTCAAATTATTAGCATCAATAGACACTTCAAAATCTTTC
TCAACAGGCTTAGCCCTTACTTTAGCAGACTTTCTAACCAGAAAGCGAACAACAGAATCT
TTGTTTTTGCATAAATCAGTTATGAGTCTCTTATCTTCAAGCTCCTCACCATCACATATC
AGTTCTTGATCCTTGAGATCAACAAGCCCTTTTCCCTTTTTAGCAATCTGCTGCTTCACA
TAACCTACATTTCTACTCCTATCGACATGAAATTCAAACACCTTACCACATACAGTCGTC
ACAGTGATGGCTTGCAGATCTGAAAGTCTAAGCACCAAATGCAAAACATTCCCATCAGCA
AGCCCATAGTCTTTGACACGAGAATTGGTCCTAGCCAGTTCCCTCCCTCCATATACCAGT
TTCTGCTTCTTCACAAAAGGCCCTTTGAAACTCTGAATCCTCATTTTCACAGAAGCAATA
GAATCTGACTCCATAACACGCATGGGAATCACAGATCCCCCAACACTCAGAAAAATAAAG
ATTGAGTCATCCGCAGACAGACCATGTCGATGGGTAACATTCCCTGGAAAGCTCAAGGAT
TCTTCAAGATACGGACTAAGAGCTACACTAGCTATTGACATTTTATCTTGTGATCAAGAA
CCTTGCGCACCGTATATCAGATTACAACTTCTATCTACAATGTCTAAACCCCTAGGCTCT
TGTTACTTTGTCATCCATCTTTAATGTCCAAAATTATTCGCAAAGGGCTAAGGGTTTATG
TAGCTTCACTGCAAATACCCTCTGGGAAGCCGAAATAGAAAGAATAAATTGAA
>tig_1272_consensus
TAAAAACTGATATCTATAAATTAAAAGTAGACAACATCCTTCAAACAATATTCTCATAAA
ACTTGTCCCAAAACATGAAAGCCAACTCAATCTATTGTAAGGTACCAAAATGTGCCCAAA
ATCATCCTTTTAACTGCTTAAGCAGAGGTGGCTGCTCCCTTGTTCCTTGGCGCTGCTTGG
GTGCCTTCTCTGAAGCCACTCTTGAACCTGCGGGGAACATGGCGGAGATACCTCATTCTG
CCTGTGCCAGTGGTCTTTCTTCGGATAGCCTTCACACTCCAGTTATACTTTCTCTTGCGA
GCAGCAGGGAACCCACAAGCGGCGCAACGGCTCTTCTGAAGATGGAAGCTACGACGGCCA
CATCGCACACACAAAGTGTGTGTCTTGTTCCTCCTTTTACCAAAACTCCCAGTACCCTTA
CCCATTGAACCTGAGACGAGTGCGAGGAATAGGCTTCTGTTGAGTTTAAAGAGTGGCGTA
GACAGAAGGTTTATATGATGAGGCTTTAAGGGTTTTTTAGGGTTGAATTGTTAGTG
>tig_1273_consensus
TGGAAAAGTCGAGGGTAGATCTGAACAGAACGGTGTAATACCGAAGAAGACAAAGTCAGA
AAAAAGAAATCACCCCGAATAAGAAAAATTATTAAATTAAAAAAAAAAAAAAACCTACAA
CAGAGAACCGTCAATAAAGACAAAAACTCATTACCCAATAATCTAAAATTTCAATTAGAA
TTCTTCAAACCCCACAGGGTTTCAAGCCCGATTCCGCTTGACTTGAATCCGCCCCTACAA
CAGATCACCGATATATTTTCGAACGACAGAAAAGTCTCTGTGTTTGGATGTCATAGCAAG
TACACAGCAAAATGTCAATGAAAAACATACCAGGCAAAGTGAAGTTGAATTTGAGATCAT
TTAAAATTGAAAAAGCATCCGATTGTTCTCTGAAATTATCCAAAAAGAAATATACATGGG
CAGGGCCTCAAGTTCTAATATTCCTCCGCCACACTTTTTGGCAATAACATTAACTTTCGC
AACAAGAAAAATAGGGAATTGTAGCTCCTGAAGTAGAAGAAAAACACTTTGGTCCCATCA
AAAAAATGAACATCATAATCATCATCATCATCATGTTTTATGACCCTGTTGCTGGAATAT
CACCCAGTACCACCTAATTAAAATTATTTGATATTTTGCAATAATCAAGAAACTGGCAAA
AGGAGGAAGGGGAATTTGTTTAGGCAGTAGATTCCTTGGCAATCTTATCAGCCTTGGCAA
TCACCTCGTCAATGCCTCCAACCATGTAAAATGACTGCTCTGGTAGGTCATCATATTTTC
CATCCAACACTCCCTGGAAACTGGCAATGCTCTCCTTCAAATCAACATACTTGCCAGGAG
CACCTGTGAAAACCTCTGCCACATGGAAAGGCTGACTCAAGAACCGTTGAATCTTACGAG
CACGAGCGACAACCAACTTGTCATCTTCACTGAGCTCATCCATACCCAAAATGGCAATAA
TATCTTGCAAATTCTTGTAGTTCTGAAGAACCTTCTGGACACCACGAGCAGTGTTGTAGT
GTTCCTCACCCAAAACAAGAGGGGAAAGCATACGAGATGTAGAATCTAGAGGATCGACAG
CAGGATAGATACCAAGCTCAGAGATCTGACGTGACAACACAGTTGTGGCATCCAAGTGAG
CAAAAGTGGTAGCAGGAGCAGGATCTGTCAAGTCATCAGCAGGCACATAGATAGCTTGGA
CAGAAGTGATGGAACCTTTCCTGGTGGTTGTAATACGCTCCTGAAGACCTCCAAGATCTG
TGGCCAAAGTTGGTTGGTAACCGACAGCAGATGGGATACGTCCAAGCAAAGCAGACACCT
CAGAGTTAGCTTGAGTGAAACGGAAAATATTGTCAATAAAGAGAAGCACATCCTGCCCCT
CAGCATCACGGAAGTGCTCAGCCACAGTAAGCCCAGTTAGACCAACTCGGGCACGAGCAC
CAGGGGACTCATTCATTTGACCATAGACAAGTGCACATTTGCTCTCAGCCTGCTTATCCC
CTAGCTTAATGACGCCACTTTCAATCATTTCTCTGTACAAGTCATTACCCTCACGAGTCC
GTTCCCCAACACCAGCAAACACTGAGAAACCACCATGAGCTTTTGCAACGTTGTTGATAA
GTTCCATAATAAGCACAGTTTTTCCGACACCAGCACCACCAAAAAGCCCGATCTTTCCTC

CTTTCTGGTATGGAGCTAGGAGATCAACAACCTTGATACCAGTAACAAGGATCTGCTGTT
CAGTTGCTTGCTCAGCAAATGTGGGAGCTTCTCTGTGGATTGGCAAGAAGTGGTCCGTTT
TGATTTCGCCTTTCTCATCAATAGGCTCTCCGAGGACATTCATGATACGGCCAAGGGTAG
CTCTACCAACAGGAATAGTGATAGGCGATCCCGTGTTGAGCACGCGCTGGCCTCTGACCA
ACCCTTCAGTTCCATCCATAGCAATAGTCCGAACCGTTCCTCCTCCCAAGTGCTGCGCCA
CTTCTAGCACCAATCTAACAGAGTGGTCGACGACTTCAAGCGCCGTCATGATCGGCGGAA
TGCCCTCTTCGAACTTCACATCGACGACGGCACCGATAACCTGACACACCTTCCCGATGG
CTCCCTTGCCAGTGAACTCATCGGTAATCTTCCCTCCGCCGCCCTTCTTGGCGTCGGATT
TGGGAGGAGCCAGAGTGGCCGCGGGAGCAGAGGATGTCGCGTACTCGGCCACGCGCGATA
AGAGGAAGTTCGAGGCAGGAGCCACCGCACGGCGCGTGACCGAAGGTGAAGTGAGAGATG
AGTGAGAGAAGGGGGATTTGGATGATGATCGGCCGAAAGAAGAGCGGAGAAGCGAGGAGG
CGAGTCTGCGAGAAGCCATCTGTCAGGCCTCGATAGAGACAAAGAGATACGGAGACTGAA
GAATTGAAGAGTTAGGGTTTCGTCTTCTGTGTGAAAGTGGCCCTTTGTATAAGCTGAGGT
AGGG
>tig_1274_consensus
GAAGTTGAATCTGAGATGAATTGAATCGTTCTTTGAAAATATCCAAAAAGAAGTATACAT
GCCGGTCGTCTATTTGGTTTGGTTTTTCCCTTATTAAATGAGAAGAAAATGAAACGCAGG
GGGCCAAGGCAAGGCTTCAAGTTGTGTTCCTCTTCCACACTTGCGGATAACATTAACATT
CAAAACAAGAAACAGGAAATTTCAGCTCCGGAAGAAGAAGAAGAAACAGTTTGGTCTCAT
CAAAAAAATGATCATCATCCTGTTGCTGGAATATCGCCCAGCACCACCTAGTTAAAATTA
TTTCATTTTTTGCAATAATCGAGAAACTGGAAAAGAGGGGTGAAGGAAGAAGAGTGATTT
GTTTAGGCAGTAGCTTCCTTGGCAATCTTATCAGCCTTGGCAATCACCTCGTCGATGCCT
CCAACCATGTAAAATGACTGTTCTGGGAGATCATCGTATTTTCCATCCAAAACTCCCTGG
AAGCTGGCGATGCTCTCCTTCAAGTCGACATACTTGCCTGGGGCACCTGTGAAAACCTCT
GCCACATGGAAAGGCTGACTCAAGAACCGTTGAATCTTACGAGCCCGAGCAACCACCAAC
TTGTCATCTTCACTGAGCTCATCCATACCCAAAATGGCAATAATATCTTGCAGATTCTTG
TAGTTTTGAAGAACCTTTTGCACACCACGAGCAGTGTTATAGTGCTCCTCACCCAAAACG
AGAGGGGAAAGCATACGAGAAGTAGAATCAAGAGGATCAACAGCAGGATAAATACCAAGC
TCAGAAATCTGACGTGACAACACAGTTGTGGCATCCAAGTGAGCAAAAGTGGTGGCAGGA
GCAGGATCTGTCAAGTCATCAGCAGGCACATAGATAGCTTGGACAGAAGTGATGGATCCT
TTCCTGGTGGTTGTAATACGCTCCTGAAGACCTCCAAGATCTGTAGCCAAAGTTGGTTGG
TAACCAACAGCAGATGGGATACGTCCAAGCAAAGCAGACACCTCAGAGTTAGCCTGAGTG
AAACGGAAAATATTGTCAATGAAGAGAAGCACATCCTGCCCCTCAGCATCACGGAAGTGC
TCAGCAACAGTAAGCCCAGTGAGACCAACACGGGCACGGGCACCAGGGGACTCATTCATT
TGACCATAAACAAGAGCACATTTGCTCTCAGCCTGCTTATCCCCTAGCTTAATGACACCA
CTTTCAATCATTTCTCTGTACAAGTCATTACCCTCACGAGTACGTTCTCCAACACCAGCA
AACACAGAGAAACCACCATGAGCTTTAGCAACATTGTTGATAAGTTCCATAATAAGCACA
GTTTTTCCAACACCAGCACCACCAAAAAGCCCAATCTTTCCTCCCTTTTGGTATGGAGCT
AGGAGATCAACAACCTTGATACCAGTAACAAGGATCTGCTGTTCAGTTGCTTGCTCAGCA
AATGTGGGAGCTTCTCTGTGAATGGGCAAGAAGTGATCGGTTTTGATTTCGCCTTTTTCG
TCAATAGGCTCTCCAAGAACATTCATGATACGGCCAAGAGTAGCCCTACCAACAGGAATA
GTGATAGGAGAGCCAGTGTTGAGCACGCGCTGGCCTCTAACCAACCCTTCAGTTCCATCC
ATAGCAATAGTCCGAACCGTCCCTCCTCCCAAGTGCTGCGCCACTTCAAGCACCAACCTA
ACGGAGTGGTCCACGACTTCAAGAGCCGTCATAATTGGTGGGATGCCCTCATCGAACTTC
ACATCGACAACGGCACCAATGACCTGACACACCTTCCCGATCGCGCCCTTCCCGGTGAAC
TCGTCGGTAATCTTTCCACCGTCTCCCTTCTTGACATCGGATTTGGGGGGAACCGGAGTG
GCCGCAGCGGCAGAGGAGGTCGCGTATTCCGCTACGCGCGAGAGGAGGAAGTTAGAGGCG
GAAATAGCAGTGCGGCGCGTGGGGGACGGTGAGGTAAGGGCGGAGTGGGAGAATGGGGAT
TTGGATGAAGATCGGCGGAGAGAGGAGCGGAGAAGCGAGGAAGCGAGCCTGCGAGAAGCC
ATCTGTTAGGGCTTCGGATGTACAGAAAGAGTGAGAGAAGAGTTGAACTTTTTAGGGTTT
GGTTTTTATGTAAAAGTGGCTTTATTCTGTGCAAGTTGCGAGAAGGTGAAGTACAACGGT
GTCGTTTCATGTTCCC
>tig_1275_consensus
CATGTAAACATCGAAATCGATTTTCTAAAAAATAATATGTATTGTATTATAACACTTCGA
CAAACAATATGGAATCAGAAAAAAAGAAAAAAAAAAAAAACACATGTTTCAACAGAGAAAC
ACTTAATTTATTTGCTACAAATTGAAACCAAAGCAGCTAGCAAGCAAGCAAGCTAGCTGT
TTGCTTTTTATTCTAGCATGACACAGTAATATAAGGTACTTACGACGTGGAAAGCGATGC
CCCTGAAATCATAAGGGCATCAATTAATTATGTTTATTGAGAGGACAATTGAAGTAGCAG

AAGCATTTCCATTTCCCATCTCTTACATGACAAGCTCCATGCTTAGCGTCTTCCCATTTC
TGGCACTGGGTGTCACAATGCCCCGTGTTCCCGCATTTCCCTGACCATGTTTGACTTGGC
TTCTCACAAAGGTTTGCTTCCACAGGCATCATCTCCCAGTTAGCAGCGAGGATGAGGATG
AAGAAGAGTGCCAAAAAGGGGAGAGACTTTAGAGTCTTGGCCATGCCTTAGCTAAAGTTT
TGCAATTCTCTCTGTGTCTGTGCTAATTGGAAAACATGATTAGGTCTATTTATAGGAGAA
GTTTGCTCGCTTTCGTGATGAAGTTGAGATACTATTTGCGATAGACGAATACGACATCAC
CTGATTGGAGATGGATGATGAGCTAATTCCAATAATCTCCCAGAACTATCAGTAGAATAT
CAAAAATGGAACACACTGCATCTATATATGGGTTCCAAATTCGCACATATTGATGCATCA
TGAGGGATTGAAAAATCC
>tig_1276_consensus
GCGATGCCCCTGAAATCATAAGGGCATCAATTAATTATGTTTATTGAGAGGACAATTGAA
GTAGCAGAAGCATTTCCATTTCCCATCTCTTACATGACAAGCTCCATGCCTAGCATTCTC
CCATTTCTGGCACTGGGTGTCGCAATGCCCCGTGTTCCCGCATTTCCCTGACCATGTTTG
ACTTGGCTTCTCACAAAGGTTTGCTTCCACAGGCATCATCTCCCAGTTGGCAGCGAGGAT
GAGGATGAAGAAGAGTGC
>tig_1277_consensus
CTGGGTAAACCTGAACTAAACTGCTGGTTTTTATTGCCAGAACTGAGTTCGCTGGTTAAT
ATATACATATTATGGAACTTTAAAACTAATGTTCCTCACTGATGCTATCCTTTTCATCCC
CTTCCTCTTTAGAAGGACCATAGGCTTTGCTACAAGCCAGGAAGTACATAAAATTAGCCA
TGCTTAAACCCGCAAGAAGCCAGTAGTAGTAATCATAACGACCCTTGTTGATATTGGTTG
AGACCCAGCTCTCCTTTCCCCCACTTTTCGTAACATCGTCAACAGTGTTCATTATGAAAC
TTGACACCAAGCTTCCTGCTGACATTCCTACTCCAGCAAGACTGGAGCCTATGCTGGACA
TGGTTTTAGGCAGTTCGGAGTAAAAGAATTGTGTCTGGCCAATGACGTTGAACGCCACGG
CCAAGCCTCCCAGGACATGATGGGGCAGTAGCCACATTGAAGACATGTGCACCACAGCTT
CTGGGTTATCCAAAAATCCTTCCTTGATTGCAAGTTCTCGCCGGAATGTCTCCACGATTG
CCCATGCTGCCAAGGATGCACAAGACAAAAGAAGTCCAATTCCCATTTTCTTCTTCAAGC
TTAGATAAACTGGTTTTCCATTGATTTTAGAAGCTAGTGGGATAATTATGCGATCATAGA
GAGCAATCCATAATATTAGAGTGATGATGGTAAATATGGTGAAAGAGCCTGGAGGGATTT
CGAAGTACGGAGTGATGTGTCGCTCCATTGTGCTCAACTGGATTACTGGAAAGGTGTTTT
GAGACATAGTCACAGCCATCATAATTCCTGCCGACCAAAGGGGCATCATCTTGATTAATG
CTTTTAGCTCTTCTACTTGATCCATTGTACAAAGACTCCATGGATTTGAGGCTCTTCCAT
CTGCTGTCAAGTCTTGCTCAGGATTTCTAACAATGCAAGCTTTGTTGAGACACCTTAACT
TTTCACTTGGTTTGAGAAGCATTGACCCCTTTCTTCGGTGGTATATTTCATTGGTGGGTT
GAGACGATAACTTCATACGTCTATTTTTGTAAGAGGCAATGACGACTTGAGCAAGCCCAG
TGAGCAAGCTTGTTTTAGCCTTCAACTTGATGTAAAAGGGAGAAGCCAGTAAGAAAGAAA
CAACTGATAAGAACATCACCACAGCCGGGACTCCCAAACCAATCTTCCACCCCAAGTTAT
CTTGAATGTACACAATACCGGTTACTGCAAACATTGATGAAACTGAGACTGAAACATAGT
ACCAGCTGAAGTAGCTCTGTAAGATGCCTGCATCTTTGAAGTTGTCTCCCCTGTTTAATT
GATCAGCACCAAAGGCCAAGGAGGATGACCTTATGCCACCTGCTCCAATAGACATAAGAC
CAAAAGACGAATACAATAACAGAAGTTGGAATGTCGTCGGCGGTTCACAATTGCTACTTC
CTTGCTCACAAGGAGGAGGCCTTGCTTGTTCAAACATGGCTGTAAACCATAGAAGAACCA
TCCCCAAGAGGGTAGCTATGGAGCCGAAACCGACCATCTGGTAACGACCCGTATAAGAAT
CGGCAATAAAGGCCCCAAGAACTGGAGTAAAATTAGTGACAGCTGACCAGATGAAGAGTA
CGTTAGATGCTTGTGTGGTTTCCATGTTATATTCTCTTGACAGATACAGTGTCATGTTTG
CTATTAAACCAATACTCGCCATCCTCTCAAATGCCTCGTTAGCTATGATGAAAGGCATGG
TTCTGAGGCCACCCTTTGGGTTCAGATTATTGCCCAACAGTGGGTCAATGATCATCTTCT
TCTCCTCTGAAGCAGAAGCCCTTTGACTTCCCATCAGATTATTGCCCAACAGTGGGTC
>tig_1278_consensus
CAACGACTTTGTATAAGCATCCAATTCCAACCTTCAATGCTACAGGATTGATCTTACTCC
CCAAAAGCTGGTTCCTCGTCCGTACCCAGCTGAATTTCTTCTAAACTAGCTACAGGAACT
GACTCCTGGATGGGAGAATCAGTTTCCAGTGCTGCATTCGATGGAGTGGTATTTTCCTCA
GTTGTTGATTTTGATGAGGCCATTTTTTTTTCCTTTCTGGAATTGGAAAAAGTGCTCCTT
TCAGAAGGCAGTTTAGGAAGGGACTTCCGCTG
>tig_1279_consensus
CATGTCTAAACAGAATCAAAACTCTAGCATGAATCAAGCTTTGTTTATCTCCTATTTGTG
CTAAAAGAAAATCGCCAGAAATCCTTCCTTCCACATCAAGCCTTCCCAGTTCTGGGGGGT
TTCTTAAGAGGCGTGGTCCGTGAATTAGAACCGCTTGAAGATGCCCGGTTATCTGCTGGC
TTCAATATCTCAAAGGAACAGACAAGAGCCTCATTGACACTCAATAAAGCACCAGCATTG

TCGAATTCCCCGCCATAGTTTGGAGCTGAAAATATTGTGACTAATCTTCGTTTAGCAAAA
AACTCGTATCCATCCTCCACCACCTGATGTCCTCGGCAAATGAGATCAAGATCATTCTTT
TCCAAAAAGTCAGCAACTACATCTGCTCCAAAAGTACATGAGACACCTCGATCACTATCT
GCCCAGCCCTCAATGTTAGCATCTGGGTCAGACCATAGCAGATCACACAGGAGACCATTT
TCAGGAATATCTGTAGGCCTAGAGATTTGCCTTATTTGATCCAAATTTTGCAAATCTGGA
GAAAGACCCCCGTGCATACAAAGTATCTTCTCATCAATGAGTGCAGCCACAGGCAAGCAA
TTAAAGCAATCAGAAAATATTTTCCAAAGCCTAACATTAAATCTCCTTTTACACTCATCA
TAAAATCCATAAACCCGATTGATCTTAGGGTCTTCATGATTTCCCCTTAAGAGGTGAATT
CTGTCTGGATATCTTATTTTATATGCCAAAAGCAA
>tig_1280_consensus
TTTCTTGCATAGAAGATGTTGCATTTTGATGATTTAGAGGTTTCCTTTTCTCTTTCGTAA
CACTTCCCATTGCAATTATATCATGCTCCGCTCGTTGAAAGGCTCAGTTCCATGTCTTAA
AAGAATAAAAAACTCTAGCATGAATCAAGCTTTCTGTATCATCTCCAGAAATTTCCATAT
CAAGCCCTCCCAGTTTTAGGGGGTTTCTTAAGAGGTGTCACCCGCAAATTAGAGCCACTT
GCAGATGCTCTATTATCAGCTGGCTTCAATATCTCAAAGGAACACACAAGTGCTTCATTG
ACACTCAATAGGGCACCAGCATTGTCAAATTCCCCACCATAGTTTGGAGCTGAAAAAATT
GTGACTAATCTTCGTTTAGCAAAAAACTCATATCCATCTTCCACCACCTGATGACCTCGG
CAAATGAGATCAAGATCATTCTTTTCCAAGAAGTCAGCAACTATATCTGGTCCAAAGGTA
CATGAAACACCTCGATCGCTATCTGCCCAGCCCTCAATGTTACCATCAGGATCAGACCAT
AGCAGATCACAGAGTAGACCATTTTCAGGAATTTCAGTAGGCCTAGTGATTTGCCTTATT
TGGTCCAAATTTTGCAACTCTGGAGAAAGTCCACCATGCATACAAAGTATCTTCTCATCA
ATGAGTGC
>tig_1281_consensus
CGTTCATGTTCATTGTACTTCCAAGGTCTTTCAAAACATCTGGATTTTGACTAAACAATG
ATGTTGGCAGAGCTGAAAGTTGCAACCCTTTGTCAGTACATGAAGAAGGATGCCAATGCG
AAGTTGGGTATTTTCATTTACTTTACATAGACTCAAAGTTTACTATACTAGTTAGGTTCC
ACCGACAAATTAAAAAGAAAAGAAAAGAAAGAAAAGATCAGTGTACAACAGGTGTTTTTT
TAGATGTCTTCACTGTTTTCCCAGCAAGCAAATTAGAGTTCATAAGCTGGAAACGTAGCT
GAGGATGGTTCAATCATATCTAGAAAAAGATAAGCCATTCCTCCGATGCCTTCAAACAGT
GAATAGGGGCGATCACCTCCATGCATCTTCCCTTCTGAAATGAGTTTTTGAGCTCTATCA
AATAGAAAGCAAGCAAATGCTTTGGCCCTGTATAAGTACTCAAGATTGCCAGTCAATCGG
CAGAGCGCCAGAAAAATATAGGTGTTTCCACTGATACCATGACAGATGCCAACTCGTTTA
AGCAGACCCCGCTGCCATACAATCTCCCCTGCATCCACAGCTGCTTGCACAAATTCTTTG
TCTCCAAAAACCTGAGCTGCTTTCACAAGGGTAAGAGTGACCCCTGGAGCACCATGGCAC
CAATGCACAAGACGGTCTGATTCACTTCCTTCACTCGAAGGATAATTGCCACTGGGGAAA
CGGTTCTTTATCATGTAGCGAAGTGTATTCTTGACATCCTCCATCTCATCAGGTTTCAAT
TCCATGTCCATCAAAACATGCATAATCCCTGCCAGGCCATGGGCAGCGCCCCAGTACTTC
TTTCCATGCCATTCATACATCAATGGACATCTTCCCTTACTAGCCAATCTTCTGCCAGCC
TTTATGATTTCATCAACAACTGCCCTCATAAAGGTGGTAGATATCGTGTCTCTACCAATA
TGCTTGTTCAAGAAAGAACAAGCCCATAAGAACCCCACTCTCCCATATAATAACTCATTA
GGCAAATCACTATGGAGTTTGATCTCTTTAAACTGTGTTATGTAGCGCTTAAGTAGCCTC
TCATCACCAGCATGCTTAGCCACAACAGCACCAAGAGCGCAAACACCAGCCCGCCCACAT
ATGAATGTCACATGCCCTGAATCTTTAGAAGCAGAATCACAAGCTTTAACAATGTCAGAG
CACAATTTAAGATCATTCTCACTATTGGTGACTTGATAAGCTTTAAAGAGCAAATAAGCA
GTGCCAAGAGCACCCGTGTACAAGGTGTAATCTTTCACACGCTTTCCACTCAATCCCCAT
GTCTCATCCAAAATGGTTTGTTTGAGTTGGAGAGCTGAAGTCTTGAGTTTATCTGAGAGA
GTTGTAAATGGAAGAGAGAGGAGGTTGGAGAGAGCGTCCTCTTGTGCTGTAGCTGTAAGG
GATGCTTCTGGTGGTATTTCATTGGGAAAGAAACGATCGGCCATGGCTGAAATCTCCCTC
GAGCTGAGCTGCCTGTTCTTGTTGCTCGATGGAGCGTGAAGTTGAAGCTCTTGGAGCTTT
GCAGAAACTGCGTAGGCCCAGAAGGACGTGACATGCCACAAGACAGA
>tig_1282_consensus
GCCACTGTGAAAGCAATTATCAATACTGGCTGGCTTAAAAAATTCCATCATTTAGATATG
CTTAAAAGTAACAATTATAACTGAAATGCTTGACCATAACTCATCAGCTAAACCAAACTT
CCAAGAGTTACAACAACCTCTAAAATGGTAAAACCATATCCAACTACAGACCAAAATCTT
GTAACATTTAAATAAAAGCATTAATGACCAAAGATAAAAAGCCTTCGTACAGCGAAAACC
AGTATTCTGATCTTAGTNNNNNNNNNNNNNNNNNNNNNNNNNNNNNNNNNNNNACACCACC
ACCAGAAGGCACAGACGCCAACTTCTCCCTTCCAGATGCAATTAGCTCAGTGATATCCTT
GCCCTTAACTTCAGACAACAGCAACTCTAGCTTGTCATCATCAGCATCGGCTCCAACTGA

TCCAAGAATATCCTTCAAATCACTAGCCGTTGGGCAGGTGTTGCCTCCCAAAACAGCAAG
CAAATATGCGGCGATGACCTTCATTTTTGAGAGTGGTGGGGCGAGATGAGAAGATTGGTA
TCGACGGCAAAGGCTGAA
>tig_1283_consensus
CCTACCTTGTTTCTGTCCGTTTCAGATAAAAATCTATTTCTACTGTATCGCACGATAAAA
ATTTATATATTCCCATTCAACAATGAATACTTAAGATAGCATAACCTATGCAATAACAGA
CTTGGGGAAACAAAAACCATCTTTAAGCCAAACTCACTCCCATTTCAGAAGTAAAATGAT
TACTAATCATCCCATGAACATAACCCACATCCTCAAAATCAGGTCTATATAATGCATAAA
CTTATAAATATGAAGTTGCAAAACTGAAAAACATTGCCATTCTGCTAGTCATTAAATAAA
GCAATCACACCCTAGTCACCAAAAATCTGTTGAGGAAGATAGTACCACCAGAGACAGATG
GTGGGGCAAATCATCGCAACCTCCTAGCTTCTCTCTCAGACTCAAGAAGGGTTAGGATAT
CACCTTCTCTCACGGGTCCCTTCACGTTCCTCATGATGAACCGATTCTGATCATCCAAGA
ACTTGACTCTAACCTGAGTCACCTGCCCTCTTGAACCTGTTCGTCCCATGACTTTCACTA
CCACAGCATGCTTAATGGCTGAATCCATTCTGCATACAAAAATAACAAGAGAAAAGCAAA
ATGTTGCTCAATAAGCAATACAGCAAACAAACGAAACATCAAACTCAGATTAACAAACTT
AACAACTATTTTCGTCACAAAATTTGCAATCCACACAAACGTACCTACTTCATACTAACA
ATAGAAACCTAAGAATTAACTCAACATTCTGGGTGCTCCTTAAGCTCACTTTGGAACAAT
AAAGAAAGAAAATCTTAAGTAAACACAAGTTTTTTTGCTTCTAGAAGATTCTAGGGTCCT
GGAGGCAGCCTATTTCATTGTCAAGTTCTCTAAGCGTATACAGAAGATCAATCCATAAAA
AAGACGCTTATTATCCACATAATCTTCAGATTAAACATCAAATATTAAATGTAATTCATT
ATTTTCTCTGCAGCCAAACAAATCATTTATGAAAATAGACAAGCAAGAGTATATAATGAA
TTACATTT
>tig_1284_consensus
TTTTTTTTTTTTAAGAGAGAACAGAAAAATGAGAAGGATTCATTCATAACTTCAATAAAC
AACATAAAGAGTGTTTCTCTATCTGCTTTTTTCTCCATTTTCTTTCAAGTAGAAAACATG
TTTCATCTTAGGCAAGGAAAGGACATCTGGGCCACAAATTCTCCAGCATATAATGATCAA
GAAAATTACACACTGTAAGGAAAAGAGTCCACTTTTACAGAGCTTGTAAAGAAGAAAAAA
TTATGGAATGAATAAGGCTACAAAAGACACCTCCCCAGAATGCATTGTTGCCGATTGCTT
GCTTATCATCTCATCTTATTCCTAGTATGTATTGCATCACTGCATATAATCTTCATAGTA
ATTGTCACTTATGACAGAGACAAACCTTTCAAGCCAAGGAAGTTGATGGAGATCCTTCAT
AGCGTCTACGTAAGAGTCTGTACAGCGGGGTGGCGATTGGTATGGATGGTGATCACTCAG
GCTGAAACCTCCGGCGAGGAATCTTTGGATGAGGGCCAGCTGAGATCTCTCTTGATCGGA
GTATGCATCCATGATCATGGGGAGAGAATTGAAAGTTTCCCACGCCCATTGATCTGATTC
CTGTGCAGATTGGGAAACTAAGTCGTCTGAAGTGTCTTCATCCCAGAAGGCACATTGGCA
AGTCTGCTGGCTGTCTCTATATTCGGCACCACATGAATAACAAAATTCATGTCCGCACCA
GCAGGTCATGTGATAGCAACCTTGGGTGAGCTCAATCATCCTCCGGCACTGCTGGCAACG
CCTCCATCTTTTATTTTGTGCTAAACGATGCAAGGTAATGTCTGAAGCATCTCTCTCCTC
CAAGGGAAGGCTCTGGTACTCTTCACAGTTCATTGAAGAATGCCAAGGAACCCCACAATC
CAAACATATAAATCTTTGACAAACTGGGCACTCAACACAGCTATTATCGGATTGACTTGA
TGAACTAGCCCTAGCTGACAAACATTCCTGAGGATCAAGCAGGACTGAACAATTTGGAAA
TGGGCAATAGATTCTATCCGAGTGCAGAACATTTGCTTCTGAAAGAGCTCTCTCAAATGA
TTCATAAGAAATAAGCGGAAGAAATGATTTGCACTCAGCAGTTGAGATGCAATATTTGCA
TTTCAACTGGGGACATCTTATTGGTACCTGAGACAACTGCACTTTCCCATCGACATAGGT
CCTCATACAATGCGAACAGAACTTGTGGGAACATTTCATTGTAATCATCATTGGAGACGG
CTTGTCCTCACAGCAGATGGAACAGCTTTCAACTGATTTGTCTCCTTTGGAAGGAGAAGA
GACAACCCCAATTGCTATTTGAGCCAATTGCAATGGCCTCTCAAGTTCGACACTTGGAAC
AAGTTTTAAAACAAAAGTTTCCAGATTACTTGTGTGTTCTAGGATTCTTTGCCTCAGCGC
CATAAGAAGCGGAATGTCAAGTCTTTCCTCATTTGTCATCTGCTCATACAACAACTCGGA
ATCGGTAAATGCATACACACTGCAGATTTTATTCTGCGTAACCTCAGCCAGACCATCCAG
TAGAGCTAAATAATCAGCAACAGATTCCTCCACATAGAAATCAAGCTTTTTCTGCACCTG
AATACGTGGAAGATTAATTGATCTCTCCATCACGACACCAATTCCAGAGAGGCCAGAATT
GGACTCTCCAGCCTCAGCTATTGACATGCCCTTGAAGAACATCTTCACTGAAAATTCATC
AAGATCTTCCTTTGCCTCTTCTTCCTTCACCACCTCCACCACTTCTTCACTCTCCTTCCA
CACCTCATCATCCTCACAACAGCTTCTGAATTCCTCTTCATCTTCCTCTAATCTCACTTC
CCTCAGTGCATTCACACAAGATTCCGCATCAACTATCTCTTGATCCTCCATTTTCCACAC
CAATTTCTTTTTTTTTTTTTAAAAAAA
>tig_1285_consensus
GGATAATTACAGCTTCCTTGCTATGGAACCGTTGTTTCTGCCCTCCCTGTTCATACCCTT

TTCTCCTCTTTTGCTCTCCCTTGGAGTTTTCTGAAAAAGATTCTATTTTGGTTTAGTTTG
GAGGTCTTAAGATCAAGATTTGTTTGTGTGTGGGGTCTTGAATATTATCTATTTTTAGCG
GTTTTTGAATTGATCTTTTGGTGGGTATTTGTTAAATCTTGGTTTTAAATTAGTAGCTGA
AAGATCGGTTTTTGGGGTGATCATTTTTTTTTTTTAAATTTGTCTTTGTTGGTTGGTTTT
GTTTGAGAAATTGTTGTGGAAGATGGAGGATCAAGAGACAGTTGGTGGGGAATCTTGTGT
GAGTACAGTGAGGGAAGTGAGATTAGAAGAAGATGAAGAGGAATTTAGAAGCTGTTGCGA
GGATGATGACGTTTGGAAGGAGAGTGAAGAAGAGGCAAAGGAAGATCTTGATGAATTTTC
CGTGAAAATGTTCTTCAAAGGCATGTCAATAGCTGAGGCTGGAGAGTCTGGTTCTGGCTT
CTCTGGAATTGGTGTTGTAATGGAGAGATCGATTAATCTTCCTGTTATTCAGGTGCAGAA
AAAGCTTGATTTCTATGTGGAAGAATCTGTGGCTGATTATTTAGCTCTAATGGATGGTCT
GGCTGAGGCTATACTGAATAAAATCCGCAGCGTCTATGCTTTTACAGATTCTGAGTTGTT
ATACGAGCAGATTATAAATGAGGAGAGACTTGATATGCCGCTTCTTATGGCACTGAGGCG
AAGGACACTAGAACATGCAAGCAATCTGGAAACTTTTGTTTTAGAACTTGTTCCAAGTGT
TGATCTTGAGAGACCATTGCGACTAGCCCAAGTAGCGATTGGGGTTGTTTCTGCTCCTGC
CAAAGGAGATAAATCAGTTGAAAACTGTTCTATATGCTGCGAGGACAAGCCGTCTCCAAT
GATGATTACAATGAAATGTTCCCACAAGTTTTGTTCGCATTGTATGAGGACCTATGTTGA
TGGGAAGGTGCAGTCCTCTCAGGTCCCAATAAGATGTCCACAGGTGAAATGTAAATATTG
CATCTCTACTGCAGAGTGCAAATCATTTCTTCCTCTTATTTCATATGAATCACTGGAGAG
AGCTCTTGCAGAAGCAAATGTTCTCCACTCAGATAGAATCTACTGCCCGTTTCCAAATTG
TTCAGTCCTGCTTGATCCTCAGGAATGTTTGTCAGCTAGGGCAAGTTCATCAAGTCAGTC
TGATAATAGCTGTGTCGAGTGCCCGGTTTGTCAAAGATTTATTTGTGTGGATTGCAGGGT
TCCCTGGCATTCTTCAATGAACTGTGAAGAGTACCAGAACCTTCCTTTAGAGGAGAGAGA
TCTGTCAGACATTACCTTGCATCGTTTAGCACAAAATAAAAGATGGAGGCGTTGCCAGCA
GTGCCGGAGGATGATTGAGCTCATGCAAGGCTGCTATCACATGACCTGCTGGTGCGGACA
TGAATTTTGTTATTCTTGTGGTGCTGAATACAGGGACAGCCAGCAGACTTGCCAANNNNN
NNNNNNATCAGATCAATGGGCTTGGGAAACTTTCAATTCTTTCCCCATGATCATGGACGC
ATACTCCGAACAAGAGAGATCTCAGCTGGCCCTCATCCAACGTTTCCTTGCTGGGGGCTT
CAGCCTGAGTGATCATCATCCTTGCCAATCCCCGCCATGCTGTACAGATTCTTATGTAGA
TGCCATGAAGGATCTCCATCAACTGCCTTGGCTCGAAAGGTTTGTCTCTGTCATAAGTGA
CAATTACTATGAAGATTATATGCAGTGATGCTGATATAAGGACTAATCCAATTCTAGGAA
TAAATTTATGTATAAGTAATATGCAACAATGCATTTGATGGAAGTGTTGAGTTTCTCGCT
CTTTGTGCTCCATGATCTTTTCTTCTGTACACGAGCGTGCAAAAAATGGACGCGCTTTGTT
ATGATGTGTAATTTTCATGATGCATTTTATGCGTGTAATTTTCATAATCCTTGCATAAGA
TGATAGCTGCTTACTCAAAGAAGTAAAAAGATAGA
>tig_1286_consensus
CCTGCATCCAGAATTGCCAGTCCGCAAAAGATCTTTAATGGCTTCATGCCTTGTTAGCAA
TGTTATTGGAGAGCCATTCCAGACCTTCATAAAGACCTTCACCAGAGGTTGCACAGGTAC
TTTGAATGTACCAGTGACGCTGCCTGAGTGAGTGGAGGCCGAGTTTATCAGTAATTTCAG
CAGCATTCATTGCATTTGGAAGATCCTGTTTGTTAGCAAATACAAGCAGGACAGCATCTC
GCAGCTCATCCTCATTCAGCATCCTATGCAATTCATCTTTTGCCTCAACAATACGATCTC
TATCATTGCTATCCACCACAAATATAAGACCCTGAGTATTCTGGAAATAGTGCCTCCACA
AAGGACGAATCTTGTCCTGACCACCAACATCCCACACTGTGAAGCTAATGTTCTTATATT
CAACAGTCTCCACATTAAACCCAATAGTAGGAATTGTGGTCACAATCTCTCCCAGCTTGA
GTTTGTATAAGATGGTGGTCTTACCAGCTGCATCAAGACCAACCATAAGAATACGCATCT
CTTTCTT
>tig_1287_consensus
TCTTTGTTCATTTTGAGACAGAAAATACGGACTTCAGAAGGCACCCCACGATCATTGAAC
AAAATGCAGGTTACAGCAACAGCTATTCGATGAGGCTGGCTTCGACTTATTATTATTATT
ATAATAGTTCTGGTTTGGAAAAACATATATAGTACAAGCCACAGAGAAGGCAAGTCCTAA
AGTAAGTTTCTCTCACACTATTTATATATTACACGCATTACTCAAGTGACATTCATATAA
ATTTCCAGAACCTCACATAAGCCTCAGTAAACTGTCACATTTTTCAAGGAAGCCTTGTAA
CTCAAGCATGTTTGTTCAGGAAGGAGAGGATTAGCTGATTCACCTGATCAGGCAATTGTT
CGTGGACAAAATGGGTCCCCTCCGACAAATATACAATCTCCAAATCCGGGACAAAATCTT
TCACCTTTCCACTTCTTATGTAGTCCTCCATCCCCGGAAATCTCAAGACATAATCTTGTT
CACCCATAATCAGCAAGGCTGGAACTTTAACTATTATATCTGATATGCCAAAATCTTGTT
GAAAACTCCTGTAAGGAACTTGCAATGCAGTTTGAAATCCAGATTTTTCATACAAAGCTC
CATATTCTGCAAGATCTTCCTCTGTAAACCAAGATGGAAGAGGAACAGAAGCATCCACCA
AATCCATGATCTCCTGGTTTTCAGAAGCTATTGGTATTTCACTTCTAGAGAAAAGTATGT

ATATGTTCCTTACTACCGTCTTTGCATCAAGGCGACCAAAATCAGCTTCTGCTCGTCCAG
GTTCCTGCCATCTTGATATATAGAAGCCTTCAGGAAGAGCTTCACGGAACCGAGGGGGAC
CTGGAGGTGAAAATGGCACTCCCATTGTTACAACTCCAGCAACCCTCTCTGGATGGA
>tig_1288_consensus
TCCAGAACCTCACATAAGCCTCATTAAACTGTTACATTTTTCAAGGAAGCCTTGTAACTC
AGACATGTTTGTTCAGGAAGGTGAGGATTAGCTGATTCACCTGATCAGGCAATTGTTCGT
GNNAATCTTTCACCTTTCCACTTCTTATGTAGTCCTCCCTCCCCGGAAATCTAAAGGCAA
AATCTTGCTCACCCACGATCAGCAAGACTGGAACTTTTACTATTATATCTGAAATGCCAG
AATCTCGTTGGAGAGTCCTGTAAGGAACTTGCAATGCAGTTTGAAATCCAGACTTTTCAT
ACAAAGCTCCATATTTTGCAAGATCTTCCTCTGTAAACCAAGGTGGAAGAGGAACAGAAG
CGTCCACCAAATCCATGATCTCCGTCTTTGTATCAAGGCGGCCAAAATCAGCTTCTGCTC
GTCCAGGTTCC
>tig_1289_consensus
GCTTCTGCTCGTCCAGGTTCCTGCCATCTTGACATATAGAAGCCTTCAGGAAGAGTTTCA
CCGAGCCGAGGGGTACCTGGAGGCATAAATGGTGCTCCCATTGTTACAATTCCTGCAACC
CTCTCCGGATGGAGAAGTGCGAGGTGATAGGCCGGCCTGGCTCCAAAATCTTTTGCAACA
AGAAAACCTTATGCCCATTGAGAAAGACCAAAATTTTTAACAACCCAATTAATCTCAATC
TAACCCTTTGCCTATTTTTATTTTTTTTTCAAAAAACATTATATCTGAAATATTGTAACC
AACTATAGGCATATACAAAAAATCCACAAAGCTGAACAC
>tig_1290_consensus
CTCTCGCCAAAACATACTTGTATAGAATAGATTATTGAAACATATTCGATAGTCCGCACC
CAAAACAATGGAAGACATTCAAACAGTCTAGTTTCCAGTTTCCACTTTTAAGGAGATGCA
ATTTCCATGCAGCCCTTAATAGCAGCAAAAAAGTGTGTCTATATATTATAAGAACTTGAG
GCAGGACCTAATATAATTGATAATTTCAGTCATGCATCTTAAGCACACAGCGGAGGCAGC
CCCCTTCATGCATCAAGTGAAATGCTTTATTGATCTCCCCAAGTGCCATATTGTGAGTTA
TGTACTCATCAACCTTAATTTCCTTCTTCAAATACTTGTCTACGAGCCAAGGCACTTGTG
AACGGCTCTTGAAACCACCAAAAGCAGTTCCTTTCCAAACACGACCAGTCACCAACTGGA
ATGGCCGGGTAGATATCTCTTGCCCAGATGCTGCAACACCCACAATAACAGATGTTCCCC
AGCCCTTGTGACAGCACTCCAGAGCAGCCCTCATTACAGAGACATTTCCAATACATTCAA
AACTATAGTCAACACCACCATCAGTGAGATCAACAATTACTTGCTGAATCGGTTTATCAT
GATCCTTTGGATTTACAAATTCTGTGACTCCAAAATTTTTTGCTATATCAAACTTTTTGC
TGTCAATATCAATGCCAATTACACGTGAAGCTCCAGCTGCTTTTGCACCCTCAGCAACTG
CAAGGCCAATAGTTCCAAGGCCAAAAATAGCAACAATAGACCCAGCCTCTACTTTTGCAG
TGTTCCAAACTGCTCCAAGACCAGTAGGAACACCACAGCCAAGGAGGCACACTTTCTCTA
AAGGTGCTTGTAGATCAATCTTTGCAACACTAACATCATGGACAACTGTGTATTGGCTAA
ATGTTGAGGTTCCCATGAAATGATAAAGAGGCTTTCCATTTATTGAGAAACGACTCTTTC
TATCATTCATCATGACTCCAACACCAGTAGCACCCCTAACCTTACCGCAGAGATTTGTCT
TTCCTGACTTACAAAACTTGCACTCTCGGCATTCAGCCTGGTAACAAGGGATAACATGAT
CTCCAGGTTGAACTTCAGTCACACCTTCACCAACACTCTCCACAATTCCAGCAGCCTCAT
GACCTAGGATACAAGGGAAGAGACCCTCAGGGTCCTTGCCACTCCAAGTATAAGCATCAG
TGTGGCAGAGAGCAGTAAAGAGTATCTTGATTCGGACTTCACCAGCCTGTGGGGGAGACA
CTTGGACATCCTCTATCACTAATGGCTTGTTGGGTTCCCAGGCAACCGCGGCTTTGCATG
TGATGACTTGACCTTGAGTAGCCATGACTACCGACTGAAGTGAGAGAGACGTGTACCTTC
ATTTAACTGCAGGTTTGCCTTCGAAATTATATAGTTGTTTTTTTGGTAAGCTTGAAATTA
TG
>tig_1291_consensus
CAGTGCAGCAAAGACAGTGCCACTCTTGCTATCATAAACAACAAAAGCAAAGCTTCCTTC
AAGATCCTTAACAACCTGATCAGCAGGGTATGGACCTCTGTCTCGAAGAGTTCGATATGC
TTCAATAATAAACATTGCCTCATTGCTTCCCTTGGACAAACCATACTGCCTATTGAGTGA
GTTCAAGTTGTTCAAGCTTCCCATGAACACACAATATATATCGTCAAACCCACAAAACAA
CCTTTGATGGTGAGAGAAAGGAGTTTCAGGGCGAACATAAGCTAGAACAGCTGCATGGCC
AAAGGTGATGGAGAAAGTGTTGTAAGGGTGATGAGAGAGGAAGTCCTGGAGAGTTTCTTC
AGGAAGTTTAGGCCTCTTGGAACCACTGTGAGATGCTGGACTGTTAAGCTCCTCTGGTGG
GTGAGCAAACGCCTTGTGGAAAATTGCCAACATCTTTGAGTAAGTTAACACGAATTCGAT
AAATGATTTCAGAAGGATGTTAATGTTATT
>tig_1292_consensus
GCATTTTGAGCTGGCATAGTTCTTAATAAATCTGCTATACAATGTTGCTCTCAATATAAA
GAATATTTCTAACCAAAGCAACACGCCTAGTTGATGCTAAAGCTAAAAACTCTTCTGCTT

ACAATTTTGTAGTTCAGAACATAGTCCCCTACCAAAATTTTGGGAGCCAGAATACAGGCT
TCCTTAAATATTAATAGCAGCAGCACCTTTGGATCACTGACCTTATCTTCAAGCATCTTT
TTCTGCTAATTCCTGCTACCAGCATCACACTGTTTGCATACACATGCTAAATGACTGGCC
GGGTGTCCTGGTATCTGCCATACTGACTATGCTTAAAAAGCAATTGTAATTAACATCTCA
CTCCATGACAACAGTCCGCTTCTCCAGTAGTGACTCTAAAAACACTGAGCACAATTTGGT
AAGCGGTGGAAGATTATGTGATGACCCATTGAAAATGGTCTCCCAAGGTTTTTCAGAGGG
CGCCGATAGGGTCCATATAGCCTGATTTTTCTTCAACTGGAAGTCCGGTAGTTCCCCATA
AATAGATGCATAACCATATTGCCCTGATTCTTCAGAGGCCAGAAGATTCTCCCGGTGAAT
CACGCTAAATTCGTCTGTTTCTTTGCCATATGGATCAAAGAGGACATACTCATGATCAGC
ATTTATATAAGCAAGCAATGTTTGAGGTGGTTTCCCATCTGAAATATACTCCTGTATAGA
AGATGGGTTTACTTGAAGAAAAGCAAGAGAACCTCCCTTTGCCTTCCTCACGGACCAGGA
AGCCACCGGAACAGGATCTGTGGCATTGAAAAGTAAGATTACACCATCTCTTCCTTGGAA
TGTTGTTCCATTTAACTCCTTGCATCTAGGTGACTCAGGAAGAAGAGCAAGAGCTGCAAA
GGATGACGTGTCTACTGCACTGGCTACAGCTTCTATCTGAAGCATATATGACCAAGATGC
AGTCAGCTGTGACATACTCCAAACAGAGAGTTGTGGTTTTGAATCTCGTGACGTAGCCAC
GAGATACTCAGACTTTCCAACAAATGACAGGCTCACAATTGGCATGAGAGTCTCTCCAAT
TACTGCAACAAGCACATTTTTATCTGGGTCCCATAATGTAATTAAAGTTTCTGCTGCAAC
AGCAAGAACAGAACCATCAGCTGAAAATGCTGCTGCTGTCAATGCCTTTTTCTTGTAAGA
TCCGACAGCATGGCAACTCCAGCCCGAATTTTGAAGCATAGGATCCTTCTGCTGAAACTC
ATCATGGTAAACCCAAACCTTGAAGTTCCCACCATAAGATGAGCTAACAGCTACACAACG
GGTAGGATGGAAAGCAATGGCTGATATGCATGCATCCCCATCATAGAGGCATCTTGGGA
>tig_1293_consensus
CTTTCTTCTTCTTCCCACCACCTTTCTTTTTAGTCTTAGTGCCTAAGGCTAACCAAGCCT
TTATTTCAGGATCATCTACCGTCTTGGTGGGCTGCAACTCCTGCAGAGGGTGAGATGTGA
TCCGGTCTGAGCCATTCGGCATTAGCAAAACAGTGAATTTGATATGAGCAACATAATCAC
CAGGCTTCTCATGAAGAACAGGATATGGCTGCAGGAGGTCATGATTCACACACTCAACTA
AACCTAATCGCGCCCTTTTTTCTTCCAAAGCCCTTGCAGAGAATGGCATGATAGGAAATT
TCTGGCTAATTTCACTAAAGATAAATCTAGAAGCTTTCATCTTCAAGTGATAGTTCTTGT
CCACAGCCCTCTTATAGATGGTCGTCTGCTTCTCATCCAACAACCTAGGCTTGCCATCCC
CTGTGCTTGTAACTATATCGATTGCATAAACTTCATTCTCCTCAAATTCTGCATCATCAA
CCCGTGTTTCTGGACTGGAGACACTGAGTACAACTTTATTTCCATCTATCACAAACTGCT
TCAGTTGGTGACTAAGAACACCTTCAACAATCTTGCAATCATATGCAGCTGCAACCTTCT
GAATTGCTTCTGTTACATCCTTGTTCTTCTTTCCTGGCCTTACAAGCCTTAAGGCAACTT
CAGCAGCAGTATTTGCTGCAGCAACAACATCAGCTGCCCTGCCTGTCACAGGCCCTTCAT
GGATAACATGAGTATGTGCAACTACTGTAATAAACCCATCTATGTGGCATCCCATATCAA
TCTTCACCATATCGCCTTCTTGCAACAATGAATCGTCACTGGCAAGCGGTGAAAAATGGC
ACACTGTGTTGTTTACAGAAACGCAAGTTGGGAAAGCAACACCCCTCTCGATCTTCTTCT
TAACATTCTTGTACATGTTCCCAGCTTGCTCTCTGATAAATGAATCTCCTTTCTCACAAA
TGTCAACAATCTTAGCTCCAGGTTTACAAGCCGGTACAACCAATTGCAATGCCTTATTGA
CAATCTCAGCAGCGTTCTTGTATTTGGTGACGACCTCAGGAGAGGTGAGATCCAACTCCT
TCTCCTCCCTCTCGTCGTCCGACATCGTTTTGATTGTGCTTCAAAAATCTAACAGCCGGT
ATAAATCGACAGCGGTGGAGCTAGCTAGGGTTTAGTGATAGAGGTGAAATGGATAGAGTG
GCTGAATACAAAGAAACAGG
>tig_1294_consensus
CATTAATCACATACAGGTAAAAGCAAGGGAGATATAGGGTTTAATCTAAAAAGTGTCTAA
CCTTTTCGTTTTTGAAGCGTGTGCGGACATTGCCAAGCTCTTTATCGACGCGAAGTCTCT
CCGCCTCTTTGTTCTGGCAATTACGAATATCGCTGATGAAAACGGAGAGACCTCTCATGC
CTGACAGAGCCATCTCGTTTCAGATCCACAAGCACTCACAAAACAAACAGAAAGAGAACG
CAATCTCTCTTCAGCTTCCAATCAATCTCAGATCCTCGCTCTCCCCTCCGCTCGATCTAA
TCTAACCTAATATAATTTTGTTGCAGCGAGATCTTGATGCACAGTGAGAGATCAACTTCA
ATATTCAAAATACAAATTCAATTGGAAGCCAACTGGAAAACAATGCGAAAGTTTAAAGC
>tig_1295_consensus
ATTGATATACATTCACTTGGACAGACAGCTAAGAGCTAACATTTCCCCTGCTCTGAATTG
CAGGAGACTAAATTTTACAGCTCTGCTATTGGTTGTGTATTTTGATATTTCAATCTTATG
CCAAAAATTGTGTTAAATTGAAAAGAATAAAACTAAATCTAACAAAAATACGATATGCAG
AAATGCGAATAGCTTGTTTCACCTCTACGAAGTCAGTGAGAATACTTCTACTTCTATGCC
AGAGTTGCCTTCTTTTTGCCTTTCGCATCTCTTGGCAGTGCAGAAGATTTTTTTGAATTA
AGAAACTGGCTGTAAATGCTATATAGTCTGTCCTTCTCAGCTTCAGAAACTGATGGTCTT

GCCTGGGAAGCGATGGACTTCAGAAGAGCATCGGTTATGACTGGCTTTTTTCCAGGACTA
ATGCTATCTGTGTTATTTAGAAGTTCATGAACCGCTGCAAGCTGTGCATCCGAGAAAAGA
GCTTGGAGGTCTG
>tig_1296_consensus
ATACACTGTTCTAAACTTGCTTTAGAAAAGGGCTCAGCAATTCATCAAGCACTTTCTGAT
TTCATAGCTGAAGCCTTGGATCATGCACCTTCAGTCGTCATCTTTGATGATCTTGATAGC
ATTATATCATCCTCATTCGACTCAGAGGGATCTCAATCTTCAACCTCTGTTATTGCACTT
ACAAAATTTCTTGCAGACATTATGGATGAATATAGGGAAAAGAGGAATAGCTCATGTGGA
ATTGGTCCGATTGCTTTTGTTGCATCTTCTCAGTCTCTAGAGAATATTCCACAGTCCTTG
ACCTCATCAGGAAGGTTTGACTTCCATGTGCAACTTCCTGCTCCTGCTGCCTCGGAACGT
AAGGCTATTTTAGAGCATGAAATTCAGAGGCGATGCTTACAATGTTCTGATGAAATTTTG
CTAGATGTGGCTTCCAAATGTGATGGATATGATGCCTATGACCTGGAAATATTGGTTGAT
AGAGCTGTTCATGCCGCTGTTGGCCGTTTTCTGCCTTCTGATTTGGGTGTTGAAAAGAAC
ACAAATCCGATATTAGTTAGGGATGACTTTACTCAAGCAATGCATGATTTCCTTCCGGTT
GCGATGCGAGATATTAGTAAACCTTCTCCTGAAGGTGGTCGGTCTGGGTGGGATGATGTT
GGTGGTCTTACTGATGTTCGGAATGTGATCAAAGAGATGATTGAATTGCCTTCAAAGTTT
CCTAATATCTTTGCCAAAGCTCCTTTACGTTTGAGGTCAAATGTTCTCTTATATGGTCCT
CCTGGTTGCGGCAAAACTCACATTGTTGGTGCTGCTGCTGCAGCTTGTTCTTTACGATTT
ATATCAGTTAAAGGGCCTGAGCTGTTGAACAAGTACATTGGTGCTTCTGAGCAAGCTGTC
AGGGATATATTCTCAAAAGCAGCTGCTGCAGCACCATGTCTCCTCTTTTTTGATGAATTT
GATTCTATTGCCCCGAAGAGGGGACATGACAATACTGGAGTAACTGATCGTGTTGTTAAT
CAGTAGTAGACCAGATCTACTTGATGCTGCACTGTTAAGGCCTGGTAGGCTTGATCGTCT
TCTTTTCTGTGATTTTCCGTCTCCACGTGAGAGGTTGGATATTCTTAAAGTTCTTTCTAG
AAAGCTACCATTGGCCAATGATGTCAATTTAGATGCCATAGCTCACATGACAGAAGGATT
TAGCGGAGCAGACCTCCAAGCTCTTTTCTCAGATGCACAGCTTGTAGCGGTTCATGAACT
TCTAAATAACACAGATAGCATTAGTCCTGGAAAAAAGCCAGTCATAACCGATGCTCTTTT
GAAGTCCATCGCTTCCCAGGCAAGACCATCAGTTTCTGAAGCTGAGAAGGACAGACTATA
TACCATTTACAGCCAGTTTCTTAATTCAAAAAAATCTTCTGCACTGCCAAGAGATGCGAA
AGGCAAAAAGAAGGCAACTCTGGCATAGAAGTAGAAGTGTTCTCACTGATTTCGTAGAGG
TCAAACAAGCTATTCGCATTTCTGCATATCGTATTTTTGTTAGATTTAGTTTTATTCTTT
TCAATTTAACACAATTTTTGGCACAAGATTGAAATATCAAAATACAGAACCAATAGCAGA
GATTAGTCTCCTGCAATTCAGAACAGGTGAAATGTTAGCTCTTAGCTGTGTCCAAGTGAA
TGTATATCAAT
>tig_1297_consensus
GCAGCCCCATCTCCTCTATTCTGTAAGCATTTCACAACAGATAGCTCATTAATAGTCAAT
TAAAGTCATGGAACAAGATCTTGACCTAAATACGCTCACATAACCCTCATCACAGGAACT
TGTATAGCAAAGACCAAAAAAAACAGAGAAAAGCAGTCTGGTGAAAATACTATAAGAAGT
ACCAACTACAGACATCATTCCTATACCTGCTCCTAATAACATCATAGCG
>tig_1298_consensus
TTTTTTTTTATCAACAAATAGAGTAACATTTTCATTAAGCAATATCACAAAAGTGCAACA
TCATGAGTGGCATTGTCAACAGGTATAAGACTTCTGCTTGAAAACAAATCAATTTCAATG
CTATCTCTTCCTTGTTTAACTTGTTTTAGGATAAAAATTATGTCATTAGCTGAAGGGATT
CGGTTATAAATGCAAAATACTAACATATTTTGGCCTAAAGATCAGAGATCTGCTCTTCCC
GGGTATCTGGGGAAAGGAATAACATCTCTAATATTCTCGATGCCAGTGGCAAAAAGTATC
ATCCGTTCAAAGCCTAAACCAAATCCAGAATGTTTCACTGTCCCAAAGCGACGCAAATCA
AGGTACCACTCATATGGCTCAAGAGGCAGCCCCATCTCCTCTATTCTGCTCCTAATAACA
TCATAGCGCTCTTCCCTTTGGCTTCCACCGATTAACTCTCCAACCTTTGGGACAAGGACA
TCCATAGCTGCCACAGTCTTTAAGTCATCATTGACCCTCATGTAAAATGCTTTAATTCCT
TTTGGGTAGTTATACACTATAACAGGCTTCTGGAATTTAACCTCCGTTAGGTATCT
>tig_1299_consensus
AGTCTGGTTATATCACCATATGAGATAGTGATAATAAAACATGTAAACAATACTTAAATC
ATATTACAATATATCATCGGAATTCTCTAGACTCTGCGTTTGTATGGAAAACAGGCAGAT
GAAATTTTACGAGAGGGTTACAAAAAACAAGTCTCCAGTATAGTTTTCAATTTTAGCTGA
ATACATGCAAAACATGACACCACAATCGGCAGGCATGGTACAGCGGAAGCAAAAGGCCCT
AATGGATAGAAAGTAACAACTAAAATTAAAAGGAAGAATTACAACCTTTAAAATAATTCC
GAGTATATACAGTAAAGCAACCGTTGGTAGTTTGAAACTGTTGAACATGAATCCTAAAGT
CTTCCAAATTCAACCCCCCAAGGACAAACCTTAATTCCATACAAGTAGACCAAGCAAATG
AAATGGGATCCTATGATAGACAACCTTGGCCAATTGCTCAGCAGAATCTGAGGTTCAGAG

TTTCCTAGAAGCCCTAAGATTGTATTCATACTTGAGAAGATTTGCCCTCAGCAACATTGC
CCTTACGTCCTGATCAAATTCATTGCCAGTCTGCAAAACCCGTTGAAAAGTTGCATTTCT
TTGATCTGCATGTCTTGCATACAGAATCTTGTTGTGTGAGTCAATGCGAGCCTGTATCTC
ATTATCAGTAATCAAGGTTTCCAGTTCTTTCTCTAACCCCGCAACGCTTGTCTTGAAAGC
ATTTGCCATCATTTGCAAATCAACAGACACAAATGGATGTGTATATTGGATAAGGGCTTT
ATTACGGATTTGATCATATAGTGTCTCAACATGGTCATGCAAATGGATATCAAGTAACAG
GTTTGTTTTAAGATTTCCAAGGTAGTCCAGACATGAAGCGTAATGGCTTGAGTAGAAATC
ATTAATAAGCTCCCTAACTTCAGGAACCAACTCCAAGATATTCCGGAAGTTCACATTGTC
TATAACTTTGCTCTTGAGCTCAGCCCGATCAAAACTTGCAAGAGCACAAAGTCCACCATA
TGTTGCAACATCTTGAGAAGCAATCACCTCATTGTAGCTACTTCCCAGATCAGGACCCAC
CTCCAAGAACTTACGAGCTGCAAGCTTGTATTTTTTAGCCTCCAAGTGAGCCAATCCTGC
AGCACAACGTAGTTTTGCAATTGTAGTAGGTTCAAGGGCCTCTGGTGTTTGCTCTGCTTT
GCTAACATAGCTTGTCACATGTGTAAATTGACCCATCTCAATGCTAACCAAAATCGCACT
CATACACATATGAATAATATGCTTCGAGGTTGTGCAATAATCACGAGTACGAACATAACT
TTTAAAAGCATCTCCAAGTGCTCCATGAGCATAGTAAAAATCTCCAAAATCATTGTATCC
CATTCTTATGCTCTCTTTAATTAAATTCGTCCTATAAGCATTGAGTTCGTTCTCAAGCTT
CTCCTTTCGCTGTTCGGCTCTGCGATCCACCGAATCACACCATGCCACGTCCATAGTATA
ATTGGGGCCCAGTCTTCCATCAATTTTCTGCACAATCTCTCTGAAGAGTTGTGTGCTCTC
GCCTTTCTTAATCTCGTCGTAGGCCATTCGTAGCGCCTCGAGCTGCATCAAAGCGTTGTC
GCAGTGTTCGGCAATAAACATAAGGCGCGTGATCTTTGTCCGCCCTTGGTAAAGGCAGGC
GTAGGCTTCGATGTCCAGCTGCTCACCGCTTATAACTGGCCTGTTACGTGCTGATTCCTC
ACCGCCGTTAGAATAGATCTCCTCCATCATTGTATTTGATTGTTCCTCTTCAGCTTCCAT
TGCGTCAGAGTGCCAATTTTAGGGTTAGGGTTTTGGTTGACGGAGAAATTTCTATCTTAT
TCTCCACGCCCACGGTAGTGACAGAGCAGCGGGGCTTTCGGTGCGTGCAGTCCTTCATTG
TAAAGGCAACATTAGGCAA
>tig_1300_consensus
CTCGGGGCCATTGACCTTAATCTCCAAAAGCCTTTCAACAGGTTGTCTGCAAATTGGACA
CCGATTTGTTTGGAACTTCAGGACCTTTGCACATCCACTGCACATACACATGTGCCGGCA
GGGAAGCACAGTTGTGTCCCGTGGTTCTGATAGGCAAATGACACAATCTTTTCCTGGGTC
ATTTGCATCAACATCACCTTCAACAGAATTCCCAATCCCATATATCTCTTGCAGCTCATA
CCTCATACCATTCACCCACAAAATCTGCCTCACTACTTTGACCTGGTATTCACCTTTCTC
TTTCTCAAATAATGCCTGTGTTATCTGAG
>tig_1301_consensus
TCTCCTTTCAATTGGTTGTCTGCAAATGGGACACCGATTTGTTTGGAACCTCAGAACCTT
TGCACATCCGCTACACATACACTGCACCACAGGAAAAGCCACATGTGGCGGCAGGGAAGT
ACAGTTGTGTCCCGCAGTTCAGATAGGCATATGACACATTCTTTTCCCGGGTCATTTGCA
TCAACATCACCGTCAACAGAATTCCCAATCCCATATATCTCTTGCAATTCATACCTTGTA
CCACTCACCCACAAAATTTGCTTCACTACTCTTACCTGGTATTCACCTTTCTCTTTCTCA
ATTACTGCCTGAGTTATCTGAGAATTTGTGGGTCCTGGAATAGAGTTTCCATCAGATCCA
TTCTGGTTCGCAGGGGCTGCCTCTGCCTTCACCGCAAGAGGATAGACATCCATGGTACCA
TCTTTCAGTAGCTCTGCCTCCTCAACCATTGTAAAGTCAATCCCTGTTCCAGATGGCTGT
CTGAACTTCTGACCGAGACCTNNNNNNNNNNNNNNNNNNNNNNNNNAAGAACCTTCCAGGA
TTTTCCTCATCACGTTCAAGCTTCAAACTCTCCTTCTTCACATTCACATCGTTCCGTATC
GTAACCGCCTTCTGGATCTCCACGTACGGCGGCGGAGCCGACGCCATGGGCCCGCACGGG
TACCTCCACTGTGGGGGGTCCACATAACCAGCTTCACGATGATGGTGATCGTATGGCGCA
GGAATCGACACCGGCATAGCTGGTGGAGGCGGCGGATAGTAGCCTTGATACTGGTAGTAC
TGCGGTGGAAAGTTAGGATTGTTATAATTTGGATTCTGCTGCGCCGGGTAGGGCGTCACC
GCCGCGAACACAAAGCGATTGGCCGAAATTTCATGCTGAGGCGGTGTGAATGGCGGCGGT
GGCGGATGATTACGTCGACTTCCGTGTCTACGGCGGCCGTTACCGCTTGTGCTACCTATG
TTTCCCATTTCTTGAGAAACTCTTTCGAAAGGGAAGATATAATGATTAAGGTAAATGAAA
CGGTATCGTTTAGTAGCTATAAAGAGCGGGTGACATGGATCGATTAACAAGAGAAAGAAG
GTGAAAGAAATGATTTAGGGGAAGAAGAAAGAAAGAAAAAAAAGGAATCTTTGGATGGGC
ACCATGGGGTTGCGTAAAGCTTCCCCCAGACCACCATTGATATTGCACAGTG
>tig_1302_consensus
AAATAATGCCTGTGTTATCTGAGAATTTGAGGATCCTGGAATATTTCCATCAGCTCCATT
CTGGTTAGCTGGGGCTGCCTCTGCCTTTACAGCAAGAGGATAGACATCCATGTTACCCTC
TTTCATTAACTCTGCTTCCTCAAACATTGAAAAGTCAATCCCTGTTCCAGAAGGCTGTCT
GAACTTCTGACCAAGACCCTGTTGAAAATTAACCGTCATTGGTGGAAGAATGTTTGC

>tig_1303_consensus
AAATAATGCCTGTGTTATCTGAGCATTTGAGGATCCCGGAATATTTCCATCAGCTCCATT
CTGGTTAGCTGGGGCTGCCTCTGCCTTCACAGCAAGAGGATAGACATCCATGTTACCCTC
TTTCATTAACTCTGCTTCCTCAAACATTGAAAAGTCAATCCCTGTTCCAGAAGGCTGTCT
GAACTTCTGACCAAGACCTTGTTGAAAATTAACTGTCATTGGTGGAAGAATGTTTGC
>tig_1304_consensus
AATGTCCCAAAAGTATCCCACCTGAATATGCATGATAAAACCCATTCAAGCGGGGGAAAA
CCATATTCTGCAACATCAGAAGAATTGAAACAGCGAAAAGAAAAAGTACCACCCACACTT
GCATAACAAAATGGCAGACCCCATAAAATTACAAATATATTTCTCTGGAATCCAAAAAAT
ATTAAACCACAAATCAGCAAACCTCAGCCGCCCTCAGATCAAAAAAACCTATNNTGCTTTC
ATTGTGAAACTTTCTAATAGAAACCATTGCCTCTCAGTCTGTTGTAGTCACCACCGTCTT
GGGTACTTCCACTGCCCAAACCACGACCACCAAAGCGCCCACCCCTAGTGGTCTCTGTTT
GGTAGTTGCTTCTGCCTCTTCCTCTTCCTCTTCGTCCTCCCCGAGAAGTACTGCTGCTGT
TTGGTCTTCGCTCCTCAATGTAAATTTGTCTTCCAGCCAACTGTATAGGAGATGCCTGGA
TAGCAATTTGAACACCAGAAATATCCTCAAACTCAACAAAAGCATAGCAGACACCAACTA
CATCCTTACGGTTTCTAACAAACACACCATCAGGTTTGATTCTGCCAAAGTTCTTAAACT
CCTGCTCAATTTCAGCAGCAGTAATAGTAGAAGGCAAGTTTCTCACATAAACAGATTTTA
CTTCACCTTCTTCCATGGTAAAGCTTTCATCTGCTGCATCAAATCCAGATTCAGGCACAT
GCGACAAAGATCCAGATTCAGGAGCATAGGAAGATGCATGATTTGACTGCTCATAAAAAG
GCTGTTGGGTGCGATTACAGTCTGATGTAATCGCGGCACTTTTGTTGAAGGATGGTTGAG
TGGCAACTGATAATGCAGACTGGTTCTTAGCTACTCGCAAAATGGATGCATAAGTTTTCT
TTGCAGGCTCGCCCACAGATTCATCAACAGCAGCAACTGGAGGGGCCTGCACATCATTCA
AATCAGTTTGATACAAGGCAGAAGTTTCCTCCACAGGATATTCCTCCACAAAAATTTCAG
CTTCCGGGTCTTCTTGCTGCTGAGGCTCTCGGAGATTGTACTGGTCAACTGAATCATCTT
CTATATGAACAGAGTTCATATATTCCCTTGCCTCTTCTTCCAAATCATAATTGGAAACTG
GCTGTTCAGGGAGAGGACTAGAAGCATCTACTTCAGCATCAAATTTGCTTTCTGACAGTG
CAGGAGCTGGAAGTTGATAAATTGTTTCCTTGTCAATGAAGTGAAAGATATCATTGAGAA
CAAAGTAACCTTTCTCCTGTGGAGCAAGGAAAAAGGTCTGCAC
>tig_1305_consensus
CACGAACGCTCTTCCGATCTGAAAATATTCTACAGTCCACTAGTTGAAATGAGAAGAACA
AAAACGGAAGCAAAAAGACACTGAGAGATTACTACACTAGAAAATTTCGCATTAGCTAGA
GCTACTACTCTCCGGCTCTGTGCGGTTGAAGCTTTGAGGAACTTGTTCATATTCGGGTTC
GGGTTCGGGTGGTTGTTGGTGTACGGATTGATGTTGGCCAGCAACGTAATCGAGGAGGTC
TTCCGCTGCGGCTTTGTGGTGACCGAGATGGGAGTCCGAGGCGGGCAGGGCGGAGGCTTT
CCCGAAGAAGACGACGGCGACGAGGAAGAGAGCAGTGGAGAGAAAGAGGGGCCAGAAATA
AGCCAAAACGGTCAAGAAACTTGGAGCGAAAACGAGCAAAGAAAACGCCAAACCCATGAC
AACTAGTGTGAACAATAAATGGAACTTGAACCTCAGAAGCTTTTGTGGGATCTCCATGGA
GGGTTTTTCTCAAGCTGCAGAGAGAGAGAGAGAGAGAGAGAGGGAGTAGGGAAGAAGGGATG
ATACTGATTGATCGATTGTGATTATTGTTTTTGTGAGTTATCTGTATGGATCTGTACAGA
GATTCTCTGAGGAAAGATGTTTGTATTCATGAGGTTTCAGAGAGAGGGGGAGTGAGAGAA
ATGTAAATTTACTGGAATTACAATGGTTAGGGCACTTTATCATTCTTTCCAGTTGTGTGT
TTTCTGTGGTGGGTAAATTTTTAGTTTCCTAACTTGCCCTTTTGGTGGATTTGTTATTTT
CTTTTTTCAGTGTAATTTGTATTTTAGTAAGTATGGCTTCTGGTCCGTATTAAAACGTAT
ACCTAAGTGCACATACTGAGAAATTTGCCTTGGTAATTGACTCAAATTTTTCAAAAAGGA
TCAGATTTAGTGGGCGAAGGGTAGTTTTGAC
>tig_1306_consensus
GCAAAAACGATAACATCAAAACAAGTGCTTTCTAGTTTCAAACTCTTCAGAGCCACAACC
AACCATAAATAGGAGATACATGAATTACAAGATCTAAACCTATGTCACTGAAAGAATATT
TGGCTTTAAATGAGTTTTGCTGGGGCCCTCAACTGTAATACAACCTTTTTATTATTTATG
CTGGCGGTAATTTGGAAAAGGTGGATGAACAATCTGTAGACGAGATGCATGAAGCAGGGA
AAGCAAGGTTTTACTGTATTCTTCATCAATTAGTAGCATCACGGCTGTAGAAAGCATCAA
GGGTCTTGGCTGGTATACGGTGGAGAAGCTCACGAGGGAAGATGCGAAGAAGTGTCCATG
CCAAATCAAGCGATTGGAAAATGTTTCTGGTATCATAAGCTCCTTGTGTCACAAACTTCC
TCTCAAACTTATCCAAGAACTCCAAGTACAGCAGGTCCTCAGAAGAAAGTGCTTCTTCTC
CAACCACAGCTTTCATTGCCTGAACATCCTTCCCAATGGCATAATTTGCATAAAGCTGGT
TAGACACATCAGAATGGTCTCTCCTAGTCATTCCTTCACCAATGGCACTCTTCATCAACC
GAGACAAGGATGGAAGGACATTGATTGGTGGATATATCTGTCTGTTATGGAGCTGCCTGT
CAATATATATCTGCCCTTCTGTGATATATCCTGTAAGATCTGGAGTGGGATGTGTAATAT

CATCATTTGGCATGGTTAAAATTGGAATCTGGGTGATAGATCCCTTTCGACCTTCAATTC
GTCCAGCACGCTCATATATTGTGGCCAAATCAGTATACATATACCCAGGATACCCTCTTC
TTCCAGGCACTTCTTCTCGAGCAGCAGACACCTCACGAAGAGCATCAGCATAAGAACTCA
TGTCTGTCAATATGACAAGAACATGCTTCCCACATTCGTATGCCAAATATTCTGCTGTAG
TAAGAGCAATACGAGGAGTGATAATACGCTCAATTGTAGGGTCATTTGCCAGATTAAGAA
AAAGGGTCACTCTCTCCATTGATCCATTTTCCTCAAAATCACGTTTGAAAAACTGTGCAG
TCTCCATGTTCACACCCATAGCTGCAAAAACAATTGCAAAATTGTCCTCTTCCCCATCCT
CAAGAAGATTTGCAGACTTCTCCAGTCGCTTTACCAAACCAGCCTGACGACAAATCTGAG
CAGCAATTTCATTATGAGGAAGACCAGCAGCAGAAAAGAGAGGAATCTTTTGTCCTCTAG
CTATGGAGTTCATGACATCAATAGTTGAAATCCCTGTCTGTATCATCTCTTCAGGATAGG
TTCTCTCACTGGGATTGATGGAGCTCCCTGAAATATCCAAGTATGCCTCAGGCAGAATAG
GAGGACCATTATCAATAGGTTTTCCAGATCCATTAAAAATGCGGCCAAGCATATCTAATG
AGACAGGAGTTTTCAGAACCTCCCCTGTAAACTGAACAGTTGTAAACTTATTGTCAATTC
CAGATGTTCCTTCAAAAACCTGAACTACAGCTTTCTCTCCATCAACTTCTAGGACCTGAC
CACGTCTCATTGTTCCATCACCTAAACGAATATTGACAATCTCTTGGTATTTGGGTCCCT
TGACTTTATCGAGGATAACTAGAGGTCCAGCAACCCCAGAGACTGTTCTATACTCCATGC
CAATTTCTAGCGTTCCCTCCTCCATATCGTTACTGTCTTTTTCCATACCCATTTCTGAGC
TTTTTCTGTGATATCTGAGTTCAGAGAAGGAATCATACGAG
>tig_1307_consensus
CCCAGCAAGCTTTCTGGCAAGATACAAGAAAGGCTTCTCAAAATTGTAGTTGCTCTTGGC
AGAGATCTCATAGTACTGCAGATTCTTCTTCCTGTGGAATGTAACCTGCTTTGCCTTGAC
TTGCCTATTCTTCACATCAACCTTGTTTCCACAGAGAACAATAGGTATATTCTCACACAC
CCGGCACAGATCACGATGCCATGTGGGAACATTCTTGTAAGTCAAGCGGGCAGTAACGTC
GAACATTATGATGGCACATTGCCCATGGATGTAGTAACCATCTCTAAGCCCACCGAATTT
TTCTTGCCCAGCAGTATCCCAGCAGTAGAAACGAATTTGACCGCAATTAGTAAAGAAATC
TAAAGGATGAACCTCCACACCAATGGTTGGTTCATACTTCTTCTCAAACTCCCCAGTCAG
ATGCCTCTTCACAAAAGTAGTCTTACCAGTCCCTCCATCACCGACGATTACAAGCTTAAA
GCTCGGGTAGTCAACAGTCTGCTGATTTGGCAAAGCCATTAGTATATAGCGATAGAGCAG
GCGATGAAGGAAGATTGCGGTGTGAGCACAGAAATGATAAACGGGGAAAACGGAGATGGG
CACGGGCTTATAAGGGAGAGAGGGATTTGAATTGAGGCTTCAGCGTGGCTTTAAGTTGAC
GCTACTCTAGTGACCATTG
>tig_1308_consensus
GAGCACAAGGTTCATCCAAATACTGAACCACTCCATTTCAATTTCAGTAGTACAAAATAA
AAACGTCCCAAATTGTAAAAAACTGAAACCACAAGCATTAAGAGACACATGAAACTACAC
AAGTATTATAATTTAAAGTCCAATAGCTAGCCACTTTATTCATATGCATGAGCAGAAAGT
CGTGGAGTGAGAAGGACCTCAAGCGGGGTGGCCTTTAGGCTTGTCAAGTCGATTCCCTCA
ACCATGTCAACTGGTTCACTGCTTGGTGATGCAAATTCAAATCCATGCAGCAAATTGGCA
AGTGTCAGTTGCATAACTTGAAGAGCAAAAGAGATTCCCGGACACATTCTTCTTCCACTA
CCAAATGGCAGCAATTCAAAATTTTGGCCCCGGACATCAAGATCTTTATGGGTCGTGAGA
AATCTTTCGGGTTGAAACTCGCAAGGATTGCACCAAATTTTGGGATCTCGATGAAGCTTT
GACAGATTGACTAAAAGGCGCGTTCTGGCTGGCACATGGTACCCACCAATGCTGCAGTCT
TCTAAGGATTCATGTGGCACAGACAGGGGGGCTGCAGGATACAGACGTAATGTTTCTTTT
AGGATAGCTTGGAGGTAAACCAAGTTTTTAACATCTGATCCCTGCACCAGCCTTCCCTCA
CCAACATGGGTGTCTAATTCATTTTGGGCTTTGTCCAATACACGACGGTTGTTGAGTAGT
AAAGCCACAGCCCATGTGAGTGTAACAGTAGTTGTGTCTGTGCCTCCCAATATCATAGTC
AGGCAGGTAGCTTTGTTGATCGTATCGGCATCATAACTGGGAAGCTCTTGAGCTTGATCA
AGAAGGGAAAGCATTATGTACATGAAATCTTTCTCACCTTTCACCTCACCTGTGCTACTC
TTCTGTTTATGCTCCTTTAACCATCCTTCCATAACTTCATCAAGTTCTTTTGCTGTCTTC
TTCATGGCCTTCTCATGTCCTCCTAAATCCACCCACCTCATAAATGGCAGTGCATCTGCA
AGTGGAAATACCCCATTTAATTCAAAAAATTTCCTTATCGCCTCTCTGCTACGTTCATTC
TCTTCACTCTCTTCCTTTGTTGTACCCTCCACGAAACTTTTACCCACAACCATCCTTAGA
ACAACGTTTAACGTTACCTTGCCGAGCCATCTCTTCATCTCCACCAACACCGGCGTCTTA
TTTTCTGTACTGCCTTTGTTCTTCATCCATAGATCATATATTTCTTTTACAGACACCTTC
ACTTCAGATTCCCGGATATTTTTAAGCATTTCGAGGCGGTGGTTTGAGAGAATTTCCAGC
GTAGCTATCTTGCGGAGTTGGCGCCAGTAAGGACCAGAAGGACTGAAACCGAACATAGCA
TAGTTGTAGCCCAAGAGTTCCGAAGCTAGAGATTTTGGGCGATTGCAAAAGACTTTATCA
TTAGTAGTAAAACACTCTTTAGCAGTCTCCCAATTGCTTACAATCAAAGCACGATATACC
CCCATCCTGATCATGAAGATCGGGCCGTACTTGTCAGCCATGTTTCCCAATGTTATATGC

GCAGGTTTTGAGCCTCCTAAGAGGTGGAGGTGGCCAATCAAAGGCCTTGCACCGCAAGCT
TCTGGTACTGCAATATTGTTGCTTGAATTTTTGTGAACACGTCTTGATATCCATAACAAA
GAGTAAAGAAAGAATAGGAACGCGAAGAAACCAGTAACTGCAGCTGGGAAGGGAAGAAGA
ATATCCATGGCTAGCTATGAGTCTGCTAATTAATTGGCGGCGAGGTGTTTTATGCTTTAG
ATGCCAAACCATCTCTTGACTGTCGAAATTGTCTAAACTTGTTTCTTTCCCATCTTTATG
ACGTATTTGACAGGCCAAACGACATGGTCCCACCCAAGCTTTATCTTTTTGAACCAAAAT
CAAATATGACGTATTTGGTAGGCCAAACGCCGCAGTCCCACC
>tig_1309_consensus
CTCAATTTTGTTAATCTTTTCAAAGATCATTAACATAATAGAATGAAATGAGTCTGGTCC
ATAGAGTAATCCAAACAGACACCACTACAGACTCAAAAGCCAGAAAAAAGCAGTGACTTC
ACTTGAATTTGAAATTAATGACATTCCCAACAGCATGAACCTTCTAGCATCCTAACCCAT
CCGTAAAGCTTTATAACCCCACTCATTCCGTCATGACAGGCTTGTTCTCATTTGCTTTGG
GTCTCTGTTCCTTTGGCCTTTGCTCTTCCATCCTCCGCTTGTCAGAATAGCCACCCTTCT
GGCTGAGGAAGGAGCGGAAGAGAGGAGAATTAACGTCGTAGATCATTGTCAGATTCAGTT
TGCATATAAAAACCCTAGCTATCCGTCCTAGCACAACTTCAATATCCTTCGATATGCATT
TATAGAGGATACTTCGGGCGATCCGGCTTACTACTTCTTGGGCTCGGGCTCCTCTTCCTC
CTGGCTTTTAAACG
>tig_1310_consensus
CACTAACCATTTAATCTTATATGTGCAAAACAAACTTTTCAAGAAAACATACTAGAGATG
GATGAGTTACGTCAGGGGACAAGCATCTCATCGAACCACATAATTACCCTGCCCTGAAGA
AAGGAAACTTTTGGAAAAGAGTAAATGCCAGATAATTCCACAGCACCAGCAGCTCTGATG
GCAACCAGTAAAATCAATTTCCGTTATGCTTCTGACATGGAATCCCAGATTTATTATTAA
TGCTGCCCGTGGCTTTTGCTCATGGACTGCCGAAGGAGCTTCCGACCAGGAGTTTGGCGA
TCATCCTTGTATAAAGAGGCCGCTGACTCAGACTGTACTGTCTG
>tig_1311_consensus
CACTAACCATTTAATCTTATATGCGCAAAACAAACTTTTCAAGAAAACATACTAGAGATG
GATGAGTTACGTCAGGGGACAAGCATCTCATCGAACCACATAATTACCCTGCCATGAAGA
AAGGAAACTTTTGGAAAAGAGTAAATGCCAGATAGTTCCACAGCACCAGCAGCTCTAATG
GCAACCAGTAAAATCAATTTTCGTTATGCTTCTGACATGGAATCCCAGATTTATTATTAA
TGCTGCCCGTGGCTTTTGCTCATGGACTGCCGCAGGAGCTTCCGACCAGGAGTTTGGCGA
TCATCCTTGTATAAAGAGGCGGCTGACTCAGACTGTACTGTCTGTATAGGAATATGCCCC
AGTGATCGTAGAGCATCCAGTGCATCCAAGGTCAGCTG
>tig_1312_consensus
TTATTCAGAGAAAAAACCTTCTCCAATTCATCAATCTTCCTAAGATCGGCCTACAAATTA
AGAAAAAAAAAAAAAAAACCCCTCAAATGCTGATTTCCATGTTATCTATGAGGCGGACCT
TCCCAAACCAGGCAGCAATGCAAAAGACAACAGGACTCTTTATCTCTTCCACCGCCTCCA
AACTTTCTTGGTCCACGATCTCAGCATAGTCAATTTTCCCCCCGGCGTCATTTATTGCTT
GGATGACTAAGCCCTTTAACTCTTTACAGTTAATTTGACCCTTCTCTGCAGAACATTTAG
CTCTGCATAATGACCTNNNNNNNNNNNNNNNNNNNNGAACCTATCACTTGTATGCCAAAA
TCAAGATCTCGAACCATTCGCTGAATAACACGAAACTGCTGATAATCTTTCTTCCCGAAG
AAAGCAAAATCAGGCTCCACAATATTAAACAACTTGCTAACAATGGTAGCCACCCCTCTG
AAGAAAACAGGTCGACTCTTCCCACATAGTCCCTTCTCCAATCTCTCGACTCTCACCCAC
GTCTCATGCCGTATCCCCTTTTCTTCAACACATGAAACAACGCCACTACCAGTATTGCCA
GACTCGCTCCTCCTACT
>tig_1313_consensus
AGGCCATATATACACAGAAATTATTTCTAACTGAAATTTTCATAACATAAAATTGGTTCC
ATTACTAAAAATGTGGCTGCTAGAGCAAATTTGATGAGAGAGCTACACAATCACAAAATA
TAGAAATACAAGTCTATGCAGGGATTCATGCACAAATCTTATTATTTTTAATCAACGCAA
AAACCGAAGAAAAACACGGTATCAACCAGGTTGAAAAAAGAGGGGGAGCATCAGCGGTAA
AGGCTTTCAGATCTGAGGTTCTCTGCAATCTCAGTCTCCCAACGATCCCCATTCTCAAGC
AGCTTCTCTTTGTCATACAGAAGCTCCTCATGAGTCTCAGTTGCAAATTCAAAGTTAGGT
CCTTCGACAATGGCATCAACAGGACAGGCCTCTTGGCAAAATCCACAGTAGATACACTTG
GTCATGTCAATATCATATCTAGTTGTCCTGCGACTTCCATCTTCTCTTTCCTCTGCCTCA
ATTGTGATAGCTTGTGCAGGACATATAGCTTCACAGAGTTTACAAGCAATGCATCGTTCC
TCTCCAGTGGGATATCGTCGCAGGGCATGCTCACCACGGAAGCGAGGGCTTAGAGGGCCC
TTCTCGAATGGATAATTGATAGTAACTTTTGGCTCAAAGAAGTATTTGAGTGTCAGCATC
AAACCCCGAACCATTTCAGTGAGAAATAGCATGTTTATGCTTCTTTCAAAAATAGAACTC
CAGTCTTTTGATATATCTTTGGCAAGTTGCTCCCTCTCTTCGTCATCTTTCTTGGAGGGA

TGATAGGAGTAAGGGTGCGCACTGAATCGCAACCCATAATGATGCGAACCCTGCAATGCT
TGTCCCGTCACCGCGAGGTGACGAGCGCGAAGAGCAGAGAGCGACTTGCGAGCTAAGATC
AAAGCCATCAGTGATCGGTATAAGTCGCTGAAAATGGATCTTCTGCCTGCGATTCGGGAG
GGCTGAGCAGAAACAATGGGTAGTACCACCTACTTAAATGGGTGATCCGATTCGGCGGTG
AAATGGCTTCCTTTG
>tig_1314_consensus
TTTCGGATTGAAAAAGAGCATACATTCCCGTAAAACAGAATAAAGCACCCATTAAACATG
AAGCAAATATGTCAAAATCTAGATTTTACACCCTCCAAGCATGGTAGAAGGGGATAATCT
CCCTAATACATGGACAAATGTAACATTAAGAAAAAACTGTCTTTTTCAGATCTAAAAACT
CAATGAAAAACCATTTCTCGAACAAAGCATGACATCTTCCCATTACATTCACTTCCTACC
CCTGATCTTCACTGCCTGTCTGTTTCTCTTCTTGACACCGGATTTGGCAACCTTAAGGCT
CCTGTTAACAGCACTAAGCCTTGCAAGGGCTGCCTTCGTCAAATCAGGCCTGTAGTAGTT
ATCAGCAACCTGATTGACAACAACCTTGGCCATCCTAGGGAACTCCTTTCTCAAGACGGA
TTTGTGAAGCACGCGAACAGGCTTGTTCTGCTTCTTGGTCTTAGTGGTAGCTAGCACCAC
AGACAGATCTTTGCCACCTGGCTGAATAGACACACTTTTGCGGTTTGCCAACCCTGAGTG
CTTGTAAGAGTTGAGATTGTAAAGGTTATTGGGTTCCTTGCTGAAGCGAACGCCCGCGGT
ACCATTGCCAAATTGCTTCACCAAGAAACAGTTGTTCTTCTTCACGATCTCCCAAATCAA
CTGACCTGGAACTGTCGCCATTCTCTTTATTACGGTGGCTGAGAGAATGCGAGGCTTTCG
GGCTGTCAAGATCGTCAAATCAGCTCATATGGTACTTATATACCCACTCGTTCAAAACCC
TAACCAGAGGAGTGGAACCCAG
>tig_1315_consensus
ACTACATTCACTTCCTACCGCTGATCTTCACAGCCTGTCTGTTTCTCTTTTTGACACCAG
ATTTGGCAACCTTAAGGCTCCTGTTAACAGCACTAAGCCTTGCAAGGGCTGCCTTCTTCA
GATCTGGTCTGTAGTAGTTATCAGCAACCTGATTGACAACAGCCTTCGCCATCCTGGGGA
ACTCCTTCTTCATAACGGACTTTTGAAGCAAGTGAGCAGGCTTGTTCTGCTTCTTGGTCT
TAGTAGTTGCCAGAACGACAGACAGATCTTTGTCACCTGGCTGAATGGACACGGTTTTGC
GGTTCGCCAACCCCGAATGCTTGTA
>tig_1316_consensus
CTTCAGACGAGTTTAGTACGGAAAATAAAAATAAGCTAAAAAAACATTTAGCACTAAAAA
AAAAATTAAATATGTTTTTATTATTTAATTAATTTTCCTTGTAATTATCGTAAGAGCTTA
TTTCCCATCCCCGTTGACAATCACATTTCTAATCTGCCTGTGTCTGAAGATGGACAAAAT
CCAGCACAAATTCATCAATGTCCAAGGACTTAACCTTCACGTTGCCGACACCGAAACGGG
TTCTAACGTGGTGGTGTTCTTGCACGGTTTTCCTGAGATATGGTACACGTGGCGCCATCA
GATGGTGGCTGCTGCACACGCGGGTCTCAGAGCTATTGCTCCTGATTATAGAGGCTACGG
CTTATCAGACCCACCACCTGAACCTGCCAAGGCTACCTTTCTAGACATCGTGGCTGATCT
TCTTGCAATTTTAGACCACCTCGGACTTGCCAAGGTTTTCCTTGTTGCAAAAGACTTTGG
AGCCAGGCCGGCCTATCAGTTTGCGCTTTTCCATCCAGAGAGGGTTGCTGGA
>tig_1317_consensus
AGTCACCAAATCAAATCAATCCAATTATCATCCAACCCTGGAAATATTGATTGAAACCAC
AAATTATGATAAATATATTAATTAGTTCACCAAATACAACAACCCAAAAAAAGCAAAAAT
TAAACTAAAATTAGACATAATTCTCTTAACAAATTACAAGTTCTGATAAACCCAGGGTAG
ATTCCTTCATATCTGCAGCTTCCAACTCTATAAACTGAGAACTCGCATGCCATTACTTGA
TATAAGCTACCATCCCACCCTTGACTACTGTTTCTTTGGTTGCCACTTTAACTTCCACTC
CTTTTCCCAACTTGATGGTGGTCGAGGTGGAGGTGGACTTGATGGCGGAGGCGGCATGTA
CCAAAGCTTGTTTAGGCTTTGGTTGAGTGAGGGCTTTTGCTGGTGCTTTATGGTGGTGGT
GGTGGTGGTGATGATGATGATAATTGTGGTGGTGGTTATGGTGGACTTGACTGGTTTGAG
ATTTTGGTGCTGATGAGAAAGTTACAACTTTCTTGTGTTTTTGACCATGGTCTGTTTTGT
TGGTGGTGCGCTTACTGGTGGACCTGGTGGTAGTGCTGGCAACGGTAGATTTGGTGGTGG
TGGCGGAGGAGG
>tig_1318_consensus
CGTTTCTAATGGGGGCAATGGATGATATGGCATTGCTGGTTATATGACAGTAGTTAATGG
CAGTACATTGGCATAGTTACTCTTTGTTGGTAGTGCAAAGGAAAAAACCAGAAAACATCA
AATATAAAAACAAATGTGGCACTCATAACCCGCAGTTTTACTAGCATTTATCAGAATGGA
AAAGAAAATTGGCATGGAGTTCATTCTATCTCCTTAGCAAAATTACACACTTTTACTGAA
TCTATGCAGAGCTTTCCTCTTGATTACCGTCACCAGGCTTCAGCTTGGATGGGGCCAATG
GTGATGGAGAACCCTCTACTCCCATCTCAGCCCTTAGATCATTAATGCTCTGCTCCAACT
CATTTATGCGGCACCCCATTTCATCAATTTTTGTAACAATGGAATCAGACATTGTCTGAA
ACCTGGACTGCATCTGCTGAAGAAGATTTTG

>tig_1319_consensus
TTTTTTTTTAAATTAAAATTTATATGTGTTCTGCGCTCACAATATTTTCACATACTGTTT
TGGAGGAAACTACGAAAACAAGTAACGAAATCATCAGCCATACTTCAAAGAAAAAGAAGC
TTAAGATTTACAATGGCCAAAAACGCCGTCCAAGTCAAACANNTAGCTAAACACAAAATC
AACCCAACCCAGTTGGCTCCCACATGCAACACAATAGGATGCCAAGCCAAACTCAATAAA
AGCTCACCCAAGCAGAAAGACAAGGCCATTGGGTTCTTGTGGAAACCGCCT
>tig_1320_consensus
AAAAAAAAAAAAAAAAAAAAAGGGAATTTCAGTCATTTCTAAAATTTTGTTGCAACATAC
AAAGTGCATTAGTAGCAATCCCAAAATCGACATTGAAATTTTGAGGCATTTCCATTATCA
GCTAACTTCTTTGCTAACCTGACACGAAATTTTATGAATTCAACTTACATTACAACCAAG
CATAAATTTGCTCTCATATCAATAGGCCTCTAGTCATGCTTATAAAATCAATTCAATTTT
CCAGCACATATCGGCTTCAAATAGGCTTCATCAGAAAATGCAGTCCCCGAATACATAACC
AATAAGCCACTCAACCACCAAGCTCAGGGAAGAGATCTAATCTGCATAGGAAACCAACCC
CCTTTGTCCAGGCGGTGAGCTTGACAGAGACTTGCCGGGAGATATAGAAGATATTCTATT
ACGATTCTTTGCTGGTGATGCCTCAGGAGATTTAGACTGAGAAGTAAAGCTCCTGGATTT
GGACCTAGACCTGGACCTCAGTCTTCTTTTGATAGGAGAGCGCCCTCTTTCAATTTTGGG
ACCCAGACGAGATTTCAACCCTTCGTTAATGGATGACCGCTTATCTCTTGGACTAGAACT
ATGTATTGGACTCCGGCTCTGACTATAGTCCCTGTAACGACCATGATAACCATCTGGACT
TCGAGATATGCTCCTAATTCGGGCTCTCCTGCGATTGTATCTGGGAGGACTCTGGAATCT
TTGTGGGCTTCCATAATATCTATATGGAGCACGATCAGTGTAACTTCTAGAATACCTATC
TCGATTCCTTTCAATGTTTCTTCCACCATATTGAAAGGATCTAGGAGGTGAACGCTCCGG
AGTACGATAACGGCGCACAAAAGAATAGCGTTCAGTAAAGCCACGACCTTTTCTAACACG
CTTTGGAGCACCATCTGGAGTTGGGCTTGGTGACAAATCCTGGCCACGGTTAGCAGCACA
AGGGTCAGGGGCTTTATGGCCAGGACTCTGAGAACTGGTCTTCTTGGGAGGACTTCTTCC
ATCATTCCTGACAATCCTTTTAGGACTCAAGCTGGGGCTATTCCTAGATCTGTTGTTAGA
CCTTTTTTTGGGGCTTGGACTCATACTCCTGGATTTGCTTGTATCATCTGAATAGGAATG
TCTATCAACAGTTCTAACCAACTTTGCTTCAGTGTCATGCCCATTATTTTGAAGCCTATC
AATCTTTAAAGATAATTCACCTTCTTCATGTGACAAGTTGTCCTTAGTTGCCTTCAGCTC
ATGATTCTTTTGCTGTTCTCTGACAGTCCCTTTTCCTGAGCGAGGGGTCAGGGATTTCTT
TTTAACATCCTTTTTGCGAGTTGAACCACGACGACCCGCTCTTTCATCATGAGAGCTGCT
GCTAGCACTCTCACTCTCAGAATCACTCAAACTGTCTGAACTCCATTTAGATTTGCGTCC
CCAGCTTCTCTTCTTCACCCTCCGTGCATCCTTCCTTTTTCTCCCATGTTCTCGCTTATC
TCTTTTTCCTGATTTCCTCTTCCTATGCCCTCCATAACTAGAGGAACTAGAATCAGACAG
TGAGGAATCTGATTCTGAAGCTGATGGTGAATCAGAATCAGATTCTGAGCTATAGGAATC
AGATGAAGAGTATCTCCTCTTTCTTTTCTTTGTTCTTTCCTTCAAAGAATTTTTGTGCTT
TTCTGCAGCTTCTCCATCAGAACTATCCTCCTCTGAAGAGATGGTCCTTGATTTTTTCTT
TTTAGCTTTCTCTTTACCATCCTGAATTTTATTTTCACTAAATTCAGCACAGTCAATGAT
TTTTACAAGTCGAGCAGGTTTGCCATCAGCTGTTCCCAACTGTTCAATTTTCTTAACTAT
GTCCAATCCCTTCACCACCTTTCCAAACACAACATGTTTCCCATCAAGATGGGGTTGCCG
CTTGAAAGTTATAAAGAACTGAGATCCATTTGTGTTCACACCACTATTGGCCATAGAGAG
AAAACCAGGTCCATCATGCCCAAGTTTAAAATTCTCATCAGCAAACTTCCCTCCATAAAT
GCTTTCCCCACCAGTGCCATTTCCCTTAGAAAAGTCACCACCTTGCGCCATAAACCCCTTT
AATTATTCGATGAAACATCGATCCCTTATAATGCAGAGGCTTTCCAGTAGATTTTCCAAT
GCCCTTCTCTCCAGTACACAGCGCCCGAAAATTCTCAGCCGTCTTAGGGACAACATCAGC
AAAAAGCTCCATAACAATTTTTTCCACGGGATCCCCATCAATTGATACATCGAAGAAAAC
AAGAGGATTCCTCCTTTTCTTACTCATTTTTGCACACTTCCAAATACTTGAGTAAGCCCA
AAATCAGACCGCGTTAACTCAGATTAGAACTCGGGAATCGAAACGGCGGAGAAAATCATT
CGCC
>tig_1321_consensus
GCCTATATCAAGTCATGAAAAACAATCTCTTTTATGATCAGCACAATGTATCTTTAGCCA
GAATAAGGCAGTCATTCCCAAAAGTGTAAATGGTACAAAATTAGTAATCATCCTTTCAAG
TTATACCAGAAGTTCTACCAAATTGCTTGAAAACAACATTTTACATATTGAATAATCCCA
AACAAAAGAACCACTCCCACAAATCTTTACAAACTTTACCTACATCATCAGTCAAGTACC
AGCGTTAGGCAAAGCAGCCATAAATGAATTGTATGACGAATCCAGATCAAAGTGTAGCTG
CCGCGCCTGCTGATCTGTTAACTCATCTGCAGCTCCCATCTTAGACAACCTAGAAATCCA
CTCCTTCATCTTGGTCTTCCCCTCAAAGTCAGGTGGCAAAATACTCAACTTATTAAGCGA
ACCCAACAGATCAGATAACAATGGATACACCTGATCAACTGCCTTAAATTCCAATTTTAA
TGTGTCCATAGCAGTTATAAAATGCTGGACACATTCTGCAACAATGGCAGCAGAGGTGGC

AGTCGATGCAGCTACAGCAGCTCGATGCTCCACTGTAGCAGGCACCCCAACGGTAACTAA
GCGATTCAGAGCAGCTGGGCAGTCCATTTTATATGTATCTGCAAACCGCTCAATGCTAGG
GACAGTATCTTTCAACGTAGAGGCTAGAGTTTTGAAATGGGCAATGAGCTTTTGGCATTC
AGCTTCATACTCACTTGAACTTAATATATCGCGAATATAAGCCTTTTCAAGCTTTTCCGT
GGCTTTGATTATAGCATATAGCTCGGCAAAGTTCTCATACATTTCTCTCTCGCGCTTGTC
GTTCCATAGCTTCACCTCCATGTGGGTAAACGCAAAACTATATCTATGAATTTATAAGTA
CTATATTGTCCGCAGTTAGATGAACAATTTTTGGTATTTTTGGATTTGAAAGTGTGGAGG
ATTGGAGAAGAGAACGGGACACGCGCAAAGAAAACTATCTTTCTTCACTGCAAGCACAAA
ACAAAGAAAAAAGCATAAACACAGAAAATCAAGCAAAGAGAGAACAATGTAATCC
>tig_1322_consensus
AAATTTTAATTAATTTTTTTTAAAAAAAAAAAATTTTGTTTAAAAAAAAAAAAAAAAAAAT
TTTTTTGTCAATAAAGGAGTCAGAATTTAGCTCCCTTCCATTACATGCCATTTACCAATG
TTTTCTATTGTTTGTAGTGGTTGAAACACCCATGTAATACTATAAAGGTACAGTCATTAT
AATTGAATAAAGAATGTGTGATCAAATTAGATCTTTTACAAATTGTTTATGTCGGTCGCA
CAAGAGCATCCATGGAGAATGTTGACACAAAACTGCCCTCTATGTTTATTGCACAGTTAC
AGAACCGTCTTGTTTCATTTTAATGAGACTGCTTTCGATATTCCGTAGCACTGGTTTATA
GAGCCGTGCAAGTCCATTATATGCTTGTGTGGCTCGCTGAAGTTGTGCTGAGAGCATCAA
TACCTGGGCTGACAGTGCCTCTGCTTTGCTCTGTTCATGCGCAAGCTGGCCCTTGAGGGA
CAGAAACTCCGGCAAACCACTCTGGCTGACAGCATCTTTCGAGATTTCTGATGATCCTCT
CATAATATCCTTATCATTCACAGACACTAAACTGGCTTGTTCATTTCTTAACTTATCTAG
AATTCCACGGAGTTCTTCATTCTGCTTAAGACAGTCTTTGACCTGATTCTCCCAGTGCTT
TGCTATGTTCATTGCCTCTTGATAAGTAGATGTGAGATTAGAATTTTCTTCAAAAAGCCT
TTCTATCTCTGAAGATTGACTATCAATCTCCATCAGAAGTTTTTGTCTGCTACTTTCTAG
CCTCTCAACCACTACAGCCATATCATGCAATTGCTTTTCAAGTTTTTCTTTTTCTTCCGG
TGAAAATGTCTTGTGAGCTGCTTTATCTGAGACTTCAGAGGCTTTTCTGGAGCTTGCAGC
AAGTTTCTCTTCCAATTCCTGCTTTTCAAATGTTAGCACAGAAATTCGAGCTTGCTCTTC
TGTCAACCGTTGTTGCTCTTTCTGTCTCAATTGTAACTCTTGCTGTAATTCATATTTTAA
AGAAGCCAATTCCTTTTCTAGTGTTTGGATATGATCAGTTGAAACTCCTATAGATGTTAT
TCCAACAGGAGGACCATTCATTGCTTGTTGCTGTATTGAGTTTAGTTCTGCTCTCAAGGC
TGCAGCATCTTCCTCAGCCCGGTACTGTTCTTCTTCTGCTTCTTTTAAACGTTTTTTCAG
AGTGCGAAGTTGAGCTGAAAGATCCTCCTACACTAAAATATTAGACTTTGTGGTGGAGAA
TAGCAATAGTCACACATGGATATGTGATGAGAATAAACAGCGCATATTGCTTAGATTAGA
TGATATCTACCCGTGCAACAACAGCTGCATCT
>tig_1323_consensus
TATTTTCCTTATTTCTCTCCAGTAATCACCATATGGTGCAAAAGAGACATCTAAAAAGTT
GTATGAGAGCTTTCCACAACCGGTTGACGGTGGCCTGCTGCAACAGTCAAGGTCATGAGT
TTTTAAGACATCCCTTGCGGCTTCAGCAGAGGAGATTACAACCAGGGACATACGACCGAG
TTTAAGAAGCATGACAGGGCCATATTGTTGGGAAAGTTTCCACAAAGATTGGTGTGGTAA
TTTTCCAAGTTGATGTAAATTGCCNNNNNTTATGGTATTAAGGAGAAGAACTATTGGGAT
TAACAGAAGAGGGAGCCAGGCCATGGATAGAGTGTCCAAAGCCATCTGAACTTCGAAGTA
CTAGGTTTTGAGATTGGTTTAGGCCCCTTAATATAATCCACCGCCACGGACTT
>tig_1324_consensus
CGCCTAATAAAATGAATAGTATTTCATATGACATAACGAACATTGATATACATACAAAAG
AAGGTTTTGTCCTGCATATATTTTTACATATCAGAGTCCTCAAGAAACATCTCCTCAATA
AGAGCATGAAGATGTCTGCAACAGACTACCCCAGGGACTATTAGACAAACTAGTTTCCGA
CTTCATATAACAAAACTACAAAACTGACATGAAAATGTCTTCAAGCCTGGAAAATTTTCC
AGTCCCACGCTGCTTAAAAGTGAATTTGAAACGAAATCAAATGCACCCAACTTGCTTGCA
TTGTCAAATTTCTGGGTGTGTTTCATTCCGCTTTCCACTAGAACTGAGATGATTGTAAAG
CTACTGTTTTAAGATATATCTAGAGTTATAGGTTTCTATGAAGGGGAGAAATATCTCTCA
AACCTTGTGATGAAACTGGTTGCTGTCGTATTCGAGACAAAAGGAAGGAAACTTGTTCAG
TTGCAAGATCTAGCCTCTCCTTTGACTTAGCAGATTCTGCAATCAGAGATGATACCATGC
TCTGAAGTAACTGAATCCGCTCAGCATGCTTGCTTGGAGAACTCTGAAGTTGCTTTATAG
AATGTGTGCTGATACGACGCCAACGGATGGGCAGATACCTTTCTGGAATCTGAAAGCAAT
TTCCTGTTGAGAGGACTCGTAAAGCATGTCGACAGAGAATCCCTGAAAACTCAAACTGAT
GACAGCTGCAACTTATGATGCCTTCCTGAGGAACCCAATACACTTTCCGGCCTCCCTCAA
GTTTGGTGTGGTGTCTCACAAGGAAAGCATCATCCATATGAAATGATGCATAATGAGCAG
CTAAAACAAATTGCTCTTGCAGCTTTGAGAATGCAAAAGGGGTGAGGACAGAAGCAGCAT
GGGATTCCATAGGAGCACCTGTCTTCAGGCAAATATTTTGGAGATTCTGCTGCATCGTCT

GTTGTTCTGCAGCTTGATCTTTAAAGTCCACAGCAACGGCCACTTGTTCTACAAAGTGCG
CAAGTCGGGTTTGAGCACTCAAAAATCTTTGAATGAAAGAATTGATTGCCTTTGAGTGGA
CAGTTGTAGTCATTCCTGCCTGTTAGTATGGAGCCCAAATGAATTGACCATGTCCCTCCA
TCCCAATTCAAAATCTTCTATAGACTCCAGATTATAGAGCCGAAAAAACTCAGTCTTCCA
CTCATTATAACGTTCTCCCAGGACAGCATTAAACCAGGAGGGAAACTTCACTACAATCAT
CCATATGCAGAGTGCATGCTTAGTTGCAGGCATTTCCATGGCTATGGCTTCTTTGAGACA
CATATTCTGGTCAGTTAATATTGTCTGTGGTGCCTTCCCATCCATAAAACCTAAGAATGC
CAACACATCAGTGATGCATCTGACTGGAAGTTGAAACTGAGAATTGTTCAAGGAATAGCC
CTAATTTGCGCAGGGAGGTGATGAGAGCCCTTTTATT
>tig_1325_consensus
CTGGTCAGCTAATTTTATATTTAACTGAGTCACTTTTACAAGAAGTGATTCTTAAGATTT
GGCAGGCAATTCTGGGCTAATAACATTGAGCCTTGAGCCCACAAATAAAAAAATTAAGAC
TTGAGTTCTGAAGCCCCATGACATGAAAAATCACCAAATAAGTTTTATAGGATAAGCACT
AGGAGAGAAAAAGGTTGGAAAATGACTTCTATTCATTACAGTCCTTGCTACTATTAAAAT
CCACGGAAGCAGAGAAGGAATTACTAGAGAATCTGCAAAGAATGATGGTCTATAAAGGCA
AGTCTTTGAAGACTGAAACTACAAACAGCAACTCCTATACGGTATAGAGAAATCTGCATA
TGAATCTCTGATTCAATCATTCCTGACCTAACTGTCAATGGTTTTGGTTGTAATCAAAGC
TCAGGGGTTGCTGACAGAAAGGAGCCTGAAAAACAGCATGTCTAAACTGCTCATATGAAT
TTGCATACATCATTGCAGCACTGGGCCATTGTACATATGCAGCTTCCATAGATGTTTGTT
GTCCGGTGGCACAATAAGGGCTCATAATAGCATTTGCTGGAGGCCTATAGTTGAGGGGAG
CTTGAGTCCTAGAGATTTGACCCACCTGGGGAAAACTAGTGTCATACTGTATGAATGGAA
GTTGAACTTTCTGCATGTTGAAGGGTGCCAAGCCAAAGCCCTGATTAGCTGGAAGGCTCC
TAACATACCTATCATAACTCCGATCATAATCAGGATCAGTACGATCCTTCTCCCTATCTC
TGAAAATAGCAACTCGAGAAGAACTACCATCTCTGATGCTAACAATCTTTTCTGAATCAA
TAACAGAATTCCTGCTACTTTCGTTCTCATCTCTGCTCAAGCTTATACCTTTCCCATCAT
TAGGGACCTGAGACAAAGTATCTTCTGAATCAGAACTGCCAGGACTTCTAAATATGCGGG
CACGTGCCCTGTCGTACTCCTCCTTCCTCTCTTCAACACTTCTGACAGGACTTCGTTTGA
CCCCAAATTCATTAGCCTCAATAATAGATCCTCTATTGGGCCTAGGCCTAATGACAATTT
TAATCTGATCAAGCTTATCACTTTCTTCAGACTGTTTAGCAGGAATTTCAGATAAACGAA
CAGCAGGATATTTGCTTTCTGCTGTTTTCCTCACCAGAATTCGATTACCCAACCCATCTA
AGCCATTCTCCTGCACCATAGTTACCAGCCCATAGTGTTGAGAAACACGATGTGCAGCTA
GTCGAAGATAAGAAGTAGGAAACTGCTGGAACTCAAAATGCTGCTGATCAGGATTTTGCA
AAAACCTCTGGATATCAAGCTCCATCCGCAGAATGGTGAGACGATGACGAGGGTTCTGGA
GAGCCTCAACCAAAAAGGGATCCACCATTGACTCTTTATCATTGACGACTGTTGTGGCGG
CTGTTGTTGTAGAATCCATGAATAGCTTCTCTCTCTCTCTCTCTCTGTTGAACTAACCCG
GTTTTCTTCCCCTTTAATCTTCTTCTCAAAATCTCTTATCTAATTCCCCCCCGACAGAAA
AAAGAATCAAAAGGAAGAAGGAAAGGGTATCTTCGATTTGGGCATTAAGATTAGAAAAGA
AGAGAAGAGAGACGGAGTGGGTTTAGATTTGGGTTGGGTTGGGTTGTTTTGATTTGGTAT
GGTTTTGTTAATTTGTTATCCTTCTCACCGAAGGAGAAAGGAAAGGAAAGGAAAAAGACA
GGAT
>tig_1326_consensus
GAGGGATAATAACCGTAACTTCTATGATGGAAAAAGTAAATAAGGTATTGATTAGGAGAT
TCTTCTGAAATTTTGGATCAGTCATGAAATTTAAAAATAGTTCATTAAGCCCCTTGAACT
TTTACGGGAGAACGATATCCACAGATAAAAATATGGCTGTATCAGAAATTGCATGAAGCC
ATTGAAGAAAACAAAACCACCCACAATGCCAATCTCAATGTCCGCCAGGATCCGAAACAG
GACAGATTCATCAACGGCAAACTTGAGCCCTTGGATCAGTCCAACGCTGGTAGCTGCAAT
ATTCCAGGGTCTGGTCCTGGTTCATCAGTTGATCAAACCAGGACTTAAGAAGTCAGTGAA
CCGGGAAAAAATGCTCAAGGGTTCGATAGATGAGTTCCAAATTTTCATGATCAGAATGCAA
ACTGAGTATAGAGAAACAGTTGAATGCAGGTATTTAATGATCAAAGGACAAAATGCGGAT
GAAAAAACCATTGAGAAGTTGATAGTGAGTGGTAAAAGTGACAGGTTTATTGCAGAAAGG
TTATAGGCCTGAGAGTTCCAGCAATAACTTATATTACTAGCATAAGGGTTTCATTGAACA
CAGACTTCAAGCTAGATCCATCTGCAACTCTTCTGTATTGACTATAATGAAAGTTTAGTA
ATATAGCAATAATTAGTAATCTTATTGTCATCGGAAAAGTCATTGCTGTATAGGCTTTTG
TATAAGACATCATTTTGTCTGACAGTTTTTTCATTATACAGAAAAAGTGATCCTGCAGAC
TGCAGCTTACACTTTTGATCCAAACACCCAATTTCAAAAAATTCACCTTCTTCCATTCAA
GTTTGATATTTTCAACCACAACCCCCTCAAACATCTCCCATCTTCCCATAGCTCGCATGT
TCAGCAACCCAATCACCTGTTCATCAACAGTGACTCTGAGATCTTTCCTCAGCCTTCCCA
GAAATCGTGGTACCAAATCATCAATCAAGTGCCAGCATTAGGCAAAGCAGCCATAAATGA

ATTGTATGAGGAGTCCAGATCGAAATGTAGTTGCCGTGCCTGCTGATCTGTCAACTCATC
TGCAGCCCCCATCTTCGATAACCTTGAAATCCAGTCCTTCATCTTGGCCTTCCCCTCAAA
GTCCGGCGGCAAAATACTCAACTTATTAAGTGAACCCAACAGATCTGATAACAGCGGATA
CACTTGATCAACTGCCTTAAATTCCAATTTTAATGTGTCCATTGCAGTTATAAAGTGCTG
GACACATTCTGCAACAATGGCAGCAGAGTTGGCAGTCAATGCAGCTGCTGCAGCTCGATG
TTCAACTGTAGCAGGCACCCCAACAGTAACTAGACGATTCAGAGCAGCCGGGCAGTCCAT
TTTATAAGTATCTACAAACCGTTCAATACTCGGGACAGTATCTTTCAATGTAGAGGCTAG
AGTTTTAAAATGAGCAATGAGTTTTTGGCATTCAGCTTCATATTCATTTGAACTCAAAAC
ATCACGAATATAAGCCTTTTCAAGCTTTTCAGTAGCTTTGATTATAGCATAAAGCTCGGC
AAAGTTCTCATACATTTCTCTCTCGCGCTTGTCATTCCATAGCTTCAACTCCATTTGGGC
AGACATAAAACTATATATAACAGTTTATAAGCAATATATATTTTCAGGTTGGATAAAATT
TTTGGTATTTTCTGATTGAAAGTGTGGAGGACTGGAGAAGAGAACGGGACACGCGCAAAG
AGAACTAACCTTCTTCAAAGAGAACTTTGAATGTCGTGAAGAGGGCTGGGCTGTCTGGTG
CTGCAGAGGAGAGAAATAATAAAAGGAAGATTGAAGAGAGAGAAGACAGGGGCCAGAG
>tig_1327_consensus
CACAATAACACCAGCATTATCTGTGCAAATAAAAACAAAACAACTGCATCAAAATCTAGC
ACCAAAAAACACATTTGTCTTCTACTAACTTAAAACATGAAATATTGTGAAAAGAAAAAT
TCAGTCTAAATATAGGGTAATGTTCAAATAACCAGCAAAACAGGGCAACATTTAAGCTTT
AAACATTGATTATTTCAGCGACTGAAAACATATGAAATATGAGACTACTAGTCACAAATA
AGTACTGTGCCCAAATGGAACAGAATGATATAAAAAAGCATCACCACTTCTGTAACGTTA
GACACAGTCATTTTTCCAAACTCGTAACTGTATCAACTGTCGAGAATTGAGGTGGACACC
TGACTTATGTCTGGTGAACAGAGATGATTAAAGCTACAAACCAGAACCATAACATGCTCA
ATATGATCCCAATACACCTTCCAAATCATTTCTTCCTAAAGTAAAAAAATTACATCTTTG
AGCCAAATGAGAACAAGAATTCATCTCTAGAGCGAAAATAACTATGACAACCTGACAGTG
ATGATAACAATTTTNNGTATGGGAGGAGCAGACAAACCTAAACAACAATCCTCAGCACCA
CATCACCATCAAAACAACCCAGCAATGGCAATTTTAAATAACATAAAATACTAATCAGAC
AGCAAAAACAAAAAAAGATGTG
>tig_1328_consensus
GTGCTCTAAGTGGTCTACTATTGAGATTAATAGAAATTTAAGTGAACGTAGCAATATCAC
AATACAATACTTCTCACATTACACACACAAAATATAAAATAAAATAGAAGAGATTACGGC
TCTGATTTTATGAGGTACATTTTGCAGATATCACCCATTACTGTAAGGCAACTCCAGAGG
CCCATGTTTAGATAGAATGGACTGCACAACCTCAGGGCAACTGCTGTATAGCCTCCACGG
CAATAATTTCTCTCTACCAATAACAAAGATATTGAACAAGGACGCATATGACAGTTGATG
AATTATGAAGCATTGAAGAACATTTACGTATTACGATCTCAATGGAAGTCAGATTCAAGG
AACATTCATAACTTGGATTGACTGCAACATGTCTGAAACGGCAATGACAAGATCACCCGA
TTTGATCATACCCCTAGCCTTAAGCAAAGAGAAAGTTTTATTCAGGTTGCTTTCCATATC
ATCAGAGAAGCTCAAGCGGAATGGTATCAAACCCCATTGTAGGTTCAGGCGCCGTCGCAC
AGATGTTGTGGTAGTAAATGCAAAGATTGGGCAGTCGGGTCGGCATCGTGACAAAAGAGA
TGCCATGTGGCCGGTCTTTGTGTACACAAAAAGCGCATCTACCTCTAGATTATTGGCCAT
CTTGGCAGCAGAAATACAAATTTCTTCTGAAATACTGTCTGCAAATGAGGATCCAATATC
TGGGAGTTCCATAGCTTCATGACGTTTCTCTTCCCTCCACCACTTCTCAATTCTTAGACT
GACAGTTCTAAGAATAGTCAATGCTTTATCTGGGAAATGGCCCATAGCTGACTCACCTGA
GAGCATCAGAGCATCTGCCCTCTGCCGAACTGCTTCAGAAACATCAGCTACTTCAGCTCT
GGTTGGTGTAGGGTACTCAATCATAGATTCAAGTAACTGAGAGGCAACAATAACTGGCTT
ATTCAGCTGCCTGCACACTTGAACAATCTTTTGCTGTGTTGATGGAACCTGTTCCAGTGG
TATCTGGGCACCCAGATCTCCTCTTGCAACCATAGCTCCATCAGATGCTCGAATAATCTC
TTCCAAGTTTTTCAACGAATCAATACTCTCAATCTTTGCAATTACTGCAATATCACTATC
ACGAGATCGTGCAGCGATATAGCTCTTAAGATGATTAATCACTTCAGCTGATTTGACAAA
TGAAATGGCAATAAAGTCAACACCCTCTGCAATTCCAAAATCAATGTCCAACCAATCCTT
AGATGAAATTGTGGGAAGCATTGCATTACGCTCCCGTACAAGGCTACCATCCCTCCAGAA
TGTTAAATTAGCTCTTGGTAGCAACAGTCCAGGATCAGTGCAACGGCATTTCACATCTGG
ACCGATCTTCTCGATCACGTCAAACCTCACCATTCCACCATCCACAAGGAGTTCATCCCC
AACCCTCACATCCTCAGTAAATCCATCATAATTAACATTGATAGTGCGTTCTGGGAGAGC
TGCATCAAAAGCTCTGACAGTAAAAGTCCAGATCTCGCCATCCTCGGCTTTAGCGGAAGG
AGCGCCACCAAAATCACCCATGTGAATCTCACTACCTTCAGTGTCCATCATAATCGCTAC
CGCATAACCTTTCTCTTCATTCAACCTCCTAACGCGTTCGATCACTCGCTTGTGCCACTC
GTGCGTGCCGTGGCACATATTAATTCTCGCCACGTTCATCCCCCCAACCGCCAAGGCCTC
CAGCTGCTCAGACCCGCACGAGGCCGGCCCAATCGTGCACACCAGTTTCGTCCGCCTAGT

GCTCCTGAAACCGTTCTCCTTCAATTCCACTTCCGTCACAGCGTCCACGTCGATCGACGA
GGAATCAGGAGAAACCGGTTGTGATTCCACCACGTGTGGCTCAGCCTCGGGCATTCCATT
GTGAGTAGTGAGTAAAACTTTCGGATCTGAAGACGATGATGCGGAAGCTCTAATTACAAC
AGGGCGTTGCTTAGTGGTGGCGGTGGTGGTGACGTGGAAGCGCCGTGCGGATGTTGTGAA
GAGAGCCTGATTGTGTTTGGGATTAAAGAGAGTAATGTTGGAGGAAGAAGTGAAGAATTG
AAGAGATTGCGACATGACAATCAATAACGGAACTCTTTAACGGCTAGGGTTTGAACTTTA
AGAAGCAAGAAATGAAGAATTTTATAGGATTCGGAGGCACGGAGGCGGACGGAGTCGACG
AAGGAAGTAAAGCACTGTGTTTGTTTTGTTAAGCTTCGAATATGTCGCAGA
>tig_1329_consensus
CCCCCACCCATGCCTTTCACGTAGTAAAGCGGTTCAGCAGTAAAAAAATTCCCTGCAAGA
ACAAATTGACACGGAGAAAAGGAGAGAGACATCCTTTCATTTAAGAGAGGCCTAAAAATT
GAATTATCCACCACATCTGCAGCATAGGGGACTGCTACGTTCATTTTAAACCTTNNNNNN
NNNNNNNNNNNNNNNNNNNNNNNNNNNNNNNNNNNNNNNNNNNNNNNNNNTGGGTTATAAA
AATTAAAGTAAAAAAGAAATTGAATGCCCACAATTTACAAATTCTCTTTGCTCTTTGAAA
TTTCTACTCTAAACATGGAGAGGGCTCAGACACATCACATGTGGCGGCCCATCCAATCAC
AGACGCAAGAACAAATGCATACCATTCACCATGCAAAAGCCGTGAAAGGCCGTCGATATT
CAGAGCAAAGCCCTTTAAGCAGTCAACGGCATTCCAGCTAAACACCGAGCAAGAACCTAA
ACACATCACTCAATGAGATGATACCTTGTACACGCTTGCTGCCAGCCTCAACAACAATAA
GTCTCCTAACCCCAGGATTTGCCAATCGATCCATCACTTTGTGTAAAGGATCAGATCGCA
AACACATCTGACATCTNNNNNTCTGAGCATAAACTCTATCCTTCGCCAAAGCAGTGATAT
CACTTCGTGAATATATATCCACCAATGAGTCATTATCATCCACAATGGGTATCGAACTGA
CTTCAGCTTGAACTAACAAAGACAGGGCAGCACCAAGCGAAGCATTTGGTCTCAACATCG
CGAATGGCCGCCCATTTGATTGCCCAATTTGTGGGACCCATGTACCCAAACGAATTGCAG
ATATAGGTTGTTGAAGAATGGGCAATGAGCTAGAAGAATGCTTAAAATGGCGACATATAC
ATTTTAGTATTCCAGACAAGGAAGCAAGATGTAGTAGCTGTGGAAATGAACCATCTGGAG
ACACTGAATTAATAATGGGAACTGTGGNNNNNTTTAGGATATCTAAAACCATTGCCTTCA
ATTTGTCGATTAAGATGGGCTTTCCCTTCCTTCCAAGCTGCTATGGTATGTGTTTCTAAT
TCCTCCTCCGTTAAATTTGATCCATGCGTCCCTAACTCTCTTAAAATCATGATGAAGTCC
AATGCAGTAAGAACTCCAACAAATTGGCCCCC
>tig_1330_consensus
TCAAGAATTTCTGTAACTCATGCAACTATAAGCAATATCAGAATTGGAAAAACAAAAAAT
TAAAGCACATAACTTTGAGGGAAGGAGCTGCATTGATCTATCCAGATTTAATTCAATAAG
ATGTCAACCACCCAAATAATCGAGATACAAGAAGCAAATAGAGAAGTAAATTTAAACAAT
GCTAGACCAAAACACACATTTCGACTGTAACTTATAAAACAAGAAAATTTAATTTTCCTT
CTAACCATCACTCAACTCAATCTAGCATCTACAGACTAGAGAGGACCTTAGATGTTGCTG
GGGTACATAAAGACCCTAACTCTATCACCCATGGACTTTGGGGGTAGGTTGGACTTGAAC
TTAGCACGAACAACACCACTATTACCATGAGGCCTGGTGACCTTGCCCCAAATGCACCGA
TAGTGTGACCCGTTCTTCTTCACCTTAGCCTTGTAAATGTAAGCCATCCGCTTTCCAGCA
TACCATGCCACCTCTTCCTTGGTGTTAACTCCCTCAATCTGGATCAAAGAGGTGTTTGGG
TACTGGTTTGCTTTTGACCTCTTGTATCCGAGGATGGTTCCTCTGACATATAATCTGACT
CGCTCTCCTTGGCGTCCCTTTACCATTTTGAGTTCAGTAGCTGAAGATCTCTGGAAGTGA
CCTGAACGGAGCTGAGGCGCTTTCTGAGAGCGAGAGAGAGATATAAACCTAAAACCCTAG
C
>tig_1331_consensus
GGACACAACTAGCGCCCAATATATAGAAACTTGGACGAAAATATACCAAAACAATAAATC
TGACACAAAACCCTTCATGTATAAAAAGGACTGCAAGCATAAATCATAAACTGGGCTTAT
CCCCTGGCACCACAAACAATCCAACAACATTTCAAGGTTGACAATTGTATAGCTCATTGG
GCTCAGCTAATAATAAGCAGCATCAGAATTGGAAAGAAGGAAGCATGCCACAGAGAGTAG
ACCTATTAAACTTAATCAGATGTCAAGCACCCAAATATTCAAAATACAAGACACAAATAG
GGCGACAAATCCTGACATTAGTAAATTTAAACAATGTTCCATTTATCATCCTAAAGAACC
AAAATTGATTCAGCTGAAAACACAAACACTTCAACTTAGCTTATAAAAACAAGTAAATTG
CATTTTCCTTCAAGTCATCACTCAACTCAATCTGGCCTCTACAGACTGAAGGACCTACCT
TAGATGTTGCTGGGGTACATAAAGACCCTAACTCTATCACCCATGGACTTTGGGGGCAGA
TTGGACTTAAACTTGGCACGTACAACACCACTGTTACCATGAGGCCTAGTGACNNNNNNN
NNNNCGCTTTCCAGCATACCATGCCACCTCTTCCTTGGTGTTAACTCCCTCAATCTGGAT
CAAAGAGGTGTTTGGGTACTGGTTTGCCTTTGACCTCTTGTAACCGAGGATGGTTCCTCT
GACATAGAGTCTGACTCGTTCTCC
>tig_1332_consensus

CTATTTATATGAATATATTGTAGTTAAATTAGTTCAAACATTGCTAGATCCCCCACTTAA
TCAACTGATCTCTGCTATCGAAACTTTATTACATAAAATATATACCACTTAATGTTACTG
CCTGGAAACGAATTATGAACTTATCCAAGCCCGAAGAACACTCTAAACTATGAATATATT
ATTATTAAATATATTACACCGGAAAATGTGCAGCGTGCGCATGGTCCTGATCATGCTCAT
TGCATTCAAGGACACTACTTGTAAGCTTTACAAAAGAAAATACATCTCAAAATTGGATTA
GGACAACATTGCAATCTGGGGCCACTTTTGTCTCAGTCTTCAACGGAGGCTTAAATGAGA
GAATGGCTCAGGGTTTCTTTTAGTCACAGGTTTAACCTCTTCAGGGGTCCCAAAGACATT
CCAGAATCTCAGAGTCTCATCTCCAGCTGCAGATGCCACTGTGTATCCATCGGGGCTCTG
AGCCATGAAAAGAACTCTAGATGTATGACCAGTAAGATCTGCCATCTTCACCATTGATGG
GTATTTCCAAAGGCTGAGCTGATTTTCTACATGGGTGTTCCAGAACTTAATGCATCGGTC
ACCCCCACCTCCACCAGAGGCTAGCAAATTCCCCTGGAATGGACACCAAGCAAGGGCTTT
TACTGCAGCTGTATGGTCTTCTAGTCTGTGAAGCCACTGGGTTGGTGAATTTGTAGAGGC
CATGGAACCGTCCCATATGAAAAGGAGATTATCATTTCCCCCACTTGCTAACTGCCGGCC
TGAAAGTGACCATCTGAGTCCACAAACCTCTAGCTGGTGTCCCCTATAGGTATCAATGAT
GTGTTCTCTCACTCTAACATCATTGTTAATGATTTTACCATCCATTCCTCCAGTAGTAAG
TATATGACTGTTCCAAGCCAGTGAATTAACTCGTAATCTATGGCCACCTCTCAATGTTCT
TAGCAGTCTTCCGGAAGTAGAATCCCATATCTGGACATCTGAATTGTTCAAGCCAATGGC
TATGCGCTGCCCATCTGGAGCCCAACTAACACTTGTAACTGGACCATCTTCATCATCAAT
GGTAATAAGTTCTGTAGTAGAACCATTTGAAGCATCCCAAAGATACACAGTGTTGGTCAG
AGCAATGGCCAGCACATTTCTGTTGCCCCAGTCTAGCAAATTCAGGTAGAAATCATCTAC
AATATCAGGGGCATCCAATGTCCTCACAGAAGACTGAGGAATGTATCGTTTGGGCTTGAG
AGGTTTGGACTGATAAACAGGAGAAGAAAAATGCTTTGGAAACAGATCAGCCACTGCAGG
CGCCTTGCTTTTGAACGTCAATATTCGAGTCCTGTTCATATTCAAAGCCTCTGCCAATTG
CCTTCGGTAAGCCTCTCTTGACGGAGAGCATAGCATGGCTGGATTTTCTTTGCCCTTTCT
TCCATGTTATCCCTGGAGCCTTTACTTTGATGAAACCGTTCTTGAAGTGGGCTTCTTGAG
TGCATCTTATTAAACGAATTCATTGGCCCTGCATCCATAAATTTACAGAAATATATAGAA
AACAAACACAAAAATCAAACAGAAACAAGAAATCAAGACTGCAAAAACACAAAAATTCAG
ATTAAACAAAGCAGGAACAATCAAAACAGAAATTTAACAAATAAAACAGAGTAGAGAACG
AGAAATTCAAATTTGAGAAGGCCCCTATTCTTGACGTTTCTTGATTTTAGAATCGTCAAA
TTGAGAAGAAATTTTATGAAACAAAGGCGTG
>tig_1333_consensus
CCCACGTTGATTTTGTTTAAAGTTTTTAATTATATGGTAACATATTATTGCACAAATTAT
GCATTATATTTATTCGACACAACCATATTTATTGGATTCATAATATACACATCACAACTT
CGAAAACCGATNNNNNNNNNNNNNNNNNNNNNGATTTAAGGGAATCTATCATTTGATGAGA
ATGCATCTGATTGGTGATCCGTCAGCACCAAACAACCTTAGAGGTAAGCAGTGGACTGAA
TATAGTCCTGCTGGCACATCGTCAAGTTTAAGGGCTTCCACCAGAATGATTTCCCTGCTT
TCTAGGAATTCATAATGAGCGGTAAGAAGATGATCAAAGGCAGCTACAGATAAGTAGTCA
ATCCCAACAAGTTTGATGTCAGTGTTCTTTGCCAGCCATCTTGCCCCATCTTTTGTAAAT
CCCACATAGCTTGTGTCGAACGCTTTATTAAACATCAGCTGCCTGTCTGTATTTAGTGTT
CTGAAAAGCACACGTTTTACTCCATTGGGAATGTGCAGGGACTGCATAACTTCAGCAGTT
ATGTTCTTATCTCTGGGAACATCAACCAGTAGCGCAGGACCATTCAAGACGTCCAAGTCA
AGTGTATCAACATCATAACCAGCGTCAAAATAGTGATCAAACACGTGGCCAGGAGCATCC
ACGTGCGTTCCAGAGTGTGCGATGAACTTCAGCTCCGAGATGTTAACGATGGAGCCGTTC
TTCATGTTCTGAGGCAGCGACAGAAACTGCCCCAGCCTTCCGTCTTCGGTGTCATACGAC
GGCATGTCGCTGGTGTAACGGTGGCTTATGTCGAATATTCGGCCACCACCGTACACCTCT
CGCCGAATCGGCTTCGGTACTTGGTCTGACGATAAGGAGCAGTCGGAAACTGATGGATAT
GCATCGGTTGCGGCGAAGGAGAGGTTGAAGCGAAGTGAAAGTAAAGCGAAGAAGAATAAT
CCGACAGCGGTTTTGGCCATTATTGGATTGGAGGTTGTTCTTGTGAAGAGGATAACCGTG
CGTAAGAATTATAGAAGCTAGTATATATTTTTTTCAAGTTGGAAAGGGACGGATTGATGA
TTCATAACCGTAAGTCTTTTCTTCACGACCTACGAGTTTGCAGTAGATGACAAAATTATA
CCGAAACACCGAAGCTC
>tig_1334_consensus
AGACCAAATTTTCTGCATGATTCTATAAAGAAATGATCTTACCATGTAAACATTGCTAGC
CTATACAAGACTCTACTCTTCGGGTGCAAAAAAATATCCTAGTCCTAGATGACGAATTCT
TCCTGTATCAAGATGAACATGTACAAGAAATAGAATCAAATAATTTTCCTCTCAACCTAT
ACAACTAAGAAAACGGCCATTACATGGCCACTACAAAATTAATTGGGGTAATCATCTAAG
CGCTGGATTTCTCCCATCATGATGCGATTACTTGTAATTTTAAAACCCAGGAGAGCAGGA
TCAATTTGCCTCCCTTTCCTACCTTTCTTTTTACCACCACGACCACCTAGGGGTACATCT

GGATCTGATGTCCCCAGTGTCGTGTGGGTGTCGGGCACAACATTTTTCTTGGTATTACTC
TTCAACATGTCTATAAATGATGGCTCTGAAACATCAGCATCACCGCCAGATGAGGTTCGG
TGAAACCTTATTTCTCTGTTCCCTGAAGCCATGTCTGACCCTTGGCATCTGACATTTCCC
CCAGGATCTTGTCTCCCATTTGTACAAGATGAAAGTTTTCCTCTCATAGCTGGATTAAAG
GTGAGATCAGTCAGTCCTTCCTGGGACGATGAAACACGTGAGACTGGTGGTCGTCTCAGC
AAAATATTTTCTTGAATTCTGGATGTGACGTGCAGCTTATCCTTGGAAATATCTTCTGTG
AAAAAATCATAAAAACCAGCATCACTACCTGCAACATGTGAATTATGTCTGCTAAGAGGA
TGAGCTGGAATTTCCATCGAATCTAAAGTAGCCAAACCTGCTTGCTTGGCCATTCCATCT
TGAATCTCAAAAATGGAACCCTTCCTTGCATCAACATCAGAGAACTCTGTGTTTACAGAA
GAATTACCAATCATGTTAGAATCAGTGTAAATTGCCTGAGCACCATCATTCACACTTGAC
AAGAATGGCTCTCCCTCAGACATTGCTCCAGATTCAGGTCTTAACCATAATTTCTCAGTG
CTATCAAAACTGCTAGCCTGCTTGTCAGCTACATAAACCTGCTGAGGGTCAGATGAATTA
GAACCATAAGATCCTACAGTAAATGAATTGGTTGGGCCTGCTTCTTGGTCTGAATGATTA
CTAAATGGGTGATAGCAAGAGTTTGACCCAGAGTAGATTCCAGATGGTGACCTCCTACCA
AAGGGTACACCATTCATATTCATGTCCATTGACTCAGCAGGGTGATTACCTGACCTCTTA
TGGAGCAACTCCATTAACAATTGCCTTGACTTGTCATCATGGGACCCATCTAACATCCAT
AAACTTGGATCTTCAGAAGGCATTTTAACTTCTGGCTCCCTTCTCTGCCGCTCAGCATTA
ATGTGCAACTGTTGAATTTGAGATTCCACCCACTCATTTGCCAGGCGCCCATTGTTATCA
GCCCGGCAACCCTCCACTGCATCCAAATGTGAAGCATTGAACTGGCTAGGGGCTAAAGGG
TGGTGTAAATTATGAGGGTGAATGCCAGAAGGAAATGTCCCTGCTTGACCAGCAGATTGC
ATACGTGAGCTCAACTCCTTTACATCCAAACCATGATGATGTGCCATAGTATTAAGTGCA
TCCAAATTCATCCTTGGAGCACAAGCAGGCAAAGACATGGACCGCTCCAATGATAGTGAA
CCAGGCTCAAAAAGTCCTTGCCGCAGCTGCTCTTGTAGTGAAAGATTCCATTCAAGCTGA
CCCAGCTGCCCCTCATGAGTTGACCTCTGTTGCTGCTGGTAAATATCTAGTGGACTAAAC
CCTGAAGAATGAGCAGCAGAAAGACTCCTATGAAATAGATTGGACTCATCAGCAGGCCAA
ATAGAATCAATACGCAGCCCTTCCTGCACATTGGTTCGCTGCCTTAATCCCATTGATAAC
TGTCTTGCTCGCATCTGGTCCTGAAGAATCTGATGCTCCAATGCTGGCATTTTCCCATGT
TGAGCACGTGACATTAACTCAAAGATTTCTCTTTGCTTATCTTGTGGTGGCACCAGACCC
AATTTCCTTTGCACAAGCTGATCTAAAGAT
>tig_1335_consensus
ACTATACACAATTTTTTCACTCATCACCCGCACACATTTAGCATTAAAGCAGGCACTGAT
TCAAAAAAGAACAAGAACATCAAGACACCTTCTCAATTGACCAAACAAGTGGCAATGGAC
ACCAGGAAAATGGGGCGGCCGAGAGCTGTCTATACACACCATCATGAATGATGAAAAGCT
TTCGGAATCTTTTTGCCTTACAATGGGTTGCTTTGTTGACTACGTTTTTAATTTCTTTTC
ATCTTCTGGAGAAGGATCTTCATGTACAAAGAAACTTGCAGCGACTATCAGATAACAAAG
AACAAGCATGAGTCCCTTAAAGTAATTAGATGTTCCTTCCTGCAGAAAGAAGGCAACAAC
TATGACAGTTATGAAAAGTGTTGCCGTCTCAAAAAGTTGAAAGTTCAAGTCCATAGTGCA
ACCCATCATCCATCCAACAACCACACAAAAAGGAATCCCAAACATGGATATTTGTGTTGA
TGATCCTATTGCCACTCCCAAAGTTATGTCAAGTTTGTCCTTCATTGCAAAAATTATAGC
ACTTGCATGCTCTGCTGCATTCCCTACAATTGGAAGTAATATAACACTAATAAATGAAAT
TGGAATGTTCCATGCGGTAGATGCTCCCTGTATAGCATCAACTAGATACTCTGAAAGGAT
AGATATCCATGCTGTCATAATTGAAAGCCAAATAATTGATTCCCACTTTGAGATCTCTGG
CTCTTCATCGTCGTCATCATCACTATCAGCAGCATCCGTAGTCTGATTCTCTTCCTCAGT
AACAGTGAAATATTGCTCTTGTTGACCTTTTAATTGGAAAACAAGAAAGGCAGCATATGC
CACTAGCATAATGCAACTGCTAAATCTTGAAAGAGCCAGCTCTGACTTTCCAAAATGCAC
CTCAGTATGTGTGTAGTGAAGAACAGCTGGAAAGAGCAGGCTCATGACTGCCATCAATAA
CAGTCCCGAATTCACAACAGCAGATGCCTGTCGCACCAGTGTAAAAGGCCAGTTGCTCTG
TAGCATAACCTAAACGCTCTGCCAAAGGAGTTATACCCAGCAAGCTTAGAAAGAAGAC
>tig_1336_consensus
AAGAGATCTTTGCCTGGTTCGATGCAGTAAAGAACACAACCACCAAAGAAATAAAAGGCA
ACTCATTGAAAAGAATAAAAGAAGAGGAATGAATTGTTATGCTCTGTAATATACACAATT
CTCTTACTCATCTTAAACTTACATTTAGCATCAAAGCAGGTACTGATATGATAAAGAACC
AAAACATCAAGACCTACTCAATCGACTGAACGAGAAGCACAGGAACACCCAGAAAATGGA
GCCGCCGAAAGCTGTCTATATACACTGCCATACGTGACGAATGCTTCAGGATTTTTTTCT
CCTTTCAATGGGTTGCTTCCATGACTATGCTTTTAGTTTCTTATTGTCTTCAGGAGAAGG
ATCTTCATGTACAAAGAAACTTGCCGCAACTATCAGATAACAAAGAATGAGCATAAGGCC
TTTAAAGTAATTAGACGTTCCTTCCTGCAGAAAGAAGGCAACGACTATGACGGTTATGAA
CAGAGTTGCTGTTTCAAAAAGCTGAAAGTTCAAGTCCATAGTACGACCCATTATCCATCC

GATAACCACAGAAAATGGAATCCCGAACATGGATATTTGTGTTGATGATCCTATTGCCAC
TCCCAAAGATAAGTCAAGTTTGTCCTTCATGGCAAACATGATGGCGCTTGCATGCTCGGC
AGCATTCCCTACAATTGGAAGTAATATAACACTGATAAATGAAATTGGAATGTCCCATGC
AGCTGACGCTCCCTCTATAGCATTGACTAGATATTCTGAAAGGATAGATATCCAAGCTGT
CATAATTGAAAGCCAAATAATTGATTCCAATTTTGAGATCTCTGGCTCTTCATCACTGTT
AACATTTTGATCAGCAGCATCCGTAGCCTGATTCTCTTCCTCAGTAAGACGGCGATATGG
CTCTTGTCGACCTTTTAATTGGAAAACAAGATAGGCGGCATATGCCACTAGCATAACACA
ACTGCTAAATCTTGAAAGAGCCAGCTCTGACTTTCCAAAATGCACTTCAGTATGTGTGTA
GTGAAGTACAGCAGGAAATAGCAGGCCCATTACTGCCATCAATAACAGTCCTGAGTTCAC
AACAGCAGATGCCT
>tig_1337_consensus
CTTCAGCACAGAATAGGTAACAAATAGATTGAAGTTTTCCAAGAATGGCAGTACAAGGAA
TGAATTCCATCCACAACCGACGGCAAATGTCTTTCTGAAATATTTACTCTTACCAATCTC
ATTTCATTTTCTTCAGATTAAGAAACGCCTCACCAAGTTCAGATTCACAAGCAGCATGAG
CATCAGGGGTTAAATCTTTAAGAAAGGACTTGGTCAATTTAAGAATTGATACCATCTGAA
AGATCTTAGAACAGAACTTGCGTACTTCAGATTTCCTTGACGGCTTCTCTGGCATATGTG
TAACCAACTCCTTCATCAAACAACTGAGATTTCGGAGGTGACTCTTGAAGGCTTTCTTTG
ATGCATCCTTGCTGGCTTCATCTGAGCTTAATGGAACCAGTGACTTCAATATCTCCATCA
CCAAATCAAGTGCCTCAACTTGACGAAATCCTAATTTTGCACTGCCACATTTTTCCAGGA
TGAAGCTGAAGAGATGATGCCCAATCCATGGTCTTCTTCGAAATATTTCTTTCAAAAATT
CAGATTTTATTTGAGACTTCTTACTATCAAAAAAACTAGCCAACACAATCTGAAAAATAT
TATAAACCTCCTGCAAGTCAGAATCTGAAAATTTTCTAGCATCAATGATCTTCAGAATCC
AAAAGGTTGAATTCTGAGCCAGGGAGACAATCATTTTGTATCTGTTCCAGGACGCTGATT
GCTTCTTCTTAGATAGATTGGCAGCAGATTTCTTTCTCTTGAATGGTCTTGCTGTCAATT
TTAAGTTTCTCTCCAACAAAGAAACAAGAGTAGGTAGTGGCACGGATTCACCCTTAGGGA
AGTCTTTTGCTTTGAATATTTTCTTCTGTAGAATTCCCCATATGCGTTGTTCAAGCTGCT
CACTACCTTCGCTGTTATGCGGGTTAACAAATGCCTGAGCCAACTTTGAGTACACCATCA
GAACCTGGGGCTTACCTGGATTTTGGTGCAGGTAAATTTCCAGCAATGATAGAACACGAA
GTTTAAACAGATTAAGCTGCGATTGAGCAGTTTCACTCCCAGCTTGATTCTTTTTCTCTC
TGAAAATCTGGGCAAGATGAGTATCCATGCGGAACATTGCCTC
>tig_1338_consensus
TCCAAGACGCAAGAGTTCAGCTTCTTTGTCATTATTCCAAATCGGAACACCTCTGGGAAA
CTGAAGGTAGCCATCATTTTGACGTTTATGCACTCGTATGAATCTTCGAGCTTTCTTATA
AACGCTAATTGAAACAAAGCGCCGATAATCATCCCAAAAGTGACGAATGTAAGGAATCCG
AACACGATCTTCATAGTGGAAGCCATGGATTTGCCCTCGACTCCTCAACTGTACGTTTCG
ATACGATGCAAATGGAGACGAAGCAAGAGACCCAGAGGGCTTCTCTTTTTTTTTTTTTTT
TCCCTCTCTCTCTCTCTCTCTCTCTCTCTCTCTCTCTCACTTGATGAGATTAAAGAATGCTA
GCATTTTTTCACAACACTAAAGAAAATTCTACCAAGAGGAACCTAAAATTCAAAATGTCA
GTCAGCCGCACAAGAAGAGATACTATCCAAGCATAAGATATTGAGCTTTTGCCTTCACAA
GCTGGAGTACAAATGACTGACAACAGCATGCAACTAGAGCGGCCCTCATTTCAAAGCTCA
CATTAAAACTTAGAACCCCTTCTGTTCAGTGGGATTTAAAGAGTTTTGTGAATTTCATCT
TACCCACAGAGCTACTAACGGGGTCGTTTAATCCAACATAATCGCTAAGAGCTGATGGAC
CACCCACATACCACTTAAATAATGGGAAAGTTAAAATTTCCGACAGGGAAACATAATATT
CTGGAAATTCAAGGAAAGTCTGAGACATAAAAGTAATACTAAAAACAGTTGTAATGCATA
CCTCTTAAAAATAATTCAACAAAACCTTCGAAGCAGTTGTTAGTCCACCATAACATTAGA
ACTGCCAAACTCACTAAAAGTATCAGAAAAATCACACAAGATCATCATACTTTTGCCTAC
ATAAGTTCACTGACCTAGACCAGAACAAAGATCCATATCCATCTGCTGCTCAAGCGCGAT
CATCTAGTACCGGTCATGTCGCCTGTGACGATCATAATCTCTTGAACGTTCTTTTGACCG
AGAGCGTGACCTCCGCCGACTTCTTGAATCATAATTGCGGCGGCGATTTGAATCCCGGTC
ACGCTCTCGATCATATCCACGGTCTCGTTCATAATACCTGTCATGATCTCGGCTACTACG
ATCATAATCCCTTACCCGGTCACGGCCGTCTCCTCGATCACGATCACCATCCCGATCCCG
ATTCACACTCACTTCCCTTTCACGATCTCTACTTCGTTCTTTTGACCTTCTATCATCATT
TCGATCTTTGCGACTCTTGTTTCTCTCTTCCTGAAGCTCTGCCAGTTTATCACGGATTTG
CATATAACCTAAATGAAGCTTCCCTCCAAAATGATCAGCTAAACGACGGTCACTGTCATA
AACACTCAAAAATGCTCCACAAATGTCACAAACACGTAGCTTCTGATCGGTAATGCGCAC
GTCTGCAGCAGTGTACTTGGTGGAATCTAGAACAGGCTCCTGTCTCGCAGGAAGCTTCTT
AAGTGCTTCAGCCTCTTCCAAAGCTTTTTGTGCCTCATCTACCATACCTTGCTCACCAAG
ATCTTCTGCCTTCTTCAGCTTCTCATTTATCATTTCTTGAGTACGCGCATCAGGTGCAGG

TATGGGCAAGGGTGCCAATGGCGGTGGGTTTGGGAGGGGTTGAGGCAAAGATGCCCTATC
TTTATTGAAGGCATCCAAAAGCAGCATAGACTGTTTGTCAGCTCTTTTTGTTCTGAGTTC
TTCCACCACTTCCAAAGCTCTGATCTTAAGATCTGTCTTGCCTTCAAAATCATATTGATC
TGCTTCTTTCAACTTCTCCTTAATCTGCTTTGACAACTCAAGTACCTCTGGTGTCTGGGT
CACCTCAGAGACTGATATTGCAATTGCAGCCTTTGCATCATCATCCTCAAGACGCTTAAG
AGCTCTGCCAATTTTCCTATCACACTCAACAATAAGTCTGTCAATTGCATCTTCTAATTC
TCTGTCATAATTATCAGTGCCCTTCGCCTTGGCTTCTTCATATTCTTTTCTCAGCTGCAA
TGAATGAACTTTTGGGCATGGTCCCATATCCATTTTCGTCAATTGGAAGAGTTCGTGGG
>tig_1339_consensus
ATTCCAATAAGTACAAAACACCGAGTTCAATGATCAAGTAAAAGGACAGCACTCCATGGA
TGAACGTTCTAGTTTCCAGACAAAGCTCATCACCTTCAACTTAAACTAACCCTCATCCAC
TGTTAGCAACCACAACATGATGACTACATTTTTCAGCTCAGCTTCAGCCTGTNNNNNNNN
NNNNNNNNNNNNNNNNNNNNNNNNNNNNNNNNNNNNNNNNNNNNNNNNNNNGAAAATTCT
ACCAAGAGGAACCTCTCAACATAATTCAACAAAATCTTCAAAGCAGTTGTTACGTCCACC
ATAACATTAGAACTGCCAAACTCACTAAAAATATCAGCTAAATCACACATGGTCATCATT
CTTTGCCTACGTAAGTTCACTGACCTAGACCAAAACACAGATCCACATCCATCCGTTGCT
CAAGCACAATCTAGTACCGATCATGTCGCCTGCGGCGATCATAATCTCTTGAACGTTCCT
TTGACCGAGAGCGTGACCTCCGCCGACTTCTTGAATCATAACTACGGTGGCGATCTGAAT
CCCTGTCTCGCTCATGATACCTATCACGATCTCGGCTACTACGGTCATAATCCCTTCCCC
GGTCACGGCTGTCTCCTCGATCATGACCACGATCCCGAATCACACTAATTTCCCTTTCGC
GGTCCTTGCTTGCTTCCCATTCATTATCTCTACTTCGTTCTTTTGATCTTCTATCATCAT
ATCGATCTTTGCGACTCTTGTTTCTCTCTTCCTGAAGCTCTGCCAGTTTATCACGGATTT
GCATATAACCTAAATGAAGCTTCCCTCCAAAATGATCTGCTAAGCGACGGTCGCTGTCAT
AAACGCTCAAAAATGCTCCACAAATGTCACAAACACGTAGCTTCTGATCAGTAATTCGAA
CATCTGCAGCAGTGTATTTGGAGGAATCTAGAACAGGTTCCTGTCTGGCAGAAAGCTTCT
TAAGTGCTTCAGCCTCTTCCAAAGCCTTTTGTGCCTCATCTACCATTCCTTGCTCACCAA
GATCTTCTGCCTTCTTCAGCTTCTCATTTATCAATTCTTGTGTGTGAGCATCAGGCGCAG
GTATAGGCAAGGGTGCCAATGCTGGTGGGTTGGGGAGGGGTTGAGGCAAAGATGCTCTAT
CTTTGTTGAAGGCATCCAAAAGCAGCATGGACTGTTTGTCAGCTCTTTTTGTTCTGAGTT
CCTCTAACACTTCTAAAGCTCGAATCTTAAGATCTGTCTTGCCTTCAAAATCATATTGAT
CAGCTTCTTTCAACTTCTCCTTGATCTGCTTAGACAACTCAAGCACCTCAGGCGTCTGAG
TCACCTCAGAGACTGATATAGCAATAGCAGCCTTTGCATCATCATCCTCGAGACGCTTAA
GAGCTCTACCAATTTTCCTATCACACTCAACAATGAGTCTGTCAATAGCATCTTCTAGTT
CTCTGTCATAATTGTCAGTGCCCTTAGCCTTTGCTGCTTCATATTCTTTTCTCAATTGCA
GCGAATGAACTTTAGGGCATGGTCCCATATCCATTTTCGTTAATTGAAAGAGCTCGTGGG
GGCAAAGTCCGGCCAA
>tig_1340_consensus
GCCATCAATAATGCAGCAACATCCAATCTATAAATAAAACAAGGGCTGAAAATCCACGGA
TTAATAAATGAACAGTGGAGATTTACATTCTAGCGGTGTCCATGTCTATATTTTATGAAC
GGAAGAAGTGTACAAAAGAACTGATGGAATTTTTACTGCCCACATCAGCTAACCCAACTT
GATTATGCTATTTGCTCATTTATGGTCTATACATACAACCAAGCTTGCTAAGCTTTCAAG
AGGCATGCCGAAAAGACAGCCTACTTGGCGAATCGATTCTTACTATGACTTCATCTTCTG
CACCCGAGTCATCGCTAGAATCATTGATCGCATGACTCGCAATTGTATCTGCATTTTGGC
CAGGGTCTGCAGATACTTCTCCGGCGGACTTCCTCAGTTCCAGTTTCTTTCTTGCAGTAG
CAGGCCCGTCTGATCTAACAGAAGGTAGTTGAGAGGGAGGAGAAGATGGAACTCTTGGAT
AGGACAGGCTATGCGACGGCTTCACCCTACTATCACTCCAAAACCTTTGCAGATTCCTTG
GGATGCCAGCCGTGTTGCCAGAGATGCTCATATCCCTTGCTTTTGCTAAGAGGCTGCTTG
CACTACAATCAGTCTCTACAGGGTTCTGCTTGTGTTGCCATCCCTTAAAGCTTTCAGGAA
AACTCATTAAGGGTGTATGCGGTTGATCACTTGAATGTGATAACAATGAGGATTTCCTTC
TAAGGAGAACACTATCTGCTTCAAAGGCTGCAATGTCAAAAATAATAACTCTTGATCTTG
TCTCAACTTGATAGCAAGATCCTTGATGAGAAAAACTGGCCAGCTTGTTACCAGATGTTT
CTACACTTAGAAAGCTTTGATTTCCTTGTGAAGGAAATAGAGCTGCAGGTGACGAAATAA
GTTCTTGGACACCATCAGTGCTTATGAATCCTTCCAACAAGAACCCAATGGAACGGTAAG
GTATTTCTATAATTTCCCCTTTAATGTATTTAGCCATAGTTGACCTCTCTGCAACAAGAG
CTCTCAAGTCTCGCAGTGTTAGCTTTTCAAATATTTTTGGAAGCAAGAGCCTAGCAAGAA
TTATAGCACTTTCCTGCCACAGGAAATCTTCTACCACAGGATCTAACCTTAGTACTGAAA
GTATTTTTTCACTTTCAATAAAGAAACACAGTACAACTGAATCTGTGACCATGTCACATA
TGTAAGGCTTTCCAATTAGTACTTCATACAGGCCCAAAGTACTCCCATGAGTGAATACTG

GATGCAATGAGTGCTTGTTTTTAATGCTCTTACTTGTCCACTTAACAACACCATTAGAGA
TAAGCCAGACTCCATTTGGCCGGGAACCCTCCTTATAGAGTGTTACACCATTAGGTCTCA
TGATTTCTTTGGCGTAAAGTTCAAATGGTTCACGAACATTAGAAGGAAGAGCCCCCAATA
AAGGATGGGAACTAATCAAATCACTTATTTTTGGGAACTTCACCAAAGGAGGATTCCTTA
GAAGCCTTTTCAAGTCAGTCTGGACACCATCATGAAGATGAAGCATCTCTTTTTCTTCTA
ACAATCCAACCTTCTCAAGATTCTGGACATACTCAATTAAATGTTTCAGCACGGAATATG
TCACTTGTCTTGTCTTTACAACGTGCAGAACCTGAGGAAACGTTACACGGACATCATCCA
AGAACATTTTTGCTTCTTTTCCTTCCACCTTGCTTTCTTCAATGACAACAGAAGCAATAT
CACTGTCACCTAAAAAGTCAAGTAATTGCTGCTGTGCAATCCTATGAGCACGAAGAAATG
CGGCACAAATGTAACAGGCAAATTCTAACCTTTCCACAATGAAATATGTAACCGCCTTTT
GCGGAAAAAATTCTTGATTGAAGGACCTTGTAATAATTTGGGAAGTGTACATTAGCTTTCA
AACCATTCCAATCACACAGAGGCTCATTAGATGCCAAATCAATTGCTTCTTCGACTGATT
GCATCAAAACATTAGCAGCAGATTGCATAATCCTTCCCTCGTCAAGCATTCCCCAGTAAG
CAGCTTGGACACCATTCAAAAGACGTATGCGTATATCTTTCAAATTTGTGGGGTCTAGAC
GACCGTCACTTTCAGATGAACTGTGAGGATGCACTTGCTGCTCTTCCAAATTATTTAAGC
TTGTAATATATCTTTTAACAGTAGGCCAGTCGGTAGGTCCCAGTTCCTCATCCTCACCAA
GATCACCGAATGCCTGTATTGCTTTGTTCAACATTTCATACTTTGTGTAGTCAAGCATAC
GCTTCTTCGCAGTTGATAGCTTATCCATCTCTAAAAGGTGTAAAATATATTGTGTAGTTG
ATCCATTCACAATTAGTGTCAAAAATACAATTCCACCAGTAAAGAACACAAACAAGATTC
CCGTTTCAGGTGTGATAAGTGGTGAGATGTCACTAGAACGCTTAACTGATAGTGAAAGTG
ACAATGCAACAGCCCCTCTCAGTCCTGACCACACAAGGATAGTAGCTTCTTTCCAATCCA
AACCGTAGCCAAAATATCGTAAAAAAGGATACAGTATTCCGACAACAAATAACCGTGAAA
CTTGCACAAACACATATAGAAGAATTAAATAGCCCCATGAATATCCATGTTTCTCAAAGA
TGTCTTCATTGCCTAGAATTCCGTCAGCAATAACAACACCACTCAAGATGAAAATCAGTG
TATTAGCAATATAAGCAACCATTTCCCAAAAATAATGCAAGCTTTGTTGACTTTCACCCG
TGAAAGCTGTTCTTGCAACTGCAGCATAAAACATTCCTAGAGTCATTACTGTCAGCACAC
CAGAAACATCAGCACCTTCCTGAGCAGTGAAGTAAGCAATGTAGCTCACAGCGAGTGTCA
GTGTAATCTCTATCACTGCGTCATTGAAGATAAATCCCAGCCACAAAACAGAAACAACCC
CAAAAGCAAGACCCATGGCTACAGCTCCAAGTGAGACCTGAAAAAGAAATTTGGCTATGG
CTTCCCAACTAAAGCTCTTTCCAAGGACCATTTGATAGAACAGCTGATAAACCACAATCG
CTGTCCCATCATTCATCAAGGATTCTCCTTCAATTATTGTACTCAGTTTTTTGCTAGCAC
CAAGCTCTTTCAGTAGAGCAACAACAGCCACAGGATCGGTAGCACTCAGGAGTCCCCCAA
GCAACAATGATGTTTTCCAACTCCATTTATATGGAAAAGTGAGCTTCAAAGCAGATCCAA
GGAAGAAGGTTGAGATCAGAACCCCTGGACCTGCTAGGAGAAGCATTTGTGCCATACACC
TCTTAATTTGGTGCAACTCCATAGCGAATGAACTCTCAAATAGAAGAGCAGGAAGAAAAA
CGGCCAATAGAAGATTGGGATCAATATTTGACCAAAGACGAATTCCATTTCCAACCTTCC
CCAAATCATGACTTGTTCCATATTCTAGAGAGCCAAGAGCGATTCCGATGATGAGAAGAG
CGACAGTATAGGGAACTCTGGTTCCACGCAGTAAATGCCTAGAAGCAATTCCAAGCACCA
AACTTATGCCGACAAAAATGACGGCGTCTGTTGCACTGCTACTTTCGTTAGAACTAGAAT
TGCTGCCGCCTCCTTGTTCGTCTAATATTCTATAGGGAAAGAACTCCTGCGTCAAGCTCT
CCATGTGAACAGCTTAGAATGCCTTTGTTCTATTCTTTCCCACCCTACTTCAGTTGATC
>tig_1341_consensus
GATGACAAAATTCCATCTCAATAAACAGTCCTATGAAACTCTTTCACTCTGTTGAGCATG
TAATTCTTCTTCAGTACGTTGCAAGAGCTCTTCATAAACTTTCACAGATTCAGCTGTGAA
TCTCTCCAAGGCCTCAAAAAAGCGTTGCAGACTACCCTGAAGACTACTACTACTAGTATC
ACTCTGATATACAATATTTCCCAATTGACTGTTACCATCCCGAGAAACCAGAATAATTTT
CTTGTCTAACGCCTGAATCTCCTTCTGTATCTTTTGCTCCTCTCTATCCAAGTTCTTAAC
TTTTCTCTCATAATCCTTATTTTCCACCATCTTTTGGTGCAGTAATGGCTTATCATGTTC
CCAGAGTTGAAGCACACTCAGGGCAAGAACGCGCATGCAATCAATCACCTCCTTCTCAGA
TATTCTGTCCAAAGCTTGTGACCACTGATTACAAATTACAAAAATAGGAGGTGCACCAAT
CCTACCAGGCGAGAAGGGAACTATTCCATCAGCTGTTTCTTCAGGTTCATACAGAAGACA
CCTCATAAGCCAACTATTCAAGGCTCTGACATAACCCTTTTG
>tig_1342_consensus
GCACCAATCCAACTAGAGAACCTGAAAATCCAATTAAGTGCCTCGTGTTCAAGCTGTGAT
GTAGCATCACCAAGTTTCTCACCAGATGCAATGGGACCTAAACCTCTGGTTTCTCTGGTT
GCTTGACATTGATTATGATGGCATTCCAGCATACTTTTCCACATTCTGGTTAACCCTTGA
ATTAATTCATTTAGTTGAGGCCATAGCTCTTCATCCCTAATCTTGTTAATTGTCACAGAG
ATCTTGTCAACAACCTGAATTGCCATTCCTATTTTTACAGACAAACTCCTAATTGAAGTC

CGAGTTGAATCTACTCTGTGAGCCTCAGCACCCCTTTCGTCCAAGCGCTTCAGCTTTCGA
ACAGTCCTGTCATGAGTTACT
>tig_1343_consensus
CAACCACGCCCACCATCTAAAACTACAATTACACTCGCCTCAAAATATTCTACGAATAAA
TTGTATATTATAGTAAATTACAACTCATGAGCCACAAATTTTGCAGAGATAGAACCCTGC
TCCCCTTCAAGATAACTATAAATGTTGACAACAAAAGTCTGGGGGCCCCTACCTTTTCTT
CTAAAAAATTAGGAACACTTAAAATTGCCTAGTGAAAATAGAAAGGTGGTATGCCATAAC
TTGCTTCCAGCATTAGCAAGATTAACGAATCAACCGGAAAATTTAGAAAGGTTGACACCA
TTGTCAAGTTCACCCATGAAATTTTGATTAACACAAAAAGTCAGCCATTTTCTGCTCGGT
CATATGCATTGGATTTGTGCTTTGGTGCAAATGTGAAGCCATCAGGAACCTTACCCAGCA
ACATCTCAGATATTCGAATCCAGTCCGCTGCAGAATCAGATGACATTAGTGTCTCAAGAT
CATCACGACCAAAGTAGGAGGGTGGCCGCCAGTTCAACCGTTGTGGAATGACATCTAAAA
GGCCAACAGGTATATATCTACATGTATAGCTAAGCCACTCTAACAAGAAATGCCTAGTAG
TTTCCACTCCTTTCTTGTCAGATCCCCAATGTTCAAGGCCAAAACGTACATAATCCTTCA
AGATATTTAATCGTTCCCCAGAACTGATGTCCCAGTGCCTCTGTTCCTTTATTTCAGTAA
ATATCCAAGGCTTAATTAGGGCTCCTCGTGCAATCATGCATGAAGTCAGTTCAGGGCAGT
CATCCATGTGCTGGTTCCAGTCTAAATATGAGAAAATATCACCATTTCCAAGAACTTGCA
GATCATTAGGCGCCTTTCTAGCACACTGATATATGTAGTCCCAATTAGCAAGCTTGCTAT
AGCGCTGCTGACGTGATCGACCATGTACAGTTACCACACTAGCTCCCCATTTGCCAATAT
CAGCAATTAATGAATCAACACGGTTTTTCCCTTCAAAGAAGCCTGTCCGCACCTTAACAG
TTATAGGTTTTCCCACAGTGCCAGAAGCTGCTTCAATTACACTTTTCATCCGCATTGGTT
TGTTGAGAAGAGATGACCCGGCACCTTTGTTAACAACAATATCAATTGGGCAACCCATAT
TGATATCAATAAAGTCCACCATACATTCTTGATCAATAAGTTCAACAGTGCGTGCCACAG
TATCTGGGTATGCCCCACATATCTGCACGCCAAACAAATCCTCAGATGAATGTCGTCTCA
GGAGAGCCCATTCTGAAGCTTGACCCTGCAAAAGATTTGTACACATTGCCATTTCACCGC
AGGTTATATCTGCTCCCAGTACTTTGCAAACCCTCCGGTAAGGAAGATTCCCTACAGTGG
TCAGAGGTGCAAGATAAAGCCTTCCACTAAAATCAATAAGCTTCTTTTCACGAGGATGTA
CTTTTAAGCTTCCATCAGTTTCTGCAAGTACATCATCAGTTGTAGTCGCAGGTTCAGCTT
GCAGAGAAATTTTCCCAGAATCTTCCTCCAACGGACCCACACCACCAGAAGCACAATTTT
CAGCACTTTCAGATTTTGCTTTCTTTAAGGGTCGAAGTTCAGCAGCTTCACACATCTCAT
CAACATTATCCTCTCCAAACTCATTTGGGCGCTCAGATTCATCCGGTGAATCACCACCAA
CTTTTGTGCAACCATTTTTGTCAGTAATGTCATGAGAAATTTTGAAATCTATTCCATTTC
CTTCCTCCTTATCCTCTGCCACTTTCATCTTATTCTTGCCATGAACCAAAAGCCCCAGAG
ATTTAAGTCTAACATCAGCTTTGGGGAACTTCATCTTATTCCTCCACAGAAGCTTCTGCA
CATCTTTGTTCAATCCATTGACCTCAGAAGTTTTCTTACGGGCATTTGAATTAATATCGC
CGTCACTAACAACAGCTCTATGAGTACCCGAAAATCTGCAGGCCAAACCATAAGGGCACG
GGCCTTCATAACTCAAAAACGGGCATTCACCTTCCAAATCAGCCGGTTTCTGAGCGTTAA
AAGCTTCTAAGTCGTGACTAAATCGGCACTTATCTTTATAAGCACAAGAACTAACATCTC
CAGTCTTAGCGATTTCCGGACACAGATTTAACGGAGATTTTTTTCCTGAAGGCGTTCAC
GTTTGAGCTGCCGTTTAGATTTCTTTTCCTTGATAATAGTTGTGGAAGACTGAGAGGGTT
TATTGCCATCATCGGCTGATGAAAGGGTATCGCTTTGAATGGTTCGGGTGGGTGGAGGAC
GAAGGAATTGTTTTTTAACGGGAGCAATGGCTTTAGCCACGAGTTGGTCTGGCGTTAGAT
TCAGTGGTTCTGGAGCTCCGTCTGAGTTCGGTTGTGGCGGAGGTGGCTCCGTCAGGGGCT
CACCAAGTTCGGTTGCCGACTCGTCCATGGAGAAGAAAGCAACTCAGATGGCTGCGAGAG
TGTTCGTTAACGGAATTAACCAG
>tig_1344_consensus
CTCATTAATAGACTTGAGAAAAAACTCAACTCGGCTTAATATGCCTTGAGCTCGAGCTTT
TAGCATTACATCGAACTGAAATCAAAACACCGGAGTGACACTCAGAATTCACAATCAAGC
TTCCCTTGGTGACAAGTAAAATAGAAAATTAGATATCAATACAACTCCTTTTTTTCATGT
CTCTGAGATAATCAACATACTCTACTACAACTTAGATGTCTACATAAACCAGGTGCTAAA
AAAATTGGAATAGCTGCAACATATTTAACGGCCACCTCGACCACGTCCGCGACCGCGTCC
ACGGCCCCGACCTCGTCCCCCAACAGGCCTCCCAGCAGTTGGCTTCTTGGGCTTGACCCT
AGGCGTCTCTTCAACCAGCAATGTTTCAAGATTCAAACTGTCAGGAAGAATGTAATAACG
AATGTTGTTGCCCCTCACACTAAGGTGATCCAAAGTTACCGGATTTTTCCCCTTCAGTGT
AAGTTTCACTGTTTTCAAATGTGTGTTCATGCTGATATCCACACCTGTGATGGTTCCGTG
AACAATGGTTCCGTTCTTAAGCTCGATCGAAACGGTTTCATTGTTCAGCTTCATCAAAAA
CCTGACCAGCTTCATTGTGGCAAAGTAAGACGTAAGCTAGGGCTTAGAAGCAAATGCGAA
GACTACCACTCTGAGAAGCGGGAACCGCTGCTCAGTGATCGGATTTAGG

>tig_1345_consensus
CGCTGCCGTTCTTTTTTACTTTTTTCAATCCATTTTGTAGCATTTTAAGAAATCAAAACA
CCGGAGTGCAACTCAGAATTCACAATCAAATATTCCCTGGGTAATTGGTGAAATAGAAAA
TTAGGTAACAATATAACTCCTTGCATGTTCCTGAGCTCACCATCATACTCTACTACGATG
TTTACATAACCATTTGCTGCAAAAAAAAATTGGAATCAGTACAACATATTTAACGGCCAC
CACGTCCACGTCCGCGACCACGTCCACGACCCCGACCGCGTCCCCCGACAGGCCTCCCAG
CAGTTGGCTTCTTGGGCTTGACCCTAGGTGTCTCTTCAACCAGCAATGTTTCAAGATTCA
AACTGTCAGGCAGGATATAATAACGAATGTTGTTGCCCCTCACACTAAGGTGATCCAAAG
TTACTGGATTTTTCCCCTTCAGCGTTAGTTTCACTGTTTTCAAATGTGTGTTCATACTGA
TATCCACACCTGTGATGGTTCCGTGAACAATGGTTCCGTTTTTAAGCTCAATGGAAACTG
TTTCATTGTTCAGTTTCATCAAAAACCTGACGAGCTTCATTGTGGCTAAGTAAGACTGGA
GCTAGTGCTTAGAAGCAACTGCAAAAATTACAACTGTAAGAAGCGCGAACCGCTGCCTTC
GGTGATCTGATTTAGGGCTCCAGTAGAGTTTTTAACAGTAACTAGCAAAGTATTAGCTAT
TTGCGAAACGGTGACGTATTAGAGTGTTTTCATTTGGACTTCCTGAGTTT
>tig_1346_consensus
GAATAATTTTTAGTCGAATCTCAAACCAAAAAGCAGCTGCTTTGATTACCATATTGTCTC
ATCAGTAAAATTTTCAAATCTGCGCCGCTAAATTGATCACAGATATGAAGAAATTCCACA
GAAGAAATTTTTTTATTCTAGTCAGGAAAAACCATAAAATATTTACATCATATGAGCAGA
TAAAAGTAGAATATCGAAATGACAATAACGATGATGACCATATCAAACGGCAACGACGAC
GGTTTCTTCGGGATCTTCATAGTTAGGATCTTTGACATCCACAACCTCTTTGTCGAAACC
GATCTCAGCCTTCGAATAACCATCGCCGGACCACGTCCACTTCCCTCCGTGACCTCCTTT
CTTCGGCGATCCTCTCAAGCCACTTCCTGATTTCTTGTCCATCTTCACCTCTGTTAATTT
TCCGTTGACCTTAACAGTGGCGACGTGGCTTTTCTCTGCCTTCTTCATTCTCACTCTCGT
TTTGCTATGAAAAATCAGTGTGC
>tig_1347_consensus
AATCTAGTTATTATAACACAAAAGCATCAACCTTAATCCAGTTTGTATTCAGTTCTTGGA
CACAAATTACAACCAAAGCTCAAGAGGCTACTAACATCTTTGTTTATAATGTGAACAAGT
ATCAGAATTATTTAACTATTATTACAACATCACCCACTTTAGAAGTGAAACCAAGCATCC
ACTCAGATACAGCAGACTTCCCTGGAAACACAGGACTATAAAACATAAAAACTTGCAATT
AACAAACCAAAAGAGAATGCAACAAGCTTTCATGTGAATGAGAGATCAAAGCAAGCATTC
AGCTCTCAGAGCTATGCAAGGTCTTGTCTGCAGATAGATTTTCTCTTTGAAATCGCTCCA
TCTCCTCATATGCCCATTCAAGACCAGGACAGTAATGAAGAGGTGGATTGAATCTGCTAT
CGTCGATGTTATCTGGCAAAAATGCTCCGTTCTCAATCAGGAATTTAACAGCTTCCCTCT
TTCTTTCTTTGGCTGCGATATGAAGCGGGGTCCAGCCACAAGCACCCTTGGTTCTAGCAT
CAATGTCAGCACCTCGATCAAGCAATTCATCCATGACTTCAAGATGGCCACCCTGAGCAG
CAAGGTGAAGTGGGGTAACCCCTTTTGATTTGGGGCCCCATGCTGACACATTCACATCCA
TTCCATCGTTAAGAAGTTGCTTCACCACTTTGAGGTCACCTTTACGAGCACTGATATGAA
GGGGAGTCCAACCCCTATCATCCCTGTCCACCACAGACCTAAAAAACCGCTTTTTGGACA
GCCTACGCTGAAAACAAAACGGACCCAGTTGCTCTTGGGGCATGCTTTCCATCATCCTTT
GCAATTTTCTTAATCAAACCACCAGAAATCAAACCCTGAAAATTCAATTATCGTTTCTGT
TCGCAACAGCAAATTGACTGAAAACAAAACAACAAACAAAGAAGCCGATTTACATGACAG
GAGAAAACAGAAATTAGCGGGAAGAAGACGAGTGATTGGCAAGGCAATAGGGAAAGAACG
GATTGAGACCAGGGCCAAAAACGCGAAGATTTTGACGCACGACGACGGAATTAACAACAG
ACGAAGATGAAATAAACATAGTAATCGATTAAAACCCTAAAAACACAAAAATTTATATGA
CTATTTGGAGATTAAAGAGTAATCAGTTAGACGAATCCGGATTTGATACGAAGAGATTTA
GGAGGAAACATTAAAAGCTAAAAACTTAAGCCCTAATTGCAAAGAGATGGAA
>tig_1348_consensus
CCCCATTTTATAGTATGGGACAATGGGGACCTTGTTACTCATGAAGTCATGGGTGGCTTG
AAGAATGTCTATGCCATTGGAGCTGGAATGGTAGCAGCTCTAACCAAGGAGAGTGCCACC
AGCAAGTCAGTATACTTTGCTCACTGTACATCTGAGATGATATTTATCACTCATCTTTTA
GCAGAGGAACCTGAAAAACTTGCTGGGCCTTTGTTGGCTGACACTTATGTGACCCTTTTG
AAAGGTCGTAATGCATGGTATGGGCAAGAATTGGCAAAAGGGAAATTGAGCCTTGACTTG
GGGGACAGCATCAAGGGCAAGGGAATGATTCAGGGGGTCTCTGCAGTGAAAGCATTTTAT
GAGCTTCTAAGTGAATCCTCTTTAAGCGTCCTACATCCAGAAGAGAACAAGGCTGTAGCT
CCTGTCGAGCTATGCCCGATCTTGAAGATGCTTTATGCTATCCTCATAAAAAGGTACCCA
ATATCCCATCCAGATCGGAAGAGCGTCGGTAGGGAAAGAGTG
>tig_1349_consensus
ATATTATTACAACACAGGCTGACTGTCACCTTCCATATTATTACAACAAAGGCTGACTGT

CACCTTCCATTGGCACCAGGACCAAACTTTTCTACACTATCTCTCCCAATAAATTCAAAA
GCCACAATTCATTAAATCAACCATCTGGTTCCTTTCGCTTCTTGTTAGATCTTCGGCTTA
CCTTGAACTTTTCTGCCAATTCCAAACTAGGCAGTGACTTAGGCTCCAGTATGCTGCTGA
GCTTCAAATTGTCAATTTCATCGAAATTGGATAAGGACTTCTCATAAACAATGGCTGCAT
CAAGGGAGTATATCCGTAAAGCAAGAGAAGACATAGTGGATGTTCTGGCAGCTGCTGAGA
ATGATGACCAATACCACCACTCATTCCTCAAAAACCCAGACTTGATCATGTCTTCTAATA
TAATTGTAGCCTGGACCATCTCATATATTGTTTCAGCAGATTTAACAAACCCACGCCAAG
CCCATCTCCTTTCAGCATGGGCCTTCGAAGGTCTCAGAGCTGCTTCAGGGAGTGCAGCTT
CCATATCTAGTAAATTAATCTTGAGCTGCCTTAATATCTGTGACACTTTACCAATTAATG
GCCTCAATGAAGACTGGGGAACTACAAAACAGTGAGCAACCCCGGACTCAGAAAATTGTC
CATCTGAGACAGATCTTTTATCTCTTTGGTTAGAACATCTCAAGGCTGTTTTATTACTTT
TAAGTACTCCACGTATCCCCATTGCAGCATATTGTCTAAAAGAATCGTCTGTGATGTTGT
TATTGCCAGCATTAGACTCAGACTTGTTTCCTTTTGCAGATAACAGTCTCTTGCTAGCCT
TCAACTCATCCCCTGCACCAACTACTCCTTTCTGAGGAGAAATTAACATTACAGTAGGAT
CGCTCAGGAAAGGAGATACAGTTGGAACAAGAGAGGGGATTCCTTCTGAACCAATGAGAC
CAATCTCCTGTATCAATTCTTCATTAGAATCTTTTGTCATAAACCTGAAACTTATTTCCT
CAAAGTCAAAAGGACACATTAACCCTTCATTTTGGAACATTGTCAATTGAGAGGCCCTTT
CATTGCACCTACTTTTAGAAGTTTCAACANNGACTGGAGGAGCTGAACAGCACCTATCAT
CATTATGATCCTCAAATTCAACATCAGACAGAAATGTTCTGTGACACAAGAGGCAGTGGT
TTCTGGAAGGCCAAATTGGTTCTAAGCATTCACAGCGATACATTTTCTCTTCGCTAGTTA
TTCTTGCCTTTTTTCCCTGCTTCTTCACAATTTCAGTGATTTCTGAAACAGAGCAAGGAC
CATAATTCTTCCCCAGTAACATGGCCGCCTTAGTAACTAGACAATCAATAAGCACAGCTT
TTTCACTATTAGTGGGTGTTCCCAAACAACTTTGGGAGTCATCCTGCACTTGGTTTTTAG
TTTGTTCAGGATCCTGGAACCTTAGCCTTCGCCAGTGCAGAAGAGACTCTTTCAGTTCCC
TTTCTTTTGGGTCATTATCCCTCAACCATTGAATCAATTCTTCAACTTCAGCATCAGTTT
GATAAAAAACCCATTGTGGACAAGCCATAGTCACATTAGGATCATAGGAAAATGGGCAGC
AGGCTTTTGAGCCCTCTGAATTCAAGAGAGTATCTGTATATGATGAAGTGGAAGTCTGCA
AGATTGAATTTTTGCCCATTTGCTTCTGAAAATCCAGCTTTTTTCTTCCCTGATGTGATG
CAAAGCTTCCATCAACAATAACCTGAGGATACATACCAGGCATAACTGAAAGCCAATATA
AACGACCGATAGAATCACTGCCCAGAAACTCCCTTCTCACATTTAGCTTCAAAAGCTGTG
ACTCAGTGGTGGTAATTGAATCTTGTAGAACAAGTGTGTCCCTCTTGATGGTGTTCAACT
CAAGATTGTAGGCTTGTGACTCATTTGTGACAACAGATGCCACTTCATGAGCTACTTGAG
GACTCATCAATTCTGAGGGGGCAGACAGTCCCTGATGATCTGAAGATGGTGTTTCAGTAG
CGACTTCCTTTGTCAACTTTTGCAAGTTTTCCTTTAAATGGATTTCTCTACTCGTATCAT
CTTGTGGCAAAGTATTTGATAAAGGGGGCTCACTCAGTTTAAGTAAGTCATTATTTTGGA
AAGGTATTTTACTGTCATCAGCAGCAGCATGATGATCTTTTCTTTTGTCTTCAGTATCTG
CAGGGTTCACATATTGGCTGTCACATTTACAACTCTTCTCTGAATACAAGAGAGATGCAT
GCTTATTGGAATCAATAAGCTCAGATTCCTGTTGACCACCGTCTGATGGATGTGCATCAT
CAGAGAAGAAAATAAACCCATTATTTCCACTACTTGAGCCCTGTGGCTGTCCTAAAAATT
TGCCATTGTTTCTAATAGTAGTAGCAGGCTCCTCAATGTTTCCAACTGCATTAAGCATGT
TTGTCTCCACCTTTGCAACTCTTGCAGCCACAATTTCTTCCCTAGACTTTAAATTTTTCC
ATTCCGCAGAAAAAGCACGTAATTTCTGCTGCAATTCAGCAGTTGTTTCAGCACATTGCT
CAAGGTGTTGACGAATAAGTGTTGAGTTAAGTAACTCATCACATAAAAACTTAAGCAGGA
AGGTTCTCTCATCAACATTGTACTCCCAATACTCTTTTTCTTCCATTACAGCTGTTAAAT
GCCTGAGACACTCCAAGTACGCGCGAGTCACTTCTCCCTGGCATTTTTTTCCTCTGCTTC
GACTACTGACTTCAGAATATCCTGATAAATCCTGAAGCATATGGTTACTGACAACACAAG
ATGGGCAGTACCAGTTTCCTTCAGGAATCCGTGCAAGTGGAGGATCCAAGCAGTAAGTAT
GATACTCAGCATCACAAGTATCACATAGAAGAACACTATCATCATCTTTATCCACACCAC
ATACTTTACAGACTCCTTCATCCCACGGAGCTTTTGGAACCTTGCTTGTGAGAGCAAGAA
TATCCTTTATTTCTTGTTTTGCTTGAGCATCTAAGTGTTCAGATTTTGCATACCCCACAA
GTTTCTGAATTAAGGGGACAACCTCCTTTTCATACAAAGCTTCAAAATTTTGGGATAATT
TCTCAGCCAATTCAACAAAATCAGGCCGATCCCCATATGCCATTCGCACATTATTCCATA
ACTCCCGTATATCCTCAAGAAAAGATTCATGTGATCCGTCATAAGCCCCAACTGCCAATC
TCATATCAATAGTCCTAAAATCCAGAGGACGAGATACCATGCCAGGCAATCCAAGTAGCC
CCTCATCATCATTATCAATAGAACTCAACTTTCTTCCCAGTAAATTACAGAAAACTTTTC
CATCATCTGCAGCAGCAGCACGACGTAACACAATACGACACTGCTTCATGATGGTGTCAG
ATATGGATAGGACAGTCTTTCTCACAGTTTTCTTATCATTTTTTTTTTCTGGCAATTCTT
CCTTCTGAACATCTGCCAGCACTGAAAGAACAGCTTTCTTTGTTGGTCCTGAAGCATTGC

CTTTATAAATTTCTTTACTGACTGAATGTTCCAATCTTTTTTTGGCCCACTCTGGTGGAT
CTTTCGATAGGGCTTCATAAACACACCTTCTGATTCTGGTTCCCACATTTGTCGGTAACT
TCCTAACAGGTTCCAGAACCTTTGCCCACTCAGGCACAGTGCCATCACTCACAACTTTCG
TTTCAATGGAACGAGTTGCTTCATATTCTTCATCTTCGATAGTCAGCATGTCATTTTGTC
TGTTCAAACAACCAAAAATTTTTTTTGTGGCCTCTGCAAGCAACAGGGCATCTGCTTCCA
TTCCAATCACTCCAGTAAGTGAGCCACAGAGAACCCCTCCATCACCTTGTAAGCAGCGAA
ATACTTTACAACATTCACGTGCAGTACTATCCACAGAGTCAAGGACACCATCCATGGATA
AGAAAGCCAAGATGTATCTCCGAGCTAATTCTGGCCAAGTTAATTCATTGATAGGAAGCA
TATTGAGCTTACCCTTCTTAAGAGGAATCAAGCTATCTGCATCTTTCTTCCTCCCCCGCT
TTGATTTCGACTCTCCAGAAGCAAAGTTTGGGTCAATAATTGCAGCCACCTTGGATTGCA
GTTCACTAATTAGTATTCCAAGTAAAGAGCTATGAGCCTTGGTCAAAACCACACCAGAAC
ATCTGCTGTAATTAACAGATGCTATTCTAGCTTGGGCTGCTTCCTTCATTGATCCAGTTT
CCATCTGCATAAATGCATGTGGATTTTCCCTTGAGGATAGCATTTGTCCACCCATACAGC
TACTTTCTTGCAAGCTTGAGGGTTCAGTTCCCTGGATTTTTTTCTTAGATTTGTCAAGAG
GACTTGAGCCAAACCATGGGTTGATAAGCTCCTCTTCAAGTTCCTCCAATGTAAAAGGTT
CCTTCAGACCCAAACTTTCATGAAAACGTCCAAAGAACTCCCATACCTGATAAAAGTCAC
CAACAATGTGAGGAGGAAGCCTCAAGCACAATGCCTTTCCAGAAGGGCAATGATCATGGT
CTTCAA
>tig_1350_consensus
TCTTTCCCCAACCCCCCCCTTTTCCTAAAAAACAAAAAATTTAACAAAAAAAAAAAAAAA
AAAAAAAAAAAAAAAAAAAAAAAAAAAAAAACCTCCTCACTGAATAATGGAGGGGCCAGCA
GAGGGGCACGAATCACAAAACTCAACAAGCTACTCAAAACCTAACTTTTCCAACGTTAGA
GAAGGAAGATCTAACTAGTAATGTTGCAGAAGTCATAACCTTCAACAATCGACCTAATTT
TGCGGTAAGAACGTATTAGTTTCCCCCCAACGCAAGATAATTAACAAGTAAAGTTGCCAA
CAAGTTCTAATATTACAAAAGCACAGAATGACATCACAATGGATTACAATCTGATCCCAT
CTCTCCCCCCTTACCTCAGTTTTGTGCACAAAATTAAGGAGCAACATCATATCACAAGAG
AAAAGAAACCACCGAAGTAAAAGCATAATGGCAGACAACAGGAATATAGTCTCTAATAAA
TGTGGTAAAAATCAGTAAGGAGTGTAGTAATACCCTGCTGGTGGTCCCATCCCATTTACT
GGTGGCATTCCGTAAGGCATCACAGGCATAGGACCTGCATTGTAGTATCCGGTACCAGTG
AGCTTGGCTAAACTAACTTCGCCTTGCATTCCATCCCTTGCATACTGGTGCCATACTTGC
TGCTCTTGCACTAGCAACTGCTGTTTCTTCTCCATATCAGACATTTGCACATATGAGGGA
GGTGGAATGCTCAATGATGCAGCAAAAGGGTCCTCGTTAACTGTCTGAACTGTTCCATCG
GGAGCCGGGAGAGCCAATACTGGTGTTGAAGTTTTCCCTGACATTGGCAATGCAACACTA
CTTGCACTTCCACCACTCAATTGAGAGATGCTCACATGCTGCCTCACAATTCCCTGATCA
TACATGCCGTTCAACAACAATGGATCAAACCCACCACCCAATGCTGCTTTCTGTCTCGAC
AAATTGCTGGCAGACTCAACCAATGCCAATTCCCAATCTGCCTTACCAGGTTCAGCGGCT
GGGGTTTGCCAAGCCGAGGTAACCTGATCCCCATTTGACGGAAATGCTTCCCATGATCCA
TTTCCACTAGCTGCAGGCCCGTTAAACAAAGCCAAGGCCAACTTATTTCCCTGATCATCT
GCTGTAACTGTATCATCCCTCAAATTCACCAGATCTTCTGTCATTTGCGGTTTCTGAGGC
TCAGATTCAGGTTTAGGCTTAGGTTCAGGTGGTGGTGGAGGAGTGTAATTCTCAGGAGGA
GGCAATGCCTTTATCTCATTCATATCTGGTACTGGTTCCTCCGGCGGAGGAGCCTCCAAT
TCTTTCCTCTCTGGACTCTTGGGCCTCTTGGCTCTGTCCCTCACAAACTCGTCCAAAGTC
TCTAATAACTTGCTTGTAATCCTCTGAACATCAGGATACTCGGAGGATCTCGCCACACCA
GTATCCTTGCACAAACTATAAAAAGCAACCAGCTCATCAATCTGCTTTGCTGCACTTACA
TATGCATCATAGGCCTTAACACAGTCTGTATACTCCATATCAAAAAACTTATCAAGCAAC
ACAGCCAAAACTTCACATATATCGGCATAAATCTGAAAACTCTCTTTCACCATCGGATAC
AAAGCAATCAAAATCATCCTATTATTCTTAGCCAGACCGGTGGGCCTGCATGACAGGAAC
CGATCCAACAATCTCTGCAAATGCCCCATTTTTCCAAATATCCTCTCAGGCTTCATTTCT
CTCAATGGAGTCACCACAGCTGATTTCTCTTCCCGACCAGTGGCCTCACTCATATCTCCA
TACGATCTCGATCTCCTCATCATTCCATAATTTCCATAACCACTTTCGCCTCTAGAGTCT
CCATAACCATACTCATGTGGTCTCGGTGGGGGAGACCGAAAATCATCCCCATATCTAGAT
CCGAATCCATGATGATGACTACAAAAGCTGAGAGGTCCCACGAGCTCGAGTGAGCTTCAT
CTCTAAAATCCGACATGTTCAAGAGTCTTGTTCCTCTTCTTGTTGCGTACAAGATCTCCT
CTTGAAATATCGGATCTCCGTCGTTTAATAGCCGGTGCACAAGAGTCAGAGCCTTCAGTG
CCACAATCCAGTCACGTGTCTTGCCCAGACGCTTTGAGATGGTCGACACACAAGCGTGAA
CGTAACCACGTGAGTATGAGGTTAAGTTGAGGATCTCGCGAATGTACTTCTCGTCGGCCG
GATCATCGTCGTGGCTCGTGGCTTTGACTATAGCAACTTCGAGGTCCGGAGCCATGTTGC
TCGCTACCTTAGCTATGCCTATGCTAGTCTGATCTTTAACGGCCCCGATCGCTTTCCGAA

TCGTGCTTGGCGCCATCTCTACCGTTGATTCAAGTCTCACACCTTTACTTTTTCACGTCA
GATCTGGAAGAAGATGCTGCAAGGAGCAAAAAAAGGTCGTCGGATCCGTCGACTTCTTTC
GAAGAAAACTAAAACTGCACAAATTTAGAAGAATCTCGAAGGAGAAAGTGGAGTGACGGC
AGACCGTCGTTGTTAGCTTGACTGAAAGCCGCTTCAGACTCTCTCTAGCGCTGAGTGGGG
AGCTCAATTAGGCCAGAGTAGGCAGAAGGTAAGAGAGTTAAAAAAAGTGAATTTAAATAG
TGAGTTGGGTCCGCCGACGTGGACCGTCGATTTGATCTAAACGAAGCGTTGGTGAGGGGC
AGCTATTTTACCGGAAGTGTCGGGCGCTGTGTGTGTTGCATTGCTTTTGTCTGAAAGAAA
TCAACCGAAGCTAAAGGCAAAAGAGAAGCGAGTTTTGATGCGGTGCGCGCTGGCATTGGG
TTGTTTATTTTACGGTACTACTACGC
>tig_1351_consensus
ATGATAAATGCCAACTATGAACATCTGATACAGATATATCTAATCACCATATGATCCCCA
ACGGGAAGAATTTCAGATTGTGAAAACAGACAACTCTTTCATGCAAAAATTCTCAGTTTC
ACTAATGTAATTGCAACTAGACCAATAAATATGTGCAAGCATCCTCAGTGCAAGTATATA
ACAGTTGAGTATACATACAAGAAGATTAGAAGTAAACATTTAAAAGGAGGAGGTACCAGG
AACAAAAATCATGTAGATAAATGGTGCCGGAGAGTAGAGAGGGAGCCCAAAGGTTAACTA
TGTCTCCCTCTATTCCTCCCTCTTCCTCTTCCTCTTCCTCTTCCTCTTCCTCTCCCTCTT
CCCCTACCTCTCCCTCTGTTTTCCAACCCATTAATCCTATCATCATCTCTTTCATAATTA
AACATGCTCATTTTAATATTCTCTGCTGCTGAATCATTCATTCCCCTTTGCACAGGAGCA
TGAGATTCTTCCAAATCAAGTTGGTTTTGAAGCTCCCATGCAAGATCTTCATCATTACTG
GTATCAGGAAGCTCATTTTTAATTGACAGCTTCACTGCCCCTTTACTCTTTTCCCACTCA
TCAAAGGTGAAAACCTGTGGCTCTTCTTGTCTTCCTTTCCCTCTATTTTTTCTACCTCTA
GAATGTTGATTATTTTGATTGGAGTTACTCATTTTCTGGAGAAGTTTCTGAGAAGCCGCT
TGATTTTGAGCAGGAAAAGACTCAACAGCTTCTTTTGGTCTTGTCTGCGAGCTACTAGGC
TTTTCTTCAAGGGAAGTCCTTTGTAGACTATTAATTGAGTTGCTTGTTCTCAGATCAGTG
CTCAGCTGACTACTTTTCAGCTGAACTTCAGCAATTCCAACAGCTTCCACAGCAATAGGC
TCATTGGTCTTTAAAGTCAACCGAGGGTCATCATAGTGCAATCTGCCACGCTGCGCAAAC
TGATGTGAAGTAGCCCTGATCTGCAACTTCTCAAACGGAGGAGGACCACCACCGTCACTT
TCTTGTGGTGGCCTTACAGAAGAACGGGAGAACACCGAGTACTTTCTACTCATCTCCCAT
TCTTCATGAAGTGATTGAACAACACCCCCCAAAATTGTAATCACTTTGGGATTCAAACAC
AATATACCACTATGTACTGCAACTTTATTCTCCAAACGGACCTTTGTACCCGGAACTACA
TCATTAGGGATAGCTGGTATATGCGAGTATTCTATTGCGGTTATTTCAGAGTGCCCATCT
GTAAGAGTTAATCTTAGTAGGCGCTCACTGCTTGAATTCCCAGAAATATCTTCTATGCTG
CTTCTAGATATGTCCCTAACTGAAGATATCTGGAGGATTTTGGGGCCTTGAATATGAGAT
AGCTTTCGGATAGAGTCAGGCAACGACTTGCCACCGATGGATCGAAGGTCCATATTAAGT
AGGTCCGGTTCAAGCGAATCGGCCACCGTACTGGTTTCCCTATCATCGGCCAAAGCGGAA
TGTATAACAATTAAGGCTCTAAGTTGCTGGATATCGCCAAAACACCAACCTCTAGTTCTT
AGGGTTTCGATGACGGCCTCAGCTTCAGTACTCGAGCCTTCTTCCATCTCCACGACTTTT
AGCGCCTGTTTTGGGTACTTTTGATTTTGAGATAAACACAGTTAG
>tig_1352_consensus
CTACTCTAATTTATGTTCATTATAAGATGCTCAAATTTATACGCTCACAACATCACTTCA
ACAATAACCTATTGAAAATCATCTAGAAAGGAGGACATCCAAAAGTCATGCAAATATATA
ATTGACTAGTCCTGTCGACCCCATATAACCAACAAGAAGCTGATGAGTTGAGTTCATACA
GACTCTGGACATCAACCACAAAGCAAAGCCACCACAAAGGAATTAAATGATATTGAGGGT
GAAGTGGGAGAACCAGAAAACTTCTTCACTACTCCATTCCCCCTATTTAATCGTGTTCAA
TATGTAGCGCAACAGGATATATTAATCACTAAATACAAGGCTCAAAGAAAATGACAAAGG
TTCCATAGTAACACCAACCATGACCAAATTACAATGTTGTTCTCATATCAGCTACATTTT
AAAAAAGGGGTAACAAATCATCCACATCCACATCCAAATACCTCCATTCATTCAAACTCA
CTCTCTTTTGTCTAGCACCATCTTTGCTTTCCTCCTAAGCCTCCTGGAAACCTGAAAACT
TGAGAGATAAGCCACTACACTTCACTGGGATTTGATTTAACCATATAAGAAACCACCCAA
CTGGAATTAACTTCTTCACTAATCATAACTACCATCTGCTGCTCAAACAGTACCACTTTG
CCTTCCATGGAAGTTCCCCCACCACGCCTTGTGTGACCCTGACCCCTCTCCCTATACCT
TCCCCCTGCCACACTATTAAGGGCATCTTTTGGTCCTTCATAGTTGTTTCCCCTGCTGCT
ATTACTGTTATTGTAAGATCCAACAGGCTGATATTCGTACTGTGAGTTGTGTCTCTGCCG
CTCCCTGTTTATAGGTTGGTTATGTTGTTTGTTATCTTGTGCAGATGAGCTCCAATCTCC
AAAAGATTCGTTGCCCCTTCCAAATCGGCTACTCTGATTTCCATTTCTACGGAACCCTGA
TGGTATGCGTTGCTCGTGTCGCATCTCTGTATTTGATGAAGCAGTTTCAAATTGGTTATC
AGGACCTTGGTGTGGGGAGTGGGGGCCTCCTTTCACTGATGGTATCCTCCTTTCTCTCTT
GGGTTCTTGATTGCCAACATTTGGTGCTTCTTCCACTTTGCTTGTATGGGATGCAGCCTG

ACCATGGTGAGATTGAGCCATAACTTCATTGGTCTCCTTGTCTGGTTGAGGTTGGTGTGG
GAACCCACCTTGAGAAGTGGAGTCCTTTTTGTTAGTCCTCCCAATTTCCACCCCTACATT
TTGACCACTGCTGGGCCGGCTTCCCCGTTGATTAGAAGCTGAAAATGCCTGTGGTTTCGG
TTGCCAGTGAGTGGTTCGAGTTTCTCTTGAAGCAGAAAGTGAGTCAGATTGACTCATCTC
AGGTACAGATACTGAAGATTGCAGGTAACTTTTGTCAGAGTCCTCGTTATTGATCTTCTT
GTGATCATGATCATGATTATTTCCTGTAACTTTTGGTCCCTTGTACTGATGCCTCTTTCC
TCTGGCTGTCACTCCCTGATCTTTTGCAACAGGTATAGTAAATGGTACAGTGTAGTCGGG
ATTTTCAGGCATGTTCCAGCCATCAGAACCCCAATCATCATAATACTTCGGCTGCTCTTT
CACCGAGCTTACATCAGGCTTTTGGAGTTGGGAATGCTCAGTTGATTTTTGAACATTCTT
ATTAGAATTTGAAGACTGTGCATCATGCAACCCTTGCACATCTGAAGAAACACGATGGCG
CCATGATCCATGCATTTTTCCCTGTTTATTCTGCCTATTATCTCCATTTTTAGACTCCAT
AACAATCCCCATCTTCCCACTGGCAACCCCAGAGGCTTGGAAACTGTCTGCACCCAGAGA
ACCAGAATCTGCTTTCACAACAATCTCATTTGGCATGGTCTGATCAACTGAAGGAGTCAA
TACATGCTGCTGAATACTACCTTGCTGCGCCATTTCTTTGGCTACAGGTTTCGGAATGTA
TCTCTCCATTTCTGCCCTCTTGTTTCTTGAATTATTTTGCACTTGATGGTCACTCTTTAC
AGATGAAGCCTCAACCATGGATTTCTCACTGACATCAGCAGCCACCTCAACTTTGTTCTG
ATTGCGCACAGGTGCCCAGACAACAGCATCATTGGTATGGAATTTCTCTCCCGATTTATT
ACTTTGTGTACTTCTTGATGTCTTACGAGGATGCTGAGATTTCCACTGGTTGGTTGCTCT
TCCATGGTTTTCATCAGTCGGTGAAGATAAGCGCATATCAGAAGACTGATTTGCATCTTT
TGGATCAGTGCATGACTGAACAGAGTTAGGATCCAACTCAGATTCAGAAGCCTTTGTCTT
AATACTTTCACTTGAAGTGTTTGTAAGTGTTTTTGTTACTACTGGTAAGGCAGCCACAGA
TGAAGTCTCTTCTACTTTGTGCTTCTTGCCACTCCTGTTATTTCTCCTCTTTTGATGTAT
GGAGGACTCAACCATGACATTTGAATTTCCTGGCAAACTTGACTCACTGCTAGGGGCAAT
TTCATTGGTTAGAACCTCAATGGATGTGGTAGCATTAACTATTAAATCAGTATGGCCAGT
GAGTATCTCAGTTGTGCTAGCACTCAAAAACTTCTCAGAGGAATTCTTTTCCAGCAGAGT
ATTCTGCTTTTGCTTATAGCTCACCCGCTTCTGTCTAGACACACTACCATCATATACCTG
TGGAGCATTGTTTCGATGAACAGAATCATTGACATCCTGTATCTTGGGAATAGATTCATT
ATGTTTATGCATCCCAACATGTTCCTTGTTGGCACTCTTTGGCCTCTCTGGAAGGAGTTC
ATTGGACGAAACAGTGGATTTCTCAACTCTACTTTTGCTGCCACTTTCACTACTTTGCGC
CACATCACTTGAGATAGAAACCAAACCTGAGGTTGATGATACTCCAGATTTGCTAGCAGC
CTTCATTGATTCAACCAGAGATTCGGAATCTTCTTGTTTCTTGGCATCAAAACTGGGGAA
AACCTCGGACTTCAGGGTTAAACCTTCCCCTGCTTGCATACGTCTATTCAACTCCTCCAA
CTTTGCAAGAGCCTTGGCCTTCTGGTCTCTCGCTCGTTCTTCCTCCTCCTTCTGGCGTTG
TTTGACGCGTTGCTTGGCTAGTTCTCTCATCTTAGCACGCTGGGCTTGGCTATCATCGGG
ATCAGACATGGGTGGCATGGACTCCCCCTCATCCTTCCCTTGAAGATATGACCCAGACTT
TTCAGCAGTCTCTTTGAGAGCCTGATTTTGAATGTGAACAATAGAATTTTCAGACTGTGT
AGTAGATTTCTTCCGCCACTCATCAACCTCTTGGGAATTAAATCTTCCTTTGCCATGATG
ATCAGTTCTACCATGCATGGCATGAGCAGATCTCCTTGGGAAAACTGTTTCACTGACAGC
TGCTAAATCAACACTCTTATCTCCAGCAGAAAAACGCCCATCATGCACAGCAGGATTATT
AATTCCAGTGGCATGACTTCTGCCAGGATATAAAGAACCAGCAGCAGCTTCATTGGCGGA
ATGATTAGCCTCATGATTACCAACTTGTGGTTCATTCTTCTGTTCCTCCTTACCAGAAAG
AGACATTAGATCATATCTACCATCTGAGGAACGTGCTTTAGCATTTAAACCCTCTATTTT
CTGAATCAAGGAAGAATCTTTTGGGGAATCATGGTTTCTGAGGAAGCAGTAGCTGGATG
TTCCAACTTCTTCACTGAAATGTCATCAAACGCCTTAGTTCCCACACTTTCAGGTGACTT
AAATTTGACAGGTACAGAAGAACCACCAATATGGTCAGATGCCTGAAAAGAAGCTTGTTC
ACCAGTGGCCTTTTGTTTAAAATTTCTCTCTTCATCTTTTCTGTAATCTCCTCTCCAGTC
ATCCTCCCATGATGACACTCTTGGTTGGTCAACCTTCTCAACACGGGATGAATCAGGTAC
AGTGTCTCCACAATTCTGTTCTTCATCTTTCCCATCCCAACCATCTTGCTGTTTTAAAAG
CATCCTGTATGGTCCTCGAACATTGAGAGGATGACCAGTAGGTTCTACTTGCTCTGAAGC
CAGGCCTTTGCCATTAGGACCATATCCACTGGATCTAGGGTGGGAATTGCTGGGATCATG
AGCATTCTGACCTGAGTACCGGTTATAAGAAGAGGGGCCAGCTGCCATTCCCATAAATGG
CACATCTCGTTCATTGGGATTACAATATCCCATGGGAGGCCTATAGTAGCCCTCATAGGC
CACTGGACCAGGATAAAAGCCAGGCCCAATTGGCATACCTGGGCGCATATAAGCATCAGG
CATAGGAGGTCTGTATATATCCCCATTTTTTGGATGGTGTCCCCTTGGTCCAGCTCCTGG
TGGGGGAACAGGTTGTGGATTGGGATGTGCAGTTCCTGGCATTGGAGGGCGATAAAAATG
AAATGGTTCCATGGGGAAGCCCCCAGGAGGAACTGGGGAATATGGAGGAACTCCTGGATT
ATTGATTGGAGGTCCTCTATACCAAACACCACCAGGATGATTATTGATTGGTGGACCATG
CCAAGACTCATAATGCTGAGGGGGCATAGCAGCATTTGGATATGGACGGGGTCCGTGAGG

ATCTGCTTGCCACTTCTCCACATTGGTCCTCCCTCCGTCTTCCCCATATGGAATATTATC
TCTTGTCCAGGAATTTGGAGTTCCACTCCTCACATTAGAGTTTACAGAAACATCAACAGA
TAGAGAAGTCTCAGTTCTTTCTTTAATTGGTGCCACTTGACCCGAAGACATTTCAGGCTG
ACCATGAGAACTGCAGTCTTGTGGCTCCGTGTTCTTTTCCACATTATCTTTCTCTGCACC
AAGTGTTGGAAAATCTCCAGATGCTAGAGAAAACTCATCATTCCGGGAAGATTTAACCCC
CAACTTTTCAGCTGTTGCAGCTGAACCCCAAGCCACCGAATTGTCAGACAATGGTTCAGC
AAACCGGGACAACAGTGAGCTACCAGGTCTTGTTTCTGCACTTCGAGGACGTAACGATGC
TGCTGATGTCTGGTTTGATGTCAATACCCCAGAAGCTGATGATGGCCTAGAATTTGAACC
CCATGCACCAGCAGTTGGTTCATGGGCTCTATCACTACTTGAAGTGGACGATCGAGTACC
ACTCCCACCAGATGAAGGGCGGCCACTTAAATGGCTTGGAGAGCCAGTACCACTGTCAGC
ATTTGGAGACAATGTTGAGGAAGCCCATGCATTTGATGCAGAAGACGATGACTTACTTCC
CCAGCTAAGTGTACCCTTGGGAACAATTTCCACATTTGGGTCCAGCCCATGATTTTCTAA
CTTCTGGCTTGGTAAGTTAATTGGTTTAGGAACAGCAACTTTGCCCAAGACAGTCATGCC
ACCTCTTCGGGCAGAAGCCCATCTTCGCTCCCCAGTCAGCATACTTGAAGTCATGGTTCA
AGTGTTGTTGCAAGGTCATTCAAACAATTTTGTAGCAATCAGAGATAACATAGAGATTTA
GGATGCGACAGACAGAAACCCTAGGGAGAAATAGAGAAGGAGAAAGAGATCGAAACGTCG
CGGTGTTCGAGCGATCACAACAACAATAGAAATAAAACAGAGATCTGTAAAGAGTGAAAG
CAACGGATTCCAGTAGGGTGATTTTTCAGATCGCGAAACGAGAGAAGCAGTGGGAGGGGG
AGGGGGGGGGGCCGAGCTTCT
>tig_1353_consensus
TCTCACTCCTCTCCCTATACCTTCCCCCTGCATTAATAGGGGCATCTTTTGGTCCTTCAT
AGTTGTTTGCCCTAATGCTATTGCTATTAGTGTAAGTTCCAACCAGCTGGAACTCATACT
GTGAATTGTGTCTCTGCCTCTCCCCGTTTACAGGTTGATTCTGTTGTCTGTTATCTTGTC
CAGATGAGATCCAATCTCCACAAGATTCATGCACCCTACCAAAACGGCCACTCTGATTTC
CATTTCTACGGAACCCTGAAGTCTTGTGTTGCTCATGTCGAATATCTATATTTGATGAAG
CTGTTTCAACTTCATCACCAGGACCTTGGTTTGGGGAGTGGGGGTCTCCTTTTACTGATG
CCATCCTGCTGTCTCTCCTGGATTCTTGATTACCAATAGTTGGTGCTTCTTCCACTTTGC
TTGTATGGGATGCAGACTGACCATGGTGAGACTGAGCCATAACTTCATTAGTCTCCATGT
CTGGTTGAGGCTGGCGTTGGAACCCACCTTGGGAACTGGAGTCCCTTTTATTAGCCCTCC
CAACTTCTGGCCCTACATTTTGATCGCTGCTGGGCCAGATTCCTTGGTCATTACAGGCTG
AAAATGCCTGTGGTTTCAGTTGCCAATGAGAGGTTCGATTTGCTTTTGAAGCAGAAGGTG
ACTCAGATTGATGCATGTGGGGTGCAGGTACTGATGATTGCAAAAAACTTTTGTCAGAGT
TCCCGCTATTTATCTTCTTGTGGTCAAAATCATGATTATTTCCTGTGCCTTTTTGTCCCT
TGTACTGATGCCGCTTTCCTCTGCCTGTCACTTCCTGATCTTTTGCAACAGGAACATTAA
GAGGCAGAACAGTGTCAGGGTTTTCAGGCATGTTCCAGCCATCAGAACCCCAATCATCAT
AATACTTACGCTGCTCTTGCACTGAGCTTACATCAGGCTTTTGGGATTGGGAAGGCTCAG
TTGATTTTTGAAAATTTCTGCTGGTATTTGAAGACTGTGCATCATGCATTTTTCCTTGCA
TATCCGAAAAAGACCGTTGGCGCCATGATCCATTCATTTTTCCATGTTTATTCTGCCTAC
TGTCTCTCATTTTTGACTCCACAGCAACCCCCATCTTCCCACTGACAATTCCAGCAGCTT
GGCAGCTGTCTGCAGCCAGAGAACCAGACTCTCTTGCAACAATCTCCTGTGGCATGGTAT
GATCAGCTGAAGGAGTTAATATATGCTGTTGAATACTGCCCTGTTGAGCCATTTCTTTGG
CTACAGGTTTCAGAATGTATCTCTCCATTTCAGCCCTCTTGTTTCTTGAATTATTTTGCA
ATTGATGGTCGCTCTTTACAGATAAAGCCTTGACCACAGATTTCTCACTGGCATCACCAG
CCATCTCAACTTTGTTTTGAGTCCGCAC
>tig_1354_consensus
CTCAGGCACATAAAATTGTACAAAAACAAGAATTTCTTCATCAAAAGATATAATTCTGCA
ATTTAATGAAGTTATAACCATAAACATCCACAAAAGTTATACTAAAATTGATCTTCAATA
GTGTACTTCAATGGGTAATTCCCCAATGAATTTTTATTCAAGTAAAATTAAAAAAAAAAA
ATTGATGTAAAACTAGAAGTCGTAACAGCGACCGTTTCGTTCCCAGTCACCATAGCGGGT
TGGCTCAGGGCCTCTTGGTCCACCGACTTCTCCTGTTTGTTTATTCACGAAATCTTCGTC
TTCCTCTCCATCTTCTTGATCTTGTTCGTCGTGACTTTCAGAGTTTGCTTTGAGAGTCTC
TTCTTGTTCTTTATTGAGAGTTTTCTCCTGGGGTTGATGATGAGGCTGTTGAGTCGAGGA
GCTAATGAGTCGGCTCACCGAGTTCGAACAGATTGACCACTCGGGCAGAGGGAAAGGCGA
GGGAAGTTTGGGTGAGACTGACGTAAATAAACGGCCAAGATTGTTTCTTGCCATAGTTAA
TGAGATCCAGAACTTGTGGAAGAAGACGAAGAAGAACACTAACAATAAAGCCCTAATTTG
ATG
>tig_1355_consensus
CATATAAACTCCAGCCTGTAGATCTTTGACTGAATACAAAATAATTGAGATTAAAGCAAT

ATATTATTGCAGTTTAGAACTGCACCCTAGACACACATAAAAAGCCCATTTGTTCAGAAA
TTTCAACATCCAAAAGATCCTGCTCTTCCCACTGAACCTAGACAGACACATAATTAGCAG
CTCAAATTCTGGAAACAAGCACAGAAAATTTGCTATAGGAACAATATGAGATATGTATGC
AATACAGCAAATTCAGAGGAATTTATAATATTAAGCAGTGTAAGTAACAAATTAAGTCAA
ATAAAAGTGTTTATTTAACTTTATTACATATCCCTAAAAATAAAATTGTTTTTATTCAGG
ATTTGCCAAACCGTCAGTGTTTAGTACGGAACGATTATGGTATCGATAAGCTCCTGAAGC
GGTGCAAGCTCCTTCTTGTCAATTAGACCAAATTCACAGGTAAATAGGATGAAGTGCTTG
AAGCATGTGTTGAGATGAGCTTCTTCCTTGAGGCTCACAATTTTCTGGAAGTGAGAGTGA
TAAATATGGGCGTATACACGGAACAATCTTTTGAATATTGTCTTCACAACCTCCTTGAAG
TTGGGAGGAAACGGAGCTCCAAGCTTTTGAGGAAATAAGGATTCATCATCAAGTTGAGAC
TCAATCCAGTCCATTAAATATTCAACATATTTTGGAGCAGAAACCTCAATAGGTTTTTTT
ATCTGTACACCATCTGCCCATCTATA
>tig_1356_consensus
GTGCAAGCTCCTTCTTGTCAATTAGACCAAATTCCTGCATACAGAAGCAGTATATTCAAT
AGAATAAAACAATAACAATACATTCTTCCATCAGTGTTGTACACATCTTGCCTAATTTTA
TAAGTTTAATTTTATAATTGAACAGAGAAACGATGGTGCAGTCTTATCTAATATCCACAG
GGCAGTGAAGAATAGGCACATAATCTGGTTTATCAATAAGGTTGGCATGAATTGTAACTT
ACACAGGTAAATAGGATGAAGTGC
>tig_1357_consensus
AACATGCAATGAATCATGATAAACATAACCTTCAATTCATGTTGTTTACAGTCACAAAAG
AATAAAAAAAGCGTTCCTTGCTTTCTGTTAGTTTGCATGATACAATCTTATAAGGAAGAA
TGATATGCATTGCATTGCTGCCCTAATCACTGTCCCAGGTTTCATCCATCTGTTTTGATG
ACTTCTTTGGTCTATGAATCACTTCACTCTTCTCATCTGTTGGTTGGTGATAATATTCTT
CACCACCGCTAATATAATTATATCTACCACCCACTGACGCTCCTGAATCTTCATCCAATT
CTGATTCAAGCATTTCAGCTTCAGATTCACTTGATAAATCTTCAGAACCGGAATCTTTCT
CTGCCATCTTACTTCTAAAGTTGGCTTCAGAATTTCTTTTGAAATGAGTATTGTAACGAG
TCTTGTGAACATCCTCCACCCTCCCATCCTCTTCATTGCTGTCACTTACATAATTAGGGC
TTGCATCTGCTCTAGAGACTCCCACATCAAGCTCTTCCTCAGAGTCAGATTGCCACATTG
CATTTTCAAGATCACTAAACATGTTTTCTGACTCAGAATCTTCTTCCACCGTGTTTCTAT
TAAAATTAGCTTCAACATTTCTCTTCCAACCTTTATTATTGCTAGCTCTGACAACCTTGT
CCCGCCTCCCTCTTTCTTGTCTAAACTCCTCATGAGCTTCATCAGCATCACTCTCAATAT
CACTCACCTCTTGAACATTATTGAAACCCCATTCATCATCTACCTCTGAATTGCTATCAT
CTTCATCATCACTGCCAATAAATCTTCTCTTGCCATGTTTATCCTTCCAACCTCTGGTAT
CATCTGGGTTGTCATTTCTAAGGTTTTCAGATCTTCTGAACCTTCCCCCAAATTCTTCCC
TGTTCCAATACCTGATTTTACCAGCCTCTTCGTTCTCTGCAGCTTCCTCTTCAGCCCATT
CTTTATCTGCTGCATCCTCTATTTCAGCTATAAATTTGTCTAGTTCCTCCTGGTCACTAT
CATCAGGAGGTTCTGAATGTTCTATATTCCTTTTGGGTTCCCCAACTAACTCTTTGTCAC
CTCGGAAGACATAAGGGCTTCCTTCCTTCTGGTCAATTGCATTGAAGAATGTTGAAGCAA
CTCTTTGTATGCTGGCCATGGCAACTGGATCCTCAGGGTTTACACCCATTCGTCGGAGCT
GCTGCTCTATCTTCTTTATATTTAACTTCTGAGATTCAAGAGCTTGTTCAAACCTGGCCT
TGAACAATGCCTTCCTTTTTGTAAGGGTGTTGAAAGGTATCAAGTTTTTGGGCTGGCGAT
AGTTCCTACCACGAAACATGATAATCGTTTTCACATTATGTATGTTAATCACAATGCCAC
CACTCAGTCTTGCAAGCATGGTTGCCATTTCCTTGATCTTTTCTTTGGGGAAATTATCAC
AACTAACTTGTACTGTCTCATGGAACTTCCAATGGAGATGCATATTTTGGACAACTCCTC
CAAAAACTCCACGAACACCAACAGGAACATAATTTTTATTTCGAAACCCTATCTTCTTAT
ATGCTTGAAGCTGCTCAGGTGTAAATAGCTCGGGATCATGCAGAGGGGCTGGTAATTCAG
GTAGCTCATATTTCTTGAGCTTTTGAAGCAGCAAAGCGACTTTCTTCTTGGCCCTTTTGA
GGTTATAGAGAAGCCTCTCCTCATCAG
>tig_1358_consensus
CCTGATACACCTTTTGCTGCTTCTCATATACTCCATGAGTATGCATCCTACTATCTCTGG
CCAACTGTTTATTAATCCAATTGGTAGGTGGCGATAGAAGTGGCTTCATGTTCTGCATAG
ACTGCTGGTTTGAATTCCCTGAGTAACCTGAATTAGCCATTCTGTAATGCATCCCTGCAG
GAGGCCTACTTCTGACACTTCCTTCTATTCTCAAATGAGATATACCCATCCTTATATCGC
CGTACTGTTCTTGGTATTGACGTCTATCCTGCATCCTAGAGTTGTGTCTGCTGCCTTGCA
TACCAGTTGATGGATTGGGAGATCTAGAGTTACTCACTATGCCTTGTGTATGAAAGTGGT
ATCCAGAATTAGAGCCTGGGCCATCACCAAATCTCCTTTCGTTACCAGATGGTCCAGCTG
GTCTAGGTCCAGTGACTGCATATGGGATATCATGGTACACAGGTTGCTCAACCAACCCAG
AAGAACCATTAGTTTTGTAGTTAAGCGTGTTCTTAAGAAGCCGATCAGCTGCTTCTCCCA

ACTTAGGGCCAGCTATTCCTCTTCGCAAATATGGCC
>tig_1359_consensus
GTTGGGTTGGGGTGGCATTCCTCCATAATTGTTGGCTGGCGGCACTGCTCCATAGTTGTT
GGCTGGTGGCATTCCACCATAGTTGTTCGCCGGTGGCACTCCTCCATAGTTGTTCCCTGA
CGGCATTCCTCCATAGTTGTTCCCTGACGGTGGCATTCCTCCATAGTTGTTTGCCGGCGG
CATTCCTCCATAGTTGTTCGGTGGCCCTCCCATGTTGCTAGGTGGCATTCTGCCCATATT
ATTGGGTGGGGGCATGTTAGTGGGAGGCGGAGGCATGTTGCTGGGAGGAGGGGACATGTT
GGTGGGAGGCGGGGGCATGTTGCTGGGAGGTGGGGGCATGTTGCTGGGAGGTGGCATGGC
ACTGTTGGAGTTCATGCCCTGGTTCTCCCTTCTTCTCTCAAAGTT
>tig_1360_consensus
GATGTTGAAAACTCAGTCAACTGATTATTTTGCAAAGCATAGCTCTGTGGTTGACCAAAT
TGGTTGTCAAATTGAGGCAAAGCAATAATGAAACTCTCTATTTTCATAGCTCAATGTATA
AATATTATCTTATAAAAACTTTGACACTGTTAAAAACATATCAATTTATCACTAGAAGTT
GCACAAAAAGGCAATATTTTCAGACCCAACTCACGTCAATATCACAAGTAAAAAGATTAC
TAATCATCCCATGAATAGCCCACATCTTAAAATCAAGTCTTACCGCAAAAACTTGTATCA
AATATGAAGTTGCAGAACTCAAAAACATAGCCATTACCACTAAACCCTAAAACAACAGTC
ACAACCCTAGTTGCTAAAAATCTGTTGAGGAAGATAGTATGACTCATACTAGAGTCGGAG
AGTCTGAAAAATCAACGCAACCTCCTAGCTTCTCTCTCAGACTCGAGAAGAGTGAGGATA
TCACCTTCTCTCACAGGTCCCTTGACATTCCTCATGATGAACCGGTTCTGATCGTCCAAG
AACTTTACTCTAACTTGAGTAACCTGCCCTCTTGAACCTGTGCGTCCCATGACCTTCACC
ACCACAGCATGTTTGATCGCAGAATCCATCCTGAGGGAAGAGACGAGTAAACAGAAACGG
CAGTAGAATCGGCTGCAAAGTCTTCCAGTGGGAAAAAGAAACCCTACCTTGATGGAACTT
TAATAGAGGCGGAGGCCAAATGACGGTTTTGCCCCGACTATTGGGCTGGAGTTTTTGGGT
CAAATCAAGAGAAAGGTTAAATGTGATGTCTTGAACTCTCAGTTGGG
>tig_1361_consensus
GATCGATAGCCTTTTTTTCAATTAATTTCCTACTAAACAGAACAATATCTTAAACCCAGA
ACCACACATCTTGAAATAACTGTTTCACTGCAAAATTATGAACTCGAGAATCATAATTAA
GATTGTAAAACTTCCAGATATAACAGGCTCCAAGTATTTCAATATCAAAAACTCAAAAAT
GGCAGCTTTGTCCAAGTCCTCGCGACAAAATCCAAACTGTGATAGTAATACCAAGCATAG
GCTACAATTATCGGTACCGACGAAGAGAGAGGGTATTGTTTCTCTTCCAGGTAGCTCTGC
GAGAAGGACGAGCCTTGTGGTGCAAGTGTCCCTTTCCTCTTAGACCTCTGTACTTCTTTC
CTGCAGAAGTGAGGCCTCGAAGTTCTCTGTGCTTGTGGACAGGATTACAGATCCAATGGA
TTCTTGGATCATTACGGATAGCACCATGAGCAGGATCAACTAGAATTACTTCATAATATT
TGTAAGTGGAATCCATGTTGATCCAGTACGAATTAAGAACCCTCAGACCTCCCAACTTAC
GACCGGCACGCTCCTCAGCAACTGATCTCTTGCTCCGCTGAAACTTCAGTTGAGTAACAC
CCTGGTTGGTCGGCTTACCATACACAATACCCTTGGGAACCGGCCTCTTACGCCCACCAC
GTCTTACCCGAACACGGTAAATGACATAACCCTGTTTGGCCTTATAACCCAAGCGACGGG
CCTTGTCAGGACGAGTAGGATGAGTGACCCGAACAATTGAAGGATGCTGGCGATACTCCC
AACACCTCACCCTCTGCAAGAACCTCATTACATCAGATTGCTTCTTCCTCCATAACTCTG
AGACATACGTGTAAGCCCCCATTGGATCAGAGCTGAAGTGCGCTGTGTGACTAGGGTTTT
CGCCCTCGGAGGAAGCTAGGATTTGTAAGCCAAAGTCTTATAAAGGAAGAGTCATCAGGA
AAAAGAGATCTGGGCCGTCTACTTAAATTGCGTGCCCTAACAGAAAAACTCGGTATTCAG
ACGTCGAGAT
>tig_1362_consensus
TCCACTGGGTTCATTGCCACACCACGTACCTTAGGCCAGCAGTTCCTCTTCACTCTAAAT
TTGTGGTAGGCATTTCCAGCTTTGAGAAGGGGCTTCTCAGTCCTTCCACCACCAGCAACC
TGCCCAATCATGGCTCTGCACCCACTTGGAACAATCTTCTTGGCACCAGATGGAAGCTTA
ATCCTGGTGGTGTCGTCATCTGGGTTATGACTGATGATGACAGCGTAATCACCAGAAGAC
CGAGCAAAGACCCCACGGTCACCGACGTGATGCTCAACGTTACAAACAACAGCACCTTCA
GGGATAGATCTAACAGGCAACACGTT
>tig_1363_consensus
CACTCTTTCCCTACACGACGCTCTTCCGGATCTGCTCCACTGGGTTCATTGCCACACCGC
GTACCTTAGGCCAGCAGTTCCTCTTCACTCTAAATTTGTGGTAGGCATTTCCAGCTTTGA
GAAGAGGCTTCTCAGTCCTTCCACCACCAGCAACCTGCCCAATCATGGCTCTGCACCCAC
TTGGAACAATCTTCTTGGCACCAGATGGAAGCTTAATCCTGGTGGTGTCCTCATCTGGGT
TATGACTGATGATGACAGCGTAATCACCAGAAGATCGAGCAAAGACCCCACGGTCACCGA
CGTGATGCTCAACGTTACAAACAACAGCACCTTCAGGGATAGATCTAACAGGTAACACAT
TTCCAACCGTAAGAGTGGCCTTNNNNNNNNNNNNNNNNNNNNNNNAAAGTGACTCGGGCAA

GAGGGGCGCCACGCCCAGGATCGTGGATGATATCAGTGATCACACCCTTGAGATAGCCAT
TGCGTTCCCCGAAATCGAGACTGCGGAATTGGGCAGGCCCCTTGCGGTGGTGGGTATGAG
ACTTGAAGACGGAACCAGCACCCTTACGCTGGGCACGAATGACTCTACCCATCTTTCCTT
GGAGAACGCTGCTGAGGCGACTTGGGTTAGAGAAT
>tig_1364_consensus
AATAGTTGCATCACAATGCACCTGGTTAAATTCAGTGAAGTGTTGGAAAAGACTCAGCAT
TACATTTTTGGTTTTGTTCCACATCCAATTAAATTCCAGTAGCACATTCATATTAGCAAG
GAAACATAACAAACAACAGATGACTGGAGGATGTGTATAGTATGCTGCAAACCTTCCAAT
TACATACCTTCACTGTCTTAGCTGAGCACCGAATTTTTCCCCATGACACAGCTTCATCAG
GACGAGCTCCAGAATCACTACCATCAAACTCTTGGGCAGTGTTGATGAAGACAGCATAAT
CTGCACCATTACGCATCATATTGGCATTGGAAATATGATGCTTAGGTAGACCACCTCCTA
AAATTATCATCCCTGTTTTCCTAGGACTTGCATGGACAGCTTCACCATTCATGGCCCTGA
TATCTATTTGAGATGGGTAGGTTTTTTCAGATAAAAAACCGAAACTATTAACTATCAGTA
TGGTATGCCATTGCAACACTGAAGCAGAAAGAACCTACCTTGCACGACATCAATAATCAA
G
>tig_1365_consensus
CATGCATCAGGTGCCTGGTATAAGGCAGATGAAGAACCAAAACATGACGCTAGCATATCA
GTGTTTATCACACCAGGATTGAGAGCAACGATTGCCATCCCATCAGGCAATTCCTTTGCC
ACTGATCTAGATAATCCTTCAACAGCCCATTTTGATGCACAATAAGGAGCAACCAGGGCA
GCAGTAGATCTTCCCCACCCAGAAGACATATTAACAATAATCCCCTGCTTTTTCATGATC
ATCAATGGAATGAAGTGTCGCAACACGTTTGCTATCCCTTTAACATTAGTATCAATAACA
GCATCAAATTCTTCTGCAGGAACCTCCCATATCTTGTTATTGTTATTTATGAATCCTGCA
TTATTTACTATAATATCAGGAACACCCTTTTTCTCCGTTACAATGCGTGCTAGTTCTTCA
ACACTGCCATTTGACTTCACGTCTACACTGAGGAAGAGATGGTTATCTGAGTTGGGGATT
TCAGATTGGAGGGAGTTGAGTTTCTCCTGCGTGCGAGAACATCCGATAACGGTGTGGCCA
CGCTTGGCCATCTCCAAAGCTAGGGCTCGCCCGAGTCCTTTACTTACTCCCGTTATCAGT
ACCGTTCGCGAAACGCCCTTCGTTATGCCATTAAAAGCTGCCTTAGCCGCCATTTGGTGT
TCGCCTTTCTGGTTCTCCCTCGATTAATCTCGCAGTCTAGCGGTTTCTGCTTGTGGCCTT
AAGAGCGTACACGCTGAT
>tig_1366_consensus
TTTTAATAGGAAATTTAGTAAAAATAATAAATTTATTAAAACCATCGTTTAATGAATTAT
ACATATATAACCAGAATCTCCGGTTAATACGCATTTTACTCTTGTCCCTATCCTTAAAAA
CACTGTGGAGGAATTCAGGTAGAATTAACCTTAACCCAAGATCCCAAAATTTATCCCACC
GATAGAGAAGCACCATTGTCTGCACCGGTAAGCTTGAGTATCATTGTAGCTGCCTTCAGA
GCCCATGCATCGGGTGCCTGGTATAAACCAGACGAAGAACCAAAACATGATGCTAGCATA
TCAGTGTTTATCACACCAGGATTTAGAGCAACGACTGCCATCCCATCAGGCAATTCCTCT
GCCACTGATCTAGATAATCCTTCAACAGCCCATTTTGATGCACAATAAGGAGCAACCAGG
GCAGCAGTAGATCTTCCCCACCCAGAAGACATATTAACAATAATCCCCCGCTTTTTCGTG
ATCATCAATGGGATGAAGTGGCGCAACATGTTTGCTATCCCTTTAACATTAGTATCAATA
ACAGCATCAAATTCTTCAGGGGAAACCTCCCATATCTTGTTATTTTTATTCATGAATCCT
GCATTATTTACTATAATATCAGGAACGCCCTTTTTCTCCATCACAATGCGTGCTAATTCT
TCTACACTGCCATTTGACTTCACGTCTACACTGAGGAAGAGATGGTTATCTGAGTTGGGG
ATTTCAGATTGGAGGGAGTTGAGTTTCTCCAGCGTGCGAGAACATCCGATAACGGTGTGG
CCACGCTTGGCCATCTCCAAAGCTAGCGCTCGCCCGAGTCCTTTACTCACTCCTGTTATC
AGTATAGTGCGGGAAACGCCCTTCGTTCTGCCATTAAAGGCCACCCTAGCCGCCATTGGG
TGTGTGCCTTTCCAGTTCTCTGTCGATGAATCTCGCAGTCTAGCGGCTCCTGTTTCCGGC
TTTAAT
>tig_1367_consensus
CAGATCTTTTGGCTCAGGTTCAACTGTTTCTCTCTTTACTCTGTTTGTTAGCGGTTTTCG
TTCATCATCCACTTCCATATCAGAATCTGGATCCCACCTCTCATCTGGATCATCTTGATC
TTCATCTGCCTCTGGAATTTCAGTATCAGGTGGTCTTTCCTGAAACGGGACGCTGGGTGC
ATGCTGAAGCTTTGAGAGATAATCAAGAAGCTTACTTCTTATTTCCTCAAGTAACTGTCT
TGAATTCTTATTCTCCATGTTACTAGGAGCAACATGAAGGGTATAGTCTGGACCAAAATA
CTCATAATACTCATGTTGTGGCATCTTGTCATCAACTTCAATACCAAGCGCAACTCCAGT
CTCATAGCACCAGCAACGAGCAACATTCCGAATGGTATAGCCACCACCTCCCAATAACAA
GAGTGGCACATTGAATGATCTCATAAACTTGACACATTCTGCATGACCTTTGATTGAAAG
GTTGAAGCATCCTAACCTATCCCCAGACAATGAGTCAGCACCGCATTGGAGAACTACAGC
ACCAGGCCTAAAAACTTCCATCACTTTTCCAATTATTGGTTTGAACAAGTAGTGATAACT

TTCATCATCAATCCCATCATCCAATGGAACATTAAGAGAATAGTACTTTCCTTTTCCATA
ACCAGTATCACGTATGTCCCCTGTACCAGGAAAATAGTCTCCGAACTTGTGGAATGAAAC
AGTCATGACCCTATCAGTTGTATAAAACGCCTCTTCAACACCATCTCCATGATGGATATC
AATATCCACATACAAGACACGCTCATGCTGCTTAAGAAGCTCCAAAATAGCCAAGACAAT
ATCATTGACATAGCAAAAGCCAGAAGCTTCACATTTCTTAGCGTGGTGCAGCCCACCAGC
CCAGTTGACAGCAATATCGCATAGACCGTGGTTCAACTTGACAGCTCCACCAACGGAGCC
GCCAGCATAAGTTTGACAAAAAGAATAAAGTCCATCAAAGACAGGGCAATCTTCACCGAC
ATTAAAGCGCTTAAGTTGCCGGAGTTGATCCTGCTGAGTTTCAGGTGTAATACTCCTGAG
AAAGGACACGTAATCGTCGGCATGAAAGCGACAAAGATCCCTATCTCTGGCAGGAAAGGG
CTTGAGGACCTGCATATGCTGAAGCAATCCATAGTGGGCGAGAAGGGCATGAGTCATCCG
AATGCGATGTGGCTTCATAGGATGACCCTGTCCGTAATAGTAATTTCCCACCTCTGGATC
ATAGAAATAACACACTTTTCTCTTAACCCCATCAGTCCCAGAGGGTAAAGAATTGCCTCC
AGTGTCCATCTAAA
>tig_1368_consensus
AGCAGGGTATTGATTAGCAAAGATGGCTGCTGACCAAAATCATCTATGAGTAGCAAACTG
AGAAGAAAACATCAAGTTCAATAAAACATAAATGGTAAAGATTCAAATAATTTCTAAACA
GATTCTACAACCTACAAGCTATAAAATAAGTTATTGAACTTAAAGAACAATCTTATTTAG
GTAATTCCACAAAGAAATGACTCAATCCAAATCCAACTACAAATTATTAGTCTTGTATTA
ACATTCATCTGTTGAGCTCATCAGCATGGAGCTAACCTAGACTATCAAATCAACCTCATC
AAAGCTGCCCTTAAACTGCATGGATAGGACTGCTACTTGAATTCATACCAGAACTGCCAG
AGGTTGGTTTAGCATGGAACGCTGATTGTCTAGAAGAACCTTCAAGAAGTTTCAATGAAA
GGGAAGACGGACCAGTCTGGCCAATAGATTCTCCCAAACTAAGTTTTGACATGCCAACCA
GTTCATCAACATTTATAGGGCTCTTCGAATGTACTGCTATTGGCTTGAGCACTTCATGTG
TTCCATTCCTACTTGGCTCTGTATTGTAACCCGGCCAGAAAGGAAAAGAAAAAGGTAAGA
ATGGTGAGACATAAGCAGGATATATAACCGGAAAAGAAGACTGTGAGCTTTCTGGCTTTG
AAGGGTCCAGTTCTCCATCATTGGAGTTGGTTGATTCCATTGATTCACATTCTTCTTCTA
AAGCAGGGGCAGCAGACAGTTGGTTATTGCTTTGTGGTTCAGCCTGGGGGTTGGCAGAGA
GTAACTCCTGAGATAACACCGCACCGTCCAATGACTCATCAGCAACAATATCGAACAGGC
TGGAACGTCTTTTTCTCCGGGAAACATTGCTTTGCCTGATAAAATATTTTTGAGCATGGC
TAGCAACTTGTGTAGGTGTTCTTGATACAACATAGTTACGAGCAATTCCACGCCAATCAC
CTTTGCCAAGCTTCTGTAGACCTAGAAGAAACATTCTATGCTCCTCTTCTGTCCATGGAA
CTCCTTTCTTCCGTTCACGACTGGATGACGACCCCGGAACGAAGTCTTCCGAGGCGTACC
CTTCAGCATGATCCGGCGTCTCACCGGGTGAAGCTTCGGGGTTTGACCCGTTCACGTTGT
TATGACTTAAATTGGAGCCAGTATAGTGGCTGAGATTGCCCATACTAGCACTTTTTCGGA
TCGAACCATCGATCAATCTGACTCCGAAGAGCTTGACACCACGGTTGGGGCATGTCCGAG
AGTTGTGGCCATTATGGCTGCAATGCGAGCACCGGCGCGTCATTTCCAATCGGGTCGAGC
AAATGGGATTCGATAGATTTGAATTGAAAAGCGAGATAAACTGTAGTTCGGATCCGGATT
TGGAGTTGGAGGGTCCTAACTTCTACATAATCCAAGACAGATGAGGATTTATTTTCCCGC
AGAAGGGAGAAAGAGAAGTAAAGATAAAGAAGAA
>tig_1369_consensus
CGAGCATCGAGCCGCAAACTTAGCAGAAAAAAGCCCGGCTCGTACAGCATCACTTTCTCC
TTGAAAAATCACATTCACATCACGCCGTTCTCACCATGTCACCAAGCAAGCATCTTGAGA
TACGGTAGGTTAAATTTAAACCACAAATCTACACAAACCAACAAGCCAATCCATAATCAT
AAATAGGAAAGACTGACAATGAAATTGTTCTTCACTGTTTATATGACCTCAAATAATGAT
TTAATCTGAGTCAGGCAGTCAGTCATATAATCGAACTCACAAGCTGAAGCAACTAAAAAA
TATTACATCCTAATCCAAGTTAAAGAGTTTTGGCATGTTATAACACACACACTGCGCCAG
ATAATAGATTGTTTTGGGCCACTTGACTAGGGAAAAACACACTCATTGATCCCATCAAAG
AAACTGAAAATTCTATCCCATGCTGCAACCGTAGCTTATGGGATTTGATATTCTGAGCTA
CAACAGCTTCATGTGCTTGGAGCAGCTTCCTTTTTTGCTTCTGAATTTTCACCTTCCTCT
TCTGGCTTTGTTTCTCCTGATGCCGAGCCAGTACCTTGTTCCCCTGGTTTTGCCACTTCN
NCACCCATGCCTCCCATGCCACCAAAATTCGAAAAGTCCATCCCTCCAAGATCCAAATCA
CCAAGACCATTATCCTCGTCTTCATCGACCCATTTGTCCCAATCAACTTTAATATAATGT
GGTGTCTTGCCATCTCCACGTAATAACTTCTTCCACCATCCCTTCTCTGCCTTTTCCAAA
ATGCAGAATATACTTCTTACTCCAATATTAATTTTACTTTCCTCCACATTGACCTTATCA
AAAAGATCCAATTTAATCTCATAAAGGTTATTTTCTGCTCCTGCACTAGCAGAGAAAGTA
AACACTCCCTCTGGCTCAAGATTGACCTTTGCATTTTTTGAATCAGGCAACAGAACAGTG
ATGAAAACCTTGTCTGCCCTCTGAGCCCACTTCACCTCAGGAACACGGCTCATGTTGTCA
AGAAATGGAAACAAAACTATGAAACCCTAGATACAAGCGTTCAG

>tig_1370_consensus
ACTATCTTCAAAGTCACCCATTCCATCGGCACCCATGCCTCCCATGCCACCCATGCCACC
CATGTCACCAAAGTTCGAAAAGTCCATCCCTCCAAGATCCAAATCACCAAGACCCTTGTC
TTCATCTTCATCCACCCATTTGTCCCAATCTACTTTGACATAATGTGGTGCCTTGCCATC
TCCACGTAATAACTTTTTCCACCATCCCTTCTCTGCCTTCTCCAAAATGCAGAATATGCT
TCTAACTCCAATATTAATTTTACTTTCCTCCACCTTGACCTTATCGAAAAGATCCAATTT
AATCTCATAAAGGTTGTTTTCAGCGCCTGCACTAGCAGAGAAAGTAAACACTCCCTCTGG
CTCAAGATTGACTTTTGCATTTTTTGAGTCAGGCAACAGAACAGTCATGAAAACCTTGTC
TGCCCTCTGGGCCCACTTTACCTCCGGTACACGGCTTACCTCATGTTGTATAGAAATGGA
AACAAAACTGTAAAACCCTAGATACAGAGTTTAGAGTTGCGACCGCTTGTATTGTAG
>tig_1371_consensus
TATTTTTTTTTTTAATGATACGGCGACCACCGAGATCTACACTCTTTCCCTACCACGACG
CTCTTCCGATCTCGTCCCTCCAAGATCCAAATCACCAAGGCCCTTGTCTTCATCTTCATC
CACCCATTTGTCCCAATCTACTTTGACATAATGTGGTGCCTTGCCATCTCCATGTAATAA
CTTTTTCCACCATCCCTTCTCTGCCTTCTCCAAAATGCAGAATATGCTTCTAACTCCAAT
ATTAATTTTACTTTCCTCCACCTTGACCGTATCGAAAAGATCCAATTTAATCTCATAAAG
GTTGTTTTCAGCTCCTGCACTAGCAGAGAAAGTAAACACTCCCTCTGGCTCAAGATTGAC
TTTTGCATTTTTTGAGTCAGGCAACAGAACAGTAATGAAAACCTTGTCTGCCCTCTGGGC
CCACTTTACCTCCGGTACACGGCTCATGTTGTATAGAAATGGAAACAAAACTGTAAAACC
CTAGATACAGAGTTTAGAGTTGCGACCGCT
>tig_1372_consensus
GCATTTTTTGAGTCAGGCAACAGAACAGTCATGAAAACCTTGTCTGCCCTCTGGGCCCAC
TTTACCTCCGGTACACGGCTACAATACATAAAGCATTTTTAAAGAAACCCCAAAAAGAAA
AACCATTGTCTACGATATGGAGACTTATTAAACGAGTAAAAGAAGACAAACTTTTAAAGA
CAAAATAATTTGAAGAAACAAGTACAAGACGAACCATAACAAACTATAAATGCATATTAT
GCACAGACATTAAACCAAGAGTAAACTGGAAAGACATAGCAACTAATATACCAACTTCAA
ACAACCATGAT
>tig_1373_consensus
CGCCGAGTATTCACAAAGATGACACTTTGGGTGATTGCCAAAGTCTCATACAGATCACAT
AATGTCTCAAGCTTCCATTCCTCCTTCTCACAATTCACATAAAACTGTTTGATACCCTCA
AGGGTAAGCTCATCACGTTTAACCAGAATCCTCACTGGTTTATTCATGAATTTCCTTGTG
ATCTCAAGGGCCTCAGGTGGCATTGTGGCAGAGAAAACCCCAACCTGAATTTTTGCTGGG
AGCAACTGAAATATATCATAGATCTGATCCTTGAAACCTCGTGAGAGCATTTCATCTGCT
TCGTCCAACACAAACATCCTAATGTGGTCAGGGCGGAGAGATTGTCTTCTCAGCATGTCA
AACACACGGCCCGGAGTACCAACCACAACATGAACCCCACTAGAAAGAATACGTTGATCT
TCACGTACGCTTGTTCCACCAACACAAGCGTGAACCTTCACACCAAGGTAGTCACCTAGG
GCTCGCATAACCTTCTCAATCTGTTGTGCCAGTTCTCGAGTTGGTGCAAGGACCAACGCC
TGGCATTCAACTAAGCTGTAATCAAGCTGCTGCAGAATTCCTGAGCAAAAAGTTGCTGTT
TTTCCAGTCCCAGATTGTGCCTGTTGAATTACATCAAGCCCC
>tig_1374_consensus
ACCCAGACAGGAAAAAGAGTAGTCAAAGTTTCAATTATTCTCTCATACTGACTCATCCTG
CTAGAAGTGTTTGTGGGAACATCTCCAGATACATCAGCAGTAGCCTTGCACAATAGTTGG
GAGTTCCCAGATGAGCCGGCAGCCTCCTAGAAGAAACTGAAGTGCCATTAATGAACGCCA
TTAAAGAAGTCTTATGTAACACCTTAATCAAAGAAAACGAGTGTTATACTGTGAATTTTT
AATGAAGATCGAACAGTTTGGCTCCGGAACGTCGGGGCTTTCATTATCCATGTGGATACA
TAGTGTGGGTCGTAGAAACCGATGAACCACACGCTCCGAGGTACCCAATCTGAATCTCAC
AGAG
>tig_1375_consensus
TGAGAAGCCTCTCCAGCAGTGCGTAAACGACCATCATTGACTTGATGGCCTCTAAGTTGG
TTCTGAGTATCAGTCCCATCTTCGTAAGGGTCACTCTCAATATCATTTAATGGGTCACTA
ATAACTTCAACTGGAACACGTCGACTTAATCTTTGTGCAGCTGTGTTCCCCAAAATTAGA
GGTTCACGTCTCCTCAAGTGTCCTTCAATCTCCTCAAATTCCTTATGCCGCAAACGATAT
TTCAACATTAGTTTGTGTATTGATGGGATAAAGATAGTGAAGTCCTCTCCAAGTGCATGA
GCCAAACAACAAAGCGCATCAACAGCAGCCTTTCGGAGCTCGTCATTTTTCCCATCCAAG
ACTAGCTTCAAGTGGTGCACAAGGGCAGATTTATGACCAGTAACCTGCACACGAGGTATC
AATCTAGTCAGAGTTTTGATAGCTGCATGCCTTATATCCACAGAAGCATCCACTTTAAAC
AATCGAATAAGAGCAGGAAGAAGCAGATGCATATGCTCATCCAGTGTCCCACCAAACACT
TCAAGGGTGTGGAGAATGTCAAGAACATAGGTGTAGTCATTGCACCGTTCAGCATCACTT

AGAACTTGAATGCAACATGGAAGAATAACAGGAAGATGTGTTCTGAACTCATCATTAAGG
GCCAAGCAAAGCTGCTCCACAAGATGCAGAACCGGAAGACCACGAGAAGTACGATTAGTA
GCTGGCAAACTGAAGGATGACCATAATTCAGAAATTAGAGAGAACAATTCTTGCAAATAT
TTACGAATATGCTGTTGTACTATAGACACAAGAGTCCCTAGCTTCCACGTTATATAATCC
TTCAGATAATCATCACATATACGAACTGTGAGAAAGAGATCAGGCAACACCTTTGGTAAG
TAAGGAACACAACCAAGGCACATTGACTTAAATATGAACATTAGAGATCC
>tig_1376_consensus
CTCACCGAGCAAGACTTCTCAGTCTCCAATGATTCTCCCTGAGCACCTACACTGTTGTTG
GAGTTAAGACCGGAACTACCTGCCAGCATTGCCATCAAACTTCCACCATGCACTCGACCT
TCCGGAATGAAAACGGACTTTGATTTAATATGGGATTTTGGGACCTTCGGGAGACCCCTTA
TTGGAATCTGAATGACTTCCGCCTGAAGTTCCTGCACTGCCCACCTTCATGGAATCCACG
TTCATGTCATCAGCAGTAACAACCTCTTTCATCTTATCAGGCTTACATTTAGGATCCTGC
ACAATAGGTCCTAAAGTGACACCACCATGTACCCTCTTATCCTGCACCAGGCCACTGTGT
AGAGCACAAAGACCTGTCTTCCCCCTCGCAAATGAGTTACATGGACCAGTAGCTTGGGGT
CCATATTCTGAACCAGGATGACCCCAGGAGCATCTTTTCCCTCCACCATGGGCCTTACAA
AAGTCAGTGCTGCCTTGTGCACTCTTGCCACACCCTTCAAACTTGCATCTCTTGCCCCCA
CCATGACGGACACAAAAGTCCGTCCTTCCCCTGGCACTCTTTGTGCACTCAGGTACAGCA
CACCTCTTACCACCACCGTGTGCGACACAAAAGTTTGTTCCTCCATGGACACTCTTTGTA
CAGACCCCACCACCTTGAAAAGCACACCTTTTTCCACCTCCATGGCCCTTGCAGAAAGGA
GTGCTGCCTTCAGCACCCTTGGTGCACCCAGCTGCTGTGCACCGTTTTCCACCACCATGA
GCCTTGCAGAACATGGTGCTTCCTTGAGCCCCTTTTGTGCAACCTAAGGCTTGGCATCGA
CGGCCACCTCCATGTGAGATGCAAAGACCTGAGAGACCTTCGGCACTCTTTGTGCAGTTT
TCTTTCTGACATCTCTTGCCACCACCATGACGGATGCACAATCCAGATTTCCCTCTAGCA
GCTCGAGTACATCCCTCATGACTGCATCTCCGACCACCACCATGGGCAATACAGAAATCA
GTGCGCCCCTCTGCACTCTTTGTACACCCAAGGGATTCACATCGCCGGCCACCCCCATGG
GCTTTACAGTACACAGTCCGGCCTTCAGCTCCCTTGTGGCAGCCAGGTTTTTGACATCGG
CGGCCACCACCATGGGATATACAACGGCCAGAAGCACCTCTTGCTCCCTTTCCACATCCT
TCAACCTGACATGTTTTCGAATTAGAGCTCCGTTGTGGTGGATGCTGCTGTGTTATCCCG
GAAGTACAGGTGACTGAGCTTCTTGGTGTGGTTATCACACTCGAAGAGTGGTCCGGAACC
TGGGTGAGAAAGAAGCCAACCTCTTTATTGGGCAAATTCTGTAATGGAGGTAAGGATATT
CCTGTTTTCCATCTGCATGATGTTAATCCTTCATCTGAATACATGCCCCCACCTACCACT
AGTGGCATATCCATGTTGAGTTGGAGTGAGCTAGAACCTGGATGAGCACAAGTAACATCA
GATTTAGAAGGCCCAGTGGAGAGACTCAACTGCAAGTCAACCTTTGGCTGCCATCCAGCT
GCTTTCTTATTTGAATTAACAAACTTTTTTGGGCTAGGCACCTTCTCATTACTAAGATGG
AGGGTGAAGTCCAATTCAAGATCCATTGAGGACTCTTCATCAGTTTCTTTGGCAGATGAC
AATGTGGTGCGGGCAGTTGCAGAACTCCCCTTGCTATCTGAGGAACTTGATGAGCGGCCA
AGCCCAAGAGACAATGTAGATCCAACTTGCTGACCCATGGATCCATCAATTAAACCCCAC
TTCCGCTTTATTCCCTTTGATGTGGGCATGTAAGGAATTGAAGTACCAGGGAAATCAAGA
CGTAAGCTCGTATCTGTGCCATACTCGGCCCCAGTTTCTCCAATCTGCATTGAACTGCTC
AAAATTTTGAATACATTTGAAGAATAATTAACACCAAAACCCAAGTTCTGGAACCTGGTG
TCCATGAAGTAATAGCTTAAATACCTCAGTGATAACCAAACCTAATCCTAATTTACAGAA
CAGTCAACCTGACTTCCGGAGAAAAGAAAATTAATTACAAAACTAGGTTAGAAGTCTCCT
GAATCATGTATCCACCCCCACTGACGAATCCTGAAGTATCACACCTATCCATTTTACTAA
AATGTAATTTAAACCAAAAACACCGATCAGTTGAAACAACAGAAGATGAAACCGAGCGGA
CTATATTCCTTCACACAAAAATCATGGAAGTTGCTCATC
>tig_1377_consensus
AAAGCCACTCACCGCCCACATTTGCAATTAAACGACCTTTCACGTCAGCTTCCACCTCTT
CCAAGACGGTGACAACCGCCCAGCTGAATTTGCAACCACTTAATTCTCCCTTCAATTTCT
TCTCCCACCGTCACACACTCATAATTCATCTCTGAGGGACGGCGTTTAAATTCTGTGGTG
TCCACAGCAATGTCAAAACTCGTAAGAAATCCAACTACCCTTTCTTCCTTATCTTCTCAA
ACTTACCTACTTTGTTTTTGTTTGTTATCACGAATGAAGTACTACAGAAAGGTAGGCCAT
CAGGATTTCCATGCCCACCGAATCTAAATTTTCATATCCATCCATCTTCCTATGCTGTTA
ATATGTCTTCAACTTCAATATTAAAACACAGAACAGAAGACAAAAAGACTAGAAAACATT
AAGTTTGATTAAGACATTGACTAATTACTAACCATACCCTAAGCATCTACCATGTTACAA
CTGAGATATTGGCTTTTTAACCTAATCAAACATAACCCTTTCTGGTTCCGGTTGAGGTAC
AAGTGGAAATAATGAAAGAAAAAAGCTGATCATTAACAGTACTGGTGATAAGTCGTATAT
TTCAATCTTCATCAGAACTTTCTGGGTTGGGATACTTGTTTAAATCGGGCTTCTCGGATA
CGCGAGTGGTAGAGTTGGAGTCTGATTGTGATGTGTGGAGAGCAGTTTGTAAGGCATCAA

CCCTAGCTCCAACTTCTGTAGCTTTCTTGCGTATGGAAGCGGCAGACATGTCTCGAAGAT
CATCGTCTTGGAAAATCAATTCAGGGAAATTAAGACGAGCAGACGGGCCTCTAAGATAGA
AGACAGCAGTGTCGTAGGCTCGTGCTGCAGCTATAGGAGAGGTGTAAGAACCAAGCCATA
TTCTAGATCTCTTGTTTGGTTCTCTAATTTCAGCCACCCATTTTCCCCACTTTCTCATCC
TTATCCCTTTGTATGGCCTTTTCTCTTGCTGTTTCCCTTGCTTCCGCTTCTCCGTGTTCA
TATTTCGTCCACAAGTCTTCGATGAACAAAATTCACCTTCCATCATAAACATTATATATA
GAGAGAGAAGCTCAGAAGCATTAGTTTCCCACTTGCCTACTAGTCTGTCGTATGAGCACC
GCCGCCACGTTGATCCTCCCAAAAGATAAATACAAATTTGACCGACATAATCCATCAGCT
CACCTCCCTCCTCCCTATTTATAGATAAACAACAACATAATATACGTAATTTATTATCTC
TTACAAGGGCATTTCCG
>tig_1378_consensus
CTCAAACCGGTACATTCCACGAATTGCACCACCTAAATCAAGAACTAACCCACGTGCACG
AATTTGATAAACCTTGGCAGTAACCATTGTTCCAAGCTTCAGATTTCTTGTAGAACTGAG
AGTCTCAACTTTGGTTTCCATATCATTCTTGCCATCATGCTTACCAGAATCAACCCCATA
AACTTGAGGCATAGTTTTTTAGACTTACTATCCCCTTCTGAACCAGCAGTTGATTTAGA
TCTACTCCTAGAACTCTGACTCAAACCAGAAGATTTTTCCTCCTCATCACACTCTGAAGC
ACTTCGAATTACTATTGCAGGGGTTGAGGAAGAATATGAGTTTGCCTCACCAGTTTCACT
TTTGCTTGCTGGGAACTCCCGGTTCTGCTCATCAGACTCATTTTCAACTGATGCCCAAAC
ATTAGTAGCTTCTTTGTCACCTGGATCACATCCTTCAACCACTTTCTTTGCCTCAGCTCC
AGGTTTAGATAAAACCGCTTTAAGGGATAGCTTAATATTACCACGAACATCTTGCCCAAT
GCACATCAAAGAAAGCTGCTGGCCAACAGTCACCATATCTGAAACTCGGGAGACCGGTTC
ATGCGACAACTCAGAAATGTGCAGCAGGCCTTGTTGACCACCATTGAACTCCACAAAGGC
ACCATATTCCTTTACTGATGTTACAACACCCTTATACACACCCCCAACCTCAATCTCACG
GCCAATTATGAAATCAACCTTATCTTGTACTTTTTCCAGCATCTCCTGATTCTTAGCTAC
TATAGTAAGCGTTCCATCACTTACAGAGATCCTCGCACCTGTTTCCTCTTCAATTTTTCT
TTTAAGAGAGCCTAGAGGGCCAATCAAGCGGCGAAGAGTGTCGTTGCTGTACTTCAAGGT
AACTAGACGAGGTGAATTTCTATCATCTTGAGTCCGCGGAGCATTAATTTGCCGTTCCAT
GTGGTCAAGGATTTGAAGACGACCTTTAAGTGCATGCTCTAAACACTCACAAATAATATC
TAGAGGAATTCCAGCAGGTTTTATATCCAACTGAATTGCTGTAACTCCTTTTCGAGTGCC
AGCAATTTTGAAGTCCATATCTCCTAAATGATCTTCCAGACCCAGTATATCAGTCAATAT
ACGATAGTCCTTAATTTCACCAGTATATGGGTCAGTTTCACTGACAAGACCAACTGAAAC
ACCAGCTACATGTTCTCGTACTGGAATACCAGCATCCATCAAAGCCATGCTGCCTCCGCA
GACAGTTGCCATGGATGTTGAACCATCAGAGGCCATGACTTCTGAATTCACACGGACAGT
ATATGGAAAGTCATATTCAGGAGGCAACACAGCAAGCAGAGCTTTCTCCGCAAGAGTTCC
ATGACCAACTTCACGCCTATTTAGGCCAACTCTTTTACCAACTTCATTGATTGAAAATGG
TGGAAAACTGTAGTGAAGCATGAATCGCTTTGTTGGGGGTCCAACCAGGGAGTCCAAGCG
TTGAGCATCTTCAGGTGCTCCAAGTGTCACAGTACAAAGAACCTGGGTATCTCCCCGAGA
AAAAAGTGCTGATCCATGCAACATAGGTAAATAACCAGCTTCACAATAAATAGGCCTAAC
TTCATTAAGATGCCTCCCATCAACCCTGAAGCCTTCCATAATAATCCTTTTGCGAACAAC
CTTCTTCCTCACAGTGTCTACTGCCTTTGGTAGGACATTTAAGCTTTCTTCATCACATTC
TTCTTCAAGGACTTTTCTCACTTCTTGCGCAATCCTTTCTAAAGCCTCTCCACGTTCAAA
CTTGCCATATGATGGATCGGTAAAGACAGCTTCAATGGGCGCTGCAGCAAGGTTACTGAC
TTTCTCCAAAGTTTTGTCTGAGATCATTGACAATTTATATTCTTTCTTATGTTTTCCTGC
TTTCGCAGCCAGTCTGATTTGAGGTTCAAGGTATTTAACTGCCTCAGGATGAGCCAGTCT
CAATCCAGCTTCTAGATCTTTCTCTGAGATCTCACGTGCCTGCACATCTATCATCAAAGT
TTTGTCCTTTGTGCATGCATATACTAAGTTGAGATCACTCAAGTTAAGCTCATCCATGGT
TGGGTTGACAATAAACTGTCCAGCAATTCTCCCAATACGTATTACTCCAATGGGACCACC
CCAAGGAATGTCTGATAACATAAGAGCAGCGGATGTTGCATTAGCTGCCATTACATCTGG
ATCTTGTTTCCCATCAGAAGAAAGAACACTTGCCATTACCTGTACTTCATGGTAAAATCC
TGCAGGAAATAGTGGTCGTATAGGTCTATCAATGAGACGACCACATAGAAGTTCTCTTTC
TTTAGGAGCACCCTCCCTCCGCATGAAGGTAGTTGGAATCATACCTTGGGCATATCGTTT
CTCTTGATAATCAAC
>tig_1379_consensus
ATTTAAACATCAGTTTCAGATAAACTACTACACAAGGAATGAAGAAAGATTTGGACAATT
GACTTACACATCTCGTGAAATGCAAATCGTTCCAGACAAAATCAACTAAAATTACCAACC
CTTCTAGTTTTTTCACCACGTTCAAACATAGAGCAAAAGTACAGCTAAAGGCTGAACATA
ACTTCCACACATCAGAATTCCTAAACACGATCATGCCACAAGCACTCAGATTGAACTGCT
CCCCCTTTCAAGAGCTCTTCAATTTCTTTGGGAGGGTTT

>tig_1380_consensus
ATTTAAACATCAGTTTCAGATAAGCTACTACACAAGGAATGAAGAAAGATTTGGACAATT
GACTTACACATCTCGTGAAATGCAAATCGTTCCAGACAAAATCAACTAAAATTACGAACC
CTTCTAGTTTTTCACCACGTTCAAACATAGAGCAAACGTACACCTAAAGGCTGAACATAA
CTTCCACACATCAGAATTCCTAAACACGATCATGCCACAAGCACTCAGATTGAACTGCTC
CCCCTTTCAAGAGCGCTTCAATTTCTTTGGGAGGGTTT
>tig_1381_consensus
GTTGCATAAAAATTCTGTCTTTCTCGATATAAAACCCTACCCATAAACCCTCATTTCCTA
TCCATCGGTTTCCTCATCTCTCGTTTTTCTCTCTTAGGGTTTTGCAGAGATGGCAACCCA
CCTCTCGAAGCTCGCTATTTCCAAAACCCTAACCGCCCTTCTCTCCCGCTCCTTTTCTTC
TGCCTCTTTCGCTTCTCATCCTCTCAAGTCCTCTCCTTTTCTCTCCCTCGCTCTCTTCAA
CCGCCTTCGCCCCTTGGCTGCTGTCAACATCGGCCGCTTTGTCACCATCGCTTCTCCTGC
TCGCGGCTTGGCCACACGCGCCACCTCGTCCTCTCTCAATGATCCCAACCCCAACTGGTC
CAACCGGCCGCCCAAAGAAACGATTCTGCTTGATGGTTGTGACTTTGAGCACTGGCTTGT
GGTTATGGAGCCACCTGAAGGAAATCCCACTAGGGATGAAATTATTGATAGCTATATCAA
AACCCTAGCCATGGTCATTGGCAGTGAAGAAGAAGCAAGGATGAAGATTTATTCAGTTTC
AACTCGGTGCTACTATGCATTTGGGGCTTTGGTTTCTGAAGAGCTCTCATACAAGATCAA
AGAATTGCCTAGAGTTCGCTGGGTTCTCCCGGATTCCTATTTGGATGTTAAGAATAAAGA
TTATGGTGGGGAACCTTTCATTAATGGGCAAGCAGTTCCTTATGACCCAAAGTATCATGA
AGAATGGATCAGGAACAACGCTCGAGCAAATGAGCGTAATCGGCGCAATGATAGGCCTCG
TAACTTTGATAGGTCTAGAAACTTTGAGAGAAGAAGGGAGA
>tig_1382_consensus
GCCATCGGGGAAGGTTCGCTAAACTCGAACGAAAAATGCCCACCGTTTTCTGTTAGTTAT
CATCTGATGTCGTAATAACAGAACTTAGGAAATTGAATATAAAATTACAAAAACTTTAGA
ATGAATGTTATAAACTGTACATGGCTCTAGTATTCCACATTTGAGCGGGGGATTATGATT
TTTCCCTTTGAGATGCTAGATGGAAGCGCAACACTCTTTGACTTGAATGTGCTTTCAGCT
GATTCTAGTGCTCGCGAAGCTTGAAGAGCCATTTCAGAATGGAGTTGGAGACATGACAAA
GCCAACGTACAACAGTCTCTATCAGTGTCTGAGTCAACTACATCAAGGGCCCATGATCGA
ATCCAGTCAAGCCCATTAGAAAGTTCAACATTCCCTTCAAGCAAGGCAGATGCTACAAAA
GAGGGATGAAGTGCAACCAACACACATGAAGCTGCAAAAAGAACAGCTTTTCTGACATAT
GCTTCTTTGTGATGGTATACCTCCCTGGATTTTAACATATCTAGAAGAGCCGGAGCCAAT
GCAGATGCTTCCGGATGCATAGATGCACATTTTATGCAAACACCAAGCATATAGACAAGT
TTTCCCAAAACAATAAAATCCCTCTCAAGCAAGTCAACACCATGCCTTTTCTTATCAAAT
CCCTGCATGGCAGGTAGCATAAACGCTGCTGCATACACAGGAAACTTGTTATGGGACAAT
TCCATCTGGTTATCTTGTATATTTGCTGATTTGAGGTTCCATCGGCGTGTCTTCCCTCTC
TTGATCAGACCAGGTTTTAATGGAAGTTCTCTCTCATAACGAGTTGACCAGTTCAGCAGA
GTTCCTGCTTCTGAGATCTCTTTCCAGGCACTGGCTCCAGGGGGTCCGCTGCTACTAGGC
AAAAACCACGGTTGAGTCTCTGAAATGGTTGAAATGAGGGCCCTGGTTTGATGTTTAGGT
TTCATAATTTTAGAATTGGCAAGCTCTTGAGCTGCTTCTGTCATGATATCAAGAATTATA
ATACGTTGGCTGACATCTAAATGAGGTGAATATAACAGTTTGTTTAGGGTCTCAAGAGAC
TCAAATGGACAAGTGACAAGCAAAGCAACCAAGGCTCTATGTCTCTTCTCTTCAGCTGAT
TCTTCTTCACCTTCTACAGCCACATCAGAGCAGCGAACTAGTACAAGAGTTTTAGCTAAA
TCACCTGCCACATGAGTAAGTTCATCAGGTGATGCTCGCACAAGCTCTTCAGCAACATCA
AGAGCCCTCTCCACCCCGTCAGCATCATCAGATTTTCGCAGAGCTCCAACTAGATCAACC
AACTGTGAAAATTTTCTTTTCAAATCATTGTCATCATCTGACAAATCATAGGGCTGTAAA
GATGAGTCTGAGGAATCAGAATCCTCACTTGCTTTTTCATCATCCTCATCTTGATCGGAG
ACTGATTCATAATTTAGAGTAGCTGGATCAATAATTTCATCAGGATCAATTGAGTTTTCA
AATAACTTGTTCTTTCCACTCTTCTTGTTGGTGAGCATCTCATCATGTGTTATGCAGTCA
AGGTCCTTTTCTGGCATTGAGGTTGAAGCCGTTATCATTTTGTTAAGATTATTTCCGTTG
GAAGTTGAGGTCTGCAGGCTCTCCTTATCACGCGTGGTTATTCCAAATTCCCAATCAATA
GTCTCCCCAGTACAGCTATCATCAAGGTATAGAGGATTTTTTGGGTCAATTAGTTTAGAG
AACATGAAAGCAACACTACTTGCCATTTTACGAACCATATCATTGGGGCTCTCCAGCCTA
CAGCTAACTCCTAGAAGAATCAAGTGCATCACATCCTTGGTCTTATCCAGTTCCTCTTTT
GACAGCTTCCCAAGGCAAAGGCCTATAGCCGCAGTAACATATGCTTGCTGCTCCATTGTG
GTAGACTGCACAAACTCTCTCTTAGACCACACCGCTACCAGCCTTTCCACTGTGTCTAAG
AGAGCTTGGGTTTCATTGTCTTTTGTGAGTGGATCAGCGACAGGTGCGCATCCAAAAACG
GCAAATTGAAGAATCCATCTCAGGCAACAAATAGGAAATACTTTCCAGAGTAAAAATTTA
TCCAGAAACATTGACCTGATTGACATATGTTGATGAAAAACCTGATGAAACAGTAACCAA

ATAATCCAATATGCTTCAGAGTCGGTCACATTATCAATTGCTAGCTGATGCAACAGCTGT
TCAGACATTCTTTCCACAGCATACGGATCTTTAATTGCTTCCATCATCTTCGACCAAACT
ATTGATTCAGGGTTAGACTCAAAAATGTCTTTGGGAGCTACATCACTATTGGAGCATAAA
ACACTCTGAATGTGTCTAAGAACACGGAGGACTACTTCACTTGAAAGCACATCAGAAGAA
CCACGCCGGCAAATGCGAGAAAAAGTTTCCCCAACAAATAGCAAAGTTCCATCTAATTCA
ATTTTATTGACGGTAGTGAATTTATCTGAAAGATTGGCAGCCCTTTCTTGCAGTCCAGAG
AGAAGTTGAATTGTTATTGTCTTGAAGAATAAATGTGATGTAAGTGAAGTTGACGCTCTA
GGCCGTGCTTTGTCTGGCACAGATGCAACAAGCTGTGCCACCCTTGAAATTGTCGGCCTC
AGCCAGTCGTTTGCAGAACCTTCACACATGCAAGAAACACCAAACTCTCTAGCTACCTGG
AAAACTCCATTATTTTCAAGCAAGCAAAGAACAACTAATCTTTCTGCATTTGAAAGGAAA
GCTTTAACTTCAATACCATCACTTCCACTCTGCTGCAGACAAGGAACCAAAGTCTGAACA
ACCTCAGACATGAGTCCATTAACAAAGAAAATATCATAGAAATGTTTCCGTGCTGAAAAA
GGGAAACAAGCAAGCCAATTTGAAGTGACATCATGTAATAAAAAGTGAGCCAATGTTGAA
AATGCAGGTCCATGGTAGAAGGACTGCCACCATTCATCCCTTTCATTTATCGATGGGGCC
TTAACATTAAGAATCTGGTACTTGTAATGCTGATGGATACAGCCTGAGATAAGGGAGGAA
TCGATTGGGAAGAGAAGCACTGCAATAGAGTGAAGGGCGGTAACAACTGAGTCAATGTGT
TTTGCATTTTTAATGGCTGTGATAACTTCTCCCACTTTTTCGAGGACTCTAGTATCAAGT
TCTCTTTTTCTTCTCTTCGAATCTTCATCCATAGTTTCCATACGATTTCCAGCGGTGGAA
GATGGCGAGCTACCCTGTAAAAATATTAAGAACCGGAACAGGAATCTGTTATGGTCGGTT
CCTTCTTGGAACCGAGGTGGAAAGTTCCGACTAGAGTCTGGTTCCTACCCGAACCGACTT
GAATTGAAAAATGTTAAAAGTTCAACCCTTGCTCAAAGCATGGAGATAGTTCAGCCACCA
ATGCAGTAGCTGTT
>tig_1383_consensus
ATTGTTGCTAAATACTTAAGACATAATTGTACAGTGCACAGAAAACTTCTCAACAGAAAT
ATTATCGAAGGCACTCACAGAATTGAACAATGAGGAATTCATTACTTACAAACATCGAAA
ACAAAATAACAATGAAGACTGGACATCAACTTTTTTGTTACAAAATAGAAATAAATCCAA
CTCACTATCTCTTGCCACATTTCTTGTTATTCGGTGTCGTTTCGCAGCAATCAACCGTCC
CAGAATTTCGCTTCTGCTTTCTCCTCAACCTCTCACACACAAAAGCCTTCTCCAACTGCT
CAACCCTATGCGATAATGAGTTCAATAACTTCGTCTGCATTTCGTTCCTCTCGAACAACT
TCGCCATCATATCCATCATCTTCTCATTGTCCTCCATCATCTTCTCCAACAGCTCTCTGT
TCCTCGAATTATCCTCTCCTGTTCTATCTTCTTTTCCACTCACTTCCCTGTCTTCTGCTT
GAGGCGTTTCAACGGCGTCGCTTTCGTCCTTTGGCGATGAATTAACATCTTCTTCACAGG
CTTCGATCCAATTCGGAGGATTTGCAGAGGAATCGGACTGTGACTCGGTCTGATTTGTTC
CTACACTTTCTTCTAAACATTCACTCACGGATGTCTTCCATTCACAATTTTCAACAACCT
CTCGCCCTACGTCGTTGTTCTGATTGTCGACGACTTCTTTAATTTCCGTATCATCCAGGG
GTTCGGCATTGTCTTCCGTTGGACCATCTCGATTTTCGCCATCTTTTGCATACCCCATCA
CCGTCTCATTAATAGCCTCCATAATCTTTTCAGCCTGATTAAATTCCACGTCGGCATTAC
CAGAAACAATGGCGTCGACAATTTCTTGCAACGATATAGCTTTCTTAATAACCGCTTTTC
TACAATCCCTGACGCCCGAATCAACGCCTCTTACCGAATCCAACTTAAAGAGTAAACTCA
TCAGCATTTCGTTAATCCTCAAACGCTCCTTGGCGTCTCTTCTAATCAAATCGACAGTCT
CCTTGTCAGAAATCCGCTTCTCAATCTCCTCGACTTGACTCCTGATCTCCACGATCTTCT
TCACGTTTTTTCTCACGAGGAAACCTCGGAACACTTTCTGGATCCTCACGGCAGAACCGG
ATCTATTTACAGGCGGCTCCGAAACGACGTAGTGGACCGGTATTTCTCTGACAATCGGTA
CCTGGCGAGGGCGAGAAGGCAAATTCCAGTAGCTTCTGTAAAATGGAGTATCCATTGTCT
CCTTGTAGAAAAAGAGATGAACAAAAGAGTTATTGCTTTTCACGAACCGCTTTCGTGCTT
TTTTTTCCTTGCGTTGAAAGAGAGAAATTATCGCCGGGGATTTATAAGGTGC
>tig_1384_consensus
AAAAAAAAAAAAAAAAAGTGAAGAAAATATGCTATATAGACCTATAATTAAAGTTCAAGAA
TGAGGTTCAGATAAAACCATTTGGTCTTTCAATTTACACGAGGAGCAATATGTGATATCC
TTTCATCTTCCAATAGTTCAGAGGGATCAACATACTGTGGTGCAGAAGTAGGGGTCTGCC
TTCCATCGTGAACGAAGGTTGACCCAAAATATAAATAGAGAGCAGAAAAAGATGCCATAG
TTATCATGATCAATGGCCAAAATCCCAAGAAAAATGTTCCACCAACAGTTAGCAGGAGTT
TAGCACGAGGAATTAGTCCCTCAAGGCCTTCACTATTCAGTGCCATAGAGCGCTCTCTTT
GATTAGGGAGATTTTTATCTAATTTGGGTGAGACAGCATTTCTTTTTGAGAAACTTATGT
CTTCTTCTCTCTGTTTTGTCTCATCATCCAATCCATAGTCCCTCCTCTTTGTGTCCTCAA
GCAATGAAGTTGGTGTCATGCCCCTTCTCTTTAGTTCCTTCATAAATAATGACTCTGGAG
GCTCTTCACCGTTGGTTCGATTGTTATCGCCGTCTTGAACTGCACAACAAACCCTAAAAT
TTCCTCCGATGGAGCAGCTCGACCGTTTATAGCTTCTAAACCGGAGCGCCATTTTCCCGG

AAGAATCCCATTTTCCGGGAAAATTAGAGTGGGAAACACAAACGGGAGTCAACAAAGTGG
ATTTCAGAATCTGGACAGAACCGGCAGCCATTTCTTAGTTGCTTTCTTGCATTAATTAGA
GCCGATTTTCAAAATTCATATCCAGAAATTTTG
>tig_1385_consensus
AAAAATTAAAAAAAAAAAAAACTCAAATTAAAAGAAATAATAGCACTACATAACAATTAT
TTTTCCATAAAATTCATAATCTTAGCTTCAGATTTTTTGTTTTGTTTATTAATCTACCAA
ATAGTTGAATTAGTTGTGAACGGAATAAAAAAAAAGATCTAATTGAAAACATTAACGCTA
AAACTTCTCTCTCCAGCTTTCATCTTTTGATGTTATTTACATTAGAGAAAAGTCAGCTAA
AAATTTGTAACTCACAACAATGAATTACCAGACAAGACTCAGTTGATGGGCCGGCGTAAT
CCAGAGTAAACTGCTCTGCGGAANNNNNNNNNNNNNNNNNNNNNNNNNNNNNNNNNNNNN
NNACACCATTGCTCTCTTTCACCTATATTGTAATCACTCCTTTGCCCACCATTTGATTGA
CTGTTTTTAGGTCTTCCACTCTCATCATCCAAGTCCATTGCTGAAGATTCTTCGACATCA
ACAGCCATGGCATTTGCAGGGACAGACTTAAGTTCCCGCAATGTACCAGCATTTGGTGCA
TCAGGCAACCGTTCACTCAATACACTCGAGAAACTTTTGTAAAGAGAAAGGAATAGTGCC
TCATTTTCATCTAAGGCTCGAGCAAAAAGAGCCTCCTTGGCCTCTAAAGATTCATGAGCT
GATAACTCCTCTTTTTTGGTTTTTTCAGCTTGTAATTTCAAATGGTTTGTCTTTGTAGGA
TTTTCACCAAGAACAGGTTCACCATCTACCAGCGTAAGCTTTGACTCAGCAGCCTCCAAA
TCTGCCTTGGCCTTCGCTGCAGCTTCTTCAGCTAATGCAACATCCTTCTTAAGAGATGCA
ATCTCTTTCCTTAGATCACAGATTCGGTTATATGTCTTGCTAACTGCATTTCTAAGGATC
TCCCATGGGCGATCTGATGTGTGAAATTGGTCAATGTTTGCTGGAGAGAAGACATATCTC
ACAATGGCCAAATTAGAAATAAGACGGTAACCCATCATTCTATCAATTGCTATGGCTGAT
ATTTGAGCATTGTTCTTCCAGTAAGAACTCACTTCTGCTATCAGCATCACTTGCTTTTCC
TGATCAGGACAAATTCTTGAAATGACTTGCCCATATCTCTCCAAGACAGTGATCAAATGA
GTGAAACTCTTGGATCCAATATCAAGAAGTGTTTGTACAATTATGCTGAGGGTTACTTCC
AAACCATGAACAGGATATACACTTTCTTCTACCCAAGCAATTATTTCGCGTGCACTTTGC
CTTCCTTTAACCTTGCTAGTAAGTTCTGTAGAAAGTTTACATTCCTCAGTTTTTTCTCGT
TCATCTTCCACACTGTACTTGAAATCTGGACTGCCTTTCGGGGGAAGCAACTCTTCTAAA
GCAGGTGCATTCTCAATGCTCTGCTTAACTTTTTCCCAGTACGATAGGCGAACCTCCCTT
TCCAACACCTCTTGGACAAACACACGTTGTGGGGCCCATTTAGGGAGTTCCAAGACATAA
GCCCATTCTTCCCAAGGCCAGATAAATTGGAAATTTGATAGGTGGTGTGAAAACCATAGT
ATGAACCGTGTCCTGCACTCCATATCCAAGTCAGCTATTTTCTCAAAAAGAGCACGGACT
GCACCAGCCACAACTGCAGGGAAGGCTCCAGGAAGAGCCTTACAGAGATCCATAATAACC
AATGTGTAATAAATTGGCTTGAAGGGGGGTTGAGGTAGCAAAAGCAACTGAGAGAAGATT
GTCTCTGCCATGAGATACTCATATCGGAAGGGCACAGGCAGACTGACCATGAAAGAGGCA
CATTCCTTTCGACTAGCAGCCATTGAAGAACAAAAGCACATCCAGCAAATACTCCTCCAC
GATAAAGCGATCAATAGGTTGCATATCCTCACTCTTACTTGTTGGAAATATATTAAGCCT
GCGTATCCTTTGAGGATACTTCAACTCTGCTTCATGCTTCTGTTTACCATAAGTTATACC
AGAAAAAGTAGATGGCGGATTAGGTTGCTCAGGGCAGCTGATTGCCCCAAATTCATGAGA
CTTTCCAGAAACCAACTGAGCTTCAAATGAAAGGTGAGGCCTTGGAACACTATCAACTTT
CCATCCACTGTTTAATAGAGTTTGAATCCGCTCCCACAAATCTTCCAAGAAATCCTTTTC
AACAACACCCTTCTCAGACTCATCATCATCATCATTCTCAAAGAAAGACAAACCACTGTC
TGAAGCATGCCTTCTAATGCTCAAATATGCTTCTAAACCAACCATGACCCTCTCAATTTC
CTCTGGAACTTGCTCAACGAGTTCTGCACCTCCCCAAGGGAGGCAAGACAAGATGCAAGT
CACGTAAAAATCAGCACATGCTTGCCATGAAGGATTTCCTTTCTCCTCATCCACTGTTGT
GGCAGCTGATGATAATAATGTTTCAAAGACAACTACCAGAGAACCAGGTTGGAGAACTTT
GCTACACATCATAGCAGTTATAAATCGCATCAAAGTACGAATTTGATCGCAGTTTCCAGA
ATTTAAGGCATCCTGAAAAGTAGCCTGAGTATTTTCCACTAGTTTCTTAACAAATTCCTC
ATTTTCCAAGTTCAACAAACCAACC
>tig_1386_consensus
CGCCTAATTGACAGCCTAGATTGTAATATGAATAAATAATAGAGAATATATATACATATA
TAAGGGAGAATCACGTTTCTACAACAGCAATCAGACAACTCTAATGGTAGACCGAGGTCC
AAGACCAACATTTGGTCTAATATGACCCCTCAGCTCTGTAAAGCTATACTAGCAACTGAA
ACACAGCCTAGACAAGTCTTGCGAGAAAACAAATATTTCAACACAGAATAATTCATTCAT
TGTTTTATCTTCTGACATTTTGTTTTGTTAAAAAATCTACATGATAGTTGAATCAGTTTT
AAAGCGAATAAGTAACAGATCTACTTGAAATGTTGCCACTAAACATGCTTTCTATCCCTT
CCATTTGTTGATGTCATTTACATTAGAGAAAAGAAGGTTAACATTTTGTAACTTATAAAA
GTGAATTACCAGACTAGACTAAGTTCAACGGCCAACGTAGTCCAGAGTAAACTGCTTTGC
GGAAAAGAGGATGCGCATCTTGAGTTAGTACCTCAGCTTCCAGCTTCTCGATATAGGGCC

ATATCTCAGAAGCATATTGCCTGGAGAAGGCCTTGACGTAGCCCAGTGTTGTTAGACACC
ATTGCTCTTTTTCACCTATATTGTAAGTATCACTCGTGTTCCCACCATTTGACTGACTAT
TTTTAGGTCTTCCGTTCTCATTGTCCAAGTCCATTGCTGATGATTCTTCAGGGTCAATGG
CCATGGCATCTGCAGGGACGGACTTTAATTCCCGCATTGTATCAGCTCTTGATGCATCAG
GCAGGCGTTCACTCAATACACTTGAGAAACTTTTGTACAGAGAAAGGAACAACAACTCAT
TCTCATCTATGGCTTGAGCTAAATGAGCCTCCTTGGCCTCTAAAGATTCTTTGGCTGATA
CCTCCTCTTTCTTTGTTTTTTCAGCTTGTAATTTCAAACGGTTCGTCCTTGCAGGGTTAT
CACCAAGAACAGGTTCACCATCTAGCAGTGTAAGTTTTGACTCAGCAGCCTCCAAATCTG
CCTTCGCCTTTGCTGCAGCCTCTTCGGTTAATGCAACATCCTTCTTAAGAGATTCAATCT
CTTTTCTTAGATCACAGATACGATTATATGTCTTGCTAACCGCATTCCTAAGGATCTCCC
ATGGGCAATCAGATGTATGAAATTGATCAATGTTTGCTGGAGAGAAGGCCCATCTCACAA
TGGCCAAATTTGAAATAAGACGGTAACCCATCATTCTATCAATTGCTATGGCTGTTATTT
GTGCATTATTCTTCCAGTAAGAACCCACTTCTGCTATCAGCATCACTTGCTTGTCCTGAT
CGGGACAAATTCTTGAAATGACTTGCCCATACCTCTCCAGGACGGTGATCAAATGAGTAA
AACTCTTGGATCCAATATCAAGAAATGTCTGCACAACTACGCTGAGGGCTACTTCCAAAC
CATGAACAGGATATATGCTTTCTTCTACCCATGCAATTATTTCACGTGCACTTTGCCTTC
CTTTGACCTTACTAGTAAGTTCTGCAGAAAGTATGTATTCCTCATTTTTTTCTCGATCAT
CTTCCACACTATACTTGAAATTAGGACTGCCTTTTGGAGGAAGCAACTCTTCTAGACCAG
GGGCATTCTCAATGCTCTGCTTAACTTTGTCCCAATAAGATAGACGAACCTCCCTTTCCA
ACACCTCTTGAACAAACACACGTTGTGGAGCCCATTTGGGGAGTTCTAAGACATAAGCCC
ATTCTTCCCAAGGCCAGATAAATTGAAAATTTGATAGGTGGTGTGAAAACCACAGAATGA
ACCGTGTCCGGCACTCCATATCTAAGTCAGCTATTTTGTCAAAAAGAGCACGGACTGCAC
CAGCAACAACTGCAGGGAAGGCACCGGGAAGAGCCTTACAGAGATCCATAATAATCAATG
TGTAATATATTGGCTTGAATGGGGGTTGAGGTAGCAAAAGCAACTGAGAGAAGATTGTCT
CTGCCATAAGATACTCATATCGGAAGGGCACAGGCAAACTGACCATGAAAGAAGCACATT
CCTTTCGACAGCCACTGAAGAACAATAGCACATCCAGCAAATACTCCTCCACGATAAAGT
GATCAATAGGTTGCATATCCTCACTCTTACTTGTCGAAAATATATTAAGCCTTCGTAACC
TTTGAGGATACTTCAACTCCGCTTCATGCTTTTGTTTACCATAACTTATCCCAGAAAAAG
TTGAAGGCGGATTAGGTTGCTCAGGGCAGCTGATTGCCCCAAATTCATGAGACTTTCCAG
AAACCAGCTGAGCTTCAAATGAAAGGTGAG
>tig_1387_consensus
GTCTTTAATGGCATTTCTTAGAAAAAGCTTAGACGATTAAGCTGTTAAACAGTCAAATGG
AACAAGAAGCCATGTTGCTTGGCTGGTTGCTTATGCAAATGGAACCCATTCATATTTGAC
ATTAACTCCATAAATAAATAACCAGCAACAATCACTCTCTGATAGGCAATCTCACGGCTG
TCATCTTCGAGAAAGAATTTACATGAACAAAATATACAAAAAGACAGAAGCAATTACAAA
CATAAGGTTCAGCCTTGGCCTGAGAAAATTAACTCAAACTGTAGTTTGACGGAAGCCTTT
AAACTGGGTATTTAGACAAGCATTTTACCCAGGTAGTTTTTTCACACTTCCACTTGGATG
ATCTCTTCTTTGTTTCAGTACTTACAGTGCCGATTCCCATCATGCATATTTCAACTTTTG
ATTTGAACAAATACCACAAACACATGCCACTTCATAATTTGTGACAAAATATACCAGTTT
AAAATTTACAACGGTAAGATTTTGGGAGTATGTCTGCTAATATTGGACCCTCTCATCATT
ATTGAAATTCAGGCTCTCAAAGCCTCTGAGAGTGGACTCAAAGTTCCGTATGTTTGAAGT
GTTTCTGCCAGAACTTATGTGACGGTTTTCTGATGACACAACAGGTGATCTTCTTTGCCC
ACTAATAATCTTTTGAAATGTTCCTGGGTTGGCATCTGTTATGTGAGGGTCAGCTTCACT
CCCAAAGACAACTGCATCCCGACTAGAGATTGCAGCTCTCCTTGATGATCCGCCTGACCT
AAAGAAACTGTAACTGGATGCCAAAGCTTCTTTAGAATGTGCGGGATCATTTGAAACTGG
ACTTCTTTGTTTTGATAAGTTTGCAGAATTCGCAAGTGGACCAGAGTTCCTCCTGCGAGT
GGGATCTGATGCAGACCAACCAGTATGCCTACTTTCTTCCCCACCTGCTTGCCTATCAAT
GTTACCAGCACCAAGAGGCATGCCAGAGCTTGGTCCAGCACCAGGAACAAGTGCACGAGC
AGGAGGATTAGCAATCTGTGCTTGTTGATACTTCAAAATTGTCCAATCAAATACATAGTC
AAACTGGAAACCTTCACGAATGAAAAGGTCACGGAAGATTCTCTTCAAATAAGGATAGTC
TGGCTTATCATCAAATCGCAGTGACCGGCAGTAGTGGAAGTAGGATGCAAATTCTGTAGG
ATAACCCCGACATAAGGCCTCAATAGAAGTTGATACTTTCTTCTCACTGATCTTCTCATA
CTTCTGCTTCTTAGTACCAGCTTTCAATCCTTGCCATGGAAGACTTCCTCTTAAGAAATA
CATGAGAACATATCCAAGTGATTCTAAGTCATCCCTGCGACTTTGTTCAATCCCAAGGTG
AGTGTTCATGCTGGCATATCTTGCAGTTCCTGTCAAATTCTTATTTTCTCTGTAAGGAAT
ATGCTGATGTGTTGCAGTGTCTCTATATCTCTTAGCAAGACCAAAGTCAATAACATAGAC
CTGATTTGCACGTCTCCCTAAACCCATGAGGAAATTGTCAGGCTTAATATCTCGATGCAG
AAATGACTTGGAATGAACAAATTCAACCCGATTGATCATTTGATCAGCAAGCATGAGAAC

AGTTTTGAGAGACAGCTTCCTACTGCAGAAGTTAAATAAATCTTCAAGGCTTGGCCCCAA
TAAATCCATTACAAGGACATTATAGTCTCCTTCGACACCATACCATTTCACATTTGGAAT
TCCAGTTCCTCCCTGAAGTATTTTATACAACTTGAATTCATACTGTAGCTGAGGATGCTT
TGTCTTGATATTTTCAAGCTTGATTGCAACTTCTTCATTAGTCTGAGTATTAGTACCCAG
ATAGATCTCCCCAAAAGAACCACTACCAATCTTCCTTCCTAGCCGGAAATTGTTCCCAAC
TCGTGGTTCCATAATTAGAATTCAAACCCTAGTTTTTATGCCACAAAAAAGTACCCTGAA
CCTTAAAAATTAAAAGTTACCCACATACAAAATTAAGACAAAAAGTACCCAGTCAATCAA
ACCGTCGAAAACAAGCCTCTACTGTGATCAATTCTGATCGCACAACTTTGAAATTCCTCC
GATTACTATCGGAGAAATCGGAGGTTTACAAAGTTTGGGGACATGGGTAGAATTAAAGTT
GAAACTTTCTCTCTCCAAACTCTTAGAGAGAGAACGATAGAAAGAGGGATGGGAAGCATA
GTTGTTGATTGAGTTTGATGGTGTTGGTAGGCATTTGATTTGTGGGCTTCGCTTTGAACC
AACCGAAAAGCGCTTTAAAAAGACAATGAGCTTTCAGCAAAACGACAAGTTATGGGATAT
TCTTCAATGTGGG
>tig_1388_consensus
TCTTTCCCTACAACGACGCTCTTCCGATCTCCACTTGATAAATAGTCTCATCTCGAACTC
GAACAGAACTATCGAACTGAAACTCCAACTCCAGTTGGACTCATTTGGGTCGAGCATAAA
TCTGGACCACTCCCATTGTTAAGGCTTATATTTAGACACATCTAACTCATCTCTCTCATA
TTTACTCCTCTTTCTTTGTAGATTCAGGATTTTCTTCATTCTTTGTCTTCCCAAACGATC
TCTGGGATTCCAAAACTGGCATGTACGCTTCACCTGATTTCTTATGCATATCGTCCCGGG
GGCTCAACATCTTCTTCTCAAGACTTTCAAATTTGGTTGTGGCCAAGGGTGCTGTGCCCA
CAATCTTTGCTGAACTTGGTCCATCAATTGTCAGTCTAGATGCCTCGTCCCTTGATGCAT
ACCAGTTATGACCGCACCCGATACACTCCAACTTATAGCGGTCTCCATGTCCTGCTTGGA
TAATGTCTCTCACACCCACTTTAAGCTCATTGCACCTTGAACAACGAGCATCTGTCATCT
GCATTCGCTCCAATTCATCAGGCTCCTTCAGTGCTATCTCCTCAGCAGTTAAACCATCCT
TTAACTCAGTAGGTGACATGTTGATCATTTTTAAAGGTTCCAACTCCCCATTTAGTAGAC
GCCGGGCTAGAACTTCTGTAGCCTTGAGATTGAACACTAGCTGCCGCATTTTTTGGTTAT
ACTTCTGAAAATCTGAGGAAAGAGCATCATGTGAAGCCTTCTCAAGAGCAGTCACTGCTG
GTACAGCAGAATCTGGCCAGATAAAGGGCTTACTGTTCTTCCGACTCTTTTCTTGGGAAC
CATTAGCAGTCCCCACACGCTTATTGTCTCCTTCGCTGTCAAACCCATCAACACCACCTT
CAACATTGTCATCCCTATTCACACTATCAGGACCATTGCACATGTACTGAATTCCTTGAA
GCAGCCTCTCCAACCATTTGTCACGAAATGTTTCTCCAGTTAATACCTTAAACTTTGTTA
AAATGGCATAATATTCAGAGGCATTGCTTGTGCAGCTTCCAGGTGTTTCAGCTTTAAGAT
GTTGATCTGACCTGGTTGTTACTTCCTCCTCCCTAGTAACATCAAGAGGTAATATGTTCT
TTTTCCGCAGGAATCTCTTAGTCTTAGACTGATCTTCCTGTTGGACCAGATCCTGAACAA
CAGTCTCTTCAGGCTCAACGTCAGGAAGTTCTCCCAAACGTGCTCGAGTTTTTTGAACAA
GAAGATCAATCTCATGTTGCTTATTATCCTCATAGTCTTTATCAGTTAGTTTCCAAAGTT
TCTTTTCCACAGTGTCATACACCTTCTGCACAATAAAACCGGGATGCTGTTTACGATTAG
GCAACTGCTTATTTATAGGGACAAAATGCACCACACACTTGTGCATCACAGATTCAGCTG
GAATTTCATCACGATGAAAACTGTAAAACAACTCTCGTGTATCTCGAGATTGCCAATTTC
CACCACCTTTTCTATCAGCCTCTTCTGGGCGATAAAACCATTGTCCAGTAACCGTCATGC
TCCCATTTTTGATATTCTGAGCAATATCCTTGAGGATTGCCACATAAGGCTTCTGCTTCT
TATCTTCAGGAACTAAAAGCACAGGATCCTCGAGTTCGTATCTATTTCCGTCAAACTCAA
ACGCTTCGTAATGCTTCCGCCGACCTCTTCCTTTACCGGTAACTCGCACGGGCTCACCAA
CCGGCTTGGCGTCCTCCTGCGGCTGCTCCTCCTCCTCATCTTCTTTCTCTTTAGCGTTTT
TGTTTCGATTTTTCTCAGCTCCTCTCTCCATGTCCTTCCCTTTCCTCTCCCTCCTCGACT
CTCTTATTACTCTATCATCTTCTTCTACTTCCTCGTCCTCCTCTTCTTCCTCTTGAAGCC
TTATTCTCTTCCTTTTGTTTTGCCTCGTGTCTTCTGATCTGCGTGTTCGCGTGGTTACGA
CTGGAACGTCGTCGTCTTCGTCGCTAGTCGAGACCTGAGCAAAACGACGGCTCCCCATTT
TTTTCTCGCCTCGTCTCAACCCACA
>tig_1389_consensus
AAATAATAATAATAATAACAAAAGAGGTAAGACAGTCATGTTAGGCTCATATTACCGACA
ACCAAGTATTAAGTTTTAGTTGATAAACTACGGCACCGTAGAAGACACCCTAGCTAGGTC
GACCCACAGATTACTATTATGAAAGGCAACACAAAGACAATCCCCAATGCTATATGCATA
ACATGCTTCTGAAATTTAAACTGTACAAAGGCAGCTATCTACCTAGCCTAACTAATACAC
TAGATTGAGAAGATTACTGAGCCAACACTCATATTCTTCATTNNTGTTAAGGCTTATATT
TAGACACATCTAACTCATCTCTCTCATATTCACTCCTCTTTCTTTGTTGATTCAGGATTT
TCTTCATTCTTTATCTTCCCAAATGATCTCTGCGCTTCTAAAACTGGCATGTATGCTTCA
CTTGACTTCTTAAGAACATCAACCCCTGACTTATCGTTCCCGCCCTGTGGACTCAACAAC

TTCTTCTCAAGACTTTCGAATTTGGCTGTGGCTAATGGTACTGTGCCCACAATTTTTGCT
GAACCTGGCCCATCGATTGTCAGTGTTGATGCCTCGTCTCTTGAGGCATACCAGTTATGA
CCGCACCCAATACACTCCAACTGATAGCGGTCTCCATGTCCTGCTTGAATAATGTCCCTA
ACACCCACTTTAAGCTCATTGCACCTAGAACACCGAGCATCTGTCATCTGCATTCGCTCC
AATTCATCAGGCTCCTTTGGTGCTATCTCTTCCGCAGTTAAACCATCCTTTAACTCATTA
GGTGACATGTTGATCAATTTTGAAGGTTCTAACTCCCCATTTAACAGACGCCGGGCTAGG
AGTTCTGTAGCCTTGAGATTGAACACTAGCTGCCGCATTTTTTGGTTATACTTTTGAAAA
TCTGAGGAAAGAGCCTCATGTGAAGCCTTCTCAAGAGCAGTCACTGCTGGTACAGCAGAA
TCTGGCCAGATAAAGGACTTGCTACTCTTCTCACCCTTTTCCTGAGAACCAGTGACTGCT
ATACGCTTATTGTCTTTTTCATGGTCAAGCCCATCAATACCACCTTCTACTTTGTCATCC
CCATTGGTGCTATCAGGAGCATAGCACATGTACTGGATTCCTTGGAGTAGCCTCTCCAAC
CATTTGTCACGATGTGTTTCTCCAGTTAATGCCTTAAAGTTTGTTAAAATGGCATAATAC
TCTGAGGTATTGCTTGTACAGCTTCCTGGTGTTTCAGCTTTATAATGTTGATCTGACCTG
GTTGTCACCTCTTCGTCCCTTGTAACATCTAGAGGTGATATGTTCTTTTTCCGGAGGAAT
CTCTTAGCCTTTGACTGATCTTCCTGTTGAACCTGATCCTGAACAACAGTCTCTTCAGGC
TCAACATCAGGAAGATCTCCCAAACGTGCACGAGTTTTTTGAACAAGAAGATCAATCTCA
TGTTGCTTATTATCCTCATAATCTTTATCAGTTAGTTTCCAAAGTTTCCTTTCCACCGTG
TCATATACCTTTTGCACAATAAAACCAGGATGCTGTTTACGATTAGGAAGCTGCTTATTT
ATGGGGACAAAATGCACCACACATTTGTGCATCACAGATTCAGCAGGAATTTCATCACGA
TGAAAACTGTAAAAGAGCTCTCGCGTATCACGAGATTGCCAATTTCCACCACCTTTTCTG
TCAGCCTCCTCTGGGCGATAAAACCATTGTCCAGTAACCGTCATGCTCCCATCCTTGATA
TTCTGAGCAATGTCCTTGAGGATTGCCACATAAGGTTTCTGCTTCTTATCTTCAGGAACT
AAAAGCACGGGATCCTCGAGTTCGTATCTATTTCCGTCAAACTCAAACGCTTCGTAATGG
TTCCTTCGACCCCTTCCTTTACCGGTAACTCTAACCGGCTCACCAACCGGCTTGGCATCC
TCCTGGGGCTGCTCCTCCTCCTCTTCCTCCTCCTCTTCTTCGTCTTTAATATTTTTCTTT
CGATTCTTCTCGGCTCCTCTCTCCATATCTTTCCCTTTCCTCTCCCTCTTGGATTATCTG
ACTCTATCATCTTCTTCTTCCTCCTCCTCTTCTTCCTCCTGAAGCTTTATCCTCTTCCTC
TTGTTTTGCCTCGGGTCTTCTGATCTGCGCGTTCGTGTGGTTACGACGGGCACGTCGTCG
TCTTCGTCGCTGGTGGAGACCTGAGCGAAACGACGGTTTCCCATTTTCAGTTGCCTATGT
TGTAC
>tig_1390_consensus
AAGTCTGTGACGACGTACAACCTAAAATAACAACTATAAACACAAAATTGCACAAAATTG
CAAACTGAACAAATATTTCAAGACTTCAGTGGAAAAGAAATCATTAAAAGAGCGGCTAGA
TTTTCATGTATATACGAAACAGACGCTGAAATTAATTACGCCAGAATTATTTGAAATCAT
TAAAATAGATAAATAAATACAAAAGCTGAAGCTTGGAACCTGAGTTATGTCACAATATT
>tig_1391_consensus
CCTTTATTCATCTGACGAAAACATAAACTGAACCAAGAACACAGGATGGAATAACAAATC
AAATATACAAAAAACATCAGTGTAGATATGGTGGACAAGCTACAGCCGCATCTGTTAACC
AAACCAGCTAATTAAACTTGGAAGAAGTCATGTACTCAGACAGACTATCATCACAAAATC
TGAGCTCCAGCGGTCCGCAAACAAGTAAGTCTGAAGCACCGGAGGCCCTAATCATCCCAT
TCCAAACTGCATGCCCTAATGTAAGTGTTGATTGTTGGTTCTAACCCCACTCCGAATCAT
CATCTTGTATATCCTCACTGCAAGAACTGGCATTTTTTGACTTGTCATCAAATTGATTAA
TGCATTGTAGGTGGACCTGTCAGGTCGAAAACCCTTTTCTTGCATGTCCTCTAACAATTT
ATAAACCAGGTCCATCCTCTTCTGATACCTAAACCCTGTGATCAAACAGGCGTAAACTGC
AGCAGCATCTAGCGGACACTGAGATTCAAGCAACTCATCAAAGTACTCTAGTACTTCTTT
CATTTTCCTTTGTTTGCAAAAATCCCAGATCATAATTTTATAGCTGCGGGCATTTGGTGA
AGGGCCCTTGGCTTTCATTATCTCATTTAACTTGATTGAATCAGATATCTTCTTACTCGT
CAGCAAACCTTCAGGCATAACACTATGGACAGCAATGTCAGTCTTAAAAACCTTATCAAT
CATATCATTCCAGACTCTCCCTGCCTCCATCAGGTTCTTCATTCTAAACCAACCATTGAG
CAACACCGAGTAAGTCGTCAAATTTAGTGTAAACGTATCTTTCAACATCTTAAAAAGTAA
CCGTGCTTCTTTACCAA
>tig_1392_consensus
CTCCCACCAAATACCACGTCCTTGTAATTATAATTTATTCCTTTCTTAGCTTGCCCTCAT
TATTAATTTTTTCACTCATATATAATAGCTTCTTCATTAACTGTCTCACCAAGTATAACC
AATACTCTTCTGAAACAACAACAACAATAATTAGGGTCACATCTGAGGTCTTGTCTGCAC
GACATAAACCTTCCCTTCCTTAATGACACCTTCAATGTCTTGTGGAGATCCATAGAATTC
CTCGATTTCATGTCCTGCACGGGCAAT
>tig_1393_consensus

GCGCTTTCTAGCCCTCCACAAGGTAAAGAGATCAACATAACATAAACATTTGAATGCGTA
ATCACTAGAAAAGCCCTAATTAGACTAGGACCAAATCCTACTACACACATCATTCTGAAA
TTAAGAATCATTTCAATTACCGTGTCTAAACCTCAACCCTACATCAGCTTCTCTGTCATA
TTGGATCAATGCAGAGCCCTGCATGTTTGAACTTCGAGTGTCGGAGGCCTCCTTTGGCTT
CTCTTGCAAAACTTCCCCGTAGTTATGAAGCCTACGGCCAATGAGATATCGATCATCGCG
GATGCTGTTATGAAGGTTAGTGAACCAGACATGGAATCTCTTTGTGCAAAACCACAATGC
ACTGAAACATAAGCATCCCAGCCAAGCAAAACGATACACTGCCGAATTAACTACTAATGG
ATACCCAAGAACAGGAAACACCCCTTTGGCTAAGACATATGGTACACACAGGGCTGTCAA
CAGCTTCATAATGATTGGGAAAACAATCTCTTTAAGCACCCATAGACTTTGCAGCCTGGA
GAAACCATCTTCTCTCACCCTCTCAAATTTTATCCGCCAGCTTTCATCTACAAGAGGCAT
CATGTGATCCAACATGACCAATCTAGTCCATATCTTCAGAAAAATGAGGCCCAATGCCCA
ATCCTGATACAGGAGGAAAACAGGGCTTTCATCCACAGGTACCCTCATTGGCACAATAAC
CAGGAGCTCAAACAGCAACCCAATTAATACCGGAATGACAAAAATCCATATTGACAATAA
TGCAGAACTCTTAAGGACAATCCCACACCACTTTGAAATTTGGGCAAACAAAACTGCTGC
CCTATTTGTCCTCAGATGATCAATGGAATATCTAGCTCCAGCTATGGCAGTCCAAATGAC
ATAGCTTCCAATGATGAAAGCATATAAATCATTGCACTTGATGCCATGAGTAATTGGAAG
AAGAGGAATTGCATTAAAAAGTGCACGTCCAAGCGAAATTGGCACCACTATCAAGGCAGA
GTTAACAACAAGCAGAGTCATCCAGGCAATCACCAGCAAGAGGACTATACGAAGAAGAAA
GCCGTACCTGTCTGAGTCAGTTTGATCATCAGCATCATACTCCTCTGATACATTAGTGTT
TCCAGTTCCTAGATTACCTCTATTTGCATCATCGACTGCTGGAAATTGAGCCAGAGCTGC
TCGTTCTTGCCCTAATGGTATCACCTGCAATCTATCAGGCCTTGGCTCAACATTTCCATT
TTCCTGGCCACCACTCTCGCCAGGCCTGGGCAGCAGAAACTTAGTCAGACCAAGTGCCCA
GCCAACTGCTGTAAACCAATAGAGAAGAAGAGACTTGATTGTTGTCCGCAATTTAAAATG
CTCAATAGCAAAAGGAATGCAGATTTGAAAGAGAAGCATATCAGCTGGTATTTCAGTAAA
TGGGTCAGATACCGATATGTCAAGAGGAAAAGTGGAGGGAGACATCCTCATAGCAAGTTT
GACAGGCAAGAAGACCAGCATCACAATCAAACTCCCATAAACTGCAACAGATAACAGCAC
TCTGCGAGCATGCTTGTGTACAGGATCATCAATCAGATCACGAAATGGATTGTAGTGGGG
ATCAGTTGGATCTCGAAGAAAATATAGAACTCCATTACGCAAAACCACTCGAAGAAGGCT
AACAAAGATACTTATTTGTAACATGTACACAATCCCAACCACCCAATGAACCAATGAGCT
TGCCAAAGGAGAGACTGAAAAGAACTGAACTCTTTGAGACATTGTCTTCCCAAACATCCT
TATGGTACAGACATCTAACCACCATCCACACATTAAAGGAAACACCCCAAGTTCAATGAC
TAGAAGGAAAGCAACCTTAATCATGGTCATCAAATGCCTCATTGCAGCCAAGAACTGCCT
GAACAGAGATGGAATTGTTTCTGCTATAGAAGCAATACCATAAAACCTTCCCATAGTCAA
AGGCTCGCCCTTGGTATACCGAATCAAGCCAACAATACCCAAGTAGAAGAAGACAAGAGA
GAATATGAACATGTATCCAATGGCAAGAGTAGTAACATCAGAAAGTCGCGATGTTCCAAT
AGTTGCACCTTTTAAGAGATCTGCAGAAAGTGATGAACTTGTACTGTTAGCTGCTTCATT
TAGTCCACTAGCATTTCCTTTTAACATTTCTGCAACAAGCATACTGTTTTCTTGACCTTC
AGATGTAAGATTTGTGACAGCACTTAACGCATTCTTCAACGTAATATTTGCTAAAGAGAG
TGTTGTATCAGTAAGTGGCATGACTGTTGACAACACTGGACCACTAGCTGAGGAGAAAAT
CCATGACATATAATATAGAATTATCCGACCTATAGAAAAGGGCACAAAGATCACAACGCC
AAGAAATATCATATTGCTAGCCAAAACAGTAAATGCATTTTCAACTAAATGGAAAACAGG
ACCCTGCATGCCAACAAGCTCATCGAAAGGTACATCCTCTGCACCATCAGCATCATCCAA
GCCATCAAACATCTGTTCAACATGGGCCTCAAGGCGAGCTGCCTGAATCTCCCATCGTGC
AGCAACATTTTCTGCATTCCTTCTGATAATTTGACCTGCTCCAGCAACAACCGGCACTCC
TCCAGCATCTTCTACATTTCCTTCACCAGCAAAATTC
>tig_1394_consensus
TTTTTTTTTTTTTTTAAGGCTAAATCTTCAATTTTTCAGAAACCGGGGTTTGGTCCAAAT
ATTGTCACCTCACTAATGCACAAAAACATGTAAATAACATAACCATTAAACATCTCCGCT
AGAAAAAATGTGCAACTTAAAACATATTTAAGTTTTCAAACAAAAAATGCTCACTAAAGA
ACAGTTAAAAGACATCTCGCAATCTTTTAAGCTCCAATTACTTCAGTAGAACCTTCAACC
ATTGTCTCATCAGCAGGCCTCTCCATTTTCACACCAACATCCTCCGAATAATCTCCATGA
ACCTCCATCAACTTTCCAAGGTCAAACTTGGGAGCTTTCAGAATCTTCACTTTGCGGATG
TAGACATTCTGCAATGGGTAGATGCTTGAAGTTGCCTTCTCAATCTCTCGTCCAATCATT
TCAGGAATGAATTTTTGGACAAGGTCCTTAAGATCGCAAGATGCTGCCTGTGCTACCATG
ATCTCCCTCATCTTGCGGCGAATCTGTCTAATTTGGCTGGACTGGGCATAGCAAGTCCTC
TTAACCTGGTTTGGACGTCTTTTGGTGAATCCAATACAGAACATCCTCAAGGTGTAGTTG
TCAGTTGTTTTGACATCCACATAGGCTTCAATCAAAGTCTGCCACTTCCTCACCAAGGAC
CTTAACTTGTCTGTGGTGAAGTCCATCCCCCAGAAATTGGTGAGGACATTCTTTCCTTGA

ACATCTTCAGCTCTCAATCTGATCTTTCTGAAAGCATGCTCCTCATCACCCTGAAGATCA
CCTAGTGATATCTCAAACACTCTATGTTTGAGTCCTTCTGAGGCAATCTTAGTACCCTGA
GTACGAGAAACCAGAGTCTTTCCAACGTTTCTGTTGTTGAAAACAGAAGGAGCCTTGATA
TCGTACCAGTCCTTCTTGGCAAATGGATCCGCAGCTTTCTTCTTTCCTCCTTTCTTTCCC
TTAGAAATTCTCTTGTTCTTTCCGACGGCCATAGTTAGGCGTGTGCAGCAGCAGAGGAGT
TTTGGGCACCAGCTGCTAAGAGACGGCTAAGTGTGTAGAAGCTCAAACCCTAATTCTGGT
TCGAGTCCTTATATAACTGTTAATTGGCTATGTTTCTGCATTGGAGGGTTGTACTTTGCG
AGACGTGGTGGTACCTTTTGGAGAATTCAGAAATTAGCAAAACTGGGCCGCTGAGGGCTT
GTGAATCTGCCAATGCAGA
>tig_1395_consensus
TGGACACCCAAGAGCAAGATTTAAATCCAATCAAAATAACGATAGGCATTAAAGGTATTA
ATGAGTTGGTCAATTCAGCACCAAAACAAGTTTAATGAGTCCAACCTCAATCATAAACAG
ATTAACATTGTTTCCCAAACTAAAATCTAGGGGTGCACGCATTGCAGTGAGCTGAAACAC
AACTGAAGCCAGCCAAGATTGTTTATAATATGATTTTCTTCTCGTGCTTATAGGCGACTA
ATGACGCCCTCCACCTCAGCAGGTGTATAGAGATGATATGTTGGAGCTACCTTCGCAATA
TCAACATTGTTCGGAGTCACCTTTTCTTCCATAACTTGCTTCAGAATGGATAGAGCAATT
GTCTCAGCTTCTTGAAGAGTAAGGTCCTTGTTAAATTGTTCTTGAAGAGAACTGTCAGCT
CCTTCTGAACCTGAACCAATAGCTTTGGCATTGCATTGCCAGAATGTGCCAGATGGATCT
GTATAGTACAAGCTGGGCCCATTCTCATCATGACCAGCAATAAGAAGAGATACTCCAAAT
GGCCGAGACATGGATTCTTCATCACCTTCACCAAAACGTAGAGCAAGATCACAAAGTGCT
TGAGTTGTAGATTCCACAGTCATTGGCTCACCATATGAGAATCTATGGTTCTGAGTTTCT
ACTCGTGCATGCTCAACAAGCGTACGGGCATCAGCAATTAATCCACTCATAGCACATCCT
ATATGGTTATCTATTTCCATAATTTTTTCAACACTGCTAGGCTCCAGGAGTGGTGAAGTA
ATCCGTTTCTCAACAGCTAGCACAACTCCTTCTTTTGTCTTCAACCCAATTGCAGTTGAA
CCTAGCTTAATGGCCTCAATAGCATACTCAACTTGAAACAACCTGCCTTCTGGAGAGAAA
GTGTTCACTCCTCTATCATACTCAGTCCCTTTGATTGGATCTGGAAACCTTGTACTTGAG
TTGAGTTGAGTTGAGTGAGTGGAATTGGATCGGGAATTATGGTGTTTTGGGAGGGGTATG
TGAGTGATGAAGTAATGGGGACGTTTGCTCCGATAGTGGTGTATTGGTTGTACGCCGGGT
TTTATCAAATGTCGTCGCCGGCGCTGGATAAGTACAGGTTGCATACGAAGCAAGAGGAGG
ATGAAAAGAACTCTGTTCCTCTTGCTGTTGTGGTCAAGGGCGTTTTGCTTCAACAACTCG
TTCAAGCTACTGTTGCTCAGGGCTTATTCTTG
>tig_1396_consensus
TTTTACCCAAGCCTTGTTAAAACCAATTTTTGAGACATTGATATGCAAGCTAGAAATGAT
AGACATCTATTTACCAACTTGAAAAGGAGTGTTAAAAAGTTGTTTGACAATTTGATCTCT
ATATCTACAAATATGTAAAGTATATTAATGTATAATATTATGTTTCCTTGATAGATTAGT
ATTCCTATTCAAGGAGCATAGAATGTTGTATATTTATCCCCTAATGTATAAT
>tig_1397_consensus
ATAATCAGTATATATCTCCAGTCAATTTCATTGGTGGCCTCTTCGAAGCATGGACAGGAG
TCCTCCGGATGATCTCTCTTTCCGACGCTCTCGTTCGCCTTCTTCTCCAACCTCCTTAGC
AATTTGCTGTAGAACCTCAATGATTTCGGAAAAGTCAGGTCTTGATGCTGGATCTTGCTG
CCAGCATTTTTCAAGCAGCTCAGTTAGCTTTGGATGAGTGTTCTTGGGGATTGTAGGCCG
TAGACCCTTTTGGACCACCCCAACAGCTGCCTGTAATGGCGTTAAGTATTCGTATGGAAG
CTTTCCGGTGAGCAATTCCCACAATACAACTCCGAAACTAAATACGTCAGCCTTGTGATC
ATATGGTTTGTGTTCAATAACCTCAGGGGCCATCCATCGATACGTCCCAGTTTCAGCAGT
CATAACTCCAGATTGAGCTTTCACTCTAGCAACACCAAAATCAGCTACTTTCACCACTTC
ATTTTCATCCATCAAAAGATTGGCAGCCTTCAAATCCCTGTGTATGATATTGTTTTGGTG
CAAGTAGTTCATTCCTTTAGAAACATCTATCGCTACTTTAATTAAGGATGGGAGTTTGAA
CACACCCTTCTGCTTGTGCAGGTAATCATACACACTTCCACCAGGCATAAATTCT
>tig_1398_consensus
ACATAGTGCATGAGTAGTATAACTTACACTTAAACAACCTCTGTACAATTATTTACACTA
CTCTTATACAAACATTGTCTGCATTCGGAAACTAATAACAGCAGGTTACTACAGTACATG
TTTGTTAATGTTTATCATAATACTCACTTCAACATGAGCCTGAGTCGACCAAGGTTAGGT
ATAGTTGACTTCAAGCTCAGCAACACTCAACCATAGTGTGACATATATCAAAACTGGAAA
GAAAAGTCTAATTTCTATCAGAAGATGAAGTTTCAAAATAGGTAGGATTCGGGGTTGAGG
GGCAACCGATAGATTGGATCAATTCTTGCACAGCAAGTACATGGAACTTTGGTGGTGAAT
TGAGAGCAATGTTGTCAACTCGGTGTTCAAAGATCTTCCCATTCTTGTCAAGTTTGTACT
CGGAAGTACCATCAAATCGACCACGACTCTCCCAGGGAACTCGTGGGACACCATGGACAG
TCCAACGAACCATTATGATGTTCTCCATAGGCTGCCAAACACTGATAATGTCAAGCCATA

ATACCCTGAAAAATATTCTGCCATGGAATCGCAAAGCCCAAAAGATTGATTTATAGTTCT
CAATGCCGATAAAAGTATTCATTGGATCTTTAAATACAATATCATCCCTGTAAATATCGA
AACTGAGCTCTTTATAGAACAGCGCTGGAAACTCTTCTCTTAGCGTCCGAATCGCATAAC
CCATGTTCACATAGTAATTCTGCTTCTCTTCTTCTTCTTTGCTCGGCTTTACGGGGGCTG
AATATTTTCCGTACAATTTAACATTTTTATCCAAACCCGAAACCCTAACCCTAGCCTGAG
ATCCAATTTTCTCATTATTGCCCAAACTAGCCAGGTTCTTGTAATTTCTAGAATTAGGGT
TAGGATTAAAGAAGATCTTGGGGGAGATTTCTGGGGTAGAGACGAGAAAGGCCATTGAAA
AGGTTATTGGGCTGAAATTTAAGGGGCACGAAGGTGAAATTAGAGAGAACTTAAACCAGT
AAACTCCATTTTGGGAAAATGAAATCCGAAGGAGATAGGAAAAATC
>tig_1399_consensus
GTTTATATATAAAAATGCATACATATATCTTACAAAATACATGGTACCAAAGCAGTTTAA
CTTGCACTTAAGCTCCCACTCTACAATATATCGACAACTCTTACACAAATACAATCTGCA
TTTGAAAGCCAATAACAGCAGGTTATTACAGTGTGTGTTTATCATATAGAAATCCATACT
AAGTTACCAGACGCCAGCAATGGTGACAAGTTCAAGCTCAGGAACACTCGACCATAGTAT
GACCATGACATGCCAAAACTGGAAAGAGAACTCTAATTTCTATCAGAAGATGAAATTTCG
AAATAGGTAGGTTTAGGGGTTGAGGGGCAACCAATTGATTGGATTAATTCTACAACAGCA
AGTACACGCAACTTTGGTGGTGAATTGAGAGCAATGTTGTCAACACGGTGCTCAAATATC
TTCCCATTCTTGTCAAGTTTGTACTCAGAAGTGCCATCAAATCGTCCACGACTTTCCCAA
GGAACCCGTGGAATGCTATGGACAGTCCAACGAACCATTATGATATTCTCCATAGGCTGC
CAAACACTAACTATGTCAAGCCACAAAGCCTTGAAAAATATTCCGCCATGGAATCGCAAA
GCCCAGAAAATTGATTTGTAGTTCTCAATGCCGATAAAAGTATTCATTGGATCTTTAAAT
ACAATATCATCTCTGTAAATATCGAAACTGAGCTCTCTATAGAAGAGCGCTGGAAACTCT
TCTCTTATAGTCCGAATCGCATAACCCATGTTCACACAGTAATTTTGCTTCTTCTCTTCT
TCATTTCTCGCCTTCACGGGAGCTGAATATTGTCCGTACAATCTAACATTTTTATCCAAT
CCCGAAACCCTAACCCTAGTCCGGGAACCAATTTTCTCATGATTGCTCAAACTAGCAAGA
TTCTTGTAATTTCTAGAATTAGGGTTAGGATTAAAAAAGATCTTGGGGGAGATTTCTGGG
GAAGACACGAGAAAGGCCATTGAAAAAGTATTTGGGTCGAAACTTAGTGTAACCGATTAG
AGGGAAGTTAAACAAGGAAACGCCATTTTTGGGAGATTTTATTATTTTAAAATTCATTCC
TTTTTAGCAACTGAGAAAATTGGTTCCC
>tig_1400_consensus
CTGAATCTAATCGTCTGAAATAAAAGTTACAGTTCATATTATCCCAGCAAGAAAAAAGGT
CATGATACATAACGTGGGGAGGCGTCCGTATCCCTCTCTTGAATTTGAATGAAGAGAAAC
TCCTATACTAAGTCTAAGAAAATTTGTTTGGATTCAGAGTCATTGAAAGCCATACGACTT
TAATACGAATGAAGACACATAACCATGGCTTTGTATTCAAAAGAAAAGACCCCCCGAAAA
TTAACAGACTGCAGCTTTGAACAGGCAACAAAAGGCTTAAGCTTAACTAAGCCTATTTAC
AACTCTCCCACACGTAATACCAAAACTAAAACGACAACACTTTGAGGACACAAGGCCGCA
CCACAACTCTCGGCCTCCATCGCTGACTCCACATTGCCTTCTTGTACAGCCTCTCAATGC
ATGCTACATACACACCGCTCGCTCAACGCCTGCGGCTCCCTGGCTCCTTCTGAGAGTTGA
AGTAAATGGTGGCCACAGGGAGGCCAAGATCCAAATGCCCTGCAAAAAGTCGGGTGTTGA
AGTTTGCACGAGACGCTGGCTGTTCCACTACCCCTAATGGACCCTTCTGCTGAAACAGCA
CGATGATGAAGCGGTGAATGCCCACTGGTGGCCGTGGACCCACGTATGGCAGAATCTCTT
TCCCTTTGGTTGGGTTAGTGCCACCGGGAATGTCAACAACGATCCAGTGAACCCATTCTC
GCATGCTGGGCTCGCTGGGGCTTGGCGCGTCAGGATCAATCATCACCAGAGCATAAAGCT
CATCAGAACGGCCAGTGATAGTGATCTTAGGGGGATTGATAGCCATGGAAGGCTTGATGT
CACACCCATTCGTCACATGCTTAGCACCATAGTAAACAGACATGTTCACACTGGGAACAA
ACATGTCAACCACATCACCAATAACCCTTCCAACCACAAGAGGGTCAACTGAAGCAGCCA
TTGCCAACCCACAAAGAAGAAGAAGAAGAAACAGACTAAGAAATTGAAATTAGAAAAGCT
GGTGCGGAACTCA
>tig_1401_consensus
GAAAGAGACAAACCAAAATCAATCTATTGCCGAAACAGTACTAGAATCTAGAAAATTTCA
TTTCAACTAATATATTACATATTTCACTTGTCAAACACAAAGAAAACACCGATTAACAAG
AATGAGATAATTTTACATGCTATGTGATATTTGAACTCCAGTGTTTTTCCTGCAAATCGC
GATGCAAACTCAGTTCTTCAATGAATGCATTGTAATCAGCGTAAGATGAACCCCCTTTCT
CAACAGCCCTCTTTGCCATCTGCCCGAGTGCCTTGGCTTTGCTTCTGATTTCTTCGGCGT
TATCACCCACCATCACCTCTTTCACCGCTTTCTCAAGCGCCTCCCTCTTGACAAAATCAC
CACCAACTCTAGACCATTGCTGAACACCAACAGCTGTACCAATCCTGAGAACCTCAGTCA
CCAGCTTTTCGTTGTAAAACTGCTCCGCAAACGCTGGCCATGTCACCATAGGCACCCCAG
CAGTCACTCCTTCAAGAGTTGAATTCCATCCACAATGTGTCACAAATCCTCCCACTGCTT

CATGATCAAGAATCAACATTTGGGGTGCCCATCCTCTTATTATAAGTCCTCTGCCTTCCA
TTCTCTTCTCAAATCCTTCAGGCAACCAATCTTCCTTTTCTTCTTCTCCGTTCTTGTTTC
TCCTCACAACCCATATGAATTGCTGCCGCGAAGCTTCGAGCCCCAGTGCAATCTCCATTA
GCTGAGCAGAACTCAAGTTGGCTAAACTTCCAAAACATACGTAAATAACTGAATTGGGTT
TCTTTGAATTTAGCCACTTTAAGCACTCGTGTTCATCAATGGAAGCCTGCTTTCCCCTTG
ATGCTTTGTCTTCAACATTCCGGTTGCATAGTGAAACAGGGCCTACATGCCATGCCCTTC
TTCCTAAAACCTTCCTGTAATGATCAGCATAAGCAGGCTCAAGCTCGTAAAAGCTGTTGA
CAATAGTACCGAAGCTCTTTACCTCTGCTTGTTGTACATCTTTCCAGAGCAGAGTAAATT
CAGATTCTTCATGTTGCAGTAAAAAATCTGGCATCTGTTTCCTTGTCAACTTAATATCGC
CAGGAAGGTTTGGCACAACAAATGGTTCTGAATCGGATGAAACTTTCTTGTGTGGCTGAT
ATTGCACTAGGCATGCTGATGTACACAAAGAGAAGAAACCTGTACCATGAAACACCAGCC
TGGGAATCCCAAATTTCGCTGCAGCAGCAGTGGCCCAAGGGAAGAACATATCGGCCACCA
AGCAGTCTGCTTTACATTCTCCAAGTAGTTCCTCAAGAGGCTCCCGGAGCAAGGTTGTGG
CCTTGAAAAACTTCGTGGCCATTTCCACACTAGCACTGACTCTGTCAAGATTTTCACATC
CTTCTGGCAAACCAGCCTCCTCAGAAGGAAGCTTGATGATCTTGACATCAATTTCAATAC
CCAAATGCTTGCTCTTCTGAATGGAATTTGCGATGAGAGGCGCGTTGGCTGGAGTAGTGA
TTATGGTTGTTTTCACGCCACGACTTCCAAAAAGCTTCGCCGTGTCCACAAACGGTATAA
TGTGGCCATGAGCCAAAAATGGAAAAAAGAACATGCGAAGCTGGGGAATTTCAACGCCCA
TATTCACACAAGCAAAAAGATGAATAAACGAAGACGATAATGCTAACAAAAAAAGCTTAT
GAACTTCTGGTTTTG
>tig_1402_consensus
CTAAAATATTCCTTCGAACTTAACATGATCTATTAAAATAATATTAGCTATGGAAATTCT
CTTGCTACTGTAAGAAACAGTATGAAGGGAGTTTACATCGATGGTCACAAATAATTTAAA
AAAGAAGAAAACCCTCCAAAACCCAAAGTACCATTTCTAGTTCTACGACGAAATTAATTT
TACTAGAAAAGTTTCCTATGCCCCGGAGGAGGGTTGCCACCTAGAGGATACGTAGGCGCA
GTGGTTTTAGGCACTAGAGCAGCGATTCCAAATTCATGGTGCTGGTGGTGTCCTTGGGTC
CTCCCTACCATCGGCTCATTCATCTGGCCATGAAGAGAAGCTGCAACATAAGGGCCGTCT
TGATAATGCTGTAATTTCTCAGCAGCATGCTCGGCGGCCTTGTCCTGGTGTAACTCCATC
TTCGCTTGAGCCTCCTTTGCCTTCCTTCGCTGGTGCGCTCTCCTTCTCTTCCTCTGTCCT
CGCCGCCGCTTGCTCCACCTTCTCTTCAACTTTGGCTAGCAGAAAAACGGTAGGAGAGCA
CCCATCTGCCACGTGTTTGGTAGTGTTAAGCTCCACCTGTCCTGCGTAGCCCGCTTCCGT
TCAGATTTGATTAGGATAATTTCCTTGTAGAAAATTGTATTACTATAATAAAAATAATTT
TTAATAAAATCGACGGTGCGTG
>tig_1403_consensus
TGCAGGAAGCATATGATATATTATAACCACGGCCATTTTCATCACAAAAAAGTAGCAACC
AAGTGCAACAAGAAGGCGGAATGAAGTAAATTACAAGGTTGAGTATCTAAACTTTTTGCC
GCACCGACGAATCTGGTCTTGCTCTTGCAAGCTCAACATAAACAAGAATTTCGTTCAAGA
CAGGCTAACGATTTTCAGAGACCAACCTCAAGCAATAATGACAAATACAGTAGCACAGAT
GTTTTCATCCCAAAAGCAGAAATGTTGCCATGAGCAAAAATGTGGCAAGTCACCTACTGC
GTCCAATGAGTCCACATTCTATTTTTTGCATTAAGACATTGGATGTCGCCTCCTCTTGGG
TCGGCTACGGTCTTCTTCATAATCCATTACTCCAC
>tig_1404_consensus
CCACAGACAACAGAAAAAGCGTGATATTATTCTAATTTAATAGAATGCCCATGAAATACA
AGCGCGGCGGCAGCTGGCAAGAGAAAGTAAGTACCAATACTGCAGAATGCTTATAATACA
CCACCCTTCAAGCTGTGTTAAAATTTGTTAAGGTACAAGAATCTAAATCAATTAGCCTTA
CAAACCTAGTCACCAAGATCAAGCATAAACACCAACCCGTATTAGGACAGTAACTCAAAA
GGAAAAACACAAATACGACAGCCCAGAAAAATCCATCACACAATTATATGGCATCACTCC
CATCATTTTCCTTCAGCTGACTCAAATCATAAACACCTACTATATCAGATCAGCAACATT
CATCGGCATCTCATCAATCTGTGTACTGTAATACTGCTCGATATCTCTAAGAATCTTGAT
ATCATCATTTTTCACAAAATTTATAGCTACACCCTTGCGTCCGAAACGACCAGAGCGTCC
AATACGGTGGATATAGAGCTCACGATTGTTTGGAAGGTCATAATTAATCACAAGAGATAC
CTGCTGAACATCAAGTCCTCGAGCCCAAACATCTGTAGTAATAAGAACACGGGTTGTACC
AGAACGAAATTCTGCCATAATTGCGTCTCTTTCTTTCTGAGGCATGTCTCCATGCATTGA
TGATACAGTAAAGTTGTAGCCACGCATCTTCTCCGTTAGCCAATCCACCTTTCGCTTTGT
GTTGCAGAAAATAACAGCTTGTGTAATTGTCAGGGTATCATAGAGATCACAGAGTGTGTC
AAACTTCCACTCCTCTCTTTCCACAGCCACAAAAAATTGCTTAATTCCCTCCAAAGTCAA
CTCATCACGCTTCACAAGAATCTTTACAGGATCTGTCATAAACTTGGTAGTCATCTCCAA
GATTTCATGAGGAAGGGTAGCAGATATCAAGACAACCTGAAGGTCCGGTGGAAGATATCG

ATACACATCATAAATTTGATCCTTGAAACCTCTGCTCAACATCTCATCAGATTCATCAAG
GACTAATAATTTAATAGCTCTGGTACGCAATGTTCTCCTCTTGATCATATCACAGACTCT
GCCCGGAGTTCCAGATACAATATGAACTCCATTCTCTAACTTTCTAATATCTTCACCAAC
ACTTTTGCCCCCAATGCAAGCATGGGCTTGTATATTTATGAAGTCACCAATTGCCAGAAT
CACTTTCTCCGTCTGAGACGCCAATTCCCTCGTAGGTGACAAAATCAATGCCTGGACCTC
TCTGGAAGCCGTGTCGACTATCTGGCAAACAGTGAGAGCAATCATAGAGGTCTTGCCGGT
ACCAGACTGGGCCTGTGCAATCACATCTCTGCCTTTGATTATAGGCATGACTGCCCTCTG
TTGAATGGCCGACGGCTTCTCGAATCCGTACTGATAAATTCCACGCAACAGATCGTCCTT
TATTCCCATCCCATCGAAACTCGTGATTGCCTCCACCCCTTCCGTCGTTTCAAACACCAT
CTTGTCTTCCTCCATTCCGCCAGCTCCCCTCCTCAACCCGCCACCGCGGCTCGTCTGCAT
CGCCGTTGATGCTGCCATCACCGCAATCTCGCAGAAAACTAACTGTGACTGTCTTCTTGA
AGATTAAGACGCACGACGGAGGAATTGGTTCGAGTCAAATTAGGGTTTTTTATGACTGAG
AGAAAGATAAAGCTAC
>tig_1405_consensus
CCATCATAAACTACCACATTGCACCCTTCAAGTTTACACATAAATCTGATAGAGAAACCC
AAATTTGCAGACTAGACATCAAAAAGTAATGTGTAAAATTTGTCAAACAAATAGGTCCAT
AACTGCATCATACCAACATGTCCTAAAAGGATCCTAACCTACACCTTAATGGCATACTTT
CTCATAGGAAAGTACATCTCCCGCTTCTTCTCCCTCTCTGTCTTCAATGATGCCTGGTGC
TTTGTAAGCCTTCTCCTGATAGCTCTGGTCTTCTTGGGACGAAGGTCAAGAGGCAAAAGC
TTCTTCTTCTTGTAAGCCTCCCTCAATGCAGCCTTCTGCTTCTGTGCAATCACTGTCAAA
ACCTGTGCAATTGACAACCTCACCACCTTGATCTTGGAGAGCTTGTTAGGAGCACCGCCG
GTGACCTTAGCAACACGAAGGAGGGAAAGCTCGGCCTTGAGCTCTTTCAACTGGTTCAAG
AGGTCTGCCTTTGATTTTTGCCTAAGCTCATGCACCTTGATCCG
>tig_1406_consensus
TTCTAACCAAAAAAAAAAAAAAAAAAAATTACAACAACCCACAATACAAGCACACTATTTA
CCACAGCAAGAGACTTAATCCCACCGTAAAGCCCATCATAAACCACACCACATGATAAAC
AAGCTAAATAAAGCAACAAANNNNNNNNNNNNNNNNNNNCGCCGTAATCCATGGATGCCTGA
ACTTGATCCACCATGGCATTCTCAGGCAGCTTAAACCGGCTCATGAACTTGCCGTCGTCA
CTGCTTATTTGAAGCACGTTGTCCTCGTCTATGTAAACTATGACTTCATCACTGGTGAGT
CCGGGAAGAAAAGCCTTGAATATATGGGCTTTTGAAGTTTCCTTCCAGTTTATTTGGGAT
CCAGAGAAGGGAAAGATTTCCCGAGTAAATGAAGGAAAATTGGTGAACGGGTTCCAAAGG
TCAAGGGAGAAGGGGAACCCTTCTGGGAAATCCTCTAATGGATCCCATGTGTCAAAGGAA
AGGGGGGTGGAGATTGTGGCATTGTGATCACTTATTGGAACAATTGACATGTCGTCTGCT
CTGGGCAAGGGAAAGCGTTTGGCTTGCAAGAAAGTAGTAAGCTCAATGAGATCAAAGGAT
GGATGATGGCACCTTCCAGAAG
>tig_1407_consensus
ATGGGCCAAATTTGCAAACACATATCCTGCTTAATTTCTTTGCTAAACTTGGACCTATAC
AATTGACAATTAAAGCCAATTGCATCTGTGATGATTCTGATGAATGACAAAGAGAGATCT
TCAACATTGTTATATTAAAAAAGTGAATCCCTCTTGCGAAACATAACGTTCATGCCATTA
AGGCCTTCTATACATGTATTGACAAGGTAGCTCAGTAGTCTGAATCTGTCTCGCGAAACG
GTAAAGGAACTGCTTTCACAGCCCTGAACTTCCCAACATTGTTCAGATGTTCATTCTCCA
ACCTGTAGAAATTCCAATGCCCTCTTCGAATTACTTCTAATGAAGCCATGAAAAATTCTA
GCATGCGCCCCTCAATATTGCTGATACGAAAACGCATTACAGTCTCCACCCAAACAATTC
TTAGAACCACATTCAAGCCAATGGACATGTAGTAGATGCCTTTGTTCTTGAGAATCAAGT
CATCTCTAAGCCAAGGGTTCTTGGATTTTGGGTCCAGGAGTCCCCAATCCTTGACAAAAT
CCCAGTATAATTGATAAATTGTTGCCCCCACTGAAGTCACCAAGACTATGCCAAACCATA
GGTGGTTGCTCTGCCTAGTGTAGGTTATCCT
>tig_1408_consensus
TTTTTTTTTTTCAAACAATCTCACATTTTATATATGGCAACAAATATAATGAAAGATTAA
AATCCAAACATCAAACATCGTTTCACCAGTAGCACAGGTAGTGTTATTTCACAATCTTAT
TTAAGATGATAAAGAAGACGACACTGTCTACTCAACCTGCAACCTCAAGTTACTAGATTG
AGGAAAATACTAATAAAGCCTAAACTAACTTAACATCAGGGTAATTACCAAAAAAAATAA
AAAAAACTTAACATGAGGGTTTTTCGTTTTTTTTCCTCCAATTATCCAGACACGACAACC
ACACAGACACTTACACCATTATATATTCTGTTTTACTTCAATTGAAACCCACCAAACCAG
CCAACTTCAGTCACCCACCCAAACTTACCTGAGAGCCAAGCCTTCCAAATCAGTAGATGA
CCACTGATGCTATAATCCGCATACAACTTATCGAATGCATTATTTTATTATCAGTTGCTG
CCAACCAATGCTGATAGCGGCAACTTCAACATCTTCACCATTAAAAAGTTAAGCACTGTG
CGTACACCCATCCTTTTATTGACAAGCTTCTAAACCCATGACAATCATTTAACACTTCTA

TTTTCCACCCCCTAACCCGGTAAGGCAATTACTTCTTACAAACTTCTCACCATAAGATGT
TTCAATGTTTCTCTTCCCCAGCCAGAAGAGCTCAATGAAATTTCTGTAGTAGCAAGTGCC
TGCAAAAGGCCAAGGCCACCAATGCTCCAACAACATAAAATTCAACACTGACAAGGCTTC
AAAGTTGGGGTTTGAACATGGTAGTAAACGAATGGGTACATCCACCAGAATCATTCTCAG
AAACAGCTATGCGTTCAACCGATATCACACGAAATAACGACTGGCCCAGATAGACTGTCC
TTGATATTGCCCAGGTAAAGGAGCAAACTGCTGGTTTCCATTAACAAATTGTTGTTGCTG
CAGCTGCTGTTCATGCTGTAATTTTAGATGATCCCGAGTTTGGAAGTCCTGCCAGGCTTT
CTGTTTTTCAAACTGGAGCTGCATAGTTGGAGCTAGTTTTCCAGGAAATGGAAAGCTGAC
TGTCCCAGTCACACCATTGCTATCACCAATATACTGAGGGTTTGCATGCGAAGAATTATT
GGTGGAACTGCCAAGGCCTCTCATTGAAGGTGGCAACTGATATCCATCCAACCAGCTGTA
ATCATCCATCAGAGGATTTTCGCTAGTCAAGTCTGAACCTGAAATCGGTGCAGTCACTTG
TTTGGAAGGAACAGGGCTGAAGCCAGGTGGAGGTCCCAATTGCCGAACAGGGCGGCTAAC
TGGATTTTTCCTCTGGCCTGCTGGCAAAGCTGATGAAGCTTTTACAGCCAAACTGTCAAA
AGTAACTCCAACACCAGTATCAATCTTGGGAGGTATCATAGCTTCTGGCCCTTTCAAATG
ACTGTAAACCAAACTACCACTACTAGCATTAACAGACCGTTGGATTGGAACCAGCCCAAC
AGCTGGAACTGAAACACCGATATTTTCTTGCAAATCATGTTTCACAACATGCCCATTTTC
TGAGACCCTTAAACCTTTAAAGCTATTAGCAAGAGAAGCTTCTTCTTCCATTAACCGGTT
TGAAGCATGCAGTTGAAATGGCTGGAGCTGCTGAGGTAGAATACTGTCAACAGATACAGG
TACTTGGGAACTATTATCAAAAGTAGCTGGCTGGCGAAGGTCATCTATAGGAGCTGAAAC
AGGACCGCCATGCAATTGCAGATCACCAGCAGATGCCCTATGGCCAGGCGTCAAGCCATC
AAAGGACATCCAGCTTGAGCTAATCACATCAGCTCTCTTCTCTGTCACTACTGGCTTAAA
AACAATCACTTCATCCTCCTCTTCCCCATCCATGTAACGCTGTGGATTCGGTTGAAAACT
CAAATTCATCTTTTTCTCAGTTTGATTTTCCTGCACTATATCATTTGATTTGGAAACATC
TGGGTAGGAGCTGAATGTTACATCATCCGAAGGCTCAACACCAATAAGAAATTTCTTCAA
CCTTGAATCAAAACATACTGTTTTCTGGTCAACAGTAACTACATTTGCCAATGCCTTCCC
AGCAGCTAAAATCCTTTTAAGACGGGCTTTCCTTTCCTTGTTGACATCACCTCCAAAGGA
ATGTTTCCTTGAAAAATCCAAGATAGTCTGTGCAGGAAGAAGCGGCAAGAATCCTCTCAA
CTCAAAATCCTCCCACAAAGCCAGTCGATTTTCAGTTTCCCCTTCAACGTACCTGCTCAT
GTTGAAAAAGCAAGTCATATCTTCATCATCATCCATGGACATTGTCCCAAGTGACAGGAT
CTTATTGAGGAAGGATATGCACTGATTCCAAAAATTTGTTCGGACTGTTGCCTGCTTCTC
ATTACCATCACTGCCAGCAGCTATTTCTGGACAGCAGGCCAACCACTCAACAAAAACCAG
AAGACCAGGTAACAGATAACTTGAAGATGGATCTCCCAGCTGAATAAATCTCTCCATGAC
ATGCCTCATTAACTTAAACACAGCAGTGAAAGCATTCTGAAGAAGCACGGCCCGCTGTAC
AATTTCCGCATAAGACTGATTTTCAGTTTCCTTTTTCAAATTATGAACTGTGAATATAAG
GATGGAGATAAGCCTAACAATAAAAAGAGCATTCTCGGCAGCATCAGATCCAAAACTCAG
CTCCTCCCCTGGTCCAGATGAAAGAAGCTCACATAATCCACTGCTAACAAAAGCAAGAAC
TTCTGCAAATGTCTCCAGACTTGTACGTGTGAAAAGAATGCCATTTAAGCGCACAAAGCG
GGTGCAAAATGATTTGAGCTTTTCCTGTACACTAGGCACAATTTCCTTCACAGAAGTGGC
CTCTTTGTCAGTATCTTTAGAAGCAAGTTTAGCTTCACCTATCCCTCTTCCTTTGCGTTG
ATGCTCCTTGGCAGTAGACAATTTAACATCCTCAGACATCTGAGAGTAAATCTGACGGTT
CTTCTCAAATAACACAATCAAGTTATCCCTGGCGGTTGTGCAGGGGGTATCAACAGCCAA
ACTCCGAAAATACCGATAAACAACAGCCAACTCATCACCAGAATAAGAAGCCAATATAGC
AAGCTGATGATGGGGGTTGCCACTTGAGGGAACAAGAGATGCAGATTGCAAATAGTAACT
TGACGCTGCTGCATATTCACGAGATTTGGAATCACCCTCCCCATATAATCCTTTGTACCT
TGCAAGATCACCCAAGTATATCAGACAACGATGGCAAGATACCAGGCCTTTCTTCACCTC
AGCAAGTTTCTTCCCATCTTTGTCCATGGTTATTTGATTCTCAGAATCCTCAGAAATACA
CCCTAAAGGAAGACCATACTTTGCTCTGATCTTCAATATAAGTTCATGATAAAATCCAGT
TGCTTCAGAAAGAAAGGTTTTGAACTGCTGTCTAATCTTTGTAACCCGATCAGGTCGTGC
AGGTACTTTCACACCCTGAGATGTGTTTGAGCTAGCTGCAGATATAGCAGCACTATAATG
TGCTCTCAGCTCCTCAATCCTCTTGTAATGTAACTGCCATAATGCAAATTCAATATTATG
CTGCTCAGAAAATGCATAGTCCTCAAGAATTATTGTTTCATAATTCTCGCGCATCAGTTG
CCATGCATTGGGATCAGATGGAACACGTGCCTGGGCCGACTTACGCCGCTTATTTTCCAA
CTCAATGTTCTTTTCATAGAGACGCTGAACACGCTCCCTTGATGAAGGAGCAGACATTTT
ATCCATCTGCACAATCATCATCCATAAAGTATATTGGACCAACCTCGAATTACATAATCG
AGTAGAATTAGATCTAGAGAGAGATGAGAATCAAAGCATCATCGGAATGAGACGAAGGGA
GATCATTAGGGTTTGGTGTGGAGAGAGAGAAGAAGAGAGGAGAAGAAGAGGTTTGTTTTG
GTATTTGGTAAAATTGGGTTTGGCCAGTTGGAGATGAGGTTTGGAGTTTCAATGTGTTTT
CCTCCCCTCTTCCGCGGATTTCC

>tig_1409_consensus
CCGGCTACAATTTAATATAAATTTGCTGAGCAATCGAGTGCAAATTTTTAAAGGTCGGCA
TCAGACACTGGTACAACACCCAAAATTAGATTGAAATTCAAATGAAACCCAATTCTTAGT
GGTCGAATTTCCTTCTGGGTCTGTTGCAGAAACTAGAAAATTGTAATGGCAACATGAAAG
TCCAGACTTTGAAGTATACAAAGAGCACTGGGTCTGCCTCCTGGTAAAGATATATACCAG
ATGAAACCCAGTCTCAAGCAAATCTGTTAAAACAAACGGAAGACACTACAAGAGCAGGTT
TATACAGGTTGAAATGTTTCTCAGTTACTCAAGTCAAGCTTTGAGTTCTGGGTGTAATGT
GAATATCCCTTGTTTGTCTATAAAAGCACTAGTGATACAATAATAATATTAGACAATGAA
GAGCCACTAAATTCTATGATATCCACCAATGGCAACAGATCGATTTCTGGATAATTGCCC
AGTTAGTTCATTGCTTGCTCCAGTTATTTTTTGTACCTATCATCCTTAGTGCTCTTGTCA
TCCCGGTTTGTCTGAGGGGTGTACTGACCAGTAGATGATGGTGAGTAGCCCGGTGCACTA
GGAGAGTATCCAGCACTTGGACTGTACGGAGGAGAACTTGGGGTATAGTCGGGGCTTACA
CCAGAATTATATGGACTGCTGGGACTAAATGAGGGGCCTGAAGGAGAATAAGATGGAGAT
GTAGGTGAATATGACGAAGATGTTGGGCTGTAATTTGGAGATGTAGGACTGTAAGGACTA
GCAGGTGACAATCTTGGGCTGCTTGGTGAATAAGCCAGAGACGGACTGTATTTGGCTGAT
TGAGGACCGTAACTTGGTGAAGTAGGGCTGTAGGTTGGTGAGGTTGGGCTGTATGTTGGT
GAAGTTGGGCTGTACCCAGGAGAAGTGGGGCTATATGCTGGTGAAGTGGGGCTATATGAT
GGAGACGTGGGGCTGTACGAGGGTGAGGTTGGACTGTAGGAAGGTGAGGTTGGGCTGTAT
GAAGGAGATGTTGGGCTGTACGAAGGAGACGTTGGGCTGTACGATGGTGACGTTGGGCTG
TATGCAGGTGAGGTGGGGCTGTACACTGGAGAAGTAGGACTATAACTAGGTGAAGTTGGA
CTGTAACTCGGAGAGGTCGGGCTGTAGCTTGGAGAGGTTGGGCTGTAGCTTGGAGAGGTT
GGGCTGTAACTAGGCGATGTAGGTGAGTATGAAGGACTAGTAGGCGAATACGCCGGACTG
GTTGGACTATAACCTGGAGAACTGGGACTATAGGTTGGTGAAGTGGGGCTATATCCGGGT
GAAGTGGGGCTATATCCGGGTGAGGTGGGGCTATATCCAGGTGAGGTGGGGCTATATCCA
GGGGATGATGGACTATATCCAGGTGATGATGGACTATATCCTGGAGATGAAGTAGGTGAG
AAGGCCATTCCACCAACATAGGGAGAAAACTGAGCATCTGTAACAGGTGACAGACGGAGA
TTTGGACTCAACAAGTAACTTGGAGACATCATGCCATCGTGATAAGGAGTTCCGGAAATG
GGAGAACGAGCAGGTGTCATGCCAAAGTCCAAACCTTCCATATAACTAGGCAGCTGAAGT
TCAATGGCATTCTTAAGCATCTCATCATTGAGATACAAGGAACAGTCTCCTGTCCCAATT
GGTGCAAGCTGGCCAAGCATTATATTCTCAGTAACACCCCTCAAATAATCAGATTCAGCA
TACACTGCAGCATCAAGAAGAATGTCCACAGTTTCTTCAAATGAGCATCTCATCATTGGC
CCAGTATCATTTCGATTGATTCCATGACGAGTGATGGCCATCAAGTGGCCGCGATAGGTC
ATTGTATCACACAGGATAGCCAAATGACGATAATTCACATAAGATCCATCAAAGGATATA
ACCACACGCAATTCATCCAGCAGTGCCCGACGAACTGCCTCAATCCCAAGAACTTCAATA
ATTTCAATTAAGTGATTGCTAGTTGTTCTCCTTGCATCAACATCTTCATGACACATAACA
GCTAAAAGATTAACACCTTCTGTATCCAGCATCCACTCATCCTCAGACTTAAATCCTTGA
TTCTCATCGAACTTGTTTGATTTACCCTTTTTAATAAATACTTTGTTGATATCTGGAATG
CCTCGTAGAGCCATTTCCGTTAGCATGTTACTCTCAATTTTCTTCAGGAAAACATCATCT
TCCGCTGATTCATCATTAAGCTCACCCTTTGGGGCTTCATCATTCATGATACGAATACGC
AGGATAAGTTTCTCAGCATTGTCATCATTAAATATACAAGTCAAATCATCATCAAACTCA
AGATTGATCTTTTCGGCAATGGCAGCCATACTTAACTTCTTATCAACCATCATTTCACGA
TTCAACTGGATACGAAGCAACCAAGGAGATATTTTTTCAGGAGCAACCTCCTCATCTGGC
ATCTCATAATAAGATTTGACAAATTCAACGTCCTCATCAATTATTGTGCTCATTGGATCC
GGATCATACCATACTTCAGTAGCTTCAGTAACACTACGAAGAGTGGTGTATTCCAATGCA
CATTGCACGTTCTTGGCCCTTTCCTTTGTTGAGTTAACTCCAGGTTTCAGATAAACTGAA
AGTGAAGGTGTTTTAATTTTCTTAGCCACATTGATAATCTCCCTCAACCTAGGGACACCA
AGAGTGACATTCTTTGCACTAACACCAGCATAGTGGAAAGTATTAAGAGTCATCTGAGTG
GCAGGTTCACCAATTGACTGTGCAGCAACACAACCAATCATCTCCCCAGGTGCCACAAGT
GATTGCAAGAATCGTGACTCTATCTCACCAATAACCCACTCAAATGCTTCACGAGTAAGC
CTATGCTCCTGCAACACTCTTTTACTGGCAAAAGTGCTGCGCAGCAAAATATTGAAGAAA
AGGGTGGCATTCTTTTGCGCTTCCACGCTCAATGCATCGTCACCAGGGACAACCTTCAGC
CGCTCCTGGAGTTTGTCAATAGCTTCCACCACTTCCATTGGGTGCATATCAGAGGGCCTT
CGTAGATCAACTTTAAAAGTCTTTTGTGCATTCCAGATAAGTCTCTTAAGGTTAACAGGC
ATAGCCCAAGAACTATCACCTGAGGTTGCAATCTCTGTTGCAAGTTGGAATCTATCTGCT
TCAAGCTTCTGAACTTCAGCATCAAACACGTCTTGTAGTTCTTTGATAGTTTTCAAATCG
TCAATAAATTCTTGCAATAGATAATTTGGGTTCCAGTTCTCCTGATCCATCTCAAATTTG
AAAGTCTTATGAAATTCTGATTTCTTCATTTTCAGAGAATCCAGTGTCTGAAATTCAATC
CAGACAGCATCCATACCATCTTCCCCATAGAGAAATTGGATTACATCGCCCAATGAGTTC

CTAACAGTGCCATCATATTTAACCATTATGTCTTCCATAGCCTTCACCAAACGCCTCTGG
ATGTACCCAGTCTCAGAAGTCTTAACTGCAGTATCTATAAGACCTTCCCTACCACCCATA
GCATGGAAAAAGAACTCTTGTGGGGTCAATCCACGAAGGTATGAGTTTTCCACAAACCCT
CGACTTTCTGGCCCATAGTCATCTTTGGTGAAATGAGGCAATGTCCGATCTATAAACCCA
AAGGGTATACGCTTACCCTCCACATTCTGCTGTCCCACACAAGCAGTCATCTGTGATATA
TTGATGAAACTTCCTTTGGATCCAGCTGTCACCATTGCCTTAAGATTATTGCTCTCTGAT
AAACTCTTCTGTGCACTGCTTCCAGCATCATCACGAGCCCTATTCAATACCTGATTGACT
TTGTTTTCAAATGACTCCATCATCGTTCGTCCAGGTTCAGGCTCTAAGCTTTTCTCTTGT
GATTTCCTGATTAGAGTTTTCACATCATCTTTTGCTTTTGAAATAGTTTCATTAATTGTT
TCCATTGTTGCGGCATCAGCAATTGTATCCCCAATTCCAATACTAAAACCATTCTGCAAA
AGCCAATAATTAACAAGCCACTGTGTATGTCCCAAAAACTTACGAGCTGCATCTGGACCA
ACCTCCTCCCAAATAACATGAATAAGACTTCCAGTTGCTGTGCCAAGTGTCTTCTTGCAA
AGGGTTCCAGTGAGAAGCTCCCCTTTTTCTATACGGACTAGAGTATCCCCTACAGTAAGT
AAGCCTTTGTCAAGATCTGAGTGCCAGGCAGCAGCTTTCATTAGATTTATCTGCTTAGGG
ATAATGAGATTAAAAACTTGTTTTCCTGTCCAAAGCGGCCGTGGTTTCAAAATGGCCGGT
TGAGGAATTTTGCCATCAAAATCTTCCCACCACATCAAGATGTTCATGAACACATCCTTC
TCTATGAAGGTGTCCCTTTTGGTGATTTTACGACATCCTAAAAGAGTATCCTGGACGATG
CCCATCACAGGCCGATTCGATTGAGGGGACACAATGCATTTAGGCACCATCATTAATTCC
AAAACTTCCGCTCTGGTTTCGAATGATTGAGGTACATGCATATTCATTTCATCACCATCA
AAATCAGCATTGTATGGTGAAGTGACAGATAGATTCAGGCGGAAAGTTGAGTATGGCATA
ATCTTGATTCTATGTCCCATAATAGACATTTTGTGAAGACTAGGTTGTCGATTAAAAAGA
ACAAAGTCTCCATCATTGAGATGTCGCTCCACCTTGTAACCAAGTTCCAAGTGGTGATCA
CTACTTTTCTTCAAGTATCGAAGATCAAGCCTTTGACCATCATCCCTTATAATATATTTG
GCACCAGTTTTGCCAGGTGGAGGATGTGGCCCATATTCAACAAGCTCTTTTAACCTTTCA
ATGTTATACGGAGTCACTGTTTCCGGATAAGTGAGATTTAATGCAATACTCCATGGTACT
CCCAACTGATCAATGTTGATGGTTGGATCTGGTGTTATAACGGTTCGTGCTGAGAAATCC
ACTCGCTTACCCATCAAGTTACCCCTGATCCGTCCTTCCTTTGCCTTCAGTCTACTACAT
ATTGATTTTATGGGTCTACCTGATCTCTGTGTAGCCCTTGGCTGCCCAGGCAATTCATTG
TCAAAGTATGTGGCTATGTGGAACTGTAACAATTGTGCGAATTCCGATATGATATGTGCA
GGTGCACCATTTCTTTCCTGTCTCCTCAAATTCTCGTTGTGCCGAATAATCATTGCCAAT
TGATGAGTTAAGTCATCCTCACTCCGGGAGGAGGTATCCATCATCACAGAGGGCCTCACA
GGAGGTGGAGGAATGGGAAGGACCTGCAGAATCATCCAATCTGGACGGGCATACTTGGGA
TTCAACCCTAACAGTTGGCAGTCCTCATCACTAATCCTCTTCAAAACACTAAGAACCCTT
TCAGCAGAAAGTGTCTGCTTCCTTTCAACAGGTTCAGGAAGTTGTTCCTGATCATCACTC
TTCTTCCTTTGAGCCTTGTACTCAGCAATCATCTTCATGCCTTCAATAGTCAGCTTAGGC
TGCTGAGCACCACAACCACCCCTACTTTTCTTAACAGGTTCTTCACCATCTTCACCTTGG
ACATTAATCTCATCATCTCCTTCACATTTAGTTTTGTTTTTGCATGCATCCAGAATCTTT
TTAAGCCTGTTCTTGGGATTCCTTATTTTCAAGGCTTGCTTAAACTTGTGATCTTCCTCA
TCAGCTAGTATTTTCGAGCAATTAAAGCAAACACAACGCATAATACTGAGAACAGTCTTC
ATAAAACCAATGTGAAACATTGGCTTAGCAAGCTCCAAATGCCCAAAATGACCCGGACAC
TCAGCCATGTTTGCAGTACAAGTTTCACACTTCATTTTCCTATCAATAGTACCAAGCCGA
GGATCACTTAGACCAGCTATCTTCGGTTTTCCCCTCTCTGTTGTTTCACTATGCTCAATC
TGTACCACTGACATTTGTCTAATCTCGTCAGGGCTGAGAATGCCGAACTGCACCAAGCGG
ACCTTGGCGACCTCCGCCGGTGAGAAAGGAAACCGCGTGTCCATGAAGATCGAAACCAAG
TAAGTTGAGCTAGGGTTTCAGAAAGAAATATGAGTAGCAATTGATTTGGAAAAAAAATTT
GTTTTTGTTTTTCTGTTTGTTAAATTACGGTTCTCTGTTTTCCCAGTTTCTATTGTCGAG
TCTGTAAGTGCTAAGCCTTGTCTATTCGAGTGTAAGAGAAATCGAGCTTTTGCCCGTGCT
TCCCTTCTCTGAAGCCTCACACAGAGAAAG
>tig_1410_consensus
GGAGCTTACTTAGAGGAGATATTTATTGCAACACAACACCCAAATAAGTATGTTTTTTTA
TGTACTCAAACTACATAATGTAATTACACAACATAATTCACTGGCATATAGTATAACATT
CTGCAACTGTAAGATTAAAATCAGATGGATTCAGCTGGGTGGGACTGGTAGGTGCGGCGT
CGAAGTTGAGAATCTTGAGTGGGCTGCTGCAAGAAGATTCTTGTTGGATCATTTGGATCA
ACATATGCATGTTTCATCATTTCATCAACTGGAGTTTGTGCTGCCAGTAAAGGATCTACT
CGAGGTTCTGCTGCCATATTTGCTTGTTGATTATACTGTCTAACTGATGAGATCAGGTTG
AAAAAGAGAGGAACGAAGGTACCACAACCCCCTTCCTTAAACAAAATCTTGAAAGAATGG
GTGGAGTATAAAGCCCTATGTTCATTTTCTGGGACCACAGGCTCCACAAGACCAGAAATA
TTGTTGCAGTGAAATATTGGCTGGTTAAATTTCTCACCATGGACGTAGAGCAGAGGCATA

TCGAAGGCAACAAAGTTTCCAACAGGTTTACTAGGAACAAAGACCATGCGAATATTCGAC
AAGTATATTGTCCCCTTTGCTTTTACGTTGCCCCCATGAGCTCCAGGAATCTTATTGAGA
TCAAA
>tig_1411_consensus
TCTTTTGATGATCCTTGATAGAGCTTGACCTACTCAAACTTTCCCTTCTCTTGCCTTCCT
CATTAGTAGCAACTGGTGCAGCATCCTCAGATGGAGCAGTTGAATCAGAGGACATAGAAT
TAATTGTCAGGTTCTCAGTAGAGGCAGCAGTGGAAACTGATGAAGACGGTTTAGTAGCTG
CTTGGAATGACTTAGAAACAGACGATTCACTACCTTCCCCAGACGGCCTTGAAGATTTTG
ACAAACCAGCCTTTCCAAAAGGTGAGGTATCCAAAAGAGAAGCGTCTAGAACCTTCTCTC
CAACAAATACTGTGCCTGGTTTAACTGTCACCTCCACAGTTCCCGAAGGAACTGAAGAAA
TTATATTATGCACATCACGAGAATGATCTGAGTTTGGTGGATCCACAACAGCAGGCATTG
CAGTGCCAGGTTTAATAACTGGCACGGAGCCAATTGTTGTTGAATTTATGAATGATATGT
TAGGGGGACCCTGGCCAACTGTATAGTTGTATCTTGGTGCCTGAGAATTGGGAGTTATCT
GGCTACTTGTTATTGGTAGAGAACCTGAAGTTGGAAAATACATGGAACTGGGACTGCACG
AATTAGAAAAATAATTGATCTGATGAGCAGGTGAATATGATTGAATTGGCTGTGATTGAG
GAATGTTGGTGTGGGACCTAGGTGCTGAAACCCCACCATCCGTATGAGTCTCTGTTCGTT
CATTGAGTTTGACCTCCATATGTGTTTTTGGATGCGTAATTTTCACTGGAGTCTTACGAG
GACTAGCAAATTTTCCTCCCTGCTGTTGCATGTATTGTGGTGTAATGCCGATTCCCATGT
TGCCCAGCTGGGGAGGACCCATCTGCGCCGTGAAATTCAGGCCCTGTCCCTGATGCATCA
TTCCCTGAGGCTGCAATGGATGCGGCTGGAGCCCAGGAACAAACATATGCTGCTGCACTT
GGGGAGCATTTCCCATGGGTAATGCAATGGGAAGTGGCAATTGAAGTGAGGTGCCAGTTA
CATTCTGAGACTGCATCTGTGGGTTATGCCCACCAAACTGCACAGATGCCTGTTTCTGGT
GAAAAGGCATTTGCATAGGAGCCACTGGCATAGGAAGAACAGAATGTTTCTGTGTTTGAT
TTGAAGTAGGAGGATGCGAGACTTGCACTTCCTTTTTGGTTTTCATCACTTGATGAACTT
CCCCAGGACTAGGTTGAGCTGTTGCACTAGTATCCTTCCTTGGCAACTGCTGCTTAGGGA
CAGATGGTGTTGGCAGATTTGGCATTGGCACTGATCTGTAAGTTTCATGGCGTGCCTGAT
CACGTTTCTGCTCATCCAAATTTGGGGGAGCTGAACTAGTTCGAGCTGGAATCGGCATCC
CAATTGAGCCAAATTGAAAATGGAATCCCTTGGATGCATCTCCTTGAGCCTTTGATTGAT
TTGCAGGTCCAGTGCTGTCAGGATTCACGGAGGCAGGTTGAGAAGTTGGAGCCTTCGGAA
CAGCTTGGGTACCTCTCTGAGTGGTGGAAGAATCAACTTGCTTGACAGCACTACTACCAA
CCGGTGCATCAGATGCTCCATGTAACTGAGACGGTAGATGTGCACCATTCTGTATATTTC
GCGATTCAGAAACATTACTGCTGGAATCCGAAGAATTGACATTAGGCACGTTTACCCTAG
ATTGACTGCCTTGTGCATTGTGGGACTTCTTAAAACCGCGGCTAGATGAAAGAGAAGGAG
AAGATGAAATAGACGGTGGTGAAGGGGCGGCCCACCGCCAGAGCCCTTAGCGTAAGAGC
CTGAGGAGGTCCGCTGCTGATTAGAGCCGGCGGATCGTCCTGATTTTCGAAACTGTGTCT
CGCTTCTGTCGGACCTTGATTGATGGAAGGACATAAATCCCAAGCGTCAAATAATTCCTT
GAATCGTTTAGCTTTTTTCTGTGAGAGTGAACAGTGAAGAGCGATAAAATTAGGGTTTTA
ATGGGTGGCAAAGACGGTGCTCGGATACAGAGAGAGAGAGAGAGGGAGAGAGAAAGAGAA
GAGACATACACTAATGCACTCATGTCAGCAAG
>tig_1412_consensus
GCAAAAGATAACATGACACTTGTTTACTAGTAGATATGTTTATGATTCATTATCACATAA
TATGGATATCAATCCAGTCCAGAAAATAGAACAGCACCAGAATTCGAGAATCTCAGTCAC
ACAACAAGATATCCAGATTAAACAAATAACACACAAACCACTCATACAGATAACAAACAT
CAAATCCTCCAAGCCATCCTTCACCAAACATAATAAGACTTCCAGAGACTGCAATACAGT
ACTGATTTATTAGTAAGGAGTGATCTTCCATAGCTGGTTATCTCCTGCAGCCCAATTATA
GAGCACAATAGTAGTGCCATCATGGACACCACCAGATTTCTTATCACCATGAAAAGCATC
AACATTCAGATGGATGTTATTAATCATCCTTACTGCTCGGTATCCTTCGCCTAAGTCCCT
GCTCTCAGACCACAGTATAGATGAGTCAAGCTCATCTGGATTATAAGGCACCAGCTGCAC
AGGATGACTAGCTCCAACAGCGTGCTTTATGGCCTGACCAGTAGCTTTGTTAACCAGAGA
AAAGCAGGGACAGCCCTCTTCATCTTTCACTCTGGTACTGTACTTCTCATCTTTGTACCA
ATGCTGGAAATCATCTGAGGGATCAGCTATTGCAAGTATCACTTGACCATCTCTGATGCT
GAGAAGGAGATCGTTCTGAGCCTTGCAGTAGACTTTGACAGTGGTTTTGTTTGCGAGGTG
AGAGAGAGGGCCAGATGCAGAATGAGAGTGATGATGAAGAAAAGACGGCAAATGAGGCAT
GAACGAATGATGTGATTGTGAAGTCTCGTGTTGATATTCTTCGTGAGCAACATGGCTCAC
TTCTGTTGGTGGGGGTGGTGTCTGTTGGTAACTGAAAGAGTTATATTCTTGTGCATCACT
GCTGTGACTGTGATACACGTGACTCACATGTGTAGACGGCGGCGCCGGTGGGACATACTC
AGGCTCCTGATAATAGGGCCGTGGTGGTGGAAATTCGTCTTCTTGGAGATACGCAGGTGG
GGGAGGTGGGGGTATAAATTCGTTGTCTTGAAAGTGTGATGATGGTGGTCGGTCAAAGGA

CGGTGGTGGGTGAGGTG
>tig_1413_consensus
TTGCCCTGATCCGAATCCATGAATTATGGCTTTTTAGATTGTGGGTTTTGGGGTTGGTG
AGGAGAAAGAGGAACCGAGTTAGTGGGCGACTCAGAGAGCAAGAGAGAGAGAGAGAGAGA
GAGAGAGAACTCAGAAACCTAACCTTTTTAAAGTAAAGAGGGGCTTGCTTTGCAATCTTT
TCGGGCAAAGGAATGCATTCAACCAGACAATGACGACGCTGTTGTGCCAAACCCATCACT
GTCTCAAGAAACACTACTTCTTTCTCTTGCTTTGCAAACATCATAATAAGGCATTTCTTG
AAATTGCGAATTTCATCCCATACATTATTGTCAACAGTTCTTGAGGATGACTCATGCTGC
ATTGTTAAGATGCAGCAATGACCAGGCACAACGGGCTGCCACTGCGGCAACATCAAGTAA
GTAAAATTTGCTAGTGCCACAACAAGATGTCTTGGCCGGTTTGGATTCTCAAAACAAAAG
AGGCATCGCTCTTGCTGAGTCAAGATACGCTTAACCATAGCCCTTTCAGAGCCCCTTATTA
TCACTTCCTCCCTTCTTTTTCCGAGCCTTTCTGCTCGGACCATCCTCAAAATCATATTCA
TCATCAGCCCGACCGGATATGCTAGATTGTTTACTCCGCATTATCTTTTGTGCTAAATGC
ATATCGGCATCATCATCTTTCTTTCGGACAGATATATCATGAATAACATATCTGCTCGAT
TTTCCTTGTAGTTCAGTTGAATTATTACCTGCATCCTGCTTCCTTTTCAAGTCGTCAGCT
TCTTGCAAAAGTTTATCAGCTTCTTCATGTTTTCCTTTCATTCGCAGCTGCATAGCCTTA
GCCGCCAACTTGTTGGTGCTCAGTCCCTCTTCAACTAATGCAAGACTTCCAGTTTGTATA
TTAGCACGTGATAATGCTGCTTCTGACTCTACATTGCCCCTGCCATTTGGATCTGAACCA
CCTGCCTCTTTACTGTCCTTGAGGTTAAATTCACGCATGAAGCTGCCATCATTTGTAAAC
TTATTTAAGCCAGACACTGCAGCAGAAATAAGTTCAGCATCCTGAGTAGACATGTTTTGA
CTTCTTCGCTTTCCCCAGGATAAGGAATCACGAACTTTAGGGGCTCTCATTTCAGGATGC
CTAAGAGACACATCCCCCAAGTAATCTCGACCAGATTTCTTGTCAATATCTCTTTCAGTT
TGATTATCTGCAAGAGTTTGGTGCTCCTCATTTAGCCCTTTTCTCCTATTTTTAATGGCA
TGCAGATGAGCACGGGATGGAGCAGCCATATGAGAAGTTGCAGATACAGCTAGTTGACCA
AGTGAGCCCCAACGCTCTTCAGCAACCTCCTCAAGTCTCCGTCCTTCTCGGGCTGCTTGC
TCTTGTGCACGCTTCAAGGCTTTAAGTCGCCAGCTTGCACCTCCATCCCCAACAAGTGAA
GAAGACAAAAGTTGGCCCCCACCTGATTTTTTATCATCTTCATTTTCTGGATAGCCACTT
CCATTATCCTTCAAATAAGGGTTCAACTCCCTGGGATTTACTTTTGGTGTCTCCTGGTCA
GCTGGTTCCTCAGGCTGGTTGTTGACAGTCACCGCAGGCCTTTTATCTGTCCTTTCTGCT
GGCTTTAACATCCACTCTAACCCCATTTCCTTCCTAACAATTTCCTGCTCCTTTGTTGAA
GATCCTCCACCATAATCAGATAAGTTATCATCTGCTATGTCCTCTTGACTCGTTCTTGCC
ATACTCTTTTTCTTTTCATCTTTCCAACTTTCTTTTCGGTGGTCATCTTCAAAATCAGAT
GAATATTCCTCAGATGAATAATATTTCCTTTTAGATTTCTTCTTTGACCTACTTCTCTTC
TCATCCGCAGAACTATCATTATTACTTCTCTTACTCCTCTTTTTCCTACTCTTTTCATCT
TGCTCAGAGCTGCCCTCATTTTCACTTGATGAATACTCATCAGATGAGTACCATTTCTTG
CTTTTTCTTGAAAATTTCTTTATTCTCTCAAGATCATCATCTGAACTACCATAACTAGGG
CTTTTCTTTTTTCTCCTATGCTTTTCCTTTCTACTATGAGATTTCGCCCTCTTTGCATCA
GAGTTTCCATCTTCTGCTTTATTGACTTGATCTCGAGGTATAAACTTCACTCCAGAAAGC
ATCTTATATCTCTCAGATCCACCCTG
>tig_1414_consensus
ACCAAATGTTCTACGTGCTACATAGCAATAATAAATAAGAATAGTATCATCGTGAGGATC
ATACAAGGAAAACCCATACCGAATTTCAAAAGACAAAATATGAAAGAATGATTATGTAGT
TTTATCCTTGTGTTCAAATTTGAATGACGACCACTTCACATGAGGAGATGATAGTGTATG
CATGCAGCAATGACAATGAGATAACTATCCCCTACACTACAATGCTTCACTCCTAAATCA
AAACAAAAGAACACCTAATCTGAAGGGCAAATCCAACCATCTGTTCAAGTCAATTGGCCA
GTTTCATTTAGTTAAATAAATCATGGCCCAGCCATATAACTTCGCTTGGTTTATACTTGC
CCTTTTCATCCAGAAAATATGCTGCAACCAAAATTATCGATTTCCGTTACCTCCTCCATG
CCCCCAGCGCCTCCTTGACCGTCCCACCAGTATCCTCGGAAGAAGACGTCTAGAAGGAGC
TGCTGAGCCAGCAAGAAAAGTACGACGCATCCCGAAATCATCCTCAACCAATGAGTCATA
ATCCATGCGATATCCCCCTTGCCAGTGGAGTCTTCGATTCTGTCCTCGATCAAATGAATC
AATATGAAAGTAGCCATCATCCGAAGATTCATCTGAATTATAATCACTATAAACACCATG
ATAACCTGGCTCAATCACATAATCTCCTAGTACCATTGCTCCAGGTGTCGATGACATTAT
TGTGCTGATCACATCATTTCGCTCTCTCTCACATTCAAGCTTCTTCCATTTTTCTTCAAG
TGCAGGGTCTACTGCTCGAGGTCGTGCCAAAGGGTGCTTTGACTTGACGTGCTTCCTGAG
TTCCTTATAAGTTCCAACAAAGGTACACTTATCTTGCATGCATGATCTTTTCTTTGCATT
GAGATATTTACGAGCTGGTTCTACCACAGTCCAACCCTTCACTTGACCACGACAAAGAGG
ACATAGGAGCTCTGGAGCTTCAGTCTTTTCATTGGCATGCCCAGCATCCAAACTGAAAAT
GGAATTCTCAACCAACCCAGTCAGTTGTTGCCCACTTTCCATTGATGACACTTTTGTATA

AGCTTTCTTGTATTGGTCAAGACAATTAGAAAAACGTCGGCTAGTGGCACACATGTAAGG
TCGGCATCCCTTGTTATAAGAGGAACAGAGTAGGAGCACAGCATTGTGAGGAAACTCAAG
ACACACTGAGCAGATTGCACCTTCCCAATCTTTCTTCTCCAAAGCTTTGGAATGTGCTTT
CTTTGTATGACCCTCTTTACCAGCCTTCTTATGGTAAGATGACAATGGATATGGTGCAAC
TCTGTGACGTCTTGGATCAGACTTGCATCGAACCTTGCTAACTTTTGCCATTTACAAATT
TAACTTGGAAATATATATATACACACGAAGAAAATAGGAAACGTTCTTTAAGATAGAGAG
GGAGGGAATGATTGAGAGGGGATATTTGAAAGTGTGACTGGAGATAGATGGAGGGAAAGG
CGGAGATGATCGGGGTGAAAAATGGGAGAAAGGAAACACCCGAAATTCAAGGGCAGGTTG
GGTCGGGTTGTGCGTTGCGTGTGCAGGTGCTCTGAGAGAGAGAGAGACAGAGAGAGAGAG
ACCGAGAGGCTTCCCCTTTTTATGTCCTGGGCTTGGAAGAAGAAACTTTGAAT
>tig_1415_consensus
GCAGGCCTCCTGTCCTCAAAATAACCTTTTCCTTGTTTCTCTGTGTCTCGCCCAACACGA
ATGGATGCACCACGGTTTGCCACACCCCATTTGAATGTGCTGATATCAGCGGTTTCATGT
CGACCAGTGAGTCTGCGTTCATTGCCTTCACCATAAGCAGCAATGTGCTCTTTGTGCCTA
CGACCAAGCTTTTCAATTGCCTTTTTGATAACCTCAAATCCTCCTTCCTTCCTCATTGAC
TTGGTGCTGTAATTTGTATGTGCACCAGCACCATTCCAATCACCCTGAATAGGCTTGGGA
TCAAAGGAAAGGACGACTCCTGCAATTTCTGTAATCCTCTCCAAAATATAGCGAGCAACC
CACAATTCATCACCAGCAGAGATACCAACAGAAGGACCTACCTGGAACTCCCACTGTCCA
GGCATAACTTCTCCATTGATACCACTAATATTAATTCCTGCGTACAAGCAAGCTTTATAA
TGGGCATCAACAATGTCCCTTCCCCAAGCTTTATCAGCACCAACGCCACAGTAGTATGGT
CCCTGAGGTCCAGGGTAACCAGCAGTGGGCCAGCCAAGGGGCCATTTAACATCTTTTTGA
AGCAGGGTGTACTCTTGCTCAATACCATACCAAGGCTCTTCAGCAAGAACATCAGGATGG
TTGAAGATCTTGGCAGCCGCATATCTCTTGTTTGTTGGAATTGGTTCTCCAGCAGGAGTA
TAAGTATCACACATAACAAGAATATTGTTGCCTCTTCTGAATGGATCCTTGAAAATGGCC
TGAGGGTATAGGATCACTTCACTGTCTTCACCGGGAGCTTGACCAGTGCTGGAACCATCA
TAGTTCCACTTGGGAAGCTTAGACGGATCACTTACAGGTCCATTAACAGTTCTTCCTTTG
CTCCTCAAATCCATTCCAGATCCGCCGACCCATATGTACTCGGCGATGATCTTGTCCGTG
GAATCTGAGAGGTTAAGGTTGATGAGATCTGTAAGAAGAGACATACTTCACCAACAAAAC
TCAAACTGAAAACGGTACGATCCCAACTCTCTTGGCTTTGCTTTCGCTCTGTAACAAGGA
TTTGTCTGGTGGAGACGTGCAGAGGGGAGAATTTCGTGTGCCTTCTTATAATGCCGAGAT
CAGGTTATAATCAAATTTTGTAAAAATATATATATTTTTTTTTCACTTTTAAAAATTCGT
GGCTGTTCGTGAAATTAATCAGAGGAGACTGGACCAAGACTTTTGCTTGTGAGAAAATCT
TACCAGAGGGTTCACGATGTAAGAAAACTTTATTTTCTGGTAAATT
>tig_1416_consensus
ACGAAACATGCCTTCACCAAAGCTGTAAGTATAACCAGATTATTTCCCAATACGTGCAAC
TAGGAGTAGTTCAGAGACATACCCCGTTGTAATGGTTACACAAGAAACATGTATTAAACC
ATTACGGGGAAACACTTGAAAACAATAAGGATCTTACAAAAGTTAAATGAGAATCAACAC
CTCCAAAGTACTCATTTTAACACAGAAGCAACGACAGCAACCAACTACTGACCAACATTC
CCAGACATCCAACATCACACAAAAAGACATGGCTTACCTAACTACTGCAACTCAGAAAAA
GACCCTAAAGACTAAAAAATGCAATCGGCCACCCATGCTTGGTGTTATTGACTTTGTCGA
ATTTGCTTTAACGATACCCGGATAGTGGATCCGCTGACTTACCAGATCTTGCATCAGTCC
AAGCCTTAGCAATGGTTCGCTTCATTTCGTCATCCCCATCCTCGTACATATTCTTCATCA
AGTCCATAATTCCAGCCATTGGATCACGTTCCTTATCCATATTTGGCTTCAGCTTGTCCT
CTTTGAAATGCAAGTCTAACCAATTCCCTTTGGAAGCTTTGATCAAGGTAATGATAACTC
TTGCGGGTTTAGCTAACACCTTACATTTTTCAGGAACAATCTCTTTGTTCAACTTTGGAA
TGGTTAACCTATAATTCTTTCCTTTGACATCGTGGAATTTGACATCAAATGACGTCTGCT
TAATGTCTGTTTCTATTTTGTCCTGCTCCACTCCTTCCAAAGAGACATATATCTTCACTT
TCTCATTGTCCTGATCCCAACTGAAGGACGCGAGTGTAACATAACTTGTGGCTGGAGTAT
AAGGCACATTAGCCGGCGCAGGAATTGGTGTTTGTGCAACGGGGCCAGGAGGAGCAGCCT
GTTTTGACAACTTCTGTAAGTTTTGAATCTCAGAGTCGATAAAAGAAAGAACTCTCGGCC
TCTTTGCGATGCTCTGTAGTTGTCTAAGCTCTTCCAAATCTAACGCCAATTCTTCTGCCA
TTTTGTTTGCTTTGTTTTCACTCAATTGGACGGGAGAGAGAATTTATAAGAGTTCTGGGT
GAACGGGGAAAAGAAAACGCTAGAAGGGCCACATTTGAACCCTTAAAAATTTAGGGCTTT
TTTCTTTTTTCATCTTATAAAGAAGGTTCTAGAGGCAGGGAGATGAAAATTCTGAGTGCA
CCCTGATCAACGGACGGCTAAGAGTTTAACGAACACGTGGCCGCATGTAGACAGATCAGG
ATTAATTCATGTACCGATTCTCATTGTTGAAACTTCTATATATCAATCCAGCCCATCAAA
ATTGCACATTCCAATTGAATTAAACTCAAGTATAATTGGATAACTGGAATACTCTGTAGA
GAATCGGTAC

>tig_1417_consensus
GTGGTTACCATCTTCAAAGTGTAGTCATGTCTATCGGTAGTAAGCCTGCTGATAAGCTGG
TGCCAAACCAGTGCCGTAGCCCCCATAGGCAGGGTAGTTCATGGGAGCTCCTGGATAGGG
TACAGCAAGAGGAGGCGCTGCTTCTGGTGAATATGCATATGGATTTGGTGGCGGTGCTTC
TGGTGAGTATGCATATGGATTTGTTGGTGGGCTCCTGTTGCCGTACACTGCAGGTGGGGA
TGCGTATGGTGAGACACCAGCAGGGTATTGACTGTGCGAAGGAGACCTGACAAAGGTGGG
TGGTGCAGGGAAGGATGAAACATATGCATTTGTCAAACGACCAGCCTTAGCAGGTGGCAT
TGGGCCACCATTGCTAGCCCGTGTGCGCTTAGTGGCAGGGACAGCAGCAGGCTTTTTCTT
CTCAATCTTGGCCTTCTCCAGCTGCTCAAGACGCTTCCTGAGATTTTCTGGAGGAAACTC
AGCTTCAAGTTTGTACTCTTCAATGCATTTAATGACTGCTCGCAGAGCTGACTGCTCCTT
GCGTGCAGCCAGGTGCCCAGCTCGGCCAGTATTATTTGGATCCTCCAAAATAGATGCAGC
AGCCTTCTTTGCATCCTTCAGAAAAGCTTTAAGTAGAGGAACTGGGGGGAACTTATCAAC
GAGGCCAACCTCATAT
>tig_1418_consensus
TTTTTTTTTGCCTCAAGTATTTTCTCACTTAATTAAAAATGAAGTTTACACACAAGAGTA
TCGTAATTGTTTTAATAAATTAAAAATTAACCAAAAACTGCCACTAGAGAAGGCATTTT
CATTACCACAGCCAACCTGGGCAGCCAGGGCATAAAGTTCCACAAACACTGCTCTAAATT
CTAATACATAAACCACTTCTTCAGCTAAAATTTCTCACTCCTAACAGTTAAATCCACGGT
TAGGCTTCCCAAGGGCTTTCTCAAATACTGCTGTACATTTACACTGCTTCGTAGGGTAAC
ATGGGGTCACCAGATGTTGCAAGCATCTGCACCTCCATTTCTTCGGCCGTCCGTTTAGGA
ATACTACTTGCCATGGATTCATNNAGCCAGTACTGTATGGCATGACCATATGAATGTTTT
AGCCCTTTAACAGCATCGAAGTTCTTGTATGTATAGCCAACAAAACTCAAATCTTTGGGA
GTTAATAGCATCTTTCGCGAAGGTCCTGAGCCAGTTCTTGCTGATGCTGGAGGATCCAAT
TCATCAAACTTCATAAAGTTCTGCGTATCCAGCTCTCCATTGACTTCAGGTTTAAATGCT
GCCTCCATTTCATAAAGTTTATCCCATGGAACATCTTTAAACCAAGGATGAGCTTTAATC
TGGTGAGCCCCTCCAGTGCCTAACCTATGTTCAACATCACACAGTAACCTAGAAATGAGA
TCCTTTGCTTCAAGTGATAGCCTCAGATCTTCTGGAAATTTTAGATGATTTCTCCAATGG
ACAATCTTTCTACATGTGGTCATTGGGTCATCTGAGTAAAATGGAGGATATCCAACAAGC
ATTTCATACATTATTGCTCCTAGCGACCACCAATCACATTCCATGCCATATCCTTTCTTT
AATAAAACCTCAGGAGCAATGTAGTCTGGTGTTCCCACAGTTGAAAATGCCAGTTTCCTT
CTGTTCATCTGCCAATGCTGAAGCTGCTCACGGGCACTTTTCCAACTACTTTTATCATCT
GTATCAGGAAAACATCCATCGATATCCATTGGTTCCACCGTATTTTCATCATCAATGGTT
TTATTTTCATGAATTGCATACAAAGTTGAACAATCAAGAGGCTTGCAAAGGCCAAAATCT
GATAACTTCATGTGACCATTTTTGTCCAGAAGAAGATTGTCAGGTTTAATATCTCTGTGA
ATGTAGTTATGTTTGTGAATTGACTCAATAGCCAGCACACTTTGAGCAATGTAAAATCTA
GCCACATTTTCAGTTAATGTGTCCTCCCTCATCAGCAAAGTCATCATATCACCACCAGGA
AGATATTCCATGATCAAATATAAGTATTCTGCATCCTGAAATGAGTAGTAGAGCTTCACT
ATGCAGTGGCTAGCAACTTCAGCCAGTAAGTTCCTCTCAGCCCTAACATGTTCTACCTGT
CCTCTCTTAAGCATTTCAGATTTCTTCAACTTCTTCATAGCATAGATGTCACCAGATTTC
TTCTCTCGACACAATCGAACCTCACCAAAGGCCCCCCTCCCTATAATGGTCAAAAGCTCA
AAATCATCAACACAAATCTTGTGCCTCTTAAGTCGCATAAACTCTGTCTCTTTCCTCTCC
AGATCTTTTATGAGATTGATCTGTTCCTCCTTGGGCACATCAGAACAAGCTAATCTTCTT
TCCAACACCCATCGTCTCTCCTTCCGTTCTTTAATATTCTTCATCTGAGCTCGATAATGA
TTCTCTATGAACTGCTTAGCTGCCGCCACCTTCTCCATCGTCAACCTGGATCCCAGCACC
TCCTCCTCCTCCTCCGTCTCCTCCATCGTGTTCATT
>tig_1419_consensus
ATCAGGATTGAATACTTCAAAAGAATGATAGAATCTAAGAACAAAGGACTGTCCAACTTC
ATTAGCTATCTTTTTTTCCTCATATTTCCCTTCTCCTCTTAAAATACAACAGTCTTGTTA
ACCTCATAAGGTAACACTCGAAGCTCACAGTATGATTTTATAGCCTTCGCAAGACACCTT
TTCTCTAGGTCCTCTGATTTCTGTACAAAACTTCGCATGTTATCTCTATGGGAAACTCTC
TCAACCATCTGTTCAATAATAGGCCCTTCATCAAGTTCTTCAGTTACAAAGTGACTTGTT
GCACCAATCATTTTAACACCAGCATCAAAAGCCTG
>tig_1420_consensus
CCGGCCAAAATCTTGGAACACATTCTTGGATCAATACGTAATCATAAAATATATTATTGT
AACCCATAATACTAAAACATGTTGAACAAGCTGTAAAATGATTGTTTGATTAAAAAGTTA
AAAATAGAATCAAGGTCAGAAGCATACAATACTAGAGTATGTGCTACAGATAAATCTCAA
GACAAACTCTTTGACGCCATATAAATTCAAGTAAAACACTAAAATGTATAACGTCTACTT
AATCTTCTTTTTGGGCCTCAGCTGGTTGCTGTGTCCACACTTCTTTTTCCTGCAGTTCAC

AGCACGAGGGTGCAGACGTGCATAGCATTTTCGACATATCATCTTGTCCTGGTTGTACTT
CCTAGCCAACGCCATCAAAGAAGGCTCAATGATACCCCCACGAAGCCTGAGAACCAGATG
CAGAGTAGATTCCTTTTGGATGTTATAGTCGGCGAGAGTACGGCCATCCTCAAGTTGCTT
CCCGGCAAAAATCAGTCTTTGCTGGTCAGGAGGAATACCTTCCTTGTCTTGAATCTTAGC
CTTTACATTGTCGATTGTGTCGCTTGATTCGACCTCGAGGGTTATGGTCTTCCCAGTTAG
GGTTTTCACGAAGATTTGCATTTTTGTTCTCTGTTTTAGCCCTCAATGGCCGCGTTTGAG
GTTTTTATTATGATTTTTCTTATATATGAGGAAACTGATGAATTAGGGTTTTTGAGAATT
TG
>tig_1421_consensus
TTAAACCAAAGGCATAGTGCCAAACTCAACAAACAATCAATAGGCAACACAGAGTTTAAA
TATGACGAAAAAAAAAATGTCATCAAACAGAGACTCAATCCAATTAACGCTATCAACATAA
TAACAAAATAGACATTTTTACTTTTTGGTTCAGAAGCACATATAAATTTGTGTACATATC
TAGACATACCCCATGGGAACTCTTTGTTGCGAATGTGCAGATATGGGTATGGAGGAGGCT
CTTCATGGTGGTGATGACCCTTTGAGAG
>tig_1422_consensus
TATATATATATATATATCTTTTTTATACACTAAATTACAGGCTTACATGTCTCAATTTCC
TTCAAAAAAAAGGCCGTTAGATGTGGAGATTTGTTGGCTCTGGTCCTAGTATTTCTTACA
CATTACAAGGAGTCTTTGCAAGTGGATTTGTTGTGTCCAAGACCCTTGCATCTGCTGCAC
TGGAGTTGCCGCTTCATTACATCCAGAGATCCAGATTTCTTTGTTGTTGGTCGGCCCGGT
GGACGACGAGTGGGAGGAGGAGTTACCATCACTGCAACCTGGGAAGGATCTGCCTGTACA
GGCCTGTCCAGATCAGGAACAGGATGTACCATATCTGAGTATGTTAGTTTATACATTTCT
GTCGTGAAATATCTGGAACAATAATCATATGCACTCCGTCCAACACAGGCAATGACAGCA
ATAGCATGGCAGCATGGCAAGCCAGTAATTTGCCACCCTTTGCAACTACAATCCCAGTGA
TCCATATCAACCACTTCACTAGTATCACCACGAACTTCACAAGTGCCCCCACCTGACAGT
AGCACTTGAAGATTATGGACCTTTAGGTTCTCCTTTTCCAGCTTTTCTTCCATGGTTGGT
GTTATTCGTGTCATCCATAGATTGGAATCTGCTTGTCTTGTATAAATCAACTCCATAATC
TTACCCTGTATCACATCAACCGTCTGTGTTATTGGTAATTCATGTGCATCTGACACCCAG
CTGTAAAACAGGTCTCCAAAGTTTGAAGTCATATGATTATATCTTTCGCCCTGAAAGAAT
GCATTTGCCCAATTCTGGGGCTCACTCTGTATGACCCAATTGTAAGCTTCCAGAGAAATA
ACTTTGATGCTTTCAATGCTCCTCTGGAATCCTTCAGGTCTAGGTGCATAAGCAGCAGCA
TAAAGATC
>tig_1423_consensus
CAGCTACTTAGCTTTCGAGCATCATTATTTGTCAAACCGATATAATTCTTAATCCACATT
CAAACAAATCGACCAAAAACAAAATTTGAGGCCTTACCAGAGGCATTGTCCCAAGAAGAG
ACGAGGGTGCACATTTCTGCAAAAGTAGAAGTCTTTAACAATCTTACCCTCCACCCTGAT
TTCGGATGACCCTTACCATTTTAGACAAGCAAGTATGGGGATCGAGGAATCTTTCTCTAG
ATATTGGATATTTTTTATTATCCAAAACGCAAACCAGTCGTCAAGTCAAATTTATACATA
AAAAAGAAATACAACATATACAATAGGTTAATTATGCCGTTTATTAATTAATTAATTACT
TAACCCTAAAAATCATAAAATTAATTTCTTTCTTTAATTGCTATCCTCATCCTGGAGTAC
TTTCTCTTCATTCCTCCGATTTAGGAGAGAAATTTTTTCTTCGCGGTGATTGTTGCCGAT
TTCTGTATAGCCATTAACAAAAATCTCATTCTTGTTGCTAATTTCAGCACTCAAATCCAA
ACCTTTCTTCCCAAAAGCCTGCTTCTTGTTGTGCATCCAAACCTTGAACACATTTTTACT
GACGCCAACTTCACTGCAGAACTCTTGTGTTAATCTTTCTTCGCCTCGGTTCATCCTCCA
CCCCAGTTTCTTCGCAAAAGCAAACATCTTTTCTTTCTGCTCTTTCGAGAATTTCGTTCT
TGATCTATTTCTTCTACTTGGGTTGACGTTCTCCATCTTTCGATTGAACCCCTGGTGGTG
AGGGTAGTGTTCATCGGACGGCCCGGAATACGCCGTGCTCAGAGACAACAGCATGTGGGG
TGCCGATGAGTAGTAAGAACAAGGAGCCGGTGATGTCACTTGGGGTTGAGGTGAGTTTGT
GTCCAGACTCGCTGCTGTGCTCGGGTCAGTACTCGGACTTAGGCTCCTCGGAAGCGCAAC
GGCTGATGATAGCAGCAGTGGTGGTGGTGGTGGTGGTGGTAGTAAATACTGAGTGAAGGC
GGAATCATAAGGGTCACGGCGATGGAAGTTGCGGTGACAGCCACAGGCGGCGCATTTGAG
GGAGGTAGGATCGGCGGAGTTGTAAGTGGAACAAGGCATGAACTCACCACAGCCATCAAG
AGCATGACCACCTAGACTTGCAGCATGGTTTTTGAGACATTCTTTGTAGGAAACCACCAT
CTGACACAAAGACTTGGTTGGTGGGAACTGAGTTTCTGTATGTGGGTTCCGTTTTGCCGA
CTTCCCTTGCCTACATTGTTCCATCGACCAGAGGCTGTTCACCTCGGAGACCTGATGCGG
TTATGAGTACGACCGGGCGCGGGCGGTTCACCTTTTTGTCCTTCTCCTC
>tig_1424_consensus
AACCAATGATCTTATTGAAAATTGGCACTTACAATTGCAGACGCATATATTATCACACAG
ACACACAGAAAATAACACAGAAGCAGGCAATTGCTGTAGCAGGGTACTACCTACAATAGC

AAAATATATGACAGGAAAAAGGATTTATACAAATTTCATTAGACTAGATTTAGGAGAAAA
CTGCAATCAAGATGATGGCTCTGCTTCCGATACCATGCTTGCAGGTGCTTGCTCCTGCCG
TTCAACATAAGCAGAGGGAAAACATCCAGCCTTGCCCTTACACTCCCCTTCCGACCATCC
GGTAGGAGCCACCTGGCGAACCACAACATAATCATCAACTGATAGACTCAGCTCCCCATC
TGCTTGAGCATCGAATGGATGTACAACTTTTGCAATGAAGTGATCATTATTCTGATTAAC
ATTCAAGTGTCTATCAGACCCGTCCGACTCAATATTCTGATTTGCAAGTTGTGAATTTAC
ATCCATTTGCATGGCAACAGATTGTGATGAAGACTCATCCGATTGCTCCTCTGCAATCAT
CTCAGCATATAGCTTCTCCAAACTAGCAATGACACATTGATGATAATTTCTCTCAGCATC
GACCATAGCAAAAAGTCGATGATATGTTATCTGTTGCTGCTGATTTTCAACTGACAACAT
GGCATCAGTTGCTTCTCTACCAAGGGCCTTCACAGAAGATTTAAGTTCTGTCAATCTCGC
TTCTGCATTTCGAAGCTTCATAGCACTCTCTGCAGATATATCAGATTCCCTGATCTTTGA
TCGACGCCTCATTACTTCTGCTGCCTGGGATTCAACCTCATGTCGGAGTCTCTCATAACG
ATGAGTCAAGTGGCGAGCATCCTCCAACGGTGCTCCATTTATTAAAGCCTTAAGAGGCTC
AGATACCTGATCACCAAGAATTCCAAGTAAAGCTTCCCTCTCATTCTCCATCAAATCATG
ACAAGAACCAAAGTGAAGAGTAGCTCTAGCAAGCCGAGAATCGTCGCTTTGATTCTCAGC
TCCGTATTTACAACAATCTTCAGCCAATCTTTTCACTATCTCCATTTGCTTCAAGCTTAC
AGCAATAAAACCTTCAACGCCACGAACAATATTCCTCTGAAAATGCTTAGCTGCCTTGGT
AGAAATATACAAATTTTGAAGTTGTTTATGACATTCAAGTTCAGCCTCATCAACCACAAC
ACCTTCATTACTAAGATGTCCCAAATGCTTCAACACCGCTTGTTGCTGTTTGGCAACTTG
CTGTCTCAGCTTGATTGCTTGCTTCCTTATAGCCTCCATAAAAACGCAATCACCAAACTG
ACTTCATCAAGCATCAAGAAAGTGACGAATCAATTAATTGACAAAGGCTGCAATCAAAAA
ATCAAGAGAAGCACACAAGCGCGTGGAGAATAAGGAAGC
>tig_1425_consensus
TACCATGCTTGCAGGTGCTTGCTCCTGCCGTTCAACATAAGCAGAGGGAAAACATCCAGC
CTTGCCCTTACACTCCCCTTCCGACCATCCGGTAGGACCCACCTTGTGATGTAATAATAA
CAAATACTTAGCCAATGGAACATTAAGATCAAACATCCCTTCACTGTCCAACTACTGTGA
GTTTCAAAGACATTAGATACACAAAATTAAGGTCAGGAGTTTGAAAAAACGAGACCTTCC
TGGACTTATCATTGATATTTTTATTCGCGTCTTTATATTCTTCTCAGGAAAGAGATCAAG
GCAAAGAAAGCAGGAATATCCGGAGGCTGGTTTTATTCCAATATCTAAAAGTAATGACCC
CAAATCCAGCTGGCTGGCTCAGACAGAATTACTAACCGAAAGAAAACCACATAACAGAGA
AAATCCTACTCTGTGAAACAACTGAGCAACAGACAATTTAAAAATAACTCAGGCTTTAAA
T
>tig_1426_consensus
GAATCAATCAGCTGGGAATGAGAATGTAGTGGTAGAGAAGCTAATAGCATTAGTATCCTC
GCAAAGAACTAGTGCTCTCTCCAAAGTAGCAACAATGTCTGTCACACTTGGCCTCTCCTT
TCCTTCTAAGTTGACACATCGAATGGCAATAGCAGCCACCATCTCCAACCCCTCCGTCTC
ATTAATATCCGGTGCCACTACCCTCTTATCTAACACGCGGTTTAGATGCCCTGCTGATAT
CTCTGGCTCTGAATAGGCCACCAGGTCGCTTGCACTCCCATGTTCATTTTTAAACACAGC
TTTTTTTCCTGTCAAAAGCTCTAATAATACAACCCCTAAGCCATAAATATCAGTTTTTGT
TGTAAGTACATTTAGTACATAGTACTCAGGATCTATGTACCCAAATGTTCCAACTGCCTT
GGTTGACACTGATTCTTGATCAGTCAATAGAGACAATCCAAAATCAGAGACTCTAGCAGT
CCAATTAGCGTCTAAAAGGATATTTGAC
>tig_1427_consensus
AGCGGGGAAAAAAAAAGCAATCAATAGCATACAACCAATTAACGTTGGCAGAGCTGAAGG
AAAAATATTTTGCTTCTAAATTCCATGCCTTACCACCAATTACCATATTGAACAGATATG
CTAACATCCTATTTATGCATAGTCGTAGAATTTTATATAAGATGTTGTAGCTCTTAGAAA
TTTACTCACCTCAAATGAATCCGTTTGGCCACAATAGAGGGTAGATCTTCAGACACAGTG
ACGTGATTCTGATATGAGAAAAGCGTTGGTGCTGCTTCGTGGTTACCCAATGGAGTTAAT
ATTTGAAGCACTTGCGTGGGGTTTGGCCTGATGGAATTCAACTGTTTCAACATCTTTAGA
AGACAGTAGAAATGTCAACAAATTAGAAAGATTCACCGCTCCATCTCAGACCAAACCAAT
TTCCATGGTCCACTTTTTGTTCTCAACCTCCAAATTCCAAATGCTACGCGCAAGGTCATG
AATAGAAATAACCCAGTCCAGACTCCAGGAAGACCAAAAACAGGAGCAGCCACAAGAAGG
AAACCTGATGAGATCACTGCAACCAGTACCATAGAGTACGCAGCATATTCATAGTCAGAA
ACCCCATAGTAGAGCCCATCCAGGACAAAGGCAAGAGCATTCACTGGCTGAGTTCCAGCA
ACAAACTGTAAGAGTCTGTAAACCACTTGGCGTGCTTCTTTGTAATTTCCTTGAGAGTAA
CCAGTAGCAAGAAGAGCCTGACCAGCCAGTGCTAGTGCATCAGTAAGCAAAGATACCGTC
AACCAAACTTGTACACAAATTTGATAACCAGCCATTGGTATGGGGCCCTCCCTAGCTGCC
ATGGATGTTGCCAGTGTCATGGTCAGAAGCACTGCTATTGTCCTGCCTATAAGAAGACCA

CCAGATTTCAGGTAACGCATTAATCTTCCACCATCAATTTCTGGGGACATCAATGATATT
TTGCCATTCAATTTCCAGAGAAGGATAAAAGCAATTAAATATTCAGAAATC
>tig_1428_consensus
GTTTTCCTCTTTGCCAATTCAAAATGCAAAAGGTACTTCCATAACCGCCTGAAGCCGTCA
AAGCTTACATGGACAGCCATGTACAGGAAACAACATAAGAAGGACATTGCTGCTGAGGCT
GTGAAGAAGAAGCGCCGAGCCACCAAGAAACCATATTCAAGGTCTATTGTTGGTGCCACC
TTGGAAGTCATTCAGAAGAGGAGGACTGAGAAGCCAGAAGTTCGAGATGCTGCAAGGGAG
GCCGCACTTCGTGAAATTAAGGAGAGGATCAAGAAGACTAAGGATGAAAAGAAGGCAAAG
AAAGCAGAGGTGATGGCCAAGACACAGAAGGTCAAGGGTAGCAATCCAAAAGGTGCTACA
CAAAAGGGCCCCAAGCTTGGTGGAGGTGGTGGAAAGCGATAATTTACTTCATCTCATGCC
TCTTTGATGAGTAGTATAAACAGTTAAGATTTTGTTTTTGTATGTCATGCATGGATCCAT
TAATTATTATGGCCAGTTTTCTTTGTTAATGATTTTTGTTGGGAGTTATATTTGCTTACA
ATTTATTTGAAAGTTTTCTGATGCATCATTTGATATGGTTTTGATTTATTATATGGTGTT
TGGTGTTTTGGTCATTGGATTTATCACATAACTAGCTTGTGTTACGGCATACAATTTTCC
CAGATGTGACTGAATAATCGCCCGTATGTTGTCATTTCAATAGGTTTTGTTTCTTCATTA
GTACATGTATTGTGAAGTCTTAATGGAAAGAATATATGGAGCATCAAGTTGTTGTTGTAA
AACTGTATGATTAATGCGGAGGCTGTGTTATTGAATTGGTGTTG
>tig_1429_consensus
CTCTATATATATCATTCTAGAAGGAACATAAATGGACAAAATTTCATGCACTAAAGAAGA
TAGTACAATCTTTTTAAAAATACGAAAAAAGGAAACACTAAATTGACAAGGAGAACTTTG
CTAAACAATTGCACCCATTTGAAAGCACATGCAAGTAACCCCAACCCTAAATGGAAGTAA
AATGTAAAGACTTGAAAGCCTTGCGGAACAAATTGAAATTCTAACTCCATTACCTGAGAT
TACAGGCCATACTTTGATCAGATCTCTTCTAACTTTATCAGGCGACCTTAAACTTTAAAG
ACCATAAAGAACCCTCAAGAACTTGCATGCAAGCTCTTCCGTAATATTTAAGGGCAATTG
CACAAGCTCTCTCAAAAATCAAACAAAAAGGGATCACCATAATCCCAAAGACTGGTTGTT
GGATACTCCTCATCATTGGTCAACGGCCACACCGTTGGCGATCCCTGTCCAACCGACACA
ATCGGATCGGGGGCAGTTGGTGCTACAATAGTCGGAGTAGAAAGGCATAAAGTTGAACCC
ATGGAACCCGCGAGAGAATTGACATCATTGTACGGGCTGACGTACGGGATTATTGCTCCA
CCAGAATCTCCGTGGTTAATAATCATTCCATCAGTCAGAGGATGCTGATACTCGTGAAAT
TGTTGCATCAGTTGTTGGCTATTATTTTGTACTATGTTCGGACAAAACATTGTGGAACCA
ACATTTGAATAAACCCCGTGGGGCATAAACCCAGTTGAAGAACCCATTCCAGCGGCCAAA
GAAATCGGCTGAGCCATAATTGGTGCGGAGCCGGAGAAAGTGAAGCCAAACCCTGACGGA
CGAGGAAGCAAAGGCCTGAGGTTTTGAGTTGAAGAAGAAGAAGAAGATGACGAACTACGA
GAGGTTGAAGATTGTGATGACGTGGAGCTGGAGGGTGTAAGATTAAGCTGAGCTCTTGAG
CCGTAGAGCATAATAGCAGCTCGATCATACGCACGCGCCGCTTCTTCGGCAGTTGCAAAA
GTTCCAAGCCATCTACGAACCCGTTTCCGAGGCTCACGGATCTCAGCTACCCATTTGCCC
CAACTTCGTTGTCTCACTCCTCTGTATCTAAACTTACTATTGTCTGGTCCACCTTTGCCT
TTGCACTTCTTGGTGTTGGTGTTGGTGTTATTATTAGCGATTGAATTGTTTTCACTGGTT
GTGGTGGTGGTGCTATCAGTGCTTTTAGTTTTAGTGTCATTGGTGGTGGTGACAGTGGTG
TTTTTATCCTGATGAGGGAGAGGAGTGTTGAGGCCTGAGTCGTCCATTTAGATTTAGGGA
TGTGCTTTAAAGATTTGAAGTGAGAGAAAGCTGGAGACTCACTCACTCACCTCGTTTATT
GGTGTTATTGTTGTTTCGAAATAGAGGATGGATGTATTTGGGTTATTTCGAGTCTGATTT
CTTAGTTAATGGTGGACTAGGGCGGTGGCCATTTTTGTGTAAGCGTATATATAATGTGAA
TGGG
>tig_1430_consensus
CCTGAGGGTTCTAAATCACTAATTTTGCAGCGATGCCTCTTCACTCCAATGAGTCCTGAG
CGCGGGCAATACATCATATTTCTCATAATTCCATAATTTAATCGGCATAGGAATTGCCTA
TTGAATGCTTGTGTCAAGCAGATCGTAATATGAACCGAAGATCCCTTAGTTGAAAAGGGA
TCAGCAGTTGCAGCAACAAAATAATGTAAAAAGAAAAAAAGAAGGCAGCAGTAAAATGCA
AGGAAAAAAGAGGTCAGTGAGCATTAAAGGGTTTCCTCAATCTTGTCATGCAAGGTGACC
ACCAAATCATGAGCATCATCTAAAAGCTTCCAAACATCCAGATGGCCAGCCATGCTGTTG
CACTCTCTCATGATCTCGTCTATGCTGCTATACTTCTCTATGAAAAGCTTCCTCCTTTGT
AATACACAGGCTGCAATTGCATAAAGCAAAAGATCATCAGTGGGAGGGGCTCTTACCCTC
ATCCTCCCCCAAGCTGATTTTGCAATCCCAGCTCGAATGGCTGCCTGGTCTGCCCACATC
ACTTCCCACAGACAAAGTGTCTGCTCAAAAGTTAACTCCCTTCTAAAAAGAACCACCACC
ATTCTATACACAAAGAAGCAATCTTCTGCTTCAAGCTTCTCCAAGTGCCGGTAGAGATGA
ATATCCTTGCCCTTGATTATCTTGGAAACAATACTCAATTGCCTTCTTATTCCAACTTCA
TCAAGCCGGAAATTATGCCTAGCCTTTTTCATGAAACCCACGAAGCACCAGAAAGCTTCA

TGGTCCTCCTCCAAAACCGATGCAATTGGTGTCAGTAGATCACTCATCCCTTGGCAATAA
CCAATCTCAGGATCATATAGTGCATAGGCTTCAAGTATAGCAACCAGGCGAGCAGCATGA
TAAATTCTGCATGGCTCCAGATGATCATAATCCTTTAAGCCAACAGCCTGAGCAAAACGT
TGTGCCTTAATTTCTGATACAGCAGCTTGAGATGGTGAATAAACAATCCATTCATCATTT
GCCCTCACAGCATCAAGCCGAATGATTCTCTGCCATGTGACAAAATCTTCATCTGTGTGG
GATTTAGAGTTACTGTTGGTCTCCTTTGAACTTTCCCTTGCATTAAGAACTTCATTTCCT
GCAGCTTCTTCTGTAGAAAGTAGAGGTACATTCTCAGTTTCTTCAGACGAGTCAGAATCA
GTAGATTCGCTATCAC
>tig_1431_consensus
CAGAGCTTTCAGCATGGAAACAAAGTAAAGACTCGTGAAAATAAAATAAAGAGAGAGGGG
AATTTTGTTTGGTAATGGTTTTAGACATTTTAGGCGTCAACAAGGATCAGGACCTAACCG
TACTTTCACTGTTGATGCTTATTCTCACCGCCGGGAACGGTGGGAAATGGATCTTGTTCG
CCGAAGGTAGCGGTGCCGGTACTGGAGTAGGCGGAGGCGGAGGCGGAGGTTTCTGGTATT
TGGCTGCTCCGTCTTCCAATGTCGGAATAGCCATCGCCGTGACGGCCATGGCCGGTCTTG
CCTTGGCCGCCACCGTCTTGTACTCTCATAGGGGTACTCTTAAATCACCATGGTCTCGTA
GGAGAAGAAAACATGCGCTTCTTGCCAAACAATGGAAGAGCTTATTTACACCAGACGGAA
GACTTATTGAAGGTGGCATCAAGTTCTTGAAGAAAGTCCGTAGGGGGGGTGTTGATCCAG
GTATTAGAGCAGAGGTTTGGCCATTCCTTCTTGGAGTATATGAGTTAAGCAGTTCCAAGG
AAGAAAGAGATTCTCTGAGCACAAAGAACAGAAACGCATATGAGAGCTTGCGGAAAGAGT
GCCGTAAAATACATCTGCGTGTCTTAAGGAGTTCTAAGTTGAAAGAAACTGCAGGAAACA
GCAATCATGAGGGCAGCGGCGATTTCAGTCAAGTTCTTGATTCTCCTGGCTTCGAAGATG
TGGCAAGTTCCAGGAGGTCCTTTTCCTCCGAGGGAGGGAGTCCGGCAGCTGAGGATCTAT
ACCCCCCTTTGTATGATGAGACCCCTCAGGGCTCTGGGTTATTAGTGGAAAGAGAAGGTG
ATAAGAGTGCACTTACCTGTGAAGACACATCTGCGGTTGATACTGAATCTACTGATTCTG
ACTCAGCTGAAGAAACTGAAAATGTGCCTCTGCTTTCCATGGAAGAATCTGAAGGGAATG
AAGTTCTTAACGAGAATGCAAAAGAAAATTTAAAGGAGACCAACAATAAGTCCAAATCCC
ACACAGACGAAGATTTTGTCACGTGGCAGAGAATCATTCGGCTTGATGCTGTGAGGGCAA
ATGATGAATGGATTGTTTATTCACCATCTCAAGCTGCAGTGTCAGAAATTAAGGCACAAC
GTCTTGCCCGGGCTGTTGGCTTAAGTGATTATGATCATCTGGAGCCGTGCAGAATTTATC
ATGCTGCTCGCCTGGTTGCTATACTTGAGGCTTATGCACTATATGATTCTGAAATTGGTT
ATTGCCAAGGGATGAGTGATCTACTGTCACCAATTGTATCAGTTTTGGAGGAAGACCATG
AAGCTTTCTGGTGCTTTGTGGGTTTCATGAAAAAGGCCAGGCATAATTTCCGGCTTGATG
AAGTTGGCATAAGAAGGCAATTGAGTATTGTTTCAAAGATAATCAAGGGCAAGGATATTC
ATCTCTACCGTCATTTGGAGAAGCTTGAAGCACAAGATTGCTTCTTTGTGTATAGAATGG
TGGTGGTGCTGTTTAGAAGGGAGTTAACTTTTGAGCAGACACTTTGTCTGTGGGAAGTCA
TGTGGGCAGACCAGGCAGCCATTCGAGCTGGGATTGCAAAGTCAGCTTGGGGGAGGATGA
GGCTAAGAGCCCCTCCAACTGATGATCTCTTGCTTTATGCAATTGCAGCCTGTGTATTAC
AGAGGAGGAAGCTGATCATAGAGAAGTATAGTAGCATGGATGAGATCATGAGAGAGTGCA
ATAGTATGGCTGGCCATCTGGATGTTTGGAAGCTTCTAGATGATGCTCATGATTTGGTGG
TCACCTTGCATGACAAGATTGAGGAAACCCTTTAATGCTCATTGACCACTTTTTCGTTTC
ATTTCACTGCTTCTTTTCTTTTTACATGTTTTTGTTGCACCAACTGCTGATCCCTTTCCA
TCTCTTTAAAAACCAAGGGGTCTTCAGCTCGCATTACACTCTGCATGACACAAGAATTTG
ATACGCAATTCCTGTACTGAAGTAAATTATGGAATTATGATACATTGCCCGCGCTCATGA
CACATTTGAGTGAAGAGGCATCACTGCAAAATTAGTGATTTAGACATTCAGGTGGCTTTA
TTGTTTCTTTCTGTACCATTGCTTTCTGTGTGGACACTGTTGGCATCTCCACTCTTTTTC
TTCTTATTATTTCTGTGAGGTGTATTCAAGCCGAGTTAATTCTAATTTAGTTTGAGAGAT
AAATTTGAAGTGAAAAAGGATAGGAG
>tig_1432_consensus
TCTCTAACTTTCTTCTGCAATGTTAACCCACCTCTGGTGTAAAGTAAATACATTTTTGTA
AAAAGAAAAAAAAAAAAAAAAAAAAAAAAAATCTGCAGATTCACCCATGAATCAAAGTTCG
GATGGGAGACCAGTCTGTCTCCAGGCAGCATACCCTCCAGCTATATCAGTAACGCCCGCA
AAACCCTGAAGTTGAAAACCAAATGCATCAGCTAGATTAAAGTAATTCAACCATTAAATG
TAATGCAGTATCAGGGCCTTCATAAAAGTTCTATTGGTTATTGGTTCTCTCTCATCCCAA
AAGCCGGCTCAAAGGTGAGGTTTTGGCTCAGGCTAATATACCTATCACAAAAAAACCCACA
CTAGAGGAATCCGGGTAATGCCCTCTGGCAACCCTAAAGCTCGTTAAAGGGTGAACCATA
CCCAGCTCAATTCCCAACCACTGTGAGATTCGGAGCACACTTGATGTTGAAAAGCAGCTA
AAAGATCCGTCGCAGCCACGAGAGATATTTT
>tig_1433_consensus

GGGCATGTTATATGAATGGTTTTTACTTGCCTTGTAAAAGAAAAAAAGCTTGTCCCATCA
AGCAGCTCTGTGAAGGCTGACCTGAATGCAGTCTTCCTGTAGAGCATTTATTCTGAGAAA
ATTTCTCGTCATTGGAAGCATACATGACTTTTCTACANNNTCTTAAGATCCCAAAATAAA
GCAAAACTGGTACATCCTGTTCCATTATGAACGACTATGACTCAAATTCTGGAACCGTGA
ACGGGCAACGGAACGGTCCCATTCACAGCGGAACAGCGATGACACATTTTAGAAAATTTG
ATAGAACATCGAACGATTAGAGCGACAGTTTCGTCACCACCTAGAACGGCAGTAACTGAA
CCCAAAACGAGACACTTCTTCCAATCCTAGAAGAAGTGCTCTGTTCAGAGATTGAAAAAA
GAAAAAGGACTACATTATTGTTATTGATTCAAACTTTCCACTAACTTTCTTCTGCAATGT
TAACCCACCTCTGGTGTAAAGTAAATACATTTTTGTAAAAAGAAAAAAAAAAATCTGCAG
ATTCACCCATGAATCAAAGTTCGGATGGGAGACCAGTCTGTCTCCAGGCAGCATACCCTC
CAGCTATATCAGTAACGCCCGCAAAACCAGCAGCTAAAAGATCCGTCGCAGCCACGAGAG
ATATTTTCCCAAGTTCACACCCAACAATAATTTCATCGTGTTTTCCGAAATGGGCTGATA
CTTCCTTCAGGAATTCTGGGTTCTTTGTCATCCC
>tig_1434_consensus
CAATCAACTTGAAACTCACAAAAATTCTAGTACATAGCATAGCAGAATGTCTCAATGCAA
AAAAGGGCAGAAAGCAGTTTCCGAGTTAAAGACTTATATAGTCCTAGTGAACAAACAAAG
CTTGTAAATAGAAAAAGTAGGTGAAATTAGTGTTCAGAANNTTTTCCACAAGCAGTCCCC
AAATCTACATTGTTCCCGTTGTAGTGATGAACCCCAACCTTAGCCAACATTGCATAGTAC
TCTATCTCAGACTTCCGAAGAGGAGGGCAGTTGTTGGCAATGATAACCAGTTTTGCTTTA
GAGCTTCTTAGGGTGTCAAGCACAGTTTTGTATCCCAAAGTGTACTTGCCACTCTTCATG
ACAAGAGCAAGCCTGCTGTTGATGCTTTCATGAGACTTCTTAGTCTTCTTGGCGGCCACC
ATCCTGTTAAGAT
>tig_1435_consensus
AGCTCACATGTATCAGCAATTCCTTCCTGATTTCATGCACAACCTTTTGAAATTTGGGAA
GATAGCTGGAGCCAGAGGATGCAGGTTTTGTTTAGACATCAGGATAGGACTTGGTTGCAA
ATTGACATGGGAACTGGGTTTTCTTTGTGCTGATGTTATGATATATAAACCCTTCAGGAA
ACAACCGTAACAAAAGATTTAAAGAGTTATCTGGAGTATCCTTTGCTTTTATGAAGGGAA
TTTTGGTGATTCATCATGAAAGCAAGTGATGTGGTGTTGATCCTTGAGGCATAGGAAAAA
GCAGTTCTTTTTACTATATTTTTTGTACACACTGTACAACACTTGAAAGCAGAGTATGTT
GCCAATCTCTTGTTTGCTACAGTGTCTATCGATTAGCAACACAATCTCTTTTCTTTTGCT
ACAGTTTCTATCGATTAGCAGCACAATCTTTTTTCATCCAAGTCACAACACTCGCAAAAT
GCAATCTAACATAACCACCTATTCCTGCCGACAATTATCTGCATACCCTTTAATTAAGCT
TCTGAATAAACCGAACTTCTCCAAAACTTTACCACTTTGCGAACATCATAAAACCGTATT
CAGATTGATATATCTTAGGGCATCTAACAACTCTTCTGAAGGCACTGACCCAGATAAACT
AGGTGCCAACTCAGAGACATCAAGGTCAGAATATTGAGGATGGAAGAGTACGTATTTAAC
TTCCTCAGCCTCTCGCTCAATCTAGTGAGGTTTGATTGGACTGGCACTTGCCATGTACTT
TCTGTAACCGGTGGAGCTTCCCCTGTGGTGGTGGTCATGGCTGCGGCTGTTGTGGGGTGC
TCACGCACGGTTGCCCTGCTAGTTTCACCTAATATGCTCTCTGTGCCTCTTCCTTCTTCT
TTCTCAACTCTCATCCTCTCGAACATCACCTTACTAGCTCGAGGCTCGAAGTACATTGAA
TTGACAAGAACCATCAAAATGGATGCTAAGAGATTACATCCCTGAAACATCTCTGCCTTG
CCTCTTACAAAAATCCGTGGCATATGTCCCAACAAAGCCAACACAACACTCGACGCCATC
CCTCTGAAATAAATTGGATATATCCTGCTTTGAACCATCCCAAATTGTTGCCTTGGCAGA
ACCTCAGCCAAAACATAACTTGAAACAAACGAAACCCAAACACACATCCCATAAGCTATT
GCAAATCCAAGCAAATTCAACACGCCCATAAGACAACCAAATGTTTCAGGGGGAACAAAT
GTATTAAAGACCTTGTATGTGCCAGTTTTAACCCTTTCTCCTGCATGTTCTTTTGCTTCT
TTTGTGTTCTCCTTTATAATATCATGACAATTAGCGACTTGCTGGGACATGTTCCTCGCT
ATATTTCCCCTGATCATCTCCCCCAAATCCTTTGTTTCTTTGATAGTGCCTTTAGCTTTC
ACCACGGGCTTTTTCACTGTTTCCTTTACCTCCTGGGCCGTTTGAGACACTCCCTCTTTC
ACTTTACCATATGCCTCCTGTGCAGCCTGTTTTGCCCCATGAGCCTTCTCCGAAAACCCT
TGTTTGGCTTCGCCGAATGCATCTTGCACAGCCTTTTTGGCCTCATGAGCTATCTCTTTA
TTCCGAGCAATGGCATCATGAGCCACCTCAGATACCTTATCTTTCGCCTCGGCTGTCTTC
TCATGAGCTGCCTCTGATAACTTCTCCTTGACTTCACTTGCCTCCTCTTTACCATGAGTT
CCCGCTTCTTTTACTTTATCCTTGGCTTTCCCAATAGTGCTTGCAATCTTATGCTTGCAA
TTCCCATAAGCATCACATAAAAGCTCTCCAGGACCATGACCGTGAGCTTCACCAGAAGAT
TTTGACCCTGAAATTCCTTGGTCAAGAACTGAAGAGGCTTCTTTGATCTTACCTTTAACA
TTGTTGTAAGCAGCAGAAGAAATCTTCTCTTCAACATCTGAAGCCGAACCAGAACCAGGC
TTCTTAATGTAATATTCATTTTCTGGAGAAACGAAAATTTCGGTGTTGCGTTGACCATAT
TCATCATTCTCTACAACAACTCTATGGCCTTCTTCGACAATAACTTGTTCTTTTTGGTTA

TCTTTTCCTGGGTTTGGAGAGAAAACTCCTGCGGCTACAAGTGAAGCAACAACAAGAGAT
ACAGCCACAAAATTCATCATTCTTTTGATGTATATTTTATGGCTCAAT
>tig_1436_consensus
GGCACTGCAATCTATCAGTATAATTTTTCAATGGCATCTAAATTAGTACGCAGGAAATCA
AAATCGATACTGCACTCAAACCAACTAAAATAGAGTAATATAAAGCTACTATGGCTTAAA
CACTATCACAGACAAGACAATTCTGCAAGTAAACAATTATAAGCATGCCGAGTTTAAACT
ACACTCAATAGCAAATACTAAATAAGATTGGCATCTAACACGCCTTGAAACTACTCTTAT
TACTATCAAAATAGTACTGCAAACTACTAGGATTCAAGCAATCCAACCAGTCCCAAAGAG
CAACAACATCAACTTGAGCTGCTCCCTTTGTTGCAGGAAACAAGAGTGTGACCTCATCCA
CAAAGAGATGGGTCTGGAAAATGGAGCTGAGGTCCAGTTGACCAAAAAAAGCAATCGAAT
CACTATTGTTGGGCTTGTCGGGCCTGAGCACCACCATATCCACCCCCATAGCCAACTTGC
CCACCATGAGGACCATTTGCTTGAGTTCCATAATTTCCAGAAGCTTGTGAAGCATCAGAT
CTCCAACCTGCATTCCCATACCCTGAATTTCCATTAGAATCACCATAGCCTCCTATATAG
TTGCTACCACCCCCTGGCAATTCTCCTGCACCCTGATTACCAGCATTACCACTTGGAACA
CTCCCAGAACGTCCACCAACAGTTCCATACACAGGGGCATTCCCGTAAGAACCTTCACTT
CCACCATACCC
>tig_1437_consensus
GCAAGATGCATTGCAAGATGCATCTATGGATTTGGTTTGATACGAAACTATTAACTTAAA
TAACAATGGTAAAATCGCCATTACACCACGAAATTGAAGGGAAAAAACTGATACAACCAG
AAAGGCCAACATTTCTGCTCCACCGCTCAAGCTTGGTAAACGTCTCGTCTGAATCCAGAT
GAAATGGTATTTAGTCCGAGGTTGGGACCAAAAACCACAGGTATTATTAATTTATATCTA
ACAGCCATTACAGAACAAGCTCATCATCTGAATCATCCTCCTCAGGCCATTCTAATTCTG
CCAGATCAAACATGTTCCCCCACAACGGCCAAGATACACTAGAGTTGACAGACCAAACCT
CAGGCTCGGTGGGTGGGGTATAACTATTAACCTTCAGCACAAATTTTCCAGAGGGTTCGA
GGGTATCAACCGCAAAGGCAGGGGACAGTGATCGTCCCACAGCCTGAACATGAGACACAC
AAATATAGTATAAGCTATCCATTTCTTGTCTCTGAACCCTACC
>tig_1438_consensus
GTTCTAGTACATAGAGTAATTCTCAGAATGCCCGGGTCGGAGGCTTTTATAAATTGTCAT
CACAACTGGAATAATGCCCAGAAGTGCGATCTGAAGTTGATCTGATGTGATGGTGCGAAC
AGTAATTTGACCGCTAAGTTTGTTGTTCAATCCCGCACGGACAGCTATCTTAGAAGCTCG
CCCAACCGAAAATTGAGATTGAAGGTTGGCCCCCAAGGCCAGGTCACCTCTCCACTTCAC
TAATGACAAACCCAAAGTTGACTGATCCTGGCCTACGGGAAAATCAGCTTCCCTCAGCCT
TACTTCCAAATTGGCTCCATATGCAGAGTCGCCCTGAGATCGGACAGTCCCAGTACTACC
CACCAACATTAAACGTTTTCCAATTGCAATCTGATCCTCAAGTTTGACTCCAGTGGCAAC
ATTTTCACCTAGAAATGTAACAGATATTCCAGCAGCTGTCTTATTCTTTCTGAAATTATT
GAATTTGGTCTCTCCTCTGATGATATATGCGAGTTGCTTCCCAACATTCTGGATGTCAAA
GCCCATCATACTTGAACCATCCTCCCCATGCTTAGCAGCTACTGAAGAATCTAAATGAAT
GTTGAACTCTTTTTTATCCTTCGTCACTTGAACAGTAACTGCTGCTGGAAACCGACTAGC
AATAGCCAAACTTTGTTCAAGGTTAACACCATCATATCCACAATCATGGTCCCAACCATG
GGTATCCAGAACTGGCCTTGCCAAGAACTGTGATGTTGGTTCCAAGAACCTGTACCTGTA
AGCTGGATTATCACTGTCAAAGGAAGGTGGCAAAACCATATCAGGTAATGGGACAGGTAC
AGCAGCTGGACTTCCATTTTCCTGATCAGCATCTTCTCCAACATACCCATAATCATCAGC
AGCAACTTTTCCTTTCTTCTTCATCTCTCTCATTCTTCTCAATTCCTCTTTCCATTGCTT
TTTTTGTAGGAGTTTCACCCGATAATCATACTCCTCAAAGTATGCCTTCCTTTGCTCCTT
GCTAAGCTTAGCAATCTGAGCTTTCCTAAGAGGTTTGAATGGTGGGAGCTGATCATATTC
ATCCCCATCTTCTTCTTGATCAGAATCAGACAAATCAGCCAAGTCGATATCAGAATCACC
ATTATCACCACCCTGTTCAGTGGGGAGTTTTGGGTGTGTACGGGACTGCAACAGCCAAGA
TAACAAATAAGGAAGAGGAGGTGAACGGACACGAAAGCCAAAGAGTTTCCGATGATCTAA
AGTATCTTGGGGTTTTGATAGAGAACTAGCTTCCGATAAAATCTTCATCGAATAGCATAA
CAGCAGTAACTGAGGTCTCCAAGCTTGACCATTAGGAAGCACCTTTTGTCCATCTCTATT
CTTTCTACAGGCAGGATGATTCTCAACAAGAGAAACTGGATTCATCAAATTTGGATTCAT
CAGACGCAAATCACCAACAGCCTGCCCAATGGATTGCTGAACAACATGGGCACGTTGGGC
AACAAAAGCTTCATAACTCAAAGGGGATCCTGATGGCCCATCCGGAGGTGCAGAAGCAGC
ATGAGTTAAAGTTACTATAACGCTTCGCCAAATTGAGGGACCAAGTGCATTACTTATTGA
CCTTAACAATGGCAGATCATTAAGATCCCTGGTCTGGCTGTCCAACCGATCCACATAGAG
GATAATATCTGGGGAGCATTTCTTTGTAAATTTCTTTATAGCAGATAAGGCCTTTCTATT
GGCACCTTGCTCCACACCAGAAGATTTCAAACCTGGAGTATCAATGACTCTGATCTTAAC
TCCATCTATTATTCCAACAATCTCTTTCACAGAAGTTGTACCAGGCTCAAAAGCATCAAT

CAAAGTCTTCTCCTCACCAAAAATAGAATTTATTGTAGCACTCTTGCCCACACCAGTTTT
TCCAAGAACTAGTATGTTCAAGGAGAAGCCCAAATCATCTTTCCCCTCTGCTTCAAGCTG
CAAGGCAGTTCTCTTTGCAGCATCAAGGTTAAATAATTGGCCTGTTTGCCTCCCAGCAGC
AAGTGACAACCGATAGAGTACTTGCCCTGCAATAGAATCTTCCAGAGAGTGACCTAGCCT
GTGAACGAGCCTCAAAAATTTCACCCTCAATTGTTGTAACTTCTCCAATTTCTTTTTCTC
TTCCTCACTCAAATTCTTGAGCTCAGTCTCTCCCCCAGTTGTGAGACTGGAGGGTGTAAA
AAGGTTAGTGCGGTTTGGTCGCGGAGCAGGTCTCAAAGTACGGAGTGAGGTCCCAAGACC
AGCTGGGTGCTCAGCAGAGAAAAGCTTTGATCCATCTTGAGAGGTTATGGTTATGCTGCC
ACCATCCGCATCAGCAGCTGTAGCTGCTTTCAATAGAGCTGCCAATGCAGCGGAATCAAA
CAACTCTTTTCCCTCCCCATCCTCATCAGTGTCAGCTTCTTCATCTGAGTCAGTAACAAT
TTGACCGTCTACTCTCTGCGAATTATCATGAGAATTCTCTGCACCTAAATGAGAACCAAC
ACC
>tig_1439_consensus
CAACCGGTCTGGCAGATTTTCAAAACACTGCTGCTAATAACAAATTTCTCTGATAGGGAT
AGAAGTTGACTAGTTTTCTGACATCAACAGCAAGACTAAAACGAACACTCGGACTAAAAA
AAATCACACTAACTTAAGCTGATAAACAATGAAGCCAAAAGGGGCACAAAACATTCTGCA
AAGCATCTGAATACTCATCCTACTGATACACTGGTTTTTACAGATATTGTATCGAGTCCC
TTCAACAAATTGCCTTTTTCATTCNNNNNNNNNNNNNNNNCAAAGTTAGAAGCTCCAGCTG
CTTTCGCTTTCCACAAATAACTTTTCAACAATGTGTGTACAGACTTGCAGTTAAAACAAT
CCTTCTCGCAAGTCCTCAAAGGATATTTTGCTGATGCTTTCCCCAGTCTTTATAAGGCGA
ATTGTCCCATCATTACGAACACATATCTCTGGGACATTCTTAACTTCACCATTAATGCTG
TCATTGTAAACTCTGGTTTCAATGTCACCCTCAACATAAACGGACGAGTTTTTAGCCAGT
TGTTGAACTGCATAAACCCCAAGTGATTCATTATGCACAGCAATTCTATGCCACTGGGCA
GGTTTCGGCAAGTCTTCTGCTCCTACAAGTCTTTGGTCAAACATTCCACCTGTTCCAACT
GAAAAGATAGTTACAGTTCGGCCATTTCTCAATATTTTTTGCACAGGAGCATCTTTTACT
CTTCCACAAATAATTGCCTTATGCACACCGCGGAAATTCCAACCCCTTTTAGGATCCACA
CCTTGGGGTTGTAACTCAGGCTTTACACGAAGATAATCATCAAACTCTTCTCCCATTTCA
TCATCCTTCTGACCATCTTTGTCATTGAAAGACTCACTCGAATAACACATCTTTGAGCTC
CTTTGCACACCCACCACTAGTGAAAAGAAGGGCTGAGCAGGAACAGAGACTCTTAATGAC
TTGGCAAACTTAGCAGCAAGAGATGTCATTTTCAGTTCAGACGAGAAGCTGATCGCTCGA
AGTTGTGTGTAAGAGAGTAGAAGGAAAGGGGGAGAACAAGGGTTTAAGAGGTTTTGTTCG
GGTCGGGTTATAGGTTGATCGGATTTTTTTTTCAGATGTTG
>tig_1440_consensus
CTCTCCATTCATCTCATATGCAAGCAAAACATCAGCATCAGGATTTGTGGCTTGGCTAAG
TGGAATAGATGCCTTATATGGGCCCCCCTTCTCCTCCTTACACTTGTCAATGCTTACAAA
TTCAACATGTTTTCCACCTGACTTGGTGACACTAGTATACTTTGGTATCCCAACAAGTTC
AAGAGCATCGGCTAATTTGGCACCAGCCCAGATGGCATTTCCTAAAGCAGAGACATCCCA
GCCTACTCCCCTCACCGTTCTGGCTTGGCTCATTGCTGTCCTTCTGTTGCCAGCACACTG
CAAAGTGGCAGTGACGTTGTATTTGGGAAGCATCCTAACATCTTTCATAAACAGATCTTT
AGGATTTTCAATCAAACCACGTATCGAAACATAATATCTGTCGACGTCATCAACCAGTGG
AATAGGACCGTGATTTCTCTTGTAAAAGAAATCAACAGGCGTCACATAAGATGAAATCAA
GGCCGACCGCGGCGGCTCAGCATTGAACGGTTCCTTGGCATTAATCTTCAGACTAGGATG
ACGAGGCGGCTCTCGAGAGTAATCGGAAGGAGCTGTGAGTCCTGGCATTCTCGAACCTTC
CTTCTTCAAACAGAATTTGTGCTTCGCAGCGAGTGATTATGACCGTGAGACGAGCATAAA
CGCAACAAAATGGTGATGATGTCAAGAGGGCGAAGTTTTCGCGGTGGAGTCCAATGTCGT
TTAATTGGAGTTGCTGTTGAGAGTGTGCCTTTTCCTC
>tig_1441_consensus
ATCTGGCTCCACGAGAAAACAATTCCTCTACTCTTTAATTCAGAGGCTTGGCGTGATTAA
ATTCATAGTCTTAATGCGCTAACAAAGAACTCTGCAGAATGGATCCAAAGTTTGGTCGCT
CACTACACCTCTTTATGATGATTTTCCAACCCAGGTTTCTTTTGAGTTAGATGAACTTGA
GACACAGAACTAAGCTGGCAGCTCTCCATATTTGCAAGTAGCTGGGCTACAGGTTTCAGA
TGAGGAACACTGTGTAATCCATATTCCATATGTGCATCCAGGGAAGAAATCACCTGAGGC
ATCTCCTCTAACACTTGCCCTGTATTCTTAATCATGAAAGTTTTTGCCAGAAGTTTTTTT
GGGAGCTTCATTAGTTGGACCTGAGAAGGCGAAGAGTCTTGCATAAGGTGAAGAAGATTT
GAGATGGACTGTATGGCTTGAGAAATCTGGTCACTAACCATCGCCTTCGCTGTTCCTTCT
CGGTTGCTTCCACCTCCACTTGCTGCAGCCATCTTTACACACTTCGCACCACCCACAAAT
AC
>tig_1442_consensus

AAACTTTATGCCCTGCCTCAACCTGAAAAACCAATCCCCAGAATAGCAAGGCCCAAGGAG
ATGAATATGTATCAAAGATCAATCAATAATCATTTCTCAATTCCTCAAGCTCTTTTGGAA
AATCTCATTAAACCCCAGAAAGTCTAGGAAAGTCAGAGAAGTTTAGATATCACTCCACTC
AAGACCTAACTAAAATTAAAAACCAGATGTGATATAAAACAAAAGTT
>tig_1443_consensus
ATCTCTTCCTCCTCCCCACTCTTCAGGGCATTCTTAGCTATGCACTGGAAAGCTTCTTCC
ACATTAACACCTTCCTTAGCAGAGGTCTCAAAATATGGGATATTTCCTTTGGAGGCACAC
CAAGCTCGAGCCTTCTTCTCTGAGACCACTCTACTGTTTCCACCATCCACATCAATCTTA
TTTCCTAGAACAACAAATGGGAAATTCTCTGGATCCGAAGGACTCGCCTGAATAAGGAAT
TCTTCTCTCCAGTTGTTGAGGTTGTCAAATGATTTCATTGAATTTACATCATAAACAAGT
ACACAGCAATCAGCACCACGATAGAAAGCAACACCTAGGCTTTGGAATCTTTCCTGACCA
GCAGTATCCCAGATCTGTAATGTGAAAAGCCTATCTTCAAACTGAACTTCCTTTGTTAGA
AAATCAGCTCCAATAGTAGCCTTGTATTGATTACTAAACTTCTTATTTACATACTGGTTC
ATCAATGAGGTCTTCCCAACCCCGCTGTCGCCGAGGATGATGACCTTCAGGAGAGTTCTT
CTACGAGACGGCATCGTTTCAACACGGACAGATGTATCTGTGAAGAGCTCGGAGGAGAGA
AAGTAGCGAGTGTGGTGTAAAACGACGGTTGCGAGTGGTCGTAGAGAGAGGAGCGCTTTT
TGTTCCCAGAGGTTGAAAAGGCTGGTCGGGCTTTTCTAGTAAATCCCTTTTGCTCTCCCG
TGGTCGCGGCAAAGCTGTAG
>tig_1444_consensus
GGGCAATACGATTGGTGGCAACTTCTAACCCTTCACCAACTCCTGCCATTGTTGCCGCCT
CTACCACCACCGGAAAATCTGTTATTTCTATTAGAAAATCTGTTATTTCTATTAGAAAAT
CCACCACCTCTGCCACCCGAAAATCTACCACTACCTCTACCAAAGCCACCACCTCCACCA
AATCTCCCCCTTGACTGGTCTCTTTCTTGCAAGCGTGGTAATGCTTTCAACACAGCTATG
CTCACATCAGCCGCATTTTCCTGACCAGAGAGGAACAAGTCCAAGTCTTCCACCGGCACA
TCAAATACAGCACCCGTTCCATCAGCTGTGAGAGTAATACCCTTCACAGACTGTACTTTC
TCCTCAGGCACAAATCTTC
>tig_1445_consensus
CTGTGATAGTGACGAGAGGGGTTGTGAGGGATGTTGCACAGGATGTAGGGGGTGTTGTAG
GGCGCGATGTTGGTGCCTGAAATAGAATAAAGATGGCGGAGGATCACTGAATTTGGATGG
TCAAATTGAGTCGTAGCAGCTGAAGGAACTGTGAAATTTGAAGATGAGAATCCTGATGAT
GCTGCAGCAGACAAGACTGGCACACAAGGGGGTCGTTTGTAAGTGGCCTCACGTTGCCAT
GCCTCTGTGAGATTTCGCCTCTCTGTCACTCTTCTCTGATAGACTGGAAAGGAAGGGGAA
TATCCACCACTCCAGGTAAGAGGGTTCCCAGATACCATAAGAGCCCCATGCTGTGATGGA
ACCTTCATTGTTGCTGGCACAGAACTCCCAATCTCGGCTCCCAAATCAGTGAGGATGTCA
TGCTGCCTATTTTGATACTGCAATCCATTTGCACTACTATTCTCCTTTTGGCAACCAGGT
GAACGAGAGGGAGCAATCCCCAAATTCAGATCAAGATTCTCATTGATGACAGCACCAGTG
TCCACAGTCATCTCTCCCTCATACAAACTTGGGGAAAAGTTGGTGACCGCATCGTTTCCA
TTGAATTTCAAGGCTGCCCTGTCATAAGCCCTCGCCGCTTCAGTCTCAGTGTCGAACAGA
CCAAGATATACATACTTCCTGCCTAGAAACTGGCCCATTCGAGCTTCCCAGCGCCCACAT
TTGTGTAGCGTAACTCCCCTGTATTTTGACGATCCTCTTGAAAAGCCGTAGCTATTGCGG
CGCAGCACATTCACAAATTCTTCTTTTGAGTAAAAACTCATCTGCAGAAAACAAAAATTA
AGCAAATGGAGAAAAATATATAAACCGATAAGCTCTTTCATTAACCCCTTATATGAGAAT
ACTCATTCAGGTCTCCAAC
>tig_1446_consensus
AGAACATATGAACACGTGCAAGAATCAGAGGGTAGGGACATGTTTGTCACAGTACAGCCT
GTGACATGAATGGTGACATTAAAATAATAGCACAGGGAAGTATAGGATGAGTGAAGAAGA
AACCAGCCAGTACCAAGAAGAAAATTCAACTACTAAAATGCGTGGTTGATGGCTTCAGAT
CACAGTAATGAAAATGATTCTTTCAAAAGTAGGAGCAATCAGGAAGCTCAAGAATTTCAA
ATACAGCACAGTCCCATGATGGGCACAGATGTCAACTTATATTATCATATATGATGTCAT
CCACGTAAGGAAACTAACAAGATATTTGCATCAAGTTTATACTGGCTGTGGCCAAGCAAG
AGAATGATACAGAAGCAAACAGACAATAAAAGCAAACCAAGGGAGCCATACAAAGCACAA
CAAAGCATACAAGAATTGGCTCCTTATTTACCAGTGACATTACTTTTACTCTATCGTAGC
AATAAATGCAAAATCATGCGGTGGCATCAATAGGATGGTCTCCAGCTTCTTTCTGCAAGA
AACCTTGAACATATTCTTTCCTTGCAGGAAGATGATCGTGGGGACGATTATATGGAGTCC
AAACAGGATCACCCACAAATAGACGCATTGCCTTAATATAGTTGTCCGTATCCAATGTAA
AGCGGTGATAACAGCCGGCAGGTAAAACAATCAATCCCCCTTTCTTTACCCATACACGAA
TCCATTTTTCATTACGGTCTCTGACATCAAAGTAACCACTTCCTGCTACAGCATAGCGTA
TCTCTTCATCAGTGTGAAGATGTTCTTCAAAGAAATTTTTTATCTTTTCTTCATAATTTG

GAAGCTTTTCTGGGCAAACCTCACAAAAGTCCATGTAAGAATATCCACGTTCTTCACGAA
TCTTCTTCAGTTCCTCATCAGTTTCATAGTTATCAGCATCTAATCGCCAGCTGAGTACTC
CAAGTTCAGAAAGTTGATCCAGGGACACAAATTCCTCAGGATCCTTGTGATGGGGAAGTC
TTTGGTCTTCATTGCTATCATCCATGTACCATGCCTGAATAACTTCCTCTCTAGGATCCT
TGCCGGAGGCACCCATGGTTTCAAAATTCTTCCAAGCTTCGAAGGAACGCCAAAGAGATG
AGAATAGGAACAGATCGAAGCCGATGGCATTTTAAACTAGGTAATCGAAG
>tig_1447_consensus
GTTAAAATGCCCCGTGGTTTGTTTTCAACTGTTACAACAGCGGAACTCATTTGAAGTTCC
AGCATCTGTTTTGTTGCCATCAAAACCGTGTCAGTTGGTGAAATTTTAACAATCCGACTT
TGTTTTCTCAGCAATGATTGTTGATAGAGATGGCTTGAACATCCGTTCACGAAGTGTCTC
AATAAATGTATCGGGACCAGATACAGATGTTCCCCAATGCTTTTCAACACCTTCAACAGC
TGCAACAATAGCCTTTCCCTTTTCAGCTGCTCTTTCCATGCGAGCAATAGCATCATATAA
ACACTTCGCTATATCTAGTATAGCAACAACCTCTCCATGTTCAACAACAGGCAGATGTCT
GAATTTTCCTTGCACCATCTTTTGGAGGGCTTCCACAGCAAGGGTATCAGATAGAACAAA
AGTCGGGTTTCTGGTCATAACCTTGGATACCGGTGTCTCTTCAAGATTAATCTCACGAGC
AATAACTCTTGTTGCTATGTCCTTGTCTGTGAGAATTCCACAGAGTAATGCATTGGAGTC
AGTTAGCAACAAAGCATCAACTCTACGAGCAGCCATTCGACGACATGCTTCTTGAATAGT
TGTATTTTCAGGTATTGTAAGGGCCTTTGACAGCCGCAACCGTTTTACCGTGCGTTCTCC
AGCTAGGCCTAAAGTACGAGAAGCTGAAAAAGATCTTCGTATAGTGTCAGGGACGCCATT
CTCGGCTTTCTTCTTCCCACCGACAGAAAAATTCGAAAAAGACGTACTTTTTCTGGAAGA
CCCTCCTTGGCTTGCCATTTTCTCCAAAACACTCCGTTAAATTGTTTGAAAGACTCGAAA
TCCAACAGTTCAATCAGAAACTACAACTCGCTAGATTCCAAAGCCCAATCTTGAACGAAG
AATTGAAGTCAAATGATACGAGAAAACAATCAAGAATAAAATTGTGAGATAATGATGATA
AGCAATCCGATGGTTAACAATTTTTCCGGGAAAATGAAACAATAGCTGCCGGAAGTTTAG
TTTTTCCCGGGAAAAAGTACCGACCGGAGGGACGGAGAGAGAAGGAAGGGAATGAGAACC
AAGGAGAGTCT
>tig_1448_consensus
ACGCATTCGGTTCTGTTCGAAAATGTGATCAATCGCCAAGCAATGTAGAACACATAAATC
AATTTGTAATCCCATCTATCGGTCACATCATATTCCCAGGTTTACAAACAGCTATATCAG
AAATAATTCATGTGAAGAAACTTATCTTCTTCTCACGGCAGAGCTTGGCCCACGTTGAAT
GGCAGCCGCTGGCTGCTGTCCTTGGCCACCTGACTGACCTGTAGCCTGTTCTTCGGGCAT
ATTCATGGCTTGTGTCACAGCATTCATGACTATAAGTCCGAGAGGAATCAAATACATCCA
ATATTTGGCCCAGAAAGACCTTTCAGGTGGTGGCAGAATTTCACCCTCTGCATTCTCCCC
ACCAAGTATATCTTCAGTGAATATAGGAGCTCTTGGTGCCTGCTCAGCATTCTTCAGTAA
TGTGTGCGAGTTAAAAGACCACTTTGCAGG
>tig_1449_consensus
CATGTGTATAACAAAAAGCTCATCCAAACCCTCCCGTGGGAGACATCTTGCCTTCACAGA
AGAAATAATGTAATCCCTTCCAGGAGGGCTCAGAGCATTGGATGGCAATCTTATCCTATA
GAAGTCATCATCTTGCAATAGTTTTTCAAATTTCTCCTTCTCCACTCCCGAAAACTGATT
TCTTGAAAACCGCAGCTTTGTAAGGGTCTGGCCACCGTGGCTGGAAGTCTTGAGGCGAGC
AGAGAAAGCACCGGCAGGTGCGAAATCGGCGTCACCAAAAGCATGTTCAAGGGAGAATTG
GATCTTGGAGTCCGAATCTGAATCGGAAAATCGTTTCCTGGTCGCGGTGGTGGTGGATCG
AGTGGCGGCCGACTCATGTGGCTTGACGACCTCAGGCTGGGCGGGCTGGACCCCGCCTTC
AATGCCGAACTCCTCGTCGTTGACGAGAAGCTCGTCCGATTCGAAAGCCGAAGAAGAAAG
AAACAAAAGAATGAAGAAAAAAGCAAAGACACACAGCTTTTTCATTTTTTTCTTGCTTCT
GTTATTAGTTTTCGAATCGGTC
>tig_1450_consensus
CCATATTCAATGACGGGGCCTCTACTCCATTCAACAACTTTGTCCCAGAACCTCAACATA
TGTAATTAAATTTCGATACATTGAGTTACAAAAGTACAGCACATAATATATGTTGAGAGT
TAGAATTTGCGAGACTTTTCTTCTTGAGGTTTCACTGTATACAAAGCAAAAACTAACTAT
ATGAACTTCCTGATCATTTCTCTTACTTCTTCAATCACAAGCCATGCTTTATGCCCTCCA
ATTACTTCTGATTTCATCTCTCCATCTTTCCATATCTGAATTGTTGGCATTTTTGAGATG
TTTCCGCGCTTGACTAAACCTTGAGGTACCTTGTTGACATCTACATAGTAAAACTTGATT
TTGGTGTTATATTCAGCTGCCAATTTCTCTAACTTGGGCTTCAAATAGATGCATTTCCGA
CACCAAGAAGCCATCCAGTCAATGAGAACAGGTTGAGAAAGTTCTTGAGCACGACCCAAA
ATCTGGTCAAGCTCATCGCCATCGTTGATGGCTTCCATTTCTACAGAAGTTGGTCTTGAT
AAATCGGGCCACAAAGCTTCGACTCTCACATCCCTTCTCACATTAATCTTCTTCTCGTGC
TTTCTACTGCTTAACCCAGAAGCACTTCTTTGCAGTAACAGCCAATTACCAGAAGCAGAA

GCACTCCATAGCTGCTGCTGCTCTCTTTGAAGAAACGATTCTTTGTACACAACACCTGAA
TTGGCAGCCAACGTTGCAGCCATTTTTCTTTTTTGTTCTACTTACTGTTGATGGGAAAAT
ATCTATAGAGAAGTTAGATATTTAGAGAGGCTTTTTAGAGTTTTGTTCGAG
>tig_1451_consensus
TTGGCCATTGCTAGTCGGTTTCCAGCAGCAGTTACTGTTCAAGTAACAAAGGATAAGAAA
GAGTTCAACATTCATCTGGATTCATCAGTTTCTGCTAAGCATGGGGAGGATGGATCAAGT
ATGATGGGCTTTGACATCCAGAATGTTGGGAAGCAACTTGCTTATATTGTCAGAGGGGAG
ACCAAATTCAAAAATTTCAGAAAGAATAAGACAGCTGCTGGAATATCTGTTACATTCTTA
GGTGAAAATGTTGCTACTGGAGTCAAACTTGAGGATCAGATTGCAATTGGAAAATGCTTA
ATGTTGGTGGGTAGTACTGGTACTGTCCGATCTCAGGGTGATTCTGCATACGGAGCCAAT
TTGGAAGTTAGGCTCAGGGAAGCTGATTTTCCAATAGGCCAGGATCAATCATCTTTTGGT
TTGTCATTAGTGAAGTGGAGAGGTGACTTGGCCTTAGGGGCTAACCTTCAATCGCAATTT
TCGATTGGTCGAGGTTCTAAGGTAGCTGTCCGTGCGGGATTGAACAACAAACTCAGTGGA
CAAATTACTGTTCGAACAAGTACATCAGATCAACTTCAGATTGCACTTCTGGGTATTGTT
CCAGTTGTGATGACAATTTATAAAAGCCTCCGACCTGGCCTTAGTGAGAATTATTCTAAG
TACTAGATCCTGTGTTAAATTTTGTTGCTAGGGACACAATTAATTAGAACTTTATCGCGA
TAATGAGGTTCCTGTTATTTTGCATTCCGGGTTATTAGGAAATTTAAATAAATTCAATTC
TCTTTTTTCCAAATAAGAAACCACGTTCTTTGTCAAGAAGAGGTGTGTATCGCTGGCTTT
GAATTTGGTGCTCTTTTGTTGTTTACTTAATTTGTTCTGTAATCTCTGTGGCTTCTATGG
TTCATGGGATATTTCCACGAACTCTCTTTCTGAAGGATGTCTGATCTGATATGCAACCGT
GAAGCCATTGATACTGTGAAGGGAGTTCCCTTTCTATGTAAATTTAGATGCAAAGGTTAT
CCATTCAAAACAGATGAGAGAATTATTATTATCGTTTTTCCACTTGGGGGAAGAAAACAG
CAAAATCATTCCAATCCAAACCCTCAAATTTCATTCCCACTACTAGAAATAAACACAAGC
CGAATGCCAGCAACTCATTTCTCATCGTGCCATCCATTGGTCGACACACTGGGTCCTCTC
TCATCCTCGTCAAAGGCTTTAGCAATTCTCTCGATTGGAATTAACGGTAGATACTCCGCC
ACCGCGTATCCTCTCTCTTCCGTATAACGAACCACCTGGTTATACTTGCCCGACCAGTAG
GCAGCCCTTGGGACAACGATCTGAGCCACTTGCGGGAAAATGGGCAGCCGATTCAGGGAC
CTCCAAGCCGAAACTGCATATTGTTCGGCAGCCGGCTCGTACCTCCAGTATAGTTCCTTA
GCCGTCGGCTCGTACTTGTAATACACACTGTTTGTAATGTGCTTTACAGTGTCCACCACA
CCGGCGCGCTGAACTTCAGAAGCCATAGCTCGAGCCTGGCTCGACGCTTGCTTCACCAGA
GAAGGCACGTGCCTTTCCAGTTCCTTGAAAGATTCGCCAACCTTGTGATCGACGAACTTG
AGAAATTCAAACGGGACGTCATGAAACTTGTCGTAAACGGGAGCAGTGACGGTTTTAACG
TTTGCTTCAACGGTTTCCACCCCGAGTTTCAGTGGACCAGAATTCTCCTTGGCGTATTCA
TATAGCGTTGAAAAGCATACTATAATGTAGATGGCTGCAACTTGGACGAAACCCAGATAC
TGAAGTCCTTCCTTCGTCTCTGAATGCTGTTTGGCATCTGTATCAGACATTTTGAAGTAG
AAATATGTCTGGAAAAGAAGCATTGCAAATGTCCGGTTTTATAGGG
>tig_1452_consensus
AATGCATCACTTCTTCTGGTATACACAACTTCTGCCGATCCACTGGGACGGCCGTTTTTG
TCATAATGAATGGCGTATCGCTTCAGTTCTCCAATCTCAGAGAAAAGTTCCCTTATATCT
TCGTTGGTTACTCCAACATGCAAGTTTGAAACATATAACTTTGCACCAACTTCTATTCCA
GATATTCCTGCAGCTCTAAGGCTGTCTTCAAACAAGTCATGTTGCCAGGGAAAATTCTTG
GTTCTGCGAAAAGACTTGGCAATAGTATATGATGATGGCCGAGCTTTCACTGTGAGAGGA
CGACCTCTACGAACAGGCCCCAGCATTCTTCCACCACCATTATTAAAAGAACTTCCTGGG
CCACCACGGCCGCGACCGCG
>tig_1453_consensus
AGCCAAATTCCACCGTATTGAACACACTGCTGTTTAACAAACTGACTGTAATTTTAAGAC
CACATTGTGAAGCAGCAGTAAGCATTATCTATCATACAAACAAATTAAATACATTTCATA
TACTATCCATCTTTTTCACACATTGGAGCTTTCAAATGTGCCTGCCACAACAGCGTCAAA
ACCTTGCCTCTTGCTCAAAAATGGCAGCCCAATCTCAACTGGCTGCTTCGCTGCTCTCTT
CTTTCACTCTATACAAGAAACTATACGCCTTGTCAATTACAATGTTGTCTTCAACAGAAT
CAAGGCTCTGTGTTTTCGCAAATACTGTCGGGCAAAGAGGGAGATGGTGAGGTTGCACCA
CTAATTCTGCTGGGTGCTGGTGTGTTTCTACGGGATAGGCGTTTTGTAGGGACTAACACG
TTTTCTTGTTCCCCGTTCCAATAGAGCGCATCATTTTCATCTTCTTCATCGCCATCGATT
TTGAAGACAGGATGGATGTATGCATTTTTAAGATAGCCTTTCAAGTTAATATTCGGTTCT
CGTGCACGTTCCAAAGTGTCTTTCATCATTGCCTCCTGTAATGGAAATTTAACAAATGCA
GATTCGTAACAGCCTGTGCAGAACCTATGAAACCATATCGTCAGTACAGGAAGAGCAATG
AGAAACGGTGTTGAATGAGCAGCTTCCTTTGTACTCAGCAATCCCATAAGAAGCAACTGT
GATATAATCAATGCAGCTATGACTCGTTTATGGACGTCAGGCCAGAAAGCTGCACCGCTC

TCGTACTCTTGGTGGTAAACATTTATGATCTGATGACGGAAAACAACATAGGCTAGTGCA
AAGAACACAATTATGAAAGGGAGCAAAAGAGGAGTCACTGTGGCATAGACAAGGCCTAGT
AGAAAATAAAACTGTATACGAGGTTCTCCAGTGTTGAAGCCAAGACTTCCTGGATCCATT
GCCTCCTCCCTATCCTTTTCAGTCTTCACCAAGAAGAAATTCTTCAAATGGTAGATTATC
AGTGGTTTCAACATTAAAATCTCTCCAGCTATACCAGCCCATCCATCAACCATTATATAA
GTTATGAAGAAAGTTGCTTTGATTGGTATAGCTACTCCAATTGTCTTGGGAATTTCGTTT
GCTGACTGCTTTATAAACGAATTCAGC
>tig_1454_consensus
CTGTAAATCACTTGACAAGTCCTTAACATTGTCTTCTGAGAGCTTCTTTTCCTTCTCAAG
TTTTTCATAGGCTTTTAATGCATCTCTTCTTATATTCCTCAATGCAACCTTTCCTTCTTC
AGCCTGTTTGGCCACAACTTTTGATAATTCCTTTCTTCTTTCAGATGTCAGTTGAGGAAG
GGTCAAACGTATTACTTCACCATCATTATTTGGAGTCAAACCAACATCAGAGCTTACTAT
GGCCTTCTCTATAGCCTTTAAGCTTGATTTGTCATATGGTTGAACCAGAAGAGAACTTGC
ATCAGGAGTAGTGATTTGAGCTATGCTTTTCAAGCTGACTGGAGTTCCATAATATTCCAC
CTCAATCTTATCTAACATGGCTGGATTTGCTCGCCCTGTCCGCACTGAATTGAAATTTGT
CCGAACCATTTCAATAGTCCTTTCCATTCTAGCTTTAACATCTTTCTCAATGGAAGATTT
CTCCGCTTCGATTTCTTCAATAGTTGCACACCTCACAAATCCTGTTCTCTTGTGAACCAA
TTGTTTTACACCGACAGATTGTACGGAGAGTTTCGTCAATCTAGCGTAGTTTGCAGCTGA
GCTCCACTTGTTCACATTCACACTGCCAGCACGGCATGTGACGCTTTCAGCTCTTCTATG
ATAAGAATCTGGAAGGGAAAGGAGGGATTTGGAAGGATTTAGTTTGGGCTGGAAAATAGA
GCGAACAGACCTCGCCGAAGAGAAGTGGCTCGCCATTGAACCTTAGACTGAAAGAAAAAG
TGTTCAAAATGCGTGGTTTTGTTAGTAGCAGTGACGGAATCAACAAGCTTGGATGCAGAA
TTGGGAAACGGTTTGGATAAGGGCTTTTCAGGGAGAATGAGGC
>tig_1455_consensus
GTACCATTGTATCGCATTACCATAAAAATGTAATTCTAGGAACATAGACAATATACACCA
TAAACTCACCTAAAAAATAAAGCTAACTCTTTTGATTCACAGAAGACATAGCTCACCATT
TACATCTGTCTGCAAACAATTATTAAACCTTCAGCAACTCCTTCTCTTTCTGTTTGTAGA
TGCTCTCAACCTTCTTCATATAATTATCTGTCAGTTTCTGTAAATCACTTGACAAGTCCT
TAACATTGTCTTCTGAGAGCTTCTTTTCCTTTCCTTCTTCAGCTTGTTTGGCCACAACTT
TTGATAAT
>tig_1456_consensus
AAGAGATGGCTTGTCAAATATGGATTGTTGTTAATCAAATTGTAAGGAATTGATCGTAAT
ATCAATTTGTGGGAAAGAGTTCTGGATTGGTAGTTTCGCTATATAACATTCCTCTTTTTC
AAGCAGGCTTGTCAAACAAGGAAGAGAAAATGGATACGTCATATTTTTTACAAAGACAT
TGATAGGGAAACATGATCTGGATATGTAAATATTCTACGCGATCAAAACCATTCACCACG
AAGCTCTCGCAAAACTCTCGAGTGTGGAAAGACATCGGGATCACCACGCATGGCATTTTG
TTTCCATATTTCAGTGGCTCTCCTCAGGGCTTCTTCAGCAGTGTCAAGCTGCTCTGCTCT
CCATCCAATCACATCTTTTCCAACCCCGTATTCTTCTCTTGCATCCGCGGTTCCATCGGT
GGCTCGAGTTATCGGTTTTCTTGTATCTGCTATCTTAATTTCCGATAATGGTGATGCCTT
GTGATGACTAAAATATTCTTTGTCATCAAGGGTTTTCTCCCCTTCATCTCTTTCTTCATA
TTCTTCGTCTTTTTTCAACGGGGTGCCATCTACACCAACGCAGATCACGTCTTCCAGTGC
TAGATTAATACTGTTGCGGCGCTTTTGTTGAAACTGAGAGTTTAACGGTTGGTTTAGCCC
AGTACTATCAAGCCTAGGAGAAGAGATATGCGGTGGTTTAGGTGTCACAAGTGGGTCGGT
TTCTATAACGTCACTACTGGGTCTGTGTTCAGATGCTAGTTTGTGCCTAGCCGGTTCAGC
CTCTGTCACCTTATCCACGCCTTGAGATTCTCCTTTGTGAACATCAGGGCCAGTTTCAAG
TTCTCCCGAGTGAATAGCAGGGTCGGCAGTTCGACCACTCGAGGCAGCAGCAGCAGAAAG
AAATTTTCTTTGAAGAGCTTGATTGTTTGCCGCCGAGAAAACCGAGCCCGATCTTGCTGC
AGTGGCTACTAATCTTGATTGCATCTTGTTCTATTTTCACAAGACTTAAGCTCTGGTTTA
TGCTCAACTTCGAGAAGTTCTTTGGTTACTTTGATGCCGATGGTCTATAAGAAAGGAACC
AAATAAAGCGATGCAGAAAAATGGAGGCCTTTCTAGAGAAGACACGTGTTAACGTGGCAA
TTGCTTAAAGTTCATCGCTGTGGTTTTCGTGTCATTCAAAGACCCGTATGAATTGGAAGC
TGCTTGATGCTTCTACGAGCAGCTAAAGGGCCCCAACGTTGAGTAGGACCAACCAGAATC
CTCTCCAAAAATCGGAGTCAAACAGTCAACTGAAATGAAATTTCAAAGTCATAGGTCAAA
GGTGAAGCAAGTGGCTGTTATGTCTGTTACGGCTTCCACTCTTTTAGCTAATGACCCTGC
ATAGAC
>tig_1457_consensus
AAGGCTTTCAGACAAATTGTGACTAATAAAAAATTGTACTAAAAGTCAATAAGCCATTCC
AATCAAAACACCAGTTAAGCACAAAAAATATAAGGAAGTATGCACCGGCTGAAACCCAGA

ATAGAAAAAAAAAATGCTTTTATAGAAATGTTACAGGAAAATAAATAGCGAGATCATAAAT
TCCCACCAAGCAAAATACCATGATATCTAAACTTAAAATCAAAGAAAAACAAGCCAAGAA
TAAAAACTCCTTATTAAAGCTAAACCCTTTACAATCAGTTTTTAACTTTTCCACAAAATT
GATATTCACCACGATTCTTGCTTGCTTTTGGGGTTCATCAACTTATGCCAAATGTCGCAT
AGGCCAGCTAAGTGGCGGAGATCCTGATCCTGATCCTAGTGCCGTTGCTTCGTGGCTGGG
AGAGAAGAGGCTCAGTTCCACATAGGGGCTACTGGAAGTCCCATCTCTTCCACACGTACG
AAAAGAGATGGAACCTCGCCGCTTTGAGGTGTCTGAGTTTCTCCCAGGGCTAATTTCAAG
GCTTTCACCGGTGTGGCACTGATTTGCAATGAGAGATAAGGTGTCATTGTGATCATCAAT
AAATCTTCTCCTAGCTTCCAAGTCCTGTAGATTCCCAAGAGCTAAAGAATCCGCAACCGG
GCAATATACAAGCATAAAAACACAGACCCCAGCACAAAATAAAA
>tig_1458_consensus
TACTCATCAATATCAGTGTAAAAATTCAATGTCGAAGCATTGTTTACGTTCTATTTCACT
GTACATAACTAGCAAGGTAAATTTTATTTCACTGTACATAACTAATATCATTGAATTAGT
AAGAATTCACTGTTGAAGCATGGTTTACCTTTTATTTACAATGGCAAAGAGTAGCGGTAG
TAAAAACTATAGAGCAGTGTCCTCTTGCGAAGCCGGAACATCTACATTGTCTCTCAATTC
CCTTCCCTTGGTGTCAAATGGAAACAGCAGAGCAGAGGCTATTGCAACAACTATTACTAC
TTCGAATAAAATCACGGCTACCGTTTGGCGACACCCTGTGACAAGTCCAACNNNNNNNNN
NNNNNNNNNNNNNNNNNNNNNNNNNNTATGGTTCCAGTGGCACTCATGCGTGCTCCAAAT
AATAAGACTGTGGTTAAAATAGCAGACTGATTGGAGACCAGTGGCAGAAGAAAAATGCAA
GCTAAGAGAAACATGGTAGCCATGGAGAATTTGCGCCCAATTTTGTCCACTAGTATTGCT
GACAAGATAAGCCCAGGAAGCTCTGCCAAACTTGTGATAAACACGTTTAGATAGAGGTTA
TCATCTGAATTCAGGATAGTTGGGCCACATTTACGTTGCCTACTACTTAGCTTTGAAGTC
AACAAGACCATACCATAATATGAAAATGCAGTTGAAAAGAACAGCACCCATAGGAGAAGA
GTTGTCCAAATTATCTTTGGTGAAAAAAGCATCACAAATGACGAGAAGCCTGACTTAAAT
TTCCTGGTAATTTGTTTTCTTGTTGAGAGCGGAAGGGTATCAAGTGATGAAGCAGATTCT
TCGTTCTGTCGGGTACTTTTATCAGAAACAAGCATGCCAGAAGGGAGTTTTGCATGGTTG
CAGCGAGCTATCTTCTCAAGAATTTGATGTGCATCGGTTGTTTTACCTTTCATACATAAA
TATCTTGGTGACTCAGGTACAAGACCGTAAAAGACAAGTAGAGCAAAGGATGGTATAGAA
GACAATGCTAATAGCCACCTCCAATTCAATCTTGGCATGACAATCCAGGCAAGTGAAGCC
TCAAAAATTGTTCCAGCAGTCCAAAAAGCAGAAAATACTACCATCCACATCCCTCTATAT
GAAATAGGAACAAACTCCAGAAAATAGGCCAAGAAAACCGGCCCCCCACCCAAACCAAAG
CCAACCAAACAACGACATATCACCAATGACGTATAGTTTGGAGAGAAAGCACTTAACAGG
CCAGCTCCACTAGTTACAATAGCTATGCTGAGAAAACTCTTCCTCCTTCCATGATTGTCA
GATATTAGGCCCCAAGAATAAGCTCCAACTAGCATACCAGCAAAAACAACAGTTGTTAAT
AAACTCTCTTTGCTCGAAGAGAGTCCCCATTTTGATTTAACTGCTGGTCCTACAAAAGAG
AGAATCATAATCTCCATTGCTTCTGAAACCCACCCTAATCCAGCATATGCTAGCACAAGA
CCTTGAAACTTCCCAAACCTCATAGCAGAAAGTGCTTCGTCCAGCGAGTAGAAAGGCTTT
CCATCTTCCATCTTTCAGAAACAGATGTTCCTTCACCAAAACACGTAACTAAAACTACAC
TAGTCTTAATTATTTTGATGTTGTTTTGCATAAAAGAAGAAAATGGTCGGCCCCACTAGA
GCGCATTATTAATAAAAAGGAGGTGTTGGAGAAAGAGGAGAGCCATCTGAAGGGCCAACT
ACAAAACTGCTTCCTGTGGTATCTGTTCGATTGATGCTATGAGTGGACAGGTAGGGTCGT
TACAGCTTAGTAGTTCAGTTGGTGTTACAG
>tig_1459_consensus
TTTGTGCTTGTGGGCTAGGGTGAATATTGCAGACCGATTATTCAGTACCAGAAGTTATAT
ACAGATGTAACATGTTTACAATTTCTCAAACTTAAACTACAGCCGAATGTCAGCTCTCAC
CATCATCCCCCCCTCCGGTCTATTTTTGGCTACTTCATCNNNNNNNNNNNNNNNNNNNNN
NAGTACTACTCTAGCTTAGATCTCAATGATAACTTCCATAATATGCCTGCGGGGCTGAGG
TCACAACAGCTGGTTGCTGCGCTGGGGCTAGTGTTGCTACTGCTGCTGCAGCCGTTGGTT
GTGCATAACTTTGGTGAGGAAAGGCGGACTGAACAGAATAGGTAGGAGGATAAGCACCAT
AAGCAGCACTTTGTGGTGTTGATGTTGGTATTTGAGGCACAGCATAACCACTACTATAAC
CACTATATGCACCGTATGCAGTCTGTTGATCATAGGCAGGGTATTGTTGTGCCTGTGCTT
GCATAGCTGGAGGCCAAGCTTGGTGCTGAACACCATAACCAGCCACCCAAGGATTTGGTG
CACTTGGATAAGCACCCATCAGAGACTGGACAGCGGACAGTGCACCAGAATATGATTTGG
CAATTTTTGCCCTCTCTGAAGCCAAGAAATCCTCTGCATGACGTTTTTTAACTCTAACA
TACTCCTATAAGGTAAGAACAATTTTGCATGCCGATCTTCAGCCCTCTTGATAGACTGTG
TATCTCCAAAGAGACTGAGAAACTCCAGAAAAATGCAAGATAACTCTTCCCTCTCAGCAG
CACTTGCAGTGTTGGAGCTATCTGAATTGCGGACAATGAACTTGTCAACTAATGATTCCA
AATAATCAGTTTGTTTTGGTGGAGACAGTATAGACTCGAAATGAATTAAGGCCTCAAGAA

GTGGTTTTGATAGTTGTACATGCTCAAGTGAATTGACAAGAATTTCCCGAGCCTTCTCTG
CATTTCCAGAAACCAAGTATACAAAACGAGAGTATTGAGCATACAACATTGGTAATGTTT
GTGAAAGTTCTTTCCCCTTTTCAATAGCAATGGCCTGTTCATATAAAGAAAAGGCATCTT
CAAGATTGCCAAGCCGACGTTCCATGTTTGCATGCTTGATAACGGCTTCAAGAAGACCCG
GAGAAATTTCAGTATGTACAACTTGATAGGCTACTCGGGCACCATCTATATCCTCATTCT
GCTCCTTGATTCGAGCAGCAAATAGGTGAATCTCTGGCACTCTCTTGACAAAAACTTGTG
TTGCACGTGCAAGTGCATTGTCGGCAAGATTGATACTTCCGCTGGCTTCCATGTGCAACA
CATACCGTATCCAGTATTCAGGATAATTTGCACAGGCTATTAAACATCTTTCATATAACT
TGACCAC
>tig_1460_consensus
GCTGGAAATCCCAAAAATGTGTAACATCAACTAGTTGTAATATGAAGTTTACCACCGACT
GATGAATTTGTACAAATTTTTGTTTCATCATCTTCCTTTCACTTGGATCACAATCTCATA
AACTTACACTTGATCACATAAAGAACTTTGGTGAAGACAAAGCCATGAACTTATATTTGC
TCTAGTTTGCCTTAAATGTTAAATCTCATAAAAGACTATTTGATACAAGATAGAAAAGAT
ATTGACTACAAGAACTTCCTCGATTTGATTGCACATGTAGTTGGAATCTACAGCAAATGA
TCCTCAAATGCATCTAGATTTATCAGAACATACATTACAAGGTAGATGAAGTCCTGGATT
TGTGCATCTTATCATCCTGTGTCGGCGAACGAGGGAGTGTTCGTGAGGAGACTGCTGCAT
GGTAAGAAGACGAAGCGAGTTGTGCTAAAGCTGGAATAGCACCACCTGCCAATCCAGCAC
AAGCTGAAGAGAATACAATCACTGGGGGTGCACCAACAAGGAAATGCAACCACATGGGTC
CCTTCACCCAACTCCGCACAACTGGCATCACATGGTTGAATCCGTAGAATGCACTCGTTG
TACCACCAACAGCAGACCCAATCATAGAAAAGCTATCCCTTATAAACGGAGCCACTGTCC
ATTCACTTGTTTCCGTCTGCATAGAGAGTGGGCCTGCACAGTGATGGCAGCGAATTGTAG
ATGGGTCGGGATTGGAATCTGCTGCCATTTTTCTGTTTGTTTTTGTTTCCACCGATTCGG
TTTGTTATTTGTCCTCTAAAACCCTTCCTTTGACCTCTCTCCAGTGAGAAATGTGTGGAA
GAGTTTTGCTGTGGCTAATCTCCACAACTACCCTGAAGATCTTTGGATTAATTAAAT
>tig_1461_consensus
CTGCAAGGGTGCTTAGTTAAATAGTAAATGTAAGTAGGATCATTTACATGTTACAACGTC
AAAACGTAATTACTCTCTTCCTTCTTCTTGTACATTTAGAGGCTTCACAATAATTGGATA
CCTCTTCTGCGTTCTAGTTGTTGGAAAGAAAACTAACCTGTCCTTCTCAGCTGGAACCCA
ACTGAATCGTGTGACTAAGTGATGCAGAAACACAGAGAGCTCAACCCTGGCAAGTTCATA
ACCAGGGCACAACCGTGGCCCTCCTCCAAACGGGGTGAACACATTCACATTCACCGAACT
TCCTCCTTCTGAATTACTCTGCCATCTCCATGGATTGAAAGTGCGAGCGTCATTGAAATG
ATCTTGATCTAAATGTACAGCTCTAAATGATGCAAAAACCCTCCATCCTTTTGGGATTGT
ATAACCTTTAATATCAATATCAGTTATTGCTCTTCTAAATACTCCACTGGTTATGTTTGC
CACTCTCAGGGTCTCATTAACAACACACTGAGTAAATGGCATTGACTTATAATCACTCCA
TTCTAGACCATCTTCCTTTTTCCTCCTTGCCCTGATCCCCTCATGCTCCTCCTTTAGTTG
AGCTAAAGCGAGAGGGGTCTCCGTGAGGAACTTGACAGCTAGAGTCATTGTGGTAGAGGT
GGTTTCATAGCCCGCCACCAATAAAGCCACCAAGAAGTCGGCAATTTCCTCGTCGGAAAA
GCCATCCTCGGCTAAAAGTGCACCCAACATGTCACTCTTTCTCTCACCCGATTCACTCTC
TTTTCTCCTTTGCCTCACTACAAAACTCAACGCCTCTGCCACCTTCGTCCTCGCTTGAAT
GGCTCTGCGGTAGGTGGCGGAGAAAATCCGCAGAGGCACCGTGAAGAAGCCTTCAATAAC
GAGAACGTATTCTTTTCTAAGGCTTTCCGTCCATTCTCCGGGATC
>tig_1462_consensus
GCTGAGGACTCAGGTGCTGGGGCAAAAGATTACGTCCATATTCGTGTTCAACAACGTAAT
GGGAGGAAAAGTCTGACAACCGTGCAAGGTTTGAAGAAGGACTACAGCTACAACAAGATT
CTCAAGGACCTCAAGAAAGAGTTCTGCTGTAATGGTACTGTTGTTCAGGACCCTGAGTTA
GGCCAGGTCATACAACTCCAGGGTGACCAAAGAAAGAACGTGTCAACCTTCCTTGTACAG
GCAGGTATTGTGAGGAAGGATTTCATCAAGATCCATGGCTTCTGAGTAGGGAAGAAGTTC
CTATATCTAGTTGGCGTTGCTTATGTATAGTTTGCAAGAAATATATGATCAGAACTTGTT
ATATTTACTTGGGCTTACAAAATATTGTGTATGTGCTTCGTCAAAAAGTCTGAAACAGCT
AGTTGGGTGTGGCGTCTTAAGCTTCTAGTCGTGTTGTTTGGGAAGGCTTATGGTTTCTTC
CAAGACTTCTCTTGATAATAAAGTTAAATATATCTGCTTTGTATTCATGTGGTCACTTGG
CACTAAATTTACAGATCGAGTTTGCGCCATGTTTGTGACAGGGGAAAAATAAAAAATTTG
CAGTACA
>tig_1463_consensus
GTTTAAAGTAAATAAACCAAAGTTACAATTATCTTAATCTAAAAAGTCAAATAATTAAAG
TATATTCTAATGTAAAATATAAATTACGGTTGGGTTGCTGATACATTTCAATGTTTTCCA
TTTTTCTTCCACGTTCTGAACTTACTTTACGCCAGCTGCGTTTCGAGGCTTGGTAAGGCT

CTTGTACACGACATTTCTATCTATGGTAAAAATTCTTAATGAATTTGCGTCCATTTTTAT
CCAGCGGAATAAGGACAGCTGAGTATGGTGACCTCACGCCAGAACCGATTAAAGGACAAG
ACTAAAAAGCTGCAATCAGGTGCACCCGATTGCTACAGTTTGGTCACAGACGGGTCTGTG
AATGCGGATCACTGCCGTTTTCATTAATCTATGACGGAGGCGTTATATTTTGTCCTTGAT
CTAAAGCTCTCAACAACTCAAGACAACTCTCGCTTTTTAAAAGGAGCCAATCTCATACCC
ATTGCAACTGAAATTTACATCTTTCTTCCTACTAAAACCTTTACCTACAAAACATGATAA
ACGAGATATCCCGTGAGAGGATAACCAGAAAGGTAGACTGAACAATAGATGAAGAAGACA
ACTGCACTAAACGCAAAAACCCAAGAAGCAAAATACCCATGTTACTGTTTATCTGTAAAT
TACACCTACTTTTCTATAGAGATGACTATTTATTGACGTTCTCTGATAATCTCTTCTTCC
CGTCTTCTCTCCTCCGCTTCCTCCTCCGTGGCAGCCGTCGCAGCAGGCTGCAACAGAAGG
GCCGTGAACGGCATGTGCCGATACACACTGCCCGCATACTGCAATCCCAAAGGTTGACAC
AAGTCCAGCCGGCAGCTCGGCGGTGGGGCCGTCACGAACATCCCCGGAGGGCCACTGCTT
ACGGGTTGAACCCGACAGGACACTGACGGCGGCGACGATGAAAGAGGAACGGGCCCTGCC
GTGGAAGAAGACACGGCAATAGCGGGAACGGTCCCGGTTCCCGTCGCGGCTATGATGGAA
GGCTCCGCTTGGCGTAGGAGCCACTCGATTGTCTCCCCGTCGGAACGGTGGCCGAGTTCT
CGAGTCAGCTGGAAGATCCGGGCGGCACAGAGAGCCGGCATCCGCACACGTCGGCCCCTG
CCGTTGACCTTAGTGTGACGGTCCTTCGAAGCAGTGGCCGAAAGAGCACGTTTTCGTGGG
TTGGCCAGTTGGTTCTTGGGTGGTGAATGGTTCGCAACAGTCATGGTGGTCGTAAGATTA
GGGTTTTGTTGGCTGTTAACTGGGATGTCTGAGGCCATTTAGGGGTTTAAAATAAAAATG
GAGGAGAAAATTTAGGGTTTGAAATCGAAGAGAAGAACAGACAGAAAGAAGAGGAGGTAA
GTTTTTTTTTTTCAAGG
>tig_1464_consensus
ACACCATTCATATTTTTTTCTTCATCTCTGCCAACAGAACCTGATTCATTTTTATCTACT
TTAGACGACTCATTTAACAGGCCATCTACAATCAGGCTACCAATTTGAACAGATGACAGC
AGCTTACTATCTCCACAGGTGTTATTTACAAAAGCATGTCCAGAATCAAGAGACATGGTG
GGTTTCATTTCTGGACTTTCATTTTCCAGTACAGTTTCAATAGGAGAAGCTTCCCAGTTG
ATGGAACCAATAATTCCAGGGCCGGATGTTAAGACCACCAGCAGAGATATGCTTAAGAGA
ATAGAGGAAGGAAAGGGTGAGCCTTCGACCTGAGGCCTATCATAGAGTGCAAAAAGATCT
CTTAGACGGTGGATAACTTGGTATGCAACCAATAGCTCTAGCAACCCATCACGCATTTGG
AGTTGTCTTTCATCAGAGCTGATATGACCAAGAATCATAGCAACAGTCCACAAAAATCCA
TCCAGTATTTCAGATACAGATTCAAAATTTTCAACTGATACTTTGCTGGACTGCAAGTTG
GTGCTGCCAGGGACATTCAAAGATGCAGCAATCTTTATATAGTTCTCAAGTGCTGCCGAC
AGCATGGGGATGATTGGGGGTAAAAGATTTTGGGCAAGAAAATAACTTCTGTTCAATGAC
ACGGATAGTATAACCCTCAAAAGTTTCAAAAGGTGTACTGTCACCTGGCAAGCTTCAGGC
TTTGATGTATGAGACGCAGGCAGGGCAGAAGCTATAAAATCAAGCAGACCAGCTTGGCGT
GAAGCCTGTAGCTCAGGGTCCTTCCCCTCTAGGAACTTAATCATTTCAGCTGTTATTAAT
CCTATACTTGCTGCTCCTTTTCTTGCTTGACGGAGTTTCTGAAGTTCTTGAAGCCACCTC
CCAATCTTGGCCCTTGCTGTCGCCACTGCTGTTCTATATCCAATACCAGCATTTTCAAAA
GCAATCGGTGGCTCAGGAAATTCATATTTTAGAGCCATAAGTTTTTGCCGAATTCTTTTA
ATCCTACACTTTAGCAAATGTTGCAAGAGAAGAACCCCCTGAACCTATAACAATATTTGC
TTGACAATCGTCACCATTGTTTATTGGAGTAAATCTTCCTTGGCCATCCTTA
>tig_1465_consensus
CACTGCAAAATAAAGCTGTTTCTGCCTACTAAAAACCACCTCAACGATAAGACAACATTA
ACAAAGAATGACAAATTTCAATCTGCAGGGCAGCTACCAATCCAATAAAACTATAATTTC
AGAAAGAATTAAGAATAGAAACAACGCCCAACATATCTAATTGGCACTCTACTACGTGGT
TTGACATTTAATCCAGGTCAACTTTGGACCTCAAGAAAAATAGTACAATGCAGCAAATCC
TCGGCAAGCCCTCTACAGCAGCCAAAAAAATGCCTCTGCTACCCCCACAAAAAAAAATATA
TATATATTATAACACATCCACACCTAATACAAAAAAAGGCCATGTTATTTCGAATAGACC
TTCACTATACGACAAAGGAACACACAAAAAAGAAAAAATTATTGCTTCAATGCCTGTGGA
AACACCACTCATTGGAAAACCTGAACCTCCTGCTGCCGAGTTCAATCTGTTTCTCTATCC
AAACAACCGAGGAATCACTCCTAATTTTCACTAGAACCGCCGAGTTAGTAGCTACCCATT
GGTGGCATGCCAAAAGGTGGCATTGGAGGCATTCCCATGCTACCCATCGGTGGTGGAGGT
GCAAAACCTCCTCCCATTGATGGTGCTCCTCCCATACCAGGCATTGGTGGCGCAGGCAAA
GCAAGAGGCAGCAACTGAGCATACATATTCTGCTGCGCAATTACATCCTTCTCTTCCTTC
TCTTTAGCCTTGACTTCATTTTGCTGCTCAATTTTGTCCTTCACCAGCTCATCAACTTTG
CCTGTGTATTCACGGATGAACTGCAAAAGATACGGGAAGGCAAAGTCAACCATGTTGTTC
ATCCAGGCAAGTTCAAGAGCAACATCGGCACGAATCAAATCATAACAAACAAAGAGACAT
GATGCAAAGCATTCCTTCTTTCCCTTTTCAATGAAATAAACAAGCAACTCCTCAGCAAGT

TCACGGTCACCAGATTGTGAGGCTGTCTCCATGGCATCTTTGTAAAGGTTGTCTTTCTTT
GACAGTGCAATAGACTGCTTCCATCTGCCTGCTTTCTTGTAAATATAAGCAGCAACTCGT
CTCATTTCCAATAGCTCATGTTTCTCAATCTTTTGCGCAAGGCCTATCTGATCAAAGTTA
TCATGCATATCAATTGATTCACGTAATCTCTCGTAGTCTTCCTCTTCTACGTAAATCTCA
TTCAAAGCCTCATTCACAGCAGAAACATTGTTGCTCTGAACTGCAACCATATATGGTTTC
ACAAGGAGCAGATGACCAGCCTTTCGCATAATGTCAACAACACGTGTATGGTCCACACGA
AGTGCGAGCACATTCAGCAGGTCGTTGATAAGATCAGGATGCTCTTGCAAGTAGAAGTGG
ACAGCCTTGTAATATAACTCCACATTAGCAACTTTGACTGCAACATCTTTGAATTGCATG
TGATCCCAAGCTTCAGGGGAATGGTTCATGATTGTAGTAGCAGCATTATCAAACTCATCA
TACTGGATATACAAATAGGTAAGTTCCTCCCAATGCTGCTGTTCATCACAAACTCGGATA
AGCTTGGGAATGTTGAGACGGGTGGAAAACAATTTGATGTGCTCCATAAGCTTTTCGTAA
CGATATCTAGCATAGAGAACTCCCAACTCAGTGAAAATGCCCATATGGGCACGTTCCAAA
CCTAAACCACTCTCCATGAGAGAGATTAGTTCATTGAAATATCCTCTGTTCTGGTAATAT
TCACTGACTTCTTCCAGGTCATCCACCTGTACGATAATGTTGAGCCCACAAATTTGAGCC
AAACGGAACTCCTCTGCATCAACACAAGCAAAGCAAACTTCCTTCCATGTCTTTGCACTG
TTGGCTTTTCGTGCTGCATCAACAGCCCCTTGATATTGTTTCAGCTTCACAAGTGTGACG
GCTAACTTCGCCCAGTTAGAAATAAATGCATATATTATTTTTGCAGCCTCATAGAGGGTG
TCATCATACAAGCGATCACCCACATTTTGAAGATTAGCAACATTTGGCATGAGAATAAAT
TCTTCAATATCACCCAGCCTATCGATCTTTGCATATGCATAAATGAGCTCACTATCCACC
TTGGGCTCTTTTACCTTTTGCCTCACCATAAGAAGGTACCTCACCAAGTCATGGTAAACA
TCTGCATCCTCTGCAGCATGAATAACATCTAGGAACTGAGTAGAATCATCAGCACGAATA
AATGACTCAATTGCATCGCTCACTAGCCCCTCCCTGAGTTGAGCCTTTGCCACCTGGCTC
CAAACTGCATCTTCTTCGACACGGAATGCAAACTCCACAGCCCGTTCAATGCTGCGAATA
TTATCCAACAAGACATTGACAGCCTGAACATTCAAATTGAACTTCTTGAAAATAGCAAAT
GCTTCTTCATATAATTGGGCTTCAACAGCCACTTCCCCTACTGCAGGTCCATCAAAGTTA
TCTAATCTATTAATATAATCCATTACTCTAGAAGGATCAGCCTTGATAGCAGTCAAGATA
AGAAGGTTTTGCAAGTTGAAGTTTCCACTGAAAGCAGAATTTTGCAATACAATCTTTTCC
AAAAGTTCAATCAGTTCATGGGGCAGATCAGCAGTCATGAAAGCTTTAACAGCAGCTGAA
ACTTGTTCTGGGCTCTTGCTCTCTGGCAAGGCTGTAGATACAACTTGATCAATGAGCTGT
CTTCTGAAGGAATTCTCAGGATTAAGAACCTTCTCCCAAAGATCAGCATCCATCCTTTCA
ACTACATATCTGGCTTGCAATTTAAATAAAGAGTTCTTATTAGTGACATTGATGAGCTCA
TCATCACATTGTCCTCTCCGATAAGCAACAACTGCCAGAGTTGGGTCCCGCTTCTCACAA
TACTTACCCACCACCCTCGAGTCATAGTATGGGTTAGTAGTCAGGAAATGCTCTGGATTG
TTATTGCTGTCAATGATAATTTTACCCAGAGCATTGTGGACATGAACATCTTGGCTTCCT
TCACTTACAAGATGCTCCAAGAACTGAGTGAGTAAACGAAGTCGATTCCTCTTCTCACAT
TCTTCAACAAGGGGCTCAACTGGAAGCAAAGACCGGACGGATAGAATGAGGCCTTTAATA
AAATCTTCTGGGCATTCATCATCCAATAGCTGCCCCACAACTAAAGGAGCATTCCCAGGG
TTTACCTTCTGAACATAACCTTCAATGTAGCGAAGCATGTTGTTTGTGTACAGGTAGTGT
GTCAGATCTGGAACGAAACCAAAACGGTCACAAACATTAATCAGCGGTCTGGCATCAGGA
AGCTTTGCTTCCATCAAAAAGTTCTTGGTCTTTTCAGCATCATAGAAATTTGATTCTCTT
GTCACTCGCTCCACCTCTTTTATTTGTCCAGTTTTAGCTGCTGCCTCTATGTACTTGAAG
TGAATATCAGGATCCTCACTTGAACTCAAATATGACCCCAAGAAAAAGTACAACCCCTCA
TACGACTTAAATTGCTCAAAAATTTTTATGCATGCATCAACACCCAATTGCTCACAGTAT
TCTTTGGCAGTCTGCACAATAATCTGAAGGTTGCCTCTCAGATTAACAAGTAAAAGGTCC
TTCATGCACTCCAAAGCCCACTCCCGTGAAAGAGTACCAAAAAATTCAACAAGGGACTGT
GGCTCAATTGCGTGAGTGTTGACAATGACCCGCTTAATATCTGGTAACTCTGTATAATGT
TGCAAAGCTCGCATATAGAGACCAGCTTTTTCACAGAGTTGGGCAATTCGAGGACGATCA
TAGTGGCTGAACATTCCATTGGCCAATATGGCATCAGCTACATTCGGAAAAGTCACTAGA
TTGATTTCCAAGACCTTTGTTTGAAGGTAACCATGTTCTGGCAGATTTGGCTTCAAAACA
TCCAACAGGAAGGCTGTTGCTTCGCGGATCAGGTTCCTCTGAAGAAAAAGATCAGTAATA
GTATTGTAATCAACAGGACATCCTCCCTCCATTTGAGACATCATTAAGGCAAAATTGACA
GCCCCCTGAGGATCTGTACGCAGAATTGTTTGCAGAAGAAACAAATAGTCAGGCATATAC
CCAACCTGCTTCGAGTAAATGAGAATTTTGTCAAACTCCCTACGTTCCGCAAAAGCAGCA
ACAACCTTTGGGGTTGCTCTAGCTTTTATATAAATTTTCAGTGCAAGATCATTATCCACA
GTCTTCACAAGGTCTCCAAGTTCCTCACTGCACTCCAACTTGTCTTCAGCCAACCAGTTC
TCAAGAAGATTCTTTTTGTTCTGATTCACAACAAGACGAGATAATTCCAAAGACTCAAAT
GCATTGAGCTTTCCTCTTGTTAAAAGTGTCCCAAAGTACTGCAATAAAGGTGGTGTCTGC
CCAGCTTGCACAGGAACACTCTGAAACTTGGCCACTGTATCAGGTGTGCGTAGTAGTCCC

TGTGGAGACTCTGCAGCAAGCTCAGCTGCCTCTTTATACTTTGTTTGCGCAAACAGCTCT
TGGAAACGTTGCACAACCAGATTCTCTGCACCAGGAAGGTTCCCTCTTTTGGCAAGATTG
ACTGCAAGTTCCAAGTTGTTTAATTGACCACTAACAAAAGGCACTATTGTTGCTTCATTC
ACAGTAGCTAACAGCACCTGGCCTCGCCTATTGATGGCATAAAAACCACCCACTGAAAAA
GCTTCAGCTGTCAAAAATATTGGATCAGGACTAATTCTATTCCTGTATACAGCAGCAGCA
GTCTCCAAATCATAAACAAACAGTAGCCCAAGCTTGGTGATAACATATATCAAACTGTAT
TTGTGGGATATCTGCATTGCCACTGGAAAGTCGTCTGCAAAATCAGGGGGGAAGAAAAGA
TCTGCCTGTTTCTTTGTAAAAGATGGCTTTCCTGGCTGGGCACCGAGCTCAATTATGTGC
AGCTTTGATGTAATCTGACCAGCATTAAAAGACTTGGTGGCAAAGGAAATAAGAATAGAA
GGATTCTCATTCCCTGGAACCTTAAATTGGGCAAATGATGCAGCATGTGCTTCAAGTGCT
TGGCTACGCTGTTGATCCACAGAGAAAAGTTGCATGTTCCCTTTCACCAATTGTGGCCTC
TCAGCTGACCCAGGAGCAATTCCAATCAAAACCAACCACTTTTCAGAAGGGTCACATTTG
TAGTTTATTATCTGATTATTGGCCAAGTTAGCTGTTCTATCAAACATCTTAACCGGTTCA
GAGTCACCTTCAATTGACCAATGATACACAGAGGTCTGGGTGACCATACCCAACATCTTG
GGGCTGATCCACTTCCAAAAGACAACTTGCTCAGGCATCTGGTGTGATTTAATTTTTGCT
TTCAACTCAATGTTAAATATTTGTAAGTGATCTTGGGTGGTCCCCGGCAGTTGAGCTTTC
AGAGCAAGGATTCGAGTATTAGGATTCATAAGTGCTGAGTCTGCAGTTATAGGCCGTCTA
AGCGGCTGCATTGGCATATTCATATCGATAATCACAACACTGTTCTGAGGGGCCGTTTCC
CGCACACATATATACTTGTCCGATTCCATCGTAACATTTGTGAACGTAATATGTTGCGGA
TTGATCCCAATACTCGGCAAAGTTAAGACCTCCTTCATGGAGATCGGTGCGTTGGCTGCC
GCCATGGTGGATCAGATTGCAACAGAAAATTGACGAATCGGAGATCTACGTTGAGATTGA
ATGATCAATTTCCGGAGTCAAAGACGCGAGATTCAAATCGCTGCGAGATCTAGAGCAGCG
ATTGAAACCTGGAAGGACGATCTAGAAAGAGGAAAGGGCGAAATGGAATTTTAAGGAAAG
AGATGTTATTTATATTCTTATCCAGGCACTCGACACACACACTACCAGTGAACGAAG
>tig_1466_consensus
GTGAAACTTTAACATTGGTTTTTTGTGTCTGCGTAAATTCTCAATACATGAAACATAATT
TTCTCATTTCAAAATAGTAGACTAGAAGGTCATGCTCAATTTTCCATGTCCCTAGATGAT
GCTGCTGCTGCTGCTATCTATATAGCCAATAACAAGACAATTGCCCTTCCAAGAAAAATG
CTCAACCAATATTTAAACCTTGCACACAGTTCTACGTCGTCCCAAGTGCCAGGGTGGTCA
CTAAAACTTCCCTTCTCCTCTTTGCCTCCTCCACTGTCAGTTTTCCTTTTTCTTTTCCTC
TTGTTTTCTCAAGAACATTGTTACACAATGAGCAGAAAGGAAATTAGGGAAAGCAAAAAG
GAGAAAAAAATTATGATGCAACAAGATCAGGTGTTTCAGCTTCGATGGATAATAAAGCTG
TGTGTACTTTGTCCAGCCAATTGTCCAGTCGATCACGTAAGGATTTGATCTGTGGGATAC
CCAAAACTCTTGGCTGTACCCAGGATACATGTACTGTTCCTTCAACTTGATCGATTATAC
CCTCAATGAGATGAACTGAGAGAGACTTCATCAGAAGATGCTCCACATCCTCAATTGAAA
GTTTTGTGCGCTCTGCAATGATGCTTAATGGAATGGTCCGGTCCTCAGAGGGCCGGCTA
>tig_1467_consensus
ACCTTTCGGCGGCGAAACTGACGGTGCGATCAGAGCAGTGTCTGGTTTTGATTTCAACGA
GAGACCCATCACTCCATTCGATGACAGATCGAAGCATGGAGTCGATTCGCCTCTCGAACT
CGGGGGTCCACCAGCGAGGCGACTCGACGAGAGACTGGAAATAAGTCTCGAGATCGAACA
CCGAGTGAAGAACCGGATCCTTGCAGGCGGAGTACCACGACTTGCATACGAACATGGGGC
CGGTCCATCGCTGGGACAGAGTGAGTCGAGAAAGGATGTTTAACAGACACACGTGAGTCA
ACTCGGCCCAGTTGGAACTCAGTGACGAGTTTTTCTCCTCTACTCTGTCGCCCATTTTTT
TTGTTTCCATGCAAGTCGCGGTA
>tig_1468_consensus
GAGTCGATTCGCCTCTCGAACTCAGGGGTCCACCAGCGAGGCGACTCGACGAGAGACTGG
AAATGAGTCTCGAGATCGAACACCGAGTGAAGAACCGGGTCCTTGCAGGCGGAGAACCAC
GACTTGCATACGAACATGGGGCCGGTCCATCGCTGGGACAGAGTGAGTCGAGAAAGGATG
TTTAACAGACACGCGTGAGTCAACTCGGCCCAGTTGGAACTCAGTGACGAGCTTTTCCCC
TCTACTCTGTCGCCCATTTTT
>tig_1469_consensus
GCTTTAATTGATTTTTCGCAAATTGAAAAGTTTATTGCACATCGAACTTATAAACTTGAA
TACCAGAGTTTGCGGGAAAAAATTCAACACAGAATTAACTACCATACTCTTTGATGAGAA
AATAAAAGCACGACAATCTCCATCTGCTTAAATCACAAATAAATATTCCTGGAATCTACT
GAAACTCAGCCAAGAATGGGAAATATGCGAGTAATGTGCTCCAACGGGATGCTATCAACT
GTGTTTTTCTTAAGCTCTGGATGATTAAATTCATTGCGAGGCAGGACAATCAGTTGACCC
CTCCCATGGCCCTTCTCAAGAACCCAGTCACAGTTGGCCTCCTGATGCTCCAGGAATTTG
TCAAGCGATAGACCTTCAATGTTGATAGCCTCAGCAAGCACAGGCCTGGGAACCTTTTGA

TAGGTCAAGGAAAGAACATGAATTGCATAGGATTGGATTGCTTGATCAAAACCTGGAACA
GATTCAACTATATGGCGACTCTTAGCTGCTTCATCCCAGAACTGACGGAATCTTCCAGTC
TCCAGATAGTGAGAGAGAACAATAAGTGTCTTGAACTGCTCCTCCATTTGCACTCGTTCT
GGAATTAGAAAAAGGCACAGGCTGAAATCTGGAGCTGGCATTGCCATGAGCGCCTTAACC
AAAATGCGAGCAACAATTTGAGTACTCATTCGTTCTGGCTCAAACTGATACAGGCGAAGT
AGGCAAAGATTTGCATCCAGACTGTAGGTTTGTGATGAAACCTGCTCGTTCACATAGTTT
TCGAGATCGGGAAGGATGTCGGGGTTGTATGGATTGACAGCAATCAGCTGTTCAACGGTA
TATGAAACCTGCTGTGGTTTCTGAGCCTCTCTTTCTCTTCCCATATTTTTTTTTCTCAGA
ACTTCTCGTAACAATACTCTGTTCGATCAATAAACACTTCTGAGAAACCCTAACTGAAGA
TTGAGAAGCGACTGCGAGGGTTTTAGTGTAATCGGTAGAAAGAAAGACTGGC
>tig_1470_consensus
TTGTATAATCATCAAGAAATACCAATGGAGCTTTCTATAAGAAAAAGCACACTGGAAAAA
AAGGATTTAGAAGCAGAATTTCCCATAATGATAAAATGATAACAGAAATCTGAACTACAG
AGCCAAATGCTTCATTTAATATACAGAATTATCAAACAAATGAGCACTAAAACAGACGAT
CCTTTTCAAGTGGAAACCATACCAATTAATTAGAAAGCATTCCGTAACAGAGCATACAAT
TTAGAAATGCCCTATGCTGCCGTACAGTGTGATTACAATTTTTTGTGCTTCTCGTCCTGT
ACCCACCAAAGCTACTCCTTAAGGTCAGCATCTTGCTTATCAGAGATCAATGGGAATTCT
TCATACAGTTCAGAGAAACCATGTCTCTCAGCCATGCTCTTCAATGACTCGTAAGCCTGA
TACATAGAAGGCCTATCCTTAGGCCTAGAAACCACACAAGCACAAGCAACCTTCAGAAAC
TGTGTAATCTCATCATCATCTCCTTTCCCACATAAAGCCTTATCAATGGCATCTTTATTT
CGACCAGTGATCACTAAATGATTAACCCAGTCAACCAAATTTCCCTTGAATCCTTCATCT
GCATTACTCACTTCAATAGGCTTCTGACCAGTAACCAACTCTAACAGCACAACTCCAAAA
CCGTAAACGTCCCCTTTCAGAGAAGCAACCATAGTGCTTGGGTACTCGGGAGCTACATAA
CCAAACTCCCCCAAATCTCCATTCACGAACGAACTATCATTCGAATCATGGGAACCGACG
AGCCTTGCCAATCCAAAATCCGTTATTCTAGCATCAAAATCATCATCAATAAGAATCACA
TTTGAACTAACATACTGATGCATTTGCGGTGGTTGGCACACCCGATGAAGCCAAACCAAT
CCTCTTGCTACACCCACACCAATCCGTAGCCTAGTAGACCAATTCAAAACACCATGCTGA
CTTTCAACAAAACAATTTCCATGTAGGAGCGAATACAACGTCCCATTGGGCATGTGCTTG
TACACCAAAAGCCTCTCTTCCTCCACAACACAAAATCCCAACAACGGCACCAAATTGGGA
TGTCTAAGCTGCCCTAACCTATTCATCTCCGACCGAAACTGCTTCTCACTAAGCTTACAA
GCACTCAACCTCTTAATCGCCAAGGCAGAGCCATCCGGCAACACCGCCTTGTAGGAAACA
CCAGTTCTTGTAGAAATAATAATATTTTCAGTATTAAAACCATTAGTAGCAGCCAACAAA
TCAGCCAATTTAATCTTAACAATAGGTTTCTGAAACAAAGAAACTTGAACAAGCTTATGT
GACCGCAACACTTGAACCCAACTACTATCATCTTTCGCATCATCAAACCCATGTCCACTT
TTCTTCCTACTAACTCTAACGAAATACCACCACCAAATCACAAACCCTAAAGTTACAGAA
CCCAAAGCACCAATAACACCAGCAACAATAATAATACCAAGACTCTTACCACTCAATCCT
CCACATTTCCCCAAAGGATTACCACAGAGTCCGTTGTTCCCATCAAAACTTGCTTCGCCA
AATTTCGCCAGATCAGACGGTATTGATCCAGACAGAGCATTCCCGGAAACAGAAAATTCT
TTTAACCGATCCAACCGGCTAACTTCGTACGGGATTGAGCCCGACAATTTATTGTTACTC
AAAACAAGTTTGTTCAGAAACTTGCACTCAACGATCTGCGAGGGGATGGGACCAGAGAGC
TGATTGTTTGAGAGATCAAGTGACACAATATAAGGCAGCCATGTGCACATTTCCGGTGGG
ATCGACCCGGAGATAGCGTTATTTGAGAGATCTAGTGTTTGCAAGGTGTGACAGAGGTTC
AAGGACTCGGGCAGTTGACCAGAAAGTTGCATTGAACTTAAAGCCAGACTGATGATCCGA
TTCTCCTTCTCGTTCCAACACGACACACCGTTTAGCTTGCAGATGGCGGTAGCGGTGGTG
TTGGCGAAGGACCAGGAGAGCTTTCCTTCAGGGTCTTGGAGAGAATTCTTGATGCCTTCA
AGGCACTTGACGTCATCTTCCCCTGAAAAAATAACAGAAAAGTAGGAAAAGACAAAGAGT
GAAGCTAAGATCAAGTTGATCTTCATGGTTTTTGTTAAGCG
>tig_1471_consensus
CTTCCAGTTTGCCGACAGATTTTGAGGTCATGACATGGCCTTCGCTAGATATTGATTTGT
ATTCCGTCGACTGCACATGAAAATCACAATCCATTCACGCGTTTTACGCTTCATGGTAAT
AAATTAACATTTGTCAAGTGCCACAAAAGACCGACACACATCTAAACCTGTCACGTATTT
ATAAGGTTTTTACATCACATAATTGCATTGCATCGCATTTCTACAAAATGTTTTCCACCC
AAAACAATAAACAAACAGGTCCAAAATTTCACTGCAGGTATGGTTAAATTATCATATGGT
ATCTCAAGTAGCTCCGGCTATTCTGAGTTACTAGCTCCTGAAGATTGCAGCAGAGACGAC
TGTGAGTCATCAAGGACTCGCATGGGCTTCCAGTCAGATTCTTCTTTGCTCTGTCTAAGG
GCAGCAAAGTGAAGGGCGTCACTTCGAACTTGATAAACATGACAAGTCCACGGGTATGAG
TCTGGCGCAGACCACACCTTTTGATCTTCAAATACCGTTTCCTCCGCATGTGACTCTGCC
ACACAGTATACCAAACTAGGTTCTGGCTCTTCAAAATTAGCCACAGTAACACATCCCAGA

GAAACGTATCCCTCCGGAGCACGAGGTTGCCAAATTGACACAGGAGCAGTGTAGTCTTCT
GCACAGTTCCTCCAAACCAGGTCATAACCCACAGGAGGTTTGAACAGTCTGTCAACATTA
CAATAAACAGCAGCCACATTTGGGGGGTGGCTTCCAACGTGAGCTATATCCCCGATAGAA
ACATATCCTTCTGGACAAATGGGTCTCCAAATGCTACATATTCCACAATCTTCTGAAACC
TGCTTTCTACACAATGTGCATCGACCTTTCAATTCTCGCTCACTGGTCCAGATCTTCTGG
AAGTTGATCGCATGTTTAACAAATCTCCTTTCATCAGATGCTGAACGGGATAATGACAAC
TCTCTAGACTTAATAATGGATTTATTTGATGTACGTGATTCTCTCCCATCAGCTTCACTC
CAGGCAAAATAAACATGTCTTTGGCTTGAAGGAACCTTCACCTTCCACATCTTCTGCACC
ACTGAACAGATCTGAACAGCTTGAGGTTCTCTCTCCACGGCTTCATCAACATTGCATTTT
ATGACTCTAACAAAATTTTCTGATTGCCTAAAAGTCTTGAGATGCAAGATCAAGTGAGAA
GGTTGTTGGCAGCCAGCTTTTGCCAACTCCACTGCCAAGAGCTCTTCCCATGGGACATCC
CACGTAATCTTGCAAGGTTTTTTATCCATTTTGTCAGGAGCTGAACACTGCAACAGCATG
ACTCTTTTATTAGTGACTAAAACAATTCGTTGGTAAGGCACAGCAAAATGTTTTTCATAA
TAATCAGACCAAGCAAATTTTGAGGGCTCCTTGAATATTTCAGTACATCCAAACTGCCTA
CTTGCTTCTGCAAGGTAAAGTATCATCTGACCAACAGCTTCCTTCTCACAATATTCTCTA
AGTATACCATCAGCACGAATGGCCCTTGGGTTTCTGATCCTCTGGGAGGTAGTTTTGCTA
TTGAGAACCTCTAAGCACTTAGAACAACTTGCACCTATGCCGTCAACAGTTAATGAGAAA
AAATCCAAAGCCCCACTCATTGGCTGCACAAAAAATCCTAGAAATGCACGACCAATCCCG
TGCGCAAGTCCAAATAGACCATTCTGCCTCGCACTCTCCATTGGCTTTGTCACAACTCCA
GATACCCCAAAAGCAACGCCTTGGGCAAGAGCTTCTGTTCCTTGAATGATTCCATCACCT
ACTCCAGTGATTCTCCTTGACCACACCTGTTTTGAACGTAACTGCAGAAACTGTCCATCA
GTTGAAAGCTCTGCAAATCCTTTACTAAGAGAAGCCAATGTTGAACTTGTCATTCCCAAC
ACATCTACTGAAAATATCAAATGCAAAGGATTATGAATCAAATCTCTCCAAATGCGATTT
CCAATTGCAGGTATAATGGAACTTTTTCTCATGAATCTGTCTCTCTGCATCACCCTCCTT
AAATGAACCTGAATTTTGAATGCATTTCCAACAGCAGAAAGTATGGGGCTCCACACTCCC
AAAACCCCATGAGGTCTCAGAGCTGGTGCTGTTTCCAATGACACCTTCAACCTCACTTCT
GAAACATCTATAAGGTCCACACGAATCTCTGGATCAACTTGAGTTACATTTGTACTTTGG
GGAACACGATTGAGCTGAAGATTGTTGTAAAAATCCACCAAAGCCCAGATAATCGGCTCA
TGAATATTAAGTCTCCACACCTTATCGGTTACCCGTATGTATACGTATGGATAAACCTGG
ATGCCATCAGCATTCTCATTGCGTATTGTAATTGTTGTCTTAAAAACTGGATGATGCATA
TCAGTGGCCTGCTCTGGTGCCAACAGCACCGGCAATAGAGTCAGAGGGAGCTGGTTATCA
ATCTGCAAATGACCAAGTATGAGCTTGAACCTGCTGATTGTTCCACCATCATATCCAGTC
AAATATGATACGGAAACCCTCTCCATGTACAGGTATGAGAGCTCCTTTGGCCTATGGTCA
ACTACAGAAACCCCAACAACTCCCAATTCAATTATTAACTCCATTGGTGTGGAGGTGGAC
TGTGTTTCTCTCTCCATTTGAGAATTTCCCCAATTTCCAGGTGTCAAAGACCGGATTTCA
TGTGAACTTGATGTCCTGTCCTCTGTGAACCTAGCAACCTTGATATCACCAATTTCTAAA
ACATGGAATTGCAATCCTAGTTCCCCCTCCACTGCAGGATATAATCCAGTGCTTTCCAAA
TCGAGTCTCCAAACCCCAATGGTATCATGACCATCAATCTTAGCATCCAAAAAATTTCTGG
CCATAAGGATCTTCCCAAGCAAAATTTGAGGTTGAAAGAGGTTCTAAGGAAATCCATGCA
TCCTCACCAAATCCAGACTGACGAATGCCAATTGTTTTGCTCATTGTTCTGTTTTCAATC
CTGATAGGACCATCAGTAGATCCAAAGCGAAATACAACAACAAAACGAGATCCTTCTTCA
TAGCCTCGTATTTCTGTCATCAAAAATCTCCGTGTATCATCTTGTCTCCTCAAAACTAGA
GAGAAGGTGTCCTCTTTCATAACATGAACAGGAAATGACCATTTTGTATCTTCCAATCGT
ACCTGTAATTTATCTGCTCCAATTGTCTCACGACAAACAAAGGAAATTCTTGAATCAGAT
GCACGAAGAACCTTTGATTCATCTTCACCATTCAGCTTAATGTATATGTCTTGACCCAGT
CTATTTGTGAAAGTAATGAACGGTCGAACAGATATCACCTTTGTAGGTATGGATGGATAT
GGGCAAGTTTTTGTAGAGATAAAAAGCCGCATGCATTTGTCATCATCATTGTATGCGCAA
AGATCCAGTGATCCATCCATGTCACCAAGGGGAGAAAGATCTTTAATAGGTCCAAAACGA
TCATTGCCAGATTGAGTGATTGATACTGAGAGACCCAACAATTTAAAATTTAAAGTCGAG
GCAATAGTGCAACCTTCATATATTTCATCTTCTGTTATTTCCTCAAGAATCACGTCACTG
TCCTTTTTAGACTGAAATGGAAGAGCAATTTTCCGTGTATGTTTTTTTCTGGTCAAATCT
AGAATTCTTAATGTAAGTGGGGGGCATCTGGCAATATCAAACCAATAGGGAGCATAGACT
CTGATCATCTTTTCCAGCAGTGGTTGTTCCTTGTCATAATTCTGTTCCAGAATGAGTTGA
ACAATCCTTCCAGATATTGAACTTCTTAAACACACTGTTTTAGATGGAACTCCGCGAGGG
TGTGATATACATACAGCTTCATGCACTGGCAGCCATCCTCTTTGGGGAAGAAGTGAAAGG
AATAAAGGGGTTCTTACATCAGCAGTGTGAACCTTGACAGTTTTTCCAGGAAAAAATATT
CCTCTAGAGGTGGCAACAAAATGACCAGTTGCTTGCATCTCAAGAACAGAATATTCAGCT
TTAAGAGGAAGATAATTGGTGATAGACAACGGAGGCTTAACTACAATACTCCAATCCTCA

ATAGGGTTAGAATGAATGTCTTTTGCAATCTCTGTTGCTCGTATGCTTACACAAAACCAC
AGTGTATGACAAACATCAGAGGATGTTCCACTTATCTGGGTGCAGTACAAGAGTTTGTCA
GACTCGGTGAGAGTTGATACACATATTTCAGAAGAACTATCTGGTCTACCAGAAACCTCT
GATTGCCCAGATCTATCCACTACAGCACTCCAAGAAAAATCATCATGACTACCAAAATTT
AAAGGTCTTAATTTCAACACAAACAATCCAGACTGCGTCAAGCCTGCTAGAGGAAGGGGC
ACAGAGTCTCCAGGTTTCAAGAGTCCAACAGATATCTCATGCTTCATATTGTCTGATATT
TGTTTTCTCTTCCTGATCCATCTCCTCTGTCTAGCTTGATTCACAAACTTTAGAGGATCA
AAAGATTCAGGCCATTTTAAACTCTCAACATCTGGGGCATATACCCAACCGTCAGCAGTT
TTAACCAATGATGTATCCAAGCACCAATCACTAATCCATTCCCAGCCTGAAGGTAACTCA
ATTCCGAAAGTTTCCTCATGTGGGCTTCCAACTTTTGCATGATCTTGAATAGGTTGAAGG
CCAACCCAGCCAAGTGTTGGGTTGTATTTTTCAGTTTCAAAGAACTCATCTATCTGAATT
CTTTCACTAACTATTTGTGAATCTCCAGGCTTACTTAAATCATTTAGTGACCTTGTCTTT
TGGTCGGAAGCTTTGAGCACAAGACACAAATCAAGTACAAAGTCTGTATTATTACATACA
GAGACCAGAGACCTAATATTCACATATCTATTGCCATCCTTCACGACTACTTCACTGGCA
ACAACATCATTGCCTAACCGCCAACATGCAGCAGGTGCAGCATAGTTTAGCCTTACAGTT
GTCCAGGGCCCTACCATGCTAGGACTGATCAGAATAAATCCAGATTTTCCGCCACCAATA
AGACTATCATTTCTGTCATCAACTTCAGGTTTGGGTGACAAGAAAACAGCACATCTAATT
CTGCCACACAGCTTCTCACATTTATTTGCTTCAGTTGCATTCATAGATTCTGCCGAGCAT
ATGTCTATCCACATTAAGCTATTTACATAATCATACGGATATGAATTGTCTTCGACATTC
CCAGCTATCTTATTCAATGGAGCTGAGAAGAATCCAATCGGATCACCTTTTCCTATATCA
GTCACTATTACTTCCATGTTGTAGTAATCCACTGAATCAACTTTAAAGAAAAATACTTCA
CTCCAATTGACGACTTCAAGCTCAGAAGATGATGAGTAGCTTGAGGTATTTCCACAGGTG
CGTGAACTCTGTTGATGGAGAGGAGAGTCGATTGAAATGCGTTGATTTGGAGTAAGTCGA
ACAGCCACAGCATACTGGTGAGGAGTCAACCCTCCGCTACTAGGGAACTGCGCGTCCACT
ATAATGAGTGTCACCATGGTTCTAACTTTCCCACAATTTGTATCCTTCAAGTGGGAGTCC
AACATATTTTTTGAAACAGGAACTTTTACAGGCTTCGTGTCCCCAGATGGCATTCTAACT
ATATTTGACCATCCTCTGATTTTAGTAGCTCGAATAAAAATATCCTGACCAAGTTTGTTT
TGTGGGATTATAAAGTAATTTCTTTTGTGATGGACATCAATGATAGATCTTCCACCATAA
GTAGGAGGAAAGGTTTCCCTTGTTTTATAGTACGCATGGA
>tig_1472_consensus
CTTCCTTTTATCACAAAATAGGTGTATAGGACAGTCATTTCCAAAATAAATTATGGCTTA
TAATAGGCTCTACAAGAATGGCCAAGATCTTAGAAATTACAGTCGCCGAGTGGAAAGAAA
ATAGATGTGATAAATTGGAGAGAGATTTCTCAGTGAGGTATTAACATAGCTAACCATGGC
[truncated: 955,227 more chars]
